# Supplementary material for: PhaBOX: a web server for identifying and characterizing phage contigs in metagenomic data
Source: Bioinform Adv. 2023 Aug 2;3(1):vbad101. doi: 10.1093/bioadv/vbad101 (PMC10460485; doi:10.1093/bioadv/vbad101)
Supplement: vbad101_Supplementary_Data [file vbad101_supplementary_data.zip › cherry_prediction.pdf]

Accession	Pred	Score	Type
DOF002_scaffold24242_3	Colwellia psychrerythraea	1	Predict
DOF002_scaffold67503_1	Staphylococcus saprophyticus	1	CRISPR
DOF002_scaffold60803_3	Bacteroides fragilis	0.98	Predict
DOF002_scaffold67436_2	Dinoroseobacter shibae	1	CRISPR
DOF002_scaffold63958_7	Bacteroides fragilis	0.97	Predict
DOF002_scaffold11952_4	Extibacter muris	1	CRISPR
DOF002_scaffold64073_3	Clostridium perfringens	1	CRISPR
DOF002_scaffold64389_1	Bacillus halmapalus	0.96	Predict
DOF002_scaffold5829_2	Lactobacillus gasseri	0.71	Predict
DOF002_scaffold386_23	Parabacteroides merdae	1	CRISPR
DOF002_scaffold45014_1	Bacteroides fragilis	0.72	Predict
DOF002_scaffold32188_6	Bacteroides fragilis	0.91	Predict
DOF002_scaffold5829_3	Lactobacillus gasseri	0.9	Predict
DOF002_scaffold65381_4	Fournierella massiliensis	1	CRISPR
DOF002_scaffold67459_1	Cellulophaga baltica	0.89	Predict
DOF002_scaffold2701_13	Micromonospora chaiyaphumensis	1	CRISPR
DOF002_scaffold67379_1	Parabacteroides merdae	1	CRISPR
DOF002_scaffold60803_2	Azospirillum brasilense	1	CRISPR
DOF002_scaffold67538_1	Bacillus halmapalus	0.81	Predict
DOF002_scaffold4671_2	Lactobacillus gasseri	0.74	Predict
DOF002_scaffold66657_1	Clostridioides difficile	1	CRISPR
DOF002_scaffold10448_2	Colwellia psychrerythraea	0.88	Predict
DOF002_scaffold11952_2	Colwellia psychrerythraea	0.74	Predict
DOF002_scaffold38225_1	Colwellia psychrerythraea	0.78	Predict
DOF002_scaffold60803_1	Colwellia psychrerythraea	0.84	Predict
DOF002_scaffold133_11	Parabacteroides distasonis	0.73	Predict
DOF002_scaffold13072_15	Colwellia psychrerythraea	0.87	Predict
DOF002_C840249_1	Candidatus Pelagibacter ubique	0.81	Predict
DOF002_scaffold67459_3	Staphylococcus hominis	0.74	Predict
DOF002_scaffold64032_6	Staphylococcus saprophyticus	0.75	Predict
DOF002_scaffold67426_3	Staphylococcus saprophyticus	1	CRISPR
DOF002_scaffold11952_3	Colwellia psychrerythraea	1	CRISPR
DOF002_scaffold39963_2	Colwellia psychrerythraea	0.84	Predict
DOF002_scaffold35792_4	Colwellia psychrerythraea	0.83	Predict
DOF002_scaffold197_4	Colwellia psychrerythraea	0.98	Predict
DOF002_scaffold4671_4	Candidatus Pelagibacter ubique	0.92	Predict
DOF002_scaffold64416_5	Bacteroides sp. A1C1	1	CRISPR
DOF002_scaffold44470_1	Candidatus Pelagibacter ubique	0.74	Predict
DOF002_scaffold9671_2	Colwellia psychrerythraea	0.83	Predict
DOF002_scaffold64032_7	Bacteroides xylanisolvens	1	CRISPR
DOF002_scaffold37947_1	Parabacteroides merdae	1	CRISPR
DOF002_scaffold20669_3	Candidatus Pelagibacter ubique	0.91	Predict
DOF003_scaffold26875_2	Colwellia psychrerythraea	1	CRISPR
DOF003_scaffold50654_1	Colwellia psychrerythraea	0.83	Predict
DOF003_scaffold19111_1	Staphylococcus hominis	0.85	Predict
DOF003_scaffold51061_3	Flavobacterium psychrophilum	1	CRISPR
DOF003_scaffold989_9	Colwellia psychrerythraea	0.94	Predict

DOF003_scaffold27483_2	Colwellia psychrerythraea	1	CRISPR
DOF003_scaffold269_12	Vibrio alginolyticus	0.83	Predict
DOF003_scaffold51115_1	Colwellia psychrerythraea	0.93	Predict
DOF003_scaffold2741_2	Colwellia psychrerythraea	0.93	Predict
DOF003_C746192_1	Colwellia psychrerythraea	0.94	Predict
DOF003_scaffold43780_1	Colwellia psychrerythraea	0.92	Predict
DOF003_scaffold48262_1	Colwellia psychrerythraea	0.79	Predict
DOF003_scaffold2826_3	Staphylococcus saprophyticus	0.91	Predict
DOF003_scaffold22191_11	Bacillus cereus	1	CRISPR
DOF003_scaffold1689_27	Clostridium perfringens	1	CRISPR
DOF003_scaffold13183_2	Colwellia psychrerythraea	0.73	Predict
DOF003_scaffold4746_1	Dinoroseobacter shibae	1	CRISPR
DOF003_scaffold23234_1	Bacteroides fragilis	1	CRISPR
DOF003_scaffold49885_1	Colwellia psychrerythraea	0.97	Predict
DOF003_scaffold50947_2	Bacteroides vulgatus	1	CRISPR
DOF003_scaffold51046_1	Bacillus halmapalus	0.94	Predict
DOF003_scaffold28472_4	Colwellia psychrerythraea	0.78	Predict
DOF003_scaffold49069_1	Staphylococcus saprophyticus	0.74	Predict
DOF003_scaffold15485_11	Colwellia psychrerythraea	0.7	Predict
DOF003_scaffold40480_7	Micromonospora chaiyaphumensis	1	CRISPR
DOF003_scaffold2826_7	Colwellia psychrerythraea	0.71	Predict
DOF003_scaffold49885_3	Bacteroides fragilis	0.91	Predict
DOF003_scaffold959_2	Colwellia psychrerythraea	0.78	Predict
DOF003_scaffold9164_2	Colwellia psychrerythraea	1	CRISPR
DOF003_scaffold22191_13	Bacillus cereus	1	CRISPR
DOF003_scaffold51075_5	Colwellia psychrerythraea	0.88	Predict
DOF003_scaffold22191_10	Bacillus cereus	1	CRISPR
DOF003_scaffold50425_1	Staphylococcus saprophyticus	0.84	Predict
DOF003_scaffold49538_2	Candidatus Pelagibacter ubique	0.93	Predict
DOF003_scaffold28914_12	Colwellia psychrerythraea	1	CRISPR
DOF003_scaffold22256_6	Staphylococcus saprophyticus	0.81	Predict
DOF003_scaffold27483_3	Candidatus Pelagibacter ubique	1	CRISPR
DOF003_scaffold35581_4	Colwellia psychrerythraea	1	CRISPR
DOF004_scaffold42185_4	Candidatus Hamiltonella defensa	0.79	Predict
DOF004_scaffold56260_1	Colwellia psychrerythraea	0.92	Predict
DOF004_scaffold22037_1	Colwellia psychrerythraea	1	CRISPR
DOF004_scaffold27880_1	Bacteroides fragilis	0.98	Predict
DOF004_scaffold37006_1	Parabacteroides merdae	1	CRISPR
DOF004_scaffold10109_6	Colwellia psychrerythraea	0.98	Predict
DOF004_scaffold35687_1	Colwellia psychrerythraea	0.98	Predict
DOF004_scaffold9090_4	Streptococcus mutans	0.99	Predict
DOF004_scaffold786_2	Colwellia psychrerythraea	0.93	Predict
DOF004_scaffold56259_1	Colwellia psychrerythraea	1	CRISPR
DOF004_scaffold28311_2	Staphylococcus saprophyticus	0.78	Predict
DOF004_scaffold148_2	Staphylococcus saprophyticus	0.8	Predict
DOF004_scaffold19258_3	Staphylococcus epidermidis	0.81	Predict
DOF004_scaffold27017_1	Colwellia psychrerythraea	0.94	Predict
DOF004_scaffold21890_2	Bacteroides fragilis	0.88	Predict

DOF004_scaffold57038_1	Parabacteroides distasonis	0.81	Predict
DOF004_C632416_1	Staphylococcus saprophyticus	0.87	Predict
DOF004_scaffold49766_4	Colwellia psychrerythraea	0.96	Predict
DOF004_scaffold7376_1	Colwellia psychrerythraea	0.95	Predict
DOF004_scaffold56952_1	Colwellia psychrerythraea	1	CRISPR
DOF004_scaffold25343_1	Bacillus halmapalus	0.97	Predict
DOF004_scaffold5206_5	Bacteroides fragilis	0.86	Predict
DOF004_scaffold24349_1	Colwellia psychrerythraea	0.82	Predict
DOF004_scaffold57121_1	Colwellia psychrerythraea	1	CRISPR
DOF004_scaffold34242_1	Colwellia psychrerythraea	0.81	Predict
DOF004_scaffold45589_1	Bacteroides fragilis	0.91	Predict
DOF006_scaffold711_1	Cronobacter sakazakii	1	CRISPR
DOF006_scaffold306_3	Parabacteroides merdae	1	CRISPR
DOF006_scaffold22817_1	Colwellia psychrerythraea	1	CRISPR
DOF006_scaffold12062_1	Colwellia psychrerythraea	0.86	Predict
DOF006_scaffold11978_4	Bacteroides fragilis	0.86	Predict
DOF006_C312135_1	Colwellia psychrerythraea	0.91	Predict
DOF006_scaffold15300_3	Colwellia psychrerythraea	1	CRISPR
DOF006_scaffold17493_1	Candidatus Pelagibacter ubique	0.73	Predict
DOF006_scaffold22996_1	Colwellia psychrerythraea	0.72	Predict
DOF006_scaffold13735_2	Bacteroides salyersiae	1	CRISPR
DOF006_scaffold11478_1	Parabacteroides distasonis	1	CRISPR
DOF006_scaffold3372_1	Staphylococcus saprophyticus	0.91	Predict
DOF006_scaffold18788_9	Cellulophaga baltica	0.86	Predict
DOF006_scaffold23004_2	Bacteroides fragilis	0.78	Predict
DOF006_scaffold15162_1	Candidatus Pelagibacter ubique	0.85	Predict
DOF006_scaffold2736_1	Colwellia psychrerythraea	1	CRISPR
DOF006_scaffold20709_1	Candidatus Pelagibacter ubique	1	CRISPR
DOF006_scaffold306_6	Parabacteroides distasonis	1	CRISPR
DOF006_scaffold22685_1	Candidatus Pelagibacter ubique	0.81	Predict
DOF006_scaffold4966_1	Helicobacter pylori	1	CRISPR
DOF006_scaffold22663_1	Colwellia psychrerythraea	0.99	Predict
DOF006_scaffold22952_1	Staphylococcus saprophyticus	0.78	Predict
DOF006_scaffold5845_1	Bacteroides fragilis	0.7	Predict
DOF006_scaffold17357_1	Staphylococcus saprophyticus	0.79	Predict
DOF006_scaffold14584_2	Staphylococcus saprophyticus	0.91	Predict
DOF006_scaffold22952_2	Flavobacterium columnare	0.9	Predict
DOF006_scaffold711_5	Colwellia psychrerythraea	1	CRISPR
DOF006_scaffold10407_1	Bacteroides fragilis	1	CRISPR
DOF006_C312621_1	Flavobacterium psychrophilum	1	CRISPR
DOF007_scaffold1171_1	Bacteroides fragilis	0.83	Predict
DOF007_scaffold35_3	Colwellia psychrerythraea	0.99	Predict
DOF007_scaffold17308_3	Candidatus Pelagibacter ubique	1	CRISPR
DOF007_C360895_1	Bacteroides fragilis	1	CRISPR
DOF007_scaffold7833_5	Colwellia psychrerythraea	0.87	Predict
DOF007_scaffold21463_1	Colwellia psychrerythraea	0.82	Predict
DOF007_scaffold2644_116	Bacillus sp. V3-13	1	CRISPR
DOF007_scaffold14864_2	Colwellia psychrerythraea	1	CRISPR

DOF007_scaffold523_7	Colwellia psychrerythraea	0.99	Predict
DOF007_scaffold15454_1	Parabacteroides distasonis	1	CRISPR
DOF007_scaffold16228_1	Bacteroides fragilis	0.73	Predict
DOF007_scaffold632_7	Colwellia psychrerythraea	0.9	Predict
DOF007_scaffold1340_5	Colwellia psychrerythraea	0.82	Predict
DOF007_scaffold2644_118	Pantoea agglomerans	0.84	Predict
DOF007_C359971_1	Parabacteroides distasonis	0.72	Predict
DOF007_scaffold4001_1	Candidatus Pelagibacter ubique	0.82	Predict
DOF007_scaffold1333_2	Colwellia psychrerythraea	0.89	Predict
DOF008_scaffold28029_2	Clostridium tetani	1	CRISPR
DOF008_scaffold567_3	Candidatus Pelagibacter ubique	0.92	Predict
DOF008_scaffold9506_2	Citrobacter rodentium	1	CRISPR
DOF008_scaffold4011_4	Ruminococcus bromii	1	CRISPR
DOF008_scaffold832_1	Colwellia psychrerythraea	0.79	Predict
DOF008_scaffold10539_9	Parabacteroides distasonis	0.96	Predict
DOF008_scaffold26020_1	Colwellia psychrerythraea	0.99	Predict
DOF008_scaffold19396_1	Candidatus Pelagibacter ubique	0.77	Predict
DOF008_scaffold6011_2	Colwellia psychrerythraea	0.84	Predict
DOF008_scaffold12257_4	Staphylococcus saprophyticus	1	CRISPR
DOF008_scaffold1028_3	Bacteroides fragilis	0.78	Predict
DOF008_C423597_1	Candidatus Pelagibacter ubique	0.78	Predict
DOF008_scaffold19808_7	Staphylococcus saprophyticus	0.82	Predict
DOF008_scaffold29015_1	Mesorhizobium loti	1	CRISPR
DOF008_scaffold12257_5	Coprococcus catus	1	CRISPR
DOF008_scaffold5856_4	Candidatus Pelagibacter ubique	0.86	Predict
DOF008_scaffold28029_1	Anaerostipes hadrus	1	CRISPR
DOF008_scaffold16092_4	Bacillus halmapalus	0.77	Predict
DOF008_scaffold27793_1	Colwellia psychrerythraea	0.87	Predict
DOF008_scaffold21540_6	Colwellia psychrerythraea	0.98	Predict
DOF008_scaffold17006_1	Acinetobacter baumannii	1	CRISPR
DOF008_scaffold15173_2	Colwellia psychrerythraea	0.83	Predict
DOF008_scaffold232_7	Xanthomonas citri	1	CRISPR
DOF008_C423975_1	Colwellia psychrerythraea	1	CRISPR
DOF009_scaffold2408_22	Colwellia psychrerythraea	0.91	Predict
DOF009_scaffold40393_12	Micromonospora chaiyaphumensis	1	CRISPR
DOF009_scaffold155_13	Staphylococcus saprophyticus	0.82	Predict
DOF009_scaffold52826_1	Staphylococcus saprophyticus	0.92	Predict
DOF009_scaffold52459_1	Colwellia psychrerythraea	0.94	Predict
DOF009_scaffold39856_9	Colwellia psychrerythraea	0.97	Predict
DOF009_scaffold43174_9	Colwellia psychrerythraea	0.78	Predict
DOF009_scaffold22475_18	Bacteroides fragilis	1	CRISPR
DOF009_scaffold47159_4	Colwellia psychrerythraea	1	CRISPR
DOF009_scaffold46839_2	Colwellia psychrerythraea	0.93	Predict
DOF009_scaffold42767_3	Bifidobacterium adolescentis	1	CRISPR
DOF009_scaffold16613_1	Parabacteroides distasonis	0.87	Predict
DOF009_scaffold36176_2	Colwellia psychrerythraea	1	CRISPR
DOF009_scaffold1_2	Bacteroides fragilis	0.8	Predict
DOF009_scaffold40125_1	Colwellia psychrerythraea	0.99	Predict

DOF009_scaffold52438_1	Bacteroides fragilis	0.85	Predict
DOF009_scaffold4516_5	Colwellia psychrerythraea	0.87	Predict
DOF009_scaffold42497_4	Bacteroides fragilis	0.93	Predict
DOF010_scaffold37104_2	Escherichia coli	1	CRISPR
DOF010_scaffold38542_4	Staphylococcus saprophyticus	0.74	Predict
DOF010_scaffold12096_1	Candidatus Pelagibacter ubique	0.83	Predict
DOF010_scaffold35610_1	Colwellia psychrerythraea	1	CRISPR
DOF010_scaffold38620_1	Colwellia psychrerythraea	1	CRISPR
DOF010_scaffold1331_7	Colwellia psychrerythraea	1	CRISPR
DOF010_scaffold31976_2	Bacillus halmapalus	1	CRISPR
DOF010_scaffold45_4	Colwellia psychrerythraea	0.83	Predict
DOF010_C495273_1	Candidatus Pelagibacter ubique	1	CRISPR
DOF010_scaffold36_4	Bacteroides fragilis	0.91	Predict
DOF010_scaffold39042_3	Cellulophaga baltica	0.81	Predict
DOF010_scaffold13814_2	Candidatus Pelagibacter ubique	0.91	Predict
DOF010_scaffold37750_5	Colwellia psychrerythraea	1	CRISPR
DOF010_scaffold65_18	Bacteroides fragilis	0.72	Predict
DOF010_scaffold11833_5	Colwellia psychrerythraea	0.78	Predict
DOF010_scaffold7607_7	Colwellia psychrerythraea	1	CRISPR
DOF010_scaffold19015_5	Coprobacillus sp. AF13-15	1	CRISPR
DOF010_scaffold27096_5	Colwellia psychrerythraea	0.87	Predict
DOF010_scaffold32482_2	Vibrio cholerae	0.87	Predict
DOF010_scaffold35610_2	Colwellia psychrerythraea	1	CRISPR
DOF010_scaffold23207_18	Streptococcus gordonii	0.77	Predict
DOF010_scaffold34_4	Colwellia psychrerythraea	0.86	Predict
DOF010_scaffold2645_5	Staphylococcus saprophyticus	1	CRISPR
DOF010_scaffold39042_1	Bacillus halmapalus	0.78	Predict
DOF010_scaffold24344_2	Colwellia psychrerythraea	1	CRISPR
DOF010_scaffold38651_2	Bacillus megaterium	0.87	Predict
DOF010_scaffold33553_4	Candidatus Pelagibacter ubique	1	Predict
DOF010_scaffold38956_2	Colwellia psychrerythraea	1	CRISPR
DOF010_scaffold19252_1	Bacillus halmapalus	0.94	Predict
DOF010_scaffold21702_2	Bacteroides fragilis	0.84	Predict
DOF010_scaffold29273_4	Colwellia psychrerythraea	1	CRISPR
DOF010_scaffold198_1	Candidatus Pelagibacter ubique	0.79	Predict
DOF010_scaffold38551_4	Clostridioides difficile	1	CRISPR
DOF010_scaffold24344_3	Colwellia psychrerythraea	1	CRISPR
DOF010_scaffold31661_1	Staphylococcus saprophyticus	0.97	Predict
DOF010_scaffold37750_4	Colwellia psychrerythraea	1	CRISPR
DOF010_scaffold39049_2	Colwellia psychrerythraea	1	CRISPR
DOF010_scaffold4723_13	Colwellia psychrerythraea	0.88	Predict
DOF010_scaffold31976_1	Staphylococcus saprophyticus	1	CRISPR
DOF011_scaffold21153_1	Megamonas funiformis	1	CRISPR
DOF011_scaffold21213_1	Colwellia psychrerythraea	1	CRISPR
DOF011_scaffold77_1	Bacillus halmapalus	0.77	Predict
DOF011_scaffold2635_5	Bacteroides fragilis	0.81	Predict
DOF011_scaffold21174_2	Colwellia psychrerythraea	0.78	Predict
DOF011_scaffold2386_1	Colwellia psychrerythraea	0.94	Predict

DOF011_scaffold12507_5	Bacteroides fragilis	0.72	Predict
DOF011_scaffold19699_2	Colwellia psychrerythraea	0.98	Predict
DOF011_scaffold20914_1	Colwellia psychrerythraea	1	CRISPR
DOF012_scaffold1710_3	Colwellia psychrerythraea	0.79	Predict
DOF012_scaffold12555_2	Veillonella sp. AF36-20BH	1	CRISPR
DOF012_scaffold463_10	Colwellia psychrerythraea	1	CRISPR
DOF012_scaffold4720_1	Colwellia psychrerythraea	1	CRISPR
DOF012_scaffold992_3	Colwellia psychrerythraea	0.72	Predict
DOF012_scaffold9844_1	Colwellia psychrerythraea	1	CRISPR
DOF012_scaffold2936_3	Colwellia psychrerythraea	0.73	Predict
DOF012_scaffold12334_1	Lactobacillus fermentum	1	CRISPR
DOF012_scaffold198_1	Colwellia psychrerythraea	1	CRISPR
DOF012_scaffold4539_3	Colwellia psychrerythraea	0.8	Predict
DOF012_scaffold381_2	Bacillus halmapalus	1	CRISPR
DOF012_scaffold3394_7	Veillonella sp. AF36-20BH	1	CRISPR
DOF012_scaffold897_10	Colwellia psychrerythraea	0.88	Predict
DOF012_scaffold4741_3	Veillonella parvula	1	CRISPR
DOF012_scaffold7_12	Colwellia psychrerythraea	1	CRISPR
DOF012_scaffold6657_1	Staphylococcus saprophyticus	0.82	Predict
DOF012_scaffold884_3	Colwellia psychrerythraea	1	CRISPR
DOF012_scaffold191_20	Staphylococcus saprophyticus	0.87	Predict
DOF012_scaffold14781_2	Colwellia psychrerythraea	0.82	Predict
DOF012_scaffold7309_6_1	Colwellia psychrerythraea	0.75	Predict
DOF012_scaffold14750_1	Lactobacillus fermentum	1	CRISPR
DOF012_scaffold22_3	Croceibacter atlanticus	0.93	Predict
DOF012_scaffold463_9_2	Parabacteroides distasonis	1	CRISPR
DOF013_scaffold2527_4	Parabacteroides merdae	1	CRISPR
DOF013_scaffold11760_13	Colwellia psychrerythraea	0.84	Predict
DOF013_scaffold12980_3	Staphylococcus saprophyticus	0.71	Predict
DOF013_scaffold5071_6	Colwellia psychrerythraea	0.77	Predict
DOF013_scaffold1588_1	Megamonas funiformis	1	CRISPR
DOF013_scaffold27644_1	Bacillus halmapalus	0.88	Predict
DOF013_scaffold4900_2	Staphylococcus saprophyticus	0.72	Predict
DOF013_scaffold12980_1	Colwellia psychrerythraea	1	CRISPR
DOF013_scaffold783_2	Bacteroides dorei	1	CRISPR
DOF013_scaffold13491_2	Bacteroides fragilis	0.8	Predict
DOF013_scaffold443_2	Parabacteroides distasonis	0.77	Predict
DOF013_scaffold2699_1	Staphylococcus saprophyticus	0.88	Predict
DOF013_scaffold4683_2	Candidatus Pelagibacter ubique	0.81	Predict
DOF013_scaffold103_6	Colwellia psychrerythraea	0.95	Predict
DOF013_scaffold392_2	Xanthomonas vesicatoria	0.71	Predict
DOF013_scaffold5240_2	Parabacteroides sp. D13	1	CRISPR
DOF013_scaffold379_1	Staphylococcus saprophyticus	0.76	Predict
DOF013_scaffold5240_9	Parabacteroides sp. D13	1	CRISPR
DOF013_scaffold21294_6	Bacteroides fragilis	0.94	Predict
DOF013_scaffold29915_1	Colwellia psychrerythraea	0.92	Predict
DOF014_scaffold49276_4	Clostridium botulinum	0.82	Predict
DOF014_scaffold46901_1	Candidatus Pelagibacter ubique	0.91	Predict

DOF014_scaffold10772_1	Candidatus Pelagibacter ubique	1	CRISPR
DOF014_scaffold30923_2	Colwellia psychrerythraea	1	CRISPR
DOF014_C612810_1	Candidatus Pelagibacter ubique	0.71	Predict
DOF014_scaffold101_2	Candidatus Pelagibacter ubique	0.82	Predict
DOF014_scaffold32658_1	Staphylococcus saprophyticus	1	CRISPR
DOF014_scaffold7189_8	Colwellia psychrerythraea	0.96	Predict
DOF014_scaffold49340_4	Colwellia psychrerythraea	1	CRISPR
DOF014_scaffold29718_2	Parabacteroides distasonis	0.99	Predict
DOF014_scaffold2821_4	Staphylococcus saprophyticus	0.81	Predict
DOF014_scaffold49604_1	Bacteroides fragilis	0.95	Predict
DOF014_scaffold49645_1	Lactobacillus gasseri	0.71	Predict
DOF014_scaffold49556_1	Clostridium perfringens	1	CRISPR
DOF014_scaffold49552_1	Colwellia psychrerythraea	1	CRISPR
DOF014_scaffold1_3	Bacteroides fragilis	0.78	Predict
DOF014_scaffold34813_4	Bacteroides fragilis	0.87	Predict
DOF014_scaffold49340_5	Colwellia psychrerythraea	0.89	Predict
DOF014_scaffold13006_1	Streptococcus mutans	0.98	Predict
DOF014_C612698_1	Bacteroides fragilis	1	CRISPR
DOF014_scaffold82_3	Colwellia psychrerythraea	0.85	Predict
DOF014_scaffold15208_5	Bacteroides fragilis	0.85	Predict
DOF002_scaffold55354_2	unknown	0	-
DOF002_scaffold65381_2	unknown	0	-
DOF002_scaffold58023_2	unknown	0	-
DOF002_scaffold39963_1	unknown	0	-
DOF002_scaffold26075_2	unknown	0	-
DOF002_C839921_1	unknown	0	-
DOF002_scaffold5030_2	unknown	0	-
DOF003_scaffold28855_2	unknown	0	-
DOF003_scaffold21246_8	unknown	0	-
DOF003_scaffold32977_2	unknown	0	-
DOF003_scaffold30739_3	unknown	0	-
DOF003_scaffold50350_1	unknown	0	-
DOF003_scaffold989_11	unknown	0	-
DOF003_scaffold197_2	unknown	0	-
DOF003_scaffold51078_6	unknown	0	-
DOF003_scaffold51078_1	unknown	0	-
DOF003_scaffold47078_1	unknown	0	-
DOF003_scaffold19871_4	unknown	0	-
DOF003_scaffold1689_28	unknown	0	-
DOF003_scaffold19201_1	unknown	0	-
DOF003_scaffold36733_1	unknown	0	-
DOF003_scaffold26587_4	unknown	0	-
DOF004_scaffold31768_1	unknown	0	-
DOF004_scaffold41981_3	unknown	0	-
DOF004_scaffold16778_14	unknown	0	-
DOF004_scaffold54702_1	unknown	0	-
DOF004_scaffold38757_1	unknown	0	-
DOF004_scaffold26721_2	unknown	0	-

DOF006_scaffold19914_2	unknown	0	-
DOF006_scaffold7307_1	unknown	0	-
DOF006_scaffold9292_2	unknown	0	-
DOF007_scaffold5443_1	unknown	0	-
DOF007_scaffold2644_113	unknown	0	-
DOF007_scaffold16914_2	unknown	0	-
DOF007_scaffold12791_4	unknown	0	-
DOF008_scaffold10567_1	unknown	0	-
DOF008_scaffold28485_2	unknown	0	-
DOF008_scaffold8861_3	unknown	0	-
DOF008_scaffold21796_1	unknown	0	-
DOF008_scaffold28698_1	unknown	0	-
DOF008_scaffold24483_1	unknown	0	-
DOF008_scaffold25427_1	unknown	0	-
DOF008_scaffold27498_1	unknown	0	-
DOF008_scaffold110_2	unknown	0	-
DOF008_scaffold2469_10	unknown	0	-
DOF009_scaffold42858_2	unknown	0	-
DOF009_scaffold3127_2	unknown	0	-
DOF009_scaffold52159_2	unknown	0	-
DOF009_scaffold31_1	unknown	0	-
DOF009_scaffold23972_2	unknown	0	-
DOF009_scaffold13901_4	unknown	0	-
DOF009_scaffold7496_2	unknown	0	-
DOF010_scaffold37194_6	unknown	0	-
DOF010_scaffold36571_1	unknown	0	-
DOF010_scaffold21978_1	unknown	0	-
DOF010_scaffold13830_4	unknown	0	-
DOF010_scaffold35846_1	unknown	0	-
DOF011_scaffold21165_1	unknown	0	-
DOF012_scaffold884_2	unknown	0	-
DOF012_scaffold9548_2	unknown	0	-
DOF013_scaffold15877_2	unknown	0	-
DOF014_scaffold15073_8	unknown	0	-
DOF014_scaffold49726_2	unknown	0	-
DOF014_scaffold49687_1	unknown	0	-
DOF014_scaffold1808_5	unknown	0	-
DOF014_scaffold90_1	unknown	0	-
NOF001_scaffold2654_4	Pseudomonas putida	0.78	Predict
NOF001_scaffold14_4	Candidatus Hamiltonella defensa	0.81	Predict
NOF001_scaffold58201_2	Parabacteroides merdae	1	CRISPR
NOF001_scaffold17586_2	Bacteroides fragilis	0.82	Predict
NOF001_scaffold58318_1	Staphylococcus saprophyticus	1	CRISPR
NOF001_scaffold5563_1	Candidatus Hamiltonella defensa	0.8	Predict
NOF001_scaffold48_1	Candidatus Hamiltonella defensa	0.9	Predict
NOF001_scaffold47705_1	Candidatus Hamiltonella defensa	1	CRISPR
NOF001_scaffold56764_1	Candidatus Hamiltonella defensa	1	CRISPR
NOF001_scaffold53059_1	Parabacteroides merdae	0.92	Predict

NOF001_scaffold58052_2	Candidatus Hamiltonella defensa	0.85	Predict
NOF001_scaffold5343_8	Bacteroides fragilis	0.9	Predict
NOF001_scaffold58235_1	Prevotella copri	1	CRISPR
NOF001_scaffold57995_2	Candidatus Hamiltonella defensa	0.86	Predict
NOF001_scaffold11560_1	Candidatus Hamiltonella defensa	0.74	Predict
NOF001_scaffold27372_1	Candidatus Hamiltonella defensa	1	CRISPR
NOF001_scaffold41855_2	Paenibacillus larvae	1	CRISPR
NOF001_scaffold24609_2	Clostridium botulinum	0.94	Predict
NOF001_scaffold41855_6	Candidatus Hamiltonella defensa	0.96	Predict
NOF001_scaffold58316_1	Candidatus Hamiltonella defensa	0.94	Predict
NOF001_scaffold41855_1	Candidatus Hamiltonella defensa	0.86	Predict
NOF001_scaffold24013_1	Candidatus Hamiltonella defensa	1	CRISPR
NOF001_scaffold58081_1	Candidatus Pelagibacter ubique	0.99	Predict
NOF001_scaffold58201_1	Parabacteroides merdae	1	CRISPR
NOF001_scaffold55226_1	Staphylococcus saprophyticus	0.84	Predict
NOF001_scaffold50021_1	Azospirillum brasilense	1	CRISPR
NOF001_scaffold58099_2	Candidatus Hamiltonella defensa	0.92	Predict
NOF002_scaffold7_6	Staphylococcus saprophyticus	0.73	Predict
NOF002_scaffold13379_1	Candidatus Hamiltonella defensa	0.91	Predict
NOF002_scaffold30763_2	Staphylococcus saprophyticus	1	CRISPR
NOF002_scaffold5730_7	Planktothrix agardhii	0.83	Predict
NOF002_scaffold3476_12	Candidatus Hamiltonella defensa	0.94	Predict
NOF002_scaffold31199_2	Bacteroides fragilis	0.89	Predict
NOF002_C425969_1	Staphylococcus saprophyticus	0.86	Predict
NOF002_scaffold31424_1	Candidatus Hamiltonella defensa	1	CRISPR
NOF002_scaffold31554_1	Candidatus Hamiltonella defensa	0.95	Predict
NOF002_scaffold3233_2	Staphylococcus saprophyticus	1	CRISPR
NOF002_scaffold6749_14	Candidatus Hamiltonella defensa	1	CRISPR
NOF002_scaffold31199_1	Bacteroides fragilis	0.77	Predict
NOF002_scaffold11620_5	Candidatus Hamiltonella defensa	0.72	Predict
NOF002_scaffold9460_1	Faecalibacterium prausnitzii	1	CRISPR
NOF002_scaffold31188_5	Bacteroides fragilis	0.88	Predict
NOF002_scaffold1584_5	Staphylococcus saprophyticus	0.81	Predict
NOF002_scaffold31615_1	Clavibacter michiganensis	0.89	Predict
NOF002_scaffold3233_5	Candidatus Hamiltonella defensa	0.98	Predict
NOF002_scaffold7_7	Candidatus Hamiltonella defensa	0.77	Predict
NOF002_scaffold30763_1	Staphylococcus saprophyticus	0.74	Predict
NOF002_scaffold27207_5	Staphylococcus pasteuri	0.97	Predict
NOF002_scaffold2745_2	Candidatus Hamiltonella defensa	0.98	Predict
NOF002_scaffold2745_3	Pasteurella multocida	0.82	Predict
NOF002_scaffold23022_4	Bacteroides fragilis	0.72	Predict
NOF002_scaffold31584_1	Candidatus Hamiltonella defensa	0.89	Predict
NOF004_scaffold5431_15	Staphylococcus saprophyticus	0.81	Predict
NOF004_scaffold25441_2	Bacteroides fragilis	0.99	Predict
NOF004_scaffold5774_1	Candidatus Hamiltonella defensa	0.89	Predict
NOF004_scaffold4703_4	Bacteroides fragilis	0.91	Predict
NOF004_scaffold19766_4	Candidatus Hamiltonella defensa	1	CRISPR
NOF004_scaffold32796_1	Candidatus Hamiltonella defensa	1	CRISPR

NOF004_scaffold30766_1	Candidatus Hamiltonella defensa	0.7	Predict
NOF004_scaffold4675_2	Bacteroides fragilis	0.79	Predict
NOF004_scaffold36774_1	Candidatus Hamiltonella defensa	1	CRISPR
NOF004_scaffold37814_4	Staphylococcus saprophyticus	0.91	Predict
NOF004_scaffold12813_30	Clostridioides difficile	1	CRISPR
NOF004_scaffold22864_4	Candidatus Hamiltonella defensa	0.75	Predict
NOF004_scaffold12390_5	Candidatus Hamiltonella defensa	0.75	Predict
NOF004_scaffold18950_2	Candidatus Hamiltonella defensa	0.9	Predict
NOF004_scaffold4703_1	Candidatus Hamiltonella defensa	0.9	Predict
NOF004_scaffold34095_2	Candidatus Hamiltonella defensa	1	Predict
NOF004_scaffold29834_1	Candidatus Hamiltonella defensa	1	CRISPR
NOF004_scaffold1680_17	Staphylococcus saprophyticus	0.86	Predict
NOF004_scaffold30605_6	Candidatus Hamiltonella defensa	0.84	Predict
NOF004_scaffold39867_3	Bacteroides fragilis	0.79	Predict
NOF004_scaffold8521_5	Candidatus Hamiltonella defensa	0.97	Predict
NOF004_scaffold8945_3	Candidatus Hamiltonella defensa	0.91	Predict
NOF004_scaffold24813_3	Candidatus Pelagibacter ubique	1	CRISPR
NOF004_C575620_1	Candidatus Hamiltonella defensa	1	CRISPR
NOF004_scaffold89_3	Bacteroides fragilis	0.85	Predict
NOF004_scaffold31703_2	Candidatus Hamiltonella defensa	1	CRISPR
NOF004_scaffold26987_2	Candidatus Hamiltonella defensa	1	CRISPR
NOF004_scaffold58_6	Candidatus Hamiltonella defensa	0.91	Predict
NOF004_scaffold4193_2	Candidatus Hamiltonella defensa	0.9	Predict
NOF004_scaffold12390_3	Enterococcus faecalis	1	CRISPR
NOF004_scaffold9875_5	Candidatus Hamiltonella defensa	0.91	Predict
NOF004_scaffold39611_1	Candidatus Pelagibacter ubique	0.84	Predict
NOF004_scaffold28322_1	Candidatus Hamiltonella defensa	1	CRISPR
NOF004_scaffold6978_23	Candidatus Hamiltonella defensa	0.77	Predict
NOF004_scaffold9355_12	Erysipelothrix rhusiopathiae	1	CRISPR
NOF004_scaffold12025_2	Candidatus Hamiltonella defensa	1	CRISPR
NOF004_scaffold14211_1	Candidatus Hamiltonella defensa	1	CRISPR
NOF004_scaffold9994_2	Candidatus Pelagibacter ubique	0.95	Predict
NOF004_scaffold2098_11	Parabacteroides merdae	0.82	Predict
NOF005_scaffold35291_5	Candidatus Hamiltonella defensa	0.9	Predict
NOF005_scaffold23396_3	Candidatus Pelagibacter ubique	1	CRISPR
NOF005_scaffold31594_11	Staphylococcus saprophyticus	1	CRISPR
NOF005_scaffold6888_3	Parabacteroides distasonis	0.82	Predict
NOF005_scaffold29221_10	Candidatus Hamiltonella defensa	0.75	Predict
NOF005_scaffold34324_4	Bacteroides fragilis	0.91	Predict
NOF005_scaffold1086_25	Candidatus Hamiltonella defensa	0.77	Predict
NOF005_scaffold604_14	Staphylococcus saprophyticus	0.86	Predict
NOF005_scaffold44749_6	Staphylococcus hominis	0.9	Predict
NOF005_scaffold51717_1	Candidatus Hamiltonella defensa	0.84	Predict
NOF005_scaffold34950_3	Candidatus Hamiltonella defensa	1	CRISPR
NOF005_scaffold51596_4	Candidatus Hamiltonella defensa	1	CRISPR
NOF005_scaffold29140_2	Staphylococcus saprophyticus	0.71	Predict
NOF005_scaffold37734_2	Candidatus Hamiltonella defensa	0.71	Predict
NOF005_scaffold165_4	Candidatus Hamiltonella defensa	0.84	Predict

NOF005_scaffold48547_9	Candidatus Hamiltonella defensa	0.97	Predict
NOF005_scaffold604_12	[Eubacterium] eligens	1	CRISPR
NOF005_scaffold52365_1	Bacteroides fragilis	0.98	Predict
NOF005_scaffold23396_2	Candidatus Hamiltonella defensa	0.8	Predict
NOF005_scaffold3465_6	Candidatus Hamiltonella defensa	1	CRISPR
NOF005_scaffold604_3	Staphylococcus saprophyticus	0.98	Predict
NOF005_scaffold52405_2	Parabacteroides distasonis	1	CRISPR
NOF005_scaffold49499_1_	Staphylococcus saprophyticus	1	CRISPR
NOF005_scaffold15268_1	Candidatus Hamiltonella defensa	0.78	Predict
NOF005_scaffold39972_17	Staphylococcus saprophyticus	0.95	Predict
NOF005_scaffold27541_5	Candidatus Hamiltonella defensa	1	CRISPR
NOF005_scaffold27426_3	Candidatus Hamiltonella defensa	0.83	Predict
NOF005_scaffold32384_3	Candidatus Hamiltonella defensa	1	CRISPR
NOF005_scaffold27198_2	Staphylococcus saprophyticus	0.87	Predict
NOF005_scaffold13462_4	Cellulophaga baltica	1	CRISPR
NOF005_scaffold52106_1	Candidatus Hamiltonella defensa	0.74	Predict
NOF005_scaffold604_13	Staphylococcus saprophyticus	0.88	Predict
NOF006_scaffold10363_1	Bacteroides fragilis	0.82	Predict
NOF006_scaffold15833_1	Candidatus Hamiltonella defensa	0.9	Predict
NOF006_scaffold6111_7	Candidatus Hamiltonella defensa	1	Predict
NOF006_scaffold4868_1	Candidatus Hamiltonella defensa	1	Predict
NOF006_scaffold12718_7	Bacteroides fragilis	0.99	Predict
NOF006_scaffold498_1	Candidatus Hamiltonella defensa	1	CRISPR
NOF006_scaffold21223_2	Candidatus Hamiltonella defensa	0.72	Predict
NOF006_scaffold121_1	Bacteroides fragilis	0.72	Predict
NOF006_scaffold21242_4	Bacteroides fragilis	0.97	Predict
NOF006_scaffold1740_2	Cellulophaga baltica	1	CRISPR
NOF006_scaffold2842_16	Candidatus Hamiltonella defensa	0.89	Predict
NOF006_scaffold19983_2	Staphylococcus saprophyticus	0.88	Predict
NOF006_scaffold14475_3	Parabacteroides distasonis	1	CRISPR
NOF006_scaffold1022_7	Staphylococcus saprophyticus	0.87	Predict
NOF006_scaffold14499_8	Candidatus Hamiltonella defensa	0.96	Predict
NOF006_scaffold21012_1	Candidatus Hamiltonella defensa	0.77	Predict
NOF006_scaffold20915_1	Staphylococcus saprophyticus	1	CRISPR
NOF006_scaffold4872_3	Candidatus Hamiltonella defensa	1	CRISPR
NOF006_C401108_1	Bacillus cereus	1	CRISPR
NOF006_scaffold20868_2	Streptococcus mutans	1	CRISPR
NOF007_scaffold27213_3	Candidatus Hamiltonella defensa	0.9	Predict
NOF007_scaffold16773_3	Bacteroides fragilis	0.84	Predict
NOF007_scaffold23774_3	Candidatus Hamiltonella defensa	1	CRISPR
NOF007_scaffold4461_7	Staphylococcus saprophyticus	0.9	Predict
NOF007_scaffold28685_4	Candidatus Hamiltonella defensa	0.94	Predict
NOF007_scaffold26160_1	Candidatus Hamiltonella defensa	0.97	Predict
NOF007_scaffold21388_2	Candidatus Hamiltonella defensa	1	CRISPR
NOF007_scaffold19207_2	Bacteroides fragilis	0.97	Predict
NOF007_scaffold8821_2	Candidatus Hamiltonella defensa	0.89	Predict
NOF007_scaffold27822_1	Candidatus Hamiltonella defensa	0.76	Predict
NOF007_scaffold23819_3	Bacteroides fragilis	0.95	Predict

NOF007_scaffold26938_2	Parabacteroides distasonis	1	CRISPR
NOF007_scaffold7559_3	Candidatus Hamiltonella defensa	0.88	Predict
NOF007_scaffold16388_6	Candidatus Hamiltonella defensa	1	CRISPR
NOF007_scaffold24483_2	Candidatus Hamiltonella defensa	1	CRISPR
NOF007_scaffold10524_1	Candidatus Hamiltonella defensa	0.97	Predict
NOF007_scaffold298_7	Parabacteroides distasonis	0.88	Predict
NOF007_scaffold505_1	Candidatus Hamiltonella defensa	0.78	Predict
NOF007_scaffold28801_1	Candidatus Hamiltonella defensa	0.75	Predict
NOF007_scaffold22039_3	Bacillus pumilus	0.72	Predict
NOF007_scaffold26224_1	Candidatus Hamiltonella defensa	0.97	Predict
NOF007_scaffold1195_9	Candidatus Hamiltonella defensa	0.92	Predict
NOF007_scaffold23774_2	Candidatus Hamiltonella defensa	1	CRISPR
NOF007_scaffold12899_1	Candidatus Hamiltonella defensa	0.76	Predict
NOF007_scaffold9468_5	Parabacteroides distasonis	0.86	Predict
NOF008_scaffold68230_1	Candidatus Hamiltonella defensa	1	CRISPR
NOF008_scaffold25367_2	Candidatus Hamiltonella defensa	0.89	Predict
NOF008_scaffold64397_1	Candidatus Pelagibacter ubique	0.73	Predict
NOF008_scaffold48782_2	Bacteroides fragilis	0.7	Predict
NOF008_scaffold5134_4	Candidatus Hamiltonella defensa	0.77	Predict
NOF008_scaffold66085_2	Candidatus Hamiltonella defensa	0.87	Predict
NOF008_scaffold11140_1	Candidatus Hamiltonella defensa	1	CRISPR
NOF008_scaffold67470_2	Candidatus Hamiltonella defensa	1	CRISPR
NOF008_scaffold28772_6	Candidatus Hamiltonella defensa	0.93	Predict
NOF008_scaffold10035_9	Candidatus Hamiltonella defensa	0.89	Predict
NOF008_scaffold30912_6	Mycobacterium persicum	1	CRISPR
NOF008_scaffold66613_1	Candidatus Hamiltonella defensa	0.94	Predict
NOF008_scaffold31488_6	Candidatus Hamiltonella defensa	1	CRISPR
NOF008_scaffold8714_2	Candidatus Hamiltonella defensa	0.78	Predict
NOF008_scaffold5616_1	Candidatus Hamiltonella defensa	0.93	Predict
NOF008_scaffold68130_1	Candidatus Hamiltonella defensa	1	CRISPR
NOF008_scaffold3062_3	Clostridioides difficile	0.84	Predict
NOF008_scaffold7421_1	Candidatus Hamiltonella defensa	1	CRISPR
NOF008_scaffold58140_1	Candidatus Hamiltonella defensa	0.93	Predict
NOF008_scaffold68203_3	Bacteroides fragilis	1	CRISPR
NOF008_scaffold36171_4	Bacteroides fragilis	0.87	Predict
NOF008_scaffold42043_1	Candidatus Pelagibacter ubique	1	CRISPR
NOF008_scaffold8066_2	Proteus mirabilis	0.81	Predict
NOF008_scaffold28625_3	Candidatus Hamiltonella defensa	0.99	Predict
NOF008_scaffold19089_7	Candidatus Hamiltonella defensa	0.8	Predict
NOF008_scaffold3708_20	Candidatus Hamiltonella defensa	0.73	Predict
NOF008_scaffold68224_1	Staphylococcus saprophyticus	0.84	Predict
NOF008_scaffold52198_1	Candidatus Hamiltonella defensa	1	CRISPR
NOF008_scaffold7904_8	Candidatus Hamiltonella defensa	0.74	Predict
NOF008_scaffold25220_3	Candidatus Hamiltonella defensa	0.79	Predict
NOF008_C865299_1	Candidatus Hamiltonella defensa	0.84	Predict
NOF008_scaffold17689_13	Lactobacillus johnsonii	0.98	Predict
NOF008_C865863_1	Candidatus Hamiltonella defensa	0.7	Predict
NOF008_scaffold68199_1	Candidatus Hamiltonella defensa	0.78	Predict

NOF008_scaffold55525_8	Roseobacter denitrificans	0.8	Predict
NOF008_scaffold29486_12	Candidatus Hamiltonella defensa	0.82	Predict
NOF009_scaffold7527_22	Candidatus Hamiltonella defensa	0.97	Predict
NOF009_scaffold43518_2	Bacteroides fragilis	0.79	Predict
NOF009_scaffold21768_4	Bacteroides fragilis	0.72	Predict
NOF009_scaffold41727_1	Staphylococcus saprophyticus	0.72	Predict
NOF009_scaffold10148_1	Candidatus Hamiltonella defensa	0.76	Predict
NOF009_scaffold19865_4	Candidatus Hamiltonella defensa	1	CRISPR
NOF009_scaffold2064_12	Bacteroides fragilis	0.73	Predict
NOF009_scaffold34567_3	Candidatus Hamiltonella defensa	1	CRISPR
NOF009_scaffold16960_2	Candidatus Hamiltonella defensa	1	CRISPR
NOF009_scaffold14075_3	Bacteroides fragilis	1	CRISPR
NOF009_scaffold67_1	Candidatus Hamiltonella defensa	1	CRISPR
NOF009_C558255_1	Candidatus Hamiltonella defensa	1	CRISPR
NOF009_scaffold42455_5	Staphylococcus saprophyticus	1	CRISPR
NOF009_scaffold2720_3	Candidatus Hamiltonella defensa	0.84	Predict
NOF009_scaffold41979_1	Microcystis aeruginosa	1	CRISPR
NOF009_scaffold30580_1	Candidatus Hamiltonella defensa	1	CRISPR
NOF009_scaffold42496_4	Bacteroides fragilis	0.81	Predict
NOF010_C699885_1	Staphylococcus saprophyticus	1	CRISPR
NOF010_scaffold33789_4	Candidatus Hamiltonella defensa	1	CRISPR
NOF010_scaffold32319_4	Staphylococcus saprophyticus	0.79	Predict
NOF010_scaffold26425_6	Candidatus Hamiltonella defensa	0.8	Predict
NOF010_scaffold14373_1	Staphylococcus saprophyticus	0.89	Predict
NOF010_scaffold51712_2	Trichormus variabilis	0.87	Predict
NOF010_scaffold16598_11	Bacteroides fragilis	0.97	Predict
NOF010_scaffold20182_2	Candidatus Hamiltonella defensa	0.73	Predict
NOF010_scaffold20459_4	Bacteroides fragilis	0.78	Predict
NOF010_scaffold9563_25	Candidatus Hamiltonella defensa	0.83	Predict
NOF010_scaffold53722_1	Candidatus Hamiltonella defensa	0.83	Predict
NOF010_scaffold6369_4	Bacteroides fragilis	1	CRISPR
NOF010_scaffold22545_2	Clostridium perfringens	0.98	Predict
NOF010_scaffold4642_2	Candidatus Hamiltonella defensa	0.74	Predict
NOF010_scaffold14618_2	Candidatus Hamiltonella defensa	1	CRISPR
NOF010_scaffold53516_6	Candidatus Hamiltonella defensa	1	CRISPR
NOF010_scaffold43806_2	Clostridium tetani	0.89	Predict
NOF010_scaffold21111_3	Candidatus Hamiltonella defensa	0.9	Predict
NOF010_scaffold27134_3	Candidatus Hamiltonella defensa	1	CRISPR
NOF010_scaffold22950_5	Candidatus Hamiltonella defensa	1	CRISPR
NOF010_scaffold54242_1	Staphylococcus saprophyticus	0.95	Predict
NOF010_scaffold54124_1	Candidatus Hamiltonella defensa	0.87	Predict
NOF010_C699039_1	Candidatus Hamiltonella defensa	1	CRISPR
NOF010_scaffold23028_3	Candidatus Hamiltonella defensa	0.76	Predict
NOF010_scaffold33103_1	Candidatus Hamiltonella defensa	0.97	Predict
NOF010_scaffold5911_1	Candidatus Hamiltonella defensa	0.74	Predict
NOF010_scaffold11305_3	Parabacteroides merdae	0.92	Predict
NOF010_scaffold21018_1	Candidatus Hamiltonella defensa	0.79	Predict
NOF011_scaffold3878_1	Bacteroides fragilis	0.98	Predict

NOF011_scaffold57558_3	Staphylococcus saprophyticus	1	CRISPR
NOF011_scaffold159_2	Parabacteroides distasonis	0.95	Predict
NOF011_scaffold56176_3	Candidatus Hamiltonella defensa	1	CRISPR
NOF011_scaffold18490_3	Bacteroides fragilis	0.82	Predict
NOF011_scaffold59088_2	Candidatus Hamiltonella defensa	0.99	Predict
NOF011_scaffold8028_19	Candidatus Hamiltonella defensa	0.78	Predict
NOF011_scaffold33258_2	Candidatus Hamiltonella defensa	0.94	Predict
NOF011_scaffold58584_1	Staphylococcus saprophyticus	0.82	Predict
NOF011_scaffold70_15	Bacteroides fragilis	0.94	Predict
NOF011_scaffold58981_1	Candidatus Hamiltonella defensa	1	CRISPR
NOF011_scaffold59056_1	Staphylococcus saprophyticus	0.8	Predict
NOF011_scaffold59100_2	Bacteroides fragilis	1	CRISPR
NOF011_scaffold27189_1	Candidatus Hamiltonella defensa	0.96	Predict
NOF011_scaffold57546_2	Parabacteroides distasonis	0.9	Predict
NOF011_scaffold59096_2	Bacteroides fragilis	0.81	Predict
NOF011_scaffold15798_1	Bacteroides fragilis	0.81	Predict
NOF011_scaffold48802_1	Candidatus Hamiltonella defensa	1	CRISPR
NOF011_scaffold57126_1	Staphylococcus saprophyticus	0.82	Predict
NOF011_scaffold52600_1	Bacteroides oleiciplenus	1	CRISPR
NOF011_scaffold56176_2	Candidatus Hamiltonella defensa	1	CRISPR
NOF011_scaffold8028_20	Candidatus Hamiltonella defensa	0.74	Predict
NOF011_scaffold2529_7	Candidatus Hamiltonella defensa	0.82	Predict
NOF011_scaffold45498_1	Candidatus Hamiltonella defensa	0.82	Predict
NOF011_scaffold40128_2	Bacteroides fragilis	1	CRISPR
NOF012_scaffold77459_1	Enterococcus faecalis	1	CRISPR
NOF012_scaffold71024_7	Bacteroides fragilis	0.96	Predict
NOF012_scaffold77267_2	Parabacteroides merdae	0.97	Predict
NOF012_scaffold12244_5	Ruminococcus sp. AF26-25AA	1	CRISPR
NOF012_scaffold5639_4	Candidatus Hamiltonella defensa	1	CRISPR
NOF012_scaffold15647_4	Candidatus Hamiltonella defensa	0.87	Predict
NOF012_scaffold8867_2	Candidatus Hamiltonella defensa	0.83	Predict
NOF012_scaffold13996_7	Candidatus Hamiltonella defensa	0.97	Predict
NOF012_scaffold77917_2	Candidatus Hamiltonella defensa	1	CRISPR
NOF012_scaffold12244_6	Clostridium botulinum	0.78	Predict
NOF012_scaffold3081_2	Enterococcus faecium	0.75	Predict
NOF012_scaffold78059_2	Bacteroides fragilis	0.98	Predict
NOF012_scaffold5639_7	Candidatus Hamiltonella defensa	1	CRISPR
NOF012_scaffold24554_12	Flavobacterium psychrophilum	0.75	Predict
NOF012_scaffold4667_6	Candidatus Hamiltonella defensa	0.92	Predict
NOF012_scaffold72024_3	Paenibacillus larvae	1	CRISPR
NOF012_scaffold19855_2	Candidatus Hamiltonella defensa	0.71	Predict
NOF012_scaffold34940_12	Micromonospora chaiyaphumensis	1	CRISPR
NOF012_scaffold8693_10	Candidatus Hamiltonella defensa	0.8	Predict
NOF012_scaffold71857_2	Butyricicoccus sp. AF24-19AC	1	CRISPR
NOF012_C833949_1	Candidatus Pelagibacter ubique	0.9	Predict
NOF012_scaffold15991_1	Candidatus Hamiltonella defensa	0.81	Predict
NOF012_scaffold71874_7	Candidatus Hamiltonella defensa	1	Predict
NOF012_scaffold339_7	Candidatus Hamiltonella defensa	0.96	Predict

NOF012_scaffold28415_3	Candidatus Hamiltonella defensa	1	CRISPR
NOF012_scaffold77196_1	Staphylococcus saprophyticus	0.81	Predict
NOF012_scaffold37026_8	Pasteurella multocida	0.94	Predict
NOF012_scaffold68565_1	Candidatus Hamiltonella defensa	0.97	Predict
NOF012_scaffold26305_22	Candidatus Hamiltonella defensa	0.96	Predict
NOF012_scaffold78081_1	Parabacteroides distasonis	0.75	Predict
NOF012_scaffold43523_1	Parabacteroides merdae	0.71	Predict
NOF012_scaffold41858_11	Acinetobacter baumannii	1	CRISPR
NOF012_scaffold28114_12	Staphylococcus xylosus	0.94	Predict
NOF013_scaffold22999_4	Bacteroides oleiciplenus	1	CRISPR
NOF013_scaffold33526_1	Bacteroides fragilis	0.98	Predict
NOF013_scaffold9359_1	Candidatus Hamiltonella defensa	0.78	Predict
NOF013_scaffold11906_1	Candidatus Hamiltonella defensa	1	CRISPR
NOF013_scaffold26437_1	Candidatus Hamiltonella defensa	0.89	Predict
NOF013_scaffold25505_1	Candidatus Hamiltonella defensa	0.93	Predict
NOF013_scaffold34730_1	Candidatus Hamiltonella defensa	1	CRISPR
NOF013_scaffold9607_2	Bacteroides fragilis	0.92	Predict
NOF013_scaffold34528_1	Candidatus Hamiltonella defensa	0.74	Predict
NOF013_C612756_1	Bacteroides fragilis	1	CRISPR
NOF013_scaffold3348_11	Bacteroides fragilis	1	CRISPR
NOF013_scaffold24565_7	Staphylococcus saprophyticus	0.76	Predict
NOF013_scaffold34630_3	Staphylococcus saprophyticus	0.81	Predict
NOF013_scaffold24565_6	Candidatus Hamiltonella defensa	0.95	Predict
NOF013_scaffold26437_2	Candidatus Hamiltonella defensa	0.98	Predict
NOF013_scaffold4750_1	Bacteroides fragilis	0.78	Predict
NOF013_scaffold316_7	Staphylococcus saprophyticus	0.97	Predict
NOF013_scaffold22999_3	Candidatus Pelagibacter ubique	1	CRISPR
NOF013_scaffold23510_11	Clostridium perfringens	1	CRISPR
NOF013_C613108_1	Staphylococcus saprophyticus	0.84	Predict
NOF013_scaffold1181_6	Acinetobacter baumannii	1	CRISPR
NOF013_scaffold131_1	Bacteroides fragilis	0.77	Predict
NOF013_C612464_1	Candidatus Hamiltonella defensa	0.9	Predict
NOF013_scaffold33363_1	Bacteroides fragilis	0.72	Predict
NOF013_scaffold33134_1	Candidatus Hamiltonella defensa	0.77	Predict
NOF013_scaffold32891_4	Candidatus Hamiltonella defensa	1	Predict
NOF013_scaffold29173_1	Parabacteroides distasonis	0.78	Predict
NOF013_scaffold24443_1	Candidatus Hamiltonella defensa	0.95	Predict
NOF013_scaffold3979_5	Bacteroides fragilis	0.94	Predict
NOF013_scaffold2241_2	Candidatus Hamiltonella defensa	0.84	Predict
NOF014_scaffold67337_2	Candidatus Hamiltonella defensa	0.8	Predict
NOF014_scaffold29077_2	Candidatus Hamiltonella defensa	0.89	Predict
NOF014_scaffold41735_1	Staphylococcus saprophyticus	0.77	Predict
NOF014_scaffold59979_2	Candidatus Hamiltonella defensa	0.75	Predict
NOF014_scaffold72132_1	Candidatus Hamiltonella defensa	0.91	Predict
NOF014_scaffold30730_5	Bacteroides fragilis	0.88	Predict
NOF014_scaffold6730_5	Staphylococcus saprophyticus	0.84	Predict
NOF014_scaffold71626_3	Streptococcus pneumoniae	1	CRISPR
NOF014_scaffold46167_2	Candidatus Hamiltonella defensa	0.85	Predict

NOF014_scaffold40347_4	Cellulophaga baltica	0.87	Predict
NOF014_scaffold72227_1	Parabacteroides merdae	0.7	Predict
NOF014_scaffold27769_8	Candidatus Hamiltonella defensa	0.72	Predict
NOF014_scaffold10135_5	Streptococcus mutans	0.95	Predict
NOF014_scaffold72263_3	Candidatus Hamiltonella defensa	0.99	Predict
NOF014_scaffold43_10	Candidatus Hamiltonella defensa	0.84	Predict
NOF014_scaffold26105_6	Candidatus Hamiltonella defensa	0.71	Predict
NOF014_scaffold63476_2	Clostridioides difficile	0.89	Predict
NOF014_scaffold72216_1	Candidatus Hamiltonella defensa	1	CRISPR
NOF014_scaffold20265_3	Veillonellaceae bacterium SB90	1	CRISPR
NOF014_scaffold5894_2	Croceibacter atlanticus	1	CRISPR
NOF014_scaffold49593_2	Candidatus Pelagibacter ubique	0.73	Predict
NOF014_scaffold71877_1	Clostridioides difficile	0.98	Predict
NOF014_scaffold72248_1	Candidatus Pelagibacter ubique	1	CRISPR
NOF014_scaffold20787_1	Candidatus Hamiltonella defensa	0.77	Predict
NOF014_scaffold66923_1	Candidatus Hamiltonella defensa	0.71	Predict
NOF014_scaffold71552_2	Candidatus Hamiltonella defensa	1	CRISPR
NOF014_scaffold71919_2	Prevotella intermedia	1	CRISPR
NOF014_scaffold66767_1	Candidatus Hamiltonella defensa	0.93	Predict
NOF014_scaffold63571_1	Candidatus Hamiltonella defensa	0.99	Predict
NOF014_scaffold65085_3	Candidatus Hamiltonella defensa	0.85	Predict
NOF014_scaffold64087_5	Staphylococcus saprophyticus	0.9	Predict
NOF014_scaffold59979_3	Candidatus Hamiltonella defensa	0.86	Predict
NOF001_scaffold1053_2	unknown	0	-
NOF001_scaffold33721_2	unknown	0	-
NOF001_scaffold13046_9	unknown	0	-
NOF001_scaffold29077_4	unknown	0	-
NOF001_scaffold13046_6	unknown	0	-
NOF001_scaffold7196_11	unknown	0	-
NOF001_scaffold2819_5	unknown	0	-
NOF001_scaffold56157_1	unknown	0	-
NOF001_scaffold49296_1	unknown	0	-
NOF001_scaffold15730_5	unknown	0	-
NOF002_scaffold4501_18	unknown	0	-
NOF002_scaffold29466_1	unknown	0	-
NOF002_scaffold12961_13	unknown	0	-
NOF002_scaffold20732_1	unknown	0	-
NOF004_scaffold39800_2	unknown	0	-
NOF004_C576082_1	unknown	0	-
NOF004_scaffold22559_2	unknown	0	-
NOF004_scaffold5741_5	unknown	0	-
NOF004_scaffold10567_2	unknown	0	-
NOF004_scaffold2367_14	unknown	0	-
NOF004_scaffold15266_9	unknown	0	-
NOF004_scaffold39800_1	unknown	0	-
NOF004_scaffold14653_3	unknown	0	-
NOF004_scaffold3847_7	unknown	0	-
NOF004_scaffold10069_19	unknown	0	-

NOF005_scaffold36104_5	unknown	0	-
NOF005_scaffold18_1	unknown	0	-
NOF005_scaffold25742_6	unknown	0	-
NOF005_scaffold35393_5	unknown	0	-
NOF005_scaffold2170_3	unknown	0	-
NOF005_scaffold24910_8	unknown	0	-
NOF005_scaffold49499_1_	unknown	0	-
NOF005_scaffold5924_1	unknown	0	-
NOF005_scaffold44821_1	unknown	0	-
NOF005_scaffold2025_2	unknown	0	-
NOF006_scaffold21352_1	unknown	0	-
NOF006_scaffold4067_5	unknown	0	-
NOF006_scaffold969_8	unknown	0	-
NOF006_scaffold21374_2	unknown	0	-
NOF006_scaffold173_3	unknown	0	-
NOF007_scaffold396_8	unknown	0	-
NOF007_scaffold11484_1	unknown	0	-
NOF007_scaffold25262_5	unknown	0	-
NOF007_scaffold1349_3	unknown	0	-
NOF007_C490687_1	unknown	0	-
NOF007_scaffold1349_2	unknown	0	-
NOF007_scaffold15189_2	unknown	0	-
NOF007_scaffold41_1	unknown	0	-
NOF007_scaffold28685_3	unknown	0	-
NOF007_scaffold25304_2	unknown	0	-
NOF007_scaffold28624_3	unknown	0	-
NOF007_scaffold3393_7	unknown	0	-
NOF007_scaffold2822_2	unknown	0	-
NOF008_scaffold34632_3	unknown	0	-
NOF008_scaffold61643_2	unknown	0	-
NOF008_scaffold1288_26	unknown	0	-
NOF008_scaffold5217_6	unknown	0	-
NOF008_scaffold1389_2	unknown	0	-
NOF008_scaffold34632_4	unknown	0	-
NOF008_scaffold48283_1	unknown	0	-
NOF008_scaffold68147_1	unknown	0	-
NOF008_scaffold25447_1	unknown	0	-
NOF008_scaffold52435_2	unknown	0	-
NOF008_scaffold35076_1	unknown	0	-
NOF008_scaffold53677_1	unknown	0	-
NOF008_scaffold68191_1	unknown	0	-
NOF008_scaffold65986_1	unknown	0	-
NOF008_scaffold63722_1	unknown	0	-
NOF008_scaffold50828_1	unknown	0	-
NOF008_scaffold41113_4	unknown	0	-
NOF008_scaffold59760_7	unknown	0	-
NOF008_scaffold13444_2	unknown	0	-
NOF008_scaffold55811_1	unknown	0	-

NOF009_C558283_1	unknown	0	-
NOF009_scaffold11228_12	unknown	0	-
NOF009_scaffold13328_11	unknown	0	-
NOF009_scaffold5136_16	unknown	0	-
NOF009_scaffold42878_5	unknown	0	-
NOF010_scaffold28531_1	unknown	0	-
NOF010_scaffold2330_4	unknown	0	-
NOF010_scaffold6485_5	unknown	0	-
NOF010_scaffold2722_9	unknown	0	-
NOF010_scaffold22545_1	unknown	0	-
NOF010_scaffold46583_3	unknown	0	-
NOF010_scaffold54281_1	unknown	0	-
NOF011_C736712_1	unknown	0	-
NOF012_scaffold24523_4	unknown	0	-
NOF012_scaffold50466_2	unknown	0	-
NOF012_scaffold44774_1	unknown	0	-
NOF012_scaffold34517_3	unknown	0	-
NOF012_scaffold34517_1	unknown	0	-
NOF012_scaffold34517_2	unknown	0	-
NOF012_scaffold77271_2	unknown	0	-
NOF012_scaffold74308_1	unknown	0	-
NOF012_scaffold9650_7	unknown	0	-
NOF013_scaffold15363_3	unknown	0	-
NOF013_scaffold24304_2	unknown	0	-
NOF013_scaffold20067_2	unknown	0	-
NOF013_scaffold6283_17	unknown	0	-
NOF013_scaffold30635_1	unknown	0	-
NOF013_scaffold28493_2	unknown	0	-
NOF013_scaffold32172_3	unknown	0	-
NOF013_scaffold15363_2	unknown	0	-
NOF013_scaffold34382_1	unknown	0	-
NOF013_scaffold1744_3	unknown	0	-
NOF013_scaffold34324_3	unknown	0	-
NOF014_scaffold72006_1	unknown	0	-
NOF014_scaffold35720_11	unknown	0	-
NOF014_scaffold4327_52	unknown	0	-
NOF014_scaffold66855_1	unknown	0	-
NOF014_scaffold20787_3	unknown	0	-
NOF014_scaffold19962_3	unknown	0	-
NOF014_scaffold26105_25	unknown	0	-
NOF014_scaffold10667_4	unknown	0	-
NOF014_scaffold52663_4	unknown	0	-
NOF014_scaffold71919_3	unknown	0	-
NOF014_scaffold2390_2	unknown	0	-
NOF014_scaffold22954_1	unknown	0	-
NOF014_scaffold72295_2	unknown	0	-
NOF014_scaffold71714_2	unknown	0	-
NOF014_scaffold71856_1	unknown	0	-

NOF014_scaffold52663_2	unknown	0	-
NOF014_scaffold54136_28	unknown	0	-
DLF001_scaffold25843_5	Bacteroides fragilis	1	CRISPR
DLF001_scaffold6028_2	Bacillus halmapalus	1	CRISPR
DLF001_scaffold16878_1	Bacteroides fragilis	0.87	Predict
DLF001_scaffold25777_2	Bacillus halmapalus	0.73	Predict
DLF001_scaffold4836_9	Bacillus halmapalus	0.93	Predict
DLF001_scaffold18617_11	Bacillus halmapalus	1	CRISPR
DLF001_scaffold24376_1	Bacillus halmapalus	0.98	Predict
DLF001_scaffold20939_1	Bacteroides fragilis	1	CRISPR
DLF001_scaffold10899_1	Lactobacillus fermentum	1	CRISPR
DLF001_scaffold10872_1	Bacteroides fragilis	1	CRISPR
DLF001_scaffold25652_2	Bacteroides fragilis	0.95	Predict
DLF001_scaffold21798_2	Bacteroides fragilis	0.74	Predict
DLF001_C323280_1	Bacteroides fragilis	0.8	Predict
DLF001_scaffold10220_3	Bacillus halmapalus	0.95	Predict
DLF001_C323242_1	Colwellia psychrerythraea	0.85	Predict
DLF001_scaffold25336_2	Parabacteroides distasonis	0.85	Predict
DLF001_scaffold46_6	Bacillus halmapalus	0.93	Predict
DLF001_scaffold80_8	Colwellia psychrerythraea	0.74	Predict
DLF001_scaffold6028_1	Bacteroides fragilis	1	CRISPR
DLF001_C323900_1	Klebsiella pneumoniae	1	CRISPR
DLF002_scaffold6624_21	Bacteroides fragilis	1	CRISPR
DLF002_scaffold7298_22	Bacteroides fragilis	1	CRISPR
DLF002_scaffold33093_1	Bacteroides vulgatus	1	CRISPR
DLF002_scaffold36414_1	Bacteroides fragilis	1	CRISPR
DLF002_scaffold85_3	Bacteroides fragilis	0.85	Predict
DLF002_scaffold32378_2	Bacteroides fragilis	0.73	Predict
DLF002_scaffold19600_3	Colwellia psychrerythraea	1	CRISPR
DLF002_scaffold24316_12	Colwellia psychrerythraea	1	CRISPR
DLF002_scaffold294_32	Colwellia psychrerythraea	0.78	Predict
DLF002_scaffold7525_1	Bacteroides fragilis	0.92	Predict
DLF002_scaffold25546_1	Bacillus halmapalus	0.95	Predict
DLF002_scaffold138_8	Prevotella intermedia	1	CRISPR
DLF002_scaffold33974_1	Bacteroides fragilis	0.89	Predict
DLF002_scaffold10343_2	Bacteroides fragilis	0.74	Predict
DLF002_scaffold36505_1	Colwellia psychrerythraea	0.81	Predict
DLF002_scaffold36298_1	Colwellia psychrerythraea	0.88	Predict
DLF002_scaffold6041_3	Bacteroides fragilis	0.88	Predict
DLF003_scaffold21732_2	Bacillus halmapalus	0.77	Predict
DLF003_scaffold29891_1	Parabacteroides distasonis	1	CRISPR
DLF003_scaffold23409_2	Colwellia psychrerythraea	0.82	Predict
DLF003_scaffold44436_1	Faecalibacterium prausnitzii	1	CRISPR
DLF003_scaffold12321_2	Acinetobacter baumannii	1	CRISPR
DLF003_C717736_1	Bacillus halmapalus	1	CRISPR
DLF003_scaffold137_1	Colwellia psychrerythraea	0.78	Predict
DLF003_scaffold3834_23	Colwellia psychrerythraea	0.73	Predict
DLF003_scaffold55800_1	Morganella morganii	0.87	Predict

DLF003_C717640_1	Ruminococcus sp. OM07-17	1	CRISPR
DLF003_scaffold56673_2	Bacillus halmapalus	0.88	Predict
DLF003_scaffold54149_1	Candidatus Pelagibacter ubique	0.99	Predict
DLF003_scaffold43976_1	Prevotella copri	1	CRISPR
DLF003_scaffold47905_1	Colwellia psychrerythraea	0.7	Predict
DLF003_scaffold57190_2	Bacillus halmapalus	0.83	Predict
DLF003_scaffold56159_4	Bacteroides fragilis	0.93	Predict
DLF003_scaffold4398_37	Flavobacterium psychrophilum	0.9	Predict
DLF003_scaffold29891_3	Colwellia psychrerythraea	0.72	Predict
DLF004_scaffold35797_1	Parabacteroides merdae	0.82	Predict
DLF004_C548546_1	Bacillus halmapalus	1	CRISPR
DLF004_scaffold18383_6	Bacillus halmapalus	1	CRISPR
DLF004_C548576_1	Bacillus halmapalus	1	CRISPR
DLF004_scaffold10854_4	Enterococcus faecium	0.7	Predict
DLF004_scaffold28880_1	Bacillus halmapalus	0.78	Predict
DLF004_scaffold25533_1	Colwellia psychrerythraea	0.73	Predict
DLF004_scaffold36320_8	Clostridioides difficile	0.98	Predict
DLF004_C548200_1	Prevotella copri	1	CRISPR
DLF004_scaffold2535_5	Colwellia psychrerythraea	0.73	Predict
DLF004_scaffold32830_2	Colwellia psychrerythraea	0.77	Predict
DLF004_scaffold27435_2	Colwellia psychrerythraea	0.9	Predict
DLF004_scaffold930_8	Bacillus halmapalus	0.71	Predict
DLF004_scaffold8102_2	Bacillus halmapalus	1	CRISPR
DLF004_scaffold6537_1	Bacillus halmapalus	0.77	Predict
DLF004_scaffold15396_3	Bacillus halmapalus	0.99	Predict
DLF004_scaffold1974_5	Bacteroides fragilis	0.73	Predict
DLF004_scaffold24525_4	Bacillus halmapalus	0.76	Predict
DLF004_scaffold15823_6	Bacillus halmapalus	0.97	Predict
DLF004_scaffold26106_5	Candidatus Hamiltonella defensa	0.71	Predict
DLF004_scaffold22703_8	Colwellia psychrerythraea	0.71	Predict
DLF004_scaffold21240_2	Bacillus halmapalus	0.87	Predict
DLF004_scaffold125_29	Bacteroides fragilis	0.85	Predict
DLF005_scaffold31168_1	Colwellia psychrerythraea	0.75	Predict
DLF005_scaffold55052_1	Candidatus Pelagibacter ubique	1	CRISPR
DLF005_scaffold31945_4	Ruminococcus sp. AM43-6	1	CRISPR
DLF005_scaffold48546_1	Bacillus halmapalus	0.81	Predict
DLF005_scaffold55770_1	Bacillus halmapalus	1	CRISPR
DLF005_scaffold56569_4	Cellulophaga baltica	1	CRISPR
DLF005_scaffold51335_1	Bacillus halmapalus	0.8	Predict
DLF005_scaffold4693_36	Colwellia psychrerythraea	0.72	Predict
DLF005_scaffold56163_1	Candidatus Pelagibacter ubique	1	CRISPR
DLF005_scaffold1570_16	Bacteroides fragilis	0.88	Predict
DLF005_scaffold13131_1	Bacillus halmapalus	0.82	Predict
DLF005_scaffold39187_4	Candidatus Hamiltonella defensa	1	CRISPR
DLF005_scaffold25785_13	Megamonas funiformis	1	CRISPR
DLF005_scaffold56894_1	Parabacteroides merdae	0.91	Predict
DLF005_scaffold2505_1	Parabacteroides distasonis	1	CRISPR
DLF005_scaffold47023_3	Colwellia psychrerythraea	1	CRISPR

DLF005_scaffold56967_1	Bacillus halmapalus	1	Predict
DLF005_scaffold13334_3	Streptococcus pneumoniae	1	CRISPR
DLF005_scaffold56817_1	Bacillus halmapalus	1	CRISPR
DLF005_scaffold56924_2	Bacillus halmapalus	0.97	Predict
DLF005_scaffold39187_3	Megamonas funiformis	1	CRISPR
DLF005_scaffold18199_1	Bacillus halmapalus	0.79	Predict
DLF005_scaffold3960_1	Bacillus halmapalus	0.79	Predict
DLF005_scaffold32479_1	Flavobacterium columnare	0.84	Predict
DLF005_scaffold41843_2	Bacteroides fragilis	0.8	Predict
DLF005_scaffold33045_1	Clostridioides difficile	0.89	Predict
DLF005_scaffold4093_2	Flavobacterium columnare	1	CRISPR
DLF005_scaffold9847_1	Microcystis aeruginosa	0.82	Predict
DLF005_scaffold31168_2	Acinetobacter baumannii	1	CRISPR
DLF005_scaffold53782_1	Flavobacterium columnare	1	CRISPR
DLF005_scaffold56569_2	Bacillus subtilis	1	CRISPR
DLF005_scaffold23342_9	Staphylococcus hominis	1	CRISPR
DLF006_scaffold52_4	Clostridium botulinum	0.76	Predict
DLF006_C667664_1	Staphylococcus hominis	0.79	Predict
DLF006_scaffold9049_10	Bacillus halmapalus	0.76	Predict
DLF006_scaffold108_5	Bacillus halmapalus	0.8	Predict
DLF006_scaffold46681_1	Bacillus halmapalus	1	CRISPR
DLF006_scaffold39883_1	Bacillus halmapalus	0.96	Predict
DLF006_scaffold32044_2	Bacteroides fragilis	0.99	Predict
DLF006_scaffold8740_3	Bacillus halmapalus	0.76	Predict
DLF006_C667862_1	Bacteroides fragilis	1	CRISPR
DLF006_scaffold19189_3	Colwellia psychrerythraea	1	CRISPR
DLF006_scaffold474_4	Colwellia psychrerythraea	1	CRISPR
DLF006_scaffold33584_4	Bacteroides fragilis	0.86	Predict
DLF006_scaffold34156_4	Colwellia psychrerythraea	0.84	Predict
DLF006_scaffold9056_18	Colwellia psychrerythraea	0.83	Predict
DLF006_scaffold11422_2	Candidatus Hamiltonella defensa	0.96	Predict
DLF006_scaffold19189_4	Colwellia psychrerythraea	0.71	Predict
DLF006_scaffold9845_3	Pseudomonas aeruginosa	0.87	Predict
DLF006_scaffold7466_5	Colwellia psychrerythraea	0.83	Predict
DLF006_scaffold22006_9	Micromonospora chaiyaphumensis	1	CRISPR
DLF006_scaffold25840_10	Colwellia psychrerythraea	0.73	Predict
DLF006_scaffold36051_1	Pectobacterium carotovorum	1	CRISPR
DLF006_scaffold9845_4	Colwellia psychrerythraea	0.73	Predict
DLF006_scaffold36051_2	Colwellia psychrerythraea	0.83	Predict
DLF006_scaffold34515_1	Colwellia psychrerythraea	0.86	Predict
DLF006_scaffold5280_1	Parabacteroides merdae	1	CRISPR
DLF006_scaffold46760_1	Bacteroides fragilis	0.9	Predict
DLF006_scaffold46848_1	Parabacteroides distasonis	0.91	Predict
DLF006_scaffold8740_4	Colwellia psychrerythraea	0.97	Predict
DLF006_scaffold27662_1	Bacteroides fragilis	0.97	Predict
DLF006_scaffold46851_2	Bacillus halmapalus	0.86	Predict
DLF006_scaffold92_1	Colwellia psychrerythraea	0.83	Predict
DLF007_scaffold15529_4	Bacillus halmapalus	0.77	Predict

DLF007_scaffold18927_1	Candidatus Hamiltonella defensa	0.99	Predict
DLF007_C306149_1	Candidatus Hamiltonella defensa	0.99	Predict
DLF007_C306343_1	Bacillus halmapalus	0.71	Predict
DLF007_scaffold1532_23	Bacillus halmapalus	1	CRISPR
DLF007_scaffold20333_2	Bacteroides fragilis	0.84	Predict
DLF007_scaffold4179_16	Bacillus halmapalus	1	CRISPR
DLF007_scaffold15763_5	Bacillus halmapalus	1	CRISPR
DLF007_scaffold2664_1	Colwellia psychrerythraea	0.87	Predict
DLF007_scaffold20109_2	Bacteroides fragilis	0.92	Predict
DLF007_scaffold18401_2	Bacteroides fragilis	1	CRISPR
DLF007_scaffold12277_1	Lactobacillus fermentum	1	CRISPR
DLF007_scaffold15181_7	Bacteroides fragilis	1	CRISPR
DLF008_scaffold7461_4	Bacteroides fragilis	0.91	Predict
DLF008_scaffold17152_1	Bacillus halmapalus	0.81	Predict
DLF008_scaffold1091_2	Bacillus halmapalus	0.93	Predict
DLF008_scaffold17625_2	Bacteroides fragilis	0.92	Predict
DLF008_scaffold17607_1	[Clostridium] clostridioforme	1	CRISPR
DLF008_scaffold17497_1	Colwellia psychrerythraea	1	CRISPR
DLF008_scaffold14160_4	Mannheimia haemolytica	0.84	Predict
DLF008_scaffold10341_2	Colwellia psychrerythraea	1	CRISPR
DLF008_scaffold16449_2	Parabacteroides merdae	0.73	Predict
DLF008_scaffold5415_1	Bacillus halmapalus	1	CRISPR
DLF008_scaffold3962_5	Colwellia psychrerythraea	0.74	Predict
DLF008_scaffold10487_1	Colwellia psychrerythraea	1	CRISPR
DLF008_C335467_1	Bacillus halmapalus	1	CRISPR
DLF008_scaffold710_21	Bacillus halmapalus	0.91	Predict
DLF008_scaffold17349_1	Candidatus Hamiltonella defensa	1	CRISPR
DLF008_scaffold8096_6	Colwellia psychrerythraea	1	CRISPR
DLF008_scaffold7166_1	Colwellia psychrerythraea	0.84	Predict
DLF008_scaffold9028_1	Bacteroides fragilis	0.79	Predict
DLF008_scaffold8047_2	Bacillus halmapalus	1	Predict
DLF008_scaffold3962_1	Bacillus halmapalus	0.88	Predict
DLF008_scaffold12889_2	Paenibacillus larvae	0.81	Predict
DLF008_scaffold2953_7	Colwellia psychrerythraea	0.9	Predict
DLF008_scaffold17084_1	Bacillus halmapalus	0.96	Predict
DLF008_scaffold14357_3	Candidatus Hamiltonella defensa	1	CRISPR
DLF008_scaffold3328_5	Staphylococcus hominis	0.78	Predict
DLF008_scaffold17350_1	Bacillus halmapalus	1	CRISPR
DLF009_scaffold34424_1	Bacillus halmapalus	0.72	Predict
DLF009_scaffold1880_30	Candidatus Hamiltonella defensa	0.94	Predict
DLF009_scaffold36221_2	Bacteroides fragilis	0.71	Predict
DLF009_C428906_1	Citrobacter freundii	0.79	Predict
DLF009_scaffold30115_1	Cellulophaga baltica	0.86	Predict
DLF009_scaffold789_3	Colwellia psychrerythraea	1	CRISPR
DLF009_scaffold37442_2	Clostridioides difficile	1	CRISPR
DLF009_scaffold36221_1	Bacteroides fragilis	0.77	Predict
DLF009_scaffold7798_1	Bacillus halmapalus	1	CRISPR
DLF009_scaffold37363_2	Colwellia psychrerythraea	0.95	Predict

DLF009_scaffold11451_17	Colwellia psychrerythraea	0.93	Predict
DLF009_scaffold21876_2	Bacillus halmapalus	1	CRISPR
DLF009_scaffold37177_1	Colwellia psychrerythraea	1	CRISPR
DLF009_scaffold37452_1	Colwellia psychrerythraea	1	CRISPR
DLF009_scaffold794_2	Bifidobacterium longum	1	CRISPR
DLF009_scaffold30115_3	Cellulophaga baltica	0.91	Predict
DLF009_scaffold25763_1	Pectobacterium carotovorum	1	CRISPR
DLF009_scaffold13354_1	Colwellia psychrerythraea	0.82	Predict
DLF009_scaffold13766_4	Bacteroides fragilis	1	CRISPR
DLF009_scaffold37443_4	Bacteroides fragilis	0.79	Predict
DLF009_scaffold37498_1	Colwellia psychrerythraea	1	CRISPR
DLF009_scaffold37448_2	Bacteroides vulgatus	1	CRISPR
DLF009_scaffold37438_1	Enterobacter cloacae	1	CRISPR
DLF009_scaffold37466_1	Bacillus halmapalus	1	CRISPR
DLF009_scaffold37020_3	Colwellia psychrerythraea	0.85	Predict
DLF009_scaffold3043_4	Colwellia psychrerythraea	1	CRISPR
DLF009_C428576_1	Colwellia psychrerythraea	1	CRISPR
DLF009_scaffold17926_1	Bacillus halmapalus	0.84	Predict
DLF009_scaffold37386_2	Bacillus halmapalus	0.79	Predict
DLF009_scaffold11949_1	Colwellia psychrerythraea	1	CRISPR
DLF009_scaffold19674_5	Colwellia psychrerythraea	0.72	Predict
DLF009_scaffold6204_7	Colwellia psychrerythraea	1	CRISPR
DLF009_scaffold37284_1	Candidatus Hamiltonella defensa	1	CRISPR
DLF009_scaffold37363_3	Colwellia psychrerythraea	0.76	Predict
DLF009_scaffold37307_2	Colwellia psychrerythraea	1	CRISPR
DLF009_scaffold2837_2	Bacillus halmapalus	1	Predict
DLF009_scaffold61_24	Rhodococcus rhodochrous	0.71	Predict
DLF009_scaffold3557_6	Colwellia psychrerythraea	0.77	Predict
DLF009_scaffold37386_1	Staphylococcus epidermidis	0.76	Predict
DLF009_scaffold30115_5	Cellulophaga baltica	0.86	Predict
DLF010_scaffold35043_4	Colwellia psychrerythraea	0.79	Predict
DLF010_scaffold55870_1	Bacillus halmapalus	0.92	Predict
DLF010_scaffold43436_5	Lachnospiraceae bacterium	1	CRISPR
DLF010_scaffold363_30	Weissella cibaria	0.88	Predict
DLF010_scaffold36638_6	Edwardsiella ictaluri	0.99	Predict
DLF010_scaffold13546_16	Colwellia psychrerythraea	0.93	Predict
DLF010_scaffold35043_3	Colwellia psychrerythraea	0.73	Predict
DLF010_scaffold46634_1	Clostridioides difficile	0.99	Predict
DLF010_scaffold49177_4	Colwellia psychrerythraea	1	CRISPR
DLF010_scaffold4302_1	Streptomyces lividans	0.9	Predict
DLF010_scaffold25454_7	Candidatus Hamiltonella defensa	0.96	Predict
DLF010_scaffold51522_2	Colwellia psychrerythraea	0.93	Predict
DLF010_scaffold8211_1	Colwellia psychrerythraea	0.82	Predict
DLF010_scaffold55918_1	Prevotella copri	1	CRISPR
DLF010_scaffold5445_2	Bacillus halmapalus	0.76	Predict
DLF010_scaffold18414_39	Clostridium botulinum	0.79	Predict
DLF010_scaffold27012_2	Aeromonas salmonicida	0.93	Predict
DLF010_scaffold14049_4	Colwellia psychrerythraea	0.94	Predict

DLF010_scaffold86_3	Staphylococcus saprophyticus	0.92	Predict
DLF010_scaffold24026_10	Bacillus cereus	0.92	Predict
DLF010_scaffold55874_1	Colwellia psychrerythraea	0.75	Predict
DLF012_scaffold4165_33	Bacillus halmapalus	1	CRISPR
DLF012_scaffold36_1	Bacillus halmapalus	0.99	Predict
DLF012_scaffold17185_5	Colwellia psychrerythraea	0.76	Predict
DLF012_scaffold15936_2	Candidatus Pelagibacter ubique	0.78	Predict
DLF012_scaffold12743_1	Bacillus halmapalus	1	CRISPR
DLF012_scaffold12838_3	Lactobacillus paracasei	1	CRISPR
DLF012_scaffold19111_1	Candidatus Hamiltonella defensa	0.97	Predict
DLF012_scaffold19146_2	Bacteroides fragilis	0.87	Predict
DLF012_scaffold571_16	Streptomyces avermitilis	1	CRISPR
DLF012_scaffold17036_1	Bacteroides fragilis	1	CRISPR
DLF012_scaffold3118_9	Flavobacterium columnare	0.74	Predict
DLF013_scaffold47429_1	Bacillus halmapalus	0.75	Predict
DLF013_scaffold18599_4	Bacteroides bouchesdurhonensis	1	CRISPR
DLF013_scaffold1520_3	Bacillus halmapalus	1	CRISPR
DLF013_scaffold34743_5	Paenibacillus larvae	0.91	Predict
DLF013_scaffold34809_3	Colwellia psychrerythraea	0.91	Predict
DLF013_scaffold14099_2	Candidatus Hamiltonella defensa	0.7	Predict
DLF013_scaffold13599_6	Cellulophaga baltica	0.84	Predict
DLF013_scaffold53908_2	Bacillus halmapalus	0.97	Predict
DLF013_scaffold45127_1	Bacillus halmapalus	0.9	Predict
DLF013_scaffold3815_1	Colwellia psychrerythraea	0.87	Predict
DLF013_scaffold53550_1	Candidatus Hamiltonella defensa	0.73	Predict
DLF013_scaffold42596_2	Clostridium perfringens	1	CRISPR
DLF013_scaffold6385_2	Parabacteroides distasonis	1	CRISPR
DLF013_scaffold42479_2	Colwellia psychrerythraea	0.94	Predict
DLF013_scaffold36273_1	Bacteroides fragilis	0.78	Predict
DLF013_scaffold31785_1	Colwellia psychrerythraea	1	CRISPR
DLF013_scaffold139_7	Bacteroides fragilis	0.97	Predict
DLF013_scaffold38734_1	Colwellia psychrerythraea	0.97	Predict
DLF013_scaffold53889_1	Bacteroides fragilis	0.7	Predict
DLF013_scaffold42888_4	Mesorhizobium loti	0.94	Predict
DLF013_scaffold19199_4	Bacillus halmapalus	1	CRISPR
DLF013_scaffold49433_5	Bacillus halmapalus	0.72	Predict
DLF013_scaffold1100_4	Colwellia psychrerythraea	1	CRISPR
DLF013_scaffold42008_2	Bacillus halmapalus	1	CRISPR
DLF013_scaffold54776_1	Cellulophaga baltica	0.76	Predict
DLF014_scaffold12062_3	Providencia stuartii	1	CRISPR
DLF014_C195831_1	Bacillus halmapalus	0.81	Predict
DLF014_scaffold12779_1	Lactobacillus fermentum	1	CRISPR
DLF014_scaffold9942_1	Clostridium perfringens	1	CRISPR
DLF014_C196781_1	Bacillus halmapalus	1	CRISPR
DLF014_scaffold12792_2	Colwellia psychrerythraea	1	CRISPR
DLF014_scaffold12718_1	Colwellia psychrerythraea	0.85	Predict
DLF014_scaffold7985_2	Colwellia psychrerythraea	0.89	Predict
DLF014_scaffold583_1	Bacillus halmapalus	1	CRISPR

DLF014_scaffold12682_1	Colwellia psychrerythraea	1	CRISPR
DLF014_scaffold2386_4	Clavibacter michiganensis	0.83	Predict
DLF014_scaffold12741_1	Bacillus halmapalus	1	CRISPR
DLF014_scaffold11967_1	Colwellia psychrerythraea	0.71	Predict
DLF014_scaffold8898_1	Bacteroides fragilis	0.7	Predict
DLF014_scaffold3999_1	Colwellia psychrerythraea	0.78	Predict
DLF014_scaffold6344_1	Bacillus halmapalus	1	CRISPR
DLF014_scaffold11623_1	Colwellia psychrerythraea	0.96	Predict
DLF014_scaffold1928_3	Lachnospiraceae bacterium AM23-7LI	1	CRISPR
DLF014_scaffold12767_1	Bacillus halmapalus	0.9	Predict
DLF014_scaffold4556_3	Colwellia psychrerythraea	1	CRISPR
DLF014_scaffold12792_1	Candidatus Hamiltonella defensa	1	CRISPR
DLF014_scaffold10166_1	Colwellia psychrerythraea	0.97	Predict
DLF014_scaffold12665_1	Bacillus halmapalus	0.73	Predict
DLF014_scaffold8_2	Colwellia psychrerythraea	0.88	Predict
DLF014_scaffold8436_2	Bacillus halmapalus	1	CRISPR
DLF014_scaffold8300_1	Colwellia psychrerythraea	1	CRISPR
DLF014_scaffold5363_6	Colwellia psychrerythraea	0.73	Predict
DLF014_scaffold5363_21	Colwellia psychrerythraea	1	CRISPR
DLF014_scaffold11967_2	Colwellia psychrerythraea	0.83	Predict
DLF014_scaffold6855_1	Candidatus Hamiltonella defensa	1	CRISPR
DLF014_scaffold2932_3	Bacillus halmapalus	0.87	Predict
DLF014_scaffold2093_4	Flavobacterium columnare	0.95	Predict
DLF014_scaffold12383_2	Bacillus halmapalus	0.96	Predict
DLF001_scaffold16608_1	unknown	0	-
DLF001_scaffold1570_1	unknown	0	-
DLF001_scaffold13_12	unknown	0	-
DLF002_scaffold28742_1	unknown	0	-
DLF002_scaffold22416_1	unknown	0	-
DLF002_scaffold36081_2	unknown	0	-
DLF002_scaffold2466_1	unknown	0	-
DLF002_scaffold21111_2	unknown	0	-
DLF002_C488685_1	unknown	0	-
DLF003_scaffold50567_2	unknown	0	-
DLF003_scaffold23403_10	unknown	0	-
DLF003_scaffold48199_1	unknown	0	-
DLF003_scaffold3834_7	unknown	0	-
DLF003_scaffold56478_1	unknown	0	-
DLF004_scaffold36527_1	unknown	0	-
DLF004_scaffold23942_9	unknown	0	-
DLF004_scaffold19957_5	unknown	0	-
DLF004_scaffold30602_2	unknown	0	-
DLF004_scaffold32187_1	unknown	0	-
DLF004_scaffold23115_1	unknown	0	-
DLF004_scaffold7918_11	unknown	0	-
DLF004_scaffold7020_1	unknown	0	-
DLF005_scaffold56707_1	unknown	0	-
DLF005_scaffold39735_4	unknown	0	-

DLF005_scaffold27529_1	unknown	0	-
DLF005_scaffold50375_1	unknown	0	-
DLF005_scaffold40395_1	unknown	0	-
DLF005_scaffold528_11	unknown	0	-
DLF005_scaffold54804_6	unknown	0	-
DLF005_scaffold2989_20	unknown	0	-
DLF005_C847538_1	unknown	0	-
DLF005_scaffold6688_21	unknown	0	-
DLF005_C847770_1	unknown	0	-
DLF005_scaffold280_2	unknown	0	-
DLF006_scaffold42885_3	unknown	0	-
DLF006_scaffold9056_20	unknown	0	-
DLF006_scaffold10687_2	unknown	0	-
DLF006_scaffold41298_2	unknown	0	-
DLF006_scaffold15736_1	unknown	0	-
DLF006_scaffold52_5	unknown	0	-
DLF006_scaffold14168_2	unknown	0	-
DLF006_scaffold46677_6	unknown	0	-
DLF006_scaffold52_6	unknown	0	-
DLF007_scaffold14424_2	unknown	0	-
DLF008_scaffold11168_2	unknown	0	-
DLF008_scaffold22_1	unknown	0	-
DLF008_scaffold1410_11	unknown	0	-
DLF008_scaffold2326_8	unknown	0	-
DLF008_scaffold17374_1	unknown	0	-
DLF008_scaffold1029_1	unknown	0	-
DLF008_scaffold222_2	unknown	0	-
DLF008_scaffold192_4	unknown	0	-
DLF009_scaffold30115_4	unknown	0	-
DLF009_scaffold7570_14	unknown	0	-
DLF010_scaffold2375_6	unknown	0	-
DLF010_scaffold24026_11	unknown	0	-
DLF010_scaffold6815_13	unknown	0	-
DLF010_scaffold13248_1	unknown	0	-
DLF010_scaffold363_33	unknown	0	-
DLF010_scaffold33244_2	unknown	0	-
DLF010_scaffold26724_1	unknown	0	-
DLF010_scaffold3276_2	unknown	0	-
DLF010_scaffold45015_1	unknown	0	-
DLF010_scaffold51387_5	unknown	0	-
DLF010_scaffold44216_5	unknown	0	-
DLF010_scaffold1405_28	unknown	0	-
DLF012_scaffold19032_1	unknown	0	-
DLF013_scaffold35295_1	unknown	0	-
DLF013_scaffold52665_1	unknown	0	-
DLF013_scaffold88_22	unknown	0	-
DLF013_scaffold46613_1	unknown	0	-
DLF013_scaffold45213_1	unknown	0	-

DLF013_scaffold31672_1	unknown	0	-
DLF013_scaffold951_1	unknown	0	-
DLF013_scaffold16307_9	unknown	0	-
DLF013_scaffold21493_3	unknown	0	-
DLF013_scaffold54798_1	unknown	0	-
DLF014_scaffold2320_1	unknown	0	-
DLF014_scaffold5471_1	unknown	0	-
DLF014_scaffold12796_1	unknown	0	-
DLF014_scaffold5330_1	unknown	0	-
DLF014_scaffold2693_2	unknown	0	-
NLF001_scaffold29251_2	Clostridioides difficile	1	CRISPR
NLF001_scaffold18524_1	Streptococcus mutans	0.72	Predict
NLF001_scaffold159_2	Streptococcus mutans	1	CRISPR
NLF001_scaffold1094_2	Bacteroides fragilis	0.94	Predict
NLF001_scaffold30907_8	Achromobacter xylosoxidans	0.82	Predict
NLF001_scaffold42470_1	Staphylococcus saprophyticus	1	Predict
NLF001_scaffold36675_1	Streptococcus mutans	0.98	Predict
NLF001_scaffold15028_2	Tannerella forsythia	1	CRISPR
NLF001_scaffold21827_1	Bacteroides fragilis	0.87	Predict
NLF001_scaffold17952_7	Staphylococcus saprophyticus	0.85	Predict
NLF001_scaffold34766_6	Clostridioides difficile	1	CRISPR
NLF001_scaffold42478_2	Streptococcus mutans	0.88	Predict
NLF001_scaffold42456_1	Bacteroides fragilis	1	CRISPR
NLF001_C572546_1	Cellulophaga baltica	1	CRISPR
NLF001_scaffold38144_4	Lactobacillus plantarum	1	CRISPR
NLF001_scaffold12289_1	Bacteroides fragilis	0.95	Predict
NLF001_scaffold34766_5	Streptococcus mutans	1	CRISPR
NLF001_scaffold33713_2	Staphylococcus saprophyticus	1	CRISPR
NLF002_scaffold10424_8	Bacteroides fragilis	0.84	Predict
NLF002_scaffold36662_1	Clostridioides difficile	0.9	Predict
NLF002_scaffold336_22	Streptococcus mutans	0.8	Predict
NLF002_scaffold16871_1	Streptococcus mutans	0.82	Predict
NLF002_scaffold28626_2	Streptococcus mutans	0.83	Predict
NLF002_scaffold34776_1	Streptococcus mutans	1	CRISPR
NLF002_scaffold22680_4	Staphylococcus saprophyticus	0.81	Predict
NLF002_scaffold6663_3	Streptococcus mutans	1	CRISPR
NLF002_scaffold3933_4	Achromobacter xylosoxidans	0.72	Predict
NLF002_scaffold14709_2	Streptococcus mutans	0.91	Predict
NLF002_scaffold27060_6	Streptococcus mutans	0.73	Predict
NLF002_scaffold15559_1	Bacteroides fragilis	0.97	Predict
NLF002_scaffold19765_4	Staphylococcus saprophyticus	0.84	Predict
NLF002_scaffold141_14	Staphylococcus saprophyticus	0.89	Predict
NLF002_scaffold2199_6	Clostridioides difficile	1	CRISPR
NLF002_scaffold36924_1	Streptococcus mutans	0.93	Predict
NLF002_scaffold115_2	Candidatus Pelagibacter ubique	0.88	Predict
NLF002_scaffold17055_1	Clostridium perfringens	1	CRISPR
NLF002_scaffold25489_2	Streptococcus mutans	1	CRISPR
NLF005_scaffold21492_3	Streptococcus mutans	1	CRISPR

NLF005_scaffold15835_5	Streptococcus mutans	0.74	Predict
NLF005_scaffold36523_1	Streptococcus mutans	0.74	Predict
NLF005_scaffold32720_4	Staphylococcus saprophyticus	1	CRISPR
NLF005_scaffold21492_6	Streptococcus mutans	0.71	Predict
NLF005_C729497_1	Staphylococcus saprophyticus	0.75	Predict
NLF005_scaffold1317_3	Streptococcus mutans	0.84	Predict
NLF005_scaffold21214_1	Streptococcus mutans	0.93	Predict
NLF005_scaffold47798_1	Staphylococcus saprophyticus	0.86	Predict
NLF005_C729695_1	Staphylococcus saprophyticus	1	CRISPR
NLF005_scaffold21492_4	Streptococcus mutans	1	CRISPR
NLF005_scaffold25196_2	Streptococcus mutans	1	CRISPR
NLF005_scaffold40967_2	Clostridium tetani	1	CRISPR
NLF005_scaffold43034_2	Streptococcus mutans	1	CRISPR
NLF005_scaffold291_42	Streptococcus mutans	0.97	Predict
NLF005_scaffold43004_3	Lactobacillus plantarum	0.78	Predict
NLF005_scaffold14516_2	Streptococcus mutans	0.71	Predict
NLF005_C729433_1	Roseburia intestinalis	1	CRISPR
NLF005_scaffold48442_3	Parabacteroides merdae	1	CRISPR
NLF005_scaffold48389_1	Clostridium perfringens	1	CRISPR
NLF005_scaffold48437_2	Microcystis aeruginosa	0.9	Predict
NLF005_scaffold291_44	Streptococcus mutans	0.98	Predict
NLF006_scaffold306_3	Staphylococcus saprophyticus	0.73	Predict
NLF006_scaffold22186_2	Streptococcus mutans	0.9	Predict
NLF006_scaffold31099_1	Clostridioides difficile	1	CRISPR
NLF006_scaffold4766_5	Streptococcus mutans	1	CRISPR
NLF006_scaffold306_1	Staphylococcus saprophyticus	1	CRISPR
NLF006_scaffold9628_2	Staphylococcus saprophyticus	1	CRISPR
NLF006_scaffold4766_2	Parabacteroides merdae	0.84	Predict
NLF006_scaffold5249_2	Streptococcus mutans	0.79	Predict
NLF006_scaffold28327_1	Bacteroides fragilis	1	CRISPR
NLF006_scaffold132_1	Myxococcus xanthus	1	CRISPR
NLF006_scaffold4358_2	Streptococcus mutans	0.8	Predict
NLF007_scaffold882_1	Cellulophaga baltica	0.97	Predict
NLF007_scaffold38312_4	Candidatus Pelagibacter ubique	0.91	Predict
NLF007_scaffold5043_8	Streptococcus mutans	0.77	Predict
NLF007_scaffold14743_4	Rhodococcus hoagii	0.71	Predict
NLF007_scaffold64602_1	Gordonia rubripertincta	0.86	Predict
NLF007_scaffold66908_2	Clostridium tetani	1	CRISPR
NLF007_scaffold8274_2	Streptococcus mutans	0.84	Predict
NLF007_scaffold22938_1	Parabacteroides distasonis	0.92	Predict
NLF007_scaffold66524_2	Streptococcus mutans	0.96	Predict
NLF007_scaffold38312_8	Staphylococcus saprophyticus	0.91	Predict
NLF007_scaffold5542_23	Staphylococcus saprophyticus	0.96	Predict
NLF007_scaffold49453_1	Streptococcus mutans	1	CRISPR
NLF007_scaffold23805_3	Bacteroides fragilis	0.79	Predict
NLF007_scaffold31937_4	Cellulophaga baltica	1	CRISPR
NLF007_scaffold110_13	Enterococcus rivorum	1	CRISPR
NLF007_scaffold23404_3	Lactobacillus plantarum	1	CRISPR

NLF007_scaffold25969_1	Streptococcus mutans	1	CRISPR
NLF007_scaffold67236_3	Bacteroides eggerthii	1	CRISPR
NLF007_scaffold27103_9	Streptococcus pneumoniae	0.73	Predict
NLF007_scaffold67413_1	Staphylococcus epidermidis	1	CRISPR
NLF007_scaffold36465_3	Cutibacterium acnes	0.95	Predict
NLF007_C1010391_1	Paenibacillus dendritiformis	1	CRISPR
NLF007_scaffold17554_50	Streptococcus mutans	0.77	Predict
NLF007_scaffold61221_1	Parabacteroides merdae	1	CRISPR
NLF007_scaffold56144_4	Streptococcus mutans	0.78	Predict
NLF007_scaffold23957_1	Dinoroseobacter shibae	1	CRISPR
NLF007_scaffold54945_9	Candidatus Pelagibacter ubique	0.77	Predict
NLF007_scaffold20693_10	Staphylococcus saprophyticus	0.99	Predict
NLF007_scaffold66908_3	Clostridioides difficile	0.78	Predict
NLF007_scaffold47325_6	Staphylococcus saprophyticus	0.87	Predict
NLF007_scaffold14743_2	Staphylococcus saprophyticus	1	CRISPR
NLF007_scaffold30358_8	Streptococcus mutans	0.98	Predict
NLF007_scaffold3779_6	Streptococcus mutans	0.77	Predict
NLF007_scaffold57652_6	Streptococcus mutans	1	CRISPR
NLF007_scaffold50111_1	Listeria monocytogenes	1	CRISPR
NLF007_scaffold57227_1	Clostridioides difficile	0.81	Predict
NLF007_scaffold48133_2	Streptococcus mutans	0.98	Predict
NLF007_scaffold22798_1	Escherichia coli	0.96	Predict
NLF007_scaffold22798_13	Streptococcus mutans	0.95	Predict
NLF007_scaffold31937_5	Staphylococcus saprophyticus	1	CRISPR
NLF007_scaffold67062_4	Staphylococcus saprophyticus	0.72	Predict
NLF007_scaffold34404_4	Bacteroides fragilis	0.79	Predict
NLF007_scaffold32046_2	Bacteroides caccae	1	CRISPR
NLF007_scaffold67062_3	Clostridium tetani	0.85	Predict
NLF007_scaffold27103_3	Streptococcus mutans	1	CRISPR
NLF008_C315574_1	Staphylococcus saprophyticus	0.76	Predict
NLF008_scaffold12997_5	Bacteroides fragilis	0.8	Predict
NLF008_scaffold18404_4	Parabacteroides merdae	1	CRISPR
NLF008_scaffold10158_1	Staphylococcus saprophyticus	0.88	Predict
NLF008_scaffold20496_1	Streptococcus mutans	0.76	Predict
NLF008_scaffold8874_2	Rhizobium leguminosarum	0.96	Predict
NLF008_scaffold18404_14	Parabacteroides merdae	1	CRISPR
NLF008_scaffold10905_2	Clostridium sp. AM43-3BH	1	CRISPR
NLF008_scaffold10905_1	Lactobacillus plantarum	1	CRISPR
NLF008_scaffold1586_11	Streptococcus mutans	0.94	Predict
NLF008_scaffold7552_2	Bacteroides fragilis	0.92	Predict
NLF008_scaffold20431_5	Cellulophaga baltica	1	CRISPR
NLF008_scaffold19473_1	Staphylococcus saprophyticus	0.81	Predict
NLF008_scaffold9366_1	Staphylococcus saprophyticus	0.99	Predict
NLF008_scaffold15389_1	Staphylococcus saprophyticus	0.94	Predict
NLF008_scaffold20125_1	Parabacteroides merdae	0.86	Predict
NLF008_scaffold17605_2	Staphylococcus saprophyticus	0.9	Predict
NLF008_scaffold6835_1	Streptococcus mutans	0.87	Predict
NLF008_scaffold9492_2	Streptococcus mutans	0.89	Predict

NLF008_scaffold12194_1	Bacteroides fragilis	0.76	Predict
NLF008_scaffold20731_1	Streptococcus mutans	0.85	Predict
NLF008_scaffold395_6	Acinetobacter baumannii	1	CRISPR
NLF008_scaffold12947_1	Bacteroides fragilis	1	CRISPR
NLF008_scaffold17931_1	Staphylococcus saprophyticus	0.84	Predict
NLF008_scaffold12834_4	Streptococcus mutans	1	CRISPR
NLF008_scaffold16324_2	Dinoroseobacter shibae	0.95	Predict
NLF008_scaffold107_1	Dinoroseobacter shibae	0.87	Predict
NLF008_scaffold98_9	Streptococcus mutans	0.87	Predict
NLF008_scaffold1813_6	Lactobacillus fermentum	0.85	Predict
NLF008_scaffold8433_4	Bacteroides fragilis	0.79	Predict
NLF008_scaffold20258_1	Streptococcus mutans	1	Predict
NLF008_scaffold18404_6	Cellulophaga baltica	0.98	Predict
NLF008_scaffold6876_9	Parabacteroides merdae	1	CRISPR
NLF008_C315880_1	Streptococcus mutans	0.72	Predict
NLF008_scaffold386_3	Streptococcus mutans	0.74	Predict
NLF008_scaffold15537_1	Parabacteroides merdae	1	CRISPR
NLF008_scaffold18404_13	Bacillus subtilis	1	CRISPR
NLF008_scaffold7896_4	Parabacteroides distasonis	1	CRISPR
NLF008_scaffold9737_2	Bacteroides fragilis	0.81	Predict
NLF008_scaffold12833_2	Streptococcus mutans	1	CRISPR
NLF008_scaffold83_1	Streptococcus mutans	0.79	Predict
NLF008_scaffold13388_2	Bacteroides fragilis	0.8	Predict
NLF008_scaffold1868_2	Streptococcus mutans	0.78	Predict
NLF009_C805905_1	Cellulophaga baltica	1	CRISPR
NLF009_scaffold3162_3	Streptococcus mutans	0.91	Predict
NLF009_scaffold17912_16	Streptococcus mutans	1	CRISPR
NLF009_scaffold30773_1	Staphylococcus saprophyticus	0.86	Predict
NLF009_scaffold21790_2	Staphylococcus saprophyticus	0.82	Predict
NLF009_scaffold7091_22	Dinoroseobacter shibae	0.87	Predict
NLF009_scaffold54553_3	Cellulophaga baltica	1	CRISPR
NLF009_scaffold4933_28	Lactobacillus fermentum	0.92	Predict
NLF009_scaffold9844_25	Bacteroides fragilis	0.83	Predict
NLF009_scaffold54499_1	Bacteroides fragilis	1	Predict
NLF009_scaffold19140_7	Bacteroides fragilis	0.74	Predict
NLF009_scaffold35663_1	Staphylococcus saprophyticus	0.87	Predict
NLF009_scaffold4106_1	Lactobacillus plantarum	0.93	Predict
NLF009_C805829_1	Streptococcus mutans	0.76	Predict
NLF009_C805873_1	Streptococcus mutans	0.88	Predict
NLF009_scaffold17912_4	Streptococcus mutans	1	CRISPR
NLF009_scaffold5429_52	Parabacteroides merdae	0.96	Predict
NLF009_scaffold40330_1	Lactobacillus fermentum	0.95	Predict
NLF009_scaffold38501_1	Bacteroides fragilis	1	CRISPR
NLF009_scaffold8414_14	Bacteroides fragilis	1	CRISPR
NLF009_scaffold17912_17	Lachnospiraceae bacterium	1	CRISPR
NLF009_scaffold52991_2	Bacteroides fragilis	0.77	Predict
NLF009_scaffold25289_5	Streptococcus mutans	0.75	Predict
NLF009_scaffold36413_1	Flavobacterium psychrophilum	1	CRISPR

NLF009_scaffold375_24	Bacteroides fragilis	0.92	Predict
NLF009_scaffold37415_3	Streptococcus mutans	1	CRISPR
NLF009_scaffold54503_1	Lactobacillus plantarum	1	CRISPR
NLF009_scaffold17912_6	Clostridiaceae bacterium	1	CRISPR
NLF010_scaffold1235_8	Streptococcus mutans	1	CRISPR
NLF010_scaffold20315_2	Lactobacillus jensenii	1	CRISPR
NLF010_scaffold1044_27	Bacteroides fragilis	0.78	Predict
NLF010_C401947_1	Staphylococcus saprophyticus	0.74	Predict
NLF010_scaffold13747_2	Staphylococcus saprophyticus	0.76	Predict
NLF010_C402235_1	Staphylococcus saprophyticus	1	CRISPR
NLF010_scaffold22417_1	Streptococcus mutans	0.72	Predict
NLF010_scaffold12295_8	Streptococcus mutans	0.85	Predict
NLF010_scaffold27728_2	Dinoroseobacter shibae	0.9	Predict
NLF010_scaffold26024_8	Staphylococcus saprophyticus	0.83	Predict
NLF010_scaffold2046_76	Coprococcus eutactus	1	CRISPR
NLF010_scaffold9747_18	Streptococcus mutans	1	CRISPR
NLF010_scaffold20591_1	Bacteroides fragilis	0.89	Predict
NLF011_scaffold4965_2	Streptococcus mutans	1	CRISPR
NLF011_scaffold42_2	Streptococcus mutans	0.87	Predict
NLF011_scaffold7214_2	Parabacteroides merdae	0.88	Predict
NLF011_scaffold10809_1	Staphylococcus saprophyticus	1	CRISPR
NLF011_scaffold4255_7	Streptococcus mutans	0.94	Predict
NLF011_scaffold10054_4	Streptococcus mutans	0.89	Predict
NLF011_scaffold1786_1	Firmicutes bacterium AM43-11BH	1	CRISPR
NLF011_scaffold4096_9	Staphylococcus saprophyticus	0.75	Predict
NLF011_scaffold2637_6_1	Streptococcus mutans	0.9	Predict
NLF011_scaffold13048_1	Streptococcus mutans	0.77	Predict
NLF011_scaffold3151_2	Streptococcus pneumoniae	0.77	Predict
NLF011_scaffold30362_5	Bacteroides fragilis	0.99	Predict
NLF011_scaffold486_7	Streptococcus mutans	0.76	Predict
NLF011_scaffold794_4	Staphylococcus saprophyticus	0.81	Predict
NLF011_scaffold22115_1	Streptococcus mutans	1	CRISPR
NLF011_scaffold803_4	Streptococcus mutans	0.81	Predict
NLF011_scaffold10943_3	Faecalibacterium prausnitzii	1	CRISPR
NLF011_scaffold6937_1	Vibrio splendidus	0.8	Predict
NLF011_scaffold21554_2	Bacteroides fragilis	0.87	Predict
NLF011_scaffold9582_1	Lactobacillus plantarum	1	CRISPR
NLF012_scaffold35219_5	Streptococcus mutans	1	CRISPR
NLF012_scaffold35219_1	Staphylococcus epidermidis	0.78	Predict
NLF012_scaffold907_13	Dinoroseobacter shibae	0.83	Predict
NLF012_scaffold2371_23	Streptococcus mutans	0.82	Predict
NLF012_scaffold22746_1	Parabacteroides merdae	0.87	Predict
NLF012_scaffold39054_1	Streptococcus mutans	0.97	Predict
NLF012_scaffold8622_2	Lactobacillus fermentum	1	CRISPR
NLF012_scaffold2172_5	Streptococcus mutans	0.77	Predict
NLF012_scaffold27405_2	Firmicutes bacterium AM43-11BH	1	CRISPR
NLF012_scaffold11833_29	Streptococcus mutans	0.81	Predict
NLF012_scaffold2371_16	Dinoroseobacter shibae	0.92	Predict

NLF012_scaffold39140_2	Bacteroides fragilis	0.87	Predict
NLF012_scaffold25728_1	Staphylococcus saprophyticus	0.72	Predict
NLF012_scaffold36675_4	Staphylococcus saprophyticus	0.99	Predict
NLF012_scaffold21675_1	Streptococcus mutans	0.82	Predict
NLF012_scaffold11833_30	Streptococcus mutans	1	CRISPR
NLF012_scaffold39159_1	Staphylococcus saprophyticus	0.94	Predict
NLF012_scaffold17264_3	Rhizobium leguminosarum	0.73	Predict
NLF012_scaffold34471_3	Bacteroides fragilis	0.8	Predict
NLF012_scaffold293_2	Staphylococcus saprophyticus	0.98	Predict
NLF012_scaffold6773_9	Staphylococcus saprophyticus	0.98	Predict
NLF012_scaffold10245_3	Streptococcus mutans	1	CRISPR
NLF012_scaffold2854_1	Streptococcus mutans	1	CRISPR
NLF012_scaffold18119_4	Lactobacillus plantarum	1	CRISPR
NLF012_scaffold38032_1	Candidatus Pelagibacter ubique	0.89	Predict
NLF012_scaffold15731_1	Bacteroides fragilis	1	CRISPR
NLF012_scaffold16348_2	Dinoroseobacter shibae	0.89	Predict
NLF012_scaffold8622_6	Pectobacterium carotovorum	0.97	Predict
NLF012_scaffold17845_1	Streptococcus mutans	0.98	Predict
NLF012_C512465_1	Staphylococcus saprophyticus	0.84	Predict
NLF012_scaffold10394_1	Bacteroides fragilis	0.94	Predict
NLF012_scaffold32276_1	Lactobacillus jensenii	0.98	Predict
NLF012_scaffold13132_1	Streptococcus mutans	1	CRISPR
NLF012_scaffold35713_1	Dinoroseobacter shibae	0.78	Predict
NLF012_scaffold10663_6	Dinoroseobacter shibae	0.92	Predict
NLF012_scaffold35670_2	Bacteroides fragilis	0.8	Predict
NLF012_scaffold15197_22	Streptococcus mutans	1	CRISPR
NLF012_scaffold37458_1	Eubacteriaceae bacterium	1	CRISPR
NLF012_scaffold39143_1	Bacteroides vulgatus	1	CRISPR
NLF012_scaffold21989_2	Streptococcus mutans	0.87	Predict
NLF012_scaffold14481_3	Streptococcus mutans	1	CRISPR
NLF012_scaffold38234_3	Bacteroides fragilis	0.82	Predict
NLF012_scaffold16451_3	Streptococcus mutans	0.88	Predict
NLF012_scaffold13372_3	Staphylococcus saprophyticus	0.88	Predict
NLF012_scaffold37376_1	[Eubacterium] eligens	1	CRISPR
NLF012_scaffold69_1	Dinoroseobacter shibae	0.78	Predict
NLF012_scaffold14206_2	Lactobacillus fermentum	0.73	Predict
NLF012_scaffold8622_3	Streptococcus mutans	1	CRISPR
NLF012_scaffold29414_2	Streptococcus mutans	0.94	Predict
NLF012_scaffold17240_1	Bacteroides fragilis	1	CRISPR
NLF013_scaffold11031_1	Streptococcus mutans	0.85	Predict
NLF013_scaffold14354_6	Geobacillus kaustophilus	0.88	Predict
NLF013_scaffold30746_1	Candidatus Pelagibacter ubique	0.92	Predict
NLF013_scaffold12177_2	Staphylococcus saprophyticus	0.72	Predict
NLF013_C659127_1	Staphylococcus saprophyticus	1	CRISPR
NLF013_scaffold328_1	Candidatus Hamiltonella defensa	0.85	Predict
NLF013_scaffold33849_1	Candidatus Pelagibacter ubique	0.99	Predict
NLF013_scaffold23543_4	Klebsiella oxytoca	0.73	Predict
NLF013_scaffold19270_4	Streptococcus mutans	0.86	Predict

NLF013_scaffold44405_3	Streptococcus mutans	0.9	Predict
NLF013_scaffold32342_11	Clostridium perfringens	0.99	Predict
NLF013_scaffold47264_1	Pseudomonas tolaasii	0.99	Predict
NLF013_scaffold33946_4	Streptococcus mutans	0.96	Predict
NLF013_scaffold11031_7	Dinoroseobacter shibae	0.89	Predict
NLF013_scaffold4561_28	Streptococcus mutans	0.76	Predict
NLF013_C659857_1	Staphylococcus saprophyticus	1	CRISPR
NLF013_scaffold19359_1	Streptococcus mutans	0.73	Predict
NLF013_scaffold26044_5	Streptococcus mutans	0.9	Predict
NLF013_scaffold43192_14	Lactobacillus delbrueckii	0.79	Predict
NLF013_scaffold48338_2	Streptococcus mutans	0.84	Predict
NLF014_scaffold5840_1	Bacteroides fragilis	0.81	Predict
NLF014_scaffold7292_1	Streptococcus mutans	0.7	Predict
NLF014_scaffold342_1	Bacteroides fragilis	0.72	Predict
NLF014_scaffold6197_2	Streptococcus mutans	0.8	Predict
NLF014_scaffold249_3	Streptococcus mutans	0.85	Predict
NLF014_scaffold819_1	Streptococcus mutans	0.73	Predict
NLF014_scaffold9408_9	Candidatus Pelagibacter ubique	0.79	Predict
NLF014_scaffold6884_2	Lactobacillus johnsonii	0.91	Predict
NLF014_scaffold73_1	Staphylococcus saprophyticus	0.79	Predict
NLF014_scaffold736_2	Streptococcus mutans	0.93	Predict
NLF015_scaffold31615_2	Dinoroseobacter shibae	0.99	Predict
NLF015_scaffold31821_3	Bacteroides fragilis	0.71	Predict
NLF015_scaffold24231_1	Staphylococcus saprophyticus	1	CRISPR
NLF015_C419895_1	Streptococcus mutans	1	CRISPR
NLF015_scaffold14491_1	Bacteroides fragilis	0.73	Predict
NLF015_scaffold22095_1	Staphylococcus saprophyticus	0.86	Predict
NLF015_scaffold591_2	Candidatus Pelagibacter ubique	0.7	Predict
NLF015_scaffold21847_3	Staphylococcus saprophyticus	0.95	Predict
NLF015_scaffold11481_6	Bacteroides fragilis	1	Predict
NLF015_scaffold22_1	Streptococcus mutans	0.8	Predict
NLF015_scaffold3859_2	Streptococcus mutans	0.81	Predict
NLF015_scaffold34057_2	Flavobacterium columnare	0.75	Predict
NLF015_scaffold24362_4	Bacteroides fragilis	0.81	Predict
NLF015_scaffold29017_1	Candidatus Liberibacter asiaticus	0.95	Predict
NLF015_scaffold31211_3	Streptococcus mutans	0.74	Predict
NLF015_scaffold12014_3	Streptococcus mutans	0.92	Predict
NLF015_scaffold8589_4	Bacteroides fragilis	0.91	Predict
NLF015_scaffold14946_1	Bacteroides fragilis	0.97	Predict
NLF015_scaffold20215_1	Streptococcus mutans	0.96	Predict
NLF015_scaffold20500_6	Bacteroides fragilis	0.82	Predict
NLF015_scaffold7628_6	Dinoroseobacter shibae	1	CRISPR
NLF015_scaffold14_1	Dinoroseobacter shibae	0.96	Predict
NLF015_scaffold3382_3	Lactobacillus fermentum	1	CRISPR
NLF015_scaffold34269_1	Streptococcus mutans	0.95	Predict
NLF015_scaffold33332_2	Streptococcus mutans	0.97	Predict
NLF015_scaffold4657_3	Lactobacillus johnsonii	0.73	Predict
NLF015_scaffold32259_1	Staphylococcus saprophyticus	1	CRISPR

NLF015_scaffold13163_7	Streptococcus mutans	1	CRISPR
NLF015_scaffold34558_2	Streptococcus mutans	0.72	Predict
NLF015_scaffold21847_1	Staphylococcus saprophyticus	1	CRISPR
NLF001_scaffold18110_3	unknown	0	-
NLF001_scaffold15921_9	unknown	0	-
NLF001_scaffold21408_7	unknown	0	-
NLF001_scaffold42472_1	unknown	0	-
NLF002_scaffold35171_2	unknown	0	-
NLF002_scaffold37757_3	unknown	0	-
NLF002_scaffold37757_4	unknown	0	-
NLF002_scaffold37757_5	unknown	0	-
NLF002_scaffold38935_2	unknown	0	-
NLF005_scaffold4391_2	unknown	0	-
NLF005_scaffold46950_1	unknown	0	-
NLF005_scaffold27522_3	unknown	0	-
NLF005_scaffold47800_1	unknown	0	-
NLF005_scaffold29618_1	unknown	0	-
NLF005_scaffold47934_7	unknown	0	-
NLF005_scaffold48237_1	unknown	0	-
NLF005_scaffold39282_7	unknown	0	-
NLF005_scaffold13387_9	unknown	0	-
NLF005_scaffold40798_1	unknown	0	-
NLF005_scaffold13387_4	unknown	0	-
NLF005_scaffold29852_1	unknown	0	-
NLF005_scaffold4391_3	unknown	0	-
NLF005_scaffold10734_4	unknown	0	-
NLF006_scaffold3814_1	unknown	0	-
NLF006_scaffold15157_3	unknown	0	-
NLF006_scaffold4466_3	unknown	0	-
NLF006_scaffold14746_1	unknown	0	-
NLF006_scaffold5891_10	unknown	0	-
NLF006_scaffold30421_2	unknown	0	-
NLF007_scaffold67260_2	unknown	0	-
NLF007_scaffold11102_4	unknown	0	-
NLF007_scaffold29510_1	unknown	0	-
NLF007_scaffold61307_5	unknown	0	-
NLF007_scaffold4118_1	unknown	0	-
NLF007_scaffold3779_4	unknown	0	-
NLF007_scaffold64205_2	unknown	0	-
NLF007_scaffold67153_2	unknown	0	-
NLF007_scaffold40076_1	unknown	0	-
NLF007_scaffold47382_8	unknown	0	-
NLF007_scaffold14743_1	unknown	0	-
NLF007_scaffold66524_3	unknown	0	-
NLF007_scaffold43991_1	unknown	0	-
NLF007_scaffold21464_1	unknown	0	-
NLF008_scaffold19700_1	unknown	0	-
NLF008_scaffold141_3	unknown	0	-

NLF008_scaffold6103_28	unknown	0	-
NLF008_scaffold9442_2	unknown	0	-
NLF008_scaffold20528_1	unknown	0	-
NLF008_scaffold1474_11	unknown	0	-
NLF008_scaffold5986_1	unknown	0	-
NLF008_scaffold14014_6	unknown	0	-
NLF008_scaffold20431_4	unknown	0	-
NLF009_scaffold54553_5	unknown	0	-
NLF009_scaffold52926_4	unknown	0	-
NLF009_scaffold52375_7	unknown	0	-
NLF009_scaffold54170_2	unknown	0	-
NLF009_scaffold1353_6	unknown	0	-
NLF009_scaffold27217_3	unknown	0	-
NLF009_scaffold18984_7	unknown	0	-
NLF009_scaffold52926_1	unknown	0	-
NLF010_scaffold24082_3	unknown	0	-
NLF010_scaffold3898_4	unknown	0	-
NLF010_scaffold29246_1	unknown	0	-
NLF010_scaffold29445_4	unknown	0	-
NLF011_scaffold5613_1	unknown	0	-
NLF011_scaffold1105_1	unknown	0	-
NLF011_scaffold25120_2	unknown	0	-
NLF012_scaffold10340_2	unknown	0	-
NLF012_scaffold39053_1	unknown	0	-
NLF012_scaffold29420_1	unknown	0	-
NLF012_scaffold16653_1	unknown	0	-
NLF012_scaffold22955_2	unknown	0	-
NLF012_scaffold13034_3	unknown	0	-
NLF012_scaffold12754_3	unknown	0	-
NLF012_scaffold30180_18	unknown	0	-
NLF012_scaffold17431_22	unknown	0	-
NLF012_scaffold4362_3	unknown	0	-
NLF012_scaffold10340_14	unknown	0	-
NLF012_scaffold17431_21	unknown	0	-
NLF012_scaffold33261_5	unknown	0	-
NLF012_scaffold2371_15	unknown	0	-
NLF013_scaffold11783_1	unknown	0	-
NLF013_scaffold48622_1	unknown	0	-
NLF013_scaffold43868_1	unknown	0	-
NLF013_scaffold34744_1	unknown	0	-
NLF013_scaffold4799_2	unknown	0	-
NLF013_scaffold25755_1	unknown	0	-
NLF013_scaffold21878_2	unknown	0	-
NLF013_scaffold23156_2	unknown	0	-
NLF013_scaffold30660_7	unknown	0	-
NLF013_scaffold20413_4	unknown	0	-
NLF014_scaffold142_6	unknown	0	-
NLF014_scaffold9741_6	unknown	0	-

NLF014_scaffold1307_2	unknown	0	-
NLF014_scaffold10772_2	unknown	0	-
NLF015_scaffold32932_4	unknown	0	-
NLF015_scaffold13173_2	unknown	0	-
NLF015_C419419_1	unknown	0	-
NLF015_scaffold6258_2	unknown	0	-
DLM001_scaffold37_3	Bacteroides fragilis	0.86	Predict
DLM001_scaffold17760_6	Candidatus Hamiltonella defensa	0.88	Predict
DLM001_scaffold57834_1	Candidatus Hamiltonella defensa	0.76	Predict
DLM001_scaffold91_6	Candidatus Hamiltonella defensa	1	CRISPR
DLM001_scaffold4307_3	Candidatus Hamiltonella defensa	0.93	Predict
DLM001_scaffold36534_1	Candidatus Hamiltonella defensa	0.82	Predict
DLM001_scaffold19551_4	Candidatus Hamiltonella defensa	1	CRISPR
DLM001_scaffold26672_2	Faecalibacterium prausnitzii	1	CRISPR
DLM001_scaffold21921_3	Candidatus Hamiltonella defensa	0.96	Predict
DLM001_scaffold23814_4	Bacteroides fragilis	1	CRISPR
DLM001_scaffold14847_5	Bacteroides fragilis	0.7	Predict
DLM001_scaffold42296_1	Candidatus Hamiltonella defensa	0.81	Predict
DLM001_scaffold4307_2	Candidatus Hamiltonella defensa	0.79	Predict
DLM001_scaffold31256_1	Rhodococcus rhodochrous	0.77	Predict
DLM001_scaffold52744_1	Candidatus Hamiltonella defensa	0.81	Predict
DLM001_scaffold20858_1	Lactobacillus gasseri	0.91	Predict
DLM001_scaffold27372_1	Candidatus Hamiltonella defensa	1	CRISPR
DLM001_scaffold57732_2	Candidatus Hamiltonella defensa	0.78	Predict
DLM001_scaffold57716_1	Candidatus Hamiltonella defensa	0.75	Predict
DLM001_scaffold54893_2	Candidatus Hamiltonella defensa	1	CRISPR
DLM001_scaffold30022_1	Candidatus Hamiltonella defensa	1	CRISPR
DLM001_scaffold58236_1	Candidatus Hamiltonella defensa	1	CRISPR
DLM001_scaffold34133_3	Bacteroides fragilis	1	CRISPR
DLM001_scaffold51940_3	Candidatus Hamiltonella defensa	0.76	Predict
DLM001_scaffold49843_4	Candidatus Hamiltonella defensa	0.78	Predict
DLM001_scaffold51928_3	Candidatus Hamiltonella defensa	0.81	Predict
DLM001_scaffold19551_5	Roseobacter denitrificans	0.92	Predict
DLM001_scaffold19465_5	Candidatus Hamiltonella defensa	0.72	Predict
DLM001_scaffold56543_4	Parabacteroides merdae	0.86	Predict
DLM001_scaffold14859_7	Bacteroides fragilis	0.7	Predict
DLM001_scaffold20858_3	Candidatus Hamiltonella defensa	0.88	Predict
DLM001_C705523_1	Bacteroides fragilis	0.86	Predict
DLM001_scaffold17051_8	Candidatus Hamiltonella defensa	0.85	Predict
DLM001_scaffold19551_1	Candidatus Hamiltonella defensa	0.81	Predict
DLM001_scaffold20817_1	Candidatus Hamiltonella defensa	0.88	Predict
DLM001_scaffold25009_5	Candidatus Hamiltonella defensa	0.83	Predict
DLM001_scaffold22953_2	Candidatus Hamiltonella defensa	0.78	Predict
DLM001_scaffold53015_2	Parabacteroides distasonis	1	CRISPR
DLM001_scaffold41842_2	Candidatus Hamiltonella defensa	0.91	Predict
DLM001_scaffold19551_3	Candidatus Hamiltonella defensa	1	CRISPR
DLM001_scaffold58253_1	Clostridium perfringens	1	Predict
DLM001_scaffold53994_4	Candidatus Hamiltonella defensa	0.72	Predict

DLM001_scaffold56486_2	Candidatus Hamiltonella defensa	0.81	Predict
DLM001_scaffold48552_3	Candidatus Hamiltonella defensa	0.71	Predict
DLM001_scaffold4106_5	Candidatus Hamiltonella defensa	0.78	Predict
DLM001_scaffold51591_2	Candidatus Hamiltonella defensa	0.84	Predict
DLM001_scaffold10420_4	Candidatus Hamiltonella defensa	0.96	Predict
DLM001_scaffold58124_4	Bifidobacterium longum	1	CRISPR
DLM001_scaffold58250_2	Candidatus Hamiltonella defensa	0.87	Predict
DLM001_scaffold23040_12	Candidatus Hamiltonella defensa	0.77	Predict
DLM001_C705425_1	Candidatus Hamiltonella defensa	0.94	Predict
DLM001_scaffold18361_2	Candidatus Hamiltonella defensa	0.75	Predict
DLM001_scaffold39252_2	Candidatus Hamiltonella defensa	0.72	Predict
DLM001_scaffold55964_1	Candidatus Hamiltonella defensa	0.82	Predict
DLM001_scaffold13671_1	Bacteroides fragilis	0.73	Predict
DLM001_scaffold277_3	Bacteroides fragilis	0.94	Predict
DLM001_scaffold39468_1	Candidatus Hamiltonella defensa	0.95	Predict
DLM001_scaffold49276_1	Candidatus Hamiltonella defensa	1	CRISPR
DLM001_scaffold21002_1	Parabacteroides distasonis	0.73	Predict
DLM001_scaffold42810_2	Candidatus Hamiltonella defensa	0.95	Predict
DLM001_scaffold49834_2	Candidatus Hamiltonella defensa	0.92	Predict
DLM001_scaffold16868_1	Candidatus Hamiltonella defensa	0.77	Predict
DLM001_scaffold52139_2	Candidatus Hamiltonella defensa	1	CRISPR
DLM001_scaffold11723_1	Candidatus Hamiltonella defensa	0.71	Predict
DLM001_scaffold19427_9	Bacteroides fragilis	0.97	Predict
DLM001_scaffold27611_5	Candidatus Hamiltonella defensa	1	Predict
DLM001_scaffold14114_7	Candidatus Hamiltonella defensa	0.92	Predict
DLM001_scaffold3576_10	Flavobacterium columnare	0.89	Predict
DLM002_scaffold52283_2	Candidatus Hamiltonella defensa	0.84	Predict
DLM002_scaffold35924_1	Candidatus Hamiltonella defensa	0.88	Predict
DLM002_scaffold39638_1	Megamonas funiformis	1	CRISPR
DLM002_scaffold58_3	Prevotella sp. P4-65	1	CRISPR
DLM002_scaffold8357_7	Candidatus Hamiltonella defensa	0.79	Predict
DLM002_scaffold37454_1	Morganella morganii	0.97	Predict
DLM002_scaffold37352_8	Candidatus Hamiltonella defensa	0.89	Predict
DLM002_scaffold4794_6	Clostridium tetani	0.9	Predict
DLM002_scaffold52713_2	Candidatus Hamiltonella defensa	0.91	Predict
DLM002_scaffold45452_2	Candidatus Hamiltonella defensa	0.95	Predict
DLM002_scaffold48724_2	Streptococcus mutans	0.85	Predict
DLM002_scaffold11013_7	Candidatus Hamiltonella defensa	0.97	Predict
DLM002_scaffold2881_3	Parabacteroides distasonis	0.72	Predict
DLM002_scaffold52526_1	Candidatus Hamiltonella defensa	1	CRISPR
DLM002_scaffold44502_1	Candidatus Hamiltonella defensa	0.73	Predict
DLM002_scaffold52526_4	Candidatus Hamiltonella defensa	1	CRISPR
DLM002_scaffold48180_13	Candidatus Hamiltonella defensa	1	CRISPR
DLM002_scaffold42939_1	Candidatus Hamiltonella defensa	0.84	Predict
DLM002_scaffold52584_1	Candidatus Hamiltonella defensa	0.78	Predict
DLM002_scaffold32192_3	Candidatus Hamiltonella defensa	1	Predict
DLM002_scaffold5336_45	Candidatus Hamiltonella defensa	0.93	Predict
DLM002_scaffold52696_1	Clostridium perfringens	1	CRISPR

DLM002_scaffold925_8	Bacteroides fragilis	0.92	Predict
DLM003_scaffold30478_2	Candidatus Hamiltonella defensa	1	CRISPR
DLM003_scaffold35975_4	Parabacteroides distasonis	0.83	Predict
DLM003_scaffold20093_1	Candidatus Hamiltonella defensa	0.93	Predict
DLM003_scaffold8802_8	Candidatus Hamiltonella defensa	0.79	Predict
DLM003_scaffold1898_14	Candidatus Hamiltonella defensa	1	CRISPR
DLM003_scaffold30928_7	Candidatus Hamiltonella defensa	0.76	Predict
DLM003_scaffold59785_6	Cellulophaga baltica	1	CRISPR
DLM003_C810975_1	Parabacteroides distasonis	1	CRISPR
DLM003_scaffold31156_2	Bacteroides vulgatus	1	CRISPR
DLM003_scaffold24702_43	Candidatus Hamiltonella defensa	0.94	Predict
DLM003_scaffold10664_1	Candidatus Hamiltonella defensa	0.93	Predict
DLM003_scaffold46929_2	Parabacteroides distasonis	1	CRISPR
DLM003_scaffold59785_5	Cellulophaga baltica	1	CRISPR
DLM003_scaffold40695_7	Candidatus Hamiltonella defensa	1	CRISPR
DLM003_scaffold4744_1	Bacteroides fragilis	0.8	Predict
DLM003_scaffold303_2	Candidatus Hamiltonella defensa	0.84	Predict
DLM003_scaffold12201_4	Candidatus Hamiltonella defensa	0.89	Predict
DLM003_scaffold49406_1	Candidatus Hamiltonella defensa	0.85	Predict
DLM003_C810753_1	Actinomyces naeslundii	1	CRISPR
DLM003_scaffold1898_11	Bacteroides salyersiae	1	CRISPR
DLM003_scaffold17135_3	Candidatus Hamiltonella defensa	0.82	Predict
DLM003_scaffold29743_2	Candidatus Hamiltonella defensa	1	CRISPR
DLM003_scaffold14015_13	Candidatus Hamiltonella defensa	0.88	Predict
DLM003_scaffold15117_2	Candidatus Hamiltonella defensa	0.88	Predict
DLM003_scaffold59785_12	Bacteroides salyersiae	1	CRISPR
DLM003_scaffold102_3	Rhizobium leguminosarum	0.8	Predict
DLM003_scaffold46929_4	Cellulophaga baltica	0.73	Predict
DLM003_scaffold40695_5	Bacteroides fragilis	0.76	Predict
DLM004_scaffold18621_3	Candidatus Hamiltonella defensa	0.71	Predict
DLM004_scaffold12500_3	Candidatus Hamiltonella defensa	0.83	Predict
DLM004_scaffold8053_11	Candidatus Hamiltonella defensa	1	CRISPR
DLM004_scaffold8053_12	Candidatus Hamiltonella defensa	1	CRISPR
DLM004_scaffold18777_1	Candidatus Hamiltonella defensa	0.99	Predict
DLM004_scaffold41_14	Candidatus Hamiltonella defensa	0.92	Predict
DLM004_scaffold12500_2	Candidatus Hamiltonella defensa	0.87	Predict
DLM004_scaffold462_3	Aeromonas media	0.7	Predict
DLM004_scaffold18776_2	Lactobacillus plantarum	0.72	Predict
DLM004_scaffold112_3	Candidatus Hamiltonella defensa	1	CRISPR
DLM004_scaffold158_30	Candidatus Hamiltonella defensa	0.83	Predict
DLM004_scaffold5647_2	Lactobacillus plantarum	0.74	Predict
DLM004_scaffold18395_2	Parabacteroides distasonis	0.78	Predict
DLM004_scaffold16125_1	Candidatus Hamiltonella defensa	0.93	Predict
DLM004_scaffold12500_5	Candidatus Hamiltonella defensa	0.72	Predict
DLM004_scaffold11715_11	Candidatus Hamiltonella defensa	0.73	Predict
DLM004_scaffold17859_1	Candidatus Hamiltonella defensa	0.9	Predict
DLM004_scaffold14310_1	Candidatus Hamiltonella defensa	0.75	Predict
DLM004_scaffold14578_1	Candidatus Hamiltonella defensa	1	CRISPR

DLM004_scaffold16036_1	Candidatus Hamiltonella defensa	0.85	Predict
DLM004_C304967_1	Candidatus Hamiltonella defensa	1	CRISPR
DLM004_scaffold12500_1	Candidatus Hamiltonella defensa	0.92	Predict
DLM004_scaffold8780_5	Candidatus Hamiltonella defensa	0.83	Predict
DLM004_scaffold14269_9	Candidatus Hamiltonella defensa	0.71	Predict
DLM005_scaffold57948_1	Candidatus Hamiltonella defensa	1	CRISPR
DLM005_scaffold44327_3	Candidatus Hamiltonella defensa	1	CRISPR
DLM005_scaffold34949_1	Candidatus Hamiltonella defensa	0.74	Predict
DLM005_scaffold48663_11	Candidatus Hamiltonella defensa	0.84	Predict
DLM005_scaffold9042_2	Candidatus Hamiltonella defensa	1	CRISPR
DLM005_scaffold3317_29	Candidatus Hamiltonella defensa	0.74	Predict
DLM005_scaffold10521_42	Candidatus Hamiltonella defensa	1	CRISPR
DLM005_scaffold39936_1	Clostridium perfringens	1	CRISPR
DLM005_scaffold21763_7	Candidatus Hamiltonella defensa	0.85	Predict
DLM005_scaffold54507_2	Candidatus Hamiltonella defensa	1	CRISPR
DLM005_scaffold55834_11	Bacteroides fragilis	0.76	Predict
DLM005_scaffold57953_2	Candidatus Hamiltonella defensa	1	CRISPR
DLM005_scaffold31312_1	Cronobacter sakazakii	1	CRISPR
DLM005_scaffold47946_1	Parabacteroides distasonis	0.77	Predict
DLM005_scaffold56278_1	Candidatus Hamiltonella defensa	0.92	Predict
DLM005_scaffold3128_1	Candidatus Hamiltonella defensa	0.98	Predict
DLM005_scaffold42596_2	Streptococcus mutans	1	CRISPR
DLM005_scaffold56545_2	Candidatus Hamiltonella defensa	0.96	Predict
DLM005_scaffold55666_1	Candidatus Hamiltonella defensa	0.88	Predict
DLM005_scaffold21763_10	Bacteroides fragilis	1	CRISPR
DLM005_scaffold21049_5	Candidatus Hamiltonella defensa	0.89	Predict
DLM005_scaffold4283_10	Candidatus Hamiltonella defensa	0.81	Predict
DLM005_scaffold25964_2	Candidatus Hamiltonella defensa	0.83	Predict
DLM005_scaffold58045_1	Candidatus Hamiltonella defensa	1	CRISPR
DLM005_scaffold56278_2	Azospirillum brasilense	0.93	Predict
DLM005_scaffold5416_6	Candidatus Hamiltonella defensa	0.97	Predict
DLM005_scaffold36491_20	Candidatus Hamiltonella defensa	0.86	Predict
DLM005_scaffold20359_5	Candidatus Hamiltonella defensa	1	CRISPR
DLM005_scaffold56832_1	Prevotella copri	1	CRISPR
DLM005_scaffold7362_2	Candidatus Hamiltonella defensa	0.82	Predict
DLM005_scaffold57077_1	Candidatus Hamiltonella defensa	1	CRISPR
DLM005_scaffold5800_5	Candidatus Hamiltonella defensa	1	CRISPR
DLM005_scaffold20359_8	Listeria monocytogenes	1	CRISPR
DLM005_scaffold27309_11	Micromonospora chaiyaphumensis	1	CRISPR
DLM005_scaffold25964_1	Candidatus Hamiltonella defensa	0.78	Predict
DLM005_scaffold57953_1	Candidatus Hamiltonella defensa	1	CRISPR
DLM005_scaffold52507_2	Bacteroides fragilis	0.83	Predict
DLM005_scaffold1593_32	Candidatus Hamiltonella defensa	0.75	Predict
DLM005_scaffold36516_2	Candidatus Hamiltonella defensa	0.86	Predict
DLM006_scaffold29093_1	Candidatus Hamiltonella defensa	0.96	Predict
DLM006_scaffold5249_12	Candidatus Hamiltonella defensa	1	CRISPR
DLM006_scaffold9115_2	Bacillus alcalophilus	1	CRISPR
DLM006_scaffold23991_4	Candidatus Hamiltonella defensa	0.87	Predict

DLM006_scaffold31867_1	Candidatus Pelagibacter ubique	0.83	Predict
DLM006_scaffold1615_27	Candidatus Hamiltonella defensa	1	CRISPR
DLM006_scaffold35098_1	Candidatus Hamiltonella defensa	1	CRISPR
DLM006_scaffold8798_11	Lactobacillus fermentum	0.71	Predict
DLM006_scaffold289_5	Candidatus Hamiltonella defensa	0.73	Predict
DLM006_scaffold15803_18	Candidatus Hamiltonella defensa	0.87	Predict
DLM006_scaffold3905_28	Candidatus Hamiltonella defensa	0.96	Predict
DLM006_scaffold23991_2	Candidatus Hamiltonella defensa	0.83	Predict
DLM006_scaffold5392_1	Candidatus Hamiltonella defensa	0.7	Predict
DLM006_scaffold19568_4	Candidatus Hamiltonella defensa	0.72	Predict
DLM006_scaffold35098_2	Candidatus Hamiltonella defensa	1	CRISPR
DLM006_scaffold3099_10	Candidatus Hamiltonella defensa	0.84	Predict
DLM006_scaffold22671_1	Candidatus Hamiltonella defensa	0.95	Predict
DLM006_scaffold12233_4	Candidatus Hamiltonella defensa	0.98	Predict
DLM006_scaffold582_5	Thermoanaerobacterium saccharolytic	0.86	Predict
DLM006_scaffold7136_55	Candidatus Hamiltonella defensa	0.98	Predict
DLM006_scaffold36079_2	Parabacteroides merdae	1	CRISPR
DLM007_scaffold30789_1	Parabacteroides merdae	0.76	Predict
DLM007_scaffold45247_1	Candidatus Hamiltonella defensa	1	CRISPR
DLM007_scaffold7436_7	Candidatus Hamiltonella defensa	0.8	Predict
DLM007_C664617_1	Actinomyces naeslundii	0.88	Predict
DLM007_scaffold45308_2	Parabacteroides sp. D13	1	CRISPR
DLM007_scaffold35599_2	Bacteroides fragilis	0.97	Predict
DLM007_scaffold28062_4	Candidatus Hamiltonella defensa	1	CRISPR
DLM007_scaffold13248_1	Candidatus Hamiltonella defensa	0.91	Predict
DLM007_scaffold31482_1	Candidatus Hamiltonella defensa	1	CRISPR
DLM007_scaffold42236_1	Candidatus Hamiltonella defensa	1	CRISPR
DLM007_scaffold5339_25	Vibrio natriegens	0.84	Predict
DLM007_scaffold41113_2	Clostridioides difficile	0.85	Predict
DLM007_scaffold4568_8	Flavobacterium columnare	0.81	Predict
DLM007_scaffold36909_2	Mycoplasma pulmonis	1	CRISPR
DLM007_scaffold45251_1	Candidatus Hamiltonella defensa	0.93	Predict
DLM008_scaffold4090_5	Candidatus Hamiltonella defensa	0.99	Predict
DLM008_scaffold251_3	Candidatus Hamiltonella defensa	1	CRISPR
DLM008_scaffold51033_29	Candidatus Hamiltonella defensa	0.94	Predict
DLM008_scaffold34307_5	Candidatus Hamiltonella defensa	0.94	Predict
DLM008_scaffold3167_2	Candidatus Hamiltonella defensa	0.93	Predict
DLM008_scaffold68109_1	Candidatus Hamiltonella defensa	0.75	Predict
DLM008_scaffold56745_2	Candidatus Hamiltonella defensa	0.9	Predict
DLM008_scaffold40955_1	Candidatus Hamiltonella defensa	0.73	Predict
DLM008_scaffold26298_3	Candidatus Hamiltonella defensa	1	CRISPR
DLM008_scaffold235_2	Bacteroides eggerthii	1	CRISPR
DLM008_scaffold19097_13	Sinorhizobium meliloti	0.8	Predict
DLM008_scaffold58161_1	Candidatus Hamiltonella defensa	0.98	Predict
DLM008_scaffold349_4	Candidatus Pelagibacter ubique	0.88	Predict
DLM008_scaffold45871_1	Candidatus Hamiltonella defensa	0.93	Predict
DLM008_scaffold62586_4	Bacteroides fragilis	0.76	Predict
DLM008_scaffold371_1	Candidatus Hamiltonella defensa	0.99	Predict

DLM008_scaffold68395_3	Candidatus Hamiltonella defensa	0.96	Predict
DLM008_scaffold58372_1	Candidatus Hamiltonella defensa	1	CRISPR
DLM008_scaffold129_3	Candidatus Hamiltonella defensa	0.88	Predict
DLM009_scaffold11883_2	Candidatus Hamiltonella defensa	0.9	Predict
DLM009_scaffold6465_2	Candidatus Hamiltonella defensa	1	CRISPR
DLM009_scaffold19658_2	Candidatus Hamiltonella defensa	1	CRISPR
DLM009_C324613_1	Candidatus Hamiltonella defensa	1	CRISPR
DLM009_scaffold17120_3	Parabacteroides merdae	0.81	Predict
DLM009_scaffold14704_1	Candidatus Hamiltonella defensa	0.93	Predict
DLM009_scaffold21367_3	Candidatus Hamiltonella defensa	0.77	Predict
DLM009_scaffold8594_4	Candidatus Hamiltonella defensa	1	CRISPR
DLM009_scaffold10681_1	Candidatus Hamiltonella defensa	1	CRISPR
DLM009_scaffold5016_13	Bacteroides fragilis	0.78	Predict
DLM009_scaffold102_1	Flavobacterium columnare	0.76	Predict
DLM009_C324441_1	Candidatus Hamiltonella defensa	0.95	Predict
DLM009_scaffold21380_1	Candidatus Hamiltonella defensa	0.97	Predict
DLM009_scaffold6465_20	Candidatus Hamiltonella defensa	1	CRISPR
DLM009_scaffold21079_1	Candidatus Hamiltonella defensa	0.91	Predict
DLM009_scaffold22_4	Candidatus Hamiltonella defensa	0.74	Predict
DLM009_scaffold21344_1	Lactobacillus fermentum	0.85	Predict
DLM009_scaffold6465_1	Candidatus Hamiltonella defensa	1	CRISPR
DLM009_scaffold21365_1	Candidatus Hamiltonella defensa	0.94	Predict
DLM009_scaffold19658_6	Candidatus Hamiltonella defensa	1	CRISPR
DLM009_scaffold2554_54	Parabacteroides merdae	0.89	Predict
DLM009_scaffold19690_1	Flavobacterium columnare	0.91	Predict
DLM009_scaffold6465_28	Bacteroides fragilis	1	CRISPR
DLM009_scaffold4728_5	Candidatus Hamiltonella defensa	0.72	Predict
DLM009_C324485_1	Candidatus Hamiltonella defensa	1	CRISPR
DLM009_scaffold18345_6	Candidatus Hamiltonella defensa	1	CRISPR
DLM009_scaffold19658_3	Salmonella enterica	1	CRISPR
DLM009_C324155_1	Candidatus Hamiltonella defensa	1	CRISPR
DLM009_scaffold6465_23	[Ruminococcus] gnavus	1	CRISPR
DLM010_scaffold41969_2	Candidatus Hamiltonella defensa	0.91	Predict
DLM010_scaffold45140_1	Candidatus Hamiltonella defensa	1	CRISPR
DLM010_scaffold179_1	Bacteroides fragilis	0.72	Predict
DLM010_scaffold503_4	Candidatus Hamiltonella defensa	0.8	Predict
DLM010_scaffold43377_3	Candidatus Hamiltonella defensa	1	CRISPR
DLM010_scaffold10158_2	Bacteroides fragilis	0.91	Predict
DLM010_scaffold18321_2	Bacteroides fragilis	0.76	Predict
DLM010_scaffold32474_2	Candidatus Hamiltonella defensa	0.82	Predict
DLM010_scaffold5445_6	Candidatus Hamiltonella defensa	0.8	Predict
DLM010_scaffold32332_1	Candidatus Hamiltonella defensa	1	CRISPR
DLM010_scaffold7753_8	Candidatus Hamiltonella defensa	0.89	Predict
DLM010_scaffold3050_2	Pasteurella multocida	1	CRISPR
DLM010_scaffold44670_1	Candidatus Hamiltonella defensa	0.84	Predict
DLM010_scaffold41095_1	Bacteroides fragilis	1	CRISPR
DLM010_scaffold16539_4	Candidatus Hamiltonella defensa	0.88	Predict
DLM010_scaffold33840_5	Candidatus Hamiltonella defensa	0.84	Predict

DLM010_scaffold6743_2	Candidatus Hamiltonella defensa	0.95	Predict
DLM010_scaffold22879_4	Candidatus Hamiltonella defensa	0.84	Predict
DLM010_scaffold45132_3	Flavobacterium columnare	0.71	Predict
DLM010_scaffold45189_1	Candidatus Hamiltonella defensa	1	CRISPR
DLM010_scaffold44889_1	Candidatus Hamiltonella defensa	1	CRISPR
DLM010_scaffold3339_9	Candidatus Hamiltonella defensa	0.89	Predict
DLM011_scaffold28392_2	Candidatus Hamiltonella defensa	1	CRISPR
DLM011_scaffold29232_2	Listeria monocytogenes	1	CRISPR
DLM011_scaffold29382_1	Parabacteroides distasonis	0.97	Predict
DLM011_scaffold21086_2	Candidatus Hamiltonella defensa	0.8	Predict
DLM011_scaffold21352_1	Candidatus Hamiltonella defensa	0.7	Predict
DLM011_scaffold29393_1	Candidatus Pelagibacter ubique	0.75	Predict
DLM011_scaffold29232_1	Candidatus Hamiltonella defensa	1	CRISPR
DLM011_scaffold28062_1	Candidatus Hamiltonella defensa	0.86	Predict
DLM011_scaffold27250_1	Candidatus Hamiltonella defensa	1	CRISPR
DLM011_scaffold29207_1	Candidatus Hamiltonella defensa	0.86	Predict
DLM011_scaffold1189_13	Parabacteroides merdae	1	CRISPR
DLM011_scaffold28659_3	Candidatus Hamiltonella defensa	1	CRISPR
DLM011_scaffold17163_7	Bacteroides fragilis	0.82	Predict
DLM011_scaffold29398_1	Candidatus Hamiltonella defensa	1	CRISPR
DLM011_scaffold1189_31	Cellulophaga baltica	1	CRISPR
DLM011_scaffold19964_1	Candidatus Hamiltonella defensa	1	CRISPR
DLM011_scaffold18867_6	Bacteroides fragilis	0.9	Predict
DLM012_scaffold11223_12	Candidatus Hamiltonella defensa	1	CRISPR
DLM012_scaffold16447_5	Candidatus Hamiltonella defensa	0.75	Predict
DLM012_scaffold19314_6	Candidatus Hamiltonella defensa	0.94	Predict
DLM012_scaffold49_1	Candidatus Hamiltonella defensa	1	CRISPR
DLM012_scaffold34010_1	Candidatus Hamiltonella defensa	1	CRISPR
DLM012_scaffold22034_2	Blautia coccoides	1	CRISPR
DLM012_scaffold7495_7	Candidatus Hamiltonella defensa	1	CRISPR
DLM012_C414271_1	Candidatus Hamiltonella defensa	0.71	Predict
DLM012_scaffold23728_1	Candidatus Hamiltonella defensa	0.74	Predict
DLM012_scaffold33026_10	Candidatus Hamiltonella defensa	1	CRISPR
DLM012_scaffold2394_3	Candidatus Hamiltonella defensa	0.97	Predict
DLM012_scaffold33913_1	Candidatus Hamiltonella defensa	1	CRISPR
DLM012_scaffold12_2	Candidatus Hamiltonella defensa	0.76	Predict
DLM012_scaffold33950_1	Candidatus Hamiltonella defensa	0.74	Predict
DLM012_scaffold29288_6	Candidatus Hamiltonella defensa	0.96	Predict
DLM012_scaffold32680_2	Candidatus Hamiltonella defensa	0.92	Predict
DLM012_scaffold26999_6	Massilioclostridium coli	1	CRISPR
DLM012_scaffold28806_1	Candidatus Hamiltonella defensa	0.88	Predict
DLM012_scaffold725_1	Candidatus Hamiltonella defensa	1	CRISPR
DLM012_scaffold33245_1	Candidatus Hamiltonella defensa	1	CRISPR
DLM012_scaffold29288_5	Thermoanaerobacterium saccharolytic	0.83	Predict
DLM013_scaffold23313_3	Candidatus Hamiltonella defensa	0.86	Predict
DLM013_scaffold17656_2	Candidatus Hamiltonella defensa	1	CRISPR
DLM013_scaffold7395_5	Candidatus Hamiltonella defensa	1	Predict
DLM013_scaffold34896_3	Lactobacillus plantarum	1	CRISPR

DLM013_scaffold21602_2	Candidatus Hamiltonella defensa	0.85	Predict
DLM013_scaffold57623_1	Candidatus Hamiltonella defensa	1	CRISPR
DLM013_scaffold55482_1	Clostridium tetani	0.97	Predict
DLM013_scaffold36790_6	Candidatus Hamiltonella defensa	0.74	Predict
DLM013_scaffold8482_4	Candidatus Hamiltonella defensa	0.71	Predict
DLM013_scaffold45817_1	Candidatus Hamiltonella defensa	0.97	Predict
DLM013_scaffold3810_8	Candidatus Hamiltonella defensa	0.91	Predict
DLM013_scaffold57456_3	Bacteroides fragilis	0.82	Predict
DLM013_scaffold3810_7	Candidatus Hamiltonella defensa	1	CRISPR
DLM013_scaffold318_1	Candidatus Hamiltonella defensa	0.9	Predict
DLM013_scaffold15355_2	Candidatus Hamiltonella defensa	0.88	Predict
DLM013_scaffold22571_2	Candidatus Hamiltonella defensa	0.96	Predict
DLM013_scaffold4430_4	Candidatus Hamiltonella defensa	0.77	Predict
DLM013_scaffold56628_1	Candidatus Hamiltonella defensa	1	CRISPR
DLM013_scaffold47801_1	Candidatus Hamiltonella defensa	0.89	Predict
DLM013_scaffold23184_12	Cellulophaga baltica	1	CRISPR
DLM013_scaffold57687_1	Actinomyces naeslundii	0.87	Predict
DLM013_scaffold23009_1	Candidatus Hamiltonella defensa	0.73	Predict
DLM013_scaffold3614_26	Flavobacterium columnare	1	CRISPR
DLM013_scaffold23737_4	Parabacteroides distasonis	0.89	Predict
DLM013_scaffold51645_2	Candidatus Hamiltonella defensa	1	CRISPR
DLM013_scaffold21436_7	Candidatus Hamiltonella defensa	0.87	Predict
DLM013_scaffold38983_1	Candidatus Hamiltonella defensa	0.85	Predict
DLM013_scaffold20700_8	Candidatus Hamiltonella defensa	0.9	Predict
DLM013_scaffold50692_1	Candidatus Hamiltonella defensa	0.74	Predict
DLM013_scaffold25009_5	Candidatus Hamiltonella defensa	1	CRISPR
DLM013_scaffold14795_2	Candidatus Hamiltonella defensa	0.88	Predict
DLM013_scaffold52105_9	Candidatus Hamiltonella defensa	1	CRISPR
DLM013_scaffold52295_2	Candidatus Hamiltonella defensa	0.88	Predict
DLM013_scaffold23184_9	Parabacteroides sp. D13	1	CRISPR
DLM013_scaffold49247_2	Candidatus Hamiltonella defensa	1	CRISPR
DLM013_scaffold56635_1	Candidatus Hamiltonella defensa	1	CRISPR
DLM013_C721558_1	Yersinia pestis	1	CRISPR
DLM013_scaffold31022_3	Candidatus Hamiltonella defensa	0.78	Predict
DLM013_scaffold30472_4	Mycoplasma pulmonis	0.87	Predict
DLM013_scaffold55003_1	Streptomyces lividans	0.71	Predict
DLM013_scaffold23574_3	Bacteroides fragilis	0.9	Predict
DLM013_scaffold3614_27	Megasphaera cerevisiae	1	CRISPR
DLM013_scaffold24100_2	Bacteroides fragilis	0.9	Predict
DLM013_scaffold2200_1	Candidatus Hamiltonella defensa	0.77	Predict
DLM014_scaffold52949_1	Candidatus Hamiltonella defensa	1	CRISPR
DLM014_scaffold14966_8	Candidatus Hamiltonella defensa	0.8	Predict
DLM014_scaffold39602_3	Bacteroides fragilis	0.83	Predict
DLM014_scaffold34561_6	Candidatus Hamiltonella defensa	0.99	Predict
DLM014_scaffold17726_11	Candidatus Hamiltonella defensa	0.93	Predict
DLM014_scaffold31737_5	Candidatus Hamiltonella defensa	0.76	Predict
DLM014_scaffold1_9	Cellulophaga baltica	0.94	Predict
DLM014_scaffold52940_1	Candidatus Hamiltonella defensa	1	CRISPR

DLM014_scaffold44624_1	Candidatus Hamiltonella defensa	1	CRISPR
DLM014_scaffold24690_1	Candidatus Hamiltonella defensa	0.84	Predict
DLM014_scaffold42199_1	Candidatus Hamiltonella defensa	0.97	Predict
DLM014_scaffold3883_2	Parabacteroides distasonis	0.99	Predict
DLM014_scaffold7847_1	Bacteroides fragilis	0.7	Predict
DLM014_scaffold46228_1	Clostridium perfringens	1	CRISPR
DLM014_scaffold35850_2	Candidatus Hamiltonella defensa	0.98	Predict
DLM014_scaffold40706_2	Bacteroides fragilis	0.71	Predict
DLM014_scaffold14317_2	Parabacteroides distasonis	0.88	Predict
DLM014_scaffold42200_1	Bacteroides fragilis	0.98	Predict
DLM014_scaffold45829_2	Bacteroides fragilis	1	CRISPR
DLM014_scaffold31737_1	Candidatus Hamiltonella defensa	0.8	Predict
DLM014_scaffold4026_7	Candidatus Hamiltonella defensa	0.76	Predict
DLM014_scaffold21553_3	Candidatus Hamiltonella defensa	0.85	Predict
DLM014_scaffold32434_6	Candidatus Hamiltonella defensa	0.91	Predict
DLM014_scaffold1942_34	Shigella boydii	0.99	Predict
DLM014_scaffold48474_2	Candidatus Hamiltonella defensa	0.81	Predict
DLM014_scaffold1_18	Candidatus Hamiltonella defensa	0.99	Predict
DLM014_scaffold52939_1	Bacteroides fragilis	0.95	Predict
DLM014_scaffold290_4	Candidatus Hamiltonella defensa	0.99	Predict
DLM014_scaffold36217_6	Parabacteroides distasonis	0.76	Predict
DLM015_scaffold18535_6	Clostridium botulinum	0.81	Predict
DLM015_scaffold5802_5	Candidatus Hamiltonella defensa	0.71	Predict
DLM015_scaffold16218_2	Candidatus Hamiltonella defensa	0.85	Predict
DLM015_scaffold50361_1	Bacteroides fragilis	0.75	Predict
DLM015_scaffold50502_1	Parabacteroides merdae	0.98	Predict
DLM015_scaffold23053_2	Candidatus Hamiltonella defensa	1	CRISPR
DLM015_scaffold1049_4	Candidatus Hamiltonella defensa	1	CRISPR
DLM015_scaffold36073_3	Clostridium perfringens	1	CRISPR
DLM015_scaffold23053_6	Candidatus Hamiltonella defensa	1	CRISPR
DLM015_scaffold47979_4	Bacteroides fragilis	0.79	Predict
DLM015_scaffold49465_1	Candidatus Hamiltonella defensa	0.75	Predict
DLM015_scaffold23554_1	Candidatus Hamiltonella defensa	1	CRISPR
DLM015_scaffold50303_1	Candidatus Hamiltonella defensa	0.72	Predict
DLM015_scaffold50189_1	Edwardsiella ictaluri	0.7	Predict
DLM015_scaffold5802_2	Lactobacillus plantarum	0.98	Predict
DLM015_scaffold14872_4	Bacteroides fragilis	0.71	Predict
DLM015_scaffold50457_1	Candidatus Hamiltonella defensa	1	CRISPR
DLM015_scaffold29903_2	Bacteroides fragilis	0.95	Predict
DLM015_scaffold36073_4	Candidatus Hamiltonella defensa	1	Predict
DLM015_scaffold145_2	Bacteroides fragilis	1	CRISPR
DLM015_scaffold17140_1	Bacteroides fragilis	0.93	Predict
DLM015_scaffold36351_2	Candidatus Hamiltonella defensa	0.84	Predict
DLM016_scaffold17586_2	Candidatus Hamiltonella defensa	0.79	Predict
DLM016_scaffold3377_13	Candidatus Hamiltonella defensa	0.9	Predict
DLM016_scaffold65085_5	Cellulophaga baltica	1	CRISPR
DLM016_scaffold64744_4	Flavobacterium columnare	0.86	Predict
DLM016_scaffold12_1	Candidatus Hamiltonella defensa	0.88	Predict

DLM016_scaffold13346_4	Candidatus Hamiltonella defensa	0.97	Predict
DLM016_scaffold27953_5	Candidatus Hamiltonella defensa	0.94	Predict
DLM016_scaffold37509_2	Candidatus Pelagibacter ubique	0.9	Predict
DLM016_scaffold46534_2	Bacteroides fragilis	0.72	Predict
DLM016_scaffold419_5	Candidatus Hamiltonella defensa	1	CRISPR
DLM016_scaffold41709_1	Parabacteroides merdae	0.97	Predict
DLM016_scaffold22143_3	Candidatus Hamiltonella defensa	1	CRISPR
DLM016_scaffold40529_1	Candidatus Hamiltonella defensa	0.82	Predict
DLM016_scaffold7191_1	Lactobacillus plantarum	0.86	Predict
DLM016_scaffold49496_1	Candidatus Hamiltonella defensa	0.72	Predict
DLM016_scaffold40187_1	Candidatus Hamiltonella defensa	0.86	Predict
DLM016_scaffold16615_5	Candidatus Pelagibacter ubique	0.94	Predict
DLM016_scaffold2806_2	Candidatus Hamiltonella defensa	0.96	Predict
DLM016_scaffold5004_5	Bacteroides fragilis	0.97	Predict
DLM016_scaffold65482_1	Candidatus Hamiltonella defensa	1	CRISPR
DLM016_scaffold65575_2	Clostridium tetani	1	CRISPR
DLM016_scaffold6713_3	Cellulophaga baltica	1	CRISPR
DLM016_scaffold6713_5	Candidatus Hamiltonella defensa	0.88	Predict
DLM016_scaffold64767_1	Lactobacillus plantarum	1	CRISPR
DLM016_scaffold6713_11	Parabacteroides merdae	1	CRISPR
DLM016_scaffold6713_2	Candidatus Hamiltonella defensa	0.82	Predict
DLM016_scaffold2366_1	Bacteroides fragilis	0.87	Predict
DLM016_scaffold4953_3	Candidatus Hamiltonella defensa	0.9	Predict
DLM016_scaffold6914_1	Candidatus Hamiltonella defensa	0.97	Predict
DLM016_scaffold58329_1	Candidatus Hamiltonella defensa	0.75	Predict
DLM016_scaffold52894_5	Bacteroides uniformis	1	CRISPR
DLM016_scaffold37873_2	Candidatus Hamiltonella defensa	1	CRISPR
DLM016_scaffold64097_2	Candidatus Hamiltonella defensa	0.86	Predict
DLM016_scaffold21785_1	Candidatus Hamiltonella defensa	0.97	Predict
DLM016_scaffold65474_2	Parabacteroides distasonis	0.72	Predict
DLM016_scaffold65085_3	Cellulophaga baltica	0.76	Predict
DLM016_scaffold6553_6	Candidatus Hamiltonella defensa	0.72	Predict
DLM016_scaffold1623_5	Candidatus Hamiltonella defensa	1	CRISPR
DLM016_scaffold63991_1	Bacteroides vulgatus	1	CRISPR
DLM016_scaffold29974_3	Candidatus Hamiltonella defensa	0.7	Predict
DLM016_scaffold53961_3	Bacteroides fragilis	0.92	Predict
DLM016_scaffold419_9	Candidatus Hamiltonella defensa	1	CRISPR
DLM017_scaffold20271_2	Faecalibacterium prausnitzii	1	CRISPR
DLM017_scaffold2813_9	Candidatus Hamiltonella defensa	0.83	Predict
DLM017_scaffold3964_2	Candidatus Hamiltonella defensa	0.76	Predict
DLM017_scaffold29996_2	Candidatus Hamiltonella defensa	1	CRISPR
DLM017_scaffold20005_2	Candidatus Hamiltonella defensa	1	CRISPR
DLM017_scaffold21266_1	Candidatus Hamiltonella defensa	1	CRISPR
DLM017_scaffold33959_3	Bacteroides fragilis	1	CRISPR
DLM017_scaffold2501_2	Candidatus Hamiltonella defensa	0.99	Predict
DLM017_scaffold43714_2	Candidatus Hamiltonella defensa	1	CRISPR
DLM017_scaffold5960_2	Candidatus Hamiltonella defensa	1	CRISPR
DLM017_scaffold28739_1	Candidatus Hamiltonella defensa	0.86	Predict

DLM017_scaffold5705_1	Candidatus Hamiltonella defensa	0.96	Predict
DLM017_scaffold42571_2	Candidatus Hamiltonella defensa	0.99	Predict
DLM017_scaffold1478_32	Candidatus Hamiltonella defensa	0.89	Predict
DLM017_scaffold10166_1	Candidatus Hamiltonella defensa	0.85	Predict
DLM017_scaffold15588_1	Candidatus Hamiltonella defensa	1	CRISPR
DLM017_scaffold31873_1	Candidatus Hamiltonella defensa	0.9	Predict
DLM017_scaffold23811_1	Candidatus Hamiltonella defensa	0.84	Predict
DLM017_scaffold18232_1	Candidatus Hamiltonella defensa	0.91	Predict
DLM018_scaffold36970_5	Candidatus Hamiltonella defensa	0.92	Predict
DLM018_scaffold38471_2	Parabacteroides distasonis	0.93	Predict
DLM018_scaffold37570_1	Candidatus Hamiltonella defensa	1	CRISPR
DLM018_scaffold197_3	Candidatus Hamiltonella defensa	0.86	Predict
DLM018_scaffold13432_9	Candidatus Hamiltonella defensa	1	CRISPR
DLM018_scaffold45347_1	Candidatus Hamiltonella defensa	0.78	Predict
DLM018_scaffold49175_1	Candidatus Hamiltonella defensa	1	CRISPR
DLM018_scaffold12094_2	Flavobacterium columnare	0.81	Predict
DLM018_scaffold3525_6	Candidatus Hamiltonella defensa	1	CRISPR
DLM018_scaffold2054_4	Candidatus Hamiltonella defensa	0.96	Predict
DLM018_scaffold82_1	Candidatus Hamiltonella defensa	0.92	Predict
DLM018_scaffold48894_1	Pantoea agglomerans	0.84	Predict
DLM018_scaffold26573_10	Roseobacter denitrificans	0.78	Predict
DLM018_scaffold45347_2	Candidatus Hamiltonella defensa	0.78	Predict
DLM018_scaffold48696_1	Actinomyces naeslundii	0.99	Predict
DLM018_scaffold15_30	Candidatus Hamiltonella defensa	0.78	Predict
DLM018_scaffold48959_3	Candidatus Hamiltonella defensa	0.71	Predict
DLM018_scaffold7847_2	Candidatus Hamiltonella defensa	0.98	Predict
DLM018_scaffold31396_2	Candidatus Hamiltonella defensa	0.9	Predict
DLM018_scaffold14900_3	Chryseobacterium carnipullorum	1	CRISPR
DLM018_scaffold48449_1	Parabacteroides merdae	0.93	Predict
DLM018_scaffold47288_3	Clostridium perfringens	1	CRISPR
DLM018_scaffold26573_4	Roseobacter denitrificans	1	CRISPR
DLM018_scaffold36970_6	Candidatus Hamiltonella defensa	1	CRISPR
DLM018_scaffold40461_1	Parabacteroides merdae	0.96	Predict
DLM018_scaffold48983_2	Clostridioides difficile	1	CRISPR
DLM018_scaffold26955_2	Candidatus Hamiltonella defensa	0.73	Predict
DLM018_scaffold3351_9	Candidatus Hamiltonella defensa	0.8	Predict
DLM018_scaffold19081_1	Candidatus Hamiltonella defensa	1	CRISPR
DLM019_scaffold19742_4	Candidatus Hamiltonella defensa	0.75	Predict
DLM019_scaffold62503_1	Candidatus Hamiltonella defensa	0.75	Predict
DLM019_scaffold22877_3	Candidatus Hamiltonella defensa	0.99	Predict
DLM019_scaffold62785_1	Candidatus Hamiltonella defensa	0.76	Predict
DLM019_scaffold53137_1	Clostridioides difficile	0.82	Predict
DLM019_scaffold20492_3	Candidatus Hamiltonella defensa	0.9	Predict
DLM019_scaffold6166_23	Candidatus Hamiltonella defensa	0.87	Predict
DLM019_scaffold8577_4	Pseudomonas tolaasii	0.72	Predict
DLM019_scaffold20492_2	Candidatus Hamiltonella defensa	0.84	Predict
DLM019_C814862_1	Candidatus Hamiltonella defensa	0.77	Predict
DLM019_scaffold45285_5	Candidatus Hamiltonella defensa	0.7	Predict

DLM019_scaffold24899_8	Candidatus Hamiltonella defensa	1	CRISPR
DLM019_scaffold63202_2	Candidatus Hamiltonella defensa	0.82	Predict
DLM019_scaffold53137_2	Candidatus Hamiltonella defensa	0.8	Predict
DLM019_scaffold61987_1	Candidatus Hamiltonella defensa	0.95	Predict
DLM019_scaffold31976_1	Flavobacterium columnare	0.75	Predict
DLM019_scaffold39_1	Candidatus Hamiltonella defensa	1	CRISPR
DLM019_scaffold38422_4	Candidatus Hamiltonella defensa	0.72	Predict
DLM019_scaffold45285_4	Candidatus Hamiltonella defensa	1	CRISPR
DLM019_scaffold4998_6	Candidatus Hamiltonella defensa	0.74	Predict
DLM019_scaffold54914_7	Candidatus Hamiltonella defensa	1	Predict
DLM019_scaffold35445_2	Candidatus Hamiltonella defensa	0.77	Predict
DLM019_scaffold37359_6	Candidatus Hamiltonella defensa	0.88	Predict
DLM019_scaffold55656_1	Candidatus Hamiltonella defensa	0.9	Predict
DLM019_scaffold38422_1	Candidatus Hamiltonella defensa	0.96	Predict
DLM019_scaffold63049_1	Candidatus Hamiltonella defensa	0.96	Predict
DLM019_scaffold102_1	Candidatus Hamiltonella defensa	1	CRISPR
DLM019_scaffold62785_2	Candidatus Hamiltonella defensa	1	CRISPR
DLM019_scaffold8817_14	Bacteroides fragilis	0.8	Predict
DLM019_scaffold55454_1	Candidatus Hamiltonella defensa	0.8	Predict
DLM019_scaffold29437_2	Candidatus Hamiltonella defensa	0.78	Predict
DLM019_scaffold12600_2	Candidatus Hamiltonella defensa	0.89	Predict
DLM019_scaffold2445_1	Candidatus Hamiltonella defensa	1	CRISPR
DLM019_scaffold44258_2	Parabacteroides distasonis	0.82	Predict
DLM019_scaffold31935_1	Candidatus Hamiltonella defensa	0.95	Predict
DLM019_scaffold8788_7	Bacteroides fragilis	0.77	Predict
DLM019_scaffold63216_1	Candidatus Hamiltonella defensa	0.79	Predict
DLM020_scaffold983_14	Lactobacillus fermentum	1	CRISPR
DLM020_scaffold8084_6	Bacteroides fragilis	0.92	Predict
DLM020_scaffold11081_1	Candidatus Hamiltonella defensa	0.7	Predict
DLM020_scaffold21144_2	Bacteroides fragilis	0.86	Predict
DLM020_scaffold19547_2	Candidatus Hamiltonella defensa	0.86	Predict
DLM020_scaffold10399_1	Candidatus Hamiltonella defensa	0.9	Predict
DLM020_scaffold3010_3	Bdellovibrio bacteriovorus	1	CRISPR
DLM020_scaffold20362_3	Candidatus Hamiltonella defensa	0.7	Predict
DLM020_scaffold8114_1	Bacteroides fragilis	0.79	Predict
DLM020_scaffold8736_5	Candidatus Hamiltonella defensa	0.87	Predict
DLM020_scaffold500_10	Bacteroides fragilis	0.93	Predict
DLM020_scaffold7279_1	Candidatus Hamiltonella defensa	1	CRISPR
DLM020_scaffold1387_1	Bacteroides fragilis	0.96	Predict
DLM020_scaffold22314_2	Candidatus Hamiltonella defensa	1	CRISPR
DLM020_scaffold8693_2	Bacteroides fragilis	0.89	Predict
DLM020_scaffold20362_2	Lactobacillus gasseri	0.83	Predict
DLM020_scaffold22182_2	Candidatus Hamiltonella defensa	1	CRISPR
DLM020_scaffold21959_1	Candidatus Hamiltonella defensa	0.91	Predict
DLM020_scaffold6157_3	Candidatus Hamiltonella defensa	0.85	Predict
DLM020_scaffold19925_2	Candidatus Hamiltonella defensa	0.87	Predict
DLM020_scaffold13039_2	Candidatus Hamiltonella defensa	1	CRISPR
DLM020_C355636_1	Candidatus Hamiltonella defensa	0.82	Predict

DLM020_scaffold20632_1	Candidatus Hamiltonella defensa	0.74	Predict
DLM020_scaffold22218_1	Bacteroides fragilis	1	CRISPR
DLM020_scaffold6936_4	Candidatus Pelagibacter ubique	0.71	Predict
DLM020_scaffold7475_1	Lactobacillus plantarum	0.89	Predict
DLM020_scaffold10415_1	Bacteroides fragilis	0.95	Predict
DLM020_scaffold10180_3	Candidatus Hamiltonella defensa	0.98	Predict
DLM020_scaffold22290_1	Candidatus Hamiltonella defensa	0.82	Predict
DLM020_scaffold5648_4	Candidatus Hamiltonella defensa	0.76	Predict
DLM020_scaffold11301_1	Candidatus Hamiltonella defensa	0.78	Predict
DLM020_scaffold21039_1	Candidatus Hamiltonella defensa	0.91	Predict
DLM020_scaffold7279_3	Roseburia inulinivorans	1	CRISPR
DLM020_scaffold13927_3	Candidatus Hamiltonella defensa	0.87	Predict
DLM020_scaffold10263_1	Cellulophaga baltica	0.91	Predict
DLM021_scaffold36034_1	Candidatus Hamiltonella defensa	0.88	Predict
DLM021_scaffold44307_1	Candidatus Hamiltonella defensa	1	CRISPR
DLM021_scaffold43159_1	Candidatus Hamiltonella defensa	0.89	Predict
DLM021_scaffold31538_5	Candidatus Hamiltonella defensa	1	CRISPR
DLM021_scaffold29140_2	Candidatus Hamiltonella defensa	0.83	Predict
DLM021_scaffold35216_1	Candidatus Hamiltonella defensa	0.81	Predict
DLM021_scaffold12971_1	Candidatus Hamiltonella defensa	0.91	Predict
DLM021_scaffold28049_1	Candidatus Hamiltonella defensa	1	CRISPR
DLM021_scaffold36642_2	Candidatus Hamiltonella defensa	0.96	Predict
DLM021_scaffold42193_7	Candidatus Hamiltonella defensa	0.85	Predict
DLM021_scaffold43501_2	Bacteroides fragilis	0.99	Predict
DLM021_scaffold554_3	Candidatus Hamiltonella defensa	0.95	Predict
DLM021_scaffold43126_6	Candidatus Hamiltonella defensa	0.88	Predict
DLM021_scaffold31475_3	Rhodococcus hoagii	0.79	Predict
DLM021_scaffold24637_8	Bacteroides fragilis	1	CRISPR
DLM021_scaffold4_54	Bacteroides fragilis	0.94	Predict
DLM021_scaffold30546_2	Candidatus Hamiltonella defensa	1	CRISPR
DLM021_scaffold10200_17	Candidatus Hamiltonella defensa	0.77	Predict
DLM021_scaffold28049_2	Bacteroides fragilis	1	CRISPR
DLM021_scaffold10200_12	Candidatus Hamiltonella defensa	0.93	Predict
DLM021_scaffold23214_1	Candidatus Hamiltonella defensa	0.79	Predict
DLM021_scaffold46_2	Candidatus Hamiltonella defensa	0.99	Predict
DLM021_scaffold24731_9	Candidatus Hamiltonella defensa	0.72	Predict
DLM021_scaffold640_7	Bacteroides fragilis	0.85	Predict
DLM021_scaffold21292_11	Bacteroides fragilis	0.88	Predict
DLM021_scaffold22868_5	Bacteroides fragilis	0.97	Predict
DLM021_scaffold2298_4	Candidatus Hamiltonella defensa	0.71	Predict
DLM021_scaffold44452_1	Candidatus Hamiltonella defensa	0.98	Predict
DLM021_scaffold8187_1	Candidatus Hamiltonella defensa	0.84	Predict
DLM021_scaffold45210_2	Candidatus Hamiltonella defensa	1	CRISPR
DLM021_scaffold381_1	Candidatus Hamiltonella defensa	1	CRISPR
DLM021_scaffold1604_4	Candidatus Hamiltonella defensa	0.84	Predict
DLM022_scaffold87335_3	Candidatus Hamiltonella defensa	0.8	Predict
DLM022_scaffold90455_1	Lactobacillus jensenii	1	CRISPR
DLM022_C1105606_1	Clostridium perfringens	0.94	Predict

DLM022_scaffold34930_3	Candidatus Hamiltonella defensa	1	CRISPR
DLM022_scaffold13671_3	Candidatus Hamiltonella defensa	1	CRISPR
DLM022_scaffold90383_1	Candidatus Hamiltonella defensa	0.75	Predict
DLM022_scaffold61396_2	Candidatus Hamiltonella defensa	0.8	Predict
DLM022_scaffold3872_14	Candidatus Hamiltonella defensa	0.71	Predict
DLM022_scaffold28531_1	Candidatus Hamiltonella defensa	0.9	Predict
DLM022_scaffold13626_15	Clostridioides difficile	0.97	Predict
DLM022_scaffold47568_2	Candidatus Hamiltonella defensa	0.93	Predict
DLM022_scaffold87188_2	Candidatus Hamiltonella defensa	0.87	Predict
DLM022_scaffold83380_1	Candidatus Hamiltonella defensa	0.75	Predict
DLM022_scaffold38614_1	Candidatus Hamiltonella defensa	0.83	Predict
DLM022_scaffold7833_6	Candidatus Hamiltonella defensa	0.73	Predict
DLM022_scaffold90441_1	Candidatus Hamiltonella defensa	1	CRISPR
DLM022_scaffold29304_11	Candidatus Hamiltonella defensa	1	CRISPR
DLM022_scaffold45845_2	Candidatus Hamiltonella defensa	0.98	Predict
DLM022_scaffold75069_1	Candidatus Hamiltonella defensa	0.85	Predict
DLM022_scaffold46815_1	Candidatus Pelagibacter ubique	0.92	Predict
DLM022_scaffold50520_4	Candidatus Hamiltonella defensa	0.87	Predict
DLM022_scaffold82276_2	Candidatus Hamiltonella defensa	0.95	Predict
DLM022_scaffold86174_3	Candidatus Hamiltonella defensa	0.98	Predict
DLM022_scaffold40951_1	Candidatus Hamiltonella defensa	0.77	Predict
DLM022_scaffold51389_1	Bacteroides vulgatus	1	CRISPR
DLM022_scaffold34373_9	Candidatus Hamiltonella defensa	0.78	Predict
DLM022_scaffold7976_5	Candidatus Hamiltonella defensa	0.77	Predict
DLM022_scaffold68876_1	Candidatus Hamiltonella defensa	0.94	Predict
DLM022_scaffold24869_7	Candidatus Hamiltonella defensa	0.75	Predict
DLM022_scaffold46210_2	Candidatus Hamiltonella defensa	1	CRISPR
DLM022_scaffold79585_1	Candidatus Hamiltonella defensa	0.94	Predict
DLM022_scaffold90471_1	Candidatus Hamiltonella defensa	0.95	Predict
DLM022_scaffold126_4	Candidatus Hamiltonella defensa	0.78	Predict
DLM022_scaffold91_3	Candidatus Hamiltonella defensa	1	CRISPR
DLM022_scaffold7197_1	Candidatus Hamiltonella defensa	0.95	Predict
DLM022_scaffold24869_6	Flavobacterium columnare	0.81	Predict
DLM022_scaffold26167_3	Parabacteroides distasonis	0.87	Predict
DLM022_scaffold8618_3	Candidatus Hamiltonella defensa	1	CRISPR
DLM022_scaffold248_3	Candidatus Hamiltonella defensa	0.95	Predict
DLM022_scaffold90464_1	Flavobacterium columnare	1	CRISPR
DLM022_scaffold63634_1	Candidatus Hamiltonella defensa	0.97	Predict
DLM022_scaffold90529_1	Clostridioides difficile	0.84	Predict
DLM022_scaffold82197_1	Mycoplasma pulmonis	1	CRISPR
DLM022_scaffold84536_3	Candidatus Hamiltonella defensa	0.87	Predict
DLM022_scaffold73260_1	Candidatus Hamiltonella defensa	1	CRISPR
DLM022_scaffold79270_1	Lactobacillus fermentum	1	CRISPR
DLM022_scaffold68020_2	Candidatus Hamiltonella defensa	0.7	Predict
DLM022_scaffold12444_5	Candidatus Hamiltonella defensa	0.85	Predict
DLM022_scaffold86174_2	Candidatus Hamiltonella defensa	0.96	Predict
DLM022_scaffold89824_2	Akkermansia muciniphila	1	CRISPR
DLM022_C1104604_1	Flavobacterium columnare	0.74	Predict

DLM022_scaffold36935_1	Candidatus Hamiltonella defensa	0.97	Predict
DLM022_scaffold85942_2	Candidatus Hamiltonella defensa	0.98	Predict
DLM022_scaffold9_2	Candidatus Hamiltonella defensa	1	CRISPR
DLM022_scaffold6448_3	Candidatus Hamiltonella defensa	0.82	Predict
DLM022_scaffold90528_2	Candidatus Hamiltonella defensa	1	CRISPR
DLM022_scaffold160_1	Clostridioides difficile	0.86	Predict
DLM022_scaffold64018_1	Selenomonas ruminantium	1	CRISPR
DLM022_scaffold89824_3	Candidatus Hamiltonella defensa	1	CRISPR
DLM022_scaffold43500_4	Candidatus Hamiltonella defensa	0.9	Predict
DLM022_scaffold90113_2	Candidatus Hamiltonella defensa	0.84	Predict
DLM022_scaffold37957_2	Candidatus Hamiltonella defensa	0.88	Predict
DLM022_scaffold11067_1	Candidatus Pelagibacter ubique	0.85	Predict
DLM022_scaffold22367_7	Candidatus Hamiltonella defensa	0.88	Predict
DLM022_scaffold24995_4	Candidatus Hamiltonella defensa	0.9	Predict
DLM022_scaffold89236_1	Candidatus Hamiltonella defensa	1	CRISPR
DLM022_scaffold30520_1	Candidatus Hamiltonella defensa	1	CRISPR
DLM022_scaffold32204_1	Candidatus Hamiltonella defensa	0.75	Predict
DLM022_scaffold46686_4	Candidatus Hamiltonella defensa	0.77	Predict
DLM022_scaffold85281_1	Bacteroides thetaiotaomicron	1	CRISPR
DLM022_scaffold90528_3	Candidatus Hamiltonella defensa	1	CRISPR
DLM022_scaffold89880_1	Candidatus Hamiltonella defensa	0.86	Predict
DLM022_scaffold89156_1	Candidatus Hamiltonella defensa	0.75	Predict
DLM022_scaffold90507_1	Candidatus Hamiltonella defensa	0.98	Predict
DLM022_scaffold62765_1	Candidatus Hamiltonella defensa	1	CRISPR
DLM022_scaffold73568_2	Actinomyces naeslundii	0.89	Predict
DLM022_scaffold88059_2	[Eubacterium] rectale	1	CRISPR
DLM022_scaffold90402_1	Cellulophaga baltica	0.98	Predict
DLM022_scaffold90509_1	Candidatus Hamiltonella defensa	0.83	Predict
DLM022_scaffold44709_3	Bacteroides fragilis	0.84	Predict
DLM022_scaffold87605_2	Bacteroides fragilis	1	CRISPR
DLM022_scaffold937_1	Croceibacter atlanticus	1	CRISPR
DLM022_scaffold30068_2	Candidatus Hamiltonella defensa	0.98	Predict
DLM022_scaffold15558_7	Candidatus Hamiltonella defensa	1	Predict
DLM023_scaffold51589_1	Candidatus Hamiltonella defensa	1	CRISPR
DLM023_scaffold25385_1	Candidatus Hamiltonella defensa	0.94	Predict
DLM023_scaffold14487_2	Candidatus Hamiltonella defensa	0.94	Predict
DLM023_scaffold48996_1	Candidatus Hamiltonella defensa	1	CRISPR
DLM023_scaffold4774_1	Roseobacter denitrificans	1	CRISPR
DLM023_scaffold27445_8	Candidatus Hamiltonella defensa	1	CRISPR
DLM023_scaffold411_39	Candidatus Hamiltonella defensa	0.89	Predict
DLM023_scaffold51795_1	Candidatus Hamiltonella defensa	0.72	Predict
DLM023_scaffold23339_1	Roseobacter denitrificans	1	CRISPR
DLM023_scaffold2126_10	Staphylococcus haemolyticus	0.75	Predict
DLM023_scaffold7323_3	Bacteroides fragilis	0.99	Predict
DLM023_scaffold44217_3	Candidatus Hamiltonella defensa	1	CRISPR
DLM023_scaffold47535_1	Ruegeria pomeroyi	0.72	Predict
DLM023_scaffold37922_1	Candidatus Hamiltonella defensa	0.76	Predict
DLM023_scaffold4774_5	Roseobacter denitrificans	0.83	Predict

DLM023_scaffold51029_1	Lactobacillus fermentum	1	CRISPR
DLM023_scaffold18407_7	Candidatus Hamiltonella defensa	0.99	Predict
DLM023_scaffold17201_4	Candidatus Hamiltonella defensa	0.76	Predict
DLM023_scaffold45333_4	Candidatus Hamiltonella defensa	0.91	Predict
DLM023_scaffold28443_5	Lactobacillus fermentum	0.85	Predict
DLM023_scaffold32334_1	Candidatus Hamiltonella defensa	1	CRISPR
DLM023_scaffold51553_3	Bacteroides fragilis	0.89	Predict
DLM023_scaffold4774_3	Roseobacter denitrificans	0.85	Predict
DLM023_scaffold58_7	Bacteroides fragilis	0.93	Predict
DLM023_scaffold51688_1	Candidatus Hamiltonella defensa	0.76	Predict
DLM023_scaffold26150_3	Candidatus Hamiltonella defensa	1	CRISPR
DLM024_scaffold4102_1	Clostridium perfringens	1	CRISPR
DLM024_scaffold6096_2	Lactobacillus fermentum	0.86	Predict
DLM024_scaffold1307_14	Candidatus Hamiltonella defensa	1	CRISPR
DLM024_scaffold6915_1	Candidatus Hamiltonella defensa	0.86	Predict
DLM024_scaffold6392_4	Candidatus Hamiltonella defensa	0.97	Predict
DLM024_scaffold6942_1	Candidatus Hamiltonella defensa	1	CRISPR
DLM024_scaffold2301_1	Candidatus Hamiltonella defensa	1	CRISPR
DLM024_scaffold6782_2	Listeria monocytogenes	1	CRISPR
DLM024_scaffold447_14	Streptococcus pneumoniae	1	CRISPR
DLM024_scaffold3270_1	Candidatus Hamiltonella defensa	0.76	Predict
DLM024_scaffold6734_2	Lactococcus garvieae	1	CRISPR
DLM024_scaffold1543_4	Candidatus Hamiltonella defensa	0.91	Predict
DLM024_scaffold5188_2	Candidatus Hamiltonella defensa	1	CRISPR
DLM024_scaffold5624_3	Candidatus Hamiltonella defensa	1	CRISPR
DLM024_scaffold6919_2	Candidatus Hamiltonella defensa	0.85	Predict
DLM024_C136347_1	Candidatus Hamiltonella defensa	0.94	Predict
DLM024_scaffold722_1	Candidatus Hamiltonella defensa	0.99	Predict
DLM024_scaffold3585_1	Candidatus Hamiltonella defensa	1	CRISPR
DLM024_scaffold2213_13	Candidatus Hamiltonella defensa	1	CRISPR
DLM024_scaffold2465_1	Flavobacterium psychrophilum	1	CRISPR
DLM024_scaffold4575_2	Candidatus Hamiltonella defensa	1	CRISPR
DLM024_scaffold1355_19	Clostridium tetani	0.79	Predict
DLM027_scaffold2040_2	Candidatus Hamiltonella defensa	0.74	Predict
DLM027_scaffold19184_4	Candidatus Hamiltonella defensa	0.9	Predict
DLM027_scaffold20206_4	Candidatus Hamiltonella defensa	1	CRISPR
DLM027_scaffold18274_3	Candidatus Hamiltonella defensa	0.85	Predict
DLM027_scaffold2427_8	Candidatus Hamiltonella defensa	0.72	Predict
DLM027_scaffold2177_5	Candidatus Hamiltonella defensa	1	CRISPR
DLM027_scaffold26586_2	Candidatus Hamiltonella defensa	1	CRISPR
DLM027_scaffold26569_1	Lactobacillus gasseri	1	CRISPR
DLM027_scaffold19716_4	Candidatus Hamiltonella defensa	0.92	Predict
DLM027_scaffold26720_1	Actinomyces naeslundii	1	CRISPR
DLM027_scaffold5447_3	Candidatus Hamiltonella defensa	0.7	Predict
DLM027_scaffold26569_2	Candidatus Hamiltonella defensa	0.92	Predict
DLM027_scaffold79_10	Candidatus Hamiltonella defensa	0.81	Predict
DLM027_scaffold19168_1	Candidatus Hamiltonella defensa	1	CRISPR
DLM027_scaffold26477_2	Candidatus Hamiltonella defensa	0.85	Predict

DLM027_scaffold9656_1	Candidatus Hamiltonella defensa	1	CRISPR
DLM027_scaffold9665_4	Candidatus Hamiltonella defensa	0.73	Predict
DLM027_scaffold8638_4	Candidatus Hamiltonella defensa	0.93	Predict
DLM027_scaffold26707_1	Candidatus Hamiltonella defensa	1	CRISPR
DLM027_scaffold14908_1	Candidatus Hamiltonella defensa	1	CRISPR
DLM028_scaffold6098_12	Vibrio natriegens	0.88	Predict
DLM028_scaffold32640_1	Candidatus Hamiltonella defensa	0.8	Predict
DLM028_scaffold21827_4	Candidatus Hamiltonella defensa	1	CRISPR
DLM028_scaffold54482_2	Candidatus Hamiltonella defensa	0.92	Predict
DLM028_scaffold33522_2	Candidatus Hamiltonella defensa	0.92	Predict
DLM028_scaffold39913_2	Candidatus Hamiltonella defensa	0.83	Predict
DLM028_scaffold30320_6	Flavobacterium columnare	0.94	Predict
DLM028_scaffold17_2	Mannheimia haemolytica	0.96	Predict
DLM028_scaffold40934_1	Candidatus Hamiltonella defensa	0.83	Predict
DLM028_scaffold30790_1	Candidatus Hamiltonella defensa	1	CRISPR
DLM028_scaffold43205_3	Bacteroides fragilis	0.87	Predict
DLM028_scaffold12478_1	Candidatus Hamiltonella defensa	0.86	Predict
DLM028_scaffold48949_2	Edwardsiella ictaluri	0.8	Predict
DLM028_scaffold28515_6	Parabacteroides merdae	1	CRISPR
DLM028_scaffold25751_8	Candidatus Hamiltonella defensa	1	CRISPR
DLM028_scaffold46719_2	Candidatus Hamiltonella defensa	0.7	Predict
DLM028_scaffold10447_6	Candidatus Hamiltonella defensa	0.99	Predict
DLM028_scaffold54580_1	Candidatus Hamiltonella defensa	0.88	Predict
DLM028_scaffold30334_1	Candidatus Hamiltonella defensa	0.88	Predict
DLM028_C675659_1	Azospirillum brasilense	0.86	Predict
DLM028_scaffold21827_3	Candidatus Hamiltonella defensa	1	CRISPR
DLM028_scaffold32640_2	Candidatus Hamiltonella defensa	0.72	Predict
DLM028_scaffold268_28	Candidatus Hamiltonella defensa	0.86	Predict
DLM028_scaffold1815_5	Candidatus Hamiltonella defensa	0.95	Predict
DLM028_scaffold29533_12	Bacteroides fragilis	0.73	Predict
DLM028_scaffold3721_11	Candidatus Hamiltonella defensa	0.82	Predict
DLM028_scaffold51026_1	Candidatus Hamiltonella defensa	0.91	Predict
DLM028_scaffold54608_1	Clostridioides difficile	0.96	Predict
DLM028_scaffold28558_6	Candidatus Hamiltonella defensa	0.94	Predict
DLM028_scaffold10666_1	Candidatus Hamiltonella defensa	0.89	Predict
DLM028_scaffold32204_5	Candidatus Hamiltonella defensa	0.77	Predict
DLM028_scaffold16942_4	Candidatus Hamiltonella defensa	0.76	Predict
DLM028_scaffold28515_4	Cellulophaga baltica	1	CRISPR
DLM028_scaffold36337_1	Candidatus Hamiltonella defensa	0.72	Predict
DLM028_scaffold13566_10	Candidatus Hamiltonella defensa	1	CRISPR
DLM028_scaffold6503_3	Candidatus Hamiltonella defensa	0.79	Predict
DLM028_scaffold1257_18	Candidatus Hamiltonella defensa	0.82	Predict
DLM028_scaffold51841_1	Candidatus Hamiltonella defensa	0.92	Predict
DLM028_scaffold37211_5	Burkholderia thailandensis	0.99	Predict
DLM028_scaffold726_8	Vibrio splendidus	1	CRISPR
DLM028_scaffold41953_1	Candidatus Hamiltonella defensa	0.91	Predict
DLM028_scaffold26265_2	Candidatus Hamiltonella defensa	1	CRISPR
DLM028_scaffold20432_3	Candidatus Hamiltonella defensa	1	CRISPR

DLM028_scaffold9236_8	Candidatus Hamiltonella defensa	0.97	Predict
DLM028_scaffold86_3	Candidatus Hamiltonella defensa	0.96	Predict
DLM028_scaffold31687_6	Parabacteroides distasonis	0.94	Predict
DLM001_scaffold2435_6	unknown	0	-
DLM001_scaffold55116_2	unknown	0	-
DLM001_scaffold17614_8	unknown	0	-
DLM001_scaffold25895_8	unknown	0	-
DLM001_scaffold21223_1	unknown	0	-
DLM001_scaffold52289_2	unknown	0	-
DLM001_scaffold55454_4	unknown	0	-
DLM001_scaffold12282_12	unknown	0	-
DLM001_scaffold50893_2	unknown	0	-
DLM001_scaffold47007_2	unknown	0	-
DLM002_scaffold5537_11	unknown	0	-
DLM003_scaffold67334_2	unknown	0	-
DLM003_scaffold36199_4	unknown	0	-
DLM003_scaffold16412_17	unknown	0	-
DLM003_scaffold66050_1	unknown	0	-
DLM003_scaffold3089_1	unknown	0	-
DLM004_scaffold2416_6	unknown	0	-
DLM004_scaffold2421_24	unknown	0	-
DLM004_scaffold15727_1	unknown	0	-
DLM004_scaffold9327_2	unknown	0	-
DLM005_scaffold2340_3	unknown	0	-
DLM005_scaffold36491_18	unknown	0	-
DLM005_scaffold10521_41	unknown	0	-
DLM006_scaffold15729_4	unknown	0	-
DLM006_scaffold10416_3	unknown	0	-
DLM006_scaffold13732_3	unknown	0	-
DLM006_scaffold13444_2	unknown	0	-
DLM006_scaffold22549_2	unknown	0	-
DLM007_scaffold20148_2	unknown	0	-
DLM007_scaffold20148_1	unknown	0	-
DLM007_scaffold45002_8	unknown	0	-
DLM008_scaffold9016_5	unknown	0	-
DLM008_scaffold54665_3	unknown	0	-
DLM008_scaffold9016_1	unknown	0	-
DLM008_scaffold9016_3	unknown	0	-
DLM008_scaffold46088_1	unknown	0	-
DLM008_scaffold43273_7	unknown	0	-
DLM008_scaffold68309_1	unknown	0	-
DLM008_scaffold129_7	unknown	0	-
DLM009_scaffold18558_3	unknown	0	-
DLM009_scaffold8439_6	unknown	0	-
DLM009_scaffold18558_6	unknown	0	-
DLM009_scaffold17086_6	unknown	0	-
DLM009_scaffold18558_4	unknown	0	-
DLM010_scaffold41867_3	unknown	0	-

DLM010_scaffold4248_8	unknown	0	-
DLM011_scaffold5932_1	unknown	0	-
DLM011_C484732_1	unknown	0	-
DLM011_scaffold1699_8	unknown	0	-
DLM011_scaffold5789_4	unknown	0	-
DLM011_scaffold24958_1	unknown	0	-
DLM013_scaffold3286_13	unknown	0	-
DLM013_scaffold23184_5	unknown	0	-
DLM013_scaffold56424_2	unknown	0	-
DLM013_scaffold51742_2	unknown	0	-
DLM013_scaffold54893_2	unknown	0	-
DLM013_scaffold8482_6	unknown	0	-
DLM013_scaffold49483_1	unknown	0	-
DLM013_scaffold11264_1	unknown	0	-
DLM013_scaffold16485_8	unknown	0	-
DLM014_scaffold26538_3	unknown	0	-
DLM014_scaffold3539_3	unknown	0	-
DLM014_scaffold51860_2	unknown	0	-
DLM014_scaffold43677_1	unknown	0	-
DLM014_scaffold23167_4	unknown	0	-
DLM014_scaffold18806_7	unknown	0	-
DLM014_scaffold18901_3	unknown	0	-
DLM014_scaffold37209_4	unknown	0	-
DLM014_scaffold7310_3	unknown	0	-
DLM015_scaffold1119_3	unknown	0	-
DLM015_scaffold34621_5	unknown	0	-
DLM015_scaffold47846_2	unknown	0	-
DLM016_scaffold61369_1	unknown	0	-
DLM016_scaffold6553_2	unknown	0	-
DLM016_scaffold53043_5	unknown	0	-
DLM016_scaffold6713_8	unknown	0	-
DLM016_scaffold6713_12	unknown	0	-
DLM016_scaffold6553_4	unknown	0	-
DLM017_scaffold1892_10	unknown	0	-
DLM018_scaffold44037_1	unknown	0	-
DLM018_scaffold1207_3	unknown	0	-
DLM018_scaffold79_28	unknown	0	-
DLM019_scaffold44099_4	unknown	0	-
DLM019_scaffold10836_2	unknown	0	-
DLM019_scaffold2743_2	unknown	0	-
DLM019_scaffold35325_15	unknown	0	-
DLM019_scaffold100_11	unknown	0	-
DLM020_scaffold15999_4	unknown	0	-
DLM020_scaffold350_1	unknown	0	-
DLM020_scaffold3936_1	unknown	0	-
DLM020_scaffold6936_3	unknown	0	-
DLM021_scaffold43126_4	unknown	0	-
DLM021_scaffold42967_2	unknown	0	-

DLM021_scaffold10545_9	unknown	0	-
DLM021_scaffold136_1	unknown	0	-
DLM022_scaffold90423_1	unknown	0	-
DLM022_scaffold25768_2	unknown	0	-
DLM022_scaffold88974_2	unknown	0	-
DLM022_scaffold37828_6	unknown	0	-
DLM022_scaffold79462_2	unknown	0	-
DLM022_scaffold20053_1	unknown	0	-
DLM022_scaffold11050_1	unknown	0	-
DLM022_scaffold47815_3	unknown	0	-
DLM022_scaffold11069_53	unknown	0	-
DLM022_scaffold80933_2	unknown	0	-
DLM022_scaffold21034_2	unknown	0	-
DLM022_scaffold88907_2	unknown	0	-
DLM022_scaffold24869_4	unknown	0	-
DLM022_scaffold27082_1	unknown	0	-
DLM023_scaffold1460_2	unknown	0	-
DLM023_scaffold13027_1	unknown	0	-
DLM024_scaffold4274_2	unknown	0	-
DLM024_scaffold1215_1	unknown	0	-
DLM024_scaffold6863_2	unknown	0	-
DLM024_scaffold3966_3	unknown	0	-
DLM027_scaffold25719_1	unknown	0	-
DLM027_scaffold10585_3	unknown	0	-
DLM027_scaffold23395_3	unknown	0	-
DLM028_scaffold35712_5	unknown	0	-
DLM028_scaffold37211_4	unknown	0	-
DLM028_C674591_1	unknown	0	-
DLM028_scaffold39849_2	unknown	0	-
DLM028_scaffold16640_1	unknown	0	-
NLM001_scaffold3386_6	Colwellia psychrerythraea	1	CRISPR
NLM001_scaffold44600_3	Parabacteroides distasonis	0.98	Predict
NLM001_scaffold38495_3	Colwellia psychrerythraea	0.75	Predict
NLM001_scaffold2478_4	Vibrio splendidus	0.83	Predict
NLM001_scaffold44595_2	Lactococcus lactis	0.83	Predict
NLM001_scaffold4227_2	Mycoplasma pulmonis	0.83	Predict
NLM001_scaffold7_9	Colwellia psychrerythraea	0.8	Predict
NLM001_scaffold43773_1	Colwellia psychrerythraea	0.96	Predict
NLM001_scaffold10910_7	Bacillus anthracis	1	CRISPR
NLM001_scaffold41698_8	Colwellia psychrerythraea	0.76	Predict
NLM001_scaffold5105_1	Colwellia psychrerythraea	0.89	Predict
NLM001_scaffold41377_1	Colwellia psychrerythraea	0.76	Predict
NLM001_scaffold43435_4	Aeromonas media	0.86	Predict
NLM001_scaffold31705_8	Colwellia psychrerythraea	0.87	Predict
NLM001_scaffold5105_4	Colwellia psychrerythraea	0.94	Predict
NLM001_scaffold2_5	Bacteroides fragilis	0.77	Predict
NLM001_scaffold44595_1	Colwellia psychrerythraea	1	CRISPR
NLM001_scaffold17693_25	Colwellia psychrerythraea	0.82	Predict

NLM001_scaffold44484_1	Colwellia psychrerythraea	1	CRISPR
NLM001_scaffold40612_7	Bacteroides fragilis	0.85	Predict
NLM002_scaffold13648_17	Colwellia psychrerythraea	1	CRISPR
NLM002_scaffold28424_2	Colwellia psychrerythraea	0.79	Predict
NLM002_scaffold15649_2	Colwellia psychrerythraea	1	CRISPR
NLM002_scaffold8112_4	Parabacteroides distasonis	0.89	Predict
NLM002_scaffold28005_2	Colwellia psychrerythraea	0.97	Predict
NLM002_scaffold13407_12	Colwellia psychrerythraea	0.78	Predict
NLM002_scaffold28119_5	Prevotella stercorea	1	CRISPR
NLM002_C361292_1	Colwellia psychrerythraea	0.89	Predict
NLM002_scaffold8657_21	Colwellia psychrerythraea	0.89	Predict
NLM002_scaffold23710_2	Colwellia psychrerythraea	0.74	Predict
NLM002_scaffold16029_2	Colwellia psychrerythraea	1	CRISPR
NLM003_scaffold10761_3	Colwellia psychrerythraea	0.96	Predict
NLM003_scaffold10922_3	Bacteroides fragilis	1	CRISPR
NLM003_scaffold261_3	Cellulophaga baltica	0.75	Predict
NLM003_scaffold977_2	Klebsiella pneumoniae	1	CRISPR
NLM003_scaffold14728_2	Bacteroides fragilis	0.81	Predict
NLM003_scaffold3763_2_2	Colwellia psychrerythraea	0.79	Predict
NLM003_scaffold189_8_1	Bacteroides fragilis	0.75	Predict
NLM003_scaffold4295_1	Colwellia psychrerythraea	0.8	Predict
NLM004_scaffold44166_11	Vibrio splendidus	1	CRISPR
NLM004_scaffold27516_1	Colwellia psychrerythraea	0.73	Predict
NLM004_scaffold815_2	Erysipelothrix rhusiopathiae	0.82	Predict
NLM004_scaffold54737_2	Colwellia psychrerythraea	0.94	Predict
NLM004_scaffold285_3	Colwellia psychrerythraea	0.73	Predict
NLM004_scaffold14733_2	Colwellia psychrerythraea	1	CRISPR
NLM004_scaffold3539_11	Colwellia psychrerythraea	0.92	Predict
NLM004_scaffold46877_2	Mycoplasma pulmonis	0.84	Predict
NLM004_scaffold217_2	Mycoplasma pulmonis	0.87	Predict
NLM004_scaffold18101_4	Colwellia psychrerythraea	0.72	Predict
NLM004_scaffold35292_1	Colwellia psychrerythraea	0.72	Predict
NLM004_scaffold13021_3	Sinorhizobium meliloti	0.89	Predict
NLM004_scaffold14042_13	Clostridioides difficile	0.9	Predict
NLM004_scaffold49362_1	Colwellia psychrerythraea	0.92	Predict
NLM004_scaffold9767_3	Colwellia psychrerythraea	0.91	Predict
NLM004_scaffold53545_1	Colwellia psychrerythraea	0.94	Predict
NLM004_scaffold20206_2	Aliivibrio fischeri	0.73	Predict
NLM004_scaffold27473_2	Colwellia psychrerythraea	0.83	Predict
NLM004_scaffold54819_1	Streptococcus parauberis	1	CRISPR
NLM004_scaffold3052_12	Streptococcus salivarius	1	CRISPR
NLM004_scaffold9622_3	Colwellia psychrerythraea	0.88	Predict
NLM005_scaffold7084_1	Bacteroides fragilis	0.95	Predict
NLM005_scaffold30374_9	Colwellia psychrerythraea	1	CRISPR
NLM005_scaffold32010_3	Colwellia psychrerythraea	1	Predict
NLM005_scaffold564_3	Bacteroides fragilis	0.99	Predict
NLM005_scaffold21805_1	Flavobacterium columnare	0.73	Predict
NLM005_scaffold7_1	Colwellia psychrerythraea	0.84	Predict

NLM005_scaffold5172_2	Bacteroides fragilis	0.77	Predict
NLM005_scaffold90_2	Bacteroides fragilis	0.93	Predict
NLM005_scaffold5999_10	Flavobacterium columnare	0.98	Predict
NLM005_scaffold11370_1	Colwellia psychrerythraea	0.91	Predict
NLM005_scaffold4896_1	Colwellia psychrerythraea	0.84	Predict
NLM005_scaffold16081_2	Colwellia psychrerythraea	1	CRISPR
NLM006_scaffold18020_1	Colwellia psychrerythraea	0.87	Predict
NLM006_scaffold30822_2	Parabacteroides distasonis	1	CRISPR
NLM006_scaffold2215_2	Salmonella enterica	1	CRISPR
NLM006_scaffold50674_1	Parabacteroides merdae	0.85	Predict
NLM006_scaffold32497_3	Colwellia psychrerythraea	0.8	Predict
NLM006_scaffold21580_3	Mycoplasma pulmonis	0.76	Predict
NLM006_scaffold43376_2	Colwellia psychrerythraea	0.83	Predict
NLM006_scaffold44414_1	Colwellia psychrerythraea	0.73	Predict
NLM006_scaffold37475_1	Clostridioides difficile	1	CRISPR
NLM006_scaffold47321_2	Colwellia psychrerythraea	0.91	Predict
NLM006_scaffold31922_8	Colwellia psychrerythraea	1	CRISPR
NLM006_scaffold43039_5	Bacteroides sp. 3_1_40A	1	CRISPR
NLM006_scaffold42358_4	Colwellia psychrerythraea	0.79	Predict
NLM006_scaffold6162_11	Hungatella hathewayi	1	CRISPR
NLM006_scaffold50021_2	Mycoplasma pulmonis	1	CRISPR
NLM006_scaffold35930_2	Colwellia psychrerythraea	1	CRISPR
NLM006_scaffold52859_1	Mycoplasma pulmonis	0.83	Predict
NLM006_scaffold36213_1	Colwellia psychrerythraea	0.9	Predict
NLM006_scaffold24429_1	Colwellia psychrerythraea	0.94	Predict
NLM006_scaffold51349_1	Colwellia psychrerythraea	1	CRISPR
NLM006_scaffold53007_3	Brevibacillus laterosporus	1	CRISPR
NLM006_scaffold22752_1	Bacteroides fragilis	1	CRISPR
NLM006_scaffold1105_10	Colwellia psychrerythraea	0.74	Predict
NLM006_scaffold21365_1	Colwellia psychrerythraea	0.88	Predict
NLM006_scaffold33813_8	Bacteroides fragilis	1	CRISPR
NLM006_scaffold26489_1	Colwellia psychrerythraea	0.96	Predict
NLM006_scaffold53154_3	Colwellia psychrerythraea	0.87	Predict
NLM006_scaffold11951_6	Colwellia psychrerythraea	0.95	Predict
NLM006_scaffold46436_2	Acinetobacter johnsonii	0.72	Predict
NLM006_scaffold4801_4	Bacteroides fragilis	0.79	Predict
NLM006_scaffold42358_1	Parabacteroides distasonis	1	CRISPR
NLM006_C734286_1	Colwellia psychrerythraea	1	CRISPR
NLM006_scaffold30260_1	Colwellia psychrerythraea	0.92	Predict
NLM006_C733934_1	Bacteroides fragilis	0.86	Predict
NLM006_scaffold37878_1	Colwellia psychrerythraea	0.72	Predict
NLM006_scaffold38154_1	Colwellia psychrerythraea	0.88	Predict
NLM006_scaffold18470_3	Cellulophaga baltica	1	CRISPR
NLM006_scaffold29091_1	Mycoplasma pulmonis	1	CRISPR
NLM006_scaffold177_4	Streptococcus parauberis	0.89	Predict
NLM006_scaffold53101_1	Colwellia psychrerythraea	0.93	Predict
NLM006_scaffold49011_1	Colwellia psychrerythraea	0.93	Predict
NLM006_scaffold12258_5	Streptococcus thermophilus	0.72	Predict

NLM006_scaffold37878_15	Citrobacter rodentium	0.98	Predict
NLM006_scaffold14681_1	Colwellia psychrerythraea	0.94	Predict
NLM006_scaffold12925_3	Colwellia psychrerythraea	0.92	Predict
NLM006_scaffold43095_1	Colwellia psychrerythraea	1	CRISPR
NLM006_scaffold17752_2	Mycoplasma pulmonis	0.99	Predict
NLM006_scaffold4407_2	Lactobacillus plantarum	0.85	Predict
NLM006_scaffold10094_3	Colwellia psychrerythraea	0.87	Predict
NLM006_scaffold1374_1	Colwellia psychrerythraea	0.79	Predict
NLM006_scaffold31193_1	Colwellia psychrerythraea	1	CRISPR
NLM006_scaffold16845_12	Mycoplasma pulmonis	0.81	Predict
NLM006_scaffold28837_2	Bacteroides fragilis	0.87	Predict
NLM006_scaffold22733_1	Bacteroides coprophilus	1	CRISPR
NLM006_C733830_1	Bacillus anthracis	0.77	Predict
NLM006_C734606_1	Colwellia psychrerythraea	0.74	Predict
NLM006_C734306_1	Cellulophaga baltica	0.78	Predict
NLM006_scaffold8324_9	Colwellia psychrerythraea	1	CRISPR
NLM006_scaffold9879_4	Colwellia psychrerythraea	1	CRISPR
NLM007_scaffold10749_4	Lachnospiraceae bacterium	1	CRISPR
NLM007_scaffold1551_2	Colwellia psychrerythraea	0.82	Predict
NLM007_scaffold25115_1	Colwellia psychrerythraea	0.83	Predict
NLM007_scaffold7202_1	Mycoplasma pulmonis	0.99	Predict
NLM007_scaffold5980_3	Mycoplasma pulmonis	0.97	Predict
NLM007_scaffold20486_1	Mycoplasma pulmonis	0.8	Predict
NLM007_scaffold17969_11	Colwellia psychrerythraea	0.87	Predict
NLM007_scaffold6647_10	Colwellia psychrerythraea	0.7	Predict
NLM007_scaffold19932_1	Colwellia psychrerythraea	0.99	Predict
NLM007_scaffold26391_1	Colwellia psychrerythraea	0.7	Predict
NLM007_scaffold2633_3	Roseobacter denitrificans	0.76	Predict
NLM007_scaffold26057_1	Colwellia psychrerythraea	0.74	Predict
NLM007_scaffold25900_1	Colwellia psychrerythraea	0.89	Predict
NLM007_scaffold20356_8	Colwellia psychrerythraea	0.79	Predict
NLM007_scaffold6392_8	Colwellia psychrerythraea	0.88	Predict
NLM007_scaffold10461_1	Colwellia psychrerythraea	0.73	Predict
NLM007_scaffold3232_7	Colwellia psychrerythraea	0.96	Predict
NLM007_scaffold22550_11	Mycoplasma pulmonis	0.81	Predict
NLM007_scaffold26394_2	Colwellia psychrerythraea	0.95	Predict
NLM007_scaffold10749_5	Colwellia psychrerythraea	1	CRISPR
NLM007_scaffold23234_1	Bacillus cereus	0.87	Predict
NLM007_scaffold10749_8	Colwellia psychrerythraea	0.95	Predict
NLM007_scaffold18350_4	Parabacteroides merdae	1	CRISPR
NLM007_scaffold21467_1	Colwellia psychrerythraea	0.9	Predict
NLM007_scaffold4022_7	Bacillus anthracis	0.85	Predict
NLM007_scaffold26378_1	Colwellia psychrerythraea	0.77	Predict
NLM007_scaffold26389_1	Colwellia psychrerythraea	0.77	Predict
NLM007_scaffold723_5	Streptococcus pneumoniae	0.85	Predict
NLM008_scaffold50164_2	Bacteroides fragilis	0.96	Predict
NLM008_scaffold12934_1	Colwellia psychrerythraea	0.71	Predict
NLM008_scaffold37575_7	Colwellia psychrerythraea	1	CRISPR

NLM008_scaffold37289_2	Colwellia psychrerythraea	0.77	Predict
NLM008_scaffold45531_1	Colwellia psychrerythraea	0.81	Predict
NLM008_scaffold22745_5	Colwellia psychrerythraea	0.75	Predict
NLM008_scaffold50138_1	Bdellovibrio bacteriovorus	0.78	Predict
NLM008_scaffold18128_9	Colwellia psychrerythraea	0.7	Predict
NLM008_scaffold49769_2	Colwellia psychrerythraea	0.74	Predict
NLM008_scaffold41985_1	Mycoplasma pulmonis	0.71	Predict
NLM008_scaffold37575_10	Clostridium tetani	1	CRISPR
NLM008_scaffold711_3	Colwellia psychrerythraea	0.9	Predict
NLM008_scaffold2753_1	Bacteroides vulgatus	1	CRISPR
NLM008_scaffold30532_8	Colwellia psychrerythraea	0.7	Predict
NLM008_scaffold2753_6	Colwellia psychrerythraea	0.71	Predict
NLM008_scaffold16329_1	Colwellia psychrerythraea	1	CRISPR
NLM008_scaffold3829_2	Colwellia psychrerythraea	0.77	Predict
NLM009_scaffold13480_1	Colwellia psychrerythraea	0.82	Predict
NLM009_scaffold8447_2	Colwellia psychrerythraea	0.78	Predict
NLM009_scaffold5257_2	Colwellia psychrerythraea	0.88	Predict
NLM009_C228882_1	Colwellia psychrerythraea	0.98	Predict
NLM010_scaffold39134_1	Colwellia psychrerythraea	0.96	Predict
NLM010_scaffold3556_1	Bacteroides fragilis	0.73	Predict
NLM010_scaffold2302_1	Achromobacter xylosoxidans	0.96	Predict
NLM010_scaffold1115_4	Colwellia psychrerythraea	1	CRISPR
NLM010_scaffold36636_1	Colwellia psychrerythraea	0.8	Predict
NLM010_scaffold23407_1	Cellulophaga baltica	1	CRISPR
NLM010_scaffold40193_1	Colwellia psychrerythraea	1	CRISPR
NLM010_scaffold18261_4	Colwellia psychrerythraea	0.93	Predict
NLM010_scaffold40145_2	Pasteurella multocida	1	CRISPR
NLM010_scaffold1890_2	Colwellia psychrerythraea	0.75	Predict
NLM010_scaffold24350_1	Colwellia psychrerythraea	0.91	Predict
NLM010_scaffold15684_10	Colwellia psychrerythraea	0.89	Predict
NLM010_scaffold13431_2	Colwellia psychrerythraea	0.74	Predict
NLM010_scaffold3952_5	Colwellia psychrerythraea	0.75	Predict
NLM010_scaffold894_1	Bacteroides fragilis	1	CRISPR
NLM010_scaffold9073_7	Clostridium botulinum	0.9	Predict
NLM010_scaffold2086_2	Mycoplasma pulmonis	0.89	Predict
NLM010_scaffold21070_2	Colwellia psychrerythraea	1	CRISPR
NLM010_scaffold32176_1	Colwellia psychrerythraea	0.71	Predict
NLM010_scaffold705_15	Colwellia psychrerythraea	0.83	Predict
NLM010_scaffold16315_3	Colwellia psychrerythraea	0.73	Predict
NLM010_scaffold39351_1	Colwellia psychrerythraea	1	CRISPR
NLM010_scaffold16684_4	Colwellia psychrerythraea	0.75	Predict
NLM010_scaffold4037_2	Actinomyces naeslundii	1	CRISPR
NLM010_scaffold10296_6	Colwellia psychrerythraea	0.94	Predict
NLM010_scaffold39268_1	Colwellia psychrerythraea	1	CRISPR
NLM010_scaffold17621_3	Aggregatibacter actinomycetemcomit	1	CRISPR
NLM010_scaffold2685_7	Colwellia psychrerythraea	0.98	Predict
NLM010_scaffold34399_5	Colwellia psychrerythraea	0.84	Predict
NLM010_scaffold10324_3	Colwellia psychrerythraea	0.77	Predict

NLM010_scaffold20986_1	Colwellia psychrerythraea	1	CRISPR
NLM010_scaffold10296_4	Colwellia psychrerythraea	0.76	Predict
NLM010_scaffold8314_1	Colwellia psychrerythraea	0.78	Predict
NLM010_scaffold15390_2	Bacteroides fragilis	1	CRISPR
NLM010_scaffold2612_4	Bacteroides fragilis	0.85	Predict
NLM010_scaffold20986_2	Colwellia psychrerythraea	0.86	Predict
NLM010_scaffold9737_35	Colwellia psychrerythraea	0.78	Predict
NLM010_scaffold28345_8	Colwellia psychrerythraea	1	CRISPR
NLM010_scaffold26505_1	Colwellia psychrerythraea	0.9	Predict
NLM010_scaffold3952_4	Flavobacterium psychrophilum	1	CRISPR
NLM010_scaffold3490_4	Parabacteroides distasonis	0.97	Predict
NLM015_scaffold34970_4	Colwellia psychrerythraea	0.85	Predict
NLM015_scaffold45_3	Colwellia psychrerythraea	0.9	Predict
NLM015_scaffold9944_1	Parabacteroides distasonis	0.9	Predict
NLM015_scaffold38949_1	Bacteroides fragilis	0.86	Predict
NLM015_scaffold38424_1	Colwellia psychrerythraea	1	CRISPR
NLM015_scaffold2372_1	Parabacteroides distasonis	1	CRISPR
NLM015_scaffold6813_1	Colwellia psychrerythraea	1	CRISPR
NLM015_scaffold7231_4	Azospirillum brasilense	0.91	Predict
NLM015_scaffold16659_2	Colwellia psychrerythraea	1	CRISPR
NLM015_scaffold10852_4	Colwellia psychrerythraea	0.73	Predict
NLM015_scaffold4740_1	Parabacteroides merdae	0.92	Predict
NLM015_scaffold1856_2	Colwellia psychrerythraea	1	CRISPR
NLM015_scaffold77_4	Colwellia psychrerythraea	0.76	Predict
NLM015_scaffold25776_2	Colwellia psychrerythraea	0.9	Predict
NLM015_scaffold21932_1	Colwellia psychrerythraea	0.9	Predict
NLM015_scaffold25867_2	Colwellia psychrerythraea	0.87	Predict
NLM015_scaffold10852_5	Colwellia psychrerythraea	0.81	Predict
NLM015_scaffold15418_1	Bacillus megaterium	0.79	Predict
NLM015_scaffold5831_6	Colwellia psychrerythraea	0.86	Predict
NLM015_scaffold104_1	Colwellia psychrerythraea	0.94	Predict
NLM016_scaffold3058_1	Colwellia psychrerythraea	0.92	Predict
NLM016_scaffold10481_1	Mycoplasma pulmonis	1	CRISPR
NLM016_scaffold19074_1	Colwellia psychrerythraea	1	CRISPR
NLM016_scaffold12933_1	Colwellia psychrerythraea	1	CRISPR
NLM016_scaffold11175_2	Colwellia psychrerythraea	1	CRISPR
NLM016_scaffold112_1	Bacteroides fragilis	0.8	Predict
NLM016_scaffold87_4	Colwellia psychrerythraea	0.76	Predict
NLM016_scaffold3275_3	Bacteroides fragilis	0.75	Predict
NLM016_scaffold11179_1	Colwellia psychrerythraea	0.76	Predict
NLM016_scaffold16587_2	Colwellia psychrerythraea	0.83	Predict
NLM016_scaffold19042_3	Prevotella copri	1	CRISPR
NLM016_scaffold16925_3	Prevotella copri	1	CRISPR
NLM017_scaffold416_2	Bacteroides fragilis	1	CRISPR
NLM017_scaffold14858_4	Colwellia psychrerythraea	0.92	Predict
NLM017_scaffold20307_2	Colwellia psychrerythraea	0.76	Predict
NLM017_scaffold27209_14	Colwellia psychrerythraea	0.73	Predict
NLM017_scaffold38835_1	Colwellia psychrerythraea	0.88	Predict

NLM017_scaffold38788_3	Colwellia psychrerythraea	0.71	Predict
NLM017_scaffold32055_2	Mycoplasma pulmonis	0.74	Predict
NLM017_scaffold8358_18	Ruminococcus bromii	1	CRISPR
NLM017_scaffold9889_3	Colwellia psychrerythraea	0.74	Predict
NLM017_scaffold20627_4	Colwellia psychrerythraea	0.73	Predict
NLM017_scaffold38145_1	Colwellia psychrerythraea	0.81	Predict
NLM017_scaffold14095_1	Colwellia psychrerythraea	0.9	Predict
NLM017_scaffold38914_3	Flavobacterium columnare	0.77	Predict
NLM017_scaffold39062_2	Colwellia psychrerythraea	0.88	Predict
NLM017_scaffold8358_21	Colwellia psychrerythraea	1	CRISPR
NLM017_scaffold35881_2	Colwellia psychrerythraea	0.99	Predict
NLM017_scaffold17839_9	Bacteroides fragilis	0.73	Predict
NLM017_scaffold24164_1	Colwellia psychrerythraea	0.83	Predict
NLM017_scaffold25009_1	Colwellia psychrerythraea	0.87	Predict
NLM017_scaffold12885_45	Parabacteroides distasonis	0.96	Predict
NLM017_scaffold24740_2	Colwellia psychrerythraea	0.91	Predict
NLM017_scaffold7452_3	Colwellia psychrerythraea	0.95	Predict
NLM017_scaffold31444_1	Colwellia psychrerythraea	0.78	Predict
NLM017_scaffold8358_65	Colwellia psychrerythraea	0.85	Predict
NLM017_scaffold38969_2	Colwellia psychrerythraea	0.71	Predict
NLM017_scaffold23004_11	Bacteroides fragilis	0.89	Predict
NLM017_scaffold174_1	Bacteroides fragilis	1	CRISPR
NLM017_scaffold38914_2	Flavobacterium columnare	0.89	Predict
NLM021_scaffold5563_6	Colwellia psychrerythraea	0.94	Predict
NLM021_scaffold562_2	Bacteroides fragilis	0.73	Predict
NLM021_scaffold42938_1	Colwellia psychrerythraea	0.94	Predict
NLM021_scaffold61286_1	Colwellia psychrerythraea	0.78	Predict
NLM021_scaffold58942_1	Colwellia psychrerythraea	1	CRISPR
NLM021_scaffold61418_1	Mycoplasma pulmonis	0.98	Predict
NLM021_scaffold5557_1	Colwellia psychrerythraea	0.88	Predict
NLM021_scaffold19029_4	Colwellia psychrerythraea	0.98	Predict
NLM021_scaffold53158_5	Colwellia psychrerythraea	0.89	Predict
NLM021_scaffold59003_3	Ruminococcus sp. OM05-7	1	CRISPR
NLM021_scaffold61366_2	Bacillus alcalophilus	0.75	Predict
NLM021_scaffold55035_3	Bacillus megaterium	0.91	Predict
NLM021_scaffold19029_1	Parabacteroides distasonis	0.72	Predict
NLM021_scaffold143_2	Colwellia psychrerythraea	0.89	Predict
NLM021_scaffold42928_1	Colwellia psychrerythraea	1	CRISPR
NLM021_scaffold61042_4	Colwellia psychrerythraea	0.76	Predict
NLM021_scaffold29192_2	Colwellia psychrerythraea	0.9	Predict
NLM021_scaffold22069_5	Colwellia psychrerythraea	0.92	Predict
NLM021_scaffold11314_5	Colwellia psychrerythraea	0.9	Predict
NLM021_scaffold23306_4	Colwellia psychrerythraea	0.94	Predict
NLM021_scaffold60054_1	Colwellia psychrerythraea	1	CRISPR
NLM021_scaffold190_8	Colwellia psychrerythraea	0.93	Predict
NLM021_scaffold15002_14	Colwellia psychrerythraea	0.76	Predict
NLM021_scaffold55417_2	Colwellia psychrerythraea	1	CRISPR
NLM021_scaffold14659_12	Colwellia psychrerythraea	0.7	Predict

NLM021_scaffold60255_1	Bacteroides fragilis	0.83	Predict
NLM021_scaffold26861_2	Colwellia psychrerythraea	0.95	Predict
NLM021_scaffold709_5	Geobacillus kaustophilus	0.94	Predict
NLM021_C812913_1	Colwellia psychrerythraea	0.81	Predict
NLM021_scaffold61257_1	Bacteroides fragilis	0.97	Predict
NLM021_scaffold46784_3	Colwellia psychrerythraea	0.85	Predict
NLM021_scaffold53021_4	Colwellia psychrerythraea	0.95	Predict
NLM021_scaffold58236_5	Lactobacillus gasseri	1	CRISPR
NLM021_scaffold46181_6	Colwellia psychrerythraea	1	CRISPR
NLM021_scaffold38431_7	Colwellia psychrerythraea	0.84	Predict
NLM021_scaffold190_7	Clostridium botulinum	0.81	Predict
NLM021_scaffold14592_1	Colwellia psychrerythraea	0.93	Predict
NLM021_scaffold49743_1	Ralstonia solanacearum	0.86	Predict
NLM021_C812807_1	Colwellia psychrerythraea	0.8	Predict
NLM021_scaffold61366_1	Colwellia psychrerythraea	0.94	Predict
NLM021_scaffold55417_5	Streptococcus pneumoniae	1	CRISPR
NLM021_scaffold45854_2	Colwellia psychrerythraea	1	CRISPR
NLM022_scaffold3577_22	Colwellia psychrerythraea	0.85	Predict
NLM022_scaffold5329_6	Colwellia psychrerythraea	0.81	Predict
NLM022_scaffold28217_1	Clostridium perfringens	1	CRISPR
NLM022_scaffold9956_5	Colwellia psychrerythraea	0.71	Predict
NLM022_scaffold17073_1	Mycoplasma pulmonis	0.74	Predict
NLM022_scaffold42567_1	Colwellia psychrerythraea	1	CRISPR
NLM022_scaffold30338_1	Colwellia psychrerythraea	1	CRISPR
NLM022_scaffold3888_1	Mycoplasma pulmonis	1	CRISPR
NLM022_scaffold16192_1	Colwellia psychrerythraea	0.87	Predict
NLM022_scaffold38630_1	Colwellia psychrerythraea	0.77	Predict
NLM022_scaffold42820_1	Colwellia psychrerythraea	0.74	Predict
NLM022_scaffold44402_1	Clostridium perfringens	0.9	Predict
NLM022_scaffold20998_1	Listeria monocytogenes	1	CRISPR
NLM022_scaffold42750_2	Clostridioides difficile	1	CRISPR
NLM022_scaffold7821_4	Colwellia psychrerythraea	0.76	Predict
NLM022_scaffold38946_1	Colwellia psychrerythraea	0.87	Predict
NLM022_scaffold7162_1	Colwellia psychrerythraea	1	CRISPR
NLM022_C640276_1	Colwellia psychrerythraea	0.94	Predict
NLM022_scaffold15285_3	Roseobacter denitrificans	0.71	Predict
NLM022_scaffold28148_1	Staphylococcus saprophyticus	1	CRISPR
NLM022_scaffold9181_1	Colwellia psychrerythraea	0.83	Predict
NLM022_scaffold35932_1	Colwellia psychrerythraea	0.81	Predict
NLM022_C641598_1	Colwellia psychrerythraea	0.91	Predict
NLM022_scaffold43025_3	Colwellia psychrerythraea	0.98	Predict
NLM022_scaffold15285_1	Paenibacillus larvae	1	CRISPR
NLM022_scaffold5329_1	Colwellia psychrerythraea	0.95	Predict
NLM022_scaffold24404_3	Colwellia psychrerythraea	0.86	Predict
NLM022_scaffold37593_2	Colwellia psychrerythraea	1	CRISPR
NLM022_scaffold17804_1	Colwellia psychrerythraea	0.98	Predict
NLM022_scaffold35358_2	Colwellia psychrerythraea	0.72	Predict
NLM022_scaffold11265_9	Colwellia psychrerythraea	1	CRISPR

NLM022_C640494_1	Bacteroides fragilis	0.76	Predict
NLM023_scaffold23138_8	Colwellia psychrerythraea	0.77	Predict
NLM023_scaffold1402_1	Colwellia psychrerythraea	0.83	Predict
NLM023_scaffold23260_2	Flavobacterium columnare	0.72	Predict
NLM023_scaffold470_13	Parabacteroides merdae	1	CRISPR
NLM023_scaffold28479_8	Colwellia psychrerythraea	0.81	Predict
NLM023_scaffold23260_3	Mycoplasma pulmonis	0.89	Predict
NLM023_scaffold21242_4	Colwellia psychrerythraea	1	CRISPR
NLM023_scaffold48941_1	Parabacteroides distasonis	1	CRISPR
NLM023_scaffold48169_1	Colwellia psychrerythraea	0.94	Predict
NLM023_C806243_1	Parabacteroides distasonis	0.74	Predict
NLM023_scaffold28479_12	Colwellia psychrerythraea	0.87	Predict
NLM023_scaffold11383_1	Parabacteroides distasonis	1	CRISPR
NLM023_scaffold911_13	Colwellia psychrerythraea	0.9	Predict
NLM023_scaffold8508_4	Colwellia psychrerythraea	0.86	Predict
NLM023_scaffold48276_1	Colwellia psychrerythraea	0.72	Predict
NLM023_scaffold43102_12	Colwellia psychrerythraea	0.74	Predict
NLM023_scaffold1348_25	Colwellia psychrerythraea	0.84	Predict
NLM023_scaffold37217_1	Colwellia psychrerythraea	0.92	Predict
NLM023_scaffold399_47	Kitasatospora aureofaciens	0.75	Predict
NLM023_scaffold260_1	Bacteroides fragilis	0.89	Predict
NLM023_scaffold253_1	Colwellia psychrerythraea	0.99	Predict
NLM023_scaffold10723_15	Colwellia psychrerythraea	0.71	Predict
NLM023_scaffold4055_10	Colwellia psychrerythraea	0.77	Predict
NLM023_scaffold1428_18	Roseobacter denitrificans	0.86	Predict
NLM023_scaffold14181_20	Colwellia psychrerythraea	0.83	Predict
NLM023_scaffold48506_1	Colwellia psychrerythraea	1	CRISPR
NLM023_scaffold33532_1	Colwellia psychrerythraea	0.89	Predict
NLM023_scaffold35604_3	Colwellia psychrerythraea	0.73	Predict
NLM023_scaffold46734_3	Colwellia psychrerythraea	0.98	Predict
NLM023_scaffold22_6	Colwellia psychrerythraea	0.81	Predict
NLM023_scaffold12109_12	Roseobacter denitrificans	0.73	Predict
NLM023_scaffold34755_1	Colwellia psychrerythraea	0.97	Predict
NLM023_scaffold48896_1	Colwellia psychrerythraea	0.95	Predict
NLM024_scaffold488_1	Bacteroides fragilis	0.75	Predict
NLM024_scaffold6119_1	Colwellia psychrerythraea	0.86	Predict
NLM024_scaffold10225_2	Sinorhizobium meliloti	0.93	Predict
NLM024_scaffold20170_1	Bacteroides fragilis	0.89	Predict
NLM024_scaffold5090_1	Colwellia psychrerythraea	1	CRISPR
NLM024_scaffold18150_1	Clostridioides difficile	0.77	Predict
NLM024_scaffold13470_1	Colwellia psychrerythraea	1	CRISPR
NLM024_scaffold14981_1	Klebsiella pneumoniae	1	CRISPR
NLM024_scaffold15487_3	Parabacteroides distasonis	0.84	Predict
NLM024_scaffold77_6	Colwellia psychrerythraea	0.9	Predict
NLM024_scaffold1371_3	Colwellia psychrerythraea	1	CRISPR
NLM024_scaffold244_5	Colwellia psychrerythraea	0.71	Predict
NLM024_scaffold9211_2	Colwellia psychrerythraea	0.96	Predict
NLM024_scaffold100_1	Colwellia psychrerythraea	0.71	Predict

NLM024_scaffold957_14	Colwellia psychrerythraea	0.93	Predict
NLM024_scaffold16628_8	Bacteroides fragilis	0.91	Predict
NLM024_scaffold11939_1	Mycoplasma pulmonis	1	CRISPR
NLM024_scaffold14981_3	Colwellia psychrerythraea	0.93	Predict
NLM025_C502105_1	Colwellia psychrerythraea	0.87	Predict
NLM025_scaffold4345_2	Parabacteroides merdae	0.72	Predict
NLM025_scaffold22167_3	Colwellia psychrerythraea	0.78	Predict
NLM025_scaffold39287_1	Colwellia psychrerythraea	0.9	Predict
NLM025_scaffold18010_7	Colwellia psychrerythraea	0.76	Predict
NLM025_scaffold39157_1	Colwellia psychrerythraea	0.93	Predict
NLM025_scaffold25589_3	Mycoplasma pulmonis	0.81	Predict
NLM025_scaffold35205_1	Colwellia psychrerythraea	0.93	Predict
NLM025_scaffold38901_1	Colwellia psychrerythraea	0.97	Predict
NLM025_scaffold12163_17	Colwellia psychrerythraea	1	CRISPR
NLM025_scaffold20016_2	Colwellia psychrerythraea	0.89	Predict
NLM025_scaffold29129_1	Colwellia psychrerythraea	0.83	Predict
NLM026_scaffold74538_1	Colwellia psychrerythraea	0.71	Predict
NLM026_scaffold29270_6	Colwellia psychrerythraea	0.96	Predict
NLM026_scaffold27749_19	Mycoplasma pulmonis	0.73	Predict
NLM026_scaffold62290_1	Colwellia psychrerythraea	0.74	Predict
NLM026_scaffold55998_10	Colwellia psychrerythraea	0.76	Predict
NLM026_scaffold57277_1	Mycoplasma pulmonis	0.71	Predict
NLM026_scaffold74568_1	Colwellia psychrerythraea	0.96	Predict
NLM026_scaffold75736_1	Colwellia psychrerythraea	0.72	Predict
NLM026_scaffold1771_11	Colwellia psychrerythraea	0.74	Predict
NLM026_scaffold258_3	Colwellia psychrerythraea	1	Predict
NLM026_scaffold75282_1	Flavobacterium columnare	0.71	Predict
NLM026_scaffold47323_1	Colwellia psychrerythraea	0.7	Predict
NLM026_scaffold65263_3	Colwellia psychrerythraea	0.88	Predict
NLM026_scaffold74983_1	Flavobacterium columnare	0.94	Predict
NLM026_scaffold58936_3	Colwellia psychrerythraea	0.88	Predict
NLM026_scaffold29289_10	Colwellia psychrerythraea	0.82	Predict
NLM026_scaffold21981_1	Colwellia psychrerythraea	0.86	Predict
NLM026_scaffold86_4	Actinomyces naeslundii	0.79	Predict
NLM026_scaffold26990_1	Colwellia psychrerythraea	0.79	Predict
NLM026_scaffold74825_1	Lactococcus lactis	0.73	Predict
NLM026_scaffold49770_2	Flavobacterium columnare	0.97	Predict
NLM026_scaffold75839_2	Colwellia psychrerythraea	0.88	Predict
NLM026_scaffold58959_4	Colwellia psychrerythraea	0.97	Predict
NLM026_scaffold74363_2	Colwellia psychrerythraea	0.96	Predict
NLM026_scaffold58139_6	Colwellia psychrerythraea	0.92	Predict
NLM026_scaffold258_2	Colwellia psychrerythraea	0.9	Predict
NLM026_scaffold75960_2	Colwellia psychrerythraea	0.98	Predict
NLM026_C924027_1	Bacillus anthracis	0.84	Predict
NLM026_scaffold9047_2	Thermoanaerobacterium saccharolytic	0.92	Predict
NLM026_scaffold6763_2	Colwellia psychrerythraea	0.84	Predict
NLM026_scaffold32844_2	Pantoea agglomerans	0.85	Predict
NLM026_scaffold13988_2	Mycoplasma pulmonis	0.72	Predict

NLM026_scaffold69595_2	Colwellia psychrerythraea	1	CRISPR
NLM026_scaffold63092_3	Colwellia psychrerythraea	0.96	Predict
NLM026_scaffold73101_2	Lactobacillus fermentum	0.8	Predict
NLM026_scaffold75674_2	Colwellia psychrerythraea	0.95	Predict
NLM026_scaffold15415_2	Mycoplasma pulmonis	0.99	Predict
NLM027_scaffold22040_4	Chlamydia pecorum	0.92	Predict
NLM027_scaffold40686_1	Colwellia psychrerythraea	0.81	Predict
NLM027_scaffold41411_1	Colwellia psychrerythraea	1	CRISPR
NLM027_scaffold41959_1	Colwellia psychrerythraea	1	CRISPR
NLM027_scaffold40329_1	Colwellia psychrerythraea	1	CRISPR
NLM027_scaffold35554_2	Colwellia psychrerythraea	1	CRISPR
NLM027_scaffold40329_3	Colwellia psychrerythraea	1	CRISPR
NLM027_scaffold39111_4	Streptococcus thermophilus	1	CRISPR
NLM027_scaffold35206_1	Colwellia psychrerythraea	1	CRISPR
NLM027_scaffold30138_3	Mycoplasma pulmonis	0.84	Predict
NLM027_scaffold14263_1	Mycoplasma pulmonis	1	CRISPR
NLM027_scaffold39770_2	Colwellia psychrerythraea	0.93	Predict
NLM027_scaffold16353_1	Colwellia psychrerythraea	0.87	Predict
NLM027_scaffold34797_1	Colwellia psychrerythraea	0.74	Predict
NLM027_scaffold8952_3	Colwellia psychrerythraea	0.81	Predict
NLM027_scaffold34798_1	Enterococcus faecium	1	CRISPR
NLM027_scaffold10002_11	Colwellia psychrerythraea	0.77	Predict
NLM027_scaffold17160_15	Enterococcus faecium	0.86	Predict
NLM027_scaffold8952_6	Colwellia psychrerythraea	0.81	Predict
NLM027_scaffold7180_15	Cellulophaga baltica	1	CRISPR
NLM027_scaffold1874_3	Colwellia psychrerythraea	1	CRISPR
NLM027_scaffold11645_1	Vibrio vulnificus	0.75	Predict
NLM027_scaffold16205_3	Clostridium sporogenes	0.9	Predict
NLM027_scaffold1886_1	Mycoplasma pulmonis	0.71	Predict
NLM027_scaffold40537_1	Mycoplasma pulmonis	0.84	Predict
NLM027_scaffold3310_5	Cronobacter sakazakii	1	CRISPR
NLM027_C668626_1	Colwellia psychrerythraea	0.93	Predict
NLM027_scaffold14181_3	Flavobacterium columnare	0.84	Predict
NLM027_scaffold34123_3	Colwellia psychrerythraea	1	CRISPR
NLM027_scaffold17357_1	Clostridioides difficile	0.88	Predict
NLM027_scaffold41891_1	Colwellia psychrerythraea	1	CRISPR
NLM027_scaffold40356_4	Clostridioides difficile	1	CRISPR
NLM027_scaffold35206_2	Colwellia psychrerythraea	0.86	Predict
NLM027_scaffold9793_1	Staphylococcus capitis	0.81	Predict
NLM027_scaffold20399_3	Colwellia psychrerythraea	0.71	Predict
NLM027_scaffold38196_4	Bacteroides fragilis	0.98	Predict
NLM028_scaffold28234_2	Colwellia psychrerythraea	0.83	Predict
NLM028_scaffold12174_3	Colwellia psychrerythraea	0.89	Predict
NLM028_scaffold54929_3	Colwellia psychrerythraea	0.94	Predict
NLM028_scaffold59756_2	Flavobacterium psychrophilum	1	CRISPR
NLM028_scaffold34514_1	Colwellia psychrerythraea	1	CRISPR
NLM028_scaffold59789_2	Bacteroides fragilis	0.75	Predict
NLM028_scaffold1532_1	Colwellia psychrerythraea	0.79	Predict

NLM028_scaffold24678_1	Colwellia psychrerythraea	1	CRISPR
NLM028_scaffold8768_2	Colwellia psychrerythraea	0.93	Predict
NLM028_scaffold34609_1	Colwellia psychrerythraea	1	CRISPR
NLM028_scaffold30720_1	Ruminococcus sp. OM05-7	1	CRISPR
NLM028_scaffold12341_5	Candidatus Hamiltonella defensa	1	CRISPR
NLM028_scaffold51949_2	Colwellia psychrerythraea	0.96	Predict
NLM028_scaffold20220_12	Clostridium perfringens	1	CRISPR
NLM028_scaffold45877_1	Bacillus megaterium	0.92	Predict
NLM028_scaffold26847_1	Colwellia psychrerythraea	0.88	Predict
NLM028_scaffold57798_4	Colwellia psychrerythraea	1	CRISPR
NLM028_scaffold15831_1	Bacteroides fragilis	0.71	Predict
NLM028_scaffold34525_2	Thermoanaerobacterium saccharolytic	1	CRISPR
NLM028_scaffold43687_1	Colwellia psychrerythraea	0.85	Predict
NLM028_scaffold20949_1	Colwellia psychrerythraea	0.98	Predict
NLM028_scaffold57543_3	Rhodovulum sp. P5	1	CRISPR
NLM028_scaffold59796_3	Parabacteroides distasonis	0.82	Predict
NLM028_scaffold57798_3	Colwellia psychrerythraea	0.8	Predict
NLM028_C652312_1	Colwellia psychrerythraea	0.83	Predict
NLM028_scaffold15831_2	Bacteroides fragilis	0.91	Predict
NLM028_scaffold42949_1	Bacteroides fragilis	1	CRISPR
NLM028_scaffold57543_2	Colwellia psychrerythraea	1	CRISPR
NLM028_scaffold37225_1	Colwellia psychrerythraea	0.85	Predict
NLM028_C652264_1	Clostridium perfringens	1	CRISPR
NLM028_scaffold13605_5	Colwellia psychrerythraea	0.92	Predict
NLM029_scaffold45341_4	Colwellia psychrerythraea	0.72	Predict
NLM029_scaffold33952_1	Bifidobacterium sp. MSTE12	1	CRISPR
NLM029_scaffold4810_3	Clostridium tetani	0.92	Predict
NLM029_C674039_1	Colwellia psychrerythraea	0.8	Predict
NLM029_scaffold2015_8	Colwellia psychrerythraea	1	CRISPR
NLM029_scaffold24024_8	Colwellia psychrerythraea	1	CRISPR
NLM029_scaffold9598_4	Colwellia psychrerythraea	0.84	Predict
NLM029_scaffold39928_1	Bacteroides fragilis	0.87	Predict
NLM029_scaffold2015_2	Colwellia psychrerythraea	1	CRISPR
NLM029_scaffold44423_10	Colwellia psychrerythraea	0.7	Predict
NLM029_scaffold3634_1	Candidatus Hamiltonella defensa	0.76	Predict
NLM029_scaffold37710_1	Colwellia psychrerythraea	1	CRISPR
NLM029_scaffold9598_2	Colwellia psychrerythraea	1	CRISPR
NLM029_scaffold24413_3	Roseobacter denitrificans	1	CRISPR
NLM029_scaffold7675_3	Colwellia psychrerythraea	1	CRISPR
NLM029_scaffold3860_9	Cellulophaga baltica	0.91	Predict
NLM029_scaffold3826_2	Colwellia psychrerythraea	1	CRISPR
NLM029_scaffold4592_2	Bacteroides fragilis	0.75	Predict
NLM029_C673977_1	Bacillus megaterium	0.9	Predict
NLM029_scaffold7768_1	Clostridioides difficile	0.96	Predict
NLM029_scaffold5035_2	Bacteroides fragilis	0.75	Predict
NLM029_scaffold45930_1	Colwellia psychrerythraea	0.85	Predict
NLM029_scaffold35112_1	Colwellia psychrerythraea	1	CRISPR
NLM029_scaffold4050_9	Colwellia psychrerythraea	1	CRISPR

NLM029_scaffold24413_4	Colwellia psychrerythraea	0.97	Predict
NLM029_scaffold9315_13	Roseobacter denitrificans	0.83	Predict
NLM029_scaffold46287_2	Flavobacterium columnare	0.84	Predict
NLM029_C674283_1	Colwellia psychrerythraea	0.93	Predict
NLM029_scaffold10574_3	Clostridioides difficile	1	CRISPR
NLM029_scaffold26243_3	Colwellia psychrerythraea	1	CRISPR
NLM029_scaffold33933_1	Mycoplasma pulmonis	0.78	Predict
NLM029_scaffold23685_2	Colwellia psychrerythraea	0.95	Predict
NLM029_scaffold8972_5	Colwellia psychrerythraea	1	CRISPR
NLM029_scaffold325_5	Colwellia psychrerythraea	0.84	Predict
NLM029_scaffold10325_2	Clostridium perfringens	1	CRISPR
NLM029_scaffold8116_24	Colwellia psychrerythraea	0.79	Predict
NLM029_scaffold20227_1	Colwellia psychrerythraea	0.77	Predict
NLM029_scaffold46752_4	Colwellia psychrerythraea	0.88	Predict
NLM031_scaffold75_4	Colwellia psychrerythraea	0.78	Predict
NLM031_scaffold75_1	Colwellia psychrerythraea	0.73	Predict
NLM031_scaffold59329_1	Bacillus megaterium	0.74	Predict
NLM031_scaffold21651_1	Paenibacillus larvae	1	CRISPR
NLM031_scaffold18436_4	Colwellia psychrerythraea	0.92	Predict
NLM031_scaffold45857_1	Bacteroides fragilis	0.81	Predict
NLM031_scaffold16685_1	Colwellia psychrerythraea	1	CRISPR
NLM031_scaffold23971_3	Colwellia psychrerythraea	0.84	Predict
NLM031_scaffold7206_4	Colwellia psychrerythraea	0.83	Predict
NLM031_scaffold27707_3	Colwellia psychrerythraea	0.92	Predict
NLM031_scaffold41310_1	Parabacteroides distasonis	0.75	Predict
NLM031_scaffold6532_6	Colwellia psychrerythraea	0.72	Predict
NLM031_scaffold1394_1	Colwellia psychrerythraea	1	CRISPR
NLM031_scaffold2847_6	Colwellia psychrerythraea	0.79	Predict
NLM031_scaffold49_2	Staphylococcus saprophyticus	0.91	Predict
NLM031_scaffold25711_6	Geobacillus kaustophilus	0.93	Predict
NLM031_scaffold18627_4	Colwellia psychrerythraea	1	CRISPR
NLM031_scaffold15300_11	Colwellia psychrerythraea	0.93	Predict
NLM031_scaffold259_8	Colwellia psychrerythraea	0.78	Predict
NLM031_scaffold36158_5	Colwellia psychrerythraea	1	CRISPR
NLM031_scaffold29823_1	Colwellia psychrerythraea	1	CRISPR
NLM031_scaffold2964_6	Colwellia psychrerythraea	1	CRISPR
NLM031_scaffold45482_5	Colwellia psychrerythraea	1	CRISPR
NLM031_scaffold107_1	Colwellia psychrerythraea	0.89	Predict
NLM031_scaffold1394_2	Colwellia psychrerythraea	0.89	Predict
NLM031_scaffold25711_7	Colwellia psychrerythraea	1	CRISPR
NLM031_scaffold23030_1	Parabacteroides distasonis	1	CRISPR
NLM031_scaffold18436_7	Colwellia psychrerythraea	0.78	Predict
NLM031_scaffold44148_4	Colwellia psychrerythraea	0.8	Predict
NLM032_scaffold68145_4	Colwellia psychrerythraea	0.96	Predict
NLM032_scaffold23012_2	Leuconostoc pseudomesenteroides	0.72	Predict
NLM032_scaffold61468_2	Colwellia psychrerythraea	0.84	Predict
NLM032_scaffold41399_1	Colwellia psychrerythraea	1	CRISPR
NLM032_scaffold58144_2	Colwellia psychrerythraea	0.99	Predict

NLM032_scaffold33461_2	Mycoplasma pulmonis	0.97	Predict
NLM032_scaffold23630_5	Colwellia psychrerythraea	1	Predict
NLM032_scaffold63260_2	Colwellia psychrerythraea	1	CRISPR
NLM032_scaffold40713_1	Mycoplasma pulmonis	0.92	Predict
NLM032_scaffold61468_1	Clostridium perfringens	0.97	Predict
NLM032_scaffold63260_1	Colwellia psychrerythraea	1	CRISPR
NLM032_scaffold69860_1	Clostridium botulinum	1	CRISPR
NLM032_scaffold10936_5	Colwellia psychrerythraea	0.88	Predict
NLM032_scaffold9843_17	Colwellia psychrerythraea	0.75	Predict
NLM032_scaffold37169_1	Colwellia psychrerythraea	0.92	Predict
NLM032_scaffold219_1	Colwellia psychrerythraea	0.74	Predict
NLM032_scaffold68980_1	Colwellia psychrerythraea	0.82	Predict
NLM032_scaffold12144_3	Clostridioides difficile	1	CRISPR
NLM032_scaffold7394_8	Ralstonia solanacearum	0.93	Predict
NLM032_scaffold69642_1	Colwellia psychrerythraea	1	CRISPR
NLM032_scaffold69111_1	Colwellia psychrerythraea	0.92	Predict
NLM032_scaffold314_2	Colwellia psychrerythraea	1	CRISPR
NLM032_scaffold3938_2	Colwellia psychrerythraea	1	CRISPR
NLM032_scaffold25961_2	Parabacteroides merdae	0.99	Predict
NLM032_scaffold60475_1	Colwellia psychrerythraea	0.87	Predict
NLM032_scaffold54796_9	Colwellia psychrerythraea	1	CRISPR
NLM032_scaffold9089_7	Faecalibacterium prausnitzii	1	CRISPR
NLM032_scaffold24196_2	Bifidobacterium pseudocatenulatum	1	CRISPR
NLM032_scaffold69594_3	Colwellia psychrerythraea	1	CRISPR
NLM032_scaffold3932_23	Colwellia psychrerythraea	0.94	Predict
NLM032_scaffold23630_7	Colwellia psychrerythraea	1	CRISPR
NLM032_scaffold50920_4	Clostridium tetani	1	CRISPR
NLM032_scaffold69179_1	Colwellia psychrerythraea	0.91	Predict
NLM032_scaffold68145_1	Colwellia psychrerythraea	1	CRISPR
NLM032_scaffold58268_2	Vibrio cholerae	0.88	Predict
NLM032_scaffold212_2	Colwellia psychrerythraea	0.9	Predict
NLM032_scaffold69594_2	Ruminococcus bromii	1	CRISPR
NLM032_scaffold40892_3	Colwellia psychrerythraea	0.99	Predict
NLM032_scaffold6383_1	Blautia sp. OM06-15AC	1	CRISPR
NLM032_scaffold49971_2	Bacteroides fragilis	0.88	Predict
NLM032_scaffold35599_1	Colwellia psychrerythraea	0.77	Predict
NLM032_scaffold11035_2	Flavobacterium columnare	1	CRISPR
NLM001_scaffold5659_21	unknown	0	-
NLM002_scaffold4211_7	unknown	0	-
NLM002_scaffold15370_2	unknown	0	-
NLM002_scaffold17510_4	unknown	0	-
NLM002_scaffold14431_4	unknown	0	-
NLM002_scaffold5906_19	unknown	0	-
NLM002_C361448_1	unknown	0	-
NLM003_scaffold4486_1	unknown	0	-
NLM004_scaffold40373_1	unknown	0	-
NLM004_scaffold2268_1	unknown	0	-
NLM004_C687357_1	unknown	0	-

NLM004_scaffold28209_1	unknown	0	-
NLM004_scaffold54430_1	unknown	0	-
NLM005_scaffold17186_2	unknown	0	-
NLM005_scaffold34767_1	unknown	0	-
NLM005_scaffold12098_1	unknown	0	-
NLM006_scaffold23522_7	unknown	0	-
NLM006_scaffold44135_1	unknown	0	-
NLM006_scaffold15317_5	unknown	0	-
NLM006_scaffold50994_2	unknown	0	-
NLM006_scaffold16491_1(	unknown	0	-
NLM006_scaffold31240_3	unknown	0	-
NLM006_scaffold4969_12	unknown	0	-
NLM007_scaffold11104_1	unknown	0	-
NLM008_scaffold70_5	unknown	0	-
NLM008_scaffold4502_17	unknown	0	-
NLM008_scaffold44104_3	unknown	0	-
NLM008_scaffold50150_1	unknown	0	-
NLM008_scaffold46068_3	unknown	0	-
NLM009_scaffold13047_6	unknown	0	-
NLM009_C228858_1	unknown	0	-
NLM009_scaffold13069_1	unknown	0	-
NLM010_scaffold15834_3	unknown	0	-
NLM010_C569179_1	unknown	0	-
NLM010_scaffold7375_4	unknown	0	-
NLM010_scaffold2398_1	unknown	0	-
NLM010_scaffold24696_6	unknown	0	-
NLM010_scaffold21133_2	unknown	0	-
NLM015_scaffold13019_2	unknown	0	-
NLM016_scaffold8626_1	unknown	0	-
NLM016_scaffold9518_15	unknown	0	-
NLM017_scaffold21461_2	unknown	0	-
NLM017_scaffold29127_2	unknown	0	-
NLM021_scaffold17568_1	unknown	0	-
NLM021_scaffold8185_4	unknown	0	-
NLM021_scaffold56233_3	unknown	0	-
NLM021_scaffold57344_1	unknown	0	-
NLM021_scaffold26944_4	unknown	0	-
NLM021_scaffold35458_5	unknown	0	-
NLM021_scaffold190_9	unknown	0	-
NLM021_scaffold57650_6	unknown	0	-
NLM021_scaffold927_6	unknown	0	-
NLM021_scaffold11116_2	unknown	0	-
NLM022_scaffold173_4	unknown	0	-
NLM022_scaffold10884_4	unknown	0	-
NLM022_scaffold43901_3	unknown	0	-
NLM022_scaffold7447_1	unknown	0	-
NLM023_scaffold33293_1	unknown	0	-
NLM023_scaffold48669_2	unknown	0	-

NLM023_scaffold28479_2(	unknown	0	-
NLM023_scaffold48946_1	unknown	0	-
NLM023_scaffold277_15	unknown	0	-
NLM023_scaffold27707_7	unknown	0	-
NLM023_scaffold17200_1!	unknown	0	-
NLM024_scaffold497_1	unknown	0	-
NLM025_scaffold78_11	unknown	0	-
NLM026_scaffold11596_2	unknown	0	-
NLM026_scaffold988_1	unknown	0	-
NLM026_scaffold68020_6	unknown	0	-
NLM026_scaffold394_5	unknown	0	-
NLM026_scaffold75724_1	unknown	0	-
NLM026_scaffold75315_1	unknown	0	-
NLM026_scaffold26189_1	unknown	0	-
NLM026_scaffold32583_1	unknown	0	-
NLM026_scaffold65585_4	unknown	0	-
NLM026_scaffold16034_7	unknown	0	-
NLM026_scaffold54916_1	unknown	0	-
NLM026_scaffold43543_3	unknown	0	-
NLM027_scaffold41690_2	unknown	0	-
NLM027_scaffold4058_19	unknown	0	-
NLM027_scaffold19284_2	unknown	0	-
NLM027_scaffold41965_1	unknown	0	-
NLM027_scaffold27969_2	unknown	0	-
NLM028_scaffold35099_2	unknown	0	-
NLM028_scaffold42575_1	unknown	0	-
NLM028_scaffold7898_1	unknown	0	-
NLM029_scaffold46834_2	unknown	0	-
NLM029_scaffold22749_1	unknown	0	-
NLM029_scaffold161_2	unknown	0	-
NLM029_scaffold9173_1	unknown	0	-
NLM029_scaffold17455_3	unknown	0	-
NLM029_scaffold25526_7	unknown	0	-
NLM029_scaffold46830_1	unknown	0	-
NLM029_scaffold35541_5	unknown	0	-
NLM029_scaffold12727_2	unknown	0	-
NLM029_scaffold15304_1	unknown	0	-
NLM029_scaffold31672_2	unknown	0	-
NLM029_scaffold17844_1	unknown	0	-
NLM029_C674325_1	unknown	0	-
NLM029_scaffold45625_1	unknown	0	-
NLM029_C674345_1	unknown	0	-
NLM031_scaffold18212_1(	unknown	0	-
NLM031_scaffold33580_2	unknown	0	-
NLM031_scaffold45482_3_	unknown	0	-
NLM031_scaffold56089_1	unknown	0	-
NLM031_scaffold20700_11	unknown	0	-
NLM031_scaffold17121_1	unknown	0	-

NLM031_C734735_1	unknown	0	-
NLM032_scaffold49689_1	unknown	0	-
NLM032_scaffold32034_2	unknown	0	-
NLM032_scaffold69647_5	unknown	0	-
DOM001_scaffold12537_8	Lactobacillus fermentum	0.81	Predict
DOM001_scaffold26527_9	Lactobacillus fermentum	0.97	Predict
DOM001_scaffold20125_2	Mycoplasma pulmonis	0.75	Predict
DOM001_scaffold6481_6	Mycoplasma pulmonis	0.94	Predict
DOM001_scaffold43_2	Candidatus Pelagibacter ubique	0.86	Predict
DOM001_scaffold2066_1	Mycoplasma pulmonis	0.99	Predict
DOM001_scaffold53704_4	Lactobacillus fermentum	0.9	Predict
DOM001_scaffold33_5	Lactobacillus fermentum	0.96	Predict
DOM001_scaffold4013_1	Roseobacter denitrificans	0.87	Predict
DOM001_scaffold9052_4	Roseobacter denitrificans	0.88	Predict
DOM001_scaffold26005_5	Mycoplasma pulmonis	0.72	Predict
DOM001_scaffold22877_4	Lactobacillus fermentum	0.83	Predict
DOM001_scaffold14492_1	Parabacteroides merdae	1	CRISPR
DOM001_scaffold39139_2	Candidatus Pelagibacter ubique	0.97	Predict
DOM001_scaffold16478_1	Parabacteroides merdae	0.82	Predict
DOM001_scaffold55189_1	Lactobacillus fermentum	0.96	Predict
DOM001_scaffold49162_1	Bacillus cereus	0.74	Predict
DOM001_scaffold5635_4	Lactobacillus fermentum	1	CRISPR
DOM001_scaffold58195_2	Bacteroides fragilis	0.71	Predict
DOM001_scaffold7274_21	Mycoplasma pulmonis	0.87	Predict
DOM001_scaffold28423_1	Candidatus Pelagibacter ubique	1	CRISPR
DOM001_scaffold26343_7	Parabacteroides distasonis	0.88	Predict
DOM001_scaffold30807_3	Francisella tularensis	1	CRISPR
DOM001_scaffold5000_5	Candidatus Pelagibacter ubique	0.72	Predict
DOM001_scaffold17386_5	Lactobacillus fermentum	1	Predict
DOM001_scaffold37685_1	Candidatus Pelagibacter ubique	0.95	Predict
DOM001_scaffold17768_4	Xanthomonas vesicatoria	0.86	Predict
DOM001_scaffold273_1	Roseobacter denitrificans	0.74	Predict
DOM001_scaffold58831_1	Mycoplasma pulmonis	1	CRISPR
DOM001_scaffold38397_1	Mycoplasma pulmonis	1	CRISPR
DOM001_scaffold19397_1	Mycoplasma pulmonis	0.72	Predict
DOM003_scaffold2919_6	[Eubacterium] eligens	1	CRISPR
DOM003_scaffold22571_7	Candidatus Pelagibacter ubique	0.93	Predict
DOM003_scaffold5840_1	Mycoplasma pulmonis	0.98	Predict
DOM003_scaffold575_3	Roseobacter denitrificans	0.87	Predict
DOM003_scaffold6007_2	Mycoplasma pulmonis	1	CRISPR
DOM003_scaffold24274_1	Lactobacillus fermentum	1	CRISPR
DOM003_scaffold24287_2	Mycoplasma pulmonis	0.99	Predict
DOM003_scaffold24307_1	Mycoplasma pulmonis	1	CRISPR
DOM003_scaffold6518_3	Mycoplasma pulmonis	1	CRISPR
DOM003_scaffold2919_2	Prochlorococcus marinus	1	CRISPR
DOM003_scaffold11839_3	Bifidobacterium breve	1	CRISPR
DOM003_scaffold19301_1	Lactobacillus johnsonii	0.71	Predict
DOM003_scaffold3648_4	Candidatus Hamiltonella defensa	1	CRISPR

DOM003_scaffold19736_6	Flavobacterium columnare	1	CRISPR
DOM003_scaffold2919_4	Candidatus Pelagibacter ubique	1	CRISPR
DOM003_scaffold18492_3	Mycoplasma pulmonis	0.81	Predict
DOM003_scaffold20665_1	Lactobacillus fermentum	1	Predict
DOM003_scaffold24151_1	Lactobacillus fermentum	0.89	Predict
DOM003_scaffold23067_1	Clostridioides difficile	1	CRISPR
DOM003_scaffold11839_7	Rhodovulum sp. P5	0.89	Predict
DOM003_scaffold24307_2	Mycoplasma pulmonis	0.73	Predict
DOM003_scaffold17266_6	Lactobacillus fermentum	0.94	Predict
DOM003_scaffold1995_7	Mycoplasma pulmonis	0.88	Predict
DOM003_scaffold24298_1	Lactobacillus fermentum	1	CRISPR
DOM003_scaffold22453_2	Mycoplasma pulmonis	0.76	Predict
DOM003_scaffold2919_11	Mycoplasma pulmonis	0.86	Predict
DOM003_scaffold11839_4	Rhizobium leguminosarum	1	CRISPR
DOM005_scaffold374_9	Lactobacillus fermentum	1	CRISPR
DOM005_scaffold920_41	Clostridium perfringens	1	CRISPR
DOM005_scaffold9501_4	Mycoplasma pulmonis	1	CRISPR
DOM005_scaffold13159_4	Mycoplasma pulmonis	1	CRISPR
DOM005_scaffold6037_1	Lactobacillus fermentum	0.75	Predict
DOM005_scaffold2259_3	Lactobacillus fermentum	1	CRISPR
DOM005_scaffold13355_2	Parabacteroides distasonis	0.94	Predict
DOM005_scaffold10392_5	Bacteroides fragilis	0.91	Predict
DOM005_scaffold10888_1	Mycoplasma pulmonis	0.92	Predict
DOM005_C258523_1	Mycoplasma pulmonis	0.95	Predict
DOM005_scaffold10888_5	Mycoplasma pulmonis	0.98	Predict
DOM005_scaffold12816_1	Bacteroides fragilis	0.92	Predict
DOM005_scaffold13159_2	Lactobacillus fermentum	1	CRISPR
DOM005_scaffold2640_50	Lactobacillus fermentum	1	CRISPR
DOM005_scaffold1509_4	Mycoplasma pulmonis	1	CRISPR
DOM005_scaffold9841_13	Lactobacillus fermentum	1	CRISPR
DOM005_scaffold168_65	Mycoplasma pulmonis	0.7	Predict
DOM005_scaffold5851_3	Candidatus Pelagibacter ubique	0.86	Predict
DOM005_scaffold374_8	Lactobacillus fermentum	1	CRISPR
DOM008_scaffold15923_2	Lactobacillus fermentum	1	CRISPR
DOM008_scaffold680_3	Mycoplasma pulmonis	0.92	Predict
DOM008_scaffold543_11	Streptomyces lividans	0.92	Predict
DOM008_scaffold4243_1	Candidatus Pelagibacter ubique	0.94	Predict
DOM008_scaffold16792_1	Mycoplasma pulmonis	0.79	Predict
DOM008_scaffold5589_2	Bacteroides fragilis	1	CRISPR
DOM008_scaffold14700_5	Lactobacillus fermentum	0.74	Predict
DOM008_scaffold3045_22	Lactobacillus fermentum	0.95	Predict
DOM008_scaffold57_7	Bacteroides fragilis	0.83	Predict
DOM008_scaffold18954_1	Bacteroides fragilis	0.95	Predict
DOM008_scaffold169_2	Mycoplasma pulmonis	0.82	Predict
DOM008_scaffold9829_1	Bacteroides fragilis	0.88	Predict
DOM008_scaffold1395_1	Candidatus Pelagibacter ubique	0.99	Predict
DOM008_scaffold8348_2	Mycoplasma pulmonis	0.76	Predict
DOM008_scaffold6987_15	Candidatus Hamiltonella defensa	0.81	Predict

DOM008_scaffold14339_1	Mycoplasma pulmonis	1	CRISPR
DOM008_scaffold11628_1	Candidatus Pelagibacter ubique	0.98	Predict
DOM008_scaffold15475_9	Mycoplasma pulmonis	0.83	Predict
DOM008_scaffold57_5	Bacteroides fragilis	0.93	Predict
DOM008_scaffold15104_4	Parabacteroides distasonis	0.88	Predict
DOM008_scaffold44_39	Lactobacillus fermentum	1	CRISPR
DOM008_scaffold18440_1	Bacteroides fragilis	0.89	Predict
DOM008_scaffold18246_2	Bacteroides fragilis	0.98	Predict
DOM008_scaffold8252_2	Mycoplasma pulmonis	0.99	Predict
DOM008_scaffold15475_4	Mycoplasma pulmonis	1	CRISPR
DOM008_scaffold17898_2	Mycoplasma pulmonis	0.87	Predict
DOM008_scaffold19226_1	Mycoplasma pulmonis	0.81	Predict
DOM008_scaffold14655_7	Roseobacter denitrificans	0.83	Predict
DOM008_scaffold11407_6	Bacteroides fragilis	0.73	Predict
DOM008_scaffold17859_3	Lactobacillus fermentum	1	CRISPR
DOM010_scaffold8807_2	Mycoplasma pulmonis	0.84	Predict
DOM010_C556729_1	Mycoplasma pulmonis	0.76	Predict
DOM010_scaffold23838_1	Enterococcus faecalis	1	CRISPR
DOM010_scaffold7398_16	Parabacteroides merdae	1	CRISPR
DOM010_scaffold11164_7	Mycoplasma pulmonis	0.85	Predict
DOM010_C557895_1	Clostridium perfringens	0.81	Predict
DOM010_scaffold29114_1	Parabacteroides distasonis	0.98	Predict
DOM010_C557123_1	Mycoplasma pulmonis	1	CRISPR
DOM010_scaffold10693_2	Candidatus Pelagibacter ubique	1	CRISPR
DOM010_scaffold42781_1	Mycoplasma pulmonis	1	CRISPR
DOM010_scaffold3905_57	Clostridium tetani	1	CRISPR
DOM010_scaffold6942_2	Bacteroides fragilis	0.86	Predict
DOM010_scaffold42298_4	Roseobacter denitrificans	0.96	Predict
DOM010_C557921_1	Clostridium perfringens	0.89	Predict
DOM010_scaffold41237_4	Streptococcus mutans	1	CRISPR
DOM010_scaffold36003_1	Bacteroides fragilis	0.93	Predict
DOM010_scaffold7_1	Bacillus alcalophilus	0.98	Predict
DOM010_scaffold19710_3	Rhizobium leguminosarum	0.91	Predict
DOM010_scaffold72_1	Candidatus Pelagibacter ubique	0.83	Predict
DOM010_scaffold25074_1	Candidatus Pelagibacter ubique	0.79	Predict
DOM010_scaffold42659_2	Bacteroides fragilis	0.85	Predict
DOM010_scaffold38114_4	Lactobacillus fermentum	1	CRISPR
DOM010_scaffold44548_1	Mycoplasma pulmonis	0.96	Predict
DOM010_scaffold44359_1	Lactobacillus fermentum	1	CRISPR
DOM010_scaffold36407_1	Mycoplasma pulmonis	0.82	Predict
DOM010_scaffold43444_3	Staphylococcus aureus	1	CRISPR
DOM010_scaffold12345_3	Lactobacillus fermentum	0.74	Predict
DOM010_scaffold27469_1	Mycoplasma pulmonis	0.96	Predict
DOM010_scaffold17754_1	Lactobacillus fermentum	1	CRISPR
DOM010_C557291_1	Mycoplasma pulmonis	0.82	Predict
DOM010_scaffold13897_1	Lactobacillus fermentum	0.82	Predict
DOM010_scaffold43785_1	Bacteroides fragilis	1	Predict
DOM010_scaffold38766_6	Mycoplasma pulmonis	0.8	Predict

DOM010_scaffold34515_1	Clostridioides difficile	1	CRISPR
DOM010_scaffold44098_1	Candidatus Pelagibacter ubique	0.94	Predict
DOM010_scaffold43222_1	Mycoplasma pulmonis	0.79	Predict
DOM010_scaffold44558_1	Staphylococcus aureus	1	CRISPR
DOM010_scaffold21662_2	Ruegeria pomeroyi	0.92	Predict
DOM010_scaffold7072_9	Mycoplasma pulmonis	0.93	Predict
DOM010_scaffold3568_1	Candidatus Pelagibacter ubique	0.75	Predict
DOM010_scaffold40874_2	Candidatus Pelagibacter ubique	0.87	Predict
DOM010_scaffold40514_1	Candidatus Pelagibacter ubique	0.82	Predict
DOM010_scaffold39715_1	Lactobacillus fermentum	1	CRISPR
DOM010_scaffold120_2	Cellulophaga baltica	0.92	Predict
DOM010_scaffold44263_3	Cellulophaga baltica	1	CRISPR
DOM012_scaffold631_3	Roseobacter denitrificans	1	CRISPR
DOM012_scaffold33660_1	Achromobacter xylosoxidans	0.83	Predict
DOM012_scaffold4248_4	Parabacteroides merdae	1	CRISPR
DOM012_scaffold33513_5	Mycoplasma pulmonis	0.9	Predict
DOM012_scaffold8289_1	Candidatus Pelagibacter ubique	0.8	Predict
DOM012_scaffold25292_2	Mycoplasma pulmonis	1	CRISPR
DOM012_scaffold9067_4	Lactobacillus fermentum	1	CRISPR
DOM012_scaffold25292_3	Mycoplasma pulmonis	0.95	Predict
DOM012_scaffold5103_5	Mycoplasma pulmonis	0.77	Predict
DOM012_scaffold631_1	Klebsiella pneumoniae	1	CRISPR
DOM012_scaffold42523_1	Mycoplasma pulmonis	0.83	Predict
DOM012_scaffold4108_5	Paenibacillus larvae	0.81	Predict
DOM012_scaffold33307_1	Staphylococcus saprophyticus	0.75	Predict
DOM012_scaffold4248_8	Mycoplasma pulmonis	0.91	Predict
DOM012_scaffold31_2	Mycoplasma pulmonis	0.78	Predict
DOM012_scaffold46434_1	Mycoplasma pulmonis	1	CRISPR
DOM012_scaffold4248_3	Mycoplasma pulmonis	0.98	Predict
DOM013_scaffold29644_2	Candidatus Pelagibacter ubique	1	Predict
DOM013_scaffold65510_2	Flavobacterium columnare	0.83	Predict
DOM013_scaffold66179_1	Mycoplasma pulmonis	1	CRISPR
DOM013_scaffold45617_1	Candidatus Pelagibacter ubique	0.81	Predict
DOM013_scaffold46653_1	Bacillus alcalophilus	1	CRISPR
DOM013_scaffold11541_3	Candidatus Pelagibacter ubique	0.88	Predict
DOM013_scaffold38557_2	Lactobacillus jensenii	0.89	Predict
DOM013_scaffold13214_2	Candidatus Pelagibacter ubique	0.86	Predict
DOM013_scaffold27634_2	Lactobacillus fermentum	0.88	Predict
DOM013_scaffold65920_1	Lactobacillus gasseri	0.99	Predict
DOM013_scaffold65670_1	Clostridium perfringens	0.88	Predict
DOM013_scaffold50516_3	Candidatus Hamiltonella defensa	0.87	Predict
DOM013_scaffold66005_2	Cellulophaga baltica	0.88	Predict
DOM013_scaffold10380_3	Roseobacter denitrificans	0.89	Predict
DOM013_scaffold34766_1	Lactobacillus fermentum	0.71	Predict
DOM013_scaffold65510_1	Flavobacterium columnare	0.96	Predict
DOM013_scaffold66036_1	Candidatus Pelagibacter ubique	0.72	Predict
DOM013_scaffold2110_2	Mycoplasma pulmonis	0.97	Predict
DOM013_scaffold9813_6	Mycoplasma pulmonis	1	CRISPR

DOM013_scaffold42751_1	Roseobacter denitrificans	1	CRISPR
DOM013_scaffold33337_2	Mycoplasma pulmonis	0.8	Predict
DOM013_scaffold3571_32	Lactobacillus fermentum	0.75	Predict
DOM013_scaffold81_8	Cellulophaga baltica	0.7	Predict
DOM013_scaffold66005_1	Cellulophaga baltica	0.94	Predict
DOM013_scaffold54135_4	Cellulophaga baltica	0.77	Predict
DOM013_scaffold66255_2	Flavobacterium columnare	1	CRISPR
DOM013_C812420_1	Lactobacillus delbrueckii	1	CRISPR
DOM013_scaffold64045_2	Lactobacillus fermentum	1	CRISPR
DOM013_scaffold63330_1	Lactobacillus fermentum	0.71	Predict
DOM013_scaffold13_2	Mannheimia haemolytica	0.86	Predict
DOM013_scaffold8272_3	Candidatus Pelagibacter ubique	0.75	Predict
DOM013_scaffold17461_1	Mycoplasma pulmonis	0.74	Predict
DOM013_scaffold23134_2(	Candidatus Pelagibacter ubique	0.95	Predict
DOM013_scaffold3755_4	Cellulophaga baltica	1	CRISPR
DOM013_scaffold62840_1	Mycoplasma pulmonis	1	CRISPR
DOM013_scaffold81_7	Mycoplasma pulmonis	0.78	Predict
DOM013_scaffold66110_1	Candidatus Pelagibacter ubique	0.71	Predict
DOM013_scaffold3571_2	Candidatus Pelagibacter ubique	1	CRISPR
DOM013_scaffold22249_3	Mycoplasma pulmonis	0.91	Predict
DOM013_scaffold54135_1(	Mycoplasma pulmonis	0.77	Predict
DOM013_scaffold16907_3	Mycoplasma pulmonis	0.92	Predict
DOM013_scaffold9359_8	Bdellovibrio bacteriovorus	0.98	Predict
DOM014_scaffold7185_19	Lactobacillus fermentum	0.7	Predict
DOM014_scaffold15062_2	Mycoplasma pulmonis	0.84	Predict
DOM014_scaffold17723_2	Bacteroides sp. 3_1_40A	1	CRISPR
DOM014_scaffold3561_14	Mycoplasma pulmonis	0.74	Predict
DOM014_scaffold9701_3	Bacillus cereus	0.96	Predict
DOM014_scaffold804_14	Flavobacterium columnare	0.81	Predict
DOM014_scaffold14238_1	Flavobacterium columnare	0.7	Predict
DOM014_scaffold283_1	Clostridioides difficile	1	CRISPR
DOM014_scaffold5325_3	Mycoplasma pulmonis	0.87	Predict
DOM014_scaffold1467_2	Flavobacterium columnare	0.79	Predict
DOM014_scaffold46104_1	Roseobacter denitrificans	0.78	Predict
DOM014_scaffold6073_4	Staphylococcus saprophyticus	0.73	Predict
DOM014_scaffold804_8	Mycoplasma pulmonis	0.78	Predict
DOM014_scaffold14238_3	Candidatus Pelagibacter ubique	0.72	Predict
DOM014_scaffold2397_7	Mycoplasma pulmonis	0.72	Predict
DOM014_scaffold804_15	Bacillus alcalophilus	1	CRISPR
DOM014_scaffold5037_9	Mycoplasma pulmonis	1	CRISPR
DOM014_scaffold8176_14	Candidatus Pelagibacter ubique	0.82	Predict
DOM014_scaffold10055_2	Mycoplasma pulmonis	0.79	Predict
DOM014_scaffold1696_5	Lactobacillus fermentum	0.72	Predict
DOM014_scaffold27264_1	Roseobacter denitrificans	1	CRISPR
DOM014_scaffold25405_4	Lactobacillus fermentum	1	CRISPR
DOM014_scaffold4337_1	Mycoplasma pulmonis	0.83	Predict
DOM015_scaffold25351_1	Candidatus Pelagibacter ubique	0.81	Predict
DOM015_scaffold21624_2	Mycoplasma pulmonis	0.98	Predict

DOM015_scaffold24987_5	Mycoplasma pulmonis	1	CRISPR
DOM015_scaffold15574_5	Lactobacillus fermentum	0.96	Predict
DOM015_scaffold226_4	Xanthomonas vesicatoria	1	CRISPR
DOM015_scaffold5854_1	Mycoplasma pulmonis	0.98	Predict
DOM015_scaffold14251_1	Candidatus Pelagibacter ubique	0.89	Predict
DOM015_scaffold22277_2	Parabacteroides merdae	0.77	Predict
DOM015_scaffold1868_1	Lactobacillus fermentum	0.9	Predict
DOM015_scaffold2034_4	Lactobacillus gasseri	1	CRISPR
DOM015_scaffold28241_1	Mycoplasma pulmonis	0.72	Predict
DOM015_scaffold16257_1	Mycoplasma pulmonis	0.77	Predict
DOM015_scaffold1673_2	Bacteroides fragilis	0.92	Predict
DOM015_scaffold4_1	Bacteroides fragilis	0.89	Predict
DOM015_scaffold21150_1	Mycoplasma pulmonis	0.88	Predict
DOM015_scaffold7250_8	Mycoplasma pulmonis	0.86	Predict
DOM015_scaffold21009_2	Lactobacillus fermentum	1	CRISPR
DOM015_scaffold10848_3	Lactobacillus fermentum	0.98	Predict
DOM015_scaffold10865_1	Lactobacillus fermentum	0.91	Predict
DOM015_C368449_1	Mycoplasma pulmonis	0.9	Predict
DOM015_scaffold5037_4	Mycoplasma pulmonis	0.78	Predict
DOM015_scaffold13677_1	Mycoplasma pulmonis	0.89	Predict
DOM015_scaffold29079_1	Mycoplasma pulmonis	0.93	Predict
DOM015_scaffold14658_2	Mycoplasma pulmonis	0.76	Predict
DOM015_scaffold28241_3	Bacteroides fragilis	0.97	Predict
DOM015_scaffold24987_1	Mycoplasma pulmonis	0.75	Predict
DOM015_C368153_1	Bacteroides fragilis	1	CRISPR
DOM016_scaffold36138_1	Mycoplasma pulmonis	0.75	Predict
DOM016_scaffold36372_1	Mycoplasma pulmonis	0.97	Predict
DOM016_scaffold28769_2	Mycoplasma pulmonis	0.9	Predict
DOM016_scaffold15209_2	Clostridioides difficile	0.81	Predict
DOM016_scaffold35635_2	Mycoplasma pulmonis	1	CRISPR
DOM016_scaffold35635_4	Clostridium sp. AF32-12BH	1	CRISPR
DOM016_scaffold27783_2	Bacteroides fragilis	0.9	Predict
DOM016_scaffold36369_1	Staphylococcus xylosus	0.77	Predict
DOM016_scaffold15402_1(	Mycoplasma pulmonis	1	CRISPR
DOM016_scaffold35922_2	Yersinia enterocolitica	1	CRISPR
DOM016_scaffold10818_1	Candidatus Pelagibacter ubique	0.97	Predict
DOM016_scaffold35524_2	Lactobacillus fermentum	0.96	Predict
DOM016_scaffold25511_1	Clostridium sporogenes	1	CRISPR
DOM016_scaffold32640_3	Flavobacterium columnare	0.76	Predict
DOM016_scaffold37071_2	Bacteroides fragilis	0.97	Predict
DOM016_scaffold701_1	Lactobacillus delbrueckii	0.96	Predict
DOM016_scaffold27658_1	Clostridioides difficile	1	CRISPR
DOM016_scaffold29662_3	Mycoplasma pulmonis	1	CRISPR
DOM016_scaffold5915_8	Clostridium perfringens	0.97	Predict
DOM016_scaffold35635_3	Mycoplasma pulmonis	1	CRISPR
DOM016_scaffold4735_5	Candidatus Pelagibacter ubique	1	CRISPR
DOM016_scaffold10222_1	Mycoplasma pulmonis	0.79	Predict
DOM016_scaffold27136_1	Mycoplasma pulmonis	1	CRISPR

DOM016_scaffold35701_7	Mycoplasma pulmonis	0.92	Predict
DOM016_scaffold35701_2	Clostridium perfringens	0.76	Predict
DOM016_scaffold29175_2	Mycoplasma pulmonis	0.72	Predict
DOM016_scaffold30030_2	Lactobacillus fermentum	0.94	Predict
DOM016_scaffold15402_6	Mycoplasma pulmonis	1	CRISPR
DOM016_scaffold31190_2	Megamonas hypermegale	1	CRISPR
DOM016_scaffold30578_2	Mycoplasma pulmonis	0.85	Predict
DOM016_scaffold65_5	Lactobacillus fermentum	1	CRISPR
DOM016_scaffold19852_1	Mycoplasma pulmonis	1	CRISPR
DOM016_scaffold11439_8	Mycoplasma pulmonis	0.72	Predict
DOM017_scaffold13265_2	Bacteroides fragilis	0.89	Predict
DOM017_scaffold7875_1	Candidatus Pelagibacter ubique	0.71	Predict
DOM017_scaffold12516_1	Bacteroides fragilis	0.78	Predict
DOM017_scaffold16028_1	Bacillus cereus	0.92	Predict
DOM017_scaffold9422_1	Mycoplasma pulmonis	0.85	Predict
DOM017_scaffold273_11	Mycoplasma pulmonis	0.94	Predict
DOM017_scaffold591_5	Staphylococcus saprophyticus	0.98	Predict
DOM017_scaffold7875_3	Staphylococcus aureus	0.92	Predict
DOM017_scaffold3771_1	Bacteroides fragilis	1	CRISPR
DOM017_scaffold35431_3	Bacteroides fragilis	0.82	Predict
DOM017_scaffold2122_1	Bacteroides fragilis	0.75	Predict
DOM017_scaffold12299_4	Lactobacillus fermentum	0.9	Predict
DOM017_scaffold30906_1	Mycoplasma pulmonis	0.91	Predict
DOM017_scaffold12432_2	Candidatus Pelagibacter ubique	1	CRISPR
DOM017_scaffold1652_4	Mycoplasma pulmonis	0.8	Predict
DOM017_scaffold28752_1	Bacillus cereus	1	CRISPR
DOM017_scaffold10632_1[illegible]	Lactobacillus fermentum	0.86	Predict
DOM018_scaffold6891_4	Candidatus Pelagibacter ubique	0.74	Predict
DOM018_scaffold41600_1	Xanthomonas vesicatoria	0.85	Predict
DOM018_scaffold27201_3	Candidatus Pelagibacter ubique	0.77	Predict
DOM018_scaffold37486_2	Bacteroides fragilis	1	Predict
DOM018_scaffold32733_2	Mycoplasma pulmonis	0.96	Predict
DOM018_scaffold35204_2	Candidatus Pelagibacter ubique	0.74	Predict
DOM018_scaffold22855_1[illegible]	Clostridioides difficile	1	CRISPR
DOM018_scaffold41384_2	Mycoplasma pulmonis	1	CRISPR
DOM018_scaffold35020_1	Lactobacillus fermentum	1	CRISPR
DOM018_scaffold41493_2	Mycoplasma pulmonis	0.75	Predict
DOM018_scaffold9337_1	Mycoplasma pulmonis	1	CRISPR
DOM018_C532545_1	Mycoplasma pulmonis	0.79	Predict
DOM018_scaffold23065_2	Candidatus Hamiltonella defensa	1	CRISPR
DOM018_scaffold6891_1	Spirosoma pollinicola	1	CRISPR
DOM018_scaffold6891_2	Faecalibacterium prausnitzii	1	CRISPR
DOM018_scaffold1832_2	Roseobacter denitrificans	1	CRISPR
DOM018_scaffold34741_1	Flavobacterium columnare	0.77	Predict
DOM018_scaffold41493_1	Candidatus Pelagibacter ubique	0.79	Predict
DOM018_C532801_1	Mycoplasma pulmonis	0.77	Predict
DOM018_scaffold32883_2	Candidatus Pelagibacter ubique	0.9	Predict
DOM018_scaffold32755_3	Candidatus Pelagibacter ubique	0.7	Predict

DOM018_scaffold35068_1	Ralstonia pickettii	1	CRISPR
DOM018_scaffold1832_4	Mycoplasma pulmonis	0.77	Predict
DOM018_scaffold18201_3	Roseobacter denitrificans	0.75	Predict
DOM018_scaffold17834_3	Mycoplasma pulmonis	0.94	Predict
DOM019_scaffold141_3	Candidatus Pelagibacter ubique	0.9	Predict
DOM019_scaffold20326_3	Mycoplasma pulmonis	0.85	Predict
DOM019_scaffold6808_2	Candidatus Pelagibacter ubique	0.9	Predict
DOM019_scaffold41546_1	Candidatus Pelagibacter ubique	0.78	Predict
DOM019_scaffold56541_1	Bacteroides fragilis	0.76	Predict
DOM019_scaffold42868_1	Streptomyces avermitilis	0.76	Predict
DOM019_scaffold5478_2	Flavobacterium columnare	0.72	Predict
DOM019_scaffold10413_1!	Lactobacillus fermentum	0.82	Predict
DOM019_scaffold3315_20	Clostridioides difficile	0.92	Predict
DOM019_scaffold1388_2	Mycoplasma pulmonis	1	CRISPR
DOM019_scaffold56605_1	Bacteroides fragilis	0.7	Predict
DOM019_scaffold6808_3	Candidatus Pelagibacter ubique	0.84	Predict
DOM019_scaffold20197_1	Candidatus Pelagibacter ubique	0.87	Predict
DOM019_scaffold56555_1	Candidatus Pelagibacter ubique	1	CRISPR
DOM019_scaffold8387_2	Parabacteroides distasonis	0.87	Predict
DOM019_scaffold56549_2	Mycoplasma pulmonis	0.86	Predict
DOM019_scaffold9440_2	Akkermansia muciniphila	1	CRISPR
DOM019_scaffold56598_2	Cellulophaga baltica	1	CRISPR
DOM019_scaffold23724_3	Candidatus Pelagibacter ubique	0.99	Predict
DOM019_scaffold56558_1	Bacillus megaterium	0.91	Predict
DOM019_scaffold56469_5	Flavobacterium columnare	1	CRISPR
DOM019_scaffold20133_1	Mycoplasma pulmonis	0.89	Predict
DOM019_scaffold34447_1	Thermoanaerobacterium saccharolytic	0.8	Predict
DOM019_scaffold56536_2	Lactobacillus fermentum	0.93	Predict
DOM019_scaffold9830_4	Mycoplasma pulmonis	0.87	Predict
DOM019_scaffold286_2	Mycoplasma pulmonis	0.99	Predict
DOM020_scaffold38403_2	Bacteroides fragilis	0.94	Predict
DOM020_scaffold38380_2	Mycoplasma pulmonis	0.84	Predict
DOM020_scaffold36128_4	Bacteroides fragilis	0.74	Predict
DOM020_scaffold8013_4	Staphylococcus saprophyticus	0.88	Predict
DOM020_scaffold1491_1	Parabacteroides merdae	0.91	Predict
DOM020_scaffold19627_1:	Mycoplasma pulmonis	0.82	Predict
DOM020_scaffold8898_2	Mycoplasma pulmonis	1	CRISPR
DOM020_scaffold16344_2	Flavobacterium columnare	1	CRISPR
DOM020_scaffold37565_2	Roseobacter denitrificans	1	CRISPR
DOM020_C483630_1	Lactobacillus fermentum	0.96	Predict
DOM020_scaffold8013_2	Mycoplasma pulmonis	0.78	Predict
DOM020_scaffold1229_1	Bacteroides fragilis	1	CRISPR
DOM020_scaffold3600_6	Candidatus Pelagibacter ubique	0.77	Predict
DOM020_scaffold7010_6	Candidatus Pelagibacter ubique	1	CRISPR
DOM021_scaffold18418_1	Mycoplasma pulmonis	0.9	Predict
DOM021_scaffold21593_8	Actinomyces naeslundii	0.81	Predict
DOM021_scaffold27752_2	Lactobacillus gasseri	0.84	Predict
DOM021_scaffold71_1	Pseudoalteromonas atlantica	0.88	Predict

DOM021_scaffold38953_1	Mycoplasma pulmonis	1	CRISPR
DOM021_scaffold39089_1	Mycoplasma pulmonis	1	CRISPR
DOM021_scaffold100_1	Lactobacillus fermentum	0.9	Predict
DOM021_scaffold39104_1	Lactobacillus fermentum	1	CRISPR
DOM021_scaffold36942_1	Lactobacillus fermentum	0.73	Predict
DOM021_scaffold23118_2	Mycoplasma pulmonis	0.8	Predict
DOM021_scaffold37667_2	Mycoplasma pulmonis	1	CRISPR
DOM021_scaffold11063_1	Lactobacillus fermentum	0.96	Predict
DOM021_scaffold39089_2	Candidatus Pelagibacter ubique	1	Predict
DOM021_scaffold36893_3	Mycoplasma pulmonis	1	CRISPR
DOM021_scaffold12359_1	Mycoplasma pulmonis	0.75	Predict
DOM021_scaffold37667_3	Candidatus Pelagibacter ubique	1	CRISPR
DOM021_scaffold37125_1	Candidatus Pelagibacter ubique	0.78	Predict
DOM022_scaffold32377_2	Mycoplasma pulmonis	0.89	Predict
DOM022_scaffold19956_4	Bacteroides fragilis	0.78	Predict
DOM022_scaffold6808_24	Lactobacillus fermentum	0.78	Predict
DOM022_scaffold28157_3	Candidatus Pelagibacter ubique	0.72	Predict
DOM022_scaffold32538_1	Mycoplasma pulmonis	0.73	Predict
DOM022_scaffold38389_3	Mycoplasma pulmonis	0.81	Predict
DOM022_scaffold2843_1	Bacillus thuringiensis	0.98	Predict
DOM022_scaffold38212_2	Mycoplasma pulmonis	1	CRISPR
DOM022_scaffold18184_9	Roseobacter denitrificans	0.82	Predict
DOM022_scaffold8882_1	Mycoplasma pulmonis	1	Predict
DOM022_scaffold38445_2	Mycoplasma pulmonis	1	CRISPR
DOM022_scaffold16044_2[illegible]	Candidatus Pelagibacter ubique	1	CRISPR
DOM022_scaffold8382_3	Bacteroides fragilis	0.87	Predict
DOM022_scaffold37182_2	Candidatus Pelagibacter ubique	0.82	Predict
DOM022_scaffold26147_1	Candidatus Pelagibacter ubique	0.81	Predict
DOM022_scaffold38016_1	Tsukamurella paurometabola	0.95	Predict
DOM022_C512095_1	Mycoplasma pulmonis	0.88	Predict
DOM022_scaffold38445_1	Candidatus Pelagibacter ubique	0.94	Predict
DOM022_scaffold35096_1	Mycoplasma pulmonis	1	CRISPR
DOM022_scaffold36652_4	Flavobacterium columnare	1	CRISPR
DOM022_scaffold38271_2	Xanthomonas vesicatoria	1	CRISPR
DOM022_scaffold21088_2	Mycoplasma pulmonis	0.77	Predict
DOM022_scaffold32837_2	Bacteroides fragilis	1	CRISPR
DOM022_scaffold37253_1	Parabacteroides merdae	0.78	Predict
DOM022_scaffold25564_3	Bacteroides sp. 3_1_40A	1	CRISPR
DOM022_scaffold35602_3	Mycoplasma pulmonis	1	CRISPR
DOM022_scaffold33506_1	Veillonella parvula	1	CRISPR
DOM022_scaffold16660_7	Bacteroides fragilis	1	CRISPR
DOM022_scaffold3588_2	Cellulophaga baltica	0.93	Predict
DOM022_scaffold9769_2	Mycoplasma pulmonis	0.96	Predict
DOM022_scaffold6474_11	Mycoplasma pulmonis	1	CRISPR
DOM022_scaffold8236_1	Bacteroides fragilis	0.85	Predict
DOM022_scaffold37834_1	Mycoplasma pulmonis	0.84	Predict
DOM022_scaffold11588_1[illegible]	Lactobacillus fermentum	0.74	Predict
DOM022_scaffold17665_1[illegible]	Mycoplasma pulmonis	0.87	Predict

DOM022_scaffold11253_14	Bacteroides fragilis	0.72	Predict
DOM022_scaffold27991_2	Mycoplasma pulmonis	1	CRISPR
DOM022_scaffold7138_11	Bacteroides fragilis	0.71	Predict
DOM022_scaffold3588_8	Candidatus Pelagibacter ubique	0.77	Predict
DOM022_scaffold38396_3	Candidatus Pelagibacter ubique	0.78	Predict
DOM022_scaffold17718_5	Mycoplasma pulmonis	0.77	Predict
DOM022_scaffold37363_3	Staphylococcus saprophyticus	1	CRISPR
DOM022_scaffold8882_2	Candidatus Pelagibacter ubique	0.96	Predict
DOM022_scaffold37244_1	Bacteroides fragilis	0.86	Predict
DOM022_scaffold37792_2	Mycoplasma pulmonis	0.88	Predict
DOM022_scaffold19876_2	Roseobacter denitrificans	0.81	Predict
DOM022_scaffold13760_14	Bacteroides fragilis	1	CRISPR
DOM022_scaffold1123_2	Lactobacillus fermentum	0.74	Predict
DOM022_scaffold28292_2	Flavobacterium columnare	1	CRISPR
DOM022_scaffold21088_3	Bacillus alcalophilus	1	CRISPR
DOM022_scaffold9987_23	Bacteroides fragilis	0.98	Predict
DOM022_scaffold36541_6	Cellulophaga baltica	1	CRISPR
DOM023_scaffold33157_2	Candidatus Pelagibacter ubique	0.81	Predict
DOM023_scaffold33236_1	Clostridium sporogenes	1	CRISPR
DOM023_scaffold33285_1	Mycoplasma pulmonis	1	CRISPR
DOM023_scaffold19886_6	Mycoplasma pulmonis	0.82	Predict
DOM023_scaffold33612_1	Mycoplasma pulmonis	0.98	Predict
DOM023_scaffold11787_6	Roseobacter denitrificans	0.75	Predict
DOM023_scaffold33611_2	Mycoplasma pulmonis	0.91	Predict
DOM023_scaffold28756_2	Citrobacter freundii	0.8	Predict
DOM023_scaffold13717_2	Mycoplasma pulmonis	0.82	Predict
DOM023_scaffold23102_8	Mycoplasma pulmonis	0.92	Predict
DOM023_scaffold33401_1	Mycoplasma pulmonis	0.85	Predict
DOM023_scaffold33622_1	Candidatus Pelagibacter ubique	1	CRISPR
DOM023_scaffold19886_5	Mycoplasma pulmonis	0.78	Predict
DOM023_scaffold2410_11	Bifidobacterium anseris	1	CRISPR
DOM023_scaffold16805_4	Candidatus Pelagibacter ubique	0.72	Predict
DOM023_scaffold33548_1	Candidatus Pelagibacter ubique	1	CRISPR
DOM023_C393870_1	Parabacteroides merdae	0.83	Predict
DOM024_scaffold35255_15	Candidatus Pelagibacter ubique	0.93	Predict
DOM024_scaffold11745_3	Parabacteroides distasonis	0.81	Predict
DOM024_scaffold34021_1	Mycoplasma pulmonis	0.87	Predict
DOM024_scaffold44466_2	Candidatus Pelagibacter ubique	0.71	Predict
DOM024_scaffold42617_1	Cellulophaga baltica	1	CRISPR
DOM024_scaffold38154_1	Candidatus Pelagibacter ubique	0.79	Predict
DOM024_scaffold56350_1	Flavobacterium columnare	1	CRISPR
DOM024_scaffold54306_4	Mycoplasma pulmonis	0.86	Predict
DOM024_scaffold42617_2	Mycoplasma pulmonis	1	CRISPR
DOM024_scaffold25752_1	Mycoplasma pulmonis	1	CRISPR
DOM024_scaffold41795_1	Candidatus Pelagibacter ubique	1	CRISPR
DOM024_scaffold56303_1	Mycoplasma pulmonis	0.94	Predict
DOM024_scaffold13072_13	Ruegeria pomeroyi	1	CRISPR
DOM024_scaffold52650_1	Bacteroides fragilis	0.71	Predict

DOM024_scaffold36750_1(	Bacteroides fragilis	1	CRISPR
DOM024_scaffold151_2	Lactobacillus fermentum	1	Predict
DOM024_scaffold55680_1	Flavobacterium columnare	1	CRISPR
DOM024_scaffold52177_5	Listeria monocytogenes	1	CRISPR
DOM024_C663872_1	Ruminococcus sp. AM43-6	1	CRISPR
DOM024_scaffold56350_2	Ruminococcus sp. AF37-20	1	CRISPR
DOM024_scaffold52366_1	Lactobacillus fermentum	1	CRISPR
DOM024_scaffold32065_2	Candidatus Hamiltonella defensa	0.82	Predict
DOM024_scaffold56206_2	Mycoplasma pulmonis	1	CRISPR
DOM024_C664368_1	Mycoplasma pulmonis	0.77	Predict
DOM024_C664620_1	Candidatus Pelagibacter ubique	0.89	Predict
DOM024_scaffold56343_2	Microcystis aeruginosa	1	CRISPR
DOM024_scaffold29589_1	Microcystis aeruginosa	0.72	Predict
DOM024_scaffold15513_1	Bacteroides fragilis	0.91	Predict
DOM024_scaffold30168_5	Lactobacillus fermentum	1	CRISPR
DOM024_scaffold4865_6	Mycoplasma pulmonis	0.9	Predict
DOM024_scaffold34021_4	Mycoplasma pulmonis	0.92	Predict
DOM024_scaffold35255_1	Cellulophaga baltica	1	CRISPR
DOM024_scaffold22388_3	Lactobacillus fermentum	1	CRISPR
DOM024_scaffold56343_3	Mycoplasma pulmonis	1	CRISPR
DOM024_scaffold15633_7	Mycoplasma pulmonis	0.95	Predict
DOM024_scaffold49650_2	Lactobacillus gasseri	1	CRISPR
DOM025_scaffold30920_4	Mycoplasma pulmonis	0.92	Predict
DOM025_scaffold44713_1	Mycoplasma pulmonis	0.86	Predict
DOM025_scaffold68_3	Mycoplasma pulmonis	1	CRISPR
DOM025_scaffold39391_1	Bacteroides fragilis	0.93	Predict
DOM025_scaffold29788_4	Lactobacillus fermentum	0.84	Predict
DOM025_scaffold44576_1	Planktothrix agardhii	0.86	Predict
DOM025_scaffold49_1	Candidatus Pelagibacter ubique	0.87	Predict
DOM025_scaffold49_3	Lactobacillus gasseri	0.86	Predict
DOM025_scaffold80_3	Aeromonas media	1	CRISPR
DOM025_scaffold300_2	Sinorhizobium meliloti	0.84	Predict
DOM025_scaffold4683_2	Lactobacillus fermentum	0.93	Predict
DOM025_scaffold9936_1	Lactobacillus fermentum	1	CRISPR
DOM025_scaffold1987_2	Azospirillum brasilense	0.73	Predict
DOM025_scaffold7455_3	Parabacteroides merdae	1	CRISPR
DOM025_scaffold41294_1	Bacteroides fragilis	0.97	Predict
DOM025_scaffold23512_1	Roseobacter denitrificans	0.7	Predict
DOM025_scaffold20212_5	Flavobacterium columnare	0.92	Predict
DOM025_scaffold5440_33	Candidatus Pelagibacter ubique	0.83	Predict
DOM025_scaffold10897_2	Lactobacillus fermentum	0.92	Predict
DOM025_scaffold12330_1	Mycoplasma pulmonis	0.96	Predict
DOM025_scaffold2257_29	Mycoplasma pulmonis	0.72	Predict
DOM026_scaffold22046_1	Mycoplasma pulmonis	0.73	Predict
DOM026_scaffold135_10	Lactobacillus fermentum	0.8	Predict
DOM026_scaffold813_3	Lactobacillus fermentum	0.87	Predict
DOM026_scaffold22251_2	Parabacteroides distasonis	0.98	Predict
DOM026_scaffold21041_1	Bacteroides faecis	1	CRISPR

DOM026_scaffold38351_1	Microcystis aeruginosa	0.84	Predict
DOM026_scaffold9255_1	Clostridioides difficile	1	CRISPR
DOM026_scaffold19764_1	Mycoplasma pulmonis	0.75	Predict
DOM026_scaffold30709_3	Mycoplasma pulmonis	0.76	Predict
DOM026_scaffold13170_6	Candidatus Hamiltonella defensa	0.8	Predict
DOM026_scaffold17576_1:	Rhodovulum sp. P5	0.91	Predict
DOM026_scaffold17476_4	Lactobacillus fermentum	0.78	Predict
DOM026_scaffold11281_2(	Roseobacter denitrificans	0.92	Predict
DOM026_scaffold10323_1	Lactobacillus fermentum	0.92	Predict
DOM026_scaffold23168_2	Lactobacillus fermentum	0.9	Predict
DOM026_scaffold28561_1	Lactobacillus fermentum	1	CRISPR
DOM026_scaffold8858_1	Clostridioides difficile	0.81	Predict
DOM026_scaffold39135_1	Mycoplasma pulmonis	0.92	Predict
DOM026_scaffold7817_3	Candidatus Pelagibacter ubique	0.74	Predict
DOM026_scaffold6782_6	Bacillus alcalophilus	0.91	Predict
DOM026_scaffold6782_3	Candidatus Pelagibacter ubique	1	CRISPR
DOM026_scaffold36555_1	Glaesserella parasuis	0.99	Predict
DOM026_scaffold11444_1	Staphylococcus pasteuri	0.93	Predict
DOM026_scaffold21142_1	Lactobacillus fermentum	1	Predict
DOM026_scaffold5666_20	Streptomyces avermitilis	0.76	Predict
DOM026_scaffold22785_1	Flavobacterium columnare	0.76	Predict
DOM026_scaffold17021_2	Mycoplasma pulmonis	1	CRISPR
DOM026_scaffold12473_1	Lactobacillus fermentum	0.77	Predict
DOM026_scaffold14353_2(	Staphylococcus simulans	1	CRISPR
DOM026_scaffold13335_3	Mycoplasma pulmonis	0.99	Predict
DOM026_scaffold28681_1	Mycoplasma pulmonis	1	CRISPR
DOM026_scaffold18755_1	Candidatus Pelagibacter ubique	0.85	Predict
DOM026_scaffold22785_2	Lactobacillus gasseri	0.84	Predict
DOM026_scaffold22404_1	Bacteroides fragilis	1	CRISPR
DOM026_scaffold4239_3	Clostridium perfringens	1	CRISPR
DOM026_scaffold26812_1	Lactobacillus fermentum	1	CRISPR
DOM026_scaffold10922_1	Mycoplasma pulmonis	0.78	Predict
DOM026_scaffold27635_1	Mycoplasma pulmonis	0.89	Predict
DOM026_scaffold21024_9	Lactobacillus fermentum	1	CRISPR
DOM026_scaffold21514_2	Candidatus Pelagibacter ubique	0.72	Predict
DOM026_scaffold35658_1	Croceibacter atlanticus	0.82	Predict
DOM026_scaffold23768_1	Candidatus Pelagibacter ubique	0.95	Predict
DOM026_scaffold17367_1	Parabacteroides distasonis	0.8	Predict
DOM001_scaffold57053_3	unknown	0	-
DOM001_scaffold21085_1(	unknown	0	-
DOM001_scaffold12537_1(	unknown	0	-
DOM001_scaffold2499_6	unknown	0	-
DOM003_scaffold11039_1	unknown	0	-
DOM003_scaffold14108_2	unknown	0	-
DOM005_scaffold1092_15	unknown	0	-
DOM005_scaffold3583_3	unknown	0	-
DOM005_scaffold10107_7	unknown	0	-
DOM005_scaffold13337_5	unknown	0	-

DOM005_scaffold8757_1	unknown	0	-
DOM008_scaffold16924_3	unknown	0	-
DOM010_scaffold2671_4	unknown	0	-
DOM010_scaffold44229_1	unknown	0	-
DOM010_scaffold20353_2	unknown	0	-
DOM010_scaffold44237_2	unknown	0	-
DOM010_scaffold44223_5	unknown	0	-
DOM010_scaffold1445_1	unknown	0	-
DOM010_scaffold5308_8	unknown	0	-
DOM010_C557197_1	unknown	0	-
DOM012_scaffold27104_2	unknown	0	-
DOM012_scaffold38243_4	unknown	0	-
DOM012_scaffold9872_1	unknown	0	-
DOM012_scaffold3937_49	unknown	0	-
DOM012_scaffold17700_1	unknown	0	-
DOM012_scaffold6197_5_2	unknown	0	-
DOM012_scaffold2137_37	unknown	0	-
DOM012_scaffold4248_15	unknown	0	-
DOM012_scaffold7054_4	unknown	0	-
DOM012_scaffold6291_4	unknown	0	-
DOM012_scaffold4007_9	unknown	0	-
DOM013_scaffold29321_1	unknown	0	-
DOM013_scaffold24958_1[illegible]	unknown	0	-
DOM013_scaffold4994_13	unknown	0	-
DOM013_scaffold63345_1	unknown	0	-
DOM013_scaffold39903_2	unknown	0	-
DOM013_scaffold46391_3	unknown	0	-
DOM013_scaffold60583_1	unknown	0	-
DOM014_scaffold43299_1	unknown	0	-
DOM014_scaffold1467_4	unknown	0	-
DOM014_scaffold804_11	unknown	0	-
DOM014_scaffold27382_3	unknown	0	-
DOM014_scaffold804_6	unknown	0	-
DOM014_scaffold25405_6	unknown	0	-
DOM015_scaffold29083_1	unknown	0	-
DOM015_scaffold8047_3	unknown	0	-
DOM016_scaffold37120_1	unknown	0	-
DOM016_scaffold714_2	unknown	0	-
DOM016_scaffold20871_2	unknown	0	-
DOM017_scaffold21083_1	unknown	0	-
DOM017_scaffold5373_1	unknown	0	-
DOM017_scaffold7344_7	unknown	0	-
DOM017_scaffold434_2	unknown	0	-
DOM017_scaffold3183_4	unknown	0	-
DOM017_scaffold12158_1	unknown	0	-
DOM017_scaffold23327_2	unknown	0	-
DOM017_scaffold35758_3	unknown	0	-
DOM017_scaffold29332_1	unknown	0	-

DOM017_scaffold2195_2	unknown	0	-
DOM018_scaffold11099_4	unknown	0	-
DOM018_scaffold88_7	unknown	0	-
DOM018_scaffold4830_3	unknown	0	-
DOM018_scaffold40717_1	unknown	0	-
DOM018_scaffold37478_3	unknown	0	-
DOM018_scaffold5182_1	unknown	0	-
DOM018_scaffold2126_7	unknown	0	-
DOM018_scaffold13608_2	unknown	0	-
DOM018_scaffold33929_1	unknown	0	-
DOM019_scaffold640_2	unknown	0	-
DOM019_scaffold33784_1	unknown	0	-
DOM019_scaffold661_7	unknown	0	-
DOM019_scaffold55994_2	unknown	0	-
DOM019_scaffold3989_6	unknown	0	-
DOM019_scaffold8084_3	unknown	0	-
DOM019_scaffold11571_1:	unknown	0	-
DOM019_scaffold35386_4	unknown	0	-
DOM020_scaffold24489_1	unknown	0	-
DOM020_scaffold1763_4	unknown	0	-
DOM021_scaffold272_1	unknown	0	-
DOM021_scaffold57_4	unknown	0	-
DOM021_scaffold5645_2	unknown	0	-
DOM021_scaffold39046_1	unknown	0	-
DOM022_scaffold847_4	unknown	0	-
DOM022_scaffold37774_1	unknown	0	-
DOM022_scaffold33500_1	unknown	0	-
DOM022_scaffold48_3	unknown	0	-
DOM022_scaffold15495_1	unknown	0	-
DOM023_scaffold12205_7	unknown	0	-
DOM023_scaffold25122_8	unknown	0	-
DOM023_scaffold31295_1	unknown	0	-
DOM023_scaffold22587_5	unknown	0	-
DOM024_scaffold52177_6	unknown	0	-
DOM024_scaffold56292_1	unknown	0	-
DOM024_scaffold18051_2	unknown	0	-
DOM024_scaffold20070_1:	unknown	0	-
DOM025_scaffold41702_1	unknown	0	-
DOM025_scaffold10571_8	unknown	0	-
DOM025_scaffold3271_23	unknown	0	-
DOM025_scaffold14033_1	unknown	0	-
DOM026_scaffold39207_2	unknown	0	-
DOM026_scaffold18742_1	unknown	0	-
DOM026_scaffold4239_7	unknown	0	-
DOM026_C539342_1	unknown	0	-
DOM026_C539214_1	unknown	0	-
NOM001_scaffold36280_1	Mycoplasma pulmonis	0.95	Predict
NOM001_scaffold22842_7	Mycoplasma pulmonis	1	CRISPR

NOM001_scaffold36050_1	Bacteroides fragilis	0.96	Predict
NOM001_scaffold22842_8	Mycoplasma pulmonis	0.88	Predict
NOM001_scaffold4297_1	Thermoanaerobacterium saccharolytic	0.92	Predict
NOM001_scaffold36892_1	Mycoplasma pulmonis	0.94	Predict
NOM001_scaffold24512_3	Mycoplasma pulmonis	0.82	Predict
NOM001_scaffold22842_6	Mycoplasma pulmonis	0.83	Predict
NOM002_scaffold4408_28	Mycoplasma pulmonis	0.72	Predict
NOM002_scaffold1807_4	Bacteroides fragilis	0.92	Predict
NOM002_scaffold5847_1	Bacillus subtilis	1	CRISPR
NOM002_scaffold139_2	Candidatus Pelagibacter ubique	0.78	Predict
NOM002_scaffold32200_2	Candidatus Pelagibacter ubique	1	CRISPR
NOM002_scaffold23302_7	Streptococcus pneumoniae	0.93	Predict
NOM002_scaffold2797_4	Bacteroides fragilis	0.72	Predict
NOM002_scaffold26702_1	Mycoplasma pulmonis	0.79	Predict
NOM002_scaffold26752_2	Candidatus Pelagibacter ubique	0.99	Predict
NOM002_scaffold65_1	Candidatus Pelagibacter ubique	0.8	Predict
NOM002_scaffold32168_1	Candidatus Pelagibacter ubique	1	CRISPR
NOM002_scaffold32212_1	Mycoplasma pulmonis	1	CRISPR
NOM002_scaffold15419_6	Candidatus Pelagibacter ubique	0.8	Predict
NOM002_scaffold7211_5	Mycoplasma pulmonis	0.92	Predict
NOM002_scaffold32093_2	Candidatus Pelagibacter ubique	1	CRISPR
NOM002_scaffold2423_2	Bacteroides fragilis	0.94	Predict
NOM002_scaffold32124_1	Colwellia psychrerythraea	1	CRISPR
NOM002_scaffold32041_1	Lactobacillus gasseri	0.91	Predict
NOM002_scaffold13215_6	Mycoplasma pulmonis	1	CRISPR
NOM002_scaffold830_1	Mycoplasma pulmonis	0.94	Predict
NOM002_scaffold3250_3	Colwellia psychrerythraea	0.96	Predict
NOM002_scaffold23876_3	Bacteroides sp. A1C1	1	CRISPR
NOM002_scaffold5995_1	Colwellia psychrerythraea	0.8	Predict
NOM002_scaffold31941_2	Candidatus Pelagibacter ubique	1	CRISPR
NOM002_scaffold30413_1	Candidatus Pelagibacter ubique	1	CRISPR
NOM002_scaffold31231_2	Mycoplasma pulmonis	0.95	Predict
NOM002_scaffold3598_9	Bacteroides fragilis	0.96	Predict
NOM002_scaffold7151_8	Mycoplasma pulmonis	0.71	Predict
NOM002_scaffold20546_3	Clostridioides difficile	1	CRISPR
NOM002_scaffold1562_8	Mycoplasma pulmonis	0.74	Predict
NOM002_scaffold27268_2	Parabacteroides distasonis	0.74	Predict
NOM002_scaffold27843_1	Francisella tularensis	1	CRISPR
NOM002_scaffold31229_1	Staphylococcus saprophyticus	0.9	Predict
NOM002_C429380_1	Trichormus variabilis	0.82	Predict
NOM002_C428718_1	Mycoplasma pulmonis	0.81	Predict
NOM002_scaffold32206_1	Bacteroides fragilis	0.74	Predict
NOM002_scaffold32205_1	Microcystis aeruginosa	0.94	Predict
NOM002_scaffold330_35	Mycoplasma pulmonis	0.82	Predict
NOM002_scaffold32135_1	Mycoplasma pulmonis	0.73	Predict
NOM002_scaffold31205_1	Mycoplasma pulmonis	1	CRISPR
NOM002_scaffold22930_2	Mycoplasma pulmonis	0.95	Predict
NOM002_scaffold3856_12	Mycoplasma pulmonis	0.84	Predict

NOM002_scaffold6808_7	Mycoplasma pulmonis	0.86	Predict
NOM002_scaffold19947_2	Candidatus Pelagibacter ubique	0.82	Predict
NOM002_scaffold12213_5	Bacteroides fragilis	0.85	Predict
NOM002_scaffold4555_2	Acinetobacter johnsonii	0.93	Predict
NOM002_scaffold6256_7	Trichormus variabilis	0.95	Predict
NOM002_scaffold24280_1	Mycoplasma pulmonis	1	CRISPR
NOM002_scaffold20040_1	Bacteroides fragilis	0.9	Predict
NOM002_scaffold7687_1	Mycoplasma pulmonis	0.85	Predict
NOM002_scaffold27308_2	Mycoplasma pulmonis	0.83	Predict
NOM002_scaffold24275_1	Colwellia psychrerythraea	1	CRISPR
NOM004_scaffold22743_2	Mycoplasma pulmonis	1	CRISPR
NOM004_scaffold14898_1	Bacteroides fragilis	0.73	Predict
NOM004_scaffold8990_22	Mycoplasma pulmonis	1	Predict
NOM004_scaffold3782_4	Mycoplasma pulmonis	0.85	Predict
NOM004_scaffold13517_2	Brevibacillus laterosporus	0.8	Predict
NOM004_scaffold22899_1	Mycoplasma pulmonis	1	CRISPR
NOM004_scaffold4741_16	Parabacteroides distasonis	1	CRISPR
NOM004_scaffold130_15	Streptomyces griseus	0.76	Predict
NOM004_C361687_1	Mycoplasma pulmonis	1	CRISPR
NOM004_scaffold11992_1	Mycoplasma pulmonis	0.96	Predict
NOM004_scaffold15695_1	Mycoplasma pulmonis	0.81	Predict
NOM004_scaffold21343_2	Mycoplasma pulmonis	0.85	Predict
NOM004_scaffold9807_9	Mycoplasma pulmonis	0.88	Predict
NOM004_scaffold21707_1	Mycoplasma pulmonis	1	CRISPR
NOM004_scaffold22694_1	Mycoplasma pulmonis	0.89	Predict
NOM004_scaffold11992_2	Mycoplasma pulmonis	0.88	Predict
NOM004_scaffold13523_7	Bacteroides fragilis	0.76	Predict
NOM004_scaffold28_10	Mycoplasma pulmonis	0.7	Predict
NOM004_scaffold6154_1	Candidatus Pelagibacter ubique	0.98	Predict
NOM004_scaffold17398_1	Mycoplasma pulmonis	0.93	Predict
NOM004_scaffold11917_4	Bacteroides fragilis	0.86	Predict
NOM004_scaffold14898_1	Bacteroides fragilis	0.78	Predict
NOM004_scaffold1169_9	Mycoplasma pulmonis	0.8	Predict
NOM004_scaffold15486_1	Parabacteroides distasonis	0.79	Predict
NOM005_scaffold53686_4	Mycoplasma pulmonis	0.89	Predict
NOM005_C715971_1	Colwellia psychrerythraea	1	CRISPR
NOM005_scaffold35434_1	Mycoplasma pulmonis	1	CRISPR
NOM005_scaffold41127_8	Candidatus Pelagibacter ubique	1	CRISPR
NOM005_scaffold563_2	Mycoplasma pulmonis	1	CRISPR
NOM005_scaffold10786_3	Mycoplasma pulmonis	0.79	Predict
NOM005_scaffold51989_2	Mycoplasma pulmonis	0.77	Predict
NOM005_scaffold55066_2	Lactobacillus jensenii	1	CRISPR
NOM005_C716463_1	Clostridium perfringens	1	CRISPR
NOM005_scaffold2849_2	Mycoplasma pulmonis	0.76	Predict
NOM005_scaffold32988_8	Mycoplasma pulmonis	1	CRISPR
NOM005_scaffold3277_10	Candidatus Pelagibacter ubique	0.76	Predict
NOM005_scaffold40041_4	Mycoplasma pulmonis	0.97	Predict
NOM005_scaffold563_4	Mycoplasma pulmonis	1	CRISPR

NOM005_scaffold37509_3	Bacteroides fragilis	0.99	Predict
NOM005_scaffold41127_7	Mycoplasma pulmonis	1	CRISPR
NOM005_scaffold11865_1	Mycoplasma pulmonis	0.79	Predict
NOM005_scaffold8808_3	Enterococcus faecalis	0.86	Predict
NOM005_scaffold130_3	Colwellia psychrerythraea	1	CRISPR
NOM005_scaffold47045_2	Mycoplasma pulmonis	0.99	Predict
NOM005_scaffold53973_2	Mycoplasma pulmonis	0.83	Predict
NOM005_scaffold16553_2	Clostridium botulinum	0.86	Predict
NOM005_scaffold563_3	Mycoplasma pulmonis	1	CRISPR
NOM005_scaffold45891_2	Mycoplasma pulmonis	0.78	Predict
NOM005_scaffold55101_1	Clostridium perfringens	1	CRISPR
NOM005_scaffold8276_2	Colwellia psychrerythraea	0.74	Predict
NOM005_C716395_1	Mycoplasma pulmonis	0.73	Predict
NOM005_scaffold47045_3	Mycoplasma pulmonis	0.71	Predict
NOM005_scaffold31514_2	Colwellia psychrerythraea	0.76	Predict
NOM005_scaffold32988_6	Mycoplasma pulmonis	1	CRISPR
NOM005_scaffold37509_5	Mycoplasma pulmonis	0.76	Predict
NOM005_scaffold11952_1	Colwellia psychrerythraea	0.97	Predict
NOM005_scaffold41127_1[illegible]	Candidatus Pelagibacter ubique	0.75	Predict
NOM005_scaffold5739_4	Aliivibrio fischeri	0.97	Predict
NOM005_scaffold5699_2	Salmonella enterica	1	CRISPR
NOM005_scaffold2279_1	Streptococcus mutans	0.9	Predict
NOM005_scaffold54732_1	Mycoplasma pulmonis	0.97	Predict
NOM005_scaffold41647_4	Candidatus Pelagibacter ubique	0.81	Predict
NOM005_scaffold41127_1[illegible]	Flavobacterium columnare	0.78	Predict
NOM005_scaffold41127_5	Mycoplasma pulmonis	1	CRISPR
NOM005_scaffold53973_3	Mycoplasma pulmonis	0.71	Predict
NOM005_scaffold47045_1	Mycoplasma pulmonis	1	CRISPR
NOM005_scaffold55068_1	Listeria monocytogenes	1	CRISPR
NOM005_scaffold20861_2	Colwellia psychrerythraea	0.77	Predict
NOM005_scaffold25180_1	Mycoplasma pulmonis	1	CRISPR
NOM005_C716365_1	Mycoplasma pulmonis	0.88	Predict
NOM007_scaffold3522_1	Mycoplasma pulmonis	0.98	Predict
NOM007_C668778_1	Flavobacterium columnare	0.9	Predict
NOM007_scaffold48877_1	Mycoplasma pulmonis	1	Predict
NOM007_scaffold46703_1	Colwellia psychrerythraea	1	CRISPR
NOM007_scaffold45023_6	Bacteroides fragilis	1	CRISPR
NOM007_scaffold15814_1	Erysipelothrix rhusiopathiae	0.77	Predict
NOM007_scaffold41225_1	Parabacteroides merdae	1	CRISPR
NOM007_scaffold35454_4	Mycoplasma pulmonis	0.93	Predict
NOM007_scaffold39468_2[illegible]	Bacteroides dorei	1	CRISPR
NOM007_C669362_1	Candidatus Pelagibacter ubique	0.78	Predict
NOM007_scaffold14193_6	Candidatus Pelagibacter ubique	0.8	Predict
NOM007_scaffold35189_1	Aeromonas hydrophila	0.85	Predict
NOM007_scaffold37589_1	Mycoplasma pulmonis	0.89	Predict
NOM007_scaffold37139_1	Colwellia psychrerythraea	0.92	Predict
NOM007_scaffold17241_4	Mannheimia haemolytica	0.72	Predict
NOM007_scaffold10787_5	Mycoplasma pulmonis	0.83	Predict

NOM007_scaffold48928_1	Mycoplasma pulmonis	0.88	Predict
NOM007_scaffold41196_1	Mycoplasma pulmonis	0.88	Predict
NOM007_scaffold4742_2	Mycoplasma pulmonis	0.97	Predict
NOM008_scaffold33378_1	Candidatus Pelagibacter ubique	0.87	Predict
NOM008_scaffold17954_2	Mycoplasma pulmonis	0.81	Predict
NOM008_scaffold18778_4	Bacteroides fragilis	0.96	Predict
NOM008_scaffold4244_38	Hymenobacteraceae bacterium SYSU	1	CRISPR
NOM008_scaffold9964_68	Mycoplasma pulmonis	0.84	Predict
NOM008_scaffold75_1	Mycoplasma pulmonis	1	CRISPR
NOM008_scaffold18096_1	Mycoplasma pulmonis	0.81	Predict
NOM008_scaffold29282_1	Parabacteroides merdae	0.79	Predict
NOM008_scaffold445_45	Colwellia psychrerythraea	0.79	Predict
NOM008_scaffold33375_1	Mycoplasma pulmonis	1	CRISPR
NOM008_scaffold24500_2	Mycoplasma pulmonis	0.92	Predict
NOM008_scaffold32938_5	Bacteroides fragilis	0.77	Predict
NOM008_scaffold6097_1	Candidatus Pelagibacter ubique	0.8	Predict
NOM008_scaffold14724_3	Candidatus Pelagibacter ubique	1	CRISPR
NOM008_scaffold33345_2	Mycoplasma pulmonis	0.8	Predict
NOM008_scaffold32941_1	Colwellia psychrerythraea	1	CRISPR
NOM008_scaffold32975_4	Parabacteroides merdae	0.72	Predict
NOM008_scaffold226_8	Mycoplasma pulmonis	0.71	Predict
NOM008_scaffold9194_1	Mycoplasma pulmonis	1	CRISPR
NOM008_scaffold30194_1	Mycoplasma pulmonis	0.78	Predict
NOM008_scaffold940_9	Mycoplasma pulmonis	0.78	Predict
NOM008_scaffold29282_6	Colwellia psychrerythraea	0.7	Predict
NOM008_scaffold1177_10	Mycoplasma pulmonis	0.9	Predict
NOM008_scaffold5454_20	Mycoplasma pulmonis	0.96	Predict
NOM008_scaffold27977_1	Mycoplasma pulmonis	0.88	Predict
NOM009_scaffold37752_3	Colwellia psychrerythraea	0.95	Predict
NOM009_scaffold15278_8	Mycoplasma pulmonis	0.78	Predict
NOM009_scaffold24584_3	Mycoplasma pulmonis	0.93	Predict
NOM009_scaffold27152_1	Bacteroides fragilis	0.85	Predict
NOM009_scaffold43294_6	Mycoplasma pulmonis	0.77	Predict
NOM009_scaffold31057_2	Mycoplasma pulmonis	0.71	Predict
NOM009_scaffold45014_3	Mycoplasma pulmonis	0.71	Predict
NOM009_scaffold41176_2	Bacteroides fragilis	0.82	Predict
NOM009_scaffold25569_1	Mycoplasma pulmonis	0.73	Predict
NOM009_scaffold19614_4	Mycoplasma pulmonis	0.91	Predict
NOM009_scaffold40704_1	Mycoplasma pulmonis	1	CRISPR
NOM009_scaffold15278_9	Mycoplasma pulmonis	0.86	Predict
NOM009_scaffold54000_1	Colwellia psychrerythraea	0.96	Predict
NOM009_scaffold1163_4	Mycoplasma pulmonis	0.99	Predict
NOM009_scaffold10818_2	Mycoplasma pulmonis	1	CRISPR
NOM009_scaffold22166_2	Mycoplasma pulmonis	0.73	Predict
NOM009_scaffold18703_1	Candidatus Pelagibacter ubique	0.85	Predict
NOM009_scaffold53866_1	Colwellia psychrerythraea	0.84	Predict
NOM009_scaffold6873_12	Mycoplasma pulmonis	0.86	Predict
NOM009_scaffold41176_6	Mycoplasma pulmonis	1	CRISPR

NOM009_scaffold337_2	Mycoplasma pulmonis	0.84	Predict
NOM009_scaffold293_1	Mycoplasma pulmonis	1	CRISPR
NOM009_scaffold37752_4	Myxococcus xanthus	0.77	Predict
NOM009_scaffold53815_1	Mycoplasma pulmonis	0.79	Predict
NOM009_scaffold14272_1	Candidatus Pelagibacter ubique	0.72	Predict
NOM009_scaffold113_2	Mycoplasma pulmonis	0.72	Predict
NOM009_scaffold14149_2	Candidatus Pelagibacter ubique	0.98	Predict
NOM009_scaffold53934_1	Mycoplasma pulmonis	0.91	Predict
NOM009_scaffold53906_1	Roseburia intestinalis	1	CRISPR
NOM009_scaffold44452_1	Bacillus thuringiensis	0.94	Predict
NOM010_scaffold16688_4	Parabacteroides merdae	0.73	Predict
NOM010_scaffold7834_6	Mycoplasma pulmonis	0.85	Predict
NOM010_scaffold36917_1	Candidatus Pelagibacter ubique	0.99	Predict
NOM010_scaffold27408_5	Mycoplasma pulmonis	0.75	Predict
NOM010_scaffold34799_1	Mycoplasma pulmonis	0.79	Predict
NOM010_scaffold7841_9	Colwellia psychrerythraea	0.87	Predict
NOM010_scaffold23144_2	Bacteroides fragilis	0.89	Predict
NOM010_scaffold6634_3	Colwellia psychrerythraea	0.87	Predict
NOM010_scaffold8017_1	Mycoplasma pulmonis	1	CRISPR
NOM010_scaffold39236_1	Bacteroides fragilis	0.79	Predict
NOM010_scaffold356_5	Mycoplasma pulmonis	1	CRISPR
NOM010_scaffold2023_1	Bacteroides fragilis	0.98	Predict
NOM010_scaffold41832_1	Clostridioides difficile	1	CRISPR
NOM010_scaffold12374_8	Bacteroides fragilis	1	CRISPR
NOM010_scaffold26994_1	Mycoplasma pulmonis	1	CRISPR
NOM010_scaffold40683_2	Croceibacter atlanticus	0.86	Predict
NOM010_scaffold26048_2	Streptococcus mutans	0.79	Predict
NOM010_scaffold19890_3	Mycoplasma pulmonis	0.89	Predict
NOM010_scaffold16391_2	Bacteroides fragilis	0.89	Predict
NOM010_scaffold19030_3	Mycoplasma pulmonis	0.74	Predict
NOM010_scaffold7692_1	Mycoplasma pulmonis	0.78	Predict
NOM010_scaffold27711_4	Candidatus Pelagibacter ubique	0.92	Predict
NOM010_scaffold13739_1	Clostridioides difficile	0.75	Predict
NOM010_scaffold27824_1	Colwellia psychrerythraea	1	CRISPR
NOM010_scaffold5951_6	Mycoplasma pulmonis	0.83	Predict
NOM010_scaffold4318_15	Colwellia psychrerythraea	0.87	Predict
NOM010_scaffold41566_2	Mycoplasma pulmonis	0.88	Predict
NOM010_scaffold30699_2	Bacteroides fragilis	0.76	Predict
NOM010_scaffold41431_1	Mycoplasma pulmonis	0.77	Predict
NOM010_scaffold29615_5	Flavobacterium psychrophilum	0.76	Predict
NOM010_scaffold7945_5	Candidatus Pelagibacter ubique	0.97	Predict
NOM010_scaffold16353_1	Mycoplasma pulmonis	0.97	Predict
NOM010_scaffold26455_8	Candidatus Pelagibacter ubique	0.82	Predict
NOM010_scaffold15321_2	Flavobacterium columnare	1	CRISPR
NOM010_scaffold27687_3	Mycoplasma pulmonis	0.76	Predict
NOM010_scaffold23538_2	Mycoplasma pulmonis	0.73	Predict
NOM010_scaffold16212_1	Mycoplasma pulmonis	0.73	Predict
NOM010_scaffold20969_4	Micromonospora chaiyaphumensis	1	CRISPR

NOM010_scaffold34_1	Colwellia psychrerythraea	1	CRISPR
NOM010_scaffold27644_3	Mycoplasma pulmonis	0.84	Predict
NOM010_scaffold24440_2	Mycoplasma pulmonis	0.73	Predict
NOM012_scaffold41765_3	Candidatus Pelagibacter ubique	1	CRISPR
NOM012_scaffold17023_1	Morganella morganii	0.72	Predict
NOM012_scaffold8044_2	Colwellia psychrerythraea	0.92	Predict
NOM012_scaffold58733_1	Mycoplasma pulmonis	1	CRISPR
NOM012_scaffold15076_8	Mycoplasma pulmonis	0.82	Predict
NOM012_scaffold8044_6	Mycoplasma pulmonis	0.82	Predict
NOM012_scaffold28597_3	Colwellia psychrerythraea	0.91	Predict
NOM012_scaffold59022_1	Mycoplasma pulmonis	0.93	Predict
NOM012_scaffold51561_1	Candidatus Pelagibacter ubique	0.99	Predict
NOM012_C822191_1	Mycoplasma pulmonis	1	CRISPR
NOM012_scaffold4060_3	Mycoplasma pulmonis	0.78	Predict
NOM012_scaffold8574_7	Mycoplasma pulmonis	0.82	Predict
NOM012_scaffold45669_3	Lactobacillus jensenii	1	CRISPR
NOM012_scaffold59297_1	Mycoplasma pulmonis	0.76	Predict
NOM012_scaffold16848_9	Lactobacillus jensenii	0.89	Predict
NOM012_scaffold19624_3	Bacteroides fragilis	0.84	Predict
NOM012_scaffold53716_1	Mycoplasma pulmonis	0.86	Predict
NOM012_scaffold15076_7	Mycoplasma pulmonis	0.94	Predict
NOM012_scaffold57636_2	Colwellia psychrerythraea	0.7	Predict
NOM012_scaffold118_12	Mycoplasma pulmonis	0.83	Predict
NOM012_scaffold6750_2	Mycoplasma pulmonis	0.93	Predict
NOM013_scaffold15784_2	Candidatus Pelagibacter ubique	0.93	Predict
NOM013_scaffold54467_1	Candidatus Pelagibacter ubique	0.93	Predict
NOM013_scaffold18789_1	Parabacteroides merdae	0.99	Predict
NOM013_scaffold47736_4	Cellulophaga baltica	1	CRISPR
NOM013_scaffold13725_4	Bacteroides fragilis	0.72	Predict
NOM013_scaffold28173_6	Mycoplasma pulmonis	0.99	Predict
NOM013_scaffold30855_5	Flavobacterium columnare	1	Predict
NOM013_scaffold283_1	Candidatus Pelagibacter ubique	0.86	Predict
NOM013_scaffold16346_5	Candidatus Pelagibacter ubique	0.94	Predict
NOM013_scaffold55293_1	Candidatus Pelagibacter ubique	1	CRISPR
NOM013_scaffold16346_8	Candidatus Pelagibacter ubique	0.79	Predict
NOM013_scaffold19917_2	Mycoplasma pulmonis	0.74	Predict
NOM013_scaffold20568_5	Candidatus Pelagibacter ubique	0.73	Predict
NOM013_scaffold42371_1	Colwellia psychrerythraea	0.84	Predict
NOM013_scaffold31636_2	Candidatus Pelagibacter ubique	0.97	Predict
NOM013_scaffold7_2	Parabacteroides merdae	0.84	Predict
NOM013_scaffold19917_6	Mycoplasma pulmonis	0.89	Predict
NOM013_scaffold47736_3	Mycoplasma pulmonis	1	CRISPR
NOM013_scaffold22501_3	Bacteroides vulgatus	1	CRISPR
NOM013_scaffold54467_2	Candidatus Pelagibacter ubique	0.95	Predict
NOM013_scaffold11200_1	Mycoplasma pulmonis	0.84	Predict
NOM013_scaffold49212_2	Mycoplasma pulmonis	0.88	Predict
NOM013_scaffold9824_2	Mycoplasma pulmonis	0.71	Predict
NOM013_scaffold4736_1	Colwellia psychrerythraea	0.81	Predict

NOM013_scaffold32541_1.	Parabacteroides merdae	0.74	Predict
NOM013_scaffold31511_2	Cellulophaga baltica	0.72	Predict
NOM013_scaffold3281_4	Clostridioides difficile	0.73	Predict
NOM013_scaffold55128_3	Cellulophaga baltica	0.78	Predict
NOM013_scaffold18200_3	Candidatus Pelagibacter ubique	0.76	Predict
NOM013_scaffold31511_3	Mycoplasma pulmonis	0.79	Predict
NOM013_scaffold38707_7	Colwellia psychrerythraea	0.97	Predict
NOM013_scaffold21734_3	Bacteroides fragilis	0.74	Predict
NOM013_scaffold16346_2	Candidatus Pelagibacter ubique	0.76	Predict
NOM013_scaffold28579_4	Cellulophaga baltica	1	CRISPR
NOM013_scaffold55291_1	Candidatus Pelagibacter ubique	0.71	Predict
NOM013_scaffold14898_7	Mycoplasma pulmonis	0.82	Predict
NOM013_scaffold16204_2	Colwellia psychrerythraea	1	CRISPR
NOM013_scaffold54010_1	Bacteroides fragilis	0.78	Predict
NOM013_scaffold31511_4	Cellulophaga baltica	0.96	Predict
NOM013_scaffold13071_1	Mycoplasma pulmonis	0.76	Predict
NOM013_scaffold42353_2	Candidatus Pelagibacter ubique	0.76	Predict
NOM014_scaffold24348_6	Flavobacterium columnare	0.71	Predict
NOM014_scaffold14255_1	Mycoplasma pulmonis	1	Predict
NOM014_scaffold15996_1	Mycoplasma pulmonis	1	Predict
NOM014_C390595_1	Eubacterium sp. am_0171	1	CRISPR
NOM014_scaffold25704_1	Mycoplasma pulmonis	0.87	Predict
NOM014_scaffold24348_5	Cellulophaga baltica	1	Predict
NOM014_scaffold28199_1	Colwellia psychrerythraea	1	Predict
NOM014_scaffold11825_3	Clostridioides difficile	0.86	Predict
NOM014_scaffold8227_3	Mycoplasma pulmonis	0.91	Predict
NOM014_scaffold28027_1	Mycoplasma pulmonis	0.77	Predict
NOM014_scaffold15825_1	Mycoplasma pulmonis	0.76	Predict
NOM014_scaffold27185_1	Mycoplasma pulmonis	0.74	Predict
NOM014_scaffold28228_1	Mycoplasma pulmonis	0.73	Predict
NOM014_scaffold14811_8	Parabacteroides distasonis	0.85	Predict
NOM014_scaffold23281_1	Mycoplasma pulmonis	1	Predict
NOM014_scaffold27301_2	Mycoplasma pulmonis	1	CRISPR
NOM014_scaffold19260_1	Bacteroides fragilis	0.81	Predict
NOM014_scaffold28329_1	Mycoplasma pulmonis	1	CRISPR
NOM014_scaffold9405_2	Candidatus Pelagibacter ubique	0.74	Predict
NOM014_scaffold3384_5	Bacteroides fragilis	0.99	Predict
NOM014_scaffold25598_1	Bacteroides fragilis	0.94	Predict
NOM014_scaffold8317_1	Mycoplasma pulmonis	0.71	Predict
NOM014_scaffold19787_2	Mycoplasma pulmonis	0.91	Predict
NOM014_scaffold28328_1	Parabacteroides merdae	0.77	Predict
NOM014_scaffold490_36	Bacteroides fragilis	0.77	Predict
NOM014_C390319_1	Mycoplasma pulmonis	1	CRISPR
NOM014_scaffold13553_1	Bacteroides fragilis	0.79	Predict
NOM014_scaffold27259_1	Bacteroides fragilis	1	Predict
NOM014_scaffold5743_3	Colwellia psychrerythraea	1	CRISPR
NOM014_scaffold28145_4	Mycoplasma pulmonis	0.99	Predict
NOM014_scaffold9372_1	Mycoplasma pulmonis	1	CRISPR

NOM014_scaffold15996_2	Lactobacillus gasseri	0.81	Predict
NOM014_scaffold13234_1	Mycoplasma pulmonis	0.73	Predict
NOM014_scaffold21899_2	Mycoplasma pulmonis	1	CRISPR
NOM014_scaffold28352_1	Colwellia psychrerythraea	1	CRISPR
NOM014_scaffold15794_3	Lactobacillus johnsonii	0.71	Predict
NOM015_scaffold35862_1	Mycoplasma pulmonis	0.88	Predict
NOM015_scaffold36658_1	Mycoplasma pulmonis	0.87	Predict
NOM015_scaffold1034_1	Lactobacillus gasseri	1	CRISPR
NOM015_scaffold16922_3	Bacteroides fragilis	0.9	Predict
NOM015_scaffold34161_4	Mycoplasma pulmonis	0.88	Predict
NOM015_scaffold226_1	Colwellia psychrerythraea	0.9	Predict
NOM015_scaffold10294_6	Mycoplasma pulmonis	0.71	Predict
NOM015_scaffold4016_5	Mycoplasma pulmonis	1	CRISPR
NOM015_scaffold4819_1	Colwellia psychrerythraea	0.92	Predict
NOM015_scaffold3437_9	Mycoplasma pulmonis	0.7	Predict
NOM015_scaffold124_1	Colwellia psychrerythraea	0.8	Predict
NOM015_scaffold18383_1	Mycoplasma pulmonis	1	CRISPR
NOM015_scaffold20240_1	Colwellia psychrerythraea	0.78	Predict
NOM015_scaffold16366_1	Flavobacterium columnare	0.94	Predict
NOM015_scaffold23464_3	Cellulophaga baltica	1	CRISPR
NOM015_scaffold8918_6	Colwellia psychrerythraea	0.72	Predict
NOM015_scaffold20333_3	Mycoplasma pulmonis	1	CRISPR
NOM015_scaffold36796_1	Bacteroides fragilis	0.85	Predict
NOM015_scaffold14266_3	Yersinia pestis	0.86	Predict
NOM015_scaffold19627_1	Mycoplasma pulmonis	0.99	Predict
NOM015_scaffold23464_2	Cellulophaga baltica	0.72	Predict
NOM015_scaffold15823_2	Mycoplasma pulmonis	1	CRISPR
NOM015_scaffold6127_1	Colwellia psychrerythraea	1	CRISPR
NOM015_scaffold11230_6	Candidatus Pelagibacter ubique	1	CRISPR
NOM015_scaffold15823_5	Mycoplasma pulmonis	1	CRISPR
NOM015_scaffold10294_4	Colwellia psychrerythraea	1	CRISPR
NOM015_scaffold5941_3	Mycoplasma pulmonis	0.86	Predict
NOM015_scaffold252_7	Candidatus Pelagibacter ubique	1	CRISPR
NOM015_scaffold749_3	Colwellia psychrerythraea	0.75	Predict
NOM015_scaffold8498_6	Azospirillum brasilense	0.98	Predict
NOM015_scaffold16067_4	Candidatus Pelagibacter ubique	0.7	Predict
NOM015_scaffold9242_9	Bacteroides fragilis	0.95	Predict
NOM015_scaffold27697_2	Cellulophaga baltica	1	CRISPR
NOM015_scaffold26753_1	Mycoplasma pulmonis	0.82	Predict
NOM016_scaffold29885_1	Mycoplasma pulmonis	1	CRISPR
NOM016_scaffold35286_4	Rhodococcus hoagii	0.9	Predict
NOM016_scaffold35286_7	Mycoplasma pulmonis	1	CRISPR
NOM016_scaffold35976_5	Mycoplasma pulmonis	1	CRISPR
NOM016_scaffold20671_4	Bacteroides fragilis	0.93	Predict
NOM016_scaffold33084_3	Candidatus Pelagibacter ubique	0.83	Predict
NOM016_scaffold16005_4	Mycoplasma pulmonis	0.76	Predict
NOM016_scaffold38252_1	Mycoplasma pulmonis	0.92	Predict
NOM016_scaffold31191_1	Mycoplasma pulmonis	0.87	Predict

NOM016_scaffold53315_1	Mycoplasma pulmonis	0.76	Predict
NOM016_scaffold37747_2	Mycoplasma pulmonis	0.95	Predict
NOM016_scaffold54950_4	Bacteroides fragilis	0.82	Predict
NOM016_scaffold55549_3	Mycoplasma pulmonis	0.93	Predict
NOM016_scaffold47449_3	Mycoplasma pulmonis	0.81	Predict
NOM016_scaffold54079_1	Candidatus Pelagibacter ubique	0.98	Predict
NOM016_scaffold12609_2	Mycoplasma pulmonis	1	CRISPR
NOM016_scaffold25995_3	Bacillus cereus	1	CRISPR
NOM016_scaffold16005_2	Mycoplasma pulmonis	0.72	Predict
NOM016_scaffold55496_1	Bacteroides fragilis	0.86	Predict
NOM016_scaffold2585_1	Parabacteroides distasonis	1	CRISPR
NOM016_scaffold55443_2	Candidatus Pelagibacter ubique	0.79	Predict
NOM016_scaffold1680_10	Mycoplasma pulmonis	1	CRISPR
NOM016_scaffold28365_4	Mycoplasma pulmonis	0.73	Predict
NOM016_scaffold35286_1	Mycoplasma pulmonis	0.83	Predict
NOM016_scaffold12609_6	Mycoplasma pulmonis	0.99	Predict
NOM016_scaffold39131_1	Parabacteroides distasonis	0.73	Predict
NOM016_scaffold55593_1	Mycoplasma pulmonis	0.76	Predict
NOM016_scaffold31927_4	Mycoplasma pulmonis	0.7	Predict
NOM016_scaffold3257_24	Colwellia psychrerythraea	1	CRISPR
NOM016_scaffold55581_3	Mycoplasma pulmonis	1	CRISPR
NOM017_scaffold43934_1	Achromobacter xylosoxidans	0.87	Predict
NOM017_scaffold40696_1	Colwellia psychrerythraea	0.96	Predict
NOM017_scaffold41715_2	Bacillus cereus	1	CRISPR
NOM017_scaffold2822_6	Klebsiella oxytoca	0.94	Predict
NOM017_scaffold4867_13	Candidatus Pelagibacter ubique	0.76	Predict
NOM017_scaffold45485_2	Mycoplasma pulmonis	0.76	Predict
NOM017_scaffold45477_1	Mycoplasma pulmonis	0.99	Predict
NOM017_scaffold42764_1	Mycoplasma pulmonis	1	CRISPR
NOM017_scaffold45435_1	Mycoplasma pulmonis	1	CRISPR
NOM017_scaffold996_22	Bacillus megaterium	0.72	Predict
NOM017_scaffold11947_1	Mycoplasma pulmonis	1	CRISPR
NOM017_scaffold22366_1	Clostridioides difficile	1	CRISPR
NOM017_scaffold845_1	Colwellia psychrerythraea	0.98	Predict
NOM017_scaffold10596_2	Lactobacillus gasseri	1	CRISPR
NOM017_scaffold6578_5	Candidatus Pelagibacter ubique	0.98	Predict
NOM017_scaffold45031_4	Aliivibrio fischeri	0.78	Predict
NOM017_scaffold44208_2	Mycoplasma pulmonis	1	CRISPR
NOM017_scaffold22040_3	Streptococcus mutans	0.75	Predict
NOM017_scaffold45430_1	Mycoplasma pulmonis	1	CRISPR
NOM017_scaffold11947_3	Colwellia psychrerythraea	1	CRISPR
NOM017_scaffold6796_4	Staphylococcus xylosus	0.75	Predict
NOM018_scaffold13094_4	Mycoplasma pulmonis	0.84	Predict
NOM018_scaffold30222_3	Mycoplasma pulmonis	0.95	Predict
NOM018_scaffold54740_2	Colwellia psychrerythraea	0.98	Predict
NOM018_scaffold5764_2	Mycoplasma pulmonis	0.7	Predict
NOM018_scaffold51296_4	Mycoplasma pulmonis	0.99	Predict
NOM018_scaffold42663_2	Flavobacterium columnare	0.82	Predict

NOM018_scaffold29911_1	Colwellia psychrerythraea	0.87	Predict
NOM018_scaffold53732_1	Mycoplasma pulmonis	0.96	Predict
NOM018_scaffold53145_1	Mycoplasma pulmonis	1	CRISPR
NOM018_scaffold1559_3	Mycoplasma pulmonis	1	CRISPR
NOM018_scaffold1296_2	Bacteroides fragilis	0.94	Predict
NOM018_scaffold37159_1	Mycoplasma pulmonis	0.97	Predict
NOM018_scaffold55244_1	Candidatus Pelagibacter ubique	0.71	Predict
NOM018_scaffold43446_1	Mycoplasma pulmonis	1	CRISPR
NOM018_scaffold18121_1	Parabacteroides merdae	0.97	Predict
NOM018_scaffold40401_5	Clostridioides difficile	0.93	Predict
NOM018_scaffold9490_1	Colwellia psychrerythraea	0.78	Predict
NOM018_scaffold191_5	Mycoplasma pulmonis	0.89	Predict
NOM018_scaffold15821_1	Thermoanaerobacterium saccharolytic	0.96	Predict
NOM018_scaffold54015_1	Mycoplasma pulmonis	0.83	Predict
NOM018_scaffold9772_2	Parabacteroides merdae	0.8	Predict
NOM018_scaffold2740_11	Mycoplasma pulmonis	1	CRISPR
NOM018_scaffold20530_1	Colwellia psychrerythraea	0.79	Predict
NOM018_scaffold11125_2	Mycoplasma pulmonis	0.72	Predict
NOM018_scaffold115_1	Sinorhizobium meliloti	0.94	Predict
NOM018_scaffold50471_1	Mycoplasma pulmonis	0.94	Predict
NOM018_scaffold30_5	Roseburia inulinivorans	1	CRISPR
NOM018_scaffold24796_3	Clostridioides difficile	1	CRISPR
NOM018_scaffold51296_6	Burkholderia cenocepacia	0.92	Predict
NOM018_scaffold1842_24	Mycoplasma pulmonis	0.96	Predict
NOM018_scaffold42663_1	Candidatus Pelagibacter ubique	1	Predict
NOM018_scaffold52711_1	Candidatus Pelagibacter ubique	0.83	Predict
NOM018_scaffold50197_1	Candidatus Pelagibacter ubique	0.77	Predict
NOM018_scaffold4393_1	Candidatus Pelagibacter ubique	0.8	Predict
NOM018_C821692_1	Mycoplasma pulmonis	0.92	Predict
NOM019_scaffold37_3	Mycoplasma pulmonis	0.88	Predict
NOM019_scaffold46040_6	Candidatus Pelagibacter ubique	0.95	Predict
NOM019_scaffold16958_6	Mycoplasma pulmonis	0.95	Predict
NOM019_scaffold52788_2	Cellulophaga baltica	1	CRISPR
NOM019_scaffold15207_2	Mycoplasma pulmonis	1	CRISPR
NOM019_scaffold52669_1	Mycoplasma pulmonis	1	CRISPR
NOM019_scaffold53126_1	Mycoplasma pulmonis	1	CRISPR
NOM019_scaffold40333_6	Mycoplasma pulmonis	1	CRISPR
NOM019_scaffold16958_5	Colwellia psychrerythraea	0.8	Predict
NOM019_scaffold50412_4	Clostridium tetani	1	CRISPR
NOM019_scaffold22541_5	Mycoplasma pulmonis	0.78	Predict
NOM019_scaffold53126_2	Clostridium tetani	1	CRISPR
NOM019_scaffold31682_1	Mycoplasma pulmonis	0.98	Predict
NOM019_scaffold22109_1	Candidatus Pelagibacter ubique	0.91	Predict
NOM019_scaffold45409_1	Parabacteroides distasonis	0.96	Predict
NOM019_scaffold50412_3	Mycoplasma pulmonis	1	CRISPR
NOM019_scaffold12477_8	Bacillus subtilis	0.95	Predict
NOM019_scaffold27076_1	Colwellia psychrerythraea	0.99	Predict
NOM019_scaffold12848_1	Candidatus Pelagibacter ubique	0.78	Predict

NOM019_scaffold46040_3	Candidatus Pelagibacter ubique	0.92	Predict
NOM019_scaffold36262_3	Colwellia psychrerythraea	1	CRISPR
NOM019_scaffold14421_2	Colwellia psychrerythraea	0.72	Predict
NOM019_scaffold24407_7	Listeria monocytogenes	0.95	Predict
NOM019_scaffold52788_3	Mycoplasma pulmonis	1	CRISPR
NOM019_scaffold22541_7	Colwellia psychrerythraea	1	CRISPR
NOM019_scaffold53127_2	Bacteroides fragilis	0.98	Predict
NOM020_scaffold5494_5	Candidatus Pelagibacter ubique	1	CRISPR
NOM020_scaffold33908_1	Mycoplasma pulmonis	0.99	Predict
NOM020_scaffold38448_1	Mycoplasma pulmonis	0.92	Predict
NOM020_scaffold7097_2	Mycoplasma pulmonis	0.92	Predict
NOM020_scaffold39245_1	Streptococcus mutans	1	CRISPR
NOM020_scaffold6778_1	Colwellia psychrerythraea	0.76	Predict
NOM020_scaffold14028_7	Mycoplasma pulmonis	0.89	Predict
NOM020_scaffold1704_2	Streptomyces sp. MUSC 125	1	CRISPR
NOM020_scaffold13829_4	Mycoplasma pulmonis	0.92	Predict
NOM020_scaffold22877_2	Candidatus Pelagibacter ubique	0.78	Predict
NOM020_scaffold32231_1	Bacteroides fragilis	0.7	Predict
NOM020_C476630_1	Colwellia psychrerythraea	1	CRISPR
NOM020_scaffold38253_1	Mycoplasma pulmonis	0.82	Predict
NOM020_scaffold12913_3	Streptococcus mutans	0.94	Predict
NOM020_scaffold37822_4	Mycoplasma pulmonis	1	CRISPR
NOM020_scaffold31685_6	Mycoplasma pulmonis	0.74	Predict
NOM020_scaffold38253_2	Rhodococcus hoagii	1	CRISPR
NOM020_scaffold35100_2	Candidatus Pelagibacter ubique	0.85	Predict
NOM020_scaffold3731_3	Mycoplasma pulmonis	0.91	Predict
NOM020_scaffold10070_1	Mycoplasma pulmonis	0.88	Predict
NOM020_scaffold26272_1	Colwellia psychrerythraea	1	CRISPR
NOM020_scaffold16941_1	Candidatus Pelagibacter ubique	0.84	Predict
NOM020_scaffold14364_1	Mycoplasma pulmonis	0.81	Predict
NOM020_scaffold39269_1	Mycoplasma pulmonis	0.74	Predict
NOM020_scaffold39193_2	Colwellia psychrerythraea	1	CRISPR
NOM022_scaffold35384_4	Mycoplasma pulmonis	0.93	Predict
NOM022_scaffold25627_6	Mycoplasma pulmonis	0.87	Predict
NOM022_scaffold2146_2	Mycoplasma pulmonis	0.93	Predict
NOM022_scaffold3633_6	Mycoplasma pulmonis	0.76	Predict
NOM022_scaffold35226_2	Mycoplasma pulmonis	0.99	Predict
NOM022_scaffold34355_1	Parabacteroides distasonis	0.85	Predict
NOM022_scaffold16867_5	Colwellia psychrerythraea	0.75	Predict
NOM022_scaffold11835_1	Mycoplasma pulmonis	0.75	Predict
NOM022_scaffold25627_3	Mycoplasma pulmonis	1	CRISPR
NOM022_scaffold1399_24	Colwellia psychrerythraea	0.96	Predict
NOM022_scaffold23992_2	Bacteroides fragilis	0.89	Predict
NOM022_C499778_1	Bacteroides fragilis	0.89	Predict
NOM022_scaffold35027_1	Mycoplasma pulmonis	1	CRISPR
NOM022_scaffold29333_3	Colwellia psychrerythraea	0.77	Predict
NOM022_scaffold9236_24	Mycoplasma pulmonis	0.76	Predict
NOM022_scaffold31238_3	Vibrio alginolyticus	0.79	Predict

NOM022_C499744_1	Mycoplasma pulmonis	1	CRISPR
NOM022_scaffold10614_1	Mycoplasma pulmonis	0.86	Predict
NOM022_C499370_1	Bacteroides cellulosilyticus	1	CRISPR
NOM022_scaffold25723_1	Mycoplasma pulmonis	0.71	Predict
NOM022_scaffold25634_1	Sinorhizobium meliloti	0.84	Predict
NOM022_scaffold30875_1	Bacteroides fragilis	0.98	Predict
NOM022_scaffold35362_2	Bacteroides fragilis	0.82	Predict
NOM022_scaffold35326_5	Mycoplasma pulmonis	0.97	Predict
NOM023_scaffold1309_14	Parabacteroides distasonis	1	CRISPR
NOM023_scaffold7114_2	Bacteroides fragilis	1	CRISPR
NOM023_scaffold2396_5	Bacteroides fragilis	0.9	Predict
NOM023_scaffold6600_10	Bacteroides fragilis	0.77	Predict
NOM023_scaffold21303_1	Mycoplasma pulmonis	1	CRISPR
NOM023_scaffold219_4	Mycoplasma pulmonis	1	CRISPR
NOM023_scaffold9577_8	Mycoplasma pulmonis	0.76	Predict
NOM023_scaffold4208_9	Mycoplasma pulmonis	1	CRISPR
NOM023_scaffold11170_3	Bacteroides vulgatus	1	CRISPR
NOM023_scaffold1634_1	Mycoplasma pulmonis	0.85	Predict
NOM023_scaffold4816_2	Parabacteroides distasonis	0.83	Predict
NOM023_scaffold6600_7	Bacteroides fragilis	1	CRISPR
NOM023_scaffold21124_2	Bacteroides fragilis	1	CRISPR
NOM023_scaffold7583_7	Parabacteroides distasonis	0.92	Predict
NOM023_scaffold67_25	Parabacteroides distasonis	1	CRISPR
NOM023_scaffold6623_2	Mycoplasma pulmonis	0.95	Predict
NOM023_scaffold16737_2	Mycoplasma pulmonis	0.98	Predict
NOM023_scaffold18365_1	Mycoplasma pulmonis	1	CRISPR
NOM023_scaffold67_27	Parabacteroides distasonis	1	CRISPR
NOM023_scaffold20012_1	Candidatus Pelagibacter ubique	0.77	Predict
NOM023_scaffold5561_1	Mycoplasma pulmonis	0.94	Predict
NOM023_scaffold5693_18	Parabacteroides distasonis	1	CRISPR
NOM025_scaffold13742_3	Colwellia psychrerythraea	0.73	Predict
NOM025_scaffold1166_3	Mycoplasma pulmonis	0.71	Predict
NOM025_scaffold24674_1	Candidatus Pelagibacter ubique	1	CRISPR
NOM025_scaffold23401_1	Clostridium tetani	1	Predict
NOM025_scaffold2064_3	Bacteroides fragilis	0.98	Predict
NOM025_scaffold173_6	Colwellia psychrerythraea	1	CRISPR
NOM025_scaffold3864_1	Candidatus Pelagibacter ubique	0.72	Predict
NOM025_scaffold6532_2	Mycoplasma pulmonis	0.88	Predict
NOM025_scaffold22402_1	Mycoplasma pulmonis	0.73	Predict
NOM025_scaffold18513_1	Mycoplasma pulmonis	1	CRISPR
NOM025_scaffold2_1	Mycoplasma pulmonis	0.99	Predict
NOM025_scaffold24512_5	Colwellia psychrerythraea	0.84	Predict
NOM025_scaffold20521_2	Colwellia psychrerythraea	0.79	Predict
NOM025_scaffold62_1	Mycoplasma pulmonis	0.71	Predict
NOM025_scaffold24601_2	Colwellia psychrerythraea	1	CRISPR
NOM025_scaffold7710_1	Colwellia psychrerythraea	0.72	Predict
NOM025_scaffold4063_4	Caulobacter vibrioides	1	CRISPR
NOM025_scaffold7973_1	Mycoplasma pulmonis	0.82	Predict

NOM025_scaffold78_1	Candidatus Pelagibacter ubique	0.77	Predict
NOM025_scaffold173_5	Mycoplasma pulmonis	1	CRISPR
NOM025_scaffold24504_2	Parabacteroides distasonis	0.99	Predict
NOM025_scaffold14182_5	Mycoplasma pulmonis	0.72	Predict
NOM025_scaffold23752_1	Mycoplasma pulmonis	0.79	Predict
NOM025_scaffold24528_3	Mycoplasma pulmonis	1	CRISPR
NOM025_scaffold24496_1	Mycoplasma pulmonis	0.82	Predict
NOM025_scaffold57_1	Mycoplasma pulmonis	0.83	Predict
NOM025_scaffold16448_2	Mycoplasma pulmonis	0.88	Predict
NOM025_scaffold24601_3	Mycoplasma pulmonis	0.99	Predict
NOM025_scaffold24362_1	Candidatus Pelagibacter ubique	0.7	Predict
NOM025_scaffold3455_1	Thermoanaerobacterium saccharolytic	0.87	Predict
NOM025_scaffold17951_2	Candidatus Pelagibacter ubique	0.79	Predict
NOM025_scaffold3831_6	Parabacteroides distasonis	0.83	Predict
NOM025_scaffold19612_1	Mycoplasma pulmonis	0.94	Predict
NOM025_scaffold3792_5	Parabacteroides distasonis	0.9	Predict
NOM025_scaffold23962_2	Colwellia psychrerythraea	1	CRISPR
NOM025_scaffold7710_3	Lactobacillus gasseri	1	CRISPR
NOM026_scaffold21771_5	Mycoplasma pulmonis	0.99	Predict
NOM026_scaffold4045_2	Mycoplasma pulmonis	0.98	Predict
NOM026_scaffold51_21	Colwellia psychrerythraea	1	CRISPR
NOM026_scaffold3975_3	Mycoplasma pulmonis	0.88	Predict
NOM026_scaffold29015_2	Flavobacterium psychrophilum	1	CRISPR
NOM026_scaffold8128_4	Mycoplasma pulmonis	0.82	Predict
NOM026_scaffold4107_22	Mycoplasma pulmonis	0.87	Predict
NOM026_scaffold29015_1	Candidatus Pelagibacter ubique	1	CRISPR
NOM026_scaffold29015_2	Mycoplasma pulmonis	0.95	Predict
NOM026_scaffold43414_4	Clostridioides difficile	0.98	Predict
NOM026_scaffold39978_1	Candidatus Pelagibacter ubique	0.9	Predict
NOM026_scaffold14437_2	Streptococcus mutans	0.86	Predict
NOM026_scaffold1436_1	Mycoplasma pulmonis	0.74	Predict
NOM026_scaffold788_2	Aliivibrio fischeri	0.79	Predict
NOM026_scaffold287_6	Colwellia psychrerythraea	0.89	Predict
NOM026_scaffold95_3_2	Colwellia psychrerythraea	0.82	Predict
NOM026_scaffold18477_4	Klebsiella pneumoniae	1	CRISPR
NOM026_scaffold273_8	Candidatus Pelagibacter ubique	0.8	Predict
NOM026_scaffold5049_5	Listeria monocytogenes	1	CRISPR
NOM026_scaffold100_1	Mycoplasma pulmonis	0.79	Predict
NOM026_scaffold2514_2	Mycoplasma pulmonis	0.73	Predict
NOM026_scaffold39163_3	Colwellia psychrerythraea	0.84	Predict
NOM026_scaffold18477_1	Colwellia psychrerythraea	1	CRISPR
NOM027_scaffold10318_1	Bacillus alcalophilus	1	CRISPR
NOM027_scaffold3843_3	Colwellia psychrerythraea	1	CRISPR
NOM027_scaffold298_5	Candidatus Pelagibacter ubique	0.96	Predict
NOM027_scaffold3057_13	Mycoplasma pulmonis	0.98	Predict
NOM027_scaffold690_9	Mycoplasma pulmonis	1	CRISPR
NOM027_scaffold233_8	Candidatus Pelagibacter ubique	1	CRISPR
NOM027_scaffold21902_2	Bacillus thuringiensis	1	CRISPR

NOM027_scaffold21753_2	Clostridium perfringens	1	CRISPR
NOM027_scaffold8220_2	Candidatus Pelagibacter ubique	1	CRISPR
NOM027_scaffold12848_6	Bacillus anthracis	0.72	Predict
NOM027_scaffold95_21	Bacteroides fragilis	0.93	Predict
NOM027_scaffold928_6	Megasphaera elsdenii	1	CRISPR
NOM027_scaffold17819_1	Colwellia psychrerythraea	1	CRISPR
NOM027_scaffold4263_1	Flavobacterium columnare	0.9	Predict
NOM027_scaffold11970_1	Mycoplasma pulmonis	0.74	Predict
NOM027_scaffold21806_1	Mycoplasma pulmonis	0.82	Predict
NOM027_scaffold21896_1	Bifidobacterium longum	1	CRISPR
NOM027_scaffold18467_1	Candidatus Pelagibacter ubique	0.86	Predict
NOM027_scaffold21932_1	Lactobacillus jensenii	0.87	Predict
NOM027_scaffold4308_5	Mycoplasma pulmonis	1	CRISPR
NOM027_scaffold16572_1	Colwellia psychrerythraea	0.81	Predict
NOM027_scaffold8270_1	Mycoplasma pulmonis	0.96	Predict
NOM027_scaffold87_1	Mycoplasma pulmonis	0.72	Predict
NOM027_scaffold13632_2	Clostridium perfringens	1	CRISPR
NOM027_scaffold21847_1	Flavobacterium columnare	1	CRISPR
NOM028_scaffold2463_21	Clavibacter michiganensis	1	CRISPR
NOM028_C573709_1	Mycoplasma pulmonis	1	CRISPR
NOM028_scaffold5766_3	Mycoplasma pulmonis	1	CRISPR
NOM028_scaffold43134_4	Mycoplasma pulmonis	0.94	Predict
NOM028_scaffold10923_1	Colwellia psychrerythraea	1	CRISPR
NOM028_scaffold39054_2	Candidatus Pelagibacter ubique	0.89	Predict
NOM028_scaffold5154_1	Mycoplasma pulmonis	1	CRISPR
NOM028_scaffold3552_4	Colwellia psychrerythraea	1	CRISPR
NOM028_scaffold11407_1	Azospirillum brasilense	1	Predict
NOM028_scaffold44706_5	Mycoplasma pulmonis	0.78	Predict
NOM028_scaffold10197_9	Clostridioides difficile	1	CRISPR
NOM028_scaffold29187_1	Colwellia psychrerythraea	1	CRISPR
NOM028_scaffold47604_1	Mycoplasma pulmonis	0.91	Predict
NOM028_scaffold10515_1	Colwellia psychrerythraea	0.97	Predict
NOM028_C573817_1	Mycoplasma pulmonis	1	CRISPR
NOM028_scaffold13734_2	Candidatus Pelagibacter ubique	1	CRISPR
NOM028_scaffold28052_1	Candidatus Pelagibacter ubique	0.84	Predict
NOM028_scaffold47465_1	Bacteroides fragilis	0.79	Predict
NOM028_scaffold46573_2	Mycoplasma pulmonis	0.71	Predict
NOM028_scaffold691_1	Vibrio alginolyticus	1	CRISPR
NOM028_C572983_1	Mycoplasma pulmonis	0.75	Predict
NOM028_scaffold36229_1	Mycoplasma pulmonis	0.99	Predict
NOM028_scaffold36508_1	Clostridium perfringens	0.89	Predict
NOM028_scaffold10515_2	Mycoplasma pulmonis	1	CRISPR
NOM028_scaffold13734_2	Mycoplasma pulmonis	0.76	Predict
NOM028_C573171_1	Clostridioides difficile	1	CRISPR
NOM028_scaffold44706_6	Anaerostipes hadrus	1	CRISPR
NOM028_scaffold8690_5	Mycoplasma pulmonis	0.92	Predict
NOM028_scaffold6524_6	Mycoplasma pulmonis	0.9	Predict
NOM028_scaffold41121_3	Mycoplasma pulmonis	1	CRISPR

NOM028_scaffold47539_2	Bacteroides fragilis	0.85	Predict
NOM028_scaffold19021_1	Colwellia psychrerythraea	0.92	Predict
NOM028_scaffold46746_1	Trichormus variabilis	1	CRISPR
NOM028_scaffold6524_7	Carboxydocella sp. JDF658	1	CRISPR
NOM028_scaffold46512_4	Bacteroides fragilis	0.71	Predict
NOM028_scaffold2529_5	Streptococcus pneumoniae	0.85	Predict
NOM028_scaffold11058_9	Clostridium perfringens	1	CRISPR
NOM028_scaffold15800_1	Candidatus Pelagibacter ubique	0.77	Predict
NOM028_scaffold10197_6	Bacillus alcalophilus	0.93	Predict
NOM028_scaffold110_3	Candidatus Pelagibacter ubique	0.74	Predict
NOM028_scaffold45529_1	Mycoplasma pulmonis	0.97	Predict
NOM028_scaffold44706_1	Clostridioides difficile	0.79	Predict
NOM028_scaffold366_38	Mycoplasma pulmonis	0.9	Predict
NOM028_scaffold37086_1	Mycoplasma pulmonis	0.84	Predict
NOM028_scaffold2922_6	Candidatus Pelagibacter ubique	0.8	Predict
NOM028_scaffold7965_13	Mycoplasma pulmonis	0.79	Predict
NOM028_scaffold6524_2	Mycoplasma pulmonis	0.84	Predict
NOM028_scaffold47589_2	Mycoplasma pulmonis	0.81	Predict
NOM028_scaffold29797_1	Mycoplasma pulmonis	1	CRISPR
NOM028_scaffold37620_3	Colwellia psychrerythraea	0.88	Predict
NOM028_scaffold39_5	Listeria monocytogenes	1	CRISPR
NOM028_scaffold47596_2	Mycoplasma pulmonis	1	CRISPR
NOM028_scaffold19223_1	Mycoplasma pulmonis	0.95	Predict
NOM028_scaffold6524_14	Mycoplasma pulmonis	0.93	Predict
NOM028_scaffold34458_5	Streptococcus oralis	1	CRISPR
NOM028_scaffold24117_2	Mycoplasma pulmonis	1	CRISPR
NOM029_scaffold15812_1	Mycoplasma pulmonis	0.89	Predict
NOM029_scaffold15613_1[illegible]	Colwellia psychrerythraea	1	CRISPR
NOM029_scaffold279_8	Candidatus Pelagibacter ubique	0.8	Predict
NOM029_scaffold27582_6	Rhodococcus hoagii	0.86	Predict
NOM029_scaffold13239_1	Colwellia psychrerythraea	1	Predict
NOM029_scaffold36387_1	Mycoplasma pulmonis	0.91	Predict
NOM029_scaffold27582_3	Mycoplasma pulmonis	0.76	Predict
NOM029_scaffold32772_2	Mycoplasma pulmonis	0.86	Predict
NOM029_scaffold1741_11	Mycoplasma pulmonis	0.97	Predict
NOM029_scaffold9689_3	Bacteroides fragilis	0.92	Predict
NOM029_scaffold23053_8	Bacteroides fragilis	0.81	Predict
NOM029_scaffold277_1	Mycoplasma pulmonis	1	CRISPR
NOM029_scaffold6303_4	Mycoplasma pulmonis	1	CRISPR
NOM029_scaffold19269_1[illegible]	Candidatus Pelagibacter ubique	1	CRISPR
NOM001_scaffold14915_2	unknown	0	-
NOM001_scaffold31539_2	unknown	0	-
NOM002_scaffold2059_12	unknown	0	-
NOM002_scaffold14897_3	unknown	0	-
NOM004_scaffold15115_6	unknown	0	-
NOM005_scaffold29223_4	unknown	0	-
NOM005_scaffold12226_1	unknown	0	-
NOM005_scaffold29223_3	unknown	0	-

NOM005_scaffold504_2	unknown	0	-
NOM005_scaffold16216_8	unknown	0	-
NOM005_scaffold50369_2	unknown	0	-
NOM005_scaffold48061_2	unknown	0	-
NOM007_scaffold37186_2	unknown	0	-
NOM007_scaffold270_1	unknown	0	-
NOM007_scaffold29869_4	unknown	0	-
NOM009_scaffold34457_1	unknown	0	-
NOM009_scaffold52880_2	unknown	0	-
NOM009_scaffold54003_3	unknown	0	-
NOM009_scaffold16373_1	unknown	0	-
NOM009_scaffold20832_3	unknown	0	-
NOM009_scaffold13490_2	unknown	0	-
NOM009_scaffold26738_4	unknown	0	-
NOM009_scaffold37752_2	unknown	0	-
NOM010_scaffold40709_2	unknown	0	-
NOM010_scaffold3371_1	unknown	0	-
NOM010_scaffold40709_1	unknown	0	-
NOM010_scaffold41425_2	unknown	0	-
NOM010_scaffold40709_6	unknown	0	-
NOM010_scaffold37895_1	unknown	0	-
NOM012_scaffold55930_5	unknown	0	-
NOM012_scaffold59314_2	unknown	0	-
NOM012_scaffold16052_1	unknown	0	-
NOM012_scaffold57810_1	unknown	0	-
NOM012_scaffold25493_7	unknown	0	-
NOM013_scaffold31636_3	unknown	0	-
NOM013_scaffold28579_1	unknown	0	-
NOM013_C706454_1	unknown	0	-
NOM013_scaffold34728_2	unknown	0	-
NOM013_scaffold11200_1	unknown	0	-
NOM013_scaffold23911_1	unknown	0	-
NOM013_scaffold53631_1	unknown	0	-
NOM013_scaffold3327_4	unknown	0	-
NOM013_scaffold19858_2	unknown	0	-
NOM013_scaffold11318_3	unknown	0	-
NOM013_scaffold36218_2	unknown	0	-
NOM014_C390531_1	unknown	0	-
NOM014_scaffold7249_2	unknown	0	-
NOM014_scaffold28353_1	unknown	0	-
NOM015_scaffold2934_14	unknown	0	-
NOM015_scaffold15823_1	unknown	0	-
NOM015_scaffold1091_31	unknown	0	-
NOM015_scaffold3765_6	unknown	0	-
NOM015_scaffold1721_3	unknown	0	-
NOM016_scaffold42933_2	unknown	0	-
NOM016_scaffold45043_2	unknown	0	-
NOM016_scaffold1263_27	unknown	0	-

NOM016_scaffold41274_5	unknown	0	-
NOM016_scaffold16783_1	unknown	0	-
NOM016_scaffold19739_3	unknown	0	-
NOM017_scaffold45482_1	unknown	0	-
NOM017_scaffold16406_3	unknown	0	-
NOM017_scaffold45496_2	unknown	0	-
NOM017_scaffold1142_45	unknown	0	-
NOM017_scaffold33562_4	unknown	0	-
NOM018_scaffold54740_4	unknown	0	-
NOM018_scaffold4478_2	unknown	0	-
NOM018_scaffold42888_2	unknown	0	-
NOM018_scaffold54709_5	unknown	0	-
NOM018_scaffold26191_1	unknown	0	-
NOM018_scaffold221_1	unknown	0	-
NOM018_scaffold16582_2	unknown	0	-
NOM018_scaffold53629_1	unknown	0	-
NOM018_scaffold54603_1	unknown	0	-
NOM019_scaffold6752_20	unknown	0	-
NOM019_scaffold52150_2	unknown	0	-
NOM019_scaffold5766_5	unknown	0	-
NOM019_scaffold53004_2	unknown	0	-
NOM019_scaffold40062_4	unknown	0	-
NOM019_scaffold38943_2	unknown	0	-
NOM019_scaffold53083_1	unknown	0	-
NOM019_scaffold53119_3	unknown	0	-
NOM019_scaffold7421_3	unknown	0	-
NOM020_scaffold1826_3	unknown	0	-
NOM020_scaffold38892_1	unknown	0	-
NOM022_scaffold11395_1	unknown	0	-
NOM022_scaffold35027_4	unknown	0	-
NOM022_scaffold33629_1	unknown	0	-
NOM023_scaffold2528_2	unknown	0	-
NOM023_scaffold272_7	unknown	0	-
NOM023_scaffold13354_4	unknown	0	-
NOM023_scaffold16737_1	unknown	0	-
NOM023_scaffold3447_6	unknown	0	-
NOM023_scaffold325_2	unknown	0	-
NOM023_scaffold6200_5	unknown	0	-
NOM025_scaffold23387_1	unknown	0	-
NOM026_scaffold15750_6	unknown	0	-
NOM026_scaffold23252_1	unknown	0	-
NOM026_scaffold32579_2	unknown	0	-
NOM027_scaffold8991_1	unknown	0	-
NOM027_scaffold3477_2	unknown	0	-
NOM028_scaffold6679_33	unknown	0	-
NOM028_scaffold5961_20	unknown	0	-
NOM028_scaffold37397_2	unknown	0	-
NOM028_scaffold47247_1	unknown	0	-

NOM028_scaffold47161_1	unknown	0	-
NOM028_scaffold6524_1	unknown	0	-
NOM029_scaffold1385_6	unknown	0	-
NOM029_scaffold2695_11	unknown	0	-
