# Supplementary material for: PhaBOX: a web server for identifying and characterizing phage contigs in metagenomic data
Source: Bioinform Adv. 2023 Aug 2;3(1):vbad101. doi: 10.1093/bioadv/vbad101 (PMC10460485; doi:10.1093/bioadv/vbad101)
Supplement: vbad101_Supplementary_Data [file vbad101_supplementary_data.zip › phagcn_prediction.pdf]

Accession	Pred	Score
DOF002_scaffold67503_1	Salasmaviridae	1
DOF002_scaffold64389_1	Herelleviridae	1
DOF002_scaffold386_23	Guelinviridae	0.32256198
DOF002_scaffold2701_13	Peduoviridae	0.35397077
DOF002_scaffold60803_2	Casjensviridae	0.6694228
DOF002_scaffold4671_2	Guelinviridae	0.37318182
DOF002_scaffold133_11	Ackermannviridae	0.37338728
DOF002_scaffold13072_15	Ackermannviridae	0.46403873
DOF002_scaffold67459_3	Schitoviridae	0.2630205
DOF002_scaffold35792_4	Peduoviridae	0.4450498
DOF002_scaffold9671_2	Ackermannviridae	0.37796977
DOF003_scaffold19111_1	Ackermannviridae	1
DOF003_scaffold989_9	Peduoviridae	1
DOF003_scaffold27483_2	Peduoviridae	1
DOF003_scaffold43780_1	Ackermannviridae	0.4324245
DOF003_scaffold13183_2	Drexlerviridae	0.39234194
DOF003_scaffold4746_1	Casjensviridae	0.6694228
DOF003_scaffold28472_4	Peduoviridae	0.4450498
DOF003_scaffold989_11	Casjensviridae	1
DOF003_scaffold15485_11	Ackermannviridae	0.4853815
DOF003_scaffold40480_7	Peduoviridae	0.35397077
DOF003_scaffold197_2	Straboviridae	0.8096964
DOF003_scaffold959_2	Straboviridae	0.4209155
DOF003_scaffold51075_5	Peduoviridae	0.3762116
DOF003_scaffold19871_4	Straboviridae	1
DOF003_scaffold50425_1	Straboviridae	0.5560466
DOF003_scaffold49538_2	Ackermannviridae	0.60703695
DOF003_scaffold19201_1	Straboviridae	0.6036092
DOF003_scaffold28914_12	Straboviridae	0.7755927
DOF003_scaffold27483_3	Drexlerviridae	0.23707402
DOF003_scaffold35581_4	Drexlerviridae	0.23707402
DOF004_scaffold56260_1	Peduoviridae	1
DOF004_scaffold16778_14	Zierdtviridae	1
DOF004_scaffold35687_1	Ackermannviridae	0.4315236
DOF004_scaffold9090_4	Peduoviridae	0.9795492
DOF004_scaffold786_2	Kyanoviridae	1
DOF004_scaffold56259_1	Straboviridae	0.7755927
DOF004_scaffold27017_1	Straboviridae	0.4209155
DOF004_scaffold21890_2	Straboviridae	0.4573948
DOF004_C632416_1	Ackermannviridae	1
DOF004_scaffold49766_4	Casjensviridae	1
DOF004_scaffold24349_1	Drexlerviridae	0.9879088
DOF004_scaffold45589_1	Straboviridae	0.48223433
DOF006_scaffold711_1	Schitoviridae	1
DOF006_scaffold306_3	Guelinviridae	0.32256198

DOF006_scaffold22817_1	Peduoviridae	1
DOF006_scaffold12062_1	Vilmaviridae	1
DOF006_C312135_1	Peduoviridae	1
DOF006_scaffold13735_2	Straboviridae	0.6036092
DOF006_scaffold3372_1	Ackermannviridae	0.37338728
DOF006_scaffold23004_2	Straboviridae	0.4573948
DOF006_scaffold2736_1	Kyanoviridae	1
DOF006_scaffold20709_1	Rountreeviridae	0.34439915
DOF006_scaffold22952_1	Guelinviridae	0.37318182
DOF006_scaffold5845_1	Straboviridae	0.48223433
DOF006_scaffold14584_2	Peduoviridae	0.18345712
DOF006_scaffold711_5	Casjensviridae	1
DOF006_scaffold10407_1	Straboviridae	0.5247502
DOF007_scaffold1171_1	Straboviridae	0.464522
DOF007_scaffold35_3	Ackermannviridae	0.49211752
DOF007_scaffold17308_3	Herelleviridae	1
DOF007_scaffold632_7	Drexlerviridae	0.30752468
DOF007_scaffold1340_5	Ackermannviridae	1
DOF007_scaffold2644_118	Drexlerviridae	0.16746306
DOF007_C359971_1	Ackermannviridae	1
DOF008_scaffold567_3	Vilmaviridae	0.20129421
DOF008_scaffold832_1	Ackermannviridae	0.4783474
DOF008_scaffold10539_9	Straboviridae	0.5668039
DOF008_scaffold21796_1	Ackermannviridae	0.39623365
DOF008_scaffold6011_2	Drexlerviridae	0.39234194
DOF008_scaffold1028_3	Straboviridae	0.4573948
DOF008_scaffold5856_4	Straboviridae	0.8034157
DOF008_scaffold17006_1	Peduoviridae	1
DOF008_scaffold232_7	Mesyanzhinovviridae	1
DOF008_C423975_1	Autographiviridae	1
DOF009_scaffold2408_22	Ackermannviridae	0.49632582
DOF009_scaffold40393_12	Peduoviridae	0.35397077
DOF009_scaffold52826_1	Salasmaviridae	1
DOF009_scaffold52459_1	Peduoviridae	0.33099112
DOF009_scaffold39856_9	Peduoviridae	1
DOF009_scaffold16613_1	Straboviridae	0.5668039
DOF009_scaffold31_1	Straboviridae	0.6022864
DOF009_scaffold1_2	Straboviridae	0.4573948
DOF009_scaffold52438_1	Herelleviridae	1
DOF009_scaffold4516_5	Ackermannviridae	0.60703695
DOF009_scaffold42497_4	Ackermannviridae	0.37338728
DOF009_scaffold7496_2	Straboviridae	0.8096964
DOF010_scaffold38620_1	Mesyanzhinovviridae	1
DOF010_scaffold1331_7	Peduoviridae	1
DOF010_scaffold45_4	Peduoviridae	1
DOF010_C495273_1	Rountreeviridae	0.3115139

DOF010_scaffold36_4	Straboviridae	0.2887408
DOF010_scaffold36571_1	Ackermannviridae	0.39623365
DOF010_scaffold37750_5	Peduoviridae	0.14169496
DOF010_scaffold65_18	Ackermannviridae	0.60703695
DOF010_scaffold11833_5	Ackermannviridae	0.4315236
DOF010_scaffold7607_7	Autographiviridae	1
DOF010_scaffold27096_5	Ackermannviridae	0.4783474
DOF010_scaffold35610_2	Herelleviridae	1
DOF010_scaffold39042_1	Casjensviridae	1
DOF010_scaffold38651_2	Herelleviridae	1
DOF010_scaffold19252_1	Straboviridae	0.56678534
DOF010_scaffold37750_4	Drexlerviridae	0.23707402
DOF010_scaffold39049_2	Casjensviridae	1
DOF010_scaffold31976_1	Rountreeviridae	0.3027439
DOF011_scaffold21213_1	Straboviridae	0.53860486
DOF011_scaffold2635_5	Straboviridae	0.4573948
DOF011_scaffold21174_2	Ackermannviridae	0.46645996
DOF011_scaffold20914_1	Straboviridae	0.32026517
DOF012_scaffold884_2	Drexlerviridae	0.39234194
DOF012_scaffold9548_2	Herelleviridae	1
DOF012_scaffold897_10	Peduoviridae	1
DOF012_scaffold4741_3	Autographiviridae	1
DOF012_scaffold6657_1	Peduoviridae	1
DOF012_scaffold191_20	Straboviridae	0.6737759
DOF012_scaffold7309_6_1	Straboviridae	0.8034157
DOF013_scaffold2527_4	Kyanoviridae	1
DOF013_scaffold5071_6	Straboviridae	0.464522
DOF013_scaffold27644_1	Straboviridae	0.374585
DOF013_scaffold13491_2	Ackermannviridae	0.31827873
DOF013_scaffold4683_2	Chaseviridae	1
DOF013_scaffold392_2	Chaseviridae	1
DOF013_scaffold379_1	Ackermannviridae	0.3173149
DOF014_scaffold10772_1	Straboviridae	0.48694646
DOF014_scaffold49726_2	Straboviridae	0.6022864
DOF014_scaffold7189_8	Peduoviridae	1
DOF014_scaffold49687_1	Kyanoviridae	1
DOF014_scaffold49645_1	Guelinviridae	0.37318182
DOF014_scaffold1_3	Straboviridae	0.4573948
DOF014_scaffold13006_1	Straboviridae	0.48694646
DOF014_scaffold82_3	Ackermannviridae	0.49632582
DOF002_scaffold24242_3	unknown	0
DOF002_scaffold60803_3	unknown	0
DOF002_scaffold67436_2	unknown	0
DOF002_scaffold63958_7	unknown	0
DOF002_scaffold11952_4	unknown	0
DOF002_scaffold64073_3	unknown	0

DOF002_scaffold45014_1	unknown	0
DOF002_scaffold32188_6	unknown	0
DOF002_scaffold65381_4	unknown	0
DOF002_scaffold67459_1	unknown	0
DOF002_scaffold67379_1	unknown	0
DOF002_scaffold67538_1	unknown	0
DOF002_scaffold55354_2	unknown	0
DOF002_scaffold66657_1	unknown	0
DOF002_scaffold10448_2	unknown	0
DOF002_scaffold11952_2	unknown	0
DOF002_scaffold38225_1	unknown	0
DOF002_scaffold65381_2	unknown	0
DOF002_scaffold58023_2	unknown	0
DOF002_scaffold60803_1	unknown	0
DOF002_scaffold39963_1	unknown	0
DOF002_C840249_1	unknown	0
DOF002_scaffold26075_2	unknown	0
DOF002_scaffold67426_3	unknown	0
DOF002_scaffold11952_3	unknown	0
DOF002_scaffold39963_2	unknown	0
DOF002_scaffold197_4	unknown	0
DOF002_scaffold64416_5	unknown	0
DOF002_C839921_1	unknown	0
DOF002_scaffold44470_1	unknown	0
DOF002_scaffold5030_2	unknown	0
DOF002_scaffold37947_1	unknown	0
DOF003_scaffold26875_2	unknown	0
DOF003_scaffold50654_1	unknown	0
DOF003_scaffold51061_3	unknown	0
DOF003_scaffold28855_2	unknown	0
DOF003_scaffold269_12	unknown	0
DOF003_scaffold21246_8	unknown	0
DOF003_scaffold51115_1	unknown	0
DOF003_scaffold2741_2	unknown	0
DOF003_C746192_1	unknown	0
DOF003_scaffold48262_1	unknown	0
DOF003_scaffold2826_3	unknown	0
DOF003_scaffold22191_11	unknown	0
DOF003_scaffold1689_27	unknown	0
DOF003_scaffold23234_1	unknown	0
DOF003_scaffold49885_1	unknown	0
DOF003_scaffold50947_2	unknown	0
DOF003_scaffold51046_1	unknown	0
DOF003_scaffold32977_2	unknown	0
DOF003_scaffold30739_3	unknown	0
DOF003_scaffold50350_1	unknown	0

DOF003_scaffold49069_1	unknown	0
DOF003_scaffold2826_7	unknown	0
DOF003_scaffold49885_3	unknown	0
DOF003_scaffold51078_6	unknown	0
DOF003_scaffold51078_1	unknown	0
DOF003_scaffold9164_2	unknown	0
DOF003_scaffold22191_13	unknown	0
DOF003_scaffold47078_1	unknown	0
DOF003_scaffold22191_10	unknown	0
DOF003_scaffold1689_28	unknown	0
DOF003_scaffold36733_1	unknown	0
DOF003_scaffold22256_6	unknown	0
DOF003_scaffold26587_4	unknown	0
DOF004_scaffold31768_1	unknown	0
DOF004_scaffold41981_3	unknown	0
DOF004_scaffold42185_4	unknown	0
DOF004_scaffold22037_1	unknown	0
DOF004_scaffold54702_1	unknown	0
DOF004_scaffold27880_1	unknown	0
DOF004_scaffold37006_1	unknown	0
DOF004_scaffold10109_6	unknown	0
DOF004_scaffold28311_2	unknown	0
DOF004_scaffold148_2	unknown	0
DOF004_scaffold19258_3	unknown	0
DOF004_scaffold57038_1	unknown	0
DOF004_scaffold38757_1	unknown	0
DOF004_scaffold7376_1	unknown	0
DOF004_scaffold56952_1	unknown	0
DOF004_scaffold25343_1	unknown	0
DOF004_scaffold5206_5	unknown	0
DOF004_scaffold57121_1	unknown	0
DOF004_scaffold26721_2	unknown	0
DOF004_scaffold34242_1	unknown	0
DOF006_scaffold11978_4	unknown	0
DOF006_scaffold15300_3	unknown	0
DOF006_scaffold17493_1	unknown	0
DOF006_scaffold19914_2	unknown	0
DOF006_scaffold22996_1	unknown	0
DOF006_scaffold11478_1	unknown	0
DOF006_scaffold15162_1	unknown	0
DOF006_scaffold306_6	unknown	0
DOF006_scaffold22685_1	unknown	0
DOF006_scaffold4966_1	unknown	0
DOF006_scaffold22663_1	unknown	0
DOF006_scaffold17357_1	unknown	0
DOF006_scaffold7307_1	unknown	0

DOF006_scaffold9292_2	unknown	0
DOF006_C312621_1	unknown	0
DOF007_C360895_1	unknown	0
DOF007_scaffold7833_5	unknown	0
DOF007_scaffold21463_1	unknown	0
DOF007_scaffold2644_116	unknown	0
DOF007_scaffold14864_2	unknown	0
DOF007_scaffold5443_1	unknown	0
DOF007_scaffold523_7	unknown	0
DOF007_scaffold2644_113	unknown	0
DOF007_scaffold15454_1	unknown	0
DOF007_scaffold16228_1	unknown	0
DOF007_scaffold16914_2	unknown	0
DOF007_scaffold4001_1	unknown	0
DOF007_scaffold12791_4	unknown	0
DOF007_scaffold1333_2	unknown	0
DOF008_scaffold28029_2	unknown	0
DOF008_scaffold10567_1	unknown	0
DOF008_scaffold9506_2	unknown	0
DOF008_scaffold4011_4	unknown	0
DOF008_scaffold28485_2	unknown	0
DOF008_scaffold8861_3	unknown	0
DOF008_scaffold28698_1	unknown	0
DOF008_scaffold26020_1	unknown	0
DOF008_scaffold19396_1	unknown	0
DOF008_scaffold24483_1	unknown	0
DOF008_scaffold12257_4	unknown	0
DOF008_scaffold25427_1	unknown	0
DOF008_scaffold27498_1	unknown	0
DOF008_scaffold110_2	unknown	0
DOF008_C423597_1	unknown	0
DOF008_scaffold2469_10	unknown	0
DOF008_scaffold19808_7	unknown	0
DOF008_scaffold29015_1	unknown	0
DOF008_scaffold12257_5	unknown	0
DOF008_scaffold28029_1	unknown	0
DOF008_scaffold16092_4	unknown	0
DOF008_scaffold27793_1	unknown	0
DOF008_scaffold21540_6	unknown	0
DOF008_scaffold15173_2	unknown	0
DOF009_scaffold42858_2	unknown	0
DOF009_scaffold155_13	unknown	0
DOF009_scaffold43174_9	unknown	0
DOF009_scaffold22475_18	unknown	0
DOF009_scaffold47159_4	unknown	0
DOF009_scaffold46839_2	unknown	0

DOF009_scaffold3127_2	unknown	0
DOF009_scaffold52159_2	unknown	0
DOF009_scaffold42767_3	unknown	0
DOF009_scaffold36176_2	unknown	0
DOF009_scaffold40125_1	unknown	0
DOF009_scaffold23972_2	unknown	0
DOF009_scaffold13901_4	unknown	0
DOF010_scaffold38542_4	unknown	0
DOF010_scaffold37194_6	unknown	0
DOF010_scaffold31976_2	unknown	0
DOF010_scaffold39042_3	unknown	0
DOF010_scaffold13814_2	unknown	0
DOF010_scaffold21978_1	unknown	0
DOF010_scaffold19015_5	unknown	0
DOF010_scaffold32482_2	unknown	0
DOF010_scaffold23207_18	unknown	0
DOF010_scaffold34_4	unknown	0
DOF010_scaffold2645_5	unknown	0
DOF010_scaffold24344_2	unknown	0
DOF010_scaffold13830_4	unknown	0
DOF010_scaffold33553_4	unknown	0
DOF010_scaffold21702_2	unknown	0
DOF010_scaffold29273_4	unknown	0
DOF010_scaffold38551_4	unknown	0
DOF010_scaffold24344_3	unknown	0
DOF010_scaffold31661_1	unknown	0
DOF010_scaffold35846_1	unknown	0
DOF010_scaffold4723_13	unknown	0
DOF011_scaffold21153_1	unknown	0
DOF011_scaffold21165_1	unknown	0
DOF011_scaffold77_1	unknown	0
DOF011_scaffold2386_1	unknown	0
DOF011_scaffold12507_5	unknown	0
DOF011_scaffold19699_2	unknown	0
DOF012_scaffold1710_3	unknown	0
DOF012_scaffold12555_2	unknown	0
DOF012_scaffold463_10	unknown	0
DOF012_scaffold4720_1	unknown	0
DOF012_scaffold992_3	unknown	0
DOF012_scaffold9844_1	unknown	0
DOF012_scaffold2936_3	unknown	0
DOF012_scaffold12334_1	unknown	0
DOF012_scaffold198_1	unknown	0
DOF012_scaffold4539_3	unknown	0
DOF012_scaffold381_2	unknown	0
DOF012_scaffold3394_7	unknown	0

DOF012_scaffold7_12	unknown	0
DOF012_scaffold884_3	unknown	0
DOF012_scaffold14750_1	unknown	0
DOF012_scaffold22_3	unknown	0
DOF012_scaffold463_9_2	unknown	0
DOF013_scaffold11760_13	unknown	0
DOF013_scaffold12980_3	unknown	0
DOF013_scaffold1588_1	unknown	0
DOF013_scaffold4900_2	unknown	0
DOF013_scaffold12980_1	unknown	0
DOF013_scaffold783_2	unknown	0
DOF013_scaffold443_2	unknown	0
DOF013_scaffold15877_2	unknown	0
DOF013_scaffold2699_1	unknown	0
DOF013_scaffold103_6	unknown	0
DOF013_scaffold5240_2	unknown	0
DOF013_scaffold5240_9	unknown	0
DOF013_scaffold21294_6	unknown	0
DOF013_scaffold29915_1	unknown	0
DOF014_scaffold15073_8	unknown	0
DOF014_scaffold49276_4	unknown	0
DOF014_scaffold30923_2	unknown	0
DOF014_C612810_1	unknown	0
DOF014_scaffold101_2	unknown	0
DOF014_scaffold32658_1	unknown	0
DOF014_scaffold49340_4	unknown	0
DOF014_scaffold29718_2	unknown	0
DOF014_scaffold1808_5	unknown	0
DOF014_scaffold49604_1	unknown	0
DOF014_scaffold90_1	unknown	0
DOF014_scaffold49556_1	unknown	0
DOF014_scaffold49552_1	unknown	0
DOF014_scaffold34813_4	unknown	0
DOF014_scaffold49340_5	unknown	0
DOF014_C612698_1	unknown	0
DOF014_scaffold15208_5	unknown	0
DOF002_scaffold5829_2	no_family_avaliable(NC_(	0.978
DOF002_scaffold5829_3	no_family_avaliable(NC_(	0.968
DOF002_scaffold64032_6	no_family_avaliable(NC_(	0.962
DOF002_scaffold4671_4	no_family_avaliable(NC_(	0.95
DOF002_scaffold64032_7	no_family_avaliable(NC_(	0.969
DOF002_scaffold20669_3	no_family_avaliable(NC_(	0.955
DOF006_scaffold18788_9	no_family_avaliable(NC_(	0.968
DOF006_scaffold22952_2	no_family_avaliable(NC_(	0.962
DOF010_scaffold37104_2	no_family_avaliable(NC_(	0.984
DOF010_scaffold12096_1	no_family_avaliable(NC_(	0.964

DOF010_scaffold35610_1	no_family_avaliable(NC_(	0.973
DOF010_scaffold38956_2	no_family_avaliable(NC_(	0.965
DOF010_scaffold198_1	no_family_avaliable(NC_(	0.978
DOF012_scaffold14781_2	no_family_avaliable(NC_(	0.98
DOF014_scaffold46901_1	no_family_avaliable(NC_(	0.971
DOF014_scaffold2821_4	no_family_avaliable(NC_(	0.97
NOF001_scaffold2654_4	Ackermannviridae	0.22812769
NOF001_scaffold14_4	Ackermannviridae	0.52144295
NOF001_scaffold58201_2	Salasmaviridae	0.43348148
NOF001_scaffold17586_2	Straboviridae	0.3021274
NOF001_scaffold1053_2	Casjensviridae	0.9785655
NOF001_scaffold58318_1	Salasmaviridae	0.45380798
NOF001_scaffold5563_1	Ackermannviridae	0.5281165
NOF001_scaffold13046_9	Kyanoviridae	1
NOF001_scaffold58235_1	Herelleviridae	0.32129836
NOF001_scaffold11560_1	Peduoviridae	0.55076057
NOF001_scaffold27372_1	Herelleviridae	1
NOF001_scaffold24609_2	Straboviridae	0.5317443
NOF001_scaffold41855_6	Peduoviridae	1
NOF001_scaffold29077_4	Casjensviridae	0.44122034
NOF001_scaffold58316_1	Salasmaviridae	1
NOF001_scaffold7196_11	Casjensviridae	0.55142415
NOF001_scaffold24013_1	Straboviridae	0.13005078
NOF001_scaffold58081_1	Autographiviridae	1
NOF001_scaffold58201_1	Herelleviridae	1
NOF001_scaffold55226_1	Straboviridae	0.3735483
NOF001_scaffold50021_1	Straboviridae	0.18451756
NOF001_scaffold58099_2	Salasmaviridae	0.26505843
NOF002_scaffold7_6	Straboviridae	0.5317443
NOF002_scaffold30763_2	Straboviridae	0.779485
NOF002_scaffold31424_1	Ackermannviridae	0.12288749
NOF002_scaffold31554_1	Ackermannviridae	0.3621296
NOF002_scaffold6749_14	Peduoviridae	1
NOF002_scaffold9460_1	Ackermannviridae	0.45842224
NOF002_scaffold1584_5	Straboviridae	0.2987051
NOF002_scaffold7_7	Casjensviridae	1
NOF002_scaffold27207_5	Herelleviridae	1
NOF002_scaffold2745_3	Ackermannviridae	0.5065008
NOF004_scaffold25441_2	Straboviridae	0.3021274
NOF004_scaffold5774_1	Straboviridae	0.69483334
NOF004_C576082_1	Demerecviridae	0.36091807
NOF004_scaffold22864_4	Ackermannviridae	0.52144295
NOF004_scaffold18950_2	Ackermannviridae	0.49674326
NOF004_scaffold22559_2	Herelleviridae	0.5537847
NOF004_scaffold10567_2	Herelleviridae	1
NOF004_scaffold8945_3	Casjensviridae	0.28481802

NOF004_scaffold39800_1	Straboviridae	0.316882
NOF004_scaffold89_3	Ackermannviridae	0.4456534
NOF004_scaffold31703_2	Herelleviridae	0.50312114
NOF004_scaffold26987_2	Casjensviridae	0.5366201
NOF004_scaffold4193_2	Casjensviridae	1
NOF004_scaffold12390_3	Casjensviridae	1
NOF004_scaffold39611_1	Straboviridae	0.86151546
NOF004_scaffold28322_1	Peduoviridae	1
NOF004_scaffold6978_23	Ackermannviridae	0.3621296
NOF005_scaffold35291_5	Straboviridae	0.25259766
NOF005_scaffold31594_11	Straboviridae	0.62966955
NOF005_scaffold1086_25	Straboviridae	0.7172016
NOF005_scaffold604_14	Straboviridae	0.19668595
NOF005_scaffold34950_3	Ackermannviridae	0.48222607
NOF005_scaffold29140_2	Straboviridae	0.69483334
NOF005_scaffold165_4	Straboviridae	0.38608253
NOF005_scaffold48547_9	Salasmaviridae	0.31764138
NOF005_scaffold604_12	Herelleviridae	1
NOF005_scaffold52365_1	Straboviridae	0.3021274
NOF005_scaffold3465_6	Straboviridae	0.45873865
NOF005_scaffold604_3	Herelleviridae	1
NOF005_scaffold49499_1_1	Straboviridae	0.63752884
NOF005_scaffold39972_17	Kyanoviridae	1
NOF005_scaffold27426_3	Ackermannviridae	0.52144295
NOF005_scaffold32384_3	Salasmaviridae	1
NOF005_scaffold27198_2	Salasmaviridae	0.9615491
NOF005_scaffold13462_4	Herelleviridae	1
NOF005_scaffold2025_2	Casjensviridae	0.9785655
NOF006_scaffold21352_1	Straboviridae	0.316882
NOF006_scaffold498_1	Ackermannviridae	0.48222607
NOF006_scaffold21223_2	Herelleviridae	1
NOF006_scaffold121_1	Vilmaviridae	1
NOF006_scaffold1740_2	Herelleviridae	1
NOF006_scaffold20915_1	Peduoviridae	1
NOF006_scaffold4872_3	Straboviridae	0.3481552
NOF006_C401108_1	Salasmaviridae	1
NOF007_scaffold16773_3	Ackermannviridae	0.4456534
NOF007_scaffold4461_7	Straboviridae	0.4051862
NOF007_scaffold396_8	Ackermannviridae	0.45842224
NOF007_scaffold21388_2	Peduoviridae	1
NOF007_scaffold1349_3	Casjensviridae	0.46178052
NOF007_scaffold1349_2	Casjensviridae	0.2757636
NOF007_scaffold8821_2	Ackermannviridae	0.5155003
NOF007_scaffold15189_2	Herelleviridae	0.3868164
NOF007_scaffold7559_3	Straboviridae	0.76637846
NOF007_scaffold16388_6	Casjensviridae	0.36081105

NOF007_scaffold24483_2	Kyanoviridae	0.20652783
NOF007_scaffold505_1	Casjensviridae	0.5581696
NOF007_scaffold28801_1	Ackermannviridae	0.5065008
NOF007_scaffold3393_7	Casjensviridae	0.38532472
NOF007_scaffold1195_9	Casjensviridae	0.1780801
NOF007_scaffold2822_2	Straboviridae	0.33762848
NOF007_scaffold23774_2	Peduoviridae	1
NOF008_scaffold68230_1	Casjensviridae	0.36081105
NOF008_scaffold64397_1	Kyanoviridae	1
NOF008_scaffold1288_26	Kyanoviridae	1
NOF008_scaffold66085_2	Ackermannviridae	0.53408796
NOF008_scaffold11140_1	Salasmaviridae	1
NOF008_scaffold1389_2	Ackermannviridae	0.34342647
NOF008_scaffold28772_6	Ackermannviridae	0.52144295
NOF008_scaffold30912_6	Peduoviridae	1
NOF008_scaffold68147_1	Salasmaviridae	0.650711
NOF008_scaffold8714_2	Casjensviridae	0.36081105
NOF008_scaffold5616_1	Ackermannviridae	0.5155003
NOF008_scaffold68130_1	Guelinviridae	0.26210135
NOF008_scaffold25447_1	Ackermannviridae	0.37941656
NOF008_scaffold52435_2	Casjensviridae	0.31979057
NOF008_scaffold36171_4	Ackermannviridae	0.4456534
NOF008_scaffold42043_1	Peduoviridae	1
NOF008_scaffold8066_2	Herelleviridae	1
NOF008_scaffold28625_3	Ackermannviridae	0.5281165
NOF008_scaffold19089_7	Peduoviridae	0.55076057
NOF008_scaffold3708_20	Casjensviridae	1
NOF008_scaffold52198_1	Casjensviridae	0.36081105
NOF008_scaffold25220_3	Ackermannviridae	0.5281165
NOF008_scaffold68199_1	Straboviridae	0.24190016
NOF009_scaffold21768_4	Straboviridae	0.6711859
NOF009_scaffold2064_12	Ackermannviridae	0.49674326
NOF009_scaffold16960_2	Straboviridae	0.3481552
NOF009_scaffold14075_3	Drexlerviridae	0.29942086
NOF009_scaffold67_1	Kyanoviridae	0.20087749
NOF009_scaffold13328_11	Straboviridae	0.37907848
NOF009_scaffold42455_5	Herelleviridae	0.40026155
NOF009_scaffold41979_1	Casjensviridae	0.36081105
NOF010_C699885_1	Salasmaviridae	0.37905717
NOF010_scaffold33789_4	Peduoviridae	1
NOF010_scaffold14373_1	Kyanoviridae	1
NOF010_scaffold2330_4	Casjensviridae	0.47887832
NOF010_scaffold16598_11	Ackermannviridae	1
NOF010_scaffold20182_2	Casjensviridae	0.5581696
NOF010_scaffold22545_1	Ackermannviridae	1
NOF010_scaffold20459_4	Ackermannviridae	0.52144295

NOF010_scaffold9563_25	Casjensviridae	0.5581696
NOF010_scaffold53722_1	Casjensviridae	0.34444138
NOF010_scaffold46583_3	Herelleviridae	1
NOF010_scaffold6369_4	Straboviridae	0.38608253
NOF010_scaffold4642_2	Straboviridae	0.76637846
NOF010_scaffold53516_6	Salasmaviridae	0.4105297
NOF010_scaffold21111_3	Straboviridae	0.3556836
NOF010_scaffold22950_5	Peduoviridae	1
NOF010_scaffold54124_1	Ackermannviridae	0.53408796
NOF010_scaffold23028_3	Ackermannviridae	0.5281165
NOF010_scaffold5911_1	Ackermannviridae	1
NOF010_scaffold54281_1	Ackermannviridae	0.45842224
NOF011_scaffold56176_3	Drexlerviridae	0.2601836
NOF011_scaffold18490_3	Ackermannviridae	0.44814733
NOF011_scaffold58981_1	Straboviridae	0.45873865
NOF011_scaffold59056_1	Herelleviridae	0.49626034
NOF011_scaffold27189_1	Casjensviridae	0.28481802
NOF011_scaffold59096_2	Ackermannviridae	0.4456534
NOF011_scaffold15798_1	Herelleviridae	1
NOF011_C736712_1	Demerecviridae	0.36091807
NOF011_scaffold48802_1	Salasmaviridae	1
NOF011_scaffold57126_1	Straboviridae	0.46207947
NOF011_scaffold2529_7	Straboviridae	0.38608253
NOF012_scaffold77459_1	Casjensviridae	1
NOF012_scaffold71024_7	Straboviridae	0.38608253
NOF012_scaffold5639_4	Peduoviridae	0.9684544
NOF012_scaffold15647_4	Ackermannviridae	0.52144295
NOF012_scaffold8867_2	Ackermannviridae	0.48947993
NOF012_scaffold78059_2	Ackermannviridae	0.4456534
NOF012_scaffold34517_3	Casjensviridae	0.2757636
NOF012_scaffold34940_12	Casjensviridae	0.55142415
NOF012_scaffold34517_1	Ackermannviridae	0.535957
NOF012_scaffold34517_2	Casjensviridae	0.46178052
NOF012_scaffold77271_2	Ackermannviridae	0.30045664
NOF012_scaffold71857_2	Casjensviridae	0.5508405
NOF012_C833949_1	Salasmaviridae	1
NOF012_scaffold15991_1	Casjensviridae	0.7678824
NOF012_scaffold339_7	Straboviridae	1
NOF012_scaffold9650_7	Zierdtviridae	1
NOF012_scaffold37026_8	Peduoviridae	1
NOF012_scaffold68565_1	Zierdtviridae	0.18320961
NOF012_scaffold28114_12	Ackermannviridae	1
NOF013_scaffold11906_1	Peduoviridae	1
NOF013_scaffold30635_1	Kyanoviridae	1
NOF013_scaffold9607_2	Straboviridae	0.37907848
NOF013_scaffold26437_2	Straboviridae	0.62966955

NOF013_scaffold4750_1	Kyanoviridae	1
NOF013_scaffold316_7	Straboviridae	0.69483334
NOF013_scaffold28493_2	Peduoviridae	1
NOF013_scaffold23510_11	Straboviridae	0.779485
NOF013_scaffold131_1	Ackermannviridae	0.52144295
NOF013_scaffold33363_1	Straboviridae	0.66659385
NOF013_scaffold1744_3	Herelleviridae	1
NOF013_scaffold29173_1	Ackermannviridae	0.39596215
NOF013_scaffold3979_5	Ackermannviridae	0.4456534
NOF013_scaffold34324_3	Straboviridae	0.5316986
NOF013_scaffold2241_2	Straboviridae	0.34966817
NOF014_scaffold59979_2	Ackermannviridae	0.5257375
NOF014_scaffold30730_5	Straboviridae	0.27278966
NOF014_scaffold72227_1	Herelleviridae	1
NOF014_scaffold27769_8	Casjensviridae	0.7678824
NOF014_scaffold43_10	Ackermannviridae	0.39596215
NOF014_scaffold19962_3	Salasmaviridae	0.650711
NOF014_scaffold72216_1	Straboviridae	0.63752884
NOF014_scaffold71919_3	Straboviridae	0.294635
NOF014_scaffold49593_2	Straboviridae	0.585123
NOF014_scaffold72248_1	Salasmaviridae	1
NOF014_scaffold20787_1	Straboviridae	0.69483334
NOF014_scaffold66923_1	Straboviridae	0.29484153
NOF014_scaffold71552_2	Guelinviridae	0.26210135
NOF014_scaffold71919_2	Straboviridae	0.32358894
NOF014_scaffold63571_1	Casjensviridae	1
NOF014_scaffold65085_3	Peduoviridae	0.55076057
NOF014_scaffold22954_1	Casjensviridae	0.26592153
NOF014_scaffold64087_5	Straboviridae	0.7172016
NOF014_scaffold71856_1	Herelleviridae	1
NOF014_scaffold59979_3	Ackermannviridae	0.53408796
NOF001_scaffold33721_2	unknown	0
NOF001_scaffold48_1	unknown	0
NOF001_scaffold47705_1	unknown	0
NOF001_scaffold56764_1	unknown	0
NOF001_scaffold53059_1	unknown	0
NOF001_scaffold58052_2	unknown	0
NOF001_scaffold5343_8	unknown	0
NOF001_scaffold57995_2	unknown	0
NOF001_scaffold41855_2	unknown	0
NOF001_scaffold13046_6	unknown	0
NOF001_scaffold41855_1	unknown	0
NOF001_scaffold2819_5	unknown	0
NOF001_scaffold56157_1	unknown	0
NOF001_scaffold49296_1	unknown	0
NOF001_scaffold15730_5	unknown	0

NOF002_scaffold13379_1	unknown	0
NOF002_scaffold4501_18	unknown	0
NOF002_scaffold29466_1	unknown	0
NOF002_scaffold5730_7	unknown	0
NOF002_scaffold3476_12	unknown	0
NOF002_scaffold31199_2	unknown	0
NOF002_C425969_1	unknown	0
NOF002_scaffold12961_13	unknown	0
NOF002_scaffold20732_1	unknown	0
NOF002_scaffold3233_2	unknown	0
NOF002_scaffold31199_1	unknown	0
NOF002_scaffold11620_5	unknown	0
NOF002_scaffold31188_5	unknown	0
NOF002_scaffold31615_1	unknown	0
NOF002_scaffold3233_5	unknown	0
NOF002_scaffold30763_1	unknown	0
NOF002_scaffold2745_2	unknown	0
NOF002_scaffold23022_4	unknown	0
NOF002_scaffold31584_1	unknown	0
NOF004_scaffold5431_15	unknown	0
NOF004_scaffold4703_4	unknown	0
NOF004_scaffold19766_4	unknown	0
NOF004_scaffold32796_1	unknown	0
NOF004_scaffold30766_1	unknown	0
NOF004_scaffold4675_2	unknown	0
NOF004_scaffold36774_1	unknown	0
NOF004_scaffold37814_4	unknown	0
NOF004_scaffold12813_30	unknown	0
NOF004_scaffold12390_5	unknown	0
NOF004_scaffold4703_1	unknown	0
NOF004_scaffold5741_5	unknown	0
NOF004_scaffold34095_2	unknown	0
NOF004_scaffold29834_1	unknown	0
NOF004_scaffold1680_17	unknown	0
NOF004_scaffold30605_6	unknown	0
NOF004_scaffold39867_3	unknown	0
NOF004_scaffold2367_14	unknown	0
NOF004_scaffold8521_5	unknown	0
NOF004_scaffold24813_3	unknown	0
NOF004_scaffold15266_9	unknown	0
NOF004_C575620_1	unknown	0
NOF004_scaffold58_6	unknown	0
NOF004_scaffold14653_3	unknown	0
NOF004_scaffold3847_7	unknown	0
NOF004_scaffold10069_19	unknown	0
NOF004_scaffold9875_5	unknown	0

NOF004_scaffold9355_12	unknown	0
NOF004_scaffold14211_1	unknown	0
NOF004_scaffold9994_2	unknown	0
NOF004_scaffold2098_11	unknown	0
NOF005_scaffold23396_3	unknown	0
NOF005_scaffold36104_5	unknown	0
NOF005_scaffold18_1	unknown	0
NOF005_scaffold25742_6	unknown	0
NOF005_scaffold6888_3	unknown	0
NOF005_scaffold29221_10	unknown	0
NOF005_scaffold34324_4	unknown	0
NOF005_scaffold44749_6	unknown	0
NOF005_scaffold51717_1	unknown	0
NOF005_scaffold35393_5	unknown	0
NOF005_scaffold37734_2	unknown	0
NOF005_scaffold2170_3	unknown	0
NOF005_scaffold24910_8	unknown	0
NOF005_scaffold23396_2	unknown	0
NOF005_scaffold52405_2	unknown	0
NOF005_scaffold49499_1_2	unknown	0
NOF005_scaffold15268_1	unknown	0
NOF005_scaffold27541_5	unknown	0
NOF005_scaffold5924_1	unknown	0
NOF005_scaffold44821_1	unknown	0
NOF005_scaffold52106_1	unknown	0
NOF005_scaffold604_13	unknown	0
NOF006_scaffold10363_1	unknown	0
NOF006_scaffold15833_1	unknown	0
NOF006_scaffold6111_7	unknown	0
NOF006_scaffold4868_1	unknown	0
NOF006_scaffold12718_7	unknown	0
NOF006_scaffold21242_4	unknown	0
NOF006_scaffold2842_16	unknown	0
NOF006_scaffold19983_2	unknown	0
NOF006_scaffold14475_3	unknown	0
NOF006_scaffold1022_7	unknown	0
NOF006_scaffold14499_8	unknown	0
NOF006_scaffold21012_1	unknown	0
NOF006_scaffold4067_5	unknown	0
NOF006_scaffold969_8	unknown	0
NOF006_scaffold21374_2	unknown	0
NOF006_scaffold20868_2	unknown	0
NOF006_scaffold173_3	unknown	0
NOF007_scaffold27213_3	unknown	0
NOF007_scaffold23774_3	unknown	0
NOF007_scaffold28685_4	unknown	0

NOF007_scaffold26160_1	unknown	0
NOF007_scaffold25262_5	unknown	0
NOF007_C490687_1	unknown	0
NOF007_scaffold19207_2	unknown	0
NOF007_scaffold27822_1	unknown	0
NOF007_scaffold23819_3	unknown	0
NOF007_scaffold26938_2	unknown	0
NOF007_scaffold10524_1	unknown	0
NOF007_scaffold298_7	unknown	0
NOF007_scaffold28685_3	unknown	0
NOF007_scaffold25304_2	unknown	0
NOF007_scaffold22039_3	unknown	0
NOF007_scaffold26224_1	unknown	0
NOF007_scaffold28624_3	unknown	0
NOF007_scaffold12899_1	unknown	0
NOF007_scaffold9468_5	unknown	0
NOF008_scaffold25367_2	unknown	0
NOF008_scaffold48782_2	unknown	0
NOF008_scaffold5134_4	unknown	0
NOF008_scaffold61643_2	unknown	0
NOF008_scaffold5217_6	unknown	0
NOF008_scaffold67470_2	unknown	0
NOF008_scaffold10035_9	unknown	0
NOF008_scaffold66613_1	unknown	0
NOF008_scaffold48283_1	unknown	0
NOF008_scaffold31488_6	unknown	0
NOF008_scaffold3062_3	unknown	0
NOF008_scaffold7421_1	unknown	0
NOF008_scaffold35076_1	unknown	0
NOF008_scaffold58140_1	unknown	0
NOF008_scaffold53677_1	unknown	0
NOF008_scaffold68203_3	unknown	0
NOF008_scaffold68191_1	unknown	0
NOF008_scaffold65986_1	unknown	0
NOF008_scaffold63722_1	unknown	0
NOF008_scaffold50828_1	unknown	0
NOF008_scaffold41113_4	unknown	0
NOF008_scaffold59760_7	unknown	0
NOF008_scaffold68224_1	unknown	0
NOF008_scaffold7904_8	unknown	0
NOF008_C865299_1	unknown	0
NOF008_scaffold17689_13	unknown	0
NOF008_C865863_1	unknown	0
NOF008_scaffold55525_8	unknown	0
NOF008_scaffold13444_2	unknown	0
NOF008_scaffold55811_1	unknown	0

NOF008_scaffold29486_12	unknown	0
NOF009_scaffold7527_22	unknown	0
NOF009_scaffold43518_2	unknown	0
NOF009_scaffold41727_1	unknown	0
NOF009_scaffold10148_1	unknown	0
NOF009_scaffold19865_4	unknown	0
NOF009_C558283_1	unknown	0
NOF009_scaffold34567_3	unknown	0
NOF009_scaffold11228_12	unknown	0
NOF009_C558255_1	unknown	0
NOF009_scaffold2720_3	unknown	0
NOF009_scaffold30580_1	unknown	0
NOF009_scaffold42878_5	unknown	0
NOF009_scaffold42496_4	unknown	0
NOF010_scaffold32319_4	unknown	0
NOF010_scaffold26425_6	unknown	0
NOF010_scaffold28531_1	unknown	0
NOF010_scaffold51712_2	unknown	0
NOF010_scaffold6485_5	unknown	0
NOF010_scaffold2722_9	unknown	0
NOF010_scaffold22545_2	unknown	0
NOF010_scaffold14618_2	unknown	0
NOF010_scaffold43806_2	unknown	0
NOF010_scaffold27134_3	unknown	0
NOF010_scaffold54242_1	unknown	0
NOF010_C699039_1	unknown	0
NOF010_scaffold33103_1	unknown	0
NOF010_scaffold11305_3	unknown	0
NOF010_scaffold21018_1	unknown	0
NOF011_scaffold3878_1	unknown	0
NOF011_scaffold57558_3	unknown	0
NOF011_scaffold159_2	unknown	0
NOF011_scaffold59088_2	unknown	0
NOF011_scaffold8028_19	unknown	0
NOF011_scaffold33258_2	unknown	0
NOF011_scaffold58584_1	unknown	0
NOF011_scaffold70_15	unknown	0
NOF011_scaffold59100_2	unknown	0
NOF011_scaffold57546_2	unknown	0
NOF011_scaffold52600_1	unknown	0
NOF011_scaffold56176_2	unknown	0
NOF011_scaffold8028_20	unknown	0
NOF011_scaffold45498_1	unknown	0
NOF011_scaffold40128_2	unknown	0
NOF012_scaffold77267_2	unknown	0
NOF012_scaffold24523_4	unknown	0

NOF012_scaffold12244_5	unknown	0
NOF012_scaffold50466_2	unknown	0
NOF012_scaffold13996_7	unknown	0
NOF012_scaffold77917_2	unknown	0
NOF012_scaffold12244_6	unknown	0
NOF012_scaffold44774_1	unknown	0
NOF012_scaffold3081_2	unknown	0
NOF012_scaffold5639_7	unknown	0
NOF012_scaffold24554_12	unknown	0
NOF012_scaffold4667_6	unknown	0
NOF012_scaffold72024_3	unknown	0
NOF012_scaffold19855_2	unknown	0
NOF012_scaffold8693_10	unknown	0
NOF012_scaffold74308_1	unknown	0
NOF012_scaffold71874_7	unknown	0
NOF012_scaffold28415_3	unknown	0
NOF012_scaffold77196_1	unknown	0
NOF012_scaffold26305_22	unknown	0
NOF012_scaffold78081_1	unknown	0
NOF012_scaffold43523_1	unknown	0
NOF012_scaffold41858_11	unknown	0
NOF013_scaffold22999_4	unknown	0
NOF013_scaffold33526_1	unknown	0
NOF013_scaffold15363_3	unknown	0
NOF013_scaffold9359_1	unknown	0
NOF013_scaffold24304_2	unknown	0
NOF013_scaffold26437_1	unknown	0
NOF013_scaffold20067_2	unknown	0
NOF013_scaffold25505_1	unknown	0
NOF013_scaffold34730_1	unknown	0
NOF013_scaffold6283_17	unknown	0
NOF013_scaffold34528_1	unknown	0
NOF013_C612756_1	unknown	0
NOF013_scaffold3348_11	unknown	0
NOF013_scaffold24565_7	unknown	0
NOF013_scaffold34630_3	unknown	0
NOF013_scaffold24565_6	unknown	0
NOF013_scaffold22999_3	unknown	0
NOF013_scaffold32172_3	unknown	0
NOF013_scaffold15363_2	unknown	0
NOF013_scaffold34382_1	unknown	0
NOF013_C613108_1	unknown	0
NOF013_scaffold1181_6	unknown	0
NOF013_C612464_1	unknown	0
NOF013_scaffold33134_1	unknown	0
NOF013_scaffold32891_4	unknown	0

NOF013_scaffold24443_1	unknown	0
NOF014_scaffold67337_2	unknown	0
NOF014_scaffold29077_2	unknown	0
NOF014_scaffold41735_1	unknown	0
NOF014_scaffold72132_1	unknown	0
NOF014_scaffold72006_1	unknown	0
NOF014_scaffold6730_5	unknown	0
NOF014_scaffold71626_3	unknown	0
NOF014_scaffold46167_2	unknown	0
NOF014_scaffold35720_11	unknown	0
NOF014_scaffold4327_52	unknown	0
NOF014_scaffold40347_4	unknown	0
NOF014_scaffold10135_5	unknown	0
NOF014_scaffold66855_1	unknown	0
NOF014_scaffold72263_3	unknown	0
NOF014_scaffold26105_6	unknown	0
NOF014_scaffold20787_3	unknown	0
NOF014_scaffold63476_2	unknown	0
NOF014_scaffold26105_25	unknown	0
NOF014_scaffold10667_4	unknown	0
NOF014_scaffold20265_3	unknown	0
NOF014_scaffold5894_2	unknown	0
NOF014_scaffold71877_1	unknown	0
NOF014_scaffold2390_2	unknown	0
NOF014_scaffold66767_1	unknown	0
NOF014_scaffold72295_2	unknown	0
NOF014_scaffold71714_2	unknown	0
NOF014_scaffold54136_28	unknown	0
NOF004_scaffold39800_2	no_family_avaliable(NC_(	0.973
NOF004_scaffold12025_2	no_family_avaliable(NC_(	0.999
NOF005_scaffold51596_4	no_family_avaliable(NC_(	0.968
NOF007_scaffold11484_1	no_family_avaliable(NC_(	0.977
NOF007_scaffold41_1	no_family_avaliable(NC_(	0.962
NOF008_scaffold34632_3	no_family_avaliable(NC_(	0.97
NOF008_scaffold34632_4	no_family_avaliable(NC_(	0.97
NOF009_scaffold5136_16	no_family_avaliable(NC_(	0.974
NOF014_scaffold52663_4	no_family_avaliable(NC_(	0.966
NOF014_scaffold52663_2	no_family_avaliable(NC_(	0.969
DLF001_scaffold6028_2	Straboviridae	0.8344107
DLF001_scaffold16878_1	Drexlerviridae	0.2793915
DLF001_scaffold4836_9	Straboviridae	0.6356147
DLF001_scaffold24376_1	Straboviridae	0.6949948
DLF001_scaffold20939_1	Straboviridae	0.2730684
DLF001_scaffold10899_1	Peduoviridae	0.5576402
DLF001_scaffold21798_2	Straboviridae	0.47796255
DLF001_C323280_1	Straboviridae	0.43901232

DLF001_scaffold80_8	Drexlerviridae	0.2793915
DLF001_scaffold6028_1	Straboviridae	0.5964493
DLF001_C323900_1	Drexlerviridae	1
DLF002_scaffold6624_21	Straboviridae	0.75750005
DLF002_scaffold85_3	Straboviridae	0.47796255
DLF002_scaffold22416_1	Salasmaviridae	1
DLF002_scaffold19600_3	Peduoviridae	1
DLF002_scaffold294_32	Drexlerviridae	0.2793915
DLF002_scaffold7525_1	Straboviridae	0.6356147
DLF002_scaffold33974_1	Straboviridae	0.43901232
DLF002_scaffold10343_2	Straboviridae	0.50703365
DLF002_scaffold2466_1	Straboviridae	0.8105539
DLF002_scaffold36298_1	Straboviridae	0.5762059
DLF002_C488685_1	Herelleviridae	0.56141484
DLF003_scaffold21732_2	Ackermannviridae	1
DLF003_scaffold23409_2	Drexlerviridae	0.2793915
DLF003_scaffold12321_2	Schitoviridae	1
DLF003_C717736_1	Salasmaviridae	0.94259506
DLF003_scaffold55800_1	Schitoviridae	0.24243324
DLF003_C717640_1	Salasmaviridae	0.936089
DLF003_scaffold56673_2	Casjensviridae	1
DLF003_scaffold54149_1	Kyanoviridae	0.19216411
DLF003_scaffold43976_1	Salasmaviridae	0.4312181
DLF003_scaffold47905_1	Drexlerviridae	0.2793915
DLF003_scaffold57190_2	Salasmaviridae	1
DLF003_scaffold29891_3	Casjensviridae	1
DLF004_scaffold18383_6	Herelleviridae	1
DLF004_C548576_1	Straboviridae	0.725355
DLF004_scaffold25533_1	Schitoviridae	0.24243324
DLF004_C548200_1	Salasmaviridae	0.4312181
DLF004_scaffold2535_5	Drexlerviridae	0.2793915
DLF004_scaffold32830_2	Drexlerviridae	0.2793915
DLF004_scaffold27435_2	Drexlerviridae	0.2793915
DLF004_scaffold8102_2	Straboviridae	0.80707747
DLF004_scaffold24525_4	Straboviridae	0.6356147
DLF004_scaffold26106_5	Casjensviridae	0.4192874
DLF004_scaffold22703_8	Peduoviridae	0.44511104
DLF004_scaffold21240_2	Straboviridae	0.6949948
DLF005_scaffold55770_1	Straboviridae	0.75948435
DLF005_scaffold56569_4	Vilmaviridae	1
DLF005_scaffold4693_36	Straboviridae	0.5399531
DLF005_scaffold47023_3	Straboviridae	0.3171625
DLF005_scaffold56967_1	Straboviridae	0.3365034
DLF005_scaffold13334_3	Straboviridae	0.35845006
DLF005_C847538_1	Straboviridae	0.72624004
DLF005_scaffold6688_21	Herelleviridae	1

DLF005_scaffold18199_1	Straboviridae	0.80707747
DLF005_scaffold3960_1	Straboviridae	0.72045255
DLF005_scaffold280_2	Straboviridae	0.6833262
DLF005_scaffold41843_2	Straboviridae	0.4287239
DLF005_scaffold4093_2	Straboviridae	0.6292496
DLF005_scaffold56569_2	Herelleviridae	1
DLF005_scaffold23342_9	Casjensviridae	1
DLF006_C667664_1	Straboviridae	0.5113246
DLF006_scaffold108_5	Schitoviridae	0.23503077
DLF006_scaffold42885_3	Herelleviridae	1
DLF006_scaffold46681_1	Straboviridae	0.725355
DLF006_scaffold32044_2	Straboviridae	0.47796255
DLF006_scaffold19189_3	Peduoviridae	1
DLF006_scaffold474_4	Herelleviridae	1
DLF006_scaffold9056_18	Peduoviridae	1
DLF006_scaffold11422_2	Ackermannviridae	0.38388076
DLF006_scaffold9845_3	Mesyanzhinovviridae	1
DLF006_scaffold7466_5	Peduoviridae	0.778437
DLF006_scaffold25840_10	Peduoviridae	0.8291513
DLF006_scaffold36051_1	Drexlerviridae	1
DLF006_scaffold52_5	Herelleviridae	1
DLF006_scaffold36051_2	Drexlerviridae	1
DLF006_scaffold34515_1	Drexlerviridae	0.2793915
DLF006_scaffold52_6	Herelleviridae	1
DLF007_scaffold18927_1	Straboviridae	0.3058537
DLF007_C306343_1	Straboviridae	0.47071996
DLF007_scaffold1532_23	Kyanoviridae	1
DLF007_scaffold15763_5	Straboviridae	0.6984924
DLF007_scaffold20109_2	Straboviridae	0.47796255
DLF007_scaffold18401_2	Straboviridae	0.72239286
DLF007_scaffold12277_1	Peduoviridae	0.5576402
DLF008_scaffold17625_2	Straboviridae	0.47796255
DLF008_scaffold17497_1	Straboviridae	0.6236477
DLF008_scaffold16449_2	Straboviridae	0.590045
DLF008_scaffold3962_5	Peduoviridae	1
DLF008_scaffold7166_1	Straboviridae	0.5762059
DLF008_scaffold9028_1	Peduoviridae	1
DLF008_scaffold8047_2	Straboviridae	0.55352753
DLF008_scaffold3962_1	Casjensviridae	1
DLF008_scaffold222_2	Herelleviridae	1
DLF008_scaffold17084_1	Straboviridae	0.5381315
DLF008_scaffold14357_3	Straboviridae	0.31066608
DLF008_scaffold3328_5	Straboviridae	0.41379508
DLF008_scaffold17350_1	Straboviridae	0.65565205
DLF009_scaffold34424_1	Straboviridae	0.8369376
DLF009_C428906_1	Drexlerviridae	1

DLF009_scaffold7798_1	Salasmaviridae	1
DLF009_scaffold37177_1	Peduoviridae	1
DLF009_scaffold37452_1	Peduoviridae	1
DLF009_scaffold794_2	Zierdtviridae	1
DLF009_scaffold30115_3	Schitoviridae	0.14546394
DLF009_scaffold13354_1	Casjensviridae	1
DLF009_scaffold37448_2	Herelleviridae	1
DLF009_scaffold37466_1	Salasmaviridae	1
DLF009_scaffold17926_1	Kyanoviridae	1
DLF009_scaffold37386_2	Straboviridae	0.19397318
DLF009_scaffold11949_1	Peduoviridae	1
DLF009_scaffold19674_5	Peduoviridae	0.778437
DLF009_scaffold6204_7	Peduoviridae	0.5576402
DLF010_scaffold55870_1	Straboviridae	0.5762059
DLF010_scaffold43436_5	Peduoviridae	1
DLF010_scaffold36638_6	Kyanoviridae	1
DLF010_scaffold46634_1	Kyanoviridae	1
DLF010_scaffold25454_7	Peduoviridae	0.61460483
DLF010_scaffold51522_2	Drexlerviridae	0.2793915
DLF010_scaffold3276_2	Straboviridae	0.7489975
DLF010_scaffold51387_5	Straboviridae	0.8105539
DLF010_scaffold5445_2	Straboviridae	0.20257144
DLF010_scaffold18414_39	Ackermannviridae	1
DLF010_scaffold27012_2	Straboviridae	0.55340713
DLF012_scaffold12743_1	Casjensviridae	1
DLF012_scaffold19111_1	Casjensviridae	1
DLF012_scaffold19146_2	Straboviridae	0.47796255
DLF012_scaffold17036_1	Ackermannviridae	1
DLF013_scaffold47429_1	Straboviridae	0.5945209
DLF013_scaffold1520_3	Straboviridae	0.6984924
DLF013_scaffold14099_2	Peduoviridae	0.44511104
DLF013_scaffold13599_6	Ackermannviridae	0.31179327
DLF013_scaffold53908_2	Straboviridae	0.68764037
DLF013_scaffold3815_1	Casjensviridae	0.4192874
DLF013_scaffold53550_1	Straboviridae	0.6949948
DLF013_scaffold42596_2	Straboviridae	0.45363978
DLF013_scaffold46613_1	Herelleviridae	1
DLF013_scaffold36273_1	Straboviridae	0.47071996
DLF013_scaffold53889_1	Peduoviridae	1
DLF013_scaffold19199_4	Straboviridae	0.4738817
DLF013_scaffold42008_2	Straboviridae	0.65565205
DLF013_scaffold54776_1	Ackermannviridae	0.31179327
DLF014_scaffold12062_3	Straboviridae	0.6495203
DLF014_C196781_1	Vilmaviridae	1
DLF014_scaffold2320_1	Herelleviridae	1
DLF014_scaffold12718_1	Vilmaviridae	1

DLF014_scaffold11967_1	Casjensviridae	1
DLF014_scaffold3999_1	Straboviridae	0.31791875
DLF014_scaffold6344_1	Straboviridae	0.3343846
DLF014_scaffold11623_1	Ackermannviridae	0.38388076
DLF014_scaffold2693_2	Herelleviridae	1
DLF014_scaffold1928_3	Straboviridae	0.6833262
DLF014_scaffold4556_3	Casjensviridae	1
DLF014_scaffold8_2	Drexlerviridae	0.27648103
DLF014_scaffold8436_2	Straboviridae	0.35845006
DLF014_scaffold11967_2	Peduoviridae	0.16271424
DLF014_scaffold6855_1	Herelleviridae	1
DLF014_scaffold2932_3	Straboviridae	0.6356147
DLF001_scaffold25843_5	unknown	0
DLF001_scaffold25777_2	unknown	0
DLF001_scaffold16608_1	unknown	0
DLF001_scaffold18617_11	unknown	0
DLF001_scaffold1570_1	unknown	0
DLF001_scaffold10872_1	unknown	0
DLF001_scaffold25652_2	unknown	0
DLF001_scaffold10220_3	unknown	0
DLF001_C323242_1	unknown	0
DLF001_scaffold13_12	unknown	0
DLF001_scaffold25336_2	unknown	0
DLF001_scaffold46_6	unknown	0
DLF002_scaffold7298_22	unknown	0
DLF002_scaffold33093_1	unknown	0
DLF002_scaffold36414_1	unknown	0
DLF002_scaffold28742_1	unknown	0
DLF002_scaffold32378_2	unknown	0
DLF002_scaffold36081_2	unknown	0
DLF002_scaffold25546_1	unknown	0
DLF002_scaffold138_8	unknown	0
DLF002_scaffold36505_1	unknown	0
DLF002_scaffold6041_3	unknown	0
DLF002_scaffold21111_2	unknown	0
DLF003_scaffold50567_2	unknown	0
DLF003_scaffold44436_1	unknown	0
DLF003_scaffold137_1	unknown	0
DLF003_scaffold3834_23	unknown	0
DLF003_scaffold23403_10	unknown	0
DLF003_scaffold48199_1	unknown	0
DLF003_scaffold3834_7	unknown	0
DLF003_scaffold56478_1	unknown	0
DLF003_scaffold56159_4	unknown	0
DLF003_scaffold4398_37	unknown	0
DLF004_scaffold35797_1	unknown	0

DLF004_scaffold36527_1	unknown	0
DLF004_C548546_1	unknown	0
DLF004_scaffold23942_9	unknown	0
DLF004_scaffold10854_4	unknown	0
DLF004_scaffold28880_1	unknown	0
DLF004_scaffold36320_8	unknown	0
DLF004_scaffold19957_5	unknown	0
DLF004_scaffold30602_2	unknown	0
DLF004_scaffold930_8	unknown	0
DLF004_scaffold32187_1	unknown	0
DLF004_scaffold23115_1	unknown	0
DLF004_scaffold6537_1	unknown	0
DLF004_scaffold15396_3	unknown	0
DLF004_scaffold7918_11	unknown	0
DLF004_scaffold7020_1	unknown	0
DLF004_scaffold1974_5	unknown	0
DLF004_scaffold15823_6	unknown	0
DLF004_scaffold125_29	unknown	0
DLF005_scaffold31168_1	unknown	0
DLF005_scaffold55052_1	unknown	0
DLF005_scaffold56707_1	unknown	0
DLF005_scaffold31945_4	unknown	0
DLF005_scaffold48546_1	unknown	0
DLF005_scaffold39735_4	unknown	0
DLF005_scaffold27529_1	unknown	0
DLF005_scaffold51335_1	unknown	0
DLF005_scaffold50375_1	unknown	0
DLF005_scaffold56163_1	unknown	0
DLF005_scaffold40395_1	unknown	0
DLF005_scaffold1570_16	unknown	0
DLF005_scaffold13131_1	unknown	0
DLF005_scaffold528_11	unknown	0
DLF005_scaffold39187_4	unknown	0
DLF005_scaffold25785_13	unknown	0
DLF005_scaffold56894_1	unknown	0
DLF005_scaffold54804_6	unknown	0
DLF005_scaffold2989_20	unknown	0
DLF005_scaffold2505_1	unknown	0
DLF005_scaffold56817_1	unknown	0
DLF005_scaffold56924_2	unknown	0
DLF005_scaffold39187_3	unknown	0
DLF005_C847770_1	unknown	0
DLF005_scaffold32479_1	unknown	0
DLF005_scaffold33045_1	unknown	0
DLF005_scaffold9847_1	unknown	0
DLF005_scaffold31168_2	unknown	0

DLF005_scaffold53782_1	unknown	0
DLF006_scaffold52_4	unknown	0
DLF006_scaffold9049_10	unknown	0
DLF006_scaffold39883_1	unknown	0
DLF006_C667862_1	unknown	0
DLF006_scaffold33584_4	unknown	0
DLF006_scaffold34156_4	unknown	0
DLF006_scaffold9056_20	unknown	0
DLF006_scaffold10687_2	unknown	0
DLF006_scaffold19189_4	unknown	0
DLF006_scaffold41298_2	unknown	0
DLF006_scaffold15736_1	unknown	0
DLF006_scaffold22006_9	unknown	0
DLF006_scaffold9845_4	unknown	0
DLF006_scaffold5280_1	unknown	0
DLF006_scaffold46760_1	unknown	0
DLF006_scaffold46848_1	unknown	0
DLF006_scaffold14168_2	unknown	0
DLF006_scaffold8740_4	unknown	0
DLF006_scaffold46677_6	unknown	0
DLF006_scaffold27662_1	unknown	0
DLF006_scaffold46851_2	unknown	0
DLF006_scaffold92_1	unknown	0
DLF007_scaffold15529_4	unknown	0
DLF007_scaffold14424_2	unknown	0
DLF007_C306149_1	unknown	0
DLF007_scaffold20333_2	unknown	0
DLF007_scaffold4179_16	unknown	0
DLF007_scaffold2664_1	unknown	0
DLF007_scaffold15181_7	unknown	0
DLF008_scaffold7461_4	unknown	0
DLF008_scaffold17152_1	unknown	0
DLF008_scaffold1091_2	unknown	0
DLF008_scaffold11168_2	unknown	0
DLF008_scaffold17607_1	unknown	0
DLF008_scaffold14160_4	unknown	0
DLF008_scaffold22_1	unknown	0
DLF008_scaffold10341_2	unknown	0
DLF008_scaffold1410_11	unknown	0
DLF008_scaffold5415_1	unknown	0
DLF008_scaffold10487_1	unknown	0
DLF008_scaffold2326_8	unknown	0
DLF008_scaffold17374_1	unknown	0
DLF008_C335467_1	unknown	0
DLF008_scaffold710_21	unknown	0
DLF008_scaffold17349_1	unknown	0

DLF008_scaffold1029_1	unknown	0
DLF008_scaffold12889_2	unknown	0
DLF008_scaffold2953_7	unknown	0
DLF008_scaffold192_4	unknown	0
DLF009_scaffold1880_30	unknown	0
DLF009_scaffold36221_2	unknown	0
DLF009_scaffold30115_1	unknown	0
DLF009_scaffold789_3	unknown	0
DLF009_scaffold37442_2	unknown	0
DLF009_scaffold36221_1	unknown	0
DLF009_scaffold37363_2	unknown	0
DLF009_scaffold11451_17	unknown	0
DLF009_scaffold21876_2	unknown	0
DLF009_scaffold13766_4	unknown	0
DLF009_scaffold37443_4	unknown	0
DLF009_scaffold30115_4	unknown	0
DLF009_scaffold37020_3	unknown	0
DLF009_scaffold3043_4	unknown	0
DLF009_scaffold37284_1	unknown	0
DLF009_scaffold37363_3	unknown	0
DLF009_scaffold2837_2	unknown	0
DLF009_scaffold61_24	unknown	0
DLF009_scaffold7570_14	unknown	0
DLF009_scaffold3557_6	unknown	0
DLF009_scaffold37386_1	unknown	0
DLF009_scaffold30115_5	unknown	0
DLF010_scaffold2375_6	unknown	0
DLF010_scaffold24026_11	unknown	0
DLF010_scaffold35043_4	unknown	0
DLF010_scaffold363_30	unknown	0
DLF010_scaffold6815_13	unknown	0
DLF010_scaffold13546_16	unknown	0
DLF010_scaffold35043_3	unknown	0
DLF010_scaffold13248_1	unknown	0
DLF010_scaffold4302_1	unknown	0
DLF010_scaffold363_33	unknown	0
DLF010_scaffold33244_2	unknown	0
DLF010_scaffold26724_1	unknown	0
DLF010_scaffold8211_1	unknown	0
DLF010_scaffold45015_1	unknown	0
DLF010_scaffold55918_1	unknown	0
DLF010_scaffold44216_5	unknown	0
DLF010_scaffold1405_28	unknown	0
DLF010_scaffold14049_4	unknown	0
DLF010_scaffold86_3	unknown	0
DLF010_scaffold24026_10	unknown	0

DLF010_scaffold55874_1	unknown	0
DLF012_scaffold4165_33	unknown	0
DLF012_scaffold36_1	unknown	0
DLF012_scaffold17185_5	unknown	0
DLF012_scaffold15936_2	unknown	0
DLF012_scaffold12838_3	unknown	0
DLF012_scaffold19032_1	unknown	0
DLF012_scaffold571_16	unknown	0
DLF012_scaffold3118_9	unknown	0
DLF013_scaffold18599_4	unknown	0
DLF013_scaffold35295_1	unknown	0
DLF013_scaffold52665_1	unknown	0
DLF013_scaffold34743_5	unknown	0
DLF013_scaffold34809_3	unknown	0
DLF013_scaffold45127_1	unknown	0
DLF013_scaffold88_22	unknown	0
DLF013_scaffold6385_2	unknown	0
DLF013_scaffold42479_2	unknown	0
DLF013_scaffold45213_1	unknown	0
DLF013_scaffold31785_1	unknown	0
DLF013_scaffold31672_1	unknown	0
DLF013_scaffold139_7	unknown	0
DLF013_scaffold38734_1	unknown	0
DLF013_scaffold951_1	unknown	0
DLF013_scaffold16307_9	unknown	0
DLF013_scaffold42888_4	unknown	0
DLF013_scaffold21493_3	unknown	0
DLF013_scaffold49433_5	unknown	0
DLF013_scaffold54798_1	unknown	0
DLF013_scaffold1100_4	unknown	0
DLF014_C195831_1	unknown	0
DLF014_scaffold12779_1	unknown	0
DLF014_scaffold9942_1	unknown	0
DLF014_scaffold12792_2	unknown	0
DLF014_scaffold7985_2	unknown	0
DLF014_scaffold5471_1	unknown	0
DLF014_scaffold583_1	unknown	0
DLF014_scaffold12796_1	unknown	0
DLF014_scaffold12682_1	unknown	0
DLF014_scaffold5330_1	unknown	0
DLF014_scaffold2386_4	unknown	0
DLF014_scaffold12741_1	unknown	0
DLF014_scaffold8898_1	unknown	0
DLF014_scaffold12767_1	unknown	0
DLF014_scaffold12792_1	unknown	0
DLF014_scaffold10166_1	unknown	0

DLF014_scaffold12665_1	unknown	0
DLF014_scaffold8300_1	unknown	0
DLF014_scaffold5363_6	unknown	0
DLF014_scaffold5363_21	unknown	0
DLF014_scaffold2093_4	unknown	0
DLF014_scaffold12383_2	unknown	0
DLF002_scaffold24316_12	no_family_avaliable(NC_(	0.977
DLF003_scaffold29891_1	no_family_avaliable(NC_(	0.968
DLF006_scaffold8740_3	no_family_avaliable(NC_(	0.98
DLF008_scaffold8096_6	no_family_avaliable(NC_(	0.977
DLF009_scaffold25763_1	no_family_avaliable(NC_(	0.956
DLF009_scaffold37498_1	no_family_avaliable(NC_(	0.966
DLF009_scaffold37438_1	no_family_avaliable(NC_(	0.98
DLF009_C428576_1	no_family_avaliable(NC_(	0.962
DLF009_scaffold37307_2	no_family_avaliable(NC_(	0.956
DLF010_scaffold49177_4	no_family_avaliable(NC_(	0.991
NLF001_scaffold29251_2	Peduoviridae	1
NLF001_scaffold30907_8	Straboviridae	0.6221042
NLF001_scaffold42470_1	Straboviridae	0.44587678
NLF001_scaffold36675_1	Peduoviridae	1
NLF001_scaffold34766_6	Straboviridae	0.66139334
NLF001_scaffold42478_2	Zierdtviridae	0.59044164
NLF001_C572546_1	Straboviridae	0.66362536
NLF001_scaffold12289_1	Straboviridae	0.60088086
NLF001_scaffold34766_5	Guelinviridae	0.14338043
NLF001_scaffold33713_2	Vilmaviridae	1
NLF002_scaffold336_22	Ackermannviridae	0.32071397
NLF002_scaffold16871_1	Casjensviridae	1
NLF002_scaffold28626_2	Straboviridae	0.35911113
NLF002_scaffold6663_3	Drexlerviridae	0.23640552
NLF002_scaffold27060_6	Ackermannviridae	0.3247123
NLF002_scaffold15559_1	Ackermannviridae	0.32062793
NLF002_scaffold19765_4	Straboviridae	0.6167804
NLF002_scaffold2199_6	Straboviridae	0.66139334
NLF005_scaffold4391_2	Demerecviridae	0.30166167
NLF005_scaffold21492_3	Ackermannviridae	0.29196933
NLF005_scaffold15835_5	Casjensviridae	1
NLF005_scaffold21492_6	Straboviridae	0.4415074
NLF005_scaffold1317_3	Ackermannviridae	0.35133132
NLF005_scaffold47798_1	Salasmaviridae	1
NLF005_C729695_1	Herelleviridae	0.3223665
NLF005_scaffold48237_1	Ackermannviridae	0.47561732
NLF005_scaffold13387_9	Casjensviridae	0.2355009
NLF005_scaffold13387_4	Ackermannviridae	0.39681083
NLF005_scaffold48437_2	Straboviridae	0.37369344
NLF006_scaffold306_3	Straboviridae	0.16758592

NLF006_scaffold306_1	Guelinviridae	0.29881853
NLF006_scaffold9628_2	Casjensviridae	1
NLF006_scaffold5249_2	Straboviridae	0.77169925
NLF006_scaffold3814_1	Ackermannviridae	0.5874024
NLF006_scaffold28327_1	Straboviridae	0.60979193
NLF006_scaffold14746_1	Kyanoviridae	0.13960746
NLF007_scaffold38312_4	Ackermannviridae	0.45278424
NLF007_scaffold66908_2	Straboviridae	0.7093209
NLF007_scaffold11102_4	Herelleviridae	1
NLF007_scaffold23805_3	Straboviridae	0.41627434
NLF007_scaffold25969_1	Casjensviridae	1
NLF007_scaffold27103_9	Straboviridae	0.7093209
NLF007_scaffold36465_3	Drexlerviridae	0.19531086
NLF007_C1010391_1	Peduoviridae	1
NLF007_scaffold67153_2	Demerecviridae	0.8270528
NLF007_scaffold23957_1	Peduoviridae	0.36351505
NLF007_scaffold66908_3	Straboviridae	0.32606444
NLF007_scaffold47382_8	Herelleviridae	0.31269833
NLF007_scaffold57652_6	Ackermannviridae	0.23807108
NLF007_scaffold22798_1	Straboviridae	0.5943833
NLF007_scaffold22798_13	Straboviridae	0.7939098
NLF007_scaffold67062_4	Straboviridae	0.35374683
NLF008_scaffold20496_1	Straboviridae	0.3779054
NLF008_scaffold8874_2	Ackermannviridae	0.45278424
NLF008_scaffold1586_11	Mesyanzhinovviridae	0.53950065
NLF008_scaffold19473_1	Straboviridae	0.40740702
NLF008_scaffold15389_1	Kyanoviridae	1
NLF008_scaffold20125_1	Straboviridae	0.35911113
NLF008_scaffold9492_2	Ackermannviridae	0.35133132
NLF008_scaffold12194_1	Herelleviridae	1
NLF008_scaffold12947_1	Straboviridae	0.5434872
NLF008_scaffold98_9	Mesyanzhinovviridae	0.28275466
NLF008_scaffold1813_6	Peduoviridae	0.6674539
NLF008_scaffold8433_4	Straboviridae	0.2508475
NLF008_scaffold18404_6	Salasmaviridae	0.19234808
NLF008_scaffold386_3	Straboviridae	0.4734725
NLF008_scaffold15537_1	Demerecviridae	0.8270528
NLF008_scaffold18404_13	Herelleviridae	1
NLF008_scaffold9737_2	Straboviridae	0.60088086
NLF008_scaffold83_1	Vilmaviridae	1
NLF008_scaffold1868_2	Ackermannviridae	0.29792926
NLF009_C805905_1	Straboviridae	0.66362536
NLF009_scaffold17912_16	Vilmaviridae	1
NLF009_scaffold7091_22	Peduoviridae	0.6674539
NLF009_scaffold54553_3	Ackermannviridae	0.12159623
NLF009_scaffold9844_25	Straboviridae	0.4734725

NLF009_scaffold52926_4	Ackermannviridae	0.3357813
NLF009_scaffold19140_7	Straboviridae	0.3779054
NLF009_scaffold35663_1	Straboviridae	0.40740702
NLF009_scaffold4106_1	Straboviridae	0.6963392
NLF009_scaffold38501_1	Demerecviridae	0.32602853
NLF009_scaffold375_24	Ackermannviridae	0.2991495
NLF009_scaffold52926_1	Casjensviridae	0.2355009
NLF009_scaffold54503_1	Peduoviridae	0.23355766
NLF009_scaffold17912_6	Herelleviridae	1
NLF010_scaffold20315_2	Peduoviridae	0.28912216
NLF010_scaffold1044_27	Straboviridae	0.35911113
NLF010_scaffold24082_3	Ackermannviridae	0.30388898
NLF010_C402235_1	Demerecviridae	1
NLF010_scaffold12295_8	Ackermannviridae	0.3644868
NLF010_scaffold26024_8	Ackermannviridae	1
NLF010_scaffold2046_76	Straboviridae	0.32298803
NLF010_scaffold9747_18	Salasmaviridae	0.4600486
NLF010_scaffold20591_1	Straboviridae	0.4734725
NLF010_scaffold29445_4	Straboviridae	0.44893572
NLF011_scaffold42_2	Straboviridae	0.3083672
NLF011_scaffold1105_1	Ackermannviridae	0.5874024
NLF011_scaffold7214_2	Straboviridae	0.61042684
NLF011_scaffold4255_7	Peduoviridae	0.3274082
NLF011_scaffold2637_6_1	Ackermannviridae	0.25874183
NLF011_scaffold13048_1	Ackermannviridae	0.35383645
NLF011_scaffold25120_2	Peduoviridae	1
NLF011_scaffold486_7	Ackermannviridae	1
NLF011_scaffold794_4	Straboviridae	0.40740702
NLF011_scaffold22115_1	Straboviridae	0.7048575
NLF011_scaffold803_4	Ackermannviridae	0.45278424
NLF012_scaffold907_13	Drexlerviridae	0.13413674
NLF012_scaffold2371_23	Peduoviridae	1
NLF012_scaffold39054_1	Peduoviridae	0.26534814
NLF012_scaffold8622_2	Peduoviridae	1
NLF012_scaffold2172_5	Straboviridae	0.44206813
NLF012_scaffold27405_2	Peduoviridae	1
NLF012_scaffold39140_2	Straboviridae	0.41627434
NLF012_scaffold39159_1	Salasmaviridae	1
NLF012_scaffold17264_3	Ackermannviridae	0.45278424
NLF012_scaffold34471_3	Straboviridae	0.6525488
NLF012_scaffold293_2	Straboviridae	0.66362536
NLF012_scaffold10245_3	Ackermannviridae	0.23807108
NLF012_scaffold13034_3	Kyanoviridae	1
NLF012_scaffold17845_1	Ackermannviridae	0.4260041
NLF012_C512465_1	Salasmaviridae	0.93962055
NLF012_scaffold32276_1	Straboviridae	0.4409281

NLF012_scaffold13132_1	Peduoviridae	0.9875101
NLF012_scaffold35713_1	Mesyanzhinovviridae	0.50958985
NLF012_scaffold10663_6	Peduoviridae	0.6674539
NLF012_scaffold35670_2	Ackermannviridae	0.3644868
NLF012_scaffold15197_22	Straboviridae	0.42601934
NLF012_scaffold37458_1	Straboviridae	0.44893572
NLF012_scaffold21989_2	Casjensviridae	0.1514813
NLF012_scaffold10340_14	Herelleviridae	1
NLF012_scaffold16451_3	Straboviridae	0.3238582
NLF012_scaffold13372_3	Herelleviridae	1
NLF012_scaffold69_1	Drexlerviridae	0.32428944
NLF012_scaffold8622_3	Casjensviridae	1
NLF012_scaffold2371_15	Casjensviridae	1
NLF013_scaffold11783_1	Straboviridae	0.42601934
NLF013_scaffold48622_1	Schitoviridae	1
NLF013_scaffold11031_1	Straboviridae	0.7939098
NLF013_C659127_1	Salasmaviridae	1
NLF013_scaffold328_1	Kyanoviridae	1
NLF013_scaffold19270_4	Ackermannviridae	0.35133132
NLF013_scaffold44405_3	Ackermannviridae	0.45278424
NLF013_scaffold11031_7	Straboviridae	0.5943833
NLF013_scaffold4561_28	Ackermannviridae	0.32071397
NLF013_scaffold20413_4	Herelleviridae	1
NLF013_scaffold48338_2	Drexlerviridae	0.30199948
NLF014_scaffold5840_1	Straboviridae	0.4415074
NLF014_scaffold7292_1	Ackermannviridae	0.45278424
NLF014_scaffold6197_2	Straboviridae	0.4734725
NLF014_scaffold819_1	Ackermannviridae	0.34401792
NLF014_scaffold6884_2	Straboviridae	0.72049403
NLF014_scaffold73_1	Straboviridae	0.34519532
NLF015_scaffold31615_2	Mesyanzhinovviridae	0.50958985
NLF015_C419895_1	Peduoviridae	0.28792256
NLF015_scaffold591_2	Straboviridae	0.4734725
NLF015_scaffold11481_6	Straboviridae	0.3779054
NLF015_scaffold3859_2	Mesyanzhinovviridae	0.28275466
NLF015_scaffold24362_4	Straboviridae	0.77169925
NLF015_scaffold29017_1	Straboviridae	0.35911113
NLF015_scaffold31211_3	Ackermannviridae	0.28982037
NLF015_scaffold20500_6	Straboviridae	0.41627434
NLF015_scaffold13173_2	Ackermannviridae	0.33847627
NLF015_scaffold7628_6	Peduoviridae	0.3274082
NLF015_scaffold14_1	Mesyanzhinovviridae	0.53950065
NLF015_scaffold3382_3	Peduoviridae	0.36351505
NLF015_scaffold34269_1	Straboviridae	0.8236184
NLF015_scaffold33332_2	Drexlerviridae	0.25311825
NLF015_scaffold4657_3	Straboviridae	0.6525488

NLF015_scaffold13163_7	Salasmaviridae	0.4600486
NLF001_scaffold18110_3	unknown	0
NLF001_scaffold18524_1	unknown	0
NLF001_scaffold159_2	unknown	0
NLF001_scaffold1094_2	unknown	0
NLF001_scaffold15921_9	unknown	0
NLF001_scaffold15028_2	unknown	0
NLF001_scaffold21827_1	unknown	0
NLF001_scaffold17952_7	unknown	0
NLF001_scaffold42456_1	unknown	0
NLF001_scaffold21408_7	unknown	0
NLF001_scaffold42472_1	unknown	0
NLF001_scaffold38144_4	unknown	0
NLF002_scaffold10424_8	unknown	0
NLF002_scaffold36662_1	unknown	0
NLF002_scaffold35171_2	unknown	0
NLF002_scaffold34776_1	unknown	0
NLF002_scaffold22680_4	unknown	0
NLF002_scaffold3933_4	unknown	0
NLF002_scaffold14709_2	unknown	0
NLF002_scaffold141_14	unknown	0
NLF002_scaffold36924_1	unknown	0
NLF002_scaffold115_2	unknown	0
NLF002_scaffold17055_1	unknown	0
NLF002_scaffold25489_2	unknown	0
NLF002_scaffold38935_2	unknown	0
NLF005_scaffold46950_1	unknown	0
NLF005_scaffold36523_1	unknown	0
NLF005_scaffold32720_4	unknown	0
NLF005_C729497_1	unknown	0
NLF005_scaffold21214_1	unknown	0
NLF005_scaffold27522_3	unknown	0
NLF005_scaffold47800_1	unknown	0
NLF005_scaffold29618_1	unknown	0
NLF005_scaffold21492_4	unknown	0
NLF005_scaffold25196_2	unknown	0
NLF005_scaffold40967_2	unknown	0
NLF005_scaffold43034_2	unknown	0
NLF005_scaffold291_42	unknown	0
NLF005_scaffold47934_7	unknown	0
NLF005_scaffold43004_3	unknown	0
NLF005_scaffold14516_2	unknown	0
NLF005_C729433_1	unknown	0
NLF005_scaffold48442_3	unknown	0
NLF005_scaffold48389_1	unknown	0
NLF005_scaffold39282_7	unknown	0

NLF005_scaffold40798_1	unknown	0
NLF005_scaffold29852_1	unknown	0
NLF005_scaffold291_44	unknown	0
NLF005_scaffold10734_4	unknown	0
NLF006_scaffold22186_2	unknown	0
NLF006_scaffold31099_1	unknown	0
NLF006_scaffold4766_5	unknown	0
NLF006_scaffold4766_2	unknown	0
NLF006_scaffold15157_3	unknown	0
NLF006_scaffold4466_3	unknown	0
NLF006_scaffold132_1	unknown	0
NLF006_scaffold5891_10	unknown	0
NLF006_scaffold30421_2	unknown	0
NLF006_scaffold4358_2	unknown	0
NLF007_scaffold882_1	unknown	0
NLF007_scaffold5043_8	unknown	0
NLF007_scaffold67260_2	unknown	0
NLF007_scaffold14743_4	unknown	0
NLF007_scaffold64602_1	unknown	0
NLF007_scaffold8274_2	unknown	0
NLF007_scaffold22938_1	unknown	0
NLF007_scaffold66524_2	unknown	0
NLF007_scaffold38312_8	unknown	0
NLF007_scaffold5542_23	unknown	0
NLF007_scaffold49453_1	unknown	0
NLF007_scaffold31937_4	unknown	0
NLF007_scaffold110_13	unknown	0
NLF007_scaffold23404_3	unknown	0
NLF007_scaffold29510_1	unknown	0
NLF007_scaffold61307_5	unknown	0
NLF007_scaffold4118_1	unknown	0
NLF007_scaffold67236_3	unknown	0
NLF007_scaffold3779_4	unknown	0
NLF007_scaffold67413_1	unknown	0
NLF007_scaffold17554_50	unknown	0
NLF007_scaffold64205_2	unknown	0
NLF007_scaffold61221_1	unknown	0
NLF007_scaffold56144_4	unknown	0
NLF007_scaffold54945_9	unknown	0
NLF007_scaffold20693_10	unknown	0
NLF007_scaffold40076_1	unknown	0
NLF007_scaffold47325_6	unknown	0
NLF007_scaffold14743_2	unknown	0
NLF007_scaffold30358_8	unknown	0
NLF007_scaffold3779_6	unknown	0
NLF007_scaffold14743_1	unknown	0

NLF007_scaffold50111_1	unknown	0
NLF007_scaffold57227_1	unknown	0
NLF007_scaffold48133_2	unknown	0
NLF007_scaffold66524_3	unknown	0
NLF007_scaffold43991_1	unknown	0
NLF007_scaffold31937_5	unknown	0
NLF007_scaffold32046_2	unknown	0
NLF007_scaffold21464_1	unknown	0
NLF007_scaffold67062_3	unknown	0
NLF007_scaffold27103_3	unknown	0
NLF008_C315574_1	unknown	0
NLF008_scaffold141_3	unknown	0
NLF008_scaffold12997_5	unknown	0
NLF008_scaffold18404_4	unknown	0
NLF008_scaffold10158_1	unknown	0
NLF008_scaffold18404_14	unknown	0
NLF008_scaffold10905_2	unknown	0
NLF008_scaffold10905_1	unknown	0
NLF008_scaffold7552_2	unknown	0
NLF008_scaffold20431_5	unknown	0
NLF008_scaffold9366_1	unknown	0
NLF008_scaffold17605_2	unknown	0
NLF008_scaffold6835_1	unknown	0
NLF008_scaffold20731_1	unknown	0
NLF008_scaffold6103_28	unknown	0
NLF008_scaffold395_6	unknown	0
NLF008_scaffold17931_1	unknown	0
NLF008_scaffold12834_4	unknown	0
NLF008_scaffold16324_2	unknown	0
NLF008_scaffold107_1	unknown	0
NLF008_scaffold20258_1	unknown	0
NLF008_scaffold6876_9	unknown	0
NLF008_scaffold9442_2	unknown	0
NLF008_C315880_1	unknown	0
NLF008_scaffold20528_1	unknown	0
NLF008_scaffold1474_11	unknown	0
NLF008_scaffold5986_1	unknown	0
NLF008_scaffold7896_4	unknown	0
NLF008_scaffold12833_2	unknown	0
NLF008_scaffold20431_4	unknown	0
NLF008_scaffold13388_2	unknown	0
NLF009_scaffold3162_3	unknown	0
NLF009_scaffold30773_1	unknown	0
NLF009_scaffold54553_5	unknown	0
NLF009_scaffold21790_2	unknown	0
NLF009_scaffold4933_28	unknown	0

NLF009_scaffold52375_7	unknown	0
NLF009_scaffold54499_1	unknown	0
NLF009_scaffold54170_2	unknown	0
NLF009_scaffold1353_6	unknown	0
NLF009_C805829_1	unknown	0
NLF009_scaffold27217_3	unknown	0
NLF009_C805873_1	unknown	0
NLF009_scaffold17912_4	unknown	0
NLF009_scaffold5429_52	unknown	0
NLF009_scaffold40330_1	unknown	0
NLF009_scaffold18984_7	unknown	0
NLF009_scaffold8414_14	unknown	0
NLF009_scaffold17912_17	unknown	0
NLF009_scaffold52991_2	unknown	0
NLF009_scaffold25289_5	unknown	0
NLF009_scaffold36413_1	unknown	0
NLF009_scaffold37415_3	unknown	0
NLF010_scaffold1235_8	unknown	0
NLF010_C401947_1	unknown	0
NLF010_scaffold13747_2	unknown	0
NLF010_scaffold22417_1	unknown	0
NLF010_scaffold3898_4	unknown	0
NLF010_scaffold27728_2	unknown	0
NLF010_scaffold29246_1	unknown	0
NLF011_scaffold4965_2	unknown	0
NLF011_scaffold5613_1	unknown	0
NLF011_scaffold10809_1	unknown	0
NLF011_scaffold10054_4	unknown	0
NLF011_scaffold1786_1	unknown	0
NLF011_scaffold4096_9	unknown	0
NLF011_scaffold3151_2	unknown	0
NLF011_scaffold30362_5	unknown	0
NLF011_scaffold10943_3	unknown	0
NLF011_scaffold6937_1	unknown	0
NLF011_scaffold21554_2	unknown	0
NLF011_scaffold9582_1	unknown	0
NLF012_scaffold10340_2	unknown	0
NLF012_scaffold35219_5	unknown	0
NLF012_scaffold35219_1	unknown	0
NLF012_scaffold39053_1	unknown	0
NLF012_scaffold22746_1	unknown	0
NLF012_scaffold11833_29	unknown	0
NLF012_scaffold2371_16	unknown	0
NLF012_scaffold25728_1	unknown	0
NLF012_scaffold36675_4	unknown	0
NLF012_scaffold21675_1	unknown	0

NLF012_scaffold11833_30	unknown	0
NLF012_scaffold29420_1	unknown	0
NLF012_scaffold6773_9	unknown	0
NLF012_scaffold2854_1	unknown	0
NLF012_scaffold18119_4	unknown	0
NLF012_scaffold38032_1	unknown	0
NLF012_scaffold16653_1	unknown	0
NLF012_scaffold22955_2	unknown	0
NLF012_scaffold15731_1	unknown	0
NLF012_scaffold16348_2	unknown	0
NLF012_scaffold8622_6	unknown	0
NLF012_scaffold12754_3	unknown	0
NLF012_scaffold10394_1	unknown	0
NLF012_scaffold30180_18	unknown	0
NLF012_scaffold17431_22	unknown	0
NLF012_scaffold39143_1	unknown	0
NLF012_scaffold14481_3	unknown	0
NLF012_scaffold4362_3	unknown	0
NLF012_scaffold38234_3	unknown	0
NLF012_scaffold37376_1	unknown	0
NLF012_scaffold14206_2	unknown	0
NLF012_scaffold17431_21	unknown	0
NLF012_scaffold33261_5	unknown	0
NLF012_scaffold29414_2	unknown	0
NLF012_scaffold17240_1	unknown	0
NLF013_scaffold43868_1	unknown	0
NLF013_scaffold14354_6	unknown	0
NLF013_scaffold30746_1	unknown	0
NLF013_scaffold12177_2	unknown	0
NLF013_scaffold34744_1	unknown	0
NLF013_scaffold33849_1	unknown	0
NLF013_scaffold23543_4	unknown	0
NLF013_scaffold4799_2	unknown	0
NLF013_scaffold32342_11	unknown	0
NLF013_scaffold47264_1	unknown	0
NLF013_scaffold33946_4	unknown	0
NLF013_scaffold25755_1	unknown	0
NLF013_scaffold21878_2	unknown	0
NLF013_scaffold23156_2	unknown	0
NLF013_scaffold30660_7	unknown	0
NLF013_C659857_1	unknown	0
NLF013_scaffold19359_1	unknown	0
NLF013_scaffold26044_5	unknown	0
NLF013_scaffold43192_14	unknown	0
NLF014_scaffold142_6	unknown	0
NLF014_scaffold342_1	unknown	0

NLF014_scaffold9741_6	unknown	0
NLF014_scaffold1307_2	unknown	0
NLF014_scaffold10772_2	unknown	0
NLF014_scaffold249_3	unknown	0
NLF014_scaffold9408_9	unknown	0
NLF014_scaffold736_2	unknown	0
NLF015_scaffold32932_4	unknown	0
NLF015_scaffold31821_3	unknown	0
NLF015_scaffold24231_1	unknown	0
NLF015_scaffold14491_1	unknown	0
NLF015_scaffold22095_1	unknown	0
NLF015_scaffold21847_3	unknown	0
NLF015_scaffold22_1	unknown	0
NLF015_scaffold34057_2	unknown	0
NLF015_scaffold12014_3	unknown	0
NLF015_scaffold8589_4	unknown	0
NLF015_scaffold14946_1	unknown	0
NLF015_scaffold20215_1	unknown	0
NLF015_C419419_1	unknown	0
NLF015_scaffold32259_1	unknown	0
NLF015_scaffold6258_2	unknown	0
NLF015_scaffold34558_2	unknown	0
NLF015_scaffold21847_1	unknown	0
NLF002_scaffold37757_3	no_family_avaliable(NC_(	0.956
NLF002_scaffold37757_4	no_family_avaliable(NC_(	0.955
NLF002_scaffold37757_5	no_family_avaliable(NC_(	0.981
NLF005_scaffold4391_3	no_family_avaliable(NC_(	0.967
NLF007_scaffold34404_4	no_family_avaliable(NC_(	0.974
NLF008_scaffold19700_1	no_family_avaliable(NC_(	0.989
NLF008_scaffold14014_6	no_family_avaliable(NC_(	0.993
DLM001_scaffold17760_6	Straboviridae	0.6455734
DLM001_scaffold91_6	Straboviridae	0.557449
DLM001_scaffold4307_3	Peduoviridae	0.5045494
DLM001_scaffold19551_4	Peduoviridae	0.46964702
DLM001_scaffold23814_4	Straboviridae	0.61924237
DLM001_scaffold14847_5	Straboviridae	0.40471888
DLM001_scaffold42296_1	Straboviridae	0.571929
DLM001_scaffold4307_2	Peduoviridae	0.5289318
DLM001_scaffold52744_1	Ackermannviridae	0.29288775
DLM001_scaffold20858_1	Straboviridae	0.18533131
DLM001_scaffold27372_1	Straboviridae	0.58128864
DLM001_scaffold57732_2	Straboviridae	0.69763845
DLM001_scaffold17614_8	Mesyanzhinovviridae	0.42145562
DLM001_scaffold57716_1	Straboviridae	0.62921655
DLM001_scaffold34133_3	Zierdtviridae	0.23121345
DLM001_scaffold49843_4	Straboviridae	0.22559838

DLM001_scaffold51928_3	Straboviridae	0.19912948
DLM001_scaffold19551_5	Mesyanzhinovviridae	0.18997274
DLM001_scaffold19465_5	Straboviridae	0.61924237
DLM001_scaffold56543_4	Drexlerviridae	0.33024517
DLM001_scaffold14859_7	Schitoviridae	0.2646486
DLM001_scaffold20858_3	Straboviridae	0.20631503
DLM001_C705523_1	Straboviridae	0.5909823
DLM001_scaffold17051_8	Ackermannviridae	0.34976166
DLM001_scaffold19551_1	Ackermannviridae	1
DLM001_scaffold20817_1	Straboviridae	0.58554715
DLM001_scaffold25009_5	Casjensviridae	0.91085446
DLM001_scaffold19551_3	Peduoviridae	0.36803198
DLM001_scaffold53994_4	Casjensviridae	0.91085446
DLM001_scaffold51591_2	Drexlerviridae	0.27567244
DLM001_scaffold10420_4	Drexlerviridae	0.1742411
DLM001_scaffold58124_4	Zierdtviridae	1
DLM001_scaffold23040_12	Straboviridae	0.6360723
DLM001_C705425_1	Straboviridae	0.64132065
DLM001_scaffold18361_2	Ackermannviridae	1
DLM001_scaffold39252_2	Straboviridae	0.38693213
DLM001_scaffold13671_1	Ackermannviridae	0.6493801
DLM001_scaffold50893_2	Herelleviridae	1
DLM001_scaffold277_3	Casjensviridae	0.65000147
DLM001_scaffold39468_1	Vilmaviridae	1
DLM001_scaffold47007_2	Drexlerviridae	0.18274732
DLM001_scaffold42810_2	Straboviridae	0.54152817
DLM001_scaffold52139_2	Casjensviridae	0.46940973
DLM001_scaffold27611_5	Ackermannviridae	0.34976166
DLM002_scaffold52283_2	Mesyanzhinovviridae	0.34989476
DLM002_scaffold8357_7	Peduoviridae	1
DLM002_scaffold37352_8	Autographiviridae	1
DLM002_scaffold52713_2	Straboviridae	0.44480026
DLM002_scaffold48724_2	Straboviridae	0.3196358
DLM002_scaffold11013_7	Zierdtviridae	0.55246586
DLM002_scaffold44502_1	Straboviridae	0.5021334
DLM002_scaffold32192_3	Drexlerviridae	0.28968555
DLM002_scaffold5336_45	Casjensviridae	1
DLM002_scaffold925_8	Ackermannviridae	0.6232304
DLM003_scaffold20093_1	Casjensviridae	0.74338967
DLM003_scaffold1898_14	Straboviridae	0.6252392
DLM003_scaffold30928_7	Ackermannviridae	0.6232304
DLM003_C810975_1	Drexlerviridae	0.33024517
DLM003_scaffold10664_1	Straboviridae	0.571929
DLM003_scaffold59785_5	Ackermannviridae	1
DLM003_scaffold40695_7	Kyanoviridae	0.20182836
DLM003_scaffold303_2	Ackermannviridae	0.21766534

DLM003_scaffold49406_1	Straboviridae	0.4553038
DLM003_C810753_1	Salasmaviridae	1
DLM003_scaffold17135_3	Ackermannviridae	1
DLM003_scaffold14015_13	Peduoviridae	0.5289318
DLM003_scaffold3089_1	Salasmaviridae	0.31627378
DLM003_scaffold15117_2	Straboviridae	0.26355055
DLM003_scaffold59785_12	Herelleviridae	1
DLM003_scaffold102_3	Ackermannviridae	0.39516526
DLM003_scaffold40695_5	Drexlerviridae	0.25272772
DLM004_scaffold12500_3	Casjensviridae	0.48759285
DLM004_scaffold8053_11	Straboviridae	0.58128864
DLM004_scaffold8053_12	Straboviridae	0.62674475
DLM004_scaffold18777_1	Casjensviridae	0.4376873
DLM004_scaffold41_14	Straboviridae	0.56166416
DLM004_scaffold158_30	Ackermannviridae	0.31015208
DLM004_scaffold16125_1	Straboviridae	0.6043284
DLM004_scaffold12500_5	Drexlerviridae	0.27603
DLM004_scaffold17859_1	Straboviridae	0.66194147
DLM004_scaffold14310_1	Straboviridae	0.62921655
DLM004_scaffold16036_1	Ackermannviridae	0.34976166
DLM004_scaffold12500_1	Casjensviridae	0.4376873
DLM004_scaffold8780_5	Straboviridae	0.6664747
DLM005_scaffold44327_3	Casjensviridae	0.811716
DLM005_scaffold21763_7	Ackermannviridae	0.6232304
DLM005_scaffold55834_11	Straboviridae	0.33227503
DLM005_scaffold56278_1	Ackermannviridae	0.3053488
DLM005_scaffold3128_1	Straboviridae	0.6360723
DLM005_scaffold55666_1	Salasmaviridae	0.45254457
DLM005_scaffold4283_10	Peduoviridae	0.5512526
DLM005_scaffold58045_1	Casjensviridae	0.73554254
DLM005_scaffold56278_2	Peduoviridae	0.43608958
DLM005_scaffold2340_3	Ackermannviridae	0.3847759
DLM005_scaffold36491_20	Peduoviridae	1
DLM005_scaffold36491_18	Casjensviridae	1
DLM005_scaffold57077_1	Peduoviridae	0.61929643
DLM005_scaffold5800_5	Peduoviridae	1
DLM005_scaffold20359_8	Peduoviridae	0.3466597
DLM005_scaffold57953_1	Peduoviridae	1
DLM005_scaffold1593_32	Straboviridae	0.5348034
DLM006_scaffold29093_1	Salasmaviridae	0.45254457
DLM006_scaffold23991_4	Straboviridae	0.44908637
DLM006_scaffold1615_27	Ackermannviridae	1
DLM006_scaffold35098_1	Casjensviridae	1
DLM006_scaffold10416_3	Peduoviridae	0.3525797
DLM006_scaffold289_5	Mesyanzhinovviridae	0.34989476
DLM006_scaffold15803_18	Casjensviridae	0.4376873

DLM006_scaffold23991_2	Straboviridae	0.38295603
DLM006_scaffold5392_1	Casjensviridae	1
DLM006_scaffold19568_4	Straboviridae	0.33227503
DLM006_scaffold582_5	Straboviridae	0.38693213
DLM007_scaffold7436_7	Peduoviridae	0.38523066
DLM007_C664617_1	Salasmaviridae	1
DLM007_scaffold45308_2	Demerecviridae	0.33583313
DLM007_scaffold35599_2	Straboviridae	0.571929
DLM007_scaffold28062_4	Straboviridae	0.58128864
DLM007_scaffold13248_1	Ackermannviridae	0.3053488
DLM007_scaffold5339_25	Straboviridae	0.31710467
DLM007_scaffold45251_1	Straboviridae	0.61924237
DLM008_scaffold34307_5	Vilmaviridae	1
DLM008_scaffold56745_2	Peduoviridae	1
DLM008_scaffold26298_3	Chaseviridae	0.49954474
DLM008_scaffold19097_13	Straboviridae	0.61924237
DLM008_scaffold45871_1	Straboviridae	0.58876103
DLM008_scaffold9016_3	Straboviridae	0.5401171
DLM008_scaffold371_1	Straboviridae	0.6741575
DLM008_scaffold68309_1	Straboviridae	0.34864473
DLM009_scaffold14704_1	Straboviridae	0.5172189
DLM009_scaffold21367_3	Drexlerviridae	0.1742411
DLM009_scaffold8594_4	Peduoviridae	1
DLM009_scaffold5016_13	Straboviridae	0.40471888
DLM009_scaffold102_1	Straboviridae	0.6931655
DLM009_C324441_1	Peduoviridae	0.28715542
DLM009_scaffold6465_20	Straboviridae	0.4652497
DLM009_scaffold21079_1	Herelleviridae	1
DLM009_scaffold22_4	Straboviridae	0.47515345
DLM009_scaffold17086_6	Ackermannviridae	1
DLM009_scaffold19690_1	Straboviridae	0.27523607
DLM009_scaffold18558_4	Straboviridae	0.5401171
DLM009_scaffold6465_28	Straboviridae	0.62464666
DLM009_C324485_1	Herelleviridae	1
DLM010_scaffold41867_3	Herelleviridae	1
DLM010_scaffold45140_1	Salasmaviridae	0.94846845
DLM010_scaffold179_1	Straboviridae	0.61924237
DLM010_scaffold503_4	Straboviridae	0.41849706
DLM010_scaffold10158_2	Straboviridae	0.40471888
DLM010_scaffold5445_6	Peduoviridae	0.46964702
DLM010_scaffold32332_1	Peduoviridae	0.63610744
DLM010_scaffold3050_2	Peduoviridae	1
DLM010_scaffold41095_1	Drexlerviridae	0.09667662
DLM010_scaffold16539_4	Drexlerviridae	0.28968555
DLM010_scaffold33840_5	Straboviridae	0.5655343
DLM010_scaffold6743_2	Straboviridae	0.6116163

DLM010_scaffold22879_4	Peduoviridae	1
DLM010_scaffold45132_3	Peduoviridae	1
DLM010_scaffold4248_8	Straboviridae	0.56166416
DLM010_scaffold45189_1	Salasmaviridae	0.969743
DLM010_scaffold44889_1	Peduoviridae	1
DLM011_scaffold29232_2	Peduoviridae	0.3466597
DLM011_scaffold29382_1	Drexlerviridae	0.28968555
DLM011_scaffold21086_2	Herelleviridae	1
DLM011_scaffold21352_1	Kyanoviridae	0.2949095
DLM011_scaffold29232_1	Peduoviridae	0.27632862
DLM011_scaffold29398_1	Straboviridae	0.62648106
DLM011_scaffold1189_31	Peduoviridae	1
DLM011_scaffold18867_6	Straboviridae	0.40471888
DLM012_scaffold11223_12	Straboviridae	0.4992557
DLM012_scaffold16447_5	Straboviridae	0.58554715
DLM012_C414271_1	Straboviridae	0.34864473
DLM012_scaffold23728_1	Peduoviridae	1
DLM012_scaffold2394_3	Peduoviridae	0.37287483
DLM012_scaffold12_2	Casjensviridae	0.46940973
DLM012_scaffold32680_2	Straboviridae	0.61910915
DLM012_scaffold28806_1	Straboviridae	0.5285559
DLM012_scaffold725_1	Peduoviridae	1
DLM012_scaffold33245_1	Schitoviridae	1
DLM013_scaffold17656_2	Drexlerviridae	0.1742411
DLM013_scaffold7395_5	Ackermannviridae	0.34976166
DLM013_scaffold34896_3	Straboviridae	0.5416722
DLM013_scaffold21602_2	Herelleviridae	1
DLM013_scaffold57623_1	Autographiviridae	1
DLM013_scaffold36790_6	Straboviridae	0.54375273
DLM013_scaffold3810_8	Straboviridae	0.52317137
DLM013_scaffold57456_3	Straboviridae	0.40471888
DLM013_scaffold22571_2	Straboviridae	0.34551933
DLM013_scaffold4430_4	Autographiviridae	1
DLM013_scaffold56628_1	Autographiviridae	1
DLM013_scaffold47801_1	Ackermannviridae	1
DLM013_scaffold51742_2	Herelleviridae	1
DLM013_scaffold23184_12	Vilmaviridae	1
DLM013_scaffold57687_1	Salasmaviridae	1
DLM013_scaffold8482_6	Herelleviridae	1
DLM013_scaffold23009_1	Straboviridae	0.5655343
DLM013_scaffold51645_2	Straboviridae	0.41326678
DLM013_scaffold20700_8	Drexlerviridae	0.199991
DLM013_scaffold50692_1	Straboviridae	0.25122714
DLM013_scaffold23184_9	Demerecviridae	0.33583313
DLM013_scaffold49247_2	Drexlerviridae	0.3231527
DLM013_scaffold56635_1	Vilmaviridae	0.11836226

DLM013_C721558_1	Autographiviridae	0.97334677
DLM013_scaffold30472_4	Ackermannviridae	0.3847759
DLM014_scaffold39602_3	Straboviridae	0.52348197
DLM014_scaffold17726_11	Peduoviridae	0.5512526
DLM014_scaffold43677_1	Kyanoviridae	1
DLM014_scaffold24690_1	Ackermannviridae	0.34976166
DLM014_scaffold35850_2	Straboviridae	0.61924237
DLM014_scaffold40706_2	Ackermannviridae	0.6232304
DLM014_scaffold42200_1	Straboviridae	0.571929
DLM014_scaffold4026_7	Ackermannviridae	0.2221647
DLM014_scaffold32434_6	Ackermannviridae	0.6232304
DLM014_scaffold290_4	Ackermannviridae	0.36708665
DLM014_scaffold7310_3	Ackermannviridae	0.26684576
DLM015_scaffold18535_6	Ackermannviridae	1
DLM015_scaffold16218_2	Straboviridae	0.6664747
DLM015_scaffold23554_1	Straboviridae	0.69763845
DLM015_scaffold14872_4	Drexlerviridae	0.1742411
DLM015_scaffold50457_1	Herelleviridae	1
DLM015_scaffold29903_2	Casjensviridae	0.4598215
DLM016_scaffold17586_2	Straboviridae	0.6816639
DLM016_scaffold3377_13	Peduoviridae	0.5512526
DLM016_scaffold27953_5	Straboviridae	0.52317137
DLM016_scaffold37509_2	Kyanoviridae	1
DLM016_scaffold419_5	Straboviridae	0.62674475
DLM016_scaffold49496_1	Straboviridae	0.46930915
DLM016_scaffold2806_2	Casjensviridae	0.8158135
DLM016_scaffold5004_5	Straboviridae	0.4380502
DLM016_scaffold6713_5	Straboviridae	0.63893926
DLM016_scaffold64767_1	Salasmaviridae	1
DLM016_scaffold2366_1	Straboviridae	0.61910915
DLM016_scaffold58329_1	Casjensviridae	0.4466287
DLM016_scaffold37873_2	Straboviridae	0.62648106
DLM016_scaffold64097_2	Straboviridae	0.5655343
DLM016_scaffold21785_1	Ackermannviridae	0.34121394
DLM016_scaffold6553_6	Herelleviridae	0.89390486
DLM017_scaffold20271_2	Straboviridae	0.3895763
DLM017_scaffold29996_2	Straboviridae	0.5416722
DLM017_scaffold2501_2	Straboviridae	1
DLM017_scaffold43714_2	Peduoviridae	0.61929643
DLM017_scaffold5960_2	Casjensviridae	0.353185
DLM017_scaffold5705_1	Straboviridae	0.6360723
DLM017_scaffold42571_2	Ackermannviridae	0.32050374
DLM017_scaffold1478_32	Peduoviridae	0.5512526
DLM017_scaffold15588_1	Peduoviridae	1
DLM017_scaffold31873_1	Casjensviridae	0.58597374
DLM017_scaffold23811_1	Straboviridae	0.40471888

DLM018_scaffold37570_1	Zierdtviridae	0.25472173
DLM018_scaffold12094_2	Straboviridae	0.431543
DLM018_scaffold2054_4	Peduoviridae	0.37287483
DLM018_scaffold48894_1	Kyanoviridae	1
DLM018_scaffold26573_10	Mesyanzhinovviridae	0.39414543
DLM018_scaffold45347_2	Straboviridae	0.5172189
DLM018_scaffold48696_1	Salasmaviridae	1
DLM018_scaffold15_30	Zierdtviridae	1
DLM018_scaffold48959_3	Straboviridae	0.27312943
DLM018_scaffold7847_2	Ackermannviridae	1
DLM018_scaffold48449_1	Straboviridae	0.4545582
DLM018_scaffold26573_4	Casjensviridae	0.38102219
DLM018_scaffold36970_6	Ackermannviridae	0.22744717
DLM018_scaffold48983_2	Straboviridae	0.42117372
DLM018_scaffold26955_2	Peduoviridae	1
DLM018_scaffold3351_9	Casjensviridae	1
DLM019_scaffold62785_1	Casjensviridae	1
DLM019_scaffold20492_3	Drexlerviridae	0.3499038
DLM019_scaffold6166_23	Drexlerviridae	0.46745822
DLM019_C814862_1	Straboviridae	0.58876103
DLM019_scaffold45285_5	Casjensviridae	1
DLM019_scaffold61987_1	Straboviridae	0.30654648
DLM019_scaffold38422_4	Chaseviridae	1
DLM019_scaffold45285_4	Drexlerviridae	0.30423322
DLM019_scaffold4998_6	Peduoviridae	0.7190166
DLM019_scaffold100_11	Peduoviridae	0.3525797
DLM019_scaffold55656_1	Drexlerviridae	0.21202745
DLM019_scaffold102_1	Peduoviridae	1
DLM019_scaffold55454_1	Straboviridae	0.5449183
DLM019_scaffold2445_1	Drexlerviridae	0.21655169
DLM019_scaffold8788_7	Straboviridae	0.40471888
DLM020_scaffold983_14	Casjensviridae	0.353185
DLM020_scaffold10399_1	Straboviridae	0.5655343
DLM020_scaffold3010_3	Peduoviridae	0.24940163
DLM020_scaffold20362_3	Straboviridae	0.20631503
DLM020_scaffold8114_1	Straboviridae	0.571929
DLM020_scaffold500_10	Straboviridae	0.40471888
DLM020_scaffold1387_1	Ackermannviridae	0.39516526
DLM020_scaffold22314_2	Peduoviridae	1
DLM020_scaffold20362_2	Drexlerviridae	0.265135
DLM020_scaffold22182_2	Straboviridae	0.3508861
DLM020_scaffold19925_2	Drexlerviridae	0.46745822
DLM020_scaffold13039_2	Straboviridae	0.5416722
DLM020_C355636_1	Straboviridae	0.6009076
DLM020_scaffold350_1	Ackermannviridae	0.21828994
DLM020_scaffold6936_4	Straboviridae	0.69947964

DLM020_scaffold10180_3	Casjensviridae	0.51866126
DLM020_scaffold22290_1	Autographiviridae	1
DLM020_scaffold21039_1	Straboviridae	0.23314427
DLM020_scaffold7279_3	Peduoviridae	1
DLM020_scaffold13927_3	Straboviridae	0.3969798
DLM020_scaffold10263_1	Straboviridae	0.5400346
DLM021_scaffold36034_1	Casjensviridae	0.4320409
DLM021_scaffold43159_1	Ackermannviridae	0.36708665
DLM021_scaffold12971_1	Peduoviridae	0.41205508
DLM021_scaffold42193_7	Straboviridae	0.3969798
DLM021_scaffold43126_6	Straboviridae	0.69947964
DLM021_scaffold24637_8	Straboviridae	0.18682745
DLM021_scaffold10545_9	Straboviridae	1
DLM021_scaffold4_54	Ackermannviridae	0.39865726
DLM021_scaffold30546_2	Straboviridae	0.4992557
DLM021_scaffold10200_17	Salasmaviridae	0.4037911
DLM021_scaffold136_1	Mesyanzhinovviridae	0.29691836
DLM021_scaffold46_2	Ackermannviridae	0.6232304
DLM021_scaffold24731_9	Casjensviridae	0.8158135
DLM021_scaffold21292_11	Ackermannviridae	0.3847759
DLM021_scaffold44452_1	Straboviridae	0.571929
DLM021_scaffold45210_2	Peduoviridae	1
DLM021_scaffold381_1	Peduoviridae	1
DLM021_scaffold1604_4	Straboviridae	0.66194147
DLM022_scaffold90455_1	Straboviridae	0.3541648
DLM022_C1105606_1	Zierdtviridae	1
DLM022_scaffold34930_3	Peduoviridae	0.22083662
DLM022_scaffold61396_2	Straboviridae	0.61910915
DLM022_scaffold3872_14	Drexlerviridae	0.23057634
DLM022_scaffold13626_15	Casjensviridae	0.4466287
DLM022_scaffold7833_6	Straboviridae	0.37812838
DLM022_scaffold25768_2	Herelleviridae	1
DLM022_scaffold45845_2	Ackermannviridae	1
DLM022_scaffold75069_1	Peduoviridae	0.35056782
DLM022_scaffold86174_3	Drexlerviridae	0.2711681
DLM022_scaffold40951_1	Straboviridae	0.61924237
DLM022_scaffold51389_1	Schitoviridae	1
DLM022_scaffold34373_9	Peduoviridae	1
DLM022_scaffold68876_1	Ackermannviridae	0.3325916
DLM022_scaffold24869_7	Herelleviridae	0.89390486
DLM022_scaffold90471_1	Straboviridae	0.36929065
DLM022_scaffold91_3	Vilmaviridae	1
DLM022_scaffold248_3	Zierdtviridae	1
DLM022_scaffold90464_1	Salasmaviridae	1
DLM022_scaffold63634_1	Straboviridae	0.38295603
DLM022_scaffold79270_1	Peduoviridae	1

DLM022_scaffold12444_5	Straboviridae	0.6043284
DLM022_scaffold86174_2	Casjensviridae	1
DLM022_scaffold11069_53	Vilmaviridae	1
DLM022_scaffold85942_2	Ackermannviridae	0.21828994
DLM022_scaffold9_2	Peduoviridae	0.46964702
DLM022_scaffold6448_3	Kyanoviridae	1
DLM022_scaffold90528_2	Peduoviridae	1
DLM022_scaffold160_1	Drexlerviridae	0.30062526
DLM022_scaffold89824_3	Mesyanzhinovviridae	1
DLM022_scaffold43500_4	Peduoviridae	1
DLM022_scaffold89236_1	Straboviridae	0.3508861
DLM022_scaffold90528_3	Peduoviridae	1
DLM022_scaffold89880_1	Drexlerviridae	0.2698334
DLM022_scaffold89156_1	Drexlerviridae	0.45531175
DLM022_scaffold73568_2	Salasmaviridae	1
DLM022_scaffold44709_3	Straboviridae	0.571929
DLM023_scaffold51589_1	Peduoviridae	1
DLM023_scaffold25385_1	Ackermannviridae	1
DLM023_scaffold14487_2	Straboviridae	0.3196358
DLM023_scaffold48996_1	Peduoviridae	1
DLM023_scaffold4774_1	Casjensviridae	0.38102219
DLM023_scaffold23339_1	Peduoviridae	1
DLM023_scaffold2126_10	Herelleviridae	1
DLM023_scaffold7323_3	Ackermannviridae	0.6493801
DLM023_scaffold44217_3	Chaseviridae	0.5610166
DLM023_scaffold37922_1	Straboviridae	0.5400346
DLM023_scaffold4774_5	Mesyanzhinovviridae	0.39414543
DLM023_scaffold17201_4	Straboviridae	0.37812838
DLM023_scaffold51553_3	Ackermannviridae	0.24887799
DLM023_scaffold26150_3	Peduoviridae	0.3214273
DLM024_scaffold6096_2	Straboviridae	0.47515345
DLM024_scaffold6392_4	Zierdtviridae	1
DLM024_scaffold6942_1	Peduoviridae	1
DLM024_scaffold6782_2	Casjensviridae	1
DLM024_scaffold6863_2	Herelleviridae	1
DLM024_scaffold5188_2	Casjensviridae	1
DLM024_C136347_1	Straboviridae	0.34864473
DLM024_scaffold2465_1	Straboviridae	0.62464666
DLM027_scaffold2040_2	Straboviridae	0.3694985
DLM027_scaffold20206_4	Herelleviridae	1
DLM027_scaffold2427_8	Ackermannviridae	1
DLM027_scaffold26569_1	Straboviridae	0.20631503
DLM027_scaffold26720_1	Salasmaviridae	1
DLM027_scaffold5447_3	Peduoviridae	0.6294716
DLM027_scaffold26569_2	Drexlerviridae	0.27153525
DLM027_scaffold9656_1	Peduoviridae	0.51119787

DLM027_scaffold14908_1	Casjensviridae	1
DLM028_scaffold6098_12	Straboviridae	0.31710467
DLM028_scaffold21827_4	Peduoviridae	0.63610744
DLM028_scaffold17_2	Straboviridae	0.3541648
DLM028_scaffold40934_1	Ackermannviridae	0.21766534
DLM028_scaffold12478_1	Salasmaviridae	0.4037911
DLM028_scaffold48949_2	Ackermannviridae	1
DLM028_scaffold37211_4	Casjensviridae	1
DLM028_scaffold25751_8	Peduoviridae	1
DLM028_C675659_1	Peduoviridae	0.33529264
DLM028_scaffold268_28	Straboviridae	0.6866557
DLM028_scaffold29533_12	Straboviridae	0.40471888
DLM028_scaffold3721_11	Peduoviridae	0.5582075
DLM028_scaffold51026_1	Peduoviridae	1
DLM028_scaffold54608_1	Kyanoviridae	1
DLM028_scaffold28515_4	Straboviridae	0.63893926
DLM028_scaffold16640_1	Mesyanzhinovviridae	0.29691836
DLM028_scaffold37211_5	Peduoviridae	1
DLM028_scaffold20432_3	Casjensviridae	0.811716
DLM028_scaffold9236_8	Peduoviridae	0.5512526
DLM028_scaffold86_3	Peduoviridae	0.38523066
DLM028_scaffold31687_6	Vilmaviridae	1
DLM001_scaffold37_3	unknown	0
DLM001_scaffold57834_1	unknown	0
DLM001_scaffold36534_1	unknown	0
DLM001_scaffold26672_2	unknown	0
DLM001_scaffold21921_3	unknown	0
DLM001_scaffold2435_6	unknown	0
DLM001_scaffold31256_1	unknown	0
DLM001_scaffold55116_2	unknown	0
DLM001_scaffold25895_8	unknown	0
DLM001_scaffold21223_1	unknown	0
DLM001_scaffold54893_2	unknown	0
DLM001_scaffold30022_1	unknown	0
DLM001_scaffold58236_1	unknown	0
DLM001_scaffold51940_3	unknown	0
DLM001_scaffold22953_2	unknown	0
DLM001_scaffold53015_2	unknown	0
DLM001_scaffold41842_2	unknown	0
DLM001_scaffold58253_1	unknown	0
DLM001_scaffold56486_2	unknown	0
DLM001_scaffold48552_3	unknown	0
DLM001_scaffold4106_5	unknown	0
DLM001_scaffold58250_2	unknown	0
DLM001_scaffold52289_2	unknown	0
DLM001_scaffold55454_4	unknown	0

DLM001_scaffold55964_1	unknown	0
DLM001_scaffold12282_12	unknown	0
DLM001_scaffold49276_1	unknown	0
DLM001_scaffold21002_1	unknown	0
DLM001_scaffold49834_2	unknown	0
DLM001_scaffold16868_1	unknown	0
DLM001_scaffold11723_1	unknown	0
DLM001_scaffold19427_9	unknown	0
DLM001_scaffold14114_7	unknown	0
DLM001_scaffold3576_10	unknown	0
DLM002_scaffold35924_1	unknown	0
DLM002_scaffold39638_1	unknown	0
DLM002_scaffold58_3	unknown	0
DLM002_scaffold37454_1	unknown	0
DLM002_scaffold4794_6	unknown	0
DLM002_scaffold45452_2	unknown	0
DLM002_scaffold5537_11	unknown	0
DLM002_scaffold2881_3	unknown	0
DLM002_scaffold52526_1	unknown	0
DLM002_scaffold52526_4	unknown	0
DLM002_scaffold42939_1	unknown	0
DLM002_scaffold52584_1	unknown	0
DLM002_scaffold52696_1	unknown	0
DLM003_scaffold35975_4	unknown	0
DLM003_scaffold8802_8	unknown	0
DLM003_scaffold59785_6	unknown	0
DLM003_scaffold67334_2	unknown	0
DLM003_scaffold31156_2	unknown	0
DLM003_scaffold24702_43	unknown	0
DLM003_scaffold4744_1	unknown	0
DLM003_scaffold36199_4	unknown	0
DLM003_scaffold12201_4	unknown	0
DLM003_scaffold16412_17	unknown	0
DLM003_scaffold1898_11	unknown	0
DLM003_scaffold66050_1	unknown	0
DLM003_scaffold29743_2	unknown	0
DLM004_scaffold2416_6	unknown	0
DLM004_scaffold18621_3	unknown	0
DLM004_scaffold2421_24	unknown	0
DLM004_scaffold12500_2	unknown	0
DLM004_scaffold462_3	unknown	0
DLM004_scaffold18776_2	unknown	0
DLM004_scaffold112_3	unknown	0
DLM004_scaffold5647_2	unknown	0
DLM004_scaffold18395_2	unknown	0
DLM004_scaffold11715_11	unknown	0

DLM004_scaffold15727_1	unknown	0
DLM004_scaffold14578_1	unknown	0
DLM004_scaffold9327_2	unknown	0
DLM004_C304967_1	unknown	0
DLM004_scaffold14269_9	unknown	0
DLM005_scaffold57948_1	unknown	0
DLM005_scaffold34949_1	unknown	0
DLM005_scaffold48663_11	unknown	0
DLM005_scaffold9042_2	unknown	0
DLM005_scaffold3317_29	unknown	0
DLM005_scaffold10521_42	unknown	0
DLM005_scaffold39936_1	unknown	0
DLM005_scaffold54507_2	unknown	0
DLM005_scaffold57953_2	unknown	0
DLM005_scaffold47946_1	unknown	0
DLM005_scaffold42596_2	unknown	0
DLM005_scaffold56545_2	unknown	0
DLM005_scaffold21763_10	unknown	0
DLM005_scaffold21049_5	unknown	0
DLM005_scaffold25964_2	unknown	0
DLM005_scaffold5416_6	unknown	0
DLM005_scaffold20359_5	unknown	0
DLM005_scaffold56832_1	unknown	0
DLM005_scaffold7362_2	unknown	0
DLM005_scaffold27309_11	unknown	0
DLM005_scaffold25964_1	unknown	0
DLM005_scaffold10521_41	unknown	0
DLM005_scaffold52507_2	unknown	0
DLM005_scaffold36516_2	unknown	0
DLM006_scaffold5249_12	unknown	0
DLM006_scaffold9115_2	unknown	0
DLM006_scaffold8798_11	unknown	0
DLM006_scaffold15729_4	unknown	0
DLM006_scaffold3905_28	unknown	0
DLM006_scaffold13732_3	unknown	0
DLM006_scaffold35098_2	unknown	0
DLM006_scaffold3099_10	unknown	0
DLM006_scaffold22671_1	unknown	0
DLM006_scaffold12233_4	unknown	0
DLM006_scaffold13444_2	unknown	0
DLM006_scaffold7136_55	unknown	0
DLM006_scaffold36079_2	unknown	0
DLM006_scaffold22549_2	unknown	0
DLM007_scaffold30789_1	unknown	0
DLM007_scaffold45247_1	unknown	0
DLM007_scaffold31482_1	unknown	0

DLM007_scaffold42236_1	unknown	0
DLM007_scaffold41113_2	unknown	0
DLM007_scaffold4568_8	unknown	0
DLM007_scaffold45002_8	unknown	0
DLM007_scaffold36909_2	unknown	0
DLM008_scaffold4090_5	unknown	0
DLM008_scaffold251_3	unknown	0
DLM008_scaffold51033_29	unknown	0
DLM008_scaffold3167_2	unknown	0
DLM008_scaffold54665_3	unknown	0
DLM008_scaffold68109_1	unknown	0
DLM008_scaffold40955_1	unknown	0
DLM008_scaffold235_2	unknown	0
DLM008_scaffold58161_1	unknown	0
DLM008_scaffold62586_4	unknown	0
DLM008_scaffold46088_1	unknown	0
DLM008_scaffold68395_3	unknown	0
DLM008_scaffold43273_7	unknown	0
DLM008_scaffold129_7	unknown	0
DLM008_scaffold58372_1	unknown	0
DLM008_scaffold129_3	unknown	0
DLM009_scaffold11883_2	unknown	0
DLM009_scaffold6465_2	unknown	0
DLM009_C324613_1	unknown	0
DLM009_scaffold17120_3	unknown	0
DLM009_scaffold18558_3	unknown	0
DLM009_scaffold10681_1	unknown	0
DLM009_scaffold21380_1	unknown	0
DLM009_scaffold21344_1	unknown	0
DLM009_scaffold8439_6	unknown	0
DLM009_scaffold18558_6	unknown	0
DLM009_scaffold6465_1	unknown	0
DLM009_scaffold21365_1	unknown	0
DLM009_scaffold2554_54	unknown	0
DLM009_scaffold4728_5	unknown	0
DLM009_C324155_1	unknown	0
DLM009_scaffold6465_23	unknown	0
DLM010_scaffold41969_2	unknown	0
DLM010_scaffold43377_3	unknown	0
DLM010_scaffold18321_2	unknown	0
DLM010_scaffold32474_2	unknown	0
DLM010_scaffold7753_8	unknown	0
DLM010_scaffold44670_1	unknown	0
DLM010_scaffold3339_9	unknown	0
DLM011_scaffold28392_2	unknown	0
DLM011_scaffold29393_1	unknown	0

DLM011_scaffold5932_1	unknown	0
DLM011_scaffold28062_1	unknown	0
DLM011_scaffold27250_1	unknown	0
DLM011_C484732_1	unknown	0
DLM011_scaffold1189_13	unknown	0
DLM011_scaffold28659_3	unknown	0
DLM011_scaffold17163_7	unknown	0
DLM011_scaffold19964_1	unknown	0
DLM011_scaffold1699_8	unknown	0
DLM011_scaffold5789_4	unknown	0
DLM011_scaffold24958_1	unknown	0
DLM012_scaffold19314_6	unknown	0
DLM012_scaffold34010_1	unknown	0
DLM012_scaffold22034_2	unknown	0
DLM012_scaffold7495_7	unknown	0
DLM012_scaffold33026_10	unknown	0
DLM012_scaffold33913_1	unknown	0
DLM012_scaffold33950_1	unknown	0
DLM012_scaffold29288_6	unknown	0
DLM012_scaffold26999_6	unknown	0
DLM012_scaffold29288_5	unknown	0
DLM013_scaffold23313_3	unknown	0
DLM013_scaffold3286_13	unknown	0
DLM013_scaffold55482_1	unknown	0
DLM013_scaffold8482_4	unknown	0
DLM013_scaffold45817_1	unknown	0
DLM013_scaffold23184_5	unknown	0
DLM013_scaffold56424_2	unknown	0
DLM013_scaffold3810_7	unknown	0
DLM013_scaffold318_1	unknown	0
DLM013_scaffold15355_2	unknown	0
DLM013_scaffold54893_2	unknown	0
DLM013_scaffold3614_26	unknown	0
DLM013_scaffold23737_4	unknown	0
DLM013_scaffold49483_1	unknown	0
DLM013_scaffold21436_7	unknown	0
DLM013_scaffold38983_1	unknown	0
DLM013_scaffold25009_5	unknown	0
DLM013_scaffold14795_2	unknown	0
DLM013_scaffold52105_9	unknown	0
DLM013_scaffold11264_1	unknown	0
DLM013_scaffold52295_2	unknown	0
DLM013_scaffold31022_3	unknown	0
DLM013_scaffold16485_8	unknown	0
DLM013_scaffold55003_1	unknown	0
DLM013_scaffold23574_3	unknown	0

DLM013_scaffold3614_27	unknown	0
DLM013_scaffold24100_2	unknown	0
DLM013_scaffold2200_1	unknown	0
DLM014_scaffold52949_1	unknown	0
DLM014_scaffold14966_8	unknown	0
DLM014_scaffold26538_3	unknown	0
DLM014_scaffold3539_3	unknown	0
DLM014_scaffold51860_2	unknown	0
DLM014_scaffold34561_6	unknown	0
DLM014_scaffold31737_5	unknown	0
DLM014_scaffold52940_1	unknown	0
DLM014_scaffold44624_1	unknown	0
DLM014_scaffold42199_1	unknown	0
DLM014_scaffold3883_2	unknown	0
DLM014_scaffold7847_1	unknown	0
DLM014_scaffold46228_1	unknown	0
DLM014_scaffold14317_2	unknown	0
DLM014_scaffold45829_2	unknown	0
DLM014_scaffold31737_1	unknown	0
DLM014_scaffold21553_3	unknown	0
DLM014_scaffold23167_4	unknown	0
DLM014_scaffold18806_7	unknown	0
DLM014_scaffold1942_34	unknown	0
DLM014_scaffold18901_3	unknown	0
DLM014_scaffold48474_2	unknown	0
DLM014_scaffold52939_1	unknown	0
DLM014_scaffold37209_4	unknown	0
DLM014_scaffold36217_6	unknown	0
DLM015_scaffold5802_5	unknown	0
DLM015_scaffold50361_1	unknown	0
DLM015_scaffold50502_1	unknown	0
DLM015_scaffold23053_2	unknown	0
DLM015_scaffold1049_4	unknown	0
DLM015_scaffold1119_3	unknown	0
DLM015_scaffold36073_3	unknown	0
DLM015_scaffold23053_6	unknown	0
DLM015_scaffold47979_4	unknown	0
DLM015_scaffold49465_1	unknown	0
DLM015_scaffold50303_1	unknown	0
DLM015_scaffold34621_5	unknown	0
DLM015_scaffold47846_2	unknown	0
DLM015_scaffold50189_1	unknown	0
DLM015_scaffold5802_2	unknown	0
DLM015_scaffold36073_4	unknown	0
DLM015_scaffold145_2	unknown	0
DLM015_scaffold17140_1	unknown	0

DLM015_scaffold36351_2	unknown	0
DLM016_scaffold65085_5	unknown	0
DLM016_scaffold61369_1	unknown	0
DLM016_scaffold64744_4	unknown	0
DLM016_scaffold12_1	unknown	0
DLM016_scaffold13346_4	unknown	0
DLM016_scaffold46534_2	unknown	0
DLM016_scaffold41709_1	unknown	0
DLM016_scaffold40529_1	unknown	0
DLM016_scaffold7191_1	unknown	0
DLM016_scaffold40187_1	unknown	0
DLM016_scaffold6553_2	unknown	0
DLM016_scaffold16615_5	unknown	0
DLM016_scaffold53043_5	unknown	0
DLM016_scaffold65482_1	unknown	0
DLM016_scaffold65575_2	unknown	0
DLM016_scaffold6713_8	unknown	0
DLM016_scaffold6713_11	unknown	0
DLM016_scaffold6713_2	unknown	0
DLM016_scaffold4953_3	unknown	0
DLM016_scaffold6914_1	unknown	0
DLM016_scaffold52894_5	unknown	0
DLM016_scaffold6713_12	unknown	0
DLM016_scaffold65474_2	unknown	0
DLM016_scaffold65085_3	unknown	0
DLM016_scaffold6553_4	unknown	0
DLM016_scaffold1623_5	unknown	0
DLM016_scaffold63991_1	unknown	0
DLM016_scaffold29974_3	unknown	0
DLM016_scaffold53961_3	unknown	0
DLM016_scaffold419_9	unknown	0
DLM017_scaffold2813_9	unknown	0
DLM017_scaffold3964_2	unknown	0
DLM017_scaffold20005_2	unknown	0
DLM017_scaffold21266_1	unknown	0
DLM017_scaffold33959_3	unknown	0
DLM017_scaffold1892_10	unknown	0
DLM017_scaffold28739_1	unknown	0
DLM017_scaffold10166_1	unknown	0
DLM017_scaffold18232_1	unknown	0
DLM018_scaffold36970_5	unknown	0
DLM018_scaffold38471_2	unknown	0
DLM018_scaffold197_3	unknown	0
DLM018_scaffold13432_9	unknown	0
DLM018_scaffold45347_1	unknown	0
DLM018_scaffold49175_1	unknown	0

DLM018_scaffold3525_6	unknown	0
DLM018_scaffold82_1	unknown	0
DLM018_scaffold44037_1	unknown	0
DLM018_scaffold1207_3	unknown	0
DLM018_scaffold79_28	unknown	0
DLM018_scaffold31396_2	unknown	0
DLM018_scaffold14900_3	unknown	0
DLM018_scaffold47288_3	unknown	0
DLM018_scaffold40461_1	unknown	0
DLM018_scaffold19081_1	unknown	0
DLM019_scaffold19742_4	unknown	0
DLM019_scaffold62503_1	unknown	0
DLM019_scaffold22877_3	unknown	0
DLM019_scaffold53137_1	unknown	0
DLM019_scaffold44099_4	unknown	0
DLM019_scaffold8577_4	unknown	0
DLM019_scaffold20492_2	unknown	0
DLM019_scaffold10836_2	unknown	0
DLM019_scaffold24899_8	unknown	0
DLM019_scaffold63202_2	unknown	0
DLM019_scaffold53137_2	unknown	0
DLM019_scaffold2743_2	unknown	0
DLM019_scaffold35325_15	unknown	0
DLM019_scaffold31976_1	unknown	0
DLM019_scaffold39_1	unknown	0
DLM019_scaffold54914_7	unknown	0
DLM019_scaffold35445_2	unknown	0
DLM019_scaffold37359_6	unknown	0
DLM019_scaffold38422_1	unknown	0
DLM019_scaffold63049_1	unknown	0
DLM019_scaffold62785_2	unknown	0
DLM019_scaffold8817_14	unknown	0
DLM019_scaffold29437_2	unknown	0
DLM019_scaffold12600_2	unknown	0
DLM019_scaffold44258_2	unknown	0
DLM019_scaffold31935_1	unknown	0
DLM019_scaffold63216_1	unknown	0
DLM020_scaffold8084_6	unknown	0
DLM020_scaffold11081_1	unknown	0
DLM020_scaffold15999_4	unknown	0
DLM020_scaffold21144_2	unknown	0
DLM020_scaffold19547_2	unknown	0
DLM020_scaffold8736_5	unknown	0
DLM020_scaffold7279_1	unknown	0
DLM020_scaffold8693_2	unknown	0
DLM020_scaffold21959_1	unknown	0

DLM020_scaffold6157_3	unknown	0
DLM020_scaffold20632_1	unknown	0
DLM020_scaffold22218_1	unknown	0
DLM020_scaffold7475_1	unknown	0
DLM020_scaffold3936_1	unknown	0
DLM020_scaffold10415_1	unknown	0
DLM020_scaffold5648_4	unknown	0
DLM020_scaffold11301_1	unknown	0
DLM020_scaffold6936_3	unknown	0
DLM021_scaffold44307_1	unknown	0
DLM021_scaffold31538_5	unknown	0
DLM021_scaffold29140_2	unknown	0
DLM021_scaffold35216_1	unknown	0
DLM021_scaffold43126_4	unknown	0
DLM021_scaffold42967_2	unknown	0
DLM021_scaffold28049_1	unknown	0
DLM021_scaffold36642_2	unknown	0
DLM021_scaffold43501_2	unknown	0
DLM021_scaffold554_3	unknown	0
DLM021_scaffold31475_3	unknown	0
DLM021_scaffold28049_2	unknown	0
DLM021_scaffold10200_12	unknown	0
DLM021_scaffold23214_1	unknown	0
DLM021_scaffold640_7	unknown	0
DLM021_scaffold22868_5	unknown	0
DLM021_scaffold2298_4	unknown	0
DLM021_scaffold8187_1	unknown	0
DLM022_scaffold87335_3	unknown	0
DLM022_scaffold13671_3	unknown	0
DLM022_scaffold28531_1	unknown	0
DLM022_scaffold47568_2	unknown	0
DLM022_scaffold87188_2	unknown	0
DLM022_scaffold83380_1	unknown	0
DLM022_scaffold38614_1	unknown	0
DLM022_scaffold90423_1	unknown	0
DLM022_scaffold90441_1	unknown	0
DLM022_scaffold29304_11	unknown	0
DLM022_scaffold46815_1	unknown	0
DLM022_scaffold50520_4	unknown	0
DLM022_scaffold82276_2	unknown	0
DLM022_scaffold88974_2	unknown	0
DLM022_scaffold37828_6	unknown	0
DLM022_scaffold7976_5	unknown	0
DLM022_scaffold46210_2	unknown	0
DLM022_scaffold79585_1	unknown	0
DLM022_scaffold126_4	unknown	0

DLM022_scaffold7197_1	unknown	0
DLM022_scaffold24869_6	unknown	0
DLM022_scaffold79462_2	unknown	0
DLM022_scaffold26167_3	unknown	0
DLM022_scaffold8618_3	unknown	0
DLM022_scaffold20053_1	unknown	0
DLM022_scaffold90529_1	unknown	0
DLM022_scaffold82197_1	unknown	0
DLM022_scaffold84536_3	unknown	0
DLM022_scaffold11050_1	unknown	0
DLM022_scaffold68020_2	unknown	0
DLM022_scaffold89824_2	unknown	0
DLM022_C1104604_1	unknown	0
DLM022_scaffold36935_1	unknown	0
DLM022_scaffold80933_2	unknown	0
DLM022_scaffold21034_2	unknown	0
DLM022_scaffold88907_2	unknown	0
DLM022_scaffold64018_1	unknown	0
DLM022_scaffold90113_2	unknown	0
DLM022_scaffold37957_2	unknown	0
DLM022_scaffold22367_7	unknown	0
DLM022_scaffold24995_4	unknown	0
DLM022_scaffold30520_1	unknown	0
DLM022_scaffold32204_1	unknown	0
DLM022_scaffold46686_4	unknown	0
DLM022_scaffold85281_1	unknown	0
DLM022_scaffold90507_1	unknown	0
DLM022_scaffold62765_1	unknown	0
DLM022_scaffold27082_1	unknown	0
DLM022_scaffold88059_2	unknown	0
DLM022_scaffold87605_2	unknown	0
DLM022_scaffold937_1	unknown	0
DLM022_scaffold30068_2	unknown	0
DLM022_scaffold15558_7	unknown	0
DLM023_scaffold1460_2	unknown	0
DLM023_scaffold27445_8	unknown	0
DLM023_scaffold411_39	unknown	0
DLM023_scaffold51795_1	unknown	0
DLM023_scaffold47535_1	unknown	0
DLM023_scaffold51029_1	unknown	0
DLM023_scaffold18407_7	unknown	0
DLM023_scaffold45333_4	unknown	0
DLM023_scaffold28443_5	unknown	0
DLM023_scaffold32334_1	unknown	0
DLM023_scaffold4774_3	unknown	0
DLM023_scaffold13027_1	unknown	0

DLM023_scaffold58_7	unknown	0
DLM023_scaffold51688_1	unknown	0
DLM024_scaffold4102_1	unknown	0
DLM024_scaffold1307_14	unknown	0
DLM024_scaffold6915_1	unknown	0
DLM024_scaffold2301_1	unknown	0
DLM024_scaffold4274_2	unknown	0
DLM024_scaffold447_14	unknown	0
DLM024_scaffold3270_1	unknown	0
DLM024_scaffold1215_1	unknown	0
DLM024_scaffold6734_2	unknown	0
DLM024_scaffold1543_4	unknown	0
DLM024_scaffold5624_3	unknown	0
DLM024_scaffold6919_2	unknown	0
DLM024_scaffold3966_3	unknown	0
DLM024_scaffold722_1	unknown	0
DLM024_scaffold3585_1	unknown	0
DLM024_scaffold2213_13	unknown	0
DLM024_scaffold1355_19	unknown	0
DLM027_scaffold19184_4	unknown	0
DLM027_scaffold25719_1	unknown	0
DLM027_scaffold18274_3	unknown	0
DLM027_scaffold2177_5	unknown	0
DLM027_scaffold10585_3	unknown	0
DLM027_scaffold26586_2	unknown	0
DLM027_scaffold23395_3	unknown	0
DLM027_scaffold19716_4	unknown	0
DLM027_scaffold79_10	unknown	0
DLM027_scaffold19168_1	unknown	0
DLM027_scaffold26477_2	unknown	0
DLM027_scaffold9665_4	unknown	0
DLM027_scaffold8638_4	unknown	0
DLM027_scaffold26707_1	unknown	0
DLM028_scaffold32640_1	unknown	0
DLM028_scaffold54482_2	unknown	0
DLM028_scaffold33522_2	unknown	0
DLM028_scaffold35712_5	unknown	0
DLM028_scaffold39913_2	unknown	0
DLM028_scaffold30320_6	unknown	0
DLM028_scaffold43205_3	unknown	0
DLM028_scaffold28515_6	unknown	0
DLM028_scaffold46719_2	unknown	0
DLM028_scaffold10447_6	unknown	0
DLM028_scaffold54580_1	unknown	0
DLM028_scaffold30334_1	unknown	0
DLM028_scaffold21827_3	unknown	0

DLM028_scaffold32640_2	unknown	0
DLM028_C674591_1	unknown	0
DLM028_scaffold1815_5	unknown	0
DLM028_scaffold39849_2	unknown	0
DLM028_scaffold28558_6	unknown	0
DLM028_scaffold10666_1	unknown	0
DLM028_scaffold32204_5	unknown	0
DLM028_scaffold16942_4	unknown	0
DLM028_scaffold36337_1	unknown	0
DLM028_scaffold6503_3	unknown	0
DLM028_scaffold1257_18	unknown	0
DLM028_scaffold51841_1	unknown	0
DLM028_scaffold726_8	unknown	0
DLM028_scaffold41953_1	unknown	0
DLM002_scaffold48180_13	no_family_avaliable(NC_(	0.954
DLM003_scaffold30478_2	no_family_avaliable(NC_(	0.974
DLM003_scaffold46929_2	no_family_avaliable(NC_(	0.985
DLM003_scaffold46929_4	no_family_avaliable(NC_(	0.964
DLM005_scaffold31312_1	no_family_avaliable(NC_(	0.981
DLM006_scaffold31867_1	no_family_avaliable(NC_(	0.966
DLM007_scaffold20148_2	no_family_avaliable(NC_(	0.963
DLM007_scaffold20148_1	no_family_avaliable(NC_(	0.971
DLM008_scaffold9016_5	no_family_avaliable(NC_(	0.955
DLM008_scaffold9016_1	no_family_avaliable(NC_(	0.953
DLM008_scaffold349_4	no_family_avaliable(NC_(	0.958
DLM009_scaffold19658_2	no_family_avaliable(NC_(	0.989
DLM009_scaffold19658_6	no_family_avaliable(NC_(	0.985
DLM009_scaffold18345_6	no_family_avaliable(NC_(	0.984
DLM009_scaffold19658_3	no_family_avaliable(NC_(	0.987
DLM011_scaffold29207_1	no_family_avaliable(NC_(	1
DLM012_scaffold49_1	no_family_avaliable(NC_(	0.979
DLM014_scaffold1_9	no_family_avaliable(NC_(	0.99
DLM014_scaffold1_18	no_family_avaliable(NC_(	0.99
DLM016_scaffold22143_3	no_family_avaliable(NC_(	0.981
DLM016_scaffold6713_3	no_family_avaliable(NC_(	0.961
DLM022_scaffold90383_1	no_family_avaliable(NC_(	0.978
DLM022_scaffold73260_1	no_family_avaliable(NC_(	0.981
DLM022_scaffold47815_3	no_family_avaliable(NC_(	0.974
DLM022_scaffold11067_1	no_family_avaliable(NC_(	0.971
DLM022_scaffold24869_4	no_family_avaliable(NC_(	0.988
DLM022_scaffold90402_1	no_family_avaliable(NC_(	0.976
DLM022_scaffold90509_1	no_family_avaliable(NC_(	0.972
DLM024_scaffold4575_2	no_family_avaliable(NC_(	0.983
DLM028_scaffold30790_1	no_family_avaliable(NC_(	0.979
DLM028_scaffold13566_10	no_family_avaliable(NC_(	0.98
DLM028_scaffold26265_2	no_family_avaliable(NC_(	0.951

NLM001_scaffold3386_6	Straboviridae	0.40241614
NLM001_scaffold44600_3	Drexlerviridae	0.39220908
NLM001_scaffold4227_2	Straboviridae	0.47494072
NLM001_scaffold7_9	Straboviridae	0.5879599
NLM001_scaffold43773_1	Straboviridae	0.4482294
NLM001_scaffold10910_7	Straboviridae	0.42512658
NLM001_scaffold5105_1	Casjensviridae	0.38493463
NLM001_scaffold5105_4	Schitoviridae	0.1449373
NLM001_scaffold2_5	Ackermannviridae	0.26196176
NLM001_scaffold44595_1	Peduoviridae	1
NLM002_scaffold15649_2	Peduoviridae	0.43805262
NLM002_scaffold8112_4	Straboviridae	0.6553155
NLM002_scaffold28005_2	Drexlerviridae	0.3840808
NLM002_scaffold13407_12	Straboviridae	0.5879599
NLM002_scaffold28119_5	Kyanoviridae	1
NLM002_scaffold8657_21	Straboviridae	0.69101197
NLM002_scaffold14431_4	Straboviridae	0.7403209
NLM002_scaffold5906_19	Straboviridae	0.47592783
NLM003_scaffold10761_3	Straboviridae	0.21159849
NLM003_scaffold261_3	Straboviridae	0.45764768
NLM003_scaffold977_2	Drexlerviridae	1
NLM003_scaffold189_8_1	Herelleviridae	1
NLM003_scaffold4295_1	Drexlerviridae	0.6004602
NLM004_scaffold285_3	Drexlerviridae	0.23599732
NLM004_scaffold46877_2	Straboviridae	0.615393
NLM004_scaffold35292_1	Straboviridae	0.45870692
NLM004_scaffold53545_1	Straboviridae	0.49783167
NLM004_scaffold27473_2	Drexlerviridae	0.3755131
NLM004_scaffold28209_1	Kyanoviridae	1
NLM004_scaffold54819_1	Herelleviridae	1
NLM004_scaffold9622_3	Mesyanzhinovviridae	0.4042104
NLM005_scaffold32010_3	Peduoviridae	1
NLM005_scaffold5172_2	Straboviridae	0.35476157
NLM005_scaffold90_2	Straboviridae	0.35521403
NLM005_scaffold5999_10	Straboviridae	0.5338497
NLM005_scaffold11370_1	Herelleviridae	0.64262027
NLM006_scaffold32497_3	Drexlerviridae	0.3840808
NLM006_scaffold21580_3	Straboviridae	0.69101197
NLM006_scaffold43376_2	Ackermannviridae	0.3039049
NLM006_scaffold31922_8	Straboviridae	0.42385665
NLM006_scaffold43039_5	Herelleviridae	1
NLM006_scaffold42358_4	Casjensviridae	1
NLM006_scaffold35930_2	Straboviridae	0.44955745
NLM006_scaffold24429_1	Straboviridae	0.24749029
NLM006_scaffold51349_1	Straboviridae	0.21516325
NLM006_scaffold53007_3	Straboviridae	0.49488285

NLM006_scaffold22752_1	Straboviridae	0.6553155
NLM006_scaffold26489_1	Straboviridae	0.5890641
NLM006_scaffold53154_3	Straboviridae	0.5852342
NLM006_scaffold11951_6	Ackermannviridae	0.35920888
NLM006_scaffold37878_1	Casjensviridae	1
NLM006_scaffold38154_1	Vilmaviridae	1
NLM006_scaffold18470_3	Herelleviridae	0.741455
NLM006_scaffold53101_1	Ackermannviridae	0.2372473
NLM006_scaffold49011_1	Peduoviridae	1
NLM006_scaffold14681_1	Straboviridae	0.7813815
NLM006_scaffold4407_2	Ackermannviridae	0.18881081
NLM006_scaffold10094_3	Straboviridae	0.4482294
NLM006_scaffold31193_1	Demerecviridae	1
NLM006_scaffold28837_2	Straboviridae	0.65579903
NLM006_C734606_1	Drexlerviridae	1
NLM006_scaffold8324_9	Straboviridae	0.4822487
NLM007_scaffold10749_4	Straboviridae	0.6418709
NLM007_scaffold1551_2	Ackermannviridae	0.42088312
NLM007_scaffold25115_1	Peduoviridae	1
NLM007_scaffold17969_11	Casjensviridae	1
NLM007_scaffold26391_1	Straboviridae	0.5454986
NLM007_scaffold2633_3	Mesyanzhinovviridae	0.7162643
NLM007_scaffold20356_8	Ackermannviridae	0.3625965
NLM007_scaffold3232_7	Straboviridae	0.69101197
NLM007_scaffold23234_1	Drexlerviridae	0.16866957
NLM007_scaffold18350_4	Kyanoviridae	1
NLM007_scaffold723_5	Ackermannviridae	1
NLM008_scaffold50164_2	Ackermannviridae	0.26196176
NLM008_scaffold37289_2	Drexlerviridae	0.32969895
NLM008_scaffold45531_1	Straboviridae	0.72108424
NLM008_scaffold22745_5	Ackermannviridae	0.26776138
NLM008_scaffold50138_1	Straboviridae	0.7403607
NLM008_scaffold18128_9	Straboviridae	0.65383214
NLM008_scaffold49769_2	Straboviridae	0.6553155
NLM008_scaffold37575_10	Straboviridae	0.73139083
NLM008_scaffold711_3	Ackermannviridae	0.5563236
NLM009_C228858_1	Straboviridae	0.5123257
NLM009_scaffold8447_2	Drexlerviridae	0.32969895
NLM009_C228882_1	Ackermannviridae	0.32887334
NLM010_scaffold39134_1	Straboviridae	0.57686263
NLM010_scaffold3556_1	Drexlerviridae	0.29855287
NLM010_scaffold2302_1	Ackermannviridae	0.42088312
NLM010_scaffold36636_1	Straboviridae	0.5890641
NLM010_scaffold23407_1	Straboviridae	0.45764768
NLM010_scaffold40193_1	Peduoviridae	1
NLM010_scaffold18261_4	Mesyanzhinovviridae	0.7162643

NLM010_scaffold1890_2	Ackermannviridae	0.24619988
NLM010_scaffold24350_1	Straboviridae	0.62909514
NLM010_scaffold2086_2	Straboviridae	0.36598924
NLM010_scaffold16315_3	Peduoviridae	0.7363866
NLM010_scaffold39351_1	Casjensviridae	0.44304314
NLM010_scaffold16684_4	Straboviridae	0.40241614
NLM010_scaffold10296_6	Drexlerviridae	0.16247487
NLM010_scaffold39268_1	Peduoviridae	0.98301136
NLM010_scaffold2685_7	Casjensviridae	0.31617516
NLM010_scaffold34399_5	Straboviridae	0.65579903
NLM010_scaffold10296_4	Casjensviridae	1
NLM010_scaffold8314_1	Straboviridae	0.45870692
NLM010_scaffold15390_2	Drexlerviridae	0.2230215
NLM015_scaffold38424_1	Ackermannviridae	0.24651025
NLM015_scaffold10852_4	Peduoviridae	0.43805262
NLM015_scaffold1856_2	Straboviridae	0.6608802
NLM015_scaffold77_4	Straboviridae	0.72108424
NLM015_scaffold25867_2	Ackermannviridae	0.42088312
NLM015_scaffold10852_5	Peduoviridae	0.26380417
NLM015_scaffold5831_6	Drexlerviridae	0.32969895
NLM015_scaffold104_1	Straboviridae	0.52144754
NLM016_scaffold10481_1	Herelleviridae	0.8111403
NLM016_scaffold9518_15	Zierdtviridae	1
NLM016_scaffold87_4	Drexlerviridae	0.3755131
NLM016_scaffold11179_1	Kyanoviridae	1
NLM016_scaffold16587_2	Drexlerviridae	0.39220908
NLM017_scaffold416_2	Herelleviridae	0.616311
NLM017_scaffold14858_4	Ackermannviridae	0.35920888
NLM017_scaffold27209_14	Straboviridae	0.42151073
NLM017_scaffold38835_1	Straboviridae	0.75188446
NLM017_scaffold14095_1	Ackermannviridae	0.3772501
NLM017_scaffold8358_21	Straboviridae	0.38791418
NLM017_scaffold7452_3	Peduoviridae	0.38545594
NLM017_scaffold38969_2	Ackermannviridae	0.25430948
NLM017_scaffold23004_11	Drexlerviridae	0.29855287
NLM017_scaffold38914_2	Guelinviridae	0.27453768
NLM021_scaffold61418_1	Straboviridae	0.2957326
NLM021_scaffold19029_4	Casjensviridae	0.9825629
NLM021_scaffold53158_5	Straboviridae	0.5338497
NLM021_scaffold8185_4	Straboviridae	1
NLM021_scaffold55035_3	Chaseviridae	0.3054243
NLM021_scaffold19029_1	Drexlerviridae	0.19249943
NLM021_scaffold42928_1	Peduoviridae	0.70607936
NLM021_scaffold61042_4	Casjensviridae	1
NLM021_scaffold29192_2	Straboviridae	0.43174168
NLM021_scaffold23306_4	Vilmaviridae	1

NLM021_scaffold60054_1	Straboviridae	0.60721505
NLM021_scaffold190_8	Straboviridae	0.34623358
NLM021_scaffold15002_14	Ackermannviridae	0.2522395
NLM021_scaffold55417_2	Herelleviridae	1
NLM021_scaffold26861_2	Ackermannviridae	0.3039049
NLM021_C812913_1	Straboviridae	0.57686263
NLM021_scaffold61257_1	Ackermannviridae	1
NLM021_scaffold46784_3	Straboviridae	0.6553155
NLM021_scaffold190_9	Herelleviridae	1
NLM021_scaffold53021_4	Straboviridae	0.5399199
NLM021_scaffold58236_5	Straboviridae	0.5006739
NLM021_scaffold49743_1	Salasmaviridae	0.2528747
NLM021_C812807_1	Casjensviridae	0.5409089
NLM021_scaffold55417_5	Straboviridae	0.4822487
NLM021_scaffold45854_2	Straboviridae	0.73139083
NLM021_scaffold927_6	Herelleviridae	0.47506937
NLM022_scaffold28217_1	Straboviridae	0.5006739
NLM022_scaffold9956_5	Straboviridae	0.43174168
NLM022_scaffold173_4	Zierdtviridae	1
NLM022_scaffold42567_1	Peduoviridae	0.43805262
NLM022_scaffold30338_1	Peduoviridae	1
NLM022_scaffold20998_1	Casjensviridae	0.44304314
NLM022_scaffold42750_2	Straboviridae	0.30859387
NLM022_C640276_1	Straboviridae	0.84417635
NLM022_scaffold9181_1	Peduoviridae	0.52169895
NLM022_scaffold35932_1	Straboviridae	0.6553155
NLM022_C641598_1	Straboviridae	0.72323537
NLM022_scaffold15285_1	Peduoviridae	1
NLM022_scaffold5329_1	Peduoviridae	1
NLM022_scaffold37593_2	Peduoviridae	1
NLM022_C640494_1	Straboviridae	0.4467759
NLM022_scaffold7447_1	Straboviridae	0.42385665
NLM023_scaffold23138_8	Ackermannviridae	0.35920888
NLM023_scaffold23260_3	Schitoviridae	1
NLM023_scaffold48669_2	Straboviridae	0.42385665
NLM023_scaffold911_13	Casjensviridae	0.52900046
NLM023_scaffold8508_4	Straboviridae	0.6553155
NLM023_scaffold48276_1	Straboviridae	0.36598924
NLM023_scaffold277_15	Straboviridae	0.58768487
NLM023_scaffold43102_12	Casjensviridae	1
NLM023_scaffold1348_25	Casjensviridae	0.22054045
NLM023_scaffold399_47	Casjensviridae	0.31617516
NLM023_scaffold260_1	Herelleviridae	0.64262027
NLM023_scaffold10723_15	Peduoviridae	0.7363866
NLM023_scaffold14181_20	Drexlerviridae	0.27454552
NLM023_scaffold33532_1	Ackermannviridae	0.31039864

NLM023_scaffold46734_3	Salasmaviridae	0.40109783
NLM023_scaffold12109_12	Peduoviridae	0.5691711
NLM023_scaffold34755_1	Straboviridae	0.57686263
NLM023_scaffold48896_1	Straboviridae	0.36598924
NLM024_scaffold488_1	Straboviridae	0.65579903
NLM024_scaffold6119_1	Kyanoviridae	1
NLM024_scaffold18150_1	Peduoviridae	1
NLM024_scaffold14981_1	Drexlerviridae	1
NLM024_scaffold77_6	Herelleviridae	1
NLM024_scaffold244_5	Straboviridae	0.6553155
NLM024_scaffold9211_2	Herelleviridae	1
NLM024_scaffold100_1	Straboviridae	0.42298242
NLM024_scaffold957_14	Ackermannviridae	0.25430948
NLM024_scaffold16628_8	Straboviridae	0.35521403
NLM024_scaffold11939_1	Straboviridae	0.22636564
NLM024_scaffold14981_3	Drexlerviridae	0.6004602
NLM025_C502105_1	Herelleviridae	0.17094114
NLM025_scaffold4345_2	Ackermannviridae	0.24692279
NLM025_scaffold35205_1	Straboviridae	0.67151016
NLM025_scaffold38901_1	Drexlerviridae	0.39220908
NLM025_scaffold12163_17	Straboviridae	0.6383465
NLM025_scaffold29129_1	Drexlerviridae	0.3840808
NLM026_scaffold27749_19	Straboviridae	0.49333465
NLM026_scaffold62290_1	Straboviridae	0.52144754
NLM026_scaffold55998_10	Salasmaviridae	0.2528747
NLM026_scaffold74568_1	Salasmaviridae	1
NLM026_scaffold75724_1	Straboviridae	0.4203055
NLM026_scaffold26990_1	Ackermannviridae	0.2372473
NLM026_scaffold75315_1	Herelleviridae	1
NLM026_scaffold49770_2	Guelinviridae	0.2955733
NLM026_scaffold75839_2	Straboviridae	0.4597352
NLM026_scaffold74363_2	Salasmaviridae	0.93296427
NLM026_scaffold58139_6	Straboviridae	0.5153456
NLM026_scaffold6763_2	Salasmaviridae	1
NLM026_scaffold32844_2	Straboviridae	0.51542896
NLM026_scaffold63092_3	Guelinviridae	1
NLM026_scaffold43543_3	Straboviridae	0.39771363
NLM026_scaffold75674_2	Ackermannviridae	0.25430948
NLM027_scaffold41411_1	Casjensviridae	1
NLM027_scaffold41959_1	Herelleviridae	1
NLM027_scaffold40329_1	Peduoviridae	0.70607936
NLM027_scaffold35554_2	Peduoviridae	1
NLM027_scaffold40329_3	Kyanoviridae	1
NLM027_scaffold39111_4	Straboviridae	0.30859387
NLM027_scaffold35206_1	Peduoviridae	0.241425
NLM027_scaffold16353_1	Straboviridae	0.33924893

NLM027_scaffold8952_3	Casjensviridae	1
NLM027_scaffold8952_6	Drexlerviridae	0.25270286
NLM027_scaffold1874_3	Chaseviridae	1
NLM027_scaffold11645_1	Straboviridae	0.42151073
NLM027_scaffold16205_3	Peduoviridae	1
NLM027_scaffold3310_5	Kyanoviridae	1
NLM027_scaffold27969_2	Straboviridae	0.6418709
NLM027_scaffold34123_3	Peduoviridae	0.14924113
NLM027_scaffold17357_1	Salasmaviridae	0.2528747
NLM027_scaffold38196_4	Straboviridae	0.57686263
NLM028_scaffold34514_1	Salasmaviridae	0.78925806
NLM028_scaffold8768_2	Peduoviridae	0.52169895
NLM028_scaffold34609_1	Casjensviridae	0.54889804
NLM028_scaffold51949_2	Ackermannviridae	1
NLM028_scaffold45877_1	Chaseviridae	0.3054243
NLM028_scaffold34525_2	Mesyanzhinovviridae	0.5455918
NLM028_scaffold20949_1	Straboviridae	0.59448063
NLM028_scaffold57543_3	Peduoviridae	1
NLM028_C652312_1	Casjensviridae	1
NLM028_scaffold13605_5	Ackermannviridae	0.2372473
NLM029_scaffold45341_4	Straboviridae	0.5399199
NLM029_scaffold33952_1	Casjensviridae	0.9217208
NLM029_scaffold4810_3	Straboviridae	0.43174168
NLM029_scaffold2015_8	Casjensviridae	0.45354968
NLM029_scaffold39928_1	Straboviridae	0.6553155
NLM029_scaffold161_2	Straboviridae	0.5123257
NLM029_scaffold2015_2	Peduoviridae	1
NLM029_scaffold3634_1	Peduoviridae	0.46395448
NLM029_scaffold37710_1	Peduoviridae	1
NLM029_scaffold7675_3	Straboviridae	0.4558217
NLM029_scaffold3860_9	Vilmaviridae	1
NLM029_scaffold25526_7	Straboviridae	0.65579903
NLM029_scaffold46830_1	Peduoviridae	1
NLM029_scaffold7768_1	Straboviridae	0.2957326
NLM029_scaffold45930_1	Straboviridae	0.5890641
NLM029_scaffold35112_1	Herelleviridae	1
NLM029_scaffold9315_13	Peduoviridae	0.5691711
NLM029_scaffold46287_2	Herelleviridae	0.858234
NLM029_scaffold31672_2	Herelleviridae	1
NLM029_scaffold17844_1	Straboviridae	0.47592783
NLM029_scaffold23685_2	Peduoviridae	0.30736294
NLM029_scaffold10325_2	Herelleviridae	1
NLM029_scaffold8116_24	Straboviridae	0.65579903
NLM029_C674345_1	Straboviridae	0.4203055
NLM031_scaffold75_1	Herelleviridae	0.53761834
NLM031_scaffold21651_1	Peduoviridae	1

NLM031_scaffold45857_1	Straboviridae	0.35521403
NLM031_scaffold23971_3	Straboviridae	0.72108424
NLM031_scaffold7206_4	Straboviridae	0.72323537
NLM031_scaffold27707_3	Kyanoviridae	1
NLM031_scaffold49_2	Straboviridae	0.6773715
NLM031_scaffold15300_11	Straboviridae	0.51102096
NLM031_scaffold259_8	Peduoviridae	0.46637547
NLM031_scaffold56089_1	Straboviridae	0.7403209
NLM031_scaffold45482_5	Straboviridae	0.6608802
NLM031_scaffold107_1	Drexlerviridae	0.25270286
NLM031_scaffold25711_7	Drexlerviridae	0.25270286
NLM031_scaffold18436_7	Casjensviridae	0.9825629
NLM032_scaffold23012_2	Schitoviridae	1
NLM032_scaffold61468_2	Straboviridae	0.5006739
NLM032_scaffold41399_1	Straboviridae	0.48703024
NLM032_scaffold58144_2	Straboviridae	0.7813815
NLM032_scaffold63260_2	Peduoviridae	1
NLM032_scaffold63260_1	Straboviridae	0.60721505
NLM032_scaffold69860_1	Straboviridae	0.47330517
NLM032_scaffold10936_5	Mesyanzhinovviridae	0.7162643
NLM032_scaffold37169_1	Casjensviridae	1
NLM032_scaffold68980_1	Herelleviridae	0.60281223
NLM032_scaffold7394_8	Peduoviridae	0.52169895
NLM032_scaffold25961_2	Salasmaviridae	0.40109783
NLM032_scaffold24196_2	Casjensviridae	0.9217208
NLM032_scaffold69594_3	Casjensviridae	1
NLM032_scaffold3932_23	Peduoviridae	0.4557199
NLM032_scaffold69179_1	Peduoviridae	0.241425
NLM032_scaffold68145_1	Peduoviridae	1
NLM032_scaffold58268_2	Ackermannviridae	0.5563236
NLM032_scaffold6383_1	Peduoviridae	1
NLM032_scaffold35599_1	Straboviridae	0.75188446
NLM001_scaffold38495_3	unknown	0
NLM001_scaffold2478_4	unknown	0
NLM001_scaffold44595_2	unknown	0
NLM001_scaffold41698_8	unknown	0
NLM001_scaffold41377_1	unknown	0
NLM001_scaffold5659_21	unknown	0
NLM001_scaffold43435_4	unknown	0
NLM001_scaffold31705_8	unknown	0
NLM001_scaffold17693_25	unknown	0
NLM001_scaffold44484_1	unknown	0
NLM001_scaffold40612_7	unknown	0
NLM002_scaffold13648_17	unknown	0
NLM002_scaffold28424_2	unknown	0
NLM002_scaffold4211_7	unknown	0

NLM002_scaffold15370_2	unknown	0
NLM002_C361292_1	unknown	0
NLM002_scaffold23710_2	unknown	0
NLM002_scaffold17510_4	unknown	0
NLM002_C361448_1	unknown	0
NLM002_scaffold16029_2	unknown	0
NLM003_scaffold4486_1	unknown	0
NLM003_scaffold10922_3	unknown	0
NLM003_scaffold14728_2	unknown	0
NLM003_scaffold3763_2_2	unknown	0
NLM004_scaffold44166_11	unknown	0
NLM004_scaffold27516_1	unknown	0
NLM004_scaffold815_2	unknown	0
NLM004_scaffold54737_2	unknown	0
NLM004_scaffold14733_2	unknown	0
NLM004_scaffold40373_1	unknown	0
NLM004_scaffold3539_11	unknown	0
NLM004_scaffold217_2	unknown	0
NLM004_scaffold18101_4	unknown	0
NLM004_scaffold13021_3	unknown	0
NLM004_scaffold14042_13	unknown	0
NLM004_scaffold49362_1	unknown	0
NLM004_scaffold2268_1	unknown	0
NLM004_C687357_1	unknown	0
NLM004_scaffold9767_3	unknown	0
NLM004_scaffold20206_2	unknown	0
NLM004_scaffold3052_12	unknown	0
NLM004_scaffold54430_1	unknown	0
NLM005_scaffold17186_2	unknown	0
NLM005_scaffold7084_1	unknown	0
NLM005_scaffold34767_1	unknown	0
NLM005_scaffold564_3	unknown	0
NLM005_scaffold21805_1	unknown	0
NLM005_scaffold7_1	unknown	0
NLM005_scaffold12098_1	unknown	0
NLM005_scaffold4896_1	unknown	0
NLM005_scaffold16081_2	unknown	0
NLM006_scaffold18020_1	unknown	0
NLM006_scaffold50674_1	unknown	0
NLM006_scaffold44414_1	unknown	0
NLM006_scaffold37475_1	unknown	0
NLM006_scaffold47321_2	unknown	0
NLM006_scaffold23522_7	unknown	0
NLM006_scaffold6162_11	unknown	0
NLM006_scaffold50021_2	unknown	0
NLM006_scaffold52859_1	unknown	0

NLM006_scaffold36213_1	unknown	0
NLM006_scaffold1105_10	unknown	0
NLM006_scaffold21365_1	unknown	0
NLM006_scaffold33813_8	unknown	0
NLM006_scaffold46436_2	unknown	0
NLM006_scaffold4801_4	unknown	0
NLM006_scaffold42358_1	unknown	0
NLM006_C734286_1	unknown	0
NLM006_scaffold30260_1	unknown	0
NLM006_C733934_1	unknown	0
NLM006_scaffold29091_1	unknown	0
NLM006_scaffold177_4	unknown	0
NLM006_scaffold44135_1	unknown	0
NLM006_scaffold15317_5	unknown	0
NLM006_scaffold12258_5	unknown	0
NLM006_scaffold37878_15	unknown	0
NLM006_scaffold50994_2	unknown	0
NLM006_scaffold12925_3	unknown	0
NLM006_scaffold16491_10	unknown	0
NLM006_scaffold43095_1	unknown	0
NLM006_scaffold17752_2	unknown	0
NLM006_scaffold31240_3	unknown	0
NLM006_scaffold1374_1	unknown	0
NLM006_scaffold4969_12	unknown	0
NLM006_scaffold16845_12	unknown	0
NLM006_scaffold22733_1	unknown	0
NLM006_C733830_1	unknown	0
NLM006_C734306_1	unknown	0
NLM006_scaffold9879_4	unknown	0
NLM007_scaffold11104_1	unknown	0
NLM007_scaffold7202_1	unknown	0
NLM007_scaffold5980_3	unknown	0
NLM007_scaffold20486_1	unknown	0
NLM007_scaffold6647_10	unknown	0
NLM007_scaffold19932_1	unknown	0
NLM007_scaffold26057_1	unknown	0
NLM007_scaffold25900_1	unknown	0
NLM007_scaffold6392_8	unknown	0
NLM007_scaffold10461_1	unknown	0
NLM007_scaffold22550_11	unknown	0
NLM007_scaffold26394_2	unknown	0
NLM007_scaffold10749_5	unknown	0
NLM007_scaffold10749_8	unknown	0
NLM007_scaffold21467_1	unknown	0
NLM007_scaffold4022_7	unknown	0
NLM007_scaffold26378_1	unknown	0

NLM007_scaffold26389_1	unknown	0
NLM008_scaffold70_5	unknown	0
NLM008_scaffold12934_1	unknown	0
NLM008_scaffold37575_7	unknown	0
NLM008_scaffold4502_17	unknown	0
NLM008_scaffold44104_3	unknown	0
NLM008_scaffold50150_1	unknown	0
NLM008_scaffold41985_1	unknown	0
NLM008_scaffold46068_3	unknown	0
NLM008_scaffold2753_1	unknown	0
NLM008_scaffold30532_8	unknown	0
NLM008_scaffold2753_6	unknown	0
NLM008_scaffold16329_1	unknown	0
NLM008_scaffold3829_2	unknown	0
NLM009_scaffold13480_1	unknown	0
NLM009_scaffold13047_6	unknown	0
NLM009_scaffold13069_1	unknown	0
NLM009_scaffold5257_2	unknown	0
NLM010_scaffold1115_4	unknown	0
NLM010_scaffold15834_3	unknown	0
NLM010_scaffold40145_2	unknown	0
NLM010_C569179_1	unknown	0
NLM010_scaffold7375_4	unknown	0
NLM010_scaffold15684_10	unknown	0
NLM010_scaffold13431_2	unknown	0
NLM010_scaffold3952_5	unknown	0
NLM010_scaffold894_1	unknown	0
NLM010_scaffold9073_7	unknown	0
NLM010_scaffold21070_2	unknown	0
NLM010_scaffold32176_1	unknown	0
NLM010_scaffold705_15	unknown	0
NLM010_scaffold4037_2	unknown	0
NLM010_scaffold17621_3	unknown	0
NLM010_scaffold10324_3	unknown	0
NLM010_scaffold20986_1	unknown	0
NLM010_scaffold2398_1	unknown	0
NLM010_scaffold24696_6	unknown	0
NLM010_scaffold21133_2	unknown	0
NLM010_scaffold2612_4	unknown	0
NLM010_scaffold20986_2	unknown	0
NLM010_scaffold9737_35	unknown	0
NLM010_scaffold28345_8	unknown	0
NLM010_scaffold26505_1	unknown	0
NLM010_scaffold3952_4	unknown	0
NLM010_scaffold3490_4	unknown	0
NLM015_scaffold34970_4	unknown	0

NLM015_scaffold45_3	unknown	0
NLM015_scaffold9944_1	unknown	0
NLM015_scaffold38949_1	unknown	0
NLM015_scaffold2372_1	unknown	0
NLM015_scaffold6813_1	unknown	0
NLM015_scaffold13019_2	unknown	0
NLM015_scaffold7231_4	unknown	0
NLM015_scaffold16659_2	unknown	0
NLM015_scaffold4740_1	unknown	0
NLM015_scaffold25776_2	unknown	0
NLM015_scaffold21932_1	unknown	0
NLM015_scaffold15418_1	unknown	0
NLM016_scaffold3058_1	unknown	0
NLM016_scaffold19074_1	unknown	0
NLM016_scaffold8626_1	unknown	0
NLM016_scaffold12933_1	unknown	0
NLM016_scaffold11175_2	unknown	0
NLM016_scaffold112_1	unknown	0
NLM016_scaffold3275_3	unknown	0
NLM016_scaffold19042_3	unknown	0
NLM016_scaffold16925_3	unknown	0
NLM017_scaffold20307_2	unknown	0
NLM017_scaffold38788_3	unknown	0
NLM017_scaffold32055_2	unknown	0
NLM017_scaffold8358_18	unknown	0
NLM017_scaffold9889_3	unknown	0
NLM017_scaffold20627_4	unknown	0
NLM017_scaffold38145_1	unknown	0
NLM017_scaffold35881_2	unknown	0
NLM017_scaffold17839_9	unknown	0
NLM017_scaffold21461_2	unknown	0
NLM017_scaffold24164_1	unknown	0
NLM017_scaffold25009_1	unknown	0
NLM017_scaffold12885_45	unknown	0
NLM017_scaffold24740_2	unknown	0
NLM017_scaffold29127_2	unknown	0
NLM017_scaffold31444_1	unknown	0
NLM017_scaffold8358_65	unknown	0
NLM021_scaffold5563_6	unknown	0
NLM021_scaffold562_2	unknown	0
NLM021_scaffold42938_1	unknown	0
NLM021_scaffold61286_1	unknown	0
NLM021_scaffold58942_1	unknown	0
NLM021_scaffold5557_1	unknown	0
NLM021_scaffold17568_1	unknown	0
NLM021_scaffold59003_3	unknown	0

NLM021_scaffold56233_3	unknown	0
NLM021_scaffold57344_1	unknown	0
NLM021_scaffold26944_4	unknown	0
NLM021_scaffold61366_2	unknown	0
NLM021_scaffold143_2	unknown	0
NLM021_scaffold35458_5	unknown	0
NLM021_scaffold22069_5	unknown	0
NLM021_scaffold11314_5	unknown	0
NLM021_scaffold14659_12	unknown	0
NLM021_scaffold60255_1	unknown	0
NLM021_scaffold709_5	unknown	0
NLM021_scaffold46181_6	unknown	0
NLM021_scaffold38431_7	unknown	0
NLM021_scaffold57650_6	unknown	0
NLM021_scaffold190_7	unknown	0
NLM021_scaffold61366_1	unknown	0
NLM021_scaffold11116_2	unknown	0
NLM022_scaffold3577_22	unknown	0
NLM022_scaffold5329_6	unknown	0
NLM022_scaffold17073_1	unknown	0
NLM022_scaffold3888_1	unknown	0
NLM022_scaffold16192_1	unknown	0
NLM022_scaffold10884_4	unknown	0
NLM022_scaffold38630_1	unknown	0
NLM022_scaffold42820_1	unknown	0
NLM022_scaffold44402_1	unknown	0
NLM022_scaffold7821_4	unknown	0
NLM022_scaffold38946_1	unknown	0
NLM022_scaffold15285_3	unknown	0
NLM022_scaffold28148_1	unknown	0
NLM022_scaffold43025_3	unknown	0
NLM022_scaffold24404_3	unknown	0
NLM022_scaffold43901_3	unknown	0
NLM022_scaffold17804_1	unknown	0
NLM022_scaffold35358_2	unknown	0
NLM022_scaffold11265_9	unknown	0
NLM023_scaffold1402_1	unknown	0
NLM023_scaffold33293_1	unknown	0
NLM023_scaffold470_13	unknown	0
NLM023_scaffold28479_8	unknown	0
NLM023_scaffold21242_4	unknown	0
NLM023_scaffold48941_1	unknown	0
NLM023_scaffold48169_1	unknown	0
NLM023_scaffold28479_20	unknown	0
NLM023_C806243_1	unknown	0
NLM023_scaffold28479_12	unknown	0

NLM023_scaffold11383_1	unknown	0
NLM023_scaffold48946_1	unknown	0
NLM023_scaffold37217_1	unknown	0
NLM023_scaffold253_1	unknown	0
NLM023_scaffold4055_10	unknown	0
NLM023_scaffold27707_7	unknown	0
NLM023_scaffold1428_18	unknown	0
NLM023_scaffold48506_1	unknown	0
NLM023_scaffold17200_15	unknown	0
NLM023_scaffold35604_3	unknown	0
NLM023_scaffold22_6	unknown	0
NLM024_scaffold497_1	unknown	0
NLM024_scaffold10225_2	unknown	0
NLM024_scaffold20170_1	unknown	0
NLM024_scaffold5090_1	unknown	0
NLM024_scaffold13470_1	unknown	0
NLM024_scaffold15487_3	unknown	0
NLM024_scaffold1371_3	unknown	0
NLM025_scaffold22167_3	unknown	0
NLM025_scaffold39287_1	unknown	0
NLM025_scaffold18010_7	unknown	0
NLM025_scaffold39157_1	unknown	0
NLM025_scaffold25589_3	unknown	0
NLM025_scaffold78_11	unknown	0
NLM025_scaffold20016_2	unknown	0
NLM026_scaffold11596_2	unknown	0
NLM026_scaffold74538_1	unknown	0
NLM026_scaffold29270_6	unknown	0
NLM026_scaffold57277_1	unknown	0
NLM026_scaffold75736_1	unknown	0
NLM026_scaffold1771_11	unknown	0
NLM026_scaffold258_3	unknown	0
NLM026_scaffold988_1	unknown	0
NLM026_scaffold75282_1	unknown	0
NLM026_scaffold47323_1	unknown	0
NLM026_scaffold65263_3	unknown	0
NLM026_scaffold68020_6	unknown	0
NLM026_scaffold58936_3	unknown	0
NLM026_scaffold394_5	unknown	0
NLM026_scaffold29289_10	unknown	0
NLM026_scaffold21981_1	unknown	0
NLM026_scaffold86_4	unknown	0
NLM026_scaffold74825_1	unknown	0
NLM026_scaffold26189_1	unknown	0
NLM026_scaffold32583_1	unknown	0
NLM026_scaffold58959_4	unknown	0

NLM026_scaffold258_2	unknown	0
NLM026_scaffold75960_2	unknown	0
NLM026_C924027_1	unknown	0
NLM026_scaffold65585_4	unknown	0
NLM026_scaffold16034_7	unknown	0
NLM026_scaffold9047_2	unknown	0
NLM026_scaffold13988_2	unknown	0
NLM026_scaffold69595_2	unknown	0
NLM026_scaffold54916_1	unknown	0
NLM026_scaffold73101_2	unknown	0
NLM026_scaffold15415_2	unknown	0
NLM027_scaffold22040_4	unknown	0
NLM027_scaffold40686_1	unknown	0
NLM027_scaffold41690_2	unknown	0
NLM027_scaffold4058_19	unknown	0
NLM027_scaffold19284_2	unknown	0
NLM027_scaffold30138_3	unknown	0
NLM027_scaffold41965_1	unknown	0
NLM027_scaffold14263_1	unknown	0
NLM027_scaffold39770_2	unknown	0
NLM027_scaffold34797_1	unknown	0
NLM027_scaffold34798_1	unknown	0
NLM027_scaffold10002_11	unknown	0
NLM027_scaffold17160_15	unknown	0
NLM027_scaffold7180_15	unknown	0
NLM027_scaffold1886_1	unknown	0
NLM027_scaffold40537_1	unknown	0
NLM027_C668626_1	unknown	0
NLM027_scaffold14181_3	unknown	0
NLM027_scaffold41891_1	unknown	0
NLM027_scaffold40356_4	unknown	0
NLM027_scaffold35206_2	unknown	0
NLM027_scaffold9793_1	unknown	0
NLM027_scaffold20399_3	unknown	0
NLM028_scaffold28234_2	unknown	0
NLM028_scaffold35099_2	unknown	0
NLM028_scaffold12174_3	unknown	0
NLM028_scaffold54929_3	unknown	0
NLM028_scaffold59756_2	unknown	0
NLM028_scaffold59789_2	unknown	0
NLM028_scaffold1532_1	unknown	0
NLM028_scaffold24678_1	unknown	0
NLM028_scaffold42575_1	unknown	0
NLM028_scaffold30720_1	unknown	0
NLM028_scaffold12341_5	unknown	0
NLM028_scaffold20220_12	unknown	0

NLM028_scaffold26847_1	unknown	0
NLM028_scaffold57798_4	unknown	0
NLM028_scaffold15831_1	unknown	0
NLM028_scaffold43687_1	unknown	0
NLM028_scaffold59796_3	unknown	0
NLM028_scaffold57798_3	unknown	0
NLM028_scaffold7898_1	unknown	0
NLM028_scaffold15831_2	unknown	0
NLM028_scaffold42949_1	unknown	0
NLM028_scaffold57543_2	unknown	0
NLM028_scaffold37225_1	unknown	0
NLM028_C652264_1	unknown	0
NLM029_scaffold46834_2	unknown	0
NLM029_C674039_1	unknown	0
NLM029_scaffold22749_1	unknown	0
NLM029_scaffold24024_8	unknown	0
NLM029_scaffold9598_4	unknown	0
NLM029_scaffold9173_1	unknown	0
NLM029_scaffold44423_10	unknown	0
NLM029_scaffold17455_3	unknown	0
NLM029_scaffold9598_2	unknown	0
NLM029_scaffold24413_3	unknown	0
NLM029_scaffold3826_2	unknown	0
NLM029_scaffold4592_2	unknown	0
NLM029_C673977_1	unknown	0
NLM029_scaffold35541_5	unknown	0
NLM029_scaffold5035_2	unknown	0
NLM029_scaffold4050_9	unknown	0
NLM029_scaffold12727_2	unknown	0
NLM029_scaffold15304_1	unknown	0
NLM029_scaffold24413_4	unknown	0
NLM029_C674283_1	unknown	0
NLM029_scaffold10574_3	unknown	0
NLM029_scaffold26243_3	unknown	0
NLM029_scaffold33933_1	unknown	0
NLM029_C674325_1	unknown	0
NLM029_scaffold8972_5	unknown	0
NLM029_scaffold325_5	unknown	0
NLM029_scaffold45625_1	unknown	0
NLM029_scaffold20227_1	unknown	0
NLM029_scaffold46752_4	unknown	0
NLM031_scaffold75_4	unknown	0
NLM031_scaffold59329_1	unknown	0
NLM031_scaffold18212_16	unknown	0
NLM031_scaffold18436_4	unknown	0
NLM031_scaffold33580_2	unknown	0

NLM031_scaffold41310_1	unknown	0
NLM031_scaffold6532_6	unknown	0
NLM031_scaffold1394_1	unknown	0
NLM031_scaffold2847_6	unknown	0
NLM031_scaffold25711_6	unknown	0
NLM031_scaffold45482_3_1	unknown	0
NLM031_scaffold36158_5	unknown	0
NLM031_scaffold29823_1	unknown	0
NLM031_scaffold2964_6	unknown	0
NLM031_scaffold20700_11	unknown	0
NLM031_scaffold17121_1	unknown	0
NLM031_scaffold1394_2	unknown	0
NLM031_scaffold23030_1	unknown	0
NLM031_C734735_1	unknown	0
NLM031_scaffold44148_4	unknown	0
NLM032_scaffold68145_4	unknown	0
NLM032_scaffold33461_2	unknown	0
NLM032_scaffold23630_5	unknown	0
NLM032_scaffold40713_1	unknown	0
NLM032_scaffold49689_1	unknown	0
NLM032_scaffold61468_1	unknown	0
NLM032_scaffold9843_17	unknown	0
NLM032_scaffold219_1	unknown	0
NLM032_scaffold12144_3	unknown	0
NLM032_scaffold69642_1	unknown	0
NLM032_scaffold69111_1	unknown	0
NLM032_scaffold314_2	unknown	0
NLM032_scaffold60475_1	unknown	0
NLM032_scaffold32034_2	unknown	0
NLM032_scaffold54796_9	unknown	0
NLM032_scaffold9089_7	unknown	0
NLM032_scaffold23630_7	unknown	0
NLM032_scaffold50920_4	unknown	0
NLM032_scaffold69647_5	unknown	0
NLM032_scaffold69594_2	unknown	0
NLM032_scaffold40892_3	unknown	0
NLM032_scaffold49971_2	unknown	0
NLM032_scaffold11035_2	unknown	0
NLM005_scaffold30374_9	no_family_avaliable(NC_(	0.954
NLM006_scaffold30822_2	no_family_avaliable(NC_(	0.972
NLM006_scaffold2215_2	no_family_avaliable(NC_(	0.955
NLM017_scaffold38914_3	no_family_avaliable(NC_(	0.97
NLM017_scaffold39062_2	no_family_avaliable(NC_(	0.981
NLM017_scaffold174_1	no_family_avaliable(NC_(	0.976
NLM021_scaffold14592_1	no_family_avaliable(NC_(	0.98
NLM022_scaffold7162_1	no_family_avaliable(NC_(	0.99

NLM023_scaffold23260_2	no_family_avaliable(NC_(	0.959
NLM026_scaffold74983_1	no_family_avaliable(NC_(	0.993
NLM031_scaffold16685_1	no_family_avaliable(NC_(	0.99
NLM031_scaffold18627_4	no_family_avaliable(NC_(	0.989
NLM032_scaffold3938_2	no_family_avaliable(NC_(	0.977
NLM032_scaffold212_2	no_family_avaliable(NC_(	0.97
DOM001_scaffold12537_8	Casjensviridae	0.9596824
DOM001_scaffold20125_2	Straboviridae	0.25507116
DOM001_scaffold57053_3	Peduoviridae	1
DOM001_scaffold43_2	Demerecviridae	0.4388233
DOM001_scaffold33_5	Ackermannviridae	0.2703984
DOM001_scaffold4013_1	Casjensviridae	0.75807345
DOM001_scaffold9052_4	Herelleviridae	1
DOM001_scaffold22877_4	Casjensviridae	0.6699858
DOM001_scaffold14492_1	Straboviridae	0.3229161
DOM001_scaffold39139_2	Casjensviridae	0.6866919
DOM001_scaffold49162_1	Straboviridae	0.52864015
DOM001_scaffold5635_4	Ackermannviridae	0.3338031
DOM001_scaffold26343_7	Salasmaviridae	0.22382887
DOM001_scaffold5000_5	Demerecviridae	0.26536036
DOM001_scaffold17386_5	Ackermannviridae	1
DOM001_scaffold17768_4	Chaseviridae	0.20182505
DOM001_scaffold38397_1	Herelleviridae	0.2461915
DOM001_scaffold19397_1	Salasmaviridae	0.4168327
DOM003_scaffold6007_2	Casjensviridae	1
DOM003_scaffold24274_1	Drexlerviridae	1
DOM003_scaffold6518_3	Autographiviridae	1
DOM003_scaffold2919_2	Straboviridae	0.26489046
DOM003_scaffold19301_1	Straboviridae	0.66039294
DOM003_scaffold19736_6	Peduoviridae	1
DOM003_scaffold20665_1	Ackermannviridae	0.3899253
DOM003_scaffold24151_1	Ackermannviridae	0.2703984
DOM003_scaffold23067_1	Straboviridae	0.5517625
DOM003_scaffold24307_2	Herelleviridae	1
DOM003_scaffold17266_6	Kyanoviridae	1
DOM003_scaffold24298_1	Peduoviridae	1
DOM003_scaffold22453_2	Peduoviridae	0.28692025
DOM005_scaffold374_9	Peduoviridae	1
DOM005_scaffold13159_4	Autographiviridae	1
DOM005_scaffold6037_1	Casjensviridae	1
DOM005_scaffold2259_3	Peduoviridae	0.33690333
DOM005_scaffold13355_2	Salasmaviridae	0.22382887
DOM005_scaffold12816_1	Ackermannviridae	0.27722943
DOM005_scaffold13159_2	Casjensviridae	1
DOM005_scaffold2640_50	Autographiviridae	1
DOM005_scaffold374_8	Peduoviridae	1

DOM008_scaffold680_3	Ackermannviridae	0.40512124
DOM008_scaffold16792_1	Salasmaviridae	0.33272234
DOM008_scaffold14700_5	Salasmaviridae	0.28656432
DOM008_scaffold1395_1	Straboviridae	0.2686186
DOM008_scaffold6987_15	Peduoviridae	1
DOM008_scaffold11628_1	Herelleviridae	1
DOM008_scaffold15475_9	Drexlerviridae	1
DOM008_scaffold44_39	Peduoviridae	1
DOM008_scaffold8252_2	Drexlerviridae	0.39265049
DOM008_scaffold15475_4	Drexlerviridae	1
DOM008_scaffold17898_2	Drexlerviridae	0.39265049
DOM008_scaffold11407_6	Ackermannviridae	0.25677204
DOM008_scaffold17859_3	Peduoviridae	0.39129964
DOM010_scaffold8807_2	Salasmaviridae	0.33272234
DOM010_scaffold23838_1	Casjensviridae	1
DOM010_scaffold20353_2	Straboviridae	0.35083705
DOM010_scaffold7398_16	Straboviridae	0.2856439
DOM010_C557895_1	Straboviridae	0.47345498
DOM010_scaffold6942_2	Ackermannviridae	0.25677204
DOM010_scaffold42298_4	Casjensviridae	0.30599892
DOM010_scaffold41237_4	Straboviridae	0.4156158
DOM010_scaffold36003_1	Ackermannviridae	0.43386555
DOM010_scaffold19710_3	Casjensviridae	0.8125134
DOM010_scaffold44548_1	Ackermannviridae	0.3611045
DOM010_scaffold44359_1	Ackermannviridae	0.3338031
DOM010_scaffold36407_1	Salasmaviridae	0.28656432
DOM010_scaffold12345_3	Ackermannviridae	0.39363876
DOM010_scaffold17754_1	Straboviridae	0.41539797
DOM010_C557291_1	Straboviridae	0.42015335
DOM010_scaffold13897_1	Casjensviridae	0.8726506
DOM010_scaffold43785_1	Ackermannviridae	0.40105554
DOM010_scaffold38766_6	Straboviridae	0.5505315
DOM010_scaffold43222_1	Ackermannviridae	1
DOM010_scaffold44558_1	Casjensviridae	0.2509271
DOM012_scaffold631_3	Drexlerviridae	0.32767826
DOM012_scaffold33513_5	Peduoviridae	1
DOM012_scaffold8289_1	Salasmaviridae	1
DOM012_scaffold9067_4	Peduoviridae	0.2989266
DOM012_scaffold3937_49	Straboviridae	0.4156158
DOM012_scaffold6197_5_2	Zierdtviridae	1
DOM012_scaffold2137_37	Zierdtviridae	1
DOM012_scaffold42523_1	Drexlerviridae	0.39265049
DOM012_scaffold6291_4	Drexlerviridae	0.14557987
DOM012_scaffold31_2	Drexlerviridae	0.13268808
DOM012_scaffold46434_1	Salasmaviridae	1
DOM013_scaffold29644_2	Straboviridae	0.6004747

DOM013_scaffold65510_2	Straboviridae	0.30413526
DOM013_scaffold66179_1	Salasmaviridae	1
DOM013_scaffold45617_1	Casjensviridae	1
DOM013_scaffold29321_1	Straboviridae	0.46327022
DOM013_scaffold4994_13	Ackermannviridae	0.2783795
DOM013_scaffold11541_3	Ackermannviridae	0.32162276
DOM013_scaffold38557_2	Herelleviridae	1
DOM013_scaffold27634_2	Ackermannviridae	0.43386555
DOM013_scaffold65920_1	Schitoviridae	1
DOM013_scaffold34766_1	Casjensviridae	0.6699858
DOM013_scaffold2110_2	Casjensviridae	1
DOM013_scaffold33337_2	Salasmaviridae	1
DOM013_scaffold3571_32	Kyanoviridae	1
DOM013_scaffold81_8	Straboviridae	0.42805165
DOM013_scaffold66005_1	Ackermannviridae	1
DOM013_scaffold54135_4	Straboviridae	0.42805165
DOM013_C812420_1	Herelleviridae	1
DOM013_scaffold64045_2	Ackermannviridae	0.3338031
DOM013_scaffold8272_3	Peduoviridae	1
DOM013_scaffold17461_1	Peduoviridae	0.34567592
DOM013_scaffold23134_20	Ackermannviridae	0.2797422
DOM013_scaffold3755_4	Herelleviridae	1
DOM013_scaffold81_7	Straboviridae	0.47816586
DOM013_scaffold66110_1	Straboviridae	0.42015335
DOM013_scaffold22249_3	Kyanoviridae	1
DOM013_scaffold16907_3	Peduoviridae	0.4545788
DOM013_scaffold9359_8	Peduoviridae	0.2989266
DOM014_scaffold7185_19	Casjensviridae	0.8125134
DOM014_scaffold17723_2	Straboviridae	0.40261546
DOM014_scaffold3561_14	Salasmaviridae	0.49816787
DOM014_scaffold9701_3	Casjensviridae	0.19781996
DOM014_scaffold283_1	Salasmaviridae	0.41580635
DOM014_scaffold5325_3	Salasmaviridae	0.4168327
DOM014_scaffold46104_1	Ackermannviridae	1
DOM014_scaffold804_11	Herelleviridae	1
DOM014_scaffold804_15	Straboviridae	1
DOM014_scaffold5037_9	Peduoviridae	1
DOM014_scaffold10055_2	Ackermannviridae	0.393936
DOM014_scaffold1696_5	Ackermannviridae	0.3338031
DOM014_scaffold27264_1	Casjensviridae	0.8726506
DOM014_scaffold25405_4	Casjensviridae	0.9596824
DOM014_scaffold4337_1	Ackermannviridae	1
DOM015_scaffold21624_2	Ackermannviridae	0.25972775
DOM015_scaffold15574_5	Ackermannviridae	0.5594898
DOM015_scaffold5854_1	Straboviridae	0.5386496
DOM015_scaffold14251_1	Peduoviridae	1

DOM015_scaffold22277_2	Straboviridae	0.5676362
DOM015_scaffold28241_1	Straboviridae	0.2686186
DOM015_scaffold1673_2	Ackermannviridae	0.27722943
DOM015_scaffold4_1	Ackermannviridae	0.43386555
DOM015_scaffold21009_2	Ackermannviridae	0.3338031
DOM015_scaffold10848_3	Ackermannviridae	0.43386555
DOM015_C368449_1	Salasmaviridae	0.49816787
DOM015_scaffold5037_4	Straboviridae	0.6004747
DOM015_scaffold24987_1	Peduoviridae	1
DOM016_scaffold36138_1	Salasmaviridae	0.28656432
DOM016_scaffold28769_2	Peduoviridae	1
DOM016_scaffold35635_4	Ackermannviridae	0.20316531
DOM016_scaffold27658_1	Straboviridae	0.5517625
DOM016_scaffold29662_3	Straboviridae	0.22926702
DOM016_scaffold35635_3	Straboviridae	0.25296193
DOM016_scaffold35701_7	Peduoviridae	1
DOM016_scaffold29175_2	Autographiviridae	1
DOM016_scaffold30030_2	Ackermannviridae	0.43386555
DOM016_scaffold15402_6	Peduoviridae	1
DOM016_scaffold31190_2	Schitoviridae	0.26457438
DOM016_scaffold20871_2	Zierdtviridae	1
DOM016_scaffold65_5	Ackermannviridae	0.3338031
DOM016_scaffold11439_8	Straboviridae	0.25507116
DOM017_scaffold9422_1	Straboviridae	0.48360676
DOM017_scaffold273_11	Ackermannviridae	0.40105554
DOM017_scaffold591_5	Ackermannviridae	0.43386555
DOM017_scaffold3771_1	Straboviridae	0.27292332
DOM017_scaffold35431_3	Herelleviridae	1
DOM017_scaffold1652_4	Ackermannviridae	0.26003572
DOM017_scaffold10632_13	Ackermannviridae	0.38445178
DOM018_scaffold41600_1	Chaseviridae	0.20182505
DOM018_scaffold35204_2	Demerecviridae	0.42195064
DOM018_scaffold4830_3	Ackermannviridae	0.28549954
DOM018_scaffold35020_1	Mesyanzhinovviridae	1
DOM018_scaffold41493_2	Casjensviridae	1
DOM018_scaffold9337_1	Straboviridae	0.66039294
DOM018_scaffold23065_2	Ackermannviridae	0.44663528
DOM018_scaffold6891_1	Herelleviridae	1
DOM018_scaffold37478_3	Salasmaviridae	0.28656432
DOM018_scaffold6891_2	Salasmaviridae	0.36038926
DOM018_scaffold1832_2	Casjensviridae	1
DOM018_C532801_1	Ackermannviridae	0.4605801
DOM018_scaffold32883_2	Straboviridae	0.5676362
DOM018_scaffold2126_7	Herelleviridae	1
DOM018_scaffold13608_2	Ackermannviridae	0.29527256
DOM018_scaffold17834_3	Straboviridae	0.6004747

DOM019_scaffold141_3	Straboviridae	0.6004747
DOM019_scaffold20326_3	Straboviridae	0.64696723
DOM019_scaffold41546_1	Ackermannviridae	0.2797422
DOM019_scaffold56541_1	Straboviridae	0.64696723
DOM019_scaffold33784_1	Herelleviridae	1
DOM019_scaffold20197_1	Straboviridae	0.3625785
DOM019_scaffold56555_1	Salasmaviridae	1
DOM019_scaffold56549_2	Ackermannviridae	0.29527256
DOM019_scaffold56598_2	Herelleviridae	1
DOM019_scaffold56469_5	Straboviridae	0.33017182
DOM019_scaffold20133_1	Ackermannviridae	0.4759637
DOM019_scaffold56536_2	Ackermannviridae	0.3338031
DOM019_scaffold9830_4	Ackermannviridae	1
DOM019_scaffold286_2	Straboviridae	0.4871907
DOM020_scaffold38403_2	Straboviridae	0.28144336
DOM020_scaffold24489_1	Salasmaviridae	0.4885404
DOM020_scaffold19627_11	Straboviridae	0.25296193
DOM020_scaffold8898_2	Peduoviridae	0.31273133
DOM020_scaffold16344_2	Vilmaviridae	1
DOM020_C483630_1	Straboviridae	0.7067733
DOM020_scaffold8013_2	Casjensviridae	0.6866919
DOM021_scaffold18418_1	Straboviridae	0.5445338
DOM021_scaffold21593_8	Peduoviridae	1
DOM021_scaffold71_1	Straboviridae	0.6004747
DOM021_scaffold38953_1	Ackermannviridae	0.28265804
DOM021_scaffold39089_1	Straboviridae	0.27696446
DOM021_scaffold100_1	Ackermannviridae	0.39984542
DOM021_scaffold39104_1	Ackermannviridae	0.3338031
DOM021_scaffold36942_1	Ackermannviridae	0.40512124
DOM021_scaffold57_4	Ackermannviridae	0.29527256
DOM021_scaffold37667_2	Casjensviridae	0.7706307
DOM021_scaffold11063_1	Ackermannviridae	0.43386555
DOM021_scaffold36893_3	Salasmaviridae	0.41580635
DOM021_scaffold37125_1	Chaseviridae	0.3020128
DOM022_scaffold6808_24	Ackermannviridae	0.43386555
DOM022_scaffold28157_3	Ackermannviridae	0.32162276
DOM022_scaffold32538_1	Straboviridae	0.6004747
DOM022_scaffold8382_3	Straboviridae	0.64696723
DOM022_scaffold37182_2	Peduoviridae	1
DOM022_scaffold38016_1	Peduoviridae	1
DOM022_scaffold35096_1	Chaseviridae	1
DOM022_scaffold36652_4	Straboviridae	0.5517625
DOM022_scaffold38271_2	Chaseviridae	0.93476343
DOM022_scaffold21088_2	Straboviridae	0.49118197
DOM022_scaffold37253_1	Peduoviridae	1
DOM022_scaffold25564_3	Straboviridae	0.40261546

DOM022_scaffold35602_3	Peduoviridae	1
DOM022_scaffold9769_2	Schitoviridae	1
DOM022_scaffold8236_1	Chaseviridae	0.28818834
DOM022_scaffold11588_18	Ackermannviridae	0.40105554
DOM022_scaffold17665_18	Casjensviridae	1
DOM022_scaffold11253_14	Ackermannviridae	0.1904263
DOM022_scaffold27991_2	Casjensviridae	1
DOM022_scaffold38396_3	Ackermannviridae	0.37239054
DOM022_scaffold48_3	Ackermannviridae	0.29527256
DOM022_scaffold37363_3	Straboviridae	0.4491102
DOM022_scaffold37792_2	Ackermannviridae	0.26003572
DOM022_scaffold1123_2	Ackermannviridae	0.43386555
DOM023_scaffold33157_2	Salasmaviridae	1
DOM023_scaffold19886_6	Autographiviridae	1
DOM023_scaffold12205_7	Straboviridae	0.35083705
DOM023_scaffold33612_1	Straboviridae	0.5505315
DOM023_scaffold33401_1	Salasmaviridae	0.45942637
DOM023_scaffold31295_1	Straboviridae	0.45292062
DOM023_scaffold19886_5	Autographiviridae	1
DOM023_scaffold33548_1	Casjensviridae	0.7706307
DOM024_scaffold35255_15	Zobellviridae	1
DOM024_scaffold54306_4	Ackermannviridae	0.40105554
DOM024_scaffold42617_2	Ackermannviridae	0.44663528
DOM024_scaffold41795_1	Straboviridae	0.4491102
DOM024_scaffold151_2	Drexlerviridae	0.15702671
DOM024_scaffold55680_1	Straboviridae	0.41539797
DOM024_scaffold56350_2	Demerecviridae	0.24218577
DOM024_scaffold56292_1	Herelleviridae	1
DOM024_scaffold32065_2	Ackermannviridae	0.38445178
DOM024_scaffold56206_2	Casjensviridae	1
DOM024_C664368_1	Salasmaviridae	1
DOM024_scaffold56343_2	Casjensviridae	0.18703812
DOM024_scaffold29589_1	Casjensviridae	0.75807345
DOM024_scaffold15513_1	Ackermannviridae	0.43386555
DOM024_scaffold20070_12	Herelleviridae	1
DOM024_scaffold30168_5	Ackermannviridae	0.3338031
DOM024_scaffold4865_6	Straboviridae	0.64696723
DOM024_scaffold35255_14	Zierdtviridae	0.49925515
DOM024_scaffold56343_3	Casjensviridae	1
DOM024_scaffold15633_7	Peduoviridae	1
DOM024_scaffold49650_2	Herelleviridae	1
DOM025_scaffold30920_4	Peduoviridae	0.4545788
DOM025_scaffold29788_4	Ackermannviridae	0.39984542
DOM025_scaffold49_3	Straboviridae	0.30413526
DOM025_scaffold4683_2	Casjensviridae	0.30599892
DOM025_scaffold10897_2	Ackermannviridae	0.40512124

DOM025_scaffold12330_13	Salasmaviridae	0.33272234
DOM026_scaffold39207_2	Salasmaviridae	0.4885404
DOM026_scaffold813_3	Ackermannviridae	0.39984542
DOM026_scaffold22251_2	Schitoviridae	0.26269838
DOM026_scaffold18742_1	Casjensviridae	0.3076411
DOM026_scaffold19764_1	Ackermannviridae	0.28265804
DOM026_scaffold30709_3	Straboviridae	0.47816586
DOM026_scaffold13170_6	Peduoviridae	1
DOM026_scaffold17576_11	Casjensviridae	0.3076411
DOM026_scaffold17476_4	Ackermannviridae	0.40512124
DOM026_scaffold6782_3	Straboviridae	0.49118197
DOM026_scaffold22785_1	Straboviridae	0.27209267
DOM026_C539214_1	Straboviridae	0.7067733
DOM026_scaffold22404_1	Straboviridae	0.37198326
DOM026_scaffold35658_1	Salasmaviridae	0.28656432
DOM001_scaffold26527_9	unknown	0
DOM001_scaffold6481_6	unknown	0
DOM001_scaffold21085_16	unknown	0
DOM001_scaffold2066_1	unknown	0
DOM001_scaffold53704_4	unknown	0
DOM001_scaffold26005_5	unknown	0
DOM001_scaffold16478_1	unknown	0
DOM001_scaffold55189_1	unknown	0
DOM001_scaffold58195_2	unknown	0
DOM001_scaffold7274_21	unknown	0
DOM001_scaffold12537_10	unknown	0
DOM001_scaffold28423_1	unknown	0
DOM001_scaffold30807_3	unknown	0
DOM001_scaffold37685_1	unknown	0
DOM001_scaffold2499_6	unknown	0
DOM001_scaffold273_1	unknown	0
DOM001_scaffold58831_1	unknown	0
DOM003_scaffold2919_6	unknown	0
DOM003_scaffold22571_7	unknown	0
DOM003_scaffold5840_1	unknown	0
DOM003_scaffold575_3	unknown	0
DOM003_scaffold24307_1	unknown	0
DOM003_scaffold11839_3	unknown	0
DOM003_scaffold3648_4	unknown	0
DOM003_scaffold2919_4	unknown	0
DOM003_scaffold18492_3	unknown	0
DOM003_scaffold11839_7	unknown	0
DOM003_scaffold11039_1	unknown	0
DOM003_scaffold1995_7	unknown	0
DOM003_scaffold14108_2	unknown	0
DOM003_scaffold2919_11	unknown	0

DOM003_scaffold11839_4	unknown	0
DOM005_scaffold920_41	unknown	0
DOM005_scaffold9501_4	unknown	0
DOM005_scaffold10392_5	unknown	0
DOM005_scaffold10888_1	unknown	0
DOM005_C258523_1	unknown	0
DOM005_scaffold1092_15	unknown	0
DOM005_scaffold10888_5	unknown	0
DOM005_scaffold3583_3	unknown	0
DOM005_scaffold10107_7	unknown	0
DOM005_scaffold13337_5	unknown	0
DOM005_scaffold168_65	unknown	0
DOM005_scaffold5851_3	unknown	0
DOM005_scaffold8757_1	unknown	0
DOM008_scaffold15923_2	unknown	0
DOM008_scaffold543_11	unknown	0
DOM008_scaffold16924_3	unknown	0
DOM008_scaffold4243_1	unknown	0
DOM008_scaffold5589_2	unknown	0
DOM008_scaffold3045_22	unknown	0
DOM008_scaffold57_7	unknown	0
DOM008_scaffold18954_1	unknown	0
DOM008_scaffold169_2	unknown	0
DOM008_scaffold9829_1	unknown	0
DOM008_scaffold8348_2	unknown	0
DOM008_scaffold14339_1	unknown	0
DOM008_scaffold57_5	unknown	0
DOM008_scaffold15104_4	unknown	0
DOM008_scaffold18440_1	unknown	0
DOM008_scaffold18246_2	unknown	0
DOM008_scaffold19226_1	unknown	0
DOM008_scaffold14655_7	unknown	0
DOM010_scaffold2671_4	unknown	0
DOM010_C556729_1	unknown	0
DOM010_scaffold44229_1	unknown	0
DOM010_scaffold11164_7	unknown	0
DOM010_scaffold44237_2	unknown	0
DOM010_scaffold29114_1	unknown	0
DOM010_scaffold44223_5	unknown	0
DOM010_C557123_1	unknown	0
DOM010_scaffold10693_2	unknown	0
DOM010_scaffold42781_1	unknown	0
DOM010_scaffold3905_57	unknown	0
DOM010_C557921_1	unknown	0
DOM010_scaffold7_1	unknown	0
DOM010_scaffold25074_1	unknown	0

DOM010_scaffold42659_2	unknown	0
DOM010_scaffold38114_4	unknown	0
DOM010_scaffold1445_1	unknown	0
DOM010_scaffold5308_8	unknown	0
DOM010_scaffold43444_3	unknown	0
DOM010_scaffold27469_1	unknown	0
DOM010_scaffold34515_1	unknown	0
DOM010_scaffold44098_1	unknown	0
DOM010_scaffold21662_2	unknown	0
DOM010_scaffold7072_9	unknown	0
DOM010_scaffold3568_1	unknown	0
DOM010_C557197_1	unknown	0
DOM010_scaffold40874_2	unknown	0
DOM010_scaffold40514_1	unknown	0
DOM010_scaffold39715_1	unknown	0
DOM010_scaffold44263_3	unknown	0
DOM012_scaffold27104_2	unknown	0
DOM012_scaffold33660_1	unknown	0
DOM012_scaffold4248_4	unknown	0
DOM012_scaffold25292_2	unknown	0
DOM012_scaffold38243_4	unknown	0
DOM012_scaffold9872_1	unknown	0
DOM012_scaffold25292_3	unknown	0
DOM012_scaffold17700_1	unknown	0
DOM012_scaffold5103_5	unknown	0
DOM012_scaffold4248_15	unknown	0
DOM012_scaffold7054_4	unknown	0
DOM012_scaffold4007_9	unknown	0
DOM012_scaffold4108_5	unknown	0
DOM012_scaffold33307_1	unknown	0
DOM012_scaffold4248_8	unknown	0
DOM012_scaffold4248_3	unknown	0
DOM013_scaffold24958_13	unknown	0
DOM013_scaffold46653_1	unknown	0
DOM013_scaffold13214_2	unknown	0
DOM013_scaffold63345_1	unknown	0
DOM013_scaffold65670_1	unknown	0
DOM013_scaffold39903_2	unknown	0
DOM013_scaffold46391_3	unknown	0
DOM013_scaffold50516_3	unknown	0
DOM013_scaffold66005_2	unknown	0
DOM013_scaffold10380_3	unknown	0
DOM013_scaffold66036_1	unknown	0
DOM013_scaffold9813_6	unknown	0
DOM013_scaffold42751_1	unknown	0
DOM013_scaffold63330_1	unknown	0

DOM013_scaffold13_2	unknown	0
DOM013_scaffold60583_1	unknown	0
DOM013_scaffold62840_1	unknown	0
DOM013_scaffold3571_2	unknown	0
DOM013_scaffold54135_10	unknown	0
DOM014_scaffold43299_1	unknown	0
DOM014_scaffold15062_27	unknown	0
DOM014_scaffold804_14	unknown	0
DOM014_scaffold6073_4	unknown	0
DOM014_scaffold804_8	unknown	0
DOM014_scaffold27382_3	unknown	0
DOM014_scaffold2397_7	unknown	0
DOM014_scaffold804_6	unknown	0
DOM014_scaffold25405_6	unknown	0
DOM014_scaffold8176_14	unknown	0
DOM015_scaffold25351_1	unknown	0
DOM015_scaffold24987_5	unknown	0
DOM015_scaffold226_4	unknown	0
DOM015_scaffold29083_1	unknown	0
DOM015_scaffold1868_1	unknown	0
DOM015_scaffold8047_3	unknown	0
DOM015_scaffold2034_4	unknown	0
DOM015_scaffold16257_1	unknown	0
DOM015_scaffold21150_1	unknown	0
DOM015_scaffold7250_8	unknown	0
DOM015_scaffold10865_1	unknown	0
DOM015_scaffold13677_1	unknown	0
DOM015_scaffold29079_1	unknown	0
DOM015_scaffold14658_2	unknown	0
DOM015_scaffold28241_3	unknown	0
DOM015_C368153_1	unknown	0
DOM016_scaffold37120_1	unknown	0
DOM016_scaffold36372_1	unknown	0
DOM016_scaffold15209_2	unknown	0
DOM016_scaffold35635_2	unknown	0
DOM016_scaffold27783_2	unknown	0
DOM016_scaffold714_2	unknown	0
DOM016_scaffold36369_1	unknown	0
DOM016_scaffold15402_10	unknown	0
DOM016_scaffold35922_2	unknown	0
DOM016_scaffold10818_1	unknown	0
DOM016_scaffold35524_2	unknown	0
DOM016_scaffold25511_1	unknown	0
DOM016_scaffold32640_3	unknown	0
DOM016_scaffold37071_2	unknown	0
DOM016_scaffold701_1	unknown	0

DOM016_scaffold5915_8	unknown	0
DOM016_scaffold4735_5	unknown	0
DOM016_scaffold10222_1	unknown	0
DOM016_scaffold27136_11	unknown	0
DOM016_scaffold35701_2	unknown	0
DOM016_scaffold30578_2	unknown	0
DOM016_scaffold19852_1	unknown	0
DOM017_scaffold13265_2	unknown	0
DOM017_scaffold7875_1	unknown	0
DOM017_scaffold12516_1	unknown	0
DOM017_scaffold21083_1	unknown	0
DOM017_scaffold16028_1	unknown	0
DOM017_scaffold5373_1	unknown	0
DOM017_scaffold7875_3	unknown	0
DOM017_scaffold7344_7	unknown	0
DOM017_scaffold434_2	unknown	0
DOM017_scaffold3183_4	unknown	0
DOM017_scaffold12158_1	unknown	0
DOM017_scaffold2122_1	unknown	0
DOM017_scaffold12299_4	unknown	0
DOM017_scaffold23327_2	unknown	0
DOM017_scaffold30906_1	unknown	0
DOM017_scaffold12432_2	unknown	0
DOM017_scaffold28752_1	unknown	0
DOM017_scaffold35758_3	unknown	0
DOM017_scaffold29332_1	unknown	0
DOM017_scaffold2195_2	unknown	0
DOM018_scaffold6891_4	unknown	0
DOM018_scaffold11099_4	unknown	0
DOM018_scaffold88_7	unknown	0
DOM018_scaffold27201_3	unknown	0
DOM018_scaffold37486_2	unknown	0
DOM018_scaffold32733_2	unknown	0
DOM018_scaffold22855_12	unknown	0
DOM018_scaffold41384_2	unknown	0
DOM018_C532545_1	unknown	0
DOM018_scaffold40717_1	unknown	0
DOM018_scaffold34741_1	unknown	0
DOM018_scaffold5182_1	unknown	0
DOM018_scaffold41493_1	unknown	0
DOM018_scaffold32755_3	unknown	0
DOM018_scaffold35068_1	unknown	0
DOM018_scaffold18201_3	unknown	0
DOM018_scaffold33929_1	unknown	0
DOM019_scaffold640_2	unknown	0
DOM019_scaffold42868_1	unknown	0

DOM019_scaffold5478_2	unknown	0
DOM019_scaffold10413_15	unknown	0
DOM019_scaffold3315_20	unknown	0
DOM019_scaffold1388_2	unknown	0
DOM019_scaffold56605_1	unknown	0
DOM019_scaffold661_7	unknown	0
DOM019_scaffold8387_2	unknown	0
DOM019_scaffold55994_2	unknown	0
DOM019_scaffold9440_2	unknown	0
DOM019_scaffold3989_6	unknown	0
DOM019_scaffold23724_3	unknown	0
DOM019_scaffold56558_1	unknown	0
DOM019_scaffold34447_1	unknown	0
DOM019_scaffold8084_3	unknown	0
DOM019_scaffold11571_11	unknown	0
DOM019_scaffold35386_4	unknown	0
DOM020_scaffold38380_2	unknown	0
DOM020_scaffold36128_4	unknown	0
DOM020_scaffold8013_4	unknown	0
DOM020_scaffold1763_4	unknown	0
DOM020_scaffold1491_1	unknown	0
DOM020_scaffold1229_1	unknown	0
DOM020_scaffold3600_6	unknown	0
DOM021_scaffold272_1	unknown	0
DOM021_scaffold23118_2	unknown	0
DOM021_scaffold39089_2	unknown	0
DOM021_scaffold12359_1	unknown	0
DOM021_scaffold37667_3	unknown	0
DOM021_scaffold5645_2	unknown	0
DOM021_scaffold39046_1	unknown	0
DOM022_scaffold32377_2	unknown	0
DOM022_scaffold19956_4	unknown	0
DOM022_scaffold38389_3	unknown	0
DOM022_scaffold2843_1	unknown	0
DOM022_scaffold38212_2	unknown	0
DOM022_scaffold18184_9	unknown	0
DOM022_scaffold8882_1	unknown	0
DOM022_scaffold38445_2	unknown	0
DOM022_scaffold16044_22	unknown	0
DOM022_scaffold847_4	unknown	0
DOM022_scaffold37774_1	unknown	0
DOM022_scaffold26147_1	unknown	0
DOM022_C512095_1	unknown	0
DOM022_scaffold38445_1	unknown	0
DOM022_scaffold33500_1	unknown	0
DOM022_scaffold33506_1	unknown	0

DOM022_scaffold16660_7	unknown	0
DOM022_scaffold3588_2	unknown	0
DOM022_scaffold6474_11	unknown	0
DOM022_scaffold37834_1	unknown	0
DOM022_scaffold7138_11	unknown	0
DOM022_scaffold3588_8	unknown	0
DOM022_scaffold17718_5	unknown	0
DOM022_scaffold15495_1	unknown	0
DOM022_scaffold8882_2	unknown	0
DOM022_scaffold37244_1	unknown	0
DOM022_scaffold19876_2	unknown	0
DOM022_scaffold13760_14	unknown	0
DOM022_scaffold28292_2	unknown	0
DOM022_scaffold21088_3	unknown	0
DOM022_scaffold9987_23	unknown	0
DOM022_scaffold36541_6	unknown	0
DOM023_scaffold33236_1	unknown	0
DOM023_scaffold33285_1	unknown	0
DOM023_scaffold25122_8	unknown	0
DOM023_scaffold11787_6	unknown	0
DOM023_scaffold33611_2	unknown	0
DOM023_scaffold28756_2	unknown	0
DOM023_scaffold13717_2	unknown	0
DOM023_scaffold23102_8	unknown	0
DOM023_scaffold33622_1	unknown	0
DOM023_scaffold2410_11	unknown	0
DOM023_scaffold16805_4	unknown	0
DOM023_C393870_1	unknown	0
DOM023_scaffold22587_5	unknown	0
DOM024_scaffold11745_3	unknown	0
DOM024_scaffold34021_1	unknown	0
DOM024_scaffold44466_2	unknown	0
DOM024_scaffold42617_1	unknown	0
DOM024_scaffold38154_1	unknown	0
DOM024_scaffold56350_1	unknown	0
DOM024_scaffold25752_1	unknown	0
DOM024_scaffold56303_1	unknown	0
DOM024_scaffold13072_13	unknown	0
DOM024_scaffold52650_1	unknown	0
DOM024_scaffold36750_16	unknown	0
DOM024_scaffold52177_5	unknown	0
DOM024_C663872_1	unknown	0
DOM024_scaffold52177_6	unknown	0
DOM024_scaffold52366_1	unknown	0
DOM024_C664620_1	unknown	0
DOM024_scaffold18051_2	unknown	0

DOM024_scaffold34021_4	unknown	0
DOM024_scaffold22388_3	unknown	0
DOM025_scaffold44713_1	unknown	0
DOM025_scaffold68_3	unknown	0
DOM025_scaffold39391_1	unknown	0
DOM025_scaffold44576_1	unknown	0
DOM025_scaffold41702_1	unknown	0
DOM025_scaffold10571_8	unknown	0
DOM025_scaffold80_3	unknown	0
DOM025_scaffold300_2	unknown	0
DOM025_scaffold9936_1	unknown	0
DOM025_scaffold1987_2	unknown	0
DOM025_scaffold3271_23	unknown	0
DOM025_scaffold7455_3	unknown	0
DOM025_scaffold41294_1	unknown	0
DOM025_scaffold14033_1	unknown	0
DOM025_scaffold23512_1	unknown	0
DOM025_scaffold5440_33	unknown	0
DOM025_scaffold2257_29	unknown	0
DOM026_scaffold22046_1	unknown	0
DOM026_scaffold135_10	unknown	0
DOM026_scaffold21041_1	unknown	0
DOM026_scaffold38351_1	unknown	0
DOM026_scaffold9255_1	unknown	0
DOM026_scaffold11281_20	unknown	0
DOM026_scaffold4239_7	unknown	0
DOM026_scaffold10323_1	unknown	0
DOM026_scaffold23168_2	unknown	0
DOM026_scaffold28561_1	unknown	0
DOM026_scaffold8858_1	unknown	0
DOM026_scaffold39135_1	unknown	0
DOM026_scaffold7817_3	unknown	0
DOM026_scaffold6782_6	unknown	0
DOM026_scaffold36555_1	unknown	0
DOM026_C539342_1	unknown	0
DOM026_scaffold11444_1	unknown	0
DOM026_scaffold21142_1	unknown	0
DOM026_scaffold5666_20	unknown	0
DOM026_scaffold17021_2	unknown	0
DOM026_scaffold12473_1	unknown	0
DOM026_scaffold14353_26	unknown	0
DOM026_scaffold13335_3	unknown	0
DOM026_scaffold28681_1	unknown	0
DOM026_scaffold18755_1	unknown	0
DOM026_scaffold4239_3	unknown	0
DOM026_scaffold26812_1	unknown	0

DOM026_scaffold10922_1	unknown	0
DOM026_scaffold27635_1	unknown	0
DOM026_scaffold21024_9	unknown	0
DOM026_scaffold21514_2	unknown	0
DOM026_scaffold23768_1	unknown	0
DOM026_scaffold17367_1	unknown	0
DOM003_scaffold24287_2	no_family_avaliable(NC_(	0.977
DOM005_scaffold1509_4	no_family_avaliable(NC_(	0.96
DOM005_scaffold9841_13	no_family_avaliable(NC_(	1
DOM010_scaffold72_1	no_family_avaliable(NC_(	0.991
DOM010_scaffold120_2	no_family_avaliable(NC_(	0.991
DOM012_scaffold631_1	no_family_avaliable(NC_(	0.952
DOM013_scaffold65510_1	no_family_avaliable(NC_(	0.971
DOM013_scaffold66255_2	no_family_avaliable(NC_(	0.979
DOM014_scaffold14238_1	no_family_avaliable(NC_(	0.973
DOM014_scaffold1467_4	no_family_avaliable(NC_(	0.968
DOM014_scaffold1467_2	no_family_avaliable(NC_(	0.981
DOM014_scaffold14238_3	no_family_avaliable(NC_(	0.969
DOM018_scaffold1832_4	no_family_avaliable(NC_(	0.98
DOM019_scaffold6808_2	no_family_avaliable(NC_(	0.969
DOM019_scaffold6808_3	no_family_avaliable(NC_(	0.979
DOM020_scaffold37565_2	no_family_avaliable(NC_(	0.983
DOM020_scaffold7010_6	no_family_avaliable(NC_(	0.992
DOM021_scaffold27752_2	no_family_avaliable(NC_(	0.974
DOM022_scaffold32837_2	no_family_avaliable(NC_(	0.981
DOM025_scaffold49_1	no_family_avaliable(NC_(	0.976
DOM025_scaffold20212_5	no_family_avaliable(NC_(	0.968
DOM026_scaffold22785_2	no_family_avaliable(NC_(	0.962
NOM001_scaffold36280_1	Peduoviridae	1
NOM001_scaffold4297_1	Straboviridae	1
NOM001_scaffold22842_6	Drexlerviridae	0.31882837
NOM002_scaffold5847_1	Zierdtviridae	0.18184917
NOM002_scaffold32200_2	Straboviridae	0.39451152
NOM002_scaffold23302_7	Ackermannviridae	1
NOM002_scaffold2059_12	Zierdtviridae	0.12534718
NOM002_scaffold32168_1	Straboviridae	0.46906093
NOM002_scaffold32212_1	Straboviridae	0.6309193
NOM002_scaffold32041_1	Kyanoviridae	1
NOM002_scaffold13215_6	Straboviridae	0.60870373
NOM002_scaffold830_1	Straboviridae	0.5261669
NOM002_scaffold3250_3	Peduoviridae	0.59965926
NOM002_scaffold5995_1	Ackermannviridae	1
NOM002_scaffold30413_1	Straboviridae	0.2870157
NOM002_scaffold3598_9	Straboviridae	0.39407468
NOM002_scaffold20546_3	Demerecviridae	0.21957879
NOM002_scaffold1562_8	Straboviridae	0.54900956

NOM002_scaffold27843_1	Straboviridae	0.4580326
NOM002_scaffold31229_1	Straboviridae	1
NOM002_C428718_1	Straboviridae	0.5692244
NOM002_scaffold32205_1	Straboviridae	0.4166439
NOM002_scaffold31205_1	Straboviridae	0.5898211
NOM002_scaffold22930_2	Straboviridae	0.31073028
NOM002_scaffold3856_12	Ackermannviridae	0.35297325
NOM002_scaffold12213_5	Straboviridae	0.3920616
NOM002_scaffold6256_7	Straboviridae	0.30048168
NOM002_scaffold7687_1	Straboviridae	0.36334187
NOM002_scaffold24275_1	Autographiviridae	1
NOM004_scaffold22743_2	Straboviridae	0.79878217
NOM004_scaffold13517_2	Peduoviridae	0.59965926
NOM004_scaffold130_15	Zierdtviridae	0.12259063
NOM004_C361687_1	Straboviridae	0.22862318
NOM004_scaffold11992_11	Straboviridae	0.5102872
NOM004_scaffold15695_1	Straboviridae	1
NOM004_scaffold9807_9	Straboviridae	0.60870373
NOM004_scaffold21707_1	Salasmaviridae	0.46300474
NOM004_scaffold22694_1	Peduoviridae	1
NOM004_scaffold11992_20	Casjensviridae	1
NOM004_scaffold13523_7	Straboviridae	0.36334187
NOM004_scaffold17398_1	Straboviridae	0.2679807
NOM004_scaffold15486_1	Straboviridae	0.375927
NOM005_scaffold53686_4	Drexlerviridae	0.27170157
NOM005_scaffold35434_1	Straboviridae	0.87696713
NOM005_scaffold41127_8	Straboviridae	0.7063348
NOM005_scaffold563_2	Casjensviridae	1
NOM005_scaffold10786_3	Straboviridae	0.55784976
NOM005_scaffold55066_2	Straboviridae	0.7000134
NOM005_scaffold2849_2	Drexlerviridae	0.36308205
NOM005_scaffold3277_10	Schitoviridae	0.2513714
NOM005_scaffold40041_4	Drexlerviridae	0.3329966
NOM005_scaffold563_4	Herelleviridae	1
NOM005_scaffold37509_3	Straboviridae	0.3920616
NOM005_scaffold41127_7	Straboviridae	0.5904167
NOM005_scaffold11865_1	Casjensviridae	1
NOM005_scaffold47045_2	Autographiviridae	1
NOM005_scaffold53973_2	Autographiviridae	1
NOM005_scaffold563_3	Zierdtviridae	1
NOM005_scaffold45891_2	Casjensviridae	1
NOM005_C716395_1	Autographiviridae	0.96155953
NOM005_scaffold47045_3	Autographiviridae	1
NOM005_scaffold31514_2	Ackermannviridae	0.34421903
NOM005_scaffold5739_4	Straboviridae	0.2870157
NOM005_scaffold41127_11	Straboviridae	0.7579115

NOM005_scaffold53973_3	Autographiviridae	1
NOM005_scaffold47045_1	Autographiviridae	1
NOM005_scaffold48061_2	Straboviridae	0.62754965
NOM005_scaffold55068_1	Peduoviridae	0.4337845
NOM005_C716365_1	Autographiviridae	0.96155393
NOM007_scaffold3522_1	Straboviridae	0.4042891
NOM007_scaffold41225_1	Straboviridae	0.57805586
NOM007_scaffold35454_4	Ackermannviridae	0.41468462
NOM007_C669362_1	Straboviridae	0.26939163
NOM007_scaffold37139_1	Straboviridae	0.28392893
NOM007_scaffold10787_5	Straboviridae	0.5013129
NOM007_scaffold48928_1	Straboviridae	0.20679401
NOM007_scaffold4742_2	Straboviridae	0.55210805
NOM008_scaffold33378_1	Chaseviridae	0.32056236
NOM008_scaffold4244_38	Straboviridae	0.48894924
NOM008_scaffold9964_68	Peduoviridae	1
NOM008_scaffold75_1	Salasmaviridae	1
NOM008_scaffold24500_2	Straboviridae	0.31073028
NOM008_scaffold32938_5	Ackermannviridae	0.30793354
NOM008_scaffold6097_1	Straboviridae	0.4709028
NOM008_scaffold14724_3	Straboviridae	0.14552358
NOM008_scaffold33345_2	Ackermannviridae	0.40345907
NOM008_scaffold226_8	Straboviridae	0.45782614
NOM008_scaffold30194_1	Straboviridae	0.69343835
NOM008_scaffold940_9	Ackermannviridae	0.35297325
NOM008_scaffold1177_10	Straboviridae	0.64330465
NOM009_scaffold31057_2	Ackermannviridae	0.41468462
NOM009_scaffold45014_3	Straboviridae	0.46134415
NOM009_scaffold25569_1	Casjensviridae	1
NOM009_scaffold19614_4	Drexlerviridae	0.25596908
NOM009_scaffold40704_1	Straboviridae	0.53671855
NOM009_scaffold54000_1	Autographiviridae	1
NOM009_scaffold10818_20	Straboviridae	0.6591823
NOM009_scaffold22166_2	Straboviridae	0.55784976
NOM009_scaffold18703_1	Schitoviridae	0.2513714
NOM009_scaffold41176_6	Straboviridae	0.7051302
NOM009_scaffold37752_2	Chaseviridae	0.26963246
NOM009_scaffold14272_1	Straboviridae	0.30048168
NOM009_scaffold44452_1	Vilmaviridae	0.24893457
NOM010_scaffold36917_1	Straboviridae	0.7514587
NOM010_scaffold40709_2	Herelleviridae	1
NOM010_scaffold27408_5	Straboviridae	0.530693
NOM010_scaffold34799_1	Straboviridae	0.7394616
NOM010_scaffold7841_9	Peduoviridae	0.3685337
NOM010_scaffold2023_1	Straboviridae	0.7514587
NOM010_scaffold41425_2	Straboviridae	0.22866157

NOM010_scaffold12374_8	Casjensviridae	0.32719818
NOM010_scaffold26048_2	Peduoviridae	1
NOM010_scaffold16391_2	Ackermannviridae	0.29854247
NOM010_scaffold19030_3	Ackermannviridae	0.28771883
NOM010_scaffold27711_4	Straboviridae	0.7514587
NOM010_scaffold30699_2	Straboviridae	0.3920616
NOM010_scaffold29615_5	Straboviridae	0.17178196
NOM010_scaffold7945_5	Peduoviridae	0.7102873
NOM010_scaffold16353_1	Straboviridae	0.6108962
NOM010_scaffold15321_2	Ackermannviridae	0.38382405
NOM010_scaffold23538_2	Straboviridae	0.39133236
NOM010_scaffold34_1	Ackermannviridae	0.31534573
NOM012_scaffold17023_1	Straboviridae	0.28039292
NOM012_scaffold8044_2	Kyanoviridae	1
NOM012_scaffold28597_33	Autographiviridae	1
NOM012_scaffold59314_2	Straboviridae	0.4580326
NOM012_scaffold59022_1	Salasmaviridae	0.39329737
NOM012_scaffold51561_1	Straboviridae	0.26939163
NOM012_C822191_1	Chaseviridae	1
NOM012_scaffold45669_3	Straboviridae	0.73880583
NOM012_scaffold53716_1	Straboviridae	0.30672264
NOM012_scaffold15076_7	Ackermannviridae	0.29854247
NOM012_scaffold57636_2	Drexlerviridae	0.39975724
NOM013_scaffold18789_1	Straboviridae	1
NOM013_scaffold47736_4	Salasmaviridae	0.44733387
NOM013_C706454_1	Straboviridae	0.48894924
NOM013_scaffold13725_4	Straboviridae	0.3920616
NOM013_scaffold30855_5	Straboviridae	0.6309193
NOM013_scaffold16346_5	Schitoviridae	1
NOM013_scaffold42371_10	Peduoviridae	0.74069476
NOM013_scaffold47736_3	Straboviridae	0.3162047
NOM013_scaffold22501_3	Straboviridae	0.23342617
NOM013_scaffold49212_2	Salasmaviridae	0.39329737
NOM013_scaffold9824_2	Peduoviridae	1
NOM013_scaffold4736_1	Straboviridae	0.78885055
NOM013_scaffold31511_2	Zobellviridae	0.5135538
NOM013_scaffold55128_3	Herelleviridae	1
NOM013_scaffold38707_7	Straboviridae	0.8097716
NOM013_scaffold55291_1	Ackermannviridae	1
NOM013_scaffold16204_2	Drexlerviridae	0.39975724
NOM013_scaffold31511_4	Schitoviridae	0.26197654
NOM014_scaffold24348_6	Schitoviridae	0.26319408
NOM014_scaffold15996_1	Straboviridae	0.2514063
NOM014_scaffold28199_1	Straboviridae	0.4166439
NOM014_scaffold27185_1	Peduoviridae	0.40998638
NOM014_scaffold19260_1	Straboviridae	0.78885055

NOM014_scaffold28329_1	Salasmaviridae	1
NOM014_scaffold9405_2	Straboviridae	0.7156085
NOM014_scaffold3384_5	Ackermannviridae	0.35297325
NOM014_scaffold25598_1	Straboviridae	0.7156085
NOM014_scaffold19787_2	Ackermannviridae	0.35297325
NOM014_C390319_1	Straboviridae	0.4356539
NOM014_scaffold27259_1	Straboviridae	0.27350757
NOM014_scaffold9372_1	Straboviridae	0.824416
NOM014_scaffold28352_1	Ackermannviridae	0.2754581
NOM014_scaffold15794_3	Straboviridae	0.31001717
NOM015_scaffold35862_1	Straboviridae	0.8097716
NOM015_scaffold36658_1	Ackermannviridae	0.177989
NOM015_scaffold1034_1	Peduoviridae	0.35915253
NOM015_scaffold226_1	Ackermannviridae	1
NOM015_scaffold16366_1	Salasmaviridae	0.96040297
NOM015_scaffold23464_3	Salasmaviridae	0.28743166
NOM015_scaffold8918_6	Ackermannviridae	0.28771883
NOM015_scaffold20333_3	Salasmaviridae	1
NOM015_scaffold36796_1	Straboviridae	0.4795738
NOM015_scaffold19627_1	Ackermannviridae	0.35297325
NOM015_scaffold23464_2	Salasmaviridae	0.31784317
NOM015_scaffold15823_2	Herelleviridae	1
NOM015_scaffold1721_3	Straboviridae	0.54900956
NOM015_scaffold11230_6	Straboviridae	0.41447464
NOM015_scaffold15823_5	Straboviridae	0.70922685
NOM015_scaffold10294_4	Mesyanzhinovviridae	0.69835234
NOM015_scaffold5941_3	Straboviridae	0.78885055
NOM015_scaffold8498_6	Casjensviridae	0.52695894
NOM015_scaffold16067_4	Straboviridae	0.7514587
NOM015_scaffold27697_2	Herelleviridae	1
NOM016_scaffold42933_2	Kyanoviridae	1
NOM016_scaffold35976_5	Salasmaviridae	0.33228892
NOM016_scaffold20671_4	Straboviridae	0.7514587
NOM016_scaffold33084_3	Straboviridae	0.26939163
NOM016_scaffold38252_1	Peduoviridae	0.7102873
NOM016_scaffold55549_3	Straboviridae	0.7514587
NOM016_scaffold47449_3	Ackermannviridae	0.28771883
NOM016_scaffold16005_2	Straboviridae	0.3003233
NOM016_scaffold55496_1	Herelleviridae	0.20963135
NOM016_scaffold55443_2	Straboviridae	0.55210805
NOM016_scaffold35286_1	Straboviridae	0.6334267
NOM016_scaffold39131_1	Straboviridae	0.43916065
NOM016_scaffold3257_24	Straboviridae	0.64330465
NOM017_scaffold43934_1	Ackermannviridae	0.41468462
NOM017_scaffold40696_1	Drexlerviridae	0.34971577
NOM017_scaffold4867_13	Straboviridae	0.8097716

NOM017_scaffold45477_1	Casjensviridae	1
NOM017_scaffold42764_1	Straboviridae	0.39451152
NOM017_scaffold996_22	Straboviridae	0.685769
NOM017_scaffold11947_1	Peduoviridae	0.17022015
NOM017_scaffold10596_2	Peduoviridae	1
NOM017_scaffold6578_5	Drexlerviridae	0.1370826
NOM017_scaffold44208_2	Straboviridae	0.5898211
NOM017_scaffold22040_3	Drexlerviridae	0.31882837
NOM018_scaffold13094_4	Straboviridae	0.47247508
NOM018_scaffold5764_2	Straboviridae	0.40843752
NOM018_scaffold29911_1	Peduoviridae	0.40998638
NOM018_scaffold53145_1	Salasmaviridae	0.9653382
NOM018_scaffold40401_5	Zierdtviridae	1
NOM018_scaffold54015_1	Straboviridae	0.29318652
NOM018_scaffold20530_1	Mesyanzhinovviridae	0.4088416
NOM018_scaffold11125_2	Straboviridae	0.47918606
NOM018_scaffold30_5	Peduoviridae	1
NOM018_scaffold53629_1	Straboviridae	0.62754965
NOM018_scaffold24796_3	Straboviridae	0.77344465
NOM018_scaffold42663_1	Straboviridae	0.26939163
NOM018_scaffold50197_1	Ackermannviridae	0.31534573
NOM018_scaffold54603_1	Straboviridae	0.40339345
NOM018_scaffold4393_1	Straboviridae	0.47193432
NOM018_C821692_1	Straboviridae	0.77971184
NOM019_scaffold6752_20	Peduoviridae	0.59965926
NOM019_scaffold52788_2	Salasmaviridae	0.42331704
NOM019_scaffold15207_20	Straboviridae	0.3162047
NOM019_scaffold52669_1	Herelleviridae	0.8982402
NOM019_scaffold22541_5	Drexlerviridae	0.23993139
NOM019_scaffold53083_1	Drexlerviridae	0.36810157
NOM019_scaffold50412_3	Straboviridae	1
NOM019_scaffold27076_10	Peduoviridae	0.74069476
NOM019_scaffold46040_3	Straboviridae	0.26783755
NOM019_scaffold14421_2	Ackermannviridae	0.20613262
NOM019_scaffold24407_7	Ackermannviridae	0.3046369
NOM019_scaffold52788_3	Straboviridae	0.3162047
NOM019_scaffold22541_7	Casjensviridae	0.6484222
NOM019_scaffold53127_2	Straboviridae	0.3920616
NOM020_scaffold5494_5	Casjensviridae	0.5695306
NOM020_scaffold39245_1	Ackermannviridae	0.31828496
NOM020_scaffold6778_1	Peduoviridae	0.74069476
NOM020_scaffold1826_3	Straboviridae	1
NOM020_scaffold14028_7	Drexlerviridae	1
NOM020_scaffold22877_2	Straboviridae	0.78885055
NOM020_scaffold32231_1	Straboviridae	0.409102
NOM020_scaffold12913_3	Straboviridae	0.36334187

NOM020_scaffold31685_6	Straboviridae	0.4640264
NOM020_scaffold35100_2	Herelleviridae	0.15467335
NOM020_scaffold3731_3	Straboviridae	0.55210805
NOM020_scaffold10070_1	Straboviridae	0.41099742
NOM020_scaffold26272_1	Casjensviridae	0.32719818
NOM020_scaffold16941_1	Straboviridae	0.81851417
NOM020_scaffold39269_1	Straboviridae	0.28039292
NOM022_scaffold35384_4	Drexlerviridae	0.38832796
NOM022_scaffold3633_6	Straboviridae	0.55784976
NOM022_scaffold35226_2	Straboviridae	0.4042891
NOM022_scaffold34355_1	Straboviridae	0.7514587
NOM022_scaffold16867_5	Ackermannviridae	0.2754581
NOM022_scaffold11835_1	Schitoviridae	0.17866491
NOM022_scaffold25627_3	Straboviridae	0.4977496
NOM022_scaffold31238_3	Straboviridae	0.7551459
NOM022_C499744_1	Straboviridae	0.71179295
NOM022_scaffold10614_11	Ackermannviridae	0.28771883
NOM022_scaffold25723_1	Ackermannviridae	0.22170894
NOM022_scaffold30875_1	Straboviridae	0.4795738
NOM022_scaffold35362_2	Straboviridae	0.4640264
NOM023_scaffold1309_14	Herelleviridae	0.31749043
NOM023_scaffold21303_1	Herelleviridae	0.8611489
NOM023_scaffold219_4	Straboviridae	0.5232858
NOM023_scaffold9577_8	Peduoviridae	0.25943342
NOM023_scaffold4208_9	Salasmaviridae	0.46300474
NOM023_scaffold1634_1	Straboviridae	0.59151065
NOM023_scaffold6600_7	Straboviridae	0.27628964
NOM023_scaffold7583_7	Ackermannviridae	0.29854247
NOM023_scaffold6623_2	Straboviridae	0.36334187
NOM023_scaffold16737_2	Salasmaviridae	0.33228892
NOM023_scaffold20012_1	Straboviridae	0.78885055
NOM023_scaffold5693_18	Drexlerviridae	0.23785804
NOM025_scaffold13742_3	Peduoviridae	1
NOM025_scaffold23401_1	Straboviridae	0.29318652
NOM025_scaffold6532_2	Straboviridae	0.45782614
NOM025_scaffold24512_5	Peduoviridae	0.6341865
NOM025_scaffold24601_2	Chaseviridae	1
NOM025_scaffold7710_1	Peduoviridae	1
NOM025_scaffold78_1	Herelleviridae	1
NOM025_scaffold173_5	Peduoviridae	1
NOM025_scaffold24496_1	Straboviridae	0.23342617
NOM025_scaffold17951_2	Ackermannviridae	1
NOM025_scaffold3831_6	Zierdtviridae	1
NOM025_scaffold23962_2	Peduoviridae	1
NOM025_scaffold7710_3	Peduoviridae	0.35915253
NOM026_scaffold21771_5	Straboviridae	0.7551459

NOM026_scaffold51_21	Peduoviridae	1
NOM026_scaffold4107_22	Straboviridae	0.47247508
NOM026_scaffold29015_1	Straboviridae	0.63267183
NOM026_scaffold43414_4	Peduoviridae	1
NOM026_scaffold39978_1	Ackermannviridae	1
NOM026_scaffold1436_1	Herelleviridae	0.16899583
NOM026_scaffold95_3_2	Straboviridae	0.6500024
NOM026_scaffold18477_4	Peduoviridae	0.24181609
NOM026_scaffold5049_5	Peduoviridae	0.4337845
NOM027_scaffold3843_3	Mesyanzhinovviridae	0.69835234
NOM027_scaffold298_5	Straboviridae	0.2902184
NOM027_scaffold21902_2	Vilmaviridae	1
NOM027_scaffold21753_2	Guelinviridae	1
NOM027_scaffold95_21	Straboviridae	0.3920616
NOM027_scaffold4263_1	Ackermannviridae	1
NOM027_scaffold16572_1	Straboviridae	0.6500024
NOM027_scaffold87_1	Straboviridae	0.7579115
NOM027_scaffold21847_1	Straboviridae	0.44452858
NOM028_C573709_1	Peduoviridae	1
NOM028_scaffold43134_4	Ackermannviridae	0.35297325
NOM028_scaffold3552_4	Peduoviridae	0.41061532
NOM028_scaffold11407_10	Chaseviridae	0.32056236
NOM028_scaffold5961_20	Straboviridae	0.23342617
NOM028_scaffold29187_1	Ackermannviridae	0.2754581
NOM028_scaffold47604_1	Straboviridae	0.79878217
NOM028_scaffold10515_18	Peduoviridae	0.6341865
NOM028_scaffold28052_1	Herelleviridae	1
NOM028_scaffold46573_2	Peduoviridae	0.7102873
NOM028_scaffold13734_22	Straboviridae	0.8292426
NOM028_scaffold8690_5	Straboviridae	0.30755553
NOM028_scaffold47539_2	Straboviridae	0.3920616
NOM028_scaffold2529_5	Ackermannviridae	1
NOM028_scaffold15800_1	Straboviridae	0.55784976
NOM028_scaffold45529_1	Straboviridae	0.4709028
NOM028_scaffold44706_1	Straboviridae	0.70313096
NOM028_scaffold366_38	Straboviridae	0.4993172
NOM028_scaffold37086_11	Straboviridae	0.685769
NOM028_scaffold29797_1	Straboviridae	0.73880583
NOM028_scaffold37620_3	Ackermannviridae	1
NOM028_scaffold39_5	Straboviridae	0.4577688
NOM028_scaffold19223_1	Drexlerviridae	0.36308205
NOM029_scaffold15812_1	Straboviridae	0.7394616
NOM029_scaffold15613_10	Peduoviridae	1
NOM029_scaffold13239_1	Ackermannviridae	0.4011864
NOM029_scaffold32772_2	Straboviridae	0.47918606
NOM029_scaffold9689_3	Straboviridae	0.3920616

NOM029_scaffold277_1	Ackermannviridae	0.34232736
NOM029_scaffold19269_12	Straboviridae	0.77344465
NOM001_scaffold14915_2	unknown	0
NOM001_scaffold22842_7	unknown	0
NOM001_scaffold36050_1	unknown	0
NOM001_scaffold22842_8	unknown	0
NOM001_scaffold36892_1	unknown	0
NOM001_scaffold31539_2	unknown	0
NOM001_scaffold24512_3	unknown	0
NOM002_scaffold4408_28	unknown	0
NOM002_scaffold1807_4	unknown	0
NOM002_scaffold139_2	unknown	0
NOM002_scaffold2797_4	unknown	0
NOM002_scaffold26702_1	unknown	0
NOM002_scaffold26752_2	unknown	0
NOM002_scaffold65_1	unknown	0
NOM002_scaffold15419_6	unknown	0
NOM002_scaffold7211_5	unknown	0
NOM002_scaffold2423_2	unknown	0
NOM002_scaffold32124_1	unknown	0
NOM002_scaffold23876_3	unknown	0
NOM002_scaffold31941_2	unknown	0
NOM002_scaffold31231_2	unknown	0
NOM002_scaffold7151_8	unknown	0
NOM002_scaffold27268_2	unknown	0
NOM002_C429380_1	unknown	0
NOM002_scaffold32206_1	unknown	0
NOM002_scaffold330_35	unknown	0
NOM002_scaffold32135_1	unknown	0
NOM002_scaffold6808_7	unknown	0
NOM002_scaffold19947_2	unknown	0
NOM002_scaffold4555_2	unknown	0
NOM002_scaffold14897_3	unknown	0
NOM002_scaffold24280_1	unknown	0
NOM002_scaffold20040_1	unknown	0
NOM002_scaffold27308_2	unknown	0
NOM004_scaffold14898_16	unknown	0
NOM004_scaffold8990_22	unknown	0
NOM004_scaffold3782_4	unknown	0
NOM004_scaffold22899_1	unknown	0
NOM004_scaffold4741_16	unknown	0
NOM004_scaffold21343_2	unknown	0
NOM004_scaffold28_10	unknown	0
NOM004_scaffold6154_1	unknown	0
NOM004_scaffold11917_4	unknown	0
NOM004_scaffold14898_14	unknown	0

NOM004_scaffold15115_6	unknown	0
NOM004_scaffold1169_9	unknown	0
NOM005_C715971_1	unknown	0
NOM005_scaffold51989_2	unknown	0
NOM005_scaffold29223_4	unknown	0
NOM005_scaffold32988_8	unknown	0
NOM005_scaffold8808_3	unknown	0
NOM005_scaffold130_3	unknown	0
NOM005_scaffold12226_1	unknown	0
NOM005_scaffold16553_2	unknown	0
NOM005_scaffold29223_3	unknown	0
NOM005_scaffold55101_1	unknown	0
NOM005_scaffold8276_2	unknown	0
NOM005_scaffold504_2	unknown	0
NOM005_scaffold32988_6	unknown	0
NOM005_scaffold37509_5	unknown	0
NOM005_scaffold16216_8	unknown	0
NOM005_scaffold11952_1	unknown	0
NOM005_scaffold41127_12	unknown	0
NOM005_scaffold50369_2	unknown	0
NOM005_scaffold2279_1	unknown	0
NOM005_scaffold54732_1	unknown	0
NOM005_scaffold41647_4	unknown	0
NOM005_scaffold41127_5	unknown	0
NOM005_scaffold20861_2	unknown	0
NOM007_C668778_1	unknown	0
NOM007_scaffold48877_1	unknown	0
NOM007_scaffold37186_2	unknown	0
NOM007_scaffold46703_1	unknown	0
NOM007_scaffold45023_6	unknown	0
NOM007_scaffold15814_1	unknown	0
NOM007_scaffold270_1	unknown	0
NOM007_scaffold39468_29	unknown	0
NOM007_scaffold14193_6	unknown	0
NOM007_scaffold35189_1	unknown	0
NOM007_scaffold37589_1	unknown	0
NOM007_scaffold17241_4	unknown	0
NOM007_scaffold41196_1	unknown	0
NOM007_scaffold29869_4	unknown	0
NOM008_scaffold17954_24	unknown	0
NOM008_scaffold18778_4	unknown	0
NOM008_scaffold18096_12	unknown	0
NOM008_scaffold29282_11	unknown	0
NOM008_scaffold445_45	unknown	0
NOM008_scaffold33375_1	unknown	0
NOM008_scaffold32941_1	unknown	0

NOM008_scaffold32975_4	unknown	0
NOM008_scaffold9194_1	unknown	0
NOM008_scaffold29282_6	unknown	0
NOM008_scaffold5454_20	unknown	0
NOM008_scaffold27977_1	unknown	0
NOM009_scaffold34457_1	unknown	0
NOM009_scaffold37752_3	unknown	0
NOM009_scaffold15278_8	unknown	0
NOM009_scaffold52880_2	unknown	0
NOM009_scaffold24584_3	unknown	0
NOM009_scaffold54003_3	unknown	0
NOM009_scaffold27152_1	unknown	0
NOM009_scaffold43294_6	unknown	0
NOM009_scaffold41176_2	unknown	0
NOM009_scaffold15278_9	unknown	0
NOM009_scaffold1163_4	unknown	0
NOM009_scaffold16373_1	unknown	0
NOM009_scaffold53866_1	unknown	0
NOM009_scaffold6873_12	unknown	0
NOM009_scaffold20832_3	unknown	0
NOM009_scaffold13490_22	unknown	0
NOM009_scaffold26738_4	unknown	0
NOM009_scaffold337_2	unknown	0
NOM009_scaffold293_1	unknown	0
NOM009_scaffold37752_4	unknown	0
NOM009_scaffold53815_1	unknown	0
NOM009_scaffold113_2	unknown	0
NOM009_scaffold14149_2	unknown	0
NOM009_scaffold53934_1	unknown	0
NOM009_scaffold53906_1	unknown	0
NOM010_scaffold16688_4	unknown	0
NOM010_scaffold7834_6	unknown	0
NOM010_scaffold23144_2	unknown	0
NOM010_scaffold6634_3	unknown	0
NOM010_scaffold8017_1	unknown	0
NOM010_scaffold39236_1	unknown	0
NOM010_scaffold356_5	unknown	0
NOM010_scaffold3371_1	unknown	0
NOM010_scaffold40709_1	unknown	0
NOM010_scaffold41832_1	unknown	0
NOM010_scaffold26994_1	unknown	0
NOM010_scaffold40683_2	unknown	0
NOM010_scaffold19890_3	unknown	0
NOM010_scaffold40709_6	unknown	0
NOM010_scaffold7692_1	unknown	0
NOM010_scaffold13739_1	unknown	0

NOM010_scaffold27824_1	unknown	0
NOM010_scaffold5951_6	unknown	0
NOM010_scaffold4318_15	unknown	0
NOM010_scaffold41566_2	unknown	0
NOM010_scaffold41431_1	unknown	0
NOM010_scaffold37895_1	unknown	0
NOM010_scaffold26455_8	unknown	0
NOM010_scaffold27687_3	unknown	0
NOM010_scaffold16212_1	unknown	0
NOM010_scaffold20969_4	unknown	0
NOM010_scaffold27644_3	unknown	0
NOM010_scaffold24440_2	unknown	0
NOM012_scaffold55930_5	unknown	0
NOM012_scaffold41765_3	unknown	0
NOM012_scaffold58733_1	unknown	0
NOM012_scaffold15076_8	unknown	0
NOM012_scaffold8044_6	unknown	0
NOM012_scaffold16052_1	unknown	0
NOM012_scaffold57810_1	unknown	0
NOM012_scaffold4060_3	unknown	0
NOM012_scaffold8574_7	unknown	0
NOM012_scaffold59297_1	unknown	0
NOM012_scaffold25493_7	unknown	0
NOM012_scaffold16848_9	unknown	0
NOM012_scaffold19624_3	unknown	0
NOM012_scaffold118_12	unknown	0
NOM012_scaffold6750_2	unknown	0
NOM013_scaffold54467_1	unknown	0
NOM013_scaffold28579_1	unknown	0
NOM013_scaffold11200_14	unknown	0
NOM013_scaffold28173_6	unknown	0
NOM013_scaffold23911_1	unknown	0
NOM013_scaffold55293_1	unknown	0
NOM013_scaffold19917_26	unknown	0
NOM013_scaffold7_2	unknown	0
NOM013_scaffold19917_6	unknown	0
NOM013_scaffold54467_2	unknown	0
NOM013_scaffold53631_1	unknown	0
NOM013_scaffold3327_4	unknown	0
NOM013_scaffold11200_16	unknown	0
NOM013_scaffold32541_12	unknown	0
NOM013_scaffold3281_4	unknown	0
NOM013_scaffold18200_3	unknown	0
NOM013_scaffold31511_3	unknown	0
NOM013_scaffold21734_30	unknown	0
NOM013_scaffold16346_2	unknown	0

NOM013_scaffold28579_4	unknown	0
NOM013_scaffold54010_1	unknown	0
NOM013_scaffold19858_2	unknown	0
NOM013_scaffold11318_3	unknown	0
NOM013_scaffold13071_1	unknown	0
NOM013_scaffold36218_2	unknown	0
NOM014_scaffold14255_16	unknown	0
NOM014_C390595_1	unknown	0
NOM014_scaffold25704_1	unknown	0
NOM014_scaffold11825_3	unknown	0
NOM014_scaffold8227_3	unknown	0
NOM014_scaffold28027_1	unknown	0
NOM014_scaffold15825_1	unknown	0
NOM014_scaffold28228_1	unknown	0
NOM014_scaffold14811_8	unknown	0
NOM014_scaffold23281_1	unknown	0
NOM014_scaffold27301_2	unknown	0
NOM014_C390531_1	unknown	0
NOM014_scaffold8317_1	unknown	0
NOM014_scaffold28328_1	unknown	0
NOM014_scaffold490_36	unknown	0
NOM014_scaffold13553_1	unknown	0
NOM014_scaffold7249_2	unknown	0
NOM014_scaffold5743_3	unknown	0
NOM014_scaffold28145_4	unknown	0
NOM014_scaffold13234_1	unknown	0
NOM014_scaffold28353_1	unknown	0
NOM014_scaffold21899_2	unknown	0
NOM015_scaffold2934_14	unknown	0
NOM015_scaffold16922_3	unknown	0
NOM015_scaffold34161_4	unknown	0
NOM015_scaffold10294_6	unknown	0
NOM015_scaffold4016_5	unknown	0
NOM015_scaffold4819_1	unknown	0
NOM015_scaffold3437_9	unknown	0
NOM015_scaffold15823_1	unknown	0
NOM015_scaffold124_1	unknown	0
NOM015_scaffold18383_1	unknown	0
NOM015_scaffold20240_1	unknown	0
NOM015_scaffold1091_31	unknown	0
NOM015_scaffold14266_3	unknown	0
NOM015_scaffold3765_6	unknown	0
NOM015_scaffold6127_1	unknown	0
NOM015_scaffold252_7	unknown	0
NOM015_scaffold749_3	unknown	0
NOM015_scaffold9242_9	unknown	0

NOM015_scaffold26753_1	unknown	0
NOM016_scaffold29885_1	unknown	0
NOM016_scaffold35286_4	unknown	0
NOM016_scaffold35286_7	unknown	0
NOM016_scaffold45043_2	unknown	0
NOM016_scaffold16005_4	unknown	0
NOM016_scaffold31191_1	unknown	0
NOM016_scaffold53315_1	unknown	0
NOM016_scaffold37747_2	unknown	0
NOM016_scaffold54950_4	unknown	0
NOM016_scaffold54079_1	unknown	0
NOM016_scaffold12609_2	unknown	0
NOM016_scaffold25995_3	unknown	0
NOM016_scaffold1263_27	unknown	0
NOM016_scaffold41274_5	unknown	0
NOM016_scaffold2585_1	unknown	0
NOM016_scaffold1680_10	unknown	0
NOM016_scaffold16783_1	unknown	0
NOM016_scaffold28365_4	unknown	0
NOM016_scaffold12609_6	unknown	0
NOM016_scaffold55593_1	unknown	0
NOM016_scaffold19739_3	unknown	0
NOM016_scaffold31927_4	unknown	0
NOM016_scaffold55581_3	unknown	0
NOM017_scaffold41715_2	unknown	0
NOM017_scaffold2822_6	unknown	0
NOM017_scaffold45485_2	unknown	0
NOM017_scaffold45482_1	unknown	0
NOM017_scaffold45435_1	unknown	0
NOM017_scaffold22366_1	unknown	0
NOM017_scaffold16406_3	unknown	0
NOM017_scaffold845_1	unknown	0
NOM017_scaffold45496_2	unknown	0
NOM017_scaffold45031_4	unknown	0
NOM017_scaffold45430_1	unknown	0
NOM017_scaffold11947_3	unknown	0
NOM017_scaffold1142_45	unknown	0
NOM017_scaffold6796_4	unknown	0
NOM017_scaffold33562_4	unknown	0
NOM018_scaffold30222_3	unknown	0
NOM018_scaffold54740_4	unknown	0
NOM018_scaffold4478_2	unknown	0
NOM018_scaffold42888_2	unknown	0
NOM018_scaffold54709_5	unknown	0
NOM018_scaffold54740_2	unknown	0
NOM018_scaffold51296_4	unknown	0

NOM018_scaffold42663_2	unknown	0
NOM018_scaffold53732_1	unknown	0
NOM018_scaffold26191_10	unknown	0
NOM018_scaffold1296_2	unknown	0
NOM018_scaffold37159_1	unknown	0
NOM018_scaffold221_1	unknown	0
NOM018_scaffold55244_1	unknown	0
NOM018_scaffold18121_1	unknown	0
NOM018_scaffold9490_1	unknown	0
NOM018_scaffold191_5	unknown	0
NOM018_scaffold15821_1	unknown	0
NOM018_scaffold16582_2	unknown	0
NOM018_scaffold9772_2	unknown	0
NOM018_scaffold2740_11	unknown	0
NOM018_scaffold115_1	unknown	0
NOM018_scaffold50471_1	unknown	0
NOM018_scaffold51296_6	unknown	0
NOM018_scaffold1842_24	unknown	0
NOM018_scaffold52711_1	unknown	0
NOM019_scaffold37_3	unknown	0
NOM019_scaffold16958_6	unknown	0
NOM019_scaffold52150_2	unknown	0
NOM019_scaffold5766_5	unknown	0
NOM019_scaffold53004_2	unknown	0
NOM019_scaffold40062_4	unknown	0
NOM019_scaffold53126_1	unknown	0
NOM019_scaffold40333_6	unknown	0
NOM019_scaffold16958_5	unknown	0
NOM019_scaffold38943_2	unknown	0
NOM019_scaffold50412_4	unknown	0
NOM019_scaffold53126_2	unknown	0
NOM019_scaffold31682_1	unknown	0
NOM019_scaffold53119_3	unknown	0
NOM019_scaffold7421_3	unknown	0
NOM019_scaffold22109_1	unknown	0
NOM019_scaffold45409_1	unknown	0
NOM019_scaffold12477_8	unknown	0
NOM019_scaffold12848_1	unknown	0
NOM019_scaffold36262_3	unknown	0
NOM020_scaffold33908_1	unknown	0
NOM020_scaffold38448_1	unknown	0
NOM020_scaffold7097_2	unknown	0
NOM020_scaffold1704_2	unknown	0
NOM020_scaffold13829_4	unknown	0
NOM020_C476630_1	unknown	0
NOM020_scaffold38253_1	unknown	0

NOM020_scaffold37822_4	unknown	0
NOM020_scaffold38253_2	unknown	0
NOM020_scaffold38892_1	unknown	0
NOM020_scaffold14364_1	unknown	0
NOM020_scaffold39193_2	unknown	0
NOM022_scaffold25627_6	unknown	0
NOM022_scaffold2146_2	unknown	0
NOM022_scaffold11395_10	unknown	0
NOM022_scaffold35027_4	unknown	0
NOM022_scaffold1399_24	unknown	0
NOM022_scaffold33629_1	unknown	0
NOM022_scaffold23992_2	unknown	0
NOM022_C499778_1	unknown	0
NOM022_scaffold35027_1	unknown	0
NOM022_scaffold29333_3	unknown	0
NOM022_scaffold9236_24	unknown	0
NOM022_C499370_1	unknown	0
NOM022_scaffold25634_1	unknown	0
NOM022_scaffold35326_5	unknown	0
NOM023_scaffold2528_2	unknown	0
NOM023_scaffold7114_2	unknown	0
NOM023_scaffold272_7	unknown	0
NOM023_scaffold2396_5	unknown	0
NOM023_scaffold6600_10	unknown	0
NOM023_scaffold13354_4	unknown	0
NOM023_scaffold16737_1	unknown	0
NOM023_scaffold11170_3	unknown	0
NOM023_scaffold4816_2	unknown	0
NOM023_scaffold67_25	unknown	0
NOM023_scaffold18365_1	unknown	0
NOM023_scaffold67_27	unknown	0
NOM023_scaffold3447_6	unknown	0
NOM023_scaffold325_2	unknown	0
NOM023_scaffold6200_5	unknown	0
NOM023_scaffold5561_1	unknown	0
NOM025_scaffold1166_3	unknown	0
NOM025_scaffold24674_1	unknown	0
NOM025_scaffold23387_1	unknown	0
NOM025_scaffold2064_3	unknown	0
NOM025_scaffold173_6	unknown	0
NOM025_scaffold3864_1	unknown	0
NOM025_scaffold22402_1	unknown	0
NOM025_scaffold18513_1	unknown	0
NOM025_scaffold2_1	unknown	0
NOM025_scaffold20521_2	unknown	0
NOM025_scaffold62_1	unknown	0

NOM025_scaffold4063_4	unknown	0
NOM025_scaffold7973_1	unknown	0
NOM025_scaffold24504_2	unknown	0
NOM025_scaffold24528_3	unknown	0
NOM025_scaffold57_1	unknown	0
NOM025_scaffold16448_2	unknown	0
NOM025_scaffold24362_1	unknown	0
NOM025_scaffold3455_1	unknown	0
NOM025_scaffold19612_1	unknown	0
NOM025_scaffold3792_5	unknown	0
NOM026_scaffold4045_2	unknown	0
NOM026_scaffold3975_3	unknown	0
NOM026_scaffold29015_25	unknown	0
NOM026_scaffold15750_6	unknown	0
NOM026_scaffold8128_4	unknown	0
NOM026_scaffold29015_22	unknown	0
NOM026_scaffold14437_2	unknown	0
NOM026_scaffold23252_1	unknown	0
NOM026_scaffold788_2	unknown	0
NOM026_scaffold287_6	unknown	0
NOM026_scaffold32579_2	unknown	0
NOM026_scaffold273_8	unknown	0
NOM026_scaffold100_1	unknown	0
NOM026_scaffold2514_2	unknown	0
NOM026_scaffold39163_3	unknown	0
NOM027_scaffold10318_1	unknown	0
NOM027_scaffold3057_13	unknown	0
NOM027_scaffold690_9	unknown	0
NOM027_scaffold233_8	unknown	0
NOM027_scaffold8220_2	unknown	0
NOM027_scaffold8991_1	unknown	0
NOM027_scaffold12848_6	unknown	0
NOM027_scaffold928_6	unknown	0
NOM027_scaffold17819_1	unknown	0
NOM027_scaffold11970_13	unknown	0
NOM027_scaffold3477_2	unknown	0
NOM027_scaffold21806_1	unknown	0
NOM027_scaffold21896_1	unknown	0
NOM027_scaffold18467_1	unknown	0
NOM027_scaffold21932_1	unknown	0
NOM027_scaffold4308_5	unknown	0
NOM027_scaffold8270_1	unknown	0
NOM027_scaffold13632_2	unknown	0
NOM028_scaffold2463_21	unknown	0
NOM028_scaffold6679_33	unknown	0
NOM028_scaffold39054_2	unknown	0

NOM028_scaffold44706_5	unknown	0
NOM028_scaffold10197_9	unknown	0
NOM028_C573817_1	unknown	0
NOM028_scaffold13734_25	unknown	0
NOM028_scaffold37397_2	unknown	0
NOM028_scaffold47465_1	unknown	0
NOM028_scaffold691_1	unknown	0
NOM028_C572983_1	unknown	0
NOM028_scaffold36229_1	unknown	0
NOM028_scaffold36508_1	unknown	0
NOM028_C573171_1	unknown	0
NOM028_scaffold44706_6	unknown	0
NOM028_scaffold47247_1	unknown	0
NOM028_scaffold6524_6	unknown	0
NOM028_scaffold41121_3	unknown	0
NOM028_scaffold19021_1	unknown	0
NOM028_scaffold46746_1	unknown	0
NOM028_scaffold6524_7	unknown	0
NOM028_scaffold46512_4	unknown	0
NOM028_scaffold11058_9	unknown	0
NOM028_scaffold47161_1	unknown	0
NOM028_scaffold10197_6	unknown	0
NOM028_scaffold110_3	unknown	0
NOM028_scaffold2922_6	unknown	0
NOM028_scaffold6524_1	unknown	0
NOM028_scaffold7965_13	unknown	0
NOM028_scaffold6524_2	unknown	0
NOM028_scaffold47589_2	unknown	0
NOM028_scaffold47596_2	unknown	0
NOM028_scaffold6524_14	unknown	0
NOM028_scaffold34458_5	unknown	0
NOM028_scaffold24117_2	unknown	0
NOM029_scaffold279_8	unknown	0
NOM029_scaffold27582_6	unknown	0
NOM029_scaffold36387_1	unknown	0
NOM029_scaffold27582_3	unknown	0
NOM029_scaffold1741_11	unknown	0
NOM029_scaffold1385_6	unknown	0
NOM029_scaffold23053_8	unknown	0
NOM029_scaffold2695_11	unknown	0
NOM029_scaffold6303_4	unknown	0
NOM002_scaffold32093_2	no_family_avaliable(NC_(	0.95
NOM005_C716463_1	no_family_avaliable(NC_(	0.986
NOM005_scaffold5699_2	no_family_avaliable(NC_(	0.987
NOM005_scaffold25180_1	no_family_avaliable(NC_(	0.964
NOM013_scaffold15784_2	no_family_avaliable(NC_(	0.99

NOM013_scaffold31636_3	no_family_avaliable(NC_(	0.97
NOM013_scaffold34728_2	no_family_avaliable(NC_(	0.983
NOM013_scaffold283_1	no_family_avaliable(NC_(	0.988
NOM013_scaffold16346_8	no_family_avaliable(NC_(	0.976
NOM013_scaffold20568_5	no_family_avaliable(NC_(	0.981
NOM013_scaffold31636_2	no_family_avaliable(NC_(	0.957
NOM013_scaffold14898_7	no_family_avaliable(NC_(	0.969
NOM013_scaffold42353_2	no_family_avaliable(NC_(	0.978
NOM014_scaffold24348_5	no_family_avaliable(NC_(	0.973
NOM014_scaffold15996_2	no_family_avaliable(NC_(	0.964
NOM018_scaffold1559_3	no_family_avaliable(NC_(	0.968
NOM018_scaffold43446_1	no_family_avaliable(NC_(	0.952
NOM019_scaffold46040_6	no_family_avaliable(NC_(	0.978
NOM023_scaffold21124_2	no_family_avaliable(NC_(	0.981
NOM025_scaffold14182_5	no_family_avaliable(NC_(	0.965
NOM025_scaffold23752_1	no_family_avaliable(NC_(	0.974
NOM025_scaffold24601_3	no_family_avaliable(NC_(	0.961
NOM026_scaffold18477_1	no_family_avaliable(NC_(	0.97
NOM028_scaffold5766_3	no_family_avaliable(NC_(	0.954
NOM028_scaffold10923_16	no_family_avaliable(NC_(	0.972
NOM028_scaffold5154_1	no_family_avaliable(NC_(	0.978
NOM028_scaffold10515_26	no_family_avaliable(NC_(	0.962
