# Supplementary material for: PhaBOX: a web server for identifying and characterizing phage contigs in metagenomic data
Source: Bioinform Adv. 2023 Aug 2;3(1):vbad101. doi: 10.1093/bioadv/vbad101 (PMC10460485; doi:10.1093/bioadv/vbad101)
Supplement: vbad101_Supplementary_Data [file vbad101_supplementary_data.zip › phatyp_prediction.pdf]

Accession	Pred	Score
DOF002_scaffold24242_3	virulent	0.99987406
DOF002_scaffold67503_1	virulent	0.9997966
DOF002_scaffold60803_3	virulent	0.99981755
DOF002_scaffold67436_2	virulent	0.9884394
DOF002_scaffold63958_7	temperate	0.9998588
DOF002_scaffold11952_4	temperate	0.9998593
DOF002_scaffold64073_3	temperate	0.99985695
DOF002_scaffold64389_1	virulent	0.999844
DOF002_scaffold5829_2	virulent	0.9998688
DOF002_scaffold386_23	virulent	0.99800384
DOF002_scaffold45014_1	temperate	0.9997769
DOF002_scaffold32188_6	temperate	0.979654
DOF002_scaffold5829_3	virulent	0.99987036
DOF002_scaffold65381_4	virulent	0.5441577
DOF002_scaffold67459_1	virulent	0.9998656
DOF002_scaffold2701_13	virulent	0.7488323
DOF002_scaffold67379_1	virulent	0.9741371
DOF002_scaffold60803_2	temperate	0.99979496
DOF002_scaffold67538_1	temperate	0.99980783
DOF002_scaffold55354_2	temperate	0.9969053
DOF002_scaffold4671_2	virulent	0.99987036
DOF002_scaffold66657_1	temperate	0.99985975
DOF002_scaffold10448_2	temperate	0.9931261
DOF002_scaffold11952_2	temperate	0.99985975
DOF002_scaffold38225_1	temperate	0.9998369
DOF002_scaffold65381_2	temperate	0.99985975
DOF002_scaffold58023_2	temperate	0.99920326
DOF002_scaffold60803_1	temperate	0.9998436
DOF002_scaffold133_11	temperate	0.77618104
DOF002_scaffold13072_15	virulent	0.99987125
DOF002_scaffold39963_1	virulent	0.99987406
DOF002_C840249_1	virulent	0.9998688
DOF002_scaffold67459_3	virulent	0.9998622
DOF002_scaffold64032_6	virulent	0.99986744
DOF002_scaffold26075_2	virulent	0.99987316
DOF002_scaffold67426_3	virulent	0.9998622
DOF002_scaffold11952_3	temperate	0.99985975
DOF002_scaffold39963_2	virulent	0.9998574
DOF002_scaffold35792_4	temperate	0.9998403
DOF002_scaffold197_4	temperate	0.9998584
DOF002_scaffold4671_4	virulent	0.99986744
DOF002_scaffold64416_5	temperate	0.9998579
DOF002_C839921_1	temperate	0.9998584
DOF002_scaffold44470_1	virulent	0.98754644
DOF002_scaffold9671_2	temperate	0.9990004

DOF002_scaffold5030_2	temperate	0.99984926
DOF002_scaffold64032_7	virulent	0.99987036
DOF002_scaffold37947_1	temperate	0.99949753
DOF002_scaffold20669_3	virulent	0.9998699
DOF003_scaffold26875_2	temperate	0.99985975
DOF003_scaffold50654_1	virulent	0.60342914
DOF003_scaffold19111_1	virulent	0.9998608
DOF003_scaffold51061_3	virulent	0.7767215
DOF003_scaffold28855_2	virulent	0.9998588
DOF003_scaffold989_9	virulent	0.92362475
DOF003_scaffold27483_2	virulent	0.99980277
DOF003_scaffold269_12	virulent	0.99985266
DOF003_scaffold21246_8	temperate	0.9962567
DOF003_scaffold51115_1	virulent	0.9983913
DOF003_scaffold2741_2	virulent	0.99986404
DOF003_C746192_1	virulent	0.99984694
DOF003_scaffold43780_1	virulent	0.9998617
DOF003_scaffold48262_1	virulent	0.9998388
DOF003_scaffold2826_3	virulent	0.9998636
DOF003_scaffold22191_11	virulent	0.99987125
DOF003_scaffold1689_27	temperate	0.9998474
DOF003_scaffold13183_2	temperate	0.9874106
DOF003_scaffold4746_1	temperate	0.9996882
DOF003_scaffold23234_1	virulent	0.99987084
DOF003_scaffold49885_1	virulent	0.9998665
DOF003_scaffold50947_2	temperate	0.999854
DOF003_scaffold51046_1	virulent	0.7838619
DOF003_scaffold32977_2	temperate	0.99985975
DOF003_scaffold30739_3	temperate	0.99985975
DOF003_scaffold50350_1	temperate	0.977706
DOF003_scaffold28472_4	virulent	0.88889205
DOF003_scaffold989_11	temperate	0.99985695
DOF003_scaffold49069_1	virulent	0.83368194
DOF003_scaffold15485_11	virulent	0.9996705
DOF003_scaffold40480_7	virulent	0.9992909
DOF003_scaffold2826_7	temperate	0.99981356
DOF003_scaffold49885_3	temperate	0.98519784
DOF003_scaffold197_2	virulent	0.99987173
DOF003_scaffold959_2	virulent	0.9998608
DOF003_scaffold51078_6	virulent	0.5876283
DOF003_scaffold51078_1	virulent	0.9998736
DOF003_scaffold9164_2	temperate	0.9998574
DOF003_scaffold22191_13	virulent	0.99985975
DOF003_scaffold51075_5	virulent	0.9998688
DOF003_scaffold47078_1	virulent	0.9998722
DOF003_scaffold22191_10	virulent	0.9998693

DOF003_scaffold19871_4	temperate	0.99983215
DOF003_scaffold1689_28	temperate	0.99985313
DOF003_scaffold50425_1	virulent	0.99960625
DOF003_scaffold49538_2	virulent	0.99987316
DOF003_scaffold19201_1	virulent	0.99987125
DOF003_scaffold28914_12	temperate	0.9998479
DOF003_scaffold36733_1	virulent	0.9746414
DOF003_scaffold22256_6	temperate	0.96187025
DOF003_scaffold27483_3	virulent	0.85946476
DOF003_scaffold35581_4	virulent	0.9997406
DOF003_scaffold26587_4	temperate	0.99604243
DOF004_scaffold31768_1	virulent	0.9718097
DOF004_scaffold41981_3	temperate	0.9998593
DOF004_scaffold42185_4	virulent	0.9998593
DOF004_scaffold56260_1	temperate	0.95989686
DOF004_scaffold22037_1	virulent	0.99984926
DOF004_scaffold16778_14	temperate	0.9662343
DOF004_scaffold54702_1	virulent	0.99987406
DOF004_scaffold27880_1	temperate	0.9998536
DOF004_scaffold37006_1	virulent	0.9274376
DOF004_scaffold10109_6	temperate	0.999094
DOF004_scaffold35687_1	virulent	0.9998722
DOF004_scaffold9090_4	temperate	0.89467365
DOF004_scaffold786_2	virulent	0.9998722
DOF004_scaffold56259_1	temperate	0.99981016
DOF004_scaffold28311_2	virulent	0.65031487
DOF004_scaffold148_2	virulent	0.9998665
DOF004_scaffold19258_3	virulent	0.99708104
DOF004_scaffold27017_1	virulent	0.9610724
DOF004_scaffold21890_2	temperate	0.9413989
DOF004_scaffold57038_1	temperate	0.9998274
DOF004_C632416_1	temperate	0.89653563
DOF004_scaffold38757_1	temperate	0.9998293
DOF004_scaffold49766_4	temperate	0.99986035
DOF004_scaffold7376_1	virulent	0.9997917
DOF004_scaffold56952_1	virulent	0.9998622
DOF004_scaffold25343_1	virulent	0.99935293
DOF004_scaffold5206_5	temperate	0.97040224
DOF004_scaffold24349_1	virulent	0.9998622
DOF004_scaffold57121_1	temperate	0.9998417
DOF004_scaffold26721_2	temperate	0.67429006
DOF004_scaffold34242_1	temperate	0.9998593
DOF004_scaffold45589_1	virulent	0.9998588
DOF006_scaffold711_1	temperate	0.99985975
DOF006_scaffold306_3	virulent	0.99968964
DOF006_scaffold22817_1	temperate	0.99985975

DOF006_scaffold12062_1	virulent	0.99987316
DOF006_scaffold11978_4	temperate	0.98519784
DOF006_C312135_1	temperate	0.99985975
DOF006_scaffold15300_3	temperate	0.99985313
DOF006_scaffold17493_1	temperate	0.99986035
DOF006_scaffold19914_2	temperate	0.9998522
DOF006_scaffold22996_1	temperate	0.9998556
DOF006_scaffold13735_2	virulent	0.99986696
DOF006_scaffold11478_1	virulent	0.9998722
DOF006_scaffold3372_1	virulent	0.99986315
DOF006_scaffold18788_9	virulent	0.99987406
DOF006_scaffold23004_2	temperate	0.9413989
DOF006_scaffold15162_1	virulent	0.99987406
DOF006_scaffold2736_1	temperate	0.978805
DOF006_scaffold20709_1	virulent	0.9998627
DOF006_scaffold306_6	virulent	0.9799923
DOF006_scaffold22685_1	temperate	0.9994771
DOF006_scaffold4966_1	virulent	0.9997132
DOF006_scaffold22663_1	temperate	0.90327543
DOF006_scaffold22952_1	virulent	0.9998688
DOF006_scaffold5845_1	virulent	0.99987125
DOF006_scaffold17357_1	temperate	0.99986035
DOF006_scaffold14584_2	virulent	0.99987173
DOF006_scaffold7307_1	temperate	0.8733236
DOF006_scaffold22952_2	virulent	0.9998656
DOF006_scaffold711_5	temperate	0.9998579
DOF006_scaffold9292_2	virulent	0.99987316
DOF006_scaffold10407_1	virulent	0.55276394
DOF006_C312621_1	virulent	0.99985975
DOF007_scaffold1171_1	virulent	0.99987125
DOF007_scaffold35_3	virulent	0.9998465
DOF007_scaffold17308_3	virulent	0.994713
DOF007_C360895_1	temperate	0.56009233
DOF007_scaffold7833_5	temperate	0.9988239
DOF007_scaffold21463_1	virulent	0.99976164
DOF007_scaffold2644_116	temperate	0.99985975
DOF007_scaffold14864_2	virulent	0.99959624
DOF007_scaffold5443_1	temperate	0.99982077
DOF007_scaffold523_7	virulent	0.9837638
DOF007_scaffold2644_113	temperate	0.9840653
DOF007_scaffold15454_1	temperate	0.99225765
DOF007_scaffold16228_1	virulent	0.99986696
DOF007_scaffold632_7	temperate	0.9997267
DOF007_scaffold16914_2	virulent	0.77566415
DOF007_scaffold1340_5	virulent	0.99957407
DOF007_scaffold2644_118	temperate	0.9869902

DOF007_C359971_1	virulent	0.99987125
DOF007_scaffold4001_1	virulent	0.9997832
DOF007_scaffold12791_4	virulent	0.9998727
DOF007_scaffold1333_2	temperate	0.9664654
DOF008_scaffold28029_2	temperate	0.9998574
DOF008_scaffold567_3	virulent	0.9933288
DOF008_scaffold10567_1	virulent	0.9998699
DOF008_scaffold9506_2	temperate	0.9997978
DOF008_scaffold4011_4	temperate	0.9998574
DOF008_scaffold832_1	virulent	0.9998727
DOF008_scaffold28485_2	temperate	0.9998593
DOF008_scaffold8861_3	temperate	0.9997135
DOF008_scaffold10539_9	virulent	0.9996882
DOF008_scaffold21796_1	temperate	0.9505956
DOF008_scaffold28698_1	virulent	0.9998727
DOF008_scaffold26020_1	virulent	0.9998551
DOF008_scaffold19396_1	temperate	0.99978536
DOF008_scaffold24483_1	temperate	0.9997587
DOF008_scaffold6011_2	virulent	0.99986124
DOF008_scaffold12257_4	temperate	0.99985605
DOF008_scaffold1028_3	temperate	0.93894786
DOF008_scaffold25427_1	temperate	0.99984884
DOF008_scaffold27498_1	temperate	0.99985975
DOF008_scaffold110_2	temperate	0.9998579
DOF008_C423597_1	temperate	0.99721605
DOF008_scaffold2469_10	virulent	0.9998608
DOF008_scaffold19808_7	temperate	0.99985695
DOF008_scaffold29015_1	temperate	0.9909741
DOF008_scaffold12257_5	temperate	0.99984837
DOF008_scaffold5856_4	temperate	0.99692744
DOF008_scaffold28029_1	temperate	0.9998513
DOF008_scaffold16092_4	temperate	0.999836
DOF008_scaffold27793_1	virulent	0.9998693
DOF008_scaffold21540_6	virulent	0.9993408
DOF008_scaffold17006_1	virulent	0.9998693
DOF008_scaffold15173_2	temperate	0.99985975
DOF008_scaffold232_7	temperate	0.99985605
DOF008_C423975_1	virulent	0.9998656
DOF009_scaffold2408_22	virulent	0.87754935
DOF009_scaffold40393_12	virulent	0.9994256
DOF009_scaffold42858_2	virulent	0.99620163
DOF009_scaffold155_13	virulent	0.9998722
DOF009_scaffold52826_1	virulent	0.99987036
DOF009_scaffold52459_1	virulent	0.9927804
DOF009_scaffold39856_9	temperate	0.9998308
DOF009_scaffold43174_9	virulent	0.9983713

DOF009_scaffold22475_18	temperate	0.9998369
DOF009_scaffold47159_4	virulent	0.9994085
DOF009_scaffold46839_2	temperate	0.99883914
DOF009_scaffold3127_2	virulent	0.7399244
DOF009_scaffold52159_2	temperate	0.9997969
DOF009_scaffold42767_3	temperate	0.99978215
DOF009_scaffold16613_1	virulent	0.9703356
DOF009_scaffold36176_2	virulent	0.99977905
DOF009_scaffold31_1	temperate	0.9998379
DOF009_scaffold1_2	temperate	0.9413989
DOF009_scaffold40125_1	temperate	0.9998026
DOF009_scaffold23972_2	temperate	0.98516726
DOF009_scaffold13901_4	temperate	0.9998593
DOF009_scaffold52438_1	virulent	0.99987406
DOF009_scaffold4516_5	virulent	0.99987406
DOF009_scaffold42497_4	virulent	0.99984217
DOF009_scaffold7496_2	virulent	0.99987173
DOF010_scaffold37104_2	temperate	0.9998545
DOF010_scaffold38542_4	temperate	0.9998593
DOF010_scaffold12096_1	virulent	0.9998722
DOF010_scaffold35610_1	virulent	0.9995016
DOF010_scaffold38620_1	virulent	0.9981961
DOF010_scaffold37194_6	virulent	0.99986696
DOF010_scaffold1331_7	temperate	0.998346
DOF010_scaffold31976_2	virulent	0.9998688
DOF010_scaffold45_4	temperate	0.99919474
DOF010_C495273_1	virulent	0.9998699
DOF010_scaffold36_4	temperate	0.635635
DOF010_scaffold39042_3	virulent	0.9997802
DOF010_scaffold13814_2	temperate	0.98312473
DOF010_scaffold36571_1	temperate	0.99985975
DOF010_scaffold37750_5	temperate	0.9997774
DOF010_scaffold65_18	virulent	0.99987316
DOF010_scaffold21978_1	virulent	0.99976045
DOF010_scaffold11833_5	virulent	0.9998727
DOF010_scaffold7607_7	virulent	0.99987036
DOF010_scaffold19015_5	temperate	0.9998556
DOF010_scaffold27096_5	virulent	0.99987173
DOF010_scaffold32482_2	virulent	0.9998736
DOF010_scaffold35610_2	virulent	0.9996372
DOF010_scaffold23207_18	temperate	0.99985975
DOF010_scaffold34_4	virulent	0.9353608
DOF010_scaffold2645_5	temperate	0.99981403
DOF010_scaffold39042_1	virulent	0.6737775
DOF010_scaffold24344_2	virulent	0.99981666
DOF010_scaffold38651_2	virulent	0.99986696

DOF010_scaffold13830_4	virulent	0.99986404
DOF010_scaffold33553_4	virulent	0.99987173
DOF010_scaffold38956_2	temperate	0.99985975
DOF010_scaffold19252_1	temperate	0.99985975
DOF010_scaffold21702_2	temperate	0.9928322
DOF010_scaffold29273_4	temperate	0.9998584
DOF010_scaffold198_1	virulent	0.9998727
DOF010_scaffold38551_4	temperate	0.9998593
DOF010_scaffold24344_3	virulent	0.99986315
DOF010_scaffold31661_1	temperate	0.9998508
DOF010_scaffold35846_1	temperate	0.9998593
DOF010_scaffold37750_4	temperate	0.9888588
DOF010_scaffold39049_2	virulent	0.9998699
DOF010_scaffold4723_13	temperate	0.9998193
DOF010_scaffold31976_1	virulent	0.99986744
DOF011_scaffold21153_1	temperate	0.9995362
DOF011_scaffold21165_1	temperate	0.8584233
DOF011_scaffold21213_1	virulent	0.6638408
DOF011_scaffold77_1	temperate	0.99985975
DOF011_scaffold2635_5	temperate	0.9413989
DOF011_scaffold21174_2	virulent	0.99987084
DOF011_scaffold2386_1	temperate	0.99985975
DOF011_scaffold12507_5	temperate	0.979654
DOF011_scaffold19699_2	temperate	0.99984217
DOF011_scaffold20914_1	temperate	0.998446
DOF012_scaffold1710_3	virulent	0.99987406
DOF012_scaffold12555_2	temperate	0.9998593
DOF012_scaffold463_10	virulent	0.9996708
DOF012_scaffold4720_1	temperate	0.99986035
DOF012_scaffold884_2	temperate	0.9998265
DOF012_scaffold9548_2	virulent	0.9998736
DOF012_scaffold992_3	temperate	0.99857235
DOF012_scaffold9844_1	temperate	0.99985695
DOF012_scaffold2936_3	virulent	0.83869
DOF012_scaffold12334_1	temperate	0.9998431
DOF012_scaffold198_1	temperate	0.9998379
DOF012_scaffold4539_3	temperate	0.99983126
DOF012_scaffold381_2	temperate	0.9998388
DOF012_scaffold3394_7	temperate	0.999854
DOF012_scaffold897_10	temperate	0.99985975
DOF012_scaffold4741_3	virulent	0.9998656
DOF012_scaffold7_12	virulent	0.61507547
DOF012_scaffold6657_1	temperate	0.9998593
DOF012_scaffold884_3	temperate	0.99984556
DOF012_scaffold191_20	temperate	0.99985975
DOF012_scaffold14781_2	virulent	0.86183524

DOF012_scaffold7309_6_1	virulent	0.9998617
DOF012_scaffold14750_1	temperate	0.934572
DOF012_scaffold22_3	virulent	0.9998679
DOF012_scaffold463_9_2	virulent	0.99987173
DOF013_scaffold2527_4	virulent	0.9998684
DOF013_scaffold11760_13	temperate	0.9627462
DOF013_scaffold12980_3	virulent	0.999671
DOF013_scaffold5071_6	temperate	0.9998556
DOF013_scaffold1588_1	virulent	0.82658577
DOF013_scaffold27644_1	temperate	0.9996058
DOF013_scaffold4900_2	virulent	0.9998545
DOF013_scaffold12980_1	temperate	0.9998545
DOF013_scaffold783_2	virulent	0.99987036
DOF013_scaffold13491_2	virulent	0.9993214
DOF013_scaffold443_2	temperate	0.997606
DOF013_scaffold15877_2	virulent	0.99987406
DOF013_scaffold2699_1	virulent	0.9998636
DOF013_scaffold4683_2	virulent	0.99978924
DOF013_scaffold103_6	temperate	0.999854
DOF013_scaffold392_2	virulent	0.99918664
DOF013_scaffold5240_2	virulent	0.9998727
DOF013_scaffold379_1	virulent	0.99985266
DOF013_scaffold5240_9	virulent	0.9998645
DOF013_scaffold21294_6	temperate	0.98519784
DOF013_scaffold29915_1	temperate	0.9998593
DOF014_scaffold15073_8	temperate	0.9998579
DOF014_scaffold49276_4	temperate	0.99985975
DOF014_scaffold46901_1	virulent	0.99987125
DOF014_scaffold10772_1	temperate	0.9998413
DOF014_scaffold49726_2	temperate	0.99980927
DOF014_scaffold30923_2	virulent	0.99986404
DOF014_C612810_1	temperate	0.9701769
DOF014_scaffold101_2	virulent	0.9998608
DOF014_scaffold32658_1	temperate	0.9987996
DOF014_scaffold7189_8	virulent	0.99944603
DOF014_scaffold49340_4	virulent	0.999671
DOF014_scaffold29718_2	virulent	0.9998536
DOF014_scaffold2821_4	virulent	0.99987173
DOF014_scaffold49687_1	virulent	0.9998722
DOF014_scaffold1808_5	temperate	0.99985975
DOF014_scaffold49604_1	temperate	0.99934953
DOF014_scaffold90_1	temperate	0.9998588
DOF014_scaffold49645_1	virulent	0.99987036
DOF014_scaffold49556_1	temperate	0.99982125
DOF014_scaffold49552_1	temperate	0.9995119
DOF014_scaffold1_3	temperate	0.93894786

DOF014_scaffold34813_4	temperate	0.979654
DOF014_scaffold49340_5	temperate	0.99984926
DOF014_scaffold13006_1	temperate	0.9585484
DOF014_C612698_1	virulent	0.9998684
DOF014_scaffold82_3	virulent	0.99966824
DOF014_scaffold15208_5	temperate	0.99985605
NOF001_scaffold2654_4	virulent	0.9997247
NOF001_scaffold14_4	virulent	0.99987406
NOF001_scaffold58201_2	virulent	0.9998679
NOF001_scaffold17586_2	temperate	0.9942528
NOF001_scaffold1053_2	temperate	0.8075163
NOF001_scaffold58318_1	temperate	0.99970055
NOF001_scaffold5563_1	virulent	0.9998556
NOF001_scaffold33721_2	temperate	0.99985975
NOF001_scaffold48_1	virulent	0.99935246
NOF001_scaffold47705_1	temperate	0.9962567
NOF001_scaffold56764_1	temperate	0.9998593
NOF001_scaffold13046_9	temperate	0.99986035
NOF001_scaffold53059_1	temperate	0.9976891
NOF001_scaffold58052_2	virulent	0.6183175
NOF001_scaffold5343_8	temperate	0.979004
NOF001_scaffold58235_1	virulent	0.9998679
NOF001_scaffold57995_2	temperate	0.99980307
NOF001_scaffold11560_1	temperate	0.99985313
NOF001_scaffold27372_1	virulent	0.9998574
NOF001_scaffold41855_2	temperate	0.999826
NOF001_scaffold24609_2	virulent	0.99987084
NOF001_scaffold41855_6	virulent	0.57410604
NOF001_scaffold29077_4	virulent	0.9986517
NOF001_scaffold13046_6	temperate	0.9998431
NOF001_scaffold58316_1	virulent	0.99987084
NOF001_scaffold7196_11	virulent	0.9994256
NOF001_scaffold41855_1	temperate	0.9998593
NOF001_scaffold2819_5	virulent	0.9998722
NOF001_scaffold24013_1	temperate	0.99144334
NOF001_scaffold58081_1	virulent	0.9998608
NOF001_scaffold56157_1	temperate	0.98633206
NOF001_scaffold58201_1	virulent	0.99987036
NOF001_scaffold55226_1	temperate	0.9998545
NOF001_scaffold50021_1	virulent	0.99876857
NOF001_scaffold49296_1	virulent	0.9998049
NOF001_scaffold15730_5	virulent	0.99987036
NOF001_scaffold58099_2	virulent	0.99987036
NOF002_scaffold7_6	temperate	0.9998579
NOF002_scaffold13379_1	temperate	0.99985975
NOF002_scaffold4501_18	temperate	0.99976397

NOF002_scaffold30763_2	temperate	0.99981403
NOF002_scaffold29466_1	virulent	0.9998071
NOF002_scaffold5730_7	temperate	0.9998236
NOF002_scaffold3476_12	virulent	0.99987316
NOF002_scaffold31199_2	virulent	0.9738244
NOF002_C425969_1	temperate	0.99966824
NOF002_scaffold31424_1	temperate	0.9998193
NOF002_scaffold12961_13	temperate	0.9998574
NOF002_scaffold20732_1	virulent	0.99986607
NOF002_scaffold31554_1	temperate	0.9998551
NOF002_scaffold3233_2	temperate	0.99885607
NOF002_scaffold6749_14	virulent	0.9900106
NOF002_scaffold31199_1	temperate	0.9529658
NOF002_scaffold11620_5	virulent	0.9788498
NOF002_scaffold9460_1	virulent	0.9675024
NOF002_scaffold31188_5	temperate	0.979654
NOF002_scaffold1584_5	temperate	0.999774
NOF002_scaffold31615_1	virulent	0.9998436
NOF002_scaffold3233_5	temperate	0.99986035
NOF002_scaffold7_7	temperate	0.99984884
NOF002_scaffold30763_1	temperate	0.99985605
NOF002_scaffold27207_5	temperate	0.9998513
NOF002_scaffold2745_2	temperate	0.9998593
NOF002_scaffold2745_3	temperate	0.9998565
NOF002_scaffold23022_4	temperate	0.997794
NOF002_scaffold31584_1	virulent	0.9996782
NOF004_scaffold5431_15	virulent	0.95118916
NOF004_scaffold25441_2	virulent	0.943157
NOF004_scaffold5774_1	virulent	0.99969965
NOF004_scaffold4703_4	virulent	0.99984694
NOF004_scaffold19766_4	virulent	0.9935199
NOF004_scaffold32796_1	virulent	0.99987406
NOF004_scaffold30766_1	virulent	0.9996992
NOF004_scaffold4675_2	temperate	0.979654
NOF004_scaffold36774_1	virulent	0.99982095
NOF004_scaffold37814_4	virulent	0.99930334
NOF004_scaffold39800_2	virulent	0.9998688
NOF004_scaffold12813_30	temperate	0.99969715
NOF004_C576082_1	virulent	0.99977165
NOF004_scaffold22864_4	virulent	0.99987173
NOF004_scaffold12390_5	virulent	0.99985695
NOF004_scaffold18950_2	virulent	0.9994799
NOF004_scaffold4703_1	virulent	0.9996036
NOF004_scaffold22559_2	virulent	0.99987036
NOF004_scaffold5741_5	virulent	0.71908617
NOF004_scaffold34095_2	temperate	0.98669714

NOF004_scaffold29834_1	temperate	0.9998588
NOF004_scaffold10567_2	virulent	0.9998693
NOF004_scaffold1680_17	virulent	0.9998565
NOF004_scaffold30605_6	virulent	0.9949012
NOF004_scaffold39867_3	virulent	0.99987173
NOF004_scaffold2367_14	temperate	0.99985033
NOF004_scaffold8521_5	virulent	0.9998336
NOF004_scaffold8945_3	virulent	0.9996477
NOF004_scaffold24813_3	virulent	0.99985695
NOF004_scaffold15266_9	virulent	0.9996546
NOF004_scaffold39800_1	virulent	0.9998684
NOF004_C575620_1	temperate	0.99985975
NOF004_scaffold89_3	temperate	0.9413989
NOF004_scaffold31703_2	temperate	0.99951804
NOF004_scaffold26987_2	temperate	0.9998374
NOF004_scaffold58_6	virulent	0.9991748
NOF004_scaffold14653_3	temperate	0.69600296
NOF004_scaffold3847_7	virulent	0.99390656
NOF004_scaffold10069_19	virulent	0.99987406
NOF004_scaffold4193_2	temperate	0.99566615
NOF004_scaffold12390_3	temperate	0.91165656
NOF004_scaffold9875_5	virulent	0.8912748
NOF004_scaffold39611_1	temperate	0.99985975
NOF004_scaffold28322_1	temperate	0.99983555
NOF004_scaffold6978_23	temperate	0.9986471
NOF004_scaffold9355_12	temperate	0.9998593
NOF004_scaffold12025_2	temperate	0.7850754
NOF004_scaffold14211_1	temperate	0.99982214
NOF004_scaffold9994_2	virulent	0.9998693
NOF004_scaffold2098_11	temperate	0.61039174
NOF005_scaffold35291_5	virulent	0.9998699
NOF005_scaffold23396_3	virulent	0.9998622
NOF005_scaffold36104_5	temperate	0.7885992
NOF005_scaffold18_1	temperate	0.99985975
NOF005_scaffold25742_6	virulent	0.9998345
NOF005_scaffold31594_11	temperate	0.9994942
NOF005_scaffold6888_3	temperate	0.974219
NOF005_scaffold29221_10	temperate	0.99985975
NOF005_scaffold34324_4	temperate	0.979654
NOF005_scaffold1086_25	temperate	0.99985695
NOF005_scaffold604_14	virulent	0.9998699
NOF005_scaffold44749_6	virulent	0.99987125
NOF005_scaffold51717_1	virulent	0.9998688
NOF005_scaffold35393_5	temperate	0.99985975
NOF005_scaffold34950_3	temperate	0.99985975
NOF005_scaffold51596_4	temperate	0.99985975

NOF005_scaffold29140_2	virulent	0.9998293
NOF005_scaffold37734_2	temperate	0.99934417
NOF005_scaffold165_4	virulent	0.9998413
NOF005_scaffold2170_3	virulent	0.9998736
NOF005_scaffold24910_8	temperate	0.99986035
NOF005_scaffold48547_9	virulent	0.99987173
NOF005_scaffold604_12	virulent	0.99915826
NOF005_scaffold52365_1	virulent	0.9998588
NOF005_scaffold23396_2	temperate	0.9925314
NOF005_scaffold3465_6	virulent	0.7216388
NOF005_scaffold604_3	temperate	0.9998593
NOF005_scaffold52405_2	temperate	0.9998593
NOF005_scaffold49499_1_1	virulent	0.6071846
NOF005_scaffold49499_1_2	virulent	0.9998736
NOF005_scaffold15268_1	temperate	0.63226247
NOF005_scaffold39972_17	virulent	0.9998736
NOF005_scaffold27541_5	virulent	0.9998131
NOF005_scaffold27426_3	virulent	0.9998727
NOF005_scaffold5924_1	virulent	0.99986315
NOF005_scaffold32384_3	virulent	0.99987316
NOF005_scaffold44821_1	temperate	0.9998551
NOF005_scaffold27198_2	virulent	0.9998684
NOF005_scaffold13462_4	virulent	0.9998608
NOF005_scaffold2025_2	temperate	0.9930787
NOF005_scaffold52106_1	virulent	0.99980336
NOF005_scaffold604_13	virulent	0.9962401
NOF006_scaffold10363_1	temperate	0.99976826
NOF006_scaffold15833_1	virulent	0.9998617
NOF006_scaffold6111_7	virulent	0.9449081
NOF006_scaffold4868_1	temperate	0.93910414
NOF006_scaffold21352_1	virulent	0.9998617
NOF006_scaffold12718_7	virulent	0.9998722
NOF006_scaffold498_1	temperate	0.9998579
NOF006_scaffold21223_2	temperate	0.9998536
NOF006_scaffold121_1	virulent	0.99987316
NOF006_scaffold21242_4	temperate	0.979654
NOF006_scaffold1740_2	virulent	0.9998608
NOF006_scaffold2842_16	virulent	0.9998722
NOF006_scaffold19983_2	virulent	0.9998736
NOF006_scaffold14475_3	virulent	0.90332973
NOF006_scaffold1022_7	virulent	0.99986744
NOF006_scaffold14499_8	virulent	0.99979323
NOF006_scaffold21012_1	virulent	0.908313
NOF006_scaffold4067_5	temperate	0.9627462
NOF006_scaffold20915_1	temperate	0.99980694
NOF006_scaffold4872_3	virulent	0.998128

NOF006_scaffold969_8	virulent	0.64794564
NOF006_C401108_1	virulent	0.9998693
NOF006_scaffold21374_2	temperate	0.9735116
NOF006_scaffold20868_2	virulent	0.9998693
NOF006_scaffold173_3	virulent	0.99987465
NOF007_scaffold27213_3	virulent	0.99987125
NOF007_scaffold16773_3	temperate	0.93894786
NOF007_scaffold23774_3	temperate	0.99871093
NOF007_scaffold4461_7	temperate	0.9998479
NOF007_scaffold28685_4	temperate	0.9998369
NOF007_scaffold396_8	temperate	0.9832063
NOF007_scaffold26160_1	virulent	0.97327614
NOF007_scaffold11484_1	virulent	0.9998693
NOF007_scaffold25262_5	virulent	0.6879927
NOF007_scaffold21388_2	temperate	0.908581
NOF007_scaffold1349_3	virulent	0.6408339
NOF007_C490687_1	virulent	0.9998722
NOF007_scaffold19207_2	virulent	0.9998736
NOF007_scaffold1349_2	temperate	0.9969053
NOF007_scaffold8821_2	virulent	0.99980664
NOF007_scaffold27822_1	virulent	0.99942935
NOF007_scaffold15189_2	virulent	0.9998679
NOF007_scaffold23819_3	temperate	0.98519784
NOF007_scaffold26938_2	temperate	0.9998565
NOF007_scaffold7559_3	virulent	0.9998545
NOF007_scaffold16388_6	temperate	0.9998417
NOF007_scaffold24483_2	virulent	0.9998627
NOF007_scaffold10524_1	temperate	0.99383783
NOF007_scaffold41_1	virulent	0.99987084
NOF007_scaffold298_7	temperate	0.9998584
NOF007_scaffold505_1	virulent	0.99987316
NOF007_scaffold28801_1	temperate	0.9998574
NOF007_scaffold28685_3	temperate	0.6311137
NOF007_scaffold25304_2	temperate	0.99769664
NOF007_scaffold22039_3	virulent	0.99986744
NOF007_scaffold26224_1	virulent	0.9998727
NOF007_scaffold28624_3	virulent	0.9998736
NOF007_scaffold3393_7	temperate	0.99985605
NOF007_scaffold1195_9	temperate	0.83707535
NOF007_scaffold2822_2	virulent	0.99987036
NOF007_scaffold23774_2	temperate	0.9997167
NOF007_scaffold12899_1	virulent	0.9998645
NOF007_scaffold9468_5	temperate	0.99984837
NOF008_scaffold34632_3	virulent	0.99987084
NOF008_scaffold68230_1	virulent	0.99928766
NOF008_scaffold25367_2	virulent	0.99956065

NOF008_scaffold64397_1	virulent	0.99979264
NOF008_scaffold48782_2	virulent	0.96857816
NOF008_scaffold5134_4	temperate	0.99985975
NOF008_scaffold61643_2	virulent	0.99692863
NOF008_scaffold1288_26	virulent	0.99982095
NOF008_scaffold66085_2	virulent	0.99986035
NOF008_scaffold11140_1	virulent	0.99983215
NOF008_scaffold5217_6	temperate	0.9998565
NOF008_scaffold1389_2	virulent	0.99987173
NOF008_scaffold34632_4	virulent	0.99987084
NOF008_scaffold67470_2	virulent	0.99987173
NOF008_scaffold28772_6	virulent	0.9998727
NOF008_scaffold10035_9	temperate	0.9998588
NOF008_scaffold30912_6	temperate	0.9998545
NOF008_scaffold66613_1	temperate	0.99985975
NOF008_scaffold48283_1	temperate	0.99958426
NOF008_scaffold31488_6	temperate	0.9998593
NOF008_scaffold68147_1	temperate	0.9998556
NOF008_scaffold8714_2	virulent	0.99982476
NOF008_scaffold5616_1	virulent	0.99987125
NOF008_scaffold68130_1	virulent	0.9998679
NOF008_scaffold25447_1	virulent	0.99973255
NOF008_scaffold3062_3	virulent	0.99964243
NOF008_scaffold7421_1	temperate	0.97783464
NOF008_scaffold52435_2	temperate	0.9972386
NOF008_scaffold35076_1	virulent	0.9998688
NOF008_scaffold58140_1	temperate	0.9925541
NOF008_scaffold53677_1	virulent	0.99987465
NOF008_scaffold68203_3	virulent	0.9998445
NOF008_scaffold36171_4	temperate	0.9413989
NOF008_scaffold68191_1	temperate	0.999854
NOF008_scaffold65986_1	temperate	0.99985975
NOF008_scaffold63722_1	temperate	0.7244286
NOF008_scaffold50828_1	virulent	0.99982554
NOF008_scaffold41113_4	virulent	0.99987173
NOF008_scaffold59760_7	virulent	0.9903983
NOF008_scaffold42043_1	temperate	0.9998593
NOF008_scaffold8066_2	temperate	0.9998584
NOF008_scaffold28625_3	virulent	0.9998684
NOF008_scaffold19089_7	temperate	0.9998403
NOF008_scaffold3708_20	temperate	0.99970675
NOF008_scaffold68224_1	temperate	0.9995376
NOF008_scaffold52198_1	virulent	0.9998574
NOF008_scaffold7904_8	temperate	0.99986035
NOF008_scaffold25220_3	virulent	0.98565406
NOF008_C865299_1	virulent	0.99987125

NOF008_scaffold17689_13	virulent	0.99981016
NOF008_C865863_1	virulent	0.96842474
NOF008_scaffold68199_1	virulent	0.99987125
NOF008_scaffold55525_8	virulent	0.9998722
NOF008_scaffold13444_2	temperate	0.9998431
NOF008_scaffold55811_1	virulent	0.9402247
NOF008_scaffold29486_12	virulent	0.99986124
NOF009_scaffold7527_22	virulent	0.99986404
NOF009_scaffold43518_2	temperate	0.99985605
NOF009_scaffold21768_4	virulent	0.9998693
NOF009_scaffold41727_1	virulent	0.9994311
NOF009_scaffold10148_1	temperate	0.99978215
NOF009_scaffold19865_4	temperate	0.99986035
NOF009_C558283_1	virulent	0.9998636
NOF009_scaffold2064_12	temperate	0.8072752
NOF009_scaffold34567_3	temperate	0.9977161
NOF009_scaffold11228_12	temperate	0.9401553
NOF009_scaffold16960_2	temperate	0.99685895
NOF009_scaffold14075_3	virulent	0.997076
NOF009_scaffold67_1	temperate	0.99984604
NOF009_C558255_1	virulent	0.99984217
NOF009_scaffold13328_11	temperate	0.9998556
NOF009_scaffold42455_5	temperate	0.9995705
NOF009_scaffold2720_3	temperate	0.7528099
NOF009_scaffold41979_1	virulent	0.6331934
NOF009_scaffold30580_1	virulent	0.66290087
NOF009_scaffold5136_16	virulent	0.9998722
NOF009_scaffold42878_5	temperate	0.9998388
NOF009_scaffold42496_4	temperate	0.979654
NOF010_C699885_1	temperate	0.9998579
NOF010_scaffold33789_4	virulent	0.9958235
NOF010_scaffold32319_4	virulent	0.99987125
NOF010_scaffold26425_6	temperate	0.5711277
NOF010_scaffold14373_1	virulent	0.99987125
NOF010_scaffold28531_1	virulent	0.9998588
NOF010_scaffold51712_2	temperate	0.99969196
NOF010_scaffold2330_4	virulent	0.9331835
NOF010_scaffold6485_5	virulent	0.9619549
NOF010_scaffold16598_11	virulent	0.99985695
NOF010_scaffold20182_2	virulent	0.99987406
NOF010_scaffold2722_9	virulent	0.99987316
NOF010_scaffold22545_1	virulent	0.98227835
NOF010_scaffold20459_4	virulent	0.99987406
NOF010_scaffold9563_25	virulent	0.99987406
NOF010_scaffold53722_1	virulent	0.99981785
NOF010_scaffold46583_3	temperate	0.9941079

NOF010_scaffold6369_4	virulent	0.9949243
NOF010_scaffold22545_2	temperate	0.99984926
NOF010_scaffold4642_2	virulent	0.9998417
NOF010_scaffold14618_2	temperate	0.99965626
NOF010_scaffold53516_6	temperate	0.9998579
NOF010_scaffold43806_2	temperate	0.9998579
NOF010_scaffold21111_3	virulent	0.9998679
NOF010_scaffold27134_3	virulent	0.9996987
NOF010_scaffold22950_5	temperate	0.9918041
NOF010_scaffold54242_1	virulent	0.9998688
NOF010_scaffold54124_1	virulent	0.9888191
NOF010_C699039_1	virulent	0.99987084
NOF010_scaffold23028_3	virulent	0.9998684
NOF010_scaffold33103_1	temperate	0.99968624
NOF010_scaffold5911_1	virulent	0.99695677
NOF010_scaffold54281_1	temperate	0.99978167
NOF010_scaffold11305_3	temperate	0.99985975
NOF010_scaffold21018_1	temperate	0.9998579
NOF011_scaffold3878_1	virulent	0.9053317
NOF011_scaffold57558_3	temperate	0.9998336
NOF011_scaffold159_2	virulent	0.99987465
NOF011_scaffold56176_3	temperate	0.99985695
NOF011_scaffold18490_3	virulent	0.99986696
NOF011_scaffold59088_2	virulent	0.99987316
NOF011_scaffold8028_19	temperate	0.9998588
NOF011_scaffold33258_2	virulent	0.9998114
NOF011_scaffold58584_1	virulent	0.9998727
NOF011_scaffold70_15	virulent	0.99987084
NOF011_scaffold58981_1	virulent	0.9996613
NOF011_scaffold59056_1	virulent	0.99984217
NOF011_scaffold59100_2	temperate	0.7812632
NOF011_scaffold27189_1	virulent	0.9996181
NOF011_scaffold57546_2	temperate	0.99984837
NOF011_scaffold59096_2	virulent	0.9171376
NOF011_scaffold15798_1	virulent	0.99987465
NOF011_C736712_1	virulent	0.9995259
NOF011_scaffold48802_1	virulent	0.9998727
NOF011_scaffold57126_1	virulent	0.9998727
NOF011_scaffold52600_1	virulent	0.9532104
NOF011_scaffold56176_2	virulent	0.84088755
NOF011_scaffold8028_20	temperate	0.9998584
NOF011_scaffold2529_7	temperate	0.78272307
NOF011_scaffold45498_1	temperate	0.999774
NOF011_scaffold40128_2	temperate	0.99894154
NOF012_scaffold77459_1	virulent	0.56465566
NOF012_scaffold71024_7	temperate	0.9998188

NOF012_scaffold77267_2	temperate	0.9998574
NOF012_scaffold24523_4	temperate	0.9994951
NOF012_scaffold12244_5	virulent	0.99973226
NOF012_scaffold50466_2	virulent	0.5916771
NOF012_scaffold5639_4	temperate	0.90100694
NOF012_scaffold15647_4	virulent	0.9998727
NOF012_scaffold8867_2	virulent	0.99978536
NOF012_scaffold13996_7	temperate	0.99985975
NOF012_scaffold77917_2	virulent	0.9998131
NOF012_scaffold12244_6	temperate	0.99985605
NOF012_scaffold44774_1	temperate	0.9998302
NOF012_scaffold3081_2	temperate	0.9076956
NOF012_scaffold78059_2	temperate	0.9413989
NOF012_scaffold34517_3	temperate	0.7887559
NOF012_scaffold5639_7	temperate	0.99948466
NOF012_scaffold24554_12	virulent	0.99695736
NOF012_scaffold4667_6	temperate	0.979654
NOF012_scaffold72024_3	temperate	0.9998513
NOF012_scaffold19855_2	temperate	0.9998593
NOF012_scaffold34940_12	virulent	0.9994256
NOF012_scaffold34517_1	virulent	0.99987036
NOF012_scaffold8693_10	temperate	0.9997774
NOF012_scaffold34517_2	virulent	0.9998202
NOF012_scaffold77271_2	virulent	0.99987316
NOF012_scaffold71857_2	temperate	0.9980795
NOF012_scaffold74308_1	virulent	0.99602866
NOF012_C833949_1	virulent	0.99987173
NOF012_scaffold15991_1	virulent	0.9998608
NOF012_scaffold71874_7	virulent	0.99987406
NOF012_scaffold339_7	virulent	0.99985033
NOF012_scaffold28415_3	virulent	0.99987316
NOF012_scaffold9650_7	virulent	0.9998656
NOF012_scaffold77196_1	virulent	0.8036844
NOF012_scaffold37026_8	temperate	0.88961107
NOF012_scaffold68565_1	temperate	0.99178636
NOF012_scaffold26305_22	temperate	0.9876869
NOF012_scaffold78081_1	virulent	0.99987465
NOF012_scaffold43523_1	virulent	0.6497936
NOF012_scaffold41858_11	temperate	0.99985975
NOF012_scaffold28114_12	virulent	0.9972729
NOF013_scaffold22999_4	virulent	0.99986124
NOF013_scaffold33526_1	virulent	0.99986696
NOF013_scaffold15363_3	temperate	0.999713
NOF013_scaffold9359_1	virulent	0.9972341
NOF013_scaffold24304_2	temperate	0.99964106
NOF013_scaffold11906_1	temperate	0.95266074

NOF013_scaffold26437_1	virulent	0.9997869
NOF013_scaffold20067_2	virulent	0.99987316
NOF013_scaffold25505_1	virulent	0.908313
NOF013_scaffold34730_1	temperate	0.9998579
NOF013_scaffold6283_17	virulent	0.9998722
NOF013_scaffold30635_1	virulent	0.99987406
NOF013_scaffold9607_2	temperate	0.99984837
NOF013_scaffold34528_1	virulent	0.99987173
NOF013_C612756_1	virulent	0.9998684
NOF013_scaffold3348_11	temperate	0.99971104
NOF013_scaffold24565_7	virulent	0.99986315
NOF013_scaffold34630_3	virulent	0.9998688
NOF013_scaffold24565_6	temperate	0.9997158
NOF013_scaffold26437_2	temperate	0.9998374
NOF013_scaffold4750_1	virulent	0.99987036
NOF013_scaffold316_7	virulent	0.9998688
NOF013_scaffold28493_2	virulent	0.99818397
NOF013_scaffold22999_3	virulent	0.99955404
NOF013_scaffold32172_3	temperate	0.9949657
NOF013_scaffold15363_2	virulent	0.99987084
NOF013_scaffold23510_11	virulent	0.99543303
NOF013_scaffold34382_1	temperate	0.99655056
NOF013_C613108_1	virulent	0.99987173
NOF013_scaffold1181_6	virulent	0.99987173
NOF013_scaffold131_1	virulent	0.99987173
NOF013_C612464_1	virulent	0.9998727
NOF013_scaffold33363_1	temperate	0.9763216
NOF013_scaffold33134_1	temperate	0.99985033
NOF013_scaffold1744_3	virulent	0.99982476
NOF013_scaffold32891_4	virulent	0.99829245
NOF013_scaffold29173_1	virulent	0.99987036
NOF013_scaffold24443_1	virulent	0.9310343
NOF013_scaffold3979_5	temperate	0.93894786
NOF013_scaffold34324_3	virulent	0.9750617
NOF013_scaffold2241_2	virulent	0.9998445
NOF014_scaffold67337_2	temperate	0.9998588
NOF014_scaffold29077_2	temperate	0.99985975
NOF014_scaffold41735_1	temperate	0.9998588
NOF014_scaffold59979_2	virulent	0.9998665
NOF014_scaffold72132_1	virulent	0.996157
NOF014_scaffold30730_5	virulent	0.99960977
NOF014_scaffold72006_1	temperate	0.99986035
NOF014_scaffold6730_5	temperate	0.99985975
NOF014_scaffold71626_3	virulent	0.99986124
NOF014_scaffold46167_2	temperate	0.9988944
NOF014_scaffold35720_11	virulent	0.9998656

NOF014_scaffold4327_52	temperate	0.9998593
NOF014_scaffold40347_4	virulent	0.99981046
NOF014_scaffold72227_1	virulent	0.99910355
NOF014_scaffold27769_8	temperate	0.9998202
NOF014_scaffold10135_5	temperate	0.99986035
NOF014_scaffold66855_1	temperate	0.99986035
NOF014_scaffold72263_3	temperate	0.99985975
NOF014_scaffold43_10	virulent	0.99987316
NOF014_scaffold26105_6	temperate	0.9998584
NOF014_scaffold20787_3	temperate	0.999541
NOF014_scaffold19962_3	virulent	0.9996329
NOF014_scaffold63476_2	temperate	0.99984694
NOF014_scaffold26105_25	temperate	0.99985975
NOF014_scaffold72216_1	virulent	0.9856357
NOF014_scaffold10667_4	temperate	0.9998593
NOF014_scaffold52663_4	virulent	0.9998693
NOF014_scaffold71919_3	virulent	0.9998684
NOF014_scaffold20265_3	virulent	0.9998388
NOF014_scaffold5894_2	virulent	0.9994251
NOF014_scaffold49593_2	temperate	0.9998593
NOF014_scaffold71877_1	temperate	0.9996805
NOF014_scaffold72248_1	virulent	0.9998727
NOF014_scaffold20787_1	virulent	0.99969965
NOF014_scaffold66923_1	virulent	0.99987406
NOF014_scaffold71552_2	virulent	0.9998693
NOF014_scaffold2390_2	temperate	0.93755734
NOF014_scaffold71919_2	virulent	0.9998684
NOF014_scaffold66767_1	virulent	0.9998556
NOF014_scaffold63571_1	temperate	0.99926966
NOF014_scaffold65085_3	temperate	0.99984264
NOF014_scaffold22954_1	virulent	0.9998693
NOF014_scaffold72295_2	temperate	0.99985975
NOF014_scaffold71714_2	temperate	0.9998565
NOF014_scaffold64087_5	virulent	0.99983406
NOF014_scaffold71856_1	virulent	0.9998684
NOF014_scaffold59979_3	virulent	0.9998722
NOF014_scaffold52663_2	virulent	0.99987316
NOF014_scaffold54136_28	virulent	0.87657833
DLF001_scaffold25843_5	temperate	0.9555723
DLF001_scaffold6028_2	temperate	0.9998365
DLF001_scaffold16878_1	virulent	0.9995294
DLF001_scaffold25777_2	virulent	0.9970052
DLF001_scaffold4836_9	virulent	0.9996882
DLF001_scaffold16608_1	temperate	0.99985975
DLF001_scaffold18617_11	virulent	0.89395195
DLF001_scaffold24376_1	virulent	0.9998636

DLF001_scaffold1570_1	virulent	0.99267465
DLF001_scaffold20939_1	temperate	0.95052063
DLF001_scaffold10899_1	temperate	0.9998417
DLF001_scaffold10872_1	temperate	0.99985975
DLF001_scaffold25652_2	virulent	0.927816
DLF001_scaffold21798_2	virulent	0.9171376
DLF001_C323280_1	virulent	0.9997661
DLF001_scaffold10220_3	virulent	0.999478
DLF001_C323242_1	temperate	0.97153795
DLF001_scaffold13_12	virulent	0.89925516
DLF001_scaffold25336_2	temperate	0.99984837
DLF001_scaffold46_6	virulent	0.9998736
DLF001_scaffold80_8	virulent	0.98799294
DLF001_scaffold6028_1	virulent	0.99986607
DLF001_C323900_1	virulent	0.9998574
DLF002_scaffold6624_21	virulent	0.99985695
DLF002_scaffold7298_22	virulent	0.99889344
DLF002_scaffold33093_1	virulent	0.87593126
DLF002_scaffold36414_1	temperate	0.99154705
DLF002_scaffold85_3	temperate	0.93894786
DLF002_scaffold28742_1	virulent	0.9746414
DLF002_scaffold22416_1	virulent	0.9048829
DLF002_scaffold32378_2	temperate	0.98519784
DLF002_scaffold19600_3	temperate	0.96093565
DLF002_scaffold24316_12	virulent	0.9998684
DLF002_scaffold36081_2	temperate	0.8272758
DLF002_scaffold294_32	virulent	0.99138457
DLF002_scaffold7525_1	virulent	0.9996882
DLF002_scaffold25546_1	virulent	0.99987406
DLF002_scaffold138_8	temperate	0.998298
DLF002_scaffold33974_1	temperate	0.9988901
DLF002_scaffold10343_2	virulent	0.984814
DLF002_scaffold36505_1	temperate	0.8868689
DLF002_scaffold2466_1	temperate	0.9998593
DLF002_scaffold36298_1	virulent	0.9870865
DLF002_scaffold6041_3	virulent	0.86695606
DLF002_scaffold21111_2	temperate	0.9998556
DLF002_C488685_1	virulent	0.9998699
DLF003_scaffold21732_2	temperate	0.99985266
DLF003_scaffold29891_1	virulent	0.9998684
DLF003_scaffold23409_2	virulent	0.9998357
DLF003_scaffold50567_2	temperate	0.9867483
DLF003_scaffold44436_1	virulent	0.9998271
DLF003_scaffold12321_2	virulent	0.99986696
DLF003_C717736_1	virulent	0.9998622
DLF003_scaffold137_1	virulent	0.99985605

DLF003_scaffold3834_23	virulent	0.9997367
DLF003_scaffold55800_1	virulent	0.99987084
DLF003_C717640_1	virulent	0.9998417
DLF003_scaffold23403_10	temperate	0.9940969
DLF003_scaffold56673_2	temperate	0.9994546
DLF003_scaffold54149_1	virulent	0.9980126
DLF003_scaffold48199_1	virulent	0.9619549
DLF003_scaffold43976_1	temperate	0.99931246
DLF003_scaffold3834_7	temperate	0.9998593
DLF003_scaffold47905_1	virulent	0.9997144
DLF003_scaffold56478_1	virulent	0.99987084
DLF003_scaffold57190_2	virulent	0.9998722
DLF003_scaffold56159_4	virulent	0.99964076
DLF003_scaffold4398_37	virulent	0.99987084
DLF003_scaffold29891_3	virulent	0.96373665
DLF004_scaffold35797_1	virulent	0.9998645
DLF004_scaffold36527_1	virulent	0.9998722
DLF004_C548546_1	virulent	0.99986696
DLF004_scaffold18383_6	temperate	0.99978584
DLF004_C548576_1	temperate	0.9998403
DLF004_scaffold23942_9	temperate	0.9998593
DLF004_scaffold10854_4	virulent	0.82519925
DLF004_scaffold28880_1	virulent	0.9791901
DLF004_scaffold25533_1	temperate	0.98823017
DLF004_scaffold36320_8	virulent	0.99987125
DLF004_C548200_1	temperate	0.9998593
DLF004_scaffold2535_5	temperate	0.9977433
DLF004_scaffold32830_2	virulent	0.99984604
DLF004_scaffold19957_5	temperate	0.99840224
DLF004_scaffold30602_2	virulent	0.99818397
DLF004_scaffold27435_2	virulent	0.99987173
DLF004_scaffold930_8	virulent	0.9998627
DLF004_scaffold8102_2	virulent	0.9998465
DLF004_scaffold32187_1	temperate	0.9997492
DLF004_scaffold23115_1	temperate	0.99985975
DLF004_scaffold6537_1	virulent	0.9998551
DLF004_scaffold15396_3	virulent	0.9998348
DLF004_scaffold7918_11	virulent	0.910048
DLF004_scaffold7020_1	virulent	0.99987036
DLF004_scaffold1974_5	virulent	0.9998727
DLF004_scaffold24525_4	virulent	0.98113614
DLF004_scaffold15823_6	virulent	0.9998656
DLF004_scaffold26106_5	virulent	0.91473454
DLF004_scaffold22703_8	temperate	0.9397936
DLF004_scaffold21240_2	virulent	0.99987173
DLF004_scaffold125_29	temperate	0.5089623

DLF005_scaffold31168_1	virulent	0.99985975
DLF005_scaffold55052_1	temperate	0.9934008
DLF005_scaffold56707_1	temperate	0.99986035
DLF005_scaffold31945_4	temperate	0.99986035
DLF005_scaffold48546_1	temperate	0.82824755
DLF005_scaffold55770_1	temperate	0.98381054
DLF005_scaffold39735_4	temperate	0.9998579
DLF005_scaffold56569_4	virulent	0.9998665
DLF005_scaffold27529_1	temperate	0.9998265
DLF005_scaffold51335_1	virulent	0.60650957
DLF005_scaffold4693_36	virulent	0.99987316
DLF005_scaffold50375_1	temperate	0.9839584
DLF005_scaffold56163_1	virulent	0.9998417
DLF005_scaffold40395_1	virulent	0.99987406
DLF005_scaffold1570_16	temperate	0.99968576
DLF005_scaffold13131_1	temperate	0.9998584
DLF005_scaffold528_11	virulent	0.9998479
DLF005_scaffold39187_4	temperate	0.9998588
DLF005_scaffold25785_13	temperate	0.99986035
DLF005_scaffold56894_1	virulent	0.98988324
DLF005_scaffold54804_6	temperate	0.5799638
DLF005_scaffold2989_20	temperate	0.94335675
DLF005_scaffold2505_1	virulent	0.62476856
DLF005_scaffold47023_3	temperate	0.9888392
DLF005_scaffold56967_1	temperate	0.7587744
DLF005_scaffold13334_3	virulent	0.7953001
DLF005_C847538_1	temperate	0.9998579
DLF005_scaffold56817_1	temperate	0.9665699
DLF005_scaffold56924_2	virulent	0.95535946
DLF005_scaffold39187_3	temperate	0.9998579
DLF005_scaffold6688_21	temperate	0.608167
DLF005_scaffold18199_1	virulent	0.9998722
DLF005_C847770_1	temperate	0.99985033
DLF005_scaffold3960_1	virulent	0.9998699
DLF005_scaffold280_2	virulent	0.99987316
DLF005_scaffold32479_1	virulent	0.9998556
DLF005_scaffold41843_2	virulent	0.94455034
DLF005_scaffold33045_1	temperate	0.9997535
DLF005_scaffold4093_2	temperate	0.9983544
DLF005_scaffold9847_1	temperate	0.9998593
DLF005_scaffold31168_2	temperate	0.99985975
DLF005_scaffold53782_1	virulent	0.9998699
DLF005_scaffold56569_2	virulent	0.99987036
DLF005_scaffold23342_9	temperate	0.97907996
DLF006_scaffold52_4	virulent	0.9998369
DLF006_C667664_1	virulent	0.9998593

DLF006_scaffold9049_10	temperate	0.9991318
DLF006_scaffold108_5	virulent	0.9949243
DLF006_scaffold42885_3	virulent	0.9998688
DLF006_scaffold46681_1	temperate	0.9998417
DLF006_scaffold39883_1	temperate	0.9998593
DLF006_scaffold32044_2	temperate	0.93894786
DLF006_scaffold8740_3	temperate	0.9998593
DLF006_C667862_1	temperate	0.99962103
DLF006_scaffold19189_3	temperate	0.9997535
DLF006_scaffold474_4	virulent	0.9998588
DLF006_scaffold33584_4	temperate	0.99836224
DLF006_scaffold34156_4	virulent	0.9983713
DLF006_scaffold9056_20	temperate	0.99977875
DLF006_scaffold9056_18	virulent	0.99741316
DLF006_scaffold10687_2	temperate	0.93755734
DLF006_scaffold11422_2	virulent	0.9998343
DLF006_scaffold19189_4	temperate	0.99985266
DLF006_scaffold9845_3	virulent	0.9998636
DLF006_scaffold41298_2	virulent	0.99987465
DLF006_scaffold7466_5	temperate	0.9998403
DLF006_scaffold15736_1	temperate	0.9998403
DLF006_scaffold22006_9	virulent	0.9992909
DLF006_scaffold25840_10	temperate	0.62177837
DLF006_scaffold36051_1	temperate	0.9997267
DLF006_scaffold52_5	virulent	0.99986744
DLF006_scaffold9845_4	virulent	0.9997515
DLF006_scaffold36051_2	temperate	0.99983126
DLF006_scaffold34515_1	virulent	0.99978536
DLF006_scaffold5280_1	temperate	0.9668182
DLF006_scaffold46760_1	virulent	0.99987316
DLF006_scaffold46848_1	virulent	0.99987465
DLF006_scaffold14168_2	virulent	0.99987036
DLF006_scaffold8740_4	temperate	0.9998022
DLF006_scaffold46677_6	temperate	0.9903336
DLF006_scaffold27662_1	temperate	0.99985975
DLF006_scaffold52_6	virulent	0.99986607
DLF006_scaffold46851_2	virulent	0.99985975
DLF006_scaffold92_1	virulent	0.9996403
DLF007_scaffold15529_4	virulent	0.9998608
DLF007_scaffold14424_2	temperate	0.9998593
DLF007_scaffold18927_1	temperate	0.99985975
DLF007_C306149_1	virulent	0.9996841
DLF007_C306343_1	virulent	0.98861355
DLF007_scaffold1532_23	virulent	0.9998688
DLF007_scaffold20333_2	temperate	0.99985975
DLF007_scaffold4179_16	temperate	0.99985975

DLF007_scaffold15763_5	virulent	0.9998722
DLF007_scaffold2664_1	virulent	0.908313
DLF007_scaffold20109_2	temperate	0.93894786
DLF007_scaffold18401_2	temperate	0.99909663
DLF007_scaffold12277_1	virulent	0.9998684
DLF007_scaffold15181_7	virulent	0.9998517
DLF008_scaffold7461_4	virulent	0.99986124
DLF008_scaffold17152_1	temperate	0.9349437
DLF008_scaffold1091_2	virulent	0.9998722
DLF008_scaffold11168_2	virulent	0.99987406
DLF008_scaffold17625_2	temperate	0.93894786
DLF008_scaffold17607_1	virulent	0.99987125
DLF008_scaffold17497_1	temperate	0.99983215
DLF008_scaffold14160_4	temperate	0.75293964
DLF008_scaffold22_1	temperate	0.99982077
DLF008_scaffold10341_2	temperate	0.9998574
DLF008_scaffold16449_2	virulent	0.9998688
DLF008_scaffold1410_11	virulent	0.99986315
DLF008_scaffold5415_1	temperate	0.9997273
DLF008_scaffold3962_5	temperate	0.9998465
DLF008_scaffold10487_1	temperate	0.9998574
DLF008_scaffold2326_8	virulent	0.99986315
DLF008_scaffold17374_1	temperate	0.9998508
DLF008_C335467_1	temperate	0.99985975
DLF008_scaffold710_21	temperate	0.94271755
DLF008_scaffold17349_1	temperate	0.62144816
DLF008_scaffold8096_6	temperate	0.99985975
DLF008_scaffold7166_1	temperate	0.99985313
DLF008_scaffold9028_1	temperate	0.9990296
DLF008_scaffold8047_2	virulent	0.6666023
DLF008_scaffold1029_1	virulent	0.99987084
DLF008_scaffold3962_1	temperate	0.9632674
DLF008_scaffold222_2	virulent	0.8016601
DLF008_scaffold12889_2	virulent	0.99987173
DLF008_scaffold2953_7	temperate	0.9911663
DLF008_scaffold17084_1	virulent	0.99987316
DLF008_scaffold14357_3	temperate	0.9998145
DLF008_scaffold192_4	temperate	0.99976826
DLF008_scaffold3328_5	virulent	0.99987406
DLF008_scaffold17350_1	virulent	0.9998656
DLF009_scaffold34424_1	virulent	0.9998688
DLF009_scaffold1880_30	virulent	0.99987406
DLF009_scaffold36221_2	temperate	0.93656045
DLF009_C428906_1	virulent	0.998085
DLF009_scaffold30115_1	virulent	0.99986607
DLF009_scaffold789_3	temperate	0.99978215

DLF009_scaffold37442_2	temperate	0.99985975
DLF009_scaffold36221_1	temperate	0.9529658
DLF009_scaffold7798_1	virulent	0.99987084
DLF009_scaffold37363_2	temperate	0.9998593
DLF009_scaffold11451_17	virulent	0.99987125
DLF009_scaffold21876_2	temperate	0.9998374
DLF009_scaffold37177_1	temperate	0.99985975
DLF009_scaffold37452_1	temperate	0.99986035
DLF009_scaffold794_2	virulent	0.99985266
DLF009_scaffold30115_3	virulent	0.999703
DLF009_scaffold25763_1	temperate	0.96952647
DLF009_scaffold13354_1	temperate	0.9982825
DLF009_scaffold13766_4	virulent	0.9985362
DLF009_scaffold37443_4	temperate	0.99985605
DLF009_scaffold37498_1	temperate	0.9072678
DLF009_scaffold37448_2	virulent	0.9998536
DLF009_scaffold37438_1	temperate	0.999796
DLF009_scaffold30115_4	virulent	0.99987173
DLF009_scaffold37466_1	virulent	0.99987125
DLF009_scaffold37020_3	virulent	0.9998688
DLF009_scaffold3043_4	temperate	0.99985975
DLF009_C428576_1	temperate	0.8476171
DLF009_scaffold17926_1	temperate	0.9998288
DLF009_scaffold37386_2	temperate	0.9998413
DLF009_scaffold11949_1	temperate	0.99985695
DLF009_scaffold19674_5	temperate	0.99984264
DLF009_scaffold6204_7	temperate	0.6889878
DLF009_scaffold37284_1	temperate	0.9998574
DLF009_scaffold37363_3	temperate	0.9910528
DLF009_scaffold37307_2	temperate	0.999854
DLF009_scaffold2837_2	temperate	0.8991208
DLF009_scaffold61_24	temperate	0.9998551
DLF009_scaffold7570_14	temperate	0.9996787
DLF009_scaffold3557_6	temperate	0.9995462
DLF009_scaffold37386_1	temperate	0.99985313
DLF009_scaffold30115_5	virulent	0.9998693
DLF010_scaffold2375_6	temperate	0.99986035
DLF010_scaffold24026_11	virulent	0.9926443
DLF010_scaffold35043_4	virulent	0.9645309
DLF010_scaffold55870_1	virulent	0.8873964
DLF010_scaffold43436_5	temperate	0.99854225
DLF010_scaffold363_30	virulent	0.99986404
DLF010_scaffold36638_6	virulent	0.99987084
DLF010_scaffold6815_13	virulent	0.9998131
DLF010_scaffold13546_16	temperate	0.9998574
DLF010_scaffold35043_3	virulent	0.8880456

DLF010_scaffold46634_1	virulent	0.8683114
DLF010_scaffold13248_1	temperate	0.8345778
DLF010_scaffold49177_4	temperate	0.9838526
DLF010_scaffold4302_1	virulent	0.9667475
DLF010_scaffold363_33	virulent	0.9989573
DLF010_scaffold33244_2	temperate	0.9998556
DLF010_scaffold26724_1	temperate	0.9998584
DLF010_scaffold25454_7	virulent	0.99983835
DLF010_scaffold51522_2	virulent	0.99987084
DLF010_scaffold3276_2	temperate	0.99985975
DLF010_scaffold8211_1	virulent	0.9660578
DLF010_scaffold45015_1	temperate	0.99985975
DLF010_scaffold51387_5	temperate	0.99985975
DLF010_scaffold55918_1	virulent	0.99953496
DLF010_scaffold5445_2	virulent	0.9998226
DLF010_scaffold18414_39	temperate	0.9200033
DLF010_scaffold27012_2	virulent	0.99979496
DLF010_scaffold44216_5	virulent	0.99987125
DLF010_scaffold1405_28	temperate	0.9998593
DLF010_scaffold14049_4	temperate	0.999844
DLF010_scaffold86_3	virulent	0.9998465
DLF010_scaffold24026_10	virulent	0.99986404
DLF010_scaffold55874_1	temperate	0.97490793
DLF012_scaffold4165_33	temperate	0.99212986
DLF012_scaffold36_1	temperate	0.9998588
DLF012_scaffold17185_5	temperate	0.99980783
DLF012_scaffold15936_2	temperate	0.9998574
DLF012_scaffold12743_1	temperate	0.99153
DLF012_scaffold12838_3	temperate	0.9998536
DLF012_scaffold19111_1	virulent	0.9626032
DLF012_scaffold19146_2	temperate	0.93894786
DLF012_scaffold19032_1	virulent	0.9977207
DLF012_scaffold571_16	virulent	0.99977875
DLF012_scaffold17036_1	virulent	0.9998645
DLF012_scaffold3118_9	temperate	0.9998574
DLF013_scaffold47429_1	virulent	0.9996779
DLF013_scaffold18599_4	virulent	0.9997595
DLF013_scaffold35295_1	virulent	0.9996255
DLF013_scaffold52665_1	temperate	0.9997906
DLF013_scaffold1520_3	virulent	0.99987036
DLF013_scaffold34743_5	temperate	0.9998588
DLF013_scaffold34809_3	temperate	0.9988944
DLF013_scaffold14099_2	virulent	0.9997367
DLF013_scaffold13599_6	virulent	0.99987173
DLF013_scaffold53908_2	virulent	0.9998474
DLF013_scaffold45127_1	virulent	0.59446454

DLF013_scaffold3815_1	temperate	0.9997206
DLF013_scaffold88_22	virulent	0.96433496
DLF013_scaffold53550_1	virulent	0.99973613
DLF013_scaffold42596_2	temperate	0.99984974
DLF013_scaffold6385_2	temperate	0.99929565
DLF013_scaffold42479_2	temperate	0.9993117
DLF013_scaffold46613_1	temperate	0.9963533
DLF013_scaffold36273_1	temperate	0.888194
DLF013_scaffold45213_1	virulent	0.9109021
DLF013_scaffold31785_1	virulent	0.9998593
DLF013_scaffold31672_1	temperate	0.98458403
DLF013_scaffold139_7	temperate	0.98519784
DLF013_scaffold38734_1	temperate	0.9998588
DLF013_scaffold951_1	temperate	0.936828
DLF013_scaffold16307_9	temperate	0.9998593
DLF013_scaffold53889_1	temperate	0.9989985
DLF013_scaffold42888_4	virulent	0.9998417
DLF013_scaffold21493_3	temperate	0.9998593
DLF013_scaffold19199_4	temperate	0.99980694
DLF013_scaffold49433_5	temperate	0.9411944
DLF013_scaffold54798_1	virulent	0.99987084
DLF013_scaffold1100_4	virulent	0.99984497
DLF013_scaffold42008_2	virulent	0.9998584
DLF013_scaffold54776_1	virulent	0.9998699
DLF014_scaffold12062_3	temperate	0.9963524
DLF014_C195831_1	temperate	0.99985695
DLF014_scaffold12779_1	temperate	0.9963427
DLF014_scaffold9942_1	temperate	0.99975306
DLF014_C196781_1	temperate	0.9998054
DLF014_scaffold12792_2	temperate	0.9998588
DLF014_scaffold2320_1	temperate	0.9989119
DLF014_scaffold12718_1	virulent	0.9809961
DLF014_scaffold7985_2	temperate	0.9998579
DLF014_scaffold5471_1	temperate	0.9998593
DLF014_scaffold583_1	temperate	0.9998584
DLF014_scaffold12796_1	virulent	0.9998436
DLF014_scaffold12682_1	temperate	0.9998202
DLF014_scaffold5330_1	temperate	0.99985975
DLF014_scaffold2386_4	virulent	0.99979997
DLF014_scaffold12741_1	temperate	0.9998556
DLF014_scaffold11967_1	temperate	0.9969512
DLF014_scaffold8898_1	virulent	0.86899716
DLF014_scaffold3999_1	temperate	0.99985975
DLF014_scaffold6344_1	temperate	0.99984604
DLF014_scaffold11623_1	temperate	0.99985605
DLF014_scaffold2693_2	temperate	0.9776519

DLF014_scaffold1928_3	virulent	0.9998608
DLF014_scaffold12767_1	virulent	0.9996782
DLF014_scaffold4556_3	temperate	0.9587585
DLF014_scaffold12792_1	temperate	0.99985605
DLF014_scaffold10166_1	temperate	0.9998588
DLF014_scaffold12665_1	temperate	0.9998579
DLF014_scaffold8_2	virulent	0.99987406
DLF014_scaffold8436_2	temperate	0.9998522
DLF014_scaffold8300_1	virulent	0.9978354
DLF014_scaffold5363_6	temperate	0.99984837
DLF014_scaffold5363_21	virulent	0.9998688
DLF014_scaffold11967_2	temperate	0.99985975
DLF014_scaffold6855_1	temperate	0.99985313
DLF014_scaffold2932_3	virulent	0.9764244
DLF014_scaffold2093_4	temperate	0.99985975
DLF014_scaffold12383_2	virulent	0.99986315
NLF001_scaffold29251_2	temperate	0.9998579
NLF001_scaffold18110_3	temperate	0.9076663
NLF001_scaffold18524_1	temperate	0.8446956
NLF001_scaffold159_2	temperate	0.9998556
NLF001_scaffold1094_2	temperate	0.979654
NLF001_scaffold30907_8	temperate	0.9998588
NLF001_scaffold42470_1	virulent	0.9998436
NLF001_scaffold15921_9	virulent	0.9998645
NLF001_scaffold36675_1	temperate	0.9671342
NLF001_scaffold15028_2	temperate	0.99986035
NLF001_scaffold21827_1	virulent	0.97969764
NLF001_scaffold17952_7	temperate	0.98458403
NLF001_scaffold34766_6	temperate	0.9998588
NLF001_scaffold42478_2	virulent	0.9998722
NLF001_scaffold42456_1	virulent	0.9998736
NLF001_scaffold21408_7	temperate	0.999804
NLF001_scaffold42472_1	temperate	0.99762505
NLF001_C572546_1	temperate	0.9998588
NLF001_scaffold38144_4	temperate	0.9998545
NLF001_scaffold12289_1	temperate	0.998079
NLF001_scaffold34766_5	temperate	0.9443795
NLF001_scaffold33713_2	temperate	0.9998465
NLF002_scaffold10424_8	temperate	0.98519784
NLF002_scaffold36662_1	temperate	0.99985975
NLF002_scaffold336_22	virulent	0.99987084
NLF002_scaffold35171_2	temperate	0.9998551
NLF002_scaffold16871_1	temperate	0.99727786
NLF002_scaffold28626_2	temperate	0.9998588
NLF002_scaffold34776_1	temperate	0.9998517
NLF002_scaffold22680_4	virulent	0.9997971

NLF002_scaffold6663_3	virulent	0.81238216
NLF002_scaffold37757_3	virulent	0.9998699
NLF002_scaffold3933_4	temperate	0.9998584
NLF002_scaffold14709_2	virulent	0.9986972
NLF002_scaffold27060_6	virulent	0.9998727
NLF002_scaffold15559_1	virulent	0.9993214
NLF002_scaffold19765_4	virulent	0.9989374
NLF002_scaffold141_14	virulent	0.9998379
NLF002_scaffold2199_6	temperate	0.9998545
NLF002_scaffold36924_1	temperate	0.99985695
NLF002_scaffold115_2	temperate	0.99986035
NLF002_scaffold37757_4	virulent	0.9998688
NLF002_scaffold37757_5	virulent	0.9998693
NLF002_scaffold17055_1	temperate	0.9996782
NLF002_scaffold25489_2	temperate	0.999816
NLF002_scaffold38935_2	virulent	0.99987173
NLF005_scaffold4391_2	virulent	0.9998688
NLF005_scaffold21492_3	temperate	0.9998593
NLF005_scaffold46950_1	temperate	0.9998388
NLF005_scaffold15835_5	temperate	0.9998145
NLF005_scaffold36523_1	temperate	0.99338275
NLF005_scaffold32720_4	temperate	0.9212118
NLF005_scaffold21492_6	virulent	0.9654628
NLF005_C729497_1	virulent	0.9995685
NLF005_scaffold1317_3	virulent	0.99980664
NLF005_scaffold21214_1	temperate	0.9998593
NLF005_scaffold47798_1	temperate	0.84956145
NLF005_C729695_1	temperate	0.9554545
NLF005_scaffold27522_3	virulent	0.9998388
NLF005_scaffold47800_1	virulent	0.9990291
NLF005_scaffold29618_1	temperate	0.9923903
NLF005_scaffold21492_4	temperate	0.9976349
NLF005_scaffold25196_2	temperate	0.9998593
NLF005_scaffold40967_2	temperate	0.9998593
NLF005_scaffold43034_2	temperate	0.837803
NLF005_scaffold291_42	virulent	0.6808251
NLF005_scaffold47934_7	temperate	0.9984386
NLF005_scaffold43004_3	temperate	0.99983126
NLF005_scaffold14516_2	temperate	0.99985975
NLF005_scaffold48237_1	virulent	0.9998636
NLF005_C729433_1	virulent	0.9998636
NLF005_scaffold48442_3	temperate	0.9997906
NLF005_scaffold48389_1	temperate	0.9998588
NLF005_scaffold39282_7	temperate	0.9998593
NLF005_scaffold13387_9	virulent	0.9998684
NLF005_scaffold40798_1	virulent	0.9998413

NLF005_scaffold13387_4	virulent	0.9970323
NLF005_scaffold48437_2	temperate	0.9981909
NLF005_scaffold29852_1	temperate	0.9998584
NLF005_scaffold4391_3	virulent	0.99986607
NLF005_scaffold291_44	virulent	0.9997163
NLF005_scaffold10734_4	temperate	0.99985975
NLF006_scaffold306_3	virulent	0.9961736
NLF006_scaffold22186_2	virulent	0.9997945
NLF006_scaffold31099_1	temperate	0.9998479
NLF006_scaffold4766_5	virulent	0.9998736
NLF006_scaffold306_1	virulent	0.99986607
NLF006_scaffold9628_2	temperate	0.999854
NLF006_scaffold4766_2	virulent	0.9998736
NLF006_scaffold5249_2	virulent	0.99952775
NLF006_scaffold3814_1	temperate	0.9960633
NLF006_scaffold15157_3	temperate	0.99962914
NLF006_scaffold28327_1	virulent	0.99986035
NLF006_scaffold4466_3	virulent	0.99987173
NLF006_scaffold14746_1	virulent	0.89459366
NLF006_scaffold132_1	temperate	0.9998545
NLF006_scaffold5891_10	temperate	0.99986035
NLF006_scaffold30421_2	virulent	0.9619549
NLF006_scaffold4358_2	temperate	0.99390066
NLF007_scaffold882_1	virulent	0.99987316
NLF007_scaffold38312_4	virulent	0.9998699
NLF007_scaffold5043_8	virulent	0.9943649
NLF007_scaffold67260_2	temperate	0.9984577
NLF007_scaffold14743_4	virulent	0.9998699
NLF007_scaffold64602_1	temperate	0.9998474
NLF007_scaffold66908_2	temperate	0.9998588
NLF007_scaffold8274_2	virulent	0.9290818
NLF007_scaffold22938_1	temperate	0.99985975
NLF007_scaffold66524_2	temperate	0.99322194
NLF007_scaffold38312_8	virulent	0.9997118
NLF007_scaffold5542_23	virulent	0.9998091
NLF007_scaffold11102_4	virulent	0.9027133
NLF007_scaffold49453_1	temperate	0.99793273
NLF007_scaffold23805_3	temperate	0.9413989
NLF007_scaffold31937_4	temperate	0.9998593
NLF007_scaffold110_13	temperate	0.9482661
NLF007_scaffold23404_3	temperate	0.9998593
NLF007_scaffold25969_1	temperate	0.9786316
NLF007_scaffold29510_1	temperate	0.99985975
NLF007_scaffold61307_5	temperate	0.9942856
NLF007_scaffold4118_1	temperate	0.99985975
NLF007_scaffold67236_3	temperate	0.9929809

NLF007_scaffold3779_4	virulent	0.9998593
NLF007_scaffold27103_9	temperate	0.99984884
NLF007_scaffold67413_1	temperate	0.9993908
NLF007_scaffold36465_3	temperate	0.9819443
NLF007_C1010391_1	temperate	0.96395195
NLF007_scaffold17554_50	temperate	0.91449815
NLF007_scaffold64205_2	temperate	0.93972445
NLF007_scaffold61221_1	virulent	0.999854
NLF007_scaffold56144_4	temperate	0.9998593
NLF007_scaffold67153_2	virulent	0.9998699
NLF007_scaffold23957_1	virulent	0.9997695
NLF007_scaffold54945_9	virulent	0.999844
NLF007_scaffold20693_10	virulent	0.9998722
NLF007_scaffold40076_1	virulent	0.99983
NLF007_scaffold66908_3	temperate	0.99985975
NLF007_scaffold47382_8	virulent	0.9998679
NLF007_scaffold47325_6	temperate	0.9998565
NLF007_scaffold14743_2	virulent	0.99986744
NLF007_scaffold30358_8	virulent	0.99987406
NLF007_scaffold3779_6	temperate	0.9998579
NLF007_scaffold14743_1	temperate	0.60106117
NLF007_scaffold57652_6	virulent	0.99964964
NLF007_scaffold50111_1	temperate	0.98840153
NLF007_scaffold57227_1	temperate	0.99985975
NLF007_scaffold48133_2	virulent	0.9997974
NLF007_scaffold66524_3	temperate	0.9998588
NLF007_scaffold22798_1	temperate	0.9997869
NLF007_scaffold43991_1	temperate	0.9941997
NLF007_scaffold22798_13	virulent	0.9993209
NLF007_scaffold31937_5	virulent	0.9998693
NLF007_scaffold67062_4	temperate	0.9998588
NLF007_scaffold34404_4	virulent	0.9994208
NLF007_scaffold32046_2	virulent	0.9998565
NLF007_scaffold21464_1	temperate	0.99985266
NLF007_scaffold67062_3	temperate	0.99985975
NLF007_scaffold27103_3	temperate	0.99985695
NLF008_C315574_1	virulent	0.9322203
NLF008_scaffold19700_1	virulent	0.99987125
NLF008_scaffold141_3	virulent	0.99987125
NLF008_scaffold12997_5	temperate	0.9998536
NLF008_scaffold18404_4	virulent	0.99987125
NLF008_scaffold10158_1	virulent	0.99984926
NLF008_scaffold20496_1	virulent	0.99987125
NLF008_scaffold8874_2	virulent	0.99987173
NLF008_scaffold18404_14	virulent	0.99976844
NLF008_scaffold10905_2	temperate	0.9998536

NLF008_scaffold10905_1	temperate	0.92832506
NLF008_scaffold1586_11	temperate	0.999836
NLF008_scaffold7552_2	temperate	0.99984837
NLF008_scaffold20431_5	virulent	0.99986696
NLF008_scaffold19473_1	virulent	0.99863577
NLF008_scaffold9366_1	virulent	0.99984926
NLF008_scaffold15389_1	virulent	0.99987084
NLF008_scaffold20125_1	virulent	0.53311074
NLF008_scaffold17605_2	temperate	0.9998593
NLF008_scaffold6835_1	virulent	0.9998651
NLF008_scaffold9492_2	virulent	0.99980664
NLF008_scaffold12194_1	virulent	0.99987406
NLF008_scaffold20731_1	temperate	0.9765676
NLF008_scaffold6103_28	temperate	0.93755734
NLF008_scaffold395_6	virulent	0.99987173
NLF008_scaffold12947_1	virulent	0.9998665
NLF008_scaffold17931_1	temperate	0.8868689
NLF008_scaffold12834_4	virulent	0.99949926
NLF008_scaffold16324_2	virulent	0.9997513
NLF008_scaffold107_1	virulent	0.91921675
NLF008_scaffold98_9	temperate	0.9996572
NLF008_scaffold1813_6	virulent	0.9998693
NLF008_scaffold8433_4	temperate	0.99984556
NLF008_scaffold20258_1	virulent	0.99987125
NLF008_scaffold18404_6	virulent	0.9998722
NLF008_scaffold6876_9	virulent	0.9998736
NLF008_scaffold9442_2	virulent	0.9998736
NLF008_C315880_1	temperate	0.99985975
NLF008_scaffold20528_1	temperate	0.9932634
NLF008_scaffold1474_11	virulent	0.9998684
NLF008_scaffold386_3	temperate	0.999854
NLF008_scaffold5986_1	virulent	0.99987036
NLF008_scaffold15537_1	virulent	0.9981073
NLF008_scaffold18404_13	virulent	0.9998699
NLF008_scaffold14014_6	virulent	0.99987125
NLF008_scaffold7896_4	temperate	0.99671596
NLF008_scaffold9737_2	temperate	0.9998593
NLF008_scaffold12833_2	virulent	0.9998226
NLF008_scaffold83_1	virulent	0.99959236
NLF008_scaffold20431_4	virulent	0.99987084
NLF008_scaffold13388_2	temperate	0.98519784
NLF008_scaffold1868_2	virulent	0.9998593
NLF009_C805905_1	temperate	0.9998579
NLF009_scaffold3162_3	temperate	0.9998593
NLF009_scaffold17912_16	temperate	0.9998593
NLF009_scaffold30773_1	temperate	0.98678803

NLF009_scaffold54553_5	virulent	0.9905057
NLF009_scaffold21790_2	temperate	0.99985975
NLF009_scaffold7091_22	virulent	0.9998474
NLF009_scaffold54553_3	virulent	0.9998699
NLF009_scaffold4933_28	virulent	0.99987125
NLF009_scaffold9844_25	temperate	0.9998379
NLF009_scaffold52926_4	temperate	0.97618574
NLF009_scaffold52375_7	virulent	0.9996982
NLF009_scaffold54499_1	temperate	0.99985975
NLF009_scaffold19140_7	virulent	0.9998727
NLF009_scaffold54170_2	temperate	0.9998513
NLF009_scaffold35663_1	virulent	0.99982476
NLF009_scaffold4106_1	temperate	0.9998593
NLF009_scaffold1353_6	temperate	0.9998593
NLF009_C805829_1	virulent	0.99960625
NLF009_scaffold27217_3	temperate	0.99986035
NLF009_C805873_1	virulent	0.8740401
NLF009_scaffold17912_4	temperate	0.99724483
NLF009_scaffold5429_52	temperate	0.9876869
NLF009_scaffold40330_1	virulent	0.9998684
NLF009_scaffold18984_7	virulent	0.9985467
NLF009_scaffold38501_1	temperate	0.99786866
NLF009_scaffold8414_14	temperate	0.9996339
NLF009_scaffold17912_17	temperate	0.9986843
NLF009_scaffold52991_2	temperate	0.979654
NLF009_scaffold25289_5	temperate	0.99985266
NLF009_scaffold36413_1	virulent	0.9193699
NLF009_scaffold375_24	virulent	0.99963766
NLF009_scaffold52926_1	virulent	0.9998688
NLF009_scaffold37415_3	temperate	0.9920004
NLF009_scaffold54503_1	virulent	0.99928766
NLF009_scaffold17912_6	temperate	0.9998336
NLF010_scaffold1235_8	virulent	0.99987084
NLF010_scaffold20315_2	virulent	0.97717094
NLF010_scaffold1044_27	virulent	0.9998736
NLF010_C401947_1	virulent	0.9998593
NLF010_scaffold13747_2	temperate	0.9958668
NLF010_scaffold24082_3	temperate	0.99985605
NLF010_C402235_1	virulent	0.9998627
NLF010_scaffold22417_1	virulent	0.9996403
NLF010_scaffold3898_4	virulent	0.9998699
NLF010_scaffold12295_8	virulent	0.97536755
NLF010_scaffold27728_2	temperate	0.999854
NLF010_scaffold29246_1	virulent	0.99987316
NLF010_scaffold26024_8	virulent	0.9998722
NLF010_scaffold2046_76	virulent	0.99987036

NLF010_scaffold9747_18	virulent	0.9953019
NLF010_scaffold20591_1	virulent	0.9484926
NLF010_scaffold29445_4	virulent	0.9995593
NLF011_scaffold4965_2	virulent	0.99987084
NLF011_scaffold5613_1	virulent	0.9998665
NLF011_scaffold42_2	virulent	0.99987036
NLF011_scaffold1105_1	temperate	0.9960633
NLF011_scaffold7214_2	virulent	0.9998727
NLF011_scaffold10809_1	virulent	0.9998699
NLF011_scaffold4255_7	temperate	0.98806924
NLF011_scaffold10054_4	temperate	0.979654
NLF011_scaffold1786_1	temperate	0.99896526
NLF011_scaffold4096_9	temperate	0.99985695
NLF011_scaffold2637_6_1	virulent	0.99985975
NLF011_scaffold13048_1	virulent	0.99978536
NLF011_scaffold3151_2	temperate	0.99986035
NLF011_scaffold30362_5	temperate	0.98519784
NLF011_scaffold25120_2	temperate	0.9799867
NLF011_scaffold486_7	virulent	0.999844
NLF011_scaffold794_4	virulent	0.99986744
NLF011_scaffold22115_1	virulent	0.9998508
NLF011_scaffold803_4	virulent	0.85896134
NLF011_scaffold10943_3	temperate	0.99985033
NLF011_scaffold6937_1	virulent	0.9998536
NLF011_scaffold21554_2	virulent	0.99987316
NLF011_scaffold9582_1	temperate	0.9989985
NLF012_scaffold10340_2	temperate	0.99985695
NLF012_scaffold35219_5	temperate	0.9980938
NLF012_scaffold35219_1	temperate	0.99985975
NLF012_scaffold907_13	temperate	0.9998403
NLF012_scaffold39053_1	temperate	0.99986035
NLF012_scaffold2371_23	virulent	0.9864348
NLF012_scaffold22746_1	temperate	0.9998593
NLF012_scaffold39054_1	virulent	0.9997444
NLF012_scaffold8622_2	temperate	0.90595436
NLF012_scaffold2172_5	virulent	0.9998369
NLF012_scaffold27405_2	virulent	0.57108474
NLF012_scaffold11833_29	temperate	0.99984884
NLF012_scaffold2371_16	temperate	0.99986035
NLF012_scaffold39140_2	temperate	0.93894786
NLF012_scaffold25728_1	temperate	0.99985975
NLF012_scaffold36675_4	virulent	0.9468337
NLF012_scaffold21675_1	virulent	0.7219442
NLF012_scaffold11833_30	temperate	0.9998593
NLF012_scaffold29420_1	temperate	0.99955946
NLF012_scaffold39159_1	virulent	0.9998679

NLF012_scaffold17264_3	virulent	0.99987173
NLF012_scaffold34471_3	temperate	0.999417
NLF012_scaffold293_2	virulent	0.99980617
NLF012_scaffold6773_9	virulent	0.9998688
NLF012_scaffold10245_3	temperate	0.99944323
NLF012_scaffold2854_1	virulent	0.99980754
NLF012_scaffold18119_4	temperate	0.9996067
NLF012_scaffold38032_1	temperate	0.99985975
NLF012_scaffold16653_1	temperate	0.99244034
NLF012_scaffold22955_2	virulent	0.99987406
NLF012_scaffold15731_1	temperate	0.9998522
NLF012_scaffold13034_3	temperate	0.9998588
NLF012_scaffold16348_2	temperate	0.9998584
NLF012_scaffold8622_6	virulent	0.9998679
NLF012_scaffold17845_1	virulent	0.99497724
NLF012_C512465_1	virulent	0.9998699
NLF012_scaffold12754_3	temperate	0.9340867
NLF012_scaffold10394_1	virulent	0.99987316
NLF012_scaffold32276_1	temperate	0.99985975
NLF012_scaffold13132_1	temperate	0.6238861
NLF012_scaffold30180_18	temperate	0.99986035
NLF012_scaffold17431_22	virulent	0.99983126
NLF012_scaffold35713_1	virulent	0.9998556
NLF012_scaffold10663_6	virulent	0.9998688
NLF012_scaffold35670_2	virulent	0.999549
NLF012_scaffold15197_22	virulent	0.9664372
NLF012_scaffold37458_1	virulent	0.99987316
NLF012_scaffold39143_1	temperate	0.9998588
NLF012_scaffold21989_2	temperate	0.9832907
NLF012_scaffold14481_3	virulent	0.99961454
NLF012_scaffold4362_3	temperate	0.93755734
NLF012_scaffold10340_14	virulent	0.99986607
NLF012_scaffold38234_3	virulent	0.9253881
NLF012_scaffold16451_3	virulent	0.9998684
NLF012_scaffold13372_3	virulent	0.99987406
NLF012_scaffold37376_1	virulent	0.99987084
NLF012_scaffold69_1	virulent	0.8038945
NLF012_scaffold14206_2	virulent	0.88010323
NLF012_scaffold17431_21	temperate	0.9952155
NLF012_scaffold8622_3	virulent	0.9804897
NLF012_scaffold33261_5	temperate	0.9998083
NLF012_scaffold29414_2	temperate	0.99985695
NLF012_scaffold2371_15	temperate	0.99982077
NLF012_scaffold17240_1	temperate	0.9997754
NLF013_scaffold11783_1	virulent	0.9998474
NLF013_scaffold48622_1	virulent	0.9998608

NLF013_scaffold11031_1	virulent	0.99969965
NLF013_scaffold43868_1	temperate	0.9998545
NLF013_scaffold14354_6	temperate	0.99983406
NLF013_scaffold30746_1	temperate	0.99985975
NLF013_scaffold12177_2	temperate	0.99984926
NLF013_C659127_1	virulent	0.9998688
NLF013_scaffold34744_1	temperate	0.9996158
NLF013_scaffold328_1	virulent	0.99982435
NLF013_scaffold33849_1	virulent	0.99777085
NLF013_scaffold23543_4	temperate	0.9994632
NLF013_scaffold19270_4	virulent	0.99980664
NLF013_scaffold4799_2	temperate	0.9991315
NLF013_scaffold44405_3	virulent	0.99987406
NLF013_scaffold32342_11	virulent	0.9998126
NLF013_scaffold47264_1	temperate	0.99986035
NLF013_scaffold33946_4	temperate	0.99867666
NLF013_scaffold25755_1	virulent	0.99987036
NLF013_scaffold11031_7	temperate	0.9998593
NLF013_scaffold21878_2	temperate	0.9998584
NLF013_scaffold4561_28	virulent	0.99981666
NLF013_scaffold23156_2	virulent	0.9905981
NLF013_scaffold30660_7	temperate	0.99921
NLF013_C659857_1	virulent	0.9998479
NLF013_scaffold20413_4	virulent	0.99986744
NLF013_scaffold19359_1	temperate	0.99693155
NLF013_scaffold26044_5	virulent	0.99800235
NLF013_scaffold43192_14	virulent	0.9998688
NLF013_scaffold48338_2	temperate	0.9998588
NLF014_scaffold142_6	virulent	0.9998722
NLF014_scaffold5840_1	temperate	0.99364245
NLF014_scaffold7292_1	virulent	0.99987316
NLF014_scaffold342_1	virulent	0.9998588
NLF014_scaffold9741_6	virulent	0.99987125
NLF014_scaffold1307_2	virulent	0.9788498
NLF014_scaffold6197_2	virulent	0.8716869
NLF014_scaffold10772_2	virulent	0.9981197
NLF014_scaffold249_3	virulent	0.9998684
NLF014_scaffold819_1	virulent	0.9998722
NLF014_scaffold9408_9	virulent	0.99987036
NLF014_scaffold6884_2	temperate	0.9998465
NLF014_scaffold73_1	virulent	0.9998736
NLF014_scaffold736_2	virulent	0.9998727
NLF015_scaffold31615_2	temperate	0.99646026
NLF015_scaffold32932_4	temperate	0.99985313
NLF015_scaffold31821_3	temperate	0.979654
NLF015_scaffold24231_1	virulent	0.99987036

NLF015_C419895_1	virulent	0.99987173
NLF015_scaffold14491_1	virulent	0.9998308
NLF015_scaffold22095_1	temperate	0.9998588
NLF015_scaffold591_2	temperate	0.89331686
NLF015_scaffold21847_3	virulent	0.99987173
NLF015_scaffold11481_6	virulent	0.9998727
NLF015_scaffold22_1	temperate	0.9607604
NLF015_scaffold3859_2	temperate	0.9998049
NLF015_scaffold34057_2	virulent	0.99987173
NLF015_scaffold24362_4	virulent	0.99984694
NLF015_scaffold29017_1	temperate	0.9998593
NLF015_scaffold31211_3	temperate	0.9995867
NLF015_scaffold12014_3	virulent	0.99984884
NLF015_scaffold8589_4	virulent	0.8963725
NLF015_scaffold14946_1	virulent	0.99985695
NLF015_scaffold20215_1	virulent	0.9700904
NLF015_scaffold20500_6	temperate	0.9413989
NLF015_scaffold13173_2	virulent	0.88146806
NLF015_scaffold7628_6	virulent	0.9998574
NLF015_scaffold14_1	temperate	0.9541461
NLF015_C419419_1	virulent	0.9998722
NLF015_scaffold3382_3	virulent	0.99587125
NLF015_scaffold34269_1	temperate	0.9998593
NLF015_scaffold33332_2	temperate	0.99976444
NLF015_scaffold4657_3	temperate	0.999417
NLF015_scaffold32259_1	temperate	0.9991372
NLF015_scaffold13163_7	virulent	0.99987173
NLF015_scaffold6258_2	virulent	0.908313
NLF015_scaffold34558_2	virulent	0.9998656
NLF015_scaffold21847_1	virulent	0.9998608
DLM001_scaffold37_3	virulent	0.99979955
DLM001_scaffold17760_6	virulent	0.9998693
DLM001_scaffold57834_1	temperate	0.9998593
DLM001_scaffold91_6	virulent	0.99764246
DLM001_scaffold4307_3	temperate	0.9987186
DLM001_scaffold36534_1	temperate	0.98458403
DLM001_scaffold19551_4	virulent	0.99984217
DLM001_scaffold26672_2	temperate	0.9998584
DLM001_scaffold21921_3	virulent	0.99956113
DLM001_scaffold2435_6	temperate	0.99985605
DLM001_scaffold23814_4	virulent	0.9996882
DLM001_scaffold14847_5	temperate	0.93894786
DLM001_scaffold42296_1	temperate	0.99985975
DLM001_scaffold4307_2	temperate	0.99954665
DLM001_scaffold31256_1	temperate	0.9868924
DLM001_scaffold52744_1	virulent	0.9997631

DLM001_scaffold20858_1	virulent	0.9995579
DLM001_scaffold27372_1	virulent	0.99987125
DLM001_scaffold55116_2	virulent	0.83690894
DLM001_scaffold57732_2	virulent	0.98364675
DLM001_scaffold17614_8	virulent	0.9998665
DLM001_scaffold57716_1	temperate	0.9800632
DLM001_scaffold25895_8	temperate	0.9998431
DLM001_scaffold21223_1	temperate	0.9390359
DLM001_scaffold54893_2	virulent	0.99987125
DLM001_scaffold30022_1	temperate	0.99984926
DLM001_scaffold58236_1	temperate	0.9080085
DLM001_scaffold34133_3	virulent	0.55847466
DLM001_scaffold51940_3	virulent	0.9998665
DLM001_scaffold49843_4	virulent	0.99987084
DLM001_scaffold51928_3	temperate	0.97777677
DLM001_scaffold19551_5	virulent	0.97540027
DLM001_scaffold19465_5	virulent	0.9998693
DLM001_scaffold56543_4	virulent	0.9489721
DLM001_scaffold14859_7	virulent	0.9998579
DLM001_scaffold20858_3	temperate	0.994922
DLM001_C705523_1	virulent	0.96739966
DLM001_scaffold17051_8	virulent	0.99986744
DLM001_scaffold19551_1	virulent	0.99560606
DLM001_scaffold20817_1	virulent	0.99987125
DLM001_scaffold25009_5	temperate	0.99862176
DLM001_scaffold22953_2	virulent	0.99861836
DLM001_scaffold53015_2	virulent	0.99987084
DLM001_scaffold41842_2	temperate	0.9939871
DLM001_scaffold19551_3	temperate	0.8985467
DLM001_scaffold58253_1	temperate	0.999836
DLM001_scaffold53994_4	temperate	0.9998308
DLM001_scaffold56486_2	virulent	0.9941044
DLM001_scaffold48552_3	virulent	0.9998128
DLM001_scaffold4106_5	temperate	0.99691886
DLM001_scaffold51591_2	virulent	0.9998688
DLM001_scaffold10420_4	virulent	0.95498294
DLM001_scaffold58124_4	virulent	0.9976206
DLM001_scaffold58250_2	temperate	0.81878257
DLM001_scaffold23040_12	temperate	0.9997654
DLM001_C705425_1	temperate	0.9704434
DLM001_scaffold52289_2	virulent	0.99987316
DLM001_scaffold18361_2	virulent	0.9998593
DLM001_scaffold55454_4	temperate	0.9998374
DLM001_scaffold39252_2	virulent	0.9997156
DLM001_scaffold55964_1	virulent	0.9998302
DLM001_scaffold12282_12	temperate	0.99980354

DLM001_scaffold13671_1	virulent	0.99987173
DLM001_scaffold50893_2	virulent	0.9998665
DLM001_scaffold277_3	virulent	0.99980354
DLM001_scaffold39468_1	virulent	0.9998736
DLM001_scaffold47007_2	virulent	0.9998688
DLM001_scaffold49276_1	temperate	0.99985975
DLM001_scaffold21002_1	virulent	0.9991016
DLM001_scaffold42810_2	temperate	0.99985695
DLM001_scaffold49834_2	virulent	0.99987406
DLM001_scaffold16868_1	temperate	0.99982554
DLM001_scaffold52139_2	virulent	0.9998288
DLM001_scaffold11723_1	temperate	0.99986035
DLM001_scaffold19427_9	virulent	0.9599575
DLM001_scaffold27611_5	virulent	0.9998636
DLM001_scaffold14114_7	virulent	0.9998645
DLM001_scaffold3576_10	virulent	0.9743186
DLM002_scaffold52283_2	virulent	0.9998727
DLM002_scaffold35924_1	temperate	0.99985975
DLM002_scaffold39638_1	temperate	0.9998593
DLM002_scaffold58_3	virulent	0.99925953
DLM002_scaffold8357_7	temperate	0.9998593
DLM002_scaffold37454_1	virulent	0.99987125
DLM002_scaffold37352_8	virulent	0.9998684
DLM002_scaffold4794_6	virulent	0.9998006
DLM002_scaffold52713_2	temperate	0.9998165
DLM002_scaffold45452_2	temperate	0.99985975
DLM002_scaffold48724_2	temperate	0.99985975
DLM002_scaffold11013_7	virulent	0.99987316
DLM002_scaffold5537_11	virulent	0.99987406
DLM002_scaffold2881_3	temperate	0.62338036
DLM002_scaffold52526_1	temperate	0.9998565
DLM002_scaffold44502_1	temperate	0.9998593
DLM002_scaffold52526_4	temperate	0.9998584
DLM002_scaffold48180_13	virulent	0.99987125
DLM002_scaffold42939_1	temperate	0.9998593
DLM002_scaffold52584_1	virulent	0.99339837
DLM002_scaffold32192_3	virulent	0.9998693
DLM002_scaffold5336_45	virulent	0.9988257
DLM002_scaffold52696_1	temperate	0.6640839
DLM002_scaffold925_8	virulent	0.9998727
DLM003_scaffold30478_2	virulent	0.9998688
DLM003_scaffold35975_4	temperate	0.9613774
DLM003_scaffold20093_1	virulent	0.9997367
DLM003_scaffold8802_8	virulent	0.9983713
DLM003_scaffold1898_14	virulent	0.94656765
DLM003_scaffold30928_7	virulent	0.9998727

DLM003_scaffold59785_6	virulent	0.99987084
DLM003_scaffold67334_2	virulent	0.99987406
DLM003_C810975_1	virulent	0.9998627
DLM003_scaffold31156_2	virulent	0.99987125
DLM003_scaffold24702_43	virulent	0.99987316
DLM003_scaffold10664_1	virulent	0.8965864
DLM003_scaffold46929_2	virulent	0.9998699
DLM003_scaffold59785_5	virulent	0.9998688
DLM003_scaffold40695_7	virulent	0.99984974
DLM003_scaffold4744_1	virulent	0.99987084
DLM003_scaffold303_2	temperate	0.9998202
DLM003_scaffold36199_4	virulent	0.9998693
DLM003_scaffold12201_4	virulent	0.89352334
DLM003_scaffold16412_17	temperate	0.9998579
DLM003_scaffold49406_1	virulent	0.9985555
DLM003_C810753_1	virulent	0.9998665
DLM003_scaffold1898_11	virulent	0.99987173
DLM003_scaffold66050_1	virulent	0.9998679
DLM003_scaffold17135_3	virulent	0.99986404
DLM003_scaffold29743_2	virulent	0.99987406
DLM003_scaffold14015_13	temperate	0.99984264
DLM003_scaffold3089_1	virulent	0.9998684
DLM003_scaffold15117_2	virulent	0.9998693
DLM003_scaffold59785_12	virulent	0.99987036
DLM003_scaffold102_3	virulent	0.99987036
DLM003_scaffold46929_4	virulent	0.9998645
DLM003_scaffold40695_5	virulent	0.999555
DLM004_scaffold2416_6	virulent	0.99987036
DLM004_scaffold18621_3	temperate	0.99957764
DLM004_scaffold12500_3	virulent	0.99986315
DLM004_scaffold8053_11	virulent	0.9998413
DLM004_scaffold2421_24	temperate	0.9998584
DLM004_scaffold8053_12	temperate	0.67867184
DLM004_scaffold18777_1	temperate	0.9998579
DLM004_scaffold41_14	temperate	0.9992274
DLM004_scaffold12500_2	virulent	0.9664789
DLM004_scaffold462_3	temperate	0.99986035
DLM004_scaffold18776_2	virulent	0.99987406
DLM004_scaffold112_3	virulent	0.9981354
DLM004_scaffold158_30	virulent	0.9998684
DLM004_scaffold5647_2	virulent	0.9998722
DLM004_scaffold18395_2	temperate	0.7185659
DLM004_scaffold16125_1	temperate	0.9998593
DLM004_scaffold12500_5	virulent	0.99987406
DLM004_scaffold11715_11	virulent	0.9998397
DLM004_scaffold15727_1	virulent	0.99987125

DLM004_scaffold17859_1	virulent	0.9998684
DLM004_scaffold14310_1	virulent	0.9985355
DLM004_scaffold14578_1	virulent	0.9997991
DLM004_scaffold9327_2	temperate	0.9942856
DLM004_scaffold16036_1	virulent	0.99985695
DLM004_C304967_1	virulent	0.6261919
DLM004_scaffold12500_1	temperate	0.9998584
DLM004_scaffold8780_5	virulent	0.9998584
DLM004_scaffold14269_9	temperate	0.99985975
DLM005_scaffold57948_1	virulent	0.92772204
DLM005_scaffold44327_3	virulent	0.9998565
DLM005_scaffold34949_1	temperate	0.9997306
DLM005_scaffold48663_11	temperate	0.9998593
DLM005_scaffold9042_2	temperate	0.9998584
DLM005_scaffold3317_29	temperate	0.96424484
DLM005_scaffold10521_42	temperate	0.9998513
DLM005_scaffold39936_1	temperate	0.9704171
DLM005_scaffold21763_7	virulent	0.99987316
DLM005_scaffold54507_2	temperate	0.99880844
DLM005_scaffold55834_11	virulent	0.5891354
DLM005_scaffold57953_2	temperate	0.9988467
DLM005_scaffold31312_1	temperate	0.9998584
DLM005_scaffold47946_1	temperate	0.995717
DLM005_scaffold56278_1	virulent	0.95286506
DLM005_scaffold3128_1	temperate	0.999248
DLM005_scaffold42596_2	virulent	0.99981785
DLM005_scaffold56545_2	virulent	0.9998736
DLM005_scaffold55666_1	virulent	0.99987084
DLM005_scaffold21763_10	temperate	0.9998288
DLM005_scaffold21049_5	temperate	0.9988944
DLM005_scaffold4283_10	temperate	0.9935788
DLM005_scaffold25964_2	virulent	0.99987036
DLM005_scaffold58045_1	temperate	0.9575632
DLM005_scaffold56278_2	virulent	0.999488
DLM005_scaffold2340_3	temperate	0.8154053
DLM005_scaffold5416_6	temperate	0.9998588
DLM005_scaffold36491_20	virulent	0.99974394
DLM005_scaffold20359_5	virulent	0.9998679
DLM005_scaffold56832_1	temperate	0.9998593
DLM005_scaffold36491_18	temperate	0.99982077
DLM005_scaffold7362_2	virulent	0.999784
DLM005_scaffold57077_1	temperate	0.99985975
DLM005_scaffold5800_5	temperate	0.99985975
DLM005_scaffold20359_8	temperate	0.9802566
DLM005_scaffold27309_11	virulent	0.9994256
DLM005_scaffold25964_1	virulent	0.9557909

DLM005_scaffold10521_41	virulent	0.8529024
DLM005_scaffold57953_1	temperate	0.8793201
DLM005_scaffold52507_2	virulent	0.9998684
DLM005_scaffold1593_32	temperate	0.9998588
DLM005_scaffold36516_2	virulent	0.9993786
DLM006_scaffold29093_1	virulent	0.99235404
DLM006_scaffold5249_12	temperate	0.977781
DLM006_scaffold9115_2	temperate	0.99984926
DLM006_scaffold23991_4	virulent	0.9998688
DLM006_scaffold31867_1	virulent	0.99986744
DLM006_scaffold1615_27	temperate	0.99984694
DLM006_scaffold35098_1	temperate	0.9997101
DLM006_scaffold8798_11	temperate	0.98404866
DLM006_scaffold15729_4	temperate	0.99985695
DLM006_scaffold10416_3	temperate	0.89246756
DLM006_scaffold289_5	virulent	0.99987125
DLM006_scaffold15803_18	virulent	0.99791527
DLM006_scaffold3905_28	temperate	0.9795305
DLM006_scaffold13732_3	virulent	0.99987084
DLM006_scaffold23991_2	temperate	0.99981356
DLM006_scaffold5392_1	temperate	0.9774004
DLM006_scaffold19568_4	temperate	0.97331256
DLM006_scaffold35098_2	virulent	0.96943474
DLM006_scaffold3099_10	virulent	0.9998508
DLM006_scaffold22671_1	virulent	0.9998688
DLM006_scaffold12233_4	temperate	0.99985975
DLM006_scaffold13444_2	temperate	0.99985975
DLM006_scaffold582_5	virulent	0.99987084
DLM006_scaffold7136_55	temperate	0.99985975
DLM006_scaffold36079_2	temperate	0.9998584
DLM006_scaffold22549_2	temperate	0.9989599
DLM007_scaffold30789_1	temperate	0.99986035
DLM007_scaffold45247_1	temperate	0.99983126
DLM007_scaffold7436_7	virulent	0.9998736
DLM007_scaffold20148_2	virulent	0.99987036
DLM007_C664617_1	virulent	0.9998699
DLM007_scaffold45308_2	virulent	0.99987173
DLM007_scaffold35599_2	virulent	0.943157
DLM007_scaffold28062_4	virulent	0.9998699
DLM007_scaffold13248_1	virulent	0.95286506
DLM007_scaffold20148_1	virulent	0.9998684
DLM007_scaffold31482_1	temperate	0.9998579
DLM007_scaffold42236_1	temperate	0.99982446
DLM007_scaffold5339_25	virulent	0.99986607
DLM007_scaffold41113_2	virulent	0.93330806
DLM007_scaffold4568_8	temperate	0.99985975

DLM007_scaffold45002_8	temperate	0.9995805
DLM007_scaffold36909_2	temperate	0.9989953
DLM007_scaffold45251_1	virulent	0.9998551
DLM008_scaffold4090_5	temperate	0.9998593
DLM008_scaffold251_3	virulent	0.9998431
DLM008_scaffold51033_29	virulent	0.9990291
DLM008_scaffold34307_5	virulent	0.9996477
DLM008_scaffold3167_2	virulent	0.9105999
DLM008_scaffold9016_5	virulent	0.99987084
DLM008_scaffold54665_3	virulent	0.9998736
DLM008_scaffold68109_1	virulent	0.9998736
DLM008_scaffold56745_2	temperate	0.9998593
DLM008_scaffold40955_1	virulent	0.99961644
DLM008_scaffold9016_1	virulent	0.9998722
DLM008_scaffold26298_3	temperate	0.92925435
DLM008_scaffold235_2	virulent	0.9998736
DLM008_scaffold19097_13	virulent	0.9949243
DLM008_scaffold58161_1	virulent	0.9986062
DLM008_scaffold349_4	virulent	0.9998693
DLM008_scaffold45871_1	temperate	0.9998588
DLM008_scaffold9016_3	virulent	0.9998699
DLM008_scaffold62586_4	temperate	0.98519784
DLM008_scaffold46088_1	temperate	0.99985975
DLM008_scaffold371_1	temperate	0.9998593
DLM008_scaffold68395_3	temperate	0.9998593
DLM008_scaffold43273_7	virulent	0.99987316
DLM008_scaffold68309_1	virulent	0.9855859
DLM008_scaffold129_7	temperate	0.99985975
DLM008_scaffold58372_1	virulent	0.9998727
DLM008_scaffold129_3	temperate	0.9697178
DLM009_scaffold11883_2	virulent	0.9997209
DLM009_scaffold6465_2	virulent	0.97065383
DLM009_scaffold19658_2	virulent	0.9420535
DLM009_C324613_1	temperate	0.99924237
DLM009_scaffold17120_3	virulent	0.9996403
DLM009_scaffold14704_1	virulent	0.9998579
DLM009_scaffold21367_3	temperate	0.9998474
DLM009_scaffold18558_3	virulent	0.9998684
DLM009_scaffold8594_4	temperate	0.99980307
DLM009_scaffold10681_1	temperate	0.8972373
DLM009_scaffold5016_13	temperate	0.9413989
DLM009_scaffold102_1	temperate	0.9998556
DLM009_C324441_1	temperate	0.99984694
DLM009_scaffold21380_1	virulent	0.99984694
DLM009_scaffold6465_20	virulent	0.9653701
DLM009_scaffold21079_1	temperate	0.99985975

DLM009_scaffold22_4	temperate	0.99985975
DLM009_scaffold21344_1	temperate	0.99981076
DLM009_scaffold8439_6	temperate	0.9993
DLM009_scaffold18558_6	virulent	0.99987173
DLM009_scaffold6465_1	temperate	0.99985695
DLM009_scaffold21365_1	virulent	0.99987173
DLM009_scaffold19658_6	virulent	0.99927014
DLM009_scaffold2554_54	temperate	0.9996577
DLM009_scaffold17086_6	virulent	0.99233663
DLM009_scaffold19690_1	temperate	0.89619565
DLM009_scaffold18558_4	virulent	0.99986744
DLM009_scaffold6465_28	virulent	0.9998183
DLM009_scaffold4728_5	temperate	0.99922544
DLM009_C324485_1	virulent	0.93833727
DLM009_scaffold18345_6	temperate	0.9989151
DLM009_scaffold19658_3	temperate	0.98952943
DLM009_C324155_1	temperate	0.9998584
DLM009_scaffold6465_23	temperate	0.9998517
DLM010_scaffold41969_2	temperate	0.99985975
DLM010_scaffold41867_3	temperate	0.99985975
DLM010_scaffold45140_1	virulent	0.99987316
DLM010_scaffold179_1	virulent	0.9484926
DLM010_scaffold503_4	temperate	0.99985975
DLM010_scaffold43377_3	temperate	0.9997669
DLM010_scaffold10158_2	temperate	0.9413989
DLM010_scaffold18321_2	temperate	0.98519784
DLM010_scaffold32474_2	temperate	0.99934417
DLM010_scaffold5445_6	virulent	0.9997258
DLM010_scaffold32332_1	temperate	0.9997897
DLM010_scaffold7753_8	virulent	0.99987125
DLM010_scaffold3050_2	temperate	0.9998517
DLM010_scaffold44670_1	virulent	0.99272764
DLM010_scaffold41095_1	temperate	0.9575748
DLM010_scaffold16539_4	virulent	0.9998699
DLM010_scaffold33840_5	virulent	0.99985975
DLM010_scaffold6743_2	temperate	0.9998397
DLM010_scaffold22879_4	virulent	0.99986404
DLM010_scaffold45132_3	temperate	0.99985975
DLM010_scaffold4248_8	temperate	0.9998588
DLM010_scaffold45189_1	virulent	0.99987036
DLM010_scaffold44889_1	virulent	0.9998693
DLM010_scaffold3339_9	virulent	0.99987173
DLM011_scaffold28392_2	virulent	0.9392347
DLM011_scaffold29232_2	temperate	0.9802566
DLM011_scaffold29382_1	virulent	0.99987036
DLM011_scaffold21086_2	virulent	0.99987406

DLM011_scaffold21352_1	virulent	0.9998465
DLM011_scaffold29393_1	virulent	0.9998722
DLM011_scaffold29232_1	temperate	0.60928476
DLM011_scaffold5932_1	virulent	0.9998699
DLM011_scaffold28062_1	temperate	0.9998556
DLM011_scaffold27250_1	temperate	0.99986035
DLM011_scaffold29207_1	virulent	0.9998656
DLM011_C484732_1	temperate	0.91957694
DLM011_scaffold1189_13	virulent	0.99960625
DLM011_scaffold28659_3	temperate	0.9998584
DLM011_scaffold17163_7	temperate	0.979654
DLM011_scaffold29398_1	virulent	0.999836
DLM011_scaffold1189_31	virulent	0.9880252
DLM011_scaffold19964_1	virulent	0.9998588
DLM011_scaffold1699_8	virulent	0.9996546
DLM011_scaffold5789_4	virulent	0.9997726
DLM011_scaffold18867_6	temperate	0.9413989
DLM011_scaffold24958_1	virulent	0.9997339
DLM012_scaffold11223_12	virulent	0.9998513
DLM012_scaffold16447_5	virulent	0.99987316
DLM012_scaffold19314_6	virulent	0.99982095
DLM012_scaffold49_1	virulent	0.93934274
DLM012_scaffold34010_1	temperate	0.99965674
DLM012_scaffold22034_2	virulent	0.7283555
DLM012_scaffold7495_7	virulent	0.999531
DLM012_C414271_1	virulent	0.99970484
DLM012_scaffold23728_1	virulent	0.9139237
DLM012_scaffold33026_10	temperate	0.9998579
DLM012_scaffold2394_3	temperate	0.92079926
DLM012_scaffold33913_1	temperate	0.99951667
DLM012_scaffold12_2	temperate	0.9997115
DLM012_scaffold33950_1	temperate	0.9998584
DLM012_scaffold29288_6	temperate	0.99985695
DLM012_scaffold32680_2	virulent	0.9996124
DLM012_scaffold26999_6	virulent	0.7405412
DLM012_scaffold28806_1	temperate	0.99986035
DLM012_scaffold725_1	virulent	0.84835416
DLM012_scaffold33245_1	temperate	0.99398
DLM012_scaffold29288_5	temperate	0.9998584
DLM013_scaffold23313_3	temperate	0.9848878
DLM013_scaffold3286_13	temperate	0.8881842
DLM013_scaffold17656_2	temperate	0.9998593
DLM013_scaffold7395_5	virulent	0.99984604
DLM013_scaffold34896_3	virulent	0.9998651
DLM013_scaffold21602_2	temperate	0.9998593
DLM013_scaffold57623_1	virulent	0.99987316

DLM013_scaffold55482_1	temperate	0.99981695
DLM013_scaffold36790_6	temperate	0.99985975
DLM013_scaffold8482_4	virulent	0.99986124
DLM013_scaffold45817_1	temperate	0.9701814
DLM013_scaffold3810_8	virulent	0.9998699
DLM013_scaffold23184_5	virulent	0.99987125
DLM013_scaffold57456_3	temperate	0.93894786
DLM013_scaffold56424_2	virulent	0.99987173
DLM013_scaffold3810_7	virulent	0.9918154
DLM013_scaffold318_1	temperate	0.99985975
DLM013_scaffold15355_2	temperate	0.9943185
DLM013_scaffold22571_2	temperate	0.99982077
DLM013_scaffold4430_4	temperate	0.6317847
DLM013_scaffold56628_1	virulent	0.9998684
DLM013_scaffold47801_1	temperate	0.9998588
DLM013_scaffold51742_2	virulent	0.99987084
DLM013_scaffold23184_12	virulent	0.99984926
DLM013_scaffold54893_2	temperate	0.85934323
DLM013_scaffold57687_1	virulent	0.99987036
DLM013_scaffold8482_6	virulent	0.9998699
DLM013_scaffold23009_1	virulent	0.9998556
DLM013_scaffold3614_26	virulent	0.67038256
DLM013_scaffold23737_4	virulent	0.9998722
DLM013_scaffold49483_1	temperate	0.99985975
DLM013_scaffold51645_2	temperate	0.99985313
DLM013_scaffold21436_7	temperate	0.9647605
DLM013_scaffold38983_1	temperate	0.9997587
DLM013_scaffold20700_8	virulent	0.99987084
DLM013_scaffold50692_1	temperate	0.9984187
DLM013_scaffold25009_5	temperate	0.99985975
DLM013_scaffold14795_2	temperate	0.99986035
DLM013_scaffold52105_9	virulent	0.9996192
DLM013_scaffold11264_1	virulent	0.99987084
DLM013_scaffold52295_2	temperate	0.9929295
DLM013_scaffold23184_9	virulent	0.99987173
DLM013_scaffold49247_2	temperate	0.99075556
DLM013_scaffold56635_1	virulent	0.99344337
DLM013_C721558_1	virulent	0.99985313
DLM013_scaffold31022_3	virulent	0.99982953
DLM013_scaffold30472_4	virulent	0.9915161
DLM013_scaffold16485_8	virulent	0.9998617
DLM013_scaffold55003_1	temperate	0.99985975
DLM013_scaffold23574_3	virulent	0.9998579
DLM013_scaffold3614_27	virulent	0.9998086
DLM013_scaffold24100_2	virulent	0.9996887
DLM013_scaffold2200_1	temperate	0.99985975

DLM014_scaffold52949_1	temperate	0.9998522
DLM014_scaffold14966_8	virulent	0.9998665
DLM014_scaffold26538_3	virulent	0.91296697
DLM014_scaffold3539_3	temperate	0.93755734
DLM014_scaffold39602_3	temperate	0.99984497
DLM014_scaffold51860_2	temperate	0.9998593
DLM014_scaffold34561_6	temperate	0.99986035
DLM014_scaffold17726_11	temperate	0.9998403
DLM014_scaffold43677_1	virulent	0.99987406
DLM014_scaffold31737_5	virulent	0.99986744
DLM014_scaffold1_9	virulent	0.99987173
DLM014_scaffold52940_1	temperate	0.99986035
DLM014_scaffold44624_1	temperate	0.99918586
DLM014_scaffold24690_1	virulent	0.99980664
DLM014_scaffold42199_1	temperate	0.9910449
DLM014_scaffold3883_2	temperate	0.99985605
DLM014_scaffold7847_1	temperate	0.99959904
DLM014_scaffold46228_1	temperate	0.99969196
DLM014_scaffold35850_2	virulent	0.9998413
DLM014_scaffold40706_2	virulent	0.99987173
DLM014_scaffold14317_2	virulent	0.9997053
DLM014_scaffold42200_1	temperate	0.9592161
DLM014_scaffold45829_2	temperate	0.9468743
DLM014_scaffold31737_1	temperate	0.99985695
DLM014_scaffold4026_7	virulent	0.99987173
DLM014_scaffold21553_3	temperate	0.99985975
DLM014_scaffold23167_4	temperate	0.550134
DLM014_scaffold32434_6	virulent	0.99987173
DLM014_scaffold18806_7	virulent	0.95832527
DLM014_scaffold1942_34	temperate	0.99985975
DLM014_scaffold18901_3	virulent	0.99980956
DLM014_scaffold48474_2	virulent	0.9406228
DLM014_scaffold1_18	virulent	0.99987084
DLM014_scaffold52939_1	virulent	0.99987173
DLM014_scaffold290_4	virulent	0.9995576
DLM014_scaffold37209_4	virulent	0.99987406
DLM014_scaffold36217_6	virulent	0.99982077
DLM014_scaffold7310_3	virulent	0.99987316
DLM015_scaffold18535_6	temperate	0.9998265
DLM015_scaffold5802_5	virulent	0.9998736
DLM015_scaffold16218_2	virulent	0.9998622
DLM015_scaffold50361_1	temperate	0.99986035
DLM015_scaffold50502_1	temperate	0.99951375
DLM015_scaffold23053_2	temperate	0.99973255
DLM015_scaffold1049_4	virulent	0.99987173
DLM015_scaffold1119_3	virulent	0.99987173

DLM015_scaffold36073_3	temperate	0.9998588
DLM015_scaffold23053_6	temperate	0.9998556
DLM015_scaffold47979_4	temperate	0.979654
DLM015_scaffold49465_1	virulent	0.9997056
DLM015_scaffold23554_1	virulent	0.99354404
DLM015_scaffold50303_1	virulent	0.8716671
DLM015_scaffold34621_5	virulent	0.99987125
DLM015_scaffold47846_2	temperate	0.99985975
DLM015_scaffold50189_1	virulent	0.99986404
DLM015_scaffold5802_2	virulent	0.9998722
DLM015_scaffold14872_4	virulent	0.9998617
DLM015_scaffold50457_1	virulent	0.99983
DLM015_scaffold29903_2	virulent	0.99896526
DLM015_scaffold36073_4	temperate	0.8653128
DLM015_scaffold145_2	virulent	0.7335834
DLM015_scaffold17140_1	virulent	0.9053317
DLM015_scaffold36351_2	temperate	0.99934417
DLM016_scaffold17586_2	temperate	0.9998551
DLM016_scaffold3377_13	virulent	0.88889205
DLM016_scaffold65085_5	virulent	0.9998665
DLM016_scaffold61369_1	virulent	0.99987173
DLM016_scaffold64744_4	temperate	0.99985975
DLM016_scaffold12_1	temperate	0.9998588
DLM016_scaffold13346_4	virulent	0.9168372
DLM016_scaffold27953_5	virulent	0.9996776
DLM016_scaffold37509_2	temperate	0.99985313
DLM016_scaffold46534_2	virulent	0.99987316
DLM016_scaffold419_5	virulent	0.9997726
DLM016_scaffold41709_1	virulent	0.88211113
DLM016_scaffold22143_3	virulent	0.9998736
DLM016_scaffold40529_1	virulent	0.9996805
DLM016_scaffold7191_1	virulent	0.9761889
DLM016_scaffold49496_1	virulent	0.9998343
DLM016_scaffold40187_1	temperate	0.9949661
DLM016_scaffold6553_2	virulent	0.9998651
DLM016_scaffold16615_5	temperate	0.9973022
DLM016_scaffold2806_2	virulent	0.9998693
DLM016_scaffold53043_5	temperate	0.9998536
DLM016_scaffold5004_5	virulent	0.9998699
DLM016_scaffold65482_1	virulent	0.99987125
DLM016_scaffold65575_2	virulent	0.999844
DLM016_scaffold6713_8	virulent	0.99987316
DLM016_scaffold6713_3	virulent	0.9998608
DLM016_scaffold6713_5	virulent	0.9998291
DLM016_scaffold64767_1	virulent	0.99987125
DLM016_scaffold6713_11	virulent	0.9998636

DLM016_scaffold6713_2	temperate	0.9998397
DLM016_scaffold2366_1	virulent	0.99978536
DLM016_scaffold4953_3	temperate	0.99985975
DLM016_scaffold6914_1	temperate	0.93755734
DLM016_scaffold58329_1	virulent	0.99986696
DLM016_scaffold52894_5	virulent	0.99987125
DLM016_scaffold6713_12	virulent	0.99986744
DLM016_scaffold37873_2	temperate	0.9998588
DLM016_scaffold64097_2	virulent	0.9998656
DLM016_scaffold21785_1	virulent	0.99980736
DLM016_scaffold65474_2	temperate	0.98817694
DLM016_scaffold65085_3	virulent	0.9998636
DLM016_scaffold6553_6	virulent	0.9998584
DLM016_scaffold6553_4	virulent	0.9998684
DLM016_scaffold1623_5	temperate	0.9998584
DLM016_scaffold63991_1	virulent	0.99987084
DLM016_scaffold29974_3	virulent	0.9997275
DLM016_scaffold53961_3	temperate	0.98519784
DLM016_scaffold419_9	virulent	0.99987125
DLM017_scaffold20271_2	temperate	0.99708074
DLM017_scaffold2813_9	virulent	0.9998699
DLM017_scaffold3964_2	virulent	0.99987316
DLM017_scaffold29996_2	virulent	0.99986744
DLM017_scaffold20005_2	temperate	0.9998308
DLM017_scaffold21266_1	temperate	0.99956053
DLM017_scaffold33959_3	temperate	0.9990059
DLM017_scaffold2501_2	temperate	0.999662
DLM017_scaffold43714_2	temperate	0.99979925
DLM017_scaffold5960_2	virulent	0.9993222
DLM017_scaffold1892_10	temperate	0.9998588
DLM017_scaffold28739_1	temperate	0.9998593
DLM017_scaffold5705_1	temperate	0.9997044
DLM017_scaffold42571_2	virulent	0.9991045
DLM017_scaffold1478_32	virulent	0.88889205
DLM017_scaffold10166_1	temperate	0.9998574
DLM017_scaffold15588_1	temperate	0.9998593
DLM017_scaffold31873_1	temperate	0.9998584
DLM017_scaffold23811_1	temperate	0.93894786
DLM017_scaffold18232_1	virulent	0.99987173
DLM018_scaffold36970_5	virulent	0.9998722
DLM018_scaffold38471_2	virulent	0.9998536
DLM018_scaffold37570_1	virulent	0.99160045
DLM018_scaffold197_3	temperate	0.81878257
DLM018_scaffold13432_9	temperate	0.99985695
DLM018_scaffold45347_1	virulent	0.99977875
DLM018_scaffold49175_1	virulent	0.9998736

DLM018_scaffold12094_2	temperate	0.9676156
DLM018_scaffold3525_6	temperate	0.9997935
DLM018_scaffold2054_4	temperate	0.9998445
DLM018_scaffold82_1	temperate	0.9975639
DLM018_scaffold48894_1	virulent	0.9998722
DLM018_scaffold26573_10	virulent	0.9998679
DLM018_scaffold45347_2	virulent	0.9998627
DLM018_scaffold48696_1	virulent	0.9998693
DLM018_scaffold44037_1	virulent	0.9998593
DLM018_scaffold1207_3	temperate	0.9988239
DLM018_scaffold79_28	virulent	0.99023
DLM018_scaffold15_30	temperate	0.94221675
DLM018_scaffold48959_3	virulent	0.99986315
DLM018_scaffold7847_2	virulent	0.999836
DLM018_scaffold31396_2	temperate	0.8019704
DLM018_scaffold14900_3	temperate	0.9025601
DLM018_scaffold48449_1	temperate	0.9998593
DLM018_scaffold47288_3	temperate	0.99981695
DLM018_scaffold26573_4	temperate	0.99986035
DLM018_scaffold36970_6	virulent	0.9998228
DLM018_scaffold40461_1	virulent	0.999322
DLM018_scaffold48983_2	temperate	0.999804
DLM018_scaffold26955_2	temperate	0.98430026
DLM018_scaffold3351_9	temperate	0.96074384
DLM018_scaffold19081_1	virulent	0.9998736
DLM019_scaffold19742_4	virulent	0.88442934
DLM019_scaffold62503_1	temperate	0.99986035
DLM019_scaffold22877_3	temperate	0.87514806
DLM019_scaffold62785_1	temperate	0.9476128
DLM019_scaffold53137_1	temperate	0.99985266
DLM019_scaffold20492_3	temperate	0.99041367
DLM019_scaffold6166_23	virulent	0.99984694
DLM019_scaffold44099_4	virulent	0.99986744
DLM019_scaffold8577_4	temperate	0.997696
DLM019_scaffold20492_2	temperate	0.9998593
DLM019_C814862_1	temperate	0.9998579
DLM019_scaffold10836_2	temperate	0.9998545
DLM019_scaffold45285_5	temperate	0.9993865
DLM019_scaffold24899_8	temperate	0.8866283
DLM019_scaffold63202_2	virulent	0.99986607
DLM019_scaffold53137_2	temperate	0.9262353
DLM019_scaffold2743_2	virulent	0.99987036
DLM019_scaffold61987_1	temperate	0.99986035
DLM019_scaffold35325_15	temperate	0.9993991
DLM019_scaffold31976_1	virulent	0.9522072
DLM019_scaffold39_1	temperate	0.99985975

DLM019_scaffold38422_4	virulent	0.99963504
DLM019_scaffold45285_4	temperate	0.99964815
DLM019_scaffold4998_6	temperate	0.99985695
DLM019_scaffold54914_7	temperate	0.9919748
DLM019_scaffold35445_2	virulent	0.9949459
DLM019_scaffold37359_6	virulent	0.9905684
DLM019_scaffold100_11	virulent	0.9998636
DLM019_scaffold55656_1	virulent	0.9997321
DLM019_scaffold38422_1	temperate	0.53554034
DLM019_scaffold63049_1	virulent	0.9998679
DLM019_scaffold102_1	temperate	0.9017353
DLM019_scaffold62785_2	virulent	0.99985695
DLM019_scaffold8817_14	virulent	0.9957946
DLM019_scaffold55454_1	temperate	0.99985975
DLM019_scaffold29437_2	temperate	0.9998574
DLM019_scaffold12600_2	virulent	0.9998608
DLM019_scaffold2445_1	temperate	0.99983495
DLM019_scaffold44258_2	temperate	0.9998574
DLM019_scaffold31935_1	temperate	0.99913585
DLM019_scaffold8788_7	temperate	0.93894786
DLM019_scaffold63216_1	virulent	0.99986744
DLM020_scaffold983_14	virulent	0.99977165
DLM020_scaffold8084_6	temperate	0.93656045
DLM020_scaffold11081_1	virulent	0.9998331
DLM020_scaffold15999_4	virulent	0.99983215
DLM020_scaffold21144_2	temperate	0.98519784
DLM020_scaffold19547_2	virulent	0.9998308
DLM020_scaffold10399_1	virulent	0.9998722
DLM020_scaffold3010_3	temperate	0.9997344
DLM020_scaffold20362_3	temperate	0.9881228
DLM020_scaffold8114_1	virulent	0.59466475
DLM020_scaffold8736_5	temperate	0.99985975
DLM020_scaffold500_10	temperate	0.9413989
DLM020_scaffold7279_1	temperate	0.9957495
DLM020_scaffold1387_1	virulent	0.99987084
DLM020_scaffold22314_2	temperate	0.95623636
DLM020_scaffold8693_2	virulent	0.9998727
DLM020_scaffold20362_2	temperate	0.99833655
DLM020_scaffold22182_2	temperate	0.98694676
DLM020_scaffold21959_1	temperate	0.9998117
DLM020_scaffold6157_3	virulent	0.99987316
DLM020_scaffold19925_2	temperate	0.9998584
DLM020_scaffold13039_2	virulent	0.9998679
DLM020_C355636_1	temperate	0.9998584
DLM020_scaffold350_1	temperate	0.9998593
DLM020_scaffold20632_1	virulent	0.908313

DLM020_scaffold22218_1	temperate	0.99985975
DLM020_scaffold6936_4	temperate	0.999854
DLM020_scaffold7475_1	virulent	0.99987084
DLM020_scaffold3936_1	temperate	0.9998188
DLM020_scaffold10415_1	virulent	0.99987173
DLM020_scaffold10180_3	virulent	0.96428293
DLM020_scaffold22290_1	virulent	0.9998627
DLM020_scaffold5648_4	virulent	0.99987465
DLM020_scaffold11301_1	temperate	0.7636316
DLM020_scaffold21039_1	virulent	0.9785579
DLM020_scaffold7279_3	temperate	0.99985975
DLM020_scaffold13927_3	virulent	0.99987465
DLM020_scaffold10263_1	temperate	0.9998593
DLM020_scaffold6936_3	virulent	0.9998012
DLM021_scaffold36034_1	temperate	0.9997206
DLM021_scaffold44307_1	virulent	0.9988832
DLM021_scaffold43159_1	virulent	0.99987036
DLM021_scaffold31538_5	temperate	0.9986814
DLM021_scaffold29140_2	virulent	0.9998608
DLM021_scaffold35216_1	virulent	0.99966675
DLM021_scaffold43126_4	virulent	0.9998012
DLM021_scaffold42967_2	temperate	0.9998369
DLM021_scaffold12971_1	temperate	0.5923225
DLM021_scaffold28049_1	virulent	0.9242962
DLM021_scaffold36642_2	virulent	0.99986696
DLM021_scaffold42193_7	virulent	0.99986035
DLM021_scaffold43501_2	temperate	0.979654
DLM021_scaffold554_3	temperate	0.9998593
DLM021_scaffold43126_6	temperate	0.99983406
DLM021_scaffold31475_3	virulent	0.99986696
DLM021_scaffold24637_8	temperate	0.99983835
DLM021_scaffold10545_9	virulent	0.99986607
DLM021_scaffold4_54	virulent	0.99982494
DLM021_scaffold30546_2	temperate	0.9998388
DLM021_scaffold10200_17	virulent	0.99972486
DLM021_scaffold28049_2	virulent	0.99892867
DLM021_scaffold10200_12	virulent	0.9998722
DLM021_scaffold23214_1	temperate	0.9998536
DLM021_scaffold136_1	virulent	0.9998651
DLM021_scaffold46_2	virulent	0.9998727
DLM021_scaffold24731_9	virulent	0.9998688
DLM021_scaffold640_7	temperate	0.97422904
DLM021_scaffold21292_11	virulent	0.9996989
DLM021_scaffold22868_5	virulent	0.9998086
DLM021_scaffold2298_4	virulent	0.99987406
DLM021_scaffold44452_1	virulent	0.9998636

DLM021_scaffold8187_1	virulent	0.9937134
DLM021_scaffold45210_2	temperate	0.99973255
DLM021_scaffold381_1	temperate	0.99985975
DLM021_scaffold1604_4	virulent	0.99987173
DLM022_scaffold87335_3	virulent	0.96945584
DLM022_scaffold90455_1	temperate	0.99954236
DLM022_C1105606_1	temperate	0.9824839
DLM022_scaffold34930_3	temperate	0.99985695
DLM022_scaffold13671_3	temperate	0.9998545
DLM022_scaffold90383_1	temperate	0.9998579
DLM022_scaffold61396_2	virulent	0.99519646
DLM022_scaffold3872_14	temperate	0.99986035
DLM022_scaffold28531_1	virulent	0.99986315
DLM022_scaffold13626_15	temperate	0.9998536
DLM022_scaffold47568_2	temperate	0.98138005
DLM022_scaffold87188_2	temperate	0.9998584
DLM022_scaffold83380_1	temperate	0.9998593
DLM022_scaffold38614_1	temperate	0.9998579
DLM022_scaffold90423_1	temperate	0.99985975
DLM022_scaffold7833_6	temperate	0.9998593
DLM022_scaffold25768_2	temperate	0.9560764
DLM022_scaffold90441_1	temperate	0.99985975
DLM022_scaffold29304_11	virulent	0.99986696
DLM022_scaffold45845_2	virulent	0.9998165
DLM022_scaffold75069_1	temperate	0.9997144
DLM022_scaffold46815_1	temperate	0.99905294
DLM022_scaffold50520_4	temperate	0.9966986
DLM022_scaffold82276_2	virulent	0.9998608
DLM022_scaffold86174_3	temperate	0.98876494
DLM022_scaffold40951_1	virulent	0.9996882
DLM022_scaffold51389_1	virulent	0.9998679
DLM022_scaffold34373_9	temperate	0.99985975
DLM022_scaffold88974_2	temperate	0.99986035
DLM022_scaffold37828_6	virulent	0.9998722
DLM022_scaffold7976_5	virulent	0.99663186
DLM022_scaffold68876_1	virulent	0.9998688
DLM022_scaffold24869_7	virulent	0.9998588
DLM022_scaffold46210_2	virulent	0.9998651
DLM022_scaffold79585_1	virulent	0.9998228
DLM022_scaffold90471_1	temperate	0.8644271
DLM022_scaffold126_4	temperate	0.9998588
DLM022_scaffold91_3	temperate	0.96827364
DLM022_scaffold7197_1	virulent	0.99984926
DLM022_scaffold24869_6	virulent	0.99986315
DLM022_scaffold79462_2	virulent	0.99987173
DLM022_scaffold26167_3	virulent	0.99987465

DLM022_scaffold8618_3	virulent	0.9998684
DLM022_scaffold20053_1	virulent	0.99987465
DLM022_scaffold248_3	temperate	0.99922353
DLM022_scaffold90464_1	virulent	0.99986744
DLM022_scaffold63634_1	temperate	0.9998308
DLM022_scaffold90529_1	temperate	0.999702
DLM022_scaffold82197_1	temperate	0.9998584
DLM022_scaffold84536_3	temperate	0.99985695
DLM022_scaffold11050_1	temperate	0.99985695
DLM022_scaffold73260_1	temperate	0.99984604
DLM022_scaffold79270_1	temperate	0.9998369
DLM022_scaffold47815_3	virulent	0.9998699
DLM022_scaffold68020_2	temperate	0.99884605
DLM022_scaffold12444_5	temperate	0.9998588
DLM022_scaffold86174_2	virulent	0.93819433
DLM022_scaffold11069_53	virulent	0.99985975
DLM022_scaffold89824_2	virulent	0.9609294
DLM022_C1104604_1	temperate	0.9998593
DLM022_scaffold36935_1	temperate	0.9998593
DLM022_scaffold80933_2	virulent	0.99983126
DLM022_scaffold21034_2	virulent	0.99987406
DLM022_scaffold85942_2	virulent	0.9971863
DLM022_scaffold9_2	virulent	0.998057
DLM022_scaffold88907_2	temperate	0.99985975
DLM022_scaffold6448_3	virulent	0.6171255
DLM022_scaffold90528_2	temperate	0.99985975
DLM022_scaffold160_1	virulent	0.97564626
DLM022_scaffold64018_1	virulent	0.9998699
DLM022_scaffold89824_3	temperate	0.9998379
DLM022_scaffold43500_4	temperate	0.9998593
DLM022_scaffold90113_2	temperate	0.999796
DLM022_scaffold37957_2	temperate	0.9998122
DLM022_scaffold11067_1	virulent	0.99987084
DLM022_scaffold22367_7	virulent	0.99986315
DLM022_scaffold24995_4	temperate	0.999016
DLM022_scaffold89236_1	temperate	0.99896556
DLM022_scaffold30520_1	temperate	0.9996515
DLM022_scaffold32204_1	temperate	0.99986035
DLM022_scaffold46686_4	virulent	0.9998645
DLM022_scaffold85281_1	virulent	0.9998736
DLM022_scaffold90528_3	temperate	0.99985975
DLM022_scaffold89880_1	temperate	0.99985975
DLM022_scaffold24869_4	virulent	0.9998627
DLM022_scaffold89156_1	temperate	0.99984264
DLM022_scaffold90507_1	temperate	0.9995376
DLM022_scaffold62765_1	temperate	0.9994489

DLM022_scaffold27082_1	temperate	0.9997535
DLM022_scaffold73568_2	virulent	0.99987125
DLM022_scaffold88059_2	virulent	0.9998699
DLM022_scaffold90402_1	virulent	0.9998651
DLM022_scaffold90509_1	virulent	0.9992649
DLM022_scaffold44709_3	temperate	0.9998388
DLM022_scaffold87605_2	virulent	0.99984884
DLM022_scaffold937_1	virulent	0.9998656
DLM022_scaffold30068_2	virulent	0.99933046
DLM022_scaffold15558_7	temperate	0.99585557
DLM023_scaffold51589_1	temperate	0.64372236
DLM023_scaffold25385_1	temperate	0.9998584
DLM023_scaffold1460_2	temperate	0.99985975
DLM023_scaffold14487_2	virulent	0.99970657
DLM023_scaffold48996_1	temperate	0.99984604
DLM023_scaffold4774_1	temperate	0.99985975
DLM023_scaffold27445_8	temperate	0.99986035
DLM023_scaffold411_39	temperate	0.9994242
DLM023_scaffold51795_1	temperate	0.9998588
DLM023_scaffold23339_1	temperate	0.8228076
DLM023_scaffold2126_10	virulent	0.9998688
DLM023_scaffold7323_3	virulent	0.9998736
DLM023_scaffold44217_3	temperate	0.9985996
DLM023_scaffold47535_1	virulent	0.9998019
DLM023_scaffold37922_1	temperate	0.9998588
DLM023_scaffold4774_5	virulent	0.99986696
DLM023_scaffold51029_1	virulent	0.9998693
DLM023_scaffold18407_7	temperate	0.74348515
DLM023_scaffold17201_4	temperate	0.9998588
DLM023_scaffold45333_4	temperate	0.9998584
DLM023_scaffold28443_5	temperate	0.99985266
DLM023_scaffold32334_1	virulent	0.9990329
DLM023_scaffold51553_3	virulent	0.99986744
DLM023_scaffold4774_3	virulent	0.9998593
DLM023_scaffold13027_1	temperate	0.9998593
DLM023_scaffold58_7	temperate	0.99984217
DLM023_scaffold51688_1	temperate	0.9998593
DLM023_scaffold26150_3	temperate	0.9998474
DLM024_scaffold4102_1	temperate	0.9998054
DLM024_scaffold6096_2	temperate	0.9998584
DLM024_scaffold1307_14	temperate	0.99985975
DLM024_scaffold6915_1	virulent	0.9997557
DLM024_scaffold6392_4	temperate	0.9998579
DLM024_scaffold6942_1	virulent	0.9849078
DLM024_scaffold2301_1	virulent	0.9992417
DLM024_scaffold6782_2	temperate	0.99939895

DLM024_scaffold4274_2	temperate	0.99947035
DLM024_scaffold447_14	temperate	0.99985975
DLM024_scaffold3270_1	temperate	0.9998584
DLM024_scaffold1215_1	temperate	0.9979246
DLM024_scaffold6863_2	virulent	0.9998556
DLM024_scaffold6734_2	temperate	0.9094732
DLM024_scaffold1543_4	virulent	0.9996913
DLM024_scaffold5188_2	virulent	0.65289533
DLM024_scaffold5624_3	temperate	0.9998588
DLM024_scaffold6919_2	virulent	0.99987316
DLM024_scaffold3966_3	temperate	0.9998522
DLM024_C136347_1	virulent	0.9998257
DLM024_scaffold722_1	virulent	0.99987125
DLM024_scaffold3585_1	virulent	0.9997592
DLM024_scaffold2213_13	temperate	0.99985975
DLM024_scaffold2465_1	virulent	0.9998574
DLM024_scaffold4575_2	temperate	0.99985033
DLM024_scaffold1355_19	temperate	0.999844
DLM027_scaffold2040_2	virulent	0.99984926
DLM027_scaffold19184_4	virulent	0.99984974
DLM027_scaffold20206_4	virulent	0.9788562
DLM027_scaffold25719_1	virulent	0.96856415
DLM027_scaffold18274_3	virulent	0.9997938
DLM027_scaffold2427_8	temperate	0.9998593
DLM027_scaffold2177_5	virulent	0.9979295
DLM027_scaffold10585_3	virulent	0.9998334
DLM027_scaffold26586_2	temperate	0.9908827
DLM027_scaffold26569_1	temperate	0.9997521
DLM027_scaffold23395_3	temperate	0.9891691
DLM027_scaffold19716_4	virulent	0.99987036
DLM027_scaffold26720_1	virulent	0.99987173
DLM027_scaffold5447_3	temperate	0.9998593
DLM027_scaffold26569_2	temperate	0.9998588
DLM027_scaffold79_10	temperate	0.99934417
DLM027_scaffold19168_1	temperate	0.9990398
DLM027_scaffold26477_2	temperate	0.8308406
DLM027_scaffold9656_1	temperate	0.9437092
DLM027_scaffold9665_4	temperate	0.9998565
DLM027_scaffold8638_4	temperate	0.64852494
DLM027_scaffold26707_1	temperate	0.646693
DLM027_scaffold14908_1	temperate	0.999806
DLM028_scaffold6098_12	virulent	0.9998699
DLM028_scaffold32640_1	virulent	0.99946034
DLM028_scaffold21827_4	temperate	0.9997997
DLM028_scaffold54482_2	temperate	0.9998556
DLM028_scaffold33522_2	virulent	0.9998617

DLM028_scaffold35712_5	temperate	0.99983835
DLM028_scaffold39913_2	virulent	0.9998727
DLM028_scaffold30320_6	virulent	0.9998679
DLM028_scaffold17_2	temperate	0.99985975
DLM028_scaffold40934_1	temperate	0.9996744
DLM028_scaffold30790_1	temperate	0.9998545
DLM028_scaffold43205_3	virulent	0.9998114
DLM028_scaffold12478_1	virulent	0.9998727
DLM028_scaffold48949_2	virulent	0.99987084
DLM028_scaffold28515_6	virulent	0.9998665
DLM028_scaffold37211_4	virulent	0.9865467
DLM028_scaffold25751_8	virulent	0.9908166
DLM028_scaffold46719_2	temperate	0.9998584
DLM028_scaffold10447_6	temperate	0.9998513
DLM028_scaffold54580_1	virulent	0.99986315
DLM028_scaffold30334_1	temperate	0.9998302
DLM028_C675659_1	virulent	0.9996658
DLM028_scaffold21827_3	virulent	0.99291134
DLM028_scaffold32640_2	virulent	0.9998688
DLM028_C674591_1	virulent	0.9998736
DLM028_scaffold268_28	temperate	0.99985975
DLM028_scaffold1815_5	virulent	0.99878967
DLM028_scaffold29533_12	temperate	0.9413989
DLM028_scaffold3721_11	temperate	0.9993922
DLM028_scaffold51026_1	temperate	0.99985313
DLM028_scaffold54608_1	temperate	0.9986798
DLM028_scaffold39849_2	temperate	0.99986035
DLM028_scaffold28558_6	temperate	0.5942064
DLM028_scaffold10666_1	virulent	0.973823
DLM028_scaffold32204_5	virulent	0.99984604
DLM028_scaffold16942_4	temperate	0.99985975
DLM028_scaffold28515_4	virulent	0.99983174
DLM028_scaffold36337_1	temperate	0.9998579
DLM028_scaffold13566_10	temperate	0.99980354
DLM028_scaffold6503_3	temperate	0.99985695
DLM028_scaffold1257_18	temperate	0.9998593
DLM028_scaffold51841_1	virulent	0.99984217
DLM028_scaffold16640_1	temperate	0.99984264
DLM028_scaffold37211_5	virulent	0.9864348
DLM028_scaffold726_8	virulent	0.99624485
DLM028_scaffold41953_1	temperate	0.99985313
DLM028_scaffold26265_2	virulent	0.9692653
DLM028_scaffold20432_3	virulent	0.99985313
DLM028_scaffold9236_8	temperate	0.9998403
DLM028_scaffold86_3	virulent	0.9998736
DLM028_scaffold31687_6	virulent	0.9967521

NLM001_scaffold3386_6	temperate	0.9468059
NLM001_scaffold44600_3	virulent	0.9997247
NLM001_scaffold38495_3	virulent	0.99987173
NLM001_scaffold2478_4	virulent	0.9998522
NLM001_scaffold44595_2	temperate	0.9998565
NLM001_scaffold4227_2	virulent	0.99961454
NLM001_scaffold7_9	virulent	0.9996124
NLM001_scaffold43773_1	virulent	0.9998684
NLM001_scaffold10910_7	temperate	0.99985975
NLM001_scaffold41698_8	temperate	0.99983925
NLM001_scaffold5105_1	virulent	0.8326442
NLM001_scaffold41377_1	temperate	0.9998508
NLM001_scaffold5659_21	virulent	0.7249233
NLM001_scaffold43435_4	virulent	0.99987316
NLM001_scaffold31705_8	virulent	0.64146
NLM001_scaffold5105_4	virulent	0.99987036
NLM001_scaffold2_5	temperate	0.9413989
NLM001_scaffold44595_1	temperate	0.99984837
NLM001_scaffold17693_25	virulent	0.9998693
NLM001_scaffold44484_1	temperate	0.9998584
NLM001_scaffold40612_7	temperate	0.9958789
NLM002_scaffold13648_17	temperate	0.9998022
NLM002_scaffold28424_2	virulent	0.99984604
NLM002_scaffold15649_2	temperate	0.98625636
NLM002_scaffold8112_4	virulent	0.9996882
NLM002_scaffold28005_2	temperate	0.8061577
NLM002_scaffold13407_12	virulent	0.99987173
NLM002_scaffold4211_7	temperate	0.8884447
NLM002_scaffold28119_5	virulent	0.8875334
NLM002_scaffold15370_2	temperate	0.8465202
NLM002_C361292_1	temperate	0.99964535
NLM002_scaffold8657_21	temperate	0.9998593
NLM002_scaffold23710_2	virulent	0.99984556
NLM002_scaffold17510_4	temperate	0.9780044
NLM002_scaffold14431_4	temperate	0.9812345
NLM002_scaffold5906_19	temperate	0.9998551
NLM002_C361448_1	temperate	0.99933356
NLM002_scaffold16029_2	temperate	0.5039522
NLM003_scaffold10761_3	virulent	0.9989405
NLM003_scaffold4486_1	virulent	0.9998722
NLM003_scaffold10922_3	virulent	0.5579255
NLM003_scaffold261_3	temperate	0.9998565
NLM003_scaffold977_2	virulent	0.9998617
NLM003_scaffold14728_2	virulent	0.9998436
NLM003_scaffold3763_2_2	temperate	0.9998588
NLM003_scaffold189_8_1	virulent	0.99987316

NLM003_scaffold4295_1	virulent	0.9998565
NLM004_scaffold44166_11	virulent	0.99987125
NLM004_scaffold27516_1	temperate	0.99978167
NLM004_scaffold815_2	virulent	0.9998727
NLM004_scaffold54737_2	virulent	0.9998688
NLM004_scaffold285_3	virulent	0.99987084
NLM004_scaffold14733_2	virulent	0.99986744
NLM004_scaffold40373_1	virulent	0.99987125
NLM004_scaffold3539_11	temperate	0.99315417
NLM004_scaffold46877_2	temperate	0.99986035
NLM004_scaffold217_2	temperate	0.99985975
NLM004_scaffold18101_4	temperate	0.9998579
NLM004_scaffold35292_1	virulent	0.99969965
NLM004_scaffold13021_3	temperate	0.9998574
NLM004_scaffold14042_13	virulent	0.8118703
NLM004_scaffold49362_1	virulent	0.99987465
NLM004_scaffold2268_1	temperate	0.9998593
NLM004_C687357_1	virulent	0.8251606
NLM004_scaffold9767_3	temperate	0.96577114
NLM004_scaffold53545_1	temperate	0.99985975
NLM004_scaffold20206_2	virulent	0.9998722
NLM004_scaffold27473_2	virulent	0.9997247
NLM004_scaffold28209_1	virulent	0.99987406
NLM004_scaffold54819_1	virulent	0.9992964
NLM004_scaffold3052_12	temperate	0.99891406
NLM004_scaffold54430_1	virulent	0.99979234
NLM004_scaffold9622_3	virulent	0.9998736
NLM005_scaffold17186_2	temperate	0.99986035
NLM005_scaffold7084_1	temperate	0.9483567
NLM005_scaffold34767_1	temperate	0.9367095
NLM005_scaffold30374_9	virulent	0.99986744
NLM005_scaffold32010_3	temperate	0.99985695
NLM005_scaffold564_3	temperate	0.99985975
NLM005_scaffold21805_1	temperate	0.99984884
NLM005_scaffold7_1	virulent	0.9998699
NLM005_scaffold5172_2	temperate	0.9960733
NLM005_scaffold12098_1	virulent	0.69228077
NLM005_scaffold90_2	temperate	0.9137359
NLM005_scaffold5999_10	virulent	0.996864
NLM005_scaffold11370_1	temperate	0.99473625
NLM005_scaffold4896_1	virulent	0.9998656
NLM005_scaffold16081_2	virulent	0.71232563
NLM006_scaffold18020_1	virulent	0.9998727
NLM006_scaffold30822_2	virulent	0.9998656
NLM006_scaffold2215_2	temperate	0.99985695
NLM006_scaffold50674_1	virulent	0.9998636

NLM006_scaffold32497_3	virulent	0.9980126
NLM006_scaffold21580_3	virulent	0.5427974
NLM006_scaffold43376_2	virulent	0.98799294
NLM006_scaffold44414_1	virulent	0.9857189
NLM006_scaffold37475_1	temperate	0.6870776
NLM006_scaffold47321_2	temperate	0.7044811
NLM006_scaffold31922_8	temperate	0.9977357
NLM006_scaffold23522_7	temperate	0.99985975
NLM006_scaffold43039_5	virulent	0.9998196
NLM006_scaffold42358_4	virulent	0.96040946
NLM006_scaffold6162_11	temperate	0.99985605
NLM006_scaffold50021_2	temperate	0.9998579
NLM006_scaffold35930_2	temperate	0.99985975
NLM006_scaffold52859_1	virulent	0.97943383
NLM006_scaffold36213_1	temperate	0.9992786
NLM006_scaffold24429_1	virulent	0.9998656
NLM006_scaffold51349_1	temperate	0.99986035
NLM006_scaffold53007_3	temperate	0.9307284
NLM006_scaffold22752_1	virulent	0.9996882
NLM006_scaffold1105_10	temperate	0.99985975
NLM006_scaffold21365_1	temperate	0.99520785
NLM006_scaffold33813_8	temperate	0.9998369
NLM006_scaffold26489_1	temperate	0.9997044
NLM006_scaffold53154_3	virulent	0.99987125
NLM006_scaffold11951_6	virulent	0.99966824
NLM006_scaffold46436_2	temperate	0.93916035
NLM006_scaffold4801_4	virulent	0.9998622
NLM006_scaffold42358_1	virulent	0.9998679
NLM006_C734286_1	virulent	0.99987406
NLM006_scaffold30260_1	temperate	0.99904966
NLM006_C733934_1	virulent	0.9884934
NLM006_scaffold37878_1	temperate	0.9968831
NLM006_scaffold38154_1	temperate	0.99986035
NLM006_scaffold18470_3	virulent	0.9998656
NLM006_scaffold29091_1	temperate	0.9998397
NLM006_scaffold177_4	virulent	0.9998699
NLM006_scaffold53101_1	temperate	0.9997587
NLM006_scaffold44135_1	temperate	0.8564934
NLM006_scaffold15317_5	virulent	0.9998331
NLM006_scaffold49011_1	virulent	0.99978524
NLM006_scaffold12258_5	virulent	0.99980384
NLM006_scaffold37878_15	temperate	0.99983555
NLM006_scaffold50994_2	virulent	0.99987465
NLM006_scaffold14681_1	temperate	0.9998593
NLM006_scaffold12925_3	virulent	0.99987406
NLM006_scaffold16491_10	virulent	0.9998722

NLM006_scaffold43095_1	temperate	0.9998345
NLM006_scaffold17752_2	temperate	0.99984926
NLM006_scaffold4407_2	virulent	0.9998693
NLM006_scaffold10094_3	virulent	0.9998593
NLM006_scaffold31240_3	virulent	0.9998574
NLM006_scaffold1374_1	temperate	0.9998379
NLM006_scaffold31193_1	virulent	0.9998688
NLM006_scaffold4969_12	temperate	0.98616534
NLM006_scaffold16845_12	temperate	0.9998165
NLM006_scaffold28837_2	virulent	0.99970657
NLM006_scaffold22733_1	virulent	0.9695475
NLM006_C733830_1	virulent	0.9998508
NLM006_C734606_1	virulent	0.9998645
NLM006_C734306_1	virulent	0.9998693
NLM006_scaffold8324_9	temperate	0.9998431
NLM006_scaffold9879_4	virulent	0.99986696
NLM007_scaffold10749_4	virulent	0.99950296
NLM007_scaffold1551_2	temperate	0.99985975
NLM007_scaffold25115_1	virulent	0.9998722
NLM007_scaffold11104_1	temperate	0.9998593
NLM007_scaffold7202_1	virulent	0.99986744
NLM007_scaffold5980_3	temperate	0.99983126
NLM007_scaffold20486_1	temperate	0.99985975
NLM007_scaffold17969_11	temperate	0.9812528
NLM007_scaffold6647_10	temperate	0.99985695
NLM007_scaffold19932_1	virulent	0.99987125
NLM007_scaffold26391_1	virulent	0.9998665
NLM007_scaffold2633_3	virulent	0.9998584
NLM007_scaffold26057_1	temperate	0.99985975
NLM007_scaffold25900_1	virulent	0.99980783
NLM007_scaffold20356_8	virulent	0.99984604
NLM007_scaffold6392_8	temperate	0.6711984
NLM007_scaffold10461_1	temperate	0.99985605
NLM007_scaffold3232_7	virulent	0.83561283
NLM007_scaffold22550_11	temperate	0.99985605
NLM007_scaffold26394_2	virulent	0.9998645
NLM007_scaffold10749_5	temperate	0.9998588
NLM007_scaffold23234_1	temperate	0.9994242
NLM007_scaffold10749_8	temperate	0.9998588
NLM007_scaffold18350_4	virulent	0.99969625
NLM007_scaffold21467_1	temperate	0.99977493
NLM007_scaffold4022_7	temperate	0.9998593
NLM007_scaffold26378_1	virulent	0.9997592
NLM007_scaffold26389_1	temperate	0.5364791
NLM007_scaffold723_5	virulent	0.76178706
NLM008_scaffold70_5	virulent	0.99939036

NLM008_scaffold50164_2	temperate	0.9413989
NLM008_scaffold12934_1	virulent	0.9994554
NLM008_scaffold37575_7	temperate	0.9998588
NLM008_scaffold37289_2	virulent	0.99987125
NLM008_scaffold45531_1	virulent	0.99986744
NLM008_scaffold22745_5	virulent	0.9786009
NLM008_scaffold50138_1	virulent	0.9998408
NLM008_scaffold4502_17	virulent	0.99984837
NLM008_scaffold18128_9	virulent	0.99986315
NLM008_scaffold49769_2	virulent	0.9949243
NLM008_scaffold44104_3	temperate	0.99986035
NLM008_scaffold50150_1	virulent	0.99942935
NLM008_scaffold41985_1	virulent	0.99969625
NLM008_scaffold37575_10	temperate	0.9998345
NLM008_scaffold46068_3	temperate	0.7353683
NLM008_scaffold711_3	virulent	0.99985975
NLM008_scaffold2753_1	virulent	0.9998699
NLM008_scaffold30532_8	temperate	0.9998593
NLM008_scaffold2753_6	virulent	0.9998722
NLM008_scaffold16329_1	virulent	0.9998622
NLM008_scaffold3829_2	temperate	0.99934417
NLM009_scaffold13480_1	temperate	0.99985605
NLM009_scaffold13047_6	temperate	0.9293613
NLM009_C228858_1	temperate	0.99986035
NLM009_scaffold13069_1	virulent	0.9976822
NLM009_scaffold8447_2	temperate	0.9987712
NLM009_scaffold5257_2	temperate	0.999322
NLM009_C228882_1	virulent	0.9968421
NLM010_scaffold39134_1	temperate	0.9998593
NLM010_scaffold3556_1	virulent	0.99973106
NLM010_scaffold2302_1	temperate	0.99977016
NLM010_scaffold1115_4	temperate	0.9998593
NLM010_scaffold36636_1	temperate	0.99985975
NLM010_scaffold23407_1	virulent	0.99986607
NLM010_scaffold40193_1	virulent	0.7885081
NLM010_scaffold15834_3	virulent	0.9998331
NLM010_scaffold18261_4	virulent	0.9998636
NLM010_scaffold40145_2	temperate	0.9050083
NLM010_scaffold1890_2	virulent	0.64273113
NLM010_C569179_1	virulent	0.9998684
NLM010_scaffold24350_1	virulent	0.99987316
NLM010_scaffold7375_4	virulent	0.9998736
NLM010_scaffold15684_10	temperate	0.9998588
NLM010_scaffold13431_2	virulent	0.9998727
NLM010_scaffold3952_5	virulent	0.9738244
NLM010_scaffold894_1	virulent	0.9998217

NLM010_scaffold9073_7	virulent	0.9998736
NLM010_scaffold2086_2	virulent	0.99987036
NLM010_scaffold21070_2	temperate	0.9993865
NLM010_scaffold32176_1	virulent	0.9849704
NLM010_scaffold705_15	temperate	0.99985975
NLM010_scaffold16315_3	virulent	0.99982435
NLM010_scaffold39351_1	virulent	0.999768
NLM010_scaffold16684_4	virulent	0.9821895
NLM010_scaffold4037_2	temperate	0.9998593
NLM010_scaffold10296_6	temperate	0.9998579
NLM010_scaffold39268_1	virulent	0.9903608
NLM010_scaffold17621_3	virulent	0.9998727
NLM010_scaffold2685_7	virulent	0.99986744
NLM010_scaffold34399_5	virulent	0.99987406
NLM010_scaffold10324_3	virulent	0.77555895
NLM010_scaffold20986_1	temperate	0.9998593
NLM010_scaffold10296_4	temperate	0.999854
NLM010_scaffold8314_1	virulent	0.99969965
NLM010_scaffold2398_1	temperate	0.99982446
NLM010_scaffold15390_2	virulent	0.9971423
NLM010_scaffold24696_6	virulent	0.940546
NLM010_scaffold21133_2	virulent	0.73562175
NLM010_scaffold2612_4	temperate	0.979654
NLM010_scaffold20986_2	temperate	0.99985605
NLM010_scaffold9737_35	temperate	0.9875896
NLM010_scaffold28345_8	virulent	0.9646963
NLM010_scaffold26505_1	virulent	0.99974734
NLM010_scaffold3952_4	virulent	0.9842398
NLM010_scaffold3490_4	temperate	0.9842014
NLM015_scaffold34970_4	temperate	0.9998517
NLM015_scaffold45_3	virulent	0.9974672
NLM015_scaffold9944_1	virulent	0.97420454
NLM015_scaffold38949_1	temperate	0.99704653
NLM015_scaffold38424_1	virulent	0.9994911
NLM015_scaffold2372_1	virulent	0.99980664
NLM015_scaffold6813_1	virulent	0.9979286
NLM015_scaffold13019_2	temperate	0.9962401
NLM015_scaffold7231_4	temperate	0.9998288
NLM015_scaffold16659_2	virulent	0.999798
NLM015_scaffold10852_4	temperate	0.68025094
NLM015_scaffold4740_1	temperate	0.9513003
NLM015_scaffold1856_2	temperate	0.9962648
NLM015_scaffold77_4	virulent	0.99986744
NLM015_scaffold25776_2	temperate	0.5775136
NLM015_scaffold21932_1	temperate	0.9998588
NLM015_scaffold25867_2	temperate	0.99985975

NLM015_scaffold10852_5	virulent	0.9998269
NLM015_scaffold15418_1	virulent	0.9998727
NLM015_scaffold5831_6	virulent	0.99703306
NLM015_scaffold104_1	virulent	0.9998699
NLM016_scaffold3058_1	temperate	0.9928458
NLM016_scaffold10481_1	virulent	0.99984926
NLM016_scaffold19074_1	temperate	0.9996615
NLM016_scaffold8626_1	virulent	0.99987406
NLM016_scaffold12933_1	temperate	0.9997874
NLM016_scaffold9518_15	temperate	0.99984884
NLM016_scaffold11175_2	temperate	0.99985975
NLM016_scaffold112_1	temperate	0.9998593
NLM016_scaffold87_4	virulent	0.9997247
NLM016_scaffold3275_3	temperate	0.9997697
NLM016_scaffold11179_1	virulent	0.9998722
NLM016_scaffold16587_2	virulent	0.99987084
NLM016_scaffold19042_3	temperate	0.9208216
NLM016_scaffold16925_3	virulent	0.9699225
NLM017_scaffold416_2	temperate	0.999816
NLM017_scaffold14858_4	virulent	0.99138457
NLM017_scaffold20307_2	virulent	0.9997209
NLM017_scaffold27209_14	virulent	0.9998736
NLM017_scaffold38835_1	virulent	0.99986607
NLM017_scaffold38788_3	temperate	0.99524623
NLM017_scaffold32055_2	virulent	0.9998417
NLM017_scaffold8358_18	virulent	0.6432086
NLM017_scaffold9889_3	temperate	0.9998593
NLM017_scaffold20627_4	temperate	0.9988693
NLM017_scaffold38145_1	virulent	0.57108474
NLM017_scaffold14095_1	virulent	0.99747163
NLM017_scaffold38914_3	virulent	0.9998693
NLM017_scaffold39062_2	virulent	0.99987036
NLM017_scaffold8358_21	virulent	0.9998465
NLM017_scaffold35881_2	virulent	0.9998736
NLM017_scaffold17839_9	virulent	0.9066871
NLM017_scaffold21461_2	virulent	0.9998736
NLM017_scaffold24164_1	temperate	0.9998274
NLM017_scaffold25009_1	virulent	0.99936676
NLM017_scaffold12885_45	virulent	0.9992214
NLM017_scaffold24740_2	virulent	0.99987084
NLM017_scaffold7452_3	temperate	0.99986035
NLM017_scaffold29127_2	virulent	0.99987316
NLM017_scaffold31444_1	temperate	0.9998593
NLM017_scaffold8358_65	temperate	0.99389774
NLM017_scaffold38969_2	virulent	0.9998727
NLM017_scaffold23004_11	virulent	0.99982494

NLM017_scaffold174_1	temperate	0.8248126
NLM017_scaffold38914_2	virulent	0.9998679
NLM021_scaffold5563_6	virulent	0.99987316
NLM021_scaffold562_2	temperate	0.979654
NLM021_scaffold42938_1	virulent	0.63364846
NLM021_scaffold61286_1	temperate	0.9998593
NLM021_scaffold58942_1	temperate	0.9998345
NLM021_scaffold61418_1	temperate	0.9998565
NLM021_scaffold5557_1	virulent	0.8075158
NLM021_scaffold17568_1	virulent	0.9998574
NLM021_scaffold19029_4	temperate	0.9605091
NLM021_scaffold53158_5	virulent	0.9998688
NLM021_scaffold59003_3	virulent	0.92176783
NLM021_scaffold8185_4	temperate	0.9022219
NLM021_scaffold56233_3	virulent	0.8864396
NLM021_scaffold57344_1	virulent	0.9908407
NLM021_scaffold26944_4	temperate	0.97423124
NLM021_scaffold61366_2	temperate	0.9998579
NLM021_scaffold55035_3	temperate	0.99983555
NLM021_scaffold19029_1	virulent	0.9998545
NLM021_scaffold143_2	virulent	0.9998699
NLM021_scaffold42928_1	temperate	0.9998588
NLM021_scaffold61042_4	temperate	0.99438643
NLM021_scaffold29192_2	temperate	0.9697178
NLM021_scaffold35458_5	virulent	0.99983126
NLM021_scaffold22069_5	temperate	0.9998584
NLM021_scaffold11314_5	virulent	0.9996798
NLM021_scaffold23306_4	virulent	0.9996477
NLM021_scaffold60054_1	temperate	0.90835065
NLM021_scaffold190_8	virulent	0.99986744
NLM021_scaffold15002_14	virulent	0.9998593
NLM021_scaffold55417_2	temperate	0.9998308
NLM021_scaffold14659_12	temperate	0.89597625
NLM021_scaffold60255_1	temperate	0.99070436
NLM021_scaffold26861_2	virulent	0.99980736
NLM021_scaffold709_5	temperate	0.9998593
NLM021_C812913_1	temperate	0.99985975
NLM021_scaffold61257_1	temperate	0.99090755
NLM021_scaffold46784_3	virulent	0.9949243
NLM021_scaffold190_9	virulent	0.9998636
NLM021_scaffold53021_4	virulent	0.9998574
NLM021_scaffold58236_5	temperate	0.9997535
NLM021_scaffold46181_6	virulent	0.9998699
NLM021_scaffold38431_7	temperate	0.99986035
NLM021_scaffold57650_6	temperate	0.99985975
NLM021_scaffold190_7	virulent	0.9998369

NLM021_scaffold14592_1	temperate	0.9998265
NLM021_scaffold49743_1	virulent	0.9998665
NLM021_C812807_1	temperate	0.8152787
NLM021_scaffold61366_1	temperate	0.99985975
NLM021_scaffold55417_5	virulent	0.9936485
NLM021_scaffold45854_2	temperate	0.9998584
NLM021_scaffold927_6	temperate	0.99977493
NLM021_scaffold11116_2	temperate	0.99985975
NLM022_scaffold3577_22	temperate	0.99985975
NLM022_scaffold5329_6	virulent	0.994396
NLM022_scaffold28217_1	temperate	0.99811184
NLM022_scaffold9956_5	virulent	0.994524
NLM022_scaffold173_4	temperate	0.99985975
NLM022_scaffold17073_1	virulent	0.6061743
NLM022_scaffold42567_1	temperate	0.99929553
NLM022_scaffold30338_1	temperate	0.99540496
NLM022_scaffold3888_1	virulent	0.9997695
NLM022_scaffold16192_1	virulent	0.9998736
NLM022_scaffold10884_4	temperate	0.9998331
NLM022_scaffold38630_1	temperate	0.99985975
NLM022_scaffold42820_1	temperate	0.99967194
NLM022_scaffold44402_1	temperate	0.99978215
NLM022_scaffold20998_1	virulent	0.69050956
NLM022_scaffold42750_2	temperate	0.99985313
NLM022_scaffold7821_4	temperate	0.9998397
NLM022_scaffold38946_1	temperate	0.9487627
NLM022_scaffold7162_1	temperate	0.99985975
NLM022_C640276_1	temperate	0.99884266
NLM022_scaffold15285_3	virulent	0.9998679
NLM022_scaffold28148_1	temperate	0.9998508
NLM022_scaffold9181_1	virulent	0.99978524
NLM022_scaffold35932_1	virulent	0.9998693
NLM022_C641598_1	virulent	0.9998688
NLM022_scaffold43025_3	temperate	0.99986035
NLM022_scaffold15285_1	virulent	0.99910206
NLM022_scaffold5329_1	temperate	0.9998593
NLM022_scaffold24404_3	virulent	0.99987036
NLM022_scaffold43901_3	temperate	0.99986035
NLM022_scaffold37593_2	temperate	0.99985605
NLM022_scaffold17804_1	temperate	0.99985975
NLM022_scaffold35358_2	temperate	0.99985975
NLM022_scaffold11265_9	virulent	0.9996532
NLM022_C640494_1	virulent	0.96739966
NLM022_scaffold7447_1	temperate	0.9998522
NLM023_scaffold23138_8	virulent	0.9997411
NLM023_scaffold1402_1	temperate	0.9998593

NLM023_scaffold23260_2	virulent	0.99987084
NLM023_scaffold33293_1	temperate	0.99985975
NLM023_scaffold470_13	temperate	0.99983925
NLM023_scaffold28479_8	temperate	0.9946079
NLM023_scaffold23260_3	virulent	0.9998651
NLM023_scaffold21242_4	temperate	0.99135226
NLM023_scaffold48669_2	temperate	0.9998522
NLM023_scaffold48941_1	virulent	0.9998651
NLM023_scaffold48169_1	temperate	0.99984926
NLM023_scaffold28479_20	virulent	0.9995251
NLM023_C806243_1	virulent	0.9354477
NLM023_scaffold28479_12	temperate	0.99986035
NLM023_scaffold11383_1	virulent	0.99987173
NLM023_scaffold911_13	virulent	0.9878793
NLM023_scaffold8508_4	virulent	0.9996882
NLM023_scaffold48946_1	virulent	0.9998736
NLM023_scaffold48276_1	virulent	0.9998545
NLM023_scaffold277_15	virulent	0.99985605
NLM023_scaffold43102_12	temperate	0.9910795
NLM023_scaffold1348_25	virulent	0.9998645
NLM023_scaffold37217_1	temperate	0.999774
NLM023_scaffold399_47	virulent	0.9998636
NLM023_scaffold260_1	virulent	0.9986079
NLM023_scaffold253_1	temperate	0.99977785
NLM023_scaffold10723_15	virulent	0.9998574
NLM023_scaffold4055_10	virulent	0.9983713
NLM023_scaffold27707_7	virulent	0.9998288
NLM023_scaffold1428_18	virulent	0.9935903
NLM023_scaffold14181_20	temperate	0.9998388
NLM023_scaffold48506_1	temperate	0.9998445
NLM023_scaffold17200_15	virulent	0.99979925
NLM023_scaffold33532_1	virulent	0.99970627
NLM023_scaffold35604_3	temperate	0.99985975
NLM023_scaffold46734_3	temperate	0.9998593
NLM023_scaffold22_6	virulent	0.99987406
NLM023_scaffold12109_12	temperate	0.99985313
NLM023_scaffold34755_1	temperate	0.9998593
NLM023_scaffold48896_1	temperate	0.9922496
NLM024_scaffold488_1	virulent	0.99970657
NLM024_scaffold497_1	temperate	0.9990155
NLM024_scaffold6119_1	virulent	0.9998722
NLM024_scaffold10225_2	virulent	0.87349826
NLM024_scaffold20170_1	temperate	0.99985975
NLM024_scaffold5090_1	virulent	0.99987084
NLM024_scaffold18150_1	temperate	0.9998588
NLM024_scaffold13470_1	virulent	0.99962884

NLM024_scaffold14981_1	virulent	0.9998579
NLM024_scaffold15487_3	temperate	0.99984837
NLM024_scaffold77_6	virulent	0.99987406
NLM024_scaffold1371_3	temperate	0.99973583
NLM024_scaffold244_5	virulent	0.9949243
NLM024_scaffold9211_2	virulent	0.99986404
NLM024_scaffold100_1	virulent	0.999768
NLM024_scaffold957_14	virulent	0.99987173
NLM024_scaffold16628_8	virulent	0.9979265
NLM024_scaffold11939_1	temperate	0.9998579
NLM024_scaffold14981_3	virulent	0.99984974
NLM025_C502105_1	temperate	0.999854
NLM025_scaffold4345_2	virulent	0.9998584
NLM025_scaffold22167_3	virulent	0.9988293
NLM025_scaffold39287_1	temperate	0.99985975
NLM025_scaffold18010_7	virulent	0.999808
NLM025_scaffold39157_1	temperate	0.6460557
NLM025_scaffold25589_3	temperate	0.99985975
NLM025_scaffold35205_1	temperate	0.99985975
NLM025_scaffold38901_1	virulent	0.9998357
NLM025_scaffold78_11	temperate	0.984522
NLM025_scaffold12163_17	virulent	0.99829227
NLM025_scaffold20016_2	temperate	0.99985313
NLM025_scaffold29129_1	temperate	0.9998517
NLM026_scaffold11596_2	virulent	0.9993334
NLM026_scaffold74538_1	temperate	0.9998579
NLM026_scaffold29270_6	temperate	0.99982554
NLM026_scaffold27749_19	temperate	0.9998331
NLM026_scaffold62290_1	virulent	0.9998556
NLM026_scaffold55998_10	temperate	0.9774005
NLM026_scaffold57277_1	temperate	0.99977446
NLM026_scaffold74568_1	virulent	0.9998722
NLM026_scaffold75736_1	temperate	0.99576545
NLM026_scaffold1771_11	virulent	0.9998736
NLM026_scaffold258_3	temperate	0.9997983
NLM026_scaffold988_1	virulent	0.99260366
NLM026_scaffold75282_1	temperate	0.99985605
NLM026_scaffold47323_1	temperate	0.99985975
NLM026_scaffold65263_3	virulent	0.9998693
NLM026_scaffold74983_1	virulent	0.9998693
NLM026_scaffold68020_6	temperate	0.99985975
NLM026_scaffold58936_3	virulent	0.9998536
NLM026_scaffold394_5	temperate	0.9998579
NLM026_scaffold29289_10	temperate	0.99985975
NLM026_scaffold21981_1	virulent	0.999579
NLM026_scaffold86_4	temperate	0.99986035

NLM026_scaffold75724_1	temperate	0.9985046
NLM026_scaffold26990_1	virulent	0.99969715
NLM026_scaffold74825_1	virulent	0.8512207
NLM026_scaffold75315_1	virulent	0.9998436
NLM026_scaffold49770_2	virulent	0.9998656
NLM026_scaffold26189_1	virulent	0.9998574
NLM026_scaffold75839_2	virulent	0.99987084
NLM026_scaffold32583_1	temperate	0.56512856
NLM026_scaffold58959_4	temperate	0.9575485
NLM026_scaffold74363_2	virulent	0.9998699
NLM026_scaffold58139_6	temperate	0.9998588
NLM026_scaffold258_2	temperate	0.9997631
NLM026_scaffold75960_2	virulent	0.99985695
NLM026_C924027_1	virulent	0.99987406
NLM026_scaffold65585_4	temperate	0.9753722
NLM026_scaffold16034_7	virulent	0.99986607
NLM026_scaffold9047_2	virulent	0.99392647
NLM026_scaffold6763_2	virulent	0.9998688
NLM026_scaffold32844_2	virulent	0.9998699
NLM026_scaffold13988_2	temperate	0.9998593
NLM026_scaffold69595_2	virulent	0.9997521
NLM026_scaffold63092_3	virulent	0.99987173
NLM026_scaffold54916_1	temperate	0.9998584
NLM026_scaffold73101_2	temperate	0.99985975
NLM026_scaffold43543_3	virulent	0.99735016
NLM026_scaffold75674_2	virulent	0.9998727
NLM026_scaffold15415_2	temperate	0.97500896
NLM027_scaffold22040_4	virulent	0.7335105
NLM027_scaffold40686_1	virulent	0.9998191
NLM027_scaffold41690_2	virulent	0.99987173
NLM027_scaffold41411_1	temperate	0.9996224
NLM027_scaffold41959_1	temperate	0.9965829
NLM027_scaffold4058_19	virulent	0.54008085
NLM027_scaffold19284_2	virulent	0.999703
NLM027_scaffold40329_1	temperate	0.9881868
NLM027_scaffold35554_2	temperate	0.99985695
NLM027_scaffold40329_3	temperate	0.99985975
NLM027_scaffold39111_4	temperate	0.99916536
NLM027_scaffold35206_1	temperate	0.9967787
NLM027_scaffold30138_3	temperate	0.9998593
NLM027_scaffold41965_1	virulent	0.99987036
NLM027_scaffold14263_1	temperate	0.9998588
NLM027_scaffold39770_2	virulent	0.99963146
NLM027_scaffold16353_1	temperate	0.9998593
NLM027_scaffold34797_1	temperate	0.99611515
NLM027_scaffold8952_3	virulent	0.8142954

NLM027_scaffold34798_1	temperate	0.99611515
NLM027_scaffold10002_11	temperate	0.99985975
NLM027_scaffold17160_15	virulent	0.99986315
NLM027_scaffold8952_6	temperate	0.9998584
NLM027_scaffold7180_15	temperate	0.9908204
NLM027_scaffold1874_3	temperate	0.97194517
NLM027_scaffold11645_1	virulent	0.99913013
NLM027_scaffold16205_3	temperate	0.99985975
NLM027_scaffold1886_1	temperate	0.9998593
NLM027_scaffold40537_1	virulent	0.9991923
NLM027_scaffold3310_5	temperate	0.99985033
NLM027_scaffold27969_2	virulent	0.99936295
NLM027_C668626_1	temperate	0.99985695
NLM027_scaffold14181_3	temperate	0.9998508
NLM027_scaffold34123_3	temperate	0.99974394
NLM027_scaffold17357_1	temperate	0.9378113
NLM027_scaffold41891_1	virulent	0.99987084
NLM027_scaffold40356_4	temperate	0.9998379
NLM027_scaffold35206_2	virulent	0.99986744
NLM027_scaffold9793_1	temperate	0.9985623
NLM027_scaffold20399_3	virulent	0.99987173
NLM027_scaffold38196_4	temperate	0.9998593
NLM028_scaffold28234_2	virulent	0.9998722
NLM028_scaffold35099_2	virulent	0.9998202
NLM028_scaffold12174_3	temperate	0.9629111
NLM028_scaffold54929_3	virulent	0.999174
NLM028_scaffold59756_2	virulent	0.9957786
NLM028_scaffold34514_1	virulent	0.9998727
NLM028_scaffold59789_2	temperate	0.9997769
NLM028_scaffold1532_1	temperate	0.9997917
NLM028_scaffold24678_1	temperate	0.99985975
NLM028_scaffold8768_2	virulent	0.99976426
NLM028_scaffold34609_1	virulent	0.9996796
NLM028_scaffold42575_1	virulent	0.9860106
NLM028_scaffold30720_1	temperate	0.5093073
NLM028_scaffold12341_5	virulent	0.88290614
NLM028_scaffold51949_2	virulent	0.9998369
NLM028_scaffold20220_12	virulent	0.6351505
NLM028_scaffold45877_1	temperate	0.99983555
NLM028_scaffold26847_1	temperate	0.9967376
NLM028_scaffold57798_4	virulent	0.9998617
NLM028_scaffold15831_1	temperate	0.97040224
NLM028_scaffold34525_2	virulent	0.99987316
NLM028_scaffold43687_1	temperate	0.9998593
NLM028_scaffold20949_1	virulent	0.999658
NLM028_scaffold57543_3	temperate	0.97810906

NLM028_scaffold59796_3	temperate	0.7185659
NLM028_scaffold57798_3	temperate	0.990775
NLM028_C652312_1	temperate	0.9977651
NLM028_scaffold7898_1	virulent	0.9998722
NLM028_scaffold15831_2	temperate	0.93888336
NLM028_scaffold42949_1	temperate	0.99978584
NLM028_scaffold57543_2	virulent	0.9758714
NLM028_scaffold37225_1	virulent	0.9997153
NLM028_C652264_1	temperate	0.99983406
NLM028_scaffold13605_5	temperate	0.99985695
NLM029_scaffold46834_2	temperate	0.9998584
NLM029_scaffold45341_4	temperate	0.59614587
NLM029_scaffold33952_1	virulent	0.9996615
NLM029_scaffold4810_3	temperate	0.9697178
NLM029_C674039_1	temperate	0.99949604
NLM029_scaffold22749_1	virulent	0.99987125
NLM029_scaffold2015_8	virulent	0.99462146
NLM029_scaffold24024_8	virulent	0.99915105
NLM029_scaffold9598_4	temperate	0.9998593
NLM029_scaffold39928_1	temperate	0.9998274
NLM029_scaffold161_2	temperate	0.99986035
NLM029_scaffold9173_1	temperate	0.9971445
NLM029_scaffold2015_2	temperate	0.98481876
NLM029_scaffold44423_10	temperate	0.99986035
NLM029_scaffold17455_3	temperate	0.99985975
NLM029_scaffold3634_1	virulent	0.9827163
NLM029_scaffold37710_1	virulent	0.99971133
NLM029_scaffold9598_2	temperate	0.99985975
NLM029_scaffold24413_3	virulent	0.9998699
NLM029_scaffold7675_3	virulent	0.96640456
NLM029_scaffold3860_9	virulent	0.9998636
NLM029_scaffold3826_2	temperate	0.97519684
NLM029_scaffold4592_2	virulent	0.99987084
NLM029_scaffold25526_7	virulent	0.9998727
NLM029_C673977_1	virulent	0.99973136
NLM029_scaffold46830_1	temperate	0.9997758
NLM029_scaffold7768_1	temperate	0.99985975
NLM029_scaffold35541_5	virulent	0.996247
NLM029_scaffold5035_2	temperate	0.98519784
NLM029_scaffold45930_1	temperate	0.9998593
NLM029_scaffold35112_1	temperate	0.9998274
NLM029_scaffold4050_9	temperate	0.9981835
NLM029_scaffold12727_2	temperate	0.9998593
NLM029_scaffold15304_1	virulent	0.99879533
NLM029_scaffold24413_4	virulent	0.9998679
NLM029_scaffold9315_13	temperate	0.999774

NLM029_scaffold46287_2	virulent	0.9998617
NLM029_scaffold31672_2	virulent	0.9998565
NLM029_C674283_1	temperate	0.99985975
NLM029_scaffold10574_3	temperate	0.95317507
NLM029_scaffold26243_3	temperate	0.9998556
NLM029_scaffold33933_1	virulent	0.7445869
NLM029_scaffold17844_1	temperate	0.99985695
NLM029_scaffold23685_2	virulent	0.9927804
NLM029_C674325_1	virulent	0.99987084
NLM029_scaffold8972_5	virulent	0.9998684
NLM029_scaffold325_5	temperate	0.5025973
NLM029_scaffold45625_1	virulent	0.99987465
NLM029_scaffold10325_2	temperate	0.9992649
NLM029_scaffold8116_24	virulent	0.99987406
NLM029_C674345_1	temperate	0.9998588
NLM029_scaffold20227_1	temperate	0.9996734
NLM029_scaffold46752_4	virulent	0.9998727
NLM031_scaffold75_4	virulent	0.99986744
NLM031_scaffold75_1	virulent	0.99986696
NLM031_scaffold59329_1	temperate	0.9998593
NLM031_scaffold21651_1	virulent	0.95285964
NLM031_scaffold18212_16	temperate	0.93755734
NLM031_scaffold18436_4	temperate	0.9476028
NLM031_scaffold45857_1	virulent	0.9979265
NLM031_scaffold33580_2	virulent	0.9998736
NLM031_scaffold16685_1	virulent	0.99986744
NLM031_scaffold23971_3	virulent	0.9998684
NLM031_scaffold7206_4	virulent	0.99987084
NLM031_scaffold27707_3	temperate	0.9965135
NLM031_scaffold41310_1	virulent	0.99939865
NLM031_scaffold6532_6	temperate	0.99985975
NLM031_scaffold1394_1	temperate	0.9998336
NLM031_scaffold2847_6	virulent	0.9997695
NLM031_scaffold49_2	temperate	0.99966246
NLM031_scaffold25711_6	virulent	0.99985313
NLM031_scaffold18627_4	temperate	0.9944285
NLM031_scaffold15300_11	temperate	0.99719846
NLM031_scaffold45482_3_1	virulent	0.9998722
NLM031_scaffold259_8	temperate	0.98147815
NLM031_scaffold36158_5	temperate	0.9998565
NLM031_scaffold29823_1	temperate	0.9998593
NLM031_scaffold56089_1	temperate	0.7586957
NLM031_scaffold2964_6	temperate	0.8352771
NLM031_scaffold45482_5	virulent	0.9629124
NLM031_scaffold107_1	temperate	0.9975632
NLM031_scaffold20700_11	temperate	0.99985975

NLM031_scaffold17121_1	temperate	0.99985975
NLM031_scaffold1394_2	virulent	0.8155634
NLM031_scaffold25711_7	temperate	0.99961764
NLM031_scaffold23030_1	temperate	0.99985975
NLM031_scaffold18436_7	temperate	0.69460785
NLM031_C734735_1	virulent	0.99987316
NLM031_scaffold44148_4	virulent	0.9988257
NLM032_scaffold68145_4	temperate	0.9998584
NLM032_scaffold23012_2	virulent	0.9998656
NLM032_scaffold61468_2	temperate	0.9113476
NLM032_scaffold41399_1	temperate	0.9728056
NLM032_scaffold58144_2	temperate	0.99873686
NLM032_scaffold33461_2	temperate	0.99985975
NLM032_scaffold23630_5	temperate	0.99983925
NLM032_scaffold63260_2	temperate	0.99985975
NLM032_scaffold40713_1	temperate	0.9997797
NLM032_scaffold49689_1	virulent	0.93356663
NLM032_scaffold61468_1	temperate	0.9996549
NLM032_scaffold63260_1	temperate	0.99984926
NLM032_scaffold69860_1	virulent	0.9998274
NLM032_scaffold10936_5	virulent	0.9998584
NLM032_scaffold9843_17	temperate	0.9998593
NLM032_scaffold37169_1	temperate	0.9998593
NLM032_scaffold219_1	temperate	0.9998588
NLM032_scaffold68980_1	virulent	0.9550464
NLM032_scaffold12144_3	temperate	0.99985975
NLM032_scaffold7394_8	temperate	0.9997606
NLM032_scaffold69642_1	temperate	0.9998574
NLM032_scaffold69111_1	virulent	0.99987406
NLM032_scaffold314_2	temperate	0.99984837
NLM032_scaffold3938_2	virulent	0.9998656
NLM032_scaffold25961_2	virulent	0.9998436
NLM032_scaffold60475_1	temperate	0.9998593
NLM032_scaffold32034_2	temperate	0.99985975
NLM032_scaffold54796_9	temperate	0.99985975
NLM032_scaffold9089_7	temperate	0.9998593
NLM032_scaffold24196_2	virulent	0.999836
NLM032_scaffold69594_3	virulent	0.5565001
NLM032_scaffold3932_23	virulent	0.9998722
NLM032_scaffold23630_7	temperate	0.8428495
NLM032_scaffold50920_4	temperate	0.9998522
NLM032_scaffold69179_1	virulent	0.9998522
NLM032_scaffold68145_1	temperate	0.9998593
NLM032_scaffold58268_2	virulent	0.9998579
NLM032_scaffold212_2	virulent	0.9998699
NLM032_scaffold69647_5	temperate	0.9996429

NLM032_scaffold69594_2	temperate	0.99985975
NLM032_scaffold40892_3	virulent	0.9998699
NLM032_scaffold6383_1	temperate	0.9998551
NLM032_scaffold49971_2	temperate	0.98519784
NLM032_scaffold35599_1	virulent	0.99982494
NLM032_scaffold11035_2	temperate	0.9997917
DOM001_scaffold12537_8	virulent	0.9745316
DOM001_scaffold26527_9	virulent	0.9142287
DOM001_scaffold20125_2	virulent	0.99973136
DOM001_scaffold6481_6	temperate	0.99985975
DOM001_scaffold57053_3	temperate	0.9998479
DOM001_scaffold21085_16	virulent	0.9998736
DOM001_scaffold43_2	virulent	0.9998699
DOM001_scaffold2066_1	temperate	0.99985975
DOM001_scaffold53704_4	temperate	0.9998408
DOM001_scaffold33_5	temperate	0.9984557
DOM001_scaffold4013_1	virulent	0.99986124
DOM001_scaffold9052_4	temperate	0.99985975
DOM001_scaffold26005_5	virulent	0.7268812
DOM001_scaffold22877_4	virulent	0.9998688
DOM001_scaffold14492_1	virulent	0.68052727
DOM001_scaffold39139_2	temperate	0.87214583
DOM001_scaffold16478_1	virulent	0.9998388
DOM001_scaffold55189_1	temperate	0.9998593
DOM001_scaffold49162_1	virulent	0.99982834
DOM001_scaffold5635_4	temperate	0.999854
DOM001_scaffold58195_2	temperate	0.979654
DOM001_scaffold7274_21	virulent	0.9998736
DOM001_scaffold12537_10	virulent	0.96556866
DOM001_scaffold28423_1	virulent	0.99979234
DOM001_scaffold26343_7	temperate	0.9972075
DOM001_scaffold30807_3	temperate	0.99985313
DOM001_scaffold5000_5	virulent	0.99986744
DOM001_scaffold17386_5	virulent	0.9996475
DOM001_scaffold37685_1	virulent	0.9761889
DOM001_scaffold17768_4	virulent	0.9730364
DOM001_scaffold2499_6	virulent	0.97140306
DOM001_scaffold273_1	temperate	0.99982214
DOM001_scaffold58831_1	temperate	0.99960196
DOM001_scaffold38397_1	virulent	0.97821456
DOM001_scaffold19397_1	virulent	0.9998656
DOM003_scaffold2919_6	temperate	0.9996587
DOM003_scaffold22571_7	temperate	0.99985975
DOM003_scaffold5840_1	temperate	0.9994154
DOM003_scaffold575_3	virulent	0.99987406
DOM003_scaffold6007_2	temperate	0.5812096

DOM003_scaffold24274_1	temperate	0.9984348
DOM003_scaffold24287_2	temperate	0.9998593
DOM003_scaffold24307_1	temperate	0.999854
DOM003_scaffold6518_3	virulent	0.99986607
DOM003_scaffold2919_2	virulent	0.99716425
DOM003_scaffold11839_3	virulent	0.9998699
DOM003_scaffold19301_1	temperate	0.99986035
DOM003_scaffold3648_4	temperate	0.99986035
DOM003_scaffold19736_6	virulent	0.9984596
DOM003_scaffold2919_4	temperate	0.9998517
DOM003_scaffold18492_3	virulent	0.9998636
DOM003_scaffold20665_1	virulent	0.9998357
DOM003_scaffold24151_1	temperate	0.89395547
DOM003_scaffold23067_1	temperate	0.9998226
DOM003_scaffold11839_7	virulent	0.9992729
DOM003_scaffold24307_2	temperate	0.9998545
DOM003_scaffold11039_1	virulent	0.9998608
DOM003_scaffold17266_6	virulent	0.99107265
DOM003_scaffold1995_7	virulent	0.8689416
DOM003_scaffold24298_1	temperate	0.99985975
DOM003_scaffold22453_2	virulent	0.9997247
DOM003_scaffold14108_2	temperate	0.9998536
DOM003_scaffold2919_11	temperate	0.99986035
DOM003_scaffold11839_4	temperate	0.9998574
DOM005_scaffold374_9	temperate	0.9998551
DOM005_scaffold920_41	temperate	0.9997626
DOM005_scaffold9501_4	virulent	0.9998722
DOM005_scaffold13159_4	temperate	0.99708104
DOM005_scaffold6037_1	temperate	0.99981695
DOM005_scaffold2259_3	temperate	0.99984926
DOM005_scaffold13355_2	temperate	0.9969182
DOM005_scaffold10392_5	virulent	0.9998727
DOM005_scaffold10888_1	virulent	0.908313
DOM005_C258523_1	temperate	0.9998579
DOM005_scaffold1092_15	temperate	0.98557746
DOM005_scaffold10888_5	temperate	0.99985975
DOM005_scaffold3583_3	virulent	0.99471563
DOM005_scaffold10107_7	virulent	0.99986035
DOM005_scaffold12816_1	virulent	0.99987173
DOM005_scaffold13159_2	temperate	0.99601823
DOM005_scaffold2640_50	virulent	0.9983449
DOM005_scaffold13337_5	virulent	0.9998727
DOM005_scaffold1509_4	temperate	0.9998593
DOM005_scaffold9841_13	temperate	0.99985975
DOM005_scaffold168_65	virulent	0.99987406
DOM005_scaffold5851_3	virulent	0.9998693

DOM005_scaffold8757_1	temperate	0.999816
DOM005_scaffold374_8	temperate	0.99985975
DOM008_scaffold15923_2	temperate	0.9961957
DOM008_scaffold680_3	virulent	0.99987173
DOM008_scaffold543_11	temperate	0.99946034
DOM008_scaffold16924_3	temperate	0.9998574
DOM008_scaffold4243_1	virulent	0.99987465
DOM008_scaffold16792_1	virulent	0.9998302
DOM008_scaffold5589_2	temperate	0.99984604
DOM008_scaffold14700_5	virulent	0.9997024
DOM008_scaffold3045_22	virulent	0.99984974
DOM008_scaffold57_7	virulent	0.9995636
DOM008_scaffold18954_1	virulent	0.9998588
DOM008_scaffold169_2	virulent	0.9998636
DOM008_scaffold9829_1	virulent	0.99987173
DOM008_scaffold1395_1	virulent	0.9998684
DOM008_scaffold8348_2	temperate	0.9998536
DOM008_scaffold6987_15	virulent	0.99987125
DOM008_scaffold14339_1	temperate	0.99984974
DOM008_scaffold11628_1	virulent	0.99987125
DOM008_scaffold15475_9	virulent	0.99986607
DOM008_scaffold57_5	temperate	0.97040224
DOM008_scaffold15104_4	virulent	0.99985975
DOM008_scaffold44_39	temperate	0.9998593
DOM008_scaffold18440_1	temperate	0.9997769
DOM008_scaffold18246_2	virulent	0.9998693
DOM008_scaffold8252_2	virulent	0.9998574
DOM008_scaffold15475_4	virulent	0.9998627
DOM008_scaffold17898_2	temperate	0.99984217
DOM008_scaffold19226_1	virulent	0.99980974
DOM008_scaffold14655_7	virulent	0.9998479
DOM008_scaffold11407_6	temperate	0.9413989
DOM008_scaffold17859_3	temperate	0.99985975
DOM010_scaffold2671_4	virulent	0.95007366
DOM010_scaffold8807_2	virulent	0.9998117
DOM010_C556729_1	virulent	0.9974672
DOM010_scaffold23838_1	virulent	0.9534296
DOM010_scaffold44229_1	virulent	0.99987406
DOM010_scaffold20353_2	temperate	0.999844
DOM010_scaffold7398_16	virulent	0.9878896
DOM010_scaffold11164_7	temperate	0.99838793
DOM010_scaffold44237_2	virulent	0.9997418
DOM010_C557895_1	virulent	0.99974227
DOM010_scaffold29114_1	virulent	0.99987125
DOM010_scaffold44223_5	temperate	0.6360983
DOM010_C557123_1	virulent	0.99962765

DOM010_scaffold10693_2	temperate	0.9998588
DOM010_scaffold42781_1	virulent	0.9998522
DOM010_scaffold3905_57	temperate	0.9998197
DOM010_scaffold6942_2	temperate	0.9413989
DOM010_scaffold42298_4	temperate	0.9996458
DOM010_C557921_1	temperate	0.9998188
DOM010_scaffold41237_4	virulent	0.99987316
DOM010_scaffold36003_1	virulent	0.99987125
DOM010_scaffold7_1	temperate	0.9998465
DOM010_scaffold19710_3	virulent	0.9992909
DOM010_scaffold72_1	virulent	0.9998308
DOM010_scaffold25074_1	temperate	0.9998579
DOM010_scaffold42659_2	temperate	0.979654
DOM010_scaffold38114_4	temperate	0.99159664
DOM010_scaffold1445_1	temperate	0.9998588
DOM010_scaffold44548_1	temperate	0.5364791
DOM010_scaffold44359_1	virulent	0.9986124
DOM010_scaffold5308_8	virulent	0.9997988
DOM010_scaffold36407_1	temperate	0.99976444
DOM010_scaffold43444_3	temperate	0.9998584
DOM010_scaffold12345_3	virulent	0.9998727
DOM010_scaffold27469_1	temperate	0.99986035
DOM010_scaffold17754_1	temperate	0.99985605
DOM010_C557291_1	virulent	0.9998297
DOM010_scaffold13897_1	virulent	0.99960005
DOM010_scaffold43785_1	virulent	0.99987173
DOM010_scaffold38766_6	virulent	0.99978536
DOM010_scaffold34515_1	temperate	0.9998388
DOM010_scaffold44098_1	temperate	0.99985975
DOM010_scaffold43222_1	virulent	0.9994699
DOM010_scaffold44558_1	temperate	0.9377786
DOM010_scaffold21662_2	virulent	0.99985695
DOM010_scaffold7072_9	temperate	0.99986035
DOM010_scaffold3568_1	virulent	0.99658924
DOM010_C557197_1	temperate	0.99986035
DOM010_scaffold40874_2	virulent	0.99987036
DOM010_scaffold40514_1	virulent	0.99986607
DOM010_scaffold39715_1	virulent	0.99987084
DOM010_scaffold120_2	virulent	0.9998513
DOM010_scaffold44263_3	temperate	0.98068535
DOM012_scaffold27104_2	virulent	0.99987316
DOM012_scaffold631_3	virulent	0.99986315
DOM012_scaffold33660_1	temperate	0.9272924
DOM012_scaffold4248_4	virulent	0.9998593
DOM012_scaffold33513_5	virulent	0.9868683
DOM012_scaffold8289_1	virulent	0.99987173

DOM012_scaffold25292_2	temperate	0.9982647
DOM012_scaffold9067_4	temperate	0.899105
DOM012_scaffold38243_4	temperate	0.9864365
DOM012_scaffold9872_1	virulent	0.9545897
DOM012_scaffold3937_49	virulent	0.9998693
DOM012_scaffold25292_3	temperate	0.9998445
DOM012_scaffold17700_1	temperate	0.999854
DOM012_scaffold6197_5_2	virulent	0.9998408
DOM012_scaffold5103_5	virulent	0.99979275
DOM012_scaffold2137_37	virulent	0.95147115
DOM012_scaffold4248_15	virulent	0.9998727
DOM012_scaffold7054_4	virulent	0.9998665
DOM012_scaffold631_1	virulent	0.9998627
DOM012_scaffold42523_1	virulent	0.99982214
DOM012_scaffold6291_4	temperate	0.6325878
DOM012_scaffold4007_9	temperate	0.99985313
DOM012_scaffold4108_5	temperate	0.9971176
DOM012_scaffold33307_1	virulent	0.7742801
DOM012_scaffold4248_8	virulent	0.99986404
DOM012_scaffold31_2	temperate	0.77475363
DOM012_scaffold46434_1	virulent	0.99987084
DOM012_scaffold4248_3	virulent	0.99987465
DOM013_scaffold29644_2	virulent	0.99986744
DOM013_scaffold65510_2	virulent	0.99986744
DOM013_scaffold66179_1	virulent	0.9998693
DOM013_scaffold45617_1	temperate	0.99986035
DOM013_scaffold29321_1	temperate	0.99980927
DOM013_scaffold24958_13	virulent	0.99987036
DOM013_scaffold4994_13	virulent	0.9998722
DOM013_scaffold46653_1	temperate	0.9992764
DOM013_scaffold11541_3	virulent	0.9995705
DOM013_scaffold38557_2	virulent	0.99987316
DOM013_scaffold13214_2	virulent	0.99987125
DOM013_scaffold63345_1	temperate	0.9997378
DOM013_scaffold27634_2	virulent	0.99980664
DOM013_scaffold65920_1	virulent	0.9998688
DOM013_scaffold65670_1	temperate	0.9857822
DOM013_scaffold39903_2	temperate	0.99985975
DOM013_scaffold46391_3	temperate	0.9998584
DOM013_scaffold50516_3	temperate	0.9585484
DOM013_scaffold66005_2	virulent	0.9998651
DOM013_scaffold10380_3	virulent	0.9998656
DOM013_scaffold34766_1	virulent	0.99987084
DOM013_scaffold65510_1	virulent	0.9998684
DOM013_scaffold66036_1	temperate	0.9998593
DOM013_scaffold2110_2	temperate	0.999784

DOM013_scaffold9813_6	temperate	0.95559967
DOM013_scaffold42751_1	temperate	0.99985266
DOM013_scaffold33337_2	virulent	0.99974734
DOM013_scaffold3571_32	virulent	0.99987316
DOM013_scaffold81_8	virulent	0.9998699
DOM013_scaffold66005_1	virulent	0.9998699
DOM013_scaffold54135_4	virulent	0.9998693
DOM013_scaffold66255_2	virulent	0.99987036
DOM013_C812420_1	virulent	0.99986035
DOM013_scaffold64045_2	temperate	0.9998417
DOM013_scaffold63330_1	virulent	0.9998736
DOM013_scaffold13_2	virulent	0.9470206
DOM013_scaffold8272_3	temperate	0.9821213
DOM013_scaffold17461_1	virulent	0.9998536
DOM013_scaffold60583_1	temperate	0.9995733
DOM013_scaffold23134_20	temperate	0.8561097
DOM013_scaffold3755_4	virulent	0.9996818
DOM013_scaffold62840_1	temperate	0.9998584
DOM013_scaffold81_7	virulent	0.99987173
DOM013_scaffold66110_1	virulent	0.999798
DOM013_scaffold3571_2	temperate	0.99985975
DOM013_scaffold22249_3	virulent	0.9998536
DOM013_scaffold54135_10	temperate	0.9998584
DOM013_scaffold16907_3	virulent	0.88889205
DOM013_scaffold9359_8	temperate	0.96828395
DOM014_scaffold7185_19	virulent	0.9994256
DOM014_scaffold43299_1	temperate	0.9998584
DOM014_scaffold15062_27	temperate	0.99985975
DOM014_scaffold17723_2	virulent	0.99956405
DOM014_scaffold3561_14	virulent	0.9998551
DOM014_scaffold9701_3	temperate	0.9998556
DOM014_scaffold804_14	temperate	0.99984884
DOM014_scaffold14238_1	virulent	0.9998693
DOM014_scaffold283_1	temperate	0.99985695
DOM014_scaffold1467_4	virulent	0.99987084
DOM014_scaffold5325_3	virulent	0.8092583
DOM014_scaffold1467_2	virulent	0.99987173
DOM014_scaffold46104_1	virulent	0.99104285
DOM014_scaffold804_11	virulent	0.9998556
DOM014_scaffold6073_4	virulent	0.9998522
DOM014_scaffold804_8	temperate	0.99098414
DOM014_scaffold27382_3	temperate	0.99984837
DOM014_scaffold14238_3	virulent	0.99987125
DOM014_scaffold2397_7	virulent	0.9998588
DOM014_scaffold804_15	virulent	0.9998369
DOM014_scaffold5037_9	virulent	0.9998086

DOM014_scaffold804_6	temperate	0.9900664
DOM014_scaffold25405_6	virulent	0.96556866
DOM014_scaffold8176_14	virulent	0.9985003
DOM014_scaffold10055_2	temperate	0.9998574
DOM014_scaffold1696_5	virulent	0.99984556
DOM014_scaffold27264_1	virulent	0.9996015
DOM014_scaffold25405_4	virulent	0.9460565
DOM014_scaffold4337_1	temperate	0.82770056
DOM015_scaffold25351_1	temperate	0.99983835
DOM015_scaffold21624_2	virulent	0.9998622
DOM015_scaffold24987_5	temperate	0.9998593
DOM015_scaffold15574_5	virulent	0.99987406
DOM015_scaffold226_4	virulent	0.9998517
DOM015_scaffold29083_1	virulent	0.9998679
DOM015_scaffold5854_1	virulent	0.9998656
DOM015_scaffold14251_1	temperate	0.9997912
DOM015_scaffold22277_2	temperate	0.9998522
DOM015_scaffold1868_1	temperate	0.99986035
DOM015_scaffold8047_3	temperate	0.99986035
DOM015_scaffold2034_4	virulent	0.99987173
DOM015_scaffold28241_1	virulent	0.9998665
DOM015_scaffold16257_1	virulent	0.83896554
DOM015_scaffold1673_2	virulent	0.9998651
DOM015_scaffold4_1	temperate	0.9996581
DOM015_scaffold21150_1	temperate	0.997755
DOM015_scaffold7250_8	virulent	0.9998517
DOM015_scaffold21009_2	virulent	0.9998684
DOM015_scaffold10848_3	virulent	0.99966824
DOM015_scaffold10865_1	virulent	0.9997092
DOM015_C368449_1	virulent	0.7615538
DOM015_scaffold5037_4	virulent	0.9998684
DOM015_scaffold13677_1	temperate	0.99383783
DOM015_scaffold29079_1	virulent	0.9996403
DOM015_scaffold14658_2	virulent	0.99912494
DOM015_scaffold28241_3	temperate	0.98519784
DOM015_scaffold24987_1	virulent	0.99870294
DOM015_C368153_1	virulent	0.9998736
DOM016_scaffold37120_1	temperate	0.9998579
DOM016_scaffold36138_1	temperate	0.99949944
DOM016_scaffold36372_1	temperate	0.99986035
DOM016_scaffold28769_2	temperate	0.9998049
DOM016_scaffold15209_2	temperate	0.99983406
DOM016_scaffold35635_2	temperate	0.9998588
DOM016_scaffold35635_4	temperate	0.999844
DOM016_scaffold27783_2	virulent	0.99977106
DOM016_scaffold714_2	temperate	0.99959195

DOM016_scaffold36369_1	virulent	0.9989985
DOM016_scaffold15402_10	temperate	0.99985975
DOM016_scaffold35922_2	temperate	0.98691773
DOM016_scaffold10818_1	virulent	0.99987084
DOM016_scaffold35524_2	virulent	0.9998727
DOM016_scaffold25511_1	virulent	0.99831057
DOM016_scaffold32640_3	temperate	0.999854
DOM016_scaffold37071_2	temperate	0.99985605
DOM016_scaffold701_1	virulent	0.99987084
DOM016_scaffold27658_1	temperate	0.9998408
DOM016_scaffold29662_3	temperate	0.9998556
DOM016_scaffold5915_8	temperate	0.99985975
DOM016_scaffold35635_3	temperate	0.9998579
DOM016_scaffold4735_5	virulent	0.9998608
DOM016_scaffold10222_1	temperate	0.99985975
DOM016_scaffold27136_11	temperate	0.99986035
DOM016_scaffold35701_7	virulent	0.9996515
DOM016_scaffold35701_2	temperate	0.9998593
DOM016_scaffold29175_2	virulent	0.9998693
DOM016_scaffold30030_2	virulent	0.99980664
DOM016_scaffold15402_6	virulent	0.98361695
DOM016_scaffold31190_2	virulent	0.8810457
DOM016_scaffold20871_2	virulent	0.99987406
DOM016_scaffold30578_2	virulent	0.99962103
DOM016_scaffold65_5	virulent	0.9997578
DOM016_scaffold19852_1	temperate	0.99111164
DOM016_scaffold11439_8	temperate	0.99985605
DOM017_scaffold13265_2	virulent	0.9998727
DOM017_scaffold7875_1	temperate	0.9998274
DOM017_scaffold12516_1	virulent	0.9053317
DOM017_scaffold21083_1	virulent	0.9998727
DOM017_scaffold16028_1	virulent	0.99986607
DOM017_scaffold5373_1	virulent	0.9998417
DOM017_scaffold9422_1	virulent	0.94220346
DOM017_scaffold273_11	virulent	0.9998727
DOM017_scaffold591_5	virulent	0.999549
DOM017_scaffold7875_3	temperate	0.9998593
DOM017_scaffold7344_7	temperate	0.5421888
DOM017_scaffold3771_1	virulent	0.9997632
DOM017_scaffold35431_3	virulent	0.99987316
DOM017_scaffold434_2	virulent	0.9962297
DOM017_scaffold3183_4	virulent	0.99934214
DOM017_scaffold12158_1	temperate	0.9989053
DOM017_scaffold2122_1	temperate	0.999084
DOM017_scaffold12299_4	virulent	0.9998302
DOM017_scaffold23327_2	virulent	0.9998736

DOM017_scaffold30906_1	virulent	0.9998371
DOM017_scaffold12432_2	temperate	0.99983925
DOM017_scaffold1652_4	temperate	0.998085
DOM017_scaffold28752_1	temperate	0.92755413
DOM017_scaffold35758_3	virulent	0.9998727
DOM017_scaffold10632_13	virulent	0.99880815
DOM017_scaffold29332_1	virulent	0.76902056
DOM017_scaffold2195_2	temperate	0.898576
DOM018_scaffold6891_4	temperate	0.9998584
DOM018_scaffold11099_4	virulent	0.9998565
DOM018_scaffold88_7	virulent	0.9998131
DOM018_scaffold41600_1	virulent	0.9871713
DOM018_scaffold27201_3	virulent	0.99987036
DOM018_scaffold37486_2	temperate	0.979654
DOM018_scaffold32733_2	virulent	0.9712672
DOM018_scaffold35204_2	temperate	0.9998474
DOM018_scaffold4830_3	virulent	0.9654628
DOM018_scaffold22855_12	temperate	0.99922955
DOM018_scaffold41384_2	temperate	0.9998579
DOM018_scaffold35020_1	virulent	0.9998627
DOM018_scaffold41493_2	temperate	0.68137324
DOM018_scaffold9337_1	temperate	0.99678105
DOM018_C532545_1	virulent	0.99986607
DOM018_scaffold40717_1	temperate	0.9997526
DOM018_scaffold23065_2	virulent	0.99486125
DOM018_scaffold6891_1	temperate	0.8910085
DOM018_scaffold37478_3	temperate	0.99985695
DOM018_scaffold6891_2	virulent	0.9998645
DOM018_scaffold1832_2	temperate	0.99276054
DOM018_scaffold34741_1	virulent	0.99985313
DOM018_scaffold5182_1	temperate	0.8945864
DOM018_scaffold41493_1	virulent	0.9998688
DOM018_C532801_1	temperate	0.9998579
DOM018_scaffold32883_2	temperate	0.99983126
DOM018_scaffold32755_3	temperate	0.99953187
DOM018_scaffold2126_7	virulent	0.99987125
DOM018_scaffold35068_1	temperate	0.99978167
DOM018_scaffold1832_4	virulent	0.8973274
DOM018_scaffold18201_3	temperate	0.99983555
DOM018_scaffold13608_2	virulent	0.99987406
DOM018_scaffold33929_1	temperate	0.99985975
DOM018_scaffold17834_3	virulent	0.9998684
DOM019_scaffold640_2	virulent	0.9998408
DOM019_scaffold141_3	virulent	0.9996124
DOM019_scaffold20326_3	virulent	0.8463101
DOM019_scaffold6808_2	virulent	0.99987173

DOM019_scaffold41546_1	virulent	0.9247743
DOM019_scaffold56541_1	virulent	0.99987316
DOM019_scaffold42868_1	temperate	0.99986035
DOM019_scaffold5478_2	temperate	0.999836
DOM019_scaffold33784_1	temperate	0.99985605
DOM019_scaffold10413_15	virulent	0.99987173
DOM019_scaffold3315_20	temperate	0.9998593
DOM019_scaffold1388_2	temperate	0.9997526
DOM019_scaffold56605_1	temperate	0.9998588
DOM019_scaffold6808_3	virulent	0.9998736
DOM019_scaffold20197_1	virulent	0.9998684
DOM019_scaffold661_7	temperate	0.99984884
DOM019_scaffold56555_1	virulent	0.9998688
DOM019_scaffold8387_2	virulent	0.9688307
DOM019_scaffold56549_2	virulent	0.9998679
DOM019_scaffold55994_2	virulent	0.9998431
DOM019_scaffold9440_2	temperate	0.9973751
DOM019_scaffold56598_2	virulent	0.9998665
DOM019_scaffold3989_6	temperate	0.9998593
DOM019_scaffold23724_3	temperate	0.99984884
DOM019_scaffold56558_1	temperate	0.99985975
DOM019_scaffold56469_5	virulent	0.9998574
DOM019_scaffold20133_1	temperate	0.9998579
DOM019_scaffold34447_1	virulent	0.9811014
DOM019_scaffold56536_2	virulent	0.99978495
DOM019_scaffold8084_3	virulent	0.999836
DOM019_scaffold11571_11	temperate	0.99947125
DOM019_scaffold35386_4	temperate	0.56512856
DOM019_scaffold9830_4	virulent	0.99987084
DOM019_scaffold286_2	virulent	0.9998656
DOM020_scaffold38403_2	temperate	0.9986213
DOM020_scaffold38380_2	virulent	0.9998574
DOM020_scaffold36128_4	temperate	0.979654
DOM020_scaffold8013_4	temperate	0.99599046
DOM020_scaffold24489_1	temperate	0.9998379
DOM020_scaffold1763_4	temperate	0.99985266
DOM020_scaffold1491_1	temperate	0.9998574
DOM020_scaffold19627_11	temperate	0.97744745
DOM020_scaffold8898_2	temperate	0.8756662
DOM020_scaffold16344_2	temperate	0.99985266
DOM020_scaffold37565_2	virulent	0.99987036
DOM020_C483630_1	temperate	0.9998593
DOM020_scaffold8013_2	temperate	0.8831192
DOM020_scaffold1229_1	temperate	0.9998217
DOM020_scaffold3600_6	temperate	0.99986035
DOM020_scaffold7010_6	virulent	0.9997811

DOM021_scaffold18418_1	virulent	0.5814494
DOM021_scaffold21593_8	temperate	0.9998579
DOM021_scaffold27752_2	virulent	0.9998679
DOM021_scaffold71_1	virulent	0.99970627
DOM021_scaffold38953_1	virulent	0.9998699
DOM021_scaffold272_1	virulent	0.99974704
DOM021_scaffold39089_1	virulent	0.9997509
DOM021_scaffold100_1	virulent	0.9998656
DOM021_scaffold39104_1	virulent	0.99986696
DOM021_scaffold36942_1	virulent	0.99987316
DOM021_scaffold23118_2	temperate	0.9959307
DOM021_scaffold57_4	virulent	0.99987173
DOM021_scaffold37667_2	virulent	0.9998684
DOM021_scaffold11063_1	virulent	0.99985695
DOM021_scaffold39089_2	temperate	0.96921164
DOM021_scaffold36893_3	temperate	0.99969774
DOM021_scaffold12359_1	temperate	0.99985975
DOM021_scaffold37667_3	virulent	0.9998693
DOM021_scaffold5645_2	virulent	0.99987316
DOM021_scaffold37125_1	temperate	0.9301825
DOM021_scaffold39046_1	virulent	0.9998293
DOM022_scaffold32377_2	virulent	0.9998645
DOM022_scaffold19956_4	temperate	0.979654
DOM022_scaffold6808_24	virulent	0.99138457
DOM022_scaffold28157_3	virulent	0.9995576
DOM022_scaffold32538_1	virulent	0.99986744
DOM022_scaffold38389_3	virulent	0.99987316
DOM022_scaffold2843_1	virulent	0.9998736
DOM022_scaffold38212_2	temperate	0.99976164
DOM022_scaffold18184_9	temperate	0.9998188
DOM022_scaffold8882_1	temperate	0.9080085
DOM022_scaffold38445_2	temperate	0.99985975
DOM022_scaffold16044_22	temperate	0.905662
DOM022_scaffold847_4	temperate	0.999816
DOM022_scaffold37774_1	virulent	0.9998699
DOM022_scaffold8382_3	temperate	0.9998508
DOM022_scaffold37182_2	virulent	0.8010681
DOM022_scaffold26147_1	temperate	0.99985313
DOM022_scaffold38016_1	temperate	0.99984694
DOM022_C512095_1	virulent	0.9764287
DOM022_scaffold38445_1	temperate	0.99985605
DOM022_scaffold35096_1	temperate	0.9996434
DOM022_scaffold33500_1	temperate	0.99985975
DOM022_scaffold36652_4	temperate	0.9998331
DOM022_scaffold38271_2	virulent	0.999398
DOM022_scaffold21088_2	virulent	0.9952504

DOM022_scaffold32837_2	virulent	0.99986696
DOM022_scaffold37253_1	virulent	0.9998722
DOM022_scaffold25564_3	temperate	0.9998388
DOM022_scaffold35602_3	virulent	0.90293515
DOM022_scaffold33506_1	temperate	0.99636406
DOM022_scaffold16660_7	temperate	0.9983715
DOM022_scaffold3588_2	virulent	0.9998688
DOM022_scaffold9769_2	virulent	0.99986404
DOM022_scaffold6474_11	virulent	0.88498414
DOM022_scaffold8236_1	temperate	0.9592161
DOM022_scaffold37834_1	virulent	0.9998617
DOM022_scaffold11588_18	virulent	0.99987173
DOM022_scaffold17665_18	temperate	0.9998397
DOM022_scaffold11253_14	virulent	0.99987406
DOM022_scaffold27991_2	temperate	0.62538254
DOM022_scaffold7138_11	virulent	0.9998736
DOM022_scaffold3588_8	virulent	0.99972224
DOM022_scaffold38396_3	virulent	0.9998197
DOM022_scaffold48_3	virulent	0.9998736
DOM022_scaffold17718_5	temperate	0.9743889
DOM022_scaffold15495_1	temperate	0.9700229
DOM022_scaffold37363_3	virulent	0.9998693
DOM022_scaffold8882_2	temperate	0.99985975
DOM022_scaffold37244_1	virulent	0.83116853
DOM022_scaffold37792_2	virulent	0.93913764
DOM022_scaffold19876_2	virulent	0.9998651
DOM022_scaffold13760_14	virulent	0.9998305
DOM022_scaffold1123_2	virulent	0.99980664
DOM022_scaffold28292_2	temperate	0.7311479
DOM022_scaffold21088_3	temperate	0.9998584
DOM022_scaffold9987_23	virulent	0.99986035
DOM022_scaffold36541_6	temperate	0.9998551
DOM023_scaffold33157_2	virulent	0.9998688
DOM023_scaffold33236_1	virulent	0.999322
DOM023_scaffold33285_1	temperate	0.9998479
DOM023_scaffold19886_6	virulent	0.9998665
DOM023_scaffold12205_7	virulent	0.9998727
DOM023_scaffold25122_8	temperate	0.9990589
DOM023_scaffold33612_1	virulent	0.99987173
DOM023_scaffold11787_6	temperate	0.9900165
DOM023_scaffold33611_2	virulent	0.99987173
DOM023_scaffold28756_2	virulent	0.9998217
DOM023_scaffold13717_2	virulent	0.99987406
DOM023_scaffold23102_8	temperate	0.9998593
DOM023_scaffold33401_1	temperate	0.9998593
DOM023_scaffold31295_1	temperate	0.9998465

DOM023_scaffold33622_1	temperate	0.9997196
DOM023_scaffold19886_5	virulent	0.99987173
DOM023_scaffold2410_11	temperate	0.9926195
DOM023_scaffold16805_4	virulent	0.9998622
DOM023_scaffold33548_1	virulent	0.9998645
DOM023_C393870_1	virulent	0.93739945
DOM023_scaffold22587_5	virulent	0.9998736
DOM024_scaffold35255_15	virulent	0.9998536
DOM024_scaffold11745_3	virulent	0.99987465
DOM024_scaffold34021_1	temperate	0.83150184
DOM024_scaffold44466_2	virulent	0.984512
DOM024_scaffold42617_1	virulent	0.9990506
DOM024_scaffold38154_1	virulent	0.99986124
DOM024_scaffold56350_1	temperate	0.99975395
DOM024_scaffold54306_4	virulent	0.9998727
DOM024_scaffold42617_2	virulent	0.99809235
DOM024_scaffold25752_1	virulent	0.9997449
DOM024_scaffold41795_1	virulent	0.99986035
DOM024_scaffold56303_1	virulent	0.9998517
DOM024_scaffold13072_13	virulent	0.99967504
DOM024_scaffold52650_1	temperate	0.99985975
DOM024_scaffold36750_16	temperate	0.98917335
DOM024_scaffold151_2	virulent	0.99519527
DOM024_scaffold55680_1	temperate	0.99985695
DOM024_scaffold52177_5	virulent	0.7548561
DOM024_C663872_1	temperate	0.9983641
DOM024_scaffold52177_6	virulent	0.7286186
DOM024_scaffold56350_2	virulent	0.9993765
DOM024_scaffold52366_1	temperate	0.99985266
DOM024_scaffold56292_1	virulent	0.9998699
DOM024_scaffold32065_2	virulent	0.9998083
DOM024_scaffold56206_2	temperate	0.93854123
DOM024_C664368_1	virulent	0.99986404
DOM024_C664620_1	virulent	0.66369885
DOM024_scaffold18051_2	virulent	0.9998699
DOM024_scaffold56343_2	temperate	0.99981695
DOM024_scaffold29589_1	temperate	0.99985695
DOM024_scaffold15513_1	virulent	0.99986035
DOM024_scaffold20070_12	virulent	0.9998736
DOM024_scaffold30168_5	temperate	0.9998417
DOM024_scaffold4865_6	temperate	0.78272307
DOM024_scaffold34021_4	temperate	0.99985975
DOM024_scaffold35255_14	virulent	0.9998679
DOM024_scaffold22388_3	temperate	0.9998556
DOM024_scaffold56343_3	temperate	0.9995614
DOM024_scaffold15633_7	virulent	0.6831714

DOM024_scaffold49650_2	temperate	0.62325543
DOM025_scaffold30920_4	virulent	0.88889205
DOM025_scaffold44713_1	temperate	0.99618447
DOM025_scaffold68_3	virulent	0.99885005
DOM025_scaffold39391_1	temperate	0.99959904
DOM025_scaffold29788_4	virulent	0.9998727
DOM025_scaffold44576_1	temperate	0.99985975
DOM025_scaffold49_1	virulent	0.9998693
DOM025_scaffold49_3	virulent	0.99986315
DOM025_scaffold41702_1	virulent	0.99987036
DOM025_scaffold10571_8	virulent	0.99987406
DOM025_scaffold80_3	temperate	0.99986035
DOM025_scaffold300_2	virulent	0.9998727
DOM025_scaffold4683_2	virulent	0.9996324
DOM025_scaffold9936_1	temperate	0.6660664
DOM025_scaffold1987_2	virulent	0.9997584
DOM025_scaffold3271_23	virulent	0.9998722
DOM025_scaffold7455_3	virulent	0.99986744
DOM025_scaffold41294_1	virulent	0.99987406
DOM025_scaffold14033_1	temperate	0.9998579
DOM025_scaffold23512_1	virulent	0.9998736
DOM025_scaffold20212_5	virulent	0.99987173
DOM025_scaffold5440_33	virulent	0.9998727
DOM025_scaffold10897_2	virulent	0.9998727
DOM025_scaffold12330_13	virulent	0.9998302
DOM025_scaffold2257_29	temperate	0.99985975
DOM026_scaffold22046_1	temperate	0.99985605
DOM026_scaffold39207_2	temperate	0.99980927
DOM026_scaffold135_10	temperate	0.9969343
DOM026_scaffold813_3	virulent	0.99978536
DOM026_scaffold22251_2	virulent	0.9469641
DOM026_scaffold21041_1	virulent	0.9998736
DOM026_scaffold38351_1	temperate	0.99986035
DOM026_scaffold9255_1	temperate	0.999194
DOM026_scaffold18742_1	virulent	0.99986607
DOM026_scaffold19764_1	virulent	0.9998722
DOM026_scaffold30709_3	virulent	0.9998699
DOM026_scaffold13170_6	temperate	0.9998593
DOM026_scaffold17576_11	temperate	0.9998545
DOM026_scaffold17476_4	virulent	0.99987316
DOM026_scaffold11281_20	virulent	0.99987406
DOM026_scaffold4239_7	temperate	0.99985605
DOM026_scaffold10323_1	temperate	0.99985975
DOM026_scaffold23168_2	virulent	0.9998257
DOM026_scaffold28561_1	temperate	0.9863652
DOM026_scaffold8858_1	temperate	0.99986035

DOM026_scaffold39135_1	temperate	0.99985975
DOM026_scaffold7817_3	temperate	0.8617855
DOM026_scaffold6782_6	temperate	0.9998584
DOM026_scaffold6782_3	temperate	0.9998413
DOM026_scaffold36555_1	virulent	0.99297863
DOM026_C539342_1	temperate	0.99949235
DOM026_scaffold11444_1	temperate	0.99985695
DOM026_scaffold21142_1	temperate	0.9998565
DOM026_scaffold5666_20	virulent	0.99987125
DOM026_scaffold22785_1	virulent	0.99986696
DOM026_scaffold17021_2	virulent	0.99987036
DOM026_scaffold12473_1	temperate	0.9998474
DOM026_C539214_1	temperate	0.9998588
DOM026_scaffold14353_26	virulent	0.9998579
DOM026_scaffold13335_3	temperate	0.9627462
DOM026_scaffold28681_1	temperate	0.9998593
DOM026_scaffold18755_1	virulent	0.99987406
DOM026_scaffold22785_2	virulent	0.99987036
DOM026_scaffold22404_1	virulent	0.9998565
DOM026_scaffold4239_3	temperate	0.9998588
DOM026_scaffold26812_1	temperate	0.99985975
DOM026_scaffold10922_1	virulent	0.9956566
DOM026_scaffold27635_1	temperate	0.9998579
DOM026_scaffold21024_9	temperate	0.99986035
DOM026_scaffold21514_2	virulent	0.9568131
DOM026_scaffold35658_1	virulent	0.9998679
DOM026_scaffold23768_1	temperate	0.98560125
DOM026_scaffold17367_1	temperate	0.995717
NOM001_scaffold14915_2	virulent	0.9998186
NOM001_scaffold36280_1	virulent	0.57108474
NOM001_scaffold22842_7	virulent	0.9998593
NOM001_scaffold36050_1	temperate	0.9333975
NOM001_scaffold22842_8	temperate	0.99986035
NOM001_scaffold4297_1	temperate	0.999723
NOM001_scaffold36892_1	temperate	0.9998593
NOM001_scaffold31539_2	virulent	0.9997986
NOM001_scaffold24512_3	virulent	0.9997156
NOM001_scaffold22842_6	virulent	0.99987406
NOM002_scaffold4408_28	virulent	0.98006254
NOM002_scaffold1807_4	temperate	0.979654
NOM002_scaffold5847_1	temperate	0.9998588
NOM002_scaffold139_2	temperate	0.99985975
NOM002_scaffold32200_2	temperate	0.9998474
NOM002_scaffold23302_7	temperate	0.7455003
NOM002_scaffold2797_4	virulent	0.9997957
NOM002_scaffold26702_1	temperate	0.99977213

NOM002_scaffold26752_2	virulent	0.9998693
NOM002_scaffold65_1	temperate	0.99985975
NOM002_scaffold2059_12	temperate	0.7585868
NOM002_scaffold32168_1	virulent	0.6105096
NOM002_scaffold32212_1	temperate	0.99986035
NOM002_scaffold15419_6	temperate	0.99985975
NOM002_scaffold7211_5	temperate	0.6142863
NOM002_scaffold32093_2	virulent	0.99987036
NOM002_scaffold2423_2	virulent	0.99987125
NOM002_scaffold32124_1	temperate	0.9998588
NOM002_scaffold32041_1	virulent	0.99846077
NOM002_scaffold13215_6	temperate	0.9998336
NOM002_scaffold830_1	temperate	0.99985975
NOM002_scaffold3250_3	virulent	0.99986696
NOM002_scaffold23876_3	temperate	0.9998574
NOM002_scaffold5995_1	temperate	0.87618214
NOM002_scaffold31941_2	temperate	0.9998517
NOM002_scaffold30413_1	temperate	0.99983495
NOM002_scaffold31231_2	virulent	0.86550814
NOM002_scaffold3598_9	virulent	0.99987173
NOM002_scaffold7151_8	virulent	0.99902534
NOM002_scaffold20546_3	temperate	0.99740297
NOM002_scaffold1562_8	temperate	0.6375706
NOM002_scaffold27268_2	temperate	0.99984837
NOM002_scaffold27843_1	temperate	0.99986035
NOM002_scaffold31229_1	virulent	0.9998693
NOM002_C429380_1	temperate	0.9998588
NOM002_C428718_1	temperate	0.99986035
NOM002_scaffold32206_1	virulent	0.9998026
NOM002_scaffold32205_1	temperate	0.9977357
NOM002_scaffold330_35	virulent	0.9998693
NOM002_scaffold32135_1	temperate	0.99984497
NOM002_scaffold31205_1	virulent	0.9991823
NOM002_scaffold22930_2	virulent	0.9998117
NOM002_scaffold3856_12	virulent	0.9998727
NOM002_scaffold6808_7	virulent	0.99987316
NOM002_scaffold19947_2	temperate	0.99985975
NOM002_scaffold12213_5	temperate	0.9413989
NOM002_scaffold4555_2	virulent	0.99983925
NOM002_scaffold14897_3	temperate	0.9119875
NOM002_scaffold6256_7	virulent	0.9998608
NOM002_scaffold24280_1	temperate	0.99986035
NOM002_scaffold20040_1	virulent	0.9998617
NOM002_scaffold7687_1	virulent	0.9627274
NOM002_scaffold27308_2	virulent	0.9989942
NOM002_scaffold24275_1	virulent	0.9998656

NOM004_scaffold22743_2	virulent	0.9454852
NOM004_scaffold14898_16	temperate	0.97422904
NOM004_scaffold8990_22	virulent	0.9322203
NOM004_scaffold3782_4	temperate	0.9996867
NOM004_scaffold13517_2	virulent	0.99987173
NOM004_scaffold22899_1	virulent	0.9998722
NOM004_scaffold4741_16	temperate	0.93755734
NOM004_scaffold130_15	virulent	0.9122659
NOM004_C361687_1	temperate	0.9368787
NOM004_scaffold11992_11	temperate	0.9998588
NOM004_scaffold15695_1	virulent	0.9998017
NOM004_scaffold21343_2	temperate	0.8460105
NOM004_scaffold9807_9	virulent	0.99973303
NOM004_scaffold21707_1	virulent	0.99736845
NOM004_scaffold22694_1	virulent	0.93689924
NOM004_scaffold11992_20	temperate	0.9516476
NOM004_scaffold13523_7	virulent	0.9998636
NOM004_scaffold28_10	virulent	0.9998651
NOM004_scaffold6154_1	virulent	0.908313
NOM004_scaffold17398_1	temperate	0.99985975
NOM004_scaffold11917_4	virulent	0.9998556
NOM004_scaffold14898_14	virulent	0.9669712
NOM004_scaffold15115_6	virulent	0.9998699
NOM004_scaffold1169_9	virulent	0.99987406
NOM004_scaffold15486_1	virulent	0.9997053
NOM005_scaffold53686_4	temperate	0.8726311
NOM005_C715971_1	temperate	0.9998574
NOM005_scaffold35434_1	virulent	0.9997797
NOM005_scaffold41127_8	virulent	0.99978644
NOM005_scaffold563_2	virulent	0.99987125
NOM005_scaffold10786_3	virulent	0.9998684
NOM005_scaffold51989_2	temperate	0.99985975
NOM005_scaffold55066_2	temperate	0.8863712
NOM005_C716463_1	temperate	0.99985695
NOM005_scaffold2849_2	virulent	0.83790475
NOM005_scaffold29223_4	virulent	0.99983126
NOM005_scaffold32988_8	virulent	0.9998727
NOM005_scaffold3277_10	temperate	0.99959195
NOM005_scaffold40041_4	virulent	0.9998722
NOM005_scaffold563_4	virulent	0.9998684
NOM005_scaffold37509_3	temperate	0.9413989
NOM005_scaffold41127_7	virulent	0.99986696
NOM005_scaffold11865_1	temperate	0.99986035
NOM005_scaffold8808_3	temperate	0.99983835
NOM005_scaffold130_3	virulent	0.99969083
NOM005_scaffold47045_2	virulent	0.99986744

NOM005_scaffold53973_2	virulent	0.9998636
NOM005_scaffold12226_1	temperate	0.51049143
NOM005_scaffold16553_2	temperate	0.99985605
NOM005_scaffold563_3	temperate	0.99985695
NOM005_scaffold45891_2	temperate	0.9998408
NOM005_scaffold29223_3	temperate	0.99962914
NOM005_scaffold55101_1	temperate	0.99980783
NOM005_scaffold8276_2	virulent	0.9998727
NOM005_C716395_1	virulent	0.9998656
NOM005_scaffold47045_3	virulent	0.9998699
NOM005_scaffold31514_2	temperate	0.9997215
NOM005_scaffold504_2	virulent	0.8143582
NOM005_scaffold32988_6	virulent	0.9998679
NOM005_scaffold37509_5	temperate	0.96727747
NOM005_scaffold16216_8	virulent	0.9998736
NOM005_scaffold11952_1	temperate	0.9997053
NOM005_scaffold41127_12	temperate	0.9998593
NOM005_scaffold5739_4	virulent	0.9998722
NOM005_scaffold50369_2	temperate	0.99985975
NOM005_scaffold5699_2	virulent	0.9814843
NOM005_scaffold2279_1	temperate	0.99652207
NOM005_scaffold54732_1	temperate	0.99986035
NOM005_scaffold41647_4	virulent	0.9998665
NOM005_scaffold41127_11	virulent	0.99979216
NOM005_scaffold41127_5	temperate	0.9998565
NOM005_scaffold53973_3	virulent	0.9998665
NOM005_scaffold47045_1	virulent	0.9998699
NOM005_scaffold48061_2	virulent	0.9983386
NOM005_scaffold55068_1	virulent	0.9918761
NOM005_scaffold20861_2	virulent	0.9996341
NOM005_scaffold25180_1	temperate	0.9998588
NOM005_C716365_1	virulent	0.9998684
NOM007_scaffold3522_1	temperate	0.999703
NOM007_C668778_1	temperate	0.9998588
NOM007_scaffold48877_1	temperate	0.99985975
NOM007_scaffold37186_2	temperate	0.99985975
NOM007_scaffold46703_1	temperate	0.98560125
NOM007_scaffold45023_6	temperate	0.999651
NOM007_scaffold15814_1	temperate	0.9998588
NOM007_scaffold41225_1	temperate	0.9901432
NOM007_scaffold270_1	virulent	0.93356663
NOM007_scaffold35454_4	temperate	0.99986035
NOM007_scaffold39468_29	virulent	0.99845034
NOM007_C669362_1	virulent	0.99986744
NOM007_scaffold14193_6	virulent	0.9992085
NOM007_scaffold35189_1	virulent	0.9998727

NOM007_scaffold37589_1	temperate	0.9998545
NOM007_scaffold37139_1	temperate	0.9991854
NOM007_scaffold17241_4	virulent	0.99211204
NOM007_scaffold10787_5	virulent	0.9998157
NOM007_scaffold48928_1	temperate	0.9995662
NOM007_scaffold41196_1	temperate	0.9998012
NOM007_scaffold29869_4	virulent	0.99987084
NOM007_scaffold4742_2	virulent	0.9998684
NOM008_scaffold33378_1	temperate	0.9998593
NOM008_scaffold17954_24	virulent	0.9997584
NOM008_scaffold18778_4	virulent	0.99986696
NOM008_scaffold4244_38	temperate	0.9998556
NOM008_scaffold9964_68	virulent	0.99974686
NOM008_scaffold75_1	virulent	0.9998727
NOM008_scaffold18096_12	virulent	0.9998736
NOM008_scaffold29282_11	virulent	0.9998736
NOM008_scaffold445_45	virulent	0.9998699
NOM008_scaffold33375_1	virulent	0.9998656
NOM008_scaffold24500_2	virulent	0.9998302
NOM008_scaffold32938_5	virulent	0.9998379
NOM008_scaffold6097_1	virulent	0.9998388
NOM008_scaffold14724_3	temperate	0.98581904
NOM008_scaffold33345_2	virulent	0.96981525
NOM008_scaffold32941_1	virulent	0.9998017
NOM008_scaffold32975_4	virulent	0.6497936
NOM008_scaffold226_8	virulent	0.99966824
NOM008_scaffold9194_1	virulent	0.96896803
NOM008_scaffold30194_1	temperate	0.9998584
NOM008_scaffold940_9	virulent	0.9998727
NOM008_scaffold29282_6	virulent	0.9998688
NOM008_scaffold1177_10	virulent	0.9181428
NOM008_scaffold5454_20	temperate	0.9998593
NOM008_scaffold27977_1	virulent	0.9998617
NOM009_scaffold34457_1	temperate	0.99985975
NOM009_scaffold37752_3	temperate	0.99985605
NOM009_scaffold15278_8	virulent	0.9998636
NOM009_scaffold52880_2	temperate	0.9839192
NOM009_scaffold24584_3	temperate	0.999784
NOM009_scaffold54003_3	temperate	0.515138
NOM009_scaffold27152_1	virulent	0.99981
NOM009_scaffold43294_6	virulent	0.798589
NOM009_scaffold31057_2	temperate	0.99986035
NOM009_scaffold45014_3	virulent	0.99986744
NOM009_scaffold41176_2	virulent	0.9998736
NOM009_scaffold25569_1	temperate	0.99984974
NOM009_scaffold19614_4	virulent	0.9998522

NOM009_scaffold40704_1	virulent	0.91393614
NOM009_scaffold15278_9	temperate	0.99985975
NOM009_scaffold54000_1	virulent	0.99986315
NOM009_scaffold1163_4	virulent	0.99986124
NOM009_scaffold16373_1	temperate	0.99986035
NOM009_scaffold10818_20	temperate	0.9998403
NOM009_scaffold22166_2	virulent	0.9998684
NOM009_scaffold18703_1	temperate	0.99986035
NOM009_scaffold53866_1	temperate	0.97646034
NOM009_scaffold6873_12	temperate	0.99985975
NOM009_scaffold20832_3	temperate	0.93755734
NOM009_scaffold13490_22	virulent	0.9998379
NOM009_scaffold26738_4	temperate	0.9998593
NOM009_scaffold41176_6	temperate	0.99985975
NOM009_scaffold37752_2	virulent	0.9995881
NOM009_scaffold337_2	virulent	0.9998736
NOM009_scaffold293_1	temperate	0.9998588
NOM009_scaffold37752_4	temperate	0.9998574
NOM009_scaffold53815_1	temperate	0.9998388
NOM009_scaffold14272_1	temperate	0.99936604
NOM009_scaffold113_2	virulent	0.9998665
NOM009_scaffold14149_2	temperate	0.99985975
NOM009_scaffold53934_1	temperate	0.99985695
NOM009_scaffold53906_1	temperate	0.9998593
NOM009_scaffold44452_1	virulent	0.9998656
NOM010_scaffold16688_4	temperate	0.9998593
NOM010_scaffold7834_6	virulent	0.9998302
NOM010_scaffold36917_1	virulent	0.99982
NOM010_scaffold40709_2	virulent	0.9998722
NOM010_scaffold27408_5	virulent	0.99987406
NOM010_scaffold34799_1	temperate	0.99986035
NOM010_scaffold7841_9	virulent	0.9998684
NOM010_scaffold23144_2	temperate	0.979654
NOM010_scaffold6634_3	virulent	0.99987173
NOM010_scaffold8017_1	virulent	0.6827767
NOM010_scaffold39236_1	temperate	0.9967525
NOM010_scaffold356_5	virulent	0.9998665
NOM010_scaffold3371_1	temperate	0.9998593
NOM010_scaffold40709_1	virulent	0.99987173
NOM010_scaffold2023_1	virulent	0.8463101
NOM010_scaffold41832_1	virulent	0.967852
NOM010_scaffold41425_2	temperate	0.8937481
NOM010_scaffold12374_8	virulent	0.99987125
NOM010_scaffold26994_1	temperate	0.9998522
NOM010_scaffold40683_2	temperate	0.9856434
NOM010_scaffold26048_2	virulent	0.9998688

NOM010_scaffold19890_3	virulent	0.99954635
NOM010_scaffold40709_6	virulent	0.9998684
NOM010_scaffold16391_2	virulent	0.9998645
NOM010_scaffold19030_3	virulent	0.99966824
NOM010_scaffold7692_1	temperate	0.99985975
NOM010_scaffold27711_4	virulent	0.9998693
NOM010_scaffold13739_1	virulent	0.9109146
NOM010_scaffold27824_1	virulent	0.99987316
NOM010_scaffold5951_6	temperate	0.9998308
NOM010_scaffold4318_15	virulent	0.99987406
NOM010_scaffold41566_2	virulent	0.99937266
NOM010_scaffold30699_2	temperate	0.9413989
NOM010_scaffold41431_1	virulent	0.99667025
NOM010_scaffold29615_5	virulent	0.9998684
NOM010_scaffold37895_1	temperate	0.99985975
NOM010_scaffold7945_5	temperate	0.98661816
NOM010_scaffold16353_1	temperate	0.9998588
NOM010_scaffold26455_8	virulent	0.99987406
NOM010_scaffold15321_2	virulent	0.99486125
NOM010_scaffold27687_3	temperate	0.99978644
NOM010_scaffold23538_2	virulent	0.9995294
NOM010_scaffold16212_1	temperate	0.9998545
NOM010_scaffold20969_4	virulent	0.9992909
NOM010_scaffold34_1	temperate	0.99985975
NOM010_scaffold27644_3	virulent	0.999038
NOM010_scaffold24440_2	temperate	0.99985975
NOM012_scaffold55930_5	virulent	0.9998684
NOM012_scaffold41765_3	temperate	0.99985313
NOM012_scaffold17023_1	virulent	0.9998736
NOM012_scaffold8044_2	temperate	0.99850935
NOM012_scaffold58733_1	temperate	0.9998474
NOM012_scaffold15076_8	virulent	0.9998684
NOM012_scaffold8044_6	virulent	0.9998684
NOM012_scaffold28597_33	virulent	0.99985266
NOM012_scaffold59314_2	temperate	0.9998584
NOM012_scaffold59022_1	virulent	0.9998565
NOM012_scaffold16052_1	virulent	0.9998688
NOM012_scaffold51561_1	virulent	0.9998645
NOM012_C822191_1	virulent	0.99867475
NOM012_scaffold57810_1	virulent	0.99987406
NOM012_scaffold4060_3	virulent	0.9998622
NOM012_scaffold8574_7	virulent	0.89126426
NOM012_scaffold45669_3	temperate	0.9997726
NOM012_scaffold59297_1	virulent	0.9998688
NOM012_scaffold25493_7	virulent	0.9998727
NOM012_scaffold16848_9	temperate	0.99985695

NOM012_scaffold19624_3	temperate	0.9998593
NOM012_scaffold53716_1	virulent	0.9998622
NOM012_scaffold15076_7	virulent	0.9998722
NOM012_scaffold57636_2	virulent	0.99987084
NOM012_scaffold118_12	temperate	0.99985033
NOM012_scaffold6750_2	temperate	0.9998474
NOM013_scaffold15784_2	virulent	0.9998722
NOM013_scaffold54467_1	virulent	0.99987036
NOM013_scaffold31636_3	virulent	0.99987316
NOM013_scaffold18789_1	virulent	0.99972486
NOM013_scaffold47736_4	virulent	0.9998645
NOM013_scaffold28579_1	virulent	0.7755035
NOM013_C706454_1	temperate	0.9998579
NOM013_scaffold34728_2	virulent	0.9998736
NOM013_scaffold13725_4	temperate	0.93894786
NOM013_scaffold11200_14	virulent	0.99987036
NOM013_scaffold28173_6	virulent	0.9997339
NOM013_scaffold30855_5	temperate	0.9998584
NOM013_scaffold283_1	virulent	0.99987173
NOM013_scaffold16346_5	virulent	0.9998688
NOM013_scaffold23911_1	virulent	0.9997732
NOM013_scaffold55293_1	virulent	0.99987125
NOM013_scaffold16346_8	virulent	0.99987173
NOM013_scaffold19917_26	virulent	0.9998522
NOM013_scaffold20568_5	virulent	0.99987084
NOM013_scaffold42371_10	temperate	0.99985313
NOM013_scaffold31636_2	virulent	0.99987125
NOM013_scaffold7_2	virulent	0.99987173
NOM013_scaffold19917_6	virulent	0.99986696
NOM013_scaffold47736_3	virulent	0.9998665
NOM013_scaffold22501_3	temperate	0.99972105
NOM013_scaffold54467_2	virulent	0.99987173
NOM013_scaffold53631_1	temperate	0.99985975
NOM013_scaffold3327_4	virulent	0.9998574
NOM013_scaffold11200_16	virulent	0.9990059
NOM013_scaffold49212_2	virulent	0.9998584
NOM013_scaffold9824_2	temperate	0.9998465
NOM013_scaffold4736_1	temperate	0.99985975
NOM013_scaffold32541_12	virulent	0.9998699
NOM013_scaffold31511_2	virulent	0.9998665
NOM013_scaffold3281_4	temperate	0.999662
NOM013_scaffold55128_3	virulent	0.99985975
NOM013_scaffold18200_3	temperate	0.9988362
NOM013_scaffold31511_3	virulent	0.99987173
NOM013_scaffold38707_7	virulent	0.99971104
NOM013_scaffold21734_30	virulent	0.56185067

NOM013_scaffold16346_2	virulent	0.9998693
NOM013_scaffold28579_4	virulent	0.9998479
NOM013_scaffold55291_1	virulent	0.99972963
NOM013_scaffold14898_7	virulent	0.9998688
NOM013_scaffold16204_2	virulent	0.9998617
NOM013_scaffold54010_1	temperate	0.9321044
NOM013_scaffold19858_2	temperate	0.99985975
NOM013_scaffold11318_3	temperate	0.99985975
NOM013_scaffold31511_4	virulent	0.99986696
NOM013_scaffold13071_1	temperate	0.9998588
NOM013_scaffold42353_2	virulent	0.99987125
NOM013_scaffold36218_2	virulent	0.93356663
NOM014_scaffold24348_6	virulent	0.99984974
NOM014_scaffold14255_16	virulent	0.99684715
NOM014_scaffold15996_1	virulent	0.99986744
NOM014_C390595_1	temperate	0.9998226
NOM014_scaffold25704_1	temperate	0.99985975
NOM014_scaffold24348_5	virulent	0.99986696
NOM014_scaffold28199_1	temperate	0.9991313
NOM014_scaffold11825_3	temperate	0.99984604
NOM014_scaffold8227_3	temperate	0.6361202
NOM014_scaffold28027_1	temperate	0.9998579
NOM014_scaffold15825_1	virulent	0.999784
NOM014_scaffold27185_1	virulent	0.99987036
NOM014_scaffold28228_1	virulent	0.9998123
NOM014_scaffold14811_8	virulent	0.9998722
NOM014_scaffold23281_1	virulent	0.9912987
NOM014_scaffold27301_2	virulent	0.99986744
NOM014_C390531_1	temperate	0.99981403
NOM014_scaffold19260_1	temperate	0.99963766
NOM014_scaffold28329_1	virulent	0.9998699
NOM014_scaffold9405_2	temperate	0.9998536
NOM014_scaffold3384_5	virulent	0.99987173
NOM014_scaffold25598_1	temperate	0.99985975
NOM014_scaffold8317_1	virulent	0.9995841
NOM014_scaffold19787_2	virulent	0.9998727
NOM014_scaffold28328_1	temperate	0.9998593
NOM014_scaffold490_36	temperate	0.99021435
NOM014_C390319_1	virulent	0.9911031
NOM014_scaffold13553_1	virulent	0.9998627
NOM014_scaffold7249_2	virulent	0.95055354
NOM014_scaffold27259_1	temperate	0.99985695
NOM014_scaffold5743_3	temperate	0.92179525
NOM014_scaffold28145_4	temperate	0.99986035
NOM014_scaffold9372_1	temperate	0.9998508
NOM014_scaffold15996_2	virulent	0.9997909

NOM014_scaffold13234_1	virulent	0.9985284
NOM014_scaffold28353_1	temperate	0.99985975
NOM014_scaffold21899_2	temperate	0.99985975
NOM014_scaffold28352_1	temperate	0.9998417
NOM014_scaffold15794_3	virulent	0.99682593
NOM015_scaffold35862_1	virulent	0.9998417
NOM015_scaffold36658_1	virulent	0.99980336
NOM015_scaffold2934_14	temperate	0.9998513
NOM015_scaffold1034_1	virulent	0.99987084
NOM015_scaffold16922_3	virulent	0.9998508
NOM015_scaffold34161_4	virulent	0.9998736
NOM015_scaffold226_1	virulent	0.9998584
NOM015_scaffold10294_6	temperate	0.9998574
NOM015_scaffold4016_5	virulent	0.99973303
NOM015_scaffold4819_1	virulent	0.98910916
NOM015_scaffold3437_9	virulent	0.9998699
NOM015_scaffold15823_1	temperate	0.9998536
NOM015_scaffold124_1	temperate	0.99985975
NOM015_scaffold18383_1	virulent	0.9998348
NOM015_scaffold20240_1	temperate	0.99985975
NOM015_scaffold16366_1	virulent	0.9998684
NOM015_scaffold1091_31	temperate	0.65716165
NOM015_scaffold23464_3	virulent	0.9998684
NOM015_scaffold8918_6	virulent	0.99642706
NOM015_scaffold20333_3	virulent	0.99987316
NOM015_scaffold36796_1	virulent	0.9998588
NOM015_scaffold14266_3	temperate	0.99985975
NOM015_scaffold19627_1	virulent	0.99987173
NOM015_scaffold23464_2	virulent	0.9998693
NOM015_scaffold15823_2	temperate	0.9998588
NOM015_scaffold3765_6	virulent	0.99935955
NOM015_scaffold1721_3	temperate	0.70776147
NOM015_scaffold6127_1	temperate	0.9998593
NOM015_scaffold11230_6	virulent	0.98540735
NOM015_scaffold15823_5	virulent	0.9997554
NOM015_scaffold10294_4	virulent	0.9954039
NOM015_scaffold5941_3	temperate	0.99985975
NOM015_scaffold252_7	temperate	0.99983925
NOM015_scaffold749_3	temperate	0.9998017
NOM015_scaffold8498_6	virulent	0.99003845
NOM015_scaffold16067_4	virulent	0.9998413
NOM015_scaffold9242_9	temperate	0.98519784
NOM015_scaffold27697_2	virulent	0.9998622
NOM015_scaffold26753_1	virulent	0.9998727
NOM016_scaffold29885_1	virulent	0.99981046
NOM016_scaffold42933_2	virulent	0.929879

NOM016_scaffold35286_4	virulent	0.9998688
NOM016_scaffold35286_7	virulent	0.9998679
NOM016_scaffold35976_5	temperate	0.9968298
NOM016_scaffold20671_4	virulent	0.9949243
NOM016_scaffold33084_3	virulent	0.99986315
NOM016_scaffold45043_2	virulent	0.99985266
NOM016_scaffold16005_4	virulent	0.9998699
NOM016_scaffold38252_1	virulent	0.9998693
NOM016_scaffold31191_1	temperate	0.9998274
NOM016_scaffold53315_1	temperate	0.98064613
NOM016_scaffold37747_2	virulent	0.9998408
NOM016_scaffold54950_4	temperate	0.979654
NOM016_scaffold55549_3	virulent	0.9996882
NOM016_scaffold47449_3	virulent	0.99855703
NOM016_scaffold54079_1	temperate	0.99985975
NOM016_scaffold12609_2	temperate	0.9992678
NOM016_scaffold25995_3	virulent	0.9998665
NOM016_scaffold1263_27	virulent	0.9998722
NOM016_scaffold16005_2	virulent	0.8753107
NOM016_scaffold41274_5	temperate	0.9997906
NOM016_scaffold55496_1	virulent	0.9998128
NOM016_scaffold2585_1	virulent	0.9998693
NOM016_scaffold55443_2	virulent	0.9998679
NOM016_scaffold1680_10	temperate	0.90217596
NOM016_scaffold16783_1	temperate	0.9998579
NOM016_scaffold28365_4	virulent	0.9996403
NOM016_scaffold35286_1	virulent	0.9998465
NOM016_scaffold12609_6	temperate	0.99985975
NOM016_scaffold39131_1	temperate	0.9880092
NOM016_scaffold55593_1	temperate	0.90639764
NOM016_scaffold19739_3	temperate	0.999854
NOM016_scaffold31927_4	virulent	0.99986744
NOM016_scaffold3257_24	temperate	0.99800956
NOM016_scaffold55581_3	temperate	0.9998388
NOM017_scaffold43934_1	temperate	0.99985975
NOM017_scaffold40696_1	virulent	0.9997144
NOM017_scaffold41715_2	virulent	0.9998019
NOM017_scaffold2822_6	temperate	0.9514629
NOM017_scaffold4867_13	virulent	0.9997883
NOM017_scaffold45485_2	temperate	0.99986035
NOM017_scaffold45477_1	virulent	0.82531583
NOM017_scaffold45482_1	temperate	0.9998202
NOM017_scaffold42764_1	virulent	0.99947464
NOM017_scaffold45435_1	temperate	0.9998579
NOM017_scaffold996_22	virulent	0.9998565
NOM017_scaffold11947_1	virulent	0.9815634

NOM017_scaffold22366_1	temperate	0.99985975
NOM017_scaffold16406_3	virulent	0.9998656
NOM017_scaffold845_1	temperate	0.99985975
NOM017_scaffold10596_2	virulent	0.9998736
NOM017_scaffold45496_2	temperate	0.9997544
NOM017_scaffold6578_5	temperate	0.82747394
NOM017_scaffold45031_4	virulent	0.99987465
NOM017_scaffold44208_2	temperate	0.9997267
NOM017_scaffold22040_3	virulent	0.99986696
NOM017_scaffold45430_1	virulent	0.9797661
NOM017_scaffold11947_3	virulent	0.9492285
NOM017_scaffold1142_45	virulent	0.9998736
NOM017_scaffold6796_4	virulent	0.9989985
NOM017_scaffold33562_4	temperate	0.9997874
NOM018_scaffold13094_4	virulent	0.9998308
NOM018_scaffold30222_3	temperate	0.99986035
NOM018_scaffold54740_4	temperate	0.93755734
NOM018_scaffold4478_2	virulent	0.999458
NOM018_scaffold42888_2	virulent	0.9998608
NOM018_scaffold54709_5	virulent	0.99908036
NOM018_scaffold54740_2	virulent	0.9998736
NOM018_scaffold5764_2	virulent	0.9998699
NOM018_scaffold51296_4	temperate	0.9617509
NOM018_scaffold42663_2	virulent	0.9998693
NOM018_scaffold29911_1	virulent	0.99982554
NOM018_scaffold53732_1	temperate	0.9998593
NOM018_scaffold26191_10	virulent	0.99979305
NOM018_scaffold53145_1	virulent	0.99987125
NOM018_scaffold1559_3	virulent	0.99987125
NOM018_scaffold1296_2	virulent	0.9988333
NOM018_scaffold37159_1	virulent	0.99979264
NOM018_scaffold221_1	virulent	0.99987173
NOM018_scaffold55244_1	virulent	0.99987406
NOM018_scaffold43446_1	virulent	0.99986744
NOM018_scaffold18121_1	temperate	0.99985975
NOM018_scaffold40401_5	temperate	0.99398565
NOM018_scaffold9490_1	temperate	0.9998588
NOM018_scaffold191_5	virulent	0.99959284
NOM018_scaffold15821_1	temperate	0.96378285
NOM018_scaffold16582_2	temperate	0.99985975
NOM018_scaffold54015_1	temperate	0.9944814
NOM018_scaffold9772_2	virulent	0.999854
NOM018_scaffold2740_11	temperate	0.9998574
NOM018_scaffold20530_1	temperate	0.99964815
NOM018_scaffold11125_2	virulent	0.9998699
NOM018_scaffold115_1	temperate	0.9948197

NOM018_scaffold50471_1	virulent	0.9982585
NOM018_scaffold30_5	temperate	0.9998545
NOM018_scaffold53629_1	virulent	0.56305987
NOM018_scaffold24796_3	temperate	0.9998579
NOM018_scaffold51296_6	virulent	0.99987084
NOM018_scaffold1842_24	temperate	0.9992
NOM018_scaffold42663_1	virulent	0.99986744
NOM018_scaffold52711_1	virulent	0.9997449
NOM018_scaffold50197_1	temperate	0.99985975
NOM018_scaffold54603_1	temperate	0.9960603
NOM018_scaffold4393_1	temperate	0.99963623
NOM018_C821692_1	temperate	0.99985975
NOM019_scaffold37_3	virulent	0.9998699
NOM019_scaffold6752_20	temperate	0.97468555
NOM019_scaffold46040_6	virulent	0.99987125
NOM019_scaffold16958_6	virulent	0.99986744
NOM019_scaffold52788_2	virulent	0.99986744
NOM019_scaffold52150_2	virulent	0.9989734
NOM019_scaffold15207_20	virulent	0.9998722
NOM019_scaffold5766_5	temperate	0.99986035
NOM019_scaffold53004_2	virulent	0.9137901
NOM019_scaffold40062_4	virulent	0.99987036
NOM019_scaffold52669_1	virulent	0.99699116
NOM019_scaffold53126_1	temperate	0.9998593
NOM019_scaffold40333_6	virulent	0.99983174
NOM019_scaffold16958_5	virulent	0.99965626
NOM019_scaffold38943_2	temperate	0.8927256
NOM019_scaffold50412_4	temperate	0.9998565
NOM019_scaffold22541_5	virulent	0.99987084
NOM019_scaffold53083_1	virulent	0.9997321
NOM019_scaffold53126_2	temperate	0.99978536
NOM019_scaffold31682_1	temperate	0.99984884
NOM019_scaffold53119_3	virulent	0.9998684
NOM019_scaffold7421_3	virulent	0.99987406
NOM019_scaffold22109_1	temperate	0.99985313
NOM019_scaffold45409_1	temperate	0.9987781
NOM019_scaffold50412_3	temperate	0.89173055
NOM019_scaffold12477_8	temperate	0.77741015
NOM019_scaffold27076_10	temperate	0.9998403
NOM019_scaffold12848_1	virulent	0.9761889
NOM019_scaffold46040_3	virulent	0.9998699
NOM019_scaffold36262_3	temperate	0.9949765
NOM019_scaffold14421_2	temperate	0.99949235
NOM019_scaffold24407_7	temperate	0.99985975
NOM019_scaffold52788_3	virulent	0.9998699
NOM019_scaffold22541_7	temperate	0.9998556

NOM019_scaffold53127_2	temperate	0.9413989
NOM020_scaffold5494_5	virulent	0.9997452
NOM020_scaffold33908_1	temperate	0.88362616
NOM020_scaffold38448_1	temperate	0.99986035
NOM020_scaffold7097_2	virulent	0.9998727
NOM020_scaffold39245_1	virulent	0.50469756
NOM020_scaffold6778_1	temperate	0.99984264
NOM020_scaffold1826_3	temperate	0.86272585
NOM020_scaffold14028_7	virulent	0.99986607
NOM020_scaffold1704_2	virulent	0.96948117
NOM020_scaffold13829_4	virulent	0.7615538
NOM020_scaffold22877_2	temperate	0.9998593
NOM020_scaffold32231_1	virulent	0.99987173
NOM020_C476630_1	virulent	0.99987036
NOM020_scaffold38253_1	virulent	0.9998699
NOM020_scaffold12913_3	virulent	0.9998593
NOM020_scaffold37822_4	virulent	0.9998188
NOM020_scaffold31685_6	virulent	0.99987036
NOM020_scaffold38253_2	virulent	0.99985266
NOM020_scaffold35100_2	temperate	0.999854
NOM020_scaffold38892_1	temperate	0.9992391
NOM020_scaffold3731_3	virulent	0.9998617
NOM020_scaffold10070_1	temperate	0.9998574
NOM020_scaffold26272_1	temperate	0.9261822
NOM020_scaffold16941_1	temperate	0.9998593
NOM020_scaffold14364_1	virulent	0.99964935
NOM020_scaffold39269_1	temperate	0.9997974
NOM020_scaffold39193_2	temperate	0.95171803
NOM022_scaffold35384_4	virulent	0.99987173
NOM022_scaffold25627_6	temperate	0.9998574
NOM022_scaffold2146_2	temperate	0.9993753
NOM022_scaffold3633_6	virulent	0.9998565
NOM022_scaffold11395_10	temperate	0.9998593
NOM022_scaffold35226_2	temperate	0.99976444
NOM022_scaffold35027_4	temperate	0.93755734
NOM022_scaffold34355_1	virulent	0.9644928
NOM022_scaffold16867_5	temperate	0.9997587
NOM022_scaffold11835_1	virulent	0.9998722
NOM022_scaffold25627_3	temperate	0.9988508
NOM022_scaffold1399_24	virulent	0.9997912
NOM022_scaffold33629_1	temperate	0.99985033
NOM022_scaffold23992_2	temperate	0.9998584
NOM022_C499778_1	virulent	0.9681861
NOM022_scaffold35027_1	virulent	0.71578294
NOM022_scaffold29333_3	temperate	0.9446985
NOM022_scaffold9236_24	virulent	0.9998517

NOM022_scaffold31238_3	temperate	0.6280358
NOM022_C499744_1	temperate	0.9997349
NOM022_scaffold10614_11	virulent	0.99971485
NOM022_C499370_1	temperate	0.9953512
NOM022_scaffold25723_1	virulent	0.99978644
NOM022_scaffold25634_1	temperate	0.99898344
NOM022_scaffold30875_1	temperate	0.99984604
NOM022_scaffold35362_2	virulent	0.99980617
NOM022_scaffold35326_5	virulent	0.9998736
NOM023_scaffold1309_14	temperate	0.99984556
NOM023_scaffold2528_2	virulent	0.99322486
NOM023_scaffold7114_2	virulent	0.60374457
NOM023_scaffold272_7	virulent	0.99987406
NOM023_scaffold2396_5	virulent	0.99947387
NOM023_scaffold6600_10	temperate	0.97422904
NOM023_scaffold21303_1	temperate	0.99985266
NOM023_scaffold219_4	temperate	0.99985975
NOM023_scaffold9577_8	virulent	0.9959083
NOM023_scaffold4208_9	virulent	0.95604503
NOM023_scaffold13354_4	virulent	0.9998727
NOM023_scaffold16737_1	temperate	0.99985975
NOM023_scaffold11170_3	virulent	0.99987036
NOM023_scaffold1634_1	temperate	0.99980694
NOM023_scaffold4816_2	virulent	0.99987465
NOM023_scaffold6600_7	temperate	0.9998565
NOM023_scaffold21124_2	virulent	0.99985975
NOM023_scaffold7583_7	virulent	0.99986744
NOM023_scaffold67_25	temperate	0.93755734
NOM023_scaffold6623_2	virulent	0.9998688
NOM023_scaffold16737_2	temperate	0.9987917
NOM023_scaffold18365_1	temperate	0.9997506
NOM023_scaffold67_27	virulent	0.99987125
NOM023_scaffold3447_6	temperate	0.98343426
NOM023_scaffold325_2	virulent	0.99710935
NOM023_scaffold20012_1	temperate	0.9998593
NOM023_scaffold6200_5	virulent	0.99987316
NOM023_scaffold5561_1	virulent	0.93865997
NOM023_scaffold5693_18	virulent	0.88076085
NOM025_scaffold13742_3	temperate	0.99985975
NOM025_scaffold1166_3	virulent	0.9975544
NOM025_scaffold24674_1	virulent	0.9998627
NOM025_scaffold23401_1	temperate	0.99986035
NOM025_scaffold23387_1	temperate	0.9998593
NOM025_scaffold2064_3	virulent	0.99986315
NOM025_scaffold173_6	temperate	0.99985975
NOM025_scaffold3864_1	virulent	0.908313

NOM025_scaffold6532_2	virulent	0.9998688
NOM025_scaffold22402_1	virulent	0.9971647
NOM025_scaffold18513_1	temperate	0.9996086
NOM025_scaffold2_1	virulent	0.9978972
NOM025_scaffold24512_5	virulent	0.9998722
NOM025_scaffold20521_2	temperate	0.9998574
NOM025_scaffold62_1	temperate	0.9998593
NOM025_scaffold24601_2	temperate	0.9998556
NOM025_scaffold7710_1	virulent	0.9998736
NOM025_scaffold4063_4	virulent	0.99986404
NOM025_scaffold7973_1	temperate	0.9934034
NOM025_scaffold78_1	virulent	0.99986124
NOM025_scaffold173_5	virulent	0.9995521
NOM025_scaffold24504_2	virulent	0.9688307
NOM025_scaffold14182_5	virulent	0.9998727
NOM025_scaffold23752_1	virulent	0.9998736
NOM025_scaffold24528_3	temperate	0.9998556
NOM025_scaffold24496_1	temperate	0.99982834
NOM025_scaffold57_1	virulent	0.9998665
NOM025_scaffold16448_2	temperate	0.9974352
NOM025_scaffold24601_3	temperate	0.99985975
NOM025_scaffold24362_1	temperate	0.9997912
NOM025_scaffold3455_1	temperate	0.99985975
NOM025_scaffold17951_2	virulent	0.99977446
NOM025_scaffold3831_6	temperate	0.87870395
NOM025_scaffold19612_1	temperate	0.9982116
NOM025_scaffold3792_5	virulent	0.51043385
NOM025_scaffold23962_2	temperate	0.99985975
NOM025_scaffold7710_3	virulent	0.99987316
NOM026_scaffold21771_5	virulent	0.99987084
NOM026_scaffold4045_2	virulent	0.9998226
NOM026_scaffold51_21	temperate	0.99779725
NOM026_scaffold3975_3	temperate	0.96546066
NOM026_scaffold29015_25	virulent	0.9998522
NOM026_scaffold15750_6	virulent	0.99985266
NOM026_scaffold8128_4	temperate	0.99985975
NOM026_scaffold4107_22	temperate	0.99985975
NOM026_scaffold29015_1	temperate	0.99984837
NOM026_scaffold29015_22	temperate	0.9998536
NOM026_scaffold43414_4	virulent	0.9961104
NOM026_scaffold39978_1	virulent	0.97466457
NOM026_scaffold14437_2	temperate	0.99869525
NOM026_scaffold23252_1	temperate	0.9616671
NOM026_scaffold1436_1	virulent	0.8414109
NOM026_scaffold788_2	virulent	0.99987465
NOM026_scaffold287_6	temperate	0.99980307

NOM026_scaffold32579_2	temperate	0.99794453
NOM026_scaffold95_3_2	virulent	0.9998727
NOM026_scaffold18477_4	virulent	0.9998408
NOM026_scaffold273_8	virulent	0.9962353
NOM026_scaffold5049_5	temperate	0.6546375
NOM026_scaffold100_1	temperate	0.99985975
NOM026_scaffold2514_2	temperate	0.9968264
NOM026_scaffold39163_3	virulent	0.99983263
NOM026_scaffold18477_1	temperate	0.98942244
NOM027_scaffold10318_1	temperate	0.99984556
NOM027_scaffold3843_3	temperate	0.99982494
NOM027_scaffold298_5	virulent	0.97201097
NOM027_scaffold3057_13	virulent	0.7218347
NOM027_scaffold690_9	temperate	0.9998593
NOM027_scaffold233_8	temperate	0.9998588
NOM027_scaffold21902_2	temperate	0.957053
NOM027_scaffold21753_2	virulent	0.9998688
NOM027_scaffold8220_2	temperate	0.99976397
NOM027_scaffold8991_1	temperate	0.9996067
NOM027_scaffold12848_6	temperate	0.9998522
NOM027_scaffold95_21	temperate	0.93894786
NOM027_scaffold928_6	virulent	0.69336796
NOM027_scaffold17819_1	temperate	0.999854
NOM027_scaffold4263_1	virulent	0.9998622
NOM027_scaffold11970_13	temperate	0.99985975
NOM027_scaffold3477_2	temperate	0.86199474
NOM027_scaffold21806_1	temperate	0.9998593
NOM027_scaffold21896_1	virulent	0.99958265
NOM027_scaffold18467_1	temperate	0.99985975
NOM027_scaffold21932_1	temperate	0.9998593
NOM027_scaffold4308_5	temperate	0.99982554
NOM027_scaffold16572_1	virulent	0.99987125
NOM027_scaffold8270_1	temperate	0.9998593
NOM027_scaffold87_1	virulent	0.9998645
NOM027_scaffold13632_2	temperate	0.999844
NOM027_scaffold21847_1	temperate	0.9997521
NOM028_scaffold2463_21	temperate	0.99608976
NOM028_C573709_1	temperate	0.9998579
NOM028_scaffold5766_3	temperate	0.99985975
NOM028_scaffold43134_4	virulent	0.99987173
NOM028_scaffold6679_33	virulent	0.99987316
NOM028_scaffold10923_16	temperate	0.99985975
NOM028_scaffold39054_2	temperate	0.99985695
NOM028_scaffold5154_1	temperate	0.9997335
NOM028_scaffold3552_4	temperate	0.9997044
NOM028_scaffold11407_10	virulent	0.9823756

NOM028_scaffold5961_20	virulent	0.99959546
NOM028_scaffold44706_5	temperate	0.9998517
NOM028_scaffold10197_9	temperate	0.9998584
NOM028_scaffold29187_1	temperate	0.999854
NOM028_scaffold47604_1	virulent	0.8595383
NOM028_scaffold10515_18	virulent	0.9998722
NOM028_C573817_1	temperate	0.9981446
NOM028_scaffold13734_25	virulent	0.9998645
NOM028_scaffold28052_1	virulent	0.99987125
NOM028_scaffold37397_2	temperate	0.99985975
NOM028_scaffold47465_1	temperate	0.99959904
NOM028_scaffold46573_2	virulent	0.9998679
NOM028_scaffold691_1	temperate	0.9998593
NOM028_C572983_1	virulent	0.9998736
NOM028_scaffold36229_1	temperate	0.9998508
NOM028_scaffold36508_1	virulent	0.5996757
NOM028_scaffold10515_26	temperate	0.99985695
NOM028_scaffold13734_22	virulent	0.9993722
NOM028_C573171_1	temperate	0.9998474
NOM028_scaffold44706_6	temperate	0.9886789
NOM028_scaffold8690_5	temperate	0.99981356
NOM028_scaffold47247_1	virulent	0.99976444
NOM028_scaffold6524_6	temperate	0.99986035
NOM028_scaffold41121_3	temperate	0.9998574
NOM028_scaffold47539_2	temperate	0.9413989
NOM028_scaffold19021_1	temperate	0.94768286
NOM028_scaffold46746_1	temperate	0.99986035
NOM028_scaffold6524_7	temperate	0.99983925
NOM028_scaffold46512_4	temperate	0.98519784
NOM028_scaffold2529_5	temperate	0.7455003
NOM028_scaffold11058_9	temperate	0.9998574
NOM028_scaffold15800_1	virulent	0.9994517
NOM028_scaffold47161_1	virulent	0.98588747
NOM028_scaffold10197_6	temperate	0.98964936
NOM028_scaffold110_3	virulent	0.9998699
NOM028_scaffold45529_1	virulent	0.9998622
NOM028_scaffold44706_1	temperate	0.999794
NOM028_scaffold366_38	temperate	0.99985695
NOM028_scaffold37086_11	temperate	0.9975716
NOM028_scaffold2922_6	virulent	0.99916154
NOM028_scaffold6524_1	virulent	0.6240467
NOM028_scaffold7965_13	temperate	0.99985605
NOM028_scaffold6524_2	temperate	0.9998584
NOM028_scaffold47589_2	temperate	0.99986035
NOM028_scaffold29797_1	temperate	0.9997569
NOM028_scaffold37620_3	virulent	0.9998369

NOM028_scaffold39_5	temperate	0.9998403
NOM028_scaffold47596_2	temperate	0.99985975
NOM028_scaffold19223_1	temperate	0.99985033
NOM028_scaffold6524_14	temperate	0.99986035
NOM028_scaffold34458_5	temperate	0.9998593
NOM028_scaffold24117_2	temperate	0.9998584
NOM029_scaffold15812_1	temperate	0.99985975
NOM029_scaffold15613_10	temperate	0.9998593
NOM029_scaffold279_8	temperate	0.9998403
NOM029_scaffold27582_6	virulent	0.9998679
NOM029_scaffold13239_1	virulent	0.99985975
NOM029_scaffold36387_1	temperate	0.9998183
NOM029_scaffold27582_3	virulent	0.99986696
NOM029_scaffold32772_2	virulent	0.9998693
NOM029_scaffold1741_11	virulent	0.9998722
NOM029_scaffold1385_6	virulent	0.99978584
NOM029_scaffold9689_3	temperate	0.93894786
NOM029_scaffold23053_8	virulent	0.9738244
NOM029_scaffold2695_11	virulent	0.9750174
NOM029_scaffold277_1	virulent	0.9856595
NOM029_scaffold6303_4	temperate	0.99984556
NOM029_scaffold19269_12	temperate	0.99985975
