# Supplementary material for: PhaBOX: a web server for identifying and characterizing phage contigs in metagenomic data
Source: Bioinform Adv. 2023 Aug 2;3(1):vbad101. doi: 10.1093/bioadv/vbad101 (PMC10460485; doi:10.1093/bioadv/vbad101)
Supplement: vbad101_Supplementary_Data [file vbad101_supplementary_data.zip › prediction_summary.pdf]

Accession	Length	phamer_pred	phamer_score	phatyp_pred	phatyp_score	phagcn_pred	phagcn_score	cherry_pred	cherry_score	Type
DOF002_scaffold24242_3	11009	phage	0.998	virulent	0.99987406	unknown	0	Colwellia psychrerythraea	1	Predict
DOF002_scaffold67503_1	12714	phage	0.999	virulent	0.9997966	Salasmaviridae	1	Staphylococcus saprophyticus	1	CRISPR
DOF002_scaffold60803_3	51884	phage	0.997	virulent	0.99981755	unknown	0	Bacteroides fragilis	0.98	Predict
DOF002_scaffold67436_2	15379	phage	1	virulent	0.9884394	unknown	0	Dinoroseobacter shibae	1	CRISPR
DOF002_scaffold63958_7	22031	phage	0.999	temperate	0.9998588	unknown	0	Bacteroides fragilis	0.97	Predict
DOF002_scaffold11952_4	16674	phage	0.999	temperate	0.9998593	unknown	0	Extibacter muris	1	CRISPR
DOF002_scaffold64073_3	17558	phage	0.999	temperate	0.99985695	unknown	0	Clostridium perfringens	1	CRISPR
DOF002_scaffold64389_1	17897	phage	0.999	virulent	0.999844	Herelleviridae	1	Bacillus halmapalus	0.96	Predict
DOF002_scaffold5829_2	48579	phage	0.999	virulent	0.9998688	no_family_avaliable(NC_062779)	0.978	Lactobacillus gasseri	0.71	Predict
DOF002_scaffold386_23	12272	phage	0.995	virulent	0.99800384	Guelinviridae	0.32256198	Parabacteroides merdae	1	CRISPR
DOF002_scaffold45014_1	11151	phage	0.998	temperate	0.9997769	unknown	0	Bacteroides fragilis	0.72	Predict
DOF002_scaffold32188_6	11276	phage	0.961	temperate	0.979654	unknown	0	Bacteroides fragilis	0.91	Predict
DOF002_scaffold5829_3	30164	phage	0.998	virulent	0.99987036	no_family_avaliable(NC_062779)	0.968	Lactobacillus gasseri	0.9	Predict
DOF002_scaffold65381_4	11509	phage	0.997	virulent	0.5441577	unknown	0	Fournierella massiliensis	1	CRISPR
DOF002_scaffold67459_1	46393	phage	0.999	virulent	0.9998656	unknown	0	Cellulophaga baltica	0.89	Predict
DOF002_scaffold2701_13	10458	phage	0.965	virulent	0.7488323	Peduoviridae	0.35397077	Micromonospora chaiyaphumensis	1	CRISPR
DOF002_scaffold67379_1	10376	phage	0.998	virulent	0.9741371	unknown	0	Parabacteroides merdae	1	CRISPR
DOF002_scaffold60803_2	57966	phage	0.988	temperate	0.99979496	Casjensviridae	0.6694228	Azospirillum brasilense	1	CRISPR
DOF002_scaffold67538_1	18958	phage	0.602	temperate	0.99980783	unknown	0	Bacillus halmapalus	0.81	Predict
DOF002_scaffold55354_2	13879	phage	0.989	temperate	0.9969053	unknown	0	unknown	0	-
DOF002_scaffold4671_2	17617	phage	0.999	virulent	0.99987036	Guelinviridae	0.37318182	Lactobacillus gasseri	0.74	Predict
DOF002_scaffold66657_1	13420	phage	1	temperate	0.99985975	unknown	0	Clostridioides difficile	1	CRISPR
DOF002_scaffold10448_2	17325	phage	0.999	temperate	0.9931261	unknown	0	Colwellia psychrerythraea	0.88	Predict
DOF002_scaffold11952_2	12686	phage	0.999	temperate	0.99985975	unknown	0	Colwellia psychrerythraea	0.74	Predict
DOF002_scaffold38225_1	13168	phage	0.999	temperate	0.9998369	unknown	0	Colwellia psychrerythraea	0.78	Predict
DOF002_scaffold65381_2	11556	phage	0.999	temperate	0.99985975	unknown	0	unknown	0	-
DOF002_scaffold58023_2	11900	phage	0.998	temperate	0.99920326	unknown	0	unknown	0	-
DOF002_scaffold60803_1	11474	phage	0.999	temperate	0.9998436	unknown	0	Colwellia psychrerythraea	0.84	Predict
DOF002_scaffold133_11	12531	phage	0.996	temperate	0.77618104	Ackermannviridae	0.37338728	Parabacteroides distasonis	0.73	Predict

DOF002_scaffold13072_15	17788	phage	0.998	virulent	0.99987125	Ackermannviridae	0.46403873	Colwellia psychrerythraea	0.87	Predict
DOF002_scaffold39963_1	10720	phage	0.999	virulent	0.99987406	unknown	0	unknown	0	-
DOF002_C840249_1	16509	phage	0.999	virulent	0.9998688	unknown	0	Candidatus Pelagibacter ubique	0.81	Predict
DOF002_scaffold67459_3	40855	phage	0.999	virulent	0.9998622	Schitoviridae	0.2630205	Staphylococcus hominis	0.74	Predict
DOF002_scaffold64032_6	30024	phage	0.999	virulent	0.99986744	no_family_avaliable(NC_067214)	0.962	Staphylococcus saprophyticus	0.75	Predict
DOF002_scaffold26075_2	11968	phage	0.99	virulent	0.99987316	unknown	0	unknown	0	-
DOF002_scaffold67426_3	14294	phage	0.999	virulent	0.9998622	unknown	0	Staphylococcus saprophyticus	1	CRISPR
DOF002_scaffold11952_3	10863	phage	1	temperate	0.99985975	unknown	0	Colwellia psychrerythraea	1	CRISPR
DOF002_scaffold39963_2	17162	phage	0.999	virulent	0.9998574	unknown	0	Colwellia psychrerythraea	0.84	Predict
DOF002_scaffold35792_4	17488	phage	0.849	temperate	0.9998403	Peduoviridae	0.4450498	Colwellia psychrerythraea	0.83	Predict
DOF002_scaffold197_4	14192	phage	0.968	temperate	0.9998584	unknown	0	Colwellia psychrerythraea	0.98	Predict
DOF002_scaffold4671_4	59017	phage	0.999	virulent	0.99986744	no_family_avaliable(NC_062776)	0.95	Candidatus Pelagibacter ubique	0.92	Predict
DOF002_scaffold64416_5	10325	phage	1	temperate	0.9998579	unknown	0	Bacteroides sp. A1C1	1	CRISPR
DOF002_C839921_1	10590	phage	0.992	temperate	0.9998584	unknown	0	unknown	0	-
DOF002_scaffold44470_1	12303	phage	0.991	virulent	0.98754644	unknown	0	Candidatus Pelagibacter ubique	0.74	Predict
DOF002_scaffold9671_2	24686	phage	0.971	temperate	0.9990004	Ackermannviridae	0.37796977	Colwellia psychrerythraea	0.83	Predict
DOF002_scaffold5030_2	13611	phage	0.608	temperate	0.99984926	unknown	0	unknown	0	-
DOF002_scaffold64032_7	13699	phage	0.999	virulent	0.99987036	no_family_avaliable(NC_067216)	0.969	Bacteroides xylanisolvens	1	CRISPR
DOF002_scaffold37947_1	17172	phage	0.999	temperate	0.99949753	unknown	0	Parabacteroides merdae	1	CRISPR
DOF002_scaffold20669_3	38067	phage	0.999	virulent	0.9998699	no_family_avaliable(NC_055877)	0.955	Candidatus Pelagibacter ubique	0.91	Predict
DOF003_scaffold26875_2	10516	phage	0.998	temperate	0.99985975	unknown	0	Colwellia psychrerythraea	1	CRISPR
DOF003_scaffold50654_1	22062	phage	1	virulent	0.60342914	unknown	0	Colwellia psychrerythraea	0.83	Predict
DOF003_scaffold19111_1	19016	phage	0.947	virulent	0.9998608	Ackermannviridae	1	Staphylococcus hominis	0.85	Predict
DOF003_scaffold51061_3	23281	phage	0.999	virulent	0.7767215	unknown	0	Flavobacterium psychrophilum	1	CRISPR
DOF003_scaffold28855_2	23382	phage	0.974	virulent	0.9998588	unknown	0	unknown	0	-
DOF003_scaffold989_9	12865	phage	0.999	virulent	0.92362475	Peduoviridae	1	Colwellia psychrerythraea	0.94	Predict
DOF003_scaffold27483_2	10069	phage	0.999	virulent	0.99980277	Peduoviridae	1	Colwellia psychrerythraea	1	CRISPR
DOF003_scaffold269_12	12233	phage	0.974	virulent	0.99985266	unknown	0	Vibrio alginolyticus	0.83	Predict
DOF003_scaffold21246_8	11444	phage	0.697	temperate	0.9962567	unknown	0	unknown	0	-
DOF003_scaffold51115_1	15464	phage	0.998	virulent	0.9983913	unknown	0	Colwellia psychrerythraea	0.93	Predict

DOF003_scaffold2741_2	11377	phage	0.998	virulent	0.99986404	unknown	0	Colwellia psychrerythraea	0.93	Predict
DOF003_C746192_1	15953	phage	0.999	virulent	0.99984694	unknown	0	Colwellia psychrerythraea	0.94	Predict
DOF003_scaffold43780_1	22828	phage	0.997	virulent	0.9998617	Ackermannviridae	0.4324245	Colwellia psychrerythraea	0.92	Predict
DOF003_scaffold48262_1	10727	phage	0.998	virulent	0.9998388	unknown	0	Colwellia psychrerythraea	0.79	Predict
DOF003_scaffold2826_3	36026	phage	0.999	virulent	0.9998636	unknown	0	Staphylococcus saprophyticus	0.91	Predict
DOF003_scaffold22191_11	11195	phage	0.998	virulent	0.99987125	unknown	0	Bacillus cereus	1	CRISPR
DOF003_scaffold1689_27	24634	phage	0.999	temperate	0.9998474	unknown	0	Clostridium perfringens	1	CRISPR
DOF003_scaffold13183_2	13383	phage	0.999	temperate	0.9874106	Drexlerviridae	0.39234194	Colwellia psychrerythraea	0.73	Predict
DOF003_scaffold4746_1	59400	phage	0.71	temperate	0.9996882	Casjensviridae	0.6694228	Dinoroseobacter shibae	1	CRISPR
DOF003_scaffold23234_1	12413	phage	0.997	virulent	0.99987084	unknown	0	Bacteroides fragilis	1	CRISPR
DOF003_scaffold49885_1	12696	phage	0.99	virulent	0.9998665	unknown	0	Colwellia psychrerythraea	0.97	Predict
DOF003_scaffold50947_2	14126	phage	0.998	temperate	0.999854	unknown	0	Bacteroides vulgatus	1	CRISPR
DOF003_scaffold51046_1	18403	phage	0.782	virulent	0.7838619	unknown	0	Bacillus halmapalus	0.94	Predict
DOF003_scaffold32977_2	14385	phage	0.999	temperate	0.99985975	unknown	0	unknown	0	-
DOF003_scaffold30739_3	13205	phage	0.999	temperate	0.99985975	unknown	0	unknown	0	-
DOF003_scaffold50350_1	30001	phage	0.999	temperate	0.977706	unknown	0	unknown	0	-
DOF003_scaffold28472_4	17486	phage	0.954	virulent	0.88889205	Peduoviridae	0.4450498	Colwellia psychrerythraea	0.78	Predict
DOF003_scaffold989_11	16918	phage	0.999	temperate	0.99985695	Casjensviridae	1	unknown	0	-
DOF003_scaffold49069_1	25906	phage	0.997	virulent	0.83368194	unknown	0	Staphylococcus saprophyticus	0.74	Predict
DOF003_scaffold15485_11	12921	phage	0.994	virulent	0.9996705	Ackermannviridae	0.4853815	Colwellia psychrerythraea	0.7	Predict
DOF003_scaffold40480_7	13758	phage	0.994	virulent	0.9992909	Peduoviridae	0.35397077	Micromonospora chaiyaphumensis	1	CRISPR
DOF003_scaffold2826_7	25451	phage	0.999	temperate	0.99981356	unknown	0	Colwellia psychrerythraea	0.71	Predict
DOF003_scaffold49885_3	11260	phage	0.974	temperate	0.98519784	unknown	0	Bacteroides fragilis	0.91	Predict
DOF003_scaffold197_2	11816	phage	0.998	virulent	0.99987173	Straboviridae	0.8096964	unknown	0	-
DOF003_scaffold959_2	14611	phage	0.995	virulent	0.9998608	Straboviridae	0.4209155	Colwellia psychrerythraea	0.78	Predict
DOF003_scaffold51078_6	34179	phage	0.999	virulent	0.5876283	unknown	0	unknown	0	-
DOF003_scaffold51078_1	11579	phage	0.994	virulent	0.9998736	unknown	0	unknown	0	-
DOF003_scaffold9164_2	18996	phage	0.999	temperate	0.9998574	unknown	0	Colwellia psychrerythraea	1	CRISPR
DOF003_scaffold22191_13	28259	phage	1	virulent	0.99985975	unknown	0	Bacillus cereus	1	CRISPR
DOF003_scaffold51075_5	31248	phage	0.521	virulent	0.9998688	Peduoviridae	0.3762116	Colwellia psychrerythraea	0.88	Predict

DOF003_scaffold47078_1	16196	phage	0.998	virulent	0.9998722	unknown	0	unknown	0 -
DOF003_scaffold22191_10	15070	phage	0.998	virulent	0.9998693	unknown	0	Bacillus cereus	1 CRISPR
DOF003_scaffold19871_4	17454	phage	0.969	temperate	0.99983215	Straboviridae	1	unknown	0 -
DOF003_scaffold1689_28	13753	phage	0.997	temperate	0.99985313	unknown	0	unknown	0 -
DOF003_scaffold50425_1	19954	phage	0.998	virulent	0.99960625	Straboviridae	0.5560466	Staphylococcus saprophyticus	0.84 Predict
DOF003_scaffold49538_2	12424	phage	0.986	virulent	0.99987316	Ackermannviridae	0.60703695	Candidatus Pelagibacter ubique	0.93 Predict
DOF003_scaffold19201_1	15182	phage	0.994	virulent	0.99987125	Straboviridae	0.6036092	unknown	0 -
DOF003_scaffold28914_12	23423	phage	0.988	temperate	0.9998479	Straboviridae	0.7755927	Colwellia psychrerythraea	1 CRISPR
DOF003_scaffold36733_1	11173	phage	0.999	virulent	0.9746414	unknown	0	unknown	0 -
DOF003_scaffold22256_6	35834	phage	0.993	temperate	0.96187025	unknown	0	Staphylococcus saprophyticus	0.81 Predict
DOF003_scaffold27483_3	33103	phage	1	virulent	0.85946476	Drexlerviridae	0.23707402	Candidatus Pelagibacter ubique	1 CRISPR
DOF003_scaffold35581_4	15679	phage	0.999	virulent	0.9997406	Drexlerviridae	0.23707402	Colwellia psychrerythraea	1 CRISPR
DOF003_scaffold26587_4	17535	phage	0.999	temperate	0.99604243	unknown	0	unknown	0 -
DOF004_scaffold31768_1	16798	phage	0.98	virulent	0.9718097	unknown	0	unknown	0 -
DOF004_scaffold41981_3	13499	phage	0.998	temperate	0.9998593	unknown	0	unknown	0 -
DOF004_scaffold42185_4	11256	phage	0.995	virulent	0.9998593	unknown	0	Candidatus Hamiltonella defensa	0.79 Predict
DOF004_scaffold56260_1	15410	phage	0.999	temperate	0.95989686	Peduoviridae	1	Colwellia psychrerythraea	0.92 Predict
DOF004_scaffold22037_1	14925	phage	0.728	virulent	0.99984926	unknown	0	Colwellia psychrerythraea	1 CRISPR
DOF004_scaffold16778_14	10392	phage	0.996	temperate	0.9662343	Zierdtviridae	1	unknown	0 -
DOF004_scaffold54702_1	13670	phage	0.789	virulent	0.99987406	unknown	0	unknown	0 -
DOF004_scaffold27880_1	13148	phage	0.988	temperate	0.9998536	unknown	0	Bacteroides fragilis	0.98 Predict
DOF004_scaffold37006_1	34801	phage	0.999	virulent	0.9274376	unknown	0	Parabacteroides merdae	1 CRISPR
DOF004_scaffold10109_6	16142	phage	1	temperate	0.999094	unknown	0	Colwellia psychrerythraea	0.98 Predict
DOF004_scaffold35687_1	16030	phage	0.751	virulent	0.9998722	Ackermannviridae	0.4315236	Colwellia psychrerythraea	0.98 Predict
DOF004_scaffold9090_4	19347	phage	0.999	temperate	0.89467365	Peduoviridae	0.9795492	Streptococcus mutans	0.99 Predict
DOF004_scaffold786_2	14467	phage	0.99	virulent	0.9998722	Kyanoviridae	1	Colwellia psychrerythraea	0.93 Predict
DOF004_scaffold56259_1	40795	phage	0.996	temperate	0.99981016	Straboviridae	0.7755927	Colwellia psychrerythraea	1 CRISPR
DOF004_scaffold28311_2	11812	phage	0.988	virulent	0.65031487	unknown	0	Staphylococcus saprophyticus	0.78 Predict
DOF004_scaffold148_2	16092	phage	0.994	virulent	0.9998665	unknown	0	Staphylococcus saprophyticus	0.8 Predict
DOF004_scaffold19258_3	10455	phage	0.645	virulent	0.99708104	unknown	0	Staphylococcus epidermidis	0.81 Predict

DOF004_scaffold27017_1	14127	phage	0.858	virulent	0.9610724	Straboviridae	0.4209155	Colwellia psychrerythraea	0.94	Predict
DOF004_scaffold21890_2	15885	phage	0.919	temperate	0.9413989	Straboviridae	0.4573948	Bacteroides fragilis	0.88	Predict
DOF004_scaffold57038_1	14566	phage	0.749	temperate	0.9998274	unknown	0	Parabacteroides distasonis	0.81	Predict
DOF004_C632416_1	12736	phage	0.759	temperate	0.89653563	Ackermannviridae	1	Staphylococcus saprophyticus	0.87	Predict
DOF004_scaffold38757_1	25802	phage	0.992	temperate	0.9998293	unknown	0	unknown	0	-
DOF004_scaffold49766_4	15776	phage	0.992	temperate	0.99986035	Casjensviridae	1	Colwellia psychrerythraea	0.96	Predict
DOF004_scaffold7376_1	13903	phage	0.999	virulent	0.9997917	unknown	0	Colwellia psychrerythraea	0.95	Predict
DOF004_scaffold56952_1	10268	phage	0.999	virulent	0.9998622	unknown	0	Colwellia psychrerythraea	1	CRISPR
DOF004_scaffold25343_1	16856	phage	0.996	virulent	0.99935293	unknown	0	Bacillus halmapalus	0.97	Predict
DOF004_scaffold5206_5	15060	phage	0.999	temperate	0.97040224	unknown	0	Bacteroides fragilis	0.86	Predict
DOF004_scaffold24349_1	44716	phage	0.999	virulent	0.9998622	Drexlerviridae	0.9879088	Colwellia psychrerythraea	0.82	Predict
DOF004_scaffold57121_1	28681	phage	0.999	temperate	0.9998417	unknown	0	Colwellia psychrerythraea	1	CRISPR
DOF004_scaffold26721_2	10860	phage	0.958	temperate	0.67429006	unknown	0	unknown	0	-
DOF004_scaffold34242_1	16076	phage	0.999	temperate	0.9998593	unknown	0	Colwellia psychrerythraea	0.81	Predict
DOF004_scaffold45589_1	11456	phage	0.988	virulent	0.9998588	Straboviridae	0.48223433	Bacteroides fragilis	0.91	Predict
DOF006_scaffold711_1	10365	phage	0.999	temperate	0.99985975	Schitoviridae	1	Cronobacter sakazakii	1	CRISPR
DOF006_scaffold306_3	10347	phage	0.996	virulent	0.99968964	Guelinviridae	0.32256198	Parabacteroides merdae	1	CRISPR
DOF006_scaffold22817_1	12224	phage	0.999	temperate	0.99985975	Peduoviridae	1	Colwellia psychrerythraea	1	CRISPR
DOF006_scaffold12062_1	11319	phage	0.995	virulent	0.99987316	Vilmaviridae	1	Colwellia psychrerythraea	0.86	Predict
DOF006_scaffold11978_4	11261	phage	0.974	temperate	0.98519784	unknown	0	Bacteroides fragilis	0.86	Predict
DOF006_C312135_1	14111	phage	0.999	temperate	0.99985975	Peduoviridae	1	Colwellia psychrerythraea	0.91	Predict
DOF006_scaffold15300_3	53223	phage	0.997	temperate	0.99985313	unknown	0	Colwellia psychrerythraea	1	CRISPR
DOF006_scaffold17493_1	19187	phage	0.999	temperate	0.99986035	unknown	0	Candidatus Pelagibacter ubique	0.73	Predict
DOF006_scaffold19914_2	16088	phage	0.993	temperate	0.9998522	unknown	0	unknown	0	-
DOF006_scaffold22996_1	11111	phage	0.997	temperate	0.9998556	unknown	0	Colwellia psychrerythraea	0.72	Predict
DOF006_scaffold13735_2	37022	phage	0.994	virulent	0.99986696	Straboviridae	0.6036092	Bacteroides salyersiae	1	CRISPR
DOF006_scaffold11478_1	10429	phage	0.996	virulent	0.9998722	unknown	0	Parabacteroides distasonis	1	CRISPR
DOF006_scaffold3372_1	18731	phage	0.998	virulent	0.99986315	Ackermannviridae	0.37338728	Staphylococcus saprophyticus	0.91	Predict
DOF006_scaffold18788_9	20471	phage	0.999	virulent	0.99987406	no_family_avaliable(NC_062765)	0.968	Cellulophaga baltica	0.86	Predict
DOF006_scaffold23004_2	15901	phage	0.919	temperate	0.9413989	Straboviridae	0.4573948	Bacteroides fragilis	0.78	Predict

DOF006_scaffold15162_1	17004	phage	0.999	virulent	0.99987406	unknown	0	Candidatus Pelagibacter ubique	0.85	Predict
DOF006_scaffold2736_1	21286	phage	0.992	temperate	0.978805	Kyanoviridae	1	Colwellia psychrerythraea	1	CRISPR
DOF006_scaffold20709_1	48036	phage	1	virulent	0.9998627	Rountreeviridae	0.34439915	Candidatus Pelagibacter ubique	1	CRISPR
DOF006_scaffold306_6	14517	phage	0.999	virulent	0.9799923	unknown	0	Parabacteroides distasonis	1	CRISPR
DOF006_scaffold22685_1	19251	phage	0.999	temperate	0.9994771	unknown	0	Candidatus Pelagibacter ubique	0.81	Predict
DOF006_scaffold4966_1	35634	phage	0.999	virulent	0.9997132	unknown	0	Helicobacter pylori	1	CRISPR
DOF006_scaffold22663_1	14455	phage	0.997	temperate	0.90327543	unknown	0	Colwellia psychrerythraea	0.99	Predict
DOF006_scaffold22952_1	11860	phage	0.994	virulent	0.9998688	Guelinviridae	0.37318182	Staphylococcus saprophyticus	0.78	Predict
DOF006_scaffold5845_1	15463	phage	0.615	virulent	0.99987125	Straboviridae	0.48223433	Bacteroides fragilis	0.7	Predict
DOF006_scaffold17357_1	13344	phage	0.996	temperate	0.99986035	unknown	0	Staphylococcus saprophyticus	0.79	Predict
DOF006_scaffold14584_2	10361	phage	0.925	virulent	0.99987173	Peduoviridae	0.18345712	Staphylococcus saprophyticus	0.91	Predict
DOF006_scaffold7307_1	11550	phage	0.997	temperate	0.8733236	unknown	0	unknown	0	-
DOF006_scaffold22952_2	84727	phage	0.999	virulent	0.9998656	no_family_avaliable(NC_062774)	0.962	Flavobacterium columnare	0.9	Predict
DOF006_scaffold711_5	13376	phage	0.999	temperate	0.9998579	Casjensviridae	1	Colwellia psychrerythraea	1	CRISPR
DOF006_scaffold9292_2	10891	phage	0.998	virulent	0.99987316	unknown	0	unknown	0	-
DOF006_scaffold10407_1	64150	phage	0.999	virulent	0.55276394	Straboviridae	0.5247502	Bacteroides fragilis	1	CRISPR
DOF006_C312621_1	20454	phage	0.998	virulent	0.99985975	unknown	0	Flavobacterium psychrophilum	1	CRISPR
DOF007_scaffold1171_1	11972	phage	0.991	virulent	0.99987125	Straboviridae	0.464522	Bacteroides fragilis	0.83	Predict
DOF007_scaffold35_3	18898	phage	0.822	virulent	0.9998465	Ackermannviridae	0.49211752	Colwellia psychrerythraea	0.99	Predict
DOF007_scaffold17308_3	24472	phage	0.999	virulent	0.994713	Herelleviridae	1	Candidatus Pelagibacter ubique	1	CRISPR
DOF007_C360895_1	33886	phage	1	temperate	0.56009233	unknown	0	Bacteroides fragilis	1	CRISPR
DOF007_scaffold7833_5	35459	phage	0.956	temperate	0.9988239	unknown	0	Colwellia psychrerythraea	0.87	Predict
DOF007_scaffold21463_1	14652	phage	0.958	virulent	0.99976164	unknown	0	Colwellia psychrerythraea	0.82	Predict
DOF007_scaffold2644_116	11576	phage	0.998	temperate	0.99985975	unknown	0	Bacillus sp. V3-13	1	CRISPR
DOF007_scaffold14864_2	10513	phage	0.999	virulent	0.99959624	unknown	0	Colwellia psychrerythraea	1	CRISPR
DOF007_scaffold5443_1	11898	phage	0.979	temperate	0.99982077	unknown	0	unknown	0	-
DOF007_scaffold523_7	13220	phage	0.96	virulent	0.9837638	unknown	0	Colwellia psychrerythraea	0.99	Predict
DOF007_scaffold2644_113	17108	phage	0.829	temperate	0.9840653	unknown	0	unknown	0	-
DOF007_scaffold15454_1	13046	phage	0.997	temperate	0.99225765	unknown	0	Parabacteroides distasonis	1	CRISPR
DOF007_scaffold16228_1	24188	phage	0.999	virulent	0.99986696	unknown	0	Bacteroides fragilis	0.73	Predict

DOF007_scaffold632_7	10865	phage	0.936	temperate	0.9997267	Drexlerviridae	0.30752468	Colwellia psychrerythraea	0.9	Predict
DOF007_scaffold16914_2	12118	phage	1	virulent	0.77566415	unknown	0	unknown	0	-
DOF007_scaffold1340_5	19657	phage	0.999	virulent	0.99957407	Ackermannviridae	1	Colwellia psychrerythraea	0.82	Predict
DOF007_scaffold2644_118	20512	phage	0.999	temperate	0.9869902	Drexlerviridae	0.16746306	Pantoea agglomerans	0.84	Predict
DOF007_C359971_1	10777	phage	0.966	virulent	0.99987125	Ackermannviridae	1	Parabacteroides distasonis	0.72	Predict
DOF007_scaffold4001_1	17178	phage	0.99	virulent	0.9997832	unknown	0	Candidatus Pelagibacter ubique	0.82	Predict
DOF007_scaffold12791_4	11702	phage	0.642	virulent	0.9998727	unknown	0	unknown	0	-
DOF007_scaffold1333_2	32550	phage	0.991	temperate	0.9664654	unknown	0	Colwellia psychrerythraea	0.89	Predict
DOF008_scaffold28029_2	12348	phage	0.999	temperate	0.9998574	unknown	0	Clostridium tetani	1	CRISPR
DOF008_scaffold567_3	10501	phage	0.882	virulent	0.9933288	Vilmaviridae	0.20129421	Candidatus Pelagibacter ubique	0.92	Predict
DOF008_scaffold10567_1	11711	phage	0.993	virulent	0.9998699	unknown	0	unknown	0	-
DOF008_scaffold9506_2	10634	phage	0.999	temperate	0.9997978	unknown	0	Citrobacter rodentium	1	CRISPR
DOF008_scaffold4011_4	27447	phage	0.997	temperate	0.9998574	unknown	0	Ruminococcus bromii	1	CRISPR
DOF008_scaffold832_1	10558	phage	0.883	virulent	0.9998727	Ackermannviridae	0.4783474	Colwellia psychrerythraea	0.79	Predict
DOF008_scaffold28485_2	10685	phage	0.997	temperate	0.9998593	unknown	0	unknown	0	-
DOF008_scaffold8861_3	12278	phage	0.991	temperate	0.9997135	unknown	0	unknown	0	-
DOF008_scaffold10539_9	15725	phage	0.941	virulent	0.9996882	Straboviridae	0.5668039	Parabacteroides distasonis	0.96	Predict
DOF008_scaffold21796_1	21727	phage	0.997	temperate	0.9505956	Ackermannviridae	0.39623365	unknown	0	-
DOF008_scaffold28698_1	10510	phage	0.991	virulent	0.9998727	unknown	0	unknown	0	-
DOF008_scaffold26020_1	12026	phage	0.997	virulent	0.9998551	unknown	0	Colwellia psychrerythraea	0.99	Predict
DOF008_scaffold19396_1	12934	phage	0.999	temperate	0.99978536	unknown	0	Candidatus Pelagibacter ubique	0.77	Predict
DOF008_scaffold24483_1	11377	phage	0.999	temperate	0.9997587	unknown	0	unknown	0	-
DOF008_scaffold6011_2	16150	phage	0.95	virulent	0.99986124	Drexlerviridae	0.39234194	Colwellia psychrerythraea	0.84	Predict
DOF008_scaffold12257_4	37032	phage	0.999	temperate	0.99985605	unknown	0	Staphylococcus saprophyticus	1	CRISPR
DOF008_scaffold1028_3	15893	phage	0.856	temperate	0.93894786	Straboviridae	0.4573948	Bacteroides fragilis	0.78	Predict
DOF008_scaffold25427_1	10252	phage	0.999	temperate	0.99984884	unknown	0	unknown	0	-
DOF008_scaffold27498_1	10182	phage	0.987	temperate	0.99985975	unknown	0	unknown	0	-
DOF008_scaffold110_2	12354	phage	0.999	temperate	0.9998579	unknown	0	unknown	0	-
DOF008_C423597_1	16040	phage	0.83	temperate	0.99721605	unknown	0	Candidatus Pelagibacter ubique	0.78	Predict
DOF008_scaffold2469_10	11105	phage	0.937	virulent	0.9998608	unknown	0	unknown	0	-

DOF008_scaffold19808_7	15251	phage	0.936	temperate	0.99985695	unknown	0	Staphylococcus saprophyticus	0.82	Predict
DOF008_scaffold29015_1	31159	phage	0.999	temperate	0.9909741	unknown	0	Mesorhizobium loti	1	CRISPR
DOF008_scaffold12257_5	39209	phage	0.999	temperate	0.99984837	unknown	0	Coprococcus catus	1	CRISPR
DOF008_scaffold5856_4	12458	phage	0.998	temperate	0.99692744	Straboviridae	0.8034157	Candidatus Pelagibacter ubique	0.86	Predict
DOF008_scaffold28029_1	16914	phage	1	temperate	0.9998513	unknown	0	Anaerostipes hadrus	1	CRISPR
DOF008_scaffold16092_4	11504	phage	0.982	temperate	0.999836	unknown	0	Bacillus halmapalus	0.77	Predict
DOF008_scaffold27793_1	33974	phage	0.999	virulent	0.9998693	unknown	0	Colwellia psychrerythraea	0.87	Predict
DOF008_scaffold21540_6	11981	phage	0.796	virulent	0.9993408	unknown	0	Colwellia psychrerythraea	0.98	Predict
DOF008_scaffold17006_1	24085	phage	0.827	virulent	0.9998693	Peduoviridae	1	Acinetobacter baumannii	1	CRISPR
DOF008_scaffold15173_2	11655	phage	0.988	temperate	0.99985975	unknown	0	Colwellia psychrerythraea	0.83	Predict
DOF008_scaffold232_7	43850	phage	0.997	temperate	0.99985605	Mesyanzhinovviridae	1	Xanthomonas citri	1	CRISPR
DOF008_C423975_1	36114	phage	0.999	virulent	0.9998656	Autographiviridae	1	Colwellia psychrerythraea	1	CRISPR
DOF009_scaffold2408_22	14013	phage	0.998	virulent	0.87754935	Ackermannviridae	0.49632582	Colwellia psychrerythraea	0.91	Predict
DOF009_scaffold40393_12	12474	phage	0.99	virulent	0.9994256	Peduoviridae	0.35397077	Micromonospora chaiyaphumensis	1	CRISPR
DOF009_scaffold42858_2	16179	phage	0.997	virulent	0.99620163	unknown	0	unknown	0	-
DOF009_scaffold155_13	11880	phage	0.999	virulent	0.9998722	unknown	0	Staphylococcus saprophyticus	0.82	Predict
DOF009_scaffold52826_1	16105	phage	1	virulent	0.99987036	Salasmaviridae	1	Staphylococcus saprophyticus	0.92	Predict
DOF009_scaffold52459_1	12095	phage	0.995	virulent	0.9927804	Peduoviridae	0.33099112	Colwellia psychrerythraea	0.94	Predict
DOF009_scaffold39856_9	10447	phage	0.987	temperate	0.9998308	Peduoviridae	1	Colwellia psychrerythraea	0.97	Predict
DOF009_scaffold43174_9	10544	phage	0.936	virulent	0.9983713	unknown	0	Colwellia psychrerythraea	0.78	Predict
DOF009_scaffold22475_18	14036	phage	0.988	temperate	0.9998369	unknown	0	Bacteroides fragilis	1	CRISPR
DOF009_scaffold47159_4	23709	phage	0.999	virulent	0.9994085	unknown	0	Colwellia psychrerythraea	1	CRISPR
DOF009_scaffold46839_2	13115	phage	0.997	temperate	0.99883914	unknown	0	Colwellia psychrerythraea	0.93	Predict
DOF009_scaffold3127_2	14384	phage	1	virulent	0.7399244	unknown	0	unknown	0	-
DOF009_scaffold52159_2	16920	phage	0.782	temperate	0.9997969	unknown	0	unknown	0	-
DOF009_scaffold42767_3	13687	phage	0.622	temperate	0.99978215	unknown	0	Bifidobacterium adolescentis	1	CRISPR
DOF009_scaffold16613_1	13637	phage	0.993	virulent	0.9703356	Straboviridae	0.5668039	Parabacteroides distasonis	0.87	Predict
DOF009_scaffold36176_2	66313	phage	0.999	virulent	0.99977905	unknown	0	Colwellia psychrerythraea	1	CRISPR
DOF009_scaffold31_1	12710	phage	0.538	temperate	0.9998379	Straboviridae	0.6022864	unknown	0	-
DOF009_scaffold1_2	15905	phage	0.919	temperate	0.9413989	Straboviridae	0.4573948	Bacteroides fragilis	0.8	Predict

DOF009_scaffold40125_1	10284	phage	0.998	temperate	0.9998026	unknown	0	Colwellia psychrerythraea	0.99	Predict
DOF009_scaffold23972_2	10805	phage	0.998	temperate	0.98516726	unknown	0	unknown	0	-
DOF009_scaffold13901_4	20310	phage	0.952	temperate	0.9998593	unknown	0	unknown	0	-
DOF009_scaffold52438_1	11388	phage	0.999	virulent	0.99987406	Herelleviridae	1	Bacteroides fragilis	0.85	Predict
DOF009_scaffold4516_5	11802	phage	0.989	virulent	0.99987406	Ackermannviridae	0.60703695	Colwellia psychrerythraea	0.87	Predict
DOF009_scaffold42497_4	29781	phage	0.998	virulent	0.99984217	Ackermannviridae	0.37338728	Bacteroides fragilis	0.93	Predict
DOF009_scaffold7496_2	13708	phage	0.998	virulent	0.99987173	Straboviridae	0.8096964	unknown	0	-
DOF010_scaffold37104_2	11349	phage	0.999	temperate	0.9998545	no_family_avaliable(NC_031129)	0.984	Escherichia coli	1	CRISPR
DOF010_scaffold38542_4	12782	phage	0.998	temperate	0.9998593	unknown	0	Staphylococcus saprophyticus	0.74	Predict
DOF010_scaffold12096_1	15990	phage	0.999	virulent	0.9998722	no_family_avaliable(NC_024711)	0.964	Candidatus Pelagibacter ubique	0.83	Predict
DOF010_scaffold35610_1	11132	phage	0.999	virulent	0.9995016	no_family_avaliable(NC_019710)	0.973	Colwellia psychrerythraea	1	CRISPR
DOF010_scaffold38620_1	15868	phage	0.999	virulent	0.9981961	Mesyanzhinovviridae	1	Colwellia psychrerythraea	1	CRISPR
DOF010_scaffold37194_6	11724	phage	0.996	virulent	0.99986696	unknown	0	unknown	0	-
DOF010_scaffold1331_7	13689	phage	0.978	temperate	0.998346	Peduoviridae	1	Colwellia psychrerythraea	1	CRISPR
DOF010_scaffold31976_2	15976	phage	0.999	virulent	0.9998688	unknown	0	Bacillus halmapalus	1	CRISPR
DOF010_scaffold45_4	10316	phage	0.677	temperate	0.99919474	Peduoviridae	1	Colwellia psychrerythraea	0.83	Predict
DOF010_C495273_1	10751	phage	0.999	virulent	0.9998699	Rountreeviridae	0.3115139	Candidatus Pelagibacter ubique	1	CRISPR
DOF010_scaffold36_4	14876	phage	0.952	temperate	0.635635	Straboviridae	0.2887408	Bacteroides fragilis	0.91	Predict
DOF010_scaffold39042_3	20411	phage	0.999	virulent	0.9997802	unknown	0	Cellulophaga baltica	0.81	Predict
DOF010_scaffold13814_2	24648	phage	0.602	temperate	0.98312473	unknown	0	Candidatus Pelagibacter ubique	0.91	Predict
DOF010_scaffold36571_1	20322	phage	0.999	temperate	0.99985975	Ackermannviridae	0.39623365	unknown	0	-
DOF010_scaffold37750_5	24116	phage	0.999	temperate	0.9997774	Peduoviridae	0.14169496	Colwellia psychrerythraea	1	CRISPR
DOF010_scaffold65_18	10808	phage	0.986	virulent	0.99987316	Ackermannviridae	0.60703695	Bacteroides fragilis	0.72	Predict
DOF010_scaffold21978_1	12765	phage	0.998	virulent	0.99976045	unknown	0	unknown	0	-
DOF010_scaffold11833_5	12616	phage	0.932	virulent	0.9998727	Ackermannviridae	0.4315236	Colwellia psychrerythraea	0.78	Predict
DOF010_scaffold7607_7	15854	phage	0.999	virulent	0.99987036	Autographiviridae	1	Colwellia psychrerythraea	1	CRISPR
DOF010_scaffold19015_5	16455	phage	0.987	temperate	0.9998556	unknown	0	Coprobacillus sp. AF13-15	1	CRISPR
DOF010_scaffold27096_5	13630	phage	0.997	virulent	0.99987173	Ackermannviridae	0.4783474	Colwellia psychrerythraea	0.87	Predict
DOF010_scaffold32482_2	13655	phage	0.996	virulent	0.9998736	unknown	0	Vibrio cholerae	0.87	Predict
DOF010_scaffold35610_2	36293	phage	0.998	virulent	0.9996372	Herelleviridae	1	Colwellia psychrerythraea	1	CRISPR

DOF010_scaffold23207_18	13238	phage	0.998	temperate	0.99985975	unknown	0	Streptococcus gordonii	0.77	Predict
DOF010_scaffold34_4	10525	phage	0.581	virulent	0.9353608	unknown	0	Colwellia psychrerythraea	0.86	Predict
DOF010_scaffold2645_5	37264	phage	0.999	temperate	0.99981403	unknown	0	Staphylococcus saprophyticus	1	CRISPR
DOF010_scaffold39042_1	15804	phage	0.993	virulent	0.6737775	Casjensviridae	1	Bacillus halmapalus	0.78	Predict
DOF010_scaffold24344_2	19077	phage	1	virulent	0.99981666	unknown	0	Colwellia psychrerythraea	1	CRISPR
DOF010_scaffold38651_2	16516	phage	0.798	virulent	0.99986696	Herelleviridae	1	Bacillus megaterium	0.87	Predict
DOF010_scaffold13830_4	13022	phage	0.935	virulent	0.99986404	unknown	0	unknown	0	-
DOF010_scaffold33553_4	12486	phage	0.999	virulent	0.99987173	unknown	0	Candidatus Pelagibacter ubique	1	Predict
DOF010_scaffold38956_2	12812	phage	0.999	temperate	0.99985975	no_family_avaliable(NC_049946)	0.965	Colwellia psychrerythraea	1	CRISPR
DOF010_scaffold19252_1	13202	phage	0.897	temperate	0.99985975	Straboviridae	0.56678534	Bacillus halmapalus	0.94	Predict
DOF010_scaffold21702_2	12214	phage	1	temperate	0.9928322	unknown	0	Bacteroides fragilis	0.84	Predict
DOF010_scaffold29273_4	10526	phage	0.999	temperate	0.9998584	unknown	0	Colwellia psychrerythraea	1	CRISPR
DOF010_scaffold198_1	11144	phage	0.998	virulent	0.9998727	no_family_avaliable(NC_024711)	0.978	Candidatus Pelagibacter ubique	0.79	Predict
DOF010_scaffold38551_4	13475	phage	0.945	temperate	0.9998593	unknown	0	Clostridioides difficile	1	CRISPR
DOF010_scaffold24344_3	19128	phage	1	virulent	0.99986315	unknown	0	Colwellia psychrerythraea	1	CRISPR
DOF010_scaffold31661_1	14129	phage	0.981	temperate	0.9998508	unknown	0	Staphylococcus saprophyticus	0.97	Predict
DOF010_scaffold35846_1	11281	phage	0.927	temperate	0.9998593	unknown	0	unknown	0	-
DOF010_scaffold37750_4	10838	phage	0.999	temperate	0.9888588	Drexlerviridae	0.23707402	Colwellia psychrerythraea	1	CRISPR
DOF010_scaffold39049_2	13488	phage	0.999	virulent	0.9998699	Casjensviridae	1	Colwellia psychrerythraea	1	CRISPR
DOF010_scaffold4723_13	11758	phage	0.769	temperate	0.9998193	unknown	0	Colwellia psychrerythraea	0.88	Predict
DOF010_scaffold31976_1	19018	phage	0.999	virulent	0.99986744	Rountreeviridae	0.3027439	Staphylococcus saprophyticus	1	CRISPR
DOF011_scaffold21153_1	52642	phage	0.999	temperate	0.9995362	unknown	0	Megamonas funiformis	1	CRISPR
DOF011_scaffold21165_1	11773	phage	0.997	temperate	0.8584233	unknown	0	unknown	0	-
DOF011_scaffold21213_1	24796	phage	1	virulent	0.6638408	Straboviridae	0.53860486	Colwellia psychrerythraea	1	CRISPR
DOF011_scaffold77_1	18460	phage	0.999	temperate	0.99985975	unknown	0	Bacillus halmapalus	0.77	Predict
DOF011_scaffold2635_5	15909	phage	0.919	temperate	0.9413989	Straboviridae	0.4573948	Bacteroides fragilis	0.81	Predict
DOF011_scaffold21174_2	14558	phage	0.585	virulent	0.99987084	Ackermannviridae	0.46645996	Colwellia psychrerythraea	0.78	Predict
DOF011_scaffold2386_1	15061	phage	0.993	temperate	0.99985975	unknown	0	Colwellia psychrerythraea	0.94	Predict
DOF011_scaffold12507_5	12384	phage	0.961	temperate	0.979654	unknown	0	Bacteroides fragilis	0.72	Predict
DOF011_scaffold19699_2	11420	phage	0.999	temperate	0.99984217	unknown	0	Colwellia psychrerythraea	0.98	Predict

DOF011_scaffold20914_1	14610	phage	0.999	temperate	0.998446	Straboviridae	0.32026517	Colwellia psychrerythraea	1	CRISPR
DOF012_scaffold1710_3	10147	phage	0.516	virulent	0.99987406	unknown	0	Colwellia psychrerythraea	0.79	Predict
DOF012_scaffold12555_2	12076	phage	0.996	temperate	0.9998593	unknown	0	Veillonella sp. AF36-20BH	1	CRISPR
DOF012_scaffold463_10	40944	phage	0.999	virulent	0.9996708	unknown	0	Colwellia psychrerythraea	1	CRISPR
DOF012_scaffold4720_1	14663	phage	0.996	temperate	0.99986035	unknown	0	Colwellia psychrerythraea	1	CRISPR
DOF012_scaffold884_2	11146	phage	1	temperate	0.9998265	Drexlerviridae	0.39234194	unknown	0	-
DOF012_scaffold9548_2	15046	phage	0.995	virulent	0.9998736	Herelleviridae	1	unknown	0	-
DOF012_scaffold992_3	12599	phage	0.964	temperate	0.99857235	unknown	0	Colwellia psychrerythraea	0.72	Predict
DOF012_scaffold9844_1	25721	phage	0.999	temperate	0.99985695	unknown	0	Colwellia psychrerythraea	1	CRISPR
DOF012_scaffold2936_3	10288	phage	0.998	virulent	0.83869	unknown	0	Colwellia psychrerythraea	0.73	Predict
DOF012_scaffold12334_1	13563	phage	0.998	temperate	0.9998431	unknown	0	Lactobacillus fermentum	1	CRISPR
DOF012_scaffold198_1	15018	phage	0.973	temperate	0.9998379	unknown	0	Colwellia psychrerythraea	1	CRISPR
DOF012_scaffold4539_3	26993	phage	0.775	temperate	0.99983126	unknown	0	Colwellia psychrerythraea	0.8	Predict
DOF012_scaffold381_2	21216	phage	0.999	temperate	0.9998388	unknown	0	Bacillus halmapalus	1	CRISPR
DOF012_scaffold3394_7	11840	phage	0.999	temperate	0.999854	unknown	0	Veillonella sp. AF36-20BH	1	CRISPR
DOF012_scaffold897_10	20920	phage	0.988	temperate	0.99985975	Peduoviridae	1	Colwellia psychrerythraea	0.88	Predict
DOF012_scaffold4741_3	10641	phage	1	virulent	0.9998656	Autographiviridae	1	Veillonella parvula	1	CRISPR
DOF012_scaffold7_12	45634	phage	0.999	virulent	0.61507547	unknown	0	Colwellia psychrerythraea	1	CRISPR
DOF012_scaffold6657_1	10964	phage	0.944	temperate	0.9998593	Peduoviridae	1	Staphylococcus saprophyticus	0.82	Predict
DOF012_scaffold884_3	26490	phage	0.999	temperate	0.99984556	unknown	0	Colwellia psychrerythraea	1	CRISPR
DOF012_scaffold191_20	14160	phage	0.878	temperate	0.99985975	Straboviridae	0.6737759	Staphylococcus saprophyticus	0.87	Predict
DOF012_scaffold14781_2	11638	phage	0.977	virulent	0.86183524	no_family_avaliable(NC_049950)	0.98	Colwellia psychrerythraea	0.82	Predict
DOF012_scaffold7309_6_1	22312	phage	0.934	virulent	0.9998617	Straboviridae	0.8034157	Colwellia psychrerythraea	0.75	Predict
DOF012_scaffold14750_1	19862	phage	0.999	temperate	0.934572	unknown	0	Lactobacillus fermentum	1	CRISPR
DOF012_scaffold22_3	32048	phage	0.999	virulent	0.9998679	unknown	0	Croceibacter atlanticus	0.93	Predict
DOF012_scaffold463_9_2	32157	phage	1	virulent	0.99987173	unknown	0	Parabacteroides distasonis	1	CRISPR
DOF013_scaffold2527_4	11526	phage	0.999	virulent	0.9998684	Kyanoviridae	1	Parabacteroides merdae	1	CRISPR
DOF013_scaffold11760_13	11955	phage	0.999	temperate	0.9627462	unknown	0	Colwellia psychrerythraea	0.84	Predict
DOF013_scaffold12980_3	10296	phage	0.998	virulent	0.999671	unknown	0	Staphylococcus saprophyticus	0.71	Predict
DOF013_scaffold5071_6	13642	phage	0.94	temperate	0.9998556	Straboviridae	0.464522	Colwellia psychrerythraea	0.77	Predict

DOF013_scaffold1588_1	15157	phage	0.999	virulent	0.82658577	unknown	0	Megamonas funiformis	1	CRISPR
DOF013_scaffold27644_1	10024	phage	0.994	temperate	0.9996058	Straboviridae	0.374585	Bacillus halmapalus	0.88	Predict
DOF013_scaffold4900_2	12538	phage	0.982	virulent	0.9998545	unknown	0	Staphylococcus saprophyticus	0.72	Predict
DOF013_scaffold12980_1	13927	phage	1	temperate	0.9998545	unknown	0	Colwellia psychrerythraea	1	CRISPR
DOF013_scaffold783_2	11738	phage	0.997	virulent	0.99987036	unknown	0	Bacteroides dorei	1	CRISPR
DOF013_scaffold13491_2	13016	phage	0.986	virulent	0.9993214	Ackermannviridae	0.31827873	Bacteroides fragilis	0.8	Predict
DOF013_scaffold443_2	10043	phage	1	temperate	0.997606	unknown	0	Parabacteroides distasonis	0.77	Predict
DOF013_scaffold15877_2	10781	phage	0.996	virulent	0.99987406	unknown	0	unknown	0	-
DOF013_scaffold2699_1	20235	phage	0.999	virulent	0.9998636	unknown	0	Staphylococcus saprophyticus	0.88	Predict
DOF013_scaffold4683_2	10578	phage	0.999	virulent	0.99978924	Chaseviridae	1	Candidatus Pelagibacter ubique	0.81	Predict
DOF013_scaffold103_6	13689	phage	0.993	temperate	0.999854	unknown	0	Colwellia psychrerythraea	0.95	Predict
DOF013_scaffold392_2	11282	phage	0.998	virulent	0.99918664	Chaseviridae	1	Xanthomonas vesicatoria	0.71	Predict
DOF013_scaffold5240_2	25902	phage	0.999	virulent	0.9998727	unknown	0	Parabacteroides sp. D13	1	CRISPR
DOF013_scaffold379_1	22657	phage	0.994	virulent	0.99985266	Ackermannviridae	0.3173149	Staphylococcus saprophyticus	0.76	Predict
DOF013_scaffold5240_9	31144	phage	0.999	virulent	0.9998645	unknown	0	Parabacteroides sp. D13	1	CRISPR
DOF013_scaffold21294_6	11097	phage	0.974	temperate	0.98519784	unknown	0	Bacteroides fragilis	0.94	Predict
DOF013_scaffold29915_1	10285	phage	0.999	temperate	0.9998593	unknown	0	Colwellia psychrerythraea	0.92	Predict
DOF014_scaffold15073_8	13003	phage	0.999	temperate	0.9998579	unknown	0	unknown	0	-
DOF014_scaffold49276_4	10223	phage	0.958	temperate	0.99985975	unknown	0	Clostridium botulinum	0.82	Predict
DOF014_scaffold46901_1	25493	phage	0.999	virulent	0.99987125	no_family_avaliable(NC_024711)	0.971	Candidatus Pelagibacter ubique	0.91	Predict
DOF014_scaffold10772_1	20194	phage	0.997	temperate	0.9998413	Straboviridae	0.48694646	Candidatus Pelagibacter ubique	1	CRISPR
DOF014_scaffold49726_2	12680	phage	0.872	temperate	0.99980927	Straboviridae	0.6022864	unknown	0	-
DOF014_scaffold30923_2	26138	phage	1	virulent	0.99986404	unknown	0	Colwellia psychrerythraea	1	CRISPR
DOF014_C612810_1	11084	phage	0.999	temperate	0.9701769	unknown	0	Candidatus Pelagibacter ubique	0.71	Predict
DOF014_scaffold101_2	14847	phage	1	virulent	0.9998608	unknown	0	Candidatus Pelagibacter ubique	0.82	Predict
DOF014_scaffold32658_1	40909	phage	0.96	temperate	0.9987996	unknown	0	Staphylococcus saprophyticus	1	CRISPR
DOF014_scaffold7189_8	10757	phage	0.91	virulent	0.99944603	Peduoviridae	1	Colwellia psychrerythraea	0.96	Predict
DOF014_scaffold49340_4	10342	phage	0.999	virulent	0.999671	unknown	0	Colwellia psychrerythraea	1	CRISPR
DOF014_scaffold29718_2	12249	phage	0.867	virulent	0.9998536	unknown	0	Parabacteroides distasonis	0.99	Predict
DOF014_scaffold2821_4	12858	phage	0.999	virulent	0.99987173	no_family_avaliable(NC_024711)	0.97	Staphylococcus saprophyticus	0.81	Predict

DOF014_scaffold49687_1	16092	phage	0.999	virulent	0.9998722	Kyanoviridae	1	unknown	0	-
DOF014_scaffold1808_5	11042	phage	0.999	temperate	0.99985975	unknown	0	unknown	0	-
DOF014_scaffold49604_1	10541	phage	0.999	temperate	0.99934953	unknown	0	Bacteroides fragilis	0.95	Predict
DOF014_scaffold90_1	11861	phage	0.906	temperate	0.9998588	unknown	0	unknown	0	-
DOF014_scaffold49645_1	43382	phage	0.999	virulent	0.99987036	Guelinviridae	0.37318182	Lactobacillus gasseri	0.71	Predict
DOF014_scaffold49556_1	42290	phage	0.999	temperate	0.99982125	unknown	0	Clostridium perfringens	1	CRISPR
DOF014_scaffold49552_1	42965	phage	0.998	temperate	0.9995119	unknown	0	Colwellia psychrerythraea	1	CRISPR
DOF014_scaffold1_3	15903	phage	0.856	temperate	0.93894786	Straboviridae	0.4573948	Bacteroides fragilis	0.78	Predict
DOF014_scaffold34813_4	11269	phage	0.961	temperate	0.979654	unknown	0	Bacteroides fragilis	0.87	Predict
DOF014_scaffold49340_5	14214	phage	0.993	temperate	0.99984926	unknown	0	Colwellia psychrerythraea	0.89	Predict
DOF014_scaffold13006_1	11473	phage	0.987	temperate	0.9585484	Straboviridae	0.48694646	Streptococcus mutans	0.98	Predict
DOF014_C612698_1	10296	phage	0.998	virulent	0.9998684	unknown	0	Bacteroides fragilis	1	CRISPR
DOF014_scaffold82_3	13723	phage	0.999	virulent	0.99966824	Ackermannviridae	0.49632582	Colwellia psychrerythraea	0.85	Predict
DOF014_scaffold15208_5	20410	phage	0.997	temperate	0.99985605	unknown	0	Bacteroides fragilis	0.85	Predict
NOF001_scaffold2654_4	15052	phage	0.737	virulent	0.9997247	Ackermannviridae	0.22812769	Pseudomonas putida	0.78	Predict
NOF001_scaffold14_4	11643	phage	0.995	virulent	0.99987406	Ackermannviridae	0.52144295	Candidatus Hamiltonella defensa	0.81	Predict
NOF001_scaffold58201_2	46717	phage	0.999	virulent	0.9998679	Salasmaviridae	0.43348148	Parabacteroides merdae	1	CRISPR
NOF001_scaffold17586_2	11626	phage	0.996	temperate	0.9942528	Straboviridae	0.3021274	Bacteroides fragilis	0.82	Predict
NOF001_scaffold1053_2	23374	phage	0.999	temperate	0.8075163	Casjensviridae	0.9785655	unknown	0	-
NOF001_scaffold58318_1	19887	phage	1	temperate	0.99970055	Salasmaviridae	0.45380798	Staphylococcus saprophyticus	1	CRISPR
NOF001_scaffold5563_1	36742	phage	0.952	virulent	0.9998556	Ackermannviridae	0.5281165	Candidatus Hamiltonella defensa	0.8	Predict
NOF001_scaffold33721_2	10617	phage	0.95	temperate	0.99985975	unknown	0	unknown	0	-
NOF001_scaffold48_1	10590	phage	0.77	virulent	0.99935246	unknown	0	Candidatus Hamiltonella defensa	0.9	Predict
NOF001_scaffold47705_1	36007	phage	0.998	temperate	0.9962567	unknown	0	Candidatus Hamiltonella defensa	1	CRISPR
NOF001_scaffold56764_1	12151	phage	0.969	temperate	0.9998593	unknown	0	Candidatus Hamiltonella defensa	1	CRISPR
NOF001_scaffold13046_9	10712	phage	0.995	temperate	0.99986035	Kyanoviridae	1	unknown	0	-
NOF001_scaffold53059_1	15723	phage	0.995	temperate	0.9976891	unknown	0	Parabacteroides merdae	0.92	Predict
NOF001_scaffold58052_2	11692	phage	0.996	virulent	0.6183175	unknown	0	Candidatus Hamiltonella defensa	0.85	Predict
NOF001_scaffold5343_8	11037	phage	0.986	temperate	0.979004	unknown	0	Bacteroides fragilis	0.9	Predict
NOF001_scaffold58235_1	91845	phage	0.999	virulent	0.9998679	Herelleviridae	0.32129836	Prevotella copri	1	CRISPR

NOF001_scaffold57995_2	31360	phage	0.999	temperate	0.99980307	unknown	0	Candidatus Hamiltonella defensa	0.86	Predict
NOF001_scaffold11560_1	10001	phage	0.942	temperate	0.99985313	Peduoviridae	0.55076057	Candidatus Hamiltonella defensa	0.74	Predict
NOF001_scaffold27372_1	40385	phage	0.998	virulent	0.9998574	Herelleviridae	1	Candidatus Hamiltonella defensa	1	CRISPR
NOF001_scaffold41855_2	11440	phage	0.999	temperate	0.999826	unknown	0	Paenibacillus larvae	1	CRISPR
NOF001_scaffold24609_2	16184	phage	0.502	virulent	0.99987084	Straboviridae	0.5317443	Clostridium botulinum	0.94	Predict
NOF001_scaffold41855_6	12977	phage	0.999	virulent	0.57410604	Peduoviridae	1	Candidatus Hamiltonella defensa	0.96	Predict
NOF001_scaffold29077_4	10317	phage	0.999	virulent	0.9986517	Casjensviridae	0.44122034	unknown	0	-
NOF001_scaffold13046_6	14009	phage	0.999	temperate	0.9998431	unknown	0	unknown	0	-
NOF001_scaffold58316_1	15570	phage	1	virulent	0.99987084	Salasmaviridae	1	Candidatus Hamiltonella defensa	0.94	Predict
NOF001_scaffold7196_11	12404	phage	0.99	virulent	0.9994256	Casjensviridae	0.55142415	unknown	0	-
NOF001_scaffold41855_1	11502	phage	0.999	temperate	0.9998593	unknown	0	Candidatus Hamiltonella defensa	0.86	Predict
NOF001_scaffold2819_5	11916	phage	0.999	virulent	0.9998722	unknown	0	unknown	0	-
NOF001_scaffold24013_1	18717	phage	1	temperate	0.99144334	Straboviridae	0.13005078	Candidatus Hamiltonella defensa	1	CRISPR
NOF001_scaffold58081_1	101574	phage	0.999	virulent	0.9998608	Autographiviridae	1	Candidatus Pelagibacter ubique	0.99	Predict
NOF001_scaffold56157_1	11810	phage	0.924	temperate	0.98633206	unknown	0	unknown	0	-
NOF001_scaffold58201_1	16227	phage	0.998	virulent	0.99987036	Herelleviridae	1	Parabacteroides merdae	1	CRISPR
NOF001_scaffold55226_1	14053	phage	0.755	temperate	0.9998545	Straboviridae	0.3735483	Staphylococcus saprophyticus	0.84	Predict
NOF001_scaffold50021_1	65648	phage	0.998	virulent	0.99876857	Straboviridae	0.18451756	Azospirillum brasilense	1	CRISPR
NOF001_scaffold49296_1	12566	phage	0.999	virulent	0.9998049	unknown	0	unknown	0	-
NOF001_scaffold15730_5	22588	phage	0.999	virulent	0.99987036	unknown	0	unknown	0	-
NOF001_scaffold58099_2	18681	phage	0.999	virulent	0.99987036	Salasmaviridae	0.26505843	Candidatus Hamiltonella defensa	0.92	Predict
NOF002_scaffold7_6	16618	phage	0.999	temperate	0.9998579	Straboviridae	0.5317443	Staphylococcus saprophyticus	0.73	Predict
NOF002_scaffold13379_1	12447	phage	0.877	temperate	0.99985975	unknown	0	Candidatus Hamiltonella defensa	0.91	Predict
NOF002_scaffold4501_18	15830	phage	0.999	temperate	0.99976397	unknown	0	unknown	0	-
NOF002_scaffold30763_2	48079	phage	0.999	temperate	0.99981403	Straboviridae	0.779485	Staphylococcus saprophyticus	1	CRISPR
NOF002_scaffold29466_1	10507	phage	0.999	virulent	0.9998071	unknown	0	unknown	0	-
NOF002_scaffold5730_7	16814	phage	0.999	temperate	0.9998236	unknown	0	Planktothrix agardhii	0.83	Predict
NOF002_scaffold3476_12	11333	phage	0.998	virulent	0.99987316	unknown	0	Candidatus Hamiltonella defensa	0.94	Predict
NOF002_scaffold31199_2	12582	phage	0.997	virulent	0.9738244	unknown	0	Bacteroides fragilis	0.89	Predict
NOF002_C425969_1	16228	phage	1	temperate	0.99966824	unknown	0	Staphylococcus saprophyticus	0.86	Predict

NOF002_scaffold31424_1	46179	phage	0.999	temperate	0.9998193	Ackermannviridae	0.12288749	Candidatus Hamiltonella defensa	1 CRISPR
NOF002_scaffold12961_13	10073	phage	0.998	temperate	0.9998574	unknown	0	unknown	0 -
NOF002_scaffold20732_1	20530	phage	0.806	virulent	0.99986607	unknown	0	unknown	0 -
NOF002_scaffold31554_1	26928	phage	0.926	temperate	0.9998551	Ackermannviridae	0.3621296	Candidatus Hamiltonella defensa	0.95 Predict
NOF002_scaffold3233_2	16755	phage	0.999	temperate	0.99885607	unknown	0	Staphylococcus saprophyticus	1 CRISPR
NOF002_scaffold6749_14	10062	phage	0.999	virulent	0.9900106	Peduoviridae	1	Candidatus Hamiltonella defensa	1 CRISPR
NOF002_scaffold31199_1	12537	phage	0.999	temperate	0.9529658	unknown	0	Bacteroides fragilis	0.77 Predict
NOF002_scaffold11620_5	15309	phage	0.999	virulent	0.9788498	unknown	0	Candidatus Hamiltonella defensa	0.72 Predict
NOF002_scaffold9460_1	18849	phage	0.999	virulent	0.9675024	Ackermannviridae	0.45842224	Faecalibacterium prausnitzii	1 CRISPR
NOF002_scaffold31188_5	11280	phage	0.961	temperate	0.979654	unknown	0	Bacteroides fragilis	0.88 Predict
NOF002_scaffold1584_5	23545	phage	0.877	temperate	0.999774	Straboviridae	0.2987051	Staphylococcus saprophyticus	0.81 Predict
NOF002_scaffold31615_1	25737	phage	0.992	virulent	0.9998436	unknown	0	Clavibacter michiganensis	0.89 Predict
NOF002_scaffold3233_5	11465	phage	0.986	temperate	0.99986035	unknown	0	Candidatus Hamiltonella defensa	0.98 Predict
NOF002_scaffold7_7	51783	phage	0.968	temperate	0.99984884	Casjensviridae	1	Candidatus Hamiltonella defensa	0.77 Predict
NOF002_scaffold30763_1	12019	phage	0.999	temperate	0.99985605	unknown	0	Staphylococcus saprophyticus	0.74 Predict
NOF002_scaffold27207_5	15918	phage	0.592	temperate	0.9998513	Herelleviridae	1	Staphylococcus pasteuri	0.97 Predict
NOF002_scaffold2745_2	15080	phage	0.998	temperate	0.9998593	unknown	0	Candidatus Hamiltonella defensa	0.98 Predict
NOF002_scaffold2745_3	23598	phage	0.987	temperate	0.9998565	Ackermannviridae	0.5065008	Pasteurella multocida	0.82 Predict
NOF002_scaffold23022_4	16042	phage	0.986	temperate	0.997794	unknown	0	Bacteroides fragilis	0.72 Predict
NOF002_scaffold31584_1	30628	phage	0.831	virulent	0.9996782	unknown	0	Candidatus Hamiltonella defensa	0.89 Predict
NOF004_scaffold5431_15	12054	phage	0.824	virulent	0.95118916	unknown	0	Staphylococcus saprophyticus	0.81 Predict
NOF004_scaffold25441_2	19517	phage	0.997	virulent	0.943157	Straboviridae	0.3021274	Bacteroides fragilis	0.99 Predict
NOF004_scaffold5774_1	14447	phage	0.98	virulent	0.99969965	Straboviridae	0.69483334	Candidatus Hamiltonella defensa	0.89 Predict
NOF004_scaffold4703_4	21721	phage	1	virulent	0.99984694	unknown	0	Bacteroides fragilis	0.91 Predict
NOF004_scaffold19766_4	17263	phage	1	virulent	0.9935199	unknown	0	Candidatus Hamiltonella defensa	1 CRISPR
NOF004_scaffold32796_1	11377	phage	0.999	virulent	0.99987406	unknown	0	Candidatus Hamiltonella defensa	1 CRISPR
NOF004_scaffold30766_1	10547	phage	1	virulent	0.9996992	unknown	0	Candidatus Hamiltonella defensa	0.7 Predict
NOF004_scaffold4675_2	11303	phage	0.961	temperate	0.979654	unknown	0	Bacteroides fragilis	0.79 Predict
NOF004_scaffold36774_1	15704	phage	0.999	virulent	0.99982095	unknown	0	Candidatus Hamiltonella defensa	1 CRISPR
NOF004_scaffold37814_4	17106	phage	0.991	virulent	0.99930334	unknown	0	Staphylococcus saprophyticus	0.91 Predict

NOF004_scaffold39800_2	33097	phage	0.999	virulent	0.9998688	no_family_avaliable(NC_024711)	0.973	unknown	0	-
NOF004_scaffold12813_30	15141	phage	0.999	temperate	0.99969715	unknown	0	Clostridioides difficile	1	CRISPR
NOF004_C576082_1	17081	phage	0.999	virulent	0.99977165	Demerecviridae	0.36091807	unknown	0	-
NOF004_scaffold22864_4	17248	phage	0.99	virulent	0.99987173	Ackermannviridae	0.52144295	Candidatus Hamiltonella defensa	0.75	Predict
NOF004_scaffold12390_5	17312	phage	0.999	virulent	0.99985695	unknown	0	Candidatus Hamiltonella defensa	0.75	Predict
NOF004_scaffold18950_2	17356	phage	0.998	virulent	0.9994799	Ackermannviridae	0.49674326	Candidatus Hamiltonella defensa	0.9	Predict
NOF004_scaffold4703_1	19794	phage	0.999	virulent	0.9996036	unknown	0	Candidatus Hamiltonella defensa	0.9	Predict
NOF004_scaffold22559_2	17478	phage	0.999	virulent	0.99987036	Herelleviridae	0.5537847	unknown	0	-
NOF004_scaffold5741_5	10933	phage	0.999	virulent	0.71908617	unknown	0	unknown	0	-
NOF004_scaffold34095_2	11192	phage	0.994	temperate	0.98669714	unknown	0	Candidatus Hamiltonella defensa	1	Predict
NOF004_scaffold29834_1	43287	phage	0.999	temperate	0.9998588	unknown	0	Candidatus Hamiltonella defensa	1	CRISPR
NOF004_scaffold10567_2	26489	phage	0.996	virulent	0.9998693	Herelleviridae	1	unknown	0	-
NOF004_scaffold1680_17	17879	phage	0.992	virulent	0.9998565	unknown	0	Staphylococcus saprophyticus	0.86	Predict
NOF004_scaffold30605_6	14052	phage	0.999	virulent	0.9949012	unknown	0	Candidatus Hamiltonella defensa	0.84	Predict
NOF004_scaffold39867_3	18638	phage	0.973	virulent	0.99987173	unknown	0	Bacteroides fragilis	0.79	Predict
NOF004_scaffold2367_14	22016	phage	0.827	temperate	0.99985033	unknown	0	unknown	0	-
NOF004_scaffold8521_5	12533	phage	0.992	virulent	0.9998336	unknown	0	Candidatus Hamiltonella defensa	0.97	Predict
NOF004_scaffold8945_3	12664	phage	0.922	virulent	0.9996477	Casjensviridae	0.28481802	Candidatus Hamiltonella defensa	0.91	Predict
NOF004_scaffold24813_3	46602	phage	0.993	virulent	0.99985695	unknown	0	Candidatus Pelagibacter ubique	1	CRISPR
NOF004_scaffold15266_9	11305	phage	0.997	virulent	0.9996546	unknown	0	unknown	0	-
NOF004_scaffold39800_1	63441	phage	0.999	virulent	0.9998684	Straboviridae	0.316882	unknown	0	-
NOF004_C575620_1	10852	phage	0.998	temperate	0.99985975	unknown	0	Candidatus Hamiltonella defensa	1	CRISPR
NOF004_scaffold89_3	17893	phage	0.919	temperate	0.9413989	Ackermannviridae	0.4456534	Bacteroides fragilis	0.85	Predict
NOF004_scaffold31703_2	10441	phage	0.999	temperate	0.99951804	Herelleviridae	0.50312114	Candidatus Hamiltonella defensa	1	CRISPR
NOF004_scaffold26987_2	16797	phage	0.999	temperate	0.9998374	Casjensviridae	0.5366201	Candidatus Hamiltonella defensa	1	CRISPR
NOF004_scaffold58_6	14427	phage	0.999	virulent	0.9991748	unknown	0	Candidatus Hamiltonella defensa	0.91	Predict
NOF004_scaffold14653_3	13064	phage	0.978	temperate	0.69600296	unknown	0	unknown	0	-
NOF004_scaffold3847_7	13953	phage	0.634	virulent	0.99390656	unknown	0	unknown	0	-
NOF004_scaffold10069_19	15340	phage	0.999	virulent	0.99987406	unknown	0	unknown	0	-
NOF004_scaffold4193_2	14769	phage	0.998	temperate	0.99566615	Casjensviridae	1	Candidatus Hamiltonella defensa	0.9	Predict

NOF004_scaffold12390_3	11167	phage	0.994	temperate	0.91165656	Casjensviridae	1	Enterococcus faecalis	1 CRISPR
NOF004_scaffold9875_5	21880	phage	1	virulent	0.8912748	unknown	0	Candidatus Hamiltonella defensa	0.91 Predict
NOF004_scaffold39611_1	14565	phage	0.998	temperate	0.99985975	Straboviridae	0.86151546	Candidatus Pelagibacter ubique	0.84 Predict
NOF004_scaffold28322_1	13958	phage	0.999	temperate	0.99983555	Peduoviridae	1	Candidatus Hamiltonella defensa	1 CRISPR
NOF004_scaffold6978_23	10883	phage	0.997	temperate	0.9986471	Ackermannviridae	0.3621296	Candidatus Hamiltonella defensa	0.77 Predict
NOF004_scaffold9355_12	11197	phage	0.999	temperate	0.9998593	unknown	0	Erysipelothrix rhusiopathiae	1 CRISPR
NOF004_scaffold12025_2	15491	phage	1	temperate	0.7850754	no_family_avaliable(NC_047915)	0.999	Candidatus Hamiltonella defensa	1 CRISPR
NOF004_scaffold14211_1	32161	phage	0.997	temperate	0.99982214	unknown	0	Candidatus Hamiltonella defensa	1 CRISPR
NOF004_scaffold9994_2	18981	phage	0.924	virulent	0.9998693	unknown	0	Candidatus Pelagibacter ubique	0.95 Predict
NOF004_scaffold2098_11	15996	phage	0.999	temperate	0.61039174	unknown	0	Parabacteroides merdae	0.82 Predict
NOF005_scaffold35291_5	17098	phage	0.995	virulent	0.9998699	Straboviridae	0.25259766	Candidatus Hamiltonella defensa	0.9 Predict
NOF005_scaffold23396_3	68091	phage	0.999	virulent	0.9998622	unknown	0	Candidatus Pelagibacter ubique	1 CRISPR
NOF005_scaffold36104_5	11284	phage	0.878	temperate	0.7885992	unknown	0	unknown	0 -
NOF005_scaffold18_1	10900	phage	0.999	temperate	0.99985975	unknown	0	unknown	0 -
NOF005_scaffold25742_6	14359	phage	0.999	virulent	0.9998345	unknown	0	unknown	0 -
NOF005_scaffold31594_11	47577	phage	0.995	temperate	0.9994942	Straboviridae	0.62966955	Staphylococcus saprophyticus	1 CRISPR
NOF005_scaffold6888_3	14696	phage	0.998	temperate	0.974219	unknown	0	Parabacteroides distasonis	0.82 Predict
NOF005_scaffold29221_10	11735	phage	0.999	temperate	0.99985975	unknown	0	Candidatus Hamiltonella defensa	0.75 Predict
NOF005_scaffold34324_4	11268	phage	0.961	temperate	0.979654	unknown	0	Bacteroides fragilis	0.91 Predict
NOF005_scaffold1086_25	13040	phage	0.981	temperate	0.99985695	Straboviridae	0.7172016	Candidatus Hamiltonella defensa	0.77 Predict
NOF005_scaffold604_14	14954	phage	0.551	virulent	0.9998699	Straboviridae	0.19668595	Staphylococcus saprophyticus	0.86 Predict
NOF005_scaffold44749_6	18393	phage	0.546	virulent	0.99987125	unknown	0	Staphylococcus hominis	0.9 Predict
NOF005_scaffold51717_1	11280	phage	0.987	virulent	0.9998688	unknown	0	Candidatus Hamiltonella defensa	0.84 Predict
NOF005_scaffold35393_5	12397	phage	0.998	temperate	0.99985975	unknown	0	unknown	0 -
NOF005_scaffold34950_3	30440	phage	0.789	temperate	0.99985975	Ackermannviridae	0.48222607	Candidatus Hamiltonella defensa	1 CRISPR
NOF005_scaffold51596_4	16699	phage	0.998	temperate	0.99985975	no_family_avaliable(NC_047912)	0.968	Candidatus Hamiltonella defensa	1 CRISPR
NOF005_scaffold29140_2	15359	phage	0.995	virulent	0.9998293	Straboviridae	0.69483334	Staphylococcus saprophyticus	0.71 Predict
NOF005_scaffold37734_2	13542	phage	0.845	temperate	0.99934417	unknown	0	Candidatus Hamiltonella defensa	0.71 Predict
NOF005_scaffold165_4	18597	phage	0.978	virulent	0.9998413	Straboviridae	0.38608253	Candidatus Hamiltonella defensa	0.84 Predict
NOF005_scaffold2170_3	10220	phage	0.991	virulent	0.9998736	unknown	0	unknown	0 -

NOF005_scaffold24910_8	12072	phage	0.993	temperate	0.99986035	unknown	0	unknown	0	-
NOF005_scaffold48547_9	11914	phage	0.999	virulent	0.99987173	Salasmaviridae	0.31764138	Candidatus Hamiltonella defensa	0.97	Predict
NOF005_scaffold604_12	25233	phage	0.998	virulent	0.99915826	Herelleviridae	1	[Eubacterium] eligens	1	CRISPR
NOF005_scaffold52365_1	10304	phage	0.984	virulent	0.9998588	Straboviridae	0.3021274	Bacteroides fragilis	0.98	Predict
NOF005_scaffold23396_2	31920	phage	0.812	temperate	0.9925314	unknown	0	Candidatus Hamiltonella defensa	0.8	Predict
NOF005_scaffold3465_6	13403	phage	0.998	virulent	0.7216388	Straboviridae	0.45873865	Candidatus Hamiltonella defensa	1	CRISPR
NOF005_scaffold604_3	10777	phage	0.999	temperate	0.9998593	Herelleviridae	1	Staphylococcus saprophyticus	0.98	Predict
NOF005_scaffold52405_2	11301	phage	0.999	temperate	0.9998593	unknown	0	Parabacteroides distasonis	1	CRISPR
NOF005_scaffold49499_1_1	22744	phage	0.996	virulent	0.6071846	Straboviridae	0.63752884	Staphylococcus saprophyticus	1	CRISPR
NOF005_scaffold49499_1_2	34121	phage	0.999	virulent	0.9998736	unknown	0	unknown	0	-
NOF005_scaffold15268_1	10309	phage	0.978	temperate	0.63226247	unknown	0	Candidatus Hamiltonella defensa	0.78	Predict
NOF005_scaffold39972_17	10249	phage	0.831	virulent	0.9998736	Kyanoviridae	1	Staphylococcus saprophyticus	0.95	Predict
NOF005_scaffold27541_5	18036	phage	0.994	virulent	0.9998131	unknown	0	Candidatus Hamiltonella defensa	1	CRISPR
NOF005_scaffold27426_3	17288	phage	0.932	virulent	0.9998727	Ackermannviridae	0.52144295	Candidatus Hamiltonella defensa	0.83	Predict
NOF005_scaffold5924_1	12450	phage	0.997	virulent	0.99986315	unknown	0	unknown	0	-
NOF005_scaffold32384_3	10409	phage	0.999	virulent	0.99987316	Salasmaviridae	1	Candidatus Hamiltonella defensa	1	CRISPR
NOF005_scaffold44821_1	21852	phage	0.998	temperate	0.9998551	unknown	0	unknown	0	-
NOF005_scaffold27198_2	18679	phage	0.999	virulent	0.9998684	Salasmaviridae	0.9615491	Staphylococcus saprophyticus	0.87	Predict
NOF005_scaffold13462_4	67436	phage	0.998	virulent	0.9998608	Herelleviridae	1	Cellulophaga baltica	1	CRISPR
NOF005_scaffold2025_2	10304	phage	0.998	temperate	0.9930787	Casjensviridae	0.9785655	unknown	0	-
NOF005_scaffold52106_1	22018	phage	0.995	virulent	0.99980336	unknown	0	Candidatus Hamiltonella defensa	0.74	Predict
NOF005_scaffold604_13	18066	phage	0.999	virulent	0.9962401	unknown	0	Staphylococcus saprophyticus	0.88	Predict
NOF006_scaffold10363_1	11025	phage	0.999	temperate	0.99976826	unknown	0	Bacteroides fragilis	0.82	Predict
NOF006_scaffold15833_1	13816	phage	0.992	virulent	0.9998617	unknown	0	Candidatus Hamiltonella defensa	0.9	Predict
NOF006_scaffold6111_7	10054	phage	0.999	virulent	0.9449081	unknown	0	Candidatus Hamiltonella defensa	1	Predict
NOF006_scaffold4868_1	11775	phage	0.999	temperate	0.93910414	unknown	0	Candidatus Hamiltonella defensa	1	Predict
NOF006_scaffold21352_1	95059	phage	0.999	virulent	0.9998617	Straboviridae	0.316882	unknown	0	-
NOF006_scaffold12718_7	11425	phage	0.86	virulent	0.9998722	unknown	0	Bacteroides fragilis	0.99	Predict
NOF006_scaffold498_1	12539	phage	1	temperate	0.9998579	Ackermannviridae	0.48222607	Candidatus Hamiltonella defensa	1	CRISPR
NOF006_scaffold21223_2	53388	phage	1	temperate	0.9998536	Herelleviridae	1	Candidatus Hamiltonella defensa	0.72	Predict

NOF006_scaffold121_1	10318	phage	0.995	virulent	0.99987316	Vilmaviridae	1	Bacteroides fragilis	0.72	Predict
NOF006_scaffold21242_4	11276	phage	0.961	temperate	0.979654	unknown	0	Bacteroides fragilis	0.97	Predict
NOF006_scaffold1740_2	67449	phage	0.998	virulent	0.9998608	Herelleviridae	1	Cellulophaga baltica	1	CRISPR
NOF006_scaffold2842_16	12637	phage	0.917	virulent	0.9998722	unknown	0	Candidatus Hamiltonella defensa	0.89	Predict
NOF006_scaffold19983_2	11463	phage	0.988	virulent	0.9998736	unknown	0	Staphylococcus saprophyticus	0.88	Predict
NOF006_scaffold14475_3	13397	phage	0.999	virulent	0.90332973	unknown	0	Parabacteroides distasonis	1	CRISPR
NOF006_scaffold1022_7	10012	phage	0.999	virulent	0.99986744	unknown	0	Staphylococcus saprophyticus	0.87	Predict
NOF006_scaffold14499_8	11596	phage	0.995	virulent	0.99979323	unknown	0	Candidatus Hamiltonella defensa	0.96	Predict
NOF006_scaffold21012_1	12001	phage	0.996	virulent	0.908313	unknown	0	Candidatus Hamiltonella defensa	0.77	Predict
NOF006_scaffold4067_5	12030	phage	0.999	temperate	0.9627462	unknown	0	unknown	0	-
NOF006_scaffold20915_1	20737	phage	0.999	temperate	0.99980694	Peduoviridae	1	Staphylococcus saprophyticus	1	CRISPR
NOF006_scaffold4872_3	12433	phage	0.997	virulent	0.998128	Straboviridae	0.3481552	Candidatus Hamiltonella defensa	1	CRISPR
NOF006_scaffold969_8	10679	phage	0.998	virulent	0.64794564	unknown	0	unknown	0	-
NOF006_C401108_1	14501	phage	0.999	virulent	0.9998693	Salasmaviridae	1	Bacillus cereus	1	CRISPR
NOF006_scaffold21374_2	11791	phage	0.902	temperate	0.9735116	unknown	0	unknown	0	-
NOF006_scaffold20868_2	12484	phage	0.999	virulent	0.9998693	unknown	0	Streptococcus mutans	1	CRISPR
NOF006_scaffold173_3	10501	phage	0.66	virulent	0.99987465	unknown	0	unknown	0	-
NOF007_scaffold27213_3	17110	phage	0.998	virulent	0.99987125	unknown	0	Candidatus Hamiltonella defensa	0.9	Predict
NOF007_scaffold16773_3	15905	phage	0.856	temperate	0.93894786	Ackermannviridae	0.4456534	Bacteroides fragilis	0.84	Predict
NOF007_scaffold23774_3	21399	phage	0.999	temperate	0.99871093	unknown	0	Candidatus Hamiltonella defensa	1	CRISPR
NOF007_scaffold4461_7	12672	phage	0.984	temperate	0.9998479	Straboviridae	0.4051862	Staphylococcus saprophyticus	0.9	Predict
NOF007_scaffold28685_4	67920	phage	0.585	temperate	0.9998369	unknown	0	Candidatus Hamiltonella defensa	0.94	Predict
NOF007_scaffold396_8	13385	phage	0.999	temperate	0.9832063	Ackermannviridae	0.45842224	unknown	0	-
NOF007_scaffold26160_1	13381	phage	0.999	virulent	0.97327614	unknown	0	Candidatus Hamiltonella defensa	0.97	Predict
NOF007_scaffold11484_1	16458	phage	0.999	virulent	0.9998693	no_family_avaliable(NC_024711)	0.977	unknown	0	-
NOF007_scaffold25262_5	11411	phage	0.997	virulent	0.6879927	unknown	0	unknown	0	-
NOF007_scaffold21388_2	28933	phage	0.999	temperate	0.908581	Peduoviridae	1	Candidatus Hamiltonella defensa	1	CRISPR
NOF007_scaffold1349_3	21748	phage	0.999	virulent	0.6408339	Casjensviridae	0.46178052	unknown	0	-
NOF007_C490687_1	14253	phage	0.998	virulent	0.9998722	unknown	0	unknown	0	-
NOF007_scaffold19207_2	11792	phage	0.996	virulent	0.9998736	unknown	0	Bacteroides fragilis	0.97	Predict

NOF007_scaffold1349_2	13881	phage	0.989	temperate	0.9969053	Casjensviridae	0.2757636	unknown	0 -
NOF007_scaffold8821_2	20128	phage	0.998	virulent	0.99980664	Ackermannviridae	0.5155003	Candidatus Hamiltonella defensa	0.89 Predict
NOF007_scaffold27822_1	11785	phage	0.97	virulent	0.99942935	unknown	0	Candidatus Hamiltonella defensa	0.76 Predict
NOF007_scaffold15189_2	35937	phage	1	virulent	0.9998679	Herelleviridae	0.3868164	unknown	0 -
NOF007_scaffold23819_3	11283	phage	0.974	temperate	0.98519784	unknown	0	Bacteroides fragilis	0.95 Predict
NOF007_scaffold26938_2	20192	phage	0.999	temperate	0.9998565	unknown	0	Parabacteroides distasonis	1 CRISPR
NOF007_scaffold7559_3	38501	phage	1	virulent	0.9998545	Straboviridae	0.76637846	Candidatus Hamiltonella defensa	0.88 Predict
NOF007_scaffold16388_6	28390	phage	0.999	temperate	0.9998417	Casjensviridae	0.36081105	Candidatus Hamiltonella defensa	1 CRISPR
NOF007_scaffold24483_2	40196	phage	0.999	virulent	0.9998627	Kyanoviridae	0.20652783	Candidatus Hamiltonella defensa	1 CRISPR
NOF007_scaffold10524_1	12379	phage	0.988	temperate	0.99383783	unknown	0	Candidatus Hamiltonella defensa	0.97 Predict
NOF007_scaffold41_1	23420	phage	0.999	virulent	0.99987084	no_family_avaliable(NC_024711)	0.962	unknown	0 -
NOF007_scaffold298_7	10775	phage	0.999	temperate	0.9998584	unknown	0	Parabacteroides distasonis	0.88 Predict
NOF007_scaffold505_1	10280	phage	0.986	virulent	0.99987316	Casjensviridae	0.5581696	Candidatus Hamiltonella defensa	0.78 Predict
NOF007_scaffold28801_1	13484	phage	0.998	temperate	0.9998574	Ackermannviridae	0.5065008	Candidatus Hamiltonella defensa	0.75 Predict
NOF007_scaffold28685_3	12569	phage	0.999	temperate	0.6311137	unknown	0	unknown	0 -
NOF007_scaffold25304_2	20443	phage	0.652	temperate	0.99769664	unknown	0	unknown	0 -
NOF007_scaffold22039_3	11206	phage	0.999	virulent	0.99986744	unknown	0	Bacillus pumilus	0.72 Predict
NOF007_scaffold26224_1	11059	phage	0.999	virulent	0.9998727	unknown	0	Candidatus Hamiltonella defensa	0.97 Predict
NOF007_scaffold28624_3	10873	phage	0.567	virulent	0.9998736	unknown	0	unknown	0 -
NOF007_scaffold3393_7	10134	phage	0.999	temperate	0.99985605	Casjensviridae	0.38532472	unknown	0 -
NOF007_scaffold1195_9	69672	phage	0.965	temperate	0.83707535	Casjensviridae	0.1780801	Candidatus Hamiltonella defensa	0.92 Predict
NOF007_scaffold2822_2	18146	phage	1	virulent	0.99987036	Straboviridae	0.33762848	unknown	0 -
NOF007_scaffold23774_2	18771	phage	1	temperate	0.9997167	Peduoviridae	1	Candidatus Hamiltonella defensa	1 CRISPR
NOF007_scaffold12899_1	14398	phage	0.578	virulent	0.9998645	unknown	0	Candidatus Hamiltonella defensa	0.76 Predict
NOF007_scaffold9468_5	10817	phage	0.997	temperate	0.99984837	unknown	0	Parabacteroides distasonis	0.86 Predict
NOF008_scaffold34632_3	31348	phage	0.999	virulent	0.99987084	no_family_avaliable(NC_062779)	0.97	unknown	0 -
NOF008_scaffold68230_1	28352	phage	1	virulent	0.99928766	Casjensviridae	0.36081105	Candidatus Hamiltonella defensa	1 CRISPR
NOF008_scaffold25367_2	10543	phage	1	virulent	0.99956065	unknown	0	Candidatus Hamiltonella defensa	0.89 Predict
NOF008_scaffold64397_1	24319	phage	0.997	virulent	0.99979264	Kyanoviridae	1	Candidatus Pelagibacter ubique	0.73 Predict
NOF008_scaffold48782_2	29716	phage	0.999	virulent	0.96857816	unknown	0	Bacteroides fragilis	0.7 Predict

NOF008_scaffold5134_4	10562	phage	0.997	temperate	0.99985975	unknown	0	Candidatus Hamiltonella defensa	0.77	Predict
NOF008_scaffold61643_2	19078	phage	0.956	virulent	0.99692863	unknown	0	unknown	0	-
NOF008_scaffold1288_26	11053	phage	0.998	virulent	0.99982095	Kyanoviridae	1	unknown	0	-
NOF008_scaffold66085_2	10418	phage	0.997	virulent	0.99986035	Ackermannviridae	0.53408796	Candidatus Hamiltonella defensa	0.87	Predict
NOF008_scaffold11140_1	10488	phage	1	virulent	0.99983215	Salasmaviridae	1	Candidatus Hamiltonella defensa	1	CRISPR
NOF008_scaffold5217_6	16628	phage	0.593	temperate	0.9998565	unknown	0	unknown	0	-
NOF008_scaffold1389_2	14325	phage	0.998	virulent	0.99987173	Ackermannviridae	0.34342647	unknown	0	-
NOF008_scaffold34632_4	15837	phage	0.999	virulent	0.99987084	no_family_avaliable(NC_062779)	0.97	unknown	0	-
NOF008_scaffold67470_2	16967	phage	0.999	virulent	0.99987173	unknown	0	Candidatus Hamiltonella defensa	1	CRISPR
NOF008_scaffold28772_6	15069	phage	0.932	virulent	0.9998727	Ackermannviridae	0.52144295	Candidatus Hamiltonella defensa	0.93	Predict
NOF008_scaffold10035_9	12393	phage	0.997	temperate	0.9998588	unknown	0	Candidatus Hamiltonella defensa	0.89	Predict
NOF008_scaffold30912_6	15544	phage	0.999	temperate	0.9998545	Peduoviridae	1	Mycobacterium persicum	1	CRISPR
NOF008_scaffold66613_1	11413	phage	0.999	temperate	0.99985975	unknown	0	Candidatus Hamiltonella defensa	0.94	Predict
NOF008_scaffold48283_1	12401	phage	0.999	temperate	0.99958426	unknown	0	unknown	0	-
NOF008_scaffold31488_6	12580	phage	0.999	temperate	0.9998593	unknown	0	Candidatus Hamiltonella defensa	1	CRISPR
NOF008_scaffold68147_1	13008	phage	0.999	temperate	0.9998556	Salasmaviridae	0.650711	unknown	0	-
NOF008_scaffold8714_2	18481	phage	0.999	virulent	0.99982476	Casjensviridae	0.36081105	Candidatus Hamiltonella defensa	0.78	Predict
NOF008_scaffold5616_1	22520	phage	0.998	virulent	0.99987125	Ackermannviridae	0.5155003	Candidatus Hamiltonella defensa	0.93	Predict
NOF008_scaffold68130_1	19718	phage	1	virulent	0.9998679	Guelinviridae	0.26210135	Candidatus Hamiltonella defensa	1	CRISPR
NOF008_scaffold25447_1	70276	phage	0.999	virulent	0.99973255	Ackermannviridae	0.37941656	unknown	0	-
NOF008_scaffold3062_3	13745	phage	0.988	virulent	0.99964243	unknown	0	Clostridioides difficile	0.84	Predict
NOF008_scaffold7421_1	13996	phage	1	temperate	0.97783464	unknown	0	Candidatus Hamiltonella defensa	1	CRISPR
NOF008_scaffold52435_2	12332	phage	0.997	temperate	0.9972386	Casjensviridae	0.31979057	unknown	0	-
NOF008_scaffold35076_1	16314	phage	0.999	virulent	0.9998688	unknown	0	unknown	0	-
NOF008_scaffold58140_1	22260	phage	0.992	temperate	0.9925541	unknown	0	Candidatus Hamiltonella defensa	0.93	Predict
NOF008_scaffold53677_1	10250	phage	0.962	virulent	0.99987465	unknown	0	unknown	0	-
NOF008_scaffold68203_3	13931	phage	0.981	virulent	0.9998445	unknown	0	Bacteroides fragilis	1	CRISPR
NOF008_scaffold36171_4	15900	phage	0.919	temperate	0.9413989	Ackermannviridae	0.4456534	Bacteroides fragilis	0.87	Predict
NOF008_scaffold68191_1	19846	phage	0.993	temperate	0.999854	unknown	0	unknown	0	-
NOF008_scaffold65986_1	12274	phage	0.968	temperate	0.99985975	unknown	0	unknown	0	-

NOF008_scaffold63722_1	11950	phage	0.999	temperate	0.7244286	unknown	0	unknown	0	-
NOF008_scaffold50828_1	16242	phage	0.953	virulent	0.99982554	unknown	0	unknown	0	-
NOF008_scaffold41113_4	11522	phage	0.639	virulent	0.99987173	unknown	0	unknown	0	-
NOF008_scaffold59760_7	11672	phage	0.999	virulent	0.9903983	unknown	0	unknown	0	-
NOF008_scaffold42043_1	28258	phage	0.999	temperate	0.9998593	Peduoviridae	1	Candidatus Pelagibacter ubique	1	CRISPR
NOF008_scaffold8066_2	10265	phage	0.596	temperate	0.9998584	Herelleviridae	1	Proteus mirabilis	0.81	Predict
NOF008_scaffold28625_3	17451	phage	0.996	virulent	0.9998684	Ackermannviridae	0.5281165	Candidatus Hamiltonella defensa	0.99	Predict
NOF008_scaffold19089_7	17476	phage	0.849	temperate	0.9998403	Peduoviridae	0.55076057	Candidatus Hamiltonella defensa	0.8	Predict
NOF008_scaffold3708_20	25031	phage	0.999	temperate	0.99970675	Casjensviridae	1	Candidatus Hamiltonella defensa	0.73	Predict
NOF008_scaffold68224_1	24371	phage	0.642	temperate	0.9995376	unknown	0	Staphylococcus saprophyticus	0.84	Predict
NOF008_scaffold52198_1	12086	phage	0.999	virulent	0.9998574	Casjensviridae	0.36081105	Candidatus Hamiltonella defensa	1	CRISPR
NOF008_scaffold7904_8	14287	phage	0.995	temperate	0.99986035	unknown	0	Candidatus Hamiltonella defensa	0.74	Predict
NOF008_scaffold25220_3	11931	phage	0.999	virulent	0.98565406	Ackermannviridae	0.5281165	Candidatus Hamiltonella defensa	0.79	Predict
NOF008_C865299_1	11361	phage	0.999	virulent	0.99987125	unknown	0	Candidatus Hamiltonella defensa	0.84	Predict
NOF008_scaffold17689_13	14788	phage	0.819	virulent	0.99981016	unknown	0	Lactobacillus johnsonii	0.98	Predict
NOF008_C865863_1	17035	phage	0.999	virulent	0.96842474	unknown	0	Candidatus Hamiltonella defensa	0.7	Predict
NOF008_scaffold68199_1	13390	phage	0.995	virulent	0.99987125	Straboviridae	0.24190016	Candidatus Hamiltonella defensa	0.78	Predict
NOF008_scaffold55525_8	12136	phage	0.928	virulent	0.9998722	unknown	0	Roseobacter denitrificans	0.8	Predict
NOF008_scaffold13444_2	10569	phage	0.999	temperate	0.9998431	unknown	0	unknown	0	-
NOF008_scaffold55811_1	16066	phage	0.999	virulent	0.9402247	unknown	0	unknown	0	-
NOF008_scaffold29486_12	15102	phage	0.999	virulent	0.99986124	unknown	0	Candidatus Hamiltonella defensa	0.82	Predict
NOF009_scaffold7527_22	26831	phage	0.998	virulent	0.99986404	unknown	0	Candidatus Hamiltonella defensa	0.97	Predict
NOF009_scaffold43518_2	19358	phage	0.999	temperate	0.99985605	unknown	0	Bacteroides fragilis	0.79	Predict
NOF009_scaffold21768_4	15420	phage	0.88	virulent	0.9998693	Straboviridae	0.6711859	Bacteroides fragilis	0.72	Predict
NOF009_scaffold41727_1	20880	phage	1	virulent	0.9994311	unknown	0	Staphylococcus saprophyticus	0.72	Predict
NOF009_scaffold10148_1	10721	phage	0.999	temperate	0.99978215	unknown	0	Candidatus Hamiltonella defensa	0.76	Predict
NOF009_scaffold19865_4	11989	phage	0.519	temperate	0.99986035	unknown	0	Candidatus Hamiltonella defensa	1	CRISPR
NOF009_C558283_1	10880	phage	0.997	virulent	0.9998636	unknown	0	unknown	0	-
NOF009_scaffold2064_12	10623	phage	0.993	temperate	0.8072752	Ackermannviridae	0.49674326	Bacteroides fragilis	0.73	Predict
NOF009_scaffold34567_3	28369	phage	0.999	temperate	0.9977161	unknown	0	Candidatus Hamiltonella defensa	1	CRISPR

NOF009_scaffold11228_12	10398	phage	0.996	temperate	0.9401553	unknown	0	unknown	0	-
NOF009_scaffold16960_2	44044	phage	0.759	temperate	0.99685895	Straboviridae	0.3481552	Candidatus Hamiltonella defensa	1	CRISPR
NOF009_scaffold14075_3	13692	phage	0.999	virulent	0.997076	Drexlerviridae	0.29942086	Bacteroides fragilis	1	CRISPR
NOF009_scaffold67_1	16822	phage	0.999	temperate	0.99984604	Kyanoviridae	0.20087749	Candidatus Hamiltonella defensa	1	CRISPR
NOF009_C558255_1	10607	phage	0.999	virulent	0.99984217	unknown	0	Candidatus Hamiltonella defensa	1	CRISPR
NOF009_scaffold13328_11	12740	phage	0.94	temperate	0.9998556	Straboviridae	0.37907848	unknown	0	-
NOF009_scaffold42455_5	31552	phage	0.999	temperate	0.9995705	Herelleviridae	0.40026155	Staphylococcus saprophyticus	1	CRISPR
NOF009_scaffold2720_3	10070	phage	1	temperate	0.7528099	unknown	0	Candidatus Hamiltonella defensa	0.84	Predict
NOF009_scaffold41979_1	41776	phage	0.988	virulent	0.6331934	Casjensviridae	0.36081105	Microcystis aeruginosa	1	CRISPR
NOF009_scaffold30580_1	26926	phage	0.993	virulent	0.66290087	unknown	0	Candidatus Hamiltonella defensa	1	CRISPR
NOF009_scaffold5136_16	10709	phage	0.999	virulent	0.9998722	no_family_avaliable(NC_067211)	0.974	unknown	0	-
NOF009_scaffold42878_5	12702	phage	1	temperate	0.9998388	unknown	0	unknown	0	-
NOF009_scaffold42496_4	10613	phage	0.961	temperate	0.979654	unknown	0	Bacteroides fragilis	0.81	Predict
NOF010_C699885_1	19978	phage	0.999	temperate	0.9998579	Salasmaviridae	0.37905717	Staphylococcus saprophyticus	1	CRISPR
NOF010_scaffold33789_4	48665	phage	0.998	virulent	0.9958235	Peduoviridae	1	Candidatus Hamiltonella defensa	1	CRISPR
NOF010_scaffold32319_4	23854	phage	0.998	virulent	0.99987125	unknown	0	Staphylococcus saprophyticus	0.79	Predict
NOF010_scaffold26425_6	10683	phage	0.924	temperate	0.5711277	unknown	0	Candidatus Hamiltonella defensa	0.8	Predict
NOF010_scaffold14373_1	19765	phage	0.993	virulent	0.99987125	Kyanoviridae	1	Staphylococcus saprophyticus	0.89	Predict
NOF010_scaffold28531_1	13805	phage	0.976	virulent	0.9998588	unknown	0	unknown	0	-
NOF010_scaffold51712_2	50893	phage	0.983	temperate	0.99969196	unknown	0	Trichormus variabilis	0.87	Predict
NOF010_scaffold2330_4	22702	phage	0.974	virulent	0.9331835	Casjensviridae	0.47887832	unknown	0	-
NOF010_scaffold6485_5	12350	phage	0.998	virulent	0.9619549	unknown	0	unknown	0	-
NOF010_scaffold16598_11	10668	phage	0.972	virulent	0.99985695	Ackermannviridae	1	Bacteroides fragilis	0.97	Predict
NOF010_scaffold20182_2	14242	phage	0.989	virulent	0.99987406	Casjensviridae	0.5581696	Candidatus Hamiltonella defensa	0.73	Predict
NOF010_scaffold2722_9	12543	phage	0.999	virulent	0.99987316	unknown	0	unknown	0	-
NOF010_scaffold22545_1	24149	phage	0.859	virulent	0.98227835	Ackermannviridae	1	unknown	0	-
NOF010_scaffold20459_4	11293	phage	0.994	virulent	0.99987406	Ackermannviridae	0.52144295	Bacteroides fragilis	0.78	Predict
NOF010_scaffold9563_25	10528	phage	0.989	virulent	0.99987406	Casjensviridae	0.5581696	Candidatus Hamiltonella defensa	0.83	Predict
NOF010_scaffold53722_1	23963	phage	0.949	virulent	0.99981785	Casjensviridae	0.34444138	Candidatus Hamiltonella defensa	0.83	Predict
NOF010_scaffold46583_3	13700	phage	0.803	temperate	0.9941079	Herelleviridae	1	unknown	0	-

NOF010_scaffold6369_4	12861	phage	0.947	virulent	0.9949243	Straboviridae	0.38608253	Bacteroides fragilis	1	CRISPR
NOF010_scaffold22545_2	34942	phage	0.999	temperate	0.99984926	unknown	0	Clostridium perfringens	0.98	Predict
NOF010_scaffold4642_2	21930	phage	1	virulent	0.9998417	Straboviridae	0.76637846	Candidatus Hamiltonella defensa	0.74	Predict
NOF010_scaffold14618_2	10280	phage	0.998	temperate	0.99965626	unknown	0	Candidatus Hamiltonella defensa	1	CRISPR
NOF010_scaffold53516_6	18410	phage	1	temperate	0.9998579	Salasmaviridae	0.4105297	Candidatus Hamiltonella defensa	1	CRISPR
NOF010_scaffold43806_2	11358	phage	0.993	temperate	0.9998579	unknown	0	Clostridium tetani	0.89	Predict
NOF010_scaffold21111_3	35265	phage	0.999	virulent	0.9998679	Straboviridae	0.3556836	Candidatus Hamiltonella defensa	0.9	Predict
NOF010_scaffold27134_3	11031	phage	0.993	virulent	0.9996987	unknown	0	Candidatus Hamiltonella defensa	1	CRISPR
NOF010_scaffold22950_5	39888	phage	0.998	temperate	0.9918041	Peduoviridae	1	Candidatus Hamiltonella defensa	1	CRISPR
NOF010_scaffold54242_1	11444	phage	0.984	virulent	0.9998688	unknown	0	Staphylococcus saprophyticus	0.95	Predict
NOF010_scaffold54124_1	10658	phage	0.97	virulent	0.9888191	Ackermannviridae	0.53408796	Candidatus Hamiltonella defensa	0.87	Predict
NOF010_C699039_1	10389	phage	0.999	virulent	0.99987084	unknown	0	Candidatus Hamiltonella defensa	1	CRISPR
NOF010_scaffold23028_3	17449	phage	0.996	virulent	0.9998684	Ackermannviridae	0.5281165	Candidatus Hamiltonella defensa	0.76	Predict
NOF010_scaffold33103_1	11763	phage	0.996	temperate	0.99968624	unknown	0	Candidatus Hamiltonella defensa	0.97	Predict
NOF010_scaffold5911_1	17399	phage	0.994	virulent	0.99695677	Ackermannviridae	1	Candidatus Hamiltonella defensa	0.74	Predict
NOF010_scaffold54281_1	11444	phage	1	temperate	0.99978167	Ackermannviridae	0.45842224	unknown	0	-
NOF010_scaffold11305_3	14288	phage	0.985	temperate	0.99985975	unknown	0	Parabacteroides merdae	0.92	Predict
NOF010_scaffold21018_1	20410	phage	0.999	temperate	0.9998579	unknown	0	Candidatus Hamiltonella defensa	0.79	Predict
NOF011_scaffold3878_1	14526	phage	0.999	virulent	0.9053317	unknown	0	Bacteroides fragilis	0.98	Predict
NOF011_scaffold57558_3	40211	phage	0.999	temperate	0.9998336	unknown	0	Staphylococcus saprophyticus	1	CRISPR
NOF011_scaffold159_2	11928	phage	0.999	virulent	0.99987465	unknown	0	Parabacteroides distasonis	0.95	Predict
NOF011_scaffold56176_3	18470	phage	1	temperate	0.99985695	Drexlerviridae	0.2601836	Candidatus Hamiltonella defensa	1	CRISPR
NOF011_scaffold18490_3	12769	phage	0.999	virulent	0.99986696	Ackermannviridae	0.44814733	Bacteroides fragilis	0.82	Predict
NOF011_scaffold59088_2	11064	phage	0.996	virulent	0.99987316	unknown	0	Candidatus Hamiltonella defensa	0.99	Predict
NOF011_scaffold8028_19	10745	phage	0.999	temperate	0.9998588	unknown	0	Candidatus Hamiltonella defensa	0.78	Predict
NOF011_scaffold33258_2	10443	phage	0.871	virulent	0.9998114	unknown	0	Candidatus Hamiltonella defensa	0.94	Predict
NOF011_scaffold58584_1	15596	phage	0.942	virulent	0.9998727	unknown	0	Staphylococcus saprophyticus	0.82	Predict
NOF011_scaffold70_15	12036	phage	0.994	virulent	0.99987084	unknown	0	Bacteroides fragilis	0.94	Predict
NOF011_scaffold58981_1	15584	phage	0.998	virulent	0.9996613	Straboviridae	0.45873865	Candidatus Hamiltonella defensa	1	CRISPR
NOF011_scaffold59056_1	28501	phage	0.999	virulent	0.99984217	Herelleviridae	0.49626034	Staphylococcus saprophyticus	0.8	Predict

NOF011_scaffold59100_2	26470	phage	0.999	temperate	0.7812632	unknown	0	Bacteroides fragilis	1	CRISPR
NOF011_scaffold27189_1	12718	phage	0.964	virulent	0.9996181	Casjensviridae	0.28481802	Candidatus Hamiltonella defensa	0.96	Predict
NOF011_scaffold57546_2	10815	phage	0.997	temperate	0.99984837	unknown	0	Parabacteroides distasonis	0.9	Predict
NOF011_scaffold59096_2	15893	phage	0.743	virulent	0.9171376	Ackermannviridae	0.4456534	Bacteroides fragilis	0.81	Predict
NOF011_scaffold15798_1	15299	phage	1	virulent	0.99987465	Herelleviridae	1	Bacteroides fragilis	0.81	Predict
NOF011_C736712_1	16937	phage	0.999	virulent	0.9995259	Demerecviridae	0.36091807	unknown	0	-
NOF011_scaffold48802_1	14169	phage	1	virulent	0.9998727	Salasmaviridae	1	Candidatus Hamiltonella defensa	1	CRISPR
NOF011_scaffold57126_1	19430	phage	0.989	virulent	0.9998727	Straboviridae	0.46207947	Staphylococcus saprophyticus	0.82	Predict
NOF011_scaffold52600_1	13568	phage	0.997	virulent	0.9532104	unknown	0	Bacteroides oleiciplenus	1	CRISPR
NOF011_scaffold56176_2	20554	phage	0.999	virulent	0.84088755	unknown	0	Candidatus Hamiltonella defensa	1	CRISPR
NOF011_scaffold8028_20	11788	phage	0.997	temperate	0.9998584	unknown	0	Candidatus Hamiltonella defensa	0.74	Predict
NOF011_scaffold2529_7	16198	phage	0.882	temperate	0.78272307	Straboviridae	0.38608253	Candidatus Hamiltonella defensa	0.82	Predict
NOF011_scaffold45498_1	11329	phage	0.815	temperate	0.999774	unknown	0	Candidatus Hamiltonella defensa	0.82	Predict
NOF011_scaffold40128_2	12415	phage	0.953	temperate	0.99894154	unknown	0	Bacteroides fragilis	1	CRISPR
NOF012_scaffold77459_1	16114	phage	0.998	virulent	0.56465566	Casjensviridae	1	Enterococcus faecalis	1	CRISPR
NOF012_scaffold71024_7	15473	phage	0.983	temperate	0.9998188	Straboviridae	0.38608253	Bacteroides fragilis	0.96	Predict
NOF012_scaffold77267_2	17164	phage	0.997	temperate	0.9998574	unknown	0	Parabacteroides merdae	0.97	Predict
NOF012_scaffold24523_4	13118	phage	0.998	temperate	0.9994951	unknown	0	unknown	0	-
NOF012_scaffold12244_5	21820	phage	1	virulent	0.99973226	unknown	0	Ruminococcus sp. AF26-25AA	1	CRISPR
NOF012_scaffold50466_2	10164	phage	1	virulent	0.5916771	unknown	0	unknown	0	-
NOF012_scaffold5639_4	25079	phage	1	temperate	0.90100694	Peduoviridae	0.9684544	Candidatus Hamiltonella defensa	1	CRISPR
NOF012_scaffold15647_4	15073	phage	0.932	virulent	0.9998727	Ackermannviridae	0.52144295	Candidatus Hamiltonella defensa	0.87	Predict
NOF012_scaffold8867_2	12066	phage	0.995	virulent	0.99978536	Ackermannviridae	0.48947993	Candidatus Hamiltonella defensa	0.83	Predict
NOF012_scaffold13996_7	15958	phage	0.999	temperate	0.99985975	unknown	0	Candidatus Hamiltonella defensa	0.97	Predict
NOF012_scaffold77917_2	18054	phage	0.994	virulent	0.9998131	unknown	0	Candidatus Hamiltonella defensa	1	CRISPR
NOF012_scaffold12244_6	14581	phage	0.999	temperate	0.99985605	unknown	0	Clostridium botulinum	0.78	Predict
NOF012_scaffold44774_1	10966	phage	0.597	temperate	0.9998302	unknown	0	unknown	0	-
NOF012_scaffold3081_2	12260	phage	0.996	temperate	0.9076956	unknown	0	Enterococcus faecium	0.75	Predict
NOF012_scaffold78059_2	15894	phage	0.919	temperate	0.9413989	Ackermannviridae	0.4456534	Bacteroides fragilis	0.98	Predict
NOF012_scaffold34517_3	13880	phage	0.983	temperate	0.7887559	Casjensviridae	0.2757636	unknown	0	-

NOF012_scaffold5639_7	12639	phage	0.983	temperate	0.99948466	unknown	0	Candidatus Hamiltonella defensa	1	CRISPR
NOF012_scaffold24554_12	12253	phage	0.996	virulent	0.99695736	unknown	0	Flavobacterium psychrophilum	0.75	Predict
NOF012_scaffold4667_6	15924	phage	0.961	temperate	0.979654	unknown	0	Candidatus Hamiltonella defensa	0.92	Predict
NOF012_scaffold72024_3	18680	phage	1	temperate	0.9998513	unknown	0	Paenibacillus larvae	1	CRISPR
NOF012_scaffold19855_2	12273	phage	0.998	temperate	0.9998593	unknown	0	Candidatus Hamiltonella defensa	0.71	Predict
NOF012_scaffold34940_12	13777	phage	0.99	virulent	0.9994256	Casjensviridae	0.55142415	Micromonospora chaiyaphumensis	1	CRISPR
NOF012_scaffold34517_1	28871	phage	0.846	virulent	0.99987036	Ackermannviridae	0.535957	unknown	0	-
NOF012_scaffold8693_10	12996	phage	0.999	temperate	0.9997774	unknown	0	Candidatus Hamiltonella defensa	0.8	Predict
NOF012_scaffold34517_2	26753	phage	0.999	virulent	0.9998202	Casjensviridae	0.46178052	unknown	0	-
NOF012_scaffold77271_2	10321	phage	0.998	virulent	0.99987316	Ackermannviridae	0.30045664	unknown	0	-
NOF012_scaffold71857_2	13451	phage	0.997	temperate	0.9980795	Casjensviridae	0.5508405	Butyricicoccus sp. AF24-19AC	1	CRISPR
NOF012_scaffold74308_1	16975	phage	0.989	virulent	0.99602866	unknown	0	unknown	0	-
NOF012_C833949_1	14212	phage	1	virulent	0.99987173	Salasmaviridae	1	Candidatus Pelagibacter ubique	0.9	Predict
NOF012_scaffold15991_1	24856	phage	0.999	virulent	0.9998608	Casjensviridae	0.7678824	Candidatus Hamiltonella defensa	0.81	Predict
NOF012_scaffold71874_7	10657	phage	0.848	virulent	0.99987406	unknown	0	Candidatus Hamiltonella defensa	1	Predict
NOF012_scaffold339_7	13514	phage	0.994	virulent	0.99985033	Straboviridae	1	Candidatus Hamiltonella defensa	0.96	Predict
NOF012_scaffold28415_3	13120	phage	0.999	virulent	0.99987316	unknown	0	Candidatus Hamiltonella defensa	1	CRISPR
NOF012_scaffold9650_7	14376	phage	0.991	virulent	0.9998656	Zierdtviridae	1	unknown	0	-
NOF012_scaffold77196_1	12110	phage	0.998	virulent	0.8036844	unknown	0	Staphylococcus saprophyticus	0.81	Predict
NOF012_scaffold37026_8	25267	phage	0.999	temperate	0.88961107	Peduoviridae	1	Pasteurella multocida	0.94	Predict
NOF012_scaffold68565_1	26569	phage	0.952	temperate	0.99178636	Zierdtviridae	0.18320961	Candidatus Hamiltonella defensa	0.97	Predict
NOF012_scaffold26305_22	19404	phage	0.999	temperate	0.9876869	unknown	0	Candidatus Hamiltonella defensa	0.96	Predict
NOF012_scaffold78081_1	15163	phage	0.999	virulent	0.99987465	unknown	0	Parabacteroides distasonis	0.75	Predict
NOF012_scaffold43523_1	18172	phage	0.529	virulent	0.6497936	unknown	0	Parabacteroides merdae	0.71	Predict
NOF012_scaffold41858_11	11193	phage	0.999	temperate	0.99985975	unknown	0	Acinetobacter baumannii	1	CRISPR
NOF012_scaffold28114_12	10891	phage	0.975	virulent	0.9972729	Ackermannviridae	1	Staphylococcus xylosus	0.94	Predict
NOF013_scaffold22999_4	17230	phage	0.578	virulent	0.99986124	unknown	0	Bacteroides oleiciplenus	1	CRISPR
NOF013_scaffold33526_1	23452	phage	0.999	virulent	0.99986696	unknown	0	Bacteroides fragilis	0.98	Predict
NOF013_scaffold15363_3	31613	phage	0.999	temperate	0.999713	unknown	0	unknown	0	-
NOF013_scaffold9359_1	13199	phage	0.998	virulent	0.9972341	unknown	0	Candidatus Hamiltonella defensa	0.78	Predict

NOF013_scaffold24304_2	10109	phage	0.986	temperate	0.99964106	unknown	0	unknown	0	-
NOF013_scaffold11906_1	20627	phage	0.999	temperate	0.95266074	Peduoviridae	1	Candidatus Hamiltonella defensa	1	CRISPR
NOF013_scaffold26437_1	13557	phage	0.995	virulent	0.9997869	unknown	0	Candidatus Hamiltonella defensa	0.89	Predict
NOF013_scaffold20067_2	10174	phage	0.996	virulent	0.99987316	unknown	0	unknown	0	-
NOF013_scaffold25505_1	15163	phage	0.996	virulent	0.908313	unknown	0	Candidatus Hamiltonella defensa	0.93	Predict
NOF013_scaffold34730_1	24277	phage	0.999	temperate	0.9998579	unknown	0	Candidatus Hamiltonella defensa	1	CRISPR
NOF013_scaffold6283_17	15626	phage	0.998	virulent	0.9998722	unknown	0	unknown	0	-
NOF013_scaffold30635_1	12865	phage	0.967	virulent	0.99987406	Kyanoviridae	1	unknown	0	-
NOF013_scaffold9607_2	13150	phage	0.998	temperate	0.99984837	Straboviridae	0.37907848	Bacteroides fragilis	0.92	Predict
NOF013_scaffold34528_1	13600	phage	0.986	virulent	0.99987173	unknown	0	Candidatus Hamiltonella defensa	0.74	Predict
NOF013_C612756_1	11648	phage	0.998	virulent	0.9998684	unknown	0	Bacteroides fragilis	1	CRISPR
NOF013_scaffold3348_11	12204	phage	0.999	temperate	0.99971104	unknown	0	Bacteroides fragilis	1	CRISPR
NOF013_scaffold24565_7	35494	phage	0.999	virulent	0.99986315	unknown	0	Staphylococcus saprophyticus	0.76	Predict
NOF013_scaffold34630_3	17425	phage	0.913	virulent	0.9998688	unknown	0	Staphylococcus saprophyticus	0.81	Predict
NOF013_scaffold24565_6	29084	phage	0.999	temperate	0.9997158	unknown	0	Candidatus Hamiltonella defensa	0.95	Predict
NOF013_scaffold26437_2	34897	phage	0.999	temperate	0.9998374	Straboviridae	0.62966955	Candidatus Hamiltonella defensa	0.98	Predict
NOF013_scaffold4750_1	19148	phage	0.814	virulent	0.99987036	Kyanoviridae	1	Bacteroides fragilis	0.78	Predict
NOF013_scaffold316_7	20553	phage	0.988	virulent	0.9998688	Straboviridae	0.69483334	Staphylococcus saprophyticus	0.97	Predict
NOF013_scaffold28493_2	12801	phage	0.891	virulent	0.99818397	Peduoviridae	1	unknown	0	-
NOF013_scaffold22999_3	46536	phage	0.997	virulent	0.99955404	unknown	0	Candidatus Pelagibacter ubique	1	CRISPR
NOF013_scaffold32172_3	10175	phage	0.997	temperate	0.9949657	unknown	0	unknown	0	-
NOF013_scaffold15363_2	10031	phage	0.999	virulent	0.99987084	unknown	0	unknown	0	-
NOF013_scaffold23510_11	42685	phage	1	virulent	0.99543303	Straboviridae	0.779485	Clostridium perfringens	1	CRISPR
NOF013_scaffold34382_1	10928	phage	0.996	temperate	0.99655056	unknown	0	unknown	0	-
NOF013_C613108_1	15087	phage	0.999	virulent	0.99987173	unknown	0	Staphylococcus saprophyticus	0.84	Predict
NOF013_scaffold1181_6	10752	phage	0.999	virulent	0.99987173	unknown	0	Acinetobacter baumannii	1	CRISPR
NOF013_scaffold131_1	12775	phage	0.99	virulent	0.99987173	Ackermannviridae	0.52144295	Bacteroides fragilis	0.77	Predict
NOF013_C612464_1	10076	phage	0.9	virulent	0.9998727	unknown	0	Candidatus Hamiltonella defensa	0.9	Predict
NOF013_scaffold33363_1	17320	phage	0.996	temperate	0.9763216	Straboviridae	0.66659385	Bacteroides fragilis	0.72	Predict
NOF013_scaffold33134_1	19848	phage	0.959	temperate	0.99985033	unknown	0	Candidatus Hamiltonella defensa	0.77	Predict

NOF013_scaffold1744_3	14440	phage	0.996	virulent	0.99982476	Herelleviridae	1	unknown	0	-
NOF013_scaffold32891_4	13745	phage	0.999	virulent	0.99829245	unknown	0	Candidatus Hamiltonella defensa	1	Predict
NOF013_scaffold29173_1	22780	phage	0.986	virulent	0.99987036	Ackermannviridae	0.39596215	Parabacteroides distasonis	0.78	Predict
NOF013_scaffold24443_1	12547	phage	0.962	virulent	0.9310343	unknown	0	Candidatus Hamiltonella defensa	0.95	Predict
NOF013_scaffold3979_5	15907	phage	0.856	temperate	0.93894786	Ackermannviridae	0.4456534	Bacteroides fragilis	0.94	Predict
NOF013_scaffold34324_3	10248	phage	0.851	virulent	0.9750617	Straboviridae	0.5316986	unknown	0	-
NOF013_scaffold2241_2	12009	phage	0.998	virulent	0.9998445	Straboviridae	0.34966817	Candidatus Hamiltonella defensa	0.84	Predict
NOF014_scaffold67337_2	12298	phage	0.996	temperate	0.9998588	unknown	0	Candidatus Hamiltonella defensa	0.8	Predict
NOF014_scaffold29077_2	10732	phage	0.999	temperate	0.99985975	unknown	0	Candidatus Hamiltonella defensa	0.89	Predict
NOF014_scaffold41735_1	14557	phage	0.988	temperate	0.9998588	unknown	0	Staphylococcus saprophyticus	0.77	Predict
NOF014_scaffold59979_2	11310	phage	0.997	virulent	0.9998665	Ackermannviridae	0.5257375	Candidatus Hamiltonella defensa	0.75	Predict
NOF014_scaffold72132_1	12141	phage	0.997	virulent	0.996157	unknown	0	Candidatus Hamiltonella defensa	0.91	Predict
NOF014_scaffold30730_5	16359	phage	0.998	virulent	0.99960977	Straboviridae	0.27278966	Bacteroides fragilis	0.88	Predict
NOF014_scaffold72006_1	13019	phage	0.999	temperate	0.99986035	unknown	0	unknown	0	-
NOF014_scaffold6730_5	17122	phage	0.996	temperate	0.99985975	unknown	0	Staphylococcus saprophyticus	0.84	Predict
NOF014_scaffold71626_3	14226	phage	0.996	virulent	0.99986124	unknown	0	Streptococcus pneumoniae	1	CRISPR
NOF014_scaffold46167_2	14636	phage	0.998	temperate	0.9988944	unknown	0	Candidatus Hamiltonella defensa	0.85	Predict
NOF014_scaffold35720_11	13143	phage	0.999	virulent	0.9998656	unknown	0	unknown	0	-
NOF014_scaffold4327_52	10432	phage	0.999	temperate	0.9998593	unknown	0	unknown	0	-
NOF014_scaffold40347_4	45747	phage	1	virulent	0.99981046	unknown	0	Cellulophaga baltica	0.87	Predict
NOF014_scaffold72227_1	12532	phage	0.999	virulent	0.99910355	Herelleviridae	1	Parabacteroides merdae	0.7	Predict
NOF014_scaffold27769_8	22448	phage	0.999	temperate	0.9998202	Casjensviridae	0.7678824	Candidatus Hamiltonella defensa	0.72	Predict
NOF014_scaffold10135_5	11205	phage	0.999	temperate	0.99986035	unknown	0	Streptococcus mutans	0.95	Predict
NOF014_scaffold66855_1	12786	phage	0.998	temperate	0.99986035	unknown	0	unknown	0	-
NOF014_scaffold72263_3	11952	phage	0.965	temperate	0.99985975	unknown	0	Candidatus Hamiltonella defensa	0.99	Predict
NOF014_scaffold43_10	10313	phage	0.997	virulent	0.99987316	Ackermannviridae	0.39596215	Candidatus Hamiltonella defensa	0.84	Predict
NOF014_scaffold26105_6	16018	phage	0.999	temperate	0.9998584	unknown	0	Candidatus Hamiltonella defensa	0.71	Predict
NOF014_scaffold20787_3	10876	phage	0.977	temperate	0.999541	unknown	0	unknown	0	-
NOF014_scaffold19962_3	11972	phage	0.998	virulent	0.9996329	Salasmaviridae	0.650711	unknown	0	-
NOF014_scaffold63476_2	18997	phage	0.998	temperate	0.99984694	unknown	0	Clostridioides difficile	0.89	Predict

NOF014_scaffold26105_25	17390	phage	0.972	temperate	0.99985975	unknown	0	unknown	0	-
NOF014_scaffold72216_1	12263	phage	0.999	virulent	0.9856357	Straboviridae	0.63752884	Candidatus Hamiltonella defensa	1	CRISPR
NOF014_scaffold10667_4	13105	phage	0.883	temperate	0.9998593	unknown	0	unknown	0	-
NOF014_scaffold52663_4	40643	phage	0.999	virulent	0.9998693	no_family_avaliable(NC_062779)	0.966	unknown	0	-
NOF014_scaffold71919_3	46147	phage	0.999	virulent	0.9998684	Straboviridae	0.294635	unknown	0	-
NOF014_scaffold20265_3	12364	phage	0.999	virulent	0.9998388	unknown	0	Veillonellaceae bacterium SB90	1	CRISPR
NOF014_scaffold5894_2	12884	phage	0.999	virulent	0.9994251	unknown	0	Croceibacter atlanticus	1	CRISPR
NOF014_scaffold49593_2	10298	phage	0.979	temperate	0.9998593	Straboviridae	0.585123	Candidatus Pelagibacter ubique	0.73	Predict
NOF014_scaffold71877_1	16373	phage	0.999	temperate	0.9996805	unknown	0	Clostridioides difficile	0.98	Predict
NOF014_scaffold72248_1	15089	phage	1	virulent	0.9998727	Salasmaviridae	1	Candidatus Pelagibacter ubique	1	CRISPR
NOF014_scaffold20787_1	18960	phage	0.98	virulent	0.99969965	Straboviridae	0.69483334	Candidatus Hamiltonella defensa	0.77	Predict
NOF014_scaffold66923_1	11892	phage	0.641	virulent	0.99987406	Straboviridae	0.29484153	Candidatus Hamiltonella defensa	0.71	Predict
NOF014_scaffold71552_2	13683	phage	0.999	virulent	0.9998693	Guelinviridae	0.26210135	Candidatus Hamiltonella defensa	1	CRISPR
NOF014_scaffold2390_2	11529	phage	0.994	temperate	0.93755734	unknown	0	unknown	0	-
NOF014_scaffold71919_2	43010	phage	0.999	virulent	0.9998684	Straboviridae	0.32358894	Prevotella intermedia	1	CRISPR
NOF014_scaffold66767_1	13008	phage	0.999	virulent	0.9998556	unknown	0	Candidatus Hamiltonella defensa	0.93	Predict
NOF014_scaffold63571_1	11995	phage	0.996	temperate	0.99926966	Casjensviridae	1	Candidatus Hamiltonella defensa	0.99	Predict
NOF014_scaffold65085_3	11190	phage	0.749	temperate	0.99984264	Peduoviridae	0.55076057	Candidatus Hamiltonella defensa	0.85	Predict
NOF014_scaffold22954_1	10914	phage	0.999	virulent	0.9998693	Casjensviridae	0.26592153	unknown	0	-
NOF014_scaffold72295_2	11554	phage	0.999	temperate	0.99985975	unknown	0	unknown	0	-
NOF014_scaffold71714_2	10162	phage	0.996	temperate	0.9998565	unknown	0	unknown	0	-
NOF014_scaffold64087_5	16926	phage	0.999	virulent	0.99983406	Straboviridae	0.7172016	Staphylococcus saprophyticus	0.9	Predict
NOF014_scaffold71856_1	23114	phage	0.998	virulent	0.9998684	Herelleviridae	1	unknown	0	-
NOF014_scaffold59979_3	13794	phage	0.995	virulent	0.9998722	Ackermannviridae	0.53408796	Candidatus Hamiltonella defensa	0.86	Predict
NOF014_scaffold52663_2	10102	phage	0.999	virulent	0.99987316	no_family_avaliable(NC_062779)	0.969	unknown	0	-
NOF014_scaffold54136_28	14550	phage	0.631	virulent	0.87657833	unknown	0	unknown	0	-
DLF001_scaffold25843_5	11910	phage	0.998	temperate	0.9555723	unknown	0	Bacteroides fragilis	1	CRISPR
DLF001_scaffold6028_2	26300	phage	0.995	temperate	0.9998365	Straboviridae	0.8344107	Bacillus halmapalus	1	CRISPR
DLF001_scaffold16878_1	12659	phage	0.999	virulent	0.9995294	Drexlerviridae	0.2793915	Bacteroides fragilis	0.87	Predict
DLF001_scaffold25777_2	22615	phage	1	virulent	0.9970052	unknown	0	Bacillus halmapalus	0.73	Predict

DLF001_scaffold4836_9	11384	phage	0.941	virulent	0.9996882	Straboviridae	0.6356147	Bacillus halmapalus	0.93	Predict
DLF001_scaffold16608_1	12836	phage	0.996	temperate	0.99985975	unknown	0	unknown	0	-
DLF001_scaffold18617_11	11225	phage	0.997	virulent	0.89395195	unknown	0	Bacillus halmapalus	1	CRISPR
DLF001_scaffold24376_1	18202	phage	0.623	virulent	0.9998636	Straboviridae	0.6949948	Bacillus halmapalus	0.98	Predict
DLF001_scaffold1570_1	10411	phage	0.998	virulent	0.99267465	unknown	0	unknown	0	-
DLF001_scaffold20939_1	21627	phage	1	temperate	0.95052063	Straboviridae	0.2730684	Bacteroides fragilis	1	CRISPR
DLF001_scaffold10899_1	28755	phage	0.999	temperate	0.9998417	Peduoviridae	0.5576402	Lactobacillus fermentum	1	CRISPR
DLF001_scaffold10872_1	12350	phage	0.998	temperate	0.99985975	unknown	0	Bacteroides fragilis	1	CRISPR
DLF001_scaffold25652_2	10281	phage	0.996	virulent	0.927816	unknown	0	Bacteroides fragilis	0.95	Predict
DLF001_scaffold21798_2	15908	phage	0.743	virulent	0.9171376	Straboviridae	0.47796255	Bacteroides fragilis	0.74	Predict
DLF001_C323280_1	13382	phage	0.978	virulent	0.9997661	Straboviridae	0.43901232	Bacteroides fragilis	0.8	Predict
DLF001_scaffold10220_3	18913	phage	0.999	virulent	0.999478	unknown	0	Bacillus halmapalus	0.95	Predict
DLF001_C323242_1	12992	phage	0.999	temperate	0.97153795	unknown	0	Colwellia psychrerythraea	0.85	Predict
DLF001_scaffold13_12	15646	phage	0.999	virulent	0.89925516	unknown	0	unknown	0	-
DLF001_scaffold25336_2	10820	phage	0.997	temperate	0.99984837	unknown	0	Parabacteroides distasonis	0.85	Predict
DLF001_scaffold46_6	13961	phage	0.997	virulent	0.9998736	unknown	0	Bacillus halmapalus	0.93	Predict
DLF001_scaffold80_8	14459	phage	0.998	virulent	0.98799294	Drexlerviridae	0.2793915	Colwellia psychrerythraea	0.74	Predict
DLF001_scaffold6028_1	35876	phage	0.979	virulent	0.99986607	Straboviridae	0.5964493	Bacteroides fragilis	1	CRISPR
DLF001_C323900_1	47021	phage	0.999	virulent	0.9998574	Drexlerviridae	1	Klebsiella pneumoniae	1	CRISPR
DLF002_scaffold6624_21	32626	phage	1	virulent	0.99985695	Straboviridae	0.75750005	Bacteroides fragilis	1	CRISPR
DLF002_scaffold7298_22	13280	phage	0.999	virulent	0.99889344	unknown	0	Bacteroides fragilis	1	CRISPR
DLF002_scaffold33093_1	12111	phage	0.999	virulent	0.87593126	unknown	0	Bacteroides vulgatus	1	CRISPR
DLF002_scaffold36414_1	25197	phage	1	temperate	0.99154705	unknown	0	Bacteroides fragilis	1	CRISPR
DLF002_scaffold85_3	15896	phage	0.856	temperate	0.93894786	Straboviridae	0.47796255	Bacteroides fragilis	0.85	Predict
DLF002_scaffold28742_1	10575	phage	0.999	virulent	0.9746414	unknown	0	unknown	0	-
DLF002_scaffold22416_1	13352	phage	0.811	virulent	0.9048829	Salasmaviridae	1	unknown	0	-
DLF002_scaffold32378_2	11294	phage	0.974	temperate	0.98519784	unknown	0	Bacteroides fragilis	0.73	Predict
DLF002_scaffold19600_3	12444	phage	0.999	temperate	0.96093565	Peduoviridae	1	Colwellia psychrerythraea	1	CRISPR
DLF002_scaffold24316_12	16580	phage	0.998	virulent	0.9998684	no_family_avaliable(NC_047916)	0.977	Colwellia psychrerythraea	1	CRISPR
DLF002_scaffold36081_2	13353	phage	0.979	temperate	0.8272758	unknown	0	unknown	0	-

DLF002_scaffold294_32	11985	phage	0.935	virulent	0.99138457	Drexlerviridae	0.2793915	Colwellia psychrerythraea	0.78	Predict
DLF002_scaffold7525_1	12508	phage	0.941	virulent	0.9996882	Straboviridae	0.6356147	Bacteroides fragilis	0.92	Predict
DLF002_scaffold25546_1	19765	phage	0.995	virulent	0.99987406	unknown	0	Bacillus halmapalus	0.95	Predict
DLF002_scaffold138_8	15451	phage	0.999	temperate	0.998298	unknown	0	Prevotella intermedia	1	CRISPR
DLF002_scaffold33974_1	22517	phage	0.999	temperate	0.9988901	Straboviridae	0.43901232	Bacteroides fragilis	0.89	Predict
DLF002_scaffold10343_2	10745	phage	0.537	virulent	0.984814	Straboviridae	0.50703365	Bacteroides fragilis	0.74	Predict
DLF002_scaffold36505_1	10101	phage	0.998	temperate	0.8868689	unknown	0	Colwellia psychrerythraea	0.81	Predict
DLF002_scaffold2466_1	12732	phage	0.998	temperate	0.9998593	Straboviridae	0.8105539	unknown	0	-
DLF002_scaffold36298_1	11637	phage	0.973	virulent	0.9870865	Straboviridae	0.5762059	Colwellia psychrerythraea	0.88	Predict
DLF002_scaffold6041_3	10460	phage	0.999	virulent	0.86695606	unknown	0	Bacteroides fragilis	0.88	Predict
DLF002_scaffold21111_2	10377	phage	0.94	temperate	0.9998556	unknown	0	unknown	0	-
DLF002_C488685_1	17035	phage	0.999	virulent	0.9998699	Herelleviridae	0.56141484	unknown	0	-
DLF003_scaffold21732_2	25748	phage	0.984	temperate	0.99985266	Ackermannviridae	1	Bacillus halmapalus	0.77	Predict
DLF003_scaffold29891_1	26874	phage	1	virulent	0.9998684	no_family_avaliable(NC_062773)	0.968	Parabacteroides distasonis	1	CRISPR
DLF003_scaffold23409_2	12897	phage	0.5	virulent	0.9998357	Drexlerviridae	0.2793915	Colwellia psychrerythraea	0.82	Predict
DLF003_scaffold50567_2	14815	phage	1	temperate	0.9867483	unknown	0	unknown	0	-
DLF003_scaffold44436_1	11734	phage	0.913	virulent	0.9998271	unknown	0	Faecalibacterium prausnitzii	1	CRISPR
DLF003_scaffold12321_2	76940	phage	0.997	virulent	0.99986696	Schitoviridae	1	Acinetobacter baumannii	1	CRISPR
DLF003_C717736_1	17944	phage	0.999	virulent	0.9998622	Salasmaviridae	0.94259506	Bacillus halmapalus	1	CRISPR
DLF003_scaffold137_1	13054	phage	0.921	virulent	0.99985605	unknown	0	Colwellia psychrerythraea	0.78	Predict
DLF003_scaffold3834_23	10944	phage	0.999	virulent	0.9997367	unknown	0	Colwellia psychrerythraea	0.73	Predict
DLF003_scaffold55800_1	10681	phage	0.994	virulent	0.99987084	Schitoviridae	0.24243324	Morganella morganii	0.87	Predict
DLF003_C717640_1	12548	phage	0.999	virulent	0.9998417	Salasmaviridae	0.936089	Ruminococcus sp. OM07-17	1	CRISPR
DLF003_scaffold23403_10	10622	phage	0.999	temperate	0.9940969	unknown	0	unknown	0	-
DLF003_scaffold56673_2	51717	phage	0.999	temperate	0.9994546	Casjensviridae	1	Bacillus halmapalus	0.88	Predict
DLF003_scaffold54149_1	10680	phage	0.999	virulent	0.9980126	Kyanoviridae	0.19216411	Candidatus Pelagibacter ubique	0.99	Predict
DLF003_scaffold48199_1	11393	phage	0.998	virulent	0.9619549	unknown	0	unknown	0	-
DLF003_scaffold43976_1	16931	phage	0.989	temperate	0.99931246	Salasmaviridae	0.4312181	Prevotella copri	1	CRISPR
DLF003_scaffold3834_7	14705	phage	0.927	temperate	0.9998593	unknown	0	unknown	0	-
DLF003_scaffold47905_1	11507	phage	0.986	virulent	0.9997144	Drexlerviridae	0.2793915	Colwellia psychrerythraea	0.7	Predict

DLF003_scaffold56478_1	13512	phage	0.99	virulent	0.99987084	unknown	0	unknown	0	-
DLF003_scaffold57190_2	11386	phage	1	virulent	0.9998722	Salasmaviridae	1	Bacillus halmapalus	0.83	Predict
DLF003_scaffold56159_4	23429	phage	0.998	virulent	0.99964076	unknown	0	Bacteroides fragilis	0.93	Predict
DLF003_scaffold4398_37	12455	phage	0.999	virulent	0.99987084	unknown	0	Flavobacterium psychrophilum	0.9	Predict
DLF003_scaffold29891_3	14298	phage	0.997	virulent	0.96373665	Casjensviridae	1	Colwellia psychrerythraea	0.72	Predict
DLF004_scaffold35797_1	12383	phage	0.997	virulent	0.9998645	unknown	0	Parabacteroides merdae	0.82	Predict
DLF004_scaffold36527_1	18475	phage	1	virulent	0.9998722	unknown	0	unknown	0	-
DLF004_C548546_1	26794	phage	1	virulent	0.99986696	unknown	0	Bacillus halmapalus	1	CRISPR
DLF004_scaffold18383_6	31288	phage	0.999	temperate	0.99978584	Herelleviridae	1	Bacillus halmapalus	1	CRISPR
DLF004_C548576_1	37607	phage	0.999	temperate	0.9998403	Straboviridae	0.725355	Bacillus halmapalus	1	CRISPR
DLF004_scaffold23942_9	12211	phage	0.993	temperate	0.9998593	unknown	0	unknown	0	-
DLF004_scaffold10854_4	11394	phage	0.999	virulent	0.82519925	unknown	0	Enterococcus faecium	0.7	Predict
DLF004_scaffold28880_1	12883	phage	1	virulent	0.9791901	unknown	0	Bacillus halmapalus	0.78	Predict
DLF004_scaffold25533_1	18229	phage	0.832	temperate	0.98823017	Schitoviridae	0.24243324	Colwellia psychrerythraea	0.73	Predict
DLF004_scaffold36320_8	22275	phage	0.995	virulent	0.99987125	unknown	0	Clostridioides difficile	0.98	Predict
DLF004_C548200_1	13410	phage	0.902	temperate	0.9998593	Salasmaviridae	0.4312181	Prevotella copri	1	CRISPR
DLF004_scaffold2535_5	29552	phage	0.996	temperate	0.9977433	Drexlerviridae	0.2793915	Colwellia psychrerythraea	0.73	Predict
DLF004_scaffold32830_2	17279	phage	0.997	virulent	0.99984604	Drexlerviridae	0.2793915	Colwellia psychrerythraea	0.77	Predict
DLF004_scaffold19957_5	10724	phage	0.99	temperate	0.99840224	unknown	0	unknown	0	-
DLF004_scaffold30602_2	11421	phage	0.891	virulent	0.99818397	unknown	0	unknown	0	-
DLF004_scaffold27435_2	17643	phage	0.949	virulent	0.99987173	Drexlerviridae	0.2793915	Colwellia psychrerythraea	0.9	Predict
DLF004_scaffold930_8	20485	phage	0.598	virulent	0.9998627	unknown	0	Bacillus halmapalus	0.71	Predict
DLF004_scaffold8102_2	40681	phage	1	virulent	0.9998465	Straboviridae	0.80707747	Bacillus halmapalus	1	CRISPR
DLF004_scaffold32187_1	10597	phage	0.603	temperate	0.9997492	unknown	0	unknown	0	-
DLF004_scaffold23115_1	11105	phage	0.987	temperate	0.99985975	unknown	0	unknown	0	-
DLF004_scaffold6537_1	16331	phage	0.987	virulent	0.9998551	unknown	0	Bacillus halmapalus	0.77	Predict
DLF004_scaffold15396_3	10225	phage	0.999	virulent	0.9998348	unknown	0	Bacillus halmapalus	0.99	Predict
DLF004_scaffold7918_11	10314	phage	0.998	virulent	0.910048	unknown	0	unknown	0	-
DLF004_scaffold7020_1	11412	phage	0.995	virulent	0.99987036	unknown	0	unknown	0	-
DLF004_scaffold1974_5	11005	phage	0.997	virulent	0.9998727	unknown	0	Bacteroides fragilis	0.73	Predict

DLF004_scaffold24525_4	19559	phage	0.904	virulent	0.98113614	Straboviridae	0.6356147	Bacillus halmapalus	0.76	Predict
DLF004_scaffold15823_6	34853	phage	0.996	virulent	0.9998656	unknown	0	Bacillus halmapalus	0.97	Predict
DLF004_scaffold26106_5	17411	phage	0.96	virulent	0.91473454	Casjensviridae	0.4192874	Candidatus Hamiltonella defensa	0.71	Predict
DLF004_scaffold22703_8	10480	phage	0.905	temperate	0.9397936	Peduoviridae	0.44511104	Colwellia psychrerythraea	0.71	Predict
DLF004_scaffold21240_2	15707	phage	0.884	virulent	0.99987173	Straboviridae	0.6949948	Bacillus halmapalus	0.87	Predict
DLF004_scaffold125_29	11145	phage	0.998	temperate	0.5089623	unknown	0	Bacteroides fragilis	0.85	Predict
DLF005_scaffold31168_1	16161	phage	0.999	virulent	0.99985975	unknown	0	Colwellia psychrerythraea	0.75	Predict
DLF005_scaffold55052_1	14034	phage	0.999	temperate	0.9934008	unknown	0	Candidatus Pelagibacter ubique	1	CRISPR
DLF005_scaffold56707_1	12863	phage	0.998	temperate	0.99986035	unknown	0	unknown	0	-
DLF005_scaffold31945_4	11999	phage	0.999	temperate	0.99986035	unknown	0	Ruminococcus sp. AM43-6	1	CRISPR
DLF005_scaffold48546_1	11534	phage	0.991	temperate	0.82824755	unknown	0	Bacillus halmapalus	0.81	Predict
DLF005_scaffold55770_1	58620	phage	0.999	temperate	0.98381054	Straboviridae	0.75948435	Bacillus halmapalus	1	CRISPR
DLF005_scaffold39735_4	13832	phage	0.961	temperate	0.9998579	unknown	0	unknown	0	-
DLF005_scaffold56569_4	39195	phage	0.951	virulent	0.9998665	Vilmaviridae	1	Cellulophaga baltica	1	CRISPR
DLF005_scaffold27529_1	10691	phage	0.721	temperate	0.9998265	unknown	0	unknown	0	-
DLF005_scaffold51335_1	19434	phage	0.993	virulent	0.60650957	unknown	0	Bacillus halmapalus	0.8	Predict
DLF005_scaffold4693_36	10048	phage	0.871	virulent	0.99987316	Straboviridae	0.5399531	Colwellia psychrerythraea	0.72	Predict
DLF005_scaffold50375_1	10395	phage	0.99	temperate	0.9839584	unknown	0	unknown	0	-
DLF005_scaffold56163_1	60837	phage	0.999	virulent	0.9998417	unknown	0	Candidatus Pelagibacter ubique	1	CRISPR
DLF005_scaffold40395_1	10572	phage	0.999	virulent	0.99987406	unknown	0	unknown	0	-
DLF005_scaffold1570_16	18727	phage	0.99	temperate	0.99968576	unknown	0	Bacteroides fragilis	0.88	Predict
DLF005_scaffold13131_1	11502	phage	0.999	temperate	0.9998584	unknown	0	Bacillus halmapalus	0.82	Predict
DLF005_scaffold528_11	14538	phage	0.999	virulent	0.9998479	unknown	0	unknown	0	-
DLF005_scaffold39187_4	21423	phage	0.999	temperate	0.9998588	unknown	0	Candidatus Hamiltonella defensa	1	CRISPR
DLF005_scaffold25785_13	14283	phage	0.995	temperate	0.99986035	unknown	0	Megamonas funiformis	1	CRISPR
DLF005_scaffold56894_1	13304	phage	0.919	virulent	0.98988324	unknown	0	Parabacteroides merdae	0.91	Predict
DLF005_scaffold54804_6	18712	phage	0.997	temperate	0.5799638	unknown	0	unknown	0	-
DLF005_scaffold2989_20	12385	phage	0.996	temperate	0.94335675	unknown	0	unknown	0	-
DLF005_scaffold2505_1	26512	phage	1	virulent	0.62476856	unknown	0	Parabacteroides distasonis	1	CRISPR
DLF005_scaffold47023_3	53553	phage	0.943	temperate	0.9888392	Straboviridae	0.3171625	Colwellia psychrerythraea	1	CRISPR

DLF005_scaffold56967_1	40643	phage	0.999	temperate	0.7587744	Straboviridae	0.3365034	Bacillus halmapalus	1	Predict
DLF005_scaffold13334_3	48829	phage	0.999	virulent	0.7953001	Straboviridae	0.35845006	Streptococcus pneumoniae	1	CRISPR
DLF005_C847538_1	10992	phage	0.997	temperate	0.9998579	Straboviridae	0.72624004	unknown	0	-
DLF005_scaffold56817_1	23003	phage	0.999	temperate	0.9665699	unknown	0	Bacillus halmapalus	1	CRISPR
DLF005_scaffold56924_2	16152	phage	0.575	virulent	0.95535946	unknown	0	Bacillus halmapalus	0.97	Predict
DLF005_scaffold39187_3	15757	phage	0.999	temperate	0.9998579	unknown	0	Megamonas funiformis	1	CRISPR
DLF005_scaffold6688_21	11319	phage	0.655	temperate	0.608167	Herelleviridae	1	unknown	0	-
DLF005_scaffold18199_1	11135	phage	0.999	virulent	0.9998722	Straboviridae	0.80707747	Bacillus halmapalus	0.79	Predict
DLF005_C847770_1	13569	phage	0.944	temperate	0.99985033	unknown	0	unknown	0	-
DLF005_scaffold3960_1	21248	phage	0.919	virulent	0.9998699	Straboviridae	0.72045255	Bacillus halmapalus	0.79	Predict
DLF005_scaffold280_2	20033	phage	0.987	virulent	0.99987316	Straboviridae	0.6833262	unknown	0	-
DLF005_scaffold32479_1	15112	phage	0.999	virulent	0.9998556	unknown	0	Flavobacterium columnare	0.84	Predict
DLF005_scaffold41843_2	11161	phage	0.755	virulent	0.94455034	Straboviridae	0.4287239	Bacteroides fragilis	0.8	Predict
DLF005_scaffold33045_1	11477	phage	0.999	temperate	0.9997535	unknown	0	Clostridioides difficile	0.89	Predict
DLF005_scaffold4093_2	73009	phage	1	temperate	0.9983544	Straboviridae	0.6292496	Flavobacterium columnare	1	CRISPR
DLF005_scaffold9847_1	12426	phage	0.991	temperate	0.9998593	unknown	0	Microcystis aeruginosa	0.82	Predict
DLF005_scaffold31168_2	14530	phage	0.664	temperate	0.99985975	unknown	0	Acinetobacter baumannii	1	CRISPR
DLF005_scaffold53782_1	23087	phage	0.999	virulent	0.9998699	unknown	0	Flavobacterium columnare	1	CRISPR
DLF005_scaffold56569_2	20540	phage	0.998	virulent	0.99987036	Herelleviridae	1	Bacillus subtilis	1	CRISPR
DLF005_scaffold23342_9	27476	phage	0.999	temperate	0.97907996	Casjensviridae	1	Staphylococcus hominis	1	CRISPR
DLF006_scaffold52_4	17677	phage	0.999	virulent	0.9998369	unknown	0	Clostridium botulinum	0.76	Predict
DLF006_C667664_1	12710	phage	0.829	virulent	0.9998593	Straboviridae	0.5113246	Staphylococcus hominis	0.79	Predict
DLF006_scaffold9049_10	12655	phage	0.998	temperate	0.9991318	unknown	0	Bacillus halmapalus	0.76	Predict
DLF006_scaffold108_5	12350	phage	0.947	virulent	0.9949243	Schitoviridae	0.23503077	Bacillus halmapalus	0.8	Predict
DLF006_scaffold42885_3	34282	phage	0.996	virulent	0.9998688	Herelleviridae	1	unknown	0	-
DLF006_scaffold46681_1	48777	phage	0.999	temperate	0.9998417	Straboviridae	0.725355	Bacillus halmapalus	1	CRISPR
DLF006_scaffold39883_1	12246	phage	0.999	temperate	0.9998593	unknown	0	Bacillus halmapalus	0.96	Predict
DLF006_scaffold32044_2	15847	phage	0.856	temperate	0.93894786	Straboviridae	0.47796255	Bacteroides fragilis	0.99	Predict
DLF006_scaffold8740_3	18182	phage	1	temperate	0.9998593	no_family_avaliable(NC_062582)	0.98	Bacillus halmapalus	0.76	Predict
DLF006_C667862_1	29324	phage	1	temperate	0.99962103	unknown	0	Bacteroides fragilis	1	CRISPR

DLF006_scaffold19189_3	17371	phage	0.999	temperate	0.9997535	Peduoviridae	1	Colwellia psychrerythraea	1 CRISPR
DLF006_scaffold474_4	37752	phage	0.989	virulent	0.9998588	Herelleviridae	1	Colwellia psychrerythraea	1 CRISPR
DLF006_scaffold33584_4	14808	phage	0.944	temperate	0.99836224	unknown	0	Bacteroides fragilis	0.86 Predict
DLF006_scaffold34156_4	10545	phage	0.936	virulent	0.9983713	unknown	0	Colwellia psychrerythraea	0.84 Predict
DLF006_scaffold9056_20	15475	phage	0.999	temperate	0.99977875	unknown	0	unknown	0 -
DLF006_scaffold9056_18	34632	phage	0.999	virulent	0.99741316	Peduoviridae	1	Colwellia psychrerythraea	0.83 Predict
DLF006_scaffold10687_2	14220	phage	0.994	temperate	0.93755734	unknown	0	unknown	0 -
DLF006_scaffold11422_2	11529	phage	0.999	virulent	0.9998343	Ackermannviridae	0.38388076	Candidatus Hamiltonella defensa	0.96 Predict
DLF006_scaffold19189_4	11326	phage	0.866	temperate	0.99985266	unknown	0	Colwellia psychrerythraea	0.71 Predict
DLF006_scaffold9845_3	18769	phage	0.997	virulent	0.9998636	Mesyanzhinovviridae	1	Pseudomonas aeruginosa	0.87 Predict
DLF006_scaffold41298_2	14028	phage	0.88	virulent	0.99987465	unknown	0	unknown	0 -
DLF006_scaffold7466_5	17484	phage	0.849	temperate	0.9998403	Peduoviridae	0.778437	Colwellia psychrerythraea	0.83 Predict
DLF006_scaffold15736_1	14934	phage	0.998	temperate	0.9998403	unknown	0	unknown	0 -
DLF006_scaffold22006_9	12470	phage	0.994	virulent	0.9992909	unknown	0	Micromonospora chaiyaphumensis	1 CRISPR
DLF006_scaffold25840_10	38763	phage	0.999	temperate	0.62177837	Peduoviridae	0.8291513	Colwellia psychrerythraea	0.73 Predict
DLF006_scaffold36051_1	39097	phage	0.999	temperate	0.9997267	Drexlerviridae	1	Pectobacterium carotovorum	1 CRISPR
DLF006_scaffold52_5	22296	phage	0.998	virulent	0.99986744	Herelleviridae	1	unknown	0 -
DLF006_scaffold9845_4	15505	phage	0.999	virulent	0.9997515	unknown	0	Colwellia psychrerythraea	0.73 Predict
DLF006_scaffold36051_2	12822	phage	0.998	temperate	0.99983126	Drexlerviridae	1	Colwellia psychrerythraea	0.83 Predict
DLF006_scaffold34515_1	14541	phage	0.995	virulent	0.99978536	Drexlerviridae	0.2793915	Colwellia psychrerythraea	0.86 Predict
DLF006_scaffold5280_1	24141	phage	0.999	temperate	0.9668182	unknown	0	Parabacteroides merdae	1 CRISPR
DLF006_scaffold46760_1	10561	phage	0.861	virulent	0.99987316	unknown	0	Bacteroides fragilis	0.9 Predict
DLF006_scaffold46848_1	15009	phage	0.999	virulent	0.99987465	unknown	0	Parabacteroides distasonis	0.91 Predict
DLF006_scaffold14168_2	12855	phage	0.997	virulent	0.99987036	unknown	0	unknown	0 -
DLF006_scaffold8740_4	37402	phage	1	temperate	0.9998022	unknown	0	Colwellia psychrerythraea	0.97 Predict
DLF006_scaffold46677_6	12826	phage	1	temperate	0.9903336	unknown	0	unknown	0 -
DLF006_scaffold27662_1	20742	phage	1	temperate	0.99985975	unknown	0	Bacteroides fragilis	0.97 Predict
DLF006_scaffold52_6	19187	phage	0.999	virulent	0.99986607	Herelleviridae	1	unknown	0 -
DLF006_scaffold46851_2	25658	phage	1	virulent	0.99985975	unknown	0	Bacillus halmapalus	0.86 Predict
DLF006_scaffold92_1	12622	phage	0.998	virulent	0.9996403	unknown	0	Colwellia psychrerythraea	0.83 Predict

DLF007_scaffold15529_4	13122	phage	0.989	virulent	0.9998608	unknown	0	Bacillus halmapalus	0.77	Predict
DLF007_scaffold14424_2	11839	phage	0.999	temperate	0.9998593	unknown	0	unknown	0	-
DLF007_scaffold18927_1	18734	phage	0.979	temperate	0.99985975	Straboviridae	0.3058537	Candidatus Hamiltonella defensa	0.99	Predict
DLF007_C306149_1	12711	phage	0.997	virulent	0.9996841	unknown	0	Candidatus Hamiltonella defensa	0.99	Predict
DLF007_C306343_1	15041	phage	0.996	virulent	0.98861355	Straboviridae	0.47071996	Bacillus halmapalus	0.71	Predict
DLF007_scaffold1532_23	15464	phage	0.948	virulent	0.9998688	Kyanoviridae	1	Bacillus halmapalus	1	CRISPR
DLF007_scaffold20333_2	13461	phage	0.994	temperate	0.99985975	unknown	0	Bacteroides fragilis	0.84	Predict
DLF007_scaffold4179_16	12450	phage	0.998	temperate	0.99985975	unknown	0	Bacillus halmapalus	1	CRISPR
DLF007_scaffold15763_5	10601	phage	0.999	virulent	0.9998722	Straboviridae	0.6984924	Bacillus halmapalus	1	CRISPR
DLF007_scaffold2664_1	15227	phage	0.996	virulent	0.908313	unknown	0	Colwellia psychrerythraea	0.87	Predict
DLF007_scaffold20109_2	15908	phage	0.856	temperate	0.93894786	Straboviridae	0.47796255	Bacteroides fragilis	0.92	Predict
DLF007_scaffold18401_2	32443	phage	1	temperate	0.99909663	Straboviridae	0.72239286	Bacteroides fragilis	1	CRISPR
DLF007_scaffold12277_1	30053	phage	1	virulent	0.9998684	Peduoviridae	0.5576402	Lactobacillus fermentum	1	CRISPR
DLF007_scaffold15181_7	15053	phage	0.985	virulent	0.9998517	unknown	0	Bacteroides fragilis	1	CRISPR
DLF008_scaffold7461_4	10663	phage	0.949	virulent	0.99986124	unknown	0	Bacteroides fragilis	0.91	Predict
DLF008_scaffold17152_1	10656	phage	0.998	temperate	0.9349437	unknown	0	Bacillus halmapalus	0.81	Predict
DLF008_scaffold1091_2	12231	phage	0.998	virulent	0.9998722	unknown	0	Bacillus halmapalus	0.93	Predict
DLF008_scaffold11168_2	14068	phage	0.692	virulent	0.99987406	unknown	0	unknown	0	-
DLF008_scaffold17625_2	15902	phage	0.856	temperate	0.93894786	Straboviridae	0.47796255	Bacteroides fragilis	0.92	Predict
DLF008_scaffold17607_1	10431	phage	0.992	virulent	0.99987125	unknown	0	[Clostridium] clostridioforme	1	CRISPR
DLF008_scaffold17497_1	18312	phage	0.998	temperate	0.99983215	Straboviridae	0.6236477	Colwellia psychrerythraea	1	CRISPR
DLF008_scaffold14160_4	36007	phage	0.997	temperate	0.75293964	unknown	0	Mannheimia haemolytica	0.84	Predict
DLF008_scaffold22_1	10730	phage	1	temperate	0.99982077	unknown	0	unknown	0	-
DLF008_scaffold10341_2	13870	phage	0.999	temperate	0.9998574	unknown	0	Colwellia psychrerythraea	1	CRISPR
DLF008_scaffold16449_2	20225	phage	0.843	virulent	0.9998688	Straboviridae	0.590045	Parabacteroides merdae	0.73	Predict
DLF008_scaffold1410_11	10827	phage	0.999	virulent	0.99986315	unknown	0	unknown	0	-
DLF008_scaffold5415_1	32388	phage	0.998	temperate	0.9997273	unknown	0	Bacillus halmapalus	1	CRISPR
DLF008_scaffold3962_5	15043	phage	0.982	temperate	0.9998465	Peduoviridae	1	Colwellia psychrerythraea	0.74	Predict
DLF008_scaffold10487_1	19192	phage	1	temperate	0.9998574	unknown	0	Colwellia psychrerythraea	1	CRISPR
DLF008_scaffold2326_8	10995	phage	0.944	virulent	0.99986315	unknown	0	unknown	0	-

DLF008_scaffold17374_1	13716	phage	0.999	temperate	0.9998508	unknown	0	unknown	0	-
DLF008_C335467_1	11047	phage	1	temperate	0.99985975	unknown	0	Bacillus halmapalus	1	CRISPR
DLF008_scaffold710_21	11872	phage	0.999	temperate	0.94271755	unknown	0	Bacillus halmapalus	0.91	Predict
DLF008_scaffold17349_1	30650	phage	0.999	temperate	0.62144816	unknown	0	Candidatus Hamiltonella defensa	1	CRISPR
DLF008_scaffold8096_6	10824	phage	0.987	temperate	0.99985975	no_family_avaliable(NC_049955)	0.977	Colwellia psychrerythraea	1	CRISPR
DLF008_scaffold7166_1	20831	phage	0.996	temperate	0.99985313	Straboviridae	0.5762059	Colwellia psychrerythraea	0.84	Predict
DLF008_scaffold9028_1	10819	phage	0.994	temperate	0.9990296	Peduoviridae	1	Bacteroides fragilis	0.79	Predict
DLF008_scaffold8047_2	11794	phage	0.997	virulent	0.6666023	Straboviridae	0.55352753	Bacillus halmapalus	1	Predict
DLF008_scaffold1029_1	11424	phage	0.99	virulent	0.99987084	unknown	0	unknown	0	-
DLF008_scaffold3962_1	52105	phage	0.923	temperate	0.9632674	Casjensviridae	1	Bacillus halmapalus	0.88	Predict
DLF008_scaffold222_2	11814	phage	0.999	virulent	0.8016601	Herelleviridae	1	unknown	0	-
DLF008_scaffold12889_2	13425	phage	0.986	virulent	0.99987173	unknown	0	Paenibacillus larvae	0.81	Predict
DLF008_scaffold2953_7	12177	phage	0.716	temperate	0.9911663	unknown	0	Colwellia psychrerythraea	0.9	Predict
DLF008_scaffold17084_1	10068	phage	0.997	virulent	0.99987316	Straboviridae	0.5381315	Bacillus halmapalus	0.96	Predict
DLF008_scaffold14357_3	68800	phage	0.998	temperate	0.9998145	Straboviridae	0.31066608	Candidatus Hamiltonella defensa	1	CRISPR
DLF008_scaffold192_4	10011	phage	0.998	temperate	0.99976826	unknown	0	unknown	0	-
DLF008_scaffold3328_5	10827	phage	0.997	virulent	0.99987406	Straboviridae	0.41379508	Staphylococcus hominis	0.78	Predict
DLF008_scaffold17350_1	25603	phage	0.999	virulent	0.9998656	Straboviridae	0.65565205	Bacillus halmapalus	1	CRISPR
DLF009_scaffold34424_1	11353	phage	0.996	virulent	0.9998688	Straboviridae	0.8369376	Bacillus halmapalus	0.72	Predict
DLF009_scaffold1880_30	10382	phage	0.981	virulent	0.99987406	unknown	0	Candidatus Hamiltonella defensa	0.94	Predict
DLF009_scaffold36221_2	19802	phage	0.999	temperate	0.93656045	unknown	0	Bacteroides fragilis	0.71	Predict
DLF009_C428906_1	22077	phage	0.999	virulent	0.998085	Drexlerviridae	1	Citrobacter freundii	0.79	Predict
DLF009_scaffold30115_1	38314	phage	0.999	virulent	0.99986607	unknown	0	Cellulophaga baltica	0.86	Predict
DLF009_scaffold789_3	16147	phage	0.622	temperate	0.99978215	unknown	0	Colwellia psychrerythraea	1	CRISPR
DLF009_scaffold37442_2	16502	phage	1	temperate	0.99985975	unknown	0	Clostridioides difficile	1	CRISPR
DLF009_scaffold36221_1	12639	phage	0.999	temperate	0.9529658	unknown	0	Bacteroides fragilis	0.77	Predict
DLF009_scaffold7798_1	13815	phage	0.999	virulent	0.99987084	Salasmaviridae	1	Bacillus halmapalus	1	CRISPR
DLF009_scaffold37363_2	11097	phage	0.999	temperate	0.9998593	unknown	0	Colwellia psychrerythraea	0.95	Predict
DLF009_scaffold11451_17	13333	phage	0.837	virulent	0.99987125	unknown	0	Colwellia psychrerythraea	0.93	Predict
DLF009_scaffold21876_2	11879	phage	0.999	temperate	0.9998374	unknown	0	Bacillus halmapalus	1	CRISPR

DLF009_scaffold37177_1	10319	phage	0.699	temperate	0.99985975	Peduoviridae	1	Colwellia psychrerythraea	1	CRISPR
DLF009_scaffold37452_1	19460	phage	0.993	temperate	0.99986035	Peduoviridae	1	Colwellia psychrerythraea	1	CRISPR
DLF009_scaffold794_2	10296	phage	0.998	virulent	0.99985266	Zierdtviridae	1	Bifidobacterium longum	1	CRISPR
DLF009_scaffold30115_3	20519	phage	0.999	virulent	0.999703	Schitoviridae	0.14546394	Cellulophaga baltica	0.91	Predict
DLF009_scaffold25763_1	11512	phage	0.999	temperate	0.96952647	no_family_avaliable(NC_025434)	0.956	Pectobacterium carotovorum	1	CRISPR
DLF009_scaffold13354_1	13049	phage	0.999	temperate	0.9982825	Casjensviridae	1	Colwellia psychrerythraea	0.82	Predict
DLF009_scaffold13766_4	12624	phage	0.999	virulent	0.9985362	unknown	0	Bacteroides fragilis	1	CRISPR
DLF009_scaffold37443_4	10560	phage	0.922	temperate	0.99985605	unknown	0	Bacteroides fragilis	0.79	Predict
DLF009_scaffold37498_1	13403	phage	0.998	temperate	0.9072678	no_family_avaliable(NC_025434)	0.966	Colwellia psychrerythraea	1	CRISPR
DLF009_scaffold37448_2	67298	phage	0.999	virulent	0.9998536	Herelleviridae	1	Bacteroides vulgatus	1	CRISPR
DLF009_scaffold37438_1	53971	phage	0.999	temperate	0.999796	no_family_avaliable(NC_019721)	0.98	Enterobacter cloacae	1	CRISPR
DLF009_scaffold30115_4	18456	phage	0.998	virulent	0.99987173	unknown	0	unknown	0	-
DLF009_scaffold37466_1	15357	phage	1	virulent	0.99987125	Salasmaviridae	1	Bacillus halmapalus	1	CRISPR
DLF009_scaffold37020_3	16450	phage	0.999	virulent	0.9998688	unknown	0	Colwellia psychrerythraea	0.85	Predict
DLF009_scaffold3043_4	16144	phage	0.888	temperate	0.99985975	unknown	0	Colwellia psychrerythraea	1	CRISPR
DLF009_C428576_1	13736	phage	0.999	temperate	0.8476171	no_family_avaliable(NC_041935)	0.962	Colwellia psychrerythraea	1	CRISPR
DLF009_scaffold17926_1	35015	phage	0.999	temperate	0.9998288	Kyanoviridae	1	Bacillus halmapalus	0.84	Predict
DLF009_scaffold37386_2	50793	phage	0.999	temperate	0.9998413	Straboviridae	0.19397318	Bacillus halmapalus	0.79	Predict
DLF009_scaffold11949_1	20631	phage	0.999	temperate	0.99985695	Peduoviridae	1	Colwellia psychrerythraea	1	CRISPR
DLF009_scaffold19674_5	11166	phage	0.749	temperate	0.99984264	Peduoviridae	0.778437	Colwellia psychrerythraea	0.72	Predict
DLF009_scaffold6204_7	23240	phage	0.999	temperate	0.6889878	Peduoviridae	0.5576402	Colwellia psychrerythraea	1	CRISPR
DLF009_scaffold37284_1	29973	phage	1	temperate	0.9998574	unknown	0	Candidatus Hamiltonella defensa	1	CRISPR
DLF009_scaffold37363_3	13472	phage	0.992	temperate	0.9910528	unknown	0	Colwellia psychrerythraea	0.76	Predict
DLF009_scaffold37307_2	13509	phage	0.613	temperate	0.999854	no_family_avaliable(NC_050152)	0.956	Colwellia psychrerythraea	1	CRISPR
DLF009_scaffold2837_2	10578	phage	0.999	temperate	0.8991208	unknown	0	Bacillus halmapalus	1	Predict
DLF009_scaffold61_24	10696	phage	0.909	temperate	0.9998551	unknown	0	Rhodococcus rhodochrous	0.71	Predict
DLF009_scaffold7570_14	11187	phage	0.988	temperate	0.9996787	unknown	0	unknown	0	-
DLF009_scaffold3557_6	11655	phage	0.988	temperate	0.9995462	unknown	0	Colwellia psychrerythraea	0.77	Predict
DLF009_scaffold37386_1	12799	phage	0.998	temperate	0.99985313	unknown	0	Staphylococcus epidermidis	0.76	Predict
DLF009_scaffold30115_5	19265	phage	0.999	virulent	0.9998693	unknown	0	Cellulophaga baltica	0.86	Predict

DLF010_scaffold2375_6	14454	phage	0.965	temperate	0.99986035	unknown	0	unknown	0	-
DLF010_scaffold24026_11	12430	phage	0.999	virulent	0.9926443	unknown	0	unknown	0	-
DLF010_scaffold35043_4	18197	phage	0.997	virulent	0.9645309	unknown	0	Colwellia psychrerythraea	0.79	Predict
DLF010_scaffold55870_1	12328	phage	0.992	virulent	0.8873964	Straboviridae	0.5762059	Bacillus halmapalus	0.92	Predict
DLF010_scaffold43436_5	13120	phage	1	temperate	0.99854225	Peduoviridae	1	Lachnospiraceae bacterium	1	CRISPR
DLF010_scaffold363_30	13003	phage	0.689	virulent	0.99986404	unknown	0	Weissella cibaria	0.88	Predict
DLF010_scaffold36638_6	13726	phage	0.937	virulent	0.99987084	Kyanoviridae	1	Edwardsiella ictaluri	0.99	Predict
DLF010_scaffold6815_13	11532	phage	0.997	virulent	0.9998131	unknown	0	unknown	0	-
DLF010_scaffold13546_16	21370	phage	0.995	temperate	0.9998574	unknown	0	Colwellia psychrerythraea	0.93	Predict
DLF010_scaffold35043_3	14369	phage	1	virulent	0.8880456	unknown	0	Colwellia psychrerythraea	0.73	Predict
DLF010_scaffold46634_1	12687	phage	0.992	virulent	0.8683114	Kyanoviridae	1	Clostridioides difficile	0.99	Predict
DLF010_scaffold13248_1	10299	phage	0.985	temperate	0.8345778	unknown	0	unknown	0	-
DLF010_scaffold49177_4	10364	phage	0.999	temperate	0.9838526	no_family_avaliable(NC_047913)	0.991	Colwellia psychrerythraea	1	CRISPR
DLF010_scaffold4302_1	12742	phage	0.999	virulent	0.9667475	unknown	0	Streptomyces lividans	0.9	Predict
DLF010_scaffold363_33	22896	phage	0.533	virulent	0.9989573	unknown	0	unknown	0	-
DLF010_scaffold33244_2	15767	phage	0.999	temperate	0.9998556	unknown	0	unknown	0	-
DLF010_scaffold26724_1	10550	phage	0.964	temperate	0.9998584	unknown	0	unknown	0	-
DLF010_scaffold25454_7	13120	phage	0.997	virulent	0.99983835	Peduoviridae	0.61460483	Candidatus Hamiltonella defensa	0.96	Predict
DLF010_scaffold51522_2	13515	phage	0.585	virulent	0.99987084	Drexlerviridae	0.2793915	Colwellia psychrerythraea	0.93	Predict
DLF010_scaffold3276_2	11708	phage	0.914	temperate	0.99985975	Straboviridae	0.7489975	unknown	0	-
DLF010_scaffold8211_1	10908	phage	0.805	virulent	0.9660578	unknown	0	Colwellia psychrerythraea	0.82	Predict
DLF010_scaffold45015_1	12720	phage	0.999	temperate	0.99985975	unknown	0	unknown	0	-
DLF010_scaffold51387_5	10368	phage	0.999	temperate	0.99985975	Straboviridae	0.8105539	unknown	0	-
DLF010_scaffold55918_1	10844	phage	0.999	virulent	0.99953496	unknown	0	Prevotella copri	1	CRISPR
DLF010_scaffold5445_2	14027	phage	0.988	virulent	0.9998226	Straboviridae	0.20257144	Bacillus halmapalus	0.76	Predict
DLF010_scaffold18414_39	13611	phage	0.987	temperate	0.9200033	Ackermannviridae	1	Clostridium botulinum	0.79	Predict
DLF010_scaffold27012_2	16397	phage	0.812	virulent	0.99979496	Straboviridae	0.55340713	Aeromonas salmonicida	0.93	Predict
DLF010_scaffold44216_5	12078	phage	0.539	virulent	0.99987125	unknown	0	unknown	0	-
DLF010_scaffold1405_28	12339	phage	0.999	temperate	0.9998593	unknown	0	unknown	0	-
DLF010_scaffold14049_4	10962	phage	0.69	temperate	0.999844	unknown	0	Colwellia psychrerythraea	0.94	Predict

DLF010_scaffold86_3	11490	phage	0.996	virulent	0.9998465	unknown	0	Staphylococcus saprophyticus	0.92	Predict
DLF010_scaffold24026_10	29868	phage	1	virulent	0.99986404	unknown	0	Bacillus cereus	0.92	Predict
DLF010_scaffold55874_1	11663	phage	0.629	temperate	0.97490793	unknown	0	Colwellia psychrerythraea	0.75	Predict
DLF012_scaffold4165_33	12120	phage	1	temperate	0.99212986	unknown	0	Bacillus halmapalus	1	CRISPR
DLF012_scaffold36_1	18139	phage	0.998	temperate	0.9998588	unknown	0	Bacillus halmapalus	0.99	Predict
DLF012_scaffold17185_5	12143	phage	0.995	temperate	0.99980783	unknown	0	Colwellia psychrerythraea	0.76	Predict
DLF012_scaffold15936_2	39203	phage	0.999	temperate	0.9998574	unknown	0	Candidatus Pelagibacter ubique	0.78	Predict
DLF012_scaffold12743_1	51197	phage	0.998	temperate	0.99153	Casjensviridae	1	Bacillus halmapalus	1	CRISPR
DLF012_scaffold12838_3	11471	phage	0.999	temperate	0.9998536	unknown	0	Lactobacillus paracasei	1	CRISPR
DLF012_scaffold19111_1	97492	phage	0.993	virulent	0.9626032	Casjensviridae	1	Candidatus Hamiltonella defensa	0.97	Predict
DLF012_scaffold19146_2	15902	phage	0.856	temperate	0.93894786	Straboviridae	0.47796255	Bacteroides fragilis	0.87	Predict
DLF012_scaffold19032_1	10598	phage	0.999	virulent	0.9977207	unknown	0	unknown	0	-
DLF012_scaffold571_16	14034	phage	0.999	virulent	0.99977875	unknown	0	Streptomyces avermitilis	1	CRISPR
DLF012_scaffold17036_1	44226	phage	0.968	virulent	0.9998645	Ackermannviridae	1	Bacteroides fragilis	1	CRISPR
DLF012_scaffold3118_9	13343	phage	0.858	temperate	0.9998574	unknown	0	Flavobacterium columnare	0.74	Predict
DLF013_scaffold47429_1	13635	phage	0.901	virulent	0.9996779	Straboviridae	0.5945209	Bacillus halmapalus	0.75	Predict
DLF013_scaffold18599_4	13549	phage	0.849	virulent	0.9997595	unknown	0	Bacteroides bouchesdurhonensis	1	CRISPR
DLF013_scaffold35295_1	11642	phage	0.979	virulent	0.9996255	unknown	0	unknown	0	-
DLF013_scaffold52665_1	12899	phage	0.995	temperate	0.9997906	unknown	0	unknown	0	-
DLF013_scaffold1520_3	18647	phage	0.998	virulent	0.99987036	Straboviridae	0.6984924	Bacillus halmapalus	1	CRISPR
DLF013_scaffold34743_5	14142	phage	0.997	temperate	0.9998588	unknown	0	Paenibacillus larvae	0.91	Predict
DLF013_scaffold34809_3	12695	phage	0.998	temperate	0.9988944	unknown	0	Colwellia psychrerythraea	0.91	Predict
DLF013_scaffold14099_2	12463	phage	0.961	virulent	0.9997367	Peduoviridae	0.44511104	Candidatus Hamiltonella defensa	0.7	Predict
DLF013_scaffold13599_6	15472	phage	0.999	virulent	0.99987173	Ackermannviridae	0.31179327	Cellulophaga baltica	0.84	Predict
DLF013_scaffold53908_2	10231	phage	0.988	virulent	0.9998474	Straboviridae	0.68764037	Bacillus halmapalus	0.97	Predict
DLF013_scaffold45127_1	13669	phage	0.999	virulent	0.59446454	unknown	0	Bacillus halmapalus	0.9	Predict
DLF013_scaffold3815_1	11511	phage	0.999	temperate	0.9997206	Casjensviridae	0.4192874	Colwellia psychrerythraea	0.87	Predict
DLF013_scaffold88_22	11378	phage	0.998	virulent	0.96433496	unknown	0	unknown	0	-
DLF013_scaffold53550_1	20533	phage	0.996	virulent	0.99973613	Straboviridae	0.6949948	Candidatus Hamiltonella defensa	0.73	Predict
DLF013_scaffold42596_2	46600	phage	0.999	temperate	0.99984974	Straboviridae	0.45363978	Clostridium perfringens	1	CRISPR

DLF013_scaffold6385_2	14860	phage	0.999	temperate	0.99929565	unknown	0	Parabacteroides distasonis	1	CRISPR
DLF013_scaffold42479_2	13678	phage	0.999	temperate	0.9993117	unknown	0	Colwellia psychrerythraea	0.94	Predict
DLF013_scaffold46613_1	11336	phage	0.999	temperate	0.9963533	Herelleviridae	1	unknown	0	-
DLF013_scaffold36273_1	30273	phage	0.994	temperate	0.888194	Straboviridae	0.47071996	Bacteroides fragilis	0.78	Predict
DLF013_scaffold45213_1	22076	phage	0.998	virulent	0.9109021	unknown	0	unknown	0	-
DLF013_scaffold31785_1	17235	phage	0.999	virulent	0.9998593	unknown	0	Colwellia psychrerythraea	1	CRISPR
DLF013_scaffold31672_1	14241	phage	1	temperate	0.98458403	unknown	0	unknown	0	-
DLF013_scaffold139_7	11235	phage	0.974	temperate	0.98519784	unknown	0	Bacteroides fragilis	0.97	Predict
DLF013_scaffold38734_1	10013	phage	0.99	temperate	0.9998588	unknown	0	Colwellia psychrerythraea	0.97	Predict
DLF013_scaffold951_1	10107	phage	0.662	temperate	0.936828	unknown	0	unknown	0	-
DLF013_scaffold16307_9	13512	phage	0.999	temperate	0.9998593	unknown	0	unknown	0	-
DLF013_scaffold53889_1	10431	phage	0.996	temperate	0.9989985	Peduoviridae	1	Bacteroides fragilis	0.7	Predict
DLF013_scaffold42888_4	10263	phage	0.943	virulent	0.9998417	unknown	0	Mesorhizobium loti	0.94	Predict
DLF013_scaffold21493_3	11516	phage	0.989	temperate	0.9998593	unknown	0	unknown	0	-
DLF013_scaffold19199_4	41704	phage	0.999	temperate	0.99980694	Straboviridae	0.4738817	Bacillus halmapalus	1	CRISPR
DLF013_scaffold49433_5	14669	phage	0.999	temperate	0.9411944	unknown	0	Bacillus halmapalus	0.72	Predict
DLF013_scaffold54798_1	13835	phage	0.998	virulent	0.99987084	unknown	0	unknown	0	-
DLF013_scaffold1100_4	20246	phage	0.998	virulent	0.99984497	unknown	0	Colwellia psychrerythraea	1	CRISPR
DLF013_scaffold42008_2	24663	phage	0.998	virulent	0.9998584	Straboviridae	0.65565205	Bacillus halmapalus	1	CRISPR
DLF013_scaffold54776_1	14708	phage	0.999	virulent	0.9998699	Ackermannviridae	0.31179327	Cellulophaga baltica	0.76	Predict
DLF014_scaffold12062_3	25534	phage	0.999	temperate	0.9963524	Straboviridae	0.6495203	Providencia stuartii	1	CRISPR
DLF014_C195831_1	15232	phage	0.984	temperate	0.99985695	unknown	0	Bacillus halmapalus	0.81	Predict
DLF014_scaffold12779_1	38865	phage	0.998	temperate	0.9963427	unknown	0	Lactobacillus fermentum	1	CRISPR
DLF014_scaffold9942_1	36767	phage	0.999	temperate	0.99975306	unknown	0	Clostridium perfringens	1	CRISPR
DLF014_C196781_1	64578	phage	0.999	temperate	0.9998054	Vilmaviridae	1	Bacillus halmapalus	1	CRISPR
DLF014_scaffold12792_2	15693	phage	0.988	temperate	0.9998588	unknown	0	Colwellia psychrerythraea	1	CRISPR
DLF014_scaffold2320_1	12730	phage	0.999	temperate	0.9989119	Herelleviridae	1	unknown	0	-
DLF014_scaffold12718_1	17136	phage	0.994	virulent	0.9809961	Vilmaviridae	1	Colwellia psychrerythraea	0.85	Predict
DLF014_scaffold7985_2	35275	phage	0.999	temperate	0.9998579	unknown	0	Colwellia psychrerythraea	0.89	Predict
DLF014_scaffold5471_1	10411	phage	0.976	temperate	0.9998593	unknown	0	unknown	0	-

DLF014_scaffold583_1	50145	phage	0.999	temperate	0.9998584	unknown	0	Bacillus halmapalus	1 CRISPR
DLF014_scaffold12796_1	13899	phage	0.998	virulent	0.9998436	unknown	0	unknown	0 -
DLF014_scaffold12682_1	31634	phage	0.999	temperate	0.9998202	unknown	0	Colwellia psychrerythraea	1 CRISPR
DLF014_scaffold5330_1	10226	phage	0.988	temperate	0.99985975	unknown	0	unknown	0 -
DLF014_scaffold2386_4	29927	phage	0.989	virulent	0.99979997	unknown	0	Clavibacter michiganensis	0.83 Predict
DLF014_scaffold12741_1	18380	phage	0.999	temperate	0.9998556	unknown	0	Bacillus halmapalus	1 CRISPR
DLF014_scaffold11967_1	18185	phage	0.993	temperate	0.9969512	Casjensviridae	1	Colwellia psychrerythraea	0.71 Predict
DLF014_scaffold8898_1	28992	phage	0.999	virulent	0.86899716	unknown	0	Bacteroides fragilis	0.7 Predict
DLF014_scaffold3999_1	14319	phage	0.998	temperate	0.99985975	Straboviridae	0.31791875	Colwellia psychrerythraea	0.78 Predict
DLF014_scaffold6344_1	57621	phage	0.987	temperate	0.99984604	Straboviridae	0.3343846	Bacillus halmapalus	1 CRISPR
DLF014_scaffold11623_1	11496	phage	0.999	temperate	0.99985605	Ackermannviridae	0.38388076	Colwellia psychrerythraea	0.96 Predict
DLF014_scaffold2693_2	18548	phage	0.988	temperate	0.9776519	Herelleviridae	1	unknown	0 -
DLF014_scaffold1928_3	14315	phage	0.997	virulent	0.9998608	Straboviridae	0.6833262	Lachnospiraceae bacterium AM23-7L	1 CRISPR
DLF014_scaffold12767_1	30312	phage	0.831	virulent	0.9996782	unknown	0	Bacillus halmapalus	0.9 Predict
DLF014_scaffold4556_3	49943	phage	0.998	temperate	0.9587585	Casjensviridae	1	Colwellia psychrerythraea	1 CRISPR
DLF014_scaffold12792_1	20221	phage	0.999	temperate	0.99985605	unknown	0	Candidatus Hamiltonella defensa	1 CRISPR
DLF014_scaffold10166_1	18780	phage	0.982	temperate	0.9998588	unknown	0	Colwellia psychrerythraea	0.97 Predict
DLF014_scaffold12665_1	23292	phage	0.997	temperate	0.9998579	unknown	0	Bacillus halmapalus	0.73 Predict
DLF014_scaffold8_2	14763	phage	0.998	virulent	0.99987406	Drexlerviridae	0.27648103	Colwellia psychrerythraea	0.88 Predict
DLF014_scaffold8436_2	107039	phage	0.99	temperate	0.9998522	Straboviridae	0.35845006	Bacillus halmapalus	1 CRISPR
DLF014_scaffold8300_1	21193	phage	0.999	virulent	0.9978354	unknown	0	Colwellia psychrerythraea	1 CRISPR
DLF014_scaffold5363_6	13462	phage	0.99	temperate	0.99984837	unknown	0	Colwellia psychrerythraea	0.73 Predict
DLF014_scaffold5363_21	11130	phage	0.998	virulent	0.9998688	unknown	0	Colwellia psychrerythraea	1 CRISPR
DLF014_scaffold11967_2	10531	phage	0.999	temperate	0.99985975	Peduoviridae	0.16271424	Colwellia psychrerythraea	0.83 Predict
DLF014_scaffold6855_1	36903	phage	0.998	temperate	0.99985313	Herelleviridae	1	Candidatus Hamiltonella defensa	1 CRISPR
DLF014_scaffold2932_3	13123	phage	0.997	virulent	0.9764244	Straboviridae	0.6356147	Bacillus halmapalus	0.87 Predict
DLF014_scaffold2093_4	10558	phage	0.997	temperate	0.99985975	unknown	0	Flavobacterium columnare	0.95 Predict
DLF014_scaffold12383_2	10821	phage	0.998	virulent	0.99986315	unknown	0	Bacillus halmapalus	0.96 Predict
NLF001_scaffold29251_2	16421	phage	1	temperate	0.9998579	Peduoviridae	1	Clostridioides difficile	1 CRISPR
NLF001_scaffold18110_3	17569	phage	0.999	temperate	0.9076663	unknown	0	unknown	0 -

NLF001_scaffold18524_1	14433	phage	0.986	temperate	0.8446956	unknown	0	Streptococcus mutans	0.72	Predict
NLF001_scaffold159_2	22787	phage	0.999	temperate	0.9998556	unknown	0	Streptococcus mutans	1	CRISPR
NLF001_scaffold1094_2	11292	phage	0.961	temperate	0.979654	unknown	0	Bacteroides fragilis	0.94	Predict
NLF001_scaffold30907_8	18626	phage	0.976	temperate	0.9998588	Straboviridae	0.6221042	Achromobacter xylosoxidans	0.82	Predict
NLF001_scaffold42470_1	25803	phage	0.992	virulent	0.9998436	Straboviridae	0.44587678	Staphylococcus saprophyticus	1	Predict
NLF001_scaffold15921_9	10175	phage	0.996	virulent	0.9998645	unknown	0	unknown	0	-
NLF001_scaffold36675_1	18792	phage	0.998	temperate	0.9671342	Peduoviridae	1	Streptococcus mutans	0.98	Predict
NLF001_scaffold15028_2	12019	phage	0.519	temperate	0.99986035	unknown	0	Tannerella forsythia	1	CRISPR
NLF001_scaffold21827_1	13016	phage	0.999	virulent	0.97969764	unknown	0	Bacteroides fragilis	0.87	Predict
NLF001_scaffold17952_7	11900	phage	1	temperate	0.98458403	unknown	0	Staphylococcus saprophyticus	0.85	Predict
NLF001_scaffold34766_6	18827	phage	0.999	temperate	0.9998588	Straboviridae	0.66139334	Clostridioides difficile	1	CRISPR
NLF001_scaffold42478_2	11699	phage	0.513	virulent	0.9998722	Zierdtviridae	0.59044164	Streptococcus mutans	0.88	Predict
NLF001_scaffold42456_1	11681	phage	0.998	virulent	0.9998736	unknown	0	Bacteroides fragilis	1	CRISPR
NLF001_scaffold21408_7	11618	phage	0.999	temperate	0.999804	unknown	0	unknown	0	-
NLF001_scaffold42472_1	12562	phage	0.996	temperate	0.99762505	unknown	0	unknown	0	-
NLF001_C572546_1	20808	phage	0.999	temperate	0.9998588	Straboviridae	0.66362536	Cellulophaga baltica	1	CRISPR
NLF001_scaffold38144_4	31937	phage	0.999	temperate	0.9998545	unknown	0	Lactobacillus plantarum	1	CRISPR
NLF001_scaffold12289_1	16745	phage	0.999	temperate	0.998079	Straboviridae	0.60088086	Bacteroides fragilis	0.95	Predict
NLF001_scaffold34766_5	13063	phage	0.999	temperate	0.9443795	Guelinviridae	0.14338043	Streptococcus mutans	1	CRISPR
NLF001_scaffold33713_2	24342	phage	0.999	temperate	0.9998465	Vilmaviridae	1	Staphylococcus saprophyticus	1	CRISPR
NLF002_scaffold10424_8	11285	phage	0.974	temperate	0.98519784	unknown	0	Bacteroides fragilis	0.84	Predict
NLF002_scaffold36662_1	10216	phage	0.999	temperate	0.99985975	unknown	0	Clostridioides difficile	0.9	Predict
NLF002_scaffold336_22	12941	phage	0.999	virulent	0.99987084	Ackermannviridae	0.32071397	Streptococcus mutans	0.8	Predict
NLF002_scaffold35171_2	12111	phage	0.95	temperate	0.9998551	unknown	0	unknown	0	-
NLF002_scaffold16871_1	32608	phage	0.991	temperate	0.99727786	Casjensviridae	1	Streptococcus mutans	0.82	Predict
NLF002_scaffold28626_2	11637	phage	0.971	temperate	0.9998588	Straboviridae	0.35911113	Streptococcus mutans	0.83	Predict
NLF002_scaffold34776_1	11773	phage	0.999	temperate	0.9998517	unknown	0	Streptococcus mutans	1	CRISPR
NLF002_scaffold22680_4	12102	phage	0.945	virulent	0.9997971	unknown	0	Staphylococcus saprophyticus	0.81	Predict
NLF002_scaffold6663_3	10249	phage	0.999	virulent	0.81238216	Drexlerviridae	0.23640552	Streptococcus mutans	1	CRISPR
NLF002_scaffold37757_3	16687	phage	0.999	virulent	0.9998699	no_family_avaliable(NC_055870)	0.956	unknown	0	-

NLF002_scaffold3933_4	17889	phage	0.999	temperate	0.9998584	unknown	0	Achromobacter xylosoxidans	0.72	Predict
NLF002_scaffold14709_2	26196	phage	0.999	virulent	0.9986972	unknown	0	Streptococcus mutans	0.91	Predict
NLF002_scaffold27060_6	10177	phage	0.937	virulent	0.9998727	Ackermannviridae	0.3247123	Streptococcus mutans	0.73	Predict
NLF002_scaffold15559_1	13092	phage	0.986	virulent	0.9993214	Ackermannviridae	0.32062793	Bacteroides fragilis	0.97	Predict
NLF002_scaffold19765_4	15659	phage	0.974	virulent	0.9989374	Straboviridae	0.6167804	Staphylococcus saprophyticus	0.84	Predict
NLF002_scaffold141_14	14530	phage	0.999	virulent	0.9998379	unknown	0	Staphylococcus saprophyticus	0.89	Predict
NLF002_scaffold2199_6	28963	phage	0.999	temperate	0.9998545	Straboviridae	0.66139334	Clostridioides difficile	1	CRISPR
NLF002_scaffold36924_1	14259	phage	0.996	temperate	0.99985695	unknown	0	Streptococcus mutans	0.93	Predict
NLF002_scaffold115_2	12334	phage	0.999	temperate	0.99986035	unknown	0	Candidatus Pelagibacter ubique	0.88	Predict
NLF002_scaffold37757_4	47713	phage	0.999	virulent	0.9998688	no_family_avaliable(NC_055870)	0.955	unknown	0	-
NLF002_scaffold37757_5	12840	phage	0.999	virulent	0.9998693	no_family_avaliable(NC_062769)	0.981	unknown	0	-
NLF002_scaffold17055_1	10545	phage	0.991	temperate	0.9996782	unknown	0	Clostridium perfringens	1	CRISPR
NLF002_scaffold25489_2	25064	phage	0.999	temperate	0.999816	unknown	0	Streptococcus mutans	1	CRISPR
NLF002_scaffold38935_2	12832	phage	0.998	virulent	0.99987173	unknown	0	unknown	0	-
NLF005_scaffold4391_2	53699	phage	1	virulent	0.9998688	Demerecviridae	0.30166167	unknown	0	-
NLF005_scaffold21492_3	14868	phage	0.999	temperate	0.9998593	Ackermannviridae	0.29196933	Streptococcus mutans	1	CRISPR
NLF005_scaffold46950_1	24160	phage	0.974	temperate	0.9998388	unknown	0	unknown	0	-
NLF005_scaffold15835_5	17441	phage	0.997	temperate	0.9998145	Casjensviridae	1	Streptococcus mutans	0.74	Predict
NLF005_scaffold36523_1	10452	phage	0.996	temperate	0.99338275	unknown	0	Streptococcus mutans	0.74	Predict
NLF005_scaffold32720_4	18787	phage	0.999	temperate	0.9212118	unknown	0	Staphylococcus saprophyticus	1	CRISPR
NLF005_scaffold21492_6	12744	phage	0.824	virulent	0.9654628	Straboviridae	0.4415074	Streptococcus mutans	0.71	Predict
NLF005_C729497_1	12721	phage	0.999	virulent	0.9995685	unknown	0	Staphylococcus saprophyticus	0.75	Predict
NLF005_scaffold1317_3	17269	phage	0.998	virulent	0.99980664	Ackermannviridae	0.35133132	Streptococcus mutans	0.84	Predict
NLF005_scaffold21214_1	10323	phage	0.999	temperate	0.9998593	unknown	0	Streptococcus mutans	0.93	Predict
NLF005_scaffold47798_1	14288	phage	0.999	temperate	0.84956145	Salasmaviridae	1	Staphylococcus saprophyticus	0.86	Predict
NLF005_C729695_1	14839	phage	0.999	temperate	0.9554545	Herelleviridae	0.3223665	Staphylococcus saprophyticus	1	CRISPR
NLF005_scaffold27522_3	13361	phage	0.99	virulent	0.9998388	unknown	0	unknown	0	-
NLF005_scaffold47800_1	10279	phage	0.75	virulent	0.9990291	unknown	0	unknown	0	-
NLF005_scaffold29618_1	11499	phage	0.782	temperate	0.9923903	unknown	0	unknown	0	-
NLF005_scaffold21492_4	17230	phage	0.996	temperate	0.9976349	unknown	0	Streptococcus mutans	1	CRISPR

NLF005_scaffold25196_2	15785	phage	0.995	temperate	0.9998593	unknown	0	Streptococcus mutans	1	CRISPR
NLF005_scaffold40967_2	21446	phage	0.999	temperate	0.9998593	unknown	0	Clostridium tetani	1	CRISPR
NLF005_scaffold43034_2	37404	phage	0.991	temperate	0.837803	unknown	0	Streptococcus mutans	1	CRISPR
NLF005_scaffold291_42	10894	phage	1	virulent	0.6808251	unknown	0	Streptococcus mutans	0.97	Predict
NLF005_scaffold47934_7	10129	phage	0.99	temperate	0.9984386	unknown	0	unknown	0	-
NLF005_scaffold43004_3	50838	phage	1	temperate	0.99983126	unknown	0	Lactobacillus plantarum	0.78	Predict
NLF005_scaffold14516_2	11999	phage	0.999	temperate	0.99985975	unknown	0	Streptococcus mutans	0.71	Predict
NLF005_scaffold48237_1	54058	phage	0.999	virulent	0.9998636	Ackermannviridae	0.47561732	unknown	0	-
NLF005_C729433_1	12317	phage	0.995	virulent	0.9998636	unknown	0	Roseburia intestinalis	1	CRISPR
NLF005_scaffold48442_3	14584	phage	0.999	temperate	0.9997906	unknown	0	Parabacteroides merdae	1	CRISPR
NLF005_scaffold48389_1	16383	phage	0.999	temperate	0.9998588	unknown	0	Clostridium perfringens	1	CRISPR
NLF005_scaffold39282_7	20214	phage	0.94	temperate	0.9998593	unknown	0	unknown	0	-
NLF005_scaffold13387_9	11929	phage	0.999	virulent	0.9998684	Casjensviridae	0.2355009	unknown	0	-
NLF005_scaffold40798_1	11947	phage	0.996	virulent	0.9998413	unknown	0	unknown	0	-
NLF005_scaffold13387_4	18142	phage	0.998	virulent	0.9970323	Ackermannviridae	0.39681083	unknown	0	-
NLF005_scaffold48437_2	10884	phage	0.999	temperate	0.9981909	Straboviridae	0.37369344	Microcystis aeruginosa	0.9	Predict
NLF005_scaffold29852_1	12021	phage	0.999	temperate	0.9998584	unknown	0	unknown	0	-
NLF005_scaffold4391_3	43456	phage	0.999	virulent	0.99986607	no_family_avaliable(NC_024711)	0.967	unknown	0	-
NLF005_scaffold291_44	13065	phage	0.993	virulent	0.9997163	unknown	0	Streptococcus mutans	0.98	Predict
NLF005_scaffold10734_4	15645	phage	0.988	temperate	0.99985975	unknown	0	unknown	0	-
NLF006_scaffold306_3	40090	phage	0.999	virulent	0.9961736	Straboviridae	0.16758592	Staphylococcus saprophyticus	0.73	Predict
NLF006_scaffold22186_2	13442	phage	0.971	virulent	0.9997945	unknown	0	Streptococcus mutans	0.9	Predict
NLF006_scaffold31099_1	26215	phage	1	temperate	0.9998479	unknown	0	Clostridioides difficile	1	CRISPR
NLF006_scaffold4766_5	10293	phage	0.999	virulent	0.9998736	unknown	0	Streptococcus mutans	1	CRISPR
NLF006_scaffold306_1	22571	phage	0.999	virulent	0.99986607	Guelinviridae	0.29881853	Staphylococcus saprophyticus	1	CRISPR
NLF006_scaffold9628_2	26496	phage	1	temperate	0.999854	Casjensviridae	1	Staphylococcus saprophyticus	1	CRISPR
NLF006_scaffold4766_2	12153	phage	1	virulent	0.9998736	unknown	0	Parabacteroides merdae	0.84	Predict
NLF006_scaffold5249_2	14860	phage	0.921	virulent	0.99952775	Straboviridae	0.77169925	Streptococcus mutans	0.79	Predict
NLF006_scaffold3814_1	12210	phage	0.942	temperate	0.9960633	Ackermannviridae	0.5874024	unknown	0	-
NLF006_scaffold15157_3	10027	phage	0.995	temperate	0.99962914	unknown	0	unknown	0	-

NLF006_scaffold28327_1	22514	phage	0.993	virulent	0.99986035	Straboviridae	0.60979193	Bacteroides fragilis	1 CRISPR
NLF006_scaffold4466_3	12296	phage	0.999	virulent	0.99987173	unknown	0	unknown	0 -
NLF006_scaffold14746_1	11291	phage	0.98	virulent	0.89459366	Kyanoviridae	0.13960746	unknown	0 -
NLF006_scaffold132_1	10216	phage	0.999	temperate	0.9998545	unknown	0	Myxococcus xanthus	1 CRISPR
NLF006_scaffold5891_10	10307	phage	0.845	temperate	0.99986035	unknown	0	unknown	0 -
NLF006_scaffold30421_2	12471	phage	0.998	virulent	0.9619549	unknown	0	unknown	0 -
NLF006_scaffold4358_2	10395	phage	0.51	temperate	0.99390066	unknown	0	Streptococcus mutans	0.8 Predict
NLF007_scaffold882_1	12850	phage	0.999	virulent	0.99987316	unknown	0	Cellulophaga baltica	0.97 Predict
NLF007_scaffold38312_4	10438	phage	0.617	virulent	0.9998699	Ackermannviridae	0.45278424	Candidatus Pelagibacter ubique	0.91 Predict
NLF007_scaffold5043_8	16830	phage	0.958	virulent	0.9943649	unknown	0	Streptococcus mutans	0.77 Predict
NLF007_scaffold67260_2	10961	phage	0.989	temperate	0.9984577	unknown	0	unknown	0 -
NLF007_scaffold14743_4	11263	phage	0.999	virulent	0.9998699	unknown	0	Rhodococcus hoagii	0.71 Predict
NLF007_scaffold64602_1	14215	phage	0.998	temperate	0.9998474	unknown	0	Gordonia rubripertincta	0.86 Predict
NLF007_scaffold66908_2	21703	phage	0.999	temperate	0.9998588	Straboviridae	0.7093209	Clostridium tetani	1 CRISPR
NLF007_scaffold8274_2	19726	phage	0.998	virulent	0.9290818	unknown	0	Streptococcus mutans	0.84 Predict
NLF007_scaffold22938_1	10311	phage	0.944	temperate	0.99985975	unknown	0	Parabacteroides distasonis	0.92 Predict
NLF007_scaffold66524_2	15013	phage	1	temperate	0.99322194	unknown	0	Streptococcus mutans	0.96 Predict
NLF007_scaffold38312_8	93112	phage	0.933	virulent	0.9997118	unknown	0	Staphylococcus saprophyticus	0.91 Predict
NLF007_scaffold5542_23	12533	phage	0.638	virulent	0.9998091	unknown	0	Staphylococcus saprophyticus	0.96 Predict
NLF007_scaffold11102_4	10071	phage	0.998	virulent	0.9027133	Herelleviridae	1	unknown	0 -
NLF007_scaffold49453_1	32139	phage	0.998	temperate	0.99793273	unknown	0	Streptococcus mutans	1 CRISPR
NLF007_scaffold23805_3	15894	phage	0.919	temperate	0.9413989	Straboviridae	0.41627434	Bacteroides fragilis	0.79 Predict
NLF007_scaffold31937_4	12250	phage	0.998	temperate	0.9998593	unknown	0	Cellulophaga baltica	1 CRISPR
NLF007_scaffold110_13	15641	phage	0.987	temperate	0.9482661	unknown	0	Enterococcus rivorum	1 CRISPR
NLF007_scaffold23404_3	11177	phage	1	temperate	0.9998593	unknown	0	Lactobacillus plantarum	1 CRISPR
NLF007_scaffold25969_1	20397	phage	0.99	temperate	0.9786316	Casjensviridae	1	Streptococcus mutans	1 CRISPR
NLF007_scaffold29510_1	18570	phage	0.994	temperate	0.99985975	unknown	0	unknown	0 -
NLF007_scaffold61307_5	11150	phage	0.999	temperate	0.9942856	unknown	0	unknown	0 -
NLF007_scaffold4118_1	10653	phage	0.992	temperate	0.99985975	unknown	0	unknown	0 -
NLF007_scaffold67236_3	11412	phage	0.998	temperate	0.9929809	unknown	0	Bacteroides eggerthii	1 CRISPR

NLF007_scaffold3779_4	11767	phage	0.999	virulent	0.9998593	unknown	0	unknown	0	-
NLF007_scaffold27103_9	15845	phage	0.999	temperate	0.99984884	Straboviridae	0.7093209	Streptococcus pneumoniae	0.73	Predict
NLF007_scaffold67413_1	31694	phage	0.999	temperate	0.9993908	unknown	0	Staphylococcus epidermidis	1	CRISPR
NLF007_scaffold36465_3	15832	phage	0.989	temperate	0.9819443	Drexlerviridae	0.19531086	Cutibacterium acnes	0.95	Predict
NLF007_C1010391_1	11100	phage	0.998	temperate	0.96395195	Peduoviridae	1	Paenibacillus dendritiformis	1	CRISPR
NLF007_scaffold17554_50	16630	phage	0.992	temperate	0.91449815	unknown	0	Streptococcus mutans	0.77	Predict
NLF007_scaffold64205_2	14966	phage	0.995	temperate	0.93972445	unknown	0	unknown	0	-
NLF007_scaffold61221_1	11586	phage	0.999	virulent	0.999854	unknown	0	Parabacteroides merdae	1	CRISPR
NLF007_scaffold56144_4	11074	phage	0.991	temperate	0.9998593	unknown	0	Streptococcus mutans	0.78	Predict
NLF007_scaffold67153_2	25986	phage	0.999	virulent	0.9998699	Demerecviridae	0.8270528	unknown	0	-
NLF007_scaffold23957_1	22870	phage	0.999	virulent	0.9997695	Peduoviridae	0.36351505	Dinoroseobacter shibae	1	CRISPR
NLF007_scaffold54945_9	11011	phage	0.998	virulent	0.999844	unknown	0	Candidatus Pelagibacter ubique	0.77	Predict
NLF007_scaffold20693_10	10878	phage	0.999	virulent	0.9998722	unknown	0	Staphylococcus saprophyticus	0.99	Predict
NLF007_scaffold40076_1	10050	phage	0.999	virulent	0.99983	unknown	0	unknown	0	-
NLF007_scaffold66908_3	14909	phage	0.999	temperate	0.99985975	Straboviridae	0.32606444	Clostridioides difficile	0.78	Predict
NLF007_scaffold47382_8	17803	phage	0.997	virulent	0.9998679	Herelleviridae	0.31269833	unknown	0	-
NLF007_scaffold47325_6	12227	phage	0.922	temperate	0.9998565	unknown	0	Staphylococcus saprophyticus	0.87	Predict
NLF007_scaffold14743_2	21593	phage	0.999	virulent	0.99986744	unknown	0	Staphylococcus saprophyticus	1	CRISPR
NLF007_scaffold30358_8	10237	phage	0.627	virulent	0.99987406	unknown	0	Streptococcus mutans	0.98	Predict
NLF007_scaffold3779_6	12926	phage	0.999	temperate	0.9998579	unknown	0	Streptococcus mutans	0.77	Predict
NLF007_scaffold14743_1	17874	phage	0.998	temperate	0.60106117	unknown	0	unknown	0	-
NLF007_scaffold57652_6	26215	phage	0.999	virulent	0.99964964	Ackermannviridae	0.23807108	Streptococcus mutans	1	CRISPR
NLF007_scaffold50111_1	17113	phage	0.999	temperate	0.98840153	unknown	0	Listeria monocytogenes	1	CRISPR
NLF007_scaffold57227_1	11376	phage	0.999	temperate	0.99985975	unknown	0	Clostridioides difficile	0.81	Predict
NLF007_scaffold48133_2	10132	phage	0.676	virulent	0.9997974	unknown	0	Streptococcus mutans	0.98	Predict
NLF007_scaffold66524_3	11931	phage	0.997	temperate	0.9998588	unknown	0	unknown	0	-
NLF007_scaffold22798_1	30453	phage	0.992	temperate	0.9997869	Straboviridae	0.5943833	Escherichia coli	0.96	Predict
NLF007_scaffold43991_1	12694	phage	0.999	temperate	0.9941997	unknown	0	unknown	0	-
NLF007_scaffold22798_13	16979	phage	0.986	virulent	0.9993209	Straboviridae	0.7939098	Streptococcus mutans	0.95	Predict
NLF007_scaffold31937_5	11967	phage	1	virulent	0.9998693	unknown	0	Staphylococcus saprophyticus	1	CRISPR

NLF007_scaffold67062_4	11471	phage	0.999	temperate	0.9998588	Straboviridae	0.35374683	Staphylococcus saprophyticus	0.72	Predict
NLF007_scaffold34404_4	11233	phage	0.999	virulent	0.9994208	no_family_avaliable(NC_055904)	0.974	Bacteroides fragilis	0.79	Predict
NLF007_scaffold32046_2	10684	phage	0.999	virulent	0.9998565	unknown	0	Bacteroides caccae	1	CRISPR
NLF007_scaffold21464_1	10691	phage	0.952	temperate	0.99985266	unknown	0	unknown	0	-
NLF007_scaffold67062_3	14092	phage	1	temperate	0.99985975	unknown	0	Clostridium tetani	0.85	Predict
NLF007_scaffold27103_3	10755	phage	0.998	temperate	0.99985695	unknown	0	Streptococcus mutans	1	CRISPR
NLF008_C315574_1	11651	phage	0.741	virulent	0.9322203	unknown	0	Staphylococcus saprophyticus	0.76	Predict
NLF008_scaffold19700_1	16872	phage	0.999	virulent	0.99987125	no_family_avaliable(NC_062778)	0.989	unknown	0	-
NLF008_scaffold141_3	11447	phage	0.997	virulent	0.99987125	unknown	0	unknown	0	-
NLF008_scaffold12997_5	10356	phage	0.653	temperate	0.9998536	unknown	0	Bacteroides fragilis	0.8	Predict
NLF008_scaffold18404_4	10687	phage	0.996	virulent	0.99987125	unknown	0	Parabacteroides merdae	1	CRISPR
NLF008_scaffold10158_1	10778	phage	0.985	virulent	0.99984926	unknown	0	Staphylococcus saprophyticus	0.88	Predict
NLF008_scaffold20496_1	25081	phage	0.712	virulent	0.99987125	Straboviridae	0.3779054	Streptococcus mutans	0.76	Predict
NLF008_scaffold8874_2	11731	phage	0.995	virulent	0.99987173	Ackermannviridae	0.45278424	Rhizobium leguminosarum	0.96	Predict
NLF008_scaffold18404_14	12447	phage	0.998	virulent	0.99976844	unknown	0	Parabacteroides merdae	1	CRISPR
NLF008_scaffold10905_2	10779	phage	0.999	temperate	0.9998536	unknown	0	Clostridium sp. AM43-3BH	1	CRISPR
NLF008_scaffold10905_1	34466	phage	1	temperate	0.92832506	unknown	0	Lactobacillus plantarum	1	CRISPR
NLF008_scaffold1586_11	11646	phage	0.94	temperate	0.999836	Mesyanzhinovviridae	0.53950065	Streptococcus mutans	0.94	Predict
NLF008_scaffold7552_2	11915	phage	0.827	temperate	0.99984837	unknown	0	Bacteroides fragilis	0.92	Predict
NLF008_scaffold20431_5	19686	phage	0.999	virulent	0.99986696	unknown	0	Cellulophaga baltica	1	CRISPR
NLF008_scaffold19473_1	59901	phage	0.998	virulent	0.99863577	Straboviridae	0.40740702	Staphylococcus saprophyticus	0.81	Predict
NLF008_scaffold9366_1	10792	phage	0.985	virulent	0.99984926	unknown	0	Staphylococcus saprophyticus	0.99	Predict
NLF008_scaffold15389_1	13461	phage	0.935	virulent	0.99987084	Kyanoviridae	1	Staphylococcus saprophyticus	0.94	Predict
NLF008_scaffold20125_1	15961	phage	0.711	virulent	0.53311074	Straboviridae	0.35911113	Parabacteroides merdae	0.86	Predict
NLF008_scaffold17605_2	10996	phage	0.997	temperate	0.9998593	unknown	0	Staphylococcus saprophyticus	0.9	Predict
NLF008_scaffold6835_1	12913	phage	0.998	virulent	0.9998651	unknown	0	Streptococcus mutans	0.87	Predict
NLF008_scaffold9492_2	17252	phage	0.998	virulent	0.99980664	Ackermannviridae	0.35133132	Streptococcus mutans	0.89	Predict
NLF008_scaffold12194_1	18067	phage	0.998	virulent	0.99987406	Herelleviridae	1	Bacteroides fragilis	0.76	Predict
NLF008_scaffold20731_1	11650	phage	0.999	temperate	0.9765676	unknown	0	Streptococcus mutans	0.85	Predict
NLF008_scaffold6103_28	14628	phage	0.994	temperate	0.93755734	unknown	0	unknown	0	-

NLF008_scaffold395_6	11524	phage	0.999	virulent	0.99987173	unknown	0	Acinetobacter baumannii	1	CRISPR
NLF008_scaffold12947_1	10549	phage	0.978	virulent	0.9998665	Straboviridae	0.5434872	Bacteroides fragilis	1	CRISPR
NLF008_scaffold17931_1	10067	phage	0.998	temperate	0.8868689	unknown	0	Staphylococcus saprophyticus	0.84	Predict
NLF008_scaffold12834_4	10165	phage	0.656	virulent	0.99949926	unknown	0	Streptococcus mutans	1	CRISPR
NLF008_scaffold16324_2	12419	phage	0.872	virulent	0.9997513	unknown	0	Dinoroseobacter shibae	0.95	Predict
NLF008_scaffold107_1	11255	phage	0.935	virulent	0.91921675	unknown	0	Dinoroseobacter shibae	0.87	Predict
NLF008_scaffold98_9	50009	phage	0.999	temperate	0.9996572	Mesyanzhinovviridae	0.28275466	Streptococcus mutans	0.87	Predict
NLF008_scaffold1813_6	13628	phage	0.996	virulent	0.9998693	Peduoviridae	0.6674539	Lactobacillus fermentum	0.85	Predict
NLF008_scaffold8433_4	29785	phage	0.999	temperate	0.99984556	Straboviridae	0.2508475	Bacteroides fragilis	0.79	Predict
NLF008_scaffold20258_1	14264	phage	0.998	virulent	0.99987125	unknown	0	Streptococcus mutans	1	Predict
NLF008_scaffold18404_6	12880	phage	0.995	virulent	0.9998722	Salasmaviridae	0.19234808	Cellulophaga baltica	0.98	Predict
NLF008_scaffold6876_9	14496	phage	0.999	virulent	0.9998736	unknown	0	Parabacteroides merdae	1	CRISPR
NLF008_scaffold9442_2	17076	phage	0.998	virulent	0.9998736	unknown	0	unknown	0	-
NLF008_C315880_1	14622	phage	0.987	temperate	0.99985975	unknown	0	Streptococcus mutans	0.72	Predict
NLF008_scaffold20528_1	10887	phage	0.818	temperate	0.9932634	unknown	0	unknown	0	-
NLF008_scaffold1474_11	23511	phage	0.953	virulent	0.9998684	unknown	0	unknown	0	-
NLF008_scaffold386_3	10053	phage	0.902	temperate	0.999854	Straboviridae	0.4734725	Streptococcus mutans	0.74	Predict
NLF008_scaffold5986_1	37605	phage	1	virulent	0.99987036	unknown	0	unknown	0	-
NLF008_scaffold15537_1	10109	phage	0.997	virulent	0.9981073	Demerecviridae	0.8270528	Parabacteroides merdae	1	CRISPR
NLF008_scaffold18404_13	21278	phage	0.999	virulent	0.9998699	Herelleviridae	1	Bacillus subtilis	1	CRISPR
NLF008_scaffold14014_6	10069	phage	0.999	virulent	0.99987125	no_family_avaliable(NC_062778)	0.993	unknown	0	-
NLF008_scaffold7896_4	17979	phage	0.999	temperate	0.99671596	unknown	0	Parabacteroides distasonis	1	CRISPR
NLF008_scaffold9737_2	10699	phage	0.582	temperate	0.9998593	Straboviridae	0.60088086	Bacteroides fragilis	0.81	Predict
NLF008_scaffold12833_2	14417	phage	0.999	virulent	0.9998226	unknown	0	Streptococcus mutans	1	CRISPR
NLF008_scaffold83_1	12688	phage	0.998	virulent	0.99959236	Vilmaviridae	1	Streptococcus mutans	0.79	Predict
NLF008_scaffold20431_4	14900	phage	0.999	virulent	0.99987084	unknown	0	unknown	0	-
NLF008_scaffold13388_2	11010	phage	0.974	temperate	0.98519784	unknown	0	Bacteroides fragilis	0.8	Predict
NLF008_scaffold1868_2	17385	phage	0.998	virulent	0.9998593	Ackermannviridae	0.29792926	Streptococcus mutans	0.78	Predict
NLF009_C805905_1	25153	phage	0.998	temperate	0.9998579	Straboviridae	0.66362536	Cellulophaga baltica	1	CRISPR
NLF009_scaffold3162_3	11102	phage	0.988	temperate	0.9998593	unknown	0	Streptococcus mutans	0.91	Predict

NLF009_scaffold17912_16	26561	phage	0.997	temperate	0.9998593	Vilmaviridae	1	Streptococcus mutans	1	CRISPR
NLF009_scaffold30773_1	10767	phage	0.991	temperate	0.98678803	unknown	0	Staphylococcus saprophyticus	0.86	Predict
NLF009_scaffold54553_5	16641	phage	0.999	virulent	0.9905057	unknown	0	unknown	0	-
NLF009_scaffold21790_2	17115	phage	0.998	temperate	0.99985975	unknown	0	Staphylococcus saprophyticus	0.82	Predict
NLF009_scaffold7091_22	13178	phage	0.982	virulent	0.9998474	Peduoviridae	0.6674539	Dinoroseobacter shibae	0.87	Predict
NLF009_scaffold54553_3	20135	phage	0.999	virulent	0.9998699	Ackermannviridae	0.12159623	Cellulophaga baltica	1	CRISPR
NLF009_scaffold4933_28	11895	phage	0.663	virulent	0.99987125	unknown	0	Lactobacillus fermentum	0.92	Predict
NLF009_scaffold9844_25	11075	phage	0.953	temperate	0.9998379	Straboviridae	0.4734725	Bacteroides fragilis	0.83	Predict
NLF009_scaffold52926_4	15290	phage	0.999	temperate	0.97618574	Ackermannviridae	0.3357813	unknown	0	-
NLF009_scaffold52375_7	17246	phage	0.979	virulent	0.9996982	unknown	0	unknown	0	-
NLF009_scaffold54499_1	12544	phage	0.816	temperate	0.99985975	unknown	0	Bacteroides fragilis	1	Predict
NLF009_scaffold19140_7	17244	phage	0.932	virulent	0.9998727	Straboviridae	0.3779054	Bacteroides fragilis	0.74	Predict
NLF009_scaffold54170_2	17995	phage	0.997	temperate	0.9998513	unknown	0	unknown	0	-
NLF009_scaffold35663_1	65533	phage	0.999	virulent	0.99982476	Straboviridae	0.40740702	Staphylococcus saprophyticus	0.87	Predict
NLF009_scaffold4106_1	12031	phage	0.999	temperate	0.9998593	Straboviridae	0.6963392	Lactobacillus plantarum	0.93	Predict
NLF009_scaffold1353_6	10526	phage	0.99	temperate	0.9998593	unknown	0	unknown	0	-
NLF009_C805829_1	17652	phage	0.998	virulent	0.99960625	unknown	0	Streptococcus mutans	0.76	Predict
NLF009_scaffold27217_3	10110	phage	0.566	temperate	0.99986035	unknown	0	unknown	0	-
NLF009_C805873_1	20669	phage	0.998	virulent	0.8740401	unknown	0	Streptococcus mutans	0.88	Predict
NLF009_scaffold17912_4	11278	phage	0.997	temperate	0.99724483	unknown	0	Streptococcus mutans	1	CRISPR
NLF009_scaffold5429_52	17094	phage	0.999	temperate	0.9876869	unknown	0	Parabacteroides merdae	0.96	Predict
NLF009_scaffold40330_1	10765	phage	0.778	virulent	0.9998684	unknown	0	Lactobacillus fermentum	0.95	Predict
NLF009_scaffold18984_7	15729	phage	0.998	virulent	0.9985467	unknown	0	unknown	0	-
NLF009_scaffold38501_1	37159	phage	0.999	temperate	0.99786866	Demerecviridae	0.32602853	Bacteroides fragilis	1	CRISPR
NLF009_scaffold8414_14	11545	phage	0.999	temperate	0.9996339	unknown	0	Bacteroides fragilis	1	CRISPR
NLF009_scaffold17912_17	10814	phage	0.999	temperate	0.9986843	unknown	0	Lachnospiraceae bacterium	1	CRISPR
NLF009_scaffold52991_2	11253	phage	0.961	temperate	0.979654	unknown	0	Bacteroides fragilis	0.77	Predict
NLF009_scaffold25289_5	22521	phage	0.997	temperate	0.99985266	unknown	0	Streptococcus mutans	0.75	Predict
NLF009_scaffold36413_1	32823	phage	0.999	virulent	0.9193699	unknown	0	Flavobacterium psychrophilum	1	CRISPR
NLF009_scaffold375_24	10578	phage	0.997	virulent	0.99963766	Ackermannviridae	0.2991495	Bacteroides fragilis	0.92	Predict

NLF009_scaffold52926_1	11507	phage	0.999	virulent	0.9998688	Casjensviridae	0.2355009	unknown	0	-
NLF009_scaffold37415_3	10416	phage	0.998	temperate	0.9920004	unknown	0	Streptococcus mutans	1	CRISPR
NLF009_scaffold54503_1	29206	phage	1	virulent	0.99928766	Peduoviridae	0.23355766	Lactobacillus plantarum	1	CRISPR
NLF009_scaffold17912_6	13102	phage	0.999	temperate	0.9998336	Herelleviridae	1	Clostridiaceae bacterium	1	CRISPR
NLF010_scaffold1235_8	23524	phage	0.999	virulent	0.99987084	unknown	0	Streptococcus mutans	1	CRISPR
NLF010_scaffold20315_2	41105	phage	1	virulent	0.97717094	Peduoviridae	0.28912216	Lactobacillus jensenii	1	CRISPR
NLF010_scaffold1044_27	12219	phage	0.99	virulent	0.9998736	Straboviridae	0.35911113	Bacteroides fragilis	0.78	Predict
NLF010_C401947_1	12712	phage	0.829	virulent	0.9998593	unknown	0	Staphylococcus saprophyticus	0.74	Predict
NLF010_scaffold13747_2	14826	phage	0.992	temperate	0.9958668	unknown	0	Staphylococcus saprophyticus	0.76	Predict
NLF010_scaffold24082_3	10036	phage	0.999	temperate	0.99985605	Ackermannviridae	0.30388898	unknown	0	-
NLF010_C402235_1	45796	phage	0.999	virulent	0.9998627	Demerecviridae	1	Staphylococcus saprophyticus	1	CRISPR
NLF010_scaffold22417_1	11930	phage	0.998	virulent	0.9996403	unknown	0	Streptococcus mutans	0.72	Predict
NLF010_scaffold3898_4	13000	phage	0.986	virulent	0.9998699	unknown	0	unknown	0	-
NLF010_scaffold12295_8	11280	phage	0.999	virulent	0.97536755	Ackermannviridae	0.3644868	Streptococcus mutans	0.85	Predict
NLF010_scaffold27728_2	13566	phage	0.993	temperate	0.999854	unknown	0	Dinoroseobacter shibae	0.9	Predict
NLF010_scaffold29246_1	10118	phage	1	virulent	0.99987316	unknown	0	unknown	0	-
NLF010_scaffold26024_8	11869	phage	0.996	virulent	0.9998722	Ackermannviridae	1	Staphylococcus saprophyticus	0.83	Predict
NLF010_scaffold2046_76	13877	phage	0.998	virulent	0.99987036	Straboviridae	0.32298803	Coprococcus eutactus	1	CRISPR
NLF010_scaffold9747_18	17494	phage	0.997	virulent	0.9953019	Salasmaviridae	0.4600486	Streptococcus mutans	1	CRISPR
NLF010_scaffold20591_1	12874	phage	0.998	virulent	0.9484926	Straboviridae	0.4734725	Bacteroides fragilis	0.89	Predict
NLF010_scaffold29445_4	22116	phage	0.998	virulent	0.9995593	Straboviridae	0.44893572	unknown	0	-
NLF011_scaffold4965_2	10589	phage	0.999	virulent	0.99987084	unknown	0	Streptococcus mutans	1	CRISPR
NLF011_scaffold5613_1	14770	phage	0.651	virulent	0.9998665	unknown	0	unknown	0	-
NLF011_scaffold42_2	17123	phage	0.999	virulent	0.99987036	Straboviridae	0.3083672	Streptococcus mutans	0.87	Predict
NLF011_scaffold1105_1	12237	phage	0.942	temperate	0.9960633	Ackermannviridae	0.5874024	unknown	0	-
NLF011_scaffold7214_2	10059	phage	0.883	virulent	0.9998727	Straboviridae	0.61042684	Parabacteroides merdae	0.88	Predict
NLF011_scaffold10809_1	11354	phage	0.993	virulent	0.9998699	unknown	0	Staphylococcus saprophyticus	1	CRISPR
NLF011_scaffold4255_7	11349	phage	0.986	temperate	0.98806924	Peduoviridae	0.3274082	Streptococcus mutans	0.94	Predict
NLF011_scaffold10054_4	10942	phage	0.961	temperate	0.979654	unknown	0	Streptococcus mutans	0.89	Predict
NLF011_scaffold1786_1	11960	phage	0.999	temperate	0.99896526	unknown	0	Firmicutes bacterium AM43-11BH	1	CRISPR

NLF011_scaffold4096_9	30634	phage	0.992	temperate	0.99985695	unknown	0	Staphylococcus saprophyticus	0.75	Predict
NLF011_scaffold2637_6_1	13403	phage	0.999	virulent	0.99985975	Ackermannviridae	0.25874183	Streptococcus mutans	0.9	Predict
NLF011_scaffold13048_1	17246	phage	0.995	virulent	0.99978536	Ackermannviridae	0.35383645	Streptococcus mutans	0.77	Predict
NLF011_scaffold3151_2	14320	phage	0.998	temperate	0.99986035	unknown	0	Streptococcus pneumoniae	0.77	Predict
NLF011_scaffold30362_5	15767	phage	0.974	temperate	0.98519784	unknown	0	Bacteroides fragilis	0.99	Predict
NLF011_scaffold25120_2	11515	phage	0.998	temperate	0.9799867	Peduoviridae	1	unknown	0	-
NLF011_scaffold486_7	13422	phage	0.938	virulent	0.999844	Ackermannviridae	1	Streptococcus mutans	0.76	Predict
NLF011_scaffold794_4	17705	phage	0.998	virulent	0.99986744	Straboviridae	0.40740702	Staphylococcus saprophyticus	0.81	Predict
NLF011_scaffold22115_1	12222	phage	0.998	virulent	0.9998508	Straboviridae	0.7048575	Streptococcus mutans	1	CRISPR
NLF011_scaffold803_4	10482	phage	0.955	virulent	0.85896134	Ackermannviridae	0.45278424	Streptococcus mutans	0.81	Predict
NLF011_scaffold10943_3	18513	phage	0.999	temperate	0.99985033	unknown	0	Faecalibacterium prausnitzii	1	CRISPR
NLF011_scaffold6937_1	13037	phage	0.867	virulent	0.9998536	unknown	0	Vibrio splendidus	0.8	Predict
NLF011_scaffold21554_2	11937	phage	0.542	virulent	0.99987316	unknown	0	Bacteroides fragilis	0.87	Predict
NLF011_scaffold9582_1	39130	phage	0.999	temperate	0.9989985	unknown	0	Lactobacillus plantarum	1	CRISPR
NLF012_scaffold10340_2	19186	phage	0.998	temperate	0.99985695	unknown	0	unknown	0	-
NLF012_scaffold35219_5	13298	phage	0.983	temperate	0.9980938	unknown	0	Streptococcus mutans	1	CRISPR
NLF012_scaffold35219_1	10878	phage	0.999	temperate	0.99985975	unknown	0	Staphylococcus epidermidis	0.78	Predict
NLF012_scaffold907_13	17237	phage	0.849	temperate	0.9998403	Drexlerviridae	0.13413674	Dinoroseobacter shibae	0.83	Predict
NLF012_scaffold39053_1	11089	phage	0.945	temperate	0.99986035	unknown	0	unknown	0	-
NLF012_scaffold2371_23	15684	phage	0.999	virulent	0.9864348	Peduoviridae	1	Streptococcus mutans	0.82	Predict
NLF012_scaffold22746_1	16741	phage	0.974	temperate	0.9998593	unknown	0	Parabacteroides merdae	0.87	Predict
NLF012_scaffold39054_1	16529	phage	0.995	virulent	0.9997444	Peduoviridae	0.26534814	Streptococcus mutans	0.97	Predict
NLF012_scaffold8622_2	37540	phage	0.949	temperate	0.90595436	Peduoviridae	1	Lactobacillus fermentum	1	CRISPR
NLF012_scaffold2172_5	15797	phage	0.983	virulent	0.9998369	Straboviridae	0.44206813	Streptococcus mutans	0.77	Predict
NLF012_scaffold27405_2	10624	phage	0.999	virulent	0.57108474	Peduoviridae	1	Firmicutes bacterium AM43-11BH	1	CRISPR
NLF012_scaffold11833_29	10953	phage	0.994	temperate	0.99984884	unknown	0	Streptococcus mutans	0.81	Predict
NLF012_scaffold2371_16	11187	phage	0.998	temperate	0.99986035	unknown	0	Dinoroseobacter shibae	0.92	Predict
NLF012_scaffold39140_2	15899	phage	0.856	temperate	0.93894786	Straboviridae	0.41627434	Bacteroides fragilis	0.87	Predict
NLF012_scaffold25728_1	19039	phage	0.998	temperate	0.99985975	unknown	0	Staphylococcus saprophyticus	0.72	Predict
NLF012_scaffold36675_4	10189	phage	0.993	virulent	0.9468337	unknown	0	Staphylococcus saprophyticus	0.99	Predict

NLF012_scaffold21675_1	18220	phage	1	virulent	0.7219442	unknown	0	Streptococcus mutans	0.82	Predict
NLF012_scaffold11833_30	14267	phage	0.999	temperate	0.9998593	unknown	0	Streptococcus mutans	1	CRISPR
NLF012_scaffold29420_1	11838	phage	0.932	temperate	0.99955946	unknown	0	unknown	0	-
NLF012_scaffold39159_1	14938	phage	1	virulent	0.9998679	Salasmaviridae	1	Staphylococcus saprophyticus	0.94	Predict
NLF012_scaffold17264_3	11734	phage	0.995	virulent	0.99987173	Ackermannviridae	0.45278424	Rhizobium leguminosarum	0.73	Predict
NLF012_scaffold34471_3	11144	phage	0.965	temperate	0.999417	Straboviridae	0.6525488	Bacteroides fragilis	0.8	Predict
NLF012_scaffold293_2	21457	phage	0.999	virulent	0.99980617	Straboviridae	0.66362536	Staphylococcus saprophyticus	0.98	Predict
NLF012_scaffold6773_9	13468	phage	0.964	virulent	0.9998688	unknown	0	Staphylococcus saprophyticus	0.98	Predict
NLF012_scaffold10245_3	13810	phage	1	temperate	0.99944323	Ackermannviridae	0.23807108	Streptococcus mutans	1	CRISPR
NLF012_scaffold2854_1	29133	phage	0.983	virulent	0.99980754	unknown	0	Streptococcus mutans	1	CRISPR
NLF012_scaffold18119_4	28285	phage	0.999	temperate	0.9996067	unknown	0	Lactobacillus plantarum	1	CRISPR
NLF012_scaffold38032_1	11715	phage	0.998	temperate	0.99985975	unknown	0	Candidatus Pelagibacter ubique	0.89	Predict
NLF012_scaffold16653_1	10851	phage	0.913	temperate	0.99244034	unknown	0	unknown	0	-
NLF012_scaffold22955_2	17070	phage	0.998	virulent	0.99987406	unknown	0	unknown	0	-
NLF012_scaffold15731_1	13596	phage	0.999	temperate	0.9998522	unknown	0	Bacteroides fragilis	1	CRISPR
NLF012_scaffold13034_3	16717	phage	0.999	temperate	0.9998588	Kyanoviridae	1	unknown	0	-
NLF012_scaffold16348_2	15299	phage	0.99	temperate	0.9998584	unknown	0	Dinoroseobacter shibae	0.89	Predict
NLF012_scaffold8622_6	10072	phage	0.916	virulent	0.9998679	unknown	0	Pectobacterium carotovorum	0.97	Predict
NLF012_scaffold17845_1	15207	phage	0.999	virulent	0.99497724	Ackermannviridae	0.4260041	Streptococcus mutans	0.98	Predict
NLF012_C512465_1	18409	phage	0.998	virulent	0.9998699	Salasmaviridae	0.93962055	Staphylococcus saprophyticus	0.84	Predict
NLF012_scaffold12754_3	10803	phage	0.998	temperate	0.9340867	unknown	0	unknown	0	-
NLF012_scaffold10394_1	16798	phage	0.833	virulent	0.99987316	unknown	0	Bacteroides fragilis	0.94	Predict
NLF012_scaffold32276_1	17951	phage	0.996	temperate	0.99985975	Straboviridae	0.4409281	Lactobacillus jensenii	0.98	Predict
NLF012_scaffold13132_1	26660	phage	0.999	temperate	0.6238861	Peduoviridae	0.9875101	Streptococcus mutans	1	CRISPR
NLF012_scaffold30180_18	10679	phage	1	temperate	0.99986035	unknown	0	unknown	0	-
NLF012_scaffold17431_22	10798	phage	0.636	virulent	0.99983126	unknown	0	unknown	0	-
NLF012_scaffold35713_1	25569	phage	0.997	virulent	0.9998556	Mesyanzhinovviridae	0.50958985	Dinoroseobacter shibae	0.78	Predict
NLF012_scaffold10663_6	16216	phage	0.521	virulent	0.9998688	Peduoviridae	0.6674539	Dinoroseobacter shibae	0.92	Predict
NLF012_scaffold35670_2	10839	phage	0.971	virulent	0.999549	Ackermannviridae	0.3644868	Bacteroides fragilis	0.8	Predict
NLF012_scaffold15197_22	15988	phage	0.606	virulent	0.9664372	Straboviridae	0.42601934	Streptococcus mutans	1	CRISPR

NLF012_scaffold37458_1	11401	phage	0.997	virulent	0.99987316	Straboviridae	0.44893572	Eubacteriaceae bacterium	1	CRISPR
NLF012_scaffold39143_1	12964	phage	0.999	temperate	0.9998588	unknown	0	Bacteroides vulgatus	1	CRISPR
NLF012_scaffold21989_2	23592	phage	0.984	temperate	0.9832907	Casjensviridae	0.1514813	Streptococcus mutans	0.87	Predict
NLF012_scaffold14481_3	15059	phage	0.999	virulent	0.99961454	unknown	0	Streptococcus mutans	1	CRISPR
NLF012_scaffold4362_3	11525	phage	0.994	temperate	0.93755734	unknown	0	unknown	0	-
NLF012_scaffold10340_14	15260	phage	0.998	virulent	0.99986607	Herelleviridae	1	unknown	0	-
NLF012_scaffold38234_3	12455	phage	0.997	virulent	0.9253881	unknown	0	Bacteroides fragilis	0.82	Predict
NLF012_scaffold16451_3	17451	phage	0.996	virulent	0.9998684	Straboviridae	0.3238582	Streptococcus mutans	0.88	Predict
NLF012_scaffold13372_3	12956	phage	0.999	virulent	0.99987406	Herelleviridae	1	Staphylococcus saprophyticus	0.88	Predict
NLF012_scaffold37376_1	11111	phage	0.999	virulent	0.99987084	unknown	0	[Eubacterium] eligens	1	CRISPR
NLF012_scaffold69_1	13003	phage	0.897	virulent	0.8038945	Drexlerviridae	0.32428944	Dinoroseobacter shibae	0.78	Predict
NLF012_scaffold14206_2	10498	phage	0.863	virulent	0.88010323	unknown	0	Lactobacillus fermentum	0.73	Predict
NLF012_scaffold17431_21	16638	phage	0.996	temperate	0.9952155	unknown	0	unknown	0	-
NLF012_scaffold8622_3	10309	phage	1	virulent	0.9804897	Casjensviridae	1	Streptococcus mutans	1	CRISPR
NLF012_scaffold33261_5	11272	phage	0.82	temperate	0.9998083	unknown	0	unknown	0	-
NLF012_scaffold29414_2	10228	phage	0.999	temperate	0.99985695	unknown	0	Streptococcus mutans	0.94	Predict
NLF012_scaffold2371_15	17977	phage	0.999	temperate	0.99982077	Casjensviridae	1	unknown	0	-
NLF012_scaffold17240_1	55853	phage	0.999	temperate	0.9997754	unknown	0	Bacteroides fragilis	1	CRISPR
NLF013_scaffold11783_1	13457	phage	0.92	virulent	0.9998474	Straboviridae	0.42601934	unknown	0	-
NLF013_scaffold48622_1	96674	phage	0.999	virulent	0.9998608	Schitoviridae	1	unknown	0	-
NLF013_scaffold11031_1	16869	phage	0.98	virulent	0.99969965	Straboviridae	0.7939098	Streptococcus mutans	0.85	Predict
NLF013_scaffold43868_1	13232	phage	0.995	temperate	0.9998545	unknown	0	unknown	0	-
NLF013_scaffold14354_6	12994	phage	0.999	temperate	0.99983406	unknown	0	Geobacillus kaustophilus	0.88	Predict
NLF013_scaffold30746_1	13932	phage	0.998	temperate	0.99985975	unknown	0	Candidatus Pelagibacter ubique	0.92	Predict
NLF013_scaffold12177_2	11552	phage	0.517	temperate	0.99984926	unknown	0	Staphylococcus saprophyticus	0.72	Predict
NLF013_C659127_1	14142	phage	0.999	virulent	0.9998688	Salasmaviridae	1	Staphylococcus saprophyticus	1	CRISPR
NLF013_scaffold34744_1	10220	phage	0.958	temperate	0.9996158	unknown	0	unknown	0	-
NLF013_scaffold328_1	11815	phage	0.996	virulent	0.99982435	Kyanoviridae	1	Candidatus Hamiltonella defensa	0.85	Predict
NLF013_scaffold33849_1	36715	phage	0.999	virulent	0.99777085	unknown	0	Candidatus Pelagibacter ubique	0.99	Predict
NLF013_scaffold23543_4	18763	phage	0.998	temperate	0.9994632	unknown	0	Klebsiella oxytoca	0.73	Predict

NLF013_scaffold19270_4	18485	phage	0.998	virulent	0.99980664	Ackermannviridae	0.35133132	Streptococcus mutans	0.86	Predict
NLF013_scaffold4799_2	13492	phage	0.999	temperate	0.9991315	unknown	0	unknown	0	-
NLF013_scaffold44405_3	10443	phage	0.989	virulent	0.99987406	Ackermannviridae	0.45278424	Streptococcus mutans	0.9	Predict
NLF013_scaffold32342_11	12264	phage	0.991	virulent	0.9998126	unknown	0	Clostridium perfringens	0.99	Predict
NLF013_scaffold47264_1	14809	phage	0.999	temperate	0.99986035	unknown	0	Pseudomonas tolaasii	0.99	Predict
NLF013_scaffold33946_4	24420	phage	1	temperate	0.99867666	unknown	0	Streptococcus mutans	0.96	Predict
NLF013_scaffold25755_1	11083	phage	0.87	virulent	0.99987036	unknown	0	unknown	0	-
NLF013_scaffold11031_7	15417	phage	0.999	temperate	0.9998593	Straboviridae	0.5943833	Dinoroseobacter shibae	0.89	Predict
NLF013_scaffold21878_2	13110	phage	0.998	temperate	0.9998584	unknown	0	unknown	0	-
NLF013_scaffold4561_28	12635	phage	0.997	virulent	0.99981666	Ackermannviridae	0.32071397	Streptococcus mutans	0.76	Predict
NLF013_scaffold23156_2	10828	phage	0.992	virulent	0.9905981	unknown	0	unknown	0	-
NLF013_scaffold30660_7	15685	phage	0.998	temperate	0.99921	unknown	0	unknown	0	-
NLF013_C659857_1	38936	phage	1	virulent	0.9998479	unknown	0	Staphylococcus saprophyticus	1	CRISPR
NLF013_scaffold20413_4	31272	phage	0.999	virulent	0.99986744	Herelleviridae	1	unknown	0	-
NLF013_scaffold19359_1	13169	phage	0.993	temperate	0.99693155	unknown	0	Streptococcus mutans	0.73	Predict
NLF013_scaffold26044_5	10580	phage	1	virulent	0.99800235	unknown	0	Streptococcus mutans	0.9	Predict
NLF013_scaffold43192_14	14225	phage	1	virulent	0.9998688	unknown	0	Lactobacillus delbrueckii	0.79	Predict
NLF013_scaffold48338_2	23841	phage	0.991	temperate	0.9998588	Drexlerviridae	0.30199948	Streptococcus mutans	0.84	Predict
NLF014_scaffold142_6	13105	phage	0.998	virulent	0.9998722	unknown	0	unknown	0	-
NLF014_scaffold5840_1	11268	phage	0.949	temperate	0.99364245	Straboviridae	0.4415074	Bacteroides fragilis	0.81	Predict
NLF014_scaffold7292_1	11640	phage	0.986	virulent	0.99987316	Ackermannviridae	0.45278424	Streptococcus mutans	0.7	Predict
NLF014_scaffold342_1	11749	phage	0.62	virulent	0.9998588	unknown	0	Bacteroides fragilis	0.72	Predict
NLF014_scaffold9741_6	10823	phage	0.682	virulent	0.99987125	unknown	0	unknown	0	-
NLF014_scaffold1307_2	14395	phage	0.999	virulent	0.9788498	unknown	0	unknown	0	-
NLF014_scaffold6197_2	11956	phage	0.996	virulent	0.8716869	Straboviridae	0.4734725	Streptococcus mutans	0.8	Predict
NLF014_scaffold10772_2	10611	phage	0.992	virulent	0.9981197	unknown	0	unknown	0	-
NLF014_scaffold249_3	11757	phage	0.525	virulent	0.9998684	unknown	0	Streptococcus mutans	0.85	Predict
NLF014_scaffold819_1	16305	phage	0.92	virulent	0.9998722	Ackermannviridae	0.34401792	Streptococcus mutans	0.73	Predict
NLF014_scaffold9408_9	12375	phage	0.997	virulent	0.99987036	unknown	0	Candidatus Pelagibacter ubique	0.79	Predict
NLF014_scaffold6884_2	18228	phage	0.993	temperate	0.9998465	Straboviridae	0.72049403	Lactobacillus johnsonii	0.91	Predict

NLF014_scaffold73_1	15165	phage	0.998	virulent	0.9998736	Straboviridae	0.34519532	Staphylococcus saprophyticus	0.79	Predict
NLF014_scaffold736_2	13993	phage	0.999	virulent	0.9998727	unknown	0	Streptococcus mutans	0.93	Predict
NLF015_scaffold31615_2	10464	phage	0.993	temperate	0.99646026	Mesyanzhinovviridae	0.50958985	Dinoroseobacter shibae	0.99	Predict
NLF015_scaffold32932_4	12289	phage	0.984	temperate	0.99985313	unknown	0	unknown	0	-
NLF015_scaffold31821_3	11275	phage	0.961	temperate	0.979654	unknown	0	Bacteroides fragilis	0.71	Predict
NLF015_scaffold24231_1	18735	phage	0.998	virulent	0.99987036	unknown	0	Staphylococcus saprophyticus	1	CRISPR
NLF015_C419895_1	21438	phage	0.999	virulent	0.99987173	Peduoviridae	0.28792256	Streptococcus mutans	1	CRISPR
NLF015_scaffold14491_1	13242	phage	1	virulent	0.9998308	unknown	0	Bacteroides fragilis	0.73	Predict
NLF015_scaffold22095_1	17233	phage	0.935	temperate	0.9998588	unknown	0	Staphylococcus saprophyticus	0.86	Predict
NLF015_scaffold591_2	23899	phage	0.977	temperate	0.89331686	Straboviridae	0.4734725	Candidatus Pelagibacter ubique	0.7	Predict
NLF015_scaffold21847_3	15424	phage	0.939	virulent	0.99987173	unknown	0	Staphylococcus saprophyticus	0.95	Predict
NLF015_scaffold11481_6	10297	phage	0.932	virulent	0.9998727	Straboviridae	0.3779054	Bacteroides fragilis	1	Predict
NLF015_scaffold22_1	21073	phage	0.999	temperate	0.9607604	unknown	0	Streptococcus mutans	0.8	Predict
NLF015_scaffold3859_2	61904	phage	0.544	temperate	0.9998049	Mesyanzhinovviridae	0.28275466	Streptococcus mutans	0.81	Predict
NLF015_scaffold34057_2	15068	phage	0.997	virulent	0.99987173	unknown	0	Flavobacterium columnare	0.75	Predict
NLF015_scaffold24362_4	11724	phage	0.686	virulent	0.99984694	Straboviridae	0.77169925	Bacteroides fragilis	0.81	Predict
NLF015_scaffold29017_1	14367	phage	0.983	temperate	0.9998593	Straboviridae	0.35911113	Candidatus Liberibacter asiaticus	0.95	Predict
NLF015_scaffold31211_3	13645	phage	0.999	temperate	0.9995867	Ackermannviridae	0.28982037	Streptococcus mutans	0.74	Predict
NLF015_scaffold12014_3	17261	phage	0.999	virulent	0.99984884	unknown	0	Streptococcus mutans	0.92	Predict
NLF015_scaffold8589_4	10494	phage	0.779	virulent	0.8963725	unknown	0	Bacteroides fragilis	0.91	Predict
NLF015_scaffold14946_1	12481	phage	0.675	virulent	0.99985695	unknown	0	Bacteroides fragilis	0.97	Predict
NLF015_scaffold20215_1	27132	phage	0.995	virulent	0.9700904	unknown	0	Streptococcus mutans	0.96	Predict
NLF015_scaffold20500_6	15898	phage	0.919	temperate	0.9413989	Straboviridae	0.41627434	Bacteroides fragilis	0.82	Predict
NLF015_scaffold13173_2	45408	phage	0.988	virulent	0.88146806	Ackermannviridae	0.33847627	unknown	0	-
NLF015_scaffold7628_6	10517	phage	0.672	virulent	0.9998574	Peduoviridae	0.3274082	Dinoroseobacter shibae	1	CRISPR
NLF015_scaffold14_1	10553	phage	0.976	temperate	0.9541461	Mesyanzhinovviridae	0.53950065	Dinoroseobacter shibae	0.96	Predict
NLF015_C419419_1	12316	phage	0.998	virulent	0.9998722	unknown	0	unknown	0	-
NLF015_scaffold3382_3	61525	phage	0.995	virulent	0.99587125	Peduoviridae	0.36351505	Lactobacillus fermentum	1	CRISPR
NLF015_scaffold34269_1	12510	phage	0.916	temperate	0.9998593	Straboviridae	0.8236184	Streptococcus mutans	0.95	Predict
NLF015_scaffold33332_2	13047	phage	0.995	temperate	0.99976444	Drexlerviridae	0.25311825	Streptococcus mutans	0.97	Predict

NLF015_scaffold4657_3	10779	phage	0.965	temperate	0.999417	Straboviridae	0.6525488	Lactobacillus johnsonii	0.73	Predict
NLF015_scaffold32259_1	23878	phage	0.999	temperate	0.9991372	unknown	0	Staphylococcus saprophyticus	1	CRISPR
NLF015_scaffold13163_7	10913	phage	0.999	virulent	0.99987173	Salasmaviridae	0.4600486	Streptococcus mutans	1	CRISPR
NLF015_scaffold6258_2	13515	phage	0.996	virulent	0.908313	unknown	0	unknown	0	-
NLF015_scaffold34558_2	23543	phage	0.996	virulent	0.9998656	unknown	0	Streptococcus mutans	0.72	Predict
NLF015_scaffold21847_1	10706	phage	0.997	virulent	0.9998608	unknown	0	Staphylococcus saprophyticus	1	CRISPR
DLM001_scaffold37_3	10138	phage	0.988	virulent	0.99979955	unknown	0	Bacteroides fragilis	0.86	Predict
DLM001_scaffold17760_6	20020	phage	0.981	virulent	0.9998693	Straboviridae	0.6455734	Candidatus Hamiltonella defensa	0.88	Predict
DLM001_scaffold57834_1	11731	phage	0.999	temperate	0.9998593	unknown	0	Candidatus Hamiltonella defensa	0.76	Predict
DLM001_scaffold91_6	41688	phage	1	virulent	0.99764246	Straboviridae	0.557449	Candidatus Hamiltonella defensa	1	CRISPR
DLM001_scaffold4307_3	15053	phage	0.997	temperate	0.9987186	Peduoviridae	0.5045494	Candidatus Hamiltonella defensa	0.93	Predict
DLM001_scaffold36534_1	15541	phage	1	temperate	0.98458403	unknown	0	Candidatus Hamiltonella defensa	0.82	Predict
DLM001_scaffold19551_4	15052	phage	0.999	virulent	0.99984217	Peduoviridae	0.46964702	Candidatus Hamiltonella defensa	1	CRISPR
DLM001_scaffold26672_2	14778	phage	0.824	temperate	0.9998584	unknown	0	Faecalibacterium prausnitzii	1	CRISPR
DLM001_scaffold21921_3	14336	phage	0.995	virulent	0.99956113	unknown	0	Candidatus Hamiltonella defensa	0.96	Predict
DLM001_scaffold2435_6	13748	phage	0.982	temperate	0.99985605	unknown	0	unknown	0	-
DLM001_scaffold23814_4	10075	phage	0.941	virulent	0.9996882	Straboviridae	0.61924237	Bacteroides fragilis	1	CRISPR
DLM001_scaffold14847_5	15869	phage	0.856	temperate	0.93894786	Straboviridae	0.40471888	Bacteroides fragilis	0.7	Predict
DLM001_scaffold42296_1	13564	phage	0.678	temperate	0.99985975	Straboviridae	0.571929	Candidatus Hamiltonella defensa	0.81	Predict
DLM001_scaffold4307_2	10139	phage	0.955	temperate	0.99954665	Peduoviridae	0.5289318	Candidatus Hamiltonella defensa	0.79	Predict
DLM001_scaffold31256_1	14890	phage	0.746	temperate	0.9868924	unknown	0	Rhodococcus rhodochrous	0.77	Predict
DLM001_scaffold52744_1	17562	phage	0.998	virulent	0.9997631	Ackermannviridae	0.29288775	Candidatus Hamiltonella defensa	0.81	Predict
DLM001_scaffold20858_1	10825	phage	0.997	virulent	0.9995579	Straboviridae	0.18533131	Lactobacillus gasseri	0.91	Predict
DLM001_scaffold27372_1	10220	phage	0.998	virulent	0.99987125	Straboviridae	0.58128864	Candidatus Hamiltonella defensa	1	CRISPR
DLM001_scaffold55116_2	10035	phage	1	virulent	0.83690894	unknown	0	unknown	0	-
DLM001_scaffold57732_2	21863	phage	0.999	virulent	0.98364675	Straboviridae	0.69763845	Candidatus Hamiltonella defensa	0.78	Predict
DLM001_scaffold17614_8	10799	phage	0.998	virulent	0.9998665	Mesyanzhinovviridae	0.42145562	unknown	0	-
DLM001_scaffold57716_1	20615	phage	0.994	temperate	0.9800632	Straboviridae	0.62921655	Candidatus Hamiltonella defensa	0.75	Predict
DLM001_scaffold25895_8	13269	phage	1	temperate	0.9998431	unknown	0	unknown	0	-
DLM001_scaffold21223_1	13962	phage	0.992	temperate	0.9390359	unknown	0	unknown	0	-

DLM001_scaffold54893_2	12328	phage	0.999	virulent	0.99987125	unknown	0	Candidatus Hamiltonella defensa	1 CRISPR
DLM001_scaffold30022_1	31255	phage	0.998	temperate	0.99984926	unknown	0	Candidatus Hamiltonella defensa	1 CRISPR
DLM001_scaffold58236_1	12503	phage	0.996	temperate	0.9080085	unknown	0	Candidatus Hamiltonella defensa	1 CRISPR
DLM001_scaffold34133_3	29709	phage	0.999	virulent	0.55847466	Zierdtviridae	0.23121345	Bacteroides fragilis	1 CRISPR
DLM001_scaffold51940_3	16275	phage	0.997	virulent	0.9998665	unknown	0	Candidatus Hamiltonella defensa	0.76 Predict
DLM001_scaffold49843_4	12597	phage	0.585	virulent	0.99987084	Straboviridae	0.22559838	Candidatus Hamiltonella defensa	0.78 Predict
DLM001_scaffold51928_3	10290	phage	0.994	temperate	0.97777677	Straboviridae	0.19912948	Candidatus Hamiltonella defensa	0.81 Predict
DLM001_scaffold19551_5	20800	phage	0.999	virulent	0.97540027	Mesyanzhinovviridae	0.18997274	Roseobacter denitrificans	0.92 Predict
DLM001_scaffold19465_5	18589	phage	0.911	virulent	0.9998693	Straboviridae	0.61924237	Candidatus Hamiltonella defensa	0.72 Predict
DLM001_scaffold56543_4	86366	phage	0.999	virulent	0.9489721	Drexlerviridae	0.33024517	Parabacteroides merdae	0.86 Predict
DLM001_scaffold14859_7	13630	phage	0.98	virulent	0.9998579	Schitoviridae	0.2646486	Bacteroides fragilis	0.7 Predict
DLM001_scaffold20858_3	12242	phage	0.996	temperate	0.994922	Straboviridae	0.20631503	Candidatus Hamiltonella defensa	0.88 Predict
DLM001_C705523_1	11555	phage	0.891	virulent	0.96739966	Straboviridae	0.5909823	Bacteroides fragilis	0.86 Predict
DLM001_scaffold17051_8	14437	phage	0.977	virulent	0.99986744	Ackermannviridae	0.34976166	Candidatus Hamiltonella defensa	0.85 Predict
DLM001_scaffold19551_1	21534	phage	0.855	virulent	0.99560606	Ackermannviridae	1	Candidatus Hamiltonella defensa	0.81 Predict
DLM001_scaffold20817_1	12697	phage	0.995	virulent	0.99987125	Straboviridae	0.58554715	Candidatus Hamiltonella defensa	0.88 Predict
DLM001_scaffold25009_5	11315	phage	0.996	temperate	0.99862176	Casjensviridae	0.91085446	Candidatus Hamiltonella defensa	0.83 Predict
DLM001_scaffold22953_2	21445	phage	0.999	virulent	0.99861836	unknown	0	Candidatus Hamiltonella defensa	0.78 Predict
DLM001_scaffold53015_2	34877	phage	0.999	virulent	0.99987084	unknown	0	Parabacteroides distasonis	1 CRISPR
DLM001_scaffold41842_2	13682	phage	0.997	temperate	0.9939871	unknown	0	Candidatus Hamiltonella defensa	0.91 Predict
DLM001_scaffold19551_3	18949	phage	0.999	temperate	0.8985467	Peduoviridae	0.36803198	Candidatus Hamiltonella defensa	1 CRISPR
DLM001_scaffold58253_1	28955	phage	0.998	temperate	0.999836	unknown	0	Clostridium perfringens	1 Predict
DLM001_scaffold53994_4	10216	phage	0.987	temperate	0.9998308	Casjensviridae	0.91085446	Candidatus Hamiltonella defensa	0.72 Predict
DLM001_scaffold56486_2	30648	phage	0.999	virulent	0.9941044	unknown	0	Candidatus Hamiltonella defensa	0.81 Predict
DLM001_scaffold48552_3	27133	phage	0.845	virulent	0.9998128	unknown	0	Candidatus Hamiltonella defensa	0.71 Predict
DLM001_scaffold4106_5	11971	phage	0.989	temperate	0.99691886	unknown	0	Candidatus Hamiltonella defensa	0.78 Predict
DLM001_scaffold51591_2	39852	phage	0.996	virulent	0.9998688	Drexlerviridae	0.27567244	Candidatus Hamiltonella defensa	0.84 Predict
DLM001_scaffold10420_4	14915	phage	0.995	virulent	0.95498294	Drexlerviridae	0.1742411	Candidatus Hamiltonella defensa	0.96 Predict
DLM001_scaffold58124_4	21230	phage	0.999	virulent	0.9976206	Zierdtviridae	1	Bifidobacterium longum	1 CRISPR
DLM001_scaffold58250_2	11280	phage	0.98	temperate	0.81878257	unknown	0	Candidatus Hamiltonella defensa	0.87 Predict

DLM001_scaffold23040_12	12866	phage	0.992	temperate	0.9997654	Straboviridae	0.6360723	Candidatus Hamiltonella defensa	0.77	Predict
DLM001_C705425_1	11227	phage	0.686	temperate	0.9704434	Straboviridae	0.64132065	Candidatus Hamiltonella defensa	0.94	Predict
DLM001_scaffold52289_2	17071	phage	0.994	virulent	0.99987316	unknown	0	unknown	0	-
DLM001_scaffold18361_2	11461	phage	0.84	virulent	0.9998593	Ackermannviridae	1	Candidatus Hamiltonella defensa	0.75	Predict
DLM001_scaffold55454_4	14826	phage	0.999	temperate	0.9998374	unknown	0	unknown	0	-
DLM001_scaffold39252_2	10163	phage	0.996	virulent	0.9997156	Straboviridae	0.38693213	Candidatus Hamiltonella defensa	0.72	Predict
DLM001_scaffold55964_1	11551	phage	0.995	virulent	0.9998302	unknown	0	Candidatus Hamiltonella defensa	0.82	Predict
DLM001_scaffold12282_12	11929	phage	0.984	temperate	0.99980354	unknown	0	unknown	0	-
DLM001_scaffold13671_1	16209	phage	0.902	virulent	0.99987173	Ackermannviridae	0.6493801	Bacteroides fragilis	0.73	Predict
DLM001_scaffold50893_2	12371	phage	0.999	virulent	0.9998665	Herelleviridae	1	unknown	0	-
DLM001_scaffold277_3	32610	phage	0.994	virulent	0.99980354	Casjensviridae	0.65000147	Bacteroides fragilis	0.94	Predict
DLM001_scaffold39468_1	10961	phage	0.992	virulent	0.9998736	Vilmaviridae	1	Candidatus Hamiltonella defensa	0.95	Predict
DLM001_scaffold47007_2	14893	phage	0.963	virulent	0.9998688	Drexlerviridae	0.18274732	unknown	0	-
DLM001_scaffold49276_1	13348	phage	0.998	temperate	0.99985975	unknown	0	Candidatus Hamiltonella defensa	1	CRISPR
DLM001_scaffold21002_1	12797	phage	0.999	virulent	0.9991016	unknown	0	Parabacteroides distasonis	0.73	Predict
DLM001_scaffold42810_2	31211	phage	0.996	temperate	0.99985695	Straboviridae	0.54152817	Candidatus Hamiltonella defensa	0.95	Predict
DLM001_scaffold49834_2	14079	phage	0.902	virulent	0.99987406	unknown	0	Candidatus Hamiltonella defensa	0.92	Predict
DLM001_scaffold16868_1	12467	phage	0.993	temperate	0.99982554	unknown	0	Candidatus Hamiltonella defensa	0.77	Predict
DLM001_scaffold52139_2	19537	phage	0.999	virulent	0.9998288	Casjensviridae	0.46940973	Candidatus Hamiltonella defensa	1	CRISPR
DLM001_scaffold11723_1	13689	phage	0.998	temperate	0.99986035	unknown	0	Candidatus Hamiltonella defensa	0.71	Predict
DLM001_scaffold19427_9	12730	phage	0.998	virulent	0.9599575	unknown	0	Bacteroides fragilis	0.97	Predict
DLM001_scaffold27611_5	14735	phage	0.993	virulent	0.9998636	Ackermannviridae	0.34976166	Candidatus Hamiltonella defensa	1	Predict
DLM001_scaffold14114_7	17341	phage	0.996	virulent	0.9998645	unknown	0	Candidatus Hamiltonella defensa	0.92	Predict
DLM001_scaffold3576_10	14949	phage	0.997	virulent	0.9743186	unknown	0	Flavobacterium columnare	0.89	Predict
DLM002_scaffold52283_2	11734	phage	0.96	virulent	0.9998727	Mesyanzhinovviridae	0.34989476	Candidatus Hamiltonella defensa	0.84	Predict
DLM002_scaffold35924_1	21533	phage	0.998	temperate	0.99985975	unknown	0	Candidatus Hamiltonella defensa	0.88	Predict
DLM002_scaffold39638_1	14432	phage	0.998	temperate	0.9998593	unknown	0	Megamonas funiformis	1	CRISPR
DLM002_scaffold58_3	22043	phage	0.998	virulent	0.99925953	unknown	0	Prevotella sp. P4-65	1	CRISPR
DLM002_scaffold8357_7	13212	phage	0.999	temperate	0.9998593	Peduoviridae	1	Candidatus Hamiltonella defensa	0.79	Predict
DLM002_scaffold37454_1	16127	phage	0.989	virulent	0.99987125	unknown	0	Morganella morganii	0.97	Predict

DLM002_scaffold37352_8	14683	phage	1	virulent	0.9998684	Autographiviridae	1	Candidatus Hamiltonella defensa	0.89	Predict
DLM002_scaffold4794_6	14102	phage	0.998	virulent	0.9998006	unknown	0	Clostridium tetani	0.9	Predict
DLM002_scaffold52713_2	30736	phage	0.998	temperate	0.9998165	Straboviridae	0.44480026	Candidatus Hamiltonella defensa	0.91	Predict
DLM002_scaffold45452_2	13872	phage	0.983	temperate	0.99985975	unknown	0	Candidatus Hamiltonella defensa	0.95	Predict
DLM002_scaffold48724_2	21004	phage	0.989	temperate	0.99985975	Straboviridae	0.3196358	Streptococcus mutans	0.85	Predict
DLM002_scaffold11013_7	11127	phage	0.949	virulent	0.99987316	Zierdtviridae	0.55246586	Candidatus Hamiltonella defensa	0.97	Predict
DLM002_scaffold5537_11	12077	phage	0.999	virulent	0.99987406	unknown	0	unknown	0	-
DLM002_scaffold2881_3	10804	phage	0.996	temperate	0.62338036	unknown	0	Parabacteroides distasonis	0.72	Predict
DLM002_scaffold52526_1	23854	phage	0.999	temperate	0.9998565	unknown	0	Candidatus Hamiltonella defensa	1	CRISPR
DLM002_scaffold44502_1	21399	phage	0.996	temperate	0.9998593	Straboviridae	0.5021334	Candidatus Hamiltonella defensa	0.73	Predict
DLM002_scaffold52526_4	11284	phage	0.999	temperate	0.9998584	unknown	0	Candidatus Hamiltonella defensa	1	CRISPR
DLM002_scaffold48180_13	18494	phage	0.999	virulent	0.99987125	no_family_avaliable(NC_062765)	0.954	Candidatus Hamiltonella defensa	1	CRISPR
DLM002_scaffold42939_1	12424	phage	0.991	temperate	0.9998593	unknown	0	Candidatus Hamiltonella defensa	0.84	Predict
DLM002_scaffold52584_1	15017	phage	0.999	virulent	0.99339837	unknown	0	Candidatus Hamiltonella defensa	0.78	Predict
DLM002_scaffold32192_3	14937	phage	0.999	virulent	0.9998693	Drexlerviridae	0.28968555	Candidatus Hamiltonella defensa	1	Predict
DLM002_scaffold5336_45	11008	phage	0.999	virulent	0.9988257	Casjensviridae	1	Candidatus Hamiltonella defensa	0.93	Predict
DLM002_scaffold52696_1	40967	phage	0.999	temperate	0.6640839	unknown	0	Clostridium perfringens	1	CRISPR
DLM002_scaffold925_8	11223	phage	0.932	virulent	0.9998727	Ackermannviridae	0.6232304	Bacteroides fragilis	0.92	Predict
DLM003_scaffold30478_2	16288	phage	0.999	virulent	0.9998688	no_family_avaliable(NC_067216)	0.974	Candidatus Hamiltonella defensa	1	CRISPR
DLM003_scaffold35975_4	15476	phage	0.997	temperate	0.9613774	unknown	0	Parabacteroides distasonis	0.83	Predict
DLM003_scaffold20093_1	12373	phage	0.961	virulent	0.9997367	Casjensviridae	0.74338967	Candidatus Hamiltonella defensa	0.93	Predict
DLM003_scaffold8802_8	10542	phage	0.936	virulent	0.9983713	unknown	0	Candidatus Hamiltonella defensa	0.79	Predict
DLM003_scaffold1898_14	23378	phage	0.999	virulent	0.94656765	Straboviridae	0.6252392	Candidatus Hamiltonella defensa	1	CRISPR
DLM003_scaffold30928_7	12195	phage	0.932	virulent	0.9998727	Ackermannviridae	0.6232304	Candidatus Hamiltonella defensa	0.76	Predict
DLM003_scaffold59785_6	15230	phage	0.998	virulent	0.99987084	unknown	0	Cellulophaga baltica	1	CRISPR
DLM003_scaffold67334_2	21358	phage	0.999	virulent	0.99987406	unknown	0	unknown	0	-
DLM003_C810975_1	111799	phage	0.998	virulent	0.9998627	Drexlerviridae	0.33024517	Parabacteroides distasonis	1	CRISPR
DLM003_scaffold31156_2	13370	phage	0.999	virulent	0.99987125	unknown	0	Bacteroides vulgatus	1	CRISPR
DLM003_scaffold24702_43	10725	phage	0.998	virulent	0.99987316	unknown	0	Candidatus Hamiltonella defensa	0.94	Predict
DLM003_scaffold10664_1	14986	phage	0.996	virulent	0.8965864	Straboviridae	0.571929	Candidatus Hamiltonella defensa	0.93	Predict

DLM003_scaffold46929_2	29872	phage	0.999	virulent	0.9998699	no_family_avaliable(NC_062765)	0.985	Parabacteroides distasonis	1	CRISPR
DLM003_scaffold59785_5	15974	phage	0.997	virulent	0.9998688	Ackermannviridae	1	Cellulophaga baltica	1	CRISPR
DLM003_scaffold40695_7	29544	phage	0.999	virulent	0.99984974	Kyanoviridae	0.20182836	Candidatus Hamiltonella defensa	1	CRISPR
DLM003_scaffold4744_1	12135	phage	0.994	virulent	0.99987084	unknown	0	Bacteroides fragilis	0.8	Predict
DLM003_scaffold303_2	13419	phage	0.856	temperate	0.9998202	Ackermannviridae	0.21766534	Candidatus Hamiltonella defensa	0.84	Predict
DLM003_scaffold36199_4	10106	phage	0.563	virulent	0.9998693	unknown	0	unknown	0	-
DLM003_scaffold12201_4	10976	phage	0.999	virulent	0.89352334	unknown	0	Candidatus Hamiltonella defensa	0.89	Predict
DLM003_scaffold16412_17	12383	phage	0.999	temperate	0.9998579	unknown	0	unknown	0	-
DLM003_scaffold49406_1	26722	phage	0.998	virulent	0.9985555	Straboviridae	0.4553038	Candidatus Hamiltonella defensa	0.85	Predict
DLM003_C810753_1	16312	phage	0.999	virulent	0.9998665	Salasmaviridae	1	Actinomyces naeslundii	1	CRISPR
DLM003_scaffold1898_11	11420	phage	0.999	virulent	0.99987173	unknown	0	Bacteroides salyersiae	1	CRISPR
DLM003_scaffold66050_1	31770	phage	0.997	virulent	0.9998679	unknown	0	unknown	0	-
DLM003_scaffold17135_3	10110	phage	0.827	virulent	0.99986404	Ackermannviridae	1	Candidatus Hamiltonella defensa	0.82	Predict
DLM003_scaffold29743_2	10335	phage	0.999	virulent	0.99987406	unknown	0	Candidatus Hamiltonella defensa	1	CRISPR
DLM003_scaffold14015_13	15158	phage	0.749	temperate	0.99984264	Peduoviridae	0.5289318	Candidatus Hamiltonella defensa	0.88	Predict
DLM003_scaffold3089_1	10243	phage	0.999	virulent	0.9998684	Salasmaviridae	0.31627378	unknown	0	-
DLM003_scaffold15117_2	12083	phage	0.999	virulent	0.9998693	Straboviridae	0.26355055	Candidatus Hamiltonella defensa	0.88	Predict
DLM003_scaffold59785_12	17059	phage	0.998	virulent	0.99987036	Herelleviridae	1	Bacteroides salyersiae	1	CRISPR
DLM003_scaffold102_3	11734	phage	0.996	virulent	0.99987036	Ackermannviridae	0.39516526	Rhizobium leguminosarum	0.8	Predict
DLM003_scaffold46929_4	41685	phage	0.999	virulent	0.9998645	no_family_avaliable(NC_062765)	0.964	Cellulophaga baltica	0.73	Predict
DLM003_scaffold40695_5	12359	phage	0.993	virulent	0.999555	Drexlerviridae	0.25272772	Bacteroides fragilis	0.76	Predict
DLM004_scaffold2416_6	13763	phage	0.989	virulent	0.99987036	unknown	0	unknown	0	-
DLM004_scaffold18621_3	14307	phage	0.999	temperate	0.99957764	unknown	0	Candidatus Hamiltonella defensa	0.71	Predict
DLM004_scaffold12500_3	11405	phage	0.999	virulent	0.99986315	Casjensviridae	0.48759285	Candidatus Hamiltonella defensa	0.83	Predict
DLM004_scaffold8053_11	18431	phage	0.999	virulent	0.9998413	Straboviridae	0.58128864	Candidatus Hamiltonella defensa	1	CRISPR
DLM004_scaffold2421_24	13261	phage	0.999	temperate	0.9998584	unknown	0	unknown	0	-
DLM004_scaffold8053_12	12658	phage	0.998	temperate	0.67867184	Straboviridae	0.62674475	Candidatus Hamiltonella defensa	1	CRISPR
DLM004_scaffold18777_1	29401	phage	0.998	temperate	0.9998579	Casjensviridae	0.4376873	Candidatus Hamiltonella defensa	0.99	Predict
DLM004_scaffold41_14	10062	phage	0.556	temperate	0.9992274	Straboviridae	0.56166416	Candidatus Hamiltonella defensa	0.92	Predict
DLM004_scaffold12500_2	11046	phage	0.998	virulent	0.9664789	unknown	0	Candidatus Hamiltonella defensa	0.87	Predict

DLM004_scaffold462_3	20356	phage	0.971	temperate	0.99986035	unknown	0	Aeromonas media	0.7	Predict
DLM004_scaffold18776_2	19732	phage	0.998	virulent	0.99987406	unknown	0	Lactobacillus plantarum	0.72	Predict
DLM004_scaffold112_3	17407	phage	0.999	virulent	0.9981354	unknown	0	Candidatus Hamiltonella defensa	1	CRISPR
DLM004_scaffold158_30	18012	phage	0.996	virulent	0.9998684	Ackermannviridae	0.31015208	Candidatus Hamiltonella defensa	0.83	Predict
DLM004_scaffold5647_2	16327	phage	0.998	virulent	0.9998722	unknown	0	Lactobacillus plantarum	0.74	Predict
DLM004_scaffold18395_2	13220	phage	0.996	temperate	0.7185659	unknown	0	Parabacteroides distasonis	0.78	Predict
DLM004_scaffold16125_1	10299	phage	0.997	temperate	0.9998593	Straboviridae	0.6043284	Candidatus Hamiltonella defensa	0.93	Predict
DLM004_scaffold12500_5	11562	phage	0.998	virulent	0.99987406	Drexlerviridae	0.27603	Candidatus Hamiltonella defensa	0.72	Predict
DLM004_scaffold11715_11	10454	phage	0.589	virulent	0.9998397	unknown	0	Candidatus Hamiltonella defensa	0.73	Predict
DLM004_scaffold15727_1	12099	phage	0.969	virulent	0.99987125	unknown	0	unknown	0	-
DLM004_scaffold17859_1	14003	phage	0.996	virulent	0.9998684	Straboviridae	0.66194147	Candidatus Hamiltonella defensa	0.9	Predict
DLM004_scaffold14310_1	28961	phage	0.973	virulent	0.9985355	Straboviridae	0.62921655	Candidatus Hamiltonella defensa	0.75	Predict
DLM004_scaffold14578_1	17682	phage	1	virulent	0.9997991	unknown	0	Candidatus Hamiltonella defensa	1	CRISPR
DLM004_scaffold9327_2	10479	phage	0.999	temperate	0.9942856	unknown	0	unknown	0	-
DLM004_scaffold16036_1	18380	phage	0.997	virulent	0.99985695	Ackermannviridae	0.34976166	Candidatus Hamiltonella defensa	0.85	Predict
DLM004_C304967_1	32491	phage	0.999	virulent	0.6261919	unknown	0	Candidatus Hamiltonella defensa	1	CRISPR
DLM004_scaffold12500_1	20763	phage	0.994	temperate	0.9998584	Casjensviridae	0.4376873	Candidatus Hamiltonella defensa	0.92	Predict
DLM004_scaffold8780_5	35963	phage	0.996	virulent	0.9998584	Straboviridae	0.6664747	Candidatus Hamiltonella defensa	0.83	Predict
DLM004_scaffold14269_9	10221	phage	0.998	temperate	0.99985975	unknown	0	Candidatus Hamiltonella defensa	0.71	Predict
DLM005_scaffold57948_1	49349	phage	0.999	virulent	0.92772204	unknown	0	Candidatus Hamiltonella defensa	1	CRISPR
DLM005_scaffold44327_3	24684	phage	0.998	virulent	0.9998565	Casjensviridae	0.811716	Candidatus Hamiltonella defensa	1	CRISPR
DLM005_scaffold34949_1	10474	phage	0.99	temperate	0.9997306	unknown	0	Candidatus Hamiltonella defensa	0.74	Predict
DLM005_scaffold48663_11	10088	phage	0.99	temperate	0.9998593	unknown	0	Candidatus Hamiltonella defensa	0.84	Predict
DLM005_scaffold9042_2	10572	phage	1	temperate	0.9998584	unknown	0	Candidatus Hamiltonella defensa	1	CRISPR
DLM005_scaffold3317_29	12016	phage	0.999	temperate	0.96424484	unknown	0	Candidatus Hamiltonella defensa	0.74	Predict
DLM005_scaffold10521_42	26343	phage	1	temperate	0.9998513	unknown	0	Candidatus Hamiltonella defensa	1	CRISPR
DLM005_scaffold39936_1	36078	phage	0.999	temperate	0.9704171	unknown	0	Clostridium perfringens	1	CRISPR
DLM005_scaffold21763_7	12233	phage	0.984	virulent	0.99987316	Ackermannviridae	0.6232304	Candidatus Hamiltonella defensa	0.85	Predict
DLM005_scaffold54507_2	35601	phage	1	temperate	0.99880844	unknown	0	Candidatus Hamiltonella defensa	1	CRISPR
DLM005_scaffold55834_11	10255	phage	0.987	virulent	0.5891354	Straboviridae	0.33227503	Bacteroides fragilis	0.76	Predict

DLM005_scaffold57953_2	17111	phage	0.878	temperate	0.9988467	unknown	0	Candidatus Hamiltonella defensa	1	CRISPR
DLM005_scaffold31312_1	12883	phage	1	temperate	0.9998584	no_family_avaliable(NC_027984)	0.981	Cronobacter sakazakii	1	CRISPR
DLM005_scaffold47946_1	10950	phage	0.996	temperate	0.995717	unknown	0	Parabacteroides distasonis	0.77	Predict
DLM005_scaffold56278_1	14909	phage	0.997	virulent	0.95286506	Ackermannviridae	0.3053488	Candidatus Hamiltonella defensa	0.92	Predict
DLM005_scaffold3128_1	13360	phage	0.992	temperate	0.999248	Straboviridae	0.6360723	Candidatus Hamiltonella defensa	0.98	Predict
DLM005_scaffold42596_2	35529	phage	0.994	virulent	0.99981785	unknown	0	Streptococcus mutans	1	CRISPR
DLM005_scaffold56545_2	14850	phage	0.988	virulent	0.9998736	unknown	0	Candidatus Hamiltonella defensa	0.96	Predict
DLM005_scaffold55666_1	14548	phage	0.998	virulent	0.99987084	Salasmaviridae	0.45254457	Candidatus Hamiltonella defensa	0.88	Predict
DLM005_scaffold21763_10	14038	phage	1	temperate	0.9998288	unknown	0	Bacteroides fragilis	1	CRISPR
DLM005_scaffold21049_5	10421	phage	0.998	temperate	0.9988944	unknown	0	Candidatus Hamiltonella defensa	0.89	Predict
DLM005_scaffold4283_10	22410	phage	0.947	temperate	0.9935788	Peduoviridae	0.5512526	Candidatus Hamiltonella defensa	0.81	Predict
DLM005_scaffold25964_2	12106	phage	0.996	virulent	0.99987036	unknown	0	Candidatus Hamiltonella defensa	0.83	Predict
DLM005_scaffold58045_1	39265	phage	0.999	temperate	0.9575632	Casjensviridae	0.73554254	Candidatus Hamiltonella defensa	1	CRISPR
DLM005_scaffold56278_2	14100	phage	0.933	virulent	0.999488	Peduoviridae	0.43608958	Azospirillum brasilense	0.93	Predict
DLM005_scaffold2340_3	10728	phage	0.998	temperate	0.8154053	Ackermannviridae	0.3847759	unknown	0	-
DLM005_scaffold5416_6	13214	phage	0.86	temperate	0.9998588	unknown	0	Candidatus Hamiltonella defensa	0.97	Predict
DLM005_scaffold36491_20	34680	phage	0.999	virulent	0.99974394	Peduoviridae	1	Candidatus Hamiltonella defensa	0.86	Predict
DLM005_scaffold20359_5	10776	phage	0.999	virulent	0.9998679	unknown	0	Candidatus Hamiltonella defensa	1	CRISPR
DLM005_scaffold56832_1	20612	phage	0.977	temperate	0.9998593	unknown	0	Prevotella copri	1	CRISPR
DLM005_scaffold36491_18	17170	phage	0.999	temperate	0.99982077	Casjensviridae	1	unknown	0	-
DLM005_scaffold7362_2	11712	phage	0.99	virulent	0.999784	unknown	0	Candidatus Hamiltonella defensa	0.82	Predict
DLM005_scaffold57077_1	11606	phage	0.994	temperate	0.99985975	Peduoviridae	0.61929643	Candidatus Hamiltonella defensa	1	CRISPR
DLM005_scaffold5800_5	13271	phage	1	temperate	0.99985975	Peduoviridae	1	Candidatus Hamiltonella defensa	1	CRISPR
DLM005_scaffold20359_8	14646	phage	1	temperate	0.9802566	Peduoviridae	0.3466597	Listeria monocytogenes	1	CRISPR
DLM005_scaffold27309_11	12486	phage	0.99	virulent	0.9994256	unknown	0	Micromonospora chaiyaphumensis	1	CRISPR
DLM005_scaffold25964_1	13330	phage	0.985	virulent	0.9557909	unknown	0	Candidatus Hamiltonella defensa	0.78	Predict
DLM005_scaffold10521_41	10124	phage	0.999	virulent	0.8529024	unknown	0	unknown	0	-
DLM005_scaffold57953_1	13961	phage	0.999	temperate	0.8793201	Peduoviridae	1	Candidatus Hamiltonella defensa	1	CRISPR
DLM005_scaffold52507_2	22027	phage	0.999	virulent	0.9998684	unknown	0	Bacteroides fragilis	0.83	Predict
DLM005_scaffold1593_32	16342	phage	0.995	temperate	0.9998588	Straboviridae	0.5348034	Candidatus Hamiltonella defensa	0.75	Predict

DLM005_scaffold36516_2	12407	phage	0.999	virulent	0.9993786	unknown	0	Candidatus Hamiltonella defensa	0.86 Predict
DLM006_scaffold29093_1	15174	phage	0.999	virulent	0.99235404	Salasmaviridae	0.45254457	Candidatus Hamiltonella defensa	0.96 Predict
DLM006_scaffold5249_12	11026	phage	0.999	temperate	0.977781	unknown	0	Candidatus Hamiltonella defensa	1 CRISPR
DLM006_scaffold9115_2	20142	phage	0.999	temperate	0.99984926	unknown	0	Bacillus alcalophilus	1 CRISPR
DLM006_scaffold23991_4	14698	phage	0.999	virulent	0.9998688	Straboviridae	0.44908637	Candidatus Hamiltonella defensa	0.87 Predict
DLM006_scaffold31867_1	38951	phage	0.999	virulent	0.99986744	no_family_avaliable(NC_067216)	0.966	Candidatus Pelagibacter ubique	0.83 Predict
DLM006_scaffold1615_27	35323	phage	0.999	temperate	0.99984694	Ackermannviridae	1	Candidatus Hamiltonella defensa	1 CRISPR
DLM006_scaffold35098_1	33393	phage	0.975	temperate	0.9997101	Casjensviridae	1	Candidatus Hamiltonella defensa	1 CRISPR
DLM006_scaffold8798_11	15736	phage	0.852	temperate	0.98404866	unknown	0	Lactobacillus fermentum	0.71 Predict
DLM006_scaffold15729_4	10385	phage	0.918	temperate	0.99985695	unknown	0	unknown	0 -
DLM006_scaffold10416_3	14673	phage	0.999	temperate	0.89246756	Peduoviridae	0.3525797	unknown	0 -
DLM006_scaffold289_5	11815	phage	0.999	virulent	0.99987125	Mesyanzhinovviridae	0.34989476	Candidatus Hamiltonella defensa	0.73 Predict
DLM006_scaffold15803_18	17589	phage	0.998	virulent	0.99791527	Casjensviridae	0.4376873	Candidatus Hamiltonella defensa	0.87 Predict
DLM006_scaffold3905_28	10110	phage	0.998	temperate	0.9795305	unknown	0	Candidatus Hamiltonella defensa	0.96 Predict
DLM006_scaffold13732_3	12277	phage	0.781	virulent	0.99987084	unknown	0	unknown	0 -
DLM006_scaffold23991_2	33321	phage	0.999	temperate	0.99981356	Straboviridae	0.38295603	Candidatus Hamiltonella defensa	0.83 Predict
DLM006_scaffold5392_1	55913	phage	0.999	temperate	0.9774004	Casjensviridae	1	Candidatus Hamiltonella defensa	0.7 Predict
DLM006_scaffold19568_4	13416	phage	0.906	temperate	0.97331256	Straboviridae	0.33227503	Candidatus Hamiltonella defensa	0.72 Predict
DLM006_scaffold35098_2	15946	phage	0.999	virulent	0.96943474	unknown	0	Candidatus Hamiltonella defensa	1 CRISPR
DLM006_scaffold3099_10	18901	phage	0.604	virulent	0.9998508	unknown	0	Candidatus Hamiltonella defensa	0.84 Predict
DLM006_scaffold22671_1	15326	phage	0.999	virulent	0.9998688	unknown	0	Candidatus Hamiltonella defensa	0.95 Predict
DLM006_scaffold12233_4	11151	phage	0.998	temperate	0.99985975	unknown	0	Candidatus Hamiltonella defensa	0.98 Predict
DLM006_scaffold13444_2	10799	phage	1	temperate	0.99985975	unknown	0	unknown	0 -
DLM006_scaffold582_5	20266	phage	0.913	virulent	0.99987084	Straboviridae	0.38693213	Thermoanaerobacterium saccharolyti	0.86 Predict
DLM006_scaffold7136_55	10887	phage	0.998	temperate	0.99985975	unknown	0	Candidatus Hamiltonella defensa	0.98 Predict
DLM006_scaffold36079_2	14889	phage	0.686	temperate	0.9998584	unknown	0	Parabacteroides merdae	1 CRISPR
DLM006_scaffold22549_2	14487	phage	0.583	temperate	0.9989599	unknown	0	unknown	0 -
DLM007_scaffold30789_1	10237	phage	0.91	temperate	0.99986035	unknown	0	Parabacteroides merdae	0.76 Predict
DLM007_scaffold45247_1	12897	phage	0.999	temperate	0.99983126	unknown	0	Candidatus Hamiltonella defensa	1 CRISPR
DLM007_scaffold7436_7	10480	phage	1	virulent	0.9998736	Peduoviridae	0.38523066	Candidatus Hamiltonella defensa	0.8 Predict

DLM007_scaffold20148_2	34505	phage	0.999	virulent	0.99987036	no_family_avaliable(NC_024711)	0.963	unknown	0	-
DLM007_C664617_1	13634	phage	1	virulent	0.9998699	Salasmaviridae	1	Actinomyces naeslundii	0.88	Predict
DLM007_scaffold45308_2	26803	phage	0.999	virulent	0.99987173	Demerecviridae	0.33583313	Parabacteroides sp. D13	1	CRISPR
DLM007_scaffold35599_2	21504	phage	0.997	virulent	0.943157	Straboviridae	0.571929	Bacteroides fragilis	0.97	Predict
DLM007_scaffold28062_4	16050	phage	0.999	virulent	0.9998699	Straboviridae	0.58128864	Candidatus Hamiltonella defensa	1	CRISPR
DLM007_scaffold13248_1	13437	phage	0.997	virulent	0.95286506	Ackermannviridae	0.3053488	Candidatus Hamiltonella defensa	0.91	Predict
DLM007_scaffold20148_1	60836	phage	1	virulent	0.9998684	no_family_avaliable(NC_024711)	0.971	unknown	0	-
DLM007_scaffold31482_1	21688	phage	0.999	temperate	0.9998579	unknown	0	Candidatus Hamiltonella defensa	1	CRISPR
DLM007_scaffold42236_1	31648	phage	0.999	temperate	0.99982446	unknown	0	Candidatus Hamiltonella defensa	1	CRISPR
DLM007_scaffold5339_25	16637	phage	0.77	virulent	0.99986607	Straboviridae	0.31710467	Vibrio natriegens	0.84	Predict
DLM007_scaffold41113_2	10650	phage	0.999	virulent	0.93330806	unknown	0	Clostridioides difficile	0.85	Predict
DLM007_scaffold4568_8	22519	phage	0.992	temperate	0.99985975	unknown	0	Flavobacterium columnare	0.81	Predict
DLM007_scaffold45002_8	18372	phage	0.999	temperate	0.9995805	unknown	0	unknown	0	-
DLM007_scaffold36909_2	13503	phage	0.984	temperate	0.9989953	unknown	0	Mycoplasma pulmonis	1	CRISPR
DLM007_scaffold45251_1	19177	phage	0.996	virulent	0.9998551	Straboviridae	0.61924237	Candidatus Hamiltonella defensa	0.93	Predict
DLM008_scaffold4090_5	11022	phage	0.989	temperate	0.9998593	unknown	0	Candidatus Hamiltonella defensa	0.99	Predict
DLM008_scaffold251_3	18355	phage	1	virulent	0.9998431	unknown	0	Candidatus Hamiltonella defensa	1	CRISPR
DLM008_scaffold51033_29	10254	phage	0.75	virulent	0.9990291	unknown	0	Candidatus Hamiltonella defensa	0.94	Predict
DLM008_scaffold34307_5	12555	phage	0.922	virulent	0.9996477	Vilmaviridae	1	Candidatus Hamiltonella defensa	0.94	Predict
DLM008_scaffold3167_2	11322	phage	0.999	virulent	0.9105999	unknown	0	Candidatus Hamiltonella defensa	0.93	Predict
DLM008_scaffold9016_5	12418	phage	0.998	virulent	0.99987084	no_family_avaliable(NC_055879)	0.955	unknown	0	-
DLM008_scaffold54665_3	17199	phage	0.998	virulent	0.9998736	unknown	0	unknown	0	-
DLM008_scaffold68109_1	10928	phage	0.995	virulent	0.9998736	unknown	0	Candidatus Hamiltonella defensa	0.75	Predict
DLM008_scaffold56745_2	12440	phage	0.976	temperate	0.9998593	Peduoviridae	1	Candidatus Hamiltonella defensa	0.9	Predict
DLM008_scaffold40955_1	10415	phage	0.999	virulent	0.99961644	unknown	0	Candidatus Hamiltonella defensa	0.73	Predict
DLM008_scaffold9016_1	10364	phage	0.998	virulent	0.9998722	no_family_avaliable(NC_055876)	0.953	unknown	0	-
DLM008_scaffold26298_3	25070	phage	0.998	temperate	0.92925435	Chaseviridae	0.49954474	Candidatus Hamiltonella defensa	1	CRISPR
DLM008_scaffold235_2	15574	phage	0.999	virulent	0.9998736	unknown	0	Bacteroides eggerthii	1	CRISPR
DLM008_scaffold19097_13	12842	phage	0.947	virulent	0.9949243	Straboviridae	0.61924237	Sinorhizobium meliloti	0.8	Predict
DLM008_scaffold58161_1	12353	phage	0.994	virulent	0.9986062	unknown	0	Candidatus Hamiltonella defensa	0.98	Predict

DLM008_scaffold349_4	14769	phage	0.999	virulent	0.9998693	no_family_avaliable(NC_067211)	0.958	Candidatus Pelagibacter ubique	0.88 Predict
DLM008_scaffold45871_1	10652	phage	0.749	temperate	0.9998588	Straboviridae	0.58876103	Candidatus Hamiltonella defensa	0.93 Predict
DLM008_scaffold9016_3	18739	phage	0.999	virulent	0.9998699	Straboviridae	0.5401171	unknown	0 -
DLM008_scaffold62586_4	11276	phage	0.974	temperate	0.98519784	unknown	0	Bacteroides fragilis	0.76 Predict
DLM008_scaffold46088_1	11660	phage	0.994	temperate	0.99985975	unknown	0	unknown	0 -
DLM008_scaffold371_1	15253	phage	0.642	temperate	0.9998593	Straboviridae	0.6741575	Candidatus Hamiltonella defensa	0.99 Predict
DLM008_scaffold68395_3	11591	phage	0.985	temperate	0.9998593	unknown	0	Candidatus Hamiltonella defensa	0.96 Predict
DLM008_scaffold43273_7	10287	phage	0.956	virulent	0.99987316	unknown	0	unknown	0 -
DLM008_scaffold68309_1	15130	phage	0.988	virulent	0.9855859	Straboviridae	0.34864473	unknown	0 -
DLM008_scaffold129_7	11957	phage	0.998	temperate	0.99985975	unknown	0	unknown	0 -
DLM008_scaffold58372_1	11223	phage	0.861	virulent	0.9998727	unknown	0	Candidatus Hamiltonella defensa	1 CRISPR
DLM008_scaffold129_3	12957	phage	0.692	temperate	0.9697178	unknown	0	Candidatus Hamiltonella defensa	0.88 Predict
DLM009_scaffold11883_2	24280	phage	0.569	virulent	0.9997209	unknown	0	Candidatus Hamiltonella defensa	0.9 Predict
DLM009_scaffold6465_2	10166	phage	0.998	virulent	0.97065383	unknown	0	Candidatus Hamiltonella defensa	1 CRISPR
DLM009_scaffold19658_2	15287	phage	0.999	virulent	0.9420535	no_family_avaliable(NC_005856)	0.989	Candidatus Hamiltonella defensa	1 CRISPR
DLM009_C324613_1	21823	phage	0.999	temperate	0.99924237	unknown	0	Candidatus Hamiltonella defensa	1 CRISPR
DLM009_scaffold17120_3	11259	phage	0.998	virulent	0.9996403	unknown	0	Parabacteroides merdae	0.81 Predict
DLM009_scaffold14704_1	94394	phage	0.759	virulent	0.9998579	Straboviridae	0.5172189	Candidatus Hamiltonella defensa	0.93 Predict
DLM009_scaffold21367_3	14570	phage	0.999	temperate	0.9998474	Drexlerviridae	0.1742411	Candidatus Hamiltonella defensa	0.77 Predict
DLM009_scaffold18558_3	19436	phage	0.999	virulent	0.9998684	unknown	0	unknown	0 -
DLM009_scaffold8594_4	26100	phage	1	temperate	0.99980307	Peduoviridae	1	Candidatus Hamiltonella defensa	1 CRISPR
DLM009_scaffold10681_1	20723	phage	0.999	temperate	0.8972373	unknown	0	Candidatus Hamiltonella defensa	1 CRISPR
DLM009_scaffold5016_13	15900	phage	0.919	temperate	0.9413989	Straboviridae	0.40471888	Bacteroides fragilis	0.78 Predict
DLM009_scaffold102_1	27916	phage	0.585	temperate	0.9998556	Straboviridae	0.6931655	Flavobacterium columnare	0.76 Predict
DLM009_C324441_1	17694	phage	0.999	temperate	0.99984694	Peduoviridae	0.28715542	Candidatus Hamiltonella defensa	0.95 Predict
DLM009_scaffold21380_1	11326	phage	0.988	virulent	0.99984694	unknown	0	Candidatus Hamiltonella defensa	0.97 Predict
DLM009_scaffold6465_20	22398	phage	0.997	virulent	0.9653701	Straboviridae	0.4652497	Candidatus Hamiltonella defensa	1 CRISPR
DLM009_scaffold21079_1	11276	phage	0.895	temperate	0.99985975	Herelleviridae	1	Candidatus Hamiltonella defensa	0.91 Predict
DLM009_scaffold22_4	23435	phage	0.999	temperate	0.99985975	Straboviridae	0.47515345	Candidatus Hamiltonella defensa	0.74 Predict
DLM009_scaffold21344_1	18730	phage	0.999	temperate	0.99981076	unknown	0	Lactobacillus fermentum	0.85 Predict

DLM009_scaffold8439_6	15226	phage	0.999	temperate	0.9993	unknown	0	unknown	0 -
DLM009_scaffold18558_6	17545	phage	0.999	virulent	0.99987173	unknown	0	unknown	0 -
DLM009_scaffold6465_1	45509	phage	0.999	temperate	0.99985695	unknown	0	Candidatus Hamiltonella defensa	1 CRISPR
DLM009_scaffold21365_1	17001	phage	0.824	virulent	0.99987173	unknown	0	Candidatus Hamiltonella defensa	0.94 Predict
DLM009_scaffold19658_6	10746	phage	0.999	virulent	0.99927014	no_family_avaliable(NC_042128)	0.985	Candidatus Hamiltonella defensa	1 CRISPR
DLM009_scaffold2554_54	13925	phage	0.932	temperate	0.9996577	unknown	0	Parabacteroides merdae	0.89 Predict
DLM009_scaffold17086_6	22353	phage	0.98	virulent	0.99233663	Ackermannviridae	1	unknown	0 -
DLM009_scaffold19690_1	33132	phage	0.916	temperate	0.89619565	Straboviridae	0.27523607	Flavobacterium columnare	0.91 Predict
DLM009_scaffold18558_4	46125	phage	0.999	virulent	0.99986744	Straboviridae	0.5401171	unknown	0 -
DLM009_scaffold6465_28	62894	phage	0.999	virulent	0.9998183	Straboviridae	0.62464666	Bacteroides fragilis	1 CRISPR
DLM009_scaffold4728_5	14496	phage	0.895	temperate	0.99922544	unknown	0	Candidatus Hamiltonella defensa	0.72 Predict
DLM009_C324485_1	18420	phage	0.999	virulent	0.93833727	Herelleviridae	1	Candidatus Hamiltonella defensa	1 CRISPR
DLM009_scaffold18345_6	13310	phage	0.999	temperate	0.9989151	no_family_avaliable(NC_031129)	0.984	Candidatus Hamiltonella defensa	1 CRISPR
DLM009_scaffold19658_3	11458	phage	0.999	temperate	0.98952943	no_family_avaliable(NC_031129)	0.987	Salmonella enterica	1 CRISPR
DLM009_C324155_1	14063	phage	0.993	temperate	0.9998584	unknown	0	Candidatus Hamiltonella defensa	1 CRISPR
DLM009_scaffold6465_23	12097	phage	0.999	temperate	0.9998517	unknown	0	[Ruminococcus] gnavus	1 CRISPR
DLM010_scaffold41969_2	15521	phage	0.674	temperate	0.99985975	unknown	0	Candidatus Hamiltonella defensa	0.91 Predict
DLM010_scaffold41867_3	13093	phage	0.991	temperate	0.99985975	Herelleviridae	1	unknown	0 -
DLM010_scaffold45140_1	16343	phage	0.999	virulent	0.99987316	Salasmaviridae	0.94846845	Candidatus Hamiltonella defensa	1 CRISPR
DLM010_scaffold179_1	16319	phage	0.998	virulent	0.9484926	Straboviridae	0.61924237	Bacteroides fragilis	0.72 Predict
DLM010_scaffold503_4	10179	phage	0.947	temperate	0.99985975	Straboviridae	0.41849706	Candidatus Hamiltonella defensa	0.8 Predict
DLM010_scaffold43377_3	21466	phage	1	temperate	0.9997669	unknown	0	Candidatus Hamiltonella defensa	1 CRISPR
DLM010_scaffold10158_2	15897	phage	0.919	temperate	0.9413989	Straboviridae	0.40471888	Bacteroides fragilis	0.91 Predict
DLM010_scaffold18321_2	10880	phage	0.974	temperate	0.98519784	unknown	0	Bacteroides fragilis	0.76 Predict
DLM010_scaffold32474_2	13542	phage	0.845	temperate	0.99934417	unknown	0	Candidatus Hamiltonella defensa	0.82 Predict
DLM010_scaffold5445_6	13301	phage	0.999	virulent	0.9997258	Peduoviridae	0.46964702	Candidatus Hamiltonella defensa	0.8 Predict
DLM010_scaffold32332_1	23035	phage	0.999	temperate	0.9997897	Peduoviridae	0.63610744	Candidatus Hamiltonella defensa	1 CRISPR
DLM010_scaffold7753_8	12203	phage	0.985	virulent	0.99987125	unknown	0	Candidatus Hamiltonella defensa	0.89 Predict
DLM010_scaffold3050_2	64700	phage	0.998	temperate	0.9998517	Peduoviridae	1	Pasteurella multocida	1 CRISPR
DLM010_scaffold44670_1	11351	phage	0.922	virulent	0.99272764	unknown	0	Candidatus Hamiltonella defensa	0.84 Predict

DLM010_scaffold41095_1	12834	phage	0.995	temperate	0.9575748	Drexlerviridae	0.09667662	Bacteroides fragilis	1	CRISPR
DLM010_scaffold16539_4	11441	phage	0.999	virulent	0.9998699	Drexlerviridae	0.28968555	Candidatus Hamiltonella defensa	0.88	Predict
DLM010_scaffold33840_5	20058	phage	0.999	virulent	0.99985975	Straboviridae	0.5655343	Candidatus Hamiltonella defensa	0.84	Predict
DLM010_scaffold6743_2	12373	phage	0.901	temperate	0.9998397	Straboviridae	0.6116163	Candidatus Hamiltonella defensa	0.95	Predict
DLM010_scaffold22879_4	36503	phage	0.98	virulent	0.99986404	Peduoviridae	1	Candidatus Hamiltonella defensa	0.84	Predict
DLM010_scaffold45132_3	10936	phage	0.997	temperate	0.99985975	Peduoviridae	1	Flavobacterium columnare	0.71	Predict
DLM010_scaffold4248_8	10630	phage	0.707	temperate	0.9998588	Straboviridae	0.56166416	unknown	0	-
DLM010_scaffold45189_1	14402	phage	1	virulent	0.99987036	Salasmaviridae	0.969743	Candidatus Hamiltonella defensa	1	CRISPR
DLM010_scaffold44889_1	12193	phage	0.968	virulent	0.9998693	Peduoviridae	1	Candidatus Hamiltonella defensa	1	CRISPR
DLM010_scaffold3339_9	11886	phage	0.999	virulent	0.99987173	unknown	0	Candidatus Hamiltonella defensa	0.89	Predict
DLM011_scaffold28392_2	10374	phage	0.999	virulent	0.9392347	unknown	0	Candidatus Hamiltonella defensa	1	CRISPR
DLM011_scaffold29232_2	14630	phage	1	temperate	0.9802566	Peduoviridae	0.3466597	Listeria monocytogenes	1	CRISPR
DLM011_scaffold29382_1	36066	phage	0.999	virulent	0.99987036	Drexlerviridae	0.28968555	Parabacteroides distasonis	0.97	Predict
DLM011_scaffold21086_2	10183	phage	0.999	virulent	0.99987406	Herelleviridae	1	Candidatus Hamiltonella defensa	0.8	Predict
DLM011_scaffold21352_1	15467	phage	0.985	virulent	0.9998465	Kyanoviridae	0.2949095	Candidatus Hamiltonella defensa	0.7	Predict
DLM011_scaffold29393_1	14181	phage	0.998	virulent	0.9998722	unknown	0	Candidatus Pelagibacter ubique	0.75	Predict
DLM011_scaffold29232_1	36896	phage	0.999	temperate	0.60928476	Peduoviridae	0.27632862	Candidatus Hamiltonella defensa	1	CRISPR
DLM011_scaffold5932_1	17171	phage	0.943	virulent	0.9998699	unknown	0	unknown	0	-
DLM011_scaffold28062_1	10695	phage	0.605	temperate	0.9998556	unknown	0	Candidatus Hamiltonella defensa	0.86	Predict
DLM011_scaffold27250_1	16264	phage	0.999	temperate	0.99986035	unknown	0	Candidatus Hamiltonella defensa	1	CRISPR
DLM011_scaffold29207_1	26105	phage	0.999	virulent	0.9998656	no_family_avaliable(NC_055876)	1	Candidatus Hamiltonella defensa	0.86	Predict
DLM011_C484732_1	17742	phage	0.999	temperate	0.91957694	unknown	0	unknown	0	-
DLM011_scaffold1189_13	14949	phage	0.998	virulent	0.99960625	unknown	0	Parabacteroides merdae	1	CRISPR
DLM011_scaffold28659_3	23774	phage	0.997	temperate	0.9998584	unknown	0	Candidatus Hamiltonella defensa	1	CRISPR
DLM011_scaffold17163_7	11293	phage	0.961	temperate	0.979654	unknown	0	Bacteroides fragilis	0.82	Predict
DLM011_scaffold29398_1	16173	phage	0.999	virulent	0.999836	Straboviridae	0.62648106	Candidatus Hamiltonella defensa	1	CRISPR
DLM011_scaffold1189_31	18297	phage	1	virulent	0.9880252	Peduoviridae	1	Cellulophaga baltica	1	CRISPR
DLM011_scaffold19964_1	20570	phage	0.999	virulent	0.9998588	unknown	0	Candidatus Hamiltonella defensa	1	CRISPR
DLM011_scaffold1699_8	10663	phage	0.997	virulent	0.9996546	unknown	0	unknown	0	-
DLM011_scaffold5789_4	10303	phage	0.992	virulent	0.9997726	unknown	0	unknown	0	-

DLM011_scaffold18867_6	15910	phage	0.919	temperate	0.9413989	Straboviridae	0.40471888	Bacteroides fragilis	0.9	Predict
DLM011_scaffold24958_1	11905	phage	0.998	virulent	0.9997339	unknown	0	unknown	0	-
DLM012_scaffold11223_12	10508	phage	0.998	virulent	0.9998513	Straboviridae	0.4992557	Candidatus Hamiltonella defensa	1	CRISPR
DLM012_scaffold16447_5	10064	phage	0.963	virulent	0.99987316	Straboviridae	0.58554715	Candidatus Hamiltonella defensa	0.75	Predict
DLM012_scaffold19314_6	25613	phage	0.995	virulent	0.99982095	unknown	0	Candidatus Hamiltonella defensa	0.94	Predict
DLM012_scaffold49_1	15580	phage	0.999	virulent	0.93934274	no_family_avaliable(NC_005856)	0.979	Candidatus Hamiltonella defensa	1	CRISPR
DLM012_scaffold34010_1	32818	phage	0.999	temperate	0.99965674	unknown	0	Candidatus Hamiltonella defensa	1	CRISPR
DLM012_scaffold22034_2	11272	phage	0.999	virulent	0.7283555	unknown	0	Blautia coccoides	1	CRISPR
DLM012_scaffold7495_7	14231	phage	0.993	virulent	0.999531	unknown	0	Candidatus Hamiltonella defensa	1	CRISPR
DLM012_C414271_1	17335	phage	0.999	virulent	0.99970484	Straboviridae	0.34864473	Candidatus Hamiltonella defensa	0.71	Predict
DLM012_scaffold23728_1	10223	phage	0.999	virulent	0.9139237	Peduoviridae	1	Candidatus Hamiltonella defensa	0.74	Predict
DLM012_scaffold33026_10	10921	phage	0.998	temperate	0.9998579	unknown	0	Candidatus Hamiltonella defensa	1	CRISPR
DLM012_scaffold2394_3	18036	phage	0.962	temperate	0.92079926	Peduoviridae	0.37287483	Candidatus Hamiltonella defensa	0.97	Predict
DLM012_scaffold33913_1	22040	phage	0.999	temperate	0.99951667	unknown	0	Candidatus Hamiltonella defensa	1	CRISPR
DLM012_scaffold12_2	15924	phage	0.999	temperate	0.9997115	Casjensviridae	0.46940973	Candidatus Hamiltonella defensa	0.76	Predict
DLM012_scaffold33950_1	15699	phage	0.908	temperate	0.9998584	unknown	0	Candidatus Hamiltonella defensa	0.74	Predict
DLM012_scaffold29288_6	25269	phage	0.999	temperate	0.99985695	unknown	0	Candidatus Hamiltonella defensa	0.96	Predict
DLM012_scaffold32680_2	12046	phage	0.998	virulent	0.9996124	Straboviridae	0.61910915	Candidatus Hamiltonella defensa	0.92	Predict
DLM012_scaffold26999_6	11728	phage	0.998	virulent	0.7405412	unknown	0	Massilioclostridium coli	1	CRISPR
DLM012_scaffold28806_1	15484	phage	0.998	temperate	0.99986035	Straboviridae	0.5285559	Candidatus Hamiltonella defensa	0.88	Predict
DLM012_scaffold725_1	38777	phage	0.999	virulent	0.84835416	Peduoviridae	1	Candidatus Hamiltonella defensa	1	CRISPR
DLM012_scaffold33245_1	36347	phage	0.999	temperate	0.99398	Schitoviridae	1	Candidatus Hamiltonella defensa	1	CRISPR
DLM012_scaffold29288_5	13899	phage	0.995	temperate	0.9998584	unknown	0	Thermoanaerobacterium saccharolyti	0.83	Predict
DLM013_scaffold23313_3	14460	phage	0.998	temperate	0.9848878	unknown	0	Candidatus Hamiltonella defensa	0.86	Predict
DLM013_scaffold3286_13	11121	phage	0.997	temperate	0.8881842	unknown	0	unknown	0	-
DLM013_scaffold17656_2	10664	phage	0.999	temperate	0.9998593	Drexlerviridae	0.1742411	Candidatus Hamiltonella defensa	1	CRISPR
DLM013_scaffold7395_5	17259	phage	0.997	virulent	0.99984604	Ackermannviridae	0.34976166	Candidatus Hamiltonella defensa	1	Predict
DLM013_scaffold34896_3	48088	phage	0.994	virulent	0.9998651	Straboviridae	0.5416722	Lactobacillus plantarum	1	CRISPR
DLM013_scaffold21602_2	12834	phage	0.999	temperate	0.9998593	Herelleviridae	1	Candidatus Hamiltonella defensa	0.85	Predict
DLM013_scaffold57623_1	11586	phage	0.996	virulent	0.99987316	Autographiviridae	1	Candidatus Hamiltonella defensa	1	CRISPR

DLM013_scaffold55482_1	11425	phage	0.998	temperate	0.99981695	unknown	0	Clostridium tetani	0.97	Predict
DLM013_scaffold36790_6	10370	phage	0.947	temperate	0.99985975	Straboviridae	0.54375273	Candidatus Hamiltonella defensa	0.74	Predict
DLM013_scaffold8482_4	12194	phage	0.999	virulent	0.99986124	unknown	0	Candidatus Hamiltonella defensa	0.71	Predict
DLM013_scaffold45817_1	23051	phage	0.876	temperate	0.9701814	unknown	0	Candidatus Hamiltonella defensa	0.97	Predict
DLM013_scaffold3810_8	17079	phage	0.999	virulent	0.9998699	Straboviridae	0.52317137	Candidatus Hamiltonella defensa	0.91	Predict
DLM013_scaffold23184_5	10376	phage	0.997	virulent	0.99987125	unknown	0	unknown	0	-
DLM013_scaffold57456_3	15174	phage	0.856	temperate	0.93894786	Straboviridae	0.40471888	Bacteroides fragilis	0.82	Predict
DLM013_scaffold56424_2	12405	phage	0.999	virulent	0.99987173	unknown	0	unknown	0	-
DLM013_scaffold3810_7	25973	phage	0.999	virulent	0.9918154	unknown	0	Candidatus Hamiltonella defensa	1	CRISPR
DLM013_scaffold318_1	11702	phage	0.999	temperate	0.99985975	unknown	0	Candidatus Hamiltonella defensa	0.9	Predict
DLM013_scaffold15355_2	10898	phage	0.898	temperate	0.9943185	unknown	0	Candidatus Hamiltonella defensa	0.88	Predict
DLM013_scaffold22571_2	15135	phage	0.999	temperate	0.99982077	Straboviridae	0.34551933	Candidatus Hamiltonella defensa	0.96	Predict
DLM013_scaffold4430_4	12408	phage	0.999	temperate	0.6317847	Autographiviridae	1	Candidatus Hamiltonella defensa	0.77	Predict
DLM013_scaffold56628_1	12520	phage	1	virulent	0.9998684	Autographiviridae	1	Candidatus Hamiltonella defensa	1	CRISPR
DLM013_scaffold47801_1	13513	phage	0.992	temperate	0.9998588	Ackermannviridae	1	Candidatus Hamiltonella defensa	0.89	Predict
DLM013_scaffold51742_2	15644	phage	0.998	virulent	0.99987084	Herelleviridae	1	unknown	0	-
DLM013_scaffold23184_12	63610	phage	0.999	virulent	0.99984926	Vilmaviridae	1	Cellulophaga baltica	1	CRISPR
DLM013_scaffold54893_2	12097	phage	0.998	temperate	0.85934323	unknown	0	unknown	0	-
DLM013_scaffold57687_1	15815	phage	1	virulent	0.99987036	Salasmaviridae	1	Actinomyces naeslundii	0.87	Predict
DLM013_scaffold8482_6	15220	phage	0.994	virulent	0.9998699	Herelleviridae	1	unknown	0	-
DLM013_scaffold23009_1	10882	phage	0.996	virulent	0.9998556	Straboviridae	0.5655343	Candidatus Hamiltonella defensa	0.73	Predict
DLM013_scaffold3614_26	30968	phage	0.999	virulent	0.67038256	unknown	0	Flavobacterium columnare	1	CRISPR
DLM013_scaffold23737_4	13802	phage	0.998	virulent	0.9998722	unknown	0	Parabacteroides distasonis	0.89	Predict
DLM013_scaffold49483_1	10128	phage	0.971	temperate	0.99985975	unknown	0	unknown	0	-
DLM013_scaffold51645_2	12338	phage	0.999	temperate	0.99985313	Straboviridae	0.41326678	Candidatus Hamiltonella defensa	1	CRISPR
DLM013_scaffold21436_7	11529	phage	0.9	temperate	0.9647605	unknown	0	Candidatus Hamiltonella defensa	0.87	Predict
DLM013_scaffold38983_1	10823	phage	0.949	temperate	0.9997587	unknown	0	Candidatus Hamiltonella defensa	0.85	Predict
DLM013_scaffold20700_8	11943	phage	0.554	virulent	0.99987084	Drexlerviridae	0.199991	Candidatus Hamiltonella defensa	0.9	Predict
DLM013_scaffold50692_1	19385	phage	0.965	temperate	0.9984187	Straboviridae	0.25122714	Candidatus Hamiltonella defensa	0.74	Predict
DLM013_scaffold25009_5	15408	phage	0.999	temperate	0.99985975	unknown	0	Candidatus Hamiltonella defensa	1	CRISPR

DLM013_scaffold14795_2	12220	phage	1	temperate	0.99986035	unknown	0	Candidatus Hamiltonella defensa	0.88	Predict
DLM013_scaffold52105_9	18930	phage	0.999	virulent	0.9996192	unknown	0	Candidatus Hamiltonella defensa	1	CRISPR
DLM013_scaffold11264_1	15664	phage	0.988	virulent	0.99987084	unknown	0	unknown	0	-
DLM013_scaffold52295_2	12239	phage	0.996	temperate	0.9929295	unknown	0	Candidatus Hamiltonella defensa	0.88	Predict
DLM013_scaffold23184_9	26650	phage	0.999	virulent	0.99987173	Demerecviridae	0.33583313	Parabacteroides sp. D13	1	CRISPR
DLM013_scaffold49247_2	39700	phage	0.999	temperate	0.99075556	Drexlerviridae	0.3231527	Candidatus Hamiltonella defensa	1	CRISPR
DLM013_scaffold56635_1	52703	phage	0.996	virulent	0.99344337	Vilmaviridae	0.11836226	Candidatus Hamiltonella defensa	1	CRISPR
DLM013_C721558_1	45355	phage	0.996	virulent	0.99985313	Autographiviridae	0.97334677	Yersinia pestis	1	CRISPR
DLM013_scaffold31022_3	11510	phage	0.985	virulent	0.99982953	unknown	0	Candidatus Hamiltonella defensa	0.78	Predict
DLM013_scaffold30472_4	12903	phage	0.998	virulent	0.9915161	Ackermannviridae	0.3847759	Mycoplasma pulmonis	0.87	Predict
DLM013_scaffold16485_8	11929	phage	0.999	virulent	0.9998617	unknown	0	unknown	0	-
DLM013_scaffold55003_1	14510	phage	0.997	temperate	0.99985975	unknown	0	Streptomyces lividans	0.71	Predict
DLM013_scaffold23574_3	13891	phage	0.988	virulent	0.9998579	unknown	0	Bacteroides fragilis	0.9	Predict
DLM013_scaffold3614_27	11554	phage	0.999	virulent	0.9998086	unknown	0	Megasphaera cerevisiae	1	CRISPR
DLM013_scaffold24100_2	10354	phage	0.995	virulent	0.9996887	unknown	0	Bacteroides fragilis	0.9	Predict
DLM013_scaffold2200_1	14274	phage	0.994	temperate	0.99985975	unknown	0	Candidatus Hamiltonella defensa	0.77	Predict
DLM014_scaffold52949_1	15771	phage	0.999	temperate	0.9998522	unknown	0	Candidatus Hamiltonella defensa	1	CRISPR
DLM014_scaffold14966_8	12854	phage	0.54	virulent	0.9998665	unknown	0	Candidatus Hamiltonella defensa	0.8	Predict
DLM014_scaffold26538_3	10440	phage	0.999	virulent	0.91296697	unknown	0	unknown	0	-
DLM014_scaffold3539_3	11946	phage	0.994	temperate	0.93755734	unknown	0	unknown	0	-
DLM014_scaffold39602_3	10515	phage	0.762	temperate	0.99984497	Straboviridae	0.52348197	Bacteroides fragilis	0.83	Predict
DLM014_scaffold51860_2	12395	phage	0.927	temperate	0.9998593	unknown	0	unknown	0	-
DLM014_scaffold34561_6	12708	phage	0.824	temperate	0.99986035	unknown	0	Candidatus Hamiltonella defensa	0.99	Predict
DLM014_scaffold17726_11	15083	phage	0.849	temperate	0.9998403	Peduoviridae	0.5512526	Candidatus Hamiltonella defensa	0.93	Predict
DLM014_scaffold43677_1	12852	phage	0.967	virulent	0.99987406	Kyanoviridae	1	unknown	0	-
DLM014_scaffold31737_5	20524	phage	0.999	virulent	0.99986744	unknown	0	Candidatus Hamiltonella defensa	0.76	Predict
DLM014_scaffold1_9	13967	phage	0.999	virulent	0.99987173	no_family_avaliable(NC_055875)	0.99	Cellulophaga baltica	0.94	Predict
DLM014_scaffold52940_1	12433	phage	0.999	temperate	0.99986035	unknown	0	Candidatus Hamiltonella defensa	1	CRISPR
DLM014_scaffold44624_1	16059	phage	0.999	temperate	0.99918586	unknown	0	Candidatus Hamiltonella defensa	1	CRISPR
DLM014_scaffold24690_1	18080	phage	0.998	virulent	0.99980664	Ackermannviridae	0.34976166	Candidatus Hamiltonella defensa	0.84	Predict

DLM014_scaffold42199_1	11267	phage	0.988	temperate	0.9910449	unknown	0	Candidatus Hamiltonella defensa	0.97	Predict
DLM014_scaffold3883_2	12700	phage	0.994	temperate	0.99985605	unknown	0	Parabacteroides distasonis	0.99	Predict
DLM014_scaffold7847_1	10595	phage	0.997	temperate	0.99959904	unknown	0	Bacteroides fragilis	0.7	Predict
DLM014_scaffold46228_1	37935	phage	0.999	temperate	0.99969196	unknown	0	Clostridium perfringens	1	CRISPR
DLM014_scaffold35850_2	17717	phage	0.978	virulent	0.9998413	Straboviridae	0.61924237	Candidatus Hamiltonella defensa	0.98	Predict
DLM014_scaffold40706_2	15554	phage	0.99	virulent	0.99987173	Ackermannviridae	0.6232304	Bacteroides fragilis	0.71	Predict
DLM014_scaffold14317_2	12774	phage	0.965	virulent	0.9997053	unknown	0	Parabacteroides distasonis	0.88	Predict
DLM014_scaffold42200_1	16225	phage	0.989	temperate	0.9592161	Straboviridae	0.571929	Bacteroides fragilis	0.98	Predict
DLM014_scaffold45829_2	29936	phage	1	temperate	0.9468743	unknown	0	Bacteroides fragilis	1	CRISPR
DLM014_scaffold31737_1	10448	phage	1	temperate	0.99985695	unknown	0	Candidatus Hamiltonella defensa	0.8	Predict
DLM014_scaffold4026_7	22287	phage	0.996	virulent	0.99987173	Ackermannviridae	0.2221647	Candidatus Hamiltonella defensa	0.76	Predict
DLM014_scaffold21553_3	14455	phage	0.996	temperate	0.99985975	unknown	0	Candidatus Hamiltonella defensa	0.85	Predict
DLM014_scaffold23167_4	12262	phage	0.999	temperate	0.550134	unknown	0	unknown	0	-
DLM014_scaffold32434_6	11694	phage	0.99	virulent	0.99987173	Ackermannviridae	0.6232304	Candidatus Hamiltonella defensa	0.91	Predict
DLM014_scaffold18806_7	11042	phage	0.867	virulent	0.95832527	unknown	0	unknown	0	-
DLM014_scaffold1942_34	12365	phage	0.989	temperate	0.99985975	unknown	0	Shigella boydii	0.99	Predict
DLM014_scaffold18901_3	11053	phage	0.871	virulent	0.99980956	unknown	0	unknown	0	-
DLM014_scaffold48474_2	14132	phage	0.999	virulent	0.9406228	unknown	0	Candidatus Hamiltonella defensa	0.81	Predict
DLM014_scaffold1_18	11568	phage	0.999	virulent	0.99987084	no_family_avaliable(NC_055875)	0.99	Candidatus Hamiltonella defensa	0.99	Predict
DLM014_scaffold52939_1	12808	phage	0.634	virulent	0.99987173	unknown	0	Bacteroides fragilis	0.95	Predict
DLM014_scaffold290_4	22685	phage	0.994	virulent	0.9995576	Ackermannviridae	0.36708665	Candidatus Hamiltonella defensa	0.99	Predict
DLM014_scaffold37209_4	17066	phage	0.998	virulent	0.99987406	unknown	0	unknown	0	-
DLM014_scaffold36217_6	10117	phage	0.986	virulent	0.99982077	unknown	0	Parabacteroides distasonis	0.76	Predict
DLM014_scaffold7310_3	11546	phage	0.999	virulent	0.99987316	Ackermannviridae	0.26684576	unknown	0	-
DLM015_scaffold18535_6	11323	phage	0.542	temperate	0.9998265	Ackermannviridae	1	Clostridium botulinum	0.81	Predict
DLM015_scaffold5802_5	11241	phage	0.983	virulent	0.9998736	unknown	0	Candidatus Hamiltonella defensa	0.71	Predict
DLM015_scaffold16218_2	29221	phage	0.984	virulent	0.9998622	Straboviridae	0.6664747	Candidatus Hamiltonella defensa	0.85	Predict
DLM015_scaffold50361_1	11182	phage	0.999	temperate	0.99986035	unknown	0	Bacteroides fragilis	0.75	Predict
DLM015_scaffold50502_1	10343	phage	0.999	temperate	0.99951375	unknown	0	Parabacteroides merdae	0.98	Predict
DLM015_scaffold23053_2	11030	phage	0.999	temperate	0.99973255	unknown	0	Candidatus Hamiltonella defensa	1	CRISPR

DLM015_scaffold1049_4	21487	phage	0.998	virulent	0.99987173	unknown	0	Candidatus Hamiltonella defensa	1	CRISPR
DLM015_scaffold1119_3	12112	phage	0.996	virulent	0.99987173	unknown	0	unknown	0	-
DLM015_scaffold36073_3	22015	phage	1	temperate	0.9998588	unknown	0	Clostridium perfringens	1	CRISPR
DLM015_scaffold23053_6	20274	phage	0.999	temperate	0.9998556	unknown	0	Candidatus Hamiltonella defensa	1	CRISPR
DLM015_scaffold47979_4	11266	phage	0.961	temperate	0.979654	unknown	0	Bacteroides fragilis	0.79	Predict
DLM015_scaffold49465_1	10590	phage	0.999	virulent	0.9997056	unknown	0	Candidatus Hamiltonella defensa	0.75	Predict
DLM015_scaffold23554_1	48818	phage	0.999	virulent	0.99354404	Straboviridae	0.69763845	Candidatus Hamiltonella defensa	1	CRISPR
DLM015_scaffold50303_1	17945	phage	0.999	virulent	0.8716671	unknown	0	Candidatus Hamiltonella defensa	0.72	Predict
DLM015_scaffold34621_5	11176	phage	0.539	virulent	0.99987125	unknown	0	unknown	0	-
DLM015_scaffold47846_2	10862	phage	0.946	temperate	0.99985975	unknown	0	unknown	0	-
DLM015_scaffold50189_1	10196	phage	0.79	virulent	0.99986404	unknown	0	Edwardsiella ictaluri	0.7	Predict
DLM015_scaffold5802_2	14908	phage	0.997	virulent	0.9998722	unknown	0	Lactobacillus plantarum	0.98	Predict
DLM015_scaffold14872_4	28522	phage	0.999	virulent	0.9998617	Drexlerviridae	0.1742411	Bacteroides fragilis	0.71	Predict
DLM015_scaffold50457_1	53207	phage	0.999	virulent	0.99983	Herelleviridae	1	Candidatus Hamiltonella defensa	1	CRISPR
DLM015_scaffold29903_2	67716	phage	0.992	virulent	0.99896526	Casjensviridae	0.4598215	Bacteroides fragilis	0.95	Predict
DLM015_scaffold36073_4	12981	phage	1	temperate	0.8653128	unknown	0	Candidatus Hamiltonella defensa	1	Predict
DLM015_scaffold145_2	29453	phage	0.999	virulent	0.7335834	unknown	0	Bacteroides fragilis	1	CRISPR
DLM015_scaffold17140_1	14301	phage	0.999	virulent	0.9053317	unknown	0	Bacteroides fragilis	0.93	Predict
DLM015_scaffold36351_2	13680	phage	0.845	temperate	0.99934417	unknown	0	Candidatus Hamiltonella defensa	0.84	Predict
DLM016_scaffold17586_2	43766	phage	0.997	temperate	0.9998551	Straboviridae	0.6816639	Candidatus Hamiltonella defensa	0.79	Predict
DLM016_scaffold3377_13	17465	phage	0.954	virulent	0.88889205	Peduoviridae	0.5512526	Candidatus Hamiltonella defensa	0.9	Predict
DLM016_scaffold65085_5	35671	phage	0.998	virulent	0.9998665	unknown	0	Cellulophaga baltica	1	CRISPR
DLM016_scaffold61369_1	11037	phage	0.997	virulent	0.99987173	unknown	0	unknown	0	-
DLM016_scaffold64744_4	27376	phage	0.971	temperate	0.99985975	unknown	0	Flavobacterium columnare	0.86	Predict
DLM016_scaffold12_1	10866	phage	0.997	temperate	0.9998588	unknown	0	Candidatus Hamiltonella defensa	0.88	Predict
DLM016_scaffold13346_4	12441	phage	0.999	virulent	0.9168372	unknown	0	Candidatus Hamiltonella defensa	0.97	Predict
DLM016_scaffold27953_5	16583	phage	0.999	virulent	0.9996776	Straboviridae	0.52317137	Candidatus Hamiltonella defensa	0.94	Predict
DLM016_scaffold37509_2	12493	phage	0.998	temperate	0.99985313	Kyanoviridae	1	Candidatus Pelagibacter ubique	0.9	Predict
DLM016_scaffold46534_2	10670	phage	0.833	virulent	0.99987316	unknown	0	Bacteroides fragilis	0.72	Predict
DLM016_scaffold419_5	10975	phage	0.998	virulent	0.9997726	Straboviridae	0.62674475	Candidatus Hamiltonella defensa	1	CRISPR

DLM016_scaffold41709_1	14599	phage	0.985	virulent	0.88211113	unknown	0	Parabacteroides merdae	0.97	Predict
DLM016_scaffold22143_3	17070	phage	0.999	virulent	0.9998736	no_family_avaliable(NC_055875)	0.981	Candidatus Hamiltonella defensa	1	CRISPR
DLM016_scaffold40529_1	12485	phage	0.999	virulent	0.9996805	unknown	0	Candidatus Hamiltonella defensa	0.82	Predict
DLM016_scaffold7191_1	10406	phage	0.965	virulent	0.9761889	unknown	0	Lactobacillus plantarum	0.86	Predict
DLM016_scaffold49496_1	66538	phage	0.999	virulent	0.9998343	Straboviridae	0.46930915	Candidatus Hamiltonella defensa	0.72	Predict
DLM016_scaffold40187_1	12419	phage	0.998	temperate	0.9949661	unknown	0	Candidatus Hamiltonella defensa	0.86	Predict
DLM016_scaffold6553_2	25373	phage	0.999	virulent	0.9998651	unknown	0	unknown	0	-
DLM016_scaffold16615_5	14599	phage	0.998	temperate	0.9973022	unknown	0	Candidatus Pelagibacter ubique	0.94	Predict
DLM016_scaffold2806_2	16264	phage	0.82	virulent	0.9998693	Casjensviridae	0.8158135	Candidatus Hamiltonella defensa	0.96	Predict
DLM016_scaffold53043_5	16066	phage	0.999	temperate	0.9998536	unknown	0	unknown	0	-
DLM016_scaffold5004_5	11754	phage	0.997	virulent	0.9998699	Straboviridae	0.4380502	Bacteroides fragilis	0.97	Predict
DLM016_scaffold65482_1	11568	phage	0.999	virulent	0.99987125	unknown	0	Candidatus Hamiltonella defensa	1	CRISPR
DLM016_scaffold65575_2	12461	phage	0.999	virulent	0.999844	unknown	0	Clostridium tetani	1	CRISPR
DLM016_scaffold6713_8	11300	phage	0.985	virulent	0.99987316	unknown	0	unknown	0	-
DLM016_scaffold6713_3	29364	phage	1	virulent	0.9998608	no_family_avaliable(NC_055873)	0.961	Cellulophaga baltica	1	CRISPR
DLM016_scaffold6713_5	10478	phage	0.979	virulent	0.9998291	Straboviridae	0.63893926	Candidatus Hamiltonella defensa	0.88	Predict
DLM016_scaffold64767_1	15261	phage	1	virulent	0.99987125	Salasmaviridae	1	Lactobacillus plantarum	1	CRISPR
DLM016_scaffold6713_11	21757	phage	0.999	virulent	0.9998636	unknown	0	Parabacteroides merdae	1	CRISPR
DLM016_scaffold6713_2	21845	phage	0.99	temperate	0.9998397	unknown	0	Candidatus Hamiltonella defensa	0.82	Predict
DLM016_scaffold2366_1	10736	phage	0.995	virulent	0.99978536	Straboviridae	0.61910915	Bacteroides fragilis	0.87	Predict
DLM016_scaffold4953_3	11906	phage	0.999	temperate	0.99985975	unknown	0	Candidatus Hamiltonella defensa	0.9	Predict
DLM016_scaffold6914_1	11759	phage	0.994	temperate	0.93755734	unknown	0	Candidatus Hamiltonella defensa	0.97	Predict
DLM016_scaffold58329_1	25940	phage	0.999	virulent	0.99986696	Casjensviridae	0.4466287	Candidatus Hamiltonella defensa	0.75	Predict
DLM016_scaffold52894_5	13459	phage	1	virulent	0.99987125	unknown	0	Bacteroides uniformis	1	CRISPR
DLM016_scaffold6713_12	12258	phage	0.997	virulent	0.99986744	unknown	0	unknown	0	-
DLM016_scaffold37873_2	13747	phage	0.999	temperate	0.9998588	Straboviridae	0.62648106	Candidatus Hamiltonella defensa	1	CRISPR
DLM016_scaffold64097_2	12236	phage	0.997	virulent	0.9998656	Straboviridae	0.5655343	Candidatus Hamiltonella defensa	0.86	Predict
DLM016_scaffold21785_1	16481	phage	0.999	virulent	0.99980736	Ackermannviridae	0.34121394	Candidatus Hamiltonella defensa	0.97	Predict
DLM016_scaffold65474_2	12130	phage	0.999	temperate	0.98817694	unknown	0	Parabacteroides distasonis	0.72	Predict
DLM016_scaffold65085_3	11532	phage	0.999	virulent	0.9998636	unknown	0	Cellulophaga baltica	0.76	Predict

DLM016_scaffold6553_6	27686	phage	0.998	virulent	0.9998584	Herelleviridae	0.89390486	Candidatus Hamiltonella defensa	0.72	Predict
DLM016_scaffold6553_4	24810	phage	0.989	virulent	0.9998684	unknown	0	unknown	0	-
DLM016_scaffold1623_5	19711	phage	0.999	temperate	0.9998584	unknown	0	Candidatus Hamiltonella defensa	1	CRISPR
DLM016_scaffold63991_1	10721	phage	0.999	virulent	0.99987084	unknown	0	Bacteroides vulgatus	1	CRISPR
DLM016_scaffold29974_3	15862	phage	0.999	virulent	0.9997275	unknown	0	Candidatus Hamiltonella defensa	0.7	Predict
DLM016_scaffold53961_3	11272	phage	0.974	temperate	0.98519784	unknown	0	Bacteroides fragilis	0.92	Predict
DLM016_scaffold419_9	12683	phage	0.955	virulent	0.99987125	unknown	0	Candidatus Hamiltonella defensa	1	CRISPR
DLM017_scaffold20271_2	11732	phage	0.999	temperate	0.99708074	Straboviridae	0.3895763	Faecalibacterium prausnitzii	1	CRISPR
DLM017_scaffold2813_9	17887	phage	0.8	virulent	0.9998699	unknown	0	Candidatus Hamiltonella defensa	0.83	Predict
DLM017_scaffold3964_2	10008	phage	0.998	virulent	0.99987316	unknown	0	Candidatus Hamiltonella defensa	0.76	Predict
DLM017_scaffold29996_2	23777	phage	0.999	virulent	0.99986744	Straboviridae	0.5416722	Candidatus Hamiltonella defensa	1	CRISPR
DLM017_scaffold20005_2	36679	phage	0.999	temperate	0.9998308	unknown	0	Candidatus Hamiltonella defensa	1	CRISPR
DLM017_scaffold21266_1	42776	phage	0.999	temperate	0.99956053	unknown	0	Candidatus Hamiltonella defensa	1	CRISPR
DLM017_scaffold33959_3	11652	phage	0.938	temperate	0.9990059	unknown	0	Bacteroides fragilis	1	CRISPR
DLM017_scaffold2501_2	10314	phage	0.878	temperate	0.999662	Straboviridae	1	Candidatus Hamiltonella defensa	0.99	Predict
DLM017_scaffold43714_2	19711	phage	0.997	temperate	0.99979925	Peduoviridae	0.61929643	Candidatus Hamiltonella defensa	1	CRISPR
DLM017_scaffold5960_2	59120	phage	0.998	virulent	0.9993222	Casjensviridae	0.353185	Candidatus Hamiltonella defensa	1	CRISPR
DLM017_scaffold1892_10	23204	phage	0.997	temperate	0.9998588	unknown	0	unknown	0	-
DLM017_scaffold28739_1	15182	phage	0.998	temperate	0.9998593	unknown	0	Candidatus Hamiltonella defensa	0.86	Predict
DLM017_scaffold5705_1	10100	phage	0.609	temperate	0.9997044	Straboviridae	0.6360723	Candidatus Hamiltonella defensa	0.96	Predict
DLM017_scaffold42571_2	14373	phage	0.996	virulent	0.9991045	Ackermannviridae	0.32050374	Candidatus Hamiltonella defensa	0.99	Predict
DLM017_scaffold1478_32	13176	phage	0.954	virulent	0.88889205	Peduoviridae	0.5512526	Candidatus Hamiltonella defensa	0.89	Predict
DLM017_scaffold10166_1	26284	phage	0.877	temperate	0.9998574	unknown	0	Candidatus Hamiltonella defensa	0.85	Predict
DLM017_scaffold15588_1	10128	phage	0.779	temperate	0.9998593	Peduoviridae	1	Candidatus Hamiltonella defensa	1	CRISPR
DLM017_scaffold31873_1	30465	phage	0.998	temperate	0.9998584	Casjensviridae	0.58597374	Candidatus Hamiltonella defensa	0.9	Predict
DLM017_scaffold23811_1	11913	phage	0.856	temperate	0.93894786	Straboviridae	0.40471888	Candidatus Hamiltonella defensa	0.84	Predict
DLM017_scaffold18232_1	23259	phage	0.997	virulent	0.99987173	unknown	0	Candidatus Hamiltonella defensa	0.91	Predict
DLM018_scaffold36970_5	33698	phage	0.999	virulent	0.9998722	unknown	0	Candidatus Hamiltonella defensa	0.92	Predict
DLM018_scaffold38471_2	12247	phage	0.867	virulent	0.9998536	unknown	0	Parabacteroides distasonis	0.93	Predict
DLM018_scaffold37570_1	23618	phage	0.999	virulent	0.99160045	Zierdtviridae	0.25472173	Candidatus Hamiltonella defensa	1	CRISPR

DLM018_scaffold197_3	10358	phage	0.98	temperate	0.81878257	unknown	0	Candidatus Hamiltonella defensa	0.86	Predict
DLM018_scaffold13432_9	12424	phage	0.999	temperate	0.99985695	unknown	0	Candidatus Hamiltonella defensa	1	CRISPR
DLM018_scaffold45347_1	32704	phage	0.988	virulent	0.99977875	unknown	0	Candidatus Hamiltonella defensa	0.78	Predict
DLM018_scaffold49175_1	11550	phage	0.995	virulent	0.9998736	unknown	0	Candidatus Hamiltonella defensa	1	CRISPR
DLM018_scaffold12094_2	58085	phage	0.987	temperate	0.9676156	Straboviridae	0.431543	Flavobacterium columnare	0.81	Predict
DLM018_scaffold3525_6	11816	phage	0.99	temperate	0.9997935	unknown	0	Candidatus Hamiltonella defensa	1	CRISPR
DLM018_scaffold2054_4	14032	phage	0.999	temperate	0.9998445	Peduoviridae	0.37287483	Candidatus Hamiltonella defensa	0.96	Predict
DLM018_scaffold82_1	22218	phage	0.999	temperate	0.9975639	unknown	0	Candidatus Hamiltonella defensa	0.92	Predict
DLM018_scaffold48894_1	12352	phage	0.999	virulent	0.9998722	Kyanoviridae	1	Pantoea agglomerans	0.84	Predict
DLM018_scaffold26573_10	30806	phage	1	virulent	0.9998679	Mesyanzhinovviridae	0.39414543	Roseobacter denitrificans	0.78	Predict
DLM018_scaffold45347_2	48799	phage	0.999	virulent	0.9998627	Straboviridae	0.5172189	Candidatus Hamiltonella defensa	0.78	Predict
DLM018_scaffold48696_1	16682	phage	1	virulent	0.9998693	Salasmaviridae	1	Actinomyces naeslundii	0.99	Predict
DLM018_scaffold44037_1	10460	phage	0.968	virulent	0.9998593	unknown	0	unknown	0	-
DLM018_scaffold1207_3	12755	phage	0.978	temperate	0.9988239	unknown	0	unknown	0	-
DLM018_scaffold79_28	14060	phage	0.995	virulent	0.99023	unknown	0	unknown	0	-
DLM018_scaffold15_30	12323	phage	0.989	temperate	0.94221675	Zierdtviridae	1	Candidatus Hamiltonella defensa	0.78	Predict
DLM018_scaffold48959_3	13267	phage	0.997	virulent	0.99986315	Straboviridae	0.27312943	Candidatus Hamiltonella defensa	0.71	Predict
DLM018_scaffold7847_2	14077	phage	0.937	virulent	0.999836	Ackermannviridae	1	Candidatus Hamiltonella defensa	0.98	Predict
DLM018_scaffold31396_2	11744	phage	0.987	temperate	0.8019704	unknown	0	Candidatus Hamiltonella defensa	0.9	Predict
DLM018_scaffold14900_3	13852	phage	0.998	temperate	0.9025601	unknown	0	Chryseobacterium carnipullorum	1	CRISPR
DLM018_scaffold48449_1	25671	phage	0.999	temperate	0.9998593	Straboviridae	0.4545582	Parabacteroides merdae	0.93	Predict
DLM018_scaffold47288_3	46727	phage	0.989	temperate	0.99981695	unknown	0	Clostridium perfringens	1	CRISPR
DLM018_scaffold26573_4	10354	phage	0.999	temperate	0.99986035	Casjensviridae	0.38102219	Roseobacter denitrificans	1	CRISPR
DLM018_scaffold36970_6	20117	phage	1	virulent	0.9998228	Ackermannviridae	0.22744717	Candidatus Hamiltonella defensa	1	CRISPR
DLM018_scaffold40461_1	13277	phage	0.92	virulent	0.999322	unknown	0	Parabacteroides merdae	0.96	Predict
DLM018_scaffold48983_2	43341	phage	0.999	temperate	0.999804	Straboviridae	0.42117372	Clostridioides difficile	1	CRISPR
DLM018_scaffold26955_2	11280	phage	0.999	temperate	0.98430026	Peduoviridae	1	Candidatus Hamiltonella defensa	0.73	Predict
DLM018_scaffold3351_9	10956	phage	0.998	temperate	0.96074384	Casjensviridae	1	Candidatus Hamiltonella defensa	0.8	Predict
DLM018_scaffold19081_1	12271	phage	0.961	virulent	0.9998736	unknown	0	Candidatus Hamiltonella defensa	1	CRISPR
DLM019_scaffold19742_4	14313	phage	0.999	virulent	0.88442934	unknown	0	Candidatus Hamiltonella defensa	0.75	Predict

DLM019_scaffold62503_1	18906	phage	0.984	temperate	0.99986035	unknown	0	Candidatus Hamiltonella defensa	0.75	Predict
DLM019_scaffold22877_3	15289	phage	1	temperate	0.87514806	unknown	0	Candidatus Hamiltonella defensa	0.99	Predict
DLM019_scaffold62785_1	11949	phage	0.984	temperate	0.9476128	Casjensviridae	1	Candidatus Hamiltonella defensa	0.76	Predict
DLM019_scaffold53137_1	27806	phage	1	temperate	0.99985266	unknown	0	Clostridioides difficile	0.82	Predict
DLM019_scaffold20492_3	21157	phage	0.999	temperate	0.99041367	Drexlerviridae	0.3499038	Candidatus Hamiltonella defensa	0.9	Predict
DLM019_scaffold6166_23	10187	phage	0.744	virulent	0.99984694	Drexlerviridae	0.46745822	Candidatus Hamiltonella defensa	0.87	Predict
DLM019_scaffold44099_4	13770	phage	0.996	virulent	0.99986744	unknown	0	unknown	0	-
DLM019_scaffold8577_4	18267	phage	0.797	temperate	0.997696	unknown	0	Pseudomonas tolaasii	0.72	Predict
DLM019_scaffold20492_2	10251	phage	0.999	temperate	0.9998593	unknown	0	Candidatus Hamiltonella defensa	0.84	Predict
DLM019_C814862_1	13434	phage	0.954	temperate	0.9998579	Straboviridae	0.58876103	Candidatus Hamiltonella defensa	0.77	Predict
DLM019_scaffold10836_2	17803	phage	1	temperate	0.9998545	unknown	0	unknown	0	-
DLM019_scaffold45285_5	14282	phage	0.999	temperate	0.9993865	Casjensviridae	1	Candidatus Hamiltonella defensa	0.7	Predict
DLM019_scaffold24899_8	13903	phage	0.999	temperate	0.8866283	unknown	0	Candidatus Hamiltonella defensa	1	CRISPR
DLM019_scaffold63202_2	15465	phage	0.765	virulent	0.99986607	unknown	0	Candidatus Hamiltonella defensa	0.82	Predict
DLM019_scaffold53137_2	14414	phage	0.999	temperate	0.9262353	unknown	0	Candidatus Hamiltonella defensa	0.8	Predict
DLM019_scaffold2743_2	14394	phage	0.997	virulent	0.99987036	unknown	0	unknown	0	-
DLM019_scaffold61987_1	18140	phage	0.998	temperate	0.99986035	Straboviridae	0.30654648	Candidatus Hamiltonella defensa	0.95	Predict
DLM019_scaffold35325_15	12269	phage	0.997	temperate	0.9993991	unknown	0	unknown	0	-
DLM019_scaffold31976_1	26102	phage	0.999	virulent	0.9522072	unknown	0	Flavobacterium columnare	0.75	Predict
DLM019_scaffold39_1	12121	phage	0.999	temperate	0.99985975	unknown	0	Candidatus Hamiltonella defensa	1	CRISPR
DLM019_scaffold38422_4	78393	phage	0.999	virulent	0.99963504	Chaseviridae	1	Candidatus Hamiltonella defensa	0.72	Predict
DLM019_scaffold45285_4	25943	phage	0.998	temperate	0.99964815	Drexlerviridae	0.30423322	Candidatus Hamiltonella defensa	1	CRISPR
DLM019_scaffold4998_6	28807	phage	0.999	temperate	0.99985695	Peduoviridae	0.7190166	Candidatus Hamiltonella defensa	0.74	Predict
DLM019_scaffold54914_7	26110	phage	0.999	temperate	0.9919748	unknown	0	Candidatus Hamiltonella defensa	1	Predict
DLM019_scaffold35445_2	14187	phage	0.999	virulent	0.9949459	unknown	0	Candidatus Hamiltonella defensa	0.77	Predict
DLM019_scaffold37359_6	10991	phage	0.836	virulent	0.9905684	unknown	0	Candidatus Hamiltonella defensa	0.88	Predict
DLM019_scaffold100_11	12325	phage	0.998	virulent	0.9998636	Peduoviridae	0.3525797	unknown	0	-
DLM019_scaffold55656_1	11408	phage	0.997	virulent	0.9997321	Drexlerviridae	0.21202745	Candidatus Hamiltonella defensa	0.9	Predict
DLM019_scaffold38422_1	10950	phage	0.999	temperate	0.53554034	unknown	0	Candidatus Hamiltonella defensa	0.96	Predict
DLM019_scaffold63049_1	33317	phage	0.999	virulent	0.9998679	unknown	0	Candidatus Hamiltonella defensa	0.96	Predict

DLM019_scaffold102_1	26038	phage	0.999	temperate	0.9017353	Peduoviridae	1	Candidatus Hamiltonella defensa	1	CRISPR
DLM019_scaffold62785_2	23560	phage	0.999	virulent	0.99985695	unknown	0	Candidatus Hamiltonella defensa	1	CRISPR
DLM019_scaffold8817_14	11465	phage	0.981	virulent	0.9957946	unknown	0	Bacteroides fragilis	0.8	Predict
DLM019_scaffold55454_1	20080	phage	0.999	temperate	0.99985975	Straboviridae	0.5449183	Candidatus Hamiltonella defensa	0.8	Predict
DLM019_scaffold29437_2	16926	phage	0.998	temperate	0.9998574	unknown	0	Candidatus Hamiltonella defensa	0.78	Predict
DLM019_scaffold12600_2	10320	phage	0.999	virulent	0.9998608	unknown	0	Candidatus Hamiltonella defensa	0.89	Predict
DLM019_scaffold2445_1	36460	phage	0.999	temperate	0.99983495	Drexlerviridae	0.21655169	Candidatus Hamiltonella defensa	1	CRISPR
DLM019_scaffold44258_2	13706	phage	0.958	temperate	0.9998574	unknown	0	Parabacteroides distasonis	0.82	Predict
DLM019_scaffold31935_1	12146	phage	0.979	temperate	0.99913585	unknown	0	Candidatus Hamiltonella defensa	0.95	Predict
DLM019_scaffold8788_7	15878	phage	0.856	temperate	0.93894786	Straboviridae	0.40471888	Bacteroides fragilis	0.77	Predict
DLM019_scaffold63216_1	25682	phage	0.999	virulent	0.99986744	unknown	0	Candidatus Hamiltonella defensa	0.79	Predict
DLM020_scaffold983_14	75380	phage	0.954	virulent	0.99977165	Casjensviridae	0.353185	Lactobacillus fermentum	1	CRISPR
DLM020_scaffold8084_6	19772	phage	0.999	temperate	0.93656045	unknown	0	Bacteroides fragilis	0.92	Predict
DLM020_scaffold11081_1	14317	phage	0.999	virulent	0.9998331	unknown	0	Candidatus Hamiltonella defensa	0.7	Predict
DLM020_scaffold15999_4	17520	phage	0.999	virulent	0.99983215	unknown	0	unknown	0	-
DLM020_scaffold21144_2	11225	phage	0.974	temperate	0.98519784	unknown	0	Bacteroides fragilis	0.86	Predict
DLM020_scaffold19547_2	10582	phage	0.998	virulent	0.9998308	unknown	0	Candidatus Hamiltonella defensa	0.86	Predict
DLM020_scaffold10399_1	10104	phage	0.998	virulent	0.9998722	Straboviridae	0.5655343	Candidatus Hamiltonella defensa	0.9	Predict
DLM020_scaffold3010_3	41865	phage	0.94	temperate	0.9997344	Peduoviridae	0.24940163	Bdellovibrio bacteriovorus	1	CRISPR
DLM020_scaffold20362_3	26903	phage	0.997	temperate	0.9881228	Straboviridae	0.20631503	Candidatus Hamiltonella defensa	0.7	Predict
DLM020_scaffold8114_1	30009	phage	0.552	virulent	0.59466475	Straboviridae	0.571929	Bacteroides fragilis	0.79	Predict
DLM020_scaffold8736_5	12869	phage	0.999	temperate	0.99985975	unknown	0	Candidatus Hamiltonella defensa	0.87	Predict
DLM020_scaffold500_10	15898	phage	0.919	temperate	0.9413989	Straboviridae	0.40471888	Bacteroides fragilis	0.93	Predict
DLM020_scaffold7279_1	20480	phage	0.999	temperate	0.9957495	unknown	0	Candidatus Hamiltonella defensa	1	CRISPR
DLM020_scaffold1387_1	11248	phage	0.965	virulent	0.99987084	Ackermannviridae	0.39516526	Bacteroides fragilis	0.96	Predict
DLM020_scaffold22314_2	38755	phage	1	temperate	0.95623636	Peduoviridae	1	Candidatus Hamiltonella defensa	1	CRISPR
DLM020_scaffold8693_2	11477	phage	0.992	virulent	0.9998727	unknown	0	Bacteroides fragilis	0.89	Predict
DLM020_scaffold20362_2	27848	phage	0.999	temperate	0.99833655	Drexlerviridae	0.265135	Lactobacillus gasseri	0.83	Predict
DLM020_scaffold22182_2	14590	phage	0.994	temperate	0.98694676	Straboviridae	0.3508861	Candidatus Hamiltonella defensa	1	CRISPR
DLM020_scaffold21959_1	13669	phage	0.999	temperate	0.9998117	unknown	0	Candidatus Hamiltonella defensa	0.91	Predict

DLM020_scaffold6157_3	12100	phage	0.998	virulent	0.99987316	unknown	0	Candidatus Hamiltonella defensa	0.85	Predict
DLM020_scaffold19925_2	13093	phage	0.992	temperate	0.9998584	Drexlerviridae	0.46745822	Candidatus Hamiltonella defensa	0.87	Predict
DLM020_scaffold13039_2	33347	phage	0.999	virulent	0.9998679	Straboviridae	0.5416722	Candidatus Hamiltonella defensa	1	CRISPR
DLM020_C355636_1	10184	phage	0.998	temperate	0.9998584	Straboviridae	0.6009076	Candidatus Hamiltonella defensa	0.82	Predict
DLM020_scaffold350_1	23743	phage	0.938	temperate	0.9998593	Ackermannviridae	0.21828994	unknown	0	-
DLM020_scaffold20632_1	13343	phage	0.996	virulent	0.908313	unknown	0	Candidatus Hamiltonella defensa	0.74	Predict
DLM020_scaffold22218_1	16108	phage	0.545	temperate	0.99985975	unknown	0	Bacteroides fragilis	1	CRISPR
DLM020_scaffold6936_4	11577	phage	0.998	temperate	0.999854	Straboviridae	0.69947964	Candidatus Pelagibacter ubique	0.71	Predict
DLM020_scaffold7475_1	19167	phage	0.997	virulent	0.99987084	unknown	0	Lactobacillus plantarum	0.89	Predict
DLM020_scaffold3936_1	15824	phage	0.929	temperate	0.9998188	unknown	0	unknown	0	-
DLM020_scaffold10415_1	16222	phage	0.991	virulent	0.99987173	unknown	0	Bacteroides fragilis	0.95	Predict
DLM020_scaffold10180_3	40654	phage	0.998	virulent	0.96428293	Casjensviridae	0.51866126	Candidatus Hamiltonella defensa	0.98	Predict
DLM020_scaffold22290_1	69771	phage	0.999	virulent	0.9998627	Autographiviridae	1	Candidatus Hamiltonella defensa	0.82	Predict
DLM020_scaffold5648_4	10206	phage	0.998	virulent	0.99987465	unknown	0	Candidatus Hamiltonella defensa	0.76	Predict
DLM020_scaffold11301_1	13665	phage	0.546	temperate	0.7636316	unknown	0	Candidatus Hamiltonella defensa	0.78	Predict
DLM020_scaffold21039_1	17725	phage	0.994	virulent	0.9785579	Straboviridae	0.23314427	Candidatus Hamiltonella defensa	0.91	Predict
DLM020_scaffold7279_3	18234	phage	0.949	temperate	0.99985975	Peduoviridae	1	Roseburia inulinivorans	1	CRISPR
DLM020_scaffold13927_3	12037	phage	0.999	virulent	0.99987465	Straboviridae	0.3969798	Candidatus Hamiltonella defensa	0.87	Predict
DLM020_scaffold10263_1	15995	phage	0.999	temperate	0.9998593	Straboviridae	0.5400346	Cellulophaga baltica	0.91	Predict
DLM020_scaffold6936_3	10992	phage	0.955	virulent	0.9998012	unknown	0	unknown	0	-
DLM021_scaffold36034_1	11493	phage	0.999	temperate	0.9997206	Casjensviridae	0.4320409	Candidatus Hamiltonella defensa	0.88	Predict
DLM021_scaffold44307_1	19974	phage	0.999	virulent	0.9988832	unknown	0	Candidatus Hamiltonella defensa	1	CRISPR
DLM021_scaffold43159_1	22514	phage	0.988	virulent	0.99987036	Ackermannviridae	0.36708665	Candidatus Hamiltonella defensa	0.89	Predict
DLM021_scaffold31538_5	16255	phage	0.999	temperate	0.9986814	unknown	0	Candidatus Hamiltonella defensa	1	CRISPR
DLM021_scaffold29140_2	14831	phage	0.993	virulent	0.9998608	unknown	0	Candidatus Hamiltonella defensa	0.83	Predict
DLM021_scaffold35216_1	14847	phage	0.998	virulent	0.99966675	unknown	0	Candidatus Hamiltonella defensa	0.81	Predict
DLM021_scaffold43126_4	10793	phage	0.955	virulent	0.9998012	unknown	0	unknown	0	-
DLM021_scaffold42967_2	10443	phage	0.999	temperate	0.9998369	unknown	0	unknown	0	-
DLM021_scaffold12971_1	12282	phage	0.806	temperate	0.5923225	Peduoviridae	0.41205508	Candidatus Hamiltonella defensa	0.91	Predict
DLM021_scaffold28049_1	16713	phage	0.997	virulent	0.9242962	unknown	0	Candidatus Hamiltonella defensa	1	CRISPR

DLM021_scaffold36642_2	11521	phage	0.771	virulent	0.99986696	unknown	0	Candidatus Hamiltonella defensa	0.96	Predict
DLM021_scaffold42193_7	10186	phage	0.998	virulent	0.99986035	Straboviridae	0.3969798	Candidatus Hamiltonella defensa	0.85	Predict
DLM021_scaffold43501_2	11266	phage	0.961	temperate	0.979654	unknown	0	Bacteroides fragilis	0.99	Predict
DLM021_scaffold554_3	14849	phage	0.998	temperate	0.9998593	unknown	0	Candidatus Hamiltonella defensa	0.95	Predict
DLM021_scaffold43126_6	13113	phage	0.998	temperate	0.99983406	Straboviridae	0.69947964	Candidatus Hamiltonella defensa	0.88	Predict
DLM021_scaffold31475_3	14424	phage	0.996	virulent	0.99986696	unknown	0	Rhodococcus hoagii	0.79	Predict
DLM021_scaffold24637_8	21713	phage	1	temperate	0.99983835	Straboviridae	0.18682745	Bacteroides fragilis	1	CRISPR
DLM021_scaffold10545_9	18740	phage	0.809	virulent	0.99986607	Straboviridae	1	unknown	0	-
DLM021_scaffold4_54	11531	phage	0.98	virulent	0.99982494	Ackermannviridae	0.39865726	Bacteroides fragilis	0.94	Predict
DLM021_scaffold30546_2	15794	phage	0.998	temperate	0.9998388	Straboviridae	0.4992557	Candidatus Hamiltonella defensa	1	CRISPR
DLM021_scaffold10200_17	11980	phage	0.999	virulent	0.99972486	Salasmaviridae	0.4037911	Candidatus Hamiltonella defensa	0.77	Predict
DLM021_scaffold28049_2	23499	phage	0.998	virulent	0.99892867	unknown	0	Bacteroides fragilis	1	CRISPR
DLM021_scaffold10200_12	14260	phage	0.999	virulent	0.9998722	unknown	0	Candidatus Hamiltonella defensa	0.93	Predict
DLM021_scaffold23214_1	10515	phage	0.653	temperate	0.9998536	unknown	0	Candidatus Hamiltonella defensa	0.79	Predict
DLM021_scaffold136_1	12928	phage	0.989	virulent	0.9998651	Mesyanzhinovviridae	0.29691836	unknown	0	-
DLM021_scaffold46_2	10108	phage	0.932	virulent	0.9998727	Ackermannviridae	0.6232304	Candidatus Hamiltonella defensa	0.99	Predict
DLM021_scaffold24731_9	22118	phage	0.521	virulent	0.9998688	Casjensviridae	0.8158135	Candidatus Hamiltonella defensa	0.72	Predict
DLM021_scaffold640_7	10370	phage	0.999	temperate	0.97422904	unknown	0	Bacteroides fragilis	0.85	Predict
DLM021_scaffold21292_11	17866	phage	0.993	virulent	0.9996989	Ackermannviridae	0.3847759	Bacteroides fragilis	0.88	Predict
DLM021_scaffold22868_5	15501	phage	1	virulent	0.9998086	unknown	0	Bacteroides fragilis	0.97	Predict
DLM021_scaffold2298_4	12523	phage	0.999	virulent	0.99987406	unknown	0	Candidatus Hamiltonella defensa	0.71	Predict
DLM021_scaffold44452_1	17673	phage	0.623	virulent	0.9998636	Straboviridae	0.571929	Candidatus Hamiltonella defensa	0.98	Predict
DLM021_scaffold8187_1	13303	phage	0.979	virulent	0.9937134	unknown	0	Candidatus Hamiltonella defensa	0.84	Predict
DLM021_scaffold45210_2	36586	phage	0.999	temperate	0.99973255	Peduoviridae	1	Candidatus Hamiltonella defensa	1	CRISPR
DLM021_scaffold381_1	20295	phage	1	temperate	0.99985975	Peduoviridae	1	Candidatus Hamiltonella defensa	1	CRISPR
DLM021_scaffold1604_4	25344	phage	0.997	virulent	0.99987173	Straboviridae	0.66194147	Candidatus Hamiltonella defensa	0.84	Predict
DLM022_scaffold87335_3	10721	phage	0.999	virulent	0.96945584	unknown	0	Candidatus Hamiltonella defensa	0.8	Predict
DLM022_scaffold90455_1	11519	phage	0.998	temperate	0.99954236	Straboviridae	0.3541648	Lactobacillus jensenii	1	CRISPR
DLM022_C1105606_1	18755	phage	0.995	temperate	0.9824839	Zierdtviridae	1	Clostridium perfringens	0.94	Predict
DLM022_scaffold34930_3	15488	phage	0.999	temperate	0.99985695	Peduoviridae	0.22083662	Candidatus Hamiltonella defensa	1	CRISPR

DLM022_scaffold13671_3	26392	phage	0.999	temperate	0.9998545	unknown	0	Candidatus Hamiltonella defensa	1	CRISPR
DLM022_scaffold90383_1	16944	phage	0.974	temperate	0.9998579	no_family_avaliable(NC_000924)	0.978	Candidatus Hamiltonella defensa	0.75	Predict
DLM022_scaffold61396_2	16516	phage	0.912	virulent	0.99519646	Straboviridae	0.61910915	Candidatus Hamiltonella defensa	0.8	Predict
DLM022_scaffold3872_14	10694	phage	0.997	temperate	0.99986035	Drexlerviridae	0.23057634	Candidatus Hamiltonella defensa	0.71	Predict
DLM022_scaffold28531_1	15569	phage	0.999	virulent	0.99986315	unknown	0	Candidatus Hamiltonella defensa	0.9	Predict
DLM022_scaffold13626_15	10922	phage	1	temperate	0.9998536	Casjensviridae	0.4466287	Clostridioides difficile	0.97	Predict
DLM022_scaffold47568_2	11118	phage	0.999	temperate	0.98138005	unknown	0	Candidatus Hamiltonella defensa	0.93	Predict
DLM022_scaffold87188_2	11612	phage	0.999	temperate	0.9998584	unknown	0	Candidatus Hamiltonella defensa	0.87	Predict
DLM022_scaffold83380_1	11415	phage	0.995	temperate	0.9998593	unknown	0	Candidatus Hamiltonella defensa	0.75	Predict
DLM022_scaffold38614_1	32226	phage	0.999	temperate	0.9998579	unknown	0	Candidatus Hamiltonella defensa	0.83	Predict
DLM022_scaffold90423_1	11633	phage	0.876	temperate	0.99985975	unknown	0	unknown	0	-
DLM022_scaffold7833_6	10200	phage	0.999	temperate	0.9998593	Straboviridae	0.37812838	Candidatus Hamiltonella defensa	0.73	Predict
DLM022_scaffold25768_2	10267	phage	0.96	temperate	0.9560764	Herelleviridae	1	unknown	0	-
DLM022_scaffold90441_1	14449	phage	0.998	temperate	0.99985975	unknown	0	Candidatus Hamiltonella defensa	1	CRISPR
DLM022_scaffold29304_11	15733	phage	0.997	virulent	0.99986696	unknown	0	Candidatus Hamiltonella defensa	1	CRISPR
DLM022_scaffold45845_2	17200	phage	0.892	virulent	0.9998165	Ackermannviridae	1	Candidatus Hamiltonella defensa	0.98	Predict
DLM022_scaffold75069_1	18896	phage	1	temperate	0.9997144	Peduoviridae	0.35056782	Candidatus Hamiltonella defensa	0.85	Predict
DLM022_scaffold46815_1	34877	phage	0.999	temperate	0.99905294	unknown	0	Candidatus Pelagibacter ubique	0.92	Predict
DLM022_scaffold50520_4	21707	phage	0.983	temperate	0.9966986	unknown	0	Candidatus Hamiltonella defensa	0.87	Predict
DLM022_scaffold82276_2	11705	phage	0.751	virulent	0.9998608	unknown	0	Candidatus Hamiltonella defensa	0.95	Predict
DLM022_scaffold86174_3	15775	phage	0.999	temperate	0.98876494	Drexlerviridae	0.2711681	Candidatus Hamiltonella defensa	0.98	Predict
DLM022_scaffold40951_1	10660	phage	0.941	virulent	0.9996882	Straboviridae	0.61924237	Candidatus Hamiltonella defensa	0.77	Predict
DLM022_scaffold51389_1	48777	phage	0.997	virulent	0.9998679	Schitoviridae	1	Bacteroides vulgatus	1	CRISPR
DLM022_scaffold34373_9	24649	phage	0.997	temperate	0.99985975	Peduoviridae	1	Candidatus Hamiltonella defensa	0.78	Predict
DLM022_scaffold88974_2	13408	phage	0.86	temperate	0.99986035	unknown	0	unknown	0	-
DLM022_scaffold37828_6	21319	phage	0.999	virulent	0.9998722	unknown	0	unknown	0	-
DLM022_scaffold7976_5	23390	phage	0.999	virulent	0.99663186	unknown	0	Candidatus Hamiltonella defensa	0.77	Predict
DLM022_scaffold68876_1	34054	phage	0.838	virulent	0.9998688	Ackermannviridae	0.3325916	Candidatus Hamiltonella defensa	0.94	Predict
DLM022_scaffold24869_7	41768	phage	0.999	virulent	0.9998588	Herelleviridae	0.89390486	Candidatus Hamiltonella defensa	0.75	Predict
DLM022_scaffold46210_2	23374	phage	1	virulent	0.9998651	unknown	0	Candidatus Hamiltonella defensa	1	CRISPR

DLM022_scaffold79585_1	13014	phage	0.999	virulent	0.9998228	unknown	0	Candidatus Hamiltonella defensa	0.94 Predict
DLM022_scaffold90471_1	55486	phage	0.999	temperate	0.8644271	Straboviridae	0.36929065	Candidatus Hamiltonella defensa	0.95 Predict
DLM022_scaffold126_4	15014	phage	0.999	temperate	0.9998588	unknown	0	Candidatus Hamiltonella defensa	0.78 Predict
DLM022_scaffold91_3	16021	phage	0.899	temperate	0.96827364	Vilmaviridae	1	Candidatus Hamiltonella defensa	1 CRISPR
DLM022_scaffold7197_1	10900	phage	0.999	virulent	0.99984926	unknown	0	Candidatus Hamiltonella defensa	0.95 Predict
DLM022_scaffold24869_6	17490	phage	0.999	virulent	0.99986315	unknown	0	Flavobacterium columnare	0.81 Predict
DLM022_scaffold79462_2	22106	phage	0.997	virulent	0.99987173	unknown	0	unknown	0 -
DLM022_scaffold26167_3	11357	phage	0.999	virulent	0.99987465	unknown	0	Parabacteroides distasonis	0.87 Predict
DLM022_scaffold8618_3	15554	phage	1	virulent	0.9998684	unknown	0	Candidatus Hamiltonella defensa	1 CRISPR
DLM022_scaffold20053_1	10986	phage	0.754	virulent	0.99987465	unknown	0	unknown	0 -
DLM022_scaffold248_3	12604	phage	0.998	temperate	0.99922353	Zierdtviridae	1	Candidatus Hamiltonella defensa	0.95 Predict
DLM022_scaffold90464_1	13500	phage	0.999	virulent	0.99986744	Salasmaviridae	1	Flavobacterium columnare	1 CRISPR
DLM022_scaffold63634_1	24137	phage	0.999	temperate	0.9998308	Straboviridae	0.38295603	Candidatus Hamiltonella defensa	0.97 Predict
DLM022_scaffold90529_1	36413	phage	0.999	temperate	0.999702	unknown	0	Clostridioides difficile	0.84 Predict
DLM022_scaffold82197_1	19577	phage	0.999	temperate	0.9998584	unknown	0	Mycoplasma pulmonis	1 CRISPR
DLM022_scaffold84536_3	20081	phage	0.997	temperate	0.99985695	unknown	0	Candidatus Hamiltonella defensa	0.87 Predict
DLM022_scaffold11050_1	10927	phage	0.996	temperate	0.99985695	unknown	0	unknown	0 -
DLM022_scaffold73260_1	11336	phage	0.998	temperate	0.99984604	no_family_avaliable(NC_025434)	0.981	Candidatus Hamiltonella defensa	1 CRISPR
DLM022_scaffold79270_1	61706	phage	0.997	temperate	0.9998369	Peduoviridae	1	Lactobacillus fermentum	1 CRISPR
DLM022_scaffold47815_3	15000	phage	0.999	virulent	0.9998699	no_family_avaliable(NC_062777)	0.974	unknown	0 -
DLM022_scaffold68020_2	15598	phage	1	temperate	0.99884605	unknown	0	Candidatus Hamiltonella defensa	0.7 Predict
DLM022_scaffold12444_5	12336	phage	0.999	temperate	0.9998588	Straboviridae	0.6043284	Candidatus Hamiltonella defensa	0.85 Predict
DLM022_scaffold86174_2	23584	phage	1	virulent	0.93819433	Casjensviridae	1	Candidatus Hamiltonella defensa	0.96 Predict
DLM022_scaffold11069_53	10964	phage	0.989	virulent	0.99985975	Vilmaviridae	1	unknown	0 -
DLM022_scaffold89824_2	10978	phage	0.999	virulent	0.9609294	unknown	0	Akkermansia muciniphila	1 CRISPR
DLM022_C1104604_1	11143	phage	0.999	temperate	0.9998593	unknown	0	Flavobacterium columnare	0.74 Predict
DLM022_scaffold36935_1	26269	phage	0.999	temperate	0.9998593	unknown	0	Candidatus Hamiltonella defensa	0.97 Predict
DLM022_scaffold80933_2	15226	phage	0.842	virulent	0.99983126	unknown	0	unknown	0 -
DLM022_scaffold21034_2	12369	phage	0.998	virulent	0.99987406	unknown	0	unknown	0 -
DLM022_scaffold85942_2	10932	phage	0.916	virulent	0.9971863	Ackermannviridae	0.21828994	Candidatus Hamiltonella defensa	0.98 Predict

DLM022_scaffold9_2	15101	phage	1	virulent	0.998057	Peduoviridae	0.46964702	Candidatus Hamiltonella defensa	1	CRISPR
DLM022_scaffold88907_2	10782	phage	0.998	temperate	0.99985975	unknown	0	unknown	0	-
DLM022_scaffold6448_3	34562	phage	0.999	virulent	0.6171255	Kyanoviridae	1	Candidatus Hamiltonella defensa	0.82	Predict
DLM022_scaffold90528_2	11481	phage	0.999	temperate	0.99985975	Peduoviridae	1	Candidatus Hamiltonella defensa	1	CRISPR
DLM022_scaffold160_1	11335	phage	0.999	virulent	0.97564626	Drexlerviridae	0.30062526	Clostridioides difficile	0.86	Predict
DLM022_scaffold64018_1	13131	phage	0.998	virulent	0.9998699	unknown	0	Selenomonas ruminantium	1	CRISPR
DLM022_scaffold89824_3	38755	phage	0.998	temperate	0.9998379	Mesyanzhinovviridae	1	Candidatus Hamiltonella defensa	1	CRISPR
DLM022_scaffold43500_4	13783	phage	0.989	temperate	0.9998593	Peduoviridae	1	Candidatus Hamiltonella defensa	0.9	Predict
DLM022_scaffold90113_2	22601	phage	0.997	temperate	0.999796	unknown	0	Candidatus Hamiltonella defensa	0.84	Predict
DLM022_scaffold37957_2	13447	phage	0.997	temperate	0.9998122	unknown	0	Candidatus Hamiltonella defensa	0.88	Predict
DLM022_scaffold11067_1	27225	phage	0.999	virulent	0.99987084	no_family_avaliable(NC_067213)	0.971	Candidatus Pelagibacter ubique	0.85	Predict
DLM022_scaffold22367_7	11306	phage	0.998	virulent	0.99986315	unknown	0	Candidatus Hamiltonella defensa	0.88	Predict
DLM022_scaffold24995_4	13667	phage	0.998	temperate	0.999016	unknown	0	Candidatus Hamiltonella defensa	0.9	Predict
DLM022_scaffold89236_1	11783	phage	0.999	temperate	0.99896556	Straboviridae	0.3508861	Candidatus Hamiltonella defensa	1	CRISPR
DLM022_scaffold30520_1	25186	phage	0.999	temperate	0.9996515	unknown	0	Candidatus Hamiltonella defensa	1	CRISPR
DLM022_scaffold32204_1	22893	phage	0.999	temperate	0.99986035	unknown	0	Candidatus Hamiltonella defensa	0.75	Predict
DLM022_scaffold46686_4	14234	phage	0.96	virulent	0.9998645	unknown	0	Candidatus Hamiltonella defensa	0.77	Predict
DLM022_scaffold85281_1	10362	phage	0.998	virulent	0.9998736	unknown	0	Bacteroides thetaiotaomicron	1	CRISPR
DLM022_scaffold90528_3	14817	phage	0.999	temperate	0.99985975	Peduoviridae	1	Candidatus Hamiltonella defensa	1	CRISPR
DLM022_scaffold89880_1	23695	phage	0.999	temperate	0.99985975	Drexlerviridae	0.2698334	Candidatus Hamiltonella defensa	0.86	Predict
DLM022_scaffold24869_4	16092	phage	0.999	virulent	0.9998627	no_family_avaliable(NC_055881)	0.988	unknown	0	-
DLM022_scaffold89156_1	30554	phage	0.999	temperate	0.99984264	Drexlerviridae	0.45531175	Candidatus Hamiltonella defensa	0.75	Predict
DLM022_scaffold90507_1	24371	phage	0.642	temperate	0.9995376	unknown	0	Candidatus Hamiltonella defensa	0.98	Predict
DLM022_scaffold62765_1	20534	phage	1	temperate	0.9994489	unknown	0	Candidatus Hamiltonella defensa	1	CRISPR
DLM022_scaffold27082_1	11504	phage	0.991	temperate	0.9997535	unknown	0	unknown	0	-
DLM022_scaffold73568_2	16293	phage	0.999	virulent	0.99987125	Salasmaviridae	1	Actinomyces naeslundii	0.89	Predict
DLM022_scaffold88059_2	23353	phage	0.999	virulent	0.9998699	unknown	0	[Eubacterium] rectale	1	CRISPR
DLM022_scaffold90402_1	101635	phage	0.999	virulent	0.9998651	no_family_avaliable(NC_062767)	0.976	Cellulophaga baltica	0.98	Predict
DLM022_scaffold90509_1	13724	phage	0.983	virulent	0.9992649	no_family_avaliable(NC_019445)	0.972	Candidatus Hamiltonella defensa	0.83	Predict
DLM022_scaffold44709_3	19402	phage	0.987	temperate	0.9998388	Straboviridae	0.571929	Bacteroides fragilis	0.84	Predict

DLM022_scaffold87605_2	18600	phage	0.999	virulent	0.99984884	unknown	0	Bacteroides fragilis	1	CRISPR
DLM022_scaffold937_1	13845	phage	0.999	virulent	0.9998656	unknown	0	Croceibacter atlanticus	1	CRISPR
DLM022_scaffold30068_2	11650	phage	0.989	virulent	0.99933046	unknown	0	Candidatus Hamiltonella defensa	0.98	Predict
DLM022_scaffold15558_7	10537	phage	0.889	temperate	0.99585557	unknown	0	Candidatus Hamiltonella defensa	1	Predict
DLM023_scaffold51589_1	52512	phage	0.999	temperate	0.64372236	Peduoviridae	1	Candidatus Hamiltonella defensa	1	CRISPR
DLM023_scaffold25385_1	11193	phage	0.996	temperate	0.9998584	Ackermannviridae	1	Candidatus Hamiltonella defensa	0.94	Predict
DLM023_scaffold1460_2	10598	phage	0.773	temperate	0.99985975	unknown	0	unknown	0	-
DLM023_scaffold14487_2	24280	phage	0.999	virulent	0.99970657	Straboviridae	0.3196358	Candidatus Hamiltonella defensa	0.94	Predict
DLM023_scaffold48996_1	40655	phage	0.999	temperate	0.99984604	Peduoviridae	1	Candidatus Hamiltonella defensa	1	CRISPR
DLM023_scaffold4774_1	16495	phage	0.999	temperate	0.99985975	Casjensviridae	0.38102219	Roseobacter denitrificans	1	CRISPR
DLM023_scaffold27445_8	12789	phage	0.998	temperate	0.99986035	unknown	0	Candidatus Hamiltonella defensa	1	CRISPR
DLM023_scaffold411_39	10058	phage	0.999	temperate	0.9994242	unknown	0	Candidatus Hamiltonella defensa	0.89	Predict
DLM023_scaffold51795_1	10829	phage	1	temperate	0.9998588	unknown	0	Candidatus Hamiltonella defensa	0.72	Predict
DLM023_scaffold23339_1	30623	phage	0.999	temperate	0.8228076	Peduoviridae	1	Roseobacter denitrificans	1	CRISPR
DLM023_scaffold2126_10	12133	phage	0.998	virulent	0.9998688	Herelleviridae	1	Staphylococcus haemolyticus	0.75	Predict
DLM023_scaffold7323_3	10041	phage	0.991	virulent	0.9998736	Ackermannviridae	0.6493801	Bacteroides fragilis	0.99	Predict
DLM023_scaffold44217_3	35812	phage	0.997	temperate	0.9985996	Chaseviridae	0.5610166	Candidatus Hamiltonella defensa	1	CRISPR
DLM023_scaffold47535_1	14434	phage	0.999	virulent	0.9998019	unknown	0	Ruegeria pomeroyi	0.72	Predict
DLM023_scaffold37922_1	15399	phage	0.999	temperate	0.9998588	Straboviridae	0.5400346	Candidatus Hamiltonella defensa	0.76	Predict
DLM023_scaffold4774_5	10175	phage	0.999	virulent	0.99986696	Mesyanzhinovviridae	0.39414543	Roseobacter denitrificans	0.83	Predict
DLM023_scaffold51029_1	29946	phage	0.998	virulent	0.9998693	unknown	0	Lactobacillus fermentum	1	CRISPR
DLM023_scaffold18407_7	35180	phage	0.998	temperate	0.74348515	unknown	0	Candidatus Hamiltonella defensa	0.99	Predict
DLM023_scaffold17201_4	11190	phage	0.999	temperate	0.9998588	Straboviridae	0.37812838	Candidatus Hamiltonella defensa	0.76	Predict
DLM023_scaffold45333_4	12993	phage	0.999	temperate	0.9998584	unknown	0	Candidatus Hamiltonella defensa	0.91	Predict
DLM023_scaffold28443_5	20462	phage	0.985	temperate	0.99985266	unknown	0	Lactobacillus fermentum	0.85	Predict
DLM023_scaffold32334_1	78945	phage	0.975	virulent	0.9990329	unknown	0	Candidatus Hamiltonella defensa	1	CRISPR
DLM023_scaffold51553_3	13249	phage	0.981	virulent	0.99986744	Ackermannviridae	0.24887799	Bacteroides fragilis	0.89	Predict
DLM023_scaffold4774_3	14762	phage	0.999	virulent	0.9998593	unknown	0	Roseobacter denitrificans	0.85	Predict
DLM023_scaffold13027_1	10206	phage	0.982	temperate	0.9998593	unknown	0	unknown	0	-
DLM023_scaffold58_7	15932	phage	0.997	temperate	0.99984217	unknown	0	Bacteroides fragilis	0.93	Predict

DLM023_scaffold51688_1	11515	phage	0.905	temperate	0.9998593	unknown	0	Candidatus Hamiltonella defensa	0.76	Predict
DLM023_scaffold26150_3	27669	phage	1	temperate	0.9998474	Peduoviridae	0.3214273	Candidatus Hamiltonella defensa	1	CRISPR
DLM024_scaffold4102_1	39728	phage	0.999	temperate	0.9998054	unknown	0	Clostridium perfringens	1	CRISPR
DLM024_scaffold6096_2	14701	phage	0.999	temperate	0.9998584	Straboviridae	0.47515345	Lactobacillus fermentum	0.86	Predict
DLM024_scaffold1307_14	12553	phage	0.997	temperate	0.99985975	unknown	0	Candidatus Hamiltonella defensa	1	CRISPR
DLM024_scaffold6915_1	14891	phage	0.997	virulent	0.9997557	unknown	0	Candidatus Hamiltonella defensa	0.86	Predict
DLM024_scaffold6392_4	16656	phage	0.999	temperate	0.9998579	Zierdtviridae	1	Candidatus Hamiltonella defensa	0.97	Predict
DLM024_scaffold6942_1	23726	phage	0.999	virulent	0.9849078	Peduoviridae	1	Candidatus Hamiltonella defensa	1	CRISPR
DLM024_scaffold2301_1	39584	phage	1	virulent	0.9992417	unknown	0	Candidatus Hamiltonella defensa	1	CRISPR
DLM024_scaffold6782_2	54919	phage	0.988	temperate	0.99939895	Casjensviridae	1	Listeria monocytogenes	1	CRISPR
DLM024_scaffold4274_2	20842	phage	0.987	temperate	0.99947035	unknown	0	unknown	0	-
DLM024_scaffold447_14	14472	phage	0.999	temperate	0.99985975	unknown	0	Streptococcus pneumoniae	1	CRISPR
DLM024_scaffold3270_1	11116	phage	0.831	temperate	0.9998584	unknown	0	Candidatus Hamiltonella defensa	0.76	Predict
DLM024_scaffold1215_1	18962	phage	0.996	temperate	0.9979246	unknown	0	unknown	0	-
DLM024_scaffold6863_2	14838	phage	0.999	virulent	0.9998556	Herelleviridae	1	unknown	0	-
DLM024_scaffold6734_2	16723	phage	0.999	temperate	0.9094732	unknown	0	Lactococcus garvieae	1	CRISPR
DLM024_scaffold1543_4	16974	phage	0.971	virulent	0.9996913	unknown	0	Candidatus Hamiltonella defensa	0.91	Predict
DLM024_scaffold5188_2	35067	phage	0.999	virulent	0.65289533	Casjensviridae	1	Candidatus Hamiltonella defensa	1	CRISPR
DLM024_scaffold5624_3	12336	phage	0.999	temperate	0.9998588	unknown	0	Candidatus Hamiltonella defensa	1	CRISPR
DLM024_scaffold6919_2	10441	phage	0.891	virulent	0.99987316	unknown	0	Candidatus Hamiltonella defensa	0.85	Predict
DLM024_scaffold3966_3	10349	phage	1	temperate	0.9998522	unknown	0	unknown	0	-
DLM024_C136347_1	15453	phage	0.997	virulent	0.9998257	Straboviridae	0.34864473	Candidatus Hamiltonella defensa	0.94	Predict
DLM024_scaffold722_1	26425	phage	0.997	virulent	0.99987125	unknown	0	Candidatus Hamiltonella defensa	0.99	Predict
DLM024_scaffold3585_1	34683	phage	0.993	virulent	0.9997592	unknown	0	Candidatus Hamiltonella defensa	1	CRISPR
DLM024_scaffold2213_13	11746	phage	1	temperate	0.99985975	unknown	0	Candidatus Hamiltonella defensa	1	CRISPR
DLM024_scaffold2465_1	50569	phage	0.999	virulent	0.9998574	Straboviridae	0.62464666	Flavobacterium psychrophilum	1	CRISPR
DLM024_scaffold4575_2	15273	phage	0.997	temperate	0.99985033	no_family_avaliable(NC_049918)	0.983	Candidatus Hamiltonella defensa	1	CRISPR
DLM024_scaffold1355_19	25140	phage	0.979	temperate	0.999844	unknown	0	Clostridium tetani	0.79	Predict
DLM027_scaffold2040_2	15267	phage	0.995	virulent	0.99984926	Straboviridae	0.3694985	Candidatus Hamiltonella defensa	0.74	Predict
DLM027_scaffold19184_4	13031	phage	0.998	virulent	0.99984974	unknown	0	Candidatus Hamiltonella defensa	0.9	Predict

DLM027_scaffold20206_4	69251	phage	0.999	virulent	0.9788562	Herelleviridae	1	Candidatus Hamiltonella defensa	1	CRISPR
DLM027_scaffold25719_1	13091	phage	0.998	virulent	0.96856415	unknown	0	unknown	0	-
DLM027_scaffold18274_3	23561	phage	0.999	virulent	0.9997938	unknown	0	Candidatus Hamiltonella defensa	0.85	Predict
DLM027_scaffold2427_8	10133	phage	0.994	temperate	0.9998593	Ackermannviridae	1	Candidatus Hamiltonella defensa	0.72	Predict
DLM027_scaffold2177_5	15245	phage	0.96	virulent	0.9979295	unknown	0	Candidatus Hamiltonella defensa	1	CRISPR
DLM027_scaffold10585_3	12320	phage	0.992	virulent	0.9998334	unknown	0	unknown	0	-
DLM027_scaffold26586_2	19404	phage	0.998	temperate	0.9908827	unknown	0	Candidatus Hamiltonella defensa	1	CRISPR
DLM027_scaffold26569_1	23556	phage	0.993	temperate	0.9997521	Straboviridae	0.20631503	Lactobacillus gasseri	1	CRISPR
DLM027_scaffold23395_3	10825	phage	0.995	temperate	0.9891691	unknown	0	unknown	0	-
DLM027_scaffold19716_4	10816	phage	0.986	virulent	0.99987036	unknown	0	Candidatus Hamiltonella defensa	0.92	Predict
DLM027_scaffold26720_1	15253	phage	1	virulent	0.99987173	Salasmaviridae	1	Actinomyces naeslundii	1	CRISPR
DLM027_scaffold5447_3	13877	phage	0.998	temperate	0.9998593	Peduoviridae	0.6294716	Candidatus Hamiltonella defensa	0.7	Predict
DLM027_scaffold26569_2	19518	phage	0.999	temperate	0.9998588	Drexlerviridae	0.27153525	Candidatus Hamiltonella defensa	0.92	Predict
DLM027_scaffold79_10	13541	phage	0.845	temperate	0.99934417	unknown	0	Candidatus Hamiltonella defensa	0.81	Predict
DLM027_scaffold19168_1	18381	phage	0.999	temperate	0.9990398	unknown	0	Candidatus Hamiltonella defensa	1	CRISPR
DLM027_scaffold26477_2	17387	phage	0.998	temperate	0.8308406	unknown	0	Candidatus Hamiltonella defensa	0.85	Predict
DLM027_scaffold9656_1	20477	phage	0.999	temperate	0.9437092	Peduoviridae	0.51119787	Candidatus Hamiltonella defensa	1	CRISPR
DLM027_scaffold9665_4	14958	phage	1	temperate	0.9998565	unknown	0	Candidatus Hamiltonella defensa	0.73	Predict
DLM027_scaffold8638_4	10645	phage	0.622	temperate	0.64852494	unknown	0	Candidatus Hamiltonella defensa	0.93	Predict
DLM027_scaffold26707_1	20095	phage	1	temperate	0.646693	unknown	0	Candidatus Hamiltonella defensa	1	CRISPR
DLM027_scaffold14908_1	53369	phage	0.999	temperate	0.999806	Casjensviridae	1	Candidatus Hamiltonella defensa	1	CRISPR
DLM028_scaffold6098_12	18694	phage	0.785	virulent	0.9998699	Straboviridae	0.31710467	Vibrio natriegens	0.88	Predict
DLM028_scaffold32640_1	12488	phage	0.999	virulent	0.99946034	unknown	0	Candidatus Hamiltonella defensa	0.8	Predict
DLM028_scaffold21827_4	15058	phage	0.999	temperate	0.9997997	Peduoviridae	0.63610744	Candidatus Hamiltonella defensa	1	CRISPR
DLM028_scaffold54482_2	24490	phage	0.997	temperate	0.9998556	unknown	0	Candidatus Hamiltonella defensa	0.92	Predict
DLM028_scaffold33522_2	15525	phage	0.998	virulent	0.9998617	unknown	0	Candidatus Hamiltonella defensa	0.92	Predict
DLM028_scaffold35712_5	10259	phage	0.998	temperate	0.99983835	unknown	0	unknown	0	-
DLM028_scaffold39913_2	11071	phage	0.916	virulent	0.9998727	unknown	0	Candidatus Hamiltonella defensa	0.83	Predict
DLM028_scaffold30320_6	13559	phage	0.738	virulent	0.9998679	unknown	0	Flavobacterium columnare	0.94	Predict
DLM028_scaffold17_2	20989	phage	0.998	temperate	0.99985975	Straboviridae	0.3541648	Mannheimia haemolytica	0.96	Predict

DLM028_scaffold40934_1	15672	phage	0.99	temperate	0.9996744	Ackermannviridae	0.21766534	Candidatus Hamiltonella defensa	0.83	Predict
DLM028_scaffold30790_1	14378	phage	0.999	temperate	0.9998545	no_family_avaliable(NC_009514)	0.979	Candidatus Hamiltonella defensa	1	CRISPR
DLM028_scaffold43205_3	11607	phage	0.999	virulent	0.9998114	unknown	0	Bacteroides fragilis	0.87	Predict
DLM028_scaffold12478_1	13252	phage	0.999	virulent	0.9998727	Salasmaviridae	0.4037911	Candidatus Hamiltonella defensa	0.86	Predict
DLM028_scaffold48949_2	11595	phage	0.992	virulent	0.99987084	Ackermannviridae	1	Edwardsiella ictaluri	0.8	Predict
DLM028_scaffold28515_6	44245	phage	0.999	virulent	0.9998665	unknown	0	Parabacteroides merdae	1	CRISPR
DLM028_scaffold37211_4	23846	phage	1	virulent	0.9865467	Casjensviridae	1	unknown	0	-
DLM028_scaffold25751_8	10092	phage	0.999	virulent	0.9908166	Peduoviridae	1	Candidatus Hamiltonella defensa	1	CRISPR
DLM028_scaffold46719_2	13044	phage	1	temperate	0.9998584	unknown	0	Candidatus Hamiltonella defensa	0.7	Predict
DLM028_scaffold10447_6	12010	phage	0.97	temperate	0.9998513	unknown	0	Candidatus Hamiltonella defensa	0.99	Predict
DLM028_scaffold54580_1	10301	phage	0.726	virulent	0.99986315	unknown	0	Candidatus Hamiltonella defensa	0.88	Predict
DLM028_scaffold30334_1	20151	phage	0.999	temperate	0.9998302	unknown	0	Candidatus Hamiltonella defensa	0.88	Predict
DLM028_C675659_1	25933	phage	0.967	virulent	0.9996658	Peduoviridae	0.33529264	Azospirillum brasilense	0.86	Predict
DLM028_scaffold21827_3	13009	phage	0.997	virulent	0.99291134	unknown	0	Candidatus Hamiltonella defensa	1	CRISPR
DLM028_scaffold32640_2	11813	phage	0.999	virulent	0.9998688	unknown	0	Candidatus Hamiltonella defensa	0.72	Predict
DLM028_C674591_1	11302	phage	0.999	virulent	0.9998736	unknown	0	unknown	0	-
DLM028_scaffold268_28	25178	phage	0.699	temperate	0.99985975	Straboviridae	0.6866557	Candidatus Hamiltonella defensa	0.86	Predict
DLM028_scaffold1815_5	24146	phage	0.948	virulent	0.99878967	unknown	0	Candidatus Hamiltonella defensa	0.95	Predict
DLM028_scaffold29533_12	15899	phage	0.919	temperate	0.9413989	Straboviridae	0.40471888	Bacteroides fragilis	0.73	Predict
DLM028_scaffold3721_11	11223	phage	0.995	temperate	0.9993922	Peduoviridae	0.5582075	Candidatus Hamiltonella defensa	0.82	Predict
DLM028_scaffold51026_1	10552	phage	0.989	temperate	0.99985313	Peduoviridae	1	Candidatus Hamiltonella defensa	0.91	Predict
DLM028_scaffold54608_1	20400	phage	0.998	temperate	0.9986798	Kyanoviridae	1	Clostridioides difficile	0.96	Predict
DLM028_scaffold39849_2	11628	phage	0.994	temperate	0.99986035	unknown	0	unknown	0	-
DLM028_scaffold28558_6	11483	phage	0.994	temperate	0.5942064	unknown	0	Candidatus Hamiltonella defensa	0.94	Predict
DLM028_scaffold10666_1	13655	phage	0.999	virulent	0.973823	unknown	0	Candidatus Hamiltonella defensa	0.89	Predict
DLM028_scaffold32204_5	14444	phage	0.998	virulent	0.99984604	unknown	0	Candidatus Hamiltonella defensa	0.77	Predict
DLM028_scaffold16942_4	15049	phage	0.679	temperate	0.99985975	unknown	0	Candidatus Hamiltonella defensa	0.76	Predict
DLM028_scaffold28515_4	64626	phage	0.996	virulent	0.99983174	Straboviridae	0.63893926	Cellulophaga baltica	1	CRISPR
DLM028_scaffold36337_1	13919	phage	0.999	temperate	0.9998579	unknown	0	Candidatus Hamiltonella defensa	0.72	Predict
DLM028_scaffold13566_10	21243	phage	0.997	temperate	0.99980354	no_family_avaliable(NC_049950)	0.98	Candidatus Hamiltonella defensa	1	CRISPR

DLM028_scaffold6503_3	15031	phage	0.998	temperate	0.99985695	unknown	0	Candidatus Hamiltonella defensa	0.79	Predict
DLM028_scaffold1257_18	15348	phage	0.998	temperate	0.9998593	unknown	0	Candidatus Hamiltonella defensa	0.82	Predict
DLM028_scaffold51841_1	12454	phage	0.996	virulent	0.99984217	unknown	0	Candidatus Hamiltonella defensa	0.92	Predict
DLM028_scaffold16640_1	21484	phage	0.999	temperate	0.99984264	Mesyanzhinovviridae	0.29691836	unknown	0	-
DLM028_scaffold37211_5	12850	phage	0.999	virulent	0.9864348	Peduoviridae	1	Burkholderia thailandensis	0.99	Predict
DLM028_scaffold726_8	10099	phage	0.998	virulent	0.99624485	unknown	0	Vibrio splendidus	1	CRISPR
DLM028_scaffold41953_1	11826	phage	0.956	temperate	0.99985313	unknown	0	Candidatus Hamiltonella defensa	0.91	Predict
DLM028_scaffold26265_2	14831	phage	0.998	virulent	0.9692653	no_family_avaliable(NC_047914)	0.951	Candidatus Hamiltonella defensa	1	CRISPR
DLM028_scaffold20432_3	31521	phage	0.999	virulent	0.99985313	Casjensviridae	0.811716	Candidatus Hamiltonella defensa	1	CRISPR
DLM028_scaffold9236_8	17480	phage	0.849	temperate	0.9998403	Peduoviridae	0.5512526	Candidatus Hamiltonella defensa	0.97	Predict
DLM028_scaffold86_3	12194	phage	1	virulent	0.9998736	Peduoviridae	0.38523066	Candidatus Hamiltonella defensa	0.96	Predict
DLM028_scaffold31687_6	20049	phage	0.995	virulent	0.9967521	Vilmaviridae	1	Parabacteroides distasonis	0.94	Predict
NLM001_scaffold3386_6	19731	phage	1	temperate	0.9468059	Straboviridae	0.40241614	Colwellia psychrerythraea	1	CRISPR
NLM001_scaffold44600_3	17015	phage	0.737	virulent	0.9997247	Drexlerviridae	0.39220908	Parabacteroides distasonis	0.98	Predict
NLM001_scaffold38495_3	18721	phage	0.99	virulent	0.99987173	unknown	0	Colwellia psychrerythraea	0.75	Predict
NLM001_scaffold2478_4	14989	phage	0.745	virulent	0.9998522	unknown	0	Vibrio splendidus	0.83	Predict
NLM001_scaffold44595_2	13018	phage	1	temperate	0.9998565	unknown	0	Lactococcus lactis	0.83	Predict
NLM001_scaffold4227_2	18071	phage	0.997	virulent	0.99961454	Straboviridae	0.47494072	Mycoplasma pulmonis	0.83	Predict
NLM001_scaffold7_9	10962	phage	0.998	virulent	0.9996124	Straboviridae	0.5879599	Colwellia psychrerythraea	0.8	Predict
NLM001_scaffold43773_1	12444	phage	0.94	virulent	0.9998684	Straboviridae	0.4482294	Colwellia psychrerythraea	0.96	Predict
NLM001_scaffold10910_7	23637	phage	1	temperate	0.99985975	Straboviridae	0.42512658	Bacillus anthracis	1	CRISPR
NLM001_scaffold41698_8	14998	phage	0.999	temperate	0.99983925	unknown	0	Colwellia psychrerythraea	0.76	Predict
NLM001_scaffold5105_1	12062	phage	0.999	virulent	0.8326442	Casjensviridae	0.38493463	Colwellia psychrerythraea	0.89	Predict
NLM001_scaffold41377_1	18889	phage	0.993	temperate	0.9998508	unknown	0	Colwellia psychrerythraea	0.76	Predict
NLM001_scaffold5659_21	10071	phage	0.987	virulent	0.7249233	unknown	0	unknown	0	-
NLM001_scaffold43435_4	15735	phage	0.867	virulent	0.99987316	unknown	0	Aeromonas media	0.86	Predict
NLM001_scaffold31705_8	23480	phage	0.999	virulent	0.64146	unknown	0	Colwellia psychrerythraea	0.87	Predict
NLM001_scaffold5105_4	22501	phage	0.999	virulent	0.99987036	Schitoviridae	0.1449373	Colwellia psychrerythraea	0.94	Predict
NLM001_scaffold2_5	15894	phage	0.919	temperate	0.9413989	Ackermannviridae	0.26196176	Bacteroides fragilis	0.77	Predict
NLM001_scaffold44595_1	24679	phage	0.999	temperate	0.99984837	Peduoviridae	1	Colwellia psychrerythraea	1	CRISPR

NLM001_scaffold17693_25	11521	phage	1	virulent	0.9998693	unknown	0	Colwellia psychrerythraea	0.82	Predict
NLM001_scaffold44484_1	23084	phage	0.999	temperate	0.9998584	unknown	0	Colwellia psychrerythraea	1	CRISPR
NLM001_scaffold40612_7	23410	phage	1	temperate	0.9958789	unknown	0	Bacteroides fragilis	0.85	Predict
NLM002_scaffold13648_17	10129	phage	0.999	temperate	0.9998022	unknown	0	Colwellia psychrerythraea	1	CRISPR
NLM002_scaffold28424_2	15130	phage	1	virulent	0.99984604	unknown	0	Colwellia psychrerythraea	0.79	Predict
NLM002_scaffold15649_2	10802	phage	0.999	temperate	0.98625636	Peduoviridae	0.43805262	Colwellia psychrerythraea	1	CRISPR
NLM002_scaffold8112_4	11804	phage	0.941	virulent	0.9996882	Straboviridae	0.6553155	Parabacteroides distasonis	0.89	Predict
NLM002_scaffold28005_2	11375	phage	0.886	temperate	0.8061577	Drexlerviridae	0.3840808	Colwellia psychrerythraea	0.97	Predict
NLM002_scaffold13407_12	10471	phage	0.999	virulent	0.99987173	Straboviridae	0.5879599	Colwellia psychrerythraea	0.78	Predict
NLM002_scaffold4211_7	20820	phage	0.999	temperate	0.8884447	unknown	0	unknown	0	-
NLM002_scaffold28119_5	15294	phage	1	virulent	0.8875334	Kyanoviridae	1	Prevotella stercorea	1	CRISPR
NLM002_scaffold15370_2	15821	phage	0.993	temperate	0.8465202	unknown	0	unknown	0	-
NLM002_C361292_1	12894	phage	0.911	temperate	0.99964535	unknown	0	Colwellia psychrerythraea	0.89	Predict
NLM002_scaffold8657_21	13453	phage	0.998	temperate	0.9998593	Straboviridae	0.69101197	Colwellia psychrerythraea	0.89	Predict
NLM002_scaffold23710_2	26306	phage	0.998	virulent	0.99984556	unknown	0	Colwellia psychrerythraea	0.74	Predict
NLM002_scaffold17510_4	12700	phage	0.859	temperate	0.9780044	unknown	0	unknown	0	-
NLM002_scaffold14431_4	14232	phage	0.999	temperate	0.9812345	Straboviridae	0.7403209	unknown	0	-
NLM002_scaffold5906_19	20539	phage	0.999	temperate	0.9998551	Straboviridae	0.47592783	unknown	0	-
NLM002_C361448_1	16389	phage	0.99	temperate	0.99933356	unknown	0	unknown	0	-
NLM002_scaffold16029_2	16409	phage	1	temperate	0.5039522	unknown	0	Colwellia psychrerythraea	1	CRISPR
NLM003_scaffold10761_3	16079	phage	0.999	virulent	0.9989405	Straboviridae	0.21159849	Colwellia psychrerythraea	0.96	Predict
NLM003_scaffold4486_1	13618	phage	0.998	virulent	0.9998722	unknown	0	unknown	0	-
NLM003_scaffold10922_3	10054	phage	0.928	virulent	0.5579255	unknown	0	Bacteroides fragilis	1	CRISPR
NLM003_scaffold261_3	17259	phage	0.999	temperate	0.9998565	Straboviridae	0.45764768	Cellulophaga baltica	0.75	Predict
NLM003_scaffold977_2	34321	phage	0.999	virulent	0.9998617	Drexlerviridae	1	Klebsiella pneumoniae	1	CRISPR
NLM003_scaffold14728_2	11612	phage	0.555	virulent	0.9998436	unknown	0	Bacteroides fragilis	0.81	Predict
NLM003_scaffold3763_2_2	14028	phage	0.998	temperate	0.9998588	unknown	0	Colwellia psychrerythraea	0.79	Predict
NLM003_scaffold189_8_1	11074	phage	0.999	virulent	0.99987316	Herelleviridae	1	Bacteroides fragilis	0.75	Predict
NLM003_scaffold4295_1	10011	phage	0.998	virulent	0.9998565	Drexlerviridae	0.6004602	Colwellia psychrerythraea	0.8	Predict
NLM004_scaffold44166_11	21477	phage	0.993	virulent	0.99987125	unknown	0	Vibrio splendidus	1	CRISPR

NLM004_scaffold27516_1	25374	phage	0.995	temperate	0.99978167	unknown	0	Colwellia psychrerythraea	0.73	Predict
NLM004_scaffold815_2	10052	phage	0.949	virulent	0.9998727	unknown	0	Erysipelothrix rhusiopathiae	0.82	Predict
NLM004_scaffold54737_2	17439	phage	0.913	virulent	0.9998688	unknown	0	Colwellia psychrerythraea	0.94	Predict
NLM004_scaffold285_3	14283	phage	0.585	virulent	0.99987084	Drexlerviridae	0.23599732	Colwellia psychrerythraea	0.73	Predict
NLM004_scaffold14733_2	14268	phage	0.999	virulent	0.99986744	unknown	0	Colwellia psychrerythraea	1	CRISPR
NLM004_scaffold40373_1	15307	phage	0.998	virulent	0.99987125	unknown	0	unknown	0	-
NLM004_scaffold3539_11	11766	phage	0.997	temperate	0.99315417	unknown	0	Colwellia psychrerythraea	0.92	Predict
NLM004_scaffold46877_2	13524	phage	0.998	temperate	0.99986035	Straboviridae	0.615393	Mycoplasma pulmonis	0.84	Predict
NLM004_scaffold217_2	16771	phage	0.992	temperate	0.99985975	unknown	0	Mycoplasma pulmonis	0.87	Predict
NLM004_scaffold18101_4	15293	phage	0.999	temperate	0.9998579	unknown	0	Colwellia psychrerythraea	0.72	Predict
NLM004_scaffold35292_1	13929	phage	0.98	virulent	0.99969965	Straboviridae	0.45870692	Colwellia psychrerythraea	0.72	Predict
NLM004_scaffold13021_3	17793	phage	0.536	temperate	0.9998574	unknown	0	Sinorhizobium meliloti	0.89	Predict
NLM004_scaffold14042_13	19789	phage	0.996	virulent	0.8118703	unknown	0	Clostridioides difficile	0.9	Predict
NLM004_scaffold49362_1	12311	phage	0.999	virulent	0.99987465	unknown	0	Colwellia psychrerythraea	0.92	Predict
NLM004_scaffold2268_1	12544	phage	0.998	temperate	0.9998593	unknown	0	unknown	0	-
NLM004_C687357_1	11307	phage	0.763	virulent	0.8251606	unknown	0	unknown	0	-
NLM004_scaffold9767_3	10063	phage	0.986	temperate	0.96577114	unknown	0	Colwellia psychrerythraea	0.91	Predict
NLM004_scaffold53545_1	20702	phage	0.993	temperate	0.99985975	Straboviridae	0.49783167	Colwellia psychrerythraea	0.94	Predict
NLM004_scaffold20206_2	14542	phage	0.911	virulent	0.9998722	unknown	0	Aliivibrio fischeri	0.73	Predict
NLM004_scaffold27473_2	15524	phage	0.737	virulent	0.9997247	Drexlerviridae	0.3755131	Colwellia psychrerythraea	0.83	Predict
NLM004_scaffold28209_1	12825	phage	0.967	virulent	0.99987406	Kyanoviridae	1	unknown	0	-
NLM004_scaffold54819_1	28800	phage	0.881	virulent	0.9992964	Herelleviridae	1	Streptococcus parauberis	1	CRISPR
NLM004_scaffold3052_12	15103	phage	1	temperate	0.99891406	unknown	0	Streptococcus salivarius	1	CRISPR
NLM004_scaffold54430_1	10066	phage	0.982	virulent	0.99979234	unknown	0	unknown	0	-
NLM004_scaffold9622_3	10300	phage	0.998	virulent	0.9998736	Mesyanzhinovviridae	0.4042104	Colwellia psychrerythraea	0.88	Predict
NLM005_scaffold17186_2	10612	phage	0.999	temperate	0.99986035	unknown	0	unknown	0	-
NLM005_scaffold7084_1	15876	phage	0.995	temperate	0.9483567	unknown	0	Bacteroides fragilis	0.95	Predict
NLM005_scaffold34767_1	10184	phage	0.997	temperate	0.9367095	unknown	0	unknown	0	-
NLM005_scaffold30374_9	11749	phage	0.999	virulent	0.99986744	no_family_avaliable(NC_047916)	0.954	Colwellia psychrerythraea	1	CRISPR
NLM005_scaffold32010_3	15535	phage	0.999	temperate	0.99985695	Peduoviridae	1	Colwellia psychrerythraea	1	Predict

NLM005_scaffold564_3	12122	phage	0.697	temperate	0.99985975	unknown	0	Bacteroides fragilis	0.99	Predict
NLM005_scaffold21805_1	17525	phage	0.997	temperate	0.99984884	unknown	0	Flavobacterium columnare	0.73	Predict
NLM005_scaffold7_1	10617	phage	0.999	virulent	0.9998699	unknown	0	Colwellia psychrerythraea	0.84	Predict
NLM005_scaffold5172_2	11621	phage	0.959	temperate	0.9960733	Straboviridae	0.35476157	Bacteroides fragilis	0.77	Predict
NLM005_scaffold12098_1	10605	phage	1	virulent	0.69228077	unknown	0	unknown	0	-
NLM005_scaffold90_2	12634	phage	0.998	temperate	0.9137359	Straboviridae	0.35521403	Bacteroides fragilis	0.93	Predict
NLM005_scaffold5999_10	33632	phage	0.997	virulent	0.996864	Straboviridae	0.5338497	Flavobacterium columnare	0.98	Predict
NLM005_scaffold11370_1	21775	phage	0.999	temperate	0.99473625	Herelleviridae	0.64262027	Colwellia psychrerythraea	0.91	Predict
NLM005_scaffold4896_1	11280	phage	0.997	virulent	0.9998656	unknown	0	Colwellia psychrerythraea	0.84	Predict
NLM005_scaffold16081_2	14188	phage	0.998	virulent	0.71232563	unknown	0	Colwellia psychrerythraea	1	CRISPR
NLM006_scaffold18020_1	11081	phage	0.994	virulent	0.9998727	unknown	0	Colwellia psychrerythraea	0.87	Predict
NLM006_scaffold30822_2	53365	phage	1	virulent	0.9998656	no_family_avaliable(NC_062765)	0.972	Parabacteroides distasonis	1	CRISPR
NLM006_scaffold2215_2	10543	phage	0.993	temperate	0.99985695	no_family_avaliable(NC_003356)	0.955	Salmonella enterica	1	CRISPR
NLM006_scaffold50674_1	13345	phage	1	virulent	0.9998636	unknown	0	Parabacteroides merdae	0.85	Predict
NLM006_scaffold32497_3	12738	phage	0.999	virulent	0.9980126	Drexlerviridae	0.3840808	Colwellia psychrerythraea	0.8	Predict
NLM006_scaffold21580_3	11377	phage	0.996	virulent	0.5427974	Straboviridae	0.69101197	Mycoplasma pulmonis	0.76	Predict
NLM006_scaffold43376_2	19096	phage	0.998	virulent	0.98799294	Ackermannviridae	0.3039049	Colwellia psychrerythraea	0.83	Predict
NLM006_scaffold44414_1	11754	phage	0.532	virulent	0.9857189	unknown	0	Colwellia psychrerythraea	0.73	Predict
NLM006_scaffold37475_1	19272	phage	0.999	temperate	0.6870776	unknown	0	Clostridioides difficile	1	CRISPR
NLM006_scaffold47321_2	18479	phage	0.999	temperate	0.7044811	unknown	0	Colwellia psychrerythraea	0.91	Predict
NLM006_scaffold31922_8	17916	phage	0.992	temperate	0.9977357	Straboviridae	0.42385665	Colwellia psychrerythraea	1	CRISPR
NLM006_scaffold23522_7	13356	phage	0.725	temperate	0.99985975	unknown	0	unknown	0	-
NLM006_scaffold43039_5	11604	phage	0.999	virulent	0.9998196	Herelleviridae	1	Bacteroides sp. 3_1_40A	1	CRISPR
NLM006_scaffold42358_4	10725	phage	0.993	virulent	0.96040946	Casjensviridae	1	Colwellia psychrerythraea	0.79	Predict
NLM006_scaffold6162_11	12773	phage	0.999	temperate	0.99985605	unknown	0	Hungatella hathewayi	1	CRISPR
NLM006_scaffold50021_2	11288	phage	0.989	temperate	0.9998579	unknown	0	Mycoplasma pulmonis	1	CRISPR
NLM006_scaffold35930_2	18003	phage	0.999	temperate	0.99985975	Straboviridae	0.44955745	Colwellia psychrerythraea	1	CRISPR
NLM006_scaffold52859_1	12116	phage	0.998	virulent	0.97943383	unknown	0	Mycoplasma pulmonis	0.83	Predict
NLM006_scaffold36213_1	19112	phage	0.997	temperate	0.9992786	unknown	0	Colwellia psychrerythraea	0.9	Predict
NLM006_scaffold24429_1	18996	phage	0.998	virulent	0.9998656	Straboviridae	0.24749029	Colwellia psychrerythraea	0.94	Predict

NLM006_scaffold51349_1	12422	phage	0.981	temperate	0.99986035	Straboviridae	0.21516325	Colwellia psychrerythraea	1	CRISPR
NLM006_scaffold53007_3	21260	phage	0.999	temperate	0.9307284	Straboviridae	0.49488285	Brevibacillus laterosporus	1	CRISPR
NLM006_scaffold22752_1	12175	phage	0.941	virulent	0.9996882	Straboviridae	0.6553155	Bacteroides fragilis	1	CRISPR
NLM006_scaffold1105_10	14780	phage	0.983	temperate	0.99985975	unknown	0	Colwellia psychrerythraea	0.74	Predict
NLM006_scaffold21365_1	11550	phage	0.985	temperate	0.99520785	unknown	0	Colwellia psychrerythraea	0.88	Predict
NLM006_scaffold33813_8	14707	phage	0.988	temperate	0.9998369	unknown	0	Bacteroides fragilis	1	CRISPR
NLM006_scaffold26489_1	10722	phage	0.609	temperate	0.9997044	Straboviridae	0.5890641	Colwellia psychrerythraea	0.96	Predict
NLM006_scaffold53154_3	17030	phage	0.962	virulent	0.99987125	Straboviridae	0.5852342	Colwellia psychrerythraea	0.87	Predict
NLM006_scaffold11951_6	11657	phage	0.999	virulent	0.99966824	Ackermannviridae	0.35920888	Colwellia psychrerythraea	0.95	Predict
NLM006_scaffold46436_2	14081	phage	0.991	temperate	0.93916035	unknown	0	Acinetobacter johnsonii	0.72	Predict
NLM006_scaffold4801_4	14477	phage	0.641	virulent	0.9998622	unknown	0	Bacteroides fragilis	0.79	Predict
NLM006_scaffold42358_1	31790	phage	1	virulent	0.9998679	unknown	0	Parabacteroides distasonis	1	CRISPR
NLM006_C734286_1	17033	phage	0.999	virulent	0.99987406	unknown	0	Colwellia psychrerythraea	1	CRISPR
NLM006_scaffold30260_1	12721	phage	0.981	temperate	0.99904966	unknown	0	Colwellia psychrerythraea	0.92	Predict
NLM006_C733934_1	12191	phage	0.996	virulent	0.9884934	unknown	0	Bacteroides fragilis	0.86	Predict
NLM006_scaffold37878_1	27096	phage	0.986	temperate	0.9968831	Casjensviridae	1	Colwellia psychrerythraea	0.72	Predict
NLM006_scaffold38154_1	20040	phage	0.997	temperate	0.99986035	Vilmaviridae	1	Colwellia psychrerythraea	0.88	Predict
NLM006_scaffold18470_3	84953	phage	0.999	virulent	0.9998656	Herelleviridae	0.741455	Cellulophaga baltica	1	CRISPR
NLM006_scaffold29091_1	17223	phage	0.999	temperate	0.9998397	unknown	0	Mycoplasma pulmonis	1	CRISPR
NLM006_scaffold177_4	11779	phage	0.998	virulent	0.9998699	unknown	0	Streptococcus parauberis	0.89	Predict
NLM006_scaffold53101_1	12175	phage	0.999	temperate	0.9997587	Ackermannviridae	0.2372473	Colwellia psychrerythraea	0.93	Predict
NLM006_scaffold44135_1	11247	phage	0.998	temperate	0.8564934	unknown	0	unknown	0	-
NLM006_scaffold15317_5	16136	phage	0.951	virulent	0.9998331	unknown	0	unknown	0	-
NLM006_scaffold49011_1	11638	phage	0.878	virulent	0.99978524	Peduoviridae	1	Colwellia psychrerythraea	0.93	Predict
NLM006_scaffold12258_5	11954	phage	0.999	virulent	0.99980384	unknown	0	Streptococcus thermophilus	0.72	Predict
NLM006_scaffold37878_15	12977	phage	0.999	temperate	0.99983555	unknown	0	Citrobacter rodentium	0.98	Predict
NLM006_scaffold50994_2	10005	phage	0.709	virulent	0.99987465	unknown	0	unknown	0	-
NLM006_scaffold14681_1	11418	phage	0.999	temperate	0.9998593	Straboviridae	0.7813815	Colwellia psychrerythraea	0.94	Predict
NLM006_scaffold12925_3	11336	phage	0.989	virulent	0.99987406	unknown	0	Colwellia psychrerythraea	0.92	Predict
NLM006_scaffold16491_10	16080	phage	0.998	virulent	0.9998722	unknown	0	unknown	0	-

NLM006_scaffold43095_1	31719	phage	0.999	temperate	0.9998345	unknown	0	Colwellia psychrerythraea	1	CRISPR
NLM006_scaffold17752_2	19191	phage	0.984	temperate	0.99984926	unknown	0	Mycoplasma pulmonis	0.99	Predict
NLM006_scaffold4407_2	35558	phage	0.766	virulent	0.9998693	Ackermannviridae	0.18881081	Lactobacillus plantarum	0.85	Predict
NLM006_scaffold10094_3	12033	phage	0.84	virulent	0.9998593	Straboviridae	0.4482294	Colwellia psychrerythraea	0.87	Predict
NLM006_scaffold31240_3	13241	phage	0.961	virulent	0.9998574	unknown	0	unknown	0	-
NLM006_scaffold1374_1	16618	phage	0.999	temperate	0.9998379	unknown	0	Colwellia psychrerythraea	0.79	Predict
NLM006_scaffold31193_1	23181	phage	1	virulent	0.9998688	Demerecviridae	1	Colwellia psychrerythraea	1	CRISPR
NLM006_scaffold4969_12	10284	phage	0.989	temperate	0.98616534	unknown	0	unknown	0	-
NLM006_scaffold16845_12	22059	phage	0.981	temperate	0.9998165	unknown	0	Mycoplasma pulmonis	0.81	Predict
NLM006_scaffold28837_2	17435	phage	0.918	virulent	0.99970657	Straboviridae	0.65579903	Bacteroides fragilis	0.87	Predict
NLM006_scaffold22733_1	17358	phage	0.555	virulent	0.9695475	unknown	0	Bacteroides coprophilus	1	CRISPR
NLM006_C733830_1	11480	phage	0.993	virulent	0.9998508	unknown	0	Bacillus anthracis	0.77	Predict
NLM006_C734606_1	45069	phage	0.999	virulent	0.9998645	Drexlerviridae	1	Colwellia psychrerythraea	0.74	Predict
NLM006_C734306_1	17313	phage	0.998	virulent	0.9998693	unknown	0	Cellulophaga baltica	0.78	Predict
NLM006_scaffold8324_9	15930	phage	0.999	temperate	0.9998431	Straboviridae	0.4822487	Colwellia psychrerythraea	1	CRISPR
NLM006_scaffold9879_4	12938	phage	0.999	virulent	0.99986696	unknown	0	Colwellia psychrerythraea	1	CRISPR
NLM007_scaffold10749_4	21083	phage	0.998	virulent	0.99950296	Straboviridae	0.6418709	Lachnospiraceae bacterium	1	CRISPR
NLM007_scaffold1551_2	12804	phage	0.909	temperate	0.99985975	Ackermannviridae	0.42088312	Colwellia psychrerythraea	0.82	Predict
NLM007_scaffold25115_1	11044	phage	0.996	virulent	0.9998722	Peduoviridae	1	Colwellia psychrerythraea	0.83	Predict
NLM007_scaffold11104_1	10415	phage	0.982	temperate	0.9998593	unknown	0	unknown	0	-
NLM007_scaffold7202_1	13927	phage	0.607	virulent	0.99986744	unknown	0	Mycoplasma pulmonis	0.99	Predict
NLM007_scaffold5980_3	12619	phage	0.998	temperate	0.99983126	unknown	0	Mycoplasma pulmonis	0.97	Predict
NLM007_scaffold20486_1	16125	phage	0.989	temperate	0.99985975	unknown	0	Mycoplasma pulmonis	0.8	Predict
NLM007_scaffold17969_11	10230	phage	0.998	temperate	0.9812528	Casjensviridae	1	Colwellia psychrerythraea	0.87	Predict
NLM007_scaffold6647_10	10046	phage	0.999	temperate	0.99985695	unknown	0	Colwellia psychrerythraea	0.7	Predict
NLM007_scaffold19932_1	23308	phage	1	virulent	0.99987125	unknown	0	Colwellia psychrerythraea	0.99	Predict
NLM007_scaffold26391_1	40239	phage	0.874	virulent	0.9998665	Straboviridae	0.5454986	Colwellia psychrerythraea	0.7	Predict
NLM007_scaffold2633_3	12294	phage	0.544	virulent	0.9998584	Mesyanzhinovviridae	0.7162643	Roseobacter denitrificans	0.76	Predict
NLM007_scaffold26057_1	11296	phage	0.994	temperate	0.99985975	unknown	0	Colwellia psychrerythraea	0.74	Predict
NLM007_scaffold25900_1	14304	phage	0.519	virulent	0.99980783	unknown	0	Colwellia psychrerythraea	0.89	Predict

NLM007_scaffold20356_8	17268	phage	0.997	virulent	0.99984604	Ackermannviridae	0.3625965	Colwellia psychrerythraea	0.79	Predict
NLM007_scaffold6392_8	12314	phage	0.754	temperate	0.6711984	unknown	0	Colwellia psychrerythraea	0.88	Predict
NLM007_scaffold10461_1	20275	phage	0.999	temperate	0.99985605	unknown	0	Colwellia psychrerythraea	0.73	Predict
NLM007_scaffold3232_7	11155	phage	0.996	virulent	0.83561283	Straboviridae	0.69101197	Colwellia psychrerythraea	0.96	Predict
NLM007_scaffold22550_11	11550	phage	0.999	temperate	0.99985605	unknown	0	Mycoplasma pulmonis	0.81	Predict
NLM007_scaffold26394_2	13045	phage	0.993	virulent	0.9998645	unknown	0	Colwellia psychrerythraea	0.95	Predict
NLM007_scaffold10749_5	13586	phage	0.999	temperate	0.9998588	unknown	0	Colwellia psychrerythraea	1	CRISPR
NLM007_scaffold23234_1	19825	phage	0.998	temperate	0.9994242	Drexlerviridae	0.16866957	Bacillus cereus	0.87	Predict
NLM007_scaffold10749_8	12787	phage	0.934	temperate	0.9998588	unknown	0	Colwellia psychrerythraea	0.95	Predict
NLM007_scaffold18350_4	43727	phage	0.977	virulent	0.99969625	Kyanoviridae	1	Parabacteroides merdae	1	CRISPR
NLM007_scaffold21467_1	18756	phage	0.999	temperate	0.99977493	unknown	0	Colwellia psychrerythraea	0.9	Predict
NLM007_scaffold4022_7	11736	phage	0.99	temperate	0.9998593	unknown	0	Bacillus anthracis	0.85	Predict
NLM007_scaffold26378_1	14157	phage	0.999	virulent	0.9997592	unknown	0	Colwellia psychrerythraea	0.77	Predict
NLM007_scaffold26389_1	12140	phage	0.941	temperate	0.5364791	unknown	0	Colwellia psychrerythraea	0.77	Predict
NLM007_scaffold723_5	12698	phage	0.974	virulent	0.76178706	Ackermannviridae	1	Streptococcus pneumoniae	0.85	Predict
NLM008_scaffold70_5	18361	phage	0.729	virulent	0.99939036	unknown	0	unknown	0	-
NLM008_scaffold50164_2	15902	phage	0.919	temperate	0.9413989	Ackermannviridae	0.26196176	Bacteroides fragilis	0.96	Predict
NLM008_scaffold12934_1	10012	phage	0.992	virulent	0.9994554	unknown	0	Colwellia psychrerythraea	0.71	Predict
NLM008_scaffold37575_7	12546	phage	0.999	temperate	0.9998588	unknown	0	Colwellia psychrerythraea	1	CRISPR
NLM008_scaffold37289_2	17439	phage	0.989	virulent	0.99987125	Drexlerviridae	0.32969895	Colwellia psychrerythraea	0.77	Predict
NLM008_scaffold45531_1	13964	phage	0.977	virulent	0.99986744	Straboviridae	0.72108424	Colwellia psychrerythraea	0.81	Predict
NLM008_scaffold22745_5	12897	phage	0.999	virulent	0.9786009	Ackermannviridae	0.26776138	Colwellia psychrerythraea	0.75	Predict
NLM008_scaffold50138_1	21650	phage	0.954	virulent	0.9998408	Straboviridae	0.7403607	Bdellovibrio bacteriovorus	0.78	Predict
NLM008_scaffold4502_17	10721	phage	0.999	virulent	0.99984837	unknown	0	unknown	0	-
NLM008_scaffold18128_9	28156	phage	0.987	virulent	0.99986315	Straboviridae	0.65383214	Colwellia psychrerythraea	0.7	Predict
NLM008_scaffold49769_2	10370	phage	0.947	virulent	0.9949243	Straboviridae	0.6553155	Colwellia psychrerythraea	0.74	Predict
NLM008_scaffold44104_3	14863	phage	0.999	temperate	0.99986035	unknown	0	unknown	0	-
NLM008_scaffold50150_1	11969	phage	0.994	virulent	0.99942935	unknown	0	unknown	0	-
NLM008_scaffold41985_1	12052	phage	0.996	virulent	0.99969625	unknown	0	Mycoplasma pulmonis	0.71	Predict
NLM008_scaffold37575_10	41650	phage	0.993	temperate	0.9998345	Straboviridae	0.73139083	Clostridium tetani	1	CRISPR

NLM008_scaffold46068_3	12287	phage	0.999	temperate	0.7353683	unknown	0	unknown	0	-
NLM008_scaffold711_3	12117	phage	0.999	virulent	0.99985975	Ackermannviridae	0.5563236	Colwellia psychrerythraea	0.9	Predict
NLM008_scaffold2753_1	10739	phage	1	virulent	0.9998699	unknown	0	Bacteroides vulgatus	1	CRISPR
NLM008_scaffold30532_8	17355	phage	0.988	temperate	0.9998593	unknown	0	Colwellia psychrerythraea	0.7	Predict
NLM008_scaffold2753_6	14107	phage	0.999	virulent	0.9998722	unknown	0	Colwellia psychrerythraea	0.71	Predict
NLM008_scaffold16329_1	37062	phage	0.999	virulent	0.9998622	unknown	0	Colwellia psychrerythraea	1	CRISPR
NLM008_scaffold3829_2	13625	phage	0.845	temperate	0.99934417	unknown	0	Colwellia psychrerythraea	0.77	Predict
NLM009_scaffold13480_1	10689	phage	0.922	temperate	0.99985605	unknown	0	Colwellia psychrerythraea	0.82	Predict
NLM009_scaffold13047_6	10409	phage	0.971	temperate	0.9293613	unknown	0	unknown	0	-
NLM009_C228858_1	14375	phage	0.995	temperate	0.99986035	Straboviridae	0.5123257	unknown	0	-
NLM009_scaffold13069_1	36970	phage	1	virulent	0.9976822	unknown	0	unknown	0	-
NLM009_scaffold8447_2	17980	phage	0.877	temperate	0.9987712	Drexlerviridae	0.32969895	Colwellia psychrerythraea	0.78	Predict
NLM009_scaffold5257_2	10319	phage	0.953	temperate	0.999322	unknown	0	Colwellia psychrerythraea	0.88	Predict
NLM009_C228882_1	14936	phage	0.98	virulent	0.9968421	Ackermannviridae	0.32887334	Colwellia psychrerythraea	0.98	Predict
NLM010_scaffold39134_1	10896	phage	0.998	temperate	0.9998593	Straboviridae	0.57686263	Colwellia psychrerythraea	0.96	Predict
NLM010_scaffold3556_1	12117	phage	0.999	virulent	0.99973106	Drexlerviridae	0.29855287	Bacteroides fragilis	0.73	Predict
NLM010_scaffold2302_1	18485	phage	0.985	temperate	0.99977016	Ackermannviridae	0.42088312	Achromobacter xylosoxidans	0.96	Predict
NLM010_scaffold1115_4	12111	phage	0.999	temperate	0.9998593	unknown	0	Colwellia psychrerythraea	1	CRISPR
NLM010_scaffold36636_1	13520	phage	0.897	temperate	0.99985975	Straboviridae	0.5890641	Colwellia psychrerythraea	0.8	Predict
NLM010_scaffold23407_1	26733	phage	0.999	virulent	0.99986607	Straboviridae	0.45764768	Cellulophaga baltica	1	CRISPR
NLM010_scaffold40193_1	34902	phage	0.999	virulent	0.7885081	Peduoviridae	1	Colwellia psychrerythraea	1	CRISPR
NLM010_scaffold15834_3	11158	phage	0.996	virulent	0.9998331	unknown	0	unknown	0	-
NLM010_scaffold18261_4	10011	phage	0.666	virulent	0.9998636	Mesyanzhinovviridae	0.7162643	Colwellia psychrerythraea	0.93	Predict
NLM010_scaffold40145_2	24696	phage	0.999	temperate	0.9050083	unknown	0	Pasteurella multocida	1	CRISPR
NLM010_scaffold1890_2	24532	phage	0.998	virulent	0.64273113	Ackermannviridae	0.24619988	Colwellia psychrerythraea	0.75	Predict
NLM010_C569179_1	23385	phage	0.996	virulent	0.9998684	unknown	0	unknown	0	-
NLM010_scaffold24350_1	10232	phage	0.998	virulent	0.99987316	Straboviridae	0.62909514	Colwellia psychrerythraea	0.91	Predict
NLM010_scaffold7375_4	10192	phage	0.991	virulent	0.9998736	unknown	0	unknown	0	-
NLM010_scaffold15684_10	15421	phage	1	temperate	0.9998588	unknown	0	Colwellia psychrerythraea	0.89	Predict
NLM010_scaffold13431_2	12511	phage	0.885	virulent	0.9998727	unknown	0	Colwellia psychrerythraea	0.74	Predict

NLM010_scaffold3952_5	11271	phage	0.997	virulent	0.9738244	unknown	0	Colwellia psychrerythraea	0.75	Predict
NLM010_scaffold894_1	46083	phage	0.996	virulent	0.9998217	unknown	0	Bacteroides fragilis	1	CRISPR
NLM010_scaffold9073_7	11464	phage	0.993	virulent	0.9998736	unknown	0	Clostridium botulinum	0.9	Predict
NLM010_scaffold2086_2	11252	phage	0.987	virulent	0.99987036	Straboviridae	0.36598924	Mycoplasma pulmonis	0.89	Predict
NLM010_scaffold21070_2	22139	phage	0.999	temperate	0.9993865	unknown	0	Colwellia psychrerythraea	1	CRISPR
NLM010_scaffold32176_1	14710	phage	0.576	virulent	0.9849704	unknown	0	Colwellia psychrerythraea	0.71	Predict
NLM010_scaffold705_15	10498	phage	0.994	temperate	0.99985975	unknown	0	Colwellia psychrerythraea	0.83	Predict
NLM010_scaffold16315_3	30751	phage	0.999	virulent	0.99982435	Peduoviridae	0.7363866	Colwellia psychrerythraea	0.73	Predict
NLM010_scaffold39351_1	45368	phage	0.999	virulent	0.999768	Casjensviridae	0.44304314	Colwellia psychrerythraea	1	CRISPR
NLM010_scaffold16684_4	10560	phage	0.999	virulent	0.9821895	Straboviridae	0.40241614	Colwellia psychrerythraea	0.75	Predict
NLM010_scaffold4037_2	11193	phage	0.81	temperate	0.9998593	unknown	0	Actinomyces naeslundii	1	CRISPR
NLM010_scaffold10296_6	19915	phage	0.999	temperate	0.9998579	Drexlerviridae	0.16247487	Colwellia psychrerythraea	0.94	Predict
NLM010_scaffold39268_1	44037	phage	0.999	virulent	0.9903608	Peduoviridae	0.98301136	Colwellia psychrerythraea	1	CRISPR
NLM010_scaffold17621_3	20396	phage	0.999	virulent	0.9998727	unknown	0	Aggregatibacter actinomycetemcom	1	CRISPR
NLM010_scaffold2685_7	15801	phage	0.912	virulent	0.99986744	Casjensviridae	0.31617516	Colwellia psychrerythraea	0.98	Predict
NLM010_scaffold34399_5	12329	phage	0.989	virulent	0.99987406	Straboviridae	0.65579903	Colwellia psychrerythraea	0.84	Predict
NLM010_scaffold10324_3	16444	phage	0.999	virulent	0.77555895	unknown	0	Colwellia psychrerythraea	0.77	Predict
NLM010_scaffold20986_1	14000	phage	0.999	temperate	0.9998593	unknown	0	Colwellia psychrerythraea	1	CRISPR
NLM010_scaffold10296_4	45647	phage	0.898	temperate	0.999854	Casjensviridae	1	Colwellia psychrerythraea	0.76	Predict
NLM010_scaffold8314_1	17050	phage	0.98	virulent	0.99969965	Straboviridae	0.45870692	Colwellia psychrerythraea	0.78	Predict
NLM010_scaffold2398_1	10261	phage	0.998	temperate	0.99982446	unknown	0	unknown	0	-
NLM010_scaffold15390_2	36162	phage	1	virulent	0.9971423	Drexlerviridae	0.2230215	Bacteroides fragilis	1	CRISPR
NLM010_scaffold24696_6	11799	phage	0.821	virulent	0.940546	unknown	0	unknown	0	-
NLM010_scaffold21133_2	11006	phage	0.998	virulent	0.73562175	unknown	0	unknown	0	-
NLM010_scaffold2612_4	12346	phage	0.961	temperate	0.979654	unknown	0	Bacteroides fragilis	0.85	Predict
NLM010_scaffold20986_2	13313	phage	0.999	temperate	0.99985605	unknown	0	Colwellia psychrerythraea	0.86	Predict
NLM010_scaffold9737_35	49976	phage	0.999	temperate	0.9875896	unknown	0	Colwellia psychrerythraea	0.78	Predict
NLM010_scaffold28345_8	29566	phage	1	virulent	0.9646963	unknown	0	Colwellia psychrerythraea	1	CRISPR
NLM010_scaffold26505_1	15379	phage	0.999	virulent	0.99974734	unknown	0	Colwellia psychrerythraea	0.9	Predict
NLM010_scaffold3952_4	12000	phage	0.999	virulent	0.9842398	unknown	0	Flavobacterium psychrophilum	1	CRISPR

NLM010_scaffold3490_4	17160	phage	0.997	temperate	0.9842014	unknown	0	Parabacteroides distasonis	0.97	Predict
NLM015_scaffold34970_4	10510	phage	0.988	temperate	0.9998517	unknown	0	Colwellia psychrerythraea	0.85	Predict
NLM015_scaffold45_3	12715	phage	0.983	virulent	0.9974672	unknown	0	Colwellia psychrerythraea	0.9	Predict
NLM015_scaffold9944_1	13457	phage	0.999	virulent	0.97420454	unknown	0	Parabacteroides distasonis	0.9	Predict
NLM015_scaffold38949_1	11959	phage	0.998	temperate	0.99704653	unknown	0	Bacteroides fragilis	0.86	Predict
NLM015_scaffold38424_1	33641	phage	1	virulent	0.9994911	Ackermannviridae	0.24651025	Colwellia psychrerythraea	1	CRISPR
NLM015_scaffold2372_1	28316	phage	1	virulent	0.99980664	unknown	0	Parabacteroides distasonis	1	CRISPR
NLM015_scaffold6813_1	13123	phage	1	virulent	0.9979286	unknown	0	Colwellia psychrerythraea	1	CRISPR
NLM015_scaffold13019_2	12643	phage	0.995	temperate	0.9962401	unknown	0	unknown	0	-
NLM015_scaffold7231_4	18738	phage	0.998	temperate	0.9998288	unknown	0	Azospirillum brasilense	0.91	Predict
NLM015_scaffold16659_2	13569	phage	0.998	virulent	0.999798	unknown	0	Colwellia psychrerythraea	1	CRISPR
NLM015_scaffold10852_4	17222	phage	0.999	temperate	0.68025094	Peduoviridae	0.43805262	Colwellia psychrerythraea	0.73	Predict
NLM015_scaffold4740_1	11292	phage	0.998	temperate	0.9513003	unknown	0	Parabacteroides merdae	0.92	Predict
NLM015_scaffold1856_2	16173	phage	1	temperate	0.9962648	Straboviridae	0.6608802	Colwellia psychrerythraea	1	CRISPR
NLM015_scaffold77_4	13674	phage	0.977	virulent	0.99986744	Straboviridae	0.72108424	Colwellia psychrerythraea	0.76	Predict
NLM015_scaffold25776_2	13751	phage	0.999	temperate	0.5775136	unknown	0	Colwellia psychrerythraea	0.9	Predict
NLM015_scaffold21932_1	15023	phage	0.999	temperate	0.9998588	unknown	0	Colwellia psychrerythraea	0.9	Predict
NLM015_scaffold25867_2	12361	phage	0.909	temperate	0.99985975	Ackermannviridae	0.42088312	Colwellia psychrerythraea	0.87	Predict
NLM015_scaffold10852_5	14353	phage	0.999	virulent	0.9998269	Peduoviridae	0.26380417	Colwellia psychrerythraea	0.81	Predict
NLM015_scaffold15418_1	19728	phage	0.997	virulent	0.9998727	unknown	0	Bacillus megaterium	0.79	Predict
NLM015_scaffold5831_6	13054	phage	0.95	virulent	0.99703306	Drexlerviridae	0.32969895	Colwellia psychrerythraea	0.86	Predict
NLM015_scaffold104_1	19678	phage	0.998	virulent	0.9998699	Straboviridae	0.52144754	Colwellia psychrerythraea	0.94	Predict
NLM016_scaffold3058_1	13178	phage	0.999	temperate	0.9928458	unknown	0	Colwellia psychrerythraea	0.92	Predict
NLM016_scaffold10481_1	94908	phage	0.999	virulent	0.99984926	Herelleviridae	0.8111403	Mycoplasma pulmonis	1	CRISPR
NLM016_scaffold19074_1	16490	phage	0.983	temperate	0.9996615	unknown	0	Colwellia psychrerythraea	1	CRISPR
NLM016_scaffold8626_1	11808	phage	0.999	virulent	0.99987406	unknown	0	unknown	0	-
NLM016_scaffold12933_1	11620	phage	0.999	temperate	0.9997874	unknown	0	Colwellia psychrerythraea	1	CRISPR
NLM016_scaffold9518_15	10103	phage	0.999	temperate	0.99984884	Zierdtviridae	1	unknown	0	-
NLM016_scaffold11175_2	12010	phage	1	temperate	0.99985975	unknown	0	Colwellia psychrerythraea	1	CRISPR
NLM016_scaffold112_1	15139	phage	0.999	temperate	0.9998593	unknown	0	Bacteroides fragilis	0.8	Predict

NLM016_scaffold87_4	14242	phage	0.737	virulent	0.9997247	Drexlerviridae	0.3755131	Colwellia psychrerythraea	0.76	Predict
NLM016_scaffold3275_3	17745	phage	0.998	temperate	0.9997697	unknown	0	Bacteroides fragilis	0.75	Predict
NLM016_scaffold11179_1	18618	phage	0.546	virulent	0.9998722	Kyanoviridae	1	Colwellia psychrerythraea	0.76	Predict
NLM016_scaffold16587_2	14364	phage	0.585	virulent	0.99987084	Drexlerviridae	0.39220908	Colwellia psychrerythraea	0.83	Predict
NLM016_scaffold19042_3	13129	phage	0.994	temperate	0.9208216	unknown	0	Prevotella copri	1	CRISPR
NLM016_scaffold16925_3	13675	phage	0.998	virulent	0.9699225	unknown	0	Prevotella copri	1	CRISPR
NLM017_scaffold416_2	24370	phage	0.999	temperate	0.999816	Herelleviridae	0.616311	Bacteroides fragilis	1	CRISPR
NLM017_scaffold14858_4	10835	phage	0.935	virulent	0.99138457	Ackermannviridae	0.35920888	Colwellia psychrerythraea	0.92	Predict
NLM017_scaffold20307_2	16891	phage	0.997	virulent	0.9997209	unknown	0	Colwellia psychrerythraea	0.76	Predict
NLM017_scaffold27209_14	11752	phage	0.991	virulent	0.9998736	Straboviridae	0.42151073	Colwellia psychrerythraea	0.73	Predict
NLM017_scaffold38835_1	14884	phage	0.77	virulent	0.99986607	Straboviridae	0.75188446	Colwellia psychrerythraea	0.88	Predict
NLM017_scaffold38788_3	23961	phage	0.999	temperate	0.99524623	unknown	0	Colwellia psychrerythraea	0.71	Predict
NLM017_scaffold32055_2	13534	phage	0.997	virulent	0.9998417	unknown	0	Mycoplasma pulmonis	0.74	Predict
NLM017_scaffold8358_18	12865	phage	0.996	virulent	0.6432086	unknown	0	Ruminococcus bromii	1	CRISPR
NLM017_scaffold9889_3	11159	phage	0.978	temperate	0.9998593	unknown	0	Colwellia psychrerythraea	0.74	Predict
NLM017_scaffold20627_4	11128	phage	0.997	temperate	0.9988693	unknown	0	Colwellia psychrerythraea	0.73	Predict
NLM017_scaffold38145_1	13526	phage	0.999	virulent	0.57108474	unknown	0	Colwellia psychrerythraea	0.81	Predict
NLM017_scaffold14095_1	14215	phage	0.763	virulent	0.99747163	Ackermannviridae	0.3772501	Colwellia psychrerythraea	0.9	Predict
NLM017_scaffold38914_3	30962	phage	0.999	virulent	0.9998693	no_family_avaliable(NC_024711)	0.97	Flavobacterium columnare	0.77	Predict
NLM017_scaffold39062_2	14982	phage	0.999	virulent	0.99987036	no_family_avaliable(NC_067210)	0.981	Colwellia psychrerythraea	0.88	Predict
NLM017_scaffold8358_21	14371	phage	0.982	virulent	0.9998465	Straboviridae	0.38791418	Colwellia psychrerythraea	1	CRISPR
NLM017_scaffold35881_2	13380	phage	0.589	virulent	0.9998736	unknown	0	Colwellia psychrerythraea	0.99	Predict
NLM017_scaffold17839_9	12431	phage	0.999	virulent	0.9066871	unknown	0	Bacteroides fragilis	0.73	Predict
NLM017_scaffold21461_2	19848	phage	0.998	virulent	0.9998736	unknown	0	unknown	0	-
NLM017_scaffold24164_1	10383	phage	0.999	temperate	0.9998274	unknown	0	Colwellia psychrerythraea	0.83	Predict
NLM017_scaffold25009_1	13210	phage	0.998	virulent	0.99936676	unknown	0	Colwellia psychrerythraea	0.87	Predict
NLM017_scaffold12885_45	14093	phage	0.998	virulent	0.9992214	unknown	0	Parabacteroides distasonis	0.96	Predict
NLM017_scaffold24740_2	11833	phage	0.979	virulent	0.99987084	unknown	0	Colwellia psychrerythraea	0.91	Predict
NLM017_scaffold7452_3	13479	phage	0.993	temperate	0.99986035	Peduoviridae	0.38545594	Colwellia psychrerythraea	0.95	Predict
NLM017_scaffold29127_2	10174	phage	0.996	virulent	0.99987316	unknown	0	unknown	0	-

NLM017_scaffold31444_1	14577	phage	0.593	temperate	0.9998593	unknown	0	Colwellia psychrerythraea	0.78	Predict
NLM017_scaffold8358_65	11833	phage	0.95	temperate	0.99389774	unknown	0	Colwellia psychrerythraea	0.85	Predict
NLM017_scaffold38969_2	14918	phage	0.932	virulent	0.9998727	Ackermannviridae	0.25430948	Colwellia psychrerythraea	0.71	Predict
NLM017_scaffold23004_11	11156	phage	0.98	virulent	0.99982494	Drexlerviridae	0.29855287	Bacteroides fragilis	0.89	Predict
NLM017_scaffold174_1	48989	phage	0.999	temperate	0.8248126	no_family_avaliable(NC_016770)	0.976	Bacteroides fragilis	1	CRISPR
NLM017_scaffold38914_2	56303	phage	0.999	virulent	0.9998679	Guelinviridae	0.27453768	Flavobacterium columnare	0.89	Predict
NLM021_scaffold5563_6	16577	phage	0.989	virulent	0.99987316	unknown	0	Colwellia psychrerythraea	0.94	Predict
NLM021_scaffold562_2	11260	phage	0.961	temperate	0.979654	unknown	0	Bacteroides fragilis	0.73	Predict
NLM021_scaffold42938_1	17587	phage	0.976	virulent	0.63364846	unknown	0	Colwellia psychrerythraea	0.94	Predict
NLM021_scaffold61286_1	12517	phage	0.566	temperate	0.9998593	unknown	0	Colwellia psychrerythraea	0.78	Predict
NLM021_scaffold58942_1	15372	phage	0.999	temperate	0.9998345	unknown	0	Colwellia psychrerythraea	1	CRISPR
NLM021_scaffold61418_1	26732	phage	0.99	temperate	0.9998565	Straboviridae	0.2957326	Mycoplasma pulmonis	0.98	Predict
NLM021_scaffold5557_1	16512	phage	0.999	virulent	0.8075158	unknown	0	Colwellia psychrerythraea	0.88	Predict
NLM021_scaffold17568_1	14917	phage	0.997	virulent	0.9998574	unknown	0	unknown	0	-
NLM021_scaffold19029_4	57862	phage	0.999	temperate	0.9605091	Casjensviridae	0.9825629	Colwellia psychrerythraea	0.98	Predict
NLM021_scaffold53158_5	29853	phage	0.998	virulent	0.9998688	Straboviridae	0.5338497	Colwellia psychrerythraea	0.89	Predict
NLM021_scaffold59003_3	13202	phage	0.942	virulent	0.92176783	unknown	0	Ruminococcus sp. OM05-7	1	CRISPR
NLM021_scaffold8185_4	27402	phage	0.984	temperate	0.9022219	Straboviridae	1	unknown	0	-
NLM021_scaffold56233_3	11510	phage	0.995	virulent	0.8864396	unknown	0	unknown	0	-
NLM021_scaffold57344_1	14529	phage	0.998	virulent	0.9908407	unknown	0	unknown	0	-
NLM021_scaffold26944_4	10036	phage	0.992	temperate	0.97423124	unknown	0	unknown	0	-
NLM021_scaffold61366_2	16140	phage	0.997	temperate	0.9998579	unknown	0	Bacillus alcalophilus	0.75	Predict
NLM021_scaffold55035_3	12003	phage	0.987	temperate	0.99983555	Chaseviridae	0.3054243	Bacillus megaterium	0.91	Predict
NLM021_scaffold19029_1	29122	phage	0.848	virulent	0.9998545	Drexlerviridae	0.19249943	Parabacteroides distasonis	0.72	Predict
NLM021_scaffold143_2	18327	phage	0.998	virulent	0.9998699	unknown	0	Colwellia psychrerythraea	0.89	Predict
NLM021_scaffold42928_1	16274	phage	0.999	temperate	0.9998588	Peduoviridae	0.70607936	Colwellia psychrerythraea	1	CRISPR
NLM021_scaffold61042_4	60769	phage	0.999	temperate	0.99438643	Casjensviridae	1	Colwellia psychrerythraea	0.76	Predict
NLM021_scaffold29192_2	14143	phage	0.692	temperate	0.9697178	Straboviridae	0.43174168	Colwellia psychrerythraea	0.9	Predict
NLM021_scaffold35458_5	10029	phage	0.636	virulent	0.99983126	unknown	0	unknown	0	-
NLM021_scaffold22069_5	17886	phage	0.999	temperate	0.9998584	unknown	0	Colwellia psychrerythraea	0.92	Predict

NLM021_scaffold11314_5	12539	phage	0.812	virulent	0.9996798	unknown	0	Colwellia psychrerythraea	0.9 Predict
NLM021_scaffold23306_4	10950	phage	0.922	virulent	0.9996477	Vilmaviridae	1	Colwellia psychrerythraea	0.94 Predict
NLM021_scaffold60054_1	19744	phage	0.998	temperate	0.90835065	Straboviridae	0.60721505	Colwellia psychrerythraea	1 CRISPR
NLM021_scaffold190_8	22179	phage	0.998	virulent	0.99986744	Straboviridae	0.34623358	Colwellia psychrerythraea	0.93 Predict
NLM021_scaffold15002_14	17373	phage	0.998	virulent	0.9998593	Ackermannviridae	0.2522395	Colwellia psychrerythraea	0.76 Predict
NLM021_scaffold55417_2	48176	phage	0.999	temperate	0.9998308	Herelleviridae	1	Colwellia psychrerythraea	1 CRISPR
NLM021_scaffold14659_12	12955	phage	0.999	temperate	0.89597625	unknown	0	Colwellia psychrerythraea	0.7 Predict
NLM021_scaffold60255_1	50476	phage	0.998	temperate	0.99070436	unknown	0	Bacteroides fragilis	0.83 Predict
NLM021_scaffold26861_2	19100	phage	0.999	virulent	0.99980736	Ackermannviridae	0.3039049	Colwellia psychrerythraea	0.95 Predict
NLM021_scaffold709_5	16053	phage	0.778	temperate	0.9998593	unknown	0	Geobacillus kaustophilus	0.94 Predict
NLM021_C812913_1	13113	phage	0.998	temperate	0.99985975	Straboviridae	0.57686263	Colwellia psychrerythraea	0.81 Predict
NLM021_scaffold61257_1	11374	phage	0.677	temperate	0.99090755	Ackermannviridae	1	Bacteroides fragilis	0.97 Predict
NLM021_scaffold46784_3	11501	phage	0.947	virulent	0.9949243	Straboviridae	0.6553155	Colwellia psychrerythraea	0.85 Predict
NLM021_scaffold190_9	28061	phage	0.999	virulent	0.9998636	Herelleviridae	1	unknown	0 -
NLM021_scaffold53021_4	11581	phage	0.695	virulent	0.9998574	Straboviridae	0.5399199	Colwellia psychrerythraea	0.95 Predict
NLM021_scaffold58236_5	38364	phage	0.996	temperate	0.9997535	Straboviridae	0.5006739	Lactobacillus gasseri	1 CRISPR
NLM021_scaffold46181_6	21835	phage	0.999	virulent	0.9998699	unknown	0	Colwellia psychrerythraea	1 CRISPR
NLM021_scaffold38431_7	10225	phage	0.999	temperate	0.99986035	unknown	0	Colwellia psychrerythraea	0.84 Predict
NLM021_scaffold57650_6	10895	phage	0.991	temperate	0.99985975	unknown	0	unknown	0 -
NLM021_scaffold190_7	17846	phage	0.999	virulent	0.9998369	unknown	0	Clostridium botulinum	0.81 Predict
NLM021_scaffold14592_1	53820	phage	0.999	temperate	0.9998265	no_family_avaliable(NC_062582)	0.98	Colwellia psychrerythraea	0.93 Predict
NLM021_scaffold49743_1	25594	phage	0.994	virulent	0.9998665	Salasmaviridae	0.2528747	Ralstonia solanacearum	0.86 Predict
NLM021_C812807_1	12320	phage	0.999	temperate	0.8152787	Casjensviridae	0.5409089	Colwellia psychrerythraea	0.8 Predict
NLM021_scaffold61366_1	10941	phage	0.999	temperate	0.99985975	unknown	0	Colwellia psychrerythraea	0.94 Predict
NLM021_scaffold55417_5	16152	phage	0.999	virulent	0.9936485	Straboviridae	0.4822487	Streptococcus pneumoniae	1 CRISPR
NLM021_scaffold45854_2	12899	phage	0.999	temperate	0.9998584	Straboviridae	0.73139083	Colwellia psychrerythraea	1 CRISPR
NLM021_scaffold927_6	23004	phage	0.999	temperate	0.99977493	Herelleviridae	0.47506937	unknown	0 -
NLM021_scaffold11116_2	11034	phage	0.998	temperate	0.99985975	unknown	0	unknown	0 -
NLM022_scaffold3577_22	10509	phage	0.999	temperate	0.99985975	unknown	0	Colwellia psychrerythraea	0.85 Predict
NLM022_scaffold5329_6	21684	phage	0.999	virulent	0.994396	unknown	0	Colwellia psychrerythraea	0.81 Predict

NLM022_scaffold28217_1	37176	phage	0.997	temperate	0.99811184	Straboviridae	0.5006739	Clostridium perfringens	1	CRISPR
NLM022_scaffold9956_5	20007	phage	0.962	virulent	0.994524	Straboviridae	0.43174168	Colwellia psychrerythraea	0.71	Predict
NLM022_scaffold173_4	13231	phage	0.646	temperate	0.99985975	Zierdtviridae	1	unknown	0	-
NLM022_scaffold17073_1	19677	phage	0.787	virulent	0.6061743	unknown	0	Mycoplasma pulmonis	0.74	Predict
NLM022_scaffold42567_1	18187	phage	0.999	temperate	0.99929553	Peduoviridae	0.43805262	Colwellia psychrerythraea	1	CRISPR
NLM022_scaffold30338_1	35349	phage	0.997	temperate	0.99540496	Peduoviridae	1	Colwellia psychrerythraea	1	CRISPR
NLM022_scaffold3888_1	11570	phage	0.993	virulent	0.9997695	unknown	0	Mycoplasma pulmonis	1	CRISPR
NLM022_scaffold16192_1	13727	phage	0.888	virulent	0.9998736	unknown	0	Colwellia psychrerythraea	0.87	Predict
NLM022_scaffold10884_4	20163	phage	0.998	temperate	0.9998331	unknown	0	unknown	0	-
NLM022_scaffold38630_1	11392	phage	0.999	temperate	0.99985975	unknown	0	Colwellia psychrerythraea	0.77	Predict
NLM022_scaffold42820_1	21665	phage	0.897	temperate	0.99967194	unknown	0	Colwellia psychrerythraea	0.74	Predict
NLM022_scaffold44402_1	26655	phage	0.999	temperate	0.99978215	unknown	0	Clostridium perfringens	0.9	Predict
NLM022_scaffold20998_1	27689	phage	0.999	virulent	0.69050956	Casjensviridae	0.44304314	Listeria monocytogenes	1	CRISPR
NLM022_scaffold42750_2	47468	phage	0.999	temperate	0.99985313	Straboviridae	0.30859387	Clostridioides difficile	1	CRISPR
NLM022_scaffold7821_4	38333	phage	0.998	temperate	0.9998397	unknown	0	Colwellia psychrerythraea	0.76	Predict
NLM022_scaffold38946_1	11220	phage	0.999	temperate	0.9487627	unknown	0	Colwellia psychrerythraea	0.87	Predict
NLM022_scaffold7162_1	10248	phage	1	temperate	0.99985975	no_family_avaliable(NC_021857)	0.99	Colwellia psychrerythraea	1	CRISPR
NLM022_C640276_1	10411	phage	0.968	temperate	0.99884266	Straboviridae	0.84417635	Colwellia psychrerythraea	0.94	Predict
NLM022_scaffold15285_3	12729	phage	0.919	virulent	0.9998679	unknown	0	Roseobacter denitrificans	0.71	Predict
NLM022_scaffold28148_1	55222	phage	0.999	temperate	0.9998508	unknown	0	Staphylococcus saprophyticus	1	CRISPR
NLM022_scaffold9181_1	10114	phage	0.993	virulent	0.99978524	Peduoviridae	0.52169895	Colwellia psychrerythraea	0.83	Predict
NLM022_scaffold35932_1	17588	phage	0.911	virulent	0.9998693	Straboviridae	0.6553155	Colwellia psychrerythraea	0.81	Predict
NLM022_C641598_1	30877	phage	0.995	virulent	0.9998688	Straboviridae	0.72323537	Colwellia psychrerythraea	0.91	Predict
NLM022_scaffold43025_3	12593	phage	0.998	temperate	0.99986035	unknown	0	Colwellia psychrerythraea	0.98	Predict
NLM022_scaffold15285_1	23481	phage	0.999	virulent	0.99910206	Peduoviridae	1	Paenibacillus larvae	1	CRISPR
NLM022_scaffold5329_1	18089	phage	0.997	temperate	0.9998593	Peduoviridae	1	Colwellia psychrerythraea	0.95	Predict
NLM022_scaffold24404_3	10874	phage	0.992	virulent	0.99987036	unknown	0	Colwellia psychrerythraea	0.86	Predict
NLM022_scaffold43901_3	16711	phage	0.84	temperate	0.99986035	unknown	0	unknown	0	-
NLM022_scaffold37593_2	18001	phage	0.998	temperate	0.99985605	Peduoviridae	1	Colwellia psychrerythraea	1	CRISPR
NLM022_scaffold17804_1	12579	phage	0.998	temperate	0.99985975	unknown	0	Colwellia psychrerythraea	0.98	Predict

NLM022_scaffold35358_2	13722	phage	0.993	temperate	0.99985975	unknown	0	Colwellia psychrerythraea	0.72	Predict
NLM022_scaffold11265_9	18375	phage	0.999	virulent	0.9996532	unknown	0	Colwellia psychrerythraea	1	CRISPR
NLM022_C640494_1	11554	phage	0.891	virulent	0.96739966	Straboviridae	0.4467759	Bacteroides fragilis	0.76	Predict
NLM022_scaffold7447_1	16168	phage	0.985	temperate	0.9998522	Straboviridae	0.42385665	unknown	0	-
NLM023_scaffold23138_8	10839	phage	0.949	virulent	0.9997411	Ackermannviridae	0.35920888	Colwellia psychrerythraea	0.77	Predict
NLM023_scaffold1402_1	31939	phage	0.998	temperate	0.9998593	unknown	0	Colwellia psychrerythraea	0.83	Predict
NLM023_scaffold23260_2	19585	phage	0.999	virulent	0.99987084	no_family_avaliable(NC_024711)	0.959	Flavobacterium columnare	0.72	Predict
NLM023_scaffold33293_1	12525	phage	0.99	temperate	0.99985975	unknown	0	unknown	0	-
NLM023_scaffold470_13	15413	phage	0.999	temperate	0.99983925	unknown	0	Parabacteroides merdae	1	CRISPR
NLM023_scaffold28479_8	14799	phage	0.956	temperate	0.9946079	unknown	0	Colwellia psychrerythraea	0.81	Predict
NLM023_scaffold23260_3	72849	phage	1	virulent	0.9998651	Schitoviridae	1	Mycoplasma pulmonis	0.89	Predict
NLM023_scaffold21242_4	13967	phage	0.998	temperate	0.99135226	unknown	0	Colwellia psychrerythraea	1	CRISPR
NLM023_scaffold48669_2	12532	phage	0.985	temperate	0.9998522	Straboviridae	0.42385665	unknown	0	-
NLM023_scaffold48941_1	46775	phage	1	virulent	0.9998651	unknown	0	Parabacteroides distasonis	1	CRISPR
NLM023_scaffold48169_1	24439	phage	1	temperate	0.99984926	unknown	0	Colwellia psychrerythraea	0.94	Predict
NLM023_scaffold28479_20	11905	phage	0.998	virulent	0.9995251	unknown	0	unknown	0	-
NLM023_C806243_1	10699	phage	0.998	virulent	0.9354477	unknown	0	Parabacteroides distasonis	0.74	Predict
NLM023_scaffold28479_12	12371	phage	0.999	temperate	0.99986035	unknown	0	Colwellia psychrerythraea	0.87	Predict
NLM023_scaffold11383_1	17434	phage	0.574	virulent	0.99987173	unknown	0	Parabacteroides distasonis	1	CRISPR
NLM023_scaffold911_13	10301	phage	0.999	virulent	0.9878793	Casjensviridae	0.52900046	Colwellia psychrerythraea	0.9	Predict
NLM023_scaffold8508_4	10221	phage	0.941	virulent	0.9996882	Straboviridae	0.6553155	Colwellia psychrerythraea	0.86	Predict
NLM023_scaffold48946_1	11520	phage	0.999	virulent	0.9998736	unknown	0	unknown	0	-
NLM023_scaffold48276_1	19137	phage	0.999	virulent	0.9998545	Straboviridae	0.36598924	Colwellia psychrerythraea	0.72	Predict
NLM023_scaffold277_15	10075	phage	0.997	virulent	0.99985605	Straboviridae	0.58768487	unknown	0	-
NLM023_scaffold43102_12	33483	phage	0.999	temperate	0.9910795	Casjensviridae	1	Colwellia psychrerythraea	0.74	Predict
NLM023_scaffold1348_25	11084	phage	0.967	virulent	0.9998645	Casjensviridae	0.22054045	Colwellia psychrerythraea	0.84	Predict
NLM023_scaffold37217_1	11231	phage	0.815	temperate	0.999774	unknown	0	Colwellia psychrerythraea	0.92	Predict
NLM023_scaffold399_47	12529	phage	0.624	virulent	0.9998636	Casjensviridae	0.31617516	Kitasatospora aureofaciens	0.75	Predict
NLM023_scaffold260_1	22119	phage	0.999	virulent	0.9986079	Herelleviridae	0.64262027	Bacteroides fragilis	0.89	Predict
NLM023_scaffold253_1	13515	phage	0.999	temperate	0.99977785	unknown	0	Colwellia psychrerythraea	0.99	Predict

NLM023_scaffold10723_15	21742	phage	0.998	virulent	0.9998574	Peduoviridae	0.7363866	Colwellia psychrerythraea	0.71	Predict
NLM023_scaffold4055_10	10545	phage	0.936	virulent	0.9983713	unknown	0	Colwellia psychrerythraea	0.77	Predict
NLM023_scaffold27707_7	16883	phage	0.997	virulent	0.9998288	unknown	0	unknown	0	-
NLM023_scaffold1428_18	12077	phage	0.993	virulent	0.9935903	unknown	0	Roseobacter denitrificans	0.86	Predict
NLM023_scaffold14181_20	31305	phage	0.999	temperate	0.9998388	Drexlerviridae	0.27454552	Colwellia psychrerythraea	0.83	Predict
NLM023_scaffold48506_1	39326	phage	0.998	temperate	0.9998445	unknown	0	Colwellia psychrerythraea	1	CRISPR
NLM023_scaffold17200_15	17300	phage	0.999	virulent	0.99979925	unknown	0	unknown	0	-
NLM023_scaffold33532_1	10466	phage	0.997	virulent	0.99970627	Ackermannviridae	0.31039864	Colwellia psychrerythraea	0.89	Predict
NLM023_scaffold35604_3	14033	phage	0.986	temperate	0.99985975	unknown	0	Colwellia psychrerythraea	0.73	Predict
NLM023_scaffold46734_3	14124	phage	0.999	temperate	0.9998593	Salasmaviridae	0.40109783	Colwellia psychrerythraea	0.98	Predict
NLM023_scaffold22_6	11130	phage	0.957	virulent	0.99987406	unknown	0	Colwellia psychrerythraea	0.81	Predict
NLM023_scaffold12109_12	11184	phage	0.942	temperate	0.99985313	Peduoviridae	0.5691711	Roseobacter denitrificans	0.73	Predict
NLM023_scaffold34755_1	14507	phage	0.997	temperate	0.9998593	Straboviridae	0.57686263	Colwellia psychrerythraea	0.97	Predict
NLM023_scaffold48896_1	12812	phage	0.743	temperate	0.9922496	Straboviridae	0.36598924	Colwellia psychrerythraea	0.95	Predict
NLM024_scaffold488_1	13160	phage	0.918	virulent	0.99970657	Straboviridae	0.65579903	Bacteroides fragilis	0.75	Predict
NLM024_scaffold497_1	11105	phage	0.974	temperate	0.9990155	unknown	0	unknown	0	-
NLM024_scaffold6119_1	14990	phage	0.99	virulent	0.9998722	Kyanoviridae	1	Colwellia psychrerythraea	0.86	Predict
NLM024_scaffold10225_2	13799	phage	0.986	virulent	0.87349826	unknown	0	Sinorhizobium meliloti	0.93	Predict
NLM024_scaffold20170_1	13229	phage	0.999	temperate	0.99985975	unknown	0	Bacteroides fragilis	0.89	Predict
NLM024_scaffold5090_1	13275	phage	0.999	virulent	0.99987084	unknown	0	Colwellia psychrerythraea	1	CRISPR
NLM024_scaffold18150_1	11312	phage	0.992	temperate	0.9998588	Peduoviridae	1	Clostridioides difficile	0.77	Predict
NLM024_scaffold13470_1	12152	phage	0.999	virulent	0.99962884	unknown	0	Colwellia psychrerythraea	1	CRISPR
NLM024_scaffold14981_1	26797	phage	0.999	virulent	0.9998579	Drexlerviridae	1	Klebsiella pneumoniae	1	CRISPR
NLM024_scaffold15487_3	10814	phage	0.997	temperate	0.99984837	unknown	0	Parabacteroides distasonis	0.84	Predict
NLM024_scaffold77_6	10794	phage	0.999	virulent	0.99987406	Herelleviridae	1	Colwellia psychrerythraea	0.9	Predict
NLM024_scaffold1371_3	10316	phage	0.998	temperate	0.99973583	unknown	0	Colwellia psychrerythraea	1	CRISPR
NLM024_scaffold244_5	10289	phage	0.947	virulent	0.9949243	Straboviridae	0.6553155	Colwellia psychrerythraea	0.71	Predict
NLM024_scaffold9211_2	12110	phage	0.891	virulent	0.99986404	Herelleviridae	1	Colwellia psychrerythraea	0.96	Predict
NLM024_scaffold100_1	11891	phage	0.999	virulent	0.999768	Straboviridae	0.42298242	Colwellia psychrerythraea	0.71	Predict
NLM024_scaffold957_14	14427	phage	0.99	virulent	0.99987173	Ackermannviridae	0.25430948	Colwellia psychrerythraea	0.93	Predict

NLM024_scaffold16628_8	21897	phage	0.993	virulent	0.9979265	Straboviridae	0.35521403	Bacteroides fragilis	0.91	Predict
NLM024_scaffold11939_1	19270	phage	0.999	temperate	0.9998579	Straboviridae	0.22636564	Mycoplasma pulmonis	1	CRISPR
NLM024_scaffold14981_3	14966	phage	0.999	virulent	0.99984974	Drexlerviridae	0.6004602	Colwellia psychrerythraea	0.93	Predict
NLM025_C502105_1	10354	phage	0.949	temperate	0.999854	Herelleviridae	0.17094114	Colwellia psychrerythraea	0.87	Predict
NLM025_scaffold4345_2	56819	phage	0.993	virulent	0.9998584	Ackermannviridae	0.24692279	Parabacteroides merdae	0.72	Predict
NLM025_scaffold22167_3	11113	phage	0.911	virulent	0.9988293	unknown	0	Colwellia psychrerythraea	0.78	Predict
NLM025_scaffold39287_1	15133	phage	0.999	temperate	0.99985975	unknown	0	Colwellia psychrerythraea	0.9	Predict
NLM025_scaffold18010_7	11076	phage	0.706	virulent	0.999808	unknown	0	Colwellia psychrerythraea	0.76	Predict
NLM025_scaffold39157_1	11030	phage	0.998	temperate	0.6460557	unknown	0	Colwellia psychrerythraea	0.93	Predict
NLM025_scaffold25589_3	11038	phage	0.932	temperate	0.99985975	unknown	0	Mycoplasma pulmonis	0.81	Predict
NLM025_scaffold35205_1	10034	phage	0.673	temperate	0.99985975	Straboviridae	0.67151016	Colwellia psychrerythraea	0.93	Predict
NLM025_scaffold38901_1	13523	phage	0.5	virulent	0.9998357	Drexlerviridae	0.39220908	Colwellia psychrerythraea	0.97	Predict
NLM025_scaffold78_11	13115	phage	0.999	temperate	0.984522	unknown	0	unknown	0	-
NLM025_scaffold12163_17	84125	phage	0.982	virulent	0.99829227	Straboviridae	0.6383465	Colwellia psychrerythraea	1	CRISPR
NLM025_scaffold20016_2	17453	phage	0.8	temperate	0.99985313	unknown	0	Colwellia psychrerythraea	0.89	Predict
NLM025_scaffold29129_1	10848	phage	0.999	temperate	0.9998517	Drexlerviridae	0.3840808	Colwellia psychrerythraea	0.83	Predict
NLM026_scaffold11596_2	10152	phage	0.999	virulent	0.9993334	unknown	0	unknown	0	-
NLM026_scaffold74538_1	12956	phage	0.999	temperate	0.9998579	unknown	0	Colwellia psychrerythraea	0.71	Predict
NLM026_scaffold29270_6	10046	phage	0.999	temperate	0.99982554	unknown	0	Colwellia psychrerythraea	0.96	Predict
NLM026_scaffold27749_19	26934	phage	0.999	temperate	0.9998331	Straboviridae	0.49333465	Mycoplasma pulmonis	0.73	Predict
NLM026_scaffold62290_1	14700	phage	0.996	virulent	0.9998556	Straboviridae	0.52144754	Colwellia psychrerythraea	0.74	Predict
NLM026_scaffold55998_10	13744	phage	0.991	temperate	0.9774005	Salasmaviridae	0.2528747	Colwellia psychrerythraea	0.76	Predict
NLM026_scaffold57277_1	21550	phage	0.856	temperate	0.99977446	unknown	0	Mycoplasma pulmonis	0.71	Predict
NLM026_scaffold74568_1	12762	phage	0.999	virulent	0.9998722	Salasmaviridae	1	Colwellia psychrerythraea	0.96	Predict
NLM026_scaffold75736_1	27065	phage	0.999	temperate	0.99576545	unknown	0	Colwellia psychrerythraea	0.72	Predict
NLM026_scaffold1771_11	11720	phage	0.998	virulent	0.9998736	unknown	0	Colwellia psychrerythraea	0.74	Predict
NLM026_scaffold258_3	15836	phage	0.999	temperate	0.9997983	unknown	0	Colwellia psychrerythraea	1	Predict
NLM026_scaffold988_1	23193	phage	0.994	virulent	0.99260366	unknown	0	unknown	0	-
NLM026_scaffold75282_1	20179	phage	0.999	temperate	0.99985605	unknown	0	Flavobacterium columnare	0.71	Predict
NLM026_scaffold47323_1	10304	phage	0.984	temperate	0.99985975	unknown	0	Colwellia psychrerythraea	0.7	Predict

NLM026_scaffold65263_3	16213	phage	0.778	virulent	0.9998693	unknown	0	Colwellia psychrerythraea	0.88	Predict
NLM026_scaffold74983_1	16672	phage	0.999	virulent	0.9998693	no_family_avaliable(NC_062778)	0.993	Flavobacterium columnare	0.94	Predict
NLM026_scaffold68020_6	10206	phage	0.998	temperate	0.99985975	unknown	0	unknown	0	-
NLM026_scaffold58936_3	11426	phage	0.867	virulent	0.9998536	unknown	0	Colwellia psychrerythraea	0.88	Predict
NLM026_scaffold394_5	12674	phage	0.999	temperate	0.9998579	unknown	0	unknown	0	-
NLM026_scaffold29289_10	10390	phage	0.998	temperate	0.99985975	unknown	0	Colwellia psychrerythraea	0.82	Predict
NLM026_scaffold21981_1	11548	phage	0.998	virulent	0.999579	unknown	0	Colwellia psychrerythraea	0.86	Predict
NLM026_scaffold86_4	16762	phage	0.992	temperate	0.99986035	unknown	0	Actinomyces naeslundii	0.79	Predict
NLM026_scaffold75724_1	18758	phage	0.727	temperate	0.9985046	Straboviridae	0.4203055	unknown	0	-
NLM026_scaffold26990_1	10612	phage	1	virulent	0.99969715	Ackermannviridae	0.2372473	Colwellia psychrerythraea	0.79	Predict
NLM026_scaffold74825_1	29190	phage	0.998	virulent	0.8512207	unknown	0	Lactococcus lactis	0.73	Predict
NLM026_scaffold75315_1	16846	phage	0.999	virulent	0.9998436	Herelleviridae	1	unknown	0	-
NLM026_scaffold49770_2	62798	phage	0.999	virulent	0.9998656	Guelinviridae	0.2955733	Flavobacterium columnare	0.97	Predict
NLM026_scaffold26189_1	38714	phage	0.982	virulent	0.9998574	unknown	0	unknown	0	-
NLM026_scaffold75839_2	13382	phage	0.81	virulent	0.99987084	Straboviridae	0.4597352	Colwellia psychrerythraea	0.88	Predict
NLM026_scaffold32583_1	11219	phage	0.939	temperate	0.56512856	unknown	0	unknown	0	-
NLM026_scaffold58959_4	12007	phage	0.997	temperate	0.9575485	unknown	0	Colwellia psychrerythraea	0.97	Predict
NLM026_scaffold74363_2	13785	phage	1	virulent	0.9998699	Salasmaviridae	0.93296427	Colwellia psychrerythraea	0.96	Predict
NLM026_scaffold58139_6	21846	phage	0.808	temperate	0.9998588	Straboviridae	0.5153456	Colwellia psychrerythraea	0.92	Predict
NLM026_scaffold258_2	19642	phage	0.999	temperate	0.9997631	unknown	0	Colwellia psychrerythraea	0.9	Predict
NLM026_scaffold75960_2	11350	phage	0.999	virulent	0.99985695	unknown	0	Colwellia psychrerythraea	0.98	Predict
NLM026_C924027_1	10453	phage	0.968	virulent	0.99987406	unknown	0	Bacillus anthracis	0.84	Predict
NLM026_scaffold65585_4	11821	phage	0.997	temperate	0.9753722	unknown	0	unknown	0	-
NLM026_scaffold16034_7	10751	phage	1	virulent	0.99986607	unknown	0	unknown	0	-
NLM026_scaffold9047_2	11529	phage	0.814	virulent	0.99392647	unknown	0	Thermoanaerobacterium saccharolyti	0.92	Predict
NLM026_scaffold6763_2	13408	phage	0.999	virulent	0.9998688	Salasmaviridae	1	Colwellia psychrerythraea	0.84	Predict
NLM026_scaffold32844_2	21089	phage	0.919	virulent	0.9998699	Straboviridae	0.51542896	Pantoea agglomerans	0.85	Predict
NLM026_scaffold13988_2	10430	phage	0.998	temperate	0.9998593	unknown	0	Mycoplasma pulmonis	0.72	Predict
NLM026_scaffold69595_2	13806	phage	0.999	virulent	0.9997521	unknown	0	Colwellia psychrerythraea	1	CRISPR
NLM026_scaffold63092_3	11289	phage	0.989	virulent	0.99987173	Guelinviridae	1	Colwellia psychrerythraea	0.96	Predict

NLM026_scaffold54916_1	12133	phage	0.996	temperate	0.9998584	unknown	0	unknown	0	-
NLM026_scaffold73101_2	19639	phage	0.997	temperate	0.99985975	unknown	0	Lactobacillus fermentum	0.8	Predict
NLM026_scaffold43543_3	13495	phage	0.997	virulent	0.99735016	Straboviridae	0.39771363	unknown	0	-
NLM026_scaffold75674_2	16165	phage	0.932	virulent	0.9998727	Ackermannviridae	0.25430948	Colwellia psychrerythraea	0.95	Predict
NLM026_scaffold15415_2	33307	phage	0.938	temperate	0.97500896	unknown	0	Mycoplasma pulmonis	0.99	Predict
NLM027_scaffold22040_4	13439	phage	0.965	virulent	0.7335105	unknown	0	Chlamydia pecorum	0.92	Predict
NLM027_scaffold40686_1	14007	phage	0.999	virulent	0.9998191	unknown	0	Colwellia psychrerythraea	0.81	Predict
NLM027_scaffold41690_2	12335	phage	0.864	virulent	0.99987173	unknown	0	unknown	0	-
NLM027_scaffold41411_1	17267	phage	0.99	temperate	0.9996224	Casjensviridae	1	Colwellia psychrerythraea	1	CRISPR
NLM027_scaffold41959_1	42090	phage	0.998	temperate	0.9965829	Herelleviridae	1	Colwellia psychrerythraea	1	CRISPR
NLM027_scaffold4058_19	11193	phage	0.603	virulent	0.54008085	unknown	0	unknown	0	-
NLM027_scaffold19284_2	12830	phage	0.999	virulent	0.999703	unknown	0	unknown	0	-
NLM027_scaffold40329_1	41938	phage	0.998	temperate	0.9881868	Peduoviridae	0.70607936	Colwellia psychrerythraea	1	CRISPR
NLM027_scaffold35554_2	43198	phage	0.998	temperate	0.99985695	Peduoviridae	1	Colwellia psychrerythraea	1	CRISPR
NLM027_scaffold40329_3	16087	phage	0.999	temperate	0.99985975	Kyanoviridae	1	Colwellia psychrerythraea	1	CRISPR
NLM027_scaffold39111_4	28084	phage	0.999	temperate	0.99916536	Straboviridae	0.30859387	Streptococcus thermophilus	1	CRISPR
NLM027_scaffold35206_1	19714	phage	0.996	temperate	0.9967787	Peduoviridae	0.241425	Colwellia psychrerythraea	1	CRISPR
NLM027_scaffold30138_3	10817	phage	0.985	temperate	0.9998593	unknown	0	Mycoplasma pulmonis	0.84	Predict
NLM027_scaffold41965_1	11913	phage	0.865	virulent	0.99987036	unknown	0	unknown	0	-
NLM027_scaffold14263_1	11687	phage	0.997	temperate	0.9998588	unknown	0	Mycoplasma pulmonis	1	CRISPR
NLM027_scaffold39770_2	15302	phage	1	virulent	0.99963146	unknown	0	Colwellia psychrerythraea	0.93	Predict
NLM027_scaffold16353_1	20708	phage	0.993	temperate	0.9998593	Straboviridae	0.33924893	Colwellia psychrerythraea	0.87	Predict
NLM027_scaffold34797_1	12393	phage	0.873	temperate	0.99611515	unknown	0	Colwellia psychrerythraea	0.74	Predict
NLM027_scaffold8952_3	11510	phage	0.981	virulent	0.8142954	Casjensviridae	1	Colwellia psychrerythraea	0.81	Predict
NLM027_scaffold34798_1	12628	phage	0.873	temperate	0.99611515	unknown	0	Enterococcus faecium	1	CRISPR
NLM027_scaffold10002_11	10047	phage	0.998	temperate	0.99985975	unknown	0	Colwellia psychrerythraea	0.77	Predict
NLM027_scaffold17160_15	12951	phage	0.998	virulent	0.99986315	unknown	0	Enterococcus faecium	0.86	Predict
NLM027_scaffold8952_6	11720	phage	0.999	temperate	0.9998584	Drexlerviridae	0.25270286	Colwellia psychrerythraea	0.81	Predict
NLM027_scaffold7180_15	11354	phage	0.986	temperate	0.9908204	unknown	0	Cellulophaga baltica	1	CRISPR
NLM027_scaffold1874_3	27078	phage	0.999	temperate	0.97194517	Chaseviridae	1	Colwellia psychrerythraea	1	CRISPR

NLM027_scaffold11645_1	16224	phage	0.996	virulent	0.99913013	Straboviridae	0.42151073	Vibrio vulnificus	0.75	Predict
NLM027_scaffold16205_3	11750	phage	0.997	temperate	0.99985975	Peduoviridae	1	Clostridium sporogenes	0.9	Predict
NLM027_scaffold1886_1	10338	phage	0.984	temperate	0.9998593	unknown	0	Mycoplasma pulmonis	0.71	Predict
NLM027_scaffold40537_1	11567	phage	0.998	virulent	0.9991923	unknown	0	Mycoplasma pulmonis	0.84	Predict
NLM027_scaffold3310_5	24584	phage	0.999	temperate	0.99985033	Kyanoviridae	1	Cronobacter sakazakii	1	CRISPR
NLM027_scaffold27969_2	11433	phage	0.775	virulent	0.99936295	Straboviridae	0.6418709	unknown	0	-
NLM027_C668626_1	15217	phage	0.984	temperate	0.99985695	unknown	0	Colwellia psychrerythraea	0.93	Predict
NLM027_scaffold14181_3	39783	phage	0.999	temperate	0.9998508	unknown	0	Flavobacterium columnare	0.84	Predict
NLM027_scaffold34123_3	26798	phage	0.999	temperate	0.99974394	Peduoviridae	0.14924113	Colwellia psychrerythraea	1	CRISPR
NLM027_scaffold17357_1	13446	phage	0.993	temperate	0.9378113	Salasmaviridae	0.2528747	Clostridioides difficile	0.88	Predict
NLM027_scaffold41891_1	11065	phage	0.999	virulent	0.99987084	unknown	0	Colwellia psychrerythraea	1	CRISPR
NLM027_scaffold40356_4	38806	phage	0.999	temperate	0.9998379	unknown	0	Clostridioides difficile	1	CRISPR
NLM027_scaffold35206_2	16953	phage	0.999	virulent	0.99986744	unknown	0	Colwellia psychrerythraea	0.86	Predict
NLM027_scaffold9793_1	21930	phage	0.956	temperate	0.9985623	unknown	0	Staphylococcus capitis	0.81	Predict
NLM027_scaffold20399_3	11351	phage	0.762	virulent	0.99987173	unknown	0	Colwellia psychrerythraea	0.71	Predict
NLM027_scaffold38196_4	10025	phage	0.582	temperate	0.9998593	Straboviridae	0.57686263	Bacteroides fragilis	0.98	Predict
NLM028_scaffold28234_2	10654	phage	0.993	virulent	0.9998722	unknown	0	Colwellia psychrerythraea	0.83	Predict
NLM028_scaffold35099_2	10968	phage	0.793	virulent	0.9998202	unknown	0	unknown	0	-
NLM028_scaffold12174_3	11146	phage	0.778	temperate	0.9629111	unknown	0	Colwellia psychrerythraea	0.89	Predict
NLM028_scaffold54929_3	12362	phage	0.595	virulent	0.999174	unknown	0	Colwellia psychrerythraea	0.94	Predict
NLM028_scaffold59756_2	22999	phage	0.999	virulent	0.9957786	unknown	0	Flavobacterium psychrophilum	1	CRISPR
NLM028_scaffold34514_1	12847	phage	0.999	virulent	0.9998727	Salasmaviridae	0.78925806	Colwellia psychrerythraea	1	CRISPR
NLM028_scaffold59789_2	10085	phage	0.998	temperate	0.9997769	unknown	0	Bacteroides fragilis	0.75	Predict
NLM028_scaffold1532_1	12915	phage	0.623	temperate	0.9997917	unknown	0	Colwellia psychrerythraea	0.79	Predict
NLM028_scaffold24678_1	29305	phage	0.998	temperate	0.99985975	unknown	0	Colwellia psychrerythraea	1	CRISPR
NLM028_scaffold8768_2	20079	phage	0.902	virulent	0.99976426	Peduoviridae	0.52169895	Colwellia psychrerythraea	0.93	Predict
NLM028_scaffold34609_1	61603	phage	0.999	virulent	0.9996796	Casjensviridae	0.54889804	Colwellia psychrerythraea	1	CRISPR
NLM028_scaffold42575_1	13429	phage	0.967	virulent	0.9860106	unknown	0	unknown	0	-
NLM028_scaffold30720_1	15734	phage	0.999	temperate	0.5093073	unknown	0	Ruminococcus sp. OM05-7	1	CRISPR
NLM028_scaffold12341_5	24750	phage	0.996	virulent	0.88290614	unknown	0	Candidatus Hamiltonella defensa	1	CRISPR

NLM028_scaffold51949_2	15807	phage	0.892	virulent	0.9998369	Ackermannviridae	1	Colwellia psychrerythraea	0.96	Predict
NLM028_scaffold20220_12	16481	phage	0.999	virulent	0.6351505	unknown	0	Clostridium perfringens	1	CRISPR
NLM028_scaffold45877_1	13775	phage	0.987	temperate	0.99983555	Chaseviridae	0.3054243	Bacillus megaterium	0.92	Predict
NLM028_scaffold26847_1	23036	phage	0.999	temperate	0.9967376	unknown	0	Colwellia psychrerythraea	0.88	Predict
NLM028_scaffold57798_4	46364	phage	0.999	virulent	0.9998617	unknown	0	Colwellia psychrerythraea	1	CRISPR
NLM028_scaffold15831_1	13715	phage	0.999	temperate	0.97040224	unknown	0	Bacteroides fragilis	0.71	Predict
NLM028_scaffold34525_2	10415	phage	0.999	virulent	0.99987316	Mesyanzhinovviridae	0.5455918	Thermoanaerobacterium saccharolyti	1	CRISPR
NLM028_scaffold43687_1	16295	phage	0.999	temperate	0.9998593	unknown	0	Colwellia psychrerythraea	0.85	Predict
NLM028_scaffold20949_1	16228	phage	0.974	virulent	0.999658	Straboviridae	0.59448063	Colwellia psychrerythraea	0.98	Predict
NLM028_scaffold57543_3	13925	phage	0.999	temperate	0.97810906	Peduoviridae	1	Rhodovulum sp. P5	1	CRISPR
NLM028_scaffold59796_3	10642	phage	0.996	temperate	0.7185659	unknown	0	Parabacteroides distasonis	0.82	Predict
NLM028_scaffold57798_3	32526	phage	0.999	temperate	0.990775	unknown	0	Colwellia psychrerythraea	0.8	Predict
NLM028_C652312_1	54494	phage	0.999	temperate	0.9977651	Casjensviridae	1	Colwellia psychrerythraea	0.83	Predict
NLM028_scaffold7898_1	10249	phage	0.995	virulent	0.9998722	unknown	0	unknown	0	-
NLM028_scaffold15831_2	21150	phage	0.999	temperate	0.93888336	unknown	0	Bacteroides fragilis	0.91	Predict
NLM028_scaffold42949_1	28623	phage	0.975	temperate	0.99978584	unknown	0	Bacteroides fragilis	1	CRISPR
NLM028_scaffold57543_2	10447	phage	0.997	virulent	0.9758714	unknown	0	Colwellia psychrerythraea	1	CRISPR
NLM028_scaffold37225_1	16038	phage	0.999	virulent	0.9997153	unknown	0	Colwellia psychrerythraea	0.85	Predict
NLM028_C652264_1	33034	phage	0.999	temperate	0.99983406	unknown	0	Clostridium perfringens	1	CRISPR
NLM028_scaffold13605_5	15796	phage	0.999	temperate	0.99985695	Ackermannviridae	0.2372473	Colwellia psychrerythraea	0.92	Predict
NLM029_scaffold46834_2	12051	phage	0.999	temperate	0.9998584	unknown	0	unknown	0	-
NLM029_scaffold45341_4	15578	phage	0.996	temperate	0.59614587	Straboviridae	0.5399199	Colwellia psychrerythraea	0.72	Predict
NLM029_scaffold33952_1	16769	phage	0.999	virulent	0.9996615	Casjensviridae	0.9217208	Bifidobacterium sp. MSTE12	1	CRISPR
NLM029_scaffold4810_3	12364	phage	0.692	temperate	0.9697178	Straboviridae	0.43174168	Clostridium tetani	0.92	Predict
NLM029_C674039_1	14587	phage	0.999	temperate	0.99949604	unknown	0	Colwellia psychrerythraea	0.8	Predict
NLM029_scaffold22749_1	13358	phage	0.95	virulent	0.99987125	unknown	0	unknown	0	-
NLM029_scaffold2015_8	21159	phage	0.998	virulent	0.99462146	Casjensviridae	0.45354968	Colwellia psychrerythraea	1	CRISPR
NLM029_scaffold24024_8	17833	phage	0.957	virulent	0.99915105	unknown	0	Colwellia psychrerythraea	1	CRISPR
NLM029_scaffold9598_4	19047	phage	0.999	temperate	0.9998593	unknown	0	Colwellia psychrerythraea	0.84	Predict
NLM029_scaffold39928_1	16122	phage	0.989	temperate	0.9998274	Straboviridae	0.6553155	Bacteroides fragilis	0.87	Predict

NLM029_scaffold161_2	10417	phage	0.991	temperate	0.99986035	Straboviridae	0.5123257	unknown	0	-
NLM029_scaffold9173_1	10269	phage	0.999	temperate	0.9971445	unknown	0	unknown	0	-
NLM029_scaffold2015_2	12030	phage	0.998	temperate	0.98481876	Peduoviridae	1	Colwellia psychrerythraea	1	CRISPR
NLM029_scaffold44423_10	19444	phage	0.999	temperate	0.99986035	unknown	0	Colwellia psychrerythraea	0.7	Predict
NLM029_scaffold17455_3	11447	phage	0.823	temperate	0.99985975	unknown	0	unknown	0	-
NLM029_scaffold3634_1	22242	phage	0.872	virulent	0.9827163	Peduoviridae	0.46395448	Candidatus Hamiltonella defensa	0.76	Predict
NLM029_scaffold37710_1	14419	phage	0.999	virulent	0.99971133	Peduoviridae	1	Colwellia psychrerythraea	1	CRISPR
NLM029_scaffold9598_2	11683	phage	1	temperate	0.99985975	unknown	0	Colwellia psychrerythraea	1	CRISPR
NLM029_scaffold24413_3	11742	phage	0.997	virulent	0.9998699	unknown	0	Roseobacter denitrificans	1	CRISPR
NLM029_scaffold7675_3	52620	phage	0.993	virulent	0.96640456	Straboviridae	0.4558217	Colwellia psychrerythraea	1	CRISPR
NLM029_scaffold3860_9	25836	phage	0.999	virulent	0.9998636	Vilmaviridae	1	Cellulophaga baltica	0.91	Predict
NLM029_scaffold3826_2	46431	phage	0.999	temperate	0.97519684	unknown	0	Colwellia psychrerythraea	1	CRISPR
NLM029_scaffold4592_2	14675	phage	0.801	virulent	0.99987084	unknown	0	Bacteroides fragilis	0.75	Predict
NLM029_scaffold25526_7	11246	phage	0.946	virulent	0.9998727	Straboviridae	0.65579903	unknown	0	-
NLM029_C673977_1	13802	phage	0.989	virulent	0.99973136	unknown	0	Bacillus megaterium	0.9	Predict
NLM029_scaffold46830_1	13320	phage	0.996	temperate	0.9997758	Peduoviridae	1	unknown	0	-
NLM029_scaffold7768_1	11575	phage	0.992	temperate	0.99985975	Straboviridae	0.2957326	Clostridioides difficile	0.96	Predict
NLM029_scaffold35541_5	14184	phage	0.69	virulent	0.996247	unknown	0	unknown	0	-
NLM029_scaffold5035_2	11286	phage	0.974	temperate	0.98519784	unknown	0	Bacteroides fragilis	0.75	Predict
NLM029_scaffold45930_1	10218	phage	0.944	temperate	0.9998593	Straboviridae	0.5890641	Colwellia psychrerythraea	0.85	Predict
NLM029_scaffold35112_1	58981	phage	0.999	temperate	0.9998274	Herelleviridae	1	Colwellia psychrerythraea	1	CRISPR
NLM029_scaffold4050_9	52599	phage	0.998	temperate	0.9981835	unknown	0	Colwellia psychrerythraea	1	CRISPR
NLM029_scaffold12727_2	11381	phage	0.999	temperate	0.9998593	unknown	0	unknown	0	-
NLM029_scaffold15304_1	10599	phage	0.999	virulent	0.99879533	unknown	0	unknown	0	-
NLM029_scaffold24413_4	21114	phage	0.999	virulent	0.9998679	unknown	0	Colwellia psychrerythraea	0.97	Predict
NLM029_scaffold9315_13	10296	phage	0.998	temperate	0.999774	Peduoviridae	0.5691711	Roseobacter denitrificans	0.83	Predict
NLM029_scaffold46287_2	95583	phage	0.998	virulent	0.9998617	Herelleviridae	0.858234	Flavobacterium columnare	0.84	Predict
NLM029_scaffold31672_2	51341	phage	0.999	virulent	0.9998565	Herelleviridae	1	unknown	0	-
NLM029_C674283_1	17944	phage	0.999	temperate	0.99985975	unknown	0	Colwellia psychrerythraea	0.93	Predict
NLM029_scaffold10574_3	21530	phage	0.999	temperate	0.95317507	unknown	0	Clostridioides difficile	1	CRISPR

NLM029_scaffold26243_3	22530	phage	1	temperate	0.9998556	unknown	0	Colwellia psychrerythraea	1 CRISPR
NLM029_scaffold33933_1	10484	phage	1	virulent	0.7445869	unknown	0	Mycoplasma pulmonis	0.78 Predict
NLM029_scaffold17844_1	22171	phage	0.999	temperate	0.99985695	Straboviridae	0.47592783	unknown	0 -
NLM029_scaffold23685_2	11530	phage	0.995	virulent	0.9927804	Peduoviridae	0.30736294	Colwellia psychrerythraea	0.95 Predict
NLM029_C674325_1	18971	phage	0.999	virulent	0.99987084	unknown	0	unknown	0 -
NLM029_scaffold8972_5	12318	phage	0.998	virulent	0.9998684	unknown	0	Colwellia psychrerythraea	1 CRISPR
NLM029_scaffold325_5	10126	phage	0.994	temperate	0.5025973	unknown	0	Colwellia psychrerythraea	0.84 Predict
NLM029_scaffold45625_1	10192	phage	0.992	virulent	0.99987465	unknown	0	unknown	0 -
NLM029_scaffold10325_2	29430	phage	0.999	temperate	0.9992649	Herelleviridae	1	Clostridium perfringens	1 CRISPR
NLM029_scaffold8116_24	12252	phage	0.989	virulent	0.99987406	Straboviridae	0.65579903	Colwellia psychrerythraea	0.79 Predict
NLM029_C674345_1	19845	phage	0.606	temperate	0.9998588	Straboviridae	0.4203055	unknown	0 -
NLM029_scaffold20227_1	11746	phage	0.998	temperate	0.9996734	unknown	0	Colwellia psychrerythraea	0.77 Predict
NLM029_scaffold46752_4	11742	phage	0.96	virulent	0.9998727	unknown	0	Colwellia psychrerythraea	0.88 Predict
NLM031_scaffold75_4	106586	phage	0.998	virulent	0.99986744	unknown	0	Colwellia psychrerythraea	0.78 Predict
NLM031_scaffold75_1	38028	phage	0.999	virulent	0.99986696	Herelleviridae	0.53761834	Colwellia psychrerythraea	0.73 Predict
NLM031_scaffold59329_1	10082	phage	0.996	temperate	0.9998593	unknown	0	Bacillus megaterium	0.74 Predict
NLM031_scaffold21651_1	32306	phage	0.998	virulent	0.95285964	Peduoviridae	1	Paenibacillus larvae	1 CRISPR
NLM031_scaffold18212_16	10989	phage	0.994	temperate	0.93755734	unknown	0	unknown	0 -
NLM031_scaffold18436_4	10312	phage	0.999	temperate	0.9476028	unknown	0	Colwellia psychrerythraea	0.92 Predict
NLM031_scaffold45857_1	19554	phage	0.993	virulent	0.9979265	Straboviridae	0.35521403	Bacteroides fragilis	0.81 Predict
NLM031_scaffold33580_2	17069	phage	0.998	virulent	0.9998736	unknown	0	unknown	0 -
NLM031_scaffold16685_1	14017	phage	0.998	virulent	0.99986744	no_family_avaliable(NC_047914)	0.99	Colwellia psychrerythraea	1 CRISPR
NLM031_scaffold23971_3	13983	phage	0.996	virulent	0.9998684	Straboviridae	0.72108424	Colwellia psychrerythraea	0.84 Predict
NLM031_scaffold7206_4	32557	phage	0.97	virulent	0.99987084	Straboviridae	0.72323537	Colwellia psychrerythraea	0.83 Predict
NLM031_scaffold27707_3	11009	phage	0.994	temperate	0.9965135	Kyanoviridae	1	Colwellia psychrerythraea	0.92 Predict
NLM031_scaffold41310_1	10831	phage	0.999	virulent	0.99939865	unknown	0	Parabacteroides distasonis	0.75 Predict
NLM031_scaffold6532_6	11441	phage	0.562	temperate	0.99985975	unknown	0	Colwellia psychrerythraea	0.72 Predict
NLM031_scaffold1394_1	38352	phage	1	temperate	0.9998336	unknown	0	Colwellia psychrerythraea	1 CRISPR
NLM031_scaffold2847_6	16260	phage	0.999	virulent	0.9997695	unknown	0	Colwellia psychrerythraea	0.79 Predict
NLM031_scaffold49_2	105992	phage	0.912	temperate	0.99966246	Straboviridae	0.6773715	Staphylococcus saprophyticus	0.91 Predict

NLM031_scaffold25711_6	12701	phage	0.999	virulent	0.99985313	unknown	0	Geobacillus kaustophilus	0.93	Predict
NLM031_scaffold18627_4	17281	phage	0.998	temperate	0.9944285	no_family_avaliable(NC_047913)	0.989	Colwellia psychrerythraea	1	CRISPR
NLM031_scaffold15300_11	10814	phage	0.774	temperate	0.99719846	Straboviridae	0.51102096	Colwellia psychrerythraea	0.93	Predict
NLM031_scaffold45482_3_1	34071	phage	1	virulent	0.9998722	unknown	0	unknown	0	-
NLM031_scaffold259_8	15105	phage	0.959	temperate	0.98147815	Peduoviridae	0.46637547	Colwellia psychrerythraea	0.78	Predict
NLM031_scaffold36158_5	11127	phage	0.997	temperate	0.9998565	unknown	0	Colwellia psychrerythraea	1	CRISPR
NLM031_scaffold29823_1	14336	phage	0.996	temperate	0.9998593	unknown	0	Colwellia psychrerythraea	1	CRISPR
NLM031_scaffold56089_1	10547	phage	0.996	temperate	0.7586957	Straboviridae	0.7403209	unknown	0	-
NLM031_scaffold2964_6	11159	phage	0.999	temperate	0.8352771	unknown	0	Colwellia psychrerythraea	1	CRISPR
NLM031_scaffold45482_5	32779	phage	0.516	virulent	0.9629124	Straboviridae	0.6608802	Colwellia psychrerythraea	1	CRISPR
NLM031_scaffold107_1	11623	phage	0.999	temperate	0.9975632	Drexlerviridae	0.25270286	Colwellia psychrerythraea	0.89	Predict
NLM031_scaffold20700_11	10525	phage	0.936	temperate	0.99985975	unknown	0	unknown	0	-
NLM031_scaffold17121_1	11364	phage	0.995	temperate	0.99985975	unknown	0	unknown	0	-
NLM031_scaffold1394_2	18948	phage	0.998	virulent	0.8155634	unknown	0	Colwellia psychrerythraea	0.89	Predict
NLM031_scaffold25711_7	14290	phage	1	temperate	0.99961764	Drexlerviridae	0.25270286	Colwellia psychrerythraea	1	CRISPR
NLM031_scaffold23030_1	10559	phage	0.998	temperate	0.99985975	unknown	0	Parabacteroides distasonis	1	CRISPR
NLM031_scaffold18436_7	18514	phage	1	temperate	0.69460785	Casjensviridae	0.9825629	Colwellia psychrerythraea	0.78	Predict
NLM031_C734735_1	12749	phage	0.998	virulent	0.99987316	unknown	0	unknown	0	-
NLM031_scaffold44148_4	11170	phage	0.967	virulent	0.9988257	unknown	0	Colwellia psychrerythraea	0.8	Predict
NLM032_scaffold68145_4	12235	phage	0.999	temperate	0.9998584	unknown	0	Colwellia psychrerythraea	0.96	Predict
NLM032_scaffold23012_2	98915	phage	0.999	virulent	0.9998656	Schitoviridae	1	Leuconostoc pseudomesenteroides	0.72	Predict
NLM032_scaffold61468_2	18818	phage	0.998	temperate	0.9113476	Straboviridae	0.5006739	Colwellia psychrerythraea	0.84	Predict
NLM032_scaffold41399_1	65760	phage	0.998	temperate	0.9728056	Straboviridae	0.48703024	Colwellia psychrerythraea	1	CRISPR
NLM032_scaffold58144_2	10545	phage	0.991	temperate	0.99873686	Straboviridae	0.7813815	Colwellia psychrerythraea	0.99	Predict
NLM032_scaffold33461_2	13781	phage	0.999	temperate	0.99985975	unknown	0	Mycoplasma pulmonis	0.97	Predict
NLM032_scaffold23630_5	18063	phage	0.999	temperate	0.99983925	unknown	0	Colwellia psychrerythraea	1	Predict
NLM032_scaffold63260_2	15327	phage	0.997	temperate	0.99985975	Peduoviridae	1	Colwellia psychrerythraea	1	CRISPR
NLM032_scaffold40713_1	14510	phage	0.993	temperate	0.9997797	unknown	0	Mycoplasma pulmonis	0.92	Predict
NLM032_scaffold49689_1	10759	phage	0.562	virulent	0.93356663	unknown	0	unknown	0	-
NLM032_scaffold61468_1	12840	phage	0.999	temperate	0.9996549	unknown	0	Clostridium perfringens	0.97	Predict

NLM032_scaffold63260_1	23989	phage	0.945	temperate	0.99984926	Straboviridae	0.60721505	Colwellia psychrerythraea	1	CRISPR
NLM032_scaffold69860_1	50946	phage	0.978	virulent	0.9998274	Straboviridae	0.47330517	Clostridium botulinum	1	CRISPR
NLM032_scaffold10936_5	11612	phage	0.544	virulent	0.9998584	Mesyanzhinovviridae	0.7162643	Colwellia psychrerythraea	0.88	Predict
NLM032_scaffold9843_17	10936	phage	0.993	temperate	0.9998593	unknown	0	Colwellia psychrerythraea	0.75	Predict
NLM032_scaffold37169_1	12224	phage	0.996	temperate	0.9998593	Casjensviridae	1	Colwellia psychrerythraea	0.92	Predict
NLM032_scaffold219_1	17036	phage	0.999	temperate	0.9998588	unknown	0	Colwellia psychrerythraea	0.74	Predict
NLM032_scaffold68980_1	30586	phage	0.999	virulent	0.9550464	Herelleviridae	0.60281223	Colwellia psychrerythraea	0.82	Predict
NLM032_scaffold12144_3	14517	phage	0.999	temperate	0.99985975	unknown	0	Clostridioides difficile	1	CRISPR
NLM032_scaffold7394_8	14891	phage	0.997	temperate	0.9997606	Peduoviridae	0.52169895	Ralstonia solanacearum	0.93	Predict
NLM032_scaffold69642_1	25690	phage	1	temperate	0.9998574	unknown	0	Colwellia psychrerythraea	1	CRISPR
NLM032_scaffold69111_1	14173	phage	0.991	virulent	0.99987406	unknown	0	Colwellia psychrerythraea	0.92	Predict
NLM032_scaffold314_2	26429	phage	0.999	temperate	0.99984837	unknown	0	Colwellia psychrerythraea	1	CRISPR
NLM032_scaffold3938_2	15974	phage	0.999	virulent	0.9998656	no_family_avaliable(NC_023498)	0.977	Colwellia psychrerythraea	1	CRISPR
NLM032_scaffold25961_2	17530	phage	0.995	virulent	0.9998436	Salasmaviridae	0.40109783	Parabacteroides merdae	0.99	Predict
NLM032_scaffold60475_1	12439	phage	0.996	temperate	0.9998593	unknown	0	Colwellia psychrerythraea	0.87	Predict
NLM032_scaffold32034_2	10326	phage	0.559	temperate	0.99985975	unknown	0	unknown	0	-
NLM032_scaffold54796_9	16317	phage	0.998	temperate	0.99985975	unknown	0	Colwellia psychrerythraea	1	CRISPR
NLM032_scaffold9089_7	13547	phage	0.998	temperate	0.9998593	unknown	0	Faecalibacterium prausnitzii	1	CRISPR
NLM032_scaffold24196_2	32927	phage	0.999	virulent	0.999836	Casjensviridae	0.9217208	Bifidobacterium pseudocatenulatum	1	CRISPR
NLM032_scaffold69594_3	27330	phage	1	virulent	0.5565001	Casjensviridae	1	Colwellia psychrerythraea	1	CRISPR
NLM032_scaffold3932_23	14137	phage	0.611	virulent	0.9998722	Peduoviridae	0.4557199	Colwellia psychrerythraea	0.94	Predict
NLM032_scaffold23630_7	11501	phage	0.996	temperate	0.8428495	unknown	0	Colwellia psychrerythraea	1	CRISPR
NLM032_scaffold50920_4	10223	phage	0.999	temperate	0.9998522	unknown	0	Clostridium tetani	1	CRISPR
NLM032_scaffold69179_1	47800	phage	0.999	virulent	0.9998522	Peduoviridae	0.241425	Colwellia psychrerythraea	0.91	Predict
NLM032_scaffold68145_1	12803	phage	1	temperate	0.9998593	Peduoviridae	1	Colwellia psychrerythraea	1	CRISPR
NLM032_scaffold58268_2	11095	phage	0.999	virulent	0.9998579	Ackermannviridae	0.5563236	Vibrio cholerae	0.88	Predict
NLM032_scaffold212_2	13881	phage	0.998	virulent	0.9998699	no_family_avaliable(NC_067218)	0.97	Colwellia psychrerythraea	0.9	Predict
NLM032_scaffold69647_5	10378	phage	0.998	temperate	0.9996429	unknown	0	unknown	0	-
NLM032_scaffold69594_2	11094	phage	0.999	temperate	0.99985975	unknown	0	Ruminococcus bromii	1	CRISPR
NLM032_scaffold40892_3	14464	phage	0.984	virulent	0.9998699	unknown	0	Colwellia psychrerythraea	0.99	Predict

NLM032_scaffold6383_1	23620	phage	0.999	temperate	0.9998551	Peduoviridae	1	Blautia sp. OM06-15AC	1	CRISPR
NLM032_scaffold49971_2	11289	phage	0.974	temperate	0.98519784	unknown	0	Bacteroides fragilis	0.88	Predict
NLM032_scaffold35599_1	23984	phage	0.985	virulent	0.99982494	Straboviridae	0.75188446	Colwellia psychrerythraea	0.77	Predict
NLM032_scaffold11035_2	59207	phage	0.999	temperate	0.9997917	unknown	0	Flavobacterium columnare	1	CRISPR
DOM001_scaffold12537_8	17389	phage	0.999	virulent	0.9745316	Casjensviridae	0.9596824	Lactobacillus fermentum	0.81	Predict
DOM001_scaffold26527_9	56634	phage	0.999	virulent	0.9142287	unknown	0	Lactobacillus fermentum	0.97	Predict
DOM001_scaffold20125_2	12596	phage	0.989	virulent	0.99973136	Straboviridae	0.25507116	Mycoplasma pulmonis	0.75	Predict
DOM001_scaffold6481_6	10031	phage	0.989	temperate	0.99985975	unknown	0	Mycoplasma pulmonis	0.94	Predict
DOM001_scaffold57053_3	10440	phage	0.983	temperate	0.9998479	Peduoviridae	1	unknown	0	-
DOM001_scaffold21085_16	10828	phage	0.567	virulent	0.9998736	unknown	0	unknown	0	-
DOM001_scaffold43_2	16969	phage	0.999	virulent	0.9998699	Demerecviridae	0.4388233	Candidatus Pelagibacter ubique	0.86	Predict
DOM001_scaffold2066_1	11082	phage	0.858	temperate	0.99985975	unknown	0	Mycoplasma pulmonis	0.99	Predict
DOM001_scaffold53704_4	10968	phage	0.999	temperate	0.9998408	unknown	0	Lactobacillus fermentum	0.9	Predict
DOM001_scaffold33_5	18469	phage	0.999	temperate	0.9984557	Ackermannviridae	0.2703984	Lactobacillus fermentum	0.96	Predict
DOM001_scaffold4013_1	13144	phage	0.993	virulent	0.99986124	Casjensviridae	0.75807345	Roseobacter denitrificans	0.87	Predict
DOM001_scaffold9052_4	10798	phage	0.998	temperate	0.99985975	Herelleviridae	1	Roseobacter denitrificans	0.88	Predict
DOM001_scaffold26005_5	10665	phage	0.999	virulent	0.7268812	unknown	0	Mycoplasma pulmonis	0.72	Predict
DOM001_scaffold22877_4	19432	phage	0.521	virulent	0.9998688	Casjensviridae	0.6699858	Lactobacillus fermentum	0.83	Predict
DOM001_scaffold14492_1	11837	phage	0.996	virulent	0.68052727	Straboviridae	0.3229161	Parabacteroides merdae	1	CRISPR
DOM001_scaffold39139_2	43981	phage	0.999	temperate	0.87214583	Casjensviridae	0.6866919	Candidatus Pelagibacter ubique	0.97	Predict
DOM001_scaffold16478_1	10413	phage	0.857	virulent	0.9998388	unknown	0	Parabacteroides merdae	0.82	Predict
DOM001_scaffold55189_1	17732	phage	0.998	temperate	0.9998593	unknown	0	Lactobacillus fermentum	0.96	Predict
DOM001_scaffold49162_1	30403	phage	0.999	virulent	0.99982834	Straboviridae	0.52864015	Bacillus cereus	0.74	Predict
DOM001_scaffold5635_4	23747	phage	0.999	temperate	0.999854	Ackermannviridae	0.3338031	Lactobacillus fermentum	1	CRISPR
DOM001_scaffold58195_2	11234	phage	0.961	temperate	0.979654	unknown	0	Bacteroides fragilis	0.71	Predict
DOM001_scaffold7274_21	10475	phage	0.994	virulent	0.9998736	unknown	0	Mycoplasma pulmonis	0.87	Predict
DOM001_scaffold12537_10	10073	phage	0.998	virulent	0.96556866	unknown	0	unknown	0	-
DOM001_scaffold28423_1	12682	phage	0.962	virulent	0.99979234	unknown	0	Candidatus Pelagibacter ubique	1	CRISPR
DOM001_scaffold26343_7	16704	phage	0.994	temperate	0.9972075	Salasmaviridae	0.22382887	Parabacteroides distasonis	0.88	Predict
DOM001_scaffold30807_3	10819	phage	0.999	temperate	0.99985313	unknown	0	Francisella tularensis	1	CRISPR

DOM001_scaffold5000_5	16543	phage	0.998	virulent	0.99986744	Demerecviridae	0.26536036	Candidatus Pelagibacter ubique	0.72	Predict
DOM001_scaffold17386_5	16093	phage	0.992	virulent	0.9996475	Ackermannviridae	1	Lactobacillus fermentum	1	Predict
DOM001_scaffold37685_1	10419	phage	0.965	virulent	0.9761889	unknown	0	Candidatus Pelagibacter ubique	0.95	Predict
DOM001_scaffold17768_4	26312	phage	0.999	virulent	0.9730364	Chaseviridae	0.20182505	Xanthomonas vesicatoria	0.86	Predict
DOM001_scaffold2499_6	13626	phage	0.99	virulent	0.97140306	unknown	0	unknown	0	-
DOM001_scaffold273_1	17754	phage	0.875	temperate	0.99982214	unknown	0	Roseobacter denitrificans	0.74	Predict
DOM001_scaffold58831_1	21525	phage	0.999	temperate	0.99960196	unknown	0	Mycoplasma pulmonis	1	CRISPR
DOM001_scaffold38397_1	24506	phage	0.999	virulent	0.97821456	Herelleviridae	0.2461915	Mycoplasma pulmonis	1	CRISPR
DOM001_scaffold19397_1	16869	phage	0.999	virulent	0.9998656	Salasmaviridae	0.4168327	Mycoplasma pulmonis	0.72	Predict
DOM003_scaffold2919_6	17357	phage	0.958	temperate	0.9996587	unknown	0	[Eubacterium] eligens	1	CRISPR
DOM003_scaffold22571_7	12535	phage	0.998	temperate	0.99985975	unknown	0	Candidatus Pelagibacter ubique	0.93	Predict
DOM003_scaffold5840_1	14142	phage	0.985	temperate	0.9994154	unknown	0	Mycoplasma pulmonis	0.98	Predict
DOM003_scaffold575_3	10244	phage	0.987	virulent	0.99987406	unknown	0	Roseobacter denitrificans	0.87	Predict
DOM003_scaffold6007_2	14666	phage	0.998	temperate	0.5812096	Casjensviridae	1	Mycoplasma pulmonis	1	CRISPR
DOM003_scaffold24274_1	28312	phage	0.999	temperate	0.9984348	Drexlerviridae	1	Lactobacillus fermentum	1	CRISPR
DOM003_scaffold24287_2	10905	phage	0.998	temperate	0.9998593	no_family_avaliable(NC_049953)	0.977	Mycoplasma pulmonis	0.99	Predict
DOM003_scaffold24307_1	12688	phage	0.999	temperate	0.999854	unknown	0	Mycoplasma pulmonis	1	CRISPR
DOM003_scaffold6518_3	37513	phage	1	virulent	0.99986607	Autographiviridae	1	Mycoplasma pulmonis	1	CRISPR
DOM003_scaffold2919_2	18280	phage	0.906	virulent	0.99716425	Straboviridae	0.26489046	Prochlorococcus marinus	1	CRISPR
DOM003_scaffold11839_3	11471	phage	0.998	virulent	0.9998699	unknown	0	Bifidobacterium breve	1	CRISPR
DOM003_scaffold19301_1	14463	phage	0.997	temperate	0.99986035	Straboviridae	0.66039294	Lactobacillus johnsonii	0.71	Predict
DOM003_scaffold3648_4	11924	phage	0.519	temperate	0.99986035	unknown	0	Candidatus Hamiltonella defensa	1	CRISPR
DOM003_scaffold19736_6	24048	phage	0.568	virulent	0.9984596	Peduoviridae	1	Flavobacterium columnare	1	CRISPR
DOM003_scaffold2919_4	33014	phage	0.999	temperate	0.9998517	unknown	0	Candidatus Pelagibacter ubique	1	CRISPR
DOM003_scaffold18492_3	15212	phage	0.987	virulent	0.9998636	unknown	0	Mycoplasma pulmonis	0.81	Predict
DOM003_scaffold20665_1	14157	phage	0.5	virulent	0.9998357	Ackermannviridae	0.3899253	Lactobacillus fermentum	1	Predict
DOM003_scaffold24151_1	10155	phage	0.999	temperate	0.89395547	Ackermannviridae	0.2703984	Lactobacillus fermentum	0.89	Predict
DOM003_scaffold23067_1	31601	phage	0.999	temperate	0.9998226	Straboviridae	0.5517625	Clostridioides difficile	1	CRISPR
DOM003_scaffold11839_7	10619	phage	0.998	virulent	0.9992729	unknown	0	Rhodovulum sp. P5	0.89	Predict
DOM003_scaffold24307_2	22308	phage	1	temperate	0.9998545	Herelleviridae	1	Mycoplasma pulmonis	0.73	Predict

DOM003_scaffold11039_1	10882	phage	0.931	virulent	0.9998608	unknown	0	unknown	0	-
DOM003_scaffold17266_6	10097	phage	0.999	virulent	0.99107265	Kyanoviridae	1	Lactobacillus fermentum	0.94	Predict
DOM003_scaffold1995_7	10338	phage	0.987	virulent	0.8689416	unknown	0	Mycoplasma pulmonis	0.88	Predict
DOM003_scaffold24298_1	16419	phage	0.999	temperate	0.99985975	Peduoviridae	1	Lactobacillus fermentum	1	CRISPR
DOM003_scaffold22453_2	13554	phage	0.737	virulent	0.9997247	Peduoviridae	0.28692025	Mycoplasma pulmonis	0.76	Predict
DOM003_scaffold14108_2	10348	phage	0.986	temperate	0.9998536	unknown	0	unknown	0	-
DOM003_scaffold2919_11	23881	phage	0.999	temperate	0.99986035	unknown	0	Mycoplasma pulmonis	0.86	Predict
DOM003_scaffold11839_4	14112	phage	0.999	temperate	0.9998574	unknown	0	Rhizobium leguminosarum	1	CRISPR
DOM005_scaffold374_9	14196	phage	0.986	temperate	0.9998551	Peduoviridae	1	Lactobacillus fermentum	1	CRISPR
DOM005_scaffold920_41	16642	phage	0.999	temperate	0.9997626	unknown	0	Clostridium perfringens	1	CRISPR
DOM005_scaffold9501_4	12730	phage	0.985	virulent	0.9998722	unknown	0	Mycoplasma pulmonis	1	CRISPR
DOM005_scaffold13159_4	18874	phage	0.999	temperate	0.99708104	Autographiviridae	1	Mycoplasma pulmonis	1	CRISPR
DOM005_scaffold6037_1	10760	phage	0.948	temperate	0.99981695	Casjensviridae	1	Lactobacillus fermentum	0.75	Predict
DOM005_scaffold2259_3	43717	phage	0.999	temperate	0.99984926	Peduoviridae	0.33690333	Lactobacillus fermentum	1	CRISPR
DOM005_scaffold13355_2	15888	phage	0.996	temperate	0.9969182	Salasmaviridae	0.22382887	Parabacteroides distasonis	0.94	Predict
DOM005_scaffold10392_5	10981	phage	0.998	virulent	0.9998727	unknown	0	Bacteroides fragilis	0.91	Predict
DOM005_scaffold10888_1	15177	phage	0.996	virulent	0.908313	unknown	0	Mycoplasma pulmonis	0.92	Predict
DOM005_C258523_1	12037	phage	0.875	temperate	0.9998579	unknown	0	Mycoplasma pulmonis	0.95	Predict
DOM005_scaffold1092_15	14231	phage	0.998	temperate	0.98557746	unknown	0	unknown	0	-
DOM005_scaffold10888_5	11707	phage	0.999	temperate	0.99985975	unknown	0	Mycoplasma pulmonis	0.98	Predict
DOM005_scaffold3583_3	11449	phage	0.998	virulent	0.99471563	unknown	0	unknown	0	-
DOM005_scaffold10107_7	10379	phage	1	virulent	0.99986035	unknown	0	unknown	0	-
DOM005_scaffold12816_1	18769	phage	0.624	virulent	0.99987173	Ackermannviridae	0.27722943	Bacteroides fragilis	0.92	Predict
DOM005_scaffold13159_2	35200	phage	0.993	temperate	0.99601823	Casjensviridae	1	Lactobacillus fermentum	1	CRISPR
DOM005_scaffold2640_50	31637	phage	0.998	virulent	0.9983449	Autographiviridae	1	Lactobacillus fermentum	1	CRISPR
DOM005_scaffold13337_5	11749	phage	0.946	virulent	0.9998727	unknown	0	unknown	0	-
DOM005_scaffold1509_4	10397	phage	0.999	temperate	0.9998593	no_family_avaliable(NC_016158)	0.96	Mycoplasma pulmonis	1	CRISPR
DOM005_scaffold9841_13	13459	phage	0.999	temperate	0.99985975	no_family_avaliable(NC_021190)	1	Lactobacillus fermentum	1	CRISPR
DOM005_scaffold168_65	11821	phage	0.992	virulent	0.99987406	unknown	0	Mycoplasma pulmonis	0.7	Predict
DOM005_scaffold5851_3	14253	phage	0.999	virulent	0.9998693	unknown	0	Candidatus Pelagibacter ubique	0.86	Predict

DOM005_scaffold8757_1	10577	phage	1	temperate	0.999816	unknown	0	unknown	0	-
DOM005_scaffold374_8	17100	phage	0.999	temperate	0.99985975	Peduoviridae	1	Lactobacillus fermentum	1	CRISPR
DOM008_scaffold15923_2	14077	phage	0.995	temperate	0.9961957	unknown	0	Lactobacillus fermentum	1	CRISPR
DOM008_scaffold680_3	20762	phage	0.986	virulent	0.99987173	Ackermannviridae	0.40512124	Mycoplasma pulmonis	0.92	Predict
DOM008_scaffold543_11	10733	phage	0.998	temperate	0.99946034	unknown	0	Streptomyces lividans	0.92	Predict
DOM008_scaffold16924_3	10562	phage	0.993	temperate	0.9998574	unknown	0	unknown	0	-
DOM008_scaffold4243_1	11925	phage	0.999	virulent	0.99987465	unknown	0	Candidatus Pelagibacter ubique	0.94	Predict
DOM008_scaffold16792_1	13302	phage	0.995	virulent	0.9998302	Salasmaviridae	0.33272234	Mycoplasma pulmonis	0.79	Predict
DOM008_scaffold5589_2	11004	phage	1	temperate	0.99984604	unknown	0	Bacteroides fragilis	1	CRISPR
DOM008_scaffold14700_5	11985	phage	0.975	virulent	0.9997024	Salasmaviridae	0.28656432	Lactobacillus fermentum	0.74	Predict
DOM008_scaffold3045_22	11399	phage	0.934	virulent	0.99984974	unknown	0	Lactobacillus fermentum	0.95	Predict
DOM008_scaffold57_7	18555	phage	0.999	virulent	0.9995636	unknown	0	Bacteroides fragilis	0.83	Predict
DOM008_scaffold18954_1	11478	phage	0.988	virulent	0.9998588	unknown	0	Bacteroides fragilis	0.95	Predict
DOM008_scaffold169_2	22202	phage	0.999	virulent	0.9998636	unknown	0	Mycoplasma pulmonis	0.82	Predict
DOM008_scaffold9829_1	17108	phage	0.995	virulent	0.99987173	unknown	0	Bacteroides fragilis	0.88	Predict
DOM008_scaffold1395_1	25604	phage	0.989	virulent	0.9998684	Straboviridae	0.2686186	Candidatus Pelagibacter ubique	0.99	Predict
DOM008_scaffold8348_2	11045	phage	0.653	temperate	0.9998536	unknown	0	Mycoplasma pulmonis	0.76	Predict
DOM008_scaffold6987_15	20988	phage	0.812	virulent	0.99987125	Peduoviridae	1	Candidatus Hamiltonella defensa	0.81	Predict
DOM008_scaffold14339_1	19053	phage	1	temperate	0.99984974	unknown	0	Mycoplasma pulmonis	1	CRISPR
DOM008_scaffold11628_1	10872	phage	0.998	virulent	0.99987125	Herelleviridae	1	Candidatus Pelagibacter ubique	0.98	Predict
DOM008_scaffold15475_9	13781	phage	0.999	virulent	0.99986607	Drexlerviridae	1	Mycoplasma pulmonis	0.83	Predict
DOM008_scaffold57_5	14585	phage	0.999	temperate	0.97040224	unknown	0	Bacteroides fragilis	0.93	Predict
DOM008_scaffold15104_4	11239	phage	0.996	virulent	0.99985975	unknown	0	Parabacteroides distasonis	0.88	Predict
DOM008_scaffold44_39	14871	phage	1	temperate	0.9998593	Peduoviridae	1	Lactobacillus fermentum	1	CRISPR
DOM008_scaffold18440_1	11140	phage	0.998	temperate	0.9997769	unknown	0	Bacteroides fragilis	0.89	Predict
DOM008_scaffold18246_2	13580	phage	0.998	virulent	0.9998693	unknown	0	Bacteroides fragilis	0.98	Predict
DOM008_scaffold8252_2	22279	phage	0.986	virulent	0.9998574	Drexlerviridae	0.39265049	Mycoplasma pulmonis	0.99	Predict
DOM008_scaffold15475_4	18560	phage	0.999	virulent	0.9998627	Drexlerviridae	1	Mycoplasma pulmonis	1	CRISPR
DOM008_scaffold17898_2	17227	phage	0.995	temperate	0.99984217	Drexlerviridae	0.39265049	Mycoplasma pulmonis	0.87	Predict
DOM008_scaffold19226_1	10685	phage	0.989	virulent	0.99980974	unknown	0	Mycoplasma pulmonis	0.81	Predict

DOM008_scaffold14655_7	11829	phage	0.996	virulent	0.9998479	unknown	0	Roseobacter denitrificans	0.83	Predict
DOM008_scaffold11407_6	15896	phage	0.919	temperate	0.9413989	Ackermannviridae	0.25677204	Bacteroides fragilis	0.73	Predict
DOM008_scaffold17859_3	15997	phage	0.915	temperate	0.99985975	Peduoviridae	0.39129964	Lactobacillus fermentum	1	CRISPR
DOM010_scaffold2671_4	10974	phage	0.748	virulent	0.95007366	unknown	0	unknown	0	-
DOM010_scaffold8807_2	10612	phage	0.995	virulent	0.9998117	Salasmaviridae	0.33272234	Mycoplasma pulmonis	0.84	Predict
DOM010_C556729_1	12030	phage	0.983	virulent	0.9974672	unknown	0	Mycoplasma pulmonis	0.76	Predict
DOM010_scaffold23838_1	15928	phage	0.999	virulent	0.9534296	Casjensviridae	1	Enterococcus faecalis	1	CRISPR
DOM010_scaffold44229_1	10403	phage	0.921	virulent	0.99987406	unknown	0	unknown	0	-
DOM010_scaffold20353_2	11561	phage	0.992	temperate	0.999844	Straboviridae	0.35083705	unknown	0	-
DOM010_scaffold7398_16	15367	phage	0.998	virulent	0.9878896	Straboviridae	0.2856439	Parabacteroides merdae	1	CRISPR
DOM010_scaffold11164_7	10695	phage	0.999	temperate	0.99838793	unknown	0	Mycoplasma pulmonis	0.85	Predict
DOM010_scaffold44237_2	11009	phage	0.997	virulent	0.9997418	unknown	0	unknown	0	-
DOM010_C557895_1	24616	phage	0.999	virulent	0.99974227	Straboviridae	0.47345498	Clostridium perfringens	0.81	Predict
DOM010_scaffold29114_1	12567	phage	1	virulent	0.99987125	unknown	0	Parabacteroides distasonis	0.98	Predict
DOM010_scaffold44223_5	26742	phage	0.996	temperate	0.6360983	unknown	0	unknown	0	-
DOM010_C557123_1	14229	phage	0.999	virulent	0.99962765	unknown	0	Mycoplasma pulmonis	1	CRISPR
DOM010_scaffold10693_2	39795	phage	0.999	temperate	0.9998588	unknown	0	Candidatus Pelagibacter ubique	1	CRISPR
DOM010_scaffold42781_1	17656	phage	1	virulent	0.9998522	unknown	0	Mycoplasma pulmonis	1	CRISPR
DOM010_scaffold3905_57	11856	phage	1	temperate	0.9998197	unknown	0	Clostridium tetani	1	CRISPR
DOM010_scaffold6942_2	15898	phage	0.919	temperate	0.9413989	Ackermannviridae	0.25677204	Bacteroides fragilis	0.86	Predict
DOM010_scaffold42298_4	14428	phage	0.998	temperate	0.9996458	Casjensviridae	0.30599892	Roseobacter denitrificans	0.96	Predict
DOM010_C557921_1	25416	phage	0.999	temperate	0.9998188	unknown	0	Clostridium perfringens	0.89	Predict
DOM010_scaffold41237_4	11473	phage	0.859	virulent	0.99987316	Straboviridae	0.4156158	Streptococcus mutans	1	CRISPR
DOM010_scaffold36003_1	17448	phage	0.998	virulent	0.99987125	Ackermannviridae	0.43386555	Bacteroides fragilis	0.93	Predict
DOM010_scaffold7_1	34254	phage	0.999	temperate	0.9998465	unknown	0	Bacillus alcalophilus	0.98	Predict
DOM010_scaffold19710_3	13728	phage	0.994	virulent	0.9992909	Casjensviridae	0.8125134	Rhizobium leguminosarum	0.91	Predict
DOM010_scaffold72_1	23990	phage	0.999	virulent	0.9998308	no_family_avaliable(NC_055883)	0.991	Candidatus Pelagibacter ubique	0.83	Predict
DOM010_scaffold25074_1	18486	phage	0.996	temperate	0.9998579	unknown	0	Candidatus Pelagibacter ubique	0.79	Predict
DOM010_scaffold42659_2	11267	phage	0.961	temperate	0.979654	unknown	0	Bacteroides fragilis	0.85	Predict
DOM010_scaffold38114_4	13237	phage	0.998	temperate	0.99159664	unknown	0	Lactobacillus fermentum	1	CRISPR

DOM010_scaffold1445_1	11823	phage	0.996	temperate	0.9998588	unknown	0	unknown	0	-
DOM010_scaffold44548_1	12112	phage	0.941	temperate	0.5364791	Ackermannviridae	0.3611045	Mycoplasma pulmonis	0.96	Predict
DOM010_scaffold44359_1	22449	phage	0.999	virulent	0.9986124	Ackermannviridae	0.3338031	Lactobacillus fermentum	1	CRISPR
DOM010_scaffold5308_8	10044	phage	0.999	virulent	0.9997988	unknown	0	unknown	0	-
DOM010_scaffold36407_1	14182	phage	0.995	temperate	0.99976444	Salasmaviridae	0.28656432	Mycoplasma pulmonis	0.82	Predict
DOM010_scaffold43444_3	27825	phage	0.996	temperate	0.9998584	unknown	0	Staphylococcus aureus	1	CRISPR
DOM010_scaffold12345_3	24769	phage	0.976	virulent	0.9998727	Ackermannviridae	0.39363876	Lactobacillus fermentum	0.74	Predict
DOM010_scaffold27469_1	10069	phage	0.987	temperate	0.99986035	unknown	0	Mycoplasma pulmonis	0.96	Predict
DOM010_scaffold17754_1	16593	phage	0.995	temperate	0.99985605	Straboviridae	0.41539797	Lactobacillus fermentum	1	CRISPR
DOM010_C557291_1	15354	phage	0.999	virulent	0.9998297	Straboviridae	0.42015335	Mycoplasma pulmonis	0.82	Predict
DOM010_scaffold13897_1	30453	phage	0.987	virulent	0.99960005	Casjensviridae	0.8726506	Lactobacillus fermentum	0.82	Predict
DOM010_scaffold43785_1	16086	phage	0.99	virulent	0.99987173	Ackermannviridae	0.40105554	Bacteroides fragilis	1	Predict
DOM010_scaffold38766_6	12957	phage	0.995	virulent	0.99978536	Straboviridae	0.5505315	Mycoplasma pulmonis	0.8	Predict
DOM010_scaffold34515_1	33747	phage	0.998	temperate	0.9998388	unknown	0	Clostridioides difficile	1	CRISPR
DOM010_scaffold44098_1	10035	phage	0.932	temperate	0.99985975	unknown	0	Candidatus Pelagibacter ubique	0.94	Predict
DOM010_scaffold43222_1	13752	phage	0.972	virulent	0.9994699	Ackermannviridae	1	Mycoplasma pulmonis	0.79	Predict
DOM010_scaffold44558_1	10573	phage	0.916	temperate	0.9377786	Casjensviridae	0.2509271	Staphylococcus aureus	1	CRISPR
DOM010_scaffold21662_2	16823	phage	0.995	virulent	0.99985695	unknown	0	Ruegeria pomeroyi	0.92	Predict
DOM010_scaffold7072_9	10271	phage	0.999	temperate	0.99986035	unknown	0	Mycoplasma pulmonis	0.93	Predict
DOM010_scaffold3568_1	14280	phage	0.999	virulent	0.99658924	unknown	0	Candidatus Pelagibacter ubique	0.75	Predict
DOM010_C557197_1	14657	phage	0.999	temperate	0.99986035	unknown	0	unknown	0	-
DOM010_scaffold40874_2	13847	phage	0.998	virulent	0.99987036	unknown	0	Candidatus Pelagibacter ubique	0.87	Predict
DOM010_scaffold40514_1	37804	phage	0.999	virulent	0.99986607	unknown	0	Candidatus Pelagibacter ubique	0.82	Predict
DOM010_scaffold39715_1	17899	phage	0.999	virulent	0.99987084	unknown	0	Lactobacillus fermentum	1	CRISPR
DOM010_scaffold120_2	62933	phage	0.999	virulent	0.9998513	no_family_avaliable(NC_055883)	0.991	Cellulophaga baltica	0.92	Predict
DOM010_scaffold44263_3	14323	phage	0.999	temperate	0.98068535	unknown	0	Cellulophaga baltica	1	CRISPR
DOM012_scaffold27104_2	12740	phage	0.791	virulent	0.99987316	unknown	0	unknown	0	-
DOM012_scaffold631_3	23309	phage	0.999	virulent	0.99986315	Drexlerviridae	0.32767826	Roseobacter denitrificans	1	CRISPR
DOM012_scaffold33660_1	14308	phage	0.998	temperate	0.9272924	unknown	0	Achromobacter xylosoxidans	0.83	Predict
DOM012_scaffold4248_4	34514	phage	1	virulent	0.9998593	unknown	0	Parabacteroides merdae	1	CRISPR

DOM012_scaffold33513_5	13735	phage	0.993	virulent	0.9868683	Peduoviridae	1	Mycoplasma pulmonis	0.9 Predict
DOM012_scaffold8289_1	13212	phage	0.999	virulent	0.99987173	Salasmaviridae	1	Candidatus Pelagibacter ubique	0.8 Predict
DOM012_scaffold25292_2	16611	phage	1	temperate	0.9982647	unknown	0	Mycoplasma pulmonis	1 CRISPR
DOM012_scaffold9067_4	14065	phage	0.999	temperate	0.899105	Peduoviridae	0.2989266	Lactobacillus fermentum	1 CRISPR
DOM012_scaffold38243_4	12693	phage	0.998	temperate	0.9864365	unknown	0	unknown	0 -
DOM012_scaffold9872_1	17966	phage	0.996	virulent	0.9545897	unknown	0	unknown	0 -
DOM012_scaffold3937_49	25225	phage	0.59	virulent	0.9998693	Straboviridae	0.4156158	unknown	0 -
DOM012_scaffold25292_3	10548	phage	0.999	temperate	0.9998445	unknown	0	Mycoplasma pulmonis	0.95 Predict
DOM012_scaffold17700_1	12679	phage	0.969	temperate	0.999854	unknown	0	unknown	0 -
DOM012_scaffold6197_5_2	19663	phage	0.999	virulent	0.9998408	Zierdtviridae	1	unknown	0 -
DOM012_scaffold5103_5	10204	phage	0.723	virulent	0.99979275	unknown	0	Mycoplasma pulmonis	0.77 Predict
DOM012_scaffold2137_37	14181	phage	0.961	virulent	0.95147115	Zierdtviridae	1	unknown	0 -
DOM012_scaffold4248_15	15523	phage	0.996	virulent	0.9998727	unknown	0	unknown	0 -
DOM012_scaffold7054_4	10766	phage	0.998	virulent	0.9998665	unknown	0	unknown	0 -
DOM012_scaffold631_1	10222	phage	0.999	virulent	0.9998627	no_family_avaliable(NC_021534)	0.952	Klebsiella pneumoniae	1 CRISPR
DOM012_scaffold42523_1	13118	phage	0.999	virulent	0.99982214	Drexlerviridae	0.39265049	Mycoplasma pulmonis	0.83 Predict
DOM012_scaffold6291_4	13567	phage	0.746	temperate	0.6325878	Drexlerviridae	0.14557987	unknown	0 -
DOM012_scaffold4007_9	15449	phage	0.992	temperate	0.99985313	unknown	0	unknown	0 -
DOM012_scaffold4108_5	15562	phage	0.999	temperate	0.9971176	unknown	0	Paenibacillus larvae	0.81 Predict
DOM012_scaffold33307_1	25810	phage	1	virulent	0.7742801	unknown	0	Staphylococcus saprophyticus	0.75 Predict
DOM012_scaffold4248_8	11896	phage	0.989	virulent	0.99986404	unknown	0	Mycoplasma pulmonis	0.91 Predict
DOM012_scaffold31_2	10481	phage	0.942	temperate	0.77475363	Drexlerviridae	0.13268808	Mycoplasma pulmonis	0.78 Predict
DOM012_scaffold46434_1	15044	phage	1	virulent	0.99987084	Salasmaviridae	1	Mycoplasma pulmonis	1 CRISPR
DOM012_scaffold4248_3	15615	phage	0.999	virulent	0.99987465	unknown	0	Mycoplasma pulmonis	0.98 Predict
DOM013_scaffold29644_2	14219	phage	0.977	virulent	0.99986744	Straboviridae	0.6004747	Candidatus Pelagibacter ubique	1 Predict
DOM013_scaffold65510_2	61264	phage	0.999	virulent	0.99986744	Straboviridae	0.30413526	Flavobacterium columnare	0.83 Predict
DOM013_scaffold66179_1	14852	phage	1	virulent	0.9998693	Salasmaviridae	1	Mycoplasma pulmonis	1 CRISPR
DOM013_scaffold45617_1	14369	phage	0.998	temperate	0.99986035	Casjensviridae	1	Candidatus Pelagibacter ubique	0.81 Predict
DOM013_scaffold29321_1	13590	phage	0.913	temperate	0.99980927	Straboviridae	0.46327022	unknown	0 -
DOM013_scaffold24958_13	13633	phage	0.971	virulent	0.99987036	unknown	0	unknown	0 -

DOM013_scaffold4994_13	13419	phage	0.998	virulent	0.9998722	Ackermannviridae	0.2783795	unknown	0	-
DOM013_scaffold46653_1	11167	phage	0.999	temperate	0.9992764	unknown	0	Bacillus alcalophilus	1	CRISPR
DOM013_scaffold11541_3	22544	phage	0.985	virulent	0.9995705	Ackermannviridae	0.32162276	Candidatus Pelagibacter ubique	0.88	Predict
DOM013_scaffold38557_2	19071	phage	0.986	virulent	0.99987316	Herelleviridae	1	Lactobacillus jensenii	0.89	Predict
DOM013_scaffold13214_2	11405	phage	0.999	virulent	0.99987125	unknown	0	Candidatus Pelagibacter ubique	0.86	Predict
DOM013_scaffold63345_1	20591	phage	0.997	temperate	0.9997378	unknown	0	unknown	0	-
DOM013_scaffold27634_2	18498	phage	0.998	virulent	0.99980664	Ackermannviridae	0.43386555	Lactobacillus fermentum	0.88	Predict
DOM013_scaffold65920_1	65829	phage	0.999	virulent	0.9998688	Schitoviridae	1	Lactobacillus gasseri	0.99	Predict
DOM013_scaffold65670_1	23535	phage	0.999	temperate	0.9857822	unknown	0	Clostridium perfringens	0.88	Predict
DOM013_scaffold39903_2	11833	phage	0.999	temperate	0.99985975	unknown	0	unknown	0	-
DOM013_scaffold46391_3	12314	phage	0.999	temperate	0.9998584	unknown	0	unknown	0	-
DOM013_scaffold50516_3	11908	phage	0.987	temperate	0.9585484	unknown	0	Candidatus Hamiltonella defensa	0.87	Predict
DOM013_scaffold66005_2	67891	phage	0.999	virulent	0.9998651	unknown	0	Cellulophaga baltica	0.88	Predict
DOM013_scaffold10380_3	10486	phage	0.942	virulent	0.9998656	unknown	0	Roseobacter denitrificans	0.89	Predict
DOM013_scaffold34766_1	31563	phage	0.705	virulent	0.99987084	Casjensviridae	0.6699858	Lactobacillus fermentum	0.71	Predict
DOM013_scaffold65510_1	30983	phage	0.999	virulent	0.9998684	no_family_avaliable(NC_024711)	0.971	Flavobacterium columnare	0.96	Predict
DOM013_scaffold66036_1	12339	phage	0.98	temperate	0.9998593	unknown	0	Candidatus Pelagibacter ubique	0.72	Predict
DOM013_scaffold2110_2	18825	phage	0.988	temperate	0.999784	Casjensviridae	1	Mycoplasma pulmonis	0.97	Predict
DOM013_scaffold9813_6	12340	phage	0.999	temperate	0.95559967	unknown	0	Mycoplasma pulmonis	1	CRISPR
DOM013_scaffold42751_1	16652	phage	0.999	temperate	0.99985266	unknown	0	Roseobacter denitrificans	1	CRISPR
DOM013_scaffold33337_2	12525	phage	0.998	virulent	0.99974734	Salasmaviridae	1	Mycoplasma pulmonis	0.8	Predict
DOM013_scaffold3571_32	11610	phage	0.764	virulent	0.99987316	Kyanoviridae	1	Lactobacillus fermentum	0.75	Predict
DOM013_scaffold81_8	13749	phage	0.999	virulent	0.9998699	Straboviridae	0.42805165	Cellulophaga baltica	0.7	Predict
DOM013_scaffold66005_1	25622	phage	0.999	virulent	0.9998699	Ackermannviridae	1	Cellulophaga baltica	0.94	Predict
DOM013_scaffold54135_4	16247	phage	0.999	virulent	0.9998693	Straboviridae	0.42805165	Cellulophaga baltica	0.77	Predict
DOM013_scaffold66255_2	22521	phage	0.999	virulent	0.99987036	no_family_avaliable(NC_055876)	0.979	Flavobacterium columnare	1	CRISPR
DOM013_C812420_1	28352	phage	0.999	virulent	0.99986035	Herelleviridae	1	Lactobacillus delbrueckii	1	CRISPR
DOM013_scaffold64045_2	28632	phage	0.999	temperate	0.9998417	Ackermannviridae	0.3338031	Lactobacillus fermentum	1	CRISPR
DOM013_scaffold63330_1	12929	phage	0.998	virulent	0.9998736	unknown	0	Lactobacillus fermentum	0.71	Predict
DOM013_scaffold13_2	13428	phage	0.993	virulent	0.9470206	unknown	0	Mannheimia haemolytica	0.86	Predict

DOM013_scaffold8272_3	57250	phage	0.998	temperate	0.9821213	Peduoviridae	1	Candidatus Pelagibacter ubique	0.75	Predict
DOM013_scaffold17461_1	14880	phage	0.999	virulent	0.9998536	Peduoviridae	0.34567592	Mycoplasma pulmonis	0.74	Predict
DOM013_scaffold60583_1	14536	phage	0.714	temperate	0.9995733	unknown	0	unknown	0	-
DOM013_scaffold23134_20	10973	phage	0.823	temperate	0.8561097	Ackermannviridae	0.2797422	Candidatus Pelagibacter ubique	0.95	Predict
DOM013_scaffold3755_4	62765	phage	0.997	virulent	0.9996818	Herelleviridae	1	Cellulophaga baltica	1	CRISPR
DOM013_scaffold62840_1	10653	phage	0.999	temperate	0.9998584	unknown	0	Mycoplasma pulmonis	1	CRISPR
DOM013_scaffold81_7	12575	phage	0.995	virulent	0.99987173	Straboviridae	0.47816586	Mycoplasma pulmonis	0.78	Predict
DOM013_scaffold66110_1	30835	phage	0.999	virulent	0.999798	Straboviridae	0.42015335	Candidatus Pelagibacter ubique	0.71	Predict
DOM013_scaffold3571_2	13689	phage	1	temperate	0.99985975	unknown	0	Candidatus Pelagibacter ubique	1	CRISPR
DOM013_scaffold22249_3	10160	phage	0.999	virulent	0.9998536	Kyanoviridae	1	Mycoplasma pulmonis	0.91	Predict
DOM013_scaffold54135_10	11540	phage	1	temperate	0.9998584	unknown	0	Mycoplasma pulmonis	0.77	Predict
DOM013_scaffold16907_3	17473	phage	0.954	virulent	0.88889205	Peduoviridae	0.4545788	Mycoplasma pulmonis	0.92	Predict
DOM013_scaffold9359_8	16596	phage	0.996	temperate	0.96828395	Peduoviridae	0.2989266	Bdellovibrio bacteriovorus	0.98	Predict
DOM014_scaffold7185_19	11630	phage	0.99	virulent	0.9994256	Casjensviridae	0.8125134	Lactobacillus fermentum	0.7	Predict
DOM014_scaffold43299_1	11116	phage	0.999	temperate	0.9998584	unknown	0	unknown	0	-
DOM014_scaffold15062_27	11428	phage	0.969	temperate	0.99985975	unknown	0	Mycoplasma pulmonis	0.84	Predict
DOM014_scaffold17723_2	10635	phage	0.996	virulent	0.99956405	Straboviridae	0.40261546	Bacteroides sp. 3_1_40A	1	CRISPR
DOM014_scaffold3561_14	12898	phage	0.983	virulent	0.9998551	Salasmaviridae	0.49816787	Mycoplasma pulmonis	0.74	Predict
DOM014_scaffold9701_3	12694	phage	1	temperate	0.9998556	Casjensviridae	0.19781996	Bacillus cereus	0.96	Predict
DOM014_scaffold804_14	17349	phage	0.998	temperate	0.99984884	unknown	0	Flavobacterium columnare	0.81	Predict
DOM014_scaffold14238_1	38499	phage	0.999	virulent	0.9998693	no_family_avaliable(NC_062779)	0.973	Flavobacterium columnare	0.7	Predict
DOM014_scaffold283_1	12696	phage	0.999	temperate	0.99985695	Salasmaviridae	0.41580635	Clostridioides difficile	1	CRISPR
DOM014_scaffold1467_4	17964	phage	0.999	virulent	0.99987084	no_family_avaliable(NC_062780)	0.968	unknown	0	-
DOM014_scaffold5325_3	15655	phage	0.999	virulent	0.8092583	Salasmaviridae	0.4168327	Mycoplasma pulmonis	0.87	Predict
DOM014_scaffold1467_2	12657	phage	0.999	virulent	0.99987173	no_family_avaliable(NC_062780)	0.981	Flavobacterium columnare	0.79	Predict
DOM014_scaffold46104_1	10145	phage	0.939	virulent	0.99104285	Ackermannviridae	1	Roseobacter denitrificans	0.78	Predict
DOM014_scaffold804_11	10045	phage	0.995	virulent	0.9998556	Herelleviridae	1	unknown	0	-
DOM014_scaffold6073_4	16027	phage	0.998	virulent	0.9998522	unknown	0	Staphylococcus saprophyticus	0.73	Predict
DOM014_scaffold804_8	13631	phage	0.999	temperate	0.99098414	unknown	0	Mycoplasma pulmonis	0.78	Predict
DOM014_scaffold27382_3	10609	phage	0.59	temperate	0.99984837	unknown	0	unknown	0	-

DOM014_scaffold14238_3	23382	phage	0.998	virulent	0.99987125	no_family_avaliable(NC_062779)	0.969	Candidatus Pelagibacter ubique	0.72	Predict
DOM014_scaffold2397_7	13186	phage	0.997	virulent	0.9998588	unknown	0	Mycoplasma pulmonis	0.72	Predict
DOM014_scaffold804_15	29294	phage	0.995	virulent	0.9998369	Straboviridae	1	Bacillus alcalophilus	1	CRISPR
DOM014_scaffold5037_9	21757	phage	0.999	virulent	0.9998086	Peduoviridae	1	Mycoplasma pulmonis	1	CRISPR
DOM014_scaffold804_6	13022	phage	1	temperate	0.9900664	unknown	0	unknown	0	-
DOM014_scaffold25405_6	10539	phage	0.998	virulent	0.96556866	unknown	0	unknown	0	-
DOM014_scaffold8176_14	12745	phage	0.997	virulent	0.9985003	unknown	0	Candidatus Pelagibacter ubique	0.82	Predict
DOM014_scaffold10055_2	17969	phage	0.98	temperate	0.9998574	Ackermannviridae	0.393936	Mycoplasma pulmonis	0.79	Predict
DOM014_scaffold1696_5	19168	phage	0.999	virulent	0.99984556	Ackermannviridae	0.3338031	Lactobacillus fermentum	0.72	Predict
DOM014_scaffold27264_1	10559	phage	0.999	virulent	0.9996015	Casjensviridae	0.8726506	Roseobacter denitrificans	1	CRISPR
DOM014_scaffold25405_4	24117	phage	0.998	virulent	0.9460565	Casjensviridae	0.9596824	Lactobacillus fermentum	1	CRISPR
DOM014_scaffold4337_1	49637	phage	0.999	temperate	0.82770056	Ackermannviridae	1	Mycoplasma pulmonis	0.83	Predict
DOM015_scaffold25351_1	12569	phage	0.997	temperate	0.99983835	unknown	0	Candidatus Pelagibacter ubique	0.81	Predict
DOM015_scaffold21624_2	13256	phage	0.999	virulent	0.9998622	Ackermannviridae	0.25972775	Mycoplasma pulmonis	0.98	Predict
DOM015_scaffold24987_5	15456	phage	1	temperate	0.9998593	unknown	0	Mycoplasma pulmonis	1	CRISPR
DOM015_scaffold15574_5	11993	phage	0.994	virulent	0.99987406	Ackermannviridae	0.5594898	Lactobacillus fermentum	0.96	Predict
DOM015_scaffold226_4	11204	phage	0.998	virulent	0.9998517	unknown	0	Xanthomonas vesicatoria	1	CRISPR
DOM015_scaffold29083_1	12725	phage	0.738	virulent	0.9998679	unknown	0	unknown	0	-
DOM015_scaffold5854_1	12628	phage	0.997	virulent	0.9998656	Straboviridae	0.5386496	Mycoplasma pulmonis	0.98	Predict
DOM015_scaffold14251_1	83482	phage	0.999	temperate	0.9997912	Peduoviridae	1	Candidatus Pelagibacter ubique	0.89	Predict
DOM015_scaffold22277_2	11371	phage	0.86	temperate	0.9998522	Straboviridae	0.5676362	Parabacteroides merdae	0.77	Predict
DOM015_scaffold1868_1	10364	phage	0.998	temperate	0.99986035	unknown	0	Lactobacillus fermentum	0.9	Predict
DOM015_scaffold8047_3	17505	phage	0.756	temperate	0.99986035	unknown	0	unknown	0	-
DOM015_scaffold2034_4	14052	phage	0.999	virulent	0.99987173	unknown	0	Lactobacillus gasseri	1	CRISPR
DOM015_scaffold28241_1	12692	phage	0.99	virulent	0.9998665	Straboviridae	0.2686186	Mycoplasma pulmonis	0.72	Predict
DOM015_scaffold16257_1	19304	phage	0.939	virulent	0.83896554	unknown	0	Mycoplasma pulmonis	0.77	Predict
DOM015_scaffold1673_2	11868	phage	0.69	virulent	0.9998651	Ackermannviridae	0.27722943	Bacteroides fragilis	0.92	Predict
DOM015_scaffold4_1	11717	phage	0.994	temperate	0.9996581	Ackermannviridae	0.43386555	Bacteroides fragilis	0.89	Predict
DOM015_scaffold21150_1	13165	phage	0.798	temperate	0.997755	unknown	0	Mycoplasma pulmonis	0.88	Predict
DOM015_scaffold7250_8	13418	phage	0.993	virulent	0.9998517	unknown	0	Mycoplasma pulmonis	0.86	Predict

DOM015_scaffold21009_2	29281	phage	1	virulent	0.9998684	Ackermannviridae	0.3338031	Lactobacillus fermentum	1	CRISPR
DOM015_scaffold10848_3	11250	phage	0.999	virulent	0.99966824	Ackermannviridae	0.43386555	Lactobacillus fermentum	0.98	Predict
DOM015_scaffold10865_1	12748	phage	0.512	virulent	0.9997092	unknown	0	Lactobacillus fermentum	0.91	Predict
DOM015_C368449_1	14319	phage	0.573	virulent	0.7615538	Salasmaviridae	0.49816787	Mycoplasma pulmonis	0.9	Predict
DOM015_scaffold5037_4	20033	phage	0.996	virulent	0.9998684	Straboviridae	0.6004747	Mycoplasma pulmonis	0.78	Predict
DOM015_scaffold13677_1	10139	phage	0.988	temperate	0.99383783	unknown	0	Mycoplasma pulmonis	0.89	Predict
DOM015_scaffold29079_1	10814	phage	0.998	virulent	0.9996403	unknown	0	Mycoplasma pulmonis	0.93	Predict
DOM015_scaffold14658_2	13866	phage	0.797	virulent	0.99912494	unknown	0	Mycoplasma pulmonis	0.76	Predict
DOM015_scaffold28241_3	11263	phage	0.974	temperate	0.98519784	unknown	0	Bacteroides fragilis	0.97	Predict
DOM015_scaffold24987_1	12390	phage	0.999	virulent	0.99870294	Peduoviridae	1	Mycoplasma pulmonis	0.75	Predict
DOM015_C368153_1	11654	phage	0.998	virulent	0.9998736	unknown	0	Bacteroides fragilis	1	CRISPR
DOM016_scaffold37120_1	22132	phage	0.999	temperate	0.9998579	unknown	0	unknown	0	-
DOM016_scaffold36138_1	16137	phage	0.994	temperate	0.99949944	Salasmaviridae	0.28656432	Mycoplasma pulmonis	0.75	Predict
DOM016_scaffold36372_1	11116	phage	0.999	temperate	0.99986035	unknown	0	Mycoplasma pulmonis	0.97	Predict
DOM016_scaffold28769_2	10647	phage	0.985	temperate	0.9998049	Peduoviridae	1	Mycoplasma pulmonis	0.9	Predict
DOM016_scaffold15209_2	16625	phage	1	temperate	0.99983406	unknown	0	Clostridioides difficile	0.81	Predict
DOM016_scaffold35635_2	12093	phage	0.999	temperate	0.9998588	unknown	0	Mycoplasma pulmonis	1	CRISPR
DOM016_scaffold35635_4	17449	phage	0.999	temperate	0.999844	Ackermannviridae	0.20316531	Clostridium sp. AF32-12BH	1	CRISPR
DOM016_scaffold27783_2	13654	phage	0.999	virulent	0.99977106	unknown	0	Bacteroides fragilis	0.9	Predict
DOM016_scaffold714_2	19311	phage	0.986	temperate	0.99959195	unknown	0	unknown	0	-
DOM016_scaffold36369_1	11129	phage	0.999	virulent	0.9989985	unknown	0	Staphylococcus xylosus	0.77	Predict
DOM016_scaffold15402_10	11643	phage	0.999	temperate	0.99985975	unknown	0	Mycoplasma pulmonis	1	CRISPR
DOM016_scaffold35922_2	12091	phage	0.999	temperate	0.98691773	unknown	0	Yersinia enterocolitica	1	CRISPR
DOM016_scaffold10818_1	14355	phage	0.998	virulent	0.99987084	unknown	0	Candidatus Pelagibacter ubique	0.97	Predict
DOM016_scaffold35524_2	11761	phage	0.96	virulent	0.9998727	unknown	0	Lactobacillus fermentum	0.96	Predict
DOM016_scaffold25511_1	11175	phage	0.999	virulent	0.99831057	unknown	0	Clostridium sporogenes	1	CRISPR
DOM016_scaffold32640_3	18301	phage	0.999	temperate	0.999854	unknown	0	Flavobacterium columnare	0.76	Predict
DOM016_scaffold37071_2	10822	phage	0.922	temperate	0.99985605	unknown	0	Bacteroides fragilis	0.97	Predict
DOM016_scaffold701_1	12355	phage	0.999	virulent	0.99987084	unknown	0	Lactobacillus delbrueckii	0.96	Predict
DOM016_scaffold27658_1	25137	phage	0.999	temperate	0.9998408	Straboviridae	0.5517625	Clostridioides difficile	1	CRISPR

DOM016_scaffold29662_3	44031	phage	0.613	temperate	0.9998556	Straboviridae	0.22926702	Mycoplasma pulmonis	1	CRISPR
DOM016_scaffold5915_8	10966	phage	0.995	temperate	0.99985975	unknown	0	Clostridium perfringens	0.97	Predict
DOM016_scaffold35635_3	12569	phage	0.999	temperate	0.9998579	Straboviridae	0.25296193	Mycoplasma pulmonis	1	CRISPR
DOM016_scaffold4735_5	20857	phage	0.778	virulent	0.9998608	unknown	0	Candidatus Pelagibacter ubique	1	CRISPR
DOM016_scaffold10222_1	10081	phage	1	temperate	0.99985975	unknown	0	Mycoplasma pulmonis	0.79	Predict
DOM016_scaffold27136_11	11431	phage	1	temperate	0.99986035	unknown	0	Mycoplasma pulmonis	1	CRISPR
DOM016_scaffold35701_7	10126	phage	0.998	virulent	0.9996515	Peduoviridae	1	Mycoplasma pulmonis	0.92	Predict
DOM016_scaffold35701_2	18116	phage	0.989	temperate	0.9998593	unknown	0	Clostridium perfringens	0.76	Predict
DOM016_scaffold29175_2	33847	phage	1	virulent	0.9998693	Autographiviridae	1	Mycoplasma pulmonis	0.72	Predict
DOM016_scaffold30030_2	18736	phage	0.998	virulent	0.99980664	Ackermannviridae	0.43386555	Lactobacillus fermentum	0.94	Predict
DOM016_scaffold15402_6	10981	phage	0.999	virulent	0.98361695	Peduoviridae	1	Mycoplasma pulmonis	1	CRISPR
DOM016_scaffold31190_2	18992	phage	0.997	virulent	0.8810457	Schitoviridae	0.26457438	Megamonas hypermegale	1	CRISPR
DOM016_scaffold20871_2	10749	phage	0.998	virulent	0.99987406	Zierdtviridae	1	unknown	0	-
DOM016_scaffold30578_2	42179	phage	0.998	virulent	0.99962103	unknown	0	Mycoplasma pulmonis	0.85	Predict
DOM016_scaffold65_5	18325	phage	0.999	virulent	0.9997578	Ackermannviridae	0.3338031	Lactobacillus fermentum	1	CRISPR
DOM016_scaffold19852_1	34031	phage	0.999	temperate	0.99111164	unknown	0	Mycoplasma pulmonis	1	CRISPR
DOM016_scaffold11439_8	12911	phage	0.988	temperate	0.99985605	Straboviridae	0.25507116	Mycoplasma pulmonis	0.72	Predict
DOM017_scaffold13265_2	17926	phage	1	virulent	0.9998727	unknown	0	Bacteroides fragilis	0.89	Predict
DOM017_scaffold7875_1	21109	phage	0.999	temperate	0.9998274	unknown	0	Candidatus Pelagibacter ubique	0.71	Predict
DOM017_scaffold12516_1	13651	phage	0.999	virulent	0.9053317	unknown	0	Bacteroides fragilis	0.78	Predict
DOM017_scaffold21083_1	19146	phage	0.999	virulent	0.9998727	unknown	0	unknown	0	-
DOM017_scaffold16028_1	19193	phage	0.998	virulent	0.99986607	unknown	0	Bacillus cereus	0.92	Predict
DOM017_scaffold5373_1	12241	phage	0.999	virulent	0.9998417	unknown	0	unknown	0	-
DOM017_scaffold9422_1	25562	phage	0.999	virulent	0.94220346	Straboviridae	0.48360676	Mycoplasma pulmonis	0.85	Predict
DOM017_scaffold273_11	17061	phage	0.932	virulent	0.9998727	Ackermannviridae	0.40105554	Mycoplasma pulmonis	0.94	Predict
DOM017_scaffold591_5	13691	phage	0.971	virulent	0.999549	Ackermannviridae	0.43386555	Staphylococcus saprophyticus	0.98	Predict
DOM017_scaffold7875_3	10941	phage	0.999	temperate	0.9998593	unknown	0	Staphylococcus aureus	0.92	Predict
DOM017_scaffold7344_7	10984	phage	0.761	temperate	0.5421888	unknown	0	unknown	0	-
DOM017_scaffold3771_1	24283	phage	0.979	virulent	0.9997632	Straboviridae	0.27292332	Bacteroides fragilis	1	CRISPR
DOM017_scaffold35431_3	11034	phage	0.999	virulent	0.99987316	Herelleviridae	1	Bacteroides fragilis	0.82	Predict

DOM017_scaffold434_2	12504	phage	0.999	virulent	0.9962297	unknown	0	unknown	0	-
DOM017_scaffold3183_4	11044	phage	0.802	virulent	0.99934214	unknown	0	unknown	0	-
DOM017_scaffold12158_1	18760	phage	0.894	temperate	0.9989053	unknown	0	unknown	0	-
DOM017_scaffold2122_1	12978	phage	0.94	temperate	0.999084	unknown	0	Bacteroides fragilis	0.75	Predict
DOM017_scaffold12299_4	13359	phage	0.838	virulent	0.9998302	unknown	0	Lactobacillus fermentum	0.9	Predict
DOM017_scaffold23327_2	10767	phage	0.998	virulent	0.9998736	unknown	0	unknown	0	-
DOM017_scaffold30906_1	10139	phage	0.999	virulent	0.9998371	unknown	0	Mycoplasma pulmonis	0.91	Predict
DOM017_scaffold12432_2	22631	phage	0.999	temperate	0.99983925	unknown	0	Candidatus Pelagibacter ubique	1	CRISPR
DOM017_scaffold1652_4	11086	phage	0.625	temperate	0.998085	Ackermannviridae	0.26003572	Mycoplasma pulmonis	0.8	Predict
DOM017_scaffold28752_1	12485	phage	0.999	temperate	0.92755413	unknown	0	Bacillus cereus	1	CRISPR
DOM017_scaffold35758_3	11143	phage	0.998	virulent	0.9998727	unknown	0	unknown	0	-
DOM017_scaffold10632_13	13849	phage	0.996	virulent	0.99880815	Ackermannviridae	0.38445178	Lactobacillus fermentum	0.86	Predict
DOM017_scaffold29332_1	10256	phage	0.995	virulent	0.76902056	unknown	0	unknown	0	-
DOM017_scaffold2195_2	11468	phage	0.998	temperate	0.898576	unknown	0	unknown	0	-
DOM018_scaffold6891_4	23249	phage	0.999	temperate	0.9998584	unknown	0	Candidatus Pelagibacter ubique	0.74	Predict
DOM018_scaffold11099_4	15110	phage	0.977	virulent	0.9998565	unknown	0	unknown	0	-
DOM018_scaffold88_7	10655	phage	0.982	virulent	0.9998131	unknown	0	unknown	0	-
DOM018_scaffold41600_1	15313	phage	1	virulent	0.9871713	Chaseviridae	0.20182505	Xanthomonas vesicatoria	0.85	Predict
DOM018_scaffold27201_3	20756	phage	1	virulent	0.99987036	unknown	0	Candidatus Pelagibacter ubique	0.77	Predict
DOM018_scaffold37486_2	11262	phage	0.961	temperate	0.979654	unknown	0	Bacteroides fragilis	1	Predict
DOM018_scaffold32733_2	12067	phage	0.997	virulent	0.9712672	unknown	0	Mycoplasma pulmonis	0.96	Predict
DOM018_scaffold35204_2	25432	phage	0.999	temperate	0.9998474	Demerecviridae	0.42195064	Candidatus Pelagibacter ubique	0.74	Predict
DOM018_scaffold4830_3	12735	phage	0.824	virulent	0.9654628	Ackermannviridae	0.28549954	unknown	0	-
DOM018_scaffold22855_12	27256	phage	0.999	temperate	0.99922955	unknown	0	Clostridioides difficile	1	CRISPR
DOM018_scaffold41384_2	17496	phage	0.999	temperate	0.9998579	unknown	0	Mycoplasma pulmonis	1	CRISPR
DOM018_scaffold35020_1	40028	phage	0.999	virulent	0.9998627	Mesyanzhinovviridae	1	Lactobacillus fermentum	1	CRISPR
DOM018_scaffold41493_2	21272	phage	0.999	temperate	0.68137324	Casjensviridae	1	Mycoplasma pulmonis	0.75	Predict
DOM018_scaffold9337_1	19285	phage	0.996	temperate	0.99678105	Straboviridae	0.66039294	Mycoplasma pulmonis	1	CRISPR
DOM018_C532545_1	10980	phage	0.999	virulent	0.99986607	unknown	0	Mycoplasma pulmonis	0.79	Predict
DOM018_scaffold40717_1	10149	phage	0.977	temperate	0.9997526	unknown	0	unknown	0	-

DOM018_scaffold23065_2	58455	phage	0.999	virulent	0.99486125	Ackermannviridae	0.44663528	Candidatus Hamiltonella defensa	1	CRISPR
DOM018_scaffold6891_1	10983	phage	0.983	temperate	0.8910085	Herelleviridae	1	Spirosoma pollinicola	1	CRISPR
DOM018_scaffold37478_3	12683	phage	0.994	temperate	0.99985695	Salasmaviridae	0.28656432	unknown	0	-
DOM018_scaffold6891_2	10497	phage	0.999	virulent	0.9998645	Salasmaviridae	0.36038926	Faecalibacterium prausnitzii	1	CRISPR
DOM018_scaffold1832_2	22884	phage	0.998	temperate	0.99276054	Casjensviridae	1	Roseobacter denitrificans	1	CRISPR
DOM018_scaffold34741_1	18299	phage	0.997	virulent	0.99985313	unknown	0	Flavobacterium columnare	0.77	Predict
DOM018_scaffold5182_1	10478	phage	0.988	temperate	0.8945864	unknown	0	unknown	0	-
DOM018_scaffold41493_1	29217	phage	1	virulent	0.9998688	unknown	0	Candidatus Pelagibacter ubique	0.79	Predict
DOM018_C532801_1	12727	phage	0.998	temperate	0.9998579	Ackermannviridae	0.4605801	Mycoplasma pulmonis	0.77	Predict
DOM018_scaffold32883_2	15850	phage	0.953	temperate	0.99983126	Straboviridae	0.5676362	Candidatus Pelagibacter ubique	0.9	Predict
DOM018_scaffold32755_3	45527	phage	0.999	temperate	0.99953187	unknown	0	Candidatus Pelagibacter ubique	0.7	Predict
DOM018_scaffold2126_7	11864	phage	0.993	virulent	0.99987125	Herelleviridae	1	unknown	0	-
DOM018_scaffold35068_1	18180	phage	1	temperate	0.99978167	unknown	0	Ralstonia pickettii	1	CRISPR
DOM018_scaffold1832_4	12385	phage	0.999	virulent	0.8973274	no_family_avaliable(NC_047911)	0.98	Mycoplasma pulmonis	0.77	Predict
DOM018_scaffold18201_3	10477	phage	0.999	temperate	0.99983555	unknown	0	Roseobacter denitrificans	0.75	Predict
DOM018_scaffold13608_2	12564	phage	0.996	virulent	0.99987406	Ackermannviridae	0.29527256	unknown	0	-
DOM018_scaffold33929_1	11890	phage	0.994	temperate	0.99985975	unknown	0	unknown	0	-
DOM018_scaffold17834_3	19799	phage	0.996	virulent	0.9998684	Straboviridae	0.6004747	Mycoplasma pulmonis	0.94	Predict
DOM019_scaffold640_2	10339	phage	0.995	virulent	0.9998408	unknown	0	unknown	0	-
DOM019_scaffold141_3	16838	phage	0.998	virulent	0.9996124	Straboviridae	0.6004747	Candidatus Pelagibacter ubique	0.9	Predict
DOM019_scaffold20326_3	12730	phage	0.983	virulent	0.8463101	Straboviridae	0.64696723	Mycoplasma pulmonis	0.85	Predict
DOM019_scaffold6808_2	10620	phage	0.999	virulent	0.99987173	no_family_avaliable(NC_062779)	0.969	Candidatus Pelagibacter ubique	0.9	Predict
DOM019_scaffold41546_1	11866	phage	0.993	virulent	0.9247743	Ackermannviridae	0.2797422	Candidatus Pelagibacter ubique	0.78	Predict
DOM019_scaffold56541_1	18193	phage	0.887	virulent	0.99987316	Straboviridae	0.64696723	Bacteroides fragilis	0.76	Predict
DOM019_scaffold42868_1	12789	phage	0.997	temperate	0.99986035	unknown	0	Streptomyces avermitilis	0.76	Predict
DOM019_scaffold5478_2	12208	phage	0.999	temperate	0.999836	unknown	0	Flavobacterium columnare	0.72	Predict
DOM019_scaffold33784_1	13770	phage	0.991	temperate	0.99985605	Herelleviridae	1	unknown	0	-
DOM019_scaffold10413_15	10718	phage	0.844	virulent	0.99987173	unknown	0	Lactobacillus fermentum	0.82	Predict
DOM019_scaffold3315_20	11343	phage	1	temperate	0.9998593	unknown	0	Clostridioides difficile	0.92	Predict
DOM019_scaffold1388_2	10168	phage	0.998	temperate	0.9997526	unknown	0	Mycoplasma pulmonis	1	CRISPR

DOM019_scaffold56605_1	17046	phage	0.998	temperate	0.9998588	unknown	0	Bacteroides fragilis	0.7	Predict
DOM019_scaffold6808_3	31096	phage	0.999	virulent	0.9998736	no_family_avaliable(NC_062779)	0.979	Candidatus Pelagibacter ubique	0.84	Predict
DOM019_scaffold20197_1	88160	phage	0.999	virulent	0.9998684	Straboviridae	0.3625785	Candidatus Pelagibacter ubique	0.87	Predict
DOM019_scaffold661_7	10650	phage	0.953	temperate	0.99984884	unknown	0	unknown	0	-
DOM019_scaffold56555_1	14169	phage	0.999	virulent	0.9998688	Salasmaviridae	1	Candidatus Pelagibacter ubique	1	CRISPR
DOM019_scaffold8387_2	10894	phage	0.995	virulent	0.9688307	unknown	0	Parabacteroides distasonis	0.87	Predict
DOM019_scaffold56549_2	12827	phage	0.999	virulent	0.9998679	Ackermannviridae	0.29527256	Mycoplasma pulmonis	0.86	Predict
DOM019_scaffold55994_2	12612	phage	0.898	virulent	0.9998431	unknown	0	unknown	0	-
DOM019_scaffold9440_2	10250	phage	0.999	temperate	0.9973751	unknown	0	Akkermansia muciniphila	1	CRISPR
DOM019_scaffold56598_2	62199	phage	0.998	virulent	0.9998665	Herelleviridae	1	Cellulophaga baltica	1	CRISPR
DOM019_scaffold3989_6	10604	phage	0.999	temperate	0.9998593	unknown	0	unknown	0	-
DOM019_scaffold23724_3	20374	phage	0.999	temperate	0.99984884	unknown	0	Candidatus Pelagibacter ubique	0.99	Predict
DOM019_scaffold56558_1	10109	phage	0.991	temperate	0.99985975	unknown	0	Bacillus megaterium	0.91	Predict
DOM019_scaffold56469_5	95806	phage	0.999	virulent	0.9998574	Straboviridae	0.33017182	Flavobacterium columnare	1	CRISPR
DOM019_scaffold20133_1	25703	phage	0.998	temperate	0.9998579	Ackermannviridae	0.4759637	Mycoplasma pulmonis	0.89	Predict
DOM019_scaffold34447_1	11436	phage	0.997	virulent	0.9811014	unknown	0	Thermoanaerobacterium saccharolyti	0.8	Predict
DOM019_scaffold56536_2	10178	phage	1	virulent	0.99978495	Ackermannviridae	0.3338031	Lactobacillus fermentum	0.93	Predict
DOM019_scaffold8084_3	10422	phage	0.985	virulent	0.999836	unknown	0	unknown	0	-
DOM019_scaffold11571_11	10192	phage	0.999	temperate	0.99947125	unknown	0	unknown	0	-
DOM019_scaffold35386_4	10541	phage	0.939	temperate	0.56512856	unknown	0	unknown	0	-
DOM019_scaffold9830_4	10262	phage	0.992	virulent	0.99987084	Ackermannviridae	1	Mycoplasma pulmonis	0.87	Predict
DOM019_scaffold286_2	22666	phage	0.998	virulent	0.9998656	Straboviridae	0.4871907	Mycoplasma pulmonis	0.99	Predict
DOM020_scaffold38403_2	14135	phage	0.747	temperate	0.9986213	Straboviridae	0.28144336	Bacteroides fragilis	0.94	Predict
DOM020_scaffold38380_2	14834	phage	0.548	virulent	0.9998574	unknown	0	Mycoplasma pulmonis	0.84	Predict
DOM020_scaffold36128_4	11278	phage	0.961	temperate	0.979654	unknown	0	Bacteroides fragilis	0.74	Predict
DOM020_scaffold8013_4	17894	phage	0.999	temperate	0.99599046	unknown	0	Staphylococcus saprophyticus	0.88	Predict
DOM020_scaffold24489_1	12670	phage	0.538	temperate	0.9998379	Salasmaviridae	0.4885404	unknown	0	-
DOM020_scaffold1763_4	12682	phage	0.998	temperate	0.99985266	unknown	0	unknown	0	-
DOM020_scaffold1491_1	15238	phage	0.997	temperate	0.9998574	unknown	0	Parabacteroides merdae	0.91	Predict
DOM020_scaffold19627_11	14637	phage	0.999	temperate	0.97744745	Straboviridae	0.25296193	Mycoplasma pulmonis	0.82	Predict

DOM020_scaffold8898_2	11857	phage	1	temperate	0.8756662	Peduoviridae	0.31273133	Mycoplasma pulmonis	1	CRISPR
DOM020_scaffold16344_2	16419	phage	0.999	temperate	0.99985266	Vilmaviridae	1	Flavobacterium columnare	1	CRISPR
DOM020_scaffold37565_2	10978	phage	0.999	virulent	0.99987036	no_family_avaliable(NC_047910)	0.983	Roseobacter denitrificans	1	CRISPR
DOM020_C483630_1	10763	phage	0.998	temperate	0.9998593	Straboviridae	0.7067733	Lactobacillus fermentum	0.96	Predict
DOM020_scaffold8013_2	19836	phage	0.999	temperate	0.8831192	Casjensviridae	0.6866919	Mycoplasma pulmonis	0.78	Predict
DOM020_scaffold1229_1	29441	phage	1	temperate	0.9998217	unknown	0	Bacteroides fragilis	1	CRISPR
DOM020_scaffold3600_6	18883	phage	0.623	temperate	0.99986035	unknown	0	Candidatus Pelagibacter ubique	0.77	Predict
DOM020_scaffold7010_6	10279	phage	0.998	virulent	0.9997811	no_family_avaliable(NC_062768)	0.992	Candidatus Pelagibacter ubique	1	CRISPR
DOM021_scaffold18418_1	12985	phage	0.999	virulent	0.5814494	Straboviridae	0.5445338	Mycoplasma pulmonis	0.9	Predict
DOM021_scaffold21593_8	10174	phage	0.989	temperate	0.9998579	Peduoviridae	1	Actinomyces naeslundii	0.81	Predict
DOM021_scaffold27752_2	72303	phage	0.999	virulent	0.9998679	no_family_avaliable(NC_062779)	0.974	Lactobacillus gasseri	0.84	Predict
DOM021_scaffold71_1	10550	phage	0.997	virulent	0.99970627	Straboviridae	0.6004747	Pseudoalteromonas atlantica	0.88	Predict
DOM021_scaffold38953_1	17057	phage	0.986	virulent	0.9998699	Ackermannviridae	0.28265804	Mycoplasma pulmonis	1	CRISPR
DOM021_scaffold272_1	33801	phage	0.999	virulent	0.99974704	unknown	0	unknown	0	-
DOM021_scaffold39089_1	30840	phage	0.997	virulent	0.9997509	Straboviridae	0.27696446	Mycoplasma pulmonis	1	CRISPR
DOM021_scaffold100_1	19586	phage	0.991	virulent	0.9998656	Ackermannviridae	0.39984542	Lactobacillus fermentum	0.9	Predict
DOM021_scaffold39104_1	26248	phage	1	virulent	0.99986696	Ackermannviridae	0.3338031	Lactobacillus fermentum	1	CRISPR
DOM021_scaffold36942_1	11829	phage	0.986	virulent	0.99987316	Ackermannviridae	0.40512124	Lactobacillus fermentum	0.73	Predict
DOM021_scaffold23118_2	22277	phage	0.999	temperate	0.9959307	unknown	0	Mycoplasma pulmonis	0.8	Predict
DOM021_scaffold57_4	14449	phage	0.99	virulent	0.99987173	Ackermannviridae	0.29527256	unknown	0	-
DOM021_scaffold37667_2	23159	phage	1	virulent	0.9998684	Casjensviridae	0.7706307	Mycoplasma pulmonis	1	CRISPR
DOM021_scaffold11063_1	18089	phage	0.997	virulent	0.99985695	Ackermannviridae	0.43386555	Lactobacillus fermentum	0.96	Predict
DOM021_scaffold39089_2	10127	phage	1	temperate	0.96921164	unknown	0	Candidatus Pelagibacter ubique	1	Predict
DOM021_scaffold36893_3	30527	phage	0.999	temperate	0.99969774	Salasmaviridae	0.41580635	Mycoplasma pulmonis	1	CRISPR
DOM021_scaffold12359_1	11240	phage	0.962	temperate	0.99985975	unknown	0	Mycoplasma pulmonis	0.75	Predict
DOM021_scaffold37667_3	17998	phage	1	virulent	0.9998693	unknown	0	Candidatus Pelagibacter ubique	1	CRISPR
DOM021_scaffold5645_2	10983	phage	0.998	virulent	0.99987316	unknown	0	unknown	0	-
DOM021_scaffold37125_1	11408	phage	0.955	temperate	0.9301825	Chaseviridae	0.3020128	Candidatus Pelagibacter ubique	0.78	Predict
DOM021_scaffold39046_1	11800	phage	0.999	virulent	0.9998293	unknown	0	unknown	0	-
DOM022_scaffold32377_2	15226	phage	0.995	virulent	0.9998645	unknown	0	Mycoplasma pulmonis	0.89	Predict

DOM022_scaffold19956_4	11282	phage	0.961	temperate	0.979654	unknown	0	Bacteroides fragilis	0.78	Predict
DOM022_scaffold6808_24	11569	phage	0.935	virulent	0.99138457	Ackermannviridae	0.43386555	Lactobacillus fermentum	0.78	Predict
DOM022_scaffold28157_3	22534	phage	0.994	virulent	0.9995576	Ackermannviridae	0.32162276	Candidatus Pelagibacter ubique	0.72	Predict
DOM022_scaffold32538_1	13974	phage	0.977	virulent	0.99986744	Straboviridae	0.6004747	Mycoplasma pulmonis	0.73	Predict
DOM022_scaffold38389_3	14441	phage	0.998	virulent	0.99987316	unknown	0	Mycoplasma pulmonis	0.81	Predict
DOM022_scaffold2843_1	13551	phage	0.996	virulent	0.9998736	unknown	0	Bacillus thuringiensis	0.98	Predict
DOM022_scaffold38212_2	15040	phage	0.999	temperate	0.99976164	unknown	0	Mycoplasma pulmonis	1	CRISPR
DOM022_scaffold18184_9	14975	phage	0.945	temperate	0.9998188	unknown	0	Roseobacter denitrificans	0.82	Predict
DOM022_scaffold8882_1	11882	phage	0.996	temperate	0.9080085	unknown	0	Mycoplasma pulmonis	1	Predict
DOM022_scaffold38445_2	14869	phage	0.999	temperate	0.99985975	unknown	0	Mycoplasma pulmonis	1	CRISPR
DOM022_scaffold16044_22	10350	phage	0.998	temperate	0.905662	unknown	0	Candidatus Pelagibacter ubique	1	CRISPR
DOM022_scaffold847_4	11398	phage	1	temperate	0.999816	unknown	0	unknown	0	-
DOM022_scaffold37774_1	15480	phage	0.889	virulent	0.9998699	unknown	0	unknown	0	-
DOM022_scaffold8382_3	16219	phage	0.953	temperate	0.9998508	Straboviridae	0.64696723	Bacteroides fragilis	0.87	Predict
DOM022_scaffold37182_2	28911	phage	0.999	virulent	0.8010681	Peduoviridae	1	Candidatus Pelagibacter ubique	0.82	Predict
DOM022_scaffold26147_1	23261	phage	0.998	temperate	0.99985313	unknown	0	Candidatus Pelagibacter ubique	0.81	Predict
DOM022_scaffold38016_1	15644	phage	0.999	temperate	0.99984694	Peduoviridae	1	Tsukamurella paurometabola	0.95	Predict
DOM022_C512095_1	20031	phage	0.999	virulent	0.9764287	unknown	0	Mycoplasma pulmonis	0.88	Predict
DOM022_scaffold38445_1	14590	phage	0.999	temperate	0.99985605	unknown	0	Candidatus Pelagibacter ubique	0.94	Predict
DOM022_scaffold35096_1	47745	phage	0.991	temperate	0.9996434	Chaseviridae	1	Mycoplasma pulmonis	1	CRISPR
DOM022_scaffold33500_1	13566	phage	0.519	temperate	0.99985975	unknown	0	unknown	0	-
DOM022_scaffold36652_4	34100	phage	0.999	temperate	0.9998331	Straboviridae	0.5517625	Flavobacterium columnare	1	CRISPR
DOM022_scaffold38271_2	16582	phage	0.999	virulent	0.999398	Chaseviridae	0.93476343	Xanthomonas vesicatoria	1	CRISPR
DOM022_scaffold21088_2	35628	phage	0.999	virulent	0.9952504	Straboviridae	0.49118197	Mycoplasma pulmonis	0.77	Predict
DOM022_scaffold32837_2	15902	phage	1	virulent	0.99986696	no_family_avaliable(NC_011222)	0.981	Bacteroides fragilis	1	CRISPR
DOM022_scaffold37253_1	12519	phage	0.999	virulent	0.9998722	Peduoviridae	1	Parabacteroides merdae	0.78	Predict
DOM022_scaffold25564_3	15793	phage	0.998	temperate	0.9998388	Straboviridae	0.40261546	Bacteroides sp. 3_1_40A	1	CRISPR
DOM022_scaffold35602_3	18616	phage	0.999	virulent	0.90293515	Peduoviridae	1	Mycoplasma pulmonis	1	CRISPR
DOM022_scaffold33506_1	30786	phage	0.998	temperate	0.99636406	unknown	0	Veillonella parvula	1	CRISPR
DOM022_scaffold16660_7	10813	phage	0.906	temperate	0.9983715	unknown	0	Bacteroides fragilis	1	CRISPR

DOM022_scaffold3588_2	36756	phage	0.998	virulent	0.9998688	unknown	0	Cellulophaga baltica	0.93	Predict
DOM022_scaffold9769_2	18966	phage	0.999	virulent	0.99986404	Schitoviridae	1	Mycoplasma pulmonis	0.96	Predict
DOM022_scaffold6474_11	11880	phage	0.984	virulent	0.88498414	unknown	0	Mycoplasma pulmonis	1	CRISPR
DOM022_scaffold8236_1	16855	phage	0.989	temperate	0.9592161	Chaseviridae	0.28818834	Bacteroides fragilis	0.85	Predict
DOM022_scaffold37834_1	10349	phage	0.996	virulent	0.9998617	unknown	0	Mycoplasma pulmonis	0.84	Predict
DOM022_scaffold11588_18	10723	phage	0.99	virulent	0.99987173	Ackermannviridae	0.40105554	Lactobacillus fermentum	0.74	Predict
DOM022_scaffold17665_18	20485	phage	0.999	temperate	0.9998397	Casjensviridae	1	Mycoplasma pulmonis	0.87	Predict
DOM022_scaffold11253_14	10609	phage	1	virulent	0.99987406	Ackermannviridae	0.1904263	Bacteroides fragilis	0.72	Predict
DOM022_scaffold27991_2	16994	phage	0.998	temperate	0.62538254	Casjensviridae	1	Mycoplasma pulmonis	1	CRISPR
DOM022_scaffold7138_11	12644	phage	0.996	virulent	0.9998736	unknown	0	Bacteroides fragilis	0.71	Predict
DOM022_scaffold3588_8	11967	phage	0.999	virulent	0.99972224	unknown	0	Candidatus Pelagibacter ubique	0.77	Predict
DOM022_scaffold38396_3	17080	phage	0.942	virulent	0.9998197	Ackermannviridae	0.37239054	Candidatus Pelagibacter ubique	0.78	Predict
DOM022_scaffold48_3	15329	phage	0.996	virulent	0.9998736	Ackermannviridae	0.29527256	unknown	0	-
DOM022_scaffold17718_5	10600	phage	0.973	temperate	0.9743889	unknown	0	Mycoplasma pulmonis	0.77	Predict
DOM022_scaffold15495_1	10563	phage	0.842	temperate	0.9700229	unknown	0	unknown	0	-
DOM022_scaffold37363_3	29557	phage	0.862	virulent	0.9998693	Straboviridae	0.4491102	Staphylococcus saprophyticus	1	CRISPR
DOM022_scaffold8882_2	10595	phage	0.999	temperate	0.99985975	unknown	0	Candidatus Pelagibacter ubique	0.96	Predict
DOM022_scaffold37244_1	11918	phage	0.993	virulent	0.83116853	unknown	0	Bacteroides fragilis	0.86	Predict
DOM022_scaffold37792_2	10169	phage	0.995	virulent	0.93913764	Ackermannviridae	0.26003572	Mycoplasma pulmonis	0.88	Predict
DOM022_scaffold19876_2	13327	phage	0.997	virulent	0.9998651	unknown	0	Roseobacter denitrificans	0.81	Predict
DOM022_scaffold13760_14	10345	phage	0.997	virulent	0.9998305	unknown	0	Bacteroides fragilis	1	CRISPR
DOM022_scaffold1123_2	17275	phage	0.998	virulent	0.99980664	Ackermannviridae	0.43386555	Lactobacillus fermentum	0.74	Predict
DOM022_scaffold28292_2	63724	phage	0.999	temperate	0.7311479	unknown	0	Flavobacterium columnare	1	CRISPR
DOM022_scaffold21088_3	19308	phage	0.998	temperate	0.9998584	unknown	0	Bacillus alcalophilus	1	CRISPR
DOM022_scaffold9987_23	15226	phage	0.866	virulent	0.99986035	unknown	0	Bacteroides fragilis	0.98	Predict
DOM022_scaffold36541_6	10627	phage	0.999	temperate	0.9998551	unknown	0	Cellulophaga baltica	1	CRISPR
DOM023_scaffold33157_2	13372	phage	0.999	virulent	0.9998688	Salasmaviridae	1	Candidatus Pelagibacter ubique	0.81	Predict
DOM023_scaffold33236_1	10672	phage	0.999	virulent	0.999322	unknown	0	Clostridium sporogenes	1	CRISPR
DOM023_scaffold33285_1	19298	phage	0.638	temperate	0.9998479	unknown	0	Mycoplasma pulmonis	1	CRISPR
DOM023_scaffold19886_6	14319	phage	1	virulent	0.9998665	Autographiviridae	1	Mycoplasma pulmonis	0.82	Predict

DOM023_scaffold12205_7	14308	phage	0.674	virulent	0.9998727	Straboviridae	0.35083705	unknown	0	-
DOM023_scaffold25122_8	10410	phage	1	temperate	0.9990589	unknown	0	unknown	0	-
DOM023_scaffold33612_1	24443	phage	0.997	virulent	0.99987173	Straboviridae	0.5505315	Mycoplasma pulmonis	0.98	Predict
DOM023_scaffold11787_6	10130	phage	0.998	temperate	0.9900165	unknown	0	Roseobacter denitrificans	0.75	Predict
DOM023_scaffold33611_2	12955	phage	0.994	virulent	0.99987173	unknown	0	Mycoplasma pulmonis	0.91	Predict
DOM023_scaffold28756_2	13533	phage	0.989	virulent	0.9998217	unknown	0	Citrobacter freundii	0.8	Predict
DOM023_scaffold13717_2	12483	phage	0.999	virulent	0.99987406	unknown	0	Mycoplasma pulmonis	0.82	Predict
DOM023_scaffold23102_8	11456	phage	0.989	temperate	0.9998593	unknown	0	Mycoplasma pulmonis	0.92	Predict
DOM023_scaffold33401_1	10932	phage	0.97	temperate	0.9998593	Salasmaviridae	0.45942637	Mycoplasma pulmonis	0.85	Predict
DOM023_scaffold31295_1	14190	phage	0.998	temperate	0.9998465	Straboviridae	0.45292062	unknown	0	-
DOM023_scaffold33622_1	11645	phage	0.998	temperate	0.9997196	unknown	0	Candidatus Pelagibacter ubique	1	CRISPR
DOM023_scaffold19886_5	11283	phage	0.999	virulent	0.99987173	Autographiviridae	1	Mycoplasma pulmonis	0.78	Predict
DOM023_scaffold2410_11	13940	phage	0.999	temperate	0.9926195	unknown	0	Bifidobacterium anseris	1	CRISPR
DOM023_scaffold16805_4	35025	phage	0.984	virulent	0.9998622	unknown	0	Candidatus Pelagibacter ubique	0.72	Predict
DOM023_scaffold33548_1	37225	phage	0.999	virulent	0.9998645	Casjensviridae	0.7706307	Candidatus Pelagibacter ubique	1	CRISPR
DOM023_C393870_1	10330	phage	0.885	virulent	0.93739945	unknown	0	Parabacteroides merdae	0.83	Predict
DOM023_scaffold22587_5	10912	phage	0.567	virulent	0.9998736	unknown	0	unknown	0	-
DOM024_scaffold35255_15	10926	phage	0.999	virulent	0.9998536	Zobellviridae	1	Candidatus Pelagibacter ubique	0.93	Predict
DOM024_scaffold11745_3	12804	phage	0.999	virulent	0.99987465	unknown	0	Parabacteroides distasonis	0.81	Predict
DOM024_scaffold34021_1	16846	phage	0.988	temperate	0.83150184	unknown	0	Mycoplasma pulmonis	0.87	Predict
DOM024_scaffold44466_2	14064	phage	0.999	virulent	0.984512	unknown	0	Candidatus Pelagibacter ubique	0.71	Predict
DOM024_scaffold42617_1	24992	phage	0.999	virulent	0.9990506	unknown	0	Cellulophaga baltica	1	CRISPR
DOM024_scaffold38154_1	18257	phage	0.999	virulent	0.99986124	unknown	0	Candidatus Pelagibacter ubique	0.79	Predict
DOM024_scaffold56350_1	78266	phage	0.999	temperate	0.99975395	unknown	0	Flavobacterium columnare	1	CRISPR
DOM024_scaffold54306_4	16162	phage	0.932	virulent	0.9998727	Ackermannviridae	0.40105554	Mycoplasma pulmonis	0.86	Predict
DOM024_scaffold42617_2	42000	phage	0.999	virulent	0.99809235	Ackermannviridae	0.44663528	Mycoplasma pulmonis	1	CRISPR
DOM024_scaffold25752_1	11251	phage	0.997	virulent	0.9997449	unknown	0	Mycoplasma pulmonis	1	CRISPR
DOM024_scaffold41795_1	29653	phage	0.999	virulent	0.99986035	Straboviridae	0.4491102	Candidatus Pelagibacter ubique	1	CRISPR
DOM024_scaffold56303_1	62411	phage	0.999	virulent	0.9998517	unknown	0	Mycoplasma pulmonis	0.94	Predict
DOM024_scaffold13072_13	11451	phage	1	virulent	0.99967504	unknown	0	Ruegeria pomeroyi	1	CRISPR

DOM024_scaffold52650_1	14399	phage	0.993	temperate	0.99985975	unknown	0	Bacteroides fragilis	0.71	Predict
DOM024_scaffold36750_16	16053	phage	0.815	temperate	0.98917335	unknown	0	Bacteroides fragilis	1	CRISPR
DOM024_scaffold151_2	13798	phage	0.999	virulent	0.99519527	Drexlerviridae	0.15702671	Lactobacillus fermentum	1	Predict
DOM024_scaffold55680_1	56118	phage	1	temperate	0.99985695	Straboviridae	0.41539797	Flavobacterium columnare	1	CRISPR
DOM024_scaffold52177_5	14662	phage	0.999	virulent	0.7548561	unknown	0	Listeria monocytogenes	1	CRISPR
DOM024_C663872_1	10238	phage	0.982	temperate	0.9983641	unknown	0	Ruminococcus sp. AM43-6	1	CRISPR
DOM024_scaffold52177_6	13338	phage	0.999	virulent	0.7286186	unknown	0	unknown	0	-
DOM024_scaffold56350_2	48410	phage	0.998	virulent	0.9993765	Demerecviridae	0.24218577	Ruminococcus sp. AF37-20	1	CRISPR
DOM024_scaffold52366_1	11802	phage	0.999	temperate	0.99985266	unknown	0	Lactobacillus fermentum	1	CRISPR
DOM024_scaffold56292_1	12236	phage	0.998	virulent	0.9998699	Herelleviridae	1	unknown	0	-
DOM024_scaffold32065_2	24551	phage	0.996	virulent	0.9998083	Ackermannviridae	0.38445178	Candidatus Hamiltonella defensa	0.82	Predict
DOM024_scaffold56206_2	23176	phage	0.999	temperate	0.93854123	Casjensviridae	1	Mycoplasma pulmonis	1	CRISPR
DOM024_C664368_1	17198	phage	1	virulent	0.99986404	Salasmaviridae	1	Mycoplasma pulmonis	0.77	Predict
DOM024_C664620_1	34141	phage	0.999	virulent	0.66369885	unknown	0	Candidatus Pelagibacter ubique	0.89	Predict
DOM024_scaffold18051_2	10241	phage	0.989	virulent	0.9998699	unknown	0	unknown	0	-
DOM024_scaffold56343_2	26109	phage	0.999	temperate	0.99981695	Casjensviridae	0.18703812	Microcystis aeruginosa	1	CRISPR
DOM024_scaffold29589_1	26713	phage	0.999	temperate	0.99985695	Casjensviridae	0.75807345	Microcystis aeruginosa	0.72	Predict
DOM024_scaffold15513_1	14919	phage	0.999	virulent	0.99986035	Ackermannviridae	0.43386555	Bacteroides fragilis	0.91	Predict
DOM024_scaffold20070_12	11162	phage	0.998	virulent	0.9998736	Herelleviridae	1	unknown	0	-
DOM024_scaffold30168_5	28648	phage	0.999	temperate	0.9998417	Ackermannviridae	0.3338031	Lactobacillus fermentum	1	CRISPR
DOM024_scaffold4865_6	16197	phage	0.882	temperate	0.78272307	Straboviridae	0.64696723	Mycoplasma pulmonis	0.9	Predict
DOM024_scaffold34021_4	11736	phage	0.999	temperate	0.99985975	unknown	0	Mycoplasma pulmonis	0.92	Predict
DOM024_scaffold35255_14	14421	phage	0.999	virulent	0.9998679	Zierdtviridae	0.49925515	Cellulophaga baltica	1	CRISPR
DOM024_scaffold22388_3	22015	phage	0.999	temperate	0.9998556	unknown	0	Lactobacillus fermentum	1	CRISPR
DOM024_scaffold56343_3	23598	phage	0.999	temperate	0.9995614	Casjensviridae	1	Mycoplasma pulmonis	1	CRISPR
DOM024_scaffold15633_7	11260	phage	0.999	virulent	0.6831714	Peduoviridae	1	Mycoplasma pulmonis	0.95	Predict
DOM024_scaffold49650_2	76624	phage	0.999	temperate	0.62325543	Herelleviridae	1	Lactobacillus gasseri	1	CRISPR
DOM025_scaffold30920_4	13534	phage	0.954	virulent	0.88889205	Peduoviridae	0.4545788	Mycoplasma pulmonis	0.92	Predict
DOM025_scaffold44713_1	12723	phage	0.996	temperate	0.99618447	unknown	0	Mycoplasma pulmonis	0.86	Predict
DOM025_scaffold68_3	11282	phage	0.999	virulent	0.99885005	unknown	0	Mycoplasma pulmonis	1	CRISPR

DOM025_scaffold39391_1	10915	phage	0.997	temperate	0.99959904	unknown	0	Bacteroides fragilis	0.93	Predict
DOM025_scaffold29788_4	19155	phage	0.991	virulent	0.9998727	Ackermannviridae	0.39984542	Lactobacillus fermentum	0.84	Predict
DOM025_scaffold44576_1	17066	phage	1	temperate	0.99985975	unknown	0	Planktothrix agardhii	0.86	Predict
DOM025_scaffold49_1	16818	phage	0.999	virulent	0.9998693	no_family_avaliable(NC_024711)	0.976	Candidatus Pelagibacter ubique	0.87	Predict
DOM025_scaffold49_3	71668	phage	1	virulent	0.99986315	Straboviridae	0.30413526	Lactobacillus gasseri	0.86	Predict
DOM025_scaffold41702_1	18109	phage	0.994	virulent	0.99987036	unknown	0	unknown	0	-
DOM025_scaffold10571_8	15444	phage	0.932	virulent	0.99987406	unknown	0	unknown	0	-
DOM025_scaffold80_3	10974	phage	0.999	temperate	0.99986035	unknown	0	Aeromonas media	1	CRISPR
DOM025_scaffold300_2	10360	phage	0.996	virulent	0.9998727	unknown	0	Sinorhizobium meliloti	0.84	Predict
DOM025_scaffold4683_2	25480	phage	0.999	virulent	0.9996324	Casjensviridae	0.30599892	Lactobacillus fermentum	0.93	Predict
DOM025_scaffold9936_1	12661	phage	0.999	temperate	0.6660664	unknown	0	Lactobacillus fermentum	1	CRISPR
DOM025_scaffold1987_2	10594	phage	0.95	virulent	0.9997584	unknown	0	Azospirillum brasilense	0.73	Predict
DOM025_scaffold3271_23	15226	phage	0.997	virulent	0.9998722	unknown	0	unknown	0	-
DOM025_scaffold7455_3	31081	phage	1	virulent	0.99986744	unknown	0	Parabacteroides merdae	1	CRISPR
DOM025_scaffold41294_1	20015	phage	0.999	virulent	0.99987406	unknown	0	Bacteroides fragilis	0.97	Predict
DOM025_scaffold14033_1	10922	phage	0.981	temperate	0.9998579	unknown	0	unknown	0	-
DOM025_scaffold23512_1	11745	phage	0.997	virulent	0.9998736	unknown	0	Roseobacter denitrificans	0.7	Predict
DOM025_scaffold20212_5	12053	phage	0.999	virulent	0.99987173	no_family_avaliable(NC_062779)	0.968	Flavobacterium columnare	0.92	Predict
DOM025_scaffold5440_33	10040	phage	0.941	virulent	0.9998727	unknown	0	Candidatus Pelagibacter ubique	0.83	Predict
DOM025_scaffold10897_2	10386	phage	0.985	virulent	0.9998727	Ackermannviridae	0.40512124	Lactobacillus fermentum	0.92	Predict
DOM025_scaffold12330_13	10984	phage	0.995	virulent	0.9998302	Salasmaviridae	0.33272234	Mycoplasma pulmonis	0.96	Predict
DOM025_scaffold2257_29	10739	phage	1	temperate	0.99985975	unknown	0	Mycoplasma pulmonis	0.72	Predict
DOM026_scaffold22046_1	17489	phage	0.998	temperate	0.99985605	unknown	0	Mycoplasma pulmonis	0.73	Predict
DOM026_scaffold39207_2	12699	phage	0.872	temperate	0.99980927	Salasmaviridae	0.4885404	unknown	0	-
DOM026_scaffold135_10	17173	phage	0.985	temperate	0.9969343	unknown	0	Lactobacillus fermentum	0.8	Predict
DOM026_scaffold813_3	12799	phage	0.995	virulent	0.99978536	Ackermannviridae	0.39984542	Lactobacillus fermentum	0.87	Predict
DOM026_scaffold22251_2	12774	phage	0.997	virulent	0.9469641	Schitoviridae	0.26269838	Parabacteroides distasonis	0.98	Predict
DOM026_scaffold21041_1	15366	phage	1	virulent	0.9998736	unknown	0	Bacteroides faecis	1	CRISPR
DOM026_scaffold38351_1	14268	phage	0.987	temperate	0.99986035	unknown	0	Microcystis aeruginosa	0.84	Predict
DOM026_scaffold9255_1	86462	phage	0.999	temperate	0.999194	unknown	0	Clostridioides difficile	1	CRISPR

DOM026_scaffold18742_1	12442	phage	0.806	virulent	0.99986607	Casjensviridae	0.3076411	unknown	0 -
DOM026_scaffold19764_1	10649	phage	0.996	virulent	0.9998722	Ackermannviridae	0.28265804	Mycoplasma pulmonis	0.75 Predict
DOM026_scaffold30709_3	17141	phage	0.995	virulent	0.9998699	Straboviridae	0.47816586	Mycoplasma pulmonis	0.76 Predict
DOM026_scaffold13170_6	13381	phage	0.991	temperate	0.9998593	Peduoviridae	1	Candidatus Hamiltonella defensa	0.8 Predict
DOM026_scaffold17576_11	12578	phage	0.637	temperate	0.9998545	Casjensviridae	0.3076411	Rhodovulum sp. P5	0.91 Predict
DOM026_scaffold17476_4	11113	phage	0.986	virulent	0.99987316	Ackermannviridae	0.40512124	Lactobacillus fermentum	0.78 Predict
DOM026_scaffold11281_20	13323	phage	0.516	virulent	0.99987406	unknown	0	Roseobacter denitrificans	0.92 Predict
DOM026_scaffold4239_7	19213	phage	0.996	temperate	0.99985605	unknown	0	unknown	0 -
DOM026_scaffold10323_1	10712	phage	0.966	temperate	0.99985975	unknown	0	Lactobacillus fermentum	0.92 Predict
DOM026_scaffold23168_2	16429	phage	0.997	virulent	0.9998257	unknown	0	Lactobacillus fermentum	0.9 Predict
DOM026_scaffold28561_1	11640	phage	0.999	temperate	0.9863652	unknown	0	Lactobacillus fermentum	1 CRISPR
DOM026_scaffold8858_1	10832	phage	0.987	temperate	0.99986035	unknown	0	Clostridioides difficile	0.81 Predict
DOM026_scaffold39135_1	13179	phage	0.995	temperate	0.99985975	unknown	0	Mycoplasma pulmonis	0.92 Predict
DOM026_scaffold7817_3	11683	phage	0.974	temperate	0.8617855	unknown	0	Candidatus Pelagibacter ubique	0.74 Predict
DOM026_scaffold6782_6	13522	phage	0.999	temperate	0.9998584	unknown	0	Bacillus alcalophilus	0.91 Predict
DOM026_scaffold6782_3	47097	phage	0.989	temperate	0.9998413	Straboviridae	0.49118197	Candidatus Pelagibacter ubique	1 CRISPR
DOM026_scaffold36555_1	42995	phage	0.983	virulent	0.99297863	unknown	0	Glaesserella parasuis	0.99 Predict
DOM026_C539342_1	15252	phage	0.999	temperate	0.99949235	unknown	0	unknown	0 -
DOM026_scaffold11444_1	16762	phage	0.997	temperate	0.99985695	unknown	0	Staphylococcus pasteuri	0.93 Predict
DOM026_scaffold21142_1	12073	phage	0.994	temperate	0.9998565	unknown	0	Lactobacillus fermentum	1 Predict
DOM026_scaffold5666_20	16158	phage	0.999	virulent	0.99987125	unknown	0	Streptomyces avermitilis	0.76 Predict
DOM026_scaffold22785_1	60884	phage	0.999	virulent	0.99986696	Straboviridae	0.27209267	Flavobacterium columnare	0.76 Predict
DOM026_scaffold17021_2	11991	phage	0.994	virulent	0.99987036	unknown	0	Mycoplasma pulmonis	1 CRISPR
DOM026_scaffold12473_1	13937	phage	0.996	temperate	0.9998474	unknown	0	Lactobacillus fermentum	0.77 Predict
DOM026_C539214_1	13824	phage	0.961	temperate	0.9998588	Straboviridae	0.7067733	unknown	0 -
DOM026_scaffold14353_26	11066	phage	0.998	virulent	0.9998579	unknown	0	Staphylococcus simulans	1 CRISPR
DOM026_scaffold13335_3	11977	phage	0.999	temperate	0.9627462	unknown	0	Mycoplasma pulmonis	0.99 Predict
DOM026_scaffold28681_1	11821	phage	0.998	temperate	0.9998593	unknown	0	Mycoplasma pulmonis	1 CRISPR
DOM026_scaffold18755_1	13879	phage	0.999	virulent	0.99987406	unknown	0	Candidatus Pelagibacter ubique	0.85 Predict
DOM026_scaffold22785_2	36713	phage	0.999	virulent	0.99987036	no_family_avaliable(NC_024711)	0.962	Lactobacillus gasseri	0.84 Predict

DOM026_scaffold22404_1	28677	phage	0.975	virulent	0.9998565	Straboviridae	0.37198326	Bacteroides fragilis	1	CRISPR
DOM026_scaffold4239_3	10480	phage	1	temperate	0.9998588	unknown	0	Clostridium perfringens	1	CRISPR
DOM026_scaffold26812_1	10885	phage	0.963	temperate	0.99985975	unknown	0	Lactobacillus fermentum	1	CRISPR
DOM026_scaffold10922_1	13201	phage	0.861	virulent	0.9956566	unknown	0	Mycoplasma pulmonis	0.78	Predict
DOM026_scaffold27635_1	11594	phage	0.798	temperate	0.9998579	unknown	0	Mycoplasma pulmonis	0.89	Predict
DOM026_scaffold21024_9	13364	phage	0.519	temperate	0.99986035	unknown	0	Lactobacillus fermentum	1	CRISPR
DOM026_scaffold21514_2	16704	phage	0.998	virulent	0.9568131	unknown	0	Candidatus Pelagibacter ubique	0.72	Predict
DOM026_scaffold35658_1	15803	phage	0.616	virulent	0.9998679	Salasmaviridae	0.28656432	Croceibacter atlanticus	0.82	Predict
DOM026_scaffold23768_1	14539	phage	0.999	temperate	0.98560125	unknown	0	Candidatus Pelagibacter ubique	0.95	Predict
DOM026_scaffold17367_1	10858	phage	0.996	temperate	0.995717	unknown	0	Parabacteroides distasonis	0.8	Predict
NOM001_scaffold14915_2	12055	phage	0.98	virulent	0.9998186	unknown	0	unknown	0	-
NOM001_scaffold36280_1	13209	phage	0.999	virulent	0.57108474	Peduoviridae	1	Mycoplasma pulmonis	0.95	Predict
NOM001_scaffold22842_7	37603	phage	0.999	virulent	0.9998593	unknown	0	Mycoplasma pulmonis	1	CRISPR
NOM001_scaffold36050_1	13760	phage	0.983	temperate	0.9333975	unknown	0	Bacteroides fragilis	0.96	Predict
NOM001_scaffold22842_8	10055	phage	0.999	temperate	0.99986035	unknown	0	Mycoplasma pulmonis	0.88	Predict
NOM001_scaffold4297_1	11905	phage	0.998	temperate	0.999723	Straboviridae	1	Thermoanaerobacterium saccharolyti	0.92	Predict
NOM001_scaffold36892_1	10494	phage	0.933	temperate	0.9998593	unknown	0	Mycoplasma pulmonis	0.94	Predict
NOM001_scaffold31539_2	10471	phage	0.602	virulent	0.9997986	unknown	0	unknown	0	-
NOM001_scaffold24512_3	11460	phage	0.937	virulent	0.9997156	unknown	0	Mycoplasma pulmonis	0.82	Predict
NOM001_scaffold22842_6	12627	phage	0.844	virulent	0.99987406	Drexlerviridae	0.31882837	Mycoplasma pulmonis	0.83	Predict
NOM002_scaffold4408_28	10428	phage	0.999	virulent	0.98006254	unknown	0	Mycoplasma pulmonis	0.72	Predict
NOM002_scaffold1807_4	11281	phage	0.961	temperate	0.979654	unknown	0	Bacteroides fragilis	0.92	Predict
NOM002_scaffold5847_1	11569	phage	0.98	temperate	0.9998588	Zierdtviridae	0.18184917	Bacillus subtilis	1	CRISPR
NOM002_scaffold139_2	34226	phage	0.999	temperate	0.99985975	unknown	0	Candidatus Pelagibacter ubique	0.78	Predict
NOM002_scaffold32200_2	55273	phage	0.998	temperate	0.9998474	Straboviridae	0.39451152	Candidatus Pelagibacter ubique	1	CRISPR
NOM002_scaffold23302_7	11659	phage	0.987	temperate	0.7455003	Ackermannviridae	1	Streptococcus pneumoniae	0.93	Predict
NOM002_scaffold2797_4	16170	phage	0.994	virulent	0.9997957	unknown	0	Bacteroides fragilis	0.72	Predict
NOM002_scaffold26702_1	12568	phage	0.999	temperate	0.99977213	unknown	0	Mycoplasma pulmonis	0.79	Predict
NOM002_scaffold26752_2	17336	phage	0.989	virulent	0.9998693	unknown	0	Candidatus Pelagibacter ubique	0.99	Predict
NOM002_scaffold65_1	12812	phage	0.995	temperate	0.99985975	unknown	0	Candidatus Pelagibacter ubique	0.8	Predict

NOM002_scaffold2059_12	11150	phage	0.992	temperate	0.7585868	Zierdtviridae	0.12534718	unknown	0	-
NOM002_scaffold32168_1	44006	phage	1	virulent	0.6105096	Straboviridae	0.46906093	Candidatus Pelagibacter ubique	1	CRISPR
NOM002_scaffold32212_1	10573	phage	0.999	temperate	0.99986035	Straboviridae	0.6309193	Mycoplasma pulmonis	1	CRISPR
NOM002_scaffold15419_6	12074	phage	0.999	temperate	0.99985975	unknown	0	Candidatus Pelagibacter ubique	0.8	Predict
NOM002_scaffold7211_5	11009	phage	0.999	temperate	0.6142863	unknown	0	Mycoplasma pulmonis	0.92	Predict
NOM002_scaffold32093_2	14171	phage	0.999	virulent	0.99987036	no_family_avaliable(NC_067210)	0.95	Candidatus Pelagibacter ubique	1	CRISPR
NOM002_scaffold2423_2	10282	phage	0.991	virulent	0.99987125	unknown	0	Bacteroides fragilis	0.94	Predict
NOM002_scaffold32124_1	12363	phage	0.999	temperate	0.9998588	unknown	0	Colwellia psychrerythraea	1	CRISPR
NOM002_scaffold32041_1	24319	phage	0.997	virulent	0.99846077	Kyanoviridae	1	Lactobacillus gasseri	0.91	Predict
NOM002_scaffold13215_6	14255	phage	0.541	temperate	0.9998336	Straboviridae	0.60870373	Mycoplasma pulmonis	1	CRISPR
NOM002_scaffold830_1	11363	phage	0.998	temperate	0.99985975	Straboviridae	0.5261669	Mycoplasma pulmonis	0.94	Predict
NOM002_scaffold3250_3	11252	phage	0.966	virulent	0.99986696	Peduoviridae	0.59965926	Colwellia psychrerythraea	0.96	Predict
NOM002_scaffold23876_3	10332	phage	0.999	temperate	0.9998574	unknown	0	Bacteroides sp. A1C1	1	CRISPR
NOM002_scaffold5995_1	31630	phage	0.956	temperate	0.87618214	Ackermannviridae	1	Colwellia psychrerythraea	0.8	Predict
NOM002_scaffold31941_2	25497	phage	0.999	temperate	0.9998517	unknown	0	Candidatus Pelagibacter ubique	1	CRISPR
NOM002_scaffold30413_1	77882	phage	0.987	temperate	0.99983495	Straboviridae	0.2870157	Candidatus Pelagibacter ubique	1	CRISPR
NOM002_scaffold31231_2	11209	phage	0.923	virulent	0.86550814	unknown	0	Mycoplasma pulmonis	0.95	Predict
NOM002_scaffold3598_9	10844	phage	0.966	virulent	0.99987173	Straboviridae	0.39407468	Bacteroides fragilis	0.96	Predict
NOM002_scaffold7151_8	11058	phage	0.999	virulent	0.99902534	unknown	0	Mycoplasma pulmonis	0.71	Predict
NOM002_scaffold20546_3	70820	phage	0.999	temperate	0.99740297	Demerecviridae	0.21957879	Clostridioides difficile	1	CRISPR
NOM002_scaffold1562_8	14377	phage	0.999	temperate	0.6375706	Straboviridae	0.54900956	Mycoplasma pulmonis	0.74	Predict
NOM002_scaffold27268_2	10813	phage	0.997	temperate	0.99984837	unknown	0	Parabacteroides distasonis	0.74	Predict
NOM002_scaffold27843_1	10535	phage	0.964	temperate	0.99986035	Straboviridae	0.4580326	Francisella tularensis	1	CRISPR
NOM002_scaffold31229_1	10432	phage	0.964	virulent	0.9998693	Straboviridae	1	Staphylococcus saprophyticus	0.9	Predict
NOM002_C429380_1	29894	phage	0.736	temperate	0.9998588	unknown	0	Trichormus variabilis	0.82	Predict
NOM002_C428718_1	15123	phage	0.998	temperate	0.99986035	Straboviridae	0.5692244	Mycoplasma pulmonis	0.81	Predict
NOM002_scaffold32206_1	12012	phage	0.997	virulent	0.9998026	unknown	0	Bacteroides fragilis	0.74	Predict
NOM002_scaffold32205_1	21428	phage	0.992	temperate	0.9977357	Straboviridae	0.4166439	Microcystis aeruginosa	0.94	Predict
NOM002_scaffold330_35	11055	phage	0.997	virulent	0.9998693	unknown	0	Mycoplasma pulmonis	0.82	Predict
NOM002_scaffold32135_1	22214	phage	0.999	temperate	0.99984497	unknown	0	Mycoplasma pulmonis	0.73	Predict

NOM002_scaffold31205_1	17002	phage	0.883	virulent	0.9991823	Straboviridae	0.5898211	Mycoplasma pulmonis	1	CRISPR
NOM002_scaffold22930_2	10904	phage	0.995	virulent	0.9998117	Straboviridae	0.31073028	Mycoplasma pulmonis	0.95	Predict
NOM002_scaffold3856_12	14942	phage	0.932	virulent	0.9998727	Ackermannviridae	0.35297325	Mycoplasma pulmonis	0.84	Predict
NOM002_scaffold6808_7	10742	phage	0.72	virulent	0.99987316	unknown	0	Mycoplasma pulmonis	0.86	Predict
NOM002_scaffold19947_2	22240	phage	0.998	temperate	0.99985975	unknown	0	Candidatus Pelagibacter ubique	0.82	Predict
NOM002_scaffold12213_5	15900	phage	0.919	temperate	0.9413989	Straboviridae	0.3920616	Bacteroides fragilis	0.85	Predict
NOM002_scaffold4555_2	14355	phage	0.972	virulent	0.99983925	unknown	0	Acinetobacter johnsonii	0.93	Predict
NOM002_scaffold14897_3	10575	phage	0.94	temperate	0.9119875	unknown	0	unknown	0	-
NOM002_scaffold6256_7	28777	phage	0.924	virulent	0.9998608	Straboviridae	0.30048168	Trichormus variabilis	0.95	Predict
NOM002_scaffold24280_1	15846	phage	0.998	temperate	0.99986035	unknown	0	Mycoplasma pulmonis	1	CRISPR
NOM002_scaffold20040_1	13909	phage	0.992	virulent	0.9998617	unknown	0	Bacteroides fragilis	0.9	Predict
NOM002_scaffold7687_1	24143	phage	0.796	virulent	0.9627274	Straboviridae	0.36334187	Mycoplasma pulmonis	0.85	Predict
NOM002_scaffold27308_2	13196	phage	0.996	virulent	0.9989942	unknown	0	Mycoplasma pulmonis	0.83	Predict
NOM002_scaffold24275_1	40597	phage	0.999	virulent	0.9998656	Autographiviridae	1	Colwellia psychrerythraea	1	CRISPR
NOM004_scaffold22743_2	30219	phage	0.998	virulent	0.9454852	Straboviridae	0.79878217	Mycoplasma pulmonis	1	CRISPR
NOM004_scaffold14898_16	10996	phage	0.999	temperate	0.97422904	unknown	0	Bacteroides fragilis	0.73	Predict
NOM004_scaffold8990_22	11958	phage	0.741	virulent	0.9322203	unknown	0	Mycoplasma pulmonis	1	Predict
NOM004_scaffold3782_4	12000	phage	0.991	temperate	0.9996867	unknown	0	Mycoplasma pulmonis	0.85	Predict
NOM004_scaffold13517_2	11499	phage	0.986	virulent	0.99987173	Peduoviridae	0.59965926	Brevibacillus laterosporus	0.8	Predict
NOM004_scaffold22899_1	18380	phage	0.999	virulent	0.9998722	unknown	0	Mycoplasma pulmonis	1	CRISPR
NOM004_scaffold4741_16	15348	phage	0.994	temperate	0.93755734	unknown	0	Parabacteroides distasonis	1	CRISPR
NOM004_scaffold130_15	10761	phage	0.933	virulent	0.9122659	Zierdtviridae	0.12259063	Streptomyces griseus	0.76	Predict
NOM004_C361687_1	10570	phage	0.996	temperate	0.9368787	Straboviridae	0.22862318	Mycoplasma pulmonis	1	CRISPR
NOM004_scaffold11992_11	10481	phage	0.999	temperate	0.9998588	Straboviridae	0.5102872	Mycoplasma pulmonis	0.96	Predict
NOM004_scaffold15695_1	26410	phage	0.998	virulent	0.9998017	Straboviridae	1	Mycoplasma pulmonis	0.81	Predict
NOM004_scaffold21343_2	16111	phage	1	temperate	0.8460105	unknown	0	Mycoplasma pulmonis	0.85	Predict
NOM004_scaffold9807_9	23943	phage	0.957	virulent	0.99973303	Straboviridae	0.60870373	Mycoplasma pulmonis	0.88	Predict
NOM004_scaffold21707_1	20433	phage	0.999	virulent	0.99736845	Salasmaviridae	0.46300474	Mycoplasma pulmonis	1	CRISPR
NOM004_scaffold22694_1	22298	phage	0.939	virulent	0.93689924	Peduoviridae	1	Mycoplasma pulmonis	0.89	Predict
NOM004_scaffold11992_20	16001	phage	0.94	temperate	0.9516476	Casjensviridae	1	Mycoplasma pulmonis	0.88	Predict

NOM004_scaffold13523_7	14462	phage	0.993	virulent	0.9998636	Straboviridae	0.36334187	Bacteroides fragilis	0.76	Predict
NOM004_scaffold28_10	13324	phage	0.991	virulent	0.9998651	unknown	0	Mycoplasma pulmonis	0.7	Predict
NOM004_scaffold6154_1	15339	phage	0.996	virulent	0.908313	unknown	0	Candidatus Pelagibacter ubique	0.98	Predict
NOM004_scaffold17398_1	11700	phage	0.601	temperate	0.99985975	Straboviridae	0.2679807	Mycoplasma pulmonis	0.93	Predict
NOM004_scaffold11917_4	11442	phage	0.995	virulent	0.9998556	unknown	0	Bacteroides fragilis	0.86	Predict
NOM004_scaffold14898_14	20812	phage	0.999	virulent	0.9669712	unknown	0	Bacteroides fragilis	0.78	Predict
NOM004_scaffold15115_6	11510	phage	0.993	virulent	0.9998699	unknown	0	unknown	0	-
NOM004_scaffold1169_9	13521	phage	0.957	virulent	0.99987406	unknown	0	Mycoplasma pulmonis	0.8	Predict
NOM004_scaffold15486_1	13022	phage	0.965	virulent	0.9997053	Straboviridae	0.375927	Parabacteroides distasonis	0.79	Predict
NOM005_scaffold53686_4	15735	phage	0.994	temperate	0.8726311	Drexlerviridae	0.27170157	Mycoplasma pulmonis	0.89	Predict
NOM005_C715971_1	13573	phage	0.999	temperate	0.9998574	unknown	0	Colwellia psychrerythraea	1	CRISPR
NOM005_scaffold35434_1	32046	phage	0.508	virulent	0.9997797	Straboviridae	0.87696713	Mycoplasma pulmonis	1	CRISPR
NOM005_scaffold41127_8	42296	phage	0.998	virulent	0.99978644	Straboviridae	0.7063348	Candidatus Pelagibacter ubique	1	CRISPR
NOM005_scaffold563_2	10712	phage	0.998	virulent	0.99987125	Casjensviridae	1	Mycoplasma pulmonis	1	CRISPR
NOM005_scaffold10786_3	13805	phage	0.996	virulent	0.9998684	Straboviridae	0.55784976	Mycoplasma pulmonis	0.79	Predict
NOM005_scaffold51989_2	13206	phage	0.951	temperate	0.99985975	unknown	0	Mycoplasma pulmonis	0.77	Predict
NOM005_scaffold55066_2	58623	phage	0.998	temperate	0.8863712	Straboviridae	0.7000134	Lactobacillus jensenii	1	CRISPR
NOM005_C716463_1	31483	phage	0.998	temperate	0.99985695	no_family_avaliable(NC_019496)	0.986	Clostridium perfringens	1	CRISPR
NOM005_scaffold2849_2	23004	phage	0.99	virulent	0.83790475	Drexlerviridae	0.36308205	Mycoplasma pulmonis	0.76	Predict
NOM005_scaffold29223_4	10998	phage	0.636	virulent	0.99983126	unknown	0	unknown	0	-
NOM005_scaffold32988_8	12641	phage	0.999	virulent	0.9998727	unknown	0	Mycoplasma pulmonis	1	CRISPR
NOM005_scaffold3277_10	22518	phage	0.997	temperate	0.99959195	Schitoviridae	0.2513714	Candidatus Pelagibacter ubique	0.76	Predict
NOM005_scaffold40041_4	15973	phage	0.611	virulent	0.9998722	Drexlerviridae	0.3329966	Mycoplasma pulmonis	0.97	Predict
NOM005_scaffold563_4	17636	phage	0.999	virulent	0.9998684	Herelleviridae	1	Mycoplasma pulmonis	1	CRISPR
NOM005_scaffold37509_3	15879	phage	0.919	temperate	0.9413989	Straboviridae	0.3920616	Bacteroides fragilis	0.99	Predict
NOM005_scaffold41127_7	27412	phage	0.991	virulent	0.99986696	Straboviridae	0.5904167	Mycoplasma pulmonis	1	CRISPR
NOM005_scaffold11865_1	10498	phage	0.998	temperate	0.99986035	Casjensviridae	1	Mycoplasma pulmonis	0.79	Predict
NOM005_scaffold8808_3	13454	phage	0.999	temperate	0.99983835	unknown	0	Enterococcus faecalis	0.86	Predict
NOM005_scaffold130_3	10329	phage	0.882	virulent	0.99969083	unknown	0	Colwellia psychrerythraea	1	CRISPR
NOM005_scaffold47045_2	13596	phage	0.999	virulent	0.99986744	Autographiviridae	1	Mycoplasma pulmonis	0.99	Predict

NOM005_scaffold53973_2	18244	phage	0.999	virulent	0.9998636	Autographiviridae	1	Mycoplasma pulmonis	0.83	Predict
NOM005_scaffold12226_1	10521	phage	0.996	temperate	0.51049143	unknown	0	unknown	0	-
NOM005_scaffold16553_2	47136	phage	1	temperate	0.99985605	unknown	0	Clostridium botulinum	0.86	Predict
NOM005_scaffold563_3	10676	phage	0.989	temperate	0.99985695	Zierdtviridae	1	Mycoplasma pulmonis	1	CRISPR
NOM005_scaffold45891_2	53714	phage	0.992	temperate	0.9998408	Casjensviridae	1	Mycoplasma pulmonis	0.78	Predict
NOM005_scaffold29223_3	12648	phage	0.998	temperate	0.99962914	unknown	0	unknown	0	-
NOM005_scaffold55101_1	42656	phage	0.999	temperate	0.99980783	unknown	0	Clostridium perfringens	1	CRISPR
NOM005_scaffold8276_2	10088	phage	0.885	virulent	0.9998727	unknown	0	Colwellia psychrerythraea	0.74	Predict
NOM005_C716395_1	24274	phage	0.999	virulent	0.9998656	Autographiviridae	0.96155953	Mycoplasma pulmonis	0.73	Predict
NOM005_scaffold47045_3	12715	phage	0.999	virulent	0.9998699	Autographiviridae	1	Mycoplasma pulmonis	0.71	Predict
NOM005_scaffold31514_2	15221	phage	0.999	temperate	0.9997215	Ackermannviridae	0.34421903	Colwellia psychrerythraea	0.76	Predict
NOM005_scaffold504_2	10359	phage	0.937	virulent	0.8143582	unknown	0	unknown	0	-
NOM005_scaffold32988_6	11659	phage	1	virulent	0.9998679	unknown	0	Mycoplasma pulmonis	1	CRISPR
NOM005_scaffold37509_5	11301	phage	0.999	temperate	0.96727747	unknown	0	Mycoplasma pulmonis	0.76	Predict
NOM005_scaffold16216_8	10368	phage	0.998	virulent	0.9998736	unknown	0	unknown	0	-
NOM005_scaffold11952_1	11109	phage	0.968	temperate	0.9997053	unknown	0	Colwellia psychrerythraea	0.97	Predict
NOM005_scaffold41127_12	40959	phage	0.999	temperate	0.9998593	unknown	0	Candidatus Pelagibacter ubique	0.75	Predict
NOM005_scaffold5739_4	14539	phage	0.911	virulent	0.9998722	Straboviridae	0.2870157	Aliivibrio fischeri	0.97	Predict
NOM005_scaffold50369_2	10106	phage	0.998	temperate	0.99985975	unknown	0	unknown	0	-
NOM005_scaffold5699_2	11986	phage	0.999	virulent	0.9814843	no_family_avaliable(NC_031129)	0.987	Salmonella enterica	1	CRISPR
NOM005_scaffold2279_1	17914	phage	0.998	temperate	0.99652207	unknown	0	Streptococcus mutans	0.9	Predict
NOM005_scaffold54732_1	15925	phage	0.998	temperate	0.99986035	unknown	0	Mycoplasma pulmonis	0.97	Predict
NOM005_scaffold41647_4	17544	phage	0.999	virulent	0.9998665	unknown	0	Candidatus Pelagibacter ubique	0.81	Predict
NOM005_scaffold41127_11	15553	phage	0.997	virulent	0.99979216	Straboviridae	0.7579115	Flavobacterium columnare	0.78	Predict
NOM005_scaffold41127_5	15272	phage	0.982	temperate	0.9998565	unknown	0	Mycoplasma pulmonis	1	CRISPR
NOM005_scaffold53973_3	14450	phage	1	virulent	0.9998665	Autographiviridae	1	Mycoplasma pulmonis	0.71	Predict
NOM005_scaffold47045_1	13373	phage	0.999	virulent	0.9998699	Autographiviridae	1	Mycoplasma pulmonis	1	CRISPR
NOM005_scaffold48061_2	13977	phage	0.994	virulent	0.9983386	Straboviridae	0.62754965	unknown	0	-
NOM005_scaffold55068_1	39341	phage	0.999	virulent	0.9918761	Peduoviridae	0.4337845	Listeria monocytogenes	1	CRISPR
NOM005_scaffold20861_2	15631	phage	0.999	virulent	0.9996341	unknown	0	Colwellia psychrerythraea	0.77	Predict

NOM005_scaffold25180_1	14533	phage	0.999	temperate	0.9998588	no_family_avaliable(NC_019501)	0.964	Mycoplasma pulmonis	1	CRISPR
NOM005_C716365_1	22350	phage	0.999	virulent	0.9998684	Autographiviridae	0.96155393	Mycoplasma pulmonis	0.88	Predict
NOM007_scaffold3522_1	12682	phage	0.996	temperate	0.999703	Straboviridae	0.4042891	Mycoplasma pulmonis	0.98	Predict
NOM007_C668778_1	11528	phage	0.999	temperate	0.9998588	unknown	0	Flavobacterium columnare	0.9	Predict
NOM007_scaffold48877_1	15190	phage	0.992	temperate	0.99985975	unknown	0	Mycoplasma pulmonis	1	Predict
NOM007_scaffold37186_2	10320	phage	0.559	temperate	0.99985975	unknown	0	unknown	0	-
NOM007_scaffold46703_1	14774	phage	0.999	temperate	0.98560125	unknown	0	Colwellia psychrerythraea	1	CRISPR
NOM007_scaffold45023_6	12368	phage	0.985	temperate	0.999651	unknown	0	Bacteroides fragilis	1	CRISPR
NOM007_scaffold15814_1	21257	phage	0.999	temperate	0.9998588	unknown	0	Erysipelothrix rhusiopathiae	0.77	Predict
NOM007_scaffold41225_1	22149	phage	1	temperate	0.9901432	Straboviridae	0.57805586	Parabacteroides merdae	1	CRISPR
NOM007_scaffold270_1	11027	phage	0.562	virulent	0.93356663	unknown	0	unknown	0	-
NOM007_scaffold35454_4	11174	phage	0.981	temperate	0.99986035	Ackermannviridae	0.41468462	Mycoplasma pulmonis	0.93	Predict
NOM007_scaffold39468_29	10883	phage	0.999	virulent	0.99845034	unknown	0	Bacteroides dorei	1	CRISPR
NOM007_C669362_1	92011	phage	0.999	virulent	0.99986744	Straboviridae	0.26939163	Candidatus Pelagibacter ubique	0.78	Predict
NOM007_scaffold14193_6	15167	phage	0.999	virulent	0.9992085	unknown	0	Candidatus Pelagibacter ubique	0.8	Predict
NOM007_scaffold35189_1	11713	phage	0.86	virulent	0.9998727	unknown	0	Aeromonas hydrophila	0.85	Predict
NOM007_scaffold37589_1	16491	phage	1	temperate	0.9998545	unknown	0	Mycoplasma pulmonis	0.89	Predict
NOM007_scaffold37139_1	17258	phage	0.999	temperate	0.9991854	Straboviridae	0.28392893	Colwellia psychrerythraea	0.92	Predict
NOM007_scaffold17241_4	10843	phage	0.876	virulent	0.99211204	unknown	0	Mannheimia haemolytica	0.72	Predict
NOM007_scaffold10787_5	13313	phage	0.999	virulent	0.9998157	Straboviridae	0.5013129	Mycoplasma pulmonis	0.83	Predict
NOM007_scaffold48928_1	33666	phage	0.983	temperate	0.9995662	Straboviridae	0.20679401	Mycoplasma pulmonis	0.88	Predict
NOM007_scaffold41196_1	10513	phage	0.999	temperate	0.9998012	unknown	0	Mycoplasma pulmonis	0.88	Predict
NOM007_scaffold29869_4	11420	phage	0.77	virulent	0.99987084	unknown	0	unknown	0	-
NOM007_scaffold4742_2	16217	phage	0.994	virulent	0.9998684	Straboviridae	0.55210805	Mycoplasma pulmonis	0.97	Predict
NOM008_scaffold33378_1	16422	phage	0.999	temperate	0.9998593	Chaseviridae	0.32056236	Candidatus Pelagibacter ubique	0.87	Predict
NOM008_scaffold17954_24	10919	phage	0.95	virulent	0.9997584	unknown	0	Mycoplasma pulmonis	0.81	Predict
NOM008_scaffold18778_4	10219	phage	0.999	virulent	0.99986696	unknown	0	Bacteroides fragilis	0.96	Predict
NOM008_scaffold4244_38	10710	phage	0.999	temperate	0.9998556	Straboviridae	0.48894924	Hymenobacteraceae bacterium SYSU	1	CRISPR
NOM008_scaffold9964_68	11819	phage	0.999	virulent	0.99974686	Peduoviridae	1	Mycoplasma pulmonis	0.84	Predict
NOM008_scaffold75_1	14408	phage	1	virulent	0.9998727	Salasmaviridae	1	Mycoplasma pulmonis	1	CRISPR

NOM008_scaffold18096_12	10916	phage	0.989	virulent	0.9998736	unknown	0	Mycoplasma pulmonis	0.81 Predict
NOM008_scaffold29282_11	10545	phage	0.998	virulent	0.9998736	unknown	0	Parabacteroides merdae	0.79 Predict
NOM008_scaffold445_45	14777	phage	0.994	virulent	0.9998699	unknown	0	Colwellia psychrerythraea	0.79 Predict
NOM008_scaffold33375_1	16373	phage	0.999	virulent	0.9998656	unknown	0	Mycoplasma pulmonis	1 CRISPR
NOM008_scaffold24500_2	11264	phage	0.995	virulent	0.9998302	Straboviridae	0.31073028	Mycoplasma pulmonis	0.92 Predict
NOM008_scaffold32938_5	23457	phage	0.995	virulent	0.9998379	Ackermannviridae	0.30793354	Bacteroides fragilis	0.77 Predict
NOM008_scaffold6097_1	28853	phage	0.996	virulent	0.9998388	Straboviridae	0.4709028	Candidatus Pelagibacter ubique	0.8 Predict
NOM008_scaffold14724_3	24215	phage	1	temperate	0.98581904	Straboviridae	0.14552358	Candidatus Pelagibacter ubique	1 CRISPR
NOM008_scaffold33345_2	10852	phage	0.998	virulent	0.96981525	Ackermannviridae	0.40345907	Mycoplasma pulmonis	0.8 Predict
NOM008_scaffold32941_1	15827	phage	0.999	virulent	0.9998017	unknown	0	Colwellia psychrerythraea	1 CRISPR
NOM008_scaffold32975_4	18218	phage	0.529	virulent	0.6497936	unknown	0	Parabacteroides merdae	0.72 Predict
NOM008_scaffold226_8	15206	phage	0.998	virulent	0.99966824	Straboviridae	0.45782614	Mycoplasma pulmonis	0.71 Predict
NOM008_scaffold9194_1	10019	phage	0.79	virulent	0.96896803	unknown	0	Mycoplasma pulmonis	1 CRISPR
NOM008_scaffold30194_1	16786	phage	0.996	temperate	0.9998584	Straboviridae	0.69343835	Mycoplasma pulmonis	0.78 Predict
NOM008_scaffold940_9	12991	phage	0.932	virulent	0.9998727	Ackermannviridae	0.35297325	Mycoplasma pulmonis	0.78 Predict
NOM008_scaffold29282_6	20611	phage	0.999	virulent	0.9998688	unknown	0	Colwellia psychrerythraea	0.7 Predict
NOM008_scaffold1177_10	11154	phage	0.999	virulent	0.9181428	Straboviridae	0.64330465	Mycoplasma pulmonis	0.9 Predict
NOM008_scaffold5454_20	10649	phage	0.998	temperate	0.9998593	unknown	0	Mycoplasma pulmonis	0.96 Predict
NOM008_scaffold27977_1	11183	phage	0.995	virulent	0.9998617	unknown	0	Mycoplasma pulmonis	0.88 Predict
NOM009_scaffold34457_1	12460	phage	0.807	temperate	0.99985975	unknown	0	unknown	0 -
NOM009_scaffold37752_3	17193	phage	0.999	temperate	0.99985605	unknown	0	Colwellia psychrerythraea	0.95 Predict
NOM009_scaffold15278_8	11501	phage	0.999	virulent	0.9998636	unknown	0	Mycoplasma pulmonis	0.78 Predict
NOM009_scaffold52880_2	11354	phage	0.997	temperate	0.9839192	unknown	0	unknown	0 -
NOM009_scaffold24584_3	10677	phage	0.999	temperate	0.999784	unknown	0	Mycoplasma pulmonis	0.93 Predict
NOM009_scaffold54003_3	10639	phage	0.999	temperate	0.515138	unknown	0	unknown	0 -
NOM009_scaffold27152_1	12355	phage	0.998	virulent	0.99981	unknown	0	Bacteroides fragilis	0.85 Predict
NOM009_scaffold43294_6	12643	phage	0.793	virulent	0.798589	unknown	0	Mycoplasma pulmonis	0.77 Predict
NOM009_scaffold31057_2	10084	phage	0.981	temperate	0.99986035	Ackermannviridae	0.41468462	Mycoplasma pulmonis	0.71 Predict
NOM009_scaffold45014_3	14414	phage	0.998	virulent	0.99986744	Straboviridae	0.46134415	Mycoplasma pulmonis	0.71 Predict
NOM009_scaffold41176_2	17589	phage	0.808	virulent	0.9998736	unknown	0	Bacteroides fragilis	0.82 Predict

NOM009_scaffold25569_1	28171	phage	0.999	temperate	0.99984974	Casjensviridae	1	Mycoplasma pulmonis	0.73	Predict
NOM009_scaffold19614_4	11111	phage	0.928	virulent	0.9998522	Drexlerviridae	0.25596908	Mycoplasma pulmonis	0.91	Predict
NOM009_scaffold40704_1	18633	phage	0.999	virulent	0.91393614	Straboviridae	0.53671855	Mycoplasma pulmonis	1	CRISPR
NOM009_scaffold15278_9	10304	phage	0.999	temperate	0.99985975	unknown	0	Mycoplasma pulmonis	0.86	Predict
NOM009_scaffold54000_1	44133	phage	0.999	virulent	0.99986315	Autographiviridae	1	Colwellia psychrerythraea	0.96	Predict
NOM009_scaffold1163_4	18180	phage	1	virulent	0.99986124	unknown	0	Mycoplasma pulmonis	0.99	Predict
NOM009_scaffold16373_1	10494	phage	0.986	temperate	0.99986035	unknown	0	unknown	0	-
NOM009_scaffold10818_20	13696	phage	1	temperate	0.9998403	Straboviridae	0.6591823	Mycoplasma pulmonis	1	CRISPR
NOM009_scaffold22166_2	17450	phage	0.996	virulent	0.9998684	Straboviridae	0.55784976	Mycoplasma pulmonis	0.73	Predict
NOM009_scaffold18703_1	14520	phage	0.998	temperate	0.99986035	Schitoviridae	0.2513714	Candidatus Pelagibacter ubique	0.85	Predict
NOM009_scaffold53866_1	11100	phage	0.997	temperate	0.97646034	unknown	0	Colwellia psychrerythraea	0.84	Predict
NOM009_scaffold6873_12	16398	phage	0.999	temperate	0.99985975	unknown	0	Mycoplasma pulmonis	0.86	Predict
NOM009_scaffold20832_3	13915	phage	0.994	temperate	0.93755734	unknown	0	unknown	0	-
NOM009_scaffold13490_22	10606	phage	0.996	virulent	0.9998379	unknown	0	unknown	0	-
NOM009_scaffold26738_4	10426	phage	0.999	temperate	0.9998593	unknown	0	unknown	0	-
NOM009_scaffold41176_6	14593	phage	1	temperate	0.99985975	Straboviridae	0.7051302	Mycoplasma pulmonis	1	CRISPR
NOM009_scaffold37752_2	19860	phage	0.999	virulent	0.9995881	Chaseviridae	0.26963246	unknown	0	-
NOM009_scaffold337_2	13377	phage	0.528	virulent	0.9998736	unknown	0	Mycoplasma pulmonis	0.84	Predict
NOM009_scaffold293_1	12955	phage	1	temperate	0.9998588	unknown	0	Mycoplasma pulmonis	1	CRISPR
NOM009_scaffold37752_4	20578	phage	0.999	temperate	0.9998574	unknown	0	Myxococcus xanthus	0.77	Predict
NOM009_scaffold53815_1	14816	phage	0.998	temperate	0.9998388	unknown	0	Mycoplasma pulmonis	0.79	Predict
NOM009_scaffold14272_1	10719	phage	0.995	temperate	0.99936604	Straboviridae	0.30048168	Candidatus Pelagibacter ubique	0.72	Predict
NOM009_scaffold113_2	16232	phage	0.999	virulent	0.9998665	unknown	0	Mycoplasma pulmonis	0.72	Predict
NOM009_scaffold14149_2	10956	phage	0.974	temperate	0.99985975	unknown	0	Candidatus Pelagibacter ubique	0.98	Predict
NOM009_scaffold53934_1	15220	phage	0.936	temperate	0.99985695	unknown	0	Mycoplasma pulmonis	0.91	Predict
NOM009_scaffold53906_1	10120	phage	0.999	temperate	0.9998593	unknown	0	Roseburia intestinalis	1	CRISPR
NOM009_scaffold44452_1	24963	phage	0.794	virulent	0.9998656	Vilmaviridae	0.24893457	Bacillus thuringiensis	0.94	Predict
NOM010_scaffold16688_4	10241	phage	0.979	temperate	0.9998593	unknown	0	Parabacteroides merdae	0.73	Predict
NOM010_scaffold7834_6	19172	phage	0.838	virulent	0.9998302	unknown	0	Mycoplasma pulmonis	0.85	Predict
NOM010_scaffold36917_1	16772	phage	0.995	virulent	0.99982	Straboviridae	0.7514587	Candidatus Pelagibacter ubique	0.99	Predict

NOM010_scaffold40709_2	14251	phage	0.997	virulent	0.9998722	Herelleviridae	1	unknown	0	-
NOM010_scaffold27408_5	10400	phage	0.989	virulent	0.99987406	Straboviridae	0.530693	Mycoplasma pulmonis	0.75	Predict
NOM010_scaffold34799_1	15495	phage	0.998	temperate	0.99986035	Straboviridae	0.7394616	Mycoplasma pulmonis	0.79	Predict
NOM010_scaffold7841_9	19561	phage	0.997	virulent	0.9998684	Peduoviridae	0.3685337	Colwellia psychrerythraea	0.87	Predict
NOM010_scaffold23144_2	11294	phage	0.961	temperate	0.979654	unknown	0	Bacteroides fragilis	0.89	Predict
NOM010_scaffold6634_3	12198	phage	0.996	virulent	0.99987173	unknown	0	Colwellia psychrerythraea	0.87	Predict
NOM010_scaffold8017_1	16217	phage	0.992	virulent	0.6827767	unknown	0	Mycoplasma pulmonis	1	CRISPR
NOM010_scaffold39236_1	10965	phage	0.994	temperate	0.9967525	unknown	0	Bacteroides fragilis	0.79	Predict
NOM010_scaffold356_5	27977	phage	0.992	virulent	0.9998665	unknown	0	Mycoplasma pulmonis	1	CRISPR
NOM010_scaffold3371_1	15993	phage	0.756	temperate	0.9998593	unknown	0	unknown	0	-
NOM010_scaffold40709_1	11929	phage	0.998	virulent	0.99987173	unknown	0	unknown	0	-
NOM010_scaffold2023_1	10246	phage	0.983	virulent	0.8463101	Straboviridae	0.7514587	Bacteroides fragilis	0.98	Predict
NOM010_scaffold41832_1	23936	phage	0.998	virulent	0.967852	unknown	0	Clostridioides difficile	1	CRISPR
NOM010_scaffold41425_2	14755	phage	0.997	temperate	0.8937481	Straboviridae	0.22866157	unknown	0	-
NOM010_scaffold12374_8	23010	phage	0.999	virulent	0.99987125	Casjensviridae	0.32719818	Bacteroides fragilis	1	CRISPR
NOM010_scaffold26994_1	13732	phage	0.998	temperate	0.9998522	unknown	0	Mycoplasma pulmonis	1	CRISPR
NOM010_scaffold40683_2	12672	phage	0.998	temperate	0.9856434	unknown	0	Croceibacter atlanticus	0.86	Predict
NOM010_scaffold26048_2	22801	phage	0.998	virulent	0.9998688	Peduoviridae	1	Streptococcus mutans	0.79	Predict
NOM010_scaffold19890_3	19396	phage	0.999	virulent	0.99954635	unknown	0	Mycoplasma pulmonis	0.89	Predict
NOM010_scaffold40709_6	20475	phage	0.999	virulent	0.9998684	unknown	0	unknown	0	-
NOM010_scaffold16391_2	47744	phage	0.967	virulent	0.9998645	Ackermannviridae	0.29854247	Bacteroides fragilis	0.89	Predict
NOM010_scaffold19030_3	11234	phage	0.999	virulent	0.99966824	Ackermannviridae	0.28771883	Mycoplasma pulmonis	0.74	Predict
NOM010_scaffold7692_1	14542	phage	0.999	temperate	0.99985975	unknown	0	Mycoplasma pulmonis	0.78	Predict
NOM010_scaffold27711_4	18590	phage	0.911	virulent	0.9998693	Straboviridae	0.7514587	Candidatus Pelagibacter ubique	0.92	Predict
NOM010_scaffold13739_1	11385	phage	0.91	virulent	0.9109146	unknown	0	Clostridioides difficile	0.75	Predict
NOM010_scaffold27824_1	13997	phage	0.999	virulent	0.99987316	unknown	0	Colwellia psychrerythraea	1	CRISPR
NOM010_scaffold5951_6	22339	phage	0.999	temperate	0.9998308	unknown	0	Mycoplasma pulmonis	0.83	Predict
NOM010_scaffold4318_15	11365	phage	0.932	virulent	0.99987406	unknown	0	Colwellia psychrerythraea	0.87	Predict
NOM010_scaffold41566_2	13530	phage	1	virulent	0.99937266	unknown	0	Mycoplasma pulmonis	0.88	Predict
NOM010_scaffold30699_2	15898	phage	0.919	temperate	0.9413989	Straboviridae	0.3920616	Bacteroides fragilis	0.76	Predict

NOM010_scaffold41431_1	13159	phage	0.991	virulent	0.99667025	unknown	0	Mycoplasma pulmonis	0.77	Predict
NOM010_scaffold29615_5	12504	phage	0.986	virulent	0.9998684	Straboviridae	0.17178196	Flavobacterium psychrophilum	0.76	Predict
NOM010_scaffold37895_1	21778	phage	0.909	temperate	0.99985975	unknown	0	unknown	0	-
NOM010_scaffold7945_5	13222	phage	0.988	temperate	0.98661816	Peduoviridae	0.7102873	Candidatus Pelagibacter ubique	0.97	Predict
NOM010_scaffold16353_1	24439	phage	0.999	temperate	0.9998588	Straboviridae	0.6108962	Mycoplasma pulmonis	0.97	Predict
NOM010_scaffold26455_8	10143	phage	0.999	virulent	0.99987406	unknown	0	Candidatus Pelagibacter ubique	0.82	Predict
NOM010_scaffold15321_2	58455	phage	0.999	virulent	0.99486125	Ackermannviridae	0.38382405	Flavobacterium columnare	1	CRISPR
NOM010_scaffold27687_3	24127	phage	0.986	temperate	0.99978644	unknown	0	Mycoplasma pulmonis	0.76	Predict
NOM010_scaffold23538_2	13799	phage	0.999	virulent	0.9995294	Straboviridae	0.39133236	Mycoplasma pulmonis	0.73	Predict
NOM010_scaffold16212_1	11869	phage	0.967	temperate	0.9998545	unknown	0	Mycoplasma pulmonis	0.73	Predict
NOM010_scaffold20969_4	13759	phage	0.994	virulent	0.9992909	unknown	0	Micromonospora chaiyaphumensis	1	CRISPR
NOM010_scaffold34_1	11373	phage	0.999	temperate	0.99985975	Ackermannviridae	0.31534573	Colwellia psychrerythraea	1	CRISPR
NOM010_scaffold27644_3	12742	phage	0.996	virulent	0.999038	unknown	0	Mycoplasma pulmonis	0.84	Predict
NOM010_scaffold24440_2	12728	phage	0.962	temperate	0.99985975	unknown	0	Mycoplasma pulmonis	0.73	Predict
NOM012_scaffold55930_5	10277	phage	0.989	virulent	0.9998684	unknown	0	unknown	0	-
NOM012_scaffold41765_3	10526	phage	1	temperate	0.99985313	unknown	0	Candidatus Pelagibacter ubique	1	CRISPR
NOM012_scaffold17023_1	12318	phage	0.913	virulent	0.9998736	Straboviridae	0.28039292	Morganella morganii	0.72	Predict
NOM012_scaffold8044_2	10062	phage	0.994	temperate	0.99850935	Kyanoviridae	1	Colwellia psychrerythraea	0.92	Predict
NOM012_scaffold58733_1	33879	phage	0.998	temperate	0.9998474	unknown	0	Mycoplasma pulmonis	1	CRISPR
NOM012_scaffold15076_8	11261	phage	0.994	virulent	0.9998684	unknown	0	Mycoplasma pulmonis	0.82	Predict
NOM012_scaffold8044_6	33557	phage	0.904	virulent	0.9998684	unknown	0	Mycoplasma pulmonis	0.82	Predict
NOM012_scaffold28597_33	49553	phage	1	virulent	0.99985266	Autographiviridae	1	Colwellia psychrerythraea	0.91	Predict
NOM012_scaffold59314_2	14855	phage	0.997	temperate	0.9998584	Straboviridae	0.4580326	unknown	0	-
NOM012_scaffold59022_1	41113	phage	0.999	virulent	0.9998565	Salasmaviridae	0.39329737	Mycoplasma pulmonis	0.93	Predict
NOM012_scaffold16052_1	18784	phage	0.998	virulent	0.9998688	unknown	0	unknown	0	-
NOM012_scaffold51561_1	94582	phage	0.999	virulent	0.9998645	Straboviridae	0.26939163	Candidatus Pelagibacter ubique	0.99	Predict
NOM012_C822191_1	46483	phage	0.998	virulent	0.99867475	Chaseviridae	1	Mycoplasma pulmonis	1	CRISPR
NOM012_scaffold57810_1	14296	phage	0.991	virulent	0.99987406	unknown	0	unknown	0	-
NOM012_scaffold4060_3	11791	phage	0.996	virulent	0.9998622	unknown	0	Mycoplasma pulmonis	0.78	Predict
NOM012_scaffold8574_7	13098	phage	0.996	virulent	0.89126426	unknown	0	Mycoplasma pulmonis	0.82	Predict

NOM012_scaffold45669_3	37418	phage	0.998	temperate	0.9997726	Straboviridae	0.73880583	Lactobacillus jensenii	1	CRISPR
NOM012_scaffold59297_1	10753	phage	0.97	virulent	0.9998688	unknown	0	Mycoplasma pulmonis	0.76	Predict
NOM012_scaffold25493_7	17001	phage	0.994	virulent	0.9998727	unknown	0	unknown	0	-
NOM012_scaffold16848_9	21659	phage	0.966	temperate	0.99985695	unknown	0	Lactobacillus jensenii	0.89	Predict
NOM012_scaffold19624_3	10151	phage	0.998	temperate	0.9998593	unknown	0	Bacteroides fragilis	0.84	Predict
NOM012_scaffold53716_1	32188	phage	0.999	virulent	0.9998622	Straboviridae	0.30672264	Mycoplasma pulmonis	0.86	Predict
NOM012_scaffold15076_7	11492	phage	0.999	virulent	0.9998722	Ackermannviridae	0.29854247	Mycoplasma pulmonis	0.94	Predict
NOM012_scaffold57636_2	13515	phage	0.585	virulent	0.99987084	Drexlerviridae	0.39975724	Colwellia psychrerythraea	0.7	Predict
NOM012_scaffold118_12	10239	phage	0.996	temperate	0.99985033	unknown	0	Mycoplasma pulmonis	0.83	Predict
NOM012_scaffold6750_2	10794	phage	0.889	temperate	0.9998474	unknown	0	Mycoplasma pulmonis	0.93	Predict
NOM013_scaffold15784_2	17708	phage	0.999	virulent	0.9998722	no_family_avaliable(NC_062778)	0.99	Candidatus Pelagibacter ubique	0.93	Predict
NOM013_scaffold54467_1	28862	phage	0.998	virulent	0.99987036	unknown	0	Candidatus Pelagibacter ubique	0.93	Predict
NOM013_scaffold31636_3	26196	phage	0.999	virulent	0.99987316	no_family_avaliable(NC_062779)	0.97	unknown	0	-
NOM013_scaffold18789_1	31156	phage	0.882	virulent	0.99972486	Straboviridae	1	Parabacteroides merdae	0.99	Predict
NOM013_scaffold47736_4	52977	phage	0.999	virulent	0.9998645	Salasmaviridae	0.44733387	Cellulophaga baltica	1	CRISPR
NOM013_scaffold28579_1	20547	phage	0.897	virulent	0.7755035	unknown	0	unknown	0	-
NOM013_C706454_1	22155	phage	0.999	temperate	0.9998579	Straboviridae	0.48894924	unknown	0	-
NOM013_scaffold34728_2	21503	phage	0.999	virulent	0.9998736	no_family_avaliable(NC_062765)	0.983	unknown	0	-
NOM013_scaffold13725_4	15901	phage	0.856	temperate	0.93894786	Straboviridae	0.3920616	Bacteroides fragilis	0.72	Predict
NOM013_scaffold11200_14	10116	phage	0.99	virulent	0.99987036	unknown	0	unknown	0	-
NOM013_scaffold28173_6	10730	phage	0.993	virulent	0.9997339	unknown	0	Mycoplasma pulmonis	0.99	Predict
NOM013_scaffold30855_5	14169	phage	0.998	temperate	0.9998584	Straboviridae	0.6309193	Flavobacterium columnare	1	Predict
NOM013_scaffold283_1	18072	phage	0.999	virulent	0.99987173	no_family_avaliable(NC_062778)	0.988	Candidatus Pelagibacter ubique	0.86	Predict
NOM013_scaffold16346_5	28044	phage	1	virulent	0.9998688	Schitoviridae	1	Candidatus Pelagibacter ubique	0.94	Predict
NOM013_scaffold23911_1	30096	phage	0.994	virulent	0.9997732	unknown	0	unknown	0	-
NOM013_scaffold55293_1	25726	phage	0.999	virulent	0.99987125	unknown	0	Candidatus Pelagibacter ubique	1	CRISPR
NOM013_scaffold16346_8	16639	phage	0.999	virulent	0.99987173	no_family_avaliable(NC_055876)	0.976	Candidatus Pelagibacter ubique	0.79	Predict
NOM013_scaffold19917_26	19169	phage	0.999	virulent	0.9998522	unknown	0	Mycoplasma pulmonis	0.74	Predict
NOM013_scaffold20568_5	15274	phage	0.999	virulent	0.99987084	no_family_avaliable(NC_062778)	0.981	Candidatus Pelagibacter ubique	0.73	Predict
NOM013_scaffold42371_10	11918	phage	0.942	temperate	0.99985313	Peduoviridae	0.74069476	Colwellia psychrerythraea	0.84	Predict

NOM013_scaffold31636_2	10418	phage	0.999	virulent	0.99987125	no_family_avaliable(NC_062779)	0.957	Candidatus Pelagibacter ubique	0.97	Predict
NOM013_scaffold7_2	10808	phage	0.998	virulent	0.99987173	unknown	0	Parabacteroides merdae	0.84	Predict
NOM013_scaffold19917_6	13145	phage	1	virulent	0.99986696	unknown	0	Mycoplasma pulmonis	0.89	Predict
NOM013_scaffold47736_3	19931	phage	0.998	virulent	0.9998665	Straboviridae	0.3162047	Mycoplasma pulmonis	1	CRISPR
NOM013_scaffold22501_3	10430	phage	0.991	temperate	0.99972105	Straboviridae	0.23342617	Bacteroides vulgatus	1	CRISPR
NOM013_scaffold54467_2	17134	phage	0.999	virulent	0.99987173	unknown	0	Candidatus Pelagibacter ubique	0.95	Predict
NOM013_scaffold53631_1	12134	phage	0.965	temperate	0.99985975	unknown	0	unknown	0	-
NOM013_scaffold3327_4	10776	phage	0.996	virulent	0.9998574	unknown	0	unknown	0	-
NOM013_scaffold11200_16	18200	phage	0.996	virulent	0.9990059	unknown	0	Mycoplasma pulmonis	0.84	Predict
NOM013_scaffold49212_2	36298	phage	1	virulent	0.9998584	Salasmaviridae	0.39329737	Mycoplasma pulmonis	0.88	Predict
NOM013_scaffold9824_2	15032	phage	0.982	temperate	0.9998465	Peduoviridae	1	Mycoplasma pulmonis	0.71	Predict
NOM013_scaffold4736_1	16553	phage	0.993	temperate	0.99985975	Straboviridae	0.78885055	Colwellia psychrerythraea	0.81	Predict
NOM013_scaffold32541_12	16365	phage	0.999	virulent	0.9998699	unknown	0	Parabacteroides merdae	0.74	Predict
NOM013_scaffold31511_2	26331	phage	0.999	virulent	0.9998665	Zobellviridae	0.5135538	Cellulophaga baltica	0.72	Predict
NOM013_scaffold3281_4	19412	phage	0.959	temperate	0.999662	unknown	0	Clostridioides difficile	0.73	Predict
NOM013_scaffold55128_3	70107	phage	0.996	virulent	0.99985975	Herelleviridae	1	Cellulophaga baltica	0.78	Predict
NOM013_scaffold18200_3	21415	phage	1	temperate	0.9988362	unknown	0	Candidatus Pelagibacter ubique	0.76	Predict
NOM013_scaffold31511_3	18337	phage	0.998	virulent	0.99987173	unknown	0	Mycoplasma pulmonis	0.79	Predict
NOM013_scaffold38707_7	14621	phage	0.953	virulent	0.99971104	Straboviridae	0.8097716	Colwellia psychrerythraea	0.97	Predict
NOM013_scaffold21734_30	13341	phage	0.999	virulent	0.56185067	unknown	0	Bacteroides fragilis	0.74	Predict
NOM013_scaffold16346_2	18732	phage	0.999	virulent	0.9998693	unknown	0	Candidatus Pelagibacter ubique	0.76	Predict
NOM013_scaffold28579_4	42717	phage	0.999	virulent	0.9998479	unknown	0	Cellulophaga baltica	1	CRISPR
NOM013_scaffold55291_1	11103	phage	0.932	virulent	0.99972963	Ackermannviridae	1	Candidatus Pelagibacter ubique	0.71	Predict
NOM013_scaffold14898_7	19821	phage	0.999	virulent	0.9998688	no_family_avaliable(NC_055894)	0.969	Mycoplasma pulmonis	0.82	Predict
NOM013_scaffold16204_2	12200	phage	0.669	virulent	0.9998617	Drexlerviridae	0.39975724	Colwellia psychrerythraea	1	CRISPR
NOM013_scaffold54010_1	22603	phage	0.577	temperate	0.9321044	unknown	0	Bacteroides fragilis	0.78	Predict
NOM013_scaffold19858_2	10962	phage	0.936	temperate	0.99985975	unknown	0	unknown	0	-
NOM013_scaffold11318_3	10155	phage	0.731	temperate	0.99985975	unknown	0	unknown	0	-
NOM013_scaffold31511_4	21904	phage	0.999	virulent	0.99986696	Schitoviridae	0.26197654	Cellulophaga baltica	0.96	Predict
NOM013_scaffold13071_1	10003	phage	0.99	temperate	0.9998588	unknown	0	Mycoplasma pulmonis	0.76	Predict

NOM013_scaffold42353_2	20929	phage	0.999	virulent	0.99987125	no_family_avaliable(NC_062765)	0.978	Candidatus Pelagibacter ubique	0.76	Predict
NOM013_scaffold36218_2	15530	phage	0.562	virulent	0.93356663	unknown	0	unknown	0	-
NOM014_scaffold24348_6	52864	phage	0.999	virulent	0.99984974	Schitoviridae	0.26319408	Flavobacterium columnare	0.71	Predict
NOM014_scaffold14255_16	16639	phage	0.955	virulent	0.99684715	unknown	0	Mycoplasma pulmonis	1	Predict
NOM014_scaffold15996_1	56016	phage	0.999	virulent	0.99986744	Straboviridae	0.2514063	Mycoplasma pulmonis	1	Predict
NOM014_C390595_1	16671	phage	0.999	temperate	0.9998226	unknown	0	Eubacterium sp. am_0171	1	CRISPR
NOM014_scaffold25704_1	12216	phage	0.999	temperate	0.99985975	unknown	0	Mycoplasma pulmonis	0.87	Predict
NOM014_scaffold24348_5	41922	phage	0.998	virulent	0.99986696	no_family_avaliable(NC_055884)	0.973	Cellulophaga baltica	1	Predict
NOM014_scaffold28199_1	12463	phage	0.995	temperate	0.9991313	Straboviridae	0.4166439	Colwellia psychrerythraea	1	Predict
NOM014_scaffold11825_3	19071	phage	0.999	temperate	0.99984604	unknown	0	Clostridioides difficile	0.86	Predict
NOM014_scaffold8227_3	22382	phage	1	temperate	0.6361202	unknown	0	Mycoplasma pulmonis	0.91	Predict
NOM014_scaffold28027_1	10908	phage	0.706	temperate	0.9998579	unknown	0	Mycoplasma pulmonis	0.77	Predict
NOM014_scaffold15825_1	10166	phage	0.995	virulent	0.999784	unknown	0	Mycoplasma pulmonis	0.76	Predict
NOM014_scaffold27185_1	22534	phage	0.988	virulent	0.99987036	Peduoviridae	0.40998638	Mycoplasma pulmonis	0.74	Predict
NOM014_scaffold28228_1	14067	phage	0.999	virulent	0.9998123	unknown	0	Mycoplasma pulmonis	0.73	Predict
NOM014_scaffold14811_8	16102	phage	0.978	virulent	0.9998722	unknown	0	Parabacteroides distasonis	0.85	Predict
NOM014_scaffold23281_1	10516	phage	0.998	virulent	0.9912987	unknown	0	Mycoplasma pulmonis	1	Predict
NOM014_scaffold27301_2	16839	phage	0.999	virulent	0.99986744	unknown	0	Mycoplasma pulmonis	1	CRISPR
NOM014_C390531_1	15563	phage	0.858	temperate	0.99981403	unknown	0	unknown	0	-
NOM014_scaffold19260_1	18155	phage	0.998	temperate	0.99963766	Straboviridae	0.78885055	Bacteroides fragilis	0.81	Predict
NOM014_scaffold28329_1	16052	phage	0.999	virulent	0.9998699	Salasmaviridae	1	Mycoplasma pulmonis	1	CRISPR
NOM014_scaffold9405_2	13592	phage	0.994	temperate	0.9998536	Straboviridae	0.7156085	Candidatus Pelagibacter ubique	0.74	Predict
NOM014_scaffold3384_5	14377	phage	0.99	virulent	0.99987173	Ackermannviridae	0.35297325	Bacteroides fragilis	0.99	Predict
NOM014_scaffold25598_1	10057	phage	0.972	temperate	0.99985975	Straboviridae	0.7156085	Bacteroides fragilis	0.94	Predict
NOM014_scaffold8317_1	10834	phage	0.961	virulent	0.9995841	unknown	0	Mycoplasma pulmonis	0.71	Predict
NOM014_scaffold19787_2	11860	phage	0.932	virulent	0.9998727	Ackermannviridae	0.35297325	Mycoplasma pulmonis	0.91	Predict
NOM014_scaffold28328_1	10160	phage	0.927	temperate	0.9998593	unknown	0	Parabacteroides merdae	0.77	Predict
NOM014_scaffold490_36	12256	phage	0.995	temperate	0.99021435	unknown	0	Bacteroides fragilis	0.77	Predict
NOM014_C390319_1	12372	phage	0.998	virulent	0.9911031	Straboviridae	0.4356539	Mycoplasma pulmonis	1	CRISPR
NOM014_scaffold13553_1	13961	phage	0.998	virulent	0.9998627	unknown	0	Bacteroides fragilis	0.79	Predict

NOM014_scaffold7249_2	10564	phage	0.999	virulent	0.95055354	unknown	0	unknown	0	-
NOM014_scaffold27259_1	15522	phage	0.976	temperate	0.99985695	Straboviridae	0.27350757	Bacteroides fragilis	1	Predict
NOM014_scaffold5743_3	11705	phage	0.999	temperate	0.92179525	unknown	0	Colwellia psychrerythraea	1	CRISPR
NOM014_scaffold28145_4	11795	phage	0.904	temperate	0.99986035	unknown	0	Mycoplasma pulmonis	0.99	Predict
NOM014_scaffold9372_1	29228	phage	0.999	temperate	0.9998508	Straboviridae	0.824416	Mycoplasma pulmonis	1	CRISPR
NOM014_scaffold15996_2	39742	phage	0.999	virulent	0.9997909	no_family_avaliable(NC_024711)	0.964	Lactobacillus gasseri	0.81	Predict
NOM014_scaffold13234_1	10181	phage	1	virulent	0.9985284	unknown	0	Mycoplasma pulmonis	0.73	Predict
NOM014_scaffold28353_1	16729	phage	1	temperate	0.99985975	unknown	0	unknown	0	-
NOM014_scaffold21899_2	10824	phage	0.998	temperate	0.99985975	unknown	0	Mycoplasma pulmonis	1	CRISPR
NOM014_scaffold28352_1	28671	phage	0.999	temperate	0.9998417	Ackermannviridae	0.2754581	Colwellia psychrerythraea	1	CRISPR
NOM014_scaffold15794_3	10979	phage	0.949	virulent	0.99682593	Straboviridae	0.31001717	Lactobacillus johnsonii	0.71	Predict
NOM015_scaffold35862_1	17374	phage	0.981	virulent	0.9998417	Straboviridae	0.8097716	Mycoplasma pulmonis	0.88	Predict
NOM015_scaffold36658_1	22530	phage	0.995	virulent	0.99980336	Ackermannviridae	0.177989	Mycoplasma pulmonis	0.87	Predict
NOM015_scaffold2934_14	12554	phage	0.998	temperate	0.9998513	unknown	0	unknown	0	-
NOM015_scaffold1034_1	17353	phage	0.998	virulent	0.99987084	Peduoviridae	0.35915253	Lactobacillus gasseri	1	CRISPR
NOM015_scaffold16922_3	10082	phage	0.958	virulent	0.9998508	unknown	0	Bacteroides fragilis	0.9	Predict
NOM015_scaffold34161_4	10873	phage	0.567	virulent	0.9998736	unknown	0	Mycoplasma pulmonis	0.88	Predict
NOM015_scaffold226_1	12779	phage	0.544	virulent	0.9998584	Ackermannviridae	1	Colwellia psychrerythraea	0.9	Predict
NOM015_scaffold10294_6	10114	phage	0.994	temperate	0.9998574	unknown	0	Mycoplasma pulmonis	0.71	Predict
NOM015_scaffold4016_5	12727	phage	0.998	virulent	0.99973303	unknown	0	Mycoplasma pulmonis	1	CRISPR
NOM015_scaffold4819_1	17747	phage	0.999	virulent	0.98910916	unknown	0	Colwellia psychrerythraea	0.92	Predict
NOM015_scaffold3437_9	12296	phage	0.999	virulent	0.9998699	unknown	0	Mycoplasma pulmonis	0.7	Predict
NOM015_scaffold15823_1	13198	phage	0.999	temperate	0.9998536	unknown	0	unknown	0	-
NOM015_scaffold124_1	13561	phage	0.995	temperate	0.99985975	unknown	0	Colwellia psychrerythraea	0.8	Predict
NOM015_scaffold18383_1	18065	phage	0.997	virulent	0.9998348	unknown	0	Mycoplasma pulmonis	1	CRISPR
NOM015_scaffold20240_1	11491	phage	0.988	temperate	0.99985975	unknown	0	Colwellia psychrerythraea	0.78	Predict
NOM015_scaffold16366_1	18892	phage	0.999	virulent	0.9998684	Salasmaviridae	0.96040297	Flavobacterium columnare	0.94	Predict
NOM015_scaffold1091_31	15445	phage	0.999	temperate	0.65716165	unknown	0	unknown	0	-
NOM015_scaffold23464_3	13388	phage	0.999	virulent	0.9998684	Salasmaviridae	0.28743166	Cellulophaga baltica	1	CRISPR
NOM015_scaffold8918_6	11828	phage	0.999	virulent	0.99642706	Ackermannviridae	0.28771883	Colwellia psychrerythraea	0.72	Predict

NOM015_scaffold20333_3	10456	phage	0.999	virulent	0.99987316	Salasmaviridae	1	Mycoplasma pulmonis	1	CRISPR
NOM015_scaffold36796_1	10305	phage	0.984	virulent	0.9998588	Straboviridae	0.4795738	Bacteroides fragilis	0.85	Predict
NOM015_scaffold14266_3	11593	phage	0.994	temperate	0.99985975	unknown	0	Yersinia pestis	0.86	Predict
NOM015_scaffold19627_1	17420	phage	0.99	virulent	0.99987173	Ackermannviridae	0.35297325	Mycoplasma pulmonis	0.99	Predict
NOM015_scaffold23464_2	17118	phage	0.993	virulent	0.9998693	Salasmaviridae	0.31784317	Cellulophaga baltica	0.72	Predict
NOM015_scaffold15823_2	29671	phage	0.999	temperate	0.9998588	Herelleviridae	1	Mycoplasma pulmonis	1	CRISPR
NOM015_scaffold3765_6	11217	phage	0.999	virulent	0.99935955	unknown	0	unknown	0	-
NOM015_scaffold1721_3	10457	phage	0.997	temperate	0.70776147	Straboviridae	0.54900956	unknown	0	-
NOM015_scaffold6127_1	11607	phage	0.998	temperate	0.9998593	unknown	0	Colwellia psychrerythraea	1	CRISPR
NOM015_scaffold11230_6	60891	phage	0.999	virulent	0.98540735	Straboviridae	0.41447464	Candidatus Pelagibacter ubique	1	CRISPR
NOM015_scaffold15823_5	45977	phage	0.996	virulent	0.9997554	Straboviridae	0.70922685	Mycoplasma pulmonis	1	CRISPR
NOM015_scaffold10294_4	13244	phage	0.999	virulent	0.9954039	Mesyanzhinovviridae	0.69835234	Colwellia psychrerythraea	1	CRISPR
NOM015_scaffold5941_3	10772	phage	0.998	temperate	0.99985975	Straboviridae	0.78885055	Mycoplasma pulmonis	0.86	Predict
NOM015_scaffold252_7	49572	phage	0.997	temperate	0.99983925	unknown	0	Candidatus Pelagibacter ubique	1	CRISPR
NOM015_scaffold749_3	26270	phage	0.999	temperate	0.9998017	unknown	0	Colwellia psychrerythraea	0.75	Predict
NOM015_scaffold8498_6	13915	phage	0.837	virulent	0.99003845	Casjensviridae	0.52695894	Azospirillum brasilense	0.98	Predict
NOM015_scaffold16067_4	17718	phage	0.978	virulent	0.9998413	Straboviridae	0.7514587	Candidatus Pelagibacter ubique	0.7	Predict
NOM015_scaffold9242_9	11272	phage	0.974	temperate	0.98519784	unknown	0	Bacteroides fragilis	0.95	Predict
NOM015_scaffold27697_2	67439	phage	0.999	virulent	0.9998622	Herelleviridae	1	Cellulophaga baltica	1	CRISPR
NOM015_scaffold26753_1	10033	phage	0.999	virulent	0.9998727	unknown	0	Mycoplasma pulmonis	0.82	Predict
NOM016_scaffold29885_1	11249	phage	0.983	virulent	0.99981046	unknown	0	Mycoplasma pulmonis	1	CRISPR
NOM016_scaffold42933_2	10051	phage	0.999	virulent	0.929879	Kyanoviridae	1	unknown	0	-
NOM016_scaffold35286_4	18302	phage	0.999	virulent	0.9998688	unknown	0	Rhodococcus hoagii	0.9	Predict
NOM016_scaffold35286_7	12384	phage	0.999	virulent	0.9998679	unknown	0	Mycoplasma pulmonis	1	CRISPR
NOM016_scaffold35976_5	11548	phage	0.999	temperate	0.9968298	Salasmaviridae	0.33228892	Mycoplasma pulmonis	1	CRISPR
NOM016_scaffold20671_4	11594	phage	0.947	virulent	0.9949243	Straboviridae	0.7514587	Bacteroides fragilis	0.93	Predict
NOM016_scaffold33084_3	97682	phage	0.999	virulent	0.99986315	Straboviridae	0.26939163	Candidatus Pelagibacter ubique	0.83	Predict
NOM016_scaffold45043_2	20130	phage	0.999	virulent	0.99985266	unknown	0	unknown	0	-
NOM016_scaffold16005_4	15495	phage	0.999	virulent	0.9998699	unknown	0	Mycoplasma pulmonis	0.76	Predict
NOM016_scaffold38252_1	15545	phage	0.931	virulent	0.9998693	Peduoviridae	0.7102873	Mycoplasma pulmonis	0.92	Predict

NOM016_scaffold31191_1	11132	phage	0.999	temperate	0.9998274	unknown	0	Mycoplasma pulmonis	0.87	Predict
NOM016_scaffold53315_1	10249	phage	0.945	temperate	0.98064613	unknown	0	Mycoplasma pulmonis	0.76	Predict
NOM016_scaffold37747_2	12087	phage	0.995	virulent	0.9998408	unknown	0	Mycoplasma pulmonis	0.95	Predict
NOM016_scaffold54950_4	11277	phage	0.961	temperate	0.979654	unknown	0	Bacteroides fragilis	0.82	Predict
NOM016_scaffold55549_3	10978	phage	0.941	virulent	0.9996882	Straboviridae	0.7514587	Mycoplasma pulmonis	0.93	Predict
NOM016_scaffold47449_3	10346	phage	0.997	virulent	0.99855703	Ackermannviridae	0.28771883	Mycoplasma pulmonis	0.81	Predict
NOM016_scaffold54079_1	13144	phage	0.897	temperate	0.99985975	unknown	0	Candidatus Pelagibacter ubique	0.98	Predict
NOM016_scaffold12609_2	15550	phage	0.999	temperate	0.9992678	unknown	0	Mycoplasma pulmonis	1	CRISPR
NOM016_scaffold25995_3	54678	phage	0.999	virulent	0.9998665	unknown	0	Bacillus cereus	1	CRISPR
NOM016_scaffold1263_27	15451	phage	0.998	virulent	0.9998722	unknown	0	unknown	0	-
NOM016_scaffold16005_2	12927	phage	0.999	virulent	0.8753107	Straboviridae	0.3003233	Mycoplasma pulmonis	0.72	Predict
NOM016_scaffold41274_5	13713	phage	0.974	temperate	0.9997906	unknown	0	unknown	0	-
NOM016_scaffold55496_1	10412	phage	0.918	virulent	0.9998128	Herelleviridae	0.20963135	Bacteroides fragilis	0.86	Predict
NOM016_scaffold2585_1	12659	phage	0.999	virulent	0.9998693	unknown	0	Parabacteroides distasonis	1	CRISPR
NOM016_scaffold55443_2	32983	phage	0.894	virulent	0.9998679	Straboviridae	0.55210805	Candidatus Pelagibacter ubique	0.79	Predict
NOM016_scaffold1680_10	12619	phage	0.999	temperate	0.90217596	unknown	0	Mycoplasma pulmonis	1	CRISPR
NOM016_scaffold16783_1	10123	phage	0.774	temperate	0.9998579	unknown	0	unknown	0	-
NOM016_scaffold28365_4	11946	phage	0.998	virulent	0.9996403	unknown	0	Mycoplasma pulmonis	0.73	Predict
NOM016_scaffold35286_1	15796	phage	0.998	virulent	0.9998465	Straboviridae	0.6334267	Mycoplasma pulmonis	0.83	Predict
NOM016_scaffold12609_6	10193	phage	0.967	temperate	0.99985975	unknown	0	Mycoplasma pulmonis	0.99	Predict
NOM016_scaffold39131_1	11606	phage	0.825	temperate	0.9880092	Straboviridae	0.43916065	Parabacteroides distasonis	0.73	Predict
NOM016_scaffold55593_1	10575	phage	0.998	temperate	0.90639764	unknown	0	Mycoplasma pulmonis	0.76	Predict
NOM016_scaffold19739_3	10887	phage	0.814	temperate	0.999854	unknown	0	unknown	0	-
NOM016_scaffold31927_4	12144	phage	0.974	virulent	0.99986744	unknown	0	Mycoplasma pulmonis	0.7	Predict
NOM016_scaffold3257_24	10254	phage	0.998	temperate	0.99800956	Straboviridae	0.64330465	Colwellia psychrerythraea	1	CRISPR
NOM016_scaffold55581_3	27770	phage	0.999	temperate	0.9998388	unknown	0	Mycoplasma pulmonis	1	CRISPR
NOM017_scaffold43934_1	18221	phage	0.997	temperate	0.99985975	Ackermannviridae	0.41468462	Achromobacter xylosoxidans	0.87	Predict
NOM017_scaffold40696_1	10633	phage	0.986	virulent	0.9997144	Drexlerviridae	0.34971577	Colwellia psychrerythraea	0.96	Predict
NOM017_scaffold41715_2	11364	phage	0.999	virulent	0.9998019	unknown	0	Bacillus cereus	1	CRISPR
NOM017_scaffold2822_6	20930	phage	0.623	temperate	0.9514629	unknown	0	Klebsiella oxytoca	0.94	Predict

NOM017_scaffold4867_13	16345	phage	0.78	virulent	0.9997883	Straboviridae	0.8097716	Candidatus Pelagibacter ubique	0.76	Predict
NOM017_scaffold45485_2	11935	phage	0.999	temperate	0.99986035	unknown	0	Mycoplasma pulmonis	0.76	Predict
NOM017_scaffold45477_1	13546	phage	0.999	virulent	0.82531583	Casjensviridae	1	Mycoplasma pulmonis	0.99	Predict
NOM017_scaffold45482_1	10182	phage	0.994	temperate	0.9998202	unknown	0	unknown	0	-
NOM017_scaffold42764_1	13741	phage	0.877	virulent	0.99947464	Straboviridae	0.39451152	Mycoplasma pulmonis	1	CRISPR
NOM017_scaffold45435_1	16473	phage	0.999	temperate	0.9998579	unknown	0	Mycoplasma pulmonis	1	CRISPR
NOM017_scaffold996_22	10865	phage	0.555	virulent	0.9998565	Straboviridae	0.685769	Bacillus megaterium	0.72	Predict
NOM017_scaffold11947_1	10864	phage	1	virulent	0.9815634	Peduoviridae	0.17022015	Mycoplasma pulmonis	1	CRISPR
NOM017_scaffold22366_1	13842	phage	0.999	temperate	0.99985975	unknown	0	Clostridioides difficile	1	CRISPR
NOM017_scaffold16406_3	10444	phage	0.999	virulent	0.9998656	unknown	0	unknown	0	-
NOM017_scaffold845_1	10733	phage	0.998	temperate	0.99985975	unknown	0	Colwellia psychrerythraea	0.98	Predict
NOM017_scaffold10596_2	10420	phage	0.999	virulent	0.9998736	Peduoviridae	1	Lactobacillus gasseri	1	CRISPR
NOM017_scaffold45496_2	15196	phage	0.93	temperate	0.9997544	unknown	0	unknown	0	-
NOM017_scaffold6578_5	24932	phage	0.999	temperate	0.82747394	Drexlerviridae	0.1370826	Candidatus Pelagibacter ubique	0.98	Predict
NOM017_scaffold45031_4	11577	phage	0.806	virulent	0.99987465	unknown	0	Aliivibrio fischeri	0.78	Predict
NOM017_scaffold44208_2	14890	phage	0.994	temperate	0.9997267	Straboviridae	0.5898211	Mycoplasma pulmonis	1	CRISPR
NOM017_scaffold22040_3	16956	phage	0.711	virulent	0.99986696	Drexlerviridae	0.31882837	Streptococcus mutans	0.75	Predict
NOM017_scaffold45430_1	16523	phage	0.999	virulent	0.9797661	unknown	0	Mycoplasma pulmonis	1	CRISPR
NOM017_scaffold11947_3	10124	phage	0.988	virulent	0.9492285	unknown	0	Colwellia psychrerythraea	1	CRISPR
NOM017_scaffold1142_45	11270	phage	0.992	virulent	0.9998736	unknown	0	unknown	0	-
NOM017_scaffold6796_4	11039	phage	0.999	virulent	0.9989985	unknown	0	Staphylococcus xylosus	0.75	Predict
NOM017_scaffold33562_4	10585	phage	0.999	temperate	0.9997874	unknown	0	unknown	0	-
NOM018_scaffold13094_4	11725	phage	0.998	virulent	0.9998308	Straboviridae	0.47247508	Mycoplasma pulmonis	0.84	Predict
NOM018_scaffold30222_3	14215	phage	0.999	temperate	0.99986035	unknown	0	Mycoplasma pulmonis	0.95	Predict
NOM018_scaffold54740_4	11271	phage	0.994	temperate	0.93755734	unknown	0	unknown	0	-
NOM018_scaffold4478_2	11769	phage	0.994	virulent	0.999458	unknown	0	unknown	0	-
NOM018_scaffold42888_2	21641	phage	0.999	virulent	0.9998608	unknown	0	unknown	0	-
NOM018_scaffold54709_5	10120	phage	0.77	virulent	0.99908036	unknown	0	unknown	0	-
NOM018_scaffold54740_2	11887	phage	0.999	virulent	0.9998736	unknown	0	Colwellia psychrerythraea	0.98	Predict
NOM018_scaffold5764_2	11321	phage	0.996	virulent	0.9998699	Straboviridae	0.40843752	Mycoplasma pulmonis	0.7	Predict

NOM018_scaffold51296_4	14319	phage	0.998	temperate	0.9617509	unknown	0	Mycoplasma pulmonis	0.99	Predict
NOM018_scaffold42663_2	28517	phage	0.999	virulent	0.9998693	unknown	0	Flavobacterium columnare	0.82	Predict
NOM018_scaffold29911_1	13701	phage	0.988	virulent	0.99982554	Peduoviridae	0.40998638	Colwellia psychrerythraea	0.87	Predict
NOM018_scaffold53732_1	10268	phage	0.999	temperate	0.9998593	unknown	0	Mycoplasma pulmonis	0.96	Predict
NOM018_scaffold26191_10	12560	phage	0.952	virulent	0.99979305	unknown	0	unknown	0	-
NOM018_scaffold53145_1	11438	phage	0.998	virulent	0.99987125	Salasmaviridae	0.9653382	Mycoplasma pulmonis	1	CRISPR
NOM018_scaffold1559_3	10019	phage	0.998	virulent	0.99987125	no_family_avaliable(NC_047916)	0.968	Mycoplasma pulmonis	1	CRISPR
NOM018_scaffold1296_2	13526	phage	0.999	virulent	0.9988333	unknown	0	Bacteroides fragilis	0.94	Predict
NOM018_scaffold37159_1	12471	phage	0.82	virulent	0.99979264	unknown	0	Mycoplasma pulmonis	0.97	Predict
NOM018_scaffold221_1	10546	phage	0.996	virulent	0.99987173	unknown	0	unknown	0	-
NOM018_scaffold55244_1	13209	phage	0.999	virulent	0.99987406	unknown	0	Candidatus Pelagibacter ubique	0.71	Predict
NOM018_scaffold43446_1	31642	phage	0.998	virulent	0.99986744	no_family_avaliable(NC_047916)	0.952	Mycoplasma pulmonis	1	CRISPR
NOM018_scaffold18121_1	10175	phage	0.944	temperate	0.99985975	unknown	0	Parabacteroides merdae	0.97	Predict
NOM018_scaffold40401_5	12391	phage	0.99	temperate	0.99398565	Zierdtviridae	1	Clostridioides difficile	0.93	Predict
NOM018_scaffold9490_1	17491	phage	0.999	temperate	0.9998588	unknown	0	Colwellia psychrerythraea	0.78	Predict
NOM018_scaffold191_5	11801	phage	0.618	virulent	0.99959284	unknown	0	Mycoplasma pulmonis	0.89	Predict
NOM018_scaffold15821_1	17263	phage	0.999	temperate	0.96378285	unknown	0	Thermoanaerobacterium saccharolyti	0.96	Predict
NOM018_scaffold16582_2	13682	phage	0.924	temperate	0.99985975	unknown	0	unknown	0	-
NOM018_scaffold54015_1	12845	phage	1	temperate	0.9944814	Straboviridae	0.29318652	Mycoplasma pulmonis	0.83	Predict
NOM018_scaffold9772_2	11040	phage	0.995	virulent	0.999854	unknown	0	Parabacteroides merdae	0.8	Predict
NOM018_scaffold2740_11	11389	phage	0.999	temperate	0.9998574	unknown	0	Mycoplasma pulmonis	1	CRISPR
NOM018_scaffold20530_1	17486	phage	0.923	temperate	0.99964815	Mesyanzhinovviridae	0.4088416	Colwellia psychrerythraea	0.79	Predict
NOM018_scaffold11125_2	21215	phage	0.919	virulent	0.9998699	Straboviridae	0.47918606	Mycoplasma pulmonis	0.72	Predict
NOM018_scaffold115_1	16375	phage	0.987	temperate	0.9948197	unknown	0	Sinorhizobium meliloti	0.94	Predict
NOM018_scaffold50471_1	12151	phage	0.999	virulent	0.9982585	unknown	0	Mycoplasma pulmonis	0.94	Predict
NOM018_scaffold30_5	21644	phage	0.987	temperate	0.9998545	Peduoviridae	1	Roseburia inulinivorans	1	CRISPR
NOM018_scaffold53629_1	10415	phage	0.997	virulent	0.56305987	Straboviridae	0.62754965	unknown	0	-
NOM018_scaffold24796_3	23572	phage	1	temperate	0.9998579	Straboviridae	0.77344465	Clostridioides difficile	1	CRISPR
NOM018_scaffold51296_6	12397	phage	0.999	virulent	0.99987084	unknown	0	Burkholderia cenocepacia	0.92	Predict
NOM018_scaffold1842_24	11277	phage	0.999	temperate	0.9992	unknown	0	Mycoplasma pulmonis	0.96	Predict

NOM018_scaffold42663_1	69957	phage	1	virulent	0.99986744	Straboviridae	0.26939163	Candidatus Pelagibacter ubique	1	Predict
NOM018_scaffold52711_1	12996	phage	0.995	virulent	0.9997449	unknown	0	Candidatus Pelagibacter ubique	0.83	Predict
NOM018_scaffold50197_1	20136	phage	0.978	temperate	0.99985975	Ackermannviridae	0.31534573	Candidatus Pelagibacter ubique	0.77	Predict
NOM018_scaffold54603_1	10125	phage	0.98	temperate	0.9960603	Straboviridae	0.40339345	unknown	0	-
NOM018_scaffold4393_1	11528	phage	0.998	temperate	0.99963623	Straboviridae	0.47193432	Candidatus Pelagibacter ubique	0.8	Predict
NOM018_C821692_1	12876	phage	0.996	temperate	0.99985975	Straboviridae	0.77971184	Mycoplasma pulmonis	0.92	Predict
NOM019_scaffold37_3	13458	phage	0.999	virulent	0.9998699	unknown	0	Mycoplasma pulmonis	0.88	Predict
NOM019_scaffold6752_20	11435	phage	0.996	temperate	0.97468555	Peduoviridae	0.59965926	unknown	0	-
NOM019_scaffold46040_6	10454	phage	0.999	virulent	0.99987125	no_family_avaliable(NC_062780)	0.978	Candidatus Pelagibacter ubique	0.95	Predict
NOM019_scaffold16958_6	25580	phage	0.999	virulent	0.99986744	unknown	0	Mycoplasma pulmonis	0.95	Predict
NOM019_scaffold52788_2	42629	phage	0.997	virulent	0.99986744	Salasmaviridae	0.42331704	Cellulophaga baltica	1	CRISPR
NOM019_scaffold52150_2	10429	phage	0.986	virulent	0.9989734	unknown	0	unknown	0	-
NOM019_scaffold15207_20	12471	phage	0.999	virulent	0.9998722	Straboviridae	0.3162047	Mycoplasma pulmonis	1	CRISPR
NOM019_scaffold5766_5	14707	phage	0.998	temperate	0.99986035	unknown	0	unknown	0	-
NOM019_scaffold53004_2	16044	phage	0.998	virulent	0.9137901	unknown	0	unknown	0	-
NOM019_scaffold40062_4	12985	phage	0.999	virulent	0.99987036	unknown	0	unknown	0	-
NOM019_scaffold52669_1	14623	phage	0.999	virulent	0.99699116	Herelleviridae	0.8982402	Mycoplasma pulmonis	1	CRISPR
NOM019_scaffold53126_1	10910	phage	0.999	temperate	0.9998593	unknown	0	Mycoplasma pulmonis	1	CRISPR
NOM019_scaffold40333_6	25407	phage	0.999	virulent	0.99983174	unknown	0	Mycoplasma pulmonis	1	CRISPR
NOM019_scaffold16958_5	10418	phage	0.999	virulent	0.99965626	unknown	0	Colwellia psychrerythraea	0.8	Predict
NOM019_scaffold38943_2	10343	phage	0.99	temperate	0.8927256	unknown	0	unknown	0	-
NOM019_scaffold50412_4	27319	phage	0.999	temperate	0.9998565	unknown	0	Clostridium tetani	1	CRISPR
NOM019_scaffold22541_5	21734	phage	0.995	virulent	0.99987084	Drexlerviridae	0.23993139	Mycoplasma pulmonis	0.78	Predict
NOM019_scaffold53083_1	11409	phage	0.997	virulent	0.9997321	Drexlerviridae	0.36810157	unknown	0	-
NOM019_scaffold53126_2	24697	phage	0.998	temperate	0.99978536	unknown	0	Clostridium tetani	1	CRISPR
NOM019_scaffold31682_1	25032	phage	0.55	temperate	0.99984884	unknown	0	Mycoplasma pulmonis	0.98	Predict
NOM019_scaffold53119_3	14172	phage	0.998	virulent	0.9998684	unknown	0	unknown	0	-
NOM019_scaffold7421_3	12206	phage	0.999	virulent	0.99987406	unknown	0	unknown	0	-
NOM019_scaffold22109_1	11831	phage	0.956	temperate	0.99985313	unknown	0	Candidatus Pelagibacter ubique	0.91	Predict
NOM019_scaffold45409_1	16784	phage	0.999	temperate	0.9987781	unknown	0	Parabacteroides distasonis	0.96	Predict

NOM019_scaffold50412_3	27823	phage	0.999	temperate	0.89173055	Straboviridae	1	Mycoplasma pulmonis	1 CRISPR
NOM019_scaffold12477_8	11207	phage	0.999	temperate	0.77741015	unknown	0	Bacillus subtilis	0.95 Predict
NOM019_scaffold27076_10	17477	phage	0.849	temperate	0.9998403	Peduoviridae	0.74069476	Colwellia psychrerythraea	0.99 Predict
NOM019_scaffold12848_1	10372	phage	0.965	virulent	0.9761889	unknown	0	Candidatus Pelagibacter ubique	0.78 Predict
NOM019_scaffold46040_3	36745	phage	0.999	virulent	0.9998699	Straboviridae	0.26783755	Candidatus Pelagibacter ubique	0.92 Predict
NOM019_scaffold36262_3	10945	phage	0.965	temperate	0.9949765	unknown	0	Colwellia psychrerythraea	1 CRISPR
NOM019_scaffold14421_2	10519	phage	0.925	temperate	0.99949235	Ackermannviridae	0.20613262	Colwellia psychrerythraea	0.72 Predict
NOM019_scaffold24407_7	11545	phage	0.999	temperate	0.99985975	Ackermannviridae	0.3046369	Listeria monocytogenes	0.95 Predict
NOM019_scaffold52788_3	19923	phage	0.999	virulent	0.9998699	Straboviridae	0.3162047	Mycoplasma pulmonis	1 CRISPR
NOM019_scaffold22541_7	47364	phage	0.998	temperate	0.9998556	Casjensviridae	0.6484222	Colwellia psychrerythraea	1 CRISPR
NOM019_scaffold53127_2	15891	phage	0.919	temperate	0.9413989	Straboviridae	0.3920616	Bacteroides fragilis	0.98 Predict
NOM020_scaffold5494_5	35951	phage	0.998	virulent	0.9997452	Casjensviridae	0.5695306	Candidatus Pelagibacter ubique	1 CRISPR
NOM020_scaffold33908_1	10175	phage	0.935	temperate	0.88362616	unknown	0	Mycoplasma pulmonis	0.99 Predict
NOM020_scaffold38448_1	10385	phage	0.997	temperate	0.99986035	unknown	0	Mycoplasma pulmonis	0.92 Predict
NOM020_scaffold7097_2	10467	phage	0.999	virulent	0.9998727	unknown	0	Mycoplasma pulmonis	0.92 Predict
NOM020_scaffold39245_1	30314	phage	0.999	virulent	0.50469756	Ackermannviridae	0.31828496	Streptococcus mutans	1 CRISPR
NOM020_scaffold6778_1	11168	phage	0.749	temperate	0.99984264	Peduoviridae	0.74069476	Colwellia psychrerythraea	0.76 Predict
NOM020_scaffold1826_3	21618	phage	0.871	temperate	0.86272585	Straboviridae	1	unknown	0 -
NOM020_scaffold14028_7	12704	phage	0.999	virulent	0.99986607	Drexlerviridae	1	Mycoplasma pulmonis	0.89 Predict
NOM020_scaffold1704_2	10167	phage	0.999	virulent	0.96948117	unknown	0	Streptomyces sp. MUSC 125	1 CRISPR
NOM020_scaffold13829_4	12430	phage	0.573	virulent	0.7615538	unknown	0	Mycoplasma pulmonis	0.92 Predict
NOM020_scaffold22877_2	16728	phage	0.99	temperate	0.9998593	Straboviridae	0.78885055	Candidatus Pelagibacter ubique	0.78 Predict
NOM020_scaffold32231_1	12567	phage	0.999	virulent	0.99987173	Straboviridae	0.409102	Bacteroides fragilis	0.7 Predict
NOM020_C476630_1	19352	phage	0.999	virulent	0.99987036	unknown	0	Colwellia psychrerythraea	1 CRISPR
NOM020_scaffold38253_1	12935	phage	1	virulent	0.9998699	unknown	0	Mycoplasma pulmonis	0.82 Predict
NOM020_scaffold12913_3	21122	phage	0.997	virulent	0.9998593	Straboviridae	0.36334187	Streptococcus mutans	0.94 Predict
NOM020_scaffold37822_4	14979	phage	0.995	virulent	0.9998188	unknown	0	Mycoplasma pulmonis	1 CRISPR
NOM020_scaffold31685_6	12918	phage	0.915	virulent	0.99987036	Straboviridae	0.4640264	Mycoplasma pulmonis	0.74 Predict
NOM020_scaffold38253_2	28119	phage	0.999	virulent	0.99985266	unknown	0	Rhodococcus hoagii	1 CRISPR
NOM020_scaffold35100_2	10318	phage	0.949	temperate	0.999854	Herelleviridae	0.15467335	Candidatus Pelagibacter ubique	0.85 Predict

NOM020_scaffold38892_1	12272	phage	0.721	temperate	0.9992391	unknown	0	unknown	0	-
NOM020_scaffold3731_3	21063	phage	0.997	virulent	0.9998617	Straboviridae	0.55210805	Mycoplasma pulmonis	0.91	Predict
NOM020_scaffold10070_1	13646	phage	0.998	temperate	0.9998574	Straboviridae	0.41099742	Mycoplasma pulmonis	0.88	Predict
NOM020_scaffold26272_1	32455	phage	1	temperate	0.9261822	Casjensviridae	0.32719818	Colwellia psychrerythraea	1	CRISPR
NOM020_scaffold16941_1	13785	phage	0.993	temperate	0.9998593	Straboviridae	0.81851417	Candidatus Pelagibacter ubique	0.84	Predict
NOM020_scaffold14364_1	10464	phage	0.548	virulent	0.99964935	unknown	0	Mycoplasma pulmonis	0.81	Predict
NOM020_scaffold39269_1	24019	phage	0.996	temperate	0.9997974	Straboviridae	0.28039292	Mycoplasma pulmonis	0.74	Predict
NOM020_scaffold39193_2	28334	phage	0.999	temperate	0.95171803	unknown	0	Colwellia psychrerythraea	1	CRISPR
NOM022_scaffold35384_4	19703	phage	0.949	virulent	0.99987173	Drexlerviridae	0.38832796	Mycoplasma pulmonis	0.93	Predict
NOM022_scaffold25627_6	26832	phage	0.999	temperate	0.9998574	unknown	0	Mycoplasma pulmonis	0.87	Predict
NOM022_scaffold2146_2	11584	phage	0.991	temperate	0.9993753	unknown	0	Mycoplasma pulmonis	0.93	Predict
NOM022_scaffold3633_6	17049	phage	0.987	virulent	0.9998565	Straboviridae	0.55784976	Mycoplasma pulmonis	0.76	Predict
NOM022_scaffold11395_10	10097	phage	0.844	temperate	0.9998593	unknown	0	unknown	0	-
NOM022_scaffold35226_2	14772	phage	0.995	temperate	0.99976444	Straboviridae	0.4042891	Mycoplasma pulmonis	0.99	Predict
NOM022_scaffold35027_4	11271	phage	0.994	temperate	0.93755734	unknown	0	unknown	0	-
NOM022_scaffold34355_1	18064	phage	0.994	virulent	0.9644928	Straboviridae	0.7514587	Parabacteroides distasonis	0.85	Predict
NOM022_scaffold16867_5	12106	phage	0.999	temperate	0.9997587	Ackermannviridae	0.2754581	Colwellia psychrerythraea	0.75	Predict
NOM022_scaffold11835_1	24298	phage	0.969	virulent	0.9998722	Schitoviridae	0.17866491	Mycoplasma pulmonis	0.75	Predict
NOM022_scaffold25627_3	53134	phage	0.999	temperate	0.9988508	Straboviridae	0.4977496	Mycoplasma pulmonis	1	CRISPR
NOM022_scaffold1399_24	11450	phage	0.999	virulent	0.9997912	unknown	0	Colwellia psychrerythraea	0.96	Predict
NOM022_scaffold33629_1	15918	phage	0.999	temperate	0.99985033	unknown	0	unknown	0	-
NOM022_scaffold23992_2	15694	phage	0.945	temperate	0.9998584	unknown	0	Bacteroides fragilis	0.89	Predict
NOM022_C499778_1	20966	phage	0.978	virulent	0.9681861	unknown	0	Bacteroides fragilis	0.89	Predict
NOM022_scaffold35027_1	11099	phage	0.999	virulent	0.71578294	unknown	0	Mycoplasma pulmonis	1	CRISPR
NOM022_scaffold29333_3	14039	phage	0.999	temperate	0.9446985	unknown	0	Colwellia psychrerythraea	0.77	Predict
NOM022_scaffold9236_24	14450	phage	0.747	virulent	0.9998517	unknown	0	Mycoplasma pulmonis	0.76	Predict
NOM022_scaffold31238_3	11580	phage	0.993	temperate	0.6280358	Straboviridae	0.7551459	Vibrio alginolyticus	0.79	Predict
NOM022_C499744_1	18558	phage	0.999	temperate	0.9997349	Straboviridae	0.71179295	Mycoplasma pulmonis	1	CRISPR
NOM022_scaffold10614_11	10439	phage	1	virulent	0.99971485	Ackermannviridae	0.28771883	Mycoplasma pulmonis	0.86	Predict
NOM022_C499370_1	11776	phage	1	temperate	0.9953512	unknown	0	Bacteroides cellulosilyticus	1	CRISPR

NOM022_scaffold25723_1	10162	phage	0.999	virulent	0.99978644	Ackermannviridae	0.22170894	Mycoplasma pulmonis	0.71	Predict
NOM022_scaffold25634_1	10926	phage	0.997	temperate	0.99898344	unknown	0	Sinorhizobium meliloti	0.84	Predict
NOM022_scaffold30875_1	16461	phage	0.991	temperate	0.99984604	Straboviridae	0.4795738	Bacteroides fragilis	0.98	Predict
NOM022_scaffold35362_2	20453	phage	0.937	virulent	0.99980617	Straboviridae	0.4640264	Bacteroides fragilis	0.82	Predict
NOM022_scaffold35326_5	11112	phage	0.997	virulent	0.9998736	unknown	0	Mycoplasma pulmonis	0.97	Predict
NOM023_scaffold1309_14	18743	phage	0.999	temperate	0.99984556	Herelleviridae	0.31749043	Parabacteroides distasonis	1	CRISPR
NOM023_scaffold2528_2	10887	phage	0.999	virulent	0.99322486	unknown	0	unknown	0	-
NOM023_scaffold7114_2	12124	phage	0.959	virulent	0.60374457	unknown	0	Bacteroides fragilis	1	CRISPR
NOM023_scaffold272_7	10558	phage	0.995	virulent	0.99987406	unknown	0	unknown	0	-
NOM023_scaffold2396_5	10154	phage	0.952	virulent	0.99947387	unknown	0	Bacteroides fragilis	0.9	Predict
NOM023_scaffold6600_10	13226	phage	0.999	temperate	0.97422904	unknown	0	Bacteroides fragilis	0.77	Predict
NOM023_scaffold21303_1	11626	phage	1	temperate	0.99985266	Herelleviridae	0.8611489	Mycoplasma pulmonis	1	CRISPR
NOM023_scaffold219_4	16345	phage	0.999	temperate	0.99985975	Straboviridae	0.5232858	Mycoplasma pulmonis	1	CRISPR
NOM023_scaffold9577_8	16104	phage	0.999	virulent	0.9959083	Peduoviridae	0.25943342	Mycoplasma pulmonis	0.76	Predict
NOM023_scaffold4208_9	21077	phage	0.999	virulent	0.95604503	Salasmaviridae	0.46300474	Mycoplasma pulmonis	1	CRISPR
NOM023_scaffold13354_4	20644	phage	0.998	virulent	0.9998727	unknown	0	unknown	0	-
NOM023_scaffold16737_1	15344	phage	0.997	temperate	0.99985975	unknown	0	unknown	0	-
NOM023_scaffold11170_3	10549	phage	0.999	virulent	0.99987036	unknown	0	Bacteroides vulgatus	1	CRISPR
NOM023_scaffold1634_1	16533	phage	0.999	temperate	0.99980694	Straboviridae	0.59151065	Mycoplasma pulmonis	0.85	Predict
NOM023_scaffold4816_2	11678	phage	0.999	virulent	0.99987465	unknown	0	Parabacteroides distasonis	0.83	Predict
NOM023_scaffold6600_7	18111	phage	0.999	temperate	0.9998565	Straboviridae	0.27628964	Bacteroides fragilis	1	CRISPR
NOM023_scaffold21124_2	17090	phage	1	virulent	0.99985975	no_family_avaliable(NC_011222)	0.981	Bacteroides fragilis	1	CRISPR
NOM023_scaffold7583_7	12895	phage	0.999	virulent	0.99986744	Ackermannviridae	0.29854247	Parabacteroides distasonis	0.92	Predict
NOM023_scaffold67_25	15133	phage	0.994	temperate	0.93755734	unknown	0	Parabacteroides distasonis	1	CRISPR
NOM023_scaffold6623_2	23690	phage	0.998	virulent	0.9998688	Straboviridae	0.36334187	Mycoplasma pulmonis	0.95	Predict
NOM023_scaffold16737_2	22007	phage	0.999	temperate	0.9987917	Salasmaviridae	0.33228892	Mycoplasma pulmonis	0.98	Predict
NOM023_scaffold18365_1	12533	phage	0.992	temperate	0.9997506	unknown	0	Mycoplasma pulmonis	1	CRISPR
NOM023_scaffold67_27	12245	phage	0.999	virulent	0.99987125	unknown	0	Parabacteroides distasonis	1	CRISPR
NOM023_scaffold3447_6	20655	phage	0.946	temperate	0.98343426	unknown	0	unknown	0	-
NOM023_scaffold325_2	27396	phage	0.999	virulent	0.99710935	unknown	0	unknown	0	-

NOM023_scaffold20012_1	12518	phage	0.998	temperate	0.9998593	Straboviridae	0.78885055	Candidatus Pelagibacter ubique	0.77	Predict
NOM023_scaffold6200_5	10302	phage	0.998	virulent	0.99987316	unknown	0	unknown	0	-
NOM023_scaffold5561_1	20011	phage	0.988	virulent	0.93865997	unknown	0	Mycoplasma pulmonis	0.94	Predict
NOM023_scaffold5693_18	36555	phage	0.996	virulent	0.88076085	Drexlerviridae	0.23785804	Parabacteroides distasonis	1	CRISPR
NOM025_scaffold13742_3	11418	phage	0.999	temperate	0.99985975	Peduoviridae	1	Colwellia psychrerythraea	0.73	Predict
NOM025_scaffold1166_3	10377	phage	0.941	virulent	0.9975544	unknown	0	Mycoplasma pulmonis	0.71	Predict
NOM025_scaffold24674_1	58888	phage	0.999	virulent	0.9998627	unknown	0	Candidatus Pelagibacter ubique	1	CRISPR
NOM025_scaffold23401_1	15932	phage	0.999	temperate	0.99986035	Straboviridae	0.29318652	Clostridium tetani	1	Predict
NOM025_scaffold23387_1	12048	phage	0.998	temperate	0.9998593	unknown	0	unknown	0	-
NOM025_scaffold2064_3	12381	phage	0.998	virulent	0.99986315	unknown	0	Bacteroides fragilis	0.98	Predict
NOM025_scaffold173_6	14953	phage	0.988	temperate	0.99985975	unknown	0	Colwellia psychrerythraea	1	CRISPR
NOM025_scaffold3864_1	15406	phage	0.996	virulent	0.908313	unknown	0	Candidatus Pelagibacter ubique	0.72	Predict
NOM025_scaffold6532_2	12247	phage	0.965	virulent	0.9998688	Straboviridae	0.45782614	Mycoplasma pulmonis	0.88	Predict
NOM025_scaffold22402_1	17201	phage	0.982	virulent	0.9971647	unknown	0	Mycoplasma pulmonis	0.73	Predict
NOM025_scaffold18513_1	12411	phage	1	temperate	0.9996086	unknown	0	Mycoplasma pulmonis	1	CRISPR
NOM025_scaffold2_1	11238	phage	0.978	virulent	0.9978972	unknown	0	Mycoplasma pulmonis	0.99	Predict
NOM025_scaffold24512_5	17743	phage	0.99	virulent	0.9998722	Peduoviridae	0.6341865	Colwellia psychrerythraea	0.84	Predict
NOM025_scaffold20521_2	13891	phage	0.887	temperate	0.9998574	unknown	0	Colwellia psychrerythraea	0.79	Predict
NOM025_scaffold62_1	10271	phage	0.998	temperate	0.9998593	unknown	0	Mycoplasma pulmonis	0.71	Predict
NOM025_scaffold24601_2	31870	phage	0.999	temperate	0.9998556	Chaseviridae	1	Colwellia psychrerythraea	1	CRISPR
NOM025_scaffold7710_1	11948	phage	0.902	virulent	0.9998736	Peduoviridae	1	Colwellia psychrerythraea	0.72	Predict
NOM025_scaffold4063_4	14653	phage	0.999	virulent	0.99986404	unknown	0	Caulobacter vibrioides	1	CRISPR
NOM025_scaffold7973_1	10726	phage	0.99	temperate	0.9934034	unknown	0	Mycoplasma pulmonis	0.82	Predict
NOM025_scaffold78_1	11204	phage	0.999	virulent	0.99986124	Herelleviridae	1	Candidatus Pelagibacter ubique	0.77	Predict
NOM025_scaffold173_5	48547	phage	0.999	virulent	0.9995521	Peduoviridae	1	Mycoplasma pulmonis	1	CRISPR
NOM025_scaffold24504_2	10905	phage	0.995	virulent	0.9688307	unknown	0	Parabacteroides distasonis	0.99	Predict
NOM025_scaffold14182_5	10075	phage	0.998	virulent	0.9998727	no_family_avaliable(NC_067210)	0.965	Mycoplasma pulmonis	0.72	Predict
NOM025_scaffold23752_1	11225	phage	0.954	virulent	0.9998736	no_family_avaliable(NC_055709)	0.974	Mycoplasma pulmonis	0.79	Predict
NOM025_scaffold24528_3	24989	phage	0.997	temperate	0.9998556	unknown	0	Mycoplasma pulmonis	1	CRISPR
NOM025_scaffold24496_1	18901	phage	0.991	temperate	0.99982834	Straboviridae	0.23342617	Mycoplasma pulmonis	0.82	Predict

NOM025_scaffold57_1	10003	phage	0.612	virulent	0.9998665	unknown	0	Mycoplasma pulmonis	0.83	Predict
NOM025_scaffold16448_2	21007	phage	0.991	temperate	0.9974352	unknown	0	Mycoplasma pulmonis	0.88	Predict
NOM025_scaffold24601_3	17684	phage	0.998	temperate	0.99985975	no_family_avaliable(NC_028656)	0.961	Mycoplasma pulmonis	0.99	Predict
NOM025_scaffold24362_1	43906	phage	0.998	temperate	0.9997912	unknown	0	Candidatus Pelagibacter ubique	0.7	Predict
NOM025_scaffold3455_1	19837	phage	0.986	temperate	0.99985975	unknown	0	Thermoanaerobacterium saccharolyti	0.87	Predict
NOM025_scaffold17951_2	13633	phage	0.937	virulent	0.99977446	Ackermannviridae	1	Candidatus Pelagibacter ubique	0.79	Predict
NOM025_scaffold3831_6	13177	phage	0.998	temperate	0.87870395	Zierdtviridae	1	Parabacteroides distasonis	0.83	Predict
NOM025_scaffold19612_1	17730	phage	0.999	temperate	0.9982116	unknown	0	Mycoplasma pulmonis	0.94	Predict
NOM025_scaffold3792_5	16065	phage	0.851	virulent	0.51043385	unknown	0	Parabacteroides distasonis	0.9	Predict
NOM025_scaffold23962_2	15919	phage	0.999	temperate	0.99985975	Peduoviridae	1	Colwellia psychrerythraea	1	CRISPR
NOM025_scaffold7710_3	17530	phage	0.998	virulent	0.99987316	Peduoviridae	0.35915253	Lactobacillus gasseri	1	CRISPR
NOM026_scaffold21771_5	17554	phage	0.995	virulent	0.99987084	Straboviridae	0.7551459	Mycoplasma pulmonis	0.99	Predict
NOM026_scaffold4045_2	20742	phage	0.987	virulent	0.9998226	unknown	0	Mycoplasma pulmonis	0.98	Predict
NOM026_scaffold51_21	16559	phage	0.762	temperate	0.99779725	Peduoviridae	1	Colwellia psychrerythraea	1	CRISPR
NOM026_scaffold3975_3	12965	phage	0.989	temperate	0.96546066	unknown	0	Mycoplasma pulmonis	0.88	Predict
NOM026_scaffold29015_25	15745	phage	0.999	virulent	0.9998522	unknown	0	Flavobacterium psychrophilum	1	CRISPR
NOM026_scaffold15750_6	10370	phage	0.962	virulent	0.99985266	unknown	0	unknown	0	-
NOM026_scaffold8128_4	11552	phage	0.977	temperate	0.99985975	unknown	0	Mycoplasma pulmonis	0.82	Predict
NOM026_scaffold4107_22	10818	phage	0.996	temperate	0.99985975	Straboviridae	0.47247508	Mycoplasma pulmonis	0.87	Predict
NOM026_scaffold29015_1	19479	phage	0.997	temperate	0.99984837	Straboviridae	0.63267183	Candidatus Pelagibacter ubique	1	CRISPR
NOM026_scaffold29015_22	10590	phage	0.999	temperate	0.9998536	unknown	0	Mycoplasma pulmonis	0.95	Predict
NOM026_scaffold43414_4	11552	phage	0.999	virulent	0.9961104	Peduoviridae	1	Clostridioides difficile	0.98	Predict
NOM026_scaffold39978_1	22711	phage	0.937	virulent	0.97466457	Ackermannviridae	1	Candidatus Pelagibacter ubique	0.9	Predict
NOM026_scaffold14437_2	10155	phage	0.997	temperate	0.99869525	unknown	0	Streptococcus mutans	0.86	Predict
NOM026_scaffold23252_1	12922	phage	0.986	temperate	0.9616671	unknown	0	unknown	0	-
NOM026_scaffold1436_1	12202	phage	0.994	virulent	0.8414109	Herelleviridae	0.16899583	Mycoplasma pulmonis	0.74	Predict
NOM026_scaffold788_2	10093	phage	0.806	virulent	0.99987465	unknown	0	Aliivibrio fischeri	0.79	Predict
NOM026_scaffold287_6	15608	phage	0.993	temperate	0.99980307	unknown	0	Colwellia psychrerythraea	0.89	Predict
NOM026_scaffold32579_2	11068	phage	0.998	temperate	0.99794453	unknown	0	unknown	0	-
NOM026_scaffold95_3_2	11259	phage	0.994	virulent	0.9998727	Straboviridae	0.6500024	Colwellia psychrerythraea	0.82	Predict

NOM026_scaffold18477_4	21327	phage	0.999	virulent	0.9998408	Peduoviridae	0.24181609	Klebsiella pneumoniae	1 CRISPR
NOM026_scaffold273_8	13066	phage	0.996	virulent	0.9962353	unknown	0	Candidatus Pelagibacter ubique	0.8 Predict
NOM026_scaffold5049_5	11537	phage	1	temperate	0.6546375	Peduoviridae	0.4337845	Listeria monocytogenes	1 CRISPR
NOM026_scaffold100_1	13610	phage	0.958	temperate	0.99985975	unknown	0	Mycoplasma pulmonis	0.79 Predict
NOM026_scaffold2514_2	10628	phage	0.992	temperate	0.9968264	unknown	0	Mycoplasma pulmonis	0.73 Predict
NOM026_scaffold39163_3	11943	phage	0.999	virulent	0.99983263	unknown	0	Colwellia psychrerythraea	0.84 Predict
NOM026_scaffold18477_1	27724	phage	0.998	temperate	0.98942244	no_family_avaliable(NC_016158)	0.97	Colwellia psychrerythraea	1 CRISPR
NOM027_scaffold10318_1	33209	phage	0.999	temperate	0.99984556	unknown	0	Bacillus alcalophilus	1 CRISPR
NOM027_scaffold3843_3	33590	phage	0.989	temperate	0.99982494	Mesyanzhinovviridae	0.69835234	Colwellia psychrerythraea	1 CRISPR
NOM027_scaffold298_5	10925	phage	0.999	virulent	0.97201097	Straboviridae	0.2902184	Candidatus Pelagibacter ubique	0.96 Predict
NOM027_scaffold3057_13	15174	phage	0.999	virulent	0.7218347	unknown	0	Mycoplasma pulmonis	0.98 Predict
NOM027_scaffold690_9	10722	phage	0.997	temperate	0.9998593	unknown	0	Mycoplasma pulmonis	1 CRISPR
NOM027_scaffold233_8	12836	phage	0.997	temperate	0.9998588	unknown	0	Candidatus Pelagibacter ubique	1 CRISPR
NOM027_scaffold21902_2	40229	phage	0.999	temperate	0.957053	Vilmaviridae	1	Bacillus thuringiensis	1 CRISPR
NOM027_scaffold21753_2	12458	phage	0.985	virulent	0.9998688	Guelinviridae	1	Clostridium perfringens	1 CRISPR
NOM027_scaffold8220_2	22521	phage	0.999	temperate	0.99976397	unknown	0	Candidatus Pelagibacter ubique	1 CRISPR
NOM027_scaffold8991_1	13717	phage	0.998	temperate	0.9996067	unknown	0	unknown	0 -
NOM027_scaffold12848_6	11258	phage	0.99	temperate	0.9998522	unknown	0	Bacillus anthracis	0.72 Predict
NOM027_scaffold95_21	15848	phage	0.856	temperate	0.93894786	Straboviridae	0.3920616	Bacteroides fragilis	0.93 Predict
NOM027_scaffold928_6	12544	phage	0.999	virulent	0.69336796	unknown	0	Megasphaera elsdenii	1 CRISPR
NOM027_scaffold17819_1	19695	phage	0.999	temperate	0.999854	unknown	0	Colwellia psychrerythraea	1 CRISPR
NOM027_scaffold4263_1	26062	phage	0.983	virulent	0.9998622	Ackermannviridae	1	Flavobacterium columnare	0.9 Predict
NOM027_scaffold11970_13	12209	phage	0.841	temperate	0.99985975	unknown	0	Mycoplasma pulmonis	0.74 Predict
NOM027_scaffold3477_2	14371	phage	0.977	temperate	0.86199474	unknown	0	unknown	0 -
NOM027_scaffold21806_1	11086	phage	0.999	temperate	0.9998593	unknown	0	Mycoplasma pulmonis	0.82 Predict
NOM027_scaffold21896_1	29667	phage	0.997	virulent	0.99958265	unknown	0	Bifidobacterium longum	1 CRISPR
NOM027_scaffold18467_1	10793	phage	0.924	temperate	0.99985975	unknown	0	Candidatus Pelagibacter ubique	0.86 Predict
NOM027_scaffold21932_1	12995	phage	0.964	temperate	0.9998593	unknown	0	Lactobacillus jensenii	0.87 Predict
NOM027_scaffold4308_5	11512	phage	0.999	temperate	0.99982554	unknown	0	Mycoplasma pulmonis	1 CRISPR
NOM027_scaffold16572_1	13760	phage	0.946	virulent	0.99987125	Straboviridae	0.6500024	Colwellia psychrerythraea	0.81 Predict

NOM027_scaffold8270_1	19484	phage	0.998	temperate	0.9998593	unknown	0	Mycoplasma pulmonis	0.96	Predict
NOM027_scaffold87_1	31009	phage	0.731	virulent	0.9998645	Straboviridae	0.7579115	Mycoplasma pulmonis	0.72	Predict
NOM027_scaffold13632_2	29795	phage	1	temperate	0.999844	unknown	0	Clostridium perfringens	1	CRISPR
NOM027_scaffold21847_1	39070	phage	0.997	temperate	0.9997521	Straboviridae	0.44452858	Flavobacterium columnare	1	CRISPR
NOM028_scaffold2463_21	11098	phage	0.894	temperate	0.99608976	unknown	0	Clavibacter michiganensis	1	CRISPR
NOM028_C573709_1	19622	phage	0.995	temperate	0.9998579	Peduoviridae	1	Mycoplasma pulmonis	1	CRISPR
NOM028_scaffold5766_3	13435	phage	0.999	temperate	0.99985975	no_family_avaliable(NC_004313)	0.954	Mycoplasma pulmonis	1	CRISPR
NOM028_scaffold43134_4	14885	phage	0.99	virulent	0.99987173	Ackermannviridae	0.35297325	Mycoplasma pulmonis	0.94	Predict
NOM028_scaffold6679_33	10463	phage	0.996	virulent	0.99987316	unknown	0	unknown	0	-
NOM028_scaffold10923_16	19609	phage	0.998	temperate	0.99985975	no_family_avaliable(NC_049955)	0.972	Colwellia psychrerythraea	1	CRISPR
NOM028_scaffold39054_2	15185	phage	0.984	temperate	0.99985695	unknown	0	Candidatus Pelagibacter ubique	0.89	Predict
NOM028_scaffold5154_1	12982	phage	0.999	temperate	0.9997335	no_family_avaliable(NC_028449)	0.978	Mycoplasma pulmonis	1	CRISPR
NOM028_scaffold3552_4	34611	phage	0.995	temperate	0.9997044	Peduoviridae	0.41061532	Colwellia psychrerythraea	1	CRISPR
NOM028_scaffold11407_10	11229	phage	0.994	virulent	0.9823756	Chaseviridae	0.32056236	Azospirillum brasilense	1	Predict
NOM028_scaffold5961_20	11935	phage	0.971	virulent	0.99959546	Straboviridae	0.23342617	unknown	0	-
NOM028_scaffold44706_5	25393	phage	0.999	temperate	0.9998517	unknown	0	Mycoplasma pulmonis	0.78	Predict
NOM028_scaffold10197_9	16454	phage	0.999	temperate	0.9998584	unknown	0	Clostridioides difficile	1	CRISPR
NOM028_scaffold29187_1	21471	phage	0.999	temperate	0.999854	Ackermannviridae	0.2754581	Colwellia psychrerythraea	1	CRISPR
NOM028_scaffold47604_1	22785	phage	0.999	virulent	0.8595383	Straboviridae	0.79878217	Mycoplasma pulmonis	0.91	Predict
NOM028_scaffold10515_18	10744	phage	0.99	virulent	0.9998722	Peduoviridae	0.6341865	Colwellia psychrerythraea	0.97	Predict
NOM028_C573817_1	23286	phage	1	temperate	0.9981446	unknown	0	Mycoplasma pulmonis	1	CRISPR
NOM028_scaffold13734_25	33297	phage	0.999	virulent	0.9998645	unknown	0	Candidatus Pelagibacter ubique	1	CRISPR
NOM028_scaffold28052_1	10766	phage	0.998	virulent	0.99987125	Herelleviridae	1	Candidatus Pelagibacter ubique	0.84	Predict
NOM028_scaffold37397_2	12774	phage	0.59	temperate	0.99985975	unknown	0	unknown	0	-
NOM028_scaffold47465_1	10661	phage	0.997	temperate	0.99959904	unknown	0	Bacteroides fragilis	0.79	Predict
NOM028_scaffold46573_2	15820	phage	0.995	virulent	0.9998679	Peduoviridae	0.7102873	Mycoplasma pulmonis	0.71	Predict
NOM028_scaffold691_1	11295	phage	0.999	temperate	0.9998593	unknown	0	Vibrio alginolyticus	1	CRISPR
NOM028_C572983_1	10917	phage	0.997	virulent	0.9998736	unknown	0	Mycoplasma pulmonis	0.75	Predict
NOM028_scaffold36229_1	10577	phage	0.999	temperate	0.9998508	unknown	0	Mycoplasma pulmonis	0.99	Predict
NOM028_scaffold36508_1	21404	phage	0.927	virulent	0.5996757	unknown	0	Clostridium perfringens	0.89	Predict

NOM028_scaffold10515_26	46766	phage	0.999	temperate	0.99985695	no_family_avaliable(NC_019501)	0.962	Mycoplasma pulmonis	1	CRISPR
NOM028_scaffold13734_22	24670	phage	0.997	virulent	0.9993722	Straboviridae	0.8292426	Mycoplasma pulmonis	0.76	Predict
NOM028_C573171_1	12044	phage	0.999	temperate	0.9998474	unknown	0	Clostridioides difficile	1	CRISPR
NOM028_scaffold44706_6	13007	phage	0.997	temperate	0.9886789	unknown	0	Anaerostipes hadrus	1	CRISPR
NOM028_scaffold8690_5	26216	phage	1	temperate	0.99981356	Straboviridae	0.30755553	Mycoplasma pulmonis	0.92	Predict
NOM028_scaffold47247_1	14929	phage	0.997	virulent	0.99976444	unknown	0	unknown	0	-
NOM028_scaffold6524_6	14925	phage	0.998	temperate	0.99986035	unknown	0	Mycoplasma pulmonis	0.9	Predict
NOM028_scaffold41121_3	13714	phage	1	temperate	0.9998574	unknown	0	Mycoplasma pulmonis	1	CRISPR
NOM028_scaffold47539_2	15897	phage	0.919	temperate	0.9413989	Straboviridae	0.3920616	Bacteroides fragilis	0.85	Predict
NOM028_scaffold19021_1	11873	phage	1	temperate	0.94768286	unknown	0	Colwellia psychrerythraea	0.92	Predict
NOM028_scaffold46746_1	10974	phage	0.519	temperate	0.99986035	unknown	0	Trichormus variabilis	1	CRISPR
NOM028_scaffold6524_7	10057	phage	0.995	temperate	0.99983925	unknown	0	Carboxydocella sp. JDF658	1	CRISPR
NOM028_scaffold46512_4	10678	phage	0.974	temperate	0.98519784	unknown	0	Bacteroides fragilis	0.71	Predict
NOM028_scaffold2529_5	12912	phage	0.987	temperate	0.7455003	Ackermannviridae	1	Streptococcus pneumoniae	0.85	Predict
NOM028_scaffold11058_9	20902	phage	0.999	temperate	0.9998574	unknown	0	Clostridium perfringens	1	CRISPR
NOM028_scaffold15800_1	23708	phage	0.981	virulent	0.9994517	Straboviridae	0.55784976	Candidatus Pelagibacter ubique	0.77	Predict
NOM028_scaffold47161_1	12720	phage	0.781	virulent	0.98588747	unknown	0	unknown	0	-
NOM028_scaffold10197_6	11516	phage	0.995	temperate	0.98964936	unknown	0	Bacillus alcalophilus	0.93	Predict
NOM028_scaffold110_3	12374	phage	0.993	virulent	0.9998699	unknown	0	Candidatus Pelagibacter ubique	0.74	Predict
NOM028_scaffold45529_1	35115	phage	0.984	virulent	0.9998622	Straboviridae	0.4709028	Mycoplasma pulmonis	0.97	Predict
NOM028_scaffold44706_1	21623	phage	0.777	temperate	0.999794	Straboviridae	0.70313096	Clostridioides difficile	0.79	Predict
NOM028_scaffold366_38	23791	phage	0.999	temperate	0.99985695	Straboviridae	0.4993172	Mycoplasma pulmonis	0.9	Predict
NOM028_scaffold37086_11	11692	phage	0.926	temperate	0.9975716	Straboviridae	0.685769	Mycoplasma pulmonis	0.84	Predict
NOM028_scaffold2922_6	10675	phage	0.999	virulent	0.99916154	unknown	0	Candidatus Pelagibacter ubique	0.8	Predict
NOM028_scaffold6524_1	57560	phage	0.99	virulent	0.6240467	unknown	0	unknown	0	-
NOM028_scaffold7965_13	33498	phage	0.998	temperate	0.99985605	unknown	0	Mycoplasma pulmonis	0.79	Predict
NOM028_scaffold6524_2	23613	phage	0.992	temperate	0.9998584	unknown	0	Mycoplasma pulmonis	0.84	Predict
NOM028_scaffold47589_2	19039	phage	0.999	temperate	0.99986035	unknown	0	Mycoplasma pulmonis	0.81	Predict
NOM028_scaffold29797_1	50941	phage	0.999	temperate	0.9997569	Straboviridae	0.73880583	Mycoplasma pulmonis	1	CRISPR
NOM028_scaffold37620_3	15886	phage	0.892	virulent	0.9998369	Ackermannviridae	1	Colwellia psychrerythraea	0.88	Predict

NOM028_scaffold39_5	41411	phage	0.999	temperate	0.9998403	Straboviridae	0.4577688	Listeria monocytogenes	1	CRISPR
NOM028_scaffold47596_2	17354	phage	0.999	temperate	0.99985975	unknown	0	Mycoplasma pulmonis	1	CRISPR
NOM028_scaffold19223_1	16072	phage	0.972	temperate	0.99985033	Drexlerviridae	0.36308205	Mycoplasma pulmonis	0.95	Predict
NOM028_scaffold6524_14	10441	phage	0.999	temperate	0.99986035	unknown	0	Mycoplasma pulmonis	0.93	Predict
NOM028_scaffold34458_5	14802	phage	0.999	temperate	0.9998593	unknown	0	Streptococcus oralis	1	CRISPR
NOM028_scaffold24117_2	14445	phage	0.967	temperate	0.9998584	unknown	0	Mycoplasma pulmonis	1	CRISPR
NOM029_scaffold15812_1	13532	phage	0.997	temperate	0.99985975	Straboviridae	0.7394616	Mycoplasma pulmonis	0.89	Predict
NOM029_scaffold15613_10	16058	phage	0.998	temperate	0.9998593	Peduoviridae	1	Colwellia psychrerythraea	1	CRISPR
NOM029_scaffold279_8	13614	phage	0.722	temperate	0.9998403	unknown	0	Candidatus Pelagibacter ubique	0.8	Predict
NOM029_scaffold27582_6	15426	phage	0.999	virulent	0.9998679	unknown	0	Rhodococcus hoagii	0.86	Predict
NOM029_scaffold13239_1	10478	phage	0.994	virulent	0.99985975	Ackermannviridae	0.4011864	Colwellia psychrerythraea	1	Predict
NOM029_scaffold36387_1	17728	phage	0.7	temperate	0.9998183	unknown	0	Mycoplasma pulmonis	0.91	Predict
NOM029_scaffold27582_3	24692	phage	0.999	virulent	0.99986696	unknown	0	Mycoplasma pulmonis	0.76	Predict
NOM029_scaffold32772_2	35148	phage	0.947	virulent	0.9998693	Straboviridae	0.47918606	Mycoplasma pulmonis	0.86	Predict
NOM029_scaffold1741_11	11456	phage	0.919	virulent	0.9998722	unknown	0	Mycoplasma pulmonis	0.97	Predict
NOM029_scaffold1385_6	14052	phage	0.995	virulent	0.99978584	unknown	0	unknown	0	-
NOM029_scaffold9689_3	14251	phage	0.856	temperate	0.93894786	Straboviridae	0.3920616	Bacteroides fragilis	0.92	Predict
NOM029_scaffold23053_8	12583	phage	0.997	virulent	0.9738244	unknown	0	Bacteroides fragilis	0.81	Predict
NOM029_scaffold2695_11	10093	phage	0.998	virulent	0.9750174	unknown	0	unknown	0	-
NOM029_scaffold277_1	32238	phage	0.998	virulent	0.9856595	Ackermannviridae	0.34232736	Mycoplasma pulmonis	1	CRISPR
NOM029_scaffold6303_4	12981	phage	1	temperate	0.99984556	unknown	0	Mycoplasma pulmonis	1	CRISPR
NOM029_scaffold19269_12	13483	phage	1	temperate	0.99985975	Straboviridae	0.77344465	Candidatus Pelagibacter ubique	1	CRISPR
