# Supplementary material for: PhaBOX: a web server for identifying and characterizing phage contigs in metagenomic data
Source: Bioinform Adv. 2023 Aug 2;3(1):vbad101. doi: 10.1093/bioadv/vbad101 (PMC10460485; doi:10.1093/bioadv/vbad101)
Supplement: vbad101_Supplementary_Data [file phamer_prediction.pdf › phamer_prediction.pdf]

Accession	Pred	Score
DOF002_scaffold67513_1	non-phage	0
DOF002_scaffold49199_3	non-phage	0
DOF002_scaffold30822_2	non-phage	0
DOF002_scaffold67460_1	non-phage	0
DOF002_scaffold5450_2	non-phage	0
DOF002_scaffold7970_8	non-phage	0
DOF002_scaffold2701_18	non-phage	0
DOF002_scaffold11259_5	non-phage	0
DOF002_scaffold62654_2	non-phage	0
DOF002_scaffold56110_6	non-phage	0
DOF002_scaffold21798_1	non-phage	0
DOF002_scaffold24242_3	phage	0.998
DOF002_scaffold20176_6	non-phage	0
DOF002_scaffold60582_1	non-phage	0
DOF002_scaffold13525_16	non-phage	0
DOF002_scaffold6112_22	non-phage	0
DOF002_scaffold13673_11	non-phage	0
DOF002_scaffold30256_21	non-phage	0
DOF002_scaffold66235_4	non-phage	0
DOF002_scaffold67503_1	phage	0.999
DOF002_scaffold14614_1	non-phage	0
DOF002_scaffold60803_3	phage	0.997
DOF002_scaffold133_7	non-phage	0
DOF002_scaffold67436_2	phage	1
DOF002_scaffold20176_3	non-phage	0
DOF002_scaffold12576_2	non-phage	0
DOF002_scaffold63958_7	phage	0.999
DOF002_scaffold20976_3	non-phage	0
DOF002_scaffold11952_4	phage	0.999
DOF002_scaffold6112_14	non-phage	0
DOF002_scaffold1696_2	non-phage	0
DOF002_scaffold13215_2	non-phage	0
DOF002_scaffold40193_28	non-phage	0
DOF002_scaffold38019_10	non-phage	0
DOF002_scaffold25411_12	non-phage	0
DOF002_scaffold67233_2	non-phage	0
DOF002_scaffold46802_1	non-phage	0
DOF002_scaffold2756_8	non-phage	0
DOF002_scaffold64073_3	phage	0.999
DOF002_scaffold67049_3	non-phage	0
DOF002_scaffold19565_4	non-phage	0
DOF002_scaffold3257_3	non-phage	0
DOF002_scaffold64389_1	phage	0.999
DOF002_scaffold55185_4	non-phage	0
DOF002_scaffold41602_2	non-phage	0

DOF002_scaffold5829_2	phage	0.999
DOF002_scaffold66235_11	non-phage	0
DOF002_scaffold24298_2	non-phage	0
DOF002_scaffold11_1	non-phage	0
DOF002_scaffold4650_19	non-phage	0
DOF002_scaffold130_2	non-phage	0
DOF002_scaffold20_41	non-phage	0
DOF002_scaffold386_23	phage	0.995
DOF002_scaffold45014_1	phage	0.998
DOF002_scaffold17843_4	non-phage	0
DOF002_scaffold33414_3	non-phage	0
DOF002_scaffold43700_1	non-phage	0
DOF002_scaffold22882_6	non-phage	0
DOF002_scaffold26487_26	non-phage	0
DOF002_scaffold32921_5	non-phage	0
DOF002_scaffold30859_15	non-phage	0
DOF002_scaffold32188_6	phage	0.961
DOF002_scaffold15901_3	non-phage	0
DOF002_scaffold11991_9	non-phage	0
DOF002_scaffold67435_1	non-phage	0
DOF002_scaffold260_29	non-phage	0
DOF002_scaffold60280_1	non-phage	0
DOF002_scaffold31506_2	non-phage	0
DOF002_scaffold16555_7	non-phage	0
DOF002_scaffold49_5	non-phage	0
DOF002_scaffold34213_6	non-phage	0
DOF002_scaffold20_14	non-phage	0
DOF002_scaffold45038_2	non-phage	0
DOF002_scaffold62507_2	non-phage	0
DOF002_scaffold13673_15	non-phage	0
DOF002_scaffold13673_12	non-phage	0
DOF002_scaffold5829_3	phage	0.998
DOF002_scaffold65381_4	phage	0.997
DOF002_scaffold44470_2	non-phage	0
DOF002_scaffold2365_1	non-phage	0
DOF002_scaffold21134_4	non-phage	0
DOF002_scaffold4400_1	non-phage	0
DOF002_scaffold52455_2	non-phage	0
DOF002_scaffold54454_2	non-phage	0
DOF002_scaffold14614_6	non-phage	0
DOF002_scaffold35792_6	non-phage	0
DOF002_scaffold8903_16	non-phage	0
DOF002_scaffold2026_3	non-phage	0
DOF002_scaffold23924_11	non-phage	0
DOF002_scaffold34441_21	non-phage	0
DOF002_scaffold11991_4	non-phage	0

DOF002_scaffold4650_7	non-phage	0
DOF002_scaffold9976_2	non-phage	0
DOF002_scaffold3970_18	non-phage	0
DOF002_scaffold67494_4	non-phage	0
DOF002_scaffold22865_1	non-phage	0
DOF002_scaffold67459_1	phage	0.999
DOF002_scaffold52911_1	non-phage	0
DOF002_scaffold64032_4	non-phage	0
DOF002_scaffold66895_2	non-phage	0
DOF002_scaffold136_6	non-phage	0
DOF002_scaffold5871_17	non-phage	0
DOF002_scaffold9633_1	non-phage	0
DOF002_scaffold25301_4	non-phage	0
DOF002_scaffold23810_3	non-phage	0
DOF002_scaffold51663_2	non-phage	0
DOF002_scaffold5567_14	non-phage	0
DOF002_scaffold310_3	non-phage	0
DOF002_scaffold67431_2	non-phage	0
DOF002_scaffold66_1	non-phage	0
DOF002_scaffold55953_3	non-phage	0
DOF002_scaffold20937_1	non-phage	0
DOF002_scaffold49199_6	non-phage	0
DOF002_scaffold2701_13	phage	0.965
DOF002_scaffold67379_1	phage	0.998
DOF002_scaffold47914_1	non-phage	0
DOF002_scaffold51733_4	non-phage	0
DOF002_scaffold2_2	non-phage	0
DOF002_scaffold260_11	non-phage	0
DOF002_scaffold11946_3	non-phage	0
DOF002_scaffold4977_4	non-phage	0
DOF002_scaffold4498_2	non-phage	0
DOF002_scaffold24_23	non-phage	0
DOF002_scaffold13766_3	non-phage	0
DOF002_scaffold26000_12	non-phage	0
DOF002_scaffold67426_1	non-phage	0
DOF002_scaffold12811_2	non-phage	0
DOF002_scaffold5871_10	non-phage	0
DOF002_scaffold67490_1	non-phage	0
DOF002_scaffold16971_2	non-phage	0
DOF002_scaffold67462_1	non-phage	0
DOF002_scaffold59673_1	non-phage	0
DOF002_scaffold39978_2	non-phage	0
DOF002_scaffold57771_3	non-phage	0
DOF002_scaffold51733_7	non-phage	0
DOF002_scaffold36750_4	non-phage	0
DOF002_scaffold67436_1	non-phage	0

DOF002_scaffold11799_1	non-phage	0
DOF002_scaffold60803_2	phage	0.988
DOF002_scaffold57185_1	non-phage	0
DOF002_scaffold40193_6	non-phage	0
DOF002_scaffold12_1	non-phage	0
DOF002_scaffold3638_4	non-phage	0
DOF002_scaffold67365_1	non-phage	0
DOF002_scaffold65401_1	non-phage	0
DOF002_scaffold32748_1	non-phage	0
DOF002_scaffold286_1	non-phage	0
DOF002_scaffold32276_4	non-phage	0
DOF002_scaffold6551_2	non-phage	0
DOF002_scaffold20669_5	non-phage	0
DOF002_scaffold32188_7	non-phage	0
DOF002_scaffold9671_1	non-phage	0
DOF002_scaffold57172_1	non-phage	0
DOF002_scaffold63958_10	non-phage	0
DOF002_scaffold67512_1	non-phage	0
DOF002_scaffold48171_3	non-phage	0
DOF002_scaffold53785_2	non-phage	0
DOF002_scaffold41359_6	non-phage	0
DOF002_scaffold323_4	non-phage	0
DOF002_scaffold60531_1	non-phage	0
DOF002_scaffold43917_1	non-phage	0
DOF002_scaffold44470_5	non-phage	0
DOF002_scaffold11259_4	non-phage	0
DOF002_scaffold17574_2	non-phage	0
DOF002_scaffold64965_1	non-phage	0
DOF002_scaffold35264_12	non-phage	0
DOF002_scaffold67538_1	phage	0.602
DOF002_scaffold266_6	non-phage	0
DOF002_scaffold2170_42	non-phage	0
DOF002_scaffold55354_2	phage	0.989
DOF002_scaffold5404_24	non-phage	0
DOF002_scaffold4671_2	phage	0.999
DOF002_scaffold66895_1	non-phage	0
DOF002_scaffold63348_1	non-phage	0
DOF002_scaffold31742_1	non-phage	0
DOF002_scaffold48931_1	non-phage	0
DOF002_scaffold52585_1	non-phage	0
DOF002_scaffold24_7	non-phage	0
DOF002_scaffold67203_2	non-phage	0
DOF002_scaffold66657_1	phage	1
DOF002_scaffold50248_1	non-phage	0
DOF002_scaffold15901_8	non-phage	0
DOF002_scaffold24_26	non-phage	0

DOF002_scaffold49143_2	non-phage	0
DOF002_scaffold44506_1	non-phage	0
DOF002_scaffold55053_5	non-phage	0
DOF002_scaffold16555_1	non-phage	0
DOF002_scaffold58349_2	non-phage	0
DOF002_scaffold67233_1	non-phage	0
DOF002_scaffold61825_1	non-phage	0
DOF002_scaffold31701_5	non-phage	0
DOF002_scaffold88_2	non-phage	0
DOF002_scaffold5192_11	non-phage	0
DOF002_scaffold8507_2	non-phage	0
DOF002_scaffold4136_1	non-phage	0
DOF002_scaffold5829_1	non-phage	0
DOF002_scaffold57238_1	non-phage	0
DOF002_scaffold66729_1	non-phage	0
DOF002_scaffold559_6	non-phage	0
DOF002_scaffold37389_1	non-phage	0
DOF002_scaffold66988_2	non-phage	0
DOF002_scaffold1739_2	non-phage	0
DOF002_scaffold49_6	non-phage	0
DOF002_scaffold18_2	non-phage	0
DOF002_scaffold32748_4	non-phage	0
DOF002_scaffold14231_6	non-phage	0
DOF002_scaffold15628_1	non-phage	0
DOF002_scaffold136_3	non-phage	0
DOF002_scaffold67551_2	non-phage	0
DOF002_scaffold67549_1	non-phage	0
DOF002_scaffold7652_4	non-phage	0
DOF002_scaffold67245_2	non-phage	0
DOF002_scaffold6095_2	non-phage	0
DOF002_scaffold23823_2	non-phage	0
DOF002_scaffold48931_12	non-phage	0
DOF002_scaffold4650_12	non-phage	0
DOF002_scaffold67472_1	non-phage	0
DOF002_scaffold19376_6	non-phage	0
DOF002_scaffold4650_4	non-phage	0
DOF002_scaffold47656_5	non-phage	0
DOF002_scaffold66033_2	non-phage	0
DOF002_scaffold386_21	non-phage	0
DOF002_scaffold64679_2	non-phage	0
DOF002_scaffold9533_28	non-phage	0
DOF002_scaffold36989_8	non-phage	0
DOF002_scaffold57516_3	non-phage	0
DOF002_scaffold6112_16	non-phage	0
DOF002_scaffold9701_2	non-phage	0
DOF002_scaffold10448_2	phage	0.999

DOF002_scaffold49755_2	non-phage	0
DOF002_scaffold11510_13	non-phage	0
DOF002_scaffold57516_6	non-phage	0
DOF002_scaffold56110_8	non-phage	0
DOF002_scaffold19740_14	non-phage	0
DOF002_scaffold302_2	non-phage	0
DOF002_scaffold61808_4	non-phage	0
DOF002_scaffold18769_5	non-phage	0
DOF002_scaffold67257_3	non-phage	0
DOF002_scaffold15996_13	non-phage	0
DOF002_scaffold34441_22	non-phage	0
DOF002_scaffold31579_2	non-phage	0
DOF002_scaffold60672_2	non-phage	0
DOF002_scaffold48171_8	non-phage	0
DOF002_scaffold15901_4	non-phage	0
DOF002_scaffold65140_5	non-phage	0
DOF002_scaffold48931_5	non-phage	0
DOF002_scaffold48931_2	non-phage	0
DOF002_scaffold21798_20	non-phage	0
DOF002_scaffold23030_3	non-phage	0
DOF002_scaffold11991_5	non-phage	0
DOF002_scaffold53953_3	non-phage	0
DOF002_scaffold42121_6	non-phage	0
DOF002_scaffold61262_2	non-phage	0
DOF002_scaffold6112_12	non-phage	0
DOF002_scaffold42030_1	non-phage	0
DOF002_scaffold1739_1	non-phage	0
DOF002_scaffold16825_1	non-phage	0
DOF002_scaffold17374_3	non-phage	0
DOF002_scaffold55053_11	non-phage	0
DOF002_scaffold105_3	non-phage	0
DOF002_scaffold22057_1	non-phage	0
DOF002_scaffold61955_1	non-phage	0
DOF002_scaffold44470_3	non-phage	0
DOF002_scaffold18312_3	non-phage	0
DOF002_scaffold34644_3	non-phage	0
DOF002_scaffold62842_4	non-phage	0
DOF002_scaffold214_2	non-phage	0
DOF002_scaffold41614_7	non-phage	0
DOF002_scaffold19740_5	non-phage	0
DOF002_scaffold35264_1	non-phage	0
DOF002_scaffold13378_6	non-phage	0
DOF002_scaffold27678_2	non-phage	0
DOF002_scaffold66375_1	non-phage	0
DOF002_scaffold5450_5	non-phage	0
DOF002_scaffold62906_8	non-phage	0

DOF002_scaffold67434_1	non-phage	0
DOF002_scaffold13673_23	non-phage	0
DOF002_scaffold8507_4	non-phage	0
DOF002_scaffold41853_9	non-phage	0
DOF002_scaffold2170_24	non-phage	0
DOF002_scaffold160_1	non-phage	0
DOF002_scaffold57771_2	non-phage	0
DOF002_scaffold30822_1	non-phage	0
DOF002_scaffold67113_1	non-phage	0
DOF002_scaffold11170_1	non-phage	0
DOF002_scaffold19565_7	non-phage	0
DOF002_scaffold56552_1	non-phage	0
DOF002_scaffold67294_2	non-phage	0
DOF002_scaffold67547_2	non-phage	0
DOF002_scaffold11952_2	phage	0.999
DOF002_scaffold38225_1	phage	0.999
DOF002_scaffold11170_2	non-phage	0
DOF002_scaffold65654_2	non-phage	0
DOF002_scaffold3031_4	non-phage	0
DOF002_scaffold58935_1	non-phage	0
DOF002_scaffold60758_7	non-phage	0
DOF002_scaffold40683_5	non-phage	0.005
DOF002_scaffold60672_1	non-phage	0
DOF002_scaffold67273_1	non-phage	0
DOF002_scaffold59077_3	non-phage	0
DOF002_scaffold65381_2	phage	0.999
DOF002_scaffold67038_2	non-phage	0
DOF002_scaffold56033_1	non-phage	0
DOF002_scaffold64449_1	non-phage	0
DOF002_scaffold266_7	non-phage	0
DOF002_scaffold17538_3	non-phage	0
DOF002_scaffold15901_9	non-phage	0
DOF002_scaffold31506_4	non-phage	0
DOF002_scaffold11946_1	non-phage	0
DOF002_scaffold13525_4	non-phage	0
DOF002_scaffold19180_2	non-phage	0
DOF002_scaffold67541_1	non-phage	0
DOF002_scaffold49199_5	non-phage	0
DOF002_scaffold323_3	non-phage	0
DOF002_scaffold11552_1	non-phage	0
DOF002_scaffold13072_33	non-phage	0
DOF002_scaffold67551_1	non-phage	0
DOF002_scaffold67203_1	non-phage	0
DOF002_scaffold63958_9	non-phage	0
DOF002_scaffold34441_26	non-phage	0
DOF002_scaffold58023_2	phage	0.998

DOF002_scaffold49143_1	non-phage	0
DOF002_scaffold25411_1	non-phage	0
DOF002_scaffold2701_9	non-phage	0
DOF002_scaffold31579_1	non-phage	0.001
DOF002_scaffold63115_2	non-phage	0
DOF002_scaffold4498_1	non-phage	0
DOF002_scaffold13525_14	non-phage	0
DOF002_scaffold17241_28	non-phage	0
DOF002_scaffold67521_1	non-phage	0
DOF002_scaffold60803_1	phage	0.999
DOF002_scaffold136_5	non-phage	0
DOF002_scaffold133_11	phage	0.996
DOF002_scaffold67539_2	non-phage	0
DOF002_scaffold71_7	non-phage	0
DOF002_scaffold3638_12	non-phage	0
DOF002_scaffold14614_8	non-phage	0
DOF002_scaffold67063_6	non-phage	0
DOF002_scaffold9730_3	non-phage	0
DOF002_scaffold67510_2	non-phage	0
DOF002_scaffold13072_15	phage	0.998
DOF002_scaffold2935_1	non-phage	0
DOF002_scaffold66842_1	non-phage	0
DOF002_scaffold23924_10	non-phage	0
DOF002_scaffold55053_10	non-phage	0
DOF002_scaffold64679_1	non-phage	0
DOF002_scaffold2659_3	non-phage	0
DOF002_scaffold67481_2	non-phage	0
DOF002_scaffold48925_1	non-phage	0
DOF002_scaffold39963_1	phage	0.999
DOF002_scaffold7341_4	non-phage	0
DOF002_scaffold4136_2	non-phage	0
DOF002_scaffold11991_6	non-phage	0
DOF002_scaffold45399_1	non-phage	0
DOF002_scaffold399_13	non-phage	0
DOF002_C840249_1	phage	0.999
DOF002_scaffold7008_1	non-phage	0
DOF002_scaffold37389_2	non-phage	0.03
DOF002_scaffold67459_3	phage	0.999
DOF002_scaffold38336_1	non-phage	0
DOF002_scaffold67137_1	non-phage	0
DOF002_scaffold15901_5	non-phage	0
DOF002_scaffold67172_1	non-phage	0
DOF002_scaffold54454_7	non-phage	0
DOF002_scaffold64032_6	phage	0.999
DOF002_scaffold67408_1	non-phage	0
DOF002_scaffold26075_2	phage	0.99

DOF002_scaffold66669_1	non-phage	0
DOF002_scaffold59113_2	non-phage	0.273
DOF002_scaffold63393_1	non-phage	0.066
DOF002_scaffold45193_1	non-phage	0
DOF002_scaffold20134_3	non-phage	0
DOF002_scaffold13072_14	non-phage	0
DOF002_scaffold32689_1	non-phage	0
DOF002_scaffold28318_5	non-phage	0
DOF002_scaffold11184_5	non-phage	0
DOF002_scaffold30129_1	non-phage	0
DOF002_scaffold67426_3	phage	0.999
DOF002_scaffold260_7	non-phage	0
DOF002_scaffold36750_6	non-phage	0
DOF002_scaffold38013_1	non-phage	0
DOF002_scaffold17843_5	non-phage	0
DOF002_scaffold53953_4	non-phage	0
DOF002_scaffold1739_13	non-phage	0
DOF002_scaffold302_1	non-phage	0
DOF002_scaffold14614_3	non-phage	0
DOF002_scaffold20176_4	non-phage	0
DOF002_scaffold25411_5	non-phage	0
DOF002_scaffold9065_20	non-phage	0
DOF002_scaffold34441_3	non-phage	0
DOF002_scaffold13766_1	non-phage	0
DOF002_scaffold58023_6	non-phage	0
DOF002_scaffold39237_1	non-phage	0
DOF002_scaffold67481_1	non-phage	0
DOF002_scaffold67071_1	non-phage	0
DOF002_scaffold62363_5	non-phage	0
DOF002_scaffold13673_5	non-phage	0
DOF002_scaffold38859_1	non-phage	0
DOF002_scaffold7970_10	non-phage	0
DOF002_scaffold48171_2	non-phage	0
DOF002_scaffold63958_3	non-phage	0
DOF002_scaffold19717_1	non-phage	0
DOF002_scaffold47656_3	non-phage	0
DOF002_scaffold260_31	non-phage	0
DOF002_scaffold1739_7	non-phage	0
DOF002_scaffold11259_3	non-phage	0
DOF002_scaffold2701_11	non-phage	0
DOF002_scaffold38430_3	non-phage	0
DOF002_scaffold42671_1	non-phage	0
DOF002_scaffold61161_3	non-phage	0
DOF002_scaffold54240_2	non-phage	0
DOF002_scaffold1696_5	non-phage	0
DOF002_scaffold35264_9	non-phage	0

DOF002_scaffold10363_3	non-phage	0
DOF002_scaffold22882_8	non-phage	0
DOF002_scaffold38008_2	non-phage	0
DOF002_scaffold49600_1	non-phage	0
DOF002_scaffold67553_1	non-phage	0
DOF002_scaffold136_4	non-phage	0
DOF002_scaffold67294_3	non-phage	0
DOF002_scaffold11946_2	non-phage	0
DOF002_scaffold11170_3	non-phage	0
DOF002_scaffold59077_2	non-phage	0
DOF002_scaffold6112_15	non-phage	0
DOF002_scaffold21134_13	non-phage	0
DOF002_scaffold62842_3	non-phage	0
DOF002_scaffold36989_5	non-phage	0
DOF002_scaffold11952_3	phage	1
DOF002_scaffold39963_2	phage	0.999
DOF002_scaffold266_4	non-phage	0
DOF002_scaffold67225_2	non-phage	0
DOF002_scaffold24242_9	non-phage	0
DOF002_scaffold19740_7	non-phage	0
DOF002_scaffold17272_2	non-phage	0
DOF002_scaffold32491_2	non-phage	0
DOF002_scaffold26000_4	non-phage	0
DOF002_scaffold57771_1	non-phage	0
DOF002_scaffold35792_4	phage	0.849
DOF002_scaffold3638_11	non-phage	0
DOF002_scaffold9976_5	non-phage	0
DOF002_scaffold31506_1	non-phage	0
DOF002_scaffold61262_1	non-phage	0
DOF002_scaffold55953_4	non-phage	0
DOF002_scaffold62843_5	non-phage	0
DOF002_scaffold67293_2	non-phage	0
DOF002_scaffold51742_1	non-phage	0
DOF002_scaffold197_4	phage	0.968
DOF002_scaffold13072_21	non-phage	0
DOF002_scaffold64073_2	non-phage	0
DOF002_scaffold66657_3	non-phage	0
DOF002_scaffold1739_9	non-phage	0
DOF002_scaffold63_3	non-phage	0
DOF002_scaffold19740_19	non-phage	0
DOF002_scaffold13962_7	non-phage	0
DOF002_scaffold60758_6	non-phage	0
DOF002_scaffold40683_4	non-phage	0
DOF002_scaffold30256_18	non-phage	0
DOF002_scaffold67519_1	non-phage	0
DOF002_scaffold37389_3	non-phage	0

DOF002_scaffold2365_4	non-phage	0
DOF002_scaffold1739_4	non-phage	0
DOF002_scaffold20_42	non-phage	0
DOF002_scaffold23810_9	non-phage	0
DOF002_scaffold43335_3	non-phage	0
DOF002_scaffold11991_8	non-phage	0
DOF002_scaffold67063_3	non-phage	0
DOF002_scaffold49_4	non-phage	0
DOF002_scaffold63968_2	non-phage	0
DOF002_scaffold11991_1	non-phage	0
DOF002_scaffold50571_3	non-phage	0
DOF002_scaffold7341_5	non-phage	0
DOF002_scaffold34_1	non-phage	0
DOF002_scaffold5838_2	non-phage	0
DOF002_scaffold6112_4	non-phage	0
DOF002_scaffold67414_2	non-phage	0
DOF002_scaffold57516_4	non-phage	0
DOF002_scaffold10729_1	non-phage	0
DOF002_scaffold67521_2	non-phage	0
DOF002_scaffold48171_5	non-phage	0
DOF002_scaffold4671_4	phage	0.999
DOF002_scaffold67520_2	non-phage	0
DOF002_scaffold59680_6	non-phage	0
DOF002_scaffold4650_18	non-phage	0
DOF002_scaffold66725_5	non-phage	0.235
DOF002_scaffold399_9	non-phage	0
DOF002_scaffold24298_3	non-phage	0
DOF002_scaffold17538_4	non-phage	0
DOF002_scaffold20_17	non-phage	0
DOF002_scaffold67540_1	non-phage	0
DOF002_scaffold35801_3	non-phage	0
DOF002_scaffold266_10	non-phage	0
DOF002_scaffold12782_1	non-phage	0
DOF002_scaffold1696_6	non-phage	0
DOF002_scaffold30859_19	non-phage	0
DOF002_scaffold62565_1	non-phage	0
DOF002_scaffold67049_2	non-phage	0
DOF002_scaffold24_10	non-phage	0
DOF002_scaffold22882_7	non-phage	0
DOF002_scaffold64416_5	phage	1
DOF002_C839921_1	phage	0.992
DOF002_scaffold764_8	non-phage	0
DOF002_scaffold55053_1	non-phage	0
DOF002_scaffold44470_1	phage	0.991
DOF002_scaffold11_9	non-phage	0
DOF002_scaffold41853_11	non-phage	0

DOF002_scaffold60287_1	non-phage	0
DOF002_scaffold8507_1	non-phage	0
DOF002_scaffold50248_3	non-phage	0
DOF002_scaffold51343_5	non-phage	0
DOF002_scaffold25411_4	non-phage	0
DOF002_scaffold9671_2	phage	0.971
DOF002_scaffold50140_1	non-phage	0
DOF002_scaffold9671_3	non-phage	0
DOF002_scaffold8213_1	non-phage	0
DOF002_scaffold6095_4	non-phage	0
DOF002_scaffold5030_2	phage	0.608
DOF002_scaffold28975_1	non-phage	0
DOF002_scaffold136_2	non-phage	0
DOF002_scaffold67245_4	non-phage	0
DOF002_scaffold6112_18	non-phage	0
DOF002_scaffold64032_7	phage	0.999
DOF002_scaffold15628_3	non-phage	0
DOF002_scaffold37947_1	phage	0.999
DOF002_scaffold39978_1	non-phage	0
DOF002_scaffold64759_1	non-phage	0
DOF002_scaffold58541_2	non-phage	0
DOF002_scaffold20176_1	non-phage	0
DOF002_scaffold14614_2	non-phage	0
DOF002_scaffold23924_14	non-phage	0
DOF002_scaffold20669_3	phage	0.999
DOF002_scaffold67415_1	non-phage	0
DOF002_scaffold5192_9	non-phage	0
DOF002_scaffold67193_1	non-phage	0
DOF002_scaffold50014_1	non-phage	0.004
DOF002_scaffold28885_4	non-phage	0
DOF002_scaffold45052_1	non-phage	0
DOF002_scaffold136_11	non-phage	0
DOF002_scaffold66283_5	non-phage	0
DOF002_scaffold61847_1	non-phage	0
DOF002_scaffold54454_3	non-phage	0
DOF002_scaffold28318_4	non-phage	0
DOF002_scaffold36586_3	non-phage	0
DOF002_scaffold32188_13	non-phage	0
DOF002_scaffold67257_1	non-phage	0
DOF002_scaffold19717_2	non-phage	0
DOF002_scaffold2701_3	non-phage	0
DOF003_scaffold15538_1	non-phage	0
DOF003_scaffold11546_2	non-phage	0
DOF003_scaffold50819_5	non-phage	0
DOF003_scaffold2523_5	non-phage	0
DOF003_scaffold34804_2	non-phage	0

DOF003_scaffold49410_1	non-phage	0
DOF003_scaffold3052_13	non-phage	0
DOF003_scaffold50707_3	non-phage	0
DOF003_scaffold1813_8	non-phage	0
DOF003_scaffold36372_1	non-phage	0
DOF003_scaffold40480_8	non-phage	0
DOF003_scaffold33283_1	non-phage	0
DOF003_scaffold51187_1	non-phage	0
DOF003_scaffold1220_2	non-phage	0
DOF003_scaffold29431_1	non-phage	0
DOF003_scaffold11899_3	non-phage	0
DOF003_scaffold50705_1	non-phage	0
DOF003_scaffold18400_6	non-phage	0
DOF003_scaffold36266_3	non-phage	0
DOF003_scaffold2072_14	non-phage	0
DOF003_scaffold47194_2	non-phage	0
DOF003_scaffold37727_1	non-phage	0
DOF003_scaffold48198_1	non-phage	0
DOF003_scaffold122_2	non-phage	0
DOF003_scaffold41583_1	non-phage	0
DOF003_scaffold12598_4	non-phage	0
DOF003_scaffold40042_4	non-phage	0
DOF003_scaffold31822_1	non-phage	0
DOF003_scaffold35923_1	non-phage	0
DOF003_scaffold43625_5	non-phage	0
DOF003_scaffold39188_3	non-phage	0
DOF003_scaffold12256_11	non-phage	0
DOF003_scaffold7744_1	non-phage	0
DOF003_scaffold35168_3	non-phage	0
DOF003_scaffold39195_3	non-phage	0
DOF003_scaffold27253_1	non-phage	0
DOF003_scaffold43170_1	non-phage	0
DOF003_scaffold26875_2	phage	0.998
DOF003_scaffold45089_3	non-phage	0
DOF003_scaffold34149_1	non-phage	0
DOF003_scaffold51188_1	non-phage	0
DOF003_scaffold47911_2	non-phage	0
DOF003_scaffold45500_1	non-phage	0
DOF003_scaffold49885_2	non-phage	0
DOF003_scaffold138_1	non-phage	0
DOF003_scaffold51075_1	non-phage	0
DOF003_scaffold49071_1	non-phage	0
DOF003_scaffold989_3	non-phage	0
DOF003_C746654_1	non-phage	0
DOF003_scaffold29103_3	non-phage	0
DOF003_scaffold54_4	non-phage	0

DOF003_scaffold33528_5	non-phage	0
DOF003_scaffold8588_8	non-phage	0
DOF003_scaffold5983_1	non-phage	0
DOF003_scaffold38789_2	non-phage	0
DOF003_scaffold51127_1	non-phage	0
DOF003_scaffold38696_9	non-phage	0
DOF003_scaffold51078_5	non-phage	0
DOF003_scaffold47494_4	non-phage	0
DOF003_scaffold27202_3	non-phage	0
DOF003_scaffold47922_2	non-phage	0
DOF003_scaffold34635_1	non-phage	0
DOF003_scaffold47922_4	non-phage	0
DOF003_scaffold41050_5	non-phage	0
DOF003_scaffold1813_19	non-phage	0
DOF003_scaffold24985_2	non-phage	0
DOF003_scaffold29613_1	non-phage	0
DOF003_scaffold35161_6	non-phage	0
DOF003_scaffold27195_4	non-phage	0
DOF003_scaffold37184_1	non-phage	0
DOF003_scaffold22171_1	non-phage	0
DOF003_scaffold673_5	non-phage	0
DOF003_scaffold28855_1	non-phage	0
DOF003_scaffold210_9	non-phage	0
DOF003_scaffold47226_1	non-phage	0
DOF003_scaffold25701_11	non-phage	0
DOF003_scaffold13313_41	non-phage	0
DOF003_scaffold49282_2	non-phage	0
DOF003_scaffold9809_1	non-phage	0
DOF003_scaffold37804_1	non-phage	0
DOF003_scaffold6937_3	non-phage	0
DOF003_scaffold37461_1	non-phage	0
DOF003_scaffold1689_11	non-phage	0
DOF003_scaffold27119_1	non-phage	0
DOF003_scaffold50819_6	non-phage	0
DOF003_scaffold9209_1	non-phage	0
DOF003_scaffold24797_2	non-phage	0
DOF003_scaffold3054_15	non-phage	0
DOF003_scaffold42726_1	non-phage	0
DOF003_scaffold26705_2	non-phage	0
DOF003_scaffold49480_1	non-phage	0
DOF003_scaffold20008_1	non-phage	0
DOF003_scaffold230_2	non-phage	0
DOF003_scaffold25702_1	non-phage	0
DOF003_scaffold29431_5	non-phage	0
DOF003_scaffold40167_4	non-phage	0
DOF003_scaffold51214_1	non-phage	0

DOF003_scaffold6902_25	non-phage	0
DOF003_scaffold34842_1	non-phage	0
DOF003_scaffold47911_5	non-phage	0
DOF003_scaffold25816_1	non-phage	0
DOF003_scaffold51185_1	non-phage	0
DOF003_scaffold40096_1	non-phage	0
DOF003_scaffold51179_1	non-phage	0
DOF003_scaffold24933_1	non-phage	0
DOF003_scaffold37299_1	non-phage	0
DOF003_scaffold19522_1	non-phage	0
DOF003_scaffold48402_2	non-phage	0
DOF003_scaffold13313_47	non-phage	0
DOF003_scaffold50654_1	phage	1
DOF003_scaffold51024_1	non-phage	0
DOF003_scaffold23724_2	non-phage	0
DOF003_scaffold50098_1	non-phage	0
DOF003_scaffold54_1	non-phage	0
DOF003_scaffold51177_1	non-phage	0
DOF003_scaffold48163_3	non-phage	0
DOF003_scaffold10514_5	non-phage	0
DOF003_scaffold37452_1	non-phage	0
DOF003_scaffold46765_2	non-phage	0
DOF003_scaffold51211_2	non-phage	0
DOF003_scaffold17362_1	non-phage	0
DOF003_scaffold34041_4	non-phage	0
DOF003_scaffold26705_1	non-phage	0
DOF003_scaffold6902_34	non-phage	0
DOF003_scaffold13313_33	non-phage	0
DOF003_scaffold10794_10	non-phage	0.282
DOF003_scaffold8819_1	non-phage	0
DOF003_scaffold19111_1	phage	0.947
DOF003_scaffold4791_11	non-phage	0
DOF003_scaffold37547_4	non-phage	0
DOF003_scaffold22736_1	non-phage	0
DOF003_scaffold40696_2	non-phage	0
DOF003_scaffold51101_2	non-phage	0
DOF003_scaffold6006_1	non-phage	0
DOF003_scaffold32234_2	non-phage	0
DOF003_scaffold3659_1	non-phage	0
DOF003_scaffold40734_2	non-phage	0.008
DOF003_scaffold50693_1	non-phage	0
DOF003_scaffold51042_2	non-phage	0
DOF003_scaffold38669_1	non-phage	0
DOF003_scaffold1019_3	non-phage	0
DOF003_scaffold31275_5	non-phage	0
DOF003_scaffold2007_28	non-phage	0

DOF003_scaffold25531_1	non-phage	0
DOF003_scaffold27202_1	non-phage	0
DOF003_scaffold33177_1	non-phage	0
DOF003_scaffold50812_2	non-phage	0
DOF003_scaffold11_2	non-phage	0
DOF003_scaffold13313_54	non-phage	0
DOF003_scaffold24449_5	non-phage	0
DOF003_scaffold21753_2	non-phage	0
DOF003_scaffold337_3	non-phage	0
DOF003_scaffold51203_1	non-phage	0
DOF003_scaffold27079_1	non-phage	0
DOF003_scaffold1480_1	non-phage	0
DOF003_scaffold51186_1	non-phage	0
DOF003_scaffold40969_2	non-phage	0
DOF003_scaffold51061_3	phage	0.999
DOF003_scaffold1220_10	non-phage	0
DOF003_scaffold3054_20	non-phage	0
DOF003_scaffold4016_5	non-phage	0
DOF003_scaffold6062_2	non-phage	0
DOF003_scaffold13343_1	non-phage	0
DOF003_scaffold1257_23	non-phage	0
DOF003_scaffold25488_2	non-phage	0
DOF003_scaffold39747_2	non-phage	0
DOF003_scaffold1441_11	non-phage	0
DOF003_scaffold51198_1	non-phage	0
DOF003_scaffold51217_1	non-phage	0
DOF003_scaffold27202_2	non-phage	0
DOF003_scaffold36470_1	non-phage	0
DOF003_scaffold28855_2	phage	0.974
DOF003_C745514_1	non-phage	0
DOF003_scaffold21715_2	non-phage	0
DOF003_scaffold10178_3	non-phage	0
DOF003_scaffold34492_2	non-phage	0
DOF003_scaffold989_9	phage	0.999
DOF003_scaffold42160_2	non-phage	0
DOF003_scaffold46994_4	non-phage	0
DOF003_scaffold3054_5	non-phage	0
DOF003_scaffold42936_2	non-phage	0
DOF003_scaffold1019_8	non-phage	0
DOF003_scaffold21438_1	non-phage	0
DOF003_scaffold28472_6	non-phage	0
DOF003_scaffold49282_4	non-phage	0
DOF003_scaffold36373_1	non-phage	0
DOF003_scaffold51172_1	non-phage	0
DOF003_scaffold42010_1	non-phage	0
DOF003_scaffold46844_7	non-phage	0

DOF003_scaffold8760_5	non-phage	0
DOF003_scaffold44416_1	non-phage	0
DOF003_scaffold26383_1	non-phage	0
DOF003_scaffold36505_2	non-phage	0
DOF003_scaffold29431_2	non-phage	0
DOF003_scaffold27483_2	phage	0.999
DOF003_scaffold1257_31	non-phage	0
DOF003_scaffold237_11	non-phage	0
DOF003_scaffold34041_5	non-phage	0
DOF003_scaffold51192_1	non-phage	0
DOF003_scaffold40480_3	non-phage	0
DOF003_scaffold43886_1	non-phage	0
DOF003_scaffold34712_1	non-phage	0
DOF003_scaffold27906_2	non-phage	0
DOF003_scaffold50806_3	non-phage	0
DOF003_scaffold31162_1	non-phage	0
DOF003_scaffold39612_2	non-phage	0
DOF003_scaffold33769_1	non-phage	0
DOF003_scaffold40096_5	non-phage	0
DOF003_scaffold21715_1	non-phage	0
DOF003_scaffold33528_1	non-phage	0
DOF003_scaffold18939_1	non-phage	0
DOF003_scaffold673_4	non-phage	0
DOF003_scaffold6937_4	non-phage	0
DOF003_scaffold3052_10	non-phage	0
DOF003_scaffold495_1	non-phage	0
DOF003_scaffold13715_7	non-phage	0
DOF003_scaffold40114_3	non-phage	0
DOF003_scaffold37610_2	non-phage	0
DOF003_scaffold51001_6	non-phage	0
DOF003_scaffold23331_1	non-phage	0
DOF003_scaffold16021_1	non-phage	0
DOF003_scaffold23953_5	non-phage	0
DOF003_scaffold54_3	non-phage	0
DOF003_scaffold33691_1	non-phage	0
DOF003_scaffold21247_2	non-phage	0
DOF003_scaffold7886_6	non-phage	0
DOF003_scaffold46994_1	non-phage	0
DOF003_scaffold19011_9	non-phage	0
DOF003_scaffold13943_5	non-phage	0
DOF003_scaffold38696_31	non-phage	0
DOF003_scaffold51135_1	non-phage	0
DOF003_scaffold50147_4	non-phage	0
DOF003_scaffold29612_1	non-phage	0
DOF003_scaffold21246_7	non-phage	0
DOF003_scaffold51078_2	non-phage	0

DOF003_scaffold21753_1	non-phage	0
DOF003_scaffold37804_2	non-phage	0
DOF003_scaffold38258_3	non-phage	0
DOF003_scaffold37803_1	non-phage	0
DOF003_scaffold29424_2	non-phage	0
DOF003_scaffold51222_2	non-phage	0
DOF003_scaffold14466_2	non-phage	0
DOF003_scaffold50455_1	non-phage	0
DOF003_scaffold44561_2	non-phage	0
DOF003_scaffold50676_1	non-phage	0
DOF003_scaffold40096_3	non-phage	0
DOF003_scaffold39822_1	non-phage	0
DOF003_scaffold40480_12	non-phage	0
DOF003_scaffold31275_4	non-phage	0
DOF003_scaffold10907_1	non-phage	0
DOF003_scaffold3054_4	non-phage	0
DOF003_scaffold51002_1	non-phage	0
DOF003_scaffold29469_3	non-phage	0
DOF003_scaffold41336_1	non-phage	0
DOF003_scaffold51039_2	non-phage	0
DOF003_scaffold2196_1	non-phage	0
DOF003_scaffold50729_4	non-phage	0
DOF003_scaffold19131_3	non-phage	0
DOF003_scaffold45903_2	non-phage	0
DOF003_scaffold46844_8	non-phage	0
DOF003_scaffold269_12	phage	0.974
DOF003_scaffold16509_4	non-phage	0
DOF003_scaffold51143_1	non-phage	0
DOF003_scaffold21246_8	phage	0.697
DOF003_scaffold13313_18	non-phage	0
DOF003_scaffold51197_1	non-phage	0
DOF003_scaffold40014_1	non-phage	0
DOF003_scaffold18939_5	non-phage	0
DOF003_scaffold26969_2	non-phage	0
DOF003_scaffold51126_1	non-phage	0
DOF003_scaffold3052_29	non-phage	0
DOF003_scaffold13715_2	non-phage	0
DOF003_scaffold51212_1	non-phage	0
DOF003_scaffold2045_6	non-phage	0
DOF003_scaffold25701_8	non-phage	0
DOF003_scaffold40734_3	non-phage	0
DOF003_scaffold43600_1	non-phage	0
DOF003_scaffold26430_1	non-phage	0
DOF003_scaffold37081_1	non-phage	0
DOF003_scaffold2523_7	non-phage	0
DOF003_scaffold33873_1	non-phage	0

DOF003_scaffold34804_4	non-phage	0
DOF003_scaffold15976_2	non-phage	0
DOF003_scaffold43543_2	non-phage	0
DOF003_scaffold989_13	non-phage	0
DOF003_scaffold28272_1	non-phage	0
DOF003_scaffold4136_17	non-phage	0
DOF003_scaffold32235_1	non-phage	0
DOF003_scaffold6665_1	non-phage	0
DOF003_scaffold28621_1	non-phage	0
DOF003_scaffold23026_1	non-phage	0
DOF003_scaffold42935_1	non-phage	0
DOF003_scaffold51001_2	non-phage	0
DOF003_scaffold47414_1	non-phage	0
DOF003_scaffold37611_1	non-phage	0
DOF003_scaffold49969_1	non-phage	0
DOF003_scaffold26962_1	non-phage	0
DOF003_scaffold51115_1	phage	0.998
DOF003_scaffold2741_2	phage	0.998
DOF003_scaffold41209_3	non-phage	0
DOF003_scaffold25701_1	non-phage	0
DOF003_scaffold42918_1	non-phage	0
DOF003_scaffold34884_1	non-phage	0
DOF003_scaffold19495_2	non-phage	0
DOF003_scaffold31192_1	non-phage	0
DOF003_scaffold49228_2	non-phage	0
DOF003_scaffold40082_2	non-phage	0
DOF003_scaffold7750_3	non-phage	0
DOF003_scaffold48530_1	non-phage	0
DOF003_scaffold18036_2	non-phage	0
DOF003_scaffold33838_1	non-phage	0
DOF003_scaffold3054_1	non-phage	0
DOF003_scaffold43958_1	non-phage	0
DOF003_scaffold28914_6	non-phage	0
DOF003_scaffold40096_6	non-phage	0
DOF003_scaffold1311_17	non-phage	0
DOF003_scaffold35883_1	non-phage	0
DOF003_scaffold27202_6	non-phage	0
DOF003_scaffold42623_1	non-phage	0
DOF003_C746192_1	phage	0.999
DOF003_scaffold6902_23	non-phage	0
DOF003_scaffold50728_1	non-phage	0
DOF003_scaffold10178_1	non-phage	0
DOF003_scaffold48548_2	non-phage	0
DOF003_scaffold27400_1	non-phage	0
DOF003_scaffold54_2_1	non-phage	0
DOF003_scaffold38789_4	non-phage	0

DOF003_scaffold46904_2	non-phage	0
DOF003_scaffold15538_3	non-phage	0
DOF003_scaffold1220_8	non-phage	0
DOF003_scaffold43780_1	phage	0.997
DOF003_scaffold1019_2	non-phage	0
DOF003_scaffold11933_1	non-phage	0
DOF003_scaffold27230_3	non-phage	0
DOF003_scaffold51078_3	non-phage	0
DOF003_scaffold40042_3	non-phage	0
DOF003_scaffold50715_1	non-phage	0
DOF003_scaffold48529_1	non-phage	0
DOF003_scaffold36266_2	non-phage	0
DOF003_scaffold35993_2	non-phage	0
DOF003_scaffold31964_1	non-phage	0
DOF003_scaffold39018_4	non-phage	0
DOF003_scaffold38892_10	non-phage	0
DOF003_scaffold22117_1	non-phage	0
DOF003_scaffold48262_1	phage	0.998
DOF003_scaffold33722_3	non-phage	0
DOF003_scaffold9164_4	non-phage	0
DOF003_scaffold28472_2	non-phage	0
DOF003_scaffold29185_1	non-phage	0
DOF003_scaffold51211_3	non-phage	0
DOF003_scaffold51181_2	non-phage	0
DOF003_scaffold1689_1	non-phage	0
DOF003_scaffold50812_1	non-phage	0
DOF003_scaffold376_1	non-phage	0
DOF003_scaffold2826_3	phage	0.999
DOF003_scaffold13715_6	non-phage	0
DOF003_scaffold50999_1	non-phage	0
DOF003_scaffold37885_2	non-phage	0
DOF003_scaffold6902_32	non-phage	0
DOF003_scaffold49622_1	non-phage	0
DOF003_scaffold194_1	non-phage	0
DOF003_C745668_1	non-phage	0
DOF003_scaffold37298_1	non-phage	0
DOF003_scaffold47559_1	non-phage	0
DOF003_scaffold49243_1	non-phage	0
DOF003_scaffold1329_21	non-phage	0
DOF003_scaffold31205_1	non-phage	0
DOF003_scaffold51162_1	non-phage	0
DOF003_scaffold1689_30	non-phage	0
DOF003_scaffold48947_1	non-phage	0
DOF003_scaffold40371_1	non-phage	0
DOF003_scaffold51190_1	non-phage	0
DOF003_scaffold31599_5	non-phage	0

DOF003_scaffold7052_1	non-phage	0
DOF003_scaffold38789_1	non-phage	0
DOF003_scaffold9943_5	non-phage	0
DOF003_scaffold22191_11	phage	0.998
DOF003_scaffold46924_1	non-phage	0
DOF003_scaffold29010_1	non-phage	0
DOF003_scaffold23953_10	non-phage	0
DOF003_scaffold15976_3	non-phage	0
DOF003_scaffold51131_1	non-phage	0
DOF003_scaffold35923_2	non-phage	0
DOF003_scaffold26170_1	non-phage	0
DOF003_scaffold50098_3	non-phage	0
DOF003_scaffold51222_1	non-phage	0
DOF003_scaffold50401_1	non-phage	0
DOF003_scaffold47680_1	non-phage	0
DOF003_scaffold29668_4	non-phage	0
DOF003_scaffold13943_2	non-phage	0
DOF003_scaffold230_1	non-phage	0
DOF003_scaffold4785_1	non-phage	0
DOF003_scaffold2865_2	non-phage	0
DOF003_scaffold51219_1	non-phage	0
DOF003_scaffold40075_2	non-phage	0
DOF003_scaffold6937_7	non-phage	0
DOF003_scaffold47175_2	non-phage	0
DOF003_scaffold1689_27	phage	0.999
DOF003_scaffold12598_1	non-phage	0
DOF003_scaffold45814_1	non-phage	0
DOF003_scaffold26623_1	non-phage	0
DOF003_C746300_1	non-phage	0
DOF003_scaffold3052_28	non-phage	0
DOF003_scaffold17362_3	non-phage	0
DOF003_scaffold28347_2	non-phage	0
DOF003_scaffold13183_2	phage	0.999
DOF003_scaffold35161_1	non-phage	0
DOF003_scaffold34182_13	non-phage	0
DOF003_scaffold12256_3	non-phage	0
DOF003_scaffold4746_1	phage	0.71
DOF003_scaffold11546_5	non-phage	0
DOF003_scaffold35168_2	non-phage	0
DOF003_scaffold23234_1	phage	0.997
DOF003_scaffold50310_1	non-phage	0
DOF003_scaffold50079_3	non-phage	0
DOF003_scaffold37461_3	non-phage	0
DOF003_scaffold24365_1	non-phage	0
DOF003_scaffold50890_1	non-phage	0
DOF003_scaffold13351_4	non-phage	0

DOF003_scaffold51218_2	non-phage	0
DOF003_scaffold8511_1	non-phage	0
DOF003_scaffold44902_1	non-phage	0
DOF003_scaffold164_1	non-phage	0
DOF003_scaffold3136_28	non-phage	0
DOF003_scaffold47636_2	non-phage	0
DOF003_scaffold14239_4	non-phage	0
DOF003_scaffold41050_2	non-phage	0
DOF003_scaffold376_2	non-phage	0
DOF003_scaffold50542_2	non-phage	0
DOF003_scaffold49885_1	phage	0.99
DOF003_scaffold45903_1	non-phage	0
DOF003_scaffold1220_12	non-phage	0
DOF003_scaffold51206_1	non-phage	0
DOF003_scaffold13943_3	non-phage	0
DOF003_scaffold50826_2	non-phage	0
DOF003_scaffold49515_1	non-phage	0
DOF003_scaffold21971_2	non-phage	0
DOF003_scaffold42346_2	non-phage	0
DOF003_scaffold36334_3	non-phage	0
DOF003_scaffold7734_1	non-phage	0
DOF003_scaffold39302_1	non-phage	0
DOF003_scaffold2697_1	non-phage	0
DOF003_scaffold50835_1	non-phage	0
DOF003_scaffold24766_10	non-phage	0
DOF003_scaffold35715_1	non-phage	0
DOF003_scaffold49369_2	non-phage	0
DOF003_scaffold34411_1	non-phage	0
DOF003_scaffold29011_1	non-phage	0
DOF003_scaffold3052_17	non-phage	0
DOF003_scaffold50147_2	non-phage	0
DOF003_scaffold35954_3	non-phage	0
DOF003_scaffold48548_1	non-phage	0
DOF003_scaffold44721_3	non-phage	0
DOF003_scaffold9209_2	non-phage	0
DOF003_scaffold47922_1	non-phage	0
DOF003_scaffold50728_2	non-phage	0
DOF003_scaffold37610_1	non-phage	0
DOF003_scaffold15485_18	non-phage	0
DOF003_scaffold1220_7	non-phage	0
DOF003_scaffold24797_1	non-phage	0
DOF003_scaffold22839_2	non-phage	0
DOF003_scaffold50670_1	non-phage	0
DOF003_scaffold7276_5	non-phage	0
DOF003_scaffold45075_4	non-phage	0
DOF003_scaffold10763_7	non-phage	0

DOF003_scaffold50947_2	phage	0.998
DOF003_scaffold40096_7	non-phage	0
DOF003_scaffold50601_2	non-phage	0
DOF003_scaffold40408_2	non-phage	0
DOF003_scaffold36457_1	non-phage	0.001
DOF003_scaffold25701_3	non-phage	0
DOF003_scaffold9402_6	non-phage	0
DOF003_scaffold47283_1	non-phage	0
DOF003_C746480_1	non-phage	0
DOF003_scaffold4746_2	non-phage	0
DOF003_scaffold12401_22	non-phage	0
DOF003_scaffold18004_2	non-phage	0
DOF003_scaffold42641_2	non-phage	0
DOF003_scaffold44438_1	non-phage	0
DOF003_scaffold482_1	non-phage	0
DOF003_scaffold48276_1	non-phage	0
DOF003_scaffold29431_6	non-phage	0
DOF003_scaffold51205_1	non-phage	0
DOF003_scaffold46765_3	non-phage	0
DOF003_scaffold47922_8	non-phage	0
DOF003_scaffold6693_2	non-phage	0
DOF003_scaffold40608_2	non-phage	0
DOF003_scaffold15024_2	non-phage	0
DOF003_scaffold48102_1	non-phage	0
DOF003_scaffold14831_3	non-phage	0.005
DOF003_scaffold25531_2	non-phage	0
DOF003_scaffold23071_2	non-phage	0
DOF003_scaffold2391_3	non-phage	0
DOF003_scaffold50786_2	non-phage	0
DOF003_scaffold6902_28	non-phage	0
DOF003_scaffold35161_4	non-phage	0
DOF003_scaffold50687_1	non-phage	0
DOF003_scaffold51046_1	phage	0.782
DOF003_scaffold4224_21	non-phage	0
DOF003_scaffold13677_7	non-phage	0
DOF003_scaffold39313_1	non-phage	0
DOF003_scaffold3054_2	non-phage	0
DOF003_scaffold34149_5	non-phage	0
DOF003_scaffold33447_1	non-phage	0
DOF003_scaffold40480_10	non-phage	0
DOF003_scaffold18305_4	non-phage	0
DOF003_scaffold4_5	non-phage	0
DOF003_scaffold51207_1	non-phage	0
DOF003_scaffold39876_1	non-phage	0
DOF003_scaffold31989_1	non-phage	0
DOF003_scaffold21576_2	non-phage	0

DOF003_scaffold35662_1	non-phage	0
DOF003_C745734_1	non-phage	0
DOF003_scaffold8511_2	non-phage	0
DOF003_scaffold13943_1	non-phage	0
DOF003_scaffold6902_21	non-phage	0
DOF003_scaffold19132_1	non-phage	0
DOF003_scaffold27693_1	non-phage	0
DOF003_scaffold37326_1	non-phage	0
DOF003_scaffold8510_44	non-phage	0
DOF003_scaffold12191_5	non-phage	0
DOF003_scaffold51200_1	non-phage	0
DOF003_scaffold36032_4	non-phage	0
DOF003_scaffold14982_2	non-phage	0
DOF003_scaffold6902_19	non-phage	0
DOF003_scaffold26394_1	non-phage	0
DOF003_scaffold50909_1	non-phage	0
DOF003_scaffold32977_2	phage	0.999
DOF003_scaffold12256_16	non-phage	0
DOF003_scaffold26431_1	non-phage	0
DOF003_scaffold30739_3	phage	0.999
DOF003_scaffold50350_1	phage	0.999
DOF003_scaffold9809_5	non-phage	0
DOF003_scaffold29542_1	non-phage	0
DOF003_scaffold18996_2	non-phage	0
DOF003_scaffold28107_3	non-phage	0
DOF003_scaffold26393_2	non-phage	0
DOF003_scaffold51040_3	non-phage	0
DOF003_scaffold34454_3	non-phage	0
DOF003_scaffold7256_1	non-phage	0
DOF003_scaffold42629_1	non-phage	0
DOF003_scaffold37600_6	non-phage	0
DOF003_scaffold47283_2	non-phage	0
DOF003_scaffold2698_1	non-phage	0
DOF003_scaffold242_1	non-phage	0
DOF003_scaffold28472_4	phage	0.954
DOF003_scaffold15538_5	non-phage	0
DOF003_scaffold41798_1	non-phage	0
DOF003_scaffold42918_3	non-phage	0
DOF003_scaffold6902_30	non-phage	0
DOF003_scaffold51054_1	non-phage	0
DOF003_scaffold27181_1	non-phage	0
DOF003_scaffold33528_8	non-phage	0
DOF003_scaffold28621_2	non-phage	0
DOF003_scaffold24449_1	non-phage	0
DOF003_scaffold48552_3	non-phage	0
DOF003_scaffold27512_3	non-phage	0

DOF003_scaffold2260_1	non-phage	0
DOF003_scaffold20370_16	non-phage	0
DOF003_scaffold47922_10	non-phage	0
DOF003_scaffold40146_1	non-phage	0
DOF003_scaffold13475_1	non-phage	0
DOF003_scaffold8_1	non-phage	0
DOF003_scaffold51219_2	non-phage	0
DOF003_scaffold22150_1	non-phage	0
DOF003_scaffold14466_4	non-phage	0
DOF003_scaffold31205_5	non-phage	0
DOF003_scaffold6693_1	non-phage	0
DOF003_scaffold50128_2	non-phage	0
DOF003_scaffold34182_8	non-phage	0
DOF003_scaffold1237_16	non-phage	0
DOF003_scaffold989_7	non-phage	0
DOF003_scaffold21247_1	non-phage	0
DOF003_scaffold40014_2	non-phage	0
DOF003_scaffold45075_2	non-phage	0
DOF003_scaffold6902_14	non-phage	0
DOF003_scaffold22256_4	non-phage	0
DOF003_scaffold48559_1	non-phage	0
DOF003_scaffold40096_8	non-phage	0
DOF003_scaffold51029_1	non-phage	0
DOF003_scaffold42346_1	non-phage	0
DOF003_scaffold46799_2	non-phage	0
DOF003_scaffold29469_1	non-phage	0
DOF003_scaffold989_11	phage	0.999
DOF003_scaffold34101_2	non-phage	0
DOF003_scaffold7276_1	non-phage	0
DOF003_scaffold48394_1	non-phage	0
DOF003_scaffold112_7	non-phage	0
DOF003_scaffold35954_2	non-phage	0
DOF003_scaffold5719_1	non-phage	0
DOF003_scaffold37826_1	non-phage	0
DOF003_scaffold24515_2	non-phage	0
DOF003_scaffold38246_1	non-phage	0
DOF003_C745894_1	non-phage	0
DOF003_scaffold40042_1	non-phage	0
DOF003_scaffold43216_1	non-phage	0
DOF003_scaffold980_1	non-phage	0
DOF003_scaffold16930_8	non-phage	0
DOF003_scaffold51001_5	non-phage	0
DOF003_scaffold25908_2	non-phage	0
DOF003_scaffold13782_1	non-phage	0
DOF003_scaffold9466_4	non-phage	0
DOF003_scaffold49069_1	phage	0.997

DOF003_scaffold33283_2	non-phage	0
DOF003_scaffold122_5	non-phage	0
DOF003_scaffold32724_1	non-phage	0
DOF003_scaffold18996_3	non-phage	0
DOF003_scaffold47161_2	non-phage	0
DOF003_scaffold989_5	non-phage	0
DOF003_scaffold50225_1	non-phage	0
DOF003_scaffold36266_4	non-phage	0
DOF003_scaffold39018_3	non-phage	0
DOF003_scaffold32457_2	non-phage	0
DOF003_scaffold9809_4	non-phage	0
DOF003_scaffold25701_9	non-phage	0
DOF003_scaffold11546_3	non-phage	0
DOF003_scaffold6671_1	non-phage	0
DOF003_scaffold65_7	non-phage	0
DOF003_scaffold41050_1	non-phage	0
DOF003_scaffold38134_1	non-phage	0
DOF003_scaffold37686_3	non-phage	0
DOF003_scaffold1689_2	non-phage	0
DOF003_scaffold15485_11	phage	0.994
DOF003_scaffold30085_1	non-phage	0
DOF003_scaffold8511_3	non-phage	0
DOF003_scaffold39195_2	non-phage	0
DOF003_scaffold27196_1	non-phage	0
DOF003_scaffold40014_4	non-phage	0
DOF003_scaffold11899_2	non-phage	0
DOF003_scaffold390_1	non-phage	0
DOF003_scaffold40480_7	phage	0.994
DOF003_scaffold45770_2	non-phage	0
DOF003_scaffold29570_2	non-phage	0
DOF003_scaffold46812_2	non-phage	0
DOF003_scaffold42921_1	non-phage	0
DOF003_scaffold7439_1	non-phage	0
DOF003_scaffold51175_2	non-phage	0
DOF003_scaffold2826_7	phage	0.999
DOF003_scaffold49694_1	non-phage	0
DOF003_scaffold6062_4	non-phage	0
DOF003_scaffold49885_3	phage	0.974
DOF003_scaffold197_2	phage	0.998
DOF003_scaffold11933_4	non-phage	0
DOF003_scaffold959_2	phage	0.995
DOF003_scaffold29102_1	non-phage	0
DOF003_scaffold3207_2	non-phage	0
DOF003_scaffold44416_2	non-phage	0
DOF003_scaffold5983_2	non-phage	0
DOF003_scaffold50994_2	non-phage	0

DOF003_scaffold42721_1	non-phage	0
DOF003_scaffold47911_1	non-phage	0
DOF003_scaffold24985_1	non-phage	0
DOF003_scaffold51142_2	non-phage	0
DOF003_scaffold7886_2	non-phage	0
DOF003_C746560_1	non-phage	0
DOF003_scaffold51202_1	non-phage	0
DOF003_scaffold51078_6	phage	0.999
DOF003_scaffold42641_1	non-phage	0
DOF003_scaffold11786_1	non-phage	0
DOF003_scaffold14239_2	non-phage	0
DOF003_scaffold51078_1	phage	0.994
DOF003_scaffold35788_1	non-phage	0
DOF003_scaffold28401_2	non-phage	0
DOF003_scaffold51006_2	non-phage	0
DOF003_scaffold41737_1	non-phage	0
DOF003_scaffold1220_9	non-phage	0
DOF003_scaffold36508_2	non-phage	0
DOF003_scaffold50729_1	non-phage	0
DOF003_scaffold27669_4	non-phage	0
DOF003_scaffold50553_3	non-phage	0
DOF003_scaffold51004_2	non-phage	0
DOF003_scaffold34182_7	non-phage	0
DOF003_scaffold33690_1	non-phage	0
DOF003_scaffold47093_1	non-phage	0
DOF003_scaffold40167_1	non-phage	0
DOF003_scaffold989_14	non-phage	0
DOF003_scaffold37664_8	non-phage	0
DOF003_scaffold26004_1	non-phage	0
DOF003_scaffold32723_1	non-phage	0
DOF003_scaffold35161_5	non-phage	0
DOF003_scaffold9164_2	phage	0.999
DOF003_scaffold48152_3	non-phage	0
DOF003_C745586_1	non-phage	0
DOF003_scaffold51045_1	non-phage	0
DOF003_scaffold6299_18	non-phage	0
DOF003_scaffold2865_5	non-phage	0
DOF003_scaffold50800_1	non-phage	0
DOF003_scaffold6208_1	non-phage	0
DOF003_scaffold22191_13	phage	1
DOF003_scaffold23724_3	non-phage	0
DOF003_scaffold17362_6	non-phage	0
DOF003_scaffold48163_1	non-phage	0
DOF003_scaffold64_7	non-phage	0
DOF003_scaffold1689_16	non-phage	0
DOF003_C746342_1	non-phage	0

DOF003_scaffold13715_8	non-phage	0
DOF003_scaffold37484_3	non-phage	0
DOF003_scaffold51208_1	non-phage	0
DOF003_scaffold19459_3	non-phage	0
DOF003_scaffold50746_1	non-phage	0
DOF003_scaffold49281_1	non-phage	0
DOF003_scaffold34805_1	non-phage	0
DOF003_scaffold102_6	non-phage	0
DOF003_scaffold34026_1	non-phage	0
DOF003_scaffold45063_2	non-phage	0
DOF003_scaffold400_4	non-phage	0
DOF003_scaffold29530_2	non-phage	0
DOF003_scaffold31965_1	non-phage	0
DOF003_scaffold242_6	non-phage	0
DOF003_scaffold51196_1	non-phage	0
DOF003_scaffold51185_2	non-phage	0
DOF003_scaffold13829_1	non-phage	0
DOF003_scaffold15538_4	non-phage	0
DOF003_scaffold37883_2	non-phage	0
DOF003_scaffold33153_1	non-phage	0
DOF003_scaffold17295_1	non-phage	0
DOF003_scaffold51075_5	phage	0.521
DOF003_scaffold7334_1	non-phage	0
DOF003_scaffold3054_9	non-phage	0
DOF003_scaffold36334_1	non-phage	0
DOF003_scaffold39188_6	non-phage	0
DOF003_scaffold12401_15	non-phage	0
DOF003_scaffold43441_1	non-phage	0
DOF003_scaffold6902_9	non-phage	0
DOF003_scaffold22325_1	non-phage	0
DOF003_scaffold20444_1	non-phage	0
DOF003_scaffold29668_8	non-phage	0
DOF003_scaffold22256_2	non-phage	0
DOF003_scaffold51178_1	non-phage	0
DOF003_scaffold65_4	non-phage	0
DOF003_scaffold8511_4	non-phage	0
DOF003_scaffold32452_2	non-phage	0
DOF003_scaffold47078_1	phage	0.998
DOF003_scaffold6902_12	non-phage	0
DOF003_scaffold29176_5	non-phage	0
DOF003_scaffold14795_4	non-phage	0
DOF003_scaffold22191_10	phage	0.998
DOF003_scaffold19871_4	phage	0.969
DOF003_scaffold29010_2	non-phage	0
DOF003_scaffold34182_6	non-phage	0
DOF003_scaffold1311_24	non-phage	0

DOF003_C746536_1	non-phage	0
DOF003_scaffold43232_1	non-phage	0
DOF003_scaffold24422_1	non-phage	0
DOF003_scaffold16207_5	non-phage	0.212
DOF003_scaffold37253_1	non-phage	0
DOF003_scaffold24449_2	non-phage	0
DOF003_scaffold31205_6	non-phage	0
DOF003_scaffold24721_3	non-phage	0
DOF003_scaffold16736_1	non-phage	0
DOF003_scaffold34438_1	non-phage	0
DOF003_scaffold40969_1	non-phage	0
DOF003_scaffold49282_3	non-phage	0
DOF003_scaffold11546_6	non-phage	0
DOF003_scaffold46844_6	non-phage	0
DOF003_scaffold11899_1	non-phage	0
DOF003_scaffold30271_1	non-phage	0
DOF003_scaffold35050_4	non-phage	0
DOF003_scaffold46070_1	non-phage	0
DOF003_scaffold51040_2	non-phage	0
DOF003_scaffold24089_5	non-phage	0
DOF003_scaffold50553_1	non-phage	0
DOF003_scaffold28401_1	non-phage	0
DOF003_scaffold1689_28	phage	0.997
DOF003_scaffold19871_2	non-phage	0
DOF003_scaffold16253_1	non-phage	0
DOF003_scaffold27373_1	non-phage	0
DOF003_scaffold10873_4	non-phage	0
DOF003_scaffold47609_2	non-phage	0
DOF003_scaffold1237_11	non-phage	0
DOF003_scaffold40167_2	non-phage	0
DOF003_scaffold50425_1	phage	0.998
DOF003_scaffold41738_1	non-phage	0
DOF003_scaffold1689_33	non-phage	0
DOF003_scaffold10514_2	non-phage	0
DOF003_scaffold40679_8	non-phage	0
DOF003_scaffold48690_1	non-phage	0
DOF003_scaffold35954_1	non-phage	0
DOF003_scaffold45770_1	non-phage	0
DOF003_scaffold24515_4	non-phage	0
DOF003_scaffold49538_2	phage	0.986
DOF003_scaffold22839_1	non-phage	0
DOF003_scaffold51154_1	non-phage	0
DOF003_scaffold42183_2	non-phage	0
DOF003_scaffold19144_1	non-phage	0
DOF003_scaffold51087_1	non-phage	0
DOF003_scaffold5465_1	non-phage	0

DOF003_scaffold51221_1	non-phage	0
DOF003_scaffold36939_1	non-phage	0
DOF003_scaffold36032_2	non-phage	0
DOF003_scaffold11899_4	non-phage	0
DOF003_scaffold102_5	non-phage	0
DOF003_scaffold51040_1	non-phage	0
DOF003_scaffold8511_5	non-phage	0
DOF003_scaffold46844_1	non-phage	0
DOF003_scaffold40480_6	non-phage	0
DOF003_scaffold198_1	non-phage	0
DOF003_scaffold3052_14	non-phage	0
DOF003_scaffold29530_1	non-phage	0
DOF003_scaffold49519_1	non-phage	0
DOF003_scaffold28472_1	non-phage	0
DOF003_scaffold50601_1	non-phage	0
DOF003_C745536_1	non-phage	0
DOF003_scaffold4746_3	non-phage	0
DOF003_C746212_1	non-phage	0
DOF003_scaffold51165_1	non-phage	0
DOF003_scaffold24873_1	non-phage	0
DOF003_scaffold3052_11	non-phage	0
DOF003_scaffold19201_1	phage	0.994
DOF003_scaffold48552_1	non-phage	0
DOF003_scaffold38_1	non-phage	0
DOF003_scaffold20624_15	non-phage	0
DOF003_scaffold40480_1	non-phage	0
DOF003_scaffold12401_8	non-phage	0
DOF003_scaffold25902_1	non-phage	0
DOF003_scaffold29683_8	non-phage	0
DOF003_scaffold24721_2	non-phage	0
DOF003_scaffold37326_2	non-phage	0
DOF003_scaffold9209_5	non-phage	0
DOF003_scaffold28914_12	phage	0.988
DOF003_scaffold36733_1	phage	0.999
DOF003_scaffold10514_8	non-phage	0
DOF003_scaffold46994_2	non-phage	0
DOF003_scaffold38008_1	non-phage	0
DOF003_scaffold25851_1	non-phage	0
DOF003_scaffold51155_1	non-phage	0
DOF003_scaffold44547_2	non-phage	0
DOF003_scaffold19131_2	non-phage	0
DOF003_scaffold51087_4	non-phage	0
DOF003_scaffold17362_8	non-phage	0
DOF003_scaffold27230_1	non-phage	0
DOF003_scaffold33722_1	non-phage	0
DOF003_scaffold37727_2	non-phage	0

DOF003_scaffold34870_1	non-phage	0
DOF003_scaffold14663_4	non-phage	0
DOF003_scaffold29356_1	non-phage	0
DOF003_scaffold12256_5	non-phage	0
DOF003_scaffold34492_4	non-phage	0
DOF003_scaffold34149_4	non-phage	0
DOF003_scaffold47911_3	non-phage	0
DOF003_scaffold8510_28	non-phage	0
DOF003_scaffold40841_2	non-phage	0
DOF003_scaffold37453_1	non-phage	0
DOF003_scaffold48545_1	non-phage	0
DOF003_scaffold46844_5	non-phage	0
DOF003_scaffold41050_6	non-phage	0
DOF003_scaffold40114_1	non-phage	0
DOF003_scaffold38135_1	non-phage	0
DOF003_scaffold3136_18	non-phage	0
DOF003_scaffold37664_3	non-phage	0
DOF003_scaffold1689_26	non-phage	0
DOF003_scaffold27373_3	non-phage	0
DOF003_scaffold29848_1	non-phage	0
DOF003_scaffold23809_1	non-phage	0
DOF003_scaffold25431_2	non-phage	0
DOF003_scaffold22150_2	non-phage	0.216
DOF003_scaffold6937_12	non-phage	0
DOF003_scaffold50826_1	non-phage	0
DOF003_scaffold22256_6	phage	0.993
DOF003_scaffold34627_1	non-phage	0
DOF003_scaffold43814_1	non-phage	0
DOF003_scaffold1310_1	non-phage	0
DOF003_scaffold47404_2	non-phage	0
DOF003_scaffold40480_9	non-phage	0
DOF003_scaffold33608_1	non-phage	0
DOF003_scaffold989_12	non-phage	0
DOF003_scaffold102_3	non-phage	0
DOF003_scaffold24010_1	non-phage	0
DOF003_scaffold39195_4	non-phage	0
DOF003_scaffold26970_2	non-phage	0
DOF003_scaffold2965_1	non-phage	0
DOF003_scaffold8559_1	non-phage	0
DOF003_scaffold48552_2	non-phage	0
DOF003_scaffold51191_1	non-phage	0
DOF003_scaffold7276_2	non-phage	0
DOF003_scaffold34182_9	non-phage	0
DOF003_scaffold25488_1	non-phage	0
DOF003_scaffold27483_3	phage	1
DOF003_scaffold8003_1	non-phage	0

DOF003_scaffold50949_1	non-phage	0
DOF003_scaffold54_10	non-phage	0
DOF003_scaffold51195_1	non-phage	0
DOF003_scaffold50542_1	non-phage	0
DOF003_scaffold989_4	non-phage	0
DOF003_scaffold51004_1	non-phage	0
DOF003_scaffold15538_2	non-phage	0
DOF003_scaffold198_2	non-phage	0
DOF003_scaffold7372_1	non-phage	0
DOF003_C746168_1	non-phage	0
DOF003_scaffold38258_5	non-phage	0
DOF003_scaffold38708_3	non-phage	0
DOF003_scaffold36783_1	non-phage	0
DOF003_scaffold7886_4	non-phage	0
DOF003_scaffold41738_2	non-phage	0
DOF003_scaffold10178_2	non-phage	0
DOF003_scaffold46799_1	non-phage	0
DOF003_scaffold673_6	non-phage	0
DOF003_scaffold5719_4	non-phage	0
DOF003_scaffold12401_12	non-phage	0.021
DOF003_scaffold6902_17	non-phage	0
DOF003_scaffold35581_4	phage	0.999
DOF003_scaffold50671_1	non-phage	0
DOF003_scaffold39219_2	non-phage	0
DOF003_scaffold49321_1	non-phage	0
DOF003_scaffold27906_10	non-phage	0
DOF003_scaffold51146_1	non-phage	0
DOF003_scaffold1689_23	non-phage	0
DOF003_scaffold50730_1	non-phage	0
DOF003_scaffold37325_1	non-phage	0
DOF003_scaffold46765_4	non-phage	0
DOF003_scaffold40042_2	non-phage	0
DOF003_scaffold2391_1	non-phage	0
DOF003_scaffold40167_3	non-phage	0
DOF003_scaffold5236_4	non-phage	0
DOF003_scaffold7447_1	non-phage	0
DOF003_scaffold16172_3	non-phage	0.013
DOF003_scaffold39809_1	non-phage	0
DOF003_scaffold48545_3	non-phage	0
DOF003_scaffold38696_1	non-phage	0
DOF003_scaffold13782_2	non-phage	0
DOF003_scaffold51047_1	non-phage	0
DOF003_scaffold26970_1	non-phage	0
DOF003_scaffold7256_2	non-phage	0
DOF003_scaffold26587_4	phage	0.999
DOF003_scaffold16930_3	non-phage	0

DOF003_scaffold989_8	non-phage	0
DOF003_scaffold42494_1	non-phage	0
DOF003_scaffold6566_5	non-phage	0
DOF003_scaffold22256_3	non-phage	0
DOF003_scaffold6902_5	non-phage	0
DOF003_scaffold29668_9	non-phage	0
DOF003_scaffold29121_3	non-phage	0
DOF003_scaffold28472_3	non-phage	0
DOF003_scaffold1813_54	non-phage	0
DOF003_scaffold25532_1	non-phage	0
DOF003_scaffold48152_1	non-phage	0
DOF004_scaffold44938_4	non-phage	0
DOF004_scaffold57130_1	non-phage	0
DOF004_scaffold2524_1	non-phage	0
DOF004_scaffold28008_5	non-phage	0
DOF004_scaffold35113_1	non-phage	0
DOF004_scaffold14724_2	non-phage	0
DOF004_scaffold57147_1	non-phage	0
DOF004_scaffold25640_1	non-phage	0
DOF004_scaffold55128_1	non-phage	0
DOF004_scaffold36304_3	non-phage	0
DOF004_scaffold56916_1	non-phage	0
DOF004_scaffold57068_1	non-phage	0
DOF004_scaffold55925_1	non-phage	0
DOF004_scaffold20907_6	non-phage	0
DOF004_scaffold57048_2	non-phage	0
DOF004_scaffold32654_1	non-phage	0
DOF004_scaffold35625_1	non-phage	0
DOF004_scaffold57049_1	non-phage	0
DOF004_scaffold42_2	non-phage	0
DOF004_scaffold41076_3	non-phage	0
DOF004_scaffold7632_2	non-phage	0
DOF004_scaffold24196_1	non-phage	0
DOF004_scaffold57088_1	non-phage	0
DOF004_scaffold39753_5	non-phage	0
DOF004_scaffold57123_1	non-phage	0
DOF004_scaffold36700_1	non-phage	0
DOF004_scaffold34544_8	non-phage	0
DOF004_scaffold3658_9	non-phage	0
DOF004_scaffold56801_1	non-phage	0
DOF004_scaffold24837_14	non-phage	0
DOF004_scaffold55029_2	non-phage	0
DOF004_scaffold44040_3	non-phage	0
DOF004_scaffold10332_2	non-phage	0
DOF004_scaffold30259_8	non-phage	0
DOF004_scaffold57081_1	non-phage	0

DOF004_scaffold52501_11	non-phage	0
DOF004_scaffold34646_1	non-phage	0
DOF004_scaffold51602_1	non-phage	0.034
DOF004_scaffold31456_4	non-phage	0
DOF004_scaffold50836_1	non-phage	0
DOF004_scaffold57075_1	non-phage	0
DOF004_scaffold19620_9	non-phage	0
DOF004_scaffold56982_3	non-phage	0
DOF004_scaffold13584_4	non-phage	0
DOF004_scaffold5206_6	non-phage	0
DOF004_scaffold20326_7	non-phage	0
DOF004_scaffold20691_1	non-phage	0
DOF004_scaffold38556_1	non-phage	0
DOF004_scaffold15795_2	non-phage	0
DOF004_scaffold16813_2	non-phage	0
DOF004_scaffold12190_2	non-phage	0
DOF004_scaffold56212_5	non-phage	0
DOF004_scaffold56799_1	non-phage	0
DOF004_scaffold48224_2	non-phage	0
DOF004_scaffold20624_3	non-phage	0
DOF004_scaffold5486_1	non-phage	0
DOF004_scaffold57149_2	non-phage	0
DOF004_scaffold14452_2	non-phage	0
DOF004_scaffold10707_1	non-phage	0
DOF004_scaffold36637_3	non-phage	0
DOF004_scaffold34545_1	non-phage	0
DOF004_scaffold20587_2	non-phage	0
DOF004_scaffold15981_5	non-phage	0
DOF004_scaffold45590_4	non-phage	0
DOF004_scaffold56672_1	non-phage	0
DOF004_scaffold54566_1	non-phage	0
DOF004_scaffold49853_2	non-phage	0
DOF004_scaffold8260_1	non-phage	0
DOF004_scaffold23545_1	non-phage	0
DOF004_scaffold20782_4	non-phage	0
DOF004_scaffold34647_2	non-phage	0
DOF004_scaffold50971_1	non-phage	0
DOF004_scaffold40518_2	non-phage	0
DOF004_scaffold56995_1	non-phage	0
DOF004_scaffold57025_1	non-phage	0
DOF004_scaffold41799_1	non-phage	0
DOF004_scaffold52336_1	non-phage	0
DOF004_scaffold49006_1	non-phage	0
DOF004_scaffold5009_7	non-phage	0
DOF004_scaffold5009_10	non-phage	0
DOF004_scaffold18503_1	non-phage	0

DOF004_scaffold52853_1	non-phage	0
DOF004_scaffold11214_1	non-phage	0
DOF004_C632144_1	non-phage	0
DOF004_C632542_1	non-phage	0
DOF004_scaffold56466_3	non-phage	0
DOF004_scaffold57072_2	non-phage	0
DOF004_scaffold56942_3	non-phage	0
DOF004_scaffold17436_1	non-phage	0
DOF004_scaffold35681_1	non-phage	0
DOF004_scaffold7_3	non-phage	0
DOF004_scaffold57115_1	non-phage	0
DOF004_scaffold23806_1	non-phage	0
DOF004_scaffold99_1	non-phage	0
DOF004_scaffold26851_1	non-phage	0
DOF004_scaffold29615_2	non-phage	0
DOF004_scaffold23582_2	non-phage	0
DOF004_scaffold57082_1	non-phage	0
DOF004_scaffold57103_1	non-phage	0
DOF004_scaffold56481_4	non-phage	0
DOF004_scaffold51050_2	non-phage	0
DOF004_scaffold19620_5	non-phage	0
DOF004_scaffold210_3	non-phage	0
DOF004_scaffold56339_1	non-phage	0
DOF004_scaffold53133_1	non-phage	0
DOF004_scaffold56958_1	non-phage	0
DOF004_scaffold52644_1	non-phage	0
DOF004_scaffold55243_1	non-phage	0
DOF004_scaffold23082_1	non-phage	0
DOF004_scaffold18559_3	non-phage	0
DOF004_scaffold39823_1	non-phage	0
DOF004_scaffold22450_3	non-phage	0
DOF004_scaffold42967_2	non-phage	0
DOF004_scaffold49754_4	non-phage	0
DOF004_scaffold13315_1	non-phage	0
DOF004_scaffold23567_1	non-phage	0
DOF004_scaffold56592_1	non-phage	0
DOF004_scaffold35038_1	non-phage	0
DOF004_scaffold49261_1	non-phage	0
DOF004_scaffold25486_1	non-phage	0.091
DOF004_scaffold33568_2	non-phage	0
DOF004_scaffold37587_1	non-phage	0
DOF004_C633636_1	non-phage	0
DOF004_scaffold51566_1	non-phage	0
DOF004_scaffold38778_4	non-phage	0
DOF004_scaffold23235_1	non-phage	0
DOF004_scaffold30259_1	non-phage	0

DOF004_scaffold57077_4	non-phage	0
DOF004_scaffold53137_1	non-phage	0
DOF004_scaffold4294_1	non-phage	0
DOF004_scaffold41902_2	non-phage	0
DOF004_scaffold28415_1	non-phage	0
DOF004_scaffold40087_3	non-phage	0
DOF004_scaffold54568_6	non-phage	0
DOF004_scaffold7220_2	non-phage	0
DOF004_scaffold31768_1	phage	0.98
DOF004_scaffold13837_3	non-phage	0
DOF004_scaffold56794_1	non-phage	0
DOF004_scaffold48662_1	non-phage	0
DOF004_scaffold33568_6	non-phage	0
DOF004_scaffold13294_2	non-phage	0
DOF004_scaffold57098_2	non-phage	0
DOF004_scaffold56978_5	non-phage	0
DOF004_scaffold54568_5	non-phage	0
DOF004_scaffold25655_4	non-phage	0
DOF004_scaffold43_1	non-phage	0
DOF004_scaffold7632_7	non-phage	0
DOF004_scaffold11714_2	non-phage	0
DOF004_scaffold56923_6	non-phage	0
DOF004_scaffold6020_1	non-phage	0
DOF004_scaffold1738_1	non-phage	0
DOF004_scaffold52998_3	non-phage	0
DOF004_scaffold21518_1	non-phage	0
DOF004_scaffold52335_2	non-phage	0
DOF004_scaffold49242_2	non-phage	0
DOF004_scaffold44558_4	non-phage	0
DOF004_scaffold804_14	non-phage	0
DOF004_scaffold8307_8	non-phage	0
DOF004_scaffold55499_3	non-phage	0
DOF004_scaffold57076_1	non-phage	0
DOF004_scaffold21257_2	non-phage	0
DOF004_scaffold925_7	non-phage	0
DOF004_scaffold2524_5	non-phage	0
DOF004_scaffold23805_2	non-phage	0
DOF004_scaffold55296_3	non-phage	0
DOF004_scaffold49754_3	non-phage	0
DOF004_scaffold802_1	non-phage	0
DOF004_scaffold55422_3	non-phage	0
DOF004_scaffold57099_1	non-phage	0
DOF004_scaffold56259_5	non-phage	0
DOF004_scaffold56950_1	non-phage	0
DOF004_scaffold56921_6	non-phage	0
DOF004_scaffold57071_3	non-phage	0

DOF004_scaffold36304_4	non-phage	0
DOF004_scaffold56868_2	non-phage	0
DOF004_scaffold198_3	non-phage	0
DOF004_scaffold57077_3	non-phage	0
DOF004_scaffold56992_1	non-phage	0
DOF004_scaffold34252_4	non-phage	0
DOF004_scaffold42980_1	non-phage	0
DOF004_scaffold27569_2	non-phage	0
DOF004_scaffold137_1	non-phage	0
DOF004_scaffold5587_3	non-phage	0
DOF004_scaffold40882_1	non-phage	0
DOF004_scaffold3199_1	non-phage	0
DOF004_scaffold11402_3	non-phage	0
DOF004_scaffold21227_1	non-phage	0
DOF004_scaffold35881_1	non-phage	0
DOF004_C633358_1	non-phage	0
DOF004_scaffold24837_13	non-phage	0
DOF004_scaffold16317_4	non-phage	0
DOF004_scaffold224_5	non-phage	0
DOF004_scaffold217_1	non-phage	0
DOF004_scaffold56921_1	non-phage	0
DOF004_scaffold42898_3	non-phage	0
DOF004_scaffold44039_3	non-phage	0
DOF004_scaffold23556_2	non-phage	0
DOF004_scaffold56939_3	non-phage	0
DOF004_scaffold8172_2	non-phage	0
DOF004_scaffold24837_5	non-phage	0
DOF004_scaffold5206_2	non-phage	0
DOF004_scaffold33569_2	non-phage	0
DOF004_scaffold39133_1	non-phage	0
DOF004_scaffold149_1	non-phage	0
DOF004_scaffold8200_2	non-phage	0
DOF004_scaffold56825_1	non-phage	0
DOF004_C632744_1	non-phage	0
DOF004_scaffold57062_1	non-phage	0
DOF004_scaffold12190_1	non-phage	0
DOF004_scaffold22467_5	non-phage	0
DOF004_scaffold16708_1	non-phage	0
DOF004_scaffold27515_2	non-phage	0
DOF004_scaffold20624_8	non-phage	0
DOF004_scaffold57047_1	non-phage	0
DOF004_scaffold41981_3	phage	0.998
DOF004_scaffold49818_6	non-phage	0
DOF004_scaffold30438_1	non-phage	0
DOF004_scaffold29098_2	non-phage	0
DOF004_scaffold56212_1	non-phage	0

DOF004_scaffold53220_1	non-phage	0
DOF004_scaffold30322_2	non-phage	0
DOF004_scaffold47782_4	non-phage	0
DOF004_scaffold56070_6	non-phage	0
DOF004_scaffold56975_1	non-phage	0
DOF004_scaffold3390_14	non-phage	0
DOF004_scaffold57134_1	non-phage	0
DOF004_scaffold40087_7	non-phage	0
DOF004_scaffold19620_1	non-phage	0
DOF004_scaffold36838_2	non-phage	0
DOF004_scaffold57148_1	non-phage	0
DOF004_scaffold55420_2	non-phage	0
DOF004_scaffold56349_2	non-phage	0
DOF004_scaffold30438_3	non-phage	0
DOF004_scaffold45118_3	non-phage	0
DOF004_scaffold56013_3	non-phage	0
DOF004_scaffold42185_4	phage	0.995
DOF004_scaffold54655_3	non-phage	0
DOF004_scaffold56797_1	non-phage	0
DOF004_scaffold2686_4	non-phage	0
DOF004_scaffold56449_1	non-phage	0
DOF004_scaffold9794_3	non-phage	0
DOF004_scaffold57005_1	non-phage	0
DOF004_scaffold21597_4	non-phage	0
DOF004_scaffold49999_1	non-phage	0
DOF004_scaffold36730_1	non-phage	0
DOF004_scaffold11214_5	non-phage	0
DOF004_scaffold56696_3	non-phage	0
DOF004_scaffold33827_1	non-phage	0
DOF004_scaffold40096_1	non-phage	0
DOF004_scaffold54909_1	non-phage	0
DOF004_scaffold38895_1	non-phage	0
DOF004_scaffold16903_2	non-phage	0
DOF004_scaffold44938_1	non-phage	0
DOF004_scaffold1908_2	non-phage	0
DOF004_scaffold1849_4	non-phage	0
DOF004_scaffold8287_3	non-phage	0
DOF004_scaffold56907_1	non-phage	0
DOF004_scaffold27372_3	non-phage	0
DOF004_scaffold19620_10	non-phage	0
DOF004_scaffold45590_1	non-phage	0
DOF004_scaffold5457_6	non-phage	0
DOF004_C631760_1	non-phage	0
DOF004_scaffold16903_1	non-phage	0
DOF004_scaffold56923_2	non-phage	0
DOF004_scaffold34366_2	non-phage	0

DOF004_scaffold11147_7	non-phage	0
DOF004_scaffold27525_1	non-phage	0
DOF004_C633696_1	non-phage	0
DOF004_scaffold46109_2	non-phage	0
DOF004_scaffold55965_2	non-phage	0
DOF004_C633404_1	non-phage	0
DOF004_scaffold16195_1	non-phage	0
DOF004_scaffold21056_2	non-phage	0
DOF004_scaffold48973_2	non-phage	0
DOF004_scaffold56392_1	non-phage	0
DOF004_scaffold18645_2	non-phage	0
DOF004_scaffold48072_2	non-phage	0
DOF004_scaffold56703_2	non-phage	0
DOF004_scaffold45784_2	non-phage	0
DOF004_scaffold40_2	non-phage	0
DOF004_scaffold25176_2	non-phage	0
DOF004_scaffold57010_1	non-phage	0
DOF004_scaffold27823_6	non-phage	0
DOF004_C631804_1	non-phage	0
DOF004_scaffold43078_8	non-phage	0
DOF004_scaffold2735_1	non-phage	0
DOF004_scaffold196_3	non-phage	0
DOF004_scaffold24529_9	non-phage	0
DOF004_scaffold35070_1	non-phage	0
DOF004_scaffold22240_1	non-phage	0
DOF004_scaffold23049_4	non-phage	0
DOF004_scaffold51482_1	non-phage	0
DOF004_scaffold20624_1	non-phage	0
DOF004_scaffold5206_7	non-phage	0
DOF004_scaffold20436_2	non-phage	0
DOF004_scaffold50251_1	non-phage	0
DOF004_scaffold53532_1	non-phage	0
DOF004_scaffold42486_3	non-phage	0
DOF004_scaffold55499_6	non-phage	0
DOF004_scaffold27823_5	non-phage	0
DOF004_scaffold56703_1	non-phage	0
DOF004_scaffold55035_1	non-phage	0
DOF004_scaffold51679_1	non-phage	0
DOF004_scaffold49576_3	non-phage	0
DOF004_scaffold57149_1	non-phage	0
DOF004_scaffold57053_1	non-phage	0
DOF004_scaffold5015_1	non-phage	0
DOF004_scaffold4469_10	non-phage	0
DOF004_scaffold48973_1	non-phage	0
DOF004_scaffold10109_5	non-phage	0
DOF004_scaffold54999_1	non-phage	0

DOF004_scaffold57021_1	non-phage	0
DOF004_scaffold226_1	non-phage	0
DOF004_scaffold50517_6	non-phage	0
DOF004_scaffold57006_2	non-phage	0
DOF004_scaffold2109_3	non-phage	0
DOF004_scaffold14493_1	non-phage	0
DOF004_scaffold35654_4	non-phage	0
DOF004_scaffold8260_2	non-phage	0
DOF004_scaffold37432_2	non-phage	0
DOF004_scaffold45051_1	non-phage	0
DOF004_scaffold54882_2	non-phage	0
DOF004_scaffold56260_1	phage	0.999
DOF004_scaffold35113_2	non-phage	0
DOF004_scaffold54898_1	non-phage	0
DOF004_scaffold55792_1	non-phage	0
DOF004_scaffold27530_2	non-phage	0
DOF004_scaffold47782_2	non-phage	0
DOF004_scaffold14346_4	non-phage	0
DOF004_scaffold56614_1	non-phage	0
DOF004_scaffold40096_2	non-phage	0
DOF004_scaffold45118_4	non-phage	0
DOF004_scaffold4812_1	non-phage	0
DOF004_scaffold20276_1	non-phage	0
DOF004_scaffold33624_2	non-phage	0
DOF004_scaffold56161_1	non-phage	0
DOF004_scaffold34053_1	non-phage	0
DOF004_scaffold33242_2	non-phage	0
DOF004_scaffold57116_2	non-phage	0
DOF004_scaffold23567_2	non-phage	0
DOF004_scaffold56801_2	non-phage	0
DOF004_scaffold56880_1	non-phage	0
DOF004_scaffold36190_1	non-phage	0
DOF004_scaffold19413_4	non-phage	0
DOF004_scaffold34862_2	non-phage	0
DOF004_scaffold34646_2	non-phage	0
DOF004_scaffold802_4	non-phage	0
DOF004_scaffold2905_1	non-phage	0
DOF004_scaffold32699_1	non-phage	0
DOF004_C631678_1	non-phage	0
DOF004_scaffold32320_3	non-phage	0
DOF004_scaffold27_5	non-phage	0
DOF004_scaffold36563_2	non-phage	0
DOF004_scaffold56105_1	non-phage	0
DOF004_scaffold55905_2	non-phage	0
DOF004_scaffold44703_2	non-phage	0
DOF004_scaffold57097_1	non-phage	0

DOF004_scaffold57115_2	non-phage	0
DOF004_scaffold21_1	non-phage	0
DOF004_scaffold57056_1	non-phage	0
DOF004_scaffold24195_1	non-phage	0
DOF004_scaffold187_1	non-phage	0
DOF004_scaffold33568_1	non-phage	0
DOF004_scaffold46165_3	non-phage	0
DOF004_scaffold21227_3	non-phage	0
DOF004_scaffold45263_2	non-phage	0
DOF004_scaffold23213_1	non-phage	0
DOF004_scaffold56966_6	non-phage	0
DOF004_scaffold56848_2	non-phage	0
DOF004_scaffold26027_6	non-phage	0
DOF004_scaffold54573_1	non-phage	0
DOF004_scaffold56980_1	non-phage	0
DOF004_scaffold27823_1	non-phage	0
DOF004_scaffold33568_5	non-phage	0
DOF004_scaffold25640_4	non-phage	0
DOF004_scaffold22_1	non-phage	0
DOF004_scaffold26654_3	non-phage	0
DOF004_scaffold36700_2	non-phage	0
DOF004_scaffold38757_2	non-phage	0
DOF004_scaffold169_2	non-phage	0
DOF004_scaffold22037_1	phage	0.728
DOF004_scaffold41902_3	non-phage	0
DOF004_scaffold34647_3	non-phage	0
DOF004_scaffold13164_1	non-phage	0
DOF004_scaffold15202_2	non-phage	0
DOF004_scaffold7632_8	non-phage	0
DOF004_scaffold20782_3	non-phage	0
DOF004_scaffold45491_1	non-phage	0
DOF004_scaffold57145_1	non-phage	0
DOF004_scaffold13584_8	non-phage	0
DOF004_scaffold57116_3	non-phage	0
DOF004_scaffold7220_1_2	non-phage	0
DOF004_scaffold54568_2	non-phage	0
DOF004_scaffold51651_3	non-phage	0
DOF004_scaffold57132_3	non-phage	0
DOF004_scaffold35687_4	non-phage	0
DOF004_scaffold57072_1	non-phage	0
DOF004_scaffold56481_5	non-phage	0
DOF004_scaffold21060_2	non-phage	0
DOF004_scaffold57026_1	non-phage	0
DOF004_scaffold5009_6	non-phage	0
DOF004_scaffold92_4	non-phage	0
DOF004_scaffold11402_5	non-phage	0

DOF004_scaffold49755_3	non-phage	0
DOF004_scaffold8287_9	non-phage	0
DOF004_scaffold3072_3	non-phage	0
DOF004_scaffold8290_9	non-phage	0
DOF004_scaffold157_2	non-phage	0
DOF004_scaffold23307_1	non-phage	0
DOF004_scaffold16778_14	phage	0.996
DOF004_scaffold34483_3	non-phage	0
DOF004_scaffold17485_22	non-phage	0.05
DOF004_scaffold37909_3	non-phage	0
DOF004_scaffold56834_1	non-phage	0
DOF004_scaffold13294_10	non-phage	0
DOF004_scaffold17675_2	non-phage	0
DOF004_scaffold55299_1	non-phage	0
DOF004_scaffold57146_1	non-phage	0
DOF004_scaffold8260_4	non-phage	0
DOF004_scaffold54702_1	phage	0.789
DOF004_scaffold48162_1	non-phage	0
DOF004_scaffold35933_2	non-phage	0
DOF004_scaffold44373_1	non-phage	0
DOF004_scaffold1277_1	non-phage	0
DOF004_scaffold45590_5	non-phage	0
DOF004_scaffold26658_3	non-phage	0
DOF004_scaffold4812_3	non-phage	0
DOF004_scaffold52891_3	non-phage	0
DOF004_scaffold22450_4	non-phage	0
DOF004_scaffold27880_1	phage	0.988
DOF004_scaffold42967_1	non-phage	0
DOF004_scaffold44482_1	non-phage	0
DOF004_scaffold14990_1	non-phage	0
DOF004_scaffold17811_2	non-phage	0
DOF004_scaffold27569_1	non-phage	0
DOF004_scaffold31456_3	non-phage	0
DOF004_scaffold224_4	non-phage	0
DOF004_scaffold1738_2	non-phage	0
DOF004_scaffold37113_2	non-phage	0
DOF004_scaffold20166_6	non-phage	0
DOF004_scaffold52959_3	non-phage	0
DOF004_scaffold37006_1	phage	0.999
DOF004_scaffold42734_2	non-phage	0
DOF004_scaffold35654_2	non-phage	0
DOF004_scaffold37756_1	non-phage	0
DOF004_scaffold10668_1	non-phage	0
DOF004_scaffold12464_1	non-phage	0
DOF004_scaffold18801_2	non-phage	0
DOF004_scaffold54736_2	non-phage	0

DOF004_scaffold56741_1	non-phage	0
DOF004_scaffold23556_3	non-phage	0
DOF004_scaffold7723_14	non-phage	0
DOF004_scaffold50517_3	non-phage	0
DOF004_scaffold40107_3	non-phage	0
DOF004_scaffold54566_3	non-phage	0
DOF004_scaffold56882_1	non-phage	0
DOF004_scaffold55761_2	non-phage	0
DOF004_scaffold56152_1	non-phage	0
DOF004_scaffold55128_2	non-phage	0
DOF004_scaffold56070_5	non-phage	0
DOF004_scaffold4908_4	non-phage	0
DOF004_scaffold55806_1	non-phage	0
DOF004_scaffold20691_5	non-phage	0
DOF004_scaffold25418_1	non-phage	0
DOF004_scaffold23081_1	non-phage	0
DOF004_scaffold46416_1	non-phage	0
DOF004_scaffold2519_1	non-phage	0
DOF004_scaffold42330_2	non-phage	0
DOF004_scaffold37432_1	non-phage	0
DOF004_scaffold17879_2	non-phage	0
DOF004_scaffold22294_2	non-phage	0
DOF004_scaffold7_2	non-phage	0
DOF004_scaffold41_2	non-phage	0
DOF004_scaffold3658_14	non-phage	0
DOF004_scaffold13050_5	non-phage	0
DOF004_scaffold17610_1	non-phage	0
DOF004_scaffold21257_3	non-phage	0
DOF004_scaffold8565_1	non-phage	0
DOF004_scaffold54807_1	non-phage	0
DOF004_scaffold47782_3	non-phage	0
DOF004_scaffold92_3	non-phage	0
DOF004_scaffold56552_5	non-phage	0
DOF004_scaffold1443_1	non-phage	0
DOF004_scaffold49755_2	non-phage	0
DOF004_scaffold20524_5	non-phage	0
DOF004_scaffold10109_6	phage	1
DOF004_scaffold54655_2	non-phage	0
DOF004_scaffold57085_1	non-phage	0
DOF004_scaffold47590_2	non-phage	0
DOF004_scaffold53962_1	non-phage	0
DOF004_scaffold55499_2	non-phage	0
DOF004_scaffold42123_7	non-phage	0
DOF004_scaffold30259_2	non-phage	0
DOF004_scaffold38895_2	non-phage	0
DOF004_scaffold56026_1	non-phage	0

DOF004_scaffold32730_2	non-phage	0
DOF004_scaffold44262_3	non-phage	0
DOF004_scaffold35687_1	phage	0.751
DOF004_scaffold39753_1	non-phage	0
DOF004_scaffold2686_2	non-phage	0
DOF004_scaffold20907_5	non-phage	0
DOF004_scaffold22038_1	non-phage	0
DOF004_scaffold40096_5	non-phage	0
DOF004_C633118_1	non-phage	0
DOF004_scaffold23702_3	non-phage	0
DOF004_scaffold56870_2	non-phage	0
DOF004_scaffold27005_3	non-phage	0
DOF004_scaffold57128_2	non-phage	0
DOF004_scaffold23656_4	non-phage	0
DOF004_scaffold49739_1	non-phage	0
DOF004_scaffold9090_4	phage	0.999
DOF004_scaffold21060_3	non-phage	0
DOF004_scaffold20729_3	non-phage	0
DOF004_scaffold53206_1	non-phage	0
DOF004_scaffold55497_1	non-phage	0
DOF004_scaffold28966_2	non-phage	0
DOF004_scaffold19_2	non-phage	0.002
DOF004_scaffold18679_4	non-phage	0.038
DOF004_scaffold27372_2	non-phage	0
DOF004_scaffold13584_5	non-phage	0
DOF004_scaffold13164_5	non-phage	0
DOF004_scaffold20309_1	non-phage	0
DOF004_scaffold46109_1	non-phage	0
DOF004_scaffold55363_2	non-phage	0
DOF004_scaffold33449_3	non-phage	0
DOF004_scaffold250_3	non-phage	0
DOF004_scaffold57120_1	non-phage	0
DOF004_scaffold48558_2	non-phage	0
DOF004_scaffold20524_3	non-phage	0
DOF004_scaffold56907_2	non-phage	0
DOF004_scaffold56710_1	non-phage	0
DOF004_scaffold55964_1	non-phage	0
DOF004_scaffold56985_1	non-phage	0
DOF004_scaffold57061_1	non-phage	0
DOF004_scaffold49261_5	non-phage	0
DOF004_scaffold56834_2	non-phage	0
DOF004_scaffold12001_3	non-phage	0
DOF004_scaffold17510_2	non-phage	0
DOF004_scaffold17675_5	non-phage	0
DOF004_scaffold53873_2	non-phage	0
DOF004_scaffold56921_2	non-phage	0

DOF004_scaffold2676_5	non-phage	0
DOF004_scaffold30721_2	non-phage	0
DOF004_scaffold786_2	phage	0.99
DOF004_scaffold13315_10	non-phage	0
DOF004_scaffold53137_4	non-phage	0
DOF004_scaffold46165_1	non-phage	0
DOF004_scaffold802_2	non-phage	0
DOF004_scaffold28008_2	non-phage	0
DOF004_scaffold56259_1	phage	0.996
DOF004_scaffold33568_9	non-phage	0
DOF004_scaffold48162_4	non-phage	0
DOF004_scaffold4812_7	non-phage	0
DOF004_scaffold57065_1	non-phage	0
DOF004_scaffold36838_3	non-phage	0
DOF004_scaffold55059_2	non-phage	0
DOF004_scaffold16036_1	non-phage	0
DOF004_scaffold54103_1	non-phage	0
DOF004_scaffold49242_3	non-phage	0
DOF004_scaffold54786_1	non-phage	0
DOF004_scaffold5415_3	non-phage	0.066
DOF004_scaffold28311_2	phage	0.988
DOF004_scaffold33481_7	non-phage	0
DOF004_scaffold8307_2	non-phage	0
DOF004_scaffold57099_2	non-phage	0
DOF004_scaffold148_2	phage	0.994
DOF004_scaffold44339_1	non-phage	0
DOF004_scaffold35183_2	non-phage	0
DOF004_scaffold23787_2	non-phage	0
DOF004_scaffold34252_1	non-phage	0
DOF004_scaffold32963_1	non-phage	0
DOF004_scaffold57031_2	non-phage	0
DOF004_scaffold42_1	non-phage	0
DOF004_scaffold15795_5	non-phage	0
DOF004_scaffold2308_1	non-phage	0
DOF004_scaffold56673_2	non-phage	0
DOF004_scaffold3457_2	non-phage	0
DOF004_scaffold56476_2	non-phage	0
DOF004_scaffold44262_1	non-phage	0
DOF004_scaffold22744_5	non-phage	0
DOF004_scaffold56942_1	non-phage	0
DOF004_scaffold11214_4	non-phage	0
DOF004_scaffold50594_1	non-phage	0
DOF004_scaffold1849_6	non-phage	0
DOF004_scaffold48355_1	non-phage	0
DOF004_scaffold40096_3	non-phage	0
DOF004_scaffold55806_2	non-phage	0

DOF004_scaffold57010_2	non-phage	0
DOF004_scaffold22460_2	non-phage	0
DOF004_scaffold19258_3	phage	0.645
DOF004_scaffold56481_2	non-phage	0
DOF004_scaffold3660_2	non-phage	0
DOF004_scaffold6821_1	non-phage	0
DOF004_scaffold19620_3	non-phage	0
DOF004_scaffold19470_2	non-phage	0
DOF004_scaffold56949_1	non-phage	0
DOF004_scaffold4812_2	non-phage	0
DOF004_scaffold5009_5	non-phage	0
DOF004_scaffold4558_3	non-phage	0
DOF004_scaffold45590_2	non-phage	0
DOF004_scaffold55580_1	non-phage	0
DOF004_scaffold18645_1	non-phage	0
DOF004_scaffold31455_2	non-phage	0
DOF004_scaffold24012_6	non-phage	0
DOF004_scaffold34862_1	non-phage	0
DOF004_scaffold26046_2	non-phage	0
DOF004_scaffold56217_1	non-phage	0
DOF004_scaffold6015_1	non-phage	0
DOF004_scaffold57027_1	non-phage	0
DOF004_scaffold55590_2	non-phage	0
DOF004_scaffold57000_1	non-phage	0
DOF004_scaffold21914_3	non-phage	0
DOF004_scaffold63_2	non-phage	0
DOF004_scaffold27017_1	phage	0.858
DOF004_scaffold196_4	non-phage	0
DOF004_scaffold22516_3	non-phage	0
DOF004_scaffold15795_4	non-phage	0
DOF004_scaffold37544_1	non-phage	0
DOF004_scaffold56552_1	non-phage	0
DOF004_scaffold49576_2	non-phage	0
DOF004_scaffold26_2	non-phage	0
DOF004_scaffold48174_2	non-phage	0
DOF004_scaffold1581_6	non-phage	0
DOF004_scaffold21597_6	non-phage	0
DOF004_scaffold57089_1	non-phage	0
DOF004_scaffold4812_4	non-phage	0
DOF004_scaffold56466_1	non-phage	0
DOF004_scaffold23049_5	non-phage	0
DOF004_scaffold5476_2	non-phage	0
DOF004_C633984_1	non-phage	0
DOF004_scaffold55299_2	non-phage	0
DOF004_scaffold1382_1	non-phage	0
DOF004_scaffold39336_2	non-phage	0

DOF004_scaffold21890_2	phage	0.919
DOF004_scaffold20668_2	non-phage	0
DOF004_scaffold53137_3	non-phage	0
DOF004_scaffold13_1	non-phage	0
DOF004_scaffold23762_1	non-phage	0
DOF004_scaffold57151_1	non-phage	0
DOF004_scaffold34544_6	non-phage	0
DOF004_scaffold94_2	non-phage	0
DOF004_scaffold25640_3	non-phage	0
DOF004_scaffold53532_2	non-phage	0
DOF004_scaffold20726_1	non-phage	0
DOF004_scaffold33568_8	non-phage	0
DOF004_scaffold45579_1	non-phage	0
DOF004_scaffold18417_1	non-phage	0
DOF004_scaffold24837_2	non-phage	0
DOF004_scaffold57093_1	non-phage	0
DOF004_scaffold39132_1	non-phage	0
DOF004_scaffold57048_1	non-phage	0
DOF004_scaffold49621_1	non-phage	0
DOF004_scaffold40_3	non-phage	0
DOF004_scaffold56696_1	non-phage	0
DOF004_scaffold57058_1	non-phage	0
DOF004_scaffold19620_2	non-phage	0
DOF004_scaffold55317_2	non-phage	0
DOF004_scaffold9596_47	non-phage	0
DOF004_scaffold55945_1	non-phage	0
DOF004_scaffold33568_4	non-phage	0
DOF004_scaffold46165_2	non-phage	0
DOF004_scaffold36401_4	non-phage	0
DOF004_scaffold26658_1	non-phage	0
DOF004_scaffold55932_1	non-phage	0
DOF004_scaffold56985_2	non-phage	0
DOF004_scaffold55064_1	non-phage	0
DOF004_scaffold9377_2	non-phage	0
DOF004_scaffold32406_2	non-phage	0
DOF004_scaffold55886_1	non-phage	0
DOF004_scaffold55296_1	non-phage	0
DOF004_scaffold56923_4	non-phage	0
DOF004_scaffold56888_1	non-phage	0
DOF004_scaffold10501_6	non-phage	0
DOF004_scaffold225_2	non-phage	0
DOF004_scaffold56902_1	non-phage	0
DOF004_scaffold12001_2	non-phage	0
DOF004_scaffold49370_1	non-phage	0
DOF004_scaffold57016_2	non-phage	0
DOF004_scaffold51772_3	non-phage	0

DOF004_scaffold44481_1	non-phage	0
DOF004_scaffold51543_3	non-phage	0
DOF004_scaffold57103_3	non-phage	0
DOF004_scaffold45_1	non-phage	0
DOF004_scaffold57015_1	non-phage	0
DOF004_scaffold44938_2	non-phage	0
DOF004_scaffold38348_2	non-phage	0
DOF004_scaffold56328_1	non-phage	0
DOF004_scaffold55324_1	non-phage	0
DOF004_scaffold56408_1	non-phage	0
DOF004_scaffold55876_4	non-phage	0
DOF004_scaffold49926_2	non-phage	0
DOF004_scaffold20166_2	non-phage	0
DOF004_scaffold56100_1	non-phage	0
DOF004_scaffold23656_8	non-phage	0
DOF004_scaffold157_3	non-phage	0
DOF004_scaffold56536_1	non-phage	0
DOF004_scaffold49818_7	non-phage	0
DOF004_scaffold2109_2	non-phage	0
DOF004_scaffold19536_1	non-phage	0
DOF004_scaffold56801_3	non-phage	0
DOF004_scaffold23556_1	non-phage	0
DOF004_scaffold55059_3	non-phage	0
DOF004_scaffold42826_1	non-phage	0
DOF004_scaffold56870_1	non-phage	0
DOF004_scaffold46936_1	non-phage	0
DOF004_scaffold25313_1	non-phage	0
DOF004_scaffold27372_5	non-phage	0
DOF004_scaffold35654_1	non-phage	0
DOF004_scaffold37034_1	non-phage	0
DOF004_scaffold57108_1	non-phage	0
DOF004_scaffold28535_1	non-phage	0
DOF004_scaffold3872_1	non-phage	0
DOF004_scaffold13315_6	non-phage	0
DOF004_scaffold55499_5	non-phage	0
DOF004_scaffold55854_1	non-phage	0
DOF004_scaffold20845_1	non-phage	0
DOF004_scaffold42091_1	non-phage	0
DOF004_scaffold57127_1	non-phage	0
DOF004_scaffold37885_1	non-phage	0
DOF004_scaffold43319_6	non-phage	0
DOF004_scaffold49754_2	non-phage	0
DOF004_scaffold27055_2	non-phage	0
DOF004_scaffold56070_7	non-phage	0
DOF004_scaffold27005_2	non-phage	0
DOF004_scaffold43340_1	non-phage	0

DOF004_scaffold20311_5	non-phage	0
DOF004_scaffold46109_3	non-phage	0
DOF004_scaffold53873_1	non-phage	0
DOF004_scaffold5206_8	non-phage	0
DOF004_scaffold53298_2	non-phage	0
DOF004_scaffold52335_1	non-phage	0
DOF004_scaffold22467_3	non-phage	0
DOF004_scaffold17510_3	non-phage	0
DOF004_scaffold13584_9	non-phage	0
DOF004_scaffold20782_2	non-phage	0
DOF004_scaffold56215_2	non-phage	0
DOF004_scaffold27530_3	non-phage	0
DOF004_scaffold21103_1	non-phage	0
DOF004_scaffold771_3	non-phage	0
DOF004_scaffold12947_3	non-phage	0.002
DOF004_scaffold26624_2	non-phage	0
DOF004_scaffold57126_1	non-phage	0
DOF004_scaffold20624_5	non-phage	0
DOF004_scaffold30563_2	non-phage	0
DOF004_scaffold3658_30	non-phage	0
DOF004_scaffold25_7	non-phage	0
DOF004_scaffold40107_4	non-phage	0
DOF004_scaffold56980_2	non-phage	0
DOF004_scaffold51566_5	non-phage	0
DOF004_scaffold23214_1	non-phage	0
DOF004_scaffold27742_3	non-phage	0
DOF004_scaffold55243_3	non-phage	0
DOF004_scaffold23049_1	non-phage	0
DOF004_scaffold56978_3	non-phage	0
DOF004_scaffold6015_5	non-phage	0
DOF004_scaffold56459_1	non-phage	0
DOF004_scaffold34544_3	non-phage	0
DOF004_scaffold20783_2	non-phage	0
DOF004_scaffold40915_1	non-phage	0
DOF004_scaffold17879_3	non-phage	0
DOF004_scaffold68_6	non-phage	0
DOF004_scaffold40518_7	non-phage	0
DOF004_scaffold4294_2	non-phage	0
DOF004_scaffold36838_4	non-phage	0
DOF004_scaffold34252_6	non-phage	0
DOF004_scaffold34682_1	non-phage	0
DOF004_scaffold879_1	non-phage	0
DOF004_scaffold9794_1	non-phage	0
DOF004_scaffold49732_2	non-phage	0
DOF004_scaffold56918_1	non-phage	0
DOF004_scaffold56801_4	non-phage	0

DOF004_scaffold55654_2	non-phage	0
DOF004_scaffold49755_1	non-phage	0
DOF004_scaffold15795_1	non-phage	0
DOF004_scaffold57122_1	non-phage	0
DOF004_scaffold33679_5	non-phage	0
DOF004_scaffold44262_2	non-phage	0
DOF004_scaffold37313_1	non-phage	0
DOF004_scaffold20293_1	non-phage	0
DOF004_scaffold56481_6	non-phage	0
DOF004_scaffold32359_2	non-phage	0
DOF004_scaffold8287_7	non-phage	0
DOF004_scaffold56726_4	non-phage	0
DOF004_scaffold4466_3	non-phage	0
DOF004_scaffold57038_1	phage	0.749
DOF004_scaffold21914_2	non-phage	0
DOF004_scaffold52492_2	non-phage	0
DOF004_scaffold57127_2	non-phage	0
DOF004_scaffold52_1	non-phage	0
DOF004_scaffold52461_2	non-phage	0
DOF004_scaffold13808_2	non-phage	0
DOF004_C632416_1	phage	0.759
DOF004_scaffold41353_2	non-phage	0
DOF004_scaffold54909_4	non-phage	0
DOF004_scaffold55434_3	non-phage	0.218
DOF004_scaffold50649_3	non-phage	0
DOF004_scaffold29446_1	non-phage	0
DOF004_scaffold2924_2	non-phage	0
DOF004_scaffold5009_2	non-phage	0
DOF004_scaffold8307_1	non-phage	0
DOF004_scaffold30890_2	non-phage	0
DOF004_scaffold925_10	non-phage	0
DOF004_scaffold64_1	non-phage	0
DOF004_scaffold41269_1	non-phage	0
DOF004_scaffold25477_2	non-phage	0
DOF004_scaffold4812_8	non-phage	0
DOF004_scaffold31406_2	non-phage	0
DOF004_scaffold57114_2	non-phage	0
DOF004_scaffold26658_4	non-phage	0
DOF004_scaffold217_2	non-phage	0
DOF004_scaffold5009_9	non-phage	0
DOF004_scaffold16903_6	non-phage	0
DOF004_scaffold53363_1	non-phage	0
DOF004_scaffold38650_1	non-phage	0
DOF004_scaffold20524_2	non-phage	0
DOF004_scaffold28416_1	non-phage	0
DOF004_scaffold38757_1	phage	0.992

DOF004_scaffold50517_2	non-phage	0
DOF004_scaffold27742_1	non-phage	0
DOF004_scaffold22638_1	non-phage	0
DOF004_scaffold2431_2	non-phage	0
DOF004_scaffold13164_2	non-phage	0
DOF004_scaffold1205_5	non-phage	0
DOF004_scaffold25_2	non-phage	0
DOF004_scaffold4330_3	non-phage	0
DOF004_scaffold27757_1	non-phage	0.054
DOF004_scaffold45557_2	non-phage	0
DOF004_scaffold27823_8	non-phage	0
DOF004_scaffold56484_2	non-phage	0
DOF004_scaffold51050_1	non-phage	0
DOF004_scaffold57019_1	non-phage	0
DOF004_scaffold52501_7	non-phage	0
DOF004_scaffold49766_4	phage	0.992
DOF004_scaffold20726_5	non-phage	0
DOF004_scaffold196_5	non-phage	0
DOF004_scaffold38778_5	non-phage	0
DOF004_scaffold4075_1	non-phage	0
DOF004_scaffold55036_3	non-phage	0
DOF004_scaffold36570_2	non-phage	0
DOF004_scaffold54613_1	non-phage	0
DOF004_scaffold31_1	non-phage	0
DOF004_scaffold57103_4	non-phage	0
DOF004_scaffold56921_3	non-phage	0
DOF004_scaffold23384_4	non-phage	0
DOF004_C634064_1	non-phage	0
DOF004_scaffold41079_2	non-phage	0
DOF004_C632004_1	non-phage	0
DOF004_scaffold7376_1	phage	0.999
DOF004_scaffold56259_4	non-phage	0
DOF004_scaffold4280_1	non-phage	0
DOF004_scaffold20669_2	non-phage	0
DOF004_scaffold28311_1	non-phage	0
DOF004_scaffold8307_15	non-phage	0
DOF004_scaffold56952_1	phage	0.999
DOF004_scaffold7096_2	non-phage	0
DOF004_scaffold6592_2	non-phage	0
DOF004_scaffold13050_4	non-phage	0
DOF004_scaffold322_1	non-phage	0
DOF004_scaffold57143_1	non-phage	0
DOF004_scaffold4355_1	non-phage	0.001
DOF004_scaffold2799_3	non-phage	0
DOF004_scaffold43319_1	non-phage	0
DOF004_scaffold32093_1	non-phage	0

DOF004_scaffold25343_1	phage	0.996
DOF004_scaffold30259_3	non-phage	0
DOF004_scaffold56673_3	non-phage	0
DOF004_scaffold14346_6	non-phage	0
DOF004_scaffold3782_2	non-phage	0
DOF004_scaffold23556_4	non-phage	0
DOF004_scaffold36730_3	non-phage	0
DOF004_scaffold56995_2	non-phage	0
DOF004_scaffold30259_7	non-phage	0
DOF004_scaffold57133_1	non-phage	0
DOF004_scaffold97_1	non-phage	0
DOF004_scaffold57070_1	non-phage	0
DOF004_scaffold37756_4	non-phage	0
DOF004_scaffold56013_1	non-phage	0
DOF004_scaffold49818_4	non-phage	0
DOF004_scaffold34931_2	non-phage	0
DOF004_scaffold44039_1	non-phage	0
DOF004_scaffold49571_2	non-phage	0
DOF004_scaffold50363_2	non-phage	0
DOF004_scaffold57084_1	non-phage	0.31
DOF004_scaffold17013_1	non-phage	0
DOF004_scaffold39753_2	non-phage	0.001
DOF004_scaffold23214_2	non-phage	0
DOF004_scaffold48558_1	non-phage	0
DOF004_scaffold32406_1	non-phage	0
DOF004_scaffold57086_1	non-phage	0
DOF004_scaffold55151_1	non-phage	0
DOF004_scaffold46734_1	non-phage	0
DOF004_scaffold56294_1	non-phage	0
DOF004_scaffold37885_2	non-phage	0
DOF004_scaffold57079_1	non-phage	0
DOF004_scaffold56070_4	non-phage	0
DOF004_scaffold18679_3	non-phage	0
DOF004_scaffold21751_2	non-phage	0
DOF004_scaffold49926_1	non-phage	0
DOF004_scaffold56930_1	non-phage	0
DOF004_scaffold56700_1	non-phage	0
DOF004_scaffold18417_2	non-phage	0
DOF004_scaffold5206_5	phage	0.999
DOF004_scaffold57077_1	non-phage	0
DOF004_scaffold4908_2	non-phage	0
DOF004_scaffold30721_3	non-phage	0
DOF004_scaffold22467_7	non-phage	0
DOF004_scaffold55761_3	non-phage	0
DOF004_scaffold28787_1	non-phage	0
DOF004_scaffold9523_2	non-phage	0

DOF004_scaffold54568_4	non-phage	0
DOF004_scaffold56966_8	non-phage	0
DOF004_scaffold23593_6	non-phage	0
DOF004_scaffold34544_5	non-phage	0
DOF004_scaffold23362_2	non-phage	0
DOF004_scaffold21890_1	non-phage	0
DOF004_scaffold50184_1	non-phage	0
DOF004_scaffold3658_8	non-phage	0
DOF004_scaffold12190_3	non-phage	0
DOF004_scaffold41222_1	non-phage	0
DOF004_scaffold33568_3	non-phage	0
DOF004_scaffold52501_9	non-phage	0
DOF004_scaffold53137_2	non-phage	0
DOF004_scaffold56696_2	non-phage	0
DOF004_scaffold53502_1	non-phage	0
DOF004_scaffold57150_1	non-phage	0
DOF004_scaffold57144_1	non-phage	0
DOF004_scaffold57057_1	non-phage	0
DOF004_scaffold250_8	non-phage	0
DOF004_scaffold36578_3	non-phage	0
DOF004_scaffold7220_3	non-phage	0
DOF004_scaffold56916_2	non-phage	0
DOF004_scaffold1382_2	non-phage	0
DOF004_scaffold56481_3	non-phage	0
DOF004_scaffold4469_7	non-phage	0
DOF004_scaffold56930_2	non-phage	0
DOF004_scaffold41799_2	non-phage	0
DOF004_scaffold57105_2	non-phage	0
DOF004_scaffold49571_1	non-phage	0
DOF004_scaffold56886_1	non-phage	0.036
DOF004_scaffold20782_5	non-phage	0
DOF004_scaffold55806_3	non-phage	0
DOF004_scaffold10109_3	non-phage	0
DOF004_scaffold16903_4	non-phage	0
DOF004_scaffold25640_2	non-phage	0
DOF004_scaffold44481_2	non-phage	0
DOF004_scaffold7865_4	non-phage	0
DOF004_scaffold56962_1	non-phage	0
DOF004_scaffold49739_3	non-phage	0
DOF004_scaffold56907_3	non-phage	0
DOF004_scaffold20166_1	non-phage	0
DOF004_scaffold31455_3	non-phage	0
DOF004_scaffold23213_3	non-phage	0
DOF004_scaffold26046_1	non-phage	0
DOF004_scaffold38886_1	non-phage	0
DOF004_scaffold34660_1	non-phage	0

DOF004_scaffold20668_5	non-phage	0
DOF004_scaffold56982_2	non-phage	0
DOF004_scaffold42_3	non-phage	0
DOF004_scaffold5009_4	non-phage	0
DOF004_scaffold24349_1	phage	0.999
DOF004_scaffold55792_2	non-phage	0
DOF004_scaffold55654_4	non-phage	0
DOF004_scaffold45590_3	non-phage	0
DOF004_scaffold21900_2	non-phage	0
DOF004_scaffold19258_2	non-phage	0
DOF004_scaffold56923_1	non-phage	0
DOF004_scaffold41243_1	non-phage	0
DOF004_scaffold52891_1	non-phage	0
DOF004_scaffold2686_3	non-phage	0
DOF004_scaffold11214_2	non-phage	0
DOF004_scaffold46591_5	non-phage	0
DOF004_scaffold55932_2	non-phage	0
DOF004_scaffold8125_1	non-phage	0
DOF004_scaffold57001_2	non-phage	0
DOF004_scaffold48174_1	non-phage	0
DOF004_scaffold56259_2	non-phage	0
DOF004_scaffold55029_1	non-phage	0
DOF004_scaffold32634_1	non-phage	0
DOF004_scaffold57119_1	non-phage	0
DOF004_scaffold4812_5	non-phage	0
DOF004_scaffold44040_2	non-phage	0
DOF004_scaffold13315_5	non-phage	0
DOF004_scaffold57083_1	non-phage	0
DOF004_scaffold54866_2	non-phage	0
DOF004_scaffold92_2	non-phage	0
DOF004_scaffold30046_2	non-phage	0
DOF004_scaffold56676_2	non-phage	0
DOF004_scaffold57121_1	phage	0.999
DOF004_scaffold27005_1	non-phage	0
DOF004_scaffold25419_1	non-phage	0
DOF004_scaffold55317_1	non-phage	0
DOF004_scaffold10951_1	non-phage	0
DOF004_scaffold42476_2	non-phage	0
DOF004_scaffold23656_9	non-phage	0
DOF004_C632528_1	non-phage	0
DOF004_scaffold23644_1	non-phage	0
DOF004_scaffold33822_1	non-phage	0
DOF004_scaffold42386_6	non-phage	0
DOF004_scaffold22467_2	non-phage	0
DOF004_scaffold24653_5	non-phage	0
DOF004_scaffold26721_2	phage	0.958

DOF004_scaffold10280_6	non-phage	0
DOF004_scaffold12828_1	non-phage	0
DOF004_scaffold42486_1	non-phage	0
DOF004_scaffold44558_3	non-phage	0
DOF004_scaffold49853_3	non-phage	0
DOF004_scaffold23307_3	non-phage	0
DOF004_C632594_1	non-phage	0
DOF004_scaffold11714_1	non-phage	0
DOF004_scaffold55965_5	non-phage	0
DOF004_scaffold29615_1	non-phage	0
DOF004_scaffold56958_2	non-phage	0
DOF004_scaffold34862_3	non-phage	0
DOF004_scaffold7_4	non-phage	0
DOF004_scaffold56672_2	non-phage	0
DOF004_scaffold50245_1	non-phage	0
DOF004_scaffold42980_2	non-phage	0
DOF004_scaffold56955_3	non-phage	0
DOF004_scaffold19413_10	non-phage	0
DOF004_scaffold26152_3	non-phage	0
DOF004_scaffold56921_4	non-phage	0
DOF004_scaffold48972_1	non-phage	0
DOF004_scaffold23733_2	non-phage	0
DOF004_scaffold1075_5	non-phage	0
DOF004_scaffold21741_1	non-phage	0
DOF004_scaffold5587_1	non-phage	0
DOF004_scaffold44558_1	non-phage	0
DOF004_scaffold13294_3	non-phage	0
DOF004_scaffold49732_1	non-phage	0
DOF004_scaffold56536_2	non-phage	0
DOF004_scaffold10867_1	non-phage	0
DOF004_scaffold56064_1	non-phage	0
DOF004_scaffold37432_4	non-phage	0
DOF004_scaffold28510_4	non-phage	0
DOF004_scaffold20376_2	non-phage	0
DOF004_scaffold38267_2	non-phage	0
DOF004_scaffold47501_1	non-phage	0
DOF004_scaffold32320_1	non-phage	0
DOF004_scaffold23611_1	non-phage	0
DOF004_scaffold57125_1	non-phage	0
DOF004_scaffold29920_1	non-phage	0
DOF004_scaffold52730_3	non-phage	0
DOF004_scaffold56880_3	non-phage	0
DOF004_scaffold57050_1	non-phage	0
DOF004_scaffold12010_1	non-phage	0
DOF004_scaffold6821_2	non-phage	0
DOF004_scaffold2735_6	non-phage	0

DOF004_scaffold4530_1	non-phage	0
DOF004_scaffold3658_16	non-phage	0
DOF004_scaffold13315_2	non-phage	0
DOF004_scaffold56992_2	non-phage	0
DOF004_scaffold196_2	non-phage	0
DOF004_scaffold57113_1	non-phage	0
DOF004_scaffold4558_2	non-phage	0
DOF004_scaffold19577_1	non-phage	0
DOF004_scaffold43319_7	non-phage	0
DOF004_scaffold210_1	non-phage	0
DOF004_scaffold23214_4	non-phage	0
DOF004_scaffold56923_3	non-phage	0
DOF004_scaffold6730_1	non-phage	0
DOF004_scaffold53821_2	non-phage	0
DOF004_scaffold34242_1	phage	0.999
DOF004_scaffold42091_3	non-phage	0
DOF004_scaffold56548_3	non-phage	0
DOF004_scaffold40880_1	non-phage	0
DOF004_scaffold33569_3	non-phage	0
DOF004_scaffold55654_5	non-phage	0
DOF004_scaffold23545_4	non-phage	0
DOF004_scaffold13584_7	non-phage	0.343
DOF004_scaffold34647_5	non-phage	0
DOF004_scaffold57070_2	non-phage	0
DOF004_scaffold42201_4	non-phage	0
DOF004_scaffold23384_6	non-phage	0
DOF004_scaffold54568_1	non-phage	0
DOF004_scaffold2431_3	non-phage	0
DOF004_scaffold52501_6	non-phage	0
DOF004_scaffold56848_3	non-phage	0
DOF004_scaffold30438_2	non-phage	0
DOF004_scaffold40087_4	non-phage	0
DOF004_scaffold8307_9	non-phage	0
DOF004_scaffold55876_1	non-phage	0
DOF004_scaffold30113_1	non-phage	0
DOF004_scaffold57003_1	non-phage	0
DOF004_scaffold28_1	non-phage	0
DOF004_scaffold57055_2	non-phage	0
DOF004_scaffold34052_1	non-phage	0
DOF004_scaffold38268_2	non-phage	0
DOF004_scaffold44482_3	non-phage	0
DOF004_scaffold13315_4	non-phage	0
DOF004_scaffold56797_2	non-phage	0
DOF004_scaffold57131_2	non-phage	0
DOF004_scaffold55965_4	non-phage	0
DOF004_scaffold21280_2	non-phage	0

DOF004_scaffold2524_4	non-phage	0
DOF004_scaffold56625_1	non-phage	0
DOF004_scaffold23582_1	non-phage	0
DOF004_scaffold51566_3	non-phage	0
DOF004_scaffold36838_5	non-phage	0
DOF004_scaffold57107_1	non-phage	0
DOF004_scaffold4103_1	non-phage	0
DOF004_scaffold55654_1	non-phage	0
DOF004_C631794_1	non-phage	0
DOF004_scaffold56534_1	non-phage	0
DOF004_scaffold56481_7	non-phage	0
DOF004_scaffold27186_1	non-phage	0
DOF004_scaffold250_7	non-phage	0
DOF004_scaffold1908_1	non-phage	0
DOF004_scaffold57129_1	non-phage	0
DOF004_scaffold31456_1	non-phage	0
DOF004_scaffold42386_2	non-phage	0
DOF004_scaffold5009_8	non-phage	0
DOF004_scaffold5009_1	non-phage	0
DOF004_scaffold21914_1	non-phage	0
DOF004_scaffold33481_2	non-phage	0
DOF004_scaffold45118_2	non-phage	0
DOF004_scaffold38_2	non-phage	0
DOF004_scaffold1849_1	non-phage	0
DOF004_scaffold13584_10	non-phage	0
DOF004_scaffold46077_3	non-phage	0
DOF004_scaffold53047_1	non-phage	0
DOF004_scaffold56395_1	non-phage	0
DOF004_scaffold50517_5	non-phage	0
DOF004_scaffold36730_2	non-phage	0
DOF004_scaffold16317_1	non-phage	0.308
DOF004_scaffold33750_1	non-phage	0
DOF004_scaffold802_3	non-phage	0
DOF004_scaffold34931_1	non-phage	0
DOF004_scaffold20668_1	non-phage	0
DOF004_scaffold20933_5	non-phage	0
DOF004_scaffold37756_3	non-phage	0
DOF004_scaffold11147_8	non-phage	0
DOF004_scaffold4156_1	non-phage	0
DOF004_scaffold34164_1	non-phage	0
DOF004_scaffold39753_3	non-phage	0
DOF004_scaffold224_2	non-phage	0
DOF004_scaffold50622_1	non-phage	0
DOF004_scaffold56956_2	non-phage	0
DOF004_scaffold33679_6	non-phage	0
DOF004_scaffold42330_4	non-phage	0

DOF004_scaffold32407_1	non-phage	0
DOF004_scaffold40518_4	non-phage	0
DOF004_scaffold40_1	non-phage	0
DOF004_scaffold21056_3	non-phage	0
DOF004_scaffold32320_2	non-phage	0
DOF004_scaffold55064_2	non-phage	0
DOF004_scaffold3059_1	non-phage	0
DOF004_scaffold54566_5	non-phage	0
DOF004_scaffold56824_1	non-phage	0
DOF004_scaffold20624_9	non-phage	0
DOF004_scaffold56882_3	non-phage	0
DOF004_scaffold10109_8	non-phage	0
DOF004_scaffold27823_7	non-phage	0
DOF004_scaffold2924_1	non-phage	0
DOF004_scaffold16778_5	non-phage	0.153
DOF004_scaffold52698_1	non-phage	0
DOF004_scaffold22450_5	non-phage	0
DOF004_scaffold21257_9	non-phage	0
DOF004_scaffold35654_3	non-phage	0
DOF004_scaffold56495_1	non-phage	0
DOF004_scaffold34646_4	non-phage	0
DOF004_scaffold34544_2	non-phage	0
DOF004_scaffold45589_1	phage	0.988
DOF004_scaffold56327_1	non-phage	0
DOF004_scaffold27134_1	non-phage	0
DOF004_scaffold6388_6	non-phage	0
DOF004_scaffold20783_1	non-phage	0
DOF004_scaffold56552_3	non-phage	0
DOF004_scaffold25343_2	non-phage	0
DOF004_scaffold43319_2	non-phage	0
DOF004_scaffold55296_2	non-phage	0
DOF004_scaffold56524_1	non-phage	0
DOF004_C631982_1	non-phage	0
DOF004_scaffold22421_5	non-phage	0
DOF004_scaffold92_1	non-phage	0
DOF004_scaffold16456_4	non-phage	0
DOF004_scaffold40087_8	non-phage	0
DOF004_scaffold37432_3	non-phage	0
DOF004_scaffold3579_1	non-phage	0
DOF004_scaffold24128_1	non-phage	0
DOF004_scaffold30259_4	non-phage	0
DOF004_scaffold6015_2	non-phage	0
DOF006_scaffold6112_5	non-phage	0
DOF006_scaffold19036_2	non-phage	0
DOF006_scaffold711_1	phage	0.999
DOF006_scaffold23020_2	non-phage	0

DOF006_scaffold2415_24	non-phage	0
DOF006_scaffold10377_1	non-phage	0
DOF006_scaffold9401_1	non-phage	0
DOF006_scaffold11335_3	non-phage	0
DOF006_scaffold21547_2	non-phage	0
DOF006_scaffold3239_2	non-phage	0
DOF006_scaffold19235_1	non-phage	0
DOF006_scaffold14284_9	non-phage	0
DOF006_scaffold2415_54	non-phage	0
DOF006_scaffold306_3	phage	0.996
DOF006_scaffold19726_5	non-phage	0
DOF006_C312443_1	non-phage	0
DOF006_scaffold2415_86	non-phage	0
DOF006_scaffold15978_4	non-phage	0
DOF006_scaffold14317_2	non-phage	0
DOF006_scaffold33_1	non-phage	0
DOF006_scaffold7902_1	non-phage	0
DOF006_scaffold2886_3	non-phage	0
DOF006_scaffold22942_2	non-phage	0
DOF006_scaffold22910_1	non-phage	0
DOF006_scaffold19385_1	non-phage	0
DOF006_scaffold22817_1	phage	0.999
DOF006_scaffold22907_2	non-phage	0
DOF006_scaffold13736_6	non-phage	0
DOF006_scaffold5845_3	non-phage	0
DOF006_scaffold22281_1	non-phage	0
DOF006_scaffold10953_3	non-phage	0
DOF006_scaffold8834_7	non-phage	0
DOF006_scaffold6386_2	non-phage	0
DOF006_scaffold3490_3	non-phage	0
DOF006_scaffold19914_1	non-phage	0
DOF006_scaffold9938_1	non-phage	0
DOF006_scaffold13267_1	non-phage	0
DOF006_scaffold1547_3	non-phage	0
DOF006_scaffold12216_2	non-phage	0
DOF006_scaffold11076_1	non-phage	0
DOF006_scaffold11800_2	non-phage	0
DOF006_scaffold10651_8	non-phage	0
DOF006_scaffold22603_4	non-phage	0
DOF006_scaffold7949_1	non-phage	0
DOF006_scaffold4765_3	non-phage	0
DOF006_scaffold11073_1	non-phage	0
DOF006_scaffold22367_5	non-phage	0
DOF006_scaffold22943_1	non-phage	0
DOF006_scaffold5441_1	non-phage	0
DOF006_scaffold22600_4	non-phage	0

DOF006_scaffold15247_3	non-phage	0
DOF006_scaffold22790_1	non-phage	0
DOF006_scaffold2951_1	non-phage	0
DOF006_scaffold3032_4	non-phage	0
DOF006_scaffold3372_9	non-phage	0
DOF006_scaffold9574_1	non-phage	0
DOF006_scaffold12062_1	phage	0.995
DOF006_scaffold139_1	non-phage	0
DOF006_scaffold22913_1	non-phage	0
DOF006_scaffold22275_2	non-phage	0
DOF006_scaffold22987_5	non-phage	0
DOF006_scaffold3608_1	non-phage	0
DOF006_scaffold10858_2	non-phage	0
DOF006_scaffold14284_2	non-phage	0
DOF006_scaffold7084_2	non-phage	0
DOF006_scaffold16603_3	non-phage	0
DOF006_scaffold635_1	non-phage	0
DOF006_scaffold2415_47	non-phage	0
DOF006_scaffold4920_1	non-phage	0
DOF006_scaffold415_1	non-phage	0
DOF006_scaffold105_1	non-phage	0
DOF006_scaffold15300_7	non-phage	0
DOF006_scaffold21992_9	non-phage	0
DOF006_scaffold2415_42	non-phage	0
DOF006_scaffold14394_2	non-phage	0
DOF006_scaffold12614_1	non-phage	0
DOF006_scaffold8171_3	non-phage	0
DOF006_scaffold1420_2	non-phage	0
DOF006_scaffold11715_2	non-phage	0
DOF006_scaffold20620_1	non-phage	0
DOF006_scaffold21423_5	non-phage	0
DOF006_scaffold9214_2	non-phage	0
DOF006_scaffold16123_1	non-phage	0
DOF006_scaffold22735_2	non-phage	0
DOF006_scaffold6551_3	non-phage	0
DOF006_scaffold266_1	non-phage	0
DOF006_scaffold17068_1	non-phage	0
DOF006_scaffold4_2	non-phage	0
DOF006_scaffold22881_4	non-phage	0
DOF006_scaffold19851_2	non-phage	0
DOF006_scaffold22908_1	non-phage	0
DOF006_scaffold15978_1	non-phage	0
DOF006_scaffold22727_2	non-phage	0
DOF006_scaffold12736_2	non-phage	0
DOF006_scaffold18215_1	non-phage	0
DOF006_scaffold15503_1	non-phage	0

DOF006_scaffold3138_1	non-phage	0
DOF006_scaffold3430_3	non-phage	0
DOF006_scaffold22853_1	non-phage	0
DOF006_scaffold22710_5	non-phage	0
DOF006_scaffold22924_1	non-phage	0
DOF006_scaffold9920_3	non-phage	0
DOF006_scaffold10223_2	non-phage	0
DOF006_scaffold70_1	non-phage	0
DOF006_scaffold2415_31	non-phage	0
DOF006_scaffold19667_3	non-phage	0
DOF006_scaffold2963_1	non-phage	0
DOF006_scaffold1375_7	non-phage	0
DOF006_scaffold22738_3	non-phage	0
DOF006_scaffold2428_3	non-phage	0
DOF006_scaffold7671_5	non-phage	0
DOF006_scaffold11077_1	non-phage	0
DOF006_scaffold23006_1	non-phage	0
DOF006_scaffold10913_4	non-phage	0
DOF006_scaffold9735_1	non-phage	0
DOF006_scaffold22993_5	non-phage	0
DOF006_scaffold10651_4	non-phage	0
DOF006_scaffold7259_1	non-phage	0
DOF006_scaffold18216_2	non-phage	0
DOF006_scaffold16_5	non-phage	0
DOF006_scaffold22988_3	non-phage	0
DOF006_scaffold9214_1	non-phage	0
DOF006_C313073_1	non-phage	0
DOF006_scaffold12944_1	non-phage	0
DOF006_scaffold22889_1	non-phage	0
DOF006_scaffold5741_1	non-phage	0
DOF006_scaffold17822_3	non-phage	0
DOF006_scaffold16822_1	non-phage	0
DOF006_scaffold10913_17	non-phage	0
DOF006_scaffold21759_3	non-phage	0
DOF006_C311711_1	non-phage	0
DOF006_scaffold21099_1	non-phage	0
DOF006_scaffold306_16	non-phage	0
DOF006_scaffold2415_18	non-phage	0
DOF006_scaffold17014_1	non-phage	0
DOF006_scaffold6908_4	non-phage	0
DOF006_scaffold123_4	non-phage	0
DOF006_scaffold11978_4	phage	0.974
DOF006_C312135_1	phage	0.999
DOF006_scaffold14284_6	non-phage	0
DOF006_scaffold14317_7	non-phage	0
DOF006_scaffold18587_7	non-phage	0

DOF006_scaffold5966_3	non-phage	0
DOF006_scaffold8093_2	non-phage	0
DOF006_scaffold22845_4	non-phage	0
DOF006_scaffold415_5	non-phage	0
DOF006_scaffold15300_3	phage	0.997
DOF006_scaffold7438_5	non-phage	0
DOF006_scaffold22493_3	non-phage	0
DOF006_scaffold13_3	non-phage	0
DOF006_scaffold16979_1	non-phage	0
DOF006_scaffold10646_1	non-phage	0
DOF006_scaffold55_4	non-phage	0
DOF006_scaffold17493_1	phage	0.999
DOF006_scaffold7173_1	non-phage	0
DOF006_scaffold13697_1	non-phage	0
DOF006_scaffold10223_1	non-phage	0
DOF006_scaffold10646_3	non-phage	0
DOF006_scaffold13725_1	non-phage	0
DOF006_scaffold23000_1	non-phage	0
DOF006_scaffold13740_2	non-phage	0
DOF006_scaffold3173_6	non-phage	0
DOF006_scaffold22170_1	non-phage	0.007
DOF006_scaffold9688_5	non-phage	0
DOF006_scaffold21726_1	non-phage	0
DOF006_scaffold18054_2	non-phage	0.004
DOF006_scaffold22950_1	non-phage	0
DOF006_scaffold6692_2	non-phage	0
DOF006_scaffold7517_1	non-phage	0
DOF006_scaffold11076_3	non-phage	0
DOF006_scaffold7621_1	non-phage	0
DOF006_scaffold11336_1	non-phage	0
DOF006_scaffold4880_1	non-phage	0
DOF006_scaffold10913_3	non-phage	0
DOF006_scaffold10913_20	non-phage	0
DOF006_scaffold22995_1	non-phage	0
DOF006_scaffold2886_5	non-phage	0
DOF006_scaffold305_1	non-phage	0
DOF006_scaffold16_12	non-phage	0
DOF006_scaffold18594_2	non-phage	0
DOF006_scaffold21377_1	non-phage	0
DOF006_scaffold14838_1	non-phage	0
DOF006_scaffold22989_2	non-phage	0
DOF006_scaffold8855_1	non-phage	0
DOF006_scaffold13388_1	non-phage	0
DOF006_scaffold6491_1	non-phage	0
DOF006_scaffold22677_2	non-phage	0
DOF006_scaffold2415_83	non-phage	0

DOF006_scaffold19914_2	phage	0.993
DOF006_scaffold8744_1	non-phage	0
DOF006_scaffold22942_3	non-phage	0
DOF006_scaffold16_9	non-phage	0
DOF006_scaffold22992_3	non-phage	0
DOF006_scaffold6373_2	non-phage	0
DOF006_scaffold2415_15	non-phage	0
DOF006_scaffold16_6	non-phage	0
DOF006_scaffold3192_4	non-phage	0
DOF006_scaffold306_1	non-phage	0
DOF006_scaffold21761_1	non-phage	0
DOF006_scaffold19435_1	non-phage	0
DOF006_scaffold11230_1	non-phage	0
DOF006_scaffold22980_1	non-phage	0
DOF006_scaffold21547_3	non-phage	0
DOF006_scaffold12157_1	non-phage	0
DOF006_scaffold21547_1	non-phage	0
DOF006_scaffold22971_2	non-phage	0
DOF006_scaffold22310_1	non-phage	0
DOF006_scaffold4278_1	non-phage	0
DOF006_scaffold2886_2	non-phage	0
DOF006_scaffold3206_5	non-phage	0
DOF006_scaffold8479_1	non-phage	0
DOF006_scaffold2423_2	non-phage	0
DOF006_scaffold5658_2	non-phage	0
DOF006_scaffold1844_2	non-phage	0
DOF006_scaffold18349_2	non-phage	0
DOF006_scaffold9920_4	non-phage	0
DOF006_scaffold5845_9	non-phage	0
DOF006_scaffold123_3	non-phage	0
DOF006_scaffold17947_1	non-phage	0
DOF006_scaffold55_3	non-phage	0
DOF006_scaffold5845_2	non-phage	0
DOF006_scaffold10957_1	non-phage	0
DOF006_scaffold8232_2	non-phage	0
DOF006_scaffold10913_8	non-phage	0
DOF006_scaffold22493_4	non-phage	0
DOF006_scaffold11978_3	non-phage	0
DOF006_scaffold7307_3	non-phage	0
DOF006_scaffold8119_1	non-phage	0
DOF006_scaffold11335_2	non-phage	0
DOF006_scaffold19211_1	non-phage	0
DOF006_scaffold21224_1	non-phage	0
DOF006_scaffold15750_1	non-phage	0
DOF006_scaffold22799_1	non-phage	0
DOF006_scaffold101_2	non-phage	0

DOF006_scaffold19667_7	non-phage	0
DOF006_scaffold22735_1	non-phage	0
DOF006_scaffold22996_1	phage	0.997
DOF006_scaffold21980_1	non-phage	0
DOF006_scaffold11289_2	non-phage	0
DOF006_scaffold16152_1	non-phage	0
DOF006_scaffold23017_1	non-phage	0
DOF006_scaffold13735_2	phage	0.994
DOF006_scaffold22_1	non-phage	0
DOF006_scaffold19539_5	non-phage	0
DOF006_scaffold8838_1	non-phage	0
DOF006_scaffold5940_4	non-phage	0
DOF006_scaffold306_20	non-phage	0
DOF006_C312249_1	non-phage	0
DOF006_scaffold6236_9	non-phage	0
DOF006_scaffold11478_1	phage	0.996
DOF006_scaffold21759_4	non-phage	0
DOF006_scaffold12568_1	non-phage	0
DOF006_scaffold6197_2	non-phage	0
DOF006_scaffold8801_1	non-phage	0
DOF006_scaffold12933_5	non-phage	0
DOF006_scaffold22603_1	non-phage	0
DOF006_scaffold5174_2	non-phage	0
DOF006_scaffold18935_1	non-phage	0
DOF006_scaffold21598_3	non-phage	0
DOF006_scaffold6236_2	non-phage	0
DOF006_scaffold22910_4	non-phage	0
DOF006_scaffold18024_4	non-phage	0
DOF006_scaffold14556_1	non-phage	0
DOF006_scaffold3875_4	non-phage	0
DOF006_scaffold17014_2	non-phage	0
DOF006_scaffold23020_1	non-phage	0
DOF006_scaffold1908_7	non-phage	0
DOF006_scaffold22275_1	non-phage	0
DOF006_scaffold1547_2	non-phage	0
DOF006_scaffold12652_1	non-phage	0
DOF006_scaffold13835_2	non-phage	0
DOF006_scaffold11630_1	non-phage	0
DOF006_scaffold3349_1	non-phage	0
DOF006_scaffold18042_2	non-phage	0
DOF006_scaffold11715_1	non-phage	0
DOF006_scaffold18587_3	non-phage	0
DOF006_scaffold1908_8	non-phage	0
DOF006_scaffold27_2	non-phage	0
DOF006_scaffold3372_1	phage	0.998
DOF006_scaffold22991_1	non-phage	0

DOF006_scaffold22979_2	non-phage	0
DOF006_scaffold13835_1	non-phage	0
DOF006_scaffold17015_1	non-phage	0
DOF006_scaffold22943_2	non-phage	0
DOF006_scaffold5845_10	non-phage	0
DOF006_scaffold7544_1	non-phage	0
DOF006_scaffold21510_3	non-phage	0
DOF006_scaffold15247_2	non-phage	0
DOF006_scaffold7285_1	non-phage	0
DOF006_C311929_1	non-phage	0
DOF006_C311835_1	non-phage	0
DOF006_scaffold19945_1	non-phage	0
DOF006_scaffold11336_8	non-phage	0
DOF006_C312063_1	non-phage	0
DOF006_scaffold3115_4	non-phage	0.283
DOF006_scaffold16_3	non-phage	0
DOF006_scaffold18019_7	non-phage	0.016
DOF006_scaffold14837_1	non-phage	0
DOF006_scaffold22946_6	non-phage	0
DOF006_scaffold10116_1	non-phage	0
DOF006_scaffold4639_12	non-phage	0
DOF006_scaffold22658_1	non-phage	0
DOF006_scaffold21992_6	non-phage	0
DOF006_scaffold13519_4	non-phage	0
DOF006_scaffold7205_2	non-phage	0
DOF006_scaffold2415_58	non-phage	0
DOF006_scaffold19585_1	non-phage	0
DOF006_scaffold8232_1	non-phage	0
DOF006_scaffold3791_1	non-phage	0
DOF006_scaffold22967_1	non-phage	0
DOF006_scaffold3192_5	non-phage	0
DOF006_scaffold22722_1	non-phage	0
DOF006_scaffold22130_1	non-phage	0
DOF006_scaffold12896_2	non-phage	0
DOF006_scaffold13500_3	non-phage	0
DOF006_scaffold6346_1	non-phage	0
DOF006_scaffold11315_1	non-phage	0
DOF006_scaffold12736_1	non-phage	0
DOF006_scaffold11335_6	non-phage	0
DOF006_scaffold5845_5	non-phage	0
DOF006_scaffold10093_1	non-phage	0
DOF006_scaffold16620_5	non-phage	0
DOF006_scaffold22947_2	non-phage	0
DOF006_scaffold22872_1	non-phage	0
DOF006_scaffold2428_4	non-phage	0
DOF006_scaffold7519_1	non-phage	0

DOF006_scaffold7302_1	non-phage	0
DOF006_scaffold2963_2	non-phage	0
DOF006_scaffold22993_3	non-phage	0
DOF006_scaffold22038_1	non-phage	0
DOF006_scaffold11077_2	non-phage	0
DOF006_scaffold16979_2	non-phage	0
DOF006_scaffold7546_6	non-phage	0
DOF006_C313043_1	non-phage	0
DOF006_scaffold22711_1	non-phage	0
DOF006_scaffold10913_13	non-phage	0
DOF006_scaffold3513_6	non-phage	0
DOF006_scaffold11336_9	non-phage	0
DOF006_scaffold266_3	non-phage	0
DOF006_scaffold11403_1	non-phage	0
DOF006_scaffold8305_2	non-phage	0
DOF006_scaffold22404_1	non-phage	0
DOF006_scaffold10171_1	non-phage	0
DOF006_scaffold10037_2	non-phage	0
DOF006_scaffold11336_11	non-phage	0
DOF006_scaffold27_1	non-phage	0
DOF006_scaffold22063_5	non-phage	0
DOF006_scaffold22947_1	non-phage	0
DOF006_scaffold3875_1	non-phage	0
DOF006_scaffold8976_1	non-phage	0
DOF006_scaffold15300_2	non-phage	0
DOF006_scaffold21852_1	non-phage	0
DOF006_scaffold4639_7	non-phage	0
DOF006_scaffold7328_3	non-phage	0
DOF006_scaffold15129_1	non-phage	0
DOF006_scaffold15978_6	non-phage	0
DOF006_scaffold7617_2	non-phage	0
DOF006_scaffold14317_4	non-phage	0
DOF006_scaffold21472_1	non-phage	0
DOF006_scaffold18788_9	phage	0.999
DOF006_scaffold635_5	non-phage	0
DOF006_scaffold15043_1	non-phage	0
DOF006_scaffold14502_1	non-phage	0
DOF006_scaffold12586_1	non-phage	0
DOF006_scaffold16979_4	non-phage	0
DOF006_C312309_1	non-phage	0
DOF006_scaffold18788_12	non-phage	0
DOF006_scaffold22655_2	non-phage	0
DOF006_scaffold3173_3	non-phage	0
DOF006_scaffold1547_6	non-phage	0
DOF006_scaffold1908_3	non-phage	0
DOF006_scaffold23011_1	non-phage	0

DOF006_scaffold1547_1	non-phage	0
DOF006_scaffold4151_2	non-phage	0
DOF006_scaffold18674_3	non-phage	0
DOF006_scaffold19994_1	non-phage	0
DOF006_scaffold18572_2	non-phage	0
DOF006_scaffold7655_1	non-phage	0
DOF006_scaffold2791_2	non-phage	0
DOF006_scaffold22988_1	non-phage	0
DOF006_scaffold13547_1	non-phage	0
DOF006_scaffold1206_3	non-phage	0
DOF006_scaffold22498_3	non-phage	0
DOF006_scaffold13295_1	non-phage	0
DOF006_scaffold1908_4	non-phage	0
DOF006_scaffold23004_2	phage	0.919
DOF006_C312483_1	non-phage	0
DOF006_scaffold12368_1	non-phage	0
DOF006_scaffold15247_5	non-phage	0
DOF006_scaffold2415_74	non-phage	0
DOF006_scaffold11800_4	non-phage	0
DOF006_scaffold11858_2	non-phage	0
DOF006_scaffold15013_1	non-phage	0
DOF006_scaffold11590_2	non-phage	0
DOF006_scaffold9249_1	non-phage	0
DOF006_scaffold13034_3	non-phage	0
DOF006_scaffold18587_2	non-phage	0
DOF006_scaffold6825_1	non-phage	0
DOF006_scaffold23013_1	non-phage	0
DOF006_scaffold22615_1	non-phage	0.162
DOF006_scaffold21183_1	non-phage	0
DOF006_scaffold11336_3	non-phage	0
DOF006_scaffold16123_3	non-phage	0
DOF006_scaffold14284_7	non-phage	0
DOF006_scaffold8855_2	non-phage	0
DOF006_scaffold15162_1	phage	0.999
DOF006_scaffold7397_2	non-phage	0
DOF006_scaffold22194_1	non-phage	0
DOF006_scaffold22922_1	non-phage	0
DOF006_scaffold123_2	non-phage	0
DOF006_scaffold2886_4	non-phage	0
DOF006_scaffold11289_4	non-phage	0
DOF006_scaffold1325_1	non-phage	0
DOF006_scaffold22337_1	non-phage	0
DOF006_scaffold23015_1	non-phage	0
DOF006_scaffold907_3	non-phage	0
DOF006_scaffold18788_10	non-phage	0
DOF006_scaffold10509_1	non-phage	0

DOF006_scaffold19306_1	non-phage	0
DOF006_scaffold22711_3	non-phage	0
DOF006_scaffold21727_1	non-phage	0
DOF006_scaffold19921_4	non-phage	0
DOF006_scaffold22994_1	non-phage	0
DOF006_scaffold19539_4	non-phage	0
DOF006_scaffold14392_1	non-phage	0
DOF006_scaffold22993_4	non-phage	0
DOF006_scaffold16_14	non-phage	0
DOF006_scaffold2736_1	phage	0.992
DOF006_scaffold22038_2	non-phage	0
DOF006_scaffold12157_2	non-phage	0
DOF006_scaffold7718_2	non-phage	0
DOF006_scaffold20709_1	phage	1
DOF006_scaffold11335_9	non-phage	0
DOF006_scaffold17724_2	non-phage	0
DOF006_scaffold5658_3	non-phage	0
DOF006_scaffold1180_5	non-phage	0
DOF006_scaffold22822_1	non-phage	0
DOF006_scaffold306_6	phage	0.999
DOF006_scaffold11626_1	non-phage	0
DOF006_scaffold19726_3	non-phage	0
DOF006_scaffold22967_3	non-phage	0
DOF006_scaffold17582_4	non-phage	0
DOF006_scaffold16620_13	non-phage	0
DOF006_scaffold22845_2	non-phage	0
DOF006_scaffold16_20	non-phage	0
DOF006_scaffold1369_1	non-phage	0
DOF006_scaffold12158_1	non-phage	0
DOF006_scaffold22987_7	non-phage	0
DOF006_scaffold19689_1	non-phage	0
DOF006_scaffold4639_2	non-phage	0
DOF006_scaffold167_1	non-phage	0
DOF006_scaffold22992_4	non-phage	0
DOF006_scaffold22603_2	non-phage	0
DOF006_scaffold16716_1	non-phage	0
DOF006_scaffold21377_5	non-phage	0
DOF006_scaffold1875_1	non-phage	0
DOF006_scaffold14394_4	non-phage	0
DOF006_scaffold2415_48	non-phage	0
DOF006_scaffold15247_9	non-phage	0
DOF006_scaffold15300_5	non-phage	0
DOF006_scaffold19435_2	non-phage	0
DOF006_scaffold415_3	non-phage	0
DOF006_scaffold22411_1	non-phage	0
DOF006_scaffold18075_1	non-phage	0

DOF006_scaffold7084_4	non-phage	0
DOF006_scaffold3453_3	non-phage	0
DOF006_scaffold22600_1	non-phage	0
DOF006_scaffold16452_1	non-phage	0
DOF006_scaffold22891_2	non-phage	0
DOF006_scaffold22948_1	non-phage	0
DOF006_scaffold22655_1	non-phage	0
DOF006_scaffold22266_1	non-phage	0
DOF006_scaffold13128_1	non-phage	0
DOF006_scaffold22685_1	phage	0.999
DOF006_scaffold19539_2	non-phage	0
DOF006_scaffold22862_1	non-phage	0
DOF006_scaffold150_3	non-phage	0
DOF006_scaffold7727_2	non-phage	0
DOF006_scaffold4966_1	phage	0.999
DOF006_scaffold21423_3	non-phage	0
DOF006_scaffold1463_1	non-phage	0
DOF006_scaffold1477_4	non-phage	0
DOF006_scaffold8001_1	non-phage	0
DOF006_scaffold6236_5	non-phage	0
DOF006_scaffold9321_1	non-phage	0
DOF006_scaffold22281_3	non-phage	0
DOF006_scaffold536_3	non-phage	0
DOF006_scaffold23004_1	non-phage	0
DOF006_scaffold3173_4	non-phage	0
DOF006_scaffold22663_1	phage	0.997
DOF006_scaffold2415_51	non-phage	0
DOF006_scaffold22576_2	non-phage	0
DOF006_scaffold15748_1	non-phage	0
DOF006_scaffold11050_3	non-phage	0
DOF006_scaffold6116_2	non-phage	0
DOF006_scaffold22923_2	non-phage	0
DOF006_scaffold22689_2	non-phage	0
DOF006_C312935_1	non-phage	0
DOF006_scaffold22918_1	non-phage	0
DOF006_scaffold21382_1	non-phage	0
DOF006_scaffold415_2	non-phage	0
DOF006_scaffold17215_2	non-phage	0
DOF006_scaffold7466_1	non-phage	0
DOF006_scaffold3032_2	non-phage	0
DOF006_scaffold12448_1	non-phage	0
DOF006_C312677_1	non-phage	0
DOF006_scaffold16620_1	non-phage	0
DOF006_scaffold22367_3	non-phage	0
DOF006_scaffold16570_1	non-phage	0
DOF006_scaffold11335_5	non-phage	0

DOF006_scaffold22952_1	phage	0.994
DOF006_scaffold19667_5	non-phage	0
DOF006_scaffold22971_1	non-phage	0
DOF006_scaffold15301_1	non-phage	0
DOF006_scaffold10037_1	non-phage	0
DOF006_scaffold22985_1	non-phage	0
DOF006_scaffold22972_2	non-phage	0
DOF006_scaffold12933_1	non-phage	0
DOF006_scaffold11800_1	non-phage	0
DOF006_scaffold70_2	non-phage	0
DOF006_scaffold13612_2	non-phage	0
DOF006_scaffold10290_1	non-phage	0
DOF006_scaffold17090_1	non-phage	0
DOF006_scaffold22954_1	non-phage	0
DOF006_scaffold5845_1	phage	0.615
DOF006_scaffold23007_1	non-phage	0
DOF006_scaffold7501_3	non-phage	0
DOF006_scaffold13429_1	non-phage	0
DOF006_scaffold8855_3	non-phage	0
DOF006_C313075_1	non-phage	0
DOF006_scaffold12158_5	non-phage	0
DOF006_scaffold21787_1	non-phage	0
DOF006_scaffold22872_4	non-phage	0
DOF006_scaffold90_2	non-phage	0
DOF006_scaffold19914_3	non-phage	0
DOF006_scaffold13547_5	non-phage	0
DOF006_scaffold22138_1	non-phage	0
DOF006_scaffold5658_4	non-phage	0
DOF006_scaffold17357_1	phage	0.996
DOF006_scaffold23009_1	non-phage	0
DOF006_scaffold15052_2	non-phage	0
DOF006_scaffold18675_2	non-phage	0
DOF006_scaffold11077_3	non-phage	0
DOF006_scaffold22967_2	non-phage	0
DOF006_scaffold150_6	non-phage	0
DOF006_scaffold10651_6	non-phage	0
DOF006_scaffold8738_5	non-phage	0
DOF006_scaffold6190_4	non-phage	0
DOF006_scaffold17419_1	non-phage	0
DOF006_scaffold12210_1	non-phage	0
DOF006_scaffold3192_9	non-phage	0
DOF006_scaffold17822_1	non-phage	0
DOF006_scaffold13736_5	non-phage	0
DOF006_scaffold3513_11	non-phage	0
DOF006_scaffold22063_4	non-phage	0.025
DOF006_scaffold21992_3	non-phage	0

DOF006_scaffold10913_6	non-phage	0
DOF006_scaffold7501_2	non-phage	0
DOF006_scaffold22845_1	non-phage	0
DOF006_scaffold23002_1	non-phage	0
DOF006_scaffold21598_1	non-phage	0
DOF006_scaffold14317_5	non-phage	0
DOF006_scaffold22922_2	non-phage	0
DOF006_scaffold2415_20	non-phage	0
DOF006_scaffold14584_2	phage	0.925
DOF006_scaffold9574_3	non-phage	0
DOF006_scaffold16712_1	non-phage	0
DOF006_scaffold14_1	non-phage	0
DOF006_scaffold2415_72	non-phage	0
DOF006_scaffold18630_1	non-phage	0
DOF006_scaffold11800_3	non-phage	0
DOF006_scaffold10913_18	non-phage	0
DOF006_scaffold7771_1	non-phage	0
DOF006_scaffold21224_3	non-phage	0
DOF006_scaffold5940_2	non-phage	0
DOF006_scaffold23008_1	non-phage	0
DOF006_scaffold22981_2	non-phage	0
DOF006_scaffold5732_2	non-phage	0
DOF006_scaffold22835_1	non-phage	0
DOF006_scaffold111_1	non-phage	0
DOF006_C311567_1	non-phage	0
DOF006_C311957_1	non-phage	0
DOF006_scaffold15293_2	non-phage	0
DOF006_scaffold20892_1	non-phage	0
DOF006_scaffold15161_1	non-phage	0
DOF006_scaffold12062_2	non-phage	0
DOF006_scaffold1908_5	non-phage	0
DOF006_scaffold8103_1	non-phage	0
DOF006_scaffold21377_4	non-phage	0
DOF006_scaffold8817_1	non-phage	0
DOF006_scaffold8033_2	non-phage	0
DOF006_scaffold23019_1	non-phage	0
DOF006_scaffold3140_1	non-phage	0
DOF006_scaffold2415_41	non-phage	0
DOF006_scaffold3875_2	non-phage	0
DOF006_scaffold9406_1	non-phage	0
DOF006_scaffold15300_1	non-phage	0
DOF006_scaffold18922_1	non-phage	0
DOF006_scaffold23016_1	non-phage	0
DOF006_scaffold14284_8	non-phage	0
DOF006_scaffold20769_1	non-phage	0
DOF006_scaffold13034_2	non-phage	0

DOF006_scaffold22842_1	non-phage	0
DOF006_scaffold1377_2	non-phage	0
DOF006_scaffold11008_3	non-phage	0
DOF006_scaffold22336_1	non-phage	0
DOF006_scaffold9938_2	non-phage	0
DOF006_scaffold19921_11	non-phage	0
DOF006_scaffold16563_1	non-phage	0
DOF006_scaffold22986_1	non-phage	0
DOF006_scaffold21992_8	non-phage	0
DOF006_scaffold2073_2	non-phage	0
DOF006_scaffold22575_1	non-phage	0
DOF006_scaffold22999_1	non-phage	0
DOF006_scaffold22946_8	non-phage	0
DOF006_scaffold22956_2	non-phage	0
DOF006_scaffold22434_1	non-phage	0
DOF006_scaffold1325_2	non-phage	0
DOF006_scaffold9647_1	non-phage	0
DOF006_scaffold22908_2	non-phage	0
DOF006_scaffold2415_32	non-phage	0
DOF006_scaffold8723_1	non-phage	0
DOF006_scaffold22983_1	non-phage	0
DOF006_scaffold22993_2	non-phage	0
DOF006_scaffold22367_4	non-phage	0
DOF006_scaffold17200_1	non-phage	0
DOF006_scaffold13642_1	non-phage	0
DOF006_scaffold13350_1	non-phage	0
DOF006_scaffold8420_1	non-phage	0
DOF006_scaffold20271_1	non-phage	0
DOF006_scaffold2415_60	non-phage	0
DOF006_scaffold12158_2	non-phage	0
DOF006_scaffold7307_1	phage	0.997
DOF006_scaffold20770_1	non-phage	0
DOF006_scaffold7546_8	non-phage	0
DOF006_scaffold22281_2	non-phage	0
DOF006_scaffold22952_2	phage	0.999
DOF006_scaffold19496_1	non-phage	0
DOF006_scaffold7084_3	non-phage	0
DOF006_scaffold16620_7	non-phage	0
DOF006_scaffold17724_1	non-phage	0
DOF006_scaffold18349_4	non-phage	0
DOF006_scaffold6238_1	non-phage	0
DOF006_scaffold5845_4	non-phage	0
DOF006_scaffold19921_9	non-phage	0
DOF006_scaffold124_3	non-phage	0
DOF006_scaffold3777_5	non-phage	0
DOF006_scaffold16_15	non-phage	0

DOF006_C311659_1	non-phage	0
DOF006_scaffold4202_1	non-phage	0
DOF006_C312389_1	non-phage	0
DOF006_scaffold14723_1	non-phage	0
DOF006_scaffold19036_1	non-phage	0
DOF006_scaffold21992_12	non-phage	0
DOF006_scaffold19921_10	non-phage	0
DOF006_scaffold23000_2	non-phage	0
DOF006_scaffold9688_1	non-phage	0
DOF006_scaffold14053_1	non-phage	0
DOF006_scaffold8018_1	non-phage	0
DOF006_scaffold13726_1	non-phage	0
DOF006_scaffold18575_1	non-phage	0
DOF006_scaffold15748_2	non-phage	0
DOF006_scaffold22527_2	non-phage	0
DOF006_scaffold8838_2	non-phage	0
DOF006_scaffold3513_9	non-phage	0
DOF006_scaffold22946_1	non-phage	0
DOF006_scaffold3173_5	non-phage	0
DOF006_scaffold21981_2	non-phage	0
DOF006_scaffold22576_3	non-phage	0
DOF006_scaffold3513_4	non-phage	0
DOF006_scaffold19244_1	non-phage	0
DOF006_scaffold5945_2	non-phage	0
DOF006_scaffold17582_3	non-phage	0
DOF006_C312039_1	non-phage	0
DOF006_scaffold11077_4	non-phage	0
DOF006_scaffold11289_3	non-phage	0
DOF006_scaffold21842_1	non-phage	0
DOF006_scaffold21472_3	non-phage	0
DOF006_scaffold13541_1	non-phage	0
DOF006_scaffold22901_1	non-phage	0
DOF006_scaffold150_7	non-phage	0
DOF006_scaffold11978_5	non-phage	0
DOF006_scaffold22978_1	non-phage	0
DOF006_scaffold22359_2	non-phage	0
DOF006_scaffold711_5	phage	0.999
DOF006_scaffold2415_45	non-phage	0
DOF006_scaffold7212_1	non-phage	0
DOF006_C312973_1	non-phage	0
DOF006_scaffold13597_1	non-phage	0
DOF006_scaffold9522_2	non-phage	0
DOF006_scaffold7621_2	non-phage	0
DOF006_scaffold18075_2	non-phage	0
DOF006_scaffold3430_2	non-phage	0
DOF006_scaffold9292_2	phage	0.998

DOF006_scaffold19539_3	non-phage	0
DOF006_scaffold1770_1	non-phage	0
DOF006_scaffold22037_2	non-phage	0
DOF006_scaffold7328_1	non-phage	0
DOF006_scaffold9921_1	non-phage	0
DOF006_scaffold22987_1	non-phage	0
DOF006_scaffold1908_1	non-phage	0
DOF006_scaffold22600_2	non-phage	0
DOF006_scaffold2415_6	non-phage	0
DOF006_scaffold8249_2	non-phage	0
DOF006_scaffold12933_4	non-phage	0
DOF006_scaffold16979_6	non-phage	0
DOF006_scaffold8033_6	non-phage	0
DOF006_scaffold22976_1	non-phage	0
DOF006_scaffold13547_4	non-phage	0
DOF006_scaffold22891_3	non-phage	0
DOF006_scaffold18677_1	non-phage	0
DOF006_scaffold18216_1	non-phage	0
DOF006_scaffold2415_82	non-phage	0
DOF006_scaffold7677_1	non-phage	0
DOF006_scaffold4639_10	non-phage	0
DOF006_scaffold11076_4	non-phage	0
DOF006_scaffold22310_2	non-phage	0
DOF006_scaffold13035_1	non-phage	0
DOF006_scaffold11362_2	non-phage	0
DOF006_scaffold18349_1	non-phage	0
DOF006_scaffold6493_1	non-phage	0
DOF006_scaffold15273_1	non-phage	0
DOF006_scaffold22942_1	non-phage	0
DOF006_scaffold305_8	non-phage	0
DOF006_scaffold10651_5	non-phage	0
DOF006_scaffold9510_2	non-phage	0
DOF006_scaffold13500_1	non-phage	0
DOF006_scaffold6190_3	non-phage	0
DOF006_scaffold6692_3	non-phage	0
DOF006_scaffold21036_1	non-phage	0
DOF006_scaffold13519_2	non-phage	0
DOF006_scaffold3032_1	non-phage	0
DOF006_scaffold20101_4	non-phage	0
DOF006_scaffold23010_2	non-phage	0
DOF006_scaffold11336_2	non-phage	0
DOF006_scaffold10_2_1	non-phage	0
DOF006_scaffold22987_2	non-phage	0
DOF006_scaffold23014_1	non-phage	0
DOF006_scaffold14284_5	non-phage	0
DOF006_scaffold13945_1	non-phage	0

DOF006_scaffold15301_2	non-phage	0
DOF006_scaffold13847_3	non-phage	0
DOF006_scaffold16894_1	non-phage	0
DOF006_scaffold2999_1	non-phage	0
DOF006_scaffold6294_1	non-phage	0
DOF006_C312477_1	non-phage	0
DOF006_scaffold55_1	non-phage	0
DOF006_scaffold22962_1	non-phage	0
DOF006_scaffold3875_7	non-phage	0
DOF006_scaffold212_1	non-phage	0
DOF006_scaffold13176_1	non-phage	0
DOF006_scaffold10254_3	non-phage	0
DOF006_scaffold22710_4	non-phage	0
DOF006_scaffold7702_2	non-phage	0
DOF006_scaffold22988_2	non-phage	0
DOF006_scaffold15940_1	non-phage	0
DOF006_scaffold11473_1	non-phage	0
DOF006_scaffold10407_1	phage	0.999
DOF006_scaffold17090_2	non-phage	0
DOF006_scaffold2504_5	non-phage	0
DOF006_C312621_1	phage	0.998
DOF006_scaffold18868_1	non-phage	0
DOF006_scaffold22493_2	non-phage	0
DOF006_scaffold16620_16	non-phage	0
DOF006_scaffold8902_1	non-phage	0
DOF006_scaffold8855_4	non-phage	0
DOF006_scaffold16_19	non-phage	0
DOF006_scaffold8152_4	non-phage	0
DOF006_scaffold18252_1	non-phage	0
DOF006_scaffold7860_1	non-phage	0
DOF006_scaffold15247_12	non-phage	0
DOF006_scaffold19494_3	non-phage	0
DOF006_scaffold22973_1	non-phage	0
DOF006_scaffold284_1	non-phage	0
DOF006_scaffold22851_1	non-phage	0
DOF006_scaffold4232_3	non-phage	0
DOF007_scaffold23244_1	non-phage	0
DOF007_scaffold11132_1	non-phage	0
DOF007_scaffold24963_1	non-phage	0
DOF007_scaffold16546_1	non-phage	0
DOF007_scaffold15099_9	non-phage	0
DOF007_scaffold8643_2	non-phage	0
DOF007_scaffold20313_1	non-phage	0
DOF007_scaffold24137_4	non-phage	0
DOF007_scaffold37_3	non-phage	0
DOF007_scaffold13359_1	non-phage	0

DOF007_scaffold11274_8	non-phage	0.003
DOF007_scaffold950_4	non-phage	0
DOF007_scaffold106_1	non-phage	0
DOF007_scaffold26109_1	non-phage	0
DOF007_scaffold20898_2	non-phage	0
DOF007_scaffold8_5	non-phage	0
DOF007_scaffold22134_1	non-phage	0
DOF007_scaffold17439_6	non-phage	0
DOF007_scaffold26105_2	non-phage	0
DOF007_scaffold6373_16	non-phage	0
DOF007_scaffold1395_3	non-phage	0
DOF007_scaffold20897_3	non-phage	0
DOF007_scaffold16114_2	non-phage	0
DOF007_scaffold4053_3	non-phage	0
DOF007_scaffold4808_4	non-phage	0
DOF007_scaffold23208_3	non-phage	0
DOF007_scaffold16928_1	non-phage	0.007
DOF007_scaffold11861_2	non-phage	0
DOF007_scaffold16942_1	non-phage	0
DOF007_scaffold1340_6	non-phage	0
DOF007_scaffold8670_5	non-phage	0
DOF007_scaffold26040_1	non-phage	0
DOF007_scaffold950_1	non-phage	0
DOF007_scaffold20312_2	non-phage	0.021
DOF007_scaffold8620_3	non-phage	0
DOF007_scaffold3897_1	non-phage	0
DOF007_scaffold16356_1	non-phage	0
DOF007_scaffold23537_1	non-phage	0
DOF007_scaffold6373_1	non-phage	0
DOF007_scaffold10958_3	non-phage	0
DOF007_scaffold1556_3	non-phage	0
DOF007_scaffold6373_4	non-phage	0
DOF007_scaffold8260_1	non-phage	0
DOF007_scaffold21584_9	non-phage	0
DOF007_scaffold1171_1	phage	0.991
DOF007_scaffold8091_1	non-phage	0
DOF007_scaffold6664_6	non-phage	0
DOF007_scaffold21497_4	non-phage	0
DOF007_scaffold35_3	phage	0.822
DOF007_C360731_1	non-phage	0
DOF007_scaffold95_2	non-phage	0
DOF007_scaffold5221_2	non-phage	0
DOF007_scaffold17439_2	non-phage	0
DOF007_scaffold19257_1	non-phage	0
DOF007_scaffold17252_1	non-phage	0
DOF007_scaffold17308_3	phage	0.999

DOF007_scaffold7049_4	non-phage	0
DOF007_scaffold19476_1	non-phage	0
DOF007_scaffold6128_2	non-phage	0
DOF007_scaffold26113_1	non-phage	0
DOF007_scaffold3003_5	non-phage	0
DOF007_scaffold19564_9	non-phage	0
DOF007_scaffold24301_1	non-phage	0
DOF007_scaffold20167_3	non-phage	0
DOF007_scaffold23990_1	non-phage	0
DOF007_scaffold16963_1	non-phage	0
DOF007_scaffold17021_1	non-phage	0
DOF007_scaffold26071_1	non-phage	0
DOF007_scaffold19132_1	non-phage	0
DOF007_scaffold25882_2	non-phage	0
DOF007_scaffold3763_1	non-phage	0
DOF007_scaffold844_3	non-phage	0
DOF007_scaffold2055_6	non-phage	0
DOF007_scaffold6717_1	non-phage	0
DOF007_C360895_1	phage	1
DOF007_scaffold1503_27	non-phage	0
DOF007_scaffold26120_1	non-phage	0
DOF007_scaffold20396_2	non-phage	0
DOF007_scaffold3630_1	non-phage	0
DOF007_scaffold7833_5	phage	0.956
DOF007_scaffold20612_2	non-phage	0
DOF007_scaffold6790_4	non-phage	0
DOF007_scaffold19832_1	non-phage	0
DOF007_scaffold20758_1	non-phage	0
DOF007_scaffold11172_4	non-phage	0
DOF007_scaffold11012_2	non-phage	0
DOF007_scaffold17567_3	non-phage	0
DOF007_scaffold26030_1	non-phage	0
DOF007_scaffold23623_1	non-phage	0
DOF007_scaffold18342_1	non-phage	0
DOF007_scaffold22682_2	non-phage	0
DOF007_scaffold25582_1	non-phage	0
DOF007_scaffold23267_5	non-phage	0
DOF007_scaffold23504_1	non-phage	0
DOF007_scaffold14521_10	non-phage	0
DOF007_scaffold2931_2	non-phage	0
DOF007_scaffold8307_8	non-phage	0
DOF007_scaffold23403_3	non-phage	0
DOF007_scaffold2330_1	non-phage	0
DOF007_scaffold6294_14	non-phage	0
DOF007_scaffold25839_2	non-phage	0
DOF007_scaffold3765_4	non-phage	0

DOF007_C360365_1	non-phage	0
DOF007_scaffold9805_1	non-phage	0
DOF007_scaffold20113_2	non-phage	0
DOF007_scaffold4400_1	non-phage	0
DOF007_scaffold6664_5	non-phage	0
DOF007_scaffold8699_4	non-phage	0
DOF007_scaffold25060_1	non-phage	0
DOF007_scaffold23990_3	non-phage	0
DOF007_scaffold1416_4	non-phage	0
DOF007_scaffold9808_8	non-phage	0
DOF007_scaffold14603_1	non-phage	0
DOF007_scaffold12822_1	non-phage	0
DOF007_scaffold8974_8	non-phage	0
DOF007_scaffold23284_2	non-phage	0
DOF007_scaffold14578_2	non-phage	0
DOF007_scaffold21810_3	non-phage	0
DOF007_scaffold24388_1	non-phage	0
DOF007_scaffold95_7	non-phage	0
DOF007_scaffold5360_1	non-phage	0
DOF007_scaffold12822_6	non-phage	0
DOF007_scaffold15992_5	non-phage	0
DOF007_scaffold21463_1	phage	0.958
DOF007_scaffold11345_3	non-phage	0
DOF007_scaffold17413_2	non-phage	0
DOF007_scaffold12822_2	non-phage	0
DOF007_scaffold25541_1	non-phage	0
DOF007_scaffold18300_1	non-phage	0
DOF007_scaffold6790_11	non-phage	0
DOF007_scaffold21_27	non-phage	0
DOF007_scaffold18100_3	non-phage	0
DOF007_scaffold25558_3	non-phage	0
DOF007_scaffold1262_3	non-phage	0
DOF007_scaffold11000_2	non-phage	0
DOF007_scaffold1503_32	non-phage	0
DOF007_scaffold21498_5	non-phage	0
DOF007_scaffold16391_1	non-phage	0
DOF007_scaffold24216_1	non-phage	0
DOF007_scaffold4612_7	non-phage	0
DOF007_scaffold2644_116	phage	0.998
DOF007_scaffold267_1	non-phage	0
DOF007_scaffold26087_1	non-phage	0
DOF007_scaffold8388_9	non-phage	0
DOF007_scaffold25043_1	non-phage	0
DOF007_scaffold13340_1	non-phage	0
DOF007_scaffold4894_3	non-phage	0
DOF007_scaffold11068_2	non-phage	0

DOF007_scaffold11132_4	non-phage	0
DOF007_scaffold24562_1	non-phage	0
DOF007_scaffold17899_1	non-phage	0
DOF007_scaffold10273_3	non-phage	0
DOF007_scaffold6988_3	non-phage	0
DOF007_scaffold25162_4	non-phage	0
DOF007_scaffold19676_4	non-phage	0
DOF007_scaffold13023_2	non-phage	0
DOF007_scaffold23868_2	non-phage	0
DOF007_scaffold16963_3	non-phage	0
DOF007_scaffold13738_6	non-phage	0
DOF007_scaffold26101_1	non-phage	0
DOF007_scaffold1395_2	non-phage	0
DOF007_C360637_1	non-phage	0
DOF007_scaffold22005_2	non-phage	0
DOF007_scaffold7833_7	non-phage	0
DOF007_scaffold3765_2	non-phage	0
DOF007_scaffold26066_1	non-phage	0
DOF007_scaffold25746_1	non-phage	0
DOF007_scaffold23108_2	non-phage	0
DOF007_scaffold3832_1	non-phage	0
DOF007_scaffold26024_2	non-phage	0
DOF007_scaffold25224_1	non-phage	0
DOF007_scaffold20897_1	non-phage	0
DOF007_scaffold17922_5	non-phage	0
DOF007_scaffold14864_9	non-phage	0
DOF007_scaffold17121_4	non-phage	0
DOF007_scaffold20173_1	non-phage	0
DOF007_scaffold19380_1	non-phage	0
DOF007_scaffold18563_3	non-phage	0
DOF007_scaffold25496_1	non-phage	0
DOF007_scaffold25987_1	non-phage	0
DOF007_scaffold13072_2	non-phage	0
DOF007_scaffold6373_2	non-phage	0
DOF007_scaffold21584_1	non-phage	0
DOF007_scaffold14864_2	phage	0.999
DOF007_scaffold95_4	non-phage	0
DOF007_scaffold18159_1	non-phage	0
DOF007_scaffold26018_1	non-phage	0
DOF007_scaffold11684_4	non-phage	0
DOF007_scaffold11262_2	non-phage	0
DOF007_scaffold373_5	non-phage	0
DOF007_scaffold25997_5	non-phage	0
DOF007_scaffold5443_1	phage	0.979
DOF007_scaffold20764_1	non-phage	0
DOF007_scaffold6143_13	non-phage	0

DOF007_C360375_1	non-phage	0
DOF007_scaffold19381_1	non-phage	0
DOF007_scaffold30_2	non-phage	0
DOF007_scaffold11868_2	non-phage	0
DOF007_scaffold4946_3	non-phage	0
DOF007_scaffold20898_1	non-phage	0
DOF007_scaffold2931_5	non-phage	0
DOF007_scaffold2934_6	non-phage	0
DOF007_scaffold8699_2	non-phage	0
DOF007_scaffold21434_16	non-phage	0
DOF007_scaffold6294_18	non-phage	0
DOF007_scaffold25056_3	non-phage	0
DOF007_scaffold21_1	non-phage	0
DOF007_scaffold23828_1	non-phage	0
DOF007_scaffold21359_1	non-phage	0
DOF007_scaffold64_2	non-phage	0
DOF007_scaffold3888_3	non-phage	0
DOF007_scaffold20167_5	non-phage	0
DOF007_scaffold25760_4	non-phage	0
DOF007_scaffold15266_2	non-phage	0
DOF007_scaffold18766_1	non-phage	0
DOF007_scaffold4053_2	non-phage	0
DOF007_scaffold11172_3	non-phage	0
DOF007_scaffold11172_2	non-phage	0
DOF007_scaffold23539_2	non-phage	0
DOF007_scaffold13203_1	non-phage	0
DOF007_scaffold26069_1	non-phage	0
DOF007_scaffold8388_2	non-phage	0
DOF007_scaffold2330_4	non-phage	0
DOF007_scaffold23121_1	non-phage	0
DOF007_scaffold9808_1	non-phage	0
DOF007_scaffold13023_6	non-phage	0
DOF007_scaffold11068_6	non-phage	0
DOF007_scaffold551_1	non-phage	0
DOF007_scaffold25827_1	non-phage	0
DOF007_scaffold5983_2	non-phage	0
DOF007_scaffold523_7	phage	0.96
DOF007_scaffold950_16	non-phage	0
DOF007_scaffold25655_1	non-phage	0
DOF007_scaffold38_4	non-phage	0
DOF007_scaffold26122_2	non-phage	0
DOF007_scaffold11274_7	non-phage	0
DOF007_scaffold36_2	non-phage	0
DOF007_scaffold4762_1	non-phage	0
DOF007_scaffold25990_3	non-phage	0
DOF007_scaffold22223_1	non-phage	0

DOF007_scaffold20577_1	non-phage	0
DOF007_scaffold4946_11	non-phage	0
DOF007_scaffold8768_6	non-phage	0
DOF007_scaffold26124_1	non-phage	0
DOF007_scaffold20517_2	non-phage	0
DOF007_scaffold17149_1	non-phage	0
DOF007_scaffold17439_7	non-phage	0
DOF007_scaffold25618_1	non-phage	0
DOF007_scaffold1699_3	non-phage	0
DOF007_scaffold20083_1	non-phage	0
DOF007_scaffold5221_10	non-phage	0
DOF007_scaffold25768_2	non-phage	0
DOF007_scaffold2683_1	non-phage	0
DOF007_scaffold24905_1	non-phage	0
DOF007_scaffold24_2	non-phage	0
DOF007_scaffold23712_4	non-phage	0
DOF007_scaffold8768_10	non-phage	0
DOF007_scaffold7999_3	non-phage	0
DOF007_scaffold11861_1	non-phage	0
DOF007_scaffold19616_1	non-phage	0
DOF007_scaffold8599_1	non-phage	0
DOF007_scaffold563_6	non-phage	0
DOF007_scaffold38_19	non-phage	0
DOF007_scaffold25046_1	non-phage	0
DOF007_scaffold26054_1	non-phage	0
DOF007_scaffold25954_1	non-phage	0
DOF007_scaffold14405_1	non-phage	0
DOF007_scaffold19760_3	non-phage	0
DOF007_scaffold22032_3	non-phage	0
DOF007_scaffold26025_3	non-phage	0
DOF007_scaffold8670_4	non-phage	0
DOF007_scaffold1040_10	non-phage	0
DOF007_scaffold16654_5	non-phage	0
DOF007_scaffold1395_1	non-phage	0
DOF007_scaffold38_20	non-phage	0
DOF007_scaffold20121_1	non-phage	0
DOF007_scaffold25811_1	non-phage	0
DOF007_scaffold2931_1	non-phage	0
DOF007_scaffold18787_2	non-phage	0
DOF007_scaffold25169_3	non-phage	0
DOF007_scaffold59_2	non-phage	0
DOF007_scaffold21422_4	non-phage	0
DOF007_scaffold17888_2	non-phage	0
DOF007_scaffold22738_2	non-phage	0
DOF007_scaffold25111_1	non-phage	0
DOF007_scaffold4612_4	non-phage	0

DOF007_scaffold6294_6	non-phage	0
DOF007_scaffold26082_1	non-phage	0
DOF007_scaffold25348_1	non-phage	0
DOF007_scaffold17439_3	non-phage	0
DOF007_scaffold15324_1	non-phage	0
DOF007_scaffold25017_1	non-phage	0
DOF007_scaffold10273_4	non-phage	0
DOF007_scaffold7271_12	non-phage	0
DOF007_scaffold20235_2	non-phage	0
DOF007_scaffold246_3	non-phage	0
DOF007_scaffold6703_1	non-phage	0
DOF007_C359965_1	non-phage	0
DOF007_scaffold23270_1	non-phage	0
DOF007_scaffold523_6	non-phage	0
DOF007_scaffold7271_26	non-phage	0
DOF007_scaffold2994_3	non-phage	0
DOF007_scaffold2636_1	non-phage	0
DOF007_scaffold7271_20	non-phage	0
DOF007_scaffold26079_1	non-phage	0
DOF007_scaffold15366_2	non-phage	0
DOF007_scaffold6127_2	non-phage	0
DOF007_scaffold16218_9	non-phage	0
DOF007_scaffold2701_1	non-phage	0
DOF007_scaffold18477_3	non-phage	0
DOF007_scaffold1340_7	non-phage	0
DOF007_scaffold25060_5	non-phage	0
DOF007_scaffold25045_2	non-phage	0
DOF007_scaffold2655_7	non-phage	0
DOF007_scaffold25760_3	non-phage	0
DOF007_scaffold25839_1	non-phage	0
DOF007_scaffold3556_4	non-phage	0
DOF007_scaffold8307_2	non-phage	0
DOF007_scaffold1796_2	non-phage	0
DOF007_scaffold11783_2	non-phage	0
DOF007_scaffold7727_8	non-phage	0
DOF007_scaffold23990_4	non-phage	0
DOF007_scaffold16636_1	non-phage	0
DOF007_scaffold12260_9	non-phage	0
DOF007_scaffold5407_2	non-phage	0
DOF007_scaffold17087_1	non-phage	0
DOF007_scaffold950_7	non-phage	0
DOF007_scaffold20972_1	non-phage	0
DOF007_scaffold22333_1	non-phage	0
DOF007_scaffold7727_4	non-phage	0
DOF007_scaffold25997_6	non-phage	0
DOF007_scaffold16636_5	non-phage	0

DOF007_scaffold17046_1	non-phage	0
DOF007_scaffold16391_10	non-phage	0
DOF007_scaffold373_4	non-phage	0
DOF007_scaffold1556_1	non-phage	0
DOF007_scaffold17888_1	non-phage	0
DOF007_scaffold19257_3	non-phage	0
DOF007_scaffold25266_2	non-phage	0
DOF007_scaffold12822_4	non-phage	0
DOF007_scaffold17308_1	non-phage	0
DOF007_scaffold2208_4	non-phage	0
DOF007_scaffold9039_1	non-phage	0
DOF007_scaffold5221_4	non-phage	0
DOF007_scaffold63_2	non-phage	0
DOF007_scaffold4946_2	non-phage	0
DOF007_scaffold23623_2	non-phage	0
DOF007_scaffold24570_1	non-phage	0
DOF007_scaffold11974_2	non-phage	0
DOF007_scaffold12260_8	non-phage	0
DOF007_scaffold25598_1	non-phage	0
DOF007_scaffold6068_2	non-phage	0
DOF007_scaffold37_4	non-phage	0
DOF007_scaffold26118_1	non-phage	0
DOF007_scaffold25676_3	non-phage	0
DOF007_scaffold9121_9	non-phage	0
DOF007_scaffold26024_1	non-phage	0
DOF007_scaffold15266_1	non-phage	0
DOF007_scaffold25675_3	non-phage	0
DOF007_scaffold23124_2	non-phage	0
DOF007_scaffold23284_3	non-phage	0
DOF007_scaffold563_1	non-phage	0
DOF007_scaffold11491_6	non-phage	0
DOF007_scaffold55_6	non-phage	0
DOF007_scaffold25074_1	non-phage	0
DOF007_C359941_1	non-phage	0
DOF007_scaffold7271_28	non-phage	0
DOF007_scaffold22174_1	non-phage	0
DOF007_scaffold17241_1	non-phage	0
DOF007_scaffold6988_1	non-phage	0
DOF007_scaffold2823_1	non-phage	0
DOF007_scaffold37_1	non-phage	0
DOF007_scaffold2644_113	phage	0.829
DOF007_scaffold110_2	non-phage	0
DOF007_scaffold15099_2	non-phage	0
DOF007_scaffold8768_7	non-phage	0
DOF007_scaffold8069_14	non-phage	0
DOF007_scaffold1300_2	non-phage	0

DOF007_scaffold25471_3	non-phage	0
DOF007_scaffold11901_1	non-phage	0
DOF007_scaffold18361_5	non-phage	0
DOF007_scaffold26053_2	non-phage	0
DOF007_scaffold8327_2_1	non-phage	0
DOF007_scaffold25900_1	non-phage	0
DOF007_scaffold6790_14	non-phage	0
DOF007_scaffold2655_3	non-phage	0
DOF007_scaffold1395_4	non-phage	0
DOF007_scaffold2931_8	non-phage	0
DOF007_scaffold776_2	non-phage	0
DOF007_scaffold12260_2	non-phage	0
DOF007_scaffold8288_3	non-phage	0
DOF007_scaffold21314_1	non-phage	0
DOF007_scaffold25688_1	non-phage	0
DOF007_scaffold4400_3	non-phage	0
DOF007_scaffold23712_6	non-phage	0
DOF007_scaffold20511_2	non-phage	0
DOF007_scaffold11274_4	non-phage	0
DOF007_scaffold2644_140	non-phage	0
DOF007_scaffold35_1	non-phage	0
DOF007_scaffold1040_28	non-phage	0
DOF007_scaffold4251_1	non-phage	0
DOF007_scaffold8_3	non-phage	0
DOF007_scaffold23208_1	non-phage	0
DOF007_scaffold17253_2	non-phage	0
DOF007_scaffold15454_1	phage	0.997
DOF007_scaffold2379_4	non-phage	0
DOF007_scaffold25742_1	non-phage	0
DOF007_scaffold16228_1	phage	0.999
DOF007_scaffold21810_6	non-phage	0
DOF007_scaffold23787_1	non-phage	0
DOF007_scaffold16054_4	non-phage	0
DOF007_scaffold9380_3	non-phage	0
DOF007_scaffold23313_1	non-phage	0
DOF007_scaffold3897_3	non-phage	0
DOF007_scaffold22440_1	non-phage	0
DOF007_scaffold26018_2	non-phage	0
DOF007_scaffold2297_2	non-phage	0
DOF007_scaffold25045_1	non-phage	0
DOF007_scaffold1812_1	non-phage	0
DOF007_scaffold2330_3	non-phage	0
DOF007_scaffold1556_5	non-phage	0
DOF007_scaffold8670_3	non-phage	0
DOF007_scaffold20006_1	non-phage	0
DOF007_scaffold18100_1	non-phage	0

DOF007_scaffold24818_2	non-phage	0
DOF007_scaffold25558_1	non-phage	0
DOF007_scaffold20083_8	non-phage	0
DOF007_scaffold4612_5	non-phage	0
DOF007_scaffold7049_5	non-phage	0
DOF007_scaffold14162_3	non-phage	0
DOF007_scaffold11132_2	non-phage	0
DOF007_scaffold20235_3	non-phage	0
DOF007_scaffold632_7	phage	0.936
DOF007_scaffold20083_4	non-phage	0
DOF007_scaffold55_2	non-phage	0
DOF007_scaffold1699_2	non-phage	0
DOF007_scaffold21_25	non-phage	0
DOF007_scaffold3447_2	non-phage	0
DOF007_scaffold1333_1	non-phage	0
DOF007_scaffold20873_1	non-phage	0
DOF007_scaffold23429_2	non-phage	0
DOF007_scaffold18890_3	non-phage	0
DOF007_scaffold38_17	non-phage	0
DOF007_scaffold23990_2	non-phage	0
DOF007_scaffold6988_5	non-phage	0
DOF007_scaffold21584_6	non-phage	0
DOF007_scaffold18410_1	non-phage	0
DOF007_scaffold11000_5	non-phage	0
DOF007_scaffold13738_1	non-phage	0
DOF007_scaffold4405_1	non-phage	0
DOF007_scaffold6790_6	non-phage	0
DOF007_scaffold11259_6	non-phage	0
DOF007_scaffold16636_4	non-phage	0
DOF007_scaffold25469_1	non-phage	0
DOF007_scaffold7758_3	non-phage	0
DOF007_scaffold6957_3	non-phage	0
DOF007_scaffold17728_2	non-phage	0
DOF007_scaffold4724_4	non-phage	0
DOF007_scaffold17663_2	non-phage	0
DOF007_scaffold22924_3	non-phage	0
DOF007_scaffold21406_1	non-phage	0
DOF007_scaffold26121_2	non-phage	0.315
DOF007_scaffold13293_1	non-phage	0
DOF007_scaffold18361_1	non-phage	0
DOF007_scaffold16914_2	phage	1
DOF007_C360213_1	non-phage	0
DOF007_scaffold16217_3	non-phage	0
DOF007_scaffold24072_1	non-phage	0
DOF007_scaffold24902_2	non-phage	0
DOF007_scaffold11068_1	non-phage	0

DOF007_scaffold17663_1	non-phage	0
DOF007_scaffold18322_2	non-phage	0
DOF007_scaffold15160_3	non-phage	0
DOF007_scaffold6294_9	non-phage	0
DOF007_scaffold5846_1	non-phage	0
DOF007_scaffold35_5	non-phage	0
DOF007_scaffold25954_3	non-phage	0
DOF007_scaffold409_2	non-phage	0
DOF007_scaffold24869_2	non-phage	0
DOF007_scaffold11983_1	non-phage	0
DOF007_scaffold95_5	non-phage	0
DOF007_scaffold3765_1	non-phage	0
DOF007_scaffold2636_3	non-phage	0
DOF007_scaffold2636_2	non-phage	0
DOF007_scaffold16654_3	non-phage	0
DOF007_scaffold11259_3	non-phage	0
DOF007_scaffold38_1	non-phage	0
DOF007_scaffold8974_2	non-phage	0
DOF007_scaffold26089_1	non-phage	0
DOF007_scaffold26065_2	non-phage	0
DOF007_scaffold6127_1	non-phage	0
DOF007_scaffold20897_4	non-phage	0
DOF007_scaffold21341_2	non-phage	0
DOF007_scaffold3888_1	non-phage	0
DOF007_scaffold12260_6	non-phage	0
DOF007_scaffold4088_1	non-phage	0
DOF007_scaffold21721_1	non-phage	0
DOF007_scaffold16218_8	non-phage	0
DOF007_scaffold22355_1	non-phage	0
DOF007_scaffold26114_1	non-phage	0
DOF007_scaffold1340_5	phage	0.999
DOF007_scaffold6621_2	non-phage	0
DOF007_scaffold2644_118	phage	0.999
DOF007_scaffold20665_1	non-phage	0
DOF007_scaffold2055_5	non-phage	0
DOF007_scaffold1282_5	non-phage	0
DOF007_scaffold22732_1	non-phage	0
DOF007_scaffold6138_2	non-phage	0
DOF007_scaffold25997_3	non-phage	0
DOF007_scaffold2655_6	non-phage	0
DOF007_scaffold8288_2	non-phage	0
DOF007_scaffold3327_5	non-phage	0
DOF007_scaffold7608_2	non-phage	0
DOF007_scaffold21498_2	non-phage	0
DOF007_scaffold551_3	non-phage	0
DOF007_scaffold16543_22	non-phage	0

DOF007_scaffold1333_6	non-phage	0
DOF007_scaffold1556_2	non-phage	0
DOF007_scaffold23267_6	non-phage	0
DOF007_scaffold20394_1	non-phage	0
DOF007_scaffold11172_5	non-phage	0
DOF007_scaffold21810_5	non-phage	0
DOF007_scaffold24879_1	non-phage	0
DOF007_scaffold24709_1	non-phage	0
DOF007_scaffold23616_1	non-phage	0
DOF007_scaffold844_2	non-phage	0
DOF007_scaffold19338_1	non-phage	0
DOF007_scaffold19564_1	non-phage	0
DOF007_scaffold19773_1	non-phage	0
DOF007_scaffold21498_7	non-phage	0
DOF007_scaffold26083_2	non-phage	0
DOF007_scaffold26046_1	non-phage	0
DOF007_scaffold16356_2	non-phage	0
DOF007_scaffold26090_1	non-phage	0
DOF007_scaffold15099_8	non-phage	0
DOF007_scaffold20113_5	non-phage	0
DOF007_scaffold1699_1	non-phage	0
DOF007_scaffold63_3	non-phage	0
DOF007_scaffold6414_1	non-phage	0
DOF007_scaffold10277_1	non-phage	0
DOF007_C359971_1	phage	0.966
DOF007_scaffold17439_8	non-phage	0
DOF007_scaffold25679_1	non-phage	0
DOF007_scaffold563_4	non-phage	0
DOF007_scaffold8069_6	non-phage	0
DOF007_scaffold8642_2	non-phage	0
DOF007_scaffold14521_2	non-phage	0
DOF007_scaffold11861_3	non-phage	0
DOF007_scaffold25768_3	non-phage	0
DOF007_scaffold16654_6	non-phage	0
DOF007_scaffold11274_3	non-phage	0
DOF007_scaffold5000_1	non-phage	0
DOF007_scaffold38_18	non-phage	0
DOF007_scaffold26025_1	non-phage	0
DOF007_scaffold8670_6	non-phage	0
DOF007_scaffold25874_1	non-phage	0
DOF007_scaffold950_9	non-phage	0
DOF007_scaffold25684_1	non-phage	0
DOF007_scaffold8_2	non-phage	0
DOF007_scaffold25611_2	non-phage	0
DOF007_scaffold25725_1	non-phage	0
DOF007_scaffold25890_1	non-phage	0

DOF007_scaffold17253_1	non-phage	0
DOF007_scaffold3447_3	non-phage	0
DOF007_C359997_1	non-phage	0
DOF007_scaffold24818_3	non-phage	0
DOF007_scaffold4001_1	phage	0.99
DOF007_scaffold3356_4	non-phage	0
DOF007_C360901_1	non-phage	0
DOF007_scaffold24_1	non-phage	0
DOF007_scaffold21558_2	non-phage	0
DOF007_scaffold25169_1	non-phage	0
DOF007_scaffold3551_1	non-phage	0
DOF007_scaffold2450_3	non-phage	0
DOF007_scaffold19017_4	non-phage	0
DOF007_scaffold19761_1	non-phage	0
DOF007_scaffold26015_1	non-phage	0.455
DOF007_scaffold16471_4	non-phage	0
DOF007_scaffold17202_2	non-phage	0
DOF007_scaffold12791_4	phage	0.642
DOF007_scaffold25471_1	non-phage	0.18
DOF007_scaffold21831_1	non-phage	0
DOF007_scaffold8303_1	non-phage	0
DOF007_scaffold18361_4	non-phage	0
DOF007_scaffold7758_1	non-phage	0
DOF007_scaffold6294_16	non-phage	0
DOF007_scaffold26119_1	non-phage	0
DOF007_scaffold1333_3	non-phage	0
DOF007_scaffold26015_2	non-phage	0
DOF007_scaffold20121_3	non-phage	0
DOF007_scaffold26073_1	non-phage	0
DOF007_scaffold9779_4	non-phage	0
DOF007_scaffold19102_1	non-phage	0
DOF007_scaffold19381_3	non-phage	0
DOF007_scaffold15160_2	non-phage	0
DOF007_scaffold24508_1	non-phage	0
DOF007_scaffold23313_7	non-phage	0
DOF007_scaffold21434_3	non-phage	0
DOF007_scaffold958_30	non-phage	0
DOF007_scaffold20322_1	non-phage	0
DOF007_scaffold25849_1	non-phage	0
DOF007_scaffold24580_1	non-phage	0
DOF007_scaffold8388_1	non-phage	0
DOF007_scaffold11787_1	non-phage	0
DOF007_scaffold20395_1	non-phage	0
DOF007_scaffold11259_2	non-phage	0
DOF007_scaffold1333_5	non-phage	0
DOF007_scaffold20918_1	non-phage	0

DOF007_scaffold8642_3	non-phage	0
DOF007_scaffold2636_4	non-phage	0
DOF007_scaffold5854_2	non-phage	0
DOF007_scaffold25820_1	non-phage	0
DOF007_scaffold11172_1	non-phage	0
DOF007_scaffold776_1	non-phage	0
DOF007_scaffold989_4	non-phage	0
DOF007_scaffold19980_1	non-phage	0
DOF007_scaffold4718_8	non-phage	0
DOF007_scaffold1333_2	phage	0.991
DOF007_scaffold24869_1	non-phage	0
DOF007_scaffold241_25	non-phage	0
DOF007_scaffold14521_18	non-phage	0
DOF007_scaffold25558_2	non-phage	0
DOF007_scaffold11259_1	non-phage	0
DOF007_scaffold13738_7	non-phage	0
DOF007_scaffold21434_11	non-phage	0
DOF007_scaffold16054_3	non-phage	0
DOF007_scaffold85_2	non-phage	0
DOF007_scaffold21407_1	non-phage	0
DOF007_scaffold26074_1	non-phage	0
DOF007_scaffold25266_1	non-phage	0
DOF007_scaffold1340_2	non-phage	0
DOF007_scaffold25571_1	non-phage	0
DOF007_scaffold25496_2	non-phage	0
DOF007_scaffold14162_7	non-phage	0
DOF007_scaffold3046_1	non-phage	0
DOF007_scaffold2013_2	non-phage	0
DOF007_scaffold11132_3	non-phage	0
DOF007_scaffold17439_5	non-phage	0
DOF007_scaffold17350_1	non-phage	0
DOF007_scaffold7833_1	non-phage	0
DOF007_scaffold11684_6	non-phage	0
DOF007_scaffold26041_1	non-phage	0
DOF007_scaffold24023_1	non-phage	0
DOF007_scaffold8388_3	non-phage	0
DOF007_scaffold73_2	non-phage	0
DOF007_scaffold95_6	non-phage	0
DOF007_scaffold14578_1	non-phage	0
DOF007_scaffold2379_2	non-phage	0
DOF007_scaffold16_1	non-phage	0
DOF007_scaffold8699_5	non-phage	0
DOF007_scaffold8401_1	non-phage	0
DOF007_scaffold6068_1	non-phage	0
DOF007_scaffold26092_1	non-phage	0
DOF007_scaffold13611_1	non-phage	0

DOF007_scaffold22005_4	non-phage	0
DOF007_scaffold20411_1	non-phage	0
DOF007_scaffold16173_1	non-phage	0
DOF007_scaffold6790_8	non-phage	0
DOF007_scaffold7999_9	non-phage	0
DOF007_scaffold25903_1	non-phage	0
DOF007_scaffold36_3	non-phage	0
DOF007_scaffold17439_1	non-phage	0
DOF007_scaffold76_2	non-phage	0
DOF007_scaffold55_1	non-phage	0
DOF007_scaffold921_4	non-phage	0
DOF007_scaffold110_1	non-phage	0
DOF007_scaffold6790_1	non-phage	0
DOF007_scaffold24069_1	non-phage	0
DOF007_scaffold21502_1	non-phage	0
DOF007_scaffold22924_4	non-phage	0
DOF007_C359869_1	non-phage	0
DOF007_scaffold25224_2	non-phage	0
DOF007_scaffold950_11	non-phage	0
DOF007_scaffold17463_1	non-phage	0
DOF007_scaffold4612_6	non-phage	0
DOF007_scaffold2934_12	non-phage	0
DOF008_scaffold6870_3	non-phage	0
DOF008_scaffold26266_6	non-phage	0
DOF008_C424045_1	non-phage	0
DOF008_scaffold20875_3	non-phage	0
DOF008_scaffold7905_4	non-phage	0
DOF008_scaffold7306_27	non-phage	0
DOF008_scaffold24126_2	non-phage	0
DOF008_scaffold1497_1	non-phage	0
DOF008_scaffold28862_1	non-phage	0.426
DOF008_scaffold20753_2	non-phage	0
DOF008_scaffold8744_2	non-phage	0
DOF008_scaffold17229_1	non-phage	0
DOF008_scaffold106_1	non-phage	0.001
DOF008_scaffold21913_1	non-phage	0
DOF008_scaffold27968_2	non-phage	0
DOF008_scaffold7961_1	non-phage	0
DOF008_scaffold27696_2	non-phage	0
DOF008_scaffold28029_2	phage	0.999
DOF008_C423573_1	non-phage	0
DOF008_scaffold9116_13	non-phage	0
DOF008_scaffold18392_4	non-phage	0
DOF008_scaffold14614_1	non-phage	0
DOF008_scaffold28963_1	non-phage	0
DOF008_scaffold7905_2	non-phage	0

DOF008_scaffold17289_6	non-phage	0
DOF008_scaffold235_1	non-phage	0
DOF008_scaffold27942_3	non-phage	0
DOF008_scaffold17121_3	non-phage	0
DOF008_scaffold413_3	non-phage	0
DOF008_scaffold11192_1	non-phage	0
DOF008_scaffold6646_1	non-phage	0
DOF008_scaffold5805_3	non-phage	0
DOF008_scaffold6870_4	non-phage	0
DOF008_scaffold4004_6	non-phage	0
DOF008_scaffold12563_3	non-phage	0
DOF008_scaffold11513_1	non-phage	0
DOF008_scaffold14204_2	non-phage	0
DOF008_scaffold62_5	non-phage	0
DOF008_scaffold20000_1	non-phage	0
DOF008_scaffold29025_1	non-phage	0
DOF008_scaffold2987_2	non-phage	0
DOF008_scaffold24351_1	non-phage	0
DOF008_scaffold9877_17	non-phage	0
DOF008_scaffold295_3	non-phage	0
DOF008_scaffold180_1	non-phage	0
DOF008_scaffold10762_1	non-phage	0
DOF008_scaffold26113_1	non-phage	0
DOF008_scaffold20326_4	non-phage	0
DOF008_scaffold15173_3	non-phage	0
DOF008_scaffold567_3	phage	0.882
DOF008_scaffold28948_2	non-phage	0
DOF008_scaffold13396_2	non-phage	0
DOF008_scaffold11489_5	non-phage	0
DOF008_scaffold27965_6	non-phage	0
DOF008_scaffold27123_3	non-phage	0
DOF008_scaffold28974_1	non-phage	0
DOF008_scaffold9416_1	non-phage	0
DOF008_scaffold17099_3	non-phage	0
DOF008_scaffold105_1	non-phage	0
DOF008_scaffold10823_4	non-phage	0
DOF008_scaffold1335_1	non-phage	0
DOF008_scaffold19294_1	non-phage	0
DOF008_scaffold10950_5	non-phage	0
DOF008_scaffold24510_2	non-phage	0
DOF008_scaffold1028_7	non-phage	0
DOF008_scaffold5495_1	non-phage	0
DOF008_scaffold232_5	non-phage	0
DOF008_scaffold14600_1	non-phage	0
DOF008_scaffold16005_2	non-phage	0
DOF008_scaffold19347_1	non-phage	0

DOF008_scaffold12283_12	non-phage	0
DOF008_scaffold138_1	non-phage	0
DOF008_scaffold10539_10	non-phage	0
DOF008_scaffold13657_3	non-phage	0
DOF008_scaffold140_2	non-phage	0
DOF008_scaffold11881_3	non-phage	0
DOF008_scaffold28702_2	non-phage	0
DOF008_scaffold15977_1	non-phage	0
DOF008_scaffold10372_6	non-phage	0
DOF008_scaffold734_6	non-phage	0
DOF008_scaffold20554_3	non-phage	0
DOF008_scaffold27940_2	non-phage	0
DOF008_scaffold4787_8	non-phage	0
DOF008_scaffold16025_6	non-phage	0
DOF008_scaffold19424_2	non-phage	0
DOF008_scaffold7816_9	non-phage	0
DOF008_scaffold1036_53	non-phage	0
DOF008_scaffold23551_1	non-phage	0
DOF008_scaffold5044_8	non-phage	0
DOF008_scaffold2744_1	non-phage	0
DOF008_scaffold21847_24	non-phage	0
DOF008_scaffold4846_9	non-phage	0
DOF008_C423151_1	non-phage	0.032
DOF008_scaffold2853_6	non-phage	0
DOF008_scaffold2177_4	non-phage	0
DOF008_scaffold18361_12	non-phage	0
DOF008_scaffold11362_6	non-phage	0
DOF008_C423815_1	non-phage	0
DOF008_scaffold65_1	non-phage	0
DOF008_scaffold114_2	non-phage	0
DOF008_scaffold29014_3	non-phage	0
DOF008_scaffold23723_1	non-phage	0
DOF008_scaffold24514_1	non-phage	0
DOF008_scaffold12743_2	non-phage	0
DOF008_scaffold10567_1	phage	0.993
DOF008_scaffold27965_2	non-phage	0
DOF008_scaffold28605_2	non-phage	0
DOF008_scaffold628_4	non-phage	0
DOF008_scaffold14777_6	non-phage	0
DOF008_scaffold28406_1	non-phage	0
DOF008_scaffold132_2	non-phage	0
DOF008_scaffold23440_4	non-phage	0
DOF008_scaffold27186_2	non-phage	0
DOF008_C423737_1	non-phage	0
DOF008_scaffold28831_1	non-phage	0
DOF008_scaffold9506_2	phage	0.999

DOF008_scaffold22139_3	non-phage	0
DOF008_scaffold26266_3	non-phage	0
DOF008_scaffold137_1	non-phage	0
DOF008_scaffold18003_1	non-phage	0
DOF008_scaffold13468_5	non-phage	0
DOF008_scaffold21871_4	non-phage	0
DOF008_scaffold122_1	non-phage	0
DOF008_scaffold232_8	non-phage	0
DOF008_scaffold4982_12	non-phage	0
DOF008_scaffold20472_1	non-phage	0
DOF008_scaffold159_5	non-phage	0
DOF008_scaffold13722_1	non-phage	0
DOF008_scaffold7802_5	non-phage	0
DOF008_scaffold552_5	non-phage	0
DOF008_scaffold4667_3	non-phage	0
DOF008_scaffold24470_3	non-phage	0
DOF008_scaffold19905_8	non-phage	0
DOF008_scaffold28459_1	non-phage	0
DOF008_scaffold28950_1	non-phage	0
DOF008_scaffold2455_1	non-phage	0
DOF008_scaffold8259_3	non-phage	0
DOF008_scaffold28975_2	non-phage	0
DOF008_scaffold25607_1	non-phage	0
DOF008_scaffold21335_1	non-phage	0
DOF008_scaffold22180_3	non-phage	0
DOF008_scaffold634_3	non-phage	0
DOF008_scaffold7905_5	non-phage	0
DOF008_scaffold1953_2	non-phage	0
DOF008_scaffold19808_5	non-phage	0
DOF008_scaffold8_1	non-phage	0
DOF008_scaffold19921_1	non-phage	0
DOF008_scaffold28967_1	non-phage	0
DOF008_scaffold628_1	non-phage	0
DOF008_scaffold15544_1	non-phage	0
DOF008_C422985_1	non-phage	0
DOF008_scaffold26865_3	non-phage	0
DOF008_scaffold22148_1	non-phage	0
DOF008_scaffold149_7	non-phage	0
DOF008_scaffold28029_3	non-phage	0.001
DOF008_scaffold14526_1	non-phage	0
DOF008_scaffold24939_1	non-phage	0
DOF008_scaffold17369_4	non-phage	0
DOF008_scaffold10317_6	non-phage	0
DOF008_scaffold9116_6	non-phage	0
DOF008_scaffold2853_2	non-phage	0
DOF008_scaffold7828_21	non-phage	0

DOF008_scaffold24993_7	non-phage	0
DOF008_scaffold15661_1	non-phage	0
DOF008_scaffold7444_2	non-phage	0
DOF008_scaffold10823_6	non-phage	0
DOF008_scaffold1802_1	non-phage	0.005
DOF008_scaffold7005_4	non-phage	0
DOF008_scaffold4011_4	phage	0.997
DOF008_scaffold26646_2	non-phage	0
DOF008_scaffold8384_2	non-phage	0
DOF008_scaffold28329_2	non-phage	0
DOF008_scaffold232_1	non-phage	0
DOF008_scaffold9116_10	non-phage	0
DOF008_scaffold629_6	non-phage	0
DOF008_scaffold21052_1	non-phage	0
DOF008_scaffold6757_2	non-phage	0
DOF008_scaffold15173_4	non-phage	0
DOF008_scaffold17343_1	non-phage	0
DOF008_scaffold9116_5	non-phage	0
DOF008_scaffold7021_6	non-phage	0
DOF008_scaffold19808_2	non-phage	0
DOF008_scaffold20890_1	non-phage	0
DOF008_scaffold13396_7	non-phage	0
DOF008_scaffold32_4	non-phage	0
DOF008_scaffold28965_1	non-phage	0
DOF008_scaffold20875_2	non-phage	0
DOF008_scaffold9444_8	non-phage	0
DOF008_scaffold18416_3	non-phage	0
DOF008_scaffold21275_2	non-phage	0
DOF008_scaffold28260_1	non-phage	0
DOF008_scaffold832_1	phage	0.883
DOF008_scaffold28708_2	non-phage	0
DOF008_scaffold13340_1	non-phage	0
DOF008_scaffold147_4	non-phage	0.006
DOF008_scaffold11628_2	non-phage	0
DOF008_scaffold1028_6	non-phage	0
DOF008_scaffold839_12	non-phage	0
DOF008_scaffold4846_5	non-phage	0
DOF008_scaffold10823_1	non-phage	0
DOF008_scaffold15345_6	non-phage	0
DOF008_scaffold17348_1	non-phage	0
DOF008_scaffold6554_3	non-phage	0
DOF008_scaffold19275_1	non-phage	0
DOF008_scaffold7799_3	non-phage	0
DOF008_scaffold6223_1	non-phage	0
DOF008_scaffold12318_1	non-phage	0
DOF008_scaffold26376_2	non-phage	0

DOF008_scaffold4717_3	non-phage	0
DOF008_scaffold20044_1	non-phage	0
DOF008_scaffold10883_2	non-phage	0.015
DOF008_scaffold28667_2	non-phage	0
DOF008_scaffold18581_2	non-phage	0
DOF008_scaffold21540_4	non-phage	0
DOF008_C423155_1	non-phage	0
DOF008_scaffold26197_1	non-phage	0
DOF008_scaffold10_1	non-phage	0
DOF008_scaffold28638_1	non-phage	0
DOF008_scaffold9444_18	non-phage	0
DOF008_scaffold13926_3	non-phage	0
DOF008_scaffold14401_5	non-phage	0
DOF008_scaffold94_1	non-phage	0
DOF008_scaffold27696_5	non-phage	0
DOF008_scaffold14400_1	non-phage	0
DOF008_scaffold7828_6	non-phage	0
DOF008_scaffold19395_5	non-phage	0
DOF008_scaffold2839_1	non-phage	0
DOF008_scaffold28982_3	non-phage	0
DOF008_scaffold8744_1	non-phage	0
DOF008_scaffold26277_2	non-phage	0
DOF008_scaffold29027_1	non-phage	0
DOF008_scaffold28485_2	phage	0.997
DOF008_scaffold5596_25	non-phage	0
DOF008_scaffold29028_1	non-phage	0
DOF008_scaffold8861_3	phage	0.991
DOF008_scaffold7828_4	non-phage	0
DOF008_scaffold27123_5	non-phage	0
DOF008_scaffold634_1	non-phage	0
DOF008_scaffold23951_1	non-phage	0
DOF008_scaffold20875_4	non-phage	0
DOF008_scaffold21339_3	non-phage	0
DOF008_scaffold3830_1	non-phage	0
DOF008_scaffold295_2	non-phage	0
DOF008_scaffold13535_1	non-phage	0
DOF008_scaffold901_1	non-phage	0
DOF008_C423957_1	non-phage	0
DOF008_scaffold20980_2	non-phage	0
DOF008_scaffold9915_1	non-phage	0
DOF008_scaffold23951_3	non-phage	0
DOF008_scaffold734_5	non-phage	0
DOF008_scaffold27968_1	non-phage	0
DOF008_scaffold20936_1	non-phage	0
DOF008_scaffold16171_4	non-phage	0
DOF008_scaffold11362_4	non-phage	0

DOF008_C423557_1	non-phage	0
DOF008_scaffold29022_2	non-phage	0
DOF008_scaffold16893_1	non-phage	0
DOF008_scaffold17367_2	non-phage	0.036
DOF008_scaffold19275_3	non-phage	0
DOF008_scaffold21718_1	non-phage	0
DOF008_scaffold18361_6	non-phage	0
DOF008_scaffold22148_2	non-phage	0
DOF008_scaffold13396_1	non-phage	0
DOF008_scaffold24991_2	non-phage	0
DOF008_scaffold19182_3	non-phage	0
DOF008_scaffold16566_3	non-phage	0
DOF008_scaffold21847_25	non-phage	0
DOF008_scaffold29013_1	non-phage	0
DOF008_C423887_1	non-phage	0
DOF008_scaffold40_5	non-phage	0
DOF008_scaffold15917_1	non-phage	0
DOF008_scaffold1_1	non-phage	0
DOF008_scaffold14818_12	non-phage	0
DOF008_scaffold14170_2	non-phage	0
DOF008_scaffold26906_2	non-phage	0
DOF008_scaffold11362_5	non-phage	0
DOF008_scaffold5495_2	non-phage	0
DOF008_scaffold28090_2	non-phage	0
DOF008_scaffold22139_2	non-phage	0
DOF008_scaffold19419_2	non-phage	0
DOF008_scaffold28579_2	non-phage	0
DOF008_scaffold832_11	non-phage	0
DOF008_scaffold18582_2	non-phage	0
DOF008_scaffold7518_1	non-phage	0
DOF008_scaffold10539_9	phage	0.941
DOF008_scaffold8866_2	non-phage	0
DOF008_scaffold17675_2	non-phage	0
DOF008_scaffold4846_4	non-phage	0
DOF008_scaffold12639_2	non-phage	0
DOF008_scaffold18733_2	non-phage	0
DOF008_scaffold15047_1	non-phage	0
DOF008_scaffold628_6	non-phage	0
DOF008_scaffold839_11	non-phage	0
DOF008_scaffold9116_12	non-phage	0
DOF008_scaffold7005_5	non-phage	0
DOF008_scaffold22144_5	non-phage	0
DOF008_scaffold21796_1	phage	0.997
DOF008_scaffold3750_2	non-phage	0
DOF008_scaffold27605_3	non-phage	0
DOF008_scaffold28325_2	non-phage	0

DOF008_scaffold10823_2	non-phage	0
DOF008_scaffold28698_1	phage	0.991
DOF008_scaffold20960_3	non-phage	0
DOF008_scaffold1953_3	non-phage	0
DOF008_scaffold28924_1	non-phage	0
DOF008_scaffold28638_4	non-phage	0
DOF008_scaffold10803_2	non-phage	0
DOF008_scaffold1886_1	non-phage	0
DOF008_scaffold13196_16	non-phage	0
DOF008_scaffold25628_4	non-phage	0
DOF008_scaffold20953_3	non-phage	0
DOF008_scaffold26020_1	phage	0.997
DOF008_scaffold1028_16	non-phage	0
DOF008_scaffold7021_3	non-phage	0
DOF008_scaffold29027_2	non-phage	0
DOF008_scaffold4846_13	non-phage	0
DOF008_scaffold2019_7	non-phage	0
DOF008_scaffold28968_1	non-phage	0
DOF008_scaffold18017_1	non-phage	0
DOF008_scaffold7961_4	non-phage	0
DOF008_scaffold629_7	non-phage	0
DOF008_scaffold7799_2	non-phage	0
DOF008_scaffold1_4	non-phage	0
DOF008_scaffold13955_1	non-phage	0
DOF008_scaffold11269_1	non-phage	0
DOF008_scaffold2448_1	non-phage	0
DOF008_scaffold901_4	non-phage	0
DOF008_scaffold21602_1	non-phage	0
DOF008_scaffold28624_1	non-phage	0
DOF008_scaffold18361_2	non-phage	0
DOF008_scaffold206_3	non-phage	0
DOF008_scaffold3_1	non-phage	0
DOF008_scaffold28543_1	non-phage	0
DOF008_scaffold2839_4	non-phage	0
DOF008_scaffold19396_1	phage	0.999
DOF008_scaffold21540_3	non-phage	0
DOF008_scaffold5596_2	non-phage	0
DOF008_C422951_1	non-phage	0
DOF008_scaffold16750_1	non-phage	0
DOF008_scaffold16025_4	non-phage	0
DOF008_scaffold4666_2	non-phage	0
DOF008_scaffold839_33	non-phage	0
DOF008_scaffold15173_5	non-phage	0
DOF008_scaffold29002_1	non-phage	0
DOF008_scaffold29012_1	non-phage	0
DOF008_scaffold13196_6	non-phage	0

DOF008_scaffold147_3	non-phage	0
DOF008_scaffold18361_10	non-phage	0
DOF008_scaffold28416_2	non-phage	0
DOF008_scaffold4846_6	non-phage	0
DOF008_scaffold28748_1	non-phage	0
DOF008_scaffold15850_3	non-phage	0
DOF008_scaffold628_2	non-phage	0
DOF008_scaffold4855_1	non-phage	0
DOF008_scaffold25859_1	non-phage	0
DOF008_scaffold16668_1	non-phage	0
DOF008_scaffold22562_6	non-phage	0
DOF008_scaffold3_4	non-phage	0
DOF008_scaffold26376_1	non-phage	0
DOF008_scaffold24483_1	phage	0.999
DOF008_scaffold19808_6	non-phage	0
DOF008_scaffold7212_3	non-phage	0
DOF008_scaffold28912_1	non-phage	0
DOF008_scaffold6554_2	non-phage	0
DOF008_scaffold11622_13	non-phage	0
DOF008_scaffold17347_1	non-phage	0
DOF008_scaffold22947_1	non-phage	0
DOF008_scaffold28667_1	non-phage	0
DOF008_scaffold18581_3	non-phage	0
DOF008_scaffold28374_2	non-phage	0
DOF008_scaffold14_3	non-phage	0
DOF008_C423725_1	non-phage	0
DOF008_scaffold5581_37	non-phage	0
DOF008_scaffold11130_3	non-phage	0
DOF008_scaffold29016_1	non-phage	0
DOF008_scaffold181_1	non-phage	0
DOF008_scaffold16171_1	non-phage	0
DOF008_scaffold1954_1	non-phage	0
DOF008_scaffold1859_9	non-phage	0
DOF008_scaffold20759_3	non-phage	0
DOF008_scaffold1640_6	non-phage	0
DOF008_scaffold19430_9	non-phage	0
DOF008_scaffold155_2	non-phage	0
DOF008_scaffold28756_1	non-phage	0
DOF008_scaffold10823_7	non-phage	0.001
DOF008_scaffold17099_2	non-phage	0
DOF008_C423667_1	non-phage	0
DOF008_scaffold25802_1	non-phage	0
DOF008_scaffold28749_1	non-phage	0
DOF008_scaffold9444_7	non-phage	0
DOF008_scaffold22778_1	non-phage	0
DOF008_scaffold16867_1	non-phage	0

DOF008_scaffold3293_23	non-phage	0
DOF008_scaffold19419_1	non-phage	0
DOF008_scaffold28504_3	non-phage	0
DOF008_scaffold23440_1	non-phage	0
DOF008_scaffold26757_1	non-phage	0
DOF008_scaffold28946_1	non-phage	0
DOF008_scaffold22139_5	non-phage	0
DOF008_scaffold769_3	non-phage	0
DOF008_scaffold29003_2	non-phage	0
DOF008_scaffold9877_19	non-phage	0
DOF008_scaffold6011_2	phage	0.95
DOF008_C423869_1	non-phage	0
DOF008_scaffold3252_4	non-phage	0
DOF008_scaffold26281_1	non-phage	0
DOF008_scaffold28964_2	non-phage	0
DOF008_scaffold7444_4	non-phage	0
DOF008_scaffold10950_2	non-phage	0
DOF008_scaffold29008_1	non-phage	0
DOF008_scaffold22927_4	non-phage	0
DOF008_scaffold21339_2	non-phage	0
DOF008_scaffold23747_1	non-phage	0
DOF008_scaffold14917_1	non-phage	0
DOF008_scaffold15887_1	non-phage	0
DOF008_scaffold11635_2	non-phage	0
DOF008_scaffold27754_4	non-phage	0
DOF008_scaffold19947_1	non-phage	0
DOF008_scaffold11084_1	non-phage	0
DOF008_scaffold9116_8	non-phage	0
DOF008_scaffold7828_15	non-phage	0
DOF008_scaffold12257_4	phage	0.999
DOF008_scaffold20759_1	non-phage	0
DOF008_scaffold15610_1	non-phage	0
DOF008_scaffold27696_4	non-phage	0
DOF008_scaffold18610_4	non-phage	0
DOF008_scaffold7520_1	non-phage	0
DOF008_scaffold29018_2	non-phage	0
DOF008_scaffold27431_1	non-phage	0
DOF008_scaffold26266_1	non-phage	0
DOF008_scaffold27028_1	non-phage	0
DOF008_scaffold17641_1	non-phage	0
DOF008_scaffold232_3	non-phage	0
DOF008_scaffold28825_1	non-phage	0
DOF008_scaffold13939_5	non-phage	0
DOF008_scaffold1028_3	phage	0.856
DOF008_scaffold29019_1	non-phage	0
DOF008_scaffold15898_4	non-phage	0

DOF008_scaffold29021_1	non-phage	0
DOF008_scaffold25427_1	phage	0.999
DOF008_scaffold8346_2	non-phage	0
DOF008_scaffold1181_5	non-phage	0
DOF008_scaffold629_5	non-phage	0
DOF008_scaffold1181_3	non-phage	0
DOF008_scaffold26080_1	non-phage	0
DOF008_scaffold913_7	non-phage	0
DOF008_scaffold27498_1	phage	0.987
DOF008_C423629_1	non-phage	0
DOF008_scaffold6312_10	non-phage	0
DOF008_scaffold24879_2	non-phage	0
DOF008_scaffold11345_1	non-phage	0
DOF008_scaffold28259_1	non-phage	0
DOF008_scaffold3830_3	non-phage	0
DOF008_scaffold22139_1	non-phage	0
DOF008_scaffold4013_1	non-phage	0
DOF008_scaffold171_2	non-phage	0
DOF008_scaffold8568_3	non-phage	0
DOF008_scaffold28995_1	non-phage	0
DOF008_scaffold4011_15	non-phage	0
DOF008_scaffold18582_1	non-phage	0
DOF008_scaffold2592_2	non-phage	0
DOF008_scaffold28694_1	non-phage	0
DOF008_scaffold110_2	phage	0.999
DOF008_scaffold28090_3	non-phage	0
DOF008_scaffold26807_2	non-phage	0
DOF008_scaffold26266_4	non-phage	0
DOF008_scaffold3293_11	non-phage	0
DOF008_scaffold28969_1	non-phage	0
DOF008_scaffold9116_11	non-phage	0
DOF008_scaffold10983_2	non-phage	0
DOF008_scaffold15835_3	non-phage	0
DOF008_scaffold18361_5	non-phage	0
DOF008_scaffold18613_12	non-phage	0
DOF008_scaffold17005_2	non-phage	0
DOF008_scaffold21630_2	non-phage	0
DOF008_scaffold9152_4	non-phage	0
DOF008_scaffold27372_5	non-phage	0
DOF008_scaffold28730_1	non-phage	0
DOF008_scaffold28605_1	non-phage	0
DOF008_scaffold154_2	non-phage	0
DOF008_scaffold18501_1	non-phage	0
DOF008_scaffold29022_3	non-phage	0
DOF008_scaffold3955_1	non-phage	0
DOF008_scaffold1859_7	non-phage	0

DOF008_scaffold4322_3	non-phage	0
DOF008_scaffold628_3	non-phage	0
DOF008_scaffold13926_1	non-phage	0
DOF008_scaffold629_1	non-phage	0
DOF008_scaffold28992_1	non-phage	0
DOF008_scaffold4413_1	non-phage	0
DOF008_scaffold4736_1	non-phage	0
DOF008_scaffold9444_19	non-phage	0
DOF008_scaffold17675_1	non-phage	0
DOF008_scaffold26483_2	non-phage	0
DOF008_scaffold26132_1	non-phage	0
DOF008_scaffold29009_1	non-phage	0
DOF008_scaffold131_16	non-phage	0
DOF008_scaffold29014_2	non-phage	0
DOF008_scaffold21275_4	non-phage	0
DOF008_scaffold10002_4	non-phage	0
DOF008_scaffold27605_2	non-phage	0
DOF008_scaffold28875_2	non-phage	0
DOF008_scaffold16596_2	non-phage	0
DOF008_scaffold29028_2	non-phage	0
DOF008_scaffold12563_5	non-phage	0
DOF008_scaffold3899_2	non-phage	0
DOF008_C423597_1	phage	0.83
DOF008_scaffold28614_1	non-phage	0
DOF008_scaffold13955_3	non-phage	0
DOF008_scaffold6947_2	non-phage	0.001
DOF008_scaffold201_2	non-phage	0
DOF008_C423775_1	non-phage	0
DOF008_scaffold10823_8	non-phage	0.004
DOF008_scaffold15073_1	non-phage	0
DOF008_scaffold16198_6	non-phage	0
DOF008_scaffold6885_2	non-phage	0
DOF008_scaffold2469_10	phage	0.937
DOF008_scaffold734_3	non-phage	0.2
DOF008_scaffold3208_2	non-phage	0
DOF008_scaffold29006_1	non-phage	0
DOF008_scaffold22927_8	non-phage	0
DOF008_scaffold29011_2	non-phage	0
DOF008_scaffold22286_2	non-phage	0
DOF008_scaffold67_1	non-phage	0
DOF008_C423489_1	non-phage	0
DOF008_scaffold78_2	non-phage	0
DOF008_scaffold19808_7	phage	0.936
DOF008_scaffold2539_2	non-phage	0
DOF008_scaffold22834_2	non-phage	0
DOF008_scaffold34_5	non-phage	0

DOF008_scaffold162_2	non-phage	0
DOF008_scaffold5851_1	non-phage	0
DOF008_scaffold25170_1	non-phage	0
DOF008_scaffold15962_2	non-phage	0
DOF008_scaffold24092_1	non-phage	0
DOF008_scaffold6808_22	non-phage	0
DOF008_scaffold4004_10	non-phage	0
DOF008_scaffold84_1	non-phage	0
DOF008_scaffold24436_5	non-phage	0
DOF008_scaffold3885_1	non-phage	0
DOF008_scaffold11521_3	non-phage	0
DOF008_scaffold9550_1	non-phage	0
DOF008_scaffold25859_2	non-phage	0
DOF008_scaffold10823_3	non-phage	0
DOF008_scaffold4409_4	non-phage	0
DOF008_scaffold20004_1	non-phage	0
DOF008_scaffold7799_1	non-phage	0
DOF008_scaffold28029_5	non-phage	0
DOF008_scaffold4666_1	non-phage	0
DOF008_scaffold11299_2	non-phage	0
DOF008_scaffold15067_1	non-phage	0
DOF008_scaffold20955_2	non-phage	0
DOF008_scaffold7021_2	non-phage	0
DOF008_scaffold29015_1	phage	0.999
DOF008_scaffold26906_1	non-phage	0
DOF008_scaffold28104_1	non-phage	0
DOF008_scaffold9550_5	non-phage	0
DOF008_scaffold1640_3	non-phage	0
DOF008_scaffold20347_2	non-phage	0
DOF008_scaffold901_5	non-phage	0
DOF008_scaffold87_2	non-phage	0
DOF008_scaffold16005_1	non-phage	0
DOF008_scaffold13468_7	non-phage	0
DOF008_scaffold28910_1	non-phage	0
DOF008_scaffold18361_1	non-phage	0
DOF008_C422959_1	non-phage	0
DOF008_scaffold132_4	non-phage	0
DOF008_scaffold9766_5	non-phage	0
DOF008_scaffold7961_3	non-phage	0
DOF008_scaffold10470_2	non-phage	0
DOF008_scaffold3249_1	non-phage	0
DOF008_scaffold6055_6	non-phage	0
DOF008_scaffold18487_4	non-phage	0
DOF008_scaffold28472_1	non-phage	0
DOF008_scaffold25802_2	non-phage	0
DOF008_scaffold12563_2	non-phage	0

DOF008_scaffold17695_1	non-phage	0
DOF008_scaffold32_1	non-phage	0
DOF008_scaffold4846_7	non-phage	0
DOF008_scaffold17995_1	non-phage	0
DOF008_scaffold4322_11	non-phage	0
DOF008_scaffold26266_8	non-phage	0
DOF008_scaffold28293_3	non-phage	0
DOF008_scaffold20980_3	non-phage	0
DOF008_scaffold5778_9	non-phage	0
DOF008_scaffold19430_10	non-phage	0
DOF008_scaffold12257_5	phage	0.999
DOF008_scaffold7816_15	non-phage	0
DOF008_scaffold10950_3	non-phage	0
DOF008_scaffold3899_3	non-phage	0
DOF008_scaffold2071_1	non-phage	0
DOF008_scaffold5856_4	phage	0.998
DOF008_scaffold1_2	non-phage	0
DOF008_scaffold6033_1	non-phage	0
DOF008_scaffold7905_3	non-phage	0
DOF008_scaffold9444_11	non-phage	0
DOF008_scaffold644_1	non-phage	0
DOF008_scaffold22927_1	non-phage	0
DOF008_scaffold28029_1	phage	1
DOF008_scaffold13318_2	non-phage	0
DOF008_scaffold13396_8	non-phage	0
DOF008_scaffold7805_1	non-phage	0
DOF008_scaffold16092_4	phage	0.982
DOF008_C423647_1	non-phage	0
DOF008_scaffold14245_2	non-phage	0
DOF008_scaffold18581_1	non-phage	0
DOF008_scaffold14424_1	non-phage	0
DOF008_scaffold27793_1	phage	0.999
DOF008_scaffold23661_3	non-phage	0
DOF008_scaffold28963_2	non-phage	0
DOF008_scaffold28801_1	non-phage	0
DOF008_scaffold12563_4	non-phage	0
DOF008_scaffold1_3	non-phage	0
DOF008_scaffold6142_1	non-phage	0
DOF008_scaffold7914_3	non-phage	0
DOF008_scaffold19543_1	non-phage	0
DOF008_scaffold9444_4	non-phage	0
DOF008_scaffold469_1	non-phage	0
DOF008_scaffold27942_2	non-phage	0
DOF008_scaffold4004_5	non-phage	0
DOF008_scaffold24774_6	non-phage	0
DOF008_scaffold4720_2	non-phage	0

DOF008_scaffold21275_5	non-phage	0
DOF008_scaffold629_2	non-phage	0
DOF008_scaffold5122_5	non-phage	0
DOF008_scaffold2400_2	non-phage	0.007
DOF008_scaffold147_1	non-phage	0
DOF008_scaffold28906_1	non-phage	0
DOF008_scaffold29010_1	non-phage	0
DOF008_scaffold1859_22	non-phage	0
DOF008_scaffold24013_1	non-phage	0
DOF008_scaffold28966_1	non-phage	0
DOF008_scaffold62_4	non-phage	0
DOF008_scaffold9877_23	non-phage	0
DOF008_scaffold28983_1	non-phage	0
DOF008_scaffold21540_6	phage	0.796
DOF008_scaffold19978_15	non-phage	0
DOF008_scaffold3208_6	non-phage	0
DOF008_scaffold17006_1	phage	0.827
DOF008_scaffold22927_3	non-phage	0
DOF008_scaffold19978_2	non-phage	0
DOF008_scaffold15837_4	non-phage	0
DOF008_C423795_1	non-phage	0
DOF008_C423835_1	non-phage	0
DOF008_scaffold14600_2	non-phage	0
DOF008_scaffold21590_1	non-phage	0
DOF008_scaffold22972_4	non-phage	0
DOF008_scaffold913_5	non-phage	0
DOF008_scaffold2853_5	non-phage	0.326
DOF008_scaffold27123_2	non-phage	0
DOF008_scaffold22834_4	non-phage	0
DOF008_scaffold24599_1	non-phage	0
DOF008_scaffold23691_1	non-phage	0
DOF008_scaffold13196_8	non-phage	0
DOF008_scaffold24697_8	non-phage	0
DOF008_scaffold20283_2	non-phage	0
DOF008_scaffold5778_21	non-phage	0
DOF008_scaffold11489_6	non-phage	0
DOF008_scaffold3173_10	non-phage	0
DOF008_scaffold3454_1	non-phage	0
DOF008_scaffold9116_14	non-phage	0.014
DOF008_scaffold26483_1	non-phage	0
DOF008_scaffold3454_3	non-phage	0
DOF008_scaffold14484_1	non-phage	0
DOF008_scaffold13652_2	non-phage	0
DOF008_scaffold13870_6	non-phage	0
DOF008_scaffold10818_2	non-phage	0
DOF008_scaffold27696_3	non-phage	0

DOF008_scaffold629_9	non-phage	0
DOF008_scaffold302_4	non-phage	0
DOF008_scaffold10484_11	non-phage	0
DOF008_scaffold25689_3	non-phage	0
DOF008_scaffold295_1	non-phage	0
DOF008_scaffold19129_1	non-phage	0
DOF008_scaffold11881_4	non-phage	0
DOF008_C423463_1	non-phage	0
DOF008_scaffold24727_1	non-phage	0
DOF008_scaffold28933_1	non-phage	0
DOF008_scaffold21275_1	non-phage	0
DOF008_scaffold27605_1	non-phage	0
DOF008_scaffold26336_1	non-phage	0
DOF008_scaffold22180_2	non-phage	0
DOF008_scaffold29026_1	non-phage	0
DOF008_scaffold18613_2	non-phage	0
DOF008_scaffold29024_1	non-phage	0
DOF008_scaffold19175_1	non-phage	0
DOF008_scaffold26756_1	non-phage	0
DOF008_scaffold3759_2	non-phage	0
DOF008_scaffold28614_2	non-phage	0
DOF008_scaffold26647_1	non-phage	0
DOF008_scaffold7816_11	non-phage	0
DOF008_scaffold34_1	non-phage	0
DOF008_scaffold4011_8	non-phage	0
DOF008_scaffold9372_1	non-phage	0
DOF008_scaffold9752_1	non-phage	0
DOF008_scaffold23655_2	non-phage	0
DOF008_scaffold15173_2	phage	0.988
DOF008_scaffold23431_1	non-phage	0
DOF008_scaffold28997_1	non-phage	0
DOF008_scaffold26266_5	non-phage	0
DOF008_scaffold12840_2	non-phage	0
DOF008_scaffold11084_2	non-phage	0
DOF008_scaffold1869_1	non-phage	0
DOF008_scaffold19430_6	non-phage	0
DOF008_scaffold4846_12	non-phage	0
DOF008_scaffold24168_1	non-phage	0
DOF008_scaffold15835_2	non-phage	0
DOF008_scaffold5558_5	non-phage	0
DOF008_C423583_1	non-phage	0
DOF008_scaffold21339_1	non-phage	0
DOF008_scaffold18362_6	non-phage	0
DOF008_scaffold25859_3	non-phage	0
DOF008_scaffold8713_1	non-phage	0
DOF008_scaffold28871_1	non-phage	0

DOF008_scaffold28955_1	non-phage	0
DOF008_scaffold19430_11	non-phage	0
DOF008_scaffold189_2	non-phage	0
DOF008_scaffold9116_4	non-phage	0
DOF008_scaffold14701_2	non-phage	0
DOF008_scaffold27896_3	non-phage	0
DOF008_scaffold3469_8	non-phage	0
DOF008_scaffold7349_1	non-phage	0
DOF008_scaffold9116_1	non-phage	0
DOF008_scaffold9668_3	non-phage	0
DOF008_scaffold12370_3	non-phage	0
DOF008_scaffold29023_2	non-phage	0
DOF008_scaffold12610_2	non-phage	0
DOF008_scaffold27123_1	non-phage	0
DOF008_scaffold13657_1	non-phage	0
DOF008_scaffold14818_1	non-phage	0
DOF008_scaffold2839_2	non-phage	0
DOF008_scaffold263_2	non-phage	0
DOF008_scaffold15965_1	non-phage	0
DOF008_scaffold5044_9	non-phage	0
DOF008_scaffold19808_4	non-phage	0
DOF008_scaffold24192_1	non-phage	0
DOF008_scaffold4353_10	non-phage	0
DOF008_scaffold28492_2	non-phage	0
DOF008_scaffold628_7	non-phage	0
DOF008_scaffold28431_1	non-phage	0
DOF008_scaffold2068_1	non-phage	0
DOF008_scaffold10_3	non-phage	0
DOF008_scaffold23723_2	non-phage	0
DOF008_scaffold232_7	phage	0.997
DOF008_scaffold13923_4	non-phage	0
DOF008_C423975_1	phage	0.999
DOF008_scaffold41_10	non-phage	0
DOF008_scaffold4004_9	non-phage	0
DOF008_scaffold25346_2	non-phage	0
DOF008_scaffold6055_2	non-phage	0
DOF008_scaffold15850_2	non-phage	0
DOF008_scaffold7388_2	non-phage	0
DOF008_scaffold5122_2	non-phage	0
DOF008_scaffold635_3	non-phage	0
DOF008_scaffold17096_2	non-phage	0
DOF008_scaffold12350_1	non-phage	0
DOF008_scaffold16116_1	non-phage	0
DOF008_scaffold3208_7	non-phage	0
DOF008_C423181_1	non-phage	0
DOF008_scaffold162_1	non-phage	0

DOF008_scaffold101_1	non-phage	0
DOF008_scaffold19028_3	non-phage	0
DOF008_scaffold9444_1	non-phage	0
DOF008_scaffold25233_1	non-phage	0
DOF008_scaffold19808_1	non-phage	0
DOF008_scaffold17695_2	non-phage	0
DOF008_scaffold11995_2	non-phage	0
DOF008_scaffold28998_1	non-phage	0
DOF008_scaffold7828_2	non-phage	0
DOF008_scaffold28970_1	non-phage	0
DOF008_scaffold147_5	non-phage	0.127
DOF008_scaffold245_1	non-phage	0
DOF008_scaffold28975_1	non-phage	0
DOF008_scaffold13691_6	non-phage	0
DOF008_C424023_1	non-phage	0
DOF008_scaffold12563_1	non-phage	0
DOF008_scaffold24697_4	non-phage	0
DOF008_scaffold24952_1	non-phage	0
DOF008_scaffold29011_1	non-phage	0
DOF008_scaffold27422_2	non-phage	0
DOF008_scaffold14739_1	non-phage	0
DOF008_scaffold4218_2	non-phage	0
DOF008_scaffold28977_1	non-phage	0
DOF008_scaffold21847_18	non-phage	0
DOF008_scaffold11874_3	non-phage	0
DOF008_scaffold13519_2	non-phage	0
DOF008_scaffold16025_2	non-phage	0
DOF008_scaffold21170_2	non-phage	0
DOF008_scaffold21540_5	non-phage	0
DOF008_scaffold16092_3	non-phage	0
DOF008_scaffold113_1	non-phage	0
DOF008_scaffold27696_1	non-phage	0
DOF008_scaffold562_3	non-phage	0
DOF008_scaffold132_3	non-phage	0
DOF008_scaffold28708_4	non-phage	0
DOF008_scaffold4312_3	non-phage	0
DOF008_scaffold13318_1	non-phage	0
DOF008_scaffold12840_1	non-phage	0
DOF008_scaffold20980_4	non-phage	0
DOF008_scaffold7961_2	non-phage	0
DOF008_scaffold4220_3	non-phage	0
DOF008_scaffold28791_2	non-phage	0
DOF008_scaffold24939_2	non-phage	0
DOF008_scaffold7487_2	non-phage	0
DOF008_scaffold26970_1	non-phage	0
DOF008_scaffold6011_1	non-phage	0

DOF008_scaffold3252_3	non-phage	0
DOF008_scaffold29000_1	non-phage	0
DOF008_scaffold24092_2	non-phage	0
DOF008_scaffold23951_2	non-phage	0
DOF008_scaffold17645_1	non-phage	0
DOF008_scaffold26646_1	non-phage	0
DOF008_scaffold28815_2	non-phage	0
DOF008_scaffold2177_22	non-phage	0
DOF008_scaffold29020_1	non-phage	0
DOF008_scaffold23661_4	non-phage	0
DOF008_scaffold62_3	non-phage	0
DOF008_scaffold1481_7	non-phage	0
DOF008_scaffold6066_1	non-phage	0
DOF008_scaffold28194_3	non-phage	0
DOF008_scaffold4416_4	non-phage	0
DOF008_scaffold9675_26	non-phage	0
DOF009_scaffold52603_2	non-phage	0
DOF009_scaffold206_8	non-phage	0.01
DOF009_scaffold33479_2	non-phage	0
DOF009_scaffold32815_3	non-phage	0
DOF009_scaffold32367_15	non-phage	0
DOF009_scaffold25500_1	non-phage	0
DOF009_scaffold43577_1	non-phage	0
DOF009_scaffold20563_4	non-phage	0
DOF009_scaffold12352_14	non-phage	0
DOF009_scaffold25733_2	non-phage	0
DOF009_scaffold28090_1	non-phage	0
DOF009_scaffold1421_1	non-phage	0
DOF009_scaffold12961_4	non-phage	0
DOF009_scaffold8510_12	non-phage	0
DOF009_scaffold329_26	non-phage	0.001
DOF009_scaffold23855_4	non-phage	0
DOF009_scaffold13576_13	non-phage	0
DOF009_scaffold51602_1	non-phage	0
DOF009_scaffold25733_9	non-phage	0
DOF009_scaffold29425_4	non-phage	0
DOF009_scaffold46834_2	non-phage	0
DOF009_scaffold8299_14	non-phage	0
DOF009_scaffold43955_2	non-phage	0
DOF009_scaffold483_6	non-phage	0
DOF009_scaffold52084_1	non-phage	0
DOF009_scaffold2829_1	non-phage	0
DOF009_scaffold49130_7	non-phage	0
DOF009_scaffold10528_4	non-phage	0
DOF009_scaffold47785_2	non-phage	0
DOF009_scaffold46251_1	non-phage	0

DOF009_scaffold2408_22	phage	0.998
DOF009_scaffold48_9	non-phage	0
DOF009_scaffold43203_3	non-phage	0
DOF009_scaffold50496_1	non-phage	0
DOF009_scaffold8280_3	non-phage	0
DOF009_scaffold39557_1	non-phage	0
DOF009_scaffold48_2	non-phage	0
DOF009_scaffold31_8	non-phage	0
DOF009_scaffold37373_4	non-phage	0
DOF009_scaffold30444_1	non-phage	0
DOF009_scaffold40393_10	non-phage	0
DOF009_scaffold49230_3	non-phage	0
DOF009_scaffold52682_2	non-phage	0
DOF009_scaffold1543_10	non-phage	0
DOF009_scaffold201_54	non-phage	0
DOF009_scaffold52853_1	non-phage	0
DOF009_scaffold7284_4	non-phage	0
DOF009_scaffold4229_6	non-phage	0
DOF009_scaffold31368_2	non-phage	0
DOF009_scaffold12352_15	non-phage	0
DOF009_scaffold37864_3	non-phage	0
DOF009_scaffold47084_2	non-phage	0
DOF009_scaffold1543_11	non-phage	0
DOF009_scaffold21_2	non-phage	0
DOF009_scaffold24832_2	non-phage	0
DOF009_scaffold2526_1	non-phage	0
DOF009_scaffold9141_20	non-phage	0
DOF009_scaffold1543_9	non-phage	0
DOF009_scaffold6348_1	non-phage	0
DOF009_scaffold41189_5	non-phage	0
DOF009_scaffold52016_4	non-phage	0
DOF009_scaffold82_2	non-phage	0
DOF009_scaffold10076_2	non-phage	0
DOF009_scaffold44041_2	non-phage	0
DOF009_scaffold6515_15	non-phage	0
DOF009_scaffold39698_2	non-phage	0
DOF009_scaffold51777_1	non-phage	0
DOF009_scaffold142_9	non-phage	0
DOF009_scaffold51062_1	non-phage	0
DOF009_scaffold35950_2	non-phage	0
DOF009_scaffold29_8	non-phage	0
DOF009_scaffold46390_3	non-phage	0
DOF009_scaffold61_1	non-phage	0
DOF009_scaffold42486_10	non-phage	0
DOF009_scaffold34694_2	non-phage	0
DOF009_scaffold16613_3	non-phage	0

DOF009_scaffold22475_10	non-phage	0
DOF009_scaffold52340_2	non-phage	0
DOF009_scaffold42774_5	non-phage	0
DOF009_scaffold52436_3	non-phage	0
DOF009_scaffold8571_3	non-phage	0
DOF009_scaffold42497_3	non-phage	0.243
DOF009_scaffold52814_1	non-phage	0
DOF009_scaffold40522_2	non-phage	0
DOF009_scaffold52805_1	non-phage	0
DOF009_scaffold48067_2	non-phage	0
DOF009_scaffold52831_2	non-phage	0
DOF009_scaffold51915_1	non-phage	0
DOF009_scaffold44695_2	non-phage	0
DOF009_scaffold52770_1	non-phage	0
DOF009_scaffold37749_1	non-phage	0
DOF009_scaffold47963_3	non-phage	0
DOF009_scaffold240_1	non-phage	0
DOF009_scaffold50495_2	non-phage	0
DOF009_scaffold31_4	non-phage	0
DOF009_scaffold42629_4	non-phage	0
DOF009_scaffold50496_5	non-phage	0
DOF009_scaffold45042_3	non-phage	0
DOF009_scaffold25733_5	non-phage	0
DOF009_scaffold52827_3	non-phage	0
DOF009_scaffold42551_2	non-phage	0
DOF009_scaffold40393_12	phage	0.99
DOF009_scaffold42858_2	phage	0.997
DOF009_scaffold31038_1	non-phage	0
DOF009_scaffold3896_3	non-phage	0
DOF009_scaffold38944_2	non-phage	0
DOF009_scaffold52303_1	non-phage	0
DOF009_scaffold46262_2	non-phage	0
DOF009_scaffold52593_1	non-phage	0
DOF009_scaffold39557_5	non-phage	0
DOF009_scaffold41372_1	non-phage	0
DOF009_scaffold334_1	non-phage	0
DOF009_scaffold9686_1	non-phage	0
DOF009_scaffold2526_4	non-phage	0
DOF009_scaffold2829_4	non-phage	0
DOF009_scaffold229_3	non-phage	0
DOF009_scaffold47008_1	non-phage	0
DOF009_scaffold19796_7	non-phage	0
DOF009_scaffold33552_6	non-phage	0
DOF009_scaffold53_4	non-phage	0
DOF009_scaffold15880_7	non-phage	0
DOF009_scaffold3536_3	non-phage	0

DOF009_scaffold25904_1	non-phage	0
DOF009_scaffold7772_40	non-phage	0
DOF009_scaffold40047_2	non-phage	0.012
DOF009_scaffold3896_7	non-phage	0
DOF009_scaffold4008_23	non-phage	0
DOF009_scaffold16216_2	non-phage	0
DOF009_scaffold41357_2	non-phage	0
DOF009_scaffold43234_1	non-phage	0
DOF009_scaffold35271_5	non-phage	0
DOF009_scaffold50193_2	non-phage	0
DOF009_scaffold3896_11	non-phage	0
DOF009_scaffold50007_1	non-phage	0
DOF009_scaffold44542_3	non-phage	0
DOF009_scaffold47435_1	non-phage	0
DOF009_scaffold970_22	non-phage	0
DOF009_scaffold21950_2	non-phage	0
DOF009_scaffold22475_14	non-phage	0
DOF009_scaffold4073_1	non-phage	0
DOF009_scaffold155_13	phage	0.999
DOF009_scaffold33347_1	non-phage	0
DOF009_scaffold43460_1	non-phage	0
DOF009_scaffold52796_3	non-phage	0
DOF009_scaffold52243_1	non-phage	0
DOF009_scaffold52243_3	non-phage	0
DOF009_scaffold184_2	non-phage	0
DOF009_scaffold47084_6	non-phage	0
DOF009_scaffold29_4	non-phage	0
DOF009_scaffold51162_4	non-phage	0
DOF009_scaffold49400_2	non-phage	0
DOF009_scaffold52604_1	non-phage	0
DOF009_scaffold52042_2	non-phage	0
DOF009_scaffold22634_3	non-phage	0
DOF009_scaffold19744_1	non-phage	0
DOF009_scaffold7219_4	non-phage	0
DOF009_scaffold22404_7	non-phage	0
DOF009_scaffold29566_1	non-phage	0
DOF009_scaffold23505_1	non-phage	0
DOF009_scaffold52826_1	phage	1
DOF009_scaffold42567_2	non-phage	0
DOF009_scaffold38608_1	non-phage	0
DOF009_scaffold52777_1	non-phage	0
DOF009_scaffold6375_1	non-phage	0
DOF009_scaffold71_1	non-phage	0
DOF009_scaffold40864_2	non-phage	0
DOF009_scaffold1543_12	non-phage	0
DOF009_scaffold49497_1	non-phage	0

DOF009_scaffold13271_2	non-phage	0
DOF009_scaffold50900_3	non-phage	0
DOF009_scaffold42486_11	non-phage	0
DOF009_scaffold31975_2	non-phage	0
DOF009_scaffold17003_2	non-phage	0
DOF009_scaffold52459_1	phage	0.995
DOF009_scaffold42774_4	non-phage	0
DOF009_scaffold13072_2	non-phage	0
DOF009_scaffold24588_2	non-phage	0
DOF009_scaffold4754_9	non-phage	0
DOF009_scaffold246_4	non-phage	0
DOF009_scaffold47758_1	non-phage	0
DOF009_scaffold47084_5	non-phage	0
DOF009_scaffold16932_1	non-phage	0
DOF009_scaffold1896_4	non-phage	0
DOF009_scaffold52603_1	non-phage	0
DOF009_scaffold33616_3	non-phage	0
DOF009_scaffold295_2	non-phage	0
DOF009_scaffold13964_1	non-phage	0
DOF009_scaffold30444_2	non-phage	0
DOF009_scaffold52682_1	non-phage	0
DOF009_scaffold36280_2	non-phage	0
DOF009_scaffold41189_2	non-phage	0
DOF009_scaffold36176_3	non-phage	0
DOF009_scaffold52145_1	non-phage	0
DOF009_scaffold47876_1	non-phage	0
DOF009_scaffold39785_5	non-phage	0
DOF009_scaffold17458_2	non-phage	0
DOF009_scaffold35866_1	non-phage	0
DOF009_scaffold2252_6	non-phage	0
DOF009_scaffold38260_3	non-phage	0
DOF009_scaffold48940_3	non-phage	0
DOF009_scaffold8571_19	non-phage	0
DOF009_scaffold38877_1	non-phage	0
DOF009_scaffold38271_1	non-phage	0
DOF009_scaffold37444_3	non-phage	0
DOF009_scaffold48797_2	non-phage	0
DOF009_scaffold39856_9	phage	0.987
DOF009_scaffold47785_1	non-phage	0
DOF009_scaffold8713_8	non-phage	0
DOF009_scaffold6167_7	non-phage	0
DOF009_scaffold142_1	non-phage	0
DOF009_scaffold5974_1	non-phage	0
DOF009_scaffold3899_14	non-phage	0
DOF009_scaffold43174_9	phage	0.936
DOF009_scaffold47240_1	non-phage	0

DOF009_scaffold38516_3	non-phage	0
DOF009_scaffold43174_2	non-phage	0
DOF009_scaffold1_1	non-phage	0
DOF009_scaffold5133_9	non-phage	0
DOF009_scaffold51088_7	non-phage	0
DOF009_scaffold13576_7	non-phage	0
DOF009_scaffold4229_7	non-phage	0
DOF009_scaffold22475_18	phage	0.988
DOF009_scaffold206_2	non-phage	0
DOF009_scaffold52607_1	non-phage	0
DOF009_scaffold12630_10	non-phage	0
DOF009_scaffold336_1	non-phage	0
DOF009_scaffold18399_6	non-phage	0
DOF009_scaffold51124_3	non-phage	0
DOF009_scaffold3176_5	non-phage	0
DOF009_scaffold52799_1	non-phage	0
DOF009_scaffold47159_4	phage	0.999
DOF009_scaffold46992_2	non-phage	0
DOF009_scaffold1543_1	non-phage	0
DOF009_scaffold31_6	non-phage	0
DOF009_scaffold328_1	non-phage	0
DOF009_scaffold44041_3	non-phage	0
DOF009_scaffold12352_16	non-phage	0
DOF009_scaffold1543_6	non-phage	0
DOF009_scaffold19059_3	non-phage	0
DOF009_scaffold46079_1	non-phage	0
DOF009_scaffold19751_10	non-phage	0
DOF009_scaffold52423_2	non-phage	0
DOF009_scaffold52756_2	non-phage	0
DOF009_scaffold25733_14	non-phage	0
DOF009_scaffold53_1	non-phage	0
DOF009_scaffold10519_2	non-phage	0
DOF009_scaffold52016_3	non-phage	0
DOF009_scaffold38730_3	non-phage	0
DOF009_scaffold3127_5	non-phage	0
DOF009_scaffold3896_9	non-phage	0
DOF009_scaffold35866_2	non-phage	0
DOF009_scaffold46839_2	phage	0.997
DOF009_scaffold16_3	non-phage	0
DOF009_scaffold229_2	non-phage	0
DOF009_scaffold38944_3	non-phage	0
DOF009_scaffold48035_5	non-phage	0
DOF009_scaffold42497_2	non-phage	0
DOF009_scaffold17003_1	non-phage	0
DOF009_scaffold65_3	non-phage	0
DOF009_scaffold50997_3	non-phage	0

DOF009_scaffold48067_1	non-phage	0
DOF009_scaffold17248_18	non-phage	0
DOF009_scaffold52161_1	non-phage	0
DOF009_scaffold3127_2	phage	1
DOF009_scaffold30444_3	non-phage	0
DOF009_scaffold18060_6	non-phage	0
DOF009_scaffold22634_4	non-phage	0
DOF009_scaffold124_1	non-phage	0
DOF009_scaffold29_1	non-phage	0
DOF009_scaffold48247_1	non-phage	0
DOF009_scaffold32471_3	non-phage	0
DOF009_scaffold38730_6	non-phage	0
DOF009_scaffold49894_1	non-phage	0
DOF009_scaffold22475_21	non-phage	0
DOF009_scaffold25970_1	non-phage	0
DOF009_scaffold45315_1	non-phage	0
DOF009_scaffold52159_2	phage	0.782
DOF009_scaffold48543_2	non-phage	0
DOF009_scaffold31368_4	non-phage	0
DOF009_scaffold50496_4	non-phage	0
DOF009_scaffold51963_1	non-phage	0
DOF009_scaffold45109_14	non-phage	0
DOF009_scaffold22874_5	non-phage	0
DOF009_scaffold31104_2	non-phage	0
DOF009_scaffold50056_1	non-phage	0
DOF009_scaffold52722_1	non-phage	0
DOF009_scaffold52581_2	non-phage	0
DOF009_scaffold51879_1	non-phage	0
DOF009_scaffold52852_2	non-phage	0
DOF009_scaffold3896_2	non-phage	0
DOF009_scaffold12228_13	non-phage	0
DOF009_scaffold52243_2	non-phage	0
DOF009_scaffold3480_6	non-phage	0
DOF009_scaffold2426_10	non-phage	0
DOF009_scaffold52042_1	non-phage	0
DOF009_scaffold39122_2	non-phage	0
DOF009_scaffold42566_2	non-phage	0
DOF009_scaffold51162_3	non-phage	0
DOF009_scaffold18712_11	non-phage	0
DOF009_scaffold37865_1	non-phage	0
DOF009_scaffold261_1	non-phage	0
DOF009_scaffold270_1	non-phage	0
DOF009_scaffold14859_9	non-phage	0
DOF009_scaffold48_12	non-phage	0
DOF009_scaffold50960_2	non-phage	0
DOF009_scaffold1907_10	non-phage	0

DOF009_scaffold7219_5	non-phage	0
DOF009_scaffold32106_1	non-phage	0
DOF009_scaffold33479_3	non-phage	0
DOF009_scaffold52044_3	non-phage	0
DOF009_scaffold3423_1	non-phage	0
DOF009_scaffold53_5	non-phage	0
DOF009_scaffold206_7	non-phage	0
DOF009_scaffold5711_4	non-phage	0
DOF009_scaffold32812_4	non-phage	0
DOF009_scaffold329_34	non-phage	0
DOF009_scaffold52759_2	non-phage	0
DOF009_scaffold17931_1	non-phage	0
DOF009_scaffold47485_1	non-phage	0
DOF009_scaffold29226_15	non-phage	0
DOF009_scaffold7284_1	non-phage	0
DOF009_scaffold3896_10	non-phage	0
DOF009_scaffold42551_3	non-phage	0
DOF009_scaffold52743_1	non-phage	0
DOF009_scaffold42767_3	phage	0.622
DOF009_scaffold24588_5	non-phage	0
DOF009_scaffold970_38	non-phage	0
DOF009_scaffold30444_7	non-phage	0
DOF009_scaffold21307_1	non-phage	0
DOF009_scaffold25904_9	non-phage	0
DOF009_scaffold9001_9	non-phage	0
DOF009_scaffold20848_4	non-phage	0
DOF009_scaffold23914_3	non-phage	0
DOF009_scaffold25555_3	non-phage	0
DOF009_scaffold29382_2	non-phage	0
DOF009_scaffold51698_5	non-phage	0
DOF009_scaffold43425_1	non-phage	0
DOF009_scaffold14185_1	non-phage	0
DOF009_scaffold48543_5	non-phage	0
DOF009_scaffold24782_1	non-phage	0
DOF009_scaffold16613_1	phage	0.993
DOF009_scaffold36593_2	non-phage	0
DOF009_scaffold3711_2	non-phage	0
DOF009_scaffold18302_1	non-phage	0
DOF009_scaffold4229_5	non-phage	0
DOF009_scaffold52760_2	non-phage	0
DOF009_scaffold46992_3	non-phage	0
DOF009_scaffold53_2	non-phage	0
DOF009_scaffold5711_2	non-phage	0
DOF009_scaffold13271_3	non-phage	0
DOF009_scaffold23709_4	non-phage	0
DOF009_scaffold36176_2	phage	0.999

DOF009_scaffold32367_9	non-phage	0
DOF009_scaffold15281_4	non-phage	0
DOF009_scaffold29566_3	non-phage	0
DOF009_scaffold41459_2	non-phage	0
DOF009_scaffold50007_2	non-phage	0
DOF009_scaffold32712_5	non-phage	0
DOF009_scaffold42495_5	non-phage	0
DOF009_scaffold22475_6	non-phage	0
DOF009_scaffold51779_2	non-phage	0
DOF009_scaffold10873_1	non-phage	0
DOF009_scaffold52044_1	non-phage	0
DOF009_scaffold49454_2	non-phage	0
DOF009_scaffold47084_4	non-phage	0
DOF009_scaffold18403_2	non-phage	0
DOF009_scaffold46144_5	non-phage	0
DOF009_scaffold13_7	non-phage	0
DOF009_scaffold45_1	non-phage	0
DOF009_scaffold4754_1	non-phage	0
DOF009_scaffold48_11	non-phage	0
DOF009_scaffold43575_4	non-phage	0
DOF009_scaffold52820_2	non-phage	0
DOF009_scaffold26435_3	non-phage	0
DOF009_scaffold52836_1	non-phage	0
DOF009_scaffold40393_9	non-phage	0
DOF009_scaffold33479_4	non-phage	0
DOF009_scaffold52603_4	non-phage	0
DOF009_scaffold3423_2	non-phage	0
DOF009_scaffold37470_1	non-phage	0
DOF009_scaffold21736_1	non-phage	0
DOF009_scaffold1543_7	non-phage	0
DOF009_scaffold50051_1	non-phage	0
DOF009_scaffold37864_1	non-phage	0
DOF009_scaffold46769_1	non-phage	0
DOF009_scaffold27295_3	non-phage	0
DOF009_scaffold8642_1	non-phage	0
DOF009_scaffold51963_2	non-phage	0
DOF009_scaffold40864_1	non-phage	0
DOF009_scaffold52791_2	non-phage	0
DOF009_scaffold15236_4	non-phage	0
DOF009_scaffold28224_2	non-phage	0
DOF009_scaffold10528_6	non-phage	0
DOF009_scaffold52729_1	non-phage	0
DOF009_scaffold41006_3	non-phage	0
DOF009_scaffold39856_1	non-phage	0
DOF009_scaffold31_3	non-phage	0
DOF009_scaffold18712_7	non-phage	0

DOF009_scaffold39_4	non-phage	0
DOF009_scaffold2526_3	non-phage	0
DOF009_scaffold51879_2	non-phage	0
DOF009_scaffold24213_2	non-phage	0
DOF009_scaffold5223_15	non-phage	0
DOF009_scaffold52638_2	non-phage	0
DOF009_scaffold43630_2	non-phage	0
DOF009_scaffold6060_16	non-phage	0.239
DOF009_scaffold3667_4	non-phage	0
DOF009_scaffold39961_1	non-phage	0
DOF009_scaffold8299_16	non-phage	0
DOF009_scaffold9642_12	non-phage	0
DOF009_scaffold20304_10	non-phage	0
DOF009_scaffold24774_2	non-phage	0
DOF009_scaffold24588_1	non-phage	0
DOF009_scaffold52508_1	non-phage	0
DOF009_scaffold12228_14	non-phage	0
DOF009_scaffold35327_6	non-phage	0
DOF009_scaffold25733_12	non-phage	0
DOF009_scaffold8588_1	non-phage	0
DOF009_scaffold52057_1	non-phage	0
DOF009_scaffold45703_3	non-phage	0
DOF009_scaffold52612_2	non-phage	0
DOF009_scaffold329_32	non-phage	0
DOF009_scaffold38516_1	non-phage	0
DOF009_scaffold10519_5	non-phage	0
DOF009_scaffold483_9	non-phage	0
DOF009_scaffold41297_2	non-phage	0
DOF009_scaffold6167_2	non-phage	0
DOF009_scaffold39297_1	non-phage	0
DOF009_scaffold184_4	non-phage	0
DOF009_scaffold2468_9	non-phage	0
DOF009_scaffold44640_2	non-phage	0
DOF009_scaffold25555_4	non-phage	0
DOF009_scaffold10879_2	non-phage	0
DOF009_scaffold31975_1	non-phage	0
DOF009_scaffold38638_3	non-phage	0
DOF009_scaffold52340_1	non-phage	0
DOF009_scaffold42271_1	non-phage	0
DOF009_scaffold46390_1	non-phage	0
DOF009_scaffold13576_10	non-phage	0
DOF009_scaffold32665_1	non-phage	0
DOF009_scaffold47963_1	non-phage	0
DOF009_scaffold4754_10	non-phage	0
DOF009_scaffold20304_4	non-phage	0
DOF009_scaffold47230_12	non-phage	0

DOF009_scaffold28609_1	non-phage	0
DOF009_scaffold180_18	non-phage	0
DOF009_scaffold19543_2	non-phage	0
DOF009_scaffold229_11	non-phage	0
DOF009_scaffold5133_14	non-phage	0
DOF009_scaffold10880_1	non-phage	0
DOF009_scaffold52601_3	non-phage	0
DOF009_scaffold29_2	non-phage	0
DOF009_scaffold31_1	phage	0.538
DOF009_scaffold12050_3	non-phage	0
DOF009_scaffold36633_9	non-phage	0
DOF009_scaffold48940_4	non-phage	0
DOF009_scaffold14237_2	non-phage	0
DOF009_scaffold39785_1	non-phage	0
DOF009_scaffold38260_2	non-phage	0
DOF009_scaffold148_1	non-phage	0
DOF009_scaffold45109_1	non-phage	0
DOF009_scaffold12604_8	non-phage	0
DOF009_scaffold47963_5	non-phage	0
DOF009_scaffold2426_2	non-phage	0
DOF009_scaffold52837_1	non-phage	0
DOF009_scaffold51752_1	non-phage	0
DOF009_scaffold38944_5	non-phage	0
DOF009_scaffold50964_2	non-phage	0
DOF009_scaffold52832_1	non-phage	0
DOF009_scaffold7470_12	non-phage	0
DOF009_scaffold7182_4	non-phage	0
DOF009_scaffold52827_1	non-phage	0
DOF009_scaffold41601_3	non-phage	0
DOF009_scaffold483_3	non-phage	0
DOF009_scaffold41464_1	non-phage	0
DOF009_scaffold970_6	non-phage	0
DOF009_scaffold12593_4	non-phage	0
DOF009_scaffold47864_1	non-phage	0
DOF009_scaffold67_14	non-phage	0
DOF009_scaffold194_11	non-phage	0
DOF009_scaffold97_1	non-phage	0
DOF009_scaffold13307_3	non-phage	0
DOF009_scaffold1237_1	non-phage	0
DOF009_scaffold1_2	phage	0.919
DOF009_scaffold42567_1	non-phage	0
DOF009_scaffold40125_1	phage	0.998
DOF009_scaffold16216_4	non-phage	0
DOF009_scaffold5047_3	non-phage	0
DOF009_scaffold3636_17	non-phage	0
DOF009_scaffold52753_2	non-phage	0

DOF009_scaffold10985_5	non-phage	0
DOF009_scaffold23972_2	phage	0.998
DOF009_scaffold52855_1	non-phage	0
DOF009_scaffold50960_1	non-phage	0
DOF009_scaffold45042_4	non-phage	0
DOF009_scaffold39912_4	non-phage	0
DOF009_scaffold12352_6	non-phage	0
DOF009_scaffold52084_3	non-phage	0
DOF009_C722696_1	non-phage	0
DOF009_scaffold37948_2	non-phage	0
DOF009_scaffold51602_2	non-phage	0
DOF009_scaffold39901_1	non-phage	0
DOF009_scaffold47638_4	non-phage	0
DOF009_scaffold52600_1	non-phage	0
DOF009_scaffold30071_4	non-phage	0
DOF009_scaffold13544_1	non-phage	0
DOF009_scaffold3896_8	non-phage	0
DOF009_scaffold16157_1	non-phage	0
DOF009_scaffold297_10	non-phage	0
DOF009_scaffold52831_1	non-phage	0
DOF009_scaffold41459_1	non-phage	0
DOF009_scaffold12802_11	non-phage	0
DOF009_scaffold15429_2	non-phage	0
DOF009_scaffold31_7	non-phage	0
DOF009_scaffold34644_8	non-phage	0
DOF009_scaffold206_5	non-phage	0
DOF009_scaffold52617_4	non-phage	0
DOF009_scaffold25555_1	non-phage	0
DOF009_scaffold47084_3	non-phage	0
DOF009_scaffold18060_3	non-phage	0
DOF009_scaffold5667_16	non-phage	0
DOF009_scaffold52799_3	non-phage	0
DOF009_scaffold51162_2	non-phage	0
DOF009_scaffold3636_29	non-phage	0
DOF009_scaffold25904_7	non-phage	0
DOF009_scaffold38736_1	non-phage	0
DOF009_scaffold13901_4	phage	0.952
DOF009_scaffold52_3	non-phage	0
DOF009_scaffold30281_1	non-phage	0
DOF009_scaffold53_3	non-phage	0
DOF009_scaffold52847_1	non-phage	0
DOF009_scaffold52829_1	non-phage	0
DOF009_scaffold39698_3	non-phage	0
DOF009_scaffold13576_4	non-phage	0
DOF009_scaffold31104_1	non-phage	0
DOF009_scaffold25970_2	non-phage	0

DOF009_scaffold25555_5	non-phage	0
DOF009_scaffold201_4	non-phage	0
DOF009_scaffold52438_1	phage	0.999
DOF009_scaffold45561_2	non-phage	0.003
DOF009_scaffold24115_4	non-phage	0
DOF009_scaffold43575_3	non-phage	0
DOF009_scaffold30475_4	non-phage	0.236
DOF009_scaffold39489_6	non-phage	0
DOF009_scaffold52652_3	non-phage	0
DOF009_scaffold229_8	non-phage	0
DOF009_scaffold2788_26	non-phage	0
DOF009_scaffold129_60	non-phage	0
DOF009_scaffold5667_31	non-phage	0
DOF009_scaffold40393_21	non-phage	0
DOF009_scaffold48490_1	non-phage	0
DOF009_scaffold39557_2	non-phage	0
DOF009_scaffold42495_2	non-phage	0
DOF009_scaffold19621_2	non-phage	0.321
DOF009_scaffold39728_1	non-phage	0
DOF009_scaffold48732_1	non-phage	0
DOF009_scaffold18215_7	non-phage	0
DOF009_scaffold189_6	non-phage	0
DOF009_scaffold52851_1	non-phage	0
DOF009_scaffold201_24	non-phage	0
DOF009_scaffold44175_1	non-phage	0
DOF009_scaffold52646_1	non-phage	0
DOF009_scaffold5667_46	non-phage	0
DOF009_scaffold1543_8	non-phage	0
DOF009_scaffold51915_3	non-phage	0
DOF009_scaffold48940_1	non-phage	0
DOF009_C722162_1	non-phage	0
DOF009_scaffold297_38	non-phage	0
DOF009_scaffold18148_3	non-phage	0
DOF009_scaffold194_5	non-phage	0
DOF009_scaffold201_3	non-phage	0
DOF009_scaffold40047_1	non-phage	0
DOF009_scaffold52854_1	non-phage	0
DOF009_scaffold48_14	non-phage	0
DOF009_scaffold45109_12	non-phage	0
DOF009_scaffold52823_1	non-phage	0
DOF009_scaffold5133_3	non-phage	0
DOF009_scaffold18712_2	non-phage	0
DOF009_scaffold52805_3	non-phage	0
DOF009_scaffold46772_1	non-phage	0
DOF009_scaffold52423_4	non-phage	0
DOF009_scaffold25970_3	non-phage	0

DOF009_scaffold8642_3	non-phage	0
DOF009_scaffold13579_2	non-phage	0
DOF009_scaffold52243_4	non-phage	0
DOF009_scaffold52816_1	non-phage	0
DOF009_scaffold44417_1	non-phage	0
DOF009_scaffold12551_6	non-phage	0
DOF009_scaffold22475_3	non-phage	0
DOF009_scaffold3246_11	non-phage	0
DOF009_scaffold128_4	non-phage	0
DOF009_scaffold52840_1	non-phage	0
DOF009_scaffold44695_1	non-phage	0
DOF009_scaffold33065_5	non-phage	0
DOF009_scaffold43762_5	non-phage	0
DOF009_scaffold67_2	non-phage	0
DOF009_scaffold29624_1	non-phage	0
DOF009_scaffold29903_4	non-phage	0
DOF009_scaffold46233_2	non-phage	0
DOF009_scaffold30444_5	non-phage	0
DOF009_scaffold32812_2	non-phage	0
DOF009_scaffold42135_2	non-phage	0
DOF009_scaffold52798_1	non-phage	0
DOF009_scaffold4516_5	phage	0.989
DOF009_scaffold5667_12	non-phage	0
DOF009_scaffold4229_1	non-phage	0
DOF009_scaffold52616_1	non-phage	0
DOF009_scaffold52825_1	non-phage	0
DOF009_scaffold229_12	non-phage	0
DOF009_scaffold31_2	non-phage	0
DOF009_scaffold173_3	non-phage	0
DOF009_scaffold229_4	non-phage	0
DOF009_scaffold24063_2	non-phage	0
DOF009_scaffold39901_3	non-phage	0
DOF009_scaffold49130_5	non-phage	0
DOF009_scaffold22634_2	non-phage	0
DOF009_scaffold13271_1	non-phage	0
DOF009_scaffold10462_1	non-phage	0
DOF009_scaffold42497_4	phage	0.998
DOF009_scaffold24115_7	non-phage	0
DOF009_scaffold21489_6	non-phage	0
DOF009_scaffold194_4	non-phage	0
DOF009_scaffold48940_5	non-phage	0
DOF009_scaffold10076_1	non-phage	0
DOF009_scaffold39_1	non-phage	0
DOF009_scaffold52796_2	non-phage	0
DOF009_scaffold39856_11	non-phage	0
DOF009_scaffold28495_3	non-phage	0

DOF009_scaffold2700_2	non-phage	0
DOF009_scaffold52171_1	non-phage	0
DOF009_scaffold36907_1	non-phage	0
DOF009_scaffold10879_3	non-phage	0
DOF009_scaffold52729_2	non-phage	0
DOF009_scaffold47963_2	non-phage	0
DOF009_scaffold28690_1	non-phage	0
DOF009_scaffold7496_2	phage	0.998
DOF009_scaffold9589_1	non-phage	0
DOF009_scaffold17322_2	non-phage	0
DOF009_scaffold52827_2	non-phage	0
DOF009_scaffold12604_4	non-phage	0
DOF009_scaffold39193_2	non-phage	0
DOF009_scaffold50038_3	non-phage	0
DOF009_scaffold52717_2	non-phage	0
DOF009_scaffold38795_1	non-phage	0
DOF009_scaffold41601_2	non-phage	0
DOF009_scaffold15281_11	non-phage	0
DOF009_scaffold201_62	non-phage	0
DOF010_scaffold3455_3	non-phage	0
DOF010_scaffold34954_2	non-phage	0
DOF010_scaffold38126_1	non-phage	0
DOF010_scaffold7400_11	non-phage	0
DOF010_scaffold8434_5	non-phage	0
DOF010_scaffold5873_24	non-phage	0
DOF010_scaffold112_2	non-phage	0
DOF010_scaffold29723_3	non-phage	0
DOF010_scaffold29928_3	non-phage	0
DOF010_scaffold4_11	non-phage	0
DOF010_scaffold33024_5	non-phage	0
DOF010_scaffold28420_1	non-phage	0
DOF010_scaffold6290_6	non-phage	0
DOF010_scaffold132_5	non-phage	0
DOF010_scaffold28819_15	non-phage	0
DOF010_scaffold2045_15	non-phage	0
DOF010_scaffold37104_2	phage	0.999
DOF010_scaffold38741_1	non-phage	0
DOF010_scaffold38856_3	non-phage	0
DOF010_scaffold24624_3	non-phage	0
DOF010_scaffold38853_1	non-phage	0
DOF010_scaffold8936_16	non-phage	0
DOF010_scaffold10918_3	non-phage	0
DOF010_scaffold15383_7	non-phage	0
DOF010_scaffold38728_2	non-phage	0
DOF010_scaffold48_9	non-phage	0
DOF010_scaffold30470_3	non-phage	0

DOF010_scaffold13764_3	non-phage	0
DOF010_scaffold37562_6	non-phage	0
DOF010_scaffold38173_4	non-phage	0
DOF010_scaffold8091_1	non-phage	0
DOF010_scaffold29607_7	non-phage	0
DOF010_scaffold3358_4	non-phage	0
DOF010_scaffold19015_2	non-phage	0
DOF010_scaffold38266_2	non-phage	0
DOF010_scaffold38542_4	phage	0.998
DOF010_scaffold12096_1	phage	0.999
DOF010_scaffold38527_2	non-phage	0
DOF010_scaffold1884_2	non-phage	0
DOF010_scaffold5_5_1	non-phage	0
DOF010_scaffold14828_6	non-phage	0
DOF010_scaffold67_3	non-phage	0
DOF010_scaffold4593_2	non-phage	0
DOF010_scaffold7293_12	non-phage	0
DOF010_scaffold31673_3	non-phage	0
DOF010_scaffold15983_2	non-phage	0
DOF010_scaffold33053_1	non-phage	0
DOF010_scaffold35610_1	phage	0.999
DOF010_scaffold38620_1	phage	0.999
DOF010_scaffold5110_4	non-phage	0
DOF010_scaffold171_4	non-phage	0
DOF010_scaffold5542_6	non-phage	0
DOF010_scaffold29888_2	non-phage	0
DOF010_scaffold30078_3	non-phage	0
DOF010_scaffold20638_5	non-phage	0
DOF010_scaffold38469_5	non-phage	0
DOF010_scaffold37948_3	non-phage	0
DOF010_scaffold29144_2	non-phage	0
DOF010_scaffold35355_2	non-phage	0
DOF010_scaffold12515_2	non-phage	0
DOF010_scaffold6233_31	non-phage	0
DOF010_scaffold19506_1	non-phage	0
DOF010_scaffold33037_1	non-phage	0
DOF010_scaffold18549_5	non-phage	0
DOF010_scaffold12990_26	non-phage	0
DOF010_scaffold39045_1	non-phage	0
DOF010_scaffold33106_3	non-phage	0
DOF010_scaffold36879_1	non-phage	0
DOF010_scaffold134_23	non-phage	0
DOF010_scaffold35385_1	non-phage	0
DOF010_scaffold37595_2	non-phage	0
DOF010_scaffold39053_1	non-phage	0
DOF010_scaffold17273_3	non-phage	0

DOF010_scaffold29898_7	non-phage	0
DOF010_scaffold10503_1	non-phage	0
DOF010_scaffold218_5	non-phage	0
DOF010_scaffold6233_4	non-phage	0
DOF010_scaffold18549_4	non-phage	0
DOF010_scaffold38914_1	non-phage	0
DOF010_scaffold177_4	non-phage	0
DOF010_scaffold20187_7	non-phage	0
DOF010_scaffold37194_6	phage	0.996
DOF010_scaffold17290_1	non-phage	0
DOF010_scaffold10218_6	non-phage	0
DOF010_scaffold1331_7	phage	0.978
DOF010_scaffold38622_1	non-phage	0
DOF010_scaffold34040_1	non-phage	0
DOF010_scaffold31976_2	phage	0.999
DOF010_scaffold18421_4	non-phage	0
DOF010_scaffold19543_4	non-phage	0
DOF010_scaffold8874_2	non-phage	0
DOF010_scaffold17034_4	non-phage	0
DOF010_scaffold45_4	phage	0.677
DOF010_C495589_1	non-phage	0
DOF010_scaffold22973_2	non-phage	0
DOF010_scaffold9832_3	non-phage	0
DOF010_scaffold18549_9	non-phage	0
DOF010_scaffold38495_8	non-phage	0
DOF010_scaffold8295_47	non-phage	0
DOF010_scaffold24541_2	non-phage	0.315
DOF010_scaffold24937_5	non-phage	0
DOF010_scaffold28819_11	non-phage	0
DOF010_scaffold3688_15	non-phage	0
DOF010_scaffold38703_4	non-phage	0
DOF010_scaffold35956_1	non-phage	0
DOF010_scaffold29723_6	non-phage	0
DOF010_C495273_1	phage	0.999
DOF010_scaffold32324_1	non-phage	0
DOF010_scaffold9633_1	non-phage	0
DOF010_scaffold38936_1	non-phage	0
DOF010_scaffold17669_3	non-phage	0
DOF010_scaffold26600_1	non-phage	0
DOF010_scaffold2773_5	non-phage	0
DOF010_scaffold2045_13	non-phage	0
DOF010_scaffold14_19	non-phage	0
DOF010_scaffold236_6	non-phage	0
DOF010_scaffold34810_4	non-phage	0
DOF010_scaffold36_4	phage	0.952
DOF010_scaffold5166_16	non-phage	0.001

DOF010_scaffold23207_19	non-phage	0
DOF010_scaffold29648_5	non-phage	0
DOF010_scaffold14595_4	non-phage	0
DOF010_scaffold26582_12	non-phage	0
DOF010_scaffold29410_5	non-phage	0
DOF010_scaffold37452_2	non-phage	0
DOF010_scaffold19251_1	non-phage	0
DOF010_scaffold38683_1	non-phage	0
DOF010_scaffold10923_1	non-phage	0
DOF010_scaffold2140_5	non-phage	0
DOF010_scaffold39021_1	non-phage	0
DOF010_scaffold39042_3	phage	0.999
DOF010_scaffold2045_27	non-phage	0
DOF010_scaffold112_7	non-phage	0
DOF010_scaffold11833_12	non-phage	0
DOF010_scaffold4776_2	non-phage	0
DOF010_scaffold5_4	non-phage	0
DOF010_scaffold14854_3	non-phage	0
DOF010_scaffold18661_2	non-phage	0
DOF010_scaffold8903_3	non-phage	0
DOF010_scaffold34_14	non-phage	0
DOF010_scaffold3107_10	non-phage	0
DOF010_scaffold2232_10	non-phage	0
DOF010_scaffold18288_3	non-phage	0
DOF010_scaffold5032_4	non-phage	0
DOF010_scaffold20_3	non-phage	0
DOF010_scaffold128_16	non-phage	0
DOF010_scaffold38703_1	non-phage	0
DOF010_scaffold38856_2	non-phage	0
DOF010_scaffold34_13	non-phage	0
DOF010_scaffold13814_2	phage	0.602
DOF010_scaffold84_2	non-phage	0
DOF010_scaffold38469_6	non-phage	0
DOF010_scaffold16058_6	non-phage	0
DOF010_scaffold39040_1	non-phage	0
DOF010_scaffold67_4	non-phage	0
DOF010_scaffold340_1	non-phage	0
DOF010_scaffold3916_6	non-phage	0
DOF010_scaffold36571_1	phage	0.999
DOF010_scaffold8418_16	non-phage	0
DOF010_scaffold4140_4	non-phage	0
DOF010_scaffold33267_1	non-phage	0
DOF010_scaffold25559_1	non-phage	0
DOF010_scaffold970_3	non-phage	0
DOF010_scaffold37750_5	phage	0.999
DOF010_scaffold1295_20	non-phage	0

DOF010_scaffold17459_2	non-phage	0
DOF010_scaffold33686_1	non-phage	0
DOF010_scaffold10626_1	non-phage	0
DOF010_scaffold21962_16	non-phage	0
DOF010_scaffold116_1	non-phage	0
DOF010_scaffold65_21	non-phage	0
DOF010_scaffold2960_7	non-phage	0
DOF010_scaffold65_18	phage	0.986
DOF010_scaffold14828_7	non-phage	0
DOF010_scaffold32201_2	non-phage	0
DOF010_scaffold38513_5	non-phage	0
DOF010_scaffold12019_5	non-phage	0
DOF010_scaffold123_3	non-phage	0
DOF010_scaffold21521_6	non-phage	0
DOF010_scaffold16716_3	non-phage	0
DOF010_scaffold8874_6	non-phage	0
DOF010_scaffold1983_11	non-phage	0
DOF010_scaffold10828_1	non-phage	0
DOF010_scaffold37348_2	non-phage	0
DOF010_scaffold21962_2	non-phage	0
DOF010_scaffold27480_5	non-phage	0
DOF010_scaffold24668_6	non-phage	0
DOF010_scaffold31661_3	non-phage	0
DOF010_scaffold10007_15	non-phage	0
DOF010_scaffold31586_1	non-phage	0
DOF010_scaffold23808_1	non-phage	0
DOF010_scaffold14590_1	non-phage	0
DOF010_scaffold28437_3	non-phage	0
DOF010_scaffold35997_1	non-phage	0
DOF010_scaffold24_7	non-phage	0
DOF010_scaffold5873_18	non-phage	0
DOF010_scaffold3021_21	non-phage	0
DOF010_scaffold35264_4	non-phage	0
DOF010_scaffold19015_1	non-phage	0
DOF010_scaffold8903_7	non-phage	0
DOF010_scaffold134_21	non-phage	0
DOF010_scaffold2960_15	non-phage	0
DOF010_scaffold38266_1	non-phage	0
DOF010_scaffold5166_29	non-phage	0
DOF010_scaffold38994_1	non-phage	0
DOF010_scaffold20732_1	non-phage	0
DOF010_scaffold38868_4	non-phage	0
DOF010_scaffold10958_6	non-phage	0
DOF010_scaffold38971_1	non-phage	0
DOF010_scaffold22975_3	non-phage	0
DOF010_scaffold3251_13	non-phage	0

DOF010_scaffold23207_31	non-phage	0
DOF010_scaffold8418_2	non-phage	0
DOF010_scaffold3455_2	non-phage	0
DOF010_scaffold17640_4	non-phage	0
DOF010_scaffold24199_1	non-phage	0
DOF010_scaffold23207_10	non-phage	0
DOF010_scaffold25299_3	non-phage	0
DOF010_scaffold4776_1	non-phage	0
DOF010_scaffold34622_1	non-phage	0
DOF010_scaffold11511_8	non-phage	0
DOF010_scaffold8295_48	non-phage	0
DOF010_scaffold10319_5	non-phage	0
DOF010_scaffold10849_2	non-phage	0
DOF010_scaffold177_6	non-phage	0.011
DOF010_scaffold37562_7	non-phage	0
DOF010_scaffold2157_26	non-phage	0
DOF010_scaffold25519_6	non-phage	0
DOF010_scaffold13830_3	non-phage	0
DOF010_scaffold117_1	non-phage	0
DOF010_scaffold27405_1	non-phage	0
DOF010_scaffold12075_2	non-phage	0
DOF010_scaffold38651_3	non-phage	0
DOF010_scaffold3351_2	non-phage	0
DOF010_scaffold21978_1	phage	0.998
DOF010_scaffold36618_1	non-phage	0
DOF010_scaffold23587_3	non-phage	0
DOF010_scaffold2061_3	non-phage	0
DOF010_scaffold21474_1	non-phage	0
DOF010_scaffold175_3	non-phage	0
DOF010_scaffold25299_9	non-phage	0
DOF010_scaffold27958_3	non-phage	0
DOF010_scaffold33024_2	non-phage	0
DOF010_scaffold25734_2	non-phage	0
DOF010_scaffold31777_1	non-phage	0
DOF010_scaffold11833_5	phage	0.932
DOF010_scaffold218_2	non-phage	0
DOF010_scaffold4838_1	non-phage	0
DOF010_scaffold3833_1	non-phage	0
DOF010_scaffold33165_1	non-phage	0
DOF010_scaffold25256_1	non-phage	0
DOF010_scaffold10127_2	non-phage	0
DOF010_scaffold5166_25	non-phage	0
DOF010_scaffold7607_7	phage	0.999
DOF010_scaffold31710_2	non-phage	0
DOF010_scaffold8418_9	non-phage	0
DOF010_scaffold28134_3	non-phage	0

DOF010_scaffold34265_4	non-phage	0
DOF010_scaffold39050_1	non-phage	0
DOF010_scaffold10999_17	non-phage	0
DOF010_scaffold134_24	non-phage	0
DOF010_scaffold37904_3	non-phage	0
DOF010_scaffold22478_8	non-phage	0
DOF010_scaffold9741_2	non-phage	0
DOF010_scaffold37985_2	non-phage	0
DOF010_scaffold19015_5	phage	0.987
DOF010_scaffold6386_1_1	non-phage	0
DOF010_scaffold38537_3	non-phage	0
DOF010_scaffold20924_2	non-phage	0
DOF010_scaffold29898_8	non-phage	0
DOF010_scaffold27096_5	phage	0.997
DOF010_scaffold290_8	non-phage	0
DOF010_scaffold8931_3	non-phage	0
DOF010_scaffold3251_2	non-phage	0
DOF010_scaffold29898_3	non-phage	0
DOF010_scaffold38445_1	non-phage	0
DOF010_scaffold6631_1	non-phage	0
DOF010_scaffold2045_19	non-phage	0
DOF010_scaffold38864_2	non-phage	0
DOF010_scaffold16695_2	non-phage	0
DOF010_scaffold36_5	non-phage	0
DOF010_scaffold26507_2	non-phage	0
DOF010_scaffold20638_18	non-phage	0
DOF010_scaffold32482_2	phage	0.996
DOF010_scaffold8434_16	non-phage	0
DOF010_scaffold35610_2	phage	0.998
DOF010_scaffold10047_12	non-phage	0
DOF010_scaffold236_9	non-phage	0
DOF010_scaffold23207_18	phage	0.998
DOF010_scaffold34_4	phage	0.581
DOF010_scaffold28134_8	non-phage	0
DOF010_scaffold16923_18	non-phage	0
DOF010_scaffold2960_8	non-phage	0
DOF010_scaffold4140_17	non-phage	0
DOF010_scaffold8901_6	non-phage	0
DOF010_scaffold24285_5	non-phage	0
DOF010_scaffold30261_2	non-phage	0
DOF010_scaffold20638_4	non-phage	0
DOF010_scaffold2645_5	phage	0.999
DOF010_scaffold29046_1	non-phage	0
DOF010_scaffold39052_1	non-phage	0
DOF010_scaffold16923_19	non-phage	0
DOF010_scaffold9744_1	non-phage	0

DOF010_scaffold2232_15	non-phage	0
DOF010_scaffold8434_11	non-phage	0
DOF010_scaffold28689_2	non-phage	0
DOF010_scaffold18661_1	non-phage	0
DOF010_scaffold2096_2	non-phage	0
DOF010_scaffold37960_3	non-phage	0
DOF010_scaffold19937_2	non-phage	0
DOF010_scaffold37948_1	non-phage	0
DOF010_scaffold38173_2	non-phage	0
DOF010_scaffold8295_7	non-phage	0
DOF010_scaffold4619_9	non-phage	0
DOF010_scaffold2317_3	non-phage	0
DOF010_scaffold123_2	non-phage	0
DOF010_scaffold18226_4	non-phage	0
DOF010_scaffold39042_1	phage	0.993
DOF010_scaffold128_9	non-phage	0
DOF010_scaffold38773_2	non-phage	0
DOF010_scaffold6631_5	non-phage	0
DOF010_scaffold29898_5	non-phage	0
DOF010_scaffold134_6	non-phage	0
DOF010_scaffold9741_1	non-phage	0.017
DOF010_scaffold18723_2	non-phage	0
DOF010_scaffold3173_1	non-phage	0
DOF010_scaffold27406_2	non-phage	0
DOF010_scaffold10828_5	non-phage	0
DOF010_scaffold24344_2	phage	1
DOF010_scaffold25559_7	non-phage	0
DOF010_scaffold103_2	non-phage	0
DOF010_scaffold3583_13	non-phage	0
DOF010_scaffold36156_3	non-phage	0.012
DOF010_scaffold3351_3	non-phage	0
DOF010_scaffold5542_8	non-phage	0
DOF010_scaffold17640_5	non-phage	0
DOF010_scaffold37194_1	non-phage	0
DOF010_scaffold38651_2	phage	0.798
DOF010_scaffold38726_1	non-phage	0
DOF010_scaffold236_20	non-phage	0
DOF010_scaffold15958_5	non-phage	0
DOF010_scaffold21506_7	non-phage	0
DOF010_scaffold16813_4	non-phage	0
DOF010_scaffold10626_4	non-phage	0
DOF010_scaffold17273_9	non-phage	0
DOF010_scaffold1871_12	non-phage	0
DOF010_scaffold25734_4	non-phage	0
DOF010_scaffold91_4	non-phage	0
DOF010_scaffold144_3	non-phage	0

DOF010_scaffold9486_1	non-phage	0
DOF010_scaffold10319_3	non-phage	0
DOF010_scaffold17290_4	non-phage	0
DOF010_scaffold13830_4	phage	0.935
DOF010_scaffold33553_4	phage	0.999
DOF010_scaffold3021_15	non-phage	0
DOF010_scaffold128_13	non-phage	0
DOF010_scaffold128_38	non-phage	0
DOF010_scaffold36156_4	non-phage	0
DOF010_scaffold104_3	non-phage	0
DOF010_scaffold128_35	non-phage	0
DOF010_scaffold9205_7	non-phage	0
DOF010_scaffold37690_1	non-phage	0
DOF010_scaffold7852_2	non-phage	0
DOF010_scaffold29723_1	non-phage	0
DOF010_scaffold33138_1	non-phage	0
DOF010_scaffold38303_3	non-phage	0
DOF010_scaffold21506_10	non-phage	0
DOF010_scaffold38956_2	phage	0.999
DOF010_scaffold8903_9	non-phage	0
DOF010_scaffold116_7	non-phage	0
DOF010_scaffold28819_1	non-phage	0
DOF010_scaffold37004_1	non-phage	0
DOF010_scaffold23049_1	non-phage	0
DOF010_scaffold33165_2	non-phage	0
DOF010_scaffold33267_4	non-phage	0
DOF010_scaffold25519_11	non-phage	0
DOF010_scaffold24199_3	non-phage	0
DOF010_scaffold11985_6	non-phage	0
DOF010_scaffold2170_20	non-phage	0
DOF010_scaffold19252_1	phage	0.897
DOF010_scaffold5166_41	non-phage	0
DOF010_scaffold37194_7	non-phage	0
DOF010_scaffold10007_8	non-phage	0
DOF010_scaffold8295_40	non-phage	0
DOF010_scaffold23207_17	non-phage	0
DOF010_scaffold38864_1	non-phage	0
DOF010_scaffold75_3	non-phage	0
DOF010_scaffold5542_10	non-phage	0
DOF010_scaffold26582_1	non-phage	0
DOF010_scaffold21702_2	phage	1
DOF010_scaffold29273_4	phage	0.999
DOF010_scaffold16923_1	non-phage	0
DOF010_scaffold34622_2	non-phage	0
DOF010_scaffold32482_5	non-phage	0
DOF010_scaffold175_2	non-phage	0

DOF010_scaffold10098_3	non-phage	0
DOF010_scaffold31673_2	non-phage	0
DOF010_scaffold38469_2	non-phage	0
DOF010_scaffold35797_5	non-phage	0
DOF010_scaffold35320_6	non-phage	0
DOF010_scaffold3173_8	non-phage	0
DOF010_scaffold37071_2	non-phage	0
DOF010_scaffold85_1	non-phage	0
DOF010_scaffold237_1	non-phage	0
DOF010_scaffold9232_2	non-phage	0
DOF010_scaffold2061_4	non-phage	0
DOF010_scaffold5_2	non-phage	0
DOF010_scaffold36670_1	non-phage	0
DOF010_scaffold10999_4	non-phage	0
DOF010_scaffold236_15	non-phage	0
DOF010_scaffold132_4	non-phage	0
DOF010_scaffold4140_12	non-phage	0
DOF010_scaffold3251_12	non-phage	0
DOF010_scaffold9735_3	non-phage	0
DOF010_scaffold23207_23	non-phage	0
DOF010_scaffold10769_2	non-phage	0
DOF010_scaffold32201_1	non-phage	0
DOF010_scaffold198_1	phage	0.998
DOF010_scaffold5873_41	non-phage	0
DOF010_scaffold38173_3	non-phage	0.002
DOF010_scaffold132_1	non-phage	0
DOF010_scaffold10999_14	non-phage	0
DOF010_scaffold35797_3	non-phage	0
DOF010_scaffold10397_4	non-phage	0
DOF010_scaffold8295_45	non-phage	0
DOF010_scaffold3021_11	non-phage	0
DOF010_scaffold4723_12	non-phage	0
DOF010_scaffold9687_10	non-phage	0
DOF010_scaffold1983_9	non-phage	0
DOF010_scaffold37904_2	non-phage	0
DOF010_scaffold144_7	non-phage	0
DOF010_scaffold34040_12	non-phage	0
DOF010_scaffold11511_3	non-phage	0
DOF010_scaffold25519_3	non-phage	0
DOF010_scaffold11354_5	non-phage	0
DOF010_scaffold7400_6	non-phage	0
DOF010_scaffold128_3	non-phage	0
DOF010_scaffold128_39	non-phage	0
DOF010_scaffold27958_4	non-phage	0
DOF010_scaffold16695_3	non-phage	0
DOF010_scaffold8295_51	non-phage	0

DOF010_scaffold38551_4	phage	0.945
DOF010_scaffold32482_3	non-phage	0
DOF010_scaffold35797_9	non-phage	0
DOF010_scaffold38929_1	non-phage	0
DOF010_scaffold38936_3	non-phage	0
DOF010_scaffold5166_32	non-phage	0
DOF010_scaffold19543_1	non-phage	0
DOF010_scaffold3351_5	non-phage	0
DOF010_scaffold12990_38	non-phage	0
DOF010_scaffold8434_10	non-phage	0
DOF010_scaffold14_1	non-phage	0
DOF010_scaffold1871_28	non-phage	0
DOF010_scaffold14207_2	non-phage	0
DOF010_scaffold29957_1	non-phage	0
DOF010_scaffold1983_5	non-phage	0
DOF010_scaffold8903_5	non-phage	0
DOF010_scaffold13635_3	non-phage	0
DOF010_scaffold20028_3	non-phage	0
DOF010_scaffold8931_5	non-phage	0
DOF010_scaffold3173_13	non-phage	0
DOF010_scaffold24344_3	phage	1
DOF010_scaffold5110_8	non-phage	0
DOF010_scaffold24_5	non-phage	0
DOF010_scaffold19251_4	non-phage	0
DOF010_scaffold38435_1	non-phage	0
DOF010_scaffold21742_4	non-phage	0
DOF010_scaffold29410_3	non-phage	0
DOF010_scaffold38745_1	non-phage	0
DOF010_scaffold38430_1	non-phage	0
DOF010_scaffold37985_1	non-phage	0
DOF010_scaffold34810_3	non-phage	0
DOF010_scaffold5166_46	non-phage	0
DOF010_scaffold34_6	non-phage	0
DOF010_scaffold8434_6	non-phage	0
DOF010_scaffold22478_7	non-phage	0
DOF010_scaffold37695_2	non-phage	0
DOF010_scaffold404_4	non-phage	0
DOF010_scaffold53_3	non-phage	0
DOF010_scaffold36553_1	non-phage	0
DOF010_scaffold7716_3	non-phage	0
DOF010_scaffold38266_5	non-phage	0
DOF010_scaffold18661_9	non-phage	0
DOF010_scaffold33024_6	non-phage	0
DOF010_scaffold31809_1	non-phage	0
DOF010_scaffold3173_10	non-phage	0
DOF010_scaffold33267_10	non-phage	0

DOF010_scaffold26507_1	non-phage	0
DOF010_scaffold16058_8	non-phage	0
DOF010_scaffold31661_1	phage	0.981
DOF010_scaffold38173_1	non-phage	0
DOF010_scaffold8874_1	non-phage	0
DOF010_scaffold18612_2	non-phage	0
DOF010_scaffold5166_28	non-phage	0
DOF010_scaffold3583_2	non-phage	0
DOF010_scaffold34432_1	non-phage	0
DOF010_scaffold4776_3	non-phage	0
DOF010_scaffold5032_6	non-phage	0
DOF010_scaffold34_15	non-phage	0
DOF010_scaffold24205_1	non-phage	0
DOF010_scaffold2645_8	non-phage	0
DOF010_scaffold35846_1	phage	0.927
DOF010_scaffold8112_11	non-phage	0
DOF010_scaffold48_3	non-phage	0
DOF010_scaffold33024_3	non-phage	0
DOF010_scaffold22791_5	non-phage	0
DOF010_scaffold34_1	non-phage	0
DOF010_scaffold1884_1	non-phage	0
DOF010_scaffold30_4	non-phage	0
DOF010_scaffold38542_3	non-phage	0
DOF010_scaffold39002_1	non-phage	0
DOF010_scaffold26582_5	non-phage	0
DOF010_scaffold1871_13	non-phage	0
DOF010_scaffold10484_7	non-phage	0
DOF010_scaffold22193_1	non-phage	0
DOF010_scaffold8936_26	non-phage	0
DOF010_scaffold31673_4	non-phage	0
DOF010_scaffold10999_13	non-phage	0
DOF010_scaffold5166_8	non-phage	0
DOF010_scaffold11511_20	non-phage	0
DOF010_scaffold11511_10	non-phage	0
DOF010_scaffold37750_4	phage	0.999
DOF010_scaffold12005_1	non-phage	0
DOF010_scaffold15909_3	non-phage	0
DOF010_scaffold5873_23	non-phage	0
DOF010_scaffold5_8	non-phage	0
DOF010_scaffold195_1	non-phage	0
DOF010_scaffold10918_1	non-phage	0
DOF010_scaffold1042_20	non-phage	0
DOF010_scaffold13635_7	non-phage	0
DOF010_scaffold8936_13	non-phage	0
DOF010_scaffold38481_1	non-phage	0
DOF010_scaffold6967_1	non-phage	0

DOF010_scaffold12515_3	non-phage	0
DOF010_scaffold4818_2	non-phage	0
DOF010_scaffold36156_1	non-phage	0
DOF010_scaffold31673_1	non-phage	0
DOF010_scaffold28137_6	non-phage	0
DOF010_scaffold10098_2	non-phage	0
DOF010_scaffold10007_17	non-phage	0
DOF010_scaffold35137_3	non-phage	0
DOF010_scaffold17476_1	non-phage	0
DOF010_scaffold12515_4	non-phage	0
DOF010_scaffold29410_7	non-phage	0
DOF010_scaffold27096_3	non-phage	0
DOF010_scaffold21471_1	non-phage	0
DOF010_scaffold134_22	non-phage	0
DOF010_scaffold10999_2	non-phage	0
DOF010_scaffold29759_1	non-phage	0
DOF010_scaffold37868_2	non-phage	0
DOF010_scaffold31586_2	non-phage	0
DOF010_scaffold8751_2	non-phage	0.001
DOF010_scaffold20928_1	non-phage	0
DOF010_scaffold11511_6	non-phage	0
DOF010_scaffold340_6	non-phage	0
DOF010_scaffold7400_8	non-phage	0
DOF010_scaffold128_4	non-phage	0
DOF010_scaffold38527_6	non-phage	0
DOF010_scaffold14828_2	non-phage	0
DOF010_scaffold25299_2	non-phage	0
DOF010_scaffold16923_16	non-phage	0
DOF010_scaffold39024_1	non-phage	0
DOF010_scaffold38733_1	non-phage	0
DOF010_scaffold34265_3	non-phage	0
DOF010_scaffold37985_4	non-phage	0
DOF010_scaffold35320_3	non-phage	0
DOF010_scaffold17669_12	non-phage	0
DOF010_scaffold10511_2	non-phage	0
DOF010_scaffold22182_1	non-phage	0
DOF010_scaffold25631_1	non-phage	0
DOF010_scaffold4927_5	non-phage	0
DOF010_scaffold6233_28	non-phage	0
DOF010_scaffold27874_2	non-phage	0
DOF010_scaffold39049_2	phage	0.999
DOF010_scaffold5166_17	non-phage	0
DOF010_scaffold32869_1	non-phage	0
DOF010_scaffold37194_3	non-phage	0
DOF010_scaffold3351_8	non-phage	0
DOF010_scaffold39019_1	non-phage	0

DOF010_scaffold4723_13	phage	0.769
DOF010_scaffold22931_3	non-phage	0
DOF010_scaffold30470_5	non-phage	0
DOF010_scaffold39051_1	non-phage	0
DOF010_scaffold31976_1	phage	0.999
DOF010_scaffold39036_1	non-phage	0
DOF010_scaffold8418_13	non-phage	0
DOF010_scaffold30386_4	non-phage	0
DOF010_scaffold132_3	non-phage	0
DOF010_scaffold236_16	non-phage	0
DOF010_scaffold104_1	non-phage	0
DOF010_scaffold19633_1	non-phage	0
DOF010_scaffold25734_1	non-phage	0
DOF010_C495227_1	non-phage	0
DOF010_scaffold9222_6	non-phage	0
DOF010_scaffold17273_6	non-phage	0
DOF010_scaffold26582_11	non-phage	0
DOF010_scaffold6949_1	non-phage	0
DOF010_scaffold10849_7	non-phage	0
DOF010_scaffold26145_1	non-phage	0
DOF010_scaffold10007_19	non-phage	0
DOF010_scaffold39001_1	non-phage	0
DOF010_scaffold8903_11	non-phage	0
DOF010_scaffold22791_1	non-phage	0
DOF011_scaffold21209_1	non-phage	0
DOF011_scaffold1139_8	non-phage	0
DOF011_scaffold7818_2	non-phage	0
DOF011_scaffold4132_1	non-phage	0
DOF011_scaffold16504_1	non-phage	0
DOF011_scaffold20463_1	non-phage	0
DOF011_scaffold10770_7	non-phage	0
DOF011_scaffold915_17	non-phage	0
DOF011_C265055_1	non-phage	0
DOF011_scaffold5372_16	non-phage	0
DOF011_scaffold426_17	non-phage	0
DOF011_scaffold21206_1	non-phage	0
DOF011_scaffold5176_3	non-phage	0
DOF011_scaffold12950_1	non-phage	0
DOF011_scaffold19678_1	non-phage	0
DOF011_scaffold21022_1	non-phage	0
DOF011_scaffold13029_5	non-phage	0
DOF011_scaffold16424_3	non-phage	0
DOF011_scaffold19545_3	non-phage	0
DOF011_scaffold18231_3	non-phage	0
DOF011_scaffold20374_1	non-phage	0
DOF011_scaffold14317_11	non-phage	0

DOF011_scaffold7420_3	non-phage	0
DOF011_scaffold18004_2	non-phage	0
DOF011_scaffold17136_1	non-phage	0
DOF011_scaffold2029_8	non-phage	0
DOF011_scaffold14317_2	non-phage	0
DOF011_scaffold2136_1	non-phage	0
DOF011_scaffold20315_2	non-phage	0
DOF011_scaffold21153_1	phage	0.999
DOF011_scaffold19811_2	non-phage	0
DOF011_scaffold26_2	non-phage	0
DOF011_scaffold15753_1	non-phage	0
DOF011_scaffold10456_2	non-phage	0
DOF011_scaffold21134_1	non-phage	0
DOF011_scaffold16423_3	non-phage	0
DOF011_scaffold10714_3	non-phage	0
DOF011_scaffold21041_1	non-phage	0
DOF011_scaffold21060_1	non-phage	0
DOF011_scaffold17807_1	non-phage	0
DOF011_scaffold21119_2	non-phage	0
DOF011_scaffold21219_1	non-phage	0
DOF011_C265025_1	non-phage	0
DOF011_scaffold1769_16	non-phage	0
DOF011_scaffold19024_1	non-phage	0
DOF011_scaffold19093_1	non-phage	0
DOF011_scaffold8241_4	non-phage	0
DOF011_scaffold847_3	non-phage	0
DOF011_scaffold21038_2	non-phage	0
DOF011_scaffold12507_9	non-phage	0
DOF011_scaffold1139_7	non-phage	0
DOF011_scaffold16504_2	non-phage	0
DOF011_scaffold915_18	non-phage	0
DOF011_scaffold19849_1	non-phage	0
DOF011_scaffold1665_1	non-phage	0
DOF011_scaffold19771_12	non-phage	0
DOF011_C265041_1	non-phage	0
DOF011_scaffold21193_2	non-phage	0
DOF011_scaffold4132_2	non-phage	0
DOF011_scaffold5960_3	non-phage	0
DOF011_scaffold7642_1	non-phage	0
DOF011_scaffold2633_19	non-phage	0
DOF011_scaffold15450_3	non-phage	0
DOF011_scaffold19812_1	non-phage	0
DOF011_scaffold21155_2	non-phage	0
DOF011_scaffold18004_3	non-phage	0
DOF011_C264903_1	non-phage	0
DOF011_scaffold8241_1	non-phage	0

DOF011_scaffold5660_3	non-phage	0
DOF011_scaffold19_1	non-phage	0
DOF011_scaffold20436_3	non-phage	0
DOF011_scaffold21136_1	non-phage	0
DOF011_scaffold10497_4	non-phage	0
DOF011_scaffold21102_1	non-phage	0
DOF011_scaffold14398_1	non-phage	0
DOF011_scaffold21168_2	non-phage	0
DOF011_scaffold58_1	non-phage	0
DOF011_scaffold10063_1	non-phage	0
DOF011_scaffold1390_2	non-phage	0
DOF011_scaffold7046_3	non-phage	0
DOF011_scaffold15873_3	non-phage	0
DOF011_scaffold10891_1	non-phage	0
DOF011_scaffold11521_1	non-phage	0
DOF011_scaffold410_2	non-phage	0
DOF011_scaffold17862_1	non-phage	0
DOF011_scaffold21165_1	phage	0.997
DOF011_scaffold5372_9	non-phage	0
DOF011_scaffold7389_2	non-phage	0
DOF011_scaffold13484_1	non-phage	0
DOF011_scaffold13014_4	non-phage	0
DOF011_scaffold20656_1	non-phage	0
DOF011_scaffold115_1	non-phage	0
DOF011_scaffold12335_1	non-phage	0
DOF011_scaffold19518_1	non-phage	0
DOF011_scaffold19850_2	non-phage	0
DOF011_scaffold21119_1	non-phage	0
DOF011_scaffold35_3	non-phage	0
DOF011_scaffold15745_1	non-phage	0
DOF011_scaffold14484_1	non-phage	0
DOF011_C264975_1	non-phage	0
DOF011_scaffold19644_1	non-phage	0
DOF011_scaffold9702_10	non-phage	0
DOF011_scaffold16989_5	non-phage	0
DOF011_scaffold19167_1	non-phage	0
DOF011_scaffold4928_10	non-phage	0
DOF011_scaffold21215_1	non-phage	0
DOF011_scaffold1982_4	non-phage	0
DOF011_scaffold13228_5	non-phage	0
DOF011_scaffold14986_1	non-phage	0
DOF011_scaffold17522_2	non-phage	0
DOF011_scaffold21195_1	non-phage	0
DOF011_scaffold16153_1	non-phage	0
DOF011_scaffold13287_1	non-phage	0
DOF011_scaffold18181_3	non-phage	0

DOF011_scaffold20958_1	non-phage	0
DOF011_scaffold9699_6	non-phage	0
DOF011_scaffold7543_1	non-phage	0
DOF011_scaffold21038_1	non-phage	0
DOF011_scaffold1099_2	non-phage	0
DOF011_scaffold1139_1	non-phage	0
DOF011_scaffold2900_9	non-phage	0
DOF011_scaffold1139_3	non-phage	0
DOF011_scaffold18445_2	non-phage	0
DOF011_scaffold5442_1	non-phage	0
DOF011_scaffold20065_1	non-phage	0
DOF011_scaffold10063_7	non-phage	0
DOF011_scaffold18383_2	non-phage	0
DOF011_scaffold35_2	non-phage	0
DOF011_scaffold16872_1	non-phage	0
DOF011_scaffold20290_2	non-phage	0
DOF011_scaffold8743_4	non-phage	0
DOF011_scaffold13484_5	non-phage	0
DOF011_scaffold11344_2	non-phage	0
DOF011_scaffold1769_9	non-phage	0
DOF011_scaffold14059_1	non-phage	0
DOF011_scaffold847_2	non-phage	0
DOF011_scaffold4214_1	non-phage	0
DOF011_scaffold17251_4	non-phage	0
DOF011_scaffold4270_14	non-phage	0
DOF011_scaffold128_2	non-phage	0
DOF011_scaffold9668_1	non-phage	0
DOF011_scaffold21193_1	non-phage	0
DOF011_scaffold21007_1	non-phage	0
DOF011_scaffold21202_1	non-phage	0
DOF011_scaffold1401_4	non-phage	0
DOF011_C264717_1	non-phage	0
DOF011_scaffold20584_2	non-phage	0
DOF011_scaffold16989_6	non-phage	0
DOF011_scaffold21214_1	non-phage	0
DOF011_scaffold21218_1	non-phage	0
DOF011_scaffold193_4	non-phage	0
DOF011_scaffold17136_7	non-phage	0
DOF011_scaffold21213_1	phage	1
DOF011_scaffold17136_2	non-phage	0
DOF011_scaffold18004_5	non-phage	0
DOF011_scaffold21069_1	non-phage	0
DOF011_scaffold66_6	non-phage	0
DOF011_scaffold193_6	non-phage	0
DOF011_scaffold9702_11	non-phage	0
DOF011_scaffold21185_1	non-phage	0

DOF011_scaffold21021_1	non-phage	0
DOF011_scaffold5414_1	non-phage	0
DOF011_scaffold11590_1	non-phage	0
DOF011_scaffold9702_4	non-phage	0
DOF011_scaffold6973_1	non-phage	0
DOF011_scaffold5773_44	non-phage	0
DOF011_scaffold18535_1	non-phage	0
DOF011_scaffold20286_6	non-phage	0
DOF011_scaffold21093_1	non-phage	0
DOF011_scaffold20219_2	non-phage	0
DOF011_scaffold10714_1	non-phage	0
DOF011_scaffold17964_1	non-phage	0
DOF011_scaffold17251_2	non-phage	0
DOF011_scaffold19811_3	non-phage	0
DOF011_scaffold9699_5	non-phage	0
DOF011_scaffold4342_2	non-phage	0
DOF011_scaffold19909_10	non-phage	0
DOF011_scaffold20770_1	non-phage	0
DOF011_scaffold16405_4	non-phage	0
DOF011_scaffold3916_2	non-phage	0
DOF011_scaffold7668_1	non-phage	0
DOF011_scaffold20080_5	non-phage	0
DOF011_scaffold77_1	phage	0.999
DOF011_scaffold15450_2	non-phage	0
DOF011_scaffold9783_9	non-phage	0
DOF011_scaffold21145_1	non-phage	0
DOF011_scaffold3182_1	non-phage	0
DOF011_scaffold21211_1	non-phage	0
DOF011_scaffold4270_4	non-phage	0
DOF011_scaffold15364_1	non-phage	0
DOF011_scaffold5581_1	non-phage	0
DOF011_scaffold66_7	non-phage	0
DOF011_scaffold15873_8	non-phage	0
DOF011_scaffold13876_1	non-phage	0
DOF011_scaffold5619_2	non-phage	0
DOF011_scaffold17470_2	non-phage	0
DOF011_scaffold15008_2	non-phage	0
DOF011_scaffold5442_2	non-phage	0
DOF011_scaffold2635_5	phage	0.919
DOF011_scaffold17251_5	non-phage	0
DOF011_scaffold19497_1	non-phage	0
DOF011_scaffold20120_1	non-phage	0
DOF011_scaffold18342_1	non-phage	0
DOF011_scaffold18710_2	non-phage	0
DOF011_scaffold14539_2	non-phage	0
DOF011_scaffold114_2	non-phage	0

DOF011_scaffold10770_2	non-phage	0
DOF011_scaffold17549_2	non-phage	0
DOF011_scaffold576_1	non-phage	0
DOF011_scaffold3625_3	non-phage	0
DOF011_scaffold20857_1	non-phage	0
DOF011_scaffold13888_3	non-phage	0
DOF011_scaffold12507_12	non-phage	0
DOF011_scaffold20550_1	non-phage	0
DOF011_scaffold2029_4	non-phage	0
DOF011_scaffold6470_1	non-phage	0
DOF011_scaffold10537_2	non-phage	0
DOF011_scaffold114_1	non-phage	0
DOF011_scaffold19879_1	non-phage	0
DOF011_scaffold17964_2	non-phage	0
DOF011_scaffold21086_1	non-phage	0
DOF011_scaffold1769_19	non-phage	0
DOF011_scaffold21179_1	non-phage	0
DOF011_scaffold12527_1	non-phage	0
DOF011_scaffold193_5	non-phage	0
DOF011_scaffold21217_2	non-phage	0
DOF011_scaffold4011_2	non-phage	0
DOF011_scaffold18444_2	non-phage	0
DOF011_scaffold21174_2	phage	0.585
DOF011_scaffold16154_1	non-phage	0
DOF011_scaffold21034_1	non-phage	0
DOF011_scaffold13228_3	non-phage	0
DOF011_scaffold20315_6	non-phage	0
DOF011_scaffold4928_6	non-phage	0
DOF011_scaffold21194_1	non-phage	0
DOF011_scaffold9589_5	non-phage	0
DOF011_scaffold16_11	non-phage	0
DOF011_scaffold19614_1	non-phage	0
DOF011_scaffold21016_2	non-phage	0
DOF011_scaffold2386_1	phage	0.993
DOF011_scaffold17202_1	non-phage	0
DOF011_scaffold16504_5	non-phage	0
DOF011_scaffold19231_3	non-phage	0
DOF011_scaffold17978_1	non-phage	0
DOF011_scaffold3639_4	non-phage	0
DOF011_scaffold426_14	non-phage	0
DOF011_scaffold6014_6	non-phage	0
DOF011_C265035_1	non-phage	0
DOF011_scaffold19164_2	non-phage	0
DOF011_scaffold20907_4	non-phage	0
DOF011_scaffold3904_3	non-phage	0
DOF011_C264573_1	non-phage	0

DOF011_scaffold10063_10	non-phage	0
DOF011_scaffold16308_1	non-phage	0
DOF011_scaffold14317_9	non-phage	0
DOF011_scaffold19804_1	non-phage	0
DOF011_scaffold18052_1	non-phage	0
DOF011_scaffold915_13	non-phage	0
DOF011_scaffold17428_1	non-phage	0
DOF011_scaffold19913_1	non-phage	0
DOF011_scaffold5372_11	non-phage	0
DOF011_scaffold19856_2	non-phage	0
DOF011_scaffold20735_1	non-phage	0
DOF011_scaffold13876_3	non-phage	0
DOF011_scaffold18758_1	non-phage	0
DOF011_scaffold4928_5	non-phage	0
DOF011_scaffold128_4	non-phage	0
DOF011_scaffold21013_2	non-phage	0
DOF011_scaffold11947_1	non-phage	0
DOF011_scaffold1_4	non-phage	0
DOF011_scaffold20393_1	non-phage	0
DOF011_scaffold1337_13	non-phage	0
DOF011_scaffold7389_3	non-phage	0
DOF011_scaffold2136_5	non-phage	0
DOF011_scaffold19395_2	non-phage	0
DOF011_scaffold20563_1	non-phage	0
DOF011_scaffold6014_1	non-phage	0
DOF011_scaffold21197_1	non-phage	0
DOF011_scaffold7212_1	non-phage	0
DOF011_scaffold9702_8	non-phage	0
DOF011_scaffold2136_3	non-phage	0
DOF011_scaffold12827_1	non-phage	0
DOF011_scaffold66_3	non-phage	0
DOF011_scaffold1298_1	non-phage	0
DOF011_scaffold4135_1	non-phage	0
DOF011_scaffold5372_3	non-phage	0
DOF011_scaffold8875_1	non-phage	0
DOF011_scaffold2635_4	non-phage	0
DOF011_scaffold9783_1	non-phage	0
DOF011_scaffold8633_15	non-phage	0
DOF011_scaffold20285_2	non-phage	0
DOF011_scaffold19497_3	non-phage	0
DOF011_scaffold2049_2	non-phage	0
DOF011_scaffold15291_1	non-phage	0
DOF011_scaffold21199_1	non-phage	0
DOF011_scaffold12507_5	phage	0.961
DOF011_scaffold16695_1	non-phage	0
DOF011_scaffold19164_1	non-phage	0

DOF011_scaffold17911_1	non-phage	0
DOF011_scaffold17522_4	non-phage	0
DOF011_scaffold21140_1	non-phage	0
DOF011_scaffold7818_3	non-phage	0
DOF011_scaffold10537_10	non-phage	0
DOF011_scaffold21170_1	non-phage	0
DOF011_scaffold20731_1	non-phage	0
DOF011_scaffold18535_2	non-phage	0
DOF011_scaffold915_14	non-phage	0
DOF011_scaffold9154_1	non-phage	0
DOF011_scaffold10714_4	non-phage	0
DOF011_scaffold18444_1	non-phage	0
DOF011_scaffold5372_13	non-phage	0
DOF011_scaffold16504_6	non-phage	0
DOF011_scaffold3639_3	non-phage	0
DOF011_scaffold21074_1	non-phage	0
DOF011_scaffold8241_3	non-phage	0
DOF011_scaffold14072_2	non-phage	0.008
DOF011_scaffold21204_1	non-phage	0
DOF011_scaffold15192_2	non-phage	0
DOF011_scaffold17198_1	non-phage	0
DOF011_scaffold10548_2	non-phage	0
DOF011_scaffold21173_2	non-phage	0
DOF011_scaffold15050_1	non-phage	0
DOF011_scaffold15980_1	non-phage	0
DOF011_scaffold15814_1	non-phage	0
DOF011_scaffold14398_8	non-phage	0
DOF011_scaffold9699_2	non-phage	0
DOF011_scaffold20931_3	non-phage	0
DOF011_scaffold14783_2	non-phage	0
DOF011_scaffold21137_1	non-phage	0
DOF011_scaffold5518_2	non-phage	0
DOF011_scaffold10537_6	non-phage	0
DOF011_scaffold8241_7	non-phage	0
DOF011_scaffold21189_1	non-phage	0
DOF011_scaffold2635_3	non-phage	0
DOF011_scaffold16207_2	non-phage	0
DOF011_scaffold13484_3	non-phage	0
DOF011_scaffold9699_1	non-phage	0
DOF011_scaffold548_3	non-phage	0
DOF011_scaffold4575_1	non-phage	0
DOF011_scaffold19699_2	phage	0.999
DOF011_scaffold5782_10	non-phage	0
DOF011_scaffold17011_1	non-phage	0
DOF011_scaffold5960_6	non-phage	0
DOF011_scaffold21167_2	non-phage	0

DOF011_scaffold18007_1	non-phage	0
DOF011_scaffold17389_1	non-phage	0
DOF011_scaffold21216_1	non-phage	0
DOF011_scaffold21210_1	non-phage	0
DOF011_scaffold21070_1	non-phage	0
DOF011_C264797_1	non-phage	0.044
DOF011_scaffold1371_2	non-phage	0
DOF011_scaffold410_1	non-phage	0
DOF011_scaffold7338_1	non-phage	0
DOF011_scaffold16504_4	non-phage	0
DOF011_scaffold5372_1	non-phage	0
DOF011_scaffold18383_4	non-phage	0
DOF011_scaffold11640_1	non-phage	0
DOF011_scaffold4070_2	non-phage	0
DOF011_C265045_1	non-phage	0
DOF011_scaffold10063_3	non-phage	0
DOF011_scaffold19227_5	non-phage	0
DOF011_scaffold21018_2	non-phage	0
DOF011_scaffold21184_1	non-phage	0
DOF011_scaffold6741_2	non-phage	0
DOF011_scaffold15412_1	non-phage	0
DOF011_scaffold19699_1	non-phage	0
DOF011_scaffold3904_6	non-phage	0
DOF011_scaffold21188_1	non-phage	0
DOF011_scaffold35_5	non-phage	0
DOF011_scaffold6014_8	non-phage	0
DOF011_scaffold39_1	non-phage	0
DOF011_scaffold19170_1	non-phage	0
DOF011_scaffold14985_1	non-phage	0
DOF011_scaffold1252_1	non-phage	0
DOF011_scaffold18275_1	non-phage	0
DOF011_scaffold7281_2	non-phage	0
DOF011_scaffold426_15	non-phage	0
DOF011_scaffold21205_1	non-phage	0
DOF011_scaffold1228_2	non-phage	0
DOF011_scaffold10063_4	non-phage	0
DOF011_scaffold1331_2	non-phage	0
DOF011_scaffold1013_1	non-phage	0
DOF011_scaffold8429_6	non-phage	0
DOF011_scaffold16405_1	non-phage	0
DOF011_scaffold9783_4	non-phage	0
DOF011_scaffold9702_13	non-phage	0
DOF011_scaffold7818_1	non-phage	0
DOF011_scaffold12955_1	non-phage	0
DOF011_scaffold14259_3	non-phage	0
DOF011_scaffold4575_2	non-phage	0

DOF011_scaffold20914_1	phage	0.999
DOF011_scaffold12085_1	non-phage	0
DOF011_scaffold1769_11	non-phage	0
DOF011_C264819_1	non-phage	0
DOF011_scaffold3385_1	non-phage	0
DOF011_scaffold15873_1	non-phage	0
DOF011_scaffold5372_15	non-phage	0
DOF011_C265027_1	non-phage	0
DOF011_scaffold21034_3	non-phage	0
DOF011_scaffold10537_5	non-phage	0
DOF011_scaffold17340_1	non-phage	0
DOF011_scaffold15450_1	non-phage	0
DOF011_scaffold8633_10	non-phage	0
DOF011_scaffold5582_1	non-phage	0
DOF011_scaffold16207_1	non-phage	0
DOF011_scaffold5337_3	non-phage	0
DOF011_C264509_1	non-phage	0
DOF011_scaffold1161_12	non-phage	0
DOF011_scaffold1393_6	non-phage	0
DOF011_scaffold21111_2	non-phage	0
DOF011_scaffold114_8	non-phage	0
DOF011_scaffold2900_11	non-phage	0
DOF011_scaffold21203_1	non-phage	0
DOF011_C264851_1	non-phage	0
DOF011_scaffold12085_4	non-phage	0
DOF011_scaffold9702_7	non-phage	0
DOF011_scaffold1175_2	non-phage	0
DOF011_scaffold10537_4	non-phage	0
DOF011_scaffold21171_1	non-phage	0
DOF011_scaffold20285_1	non-phage	0
DOF011_scaffold12318_1	non-phage	0
DOF011_scaffold21190_1	non-phage	0
DOF011_scaffold15664_3	non-phage	0
DOF011_scaffold426_16	non-phage	0
DOF011_scaffold21138_1	non-phage	0
DOF011_scaffold15980_3	non-phage	0
DOF011_scaffold128_3	non-phage	0
DOF011_scaffold15585_1	non-phage	0
DOF011_scaffold16419_1	non-phage	0
DOF011_scaffold2210_3	non-phage	0
DOF011_scaffold1331_1	non-phage	0
DOF011_C264847_1	non-phage	0
DOF011_scaffold14259_2	non-phage	0
DOF011_scaffold15873_11	non-phage	0
DOF011_scaffold5176_1	non-phage	0
DOF011_scaffold21212_1	non-phage	0

DOF012_scaffold5874_1	non-phage	0
DOF012_scaffold1920_5	non-phage	0
DOF012_scaffold3010_2	non-phage	0
DOF012_scaffold932_8	non-phage	0
DOF012_scaffold20_8	non-phage	0
DOF012_scaffold2141_4	non-phage	0
DOF012_scaffold409_12	non-phage	0
DOF012_scaffold932_14	non-phage	0
DOF012_scaffold3955_3	non-phage	0
DOF012_scaffold11961_3	non-phage	0
DOF012_scaffold120_3	non-phage	0.007
DOF012_scaffold5482_3	non-phage	0
DOF012_scaffold1713_4	non-phage	0
DOF012_scaffold288_1	non-phage	0
DOF012_scaffold239_5	non-phage	0
DOF012_scaffold1710_3	phage	0.516
DOF012_scaffold116_2	non-phage	0
DOF012_scaffold1231_1	non-phage	0
DOF012_scaffold347_4	non-phage	0
DOF012_scaffold1127_8	non-phage	0
DOF012_scaffold5263_5	non-phage	0
DOF012_scaffold3817_1	non-phage	0
DOF012_scaffold1436_3	non-phage	0
DOF012_scaffold4983_2	non-phage	0
DOF012_scaffold2201_1	non-phage	0
DOF012_scaffold1703_5	non-phage	0
DOF012_scaffold7331_8	non-phage	0
DOF012_scaffold1970_5	non-phage	0.042
DOF012_scaffold2697_2	non-phage	0
DOF012_scaffold2669_1	non-phage	0
DOF012_scaffold523_4	non-phage	0
DOF012_scaffold337_2	non-phage	0
DOF012_scaffold13705_1	non-phage	0
DOF012_scaffold12083_6	non-phage	0
DOF012_scaffold819_1	non-phage	0
DOF012_scaffold314_2	non-phage	0
DOF012_scaffold233_10	non-phage	0
DOF012_scaffold3470_2	non-phage	0
DOF012_scaffold1855_5	non-phage	0
DOF012_scaffold225_7	non-phage	0
DOF012_scaffold57_5	non-phage	0
DOF012_scaffold14014_1	non-phage	0
DOF012_scaffold1786_4	non-phage	0
DOF012_scaffold7884_2	non-phage	0
DOF012_scaffold5504_1	non-phage	0
DOF012_scaffold2311_5	non-phage	0

DOF012_scaffold1370_3	non-phage	0
DOF012_scaffold5859_1	non-phage	0
DOF012_scaffold4796_1	non-phage	0
DOF012_scaffold20_2	non-phage	0
DOF012_scaffold12833_1	non-phage	0
DOF012_scaffold4983_4	non-phage	0
DOF012_scaffold12555_2	phage	0.996
DOF012_scaffold4848_2	non-phage	0
DOF012_scaffold11830_2	non-phage	0
DOF012_scaffold463_10	phage	0.999
DOF012_scaffold1430_1	non-phage	0
DOF012_scaffold4720_1	phage	0.996
DOF012_scaffold604_2	non-phage	0
DOF012_scaffold13712_2	non-phage	0
DOF012_scaffold1833_2	non-phage	0
DOF012_scaffold9869_1	non-phage	0
DOF012_scaffold149_2	non-phage	0
DOF012_scaffold166_17	non-phage	0
DOF012_scaffold244_2	non-phage	0
DOF012_scaffold2936_1	non-phage	0
DOF012_scaffold10551_2	non-phage	0
DOF012_scaffold392_4	non-phage	0
DOF012_scaffold2141_12	non-phage	0
DOF012_scaffold420_11	non-phage	0
DOF012_scaffold441_2	non-phage	0
DOF012_scaffold376_7	non-phage	0
DOF012_scaffold6801_3	non-phage	0
DOF012_scaffold6428_2	non-phage	0
DOF012_scaffold6243_1	non-phage	0
DOF012_scaffold813_1	non-phage	0
DOF012_scaffold2141_1	non-phage	0
DOF012_scaffold2926_7	non-phage	0
DOF012_scaffold8331_3	non-phage	0
DOF012_scaffold884_2	phage	1
DOF012_scaffold14425_2	non-phage	0
DOF012_scaffold798_3	non-phage	0
DOF012_scaffold332_5	non-phage	0
DOF012_scaffold2843_5	non-phage	0
DOF012_scaffold463_2	non-phage	0
DOF012_scaffold6439_6	non-phage	0
DOF012_scaffold42_4	non-phage	0
DOF012_scaffold5305_2	non-phage	0
DOF012_scaffold2145_5	non-phage	0
DOF012_scaffold125_5	non-phage	0
DOF012_scaffold2926_9	non-phage	0
DOF012_scaffold288_3	non-phage	0

DOF012_scaffold9519_2	non-phage	0
DOF012_scaffold3862_3	non-phage	0
DOF012_scaffold1206_5	non-phage	0
DOF012_scaffold3203_2	non-phage	0
DOF012_scaffold9548_2	phage	0.995
DOF012_scaffold932_6	non-phage	0
DOF012_scaffold189_4	non-phage	0
DOF012_scaffold523_2	non-phage	0
DOF012_scaffold2641_2	non-phage	0
DOF012_scaffold2926_2	non-phage	0
DOF012_scaffold1970_3	non-phage	0
DOF012_scaffold7683_3	non-phage	0
DOF012_scaffold7494_9	non-phage	0
DOF012_scaffold3559_4	non-phage	0
DOF012_scaffold1229_3	non-phage	0
DOF012_scaffold215_2	non-phage	0
DOF012_scaffold8331_5	non-phage	0
DOF012_scaffold12133_1	non-phage	0
DOF012_scaffold7471_2	non-phage	0
DOF012_scaffold992_3	phage	0.964
DOF012_scaffold3214_2	non-phage	0
DOF012_scaffold198_5	non-phage	0
DOF012_scaffold10083_1	non-phage	0
DOF012_scaffold143_1	non-phage	0
DOF012_scaffold7138_1	non-phage	0
DOF012_scaffold12083_1	non-phage	0
DOF012_scaffold3619_4	non-phage	0
DOF012_scaffold5495_1	non-phage	0
DOF012_scaffold9844_1	phage	0.999
DOF012_scaffold1232_1	non-phage	0
DOF012_scaffold4796_3	non-phage	0
DOF012_scaffold1833_4	non-phage	0
DOF012_scaffold1380_9	non-phage	0
DOF012_scaffold62_7	non-phage	0
DOF012_scaffold418_7	non-phage	0
DOF012_scaffold4353_5	non-phage	0
DOF012_scaffold2731_5	non-phage	0
DOF012_scaffold1819_1	non-phage	0
DOF012_scaffold360_1	non-phage	0
DOF012_scaffold150_9	non-phage	0
DOF012_scaffold198_4	non-phage	0
DOF012_scaffold274_4	non-phage	0.001
DOF012_scaffold5414_4	non-phage	0
DOF012_scaffold1150_4	non-phage	0
DOF012_scaffold314_4	non-phage	0
DOF012_scaffold1855_3	non-phage	0

DOF012_scaffold2165_2	non-phage	0
DOF012_scaffold42_8	non-phage	0
DOF012_scaffold5764_1	non-phage	0
DOF012_scaffold999_1	non-phage	0
DOF012_scaffold1786_2	non-phage	0
DOF012_scaffold3470_4	non-phage	0
DOF012_scaffold8336_1	non-phage	0.075
DOF012_scaffold1021_1	non-phage	0
DOF012_scaffold7309_3	non-phage	0
DOF012_scaffold10000_1_2	non-phage	0
DOF012_scaffold2324_4	non-phage	0
DOF012_scaffold18_8	non-phage	0
DOF012_scaffold1847_11	non-phage	0
DOF012_scaffold469_6	non-phage	0
DOF012_scaffold1941_1	non-phage	0
DOF012_scaffold298_2	non-phage	0
DOF012_scaffold6657_3	non-phage	0
DOF012_scaffold98_2	non-phage	0
DOF012_scaffold7291_1	non-phage	0
DOF012_scaffold4335_3	non-phage	0
DOF012_scaffold2145_3	non-phage	0
DOF012_scaffold4454_3	non-phage	0
DOF012_scaffold1004_1	non-phage	0
DOF012_scaffold7291_6	non-phage	0
DOF012_scaffold2463_2	non-phage	0
DOF012_scaffold4319_5	non-phage	0
DOF012_scaffold262_2	non-phage	0
DOF012_scaffold3502_2	non-phage	0
DOF012_scaffold604_4	non-phage	0
DOF012_scaffold4068_1	non-phage	0
DOF012_scaffold4483_10	non-phage	0
DOF012_scaffold10551_4	non-phage	0
DOF012_scaffold2936_3	phage	0.998
DOF012_scaffold858_1	non-phage	0
DOF012_scaffold304_2_1	non-phage	0
DOF012_scaffold4168_2	non-phage	0
DOF012_scaffold9023_2	non-phage	0
DOF012_scaffold897_11	non-phage	0
DOF012_scaffold813_10	non-phage	0
DOF012_scaffold92_10	non-phage	0
DOF012_scaffold298_5	non-phage	0
DOF012_scaffold2224_45	non-phage	0
DOF012_scaffold8296_1	non-phage	0
DOF012_scaffold167_3	non-phage	0
DOF012_scaffold4762_2	non-phage	0
DOF012_scaffold4227_7	non-phage	0

DOF012_scaffold4324_3	non-phage	0
DOF012_scaffold10083_3	non-phage	0
DOF012_scaffold12334_1	phage	0.998
DOF012_scaffold337_4	non-phage	0
DOF012_scaffold897_15	non-phage	0
DOF012_scaffold6460_5	non-phage	0
DOF012_scaffold1286_1	non-phage	0
DOF012_scaffold906_2	non-phage	0
DOF012_scaffold2532_2	non-phage	0
DOF012_scaffold1127_5	non-phage	0
DOF012_scaffold240_1	non-phage	0
DOF012_scaffold12083_2	non-phage	0
DOF012_scaffold3056_5	non-phage	0
DOF012_scaffold1242_3	non-phage	0
DOF012_scaffold4483_8	non-phage	0
DOF012_scaffold28_7	non-phage	0
DOF012_scaffold7057_2	non-phage	0
DOF012_scaffold12133_3	non-phage	0
DOF012_scaffold4450_1	non-phage	0
DOF012_scaffold9512_1	non-phage	0
DOF012_scaffold278_14	non-phage	0
DOF012_scaffold20_4	non-phage	0
DOF012_scaffold843_2	non-phage	0
DOF012_scaffold198_3	non-phage	0
DOF012_scaffold233_4	non-phage	0
DOF012_scaffold213_3	non-phage	0
DOF012_scaffold1688_5	non-phage	0
DOF012_scaffold469_5	non-phage	0
DOF012_scaffold1833_3	non-phage	0
DOF012_scaffold178_2	non-phage	0
DOF012_scaffold897_3	non-phage	0
DOF012_scaffold2926_4	non-phage	0
DOF012_scaffold1920_2	non-phage	0
DOF012_scaffold7943_2	non-phage	0
DOF012_scaffold4090_1	non-phage	0
DOF012_scaffold7698_3	non-phage	0
DOF012_scaffold3142_3	non-phage	0
DOF012_scaffold1114_8	non-phage	0
DOF012_scaffold4226_2	non-phage	0
DOF012_scaffold463_1	non-phage	0
DOF012_scaffold150_3	non-phage	0
DOF012_scaffold64_1	non-phage	0
DOF012_scaffold7331_2	non-phage	0
DOF012_scaffold897_13	non-phage	0
DOF012_scaffold217_1	non-phage	0
DOF012_scaffold10992_2	non-phage	0

DOF012_scaffold188_5	non-phage	0
DOF012_scaffold9519_5	non-phage	0
DOF012_scaffold239_1	non-phage	0
DOF012_scaffold10555_2	non-phage	0
DOF012_scaffold48_5	non-phage	0
DOF012_scaffold2304_9	non-phage	0
DOF012_scaffold5498_5	non-phage	0
DOF012_scaffold250_4	non-phage	0
DOF012_scaffold327_11	non-phage	0
DOF012_scaffold258_7	non-phage	0
DOF012_scaffold8821_1	non-phage	0
DOF012_scaffold5780_4	non-phage	0
DOF012_scaffold4454_5	non-phage	0
DOF012_scaffold3497_2	non-phage	0
DOF012_scaffold6356_3	non-phage	0
DOF012_scaffold1349_1	non-phage	0
DOF012_scaffold1225_2	non-phage	0
DOF012_scaffold28_15	non-phage	0
DOF012_scaffold1236_1	non-phage	0
DOF012_scaffold20_7	non-phage	0
DOF012_scaffold278_2	non-phage	0
DOF012_scaffold469_4	non-phage	0
DOF012_scaffold217_6	non-phage	0
DOF012_scaffold150_8	non-phage	0
DOF012_scaffold430_2	non-phage	0
DOF012_scaffold6296_1	non-phage	0
DOF012_scaffold233_8	non-phage	0
DOF012_scaffold191_16	non-phage	0
DOF012_scaffold8331_4	non-phage	0
DOF012_scaffold523_5	non-phage	0
DOF012_scaffold11422_5	non-phage	0
DOF012_scaffold4053_6	non-phage	0
DOF012_scaffold225_10	non-phage	0
DOF012_scaffold758_2	non-phage	0
DOF012_scaffold12555_1	non-phage	0
DOF012_scaffold419_7	non-phage	0
DOF012_scaffold445_7	non-phage	0
DOF012_scaffold1842_1	non-phage	0
DOF012_scaffold932_2	non-phage	0
DOF012_scaffold1127_7	non-phage	0
DOF012_scaffold28_9	non-phage	0
DOF012_scaffold1436_6	non-phage	0
DOF012_scaffold3203_7	non-phage	0
DOF012_scaffold3619_1	non-phage	0
DOF012_scaffold248_1	non-phage	0
DOF012_scaffold4227_5	non-phage	0

DOF012_scaffold143_5	non-phage	0
DOF012_scaffold1905_1	non-phage	0
DOF012_scaffold13_3	non-phage	0
DOF012_scaffold5780_1	non-phage	0
DOF012_scaffold9792_4	non-phage	0
DOF012_scaffold198_1	phage	0.973
DOF012_scaffold70_2	non-phage	0
DOF012_scaffold4685_1	non-phage	0
DOF012_scaffold1920_4	non-phage	0
DOF012_scaffold2787_4	non-phage	0
DOF012_scaffold2141_9	non-phage	0
DOF012_scaffold5083_4	non-phage	0
DOF012_scaffold14634_1	non-phage	0
DOF012_scaffold7585_1	non-phage	0
DOF012_scaffold300_1	non-phage	0
DOF012_scaffold1713_3	non-phage	0
DOF012_scaffold4839_4	non-phage	0
DOF012_scaffold4539_3	phage	0.775
DOF012_scaffold6444_1	non-phage	0
DOF012_scaffold5961_1	non-phage	0
DOF012_scaffold7884_5	non-phage	0
DOF012_scaffold28_10	non-phage	0
DOF012_scaffold444_1	non-phage	0
DOF012_scaffold3559_7	non-phage	0
DOF012_scaffold718_3	non-phage	0
DOF012_scaffold1127_10	non-phage	0
DOF012_scaffold557_2	non-phage	0
DOF012_scaffold242_3	non-phage	0
DOF012_scaffold3862_4	non-phage	0
DOF012_scaffold409_2	non-phage	0
DOF012_scaffold8351_5	non-phage	0
DOF012_scaffold819_5	non-phage	0
DOF012_scaffold4959_1	non-phage	0
DOF012_scaffold392_6	non-phage	0
DOF012_scaffold1977_1	non-phage	0
DOF012_scaffold20_9	non-phage	0
DOF012_scaffold130_13	non-phage	0
DOF012_scaffold858_4	non-phage	0
DOF012_scaffold11961_2	non-phage	0
DOF012_scaffold8213_2	non-phage	0
DOF012_scaffold381_2	phage	0.999
DOF012_scaffold198_7	non-phage	0
DOF012_scaffold321_4	non-phage	0
DOF012_scaffold3056_8	non-phage	0
DOF012_scaffold8331_8	non-phage	0
DOF012_scaffold101_6	non-phage	0

DOF012_scaffold1874_3	non-phage	0
DOF012_scaffold780_2	non-phage	0
DOF012_scaffold2201_3	non-phage	0
DOF012_scaffold7331_4	non-phage	0
DOF012_scaffold11813_1	non-phage	0
DOF012_scaffold14126_1	non-phage	0
DOF012_scaffold4324_5	non-phage	0
DOF012_scaffold298_7	non-phage	0
DOF012_scaffold10992_4	non-phage	0
DOF012_scaffold992_7	non-phage	0
DOF012_scaffold20_16	non-phage	0
DOF012_scaffold189_1	non-phage	0
DOF012_scaffold7331_10	non-phage	0
DOF012_scaffold3686_13	non-phage	0
DOF012_scaffold4796_7	non-phage	0
DOF012_scaffold57_11	non-phage	0
DOF012_scaffold2578_15	non-phage	0
DOF012_scaffold5888_2	non-phage	0
DOF012_scaffold2451_1	non-phage	0
DOF012_scaffold1970_7	non-phage	0
DOF012_scaffold166_6	non-phage	0
DOF012_scaffold289_3	non-phage	0
DOF012_scaffold4353_1	non-phage	0
DOF012_scaffold4983_3	non-phage	0
DOF012_scaffold3394_7	phage	0.999
DOF012_scaffold6561_1	non-phage	0
DOF012_scaffold633_5	non-phage	0
DOF012_scaffold3973_3	non-phage	0
DOF012_scaffold8733_5	non-phage	0
DOF012_scaffold7309_7	non-phage	0
DOF012_scaffold1380_4	non-phage	0
DOF012_scaffold7046_5	non-phage	0
DOF012_scaffold2141_13	non-phage	0.005
DOF012_scaffold7119_1	non-phage	0
DOF012_scaffold5859_2	non-phage	0
DOF012_scaffold20_3	non-phage	0
DOF012_scaffold7884_3	non-phage	0
DOF012_scaffold314_3	non-phage	0.005
DOF012_scaffold1855_4	non-phage	0
DOF012_scaffold4324_4	non-phage	0
DOF012_scaffold813_3	non-phage	0
DOF012_scaffold12779_2	non-phage	0
DOF012_scaffold1786_5	non-phage	0
DOF012_scaffold931_1	non-phage	0
DOF012_scaffold17_8	non-phage	0
DOF012_scaffold13424_1	non-phage	0

DOF012_scaffold4796_2	non-phage	0
DOF012_scaffold1970_2	non-phage	0
DOF012_scaffold98_1	non-phage	0
DOF012_scaffold3647_2	non-phage	0
DOF012_scaffold2311_4	non-phage	0
DOF012_scaffold62_10	non-phage	0
DOF012_scaffold1133_1	non-phage	0
DOF012_scaffold835_2	non-phage	0
DOF012_scaffold14380_1	non-phage	0
DOF012_scaffold3364_1	non-phage	0
DOF012_scaffold191_19	non-phage	0
DOF012_scaffold3133_1	non-phage	0
DOF012_scaffold1568_1	non-phage	0
DOF012_scaffold11306_2	non-phage	0
DOF012_scaffold105_5	non-phage	0
DOF012_scaffold303_7	non-phage	0
DOF012_scaffold215_3	non-phage	0
DOF012_scaffold11803_1	non-phage	0
DOF012_scaffold537_3	non-phage	0
DOF012_scaffold191_12	non-phage	0
DOF012_scaffold10551_1	non-phage	0
DOF012_scaffold9953_1	non-phage	0
DOF012_scaffold125_4	non-phage	0
DOF012_scaffold8268_1	non-phage	0
DOF012_scaffold14126_6	non-phage	0
DOF012_scaffold397_2	non-phage	0
DOF012_scaffold166_20	non-phage	0
DOF012_scaffold3396_1	non-phage	0
DOF012_scaffold191_17	non-phage	0
DOF012_scaffold62_4	non-phage	0
DOF012_scaffold6819_1	non-phage	0
DOF012_scaffold5047_1	non-phage	0
DOF012_scaffold116_1	non-phage	0
DOF012_scaffold433_1	non-phage	0
DOF012_scaffold523_1	non-phage	0
DOF012_scaffold1571_5	non-phage	0
DOF012_scaffold4166_1	non-phage	0
DOF012_scaffold5482_2	non-phage	0
DOF012_scaffold9916_3	non-phage	0
DOF012_scaffold10730_2	non-phage	0
DOF012_scaffold2926_11	non-phage	0
DOF012_scaffold14410_1	non-phage	0
DOF012_scaffold718_8	non-phage	0
DOF012_scaffold2482_4	non-phage	0
DOF012_scaffold846_2	non-phage	0
DOF012_scaffold241_3	non-phage	0

DOF012_scaffold2787_5	non-phage	0
DOF012_scaffold240_6	non-phage	0
DOF012_scaffold11298_1	non-phage	0
DOF012_scaffold8351_4	non-phage	0
DOF012_scaffold195_13	non-phage	0
DOF012_scaffold12401_1	non-phage	0
DOF012_scaffold150_11	non-phage	0
DOF012_scaffold9593_3	non-phage	0
DOF012_scaffold10083_4	non-phage	0
DOF012_scaffold1887_1	non-phage	0
DOF012_scaffold13705_2	non-phage	0
DOF012_scaffold28_3	non-phage	0
DOF012_scaffold198_10	non-phage	0
DOF012_scaffold1874_2	non-phage	0
DOF012_scaffold897_10	phage	0.988
DOF012_scaffold2879_5	non-phage	0
DOF012_scaffold3311_1	non-phage	0
DOF012_scaffold78_3	non-phage	0
DOF012_scaffold1270_2	non-phage	0
DOF012_scaffold1621_2	non-phage	0
DOF012_scaffold1351_8	non-phage	0
DOF012_scaffold191_15	non-phage	0
DOF012_scaffold1004_2	non-phage	0
DOF012_scaffold884_1	non-phage	0
DOF012_scaffold463_13	non-phage	0
DOF012_scaffold166_2	non-phage	0
DOF012_scaffold1380_8	non-phage	0
DOF012_scaffold4848_1	non-phage	0
DOF012_scaffold4741_3	phage	1
DOF012_scaffold4227_3	non-phage	0
DOF012_scaffold298_1	non-phage	0
DOF012_scaffold113_3	non-phage	0
DOF012_scaffold2038_3	non-phage	0
DOF012_scaffold9466_1	non-phage	0
DOF012_scaffold3025_5	non-phage	0
DOF012_scaffold376_4	non-phage	0
DOF012_scaffold5414_3	non-phage	0
DOF012_scaffold20_5	non-phage	0
DOF012_scaffold777_29	non-phage	0
DOF012_scaffold9839_2	non-phage	0
DOF012_scaffold18_11	non-phage	0
DOF012_scaffold392_5	non-phage	0
DOF012_scaffold9333_7	non-phage	0
DOF012_scaffold4774_4	non-phage	0
DOF012_scaffold2178_1	non-phage	0
DOF012_scaffold1703_7	non-phage	0

DOF012_scaffold1206_6	non-phage	0
DOF012_scaffold3678_2	non-phage	0
DOF012_scaffold143_4	non-phage	0
DOF012_scaffold7309_9	non-phage	0
DOF012_scaffold5185_2	non-phage	0
DOF012_scaffold341_2	non-phage	0
DOF012_scaffold1351_10	non-phage	0
DOF012_scaffold7_12	phage	0.999
DOF012_scaffold5109_2	non-phage	0
DOF012_scaffold5305_1	non-phage	0
DOF012_scaffold1713_14	non-phage	0
DOF012_scaffold51_2	non-phage	0
DOF012_scaffold42_5	non-phage	0
DOF012_scaffold3619_3	non-phage	0
DOF012_scaffold6202_3	non-phage	0
DOF012_scaffold1710_4	non-phage	0
DOF012_scaffold3862_2	non-phage	0
DOF012_scaffold6516_5	non-phage	0
DOF012_scaffold5109_5	non-phage	0
DOF012_scaffold718_6	non-phage	0
DOF012_scaffold1688_2	non-phage	0
DOF012_scaffold575_1	non-phage	0
DOF012_scaffold304_1	non-phage	0
DOF012_scaffold9519_1	non-phage	0
DOF012_scaffold239_4	non-phage	0
DOF012_scaffold14378_1	non-phage	0
DOF012_scaffold11830_6	non-phage	0
DOF012_scaffold3966_3	non-phage	0
DOF012_scaffold2086_1	non-phage	0
DOF012_scaffold906_6	non-phage	0
DOF012_scaffold6993_2	non-phage	0
DOF012_scaffold2165_3	non-phage	0
DOF012_scaffold7471_3	non-phage	0
DOF012_scaffold633_2	non-phage	0
DOF012_scaffold8831_1	non-phage	0
DOF012_scaffold4168_3	non-phage	0
DOF012_scaffold20_21	non-phage	0
DOF012_scaffold7683_2	non-phage	0
DOF012_scaffold451_1	non-phage	0
DOF012_scaffold298_3	non-phage	0
DOF012_scaffold17_3	non-phage	0
DOF012_scaffold8351_2	non-phage	0
DOF012_scaffold1196_2	non-phage	0
DOF012_scaffold1351_7	non-phage	0
DOF012_scaffold4227_8	non-phage	0
DOF012_scaffold797_1	non-phage	0

DOF012_scaffold496_3	non-phage	0
DOF012_scaffold7291_2	non-phage	0
DOF012_scaffold11813_2	non-phage	0
DOF012_scaffold14639_1	non-phage	0
DOF012_scaffold4266_3	non-phage	0
DOF012_scaffold604_5	non-phage	0
DOF012_scaffold3358_9	non-phage	0
DOF012_scaffold9544_6_2	non-phage	0
DOF012_scaffold241_1	non-phage	0
DOF012_scaffold11870_2	non-phage	0
DOF012_scaffold748_5	non-phage	0
DOF012_scaffold2324_2	non-phage	0
DOF012_scaffold6657_1	phage	0.944
DOF012_scaffold884_3	phage	0.999
DOF012_scaffold2879_1	non-phage	0
DOF012_scaffold8962_2	non-phage	0
DOF012_scaffold5418_1	non-phage	0
DOF012_scaffold1847_10	non-phage	0
DOF012_scaffold1970_1	non-phage	0
DOF012_scaffold3619_7	non-phage	0
DOF012_scaffold7309_4	non-phage	0
DOF012_scaffold144_1	non-phage	0
DOF012_scaffold191_8	non-phage	0
DOF012_scaffold304_3	non-phage	0
DOF012_scaffold4454_2	non-phage	0
DOF012_scaffold409_8	non-phage	0
DOF012_scaffold117_1	non-phage	0
DOF012_scaffold2038_4	non-phage	0
DOF012_scaffold4353_2	non-phage	0
DOF012_scaffold11656_1	non-phage	0
DOF012_scaffold4539_8	non-phage	0
DOF012_scaffold1351_6	non-phage	0
DOF012_scaffold5783_6	non-phage	0
DOF012_scaffold3619_6	non-phage	0
DOF012_scaffold20_20	non-phage	0
DOF012_scaffold1713_7	non-phage	0
DOF012_scaffold8331_9	non-phage	0
DOF012_scaffold1710_2	non-phage	0
DOF012_scaffold11390_4	non-phage	0
DOF012_scaffold150_7	non-phage	0
DOF012_scaffold5673_3_1	non-phage	0
DOF012_scaffold5305_4	non-phage	0
DOF012_scaffold205_1	non-phage	0
DOF012_scaffold1887_4	non-phage	0.003
DOF012_scaffold3762_2	non-phage	0
DOF012_scaffold191_20	phage	0.878

DOF012_scaffold3973_14	non-phage	0
DOF012_scaffold298_6	non-phage	0
DOF012_scaffold4227_6	non-phage	0
DOF012_scaffold7025_1	non-phage	0
DOF012_scaffold4483_5	non-phage	0
DOF012_scaffold555_8	non-phage	0
DOF012_scaffold284_3	non-phage	0
DOF012_scaffold8331_7	non-phage	0
DOF012_scaffold14816_1	non-phage	0
DOF012_scaffold10862_1	non-phage	0
DOF012_scaffold2532_1	non-phage	0
DOF012_scaffold409_6	non-phage	0
DOF012_scaffold2470_5	non-phage	0
DOF012_scaffold1632_2	non-phage	0
DOF012_scaffold6802_2	non-phage	0
DOF012_scaffold111_3	non-phage	0
DOF012_scaffold2787_1	non-phage	0
DOF012_scaffold14781_2	phage	0.977
DOF012_scaffold992_4	non-phage	0
DOF012_scaffold13701_1	non-phage	0
DOF012_scaffold4832_26	non-phage	0
DOF012_scaffold11390_6	non-phage	0
DOF012_scaffold5109_4	non-phage	0
DOF012_scaffold1713_13	non-phage	0
DOF012_scaffold101_1	non-phage	0
DOF012_scaffold213_2	non-phage	0
DOF012_scaffold1786_1	non-phage	0
DOF012_scaffold142_2	non-phage	0
DOF012_scaffold7527_2	non-phage	0
DOF012_scaffold459_1	non-phage	0
DOF012_scaffold13482_2	non-phage	0
DOF012_scaffold8351_6	non-phage	0
DOF012_scaffold7428_1	non-phage	0
DOF012_scaffold463_7	non-phage	0
DOF012_scaffold241_8	non-phage	0
DOF012_scaffold3559_3	non-phage	0
DOF012_scaffold2141_8	non-phage	0
DOF012_scaffold630_2	non-phage	0
DOF012_scaffold147_3	non-phage	0
DOF012_scaffold537_1	non-phage	0
DOF012_scaffold8779_9_1	non-phage	0
DOF012_scaffold2470_1	non-phage	0
DOF012_scaffold11414_1	non-phage	0
DOF012_scaffold6428_1	non-phage	0
DOF012_scaffold1977_2	non-phage	0
DOF012_scaffold4762_3	non-phage	0

DOF012_scaffold8213_1	non-phage	0
DOF012_scaffold19_2	non-phage	0
DOF012_scaffold9519_4	non-phage	0
DOF012_scaffold217_5	non-phage	0
DOF012_scaffold148_7	non-phage	0
DOF012_scaffold20_15	non-phage	0
DOF012_scaffold347_2	non-phage	0
DOF012_scaffold3437_3	non-phage	0
DOF012_scaffold11504_1	non-phage	0
DOF012_scaffold758_3	non-phage	0
DOF012_scaffold2698_4	non-phage	0
DOF012_scaffold266_3	non-phage	0
DOF012_scaffold14716_1	non-phage	0
DOF012_scaffold321_5	non-phage	0
DOF012_scaffold62_8	non-phage	0
DOF012_scaffold7874_1	non-phage	0
DOF012_scaffold5548_1	non-phage	0
DOF012_scaffold14739_1	non-phage	0
DOF012_scaffold805_2	non-phage	0
DOF012_scaffold979_2	non-phage	0
DOF012_scaffold14634_2	non-phage	0
DOF012_scaffold4848_4	non-phage	0
DOF012_scaffold57_10	non-phage	0
DOF012_scaffold858_7	non-phage	0
DOF012_scaffold7309_6_1	phage	0.934
DOF012_scaffold7309_6_2	non-phage	0
DOF012_scaffold278_3	non-phage	0
DOF012_scaffold2038_5	non-phage	0
DOF012_scaffold28_12	non-phage	0
DOF012_scaffold1940_1	non-phage	0
DOF012_scaffold14750_1	phage	0.999
DOF012_scaffold813_2	non-phage	0
DOF012_scaffold445_8	non-phage	0
DOF012_scaffold10555_1	non-phage	0
DOF012_scaffold1688_3	non-phage	0
DOF012_scaffold1127_1	non-phage	0
DOF012_scaffold13906_2	non-phage	0
DOF012_scaffold6484_4	non-phage	0
DOF012_scaffold3203_8	non-phage	0
DOF012_scaffold7499_2	non-phage	0
DOF012_scaffold7683_6	non-phage	0
DOF012_scaffold4227_4	non-phage	0
DOF012_scaffold12_12	non-phage	0
DOF012_scaffold650_3	non-phage	0
DOF012_scaffold2512_6	non-phage	0
DOF012_scaffold22_3	phage	0.999

DOF012_scaffold3378_1	non-phage	0
DOF012_scaffold3559_1	non-phage	0
DOF012_scaffold2451_2	non-phage	0
DOF012_scaffold10083_2	non-phage	0
DOF012_scaffold2492_2	non-phage	0
DOF012_scaffold184_1	non-phage	0
DOF012_scaffold463_6	non-phage	0
DOF012_scaffold4539_2	non-phage	0
DOF012_scaffold1713_11	non-phage	0
DOF012_scaffold1964_16_2	non-phage	0
DOF012_scaffold390_4	non-phage	0
DOF012_scaffold2697_3	non-phage	0
DOF012_scaffold7331_3	non-phage	0
DOF012_scaffold718_2	non-phage	0
DOF012_scaffold780_3	non-phage	0
DOF012_scaffold463_9_1	non-phage	0
DOF012_scaffold463_9_2	phage	1
DOF012_scaffold7874_3	non-phage	0
DOF012_scaffold5879_3	non-phage	0
DOF012_scaffold777_47	non-phage	0
DOF012_scaffold12_17	non-phage	0
DOF012_scaffold10214_2	non-phage	0
DOF012_scaffold1887_2	non-phage	0
DOF012_scaffold7_1	non-phage	0
DOF012_scaffold3840_4	non-phage	0
DOF012_scaffold992_6	non-phage	0
DOF012_scaffold1847_9	non-phage	0
DOF012_scaffold3010_1	non-phage	0
DOF012_scaffold7884_4	non-phage	0
DOF012_scaffold1970_6	non-phage	0
DOF013_scaffold16019_1	non-phage	0
DOF013_scaffold28205_6_1	non-phage	0
DOF013_scaffold6875_10	non-phage	0
DOF013_scaffold797_12	non-phage	0
DOF013_scaffold10235_1	non-phage	0
DOF013_scaffold1529_14	non-phage	0
DOF013_scaffold1431_10	non-phage	0
DOF013_scaffold11760_3	non-phage	0
DOF013_scaffold823_2	non-phage	0
DOF013_scaffold3577_6	non-phage	0
DOF013_scaffold309_9	non-phage	0
DOF013_scaffold460_12	non-phage	0
DOF013_scaffold2790_1	non-phage	0
DOF013_scaffold14895_3	non-phage	0
DOF013_scaffold4347_6	non-phage	0
DOF013_scaffold314_2	non-phage	0

DOF013_scaffold28393_1	non-phage	0
DOF013_scaffold631_3	non-phage	0
DOF013_scaffold235_1	non-phage	0
DOF013_scaffold20700_1	non-phage	0
DOF013_scaffold2527_4	phage	0.999
DOF013_scaffold14846_1	non-phage	0
DOF013_scaffold587_3	non-phage	0
DOF013_scaffold14056_3	non-phage	0
DOF013_scaffold8447_1	non-phage	0
DOF013_scaffold11367_9	non-phage	0
DOF013_scaffold7312_4	non-phage	0
DOF013_scaffold11760_13	phage	0.999
DOF013_scaffold518_1	non-phage	0
DOF013_scaffold18657_1	non-phage	0
DOF013_scaffold672_6	non-phage	0
DOF013_scaffold11518_3	non-phage	0
DOF013_scaffold11486_3	non-phage	0
DOF013_scaffold7141_2	non-phage	0
DOF013_scaffold4653_6_2	non-phage	0
DOF013_scaffold3420_1	non-phage	0
DOF013_scaffold12980_3	phage	0.998
DOF013_scaffold1258_1	non-phage	0
DOF013_scaffold4414_3	non-phage	0
DOF013_scaffold1330_1	non-phage	0
DOF013_scaffold879_2	non-phage	0
DOF013_scaffold3670_1	non-phage	0
DOF013_scaffold103_23	non-phage	0
DOF013_scaffold20961_2	non-phage	0
DOF013_scaffold11066_1	non-phage	0
DOF013_scaffold2291_15	non-phage	0
DOF013_scaffold5071_6	phage	0.94
DOF013_scaffold614_3	non-phage	0
DOF013_scaffold21807_3	non-phage	0
DOF013_scaffold140_2	non-phage	0
DOF013_scaffold2002_2	non-phage	0
DOF013_scaffold8990_2	non-phage	0.387
DOF013_scaffold60_1	non-phage	0
DOF013_scaffold8607_15	non-phage	0
DOF013_scaffold5184_2	non-phage	0
DOF013_scaffold423_3	non-phage	0
DOF013_scaffold7581_7	non-phage	0
DOF013_scaffold14119_6	non-phage	0
DOF013_scaffold961_5	non-phage	0
DOF013_scaffold65_1	non-phage	0
DOF013_scaffold20251_1	non-phage	0
DOF013_scaffold10538_2	non-phage	0

DOF013_scaffold14250_2	non-phage	0
DOF013_scaffold1588_1	phage	0.999
DOF013_scaffold6260_4	non-phage	0
DOF013_scaffold4066_2	non-phage	0
DOF013_scaffold2176_3	non-phage	0
DOF013_scaffold27644_1	phage	0.994
DOF013_scaffold17_2	non-phage	0
DOF013_scaffold242_1	non-phage	0
DOF013_scaffold2187_2	non-phage	0
DOF013_scaffold9185_1	non-phage	0
DOF013_scaffold18856_1	non-phage	0
DOF013_scaffold13417_3	non-phage	0
DOF013_scaffold1529_10	non-phage	0.207
DOF013_scaffold2663_3	non-phage	0
DOF013_scaffold11791_1	non-phage	0
DOF013_scaffold2113_3	non-phage	0
DOF013_scaffold3897_5	non-phage	0
DOF013_scaffold9473_3	non-phage	0
DOF013_scaffold16301_3	non-phage	0
DOF013_scaffold4177_1	non-phage	0
DOF013_scaffold997_3	non-phage	0.002
DOF013_scaffold757_2	non-phage	0
DOF013_scaffold1323_2	non-phage	0
DOF013_scaffold1258_2	non-phage	0
DOF013_scaffold15479_1	non-phage	0
DOF013_scaffold4900_2	phage	0.982
DOF013_scaffold797_8	non-phage	0
DOF013_scaffold13417_14	non-phage	0
DOF013_scaffold5283_4	non-phage	0
DOF013_scaffold4347_2	non-phage	0
DOF013_scaffold13417_8	non-phage	0
DOF013_scaffold140_5	non-phage	0
DOF013_scaffold2672_2	non-phage	0
DOF013_scaffold14268_2	non-phage	0
DOF013_scaffold2176_8	non-phage	0
DOF013_scaffold9729_2	non-phage	0
DOF013_scaffold1747_2	non-phage	0
DOF013_scaffold155_3	non-phage	0
DOF013_scaffold2359_3	non-phage	0
DOF013_scaffold4132_10	non-phage	0
DOF013_scaffold460_17	non-phage	0
DOF013_scaffold2176_11	non-phage	0
DOF013_scaffold202_13	non-phage	0
DOF013_scaffold18592_2	non-phage	0
DOF013_scaffold186_4	non-phage	0
DOF013_scaffold7574_1	non-phage	0

DOF013_scaffold16911_7	non-phage	0
DOF013_scaffold202_1	non-phage	0
DOF013_scaffold19312_2	non-phage	0
DOF013_scaffold21446_2	non-phage	0
DOF013_scaffold18347_2	non-phage	0
DOF013_scaffold697_1	non-phage	0
DOF013_scaffold15541_2	non-phage	0
DOF013_scaffold14806_11	non-phage	0
DOF013_scaffold1283_1	non-phage	0
DOF013_scaffold848_2	non-phage	0
DOF013_scaffold5240_4	non-phage	0
DOF013_scaffold443_1	non-phage	0
DOF013_scaffold29209_1	non-phage	0
DOF013_scaffold22163_5	non-phage	0
DOF013_scaffold6223_1	non-phage	0
DOF013_scaffold8877_4	non-phage	0
DOF013_scaffold8607_10	non-phage	0
DOF013_scaffold12442_2	non-phage	0
DOF013_scaffold4833_3	non-phage	0
DOF013_scaffold17989_2	non-phage	0
DOF013_scaffold4044_24	non-phage	0
DOF013_scaffold9644_6	non-phage	0
DOF013_scaffold6496_8	non-phage	0
DOF013_scaffold614_4	non-phage	0
DOF013_scaffold5015_1	non-phage	0
DOF013_scaffold25752_1	non-phage	0
DOF013_scaffold6979_1	non-phage	0
DOF013_scaffold226_1	non-phage	0
DOF013_scaffold5592_3	non-phage	0
DOF013_scaffold26148_1	non-phage	0
DOF013_scaffold86_1	non-phage	0
DOF013_scaffold29661_1	non-phage	0
DOF013_scaffold13417_5	non-phage	0
DOF013_scaffold2889_2	non-phage	0
DOF013_scaffold4588_4	non-phage	0
DOF013_scaffold572_1	non-phage	0
DOF013_scaffold9692_6	non-phage	0
DOF013_scaffold797_3	non-phage	0
DOF013_scaffold1963_6	non-phage	0
DOF013_scaffold3965_11	non-phage	0
DOF013_scaffold9208_26	non-phage	0
DOF013_scaffold15098_1	non-phage	0
DOF013_scaffold82_1	non-phage	0
DOF013_scaffold1498_5	non-phage	0
DOF013_scaffold13396_1	non-phage	0
DOF013_scaffold23797_1	non-phage	0

DOF013_scaffold884_1	non-phage	0
DOF013_scaffold186_7	non-phage	0
DOF013_scaffold2791_8	non-phage	0
DOF013_scaffold21858_3	non-phage	0
DOF013_scaffold14006_13	non-phage	0
DOF013_scaffold822_19	non-phage	0
DOF013_scaffold307_37	non-phage	0
DOF013_scaffold250_6	non-phage	0
DOF013_scaffold2790_2	non-phage	0
DOF013_scaffold235_10	non-phage	0
DOF013_scaffold12980_1	phage	1
DOF013_scaffold335_3	non-phage	0
DOF013_scaffold22254_1	non-phage	0
DOF013_scaffold24880_7	non-phage	0
DOF013_scaffold13091_5	non-phage	0
DOF013_scaffold2022_27	non-phage	0
DOF013_scaffold9208_17	non-phage	0
DOF013_scaffold8713_3	non-phage	0
DOF013_scaffold29209_2	non-phage	0
DOF013_scaffold23839_3	non-phage	0
DOF013_scaffold2403_5	non-phage	0
DOF013_scaffold14070_4	non-phage	0
DOF013_scaffold11526_2	non-phage	0
DOF013_scaffold1888_1	non-phage	0
DOF013_scaffold12980_2	non-phage	0
DOF013_scaffold5240_7	non-phage	0
DOF013_scaffold6415_1	non-phage	0
DOF013_scaffold1053_1	non-phage	0
DOF013_scaffold14702_3	non-phage	0
DOF013_scaffold16301_20	non-phage	0
DOF013_scaffold6333_2	non-phage	0
DOF013_scaffold14669_1	non-phage	0
DOF013_scaffold13945_2	non-phage	0
DOF013_scaffold11521_2	non-phage	0
DOF013_scaffold202_5	non-phage	0
DOF013_scaffold1035_2	non-phage	0
DOF013_scaffold14895_4	non-phage	0
DOF013_scaffold188_4	non-phage	0
DOF013_scaffold51_1	non-phage	0
DOF013_scaffold16019_3	non-phage	0
DOF013_scaffold7312_3	non-phage	0
DOF013_scaffold783_2	phage	0.997
DOF013_scaffold8246_4	non-phage	0
DOF013_scaffold3576_4	non-phage	0
DOF013_scaffold1464_4	non-phage	0
DOF013_scaffold3490_6	non-phage	0

DOF013_scaffold13963_1	non-phage	0
DOF013_scaffold23477_2	non-phage	0
DOF013_scaffold9858_2	non-phage	0
DOF013_scaffold20556_1	non-phage	0
DOF013_scaffold4616_2	non-phage	0
DOF013_scaffold18276_6	non-phage	0
DOF013_scaffold124_1	non-phage	0
DOF013_scaffold2440_1	non-phage	0
DOF013_scaffold6276_1	non-phage	0
DOF013_scaffold9692_1	non-phage	0
DOF013_scaffold24662_1	non-phage	0
DOF013_scaffold3237_14	non-phage	0
DOF013_scaffold3094_8	non-phage	0
DOF013_scaffold9996_1	non-phage	0
DOF013_scaffold7240_5	non-phage	0
DOF013_scaffold641_2	non-phage	0
DOF013_scaffold3_1	non-phage	0
DOF013_scaffold1125_1	non-phage	0
DOF013_scaffold379_5	non-phage	0
DOF013_scaffold7927_2	non-phage	0
DOF013_scaffold5381_3	non-phage	0
DOF013_scaffold23186_2	non-phage	0
DOF013_scaffold103_7	non-phage	0
DOF013_scaffold1963_2	non-phage	0
DOF013_scaffold405_3	non-phage	0
DOF013_scaffold21446_1	non-phage	0
DOF013_scaffold26650_1	non-phage	0
DOF013_scaffold25833_5	non-phage	0
DOF013_scaffold18294_5	non-phage	0
DOF013_scaffold13491_2	phage	0.986
DOF013_scaffold19887_2	non-phage	0
DOF013_scaffold4230_14	non-phage	0
DOF013_scaffold7045_4	non-phage	0
DOF013_scaffold2618_3	non-phage	0
DOF013_scaffold907_2	non-phage	0
DOF013_scaffold423_1	non-phage	0
DOF013_scaffold4683_6	non-phage	0
DOF013_scaffold2358_10	non-phage	0
DOF013_scaffold10362_6	non-phage	0
DOF013_scaffold10538_1	non-phage	0.001
DOF013_scaffold1804_5	non-phage	0
DOF013_scaffold270_1	non-phage	0
DOF013_scaffold822_20	non-phage	0
DOF013_scaffold443_2	phage	1
DOF013_scaffold10294_2	non-phage	0
DOF013_scaffold28391_1	non-phage	0

DOF013_scaffold6335_4	non-phage	0
DOF013_scaffold202_12	non-phage	0
DOF013_scaffold4414_7	non-phage	0
DOF013_scaffold1152_1	non-phage	0
DOF013_scaffold12442_1	non-phage	0
DOF013_scaffold7376_2	non-phage	0
DOF013_scaffold2582_3	non-phage	0
DOF013_scaffold11760_10	non-phage	0
DOF013_scaffold15877_2	phage	0.996
DOF013_scaffold3069_11	non-phage	0
DOF013_scaffold19260_1	non-phage	0
DOF013_scaffold3670_5	non-phage	0
DOF013_scaffold4066_3	non-phage	0
DOF013_scaffold2152_10	non-phage	0
DOF013_scaffold202_9	non-phage	0
DOF013_scaffold11367_1	non-phage	0
DOF013_scaffold155_2	non-phage	0
DOF013_scaffold778_5	non-phage	0
DOF013_scaffold667_7	non-phage	0
DOF013_scaffold25200_1	non-phage	0
DOF013_scaffold2699_1	phage	0.999
DOF013_scaffold4411_6	non-phage	0
DOF013_scaffold4076_1	non-phage	0
DOF013_scaffold14987_7	non-phage	0
DOF013_scaffold672_8	non-phage	0
DOF013_scaffold216_1	non-phage	0
DOF013_scaffold2234_1	non-phage	0
DOF013_scaffold12957_1	non-phage	0
DOF013_scaffold21996_1	non-phage	0
DOF013_scaffold12561_2	non-phage	0
DOF013_scaffold103_30	non-phage	0
DOF013_scaffold9349_6	non-phage	0
DOF013_scaffold9181_4	non-phage	0
DOF013_scaffold241_6	non-phage	0
DOF013_scaffold118_1	non-phage	0
DOF013_scaffold202_8	non-phage	0
DOF013_scaffold202_15	non-phage	0
DOF013_scaffold3360_3	non-phage	0
DOF013_scaffold4637_4	non-phage	0
DOF013_scaffold23529_1	non-phage	0
DOF013_scaffold4627_2	non-phage	0
DOF013_scaffold1431_2	non-phage	0
DOF013_scaffold19947_1	non-phage	0
DOF013_scaffold7303_4	non-phage	0
DOF013_scaffold783_5	non-phage	0
DOF013_scaffold961_7	non-phage	0

DOF013_scaffold2401_22	non-phage	0
DOF013_scaffold8478_1	non-phage	0
DOF013_scaffold5071_9	non-phage	0
DOF013_scaffold1804_1	non-phage	0
DOF013_scaffold7930_2	non-phage	0
DOF013_scaffold12056_2	non-phage	0.124
DOF013_scaffold10071_1	non-phage	0
DOF013_scaffold3074_1	non-phage	0
DOF013_scaffold11521_1	non-phage	0
DOF013_scaffold8246_6	non-phage	0
DOF013_scaffold4683_2	phage	0.999
DOF013_scaffold387_1	non-phage	0
DOF013_scaffold14096_2	non-phage	0
DOF013_scaffold1069_15	non-phage	0
DOF013_scaffold4870_2	non-phage	0
DOF013_scaffold4347_4	non-phage	0
DOF013_scaffold6973_3	non-phage	0
DOF013_scaffold29290_1	non-phage	0
DOF013_scaffold68_28	non-phage	0
DOF013_scaffold704_1	non-phage	0
DOF013_scaffold8150_1	non-phage	0
DOF013_scaffold8750_9	non-phage	0
DOF013_scaffold2118_1	non-phage	0
DOF013_scaffold20558_1	non-phage	0
DOF013_scaffold14056_1	non-phage	0
DOF013_scaffold11508_2	non-phage	0
DOF013_scaffold314_4	non-phage	0
DOF013_scaffold10394_1	non-phage	0
DOF013_scaffold335_2	non-phage	0
DOF013_scaffold6496_9	non-phage	0
DOF013_scaffold1283_2	non-phage	0
DOF013_scaffold12304_2	non-phage	0
DOF013_scaffold167_1	non-phage	0
DOF013_scaffold3897_3_2	non-phage	0
DOF013_scaffold4817_2	non-phage	0
DOF013_scaffold17666_7_1	non-phage	0
DOF013_scaffold202_11	non-phage	0
DOF013_scaffold6573_4	non-phage	0
DOF013_scaffold16237_1	non-phage	0
DOF013_scaffold10071_6	non-phage	0
DOF013_scaffold1023_32	non-phage	0
DOF013_scaffold481_3	non-phage	0
DOF013_scaffold155_1	non-phage	0
DOF013_scaffold9996_2	non-phage	0
DOF013_scaffold338_8	non-phage	0
DOF013_scaffold103_6	phage	0.993

DOF013_scaffold111_6	non-phage	0
DOF013_scaffold5165_2	non-phage	0
DOF013_scaffold9474_2	non-phage	0
DOF013_scaffold1493_1	non-phage	0
DOF013_scaffold4411_7	non-phage	0
DOF013_scaffold100_5	non-phage	0
DOF013_scaffold30153_1	non-phage	0
DOF013_scaffold2193_6	non-phage	0
DOF013_scaffold5561_4	non-phage	0
DOF013_scaffold6310_6	non-phage	0
DOF013_scaffold1636_18	non-phage	0
DOF013_scaffold26945_1	non-phage	0
DOF013_scaffold241_2	non-phage	0
DOF013_scaffold1476_3	non-phage	0
DOF013_scaffold13424_2	non-phage	0
DOF013_scaffold742_9	non-phage	0
DOF013_scaffold4653_5	non-phage	0
DOF013_scaffold27111_3	non-phage	0
DOF013_scaffold1328_4	non-phage	0
DOF013_scaffold10461_6	non-phage	0
DOF013_scaffold392_2	phage	0.998
DOF013_scaffold562_5_1	non-phage	0
DOF013_scaffold562_5_2	non-phage	0
DOF013_scaffold10617_6	non-phage	0
DOF013_scaffold6550_3	non-phage	0
DOF013_scaffold17232_1	non-phage	0
DOF013_scaffold436_34	non-phage	0
DOF013_scaffold7240_2	non-phage	0
DOF013_scaffold4833_1	non-phage	0
DOF013_scaffold4066_4	non-phage	0
DOF013_scaffold16301_18	non-phage	0
DOF013_scaffold2176_1	non-phage	0
DOF013_scaffold11755_2	non-phage	0
DOF013_scaffold16534_2	non-phage	0
DOF013_scaffold11760_11	non-phage	0
DOF013_scaffold572_2	non-phage	0
DOF013_scaffold2600_9	non-phage	0
DOF013_scaffold5581_4	non-phage	0
DOF013_scaffold742_11	non-phage	0
DOF013_scaffold9504_1	non-phage	0
DOF013_scaffold641_5	non-phage	0
DOF013_scaffold453_5	non-phage	0
DOF013_scaffold4579_15	non-phage	0
DOF013_scaffold375_2	non-phage	0
DOF013_scaffold1907_4	non-phage	0
DOF013_scaffold7241_1	non-phage	0

DOF013_scaffold5740_1	non-phage	0
DOF013_scaffold3_3	non-phage	0
DOF013_scaffold4107_5_1	non-phage	0
DOF013_scaffold305_2	non-phage	0
DOF013_scaffold11094_1	non-phage	0
DOF013_scaffold16019_7	non-phage	0
DOF013_scaffold261_2	non-phage	0
DOF013_scaffold3121_3	non-phage	0
DOF013_scaffold850_2	non-phage	0
DOF013_scaffold2401_18	non-phage	0
DOF013_scaffold171_7	non-phage	0
DOF013_scaffold13417_6	non-phage	0
DOF013_scaffold6335_5	non-phage	0
DOF013_scaffold7540_7	non-phage	0
DOF013_scaffold1464_3	non-phage	0
DOF013_scaffold8151_1	non-phage	0
DOF013_scaffold7930_1	non-phage	0
DOF013_scaffold14581_3	non-phage	0
DOF013_scaffold133_8	non-phage	0
DOF013_scaffold16536_1	non-phage	0
DOF013_scaffold20884_1	non-phage	0
DOF013_scaffold2150_3	non-phage	0
DOF013_scaffold1983_5	non-phage	0
DOF013_scaffold667_31	non-phage	0
DOF013_scaffold25133_2	non-phage	0
DOF013_scaffold2690_4	non-phage	0
DOF013_scaffold4037_1	non-phage	0
DOF013_scaffold7278_1	non-phage	0
DOF013_scaffold9171_5	non-phage	0
DOF013_scaffold335_5	non-phage	0
DOF013_scaffold21294_1	non-phage	0
DOF013_scaffold2345_7	non-phage	0
DOF013_scaffold4411_5	non-phage	0
DOF013_scaffold5240_2	phage	0.999
DOF013_scaffold29147_1	non-phage	0
DOF013_scaffold104_2	non-phage	0
DOF013_scaffold42_3	non-phage	0
DOF013_scaffold13246_1	non-phage	0
DOF013_scaffold3069_4	non-phage	0
DOF013_scaffold379_1	phage	0.994
DOF013_scaffold19260_2	non-phage	0
DOF013_scaffold2703_4	non-phage	0
DOF013_scaffold18601_11	non-phage	0
DOF013_scaffold8364_2	non-phage	0
DOF013_scaffold9841_1	non-phage	0
DOF013_scaffold4637_5	non-phage	0

DOF013_scaffold226_7	non-phage	0
DOF013_scaffold5240_9	phage	0.999
DOF013_scaffold30017_2	non-phage	0
DOF013_scaffold353_1	non-phage	0
DOF013_scaffold4107_1	non-phage	0
DOF013_scaffold6333_6	non-phage	0
DOF013_scaffold15479_6	non-phage	0
DOF013_scaffold4407_12	non-phage	0
DOF013_scaffold23354_2	non-phage	0
DOF013_scaffold17253_1	non-phage	0
DOF013_scaffold4447_1	non-phage	0
DOF013_scaffold7912_1	non-phage	0
DOF013_scaffold21161_3	non-phage	0
DOF013_scaffold14250_3	non-phage	0
DOF013_scaffold81_3	non-phage	0
DOF013_scaffold29984_1	non-phage	0
DOF013_scaffold20984_2_1	non-phage	0
DOF013_scaffold2713_1	non-phage	0
DOF013_scaffold112_1	non-phage	0
DOF013_scaffold23641_2	non-phage	0
DOF013_scaffold7240_6	non-phage	0
DOF013_scaffold12967_2	non-phage	0
DOF013_scaffold953_6	non-phage	0
DOF013_scaffold220_1	non-phage	0
DOF013_scaffold797_1	non-phage	0
DOF013_scaffold4347_5	non-phage	0
DOF013_scaffold2463_9	non-phage	0
DOF013_scaffold2713_4	non-phage	0
DOF013_scaffold4799_3	non-phage	0
DOF013_scaffold30067_2	non-phage	0
DOF013_scaffold21294_6	phage	0.974
DOF013_scaffold7574_3	non-phage	0
DOF013_scaffold310_19	non-phage	0
DOF013_scaffold1636_24	non-phage	0
DOF013_scaffold4818_2	non-phage	0
DOF013_scaffold2771_16	non-phage	0
DOF013_scaffold9638_1	non-phage	0
DOF013_scaffold2582_1	non-phage	0
DOF013_scaffold1529_9	non-phage	0
DOF013_scaffold961_4	non-phage	0
DOF013_scaffold10570_7	non-phage	0
DOF013_scaffold9414_2	non-phage	0
DOF013_scaffold21294_2	non-phage	0
DOF013_scaffold1588_2	non-phage	0
DOF013_scaffold186_5	non-phage	0
DOF013_scaffold10071_5_1	non-phage	0

DOF013_scaffold2680_1	non-phage	0
DOF013_scaffold103_21	non-phage	0
DOF013_scaffold8168_2	non-phage	0
DOF013_scaffold1973_2	non-phage	0
DOF013_scaffold162_8	non-phage	0
DOF013_scaffold4411_8	non-phage	0
DOF013_scaffold2582_12	non-phage	0
DOF013_scaffold162_1	non-phage	0
DOF013_scaffold1044_1	non-phage	0
DOF013_scaffold9441_1	non-phage	0
DOF013_scaffold1747_1	non-phage	0
DOF013_scaffold17294_1	non-phage	0
DOF013_scaffold4646_11	non-phage	0
DOF013_scaffold254_6	non-phage	0
DOF013_scaffold25_3	non-phage	0
DOF013_scaffold11367_7	non-phage	0
DOF013_scaffold215_8	non-phage	0
DOF013_scaffold2358_6	non-phage	0
DOF013_scaffold6276_3	non-phage	0
DOF013_scaffold4087_1	non-phage	0
DOF013_scaffold18276_4	non-phage	0
DOF013_scaffold21858_2	non-phage	0
DOF013_scaffold2442_2	non-phage	0
DOF013_scaffold6294_1	non-phage	0
DOF013_scaffold29915_1	phage	0.999
DOF013_scaffold370_1	non-phage	0
DOF013_scaffold16464_14	non-phage	0
DOF013_scaffold13575_2	non-phage	0
DOF013_scaffold129_3	non-phage	0
DOF013_scaffold11114_1	non-phage	0
DOF013_scaffold235_12	non-phage	0
DOF013_scaffold20418_1	non-phage	0
DOF013_scaffold19494_3	non-phage	0
DOF013_scaffold26299_1	non-phage	0
DOF013_scaffold491_2	non-phage	0
DOF013_scaffold6288_7	non-phage	0
DOF013_scaffold103_5_1	non-phage	0
DOF013_scaffold6581_3	non-phage	0
DOF013_scaffold10906_1	non-phage	0
DOF014_scaffold10548_3	non-phage	0
DOF014_scaffold35015_1	non-phage	0
DOF014_scaffold49591_1	non-phage	0
DOF014_scaffold47396_1	non-phage	0
DOF014_scaffold45105_1	non-phage	0
DOF014_scaffold43291_2	non-phage	0
DOF014_scaffold13401_6	non-phage	0

DOF014_scaffold44636_1	non-phage	0
DOF014_scaffold29861_1	non-phage	0
DOF014_scaffold34813_3	non-phage	0
DOF014_scaffold12966_6	non-phage	0
DOF014_scaffold38966_2	non-phage	0
DOF014_scaffold47367_1	non-phage	0
DOF014_scaffold49589_1	non-phage	0
DOF014_scaffold39727_1	non-phage	0
DOF014_scaffold45904_1	non-phage	0
DOF014_scaffold12966_1	non-phage	0
DOF014_scaffold38538_5	non-phage	0
DOF014_scaffold30449_4	non-phage	0
DOF014_scaffold48161_7	non-phage	0
DOF014_scaffold46098_2	non-phage	0
DOF014_scaffold49685_3	non-phage	0
DOF014_scaffold258_12	non-phage	0
DOF014_scaffold49380_2	non-phage	0
DOF014_scaffold49655_2	non-phage	0
DOF014_scaffold17667_1	non-phage	0
DOF014_scaffold49664_2	non-phage	0
DOF014_scaffold20394_2	non-phage	0
DOF014_scaffold42369_1	non-phage	0
DOF014_scaffold15073_8	phage	0.999
DOF014_scaffold17290_6	non-phage	0
DOF014_scaffold1290_2	non-phage	0
DOF014_scaffold49589_4	non-phage	0
DOF014_scaffold49496_2	non-phage	0
DOF014_scaffold47496_8	non-phage	0
DOF014_scaffold49206_2	non-phage	0
DOF014_scaffold43839_1	non-phage	0
DOF014_scaffold47496_1	non-phage	0
DOF014_scaffold49730_1	non-phage	0
DOF014_scaffold153_3	non-phage	0
DOF014_scaffold45099_2	non-phage	0
DOF014_scaffold6168_5	non-phage	0
DOF014_scaffold9826_1	non-phage	0
DOF014_scaffold49019_2	non-phage	0
DOF014_scaffold49276_4	phage	0.958
DOF014_scaffold14696_2	non-phage	0
DOF014_scaffold49489_1	non-phage	0
DOF014_scaffold10666_2	non-phage	0
DOF014_scaffold49613_2	non-phage	0
DOF014_scaffold49639_2	non-phage	0
DOF014_scaffold46901_1	phage	0.999
DOF014_scaffold49728_1	non-phage	0
DOF014_scaffold47768_3	non-phage	0

DOF014_scaffold33044_5	non-phage	0
DOF014_scaffold49696_1	non-phage	0
DOF014_scaffold49545_5	non-phage	0
DOF014_scaffold49706_1	non-phage	0
DOF014_scaffold45437_2	non-phage	0
DOF014_scaffold49595_2	non-phage	0
DOF014_scaffold5989_10	non-phage	0
DOF014_scaffold3256_2	non-phage	0
DOF014_C613306_1	non-phage	0
DOF014_scaffold4323_2	non-phage	0
DOF014_scaffold42447_1	non-phage	0
DOF014_scaffold49716_1	non-phage	0
DOF014_scaffold5989_5	non-phage	0
DOF014_scaffold31681_2	non-phage	0
DOF014_scaffold45609_1	non-phage	0
DOF014_C613020_1	non-phage	0
DOF014_scaffold8270_7	non-phage	0
DOF014_scaffold8270_3	non-phage	0
DOF014_scaffold35452_1	non-phage	0
DOF014_scaffold40857_1	non-phage	0
DOF014_scaffold48744_1	non-phage	0
DOF014_scaffold49647_6	non-phage	0
DOF014_scaffold49724_3	non-phage	0
DOF014_scaffold10141_7	non-phage	0
DOF014_scaffold9639_4	non-phage	0
DOF014_scaffold46293_3	non-phage	0
DOF014_scaffold40604_2	non-phage	0
DOF014_scaffold44196_1	non-phage	0
DOF014_scaffold49610_2	non-phage	0
DOF014_scaffold28546_2	non-phage	0
DOF014_scaffold1290_14	non-phage	0
DOF014_scaffold37183_1	non-phage	0
DOF014_scaffold91_1	non-phage	0
DOF014_scaffold46247_1	non-phage	0
DOF014_scaffold3556_5	non-phage	0
DOF014_scaffold38538_1	non-phage	0
DOF014_scaffold41013_1	non-phage	0
DOF014_scaffold20113_2	non-phage	0
DOF014_scaffold38_3	non-phage	0
DOF014_C613360_1	non-phage	0
DOF014_scaffold19364_1	non-phage	0.047
DOF014_scaffold41944_14	non-phage	0
DOF014_scaffold10772_1	phage	0.997
DOF014_scaffold49726_2	phage	0.872
DOF014_scaffold44265_1	non-phage	0
DOF014_scaffold48642_1	non-phage	0

DOF014_scaffold12916_1	non-phage	0
DOF014_scaffold49321_2	non-phage	0
DOF014_scaffold16_2	non-phage	0
DOF014_scaffold26455_1	non-phage	0
DOF014_scaffold16_5	non-phage	0
DOF014_scaffold43973_1	non-phage	0
DOF014_scaffold3003_2	non-phage	0
DOF014_scaffold30923_2	phage	1
DOF014_scaffold97_7	non-phage	0
DOF014_scaffold49276_7	non-phage	0
DOF014_scaffold14696_6	non-phage	0
DOF014_scaffold49672_2	non-phage	0
DOF014_scaffold37065_3	non-phage	0.114
DOF014_scaffold41944_9	non-phage	0
DOF014_scaffold43789_2	non-phage	0
DOF014_scaffold49655_3	non-phage	0
DOF014_scaffold38362_2	non-phage	0
DOF014_scaffold48988_1	non-phage	0
DOF014_scaffold49239_1	non-phage	0
DOF014_scaffold38286_1	non-phage	0
DOF014_scaffold44990_5	non-phage	0
DOF014_scaffold19627_3	non-phage	0
DOF014_scaffold49671_1	non-phage	0
DOF014_scaffold49640_1	non-phage	0
DOF014_scaffold6221_3	non-phage	0
DOF014_scaffold49539_1	non-phage	0
DOF014_scaffold15073_3	non-phage	0
DOF014_scaffold49480_1	non-phage	0
DOF014_scaffold22216_4	non-phage	0
DOF014_scaffold18300_1	non-phage	0
DOF014_scaffold5989_4	non-phage	0
DOF014_scaffold49_1	non-phage	0
DOF014_scaffold5645_31	non-phage	0
DOF014_scaffold45099_1	non-phage	0
DOF014_scaffold153_4	non-phage	0
DOF014_C612810_1	phage	0.999
DOF014_scaffold34813_6	non-phage	0
DOF014_scaffold43286_1	non-phage	0
DOF014_scaffold49719_2	non-phage	0
DOF014_scaffold150_1	non-phage	0
DOF014_scaffold55_4	non-phage	0
DOF014_scaffold162_4	non-phage	0
DOF014_scaffold36011_2	non-phage	0
DOF014_scaffold1879_3	non-phage	0
DOF014_scaffold38888_1	non-phage	0
DOF014_scaffold48333_4	non-phage	0

DOF014_scaffold89_1	non-phage	0
DOF014_scaffold37532_2	non-phage	0
DOF014_scaffold13401_7	non-phage	0
DOF014_scaffold49631_1	non-phage	0
DOF014_scaffold49546_1	non-phage	0
DOF014_scaffold34937_1	non-phage	0
DOF014_scaffold31633_1	non-phage	0
DOF014_scaffold18822_3	non-phage	0
DOF014_scaffold49647_2	non-phage	0
DOF014_scaffold2347_1	non-phage	0
DOF014_scaffold39592_3	non-phage	0
DOF014_scaffold49458_2	non-phage	0
DOF014_scaffold11093_4	non-phage	0.062
DOF014_scaffold41012_1	non-phage	0
DOF014_scaffold11002_2	non-phage	0
DOF014_scaffold49568_1	non-phage	0
DOF014_scaffold33325_2	non-phage	0
DOF014_scaffold10772_7	non-phage	0
DOF014_scaffold49664_1	non-phage	0
DOF014_scaffold44304_6	non-phage	0
DOF014_scaffold37368_1	non-phage	0
DOF014_scaffold49589_5	non-phage	0
DOF014_scaffold31587_2	non-phage	0
DOF014_scaffold31681_1	non-phage	0
DOF014_scaffold41944_5	non-phage	0
DOF014_scaffold38538_6	non-phage	0
DOF014_scaffold43830_2	non-phage	0
DOF014_scaffold24258_1	non-phage	0
DOF014_scaffold49576_3	non-phage	0
DOF014_scaffold49724_4	non-phage	0
DOF014_C613220_1	non-phage	0
DOF014_scaffold49688_3	non-phage	0
DOF014_scaffold6676_5	non-phage	0
DOF014_scaffold49540_1	non-phage	0
DOF014_scaffold37685_5	non-phage	0
DOF014_scaffold32757_2	non-phage	0
DOF014_scaffold2070_1	non-phage	0
DOF014_scaffold35326_2	non-phage	0
DOF014_scaffold48333_2	non-phage	0
DOF014_scaffold28806_3	non-phage	0
DOF014_scaffold44645_2	non-phage	0
DOF014_scaffold14163_1	non-phage	0
DOF014_scaffold49699_1	non-phage	0
DOF014_scaffold8023_1	non-phage	0
DOF014_C613208_1	non-phage	0
DOF014_scaffold35015_2	non-phage	0

DOF014_scaffold49201_2	non-phage	0
DOF014_scaffold49545_6	non-phage	0.025
DOF014_scaffold2787_5	non-phage	0
DOF014_scaffold7153_1	non-phage	0
DOF014_scaffold49657_1	non-phage	0
DOF014_scaffold19446_1	non-phage	0
DOF014_scaffold29288_3	non-phage	0
DOF014_scaffold76_4	non-phage	0
DOF014_scaffold82_1	non-phage	0
DOF014_scaffold30923_1	non-phage	0
DOF014_scaffold101_2	phage	1
DOF014_scaffold255_4	non-phage	0
DOF014_scaffold32658_1	phage	0.96
DOF014_scaffold142_1	non-phage	0
DOF014_scaffold16942_2	non-phage	0
DOF014_scaffold8502_1	non-phage	0
DOF014_scaffold49352_3	non-phage	0
DOF014_scaffold1_1	non-phage	0
DOF014_scaffold3611_1	non-phage	0
DOF014_scaffold22526_1	non-phage	0
DOF014_scaffold22216_1	non-phage	0
DOF014_scaffold49332_2	non-phage	0
DOF014_scaffold43862_2	non-phage	0
DOF014_scaffold7189_8	phage	0.91
DOF014_scaffold13028_2	non-phage	0
DOF014_scaffold47768_2	non-phage	0
DOF014_scaffold38285_2	non-phage	0
DOF014_scaffold34997_2	non-phage	0
DOF014_scaffold49588_4	non-phage	0
DOF014_scaffold36423_2	non-phage	0
DOF014_scaffold33907_1	non-phage	0
DOF014_scaffold35255_1	non-phage	0
DOF014_scaffold35255_2	non-phage	0
DOF014_scaffold13401_3	non-phage	0
DOF014_scaffold49648_2	non-phage	0
DOF014_scaffold24170_6	non-phage	0
DOF014_scaffold49108_5	non-phage	0
DOF014_scaffold49685_4	non-phage	0
DOF014_scaffold41497_2	non-phage	0
DOF014_scaffold40192_1	non-phage	0
DOF014_scaffold39623_1	non-phage	0
DOF014_scaffold49407_2	non-phage	0
DOF014_C612946_1	non-phage	0
DOF014_C613224_1	non-phage	0
DOF014_scaffold9337_1	non-phage	0
DOF014_scaffold49340_4	phage	0.999

DOF014_scaffold34278_1	non-phage	0
DOF014_scaffold49302_1	non-phage	0
DOF014_scaffold16_3	non-phage	0
DOF014_scaffold29718_2	phage	0.867
DOF014_scaffold26455_2	non-phage	0
DOF014_scaffold10711_2	non-phage	0
DOF014_scaffold41944_15	non-phage	0
DOF014_scaffold45325_1	non-phage	0
DOF014_scaffold44503_1	non-phage	0
DOF014_scaffold48988_2	non-phage	0
DOF014_scaffold97_6	non-phage	0
DOF014_scaffold2738_4	non-phage	0
DOF014_scaffold49502_1	non-phage	0
DOF014_scaffold44304_2	non-phage	0
DOF014_scaffold36958_2	non-phage	0
DOF014_scaffold49681_1	non-phage	0
DOF014_scaffold49616_3	non-phage	0
DOF014_scaffold29_1	non-phage	0
DOF014_scaffold44983_3	non-phage	0
DOF014_scaffold28546_5	non-phage	0
DOF014_scaffold49371_1	non-phage	0
DOF014_scaffold41930_1	non-phage	0
DOF014_scaffold40604_3	non-phage	0
DOF014_scaffold1_4	non-phage	0
DOF014_scaffold237_2	non-phage	0
DOF014_scaffold2821_4	phage	0.999
DOF014_scaffold38538_2	non-phage	0
DOF014_scaffold45399_2	non-phage	0
DOF014_scaffold82_5	non-phage	0
DOF014_scaffold4319_3	non-phage	0
DOF014_scaffold36089_1	non-phage	0
DOF014_scaffold19364_2	non-phage	0
DOF014_scaffold49675_2	non-phage	0
DOF014_scaffold21117_2	non-phage	0
DOF014_scaffold37685_2	non-phage	0
DOF014_scaffold47893_1	non-phage	0
DOF014_scaffold35015_6	non-phage	0
DOF014_scaffold7289_7	non-phage	0
DOF014_scaffold49361_1	non-phage	0
DOF014_scaffold49545_2	non-phage	0
DOF014_scaffold1290_15	non-phage	0
DOF014_scaffold35901_1	non-phage	0
DOF014_scaffold9337_2	non-phage	0
DOF014_scaffold49687_1	phage	0.999
DOF014_C613148_1	non-phage	0
DOF014_scaffold9639_1	non-phage	0

DOF014_scaffold49091_1	non-phage	0
DOF014_scaffold44133_5	non-phage	0
DOF014_scaffold48333_5	non-phage	0
DOF014_scaffold13479_30	non-phage	0
DOF014_scaffold3943_1	non-phage	0
DOF014_scaffold22216_5	non-phage	0
DOF014_scaffold10063_3	non-phage	0
DOF014_scaffold1808_5	phage	0.999
DOF014_scaffold42358_4	non-phage	0
DOF014_scaffold49276_3	non-phage	0
DOF014_scaffold41012_2	non-phage	0
DOF014_scaffold43779_2	non-phage	0
DOF014_scaffold44990_4	non-phage	0
DOF014_scaffold34813_11	non-phage	0
DOF014_scaffold49456_2	non-phage	0
DOF014_scaffold49626_1	non-phage	0
DOF014_scaffold22516_1	non-phage	0
DOF014_scaffold26239_6	non-phage	0
DOF014_scaffold43328_1	non-phage	0
DOF014_scaffold49352_1	non-phage	0
DOF014_scaffold49685_1	non-phage	0
DOF014_scaffold49539_2	non-phage	0
DOF014_scaffold49660_1	non-phage	0
DOF014_scaffold38888_5	non-phage	0
DOF014_scaffold49604_1	phage	0.999
DOF014_scaffold48243_1	non-phage	0
DOF014_scaffold49656_2	non-phage	0
DOF014_scaffold49692_2	non-phage	0
DOF014_scaffold14163_3	non-phage	0
DOF014_scaffold89_3	non-phage	0
DOF014_scaffold48009_1	non-phage	0
DOF014_scaffold134_3	non-phage	0
DOF014_scaffold35554_2	non-phage	0
DOF014_scaffold42969_1	non-phage	0
DOF014_scaffold35326_1	non-phage	0
DOF014_scaffold37192_1	non-phage	0
DOF014_scaffold49340_3	non-phage	0
DOF014_scaffold45399_4	non-phage	0
DOF014_scaffold49576_2	non-phage	0
DOF014_scaffold162_5	non-phage	0
DOF014_scaffold33044_10	non-phage	0
DOF014_scaffold6861_2	non-phage	0
DOF014_scaffold49057_3	non-phage	0
DOF014_scaffold14850_4	non-phage	0
DOF014_scaffold42453_1	non-phage	0
DOF014_scaffold43741_1	non-phage	0

DOF014_scaffold49609_1	non-phage	0.057
DOF014_scaffold1879_2	non-phage	0
DOF014_scaffold46201_2	non-phage	0
DOF014_scaffold666_2	non-phage	0
DOF014_scaffold20077_5	non-phage	0
DOF014_scaffold29757_4	non-phage	0
DOF014_scaffold48753_1	non-phage	0
DOF014_scaffold44133_2	non-phage	0
DOF014_scaffold46651_2	non-phage	0
DOF014_scaffold30958_2	non-phage	0
DOF014_scaffold25647_4	non-phage	0
DOF014_scaffold49717_1	non-phage	0
DOF014_scaffold44990_1	non-phage	0
DOF014_scaffold16371_1	non-phage	0
DOF014_scaffold49625_1	non-phage	0
DOF014_scaffold49647_5	non-phage	0
DOF014_scaffold49633_1	non-phage	0
DOF014_scaffold48333_3	non-phage	0
DOF014_scaffold115_1	non-phage	0
DOF014_scaffold27474_2	non-phage	0
DOF014_scaffold37051_6	non-phage	0
DOF014_scaffold23348_1	non-phage	0
DOF014_scaffold45446_1	non-phage	0
DOF014_scaffold90_1	phage	0.906
DOF014_scaffold22216_2	non-phage	0
DOF014_scaffold47893_4	non-phage	0
DOF014_scaffold38_5	non-phage	0
DOF014_scaffold43254_1	non-phage	0
DOF014_scaffold49713_1	non-phage	0
DOF014_scaffold47483_2	non-phage	0
DOF014_scaffold29811_3	non-phage	0
DOF014_scaffold49734_2	non-phage	0
DOF014_scaffold26933_3	non-phage	0
DOF014_scaffold48114_2	non-phage	0
DOF014_scaffold18300_2	non-phage	0
DOF014_scaffold35015_3	non-phage	0
DOF014_scaffold49172_2	non-phage	0
DOF014_scaffold49708_1	non-phage	0
DOF014_scaffold33908_3	non-phage	0
DOF014_scaffold49620_1	non-phage	0
DOF014_scaffold49645_1	phage	0.999
DOF014_scaffold46171_4	non-phage	0
DOF014_scaffold37183_2	non-phage	0
DOF014_scaffold32658_2	non-phage	0
DOF014_scaffold10711_1	non-phage	0
DOF014_scaffold17_1	non-phage	0

DOF014_scaffold46843_1	non-phage	0
DOF014_scaffold14456_2	non-phage	0
DOF014_scaffold44990_7	non-phage	0
DOF014_scaffold49616_2	non-phage	0
DOF014_scaffold15073_6	non-phage	0
DOF014_C612722_1	non-phage	0
DOF014_scaffold39772_2	non-phage	0
DOF014_scaffold77_1	non-phage	0
DOF014_scaffold49276_2	non-phage	0
DOF014_scaffold20962_6	non-phage	0
DOF014_scaffold97_5	non-phage	0
DOF014_scaffold28546_4	non-phage	0
DOF014_scaffold9337_8	non-phage	0
DOF014_scaffold13381_7	non-phage	0
DOF014_scaffold36524_13	non-phage	0
DOF014_scaffold49494_3	non-phage	0
DOF014_scaffold24170_3	non-phage	0
DOF014_scaffold47768_1	non-phage	0
DOF014_scaffold5989_6	non-phage	0
DOF014_scaffold45399_1	non-phage	0
DOF014_scaffold49732_2	non-phage	0
DOF014_scaffold49372_1	non-phage	0
DOF014_scaffold41197_5	non-phage	0
DOF014_scaffold26455_3	non-phage	0
DOF014_scaffold35370_4	non-phage	0
DOF014_scaffold8270_6	non-phage	0
DOF014_scaffold49715_1	non-phage	0
DOF014_scaffold49529_2	non-phage	0
DOF014_scaffold30449_3	non-phage	0
DOF014_scaffold49686_2	non-phage	0
DOF014_scaffold49556_1	phage	0.999
DOF014_scaffold57_4	non-phage	0
DOF014_scaffold14850_1	non-phage	0
DOF014_scaffold49595_1	non-phage	0
DOF014_scaffold4937_2	non-phage	0
DOF014_scaffold48988_3	non-phage	0
DOF014_scaffold49276_5	non-phage	0
DOF014_scaffold18822_1	non-phage	0
DOF014_scaffold49552_1	phage	0.998
DOF014_scaffold55_2	non-phage	0
DOF014_scaffold9639_2	non-phage	0
DOF014_scaffold34813_8	non-phage	0
DOF014_scaffold49683_1	non-phage	0
DOF014_C612644_1	non-phage	0
DOF014_scaffold43428_2	non-phage	0
DOF014_scaffold4064_2	non-phage	0

DOF014_scaffold24170_4	non-phage	0
DOF014_scaffold33072_1	non-phage	0
DOF014_scaffold49679_1	non-phage	0
DOF014_scaffold43328_3	non-phage	0
DOF014_scaffold44304_5	non-phage	0
DOF014_scaffold47818_2	non-phage	0
DOF014_scaffold49646_1	non-phage	0
DOF014_scaffold49395_1	non-phage	0
DOF014_scaffold49667_2	non-phage	0
DOF014_scaffold3943_2	non-phage	0
DOF014_scaffold255_2	non-phage	0
DOF014_scaffold29_2	non-phage	0
DOF014_scaffold49019_1	non-phage	0
DOF014_scaffold19506_12	non-phage	0
DOF014_scaffold32848_6	non-phage	0
DOF014_scaffold43056_1	non-phage	0
DOF014_scaffold35015_7	non-phage	0
DOF014_scaffold10666_1	non-phage	0
DOF014_scaffold46171_3	non-phage	0
DOF014_scaffold37685_1	non-phage	0
DOF014_scaffold14163_2	non-phage	0
DOF014_scaffold36423_1	non-phage	0
DOF014_scaffold33143_2	non-phage	0
DOF014_scaffold5903_7	non-phage	0
DOF014_scaffold41944_16	non-phage	0
DOF014_scaffold5989_1	non-phage	0
DOF014_scaffold49692_1	non-phage	0
DOF014_scaffold49698_1	non-phage	0
DOF014_scaffold49455_1	non-phage	0
DOF014_scaffold90_2	non-phage	0
DOF014_scaffold97_1	non-phage	0
DOF014_scaffold47659_2	non-phage	0
DOF014_scaffold24067_1	non-phage	0
DOF014_scaffold14696_5	non-phage	0
DOF014_scaffold48333_6	non-phage	0
DOF014_scaffold17410_1	non-phage	0
DOF014_scaffold40587_2	non-phage	0
DOF014_scaffold8171_1	non-phage	0
DOF014_scaffold22216_6	non-phage	0
DOF014_scaffold25141_1	non-phage	0
DOF014_scaffold44983_4	non-phage	0
DOF014_scaffold2660_2	non-phage	0
DOF014_scaffold1290_16	non-phage	0
DOF014_scaffold36132_2	non-phage	0
DOF014_scaffold49600_1	non-phage	0
DOF014_scaffold41944_7	non-phage	0

DOF014_scaffold76_3	non-phage	0
DOF014_scaffold49618_1	non-phage	0
DOF014_scaffold49684_1	non-phage	0
DOF014_scaffold1_3	phage	0.856
DOF014_scaffold4306_1	non-phage	0
DOF014_scaffold49655_1	non-phage	0
DOF014_scaffold49523_2	non-phage	0
DOF014_scaffold34813_4	phage	0.961
DOF014_scaffold55_5	non-phage	0
DOF014_scaffold39574_1	non-phage	0
DOF014_scaffold9337_4	non-phage	0
DOF014_scaffold39592_5	non-phage	0
DOF014_scaffold49276_1	non-phage	0
DOF014_scaffold30958_1	non-phage	0
DOF014_scaffold1290_17	non-phage	0
DOF014_scaffold29757_6	non-phage	0
DOF014_scaffold37051_5	non-phage	0
DOF014_scaffold49710_1	non-phage	0
DOF014_scaffold49731_1	non-phage	0
DOF014_scaffold13401_5	non-phage	0
DOF014_scaffold47396_2	non-phage	0
DOF014_scaffold10063_2	non-phage	0
DOF014_scaffold14456_1	non-phage	0
DOF014_scaffold104_2	non-phage	0
DOF014_scaffold49661_1	non-phage	0
DOF014_scaffold49712_2	non-phage	0
DOF014_scaffold49730_2	non-phage	0
DOF014_scaffold8900_2	non-phage	0
DOF014_C612894_1	non-phage	0
DOF014_scaffold63_3	non-phage	0
DOF014_scaffold412_6	non-phage	0
DOF014_scaffold40597_5	non-phage	0
DOF014_scaffold6861_3	non-phage	0
DOF014_scaffold44304_8	non-phage	0
DOF014_scaffold49340_5	phage	0.993
DOF014_scaffold49380_1	non-phage	0
DOF014_scaffold258_11	non-phage	0
DOF014_scaffold49638_1	non-phage	0
DOF014_scaffold2738_7	non-phage	0
DOF014_scaffold49642_2	non-phage	0
DOF014_scaffold6221_1	non-phage	0
DOF014_scaffold42001_1	non-phage	0
DOF014_scaffold11405_1	non-phage	0
DOF014_scaffold29757_3	non-phage	0
DOF014_scaffold41197_3	non-phage	0
DOF014_scaffold49650_1	non-phage	0

DOF014_scaffold31681_3	non-phage	0
DOF014_C613446_1	non-phage	0
DOF014_scaffold29955_1	non-phage	0
DOF014_scaffold97_4	non-phage	0
DOF014_scaffold49647_4	non-phage	0
DOF014_scaffold49494_1	non-phage	0
DOF014_scaffold14696_3	non-phage	0
DOF014_scaffold134_2	non-phage	0
DOF014_scaffold16_4	non-phage	0
DOF014_scaffold10548_9	non-phage	0
DOF014_scaffold43508_1	non-phage	0
DOF014_scaffold6861_1	non-phage	0
DOF014_scaffold38_6	non-phage	0
DOF014_scaffold46171_7	non-phage	0
DOF014_scaffold49589_6	non-phage	0
DOF014_scaffold49701_1	non-phage	0
DOF014_scaffold35015_4	non-phage	0
DOF014_scaffold44983_1	non-phage	0
DOF014_scaffold1612_4	non-phage	0
DOF014_scaffold49591_2	non-phage	0
DOF014_scaffold48114_1	non-phage	0
DOF014_scaffold44770_1	non-phage	0
DOF014_scaffold49511_1	non-phage	0
DOF014_scaffold49172_3	non-phage	0
DOF014_scaffold26256_1	non-phage	0
DOF014_scaffold43981_2	non-phage	0
DOF014_scaffold38538_4	non-phage	0
DOF014_scaffold34813_7	non-phage	0
DOF014_scaffold3750_1	non-phage	0
DOF014_scaffold13082_2	non-phage	0
DOF014_scaffold25647_3	non-phage	0
DOF014_scaffold49733_1	non-phage	0
DOF014_scaffold38285_1	non-phage	0
DOF014_scaffold35015_8	non-phage	0
DOF014_scaffold49276_8	non-phage	0
DOF014_scaffold2711_3	non-phage	0
DOF014_scaffold43428_4	non-phage	0
DOF014_scaffold49258_1	non-phage	0
DOF014_scaffold37214_2	non-phage	0
DOF014_scaffold13006_1	phage	0.987
DOF014_scaffold29811_4	non-phage	0
DOF014_scaffold7205_9	non-phage	0
DOF014_scaffold258_15	non-phage	0
DOF014_scaffold44990_6	non-phage	0
DOF014_scaffold33907_3	non-phage	0
DOF014_scaffold28546_3	non-phage	0

DOF014_scaffold8270_1	non-phage	0
DOF014_scaffold14850_2	non-phage	0
DOF014_scaffold13381_6	non-phage	0
DOF014_scaffold42447_3	non-phage	0
DOF014_scaffold8270_5	non-phage	0
DOF014_scaffold8832_1	non-phage	0
DOF014_scaffold49559_1	non-phage	0
DOF014_scaffold26239_4	non-phage	0
DOF014_scaffold34645_2	non-phage	0
DOF014_scaffold29991_2	non-phage	0
DOF014_C613100_1	non-phage	0
DOF014_scaffold901_6	non-phage	0
DOF014_scaffold49718_2	non-phage	0
DOF014_scaffold8163_1	non-phage	0
DOF014_scaffold49240_3	non-phage	0
DOF014_scaffold38_2	non-phage	0
DOF014_scaffold24277_2	non-phage	0
DOF014_scaffold49687_3	non-phage	0
DOF014_scaffold9974_9	non-phage	0
DOF014_scaffold49321_1	non-phage	0
DOF014_scaffold16_1	non-phage	0
DOF014_scaffold245_1	non-phage	0
DOF014_C612698_1	phage	0.998
DOF014_scaffold44304_4	non-phage	0
DOF014_scaffold18822_2	non-phage	0
DOF014_scaffold14696_4	non-phage	0
DOF014_scaffold49647_3	non-phage	0
DOF014_scaffold49668_1	non-phage	0
DOF014_scaffold49458_1	non-phage	0
DOF014_scaffold5989_3	non-phage	0
DOF014_scaffold36132_3	non-phage	0
DOF014_scaffold36132_1	non-phage	0
DOF014_scaffold31587_4	non-phage	0
DOF014_scaffold47496_9	non-phage	0
DOF014_scaffold132_3	non-phage	0
DOF014_scaffold104_1	non-phage	0
DOF014_scaffold76_2	non-phage	0
DOF014_scaffold3523_1	non-phage	0
DOF014_scaffold49725_1	non-phage	0
DOF014_scaffold49494_2	non-phage	0
DOF014_scaffold13521_2	non-phage	0
DOF014_scaffold153_5	non-phage	0
DOF014_scaffold49651_1	non-phage	0
DOF014_scaffold106_3	non-phage	0
DOF014_scaffold41944_6	non-phage	0
DOF014_scaffold32996_5	non-phage	0

DOF014_scaffold82_3	phage	0.999
DOF014_scaffold49650_2	non-phage	0
DOF014_scaffold1612_8	non-phage	0
DOF014_scaffold49589_2	non-phage	0
DOF014_scaffold20582_5	non-phage	0
DOF014_scaffold48988_4	non-phage	0
DOF014_scaffold48564_1	non-phage	0
DOF014_scaffold15208_5	phage	0.997
DOF014_scaffold89_2	non-phage	0
DOF014_scaffold5989_2	non-phage	0
DOF014_scaffold15265_1	non-phage	0
DOF014_scaffold9080_2	non-phage	0
DOF014_scaffold48152_1	non-phage	0
DOF002_scaffold54454_8	non-phage	0
DOF002_scaffold13673_3	non-phage	0
DOF002_scaffold56848_6	non-phage	0
DOF002_scaffold19630_1	non-phage	0
DOF002_scaffold13481_2	non-phage	0
DOF002_scaffold35792_5	non-phage	0
DOF002_scaffold36100_4	non-phage	0
DOF002_scaffold67121_2	non-phage	0
DOF002_scaffold559_4	non-phage	0
DOF002_scaffold28274_3	non-phage	0
DOF002_scaffold60758_5	non-phage	0
DOF002_scaffold65545_1	non-phage	0
DOF002_scaffold36043_6	non-phage	0
DOF002_scaffold1739_5	non-phage	0
DOF002_scaffold3970_14	non-phage	0
DOF002_scaffold49600_2	non-phage	0
DOF002_scaffold8507_3	non-phage	0
DOF002_scaffold399_4	non-phage	0
DOF002_scaffold31579_7	non-phage	0
DOF002_scaffold41614_8	non-phage	0
DOF002_scaffold24292_6	non-phage	0
DOF002_scaffold67523_1	non-phage	0
DOF002_scaffold58314_1	non-phage	0
DOF002_scaffold36043_20	non-phage	0
DOF002_scaffold129_2	non-phage	0
DOF002_scaffold5329_3	non-phage	0
DOF002_scaffold9533_16	non-phage	0
DOF002_scaffold34441_1	non-phage	0
DOF002_scaffold8213_4	non-phage	0
DOF002_scaffold65590_5	non-phage	0
DOF002_scaffold2659_1	non-phage	0
DOF002_scaffold60526_5	non-phage	0
DOF002_scaffold30256_12	non-phage	0

DOF002_scaffold63958_1	non-phage	0
DOF002_scaffold23030_8	non-phage	0
DOF002_scaffold60182_2	non-phage	0
DOF002_scaffold5450_12	non-phage	0
DOF002_scaffold54511_1	non-phage	0
DOF002_scaffold2701_21	non-phage	0
DOF002_scaffold43504_1	non-phage	0
DOF002_scaffold4650_16	non-phage	0
DOF002_scaffold2701_2	non-phage	0
DOF002_scaffold13962_1	non-phage	0
DOF002_scaffold386_18	non-phage	0
DOF002_scaffold13072_8	non-phage	0
DOF002_scaffold13525_8	non-phage	0
DOF002_scaffold63140_2	non-phage	0
DOF002_scaffold399_21	non-phage	0
DOF002_scaffold399_3	non-phage	0
DOF002_C840011_1	non-phage	0
DOF002_scaffold58023_1	non-phage	0
DOF002_scaffold47157_11	non-phage	0
DOF002_scaffold7970_9	non-phage	0
DOF002_scaffold47157_2	non-phage	0
DOF002_scaffold62906_4	non-phage	0
DOF002_scaffold67543_1	non-phage	0
DOF002_scaffold13072_5	non-phage	0
DOF002_scaffold55053_6	non-phage	0
DOF002_scaffold62363_3	non-phage	0
DOF002_scaffold65831_1	non-phage	0
DOF002_scaffold28318_6	non-phage	0
DOF002_scaffold40355_7	non-phage	0
DOF002_scaffold28885_1	non-phage	0
DOF002_scaffold20176_7	non-phage	0
DOF002_scaffold51733_5	non-phage	0
DOF002_scaffold26977_1	non-phage	0
DOF002_scaffold64925_2	non-phage	0
DOF002_scaffold15513_3	non-phage	0
DOF002_scaffold67404_1	non-phage	0
DOF002_scaffold22882_9	non-phage	0
DOF002_scaffold37_7	non-phage	0
DOF002_scaffold19740_18	non-phage	0
DOF002_scaffold26075_1	non-phage	0
DOF002_scaffold11_7	non-phage	0
DOF002_scaffold3257_1	non-phage	0
DOF002_scaffold4977_3	non-phage	0
DOF002_scaffold60287_6	non-phage	0
DOF002_scaffold24_17	non-phage	0
DOF002_scaffold2701_23	non-phage	0

DOF002_scaffold6551_1	non-phage	0
DOF002_scaffold25301_3	non-phage	0
DOF002_scaffold67456_4	non-phage	0
DOF002_scaffold67534_1	non-phage	0
DOF002_scaffold31506_6	non-phage	0
DOF002_scaffold61808_6	non-phage	0
DOF002_scaffold17574_3	non-phage	0
DOF002_scaffold21798_16	non-phage	0
DOF002_scaffold55694_2	non-phage	0
DOF002_scaffold64032_1	non-phage	0
DOF002_scaffold67384_1	non-phage	0
DOF002_scaffold260_14	non-phage	0
DOF002_scaffold13525_7	non-phage	0
DOF002_scaffold67399_3	non-phage	0
DOF002_scaffold22882_10	non-phage	0
DOF002_scaffold17241_17	non-phage	0
DOF002_scaffold40683_9	non-phage	0
DOF002_scaffold19740_15	non-phage	0
DOF002_scaffold197_1	non-phage	0
DOF002_scaffold38019_5	non-phage	0
DOF002_scaffold10939_13	non-phage	0
DOF002_scaffold67202_1	non-phage	0
DOF002_scaffold214_1	non-phage	0
DOF002_scaffold9730_4	non-phage	0
DOF002_scaffold5192_15	non-phage	0
DOF002_scaffold60881_10	non-phage	0
DOF002_scaffold4650_9	non-phage	0
DOF002_scaffold31579_5	non-phage	0
DOF002_scaffold5404_3	non-phage	0
DOF002_scaffold3638_3	non-phage	0
DOF002_scaffold37093_3	non-phage	0
DOF002_scaffold25411_2	non-phage	0
DOF002_scaffold13072_16	non-phage	0
DOF002_scaffold2659_4	non-phage	0
DOF002_scaffold13215_1	non-phage	0
DOF002_scaffold47997_1	non-phage	0
DOF002_scaffold27678_1	non-phage	0
DOF002_scaffold59673_2	non-phage	0
DOF002_scaffold37_2	non-phage	0
DOF002_scaffold13072_6	non-phage	0
DOF002_scaffold67497_1	non-phage	0
DOF002_scaffold5450_8	non-phage	0
DOF002_scaffold32188_5	non-phage	0
DOF002_scaffold5140_7	non-phage	0
DOF002_scaffold60099_2	non-phage	0
DOF002_scaffold60280_2	non-phage	0

DOF002_scaffold13525_15	non-phage	0
DOF002_scaffold67520_1	non-phage	0
DOF002_scaffold71_11	non-phage	0
DOF002_scaffold46904_1	non-phage	0
DOF002_scaffold35792_7	non-phage	0
DOF002_scaffold67327_1	non-phage	0
DOF002_scaffold5450_7	non-phage	0
DOF002_scaffold52020_1	non-phage	0
DOF002_scaffold8903_15	non-phage	0
DOF002_scaffold11506_2	non-phage	0
DOF002_scaffold29897_2	non-phage	0
DOF002_scaffold11_5	non-phage	0
DOF002_scaffold67451_1	non-phage	0
DOF002_scaffold54226_2	non-phage	0
DOF002_scaffold65590_1	non-phage	0
DOF002_scaffold36043_11	non-phage	0
DOF002_scaffold67523_6	non-phage	0
DOF002_scaffold133_10	non-phage	0
DOF002_scaffold13072_11	non-phage	0
DOF002_scaffold21134_5	non-phage	0
DOF003_scaffold51181_1	non-phage	0
DOF003_scaffold46844_3	non-phage	0
DOF003_scaffold31324_1	non-phage	0
DOF003_scaffold48545_2	non-phage	0
DOF003_scaffold8510_16	non-phage	0
DOF003_scaffold51048_1	non-phage	0
DOF003_scaffold49294_2	non-phage	0
DOF003_scaffold18528_1	non-phage	0
DOF003_scaffold1441_9	non-phage	0
DOF003_scaffold18311_7	non-phage	0
DOF003_scaffold50718_1	non-phage	0
DOF003_scaffold4749_1	non-phage	0
DOF003_scaffold39003_1	non-phage	0
DOF003_scaffold49538_1	non-phage	0
DOF003_scaffold48898_1	non-phage	0
DOF003_scaffold35012_1	non-phage	0
DOF003_C745744_1	non-phage	0
DOF003_scaffold51174_2	non-phage	0
DOF003_scaffold36846_1	non-phage	0
DOF003_scaffold50671_2	non-phage	0
DOF003_scaffold6902_16	non-phage	0
DOF003_scaffold35529_7	non-phage	0
DOF003_scaffold2523_4	non-phage	0
DOF003_scaffold36334_2	non-phage	0
DOF003_scaffold18528_2	non-phage	0
DOF003_scaffold38302_3	non-phage	0

DOF003_scaffold23992_1	non-phage	0
DOF003_scaffold23062_1	non-phage	0
DOF003_scaffold27080_1	non-phage	0
DOF003_scaffold122_11	non-phage	0
DOF003_scaffold51204_1	non-phage	0
DOF003_scaffold568_1	non-phage	0
DOF003_scaffold44243_1	non-phage	0
DOF003_scaffold394_7	non-phage	0
DOF003_scaffold51180_1	non-phage	0
DOF003_scaffold51087_3	non-phage	0
DOF003_scaffold27092_1	non-phage	0
DOF003_scaffold27167_2	non-phage	0
DOF003_scaffold41886_1	non-phage	0
DOF003_scaffold48756_1	non-phage	0
DOF003_scaffold40096_2	non-phage	0
DOF003_scaffold19011_10	non-phage	0
DOF003_scaffold40743_1	non-phage	0
DOF003_scaffold24126_1	non-phage	0
DOF003_scaffold46361_3	non-phage	0
DOF003_scaffold36515_1	non-phage	0
DOF003_scaffold49282_5	non-phage	0
DOF003_scaffold19146_2	non-phage	0
DOF003_scaffold200_4	non-phage	0
DOF003_scaffold122_13	non-phage	0
DOF003_scaffold6902_22	non-phage	0
DOF003_scaffold48081_2	non-phage	0
DOF003_scaffold40135_1	non-phage	0
DOF003_scaffold122_9	non-phage	0
DOF003_scaffold12405_1	non-phage	0
DOF003_scaffold43367_1	non-phage	0
DOF003_scaffold29348_3	non-phage	0
DOF003_scaffold43170_2	non-phage	0
DOF003_scaffold50995_1	non-phage	0
DOF003_scaffold50975_1	non-phage	0
DOF003_scaffold27372_2	non-phage	0
DOF003_scaffold26969_1	non-phage	0
DOF003_scaffold989_2	non-phage	0
DOF003_scaffold7744_4	non-phage	0
DOF003_scaffold51089_2	non-phage	0
DOF003_scaffold27013_4	non-phage	0
DOF003_scaffold21688_2	non-phage	0
DOF003_scaffold19258_3	non-phage	0
DOF003_scaffold51139_1	non-phage	0
DOF003_scaffold129_1	non-phage	0
DOF003_scaffold30760_8	non-phage	0
DOF003_scaffold34933_2	non-phage	0

DOF003_scaffold50978_1	non-phage	0
DOF003_scaffold28876_3	non-phage	0
DOF003_scaffold33350_1	non-phage	0
DOF003_scaffold9209_4	non-phage	0
DOF003_scaffold18_41	non-phage	0
DOF003_scaffold127_1	non-phage	0
DOF003_scaffold3054_11	non-phage	0
DOF003_scaffold28914_9	non-phage	0
DOF003_scaffold18527_1	non-phage	0
DOF003_scaffold51089_5	non-phage	0
DOF003_scaffold46023_1	non-phage	0
DOF003_scaffold43058_1	non-phage	0
DOF003_scaffold51216_1	non-phage	0
DOF003_scaffold4320_7	non-phage	0
DOF003_scaffold1311_44	non-phage	0
DOF003_scaffold27512_1	non-phage	0
DOF003_scaffold51151_1	non-phage	0
DOF003_scaffold44802_2	non-phage	0
DOF003_scaffold28956_3	non-phage	0
DOF003_scaffold51174_1	non-phage	0
DOF003_scaffold17766_2	non-phage	0
DOF003_scaffold19665_4	non-phage	0
DOF003_scaffold51118_1	non-phage	0
DOF003_scaffold34411_2	non-phage	0
DOF003_scaffold45089_1	non-phage	0
DOF003_scaffold51210_1	non-phage	0
DOF003_scaffold5363_1	non-phage	0
DOF003_scaffold36508_1	non-phage	0
DOF003_scaffold11672_6	non-phage	0
DOF003_scaffold16930_2	non-phage	0
DOF003_scaffold13351_7	non-phage	0
DOF003_scaffold7239_4	non-phage	0
DOF003_scaffold25745_3	non-phage	0
DOF003_scaffold24985_4	non-phage	0
DOF003_scaffold2045_3	non-phage	0
DOF003_scaffold50774_1	non-phage	0
DOF003_scaffold237_12	non-phage	0
DOF003_scaffold16299_8	non-phage	0
DOF003_scaffold51075_4	non-phage	0
DOF003_scaffold14_1	non-phage	0
DOF003_scaffold274_8	non-phage	0
DOF003_scaffold12401_16	non-phage	0
DOF003_scaffold45387_1	non-phage	0
DOF003_scaffold37664_13	non-phage	0
DOF003_scaffold40096_4	non-phage	0
DOF003_scaffold40480_11	non-phage	0

DOF003_scaffold51132_1	non-phage	0
DOF003_scaffold9809_18	non-phage	0
DOF003_scaffold40420_4	non-phage	0
DOF003_scaffold29766_3	non-phage	0
DOF003_C746004_1	non-phage	0
DOF003_scaffold13715_1	non-phage	0
DOF003_scaffold51054_2	non-phage	0
DOF003_scaffold74_4	non-phage	0
DOF003_scaffold49678_2	non-phage	0
DOF003_scaffold39808_1	non-phage	0
DOF003_scaffold29101_1	non-phage	0
DOF003_scaffold7140_1	non-phage	0
DOF003_scaffold26402_1	non-phage	0
DOF003_scaffold2045_1	non-phage	0
DOF003_scaffold11_3	non-phage	0
DOF003_scaffold44081_3	non-phage	0
DOF003_scaffold35050_2	non-phage	0
DOF003_scaffold50649_1	non-phage	0
DOF003_scaffold7690_8	non-phage	0
DOF003_scaffold39845_1	non-phage	0
DOF003_scaffold27669_2	non-phage	0
DOF003_scaffold42346_3	non-phage	0
DOF003_scaffold11_1	non-phage	0
DOF003_scaffold14795_2	non-phage	0
DOF003_scaffold7945_4	non-phage	0
DOF003_scaffold45014_1	non-phage	0
DOF003_scaffold308_6	non-phage	0
DOF003_scaffold1441_12	non-phage	0
DOF003_scaffold18939_3	non-phage	0
DOF003_scaffold29470_4	non-phage	0
DOF003_scaffold1220_13	non-phage	0
DOF003_scaffold51220_1	non-phage	0
DOF003_scaffold4033_7	non-phage	0
DOF003_scaffold23237_1	non-phage	0
DOF003_scaffold32492_1	non-phage	0
DOF003_scaffold43036_4	non-phage	0
DOF003_scaffold42081_1	non-phage	0
DOF003_scaffold50938_1	non-phage	0
DOF003_scaffold51063_1	non-phage	0
DOF003_scaffold50883_1	non-phage	0
DOF003_scaffold16299_1	non-phage	0
DOF003_scaffold50147_3	non-phage	0
DOF003_scaffold51168_1	non-phage	0
DOF003_scaffold49745_1	non-phage	0
DOF003_scaffold31964_2	non-phage	0
DOF003_scaffold1123_3	non-phage	0

DOF003_scaffold48767_1	non-phage	0
DOF003_scaffold29668_7	non-phage	0
DOF003_scaffold8689_3	non-phage	0
DOF003_scaffold35529_3	non-phage	0
DOF003_scaffold50079_5	non-phage	0
DOF003_scaffold6257_32	non-phage	0
DOF003_scaffold278_7	non-phage	0
DOF003_scaffold12105_1	non-phage	0
DOF003_scaffold3729_8	non-phage	0
DOF003_scaffold34149_2	non-phage	0
DOF003_scaffold41036_1	non-phage	0
DOF003_scaffold49182_1	non-phage	0
DOF003_scaffold29470_5	non-phage	0
DOF003_scaffold51213_1	non-phage	0
DOF003_C745482_1	non-phage	0
DOF003_scaffold48080_1	non-phage	0
DOF003_scaffold49282_1	non-phage	0
DOF003_scaffold5437_3	non-phage	0
DOF003_scaffold17746_1	non-phage	0
DOF003_scaffold49622_2	non-phage	0
DOF003_scaffold46924_2	non-phage	0
DOF003_C746092_1	non-phage	0
DOF003_scaffold36954_1	non-phage	0
DOF003_scaffold35610_3	non-phage	0
DOF003_scaffold18305_2	non-phage	0
DOF003_scaffold48100_4	non-phage	0
DOF003_scaffold324_1	non-phage	0
DOF003_scaffold51163_1	non-phage	0
DOF003_scaffold50528_2	non-phage	0
DOF003_C745960_1	non-phage	0
DOF003_scaffold19522_3	non-phage	0
DOF003_scaffold34492_3	non-phage	0
DOF003_scaffold14239_1	non-phage	0
DOF003_scaffold24933_2	non-phage	0
DOF003_scaffold122_8	non-phage	0
DOF003_scaffold2798_2	non-phage	0
DOF003_scaffold241_2	non-phage	0
DOF003_scaffold25731_7	non-phage	0
DOF003_scaffold33848_1	non-phage	0
DOF003_scaffold29103_1	non-phage	0
DOF003_scaffold49978_1	non-phage	0
DOF003_scaffold33057_1	non-phage	0
DOF003_scaffold48276_2	non-phage	0
DOF003_scaffold31258_1	non-phage	0
DOF003_scaffold35050_1	non-phage	0
DOF003_scaffold8559_4	non-phage	0

DOF003_scaffold50258_1	non-phage	0
DOF003_scaffold1311_12	non-phage	0
DOF003_scaffold24934_1	non-phage	0
DOF003_scaffold4749_7	non-phage	0
DOF003_scaffold210_35	non-phage	0
DOF003_C746608_1	non-phage	0
DOF003_scaffold19258_2	non-phage	0
DOF003_scaffold1257_36	non-phage	0
DOF003_scaffold48163_2	non-phage	0
DOF003_scaffold122_3	non-phage	0
DOF003_scaffold40696_8	non-phage	0
DOF003_scaffold21540_1	non-phage	0
DOF003_scaffold49746_1	non-phage	0
DOF003_scaffold47892_2	non-phage	0
DOF003_scaffold5464_1	non-phage	0
DOF003_scaffold50474_1	non-phage	0
DOF003_scaffold16299_4	non-phage	0
DOF003_scaffold50514_1	non-phage	0
DOF003_scaffold31325_2	non-phage	0
DOF003_scaffold16800_1	non-phage	0
DOF003_scaffold200_3	non-phage	0
DOF003_scaffold18036_1	non-phage	0
DOF003_scaffold3543_14	non-phage	0
DOF003_scaffold21243_3	non-phage	0
DOF003_scaffold4749_4	non-phage	0
DOF003_scaffold45931_1	non-phage	0
DOF003_scaffold21246_1	non-phage	0
DOF003_scaffold33528_3	non-phage	0
DOF004_scaffold56990_3	non-phage	0
DOF004_scaffold56921_5	non-phage	0
DOF004_C631776_1	non-phage	0
DOF004_scaffold52980_1	non-phage	0
DOF004_scaffold28695_2	non-phage	0
DOF004_scaffold29672_3	non-phage	0
DOF004_scaffold20624_2	non-phage	0
DOF004_scaffold38895_8	non-phage	0
DOF004_scaffold53873_3	non-phage	0
DOF004_scaffold23120_5	non-phage	0
DOF004_scaffold1493_7	non-phage	0
DOF004_scaffold57132_2	non-phage	0
DOF004_scaffold56070_2	non-phage	0
DOF004_scaffold20668_4	non-phage	0
DOF004_scaffold169_1	non-phage	0
DOF004_scaffold36778_2	non-phage	0
DOF004_scaffold48492_1	non-phage	0
DOF004_scaffold57137_1	non-phage	0

DOF004_scaffold54951_1	non-phage	0
DOF004_scaffold31023_3	non-phage	0
DOF004_scaffold24301_1	non-phage	0
DOF004_scaffold49006_2	non-phage	0
DOF004_scaffold56998_3	non-phage	0
DOF004_scaffold2735_5	non-phage	0
DOF004_scaffold49811_7	non-phage	0
DOF004_scaffold38778_3	non-phage	0
DOF004_scaffold2120_1	non-phage	0
DOF004_scaffold40075_3	non-phage	0
DOF004_scaffold57055_1	non-phage	0
DOF004_C631662_1	non-phage	0
DOF004_scaffold1382_3	non-phage	0
DOF004_scaffold19523_4	non-phage	0
DOF004_scaffold52501_5	non-phage	0
DOF004_scaffold26658_2	non-phage	0
DOF004_scaffold3658_15	non-phage	0
DOF004_scaffold13584_1	non-phage	0
DOF004_scaffold210_2	non-phage	0
DOF004_scaffold42330_3	non-phage	0
DOF004_scaffold54566_4	non-phage	0
DOF004_scaffold10951_3	non-phage	0
DOF004_scaffold57024_2	non-phage	0
DOF004_C631934_1	non-phage	0
DOF004_scaffold57124_1	non-phage	0
DOF004_scaffold35183_1	non-phage	0
DOF004_scaffold56552_4	non-phage	0
DOF004_scaffold49596_1	non-phage	0
DOF004_scaffold41304_11	non-phage	0
DOF004_scaffold26027_2	non-phage	0
DOF004_scaffold19144_3	non-phage	0
DOF004_scaffold28829_5	non-phage	0
DOF004_scaffold21098_2	non-phage	0
DOF004_scaffold23593_2	non-phage	0
DOF004_C632286_1	non-phage	0
DOF004_scaffold25418_2	non-phage	0
DOF004_scaffold28829_1	non-phage	0
DOF004_scaffold23953_2	non-phage	0
DOF004_scaffold49006_3	non-phage	0
DOF004_scaffold2686_1	non-phage	0
DOF004_scaffold57040_1	non-phage	0
DOF004_scaffold57103_2	non-phage	0
DOF004_scaffold44040_1	non-phage	0
DOF004_scaffold55036_2	non-phage	0
DOF004_scaffold57093_2	non-phage	0
DOF004_scaffold57109_2	non-phage	0

DOF004_scaffold21281_1	non-phage	0
DOF004_scaffold56868_1	non-phage	0
DOF004_scaffold168_2	non-phage	0
DOF004_scaffold56964_1	non-phage	0
DOF004_scaffold56939_2	non-phage	0
DOF004_scaffold57100_1	non-phage	0
DOF004_scaffold24837_4	non-phage	0
DOF004_scaffold46916_1	non-phage	0
DOF004_scaffold48157_1	non-phage	0
DOF004_scaffold8200_3	non-phage	0
DOF004_scaffold56995_3	non-phage	0
DOF004_scaffold22467_8	non-phage	0
DOF004_scaffold5274_1	non-phage	0
DOF004_scaffold38495_2	non-phage	0
DOF004_scaffold6992_1	non-phage	0
DOF004_scaffold10109_1	non-phage	0
DOF004_scaffold13294_5	non-phage	0
DOF004_scaffold12410_2	non-phage	0
DOF004_scaffold43319_4	non-phage	0
DOF004_scaffold24229_2	non-phage	0
DOF004_scaffold41353_3	non-phage	0
DOF004_scaffold22467_4	non-phage	0
DOF004_scaffold57077_2	non-phage	0
DOF004_scaffold9922_2	non-phage	0
DOF004_scaffold56466_2	non-phage	0
DOF004_scaffold8568_4	non-phage	0
DOF004_scaffold54882_1	non-phage	0
DOF004_scaffold42898_2	non-phage	0
DOF004_scaffold28481_3	non-phage	0
DOF004_scaffold28120_3	non-phage	0
DOF004_scaffold5622_3	non-phage	0
DOF004_scaffold57102_1	non-phage	0
DOF004_scaffold1075_3	non-phage	0
DOF004_scaffold50555_1	non-phage	0
DOF004_scaffold9794_2	non-phage	0
DOF004_scaffold16446_4	non-phage	0
DOF004_scaffold57064_1	non-phage	0
DOF004_scaffold47501_3	non-phage	0
DOF004_scaffold56830_2	non-phage	0
DOF004_scaffold42486_2	non-phage	0
DOF004_scaffold24837_7	non-phage	0
DOF004_scaffold19129_2	non-phage	0
DOF004_scaffold56650_2	non-phage	0
DOF004_scaffold49695_1	non-phage	0
DOF004_scaffold56822_1	non-phage	0
DOF004_scaffold16903_3	non-phage	0

DOF004_scaffold57080_1	non-phage	0
DOF004_scaffold36730_4	non-phage	0
DOF004_scaffold28349_2	non-phage	0
DOF004_scaffold56990_1	non-phage	0
DOF004_scaffold52891_2	non-phage	0
DOF004_scaffold36843_1	non-phage	0
DOF004_scaffold24837_10	non-phage	0
DOF004_scaffold42330_1	non-phage	0
DOF004_scaffold56212_3	non-phage	0
DOF004_scaffold4865_2	non-phage	0
DOF004_scaffold20624_10	non-phage	0
DOF004_scaffold187_2	non-phage	0
DOF004_scaffold804_3	non-phage	0
DOF004_scaffold57139_1	non-phage	0
DOF004_scaffold55965_3	non-phage	0
DOF004_scaffold20524_4	non-phage	0
DOF004_scaffold57118_1	non-phage	0
DOF004_scaffold44930_1	non-phage	0
DOF004_scaffold7865_3	non-phage	0
DOF004_scaffold57140_1	non-phage	0
DOF004_scaffold39291_2	non-phage	0
DOF004_scaffold42091_2	non-phage	0
DOF004_scaffold1738_3	non-phage	0
DOF004_scaffold9046_3	non-phage	0
DOF004_scaffold40915_4	non-phage	0
DOF004_scaffold12128_1	non-phage	0
DOF004_scaffold20311_8	non-phage	0
DOF004_scaffold24837_8	non-phage	0
DOF004_scaffold55499_1	non-phage	0
DOF004_scaffold34646_3	non-phage	0
DOF004_scaffold42330_5	non-phage	0
DOF004_scaffold27054_1	non-phage	0
DOF004_scaffold41351_3	non-phage	0
DOF004_scaffold57049_2	non-phage	0
DOF004_scaffold54566_6	non-phage	0
DOF004_scaffold54566_7	non-phage	0
DOF004_scaffold1_2	non-phage	0
DOF004_scaffold57110_1	non-phage	0
DOF004_scaffold5206_4	non-phage	0
DOF004_scaffold13584_6	non-phage	0
DOF004_C633482_1	non-phage	0
DOF004_scaffold24712_8	non-phage	0
DOF004_scaffold39753_4	non-phage	0
DOF004_scaffold42515_1	non-phage	0
DOF004_scaffold56978_4	non-phage	0
DOF004_scaffold56726_5	non-phage	0

DOF004_scaffold25776_2	non-phage	0
DOF004_scaffold4865_1	non-phage	0
DOF004_scaffold3390_3	non-phage	0
DOF004_scaffold20729_5	non-phage	0
DOF004_scaffold57016_1	non-phage	0
DOF004_scaffold52501_2	non-phage	0
DOF004_scaffold28966_1	non-phage	0
DOF004_scaffold43985_2	non-phage	0
DOF004_scaffold10261_4	non-phage	0
DOF004_scaffold15795_3	non-phage	0
DOF004_scaffold56709_1	non-phage	0
DOF004_scaffold1514_4	non-phage	0
DOF004_scaffold56382_1	non-phage	0
DOF004_scaffold10991_3	non-phage	0
DOF004_scaffold21048_1	non-phage	0
DOF004_scaffold50517_8	non-phage	0
DOF004_scaffold24837_1	non-phage	0
DOF004_scaffold21604_1	non-phage	0
DOF004_scaffold56105_3_1	non-phage	0
DOF004_scaffold224_1	non-phage	0
DOF004_scaffold55946_1	non-phage	0
DOF004_scaffold25227_2	non-phage	0
DOF004_scaffold56882_2	non-phage	0
DOF004_scaffold41902_1	non-phage	0
DOF004_scaffold56232_1	non-phage	0
DOF004_scaffold804_7	non-phage	0
DOF004_C631806_1	non-phage	0
DOF004_scaffold13315_9	non-phage	0
DOF004_scaffold51651_4	non-phage	0
DOF004_scaffold20326_1	non-phage	0
DOF004_scaffold18503_5	non-phage	0
DOF004_scaffold23749_4	non-phage	0
DOF004_scaffold34252_3	non-phage	0
DOF004_scaffold33679_2	non-phage	0
DOF004_scaffold20742_1	non-phage	0
DOF004_scaffold55923_3	non-phage	0
DOF004_scaffold51054_2	non-phage	0
DOF004_scaffold34545_2	non-phage	0
DOF004_scaffold57118_2	non-phage	0
DOF004_scaffold56595_1	non-phage	0
DOF004_scaffold28792_2	non-phage	0
DOF006_scaffold13120_1	non-phage	0
DOF006_scaffold7328_2	non-phage	0
DOF006_scaffold22738_4	non-phage	0
DOF006_scaffold7840_1	non-phage	0
DOF006_scaffold10588_1	non-phage	0

DOF006_scaffold9931_1	non-phage	0
DOF006_scaffold13539_1	non-phage	0
DOF006_scaffold5174_1	non-phage	0
DOF006_scaffold7084_1	non-phage	0
DOF006_scaffold9943_3	non-phage	0
DOF006_scaffold7284_4	non-phage	0
DOF006_scaffold15219_1	non-phage	0
DOF006_scaffold19435_4	non-phage	0
DOF006_scaffold8794_2	non-phage	0
DOF006_scaffold10913_21	non-phage	0
DOF006_scaffold13003_2	non-phage	0
DOF006_scaffold8033_8	non-phage	0
DOF006_scaffold22723_1	non-phage	0
DOF006_scaffold76_1	non-phage	0
DOF006_scaffold22367_1	non-phage	0
DOF006_scaffold11335_8	non-phage	0
DOF006_scaffold22987_6	non-phage	0
DOF006_scaffold18019_1	non-phage	0
DOF006_scaffold2423_4	non-phage	0
DOF006_scaffold8291_1	non-phage	0
DOF006_scaffold22946_3	non-phage	0
DOF006_scaffold2415_55	non-phage	0
DOF006_scaffold5289_1	non-phage	0
DOF006_scaffold4917_2	non-phage	0
DOF006_scaffold12896_1	non-phage	0
DOF006_scaffold22955_1	non-phage	0
DOF006_scaffold1000_2	non-phage	0
DOF006_scaffold20848_1	non-phage	0
DOF006_scaffold6236_3	non-phage	0
DOF006_scaffold16300_1	non-phage	0
DOF006_scaffold15559_2	non-phage	0
DOF006_scaffold8594_1	non-phage	0
DOF006_scaffold22738_5	non-phage	0
DOF006_scaffold21084_1	non-phage	0
DOF006_scaffold10147_1	non-phage	0
DOF006_scaffold4888_1	non-phage	0
DOF006_scaffold16620_9	non-phage	0
DOF006_scaffold22965_1	non-phage	0
DOF006_scaffold6236_11	non-phage	0
DOF006_scaffold22945_1	non-phage	0
DOF006_scaffold20364_1	non-phage	0
DOF006_scaffold13247_1	non-phage	0
DOF006_scaffold12376_1	non-phage	0
DOF006_scaffold99_2	non-phage	0
DOF006_scaffold15219_2	non-phage	0
DOF006_scaffold7860_2	non-phage	0

DOF006_scaffold13945_2	non-phage	0
DOF006_scaffold9810_2	non-phage	0
DOF006_scaffold10532_1	non-phage	0
DOF006_scaffold11129_1	non-phage	0
DOF006_scaffold4232_6	non-phage	0
DOF006_scaffold21423_2	non-phage	0
DOF006_scaffold10913_11	non-phage	0
DOF006_scaffold7839_3	non-phage	0
DOF006_scaffold8737_2	non-phage	0
DOF006_scaffold22992_2	non-phage	0
DOF006_scaffold21328_1	non-phage	0
DOF006_scaffold4470_2	non-phage	0
DOF006_scaffold13736_3	non-phage	0
DOF006_scaffold10461_2	non-phage	0
DOF006_scaffold7682_1	non-phage	0
DOF006_scaffold16653_1	non-phage	0
DOF006_scaffold6386_1	non-phage	0
DOF006_scaffold6081_2	non-phage	0
DOF006_scaffold22862_2	non-phage	0
DOF006_scaffold2415_52	non-phage	0
DOF006_scaffold1101_4	non-phage	0
DOF006_scaffold9333_3	non-phage	0
DOF006_scaffold415_4	non-phage	0
DOF006_scaffold21598_2	non-phage	0
DOF006_scaffold2415_43	non-phage	0
DOF006_scaffold13755_1	non-phage	0
DOF006_scaffold22545_1	non-phage	0
DOF006_scaffold3685_2	non-phage	0
DOF006_scaffold12652_2	non-phage	0
DOF006_scaffold1825_4	non-phage	0
DOF006_scaffold13671_1	non-phage	0
DOF006_scaffold13724_2	non-phage	0
DOF006_scaffold5966_1	non-phage	0
DOF006_C311959_1	non-phage	0
DOF006_scaffold6551_1	non-phage	0
DOF006_scaffold16109_1	non-phage	0
DOF006_scaffold22738_6	non-phage	0
DOF006_scaffold3372_16	non-phage	0
DOF006_scaffold21519_1	non-phage	0
DOF006_scaffold7275_1	non-phage	0
DOF006_scaffold10651_2	non-phage	0
DOF006_scaffold17_1	non-phage	0
DOF006_scaffold12901_1	non-phage	0
DOF006_scaffold16_7	non-phage	0
DOF006_scaffold1858_1	non-phage	0
DOF006_scaffold7859_1	non-phage	0

DOF006_scaffold16378_1	non-phage	0
DOF006_scaffold7840_4	non-phage	0
DOF006_scaffold1547_5	non-phage	0
DOF006_scaffold13287_2	non-phage	0
DOF006_scaffold55_2	non-phage	0
DOF006_scaffold10913_10	non-phage	0
DOF006_scaffold4639_11	non-phage	0
DOF006_scaffold22638_1	non-phage	0
DOF006_C311575_1	non-phage	0
DOF006_scaffold11130_2	non-phage	0
DOF006_scaffold22252_1	non-phage	0
DOF006_scaffold9292_1	non-phage	0
DOF006_scaffold13539_3	non-phage	0
DOF006_scaffold1770_2	non-phage	0
DOF006_scaffold135_2	non-phage	0
DOF006_scaffold7915_3	non-phage	0
DOF006_scaffold7840_2	non-phage	0
DOF006_scaffold19188_1	non-phage	0
DOF006_scaffold18019_3	non-phage	0
DOF006_scaffold22987_3	non-phage	0
DOF006_scaffold20364_3	non-phage	0
DOF006_scaffold18838_1	non-phage	0
DOF006_scaffold8685_1	non-phage	0
DOF006_scaffold9013_1	non-phage	0
DOF006_scaffold22984_1	non-phage	0
DOF006_scaffold4232_4	non-phage	0
DOF006_scaffold7700_1	non-phage	0
DOF006_scaffold2415_85	non-phage	0
DOF006_scaffold10913_1	non-phage	0
DOF006_scaffold11658_1	non-phage	0
DOF006_scaffold305_3	non-phage	0
DOF006_scaffold16620_12	non-phage	0
DOF006_scaffold10651_1	non-phage	0
DOF006_C311481_1	non-phage	0
DOF006_scaffold23018_1	non-phage	0
DOF006_scaffold21727_2	non-phage	0
DOF006_scaffold22727_1	non-phage	0
DOF006_scaffold22992_1	non-phage	0
DOF006_scaffold22997_2	non-phage	0
DOF006_scaffold9574_2	non-phage	0
DOF006_scaffold5899_3	non-phage	0
DOF006_scaffold8257_3	non-phage	0
DOF006_scaffold3372_15	non-phage	0
DOF006_scaffold13473_1	non-phage	0
DOF006_scaffold1180_6	non-phage	0
DOF006_scaffold1206_4	non-phage	0

DOF006_scaffold154_1	non-phage	0
DOF006_scaffold7541_1	non-phage	0
DOF006_scaffold2733_4	non-phage	0
DOF006_scaffold20491_1	non-phage	0
DOF006_C312765_1	non-phage	0
DOF006_scaffold5845_7	non-phage	0
DOF006_scaffold16_16	non-phage	0
DOF006_scaffold1813_4	non-phage	0
DOF006_scaffold13984_1	non-phage	0
DOF006_scaffold10646_2	non-phage	0
DOF006_scaffold15247_8	non-phage	0
DOF006_scaffold10913_5	non-phage	0
DOF006_scaffold7084_6	non-phage	0
DOF006_scaffold11050_2	non-phage	0
DOF006_scaffold16_1	non-phage	0
DOF006_scaffold10913_9	non-phage	0
DOF006_scaffold10191_1	non-phage	0
DOF006_scaffold994_1	non-phage	0
DOF006_scaffold22961_1	non-phage	0
DOF006_scaffold16_10	non-phage	0
DOF006_scaffold9998_1	non-phage	0
DOF006_scaffold12635_1	non-phage	0
DOF006_scaffold12158_6	non-phage	0
DOF006_scaffold7840_3	non-phage	0
DOF007_scaffold22317_1	non-phage	0
DOF007_scaffold4223_2	non-phage	0
DOF007_scaffold23659_1	non-phage	0
DOF007_scaffold18262_3	non-phage	0
DOF007_scaffold23343_1	non-phage	0
DOF007_scaffold20437_2	non-phage	0
DOF007_scaffold32_10	non-phage	0
DOF007_scaffold23313_3	non-phage	0
DOF007_scaffold26085_1	non-phage	0
DOF007_scaffold26081_4	non-phage	0
DOF007_scaffold12268_2	non-phage	0
DOF007_scaffold3327_6	non-phage	0
DOF007_scaffold4718_21	non-phage	0
DOF007_scaffold8597_1	non-phage	0
DOF007_scaffold4083_1	non-phage	0
DOF007_scaffold21810_2	non-phage	0
DOF007_scaffold11262_1	non-phage	0
DOF007_scaffold23504_2	non-phage	0
DOF007_scaffold22071_1	non-phage	0
DOF007_C360471_1	non-phage	0
DOF007_scaffold16218_2	non-phage	0
DOF007_scaffold1333_4	non-phage	0

DOF007_scaffold4397_1	non-phage	0
DOF007_scaffold2171_1	non-phage	0
DOF007_scaffold19347_1	non-phage	0
DOF007_scaffold18285_2	non-phage	0
DOF007_scaffold9121_7	non-phage	0
DOF007_scaffold551_2	non-phage	0
DOF007_scaffold25165_1	non-phage	0
DOF007_scaffold16654_4	non-phage	0
DOF007_scaffold19386_1	non-phage	0
DOF007_scaffold21883_1	non-phage	0
DOF007_scaffold21407_2	non-phage	0
DOF007_scaffold6057_8	non-phage	0
DOF007_scaffold23252_5	non-phage	0
DOF007_scaffold19381_2	non-phage	0
DOF007_scaffold26106_1	non-phage	0
DOF007_scaffold35_7	non-phage	0
DOF007_scaffold18361_3	non-phage	0
DOF007_scaffold22865_1	non-phage	0
DOF007_scaffold22629_1	non-phage	0
DOF007_C360305_1	non-phage	0
DOF007_scaffold8670_2	non-phage	0
DOF007_scaffold25392_1	non-phage	0
DOF007_scaffold18_1	non-phage	0
DOF007_scaffold19337_6	non-phage	0
DOF007_scaffold21_6	non-phage	0
DOF007_scaffold25414_1	non-phage	0
DOF007_scaffold26099_2	non-phage	0
DOF007_scaffold32_4	non-phage	0
DOF007_scaffold2055_2	non-phage	0
DOF007_scaffold6717_10	non-phage	0
DOF007_scaffold12772_3	non-phage	0
DOF007_scaffold25874_2	non-phage	0
DOF007_scaffold3096_91	non-phage	0
DOF007_scaffold15_3	non-phage	0
DOF007_scaffold23559_1	non-phage	0
DOF007_scaffold18477_2	non-phage	0
DOF007_scaffold15138_2	non-phage	0
DOF007_scaffold5854_1	non-phage	0
DOF007_scaffold8_4	non-phage	0
DOF007_scaffold84_2	non-phage	0
DOF007_scaffold2655_14	non-phage	0
DOF007_scaffold26123_1	non-phage	0
DOF007_scaffold8388_5	non-phage	0
DOF007_scaffold12268_1	non-phage	0
DOF007_scaffold32_6	non-phage	0
DOF007_scaffold6128_1	non-phage	0

DOF007_scaffold20294_3	non-phage	0
DOF007_scaffold23940_1	non-phage	0
DOF007_scaffold23659_2	non-phage	0
DOF007_scaffold15353_9	non-phage	0
DOF007_scaffold14905_2	non-phage	0
DOF007_scaffold24960_3	non-phage	0
DOF007_scaffold25771_1	non-phage	0
DOF007_scaffold16942_2	non-phage	0
DOF007_scaffold24611_1	non-phage	0
DOF007_scaffold21428_1	non-phage	0
DOF007_scaffold35_2	non-phage	0
DOF007_scaffold22227_1	non-phage	0
DOF007_scaffold4083_4	non-phage	0
DOF007_scaffold8224_2	non-phage	0
DOF007_scaffold26026_1	non-phage	0
DOF007_scaffold13567_4	non-phage	0
DOF007_scaffold25726_2	non-phage	0
DOF007_scaffold104_4	non-phage	0
DOF007_scaffold18472_2	non-phage	0
DOF007_scaffold4946_7	non-phage	0
DOF007_scaffold18343_1	non-phage	0
DOF007_scaffold4397_3	non-phage	0
DOF007_scaffold19265_2	non-phage	0
DOF007_scaffold23284_1	non-phage	0
DOF007_scaffold117_1	non-phage	0
DOF007_scaffold8670_1	non-phage	0
DOF007_scaffold9121_6	non-phage	0
DOF007_scaffold8149_1	non-phage	0
DOF007_scaffold23267_3	non-phage	0
DOF007_scaffold25387_1	non-phage	0
DOF007_scaffold2655_21	non-phage	0
DOF007_scaffold19622_2	non-phage	0
DOF007_scaffold4973_3	non-phage	0
DOF007_scaffold5221_11	non-phage	0
DOF007_scaffold10382_3	non-phage	0
DOF007_scaffold11068_3	non-phage	0
DOF007_scaffold6061_2	non-phage	0
DOF007_scaffold12990_1	non-phage	0
DOF007_scaffold17201_1	non-phage	0
DOF007_scaffold36_5	non-phage	0
DOF007_scaffold25063_1	non-phage	0
DOF007_scaffold1390_4	non-phage	0
DOF007_scaffold17149_6	non-phage	0
DOF007_scaffold26050_1	non-phage	0
DOF007_scaffold100_1	non-phage	0
DOF007_scaffold4252_1	non-phage	0

DOF007_scaffold17663_5	non-phage	0
DOF007_scaffold22864_3	non-phage	0
DOF007_scaffold23252_3	non-phage	0
DOF007_scaffold16201_3	non-phage	0
DOF007_scaffold6061_1	non-phage	0
DOF007_scaffold11868_1	non-phage	0
DOF007_scaffold25033_1	non-phage	0
DOF007_scaffold24463_1	non-phage	0
DOF007_C360029_1	non-phage	0
DOF007_scaffold1812_2	non-phage	0
DOF007_scaffold26080_1	non-phage	0
DOF007_scaffold25697_1	non-phage	0
DOF007_scaffold24787_1	non-phage	0
DOF007_scaffold23313_8	non-phage	0
DOF007_scaffold9952_2	non-phage	0
DOF007_scaffold1583_15	non-phage	0
DOF007_scaffold5834_1	non-phage	0
DOF007_scaffold25990_2	non-phage	0
DOF007_scaffold6294_15	non-phage	0
DOF007_scaffold13171_3	non-phage	0
DOF007_scaffold2013_1	non-phage	0
DOF007_scaffold10192_1	non-phage	0
DOF007_scaffold8642_1	non-phage	0
DOF007_scaffold25806_1	non-phage	0
DOF007_scaffold24107_1	non-phage	0
DOF007_scaffold25611_3	non-phage	0
DOF007_scaffold7732_5	non-phage	0
DOF007_scaffold25348_2	non-phage	0
DOF007_scaffold25676_2	non-phage	0
DOF007_scaffold36_6	non-phage	0
DOF007_scaffold11684_7	non-phage	0
DOF007_scaffold11210_4	non-phage	0
DOF007_scaffold21810_1	non-phage	0
DOF007_scaffold21434_2	non-phage	0
DOF007_scaffold25969_1	non-phage	0
DOF007_scaffold8307_10	non-phage	0
DOF007_scaffold20113_10	non-phage	0
DOF007_scaffold6294_12	non-phage	0
DOF007_scaffold26013_1	non-phage	0
DOF007_scaffold14315_2	non-phage	0
DOF007_scaffold4808_5	non-phage	0
DOF007_scaffold23826_1	non-phage	0
DOF007_scaffold6294_10	non-phage	0
DOF007_scaffold25996_1	non-phage	0
DOF007_scaffold23313_4	non-phage	0
DOF007_scaffold20324_1	non-phage	0

DOF007_scaffold2655_18	non-phage	0
DOF007_scaffold4400_2	non-phage	0
DOF007_scaffold4724_6	non-phage	0
DOF007_scaffold9520_1	non-phage	0
DOF007_scaffold17219_1	non-phage	0
DOF007_scaffold23094_1	non-phage	0
DOF007_scaffold12822_5	non-phage	0
DOF007_scaffold523_1	non-phage	0
DOF007_scaffold8136_1	non-phage	0
DOF007_scaffold14247_1	non-phage	0
DOF007_scaffold25558_5	non-phage	0
DOF007_scaffold25964_1	non-phage	0
DOF007_scaffold11901_2	non-phage	0
DOF007_scaffold24179_1	non-phage	0
DOF007_scaffold6373_3	non-phage	0
DOF007_scaffold19073_3	non-phage	0
DOF007_scaffold10565_1	non-phage	0
DOF007_scaffold9380_2	non-phage	0
DOF007_scaffold8721_1	non-phage	0
DOF007_scaffold18177_1	non-phage	0
DOF007_scaffold24791_1	non-phage	0
DOF007_scaffold2931_7	non-phage	0
DOF007_scaffold35_8	non-phage	0
DOF007_scaffold5360_2	non-phage	0
DOF007_scaffold24525_1	non-phage	0
DOF007_scaffold4724_3	non-phage	0
DOF007_scaffold4724_5	non-phage	0
DOF007_scaffold2931_14	non-phage	0
DOF007_scaffold20113_1	non-phage	0
DOF007_scaffold15374_1	non-phage	0
DOF007_scaffold7812_2	non-phage	0
DOF007_scaffold16947_1	non-phage	0
DOF007_C360489_1	non-phage	0
DOF007_scaffold26117_1	non-phage	0
DOF007_scaffold3266_14	non-phage	0
DOF007_scaffold18820_1	non-phage	0
DOF007_scaffold26023_1	non-phage	0
DOF007_scaffold25810_1	non-phage	0
DOF007_scaffold25046_3	non-phage	0
DOF007_scaffold373_3	non-phage	0
DOF007_scaffold18410_2	non-phage	0
DOF007_scaffold23868_1	non-phage	0
DOF007_scaffold6294_13	non-phage	0
DOF007_scaffold26053_1	non-phage	0
DOF007_scaffold13202_2	non-phage	0
DOF007_scaffold5221_5	non-phage	0

DOF007_scaffold18322_1	non-phage	0
DOF007_scaffold26107_3	non-phage	0
DOF007_scaffold8721_5	non-phage	0
DOF007_scaffold17048_3	non-phage	0
DOF007_scaffold1871_7	non-phage	0
DOF007_scaffold32_3	non-phage	0
DOF008_scaffold3259_7	non-phage	0
DOF008_scaffold20980_1	non-phage	0
DOF008_scaffold14818_5	non-phage	0
DOF008_scaffold11521_5	non-phage	0
DOF008_scaffold13939_7	non-phage	0
DOF008_scaffold1687_4	non-phage	0
DOF008_scaffold1028_13	non-phage	0
DOF008_scaffold1028_10	non-phage	0
DOF008_scaffold24991_1	non-phage	0
DOF008_scaffold11004_1	non-phage	0
DOF008_scaffold16092_2	non-phage	0
DOF008_scaffold635_2	non-phage	0
DOF008_scaffold24269_1	non-phage	0
DOF008_scaffold19028_1	non-phage	0
DOF008_scaffold4322_1	non-phage	0
DOF008_scaffold21853_3	non-phage	0
DOF008_scaffold18361_8	non-phage	0
DOF008_scaffold15850_1	non-phage	0
DOF008_scaffold16750_2	non-phage	0
DOF008_scaffold5596_3	non-phage	0
DOF008_scaffold11218_4	non-phage	0
DOF008_scaffold3816_1	non-phage	0
DOF008_scaffold10372_3	non-phage	0
DOF008_scaffold11362_1	non-phage	0
DOF008_scaffold18017_2	non-phage	0
DOF008_scaffold10482_1	non-phage	0
DOF008_scaffold18613_6	non-phage	0
DOF008_scaffold4969_1	non-phage	0
DOF008_scaffold16092_8	non-phage	0
DOF008_scaffold58_2	non-phage	0
DOF008_scaffold9323_3	non-phage	0
DOF008_scaffold5842_3	non-phage	0
DOF008_scaffold27533_1	non-phage	0
DOF008_scaffold629_4	non-phage	0
DOF008_scaffold14917_2	non-phage	0
DOF008_scaffold28815_3	non-phage	0
DOF008_scaffold1105_7	non-phage	0
DOF008_scaffold2853_3	non-phage	0
DOF008_scaffold12344_3	non-phage	0
DOF008_scaffold2177_20	non-phage	0

DOF008_scaffold232_2	non-phage	0
DOF008_C423329_1	non-phage	0
DOF008_scaffold6808_23	non-phage	0
DOF008_scaffold1859_6	non-phage	0
DOF008_scaffold27940_1	non-phage	0
DOF008_scaffold22701_2	non-phage	0
DOF008_scaffold28702_1	non-phage	0
DOF008_scaffold5122_3	non-phage	0
DOF008_scaffold15977_3	non-phage	0
DOF008_scaffold13277_2	non-phage	0
DOF008_scaffold15345_3	non-phage	0
DOF008_scaffold6267_5	non-phage	0
DOF008_scaffold7802_4	non-phage	0
DOF008_scaffold28708_1	non-phage	0
DOF008_scaffold62_1	non-phage	0
DOF008_scaffold28656_2	non-phage	0
DOF008_scaffold13396_6	non-phage	0
DOF008_scaffold5330_9	non-phage	0
DOF008_scaffold4013_2	non-phage	0
DOF008_scaffold2592_1	non-phage	0
DOF008_scaffold734_1	non-phage	0
DOF008_scaffold22125_7	non-phage	0
DOF008_scaffold28605_3	non-phage	0
DOF008_scaffold629_10	non-phage	0
DOF008_scaffold28978_1	non-phage	0
DOF008_scaffold29017_1	non-phage	0
DOF008_scaffold24510_1	non-phage	0
DOF008_scaffold3454_2	non-phage	0
DOF008_scaffold26924_1	non-phage	0
DOF008_scaffold186_1	non-phage	0
DOF008_scaffold3293_18	non-phage	0
DOF008_scaffold7005_1	non-phage	0
DOF008_scaffold1036_60	non-phage	0
DOF008_scaffold1497_3	non-phage	0
DOF008_scaffold23359_2	non-phage	0
DOF008_scaffold899_2	non-phage	0
DOF008_scaffold7138_1	non-phage	0
DOF008_scaffold16092_6	non-phage	0
DOF008_scaffold4011_2	non-phage	0
DOF008_scaffold13720_4	non-phage	0
DOF008_scaffold8948_3	non-phage	0
DOF008_scaffold28903_1	non-phage	0
DOF008_scaffold1043_6	non-phage	0
DOF008_scaffold5581_32	non-phage	0
DOF008_scaffold19424_1	non-phage	0
DOF008_scaffold5805_2	non-phage	0

DOF008_scaffold7974_1	non-phage	0
DOF008_scaffold13923_3	non-phage	0
DOF008_scaffold24697_2	non-phage	0
DOF008_scaffold11424_1	non-phage	0
DOF008_scaffold4982_2	non-phage	0
DOF008_scaffold1953_4	non-phage	0
DOF008_scaffold12930_3	non-phage	0
DOF008_scaffold18003_6	non-phage	0
DOF008_scaffold1028_4	non-phage	0
DOF008_scaffold839_27	non-phage	0
DOF008_scaffold24157_1	non-phage	0
DOF008_scaffold10763_1	non-phage	0
DOF008_scaffold24879_1	non-phage	0
DOF008_scaffold3377_29	non-phage	0
DOF008_scaffold25628_2	non-phage	0
DOF008_scaffold11004_2	non-phage	0
DOF008_scaffold8911_1	non-phage	0
DOF008_scaffold12452_1	non-phage	0
DOF008_scaffold9668_2	non-phage	0
DOF008_scaffold4006_13	non-phage	0
DOF008_scaffold9393_5	non-phage	0
DOF008_scaffold28545_2	non-phage	0
DOF008_scaffold5596_1	non-phage	0
DOF008_scaffold28706_1	non-phage	0
DOF008_scaffold12283_1	non-phage	0
DOF008_scaffold9097_8	non-phage	0
DOF008_scaffold12743_4	non-phage	0
DOF008_scaffold14346_1	non-phage	0
DOF008_scaffold19876_7	non-phage	0
DOF008_scaffold7444_3	non-phage	0
DOF008_scaffold7802_3	non-phage	0
DOF008_scaffold164_1	non-phage	0
DOF008_scaffold9550_4	non-phage	0
DOF008_scaffold17675_3	non-phage	0
DOF008_scaffold22144_2	non-phage	0
DOF008_scaffold25802_3	non-phage	0
DOF008_scaffold5207_1	non-phage	0
DOF008_scaffold32_3	non-phage	0
DOF009_scaffold129_78	non-phage	0
DOF009_scaffold45042_5	non-phage	0
DOF009_scaffold24832_7	non-phage	0
DOF009_scaffold51124_2	non-phage	0
DOF009_scaffold50618_2	non-phage	0
DOF009_scaffold46233_1	non-phage	0
DOF009_scaffold25555_7	non-phage	0
DOF009_scaffold52845_2	non-phage	0

DOF009_scaffold23999_1	non-phage	0
DOF009_scaffold50318_1	non-phage	0
DOF009_scaffold25904_4	non-phage	0
DOF009_scaffold34417_1	non-phage	0
DOF009_scaffold20410_4	non-phage	0
DOF009_scaffold52623_1	non-phage	0
DOF009_scaffold52711_2	non-phage	0
DOF009_scaffold46474_4	non-phage	0
DOF009_scaffold33065_1	non-phage	0
DOF009_scaffold1543_17	non-phage	0
DOF009_scaffold40850_2	non-phage	0
DOF009_scaffold52380_1	non-phage	0
DOF009_scaffold52015_1	non-phage	0
DOF009_scaffold2864_17	non-phage	0
DOF009_scaffold3636_62	non-phage	0
DOF009_scaffold42431_3	non-phage	0
DOF009_scaffold52780_1	non-phage	0
DOF009_scaffold29792_9	non-phage	0
DOF009_scaffold8571_14	non-phage	0
DOF009_scaffold46624_2	non-phage	0
DOF009_scaffold46144_1	non-phage	0
DOF009_scaffold970_37	non-phage	0
DOF009_scaffold2408_19	non-phage	0
DOF009_scaffold3642_2	non-phage	0
DOF009_scaffold50997_4	non-phage	0
DOF009_scaffold52839_1	non-phage	0
DOF009_scaffold27303_1	non-phage	0
DOF009_scaffold52624_1	non-phage	0
DOF009_scaffold249_10	non-phage	0
DOF009_C722296_1	non-phage	0
DOF009_scaffold2408_12	non-phage	0
DOF009_scaffold48442_1	non-phage	0
DOF009_scaffold52549_1	non-phage	0
DOF009_scaffold48335_1	non-phage	0
DOF009_scaffold33065_6	non-phage	0
DOF009_scaffold50292_1	non-phage	0
DOF009_scaffold12976_14	non-phage	0
DOF009_scaffold25904_8	non-phage	0
DOF009_scaffold1543_14	non-phage	0
DOF009_scaffold17856_1	non-phage	0
DOF009_scaffold52409_1	non-phage	0
DOF009_scaffold52601_1	non-phage	0
DOF009_scaffold129_21	non-phage	0
DOF009_scaffold10204_1	non-phage	0
DOF009_scaffold52185_1	non-phage	0
DOF009_scaffold50997_1	non-phage	0

DOF009_scaffold50038_1	non-phage	0
DOF009_scaffold31368_3	non-phage	0
DOF009_scaffold12352_2	non-phage	0
DOF009_scaffold52797_2	non-phage	0
DOF009_scaffold23855_5	non-phage	0
DOF009_scaffold52739_1	non-phage	0
DOF009_scaffold5444_8	non-phage	0
DOF009_scaffold52629_2	non-phage	0
DOF009_scaffold46474_3	non-phage	0
DOF009_scaffold4516_4	non-phage	0
DOF009_scaffold43538_2	non-phage	0
DOF009_scaffold297_12	non-phage	0
DOF009_scaffold10528_10	non-phage	0
DOF009_scaffold41297_1	non-phage	0
DOF009_scaffold1543_16	non-phage	0
DOF009_scaffold229_10	non-phage	0
DOF009_scaffold33065_2	non-phage	0
DOF009_scaffold52303_2	non-phage	0
DOF009_scaffold3560_1	non-phage	0
DOF009_scaffold50964_4	non-phage	0
DOF009_scaffold130_1	non-phage	0
DOF009_scaffold17458_1	non-phage	0
DOF009_scaffold50398_1	non-phage	0
DOF009_scaffold45791_2	non-phage	0
DOF009_scaffold17671_5	non-phage	0
DOF009_scaffold38377_4	non-phage	0
DOF009_C722126_1	non-phage	0
DOF009_scaffold11693_1	non-phage	0
DOF009_scaffold8571_8	non-phage	0
DOF009_scaffold6235_1	non-phage	0
DOF009_scaffold5133_12	non-phage	0
DOF009_scaffold43762_3	non-phage	0
DOF009_scaffold5667_32	non-phage	0
DOF009_scaffold13964_5	non-phage	0
DOF009_scaffold20784_9	non-phage	0
DOF009_scaffold36633_16	non-phage	0
DOF009_scaffold9726_66	non-phage	0
DOF009_scaffold8510_1	non-phage	0
DOF009_scaffold51089_1	non-phage	0
DOF009_scaffold43630_12	non-phage	0
DOF009_scaffold33616_4	non-phage	0
DOF009_scaffold45109_8	non-phage	0
DOF009_scaffold47589_6	non-phage	0
DOF009_scaffold8434_1	non-phage	0
DOF009_scaffold51609_2	non-phage	0
DOF009_scaffold297_8	non-phage	0

DOF009_scaffold49621_1	non-phage	0
DOF009_scaffold297_16	non-phage	0
DOF009_scaffold8595_5	non-phage	0
DOF009_scaffold35184_1	non-phage	0
DOF009_scaffold38638_2	non-phage	0
DOF009_scaffold52848_1	non-phage	0
DOF009_scaffold17413_1	non-phage	0
DOF009_scaffold36633_6	non-phage	0
DOF009_scaffold48_4	non-phage	0
DOF009_scaffold38260_6	non-phage	0
DOF009_scaffold14859_4	non-phage	0
DOF009_scaffold31791_4	non-phage	0
DOF009_scaffold52711_3	non-phage	0
DOF009_scaffold9726_38	non-phage	0
DOF009_scaffold52581_1	non-phage	0
DOF009_scaffold43767_1	non-phage	0
DOF009_scaffold35271_3	non-phage	0
DOF009_scaffold50398_2	non-phage	0
DOF009_scaffold36633_13	non-phage	0
DOF009_scaffold33347_3	non-phage	0
DOF009_scaffold10879_1	non-phage	0
DOF009_scaffold51830_3	non-phage	0
DOF009_scaffold52452_1	non-phage	0
DOF009_scaffold46233_3	non-phage	0
DOF009_scaffold2526_7	non-phage	0
DOF009_scaffold2408_17	non-phage	0
DOF009_scaffold45264_2	non-phage	0
DOF009_scaffold38944_4	non-phage	0
DOF009_scaffold10528_2	non-phage	0
DOF009_scaffold23738_3	non-phage	0
DOF009_scaffold50026_3	non-phage	0
DOF009_scaffold22461_2	non-phage	0
DOF009_scaffold43779_1	non-phage	0
DOF009_scaffold8602_2	non-phage	0
DOF009_scaffold2476_2	non-phage	0
DOF009_scaffold49040_2	non-phage	0
DOF009_scaffold42495_4	non-phage	0
DOF009_scaffold7741_11	non-phage	0
DOF009_C722472_1	non-phage	0
DOF009_scaffold13397_5	non-phage	0
DOF009_scaffold770_1	non-phage	0
DOF009_scaffold49230_2	non-phage	0
DOF009_scaffold52717_1	non-phage	0
DOF009_scaffold19916_1	non-phage	0
DOF009_scaffold52748_2	non-phage	0
DOF009_scaffold25679_1	non-phage	0

DOF009_scaffold25231_1	non-phage	0
DOF009_scaffold4851_2	non-phage	0
DOF009_scaffold2037_23	non-phage	0
DOF009_scaffold45042_2	non-phage	0
DOF009_scaffold25132_1	non-phage	0
DOF009_scaffold34499_5	non-phage	0
DOF009_scaffold47008_3	non-phage	0
DOF009_scaffold36944_1	non-phage	0
DOF009_scaffold52817_1	non-phage	0
DOF009_scaffold32812_1	non-phage	0
DOF009_scaffold8571_18	non-phage	0
DOF009_scaffold8771_3	non-phage	0
DOF009_scaffold35271_1	non-phage	0
DOF009_scaffold48543_4	non-phage	0
DOF009_scaffold3896_4	non-phage	0
DOF009_scaffold44542_2	non-phage	0
DOF009_scaffold3176_1	non-phage	0
DOF009_scaffold52436_1	non-phage	0
DOF009_scaffold43377_2	non-phage	0
DOF009_scaffold2468_14	non-phage	0
DOF009_scaffold1543_3	non-phage	0
DOF009_scaffold141_8	non-phage	0
DOF009_scaffold23738_1	non-phage	0
DOF009_scaffold39212_1	non-phage	0
DOF009_scaffold50496_2	non-phage	0
DOF009_scaffold14859_1	non-phage	0
DOF009_scaffold43203_1	non-phage	0
DOF009_scaffold25904_11	non-phage	0
DOF009_scaffold41748_1	non-phage	0
DOF009_C722458_1	non-phage	0
DOF009_scaffold45670_1	non-phage	0
DOF009_scaffold31842_3	non-phage	0
DOF009_scaffold18228_1	non-phage	0
DOF009_scaffold11998_1	non-phage	0
DOF009_scaffold23810_2	non-phage	0
DOF009_scaffold22443_3	non-phage	0
DOF009_scaffold16677_4	non-phage	0
DOF009_scaffold43234_4	non-phage	0
DOF010_scaffold20028_2	non-phage	0
DOF010_scaffold38967_1	non-phage	0
DOF010_scaffold16923_26	non-phage	0
DOF010_scaffold34_10	non-phage	0
DOF010_scaffold1871_27	non-phage	0
DOF010_scaffold31173_3	non-phage	0
DOF010_scaffold20928_5	non-phage	0
DOF010_scaffold47_1	non-phage	0

DOF010_scaffold14_4	non-phage	0
DOF010_scaffold13830_6	non-phage	0
DOF010_scaffold3279_10	non-phage	0
DOF010_scaffold10999_10	non-phage	0
DOF010_scaffold18104_6	non-phage	0
DOF010_scaffold8434_3	non-phage	0
DOF010_scaffold14480_3	non-phage	0
DOF010_scaffold16896_2	non-phage	0
DOF010_scaffold11985_8	non-phage	0
DOF010_scaffold3251_6	non-phage	0
DOF010_scaffold27096_2	non-phage	0
DOF010_scaffold5166_3	non-phage	0
DOF010_scaffold14015_21	non-phage	0
DOF010_scaffold5873_4	non-phage	0
DOF010_scaffold3021_16	non-phage	0
DOF010_scaffold5166_2	non-phage	0
DOF010_scaffold7635_4	non-phage	0
DOF010_scaffold16695_5	non-phage	0
DOF010_scaffold37594_3	non-phage	0
DOF010_scaffold3351_1	non-phage	0
DOF010_scaffold6949_2	non-phage	0
DOF010_scaffold27958_2	non-phage	0
DOF010_scaffold34953_2	non-phage	0
DOF010_scaffold199_8	non-phage	0
DOF010_scaffold14015_12	non-phage	0
DOF010_scaffold11128_4	non-phage	0
DOF010_scaffold29273_2	non-phage	0
DOF010_scaffold11354_3	non-phage	0
DOF010_scaffold2170_9	non-phage	0
DOF010_scaffold20040_3	non-phage	0
DOF010_scaffold10999_7	non-phage	0
DOF010_scaffold65_7	non-phage	0
DOF010_scaffold5873_40	non-phage	0
DOF010_scaffold35264_2	non-phage	0
DOF010_scaffold28690_3	non-phage	0
DOF010_C495309_1	non-phage	0
DOF010_scaffold12990_29	non-phage	0
DOF010_scaffold8559_10	non-phage	0
DOF010_scaffold25946_1	non-phage	0
DOF010_scaffold53_1	non-phage	0
DOF010_scaffold16923_3	non-phage	0
DOF010_scaffold128_30	non-phage	0
DOF010_scaffold6233_34	non-phage	0
DOF010_scaffold7400_3	non-phage	0
DOF010_C495277_1	non-phage	0
DOF010_scaffold237_2	non-phage	0

DOF010_C495449_1	non-phage	0
DOF010_scaffold8903_10	non-phage	0
DOF010_scaffold20143_3	non-phage	0
DOF010_scaffold12990_1	non-phage	0
DOF010_scaffold3566_6	non-phage	0
DOF010_scaffold131_1	non-phage	0
DOF010_scaffold48_12	non-phage	0
DOF010_scaffold27032_5	non-phage	0
DOF010_scaffold2773_4	non-phage	0
DOF010_scaffold5196_3	non-phage	0
DOF010_scaffold35797_4	non-phage	0
DOF010_scaffold3173_3	non-phage	0
DOF010_scaffold290_5	non-phage	0
DOF010_scaffold6233_24	non-phage	0
DOF010_scaffold9978_3	non-phage	0
DOF010_scaffold11930_6	non-phage	0
DOF010_scaffold14015_5	non-phage	0
DOF010_scaffold1871_20	non-phage	0
DOF010_scaffold4412_11	non-phage	0
DOF010_scaffold14015_14	non-phage	0
DOF010_scaffold23674_6	non-phage	0
DOF010_scaffold28586_1	non-phage	0
DOF010_scaffold15383_5	non-phage	0
DOF010_scaffold16923_24	non-phage	0
DOF010_scaffold17911_8	non-phage	0
DOF010_scaffold38039_2	non-phage	0
DOF010_scaffold38266_7	non-phage	0
DOF010_scaffold9522_8	non-phage	0
DOF010_scaffold5166_26	non-phage	0
DOF010_scaffold29898_2	non-phage	0
DOF010_scaffold23847_3	non-phage	0
DOF010_scaffold38969_1	non-phage	0
DOF010_scaffold19506_3	non-phage	0
DOF010_scaffold38527_3	non-phage	0
DOF010_scaffold11930_10	non-phage	0
DOF010_scaffold37960_1	non-phage	0
DOF010_scaffold11511_16	non-phage	0
DOF010_scaffold28690_4	non-phage	0
DOF010_scaffold2170_1	non-phage	0
DOF010_scaffold9832_1	non-phage	0
DOF010_scaffold33156_2	non-phage	0
DOF010_scaffold28134_7	non-phage	0
DOF010_scaffold19394_3	non-phage	0
DOF010_scaffold38418_3	non-phage	0
DOF010_scaffold24082_1	non-phage	0
DOF010_scaffold29607_6	non-phage	0

DOF010_scaffold3478_1	non-phage	0
DOF010_scaffold38980_2	non-phage	0
DOF010_scaffold14015_13	non-phage	0
DOF010_scaffold38657_1	non-phage	0
DOF010_scaffold19550_2	non-phage	0
DOF010_scaffold37905_3	non-phage	0
DOF010_scaffold38849_1	non-phage	0
DOF010_scaffold22478_6	non-phage	0
DOF010_scaffold1362_6	non-phage	0
DOF010_scaffold9735_6	non-phage	0
DOF010_scaffold37594_5	non-phage	0
DOF010_scaffold5166_1	non-phage	0
DOF010_scaffold10007_29	non-phage	0
DOF010_scaffold33869_1	non-phage	0
DOF010_scaffold10999_9	non-phage	0
DOF010_scaffold21962_4	non-phage	0
DOF010_scaffold290_1	non-phage	0
DOF010_scaffold14015_20	non-phage	0
DOF010_scaffold10007_25	non-phage	0
DOF010_scaffold38733_3	non-phage	0
DOF010_scaffold24466_5	non-phage	0
DOF010_scaffold38441_8	non-phage	0
DOF010_scaffold144_13	non-phage	0
DOF010_scaffold38266_4	non-phage	0
DOF010_scaffold39039_2	non-phage	0
DOF010_scaffold2045_21	non-phage	0
DOF010_scaffold27761_2	non-phage	0
DOF010_scaffold39046_2	non-phage	0
DOF010_scaffold65_12	non-phage	0
DOF010_scaffold20028_4	non-phage	0
DOF010_scaffold20482_4	non-phage	0
DOF010_scaffold7400_1	non-phage	0
DOF010_scaffold128_32	non-phage	0
DOF010_C495425_1	non-phage	0
DOF010_scaffold37871_1	non-phage	0
DOF011_scaffold21176_1	non-phage	0
DOF011_scaffold19396_2	non-phage	0
DOF011_scaffold8233_1	non-phage	0
DOF011_C265049_1	non-phage	0
DOF011_scaffold21147_1	non-phage	0
DOF011_scaffold33_1	non-phage	0
DOF011_scaffold16851_1	non-phage	0
DOF011_scaffold14793_1	non-phage	0
DOF011_scaffold21208_1	non-phage	0
DOF011_scaffold1982_2	non-phage	0
DOF011_scaffold19811_1	non-phage	0

DOF011_scaffold15655_1	non-phage	0
DOF011_scaffold21020_2	non-phage	0
DOF011_scaffold182_4	non-phage	0
DOF011_scaffold17470_3	non-phage	0
DOF011_C264941_1	non-phage	0
DOF011_scaffold193_2	non-phage	0
DOF011_scaffold18445_1	non-phage	0
DOF011_scaffold1769_17	non-phage	0
DOF011_scaffold3209_5	non-phage	0
DOF011_scaffold7818_5	non-phage	0
DOF011_scaffold13325_3	non-phage	0
DOF011_scaffold7668_3	non-phage	0
DOF011_scaffold39_2	non-phage	0
DOF011_scaffold11521_4	non-phage	0
DOF011_scaffold20984_3	non-phage	0
DOF011_scaffold10063_8	non-phage	0
DOF011_scaffold18698_1	non-phage	0
DOF011_scaffold12924_4	non-phage	0
DOF011_scaffold4004_1	non-phage	0
DOF011_scaffold15673_3	non-phage	0
DOF011_scaffold16289_5	non-phage	0
DOF011_scaffold21162_1	non-phage	0
DOF011_scaffold16423_1	non-phage	0
DOF011_scaffold17136_8	non-phage	0
DOF011_scaffold20800_5	non-phage	0
DOF011_scaffold3194_1	non-phage	0
DOF011_scaffold6391_2	non-phage	0
DOF011_scaffold18004_7	non-phage	0
DOF011_scaffold21130_1	non-phage	0
DOF011_scaffold5372_5	non-phage	0
DOF011_scaffold19553_1	non-phage	0
DOF011_scaffold1139_6	non-phage	0
DOF011_scaffold4011_1	non-phage	0
DOF011_scaffold17042_1	non-phage	0
DOF011_scaffold21174_1	non-phage	0
DOF011_C264751_1	non-phage	0
DOF011_scaffold11521_2	non-phage	0
DOF011_scaffold14317_8	non-phage	0
DOF011_scaffold20705_2	non-phage	0
DOF011_scaffold18103_1	non-phage	0
DOF011_scaffold4575_3	non-phage	0
DOF011_scaffold426_4	non-phage	0
DOF011_scaffold21005_1	non-phage	0
DOF011_scaffold21161_1	non-phage	0
DOF011_C264629_1	non-phage	0
DOF011_scaffold14863_1	non-phage	0

DOF011_scaffold223_2	non-phage	0
DOF011_scaffold10770_3	non-phage	0
DOF011_scaffold19817_1	non-phage	0
DOF011_scaffold21198_2	non-phage	0
DOF011_scaffold21173_1	non-phage	0
DOF011_scaffold6064_1	non-phage	0
DOF011_scaffold16361_1	non-phage	0
DOF011_scaffold2633_14	non-phage	0
DOF011_C264559_1	non-phage	0
DOF011_scaffold793_1	non-phage	0
DOF011_scaffold278_1	non-phage	0
DOF011_scaffold5619_1	non-phage	0
DOF011_scaffold18115_4	non-phage	0
DOF011_scaffold17388_1	non-phage	0
DOF011_scaffold21055_6	non-phage	0
DOF011_scaffold17198_2	non-phage	0
DOF011_scaffold21192_1	non-phage	0
DOF011_scaffold4934_1	non-phage	0
DOF011_scaffold7543_4	non-phage	0
DOF011_scaffold20650_1	non-phage	0
DOF011_scaffold18004_1	non-phage	0
DOF011_scaffold12678_1	non-phage	0
DOF011_scaffold6063_1	non-phage	0
DOF011_C264665_1	non-phage	0
DOF011_scaffold14317_7	non-phage	0
DOF011_scaffold16304_2	non-phage	0
DOF011_scaffold6736_1	non-phage	0
DOF011_scaffold13014_5	non-phage	0
DOF011_scaffold19_2	non-phage	0
DOF011_scaffold2900_2	non-phage	0
DOF011_scaffold21065_2	non-phage	0
DOF011_scaffold15450_4	non-phage	0
DOF011_scaffold21157_1	non-phage	0
DOF011_scaffold2266_2	non-phage	0
DOF011_C264643_1	non-phage	0
DOF011_scaffold7543_5	non-phage	0
DOF011_scaffold16289_2	non-phage	0
DOF011_C264541_1	non-phage	0
DOF011_scaffold19850_1	non-phage	0
DOF011_scaffold9376_5	non-phage	0
DOF011_scaffold9376_1	non-phage	0
DOF011_scaffold17251_1	non-phage	0
DOF011_scaffold1337_15	non-phage	0
DOF011_scaffold14317_4	non-phage	0
DOF011_scaffold3639_5	non-phage	0
DOF011_scaffold7261_2	non-phage	0

DOF011_scaffold15754_1	non-phage	0
DOF011_scaffold21128_2	non-phage	0
DOF011_scaffold16361_2	non-phage	0
DOF011_scaffold21132_1	non-phage	0
DOF011_C264787_1	non-phage	0
DOF011_scaffold16_30	non-phage	0
DOF011_scaffold15450_5	non-phage	0
DOF011_scaffold21115_2	non-phage	0
DOF012_scaffold163_1	non-phage	0
DOF012_scaffold9792_2	non-phage	0
DOF012_scaffold2512_7	non-phage	0
DOF012_scaffold750_11	non-phage	0
DOF012_scaffold4483_2	non-phage	0
DOF012_scaffold3502_1	non-phage	0
DOF012_scaffold240_5	non-phage	0
DOF012_scaffold10254_13	non-phage	0
DOF012_scaffold8351_3	non-phage	0
DOF012_scaffold1282_6	non-phage	0
DOF012_scaffold1127_11	non-phage	0
DOF012_scaffold258_4	non-phage	0
DOF012_scaffold12975_3	non-phage	0
DOF012_scaffold3618_10	non-phage	0
DOF012_scaffold298_9	non-phage	0
DOF012_scaffold8657_1	non-phage	0
DOF012_scaffold4867_4	non-phage	0
DOF012_scaffold782_1	non-phage	0
DOF012_scaffold284_10	non-phage	0
DOF012_scaffold259_1	non-phage	0
DOF012_scaffold62_11	non-phage	0
DOF012_scaffold2430_8	non-phage	0
DOF012_scaffold2482_6	non-phage	0
DOF012_scaffold633_1	non-phage	0
DOF012_scaffold81_9	non-phage	0
DOF012_scaffold3451_2	non-phage	0
DOF012_scaffold4168_4	non-phage	0
DOF012_scaffold4227_9	non-phage	0
DOF012_scaffold10817_3	non-phage	0
DOF012_scaffold3205_2	non-phage	0
DOF012_scaffold11626_1	non-phage	0
DOF012_scaffold722_6	non-phage	0
DOF012_scaffold4483_7	non-phage	0
DOF012_scaffold8351_1	non-phage	0
DOF012_scaffold225_9	non-phage	0
DOF012_scaffold683_21	non-phage	0
DOF012_scaffold191_3	non-phage	0
DOF012_scaffold1045_13	non-phage	0

DOF012_scaffold5673_8	non-phage	0
DOF012_scaffold4079_21	non-phage	0
DOF012_scaffold10254_15	non-phage	0
DOF012_scaffold5303_5	non-phage	0
DOF012_scaffold4877_1	non-phage	0
DOF012_scaffold2446_1	non-phage	0
DOF012_scaffold1196_1	non-phage	0
DOF012_scaffold14691_1	non-phage	0
DOF012_scaffold409_7	non-phage	0
DOF012_scaffold28_6	non-phage	0
DOF012_scaffold1713_18	non-phage	0
DOF012_scaffold9951_1	non-phage	0
DOF012_scaffold347_16	non-phage	0
DOF012_scaffold2371_2	non-phage	0
DOF012_scaffold2641_1	non-phage	0
DOF012_scaffold992_5	non-phage	0
DOF012_scaffold17_5	non-phage	0
DOF012_scaffold10401_1	non-phage	0
DOF012_scaffold57_1	non-phage	0
DOF012_scaffold7291_3	non-phage	0
DOF012_scaffold20_18	non-phage	0
DOF012_scaffold191_5	non-phage	0
DOF012_scaffold7309_8	non-phage	0
DOF012_scaffold813_8	non-phage	0
DOF012_scaffold20_19	non-phage	0
DOF012_scaffold13244_1	non-phage	0
DOF012_scaffold6484_3	non-phage	0
DOF012_scaffold1713_6	non-phage	0
DOF012_scaffold7775_3	non-phage	0
DOF012_scaffold10862_2	non-phage	0
DOF012_scaffold707_6	non-phage	0
DOF012_scaffold9544_1	non-phage	0
DOF012_scaffold1874_1	non-phage	0
DOF012_scaffold1996_2	non-phage	0
DOF012_scaffold298_13	non-phage	0
DOF012_scaffold463_3	non-phage	0
DOF012_scaffold6502_3	non-phage	0
DOF012_scaffold2145_2	non-phage	0
DOF012_scaffold278_9	non-phage	0
DOF012_scaffold17_7	non-phage	0
DOF012_scaffold8962_3	non-phage	0
DOF012_scaffold14368_1	non-phage	0
DOF012_scaffold7291_5	non-phage	0
DOF012_scaffold2038_1	non-phage	0
DOF012_scaffold1127_9	non-phage	0
DOF012_scaffold2879_4	non-phage	0

DOF012_scaffold9023_1	non-phage	0
DOF012_scaffold1282_5	non-phage	0
DOF012_scaffold1150_2	non-phage	0
DOF012_scaffold14325_1	non-phage	0
DOF012_scaffold12975_4	non-phage	0
DOF012_scaffold10195_3	non-phage	0
DOF012_scaffold298_11	non-phage	0
DOF012_scaffold4865_1	non-phage	0
DOF012_scaffold5732_2	non-phage	0
DOF012_scaffold8330_1	non-phage	0
DOF012_scaffold3025_2	non-phage	0
DOF012_scaffold8733_10	non-phage	0
DOF012_scaffold1127_2	non-phage	0
DOF012_scaffold932_13	non-phage	0
DOF012_scaffold12552_1	non-phage	0
DOF012_scaffold351_5	non-phage	0
DOF012_scaffold906_1	non-phage	0
DOF012_scaffold633_4	non-phage	0
DOF012_scaffold303_1	non-phage	0
DOF012_scaffold1238_8	non-phage	0
DOF012_scaffold271_2	non-phage	0
DOF012_scaffold3656_3	non-phage	0
DOF012_scaffold7046_6	non-phage	0
DOF012_scaffold239_2	non-phage	0
DOF012_scaffold347_7	non-phage	0
DOF012_scaffold11870_3	non-phage	0
DOF012_scaffold2103_1	non-phage	0
DOF012_scaffold7331_7	non-phage	0
DOF012_scaffold229_1	non-phage	0
DOF012_scaffold1229_2	non-phage	0
DOF012_scaffold248_2	non-phage	0
DOF012_scaffold28_5	non-phage	0
DOF012_scaffold683_18	non-phage	0
DOF012_scaffold485_1	non-phage	0
DOF012_scaffold1874_6	non-phage	0
DOF012_scaffold229_2	non-phage	0
DOF012_scaffold20_17	non-phage	0
DOF012_scaffold419_2	non-phage	0
DOF012_scaffold101_7	non-phage	0
DOF012_scaffold373_1	non-phage	0
DOF012_scaffold9330_2	non-phage	0
DOF012_scaffold225_6	non-phage	0
DOF012_scaffold191_6	non-phage	0
DOF012_scaffold14557_1	non-phage	0
DOF012_scaffold3056_6	non-phage	0
DOF012_scaffold13672_1	non-phage	0

DOF012_scaffold14400_3	non-phage	0
DOF012_scaffold7331_11	non-phage	0
DOF012_scaffold2324_11	non-phage	0
DOF012_scaffold4090_2	non-phage	0
DOF012_scaffold284_8	non-phage	0
DOF012_scaffold419_4	non-phage	0
DOF012_scaffold149_5	non-phage	0
DOF012_scaffold4490_3	non-phage	0
DOF012_scaffold1237_4	non-phage	0
DOF012_scaffold372_9	non-phage	0
DOF012_scaffold166_5	non-phage	0
DOF012_scaffold2879_3	non-phage	0
DOF012_scaffold5482_4	non-phage	0
DOF012_scaffold1855_6	non-phage	0
DOF012_scaffold5504_2	non-phage	0
DOF012_scaffold12200_2	non-phage	0
DOF012_scaffold11998_1	non-phage	0
DOF012_scaffold3385_1	non-phage	0
DOF012_scaffold2482_5	non-phage	0
DOF012_scaffold819_6	non-phage	0
DOF012_scaffold14426_1	non-phage	0
DOF012_scaffold57_3	non-phage	0
DOF012_scaffold62_3	non-phage	0
DOF012_scaffold4893_5	non-phage	0
DOF012_scaffold253_1	non-phage	0
DOF012_scaffold1996_3	non-phage	0
DOF013_scaffold2002_1	non-phage	0
DOF013_scaffold6941_2	non-phage	0
DOF013_scaffold2923_9	non-phage	0
DOF013_scaffold1438_6	non-phage	0
DOF013_scaffold11367_5	non-phage	0
DOF013_scaffold29923_1	non-phage	0
DOF013_scaffold697_11_2	non-phage	0
DOF013_scaffold113_6	non-phage	0
DOF013_scaffold5922_1	non-phage	0
DOF013_scaffold1672_3	non-phage	0
DOF013_scaffold6294_4	non-phage	0
DOF013_scaffold15364_2	non-phage	0
DOF013_scaffold23282_1	non-phage	0
DOF013_scaffold12689_3	non-phage	0
DOF013_scaffold4019_3	non-phage	0
DOF013_scaffold18912_1	non-phage	0
DOF013_scaffold1035_1	non-phage	0
DOF013_scaffold4487_27	non-phage	0
DOF013_scaffold10071_8	non-phage	0
DOF013_scaffold6335_8	non-phage	0

DOF013_scaffold4411_9	non-phage	0
DOF013_scaffold22261_2	non-phage	0
DOF013_scaffold26030_1	non-phage	0
DOF013_scaffold9100_3	non-phage	0
DOF013_scaffold460_3_1	non-phage	0
DOF013_scaffold202_10	non-phage	0
DOF013_scaffold11053_1	non-phage	0
DOF013_scaffold8215_1	non-phage	0
DOF013_scaffold81_5	non-phage	0
DOF013_scaffold1172_5	non-phage	0
DOF013_scaffold4079_2	non-phage	0
DOF013_scaffold453_7_2	non-phage	0
DOF013_scaffold134_4	non-phage	0
DOF013_scaffold3953_2	non-phage	0
DOF013_scaffold4818_3	non-phage	0
DOF013_scaffold17828_2	non-phage	0
DOF013_scaffold1325_6	non-phage	0
DOF013_scaffold9644_4	non-phage	0
DOF013_scaffold6022_4	non-phage	0
DOF013_scaffold66_2	non-phage	0
DOF013_scaffold13520_8	non-phage	0
DOF013_scaffold368_4	non-phage	0
DOF013_scaffold111_4	non-phage	0
DOF013_scaffold6288_6	non-phage	0
DOF013_scaffold24641_1	non-phage	0
DOF013_scaffold1529_6	non-phage	0
DOF013_scaffold14853_2	non-phage	0
DOF013_scaffold850_12	non-phage	0
DOF013_scaffold5283_5	non-phage	0
DOF013_scaffold27646_2	non-phage	0
DOF013_scaffold18276_3	non-phage	0
DOF013_scaffold11507_4	non-phage	0
DOF013_scaffold13367_2	non-phage	0
DOF013_scaffold113_5	non-phage	0
DOF013_scaffold11066_3	non-phage	0
DOF013_scaffold17671_7	non-phage	0
DOF013_scaffold6022_8	non-phage	0
DOF013_scaffold11584_5	non-phage	0
DOF013_scaffold4044_5	non-phage	0
DOF013_scaffold18593_2	non-phage	0
DOF013_scaffold418_4	non-phage	0
DOF013_scaffold2653_7	non-phage	0
DOF013_scaffold12912_4	non-phage	0
DOF013_scaffold16072_1	non-phage	0
DOF013_scaffold1983_11	non-phage	0
DOF013_scaffold5043_16	non-phage	0

DOF013_scaffold10570_1	non-phage	0
DOF013_scaffold18835_3	non-phage	0
DOF013_scaffold12056_3	non-phage	0
DOF013_scaffold4132_3	non-phage	0
DOF013_scaffold251_5	non-phage	0
DOF013_scaffold5922_2	non-phage	0
DOF013_scaffold17671_1	non-phage	0
DOF013_scaffold1619_4	non-phage	0
DOF013_scaffold26353_1	non-phage	0
DOF013_scaffold104_4	non-phage	0
DOF013_scaffold248_2	non-phage	0
DOF013_scaffold3182_1	non-phage	0
DOF013_scaffold9100_7	non-phage	0
DOF013_scaffold3736_1	non-phage	0
DOF013_scaffold103_10	non-phage	0
DOF013_scaffold5496_1	non-phage	0
DOF013_scaffold13929_2	non-phage	0
DOF013_scaffold23462_3	non-phage	0
DOF013_scaffold3897_4	non-phage	0
DOF013_scaffold26337_1	non-phage	0
DOF013_scaffold3109_15	non-phage	0
DOF013_scaffold11729_1	non-phage	0
DOF013_scaffold1532_3	non-phage	0
DOF013_scaffold3533_3	non-phage	0
DOF013_scaffold2176_2	non-phage	0
DOF013_scaffold4984_5	non-phage	0
DOF013_scaffold4159_2	non-phage	0
DOF013_scaffold1529_18	non-phage	0
DOF013_scaffold10071_3	non-phage	0
DOF013_scaffold1328_5	non-phage	0
DOF013_scaffold4487_2	non-phage	0
DOF013_scaffold460_2	non-phage	0
DOF013_scaffold25619_1	non-phage	0
DOF013_scaffold2285_4	non-phage	0
DOF013_scaffold692_2	non-phage	0
DOF013_scaffold379_2	non-phage	0
DOF013_scaffold1013_2	non-phage	0
DOF013_scaffold798_1	non-phage	0
DOF013_scaffold27897_2	non-phage	0
DOF013_scaffold1035_3	non-phage	0
DOF013_scaffold460_5	non-phage	0
DOF013_scaffold6333_3	non-phage	0
DOF013_scaffold11796_2	non-phage	0
DOF013_scaffold797_6	non-phage	0
DOF013_scaffold18835_2	non-phage	0
DOF013_scaffold13091_4	non-phage	0

DOF013_scaffold14431_2	non-phage	0
DOF013_scaffold6310_3	non-phage	0
DOF013_scaffold3069_5	non-phage	0
DOF013_scaffold3570_2	non-phage	0
DOF013_scaffold441_2	non-phage	0
DOF013_scaffold3958_1	non-phage	0
DOF013_scaffold9208_16	non-phage	0
DOF013_scaffold4781_1	non-phage	0
DOF013_scaffold953_7	non-phage	0
DOF013_scaffold9100_6	non-phage	0
DOF013_scaffold14702_2	non-phage	0
DOF013_scaffold797_7	non-phage	0
DOF013_scaffold29920_2	non-phage	0
DOF013_scaffold19578_6	non-phage	0
DOF013_scaffold6727_1	non-phage	0
DOF013_scaffold2613_1	non-phage	0
DOF013_scaffold11253_2	non-phage	0
DOF013_scaffold1636_4	non-phage	0
DOF013_scaffold18276_1	non-phage	0
DOF013_scaffold2113_9_1	non-phage	0
DOF013_scaffold2113_9_2	non-phage	0
DOF013_scaffold24252_3	non-phage	0
DOF013_scaffold3739_1	non-phage	0
DOF013_scaffold16019_4	non-phage	0
DOF013_scaffold224_7	non-phage	0
DOF013_scaffold18569_1	non-phage	0
DOF013_scaffold11760_16	non-phage	0
DOF013_scaffold3351_19	non-phage	0
DOF013_scaffold22432_2	non-phage	0
DOF013_scaffold29169_2	non-phage	0
DOF013_scaffold13611_3	non-phage	0
DOF013_scaffold19588_1	non-phage	0
DOF013_scaffold379_4	non-phage	0
DOF013_scaffold4801_5	non-phage	0
DOF013_scaffold4138_1	non-phage	0
DOF013_scaffold1498_4	non-phage	0
DOF013_scaffold10223_4	non-phage	0
DOF013_scaffold5496_4	non-phage	0
DOF013_scaffold24037_1	non-phage	0
DOF013_scaffold24270_3	non-phage	0
DOF013_scaffold3154_2	non-phage	0
DOF013_scaffold24105_7	non-phage	0
DOF013_scaffold3109_22	non-phage	0
DOF013_scaffold8692_1	non-phage	0
DOF013_scaffold2719_5	non-phage	0
DOF013_scaffold797_11	non-phage	0

DOF013_scaffold23218_1	non-phage	0
DOF013_scaffold4716_4	non-phage	0
DOF013_scaffold1330_3	non-phage	0
DOF013_scaffold14702_1	non-phage	0
DOF013_scaffold10853_2	non-phage	0
DOF013_scaffold10570_6	non-phage	0
DOF013_scaffold28076_2	non-phage	0
DOF013_scaffold6496_1	non-phage	0
DOF013_scaffold20049_1	non-phage	0
DOF013_scaffold1672_4	non-phage	0
DOF013_scaffold2409_2	non-phage	0
DOF013_scaffold1152_17	non-phage	0
DOF013_scaffold614_2	non-phage	0
DOF013_scaffold20251_2	non-phage	0
DOF013_scaffold7374_1	non-phage	0
DOF013_scaffold38_6	non-phage	0
DOF013_scaffold667_1	non-phage	0
DOF013_scaffold1529_5	non-phage	0
DOF013_scaffold29682_1	non-phage	0
DOF013_scaffold1983_8	non-phage	0
DOF013_scaffold12122_1	non-phage	0
DOF013_scaffold13417_7	non-phage	0
DOF013_scaffold27_3	non-phage	0
DOF013_scaffold1671_5	non-phage	0
DOF013_scaffold17327_1	non-phage	0
DOF013_scaffold20083_3_2	non-phage	0
DOF013_scaffold2056_1	non-phage	0
DOF013_scaffold11367_11	non-phage	0
DOF013_scaffold23474_1	non-phage	0
DOF013_scaffold46_1	non-phage	0
DOF013_scaffold9415_2	non-phage	0
DOF013_scaffold5808_2	non-phage	0
DOF013_scaffold4044_4	non-phage	0
DOF013_scaffold8447_2	non-phage	0
DOF013_scaffold16019_8	non-phage	0
DOF013_scaffold16019_5	non-phage	0
DOF013_scaffold101_5	non-phage	0
DOF013_scaffold6022_5	non-phage	0
DOF013_scaffold8168_1	non-phage	0
DOF013_scaffold17372_3	non-phage	0
DOF013_scaffold11287_1	non-phage	0
DOF013_scaffold23186_4	non-phage	0
DOF013_scaffold23456_7	non-phage	0
DOF013_scaffold4487_42	non-phage	0
DOF013_scaffold29912_2	non-phage	0
DOF013_scaffold16462_3	non-phage	0

DOF013_scaffold4066_6	non-phage	0
DOF013_scaffold1529_1	non-phage	0
DOF013_scaffold103_5_2	non-phage	0
DOF013_scaffold25672_2	non-phage	0
DOF014_scaffold48333_1	non-phage	0
DOF014_scaffold49735_2	non-phage	0
DOF014_scaffold8286_2	non-phage	0
DOF014_scaffold28722_2	non-phage	0
DOF014_scaffold37214_1	non-phage	0
DOF014_scaffold40652_2	non-phage	0
DOF014_scaffold29901_1	non-phage	0
DOF014_scaffold49372_2	non-phage	0
DOF014_scaffold97_3	non-phage	0
DOF014_scaffold36108_1	non-phage	0
DOF014_scaffold11551_1	non-phage	0
DOF014_scaffold49013_1	non-phage	0
DOF014_scaffold47440_1	non-phage	0
DOF014_scaffold49658_1	non-phage	0
DOF014_scaffold10703_4	non-phage	0
DOF014_scaffold30707_2	non-phage	0
DOF014_scaffold47226_1	non-phage	0
DOF014_scaffold49518_1	non-phage	0
DOF014_scaffold36073_3	non-phage	0
DOF014_scaffold82_6	non-phage	0
DOF014_scaffold49559_2	non-phage	0
DOF014_scaffold24170_1	non-phage	0
DOF014_scaffold8270_4	non-phage	0
DOF014_scaffold49723_1	non-phage	0
DOF014_scaffold44645_4	non-phage	0
DOF014_scaffold46575_6	non-phage	0
DOF014_scaffold43973_2	non-phage	0
DOF014_scaffold46322_4	non-phage	0
DOF014_scaffold48564_2	non-phage	0
DOF014_scaffold10548_6	non-phage	0
DOF014_scaffold17410_3	non-phage	0
DOF014_scaffold48961_1	non-phage	0
DOF014_scaffold31587_5	non-phage	0
DOF014_C613690_1	non-phage	0
DOF014_scaffold42555_1	non-phage	0
DOF014_scaffold3003_4	non-phage	0
DOF014_scaffold13781_2	non-phage	0
DOF014_scaffold153_6	non-phage	0
DOF014_scaffold49649_1	non-phage	0
DOF014_scaffold44973_1	non-phage	0
DOF014_scaffold33325_1	non-phage	0
DOF014_scaffold44265_4	non-phage	0

DOF014_scaffold9145_3	non-phage	0
DOF014_scaffold37903_3	non-phage	0
DOF014_scaffold27486_9	non-phage	0
DOF014_scaffold49526_2	non-phage	0
DOF014_scaffold45437_4	non-phage	0
DOF014_scaffold48378_1	non-phage	0
DOF014_scaffold49659_2	non-phage	0
DOF014_scaffold258_10	non-phage	0
DOF014_scaffold46322_5	non-phage	0
DOF014_scaffold12_2	non-phage	0
DOF014_scaffold45_1	non-phage	0
DOF014_scaffold49632_2	non-phage	0
DOF014_scaffold36312_1	non-phage	0
DOF014_scaffold1612_5	non-phage	0
DOF014_scaffold1290_4	non-phage	0
DOF014_scaffold15351_6	non-phage	0
DOF014_scaffold49587_1	non-phage	0
DOF014_scaffold43428_3	non-phage	0
DOF014_C613160_1	non-phage	0
DOF014_scaffold49660_2	non-phage	0
DOF014_scaffold9145_2	non-phage	0
DOF014_scaffold49735_1	non-phage	0
DOF014_scaffold46651_1	non-phage	0
DOF014_scaffold63_6	non-phage	0
DOF014_scaffold49697_1	non-phage	0
DOF014_scaffold16371_2	non-phage	0
DOF014_scaffold36011_4	non-phage	0
DOF014_scaffold237_1	non-phage	0
DOF014_scaffold48208_1	non-phage	0
DOF014_scaffold43291_1	non-phage	0
DOF014_scaffold44133_4	non-phage	0
DOF014_scaffold10198_4	non-phage	0
DOF014_scaffold2070_2	non-phage	0
DOF014_scaffold49708_2	non-phage	0
DOF014_scaffold44990_3	non-phage	0
DOF014_scaffold49625_2	non-phage	0
DOF014_scaffold43516_9	non-phage	0
DOF014_scaffold35102_7	non-phage	0
DOF014_scaffold49468_1	non-phage	0
DOF014_scaffold13781_4	non-phage	0
DOF014_scaffold35499_3	non-phage	0
DOF014_scaffold3988_2	non-phage	0
DOF014_scaffold49647_7	non-phage	0
DOF014_scaffold49665_1	non-phage	0
DOF014_scaffold8453_1	non-phage	0
DOF014_scaffold9145_5	non-phage	0

DOF014_scaffold44265_3	non-phage	0
DOF014_scaffold35633_1	non-phage	0
DOF014_scaffold49729_1	non-phage	0
DOF014_C613352_1	non-phage	0
DOF014_scaffold43328_4	non-phage	0
DOF014_scaffold39702_2	non-phage	0
DOF014_scaffold82_7	non-phage	0
DOF014_scaffold49688_1	non-phage	0
DOF014_scaffold34990_2	non-phage	0
DOF014_scaffold45203_1	non-phage	0
DOF014_scaffold44645_1	non-phage	0
DOF014_scaffold55_1	non-phage	0
DOF014_scaffold38462_1	non-phage	0
DOF014_scaffold24170_2	non-phage	0
DOF014_scaffold45411_1	non-phage	0
DOF014_scaffold44304_7	non-phage	0
DOF014_scaffold10805_1	non-phage	0
DOF014_scaffold14113_1	non-phage	0
DOF014_scaffold7605_2	non-phage	0
DOF014_scaffold49703_1	non-phage	0
DOF014_scaffold44265_2	non-phage	0
NOF001_scaffold56968_1	non-phage	0
NOF001_scaffold30801_2	non-phage	0
NOF001_scaffold58197_1	non-phage	0
NOF001_scaffold50408_2	non-phage	0
NOF001_scaffold20222_2	non-phage	0
NOF001_scaffold20182_5	non-phage	0
NOF001_scaffold2654_4	phage	0.737
NOF001_scaffold18319_3	non-phage	0
NOF001_scaffold57081_1	non-phage	0
NOF001_scaffold35301_1	non-phage	0
NOF001_scaffold13116_8	non-phage	0
NOF001_scaffold3213_8	non-phage	0
NOF001_scaffold50943_1	non-phage	0
NOF001_scaffold45121_1	non-phage	0
NOF001_scaffold58291_1	non-phage	0
NOF001_scaffold21088_10	non-phage	0
NOF001_scaffold11178_1	non-phage	0
NOF001_scaffold24472_4	non-phage	0
NOF001_scaffold6011_10	non-phage	0
NOF001_scaffold40742_3	non-phage	0
NOF001_scaffold35427_2	non-phage	0
NOF001_scaffold44288_1	non-phage	0
NOF001_scaffold58309_1	non-phage	0
NOF001_scaffold49730_1	non-phage	0
NOF001_scaffold29077_6	non-phage	0

NOF001_scaffold41659_1	non-phage	0
NOF001_scaffold56957_2	non-phage	0
NOF001_scaffold122_2	non-phage	0
NOF001_scaffold57507_1	non-phage	0
NOF001_scaffold24075_1	non-phage	0
NOF001_scaffold14_4	phage	0.995
NOF001_scaffold58201_2	phage	0.999
NOF001_scaffold35368_3	non-phage	0
NOF001_scaffold58244_1	non-phage	0
NOF001_scaffold39423_1	non-phage	0
NOF001_scaffold57834_1	non-phage	0
NOF001_scaffold5911_2	non-phage	0
NOF001_scaffold140_9	non-phage	0
NOF001_scaffold37864_3	non-phage	0
NOF001_scaffold17586_2	phage	0.996
NOF001_scaffold35301_3	non-phage	0
NOF001_scaffold58323_2	non-phage	0
NOF001_scaffold99_1	non-phage	0
NOF001_scaffold36771_2	non-phage	0
NOF001_scaffold336_4	non-phage	0
NOF001_scaffold39519_2	non-phage	0
NOF001_scaffold18613_10	non-phage	0
NOF001_scaffold34900_2	non-phage	0
NOF001_scaffold39121_5	non-phage	0
NOF001_scaffold386_2	non-phage	0
NOF001_scaffold33564_3	non-phage	0
NOF001_scaffold9469_6	non-phage	0
NOF001_scaffold9891_1	non-phage	0
NOF001_scaffold8698_5	non-phage	0
NOF001_scaffold20093_1	non-phage	0
NOF001_scaffold14498_5	non-phage	0
NOF001_scaffold361_3	non-phage	0
NOF001_scaffold2149_3	non-phage	0
NOF001_scaffold14725_3	non-phage	0
NOF001_scaffold47620_1	non-phage	0
NOF001_scaffold45338_3	non-phage	0
NOF001_scaffold35130_3	non-phage	0
NOF001_scaffold57359_1	non-phage	0
NOF001_scaffold54525_4	non-phage	0
NOF001_scaffold14684_5	non-phage	0
NOF001_scaffold41_3	non-phage	0
NOF001_scaffold1053_2	phage	0.999
NOF001_scaffold47923_1	non-phage	0
NOF001_scaffold58049_3	non-phage	0
NOF001_scaffold32889_1	non-phage	0
NOF001_scaffold57181_4	non-phage	0

NOF001_scaffold2828_6	non-phage	0
NOF001_scaffold58150_1	non-phage	0
NOF001_scaffold58318_1	phage	1
NOF001_scaffold58099_1	non-phage	0
NOF001_scaffold7357_11	non-phage	0
NOF001_scaffold58317_1	non-phage	0
NOF001_scaffold57200_2	non-phage	0
NOF001_scaffold19308_2	non-phage	0
NOF001_scaffold122_1	non-phage	0
NOF001_scaffold24033_3	non-phage	0
NOF001_scaffold13384_2	non-phage	0
NOF001_scaffold58264_1	non-phage	0
NOF001_scaffold58322_1	non-phage	0
NOF001_scaffold18431_1	non-phage	0
NOF001_scaffold58295_1	non-phage	0
NOF001_scaffold37864_10	non-phage	0
NOF001_scaffold3979_22	non-phage	0
NOF001_scaffold58099_3	non-phage	0
NOF001_scaffold13046_10	non-phage	0
NOF001_scaffold34900_3	non-phage	0
NOF001_scaffold22764_2	non-phage	0
NOF001_scaffold57248_2	non-phage	0
NOF001_scaffold5984_2	non-phage	0
NOF001_scaffold51661_2	non-phage	0
NOF001_scaffold17580_5	non-phage	0
NOF001_scaffold29077_3	non-phage	0
NOF001_scaffold265_2	non-phage	0
NOF001_scaffold57818_2	non-phage	0
NOF001_scaffold4067_3	non-phage	0
NOF001_scaffold58319_2	non-phage	0
NOF001_scaffold58299_1	non-phage	0
NOF001_scaffold39346_1	non-phage	0
NOF001_scaffold57408_1	non-phage	0
NOF001_scaffold14847_5	non-phage	0
NOF001_scaffold58294_1	non-phage	0
NOF001_scaffold10584_2	non-phage	0
NOF001_scaffold57746_1	non-phage	0
NOF001_scaffold11178_6	non-phage	0
NOF001_scaffold30016_2	non-phage	0
NOF001_scaffold9469_1	non-phage	0
NOF001_scaffold31992_8	non-phage	0
NOF001_scaffold58225_2	non-phage	0
NOF001_scaffold8745_8	non-phage	0
NOF001_scaffold32906_1	non-phage	0
NOF001_scaffold5563_1	phage	0.952
NOF001_scaffold15478_5	non-phage	0

NOF001_scaffold122_5	non-phage	0
NOF001_scaffold42914_5	non-phage	0
NOF001_scaffold33721_2	phage	0.95
NOF001_scaffold25382_2	non-phage	0
NOF001_scaffold48_1	phage	0.77
NOF001_scaffold57802_1	non-phage	0
NOF001_scaffold37739_2	non-phage	0
NOF001_scaffold47705_1	phage	0.998
NOF001_scaffold26889_7	non-phage	0
NOF001_scaffold58089_2	non-phage	0
NOF001_scaffold160_2	non-phage	0
NOF001_scaffold58279_1	non-phage	0
NOF001_scaffold47_2	non-phage	0
NOF001_scaffold398_1	non-phage	0
NOF001_scaffold55489_5	non-phage	0
NOF001_scaffold56764_1	phage	0.969
NOF001_scaffold2763_3	non-phage	0
NOF001_scaffold30_2	non-phage	0
NOF001_scaffold16291_1	non-phage	0
NOF001_scaffold30217_1	non-phage	0
NOF001_scaffold13046_9	phage	0.995
NOF001_scaffold18613_11	non-phage	0
NOF001_scaffold14_9	non-phage	0
NOF001_C716329_1	non-phage	0
NOF001_scaffold14991_2	non-phage	0
NOF001_scaffold23449_6	non-phage	0
NOF001_scaffold53059_1	phage	0.995
NOF001_scaffold15478_11	non-phage	0
NOF001_scaffold33934_10	non-phage	0
NOF001_scaffold29077_5	non-phage	0
NOF001_scaffold50802_1	non-phage	0
NOF001_scaffold336_1	non-phage	0
NOF001_scaffold10358_2	non-phage	0
NOF001_scaffold53897_1	non-phage	0
NOF001_scaffold3065_2	non-phage	0
NOF001_scaffold58052_2	phage	0.996
NOF001_scaffold57367_1	non-phage	0
NOF001_scaffold45_2	non-phage	0
NOF001_scaffold58320_1	non-phage	0
NOF001_scaffold157_2	non-phage	0
NOF001_scaffold5343_8	phage	0.986
NOF001_scaffold58235_1	phage	0.999
NOF001_scaffold80_3	non-phage	0
NOF001_scaffold36792_1	non-phage	0
NOF001_scaffold28588_4	non-phage	0
NOF001_scaffold41975_1	non-phage	0

NOF001_scaffold34852_4	non-phage	0
NOF001_scaffold53735_2	non-phage	0
NOF001_scaffold57995_2	phage	0.999
NOF001_scaffold57906_1	non-phage	0
NOF001_scaffold51481_1	non-phage	0
NOF001_scaffold43881_9	non-phage	0
NOF001_scaffold9855_4	non-phage	0
NOF001_scaffold58308_3	non-phage	0
NOF001_scaffold58201_3	non-phage	0
NOF001_scaffold386_5	non-phage	0
NOF001_scaffold2890_6	non-phage	0
NOF001_scaffold4725_2	non-phage	0
NOF001_scaffold35368_6	non-phage	0
NOF001_scaffold20147_2	non-phage	0
NOF001_scaffold11560_1	phage	0.942
NOF001_scaffold2828_5	non-phage	0
NOF001_scaffold27372_1	phage	0.998
NOF001_scaffold37167_1	non-phage	0
NOF001_scaffold40678_2	non-phage	0
NOF001_scaffold37864_9	non-phage	0
NOF001_scaffold509_2	non-phage	0
NOF001_scaffold24942_7	non-phage	0
NOF001_scaffold41855_2	phage	0.999
NOF001_scaffold24697_1	non-phage	0
NOF001_scaffold58159_2	non-phage	0
NOF001_scaffold28168_1	non-phage	0
NOF001_scaffold16542_2	non-phage	0
NOF001_scaffold58297_1	non-phage	0
NOF001_scaffold57765_6	non-phage	0
NOF001_scaffold51674_1	non-phage	0
NOF001_scaffold24942_3	non-phage	0
NOF001_scaffold17314_4	non-phage	0
NOF001_scaffold41805_3	non-phage	0
NOF001_scaffold47143_1	non-phage	0
NOF001_scaffold4849_5	non-phage	0
NOF001_scaffold24609_2	phage	0.502
NOF001_scaffold668_18	non-phage	0
NOF001_scaffold44574_2	non-phage	0
NOF001_scaffold24013_6	non-phage	0
NOF001_scaffold57951_3	non-phage	0
NOF001_scaffold39121_1	non-phage	0
NOF001_scaffold224_15	non-phage	0
NOF001_scaffold41855_6	phage	0.999
NOF001_scaffold14684_4	non-phage	0
NOF001_scaffold29077_4	phage	0.999
NOF001_scaffold9891_4	non-phage	0

NOF001_scaffold53782_4	non-phage	0
NOF001_scaffold13046_6	phage	0.999
NOF001_scaffold57730_1	non-phage	0
NOF001_scaffold34852_9	non-phage	0
NOF001_scaffold52495_5	non-phage	0
NOF001_scaffold57997_1	non-phage	0
NOF001_scaffold29250_3	non-phage	0
NOF001_scaffold23825_2	non-phage	0
NOF001_C716211_1	non-phage	0
NOF001_scaffold336_38	non-phage	0
NOF001_scaffold43167_3	non-phage	0
NOF001_scaffold122_4	non-phage	0
NOF001_scaffold42075_1	non-phage	0
NOF001_scaffold58263_1	non-phage	0
NOF001_scaffold7196_5	non-phage	0
NOF001_scaffold35427_3	non-phage	0
NOF001_scaffold4605_10	non-phage	0
NOF001_scaffold35301_5	non-phage	0
NOF001_scaffold15069_2	non-phage	0
NOF001_scaffold94_2	non-phage	0
NOF001_scaffold53459_1	non-phage	0
NOF001_scaffold14_11	non-phage	0
NOF001_scaffold30016_3	non-phage	0
NOF001_scaffold22764_3	non-phage	0
NOF001_scaffold43645_2	non-phage	0
NOF001_scaffold58316_1	phage	1
NOF001_scaffold17927_9	non-phage	0
NOF001_scaffold19180_2	non-phage	0
NOF001_scaffold33721_1	non-phage	0
NOF001_scaffold8308_2	non-phage	0
NOF001_scaffold45_1	non-phage	0
NOF001_scaffold2907_5	non-phage	0
NOF001_scaffold2828_2	non-phage	0
NOF001_scaffold49446_2	non-phage	0
NOF001_scaffold5343_9	non-phage	0
NOF001_scaffold58101_2	non-phage	0
NOF001_scaffold39244_4	non-phage	0
NOF001_scaffold3065_1	non-phage	0
NOF001_scaffold21926_1	non-phage	0
NOF001_scaffold49266_1	non-phage	0
NOF001_scaffold53232_2	non-phage	0
NOF001_scaffold40234_2	non-phage	0
NOF001_scaffold17101_2	non-phage	0
NOF001_scaffold34652_1	non-phage	0
NOF001_scaffold37167_2	non-phage	0
NOF001_scaffold13384_3	non-phage	0

NOF001_scaffold57051_1	non-phage	0
NOF001_scaffold39449_1	non-phage	0
NOF001_scaffold5122_4	non-phage	0
NOF001_scaffold12565_8	non-phage	0
NOF001_scaffold58291_3	non-phage	0
NOF001_scaffold34852_5	non-phage	0
NOF001_scaffold26889_11	non-phage	0
NOF001_scaffold28819_1	non-phage	0
NOF001_scaffold11107_3	non-phage	0
NOF001_scaffold24742_1	non-phage	0
NOF001_scaffold24496_1	non-phage	0
NOF001_scaffold20952_1	non-phage	0
NOF001_scaffold58111_2	non-phage	0
NOF001_scaffold16499_1	non-phage	0
NOF001_scaffold7196_11	phage	0.99
NOF001_scaffold35336_1	non-phage	0
NOF001_scaffold47923_4	non-phage	0
NOF001_scaffold39121_2	non-phage	0
NOF001_scaffold55138_3	non-phage	0
NOF001_scaffold15547_3	non-phage	0
NOF001_scaffold52386_3	non-phage	0
NOF001_scaffold53319_1	non-phage	0
NOF001_scaffold16382_1	non-phage	0
NOF001_scaffold133_9	non-phage	0
NOF001_scaffold41855_1	phage	0.999
NOF001_scaffold24921_6	non-phage	0
NOF001_scaffold52495_4	non-phage	0
NOF001_scaffold824_18	non-phage	0
NOF001_scaffold18783_1	non-phage	0
NOF001_scaffold1096_2	non-phage	0
NOF001_scaffold9205_1	non-phage	0
NOF001_scaffold35620_1	non-phage	0
NOF001_scaffold24697_3	non-phage	0
NOF001_scaffold44702_9	non-phage	0
NOF001_scaffold19180_4	non-phage	0
NOF001_scaffold39478_3	non-phage	0
NOF001_scaffold6011_9	non-phage	0
NOF001_scaffold15415_1	non-phage	0
NOF001_scaffold668_15	non-phage	0
NOF001_scaffold4_4	non-phage	0
NOF001_scaffold4725_5	non-phage	0
NOF001_scaffold15676_1	non-phage	0
NOF001_scaffold58312_1	non-phage	0
NOF001_scaffold336_3	non-phage	0
NOF001_scaffold57247_3	non-phage	0
NOF001_scaffold1365_1	non-phage	0

NOF001_scaffold6275_3	non-phage	0
NOF001_scaffold17314_1	non-phage	0
NOF001_scaffold133_4	non-phage	0
NOF001_scaffold30662_2	non-phage	0
NOF001_scaffold3948_9	non-phage	0
NOF001_scaffold8141_25	non-phage	0
NOF001_scaffold14991_5	non-phage	0
NOF001_scaffold18613_16	non-phage	0
NOF001_scaffold24248_7	non-phage	0
NOF001_scaffold58049_1	non-phage	0
NOF001_scaffold32562_2	non-phage	0
NOF001_scaffold742_1	non-phage	0
NOF001_scaffold58164_1	non-phage	0
NOF001_scaffold17606_4	non-phage	0
NOF001_scaffold8089_24	non-phage	0
NOF001_scaffold398_2	non-phage	0
NOF001_scaffold5122_1	non-phage	0
NOF001_scaffold56968_3	non-phage	0
NOF001_scaffold7357_13	non-phage	0
NOF001_scaffold14758_9	non-phage	0
NOF001_scaffold16291_3	non-phage	0
NOF001_scaffold37336_1	non-phage	0
NOF001_scaffold340_2	non-phage	0
NOF001_scaffold52946_1	non-phage	0
NOF001_scaffold9376_1	non-phage	0
NOF001_scaffold17580_3	non-phage	0
NOF001_scaffold211_2	non-phage	0
NOF001_scaffold15097_4	non-phage	0
NOF001_scaffold23825_3	non-phage	0
NOF001_scaffold37644_6	non-phage	0
NOF001_scaffold120_1	non-phage	0
NOF001_scaffold14_12	non-phage	0
NOF001_C716105_1	non-phage	0
NOF001_scaffold36337_2	non-phage	0
NOF001_scaffold55111_9	non-phage	0
NOF001_scaffold113_2	non-phage	0
NOF001_scaffold36337_1	non-phage	0
NOF001_scaffold56544_2	non-phage	0
NOF001_scaffold2994_10	non-phage	0
NOF001_scaffold6011_8	non-phage	0
NOF001_scaffold52240_2	non-phage	0
NOF001_scaffold5191_13	non-phage	0
NOF001_scaffold35427_1	non-phage	0
NOF001_scaffold2819_5	phage	0.999
NOF001_scaffold7522_11	non-phage	0
NOF001_scaffold37929_4	non-phage	0

NOF001_scaffold54525_1	non-phage	0
NOF001_scaffold5683_8	non-phage	0
NOF001_scaffold34852_12	non-phage	0
NOF001_scaffold18424_12	non-phage	0
NOF001_scaffold58268_1	non-phage	0
NOF001_scaffold8692_1	non-phage	0
NOF001_scaffold58291_2	non-phage	0
NOF001_scaffold4_3	non-phage	0
NOF001_scaffold51350_1	non-phage	0
NOF001_scaffold1549_8	non-phage	0
NOF001_scaffold57341_1	non-phage	0
NOF001_scaffold24013_1	phage	1
NOF001_scaffold8751_9	non-phage	0
NOF001_scaffold58054_1	non-phage	0
NOF001_scaffold28588_6	non-phage	0
NOF001_scaffold3065_4	non-phage	0
NOF001_scaffold58227_2	non-phage	0
NOF001_scaffold43167_4	non-phage	0
NOF001_scaffold58081_1	phage	0.999
NOF001_scaffold53164_2	non-phage	0
NOF001_scaffold51156_1	non-phage	0
NOF001_scaffold11144_2	non-phage	0
NOF001_scaffold56157_1	phage	0.924
NOF001_C716191_1	non-phage	0
NOF001_scaffold42075_2	non-phage	0
NOF001_scaffold14684_2	non-phage	0
NOF001_scaffold22443_4	non-phage	0
NOF001_scaffold13926_14	non-phage	0
NOF001_scaffold35301_2	non-phage	0
NOF001_scaffold8579_4	non-phage	0
NOF001_scaffold58270_1	non-phage	0
NOF001_scaffold41975_3	non-phage	0
NOF001_scaffold33999_3	non-phage	0
NOF001_scaffold113_3	non-phage	0
NOF001_scaffold44904_3	non-phage	0
NOF001_scaffold49446_1	non-phage	0
NOF001_scaffold122_3	non-phage	0
NOF001_scaffold58141_2	non-phage	0
NOF001_scaffold58228_1	non-phage	0
NOF001_scaffold41298_1	non-phage	0
NOF001_scaffold9333_7	non-phage	0
NOF001_scaffold39398_3	non-phage	0
NOF001_scaffold44196_2	non-phage	0
NOF001_scaffold23825_5	non-phage	0
NOF001_scaffold58201_1	phage	0.998
NOF001_scaffold25232_2	non-phage	0

NOF001_scaffold57707_1	non-phage	0
NOF001_scaffold2408_2	non-phage	0
NOF001_scaffold48899_1	non-phage	0
NOF001_scaffold53331_1	non-phage	0
NOF001_scaffold15069_5	non-phage	0
NOF001_scaffold17037_3	non-phage	0
NOF001_scaffold29749_1	non-phage	0
NOF001_scaffold24697_5	non-phage	0
NOF001_scaffold496_3	non-phage	0
NOF001_scaffold58324_1	non-phage	0
NOF001_scaffold25382_1	non-phage	0
NOF001_scaffold14822_1	non-phage	0
NOF001_scaffold58173_1	non-phage	0
NOF001_scaffold58264_2	non-phage	0
NOF001_scaffold58306_1	non-phage	0
NOF001_scaffold46596_4	non-phage	0
NOF001_scaffold265_1_1	non-phage	0
NOF001_scaffold265_1_2	non-phage	0
NOF001_scaffold49482_4	non-phage	0
NOF001_scaffold14_8	non-phage	0
NOF001_scaffold31415_1	non-phage	0
NOF001_scaffold20932_2	non-phage	0
NOF001_scaffold55502_7	non-phage	0
NOF001_scaffold25232_5	non-phage	0
NOF001_scaffold55226_1	phage	0.755
NOF001_scaffold8175_1	non-phage	0
NOF001_scaffold58099_4	non-phage	0
NOF001_scaffold11383_1	non-phage	0
NOF001_scaffold24921_10	non-phage	0
NOF001_scaffold50021_1	phage	0.998
NOF001_scaffold50628_1	non-phage	0
NOF001_scaffold3979_21	non-phage	0
NOF001_scaffold17314_2	non-phage	0
NOF001_scaffold30662_3	non-phage	0
NOF001_scaffold31992_9	non-phage	0
NOF001_scaffold57248_1	non-phage	0
NOF001_scaffold198_2	non-phage	0
NOF001_scaffold33934_7	non-phage	0
NOF001_scaffold32959_1	non-phage	0
NOF001_scaffold1053_3	non-phage	0
NOF001_scaffold18905_2	non-phage	0
NOF001_scaffold54887_1	non-phage	0
NOF001_scaffold49296_1	phage	0.999
NOF001_scaffold34388_2	non-phage	0
NOF001_scaffold24013_4	non-phage	0
NOF001_scaffold51453_1	non-phage	0

NOF001_scaffold42156_5	non-phage	0
NOF001_scaffold55450_1	non-phage	0
NOF001_scaffold10172_5	non-phage	0
NOF001_C716099_1	non-phage	0
NOF001_scaffold44264_3	non-phage	0
NOF001_scaffold24496_4	non-phage	0
NOF001_scaffold56141_1	non-phage	0
NOF001_scaffold224_2	non-phage	0
NOF001_scaffold1096_1	non-phage	0
NOF001_scaffold58277_1	non-phage	0
NOF001_scaffold3081_3	non-phage	0
NOF001_scaffold20557_2	non-phage	0
NOF001_scaffold26416_1	non-phage	0
NOF001_scaffold113_1	non-phage	0
NOF001_scaffold32429_1	non-phage	0
NOF001_scaffold58225_1	non-phage	0
NOF001_scaffold133_3	non-phage	0
NOF001_scaffold20557_1	non-phage	0
NOF001_scaffold44574_4	non-phage	0
NOF001_scaffold54139_5	non-phage	0
NOF001_scaffold104_1	non-phage	0
NOF001_scaffold7196_10	non-phage	0
NOF001_scaffold44656_3	non-phage	0
NOF001_scaffold58263_3	non-phage	0
NOF001_scaffold591_11	non-phage	0
NOF001_scaffold57759_1	non-phage	0
NOF001_scaffold24472_2	non-phage	0
NOF001_scaffold24392_1	non-phage	0
NOF001_scaffold13116_9	non-phage	0
NOF001_scaffold174_1	non-phage	0
NOF001_scaffold34362_1	non-phage	0
NOF001_scaffold39398_7	non-phage	0
NOF001_scaffold48756_7	non-phage	0
NOF001_scaffold15730_5	phage	0.999
NOF001_scaffold58099_2	phage	0.999
NOF001_scaffold58314_2	non-phage	0
NOF001_scaffold744_1	non-phage	0
NOF002_scaffold26730_2	non-phage	0
NOF002_scaffold14960_1	non-phage	0
NOF002_scaffold27558_1	non-phage	0
NOF002_scaffold31198_1	non-phage	0
NOF002_scaffold25198_2	non-phage	0
NOF002_scaffold31168_1	non-phage	0
NOF002_scaffold6524_4	non-phage	0
NOF002_scaffold8188_2	non-phage	0
NOF002_scaffold8611_5	non-phage	0

NOF002_scaffold6638_27	non-phage	0
NOF002_scaffold31570_1	non-phage	0
NOF002_scaffold12129_2	non-phage	0
NOF002_scaffold31602_1	non-phage	0
NOF002_scaffold865_3	non-phage	0
NOF002_scaffold30426_2	non-phage	0
NOF002_scaffold6177_4	non-phage	0
NOF002_scaffold26729_3	non-phage	0
NOF002_scaffold13099_5	non-phage	0
NOF002_scaffold6415_34	non-phage	0
NOF002_scaffold8188_3	non-phage	0
NOF002_scaffold26729_1	non-phage	0
NOF002_scaffold25706_1	non-phage	0
NOF002_scaffold31095_5	non-phage	0
NOF002_scaffold19068_2	non-phage	0
NOF002_scaffold31152_1	non-phage	0
NOF002_scaffold29347_2	non-phage	0
NOF002_scaffold23479_8	non-phage	0
NOF002_scaffold3233_30	non-phage	0
NOF002_scaffold27721_6	non-phage	0
NOF002_scaffold13114_6	non-phage	0
NOF002_scaffold9805_2	non-phage	0
NOF002_scaffold31014_2	non-phage	0
NOF002_scaffold9194_2	non-phage	0
NOF002_scaffold6352_6	non-phage	0
NOF002_scaffold58_1	non-phage	0
NOF002_scaffold7_6	phage	0.999
NOF002_scaffold17317_1	non-phage	0
NOF002_scaffold21050_1	non-phage	0
NOF002_scaffold13379_1	phage	0.877
NOF002_scaffold8_36	non-phage	0
NOF002_scaffold31612_1	non-phage	0
NOF002_scaffold30212_1	non-phage	0
NOF002_scaffold24015_2	non-phage	0
NOF002_scaffold17211_3	non-phage	0
NOF002_scaffold8411_2	non-phage	0
NOF002_scaffold27397_1	non-phage	0
NOF002_scaffold6415_8	non-phage	0
NOF002_scaffold20110_3	non-phage	0
NOF002_scaffold31469_1	non-phage	0
NOF002_scaffold26730_3	non-phage	0
NOF002_scaffold17664_1	non-phage	0
NOF002_scaffold8_29	non-phage	0
NOF002_scaffold8494_8	non-phage	0
NOF002_scaffold31198_4	non-phage	0
NOF002_scaffold19162_2	non-phage	0

NOF002_scaffold1585_3	non-phage	0
NOF002_scaffold18160_1	non-phage	0
NOF002_scaffold29684_1	non-phage	0
NOF002_scaffold8411_1	non-phage	0
NOF002_scaffold26746_2	non-phage	0
NOF002_scaffold31188_8	non-phage	0
NOF002_scaffold12372_1	non-phage	0
NOF002_scaffold29569_5	non-phage	0
NOF002_scaffold24433_5	non-phage	0
NOF002_scaffold5948_1	non-phage	0
NOF002_scaffold10358_3	non-phage	0
NOF002_scaffold31583_1	non-phage	0
NOF002_scaffold31478_1	non-phage	0
NOF002_scaffold25166_5	non-phage	0
NOF002_scaffold9194_11	non-phage	0
NOF002_scaffold14795_2	non-phage	0
NOF002_scaffold23873_2	non-phage	0
NOF002_scaffold793_11	non-phage	0
NOF002_scaffold28417_1	non-phage	0
NOF002_scaffold31295_2	non-phage	0
NOF002_scaffold30758_2	non-phage	0
NOF002_scaffold7506_2	non-phage	0
NOF002_scaffold31578_2	non-phage	0
NOF002_scaffold17053_7	non-phage	0
NOF002_scaffold24336_1	non-phage	0
NOF002_scaffold31188_12	non-phage	0
NOF002_scaffold22806_3	non-phage	0
NOF002_scaffold1562_5	non-phage	0
NOF002_scaffold9460_3	non-phage	0.023
NOF002_scaffold4501_18	phage	0.999
NOF002_scaffold24495_7	non-phage	0
NOF002_scaffold6578_3	non-phage	0
NOF002_scaffold31515_2	non-phage	0
NOF002_scaffold31017_4	non-phage	0
NOF002_scaffold20781_1	non-phage	0
NOF002_scaffold23022_2	non-phage	0
NOF002_scaffold6749_7_1	non-phage	0
NOF002_scaffold6749_7_2	non-phage	0
NOF002_scaffold30763_2	phage	0.999
NOF002_scaffold27007_2	non-phage	0
NOF002_scaffold23544_11	non-phage	0
NOF002_scaffold8188_7	non-phage	0
NOF002_scaffold26730_7	non-phage	0
NOF002_scaffold29373_7	non-phage	0
NOF002_scaffold9194_16	non-phage	0
NOF002_scaffold26746_1	non-phage	0

NOF002_scaffold11695_2	non-phage	0
NOF002_scaffold8238_1	non-phage	0
NOF002_scaffold17053_1	non-phage	0
NOF002_scaffold9194_4	non-phage	0
NOF002_scaffold31611_1	non-phage	0
NOF002_scaffold1059_2	non-phage	0
NOF002_scaffold31511_1	non-phage	0
NOF002_C426193_1	non-phage	0
NOF002_scaffold24336_4	non-phage	0
NOF002_scaffold137_1	non-phage	0
NOF002_scaffold12_3	non-phage	0
NOF002_scaffold31300_1	non-phage	0
NOF002_scaffold31555_1	non-phage	0
NOF002_scaffold22539_1	non-phage	0
NOF002_scaffold26455_1	non-phage	0
NOF002_scaffold11544_1	non-phage	0
NOF002_scaffold27557_1	non-phage	0
NOF002_scaffold1836_7	non-phage	0
NOF002_scaffold31378_1	non-phage	0
NOF002_scaffold17433_3	non-phage	0
NOF002_scaffold31589_1	non-phage	0
NOF002_scaffold31328_1	non-phage	0
NOF002_scaffold26730_6	non-phage	0
NOF002_scaffold4501_13	non-phage	0
NOF002_scaffold6168_1	non-phage	0
NOF002_scaffold13114_3	non-phage	0
NOF002_scaffold8854_7	non-phage	0
NOF002_scaffold14199_2	non-phage	0
NOF002_scaffold28584_1	non-phage	0
NOF002_scaffold31188_7	non-phage	0
NOF002_scaffold8411_5	non-phage	0
NOF002_scaffold5663_1	non-phage	0
NOF002_scaffold10562_1	non-phage	0
NOF002_scaffold23544_1	non-phage	0
NOF002_scaffold15323_3	non-phage	0
NOF002_scaffold23749_1	non-phage	0
NOF002_scaffold25847_1	non-phage	0
NOF002_scaffold3233_12	non-phage	0.084
NOF002_scaffold31499_5	non-phage	0
NOF002_scaffold29466_1	phage	0.999
NOF002_scaffold31548_2	non-phage	0
NOF002_scaffold29980_1	non-phage	0
NOF002_scaffold5730_7	phage	0.999
NOF002_scaffold58_9	non-phage	0
NOF002_scaffold13323_1	non-phage	0
NOF002_scaffold31415_3	non-phage	0

NOF002_scaffold27888_1	non-phage	0
NOF002_scaffold2745_24	non-phage	0
NOF002_scaffold30692_4	non-phage	0
NOF002_scaffold23479_9	non-phage	0
NOF002_scaffold29929_1	non-phage	0
NOF002_scaffold31329_2	non-phage	0
NOF002_scaffold31533_2	non-phage	0
NOF002_scaffold31605_1	non-phage	0
NOF002_scaffold3476_12	phage	0.998
NOF002_scaffold31556_1	non-phage	0
NOF002_scaffold5675_1	non-phage	0
NOF002_scaffold2_2	non-phage	0
NOF002_scaffold26091_2	non-phage	0
NOF002_scaffold31328_7	non-phage	0
NOF002_scaffold31552_1	non-phage	0
NOF002_scaffold31556_2	non-phage	0
NOF002_scaffold3476_9	non-phage	0
NOF002_scaffold18540_1	non-phage	0
NOF002_scaffold27889_2	non-phage	0
NOF002_scaffold31574_2	non-phage	0
NOF002_scaffold31462_1	non-phage	0
NOF002_C426563_1	non-phage	0
NOF002_scaffold23022_5	non-phage	0
NOF002_scaffold13864_2	non-phage	0
NOF002_scaffold1584_7	non-phage	0
NOF002_scaffold17317_2	non-phage	0
NOF002_scaffold31199_2	phage	0.997
NOF002_scaffold28584_2	non-phage	0
NOF002_scaffold11544_2	non-phage	0
NOF002_scaffold12_1	non-phage	0
NOF002_scaffold865_4	non-phage	0
NOF002_scaffold17053_4	non-phage	0
NOF002_scaffold28783_1	non-phage	0
NOF002_scaffold31499_3	non-phage	0
NOF002_scaffold27207_8	non-phage	0
NOF002_scaffold28625_4	non-phage	0
NOF002_scaffold3476_6	non-phage	0
NOF002_scaffold1562_2	non-phage	0
NOF002_scaffold23479_2	non-phage	0
NOF002_scaffold22806_4	non-phage	0
NOF002_scaffold15502_2	non-phage	0
NOF002_scaffold2611_2	non-phage	0
NOF002_scaffold14764_1	non-phage	0
NOF002_scaffold9194_10	non-phage	0
NOF002_scaffold14960_2	non-phage	0
NOF002_scaffold8854_4	non-phage	0

NOF002_C425969_1	phage	1
NOF002_scaffold17760_11	non-phage	0
NOF002_scaffold30426_3	non-phage	0
NOF002_scaffold6749_10	non-phage	0
NOF002_scaffold22561_1	non-phage	0
NOF002_scaffold23456_1	non-phage	0
NOF002_scaffold29685_2	non-phage	0
NOF002_scaffold14962_4	non-phage	0
NOF002_scaffold31536_1	non-phage	0
NOF002_scaffold31424_1	phage	0.999
NOF002_scaffold31431_1	non-phage	0
NOF002_scaffold2344_2	non-phage	0
NOF002_scaffold1633_2	non-phage	0
NOF002_scaffold11544_5	non-phage	0
NOF002_scaffold31188_9	non-phage	0
NOF002_scaffold8654_3	non-phage	0
NOF002_scaffold16147_4	non-phage	0
NOF002_scaffold865_6	non-phage	0
NOF002_scaffold29373_4	non-phage	0
NOF002_scaffold6261_1	non-phage	0
NOF002_scaffold31426_1	non-phage	0
NOF002_scaffold7506_3	non-phage	0
NOF002_scaffold31613_1	non-phage	0
NOF002_scaffold12961_13	phage	0.998
NOF002_scaffold23022_3	non-phage	0
NOF002_scaffold2096_5	non-phage	0
NOF002_scaffold31609_1	non-phage	0
NOF002_scaffold2_6	non-phage	0
NOF002_scaffold26762_1	non-phage	0
NOF002_scaffold31515_1	non-phage	0
NOF002_scaffold31361_2	non-phage	0
NOF002_scaffold19847_1	non-phage	0
NOF002_scaffold31559_1	non-phage	0
NOF002_scaffold1182_4	non-phage	0
NOF002_scaffold4501_1	non-phage	0
NOF002_scaffold30692_3	non-phage	0
NOF002_scaffold26091_6	non-phage	0
NOF002_scaffold10358_2	non-phage	0
NOF002_scaffold28423_1	non-phage	0
NOF002_scaffold16147_1	non-phage	0
NOF002_scaffold22511_1	non-phage	0
NOF002_scaffold8394_2	non-phage	0
NOF002_scaffold21779_2	non-phage	0
NOF002_scaffold20732_2	non-phage	0
NOF002_scaffold24316_3	non-phage	0
NOF002_scaffold6261_2	non-phage	0

NOF002_scaffold20732_1	phage	0.806
NOF002_scaffold12444_2	non-phage	0
NOF002_scaffold8611_4	non-phage	0
NOF002_scaffold31090_1	non-phage	0
NOF002_scaffold31554_1	phage	0.926
NOF002_scaffold29569_2	non-phage	0
NOF002_scaffold8494_7	non-phage	0
NOF002_scaffold4571_11	non-phage	0
NOF002_scaffold21593_2	non-phage	0
NOF002_scaffold11695_3	non-phage	0
NOF002_scaffold30254_1	non-phage	0
NOF002_scaffold11008_5	non-phage	0
NOF002_scaffold25040_1	non-phage	0
NOF002_scaffold12_4	non-phage	0
NOF002_scaffold31530_1	non-phage	0
NOF002_scaffold2313_4	non-phage	0
NOF002_scaffold23544_10	non-phage	0
NOF002_scaffold31606_1	non-phage	0
NOF002_scaffold28625_6	non-phage	0
NOF002_scaffold31095_4	non-phage	0
NOF002_scaffold14795_3	non-phage	0
NOF002_scaffold27207_3	non-phage	0
NOF002_scaffold26455_2	non-phage	0
NOF002_scaffold27828_2	non-phage	0
NOF002_scaffold12464_1	non-phage	0
NOF002_scaffold18540_4	non-phage	0
NOF002_scaffold31531_2	non-phage	0
NOF002_scaffold2_5	non-phage	0
NOF002_scaffold31328_6	non-phage	0
NOF002_scaffold25175_8	non-phage	0
NOF002_scaffold30836_1	non-phage	0
NOF002_scaffold3233_2	phage	0.999
NOF002_scaffold31378_2	non-phage	0
NOF002_scaffold28343_2	non-phage	0
NOF002_scaffold14962_1	non-phage	0
NOF002_scaffold1584_8	non-phage	0
NOF002_scaffold26882_3	non-phage	0
NOF002_scaffold20948_5	non-phage	0
NOF002_scaffold2745_22	non-phage	0
NOF002_scaffold102_11	non-phage	0
NOF002_scaffold13864_3	non-phage	0
NOF002_scaffold11662_1	non-phage	0
NOF002_scaffold31551_1	non-phage	0
NOF002_scaffold8188_8	non-phage	0
NOF002_scaffold13099_3	non-phage	0
NOF002_scaffold18447_1	non-phage	0

NOF002_scaffold24495_1	non-phage	0
NOF002_scaffold11110_2	non-phage	0
NOF002_scaffold31017_2	non-phage	0
NOF002_scaffold13099_1	non-phage	0
NOF002_scaffold31345_2	non-phage	0
NOF002_scaffold27007_1	non-phage	0
NOF002_scaffold12372_2	non-phage	0
NOF002_scaffold7307_13	non-phage	0
NOF002_scaffold22806_5	non-phage	0
NOF002_scaffold31582_1	non-phage	0
NOF002_scaffold31576_1	non-phage	0
NOF002_scaffold19412_4	non-phage	0
NOF002_scaffold3233_33	non-phage	0
NOF002_scaffold31328_4	non-phage	0
NOF002_scaffold6749_14	phage	0.999
NOF002_scaffold24336_5	non-phage	0
NOF002_scaffold29742_3	non-phage	0
NOF002_scaffold29346_1	non-phage	0
NOF002_scaffold11008_9	non-phage	0
NOF002_scaffold27939_1	non-phage	0
NOF002_scaffold1708_1	non-phage	0
NOF002_scaffold27721_5	non-phage	0
NOF002_scaffold25166_1	non-phage	0
NOF002_scaffold23544_9	non-phage	0
NOF002_scaffold31509_1	non-phage	0
NOF002_scaffold24430_3	non-phage	0
NOF002_scaffold8494_3	non-phage	0
NOF002_scaffold2346_18	non-phage	0
NOF002_scaffold28783_2	non-phage	0
NOF002_scaffold28253_1	non-phage	0
NOF002_scaffold29742_2	non-phage	0
NOF002_scaffold30696_2	non-phage	0
NOF002_scaffold5663_2	non-phage	0
NOF002_scaffold102_6	non-phage	0
NOF002_scaffold11008_1	non-phage	0
NOF002_scaffold4968_1	non-phage	0
NOF002_scaffold6749_8	non-phage	0
NOF002_scaffold2390_2	non-phage	0
NOF002_scaffold26730_1	non-phage	0
NOF002_scaffold25007_2	non-phage	0
NOF002_scaffold9194_15	non-phage	0
NOF002_scaffold20732_6	non-phage	0
NOF002_scaffold31508_1	non-phage	0
NOF002_scaffold17666_3	non-phage	0
NOF002_scaffold8494_2	non-phage	0
NOF002_scaffold23831_3	non-phage	0

NOF002_scaffold3233_7	non-phage	0
NOF002_scaffold31344_1	non-phage	0
NOF002_scaffold15585_1	non-phage	0
NOF002_scaffold25198_1	non-phage	0
NOF002_scaffold8611_1	non-phage	0
NOF002_scaffold21799_1	non-phage	0
NOF002_scaffold20732_5	non-phage	0
NOF002_scaffold30649_1	non-phage	0
NOF002_scaffold31418_1	non-phage	0
NOF002_scaffold6749_11	non-phage	0
NOF002_scaffold31536_2	non-phage	0
NOF002_scaffold25007_1	non-phage	0
NOF002_scaffold31499_4	non-phage	0
NOF002_scaffold4637_4	non-phage	0
NOF002_scaffold26939_2	non-phage	0
NOF002_scaffold31014_4	non-phage	0
NOF002_scaffold8494_5	non-phage	0
NOF002_scaffold1850_37	non-phage	0
NOF002_scaffold2313_1	non-phage	0
NOF002_scaffold31188_6	non-phage	0
NOF002_scaffold30758_4	non-phage	0
NOF002_scaffold8494_10	non-phage	0
NOF002_scaffold8_14	non-phage	0
NOF002_C425535_1	non-phage	0
NOF002_scaffold29349_1	non-phage	0
NOF002_scaffold22247_1	non-phage	0
NOF002_scaffold20480_1	non-phage	0
NOF002_scaffold18921_6	non-phage	0
NOF002_scaffold31199_1	phage	0.999
NOF002_scaffold1562_8	non-phage	0
NOF002_scaffold31591_1	non-phage	0
NOF002_scaffold21779_1	non-phage	0
NOF002_scaffold2611_1	non-phage	0
NOF002_scaffold23348_1	non-phage	0
NOF002_scaffold2344_1	non-phage	0
NOF002_scaffold24606_1	non-phage	0
NOF002_scaffold31499_2	non-phage	0
NOF002_scaffold13654_1	non-phage	0
NOF002_scaffold1633_1	non-phage	0
NOF002_scaffold6749_16	non-phage	0
NOF002_scaffold14199_4	non-phage	0
NOF002_scaffold15101_1	non-phage	0
NOF002_scaffold583_1	non-phage	0
NOF002_scaffold27803_1	non-phage	0
NOF002_scaffold11620_5	phage	0.999
NOF002_scaffold24336_3	non-phage	0

NOF002_C425419_1	non-phage	0
NOF002_scaffold9460_1	phage	0.999
NOF002_scaffold102_9	non-phage	0
NOF002_scaffold31456_2	non-phage	0
NOF002_scaffold29047_1	non-phage	0
NOF002_scaffold11008_6	non-phage	0
NOF002_scaffold212_5	non-phage	0
NOF002_scaffold1584_10	non-phage	0
NOF002_scaffold31362_1	non-phage	0
NOF002_scaffold29047_2	non-phage	0
NOF002_scaffold25156_1	non-phage	0
NOF002_scaffold31241_5	non-phage	0
NOF002_scaffold31592_1	non-phage	0
NOF002_scaffold2745_26	non-phage	0
NOF002_scaffold23479_10	non-phage	0
NOF002_C425767_1	non-phage	0
NOF002_scaffold31496_2	non-phage	0
NOF002_scaffold28992_1	non-phage	0
NOF002_scaffold31529_2	non-phage	0
NOF002_scaffold31520_1	non-phage	0
NOF002_scaffold2745_16	non-phage	0
NOF002_scaffold31128_3	non-phage	0
NOF002_scaffold27207_7	non-phage	0
NOF002_scaffold11544_3	non-phage	0
NOF002_scaffold31511_2	non-phage	0
NOF002_scaffold26891_3	non-phage	0
NOF002_scaffold6177_2	non-phage	0
NOF002_scaffold582_1	non-phage	0
NOF002_scaffold31555_3	non-phage	0
NOF002_scaffold2_9	non-phage	0
NOF002_scaffold4501_29	non-phage	0
NOF002_scaffold3233_19	non-phage	0
NOF002_scaffold29980_3	non-phage	0
NOF002_scaffold12372_3	non-phage	0
NOF002_scaffold31598_1	non-phage	0
NOF002_scaffold12590_1	non-phage	0
NOF002_scaffold31188_5	phage	0.961
NOF002_scaffold31581_2	non-phage	0
NOF002_scaffold23544_3	non-phage	0
NOF002_scaffold1584_5	phage	0.877
NOF002_scaffold18540_3	non-phage	0
NOF002_scaffold12444_3	non-phage	0
NOF002_scaffold23347_1	non-phage	0
NOF002_scaffold19681_1	non-phage	0
NOF002_scaffold26091_4	non-phage	0
NOF002_scaffold793_26	non-phage	0

NOF002_C426513_1	non-phage	0
NOF002_scaffold27828_3	non-phage	0
NOF002_scaffold23347_2	non-phage	0
NOF002_scaffold11153_3	non-phage	0
NOF002_scaffold13114_4	non-phage	0
NOF002_scaffold31095_3	non-phage	0
NOF002_scaffold25809_1	non-phage	0
NOF002_scaffold8497_1	non-phage	0
NOF002_scaffold24430_2	non-phage	0
NOF002_scaffold22654_1	non-phage	0
NOF002_scaffold31561_1	non-phage	0
NOF002_scaffold17211_4	non-phage	0
NOF002_scaffold12_5	non-phage	0
NOF002_scaffold25166_6	non-phage	0
NOF002_scaffold27721_4	non-phage	0
NOF002_scaffold23012_1	non-phage	0
NOF002_scaffold28253_4	non-phage	0
NOF002_scaffold2_4	non-phage	0
NOF002_scaffold31580_2	non-phage	0
NOF002_scaffold19847_3	non-phage	0.03
NOF002_scaffold1584_9	non-phage	0
NOF002_scaffold31510_1	non-phage	0
NOF002_scaffold6177_6	non-phage	0
NOF002_scaffold29347_7	non-phage	0
NOF002_scaffold12484_2	non-phage	0
NOF002_scaffold20216_1	non-phage	0
NOF002_scaffold31615_1	phage	0.992
NOF002_scaffold31608_1	non-phage	0
NOF002_scaffold29392_1	non-phage	0
NOF002_scaffold3233_5	phage	0.986
NOF002_scaffold31571_2	non-phage	0
NOF002_scaffold26763_1	non-phage	0
NOF002_scaffold31613_2	non-phage	0
NOF002_scaffold19162_1	non-phage	0
NOF002_scaffold27565_3	non-phage	0
NOF002_scaffold2_10	non-phage	0
NOF002_scaffold4571_7	non-phage	0
NOF002_scaffold31345_3	non-phage	0
NOF002_scaffold11662_2	non-phage	0
NOF002_scaffold14795_1	non-phage	0
NOF002_scaffold20110_6	non-phage	0
NOF002_scaffold11544_7	non-phage	0
NOF002_scaffold17363_1	non-phage	0
NOF002_scaffold102_5	non-phage	0
NOF002_scaffold8611_7	non-phage	0
NOF002_scaffold7382_3	non-phage	0

NOF002_scaffold31241_1	non-phage	0
NOF002_scaffold17666_4	non-phage	0
NOF002_scaffold14960_4	non-phage	0
NOF002_scaffold22985_1	non-phage	0
NOF002_scaffold14962_2	non-phage	0
NOF002_scaffold20110_2	non-phage	0
NOF002_scaffold31565_2	non-phage	0
NOF002_scaffold4525_9	non-phage	0
NOF002_scaffold8188_9	non-phage	0
NOF002_scaffold31511_3	non-phage	0
NOF002_scaffold25166_2	non-phage	0
NOF002_scaffold19320_2	non-phage	0
NOF002_scaffold7_7	phage	0.968
NOF002_scaffold31529_6	non-phage	0
NOF002_scaffold9194_6	non-phage	0
NOF002_scaffold31255_3	non-phage	0
NOF002_scaffold23646_1	non-phage	0
NOF002_scaffold8188_10	non-phage	0
NOF002_scaffold23_3	non-phage	0
NOF002_scaffold21419_3	non-phage	0
NOF002_scaffold31328_3	non-phage	0
NOF002_scaffold15988_1	non-phage	0
NOF002_scaffold13708_3	non-phage	0
NOF002_scaffold31234_2	non-phage	0
NOF002_scaffold31542_1	non-phage	0
NOF002_scaffold22549_1	non-phage	0
NOF002_scaffold31039_1	non-phage	0
NOF002_scaffold25088_2	non-phage	0
NOF002_scaffold6352_8	non-phage	0
NOF002_C426339_1	non-phage	0
NOF002_scaffold31378_3	non-phage	0
NOF002_scaffold3968_1	non-phage	0
NOF002_scaffold28674_2	non-phage	0
NOF002_scaffold1692_1	non-phage	0
NOF002_scaffold31509_2	non-phage	0
NOF002_scaffold31567_2	non-phage	0
NOF002_scaffold20485_2	non-phage	0
NOF002_scaffold2346_23	non-phage	0
NOF002_scaffold30802_1	non-phage	0
NOF002_scaffold30758_1	non-phage	0
NOF002_scaffold26745_2	non-phage	0
NOF002_scaffold24432_5	non-phage	0
NOF002_scaffold28992_2	non-phage	0
NOF002_scaffold19796_3	non-phage	0
NOF002_scaffold31591_2	non-phage	0
NOF002_scaffold16376_3	non-phage	0

NOF002_scaffold2_8	non-phage	0
NOF002_scaffold27939_3	non-phage	0
NOF002_scaffold24015_1	non-phage	0
NOF002_scaffold31601_1	non-phage	0
NOF002_scaffold23449_9	non-phage	0
NOF002_scaffold17211_2	non-phage	0
NOF002_scaffold31344_3	non-phage	0
NOF002_scaffold30763_1	phage	0.999
NOF002_scaffold20110_4	non-phage	0
NOF002_scaffold26091_3	non-phage	0
NOF002_scaffold25527_1	non-phage	0
NOF002_scaffold29374_1	non-phage	0
NOF002_scaffold20732_4	non-phage	0
NOF002_scaffold24495_6	non-phage	0
NOF002_scaffold379_1	non-phage	0
NOF002_scaffold793_31	non-phage	0
NOF002_scaffold9275_1	non-phage	0
NOF002_scaffold28625_8	non-phage	0
NOF002_scaffold23544_7	non-phage	0
NOF002_scaffold29569_4	non-phage	0
NOF002_scaffold143_2	non-phage	0
NOF002_scaffold25198_3	non-phage	0
NOF002_scaffold5978_9	non-phage	0
NOF002_scaffold22347_1	non-phage	0
NOF002_scaffold30758_5	non-phage	0
NOF002_scaffold8611_6	non-phage	0
NOF002_scaffold2313_2	non-phage	0
NOF002_scaffold1584_11	non-phage	0
NOF002_scaffold17666_1	non-phage	0
NOF002_scaffold12464_3	non-phage	0
NOF002_scaffold31496_1	non-phage	0
NOF002_scaffold3233_31	non-phage	0
NOF002_scaffold31529_3	non-phage	0
NOF002_scaffold865_2	non-phage	0
NOF002_scaffold14795_5	non-phage	0
NOF002_scaffold8_2	non-phage	0
NOF002_scaffold29347_3	non-phage	0
NOF002_scaffold19068_1	non-phage	0
NOF002_scaffold22806_2	non-phage	0
NOF002_scaffold18921_5	non-phage	0
NOF002_scaffold13864_1	non-phage	0
NOF002_scaffold27207_5	phage	0.592
NOF002_scaffold31017_1	non-phage	0
NOF002_scaffold31562_1	non-phage	0
NOF002_scaffold24608_2	non-phage	0
NOF002_scaffold28253_3	non-phage	0

NOF002_scaffold31460_3	non-phage	0
NOF002_scaffold7_12	non-phage	0
NOF002_scaffold25857_2	non-phage	0
NOF002_scaffold31532_2	non-phage	0
NOF002_scaffold919_4	non-phage	0
NOF002_scaffold29685_4	non-phage	0
NOF002_scaffold2611_5	non-phage	0
NOF002_scaffold17664_2	non-phage	0
NOF002_scaffold31168_2	non-phage	0
NOF002_scaffold27207_6	non-phage	0
NOF002_scaffold22539_2	non-phage	0
NOF002_scaffold31535_1	non-phage	0
NOF002_scaffold102_3	non-phage	0
NOF002_scaffold31128_4	non-phage	0
NOF002_scaffold2745_2	phage	0.998
NOF002_scaffold26834_2	non-phage	0
NOF002_scaffold31610_1	non-phage	0
NOF002_scaffold9275_6	non-phage	0.036
NOF002_scaffold31508_2	non-phage	0
NOF002_scaffold30426_1	non-phage	0
NOF002_scaffold1836_2	non-phage	0
NOF002_scaffold31362_2	non-phage	0
NOF002_scaffold8188_6	non-phage	0
NOF002_scaffold27939_4	non-phage	0
NOF002_scaffold31599_1	non-phage	0
NOF002_scaffold3233_25	non-phage	0
NOF002_scaffold29769_1	non-phage	0
NOF002_scaffold25156_2	non-phage	0
NOF002_scaffold31555_2	non-phage	0
NOF002_scaffold6352_9	non-phage	0
NOF002_scaffold23873_1	non-phage	0
NOF002_scaffold1850_3	non-phage	0
NOF002_scaffold19847_2	non-phage	0
NOF002_scaffold2745_3	phage	0.987
NOF002_scaffold11213_2	non-phage	0
NOF002_scaffold102_8	non-phage	0
NOF002_scaffold8188_11	non-phage	0
NOF002_scaffold26091_7	non-phage	0
NOF002_scaffold10358_1	non-phage	0
NOF002_scaffold19749_1	non-phage	0
NOF002_scaffold31548_1	non-phage	0
NOF002_scaffold28_1	non-phage	0
NOF002_scaffold24430_1	non-phage	0
NOF002_scaffold21593_1	non-phage	0
NOF002_scaffold23022_4	phage	0.986
NOF002_scaffold6749_6	non-phage	0

NOF002_scaffold6638_22	non-phage	0
NOF002_scaffold1633_3	non-phage	0
NOF002_scaffold25040_2	non-phage	0.071
NOF002_scaffold31180_1	non-phage	0
NOF002_scaffold19043_1	non-phage	0
NOF002_scaffold2_7	non-phage	0
NOF002_scaffold23544_12	non-phage	0
NOF002_scaffold31509_3	non-phage	0
NOF002_scaffold31595_1	non-phage	0
NOF002_C426279_1	non-phage	0
NOF002_scaffold11620_3	non-phage	0
NOF002_scaffold31415_2	non-phage	0
NOF002_scaffold8494_9	non-phage	0
NOF002_scaffold31456_1	non-phage	0
NOF002_scaffold31096_2	non-phage	0
NOF002_scaffold28642_1	non-phage	0
NOF002_scaffold27842_1	non-phage	0
NOF002_scaffold27828_4	non-phage	0
NOF002_scaffold31234_1	non-phage	0
NOF002_scaffold25857_3	non-phage	0
NOF002_scaffold6578_6	non-phage	0
NOF002_scaffold25198_6	non-phage	0
NOF002_scaffold2_3	non-phage	0
NOF002_scaffold15164_1	non-phage	0
NOF002_scaffold137_2	non-phage	0
NOF002_C425893_1	non-phage	0
NOF002_scaffold8225_4	non-phage	0
NOF002_scaffold8854_3	non-phage	0
NOF002_scaffold30692_5	non-phage	0
NOF002_scaffold19412_2	non-phage	0
NOF002_C426477_1	non-phage	0
NOF002_scaffold11544_4	non-phage	0
NOF002_scaffold27565_4	non-phage	0
NOF002_scaffold31567_3	non-phage	0
NOF002_scaffold31441_1	non-phage	0
NOF002_scaffold20110_1	non-phage	0.008
NOF002_scaffold30696_4	non-phage	0
NOF002_scaffold8411_4	non-phage	0
NOF002_scaffold12404_2	non-phage	0
NOF002_scaffold212_1	non-phage	0
NOF002_scaffold15323_2	non-phage	0
NOF002_scaffold30906_2	non-phage	0
NOF002_C425921_1	non-phage	0
NOF002_scaffold7382_2	non-phage	0
NOF002_scaffold26012_1	non-phage	0
NOF002_scaffold19985_1	non-phage	0

NOF002_scaffold9275_9	non-phage	0
NOF002_scaffold8611_8	non-phage	0
NOF002_scaffold1836_4	non-phage	0
NOF002_scaffold27888_2	non-phage	0
NOF002_scaffold17053_2	non-phage	0
NOF002_scaffold26729_4	non-phage	0
NOF002_scaffold21593_3	non-phage	0
NOF002_scaffold31584_1	phage	0.831
NOF004_scaffold30357_5	non-phage	0
NOF004_scaffold318_5	non-phage	0.382
NOF004_scaffold33904_1	non-phage	0
NOF004_scaffold163_4	non-phage	0
NOF004_scaffold5431_15	phage	0.824
NOF004_scaffold32859_1	non-phage	0
NOF004_scaffold9553_3	non-phage	0
NOF004_scaffold9201_33	non-phage	0
NOF004_scaffold29075_4	non-phage	0
NOF004_scaffold29644_2	non-phage	0
NOF004_scaffold9307_4	non-phage	0
NOF004_scaffold69_7	non-phage	0
NOF004_scaffold9914_13	non-phage	0
NOF004_scaffold2367_11	non-phage	0
NOF004_scaffold14786_1	non-phage	0
NOF004_scaffold11215_4	non-phage	0
NOF004_scaffold11784_7	non-phage	0
NOF004_scaffold39929_2	non-phage	0
NOF004_scaffold11596_2	non-phage	0
NOF004_scaffold16908_1	non-phage	0
NOF004_scaffold10401_15	non-phage	0
NOF004_scaffold1562_22	non-phage	0
NOF004_scaffold18950_1	non-phage	0
NOF004_scaffold8811_2	non-phage	0
NOF004_scaffold20556_4	non-phage	0
NOF004_scaffold15531_3	non-phage	0
NOF004_scaffold25441_2	phage	0.997
NOF004_scaffold40177_1	non-phage	0
NOF004_scaffold9994_3	non-phage	0
NOF004_scaffold7532_1	non-phage	0
NOF004_scaffold38036_4	non-phage	0
NOF004_scaffold39843_1	non-phage	0
NOF004_scaffold235_1	non-phage	0
NOF004_scaffold31617_2	non-phage	0
NOF004_scaffold5990_4	non-phage	0
NOF004_scaffold18628_11	non-phage	0
NOF004_scaffold29785_5	non-phage	0
NOF004_scaffold23288_3	non-phage	0

NOF004_scaffold5774_1	phage	0.98
NOF004_scaffold51_8	non-phage	0
NOF004_scaffold15104_2	non-phage	0
NOF004_scaffold4506_4	non-phage	0
NOF004_scaffold4703_4	phage	1
NOF004_scaffold35800_2	non-phage	0
NOF004_scaffold4675_5	non-phage	0
NOF004_scaffold40185_2	non-phage	0.265
NOF004_scaffold48_2	non-phage	0
NOF004_scaffold22559_1	non-phage	0
NOF004_scaffold10666_2	non-phage	0
NOF004_scaffold38234_1	non-phage	0
NOF004_scaffold39760_4	non-phage	0.027
NOF004_scaffold4382_3	non-phage	0
NOF004_scaffold11990_2	non-phage	0
NOF004_scaffold6701_36	non-phage	0
NOF004_scaffold39570_4	non-phage	0
NOF004_scaffold40175_1	non-phage	0
NOF004_scaffold39_30	non-phage	0
NOF004_scaffold35295_1	non-phage	0
NOF004_scaffold33904_8	non-phage	0
NOF004_scaffold32223_6	non-phage	0
NOF004_scaffold9322_10	non-phage	0
NOF004_scaffold40262_1	non-phage	0
NOF004_scaffold40176_2	non-phage	0
NOF004_scaffold8581_2	non-phage	0
NOF004_scaffold3196_5	non-phage	0
NOF004_scaffold8521_1	non-phage	0
NOF004_scaffold30656_4	non-phage	0
NOF004_scaffold9201_2	non-phage	0
NOF004_scaffold40245_1	non-phage	0
NOF004_scaffold6760_2	non-phage	0
NOF004_scaffold25761_4	non-phage	0
NOF004_scaffold1989_3	non-phage	0
NOF004_scaffold38620_1	non-phage	0
NOF004_scaffold37326_1	non-phage	0
NOF004_scaffold19766_4	phage	1
NOF004_scaffold18250_2	non-phage	0
NOF004_scaffold278_8	non-phage	0
NOF004_scaffold4004_2	non-phage	0
NOF004_scaffold7909_7	non-phage	0
NOF004_scaffold22363_4	non-phage	0
NOF004_scaffold60_1	non-phage	0
NOF004_scaffold32832_1	non-phage	0
NOF004_scaffold40276_1	non-phage	0
NOF004_scaffold4995_1	non-phage	0

NOF004_scaffold39298_1	non-phage	0
NOF004_scaffold26752_2	non-phage	0
NOF004_scaffold22282_3	non-phage	0
NOF004_scaffold13875_4	non-phage	0
NOF004_scaffold40024_4	non-phage	0
NOF004_scaffold28192_1	non-phage	0
NOF004_scaffold17090_3	non-phage	0
NOF004_scaffold27230_5	non-phage	0
NOF004_scaffold32796_1	phage	0.999
NOF004_scaffold30766_1	phage	1
NOF004_scaffold31736_4	non-phage	0
NOF004_scaffold6915_3	non-phage	0
NOF004_scaffold4859_1	non-phage	0
NOF004_scaffold11238_8	non-phage	0
NOF004_scaffold39870_1	non-phage	0
NOF004_scaffold27996_17	non-phage	0
NOF004_scaffold76_1	non-phage	0
NOF004_scaffold10567_1	non-phage	0
NOF004_scaffold3196_34	non-phage	0
NOF004_scaffold35590_9	non-phage	0
NOF004_scaffold20_4	non-phage	0
NOF004_scaffold39349_2	non-phage	0
NOF004_scaffold32845_8	non-phage	0
NOF004_scaffold51_4	non-phage	0
NOF004_scaffold12813_24	non-phage	0
NOF004_scaffold5394_3	non-phage	0
NOF004_scaffold7629_3	non-phage	0
NOF004_scaffold11762_4	non-phage	0
NOF004_scaffold19900_3	non-phage	0
NOF004_scaffold45_4	non-phage	0
NOF004_scaffold3196_25	non-phage	0
NOF004_scaffold19900_2	non-phage	0
NOF004_scaffold34301_2	non-phage	0
NOF004_scaffold2248_12	non-phage	0
NOF004_scaffold18841_5	non-phage	0
NOF004_scaffold2248_6	non-phage	0
NOF004_scaffold35336_3	non-phage	0
NOF004_scaffold270_4	non-phage	0
NOF004_scaffold27598_1	non-phage	0
NOF004_scaffold40041_2	non-phage	0
NOF004_scaffold4675_2	phage	0.961
NOF004_scaffold18628_20	non-phage	0
NOF004_scaffold14462_1	non-phage	0
NOF004_scaffold3196_22	non-phage	0
NOF004_scaffold5489_2	non-phage	0
NOF004_scaffold5730_3	non-phage	0

NOF004_scaffold35039_2	non-phage	0
NOF004_scaffold36774_1	phage	0.999
NOF004_scaffold5774_9	non-phage	0
NOF004_scaffold6617_4	non-phage	0
NOF004_scaffold37814_4	phage	0.991
NOF004_scaffold7031_1	non-phage	0
NOF004_scaffold24512_4	non-phage	0
NOF004_scaffold8883_6	non-phage	0
NOF004_scaffold33440_6	non-phage	0
NOF004_scaffold20230_3	non-phage	0
NOF004_scaffold19516_1	non-phage	0
NOF004_scaffold36548_4	non-phage	0
NOF004_scaffold38244_2	non-phage	0
NOF004_scaffold30656_5	non-phage	0
NOF004_scaffold36373_4	non-phage	0
NOF004_scaffold28590_1	non-phage	0
NOF004_scaffold10102_12	non-phage	0
NOF004_scaffold40073_2	non-phage	0
NOF004_scaffold29806_7	non-phage	0
NOF004_scaffold39800_2	phage	0.999
NOF004_scaffold16993_1	non-phage	0
NOF004_scaffold37011_1	non-phage	0
NOF004_scaffold31446_1	non-phage	0
NOF004_scaffold32586_1	non-phage	0
NOF004_scaffold21489_7	non-phage	0
NOF004_scaffold40024_5	non-phage	0
NOF004_scaffold29644_3	non-phage	0
NOF004_scaffold20776_3	non-phage	0
NOF004_scaffold3196_12	non-phage	0
NOF004_scaffold40161_2	non-phage	0
NOF004_scaffold29758_1	non-phage	0
NOF004_scaffold3561_5	non-phage	0
NOF004_scaffold6513_1	non-phage	0
NOF004_scaffold17182_3	non-phage	0
NOF004_scaffold19900_10	non-phage	0
NOF004_scaffold213_1	non-phage	0
NOF004_scaffold678_2	non-phage	0
NOF004_scaffold28491_11	non-phage	0
NOF004_scaffold6479_2	non-phage	0
NOF004_scaffold1702_13	non-phage	0
NOF004_scaffold28158_2	non-phage	0
NOF004_scaffold2_2	non-phage	0
NOF004_scaffold19747_2	non-phage	0
NOF004_scaffold11596_1	non-phage	0
NOF004_scaffold11784_4	non-phage	0
NOF004_scaffold13093_4	non-phage	0

NOF004_C575922_1	non-phage	0
NOF004_scaffold31671_1	non-phage	0
NOF004_scaffold22066_5	non-phage	0
NOF004_scaffold164_10	non-phage	0
NOF004_scaffold9204_1	non-phage	0
NOF004_scaffold9697_10	non-phage	0
NOF004_scaffold2367_5	non-phage	0
NOF004_scaffold46_4	non-phage	0
NOF004_scaffold40267_1	non-phage	0
NOF004_scaffold4148_1	non-phage	0
NOF004_scaffold12813_30	phage	0.999
NOF004_scaffold7669_4	non-phage	0
NOF004_C576082_1	phage	0.999
NOF004_scaffold22864_4	phage	0.99
NOF004_scaffold6726_3	non-phage	0
NOF004_scaffold20176_2	non-phage	0
NOF004_scaffold25093_19	non-phage	0
NOF004_scaffold17538_9	non-phage	0
NOF004_scaffold28125_1	non-phage	0
NOF004_scaffold6247_5	non-phage	0
NOF004_scaffold12390_5	phage	0.999
NOF004_scaffold34758_3	non-phage	0
NOF004_scaffold36625_5	non-phage	0
NOF004_scaffold31805_2	non-phage	0
NOF004_scaffold170_2	non-phage	0
NOF004_scaffold40248_2	non-phage	0
NOF004_scaffold20_3	non-phage	0
NOF004_scaffold21835_3	non-phage	0
NOF004_scaffold18950_2	phage	0.998
NOF004_scaffold14786_4	non-phage	0
NOF004_scaffold33375_1	non-phage	0
NOF004_scaffold40113_3	non-phage	0
NOF004_scaffold34615_7	non-phage	0
NOF004_scaffold39872_1	non-phage	0
NOF004_scaffold9914_2	non-phage	0
NOF004_scaffold32298_1	non-phage	0
NOF004_scaffold19386_6	non-phage	0
NOF004_scaffold39349_8	non-phage	0
NOF004_scaffold30693_2	non-phage	0
NOF004_scaffold3252_5	non-phage	0
NOF004_scaffold164_4	non-phage	0
NOF004_scaffold563_2	non-phage	0
NOF004_scaffold1680_14	non-phage	0
NOF004_scaffold9964_2	non-phage	0
NOF004_scaffold4703_1	phage	0.999
NOF004_scaffold40230_1	non-phage	0

NOF004_scaffold26947_3	non-phage	0
NOF004_scaffold62_2	non-phage	0
NOF004_scaffold22559_2	phage	0.999
NOF004_scaffold51_5	non-phage	0
NOF004_scaffold6701_37	non-phage	0
NOF004_scaffold5741_5	phage	0.999
NOF004_scaffold28158_3	non-phage	0
NOF004_scaffold69_4	non-phage	0
NOF004_scaffold7949_16	non-phage	0
NOF004_scaffold29602_1	non-phage	0
NOF004_scaffold7608_4	non-phage	0
NOF004_scaffold10702_20	non-phage	0
NOF004_scaffold31872_1	non-phage	0
NOF004_scaffold34095_2	phage	0.994
NOF004_scaffold38773_3	non-phage	0
NOF004_scaffold16950_1	non-phage	0
NOF004_scaffold30404_1	non-phage	0
NOF004_scaffold25761_1	non-phage	0
NOF004_scaffold17947_1	non-phage	0
NOF004_scaffold11238_4	non-phage	0
NOF004_scaffold2927_2	non-phage	0
NOF004_scaffold37814_5	non-phage	0
NOF004_scaffold29834_1	phage	0.999
NOF004_scaffold10567_2	phage	0.996
NOF004_scaffold10102_5	non-phage	0.002
NOF004_scaffold15369_3	non-phage	0
NOF004_scaffold9127_2	non-phage	0
NOF004_scaffold27996_5	non-phage	0
NOF004_scaffold29075_1	non-phage	0
NOF004_scaffold291_3	non-phage	0
NOF004_scaffold9697_21	non-phage	0
NOF004_scaffold9201_11	non-phage	0
NOF004_scaffold13673_4	non-phage	0
NOF004_scaffold35906_1	non-phage	0
NOF004_scaffold33473_2	non-phage	0
NOF004_scaffold31316_3	non-phage	0
NOF004_scaffold9827_2	non-phage	0
NOF004_scaffold36328_1	non-phage	0
NOF004_scaffold39298_2	non-phage	0
NOF004_scaffold39570_5	non-phage	0
NOF004_scaffold14319_1	non-phage	0
NOF004_scaffold369_9	non-phage	0
NOF004_scaffold40234_1	non-phage	0
NOF004_scaffold39789_2	non-phage	0
NOF004_scaffold5990_5	non-phage	0
NOF004_scaffold31382_4	non-phage	0

NOF004_scaffold5779_1	non-phage	0
NOF004_scaffold278_7	non-phage	0
NOF004_scaffold7219_1	non-phage	0
NOF004_scaffold270_5	non-phage	0
NOF004_scaffold3545_4	non-phage	0
NOF004_scaffold24813_7	non-phage	0
NOF004_scaffold8945_2	non-phage	0
NOF004_scaffold94_8	non-phage	0
NOF004_scaffold60_2	non-phage	0
NOF004_scaffold9977_14	non-phage	0
NOF004_scaffold35295_2	non-phage	0
NOF004_scaffold40099_2	non-phage	0
NOF004_scaffold34168_3	non-phage	0
NOF004_scaffold39593_1	non-phage	0
NOF004_scaffold30605_3	non-phage	0
NOF004_scaffold40024_1	non-phage	0
NOF004_scaffold11762_5	non-phage	0
NOF004_scaffold21489_3	non-phage	0
NOF004_scaffold30537_8	non-phage	0
NOF004_scaffold19481_2	non-phage	0
NOF004_scaffold11886_2	non-phage	0
NOF004_scaffold9553_4	non-phage	0
NOF004_scaffold33943_3	non-phage	0
NOF004_scaffold30939_1	non-phage	0
NOF004_scaffold24520_1	non-phage	0
NOF004_scaffold22066_9	non-phage	0
NOF004_scaffold8083_1	non-phage	0
NOF004_scaffold32367_1	non-phage	0
NOF004_scaffold39903_5	non-phage	0
NOF004_scaffold11215_2	non-phage	0
NOF004_scaffold9201_3	non-phage	0
NOF004_scaffold1680_1	non-phage	0
NOF004_scaffold30655_1	non-phage	0
NOF004_scaffold7551_5	non-phage	0
NOF004_scaffold10227_1	non-phage	0
NOF004_scaffold4230_2	non-phage	0
NOF004_scaffold46_3	non-phage	0
NOF004_scaffold39719_3	non-phage	0
NOF004_scaffold28010_3	non-phage	0
NOF004_scaffold28776_2	non-phage	0.005
NOF004_scaffold3560_1	non-phage	0
NOF004_scaffold9977_34	non-phage	0
NOF004_scaffold32845_2	non-phage	0
NOF004_scaffold7949_5	non-phage	0
NOF004_scaffold74_1	non-phage	0
NOF004_scaffold22282_6	non-phage	0.175

NOF004_scaffold9875_4	non-phage	0
NOF004_scaffold32845_7	non-phage	0
NOF004_scaffold1680_17	phage	0.992
NOF004_scaffold8660_3	non-phage	0
NOF004_scaffold30656_1	non-phage	0
NOF004_scaffold15248_9	non-phage	0
NOF004_scaffold21630_4	non-phage	0
NOF004_scaffold27185_1	non-phage	0
NOF004_scaffold30605_6	phage	0.999
NOF004_scaffold33904_10	non-phage	0
NOF004_scaffold1680_10	non-phage	0
NOF004_scaffold18250_1	non-phage	0
NOF004_scaffold40178_1	non-phage	0
NOF004_scaffold29075_2	non-phage	0
NOF004_scaffold29_1	non-phage	0
NOF004_scaffold9201_29	non-phage	0
NOF004_scaffold1589_3	non-phage	0
NOF004_scaffold22400_2	non-phage	0
NOF004_scaffold29644_4	non-phage	0
NOF004_scaffold39867_3	phage	0.973
NOF004_scaffold36548_3	non-phage	0
NOF004_scaffold36955_2	non-phage	0
NOF004_scaffold175_3	non-phage	0
NOF004_scaffold30644_1	non-phage	0
NOF004_scaffold874_11	non-phage	0
NOF004_scaffold37814_1	non-phage	0
NOF004_scaffold92_3	non-phage	0
NOF004_scaffold10069_16	non-phage	0
NOF004_scaffold39356_1	non-phage	0
NOF004_scaffold7532_6	non-phage	0
NOF004_scaffold39927_1	non-phage	0
NOF004_scaffold28845_1	non-phage	0
NOF004_scaffold2367_14	phage	0.827
NOF004_scaffold4623_3	non-phage	0.394
NOF004_scaffold30177_3	non-phage	0
NOF004_scaffold233_2	non-phage	0
NOF004_scaffold10041_1	non-phage	0
NOF004_scaffold8060_1	non-phage	0
NOF004_scaffold40024_6	non-phage	0
NOF004_scaffold31432_2	non-phage	0
NOF004_scaffold3847_5	non-phage	0
NOF004_scaffold213_2	non-phage	0
NOF004_scaffold34035_1	non-phage	0
NOF004_scaffold33941_4	non-phage	0
NOF004_C576054_1	non-phage	0
NOF004_scaffold33943_2	non-phage	0

NOF004_scaffold13875_2	non-phage	0
NOF004_scaffold16607_3	non-phage	0
NOF004_scaffold78_4	non-phage	0
NOF004_scaffold8521_5	phage	0.992
NOF004_scaffold12829_15	non-phage	0
NOF004_scaffold8945_3	phage	0.922
NOF004_scaffold7560_10	non-phage	0
NOF004_scaffold24813_3	phage	0.993
NOF004_scaffold215_1	non-phage	0
NOF004_scaffold12813_11	non-phage	0
NOF004_scaffold34793_3	non-phage	0
NOF004_scaffold34211_3	non-phage	0
NOF004_scaffold128_5	non-phage	0
NOF004_scaffold8725_1	non-phage	0
NOF004_scaffold9697_11	non-phage	0
NOF004_scaffold7669_3	non-phage	0
NOF004_scaffold13408_2	non-phage	0
NOF004_scaffold21213_4	non-phage	0
NOF004_scaffold12813_38	non-phage	0
NOF004_scaffold4995_4	non-phage	0
NOF004_scaffold15266_9	phage	0.997
NOF004_scaffold39769_1	non-phage	0
NOF004_scaffold27_1	non-phage	0
NOF004_scaffold26947_5	non-phage	0
NOF004_scaffold6247_6	non-phage	0
NOF004_scaffold40177_2	non-phage	0
NOF004_scaffold1482_3	non-phage	0
NOF004_scaffold19011_3	non-phage	0
NOF004_scaffold37998_4	non-phage	0
NOF004_scaffold5333_1	non-phage	0
NOF004_scaffold39800_1	phage	0.999
NOF004_scaffold31588_1	non-phage	0
NOF004_scaffold21831_7	non-phage	0
NOF004_scaffold19702_1	non-phage	0
NOF004_scaffold39284_2	non-phage	0
NOF004_scaffold29075_5	non-phage	0
NOF004_scaffold25836_1	non-phage	0
NOF004_scaffold36266_1	non-phage	0
NOF004_scaffold37229_4	non-phage	0
NOF004_scaffold12934_2	non-phage	0
NOF004_scaffold16975_2	non-phage	0
NOF004_scaffold46_8	non-phage	0
NOF004_scaffold7049_9	non-phage	0
NOF004_C575664_1	non-phage	0
NOF004_scaffold11611_5	non-phage	0
NOF004_scaffold32223_8	non-phage	0

NOF004_scaffold7049_6	non-phage	0
NOF004_scaffold36089_2	non-phage	0
NOF004_scaffold26122_6	non-phage	0
NOF004_scaffold25021_1	non-phage	0
NOF004_scaffold2640_1	non-phage	0
NOF004_scaffold26947_4	non-phage	0
NOF004_scaffold3794_7	non-phage	0
NOF004_scaffold17255_5	non-phage	0
NOF004_scaffold21693_11	non-phage	0
NOF004_scaffold21353_1	non-phage	0
NOF004_C575620_1	phage	0.998
NOF004_scaffold20115_1	non-phage	0
NOF004_scaffold30537_6	non-phage	0
NOF004_scaffold30335_1	non-phage	0
NOF004_scaffold35809_1	non-phage	0
NOF004_scaffold24365_2	non-phage	0
NOF004_scaffold39470_3	non-phage	0
NOF004_scaffold89_3	phage	0.919
NOF004_scaffold11322_3	non-phage	0
NOF004_scaffold38193_1	non-phage	0
NOF004_scaffold134_3	non-phage	0
NOF004_scaffold27627_2	non-phage	0
NOF004_scaffold31703_2	phage	0.999
NOF004_scaffold34044_2	non-phage	0
NOF004_scaffold26987_2	phage	0.999
NOF004_scaffold39757_1	non-phage	0
NOF004_scaffold37998_6	non-phage	0
NOF004_scaffold32845_10	non-phage	0
NOF004_scaffold10702_12	non-phage	0
NOF004_scaffold22864_3	non-phage	0
NOF004_scaffold8811_4	non-phage	0
NOF004_scaffold29842_2	non-phage	0
NOF004_scaffold13601_1	non-phage	0
NOF004_scaffold1680_2	non-phage	0.015
NOF004_scaffold32450_1	non-phage	0
NOF004_scaffold39942_1	non-phage	0
NOF004_scaffold7219_2	non-phage	0
NOF004_scaffold58_6	phage	0.999
NOF004_scaffold30537_3	non-phage	0
NOF004_scaffold14653_3	phage	0.978
NOF004_scaffold5394_1	non-phage	0
NOF004_scaffold30513_1	non-phage	0
NOF004_scaffold1702_2	non-phage	0
NOF004_scaffold8521_6	non-phage	0
NOF004_scaffold563_1	non-phage	0
NOF004_scaffold9977_3	non-phage	0

NOF004_scaffold20_2	non-phage	0
NOF004_scaffold9697_17	non-phage	0
NOF004_scaffold11990_4	non-phage	0
NOF004_scaffold18250_4	non-phage	0
NOF004_scaffold72_1	non-phage	0
NOF004_scaffold17182_1	non-phage	0
NOF004_scaffold278_6	non-phage	0
NOF004_scaffold22215_1	non-phage	0
NOF004_scaffold26063_3	non-phage	0
NOF004_scaffold39819_2	non-phage	0
NOF004_scaffold3658_4	non-phage	0
NOF004_C575524_1	non-phage	0
NOF004_scaffold29802_1	non-phage	0
NOF004_scaffold8883_18	non-phage	0
NOF004_scaffold30605_4	non-phage	0
NOF004_scaffold20499_5	non-phage	0
NOF004_scaffold29075_6	non-phage	0
NOF004_scaffold2248_2	non-phage	0
NOF004_scaffold35380_2	non-phage	0.029
NOF004_scaffold38621_1	non-phage	0
NOF004_scaffold37133_3	non-phage	0
NOF004_scaffold40099_1	non-phage	0
NOF004_scaffold22_2	non-phage	0
NOF004_scaffold16856_2	non-phage	0
NOF004_scaffold15745_1	non-phage	0
NOF004_scaffold8883_22	non-phage	0
NOF004_scaffold40282_1	non-phage	0
NOF004_scaffold4972_4	non-phage	0
NOF004_scaffold20230_1	non-phage	0
NOF004_C575978_1	non-phage	0
NOF004_scaffold22282_1	non-phage	0
NOF004_scaffold23271_2	non-phage	0
NOF004_scaffold33473_1	non-phage	0
NOF004_scaffold38165_1	non-phage	0
NOF004_scaffold6915_1	non-phage	0
NOF004_scaffold4845_7	non-phage	0
NOF004_scaffold35518_1	non-phage	0
NOF004_scaffold5431_17	non-phage	0
NOF004_scaffold19848_3	non-phage	0
NOF004_scaffold164_3	non-phage	0
NOF004_scaffold21828_1	non-phage	0
NOF004_scaffold24494_1	non-phage	0
NOF004_scaffold32996_1	non-phage	0
NOF004_scaffold38036_6	non-phage	0
NOF004_scaffold1777_1	non-phage	0
NOF004_C575706_1	non-phage	0

NOF004_scaffold3847_7	phage	0.634
NOF004_scaffold11322_1	non-phage	0
NOF004_scaffold30693_1	non-phage	0
NOF004_scaffold10069_19	phage	0.999
NOF004_scaffold38525_6	non-phage	0
NOF004_scaffold9893_10	non-phage	0
NOF004_scaffold128_13	non-phage	0
NOF004_scaffold9697_2	non-phage	0
NOF004_scaffold37782_2	non-phage	0
NOF004_scaffold8775_5	non-phage	0
NOF004_scaffold874_14	non-phage	0
NOF004_scaffold1964_3	non-phage	0
NOF004_scaffold38615_1	non-phage	0
NOF004_scaffold36548_2	non-phage	0
NOF004_scaffold26947_2	non-phage	0
NOF004_scaffold39668_1	non-phage	0
NOF004_scaffold39969_3	non-phage	0
NOF004_scaffold33859_4	non-phage	0
NOF004_scaffold22282_4	non-phage	0
NOF004_scaffold6760_1	non-phage	0
NOF004_scaffold3405_4	non-phage	0
NOF004_scaffold39593_2	non-phage	0
NOF004_scaffold12911_2	non-phage	0
NOF004_scaffold29974_1	non-phage	0
NOF004_scaffold12813_42	non-phage	0
NOF004_scaffold10790_3	non-phage	0
NOF004_scaffold35336_1	non-phage	0
NOF004_scaffold311_3	non-phage	0
NOF004_scaffold37959_1	non-phage	0
NOF004_scaffold40285_2	non-phage	0
NOF004_scaffold111_6	non-phage	0
NOF004_scaffold10102_20	non-phage	0
NOF004_scaffold10102_17	non-phage	0
NOF004_C576128_1	non-phage	0
NOF004_scaffold6247_2	non-phage	0
NOF004_scaffold7909_5	non-phage	0
NOF004_scaffold162_2	non-phage	0
NOF004_scaffold38773_5	non-phage	0
NOF004_scaffold34944_1	non-phage	0
NOF004_scaffold31966_6	non-phage	0
NOF004_scaffold40029_1	non-phage	0
NOF004_scaffold52_1	non-phage	0
NOF004_scaffold40279_1	non-phage	0
NOF004_scaffold84_1	non-phage	0
NOF004_scaffold9441_4	non-phage	0
NOF004_scaffold7416_9	non-phage	0.002

NOF004_scaffold39797_1	non-phage	0
NOF004_scaffold46_7	non-phage	0
NOF004_scaffold33458_2	non-phage	0
NOF004_scaffold10266_1	non-phage	0
NOF004_scaffold8725_2	non-phage	0
NOF004_scaffold37229_6	non-phage	0
NOF004_scaffold58_4	non-phage	0.073
NOF004_scaffold38269_19	non-phage	0
NOF004_scaffold18414_7	non-phage	0
NOF004_scaffold4193_2	phage	0.998
NOF004_scaffold46_2	non-phage	0
NOF004_scaffold175_2	non-phage	0
NOF004_scaffold37932_3	non-phage	0
NOF004_scaffold12390_3	phage	0.994
NOF004_scaffold35809_2	non-phage	0
NOF004_scaffold22066_7	non-phage	0
NOF004_scaffold3794_4	non-phage	0
NOF004_scaffold11322_6	non-phage	0
NOF004_scaffold3081_2	non-phage	0
NOF004_scaffold27395_3	non-phage	0
NOF004_scaffold38596_1	non-phage	0
NOF004_scaffold1589_4	non-phage	0
NOF004_scaffold8521_2	non-phage	0
NOF004_scaffold30766_3	non-phage	0
NOF004_scaffold12813_1	non-phage	0
NOF004_scaffold21630_6	non-phage	0
NOF004_scaffold37998_5	non-phage	0
NOF004_scaffold20412_2	non-phage	0
NOF004_scaffold31588_2	non-phage	0
NOF004_scaffold39985_3	non-phage	0
NOF004_scaffold164_7	non-phage	0
NOF004_scaffold33440_1	non-phage	0
NOF004_scaffold39923_2	non-phage	0
NOF004_scaffold39819_3	non-phage	0
NOF004_scaffold9875_5	phage	1
NOF004_scaffold4675_10	non-phage	0
NOF004_scaffold16856_3	non-phage	0
NOF004_scaffold34883_1	non-phage	0
NOF004_scaffold2452_1	non-phage	0
NOF004_scaffold20115_2	non-phage	0
NOF004_scaffold40174_1	non-phage	0
NOF004_scaffold40200_1	non-phage	0
NOF004_scaffold28593_1	non-phage	0
NOF004_scaffold40091_1	non-phage	0
NOF004_scaffold13673_5	non-phage	0
NOF004_scaffold31114_1	non-phage	0

NOF004_scaffold20532_1	non-phage	0
NOF004_scaffold22400_1	non-phage	0
NOF004_scaffold9201_21	non-phage	0
NOF004_scaffold31966_2	non-phage	0
NOF004_scaffold128_6	non-phage	0
NOF004_scaffold28404_1	non-phage	0
NOF004_scaffold39611_1	phage	0.998
NOF004_scaffold3560_2	non-phage	0
NOF004_scaffold9378_9	non-phage	0
NOF004_scaffold28322_1	phage	0.999
NOF004_scaffold29812_4	non-phage	0
NOF004_scaffold34568_2	non-phage	0
NOF004_scaffold18841_3	non-phage	0
NOF004_scaffold9926_8	non-phage	0
NOF004_scaffold32450_5	non-phage	0
NOF004_scaffold51_6	non-phage	0
NOF004_scaffold3320_1	non-phage	0
NOF004_scaffold23288_1	non-phage	0
NOF004_scaffold1283_6	non-phage	0
NOF004_scaffold6978_23	phage	0.997
NOF004_scaffold12398_3	non-phage	0
NOF004_scaffold52_5	non-phage	0
NOF004_scaffold9355_12	phage	0.999
NOF004_scaffold40024_7	non-phage	0
NOF004_scaffold27662_2	non-phage	0
NOF004_scaffold36955_3	non-phage	0
NOF004_scaffold34975_1	non-phage	0
NOF004_scaffold32845_4	non-phage	0
NOF004_scaffold40165_1	non-phage	0
NOF004_scaffold38742_1	non-phage	0
NOF004_scaffold17182_2	non-phage	0
NOF004_scaffold5779_3	non-phage	0
NOF004_scaffold37782_1	non-phage	0
NOF004_scaffold11475_2	non-phage	0
NOF004_scaffold9148_1	non-phage	0
NOF004_scaffold39879_2	non-phage	0
NOF004_scaffold1981_2	non-phage	0
NOF004_scaffold34211_1	non-phage	0
NOF004_scaffold31710_1	non-phage	0
NOF004_scaffold12025_2	phage	1
NOF004_scaffold21489_1	non-phage	0
NOF004_scaffold29958_2	non-phage	0
NOF004_scaffold891_2	non-phage	0
NOF004_scaffold40185_3	non-phage	0
NOF004_scaffold5672_12	non-phage	0
NOF004_scaffold4382_4	non-phage	0

NOF004_scaffold7551_3	non-phage	0
NOF004_scaffold29075_7	non-phage	0
NOF004_scaffold15518_1	non-phage	0
NOF004_scaffold7560_6	non-phage	0
NOF004_scaffold8521_3	non-phage	0
NOF004_scaffold12729_1	non-phage	0
NOF004_scaffold39819_1	non-phage	0
NOF004_scaffold32223_3	non-phage	0
NOF004_scaffold7949_4	non-phage	0
NOF004_scaffold37987_2	non-phage	0
NOF004_scaffold21384_1	non-phage	0
NOF004_scaffold9386_8	non-phage	0
NOF004_scaffold8883_1	non-phage	0
NOF004_scaffold32223_5	non-phage	0
NOF004_scaffold27259_1	non-phage	0
NOF004_scaffold26063_2	non-phage	0
NOF004_scaffold9553_2	non-phage	0
NOF004_scaffold104_2	non-phage	0
NOF004_scaffold25021_3	non-phage	0
NOF004_scaffold39841_1	non-phage	0
NOF004_scaffold14355_3	non-phage	0
NOF004_scaffold21108_6	non-phage	0.454
NOF004_scaffold1487_6	non-phage	0
NOF004_scaffold30655_3	non-phage	0
NOF004_scaffold43_4	non-phage	0
NOF004_scaffold9994_11	non-phage	0.06
NOF004_scaffold45_10	non-phage	0
NOF004_scaffold2472_1	non-phage	0
NOF004_scaffold32367_3	non-phage	0
NOF004_scaffold37229_1	non-phage	0
NOF004_scaffold12813_12	non-phage	0
NOF004_scaffold16908_2	non-phage	0
NOF004_scaffold40280_1	non-phage	0
NOF004_scaffold27754_3	non-phage	0
NOF004_scaffold13601_2	non-phage	0
NOF004_scaffold8883_3	non-phage	0
NOF004_scaffold2452_2	non-phage	0
NOF004_scaffold25836_3	non-phage	0
NOF004_scaffold29785_3	non-phage	0
NOF004_scaffold92_2	non-phage	0
NOF004_scaffold40284_1	non-phage	0
NOF004_scaffold5741_3	non-phage	0
NOF004_scaffold39716_2	non-phage	0
NOF004_scaffold19900_1	non-phage	0
NOF004_scaffold16509_3	non-phage	0
NOF004_scaffold29812_5	non-phage	0

NOF004_scaffold35309_2	non-phage	0
NOF004_scaffold9322_5	non-phage	0
NOF004_scaffold4230_4	non-phage	0
NOF004_scaffold2367_7	non-phage	0
NOF004_scaffold15026_8	non-phage	0
NOF004_scaffold15248_3	non-phage	0
NOF004_scaffold39635_4	non-phage	0
NOF004_scaffold39483_6	non-phage	0
NOF004_scaffold30600_2	non-phage	0
NOF004_scaffold39_27	non-phage	0
NOF004_scaffold51_7	non-phage	0
NOF004_scaffold28438_1	non-phage	0
NOF004_scaffold39888_1	non-phage	0
NOF004_scaffold6513_7	non-phage	0
NOF004_scaffold40265_1	non-phage	0
NOF004_scaffold3658_5	non-phage	0
NOF004_scaffold8320_2	non-phage	0
NOF004_scaffold25538_1	non-phage	0
NOF004_scaffold38621_2	non-phage	0
NOF004_scaffold26947_1	non-phage	0
NOF004_scaffold5300_6	non-phage	0
NOF004_scaffold134_2	non-phage	0
NOF004_scaffold40041_7	non-phage	0
NOF004_scaffold51_2	non-phage	0
NOF004_scaffold18855_1	non-phage	0
NOF004_scaffold28116_1	non-phage	0
NOF004_scaffold28010_4	non-phage	0
NOF004_scaffold39882_5	non-phage	0
NOF004_scaffold13098_5	non-phage	0
NOF004_scaffold10924_2	non-phage	0
NOF004_scaffold3320_12	non-phage	0
NOF004_scaffold3196_33	non-phage	0
NOF004_scaffold40243_1	non-phage	0
NOF004_scaffold28404_2	non-phage	0
NOF004_scaffold5780_2	non-phage	0
NOF004_scaffold94_34	non-phage	0
NOF004_scaffold3847_18	non-phage	0
NOF004_scaffold31007_2	non-phage	0
NOF004_scaffold31736_2	non-phage	0
NOF004_scaffold35835_1	non-phage	0
NOF004_scaffold8113_2	non-phage	0
NOF004_scaffold4939_5	non-phage	0
NOF004_scaffold40131_1	non-phage	0
NOF004_scaffold18640_5	non-phage	0
NOF004_scaffold3561_7	non-phage	0
NOF004_scaffold22559_3	non-phage	0

NOF004_scaffold13098_2	non-phage	0
NOF004_scaffold40083_1	non-phage	0
NOF004_scaffold39882_3	non-phage	0
NOF004_scaffold9875_1	non-phage	0
NOF004_scaffold4130_4	non-phage	0
NOF004_scaffold39757_2	non-phage	0
NOF004_scaffold30426_1	non-phage	0
NOF004_scaffold164_1	non-phage	0
NOF004_scaffold11762_3	non-phage	0
NOF004_scaffold39893_1	non-phage	0
NOF004_scaffold39379_2	non-phage	0
NOF004_scaffold5489_9	non-phage	0
NOF004_scaffold18841_4	non-phage	0
NOF004_scaffold12398_2	non-phage	0
NOF004_scaffold33115_6	non-phage	0
NOF004_scaffold10231_1	non-phage	0
NOF004_scaffold39839_2	non-phage	0
NOF004_scaffold31834_1	non-phage	0
NOF004_scaffold35689_2	non-phage	0
NOF004_scaffold8883_7	non-phage	0
NOF004_scaffold278_1	non-phage	0
NOF004_scaffold1680_4	non-phage	0
NOF004_scaffold31642_1	non-phage	0
NOF004_scaffold594_2	non-phage	0
NOF004_scaffold8775_10	non-phage	0
NOF004_scaffold35924_2	non-phage	0
NOF004_scaffold14211_1	phage	0.997
NOF004_scaffold30766_2	non-phage	0
NOF004_scaffold38772_1	non-phage	0
NOF004_scaffold17538_10	non-phage	0
NOF004_scaffold89_5	non-phage	0
NOF004_scaffold7693_13	non-phage	0
NOF004_scaffold311_2	non-phage	0
NOF004_scaffold19900_15	non-phage	0
NOF004_scaffold40029_2	non-phage	0
NOF004_scaffold39083_4	non-phage	0
NOF004_scaffold16824_4	non-phage	0
NOF004_scaffold23286_1	non-phage	0
NOF004_scaffold30537_7	non-phage	0
NOF004_scaffold46_6	non-phage	0
NOF004_scaffold5730_19	non-phage	0
NOF004_scaffold26987_1	non-phage	0
NOF004_scaffold3405_1	non-phage	0
NOF004_scaffold10187_1	non-phage	0
NOF004_scaffold40118_1	non-phage	0
NOF004_scaffold28158_1	non-phage	0

NOF004_scaffold40125_4	non-phage	0
NOF004_scaffold28010_1	non-phage	0
NOF004_scaffold7608_5	non-phage	0
NOF004_scaffold40041_3	non-phage	0
NOF004_scaffold270_8	non-phage	0
NOF004_scaffold9994_9	non-phage	0
NOF004_scaffold9994_2	phage	0.924
NOF004_scaffold7909_14	non-phage	0
NOF004_scaffold9697_6	non-phage	0
NOF004_scaffold20_1	non-phage	0
NOF004_scaffold15026_4	non-phage	0
NOF004_scaffold4528_9	non-phage	0
NOF004_scaffold29812_1	non-phage	0
NOF004_scaffold19874_1	non-phage	0
NOF004_scaffold28352_1	non-phage	0
NOF004_scaffold39156_1	non-phage	0
NOF004_scaffold33440_9	non-phage	0
NOF004_scaffold16982_5	non-phage	0
NOF004_scaffold1702_4	non-phage	0
NOF004_scaffold40162_1	non-phage	0
NOF004_scaffold2098_11	phage	0.999
NOF004_scaffold15026_2	non-phage	0
NOF004_scaffold17549_1	non-phage	0
NOF004_scaffold1274_43	non-phage	0
NOF004_scaffold12265_2	non-phage	0
NOF004_scaffold27996_7	non-phage	0
NOF004_scaffold2434_2	non-phage	0
NOF004_scaffold39867_5	non-phage	0
NOF004_scaffold25761_7	non-phage	0
NOF004_scaffold16385_3	non-phage	0
NOF004_scaffold7360_2	non-phage	0
NOF004_scaffold5990_3	non-phage	0
NOF004_scaffold874_18	non-phage	0
NOF004_scaffold33966_3	non-phage	0
NOF004_scaffold6915_4	non-phage	0
NOF004_scaffold62_3	non-phage	0
NOF004_scaffold89_2	non-phage	0
NOF004_scaffold7230_5	non-phage	0
NOF004_scaffold11322_5	non-phage	0
NOF004_scaffold33391_2	non-phage	0
NOF004_scaffold28491_6	non-phage	0
NOF004_scaffold11784_5	non-phage	0
NOF004_scaffold10815_1	non-phage	0
NOF004_scaffold2523_3	non-phage	0
NOF005_scaffold39463_1	non-phage	0
NOF005_scaffold28694_8	non-phage	0

NOF005_scaffold52461_1	non-phage	0
NOF005_scaffold52_4	non-phage	0
NOF005_scaffold3701_6	non-phage	0
NOF005_scaffold35291_5	phage	0.995
NOF005_scaffold45131_1	non-phage	0
NOF005_scaffold26764_1	non-phage	0
NOF005_scaffold48709_1	non-phage	0
NOF005_scaffold18041_7	non-phage	0
NOF005_scaffold10087_1	non-phage	0
NOF005_scaffold43705_2	non-phage	0
NOF005_scaffold35413_1	non-phage	0
NOF005_scaffold2521_14	non-phage	0
NOF005_scaffold32354_2	non-phage	0
NOF005_scaffold41924_6	non-phage	0
NOF005_scaffold11663_3	non-phage	0
NOF005_scaffold49374_1	non-phage	0
NOF005_scaffold52033_2	non-phage	0
NOF005_scaffold29205_11	non-phage	0
NOF005_scaffold46728_1	non-phage	0
NOF005_scaffold27601_3	non-phage	0
NOF005_scaffold22925_1	non-phage	0
NOF005_scaffold58_1	non-phage	0
NOF005_scaffold16555_3	non-phage	0
NOF005_scaffold1942_2	non-phage	0
NOF005_scaffold52460_4	non-phage	0
NOF005_scaffold47_1	non-phage	0
NOF005_scaffold51824_2	non-phage	0
NOF005_scaffold11123_5	non-phage	0
NOF005_scaffold26238_3	non-phage	0
NOF005_scaffold41234_2	non-phage	0
NOF005_scaffold21670_9	non-phage	0
NOF005_scaffold23396_3	phage	0.999
NOF005_scaffold1697_3	non-phage	0
NOF005_scaffold37284_3	non-phage	0
NOF005_scaffold43163_3	non-phage	0
NOF005_scaffold13806_7	non-phage	0
NOF005_scaffold5970_2	non-phage	0
NOF005_scaffold38949_5	non-phage	0
NOF005_scaffold40618_2	non-phage	0
NOF005_scaffold6582_1	non-phage	0
NOF005_scaffold13304_2	non-phage	0
NOF005_scaffold45604_1	non-phage	0
NOF005_scaffold16457_6	non-phage	0
NOF005_scaffold12064_2	non-phage	0
NOF005_scaffold35914_11	non-phage	0
NOF005_scaffold52453_5	non-phage	0

NOF005_scaffold31594_5	non-phage	0
NOF005_scaffold26238_13	non-phage	0
NOF005_scaffold15630_3	non-phage	0
NOF005_scaffold8426_4	non-phage	0
NOF005_scaffold16100_2	non-phage	0
NOF005_scaffold52367_2	non-phage	0
NOF005_scaffold20208_3	non-phage	0
NOF005_scaffold4_2	non-phage	0
NOF005_scaffold439_1	non-phage	0
NOF005_scaffold3106_5	non-phage	0
NOF005_scaffold19160_6	non-phage	0
NOF005_scaffold52288_1	non-phage	0
NOF005_scaffold44925_1	non-phage	0
NOF005_scaffold65_1	non-phage	0
NOF005_scaffold43877_1	non-phage	0
NOF005_scaffold45351_8	non-phage	0.004
NOF005_scaffold49267_2	non-phage	0
NOF005_scaffold34035_2	non-phage	0
NOF005_scaffold16715_2	non-phage	0
NOF005_scaffold17404_4	non-phage	0
NOF005_scaffold11467_1	non-phage	0
NOF005_scaffold3_2	non-phage	0
NOF005_scaffold41161_3	non-phage	0
NOF005_scaffold35516_2	non-phage	0
NOF005_scaffold44080_1	non-phage	0
NOF005_scaffold17151_8	non-phage	0
NOF005_scaffold15950_1	non-phage	0
NOF005_scaffold26958_1	non-phage	0
NOF005_scaffold36104_5	phage	0.878
NOF005_scaffold21949_6	non-phage	0
NOF005_scaffold26660_2	non-phage	0
NOF005_scaffold8509_3	non-phage	0
NOF005_scaffold18626_1	non-phage	0
NOF005_scaffold35323_2	non-phage	0
NOF005_scaffold27002_9	non-phage	0
NOF005_scaffold10894_1	non-phage	0
NOF005_scaffold21949_1	non-phage	0
NOF005_scaffold34324_5	non-phage	0
NOF005_scaffold8708_2	non-phage	0
NOF005_scaffold48820_1	non-phage	0
NOF005_scaffold8708_14	non-phage	0
NOF005_scaffold6508_2	non-phage	0
NOF005_scaffold18_1	phage	0.999
NOF005_scaffold25494_10	non-phage	0
NOF005_scaffold14342_5	non-phage	0
NOF005_scaffold31120_1	non-phage	0

NOF005_scaffold48975_4	non-phage	0
NOF005_scaffold6983_16	non-phage	0
NOF005_scaffold30881_5	non-phage	0
NOF005_scaffold4354_2	non-phage	0
NOF005_scaffold3262_1	non-phage	0
NOF005_scaffold43163_6	non-phage	0
NOF005_scaffold35434_1	non-phage	0
NOF005_scaffold18492_3	non-phage	0
NOF005_scaffold15324_2	non-phage	0
NOF005_scaffold21300_2	non-phage	0
NOF005_scaffold202_1	non-phage	0
NOF005_scaffold44982_1	non-phage	0
NOF005_scaffold6206_12	non-phage	0
NOF005_scaffold18329_5	non-phage	0
NOF005_scaffold10223_1	non-phage	0
NOF005_scaffold13302_3	non-phage	0
NOF005_scaffold93_13	non-phage	0
NOF005_scaffold37161_2	non-phage	0
NOF005_scaffold37598_3	non-phage	0
NOF005_scaffold15523_4	non-phage	0
NOF005_scaffold23414_2	non-phage	0
NOF005_scaffold32148_1	non-phage	0
NOF005_scaffold27375_1	non-phage	0
NOF005_scaffold46_4	non-phage	0
NOF005_scaffold27426_1	non-phage	0
NOF005_scaffold25899_1	non-phage	0
NOF005_scaffold4484_4	non-phage	0
NOF005_C713226_1	non-phage	0
NOF005_scaffold52409_1	non-phage	0
NOF005_scaffold26068_2	non-phage	0
NOF005_scaffold29919_3	non-phage	0
NOF005_scaffold27332_7	non-phage	0
NOF005_scaffold71_1	non-phage	0
NOF005_scaffold29653_1	non-phage	0
NOF005_scaffold8291_18	non-phage	0
NOF005_scaffold2638_10	non-phage	0
NOF005_scaffold28197_2	non-phage	0
NOF005_scaffold37734_1	non-phage	0
NOF005_scaffold9104_3	non-phage	0
NOF005_scaffold23337_1	non-phage	0
NOF005_scaffold9577_3	non-phage	0
NOF005_scaffold4573_1	non-phage	0
NOF005_scaffold3106_10	non-phage	0
NOF005_scaffold20156_5	non-phage	0
NOF005_scaffold27690_1	non-phage	0
NOF005_scaffold52439_2	non-phage	0

NOF005_scaffold44684_3	non-phage	0
NOF005_scaffold25742_6	phage	0.999
NOF005_scaffold3288_4	non-phage	0
NOF005_scaffold13806_8	non-phage	0
NOF005_scaffold8889_1	non-phage	0
NOF005_scaffold25218_5	non-phage	0
NOF005_scaffold24211_6	non-phage	0
NOF005_scaffold51409_1	non-phage	0
NOF005_scaffold35830_1	non-phage	0
NOF005_scaffold26238_10	non-phage	0
NOF005_scaffold4573_5	non-phage	0
NOF005_scaffold39025_3	non-phage	0
NOF005_scaffold24674_2	non-phage	0
NOF005_C713404_1	non-phage	0
NOF005_scaffold26370_5	non-phage	0
NOF005_scaffold6206_1	non-phage	0
NOF005_scaffold15630_2	non-phage	0
NOF005_scaffold8082_3	non-phage	0.053
NOF005_scaffold13104_7	non-phage	0
NOF005_scaffold1167_10	non-phage	0
NOF005_scaffold16100_3	non-phage	0
NOF005_scaffold52424_1	non-phage	0
NOF005_scaffold24591_1	non-phage	0
NOF005_scaffold49857_2	non-phage	0
NOF005_scaffold24211_10	non-phage	0
NOF005_scaffold24211_9	non-phage	0
NOF005_scaffold27003_2	non-phage	0
NOF005_scaffold41924_2	non-phage	0
NOF005_scaffold8796_2	non-phage	0
NOF005_scaffold8426_2	non-phage	0
NOF005_scaffold10034_2	non-phage	0
NOF005_scaffold15523_1	non-phage	0
NOF005_scaffold43134_1	non-phage	0
NOF005_scaffold19920_3	non-phage	0
NOF005_scaffold207_2	non-phage	0
NOF005_scaffold17197_2	non-phage	0.315
NOF005_scaffold42463_1	non-phage	0
NOF005_scaffold48975_2	non-phage	0
NOF005_scaffold45_2	non-phage	0
NOF005_scaffold19831_1	non-phage	0
NOF005_scaffold249_8	non-phage	0
NOF005_scaffold27453_1	non-phage	0
NOF005_scaffold2638_1	non-phage	0
NOF005_scaffold31594_11	phage	0.995
NOF005_scaffold26764_2	non-phage	0
NOF005_scaffold230_8	non-phage	0

NOF005_scaffold831_1	non-phage	0
NOF005_scaffold35637_3	non-phage	0
NOF005_scaffold51_1	non-phage	0
NOF005_scaffold6955_1	non-phage	0
NOF005_scaffold3061_27	non-phage	0
NOF005_scaffold5258_9	non-phage	0
NOF005_scaffold16170_6	non-phage	0
NOF005_scaffold52358_1	non-phage	0
NOF005_scaffold35557_2	non-phage	0
NOF005_scaffold21300_1	non-phage	0
NOF005_scaffold27002_3	non-phage	0
NOF005_scaffold52110_2	non-phage	0
NOF005_scaffold31594_4	non-phage	0.007
NOF005_scaffold27238_2	non-phage	0
NOF005_scaffold16003_1	non-phage	0
NOF005_scaffold10087_4	non-phage	0
NOF005_scaffold28648_1	non-phage	0
NOF005_scaffold6888_3	phage	0.998
NOF005_scaffold52460_1	non-phage	0
NOF005_scaffold25082_1	non-phage	0
NOF005_scaffold28624_1	non-phage	0
NOF005_scaffold3118_11	non-phage	0
NOF005_scaffold1035_7	non-phage	0
NOF005_scaffold30515_1	non-phage	0
NOF005_scaffold30881_10	non-phage	0
NOF005_scaffold28694_5	non-phage	0
NOF005_scaffold52395_4	non-phage	0
NOF005_scaffold52468_2	non-phage	0
NOF005_scaffold18329_4	non-phage	0
NOF005_scaffold7631_2	non-phage	0
NOF005_scaffold33237_2	non-phage	0
NOF005_scaffold35914_4	non-phage	0
NOF005_scaffold7504_3	non-phage	0
NOF005_scaffold24616_14	non-phage	0
NOF005_scaffold50155_1	non-phage	0
NOF005_scaffold23406_2	non-phage	0
NOF005_scaffold16457_3	non-phage	0
NOF005_scaffold52453_2	non-phage	0
NOF005_scaffold3573_11	non-phage	0
NOF005_scaffold41917_1	non-phage	0
NOF005_scaffold8708_3	non-phage	0
NOF005_scaffold36939_3	non-phage	0
NOF005_scaffold23835_1	non-phage	0
NOF005_scaffold9151_2	non-phage	0
NOF005_scaffold49109_3	non-phage	0
NOF005_scaffold11755_1	non-phage	0

NOF005_scaffold4590_8	non-phage	0
NOF005_scaffold25564_1	non-phage	0
NOF005_scaffold150_2	non-phage	0
NOF005_scaffold27453_4	non-phage	0
NOF005_scaffold42_1	non-phage	0
NOF005_scaffold44684_7	non-phage	0
NOF005_scaffold49376_1	non-phage	0
NOF005_scaffold68_15	non-phage	0
NOF005_scaffold45604_3	non-phage	0
NOF005_scaffold45429_7	non-phage	0
NOF005_scaffold23396_1	non-phage	0
NOF005_scaffold44684_1	non-phage	0
NOF005_scaffold45002_5	non-phage	0
NOF005_scaffold13942_4	non-phage	0
NOF005_scaffold32199_6	non-phage	0
NOF005_scaffold31855_2	non-phage	0
NOF005_scaffold29221_10	phage	0.999
NOF005_scaffold26370_4	non-phage	0
NOF005_scaffold6583_3	non-phage	0
NOF005_scaffold40100_3	non-phage	0
NOF005_scaffold6626_1	non-phage	0
NOF005_scaffold24401_1	non-phage	0
NOF005_scaffold45351_6	non-phage	0
NOF005_scaffold16555_14	non-phage	0
NOF005_scaffold32056_1	non-phage	0
NOF005_scaffold27332_4	non-phage	0
NOF005_scaffold51727_1	non-phage	0
NOF005_scaffold52348_1	non-phage	0
NOF005_scaffold51620_1	non-phage	0
NOF005_scaffold41161_1	non-phage	0
NOF005_scaffold45_1	non-phage	0
NOF005_scaffold3061_25	non-phage	0
NOF005_scaffold24039_1	non-phage	0
NOF005_scaffold37206_3	non-phage	0
NOF005_scaffold16170_2	non-phage	0
NOF005_scaffold44684_8	non-phage	0
NOF005_scaffold24211_7	non-phage	0
NOF005_scaffold36589_1	non-phage	0
NOF005_scaffold49983_2	non-phage	0
NOF005_scaffold29757_9	non-phage	0
NOF005_scaffold2976_1	non-phage	0
NOF005_scaffold18127_1	non-phage	0
NOF005_scaffold50121_3	non-phage	0
NOF005_scaffold51112_5	non-phage	0
NOF005_scaffold24363_2	non-phage	0
NOF005_scaffold34324_4	phage	0.961

NOF005_scaffold4142_3	non-phage	0
NOF005_scaffold14389_2	non-phage	0
NOF005_scaffold29047_1	non-phage	0
NOF005_scaffold12676_1	non-phage	0
NOF005_scaffold27332_8	non-phage	0
NOF005_scaffold3118_1	non-phage	0
NOF005_scaffold1167_18	non-phage	0
NOF005_scaffold16100_4	non-phage	0
NOF005_scaffold45351_1	non-phage	0
NOF005_scaffold27559_1	non-phage	0
NOF005_scaffold35736_4	non-phage	0
NOF005_scaffold25494_8	non-phage	0
NOF005_scaffold51151_1	non-phage	0
NOF005_scaffold52464_1	non-phage	0
NOF005_scaffold9577_2	non-phage	0
NOF005_scaffold11121_1	non-phage	0
NOF005_scaffold52457_2	non-phage	0
NOF005_scaffold3061_33	non-phage	0
NOF005_scaffold44930_1	non-phage	0
NOF005_scaffold31199_5	non-phage	0
NOF005_scaffold45429_9	non-phage	0
NOF005_scaffold44820_1	non-phage	0
NOF005_scaffold15434_1	non-phage	0
NOF005_scaffold52398_4	non-phage	0
NOF005_scaffold2588_4	non-phage	0
NOF005_scaffold43999_1	non-phage	0
NOF005_scaffold2953_5	non-phage	0
NOF005_scaffold51549_2	non-phage	0
NOF005_scaffold22222_6	non-phage	0
NOF005_scaffold38780_4	non-phage	0
NOF005_scaffold52340_1	non-phage	0
NOF005_scaffold58_4	non-phage	0
NOF005_scaffold33564_2	non-phage	0
NOF005_scaffold3106_7	non-phage	0
NOF005_scaffold1086_25	phage	0.981
NOF005_scaffold6582_3	non-phage	0
NOF005_scaffold15823_2	non-phage	0
NOF005_scaffold31032_1	non-phage	0
NOF005_scaffold39974_1	non-phage	0
NOF005_scaffold27740_2	non-phage	0
NOF005_scaffold16170_5	non-phage	0
NOF005_scaffold7502_4	non-phage	0
NOF005_scaffold52447_1	non-phage	0
NOF005_scaffold43705_6	non-phage	0
NOF005_scaffold23281_2	non-phage	0
NOF005_scaffold6043_2	non-phage	0

NOF005_scaffold29911_1	non-phage	0
NOF005_scaffold29858_1	non-phage	0
NOF005_scaffold11420_2	non-phage	0
NOF005_scaffold22654_2	non-phage	0
NOF005_scaffold45099_8	non-phage	0
NOF005_scaffold52466_1	non-phage	0
NOF005_scaffold24476_5	non-phage	0
NOF005_scaffold52092_1	non-phage	0
NOF005_C712870_1	non-phage	0
NOF005_scaffold11420_3	non-phage	0
NOF005_scaffold5564_14	non-phage	0
NOF005_scaffold18917_1	non-phage	0
NOF005_scaffold26238_2	non-phage	0
NOF005_scaffold163_3	non-phage	0
NOF005_scaffold50121_2	non-phage	0
NOF005_scaffold604_14	phage	0.551
NOF005_scaffold51112_1	non-phage	0
NOF005_scaffold26669_2	non-phage	0
NOF005_scaffold8855_3	non-phage	0
NOF005_scaffold48705_6	non-phage	0
NOF005_scaffold50325_2	non-phage	0.012
NOF005_scaffold11432_3	non-phage	0
NOF005_scaffold8269_1	non-phage	0
NOF005_scaffold44749_6	phage	0.546
NOF005_scaffold34324_7	non-phage	0
NOF005_scaffold13603_1	non-phage	0
NOF005_scaffold11415_2	non-phage	0
NOF005_scaffold43551_6	non-phage	0
NOF005_scaffold1032_41	non-phage	0
NOF005_scaffold3465_2	non-phage	0
NOF005_scaffold12221_5	non-phage	0
NOF005_scaffold8708_12	non-phage	0
NOF005_scaffold11077_3	non-phage	0
NOF005_scaffold33384_1	non-phage	0
NOF005_scaffold52419_2	non-phage	0
NOF005_scaffold2638_8	non-phage	0
NOF005_scaffold52395_2	non-phage	0
NOF005_scaffold16555_5	non-phage	0
NOF005_scaffold24626_9	non-phage	0
NOF005_scaffold51717_1	phage	0.987
NOF005_scaffold42745_1	non-phage	0
NOF005_scaffold38743_1	non-phage	0
NOF005_scaffold8096_3	non-phage	0
NOF005_scaffold49424_1	non-phage	0
NOF005_scaffold33048_5	non-phage	0
NOF005_scaffold43705_3	non-phage	0

NOF005_scaffold34324_11	non-phage	0
NOF005_scaffold16555_4	non-phage	0
NOF005_scaffold21237_2	non-phage	0
NOF005_scaffold7771_1	non-phage	0
NOF005_scaffold23089_5	non-phage	0
NOF005_scaffold186_2	non-phage	0
NOF005_scaffold981_1	non-phage	0
NOF005_scaffold19642_1	non-phage	0
NOF005_scaffold2638_3	non-phage	0
NOF005_C713394_1	non-phage	0
NOF005_scaffold35815_8	non-phage	0
NOF005_scaffold1032_13	non-phage	0
NOF005_scaffold13943_2	non-phage	0
NOF005_scaffold52401_2	non-phage	0
NOF005_scaffold18762_2	non-phage	0
NOF005_scaffold24684_2	non-phage	0
NOF005_scaffold6221_1	non-phage	0
NOF005_scaffold32645_2	non-phage	0
NOF005_scaffold45351_9	non-phage	0
NOF005_scaffold50475_2	non-phage	0
NOF005_scaffold24179_1	non-phage	0
NOF005_scaffold6508_5	non-phage	0
NOF005_scaffold2751_6	non-phage	0
NOF005_scaffold51824_3	non-phage	0
NOF005_scaffold26669_7	non-phage	0
NOF005_scaffold51992_2	non-phage	0
NOF005_scaffold42005_4	non-phage	0
NOF005_scaffold22790_2	non-phage	0
NOF005_scaffold14692_1	non-phage	0
NOF005_scaffold45131_2	non-phage	0
NOF005_scaffold30881_14	non-phage	0
NOF005_scaffold8925_9	non-phage	0
NOF005_scaffold44511_1	non-phage	0
NOF005_scaffold23396_4	non-phage	0
NOF005_C713624_1	non-phage	0
NOF005_scaffold35393_5	phage	0.998
NOF005_scaffold14342_1	non-phage	0
NOF005_scaffold16555_15	non-phage	0
NOF005_scaffold13104_9	non-phage	0
NOF005_scaffold52446_1	non-phage	0
NOF005_scaffold26238_12	non-phage	0
NOF005_scaffold2521_18	non-phage	0
NOF005_scaffold17684_2	non-phage	0
NOF005_scaffold30881_9	non-phage	0
NOF005_scaffold981_2	non-phage	0
NOF005_scaffold18041_2	non-phage	0

NOF005_scaffold34950_3	phage	0.789
NOF005_scaffold50658_2	non-phage	0
NOF005_scaffold27003_4	non-phage	0
NOF005_scaffold52185_4	non-phage	0
NOF005_scaffold1972_7	non-phage	0
NOF005_scaffold36939_5	non-phage	0
NOF005_scaffold6824_2	non-phage	0
NOF005_scaffold52454_1	non-phage	0
NOF005_scaffold4015_8	non-phage	0
NOF005_scaffold51705_2	non-phage	0
NOF005_scaffold21949_4	non-phage	0
NOF005_scaffold9605_2	non-phage	0
NOF005_scaffold34324_6	non-phage	0
NOF005_scaffold33243_1	non-phage	0
NOF005_scaffold28197_4	non-phage	0
NOF005_scaffold19395_2	non-phage	0
NOF005_scaffold25148_4	non-phage	0
NOF005_scaffold26238_6	non-phage	0.002
NOF005_scaffold24384_4	non-phage	0
NOF005_scaffold10374_4	non-phage	0
NOF005_scaffold26764_3	non-phage	0
NOF005_scaffold49109_1	non-phage	0
NOF005_scaffold4514_1	non-phage	0
NOF005_scaffold202_2	non-phage	0
NOF005_scaffold24980_2	non-phage	0
NOF005_scaffold52412_1	non-phage	0
NOF005_scaffold23089_6	non-phage	0
NOF005_scaffold13304_6	non-phage	0
NOF005_scaffold8509_2	non-phage	0
NOF005_scaffold41569_1	non-phage	0
NOF005_scaffold51596_4	phage	0.998
NOF005_scaffold17151_9	non-phage	0
NOF005_scaffold39842_1	non-phage	0
NOF005_scaffold21064_6	non-phage	0
NOF005_scaffold42751_1	non-phage	0
NOF005_scaffold4484_1	non-phage	0
NOF005_scaffold52408_1	non-phage	0
NOF005_scaffold17952_1	non-phage	0
NOF005_scaffold21663_1	non-phage	0
NOF005_scaffold24616_6	non-phage	0
NOF005_scaffold27018_2	non-phage	0
NOF005_scaffold14342_4	non-phage	0
NOF005_scaffold28197_1	non-phage	0
NOF005_scaffold52176_3	non-phage	0
NOF005_scaffold52467_1	non-phage	0
NOF005_scaffold52031_2	non-phage	0

NOF005_scaffold226_2	non-phage	0
NOF005_scaffold13104_5	non-phage	0
NOF005_scaffold52457_1	non-phage	0
NOF005_scaffold13408_8	non-phage	0
NOF005_scaffold1896_1	non-phage	0
NOF005_scaffold40002_1	non-phage	0
NOF005_scaffold21300_3	non-phage	0
NOF005_scaffold43990_2	non-phage	0
NOF005_scaffold48570_1	non-phage	0
NOF005_scaffold6008_2	non-phage	0
NOF005_scaffold37290_1	non-phage	0
NOF005_scaffold37528_3	non-phage	0
NOF005_scaffold8925_6	non-phage	0
NOF005_scaffold49566_3	non-phage	0
NOF005_scaffold33243_12	non-phage	0
NOF005_scaffold27397_2	non-phage	0
NOF005_scaffold52324_2	non-phage	0
NOF005_C713402_1	non-phage	0
NOF005_scaffold1551_2	non-phage	0
NOF005_scaffold4573_4	non-phage	0
NOF005_scaffold35914_2	non-phage	0
NOF005_scaffold12261_8	non-phage	0
NOF005_scaffold13104_6	non-phage	0
NOF005_scaffold7606_1	non-phage	0
NOF005_scaffold833_1	non-phage	0
NOF005_scaffold34434_1	non-phage	0
NOF005_scaffold24363_4	non-phage	0
NOF005_scaffold27795_2	non-phage	0
NOF005_scaffold29140_2	phage	0.995
NOF005_scaffold6206_3	non-phage	0
NOF005_scaffold12221_7	non-phage	0
NOF005_scaffold26994_2	non-phage	0
NOF005_scaffold11472_1	non-phage	0
NOF005_scaffold26068_3	non-phage	0
NOF005_scaffold16798_4	non-phage	0
NOF005_scaffold41234_1	non-phage	0
NOF005_scaffold7653_1	non-phage	0
NOF005_scaffold37734_2	phage	0.845
NOF005_scaffold1167_13	non-phage	0
NOF005_scaffold17197_4	non-phage	0
NOF005_scaffold165_4	phage	0.978
NOF005_scaffold169_1	non-phage	0
NOF005_scaffold42005_1	non-phage	0
NOF005_scaffold27318_2	non-phage	0
NOF005_scaffold52417_1	non-phage	0
NOF005_scaffold29908_5	non-phage	0

NOF005_scaffold44221_2	non-phage	0
NOF005_scaffold22251_14	non-phage	0
NOF005_scaffold25018_1	non-phage	0
NOF005_scaffold7771_2	non-phage	0
NOF005_scaffold34100_3	non-phage	0
NOF005_scaffold2170_3	phage	0.991
NOF005_scaffold38339_1	non-phage	0
NOF005_scaffold27466_1	non-phage	0
NOF005_scaffold28416_13	non-phage	0
NOF005_scaffold36939_14	non-phage	0
NOF005_scaffold52422_1	non-phage	0
NOF005_scaffold24910_8	phage	0.993
NOF005_scaffold22066_1	non-phage	0
NOF005_scaffold7501_14	non-phage	0
NOF005_scaffold220_7	non-phage	0
NOF005_scaffold4015_1	non-phage	0
NOF005_scaffold50952_4	non-phage	0
NOF005_scaffold17404_7	non-phage	0
NOF005_scaffold38428_2	non-phage	0
NOF005_scaffold2953_2	non-phage	0
NOF005_scaffold2991_16	non-phage	0
NOF005_scaffold2638_2	non-phage	0
NOF005_scaffold13943_1	non-phage	0
NOF005_scaffold5111_5	non-phage	0
NOF005_scaffold24576_4	non-phage	0
NOF005_scaffold2638_13	non-phage	0
NOF005_C713490_1	non-phage	0
NOF005_scaffold37308_1	non-phage	0
NOF005_scaffold33510_1	non-phage	0
NOF005_scaffold2944_6	non-phage	0
NOF005_scaffold48547_9	phage	0.999
NOF005_scaffold17212_1	non-phage	0
NOF005_scaffold52340_2	non-phage	0
NOF005_scaffold3288_1	non-phage	0
NOF005_scaffold2811_1	non-phage	0
NOF005_scaffold43369_12	non-phage	0
NOF005_scaffold14654_2	non-phage	0
NOF005_scaffold26238_7	non-phage	0
NOF005_scaffold51466_3	non-phage	0
NOF005_scaffold26725_1	non-phage	0
NOF005_scaffold3118_3	non-phage	0
NOF005_scaffold42202_1	non-phage	0
NOF005_scaffold27332_11	non-phage	0
NOF005_scaffold45002_2	non-phage	0
NOF005_scaffold51958_1	non-phage	0
NOF005_scaffold38001_1	non-phage	0

NOF005_scaffold169_5	non-phage	0
NOF005_scaffold52284_1	non-phage	0
NOF005_scaffold24531_1	non-phage	0
NOF005_scaffold25494_7	non-phage	0
NOF005_scaffold47562_1	non-phage	0
NOF005_scaffold691_12	non-phage	0
NOF005_scaffold15268_2	non-phage	0
NOF005_scaffold32199_8	non-phage	0
NOF005_scaffold24164_3	non-phage	0
NOF005_scaffold51_4	non-phage	0
NOF005_scaffold16003_2	non-phage	0
NOF005_scaffold12473_1	non-phage	0
NOF005_scaffold604_12	phage	0.998
NOF005_scaffold51437_1	non-phage	0
NOF005_scaffold13408_1	non-phage	0
NOF005_scaffold46664_1	non-phage	0
NOF005_scaffold24314_3	non-phage	0
NOF005_scaffold11187_1	non-phage	0
NOF005_scaffold3654_2	non-phage	0
NOF005_scaffold50335_4	non-phage	0
NOF005_scaffold24616_3	non-phage	0
NOF005_scaffold20152_2	non-phage	0
NOF005_scaffold22790_5	non-phage	0
NOF005_scaffold25494_4	non-phage	0
NOF005_scaffold31452_3	non-phage	0
NOF005_scaffold1032_29	non-phage	0
NOF005_scaffold34607_1	non-phage	0.011
NOF005_scaffold13281_4	non-phage	0
NOF005_scaffold2175_1	non-phage	0
NOF005_scaffold51888_1	non-phage	0
NOF005_scaffold43786_1	non-phage	0
NOF005_scaffold27332_3	non-phage	0
NOF005_scaffold24008_2	non-phage	0
NOF005_scaffold52405_1	non-phage	0
NOF005_scaffold7732_10	non-phage	0
NOF005_scaffold35198_1	non-phage	0
NOF005_scaffold50445_2	non-phage	0
NOF005_scaffold12140_1	non-phage	0
NOF005_scaffold2638_5	non-phage	0
NOF005_scaffold52365_1	phage	0.984
NOF005_scaffold4006_1	non-phage	0
NOF005_scaffold51228_2	non-phage	0
NOF005_scaffold22515_1	non-phage	0
NOF005_scaffold93_8	non-phage	0
NOF005_scaffold11594_1	non-phage	0
NOF005_scaffold302_10	non-phage	0

NOF005_scaffold7906_3	non-phage	0
NOF005_scaffold150_1	non-phage	0
NOF005_scaffold7750_1	non-phage	0
NOF005_scaffold2521_2	non-phage	0
NOF005_scaffold41315_6	non-phage	0
NOF005_scaffold19003_8	non-phage	0
NOF005_scaffold51705_1	non-phage	0
NOF005_scaffold27556_1	non-phage	0
NOF005_scaffold15822_21	non-phage	0
NOF005_scaffold7770_7	non-phage	0
NOF005_scaffold37200_1	non-phage	0
NOF005_scaffold24515_2	non-phage	0
NOF005_scaffold51944_2	non-phage	0
NOF005_scaffold34335_1	non-phage	0
NOF005_scaffold48051_3	non-phage	0
NOF005_scaffold48589_3	non-phage	0
NOF005_scaffold31805_2	non-phage	0
NOF005_scaffold11253_1	non-phage	0
NOF005_scaffold34092_1	non-phage	0
NOF005_scaffold27601_4	non-phage	0
NOF005_scaffold191_2	non-phage	0
NOF005_scaffold478_9	non-phage	0
NOF005_scaffold52130_2	non-phage	0
NOF005_scaffold27002_7	non-phage	0
NOF005_scaffold45351_7	non-phage	0
NOF005_scaffold17151_4	non-phage	0
NOF005_scaffold17151_10	non-phage	0
NOF005_scaffold33721_2	non-phage	0
NOF005_scaffold51797_1	non-phage	0
NOF005_scaffold33945_2	non-phage	0
NOF005_scaffold23396_2	phage	0.812
NOF005_scaffold10741_2	non-phage	0
NOF005_scaffold2177_1	non-phage	0
NOF005_scaffold51_5	non-phage	0
NOF005_scaffold24616_1	non-phage	0
NOF005_scaffold34187_1	non-phage	0
NOF005_scaffold1896_4	non-phage	0
NOF005_scaffold4459_4	non-phage	0
NOF005_scaffold52295_1	non-phage	0
NOF005_scaffold52176_5	non-phage	0
NOF005_scaffold115_4	non-phage	0
NOF005_scaffold52048_1	non-phage	0
NOF005_scaffold15823_4	non-phage	0
NOF005_scaffold45768_2	non-phage	0
NOF005_scaffold30_2	non-phage	0
NOF005_scaffold26669_1	non-phage	0

NOF005_scaffold52456_1	non-phage	0
NOF005_scaffold32575_1	non-phage	0
NOF005_scaffold52369_1	non-phage	0
NOF005_scaffold24616_18	non-phage	0
NOF005_scaffold17404_8	non-phage	0
NOF005_scaffold25656_9	non-phage	0
NOF005_scaffold42945_1	non-phage	0
NOF005_scaffold52229_4	non-phage	0
NOF005_scaffold43705_1	non-phage	0
NOF005_scaffold3465_6	phage	0.998
NOF005_scaffold45950_1	non-phage	0
NOF005_scaffold40223_5	non-phage	0
NOF005_scaffold27559_2	non-phage	0
NOF005_scaffold12221_6	non-phage	0
NOF005_scaffold51549_1	non-phage	0
NOF005_scaffold52443_2	non-phage	0
NOF005_scaffold51571_1	non-phage	0
NOF005_scaffold52349_1	non-phage	0
NOF005_scaffold45892_2	non-phage	0
NOF005_scaffold15630_1	non-phage	0
NOF005_scaffold50325_1	non-phage	0
NOF005_scaffold3106_2	non-phage	0
NOF005_scaffold50217_1	non-phage	0
NOF005_scaffold41365_4	non-phage	0
NOF005_scaffold604_3	phage	0.999
NOF005_scaffold21670_1	non-phage	0
NOF005_scaffold33243_7	non-phage	0
NOF005_scaffold52462_1	non-phage	0
NOF005_C713114_1	non-phage	0
NOF005_scaffold3668_1	non-phage	0
NOF005_scaffold12064_3	non-phage	0
NOF005_scaffold51606_1	non-phage	0
NOF005_scaffold8426_3	non-phage	0
NOF005_scaffold26994_1	non-phage	0
NOF005_scaffold16457_7	non-phage	0
NOF005_scaffold51228_5	non-phage	0
NOF005_scaffold44373_1	non-phage	0
NOF005_scaffold16100_10	non-phage	0
NOF005_scaffold25956_4	non-phage	0
NOF005_scaffold43640_2	non-phage	0
NOF005_scaffold28624_5	non-phage	0
NOF005_scaffold11420_7	non-phage	0
NOF005_scaffold6564_1	non-phage	0
NOF005_scaffold26958_2	non-phage	0
NOF005_scaffold33455_7	non-phage	0
NOF005_scaffold6066_3	non-phage	0

NOF005_scaffold16170_3	non-phage	0
NOF005_scaffold36108_4	non-phage	0
NOF005_scaffold14441_19	non-phage	0
NOF005_scaffold33814_2	non-phage	0
NOF005_scaffold27009_2	non-phage	0
NOF005_scaffold28197_6	non-phage	0
NOF005_scaffold50581_4	non-phage	0
NOF005_scaffold33384_2	non-phage	0
NOF005_scaffold31863_5	non-phage	0
NOF005_scaffold33945_3	non-phage	0
NOF005_scaffold24809_1	non-phage	0
NOF005_scaffold10051_5	non-phage	0
NOF005_scaffold249_6	non-phage	0
NOF005_scaffold45429_11	non-phage	0
NOF005_scaffold9790_7	non-phage	0
NOF005_scaffold51504_1	non-phage	0
NOF005_scaffold4015_11	non-phage	0
NOF005_scaffold3_1	non-phage	0
NOF005_scaffold52405_2	phage	0.999
NOF005_scaffold17611_3	non-phage	0
NOF005_scaffold49499_1_1	phage	0.996
NOF005_scaffold49499_1_2	phage	0.999
NOF005_scaffold48705_5	non-phage	0
NOF005_scaffold25218_1	non-phage	0
NOF005_scaffold36586_10	non-phage	0
NOF005_scaffold26655_1	non-phage	0
NOF005_scaffold51114_1	non-phage	0
NOF005_scaffold34587_1	non-phage	0
NOF005_scaffold23414_3	non-phage	0
NOF005_scaffold2638_15	non-phage	0
NOF005_scaffold50581_1	non-phage	0
NOF005_scaffold13281_2	non-phage	0
NOF005_scaffold48051_6	non-phage	0
NOF005_scaffold33243_15	non-phage	0
NOF005_scaffold12221_3	non-phage	0
NOF005_scaffold2638_9	non-phage	0
NOF005_scaffold49374_2	non-phage	0
NOF005_scaffold51323_5	non-phage	0
NOF005_scaffold8925_14	non-phage	0
NOF005_scaffold19099_2	non-phage	0
NOF005_scaffold52323_2	non-phage	0
NOF005_scaffold50763_2	non-phage	0
NOF005_scaffold8641_6	non-phage	0
NOF005_scaffold7898_3	non-phage	0
NOF005_scaffold235_30	non-phage	0
NOF005_scaffold22639_4	non-phage	0

NOF005_scaffold29908_3	non-phage	0
NOF005_scaffold31389_1	non-phage	0
NOF005_scaffold26238_17	non-phage	0
NOF005_scaffold9290_4	non-phage	0
NOF005_scaffold2001_2	non-phage	0
NOF005_scaffold13462_2	non-phage	0
NOF005_scaffold4532_11	non-phage	0
NOF005_scaffold19642_3	non-phage	0
NOF005_scaffold51650_1	non-phage	0
NOF005_scaffold15268_1	phage	0.978
NOF005_scaffold37161_1	non-phage	0
NOF005_scaffold15412_3	non-phage	0
NOF005_scaffold24211_1	non-phage	0
NOF005_scaffold165_6	non-phage	0
NOF005_scaffold18919_1	non-phage	0
NOF005_scaffold34797_2	non-phage	0
NOF005_scaffold24476_3	non-phage	0
NOF005_scaffold34434_6	non-phage	0
NOF005_scaffold39972_17	phage	0.831
NOF005_scaffold8139_21	non-phage	0
NOF005_scaffold53_2	non-phage	0
NOF005_scaffold24515_1	non-phage	0
NOF005_scaffold13603_7	non-phage	0
NOF005_scaffold25109_1	non-phage	0
NOF005_scaffold45627_1	non-phage	0
NOF005_scaffold27541_5	phage	0.994
NOF005_scaffold24616_24	non-phage	0
NOF005_scaffold31805_1	non-phage	0
NOF005_scaffold15244_2	non-phage	0
NOF005_scaffold8855_2	non-phage	0
NOF005_scaffold27426_3	phage	0.932
NOF005_scaffold8708_10	non-phage	0
NOF005_scaffold51779_2	non-phage	0
NOF005_scaffold51228_3	non-phage	0
NOF005_scaffold1167_5	non-phage	0
NOF005_scaffold26277_3	non-phage	0
NOF005_scaffold5924_1	phage	0.997
NOF005_scaffold29757_4	non-phage	0
NOF005_scaffold48470_1	non-phage	0
NOF005_scaffold31199_1	non-phage	0
NOF005_scaffold5278_2	non-phage	0
NOF005_scaffold1035_4	non-phage	0
NOF005_scaffold2944_4	non-phage	0
NOF005_scaffold1280_10	non-phage	0
NOF005_scaffold9961_1	non-phage	0
NOF005_scaffold604_10	non-phage	0

NOF005_scaffold42_4	non-phage	0
NOF005_scaffold26669_6	non-phage	0
NOF005_scaffold43354_1	non-phage	0
NOF005_scaffold45131_3	non-phage	0
NOF005_scaffold4946_6	non-phage	0
NOF005_scaffold13218_2	non-phage	0
NOF005_scaffold33617_4	non-phage	0
NOF005_scaffold24314_4	non-phage	0
NOF005_scaffold6983_10	non-phage	0
NOF005_scaffold27740_3	non-phage	0
NOF005_scaffold41924_13	non-phage	0
NOF005_scaffold5279_5	non-phage	0
NOF005_scaffold52449_1	non-phage	0
NOF005_scaffold43695_1	non-phage	0
NOF005_scaffold23281_7	non-phage	0
NOF005_scaffold29919_2	non-phage	0
NOF005_scaffold52335_1	non-phage	0
NOF005_scaffold16265_7	non-phage	0
NOF005_scaffold15893_1	non-phage	0
NOF005_scaffold34187_2	non-phage	0
NOF005_scaffold51807_1	non-phage	0
NOF005_scaffold52169_1	non-phage	0.296
NOF005_scaffold51824_4	non-phage	0
NOF005_C713090_1	non-phage	0
NOF005_scaffold52337_2	non-phage	0
NOF005_scaffold17151_3	non-phage	0
NOF005_scaffold52414_2	non-phage	0
NOF005_scaffold22790_3	non-phage	0
NOF005_scaffold23063_1	non-phage	0
NOF005_scaffold28197_3	non-phage	0
NOF005_scaffold32384_3	phage	0.999
NOF005_scaffold39128_1	non-phage	0
NOF005_scaffold2638_7	non-phage	0
NOF005_scaffold249_7	non-phage	0
NOF005_scaffold51761_1	non-phage	0
NOF005_scaffold39599_1	non-phage	0
NOF005_scaffold46139_17	non-phage	0
NOF005_scaffold31919_1	non-phage	0
NOF005_scaffold25991_5	non-phage	0
NOF005_scaffold52404_1	non-phage	0
NOF005_scaffold21670_10	non-phage	0
NOF005_scaffold2953_4	non-phage	0
NOF005_scaffold24363_1	non-phage	0
NOF005_scaffold40618_1	non-phage	0
NOF005_scaffold1942_19	non-phage	0
NOF005_scaffold51639_1	non-phage	0

NOF005_scaffold44821_1	phage	0.998
NOF005_scaffold52412_2	non-phage	0
NOF005_scaffold3106_4	non-phage	0
NOF005_scaffold63_6	non-phage	0
NOF005_scaffold52468_1	non-phage	0
NOF005_scaffold2961_3	non-phage	0
NOF005_scaffold15630_5	non-phage	0
NOF005_scaffold6564_7	non-phage	0
NOF005_scaffold24674_3	non-phage	0
NOF005_scaffold31770_1	non-phage	0
NOF005_scaffold47965_2	non-phage	0
NOF005_scaffold6083_19	non-phage	0
NOF005_scaffold27198_2	phage	0.999
NOF005_scaffold29675_6	non-phage	0
NOF005_scaffold31594_7	non-phage	0
NOF005_scaffold8796_1	non-phage	0
NOF005_scaffold13281_1	non-phage	0
NOF005_scaffold28906_4	non-phage	0
NOF005_scaffold34820_1	non-phage	0
NOF005_scaffold29140_8	non-phage	0
NOF005_scaffold173_17	non-phage	0
NOF005_scaffold31608_1	non-phage	0
NOF005_scaffold15244_5	non-phage	0
NOF005_scaffold4_4	non-phage	0
NOF005_scaffold17404_2	non-phage	0
NOF005_scaffold14214_1	non-phage	0
NOF005_scaffold27640_3	non-phage	0
NOF005_scaffold29675_4	non-phage	0
NOF005_scaffold18361_1	non-phage	0
NOF005_scaffold14441_21	non-phage	0
NOF005_scaffold9379_3	non-phage	0
NOF005_scaffold35291_7	non-phage	0
NOF005_scaffold19213_9	non-phage	0
NOF005_scaffold17197_6	non-phage	0
NOF005_scaffold51971_1	non-phage	0
NOF005_scaffold339_1	non-phage	0
NOF005_scaffold32351_1	non-phage	0
NOF005_scaffold13825_3	non-phage	0
NOF005_scaffold3_3	non-phage	0
NOF005_scaffold23835_8	non-phage	0
NOF005_scaffold39611_1	non-phage	0
NOF005_scaffold52397_1	non-phage	0
NOF005_scaffold52460_6	non-phage	0
NOF005_scaffold52453_3	non-phage	0
NOF005_scaffold52189_1	non-phage	0
NOF005_scaffold23281_4	non-phage	0

NOF005_C713074_1	non-phage	0
NOF005_scaffold113_2	non-phage	0
NOF005_scaffold13806_3	non-phage	0
NOF005_scaffold51606_4	non-phage	0
NOF005_scaffold3253_1	non-phage	0
NOF005_scaffold31594_1	non-phage	0
NOF005_scaffold26996_1	non-phage	0
NOF005_scaffold16265_8	non-phage	0
NOF005_scaffold2521_15	non-phage	0
NOF005_scaffold4532_3	non-phage	0
NOF005_scaffold34775_5	non-phage	0
NOF005_scaffold4_3	non-phage	0
NOF005_scaffold31389_3	non-phage	0
NOF005_scaffold13942_5	non-phage	0
NOF005_scaffold12003_1	non-phage	0
NOF005_scaffold34797_4	non-phage	0
NOF005_scaffold4131_2	non-phage	0
NOF005_scaffold31189_1	non-phage	0
NOF005_scaffold4946_5	non-phage	0
NOF005_scaffold52323_1	non-phage	0
NOF005_C712924_1	non-phage	0
NOF005_scaffold31594_12	non-phage	0
NOF005_scaffold12064_1	non-phage	0
NOF005_scaffold13462_4	phage	0.998
NOF005_scaffold27002_5	non-phage	0
NOF005_scaffold31199_4	non-phage	0
NOF005_scaffold51004_3	non-phage	0
NOF005_scaffold29757_3	non-phage	0
NOF005_scaffold38135_1	non-phage	0
NOF005_scaffold34324_3	non-phage	0
NOF005_scaffold8276_1	non-phage	0
NOF005_scaffold35914_3	non-phage	0
NOF005_scaffold45429_1	non-phage	0
NOF005_scaffold24626_10	non-phage	0
NOF005_scaffold2025_2	phage	0.998
NOF005_scaffold52460_3	non-phage	0
NOF005_scaffold17404_6	non-phage	0
NOF005_scaffold13408_4	non-phage	0
NOF005_C713380_1	non-phage	0
NOF005_scaffold1167_21	non-phage	0
NOF005_scaffold15324_6	non-phage	0
NOF005_scaffold48580_5	non-phage	0
NOF005_scaffold21878_5_2	non-phage	0
NOF005_scaffold45002_3	non-phage	0
NOF005_scaffold24363_5	non-phage	0
NOF005_scaffold37284_1	non-phage	0

NOF005_scaffold5970_1	non-phage	0
NOF005_scaffold52455_1	non-phage	0
NOF005_scaffold165_9	non-phage	0
NOF005_scaffold15630_4	non-phage	0
NOF005_scaffold13104_1	non-phage	0
NOF005_scaffold35638_2	non-phage	0
NOF005_scaffold52106_1	phage	0.995
NOF005_scaffold458_5	non-phage	0
NOF005_scaffold44770_1	non-phage	0
NOF005_scaffold24616_16	non-phage	0
NOF005_scaffold24616_25	non-phage	0
NOF005_scaffold24314_2	non-phage	0
NOF005_scaffold24576_3	non-phage	0
NOF005_scaffold22290_1	non-phage	0
NOF005_scaffold34100_2	non-phage	0
NOF005_scaffold207_1	non-phage	0
NOF005_scaffold12064_4	non-phage	0
NOF005_scaffold7564_3	non-phage	0
NOF005_scaffold51004_1	non-phage	0
NOF005_scaffold1551_5	non-phage	0
NOF005_scaffold40338_1	non-phage	0
NOF005_scaffold15292_2	non-phage	0
NOF005_scaffold30863_1	non-phage	0
NOF005_scaffold8708_15	non-phage	0
NOF005_scaffold205_1	non-phage	0
NOF005_scaffold1032_53	non-phage	0
NOF005_scaffold6206_5	non-phage	0
NOF005_scaffold67_2	non-phage	0
NOF005_scaffold30881_6	non-phage	0
NOF005_scaffold7898_5	non-phage	0
NOF005_scaffold24419_3	non-phage	0
NOF005_scaffold50581_6	non-phage	0
NOF005_scaffold50658_1	non-phage	0
NOF005_scaffold26345_1	non-phage	0
NOF005_scaffold44749_5	non-phage	0
NOF005_scaffold5564_17	non-phage	0
NOF005_scaffold38960_3	non-phage	0
NOF005_scaffold15759_1	non-phage	0
NOF005_scaffold44241_2	non-phage	0
NOF005_scaffold46077_3	non-phage	0
NOF005_scaffold31_2	non-phage	0
NOF005_scaffold173_14	non-phage	0
NOF005_scaffold604_13	phage	0.999
NOF005_scaffold7652_2	non-phage	0
NOF005_scaffold32199_3	non-phage	0
NOF005_scaffold39972_12	non-phage	0

NOF005_scaffold45351_5	non-phage	0
NOF005_scaffold41924_10	non-phage	0
NOF005_scaffold35736_8	non-phage	0
NOF005_scaffold51228_1	non-phage	0
NOF005_scaffold32883_3	non-phage	0
NOF005_scaffold28275_12	non-phage	0
NOF005_scaffold27002_8	non-phage	0
NOF005_scaffold48705_1	non-phage	0
NOF005_scaffold43830_1	non-phage	0
NOF005_scaffold4532_7	non-phage	0
NOF005_scaffold50121_1	non-phage	0
NOF005_scaffold4548_1	non-phage	0
NOF005_scaffold34129_1	non-phage	0
NOF005_scaffold1046_6	non-phage	0
NOF005_scaffold394_2	non-phage	0.418
NOF005_scaffold44810_2	non-phage	0
NOF005_scaffold21663_6	non-phage	0
NOF005_scaffold12434_6	non-phage	0
NOF005_scaffold11432_2	non-phage	0
NOF006_scaffold3975_2	non-phage	0
NOF006_scaffold18125_2	non-phage	0
NOF006_scaffold232_15	non-phage	0
NOF006_scaffold20868_1	non-phage	0
NOF006_scaffold17694_1	non-phage	0
NOF006_scaffold2261_2	non-phage	0
NOF006_C401282_1	non-phage	0
NOF006_scaffold497_1	non-phage	0
NOF006_scaffold19955_1	non-phage	0
NOF006_scaffold5367_3	non-phage	0
NOF006_scaffold5526_7	non-phage	0
NOF006_scaffold18845_3	non-phage	0
NOF006_scaffold11720_1	non-phage	0
NOF006_scaffold1685_20	non-phage	0
NOF006_scaffold13887_3	non-phage	0
NOF006_scaffold21097_3	non-phage	0
NOF006_scaffold7818_8	non-phage	0
NOF006_scaffold108_1	non-phage	0
NOF006_scaffold10363_1	phage	0.999
NOF006_scaffold9997_2	non-phage	0
NOF006_scaffold13045_3	non-phage	0
NOF006_scaffold1687_4	non-phage	0
NOF006_scaffold6165_1	non-phage	0
NOF006_scaffold21296_2	non-phage	0
NOF006_scaffold6724_39	non-phage	0
NOF006_scaffold16536_2	non-phage	0
NOF006_scaffold6993_6	non-phage	0

NOF006_scaffold1270_1	non-phage	0
NOF006_scaffold11076_1	non-phage	0
NOF006_scaffold14846_1	non-phage	0
NOF006_scaffold21165_1	non-phage	0
NOF006_scaffold21223_4	non-phage	0
NOF006_scaffold969_7	non-phage	0
NOF006_scaffold365_46	non-phage	0
NOF006_scaffold13154_1	non-phage	0
NOF006_scaffold12618_2	non-phage	0
NOF006_scaffold14021_4	non-phage	0
NOF006_scaffold12627_2	non-phage	0
NOF006_scaffold4382_3	non-phage	0
NOF006_scaffold41_24	non-phage	0
NOF006_scaffold20916_2	non-phage	0
NOF006_scaffold12203_2	non-phage	0
NOF006_scaffold5428_6	non-phage	0
NOF006_scaffold21148_1	non-phage	0
NOF006_scaffold1721_5	non-phage	0
NOF006_scaffold20410_1	non-phage	0
NOF006_scaffold21215_1	non-phage	0
NOF006_scaffold10239_3	non-phage	0
NOF006_scaffold475_17	non-phage	0
NOF006_scaffold10092_1	non-phage	0
NOF006_scaffold208_7	non-phage	0
NOF006_scaffold8110_9	non-phage	0
NOF006_scaffold1580_8	non-phage	0
NOF006_scaffold249_1	non-phage	0
NOF006_scaffold20901_1	non-phage	0
NOF006_scaffold21255_2	non-phage	0
NOF006_scaffold15471_2	non-phage	0.003
NOF006_scaffold15983_2	non-phage	0
NOF006_scaffold18451_1	non-phage	0
NOF006_scaffold7367_2	non-phage	0
NOF006_scaffold232_5	non-phage	0
NOF006_scaffold19655_2	non-phage	0
NOF006_scaffold173_2	non-phage	0
NOF006_scaffold17016_1	non-phage	0
NOF006_scaffold1349_6	non-phage	0
NOF006_scaffold20990_1	non-phage	0
NOF006_scaffold16648_1	non-phage	0
NOF006_scaffold15833_1	phage	0.992
NOF006_scaffold16494_1	non-phage	0
NOF006_scaffold16944_1	non-phage	0
NOF006_scaffold10207_3	non-phage	0
NOF006_scaffold5164_1	non-phage	0
NOF006_scaffold20505_1	non-phage	0

NOF006_scaffold365_51	non-phage	0
NOF006_scaffold7931_29	non-phage	0
NOF006_scaffold475_12	non-phage	0
NOF006_scaffold114_2	non-phage	0
NOF006_scaffold93_2	non-phage	0
NOF006_scaffold1103_1	non-phage	0
NOF006_scaffold10075_3	non-phage	0
NOF006_scaffold9915_3	non-phage	0
NOF006_scaffold7257_2	non-phage	0
NOF006_scaffold1178_10	non-phage	0
NOF006_scaffold10859_3	non-phage	0
NOF006_scaffold320_43	non-phage	0
NOF006_scaffold21259_4	non-phage	0
NOF006_scaffold21287_1	non-phage	0
NOF006_scaffold1867_1	non-phage	0
NOF006_scaffold1740_4	non-phage	0
NOF006_scaffold13832_2	non-phage	0
NOF006_scaffold18451_4	non-phage	0
NOF006_scaffold14676_2	non-phage	0
NOF006_scaffold12504_3	non-phage	0
NOF006_scaffold728_2	non-phage	0
NOF006_scaffold19778_3	non-phage	0
NOF006_scaffold232_8	non-phage	0
NOF006_scaffold5526_6	non-phage	0
NOF006_scaffold13584_1	non-phage	0
NOF006_scaffold6724_18	non-phage	0
NOF006_scaffold11817_2	non-phage	0
NOF006_scaffold8622_2	non-phage	0
NOF006_scaffold7557_1	non-phage	0
NOF006_scaffold134_4	non-phage	0
NOF006_scaffold3920_4	non-phage	0
NOF006_scaffold365_42	non-phage	0
NOF006_scaffold93_3	non-phage	0
NOF006_scaffold1001_5	non-phage	0
NOF006_scaffold3079_4	non-phage	0
NOF006_scaffold21141_1	non-phage	0
NOF006_scaffold10955_1	non-phage	0
NOF006_scaffold3468_24	non-phage	0
NOF006_scaffold9104_1	non-phage	0
NOF006_scaffold21099_1	non-phage	0
NOF006_scaffold14499_10	non-phage	0
NOF006_scaffold20073_2	non-phage	0
NOF006_scaffold16610_1	non-phage	0
NOF006_scaffold9025_1	non-phage	0
NOF006_scaffold21197_2	non-phage	0
NOF006_scaffold1270_5	non-phage	0

NOF006_scaffold19869_4	non-phage	0
NOF006_scaffold6111_7	phage	0.999
NOF006_scaffold21373_1	non-phage	0
NOF006_scaffold15703_1	non-phage	0
NOF006_scaffold722_23	non-phage	0
NOF006_scaffold155_3	non-phage	0
NOF006_scaffold1905_23	non-phage	0
NOF006_scaffold1200_8	non-phage	0
NOF006_scaffold14518_2	non-phage	0
NOF006_C401092_1	non-phage	0
NOF006_C400976_1	non-phage	0
NOF006_scaffold4868_1	phage	0.999
NOF006_scaffold4162_1	non-phage	0
NOF006_scaffold7818_4	non-phage	0
NOF006_scaffold8378_13	non-phage	0
NOF006_scaffold978_29	non-phage	0
NOF006_scaffold448_23	non-phage	0
NOF006_scaffold18265_2	non-phage	0
NOF006_scaffold232_10	non-phage	0
NOF006_scaffold9547_4	non-phage	0
NOF006_scaffold915_2	non-phage	0
NOF006_scaffold6724_54	non-phage	0
NOF006_scaffold14650_6	non-phage	0
NOF006_scaffold21350_2	non-phage	0
NOF006_scaffold7033_1	non-phage	0
NOF006_scaffold19824_6	non-phage	0
NOF006_scaffold8759_6	non-phage	0
NOF006_scaffold992_36	non-phage	0
NOF006_scaffold15829_1	non-phage	0
NOF006_scaffold21364_4	non-phage	0
NOF006_scaffold7267_4	non-phage	0
NOF006_scaffold21352_1	phage	0.999
NOF006_scaffold11629_1	non-phage	0
NOF006_scaffold19461_2	non-phage	0
NOF006_scaffold21138_1	non-phage	0
NOF006_scaffold1349_3	non-phage	0
NOF006_scaffold475_13	non-phage	0
NOF006_scaffold6325_4	non-phage	0
NOF006_scaffold3056_1	non-phage	0
NOF006_scaffold3433_3	non-phage	0
NOF006_scaffold10_1	non-phage	0
NOF006_scaffold21197_4	non-phage	0
NOF006_scaffold20457_4	non-phage	0
NOF006_scaffold10298_1	non-phage	0
NOF006_scaffold21369_1	non-phage	0
NOF006_scaffold11258_1	non-phage	0

NOF006_scaffold10859_6	non-phage	0
NOF006_scaffold10569_1	non-phage	0
NOF006_scaffold21336_2	non-phage	0
NOF006_scaffold1424_1	non-phage	0
NOF006_scaffold11114_3	non-phage	0
NOF006_scaffold21088_1	non-phage	0
NOF006_scaffold21349_2	non-phage	0
NOF006_scaffold8110_4	non-phage	0
NOF006_scaffold3902_6	non-phage	0
NOF006_scaffold112_9	non-phage	0
NOF006_scaffold19824_1	non-phage	0
NOF006_scaffold17694_3	non-phage	0
NOF006_scaffold95_4	non-phage	0
NOF006_scaffold18080_2	non-phage	0
NOF006_scaffold14950_3	non-phage	0
NOF006_scaffold21334_1	non-phage	0
NOF006_scaffold365_23	non-phage	0
NOF006_scaffold12718_7	phage	0.86
NOF006_scaffold21375_1	non-phage	0
NOF006_scaffold13326_7	non-phage	0
NOF006_scaffold19133_2	non-phage	0
NOF006_scaffold16930_1	non-phage	0.05
NOF006_scaffold18869_1	non-phage	0
NOF006_scaffold18869_2	non-phage	0
NOF006_scaffold20916_1	non-phage	0
NOF006_scaffold498_1	phage	1
NOF006_C401400_1	non-phage	0
NOF006_scaffold1541_18	non-phage	0
NOF006_scaffold21223_2	phage	1
NOF006_scaffold475_11	non-phage	0
NOF006_C401544_1	non-phage	0
NOF006_scaffold21371_1	non-phage	0
NOF006_scaffold20771_3	non-phage	0
NOF006_scaffold21350_5	non-phage	0
NOF006_scaffold232_6	non-phage	0
NOF006_scaffold14489_1	non-phage	0
NOF006_scaffold131_2	non-phage	0
NOF006_scaffold14945_3	non-phage	0
NOF006_scaffold4847_3	non-phage	0
NOF006_scaffold138_2	non-phage	0
NOF006_scaffold7220_1	non-phage	0
NOF006_scaffold15936_1	non-phage	0
NOF006_scaffold121_1	phage	0.995
NOF006_scaffold15841_2	non-phage	0
NOF006_scaffold21242_4	phage	0.961
NOF006_scaffold21130_1	non-phage	0

NOF006_scaffold2261_1	non-phage	0
NOF006_scaffold1740_2	phage	0.998
NOF006_scaffold31_6	non-phage	0
NOF006_scaffold15392_1	non-phage	0
NOF006_scaffold6876_7	non-phage	0
NOF006_scaffold320_37	non-phage	0
NOF006_scaffold1680_1	non-phage	0
NOF006_scaffold9547_2	non-phage	0
NOF006_scaffold7184_5	non-phage	0
NOF006_scaffold12504_2	non-phage	0
NOF006_scaffold108_18	non-phage	0
NOF006_scaffold10075_2	non-phage	0
NOF006_scaffold26_7	non-phage	0
NOF006_scaffold15471_3	non-phage	0
NOF006_scaffold21306_1	non-phage	0
NOF006_scaffold20670_2	non-phage	0
NOF006_scaffold20073_3	non-phage	0
NOF006_scaffold19778_2	non-phage	0
NOF006_scaffold114_1	non-phage	0
NOF006_scaffold1435_2	non-phage	0
NOF006_scaffold365_41	non-phage	0
NOF006_scaffold21362_1	non-phage	0
NOF006_scaffold4340_3	non-phage	0
NOF006_scaffold21366_4	non-phage	0
NOF006_scaffold21337_1	non-phage	0
NOF006_scaffold21276_3	non-phage	0
NOF006_scaffold1685_55	non-phage	0.129
NOF006_scaffold2842_16	phage	0.917
NOF006_scaffold12207_2	non-phage	0
NOF006_scaffold1372_2	non-phage	0
NOF006_scaffold14086_9	non-phage	0
NOF006_scaffold18265_1	non-phage	0
NOF006_scaffold19983_2	phage	0.988
NOF006_scaffold3495_2	non-phage	0
NOF006_scaffold14098_1	non-phage	0
NOF006_scaffold20013_1	non-phage	0
NOF006_scaffold13772_1	non-phage	0
NOF006_scaffold7257_1	non-phage	0
NOF006_scaffold175_3	non-phage	0
NOF006_scaffold5526_3	non-phage	0
NOF006_scaffold21197_1	non-phage	0
NOF006_scaffold3597_6	non-phage	0
NOF006_scaffold14475_3	phage	0.999
NOF006_scaffold21287_2	non-phage	0
NOF006_scaffold21333_1	non-phage	0
NOF006_scaffold365_31	non-phage	0

NOF006_scaffold18646_2	non-phage	0
NOF006_scaffold5807_15	non-phage	0
NOF006_scaffold20133_2	non-phage	0
NOF006_scaffold16015_3	non-phage	0
NOF006_scaffold16915_2	non-phage	0
NOF006_scaffold6876_2	non-phage	0
NOF006_scaffold20817_2	non-phage	0
NOF006_scaffold16479_1	non-phage	0
NOF006_scaffold1541_10	non-phage	0
NOF006_scaffold21350_1	non-phage	0
NOF006_scaffold1488_2	non-phage	0
NOF006_scaffold21092_1	non-phage	0
NOF006_scaffold18779_1	non-phage	0
NOF006_scaffold18622_2	non-phage	0
NOF006_scaffold19121_1	non-phage	0
NOF006_scaffold18796_1	non-phage	0
NOF006_scaffold969_6	non-phage	0
NOF006_scaffold8498_1	non-phage	0
NOF006_scaffold13326_4	non-phage	0
NOF006_scaffold8378_14	non-phage	0
NOF006_scaffold11693_1	non-phage	0
NOF006_scaffold3597_7	non-phage	0
NOF006_scaffold1721_2	non-phage	0
NOF006_scaffold131_1	non-phage	0
NOF006_scaffold21242_3	non-phage	0
NOF006_scaffold1388_27	non-phage	0
NOF006_scaffold13887_7	non-phage	0
NOF006_scaffold10063_3	non-phage	0
NOF006_scaffold16658_1	non-phage	0
NOF006_scaffold1200_11	non-phage	0
NOF006_scaffold1022_7	phage	0.999
NOF006_scaffold10165_2	non-phage	0
NOF006_scaffold15359_1	non-phage	0
NOF006_scaffold8110_3	non-phage	0
NOF006_scaffold10075_6	non-phage	0
NOF006_scaffold18451_5	non-phage	0
NOF006_scaffold21343_1	non-phage	0
NOF006_scaffold20896_2	non-phage	0
NOF006_scaffold7696_1	non-phage	0
NOF006_scaffold16916_2	non-phage	0
NOF006_scaffold19565_1	non-phage	0
NOF006_scaffold9749_2	non-phage	0
NOF006_scaffold17698_6	non-phage	0
NOF006_scaffold1219_1	non-phage	0
NOF006_scaffold57_3	non-phage	0
NOF006_scaffold11132_5	non-phage	0

NOF006_scaffold1605_1	non-phage	0
NOF006_scaffold21353_1	non-phage	0
NOF006_scaffold13100_4	non-phage	0
NOF006_scaffold18658_4	non-phage	0
NOF006_scaffold17142_1	non-phage	0
NOF006_scaffold134_3	non-phage	0
NOF006_scaffold8110_7	non-phage	0
NOF006_scaffold3853_1	non-phage	0
NOF006_scaffold10433_1	non-phage	0
NOF006_scaffold12718_8	non-phage	0
NOF006_scaffold138_3	non-phage	0
NOF006_scaffold7655_1	non-phage	0
NOF006_scaffold365_65	non-phage	0
NOF006_scaffold21349_1	non-phage	0
NOF006_scaffold9028_6	non-phage	0
NOF006_scaffold199_2	non-phage	0
NOF006_scaffold10673_1	non-phage	0
NOF006_scaffold1200_13	non-phage	0
NOF006_scaffold19290_1	non-phage	0
NOF006_scaffold18771_1	non-phage	0
NOF006_scaffold12504_4	non-phage	0
NOF006_scaffold4067_2	non-phage	0
NOF006_scaffold18328_2	non-phage	0
NOF006_scaffold6325_6	non-phage	0
NOF006_scaffold6724_40	non-phage	0
NOF006_scaffold1372_3	non-phage	0
NOF006_scaffold5504_1	non-phage	0
NOF006_scaffold6732_6	non-phage	0
NOF006_scaffold21065_1	non-phage	0
NOF006_scaffold15271_1	non-phage	0
NOF006_scaffold3433_4	non-phage	0
NOF006_scaffold1349_4	non-phage	0
NOF006_scaffold13887_1	non-phage	0
NOF006_scaffold10063_1	non-phage	0
NOF006_scaffold15444_2	non-phage	0
NOF006_scaffold18845_5	non-phage	0
NOF006_scaffold225_2	non-phage	0
NOF006_scaffold15829_3	non-phage	0
NOF006_scaffold21364_2	non-phage	0
NOF006_scaffold2938_1	non-phage	0
NOF006_scaffold1103_3	non-phage	0
NOF006_scaffold259_1	non-phage	0
NOF006_scaffold20868_3	non-phage	0
NOF006_scaffold19167_1	non-phage	0
NOF006_scaffold6849_4	non-phage	0
NOF006_scaffold15062_1	non-phage	0

NOF006_scaffold4989_3	non-phage	0
NOF006_scaffold13100_1	non-phage	0
NOF006_scaffold9915_2	non-phage	0
NOF006_scaffold20896_1	non-phage	0
NOF006_scaffold8762_3	non-phage	0
NOF006_scaffold4214_1	non-phage	0
NOF006_scaffold21338_1	non-phage	0
NOF006_scaffold12961_2	non-phage	0
NOF006_scaffold21257_1	non-phage	0
NOF006_scaffold14508_1	non-phage	0
NOF006_scaffold10075_1	non-phage	0
NOF006_scaffold9547_3	non-phage	0
NOF006_scaffold10859_5	non-phage	0
NOF006_scaffold1349_8	non-phage	0
NOF006_scaffold6724_34	non-phage	0
NOF006_scaffold18354_1	non-phage	0
NOF006_scaffold4865_2	non-phage	0
NOF006_scaffold15457_1	non-phage	0
NOF006_scaffold1270_7	non-phage	0
NOF006_scaffold8311_1	non-phage	0
NOF006_scaffold8614_4	non-phage	0
NOF006_scaffold21197_5	non-phage	0
NOF006_scaffold13887_8	non-phage	0
NOF006_scaffold21337_2	non-phage	0
NOF006_scaffold20670_1	non-phage	0
NOF006_scaffold1200_3	non-phage	0
NOF006_scaffold1858_1	non-phage	0
NOF006_scaffold10673_3	non-phage	0
NOF006_scaffold15847_3	non-phage	0
NOF006_scaffold1135_33	non-phage	0
NOF006_scaffold20952_1	non-phage	0
NOF006_scaffold15585_2	non-phage	0
NOF006_scaffold8543_1	non-phage	0
NOF006_scaffold155_1	non-phage	0
NOF006_scaffold12693_1	non-phage	0
NOF006_scaffold11309_3	non-phage	0
NOF006_scaffold241_7	non-phage	0
NOF006_scaffold14499_8	phage	0.995
NOF006_scaffold10461_1	non-phage	0
NOF006_scaffold18561_1	non-phage	0
NOF006_scaffold5882_1	non-phage	0
NOF006_scaffold19824_4	non-phage	0
NOF006_scaffold365_61	non-phage	0
NOF006_scaffold1452_1	non-phage	0
NOF006_scaffold21372_2	non-phage	0
NOF006_scaffold9076_17	non-phage	0

NOF006_scaffold21012_1	phage	0.996
NOF006_scaffold1937_13	non-phage	0
NOF006_scaffold17430_1	non-phage	0
NOF006_C400832_1	non-phage	0
NOF006_scaffold16261_4	non-phage	0
NOF006_scaffold15425_1	non-phage	0
NOF006_scaffold8762_4	non-phage	0
NOF006_scaffold20457_5	non-phage	0
NOF006_scaffold17181_2	non-phage	0
NOF006_scaffold2230_2	non-phage	0
NOF006_scaffold1349_1	non-phage	0
NOF006_scaffold21296_1	non-phage	0
NOF006_scaffold16597_1	non-phage	0
NOF006_scaffold978_27	non-phage	0
NOF006_scaffold4067_5	phage	0.999
NOF006_scaffold304_15	non-phage	0
NOF006_scaffold9915_5	non-phage	0
NOF006_scaffold914_3	non-phage	0
NOF006_scaffold21370_1	non-phage	0
NOF006_scaffold20915_2	non-phage	0
NOF006_scaffold31_1	non-phage	0
NOF006_scaffold13887_4	non-phage	0
NOF006_scaffold15352_2	non-phage	0
NOF006_scaffold19565_2	non-phage	0
NOF006_scaffold15074_6	non-phage	0
NOF006_scaffold10075_5	non-phage	0
NOF006_scaffold10859_1	non-phage	0
NOF006_scaffold978_17	non-phage	0
NOF006_scaffold21232_1	non-phage	0
NOF006_scaffold20915_1	phage	0.999
NOF006_scaffold3468_15	non-phage	0
NOF006_scaffold132_1	non-phage	0
NOF006_scaffold8938_1	non-phage	0
NOF006_scaffold365_11	non-phage	0
NOF006_scaffold18658_5	non-phage	0
NOF006_scaffold4872_3	phage	0.997
NOF006_scaffold10422_1	non-phage	0
NOF006_scaffold978_23	non-phage	0
NOF006_scaffold121_3	non-phage	0
NOF006_scaffold12570_1	non-phage	0
NOF006_scaffold73_1	non-phage	0
NOF006_scaffold14846_3	non-phage	0.001
NOF006_scaffold10435_8	non-phage	0
NOF006_scaffold1685_54	non-phage	0
NOF006_scaffold7372_16	non-phage	0
NOF006_scaffold15449_1	non-phage	0

NOF006_scaffold6849_3	non-phage	0
NOF006_scaffold6647_1	non-phage	0
NOF006_scaffold15841_3	non-phage	0
NOF006_scaffold969_5	non-phage	0
NOF006_scaffold1001_3	non-phage	0
NOF006_scaffold4088_1	non-phage	0
NOF006_scaffold1108_8	non-phage	0
NOF006_scaffold20844_1	non-phage	0
NOF006_scaffold21278_1	non-phage	0
NOF006_scaffold21173_5	non-phage	0
NOF006_scaffold21323_1	non-phage	0
NOF006_scaffold3468_4	non-phage	0
NOF006_scaffold1685_30	non-phage	0
NOF006_scaffold9022_4	non-phage	0
NOF006_scaffold21175_1	non-phage	0
NOF006_scaffold1687_5	non-phage	0
NOF006_scaffold8755_2	non-phage	0
NOF006_scaffold15132_1	non-phage	0
NOF006_scaffold6138_2	non-phage	0
NOF006_scaffold475_21	non-phage	0
NOF006_scaffold232_4	non-phage	0
NOF006_scaffold2050_6	non-phage	0
NOF006_scaffold969_8	phage	0.998
NOF006_scaffold31_7	non-phage	0
NOF006_scaffold4865_1	non-phage	0
NOF006_scaffold9076_14	non-phage	0
NOF006_scaffold14827_2	non-phage	0
NOF006_scaffold1200_12	non-phage	0
NOF006_scaffold112_14	non-phage	0
NOF006_C401108_1	phage	0.999
NOF006_scaffold199_1	non-phage	0
NOF006_scaffold13100_2	non-phage	0
NOF006_scaffold10239_1	non-phage	0
NOF006_scaffold19258_2	non-phage	0
NOF006_scaffold12627_1	non-phage	0
NOF006_scaffold21201_1	non-phage	0
NOF006_scaffold5511_9	non-phage	0.001
NOF006_scaffold8110_17	non-phage	0
NOF006_scaffold21342_1	non-phage	0
NOF006_scaffold4865_6	non-phage	0
NOF006_scaffold7367_1	non-phage	0
NOF006_scaffold6724_27	non-phage	0
NOF006_scaffold20013_2	non-phage	0
NOF006_scaffold365_28	non-phage	0
NOF006_scaffold20619_1	non-phage	0
NOF006_scaffold21309_2	non-phage	0

NOF006_scaffold123_1	non-phage	0
NOF006_scaffold1349_7	non-phage	0
NOF006_scaffold6724_33	non-phage	0
NOF006_scaffold20555_1	non-phage	0
NOF006_scaffold2050_15	non-phage	0
NOF006_scaffold134_5	non-phage	0
NOF006_scaffold21018_1	non-phage	0
NOF006_scaffold16648_2	non-phage	0
NOF006_scaffold21374_2	phage	0.902
NOF006_scaffold21099_2	non-phage	0
NOF006_scaffold20457_2	non-phage	0
NOF006_scaffold1580_5	non-phage	0
NOF006_scaffold15661_2	non-phage	0
NOF006_scaffold19824_3	non-phage	0
NOF006_scaffold4340_1	non-phage	0
NOF006_scaffold6558_3	non-phage	0
NOF006_scaffold134_2	non-phage	0
NOF006_scaffold17603_1	non-phage	0
NOF006_scaffold1416_2	non-phage	0
NOF006_scaffold5367_1	non-phage	0
NOF006_scaffold10239_4	non-phage	0
NOF006_scaffold9028_3	non-phage	0
NOF006_scaffold20868_2	phage	0.999
NOF006_scaffold10859_4	non-phage	0
NOF006_scaffold10175_1	non-phage	0
NOF006_scaffold2369_47	non-phage	0
NOF006_scaffold5807_11	non-phage	0
NOF006_scaffold15226_1	non-phage	0
NOF006_scaffold7818_10	non-phage	0
NOF006_scaffold192_2	non-phage	0
NOF006_scaffold238_1	non-phage	0
NOF006_scaffold12165_1	non-phage	0
NOF006_scaffold3853_2	non-phage	0
NOF006_scaffold7824_1	non-phage	0
NOF006_scaffold8592_2	non-phage	0
NOF006_scaffold1200_5	non-phage	0
NOF006_scaffold6849_2	non-phage	0
NOF006_scaffold6574_1	non-phage	0
NOF006_scaffold8110_13	non-phage	0
NOF006_C401404_1	non-phage	0
NOF006_scaffold15056_1	non-phage	0
NOF006_scaffold21221_1	non-phage	0
NOF006_scaffold16879_1	non-phage	0
NOF006_scaffold1605_6	non-phage	0
NOF006_scaffold5504_4	non-phage	0
NOF006_scaffold15535_3	non-phage	0

NOF006_scaffold3433_2	non-phage	0
NOF006_scaffold10038_1	non-phage	0
NOF006_scaffold7805_2	non-phage	0
NOF006_scaffold21173_1	non-phage	0
NOF006_scaffold5537_1	non-phage	0
NOF006_scaffold21173_4	non-phage	0
NOF006_scaffold13887_5	non-phage	0
NOF006_scaffold20916_3	non-phage	0
NOF006_scaffold21242_5	non-phage	0
NOF006_scaffold1680_4	non-phage	0.154
NOF006_scaffold365_6	non-phage	0
NOF006_scaffold10075_4	non-phage	0
NOF006_scaffold205_1	non-phage	0
NOF006_scaffold1135_13	non-phage	0
NOF006_scaffold20705_1	non-phage	0
NOF006_scaffold1103_2	non-phage	0
NOF006_scaffold232_7	non-phage	0
NOF006_scaffold18451_3	non-phage	0
NOF006_scaffold21372_1	non-phage	0
NOF006_scaffold20326_1	non-phage	0
NOF006_scaffold15585_3	non-phage	0
NOF006_scaffold18658_2	non-phage	0
NOF006_scaffold6732_2	non-phage	0
NOF006_scaffold4847_2	non-phage	0
NOF006_scaffold21354_1	non-phage	0
NOF006_scaffold8622_3	non-phage	0
NOF006_scaffold241_8	non-phage	0
NOF006_scaffold2080_13	non-phage	0
NOF006_scaffold10175_4	non-phage	0
NOF006_scaffold13932_2	non-phage	0
NOF006_scaffold19824_7	non-phage	0
NOF006_scaffold31_2	non-phage	0
NOF006_scaffold173_3	phage	0.66
NOF006_scaffold73_2	non-phage	0
NOF006_scaffold16_1	non-phage	0
NOF006_scaffold20457_6	non-phage	0
NOF006_scaffold9997_6	non-phage	0
NOF006_scaffold21364_3	non-phage	0
NOF006_scaffold20960_1	non-phage	0
NOF006_scaffold20670_4	non-phage	0
NOF006_scaffold61_4	non-phage	0
NOF006_scaffold11346_1	non-phage	0
NOF006_scaffold2061_1	non-phage	0
NOF006_scaffold12718_6	non-phage	0
NOF006_scaffold19943_4	non-phage	0
NOF006_scaffold17694_5	non-phage	0

NOF006_scaffold304_14	non-phage	0
NOF006_scaffold5751_2	non-phage	0
NOF006_scaffold20073_5	non-phage	0
NOF006_scaffold4067_4	non-phage	0
NOF006_scaffold94_3	non-phage	0
NOF006_scaffold15635_6	non-phage	0
NOF006_scaffold18704_1	non-phage	0
NOF006_scaffold249_4	non-phage	0
NOF006_scaffold16894_1	non-phage	0
NOF006_scaffold15352_1	non-phage	0
NOF006_scaffold21327_1	non-phage	0
NOF006_scaffold20457_3	non-phage	0
NOF006_scaffold15703_2	non-phage	0
NOF006_scaffold9915_6	non-phage	0
NOF006_C400756_1	non-phage	0
NOF006_scaffold9547_7	non-phage	0
NOF006_scaffold9412_16	non-phage	0
NOF006_scaffold10852_2	non-phage	0
NOF006_scaffold50_1	non-phage	0
NOF006_scaffold5526_5	non-phage	0
NOF006_scaffold232_13	non-phage	0
NOF006_scaffold6359_9	non-phage	0
NOF006_scaffold3441_2	non-phage	0
NOF006_scaffold4143_1	non-phage	0
NOF007_scaffold8758_3	non-phage	0
NOF007_scaffold27213_3	phage	0.998
NOF007_scaffold16388_4	non-phage	0
NOF007_scaffold665_3	non-phage	0
NOF007_scaffold26603_1	non-phage	0
NOF007_scaffold22149_2	non-phage	0
NOF007_scaffold7681_3	non-phage	0
NOF007_scaffold13542_1	non-phage	0
NOF007_scaffold112_2	non-phage	0.002
NOF007_scaffold16936_8	non-phage	0
NOF007_scaffold12398_1	non-phage	0
NOF007_scaffold9535_5	non-phage	0
NOF007_scaffold29022_1	non-phage	0
NOF007_scaffold18376_1	non-phage	0
NOF007_scaffold26406_1	non-phage	0
NOF007_scaffold23476_3	non-phage	0
NOF007_scaffold28735_1	non-phage	0
NOF007_scaffold5752_4	non-phage	0
NOF007_scaffold16936_10	non-phage	0
NOF007_scaffold11903_11	non-phage	0
NOF007_scaffold22566_1	non-phage	0
NOF007_scaffold10423_25	non-phage	0

NOF007_scaffold27110_2	non-phage	0
NOF007_scaffold23976_2	non-phage	0
NOF007_scaffold20582_3	non-phage	0
NOF007_scaffold84_3	non-phage	0
NOF007_C490407_1	non-phage	0
NOF007_scaffold29026_2	non-phage	0
NOF007_scaffold16773_3	phage	0.856
NOF007_scaffold9936_4	non-phage	0
NOF007_scaffold25584_3	non-phage	0
NOF007_scaffold15930_3	non-phage	0
NOF007_scaffold47_1	non-phage	0
NOF007_scaffold1603_52	non-phage	0
NOF007_scaffold11157_11	non-phage	0
NOF007_C490467_1	non-phage	0
NOF007_scaffold29025_1	non-phage	0
NOF007_scaffold23774_3	phage	0.999
NOF007_scaffold1770_5	non-phage	0
NOF007_scaffold4461_7	phage	0.984
NOF007_scaffold28650_2	non-phage	0
NOF007_scaffold28991_1	non-phage	0
NOF007_scaffold8869_1	non-phage	0
NOF007_scaffold28932_1	non-phage	0
NOF007_scaffold28946_5	non-phage	0
NOF007_scaffold10169_7	non-phage	0
NOF007_scaffold28726_2	non-phage	0
NOF007_scaffold2731_3	non-phage	0
NOF007_scaffold67_3	non-phage	0
NOF007_scaffold5255_1	non-phage	0
NOF007_scaffold8553_7	non-phage	0
NOF007_scaffold18580_8	non-phage	0
NOF007_scaffold8229_3	non-phage	0
NOF007_scaffold7015_1	non-phage	0
NOF007_scaffold12493_7	non-phage	0
NOF007_scaffold8086_3	non-phage	0
NOF007_scaffold11866_1	non-phage	0
NOF007_scaffold18263_1	non-phage	0
NOF007_scaffold27210_1	non-phage	0
NOF007_scaffold105_1	non-phage	0
NOF007_scaffold7694_12	non-phage	0
NOF007_scaffold18273_2	non-phage	0
NOF007_scaffold17360_20	non-phage	0
NOF007_scaffold8821_3	non-phage	0
NOF007_scaffold6134_4	non-phage	0
NOF007_scaffold26017_8	non-phage	0
NOF007_scaffold27992_2	non-phage	0
NOF007_scaffold26933_1	non-phage	0

NOF007_scaffold14749_1	non-phage	0
NOF007_scaffold2442_17	non-phage	0
NOF007_scaffold29023_1	non-phage	0
NOF007_scaffold12708_6	non-phage	0
NOF007_scaffold60_1	non-phage	0
NOF007_scaffold61_1	non-phage	0
NOF007_scaffold13628_3	non-phage	0
NOF007_scaffold17701_3	non-phage	0
NOF007_scaffold28685_4	phage	0.585
NOF007_scaffold28955_2	non-phage	0
NOF007_scaffold12789_1	non-phage	0
NOF007_scaffold28901_2	non-phage	0
NOF007_scaffold9476_9	non-phage	0
NOF007_scaffold12524_3	non-phage	0
NOF007_scaffold28196_2	non-phage	0
NOF007_scaffold21740_3	non-phage	0
NOF007_scaffold396_8	phage	0.999
NOF007_scaffold69_3	non-phage	0
NOF007_scaffold28753_1	non-phage	0
NOF007_scaffold2442_6	non-phage	0
NOF007_scaffold3123_1	non-phage	0
NOF007_scaffold28677_2	non-phage	0
NOF007_scaffold26428_1	non-phage	0
NOF007_scaffold242_1	non-phage	0
NOF007_scaffold28537_4	non-phage	0
NOF007_scaffold6562_4	non-phage	0
NOF007_scaffold23_1	non-phage	0
NOF007_scaffold28465_1	non-phage	0
NOF007_scaffold26160_1	phage	0.999
NOF007_scaffold14333_2	non-phage	0
NOF007_scaffold1499_2	non-phage	0
NOF007_scaffold12399_1	non-phage	0
NOF007_scaffold15830_1	non-phage	0
NOF007_scaffold25027_1	non-phage	0
NOF007_scaffold14373_1	non-phage	0
NOF007_scaffold11484_1	phage	0.999
NOF007_scaffold13567_1	non-phage	0
NOF007_scaffold9136_1	non-phage	0
NOF007_scaffold28979_2	non-phage	0
NOF007_scaffold15950_1	non-phage	0
NOF007_scaffold28950_1	non-phage	0
NOF007_scaffold25262_5	phage	0.997
NOF007_scaffold1568_4	non-phage	0
NOF007_scaffold21771_1	non-phage	0
NOF007_scaffold5848_5	non-phage	0
NOF007_scaffold28931_3	non-phage	0

NOF007_scaffold19985_4	non-phage	0
NOF007_scaffold237_10	non-phage	0
NOF007_scaffold28967_1	non-phage	0
NOF007_scaffold26705_2	non-phage	0
NOF007_scaffold13558_5	non-phage	0
NOF007_scaffold18069_14	non-phage	0
NOF007_scaffold1028_14	non-phage	0
NOF007_scaffold2675_5	non-phage	0
NOF007_scaffold20452_1	non-phage	0.007
NOF007_scaffold28789_1	non-phage	0
NOF007_scaffold21373_1	non-phage	0
NOF007_scaffold2442_1	non-phage	0
NOF007_scaffold21388_2	phage	0.999
NOF007_scaffold17572_2	non-phage	0
NOF007_scaffold4886_21	non-phage	0
NOF007_scaffold23975_1	non-phage	0
NOF007_scaffold18273_7	non-phage	0
NOF007_scaffold23933_1	non-phage	0
NOF007_scaffold8553_3	non-phage	0
NOF007_scaffold12493_6	non-phage	0
NOF007_scaffold28976_2	non-phage	0
NOF007_scaffold4503_5	non-phage	0
NOF007_scaffold7015_5	non-phage	0
NOF007_scaffold5139_1	non-phage	0
NOF007_scaffold9318_2	non-phage	0
NOF007_scaffold28532_3	non-phage	0
NOF007_scaffold202_1	non-phage	0
NOF007_scaffold28965_1	non-phage	0
NOF007_scaffold3135_2	non-phage	0
NOF007_scaffold11302_1	non-phage	0
NOF007_scaffold6686_3	non-phage	0
NOF007_scaffold16724_2	non-phage	0
NOF007_scaffold19168_2	non-phage	0
NOF007_scaffold211_16	non-phage	0
NOF007_scaffold198_7	non-phage	0
NOF007_scaffold16936_1	non-phage	0
NOF007_scaffold25304_1	non-phage	0
NOF007_scaffold3181_10	non-phage	0
NOF007_scaffold9936_3	non-phage	0
NOF007_scaffold19_3	non-phage	0
NOF007_scaffold231_1	non-phage	0
NOF007_scaffold26938_1	non-phage	0
NOF007_scaffold28649_2	non-phage	0
NOF007_scaffold28830_1	non-phage	0
NOF007_scaffold15481_1	non-phage	0
NOF007_scaffold28801_4	non-phage	0

NOF007_scaffold1349_3	phage	0.999
NOF007_scaffold24268_1	non-phage	0
NOF007_C490411_1	non-phage	0
NOF007_scaffold12990_2	non-phage	0
NOF007_scaffold3492_7	non-phage	0
NOF007_scaffold22402_1	non-phage	0
NOF007_scaffold2015_1	non-phage	0
NOF007_scaffold6562_2	non-phage	0
NOF007_scaffold61_5	non-phage	0
NOF007_scaffold22039_1	non-phage	0
NOF007_scaffold17292_54	non-phage	0
NOF007_scaffold13403_2	non-phage	0
NOF007_scaffold25584_1	non-phage	0
NOF007_scaffold16773_4	non-phage	0
NOF007_scaffold1499_11	non-phage	0
NOF007_scaffold8991_5	non-phage	0
NOF007_scaffold26705_1	non-phage	0
NOF007_scaffold3135_1	non-phage	0
NOF007_scaffold21322_2	non-phage	0.044
NOF007_scaffold28532_1	non-phage	0
NOF007_scaffold21771_6	non-phage	0
NOF007_scaffold2058_8	non-phage	0
NOF007_scaffold24313_1	non-phage	0
NOF007_scaffold22741_1	non-phage	0
NOF007_scaffold27056_4	non-phage	0
NOF007_scaffold29027_1	non-phage	0
NOF007_scaffold2058_1	non-phage	0
NOF007_scaffold2015_2	non-phage	0
NOF007_scaffold145_1	non-phage	0
NOF007_scaffold16724_1	non-phage	0
NOF007_scaffold29007_1	non-phage	0
NOF007_scaffold27268_6	non-phage	0
NOF007_scaffold28948_1	non-phage	0
NOF007_scaffold8434_2	non-phage	0
NOF007_scaffold22488_1	non-phage	0
NOF007_scaffold12698_5	non-phage	0
NOF007_scaffold10730_2	non-phage	0
NOF007_scaffold11638_4	non-phage	0
NOF007_scaffold27787_2	non-phage	0
NOF007_scaffold19223_3	non-phage	0
NOF007_scaffold8528_9	non-phage	0
NOF007_scaffold19207_7	non-phage	0
NOF007_scaffold25347_1	non-phage	0
NOF007_scaffold12401_1	non-phage	0
NOF007_scaffold5275_16	non-phage	0
NOF007_scaffold1684_3	non-phage	0

NOF007_scaffold16627_4	non-phage	0
NOF007_scaffold1499_4	non-phage	0
NOF007_scaffold25518_6	non-phage	0
NOF007_scaffold25771_1	non-phage	0
NOF007_scaffold18272_1	non-phage	0
NOF007_scaffold28155_1	non-phage	0
NOF007_scaffold16936_3	non-phage	0
NOF007_scaffold29013_1	non-phage	0
NOF007_scaffold2836_54	non-phage	0
NOF007_scaffold11540_19	non-phage	0
NOF007_scaffold28408_1	non-phage	0
NOF007_scaffold181_6	non-phage	0
NOF007_scaffold18445_2	non-phage	0
NOF007_scaffold12789_2	non-phage	0
NOF007_scaffold237_6	non-phage	0
NOF007_scaffold25888_3	non-phage	0
NOF007_scaffold60_2	non-phage	0
NOF007_scaffold9468_7	non-phage	0
NOF007_scaffold1203_3	non-phage	0
NOF007_C491009_1	non-phage	0
NOF007_scaffold1608_5	non-phage	0
NOF007_scaffold558_1	non-phage	0
NOF007_scaffold13558_13	non-phage	0
NOF007_scaffold28685_5	non-phage	0
NOF007_scaffold24425_1	non-phage	0
NOF007_scaffold17023_1	non-phage	0
NOF007_scaffold321_2	non-phage	0
NOF007_scaffold27414_3	non-phage	0
NOF007_scaffold15830_2	non-phage	0
NOF007_scaffold5941_1	non-phage	0
NOF007_scaffold28896_1	non-phage	0
NOF007_scaffold10599_2	non-phage	0
NOF007_scaffold7948_5	non-phage	0
NOF007_scaffold20325_1	non-phage	0
NOF007_scaffold111_8	non-phage	0
NOF007_C490687_1	phage	0.998
NOF007_scaffold28649_4	non-phage	0
NOF007_scaffold26445_2	non-phage	0
NOF007_scaffold1973_1	non-phage	0
NOF007_scaffold15959_1	non-phage	0
NOF007_scaffold28995_2	non-phage	0
NOF007_scaffold13994_1	non-phage	0
NOF007_scaffold1770_3	non-phage	0
NOF007_scaffold22592_1	non-phage	0
NOF007_scaffold28754_4	non-phage	0
NOF007_scaffold5752_5	non-phage	0

NOF007_scaffold9575_4	non-phage	0
NOF007_scaffold198_9	non-phage	0
NOF007_scaffold22420_1	non-phage	0
NOF007_C490627_1	non-phage	0
NOF007_scaffold25262_4	non-phage	0
NOF007_scaffold19207_2	phage	0.996
NOF007_scaffold2058_5	non-phage	0
NOF007_scaffold28849_3	non-phage	0
NOF007_scaffold3482_8	non-phage	0
NOF007_scaffold6562_1	non-phage	0
NOF007_scaffold669_5	non-phage	0
NOF007_scaffold3112_2	non-phage	0
NOF007_scaffold11638_5	non-phage	0
NOF007_C490763_1	non-phage	0
NOF007_scaffold62_1	non-phage	0
NOF007_scaffold61_6	non-phage	0
NOF007_scaffold17837_7	non-phage	0
NOF007_scaffold1349_2	phage	0.989
NOF007_scaffold237_11	non-phage	0
NOF007_scaffold20582_1	non-phage	0
NOF007_scaffold4503_3	non-phage	0
NOF007_scaffold2462_5	non-phage	0
NOF007_scaffold11971_1	non-phage	0
NOF007_scaffold8821_2	phage	0.998
NOF007_scaffold9187_24	non-phage	0
NOF007_scaffold9051_5	non-phage	0
NOF007_scaffold5672_3	non-phage	0
NOF007_scaffold22149_1	non-phage	0
NOF007_scaffold237_2	non-phage	0
NOF007_scaffold24049_1	non-phage	0
NOF007_scaffold22085_1	non-phage	0
NOF007_scaffold10320_2	non-phage	0
NOF007_scaffold17766_1	non-phage	0
NOF007_C490313_1	non-phage	0
NOF007_scaffold102_2	non-phage	0
NOF007_scaffold659_1	non-phage	0
NOF007_scaffold28585_1	non-phage	0
NOF007_scaffold10169_8	non-phage	0
NOF007_scaffold12777_2	non-phage	0
NOF007_scaffold4780_3	non-phage	0
NOF007_scaffold27822_1	phage	0.97
NOF007_scaffold24025_1	non-phage	0
NOF007_scaffold471_4	non-phage	0
NOF007_scaffold29012_1	non-phage	0
NOF007_scaffold23737_2	non-phage	0
NOF007_scaffold15189_2	phage	1

NOF007_scaffold23819_3	phage	0.974
NOF007_scaffold12990_1	non-phage	0
NOF007_scaffold28826_2	non-phage	0
NOF007_scaffold19_2	non-phage	0
NOF007_scaffold10776_16	non-phage	0
NOF007_scaffold9936_2	non-phage	0
NOF007_scaffold4621_1	non-phage	0
NOF007_scaffold21664_1	non-phage	0
NOF007_scaffold27189_1	non-phage	0
NOF007_scaffold1587_2	non-phage	0
NOF007_scaffold13674_14	non-phage	0
NOF007_scaffold9136_4	non-phage	0
NOF007_C490425_1	non-phage	0
NOF007_scaffold22783_2	non-phage	0
NOF007_scaffold16936_2	non-phage	0
NOF007_scaffold14275_1	non-phage	0
NOF007_scaffold24981_2	non-phage	0
NOF007_scaffold10063_3	non-phage	0
NOF007_scaffold26102_1	non-phage	0
NOF007_scaffold21322_7	non-phage	0
NOF007_scaffold28374_2	non-phage	0
NOF007_scaffold27994_1	non-phage	0
NOF007_scaffold2058_10	non-phage	0
NOF007_scaffold15970_2	non-phage	0
NOF007_scaffold25601_2	non-phage	0.007
NOF007_scaffold181_1	non-phage	0
NOF007_scaffold11253_6	non-phage	0
NOF007_scaffold26938_2	phage	0.999
NOF007_scaffold9256_1	non-phage	0
NOF007_scaffold28879_1	non-phage	0
NOF007_scaffold24271_3	non-phage	0
NOF007_scaffold17572_3	non-phage	0
NOF007_scaffold9992_2	non-phage	0
NOF007_scaffold26159_2	non-phage	0
NOF007_scaffold25564_1	non-phage	0
NOF007_scaffold18273_1	non-phage	0
NOF007_scaffold21531_2	non-phage	0
NOF007_scaffold28756_1	non-phage	0
NOF007_scaffold2492_2	non-phage	0
NOF007_scaffold10387_4	non-phage	0
NOF007_scaffold18722_4	non-phage	0
NOF007_scaffold3704_2	non-phage	0
NOF007_scaffold13628_10	non-phage	0
NOF007_scaffold1491_2	non-phage	0
NOF007_scaffold15788_2	non-phage	0
NOF007_scaffold28749_1	non-phage	0

NOF007_scaffold28465_2	non-phage	0
NOF007_scaffold25200_1	non-phage	0
NOF007_scaffold10169_3	non-phage	0
NOF007_scaffold198_8	non-phage	0
NOF007_scaffold23_4	non-phage	0
NOF007_scaffold5848_2	non-phage	0
NOF007_scaffold3786_4	non-phage	0
NOF007_scaffold25873_2	non-phage	0
NOF007_scaffold111_2	non-phage	0
NOF007_scaffold160_1	non-phage	0
NOF007_scaffold28998_3	non-phage	0
NOF007_scaffold6694_2	non-phage	0
NOF007_scaffold29017_1	non-phage	0
NOF007_scaffold28914_2	non-phage	0
NOF007_scaffold23736_2	non-phage	0
NOF007_scaffold27268_7	non-phage	0
NOF007_scaffold8768_3	non-phage	0
NOF007_scaffold6100_2	non-phage	0
NOF007_scaffold10673_1	non-phage	0
NOF007_scaffold15604_1	non-phage	0
NOF007_scaffold28826_4	non-phage	0
NOF007_scaffold27787_1	non-phage	0
NOF007_scaffold27787_4	non-phage	0
NOF007_scaffold7015_3	non-phage	0
NOF007_scaffold28537_1	non-phage	0
NOF007_scaffold18273_4	non-phage	0
NOF007_scaffold23819_1	non-phage	0
NOF007_scaffold13_1	non-phage	0
NOF007_scaffold25033_1	non-phage	0
NOF007_scaffold21322_1	non-phage	0
NOF007_scaffold28894_1	non-phage	0
NOF007_scaffold8482_9	non-phage	0
NOF007_scaffold9523_1	non-phage	0
NOF007_scaffold16603_1	non-phage	0
NOF007_scaffold139_4	non-phage	0
NOF007_scaffold12447_1	non-phage	0
NOF007_scaffold12990_3	non-phage	0
NOF007_scaffold29019_1	non-phage	0
NOF007_scaffold8991_13	non-phage	0
NOF007_scaffold16232_1	non-phage	0
NOF007_scaffold25363_2	non-phage	0
NOF007_scaffold18678_2	non-phage	0
NOF007_scaffold27690_2	non-phage	0
NOF007_scaffold7559_3	phage	1
NOF007_scaffold1806_3	non-phage	0
NOF007_scaffold43_15	non-phage	0

NOF007_scaffold28993_1	non-phage	0
NOF007_scaffold11638_2	non-phage	0
NOF007_scaffold387_1	non-phage	0
NOF007_scaffold23424_1	non-phage	0
NOF007_scaffold26603_3	non-phage	0
NOF007_scaffold18658_1	non-phage	0
NOF007_scaffold16388_6	phage	0.999
NOF007_scaffold28685_2	non-phage	0
NOF007_scaffold21322_3	non-phage	0
NOF007_C490939_1	non-phage	0
NOF007_scaffold4780_4	non-phage	0
NOF007_scaffold27989_1	non-phage	0
NOF007_scaffold2462_7	non-phage	0
NOF007_scaffold6571_20	non-phage	0
NOF007_scaffold28605_1	non-phage	0
NOF007_scaffold12777_3	non-phage	0
NOF007_scaffold20245_1	non-phage	0
NOF007_scaffold12560_1	non-phage	0
NOF007_scaffold13902_4	non-phage	0
NOF007_scaffold20845_1	non-phage	0
NOF007_scaffold91_4	non-phage	0
NOF007_scaffold18722_5	non-phage	0
NOF007_scaffold24483_2	phage	0.999
NOF007_scaffold21314_1	non-phage	0
NOF007_scaffold28155_2	non-phage	0
NOF007_scaffold28910_2	non-phage	0
NOF007_scaffold13403_1	non-phage	0
NOF007_scaffold28087_1	non-phage	0
NOF007_scaffold8437_5	non-phage	0
NOF007_scaffold21771_3	non-phage	0
NOF007_scaffold198_4	non-phage	0
NOF007_scaffold81_24	non-phage	0
NOF007_scaffold1964_3	non-phage	0
NOF007_scaffold21550_3	non-phage	0
NOF007_scaffold77_1	non-phage	0
NOF007_scaffold237_5	non-phage	0
NOF007_scaffold28875_2	non-phage	0
NOF007_scaffold8836_1	non-phage	0
NOF007_scaffold28763_1	non-phage	0
NOF007_scaffold27268_2	non-phage	0
NOF007_scaffold8528_6	non-phage	0
NOF007_scaffold22790_3	non-phage	0
NOF007_scaffold2036_2	non-phage	0
NOF007_scaffold198_12	non-phage	0
NOF007_scaffold7977_1	non-phage	0
NOF007_scaffold28471_3	non-phage	0

NOF007_scaffold9051_4	non-phage	0
NOF007_scaffold236_1	non-phage	0
NOF007_scaffold81_22	non-phage	0
NOF007_scaffold67_1	non-phage	0
NOF007_scaffold28866_1	non-phage	0
NOF007_scaffold111_6	non-phage	0
NOF007_scaffold8553_1	non-phage	0
NOF007_scaffold18064_1	non-phage	0
NOF007_scaffold16724_4	non-phage	0
NOF007_scaffold111_5	non-phage	0
NOF007_scaffold10320_1	non-phage	0
NOF007_scaffold19852_2	non-phage	0
NOF007_scaffold10169_9	non-phage	0
NOF007_scaffold22094_2	non-phage	0
NOF007_scaffold28918_1	non-phage	0
NOF007_scaffold23774_1	non-phage	0
NOF007_scaffold22250_1	non-phage	0
NOF007_C490813_1	non-phage	0
NOF007_scaffold16966_2	non-phage	0
NOF007_scaffold3348_5	non-phage	0
NOF007_scaffold23975_3	non-phage	0
NOF007_scaffold9936_1	non-phage	0
NOF007_scaffold26467_1	non-phage	0
NOF007_scaffold24402_2	non-phage	0
NOF007_scaffold18678_3	non-phage	0
NOF007_scaffold156_8	non-phage	0
NOF007_scaffold28762_1	non-phage	0
NOF007_scaffold28903_1	non-phage	0
NOF007_scaffold6134_1	non-phage	0
NOF007_scaffold25317_2	non-phage	0
NOF007_scaffold25504_1	non-phage	0
NOF007_scaffold5472_3	non-phage	0
NOF007_scaffold984_14	non-phage	0
NOF007_scaffold7948_4	non-phage	0
NOF007_scaffold2474_2	non-phage	0
NOF007_scaffold1499_14	non-phage	0
NOF007_scaffold10524_1	phage	0.988
NOF007_C490299_1	non-phage	0.313
NOF007_scaffold13628_6	non-phage	0
NOF007_scaffold10063_6	non-phage	0
NOF007_scaffold4031_26	non-phage	0
NOF007_scaffold21383_1	non-phage	0
NOF007_scaffold20424_2	non-phage	0
NOF007_scaffold19985_7	non-phage	0
NOF007_scaffold28910_1	non-phage	0
NOF007_scaffold106_4	non-phage	0

NOF007_scaffold10571_3	non-phage	0
NOF007_scaffold27294_6	non-phage	0
NOF007_scaffold19736_2	non-phage	0
NOF007_scaffold1603_17	non-phage	0
NOF007_scaffold23323_1	non-phage	0
NOF007_scaffold24458_6	non-phage	0
NOF007_scaffold41_1	phage	0.999
NOF007_scaffold2058_2	non-phage	0
NOF007_scaffold2329_1	non-phage	0
NOF007_scaffold25493_3	non-phage	0
NOF007_scaffold18658_5	non-phage	0
NOF007_scaffold28537_6	non-phage	0
NOF007_scaffold23436_2	non-phage	0
NOF007_scaffold20501_6	non-phage	0
NOF007_scaffold23_3	non-phage	0
NOF007_scaffold10063_4	non-phage	0
NOF007_scaffold10776_3	non-phage	0
NOF007_scaffold5848_3	non-phage	0
NOF007_scaffold26501_1	non-phage	0
NOF007_scaffold27294_1	non-phage	0
NOF007_scaffold25717_1	non-phage	0
NOF007_scaffold28908_1	non-phage	0
NOF007_scaffold1_2	non-phage	0
NOF007_scaffold15189_1	non-phage	0
NOF007_scaffold298_7	phage	0.999
NOF007_scaffold18990_4	non-phage	0
NOF007_scaffold505_1	phage	0.986
NOF007_scaffold18_3	non-phage	0
NOF007_scaffold22719_3	non-phage	0
NOF007_scaffold20442_2	non-phage	0
NOF007_scaffold22018_1	non-phage	0
NOF007_scaffold22566_3	non-phage	0
NOF007_scaffold16917_1	non-phage	0
NOF007_scaffold25518_3	non-phage	0
NOF007_scaffold181_2	non-phage	0
NOF007_C490829_1	non-phage	0
NOF007_scaffold15797_6	non-phage	0
NOF007_scaffold3285_1	non-phage	0
NOF007_scaffold28875_1	non-phage	0
NOF007_scaffold11903_12	non-phage	0
NOF007_scaffold1028_4	non-phage	0
NOF007_scaffold60_3	non-phage	0
NOF007_scaffold28801_1	phage	0.998
NOF007_scaffold12591_3	non-phage	0
NOF007_scaffold166_9	non-phage	0
NOF007_scaffold10387_2	non-phage	0

NOF007_scaffold22719_2	non-phage	0
NOF007_scaffold12524_2	non-phage	0
NOF007_scaffold2604_1	non-phage	0
NOF007_scaffold8183_4	non-phage	0
NOF007_scaffold25350_3	non-phage	0
NOF007_scaffold28685_3	phage	0.999
NOF007_scaffold5255_2	non-phage	0
NOF007_scaffold25304_2	phage	0.652
NOF007_scaffold15930_4	non-phage	0
NOF007_scaffold111_1	non-phage	0
NOF007_scaffold28634_1	non-phage	0
NOF007_scaffold17572_5	non-phage	0
NOF007_scaffold28966_1	non-phage	0
NOF007_scaffold5752_3	non-phage	0
NOF007_scaffold10169_4	non-phage	0
NOF007_scaffold28745_1	non-phage	0
NOF007_scaffold1587_3	non-phage	0
NOF007_scaffold11855_5	non-phage	0
NOF007_scaffold23736_3	non-phage	0
NOF007_scaffold28865_2	non-phage	0
NOF007_scaffold22094_1	non-phage	0
NOF007_scaffold27056_2	non-phage	0
NOF007_scaffold5941_3	non-phage	0
NOF007_scaffold1964_1	non-phage	0
NOF007_scaffold143_2	non-phage	0
NOF007_scaffold25564_2	non-phage	0
NOF007_scaffold665_2	non-phage	0
NOF007_scaffold16936_11	non-phage	0
NOF007_scaffold198_6	non-phage	0
NOF007_scaffold26507_1	non-phage	0
NOF007_scaffold13902_5	non-phage	0
NOF007_scaffold13466_1	non-phage	0
NOF007_scaffold4503_1	non-phage	0
NOF007_scaffold24098_3	non-phage	0
NOF007_scaffold4368_2	non-phage	0
NOF007_scaffold9658_2	non-phage	0
NOF007_scaffold17360_8	non-phage	0
NOF007_scaffold8482_11	non-phage	0
NOF007_scaffold22039_3	phage	0.999
NOF007_scaffold9187_9	non-phage	0
NOF007_scaffold669_13	non-phage	0
NOF007_scaffold29024_1	non-phage	0
NOF007_scaffold2058_6	non-phage	0
NOF007_scaffold5152_1	non-phage	0
NOF007_scaffold24297_1	non-phage	0
NOF007_scaffold21537_1	non-phage	0

NOF007_scaffold26224_1	phage	0.999
NOF007_scaffold1174_18	non-phage	0
NOF007_scaffold669_6	non-phage	0
NOF007_scaffold7941_1	non-phage	0
NOF007_scaffold28624_3	phage	0.567
NOF007_scaffold20184_3	non-phage	0
NOF007_scaffold103_1	non-phage	0
NOF007_scaffold10169_6	non-phage	0
NOF007_scaffold8991_20	non-phage	0
NOF007_scaffold22302_2	non-phage	0
NOF007_scaffold8363_4	non-phage	0
NOF007_scaffold11121_2	non-phage	0
NOF007_scaffold28677_1	non-phage	0
NOF007_C490521_1	non-phage	0
NOF007_scaffold112_1	non-phage	0
NOF007_scaffold16936_7	non-phage	0
NOF007_scaffold21979_1	non-phage	0
NOF007_scaffold3448_2	non-phage	0
NOF007_scaffold28981_1	non-phage	0
NOF007_scaffold14373_2	non-phage	0
NOF007_C490379_1	non-phage	0
NOF007_scaffold9804_2	non-phage	0
NOF007_scaffold27927_3	non-phage	0
NOF007_C490583_1	non-phage	0
NOF007_scaffold24576_3	non-phage	0
NOF007_scaffold18990_5	non-phage	0
NOF007_scaffold5848_4	non-phage	0
NOF007_scaffold10063_5	non-phage	0.028
NOF007_scaffold13661_2	non-phage	0
NOF007_scaffold9575_8	non-phage	0
NOF007_scaffold15868_4	non-phage	0
NOF007_scaffold28901_3	non-phage	0
NOF007_scaffold7948_3	non-phage	0
NOF007_scaffold2582_8	non-phage	0
NOF007_C490775_1	non-phage	0
NOF007_scaffold8994_2	non-phage	0
NOF007_scaffold1533_1	non-phage	0
NOF007_scaffold18272_3	non-phage	0
NOF007_scaffold3393_7	phage	0.999
NOF007_scaffold198_13	non-phage	0
NOF007_scaffold20442_3	non-phage	0
NOF007_scaffold27_3	non-phage	0
NOF007_scaffold1195_9	phage	0.965
NOF007_scaffold198_2	non-phage	0
NOF007_scaffold20999_1	non-phage	0
NOF007_scaffold2773_2	non-phage	0

NOF007_scaffold2822_2	phage	1
NOF007_scaffold1973_2	non-phage	0
NOF007_scaffold23774_2	phage	1
NOF007_scaffold28837_1	non-phage	0
NOF007_scaffold22420_2	non-phage	0
NOF007_scaffold5145_10	non-phage	0
NOF007_scaffold23_2	non-phage	0
NOF007_scaffold18273_8	non-phage	0
NOF007_scaffold20639_1	non-phage	0
NOF007_scaffold28946_3	non-phage	0
NOF007_scaffold8768_4	non-phage	0
NOF007_scaffold21927_3	non-phage	0
NOF007_scaffold6474_4	non-phage	0
NOF007_scaffold8229_7	non-phage	0
NOF007_scaffold15830_4	non-phage	0
NOF007_scaffold28650_3	non-phage	0
NOF007_scaffold28779_2	non-phage	0
NOF007_scaffold15788_5	non-phage	0
NOF007_C490309_1	non-phage	0
NOF007_scaffold27689_1	non-phage	0
NOF007_scaffold11572_1	non-phage	0
NOF007_scaffold18064_2	non-phage	0
NOF007_C490669_1	non-phage	0
NOF007_scaffold6562_3	non-phage	0
NOF007_scaffold9187_46	non-phage	0
NOF007_scaffold11638_3	non-phage	0
NOF007_scaffold11943_1	non-phage	0
NOF007_scaffold28010_1	non-phage	0
NOF007_scaffold1798_9	non-phage	0
NOF007_scaffold13403_4	non-phage	0
NOF007_scaffold28975_1	non-phage	0
NOF007_scaffold7102_2	non-phage	0
NOF007_scaffold61_4	non-phage	0
NOF007_scaffold2058_3	non-phage	0
NOF007_scaffold21975_1	non-phage	0
NOF007_scaffold28908_2	non-phage	0
NOF007_scaffold16603_2	non-phage	0
NOF007_scaffold29018_1	non-phage	0
NOF007_scaffold29011_1	non-phage	0
NOF007_scaffold12899_1	phage	0.578
NOF007_scaffold25253_1	non-phage	0
NOF007_scaffold9256_3	non-phage	0
NOF007_scaffold17409_5	non-phage	0
NOF007_scaffold237_9	non-phage	0
NOF007_scaffold28683_1	non-phage	0
NOF007_scaffold16839_1	non-phage	0

NOF007_scaffold18327_10	non-phage	0
NOF007_scaffold6593_6	non-phage	0
NOF007_scaffold8553_2	non-phage	0
NOF007_scaffold26007_3	non-phage	0
NOF007_scaffold28684_2	non-phage	0
NOF007_scaffold19852_1	non-phage	0
NOF007_C490795_1	non-phage	0
NOF007_scaffold9643_1	non-phage	0
NOF007_scaffold22700_1	non-phage	0
NOF007_C490819_1	non-phage	0
NOF007_scaffold9468_5	phage	0.997
NOF007_scaffold22250_2	non-phage	0
NOF007_scaffold14039_1	non-phage	0
NOF007_scaffold13779_14	non-phage	0
NOF007_scaffold20610_4	non-phage	0
NOF007_scaffold17701_1	non-phage	0
NOF007_scaffold29007_2	non-phage	0
NOF008_scaffold16777_13	non-phage	0
NOF008_scaffold31875_6	non-phage	0
NOF008_scaffold66864_2	non-phage	0
NOF008_scaffold24829_1	non-phage	0
NOF008_scaffold29226_2	non-phage	0
NOF008_scaffold68019_2	non-phage	0
NOF008_scaffold28695_2	non-phage	0
NOF008_scaffold29243_2	non-phage	0
NOF008_scaffold30272_1	non-phage	0
NOF008_scaffold23363_1	non-phage	0
NOF008_scaffold329_1	non-phage	0
NOF008_scaffold1681_3	non-phage	0
NOF008_scaffold108_1	non-phage	0
NOF008_scaffold11843_1	non-phage	0
NOF008_scaffold11125_5	non-phage	0
NOF008_scaffold34632_3	phage	0.999
NOF008_scaffold54506_2	non-phage	0
NOF008_scaffold8428_2	non-phage	0
NOF008_scaffold32845_3	non-phage	0
NOF008_scaffold32823_5	non-phage	0
NOF008_scaffold52929_2	non-phage	0
NOF008_scaffold38962_10	non-phage	0.308
NOF008_scaffold3708_1	non-phage	0
NOF008_scaffold15546_5	non-phage	0
NOF008_scaffold19441_2	non-phage	0
NOF008_scaffold20191_1	non-phage	0
NOF008_scaffold63594_1	non-phage	0
NOF008_scaffold55525_2	non-phage	0
NOF008_scaffold29118_1	non-phage	0

NOF008_scaffold41486_4	non-phage	0
NOF008_scaffold68176_2	non-phage	0
NOF008_scaffold40777_2	non-phage	0
NOF008_scaffold43713_2	non-phage	0
NOF008_scaffold39560_5	non-phage	0
NOF008_scaffold204_1	non-phage	0
NOF008_scaffold68111_1	non-phage	0
NOF008_scaffold64536_1	non-phage	0
NOF008_scaffold139_1	non-phage	0
NOF008_scaffold68053_1	non-phage	0
NOF008_scaffold47013_1	non-phage	0
NOF008_scaffold210_3	non-phage	0
NOF008_scaffold22468_10	non-phage	0
NOF008_scaffold49915_4	non-phage	0
NOF008_scaffold58330_2	non-phage	0
NOF008_scaffold68093_1	non-phage	0
NOF008_scaffold25637_3	non-phage	0
NOF008_scaffold34837_1	non-phage	0
NOF008_scaffold62785_1	non-phage	0
NOF008_scaffold68187_1	non-phage	0
NOF008_scaffold68202_3	non-phage	0
NOF008_scaffold68230_1	phage	1
NOF008_scaffold9235_6	non-phage	0
NOF008_scaffold3548_3	non-phage	0
NOF008_scaffold29960_1	non-phage	0
NOF008_scaffold65257_2	non-phage	0
NOF008_scaffold64411_1	non-phage	0
NOF008_scaffold33528_5	non-phage	0
NOF008_scaffold298_5	non-phage	0
NOF008_scaffold46749_1	non-phage	0
NOF008_scaffold68182_2	non-phage	0
NOF008_scaffold59481_2	non-phage	0
NOF008_scaffold16175_9	non-phage	0
NOF008_C865253_1	non-phage	0
NOF008_scaffold31266_2	non-phage	0
NOF008_scaffold18168_1	non-phage	0
NOF008_scaffold65865_1	non-phage	0
NOF008_scaffold67966_2	non-phage	0
NOF008_scaffold54543_1	non-phage	0
NOF008_scaffold66641_4	non-phage	0
NOF008_scaffold68184_1	non-phage	0
NOF008_scaffold14817_4	non-phage	0
NOF008_scaffold34040_1	non-phage	0
NOF008_scaffold29176_2	non-phage	0
NOF008_scaffold505_3	non-phage	0
NOF008_scaffold43105_1	non-phage	0

NOF008_scaffold58329_2	non-phage	0
NOF008_scaffold28302_1	non-phage	0
NOF008_scaffold24989_1	non-phage	0
NOF008_scaffold23236_3	non-phage	0
NOF008_scaffold3708_13	non-phage	0
NOF008_scaffold68162_2	non-phage	0
NOF008_scaffold25367_2	phage	1
NOF008_scaffold14519_4	non-phage	0
NOF008_scaffold43357_2	non-phage	0
NOF008_scaffold22353_2	non-phage	0
NOF008_scaffold2659_1	non-phage	0
NOF008_scaffold64397_1	phage	0.997
NOF008_scaffold18668_2	non-phage	0
NOF008_scaffold17689_4	non-phage	0
NOF008_scaffold18668_24	non-phage	0
NOF008_scaffold144_2	non-phage	0
NOF008_scaffold54744_2	non-phage	0
NOF008_scaffold67298_8	non-phage	0
NOF008_scaffold25702_1	non-phage	0
NOF008_scaffold25333_2	non-phage	0
NOF008_scaffold48782_2	phage	0.999
NOF008_scaffold31875_3	non-phage	0
NOF008_scaffold67169_1	non-phage	0
NOF008_scaffold7428_3	non-phage	0
NOF008_scaffold12766_2	non-phage	0
NOF008_scaffold63731_4	non-phage	0
NOF008_scaffold10425_7	non-phage	0
NOF008_scaffold5134_4	phage	0.997
NOF008_scaffold37931_6	non-phage	0
NOF008_scaffold64071_1	non-phage	0
NOF008_scaffold42338_1	non-phage	0
NOF008_scaffold16175_7	non-phage	0
NOF008_scaffold35367_3	non-phage	0
NOF008_scaffold19074_3	non-phage	0
NOF008_scaffold13850_3	non-phage	0
NOF008_scaffold55726_5	non-phage	0
NOF008_scaffold46576_2	non-phage	0
NOF008_scaffold25583_1	non-phage	0
NOF008_scaffold21086_2	non-phage	0
NOF008_scaffold4633_2	non-phage	0
NOF008_scaffold17922_1	non-phage	0
NOF008_scaffold1031_1	non-phage	0
NOF008_scaffold46132_3	non-phage	0
NOF008_scaffold60881_1	non-phage	0
NOF008_scaffold13444_3	non-phage	0
NOF008_scaffold44209_1	non-phage	0

NOF008_scaffold18668_9	non-phage	0
NOF008_scaffold25136_2	non-phage	0
NOF008_scaffold31199_2	non-phage	0
NOF008_scaffold1747_41	non-phage	0
NOF008_scaffold11125_3	non-phage	0
NOF008_scaffold25093_2	non-phage	0
NOF008_scaffold68227_3	non-phage	0
NOF008_scaffold39575_7	non-phage	0
NOF008_C866017_1	non-phage	0
NOF008_scaffold61643_2	phage	0.956
NOF008_scaffold8268_1	non-phage	0
NOF008_C866181_1	non-phage	0
NOF008_scaffold388_1	non-phage	0
NOF008_scaffold26239_1	non-phage	0
NOF008_C865983_1	non-phage	0
NOF008_scaffold67644_2	non-phage	0
NOF008_scaffold39996_1	non-phage	0
NOF008_scaffold54731_2	non-phage	0
NOF008_scaffold42820_2	non-phage	0
NOF008_scaffold37931_5	non-phage	0
NOF008_scaffold50375_2	non-phage	0
NOF008_scaffold12902_1	non-phage	0
NOF008_scaffold1288_26	phage	0.998
NOF008_scaffold66638_3	non-phage	0
NOF008_scaffold20757_1	non-phage	0
NOF008_scaffold33337_1	non-phage	0
NOF008_scaffold66085_2	phage	0.997
NOF008_scaffold17557_1	non-phage	0
NOF008_scaffold34327_1	non-phage	0
NOF008_scaffold249_2	non-phage	0
NOF008_scaffold54140_5	non-phage	0
NOF008_scaffold67672_1	non-phage	0
NOF008_scaffold6569_3	non-phage	0
NOF008_scaffold18668_22	non-phage	0
NOF008_scaffold30369_4	non-phage	0
NOF008_scaffold23236_1	non-phage	0
NOF008_scaffold6798_3	non-phage	0
NOF008_scaffold23296_2	non-phage	0
NOF008_scaffold37051_1	non-phage	0
NOF008_scaffold33344_1	non-phage	0
NOF008_scaffold29144_1	non-phage	0
NOF008_scaffold169_2	non-phage	0
NOF008_scaffold30094_1	non-phage	0
NOF008_scaffold38254_4	non-phage	0
NOF008_scaffold57488_1	non-phage	0
NOF008_scaffold19505_1	non-phage	0

NOF008_scaffold46443_1	non-phage	0
NOF008_scaffold1905_3	non-phage	0
NOF008_scaffold120_4	non-phage	0.001
NOF008_scaffold13000_3	non-phage	0
NOF008_scaffold53048_1	non-phage	0
NOF008_scaffold8714_3	non-phage	0
NOF008_scaffold16409_1	non-phage	0
NOF008_scaffold54762_1	non-phage	0
NOF008_scaffold66837_1	non-phage	0
NOF008_scaffold9152_3	non-phage	0
NOF008_scaffold67458_1	non-phage	0
NOF008_scaffold271_5	non-phage	0
NOF008_scaffold68217_1	non-phage	0
NOF008_scaffold31199_6	non-phage	0
NOF008_scaffold17230_4	non-phage	0
NOF008_scaffold10868_1	non-phage	0
NOF008_scaffold68151_1	non-phage	0
NOF008_scaffold67807_1	non-phage	0
NOF008_scaffold13559_1	non-phage	0
NOF008_scaffold17943_1	non-phage	0
NOF008_C866171_1	non-phage	0
NOF008_scaffold25702_4	non-phage	0
NOF008_scaffold18668_8	non-phage	0
NOF008_scaffold67912_1	non-phage	0
NOF008_scaffold16560_7	non-phage	0
NOF008_scaffold67708_1	non-phage	0
NOF008_scaffold6829_1	non-phage	0
NOF008_scaffold11140_1	phage	1
NOF008_scaffold36418_1	non-phage	0
NOF008_scaffold52275_1	non-phage	0
NOF008_scaffold5217_6	phage	0.593
NOF008_scaffold41_2	non-phage	0
NOF008_scaffold37264_1	non-phage	0
NOF008_scaffold28772_4	non-phage	0
NOF008_scaffold68154_1	non-phage	0
NOF008_scaffold62521_1	non-phage	0
NOF008_scaffold49915_3	non-phage	0
NOF008_C865377_1	non-phage	0
NOF008_scaffold16641_2	non-phage	0
NOF008_scaffold15546_2	non-phage	0
NOF008_scaffold1389_2	phage	0.998
NOF008_scaffold25447_3_1	non-phage	0
NOF008_scaffold25447_3_2	non-phage	0
NOF008_scaffold12673_5	non-phage	0
NOF008_scaffold26514_2	non-phage	0
NOF008_scaffold38254_3	non-phage	0

NOF008_scaffold25135_1	non-phage	0
NOF008_scaffold9493_2	non-phage	0
NOF008_scaffold31276_2	non-phage	0
NOF008_scaffold4697_3	non-phage	0
NOF008_scaffold68168_1	non-phage	0
NOF008_scaffold66907_2	non-phage	0
NOF008_scaffold34717_1	non-phage	0
NOF008_scaffold18668_23	non-phage	0
NOF008_scaffold57578_1	non-phage	0
NOF008_scaffold45877_1	non-phage	0
NOF008_scaffold18335_2	non-phage	0
NOF008_scaffold53048_2	non-phage	0
NOF008_scaffold35323_1	non-phage	0
NOF008_scaffold62115_4	non-phage	0
NOF008_scaffold31875_4	non-phage	0
NOF008_scaffold63306_1	non-phage	0
NOF008_scaffold63933_1	non-phage	0
NOF008_scaffold67939_2	non-phage	0
NOF008_scaffold30957_1	non-phage	0
NOF008_scaffold364_2	non-phage	0
NOF008_scaffold68180_2	non-phage	0
NOF008_scaffold21410_1	non-phage	0
NOF008_scaffold67302_1	non-phage	0
NOF008_scaffold32044_16	non-phage	0
NOF008_scaffold12020_5	non-phage	0
NOF008_scaffold66738_2	non-phage	0
NOF008_scaffold34632_4	phage	0.999
NOF008_scaffold23011_1	non-phage	0
NOF008_scaffold12024_1	non-phage	0
NOF008_scaffold68101_3	non-phage	0
NOF008_scaffold2442_3	non-phage	0
NOF008_scaffold68142_2	non-phage	0
NOF008_scaffold68223_1	non-phage	0
NOF008_scaffold292_1	non-phage	0
NOF008_scaffold45486_1	non-phage	0
NOF008_scaffold67644_1	non-phage	0
NOF008_scaffold67470_2	phage	0.999
NOF008_scaffold118_1	non-phage	0
NOF008_scaffold62064_1	non-phage	0
NOF008_scaffold9176_2	non-phage	0
NOF008_scaffold32044_4	non-phage	0
NOF008_scaffold35323_5	non-phage	0
NOF008_scaffold67871_1	non-phage	0
NOF008_scaffold271_3	non-phage	0
NOF008_scaffold39836_1	non-phage	0
NOF008_scaffold401_3	non-phage	0

NOF008_scaffold134_6	non-phage	0
NOF008_scaffold55044_3	non-phage	0
NOF008_scaffold55479_4	non-phage	0
NOF008_scaffold65269_2	non-phage	0
NOF008_scaffold23554_6	non-phage	0
NOF008_scaffold67203_1	non-phage	0
NOF008_scaffold60455_1	non-phage	0
NOF008_scaffold65257_4	non-phage	0
NOF008_scaffold56963_1	non-phage	0
NOF008_scaffold34580_1	non-phage	0
NOF008_scaffold3708_8	non-phage	0
NOF008_scaffold55278_5	non-phage	0
NOF008_scaffold43105_5	non-phage	0
NOF008_scaffold15978_2	non-phage	0
NOF008_scaffold50382_1	non-phage	0
NOF008_scaffold28455_5	non-phage	0
NOF008_scaffold787_2	non-phage	0
NOF008_scaffold91_4	non-phage	0
NOF008_scaffold57608_5	non-phage	0
NOF008_scaffold23296_3	non-phage	0
NOF008_scaffold17003_3	non-phage	0
NOF008_scaffold24903_5	non-phage	0
NOF008_scaffold59102_2	non-phage	0
NOF008_scaffold4831_1	non-phage	0
NOF008_scaffold2771_8	non-phage	0
NOF008_scaffold68218_1	non-phage	0
NOF008_scaffold66842_1	non-phage	0
NOF008_scaffold2635_2	non-phage	0
NOF008_scaffold38491_1	non-phage	0
NOF008_scaffold40400_3	non-phage	0
NOF008_scaffold67384_1	non-phage	0
NOF008_scaffold28772_6	phage	0.932
NOF008_scaffold26339_4	non-phage	0
NOF008_scaffold9804_3	non-phage	0
NOF008_scaffold38986_2	non-phage	0
NOF008_scaffold34215_2	non-phage	0
NOF008_scaffold31875_1	non-phage	0
NOF008_scaffold36419_1	non-phage	0
NOF008_scaffold10035_9	phage	0.997
NOF008_scaffold31199_5	non-phage	0
NOF008_scaffold50962_3	non-phage	0
NOF008_scaffold5196_2	non-phage	0
NOF008_scaffold2226_8	non-phage	0
NOF008_scaffold68154_2	non-phage	0
NOF008_scaffold197_1	non-phage	0
NOF008_scaffold67612_2	non-phage	0

NOF008_scaffold16641_1	non-phage	0
NOF008_scaffold50552_1	non-phage	0
NOF008_scaffold64203_1	non-phage	0
NOF008_scaffold325_1	non-phage	0
NOF008_scaffold28624_2	non-phage	0
NOF008_scaffold68172_1	non-phage	0
NOF008_scaffold25367_4	non-phage	0
NOF008_scaffold38528_2	non-phage	0
NOF008_scaffold38254_2	non-phage	0
NOF008_scaffold4211_6	non-phage	0
NOF008_scaffold41614_2	non-phage	0
NOF008_scaffold10014_5	non-phage	0
NOF008_scaffold67636_1	non-phage	0
NOF008_scaffold13850_1	non-phage	0
NOF008_scaffold48151_1	non-phage	0
NOF008_scaffold10891_2	non-phage	0
NOF008_scaffold61595_1	non-phage	0
NOF008_scaffold26201_1	non-phage	0
NOF008_scaffold19089_6	non-phage	0
NOF008_scaffold11696_1	non-phage	0
NOF008_scaffold5143_2	non-phage	0
NOF008_scaffold38491_3	non-phage	0
NOF008_scaffold2635_3	non-phage	0
NOF008_scaffold35323_11	non-phage	0
NOF008_scaffold30912_6	phage	0.999
NOF008_scaffold37931_1	non-phage	0
NOF008_scaffold68227_1	non-phage	0
NOF008_scaffold3553_2	non-phage	0
NOF008_scaffold13444_1	non-phage	0
NOF008_scaffold44502_3	non-phage	0
NOF008_scaffold23387_2	non-phage	0
NOF008_scaffold68182_4	non-phage	0
NOF008_scaffold54506_1	non-phage	0
NOF008_scaffold41_1	non-phage	0
NOF008_scaffold19089_2	non-phage	0
NOF008_scaffold53174_8	non-phage	0
NOF008_scaffold50105_10	non-phage	0
NOF008_scaffold39575_5	non-phage	0
NOF008_scaffold24989_3	non-phage	0
NOF008_scaffold50287_1	non-phage	0
NOF008_scaffold50105_2	non-phage	0
NOF008_scaffold34508_1	non-phage	0
NOF008_scaffold17689_6	non-phage	0
NOF008_scaffold68059_1	non-phage	0
NOF008_scaffold68048_1	non-phage	0
NOF008_scaffold2719_10	non-phage	0

NOF008_scaffold65257_5	non-phage	0
NOF008_scaffold66613_1	phage	0.999
NOF008_scaffold36987_1	non-phage	0
NOF008_scaffold28772_7	non-phage	0
NOF008_scaffold49915_2	non-phage	0
NOF008_scaffold48599_1	non-phage	0
NOF008_scaffold68203_1	non-phage	0
NOF008_scaffold48283_1	phage	0.999
NOF008_scaffold34354_1	non-phage	0
NOF008_scaffold1563_4	non-phage	0
NOF008_scaffold67848_1	non-phage	0
NOF008_scaffold19442_1	non-phage	0
NOF008_scaffold21086_5	non-phage	0
NOF008_scaffold12195_12	non-phage	0
NOF008_scaffold31488_6	phage	0.999
NOF008_scaffold142_3	non-phage	0
NOF008_scaffold15599_3	non-phage	0
NOF008_scaffold49364_1	non-phage	0
NOF008_scaffold65896_1	non-phage	0
NOF008_scaffold2719_5	non-phage	0
NOF008_scaffold183_1	non-phage	0
NOF008_scaffold65602_1	non-phage	0
NOF008_scaffold37931_15	non-phage	0
NOF008_scaffold67298_4	non-phage	0
NOF008_scaffold31040_1	non-phage	0
NOF008_C865497_1	non-phage	0
NOF008_scaffold12335_1	non-phage	0
NOF008_scaffold34040_2	non-phage	0
NOF008_scaffold46577_1	non-phage	0
NOF008_scaffold60852_3	non-phage	0
NOF008_scaffold295_1	non-phage	0
NOF008_scaffold58690_7	non-phage	0
NOF008_scaffold35323_4	non-phage	0
NOF008_scaffold15599_5	non-phage	0
NOF008_scaffold20191_6	non-phage	0
NOF008_scaffold25583_2	non-phage	0
NOF008_scaffold37931_11	non-phage	0
NOF008_scaffold292_2	non-phage	0
NOF008_scaffold12899_4	non-phage	0
NOF008_scaffold58690_1	non-phage	0
NOF008_scaffold39575_8	non-phage	0
NOF008_scaffold67251_1	non-phage	0
NOF008_scaffold65865_2	non-phage	0.006
NOF008_scaffold65279_1	non-phage	0
NOF008_scaffold416_1	non-phage	0
NOF008_scaffold137_3	non-phage	0

NOF008_scaffold30807_1	non-phage	0
NOF008_scaffold21086_12	non-phage	0
NOF008_scaffold36171_2	non-phage	0
NOF008_scaffold68147_1	phage	0.999
NOF008_scaffold40636_1	non-phage	0
NOF008_scaffold52435_3	non-phage	0
NOF008_scaffold195_1	non-phage	0
NOF008_scaffold1078_2	non-phage	0
NOF008_scaffold5876_14	non-phage	0
NOF008_scaffold24989_2	non-phage	0
NOF008_scaffold22468_21	non-phage	0
NOF008_scaffold56878_1	non-phage	0
NOF008_scaffold22468_12	non-phage	0
NOF008_scaffold11898_1	non-phage	0
NOF008_scaffold8714_2	phage	0.999
NOF008_scaffold32823_2	non-phage	0
NOF008_scaffold66012_1	non-phage	0
NOF008_scaffold68202_1	non-phage	0
NOF008_scaffold3174_6	non-phage	0
NOF008_scaffold1563_1	non-phage	0
NOF008_scaffold35665_1	non-phage	0
NOF008_scaffold43371_3	non-phage	0
NOF008_scaffold152_1	non-phage	0
NOF008_scaffold15978_8	non-phage	0
NOF008_scaffold18168_3	non-phage	0
NOF008_scaffold5616_1	phage	0.998
NOF008_scaffold35323_12	non-phage	0
NOF008_scaffold25594_1	non-phage	0
NOF008_scaffold140_1	non-phage	0
NOF008_scaffold27880_2	non-phage	0.002
NOF008_scaffold68169_1	non-phage	0
NOF008_scaffold67298_5	non-phage	0
NOF008_scaffold2146_3	non-phage	0
NOF008_scaffold67298_7	non-phage	0
NOF008_scaffold8211_7	non-phage	0
NOF008_scaffold57632_1	non-phage	0
NOF008_scaffold2771_5	non-phage	0
NOF008_scaffold13000_5	non-phage	0
NOF008_scaffold67014_1	non-phage	0
NOF008_scaffold142_2	non-phage	0
NOF008_scaffold14519_1	non-phage	0
NOF008_scaffold68213_2	non-phage	0
NOF008_scaffold64561_1	non-phage	0
NOF008_scaffold68130_1	phage	1
NOF008_scaffold137_2	non-phage	0
NOF008_scaffold68137_1	non-phage	0

NOF008_scaffold25388_1	non-phage	0
NOF008_scaffold54744_1	non-phage	0
NOF008_scaffold25093_4	non-phage	0
NOF008_scaffold11247_4	non-phage	0
NOF008_scaffold3708_12	non-phage	0
NOF008_scaffold29750_1	non-phage	0
NOF008_scaffold14519_5	non-phage	0
NOF008_scaffold18668_3	non-phage	0
NOF008_scaffold12948_3	non-phage	0
NOF008_scaffold43840_8	non-phage	0
NOF008_scaffold41397_1	non-phage	0
NOF008_scaffold8419_7	non-phage	0
NOF008_scaffold14237_5	non-phage	0
NOF008_scaffold9423_3	non-phage	0
NOF008_scaffold370_1	non-phage	0
NOF008_scaffold5444_2	non-phage	0
NOF008_scaffold29753_1	non-phage	0
NOF008_scaffold67966_3	non-phage	0
NOF008_scaffold5876_23	non-phage	0
NOF008_scaffold25447_1	phage	0.999
NOF008_scaffold65399_2	non-phage	0
NOF008_scaffold22468_16	non-phage	0
NOF008_scaffold11006_4	non-phage	0
NOF008_scaffold42820_4	non-phage	0
NOF008_scaffold22468_17	non-phage	0
NOF008_scaffold51745_2	non-phage	0
NOF008_scaffold1078_4	non-phage	0
NOF008_scaffold58690_5	non-phage	0
NOF008_scaffold19089_11	non-phage	0
NOF008_scaffold3062_3	phage	0.988
NOF008_scaffold25637_1	non-phage	0
NOF008_scaffold7421_1	phage	1
NOF008_scaffold2608_1	non-phage	0
NOF008_scaffold18168_5	non-phage	0
NOF008_scaffold15546_8	non-phage	0
NOF008_scaffold59314_1	non-phage	0
NOF008_scaffold10014_2	non-phage	0
NOF008_scaffold27132_3	non-phage	0
NOF008_scaffold17922_2	non-phage	0
NOF008_scaffold57608_3	non-phage	0
NOF008_scaffold11006_5	non-phage	0
NOF008_scaffold53344_2	non-phage	0
NOF008_scaffold134_1	non-phage	0
NOF008_scaffold58979_2	non-phage	0
NOF008_scaffold19979_23	non-phage	0
NOF008_scaffold14035_1	non-phage	0

NOF008_scaffold152_3	non-phage	0
NOF008_scaffold44877_1	non-phage	0
NOF008_scaffold32044_6	non-phage	0
NOF008_scaffold37931_2	non-phage	0
NOF008_scaffold67107_1	non-phage	0
NOF008_scaffold25777_2	non-phage	0
NOF008_scaffold2226_6	non-phage	0
NOF008_scaffold55726_1	non-phage	0
NOF008_scaffold52435_2	phage	0.997
NOF008_scaffold50962_2	non-phage	0
NOF008_scaffold13000_6	non-phage	0
NOF008_scaffold68226_1	non-phage	0
NOF008_scaffold155_7	non-phage	0
NOF008_scaffold66602_1	non-phage	0
NOF008_scaffold37101_1	non-phage	0
NOF008_scaffold35076_1	phage	0.999
NOF008_scaffold4243_1	non-phage	0
NOF008_scaffold6569_4	non-phage	0
NOF008_scaffold67393_1	non-phage	0
NOF008_scaffold26201_2	non-phage	0
NOF008_scaffold271_4	non-phage	0
NOF008_scaffold916_5	non-phage	0
NOF008_scaffold37051_2	non-phage	0
NOF008_scaffold33247_2	non-phage	0
NOF008_scaffold58140_1	phage	0.992
NOF008_scaffold33324_4	non-phage	0
NOF008_scaffold65242_3	non-phage	0
NOF008_scaffold21086_6	non-phage	0
NOF008_scaffold16175_3	non-phage	0
NOF008_scaffold38254_5	non-phage	0
NOF008_scaffold67807_2	non-phage	0
NOF008_scaffold151_1	non-phage	0
NOF008_scaffold28624_4	non-phage	0
NOF008_scaffold53677_1	phage	0.962
NOF008_scaffold37931_18	non-phage	0
NOF008_scaffold5616_2	non-phage	0
NOF008_scaffold65497_1	non-phage	0
NOF008_scaffold37264_2	non-phage	0
NOF008_scaffold990_4	non-phage	0
NOF008_scaffold22468_13	non-phage	0
NOF008_scaffold1389_1	non-phage	0
NOF008_scaffold16520_4	non-phage	0
NOF008_scaffold58690_4	non-phage	0
NOF008_scaffold137_1	non-phage	0
NOF008_scaffold62956_1	non-phage	0
NOF008_scaffold55765_6	non-phage	0

NOF008_scaffold16434_2	non-phage	0
NOF008_scaffold23573_9	non-phage	0
NOF008_scaffold55525_6	non-phage	0
NOF008_scaffold26499_7	non-phage	0
NOF008_scaffold40477_1	non-phage	0
NOF008_scaffold24903_3	non-phage	0
NOF008_scaffold68203_3	phage	0.981
NOF008_scaffold19148_2	non-phage	0
NOF008_scaffold16560_8	non-phage	0
NOF008_scaffold36171_4	phage	0.919
NOF008_scaffold1563_2	non-phage	0
NOF008_scaffold26239_5	non-phage	0
NOF008_scaffold18168_4	non-phage	0
NOF008_scaffold32823_1	non-phage	0
NOF008_scaffold118_3	non-phage	0
NOF008_scaffold2442_1	non-phage	0
NOF008_C866205_1	non-phage	0
NOF008_scaffold24887_2	non-phage	0
NOF008_scaffold8077_2	non-phage	0
NOF008_scaffold1905_1	non-phage	0
NOF008_scaffold21086_13	non-phage	0
NOF008_scaffold63109_1	non-phage	0
NOF008_scaffold68191_1	phage	0.993
NOF008_scaffold32044_2	non-phage	0
NOF008_scaffold32291_1	non-phage	0
NOF008_scaffold39910_1	non-phage	0
NOF008_scaffold18335_1	non-phage	0
NOF008_scaffold44877_2	non-phage	0
NOF008_scaffold152_2	non-phage	0
NOF008_scaffold66931_1	non-phage	0
NOF008_scaffold37540_1	non-phage	0
NOF008_scaffold27536_7	non-phage	0
NOF008_scaffold8096_1	non-phage	0.001
NOF008_scaffold46561_3	non-phage	0
NOF008_scaffold52223_4	non-phage	0
NOF008_scaffold65986_1	phage	0.968
NOF008_scaffold2146_7	non-phage	0
NOF008_scaffold68197_1	non-phage	0
NOF008_scaffold15546_4	non-phage	0
NOF008_scaffold18668_6	non-phage	0
NOF008_scaffold3708_6	non-phage	0
NOF008_scaffold16777_2	non-phage	0
NOF008_scaffold28695_3	non-phage	0
NOF008_scaffold58485_2	non-phage	0
NOF008_C865467_1	non-phage	0
NOF008_scaffold64529_1	non-phage	0

NOF008_scaffold31040_3	non-phage	0
NOF008_scaffold66906_1	non-phage	0
NOF008_scaffold681_4	non-phage	0
NOF008_scaffold10425_3	non-phage	0
NOF008_scaffold68031_2	non-phage	0
NOF008_scaffold49459_1	non-phage	0
NOF008_scaffold34078_4	non-phage	0
NOF008_scaffold67512_1	non-phage	0
NOF008_scaffold22582_2	non-phage	0
NOF008_scaffold18834_4	non-phage	0
NOF008_scaffold59760_4	non-phage	0
NOF008_scaffold5256_2	non-phage	0
NOF008_scaffold19441_3	non-phage	0.007
NOF008_scaffold8110_1	non-phage	0
NOF008_scaffold49915_7	non-phage	0
NOF008_scaffold52317_1	non-phage	0
NOF008_scaffold60208_1	non-phage	0
NOF008_scaffold65257_3	non-phage	0
NOF008_scaffold36419_2	non-phage	0
NOF008_scaffold68134_1	non-phage	0
NOF008_scaffold13941_3	non-phage	0
NOF008_scaffold17943_2	non-phage	0
NOF008_scaffold27164_3	non-phage	0
NOF008_scaffold32044_12	non-phage	0
NOF008_scaffold38916_3	non-phage	0
NOF008_scaffold51213_1	non-phage	0
NOF008_scaffold66872_1	non-phage	0
NOF008_scaffold68053_2	non-phage	0
NOF008_scaffold33908_2	non-phage	0
NOF008_scaffold4444_2	non-phage	0
NOF008_scaffold357_1	non-phage	0
NOF008_scaffold67298_6	non-phage	0
NOF008_scaffold36294_3	non-phage	0
NOF008_scaffold50382_2	non-phage	0
NOF008_scaffold16175_4	non-phage	0
NOF008_scaffold29227_1	non-phage	0
NOF008_scaffold19505_6	non-phage	0
NOF008_scaffold66931_2	non-phage	0
NOF008_scaffold20191_5	non-phage	0
NOF008_scaffold63722_1	phage	0.999
NOF008_scaffold66299_3	non-phage	0
NOF008_scaffold30808_1	non-phage	0
NOF008_scaffold36460_1	non-phage	0
NOF008_scaffold50105_6	non-phage	0
NOF008_scaffold23296_4	non-phage	0
NOF008_scaffold40248_1	non-phage	0

NOF008_scaffold18668_5	non-phage	0
NOF008_scaffold22468_3	non-phage	0
NOF008_scaffold14519_3	non-phage	0
NOF008_scaffold41726_1	non-phage	0
NOF008_scaffold50828_1	phage	0.953
NOF008_scaffold41113_4	phage	0.639
NOF008_scaffold43357_1	non-phage	0
NOF008_scaffold33293_2	non-phage	0
NOF008_scaffold26297_1	non-phage	0
NOF008_scaffold50721_1	non-phage	0
NOF008_scaffold59760_7	phage	0.999
NOF008_scaffold65242_5	non-phage	0
NOF008_scaffold37931_10	non-phage	0
NOF008_scaffold66715_1	non-phage	0
NOF008_scaffold21086_7	non-phage	0
NOF008_scaffold2226_9	non-phage	0
NOF008_scaffold62343_2	non-phage	0
NOF008_scaffold1389_3	non-phage	0
NOF008_scaffold2361_3	non-phage	0
NOF008_scaffold67966_1	non-phage	0
NOF008_scaffold68219_1	non-phage	0
NOF008_scaffold325_2	non-phage	0
NOF008_scaffold11247_7	non-phage	0
NOF008_scaffold16434_1	non-phage	0
NOF008_scaffold24989_4	non-phage	0
NOF008_scaffold50193_1	non-phage	0
NOF008_scaffold1031_2	non-phage	0
NOF008_scaffold14862_1	non-phage	0
NOF008_scaffold131_1	non-phage	0
NOF008_scaffold64071_2	non-phage	0.001
NOF008_scaffold60455_2	non-phage	0
NOF008_scaffold66313_1	non-phage	0
NOF008_scaffold46132_2	non-phage	0
NOF008_scaffold52929_1	non-phage	0
NOF008_scaffold10994_2	non-phage	0
NOF008_scaffold46576_3	non-phage	0
NOF008_scaffold18813_1	non-phage	0
NOF008_scaffold13000_7	non-phage	0
NOF008_scaffold68203_2	non-phage	0
NOF008_scaffold10246_1	non-phage	0
NOF008_scaffold68170_1	non-phage	0
NOF008_scaffold2540_2	non-phage	0
NOF008_scaffold44390_1	non-phage	0
NOF008_scaffold15973_1	non-phage	0
NOF008_scaffold68182_3	non-phage	0
NOF008_scaffold35323_10	non-phage	0

NOF008_scaffold67470_1	non-phage	0
NOF008_scaffold14817_6	non-phage	0
NOF008_scaffold4415_4	non-phage	0
NOF008_scaffold57789_1	non-phage	0
NOF008_scaffold55726_4	non-phage	0
NOF008_scaffold46267_2	non-phage	0
NOF008_scaffold13727_2	non-phage	0
NOF008_scaffold160_1	non-phage	0
NOF008_scaffold2771_1	non-phage	0
NOF008_scaffold62547_1	non-phage	0
NOF008_scaffold2091_10	non-phage	0
NOF008_scaffold2226_4	non-phage	0
NOF008_scaffold62570_2	non-phage	0
NOF008_scaffold161_3	non-phage	0
NOF008_scaffold42043_1	phage	0.999
NOF008_scaffold364_1	non-phage	0
NOF008_scaffold25136_3	non-phage	0
NOF008_scaffold54517_1	non-phage	0
NOF008_scaffold24831_18	non-phage	0
NOF008_scaffold658_3	non-phage	0
NOF008_scaffold43189_1	non-phage	0
NOF008_scaffold66837_2	non-phage	0
NOF008_scaffold38448_12	non-phage	0
NOF008_scaffold16175_1	non-phage	0
NOF008_scaffold68215_1	non-phage	0
NOF008_scaffold4243_6	non-phage	0
NOF008_scaffold65242_1	non-phage	0
NOF008_scaffold68231_1	non-phage	0
NOF008_scaffold68210_1	non-phage	0
NOF008_scaffold64289_1	non-phage	0
NOF008_scaffold60762_6	non-phage	0
NOF008_scaffold17943_3	non-phage	0
NOF008_scaffold37931_12	non-phage	0
NOF008_scaffold29819_2	non-phage	0
NOF008_scaffold28624_6	non-phage	0
NOF008_scaffold33821_4	non-phage	0
NOF008_scaffold6792_7	non-phage	0
NOF008_scaffold46598_1	non-phage	0
NOF008_scaffold8066_2	phage	0.596
NOF008_scaffold22468_7	non-phage	0
NOF008_scaffold24749_2	non-phage	0
NOF008_scaffold11468_2	non-phage	0
NOF008_scaffold52079_2	non-phage	0
NOF008_scaffold16007_2	non-phage	0
NOF008_scaffold68225_1	non-phage	0
NOF008_scaffold33908_3	non-phage	0

NOF008_scaffold14702_2	non-phage	0
NOF008_scaffold56695_2	non-phage	0
NOF008_scaffold19089_8	non-phage	0
NOF008_scaffold56160_1	non-phage	0
NOF008_scaffold68145_1	non-phage	0
NOF008_scaffold29058_4	non-phage	0
NOF008_scaffold68214_1	non-phage	0
NOF008_scaffold12901_1	non-phage	0
NOF008_scaffold35323_3	non-phage	0
NOF008_scaffold681_6	non-phage	0
NOF008_scaffold27495_1	non-phage	0
NOF008_scaffold20833_3	non-phage	0
NOF008_scaffold12948_1	non-phage	0
NOF008_scaffold51414_3	non-phage	0
NOF008_scaffold26339_1	non-phage	0
NOF008_scaffold7970_1	non-phage	0
NOF008_scaffold2226_3	non-phage	0
NOF008_scaffold39226_1	non-phage	0
NOF008_scaffold124_2	non-phage	0
NOF008_C865841_1	non-phage	0
NOF008_scaffold3708_18	non-phage	0
NOF008_scaffold25049_1	non-phage	0
NOF008_scaffold47013_2	non-phage	0
NOF008_scaffold37101_3	non-phage	0
NOF008_scaffold53274_2	non-phage	0
NOF008_scaffold13000_8	non-phage	0
NOF008_scaffold30957_4	non-phage	0
NOF008_scaffold67189_1	non-phage	0
NOF008_scaffold67152_1	non-phage	0
NOF008_scaffold234_4	non-phage	0
NOF008_scaffold156_8	non-phage	0
NOF008_scaffold271_7	non-phage	0
NOF008_scaffold161_4	non-phage	0
NOF008_scaffold35323_9	non-phage	0
NOF008_scaffold50105_5	non-phage	0
NOF008_scaffold57868_2	non-phage	0
NOF008_scaffold61699_2	non-phage	0
NOF008_scaffold36815_1	non-phage	0
NOF008_scaffold17929_2	non-phage	0
NOF008_scaffold68057_3	non-phage	0
NOF008_scaffold15546_6	non-phage	0
NOF008_scaffold50105_9	non-phage	0
NOF008_scaffold14237_2	non-phage	0
NOF008_scaffold39560_3	non-phage	0
NOF008_scaffold38277_2	non-phage	0
NOF008_scaffold45736_5	non-phage	0

NOF008_scaffold20757_2	non-phage	0
NOF008_scaffold125_1	non-phage	0
NOF008_scaffold57789_2	non-phage	0
NOF008_scaffold68170_2	non-phage	0
NOF008_scaffold43105_3	non-phage	0
NOF008_C865561_1	non-phage	0
NOF008_scaffold3708_4	non-phage	0
NOF008_scaffold68228_1	non-phage	0
NOF008_scaffold16777_11	non-phage	0
NOF008_scaffold3708_11	non-phage	0
NOF008_scaffold10756_3	non-phage	0
NOF008_scaffold38962_8	non-phage	0
NOF008_scaffold63005_3	non-phage	0
NOF008_scaffold46505_3	non-phage	0
NOF008_scaffold59760_5	non-phage	0
NOF008_scaffold28625_3	phage	0.996
NOF008_scaffold39575_2	non-phage	0
NOF008_scaffold41928_1	non-phage	0
NOF008_scaffold67663_2	non-phage	0
NOF008_scaffold67298_2	non-phage	0
NOF008_scaffold120_1	non-phage	0
NOF008_scaffold40742_2	non-phage	0
NOF008_scaffold24903_1	non-phage	0
NOF008_scaffold67650_1	non-phage	0
NOF008_scaffold9152_1	non-phage	0
NOF008_scaffold2771_6	non-phage	0
NOF008_scaffold40142_2	non-phage	0
NOF008_scaffold52488_1	non-phage	0
NOF008_scaffold10014_1	non-phage	0
NOF008_scaffold19089_7	phage	0.849
NOF008_scaffold32823_6	non-phage	0
NOF008_scaffold3708_2	non-phage	0
NOF008_scaffold50105_7	non-phage	0
NOF008_scaffold16193_2	non-phage	0
NOF008_scaffold68193_1	non-phage	0
NOF008_scaffold65269_1	non-phage	0
NOF008_scaffold54140_3	non-phage	0
NOF008_scaffold24989_6	non-phage	0
NOF008_scaffold68128_1	non-phage	0
NOF008_scaffold34040_3	non-phage	0
NOF008_scaffold43713_1	non-phage	0
NOF008_scaffold3708_20	phage	0.999
NOF008_scaffold21086_4	non-phage	0
NOF008_scaffold36283_3	non-phage	0
NOF008_scaffold68224_1	phage	0.642
NOF008_scaffold13485_3	non-phage	0

NOF008_scaffold68194_2	non-phage	0
NOF008_scaffold23319_2	non-phage	0
NOF008_scaffold14862_2	non-phage	0
NOF008_scaffold14702_1	non-phage	0
NOF008_scaffold3708_23	non-phage	0
NOF008_scaffold33589_2	non-phage	0
NOF008_scaffold52198_1	phage	0.999
NOF008_scaffold34078_12	non-phage	0
NOF008_scaffold1270_2	non-phage	0
NOF008_scaffold7904_8	phage	0.995
NOF008_scaffold25220_3	phage	0.999
NOF008_scaffold52369_3	non-phage	0
NOF008_scaffold64168_3	non-phage	0
NOF008_scaffold16560_1	non-phage	0
NOF008_scaffold234_2	non-phage	0
NOF008_scaffold33908_4	non-phage	0
NOF008_scaffold5959_7	non-phage	0
NOF008_scaffold21540_1	non-phage	0
NOF008_scaffold54506_4	non-phage	0
NOF008_scaffold28772_3	non-phage	0
NOF008_scaffold1162_8	non-phage	0.002
NOF008_C865299_1	phage	0.999
NOF008_scaffold134_2	non-phage	0
NOF008_scaffold64084_1	non-phage	0
NOF008_scaffold35840_1	non-phage	0
NOF008_scaffold15546_7	non-phage	0
NOF008_scaffold8862_1	non-phage	0
NOF008_scaffold67629_1	non-phage	0
NOF008_scaffold32823_7	non-phage	0
NOF008_scaffold68220_1	non-phage	0
NOF008_scaffold43105_6	non-phage	0
NOF008_scaffold17689_13	phage	0.819
NOF008_C865863_1	phage	0.999
NOF008_scaffold2226_7	non-phage	0
NOF008_scaffold18834_10	non-phage	0
NOF008_scaffold28695_1	non-phage	0
NOF008_scaffold196_2	non-phage	0
NOF008_scaffold37101_2	non-phage	0
NOF008_scaffold54644_1	non-phage	0
NOF008_scaffold34837_2	non-phage	0
NOF008_scaffold63519_1	non-phage	0
NOF008_scaffold210_1	non-phage	0
NOF008_scaffold25475_1	non-phage	0
NOF008_scaffold59760_6	non-phage	0
NOF008_scaffold41614_1	non-phage	0
NOF008_scaffold26226_2	non-phage	0

NOF008_scaffold10855_1	non-phage	0
NOF008_scaffold38763_1	non-phage	0
NOF008_scaffold58786_2	non-phage	0
NOF008_scaffold25367_1	non-phage	0
NOF008_scaffold13444_5	non-phage	0
NOF008_scaffold36171_3	non-phage	0
NOF008_scaffold31875_9	non-phage	0
NOF008_scaffold39315_1	non-phage	0
NOF008_scaffold16007_1	non-phage	0
NOF008_scaffold2686_7	non-phage	0
NOF008_scaffold68199_1	phage	0.995
NOF008_scaffold25777_1	non-phage	0
NOF008_scaffold5196_1	non-phage	0
NOF008_C865297_1	non-phage	0
NOF008_scaffold101_1	non-phage	0
NOF008_scaffold2091_12	non-phage	0
NOF008_scaffold46_1	non-phage	0
NOF008_scaffold5009_1	non-phage	0
NOF008_scaffold6569_2	non-phage	0
NOF008_scaffold10846_1	non-phage	0
NOF008_scaffold64572_1	non-phage	0
NOF008_scaffold8937_20	non-phage	0
NOF008_scaffold66513_3	non-phage	0
NOF008_scaffold234_5	non-phage	0
NOF008_scaffold68199_2	non-phage	0
NOF008_scaffold66112_3	non-phage	0
NOF008_scaffold15329_4	non-phage	0
NOF008_scaffold68059_2	non-phage	0
NOF008_scaffold29520_2	non-phage	0
NOF008_scaffold197_7	non-phage	0
NOF008_scaffold7528_4	non-phage	0
NOF008_scaffold68162_1	non-phage	0
NOF008_scaffold55525_8	phage	0.928
NOF008_scaffold64561_2	non-phage	0
NOF008_scaffold19148_3	non-phage	0
NOF008_scaffold13444_2	phage	0.999
NOF008_scaffold64575_1	non-phage	0
NOF008_scaffold55811_1	phage	0.999
NOF008_scaffold35323_8	non-phage	0
NOF008_scaffold25333_1	non-phage	0
NOF008_scaffold29415_1	non-phage	0
NOF008_scaffold25702_2	non-phage	0
NOF008_scaffold15158_1	non-phage	0
NOF008_scaffold61492_1	non-phage	0.009
NOF008_scaffold68227_2	non-phage	0
NOF008_scaffold5876_10	non-phage	0

NOF008_scaffold65497_2	non-phage	0
NOF008_scaffold31266_1	non-phage	0
NOF008_scaffold40565_2	non-phage	0
NOF008_C866209_1	non-phage	0
NOF008_scaffold1681_1	non-phage	0
NOF008_scaffold29486_12	phage	0.999
NOF009_scaffold2246_1	non-phage	0
NOF009_scaffold52_4	non-phage	0
NOF009_scaffold1156_2	non-phage	0
NOF009_scaffold2011_5	non-phage	0
NOF009_scaffold43480_1	non-phage	0
NOF009_scaffold35495_1	non-phage	0
NOF009_scaffold43352_1	non-phage	0
NOF009_scaffold42645_2	non-phage	0
NOF009_scaffold37326_4	non-phage	0
NOF009_scaffold43470_1	non-phage	0
NOF009_scaffold33_1	non-phage	0
NOF009_scaffold610_2	non-phage	0
NOF009_scaffold23569_7	non-phage	0
NOF009_scaffold27945_18	non-phage	0
NOF009_scaffold33100_1	non-phage	0.105
NOF009_scaffold34471_1	non-phage	0
NOF009_scaffold43546_1	non-phage	0
NOF009_scaffold36975_2	non-phage	0
NOF009_scaffold17356_3	non-phage	0
NOF009_scaffold56_1	non-phage	0
NOF009_scaffold42968_1	non-phage	0
NOF009_scaffold29245_4	non-phage	0
NOF009_scaffold7527_22	phage	0.998
NOF009_scaffold41676_8	non-phage	0
NOF009_scaffold32280_2	non-phage	0
NOF009_scaffold29183_4	non-phage	0
NOF009_scaffold43350_1	non-phage	0
NOF009_scaffold37426_2	non-phage	0
NOF009_scaffold37707_1	non-phage	0
NOF009_scaffold16720_5	non-phage	0
NOF009_scaffold2208_2	non-phage	0
NOF009_scaffold40185_2	non-phage	0
NOF009_scaffold122_2	non-phage	0.007
NOF009_scaffold48_2	non-phage	0
NOF009_scaffold9443_11	non-phage	0
NOF009_scaffold2461_1	non-phage	0
NOF009_scaffold162_7	non-phage	0
NOF009_scaffold33144_1	non-phage	0
NOF009_scaffold12794_13	non-phage	0
NOF009_scaffold1541_8	non-phage	0

NOF009_scaffold32703_4	non-phage	0
NOF009_scaffold32_5	non-phage	0
NOF009_scaffold43402_1	non-phage	0
NOF009_scaffold41727_2	non-phage	0
NOF009_scaffold512_5	non-phage	0
NOF009_scaffold38179_1	non-phage	0
NOF009_scaffold19254_5	non-phage	0
NOF009_scaffold3760_6	non-phage	0
NOF009_scaffold25558_6	non-phage	0.001
NOF009_scaffold8317_3	non-phage	0
NOF009_scaffold14073_2	non-phage	0
NOF009_scaffold7059_16	non-phage	0
NOF009_scaffold2246_10	non-phage	0
NOF009_scaffold8723_30	non-phage	0
NOF009_scaffold85_7	non-phage	0
NOF009_scaffold21524_4	non-phage	0
NOF009_scaffold9157_3	non-phage	0
NOF009_scaffold14965_4	non-phage	0
NOF009_scaffold18741_6	non-phage	0
NOF009_scaffold29439_11	non-phage	0
NOF009_scaffold37326_1	non-phage	0
NOF009_scaffold11660_3	non-phage	0
NOF009_scaffold35038_1	non-phage	0
NOF009_scaffold12165_4	non-phage	0
NOF009_scaffold29757_2	non-phage	0
NOF009_scaffold43398_2	non-phage	0
NOF009_scaffold25928_1	non-phage	0
NOF009_scaffold41676_3	non-phage	0
NOF009_scaffold15033_4	non-phage	0
NOF009_scaffold18836_1	non-phage	0
NOF009_scaffold43376_2	non-phage	0
NOF009_scaffold2805_3	non-phage	0
NOF009_scaffold26407_7	non-phage	0
NOF009_scaffold34600_2	non-phage	0
NOF009_scaffold42636_2	non-phage	0
NOF009_scaffold33036_3	non-phage	0
NOF009_scaffold37533_1	non-phage	0
NOF009_scaffold29439_9	non-phage	0
NOF009_scaffold34657_1	non-phage	0
NOF009_scaffold39045_1	non-phage	0
NOF009_scaffold22536_3	non-phage	0
NOF009_scaffold19673_1	non-phage	0
NOF009_scaffold30432_8	non-phage	0
NOF009_scaffold27561_2	non-phage	0
NOF009_scaffold22392_2	non-phage	0
NOF009_scaffold21158_6	non-phage	0

NOF009_scaffold43306_1	non-phage	0
NOF009_scaffold1541_1	non-phage	0
NOF009_scaffold52_17	non-phage	0
NOF009_scaffold28927_2	non-phage	0
NOF009_scaffold76_1	non-phage	0
NOF009_scaffold19641_2	non-phage	0
NOF009_scaffold15699_2	non-phage	0
NOF009_scaffold512_12	non-phage	0
NOF009_scaffold32500_1	non-phage	0
NOF009_scaffold26407_3	non-phage	0
NOF009_scaffold23_1	non-phage	0
NOF009_scaffold5999_5	non-phage	0
NOF009_scaffold19904_1	non-phage	0
NOF009_scaffold38131_1	non-phage	0
NOF009_scaffold7098_1	non-phage	0
NOF009_scaffold26150_2	non-phage	0
NOF009_scaffold4063_18	non-phage	0
NOF009_scaffold1156_3	non-phage	0
NOF009_scaffold6359_11	non-phage	0
NOF009_scaffold43118_1	non-phage	0
NOF009_scaffold39043_1	non-phage	0
NOF009_scaffold43353_2	non-phage	0
NOF009_scaffold43333_1	non-phage	0
NOF009_scaffold21182_1	non-phage	0
NOF009_scaffold92_16	non-phage	0
NOF009_scaffold19618_10	non-phage	0
NOF009_scaffold43369_2	non-phage	0
NOF009_scaffold41548_3	non-phage	0
NOF009_scaffold5085_9	non-phage	0
NOF009_scaffold34600_3	non-phage	0
NOF009_scaffold40597_3	non-phage	0
NOF009_scaffold6724_2	non-phage	0
NOF009_scaffold36293_1	non-phage	0
NOF009_scaffold7754_6	non-phage	0
NOF009_scaffold18116_1	non-phage	0
NOF009_scaffold8317_6	non-phage	0
NOF009_scaffold2483_1	non-phage	0
NOF009_scaffold43502_6	non-phage	0
NOF009_scaffold7283_4	non-phage	0
NOF009_scaffold11067_3	non-phage	0
NOF009_scaffold63_1	non-phage	0
NOF009_scaffold6_4	non-phage	0
NOF009_scaffold24_16	non-phage	0
NOF009_scaffold38747_2	non-phage	0
NOF009_scaffold2534_2	non-phage	0
NOF009_scaffold43515_1	non-phage	0

NOF009_scaffold43518_2	phage	0.999
NOF009_scaffold2457_1	non-phage	0
NOF009_scaffold40883_4	non-phage	0
NOF009_scaffold162_4	non-phage	0
NOF009_scaffold21768_4	phage	0.88
NOF009_scaffold7527_13	non-phage	0
NOF009_scaffold32_4	non-phage	0
NOF009_scaffold23_8	non-phage	0
NOF009_scaffold30432_6	non-phage	0
NOF009_scaffold19618_3	non-phage	0
NOF009_scaffold12516_1	non-phage	0
NOF009_scaffold19803_1	non-phage	0
NOF009_scaffold17704_3	non-phage	0
NOF009_scaffold31458_2	non-phage	0
NOF009_scaffold43180_2	non-phage	0
NOF009_scaffold2534_12	non-phage	0
NOF009_scaffold26079_9	non-phage	0
NOF009_scaffold29332_2	non-phage	0
NOF009_scaffold43504_1	non-phage	0
NOF009_scaffold8370_3	non-phage	0
NOF009_scaffold3026_1	non-phage	0
NOF009_scaffold39576_5	non-phage	0
NOF009_scaffold36522_1	non-phage	0
NOF009_scaffold26421_3	non-phage	0
NOF009_scaffold5085_12	non-phage	0
NOF009_scaffold41192_2	non-phage	0
NOF009_scaffold32280_3	non-phage	0
NOF009_scaffold31964_2	non-phage	0
NOF009_scaffold6863_48	non-phage	0
NOF009_scaffold24402_4	non-phage	0
NOF009_scaffold29439_6	non-phage	0
NOF009_scaffold56_2	non-phage	0
NOF009_scaffold28039_2	non-phage	0
NOF009_scaffold42607_1	non-phage	0
NOF009_scaffold14847_24	non-phage	0
NOF009_scaffold19924_1	non-phage	0
NOF009_scaffold13038_4	non-phage	0
NOF009_scaffold35164_1	non-phage	0
NOF009_scaffold9020_2	non-phage	0
NOF009_scaffold377_3	non-phage	0
NOF009_scaffold34629_3	non-phage	0
NOF009_scaffold9417_2	non-phage	0
NOF009_scaffold1541_7	non-phage	0
NOF009_scaffold43376_1	non-phage	0
NOF009_scaffold23_6	non-phage	0
NOF009_scaffold11633_6	non-phage	0

NOF009_scaffold19641_1	non-phage	0
NOF009_scaffold16009_2	non-phage	0
NOF009_scaffold12165_6	non-phage	0
NOF009_scaffold41809_1	non-phage	0
NOF009_scaffold7625_4	non-phage	0
NOF009_scaffold30155_2	non-phage	0
NOF009_scaffold39_2	non-phage	0
NOF009_scaffold13900_2	non-phage	0
NOF009_scaffold2534_3	non-phage	0
NOF009_scaffold7558_2	non-phage	0
NOF009_scaffold43398_1	non-phage	0
NOF009_scaffold23569_4	non-phage	0
NOF009_scaffold43495_1	non-phage	0
NOF009_scaffold8370_1	non-phage	0
NOF009_scaffold43130_2	non-phage	0
NOF009_scaffold33036_4	non-phage	0
NOF009_scaffold4063_19	non-phage	0
NOF009_scaffold19475_9	non-phage	0
NOF009_scaffold38834_2	non-phage	0
NOF009_scaffold42787_3	non-phage	0
NOF009_scaffold23391_8	non-phage	0
NOF009_scaffold12710_3	non-phage	0
NOF009_scaffold19924_4	non-phage	0
NOF009_scaffold18741_3	non-phage	0
NOF009_scaffold52_14	non-phage	0
NOF009_scaffold43392_1	non-phage	0
NOF009_scaffold29439_12	non-phage	0
NOF009_scaffold19584_1	non-phage	0
NOF009_scaffold40185_5	non-phage	0
NOF009_scaffold22254_1	non-phage	0
NOF009_scaffold41373_1	non-phage	0
NOF009_scaffold22536_4	non-phage	0
NOF009_scaffold28727_1	non-phage	0
NOF009_scaffold40176_1	non-phage	0
NOF009_scaffold38411_7	non-phage	0
NOF009_scaffold31187_1	non-phage	0
NOF009_scaffold41036_1	non-phage	0
NOF009_scaffold43284_1	non-phage	0
NOF009_scaffold9020_1	non-phage	0
NOF009_scaffold3900_1	non-phage	0
NOF009_scaffold17545_1	non-phage	0
NOF009_scaffold17704_8	non-phage	0
NOF009_scaffold33511_1	non-phage	0
NOF009_scaffold42966_1	non-phage	0
NOF009_scaffold32904_4	non-phage	0
NOF009_scaffold25558_7	non-phage	0

NOF009_scaffold9157_2	non-phage	0
NOF009_scaffold6_5	non-phage	0
NOF009_scaffold8637_8	non-phage	0
NOF009_scaffold21524_3	non-phage	0
NOF009_scaffold21158_1	non-phage	0
NOF009_scaffold8197_7	non-phage	0
NOF009_scaffold43224_2	non-phage	0
NOF009_scaffold36091_1	non-phage	0
NOF009_scaffold38318_1	non-phage	0
NOF009_scaffold33347_2	non-phage	0
NOF009_scaffold43502_4	non-phage	0
NOF009_scaffold130_1	non-phage	0
NOF009_scaffold34600_1	non-phage	0
NOF009_scaffold2805_1	non-phage	0
NOF009_scaffold24402_1	non-phage	0
NOF009_scaffold32245_2	non-phage	0
NOF009_scaffold32329_3	non-phage	0
NOF009_scaffold33254_1	non-phage	0
NOF009_scaffold21265_2	non-phage	0
NOF009_scaffold40684_6	non-phage	0
NOF009_scaffold41328_1	non-phage	0
NOF009_scaffold18836_2	non-phage	0
NOF009_scaffold40455_1	non-phage	0
NOF009_scaffold14051_1	non-phage	0
NOF009_scaffold12165_2	non-phage	0
NOF009_scaffold30792_1	non-phage	0
NOF009_scaffold31469_2	non-phage	0
NOF009_scaffold40185_7	non-phage	0
NOF009_scaffold23391_7	non-phage	0
NOF009_scaffold22862_5	non-phage	0
NOF009_scaffold27105_2	non-phage	0
NOF009_scaffold43285_1	non-phage	0
NOF009_scaffold15033_3	non-phage	0
NOF009_scaffold37612_1	non-phage	0
NOF009_scaffold43252_1	non-phage	0
NOF009_scaffold14965_3	non-phage	0
NOF009_scaffold26562_4	non-phage	0
NOF009_scaffold14073_1	non-phage	0
NOF009_scaffold41548_4	non-phage	0
NOF009_scaffold6233_3	non-phage	0
NOF009_scaffold31105_2	non-phage	0
NOF009_scaffold5999_4	non-phage	0
NOF009_scaffold43268_1	non-phage	0
NOF009_scaffold33885_2	non-phage	0
NOF009_scaffold17339_6	non-phage	0
NOF009_scaffold3_1	non-phage	0

NOF009_scaffold43367_1	non-phage	0
NOF009_scaffold37678_3	non-phage	0
NOF009_scaffold29101_6	non-phage	0
NOF009_scaffold42496_6	non-phage	0
NOF009_scaffold41727_1	phage	1
NOF009_scaffold2632_3	non-phage	0
NOF009_scaffold17968_1	non-phage	0
NOF009_scaffold41363_4	non-phage	0
NOF009_scaffold27129_3	non-phage	0
NOF009_scaffold17834_6	non-phage	0
NOF009_scaffold7527_24	non-phage	0
NOF009_scaffold2011_3	non-phage	0
NOF009_scaffold10148_1	phage	0.999
NOF009_scaffold1678_28	non-phage	0
NOF009_scaffold20144_17	non-phage	0
NOF009_scaffold52_10	non-phage	0
NOF009_scaffold31668_1	non-phage	0
NOF009_scaffold33575_1	non-phage	0
NOF009_scaffold13038_1	non-phage	0
NOF009_scaffold15318_4	non-phage	0
NOF009_scaffold7527_23	non-phage	0
NOF009_scaffold3_4	non-phage	0
NOF009_scaffold43102_1	non-phage	0
NOF009_scaffold38700_3	non-phage	0
NOF009_scaffold32138_2	non-phage	0
NOF009_scaffold2246_14	non-phage	0
NOF009_scaffold165_2	non-phage	0
NOF009_scaffold40492_1	non-phage	0
NOF009_scaffold187_5	non-phage	0
NOF009_scaffold43419_1	non-phage	0
NOF009_scaffold43022_1	non-phage	0
NOF009_scaffold19618_8	non-phage	0
NOF009_scaffold610_4	non-phage	0
NOF009_scaffold30995_1	non-phage	0.05
NOF009_scaffold19865_4	phage	0.519
NOF009_scaffold15699_3	non-phage	0
NOF009_scaffold43502_5	non-phage	0
NOF009_scaffold10984_3	non-phage	0
NOF009_scaffold38118_1	non-phage	0
NOF009_scaffold10667_2	non-phage	0
NOF009_C558283_1	phage	0.997
NOF009_scaffold17631_1	non-phage	0
NOF009_scaffold4005_1	non-phage	0
NOF009_scaffold26242_3	non-phage	0
NOF009_scaffold40712_7	non-phage	0
NOF009_scaffold43545_3	non-phage	0

NOF009_scaffold7527_6	non-phage	0
NOF009_scaffold5085_6	non-phage	0
NOF009_scaffold2064_12	phage	0.993
NOF009_scaffold43489_1	non-phage	0
NOF009_scaffold7_1	non-phage	0
NOF009_scaffold30969_1	non-phage	0
NOF009_scaffold162_3	non-phage	0
NOF009_scaffold32565_2	non-phage	0
NOF009_scaffold17814_1	non-phage	0
NOF009_scaffold24669_3	non-phage	0
NOF009_scaffold23_7	non-phage	0
NOF009_scaffold34693_1	non-phage	0
NOF009_scaffold34681_2	non-phage	0
NOF009_scaffold34567_3	phage	0.999
NOF009_scaffold42674_1	non-phage	0
NOF009_scaffold38457_2	non-phage	0
NOF009_scaffold41195_1	non-phage	0
NOF009_scaffold43503_1	non-phage	0
NOF009_scaffold26260_4	non-phage	0
NOF009_scaffold39_7	non-phage	0
NOF009_scaffold12165_5	non-phage	0
NOF009_scaffold20444_1	non-phage	0
NOF009_scaffold21768_2	non-phage	0
NOF009_scaffold5085_7	non-phage	0
NOF009_scaffold92_14	non-phage	0
NOF009_scaffold7625_5	non-phage	0
NOF009_scaffold11228_12	phage	0.996
NOF009_scaffold39919_1	non-phage	0
NOF009_scaffold40401_1	non-phage	0
NOF009_scaffold2534_4	non-phage	0
NOF009_scaffold52_12	non-phage	0
NOF009_scaffold512_7	non-phage	0
NOF009_scaffold252_1	non-phage	0
NOF009_scaffold42496_3	non-phage	0
NOF009_scaffold40684_2	non-phage	0
NOF009_scaffold41925_1	non-phage	0
NOF009_scaffold85_9	non-phage	0
NOF009_scaffold2055_4	non-phage	0
NOF009_scaffold8302_1	non-phage	0
NOF009_scaffold25928_4	non-phage	0
NOF009_scaffold33410_1	non-phage	0
NOF009_scaffold15857_1	non-phage	0
NOF009_scaffold43186_4	non-phage	0
NOF009_scaffold38299_2	non-phage	0
NOF009_scaffold23391_2	non-phage	0
NOF009_C558345_1	non-phage	0

NOF009_scaffold2461_3	non-phage	0
NOF009_scaffold32904_3	non-phage	0
NOF009_scaffold29439_7	non-phage	0
NOF009_scaffold23509_1	non-phage	0
NOF009_scaffold24861_1	non-phage	0
NOF009_scaffold16960_2	phage	0.759
NOF009_scaffold42345_1	non-phage	0
NOF009_scaffold42597_1	non-phage	0
NOF009_C558859_1	non-phage	0
NOF009_scaffold1156_4	non-phage	0
NOF009_scaffold16009_1	non-phage	0
NOF009_scaffold5085_3	non-phage	0
NOF009_scaffold41363_3	non-phage	0
NOF009_scaffold31729_4	non-phage	0
NOF009_scaffold43543_2	non-phage	0
NOF009_scaffold38044_2	non-phage	0
NOF009_scaffold40883_2	non-phage	0
NOF009_scaffold14075_3	phage	0.999
NOF009_scaffold17778_2	non-phage	0
NOF009_scaffold32289_1	non-phage	0
NOF009_scaffold17050_1	non-phage	0
NOF009_scaffold36667_1	non-phage	0
NOF009_scaffold40969_1	non-phage	0
NOF009_scaffold11654_3	non-phage	0
NOF009_scaffold19865_6	non-phage	0
NOF009_scaffold5999_1	non-phage	0
NOF009_scaffold40597_2	non-phage	0
NOF009_scaffold2246_4	non-phage	0
NOF009_scaffold32280_6	non-phage	0
NOF009_scaffold7532_11	non-phage	0
NOF009_scaffold26670_9	non-phage	0
NOF009_scaffold48_4	non-phage	0
NOF009_scaffold28221_1	non-phage	0
NOF009_scaffold2011_4	non-phage	0
NOF009_scaffold30336_1	non-phage	0
NOF009_scaffold19899_1	non-phage	0
NOF009_scaffold38700_7	non-phage	0
NOF009_scaffold42399_6	non-phage	0
NOF009_scaffold29295_1	non-phage	0
NOF009_scaffold42111_1	non-phage	0
NOF009_scaffold39_4	non-phage	0
NOF009_scaffold8140_27	non-phage	0
NOF009_scaffold28842_1	non-phage	0
NOF009_scaffold187_2	non-phage	0
NOF009_scaffold42787_4	non-phage	0
NOF009_scaffold30973_2	non-phage	0

NOF009_scaffold31105_1	non-phage	0
NOF009_scaffold512_14	non-phage	0
NOF009_scaffold38063_1	non-phage	0
NOF009_scaffold13459_6	non-phage	0
NOF009_scaffold17631_2	non-phage	0
NOF009_scaffold24_18	non-phage	0
NOF009_scaffold33015_3	non-phage	0
NOF009_scaffold22411_1	non-phage	0
NOF009_scaffold24864_3	non-phage	0
NOF009_scaffold67_1	phage	0.999
NOF009_scaffold42496_7	non-phage	0
NOF009_scaffold32882_1	non-phage	0
NOF009_scaffold8637_7	non-phage	0
NOF009_scaffold7754_4	non-phage	0
NOF009_scaffold16960_1	non-phage	0
NOF009_scaffold38411_3	non-phage	0
NOF009_scaffold27945_15	non-phage	0
NOF009_scaffold34319_1	non-phage	0
NOF009_scaffold30524_1	non-phage	0
NOF009_scaffold162_2	non-phage	0
NOF009_scaffold38700_1	non-phage	0
NOF009_scaffold25558_8	non-phage	0
NOF009_scaffold17968_2	non-phage	0
NOF009_scaffold7527_3	non-phage	0
NOF009_scaffold34567_1	non-phage	0
NOF009_scaffold35825_1	non-phage	0
NOF009_scaffold22862_1	non-phage	0
NOF009_scaffold29332_4	non-phage	0
NOF009_scaffold35061_1	non-phage	0
NOF009_scaffold41954_1	non-phage	0
NOF009_scaffold24_25	non-phage	0
NOF009_scaffold21302_1	non-phage	0
NOF009_scaffold24402_2	non-phage	0
NOF009_scaffold43502_3	non-phage	0
NOF009_scaffold43533_2	non-phage	0
NOF009_scaffold42822_1	non-phage	0
NOF009_C558255_1	phage	0.999
NOF009_scaffold41979_3	non-phage	0
NOF009_scaffold26407_8	non-phage	0
NOF009_scaffold19331_3	non-phage	0
NOF009_scaffold42455_1	non-phage	0
NOF009_C558501_1	non-phage	0
NOF009_scaffold19254_3	non-phage	0
NOF009_scaffold29929_2	non-phage	0
NOF009_scaffold2519_2	non-phage	0
NOF009_scaffold2756_1	non-phage	0

NOF009_scaffold19803_7	non-phage	0
NOF009_scaffold15699_4	non-phage	0
NOF009_scaffold43005_1	non-phage	0
NOF009_scaffold7527_29	non-phage	0
NOF009_scaffold7029_2	non-phage	0
NOF009_scaffold2246_3	non-phage	0
NOF009_scaffold13328_11	phage	0.94
NOF009_scaffold39660_1	non-phage	0
NOF009_scaffold26150_4	non-phage	0
NOF009_scaffold31964_1	non-phage	0
NOF009_scaffold7048_3	non-phage	0
NOF009_scaffold7082_1	non-phage	0
NOF009_scaffold43118_4	non-phage	0
NOF009_scaffold7527_8	non-phage	0
NOF009_scaffold23_3	non-phage	0
NOF009_scaffold43281_2	non-phage	0
NOF009_scaffold7362_2	non-phage	0
NOF009_scaffold43159_3	non-phage	0
NOF009_scaffold43540_2	non-phage	0
NOF009_scaffold26501_1	non-phage	0
NOF009_scaffold14992_2	non-phage	0
NOF009_scaffold39553_1	non-phage	0
NOF009_scaffold43541_2	non-phage	0
NOF009_C558951_1	non-phage	0
NOF009_scaffold26079_7	non-phage	0
NOF009_scaffold42455_5	phage	0.999
NOF009_scaffold10518_12	non-phage	0
NOF009_scaffold610_3	non-phage	0
NOF009_scaffold20442_2	non-phage	0
NOF009_scaffold12039_1	non-phage	0
NOF009_scaffold32439_1	non-phage	0
NOF009_scaffold32245_1	non-phage	0
NOF009_scaffold9157_1	non-phage	0
NOF009_scaffold32280_7	non-phage	0
NOF009_scaffold3860_9	non-phage	0
NOF009_scaffold52_5	non-phage	0
NOF009_scaffold181_2	non-phage	0
NOF009_scaffold32948_1	non-phage	0
NOF009_scaffold6621_2	non-phage	0.068
NOF009_scaffold2720_3	phage	1
NOF009_scaffold31729_2	non-phage	0
NOF009_scaffold42787_5	non-phage	0
NOF009_scaffold30505_2	non-phage	0
NOF009_scaffold42265_1	non-phage	0
NOF009_scaffold42455_3	non-phage	0
NOF009_scaffold43398_3	non-phage	0

NOF009_scaffold29439_10	non-phage	0
NOF009_scaffold6_2	non-phage	0
NOF009_scaffold20192_2	non-phage	0
NOF009_scaffold29886_5	non-phage	0
NOF009_scaffold26079_3	non-phage	0
NOF009_scaffold42645_1	non-phage	0
NOF009_scaffold8197_2	non-phage	0
NOF009_scaffold15969_3	non-phage	0
NOF009_scaffold43447_1	non-phage	0
NOF009_scaffold40185_3	non-phage	0
NOF009_scaffold43347_1	non-phage	0
NOF009_scaffold10518_8	non-phage	0
NOF009_scaffold29886_10	non-phage	0
NOF009_scaffold20144_15	non-phage	0
NOF009_scaffold3990_1	non-phage	0
NOF009_scaffold40684_1	non-phage	0
NOF009_scaffold23_5	non-phage	0
NOF009_C558651_1	non-phage	0
NOF009_scaffold29439_8	non-phage	0
NOF009_scaffold29491_2	non-phage	0
NOF009_scaffold2534_7	non-phage	0
NOF009_scaffold15857_2	non-phage	0
NOF009_scaffold22254_3	non-phage	0
NOF009_scaffold2632_8	non-phage	0
NOF009_scaffold7059_11	non-phage	0
NOF009_scaffold21524_1	non-phage	0
NOF009_scaffold7754_1	non-phage	0
NOF009_scaffold25834_1	non-phage	0
NOF009_scaffold41998_1	non-phage	0
NOF009_scaffold12697_4	non-phage	0
NOF009_scaffold5085_10	non-phage	0
NOF009_scaffold19810_1	non-phage	0
NOF009_scaffold32854_1	non-phage	0
NOF009_scaffold23391_1	non-phage	0
NOF009_scaffold52_9	non-phage	0
NOF009_scaffold32280_5	non-phage	0
NOF009_scaffold43345_1	non-phage	0
NOF009_scaffold26562_3	non-phage	0
NOF009_scaffold52_2	non-phage	0
NOF009_scaffold32289_2	non-phage	0
NOF009_scaffold8637_1	non-phage	0
NOF009_scaffold11216_1	non-phage	0
NOF009_scaffold27484_1	non-phage	0
NOF009_scaffold28927_3	non-phage	0
NOF009_scaffold7625_2	non-phage	0
NOF009_scaffold39_5	non-phage	0

NOF009_scaffold4001_1	non-phage	0
NOF009_scaffold19924_3	non-phage	0
NOF009_scaffold11622_3	non-phage	0
NOF009_scaffold2136_16	non-phage	0
NOF009_scaffold26407_4	non-phage	0
NOF009_scaffold43166_1	non-phage	0.006
NOF009_scaffold43508_1	non-phage	0
NOF009_scaffold9417_4	non-phage	0
NOF009_scaffold32703_3	non-phage	0
NOF009_scaffold30524_2	non-phage	0
NOF009_scaffold19641_3	non-phage	0
NOF009_scaffold37678_1	non-phage	0
NOF009_scaffold1156_1	non-phage	0
NOF009_scaffold11067_2	non-phage	0
NOF009_scaffold32083_5	non-phage	0
NOF009_C558783_1	non-phage	0
NOF009_scaffold1532_4	non-phage	0
NOF009_scaffold41979_1	phage	0.988
NOF009_scaffold2802_2	non-phage	0
NOF009_scaffold30580_1	phage	0.993
NOF009_scaffold33456_2	non-phage	0
NOF009_scaffold30973_1	non-phage	0.395
NOF009_scaffold3037_1	non-phage	0
NOF009_scaffold30432_7	non-phage	0
NOF009_scaffold39576_2	non-phage	0
NOF009_scaffold14206_2	non-phage	0
NOF009_scaffold29289_1	non-phage	0
NOF009_scaffold2055_1	non-phage	0
NOF009_scaffold41979_2	non-phage	0
NOF009_scaffold13978_1	non-phage	0
NOF009_scaffold5136_16	phage	0.999
NOF009_scaffold40004_1	non-phage	0
NOF009_scaffold30155_1	non-phage	0
NOF009_scaffold27003_4	non-phage	0
NOF009_scaffold12340_2	non-phage	0
NOF009_scaffold9262_2	non-phage	0
NOF009_scaffold42878_5	phage	1
NOF009_scaffold43377_2	non-phage	0
NOF009_scaffold20442_3	non-phage	0
NOF009_scaffold26421_7	non-phage	0
NOF009_scaffold27_3	non-phage	0
NOF009_scaffold40903_2	non-phage	0
NOF009_scaffold8197_1	non-phage	0
NOF009_scaffold28728_1	non-phage	0
NOF009_scaffold3871_4	non-phage	0
NOF009_scaffold63_7	non-phage	0

NOF009_scaffold12501_5	non-phage	0
NOF009_scaffold43544_1	non-phage	0
NOF009_scaffold40684_5	non-phage	0
NOF009_scaffold162_8	non-phage	0
NOF009_scaffold15532_3	non-phage	0
NOF009_scaffold20116_1	non-phage	0
NOF009_scaffold41469_1	non-phage	0
NOF009_scaffold34639_3	non-phage	0
NOF009_scaffold43502_2	non-phage	0
NOF009_scaffold23_2	non-phage	0
NOF009_scaffold2246_11	non-phage	0
NOF009_scaffold7527_9	non-phage	0
NOF009_scaffold7527_10	non-phage	0
NOF009_scaffold24933_4	non-phage	0
NOF009_scaffold31419_1	non-phage	0
NOF009_scaffold15699_1	non-phage	0
NOF009_scaffold162_1	non-phage	0
NOF009_scaffold32_2	non-phage	0
NOF009_scaffold43402_2	non-phage	0
NOF009_scaffold41605_1	non-phage	0
NOF009_scaffold31458_1	non-phage	0
NOF009_scaffold9417_3	non-phage	0
NOF009_scaffold28020_2	non-phage	0
NOF009_scaffold43180_1	non-phage	0
NOF009_scaffold43281_1	non-phage	0
NOF009_scaffold13900_3	non-phage	0
NOF009_scaffold17952_1	non-phage	0
NOF009_scaffold14965_1	non-phage	0
NOF009_scaffold69_2	non-phage	0
NOF009_scaffold39_1	non-phage	0
NOF009_scaffold52_6	non-phage	0
NOF009_scaffold41129_1	non-phage	0
NOF009_scaffold3990_2	non-phage	0
NOF009_scaffold18116_2	non-phage	0
NOF009_scaffold29101_1	non-phage	0
NOF009_scaffold7527_26	non-phage	0
NOF009_scaffold2246_9	non-phage	0
NOF009_scaffold34681_3	non-phage	0
NOF009_scaffold42496_4	phage	0.961
NOF009_scaffold43369_1	non-phage	0
NOF009_scaffold22247_4	non-phage	0
NOF009_scaffold30344_1	non-phage	0
NOF009_scaffold19615_3	non-phage	0
NOF009_scaffold7337_1	non-phage	0
NOF009_scaffold12645_3	non-phage	0
NOF009_scaffold52_16	non-phage	0

NOF009_scaffold22544_4	non-phage	0
NOF009_scaffold4336_2	non-phage	0
NOF009_scaffold8197_8	non-phage	0
NOF009_scaffold24404_2	non-phage	0
NOF009_scaffold19986_2	non-phage	0
NOF009_scaffold24111_1	non-phage	0
NOF009_scaffold40221_1	non-phage	0
NOF009_scaffold43540_1	non-phage	0
NOF010_scaffold45026_1	non-phage	0
NOF010_scaffold52888_1	non-phage	0
NOF010_scaffold32872_4	non-phage	0
NOF010_scaffold20977_5	non-phage	0
NOF010_scaffold13746_2	non-phage	0
NOF010_scaffold47114_1	non-phage	0
NOF010_scaffold36010_5	non-phage	0
NOF010_scaffold24656_1	non-phage	0
NOF010_scaffold5729_16	non-phage	0
NOF010_scaffold42_2	non-phage	0
NOF010_scaffold23822_3	non-phage	0
NOF010_scaffold20091_3	non-phage	0
NOF010_scaffold30178_4	non-phage	0
NOF010_scaffold52392_1	non-phage	0
NOF010_scaffold30958_5	non-phage	0
NOF010_scaffold8_5	non-phage	0
NOF010_scaffold52043_2	non-phage	0
NOF010_scaffold53583_9	non-phage	0
NOF010_scaffold83_2	non-phage	0
NOF010_scaffold2626_3	non-phage	0
NOF010_scaffold60_4	non-phage	0
NOF010_scaffold54007_2	non-phage	0
NOF010_scaffold54302_3	non-phage	0
NOF010_scaffold22545_3	non-phage	0
NOF010_scaffold54247_2	non-phage	0
NOF010_scaffold15644_6	non-phage	0
NOF010_scaffold10806_2	non-phage	0
NOF010_scaffold153_1	non-phage	0
NOF010_scaffold19921_2	non-phage	0
NOF010_scaffold7946_2	non-phage	0
NOF010_C699885_1	phage	0.999
NOF010_scaffold15171_3	non-phage	0
NOF010_scaffold54226_1	non-phage	0
NOF010_scaffold47128_1	non-phage	0
NOF010_scaffold53615_1	non-phage	0
NOF010_scaffold5911_7	non-phage	0
NOF010_scaffold20907_1	non-phage	0
NOF010_scaffold53516_8	non-phage	0

NOF010_scaffold23947_3	non-phage	0
NOF010_scaffold33789_4	phage	0.998
NOF010_scaffold34217_4	non-phage	0
NOF010_scaffold38974_2	non-phage	0
NOF010_scaffold44020_2	non-phage	0
NOF010_scaffold15913_2	non-phage	0
NOF010_scaffold8768_1	non-phage	0
NOF010_scaffold82_2	non-phage	0
NOF010_scaffold49074_2	non-phage	0
NOF010_scaffold54301_1	non-phage	0
NOF010_scaffold49117_1	non-phage	0.112
NOF010_scaffold42506_1	non-phage	0
NOF010_scaffold29759_3	non-phage	0
NOF010_scaffold8370_2	non-phage	0
NOF010_scaffold40646_3	non-phage	0.058
NOF010_scaffold45747_7	non-phage	0
NOF010_scaffold42700_2	non-phage	0
NOF010_scaffold48727_15	non-phage	0
NOF010_scaffold16366_22	non-phage	0.295
NOF010_scaffold1583_22	non-phage	0
NOF010_scaffold45354_1	non-phage	0
NOF010_scaffold12459_6	non-phage	0
NOF010_scaffold32319_4	phage	0.998
NOF010_scaffold53380_1	non-phage	0
NOF010_scaffold22937_1	non-phage	0.396
NOF010_scaffold48703_5	non-phage	0
NOF010_scaffold23198_5	non-phage	0
NOF010_scaffold31835_3	non-phage	0
NOF010_scaffold4826_5	non-phage	0
NOF010_scaffold16897_3	non-phage	0
NOF010_scaffold53619_2	non-phage	0
NOF010_scaffold36599_1	non-phage	0
NOF010_scaffold26248_1_1	non-phage	0
NOF010_scaffold26248_1_2	non-phage	0
NOF010_scaffold26702_1	non-phage	0
NOF010_scaffold40439_2	non-phage	0
NOF010_scaffold53501_1	non-phage	0
NOF010_scaffold23102_5	non-phage	0
NOF010_scaffold46967_2	non-phage	0
NOF010_scaffold11679_12	non-phage	0
NOF010_scaffold21290_1	non-phage	0
NOF010_scaffold26425_6	phage	0.924
NOF010_scaffold54134_1	non-phage	0
NOF010_scaffold21893_37	non-phage	0
NOF010_scaffold15806_6	non-phage	0
NOF010_scaffold7431_5	non-phage	0

NOF010_scaffold21477_1	non-phage	0
NOF010_scaffold18860_1	non-phage	0
NOF010_scaffold1185_3	non-phage	0
NOF010_scaffold51939_3	non-phage	0
NOF010_scaffold32900_3	non-phage	0
NOF010_scaffold25334_2	non-phage	0
NOF010_scaffold43909_3	non-phage	0
NOF010_scaffold39067_3	non-phage	0
NOF010_scaffold24355_2	non-phage	0
NOF010_scaffold52043_1	non-phage	0
NOF010_scaffold16110_1	non-phage	0
NOF010_scaffold14373_1	phage	0.993
NOF010_scaffold33856_2	non-phage	0
NOF010_scaffold156_1	non-phage	0
NOF010_scaffold26613_2	non-phage	0
NOF010_scaffold9954_14	non-phage	0
NOF010_scaffold18502_5	non-phage	0
NOF010_scaffold28531_1	phage	0.976
NOF010_scaffold54212_1	non-phage	0
NOF010_scaffold4583_4	non-phage	0
NOF010_scaffold2119_4	non-phage	0
NOF010_scaffold31538_1	non-phage	0
NOF010_scaffold23595_2	non-phage	0
NOF010_scaffold26102_2	non-phage	0
NOF010_scaffold51712_2	phage	0.983
NOF010_scaffold24094_3	non-phage	0
NOF010_scaffold31592_3	non-phage	0
NOF010_scaffold20008_1	non-phage	0
NOF010_scaffold2722_1	non-phage	0
NOF010_scaffold25288_1	non-phage	0
NOF010_scaffold52783_3	non-phage	0
NOF010_scaffold6895_3	non-phage	0
NOF010_scaffold15258_8	non-phage	0
NOF010_scaffold9954_18	non-phage	0
NOF010_scaffold22_4	non-phage	0
NOF010_scaffold53583_2	non-phage	0
NOF010_scaffold16110_8	non-phage	0
NOF010_scaffold17353_5_1	non-phage	0
NOF010_scaffold95_10	non-phage	0
NOF010_scaffold49150_8	non-phage	0
NOF010_scaffold54211_1	non-phage	0
NOF010_scaffold7173_1	non-phage	0
NOF010_scaffold27017_5	non-phage	0
NOF010_scaffold44491_3	non-phage	0
NOF010_scaffold16934_1	non-phage	0
NOF010_scaffold6259_3	non-phage	0

NOF010_scaffold4452_1	non-phage	0
NOF010_scaffold45354_6	non-phage	0
NOF010_scaffold23102_2	non-phage	0.174
NOF010_scaffold20550_4	non-phage	0
NOF010_scaffold22545_4	non-phage	0
NOF010_scaffold46644_1	non-phage	0
NOF010_scaffold2367_5	non-phage	0
NOF010_scaffold11_4	non-phage	0
NOF010_scaffold47490_2	non-phage	0
NOF010_scaffold54_1	non-phage	0
NOF010_scaffold21893_11	non-phage	0
NOF010_scaffold21651_1	non-phage	0
NOF010_scaffold299_7	non-phage	0
NOF010_scaffold43072_10	non-phage	0
NOF010_scaffold53634_3	non-phage	0
NOF010_scaffold16110_11	non-phage	0
NOF010_scaffold18495_2	non-phage	0
NOF010_scaffold172_8	non-phage	0
NOF010_scaffold54121_1	non-phage	0
NOF010_scaffold24806_3	non-phage	0
NOF010_scaffold51638_1	non-phage	0
NOF010_scaffold5504_12	non-phage	0
NOF010_scaffold20_3	non-phage	0
NOF010_scaffold53609_1	non-phage	0
NOF010_scaffold33707_1	non-phage	0
NOF010_scaffold44702_2	non-phage	0
NOF010_scaffold32319_3	non-phage	0
NOF010_scaffold24377_1	non-phage	0
NOF010_scaffold23451_3	non-phage	0
NOF010_C699925_1	non-phage	0
NOF010_scaffold18502_2	non-phage	0
NOF010_scaffold24377_3	non-phage	0
NOF010_scaffold53892_1	non-phage	0
NOF010_scaffold3611_4	non-phage	0
NOF010_scaffold2424_2	non-phage	0
NOF010_scaffold16462_1	non-phage	0
NOF010_scaffold12274_3	non-phage	0
NOF010_scaffold9596_3	non-phage	0
NOF010_scaffold23947_2	non-phage	0
NOF010_scaffold13641_2	non-phage	0
NOF010_scaffold398_7	non-phage	0
NOF010_scaffold2119_9	non-phage	0
NOF010_scaffold25761_1	non-phage	0
NOF010_scaffold42307_2	non-phage	0
NOF010_scaffold7431_2	non-phage	0
NOF010_scaffold50238_1	non-phage	0

NOF010_scaffold2722_12	non-phage	0
NOF010_scaffold27052_4	non-phage	0
NOF010_scaffold1943_5	non-phage	0
NOF010_scaffold1222_4	non-phage	0
NOF010_scaffold43072_7	non-phage	0
NOF010_scaffold8_8	non-phage	0
NOF010_scaffold9563_11	non-phage	0
NOF010_scaffold43398_1	non-phage	0
NOF010_scaffold2885_2	non-phage	0
NOF010_scaffold43876_2	non-phage	0
NOF010_scaffold35473_1	non-phage	0
NOF010_scaffold50220_2	non-phage	0
NOF010_scaffold4510_1	non-phage	0
NOF010_scaffold2330_4	phage	0.974
NOF010_scaffold12059_10	non-phage	0
NOF010_scaffold54305_4	non-phage	0
NOF010_scaffold172_1	non-phage	0
NOF010_scaffold54273_1	non-phage	0
NOF010_scaffold33601_1	non-phage	0
NOF010_scaffold54304_1	non-phage	0
NOF010_scaffold33735_4	non-phage	0
NOF010_scaffold51222_3	non-phage	0
NOF010_scaffold6485_5	phage	0.998
NOF010_scaffold31311_2	non-phage	0
NOF010_scaffold40819_1	non-phage	0
NOF010_C699353_1	non-phage	0
NOF010_scaffold29638_2	non-phage	0
NOF010_scaffold87_4	non-phage	0
NOF010_scaffold54313_1	non-phage	0
NOF010_scaffold44040_4	non-phage	0
NOF010_scaffold54001_2	non-phage	0
NOF010_scaffold26832_3	non-phage	0
NOF010_scaffold16598_11	phage	0.972
NOF010_scaffold46346_2	non-phage	0
NOF010_scaffold9563_6	non-phage	0
NOF010_scaffold1583_5	non-phage	0
NOF010_scaffold28439_1	non-phage	0
NOF010_scaffold24182_7	non-phage	0
NOF010_scaffold54222_2	non-phage	0
NOF010_scaffold27889_1	non-phage	0
NOF010_scaffold37146_3	non-phage	0
NOF010_scaffold7673_1	non-phage	0
NOF010_scaffold52255_2	non-phage	0
NOF010_scaffold6485_3	non-phage	0
NOF010_scaffold42545_9	non-phage	0
NOF010_scaffold4826_10	non-phage	0

NOF010_scaffold9954_6	non-phage	0
NOF010_scaffold53634_2	non-phage	0
NOF010_scaffold35538_12	non-phage	0
NOF010_scaffold52710_1	non-phage	0
NOF010_scaffold237_11	non-phage	0
NOF010_scaffold2814_1	non-phage	0
NOF010_scaffold9596_4	non-phage	0
NOF010_scaffold53500_1	non-phage	0
NOF010_scaffold54282_2	non-phage	0
NOF010_scaffold53056_4	non-phage	0
NOF010_scaffold30105_1	non-phage	0
NOF010_scaffold34536_5	non-phage	0
NOF010_scaffold3611_8	non-phage	0
NOF010_scaffold44233_6	non-phage	0
NOF010_scaffold52584_2	non-phage	0
NOF010_scaffold36239_1	non-phage	0
NOF010_scaffold272_1	non-phage	0
NOF010_scaffold4494_3	non-phage	0
NOF010_scaffold3611_5	non-phage	0
NOF010_scaffold41325_1	non-phage	0.102
NOF010_scaffold51916_2	non-phage	0
NOF010_scaffold27110_9	non-phage	0
NOF010_scaffold54289_2	non-phage	0
NOF010_scaffold11431_4	non-phage	0
NOF010_scaffold52217_1	non-phage	0
NOF010_scaffold34033_5	non-phage	0
NOF010_scaffold5054_6	non-phage	0
NOF010_scaffold20182_2	phage	0.989
NOF010_scaffold19059_14	non-phage	0
NOF010_scaffold46002_1	non-phage	0
NOF010_scaffold54201_2	non-phage	0
NOF010_scaffold54314_1	non-phage	0
NOF010_scaffold54239_1	non-phage	0
NOF010_scaffold34398_1	non-phage	0
NOF010_scaffold38948_1	non-phage	0
NOF010_scaffold53939_2	non-phage	0
NOF010_scaffold52243_2	non-phage	0
NOF010_scaffold48146_1	non-phage	0
NOF010_scaffold41422_1	non-phage	0
NOF010_scaffold11404_4	non-phage	0
NOF010_scaffold45133_1	non-phage	0
NOF010_scaffold13917_7	non-phage	0
NOF010_scaffold36912_1	non-phage	0
NOF010_scaffold53966_1	non-phage	0
NOF010_scaffold31414_1	non-phage	0
NOF010_scaffold7521_3	non-phage	0

NOF010_scaffold54290_1	non-phage	0
NOF010_scaffold43393_1	non-phage	0
NOF010_scaffold5911_6	non-phage	0
NOF010_scaffold12844_1	non-phage	0
NOF010_scaffold33374_9	non-phage	0
NOF010_scaffold23664_6	non-phage	0
NOF010_scaffold2722_9	phage	0.999
NOF010_scaffold53749_2	non-phage	0
NOF010_scaffold8074_2	non-phage	0
NOF010_scaffold35262_1	non-phage	0
NOF010_scaffold38974_3	non-phage	0
NOF010_scaffold48727_2	non-phage	0
NOF010_scaffold22545_1	phage	0.859
NOF010_scaffold53516_4	non-phage	0
NOF010_scaffold50950_1	non-phage	0
NOF010_scaffold12769_3	non-phage	0
NOF010_scaffold54302_1	non-phage	0
NOF010_scaffold29701_1	non-phage	0
NOF010_scaffold44459_1	non-phage	0
NOF010_scaffold28004_2	non-phage	0
NOF010_scaffold4065_8	non-phage	0
NOF010_scaffold21289_1	non-phage	0
NOF010_scaffold16366_21	non-phage	0
NOF010_scaffold45133_5	non-phage	0
NOF010_scaffold49484_1	non-phage	0
NOF010_scaffold7521_4	non-phage	0
NOF010_scaffold37783_7	non-phage	0
NOF010_scaffold32157_5	non-phage	0
NOF010_scaffold5054_11	non-phage	0
NOF010_scaffold87_3	non-phage	0
NOF010_scaffold172_6	non-phage	0
NOF010_scaffold26707_2	non-phage	0
NOF010_scaffold44702_1	non-phage	0
NOF010_scaffold40283_8	non-phage	0
NOF010_scaffold41627_1	non-phage	0
NOF010_scaffold5784_7	non-phage	0
NOF010_scaffold1185_6	non-phage	0
NOF010_scaffold34592_1	non-phage	0
NOF010_scaffold7159_4	non-phage	0
NOF010_scaffold53180_1	non-phage	0
NOF010_scaffold45105_3	non-phage	0
NOF010_scaffold26467_2	non-phage	0
NOF010_C699667_1	non-phage	0
NOF010_scaffold54140_2	non-phage	0
NOF010_scaffold31311_1	non-phage	0
NOF010_scaffold52482_1	non-phage	0

NOF010_scaffold40646_1	non-phage	0
NOF010_scaffold13905_1	non-phage	0
NOF010_scaffold46158_1	non-phage	0
NOF010_scaffold12274_6	non-phage	0
NOF010_scaffold10874_8	non-phage	0
NOF010_scaffold53755_1	non-phage	0
NOF010_scaffold26316_5	non-phage	0
NOF010_scaffold54143_3	non-phage	0
NOF010_scaffold50864_1	non-phage	0
NOF010_scaffold29708_1	non-phage	0
NOF010_scaffold35518_1	non-phage	0
NOF010_scaffold637_5	non-phage	0
NOF010_scaffold53210_2	non-phage	0
NOF010_scaffold41594_3	non-phage	0
NOF010_scaffold8971_2	non-phage	0
NOF010_scaffold50976_1	non-phage	0
NOF010_scaffold14373_3	non-phage	0
NOF010_scaffold15644_4	non-phage	0
NOF010_scaffold53984_1	non-phage	0
NOF010_scaffold5504_17	non-phage	0
NOF010_scaffold50772_1	non-phage	0
NOF010_scaffold33039_2	non-phage	0
NOF010_scaffold30369_1	non-phage	0
NOF010_scaffold43876_1	non-phage	0
NOF010_scaffold54093_1	non-phage	0
NOF010_scaffold16521_1	non-phage	0
NOF010_scaffold43131_2	non-phage	0
NOF010_scaffold48761_2	non-phage	0
NOF010_scaffold19477_2	non-phage	0
NOF010_scaffold15601_1	non-phage	0
NOF010_scaffold5054_9	non-phage	0
NOF010_scaffold54266_4	non-phage	0
NOF010_C699119_1	non-phage	0
NOF010_scaffold20459_4	phage	0.994
NOF010_scaffold9563_25	phage	0.989
NOF010_scaffold34319_1	non-phage	0
NOF010_scaffold48161_1	non-phage	0
NOF010_scaffold6335_9	non-phage	0
NOF010_scaffold17353_8	non-phage	0
NOF010_C700069_1	non-phage	0
NOF010_scaffold44526_1	non-phage	0
NOF010_scaffold13438_5	non-phage	0
NOF010_scaffold23411_2	non-phage	0
NOF010_scaffold2865_4	non-phage	0
NOF010_scaffold46013_2	non-phage	0
NOF010_scaffold30461_1	non-phage	0

NOF010_scaffold53722_1	phage	0.949
NOF010_scaffold5911_8	non-phage	0
NOF010_scaffold4065_12	non-phage	0
NOF010_scaffold22_5	non-phage	0
NOF010_scaffold45354_4	non-phage	0
NOF010_scaffold54220_1	non-phage	0
NOF010_scaffold6340_7	non-phage	0
NOF010_scaffold11404_3	non-phage	0
NOF010_scaffold26832_1	non-phage	0
NOF010_scaffold17170_5	non-phage	0
NOF010_scaffold34398_2	non-phage	0
NOF010_scaffold53931_1	non-phage	0
NOF010_scaffold24044_1	non-phage	0
NOF010_scaffold54_2	non-phage	0
NOF010_scaffold46583_3	phage	0.803
NOF010_scaffold53819_2	non-phage	0
NOF010_scaffold54040_2	non-phage	0
NOF010_scaffold52262_4	non-phage	0
NOF010_scaffold4494_2	non-phage	0
NOF010_scaffold54315_1	non-phage	0
NOF010_scaffold6369_4	phage	0.947
NOF010_scaffold53634_1	non-phage	0
NOF010_scaffold27110_7	non-phage	0
NOF010_scaffold53479_4	non-phage	0
NOF010_scaffold9954_7	non-phage	0
NOF010_scaffold87_2	non-phage	0
NOF010_scaffold48871_1	non-phage	0
NOF010_scaffold15048_2	non-phage	0
NOF010_scaffold27877_35	non-phage	0
NOF010_C699601_1	non-phage	0
NOF010_scaffold41018_2	non-phage	0
NOF010_scaffold40643_2	non-phage	0
NOF010_scaffold8840_6	non-phage	0
NOF010_scaffold32_1	non-phage	0
NOF010_scaffold32319_2	non-phage	0
NOF010_scaffold21111_1	non-phage	0
NOF010_scaffold4274_2	non-phage	0
NOF010_scaffold10806_1	non-phage	0
NOF010_scaffold4452_3	non-phage	0
NOF010_scaffold53531_3	non-phage	0
NOF010_scaffold172_2	non-phage	0
NOF010_scaffold11431_1	non-phage	0
NOF010_scaffold36987_1	non-phage	0
NOF010_scaffold23525_12	non-phage	0
NOF010_scaffold23947_1	non-phage	0
NOF010_scaffold22_12	non-phage	0

NOF010_scaffold1191_1	non-phage	0
NOF010_scaffold6335_5	non-phage	0
NOF010_scaffold2119_6	non-phage	0
NOF010_scaffold21806_1	non-phage	0
NOF010_scaffold54227_1	non-phage	0
NOF010_scaffold6147_3	non-phage	0
NOF010_scaffold54307_1	non-phage	0.015
NOF010_scaffold4826_9	non-phage	0
NOF010_scaffold37403_2	non-phage	0
NOF010_scaffold22545_2	phage	0.999
NOF010_scaffold2119_7_2	non-phage	0
NOF010_scaffold23761_6	non-phage	0
NOF010_scaffold33668_1	non-phage	0
NOF010_scaffold26289_3	non-phage	0
NOF010_scaffold5060_1	non-phage	0
NOF010_scaffold3575_2	non-phage	0
NOF010_scaffold24005_5	non-phage	0
NOF010_scaffold24420_1	non-phage	0
NOF010_scaffold23102_8	non-phage	0
NOF010_scaffold53516_5	non-phage	0
NOF010_scaffold53533_2	non-phage	0
NOF010_scaffold54126_1	non-phage	0
NOF010_scaffold23916_1	non-phage	0
NOF010_scaffold48727_3	non-phage	0
NOF010_scaffold54105_2	non-phage	0
NOF010_scaffold40684_1	non-phage	0
NOF010_scaffold51676_2	non-phage	0
NOF010_scaffold12844_3	non-phage	0
NOF010_scaffold54299_1	non-phage	0
NOF010_scaffold3575_14	non-phage	0
NOF010_scaffold4642_2	phage	1
NOF010_scaffold54283_1	non-phage	0
NOF010_scaffold21578_1	non-phage	0
NOF010_scaffold25470_1	non-phage	0
NOF010_scaffold22_18	non-phage	0
NOF010_scaffold20511_1	non-phage	0
NOF010_scaffold18164_4	non-phage	0
NOF010_scaffold1583_23	non-phage	0
NOF010_scaffold2865_2	non-phage	0
NOF010_scaffold13918_1	non-phage	0
NOF010_scaffold49642_2	non-phage	0
NOF010_scaffold16366_12	non-phage	0
NOF010_scaffold37539_7	non-phage	0
NOF010_scaffold45528_1	non-phage	0
NOF010_scaffold34271_2	non-phage	0
NOF010_scaffold14618_2	phage	0.998

NOF010_scaffold5054_1	non-phage	0
NOF010_scaffold13641_5	non-phage	0
NOF010_scaffold30178_3	non-phage	0
NOF010_scaffold54306_1	non-phage	0
NOF010_scaffold44459_2	non-phage	0
NOF010_scaffold4590_5	non-phage	0
NOF010_scaffold46967_3	non-phage	0
NOF010_scaffold27825_1	non-phage	0
NOF010_scaffold27110_4	non-phage	0
NOF010_scaffold53850_1	non-phage	0
NOF010_scaffold49478_1	non-phage	0
NOF010_scaffold26523_4	non-phage	0
NOF010_scaffold48161_2	non-phage	0
NOF010_scaffold12460_11	non-phage	0
NOF010_scaffold28331_1	non-phage	0
NOF010_scaffold27698_3	non-phage	0
NOF010_scaffold2144_4	non-phage	0
NOF010_scaffold32157_17	non-phage	0
NOF010_scaffold20459_3	non-phage	0
NOF010_scaffold5278_1	non-phage	0
NOF010_scaffold54196_2	non-phage	0
NOF010_scaffold39200_1	non-phage	0
NOF010_scaffold53853_1	non-phage	0
NOF010_scaffold38189_1	non-phage	0
NOF010_scaffold14359_1	non-phage	0
NOF010_scaffold172_3	non-phage	0
NOF010_scaffold45354_2	non-phage	0
NOF010_scaffold7440_8	non-phage	0
NOF010_scaffold29759_1	non-phage	0
NOF010_scaffold52507_2	non-phage	0
NOF010_scaffold48274_1	non-phage	0
NOF010_scaffold7159_2	non-phage	0
NOF010_scaffold26537_1	non-phage	0
NOF010_scaffold23393_1	non-phage	0
NOF010_scaffold53492_2	non-phage	0
NOF010_scaffold32959_1	non-phage	0
NOF010_scaffold34645_2	non-phage	0
NOF010_scaffold473_7	non-phage	0
NOF010_scaffold790_2	non-phage	0
NOF010_scaffold53516_6	phage	1
NOF010_scaffold9563_8	non-phage	0
NOF010_scaffold48369_5	non-phage	0
NOF010_scaffold33103_2	non-phage	0
NOF010_scaffold43806_2	phage	0.993
NOF010_scaffold35860_1	non-phage	0
NOF010_scaffold23664_4	non-phage	0

NOF010_scaffold54274_1	non-phage	0
NOF010_scaffold54197_3	non-phage	0
NOF010_C699655_1	non-phage	0
NOF010_scaffold45915_3	non-phage	0
NOF010_scaffold95_6	non-phage	0
NOF010_scaffold36758_5	non-phage	0
NOF010_scaffold19374_2	non-phage	0
NOF010_scaffold14010_8	non-phage	0
NOF010_scaffold14359_2	non-phage	0
NOF010_scaffold52504_1	non-phage	0
NOF010_scaffold54281_2	non-phage	0
NOF010_scaffold23411_14	non-phage	0
NOF010_scaffold44702_3	non-phage	0
NOF010_scaffold1222_5	non-phage	0
NOF010_scaffold37416_2	non-phage	0
NOF010_scaffold48161_5	non-phage	0
NOF010_scaffold35836_1	non-phage	0
NOF010_scaffold223_9	non-phage	0
NOF010_scaffold23773_1	non-phage	0
NOF010_scaffold18495_1	non-phage	0
NOF010_scaffold37048_1	non-phage	0
NOF010_C699701_1	non-phage	0
NOF010_scaffold3611_3	non-phage	0
NOF010_scaffold5054_8	non-phage	0
NOF010_scaffold51040_6	non-phage	0.202
NOF010_scaffold21847_1	non-phage	0
NOF010_scaffold53596_1	non-phage	0
NOF010_scaffold4826_11	non-phage	0
NOF010_scaffold36239_3	non-phage	0
NOF010_scaffold52052_1	non-phage	0
NOF010_scaffold54225_4	non-phage	0
NOF010_scaffold37783_3	non-phage	0
NOF010_scaffold251_3	non-phage	0
NOF010_scaffold31398_3	non-phage	0
NOF010_scaffold32392_1	non-phage	0
NOF010_scaffold53879_1	non-phage	0.005
NOF010_scaffold21111_3	phage	0.999
NOF010_scaffold18664_1	non-phage	0
NOF010_scaffold24377_2	non-phage	0
NOF010_scaffold27106_1	non-phage	0
NOF010_scaffold54283_3	non-phage	0
NOF010_scaffold7421_1	non-phage	0
NOF010_scaffold31566_1	non-phage	0
NOF010_scaffold27134_3	phage	0.993
NOF010_scaffold1943_6	non-phage	0
NOF010_scaffold50663_2	non-phage	0

NOF010_scaffold22950_5	phage	0.998
NOF010_scaffold31565_1	non-phage	0
NOF010_scaffold31517_2	non-phage	0
NOF010_scaffold172_4	non-phage	0
NOF010_scaffold36539_1	non-phage	0
NOF010_scaffold3268_7	non-phage	0
NOF010_scaffold54242_1	phage	0.984
NOF010_scaffold5504_11	non-phage	0
NOF010_scaffold18995_3	non-phage	0
NOF010_scaffold17353_4	non-phage	0
NOF010_scaffold44527_1	non-phage	0
NOF010_scaffold3175_12	non-phage	0
NOF010_C700011_1	non-phage	0
NOF010_scaffold43303_9	non-phage	0
NOF010_scaffold14359_3	non-phage	0
NOF010_scaffold18502_1	non-phage	0
NOF010_scaffold20656_1	non-phage	0
NOF010_scaffold27410_5	non-phage	0
NOF010_scaffold18981_7	non-phage	0
NOF010_scaffold53948_3	non-phage	0
NOF010_scaffold54098_1	non-phage	0
NOF010_scaffold54091_1	non-phage	0
NOF010_scaffold95_2	non-phage	0
NOF010_scaffold152_3	non-phage	0
NOF010_scaffold46238_1	non-phage	0
NOF010_scaffold53366_1	non-phage	0
NOF010_scaffold53988_2	non-phage	0
NOF010_scaffold14234_8	non-phage	0
NOF010_scaffold49207_1	non-phage	0
NOF010_scaffold12460_13	non-phage	0
NOF010_scaffold25096_1	non-phage	0
NOF010_scaffold9563_7	non-phage	0
NOF010_scaffold49522_4	non-phage	0
NOF010_scaffold28519_1	non-phage	0
NOF010_scaffold6489_1	non-phage	0
NOF010_scaffold29638_1	non-phage	0
NOF010_scaffold54124_1	phage	0.97
NOF010_C699039_1	phage	0.999
NOF010_C699829_1	non-phage	0
NOF010_scaffold7946_10	non-phage	0
NOF010_scaffold43398_2	non-phage	0
NOF010_scaffold45747_2	non-phage	0
NOF010_scaffold40486_1	non-phage	0
NOF010_scaffold15806_19	non-phage	0
NOF010_scaffold23895_1	non-phage	0
NOF010_scaffold24806_1	non-phage	0

NOF010_scaffold24720_5	non-phage	0
NOF010_scaffold18357_14	non-phage	0
NOF010_scaffold54250_1	non-phage	0
NOF010_scaffold43378_1	non-phage	0
NOF010_scaffold5784_1	non-phage	0
NOF010_scaffold49325_2	non-phage	0
NOF010_scaffold29063_6	non-phage	0
NOF010_scaffold26467_4	non-phage	0
NOF010_scaffold19374_3	non-phage	0
NOF010_scaffold45725_1	non-phage	0
NOF010_scaffold30072_4	non-phage	0
NOF010_scaffold38491_5	non-phage	0
NOF010_scaffold54001_1	non-phage	0
NOF010_scaffold2330_1	non-phage	0
NOF010_scaffold34645_3	non-phage	0
NOF010_scaffold6485_10	non-phage	0
NOF010_scaffold17487_1	non-phage	0
NOF010_scaffold25090_1	non-phage	0
NOF010_scaffold15952_1	non-phage	0
NOF010_scaffold40038_1	non-phage	0
NOF010_scaffold49134_1	non-phage	0
NOF010_scaffold5819_2	non-phage	0
NOF010_scaffold23028_3	phage	0.996
NOF010_scaffold40754_2	non-phage	0
NOF010_scaffold20225_1	non-phage	0
NOF010_scaffold27110_8	non-phage	0
NOF010_scaffold6340_10	non-phage	0
NOF010_scaffold20712_3	non-phage	0
NOF010_scaffold44942_1	non-phage	0
NOF010_scaffold239_1	non-phage	0
NOF010_scaffold1943_1	non-phage	0
NOF010_scaffold23102_6	non-phage	0
NOF010_scaffold8501_22	non-phage	0
NOF010_scaffold13275_5	non-phage	0
NOF010_scaffold9563_30	non-phage	0
NOF010_scaffold52783_6	non-phage	0
NOF010_scaffold20_7	non-phage	0
NOF010_scaffold3611_7	non-phage	0
NOF010_scaffold29701_3	non-phage	0
NOF010_C699863_1	non-phage	0
NOF010_scaffold40685_1	non-phage	0
NOF010_scaffold6335_7	non-phage	0
NOF010_scaffold7521_2	non-phage	0
NOF010_scaffold53359_2	non-phage	0
NOF010_scaffold16108_4	non-phage	0
NOF010_scaffold18502_8	non-phage	0

NOF010_scaffold48161_4	non-phage	0
NOF010_scaffold30356_1	non-phage	0
NOF010_scaffold637_8	non-phage	0
NOF010_scaffold54146_1	non-phage	0
NOF010_scaffold37783_5	non-phage	0
NOF010_scaffold33103_1	phage	0.996
NOF010_scaffold26293_3	non-phage	0
NOF010_scaffold7421_5	non-phage	0
NOF010_scaffold7440_10	non-phage	0
NOF010_scaffold23761_3	non-phage	0
NOF010_scaffold54302_2	non-phage	0
NOF010_scaffold48727_4	non-phage	0
NOF010_scaffold16891_4	non-phage	0
NOF010_scaffold11404_1	non-phage	0
NOF010_C700077_1	non-phage	0
NOF010_scaffold52243_1	non-phage	0
NOF010_scaffold42586_1	non-phage	0
NOF010_scaffold7812_4	non-phage	0
NOF010_scaffold44206_4	non-phage	0
NOF010_scaffold38491_8	non-phage	0
NOF010_scaffold38939_1	non-phage	0
NOF010_scaffold51558_2	non-phage	0
NOF010_scaffold22950_1	non-phage	0
NOF010_scaffold54269_1	non-phage	0
NOF010_scaffold11110_5	non-phage	0
NOF010_scaffold21289_2	non-phage	0
NOF010_scaffold37403_1	non-phage	0
NOF010_scaffold31933_4	non-phage	0
NOF010_scaffold7811_3	non-phage	0
NOF010_scaffold38527_12	non-phage	0
NOF010_scaffold7390_1	non-phage	0
NOF010_scaffold53914_1	non-phage	0
NOF010_scaffold41310_6	non-phage	0
NOF010_scaffold54007_1	non-phage	0
NOF010_scaffold42545_11	non-phage	0
NOF010_scaffold20206_7	non-phage	0
NOF010_scaffold35787_1	non-phage	0
NOF010_scaffold14010_6	non-phage	0
NOF010_scaffold26941_1	non-phage	0
NOF010_scaffold54266_3	non-phage	0
NOF010_scaffold14234_7	non-phage	0
NOF010_scaffold14234_4	non-phage	0
NOF010_scaffold30842_1	non-phage	0
NOF010_scaffold26524_2	non-phage	0
NOF010_scaffold31923_2	non-phage	0
NOF010_scaffold33104_25	non-phage	0

NOF010_scaffold3766_18	non-phage	0
NOF010_scaffold48727_1	non-phage	0
NOF010_scaffold22861_3	non-phage	0
NOF010_scaffold53662_1	non-phage	0
NOF010_scaffold20535_3	non-phage	0
NOF010_scaffold51008_1	non-phage	0
NOF010_scaffold28004_1	non-phage	0
NOF010_scaffold35697_1	non-phage	0
NOF010_scaffold21817_1	non-phage	0
NOF010_scaffold54140_1	non-phage	0
NOF010_scaffold4826_15	non-phage	0
NOF010_scaffold12460_8	non-phage	0
NOF010_scaffold9313_1	non-phage	0
NOF010_scaffold40643_1	non-phage	0
NOF010_scaffold9341_1	non-phage	0
NOF010_scaffold83_3	non-phage	0
NOF010_scaffold8971_4	non-phage	0
NOF010_scaffold28474_6	non-phage	0
NOF010_scaffold51040_4	non-phage	0
NOF010_scaffold20616_3	non-phage	0
NOF010_scaffold15644_3	non-phage	0
NOF010_scaffold6482_1	non-phage	0
NOF010_scaffold24044_2	non-phage	0
NOF010_scaffold12198_6	non-phage	0
NOF010_scaffold39794_1	non-phage	0
NOF010_scaffold48727_14	non-phage	0
NOF010_scaffold4631_1	non-phage	0
NOF010_scaffold172_11	non-phage	0
NOF010_scaffold251_5	non-phage	0
NOF010_scaffold38970_1	non-phage	0
NOF010_scaffold54176_1	non-phage	0
NOF010_scaffold23822_1	non-phage	0
NOF010_scaffold7812_1	non-phage	0
NOF010_scaffold5198_1	non-phage	0
NOF010_scaffold24005_7	non-phage	0
NOF010_scaffold48703_1	non-phage	0
NOF010_scaffold40057_1	non-phage	0
NOF010_scaffold12769_7	non-phage	0
NOF010_scaffold54258_2	non-phage	0
NOF010_scaffold4826_4	non-phage	0
NOF010_scaffold15171_6	non-phage	0
NOF010_scaffold20514_2	non-phage	0
NOF010_scaffold5911_1	phage	0.994
NOF010_scaffold13835_1	non-phage	0
NOF010_scaffold3594_8	non-phage	0
NOF010_scaffold54312_1	non-phage	0

NOF010_scaffold35413_2	non-phage	0
NOF010_scaffold44480_2	non-phage	0
NOF010_scaffold7946_8	non-phage	0
NOF010_scaffold33789_3	non-phage	0
NOF010_scaffold22937_5	non-phage	0
NOF010_scaffold16571_1	non-phage	0
NOF010_scaffold54261_1	non-phage	0
NOF010_scaffold18502_6	non-phage	0
NOF010_scaffold28531_2	non-phage	0
NOF010_scaffold24094_2	non-phage	0
NOF010_scaffold54277_1	non-phage	0
NOF010_scaffold54257_2	non-phage	0
NOF010_scaffold24138_1	non-phage	0
NOF010_scaffold133_1	non-phage	0
NOF010_scaffold4832_10	non-phage	0
NOF010_scaffold29703_2	non-phage	0
NOF010_scaffold36758_2	non-phage	0
NOF010_scaffold2264_3	non-phage	0
NOF010_scaffold17487_7	non-phage	0
NOF010_scaffold37048_3	non-phage	0
NOF010_scaffold48398_1	non-phage	0
NOF010_scaffold20225_3	non-phage	0
NOF010_scaffold15048_1	non-phage	0
NOF010_scaffold11615_7	non-phage	0
NOF010_scaffold53506_2	non-phage	0
NOF010_scaffold15555_2	non-phage	0
NOF010_scaffold6147_6	non-phage	0
NOF010_scaffold82_5	non-phage	0
NOF010_scaffold48161_3	non-phage	0
NOF010_scaffold51916_1	non-phage	0
NOF010_scaffold268_1	non-phage	0
NOF010_scaffold31042_2	non-phage	0
NOF010_C699303_1	non-phage	0
NOF010_scaffold11306_17	non-phage	0
NOF010_scaffold26523_2	non-phage	0
NOF010_scaffold53870_1	non-phage	0
NOF010_scaffold53786_1	non-phage	0
NOF010_scaffold22792_1	non-phage	0
NOF010_scaffold18495_3	non-phage	0
NOF010_scaffold215_1	non-phage	0
NOF010_scaffold22_20	non-phage	0
NOF010_scaffold44915_1	non-phage	0
NOF010_scaffold54080_1	non-phage	0
NOF010_scaffold8370_6	non-phage	0
NOF010_scaffold47998_3	non-phage	0
NOF010_scaffold45026_4	non-phage	0

NOF010_scaffold27_1	non-phage	0
NOF010_scaffold37305_1	non-phage	0
NOF010_scaffold37539_4	non-phage	0
NOF010_scaffold40038_2	non-phage	0
NOF010_scaffold1943_8	non-phage	0
NOF010_scaffold13641_7	non-phage	0
NOF010_scaffold27017_2	non-phage	0
NOF010_scaffold52928_3	non-phage	0
NOF010_scaffold172_9	non-phage	0
NOF010_scaffold17019_3	non-phage	0
NOF010_scaffold24007_5	non-phage	0
NOF010_scaffold54037_1	non-phage	0
NOF010_scaffold12844_2	non-phage	0
NOF010_scaffold17353_6	non-phage	0
NOF010_scaffold11325_3	non-phage	0
NOF010_scaffold27126_1	non-phage	0
NOF010_scaffold54200_2	non-phage	0
NOF010_scaffold54260_1	non-phage	0
NOF010_scaffold25773_1	non-phage	0
NOF010_scaffold20535_2	non-phage	0
NOF010_scaffold43072_5	non-phage	0
NOF010_scaffold54213_1	non-phage	0
NOF010_scaffold29063_4	non-phage	0
NOF010_scaffold50067_3	non-phage	0
NOF010_C699341_1	non-phage	0
NOF010_scaffold53654_1	non-phage	0
NOF010_scaffold48727_5	non-phage	0
NOF010_scaffold53539_1	non-phage	0
NOF010_scaffold54199_2	non-phage	0
NOF010_C699001_1	non-phage	0
NOF010_scaffold40684_2	non-phage	0
NOF010_scaffold52225_1	non-phage	0
NOF010_scaffold27_4	non-phage	0
NOF010_scaffold52551_1	non-phage	0
NOF010_scaffold5504_1	non-phage	0
NOF010_scaffold3641_3	non-phage	0
NOF010_scaffold37991_1	non-phage	0
NOF010_scaffold21065_3	non-phage	0
NOF010_scaffold54189_2	non-phage	0
NOF010_scaffold52262_3	non-phage	0
NOF010_scaffold43909_1	non-phage	0
NOF010_scaffold5784_10	non-phage	0
NOF010_scaffold24720_7	non-phage	0
NOF010_scaffold47493_2	non-phage	0
NOF010_scaffold30842_2	non-phage	0
NOF010_scaffold23595_3	non-phage	0

NOF010_scaffold21032_3	non-phage	0
NOF010_scaffold19921_4	non-phage	0
NOF010_scaffold54305_3	non-phage	0
NOF010_scaffold37_1	non-phage	0
NOF010_scaffold18664_3	non-phage	0
NOF010_scaffold54168_1	non-phage	0
NOF010_scaffold15759_5	non-phage	0
NOF010_scaffold23117_1	non-phage	0
NOF010_scaffold13100_1	non-phage	0
NOF010_scaffold42381_1	non-phage	0
NOF010_scaffold22_8	non-phage	0
NOF010_scaffold27179_1	non-phage	0
NOF010_scaffold1222_1	non-phage	0
NOF010_scaffold1185_1	non-phage	0
NOF010_scaffold26537_2	non-phage	0
NOF010_scaffold54266_1	non-phage	0
NOF010_scaffold2722_15	non-phage	0
NOF010_scaffold54308_1	non-phage	0
NOF010_scaffold23658_2	non-phage	0
NOF010_scaffold13917_3	non-phage	0
NOF010_scaffold47114_3	non-phage	0
NOF010_scaffold23947_5	non-phage	0
NOF010_scaffold24094_1	non-phage	0
NOF010_scaffold53457_2	non-phage	0
NOF010_scaffold37539_6	non-phage	0
NOF010_scaffold10095_2	non-phage	0
NOF010_scaffold44862_1	non-phage	0
NOF010_scaffold54148_2	non-phage	0
NOF010_scaffold3142_9	non-phage	0
NOF010_scaffold35983_1	non-phage	0
NOF010_scaffold2865_3	non-phage	0
NOF010_C699427_1	non-phage	0
NOF010_scaffold54311_1	non-phage	0
NOF010_scaffold32959_2	non-phage	0
NOF010_scaffold5911_5	non-phage	0
NOF010_scaffold45703_3	non-phage	0
NOF010_scaffold415_3	non-phage	0
NOF010_scaffold23578_1	non-phage	0
NOF010_scaffold4890_7	non-phage	0
NOF010_scaffold54120_1	non-phage	0
NOF010_scaffold18654_2	non-phage	0
NOF010_scaffold26523_1	non-phage	0
NOF010_scaffold48369_6	non-phage	0
NOF010_scaffold10634_5	non-phage	0
NOF010_scaffold15913_1	non-phage	0
NOF010_scaffold27017_3	non-phage	0

NOF010_scaffold44306_3	non-phage	0
NOF010_scaffold12870_1	non-phage	0
NOF010_scaffold38065_1	non-phage	0
NOF010_scaffold29063_1	non-phage	0
NOF010_scaffold36061_1	non-phage	0
NOF010_scaffold22937_6	non-phage	0
NOF010_scaffold64_1	non-phage	0
NOF010_scaffold54171_1	non-phage	0
NOF010_scaffold51382_1	non-phage	0
NOF010_scaffold49484_2	non-phage	0
NOF010_scaffold12198_1	non-phage	0
NOF010_scaffold26425_4	non-phage	0
NOF010_scaffold53342_1	non-phage	0
NOF010_scaffold54300_2	non-phage	0
NOF010_scaffold2722_6	non-phage	0
NOF010_scaffold2693_2	non-phage	0
NOF010_scaffold20550_2	non-phage	0
NOF010_scaffold3575_3	non-phage	0
NOF010_scaffold26365_3	non-phage	0
NOF010_scaffold54072_2	non-phage	0
NOF010_scaffold16598_2	non-phage	0
NOF010_scaffold53492_3	non-phage	0
NOF010_scaffold31243_1	non-phage	0
NOF010_C699345_1	non-phage	0
NOF010_scaffold52888_3	non-phage	0
NOF010_scaffold53753_1	non-phage	0
NOF010_scaffold19374_1	non-phage	0
NOF010_scaffold45747_4	non-phage	0
NOF010_scaffold54281_1	phage	1
NOF010_scaffold2722_10	non-phage	0
NOF010_scaffold45133_6	non-phage	0
NOF010_scaffold4826_12	non-phage	0
NOF010_scaffold54271_1	non-phage	0
NOF010_scaffold40038_3	non-phage	0
NOF010_scaffold54201_1	non-phage	0
NOF010_scaffold6249_2	non-phage	0
NOF010_scaffold24377_6	non-phage	0
NOF010_scaffold54225_1	non-phage	0
NOF010_scaffold54288_1	non-phage	0
NOF010_scaffold30072_3	non-phage	0
NOF010_scaffold120_1	non-phage	0
NOF010_scaffold30653_1	non-phage	0
NOF010_scaffold9401_2	non-phage	0
NOF010_scaffold21936_6	non-phage	0
NOF010_scaffold52833_2	non-phage	0
NOF010_scaffold30369_9	non-phage	0

NOF010_scaffold31144_13	non-phage	0
NOF010_scaffold52414_1	non-phage	0
NOF010_scaffold22937_2	non-phage	0
NOF010_scaffold17170_9	non-phage	0
NOF010_scaffold16891_2	non-phage	0
NOF010_scaffold9347_5	non-phage	0
NOF010_scaffold1943_7	non-phage	0
NOF010_scaffold27052_1	non-phage	0
NOF010_scaffold31648_1	non-phage	0
NOF010_scaffold26467_3	non-phage	0
NOF010_scaffold54234_2	non-phage	0
NOF010_scaffold5504_9	non-phage	0
NOF010_scaffold33600_1	non-phage	0
NOF010_scaffold28938_2	non-phage	0
NOF010_scaffold5360_6	non-phage	0
NOF010_scaffold7688_1	non-phage	0
NOF010_scaffold415_6	non-phage	0
NOF010_scaffold14373_9	non-phage	0
NOF010_scaffold26293_4	non-phage	0
NOF010_scaffold16110_4	non-phage	0
NOF010_scaffold52666_1	non-phage	0
NOF010_scaffold9563_22	non-phage	0
NOF010_scaffold51156_1	non-phage	0
NOF010_scaffold52928_1	non-phage	0
NOF010_scaffold11305_3	phage	0.985
NOF010_scaffold251_4	non-phage	0
NOF010_scaffold7811_1	non-phage	0
NOF010_scaffold5353_1	non-phage	0
NOF010_scaffold15171_1	non-phage	0
NOF010_scaffold54245_1	non-phage	0
NOF010_scaffold21018_1	phage	0.999
NOF010_scaffold24806_2	non-phage	0
NOF010_scaffold36758_8	non-phage	0
NOF010_scaffold31678_1	non-phage	0
NOF010_scaffold36758_1	non-phage	0
NOF010_C699401_1	non-phage	0
NOF010_scaffold23102_4	non-phage	0
NOF010_scaffold37952_3	non-phage	0
NOF010_scaffold239_2	non-phage	0
NOF010_scaffold29063_5	non-phage	0
NOF010_scaffold23369_3	non-phage	0
NOF010_scaffold43741_2	non-phage	0
NOF010_scaffold44133_1	non-phage	0
NOF010_scaffold103_1	non-phage	0
NOF010_scaffold12460_12	non-phage	0
NOF010_scaffold52590_1	non-phage	0

NOF010_scaffold33374_2	non-phage	0
NOF010_scaffold15644_1	non-phage	0
NOF010_scaffold45869_1	non-phage	0
NOF010_scaffold32157_8	non-phage	0
NOF010_scaffold54090_2	non-phage	0
NOF010_scaffold19497_1	non-phage	0
NOF010_scaffold29888_1	non-phage	0
NOF010_scaffold53583_6	non-phage	0
NOF010_scaffold40819_2	non-phage	0
NOF010_scaffold3832_2	non-phage	0
NOF010_scaffold1943_2	non-phage	0
NOF010_scaffold5504_4	non-phage	0
NOF010_scaffold13657_8	non-phage	0
NOF010_scaffold6335_6	non-phage	0
NOF010_scaffold10874_7	non-phage	0
NOF010_scaffold40494_1	non-phage	0
NOF010_scaffold64_9	non-phage	0
NOF010_scaffold3932_1	non-phage	0
NOF010_scaffold15171_4	non-phage	0
NOF010_scaffold54303_1	non-phage	0
NOF010_scaffold52783_4	non-phage	0
NOF010_scaffold53996_3	non-phage	0
NOF010_scaffold87_1	non-phage	0
NOF010_scaffold48761_5	non-phage	0
NOF010_scaffold4065_4	non-phage	0
NOF010_scaffold24355_3	non-phage	0
NOF010_scaffold7812_2	non-phage	0
NOF010_scaffold40175_3	non-phage	0
NOF010_scaffold13917_1	non-phage	0
NOF010_scaffold2559_2	non-phage	0
NOF010_scaffold23123_1	non-phage	0
NOF010_scaffold22415_1	non-phage	0
NOF010_scaffold54026_1	non-phage	0
NOF010_scaffold10176_3	non-phage	0
NOF010_scaffold51141_1	non-phage	0
NOF010_scaffold23947_7	non-phage	0
NOF010_scaffold51222_5	non-phage	0
NOF010_scaffold54236_1	non-phage	0
NOF010_scaffold29365_2	non-phage	0
NOF010_scaffold48249_1	non-phage	0
NOF010_scaffold18502_10	non-phage	0
NOF010_scaffold9596_2	non-phage	0
NOF010_scaffold29063_2	non-phage	0
NOF010_scaffold38527_13	non-phage	0
NOF010_scaffold24720_8	non-phage	0
NOF010_scaffold54042_3	non-phage	0

NOF010_scaffold45611_1	non-phage	0
NOF010_scaffold48896_1	non-phage	0
NOF010_scaffold50365_3	non-phage	0
NOF010_scaffold16993_2	non-phage	0
NOF010_scaffold35538_9	non-phage	0
NOF010_scaffold42286_4	non-phage	0
NOF010_scaffold212_1	non-phage	0
NOF010_scaffold7431_4	non-phage	0
NOF010_scaffold83_1	non-phage	0
NOF010_scaffold6926_7	non-phage	0
NOF010_scaffold32319_1	non-phage	0
NOF010_scaffold21111_2	non-phage	0
NOF010_scaffold5784_6	non-phage	0
NOF010_scaffold53531_2	non-phage	0
NOF010_C699805_1	non-phage	0
NOF010_scaffold17487_5	non-phage	0
NOF010_scaffold20535_5	non-phage	0
NOF010_scaffold31042_1	non-phage	0
NOF010_scaffold7421_4	non-phage	0
NOF010_scaffold36010_6	non-phage	0
NOF011_scaffold48026_3	non-phage	0
NOF011_scaffold208_2	non-phage	0
NOF011_scaffold55119_1	non-phage	0
NOF011_scaffold30363_4	non-phage	0
NOF011_scaffold44314_1	non-phage	0
NOF011_scaffold21129_3	non-phage	0
NOF011_scaffold33_1	non-phage	0
NOF011_scaffold58809_2	non-phage	0
NOF011_scaffold16553_2	non-phage	0
NOF011_scaffold5432_6	non-phage	0
NOF011_scaffold10953_3	non-phage	0
NOF011_scaffold28993_53	non-phage	0
NOF011_scaffold3878_1	phage	0.999
NOF011_scaffold7058_11	non-phage	0
NOF011_scaffold11494_1	non-phage	0
NOF011_scaffold57558_3	phage	0.999
NOF011_scaffold25356_1	non-phage	0
NOF011_scaffold41004_1	non-phage	0
NOF011_scaffold35598_3	non-phage	0
NOF011_scaffold16907_23	non-phage	0
NOF011_scaffold51959_6	non-phage	0
NOF011_scaffold35226_1	non-phage	0
NOF011_scaffold58896_1	non-phage	0
NOF011_C736796_1	non-phage	0
NOF011_scaffold5432_3	non-phage	0
NOF011_scaffold44680_1	non-phage	0

NOF011_scaffold1013_4	non-phage	0
NOF011_scaffold27151_1	non-phage	0
NOF011_scaffold10693_1	non-phage	0
NOF011_scaffold49124_2	non-phage	0
NOF011_scaffold56445_1	non-phage	0
NOF011_scaffold30363_3	non-phage	0
NOF011_scaffold6890_4	non-phage	0
NOF011_scaffold54498_1	non-phage	0
NOF011_scaffold59085_1	non-phage	0
NOF011_scaffold58976_4	non-phage	0
NOF011_scaffold203_2	non-phage	0
NOF011_scaffold32261_6	non-phage	0
NOF011_scaffold27189_3	non-phage	0
NOF011_scaffold14034_7	non-phage	0
NOF011_scaffold171_4	non-phage	0
NOF011_scaffold7675_1	non-phage	0
NOF011_scaffold58361_3	non-phage	0
NOF011_scaffold40568_4	non-phage	0
NOF011_scaffold5846_39	non-phage	0.194
NOF011_scaffold58845_1	non-phage	0
NOF011_scaffold58891_2	non-phage	0
NOF011_scaffold29752_3	non-phage	0
NOF011_scaffold40870_5	non-phage	0
NOF011_scaffold110_4	non-phage	0
NOF011_scaffold32348_4	non-phage	0
NOF011_scaffold41562_2	non-phage	0
NOF011_scaffold53780_2	non-phage	0
NOF011_scaffold53780_1	non-phage	0
NOF011_scaffold1588_1	non-phage	0
NOF011_scaffold55274_1	non-phage	0
NOF011_scaffold45497_2	non-phage	0
NOF011_scaffold16334_3	non-phage	0
NOF011_scaffold57390_1	non-phage	0
NOF011_scaffold59074_1	non-phage	0
NOF011_scaffold29831_2	non-phage	0
NOF011_scaffold59067_1	non-phage	0
NOF011_scaffold56300_2	non-phage	0
NOF011_scaffold57360_1	non-phage	0
NOF011_scaffold15688_7	non-phage	0
NOF011_scaffold47105_1	non-phage	0
NOF011_scaffold45651_1	non-phage	0
NOF011_scaffold159_2	phage	0.999
NOF011_scaffold56596_1	non-phage	0
NOF011_scaffold56478_3	non-phage	0
NOF011_scaffold7058_8	non-phage	0
NOF011_scaffold41092_1	non-phage	0

NOF011_scaffold128_1	non-phage	0
NOF011_scaffold2529_5	non-phage	0
NOF011_scaffold52398_2	non-phage	0
NOF011_scaffold44488_4	non-phage	0
NOF011_scaffold54802_1	non-phage	0
NOF011_scaffold23997_2	non-phage	0.002
NOF011_scaffold59098_1	non-phage	0
NOF011_scaffold58919_1	non-phage	0
NOF011_scaffold41831_2	non-phage	0
NOF011_scaffold52517_4	non-phage	0
NOF011_scaffold22950_2	non-phage	0
NOF011_scaffold35198_1	non-phage	0
NOF011_scaffold15879_2	non-phage	0
NOF011_scaffold59091_7	non-phage	0
NOF011_scaffold2794_7	non-phage	0
NOF011_scaffold42008_3	non-phage	0
NOF011_scaffold58405_2	non-phage	0
NOF011_scaffold199_8	non-phage	0
NOF011_scaffold33767_5	non-phage	0
NOF011_scaffold17432_2	non-phage	0
NOF011_scaffold57717_1	non-phage	0
NOF011_scaffold171_5	non-phage	0
NOF011_scaffold54211_1	non-phage	0
NOF011_scaffold46447_2	non-phage	0
NOF011_scaffold7058_2	non-phage	0
NOF011_scaffold58892_1	non-phage	0
NOF011_scaffold24755_21	non-phage	0
NOF011_scaffold59099_1	non-phage	0
NOF011_scaffold161_2	non-phage	0
NOF011_scaffold59086_1	non-phage	0
NOF011_scaffold56176_3	phage	1
NOF011_scaffold28099_3	non-phage	0
NOF011_scaffold15456_2	non-phage	0
NOF011_scaffold28934_1	non-phage	0
NOF011_scaffold5846_55	non-phage	0
NOF011_scaffold45209_4	non-phage	0
NOF011_scaffold27777_5	non-phage	0
NOF011_scaffold43038_2	non-phage	0
NOF011_scaffold17142_9	non-phage	0
NOF011_scaffold59100_1	non-phage	0
NOF011_scaffold18490_3	phage	0.999
NOF011_scaffold59036_2	non-phage	0
NOF011_scaffold13821_15	non-phage	0
NOF011_scaffold37519_10	non-phage	0
NOF011_scaffold27189_4	non-phage	0
NOF011_scaffold53780_4	non-phage	0

NOF011_scaffold43733_1	non-phage	0
NOF011_scaffold26226_3	non-phage	0
NOF011_scaffold41284_4	non-phage	0
NOF011_scaffold57828_1	non-phage	0
NOF011_scaffold7058_3	non-phage	0
NOF011_scaffold59010_2	non-phage	0
NOF011_scaffold24282_3	non-phage	0
NOF011_scaffold4406_1	non-phage	0
NOF011_scaffold59088_2	phage	0.996
NOF011_scaffold52477_2	non-phage	0
NOF011_scaffold12_1	non-phage	0
NOF011_scaffold36609_2	non-phage	0
NOF011_scaffold47308_3	non-phage	0
NOF011_scaffold35022_1	non-phage	0
NOF011_scaffold48446_4	non-phage	0
NOF011_scaffold8028_19	phage	0.999
NOF011_scaffold57790_2	non-phage	0
NOF011_scaffold25_1	non-phage	0
NOF011_scaffold58896_2	non-phage	0
NOF011_scaffold28993_37	non-phage	0
NOF011_scaffold35221_1	non-phage	0
NOF011_scaffold42317_2	non-phage	0
NOF011_scaffold52477_3	non-phage	0
NOF011_scaffold40867_1	non-phage	0
NOF011_scaffold53093_1	non-phage	0
NOF011_scaffold90_4	non-phage	0
NOF011_scaffold24632_4	non-phage	0
NOF011_scaffold20477_1	non-phage	0
NOF011_scaffold5708_3	non-phage	0
NOF011_scaffold51294_1	non-phage	0
NOF011_scaffold57546_1	non-phage	0
NOF011_scaffold33258_2	phage	0.871
NOF011_scaffold58945_3	non-phage	0
NOF011_scaffold55401_1	non-phage	0
NOF011_scaffold40687_1	non-phage	0
NOF011_scaffold3620_24	non-phage	0
NOF011_scaffold30363_5	non-phage	0
NOF011_scaffold41870_6	non-phage	0
NOF011_scaffold58887_1	non-phage	0
NOF011_scaffold4407_1	non-phage	0
NOF011_scaffold30363_2	non-phage	0
NOF011_scaffold58725_1	non-phage	0
NOF011_scaffold59078_1	non-phage	0
NOF011_scaffold2794_3	non-phage	0
NOF011_scaffold51821_1	non-phage	0
NOF011_scaffold57339_1	non-phage	0

NOF011_scaffold28993_44	non-phage	0
NOF011_scaffold43841_2	non-phage	0
NOF011_scaffold58584_1	phage	0.942
NOF011_scaffold9_2	non-phage	0
NOF011_scaffold58638_1	non-phage	0
NOF011_scaffold52517_3	non-phage	0
NOF011_scaffold58361_2	non-phage	0
NOF011_C736458_1	non-phage	0
NOF011_scaffold59051_1	non-phage	0
NOF011_scaffold6564_1	non-phage	0
NOF011_scaffold26602_3	non-phage	0
NOF011_scaffold38741_5	non-phage	0
NOF011_scaffold41628_8	non-phage	0
NOF011_scaffold74_1	non-phage	0
NOF011_scaffold70_15	phage	0.994
NOF011_scaffold5846_13	non-phage	0
NOF011_scaffold44366_1	non-phage	0
NOF011_scaffold40991_1	non-phage	0
NOF011_scaffold27190_1	non-phage	0
NOF011_scaffold58651_3	non-phage	0
NOF011_scaffold55210_2	non-phage	0
NOF011_scaffold2794_6	non-phage	0
NOF011_scaffold48688_2	non-phage	0
NOF011_scaffold58981_1	phage	0.998
NOF011_scaffold7058_9	non-phage	0
NOF011_scaffold59084_1	non-phage	0
NOF011_scaffold57405_3	non-phage	0
NOF011_scaffold51959_4	non-phage	0
NOF011_scaffold41710_2	non-phage	0
NOF011_scaffold38085_3	non-phage	0
NOF011_scaffold35986_1	non-phage	0
NOF011_scaffold12194_1	non-phage	0
NOF011_scaffold48453_1	non-phage	0
NOF011_scaffold48280_1	non-phage	0
NOF011_scaffold37519_6	non-phage	0
NOF011_scaffold20814_1	non-phage	0
NOF011_scaffold59056_1	phage	0.999
NOF011_scaffold9_3	non-phage	0
NOF011_scaffold17142_7	non-phage	0
NOF011_scaffold41857_1	non-phage	0
NOF011_scaffold59034_2	non-phage	0
NOF011_scaffold37063_2	non-phage	0
NOF011_scaffold59100_2	phage	0.999
NOF011_scaffold27189_1	phage	0.964
NOF011_scaffold17142_3	non-phage	0
NOF011_scaffold15688_8	non-phage	0

NOF011_scaffold56478_2	non-phage	0
NOF011_scaffold41908_4	non-phage	0
NOF011_scaffold57546_2	phage	0.997
NOF011_scaffold15557_2	non-phage	0
NOF011_scaffold59077_1	non-phage	0
NOF011_scaffold17991_1	non-phage	0
NOF011_scaffold54967_1	non-phage	0
NOF011_scaffold46370_2	non-phage	0
NOF011_scaffold59044_1	non-phage	0
NOF011_scaffold14034_14	non-phage	0
NOF011_scaffold59095_1	non-phage	0
NOF011_scaffold59096_2	phage	0.743
NOF011_scaffold58405_5	non-phage	0
NOF011_scaffold58951_1	non-phage	0
NOF011_scaffold15798_1	phage	1
NOF011_scaffold203_3	non-phage	0
NOF011_scaffold59096_3	non-phage	0
NOF011_scaffold58172_1	non-phage	0
NOF011_scaffold51326_3	non-phage	0
NOF011_scaffold51259_1	non-phage	0
NOF011_scaffold49682_1	non-phage	0
NOF011_scaffold58976_1	non-phage	0
NOF011_scaffold319_1	non-phage	0
NOF011_scaffold45497_1	non-phage	0
NOF011_scaffold1013_2	non-phage	0
NOF011_scaffold48732_4	non-phage	0
NOF011_scaffold49608_1	non-phage	0
NOF011_C736744_1	non-phage	0
NOF011_scaffold39840_1	non-phage	0
NOF011_scaffold41562_3	non-phage	0
NOF011_scaffold1207_3	non-phage	0
NOF011_scaffold58988_1	non-phage	0
NOF011_scaffold58582_1	non-phage	0
NOF011_scaffold59093_1	non-phage	0
NOF011_scaffold19748_33	non-phage	0
NOF011_scaffold32679_1	non-phage	0
NOF011_scaffold27151_13	non-phage	0
NOF011_scaffold39840_2	non-phage	0
NOF011_scaffold9523_1	non-phage	0
NOF011_scaffold58361_5	non-phage	0
NOF011_scaffold37519_1	non-phage	0
NOF011_scaffold50811_1	non-phage	0
NOF011_C736712_1	phage	0.999
NOF011_scaffold22865_2	non-phage	0
NOF011_scaffold57790_1	non-phage	0
NOF011_scaffold48156_1	non-phage	0

NOF011_scaffold50101_1	non-phage	0
NOF011_scaffold59036_1	non-phage	0
NOF011_scaffold59069_2	non-phage	0
NOF011_scaffold22453_1	non-phage	0
NOF011_scaffold58649_1	non-phage	0
NOF011_scaffold38396_4	non-phage	0
NOF011_scaffold58752_1	non-phage	0
NOF011_scaffold30363_6	non-phage	0
NOF011_scaffold59080_1	non-phage	0
NOF011_scaffold58833_1	non-phage	0
NOF011_scaffold28301_2	non-phage	0
NOF011_scaffold7058_7	non-phage	0
NOF011_scaffold43802_2	non-phage	0
NOF011_scaffold39216_1	non-phage	0
NOF011_scaffold51645_1	non-phage	0
NOF011_scaffold2418_6	non-phage	0
NOF011_scaffold19916_2	non-phage	0
NOF011_scaffold7058_4	non-phage	0
NOF011_scaffold48802_1	phage	1
NOF011_scaffold715_2	non-phage	0
NOF011_scaffold16711_1	non-phage	0
NOF011_C736254_1	non-phage	0
NOF011_scaffold54899_2	non-phage	0
NOF011_scaffold51680_1	non-phage	0
NOF011_scaffold15034_1	non-phage	0
NOF011_scaffold56320_1	non-phage	0
NOF011_scaffold10244_1	non-phage	0
NOF011_scaffold46370_3	non-phage	0
NOF011_scaffold10196_1	non-phage	0
NOF011_scaffold57126_1	phage	0.989
NOF011_scaffold24282_7	non-phage	0
NOF011_scaffold2794_2	non-phage	0
NOF011_scaffold15688_5	non-phage	0
NOF011_scaffold34761_1	non-phage	0
NOF011_scaffold27777_2	non-phage	0
NOF011_scaffold46365_1	non-phage	0
NOF011_scaffold58331_1	non-phage	0
NOF011_scaffold2794_5	non-phage	0
NOF011_scaffold4552_1	non-phage	0
NOF011_scaffold37519_7	non-phage	0
NOF011_scaffold6040_10	non-phage	0
NOF011_scaffold15082_1	non-phage	0
NOF011_scaffold10860_4	non-phage	0
NOF011_scaffold59048_1	non-phage	0
NOF011_scaffold51892_1	non-phage	0
NOF011_scaffold3876_2	non-phage	0

NOF011_scaffold55847_1	non-phage	0
NOF011_scaffold27189_2	non-phage	0
NOF011_scaffold8241_16	non-phage	0
NOF011_scaffold14592_7	non-phage	0
NOF011_scaffold9936_1	non-phage	0
NOF011_scaffold46979_2	non-phage	0
NOF011_scaffold24755_46	non-phage	0
NOF011_scaffold19551_2	non-phage	0
NOF011_scaffold58094_2	non-phage	0
NOF011_scaffold58187_2	non-phage	0
NOF011_scaffold30363_1	non-phage	0
NOF011_scaffold57723_1	non-phage	0
NOF011_scaffold21383_1	non-phage	0
NOF011_scaffold35986_2	non-phage	0
NOF011_scaffold6564_4	non-phage	0
NOF011_scaffold54401_1	non-phage	0
NOF011_scaffold58896_3	non-phage	0
NOF011_scaffold41059_1	non-phage	0
NOF011_scaffold10953_1	non-phage	0
NOF011_scaffold45488_2	non-phage	0
NOF011_scaffold16128_2	non-phage	0
NOF011_scaffold17142_4	non-phage	0
NOF011_scaffold58633_1	non-phage	0
NOF011_scaffold35_5	non-phage	0
NOF011_scaffold44611_3	non-phage	0
NOF011_scaffold24974_1	non-phage	0
NOF011_scaffold6040_4	non-phage	0
NOF011_scaffold2529_3	non-phage	0
NOF011_scaffold37063_3	non-phage	0
NOF011_scaffold44277_2	non-phage	0
NOF011_scaffold57558_1	non-phage	0
NOF011_scaffold54211_2	non-phage	0
NOF011_scaffold33711_1	non-phage	0
NOF011_scaffold11648_1	non-phage	0
NOF011_scaffold53817_2	non-phage	0
NOF011_scaffold33589_4	non-phage	0
NOF011_scaffold45660_1	non-phage	0
NOF011_scaffold41908_2	non-phage	0
NOF011_C736944_1	non-phage	0
NOF011_scaffold21010_1	non-phage	0
NOF011_scaffold58763_1	non-phage	0
NOF011_scaffold10860_7	non-phage	0
NOF011_scaffold58945_2	non-phage	0
NOF011_scaffold44766_2	non-phage	0
NOF011_scaffold58551_1	non-phage	0
NOF011_scaffold57970_3	non-phage	0

NOF011_C736972_1	non-phage	0
NOF011_scaffold27151_9	non-phage	0
NOF011_scaffold31760_2	non-phage	0
NOF011_scaffold54930_1	non-phage	0
NOF011_scaffold185_3	non-phage	0
NOF011_scaffold58405_4	non-phage	0
NOF011_scaffold52600_1	phage	0.997
NOF011_scaffold16712_1	non-phage	0
NOF011_scaffold40291_1	non-phage	0
NOF011_scaffold14034_15	non-phage	0
NOF011_scaffold42008_1	non-phage	0
NOF011_scaffold1013_3	non-phage	0
NOF011_scaffold41599_1	non-phage	0
NOF011_scaffold16353_1	non-phage	0
NOF011_scaffold109_1	non-phage	0
NOF011_scaffold6040_3	non-phage	0
NOF011_scaffold5432_4	non-phage	0
NOF011_scaffold27701_2	non-phage	0
NOF011_scaffold56176_2	phage	0.999
NOF011_scaffold57858_1	non-phage	0
NOF011_scaffold28099_5	non-phage	0
NOF011_scaffold28301_3	non-phage	0
NOF011_scaffold58809_1	non-phage	0
NOF011_scaffold8028_20	phage	0.997
NOF011_scaffold50692_2	non-phage	0
NOF011_scaffold6890_2	non-phage	0
NOF011_scaffold16430_2	non-phage	0
NOF011_scaffold14034_12	non-phage	0
NOF011_scaffold11675_1	non-phage	0
NOF011_scaffold6282_4	non-phage	0
NOF011_scaffold43928_2	non-phage	0
NOF011_scaffold56989_1	non-phage	0
NOF011_scaffold52517_1	non-phage	0
NOF011_scaffold55798_1	non-phage	0
NOF011_scaffold59076_1	non-phage	0
NOF011_scaffold49709_1	non-phage	0
NOF011_scaffold17142_2	non-phage	0
NOF011_scaffold14034_6	non-phage	0
NOF011_scaffold21129_2	non-phage	0
NOF011_scaffold5846_45	non-phage	0
NOF011_scaffold31189_1	non-phage	0
NOF011_scaffold3620_5	non-phage	0
NOF011_scaffold9726_4	non-phage	0
NOF011_scaffold58394_1	non-phage	0
NOF011_scaffold203_1	non-phage	0
NOF011_scaffold2468_1	non-phage	0

NOF011_scaffold32208_1	non-phage	0
NOF011_scaffold51959_2	non-phage	0
NOF011_scaffold9_4	non-phage	0
NOF011_scaffold24385_1	non-phage	0
NOF011_scaffold6890_3	non-phage	0
NOF011_scaffold27151_14	non-phage	0
NOF011_scaffold7058_5	non-phage	0
NOF011_scaffold49838_1	non-phage	0
NOF011_scaffold58859_1	non-phage	0
NOF011_scaffold58752_2	non-phage	0
NOF011_scaffold13177_1	non-phage	0
NOF011_scaffold53987_1	non-phage	0
NOF011_scaffold44784_4	non-phage	0
NOF011_scaffold58710_1	non-phage	0
NOF011_scaffold19973_6	non-phage	0
NOF011_scaffold51_2	non-phage	0
NOF011_scaffold46933_1	non-phage	0
NOF011_scaffold171_1	non-phage	0
NOF011_scaffold53109_2	non-phage	0
NOF011_scaffold59049_1	non-phage	0
NOF011_scaffold50572_2	non-phage	0
NOF011_scaffold35022_3	non-phage	0
NOF011_scaffold31676_3	non-phage	0
NOF011_scaffold27150_5	non-phage	0
NOF011_scaffold10196_2	non-phage	0
NOF011_scaffold58886_2	non-phage	0
NOF011_scaffold59072_1	non-phage	0
NOF011_scaffold2529_7	phage	0.882
NOF011_scaffold45498_1	phage	0.815
NOF011_scaffold14034_1	non-phage	0
NOF011_scaffold42024_1	non-phage	0
NOF011_C736624_1	non-phage	0
NOF011_scaffold43790_3	non-phage	0
NOF011_scaffold40128_2	phage	0.953
NOF011_scaffold59033_2	non-phage	0
NOF011_scaffold59063_1	non-phage	0
NOF011_scaffold10230_2	non-phage	0
NOF011_scaffold28099_2	non-phage	0
NOF011_scaffold30474_4	non-phage	0
NOF011_scaffold57036_1	non-phage	0
NOF011_scaffold20389_2	non-phage	0
NOF011_scaffold185_1	non-phage	0
NOF011_scaffold6040_7	non-phage	0
NOF011_scaffold54420_3	non-phage	0
NOF011_scaffold28099_1	non-phage	0
NOF011_scaffold22306_4	non-phage	0

NOF011_scaffold57073_2	non-phage	0
NOF011_scaffold59007_1	non-phage	0
NOF011_scaffold11411_1	non-phage	0
NOF011_scaffold50296_2	non-phage	0
NOF011_scaffold42024_4	non-phage	0
NOF011_scaffold17321_4	non-phage	0
NOF011_scaffold57773_1	non-phage	0
NOF011_scaffold58243_1	non-phage	0
NOF011_scaffold41496_2	non-phage	0
NOF011_scaffold25352_1	non-phage	0
NOF011_scaffold59091_3	non-phage	0
NOF011_C736388_1	non-phage	0
NOF011_scaffold10860_6	non-phage	0
NOF011_scaffold56478_4	non-phage	0
NOF011_scaffold47534_3	non-phage	0
NOF011_scaffold2794_4	non-phage	0
NOF011_scaffold311_2	non-phage	0
NOF011_scaffold14034_16	non-phage	0
NOF011_scaffold15688_6	non-phage	0
NOF011_scaffold6282_2	non-phage	0
NOF011_scaffold171_6	non-phage	0
NOF011_scaffold47057_3	non-phage	0
NOF011_scaffold26088_1	non-phage	0
NOF011_C736372_1	non-phage	0
NOF011_scaffold45027_1	non-phage	0
NOF011_scaffold40656_2	non-phage	0
NOF011_scaffold6113_2	non-phage	0
NOF011_scaffold21552_2	non-phage	0
NOF011_scaffold3878_3	non-phage	0
NOF011_scaffold58094_1	non-phage	0
NOF011_scaffold53954_1	non-phage	0
NOF011_scaffold58605_1	non-phage	0
NOF011_scaffold4826_2	non-phage	0
NOF011_scaffold24731_4	non-phage	0
NOF011_scaffold44543_3	non-phage	0
NOF011_scaffold14359_2	non-phage	0
NOF011_scaffold21584_3	non-phage	0
NOF011_scaffold57970_2	non-phage	0
NOF011_scaffold2794_1	non-phage	0
NOF011_scaffold45092_6	non-phage	0.075
NOF011_scaffold10953_2	non-phage	0
NOF011_scaffold37519_8	non-phage	0
NOF011_scaffold35221_2	non-phage	0
NOF011_scaffold110_1	non-phage	0
NOF011_scaffold45411_1	non-phage	0
NOF011_C736326_1	non-phage	0.289

NOF011_scaffold47308_1	non-phage	0
NOF011_scaffold42714_1	non-phage	0
NOF011_scaffold42611_3	non-phage	0
NOF011_scaffold58662_1	non-phage	0
NOF011_scaffold58921_1	non-phage	0
NOF011_C736960_1	non-phage	0
NOF011_scaffold53597_5	non-phage	0
NOF011_scaffold11768_12	non-phage	0
NOF012_scaffold31085_8	non-phage	0
NOF012_scaffold77459_1	phage	0.998
NOF012_scaffold1013_9	non-phage	0
NOF012_scaffold71024_7	phage	0.983
NOF012_C833763_1	non-phage	0
NOF012_scaffold75991_5	non-phage	0
NOF012_scaffold66482_1	non-phage	0
NOF012_scaffold29562_22	non-phage	0
NOF012_scaffold31085_5	non-phage	0
NOF012_scaffold47172_1	non-phage	0
NOF012_scaffold70947_1	non-phage	0
NOF012_scaffold10353_13	non-phage	0
NOF012_scaffold76511_1	non-phage	0
NOF012_scaffold37731_2	non-phage	0
NOF012_scaffold77267_2	phage	0.997
NOF012_scaffold71765_4	non-phage	0
NOF012_scaffold78048_1	non-phage	0
NOF012_scaffold24523_4	phage	0.998
NOF012_scaffold68463_1	non-phage	0
NOF012_scaffold71431_1	non-phage	0
NOF012_scaffold77616_2	non-phage	0
NOF012_scaffold19933_2	non-phage	0
NOF012_scaffold43748_1	non-phage	0
NOF012_scaffold18912_1	non-phage	0
NOF012_scaffold50891_1	non-phage	0
NOF012_scaffold67_3	non-phage	0
NOF012_scaffold19351_1	non-phage	0
NOF012_scaffold23932_25	non-phage	0
NOF012_scaffold58520_1	non-phage	0
NOF012_scaffold44041_2	non-phage	0
NOF012_scaffold62924_3	non-phage	0.34
NOF012_scaffold18583_1	non-phage	0
NOF012_scaffold12244_5	phage	1
NOF012_scaffold62192_4	non-phage	0
NOF012_scaffold78074_1	non-phage	0
NOF012_scaffold71874_5	non-phage	0
NOF012_scaffold50054_1	non-phage	0
NOF012_scaffold41783_3	non-phage	0

NOF012_scaffold77898_1	non-phage	0.109
NOF012_scaffold48292_1	non-phage	0
NOF012_scaffold70924_2	non-phage	0
NOF012_scaffold46734_2	non-phage	0
NOF012_scaffold34940_17	non-phage	0
NOF012_scaffold37026_5	non-phage	0
NOF012_scaffold50466_2	phage	1
NOF012_scaffold14742_6	non-phage	0
NOF012_scaffold77982_1	non-phage	0
NOF012_scaffold74676_2	non-phage	0
NOF012_scaffold14742_8	non-phage	0
NOF012_scaffold78076_1	non-phage	0
NOF012_scaffold5639_4	phage	1
NOF012_scaffold65690_1	non-phage	0
NOF012_scaffold77771_4	non-phage	0
NOF012_scaffold40163_7	non-phage	0
NOF012_scaffold10713_2	non-phage	0
NOF012_scaffold74111_4	non-phage	0
NOF012_scaffold7935_7	non-phage	0
NOF012_C833941_1	non-phage	0
NOF012_scaffold45416_1	non-phage	0
NOF012_scaffold31286_40	non-phage	0
NOF012_scaffold44598_4	non-phage	0
NOF012_scaffold41046_6	non-phage	0
NOF012_scaffold47590_6	non-phage	0
NOF012_scaffold69941_1	non-phage	0
NOF012_scaffold76588_2	non-phage	0
NOF012_scaffold1013_11	non-phage	0
NOF012_scaffold76042_1	non-phage	0
NOF012_scaffold5305_3	non-phage	0
NOF012_scaffold78052_2	non-phage	0
NOF012_scaffold47590_16	non-phage	0
NOF012_scaffold5282_2	non-phage	0
NOF012_scaffold77999_1	non-phage	0
NOF012_scaffold65690_2	non-phage	0
NOF012_scaffold47412_2	non-phage	0
NOF012_C834301_1	non-phage	0
NOF012_scaffold72463_2	non-phage	0
NOF012_scaffold3_9	non-phage	0
NOF012_scaffold74840_1	non-phage	0
NOF012_scaffold13487_14	non-phage	0
NOF012_scaffold64464_1	non-phage	0
NOF012_scaffold78003_1	non-phage	0
NOF012_scaffold64620_2	non-phage	0
NOF012_scaffold15647_4	phage	0.932
NOF012_scaffold77974_1	non-phage	0

NOF012_scaffold32898_4	non-phage	0
NOF012_scaffold63882_2	non-phage	0
NOF012_scaffold62192_3	non-phage	0
NOF012_scaffold78070_3	non-phage	0
NOF012_scaffold3211_4	non-phage	0
NOF012_scaffold23527_2	non-phage	0
NOF012_scaffold46572_1	non-phage	0
NOF012_scaffold67365_6	non-phage	0
NOF012_scaffold17861_3	non-phage	0
NOF012_scaffold36677_1	non-phage	0
NOF012_scaffold14742_5	non-phage	0
NOF012_scaffold50973_9	non-phage	0
NOF012_scaffold73008_1	non-phage	0
NOF012_scaffold37026_10	non-phage	0
NOF012_scaffold2426_6	non-phage	0
NOF012_scaffold8867_2	phage	0.995
NOF012_scaffold41586_2	non-phage	0
NOF012_scaffold37026_4	non-phage	0
NOF012_scaffold13996_7	phage	0.999
NOF012_scaffold48296_3	non-phage	0
NOF012_scaffold61_13	non-phage	0
NOF012_scaffold27206_6	non-phage	0
NOF012_scaffold8356_10	non-phage	0
NOF012_scaffold31286_39	non-phage	0
NOF012_scaffold68694_1	non-phage	0
NOF012_scaffold77930_3	non-phage	0
NOF012_scaffold5587_2	non-phage	0
NOF012_scaffold40091_4	non-phage	0
NOF012_scaffold78058_2	non-phage	0
NOF012_C834333_1	non-phage	0
NOF012_scaffold44642_1	non-phage	0
NOF012_scaffold77362_1	non-phage	0
NOF012_scaffold38144_3	non-phage	0.006
NOF012_scaffold78032_1	non-phage	0
NOF012_scaffold5282_1	non-phage	0
NOF012_scaffold77917_2	phage	0.994
NOF012_scaffold19351_5	non-phage	0
NOF012_scaffold20150_1	non-phage	0
NOF012_scaffold7935_11	non-phage	0
NOF012_C834201_1	non-phage	0
NOF012_scaffold47590_2	non-phage	0
NOF012_C834325_1	non-phage	0
NOF012_scaffold31286_34	non-phage	0
NOF012_scaffold35798_1	non-phage	0
NOF012_scaffold23070_1	non-phage	0
NOF012_scaffold31286_18	non-phage	0

NOF012_scaffold48920_1	non-phage	0
NOF012_scaffold924_2	non-phage	0
NOF012_scaffold76961_2	non-phage	0
NOF012_scaffold21085_2	non-phage	0
NOF012_scaffold2849_2	non-phage	0
NOF012_scaffold1013_14	non-phage	0
NOF012_scaffold78080_1	non-phage	0.009
NOF012_scaffold73368_1	non-phage	0
NOF012_scaffold74676_1	non-phage	0
NOF012_scaffold78087_2	non-phage	0
NOF012_scaffold26305_5	non-phage	0
NOF012_C833791_1	non-phage	0
NOF012_scaffold960_25	non-phage	0
NOF012_scaffold6530_19	non-phage	0
NOF012_scaffold77458_2	non-phage	0
NOF012_scaffold34940_9	non-phage	0
NOF012_scaffold77968_1	non-phage	0
NOF012_scaffold12596_4	non-phage	0
NOF012_scaffold36268_4	non-phage	0
NOF012_scaffold3211_3	non-phage	0
NOF012_scaffold42179_1	non-phage	0
NOF012_scaffold68810_3	non-phage	0
NOF012_scaffold55603_2	non-phage	0
NOF012_scaffold62924_1	non-phage	0
NOF012_scaffold76931_1	non-phage	0
NOF012_scaffold72024_5	non-phage	0
NOF012_scaffold55217_3	non-phage	0
NOF012_scaffold78082_2	non-phage	0
NOF012_scaffold60516_1	non-phage	0
NOF012_scaffold19855_1	non-phage	0
NOF012_scaffold78070_2	non-phage	0
NOF012_scaffold67344_2	non-phage	0
NOF012_scaffold78062_1	non-phage	0
NOF012_scaffold76731_2	non-phage	0
NOF012_scaffold21368_3	non-phage	0
NOF012_scaffold8450_6	non-phage	0
NOF012_scaffold7715_1	non-phage	0
NOF012_C833885_1	non-phage	0
NOF012_scaffold48006_2	non-phage	0
NOF012_scaffold12244_6	phage	0.999
NOF012_scaffold13232_1	non-phage	0
NOF012_scaffold44774_1	phage	0.597
NOF012_scaffold77746_2	non-phage	0
NOF012_scaffold48964_1	non-phage	0
NOF012_scaffold77201_1	non-phage	0
NOF012_scaffold5880_15	non-phage	0

NOF012_scaffold15396_18	non-phage	0.377
NOF012_scaffold77687_1	non-phage	0
NOF012_scaffold60378_1	non-phage	0
NOF012_scaffold76588_4	non-phage	0
NOF012_scaffold28911_16	non-phage	0
NOF012_scaffold77982_2	non-phage	0
NOF012_scaffold29562_5	non-phage	0
NOF012_scaffold37026_11	non-phage	0
NOF012_scaffold57796_2	non-phage	0
NOF012_scaffold21085_1	non-phage	0
NOF012_scaffold9019_1	non-phage	0
NOF012_scaffold78001_3	non-phage	0
NOF012_scaffold11454_7	non-phage	0
NOF012_scaffold78088_1	non-phage	0
NOF012_scaffold47590_3	non-phage	0
NOF012_scaffold16215_1	non-phage	0
NOF012_C833827_1	non-phage	0
NOF012_scaffold77930_2	non-phage	0
NOF012_scaffold29562_1	non-phage	0
NOF012_scaffold44625_1	non-phage	0
NOF012_C834357_1	non-phage	0
NOF012_scaffold66348_4	non-phage	0
NOF012_scaffold29273_4	non-phage	0
NOF012_scaffold47590_10	non-phage	0
NOF012_scaffold68441_1	non-phage	0
NOF012_scaffold77994_1	non-phage	0
NOF012_scaffold5880_9	non-phage	0
NOF012_scaffold20171_9	non-phage	0
NOF012_scaffold51951_4	non-phage	0
NOF012_scaffold3081_2	phage	0.996
NOF012_scaffold69076_1	non-phage	0
NOF012_scaffold78071_1	non-phage	0
NOF012_scaffold78059_2	phage	0.919
NOF012_scaffold29562_8	non-phage	0
NOF012_scaffold58049_1	non-phage	0
NOF012_scaffold76562_1	non-phage	0
NOF012_scaffold37997_3	non-phage	0
NOF012_scaffold77992_1	non-phage	0
NOF012_scaffold60956_1	non-phage	0
NOF012_scaffold2510_1	non-phage	0
NOF012_scaffold63585_5	non-phage	0
NOF012_scaffold6364_12	non-phage	0
NOF012_scaffold17861_4	non-phage	0
NOF012_scaffold19351_3	non-phage	0
NOF012_scaffold65756_1	non-phage	0
NOF012_scaffold44598_6	non-phage	0

NOF012_scaffold61480_1	non-phage	0
NOF012_scaffold50579_1	non-phage	0
NOF012_scaffold26305_24	non-phage	0
NOF012_scaffold32950_2	non-phage	0
NOF012_scaffold46734_1	non-phage	0
NOF012_scaffold67019_4	non-phage	0
NOF012_scaffold50691_3	non-phage	0
NOF012_scaffold69941_3	non-phage	0
NOF012_scaffold34517_3	phage	0.983
NOF012_scaffold15991_4	non-phage	0
NOF012_scaffold77545_2	non-phage	0
NOF012_scaffold10353_14	non-phage	0
NOF012_C834285_1	non-phage	0
NOF012_scaffold66348_1	non-phage	0
NOF012_scaffold37997_1	non-phage	0
NOF012_scaffold23961_13	non-phage	0
NOF012_scaffold76688_1	non-phage	0
NOF012_scaffold76120_3	non-phage	0
NOF012_scaffold5639_7	phage	0.983
NOF012_scaffold20559_3	non-phage	0
NOF012_scaffold40314_3	non-phage	0
NOF012_scaffold63112_1	non-phage	0.148
NOF012_scaffold46014_3	non-phage	0
NOF012_scaffold68110_6	non-phage	0
NOF012_scaffold19351_4	non-phage	0
NOF012_scaffold26128_7	non-phage	0
NOF012_scaffold71874_9	non-phage	0
NOF012_scaffold1324_13	non-phage	0
NOF012_scaffold77127_2	non-phage	0
NOF012_scaffold44041_1	non-phage	0
NOF012_scaffold67658_1	non-phage	0
NOF012_scaffold339_20	non-phage	0
NOF012_scaffold41046_7	non-phage	0
NOF012_scaffold21374_2	non-phage	0
NOF012_scaffold31286_7	non-phage	0
NOF012_scaffold77135_2	non-phage	0
NOF012_scaffold43844_2	non-phage	0
NOF012_scaffold20233_14	non-phage	0
NOF012_scaffold77850_1	non-phage	0
NOF012_scaffold10353_15	non-phage	0
NOF012_scaffold51398_1	non-phage	0
NOF012_scaffold40088_4	non-phage	0
NOF012_scaffold71836_5	non-phage	0
NOF012_scaffold47590_12	non-phage	0
NOF012_scaffold67005_4	non-phage	0
NOF012_scaffold62117_4	non-phage	0

NOF012_scaffold46848_1	non-phage	0
NOF012_scaffold77354_2	non-phage	0
NOF012_scaffold77383_1	non-phage	0
NOF012_scaffold195_1	non-phage	0
NOF012_scaffold77490_1	non-phage	0
NOF012_scaffold41586_1	non-phage	0
NOF012_scaffold117_9	non-phage	0
NOF012_scaffold20150_7	non-phage	0
NOF012_scaffold78083_1	non-phage	0
NOF012_scaffold74698_1	non-phage	0
NOF012_scaffold24554_12	phage	0.996
NOF012_scaffold73677_1	non-phage	0
NOF012_scaffold8693_7	non-phage	0
NOF012_scaffold77869_1	non-phage	0
NOF012_scaffold32898_6	non-phage	0
NOF012_scaffold55619_3	non-phage	0
NOF012_scaffold13610_14	non-phage	0
NOF012_scaffold63693_1	non-phage	0
NOF012_scaffold47640_4	non-phage	0
NOF012_scaffold19293_1	non-phage	0
NOF012_scaffold77217_1	non-phage	0
NOF012_scaffold47590_5	non-phage	0
NOF012_scaffold77987_1	non-phage	0
NOF012_scaffold7351_1	non-phage	0
NOF012_scaffold71874_10	non-phage	0
NOF012_scaffold74603_4	non-phage	0
NOF012_scaffold960_13	non-phage	0
NOF012_scaffold62192_2	non-phage	0
NOF012_scaffold14331_7	non-phage	0
NOF012_scaffold36677_2	non-phage	0
NOF012_scaffold60524_9	non-phage	0
NOF012_scaffold32366_1	non-phage	0
NOF012_scaffold62460_3	non-phage	0
NOF012_scaffold78054_1	non-phage	0
NOF012_scaffold68722_2	non-phage	0
NOF012_scaffold41862_4	non-phage	0
NOF012_scaffold78072_3	non-phage	0
NOF012_scaffold77557_1	non-phage	0
NOF012_scaffold78019_2	non-phage	0
NOF012_scaffold33876_1	non-phage	0.001
NOF012_scaffold5639_8	non-phage	0.343
NOF012_scaffold48433_1	non-phage	0
NOF012_C834111_1	non-phage	0
NOF012_scaffold78042_1	non-phage	0
NOF012_scaffold27365_3	non-phage	0
NOF012_scaffold30002_3	non-phage	0

NOF012_scaffold73676_1	non-phage	0
NOF012_C834065_1	non-phage	0
NOF012_scaffold26717_1	non-phage	0
NOF012_scaffold24554_14	non-phage	0
NOF012_scaffold39071_1	non-phage	0
NOF012_scaffold1013_15	non-phage	0
NOF012_scaffold26128_5	non-phage	0.138
NOF012_scaffold8245_8	non-phage	0
NOF012_scaffold117_5	non-phage	0
NOF012_scaffold76646_1	non-phage	0
NOF012_scaffold4667_6	phage	0.961
NOF012_scaffold19855_15	non-phage	0.09
NOF012_scaffold78057_2	non-phage	0
NOF012_scaffold51951_5	non-phage	0
NOF012_scaffold65926_3	non-phage	0
NOF012_scaffold15396_5	non-phage	0
NOF012_scaffold78023_1	non-phage	0
NOF012_scaffold3211_5	non-phage	0
NOF012_scaffold78091_1	non-phage	0
NOF012_scaffold78044_1	non-phage	0
NOF012_scaffold72024_3	phage	1
NOF012_scaffold47768_6	non-phage	0
NOF012_scaffold8940_21	non-phage	0
NOF012_scaffold77631_2	non-phage	0
NOF012_scaffold46907_1	non-phage	0
NOF012_scaffold36834_1	non-phage	0
NOF012_scaffold71857_4	non-phage	0
NOF012_scaffold19855_2	phage	0.998
NOF012_scaffold77930_4	non-phage	0
NOF012_scaffold42302_2	non-phage	0
NOF012_scaffold75894_4	non-phage	0
NOF012_scaffold11508_3	non-phage	0
NOF012_scaffold19351_8	non-phage	0
NOF012_scaffold78057_1	non-phage	0
NOF012_scaffold75988_1	non-phage	0
NOF012_scaffold77635_1	non-phage	0
NOF012_C834151_1	non-phage	0
NOF012_scaffold31085_17	non-phage	0
NOF012_C833925_1	non-phage	0
NOF012_scaffold34940_12	phage	0.99
NOF012_scaffold77127_1	non-phage	0
NOF012_scaffold34517_1	phage	0.846
NOF012_scaffold239_1	non-phage	0
NOF012_scaffold76442_4	non-phage	0
NOF012_scaffold2463_3	non-phage	0
NOF012_scaffold62038_1	non-phage	0

NOF012_scaffold77556_1	non-phage	0
NOF012_scaffold28911_14	non-phage	0
NOF012_scaffold53110_1	non-phage	0
NOF012_scaffold8_1	non-phage	0
NOF012_scaffold78047_1	non-phage	0
NOF012_scaffold14331_8	non-phage	0
NOF012_scaffold78021_1	non-phage	0
NOF012_scaffold77156_1	non-phage	0
NOF012_scaffold63730_1	non-phage	0
NOF012_scaffold7627_7	non-phage	0
NOF012_scaffold44198_1	non-phage	0
NOF012_scaffold12361_4	non-phage	0
NOF012_scaffold19351_10	non-phage	0
NOF012_scaffold78087_1	non-phage	0
NOF012_scaffold26305_1	non-phage	0
NOF012_scaffold15991_2	non-phage	0
NOF012_C834327_1	non-phage	0
NOF012_scaffold63730_2	non-phage	0
NOF012_scaffold62623_1	non-phage	0
NOF012_scaffold29929_1	non-phage	0
NOF012_scaffold44584_1	non-phage	0
NOF012_scaffold52506_1	non-phage	0
NOF012_scaffold77208_2	non-phage	0.173
NOF012_scaffold76454_1	non-phage	0
NOF012_scaffold31286_17	non-phage	0
NOF012_scaffold78062_3	non-phage	0
NOF012_scaffold8693_10	phage	0.999
NOF012_scaffold78091_2	non-phage	0
NOF012_scaffold2849_1	non-phage	0
NOF012_scaffold34_14	non-phage	0
NOF012_scaffold34517_2	phage	0.999
NOF012_scaffold77271_2	phage	0.998
NOF012_scaffold13487_4	non-phage	0
NOF012_scaffold65721_2	non-phage	0
NOF012_scaffold42219_4	non-phage	0
NOF012_scaffold14331_10	non-phage	0
NOF012_scaffold41046_2	non-phage	0
NOF012_scaffold16635_4	non-phage	0
NOF012_scaffold77458_1	non-phage	0
NOF012_scaffold78089_2	non-phage	0
NOF012_scaffold71857_2	phage	0.997
NOF012_scaffold77970_1	non-phage	0
NOF012_scaffold3211_9	non-phage	0
NOF012_scaffold77844_1	non-phage	0
NOF012_scaffold30141_3	non-phage	0
NOF012_scaffold74308_1	phage	0.989

NOF012_scaffold10364_11	non-phage	0
NOF012_scaffold65794_1	non-phage	0
NOF012_C834181_1	non-phage	0
NOF012_scaffold58520_2	non-phage	0
NOF012_C833949_1	phage	1
NOF012_scaffold73231_1	non-phage	0
NOF012_scaffold27532_8	non-phage	0.001
NOF012_C834097_1	non-phage	0
NOF012_scaffold77994_2	non-phage	0
NOF012_scaffold31480_5	non-phage	0
NOF012_scaffold49143_2	non-phage	0
NOF012_scaffold78092_1	non-phage	0
NOF012_scaffold32366_2	non-phage	0
NOF012_scaffold77904_1	non-phage	0
NOF012_scaffold25905_3	non-phage	0
NOF012_scaffold74681_1	non-phage	0
NOF012_scaffold59498_1	non-phage	0
NOF012_scaffold19351_2	non-phage	0
NOF012_scaffold29562_21	non-phage	0
NOF012_scaffold39471_4	non-phage	0
NOF012_scaffold49971_3	non-phage	0
NOF012_scaffold30359_1	non-phage	0
NOF012_scaffold38088_3	non-phage	0
NOF012_scaffold9095_1	non-phage	0
NOF012_scaffold65926_1	non-phage	0
NOF012_scaffold53110_3	non-phage	0
NOF012_scaffold78025_1	non-phage	0.001
NOF012_scaffold15991_1	phage	0.999
NOF012_scaffold78001_2	non-phage	0
NOF012_scaffold78065_1	non-phage	0
NOF012_scaffold67344_1	non-phage	0
NOF012_scaffold44598_3	non-phage	0
NOF012_scaffold2510_5	non-phage	0
NOF012_scaffold50054_2	non-phage	0
NOF012_scaffold41862_2	non-phage	0
NOF012_scaffold8693_3	non-phage	0
NOF012_scaffold66348_7	non-phage	0
NOF012_scaffold10688_4	non-phage	0
NOF012_scaffold3120_9	non-phage	0
NOF012_scaffold78077_1	non-phage	0
NOF012_scaffold71567_1	non-phage	0
NOF012_scaffold77124_1	non-phage	0
NOF012_scaffold28911_23	non-phage	0
NOF012_scaffold28911_11	non-phage	0
NOF012_scaffold4268_2	non-phage	0
NOF012_scaffold8940_12	non-phage	0

NOF012_scaffold67142_5	non-phage	0
NOF012_scaffold30571_1	non-phage	0
NOF012_scaffold78060_1	non-phage	0
NOF012_scaffold38218_1	non-phage	0
NOF012_scaffold73676_2	non-phage	0
NOF012_scaffold59780_4	non-phage	0
NOF012_scaffold72463_1	non-phage	0
NOF012_scaffold28911_28	non-phage	0
NOF012_scaffold66013_1	non-phage	0
NOF012_scaffold48246_3	non-phage	0
NOF012_scaffold40895_2	non-phage	0
NOF012_scaffold50544_1	non-phage	0
NOF012_scaffold46014_4	non-phage	0
NOF012_scaffold5393_7	non-phage	0
NOF012_scaffold78078_1	non-phage	0
NOF012_scaffold8940_20	non-phage	0
NOF012_scaffold71874_7	phage	0.848
NOF012_scaffold32236_4	non-phage	0
NOF012_scaffold47590_14	non-phage	0
NOF012_scaffold78073_1	non-phage	0
NOF012_scaffold67659_1	non-phage	0
NOF012_scaffold7172_10	non-phage	0
NOF012_scaffold48292_4	non-phage	0
NOF012_scaffold21309_5	non-phage	0
NOF012_scaffold41340_8	non-phage	0
NOF012_scaffold29562_20	non-phage	0
NOF012_scaffold25167_5	non-phage	0
NOF012_scaffold10353_1	non-phage	0
NOF012_scaffold23932_14	non-phage	0
NOF012_scaffold42413_1	non-phage	0
NOF012_scaffold2463_2	non-phage	0
NOF012_scaffold2849_7	non-phage	0
NOF012_scaffold339_7	phage	0.994
NOF012_scaffold75991_2	non-phage	0
NOF012_scaffold1013_6	non-phage	0
NOF012_scaffold39049_1	non-phage	0
NOF012_scaffold25167_6	non-phage	0
NOF012_scaffold28415_3	phage	0.999
NOF012_scaffold9650_7	phage	0.991
NOF012_scaffold78059_1	non-phage	0
NOF012_scaffold33935_5	non-phage	0
NOF012_scaffold78090_1	non-phage	0
NOF012_scaffold33726_2	non-phage	0
NOF012_scaffold77880_1	non-phage	0
NOF012_scaffold65926_2	non-phage	0
NOF012_scaffold21085_8	non-phage	0

NOF012_scaffold57903_7	non-phage	0
NOF012_scaffold19351_6	non-phage	0
NOF012_scaffold77196_1	phage	0.998
NOF012_C834045_1	non-phage	0
NOF012_scaffold4949_7	non-phage	0
NOF012_scaffold41862_3	non-phage	0
NOF012_scaffold65542_1	non-phage	0
NOF012_scaffold63165_3	non-phage	0
NOF012_scaffold56160_11	non-phage	0
NOF012_scaffold31085_7	non-phage	0
NOF012_scaffold78072_2	non-phage	0
NOF012_scaffold34940_8	non-phage	0
NOF012_scaffold40886_5	non-phage	0
NOF012_scaffold47590_4	non-phage	0
NOF012_scaffold19871_1	non-phage	0
NOF012_scaffold76961_1	non-phage	0
NOF012_scaffold78048_2	non-phage	0
NOF012_scaffold78039_1	non-phage	0
NOF012_scaffold26305_18	non-phage	0
NOF012_scaffold76278_1	non-phage	0
NOF012_scaffold41331_1	non-phage	0
NOF012_scaffold7172_2	non-phage	0
NOF012_scaffold18670_4	non-phage	0
NOF012_scaffold77491_1	non-phage	0
NOF012_scaffold77798_1	non-phage	0
NOF012_scaffold37026_8	phage	0.999
NOF012_scaffold77767_2	non-phage	0
NOF012_scaffold65944_1	non-phage	0
NOF012_scaffold77994_4	non-phage	0
NOF012_scaffold76507_2	non-phage	0
NOF012_scaffold71836_1	non-phage	0
NOF012_scaffold76851_1	non-phage	0
NOF012_scaffold68722_1	non-phage	0
NOF012_scaffold18336_1	non-phage	0
NOF012_scaffold51398_2	non-phage	0
NOF012_scaffold25905_6	non-phage	0
NOF012_scaffold17514_5	non-phage	0
NOF012_scaffold13487_2	non-phage	0
NOF012_scaffold29562_7	non-phage	0
NOF012_scaffold29562_23	non-phage	0
NOF012_scaffold8940_1	non-phage	0
NOF012_scaffold52562_1	non-phage	0
NOF012_scaffold78068_1	non-phage	0
NOF012_scaffold68565_1	phage	0.952
NOF012_scaffold75991_4	non-phage	0
NOF012_scaffold41046_4	non-phage	0

NOF012_scaffold26305_22	phage	0.999
NOF012_scaffold29562_4	non-phage	0
NOF012_scaffold37857_1	non-phage	0
NOF012_scaffold69146_2	non-phage	0
NOF012_scaffold76827_1	non-phage	0
NOF012_scaffold19855_18	non-phage	0
NOF012_scaffold66477_1	non-phage	0
NOF012_scaffold21368_2	non-phage	0
NOF012_scaffold77948_1	non-phage	0
NOF012_scaffold78081_1	phage	0.999
NOF012_scaffold74020_2	non-phage	0
NOF012_scaffold16635_6	non-phage	0
NOF012_scaffold77766_1	non-phage	0
NOF012_scaffold18202_5	non-phage	0
NOF012_scaffold40322_1	non-phage	0
NOF012_scaffold7160_1	non-phage	0
NOF012_scaffold77746_1	non-phage	0
NOF012_scaffold61119_1	non-phage	0
NOF012_scaffold43523_1	phage	0.529
NOF012_scaffold76926_2	non-phage	0
NOF012_scaffold66136_1	non-phage	0
NOF012_scaffold28911_1	non-phage	0
NOF012_scaffold41858_11	phage	0.999
NOF012_scaffold28114_12	phage	0.975
NOF012_scaffold66949_1	non-phage	0
NOF012_scaffold45565_1	non-phage	0
NOF012_scaffold28114_24	non-phage	0
NOF012_scaffold57796_1	non-phage	0
NOF012_scaffold67_2	non-phage	0
NOF012_C834313_1	non-phage	0
NOF012_scaffold16956_4	non-phage	0
NOF012_scaffold72563_1	non-phage	0
NOF012_scaffold73194_1	non-phage	0
NOF012_scaffold59732_1	non-phage	0
NOF012_scaffold77930_1	non-phage	0
NOF012_scaffold9095_3	non-phage	0
NOF012_scaffold25137_4	non-phage	0
NOF012_scaffold5300_1	non-phage	0
NOF012_scaffold25916_4	non-phage	0
NOF012_scaffold30002_4	non-phage	0
NOF012_scaffold2007_7	non-phage	0
NOF012_scaffold73735_1	non-phage	0
NOF012_scaffold39585_2	non-phage	0
NOF012_scaffold47491_2	non-phage	0
NOF012_scaffold15971_3	non-phage	0
NOF012_scaffold25588_1	non-phage	0

NOF012_scaffold47357_1	non-phage	0
NOF012_scaffold5016_2	non-phage	0
NOF012_scaffold23527_3	non-phage	0
NOF012_scaffold27783_3	non-phage	0
NOF012_scaffold14671_1	non-phage	0
NOF012_scaffold78000_1	non-phage	0
NOF012_scaffold76511_2	non-phage	0
NOF012_scaffold21856_5	non-phage	0
NOF012_scaffold44598_5	non-phage	0
NOF013_scaffold13815_1	non-phage	0
NOF013_scaffold50_3	non-phage	0
NOF013_scaffold28391_3	non-phage	0
NOF013_scaffold8072_2	non-phage	0
NOF013_scaffold19329_3	non-phage	0
NOF013_scaffold26645_4	non-phage	0
NOF013_scaffold15223_1	non-phage	0
NOF013_scaffold3459_2	non-phage	0
NOF013_scaffold27985_1	non-phage	0
NOF013_scaffold26916_3	non-phage	0
NOF013_scaffold33770_1	non-phage	0
NOF013_scaffold6862_1	non-phage	0
NOF013_scaffold33409_1	non-phage	0
NOF013_scaffold30013_1	non-phage	0
NOF013_scaffold3570_8	non-phage	0
NOF013_scaffold12416_3	non-phage	0
NOF013_scaffold34385_2	non-phage	0
NOF013_scaffold594_61	non-phage	0
NOF013_scaffold119_8	non-phage	0
NOF013_scaffold815_42	non-phage	0
NOF013_scaffold19960_3	non-phage	0
NOF013_scaffold8022_2	non-phage	0
NOF013_scaffold34711_1	non-phage	0
NOF013_scaffold14842_2	non-phage	0
NOF013_scaffold4120_2	non-phage	0
NOF013_scaffold22999_4	phage	0.578
NOF013_scaffold5515_9	non-phage	0
NOF013_scaffold32556_1	non-phage	0
NOF013_scaffold1044_8	non-phage	0
NOF013_scaffold33871_1	non-phage	0
NOF013_scaffold47_1	non-phage	0
NOF013_scaffold8597_1	non-phage	0
NOF013_scaffold15248_2	non-phage	0
NOF013_scaffold3664_16	non-phage	0
NOF013_scaffold2512_3	non-phage	0
NOF013_scaffold32115_1	non-phage	0
NOF013_scaffold34486_3	non-phage	0

NOF013_scaffold2098_2	non-phage	0
NOF013_scaffold33522_2	non-phage	0
NOF013_scaffold15092_2	non-phage	0
NOF013_scaffold4522_5	non-phage	0
NOF013_scaffold3697_5	non-phage	0
NOF013_scaffold4934_2	non-phage	0
NOF013_scaffold12336_4	non-phage	0
NOF013_scaffold21_2	non-phage	0
NOF013_scaffold2193_4	non-phage	0
NOF013_scaffold2743_1	non-phage	0
NOF013_scaffold3033_5	non-phage	0
NOF013_scaffold33526_1	phage	0.999
NOF013_scaffold34728_2	non-phage	0
NOF013_scaffold17733_1	non-phage	0
NOF013_scaffold651_5	non-phage	0
NOF013_scaffold30978_1	non-phage	0
NOF013_scaffold24441_3	non-phage	0
NOF013_scaffold29847_1	non-phage	0
NOF013_scaffold9231_3	non-phage	0
NOF013_scaffold30707_2	non-phage	0
NOF013_scaffold34657_1	non-phage	0
NOF013_scaffold3570_4	non-phage	0
NOF013_scaffold33521_1	non-phage	0
NOF013_scaffold15363_3	phage	0.999
NOF013_scaffold14556_3	non-phage	0
NOF013_scaffold23550_2	non-phage	0
NOF013_scaffold24313_2	non-phage	0
NOF013_scaffold34327_2	non-phage	0
NOF013_scaffold33015_1	non-phage	0
NOF013_scaffold25867_4	non-phage	0
NOF013_scaffold29416_1	non-phage	0
NOF013_scaffold34635_1	non-phage	0
NOF013_scaffold28708_3	non-phage	0
NOF013_scaffold28359_1	non-phage	0
NOF013_scaffold17321_3	non-phage	0
NOF013_scaffold12910_15	non-phage	0
NOF013_scaffold9700_2	non-phage	0
NOF013_scaffold10722_3	non-phage	0
NOF013_scaffold25004_2	non-phage	0
NOF013_scaffold33894_3	non-phage	0
NOF013_C612978_1	non-phage	0
NOF013_scaffold9506_2	non-phage	0
NOF013_scaffold9372_3	non-phage	0
NOF013_scaffold32152_1	non-phage	0
NOF013_scaffold7185_1	non-phage	0
NOF013_scaffold16252_3	non-phage	0

NOF013_scaffold5920_8	non-phage	0
NOF013_scaffold34675_4	non-phage	0
NOF013_scaffold8799_4	non-phage	0
NOF013_scaffold3944_1	non-phage	0
NOF013_scaffold9359_1	phage	0.998
NOF013_scaffold29987_1	non-phage	0
NOF013_scaffold34723_2	non-phage	0
NOF013_scaffold3614_2	non-phage	0
NOF013_scaffold24304_2	phage	0.986
NOF013_scaffold9983_9	non-phage	0
NOF013_scaffold33523_4	non-phage	0.112
NOF013_scaffold32172_2	non-phage	0
NOF013_scaffold19617_1	non-phage	0
NOF013_scaffold24211_4	non-phage	0
NOF013_scaffold24804_5	non-phage	0
NOF013_scaffold22283_1	non-phage	0
NOF013_scaffold31454_1	non-phage	0
NOF013_scaffold20633_5	non-phage	0
NOF013_scaffold8597_10	non-phage	0
NOF013_scaffold34010_1	non-phage	0
NOF013_scaffold34511_1	non-phage	0
NOF013_scaffold33613_3	non-phage	0
NOF013_scaffold21711_1	non-phage	0
NOF013_scaffold33086_5	non-phage	0
NOF013_scaffold14160_2	non-phage	0
NOF013_scaffold27483_1	non-phage	0
NOF013_scaffold25409_1	non-phage	0
NOF013_scaffold4760_1	non-phage	0
NOF013_scaffold4856_2	non-phage	0
NOF013_scaffold9802_4	non-phage	0
NOF013_scaffold12467_5	non-phage	0
NOF013_scaffold33642_2	non-phage	0
NOF013_scaffold11906_1	phage	0.999
NOF013_scaffold34236_6	non-phage	0
NOF013_scaffold172_8	non-phage	0
NOF013_scaffold32212_1	non-phage	0
NOF013_scaffold23078_1	non-phage	0
NOF013_scaffold9819_2	non-phage	0
NOF013_scaffold34585_3	non-phage	0
NOF013_scaffold26437_1	phage	0.995
NOF013_scaffold20067_2	phage	0.996
NOF013_scaffold56_2	non-phage	0
NOF013_scaffold10786_3	non-phage	0
NOF013_scaffold34712_2	non-phage	0
NOF013_scaffold14837_5	non-phage	0
NOF013_scaffold9806_1	non-phage	0

NOF013_scaffold28807_1	non-phage	0
NOF013_scaffold9372_4	non-phage	0
NOF013_scaffold194_7	non-phage	0
NOF013_scaffold4307_2	non-phage	0
NOF013_scaffold30687_4	non-phage	0
NOF013_scaffold34146_1	non-phage	0
NOF013_scaffold34327_1	non-phage	0
NOF013_scaffold25505_1	phage	0.996
NOF013_scaffold33029_1	non-phage	0
NOF013_scaffold12371_2	non-phage	0
NOF013_scaffold15195_2	non-phage	0
NOF013_scaffold34730_1	phage	0.999
NOF013_scaffold13333_1	non-phage	0
NOF013_scaffold31503_6	non-phage	0
NOF013_scaffold23265_3	non-phage	0
NOF013_scaffold34606_1	non-phage	0
NOF013_scaffold33086_4	non-phage	0
NOF013_scaffold10848_9	non-phage	0
NOF013_scaffold27423_1	non-phage	0
NOF013_scaffold34675_2	non-phage	0
NOF013_scaffold31815_1	non-phage	0
NOF013_scaffold27944_1	non-phage	0
NOF013_scaffold34281_1	non-phage	0
NOF013_scaffold20041_1	non-phage	0
NOF013_scaffold594_60	non-phage	0
NOF013_scaffold1888_8	non-phage	0
NOF013_scaffold9070_17	non-phage	0
NOF013_scaffold31187_1	non-phage	0
NOF013_scaffold1888_1	non-phage	0
NOF013_scaffold17318_1	non-phage	0
NOF013_scaffold24520_1	non-phage	0
NOF013_scaffold304_1	non-phage	0
NOF013_scaffold27097_6	non-phage	0
NOF013_scaffold32191_2	non-phage	0
NOF013_scaffold34733_1	non-phage	0
NOF013_scaffold34586_2	non-phage	0.03
NOF013_scaffold30520_3	non-phage	0.071
NOF013_scaffold26171_1	non-phage	0
NOF013_scaffold594_76	non-phage	0
NOF013_scaffold8658_5	non-phage	0
NOF013_scaffold32125_2	non-phage	0
NOF013_scaffold15223_4	non-phage	0
NOF013_scaffold32460_2	non-phage	0
NOF013_scaffold51_1	non-phage	0
NOF013_scaffold28708_1	non-phage	0
NOF013_scaffold13934_1	non-phage	0

NOF013_scaffold24444_1	non-phage	0
NOF013_scaffold34617_1	non-phage	0
NOF013_scaffold9227_1	non-phage	0
NOF013_scaffold25409_2	non-phage	0
NOF013_scaffold3152_1	non-phage	0
NOF013_scaffold173_1	non-phage	0
NOF013_scaffold26502_2	non-phage	0
NOF013_scaffold14842_8	non-phage	0
NOF013_scaffold27250_1	non-phage	0
NOF013_scaffold117_1	non-phage	0
NOF013_scaffold34275_1	non-phage	0
NOF013_scaffold26645_3	non-phage	0
NOF013_scaffold10087_4	non-phage	0
NOF013_scaffold24709_2	non-phage	0
NOF013_scaffold22283_5	non-phage	0
NOF013_scaffold34712_1	non-phage	0
NOF013_scaffold16307_1	non-phage	0
NOF013_scaffold13612_1	non-phage	0
NOF013_scaffold32760_1	non-phage	0
NOF013_scaffold207_11	non-phage	0
NOF013_scaffold28807_2	non-phage	0
NOF013_scaffold8597_4	non-phage	0
NOF013_scaffold32473_1	non-phage	0
NOF013_scaffold30915_2	non-phage	0
NOF013_scaffold23006_3	non-phage	0
NOF013_scaffold4358_5	non-phage	0
NOF013_scaffold6002_4	non-phage	0
NOF013_scaffold8799_3	non-phage	0
NOF013_scaffold32659_2	non-phage	0
NOF013_scaffold33002_2	non-phage	0
NOF013_scaffold3132_4	non-phage	0
NOF013_scaffold16460_3	non-phage	0
NOF013_scaffold1608_1	non-phage	0
NOF013_scaffold12336_11	non-phage	0
NOF013_scaffold8559_5	non-phage	0
NOF013_scaffold34568_1	non-phage	0
NOF013_scaffold6283_17	phage	0.998
NOF013_scaffold34717_1	non-phage	0
NOF013_scaffold31497_3	non-phage	0
NOF013_scaffold31503_7	non-phage	0
NOF013_scaffold3071_1	non-phage	0
NOF013_scaffold21542_1	non-phage	0
NOF013_scaffold34656_1	non-phage	0
NOF013_scaffold28391_1	non-phage	0
NOF013_scaffold18866_7	non-phage	0
NOF013_scaffold1861_1	non-phage	0

NOF013_scaffold27097_3	non-phage	0
NOF013_scaffold1888_7	non-phage	0
NOF013_scaffold24804_3	non-phage	0
NOF013_scaffold1499_1	non-phage	0
NOF013_scaffold30635_1	phage	0.967
NOF013_scaffold11252_3	non-phage	0
NOF013_scaffold31397_1	non-phage	0
NOF013_scaffold22266_2	non-phage	0
NOF013_scaffold25198_1	non-phage	0
NOF013_scaffold34593_3	non-phage	0
NOF013_scaffold651_3	non-phage	0
NOF013_scaffold33969_1	non-phage	0
NOF013_scaffold9405_2	non-phage	0
NOF013_scaffold18543_1	non-phage	0
NOF013_scaffold30261_2	non-phage	0
NOF013_scaffold14160_1	non-phage	0
NOF013_scaffold7705_7	non-phage	0
NOF013_scaffold10303_2	non-phage	0
NOF013_scaffold6729_22	non-phage	0
NOF013_scaffold26019_2	non-phage	0
NOF013_scaffold16873_2	non-phage	0
NOF013_scaffold9983_5	non-phage	0
NOF013_scaffold26424_1	non-phage	0
NOF013_scaffold6362_2	non-phage	0
NOF013_scaffold7403_6	non-phage	0
NOF013_scaffold34734_1	non-phage	0
NOF013_scaffold33029_2	non-phage	0
NOF013_scaffold10030_4	non-phage	0
NOF013_scaffold2892_2	non-phage	0
NOF013_scaffold1982_2	non-phage	0
NOF013_scaffold30467_1	non-phage	0
NOF013_scaffold33523_5	non-phage	0
NOF013_scaffold27517_1	non-phage	0
NOF013_scaffold31503_5	non-phage	0
NOF013_scaffold33183_1	non-phage	0
NOF013_scaffold20_2	non-phage	0
NOF013_scaffold34329_3	non-phage	0
NOF013_scaffold5183_4	non-phage	0
NOF013_scaffold4759_1	non-phage	0
NOF013_scaffold5891_3	non-phage	0
NOF013_scaffold29042_4	non-phage	0
NOF013_scaffold9607_2	phage	0.998
NOF013_scaffold32837_1	non-phage	0
NOF013_scaffold27155_1	non-phage	0
NOF013_scaffold8522_1	non-phage	0
NOF013_scaffold33770_3	non-phage	0

NOF013_scaffold13114_2	non-phage	0
NOF013_scaffold22096_1	non-phage	0
NOF013_scaffold28482_2	non-phage	0
NOF013_scaffold34528_1	phage	0.986
NOF013_scaffold29925_1	non-phage	0
NOF013_scaffold18685_1	non-phage	0
NOF013_scaffold33395_1	non-phage	0
NOF013_scaffold34731_2	non-phage	0
NOF013_scaffold33894_5	non-phage	0
NOF013_scaffold9811_1	non-phage	0
NOF013_scaffold4136_20	non-phage	0
NOF013_scaffold34324_4	non-phage	0
NOF013_scaffold29297_3	non-phage	0
NOF013_scaffold27785_2	non-phage	0
NOF013_scaffold24519_3	non-phage	0
NOF013_scaffold34207_2	non-phage	0
NOF013_scaffold17321_2	non-phage	0
NOF013_scaffold31773_2	non-phage	0
NOF013_scaffold3621_2	non-phage	0
NOF013_scaffold6283_16	non-phage	0
NOF013_scaffold23636_5	non-phage	0
NOF013_scaffold2656_2	non-phage	0
NOF013_scaffold23160_23	non-phage	0
NOF013_scaffold34699_2	non-phage	0
NOF013_scaffold27946_7	non-phage	0
NOF013_scaffold31824_1	non-phage	0
NOF013_scaffold34548_1	non-phage	0
NOF013_scaffold32997_1	non-phage	0
NOF013_scaffold29594_1	non-phage	0
NOF013_scaffold23078_2	non-phage	0
NOF013_scaffold25534_1	non-phage	0
NOF013_scaffold24429_1	non-phage	0
NOF013_scaffold21793_8	non-phage	0
NOF013_scaffold9250_4	non-phage	0
NOF013_scaffold32954_2	non-phage	0
NOF013_scaffold24211_2	non-phage	0
NOF013_scaffold29371_1	non-phage	0
NOF013_scaffold32145_1	non-phage	0
NOF013_scaffold8558_2	non-phage	0
NOF013_scaffold32855_1	non-phage	0.04
NOF013_scaffold29652_1	non-phage	0
NOF013_C612756_1	phage	0.998
NOF013_scaffold20633_3	non-phage	0
NOF013_scaffold9829_7	non-phage	0
NOF013_scaffold32744_2	non-phage	0
NOF013_scaffold2205_1	non-phage	0

NOF013_scaffold37_19	non-phage	0
NOF013_scaffold105_53	non-phage	0
NOF013_scaffold20212_3	non-phage	0
NOF013_scaffold34342_1	non-phage	0
NOF013_scaffold2383_9	non-phage	0
NOF013_scaffold13658_1	non-phage	0
NOF013_scaffold5430_8	non-phage	0
NOF013_scaffold25505_3	non-phage	0
NOF013_scaffold30219_1	non-phage	0
NOF013_scaffold33175_2	non-phage	0
NOF013_C612740_1	non-phage	0
NOF013_scaffold34395_1	non-phage	0
NOF013_scaffold275_6	non-phage	0
NOF013_scaffold34649_1	non-phage	0
NOF013_scaffold2329_1	non-phage	0
NOF013_scaffold26916_4	non-phage	0
NOF013_scaffold34336_1	non-phage	0
NOF013_scaffold25484_3	non-phage	0
NOF013_scaffold20730_7	non-phage	0
NOF013_scaffold9518_4	non-phage	0
NOF013_scaffold4713_8	non-phage	0
NOF013_scaffold31292_3	non-phage	0
NOF013_scaffold11484_3	non-phage	0
NOF013_scaffold25538_4	non-phage	0
NOF013_scaffold22228_1	non-phage	0
NOF013_scaffold8799_2	non-phage	0
NOF013_scaffold18478_2	non-phage	0
NOF013_scaffold4120_1	non-phage	0
NOF013_scaffold12223_10	non-phage	0
NOF013_scaffold19960_2	non-phage	0
NOF013_scaffold3348_11	phage	0.999
NOF013_scaffold25305_1	non-phage	0
NOF013_scaffold34419_1	non-phage	0
NOF013_scaffold24565_7	phage	0.999
NOF013_scaffold8799_1	non-phage	0
NOF013_scaffold28482_1	non-phage	0
NOF013_scaffold16305_2	non-phage	0
NOF013_scaffold34462_1	non-phage	0
NOF013_scaffold15447_3	non-phage	0
NOF013_scaffold24804_1	non-phage	0
NOF013_scaffold34584_1	non-phage	0
NOF013_scaffold22233_11	non-phage	0
NOF013_scaffold34586_3	non-phage	0
NOF013_scaffold34630_3	phage	0.913
NOF013_scaffold24709_1	non-phage	0
NOF013_scaffold34317_1	non-phage	0

NOF013_scaffold9606_2	non-phage	0
NOF013_scaffold27097_4	non-phage	0
NOF013_scaffold31815_3	non-phage	0
NOF013_scaffold34660_1	non-phage	0
NOF013_scaffold104_2	non-phage	0
NOF013_scaffold32954_3	non-phage	0
NOF013_scaffold26459_1	non-phage	0
NOF013_scaffold6124_3	non-phage	0
NOF013_scaffold12144_1	non-phage	0
NOF013_scaffold2151_3	non-phage	0
NOF013_scaffold27668_12	non-phage	0
NOF013_scaffold12141_1	non-phage	0
NOF013_scaffold9227_7	non-phage	0
NOF013_C612784_1	non-phage	0
NOF013_scaffold20730_4	non-phage	0
NOF013_scaffold31966_1	non-phage	0
NOF013_scaffold34729_1	non-phage	0
NOF013_scaffold8022_5	non-phage	0
NOF013_scaffold20633_7	non-phage	0
NOF013_scaffold15223_2	non-phage	0
NOF013_scaffold26283_1	non-phage	0
NOF013_scaffold31065_5	non-phage	0
NOF013_scaffold14326_2	non-phage	0
NOF013_scaffold8072_3	non-phage	0
NOF013_scaffold2743_2	non-phage	0
NOF013_scaffold34273_1	non-phage	0
NOF013_scaffold33029_3	non-phage	0
NOF013_scaffold270_3	non-phage	0
NOF013_scaffold34510_1	non-phage	0
NOF013_scaffold18327_2	non-phage	0
NOF013_scaffold8812_2	non-phage	0
NOF013_scaffold32825_4	non-phage	0
NOF013_scaffold30013_2	non-phage	0
NOF013_scaffold29158_1	non-phage	0
NOF013_scaffold12658_1	non-phage	0
NOF013_scaffold33201_1	non-phage	0
NOF013_scaffold5920_9	non-phage	0
NOF013_scaffold9405_1	non-phage	0
NOF013_scaffold21911_5	non-phage	0
NOF013_scaffold22044_2	non-phage	0
NOF013_scaffold29042_1	non-phage	0
NOF013_scaffold12658_12	non-phage	0
NOF013_scaffold12416_2	non-phage	0
NOF013_scaffold6505_2	non-phage	0
NOF013_scaffold21981_2	non-phage	0
NOF013_scaffold10299_2	non-phage	0

NOF013_scaffold10269_1	non-phage	0
NOF013_scaffold1022_5	non-phage	0.002
NOF013_scaffold6505_6	non-phage	0
NOF013_scaffold25516_2	non-phage	0
NOF013_scaffold21573_3	non-phage	0
NOF013_scaffold34521_1	non-phage	0
NOF013_scaffold9983_19	non-phage	0
NOF013_scaffold7403_1	non-phage	0
NOF013_scaffold27097_1	non-phage	0
NOF013_scaffold13801_6	non-phage	0
NOF013_scaffold28205_1	non-phage	0
NOF013_scaffold24565_6	phage	0.999
NOF013_scaffold31981_2	non-phage	0
NOF013_scaffold34377_1	non-phage	0
NOF013_scaffold27004_8	non-phage	0
NOF013_scaffold16337_1	non-phage	0
NOF013_scaffold98_4	non-phage	0
NOF013_scaffold33578_1	non-phage	0
NOF013_scaffold173_3	non-phage	0
NOF013_scaffold34013_1	non-phage	0
NOF013_scaffold16252_2	non-phage	0
NOF013_C612822_1	non-phage	0
NOF013_scaffold4358_2	non-phage	0
NOF013_scaffold19904_2	non-phage	0
NOF013_scaffold5515_2	non-phage	0
NOF013_scaffold30901_1	non-phage	0.1
NOF013_scaffold30915_3	non-phage	0
NOF013_scaffold20470_1	non-phage	0
NOF013_scaffold19960_5	non-phage	0
NOF013_scaffold120_2	non-phage	0
NOF013_scaffold30260_1	non-phage	0
NOF013_scaffold18009_1	non-phage	0
NOF013_scaffold8379_2	non-phage	0
NOF013_scaffold33358_2	non-phage	0
NOF013_scaffold28708_4	non-phage	0
NOF013_scaffold104_1	non-phage	0
NOF013_scaffold13229_3	non-phage	0
NOF013_scaffold11674_1	non-phage	0
NOF013_scaffold31945_1	non-phage	0
NOF013_scaffold6299_2	non-phage	0
NOF013_scaffold316_5	non-phage	0
NOF013_scaffold15323_2	non-phage	0
NOF013_scaffold34735_1	non-phage	0
NOF013_scaffold22283_4	non-phage	0
NOF013_scaffold23323_2	non-phage	0
NOF013_scaffold13658_9	non-phage	0

NOF013_scaffold18495_1	non-phage	0
NOF013_scaffold32182_1	non-phage	0
NOF013_scaffold5993_3	non-phage	0
NOF013_scaffold6029_1	non-phage	0
NOF013_scaffold34684_1	non-phage	0
NOF013_scaffold267_2	non-phage	0
NOF013_scaffold32760_2	non-phage	0
NOF013_scaffold26159_3	non-phage	0
NOF013_scaffold32029_2	non-phage	0
NOF013_scaffold9379_6	non-phage	0
NOF013_scaffold20868_1	non-phage	0
NOF013_scaffold29291_2	non-phage	0
NOF013_scaffold10146_1	non-phage	0
NOF013_scaffold3664_2	non-phage	0.087
NOF013_scaffold15031_5	non-phage	0
NOF013_scaffold18327_3	non-phage	0
NOF013_scaffold28390_1	non-phage	0
NOF013_scaffold34585_6	non-phage	0
NOF013_scaffold6607_3	non-phage	0
NOF013_scaffold194_6	non-phage	0
NOF013_scaffold24778_7	non-phage	0
NOF013_scaffold13658_4	non-phage	0
NOF013_scaffold24710_1	non-phage	0
NOF013_scaffold29297_1	non-phage	0
NOF013_scaffold207_15	non-phage	0
NOF013_scaffold34375_1	non-phage	0
NOF013_scaffold27692_1	non-phage	0
NOF013_scaffold172_4	non-phage	0
NOF013_scaffold34732_1	non-phage	0
NOF013_scaffold34508_2	non-phage	0
NOF013_scaffold24918_2	non-phage	0
NOF013_scaffold30186_2	non-phage	0
NOF013_scaffold24_4	non-phage	0
NOF013_scaffold16460_1	non-phage	0
NOF013_scaffold34626_1	non-phage	0
NOF013_scaffold34651_1	non-phage	0
NOF013_scaffold34579_1	non-phage	0
NOF013_scaffold27004_3	non-phage	0
NOF013_scaffold26159_4	non-phage	0
NOF013_scaffold32744_4	non-phage	0
NOF013_scaffold21817_2	non-phage	0
NOF013_scaffold16111_1	non-phage	0
NOF013_scaffold34586_1	non-phage	0
NOF013_scaffold282_1	non-phage	0
NOF013_scaffold10339_1	non-phage	0
NOF013_scaffold34665_1	non-phage	0

NOF013_scaffold9606_13	non-phage	0
NOF013_scaffold33213_2	non-phage	0
NOF013_scaffold33029_4	non-phage	0
NOF013_scaffold31989_1	non-phage	0
NOF013_scaffold5183_6	non-phage	0
NOF013_scaffold34510_2	non-phage	0
NOF013_scaffold10246_6	non-phage	0
NOF013_scaffold23567_1	non-phage	0
NOF013_scaffold987_7	non-phage	0
NOF013_scaffold26437_2	phage	0.999
NOF013_scaffold29171_1	non-phage	0
NOF013_scaffold34045_1	non-phage	0
NOF013_scaffold33703_2	non-phage	0
NOF013_scaffold12371_1	non-phage	0
NOF013_scaffold12222_1	non-phage	0
NOF013_scaffold30529_1	non-phage	0
NOF013_scaffold15248_4	non-phage	0
NOF013_scaffold119_1	non-phage	0
NOF013_scaffold13801_5	non-phage	0
NOF013_scaffold10786_2	non-phage	0
NOF013_scaffold16494_1	non-phage	0
NOF013_scaffold34584_2	non-phage	0
NOF013_scaffold27279_2	non-phage	0
NOF013_scaffold1008_28	non-phage	0
NOF013_scaffold4750_1	phage	0.814
NOF013_scaffold23510_12	non-phage	0
NOF013_scaffold6020_1	non-phage	0
NOF013_scaffold23957_1	non-phage	0
NOF013_scaffold25999_1	non-phage	0
NOF013_scaffold24709_3	non-phage	0
NOF013_scaffold131_3	non-phage	0
NOF013_scaffold316_7	phage	0.988
NOF013_scaffold29849_1	non-phage	0
NOF013_scaffold132_2	non-phage	0
NOF013_scaffold32125_1	non-phage	0
NOF013_scaffold28493_2	phage	0.891
NOF013_scaffold16305_1	non-phage	0
NOF013_scaffold34721_2	non-phage	0
NOF013_scaffold25061_1	non-phage	0
NOF013_scaffold23006_1	non-phage	0
NOF013_scaffold26619_4	non-phage	0
NOF013_scaffold2329_4	non-phage	0
NOF013_scaffold25538_2	non-phage	0
NOF013_scaffold9518_1	non-phage	0
NOF013_scaffold2310_2	non-phage	0
NOF013_scaffold1943_1	non-phage	0

NOF013_scaffold6772_3	non-phage	0
NOF013_scaffold6967_2	non-phage	0
NOF013_scaffold24025_2	non-phage	0
NOF013_scaffold4136_6	non-phage	0
NOF013_scaffold8017_1	non-phage	0
NOF013_scaffold13126_1	non-phage	0
NOF013_scaffold12602_4	non-phage	0
NOF013_scaffold30915_4	non-phage	0
NOF013_scaffold33972_3	non-phage	0
NOF013_scaffold32744_10	non-phage	0
NOF013_scaffold20523_6	non-phage	0
NOF013_scaffold29368_3	non-phage	0
NOF013_scaffold29817_1	non-phage	0
NOF013_scaffold30227_1	non-phage	0
NOF013_scaffold24066_2	non-phage	0
NOF013_scaffold30530_3	non-phage	0
NOF013_scaffold11455_19	non-phage	0
NOF013_scaffold33920_3	non-phage	0
NOF013_scaffold14739_11	non-phage	0
NOF013_scaffold24918_1	non-phage	0
NOF013_scaffold22358_2	non-phage	0
NOF013_scaffold34333_1	non-phage	0
NOF013_scaffold33854_2	non-phage	0
NOF013_scaffold33280_2	non-phage	0
NOF013_scaffold3132_5	non-phage	0
NOF013_scaffold6505_1	non-phage	0
NOF013_scaffold26783_2	non-phage	0
NOF013_scaffold34414_3	non-phage	0
NOF013_scaffold23261_1	non-phage	0
NOF013_scaffold10848_1	non-phage	0
NOF013_scaffold29109_3	non-phage	0
NOF013_scaffold9389_1	non-phage	0
NOF013_scaffold34719_2	non-phage	0
NOF013_scaffold28391_2	non-phage	0
NOF013_scaffold6505_5	non-phage	0
NOF013_C612606_1	non-phage	0
NOF013_scaffold33505_1	non-phage	0
NOF013_scaffold23505_2	non-phage	0
NOF013_scaffold32806_1	non-phage	0
NOF013_scaffold14118_1	non-phage	0
NOF013_scaffold17520_4	non-phage	0
NOF013_scaffold34229_7	non-phage	0
NOF013_scaffold26718_2	non-phage	0
NOF013_scaffold12336_12	non-phage	0
NOF013_scaffold29416_4	non-phage	0
NOF013_scaffold17799_1	non-phage	0

NOF013_scaffold119_7	non-phage	0
NOF013_scaffold34536_1	non-phage	0.025
NOF013_scaffold15490_1	non-phage	0
NOF013_scaffold235_2	non-phage	0
NOF013_scaffold34546_1	non-phage	0
NOF013_scaffold9607_1	non-phage	0
NOF013_scaffold25567_4	non-phage	0
NOF013_scaffold22999_3	phage	0.997
NOF013_scaffold47_2	non-phage	0
NOF013_scaffold11455_6	non-phage	0
NOF013_scaffold6162_3	non-phage	0
NOF013_scaffold16873_3	non-phage	0
NOF013_scaffold15182_2	non-phage	0
NOF013_scaffold32474_2	non-phage	0
NOF013_scaffold33558_1	non-phage	0
NOF013_scaffold32172_3	phage	0.997
NOF013_scaffold15071_1	non-phage	0
NOF013_scaffold4_10	non-phage	0
NOF013_scaffold13792_3	non-phage	0
NOF013_scaffold7439_1	non-phage	0
NOF013_scaffold9227_8	non-phage	0
NOF013_scaffold30186_1	non-phage	0
NOF013_scaffold16069_2	non-phage	0
NOF013_scaffold33048_1	non-phage	0
NOF013_scaffold9983_25	non-phage	0
NOF013_scaffold10146_2	non-phage	0
NOF013_scaffold22526_1	non-phage	0
NOF013_scaffold1181_9	non-phage	0
NOF013_scaffold15363_2	phage	0.999
NOF013_scaffold16662_1	non-phage	0
NOF013_scaffold4934_3	non-phage	0
NOF013_scaffold33522_1	non-phage	0
NOF013_scaffold13114_1	non-phage	0
NOF013_scaffold13890_5	non-phage	0
NOF013_scaffold23510_11	phage	1
NOF013_scaffold24218_1	non-phage	0
NOF013_scaffold34563_2	non-phage	0
NOF013_scaffold2599_6	non-phage	0
NOF013_scaffold119_3	non-phage	0
NOF013_scaffold3182_1	non-phage	0
NOF013_scaffold33176_1	non-phage	0
NOF013_scaffold3621_3_1	non-phage	0
NOF013_scaffold23261_2	non-phage	0
NOF013_scaffold29458_2	non-phage	0
NOF013_scaffold10553_3	non-phage	0
NOF013_scaffold9802_1	non-phage	0

NOF013_scaffold19265_2	non-phage	0
NOF013_scaffold34222_2	non-phage	0
NOF013_scaffold1606_2	non-phage	0
NOF013_scaffold24_2	non-phage	0
NOF013_scaffold10339_2	non-phage	0
NOF013_scaffold26783_3	non-phage	0
NOF013_scaffold26323_2	non-phage	0
NOF013_scaffold16752_1	non-phage	0
NOF013_scaffold34569_1	non-phage	0
NOF013_scaffold3933_2	non-phage	0
NOF013_scaffold3979_6	non-phage	0
NOF013_C613232_1	non-phage	0
NOF013_scaffold13093_5	non-phage	0
NOF013_scaffold34382_1	phage	0.996
NOF013_scaffold25835_2	non-phage	0
NOF013_scaffold34566_2	non-phage	0
NOF013_scaffold16252_4	non-phage	0
NOF013_C613108_1	phage	0.999
NOF013_scaffold10722_4	non-phage	0
NOF013_scaffold12765_2	non-phage	0
NOF013_scaffold9723_1	non-phage	0
NOF013_scaffold3933_4	non-phage	0
NOF013_scaffold3248_5	non-phage	0
NOF013_scaffold2909_1	non-phage	0
NOF013_scaffold2329_2	non-phage	0
NOF013_scaffold12416_5	non-phage	0
NOF013_scaffold4136_14	non-phage	0
NOF013_scaffold29883_1	non-phage	0
NOF013_scaffold33575_3	non-phage	0
NOF013_scaffold13034_4	non-phage	0
NOF013_scaffold3868_3	non-phage	0
NOF013_scaffold119_6	non-phage	0
NOF013_scaffold33575_1	non-phage	0
NOF013_scaffold1181_6	phage	0.999
NOF013_scaffold131_1	phage	0.99
NOF013_scaffold24851_1	non-phage	0
NOF013_scaffold25305_2	non-phage	0
NOF013_scaffold34346_1	non-phage	0
NOF013_scaffold22228_2	non-phage	0
NOF013_C612464_1	phage	0.9
NOF013_scaffold16332_2	non-phage	0
NOF013_scaffold31248_2	non-phage	0
NOF013_scaffold17337_1	non-phage	0
NOF013_scaffold32572_1	non-phage	0
NOF013_scaffold18815_1	non-phage	0
NOF013_scaffold270_1	non-phage	0

NOF013_scaffold21844_2	non-phage	0
NOF013_scaffold172_9	non-phage	0
NOF013_scaffold27004_11	non-phage	0
NOF013_scaffold33363_1	phage	0.996
NOF013_scaffold34648_1	non-phage	0
NOF013_scaffold24804_6	non-phage	0
NOF013_scaffold33134_1	phage	0.959
NOF013_scaffold34703_5	non-phage	0
NOF013_scaffold1744_3	phage	0.996
NOF013_scaffold24211_1	non-phage	0
NOF013_scaffold32891_4	phage	0.999
NOF013_scaffold19745_1	non-phage	0
NOF013_scaffold32191_1	non-phage	0
NOF013_scaffold20730_6	non-phage	0
NOF013_scaffold9227_11	non-phage	0
NOF013_scaffold26019_3	non-phage	0
NOF013_scaffold34414_4	non-phage	0
NOF013_scaffold6401_2	non-phage	0
NOF013_scaffold33613_1	non-phage	0
NOF013_scaffold13890_1	non-phage	0
NOF013_scaffold34310_3	non-phage	0
NOF013_scaffold29322_2	non-phage	0
NOF013_scaffold34371_1	non-phage	0
NOF013_scaffold4120_3	non-phage	0
NOF013_scaffold227_5	non-phage	0
NOF013_scaffold16332_1	non-phage	0
NOF013_scaffold9518_7	non-phage	0
NOF013_scaffold18916_5	non-phage	0
NOF013_scaffold34438_5	non-phage	0
NOF013_C612530_1	non-phage	0
NOF013_scaffold31349_1	non-phage	0
NOF013_scaffold18268_3	non-phage	0
NOF013_C613234_1	non-phage	0
NOF013_scaffold10786_4	non-phage	0
NOF013_scaffold26892_1	non-phage	0
NOF013_scaffold34703_2	non-phage	0
NOF013_scaffold26429_2	non-phage	0
NOF013_scaffold33054_1	non-phage	0
NOF013_scaffold16877_1	non-phage	0
NOF013_scaffold14326_3	non-phage	0
NOF013_scaffold33642_1	non-phage	0
NOF013_scaffold25534_2	non-phage	0
NOF013_scaffold17101_2	non-phage	0
NOF013_scaffold18866_4	non-phage	0
NOF013_scaffold34325_1	non-phage	0
NOF013_scaffold30551_1	non-phage	0

NOF013_scaffold24494_1	non-phage	0
NOF013_scaffold28599_1	non-phage	0
NOF013_scaffold11774_1	non-phage	0
NOF013_scaffold19960_1	non-phage	0
NOF013_scaffold33879_1	non-phage	0
NOF013_scaffold20440_1	non-phage	0
NOF013_scaffold29251_5	non-phage	0
NOF013_scaffold8022_4	non-phage	0
NOF013_scaffold21817_4	non-phage	0
NOF013_scaffold34368_1	non-phage	0
NOF013_scaffold1440_1	non-phage	0
NOF013_scaffold28507_1	non-phage	0
NOF013_scaffold30238_2	non-phage	0
NOF013_scaffold6002_6	non-phage	0
NOF013_scaffold3456_25	non-phage	0
NOF013_scaffold9983_18	non-phage	0.09
NOF013_scaffold594_66	non-phage	0
NOF013_scaffold25811_2	non-phage	0
NOF013_scaffold26579_1	non-phage	0
NOF013_scaffold124_2	non-phage	0
NOF013_scaffold155_1	non-phage	0
NOF013_scaffold7403_2	non-phage	0
NOF013_scaffold34629_1	non-phage	0
NOF013_scaffold33523_1	non-phage	0
NOF013_scaffold7403_17	non-phage	0
NOF013_scaffold32485_1	non-phage	0
NOF013_scaffold33771_3	non-phage	0
NOF013_scaffold9227_2	non-phage	0
NOF013_scaffold29173_1	phage	0.986
NOF013_scaffold27004_7	non-phage	0
NOF013_scaffold33793_2	non-phage	0
NOF013_scaffold26592_1	non-phage	0
NOF013_scaffold2193_6	non-phage	0
NOF013_scaffold6873_7	non-phage	0
NOF013_scaffold20517_1	non-phage	0
NOF013_scaffold34686_4	non-phage	0
NOF013_scaffold9802_2	non-phage	0
NOF013_scaffold23994_2	non-phage	0
NOF013_scaffold6505_3	non-phage	0
NOF013_scaffold23399_2	non-phage	0
NOF013_scaffold207_5	non-phage	0
NOF013_scaffold10902_1	non-phage	0
NOF013_scaffold34718_3	non-phage	0
NOF013_scaffold8658_4	non-phage	0
NOF013_scaffold34522_2	non-phage	0
NOF013_scaffold11455_5	non-phage	0

NOF013_scaffold11317_2	non-phage	0
NOF013_scaffold14974_3	non-phage	0
NOF013_scaffold16752_2	non-phage	0
NOF013_scaffold31503_1	non-phage	0
NOF013_scaffold8038_4	non-phage	0
NOF013_scaffold25516_1	non-phage	0
NOF013_scaffold33848_1	non-phage	0
NOF013_scaffold4120_6	non-phage	0.051
NOF013_scaffold8597_8	non-phage	0
NOF013_scaffold10722_1	non-phage	0
NOF013_scaffold24443_1	phage	0.962
NOF013_scaffold24512_1	non-phage	0
NOF013_scaffold13658_8	non-phage	0
NOF013_scaffold30520_1	non-phage	0
NOF013_scaffold15158_3	non-phage	0
NOF013_scaffold31945_2	non-phage	0
NOF013_scaffold132_1	non-phage	0
NOF013_scaffold34485_2	non-phage	0
NOF013_scaffold10437_1	non-phage	0
NOF013_scaffold3033_3	non-phage	0
NOF013_scaffold16317_2	non-phage	0
NOF013_scaffold33575_4	non-phage	0
NOF013_scaffold34736_1	non-phage	0
NOF013_scaffold3868_4	non-phage	0
NOF013_scaffold34585_2	non-phage	0
NOF013_scaffold21859_3	non-phage	0
NOF013_scaffold14842_10	non-phage	0
NOF013_scaffold33789_2	non-phage	0
NOF013_scaffold11109_4	non-phage	0
NOF013_scaffold16252_1	non-phage	0
NOF013_scaffold19394_1	non-phage	0
NOF013_scaffold34537_2	non-phage	0
NOF013_scaffold34727_1	non-phage	0
NOF013_scaffold23796_1	non-phage	0
NOF013_C612752_1	non-phage	0
NOF013_scaffold33972_1	non-phage	0
NOF013_scaffold3979_5	phage	0.856
NOF013_scaffold23636_1	non-phage	0
NOF013_scaffold32150_1	non-phage	0
NOF013_scaffold33001_3	non-phage	0
NOF013_scaffold31567_2	non-phage	0
NOF013_scaffold17248_2	non-phage	0
NOF013_scaffold31503_4	non-phage	0
NOF013_scaffold1022_1	non-phage	0
NOF013_scaffold117_2	non-phage	0
NOF013_scaffold34329_4	non-phage	0

NOF013_scaffold27517_2	non-phage	0
NOF013_scaffold34547_1	non-phage	0
NOF013_scaffold33086_2	non-phage	0
NOF013_scaffold4522_12	non-phage	0
NOF013_scaffold13801_2	non-phage	0
NOF013_scaffold15248_1	non-phage	0
NOF013_scaffold4760_2	non-phage	0
NOF013_scaffold31206_1	non-phage	0.001
NOF013_scaffold24_5	non-phage	0
NOF013_scaffold34589_1	non-phage	0
NOF013_scaffold9478_5	non-phage	0
NOF013_scaffold16111_2	non-phage	0
NOF013_scaffold29374_1	non-phage	0
NOF013_scaffold34703_3	non-phage	0
NOF013_scaffold25061_4	non-phage	0
NOF013_scaffold18866_5	non-phage	0
NOF013_scaffold10516_5	non-phage	0
NOF013_scaffold2829_12	non-phage	0
NOF013_scaffold34553_1	non-phage	0
NOF013_scaffold31189_1	non-phage	0
NOF013_scaffold34711_2	non-phage	0
NOF013_scaffold34356_2	non-phage	0
NOF013_scaffold34706_2	non-phage	0
NOF013_scaffold651_4	non-phage	0
NOF013_scaffold33527_3	non-phage	0
NOF013_scaffold29797_1	non-phage	0
NOF013_scaffold7403_5	non-phage	0
NOF013_scaffold29297_2	non-phage	0
NOF013_scaffold2405_2	non-phage	0
NOF013_scaffold1419_22	non-phage	0
NOF013_scaffold4120_4	non-phage	0
NOF013_scaffold29730_2	non-phage	0
NOF013_scaffold26437_3	non-phage	0
NOF013_scaffold12910_12	non-phage	0
NOF013_scaffold34329_1	non-phage	0
NOF013_scaffold34324_3	phage	0.851
NOF013_scaffold25867_6	non-phage	0
NOF013_scaffold39_5	non-phage	0
NOF013_scaffold32659_1	non-phage	0
NOF013_scaffold5920_3	non-phage	0
NOF013_scaffold2331_10	non-phage	0
NOF013_scaffold30884_2	non-phage	0
NOF013_scaffold24_1	non-phage	0
NOF013_scaffold6401_1	non-phage	0
NOF013_scaffold16873_4	non-phage	0
NOF013_scaffold12141_2	non-phage	0

NOF013_scaffold34207_1	non-phage	0
NOF013_scaffold112_1	non-phage	0
NOF013_scaffold24710_2	non-phage	0
NOF013_scaffold9250_3	non-phage	0
NOF013_scaffold3996_1	non-phage	0
NOF013_scaffold4620_4	non-phage	0
NOF013_scaffold132_6	non-phage	0
NOF013_scaffold13792_1	non-phage	0
NOF013_scaffold17100_6	non-phage	0
NOF013_scaffold13658_7	non-phage	0
NOF013_scaffold28003_2	non-phage	0
NOF013_scaffold154_1	non-phage	0
NOF013_scaffold194_5	non-phage	0
NOF013_scaffold18749_1	non-phage	0
NOF013_scaffold34728_1	non-phage	0
NOF013_scaffold24298_3	non-phage	0
NOF013_scaffold23265_4	non-phage	0
NOF013_scaffold33001_2	non-phage	0
NOF013_scaffold8559_1	non-phage	0
NOF013_scaffold9976_1	non-phage	0
NOF013_C612504_1	non-phage	0
NOF013_scaffold34570_1	non-phage	0
NOF013_scaffold207_1	non-phage	0
NOF013_scaffold9793_1	non-phage	0
NOF013_scaffold12910_21	non-phage	0
NOF013_scaffold19781_3	non-phage	0
NOF013_scaffold22916_2	non-phage	0
NOF013_scaffold34364_1	non-phage	0
NOF013_scaffold11465_1	non-phage	0
NOF013_scaffold1419_6	non-phage	0
NOF013_scaffold10553_1	non-phage	0.253
NOF013_scaffold4934_1	non-phage	0
NOF013_scaffold504_8	non-phage	0
NOF013_scaffold12910_16	non-phage	0
NOF013_scaffold33613_4	non-phage	0
NOF013_scaffold33086_3	non-phage	0
NOF013_scaffold7705_1	non-phage	0
NOF013_scaffold27270_1	non-phage	0
NOF013_scaffold2241_2	phage	0.998
NOF013_scaffold9478_2	non-phage	0
NOF013_scaffold23438_4	non-phage	0
NOF013_scaffold1888_3	non-phage	0
NOF013_scaffold20633_2	non-phage	0
NOF013_scaffold19824_7	non-phage	0
NOF013_scaffold23253_1	non-phage	0
NOF013_scaffold6723_3	non-phage	0

NOF013_scaffold33087_2	non-phage	0
NOF013_scaffold24312_1	non-phage	0
NOF013_scaffold207_4	non-phage	0
NOF013_scaffold19329_1	non-phage	0
NOF013_scaffold18987_2	non-phage	0
NOF013_scaffold9802_3	non-phage	0
NOF013_scaffold2971_3	non-phage	0
NOF013_scaffold33410_2	non-phage	0
NOF013_scaffold10030_2	non-phage	0
NOF013_scaffold32474_1	non-phage	0
NOF013_scaffold194_4	non-phage	0
NOF013_scaffold32164_1	non-phage	0
NOF013_scaffold1904_100	non-phage	0
NOF013_scaffold28683_1	non-phage	0
NOF013_scaffold27070_3	non-phage	0
NOF013_scaffold15158_1	non-phage	0
NOF013_scaffold26592_2	non-phage	0
NOF013_scaffold33395_2	non-phage	0
NOF013_scaffold7403_16	non-phage	0.248
NOF013_scaffold34459_2	non-phage	0
NOF013_scaffold2383_2	non-phage	0
NOF013_scaffold275_5	non-phage	0
NOF014_scaffold68206_1	non-phage	0
NOF014_scaffold24380_4	non-phage	0
NOF014_scaffold22659_1	non-phage	0
NOF014_scaffold8296_6	non-phage	0
NOF014_scaffold54582_1	non-phage	0
NOF014_scaffold70157_4	non-phage	0
NOF014_scaffold40001_12	non-phage	0
NOF014_scaffold120_3	non-phage	0
NOF014_scaffold58643_6	non-phage	0
NOF014_scaffold26905_6	non-phage	0
NOF014_scaffold64083_3	non-phage	0
NOF014_scaffold496_2	non-phage	0
NOF014_scaffold67337_2	phage	0.996
NOF014_scaffold52637_4	non-phage	0
NOF014_scaffold37012_4	non-phage	0
NOF014_scaffold62155_2	non-phage	0
NOF014_scaffold28449_4	non-phage	0
NOF014_scaffold71890_1	non-phage	0
NOF014_scaffold54708_2	non-phage	0
NOF014_scaffold46501_10	non-phage	0
NOF014_scaffold41027_8	non-phage	0
NOF014_scaffold29077_2	phage	0.999
NOF014_scaffold177_1	non-phage	0
NOF014_scaffold43_13	non-phage	0

NOF014_scaffold6428_2	non-phage	0
NOF014_scaffold30528_10	non-phage	0
NOF014_scaffold71085_1	non-phage	0
NOF014_scaffold52954_1	non-phage	0
NOF014_scaffold216_17	non-phage	0
NOF014_scaffold4753_5	non-phage	0
NOF014_scaffold60029_3	non-phage	0
NOF014_scaffold70598_5	non-phage	0
NOF014_scaffold69592_1	non-phage	0
NOF014_scaffold249_1	non-phage	0
NOF014_scaffold20787_11	non-phage	0
NOF014_scaffold38588_15	non-phage	0
NOF014_scaffold70742_1	non-phage	0
NOF014_scaffold72236_2	non-phage	0
NOF014_scaffold18639_1	non-phage	0
NOF014_scaffold41735_1	phage	0.988
NOF014_scaffold70287_2	non-phage	0
NOF014_scaffold53546_3	non-phage	0
NOF014_scaffold4940_43	non-phage	0
NOF014_scaffold17282_6	non-phage	0
NOF014_scaffold48645_4	non-phage	0.189
NOF014_scaffold34818_2	non-phage	0
NOF014_scaffold55836_1	non-phage	0
NOF014_scaffold72277_2	non-phage	0
NOF014_scaffold59979_2	phage	0.997
NOF014_scaffold4940_5	non-phage	0
NOF014_scaffold23303_30	non-phage	0
NOF014_scaffold41204_21	non-phage	0
NOF014_scaffold72087_1	non-phage	0
NOF014_scaffold71626_2	non-phage	0
NOF014_scaffold62257_1	non-phage	0
NOF014_scaffold43540_16	non-phage	0
NOF014_scaffold15856_1	non-phage	0
NOF014_scaffold43249_2	non-phage	0
NOF014_scaffold41523_7	non-phage	0
NOF014_scaffold43_12	non-phage	0
NOF014_scaffold463_1	non-phage	0
NOF014_scaffold26440_3	non-phage	0
NOF014_scaffold39735_5	non-phage	0
NOF014_scaffold64335_2	non-phage	0
NOF014_scaffold30038_2	non-phage	0
NOF014_scaffold24447_2	non-phage	0
NOF014_scaffold55387_16	non-phage	0
NOF014_scaffold34818_10	non-phage	0
NOF014_scaffold58457_2	non-phage	0
NOF014_scaffold72169_1	non-phage	0

NOF014_scaffold4965_8	non-phage	0
NOF014_scaffold19437_10	non-phage	0
NOF014_scaffold35947_4	non-phage	0
NOF014_scaffold72132_1	phage	0.997
NOF014_C988904_1	non-phage	0
NOF014_scaffold22914_17	non-phage	0
NOF014_scaffold71853_3	non-phage	0
NOF014_scaffold37334_6	non-phage	0
NOF014_scaffold47868_3	non-phage	0
NOF014_scaffold29772_3	non-phage	0
NOF014_scaffold37012_11	non-phage	0
NOF014_scaffold71263_1	non-phage	0
NOF014_scaffold65521_1	non-phage	0
NOF014_scaffold21218_11	non-phage	0
NOF014_scaffold41283_10	non-phage	0
NOF014_scaffold62050_5	non-phage	0
NOF014_scaffold20265_9	non-phage	0
NOF014_scaffold17899_1	non-phage	0
NOF014_scaffold4528_3	non-phage	0
NOF014_scaffold2358_1	non-phage	0
NOF014_scaffold47868_1	non-phage	0
NOF014_scaffold14628_12	non-phage	0
NOF014_scaffold11234_17	non-phage	0
NOF014_scaffold30730_5	phage	0.998
NOF014_scaffold66457_1	non-phage	0
NOF014_scaffold69185_3	non-phage	0
NOF014_scaffold71448_5	non-phage	0
NOF014_scaffold39735_16	non-phage	0
NOF014_scaffold55387_3	non-phage	0
NOF014_scaffold71373_1	non-phage	0
NOF014_scaffold54610_3	non-phage	0
NOF014_scaffold20327_3	non-phage	0
NOF014_scaffold68447_1	non-phage	0
NOF014_scaffold25084_3	non-phage	0
NOF014_scaffold72006_1	phage	0.999
NOF014_scaffold20686_11	non-phage	0
NOF014_scaffold66648_1	non-phage	0
NOF014_scaffold26105_23	non-phage	0
NOF014_scaffold11234_7	non-phage	0
NOF014_scaffold19437_7	non-phage	0
NOF014_scaffold6730_5	phage	0.996
NOF014_scaffold25107_9	non-phage	0
NOF014_scaffold71626_3	phage	0.996
NOF014_scaffold30343_5	non-phage	0
NOF014_scaffold249_2	non-phage	0
NOF014_scaffold20669_10	non-phage	0

NOF014_scaffold34250_10	non-phage	0
NOF014_scaffold70385_1	non-phage	0
NOF014_scaffold46167_2	phage	0.998
NOF014_scaffold3467_2	non-phage	0
NOF014_scaffold34818_1	non-phage	0
NOF014_scaffold61340_7	non-phage	0
NOF014_scaffold72283_1	non-phage	0
NOF014_scaffold64888_4	non-phage	0
NOF014_scaffold45222_4	non-phage	0
NOF014_scaffold13862_3	non-phage	0
NOF014_scaffold168_2	non-phage	0
NOF014_scaffold70918_2	non-phage	0
NOF014_scaffold72271_3	non-phage	0
NOF014_scaffold69463_7	non-phage	0
NOF014_scaffold72293_2	non-phage	0
NOF014_scaffold53544_1	non-phage	0
NOF014_scaffold42652_11	non-phage	0
NOF014_scaffold56646_3	non-phage	0
NOF014_scaffold69964_2	non-phage	0
NOF014_scaffold69982_1	non-phage	0
NOF014_scaffold30528_2	non-phage	0
NOF014_scaffold71856_3	non-phage	0
NOF014_scaffold21218_19	non-phage	0
NOF014_scaffold24607_7	non-phage	0
NOF014_scaffold11030_8	non-phage	0.216
NOF014_scaffold38249_4	non-phage	0
NOF014_scaffold70600_3	non-phage	0
NOF014_scaffold35720_11	phage	0.999
NOF014_scaffold16488_2	non-phage	0
NOF014_scaffold63658_2	non-phage	0
NOF014_scaffold71771_2	non-phage	0
NOF014_scaffold19758_1	non-phage	0
NOF014_scaffold72065_1	non-phage	0
NOF014_scaffold70183_1	non-phage	0
NOF014_scaffold64252_2	non-phage	0
NOF014_scaffold117_1	non-phage	0
NOF014_scaffold31525_5	non-phage	0
NOF014_scaffold4327_52	phage	0.999
NOF014_scaffold37901_6	non-phage	0
NOF014_scaffold18639_2	non-phage	0
NOF014_scaffold38016_1	non-phage	0
NOF014_scaffold72161_3	non-phage	0
NOF014_scaffold55348_1	non-phage	0
NOF014_scaffold28648_1	non-phage	0
NOF014_scaffold54976_1	non-phage	0
NOF014_scaffold63888_1	non-phage	0

NOF014_scaffold69943_1	non-phage	0
NOF014_scaffold40347_4	phage	1
NOF014_scaffold41204_4	non-phage	0
NOF014_scaffold2358_4	non-phage	0
NOF014_scaffold63144_1	non-phage	0
NOF014_scaffold72227_1	phage	0.999
NOF014_scaffold6309_3	non-phage	0
NOF014_scaffold48645_1	non-phage	0
NOF014_scaffold67661_1	non-phage	0.007
NOF014_scaffold21516_6	non-phage	0
NOF014_scaffold13862_5	non-phage	0
NOF014_scaffold54928_4	non-phage	0
NOF014_scaffold9890_11	non-phage	0
NOF014_scaffold29_14	non-phage	0
NOF014_scaffold72298_1	non-phage	0
NOF014_scaffold2849_2	non-phage	0
NOF014_scaffold47868_5	non-phage	0
NOF014_scaffold7886_6	non-phage	0
NOF014_scaffold57951_4	non-phage	0
NOF014_scaffold35120_11	non-phage	0
NOF014_scaffold57951_3	non-phage	0
NOF014_scaffold72164_1	non-phage	0
NOF014_scaffold11916_4	non-phage	0
NOF014_scaffold53188_1	non-phage	0
NOF014_scaffold27848_7	non-phage	0
NOF014_scaffold43_11	non-phage	0
NOF014_scaffold70721_1	non-phage	0
NOF014_scaffold62304_2	non-phage	0
NOF014_scaffold27769_8	phage	0.999
NOF014_scaffold52372_1	non-phage	0
NOF014_scaffold129_1	non-phage	0
NOF014_scaffold22914_7	non-phage	0
NOF014_scaffold15365_6	non-phage	0
NOF014_scaffold22051_1	non-phage	0
NOF014_scaffold72288_2	non-phage	0
NOF014_scaffold67473_2	non-phage	0
NOF014_scaffold28648_14	non-phage	0
NOF014_scaffold463_10	non-phage	0
NOF014_scaffold14628_1	non-phage	0.01
NOF014_scaffold69185_4	non-phage	0
NOF014_scaffold39735_15	non-phage	0
NOF014_scaffold55387_11	non-phage	0
NOF014_scaffold37012_23	non-phage	0
NOF014_scaffold22096_1	non-phage	0
NOF014_scaffold69288_1	non-phage	0
NOF014_scaffold54976_4	non-phage	0

NOF014_scaffold13295_6	non-phage	0
NOF014_scaffold1340_4	non-phage	0
NOF014_scaffold60803_1	non-phage	0
NOF014_scaffold20787_9	non-phage	0
NOF014_scaffold10135_5	phage	0.999
NOF014_scaffold70638_1	non-phage	0
NOF014_scaffold38588_2	non-phage	0
NOF014_scaffold53987_4	non-phage	0
NOF014_scaffold66855_1	phage	0.998
NOF014_scaffold7923_7	non-phage	0
NOF014_scaffold6848_2	non-phage	0
NOF014_scaffold67473_3	non-phage	0
NOF014_scaffold37156_18	non-phage	0
NOF014_scaffold72263_3	phage	0.965
NOF014_scaffold20864_2	non-phage	0
NOF014_scaffold9380_3	non-phage	0
NOF014_scaffold156_3	non-phage	0
NOF014_scaffold20864_7	non-phage	0
NOF014_scaffold66281_5	non-phage	0
NOF014_scaffold30528_14	non-phage	0.088
NOF014_scaffold15360_2	non-phage	0
NOF014_scaffold41870_1	non-phage	0
NOF014_scaffold72161_4	non-phage	0
NOF014_scaffold41751_2	non-phage	0
NOF014_scaffold7923_6	non-phage	0
NOF014_scaffold43_10	phage	0.997
NOF014_scaffold41283_3	non-phage	0
NOF014_scaffold15_1	non-phage	0
NOF014_scaffold39775_6	non-phage	0
NOF014_scaffold72138_1	non-phage	0
NOF014_scaffold71421_1	non-phage	0
NOF014_scaffold48431_3	non-phage	0
NOF014_scaffold148_6	non-phage	0
NOF014_scaffold66371_14	non-phage	0
NOF014_scaffold26105_6	phage	0.999
NOF014_scaffold34250_28	non-phage	0
NOF014_scaffold46501_13	non-phage	0
NOF014_scaffold20787_3	phage	0.977
NOF014_scaffold4327_49	non-phage	0
NOF014_scaffold72065_2	non-phage	0
NOF014_scaffold52163_4	non-phage	0
NOF014_scaffold19962_3	phage	0.998
NOF014_scaffold34250_21	non-phage	0
NOF014_scaffold12830_6	non-phage	0
NOF014_scaffold8485_1	non-phage	0
NOF014_scaffold29595_10	non-phage	0

NOF014_scaffold19852_13	non-phage	0
NOF014_scaffold39735_8	non-phage	0
NOF014_scaffold66281_1	non-phage	0
NOF014_scaffold44142_1	non-phage	0
NOF014_scaffold44142_18	non-phage	0
NOF014_scaffold2216_4	non-phage	0
NOF014_scaffold9890_12	non-phage	0
NOF014_scaffold70315_1	non-phage	0
NOF014_scaffold4004_4	non-phage	0
NOF014_scaffold38062_4	non-phage	0
NOF014_scaffold64083_2	non-phage	0
NOF014_scaffold1188_20	non-phage	0
NOF014_scaffold46501_11	non-phage	0
NOF014_scaffold20787_8	non-phage	0
NOF014_scaffold34534_5	non-phage	0
NOF014_scaffold588_2	non-phage	0
NOF014_scaffold28383_4	non-phage	0
NOF014_scaffold70891_1	non-phage	0
NOF014_scaffold77_4	non-phage	0
NOF014_scaffold11916_11	non-phage	0
NOF014_scaffold72271_1	non-phage	0
NOF014_scaffold20265_6	non-phage	0
NOF014_scaffold13844_7	non-phage	0
NOF014_scaffold29203_20	non-phage	0
NOF014_scaffold65667_3	non-phage	0
NOF014_scaffold4710_2	non-phage	0
NOF014_scaffold62_4	non-phage	0
NOF014_scaffold309_8	non-phage	0
NOF014_scaffold26781_2	non-phage	0
NOF014_scaffold22914_9	non-phage	0
NOF014_scaffold38249_2	non-phage	0
NOF014_scaffold14549_10	non-phage	0
NOF014_scaffold49025_3	non-phage	0
NOF014_scaffold34818_11	non-phage	0
NOF014_scaffold15856_13	non-phage	0
NOF014_scaffold19264_9	non-phage	0
NOF014_scaffold6908_14	non-phage	0
NOF014_scaffold59922_2	non-phage	0
NOF014_scaffold4327_50	non-phage	0
NOF014_scaffold4528_1	non-phage	0
NOF014_scaffold31140_2	non-phage	0
NOF014_scaffold10486_4	non-phage	0
NOF014_scaffold63476_2	phage	0.998
NOF014_scaffold71785_1	non-phage	0
NOF014_scaffold72125_1	non-phage	0
NOF014_scaffold22790_2	non-phage	0

NOF014_scaffold36523_2	non-phage	0
NOF014_scaffold31065_5	non-phage	0
NOF014_scaffold55711_1	non-phage	0
NOF014_scaffold71534_3	non-phage	0
NOF014_scaffold26702_2	non-phage	0
NOF014_scaffold34818_3	non-phage	0
NOF014_scaffold71739_2	non-phage	0
NOF014_scaffold11212_10	non-phage	0
NOF014_scaffold107_5	non-phage	0
NOF014_scaffold270_3	non-phage	0
NOF014_scaffold20864_6	non-phage	0
NOF014_scaffold9890_5	non-phage	0
NOF014_scaffold72143_3	non-phage	0
NOF014_scaffold26790_6	non-phage	0
NOF014_scaffold21516_1	non-phage	0
NOF014_scaffold17886_28	non-phage	0
NOF014_scaffold64990_5	non-phage	0
NOF014_scaffold71912_1	non-phage	0
NOF014_scaffold14884_2	non-phage	0
NOF014_scaffold61486_1	non-phage	0
NOF014_scaffold21877_4	non-phage	0
NOF014_scaffold4567_11	non-phage	0
NOF014_scaffold11916_6	non-phage	0
NOF014_scaffold140_4	non-phage	0
NOF014_scaffold40978_1	non-phage	0
NOF014_scaffold26105_25	phage	0.972
NOF014_scaffold72216_1	phage	0.999
NOF014_scaffold10667_4	phage	0.883
NOF014_scaffold4731_8	non-phage	0
NOF014_scaffold55135_2	non-phage	0
NOF014_scaffold70638_6	non-phage	0
NOF014_scaffold41204_20	non-phage	0
NOF014_scaffold49291_2	non-phage	0
NOF014_scaffold50096_1	non-phage	0
NOF014_scaffold36044_2	non-phage	0
NOF014_scaffold72275_1	non-phage	0
NOF014_C988796_1	non-phage	0
NOF014_scaffold72026_1	non-phage	0
NOF014_scaffold71437_2	non-phage	0
NOF014_scaffold18358_2	non-phage	0
NOF014_scaffold25084_1	non-phage	0
NOF014_scaffold22659_5	non-phage	0
NOF014_scaffold39735_18	non-phage	0
NOF014_scaffold71443_1	non-phage	0
NOF014_scaffold59222_3	non-phage	0
NOF014_scaffold15172_3	non-phage	0

NOF014_scaffold69_2	non-phage	0
NOF014_scaffold52663_4	phage	0.999
NOF014_scaffold43537_16	non-phage	0
NOF014_scaffold249_4	non-phage	0
NOF014_scaffold51244_2	non-phage	0
NOF014_scaffold37286_1	non-phage	0
NOF014_scaffold536_11	non-phage	0
NOF014_scaffold17823_2	non-phage	0
NOF014_scaffold41047_2	non-phage	0
NOF014_scaffold9890_2	non-phage	0
NOF014_scaffold37623_2	non-phage	0
NOF014_scaffold52931_4	non-phage	0
NOF014_scaffold43540_17	non-phage	0
NOF014_scaffold54232_3	non-phage	0
NOF014_scaffold11234_18	non-phage	0
NOF014_scaffold216_6	non-phage	0
NOF014_scaffold67850_3	non-phage	0
NOF014_scaffold54610_4	non-phage	0
NOF014_scaffold43540_12	non-phage	0
NOF014_scaffold72100_1	non-phage	0
NOF014_scaffold68026_1	non-phage	0
NOF014_scaffold72070_1	non-phage	0
NOF014_scaffold19264_4	non-phage	0
NOF014_scaffold19852_6	non-phage	0
NOF014_scaffold28648_15	non-phage	0
NOF014_scaffold309_9	non-phage	0
NOF014_scaffold14549_13	non-phage	0
NOF014_scaffold50103_13	non-phage	0
NOF014_scaffold19758_3	non-phage	0
NOF014_scaffold68447_2	non-phage	0
NOF014_scaffold38249_7	non-phage	0
NOF014_scaffold72202_1	non-phage	0
NOF014_scaffold54136_18	non-phage	0
NOF014_scaffold4710_1	non-phage	0
NOF014_scaffold21516_4	non-phage	0
NOF014_scaffold71919_3	phage	0.999
NOF014_scaffold66281_7	non-phage	0
NOF014_scaffold13862_9	non-phage	0
NOF014_scaffold50103_21	non-phage	0
NOF014_scaffold38249_3	non-phage	0
NOF014_scaffold14628_7	non-phage	0
NOF014_scaffold42447_1	non-phage	0
NOF014_scaffold22318_4	non-phage	0
NOF014_scaffold38588_21	non-phage	0
NOF014_scaffold18803_1	non-phage	0
NOF014_scaffold34839_6	non-phage	0

NOF014_scaffold71994_1	non-phage	0
NOF014_scaffold30914_4	non-phage	0
NOF014_scaffold234_3	non-phage	0
NOF014_scaffold21218_16	non-phage	0
NOF014_scaffold13862_2	non-phage	0
NOF014_scaffold62_13	non-phage	0
NOF014_scaffold20265_3	phage	0.999
NOF014_scaffold20265_5	non-phage	0
NOF014_scaffold5894_2	phage	0.999
NOF014_scaffold25574_2	non-phage	0
NOF014_scaffold10667_10	non-phage	0
NOF014_scaffold37522_1	non-phage	0
NOF014_scaffold72125_2	non-phage	0
NOF014_scaffold536_5	non-phage	0
NOF014_scaffold46763_5	non-phage	0
NOF014_scaffold21327_7	non-phage	0
NOF014_scaffold45310_6	non-phage	0
NOF014_scaffold37012_7	non-phage	0
NOF014_scaffold5496_2	non-phage	0
NOF014_scaffold71448_1	non-phage	0
NOF014_scaffold64540_1	non-phage	0
NOF014_scaffold47355_4	non-phage	0
NOF014_scaffold2216_6	non-phage	0
NOF014_scaffold41283_12	non-phage	0
NOF014_scaffold11916_12	non-phage	0
NOF014_scaffold4528_5	non-phage	0
NOF014_scaffold8296_3	non-phage	0
NOF014_scaffold57951_5	non-phage	0
NOF014_scaffold71579_1	non-phage	0
NOF014_scaffold49593_2	phage	0.979
NOF014_scaffold52931_3	non-phage	0
NOF014_scaffold5043_1	non-phage	0
NOF014_C988832_1	non-phage	0
NOF014_scaffold35302_2	non-phage	0
NOF014_scaffold348_6	non-phage	0
NOF014_scaffold37012_15	non-phage	0
NOF014_scaffold38588_19	non-phage	0
NOF014_scaffold17785_3	non-phage	0
NOF014_scaffold58561_2	non-phage	0
NOF014_scaffold70745_7	non-phage	0.051
NOF014_scaffold13093_4	non-phage	0
NOF014_scaffold28765_5	non-phage	0
NOF014_scaffold463_16	non-phage	0
NOF014_scaffold36693_2	non-phage	0
NOF014_scaffold65667_6	non-phage	0
NOF014_scaffold71877_1	phage	0.999

NOF014_scaffold6964_2	non-phage	0
NOF014_scaffold52663_3	non-phage	0
NOF014_scaffold29595_1	non-phage	0
NOF014_scaffold71448_2	non-phage	0
NOF014_scaffold65721_2	non-phage	0
NOF014_scaffold64741_3	non-phage	0
NOF014_scaffold32578_4	non-phage	0
NOF014_scaffold64353_1	non-phage	0
NOF014_scaffold66533_1	non-phage	0
NOF014_scaffold4940_42	non-phage	0
NOF014_scaffold55203_3	non-phage	0
NOF014_scaffold20669_5	non-phage	0
NOF014_scaffold28970_3	non-phage	0
NOF014_C988706_1	non-phage	0
NOF014_scaffold38062_17	non-phage	0
NOF014_scaffold54976_7	non-phage	0
NOF014_scaffold41283_8	non-phage	0
NOF014_scaffold36523_4	non-phage	0
NOF014_scaffold72223_1	non-phage	0
NOF014_scaffold72248_1	phage	1
NOF014_scaffold37012_3	non-phage	0
NOF014_scaffold41494_1	non-phage	0
NOF014_scaffold20864_4	non-phage	0
NOF014_scaffold27197_2	non-phage	0
NOF014_scaffold15133_3	non-phage	0
NOF014_scaffold7923_2	non-phage	0
NOF014_scaffold20686_9	non-phage	0
NOF014_scaffold52163_3	non-phage	0
NOF014_scaffold65051_1	non-phage	0
NOF014_scaffold20787_1	phage	0.98
NOF014_scaffold46501_8	non-phage	0
NOF014_scaffold72294_1	non-phage	0
NOF014_scaffold66923_1	phage	0.641
NOF014_scaffold4516_4	non-phage	0
NOF014_scaffold60756_3	non-phage	0
NOF014_scaffold40347_3	non-phage	0
NOF014_scaffold71770_1	non-phage	0
NOF014_scaffold30733_3	non-phage	0
NOF014_scaffold15360_1	non-phage	0
NOF014_scaffold22276_1	non-phage	0
NOF014_scaffold38249_8	non-phage	0
NOF014_scaffold62292_1	non-phage	0
NOF014_scaffold96_3	non-phage	0
NOF014_scaffold64740_2	non-phage	0
NOF014_scaffold8356_7	non-phage	0
NOF014_scaffold34250_27	non-phage	0

NOF014_scaffold22223_1	non-phage	0
NOF014_scaffold216_14	non-phage	0
NOF014_scaffold10667_5	non-phage	0
NOF014_scaffold7575_7	non-phage	0
NOF014_scaffold25705_1	non-phage	0
NOF014_scaffold11212_3	non-phage	0
NOF014_scaffold71708_2	non-phage	0
NOF014_scaffold69185_7	non-phage	0
NOF014_scaffold30528_25	non-phage	0
NOF014_scaffold3181_30	non-phage	0
NOF014_scaffold52981_3	non-phage	0
NOF014_scaffold62_1	non-phage	0
NOF014_scaffold65806_1	non-phage	0
NOF014_scaffold13862_12	non-phage	0
NOF014_scaffold63325_1	non-phage	0
NOF014_scaffold536_13	non-phage	0
NOF014_scaffold19852_17	non-phage	0
NOF014_scaffold1729_2	non-phage	0
NOF014_scaffold66281_6	non-phage	0
NOF014_scaffold52405_2	non-phage	0
NOF014_scaffold45649_1	non-phage	0
NOF014_scaffold19798_6	non-phage	0
NOF014_scaffold7923_5	non-phage	0
NOF014_scaffold25284_5	non-phage	0
NOF014_scaffold70630_1	non-phage	0
NOF014_scaffold22659_13	non-phage	0
NOF014_scaffold41204_9	non-phage	0
NOF014_scaffold52931_2	non-phage	0
NOF014_scaffold57464_2	non-phage	0
NOF014_scaffold33173_2	non-phage	0
NOF014_scaffold57356_2	non-phage	0
NOF014_scaffold30528_24	non-phage	0
NOF014_scaffold72180_2	non-phage	0
NOF014_scaffold72231_1	non-phage	0
NOF014_scaffold3181_27	non-phage	0
NOF014_scaffold13844_4	non-phage	0
NOF014_scaffold38062_3	non-phage	0
NOF014_scaffold20057_1	non-phage	0
NOF014_scaffold25107_13	non-phage	0
NOF014_scaffold41027_10	non-phage	0
NOF014_scaffold47868_4	non-phage	0
NOF014_scaffold16877_5	non-phage	0
NOF014_scaffold71552_2	phage	0.999
NOF014_scaffold52572_1	non-phage	0
NOF014_scaffold64757_1	non-phage	0
NOF014_scaffold2390_2	phage	0.994

NOF014_scaffold70638_9	non-phage	0
NOF014_scaffold24380_13	non-phage	0
NOF014_scaffold72262_1	non-phage	0
NOF014_scaffold54590_2	non-phage	0
NOF014_scaffold13888_9	non-phage	0
NOF014_scaffold71919_2	phage	0.999
NOF014_scaffold85_5	non-phage	0
NOF014_scaffold216_1	non-phage	0
NOF014_scaffold43594_3	non-phage	0
NOF014_scaffold71889_1	non-phage	0
NOF014_scaffold3181_84	non-phage	0
NOF014_scaffold72213_1	non-phage	0
NOF014_scaffold18956_26	non-phage	0
NOF014_scaffold216_10	non-phage	0
NOF014_scaffold63397_2	non-phage	0
NOF014_scaffold25574_5	non-phage	0
NOF014_scaffold38249_1	non-phage	0
NOF014_scaffold20864_3	non-phage	0
NOF014_scaffold59922_3	non-phage	0
NOF014_scaffold52572_3	non-phage	0
NOF014_scaffold13888_6	non-phage	0
NOF014_scaffold10667_8	non-phage	0
NOF014_scaffold47868_2	non-phage	0
NOF014_scaffold234_1	non-phage	0
NOF014_scaffold65667_4	non-phage	0
NOF014_scaffold28765_7	non-phage	0
NOF014_scaffold71797_2	non-phage	0
NOF014_scaffold71076_1	non-phage	0
NOF014_scaffold60371_1	non-phage	0
NOF014_scaffold25107_11	non-phage	0
NOF014_scaffold60756_4	non-phage	0
NOF014_scaffold60229_2	non-phage	0
NOF014_scaffold70532_1	non-phage	0
NOF014_scaffold8_17	non-phage	0
NOF014_scaffold20265_7	non-phage	0
NOF014_scaffold41870_7	non-phage	0
NOF014_scaffold463_2	non-phage	0
NOF014_scaffold20686_13	non-phage	0
NOF014_scaffold36523_3	non-phage	0
NOF014_scaffold39953_12	non-phage	0
NOF014_scaffold41523_2	non-phage	0
NOF014_scaffold67718_6	non-phage	0
NOF014_scaffold22914_12	non-phage	0
NOF014_scaffold53712_1	non-phage	0
NOF014_scaffold46763_3	non-phage	0
NOF014_scaffold18358_6	non-phage	0

NOF014_scaffold52163_8	non-phage	0
NOF014_scaffold56870_1	non-phage	0
NOF014_scaffold107_1	non-phage	0
NOF014_scaffold71718_2	non-phage	0
NOF014_scaffold57647_2	non-phage	0
NOF014_scaffold161_7	non-phage	0
NOF014_scaffold65626_2	non-phage	0
NOF014_scaffold59039_1	non-phage	0
NOF014_scaffold67718_4	non-phage	0
NOF014_scaffold20576_25	non-phage	0
NOF014_scaffold4528_2	non-phage	0
NOF014_scaffold50103_7	non-phage	0
NOF014_scaffold77_1	non-phage	0
NOF014_scaffold67465_8	non-phage	0
NOF014_scaffold69288_2	non-phage	0
NOF014_C988758_1	non-phage	0
NOF014_scaffold41027_3	non-phage	0
NOF014_scaffold54433_2	non-phage	0
NOF014_scaffold66767_1	phage	0.999
NOF014_scaffold155_1	non-phage	0
NOF014_scaffold71942_4	non-phage	0
NOF014_scaffold71519_4	non-phage	0
NOF014_scaffold13844_3	non-phage	0
NOF014_scaffold383_2	non-phage	0
NOF014_scaffold120_8	non-phage	0
NOF014_scaffold71506_1	non-phage	0
NOF014_scaffold24607_8	non-phage	0
NOF014_scaffold27828_3	non-phage	0
NOF014_scaffold6847_3	non-phage	0
NOF014_scaffold70598_4	non-phage	0
NOF014_scaffold38062_8	non-phage	0
NOF014_scaffold55_2	non-phage	0
NOF014_scaffold71939_1	non-phage	0
NOF014_scaffold67189_1	non-phage	0
NOF014_scaffold63571_1	phage	0.996
NOF014_scaffold14884_1	non-phage	0
NOF014_scaffold69128_1	non-phage	0.001
NOF014_scaffold30528_15	non-phage	0
NOF014_scaffold9460_9	non-phage	0
NOF014_scaffold49073_8	non-phage	0
NOF014_scaffold21516_2	non-phage	0
NOF014_scaffold65085_3	phage	0.749
NOF014_scaffold36693_4	non-phage	0
NOF014_scaffold35679_6	non-phage	0
NOF014_scaffold4327_61	non-phage	0
NOF014_scaffold72212_1	non-phage	0

NOF014_scaffold37901_7	non-phage	0
NOF014_scaffold18639_3	non-phage	0
NOF014_scaffold8356_2	non-phage	0
NOF014_scaffold10667_12	non-phage	0
NOF014_scaffold52032_2	non-phage	0
NOF014_scaffold20669_2	non-phage	0
NOF014_scaffold39661_1	non-phage	0
NOF014_scaffold13658_8	non-phage	0
NOF014_scaffold11916_5	non-phage	0
NOF014_scaffold3967_1	non-phage	0
NOF014_scaffold26105_31	non-phage	0
NOF014_scaffold41523_5	non-phage	0
NOF014_scaffold22954_1	phage	0.999
NOF014_scaffold69436_2	non-phage	0
NOF014_scaffold81_7	non-phage	0
NOF014_scaffold59562_1	non-phage	0
NOF014_scaffold54816_2	non-phage	0
NOF014_scaffold27250_3	non-phage	0
NOF014_scaffold72143_2	non-phage	0
NOF014_scaffold18358_1	non-phage	0
NOF014_scaffold72295_2	phage	0.999
NOF014_scaffold13295_2	non-phage	0
NOF014_scaffold14195_5	non-phage	0
NOF014_scaffold44027_3	non-phage	0
NOF014_scaffold24182_1	non-phage	0
NOF014_scaffold69567_1	non-phage	0
NOF014_scaffold71714_2	phage	0.996
NOF014_scaffold71989_5	non-phage	0
NOF014_scaffold3899_3	non-phage	0
NOF014_scaffold60756_1	non-phage	0
NOF014_scaffold348_4	non-phage	0
NOF014_scaffold37156_2	non-phage	0
NOF014_scaffold20490_6	non-phage	0
NOF014_scaffold55387_14	non-phage	0
NOF014_scaffold19191_1	non-phage	0
NOF014_scaffold36330_3	non-phage	0
NOF014_scaffold66601_8	non-phage	0
NOF014_scaffold21910_4	non-phage	0
NOF014_scaffold42652_8	non-phage	0
NOF014_scaffold40347_1	non-phage	0
NOF014_scaffold2390_1	non-phage	0
NOF014_scaffold463_11	non-phage	0
NOF014_scaffold64087_5	phage	0.999
NOF014_scaffold29595_13	non-phage	0
NOF014_scaffold69463_9	non-phage	0
NOF014_scaffold62304_4	non-phage	0

NOF014_scaffold22051_2	non-phage	0
NOF014_scaffold46763_2	non-phage	0
NOF014_scaffold58806_2	non-phage	0
NOF014_scaffold70374_1	non-phage	0
NOF014_scaffold71856_1	phage	0.998
NOF014_scaffold309_14	non-phage	0
NOF014_scaffold30172_2	non-phage	0
NOF014_scaffold6908_5	non-phage	0
NOF014_scaffold38016_5	non-phage	0
NOF014_scaffold71989_3	non-phage	0
NOF014_scaffold67885_3	non-phage	0
NOF014_scaffold15172_5	non-phage	0
NOF014_scaffold11916_9	non-phage	0
NOF014_scaffold41283_9	non-phage	0
NOF014_scaffold4004_1	non-phage	0
NOF014_scaffold234_2	non-phage	0
NOF014_scaffold68447_3	non-phage	0
NOF014_scaffold71259_8	non-phage	0
NOF014_scaffold45416_2	non-phage	0
NOF014_scaffold8485_3	non-phage	0
NOF014_scaffold54136_9	non-phage	0
NOF014_scaffold25574_1	non-phage	0
NOF014_scaffold39735_14	non-phage	0
NOF014_scaffold55387_1	non-phage	0
NOF014_scaffold20864_1	non-phage	0
NOF014_scaffold25084_5	non-phage	0
NOF014_scaffold31460_3	non-phage	0
NOF014_C988768_1	non-phage	0
NOF014_scaffold53987_3	non-phage	0
NOF014_scaffold71932_1	non-phage	0
NOF014_scaffold71616_1	non-phage	0
NOF014_scaffold41283_6	non-phage	0
NOF014_scaffold4940_10	non-phage	0
NOF014_scaffold41204_1	non-phage	0
NOF014_scaffold3082_21	non-phage	0
NOF014_scaffold11542_4	non-phage	0
NOF014_scaffold19852_11	non-phage	0
NOF014_scaffold7923_4	non-phage	0
NOF014_scaffold18956_22	non-phage	0
NOF014_scaffold66416_1	non-phage	0
NOF014_scaffold52163_1	non-phage	0
NOF014_scaffold63519_1	non-phage	0
NOF014_scaffold22659_9	non-phage	0
NOF014_scaffold72299_1	non-phage	0
NOF014_scaffold11916_15	non-phage	0
NOF014_scaffold54371_1	non-phage	0

NOF014_scaffold4731_5	non-phage	0
NOF014_scaffold30419_3	non-phage	0
NOF014_scaffold11886_1	non-phage	0
NOF014_scaffold64335_1	non-phage	0.475
NOF014_C988438_1	non-phage	0
NOF014_scaffold20787_2	non-phage	0
NOF014_scaffold38062_5	non-phage	0
NOF014_scaffold70958_1	non-phage	0
NOF014_scaffold22318_2	non-phage	0
NOF014_scaffold54590_4	non-phage	0
NOF014_scaffold59979_3	phage	0.995
NOF014_scaffold55387_15	non-phage	0
NOF014_scaffold14969_3	non-phage	0
NOF014_scaffold15176_2	non-phage	0
NOF014_scaffold49025_2	non-phage	0
NOF014_scaffold64888_1	non-phage	0
NOF014_scaffold71970_1	non-phage	0
NOF014_scaffold229_4	non-phage	0
NOF014_scaffold29_3	non-phage	0
NOF014_scaffold40251_3	non-phage	0
NOF014_scaffold4327_54	non-phage	0
NOF014_scaffold38832_3	non-phage	0
NOF014_scaffold52663_2	phage	0.999
NOF014_scaffold66200_3	non-phage	0
NOF014_scaffold27769_10	non-phage	0
NOF014_scaffold58812_2	non-phage	0
NOF014_scaffold54610_2	non-phage	0
NOF014_scaffold9890_9	non-phage	0
NOF014_scaffold2590_1	non-phage	0
NOF014_scaffold463_9	non-phage	0
NOF014_scaffold66371_12	non-phage	0
NOF014_scaffold54136_28	phage	0.631
NOF014_scaffold62_27	non-phage	0
NOF014_scaffold4731_1	non-phage	0
NOF014_scaffold30528_7	non-phage	0
NOF001_scaffold17422_4	non-phage	0
NOF001_scaffold3065_3	non-phage	0
NOF001_scaffold2763_2	non-phage	0
NOF001_scaffold496_2	non-phage	0
NOF001_scaffold25077_2	non-phage	0
NOF001_scaffold35073_3	non-phage	0
NOF001_scaffold133_7	non-phage	0
NOF001_scaffold33934_1	non-phage	0
NOF001_scaffold305_4	non-phage	0
NOF001_scaffold58234_3	non-phage	0
NOF001_scaffold57986_2	non-phage	0

NOF001_scaffold38499_1	non-phage	0
NOF001_scaffold53334_3	non-phage	0
NOF001_scaffold51860_1	non-phage	0
NOF001_C716095_1	non-phage	0
NOF001_scaffold12565_1	non-phage	0
NOF001_scaffold57699_3	non-phage	0
NOF001_scaffold15478_4	non-phage	0
NOF001_scaffold9333_6	non-phage	0
NOF001_scaffold19940_17	non-phage	0
NOF001_scaffold36446_1	non-phage	0
NOF001_scaffold36846_1	non-phage	0
NOF001_scaffold33612_5	non-phage	0
NOF001_scaffold48844_4	non-phage	0
NOF001_scaffold8685_10	non-phage	0
NOF001_scaffold44656_2	non-phage	0
NOF001_scaffold3098_1	non-phage	0
NOF001_scaffold43855_3	non-phage	0
NOF001_scaffold9891_5	non-phage	0
NOF001_scaffold258_21	non-phage	0
NOF001_scaffold224_13	non-phage	0
NOF001_scaffold2715_2	non-phage	0
NOF001_scaffold35189_11	non-phage	0
NOF001_scaffold55965_2	non-phage	0
NOF001_scaffold58218_1	non-phage	0
NOF001_scaffold41814_7	non-phage	0
NOF001_scaffold133_6	non-phage	0
NOF001_scaffold24496_7	non-phage	0
NOF001_scaffold11379_3	non-phage	0
NOF001_scaffold16_12	non-phage	0
NOF001_scaffold58182_1	non-phage	0
NOF001_scaffold55897_4	non-phage	0
NOF001_scaffold39398_1	non-phage	0
NOF001_scaffold7357_9	non-phage	0
NOF001_scaffold7196_4	non-phage	0
NOF001_scaffold39121_6	non-phage	0
NOF001_scaffold5191_11	non-phage	0
NOF001_scaffold40234_1	non-phage	0
NOF001_scaffold41037_7	non-phage	0
NOF001_scaffold58080_1	non-phage	0
NOF001_scaffold58234_2	non-phage	0
NOF001_scaffold23267_4	non-phage	0
NOF001_scaffold58101_3	non-phage	0
NOF001_scaffold51674_4	non-phage	0
NOF001_scaffold11812_6	non-phage	0
NOF001_scaffold55489_2	non-phage	0
NOF001_scaffold29911_10	non-phage	0

NOF001_scaffold32719_2	non-phage	0
NOF001_scaffold15730_22	non-phage	0
NOF001_scaffold57746_2	non-phage	0
NOF001_scaffold58146_1	non-phage	0
NOF001_scaffold57863_1	non-phage	0
NOF001_scaffold42914_6	non-phage	0
NOF001_scaffold16309_3	non-phage	0
NOF001_scaffold824_20	non-phage	0
NOF001_scaffold57389_1	non-phage	0
NOF001_scaffold58321_1	non-phage	0
NOF001_scaffold53_5	non-phage	0
NOF001_scaffold58302_1	non-phage	0
NOF001_scaffold16559_2	non-phage	0
NOF001_scaffold35055_1	non-phage	0
NOF001_scaffold58014_1	non-phage	0
NOF001_scaffold8858_21	non-phage	0
NOF001_scaffold40241_11	non-phage	0
NOF001_scaffold57247_2	non-phage	0
NOF001_scaffold42082_3	non-phage	0
NOF001_scaffold396_9	non-phage	0
NOF001_scaffold31012_11	non-phage	0
NOF001_scaffold17_9	non-phage	0
NOF001_scaffold58171_1	non-phage	0
NOF001_scaffold4605_2	non-phage	0
NOF001_scaffold57480_3	non-phage	0
NOF001_scaffold55708_1	non-phage	0
NOF001_scaffold39915_3	non-phage	0
NOF001_scaffold48925_1	non-phage	0
NOF001_scaffold15478_8	non-phage	0
NOF001_scaffold52931_1	non-phage	0
NOF001_scaffold14822_3	non-phage	0
NOF001_scaffold26578_3	non-phage	0
NOF001_scaffold57967_2	non-phage	0
NOF001_scaffold15426_6	non-phage	0
NOF001_scaffold824_14	non-phage	0
NOF001_scaffold43384_1	non-phage	0
NOF001_scaffold57548_2	non-phage	0
NOF001_scaffold862_1	non-phage	0
NOF001_scaffold15602_1	non-phage	0
NOF001_scaffold577_3	non-phage	0
NOF001_scaffold44574_3	non-phage	0
NOF001_scaffold24425_3	non-phage	0
NOF001_scaffold32424_4	non-phage	0
NOF001_scaffold386_3	non-phage	0
NOF001_scaffold50429_6	non-phage	0
NOF001_scaffold15547_1	non-phage	0

NOF001_scaffold35427_4	non-phage	0
NOF001_scaffold16291_4	non-phage	0
NOF001_scaffold99_6	non-phage	0
NOF001_scaffold28613_1	non-phage	0
NOF001_scaffold17927_17	non-phage	0
NOF001_scaffold45670_2	non-phage	0
NOF001_scaffold51674_5	non-phage	0
NOF001_scaffold17210_1	non-phage	0
NOF001_scaffold2149_1	non-phage	0
NOF001_scaffold154_4	non-phage	0
NOF001_scaffold33934_11	non-phage	0
NOF001_scaffold44432_1	non-phage	0
NOF001_scaffold5984_1	non-phage	0
NOF001_scaffold5122_2	non-phage	0
NOF001_scaffold9229_6	non-phage	0
NOF001_scaffold26416_2	non-phage	0
NOF001_scaffold11576_41	non-phage	0
NOF001_scaffold30016_1	non-phage	0
NOF001_C716195_1	non-phage	0
NOF001_scaffold55965_1	non-phage	0
NOF001_scaffold44215_2	non-phage	0
NOF001_scaffold7357_10	non-phage	0
NOF002_scaffold10358_4	non-phage	0
NOF002_scaffold17922_24	non-phage	0
NOF002_scaffold31587_1	non-phage	0
NOF002_scaffold23012_2	non-phage	0
NOF002_scaffold31607_1	non-phage	0
NOF002_scaffold31460_2	non-phage	0
NOF002_scaffold28625_7	non-phage	0
NOF002_scaffold27803_3	non-phage	0
NOF002_scaffold31574_1	non-phage	0
NOF002_scaffold31570_2	non-phage	0
NOF002_scaffold1836_1	non-phage	0
NOF002_scaffold7382_1	non-phage	0
NOF002_scaffold31545_2	non-phage	0
NOF002_scaffold28625_1	non-phage	0
NOF002_scaffold16147_2	non-phage	0
NOF002_scaffold28343_1	non-phage	0
NOF002_C425901_1	non-phage	0
NOF002_scaffold31452_3	non-phage	0
NOF002_scaffold1584_3	non-phage	0
NOF002_scaffold3135_2	non-phage	0
NOF002_scaffold9194_8	non-phage	0
NOF002_scaffold19162_3	non-phage	0
NOF002_scaffold31361_1	non-phage	0
NOF002_scaffold24315_1	non-phage	0

NOF002_scaffold9194_12	non-phage	0
NOF002_scaffold31452_1	non-phage	0
NOF002_scaffold3135_1	non-phage	0
NOF002_scaffold31521_1	non-phage	0
NOF002_scaffold24503_3	non-phage	0
NOF002_scaffold29347_1	non-phage	0
NOF002_scaffold2_1	non-phage	0
NOF002_scaffold7177_1	non-phage	0
NOF002_scaffold31521_2	non-phage	0
NOF002_scaffold31572_4	non-phage	0
NOF002_scaffold29373_1	non-phage	0
NOF002_scaffold1562_6	non-phage	0
NOF002_scaffold31188_4	non-phage	0
NOF002_scaffold26091_5	non-phage	0
NOF002_scaffold919_5	non-phage	0
NOF002_scaffold19847_4	non-phage	0
NOF002_scaffold27558_2	non-phage	0
NOF002_C425271_1	non-phage	0
NOF002_scaffold31570_3	non-phage	0
NOF002_scaffold2313_3	non-phage	0
NOF002_scaffold102_4	non-phage	0
NOF002_scaffold793_20	non-phage	0
NOF002_scaffold16376_4	non-phage	0
NOF002_scaffold6803_2	non-phage	0
NOF002_scaffold31570_4	non-phage	0
NOF002_scaffold29684_3	non-phage	0
NOF002_scaffold20558_1	non-phage	0
NOF002_scaffold13708_1	non-phage	0
NOF002_scaffold18921_8	non-phage	0
NOF002_scaffold3176_12	non-phage	0
NOF002_scaffold30696_3	non-phage	0
NOF002_scaffold26455_3	non-phage	0
NOF002_scaffold6352_10	non-phage	0
NOF002_scaffold3233_32	non-phage	0
NOF002_scaffold29373_2	non-phage	0
NOF002_scaffold30662_2	non-phage	0
NOF002_scaffold212_4	non-phage	0
NOF002_scaffold31539_2	non-phage	0
NOF002_scaffold1836_5	non-phage	0
NOF002_scaffold21687_1	non-phage	0
NOF002_scaffold27207_2	non-phage	0
NOF002_scaffold28625_3	non-phage	0
NOF002_scaffold31614_1	non-phage	0
NOF002_scaffold533_12	non-phage	0
NOF002_scaffold583_4	non-phage	0
NOF002_scaffold29373_6	non-phage	0

NOF002_scaffold31539_1	non-phage	0
NOF002_scaffold31594_1	non-phage	0
NOF002_scaffold11439_1	non-phage	0
NOF002_scaffold24433_4	non-phage	0
NOF002_scaffold17053_6	non-phage	0
NOF002_scaffold6578_2	non-phage	0
NOF002_scaffold28270_2	non-phage	0
NOF002_scaffold1562_4	non-phage	0
NOF002_scaffold19412_6	non-phage	0
NOF002_scaffold30289_1	non-phage	0
NOF002_scaffold6415_33	non-phage	0
NOF002_scaffold31581_1	non-phage	0
NOF002_scaffold24315_2	non-phage	0
NOF002_scaffold31415_1	non-phage	0
NOF002_scaffold2346_19	non-phage	0
NOF002_scaffold582_2	non-phage	0
NOF002_scaffold28625_5	non-phage	0
NOF002_scaffold8611_3	non-phage	0
NOF002_scaffold31536_3	non-phage	0
NOF002_scaffold18540_2	non-phage	0
NOF002_scaffold28625_2	non-phage	0
NOF002_scaffold14962_3	non-phage	0
NOF002_scaffold31597_4	non-phage	0
NOF002_scaffold10358_5	non-phage	0
NOF002_scaffold7276_5	non-phage	0
NOF002_scaffold29980_2	non-phage	0
NOF002_scaffold13114_5	non-phage	0
NOF002_scaffold9194_7	non-phage	0
NOF004_scaffold3518_2	non-phage	0
NOF004_scaffold39134_1	non-phage	0
NOF004_scaffold2452_3	non-phage	0
NOF004_scaffold4506_1	non-phage	0
NOF004_scaffold27230_9	non-phage	0
NOF004_scaffold40235_1	non-phage	0
NOF004_scaffold21213_3	non-phage	0
NOF004_scaffold1482_5	non-phage	0
NOF004_scaffold1680_13	non-phage	0
NOF004_scaffold33440_2	non-phage	0
NOF004_scaffold40283_1	non-phage	0
NOF004_scaffold35309_1	non-phage	0
NOF004_scaffold40275_1	non-phage	0
NOF004_scaffold7230_1	non-phage	0
NOF004_scaffold18950_4	non-phage	0
NOF004_scaffold9378_12	non-phage	0
NOF004_scaffold32_5	non-phage	0
NOF004_scaffold29739_7	non-phage	0

NOF004_scaffold9977_9	non-phage	0
NOF004_scaffold2726_7	non-phage	0
NOF004_scaffold39712_1	non-phage	0
NOF004_scaffold39155_4	non-phage	0
NOF004_scaffold17538_5	non-phage	0
NOF004_scaffold39483_7	non-phage	0
NOF004_scaffold5730_4	non-phage	0
NOF004_scaffold7592_4	non-phage	0
NOF004_scaffold3628_2	non-phage	0
NOF004_scaffold12265_3	non-phage	0
NOF004_scaffold35809_4	non-phage	0
NOF004_scaffold40224_1	non-phage	0
NOF004_scaffold9166_5	non-phage	0
NOF004_scaffold8775_11	non-phage	0
NOF004_scaffold2273_4	non-phage	0
NOF004_scaffold104_5	non-phage	0
NOF004_scaffold21264_5	non-phage	0
NOF004_scaffold2525_1	non-phage	0
NOF004_scaffold15525_1	non-phage	0
NOF004_scaffold7693_10	non-phage	0
NOF004_scaffold22865_1	non-phage	0
NOF004_scaffold18883_3	non-phage	0
NOF004_scaffold123_4	non-phage	0
NOF004_scaffold40090_1	non-phage	0
NOF004_scaffold104_6	non-phage	0
NOF004_scaffold38745_2	non-phage	0
NOF004_scaffold9977_19	non-phage	0
NOF004_scaffold316_2	non-phage	0
NOF004_scaffold39083_3	non-phage	0
NOF004_scaffold7101_1	non-phage	0
NOF004_scaffold1589_6	non-phage	0
NOF004_scaffold30537_5	non-phage	0
NOF004_scaffold4452_1	non-phage	0
NOF004_scaffold7560_7	non-phage	0
NOF004_scaffold39867_12	non-phage	0
NOF004_scaffold58_2	non-phage	0
NOF004_scaffold7698_1	non-phage	0
NOF004_scaffold38576_2	non-phage	0
NOF004_scaffold3794_6	non-phage	0
NOF004_scaffold9166_9	non-phage	0
NOF004_scaffold128_18	non-phage	0
NOF004_scaffold16385_1	non-phage	0
NOF004_scaffold21861_6	non-phage	0
NOF004_scaffold40141_1	non-phage	0
NOF004_scaffold39994_5	non-phage	0
NOF004_scaffold45_9	non-phage	0

NOF004_scaffold7909_9	non-phage	0
NOF004_scaffold13013_1	non-phage	0
NOF004_scaffold14114_5	non-phage	0
NOF004_scaffold21835_2	non-phage	0
NOF004_scaffold40096_2	non-phage	0
NOF004_scaffold5063_1	non-phage	0
NOF004_scaffold10102_13	non-phage	0
NOF004_scaffold25636_2	non-phage	0
NOF004_scaffold10943_1	non-phage	0
NOF004_scaffold15104_1	non-phage	0
NOF004_scaffold36210_1	non-phage	0
NOF004_scaffold17937_3	non-phage	0
NOF004_scaffold9548_1	non-phage	0
NOF004_scaffold22979_2	non-phage	0
NOF004_scaffold2112_8	non-phage	0
NOF004_scaffold27230_8	non-phage	0
NOF004_scaffold9903_12	non-phage	0
NOF004_scaffold38036_5	non-phage	0
NOF004_scaffold4623_4	non-phage	0
NOF004_scaffold40281_1	non-phage	0
NOF004_scaffold18950_5	non-phage	0
NOF004_scaffold29785_1	non-phage	0
NOF004_scaffold20556_1	non-phage	0
NOF004_scaffold39699_1	non-phage	0
NOF004_scaffold26247_1	non-phage	0
NOF004_scaffold270_6	non-phage	0
NOF004_scaffold30799_1	non-phage	0
NOF004_scaffold39262_1	non-phage	0
NOF004_scaffold6915_2	non-phage	0
NOF004_scaffold24345_1	non-phage	0
NOF004_scaffold21264_2	non-phage	0
NOF004_scaffold40266_1	non-phage	0
NOF004_scaffold20386_3	non-phage	0
NOF004_scaffold32088_2	non-phage	0
NOF004_scaffold5983_3	non-phage	0
NOF004_scaffold40258_1	non-phage	0
NOF004_scaffold2640_2	non-phage	0
NOF004_scaffold22453_2	non-phage	0
NOF004_scaffold30357_1	non-phage	0
NOF004_scaffold30143_2	non-phage	0
NOF004_scaffold36625_3	non-phage	0
NOF004_scaffold34568_1	non-phage	0
NOF004_scaffold30721_2	non-phage	0
NOF004_C576016_1	non-phage	0
NOF004_scaffold39903_1	non-phage	0
NOF004_scaffold9994_7	non-phage	0

NOF004_scaffold40071_2	non-phage	0
NOF004_scaffold3771_1	non-phage	0
NOF004_C575550_1	non-phage	0
NOF004_scaffold9441_8	non-phage	0
NOF004_scaffold9166_3	non-phage	0
NOF004_scaffold29806_2	non-phage	0
NOF004_scaffold39570_2	non-phage	0
NOF004_scaffold18640_4	non-phage	0
NOF004_scaffold10474_4	non-phage	0
NOF004_scaffold291_2	non-phage	0
NOF004_scaffold12395_7	non-phage	0
NOF004_scaffold16509_4	non-phage	0
NOF004_scaffold29_6	non-phage	0
NOF004_scaffold10128_3	non-phage	0
NOF004_scaffold32917_4	non-phage	0
NOF004_scaffold7560_9	non-phage	0
NOF004_scaffold11611_1	non-phage	0
NOF004_scaffold9307_2	non-phage	0
NOF004_scaffold16982_2	non-phage	0
NOF004_scaffold2439_2	non-phage	0
NOF004_scaffold40113_2	non-phage	0
NOF004_scaffold39696_5	non-phage	0
NOF004_scaffold40173_1	non-phage	0
NOF004_scaffold40218_1	non-phage	0
NOF004_scaffold2112_3	non-phage	0
NOF004_scaffold22619_5	non-phage	0
NOF004_scaffold78_2	non-phage	0
NOF004_scaffold17538_11	non-phage	0
NOF004_scaffold9441_9	non-phage	0
NOF004_scaffold88_38	non-phage	0
NOF004_scaffold2523_2	non-phage	0
NOF004_scaffold233_4	non-phage	0
NOF004_scaffold24690_1_1	non-phage	0
NOF004_scaffold30655_2	non-phage	0
NOF004_scaffold12813_32	non-phage	0
NOF004_scaffold6208_2	non-phage	0
NOF004_scaffold7314_2	non-phage	0
NOF004_scaffold9166_7	non-phage	0
NOF004_scaffold35321_1	non-phage	0
NOF004_scaffold39839_1	non-phage	0
NOF004_scaffold17538_7	non-phage	0
NOF004_scaffold18628_2	non-phage	0
NOF004_scaffold10041_2	non-phage	0
NOF004_scaffold39903_2	non-phage	0
NOF004_scaffold34975_3	non-phage	0
NOF004_scaffold9994_8	non-phage	0

NOF004_scaffold27984_2	non-phage	0
NOF004_scaffold39058_1	non-phage	0
NOF004_scaffold18841_9	non-phage	0
NOF004_scaffold28010_2	non-phage	0
NOF004_scaffold39872_3	non-phage	0
NOF004_scaffold26_3	non-phage	0
NOF004_scaffold38_1	non-phage	0
NOF004_scaffold9136_3	non-phage	0
NOF004_scaffold29_9	non-phage	0
NOF004_C576100_1	non-phage	0
NOF004_scaffold40173_5	non-phage	0
NOF004_scaffold31834_2	non-phage	0
NOF004_scaffold26247_2	non-phage	0
NOF004_scaffold11611_2	non-phage	0
NOF004_scaffold9964_1	non-phage	0
NOF004_scaffold12458_3	non-phage	0
NOF004_scaffold3338_5	non-phage	0
NOF004_scaffold27230_1	non-phage	0
NOF004_scaffold39570_3	non-phage	0
NOF004_scaffold39606_1	non-phage	0
NOF004_scaffold29533_1	non-phage	0
NOF004_scaffold38745_1	non-phage	0
NOF004_scaffold38886_1	non-phage	0
NOF004_scaffold21689_2	non-phage	0
NOF004_scaffold39_31	non-phage	0
NOF004_scaffold37334_2	non-phage	0
NOF004_scaffold34944_2	non-phage	0
NOF004_scaffold1680_8	non-phage	0
NOF004_scaffold9148_2	non-phage	0
NOF004_scaffold22547_3	non-phage	0
NOF004_scaffold8357_1	non-phage	0
NOF004_scaffold9553_1	non-phage	0
NOF004_scaffold23288_6	non-phage	0
NOF004_scaffold20211_1	non-phage	0
NOF004_scaffold128_14	non-phage	0
NOF004_scaffold39514_1	non-phage	0
NOF004_scaffold10041_3	non-phage	0
NOF004_scaffold31562_1	non-phage	0
NOF004_scaffold28491_5	non-phage	0
NOF004_scaffold30651_2	non-phage	0
NOF004_scaffold10102_19	non-phage	0
NOF004_scaffold2940_4	non-phage	0
NOF004_scaffold40128_2	non-phage	0
NOF004_scaffold17538_4	non-phage	0
NOF004_scaffold58_5	non-phage	0
NOF004_scaffold15525_2	non-phage	0

NOF004_scaffold3290_7	non-phage	0
NOF004_scaffold40103_1	non-phage	0
NOF004_scaffold2367_2	non-phage	0
NOF004_scaffold128_4	non-phage	0
NOF004_scaffold2112_2	non-phage	0
NOF004_scaffold12946_3	non-phage	0
NOF004_scaffold28307_2	non-phage	0
NOF004_scaffold37971_2	non-phage	0
NOF004_scaffold34543_1	non-phage	0
NOF004_scaffold40183_1	non-phage	0
NOF004_scaffold23288_2	non-phage	0
NOF004_scaffold20176_1	non-phage	0
NOF004_scaffold12458_4	non-phage	0
NOF004_scaffold104_7	non-phage	0
NOF004_scaffold40148_2	non-phage	0
NOF004_scaffold34036_4	non-phage	0
NOF004_scaffold30352_2	non-phage	0
NOF004_scaffold94_13	non-phage	0
NOF004_scaffold4623_1	non-phage	0
NOF004_scaffold20386_1	non-phage	0
NOF004_scaffold39872_2	non-phage	0
NOF005_scaffold52465_1	non-phage	0
NOF005_scaffold46962_3	non-phage	0
NOF005_C712998_1	non-phage	0
NOF005_scaffold45351_4	non-phage	0
NOF005_scaffold5072_6	non-phage	0
NOF005_scaffold2021_2	non-phage	0
NOF005_scaffold9290_5	non-phage	0
NOF005_C713254_1	non-phage	0
NOF005_scaffold13029_3	non-phage	0
NOF005_scaffold21663_5	non-phage	0
NOF005_scaffold942_2	non-phage	0
NOF005_scaffold7901_4	non-phage	0
NOF005_scaffold26125_5	non-phage	0
NOF005_scaffold24616_17	non-phage	0
NOF005_scaffold47329_2	non-phage	0
NOF005_scaffold16555_12	non-phage	0
NOF005_scaffold43510_1	non-phage	0
NOF005_scaffold7148_1	non-phage	0
NOF005_scaffold18762_1	non-phage	0
NOF005_scaffold34434_4	non-phage	0
NOF005_scaffold6453_1	non-phage	0
NOF005_scaffold16555_7	non-phage	0
NOF005_scaffold48705_4	non-phage	0
NOF005_scaffold35038_14	non-phage	0
NOF005_scaffold26370_2	non-phage	0

NOF005_scaffold27003_3	non-phage	0
NOF005_scaffold44550_3	non-phage	0
NOF005_scaffold7719_1	non-phage	0
NOF005_scaffold13462_3	non-phage	0
NOF005_scaffold53_4	non-phage	0
NOF005_scaffold35947_4	non-phage	0
NOF005_scaffold13218_4	non-phage	0
NOF005_scaffold48207_1	non-phage	0
NOF005_scaffold47316_1	non-phage	0
NOF005_scaffold15589_1	non-phage	0
NOF005_scaffold51727_3	non-phage	0
NOF005_scaffold9602_1	non-phage	0
NOF005_scaffold18762_4	non-phage	0
NOF005_scaffold31634_1	non-phage	0
NOF005_scaffold49855_1	non-phage	0
NOF005_scaffold6841_2	non-phage	0
NOF005_scaffold18875_1	non-phage	0
NOF005_scaffold9602_4	non-phage	0
NOF005_scaffold24101_2	non-phage	0
NOF005_scaffold32028_1	non-phage	0
NOF005_scaffold40020_4	non-phage	0
NOF005_scaffold24616_4	non-phage	0
NOF005_scaffold52398_1	non-phage	0
NOF005_scaffold7204_10	non-phage	0
NOF005_scaffold458_1	non-phage	0
NOF005_scaffold22323_2	non-phage	0
NOF005_scaffold9602_9	non-phage	0
NOF005_scaffold2609_1	non-phage	0
NOF005_scaffold51169_6	non-phage	0
NOF005_scaffold5564_4	non-phage	0
NOF005_scaffold29757_5	non-phage	0
NOF005_scaffold13942_12	non-phage	0
NOF005_scaffold24241_5	non-phage	0
NOF005_scaffold28694_13	non-phage	0
NOF005_scaffold18762_3	non-phage	0
NOF005_scaffold45624_1	non-phage	0
NOF005_scaffold4354_4	non-phage	0
NOF005_scaffold24211_3	non-phage	0
NOF005_scaffold11660_6	non-phage	0
NOF005_scaffold49382_1	non-phage	0
NOF005_scaffold26202_1	non-phage	0
NOF005_scaffold51645_1	non-phage	0
NOF005_scaffold13816_11	non-phage	0
NOF005_scaffold42872_1	non-phage	0
NOF005_scaffold2741_2	non-phage	0
NOF005_scaffold39708_1	non-phage	0

NOF005_scaffold17404_5	non-phage	0
NOF005_scaffold3654_1	non-phage	0
NOF005_scaffold24211_2	non-phage	0
NOF005_scaffold16555_6	non-phage	0
NOF005_scaffold3061_22	non-phage	0
NOF005_scaffold13806_5	non-phage	0
NOF005_scaffold25218_2	non-phage	0
NOF005_scaffold45427_2	non-phage	0
NOF005_scaffold23189_4	non-phage	0
NOF005_scaffold2001_1	non-phage	0
NOF005_scaffold37714_10	non-phage	0
NOF005_scaffold37849_1	non-phage	0
NOF005_scaffold32635_6	non-phage	0
NOF005_scaffold52112_1	non-phage	0
NOF005_scaffold23949_1	non-phage	0
NOF005_scaffold41315_5	non-phage	0
NOF005_scaffold51411_2	non-phage	0
NOF005_scaffold15523_3	non-phage	0
NOF005_scaffold28906_2	non-phage	0
NOF005_scaffold39273_5	non-phage	0
NOF005_scaffold13104_12	non-phage	0
NOF005_scaffold52425_1	non-phage	0
NOF005_scaffold308_1	non-phage	0
NOF005_scaffold6206_15	non-phage	0
NOF005_scaffold41436_1	non-phage	0
NOF005_scaffold49908_4	non-phage	0
NOF005_scaffold51_2	non-phage	0
NOF005_scaffold30_4	non-phage	0
NOF005_C713112_1	non-phage	0
NOF005_scaffold21300_14	non-phage	0
NOF005_scaffold42097_2	non-phage	0
NOF005_scaffold32957_5	non-phage	0
NOF005_scaffold52229_3	non-phage	0
NOF005_scaffold19160_4	non-phage	0
NOF005_scaffold15330_1	non-phage	0
NOF005_scaffold16170_1	non-phage	0
NOF005_scaffold15535_5	non-phage	0
NOF005_scaffold4462_2	non-phage	0
NOF005_scaffold32366_1	non-phage	0
NOF005_scaffold22066_2	non-phage	0
NOF005_scaffold26068_1	non-phage	0
NOF005_scaffold14265_1	non-phage	0
NOF005_scaffold16053_2	non-phage	0
NOF005_scaffold37714_11	non-phage	0
NOF005_scaffold51228_4	non-phage	0
NOF005_scaffold52086_1	non-phage	0

NOF005_scaffold46683_6	non-phage	0
NOF005_scaffold14_4	non-phage	0
NOF005_scaffold36108_1	non-phage	0
NOF005_scaffold220_11	non-phage	0
NOF005_scaffold52176_6	non-phage	0
NOF005_scaffold29908_1	non-phage	0
NOF005_scaffold49013_1	non-phage	0
NOF005_scaffold44909_2	non-phage	0
NOF005_scaffold51004_4	non-phage	0
NOF005_scaffold51313_1	non-phage	0
NOF005_scaffold17684_9	non-phage	0
NOF005_scaffold28694_4	non-phage	0
NOF005_scaffold3971_3	non-phage	0
NOF005_scaffold11420_12	non-phage	0
NOF005_scaffold2782_1	non-phage	0
NOF005_scaffold34797_3	non-phage	0
NOF005_C713178_1	non-phage	0
NOF005_scaffold50332_2	non-phage	0
NOF005_scaffold2_2	non-phage	0
NOF005_scaffold27453_5	non-phage	0
NOF005_scaffold27061_2	non-phage	0
NOF005_scaffold51411_3	non-phage	0
NOF005_scaffold7488_3	non-phage	0
NOF005_scaffold48969_1	non-phage	0
NOF005_scaffold51076_1	non-phage	0
NOF005_scaffold44909_1	non-phage	0
NOF005_scaffold51112_4	non-phage	0
NOF005_scaffold35536_1	non-phage	0
NOF005_scaffold30658_1	non-phage	0
NOF005_scaffold30881_7	non-phage	0
NOF005_scaffold2687_1	non-phage	0
NOF005_scaffold51129_4	non-phage	0
NOF005_scaffold24573_9	non-phage	0
NOF005_scaffold52469_1	non-phage	0
NOF005_scaffold6453_2	non-phage	0
NOF005_scaffold14025_1	non-phage	0
NOF005_scaffold16555_1	non-phage	0
NOF005_scaffold22290_2	non-phage	0
NOF005_scaffold52423_2	non-phage	0
NOF005_scaffold29951_1	non-phage	0
NOF005_scaffold52428_1	non-phage	0
NOF005_scaffold2388_29	non-phage	0
NOF005_scaffold41365_1	non-phage	0
NOF005_scaffold29908_2	non-phage	0
NOF005_scaffold34860_1	non-phage	0
NOF005_scaffold41905_2	non-phage	0

NOF005_scaffold38780_5	non-phage	0
NOF005_scaffold16108_3	non-phage	0
NOF005_C713310_1	non-phage	0
NOF005_scaffold17830_9	non-phage	0
NOF005_scaffold4532_5	non-phage	0
NOF005_scaffold35947_3	non-phage	0
NOF005_scaffold78_1	non-phage	0
NOF005_scaffold32199_9	non-phage	0
NOF005_scaffold15630_7	non-phage	0
NOF005_scaffold52324_1	non-phage	0
NOF005_scaffold36290_1	non-phage	0
NOF005_scaffold35536_2	non-phage	0
NOF005_scaffold12205_2	non-phage	0
NOF005_scaffold52401_1	non-phage	0
NOF005_scaffold29675_1	non-phage	0
NOF005_scaffold30_5	non-phage	0
NOF005_scaffold25218_6	non-phage	0
NOF005_scaffold682_1	non-phage	0
NOF005_scaffold1586_4	non-phage	0
NOF005_scaffold24384_1	non-phage	0
NOF005_scaffold24616_15	non-phage	0
NOF005_scaffold43842_1	non-phage	0
NOF005_scaffold34306_2	non-phage	0
NOF005_scaffold52442_1	non-phage	0
NOF005_scaffold15489_2	non-phage	0
NOF005_scaffold43163_4	non-phage	0
NOF005_scaffold26775_1	non-phage	0
NOF005_scaffold27453_3	non-phage	0
NOF005_scaffold68_11	non-phage	0
NOF005_scaffold51228_6	non-phage	0
NOF005_scaffold32482_5	non-phage	0
NOF005_scaffold7501_3	non-phage	0
NOF005_scaffold50322_1	non-phage	0
NOF005_scaffold29670_2	non-phage	0
NOF005_scaffold8708_4	non-phage	0
NOF005_scaffold27189_6	non-phage	0
NOF005_scaffold28906_1	non-phage	0
NOF005_scaffold11420_9	non-phage	0
NOF005_scaffold42_3	non-phage	0
NOF005_scaffold7052_4	non-phage	0
NOF005_scaffold35914_1	non-phage	0
NOF005_scaffold35347_1	non-phage	0
NOF005_scaffold16251_20	non-phage	0
NOF005_scaffold27532_1	non-phage	0
NOF005_C713488_1	non-phage	0
NOF005_scaffold163_12	non-phage	0

NOF005_scaffold2944_5	non-phage	0
NOF005_scaffold5470_3	non-phage	0
NOF005_scaffold16003_3	non-phage	0
NOF005_scaffold169_4	non-phage	0
NOF005_scaffold19920_5	non-phage	0
NOF005_scaffold18919_4	non-phage	0
NOF005_scaffold52400_1	non-phage	0
NOF005_scaffold15_2	non-phage	0
NOF005_scaffold52443_1	non-phage	0
NOF005_scaffold25659_3	non-phage	0
NOF005_scaffold23089_1	non-phage	0
NOF005_scaffold10752_4	non-phage	0
NOF005_scaffold9349_13	non-phage	0
NOF005_scaffold30900_1	non-phage	0
NOF005_scaffold52368_2	non-phage	0
NOF005_C713116_1	non-phage	0
NOF005_scaffold25494_3	non-phage	0
NOF005_scaffold478_12	non-phage	0
NOF005_scaffold13281_5	non-phage	0
NOF005_scaffold110_1	non-phage	0
NOF005_scaffold51129_1	non-phage	0
NOF005_scaffold24660_1	non-phage	0
NOF005_scaffold6206_13	non-phage	0
NOF005_scaffold50332_1	non-phage	0
NOF005_scaffold8925_15	non-phage	0
NOF005_scaffold7770_3	non-phage	0
NOF005_scaffold43551_3	non-phage	0
NOF005_scaffold32_3	non-phage	0
NOF006_scaffold9072_1	non-phage	0
NOF006_scaffold15501_2	non-phage	0
NOF006_scaffold14086_5	non-phage	0
NOF006_scaffold163_1	non-phage	0
NOF006_scaffold1208_38	non-phage	0
NOF006_scaffold21158_2	non-phage	0
NOF006_scaffold9041_3	non-phage	0
NOF006_scaffold365_13	non-phage	0
NOF006_scaffold12718_1	non-phage	0
NOF006_scaffold31_8	non-phage	0
NOF006_scaffold139_1	non-phage	0
NOF006_scaffold3853_3	non-phage	0
NOF006_scaffold20457_7	non-phage	0
NOF006_scaffold20881_1	non-phage	0
NOF006_scaffold17694_2	non-phage	0
NOF006_scaffold18237_3	non-phage	0
NOF006_scaffold18642_1	non-phage	0
NOF006_scaffold1372_1	non-phage	0

NOF006_scaffold14344_4	non-phage	0
NOF006_scaffold21029_2	non-phage	0
NOF006_scaffold4892_1	non-phage	0
NOF006_scaffold6603_2	non-phage	0
NOF006_scaffold15841_1	non-phage	0
NOF006_scaffold235_14	non-phage	0
NOF006_scaffold853_14	non-phage	0
NOF006_scaffold5544_1	non-phage	0
NOF006_scaffold13894_1	non-phage	0
NOF006_scaffold9997_7	non-phage	0
NOF006_scaffold189_3	non-phage	0
NOF006_scaffold19603_2	non-phage	0
NOF006_scaffold478_4	non-phage	0
NOF006_scaffold14544_2	non-phage	0
NOF006_scaffold2934_2_2	non-phage	0
NOF006_scaffold1820_2	non-phage	0
NOF006_scaffold6603_7	non-phage	0
NOF006_scaffold2050_2	non-phage	0
NOF006_scaffold175_4	non-phage	0
NOF006_scaffold21028_3	non-phage	0
NOF006_scaffold75_1	non-phage	0
NOF006_scaffold1135_17	non-phage	0
NOF006_scaffold232_1	non-phage	0
NOF006_C400742_1	non-phage	0
NOF006_scaffold5396_6	non-phage	0
NOF006_scaffold1388_22	non-phage	0
NOF006_scaffold8110_15	non-phage	0
NOF006_scaffold5914_2	non-phage	0
NOF006_C401014_1	non-phage	0
NOF006_scaffold8684_2	non-phage	0
NOF006_scaffold9782_1	non-phage	0
NOF006_scaffold1108_7	non-phage	0
NOF006_scaffold11570_1	non-phage	0
NOF006_scaffold13388_1	non-phage	0
NOF006_scaffold6724_19	non-phage	0
NOF006_scaffold365_38	non-phage	0
NOF006_scaffold6724_14	non-phage	0
NOF006_scaffold100_2	non-phage	0
NOF006_scaffold21281_1	non-phage	0
NOF006_scaffold6603_6	non-phage	0
NOF006_scaffold20694_1	non-phage	0
NOF006_scaffold232_2	non-phage	0
NOF006_scaffold2050_24	non-phage	0
NOF006_scaffold9915_8	non-phage	0
NOF006_scaffold18451_2	non-phage	0
NOF006_scaffold8614_3	non-phage	0

NOF006_scaffold11150_2	non-phage	0
NOF006_scaffold3969_1	non-phage	0
NOF006_scaffold7350_2	non-phage	0
NOF006_scaffold15106_1	non-phage	0
NOF006_scaffold5396_15	non-phage	0
NOF006_scaffold1841_1	non-phage	0
NOF006_scaffold21301_1	non-phage	0
NOF006_scaffold9076_16	non-phage	0
NOF006_C400856_1	non-phage	0
NOF006_scaffold11558_1	non-phage	0
NOF006_scaffold5837_2	non-phage	0
NOF006_scaffold13130_3	non-phage	0
NOF006_scaffold93_1	non-phage	0
NOF006_scaffold1905_22	non-phage	0
NOF006_scaffold17088_1	non-phage	0
NOF006_scaffold1639_7	non-phage	0
NOF006_scaffold21330_1	non-phage	0
NOF006_scaffold1200_14	non-phage	0
NOF006_scaffold19616_1	non-phage	0
NOF006_scaffold21016_2	non-phage	0
NOF006_scaffold18673_1	non-phage	0
NOF006_scaffold18722_1	non-phage	0
NOF006_scaffold1270_4	non-phage	0
NOF006_scaffold14405_1	non-phage	0
NOF006_scaffold8110_8	non-phage	0
NOF006_scaffold13768_2	non-phage	0
NOF006_scaffold914_2	non-phage	0
NOF006_scaffold722_14	non-phage	0
NOF006_scaffold14518_3	non-phage	0
NOF006_scaffold1329_1	non-phage	0
NOF006_scaffold4067_6	non-phage	0
NOF006_scaffold232_11	non-phage	0
NOF006_scaffold2272_11	non-phage	0
NOF006_scaffold16970_3	non-phage	0
NOF006_scaffold475_16	non-phage	0
NOF006_scaffold1200_7	non-phage	0
NOF006_scaffold6325_5	non-phage	0
NOF006_scaffold9781_12	non-phage	0
NOF006_scaffold5504_2	non-phage	0
NOF006_scaffold1685_33	non-phage	0
NOF006_scaffold12627_3	non-phage	0
NOF006_scaffold15627_1	non-phage	0
NOF006_scaffold21366_1	non-phage	0
NOF006_scaffold17031_1	non-phage	0
NOF006_scaffold1934_1	non-phage	0
NOF006_scaffold6876_6	non-phage	0

NOF006_scaffold9788_2	non-phage	0
NOF006_scaffold15134_1	non-phage	0
NOF006_scaffold12148_1	non-phage	0
NOF006_scaffold5884_1	non-phage	0
NOF006_scaffold17763_1	non-phage	0
NOF006_scaffold6849_1	non-phage	0
NOF006_scaffold14668_1	non-phage	0
NOF006_scaffold14501_2	non-phage	0
NOF006_scaffold365_9	non-phage	0
NOF006_scaffold19608_1	non-phage	0
NOF006_scaffold5521_1	non-phage	0
NOF006_scaffold20457_1	non-phage	0
NOF006_scaffold15661_3	non-phage	0
NOF006_scaffold20875_1	non-phage	0
NOF006_scaffold15745_1	non-phage	0
NOF006_scaffold5793_1	non-phage	0
NOF006_scaffold14344_3	non-phage	0
NOF006_scaffold6724_13	non-phage	0
NOF006_scaffold969_1	non-phage	0
NOF006_scaffold6876_1	non-phage	0
NOF006_scaffold17088_2	non-phage	0
NOF006_scaffold17_1	non-phage	0
NOF006_scaffold2050_14	non-phage	0
NOF006_scaffold3784_1	non-phage	0
NOF006_scaffold16891_1	non-phage	0
NOF006_scaffold969_4	non-phage	0
NOF006_scaffold26_9	non-phage	0
NOF006_scaffold15603_2	non-phage	0
NOF006_scaffold8614_1	non-phage	0
NOF006_scaffold8110_12	non-phage	0
NOF006_scaffold13644_2	non-phage	0
NOF006_scaffold108_6	non-phage	0
NOF006_C400904_1	non-phage	0
NOF006_scaffold15736_1	non-phage	0
NOF006_scaffold57_1	non-phage	0
NOF006_scaffold978_16	non-phage	0
NOF006_scaffold21302_1	non-phage	0
NOF006_scaffold4977_2	non-phage	0
NOF006_scaffold7463_1	non-phage	0
NOF006_scaffold6446_26	non-phage	0
NOF006_scaffold5490_1	non-phage	0
NOF006_scaffold21314_2	non-phage	0
NOF006_scaffold20914_1	non-phage	0
NOF006_scaffold20327_1	non-phage	0
NOF006_scaffold261_2	non-phage	0
NOF006_scaffold21028_1	non-phage	0

NOF006_scaffold14933_2	non-phage	0
NOF006_scaffold6849_7	non-phage	0
NOF006_scaffold17031_2	non-phage	0
NOF006_scaffold15417_1	non-phage	0
NOF006_scaffold8999_10	non-phage	0
NOF006_scaffold12875_2	non-phage	0
NOF006_scaffold155_4	non-phage	0
NOF006_scaffold1329_6	non-phage	0
NOF006_scaffold14271_1	non-phage	0
NOF006_scaffold8950_4	non-phage	0
NOF006_scaffold3613_47	non-phage	0
NOF006_scaffold92_2	non-phage	0
NOF006_scaffold365_17	non-phage	0
NOF006_scaffold19991_1	non-phage	0
NOF006_scaffold15945_1	non-phage	0
NOF006_scaffold110_5	non-phage	0
NOF006_scaffold15506_1	non-phage	0
NOF006_scaffold8762_2	non-phage	0
NOF006_scaffold15090_5	non-phage	0
NOF006_scaffold3933_3	non-phage	0
NOF006_scaffold320_36	non-phage	0
NOF006_scaffold57_2	non-phage	0
NOF006_scaffold7669_1	non-phage	0
NOF006_scaffold11629_2	non-phage	0
NOF006_scaffold13284_6	non-phage	0
NOF006_scaffold2124_15	non-phage	0
NOF006_scaffold4636_1	non-phage	0
NOF006_scaffold4253_6	non-phage	0
NOF006_scaffold1488_4	non-phage	0
NOF006_scaffold4865_4	non-phage	0
NOF006_scaffold6876_4	non-phage	0
NOF006_scaffold722_16	non-phage	0
NOF006_scaffold11817_3	non-phage	0
NOF006_scaffold7893_2	non-phage	0
NOF006_scaffold5371_1	non-phage	0
NOF006_scaffold13080_2	non-phage	0
NOF006_scaffold8189_1	non-phage	0
NOF007_scaffold12493_2	non-phage	0
NOF007_scaffold5275_17	non-phage	0
NOF007_scaffold11637_10	non-phage	0
NOF007_scaffold1073_14	non-phage	0
NOF007_scaffold1028_13	non-phage	0
NOF007_scaffold8374_1	non-phage	0
NOF007_scaffold7694_17	non-phage	0
NOF007_scaffold11855_3	non-phage	0
NOF007_scaffold26445_1	non-phage	0

NOF007_C490255_1	non-phage	0
NOF007_scaffold28897_2	non-phage	0
NOF007_scaffold29005_1	non-phage	0
NOF007_scaffold8171_17	non-phage	0
NOF007_scaffold2518_23	non-phage	0
NOF007_scaffold25089_1	non-phage	0
NOF007_scaffold2675_3	non-phage	0
NOF007_scaffold28759_1	non-phage	0
NOF007_scaffold13466_2	non-phage	0
NOF007_scaffold9575_7	non-phage	0
NOF007_scaffold1463_2	non-phage	0
NOF007_scaffold21277_1	non-phage	0
NOF007_scaffold5145_3	non-phage	0
NOF007_scaffold321_1	non-phage	0
NOF007_scaffold16851_2	non-phage	0
NOF007_scaffold17371_1	non-phage	0
NOF007_scaffold28359_1	non-phage	0
NOF007_scaffold10320_4	non-phage	0
NOF007_scaffold8344_1	non-phage	0
NOF007_scaffold23387_1	non-phage	0
NOF007_scaffold2836_34	non-phage	0
NOF007_scaffold28824_7	non-phage	0
NOF007_scaffold9051_6	non-phage	0
NOF007_scaffold1973_3	non-phage	0
NOF007_scaffold111_4	non-phage	0
NOF007_scaffold28971_1	non-phage	0
NOF007_scaffold7460_27	non-phage	0
NOF007_scaffold28934_1	non-phage	0
NOF007_scaffold9496_13	non-phage	0
NOF007_scaffold28667_2	non-phage	0
NOF007_scaffold28892_3	non-phage	0
NOF007_scaffold2836_30	non-phage	0
NOF007_scaffold20582_2	non-phage	0
NOF007_scaffold3285_3	non-phage	0
NOF007_scaffold27268_1	non-phage	0
NOF007_scaffold69_4	non-phage	0
NOF007_scaffold21710_3	non-phage	0
NOF007_scaffold6925_2	non-phage	0
NOF007_scaffold834_15	non-phage	0
NOF007_scaffold6134_5	non-phage	0
NOF007_scaffold14358_3	non-phage	0
NOF007_scaffold24049_2	non-phage	0
NOF007_scaffold88_2	non-phage	0
NOF007_scaffold25584_6	non-phage	0
NOF007_scaffold983_21	non-phage	0
NOF007_scaffold28249_1	non-phage	0

NOF007_scaffold98_6	non-phage	0
NOF007_scaffold3285_2	non-phage	0
NOF007_scaffold1910_2	non-phage	0
NOF007_scaffold28824_3	non-phage	0
NOF007_C490473_1	non-phage	0
NOF007_scaffold5275_8	non-phage	0
NOF007_scaffold28807_2	non-phage	0
NOF007_scaffold10343_1	non-phage	0
NOF007_scaffold28738_1	non-phage	0
NOF007_scaffold6670_25	non-phage	0
NOF007_scaffold7015_4	non-phage	0
NOF007_scaffold26097_2	non-phage	0
NOF007_scaffold28772_1	non-phage	0
NOF007_scaffold28647_1	non-phage	0
NOF007_scaffold28457_5	non-phage	0
NOF007_scaffold12493_4	non-phage	0
NOF007_scaffold25072_1	non-phage	0
NOF007_scaffold26097_3	non-phage	0
NOF007_scaffold1603_64	non-phage	0
NOF007_scaffold14358_2	non-phage	0
NOF007_scaffold9256_2	non-phage	0
NOF007_C490929_1	non-phage	0
NOF007_scaffold24498_4	non-phage	0
NOF007_scaffold81_9	non-phage	0
NOF007_scaffold29004_2	non-phage	0
NOF007_scaffold28985_1	non-phage	0
NOF007_scaffold17288_1	non-phage	0
NOF007_scaffold181_7	non-phage	0
NOF007_scaffold834_33	non-phage	0
NOF007_scaffold28900_2	non-phage	0
NOF007_scaffold28824_5	non-phage	0
NOF007_scaffold6571_22	non-phage	0
NOF007_scaffold7948_1	non-phage	0
NOF007_scaffold28360_1	non-phage	0
NOF007_scaffold2422_2	non-phage	0
NOF007_scaffold1499_9	non-phage	0
NOF007_scaffold9603_1	non-phage	0
NOF007_scaffold28994_1	non-phage	0
NOF007_scaffold29015_1	non-phage	0
NOF007_scaffold28892_1	non-phage	0
NOF007_scaffold5752_2	non-phage	0
NOF007_scaffold15970_3	non-phage	0
NOF007_scaffold28893_1	non-phage	0
NOF007_scaffold81_7	non-phage	0
NOF007_scaffold15284_2	non-phage	0
NOF007_scaffold6562_6	non-phage	0

NOF007_scaffold27305_2	non-phage	0
NOF007_scaffold1544_14	non-phage	0
NOF007_scaffold25584_5	non-phage	0
NOF007_scaffold20124_1	non-phage	0
NOF007_C490293_1	non-phage	0
NOF007_scaffold27268_4	non-phage	0
NOF007_scaffold14305_2	non-phage	0
NOF007_scaffold9187_40	non-phage	0
NOF007_scaffold17701_4	non-phage	0
NOF007_scaffold21055_3	non-phage	0
NOF007_C490723_1	non-phage	0
NOF007_scaffold23819_4	non-phage	0
NOF007_scaffold198_10	non-phage	0
NOF007_scaffold23188_1	non-phage	0
NOF007_scaffold8553_6	non-phage	0
NOF007_C490325_1	non-phage	0
NOF007_scaffold9048_1	non-phage	0
NOF007_scaffold27605_1	non-phage	0
NOF007_scaffold22790_2	non-phage	0
NOF007_scaffold8758_2	non-phage	0
NOF007_scaffold14672_1	non-phage	0
NOF007_scaffold1684_1	non-phage	0
NOF007_scaffold7460_42	non-phage	0
NOF007_scaffold28874_1	non-phage	0
NOF007_scaffold220_1	non-phage	0
NOF007_scaffold21995_2	non-phage	0
NOF007_scaffold2147_17	non-phage	0
NOF007_scaffold181_3	non-phage	0
NOF007_scaffold2891_1	non-phage	0
NOF007_scaffold25888_1	non-phage	0
NOF007_scaffold28778_2	non-phage	0
NOF007_scaffold2836_35	non-phage	0
NOF007_scaffold7694_8	non-phage	0
NOF007_scaffold1499_3	non-phage	0
NOF007_scaffold597_32	non-phage	0
NOF007_scaffold10776_4	non-phage	0
NOF007_scaffold25584_4	non-phage	0
NOF007_scaffold28883_2	non-phage	0
NOF007_scaffold20327_2	non-phage	0
NOF007_scaffold608_5	non-phage	0
NOF007_scaffold13558_8	non-phage	0
NOF007_scaffold19885_3	non-phage	0
NOF007_scaffold27268_5	non-phage	0
NOF007_scaffold8575_1	non-phage	0
NOF007_scaffold5610_2	non-phage	0
NOF007_scaffold29020_1	non-phage	0

NOF007_scaffold17861_5	non-phage	0
NOF007_scaffold9136_2	non-phage	0
NOF008_scaffold238_5	non-phage	0
NOF008_scaffold34078_1	non-phage	0
NOF008_scaffold14006_12	non-phage	0
NOF008_scaffold30669_1	non-phage	0
NOF008_scaffold22341_8	non-phage	0
NOF008_scaffold53350_3	non-phage	0
NOF008_scaffold51824_2	non-phage	0
NOF008_scaffold8428_1	non-phage	0
NOF008_scaffold22635_1	non-phage	0
NOF008_scaffold3623_3	non-phage	0
NOF008_scaffold15063_4	non-phage	0
NOF008_scaffold82_2	non-phage	0
NOF008_scaffold68222_2	non-phage	0
NOF008_scaffold37888_6	non-phage	0
NOF008_scaffold67421_1	non-phage	0
NOF008_scaffold12766_1	non-phage	0
NOF008_scaffold54731_3	non-phage	0
NOF008_scaffold51808_1	non-phage	0
NOF008_scaffold2516_5	non-phage	0
NOF008_scaffold9423_4	non-phage	0
NOF008_scaffold65868_1	non-phage	0
NOF008_scaffold15953_1	non-phage	0
NOF008_scaffold11646_1	non-phage	0
NOF008_scaffold163_5	non-phage	0
NOF008_scaffold8169_1	non-phage	0
NOF008_scaffold63598_2	non-phage	0
NOF008_scaffold30807_2	non-phage	0
NOF008_scaffold21485_3	non-phage	0
NOF008_scaffold3708_25	non-phage	0
NOF008_scaffold18551_2	non-phage	0
NOF008_scaffold156_5	non-phage	0
NOF008_scaffold65073_1	non-phage	0
NOF008_scaffold10868_2	non-phage	0
NOF008_scaffold30211_4	non-phage	0
NOF008_scaffold68159_1	non-phage	0
NOF008_scaffold28124_2	non-phage	0
NOF008_scaffold67907_1	non-phage	0
NOF008_scaffold33589_1	non-phage	0
NOF008_scaffold56464_5	non-phage	0
NOF008_scaffold33616_3	non-phage	0
NOF008_scaffold65972_1_1	non-phage	0
NOF008_scaffold39396_4	non-phage	0
NOF008_scaffold161_1	non-phage	0
NOF008_scaffold3158_2	non-phage	0

NOF008_scaffold47094_4	non-phage	0
NOF008_scaffold9235_22	non-phage	0
NOF008_scaffold152_4	non-phage	0
NOF008_scaffold31875_7	non-phage	0
NOF008_scaffold21723_2	non-phage	0
NOF008_scaffold51_1	non-phage	0
NOF008_scaffold19660_2	non-phage	0
NOF008_scaffold63815_1	non-phage	0
NOF008_scaffold28624_1	non-phage	0
NOF008_scaffold27411_7	non-phage	0
NOF008_scaffold13941_7	non-phage	0
NOF008_scaffold40142_1	non-phage	0
NOF008_scaffold49915_6	non-phage	0
NOF008_scaffold37931_4	non-phage	0
NOF008_scaffold916_7	non-phage	0
NOF008_scaffold65073_3	non-phage	0
NOF008_scaffold68193_2	non-phage	0
NOF008_scaffold28163_1	non-phage	0
NOF008_scaffold10425_2	non-phage	0
NOF008_scaffold916_3	non-phage	0
NOF008_scaffold53350_1	non-phage	0
NOF008_scaffold169_3	non-phage	0
NOF008_scaffold68229_1	non-phage	0
NOF008_scaffold61744_2	non-phage	0
NOF008_scaffold1078_3	non-phage	0
NOF008_scaffold2719_9	non-phage	0
NOF008_scaffold440_2	non-phage	0
NOF008_scaffold32931_4	non-phage	0
NOF008_scaffold1563_9	non-phage	0
NOF008_scaffold163_3	non-phage	0
NOF008_scaffold2771_4	non-phage	0
NOF008_scaffold28883_1	non-phage	0
NOF008_scaffold64706_1	non-phage	0
NOF008_scaffold17230_5	non-phage	0
NOF008_scaffold53642_2	non-phage	0
NOF008_scaffold65302_1	non-phage	0
NOF008_scaffold4697_1	non-phage	0
NOF008_C865295_1	non-phage	0
NOF008_scaffold29073_1	non-phage	0
NOF008_C865429_1	non-phage	0
NOF008_scaffold14006_11	non-phage	0
NOF008_scaffold26133_1	non-phage	0
NOF008_scaffold2686_3	non-phage	0
NOF008_scaffold30418_13	non-phage	0
NOF008_scaffold44980_2	non-phage	0
NOF008_scaffold26499_8	non-phage	0

NOF008_scaffold4211_5	non-phage	0
NOF008_scaffold67785_2	non-phage	0
NOF008_scaffold11843_2	non-phage	0
NOF008_scaffold49915_5	non-phage	0
NOF008_scaffold11468_1	non-phage	0
NOF008_scaffold46598_2	non-phage	0
NOF008_scaffold46576_1	non-phage	0
NOF008_scaffold39023_2	non-phage	0
NOF008_scaffold54791_1	non-phage	0
NOF008_scaffold26239_4	non-phage	0
NOF008_scaffold30957_3	non-phage	0
NOF008_scaffold68132_1	non-phage	0
NOF008_C865849_1	non-phage	0
NOF008_scaffold64411_3	non-phage	0
NOF008_scaffold332_1	non-phage	0
NOF008_scaffold67146_1	non-phage	0
NOF008_C866013_1	non-phage	0
NOF008_scaffold16777_12	non-phage	0
NOF008_scaffold55864_1	non-phage	0
NOF008_scaffold63731_3	non-phage	0
NOF008_scaffold82_3	non-phage	0
NOF008_scaffold29594_2	non-phage	0
NOF008_scaffold163_1	non-phage	0
NOF008_scaffold68221_2	non-phage	0
NOF008_scaffold38215_6	non-phage	0
NOF008_scaffold32944_1	non-phage	0
NOF008_scaffold67797_1	non-phage	0
NOF008_scaffold47632_3	non-phage	0
NOF008_scaffold2635_1	non-phage	0
NOF008_scaffold68057_1	non-phage	0
NOF008_C866037_1	non-phage	0
NOF008_scaffold30957_2	non-phage	0
NOF008_scaffold30211_2	non-phage	0
NOF008_scaffold5959_2	non-phage	0
NOF008_scaffold29594_3	non-phage	0
NOF008_scaffold4691_3	non-phage	0
NOF008_scaffold54389_1	non-phage	0
NOF008_scaffold9493_1	non-phage	0
NOF008_C866107_1	non-phage	0
NOF008_scaffold40248_2	non-phage	0
NOF008_scaffold25220_1	non-phage	0
NOF008_scaffold16551_5	non-phage	0
NOF008_scaffold57634_1	non-phage	0
NOF008_scaffold34250_1	non-phage	0
NOF008_scaffold12195_8	non-phage	0
NOF008_scaffold52223_1	non-phage	0

NOF008_scaffold67298_10	non-phage	0
NOF008_scaffold64704_1	non-phage	0
NOF008_scaffold41859_1	non-phage	0
NOF008_scaffold39443_1	non-phage	0
NOF008_scaffold63828_2	non-phage	0
NOF008_scaffold35151_2	non-phage	0
NOF008_scaffold40827_2	non-phage	0
NOF008_scaffold51825_2	non-phage	0
NOF008_scaffold35956_2	non-phage	0
NOF008_scaffold15158_2	non-phage	0
NOF008_scaffold38010_5	non-phage	0
NOF008_scaffold53592_1	non-phage	0
NOF008_scaffold67828_1	non-phage	0
NOF008_scaffold65279_2	non-phage	0
NOF008_scaffold3708_26	non-phage	0
NOF008_scaffold11569_2	non-phage	0
NOF008_scaffold161_5	non-phage	0
NOF008_scaffold15329_24	non-phage	0
NOF008_scaffold37931_17	non-phage	0
NOF008_scaffold60618_1	non-phage	0
NOF008_scaffold234_1	non-phage	0
NOF008_scaffold29227_2	non-phage	0
NOF008_scaffold10728_2	non-phage	0
NOF008_scaffold15599_4	non-phage	0
NOF008_scaffold13444_4	non-phage	0
NOF008_scaffold388_5	non-phage	0
NOF008_scaffold18034_1	non-phage	0
NOF008_scaffold55812_1	non-phage	0
NOF008_scaffold46478_1	non-phage	0
NOF008_scaffold690_7	non-phage	0
NOF008_scaffold14515_13	non-phage	0
NOF008_scaffold34840_1	non-phage	0
NOF008_scaffold65983_1	non-phage	0
NOF008_scaffold11898_2	non-phage	0
NOF008_scaffold23750_1	non-phage	0
NOF008_scaffold5444_1	non-phage	0
NOF008_scaffold19089_9	non-phage	0
NOF008_scaffold51745_1	non-phage	0
NOF008_scaffold30286_1	non-phage	0
NOF008_scaffold8939_1	non-phage	0
NOF008_scaffold43753_1	non-phage	0
NOF008_scaffold37314_2	non-phage	0
NOF008_scaffold24602_1	non-phage	0
NOF008_scaffold15238_8	non-phage	0
NOF008_scaffold31481_4	non-phage	0
NOF008_scaffold26413_3	non-phage	0

NOF008_scaffold68205_2	non-phage	0
NOF008_scaffold31199_4	non-phage	0
NOF008_scaffold54029_1	non-phage	0
NOF008_scaffold68218_2	non-phage	0
NOF008_scaffold22970_1	non-phage	0
NOF008_scaffold6936_14	non-phage	0
NOF008_scaffold46689_4	non-phage	0
NOF008_scaffold68195_2	non-phage	0
NOF008_scaffold5505_18	non-phage	0
NOF008_scaffold56464_3	non-phage	0
NOF008_scaffold66299_1	non-phage	0
NOF008_scaffold32044_8	non-phage	0
NOF008_scaffold60429_2	non-phage	0
NOF008_scaffold156_4	non-phage	0
NOF008_scaffold5959_3	non-phage	0
NOF008_scaffold44502_2	non-phage	0
NOF008_scaffold19850_1	non-phage	0
NOF008_scaffold68032_1	non-phage	0
NOF008_scaffold22768_26	non-phage	0
NOF008_scaffold4415_1	non-phage	0
NOF008_scaffold52223_3	non-phage	0
NOF008_scaffold10305_2	non-phage	0
NOF009_scaffold27722_1	non-phage	0
NOF009_scaffold7274_2	non-phage	0
NOF009_scaffold24422_3	non-phage	0
NOF009_C558377_1	non-phage	0
NOF009_scaffold12768_1	non-phage	0
NOF009_scaffold34115_1	non-phage	0
NOF009_scaffold43251_1	non-phage	0
NOF009_scaffold34114_1	non-phage	0
NOF009_scaffold41583_1	non-phage	0
NOF009_scaffold204_1	non-phage	0
NOF009_scaffold41596_1	non-phage	0
NOF009_scaffold31105_3	non-phage	0
NOF009_scaffold8370_2	non-phage	0
NOF009_scaffold33864_1	non-phage	0
NOF009_scaffold30252_1	non-phage	0
NOF009_scaffold43496_1	non-phage	0
NOF009_scaffold41676_2	non-phage	0
NOF009_scaffold43353_3	non-phage	0
NOF009_scaffold10565_2	non-phage	0
NOF009_scaffold20144_10	non-phage	0
NOF009_scaffold43507_2	non-phage	0
NOF009_scaffold2909_13	non-phage	0
NOF009_scaffold36452_1	non-phage	0
NOF009_scaffold37604_1	non-phage	0

NOF009_scaffold43186_2	non-phage	0
NOF009_scaffold5085_5	non-phage	0
NOF009_scaffold231_23	non-phage	0
NOF009_scaffold7082_3	non-phage	0
NOF009_scaffold349_18	non-phage	0
NOF009_scaffold12022_1	non-phage	0
NOF009_scaffold43528_1	non-phage	0
NOF009_scaffold8637_5	non-phage	0
NOF009_scaffold24933_1	non-phage	0
NOF009_scaffold26421_6	non-phage	0
NOF009_scaffold8922_6	non-phage	0
NOF009_scaffold17368_3	non-phage	0
NOF009_scaffold16899_1	non-phage	0
NOF009_scaffold4604_2	non-phage	0
NOF009_scaffold39_3	non-phage	0
NOF009_scaffold5085_2	non-phage	0
NOF009_scaffold43506_1	non-phage	0
NOF009_scaffold20144_11	non-phage	0
NOF009_scaffold34681_1	non-phage	0
NOF009_scaffold2707_1	non-phage	0
NOF009_scaffold18986_3	non-phage	0
NOF009_scaffold39491_2	non-phage	0
NOF009_scaffold22581_5	non-phage	0
NOF009_scaffold366_4	non-phage	0
NOF009_scaffold15303_2	non-phage	0
NOF009_scaffold27845_4	non-phage	0
NOF009_scaffold31742_1	non-phage	0
NOF009_scaffold63_5	non-phage	0
NOF009_scaffold30843_1	non-phage	0
NOF009_scaffold512_10	non-phage	0
NOF009_scaffold13113_1	non-phage	0
NOF009_scaffold41737_1	non-phage	0
NOF009_scaffold36314_1	non-phage	0
NOF009_scaffold43496_3	non-phage	0
NOF009_scaffold16720_1	non-phage	0
NOF009_scaffold39447_1	non-phage	0
NOF009_scaffold26150_1	non-phage	0
NOF009_scaffold41192_1	non-phage	0
NOF009_C558753_1	non-phage	0
NOF009_scaffold19_2	non-phage	0
NOF009_scaffold4146_1	non-phage	0
NOF009_scaffold42145_2	non-phage	0
NOF009_scaffold24090_1	non-phage	0
NOF009_scaffold7082_2	non-phage	0
NOF009_scaffold30432_5	non-phage	0
NOF009_scaffold39576_6	non-phage	0

NOF009_scaffold20897_5	non-phage	0
NOF009_scaffold15769_5	non-phage	0
NOF009_scaffold22254_2	non-phage	0
NOF009_scaffold8197_3	non-phage	0
NOF009_scaffold35967_1	non-phage	0
NOF009_scaffold17778_1	non-phage	0
NOF009_scaffold39660_2	non-phage	0
NOF009_scaffold29183_3	non-phage	0
NOF009_scaffold7283_2	non-phage	0
NOF009_scaffold40367_5	non-phage	0
NOF009_scaffold11957_2	non-phage	0
NOF009_scaffold2756_2	non-phage	0
NOF009_scaffold8483_8	non-phage	0
NOF009_scaffold30505_1	non-phage	0
NOF009_scaffold185_2	non-phage	0
NOF009_scaffold39576_7	non-phage	0
NOF009_scaffold43085_1	non-phage	0
NOF009_scaffold31729_3	non-phage	0
NOF009_scaffold8637_2	non-phage	0
NOF009_scaffold35385_3	non-phage	0
NOF009_scaffold29273_1	non-phage	0
NOF009_scaffold25785_1	non-phage	0
NOF009_scaffold26407_5	non-phage	0
NOF009_scaffold31727_1	non-phage	0
NOF009_scaffold40185_6	non-phage	0
NOF009_C558843_1	non-phage	0
NOF009_scaffold24422_1	non-phage	0
NOF009_scaffold14088_1	non-phage	0
NOF009_scaffold33925_1	non-phage	0
NOF009_scaffold5085_11	non-phage	0
NOF009_scaffold42006_2	non-phage	0
NOF009_scaffold30792_3	non-phage	0
NOF009_scaffold43340_1	non-phage	0
NOF009_scaffold6106_9	non-phage	0
NOF009_scaffold366_2	non-phage	0
NOF009_scaffold15769_1	non-phage	0
NOF009_scaffold37501_2	non-phage	0
NOF009_scaffold1628_1	non-phage	0
NOF009_scaffold7625_1	non-phage	0
NOF009_scaffold40185_4	non-phage	0
NOF009_scaffold8317_4	non-phage	0
NOF009_scaffold33036_1	non-phage	0
NOF009_scaffold11416_1	non-phage	0
NOF009_scaffold34639_2	non-phage	0
NOF009_scaffold7232_1	non-phage	0
NOF009_scaffold16720_2	non-phage	0

NOF009_scaffold2890_4	non-phage	0
NOF009_scaffold2534_10	non-phage	0
NOF009_scaffold31037_1	non-phage	0
NOF009_C558609_1	non-phage	0
NOF009_scaffold41569_2	non-phage	0
NOF009_scaffold10964_12	non-phage	0
NOF009_scaffold25686_1	non-phage	0
NOF009_scaffold1541_3	non-phage	0
NOF009_scaffold43539_1	non-phage	0
NOF009_scaffold9422_4	non-phage	0
NOF009_scaffold43459_1	non-phage	0
NOF009_scaffold17834_2	non-phage	0
NOF009_scaffold5085_15	non-phage	0
NOF009_scaffold32_1	non-phage	0
NOF009_scaffold42710_2	non-phage	0
NOF009_scaffold17050_2	non-phage	0
NOF009_scaffold28020_3	non-phage	0
NOF009_scaffold42574_1	non-phage	0
NOF009_scaffold1678_30	non-phage	0
NOF009_scaffold11277_2	non-phage	0
NOF009_scaffold36543_1	non-phage	0
NOF009_scaffold940_11	non-phage	0
NOF009_scaffold42607_2	non-phage	0
NOF009_scaffold24933_3	non-phage	0
NOF009_scaffold32738_4	non-phage	0
NOF009_scaffold43545_2	non-phage	0
NOF009_scaffold7048_6	non-phage	0
NOF009_scaffold29029_2	non-phage	0
NOF009_scaffold32030_2	non-phage	0
NOF009_scaffold16309_1	non-phage	0
NOF009_scaffold7274_1	non-phage	0
NOF009_scaffold43453_1	non-phage	0
NOF009_scaffold40676_1	non-phage	0
NOF009_scaffold17291_1	non-phage	0
NOF009_scaffold7527_4	non-phage	0
NOF009_scaffold9505_1	non-phage	0
NOF009_scaffold38299_1	non-phage	0
NOF009_scaffold3692_5	non-phage	0
NOF009_scaffold7283_1	non-phage	0
NOF009_scaffold19254_2	non-phage	0
NOF009_scaffold78_3	non-phage	0
NOF009_scaffold43042_2	non-phage	0
NOF009_scaffold43542_1	non-phage	0
NOF009_scaffold8197_5	non-phage	0
NOF009_C558689_1	non-phage	0
NOF009_scaffold26214_1	non-phage	0

NOF009_scaffold29024_1	non-phage	0
NOF009_scaffold19158_2	non-phage	0
NOF009_scaffold15164_2	non-phage	0
NOF009_scaffold35470_1	non-phage	0
NOF009_scaffold37678_5	non-phage	0
NOF009_scaffold185_1	non-phage	0
NOF009_scaffold41676_1	non-phage	0
NOF009_scaffold35688_2	non-phage	0
NOF009_scaffold43505_1	non-phage	0
NOF009_scaffold43410_1	non-phage	0
NOF009_scaffold18741_5	non-phage	0
NOF009_scaffold4221_2	non-phage	0
NOF009_scaffold13265_1	non-phage	0
NOF009_scaffold2534_14	non-phage	0
NOF009_scaffold41440_1	non-phage	0
NOF009_scaffold26242_1	non-phage	0
NOF009_scaffold24864_4	non-phage	0
NOF009_scaffold8317_5	non-phage	0
NOF009_scaffold27228_1	non-phage	0
NOF009_scaffold2707_4	non-phage	0
NOF009_scaffold8808_3	non-phage	0
NOF009_scaffold7082_4	non-phage	0
NOF009_scaffold43545_1	non-phage	0
NOF009_scaffold32289_5	non-phage	0
NOF009_scaffold11743_3	non-phage	0
NOF009_scaffold34115_2	non-phage	0
NOF009_scaffold43203_1	non-phage	0
NOF009_scaffold7852_9	non-phage	0
NOF009_scaffold36975_1	non-phage	0
NOF009_scaffold43296_1	non-phage	0
NOF009_scaffold33296_1	non-phage	0
NOF009_scaffold33410_2	non-phage	0
NOF009_scaffold17834_3	non-phage	0
NOF009_scaffold1520_4	non-phage	0
NOF009_scaffold20144_8	non-phage	0
NOF009_scaffold19618_2	non-phage	0
NOF009_scaffold28555_1	non-phage	0
NOF010_scaffold43072_1	non-phage	0
NOF010_scaffold45105_1	non-phage	0
NOF010_scaffold25411_3	non-phage	0
NOF010_scaffold17170_3	non-phage	0
NOF010_scaffold48991_1	non-phage	0
NOF010_scaffold48534_2	non-phage	0
NOF010_scaffold3745_8	non-phage	0
NOF010_scaffold24007_2	non-phage	0
NOF010_scaffold27461_3	non-phage	0

NOF010_scaffold29108_2	non-phage	0
NOF010_scaffold20634_1	non-phage	0
NOF010_scaffold13234_8	non-phage	0
NOF010_scaffold16366_30	non-phage	0
NOF010_scaffold5204_1	non-phage	0
NOF010_scaffold43561_1	non-phage	0
NOF010_scaffold9563_12	non-phage	0
NOF010_scaffold54264_1	non-phage	0
NOF010_scaffold37334_1	non-phage	0
NOF010_scaffold49150_5	non-phage	0
NOF010_scaffold53749_1	non-phage	0
NOF010_scaffold5360_4	non-phage	0
NOF010_scaffold51188_1	non-phage	0
NOF010_scaffold223_13	non-phage	0
NOF010_scaffold25256_12	non-phage	0
NOF010_scaffold53492_1	non-phage	0
NOF010_scaffold23664_3	non-phage	0
NOF010_scaffold53531_1	non-phage	0
NOF010_scaffold53638_1	non-phage	0
NOF010_scaffold54309_1	non-phage	0
NOF010_scaffold19892_9	non-phage	0
NOF010_scaffold5384_9	non-phage	0
NOF010_scaffold41594_4	non-phage	0
NOF010_scaffold33735_9	non-phage	0
NOF010_scaffold43072_3	non-phage	0
NOF010_scaffold20466_3	non-phage	0
NOF010_scaffold44756_2	non-phage	0
NOF010_scaffold51185_3	non-phage	0
NOF010_scaffold54197_2	non-phage	0
NOF010_scaffold6340_5	non-phage	0
NOF010_scaffold56_12	non-phage	0
NOF010_scaffold47284_1	non-phage	0
NOF010_scaffold28124_2	non-phage	0
NOF010_scaffold48761_1	non-phage	0
NOF010_scaffold4119_5	non-phage	0
NOF010_scaffold53583_1	non-phage	0
NOF010_scaffold6485_14	non-phage	0
NOF010_scaffold17487_3	non-phage	0
NOF010_scaffold36303_3	non-phage	0
NOF010_scaffold2553_1	non-phage	0
NOF010_scaffold51939_1	non-phage	0
NOF010_scaffold9001_1	non-phage	0
NOF010_scaffold27940_1	non-phage	0
NOF010_scaffold49844_1	non-phage	0
NOF010_scaffold4065_9	non-phage	0
NOF010_scaffold34033_17	non-phage	0

NOF010_scaffold13835_2	non-phage	0
NOF010_scaffold53823_1	non-phage	0
NOF010_scaffold2626_8	non-phage	0
NOF010_scaffold23102_3	non-phage	0
NOF010_scaffold27565_2	non-phage	0
NOF010_scaffold31144_9	non-phage	0
NOF010_scaffold31933_1	non-phage	0
NOF010_scaffold54249_1	non-phage	0
NOF010_scaffold15393_2	non-phage	0
NOF010_scaffold7440_6	non-phage	0
NOF010_scaffold21578_2	non-phage	0
NOF010_scaffold415_4	non-phage	0
NOF010_scaffold51939_2	non-phage	0
NOF010_scaffold32157_1	non-phage	0
NOF010_scaffold9954_12	non-phage	0
NOF010_scaffold39067_2	non-phage	0
NOF010_scaffold21996_1	non-phage	0
NOF010_C699311_1	non-phage	0
NOF010_scaffold54048_1	non-phage	0
NOF010_scaffold17707_33	non-phage	0
NOF010_scaffold18559_1	non-phage	0
NOF010_scaffold49150_3	non-phage	0
NOF010_scaffold46885_1	non-phage	0
NOF010_scaffold12198_4	non-phage	0
NOF010_scaffold2865_6	non-phage	0
NOF010_scaffold20182_3	non-phage	0
NOF010_scaffold20535_1	non-phage	0
NOF010_scaffold4007_6	non-phage	0
NOF010_scaffold17487_9	non-phage	0
NOF010_scaffold20906_1	non-phage	0
NOF010_scaffold2626_9	non-phage	0
NOF010_scaffold54295_1	non-phage	0
NOF010_scaffold13438_3	non-phage	0
NOF010_scaffold20514_4	non-phage	0
NOF010_scaffold17487_13	non-phage	0
NOF010_scaffold34033_4	non-phage	0
NOF010_scaffold45105_4	non-phage	0
NOF010_C699565_1	non-phage	0
NOF010_scaffold42673_1	non-phage	0
NOF010_scaffold5504_5	non-phage	0
NOF010_scaffold7431_3	non-phage	0
NOF010_scaffold15806_8	non-phage	0
NOF010_scaffold27052_8	non-phage	0
NOF010_scaffold28985_7	non-phage	0
NOF010_scaffold35538_6	non-phage	0
NOF010_scaffold20977_3	non-phage	0

NOF010_scaffold20820_5	non-phage	0
NOF010_scaffold40995_1	non-phage	0
NOF010_scaffold2725_14	non-phage	0
NOF010_scaffold23559_8	non-phage	0
NOF010_scaffold237_12	non-phage	0
NOF010_C699275_1	non-phage	0
NOF010_scaffold32157_14	non-phage	0
NOF010_scaffold637_6	non-phage	0
NOF010_scaffold18357_11	non-phage	0
NOF010_scaffold1185_7	non-phage	0
NOF010_scaffold20278_3	non-phage	0
NOF010_scaffold23947_4	non-phage	0
NOF010_scaffold54156_1	non-phage	0
NOF010_scaffold9408_1	non-phage	0
NOF010_scaffold25931_1	non-phage	0
NOF010_scaffold2722_14	non-phage	0
NOF010_scaffold31566_2	non-phage	0
NOF010_scaffold4611_7	non-phage	0
NOF010_scaffold11750_1	non-phage	0
NOF010_scaffold5390_1	non-phage	0
NOF010_scaffold2626_13	non-phage	0
NOF010_scaffold47487_1	non-phage	0
NOF010_scaffold24377_5	non-phage	0
NOF010_scaffold12198_9	non-phage	0
NOF010_scaffold49274_1	non-phage	0
NOF010_scaffold39120_1	non-phage	0
NOF010_scaffold53996_2	non-phage	0
NOF010_scaffold4826_2	non-phage	0
NOF010_scaffold16891_5	non-phage	0
NOF010_scaffold1185_4	non-phage	0
NOF010_scaffold23761_2	non-phage	0
NOF010_scaffold44717_2	non-phage	0
NOF010_scaffold82_3	non-phage	0
NOF010_scaffold267_2	non-phage	0
NOF010_scaffold40754_1	non-phage	0
NOF010_scaffold25931_2	non-phage	0
NOF010_scaffold20_8	non-phage	0
NOF010_scaffold35538_3	non-phage	0
NOF010_scaffold15806_2	non-phage	0
NOF010_scaffold53742_2	non-phage	0
NOF010_scaffold44040_3	non-phage	0
NOF010_C699167_1	non-phage	0
NOF010_scaffold27002_13	non-phage	0
NOF010_scaffold24408_3	non-phage	0
NOF010_scaffold53988_1	non-phage	0
NOF010_scaffold54210_2	non-phage	0

NOF010_scaffold25256_7	non-phage	0
NOF010_scaffold4826_8	non-phage	0
NOF010_scaffold52592_2	non-phage	0
NOF010_scaffold37783_1	non-phage	0
NOF010_scaffold9543_8	non-phage	0
NOF010_C699075_1	non-phage	0
NOF010_scaffold46849_4	non-phage	0
NOF010_scaffold42286_2	non-phage	0
NOF010_scaffold7440_2	non-phage	0
NOF010_C699719_1	non-phage	0
NOF010_scaffold12198_10	non-phage	0
NOF010_scaffold10874_6	non-phage	0
NOF010_scaffold23947_6	non-phage	0
NOF010_scaffold43749_3	non-phage	0
NOF010_scaffold9563_19	non-phage	0
NOF010_scaffold35538_8	non-phage	0
NOF010_scaffold5390_2	non-phage	0
NOF010_scaffold95_7	non-phage	0
NOF010_scaffold30072_1	non-phage	0
NOF010_scaffold35538_11	non-phage	0
NOF010_scaffold4065_3	non-phage	0
NOF010_scaffold2786_5	non-phage	0
NOF010_scaffold9323_2	non-phage	0
NOF010_scaffold1730_3	non-phage	0
NOF010_scaffold54024_1	non-phage	0
NOF010_scaffold34033_7	non-phage	0
NOF010_C700023_1	non-phage	0
NOF010_scaffold49307_1	non-phage	0
NOF010_scaffold53674_1	non-phage	0
NOF010_scaffold27048_6	non-phage	0
NOF010_scaffold64_10	non-phage	0
NOF010_scaffold33789_6	non-phage	0
NOF010_scaffold1185_5	non-phage	0
NOF010_scaffold82_4	non-phage	0
NOF010_scaffold22159_3	non-phage	0
NOF010_scaffold22589_4	non-phage	0
NOF010_scaffold22792_2	non-phage	0
NOF010_scaffold95_4	non-phage	0
NOF010_scaffold1730_1	non-phage	0
NOF010_scaffold27363_2	non-phage	0
NOF010_scaffold53635_1	non-phage	0
NOF010_scaffold32157_6	non-phage	0
NOF010_scaffold34033_10	non-phage	0
NOF010_scaffold4826_7	non-phage	0
NOF010_scaffold32959_3	non-phage	0
NOF010_scaffold17353_2	non-phage	0

NOF010_scaffold3575_4	non-phage	0
NOF010_scaffold95_9	non-phage	0
NOF010_scaffold53964_2	non-phage	0
NOF010_scaffold38527_10	non-phage	0
NOF010_scaffold7_2	non-phage	0
NOF010_scaffold12059_6	non-phage	0
NOF010_scaffold44368_1	non-phage	0
NOF010_scaffold20466_4	non-phage	0
NOF010_scaffold33162_1	non-phage	0
NOF010_scaffold45354_5	non-phage	0
NOF010_scaffold9563_20	non-phage	0
NOF010_scaffold38527_8	non-phage	0
NOF010_scaffold54310_1	non-phage	0
NOF010_C699095_1	non-phage	0
NOF010_scaffold28679_3	non-phage	0
NOF010_scaffold37649_1	non-phage	0
NOF010_scaffold12198_7	non-phage	0
NOF010_scaffold50685_2	non-phage	0
NOF010_scaffold32409_8	non-phage	0
NOF010_scaffold25697_1	non-phage	0
NOF010_scaffold15806_12	non-phage	0
NOF010_scaffold21511_2	non-phage	0
NOF010_scaffold50017_2	non-phage	0
NOF010_scaffold46607_1	non-phage	0
NOF010_scaffold53232_2	non-phage	0
NOF010_C699071_1	non-phage	0
NOF010_C699739_1	non-phage	0
NOF010_scaffold2814_5	non-phage	0
NOF010_scaffold53516_1	non-phage	0
NOF010_scaffold38527_9	non-phage	0
NOF010_scaffold12198_11	non-phage	0
NOF010_scaffold42286_3	non-phage	0
NOF010_scaffold28462_7	non-phage	0
NOF010_scaffold20745_2	non-phage	0
NOF010_scaffold6392_1	non-phage	0
NOF010_scaffold42545_8	non-phage	0
NOF010_scaffold2142_4	non-phage	0
NOF010_scaffold53853_2	non-phage	0
NOF010_scaffold32157_16	non-phage	0
NOF010_scaffold2626_14	non-phage	0
NOF010_scaffold48727_6	non-phage	0
NOF010_scaffold38527_3	non-phage	0
NOF010_scaffold18911_2	non-phage	0
NOF010_scaffold43303_15	non-phage	0
NOF010_scaffold13234_6	non-phage	0
NOF010_scaffold23947_8	non-phage	0

NOF010_scaffold473_4	non-phage	0
NOF010_scaffold52485_1	non-phage	0
NOF010_scaffold53246_2	non-phage	0
NOF010_scaffold31422_1	non-phage	0
NOF010_scaffold31835_2	non-phage	0
NOF010_scaffold2108_15	non-phage	0
NOF010_scaffold6340_3	non-phage	0
NOF010_scaffold9598_1	non-phage	0
NOF010_scaffold35262_2	non-phage	0
NOF010_scaffold285_5	non-phage	0
NOF010_scaffold9563_16	non-phage	0
NOF010_scaffold40819_3	non-phage	0
NOF010_scaffold53516_2	non-phage	0
NOF010_scaffold33735_8	non-phage	0
NOF010_scaffold30153_5	non-phage	0
NOF010_scaffold49150_4	non-phage	0
NOF010_scaffold16231_1	non-phage	0
NOF010_scaffold27002_5	non-phage	0
NOF010_scaffold45133_3	non-phage	0
NOF010_scaffold2693_1	non-phage	0
NOF010_scaffold25256_8	non-phage	0
NOF010_scaffold43303_12	non-phage	0
NOF010_scaffold129_4	non-phage	0
NOF010_scaffold29950_4	non-phage	0
NOF010_scaffold29621_4	non-phage	0
NOF010_scaffold95_1	non-phage	0
NOF010_scaffold8971_1	non-phage	0
NOF010_scaffold20182_4	non-phage	0
NOF010_scaffold27052_6	non-phage	0
NOF010_scaffold54194_1	non-phage	0
NOF010_scaffold4065_11	non-phage	0
NOF010_C699047_1	non-phage	0
NOF010_scaffold37539_2	non-phage	0
NOF010_scaffold48727_12	non-phage	0
NOF010_scaffold35262_3	non-phage	0
NOF010_scaffold16366_28	non-phage	0
NOF010_scaffold26613_1	non-phage	0
NOF010_scaffold9563_9	non-phage	0
NOF010_scaffold37285_1	non-phage	0
NOF010_scaffold26832_2	non-phage	0
NOF010_scaffold35637_1	non-phage	0
NOF010_scaffold48727_7	non-phage	0
NOF010_scaffold53583_3	non-phage	0
NOF010_scaffold20745_6	non-phage	0
NOF010_scaffold5504_13	non-phage	0
NOF010_scaffold6485_11	non-phage	0

NOF010_scaffold35859_1	non-phage	0
NOF010_scaffold26316_1	non-phage	0
NOF011_scaffold59050_2	non-phage	0
NOF011_scaffold58771_1	non-phage	0
NOF011_scaffold59097_2	non-phage	0
NOF011_scaffold52163_2	non-phage	0
NOF011_scaffold44766_4	non-phage	0
NOF011_scaffold11876_3	non-phage	0
NOF011_scaffold58761_1	non-phage	0
NOF011_scaffold59006_1	non-phage	0
NOF011_scaffold8154_3	non-phage	0
NOF011_scaffold58817_1	non-phage	0
NOF011_scaffold48688_1	non-phage	0
NOF011_scaffold382_4	non-phage	0
NOF011_scaffold58998_1	non-phage	0
NOF011_scaffold26462_1	non-phage	0
NOF011_scaffold35735_1	non-phage	0
NOF011_scaffold55388_1	non-phage	0
NOF011_scaffold32208_2	non-phage	0
NOF011_scaffold41902_2	non-phage	0
NOF011_scaffold38396_2	non-phage	0
NOF011_scaffold56745_2	non-phage	0
NOF011_scaffold41987_1	non-phage	0
NOF011_C736408_1	non-phage	0
NOF011_scaffold59052_1	non-phage	0
NOF011_scaffold6040_6	non-phage	0
NOF011_scaffold44097_4	non-phage	0
NOF011_scaffold30363_7	non-phage	0
NOF011_scaffold47566_1	non-phage	0
NOF011_scaffold2018_3	non-phage	0
NOF011_scaffold11411_2	non-phage	0
NOF011_scaffold50146_2	non-phage	0
NOF011_scaffold48365_1	non-phage	0
NOF011_scaffold28993_70	non-phage	0
NOF011_scaffold10436_1	non-phage	0
NOF011_scaffold51557_3	non-phage	0
NOF011_scaffold25707_2	non-phage	0
NOF011_scaffold14034_17	non-phage	0
NOF011_scaffold15244_3	non-phage	0
NOF011_scaffold44097_3	non-phage	0
NOF011_scaffold58960_1	non-phage	0
NOF011_scaffold7058_1	non-phage	0
NOF011_scaffold59089_1	non-phage	0
NOF011_scaffold71_1	non-phage	0
NOF011_scaffold59087_1	non-phage	0
NOF011_scaffold58507_1	non-phage	0

NOF011_scaffold56812_1	non-phage	0
NOF011_scaffold37519_2	non-phage	0
NOF011_scaffold54143_1	non-phage	0
NOF011_scaffold41814_2	non-phage	0
NOF011_scaffold10244_2	non-phage	0
NOF011_scaffold55438_1	non-phage	0
NOF011_scaffold59081_2	non-phage	0
NOF011_scaffold3610_1	non-phage	0
NOF011_C736498_1	non-phage	0
NOF011_scaffold22346_1	non-phage	0
NOF011_scaffold36061_3	non-phage	0
NOF011_scaffold14034_8	non-phage	0
NOF011_scaffold55892_1	non-phage	0
NOF011_scaffold47786_1	non-phage	0
NOF011_scaffold58907_3	non-phage	0
NOF011_scaffold49024_2	non-phage	0
NOF011_scaffold57360_2	non-phage	0
NOF011_scaffold27189_5	non-phage	0
NOF011_scaffold17644_1	non-phage	0
NOF011_scaffold21144_1	non-phage	0
NOF011_scaffold41413_2	non-phage	0
NOF011_scaffold40480_3	non-phage	0
NOF011_scaffold58571_1	non-phage	0
NOF011_scaffold1490_3	non-phage	0
NOF011_scaffold59092_1	non-phage	0
NOF011_scaffold31898_1	non-phage	0
NOF011_scaffold58795_1	non-phage	0
NOF011_scaffold41908_3	non-phage	0
NOF011_scaffold55441_1	non-phage	0
NOF011_scaffold18490_2	non-phage	0
NOF011_scaffold20031_1	non-phage	0
NOF011_scaffold47361_4	non-phage	0
NOF011_scaffold59091_1	non-phage	0
NOF011_scaffold50262_3	non-phage	0
NOF011_scaffold59076_2	non-phage	0
NOF011_scaffold9788_2	non-phage	0
NOF011_scaffold25728_2	non-phage	0
NOF011_scaffold57023_1	non-phage	0
NOF011_scaffold44912_14	non-phage	0
NOF011_scaffold3620_3	non-phage	0
NOF011_scaffold53964_1	non-phage	0
NOF011_scaffold40100_3	non-phage	0
NOF011_scaffold5350_2	non-phage	0
NOF011_scaffold48283_2	non-phage	0
NOF011_scaffold26658_1	non-phage	0
NOF011_scaffold55650_1	non-phage	0

NOF011_scaffold56548_1	non-phage	0
NOF011_scaffold44496_4	non-phage	0
NOF011_scaffold1854_1	non-phage	0
NOF011_scaffold42611_1	non-phage	0
NOF011_scaffold28099_4	non-phage	0
NOF011_scaffold83_4	non-phage	0
NOF011_scaffold59065_1	non-phage	0
NOF011_scaffold58990_2	non-phage	0
NOF011_scaffold21129_1	non-phage	0
NOF011_scaffold58742_1	non-phage	0
NOF011_scaffold36609_1	non-phage	0
NOF011_scaffold20810_2	non-phage	0
NOF011_scaffold51383_1	non-phage	0
NOF011_scaffold98_2	non-phage	0
NOF011_scaffold2018_4	non-phage	0
NOF011_scaffold58872_1	non-phage	0
NOF011_scaffold41628_5	non-phage	0
NOF011_scaffold35986_3	non-phage	0
NOF011_scaffold59091_5	non-phage	0
NOF011_scaffold58890_1	non-phage	0
NOF011_scaffold58361_4	non-phage	0
NOF011_scaffold19674_1	non-phage	0
NOF011_scaffold47624_1	non-phage	0
NOF011_scaffold24731_3	non-phage	0
NOF011_scaffold40128_1	non-phage	0
NOF011_scaffold214_1	non-phage	0
NOF011_scaffold55233_1_1	non-phage	0
NOF011_scaffold20044_3	non-phage	0
NOF011_scaffold58444_2	non-phage	0
NOF011_scaffold59015_1	non-phage	0
NOF011_scaffold49861_1	non-phage	0
NOF011_scaffold43460_3	non-phage	0
NOF011_scaffold15688_9	non-phage	0
NOF011_scaffold55934_1	non-phage	0
NOF011_scaffold40568_2	non-phage	0
NOF011_scaffold56478_1	non-phage	0
NOF011_scaffold19785_3	non-phage	0
NOF011_scaffold26226_1	non-phage	0
NOF011_scaffold58507_2	non-phage	0
NOF011_scaffold41346_4	non-phage	0
NOF011_scaffold45524_1	non-phage	0
NOF011_scaffold37063_4	non-phage	0
NOF011_C736428_1	non-phage	0
NOF011_scaffold746_3	non-phage	0
NOF011_scaffold58799_1	non-phage	0
NOF011_scaffold33_2	non-phage	0

NOF011_scaffold35735_2	non-phage	0
NOF011_scaffold50475_2	non-phage	0
NOF011_scaffold25105_6	non-phage	0
NOF011_scaffold37292_1	non-phage	0
NOF011_scaffold35599_1	non-phage	0
NOF011_scaffold9714_6	non-phage	0
NOF011_scaffold58989_4	non-phage	0
NOF011_scaffold24222_4	non-phage	0
NOF011_scaffold25105_2	non-phage	0
NOF011_scaffold56325_1	non-phage	0
NOF011_scaffold59058_2	non-phage	0
NOF011_scaffold59083_2	non-phage	0
NOF011_scaffold59055_1	non-phage	0
NOF011_scaffold31952_10	non-phage	0
NOF011_scaffold1459_1	non-phage	0
NOF011_scaffold9788_3	non-phage	0
NOF011_scaffold57518_2	non-phage	0
NOF011_scaffold58965_1	non-phage	0
NOF011_scaffold13623_2	non-phage	0
NOF011_scaffold20094_3	non-phage	0
NOF011_scaffold57649_2	non-phage	0
NOF011_scaffold41532_2	non-phage	0
NOF011_scaffold58405_3	non-phage	0
NOF011_scaffold54088_1	non-phage	0
NOF011_scaffold45587_1	non-phage	0
NOF011_scaffold58962_3	non-phage	0
NOF011_scaffold11256_5	non-phage	0
NOF011_scaffold42008_2	non-phage	0
NOF011_scaffold57024_1	non-phage	0
NOF011_scaffold5666_3	non-phage	0
NOF012_scaffold60023_9	non-phage	0
NOF012_scaffold44498_1	non-phage	0
NOF012_scaffold63936_1	non-phage	0
NOF012_scaffold8450_4	non-phage	0
NOF012_scaffold3555_14	non-phage	0
NOF012_scaffold71582_5	non-phage	0
NOF012_scaffold8940_14	non-phage	0
NOF012_C833927_1	non-phage	0
NOF012_scaffold29046_13	non-phage	0
NOF012_scaffold31286_25	non-phage	0
NOF012_scaffold73075_1	non-phage	0
NOF012_scaffold33170_8	non-phage	0
NOF012_scaffold75929_3	non-phage	0
NOF012_scaffold75618_4	non-phage	0
NOF012_scaffold41334_6	non-phage	0
NOF012_scaffold72540_1	non-phage	0

NOF012_scaffold77700_2	non-phage	0
NOF012_scaffold67829_7	non-phage	0
NOF012_scaffold49770_3	non-phage	0
NOF012_scaffold55619_1	non-phage	0
NOF012_scaffold40307_1	non-phage	0
NOF012_scaffold17514_3	non-phage	0
NOF012_scaffold44041_3	non-phage	0
NOF012_scaffold50785_1	non-phage	0
NOF012_scaffold11216_3	non-phage	0
NOF012_scaffold60956_4	non-phage	0
NOF012_scaffold11508_4	non-phage	0
NOF012_scaffold21503_16	non-phage	0
NOF012_scaffold11638_8	non-phage	0
NOF012_scaffold10393_1	non-phage	0
NOF012_scaffold7714_1	non-phage	0
NOF012_scaffold74640_1	non-phage	0
NOF012_scaffold46349_2	non-phage	0
NOF012_scaffold18670_5	non-phage	0
NOF012_scaffold78050_1	non-phage	0
NOF012_scaffold35595_1	non-phage	0
NOF012_scaffold6364_11	non-phage	0
NOF012_scaffold1013_2	non-phage	0
NOF012_scaffold7210_4	non-phage	0
NOF012_scaffold31424_2	non-phage	0
NOF012_scaffold12030_9	non-phage	0
NOF012_scaffold7636_2	non-phage	0
NOF012_C834035_1	non-phage	0
NOF012_scaffold16820_5	non-phage	0
NOF012_scaffold34940_4	non-phage	0
NOF012_scaffold78079_1	non-phage	0
NOF012_scaffold35953_2	non-phage	0
NOF012_scaffold59498_2	non-phage	0
NOF012_scaffold52547_4	non-phage	0
NOF012_scaffold65456_1	non-phage	0
NOF012_C833853_1	non-phage	0
NOF012_scaffold30397_1	non-phage	0
NOF012_scaffold68463_2	non-phage	0
NOF012_scaffold77188_2	non-phage	0
NOF012_scaffold5820_3	non-phage	0
NOF012_scaffold52992_5	non-phage	0
NOF012_scaffold26305_4	non-phage	0
NOF012_scaffold7172_3	non-phage	0
NOF012_scaffold41587_1	non-phage	0
NOF012_scaffold65177_1	non-phage	0
NOF012_C833769_1	non-phage	0
NOF012_scaffold68797_1	non-phage	0

NOF012_scaffold27206_3	non-phage	0
NOF012_scaffold62_4	non-phage	0
NOF012_scaffold20273_6	non-phage	0
NOF012_scaffold51951_3	non-phage	0
NOF012_scaffold78028_1	non-phage	0
NOF012_scaffold2007_3	non-phage	0
NOF012_scaffold5587_1	non-phage	0
NOF012_scaffold78027_1	non-phage	0
NOF012_scaffold78086_2	non-phage	0
NOF012_scaffold39849_1	non-phage	0
NOF012_scaffold71894_1	non-phage	0
NOF012_scaffold41680_3	non-phage	0
NOF012_scaffold43711_2	non-phage	0
NOF012_scaffold15924_1	non-phage	0
NOF012_scaffold50893_1	non-phage	0
NOF012_scaffold26305_12	non-phage	0
NOF012_scaffold37518_3	non-phage	0
NOF012_scaffold26296_1	non-phage	0
NOF012_scaffold78085_1	non-phage	0
NOF012_scaffold11638_7	non-phage	0
NOF012_scaffold57608_8	non-phage	0
NOF012_scaffold78058_3	non-phage	0
NOF012_scaffold28857_4	non-phage	0
NOF012_scaffold39910_1	non-phage	0
NOF012_scaffold37623_1	non-phage	0
NOF012_scaffold38555_1	non-phage	0
NOF012_scaffold30476_3	non-phage	0
NOF012_scaffold19465_3	non-phage	0
NOF012_scaffold9422_1	non-phage	0
NOF012_scaffold26488_12	non-phage	0
NOF012_scaffold26965_1	non-phage	0
NOF012_scaffold77962_1	non-phage	0
NOF012_scaffold13742_4	non-phage	0
NOF012_scaffold75625_2	non-phage	0
NOF012_scaffold78046_1	non-phage	0
NOF012_scaffold10353_23	non-phage	0
NOF012_scaffold76044_3	non-phage	0
NOF012_scaffold4901_1	non-phage	0
NOF012_scaffold76672_1	non-phage	0
NOF012_scaffold5305_10	non-phage	0
NOF012_scaffold34_9	non-phage	0
NOF012_scaffold50856_4	non-phage	0
NOF012_scaffold68110_4	non-phage	0
NOF012_scaffold4667_15	non-phage	0
NOF012_scaffold44041_4	non-phage	0
NOF012_scaffold34940_10	non-phage	0

NOF012_scaffold26305_8	non-phage	0
NOF012_scaffold33308_2	non-phage	0
NOF012_scaffold21713_1	non-phage	0
NOF012_scaffold35709_1	non-phage	0
NOF012_scaffold34940_19	non-phage	0
NOF012_scaffold10353_25	non-phage	0
NOF012_scaffold8245_9	non-phage	0
NOF012_scaffold25882_15	non-phage	0
NOF012_scaffold76452_1	non-phage	0
NOF012_scaffold37987_2	non-phage	0
NOF012_scaffold77873_4	non-phage	0
NOF012_scaffold38179_2	non-phage	0
NOF012_scaffold65712_2	non-phage	0
NOF012_scaffold74162_1	non-phage	0
NOF012_scaffold78084_1	non-phage	0
NOF012_scaffold7210_8	non-phage	0
NOF012_scaffold71874_4	non-phage	0
NOF012_scaffold52260_1	non-phage	0
NOF012_scaffold62452_3	non-phage	0
NOF012_scaffold72304_1	non-phage	0
NOF012_scaffold5820_4	non-phage	0
NOF012_scaffold13516_1	non-phage	0
NOF012_C833745_1	non-phage	0
NOF012_scaffold36268_14	non-phage	0
NOF012_scaffold73007_1	non-phage	0
NOF013_scaffold6283_6	non-phage	0
NOF013_scaffold23244_1	non-phage	0
NOF013_scaffold549_8	non-phage	0
NOF013_scaffold32425_4	non-phage	0
NOF013_scaffold22504_1	non-phage	0
NOF013_scaffold18051_1	non-phage	0
NOF013_scaffold7004_3	non-phage	0
NOF013_scaffold4522_8	non-phage	0
NOF013_scaffold1824_1	non-phage	0
NOF013_scaffold32744_9	non-phage	0
NOF013_scaffold15195_3	non-phage	0
NOF013_scaffold207_3	non-phage	0
NOF013_scaffold4163_1	non-phage	0
NOF013_scaffold9858_1	non-phage	0
NOF013_scaffold34236_4	non-phage	0
NOF013_scaffold6873_5	non-phage	0
NOF013_scaffold34613_1	non-phage	0
NOF013_scaffold9614_4	non-phage	0
NOF013_scaffold13792_2	non-phage	0
NOF013_scaffold21414_7	non-phage	0
NOF013_scaffold2000_2	non-phage	0

NOF013_scaffold15198_9	non-phage	0
NOF013_scaffold31992_2	non-phage	0
NOF013_scaffold22526_3	non-phage	0
NOF013_scaffold34720_1	non-phage	0
NOF013_scaffold11455_12	non-phage	0
NOF013_scaffold9606_10	non-phage	0
NOF013_scaffold4687_3	non-phage	0
NOF013_scaffold34240_1	non-phage	0
NOF013_scaffold5587_3	non-phage	0
NOF013_scaffold156_1	non-phage	0
NOF013_scaffold30552_2	non-phage	0
NOF013_scaffold21741_3	non-phage	0
NOF013_scaffold17342_1	non-phage	0
NOF013_scaffold28410_1	non-phage	0
NOF013_C612924_1	non-phage	0
NOF013_scaffold27529_2	non-phage	0
NOF013_scaffold34716_2	non-phage	0
NOF013_scaffold33220_1	non-phage	0
NOF013_scaffold1671_2	non-phage	0
NOF013_scaffold10268_2	non-phage	0
NOF013_scaffold31065_1	non-phage	0
NOF013_scaffold13801_9	non-phage	0
NOF013_scaffold28158_2	non-phage	0
NOF013_scaffold22841_1	non-phage	0
NOF013_scaffold33428_1	non-phage	0
NOF013_scaffold30723_2	non-phage	0
NOF013_scaffold34467_1	non-phage	0
NOF013_scaffold34371_2	non-phage	0
NOF013_scaffold20587_8	non-phage	0
NOF013_scaffold295_2	non-phage	0
NOF013_scaffold31567_1	non-phage	0
NOF013_scaffold12415_1	non-phage	0
NOF013_scaffold22054_2	non-phage	0
NOF013_scaffold13658_5	non-phage	0
NOF013_scaffold34664_1	non-phage	0
NOF013_scaffold27079_1	non-phage	0
NOF013_scaffold18354_2	non-phage	0
NOF013_scaffold32997_2	non-phage	0
NOF013_scaffold27862_2	non-phage	0
NOF013_scaffold25581_1	non-phage	0
NOF013_scaffold20514_3	non-phage	0
NOF013_scaffold24548_1	non-phage	0
NOF013_scaffold1824_4	non-phage	0
NOF013_scaffold18366_2	non-phage	0
NOF013_scaffold6283_10	non-phage	0
NOF013_scaffold32325_1	non-phage	0

NOF013_scaffold20623_1	non-phage	0
NOF013_scaffold28714_3	non-phage	0
NOF013_scaffold32184_1	non-phage	0
NOF013_scaffold16643_1	non-phage	0
NOF013_scaffold19716_2	non-phage	0
NOF013_scaffold21269_2	non-phage	0
NOF013_scaffold34689_2	non-phage	0
NOF013_scaffold3982_3	non-phage	0
NOF013_scaffold9614_1	non-phage	0
NOF013_scaffold34610_2	non-phage	0
NOF013_scaffold12051_2	non-phage	0
NOF013_scaffold6873_2	non-phage	0
NOF013_scaffold32132_1	non-phage	0
NOF013_scaffold8034_1	non-phage	0
NOF013_scaffold18979_1	non-phage	0
NOF013_scaffold6737_10	non-phage	0
NOF013_scaffold9518_2	non-phage	0
NOF013_scaffold14806_1	non-phage	0
NOF013_scaffold6626_1	non-phage	0
NOF013_scaffold24837_2	non-phage	0
NOF013_scaffold34485_3	non-phage	0
NOF013_scaffold14556_5	non-phage	0
NOF013_scaffold27649_3	non-phage	0
NOF013_scaffold26458_2	non-phage	0
NOF013_scaffold5920_4	non-phage	0
NOF013_scaffold4136_17	non-phage	0
NOF013_scaffold549_37	non-phage	0
NOF013_scaffold18354_1	non-phage	0
NOF013_scaffold1440_2	non-phage	0
NOF013_scaffold4136_30	non-phage	0
NOF013_scaffold29602_2	non-phage	0
NOF013_scaffold32848_4	non-phage	0
NOF013_scaffold15010_3	non-phage	0
NOF013_scaffold1008_38	non-phage	0
NOF013_scaffold15092_4	non-phage	0
NOF013_scaffold12431_1	non-phage	0
NOF013_scaffold26624_4	non-phage	0
NOF013_scaffold14842_9	non-phage	0
NOF013_scaffold31503_8	non-phage	0
NOF013_scaffold1008_31	non-phage	0
NOF013_scaffold26593_1	non-phage	0
NOF013_scaffold21793_9	non-phage	0
NOF013_scaffold32786_2	non-phage	0
NOF013_scaffold34490_1	non-phage	0
NOF013_scaffold3570_7	non-phage	0
NOF013_scaffold22627_8	non-phage	0

NOF013_scaffold25724_6	non-phage	0
NOF013_scaffold11912_1	non-phage	0
NOF013_scaffold2948_6	non-phage	0
NOF013_scaffold19100_1	non-phage	0
NOF013_scaffold14609_8	non-phage	0
NOF013_scaffold24672_2	non-phage	0
NOF013_scaffold29281_1	non-phage	0
NOF013_scaffold4136_3	non-phage	0
NOF013_scaffold6162_5	non-phage	0
NOF013_scaffold22390_2	non-phage	0
NOF013_scaffold2331_2	non-phage	0
NOF013_scaffold22526_4	non-phage	0
NOF013_scaffold3933_3	non-phage	0
NOF013_scaffold25507_1	non-phage	0
NOF013_scaffold12144_3	non-phage	0
NOF013_scaffold27080_2	non-phage	0
NOF013_scaffold8658_6	non-phage	0
NOF013_scaffold27004_2	non-phage	0
NOF013_scaffold13890_3	non-phage	0
NOF013_scaffold11874_2	non-phage	0
NOF013_scaffold6723_6	non-phage	0
NOF013_scaffold31083_2	non-phage	0
NOF013_scaffold34411_1	non-phage	0
NOF013_scaffold34653_1	non-phage	0
NOF013_scaffold20523_13	non-phage	0
NOF013_scaffold4136_12	non-phage	0
NOF013_scaffold12473_2	non-phage	0
NOF013_scaffold32250_3	non-phage	0
NOF013_scaffold24672_1	non-phage	0
NOF013_scaffold23862_1	non-phage	0
NOF013_scaffold9279_1	non-phage	0
NOF013_scaffold25294_2	non-phage	0
NOF013_scaffold25409_5	non-phage	0
NOF013_scaffold101_10	non-phage	0
NOF013_scaffold15303_1	non-phage	0
NOF013_scaffold124_4	non-phage	0
NOF013_scaffold34317_2	non-phage	0
NOF013_scaffold8559_3	non-phage	0
NOF013_scaffold4136_4	non-phage	0
NOF013_scaffold12217_1	non-phage	0
NOF013_scaffold32232_2	non-phage	0
NOF013_scaffold12910_2	non-phage	0
NOF013_scaffold23413_3	non-phage	0
NOF013_scaffold15031_4	non-phage	0
NOF013_scaffold34703_1	non-phage	0
NOF013_scaffold181_5	non-phage	0

NOF013_scaffold17652_4	non-phage	0
NOF013_scaffold9983_26	non-phage	0
NOF013_scaffold25635_2	non-phage	0
NOF013_scaffold118_3	non-phage	0
NOF013_scaffold15198_5	non-phage	0
NOF013_scaffold29929_1	non-phage	0
NOF013_scaffold21403_4	non-phage	0
NOF013_scaffold2_2	non-phage	0
NOF013_scaffold30223_1	non-phage	0
NOF013_scaffold33799_4	non-phage	0
NOF013_scaffold34340_1	non-phage	0
NOF013_scaffold12217_2	non-phage	0
NOF013_scaffold25712_2	non-phage	0
NOF013_scaffold16172_1	non-phage	0
NOF013_scaffold3933_1	non-phage	0
NOF013_scaffold30333_1	non-phage	0
NOF013_scaffold13815_2	non-phage	0
NOF013_scaffold6800_2	non-phage	0
NOF013_scaffold31992_1	non-phage	0
NOF013_scaffold7233_5	non-phage	0
NOF013_scaffold34609_1	non-phage	0
NOF013_scaffold13412_2	non-phage	0
NOF013_scaffold18354_5	non-phage	0
NOF013_scaffold34063_1	non-phage	0
NOF013_scaffold34673_1	non-phage	0
NOF013_scaffold30261_1	non-phage	0
NOF013_scaffold34691_1	non-phage	0
NOF013_scaffold26356_1	non-phage	0
NOF013_scaffold22800_2	non-phage	0
NOF013_scaffold11861_1	non-phage	0
NOF013_scaffold34329_2	non-phage	0
NOF013_scaffold741_1	non-phage	0
NOF013_scaffold3526_2	non-phage	0
NOF013_scaffold20673_4	non-phage	0
NOF013_scaffold34377_3	non-phage	0
NOF013_scaffold31272_3	non-phage	0
NOF013_scaffold9132_1	non-phage	0
NOF013_scaffold10902_2	non-phage	0
NOF013_scaffold19767_4	non-phage	0
NOF013_scaffold27491_3	non-phage	0
NOF013_scaffold28785_2	non-phage	0
NOF013_scaffold34498_1	non-phage	0
NOF013_scaffold20791_3	non-phage	0
NOF013_scaffold19092_5	non-phage	0
NOF013_scaffold34693_1	non-phage	0
NOF013_scaffold17006_2	non-phage	0

NOF013_scaffold300_2	non-phage	0
NOF013_scaffold15887_1	non-phage	0
NOF013_scaffold24441_1	non-phage	0
NOF013_scaffold15348_4	non-phage	0
NOF013_scaffold29375_1	non-phage	0
NOF013_scaffold33781_1	non-phage	0
NOF013_scaffold34438_1	non-phage	0
NOF013_scaffold2331_7	non-phage	0
NOF013_scaffold4457_4	non-phage	0
NOF013_scaffold23057_2	non-phage	0
NOF013_scaffold33887_1	non-phage	0
NOF013_scaffold13923_6	non-phage	0
NOF013_scaffold14974_1	non-phage	0
NOF013_scaffold28221_2	non-phage	0
NOF013_scaffold14202_1	non-phage	0
NOF013_scaffold15223_3	non-phage	0
NOF013_scaffold30529_2	non-phage	0
NOF013_scaffold27848_1	non-phage	0
NOF013_scaffold34328_1	non-phage	0
NOF013_scaffold3099_32	non-phage	0
NOF013_scaffold33771_1	non-phage	0
NOF013_scaffold10416_13	non-phage	0
NOF013_scaffold30366_1	non-phage	0
NOF013_scaffold11918_5	non-phage	0
NOF013_scaffold34687_1	non-phage	0
NOF013_C613102_1	non-phage	0
NOF013_scaffold27638_1	non-phage	0
NOF013_scaffold24859_2	non-phage	0
NOF013_scaffold34585_4	non-phage	0
NOF013_scaffold6723_5	non-phage	0
NOF013_scaffold27491_2	non-phage	0
NOF013_scaffold1824_5	non-phage	0
NOF013_scaffold15195_4	non-phage	0
NOF013_scaffold28021_2	non-phage	0
NOF013_scaffold7705_11	non-phage	0
NOF013_scaffold25492_1	non-phage	0
NOF013_scaffold32152_2	non-phage	0
NOF013_scaffold31678_1	non-phage	0
NOF013_scaffold22878_2	non-phage	0
NOF013_scaffold34626_2	non-phage	0
NOF013_scaffold2329_5	non-phage	0
NOF013_scaffold34656_3	non-phage	0
NOF013_scaffold5970_1	non-phage	0
NOF013_scaffold34725_1	non-phage	0
NOF013_scaffold34449_1	non-phage	0
NOF013_scaffold24441_2	non-phage	0

NOF013_scaffold14025_3	non-phage	0
NOF013_scaffold25492_2	non-phage	0
NOF013_scaffold17248_3	non-phage	0
NOF013_scaffold12787_17	non-phage	0
NOF013_scaffold27819_1	non-phage	0
NOF013_scaffold3782_1	non-phage	0
NOF013_scaffold34367_2	non-phage	0
NOF013_scaffold7123_6	non-phage	0
NOF013_scaffold11484_2	non-phage	0
NOF013_scaffold29818_1	non-phage	0
NOF014_scaffold14195_3	non-phage	0
NOF014_scaffold1450_2	non-phage	0
NOF014_scaffold22763_2	non-phage	0
NOF014_scaffold5936_7	non-phage	0
NOF014_scaffold58520_1	non-phage	0
NOF014_scaffold50760_3	non-phage	0
NOF014_scaffold62324_1	non-phage	0
NOF014_scaffold13862_8	non-phage	0
NOF014_scaffold39661_3	non-phage	0
NOF014_scaffold26105_24	non-phage	0
NOF014_scaffold61702_1	non-phage	0
NOF014_scaffold22659_12	non-phage	0
NOF014_scaffold45416_1	non-phage	0
NOF014_scaffold22659_7	non-phage	0
NOF014_scaffold19798_4	non-phage	0
NOF014_scaffold348_3	non-phage	0
NOF014_scaffold59308_1	non-phage	0
NOF014_scaffold72236_1	non-phage	0
NOF014_scaffold29595_3	non-phage	0
NOF014_scaffold70923_1	non-phage	0
NOF014_scaffold72195_1	non-phage	0
NOF014_scaffold67850_2	non-phage	0
NOF014_scaffold20894_1	non-phage	0
NOF014_scaffold259_2	non-phage	0
NOF014_scaffold64990_3	non-phage	0
NOF014_scaffold15172_1	non-phage	0
NOF014_scaffold5889_9	non-phage	0
NOF014_scaffold17690_18	non-phage	0
NOF014_scaffold64313_1	non-phage	0
NOF014_scaffold63512_3	non-phage	0
NOF014_scaffold18883_6	non-phage	0
NOF014_scaffold156_2	non-phage	0
NOF014_scaffold20265_4	non-phage	0
NOF014_scaffold71685_1	non-phage	0
NOF014_scaffold21583_4	non-phage	0
NOF014_scaffold46502_3	non-phage	0

NOF014_scaffold48917_3	non-phage	0
NOF014_scaffold8889_4	non-phage	0
NOF014_scaffold38062_13	non-phage	0
NOF014_scaffold56907_2	non-phage	0
NOF014_scaffold71440_2	non-phage	0
NOF014_scaffold72090_1	non-phage	0
NOF014_scaffold6172_3	non-phage	0
NOF014_scaffold68495_2	non-phage	0
NOF014_scaffold58833_4	non-phage	0
NOF014_scaffold40001_8	non-phage	0
NOF014_scaffold22318_6	non-phage	0
NOF014_scaffold249_3	non-phage	0
NOF014_scaffold45222_3	non-phage	0
NOF014_scaffold70298_1	non-phage	0
NOF014_scaffold15856_10	non-phage	0
NOF014_scaffold55387_4	non-phage	0
NOF014_scaffold49291_1	non-phage	0
NOF014_scaffold58144_1	non-phage	0
NOF014_scaffold20686_3	non-phage	0
NOF014_scaffold17093_5	non-phage	0
NOF014_scaffold588_3	non-phage	0
NOF014_scaffold52981_4	non-phage	0
NOF014_scaffold35183_7	non-phage	0
NOF014_scaffold20586_1	non-phage	0
NOF014_scaffold71853_1	non-phage	0
NOF014_scaffold54232_2	non-phage	0
NOF014_scaffold54590_3	non-phage	0
NOF014_scaffold69341_1	non-phage	0
NOF014_C988560_1	non-phage	0
NOF014_scaffold69436_3	non-phage	0
NOF014_scaffold77_5	non-phage	0
NOF014_scaffold71448_3	non-phage	0
NOF014_scaffold25574_6	non-phage	0
NOF014_scaffold17690_8	non-phage	0
NOF014_scaffold62442_1	non-phage	0
NOF014_scaffold65667_2	non-phage	0
NOF014_scaffold13844_6	non-phage	0
NOF014_scaffold55730_1	non-phage	0
NOF014_C988864_1	non-phage	0
NOF014_scaffold27100_1	non-phage	0
NOF014_scaffold55711_4	non-phage	0
NOF014_scaffold56750_1	non-phage	0
NOF014_scaffold41027_20	non-phage	0
NOF014_scaffold4528_6	non-phage	0
NOF014_scaffold19852_19	non-phage	0
NOF014_scaffold47374_1	non-phage	0

NOF014_scaffold16488_6	non-phage	0
NOF014_scaffold13658_4	non-phage	0
NOF014_scaffold135_4	non-phage	0
NOF014_scaffold28268_1	non-phage	0
NOF014_scaffold48431_2	non-phage	0
NOF014_scaffold70945_1	non-phage	0
NOF014_scaffold168_8	non-phage	0
NOF014_scaffold55348_7	non-phage	0
NOF014_scaffold58249_2	non-phage	0
NOF014_scaffold41027_22	non-phage	0
NOF014_scaffold51886_3	non-phage	0
NOF014_scaffold13460_2	non-phage	0
NOF014_scaffold178_1	non-phage	0
NOF014_scaffold12725_5	non-phage	0
NOF014_scaffold2358_3	non-phage	0
NOF014_scaffold6908_4	non-phage	0
NOF014_scaffold61106_2	non-phage	0
NOF014_scaffold52365_1	non-phage	0
NOF014_scaffold64544_1	non-phage	0
NOF014_scaffold27848_6	non-phage	0
NOF014_scaffold4327_46	non-phage	0
NOF014_scaffold34078_16	non-phage	0
NOF014_scaffold26999_4	non-phage	0
NOF014_scaffold13844_2	non-phage	0
NOF014_scaffold72297_1	non-phage	0
NOF014_C988610_1	non-phage	0
NOF014_scaffold37901_2	non-phage	0
NOF014_scaffold61702_3	non-phage	0
NOF014_scaffold72005_1	non-phage	0
NOF014_scaffold14436_5	non-phage	0
NOF014_scaffold62387_1	non-phage	0
NOF014_scaffold64990_4	non-phage	0
NOF014_scaffold71248_2	non-phage	0
NOF014_scaffold251_1	non-phage	0
NOF014_scaffold19798_2	non-phage	0
NOF014_scaffold52730_2	non-phage	0
NOF014_scaffold8_3	non-phage	0
NOF014_scaffold35087_1	non-phage	0
NOF014_scaffold30731_2	non-phage	0
NOF014_scaffold37156_25	non-phage	0
NOF014_scaffold13862_11	non-phage	0
NOF014_scaffold71440_1	non-phage	0
NOF014_scaffold71432_1	non-phage	0
NOF014_scaffold52931_5	non-phage	0
NOF014_scaffold28449_2	non-phage	0
NOF014_scaffold11916_14	non-phage	0

NOF014_scaffold14195_15	non-phage	0
NOF014_scaffold54140_3	non-phage	0
NOF014_scaffold41282_1	non-phage	0
NOF014_scaffold71815_1	non-phage	0
NOF014_scaffold31350_4	non-phage	0
NOF014_scaffold16955_21	non-phage	0
NOF014_scaffold31065_4	non-phage	0
NOF014_scaffold55560_2	non-phage	0
NOF014_scaffold4516_5	non-phage	0
NOF014_scaffold34399_1	non-phage	0
NOF014_scaffold65718_1	non-phage	0
NOF014_scaffold46512_1	non-phage	0
NOF014_scaffold14628_14	non-phage	0
NOF014_scaffold54976_8	non-phage	0
NOF014_scaffold54136_22	non-phage	0
NOF014_scaffold33173_4	non-phage	0
NOF014_C988806_1	non-phage	0
DLF001_scaffold7821_1	non-phage	0
DLF001_scaffold25175_4	non-phage	0
DLF001_scaffold11273_8	non-phage	0
DLF001_scaffold1657_3	non-phage	0
DLF001_scaffold9142_14	non-phage	0
DLF001_scaffold21615_3	non-phage	0
DLF001_scaffold26043_2	non-phage	0
DLF001_scaffold11273_1	non-phage	0
DLF001_scaffold25804_6	non-phage	0
DLF001_scaffold18979_1	non-phage	0
DLF001_scaffold22435_1	non-phage	0
DLF001_scaffold25843_5	phage	0.998
DLF001_scaffold1440_40	non-phage	0
DLF001_scaffold42_2	non-phage	0
DLF001_scaffold5770_1	non-phage	0
DLF001_scaffold5299_1	non-phage	0
DLF001_scaffold26019_2	non-phage	0
DLF001_scaffold23412_1	non-phage	0
DLF001_scaffold22895_2	non-phage	0
DLF001_scaffold16955_5	non-phage	0
DLF001_scaffold1209_2	non-phage	0
DLF001_scaffold24721_1	non-phage	0
DLF001_scaffold19117_1	non-phage	0
DLF001_scaffold6749_2	non-phage	0
DLF001_scaffold3540_2	non-phage	0
DLF001_scaffold20624_2	non-phage	0
DLF001_scaffold22319_1	non-phage	0
DLF001_scaffold1894_6	non-phage	0
DLF001_scaffold3241_27	non-phage	0

DLF001_scaffold2186_1	non-phage	0
DLF001_scaffold9005_12	non-phage	0
DLF001_scaffold7305_34	non-phage	0
DLF001_scaffold8851_1	non-phage	0
DLF001_scaffold25172_1	non-phage	0
DLF001_scaffold19421_10	non-phage	0
DLF001_scaffold6700_2	non-phage	0
DLF001_scaffold25908_3	non-phage	0
DLF001_scaffold13529_2	non-phage	0
DLF001_scaffold25938_1	non-phage	0
DLF001_scaffold18812_1	non-phage	0
DLF001_scaffold2174_1	non-phage	0
DLF001_scaffold308_8	non-phage	0
DLF001_scaffold55_6	non-phage	0
DLF001_scaffold26040_1	non-phage	0
DLF001_scaffold26042_1	non-phage	0
DLF001_scaffold717_35	non-phage	0
DLF001_scaffold24839_3	non-phage	0
DLF001_scaffold11273_3	non-phage	0
DLF001_scaffold7839_29	non-phage	0
DLF001_scaffold6028_2	phage	0.995
DLF001_scaffold2572_25	non-phage	0
DLF001_scaffold35_16	non-phage	0
DLF001_scaffold9142_4	non-phage	0
DLF001_scaffold25793_1	non-phage	0
DLF001_scaffold3621_25	non-phage	0
DLF001_scaffold2651_3	non-phage	0
DLF001_scaffold24991_1	non-phage	0
DLF001_scaffold39_26	non-phage	0
DLF001_scaffold26022_1	non-phage	0
DLF001_scaffold14359_3	non-phage	0
DLF001_scaffold1831_3	non-phage	0
DLF001_scaffold18097_8	non-phage	0
DLF001_scaffold26029_3	non-phage	0
DLF001_scaffold4219_1	non-phage	0
DLF001_scaffold24223_2	non-phage	0
DLF001_scaffold8374_1	non-phage	0
DLF001_scaffold25759_1	non-phage	0
DLF001_scaffold2688_12	non-phage	0
DLF001_scaffold765_15	non-phage	0
DLF001_scaffold21119_1	non-phage	0
DLF001_scaffold2809_38	non-phage	0
DLF001_scaffold16878_1	phage	0.999
DLF001_scaffold38_7	non-phage	0
DLF001_scaffold25460_2	non-phage	0
DLF001_scaffold24474_14	non-phage	0

DLF001_scaffold25832_1	non-phage	0
DLF001_scaffold11332_1	non-phage	0
DLF001_scaffold25215_1	non-phage	0
DLF001_scaffold42_4	non-phage	0
DLF001_scaffold25830_1	non-phage	0
DLF001_scaffold717_19	non-phage	0
DLF001_scaffold5092_1	non-phage	0
DLF001_scaffold25923_2	non-phage	0
DLF001_scaffold25942_4	non-phage	0
DLF001_scaffold23211_2	non-phage	0
DLF001_scaffold16237_2	non-phage	0
DLF001_scaffold825_4	non-phage	0
DLF001_scaffold7013_9	non-phage	0
DLF001_scaffold24722_1	non-phage	0
DLF001_scaffold1440_27	non-phage	0
DLF001_scaffold19903_3	non-phage	0
DLF001_scaffold25777_2	phage	1
DLF001_scaffold3241_25	non-phage	0
DLF001_scaffold6699_2	non-phage	0
DLF001_scaffold25336_3	non-phage	0
DLF001_scaffold7839_13	non-phage	0.207
DLF001_scaffold18097_6	non-phage	0
DLF001_scaffold19264_2	non-phage	0
DLF001_scaffold9543_8	non-phage	0
DLF001_scaffold21828_1	non-phage	0
DLF001_scaffold14880_1	non-phage	0
DLF001_scaffold18717_1	non-phage	0
DLF001_scaffold13_15	non-phage	0
DLF001_scaffold25900_1	non-phage	0
DLF001_scaffold4496_30	non-phage	0
DLF001_scaffold2277_1	non-phage	0
DLF001_scaffold7090_1	non-phage	0
DLF001_scaffold15499_1	non-phage	0
DLF001_scaffold22418_1	non-phage	0
DLF001_scaffold3621_2	non-phage	0
DLF001_scaffold25854_2	non-phage	0
DLF001_scaffold26029_1	non-phage	0
DLF001_scaffold26009_1	non-phage	0
DLF001_scaffold8082_2	non-phage	0
DLF001_scaffold1831_5	non-phage	0
DLF001_scaffold18097_2	non-phage	0
DLF001_scaffold24840_2	non-phage	0
DLF001_scaffold17506_1	non-phage	0
DLF001_scaffold25095_2	non-phage	0
DLF001_scaffold11813_6	non-phage	0
DLF001_scaffold2174_3	non-phage	0

DLF001_scaffold11274_4	non-phage	0
DLF001_scaffold9880_3	non-phage	0
DLF001_scaffold19421_2	non-phage	0
DLF001_scaffold80_18	non-phage	0
DLF001_scaffold4002_18	non-phage	0
DLF001_scaffold14_2	non-phage	0
DLF001_scaffold19612_4	non-phage	0
DLF001_scaffold20624_10	non-phage	0
DLF001_scaffold8992_20	non-phage	0
DLF001_scaffold75_17	non-phage	0
DLF001_scaffold26039_2	non-phage	0
DLF001_scaffold16955_3	non-phage	0
DLF001_scaffold2560_1	non-phage	0
DLF001_scaffold1411_6	non-phage	0
DLF001_scaffold5935_1	non-phage	0
DLF001_scaffold3285_4	non-phage	0
DLF001_scaffold717_22	non-phage	0
DLF001_scaffold25941_1	non-phage	0
DLF001_scaffold4836_9	phage	0.941
DLF001_scaffold8487_2	non-phage	0
DLF001_scaffold9142_1	non-phage	0
DLF001_scaffold24605_3	non-phage	0
DLF001_scaffold4084_1	non-phage	0
DLF001_scaffold22267_1	non-phage	0
DLF001_scaffold13003_15	non-phage	0
DLF001_scaffold17074_3	non-phage	0
DLF001_scaffold43_1	non-phage	0
DLF001_scaffold12258_1	non-phage	0
DLF001_C323130_1	non-phage	0
DLF001_scaffold25914_1	non-phage	0
DLF001_scaffold12148_16	non-phage	0
DLF001_scaffold4799_2	non-phage	0
DLF001_scaffold24964_1	non-phage	0
DLF001_scaffold25958_1	non-phage	0
DLF001_scaffold11266_3	non-phage	0
DLF001_scaffold24400_6	non-phage	0
DLF001_scaffold3736_2	non-phage	0
DLF001_scaffold4223_5	non-phage	0
DLF001_scaffold4153_4	non-phage	0
DLF001_scaffold9005_5	non-phage	0
DLF001_scaffold8766_5	non-phage	0
DLF001_scaffold20836_1	non-phage	0
DLF001_scaffold15493_3	non-phage	0
DLF001_scaffold76_1	non-phage	0
DLF001_scaffold9563_1	non-phage	0
DLF001_scaffold41_3	non-phage	0

DLF001_scaffold25336_1	non-phage	0
DLF001_scaffold4832_1	non-phage	0
DLF001_scaffold20196_8	non-phage	0
DLF001_scaffold4132_5	non-phage	0
DLF001_scaffold5723_3	non-phage	0
DLF001_scaffold11416_1	non-phage	0
DLF001_scaffold20136_3	non-phage	0
DLF001_scaffold9901_3	non-phage	0
DLF001_C323136_1	non-phage	0
DLF001_scaffold38_3	non-phage	0
DLF001_scaffold21502_2	non-phage	0
DLF001_scaffold2215_33	non-phage	0
DLF001_scaffold15747_3	non-phage	0
DLF001_scaffold9018_44	non-phage	0
DLF001_scaffold5135_3	non-phage	0
DLF001_scaffold9142_7	non-phage	0
DLF001_scaffold7386_4	non-phage	0
DLF001_scaffold75_27	non-phage	0
DLF001_scaffold11546_1	non-phage	0
DLF001_scaffold24839_1	non-phage	0
DLF001_scaffold26017_1	non-phage	0
DLF001_scaffold1872_24	non-phage	0
DLF001_scaffold4545_3	non-phage	0
DLF001_scaffold25484_1	non-phage	0
DLF001_scaffold7_9	non-phage	0
DLF001_scaffold18104_5	non-phage	0
DLF001_scaffold6571_45	non-phage	0
DLF001_scaffold21873_1	non-phage	0
DLF001_scaffold77_12	non-phage	0
DLF001_scaffold3894_1	non-phage	0
DLF001_scaffold22_5	non-phage	0
DLF001_scaffold20617_4	non-phage	0
DLF001_scaffold17078_6	non-phage	0
DLF001_scaffold4084_21	non-phage	0
DLF001_scaffold25097_4	non-phage	0
DLF001_scaffold25768_1	non-phage	0
DLF001_scaffold16955_1	non-phage	0
DLF001_scaffold20591_2	non-phage	0
DLF001_scaffold3102_2	non-phage	0
DLF001_scaffold23823_1	non-phage	0
DLF001_scaffold25942_2	non-phage	0
DLF001_scaffold24840_4	non-phage	0
DLF001_scaffold17335_1	non-phage	0
DLF001_C323572_1	non-phage	0
DLF001_scaffold7350_1	non-phage	0
DLF001_scaffold2572_20	non-phage	0

DLF001_scaffold23418_1	non-phage	0
DLF001_scaffold25175_2	non-phage	0
DLF001_scaffold25746_4	non-phage	0
DLF001_scaffold19164_1	non-phage	0
DLF001_scaffold1410_23	non-phage	0
DLF001_scaffold16608_1	phage	0.996
DLF001_scaffold20624_8	non-phage	0
DLF001_scaffold23779_2	non-phage	0
DLF001_scaffold20675_1	non-phage	0
DLF001_scaffold4223_7	non-phage	0
DLF001_scaffold18617_11	phage	0.997
DLF001_scaffold6205_3	non-phage	0
DLF001_scaffold21842_2	non-phage	0
DLF001_scaffold14411_1	non-phage	0
DLF001_scaffold12293_1	non-phage	0
DLF001_scaffold39_21	non-phage	0
DLF001_scaffold24376_1	phage	0.623
DLF001_scaffold24609_1	non-phage	0
DLF001_scaffold3737_26	non-phage	0
DLF001_scaffold17628_6	non-phage	0
DLF001_scaffold1570_1	phage	0.998
DLF001_scaffold17143_9	non-phage	0
DLF001_scaffold19366_3	non-phage	0
DLF001_scaffold12212_1	non-phage	0
DLF001_scaffold25414_1	non-phage	0
DLF001_scaffold22978_2	non-phage	0
DLF001_scaffold2850_1	non-phage	0
DLF001_scaffold28_9	non-phage	0
DLF001_scaffold26006_1	non-phage	0
DLF001_scaffold15135_1	non-phage	0
DLF001_scaffold7839_17	non-phage	0
DLF001_scaffold25505_3	non-phage	0
DLF001_scaffold20136_1	non-phage	0
DLF001_scaffold25973_1	non-phage	0
DLF001_scaffold4132_10	non-phage	0
DLF001_scaffold23776_3	non-phage	0
DLF001_scaffold13_55	non-phage	0
DLF001_scaffold12293_5	non-phage	0
DLF001_C323518_1	non-phage	0
DLF001_scaffold41_1	non-phage	0
DLF001_scaffold26033_1	non-phage	0.003
DLF001_scaffold9159_7	non-phage	0
DLF001_scaffold18104_1	non-phage	0
DLF001_scaffold25746_3	non-phage	0
DLF001_scaffold15652_1	non-phage	0
DLF001_scaffold4153_1	non-phage	0

DLF001_scaffold22791_2	non-phage	0
DLF001_scaffold35_25	non-phage	0
DLF001_scaffold23956_1	non-phage	0
DLF001_scaffold26045_1	non-phage	0
DLF001_scaffold1571_1	non-phage	0
DLF001_scaffold9142_12	non-phage	0
DLF001_scaffold7501_3	non-phage	0
DLF001_scaffold22903_3	non-phage	0
DLF001_scaffold11273_6	non-phage	0
DLF001_scaffold20939_1	phage	1
DLF001_scaffold25982_1	non-phage	0
DLF001_scaffold5135_1	non-phage	0
DLF001_scaffold21798_3	non-phage	0
DLF001_scaffold24310_2	non-phage	0
DLF001_scaffold9668_14	non-phage	0
DLF001_scaffold3102_11	non-phage	0
DLF001_scaffold4799_6	non-phage	0
DLF001_scaffold11189_3	non-phage	0
DLF001_scaffold7501_1	non-phage	0
DLF001_scaffold4330_2	non-phage	0
DLF001_scaffold17335_3	non-phage	0
DLF001_scaffold2101_1	non-phage	0
DLF001_scaffold25840_2	non-phage	0
DLF001_scaffold18018_5	non-phage	0
DLF001_scaffold4653_19	non-phage	0
DLF001_scaffold2572_22	non-phage	0
DLF001_scaffold939_3	non-phage	0
DLF001_scaffold25843_4	non-phage	0
DLF001_scaffold26048_1	non-phage	0
DLF001_scaffold38_1	non-phage	0
DLF001_scaffold20892_3	non-phage	0
DLF001_scaffold25987_3	non-phage	0
DLF001_scaffold25097_2	non-phage	0
DLF001_scaffold23009_1	non-phage	0
DLF001_scaffold20617_2	non-phage	0
DLF001_scaffold7386_2	non-phage	0
DLF001_scaffold18894_3	non-phage	0
DLF001_scaffold20220_2	non-phage	0
DLF001_scaffold24230_1	non-phage	0
DLF001_scaffold54_1	non-phage	0
DLF001_scaffold5683_1	non-phage	0
DLF001_scaffold2688_8	non-phage	0
DLF001_scaffold18617_13	non-phage	0
DLF001_scaffold16_24	non-phage	0
DLF001_C323420_1	non-phage	0
DLF001_scaffold10899_1	phage	0.999

DLF001_scaffold717_4	non-phage	0
DLF001_scaffold25983_2	non-phage	0
DLF001_scaffold19432_1	non-phage	0
DLF001_scaffold4836_15	non-phage	0
DLF001_scaffold39_17	non-phage	0
DLF001_scaffold19334_15	non-phage	0
DLF001_scaffold7501_2	non-phage	0
DLF001_scaffold11971_2	non-phage	0
DLF001_scaffold16355_1	non-phage	0
DLF001_scaffold9142_5	non-phage	0
DLF001_scaffold26032_1	non-phage	0
DLF001_scaffold6700_3	non-phage	0
DLF001_scaffold10872_1	phage	0.998
DLF001_scaffold20253_1	non-phage	0
DLF001_scaffold13529_3	non-phage	0
DLF001_scaffold9901_16	non-phage	0
DLF001_scaffold25921_1	non-phage	0
DLF001_scaffold1378_1	non-phage	0
DLF001_scaffold25490_2	non-phage	0
DLF001_scaffold3102_25	non-phage	0
DLF001_scaffold5528_2	non-phage	0
DLF001_scaffold9297_3	non-phage	0
DLF001_scaffold13_56	non-phage	0
DLF001_scaffold18979_3	non-phage	0
DLF001_scaffold717_34	non-phage	0.001
DLF001_scaffold18097_7	non-phage	0
DLF001_scaffold11428_3	non-phage	0
DLF001_scaffold8487_5	non-phage	0
DLF001_scaffold18195_8	non-phage	0
DLF001_scaffold5910_3	non-phage	0
DLF001_scaffold25999_2	non-phage	0
DLF001_C323436_1	non-phage	0
DLF001_scaffold11273_2	non-phage	0
DLF001_scaffold4002_30	non-phage	0
DLF001_scaffold14990_3	non-phage	0
DLF001_scaffold17106_2	non-phage	0
DLF001_scaffold23009_4	non-phage	0
DLF001_scaffold2850_4	non-phage	0
DLF001_scaffold25912_2	non-phage	0
DLF001_scaffold11189_4	non-phage	0
DLF001_scaffold717_21	non-phage	0
DLF001_scaffold1767_4	non-phage	0
DLF001_scaffold24223_1	non-phage	0
DLF001_scaffold4597_12	non-phage	0
DLF001_scaffold12855_4	non-phage	0
DLF001_scaffold21936_1	non-phage	0

DLF001_scaffold21_8	non-phage	0
DLF001_scaffold4653_31	non-phage	0
DLF001_scaffold22815_1	non-phage	0
DLF001_scaffold13649_4	non-phage	0
DLF001_scaffold7013_10	non-phage	0
DLF001_scaffold2850_2	non-phage	0
DLF001_scaffold25652_2	phage	0.996
DLF001_scaffold20172_2	non-phage	0
DLF001_scaffold24400_4	non-phage	0
DLF001_scaffold7102_4	non-phage	0
DLF001_scaffold5725_2	non-phage	0
DLF001_scaffold22791_3	non-phage	0
DLF001_scaffold1597_6	non-phage	0
DLF001_scaffold5158_10	non-phage	0
DLF001_scaffold26046_1	non-phage	0
DLF001_scaffold809_8	non-phage	0
DLF001_scaffold38_8	non-phage	0
DLF001_scaffold42_3	non-phage	0
DLF001_scaffold2068_2	non-phage	0
DLF001_scaffold25341_2	non-phage	0
DLF001_scaffold4909_1	non-phage	0
DLF001_scaffold9668_2	non-phage	0
DLF001_C323154_1	non-phage	0
DLF001_scaffold18769_1	non-phage	0
DLF001_scaffold45_10	non-phage	0
DLF001_scaffold20939_2	non-phage	0
DLF001_scaffold6765_1	non-phage	0
DLF001_scaffold55_3	non-phage	0
DLF001_scaffold3038_4	non-phage	0
DLF001_scaffold23412_2	non-phage	0
DLF001_scaffold10237_27	non-phage	0
DLF001_scaffold2947_3	non-phage	0
DLF001_scaffold17143_1	non-phage	0
DLF001_scaffold24999_1	non-phage	0
DLF001_scaffold4180_10	non-phage	0
DLF001_scaffold25819_1	non-phage	0
DLF001_scaffold25843_6	non-phage	0
DLF001_scaffold23776_1	non-phage	0
DLF001_scaffold3736_5	non-phage	0
DLF001_scaffold1410_46	non-phage	0
DLF001_scaffold13003_8	non-phage	0
DLF001_scaffold26028_1	non-phage	0
DLF001_scaffold4132_7	non-phage	0
DLF001_scaffold25885_1	non-phage	0
DLF001_scaffold25505_1	non-phage	0
DLF001_scaffold18697_1	non-phage	0

DLF001_scaffold22155_1	non-phage	0
DLF001_scaffold21798_2	phage	0.743
DLF001_scaffold2816_2	non-phage	0
DLF001_C323280_1	phage	0.978
DLF001_scaffold19462_1	non-phage	0
DLF001_scaffold6205_1	non-phage	0
DLF001_scaffold17972_8	non-phage	0
DLF001_scaffold4496_21	non-phage	0
DLF001_scaffold16955_4	non-phage	0
DLF001_scaffold11274_3	non-phage	0
DLF001_scaffold2174_4	non-phage	0
DLF001_scaffold7138_11	non-phage	0
DLF001_scaffold1569_33	non-phage	0
DLF001_scaffold12961_1	non-phage	0
DLF001_scaffold24081_2	non-phage	0
DLF001_scaffold26009_2	non-phage	0
DLF001_scaffold19350_3	non-phage	0
DLF001_scaffold9746_1	non-phage	0
DLF001_scaffold8101_4	non-phage	0
DLF001_scaffold26014_1	non-phage	0
DLF001_scaffold2816_20	non-phage	0
DLF001_scaffold4673_6	non-phage	0
DLF001_scaffold1410_21	non-phage	0
DLF001_scaffold765_19	non-phage	0
DLF001_scaffold4509_2	non-phage	0
DLF001_scaffold3212_3	non-phage	0
DLF001_scaffold9302_3	non-phage	0
DLF001_scaffold19421_11	non-phage	0
DLF001_scaffold2688_6	non-phage	0
DLF001_scaffold10220_3	phage	0.999
DLF001_scaffold16868_1	non-phage	0
DLF001_scaffold2850_5	non-phage	0
DLF001_scaffold7812_1	non-phage	0
DLF001_scaffold4597_24	non-phage	0
DLF001_scaffold19948_2	non-phage	0
DLF001_scaffold22434_1	non-phage	0
DLF001_scaffold25674_1	non-phage	0
DLF001_scaffold3084_2	non-phage	0
DLF001_scaffold9403_4	non-phage	0
DLF001_scaffold9142_2	non-phage	0
DLF001_scaffold25877_2	non-phage	0
DLF001_scaffold10641_1	non-phage	0.019
DLF001_scaffold6571_6	non-phage	0
DLF001_scaffold17448_2	non-phage	0
DLF001_scaffold16_21	non-phage	0
DLF001_scaffold11135_3	non-phage	0

DLF001_scaffold42_7	non-phage	0
DLF001_scaffold18720_1	non-phage	0
DLF001_scaffold15893_2	non-phage	0
DLF001_scaffold23103_1	non-phage	0
DLF001_scaffold19421_1	non-phage	0
DLF001_scaffold717_30	non-phage	0
DLF001_scaffold2417_7	non-phage	0
DLF001_scaffold25523_2	non-phage	0
DLF001_scaffold25830_2	non-phage	0
DLF001_scaffold7189_8	non-phage	0
DLF001_scaffold26026_1	non-phage	0
DLF001_scaffold9668_4	non-phage	0
DLF001_scaffold1440_48	non-phage	0
DLF001_scaffold21615_2	non-phage	0
DLF001_scaffold23307_1	non-phage	0
DLF001_scaffold36_2	non-phage	0
DLF001_scaffold12293_15	non-phage	0
DLF001_scaffold25149_1	non-phage	0
DLF001_scaffold25671_1	non-phage	0
DLF001_scaffold4115_1	non-phage	0
DLF001_scaffold4084_30	non-phage	0
DLF001_scaffold6015_11	non-phage	0
DLF001_scaffold3737_23	non-phage	0
DLF001_scaffold25_5	non-phage	0
DLF001_scaffold28_5	non-phage	0
DLF001_scaffold20836_2	non-phage	0
DLF001_C323592_1	non-phage	0
DLF001_scaffold24605_2	non-phage	0
DLF001_scaffold25975_2	non-phage	0
DLF001_scaffold18599_5	non-phage	0
DLF001_scaffold25858_1	non-phage	0
DLF001_scaffold3736_1	non-phage	0
DLF001_scaffold21979_1	non-phage	0
DLF001_scaffold22_67	non-phage	0
DLF001_scaffold2225_13	non-phage	0
DLF001_scaffold19903_4	non-phage	0
DLF001_scaffold14990_1	non-phage	0
DLF001_scaffold19264_1	non-phage	0
DLF001_scaffold46_3	non-phage	0
DLF001_scaffold20169_1	non-phage	0
DLF001_scaffold20892_2	non-phage	0
DLF001_scaffold16_3	non-phage	0
DLF001_scaffold6484_29	non-phage	0
DLF001_scaffold25097_3	non-phage	0
DLF001_scaffold9005_13	non-phage	0
DLF001_scaffold19265_2	non-phage	0

DLF001_scaffold7386_5	non-phage	0
DLF001_scaffold2981_17	non-phage	0
DLF001_scaffold10606_8	non-phage	0
DLF001_scaffold39_13	non-phage	0
DLF001_scaffold20624_6	non-phage	0
DLF001_C323266_1	non-phage	0
DLF001_scaffold26029_2	non-phage	0
DLF001_scaffold9668_3	non-phage	0
DLF001_scaffold20591_1	non-phage	0
DLF001_scaffold14359_1	non-phage	0
DLF001_scaffold24840_3	non-phage	0
DLF001_scaffold14275_3	non-phage	0
DLF001_scaffold20624_7	non-phage	0
DLF001_scaffold25887_1	non-phage	0
DLF001_scaffold24916_2	non-phage	0
DLF001_scaffold16955_2	non-phage	0
DLF001_scaffold25746_5	non-phage	0
DLF001_scaffold23211_1	non-phage	0
DLF001_scaffold25344_10	non-phage	0
DLF001_scaffold22903_5	non-phage	0
DLF001_scaffold6028_3	non-phage	0
DLF001_scaffold4900_4	non-phage	0
DLF001_scaffold21846_1	non-phage	0
DLF001_scaffold18739_1	non-phage	0
DLF001_scaffold18097_4	non-phage	0
DLF001_scaffold25975_1	non-phage	0
DLF001_scaffold16_23	non-phage	0
DLF001_scaffold29_1	non-phage	0
DLF001_scaffold14268_3	non-phage	0
DLF001_scaffold7362_1	non-phage	0
DLF001_scaffold15389_2	non-phage	0
DLF001_C323242_1	phage	0.999
DLF001_scaffold10872_5	non-phage	0
DLF001_scaffold2680_3	non-phage	0
DLF001_scaffold5_1	non-phage	0
DLF001_scaffold15801_1	non-phage	0
DLF001_scaffold20136_2	non-phage	0
DLF001_scaffold25843_2	non-phage	0
DLF001_scaffold21492_1	non-phage	0
DLF001_scaffold17461_1	non-phage	0
DLF001_scaffold21260_2	non-phage	0
DLF001_scaffold26016_1	non-phage	0
DLF001_C323238_1	non-phage	0
DLF001_scaffold18599_7	non-phage	0
DLF001_scaffold28_8	non-phage	0
DLF001_scaffold4132_6	non-phage	0

DLF001_scaffold25953_1	non-phage	0
DLF001_scaffold59_2	non-phage	0
DLF001_scaffold3_1	non-phage	0
DLF001_scaffold6699_1	non-phage	0
DLF001_scaffold13_12	phage	0.999
DLF001_scaffold20154_1	non-phage	0
DLF001_scaffold9477_12	non-phage	0
DLF001_scaffold25777_1	non-phage	0.016
DLF001_scaffold80_6	non-phage	0
DLF001_scaffold39_14	non-phage	0
DLF001_scaffold9668_5	non-phage	0
DLF001_scaffold15215_1	non-phage	0
DLF001_scaffold11273_5	non-phage	0
DLF001_scaffold26031_2	non-phage	0
DLF001_scaffold21615_1	non-phage	0
DLF001_scaffold18424_2	non-phage	0
DLF001_scaffold26004_1	non-phage	0
DLF001_scaffold80_10	non-phage	0
DLF001_scaffold24846_1	non-phage	0
DLF001_scaffold24474_12	non-phage	0
DLF001_scaffold25336_2	phage	0.997
DLF001_scaffold4459_3	non-phage	0
DLF001_scaffold21933_1	non-phage	0
DLF001_scaffold3241_1	non-phage	0
DLF001_scaffold25800_1	non-phage	0
DLF001_scaffold2280_7	non-phage	0
DLF001_scaffold7411_7	non-phage	0
DLF001_scaffold1894_3	non-phage	0
DLF001_scaffold24910_1	non-phage	0
DLF001_scaffold5381_1	non-phage	0
DLF001_scaffold25097_5	non-phage	0
DLF001_scaffold9880_2	non-phage	0
DLF001_scaffold4909_5	non-phage	0
DLF001_scaffold46_6	phage	0.997
DLF001_scaffold2850_6	non-phage	0
DLF001_scaffold25523_4	non-phage	0
DLF001_scaffold25632_16	non-phage	0
DLF001_scaffold24473_2	non-phage	0
DLF001_scaffold6229_1	non-phage	0
DLF001_scaffold22895_4	non-phage	0
DLF001_scaffold26041_1	non-phage	0
DLF001_scaffold47_3	non-phage	0
DLF001_scaffold18195_2	non-phage	0
DLF001_scaffold25818_1	non-phage	0
DLF001_scaffold24589_1	non-phage	0
DLF001_scaffold35_6	non-phage	0

DLF001_scaffold18916_1	non-phage	0
DLF001_scaffold1440_34	non-phage	0
DLF001_scaffold25843_3	non-phage	0
DLF001_scaffold26044_1	non-phage	0
DLF001_scaffold23009_2	non-phage	0
DLF001_scaffold23103_6	non-phage	0
DLF001_scaffold29_3	non-phage	0
DLF001_scaffold21318_1	non-phage	0
DLF001_scaffold23595_1	non-phage	0
DLF001_scaffold20566_1	non-phage	0
DLF001_scaffold26023_1	non-phage	0
DLF001_scaffold80_8	phage	0.998
DLF001_scaffold1972_2	non-phage	0
DLF001_scaffold21842_3	non-phage	0
DLF001_scaffold12855_6	non-phage	0
DLF001_scaffold2555_1	non-phage	0
DLF001_scaffold11813_10	non-phage	0
DLF001_scaffold13494_3	non-phage	0
DLF001_scaffold54_3	non-phage	0
DLF001_scaffold25544_2	non-phage	0
DLF001_scaffold1410_6	non-phage	0
DLF001_scaffold6028_1	phage	0.979
DLF001_scaffold26047_1	non-phage	0
DLF001_scaffold25989_1	non-phage	0
DLF001_scaffold11793_2	non-phage	0
DLF001_scaffold10787_2	non-phage	0
DLF001_scaffold19334_14	non-phage	0
DLF001_C323900_1	phage	0.999
DLF001_scaffold20617_1	non-phage	0.299
DLF001_scaffold4132_8	non-phage	0
DLF001_scaffold25544_1	non-phage	0
DLF001_scaffold18599_9	non-phage	0
DLF001_scaffold16898_1	non-phage	0
DLF001_scaffold2850_8	non-phage	0
DLF001_scaffold11817_3	non-phage	0
DLF001_scaffold23776_2	non-phage	0
DLF001_scaffold22612_1	non-phage	0
DLF001_scaffold7090_3	non-phage	0
DLF001_scaffold25505_6	non-phage	0
DLF001_scaffold25918_3	non-phage	0
DLF001_scaffold16868_4	non-phage	0
DLF001_scaffold16_19	non-phage	0
DLF001_scaffold4223_1	non-phage	0
DLF001_scaffold42_1	non-phage	0
DLF001_scaffold25746_2	non-phage	0
DLF001_scaffold24739_1	non-phage	0

DLF001_scaffold11273_7	non-phage	0
DLF001_scaffold7_1	non-phage	0
DLF001_scaffold1999_1	non-phage	0
DLF001_scaffold717_15	non-phage	0
DLF002_scaffold20138_1	non-phage	0
DLF002_scaffold27722_1	non-phage	0
DLF002_scaffold11366_10	non-phage	0
DLF002_scaffold15360_11	non-phage	0
DLF002_scaffold36328_4	non-phage	0
DLF002_scaffold36506_2	non-phage	0
DLF002_scaffold138_50	non-phage	0
DLF002_scaffold32107_1	non-phage	0
DLF002_scaffold23591_1	non-phage	0
DLF002_scaffold8222_1	non-phage	0
DLF002_scaffold13816_21	non-phage	0
DLF002_scaffold26841_1	non-phage	0
DLF002_scaffold10636_1	non-phage	0
DLF002_scaffold32378_1	non-phage	0
DLF002_scaffold6624_21	phage	1
DLF002_scaffold30315_5	non-phage	0
DLF002_scaffold34449_2	non-phage	0
DLF002_scaffold10404_18	non-phage	0
DLF002_scaffold22326_7	non-phage	0
DLF002_scaffold8669_6	non-phage	0
DLF002_scaffold32411_1	non-phage	0
DLF002_scaffold26181_4	non-phage	0
DLF002_scaffold12595_4	non-phage	0
DLF002_scaffold134_1	non-phage	0
DLF002_scaffold178_5	non-phage	0
DLF002_scaffold28065_1	non-phage	0
DLF002_scaffold35923_1	non-phage	0
DLF002_scaffold16082_2	non-phage	0
DLF002_scaffold7298_22	phage	0.999
DLF002_scaffold289_7	non-phage	0
DLF002_scaffold152_3	non-phage	0
DLF002_scaffold11314_5	non-phage	0
DLF002_scaffold22865_4	non-phage	0
DLF002_scaffold36419_3	non-phage	0
DLF002_scaffold28066_1	non-phage	0
DLF002_scaffold36525_1	non-phage	0
DLF002_scaffold23601_5	non-phage	0
DLF002_scaffold26985_1	non-phage	0
DLF002_scaffold17786_3	non-phage	0
DLF002_scaffold36411_4	non-phage	0
DLF002_scaffold9923_1	non-phage	0
DLF002_scaffold33093_1	phage	0.999

DLF002_scaffold143_1	non-phage	0
DLF002_scaffold124_4	non-phage	0
DLF002_scaffold72_3	non-phage	0
DLF002_scaffold26934_9	non-phage	0
DLF002_scaffold12371_1	non-phage	0
DLF002_scaffold36414_1	phage	1
DLF002_scaffold27691_1	non-phage	0
DLF002_C488919_1	non-phage	0
DLF002_scaffold36496_2	non-phage	0
DLF002_scaffold35438_1	non-phage	0
DLF002_scaffold806_14	non-phage	0
DLF002_scaffold34439_1	non-phage	0
DLF002_scaffold7788_12	non-phage	0
DLF002_scaffold10079_1	non-phage	0
DLF002_scaffold28049_1	non-phage	0
DLF002_scaffold2832_26	non-phage	0
DLF002_scaffold30766_1	non-phage	0
DLF002_scaffold6624_33	non-phage	0
DLF002_scaffold26181_7	non-phage	0
DLF002_scaffold9559_9	non-phage	0
DLF002_scaffold32547_4	non-phage	0
DLF002_scaffold32224_1	non-phage	0
DLF002_scaffold27965_2	non-phage	0
DLF002_scaffold3267_3	non-phage	0
DLF002_C488281_1	non-phage	0
DLF002_scaffold36512_2	non-phage	0
DLF002_scaffold27783_2	non-phage	0
DLF002_scaffold36452_1	non-phage	0
DLF002_scaffold178_1	non-phage	0
DLF002_scaffold284_2	non-phage	0
DLF002_scaffold21186_3	non-phage	0
DLF002_scaffold32846_2	non-phage	0
DLF002_scaffold10399_1	non-phage	0
DLF002_scaffold33068_4	non-phage	0
DLF002_scaffold7788_11	non-phage	0
DLF002_scaffold28854_1	non-phage	0
DLF002_scaffold134_4	non-phage	0
DLF002_scaffold34535_1	non-phage	0
DLF002_scaffold33320_3	non-phage	0
DLF002_scaffold25942_2	non-phage	0
DLF002_scaffold9106_2	non-phage	0
DLF002_scaffold15419_1	non-phage	0
DLF002_scaffold8669_1	non-phage	0
DLF002_scaffold31214_2	non-phage	0
DLF002_scaffold21633_4	non-phage	0
DLF002_scaffold22006_2	non-phage	0

DLF002_scaffold2172_5	non-phage	0
DLF002_scaffold22326_3	non-phage	0
DLF002_scaffold36001_1	non-phage	0
DLF002_scaffold27722_2	non-phage	0
DLF002_scaffold28768_1	non-phage	0
DLF002_scaffold806_3	non-phage	0
DLF002_scaffold294_25	non-phage	0
DLF002_scaffold85_3	phage	0.856
DLF002_scaffold357_3	non-phage	0
DLF002_scaffold23927_2	non-phage	0
DLF002_scaffold33382_7	non-phage	0
DLF002_scaffold32224_4	non-phage	0
DLF002_scaffold152_2	non-phage	0
DLF002_scaffold5353_5	non-phage	0
DLF002_scaffold710_6	non-phage	0
DLF002_scaffold19097_3	non-phage	0
DLF002_scaffold875_17	non-phage	0
DLF002_scaffold9106_6	non-phage	0
DLF002_scaffold13816_12	non-phage	0
DLF002_scaffold28742_1	phage	0.999
DLF002_scaffold36524_1	non-phage	0
DLF002_scaffold16987_25	non-phage	0
DLF002_scaffold271_1	non-phage	0
DLF002_scaffold25530_1	non-phage	0
DLF002_scaffold1882_8	non-phage	0
DLF002_scaffold255_9	non-phage	0
DLF002_scaffold36522_1	non-phage	0
DLF002_scaffold24106_1	non-phage	0
DLF002_scaffold9717_9	non-phage	0
DLF002_scaffold16171_2	non-phage	0
DLF002_scaffold13023_2	non-phage	0
DLF002_C488905_1	non-phage	0
DLF002_scaffold28371_1	non-phage	0
DLF002_scaffold124_11	non-phage	0
DLF002_scaffold27799_1	non-phage	0
DLF002_scaffold5548_4	non-phage	0
DLF002_scaffold10272_1	non-phage	0
DLF002_scaffold22416_1	phage	0.811
DLF002_scaffold1824_2	non-phage	0
DLF002_scaffold32378_2	phage	0.974
DLF002_scaffold128_40	non-phage	0
DLF002_scaffold35202_2	non-phage	0
DLF002_scaffold255_10	non-phage	0
DLF002_scaffold36519_2	non-phage	0
DLF002_scaffold72_14	non-phage	0
DLF002_scaffold7713_2	non-phage	0

DLF002_scaffold8669_7	non-phage	0
DLF002_scaffold35072_2	non-phage	0
DLF002_scaffold104_8	non-phage	0
DLF002_scaffold34763_1	non-phage	0
DLF002_scaffold35941_2	non-phage	0
DLF002_scaffold30315_2	non-phage	0
DLF002_scaffold19600_3	phage	0.999
DLF002_scaffold1878_12	non-phage	0
DLF002_scaffold6624_14	non-phage	0
DLF002_scaffold35326_2	non-phage	0
DLF002_scaffold25684_4	non-phage	0
DLF002_scaffold18751_1	non-phage	0
DLF002_scaffold36510_1	non-phage	0
DLF002_scaffold36336_1	non-phage	0
DLF002_scaffold26181_3	non-phage	0
DLF002_scaffold19705_4	non-phage	0
DLF002_scaffold26841_7	non-phage	0
DLF002_scaffold6614_2	non-phage	0
DLF002_scaffold36411_1	non-phage	0
DLF002_scaffold15536_1	non-phage	0
DLF002_scaffold5353_4	non-phage	0
DLF002_scaffold64_3	non-phage	0
DLF002_scaffold8096_5	non-phage	0
DLF002_scaffold255_4	non-phage	0
DLF002_scaffold289_2	non-phage	0
DLF002_scaffold170_3	non-phage	0
DLF002_scaffold33382_2	non-phage	0
DLF002_scaffold36419_2	non-phage	0
DLF002_scaffold27722_3	non-phage	0
DLF002_scaffold19757_1	non-phage	0
DLF002_scaffold2788_4	non-phage	0
DLF002_scaffold15360_1	non-phage	0
DLF002_scaffold36507_1	non-phage	0
DLF002_scaffold24593_13	non-phage	0
DLF002_scaffold289_9	non-phage	0
DLF002_scaffold5283_3	non-phage	0
DLF002_scaffold134_28	non-phage	0
DLF002_scaffold34919_1	non-phage	0
DLF002_C488807_1	non-phage	0
DLF002_scaffold44_3	non-phage	0
DLF002_scaffold28065_2	non-phage	0
DLF002_scaffold19097_1	non-phage	0
DLF002_scaffold33068_3	non-phage	0
DLF002_scaffold138_23	non-phage	0
DLF002_scaffold134_14	non-phage	0.009
DLF002_scaffold15360_23	non-phage	0

DLF002_scaffold30311_2	non-phage	0
DLF002_scaffold24316_12	phage	0.998
DLF002_scaffold8470_3	non-phage	0
DLF002_scaffold4392_1	non-phage	0
DLF002_scaffold7340_5	non-phage	0
DLF002_scaffold11171_7	non-phage	0
DLF002_scaffold11171_4	non-phage	0
DLF002_scaffold36081_2	phage	0.979
DLF002_scaffold27574_2	non-phage	0
DLF002_scaffold114_1	non-phage	0
DLF002_scaffold36026_4	non-phage	0
DLF002_scaffold19705_1	non-phage	0
DLF002_scaffold21577_10	non-phage	0
DLF002_scaffold13023_1	non-phage	0
DLF002_scaffold72_17	non-phage	0
DLF002_scaffold28824_3	non-phage	0
DLF002_scaffold15360_5	non-phage	0
DLF002_scaffold32264_3	non-phage	0
DLF002_scaffold5353_7	non-phage	0
DLF002_scaffold4323_3	non-phage	0
DLF002_scaffold25530_2	non-phage	0
DLF002_scaffold33848_2	non-phage	0
DLF002_scaffold32818_2	non-phage	0
DLF002_scaffold36449_1	non-phage	0
DLF002_scaffold15360_17	non-phage	0
DLF002_scaffold32804_2	non-phage	0
DLF002_scaffold8669_13	non-phage	0
DLF002_scaffold30315_6	non-phage	0
DLF002_scaffold32846_3	non-phage	0
DLF002_scaffold11619_1	non-phage	0
DLF002_scaffold21633_3	non-phage	0
DLF002_scaffold18037_1	non-phage	0
DLF002_scaffold21585_3	non-phage	0
DLF002_C488431_1	non-phage	0
DLF002_scaffold47_5	non-phage	0
DLF002_scaffold36513_2	non-phage	0
DLF002_scaffold5659_11	non-phage	0
DLF002_scaffold3267_4	non-phage	0
DLF002_scaffold294_32	phage	0.935
DLF002_scaffold33382_6	non-phage	0.043
DLF002_scaffold34431_2	non-phage	0
DLF002_scaffold10343_1	non-phage	0
DLF002_scaffold134_27	non-phage	0
DLF002_scaffold5900_3	non-phage	0
DLF002_scaffold7525_1	phage	0.941
DLF002_scaffold24471_1	non-phage	0

DLF002_scaffold5548_1	non-phage	0
DLF002_scaffold22326_2	non-phage	0
DLF002_scaffold36043_3	non-phage	0
DLF002_scaffold30246_2	non-phage	0
DLF002_scaffold27_1	non-phage	0
DLF002_scaffold12838_5	non-phage	0
DLF002_scaffold2832_19	non-phage	0
DLF002_scaffold36356_1	non-phage	0
DLF002_scaffold26047_1	non-phage	0
DLF002_scaffold30164_2	non-phage	0
DLF002_scaffold23927_1	non-phage	0
DLF002_scaffold36266_1	non-phage	0
DLF002_scaffold8222_7	non-phage	0
DLF002_C488763_1	non-phage	0
DLF002_scaffold8249_7	non-phage	0
DLF002_scaffold85_4	non-phage	0
DLF002_scaffold36495_1	non-phage	0
DLF002_C488769_1	non-phage	0
DLF002_scaffold19097_4	non-phage	0
DLF002_scaffold24016_2	non-phage	0
DLF002_scaffold134_10	non-phage	0
DLF002_scaffold17786_4	non-phage	0
DLF002_scaffold2172_2	non-phage	0
DLF002_scaffold10705_2	non-phage	0
DLF002_scaffold36493_1	non-phage	0
DLF002_scaffold26586_1	non-phage	0
DLF002_scaffold2411_20	non-phage	0
DLF002_scaffold30901_3	non-phage	0
DLF002_scaffold85_5	non-phage	0
DLF002_scaffold28510_1	non-phage	0
DLF002_scaffold32129_2	non-phage	0
DLF002_scaffold19307_2	non-phage	0
DLF002_scaffold134_3	non-phage	0
DLF002_scaffold10272_2	non-phage	0
DLF002_scaffold36026_2	non-phage	0
DLF002_scaffold36521_1	non-phage	0
DLF002_scaffold8249_6	non-phage	0
DLF002_scaffold36510_2	non-phage	0
DLF002_scaffold124_6	non-phage	0
DLF002_scaffold21860_1	non-phage	0
DLF002_scaffold35326_1	non-phage	0
DLF002_scaffold36026_7	non-phage	0
DLF002_scaffold19097_5	non-phage	0
DLF002_scaffold33320_8	non-phage	0
DLF002_scaffold36411_2	non-phage	0
DLF002_scaffold8387_3	non-phage	0

DLF002_scaffold1382_1	non-phage	0
DLF002_scaffold172_6	non-phage	0
DLF002_scaffold3267_1	non-phage	0
DLF002_scaffold3397_1	non-phage	0
DLF002_scaffold806_12	non-phage	0
DLF002_scaffold17380_2	non-phage	0
DLF002_scaffold7788_1	non-phage	0
DLF002_scaffold8361_6	non-phage	0
DLF002_scaffold128_26	non-phage	0
DLF002_scaffold36180_1	non-phage	0
DLF002_scaffold35184_1	non-phage	0
DLF002_scaffold7241_4	non-phage	0
DLF002_scaffold138_52	non-phage	0
DLF002_scaffold33068_2	non-phage	0
DLF002_scaffold26750_1	non-phage	0
DLF002_scaffold6546_1	non-phage	0
DLF002_scaffold28824_1	non-phage	0
DLF002_scaffold21585_8	non-phage	0
DLF002_scaffold26181_9	non-phage	0
DLF002_scaffold29220_1	non-phage	0.087
DLF002_scaffold16987_7	non-phage	0
DLF002_scaffold35853_2	non-phage	0
DLF002_scaffold20273_3	non-phage	0
DLF002_scaffold4170_7	non-phage	0
DLF002_scaffold6650_2	non-phage	0
DLF002_scaffold154_2	non-phage	0
DLF002_scaffold32028_2	non-phage	0
DLF002_scaffold28049_2	non-phage	0
DLF002_scaffold31141_5	non-phage	0
DLF002_scaffold32841_2	non-phage	0
DLF002_scaffold5063_5	non-phage	0
DLF002_scaffold30901_6	non-phage	0
DLF002_scaffold32846_1	non-phage	0
DLF002_scaffold20703_3	non-phage	0
DLF002_scaffold17792_3	non-phage	0
DLF002_scaffold3267_2	non-phage	0
DLF002_scaffold20760_2	non-phage	0
DLF002_scaffold22326_5	non-phage	0
DLF002_scaffold32378_3	non-phage	0
DLF002_scaffold9717_8	non-phage	0
DLF002_scaffold44_4	non-phage	0
DLF002_scaffold33980_7	non-phage	0
DLF002_scaffold25876_1	non-phage	0
DLF002_scaffold36501_1	non-phage	0
DLF002_scaffold21520_1	non-phage	0
DLF002_scaffold23810_1	non-phage	0

DLF002_scaffold15360_24	non-phage	0
DLF002_scaffold4170_14	non-phage	0.245
DLF002_scaffold26675_1	non-phage	0
DLF002_scaffold36489_1	non-phage	0
DLF002_scaffold25546_1	phage	0.995
DLF002_scaffold35294_2	non-phage	0
DLF002_scaffold15360_25	non-phage	0
DLF002_scaffold33320_5	non-phage	0
DLF002_scaffold8249_3	non-phage	0
DLF002_scaffold30315_7	non-phage	0
DLF002_scaffold8669_14	non-phage	0
DLF002_scaffold21456_1	non-phage	0
DLF002_scaffold2832_16	non-phage	0
DLF002_scaffold24016_6	non-phage	0
DLF002_scaffold138_8	phage	0.999
DLF002_scaffold22387_8	non-phage	0
DLF002_scaffold7298_21	non-phage	0
DLF002_scaffold3523_18	non-phage	0
DLF002_scaffold29169_2	non-phage	0
DLF002_scaffold6441_22	non-phage	0
DLF002_scaffold6324_4	non-phage	0.001
DLF002_scaffold85_1	non-phage	0
DLF002_scaffold28892_1	non-phage	0
DLF002_scaffold36419_5	non-phage	0
DLF002_scaffold33726_2	non-phage	0
DLF002_scaffold128_21	non-phage	0
DLF002_scaffold30766_3	non-phage	0
DLF002_scaffold36279_1	non-phage	0
DLF002_scaffold9339_2	non-phage	0
DLF002_scaffold15360_18	non-phage	0
DLF002_scaffold36472_4	non-phage	0
DLF002_scaffold36169_1	non-phage	0
DLF002_scaffold24016_5	non-phage	0
DLF002_scaffold4573_2	non-phage	0
DLF002_scaffold18751_3	non-phage	0
DLF002_scaffold8470_1	non-phage	0
DLF002_scaffold33068_6	non-phage	0
DLF002_C488315_1	non-phage	0
DLF002_scaffold27324_1	non-phage	0
DLF002_scaffold7241_1	non-phage	0
DLF002_scaffold30282_2	non-phage	0
DLF002_scaffold33382_5	non-phage	0
DLF002_scaffold5328_8	non-phage	0
DLF002_scaffold172_2	non-phage	0
DLF002_scaffold24471_2	non-phage	0
DLF002_scaffold806_9	non-phage	0

DLF002_scaffold3108_1	non-phage	0
DLF002_scaffold33974_1	phage	0.999
DLF002_scaffold23601_4	non-phage	0
DLF002_scaffold18469_2	non-phage	0
DLF002_scaffold10343_2	phage	0.537
DLF002_scaffold30164_1	non-phage	0
DLF002_scaffold30745_2	non-phage	0
DLF002_scaffold255_6	non-phage	0
DLF002_scaffold13023_4	non-phage	0
DLF002_C488527_1	non-phage	0
DLF002_scaffold10404_2	non-phage	0
DLF002_scaffold5283_1	non-phage	0
DLF002_scaffold33668_1	non-phage	0
DLF002_scaffold3523_34	non-phage	0
DLF002_scaffold17360_1	non-phage	0
DLF002_scaffold31953_1	non-phage	0
DLF002_scaffold36438_2	non-phage	0
DLF002_scaffold4865_1	non-phage	0
DLF002_scaffold2788_2	non-phage	0
DLF002_scaffold19755_8	non-phage	0
DLF002_scaffold20014_18	non-phage	0
DLF002_scaffold32841_1	non-phage	0
DLF002_scaffold26232_8	non-phage	0
DLF002_scaffold32109_3	non-phage	0
DLF002_scaffold7241_3	non-phage	0
DLF002_scaffold10272_3	non-phage	0
DLF002_scaffold6546_2	non-phage	0
DLF002_scaffold11314_4	non-phage	0
DLF002_scaffold33068_1	non-phage	0
DLF002_scaffold22387_1	non-phage	0
DLF002_scaffold23927_3	non-phage	0
DLF002_scaffold1383_1	non-phage	0
DLF002_scaffold6650_1	non-phage	0
DLF002_scaffold33320_7	non-phage	0
DLF002_scaffold35056_4	non-phage	0
DLF002_scaffold34926_1	non-phage	0
DLF002_scaffold30901_5	non-phage	0
DLF002_scaffold5353_1	non-phage	0
DLF002_scaffold5548_6	non-phage	0
DLF002_scaffold449_2	non-phage	0
DLF002_scaffold20760_1	non-phage	0
DLF002_scaffold28740_1	non-phage	0
DLF002_scaffold9717_7	non-phage	0
DLF002_scaffold7277_1	non-phage	0
DLF002_scaffold32224_2	non-phage	0
DLF002_scaffold36505_1	phage	0.998

DLF002_scaffold7305_42	non-phage	0
DLF002_scaffold23591_2	non-phage	0
DLF002_scaffold16987_16	non-phage	0
DLF002_scaffold12306_4	non-phage	0
DLF002_scaffold22178_2	non-phage	0
DLF002_scaffold16987_22	non-phage	0
DLF002_scaffold34817_1	non-phage	0
DLF002_scaffold8361_1	non-phage	0
DLF002_scaffold9106_4	non-phage	0
DLF002_scaffold36411_3	non-phage	0
DLF002_scaffold28049_3	non-phage	0.005
DLF002_scaffold15360_15	non-phage	0
DLF002_scaffold26181_1	non-phage	0
DLF002_scaffold154_1	non-phage	0
DLF002_scaffold28695_1	non-phage	0
DLF002_scaffold4323_1	non-phage	0
DLF002_scaffold220_1	non-phage	0
DLF002_scaffold36511_1	non-phage	0
DLF002_scaffold44_1	non-phage	0
DLF002_scaffold11918_6	non-phage	0
DLF002_scaffold26607_1	non-phage	0
DLF002_scaffold1383_2	non-phage	0
DLF002_scaffold26328_7	non-phage	0
DLF002_scaffold34765_1	non-phage	0
DLF002_scaffold28048_2	non-phage	0
DLF002_scaffold33382_4	non-phage	0
DLF002_scaffold34431_4	non-phage	0
DLF002_scaffold17567_1	non-phage	0
DLF002_scaffold21211_2	non-phage	0
DLF002_scaffold29553_1	non-phage	0
DLF002_scaffold13816_8	non-phage	0
DLF002_scaffold72_5	non-phage	0
DLF002_scaffold19755_1	non-phage	0
DLF002_scaffold2411_19	non-phage	0
DLF002_scaffold31958_1	non-phage	0
DLF002_scaffold33093_3	non-phage	0
DLF002_scaffold2466_1	phage	0.998
DLF002_scaffold152_1	non-phage	0
DLF002_scaffold128_4	non-phage	0
DLF002_scaffold220_2	non-phage	0
DLF002_scaffold5548_10	non-phage	0
DLF002_scaffold30766_2	non-phage	0
DLF002_scaffold26934_5	non-phage	0
DLF002_scaffold19555_3	non-phage	0
DLF002_scaffold32436_6	non-phage	0
DLF002_scaffold5170_7	non-phage	0

DLF002_scaffold31693_3	non-phage	0
DLF002_scaffold9717_6	non-phage	0
DLF002_scaffold33320_4	non-phage	0
DLF002_scaffold4865_4	non-phage	0
DLF002_scaffold152_10	non-phage	0
DLF002_scaffold23_2	non-phage	0
DLF002_scaffold36026_6	non-phage	0
DLF002_scaffold35031_2	non-phage	0
DLF002_scaffold19097_2	non-phage	0
DLF002_scaffold32109_1	non-phage	0
DLF002_scaffold8249_2	non-phage	0
DLF002_scaffold21198_1	non-phage	0
DLF002_scaffold11314_8	non-phage	0
DLF002_scaffold13857_3	non-phage	0.468
DLF002_scaffold36492_2	non-phage	0
DLF002_scaffold33382_8	non-phage	0
DLF002_scaffold35941_1	non-phage	0
DLF002_scaffold36298_1	phage	0.973
DLF002_scaffold8096_12	non-phage	0
DLF002_scaffold72_10	non-phage	0
DLF002_scaffold255_8	non-phage	0
DLF002_scaffold36005_2	non-phage	0
DLF002_scaffold255_3	non-phage	0
DLF002_scaffold21585_10	non-phage	0
DLF002_scaffold8361_5	non-phage	0
DLF002_scaffold15360_19	non-phage	0
DLF002_scaffold25734_1	non-phage	0
DLF002_scaffold20138_7	non-phage	0
DLF002_scaffold23810_2	non-phage	0
DLF002_scaffold20783_1	non-phage	0
DLF002_scaffold13594_2	non-phage	0
DLF002_scaffold6041_3	phage	0.999
DLF002_scaffold34576_1	non-phage	0
DLF002_scaffold20563_3	non-phage	0
DLF002_scaffold19705_6	non-phage	0
DLF002_scaffold36491_1	non-phage	0
DLF002_scaffold21111_2	phage	0.94
DLF002_scaffold92_1	non-phage	0
DLF002_scaffold178_3	non-phage	0
DLF002_C488685_1	phage	0.999
DLF002_scaffold4802_1	non-phage	0
DLF002_scaffold21585_5	non-phage	0
DLF002_scaffold30315_4	non-phage	0
DLF002_scaffold24842_1	non-phage	0
DLF002_scaffold15360_7	non-phage	0
DLF003_scaffold40770_1	non-phage	0

DLF003_scaffold56916_1	non-phage	0
DLF003_scaffold37_3	non-phage	0
DLF003_scaffold8312_1	non-phage	0
DLF003_scaffold21732_2	phage	0.984
DLF003_scaffold29891_1	phage	1
DLF003_scaffold2206_6	non-phage	0
DLF003_scaffold23409_2	phage	0.5
DLF003_scaffold32570_1	non-phage	0
DLF003_scaffold56159_5	non-phage	0
DLF003_scaffold40017_1	non-phage	0
DLF003_scaffold10_19	non-phage	0
DLF003_scaffold41858_1	non-phage	0
DLF003_scaffold57132_2	non-phage	0
DLF003_scaffold57137_1	non-phage	0
DLF003_scaffold50567_2	phage	1
DLF003_scaffold42903_1	non-phage	0
DLF003_scaffold13463_1	non-phage	0
DLF003_scaffold13331_1	non-phage	0
DLF003_scaffold11407_1	non-phage	0
DLF003_scaffold46050_3	non-phage	0
DLF003_scaffold19665_2	non-phage	0
DLF003_scaffold44436_1	phage	0.913
DLF003_scaffold49561_1	non-phage	0
DLF003_scaffold21152_1	non-phage	0
DLF003_scaffold40102_1	non-phage	0
DLF003_scaffold10586_4	non-phage	0
DLF003_scaffold12321_2	phage	0.997
DLF003_scaffold38551_1	non-phage	0
DLF003_scaffold53_9	non-phage	0
DLF003_scaffold7511_6	non-phage	0
DLF003_scaffold130_2	non-phage	0
DLF003_scaffold5892_1	non-phage	0
DLF003_C717678_1	non-phage	0
DLF003_scaffold4936_4	non-phage	0
DLF003_scaffold14748_2	non-phage	0
DLF003_scaffold3710_2	non-phage	0
DLF003_scaffold53540_1	non-phage	0
DLF003_scaffold3492_3	non-phage	0
DLF003_scaffold133_2	non-phage	0
DLF003_scaffold37512_1	non-phage	0
DLF003_scaffold57055_1	non-phage	0
DLF003_C717736_1	phage	0.999
DLF003_scaffold28027_3	non-phage	0
DLF003_scaffold30405_3	non-phage	0
DLF003_scaffold38789_2	non-phage	0
DLF003_scaffold47957_1	non-phage	0

DLF003_scaffold32578_3	non-phage	0
DLF003_scaffold2209_1	non-phage	0
DLF003_C717592_1	non-phage	0
DLF003_scaffold370_8	non-phage	0
DLF003_scaffold229_39	non-phage	0
DLF003_scaffold12321_3	non-phage	0
DLF003_scaffold48522_1	non-phage	0
DLF003_scaffold23_1	non-phage	0
DLF003_scaffold137_1	phage	0.921
DLF003_scaffold56755_1	non-phage	0
DLF003_scaffold56507_1	non-phage	0
DLF003_scaffold25812_3	non-phage	0
DLF003_scaffold8272_1	non-phage	0
DLF003_scaffold12122_2	non-phage	0
DLF003_scaffold57047_1	non-phage	0
DLF003_scaffold35431_23	non-phage	0
DLF003_scaffold54323_1	non-phage	0
DLF003_scaffold16938_3	non-phage	0
DLF003_scaffold54717_1	non-phage	0.061
DLF003_scaffold3834_23	phage	0.999
DLF003_scaffold18845_1	non-phage	0
DLF003_scaffold23839_1	non-phage	0
DLF003_C717764_1	non-phage	0
DLF003_scaffold25587_4	non-phage	0
DLF003_scaffold54024_1	non-phage	0
DLF003_scaffold36524_1	non-phage	0
DLF003_scaffold53822_1	non-phage	0
DLF003_scaffold2267_1	non-phage	0
DLF003_scaffold37299_1	non-phage	0
DLF003_scaffold41656_1	non-phage	0
DLF003_scaffold37648_3	non-phage	0
DLF003_scaffold56994_1	non-phage	0
DLF003_scaffold17438_13	non-phage	0
DLF003_C717716_1	non-phage	0
DLF003_scaffold19_3	non-phage	0
DLF003_scaffold443_1	non-phage	0
DLF003_scaffold40330_2	non-phage	0
DLF003_scaffold26323_3	non-phage	0
DLF003_scaffold3305_5	non-phage	0
DLF003_scaffold53163_1	non-phage	0
DLF003_scaffold55800_1	phage	0.994
DLF003_scaffold3143_5	non-phage	0
DLF003_scaffold36524_2	non-phage	0
DLF003_scaffold28027_4	non-phage	0
DLF003_scaffold168_5	non-phage	0
DLF003_scaffold32624_1	non-phage	0

DLF003_scaffold57053_1	non-phage	0
DLF003_scaffold327_1	non-phage	0
DLF003_scaffold130_6	non-phage	0
DLF003_scaffold3492_4	non-phage	0
DLF003_scaffold23490_3	non-phage	0
DLF003_scaffold11408_1	non-phage	0
DLF003_scaffold31822_5	non-phage	0
DLF003_scaffold1924_5	non-phage	0
DLF003_scaffold57197_1	non-phage	0
DLF003_scaffold48074_1	non-phage	0
DLF003_scaffold48187_1	non-phage	0
DLF003_scaffold45245_2	non-phage	0
DLF003_scaffold3102_65	non-phage	0
DLF003_scaffold17867_1	non-phage	0
DLF003_scaffold35373_1	non-phage	0
DLF003_scaffold36411_1	non-phage	0
DLF003_scaffold107_7	non-phage	0
DLF003_scaffold21732_1	non-phage	0
DLF003_scaffold21_1	non-phage	0
DLF003_scaffold51633_1	non-phage	0
DLF003_scaffold36463_1	non-phage	0
DLF003_scaffold53799_1	non-phage	0
DLF003_scaffold52742_1	non-phage	0
DLF003_scaffold168_2	non-phage	0
DLF003_scaffold56980_1	non-phage	0
DLF003_scaffold10836_3	non-phage	0
DLF003_scaffold159_1	non-phage	0
DLF003_scaffold3710_3	non-phage	0
DLF003_scaffold6431_5	non-phage	0
DLF003_scaffold57016_3	non-phage	0
DLF003_scaffold77_2	non-phage	0
DLF003_scaffold47278_1	non-phage	0
DLF003_scaffold18805_1	non-phage	0
DLF003_scaffold21397_2	non-phage	0
DLF003_C717640_1	phage	0.999
DLF003_scaffold57186_2	non-phage	0
DLF003_scaffold36659_1	non-phage	0
DLF003_scaffold8762_11	non-phage	0
DLF003_scaffold49744_1	non-phage	0
DLF003_scaffold299_6	non-phage	0
DLF003_scaffold23803_1	non-phage	0
DLF003_scaffold12321_1	non-phage	0
DLF003_scaffold137_4	non-phage	0
DLF003_scaffold57200_1	non-phage	0
DLF003_scaffold44043_1	non-phage	0
DLF003_scaffold56975_2	non-phage	0

DLF003_scaffold11879_3	non-phage	0
DLF003_scaffold3102_87	non-phage	0
DLF003_scaffold2890_3	non-phage	0
DLF003_scaffold13656_2	non-phage	0
DLF003_scaffold38797_3	non-phage	0
DLF003_scaffold10_16	non-phage	0
DLF003_scaffold53_14	non-phage	0
DLF003_scaffold3094_8	non-phage	0
DLF003_scaffold35909_2	non-phage	0
DLF003_scaffold51876_1	non-phage	0
DLF003_scaffold13_6	non-phage	0
DLF003_scaffold40167_1	non-phage	0
DLF003_scaffold23403_10	phage	0.999
DLF003_C717742_1	non-phage	0
DLF003_scaffold56691_4	non-phage	0
DLF003_scaffold42485_1	non-phage	0
DLF003_scaffold7333_15	non-phage	0
DLF003_scaffold49331_1	non-phage	0
DLF003_scaffold25818_1	non-phage	0
DLF003_scaffold42864_1	non-phage	0
DLF003_scaffold21993_4	non-phage	0
DLF003_scaffold21732_5	non-phage	0
DLF003_scaffold37648_2	non-phage	0
DLF003_scaffold55964_1	non-phage	0
DLF003_scaffold56159_1	non-phage	0
DLF003_scaffold11478_2	non-phage	0
DLF003_scaffold48199_2	non-phage	0
DLF003_scaffold57102_1	non-phage	0
DLF003_scaffold57171_1	non-phage	0
DLF003_scaffold42512_1	non-phage	0
DLF003_scaffold56826_1	non-phage	0
DLF003_scaffold47117_1	non-phage	0
DLF003_scaffold19936_1	non-phage	0
DLF003_scaffold23047_1	non-phage	0
DLF003_scaffold47104_1	non-phage	0
DLF003_scaffold46501_4	non-phage	0
DLF003_scaffold32215_1	non-phage	0
DLF003_scaffold35985_1	non-phage	0
DLF003_scaffold56673_2	phage	0.999
DLF003_scaffold56251_2	non-phage	0
DLF003_scaffold16911_4	non-phage	0
DLF003_scaffold42481_1	non-phage	0
DLF003_scaffold6431_6	non-phage	0
DLF003_scaffold57177_1	non-phage	0
DLF003_scaffold53539_1	non-phage	0
DLF003_scaffold42176_1	non-phage	0

DLF003_scaffold7634_4	non-phage	0
DLF003_scaffold57188_1	non-phage	0
DLF003_scaffold698_8	non-phage	0
DLF003_scaffold29589_2	non-phage	0
DLF003_scaffold3492_5	non-phage	0
DLF003_scaffold365_2	non-phage	0
DLF003_scaffold19_1	non-phage	0
DLF003_scaffold123_2	non-phage	0
DLF003_scaffold38516_2	non-phage	0
DLF003_scaffold23328_4	non-phage	0
DLF003_scaffold51877_1	non-phage	0
DLF003_scaffold56226_1	non-phage	0
DLF003_scaffold22562_1	non-phage	0
DLF003_scaffold30405_1	non-phage	0
DLF003_scaffold265_4	non-phage	0
DLF003_scaffold48021_1	non-phage	0
DLF003_scaffold31822_4	non-phage	0
DLF003_scaffold9111_11	non-phage	0
DLF003_scaffold37_1	non-phage	0.04
DLF003_scaffold54964_1	non-phage	0
DLF003_scaffold5185_1	non-phage	0
DLF003_scaffold40770_3	non-phage	0
DLF003_scaffold294_1	non-phage	0
DLF003_scaffold56222_2	non-phage	0
DLF003_scaffold27447_8	non-phage	0
DLF003_scaffold136_5	non-phage	0
DLF003_scaffold15212_9	non-phage	0
DLF003_scaffold36874_2	non-phage	0
DLF003_scaffold37767_1	non-phage	0
DLF003_scaffold12321_4	non-phage	0
DLF003_scaffold94_9	non-phage	0
DLF003_scaffold20260_28	non-phage	0
DLF003_scaffold47100_1	non-phage	0.046
DLF003_C717660_1	non-phage	0
DLF003_scaffold33208_4	non-phage	0
DLF003_scaffold57198_1	non-phage	0
DLF003_scaffold56441_1	non-phage	0
DLF003_scaffold41929_1	non-phage	0
DLF003_scaffold22133_3	non-phage	0
DLF003_scaffold28888_2	non-phage	0
DLF003_scaffold880_1	non-phage	0
DLF003_scaffold33443_5	non-phage	0
DLF003_scaffold52113_2	non-phage	0
DLF003_scaffold5185_4	non-phage	0
DLF003_scaffold13196_1	non-phage	0
DLF003_scaffold54287_1	non-phage	0

DLF003_scaffold10852_1	non-phage	0
DLF003_scaffold38582_1	non-phage	0
DLF003_scaffold38601_1	non-phage	0
DLF003_scaffold39989_2	non-phage	0
DLF003_scaffold37648_1	non-phage	0
DLF003_scaffold130_4	non-phage	0.41
DLF003_scaffold2209_5	non-phage	0
DLF003_scaffold17438_15	non-phage	0
DLF003_scaffold56451_2	non-phage	0
DLF003_scaffold55041_1	non-phage	0
DLF003_scaffold37648_6	non-phage	0
DLF003_scaffold43843_3	non-phage	0
DLF003_scaffold13553_1	non-phage	0
DLF003_scaffold56858_1	non-phage	0.002
DLF003_scaffold51736_1	non-phage	0
DLF003_scaffold56573_1	non-phage	0.001
DLF003_scaffold54149_1	phage	0.999
DLF003_scaffold48199_1	phage	0.998
DLF003_scaffold31696_1	non-phage	0
DLF003_scaffold5952_1	non-phage	0
DLF003_scaffold9111_4	non-phage	0
DLF003_scaffold57196_2	non-phage	0
DLF003_scaffold18271_5	non-phage	0
DLF003_scaffold43976_1	phage	0.989
DLF003_scaffold10_4	non-phage	0
DLF003_scaffold26062_1	non-phage	0
DLF003_scaffold3595_3	non-phage	0
DLF003_scaffold57128_3	non-phage	0
DLF003_scaffold17048_2	non-phage	0
DLF003_scaffold29384_2	non-phage	0
DLF003_scaffold3834_7	phage	0.927
DLF003_scaffold3094_9	non-phage	0
DLF003_scaffold8271_1	non-phage	0
DLF003_scaffold40200_1	non-phage	0
DLF003_scaffold55988_1	non-phage	0
DLF003_scaffold242_3	non-phage	0.001
DLF003_scaffold1152_2	non-phage	0
DLF003_scaffold47905_1	phage	0.986
DLF003_scaffold21993_5	non-phage	0
DLF003_scaffold11885_3	non-phage	0
DLF003_scaffold56434_3	non-phage	0
DLF003_scaffold31129_3	non-phage	0
DLF003_scaffold25812_1	non-phage	0
DLF003_scaffold36987_1	non-phage	0
DLF003_scaffold53201_1	non-phage	0
DLF003_scaffold13604_1	non-phage	0

DLF003_scaffold53220_3	non-phage	0
DLF003_scaffold41928_1	non-phage	0
DLF003_scaffold38444_4	non-phage	0
DLF003_scaffold49600_1	non-phage	0
DLF003_scaffold2267_8	non-phage	0
DLF003_scaffold53034_3	non-phage	0
DLF003_scaffold11984_11	non-phage	0
DLF003_scaffold56478_1	phage	0.99
DLF003_scaffold2269_6	non-phage	0
DLF003_scaffold16241_38	non-phage	0
DLF003_scaffold10256_1	non-phage	0
DLF003_scaffold43965_1	non-phage	0
DLF003_scaffold38560_1	non-phage	0
DLF003_scaffold55562_1	non-phage	0
DLF003_scaffold276_7	non-phage	0
DLF003_scaffold3907_1	non-phage	0
DLF003_scaffold55658_3	non-phage	0
DLF003_scaffold42861_1	non-phage	0
DLF003_scaffold16911_5	non-phage	0
DLF003_scaffold56930_2	non-phage	0.395
DLF003_scaffold621_29	non-phage	0
DLF003_scaffold48911_2	non-phage	0
DLF003_scaffold12151_1	non-phage	0
DLF003_scaffold57190_2	phage	1
DLF003_scaffold3387_4	non-phage	0
DLF003_scaffold53668_1	non-phage	0
DLF003_scaffold23328_3	non-phage	0
DLF003_scaffold53_8	non-phage	0
DLF003_scaffold838_1	non-phage	0
DLF003_scaffold56951_2	non-phage	0
DLF003_scaffold41998_1	non-phage	0
DLF003_scaffold57184_1	non-phage	0
DLF003_scaffold23547_1	non-phage	0
DLF003_scaffold49590_1	non-phage	0
DLF003_scaffold123_1	non-phage	0
DLF003_scaffold56159_4	phage	0.998
DLF003_scaffold56470_3	non-phage	0
DLF003_scaffold15762_8	non-phage	0
DLF003_scaffold56754_2	non-phage	0
DLF003_scaffold22429_1	non-phage	0
DLF003_scaffold33510_2	non-phage	0
DLF003_scaffold39018_1	non-phage	0
DLF003_scaffold4093_6	non-phage	0
DLF003_scaffold6372_1	non-phage	0
DLF003_scaffold4398_37	phage	0.999
DLF003_scaffold55209_1	non-phage	0

DLF003_scaffold36400_2	non-phage	0
DLF003_scaffold12499_1	non-phage	0
DLF003_scaffold56222_3	non-phage	0
DLF003_scaffold20948_6	non-phage	0
DLF003_scaffold42292_3	non-phage	0
DLF003_scaffold7227_2	non-phage	0
DLF003_scaffold38272_1	non-phage	0
DLF003_scaffold19305_2	non-phage	0
DLF003_scaffold23873_1	non-phage	0
DLF003_scaffold23994_1	non-phage	0
DLF003_scaffold168_4	non-phage	0
DLF003_scaffold8921_1	non-phage	0
DLF003_scaffold11902_3	non-phage	0
DLF003_scaffold56709_2	non-phage	0
DLF003_C717784_1	non-phage	0
DLF003_scaffold4936_10	non-phage	0
DLF003_scaffold53800_1	non-phage	0
DLF003_scaffold53023_4	non-phage	0
DLF003_scaffold13196_4	non-phage	0
DLF003_scaffold53764_1	non-phage	0
DLF003_scaffold48238_1	non-phage	0
DLF003_scaffold45496_1	non-phage	0
DLF003_scaffold57129_1	non-phage	0
DLF003_scaffold9111_5	non-phage	0
DLF003_scaffold37151_2	non-phage	0
DLF003_scaffold29891_3	phage	0.997
DLF003_scaffold44631_1	non-phage	0
DLF003_scaffold16_1	non-phage	0
DLF003_scaffold45224_1	non-phage	0
DLF003_scaffold18271_2	non-phage	0
DLF003_scaffold13553_2	non-phage	0
DLF003_scaffold1924_4	non-phage	0
DLF003_scaffold3993_5	non-phage	0
DLF003_scaffold120_2	non-phage	0
DLF003_scaffold19227_2	non-phage	0
DLF003_scaffold54204_1	non-phage	0
DLF003_scaffold17462_3	non-phage	0
DLF003_scaffold16249_2	non-phage	0
DLF003_scaffold53023_1	non-phage	0
DLF003_scaffold35032_1	non-phage	0
DLF003_scaffold56524_1	non-phage	0
DLF003_scaffold55655_1	non-phage	0
DLF003_scaffold10795_3	non-phage	0
DLF003_scaffold55539_1	non-phage	0
DLF004_scaffold50_3	non-phage	0
DLF004_scaffold1974_1	non-phage	0

DLF004_scaffold35797_1	phage	0.997
DLF004_scaffold34288_2	non-phage	0
DLF004_scaffold10854_7	non-phage	0
DLF004_scaffold30_3	non-phage	0
DLF004_scaffold3599_14	non-phage	0
DLF004_scaffold2517_1	non-phage	0
DLF004_scaffold7918_3	non-phage	0
DLF004_scaffold36527_1	phage	1
DLF004_scaffold11847_5	non-phage	0
DLF004_scaffold11_3	non-phage	0
DLF004_scaffold35154_2	non-phage	0
DLF004_scaffold36519_1	non-phage	0
DLF004_scaffold35214_1	non-phage	0
DLF004_scaffold3599_3	non-phage	0
DLF004_scaffold27866_1	non-phage	0
DLF004_scaffold17812_1	non-phage	0
DLF004_scaffold3669_2	non-phage	0
DLF004_scaffold4371_1	non-phage	0
DLF004_scaffold930_19	non-phage	0
DLF004_scaffold4958_2	non-phage	0
DLF004_scaffold33732_7	non-phage	0
DLF004_scaffold14299_2	non-phage	0
DLF004_scaffold36372_1	non-phage	0
DLF004_scaffold36386_1	non-phage	0
DLF004_scaffold35916_2	non-phage	0
DLF004_scaffold21949_2	non-phage	0
DLF004_scaffold9484_1	non-phage	0
DLF004_scaffold27736_4	non-phage	0
DLF004_scaffold19039_7	non-phage	0
DLF004_scaffold16536_2	non-phage	0
DLF004_scaffold36375_1	non-phage	0
DLF004_scaffold14499_6	non-phage	0
DLF004_scaffold90_5	non-phage	0
DLF004_scaffold21755_2	non-phage	0
DLF004_scaffold31108_1	non-phage	0
DLF004_scaffold31365_4	non-phage	0
DLF004_scaffold33148_1	non-phage	0
DLF004_scaffold33829_6	non-phage	0
DLF004_scaffold31284_1	non-phage	0
DLF004_scaffold17377_6	non-phage	0
DLF004_scaffold12916_5	non-phage	0
DLF004_scaffold122_2	non-phage	0
DLF004_scaffold22703_3	non-phage	0
DLF004_scaffold36593_1	non-phage	0
DLF004_scaffold26106_4	non-phage	0
DLF004_scaffold36569_1	non-phage	0

DLF004_scaffold2108_1	non-phage	0
DLF004_scaffold36308_1	non-phage	0
DLF004_scaffold27539_1	non-phage	0
DLF004_scaffold1135_19	non-phage	0
DLF004_scaffold35359_8	non-phage	0
DLF004_scaffold1089_1	non-phage	0
DLF004_scaffold36595_1	non-phage	0
DLF004_scaffold36525_1	non-phage	0
DLF004_scaffold32830_1	non-phage	0
DLF004_scaffold36364_2	non-phage	0
DLF004_scaffold27866_6	non-phage	0
DLF004_scaffold22523_2	non-phage	0
DLF004_scaffold10898_10	non-phage	0
DLF004_scaffold31054_1	non-phage	0
DLF004_scaffold30047_4	non-phage	0
DLF004_scaffold34030_1	non-phage	0
DLF004_scaffold1538_3	non-phage	0
DLF004_scaffold32800_1	non-phage	0
DLF004_scaffold25533_2	non-phage	0
DLF004_scaffold31616_1	non-phage	0
DLF004_scaffold12629_1	non-phage	0
DLF004_scaffold11_1	non-phage	0
DLF004_C548546_1	phage	1
DLF004_scaffold27971_1	non-phage	0
DLF004_scaffold14294_1	non-phage	0
DLF004_scaffold36466_1	non-phage	0
DLF004_scaffold2352_2	non-phage	0
DLF004_scaffold33218_2	non-phage	0
DLF004_scaffold25579_1	non-phage	0
DLF004_scaffold10898_11	non-phage	0
DLF004_scaffold8020_4	non-phage	0
DLF004_scaffold36201_3	non-phage	0
DLF004_scaffold80_18	non-phage	0
DLF004_scaffold3599_6	non-phage	0
DLF004_scaffold35305_1	non-phage	0
DLF004_scaffold33632_1	non-phage	0
DLF004_scaffold181_8	non-phage	0
DLF004_scaffold61_1	non-phage	0
DLF004_scaffold36603_1	non-phage	0
DLF004_scaffold12069_1	non-phage	0
DLF004_scaffold5950_5	non-phage	0
DLF004_scaffold36599_1	non-phage	0
DLF004_scaffold17241_3	non-phage	0
DLF004_scaffold32559_1	non-phage	0
DLF004_scaffold16674_1	non-phage	0
DLF004_scaffold88_3	non-phage	0

DLF004_scaffold35854_4	non-phage	0
DLF004_scaffold10173_6	non-phage	0
DLF004_scaffold21638_1	non-phage	0
DLF004_scaffold34686_2	non-phage	0
DLF004_scaffold8570_4	non-phage	0
DLF004_scaffold9620_1	non-phage	0
DLF004_scaffold35527_4	non-phage	0.43
DLF004_C548254_1	non-phage	0
DLF004_scaffold19957_4	non-phage	0
DLF004_scaffold10898_4	non-phage	0.326
DLF004_scaffold31_4	non-phage	0
DLF004_scaffold32365_2	non-phage	0
DLF004_scaffold19326_3	non-phage	0
DLF004_scaffold36207_1	non-phage	0
DLF004_scaffold104_5	non-phage	0
DLF004_scaffold1765_2	non-phage	0
DLF004_scaffold3589_19	non-phage	0
DLF004_scaffold81_4	non-phage	0
DLF004_scaffold6784_2	non-phage	0
DLF004_scaffold36475_1	non-phage	0
DLF004_scaffold36010_1	non-phage	0
DLF004_scaffold35372_2	non-phage	0
DLF004_scaffold18383_6	phage	0.999
DLF004_scaffold36364_1	non-phage	0
DLF004_scaffold36500_1	non-phage	0
DLF004_scaffold174_2	non-phage	0
DLF004_scaffold19876_1	non-phage	0
DLF004_scaffold36412_1	non-phage	0
DLF004_scaffold7088_2	non-phage	0
DLF004_scaffold21819_1	non-phage	0
DLF004_C548064_1	non-phage	0
DLF004_scaffold125_28	non-phage	0
DLF004_scaffold16229_1	non-phage	0
DLF004_scaffold9667_3	non-phage	0
DLF004_scaffold21736_2	non-phage	0
DLF004_scaffold13680_2	non-phage	0
DLF004_scaffold21771_1	non-phage	0
DLF004_scaffold6254_1	non-phage	0
DLF004_scaffold17733_5	non-phage	0
DLF004_scaffold21949_1	non-phage	0
DLF004_scaffold36600_1	non-phage	0
DLF004_scaffold22865_1	non-phage	0
DLF004_scaffold181_5	non-phage	0
DLF004_scaffold3604_9	non-phage	0
DLF004_scaffold14590_8	non-phage	0
DLF004_scaffold1531_1	non-phage	0

DLF004_scaffold27283_1	non-phage	0
DLF004_scaffold8289_4	non-phage	0
DLF004_scaffold36289_1	non-phage	0
DLF004_scaffold118_3	non-phage	0
DLF004_scaffold9729_2	non-phage	0
DLF004_scaffold49_1	non-phage	0
DLF004_scaffold36356_2	non-phage	0
DLF004_scaffold34665_5	non-phage	0
DLF004_scaffold53_4	non-phage	0
DLF004_scaffold15816_3	non-phage	0
DLF004_scaffold29269_1	non-phage	0
DLF004_scaffold7282_1	non-phage	0
DLF004_scaffold3608_56	non-phage	0
DLF004_scaffold35960_2	non-phage	0
DLF004_scaffold10107_2	non-phage	0
DLF004_scaffold2534_2	non-phage	0
DLF004_scaffold1135_17	non-phage	0
DLF004_scaffold17043_13	non-phage	0
DLF004_scaffold17878_5	non-phage	0
DLF004_scaffold36334_4	non-phage	0
DLF004_scaffold4408_17	non-phage	0
DLF004_scaffold2195_1	non-phage	0
DLF004_scaffold17377_11	non-phage	0
DLF004_scaffold36572_1	non-phage	0
DLF004_scaffold8099_13	non-phage	0
DLF004_scaffold36581_1	non-phage	0
DLF004_scaffold36524_1	non-phage	0
DLF004_scaffold50_10	non-phage	0
DLF004_scaffold35854_8	non-phage	0
DLF004_scaffold8099_10	non-phage	0
DLF004_scaffold34534_4	non-phage	0
DLF004_scaffold4249_1	non-phage	0
DLF004_scaffold7386_2	non-phage	0
DLF004_scaffold29332_2	non-phage	0
DLF004_scaffold36601_3	non-phage	0
DLF004_scaffold18054_6	non-phage	0
DLF004_scaffold36319_3	non-phage	0
DLF004_scaffold29315_1	non-phage	0
DLF004_scaffold196_3	non-phage	0
DLF004_scaffold19599_3	non-phage	0
DLF004_scaffold16427_2	non-phage	0
DLF004_scaffold10_1	non-phage	0
DLF004_scaffold20262_1	non-phage	0
DLF004_scaffold13465_2	non-phage	0
DLF004_scaffold34686_3	non-phage	0
DLF004_scaffold5567_1	non-phage	0

DLF004_C548576_1	phage	0.999
DLF004_scaffold36568_1	non-phage	0
DLF004_scaffold1531_4	non-phage	0
DLF004_scaffold29329_1	non-phage	0
DLF004_scaffold19039_6	non-phage	0
DLF004_scaffold29106_15	non-phage	0
DLF004_scaffold1716_4	non-phage	0
DLF004_scaffold23337_1	non-phage	0
DLF004_scaffold33829_8	non-phage	0
DLF004_scaffold12916_6	non-phage	0
DLF004_scaffold22703_2	non-phage	0
DLF004_scaffold34372_2	non-phage	0
DLF004_scaffold13680_3	non-phage	0
DLF004_scaffold2597_4	non-phage	0
DLF004_scaffold20398_10	non-phage	0
DLF004_scaffold36287_1	non-phage	0
DLF004_scaffold178_4	non-phage	0
DLF004_scaffold23942_9	phage	0.993
DLF004_scaffold35882_1	non-phage	0
DLF004_scaffold34869_2	non-phage	0
DLF004_scaffold1675_2	non-phage	0
DLF004_scaffold2184_1	non-phage	0
DLF004_scaffold33116_1	non-phage	0
DLF004_scaffold29661_1	non-phage	0
DLF004_scaffold15823_4	non-phage	0
DLF004_scaffold24576_1	non-phage	0
DLF004_scaffold10291_5	non-phage	0
DLF004_scaffold6466_1	non-phage	0
DLF004_scaffold11_2	non-phage	0
DLF004_scaffold35519_3	non-phage	0
DLF004_scaffold4408_2	non-phage	0
DLF004_scaffold10471_2	non-phage	0
DLF004_scaffold12629_2	non-phage	0
DLF004_scaffold10854_4	phage	0.999
DLF004_scaffold29106_12	non-phage	0
DLF004_scaffold3669_3	non-phage	0
DLF004_scaffold32532_2	non-phage	0
DLF004_scaffold23452_4	non-phage	0
DLF004_scaffold18130_6	non-phage	0
DLF004_scaffold20398_1	non-phage	0
DLF004_scaffold33218_3	non-phage	0
DLF004_scaffold15929_3	non-phage	0
DLF004_scaffold32186_1	non-phage	0
DLF004_scaffold23291_3	non-phage	0
DLF004_scaffold14590_1	non-phage	0
DLF004_scaffold26319_3	non-phage	0

DLF004_scaffold26342_1	non-phage	0
DLF004_scaffold35341_1	non-phage	0
DLF004_scaffold15271_2	non-phage	0.006
DLF004_scaffold80_1	non-phage	0
DLF004_scaffold7088_6	non-phage	0
DLF004_scaffold21965_1	non-phage	0
DLF004_scaffold77_2	non-phage	0
DLF004_scaffold36365_1	non-phage	0
DLF004_scaffold29302_1	non-phage	0
DLF004_C548404_1	non-phage	0
DLF004_scaffold17377_5	non-phage	0
DLF004_scaffold28880_1	phage	1
DLF004_scaffold25579_5	non-phage	0
DLF004_scaffold36509_2	non-phage	0
DLF004_scaffold99_2	non-phage	0
DLF004_scaffold8289_2	non-phage	0
DLF004_scaffold36405_4	non-phage	0
DLF004_scaffold23723_5	non-phage	0
DLF004_scaffold25533_1	phage	0.832
DLF004_scaffold36320_8	phage	0.995
DLF004_scaffold36595_2	non-phage	0
DLF004_scaffold28_5	non-phage	0
DLF004_scaffold33732_6	non-phage	0
DLF004_scaffold9667_1	non-phage	0
DLF004_scaffold28266_11	non-phage	0
DLF004_scaffold31627_1	non-phage	0
DLF004_scaffold14809_3	non-phage	0
DLF004_scaffold17812_2	non-phage	0
DLF004_scaffold36333_1	non-phage	0
DLF004_scaffold25705_1	non-phage	0
DLF004_scaffold9079_4	non-phage	0
DLF004_C548200_1	phage	0.902
DLF004_scaffold6226_4	non-phage	0
DLF004_scaffold7918_6	non-phage	0
DLF004_scaffold36296_1	non-phage	0
DLF004_scaffold2958_1	non-phage	0
DLF004_scaffold16420_2	non-phage	0
DLF004_scaffold2869_1	non-phage	0
DLF004_scaffold2597_8	non-phage	0
DLF004_scaffold9095_1	non-phage	0
DLF004_scaffold20262_2	non-phage	0
DLF004_scaffold15711_1	non-phage	0
DLF004_scaffold35135_1	non-phage	0
DLF004_scaffold35367_1	non-phage	0
DLF004_scaffold62_1	non-phage	0
DLF004_scaffold36470_3	non-phage	0

DLF004_scaffold117_1	non-phage	0
DLF004_scaffold10947_1	non-phage	0
DLF004_scaffold10898_8	non-phage	0
DLF004_scaffold34098_1	non-phage	0
DLF004_scaffold2535_5	phage	0.996
DLF004_scaffold14370_1	non-phage	0
DLF004_scaffold32830_2	phage	0.997
DLF004_scaffold2958_8	non-phage	0.153
DLF004_scaffold15075_1	non-phage	0
DLF004_scaffold24809_1	non-phage	0
DLF004_scaffold129_10	non-phage	0
DLF004_scaffold22703_6	non-phage	0
DLF004_scaffold27410_2	non-phage	0
DLF004_scaffold1675_1	non-phage	0
DLF004_scaffold19654_3	non-phage	0
DLF004_scaffold80_6	non-phage	0
DLF004_scaffold19957_5	phage	0.99
DLF004_scaffold24000_1	non-phage	0
DLF004_scaffold4958_3	non-phage	0
DLF004_scaffold30602_2	phage	0.891
DLF004_scaffold30_7	non-phage	0
DLF004_scaffold4470_2	non-phage	0
DLF004_scaffold81_6	non-phage	0
DLF004_scaffold10635_1	non-phage	0
DLF004_scaffold36362_1	non-phage	0
DLF004_scaffold2108_2	non-phage	0
DLF004_scaffold29106_2	non-phage	0
DLF004_scaffold8570_3	non-phage	0
DLF004_scaffold7336_10	non-phage	0
DLF004_scaffold33629_1	non-phage	0
DLF004_scaffold14138_1	non-phage	0
DLF004_scaffold930_12	non-phage	0
DLF004_scaffold36475_2	non-phage	0
DLF004_scaffold22523_1	non-phage	0
DLF004_scaffold7088_3	non-phage	0
DLF004_scaffold17377_8	non-phage	0
DLF004_scaffold17043_3	non-phage	0
DLF004_scaffold18736_31	non-phage	0
DLF004_scaffold35940_3	non-phage	0
DLF004_scaffold20012_1	non-phage	0
DLF004_scaffold21209_3	non-phage	0
DLF004_scaffold5175_2	non-phage	0
DLF004_scaffold36482_1	non-phage	0
DLF004_scaffold23033_1	non-phage	0
DLF004_scaffold18527_2	non-phage	0
DLF004_scaffold35960_1	non-phage	0

DLF004_scaffold27_1	non-phage	0
DLF004_scaffold36586_1	non-phage	0
DLF004_scaffold24576_5	non-phage	0
DLF004_scaffold8390_17	non-phage	0
DLF004_scaffold129_9	non-phage	0
DLF004_scaffold36319_2	non-phage	0
DLF004_scaffold13540_1	non-phage	0
DLF004_scaffold17467_1	non-phage	0
DLF004_scaffold20496_11	non-phage	0
DLF004_scaffold11847_2	non-phage	0
DLF004_scaffold27435_2	phage	0.949
DLF004_scaffold18478_3	non-phage	0
DLF004_scaffold21102_3	non-phage	0
DLF004_scaffold12422_5	non-phage	0
DLF004_scaffold23723_4	non-phage	0
DLF004_scaffold8890_3	non-phage	0
DLF004_scaffold36582_2	non-phage	0
DLF004_scaffold10964_1	non-phage	0
DLF004_scaffold9620_2	non-phage	0
DLF004_scaffold36583_1	non-phage	0
DLF004_scaffold36321_2	non-phage	0
DLF004_scaffold114_19	non-phage	0
DLF004_scaffold31466_11	non-phage	0
DLF004_scaffold10107_3	non-phage	0
DLF004_scaffold15128_1	non-phage	0
DLF004_scaffold36402_1	non-phage	0
DLF004_scaffold162_3	non-phage	0
DLF004_scaffold31365_2	non-phage	0
DLF004_scaffold3608_54	non-phage	0
DLF004_scaffold19599_2	non-phage	0
DLF004_scaffold111_2	non-phage	0
DLF004_scaffold17382_6	non-phage	0
DLF004_scaffold16251_9	non-phage	0
DLF004_scaffold34815_1	non-phage	0
DLF004_scaffold36602_1	non-phage	0
DLF004_scaffold129_1	non-phage	0
DLF004_scaffold35326_1	non-phage	0
DLF004_scaffold4408_21	non-phage	0
DLF004_scaffold14294_3	non-phage	0
DLF004_scaffold8390_22	non-phage	0
DLF004_scaffold930_8	phage	0.598
DLF004_scaffold8102_2	phage	1
DLF004_scaffold80_2	non-phage	0
DLF004_scaffold33761_1	non-phage	0
DLF004_scaffold30047_2	non-phage	0
DLF004_scaffold31627_2	non-phage	0

DLF004_scaffold2090_4	non-phage	0
DLF004_scaffold17043_11	non-phage	0
DLF004_scaffold36319_4	non-phage	0
DLF004_scaffold32187_1	phage	0.603
DLF004_scaffold17901_17	non-phage	0
DLF004_scaffold2870_5	non-phage	0
DLF004_scaffold25438_1	non-phage	0
DLF004_scaffold196_1	non-phage	0
DLF004_scaffold31382_3	non-phage	0
DLF004_scaffold33732_5	non-phage	0
DLF004_scaffold15783_4	non-phage	0
DLF004_scaffold27816_3	non-phage	0
DLF004_scaffold29106_16	non-phage	0
DLF004_scaffold36309_1	non-phage	0
DLF004_scaffold21209_2	non-phage	0
DLF004_scaffold29329_2	non-phage	0
DLF004_scaffold30_1	non-phage	0
DLF004_scaffold36481_2	non-phage	0
DLF004_scaffold36573_1	non-phage	0
DLF004_scaffold5687_4	non-phage	0
DLF004_scaffold31813_1	non-phage	0
DLF004_scaffold21453_5	non-phage	0
DLF004_scaffold7918_10	non-phage	0
DLF004_scaffold33148_3	non-phage	0
DLF004_scaffold35854_2	non-phage	0
DLF004_scaffold13540_2	non-phage	0
DLF004_scaffold103_2	non-phage	0
DLF004_scaffold35964_6	non-phage	0
DLF004_scaffold5790_1	non-phage	0
DLF004_scaffold34845_1	non-phage	0
DLF004_scaffold90_1	non-phage	0
DLF004_scaffold30_5	non-phage	0
DLF004_scaffold18383_9	non-phage	0
DLF004_scaffold36520_1	non-phage	0
DLF004_scaffold37_1	non-phage	0
DLF004_scaffold1675_4	non-phage	0
DLF004_scaffold34230_5	non-phage	0
DLF004_scaffold11448_10	non-phage	0
DLF004_scaffold3057_1	non-phage	0
DLF004_scaffold15929_2	non-phage	0
DLF004_scaffold26136_3	non-phage	0
DLF004_scaffold23115_1	phage	0.987
DLF004_scaffold36236_2	non-phage	0
DLF004_scaffold91_4	non-phage	0
DLF004_scaffold50_6	non-phage	0
DLF004_scaffold34372_3	non-phage	0

DLF004_scaffold6537_1	phage	0.987
DLF004_scaffold26319_1	non-phage	0
DLF004_C548296_1	non-phage	0
DLF004_scaffold141_1	non-phage	0
DLF004_scaffold17377_4	non-phage	0
DLF004_scaffold1531_3	non-phage	0
DLF004_scaffold36520_2	non-phage	0
DLF004_scaffold841_6	non-phage	0
DLF004_scaffold14590_7	non-phage	0
DLF004_scaffold11997_2	non-phage	0
DLF004_scaffold104_3	non-phage	0
DLF004_scaffold15396_3	phage	0.999
DLF004_scaffold12454_2	non-phage	0
DLF004_scaffold36490_1	non-phage	0
DLF004_scaffold35341_2	non-phage	0
DLF004_scaffold1194_2	non-phage	0
DLF004_scaffold137_6	non-phage	0
DLF004_scaffold36208_1	non-phage	0
DLF004_scaffold19326_1	non-phage	0
DLF004_scaffold29380_1	non-phage	0
DLF004_scaffold8390_32	non-phage	0
DLF004_scaffold36501_1	non-phage	0
DLF004_scaffold36484_1	non-phage	0
DLF004_scaffold35061_2	non-phage	0
DLF004_scaffold201_2	non-phage	0
DLF004_scaffold6226_3	non-phage	0
DLF004_scaffold2480_1	non-phage	0
DLF004_scaffold19654_1	non-phage	0
DLF004_scaffold2352_3	non-phage	0
DLF004_scaffold13768_3	non-phage	0
DLF004_scaffold22180_1	non-phage	0
DLF004_scaffold34328_1	non-phage	0
DLF004_scaffold22703_5	non-phage	0
DLF004_scaffold33220_3	non-phage	0
DLF004_scaffold4210_2	non-phage	0
DLF004_C548182_1	non-phage	0
DLF004_scaffold35897_1	non-phage	0
DLF004_scaffold29551_1	non-phage	0
DLF004_scaffold1120_2	non-phage	0.015
DLF004_scaffold1006_4	non-phage	0
DLF004_scaffold14499_8	non-phage	0
DLF004_scaffold1135_15	non-phage	0
DLF004_scaffold5096_1	non-phage	0
DLF004_scaffold25582_3	non-phage	0
DLF004_scaffold35854_6	non-phage	0
DLF004_scaffold7918_11	phage	0.998

DLF004_scaffold26814_1	non-phage	0
DLF004_scaffold35061_1	non-phage	0
DLF004_scaffold17241_8	non-phage	0
DLF004_scaffold7163_2	non-phage	0
DLF004_scaffold17377_2	non-phage	0
DLF004_scaffold17443_5	non-phage	0
DLF004_scaffold10854_1	non-phage	0
DLF004_scaffold16420_3	non-phage	0
DLF004_scaffold9889_2	non-phage	0
DLF004_scaffold30353_2	non-phage	0
DLF004_scaffold17855_1	non-phage	0
DLF004_scaffold7020_1	phage	0.995
DLF004_scaffold27816_2	non-phage	0
DLF004_scaffold36601_1	non-phage	0
DLF004_scaffold1974_5	phage	0.997
DLF004_scaffold2870_2	non-phage	0
DLF004_scaffold19152_3	non-phage	0
DLF004_scaffold10964_3	non-phage	0
DLF004_scaffold8655_1	non-phage	0
DLF004_scaffold4408_13	non-phage	0
DLF004_scaffold27866_3	non-phage	0
DLF004_scaffold31408_1	non-phage	0
DLF004_scaffold17901_6	non-phage	0
DLF004_scaffold19957_2	non-phage	0
DLF004_scaffold12629_5	non-phage	0
DLF004_scaffold23665_2	non-phage	0
DLF004_scaffold7088_4	non-phage	0
DLF004_scaffold19876_3	non-phage	0
DLF004_scaffold22703_9	non-phage	0
DLF004_scaffold34925_2	non-phage	0
DLF004_scaffold34490_1	non-phage	0
DLF004_scaffold27725_3	non-phage	0
DLF004_scaffold15128_2	non-phage	0
DLF004_scaffold36579_1	non-phage	0
DLF004_scaffold80_13	non-phage	0
DLF004_scaffold8885_3	non-phage	0
DLF004_scaffold18130_5	non-phage	0
DLF004_scaffold32365_4	non-phage	0
DLF004_scaffold36582_3	non-phage	0
DLF004_scaffold21819_3	non-phage	0
DLF004_scaffold930_24	non-phage	0
DLF004_scaffold26580_1	non-phage	0
DLF004_scaffold36585_1	non-phage	0
DLF004_scaffold14410_3	non-phage	0
DLF004_scaffold4408_10	non-phage	0
DLF004_scaffold36543_1	non-phage	0

DLF004_scaffold24525_4	phage	0.904
DLF004_scaffold181_2	non-phage	0
DLF004_scaffold36539_6	non-phage	0
DLF004_scaffold36516_1	non-phage	0
DLF004_scaffold36242_1	non-phage	0
DLF004_scaffold24000_4	non-phage	0
DLF004_scaffold21209_1	non-phage	0
DLF004_scaffold9693_2	non-phage	0
DLF004_scaffold35175_2	non-phage	0
DLF004_scaffold14499_7	non-phage	0
DLF004_scaffold18770_8	non-phage	0
DLF004_scaffold35652_9	non-phage	0
DLF004_scaffold16674_2	non-phage	0
DLF004_scaffold15823_6	phage	0.996
DLF004_scaffold3589_23	non-phage	0
DLF004_scaffold23055_1	non-phage	0
DLF004_scaffold32013_2	non-phage	0
DLF004_scaffold16420_10	non-phage	0
DLF004_scaffold5653_2	non-phage	0
DLF004_scaffold35580_1	non-phage	0
DLF004_scaffold111_1	non-phage	0
DLF004_scaffold35352_1	non-phage	0
DLF004_scaffold3599_2	non-phage	0
DLF004_scaffold24664_2	non-phage	0
DLF004_scaffold25593_2	non-phage	0
DLF004_scaffold1120_5	non-phage	0
DLF004_scaffold114_6	non-phage	0
DLF004_scaffold23019_1	non-phage	0
DLF004_scaffold9575_2	non-phage	0
DLF004_scaffold36203_3	non-phage	0
DLF004_scaffold24809_6	non-phage	0
DLF004_scaffold20200_2	non-phage	0
DLF004_scaffold140_8	non-phage	0
DLF004_scaffold53_3	non-phage	0
DLF004_scaffold33218_5	non-phage	0
DLF004_scaffold12493_1	non-phage	0
DLF004_scaffold23115_2	non-phage	0
DLF004_scaffold73_3	non-phage	0
DLF004_scaffold35797_2	non-phage	0
DLF004_scaffold10622_1	non-phage	0
DLF004_scaffold24184_3	non-phage	0
DLF004_scaffold33294_8	non-phage	0
DLF004_scaffold34885_1	non-phage	0
DLF004_scaffold27458_1	non-phage	0
DLF004_scaffold25533_3	non-phage	0
DLF004_scaffold18736_12	non-phage	0

DLF004_scaffold35940_1	non-phage	0
DLF004_scaffold30210_3	non-phage	0
DLF004_scaffold31466_2	non-phage	0
DLF004_scaffold32754_4	non-phage	0
DLF004_scaffold6365_18	non-phage	0
DLF004_scaffold10622_5	non-phage	0
DLF004_scaffold11997_1	non-phage	0
DLF004_scaffold6226_10	non-phage	0
DLF004_scaffold17043_9	non-phage	0
DLF004_scaffold27201_2	non-phage	0
DLF004_scaffold26106_5	phage	0.96
DLF004_scaffold12069_2	non-phage	0
DLF004_scaffold4136_3	non-phage	0
DLF004_scaffold11849_1	non-phage	0
DLF004_scaffold10426_4	non-phage	0
DLF004_scaffold30812_2	non-phage	0
DLF004_scaffold13540_3	non-phage	0
DLF004_scaffold4632_20	non-phage	0
DLF004_scaffold31365_1	non-phage	0
DLF004_scaffold6537_4	non-phage	0
DLF004_scaffold129_4	non-phage	0
DLF004_scaffold24454_17	non-phage	0
DLF004_scaffold178_7	non-phage	0
DLF004_scaffold25632_1	non-phage	0
DLF004_scaffold12629_9	non-phage	0
DLF004_scaffold36580_1	non-phage	0
DLF004_scaffold23769_1	non-phage	0
DLF004_scaffold36551_2	non-phage	0
DLF004_scaffold9874_2	non-phage	0
DLF004_scaffold33294_11	non-phage	0
DLF004_scaffold36482_7	non-phage	0
DLF004_scaffold3120_1	non-phage	0
DLF004_scaffold17153_3	non-phage	0
DLF004_scaffold22703_8	phage	0.905
DLF004_scaffold4408_9	non-phage	0
DLF004_scaffold7970_15	non-phage	0
DLF004_scaffold33441_2	non-phage	0
DLF004_scaffold14294_2	non-phage	0
DLF004_scaffold31409_1	non-phage	0
DLF004_scaffold1603_1	non-phage	0
DLF004_scaffold36355_2	non-phage	0
DLF004_scaffold33467_1	non-phage	0
DLF004_scaffold27866_7	non-phage	0
DLF004_scaffold196_2	non-phage	0
DLF004_scaffold31262_2	non-phage	0
DLF004_scaffold2418_7	non-phage	0

DLF004_scaffold164_1	non-phage	0
DLF004_scaffold36511_1	non-phage	0
DLF004_scaffold9673_2	non-phage	0
DLF004_scaffold26814_2	non-phage	0
DLF004_scaffold1642_7	non-phage	0
DLF004_scaffold24576_3	non-phage	0
DLF004_scaffold31071_1	non-phage	0
DLF004_scaffold36276_1	non-phage	0
DLF004_scaffold24809_3	non-phage	0
DLF004_scaffold27778_1	non-phage	0
DLF004_scaffold36531_1	non-phage	0
DLF004_scaffold32754_1	non-phage	0
DLF004_scaffold24525_1	non-phage	0
DLF004_scaffold17377_3	non-phage	0
DLF004_scaffold36591_1	non-phage	0
DLF004_scaffold4408_12	non-phage	0
DLF004_scaffold152_1	non-phage	0
DLF004_scaffold25834_5	non-phage	0
DLF004_scaffold36356_3	non-phage	0
DLF004_scaffold23723_7	non-phage	0
DLF004_scaffold36556_1	non-phage	0
DLF004_scaffold36191_2	non-phage	0
DLF004_scaffold2480_2	non-phage	0
DLF004_scaffold8385_2	non-phage	0
DLF004_scaffold36530_1	non-phage	0
DLF004_scaffold13126_4	non-phage	0
DLF004_scaffold111_3	non-phage	0
DLF004_scaffold3694_2	non-phage	0
DLF004_scaffold21240_2	phage	0.884
DLF004_scaffold1908_1	non-phage	0
DLF004_scaffold3599_17	non-phage	0
DLF004_scaffold28836_1	non-phage	0
DLF004_scaffold91_11	non-phage	0
DLF004_scaffold36447_2	non-phage	0
DLF004_scaffold35372_1	non-phage	0
DLF004_scaffold9673_1	non-phage	0
DLF004_scaffold21755_1	non-phage	0
DLF004_scaffold35341_3	non-phage	0
DLF004_scaffold7918_1	non-phage	0
DLF004_scaffold12916_4	non-phage	0
DLF004_scaffold21755_5	non-phage	0
DLF004_scaffold1394_20	non-phage	0
DLF004_scaffold36516_2	non-phage	0
DLF004_scaffold23665_1	non-phage	0
DLF004_scaffold32134_3	non-phage	0
DLF004_scaffold20_1	non-phage	0

DLF004_scaffold36424_1	non-phage	0
DLF004_scaffold19957_3	non-phage	0
DLF004_scaffold3589_17	non-phage	0
DLF004_scaffold36597_1	non-phage	0
DLF004_scaffold32365_5	non-phage	0
DLF004_scaffold34444_3	non-phage	0
DLF004_scaffold19039_12	non-phage	0.414
DLF004_scaffold36558_3	non-phage	0
DLF004_scaffold33220_2	non-phage	0
DLF004_scaffold36216_1	non-phage	0
DLF004_scaffold3589_9	non-phage	0
DLF004_scaffold104_1	non-phage	0
DLF004_scaffold1974_4	non-phage	0
DLF004_scaffold16030_1	non-phage	0
DLF004_scaffold7163_1	non-phage	0
DLF004_scaffold10471_3	non-phage	0
DLF004_scaffold8396_2	non-phage	0
DLF004_scaffold174_1	non-phage	0
DLF004_scaffold5994_5	non-phage	0
DLF004_scaffold178_3	non-phage	0
DLF004_scaffold17878_4	non-phage	0
DLF004_scaffold7088_5	non-phage	0
DLF004_scaffold35154_1	non-phage	0
DLF004_scaffold23452_13	non-phage	0
DLF004_scaffold62_3	non-phage	0
DLF004_scaffold125_29	phage	0.998
DLF004_scaffold34534_1	non-phage	0
DLF004_scaffold35970_1	non-phage	0
DLF004_scaffold6784_1	non-phage	0
DLF004_scaffold32797_2	non-phage	0
DLF004_scaffold4958_8	non-phage	0
DLF004_scaffold19920_1	non-phage	0
DLF004_scaffold13080_2	non-phage	0
DLF004_scaffold2958_11	non-phage	0
DLF005_scaffold31168_1	phage	0.999
DLF005_scaffold2807_1	non-phage	0
DLF005_scaffold56890_1	non-phage	0
DLF005_scaffold56698_1	non-phage	0
DLF005_scaffold35811_1	non-phage	0
DLF005_scaffold4062_23	non-phage	0
DLF005_scaffold16722_5	non-phage	0
DLF005_scaffold55052_1	phage	0.999
DLF005_scaffold56993_2	non-phage	0
DLF005_scaffold9847_7	non-phage	0
DLF005_C848054_1	non-phage	0
DLF005_scaffold32585_3	non-phage	0

DLF005_scaffold588_1	non-phage	0
DLF005_scaffold41749_3	non-phage	0
DLF005_scaffold56707_1	phage	0.998
DLF005_scaffold54264_1	non-phage	0
DLF005_scaffold49696_1	non-phage	0
DLF005_scaffold56989_2	non-phage	0
DLF005_scaffold7947_8	non-phage	0
DLF005_scaffold34625_3	non-phage	0
DLF005_scaffold27755_10	non-phage	0
DLF005_scaffold48193_1	non-phage	0
DLF005_scaffold55556_2	non-phage	0
DLF005_scaffold9815_2	non-phage	0
DLF005_scaffold56474_3	non-phage	0
DLF005_scaffold21023_1	non-phage	0
DLF005_scaffold22011_11	non-phage	0
DLF005_scaffold56532_2	non-phage	0
DLF005_scaffold141_3	non-phage	0
DLF005_scaffold23342_8	non-phage	0
DLF005_scaffold4132_4	non-phage	0
DLF005_scaffold52174_5	non-phage	0
DLF005_scaffold51731_3	non-phage	0
DLF005_scaffold35078_4	non-phage	0
DLF005_scaffold56951_1	non-phage	0
DLF005_scaffold31945_4	phage	0.999
DLF005_scaffold56905_1	non-phage	0
DLF005_scaffold44507_2	non-phage	0
DLF005_scaffold34528_3	non-phage	0
DLF005_scaffold52948_4	non-phage	0
DLF005_scaffold52997_2	non-phage	0
DLF005_scaffold56853_2	non-phage	0
DLF005_scaffold47023_4	non-phage	0
DLF005_scaffold3312_2	non-phage	0
DLF005_scaffold54804_2	non-phage	0
DLF005_scaffold56924_1	non-phage	0
DLF005_scaffold11641_6	non-phage	0
DLF005_scaffold56363_3	non-phage	0
DLF005_scaffold4062_41	non-phage	0
DLF005_scaffold56358_1	non-phage	0
DLF005_scaffold93_3	non-phage	0
DLF005_scaffold2503_2	non-phage	0
DLF005_scaffold47023_9	non-phage	0
DLF005_scaffold56612_1	non-phage	0
DLF005_scaffold4919_2	non-phage	0
DLF005_scaffold56256_1	non-phage	0
DLF005_scaffold56913_3	non-phage	0
DLF005_scaffold50670_2	non-phage	0

DLF005_scaffold63_1	non-phage	0
DLF005_scaffold9815_3	non-phage	0
DLF005_scaffold250_2	non-phage	0
DLF005_scaffold56935_2	non-phage	0
DLF005_scaffold11846_31	non-phage	0
DLF005_scaffold53141_1	non-phage	0
DLF005_scaffold56683_2	non-phage	0
DLF005_scaffold25785_3	non-phage	0
DLF005_scaffold25099_8	non-phage	0
DLF005_scaffold51024_1	non-phage	0
DLF005_scaffold56392_1	non-phage	0
DLF005_scaffold43959_2	non-phage	0
DLF005_scaffold4602_3	non-phage	0
DLF005_scaffold56933_1	non-phage	0
DLF005_scaffold4062_48	non-phage	0
DLF005_scaffold52014_6	non-phage	0
DLF005_scaffold56609_1	non-phage	0
DLF005_scaffold25347_2	non-phage	0
DLF005_scaffold141_2	non-phage	0
DLF005_scaffold38424_1	non-phage	0
DLF005_scaffold28829_1	non-phage	0
DLF005_scaffold12_1	non-phage	0
DLF005_scaffold46063_1	non-phage	0
DLF005_scaffold56409_1	non-phage	0
DLF005_scaffold56849_2	non-phage	0
DLF005_scaffold49454_1	non-phage	0
DLF005_scaffold55999_1	non-phage	0
DLF005_scaffold56519_2	non-phage	0
DLF005_scaffold52919_1	non-phage	0
DLF005_scaffold52174_2	non-phage	0
DLF005_scaffold56547_2	non-phage	0
DLF005_scaffold22452_3	non-phage	0
DLF005_scaffold56868_1	non-phage	0
DLF005_scaffold35811_4	non-phage	0.099
DLF005_scaffold51294_1	non-phage	0
DLF005_scaffold946_2	non-phage	0
DLF005_scaffold4510_1	non-phage	0
DLF005_scaffold35441_4	non-phage	0
DLF005_scaffold56940_1	non-phage	0
DLF005_scaffold55521_2	non-phage	0
DLF005_scaffold53502_2	non-phage	0
DLF005_scaffold56313_5	non-phage	0
DLF005_scaffold93_1	non-phage	0
DLF005_scaffold20658_2	non-phage	0
DLF005_scaffold42015_2	non-phage	0
DLF005_scaffold46767_2	non-phage	0

DLF005_scaffold36_2	non-phage	0
DLF005_scaffold56704_1	non-phage	0
DLF005_scaffold56266_3	non-phage	0
DLF005_scaffold6871_1	non-phage	0
DLF005_scaffold14032_2	non-phage	0
DLF005_scaffold48546_1	phage	0.991
DLF005_scaffold56189_3	non-phage	0
DLF005_scaffold56532_4	non-phage	0
DLF005_scaffold56841_1	non-phage	0
DLF005_scaffold51226_6	non-phage	0
DLF005_scaffold48608_1	non-phage	0
DLF005_scaffold55770_1	phage	0.999
DLF005_scaffold52014_5	non-phage	0
DLF005_scaffold56719_5	non-phage	0
DLF005_scaffold33331_1	non-phage	0
DLF005_scaffold965_2	non-phage	0
DLF005_scaffold55567_2	non-phage	0
DLF005_scaffold12082_2	non-phage	0
DLF005_scaffold49989_1	non-phage	0
DLF005_scaffold52984_3	non-phage	0
DLF005_scaffold22452_7	non-phage	0
DLF005_scaffold50984_3	non-phage	0
DLF005_scaffold39735_4	phage	0.961
DLF005_scaffold16285_5	non-phage	0
DLF005_scaffold13191_11	non-phage	0
DLF005_scaffold41201_1	non-phage	0
DLF005_scaffold10163_9	non-phage	0
DLF005_scaffold56569_4	phage	0.951
DLF005_scaffold44335_1	non-phage	0
DLF005_scaffold4_1	non-phage	0
DLF005_scaffold31659_2	non-phage	0
DLF005_scaffold27529_1	phage	0.721
DLF005_scaffold141_6	non-phage	0
DLF005_scaffold56993_1	non-phage	0
DLF005_scaffold50670_3	non-phage	0
DLF005_scaffold51786_10	non-phage	0
DLF005_scaffold1547_6	non-phage	0
DLF005_scaffold56656_3	non-phage	0
DLF005_scaffold56987_2	non-phage	0
DLF005_scaffold13302_5	non-phage	0
DLF005_scaffold42862_31	non-phage	0
DLF005_scaffold48193_3	non-phage	0
DLF005_scaffold56936_1	non-phage	0
DLF005_scaffold34907_5	non-phage	0
DLF005_scaffold56547_3	non-phage	0
DLF005_scaffold63_2	non-phage	0

DLF005_scaffold25785_14	non-phage	0
DLF005_scaffold56743_1	non-phage	0
DLF005_scaffold42408_2	non-phage	0
DLF005_scaffold52328_1	non-phage	0
DLF005_scaffold10163_7	non-phage	0
DLF005_scaffold51250_2	non-phage	0
DLF005_scaffold55932_1	non-phage	0
DLF005_scaffold51335_1	phage	0.993
DLF005_scaffold4693_36	phage	0.871
DLF005_scaffold55999_2	non-phage	0
DLF005_scaffold50375_1	phage	0.99
DLF005_scaffold56982_1	non-phage	0
DLF005_scaffold27544_5	non-phage	0
DLF005_scaffold55521_1	non-phage	0
DLF005_scaffold14853_8	non-phage	0
DLF005_scaffold50984_6	non-phage	0
DLF005_scaffold52190_2	non-phage	0
DLF005_scaffold56465_2	non-phage	0
DLF005_scaffold11480_7	non-phage	0
DLF005_scaffold56163_1	phage	0.999
DLF005_scaffold23251_3	non-phage	0
DLF005_scaffold22763_3	non-phage	0
DLF005_scaffold34528_1	non-phage	0
DLF005_scaffold30214_2	non-phage	0
DLF005_scaffold46063_2	non-phage	0
DLF005_scaffold55606_4	non-phage	0
DLF005_scaffold52894_2	non-phage	0
DLF005_scaffold40395_1	phage	0.999
DLF005_scaffold41863_1	non-phage	0
DLF005_scaffold36843_1	non-phage	0
DLF005_scaffold6101_6	non-phage	0
DLF005_scaffold56972_2	non-phage	0
DLF005_scaffold45329_2	non-phage	0
DLF005_scaffold28550_1	non-phage	0
DLF005_scaffold35887_1	non-phage	0
DLF005_scaffold56979_1	non-phage	0.229
DLF005_scaffold33412_3	non-phage	0
DLF005_scaffold32518_1	non-phage	0
DLF005_scaffold36_6	non-phage	0
DLF005_scaffold267_3	non-phage	0
DLF005_scaffold56316_1	non-phage	0
DLF005_scaffold53283_2	non-phage	0
DLF005_scaffold44524_2	non-phage	0
DLF005_scaffold46767_4	non-phage	0
DLF005_scaffold25785_5	non-phage	0
DLF005_scaffold55688_1	non-phage	0

DLF005_scaffold56655_1	non-phage	0
DLF005_scaffold217_2	non-phage	0
DLF005_scaffold1570_16	phage	0.99
DLF005_scaffold328_3	non-phage	0
DLF005_scaffold51024_3	non-phage	0
DLF005_scaffold10163_3	non-phage	0
DLF005_scaffold30892_2	non-phage	0
DLF005_scaffold27400_1	non-phage	0
DLF005_scaffold47023_2	non-phage	0
DLF005_scaffold35441_3	non-phage	0
DLF005_scaffold56520_1	non-phage	0
DLF005_scaffold41201_4	non-phage	0
DLF005_scaffold35811_7	non-phage	0
DLF005_scaffold56952_1	non-phage	0
DLF005_scaffold13131_1	phage	0.999
DLF005_scaffold24081_9	non-phage	0
DLF005_scaffold41843_1	non-phage	0
DLF005_scaffold528_11	phage	0.999
DLF005_scaffold53386_1	non-phage	0
DLF005_scaffold56934_2	non-phage	0
DLF005_scaffold56955_2	non-phage	0
DLF005_scaffold32_1	non-phage	0
DLF005_scaffold30371_1	non-phage	0
DLF005_scaffold965_1	non-phage	0
DLF005_scaffold13579_1	non-phage	0
DLF005_scaffold56137_1	non-phage	0
DLF005_scaffold39219_1	non-phage	0
DLF005_scaffold27806_7	non-phage	0
DLF005_scaffold23251_4	non-phage	0
DLF005_scaffold32518_3	non-phage	0
DLF005_scaffold588_2	non-phage	0
DLF005_scaffold50539_2	non-phage	0
DLF005_scaffold22452_5	non-phage	0
DLF005_scaffold56137_2	non-phage	0
DLF005_scaffold56029_1	non-phage	0
DLF005_scaffold8269_17	non-phage	0
DLF005_scaffold7898_1	non-phage	0
DLF005_scaffold141_4	non-phage	0
DLF005_scaffold22452_2	non-phage	0
DLF005_scaffold34829_1	non-phage	0
DLF005_scaffold39187_4	phage	0.999
DLF005_scaffold51786_5	non-phage	0
DLF005_scaffold54306_1	non-phage	0
DLF005_scaffold56897_3	non-phage	0
DLF005_scaffold56897_1	non-phage	0
DLF005_scaffold23549_1	non-phage	0

DLF005_scaffold4919_8	non-phage	0
DLF005_scaffold46912_2	non-phage	0
DLF005_scaffold36_7	non-phage	0
DLF005_scaffold56835_1	non-phage	0
DLF005_scaffold53334_1	non-phage	0.43
DLF005_scaffold99_3	non-phage	0
DLF005_scaffold27755_8	non-phage	0
DLF005_scaffold56352_1	non-phage	0
DLF005_scaffold48951_1	non-phage	0
DLF005_scaffold11480_5	non-phage	0
DLF005_scaffold278_1	non-phage	0
DLF005_scaffold41201_3	non-phage	0
DLF005_scaffold41863_4	non-phage	0
DLF005_scaffold43959_3	non-phage	0
DLF005_scaffold7376_4	non-phage	0
DLF005_scaffold8526_1	non-phage	0
DLF005_scaffold56309_1	non-phage	0
DLF005_scaffold52014_3	non-phage	0
DLF005_scaffold4954_2	non-phage	0
DLF005_scaffold2188_3	non-phage	0
DLF005_scaffold56606_2	non-phage	0
DLF005_scaffold25785_13	phage	0.995
DLF005_scaffold45714_2	non-phage	0
DLF005_C847560_1	non-phage	0
DLF005_scaffold56894_1	phage	0.919
DLF005_scaffold49780_1	non-phage	0
DLF005_scaffold12123_1	non-phage	0
DLF005_scaffold56966_1	non-phage	0
DLF005_scaffold51024_4	non-phage	0
DLF005_scaffold34528_6	non-phage	0
DLF005_scaffold4627_3	non-phage	0
DLF005_scaffold3370_11	non-phage	0
DLF005_scaffold13010_17	non-phage	0
DLF005_scaffold56915_2	non-phage	0
DLF005_scaffold44522_1	non-phage	0
DLF005_scaffold55492_2	non-phage	0
DLF005_scaffold22763_1	non-phage	0
DLF005_scaffold41487_2	non-phage	0
DLF005_scaffold9847_5	non-phage	0
DLF005_scaffold55949_1	non-phage	0
DLF005_scaffold25615_6	non-phage	0
DLF005_scaffold54804_6	phage	0.997
DLF005_scaffold20658_1	non-phage	0
DLF005_scaffold46031_3	non-phage	0
DLF005_scaffold52895_1	non-phage	0
DLF005_scaffold46063_4	non-phage	0

DLF005_scaffold3312_5	non-phage	0
DLF005_scaffold54626_1	non-phage	0
DLF005_scaffold34065_1	non-phage	0
DLF005_scaffold35811_5	non-phage	0
DLF005_scaffold32518_6	non-phage	0
DLF005_scaffold38424_2	non-phage	0
DLF005_scaffold16285_8	non-phage	0
DLF005_scaffold4602_6	non-phage	0
DLF005_scaffold2989_20	phage	0.996
DLF005_scaffold22452_6	non-phage	0
DLF005_scaffold56808_2	non-phage	0
DLF005_scaffold56995_1	non-phage	0
DLF005_scaffold4632_8	non-phage	0
DLF005_C847548_1	non-phage	0
DLF005_scaffold19909_8	non-phage	0
DLF005_scaffold34528_21	non-phage	0
DLF005_scaffold2505_1	phage	1
DLF005_scaffold47023_3	phage	0.943
DLF005_scaffold31074_1	non-phage	0
DLF005_scaffold56967_1	phage	0.999
DLF005_scaffold56958_1	non-phage	0
DLF005_scaffold36_8	non-phage	0
DLF005_scaffold56587_1	non-phage	0
DLF005_scaffold13334_3	phage	0.999
DLF005_C847538_1	phage	0.997
DLF005_scaffold35441_5	non-phage	0
DLF005_scaffold9570_3	non-phage	0
DLF005_scaffold873_72	non-phage	0
DLF005_scaffold6183_26	non-phage	0
DLF005_scaffold36063_1	non-phage	0
DLF005_scaffold46044_8	non-phage	0
DLF005_scaffold4510_2	non-phage	0
DLF005_scaffold267_5	non-phage	0
DLF005_scaffold56950_1	non-phage	0
DLF005_scaffold56868_2	non-phage	0
DLF005_scaffold49747_1	non-phage	0
DLF005_scaffold22298_16	non-phage	0
DLF005_scaffold16722_10	non-phage	0
DLF005_scaffold294_3	non-phage	0
DLF005_scaffold56596_1	non-phage	0
DLF005_scaffold51530_1	non-phage	0
DLF005_scaffold40204_3	non-phage	0
DLF005_scaffold49989_4	non-phage	0
DLF005_scaffold17120_2	non-phage	0
DLF005_scaffold56726_2	non-phage	0
DLF005_scaffold56944_2	non-phage	0

DLF005_scaffold26644_6	non-phage	0
DLF005_scaffold49976_2	non-phage	0
DLF005_scaffold36849_1	non-phage	0
DLF005_scaffold52365_1	non-phage	0
DLF005_scaffold52174_1	non-phage	0
DLF005_scaffold56547_1	non-phage	0
DLF005_scaffold4954_1	non-phage	0
DLF005_scaffold44522_2	non-phage	0
DLF005_scaffold56906_1	non-phage	0
DLF005_scaffold13302_2	non-phage	0
DLF005_scaffold56907_1	non-phage	0
DLF005_scaffold27400_3	non-phage	0
DLF005_scaffold41617_3	non-phage	0.019
DLF005_scaffold12803_2	non-phage	0
DLF005_scaffold55688_3	non-phage	0
DLF005_scaffold56994_1	non-phage	0.008
DLF005_scaffold56923_2	non-phage	0
DLF005_scaffold271_1	non-phage	0
DLF005_scaffold16722_7	non-phage	0.068
DLF005_scaffold38445_2	non-phage	0
DLF005_scaffold4919_10	non-phage	0
DLF005_scaffold41865_1	non-phage	0
DLF005_scaffold25099_3	non-phage	0
DLF005_scaffold56656_2	non-phage	0
DLF005_scaffold398_1	non-phage	0
DLF005_C847534_1	non-phage	0
DLF005_scaffold25347_1	non-phage	0
DLF005_scaffold55606_3	non-phage	0
DLF005_scaffold13_2	non-phage	0
DLF005_scaffold48193_2	non-phage	0
DLF005_scaffold56987_1	non-phage	0
DLF005_scaffold247_1	non-phage	0
DLF005_scaffold49269_1	non-phage	0
DLF005_scaffold12078_2	non-phage	0
DLF005_scaffold271_2	non-phage	0
DLF005_scaffold41201_5	non-phage	0
DLF005_scaffold1968_3	non-phage	0
DLF005_scaffold56976_1	non-phage	0
DLF005_scaffold23948_4	non-phage	0
DLF005_scaffold23948_45	non-phage	0
DLF005_scaffold52014_2	non-phage	0
DLF005_scaffold55991_1	non-phage	0
DLF005_scaffold56980_1	non-phage	0
DLF005_scaffold51226_3	non-phage	0
DLF005_scaffold25785_6	non-phage	0
DLF005_scaffold51024_5	non-phage	0

DLF005_scaffold50535_1	non-phage	0
DLF005_scaffold11480_6	non-phage	0
DLF005_scaffold52877_1	non-phage	0
DLF005_scaffold41749_2	non-phage	0
DLF005_scaffold845_9	non-phage	0.173
DLF005_scaffold56817_1	phage	0.999
DLF005_scaffold4147_1	non-phage	0
DLF005_scaffold39175_1	non-phage	0
DLF005_scaffold129_16	non-phage	0
DLF005_scaffold40204_1	non-phage	0
DLF005_scaffold47258_1	non-phage	0
DLF005_scaffold16722_1	non-phage	0
DLF005_scaffold23549_3	non-phage	0
DLF005_scaffold30997_3	non-phage	0
DLF005_scaffold56385_1	non-phage	0
DLF005_scaffold48158_4	non-phage	0.004
DLF005_scaffold52997_1	non-phage	0
DLF005_scaffold16285_13	non-phage	0
DLF005_scaffold9570_1	non-phage	0
DLF005_scaffold47023_5	non-phage	0
DLF005_scaffold3312_1	non-phage	0
DLF005_scaffold52190_10	non-phage	0
DLF005_scaffold56924_2	phage	0.575
DLF005_scaffold39187_3	phage	0.999
DLF005_scaffold182_1	non-phage	0
DLF005_C847802_1	non-phage	0
DLF005_scaffold50401_2	non-phage	0
DLF005_scaffold56845_2	non-phage	0
DLF005_scaffold16532_1	non-phage	0
DLF005_scaffold52948_2	non-phage	0
DLF005_scaffold56609_2	non-phage	0
DLF005_scaffold56885_1	non-phage	0
DLF005_scaffold55741_1	non-phage	0
DLF005_scaffold54745_3	non-phage	0
DLF005_scaffold56827_2	non-phage	0
DLF005_scaffold28014_1	non-phage	0
DLF005_scaffold34783_3	non-phage	0.077
DLF005_scaffold46916_5	non-phage	0
DLF005_scaffold6688_21	phage	0.655
DLF005_scaffold56996_1	non-phage	0
DLF005_scaffold78_1	non-phage	0
DLF005_scaffold47714_1	non-phage	0
DLF005_scaffold18199_1	phage	0.999
DLF005_scaffold50545_1	non-phage	0
DLF005_scaffold23948_17	non-phage	0
DLF005_scaffold54804_7	non-phage	0

DLF005_scaffold2505_3	non-phage	0
DLF005_scaffold45447_5	non-phage	0
DLF005_scaffold41287_4	non-phage	0
DLF005_scaffold55909_1	non-phage	0
DLF005_scaffold26987_2	non-phage	0
DLF005_scaffold141_5	non-phage	0
DLF005_C847770_1	phage	0.944
DLF005_scaffold3960_1	phage	0.919
DLF005_scaffold280_2	phage	0.987
DLF005_scaffold56985_2	non-phage	0
DLF005_scaffold30_1	non-phage	0
DLF005_scaffold3191_18	non-phage	0
DLF005_scaffold25785_1	non-phage	0
DLF005_scaffold54790_1	non-phage	0
DLF005_scaffold56837_2	non-phage	0
DLF005_scaffold35441_2	non-phage	0
DLF005_scaffold47023_6	non-phage	0
DLF005_scaffold56897_2	non-phage	0
DLF005_scaffold3914_1	non-phage	0
DLF005_scaffold39343_1	non-phage	0
DLF005_scaffold43244_3	non-phage	0
DLF005_scaffold53529_1	non-phage	0
DLF005_scaffold25615_4	non-phage	0
DLF005_scaffold21572_9	non-phage	0
DLF005_scaffold34445_13	non-phage	0.124
DLF005_scaffold32479_1	phage	0.999
DLF005_scaffold53388_1	non-phage	0
DLF005_scaffold45485_8	non-phage	0
DLF005_scaffold24328_11	non-phage	0
DLF005_scaffold16898_8	non-phage	0
DLF005_scaffold41843_2	phage	0.755
DLF005_scaffold56787_1	non-phage	0
DLF005_scaffold14604_5	non-phage	0
DLF005_scaffold11641_5	non-phage	0
DLF005_scaffold52877_2	non-phage	0
DLF005_scaffold53378_1	non-phage	0
DLF005_scaffold47023_10	non-phage	0
DLF005_scaffold56913_1	non-phage	0
DLF005_scaffold41271_3	non-phage	0
DLF005_scaffold53228_2	non-phage	0
DLF005_scaffold1570_3	non-phage	0
DLF005_scaffold50425_1	non-phage	0
DLF005_scaffold43343_1	non-phage	0
DLF005_scaffold33394_2	non-phage	0
DLF005_scaffold56329_1	non-phage	0
DLF005_scaffold9570_2	non-phage	0

DLF005_scaffold55210_1	non-phage	0
DLF005_scaffold56444_2	non-phage	0
DLF005_scaffold12082_1	non-phage	0
DLF005_scaffold13191_2	non-phage	0
DLF005_scaffold3370_12	non-phage	0
DLF005_scaffold33045_1	phage	0.999
DLF005_scaffold50984_2	non-phage	0
DLF005_scaffold29631_1	non-phage	0
DLF005_scaffold56995_2	non-phage	0
DLF005_scaffold56363_1	non-phage	0
DLF005_scaffold51847_3	non-phage	0
DLF005_scaffold4093_2	phage	1
DLF005_scaffold49308_6	non-phage	0.168
DLF005_scaffold54512_2	non-phage	0
DLF005_scaffold48944_1	non-phage	0
DLF005_scaffold51250_3	non-phage	0
DLF005_scaffold46044_1	non-phage	0
DLF005_scaffold14604_1	non-phage	0
DLF005_scaffold46054_1	non-phage	0
DLF005_scaffold56913_2	non-phage	0
DLF005_scaffold19189_1	non-phage	0
DLF005_scaffold33394_3	non-phage	0
DLF005_scaffold54705_4	non-phage	0
DLF005_scaffold56776_1	non-phage	0
DLF005_scaffold54804_4	non-phage	0
DLF005_scaffold1695_13	non-phage	0
DLF005_scaffold21102_1	non-phage	0
DLF005_scaffold21572_17	non-phage	0
DLF005_scaffold51847_1	non-phage	0
DLF005_scaffold48193_9	non-phage	0
DLF005_scaffold63_3	non-phage	0
DLF005_scaffold36761_2	non-phage	0
DLF005_scaffold33412_1	non-phage	0
DLF005_scaffold14484_5	non-phage	0
DLF005_scaffold56971_1	non-phage	0
DLF005_scaffold52884_1	non-phage	0
DLF005_scaffold1358_4	non-phage	0
DLF005_scaffold9847_1	phage	0.991
DLF005_scaffold2379_5	non-phage	0
DLF005_scaffold39868_1	non-phage	0
DLF005_scaffold47448_3	non-phage	0
DLF005_scaffold47006_2	non-phage	0
DLF005_scaffold51731_1	non-phage	0
DLF005_scaffold56955_3	non-phage	0
DLF005_scaffold742_15	non-phage	0.057
DLF005_scaffold36135_1	non-phage	0

DLF005_scaffold31168_2	phage	0.664
DLF005_scaffold17323_1	non-phage	0
DLF005_scaffold9628_4	non-phage	0
DLF005_scaffold6101_7	non-phage	0
DLF005_scaffold30669_5	non-phage	0
DLF005_scaffold21023_2	non-phage	0
DLF005_scaffold14604_4	non-phage	0
DLF005_scaffold55688_2	non-phage	0
DLF005_scaffold56991_1	non-phage	0
DLF005_scaffold56845_4	non-phage	0
DLF005_scaffold53782_1	phage	0.999
DLF005_scaffold56714_1	non-phage	0
DLF005_scaffold56569_2	phage	0.998
DLF005_scaffold43084_1	non-phage	0
DLF005_scaffold56944_1	non-phage	0
DLF005_scaffold56845_1	non-phage	0
DLF005_scaffold19580_8	non-phage	0
DLF005_scaffold7898_5	non-phage	0
DLF005_scaffold9815_4	non-phage	0
DLF005_scaffold3566_17	non-phage	0
DLF005_scaffold50984_1	non-phage	0
DLF005_scaffold41757_2	non-phage	0
DLF005_scaffold56569_6	non-phage	0
DLF005_scaffold43244_5	non-phage	0
DLF005_scaffold4393_1	non-phage	0
DLF005_scaffold23948_15	non-phage	0
DLF005_scaffold93_4	non-phage	0
DLF005_scaffold14311_2	non-phage	0
DLF005_scaffold16544_1	non-phage	0
DLF005_scaffold51731_4	non-phage	0
DLF005_scaffold280_1	non-phage	0
DLF005_scaffold56612_2	non-phage	0
DLF005_scaffold47494_2	non-phage	0
DLF005_scaffold17120_4	non-phage	0
DLF005_scaffold16722_8	non-phage	0
DLF005_scaffold45418_2	non-phage	0
DLF005_scaffold23988_4	non-phage	0
DLF005_scaffold4062_30	non-phage	0
DLF005_scaffold23342_9	phage	0.999
DLF005_scaffold56266_9	non-phage	0.042
DLF005_scaffold35811_6	non-phage	0
DLF005_scaffold47023_7	non-phage	0
DLF005_scaffold17153_12	non-phage	0
DLF005_scaffold53141_2	non-phage	0
DLF005_scaffold52930_3	non-phage	0
DLF005_scaffold17153_7	non-phage	0

DLF006_scaffold30627_10	non-phage	0
DLF006_scaffold8107_7	non-phage	0
DLF006_scaffold33128_1	non-phage	0
DLF006_scaffold9056_6	non-phage	0
DLF006_scaffold52_4	phage	0.999
DLF006_scaffold10655_23	non-phage	0
DLF006_scaffold7805_11	non-phage	0
DLF006_scaffold27731_3	non-phage	0
DLF006_scaffold46818_1	non-phage	0
DLF006_scaffold17229_1	non-phage	0
DLF006_scaffold16517_1	non-phage	0
DLF006_scaffold25745_1	non-phage	0
DLF006_scaffold9366_5	non-phage	0
DLF006_scaffold7805_14	non-phage	0
DLF006_scaffold32459_3	non-phage	0
DLF006_scaffold1948_12	non-phage	0
DLF006_scaffold44828_1	non-phage	0
DLF006_scaffold8996_1	non-phage	0
DLF006_scaffold2524_13	non-phage	0
DLF006_scaffold18701_6	non-phage	0
DLF006_scaffold19578_4	non-phage	0
DLF006_scaffold46728_1	non-phage	0
DLF006_scaffold41_21	non-phage	0
DLF006_scaffold1108_6	non-phage	0
DLF006_scaffold7_6	non-phage	0
DLF006_C667664_1	phage	0.829
DLF006_scaffold3671_1	non-phage	0
DLF006_scaffold12572_1	non-phage	0
DLF006_scaffold19713_13	non-phage	0
DLF006_scaffold46600_1	non-phage	0
DLF006_scaffold41_4	non-phage	0
DLF006_scaffold38598_3	non-phage	0
DLF006_scaffold7_3	non-phage	0
DLF006_scaffold857_11	non-phage	0
DLF006_scaffold43316_3	non-phage	0
DLF006_scaffold5230_7	non-phage	0
DLF006_scaffold21079_8	non-phage	0
DLF006_scaffold22652_1	non-phage	0
DLF006_scaffold11481_7	non-phage	0
DLF006_scaffold11076_8	non-phage	0
DLF006_scaffold19713_7	non-phage	0
DLF006_scaffold11927_12	non-phage	0
DLF006_scaffold29751_4	non-phage	0
DLF006_scaffold41_19	non-phage	0
DLF006_scaffold9049_10	phage	0.998
DLF006_scaffold1668_12	non-phage	0

DLF006_scaffold108_5	phage	0.947
DLF006_scaffold25840_4	non-phage	0
DLF006_scaffold36308_2	non-phage	0
DLF006_scaffold10407_5	non-phage	0
DLF006_scaffold75_4	non-phage	0
DLF006_scaffold25_18	non-phage	0
DLF006_scaffold15738_3	non-phage	0
DLF006_scaffold21093_1	non-phage	0
DLF006_scaffold46738_3	non-phage	0
DLF006_scaffold60_1	non-phage	0
DLF006_scaffold42885_3	phage	0.996
DLF006_scaffold45344_3	non-phage	0
DLF006_scaffold1381_9	non-phage	0
DLF006_scaffold45997_5	non-phage	0
DLF006_scaffold15660_1	non-phage	0
DLF006_scaffold46530_1	non-phage	0
DLF006_scaffold46681_1	phage	0.999
DLF006_scaffold46826_1	non-phage	0
DLF006_scaffold34472_11	non-phage	0
DLF006_scaffold41_3	non-phage	0
DLF006_scaffold569_18	non-phage	0
DLF006_scaffold9257_3	non-phage	0
DLF006_scaffold11325_16	non-phage	0
DLF006_scaffold27078_4	non-phage	0
DLF006_scaffold46842_1	non-phage	0
DLF006_scaffold11449_4	non-phage	0
DLF006_scaffold13320_3	non-phage	0
DLF006_scaffold6312_2	non-phage	0
DLF006_scaffold9366_12	non-phage	0
DLF006_scaffold22612_2	non-phage	0
DLF006_scaffold19301_1	non-phage	0
DLF006_scaffold7_9	non-phage	0
DLF006_scaffold45140_1	non-phage	0
DLF006_scaffold7967_18	non-phage	0
DLF006_scaffold9095_9	non-phage	0
DLF006_scaffold46414_3	non-phage	0
DLF006_C667490_1	non-phage	0
DLF006_scaffold10001_1	non-phage	0
DLF006_scaffold51_9	non-phage	0
DLF006_scaffold24206_5	non-phage	0
DLF006_scaffold154_3	non-phage	0
DLF006_scaffold25693_1	non-phage	0
DLF006_scaffold40648_1	non-phage	0
DLF006_scaffold3325_27	non-phage	0
DLF006_scaffold46133_2	non-phage	0
DLF006_scaffold4378_3	non-phage	0

DLF006_scaffold39883_1	phage	0.999
DLF006_scaffold14591_3	non-phage	0
DLF006_scaffold17300_5	non-phage	0
DLF006_scaffold46798_1	non-phage	0
DLF006_scaffold2090_16	non-phage	0
DLF006_scaffold5230_1	non-phage	0
DLF006_scaffold46807_1	non-phage	0
DLF006_scaffold46487_1	non-phage	0
DLF006_scaffold2825_2	non-phage	0
DLF006_scaffold32044_3	non-phage	0
DLF006_scaffold32044_2	phage	0.856
DLF006_scaffold25950_8	non-phage	0
DLF006_scaffold8912_6	non-phage	0
DLF006_scaffold9584_1	non-phage	0
DLF006_scaffold520_7	non-phage	0
DLF006_scaffold8023_33	non-phage	0
DLF006_scaffold5042_11	non-phage	0
DLF006_scaffold46854_1	non-phage	0
DLF006_scaffold8740_3	phage	1
DLF006_scaffold2090_30	non-phage	0
DLF006_scaffold7086_21	non-phage	0
DLF006_scaffold26715_4	non-phage	0
DLF006_C667862_1	phage	1
DLF006_scaffold20374_2	non-phage	0
DLF006_scaffold39217_3	non-phage	0
DLF006_scaffold5702_3	non-phage	0
DLF006_scaffold41113_3	non-phage	0
DLF006_scaffold10001_6	non-phage	0
DLF006_scaffold11927_4	non-phage	0
DLF006_scaffold44716_1	non-phage	0
DLF006_scaffold12572_2	non-phage	0
DLF006_scaffold2524_9	non-phage	0
DLF006_scaffold192_6	non-phage	0
DLF006_scaffold5672_9	non-phage	0
DLF006_scaffold84_2	non-phage	0
DLF006_scaffold32950_1	non-phage	0
DLF006_scaffold19713_5	non-phage	0
DLF006_scaffold31260_3	non-phage	0
DLF006_scaffold23490_3	non-phage	0
DLF006_scaffold9889_3	non-phage	0
DLF006_scaffold8996_2	non-phage	0
DLF006_scaffold18225_6	non-phage	0
DLF006_scaffold25261_3	non-phage	0
DLF006_scaffold5479_1	non-phage	0
DLF006_scaffold2636_30	non-phage	0
DLF006_scaffold83_3	non-phage	0

DLF006_scaffold36652_11	non-phage	0
DLF006_scaffold23419_1	non-phage	0
DLF006_scaffold46536_1	non-phage	0
DLF006_scaffold22612_3	non-phage	0
DLF006_scaffold40174_2	non-phage	0
DLF006_scaffold5702_6	non-phage	0
DLF006_scaffold21807_2	non-phage	0
DLF006_scaffold11325_26	non-phage	0
DLF006_scaffold25950_4	non-phage	0
DLF006_scaffold19189_3	phage	0.999
DLF006_scaffold41_18	non-phage	0
DLF006_scaffold474_4	phage	0.989
DLF006_scaffold25840_7	non-phage	0
DLF006_scaffold46729_1	non-phage	0
DLF006_scaffold10221_1	non-phage	0
DLF006_scaffold41298_6	non-phage	0
DLF006_scaffold36858_1	non-phage	0
DLF006_scaffold42885_4	non-phage	0
DLF006_scaffold11481_15	non-phage	0
DLF006_scaffold36481_1	non-phage	0
DLF006_scaffold2461_2	non-phage	0
DLF006_scaffold8023_28	non-phage	0
DLF006_scaffold46801_1	non-phage	0
DLF006_scaffold42015_2	non-phage	0
DLF006_scaffold46078_1	non-phage	0
DLF006_scaffold290_22	non-phage	0
DLF006_scaffold3054_5	non-phage	0
DLF006_scaffold33584_4	phage	0.944
DLF006_scaffold8107_4	non-phage	0
DLF006_scaffold12287_2	non-phage	0
DLF006_scaffold27078_3	non-phage	0
DLF006_scaffold46793_1	non-phage	0
DLF006_scaffold18701_9	non-phage	0
DLF006_scaffold46330_2	non-phage	0
DLF006_scaffold5666_42	non-phage	0
DLF006_scaffold1948_2	non-phage	0
DLF006_scaffold75_5	non-phage	0
DLF006_scaffold5585_2	non-phage	0
DLF006_scaffold41298_3	non-phage	0
DLF006_scaffold18701_16	non-phage	0
DLF006_scaffold29387_1	non-phage	0
DLF006_scaffold46821_1	non-phage	0
DLF006_scaffold9095_7	non-phage	0
DLF006_scaffold22006_19	non-phage	0
DLF006_scaffold43316_2	non-phage	0
DLF006_scaffold25693_7	non-phage	0

DLF006_scaffold94_5	non-phage	0
DLF006_scaffold14051_1	non-phage	0
DLF006_scaffold46688_3	non-phage	0
DLF006_scaffold5702_4	non-phage	0
DLF006_scaffold8023_5	non-phage	0
DLF006_scaffold29_1	non-phage	0
DLF006_scaffold41_11	non-phage	0
DLF006_C667470_1	non-phage	0
DLF006_scaffold11076_15	non-phage	0
DLF006_scaffold35956_2	non-phage	0
DLF006_scaffold26399_1	non-phage	0
DLF006_scaffold34156_4	phage	0.936
DLF006_scaffold28241_1	non-phage	0
DLF006_scaffold7_2	non-phage	0
DLF006_scaffold41_2	non-phage	0
DLF006_scaffold9056_20	phage	0.999
DLF006_scaffold46629_1	non-phage	0
DLF006_scaffold38144_1	non-phage	0
DLF006_scaffold9056_18	phage	0.999
DLF006_scaffold2090_17	non-phage	0
DLF006_scaffold19713_17	non-phage	0
DLF006_scaffold22006_8	non-phage	0
DLF006_scaffold9366_11	non-phage	0
DLF006_scaffold23700_1	non-phage	0
DLF006_scaffold10687_2	phage	0.994
DLF006_scaffold9366_2	non-phage	0
DLF006_scaffold25327_1	non-phage	0
DLF006_scaffold23861_1	non-phage	0
DLF006_scaffold5514_7	non-phage	0
DLF006_scaffold45552_2	non-phage	0
DLF006_scaffold39576_4	non-phage	0
DLF006_scaffold474_2	non-phage	0
DLF006_scaffold474_10	non-phage	0
DLF006_scaffold474_9	non-phage	0
DLF006_scaffold41089_3	non-phage	0
DLF006_scaffold8762_1	non-phage	0
DLF006_scaffold32716_3	non-phage	0
DLF006_scaffold42348_2	non-phage	0
DLF006_scaffold22006_29	non-phage	0
DLF006_scaffold25950_3	non-phage	0
DLF006_scaffold388_9	non-phage	0
DLF006_scaffold23450_3	non-phage	0
DLF006_scaffold38464_3	non-phage	0
DLF006_scaffold7_1	non-phage	0
DLF006_scaffold24334_1	non-phage	0
DLF006_scaffold23557_2	non-phage	0

DLF006_scaffold11422_2	phage	0.999
DLF006_scaffold19376_2	non-phage	0
DLF006_scaffold129_1	non-phage	0
DLF006_scaffold17300_3	non-phage	0
DLF006_scaffold18701_13	non-phage	0
DLF006_scaffold42885_2	non-phage	0
DLF006_scaffold24979_1	non-phage	0
DLF006_scaffold36098_3	non-phage	0
DLF006_scaffold31224_2	non-phage	0
DLF006_scaffold20006_3	non-phage	0
DLF006_scaffold19189_4	phage	0.866
DLF006_scaffold23419_2	non-phage	0
DLF006_scaffold11076_14	non-phage	0
DLF006_scaffold12505_2	non-phage	0
DLF006_scaffold94_2	non-phage	0
DLF006_scaffold12005_2	non-phage	0
DLF006_scaffold9845_3	phage	0.997
DLF006_scaffold20275_7	non-phage	0
DLF006_scaffold45293_3	non-phage	0
DLF006_scaffold43687_5	non-phage	0
DLF006_scaffold46268_2	non-phage	0
DLF006_scaffold16671_22	non-phage	0
DLF006_scaffold45344_1	non-phage	0
DLF006_scaffold37577_2	non-phage	0
DLF006_scaffold8023_29	non-phage	0
DLF006_scaffold37381_5	non-phage	0
DLF006_scaffold18813_4	non-phage	0
DLF006_scaffold41298_2	phage	0.88
DLF006_scaffold46078_9	non-phage	0
DLF006_scaffold8107_5	non-phage	0
DLF006_scaffold5200_1	non-phage	0
DLF006_scaffold38044_2	non-phage	0
DLF006_scaffold27078_9	non-phage	0
DLF006_scaffold10407_3	non-phage	0
DLF006_scaffold8762_3	non-phage	0
DLF006_scaffold25716_4	non-phage	0
DLF006_scaffold46299_1	non-phage	0
DLF006_scaffold19875_5	non-phage	0
DLF006_scaffold74_8	non-phage	0
DLF006_scaffold8762_10	non-phage	0
DLF006_scaffold8905_37	non-phage	0
DLF006_scaffold7466_5	phage	0.849
DLF006_scaffold5702_7	non-phage	0
DLF006_scaffold10001_3	non-phage	0
DLF006_scaffold12603_11	non-phage	0
DLF006_scaffold46521_1	non-phage	0

DLF006_scaffold237_5	non-phage	0
DLF006_scaffold2090_27	non-phage	0
DLF006_scaffold10221_2	non-phage	0
DLF006_scaffold22006_10	non-phage	0
DLF006_scaffold7230_11	non-phage	0
DLF006_scaffold38598_1	non-phage	0
DLF006_scaffold33081_2	non-phage	0
DLF006_scaffold25840_9	non-phage	0.009
DLF006_scaffold3805_9	non-phage	0
DLF006_scaffold7164_1	non-phage	0
DLF006_scaffold108_6	non-phage	0
DLF006_scaffold15736_1	phage	0.998
DLF006_scaffold46806_1	non-phage	0
DLF006_scaffold25693_8	non-phage	0
DLF006_scaffold1668_6	non-phage	0
DLF006_scaffold15611_4	non-phage	0
DLF006_scaffold25_2	non-phage	0
DLF006_scaffold12115_1	non-phage	0
DLF006_scaffold34472_6	non-phage	0
DLF006_scaffold34370_3	non-phage	0
DLF006_scaffold22006_9	phage	0.994
DLF006_scaffold32459_1	non-phage	0
DLF006_scaffold3576_5	non-phage	0
DLF006_scaffold43914_2	non-phage	0
DLF006_scaffold16195_9	non-phage	0
DLF006_scaffold46721_2	non-phage	0
DLF006_scaffold46795_1	non-phage	0
DLF006_scaffold25840_10	phage	0.999
DLF006_scaffold43459_1	non-phage	0
DLF006_scaffold148_1	non-phage	0
DLF006_scaffold21481_1	non-phage	0
DLF006_scaffold569_6	non-phage	0
DLF006_scaffold39217_1	non-phage	0
DLF006_scaffold46414_2	non-phage	0
DLF006_scaffold22612_4	non-phage	0
DLF006_scaffold7_7	non-phage	0
DLF006_scaffold234_8	non-phage	0
DLF006_scaffold31392_1	non-phage	0
DLF006_scaffold4397_4	non-phage	0
DLF006_scaffold5848_3	non-phage	0
DLF006_scaffold43687_7	non-phage	0
DLF006_scaffold25840_2	non-phage	0
DLF006_scaffold858_54	non-phage	0
DLF006_scaffold18813_8	non-phage	0
DLF006_scaffold1923_2	non-phage	0
DLF006_scaffold31315_2	non-phage	0

DLF006_scaffold31070_1	non-phage	0
DLF006_scaffold46593_1	non-phage	0
DLF006_scaffold19713_11	non-phage	0
DLF006_scaffold46541_1	non-phage	0
DLF006_scaffold36051_1	phage	0.999
DLF006_scaffold52_5	phage	0.998
DLF006_scaffold35773_2	non-phage	0
DLF006_scaffold9095_4	non-phage	0
DLF006_scaffold10001_4	non-phage	0
DLF006_scaffold8996_10	non-phage	0
DLF006_scaffold9845_4	phage	0.999
DLF006_scaffold28779_1	non-phage	0
DLF006_scaffold6735_12	non-phage	0
DLF006_scaffold30202_2	non-phage	0
DLF006_scaffold41_9	non-phage	0
DLF006_scaffold19224_3	non-phage	0
DLF006_scaffold2042_4	non-phage	0
DLF006_scaffold10565_5	non-phage	0
DLF006_scaffold19713_14	non-phage	0
DLF006_scaffold24875_1	non-phage	0
DLF006_scaffold234_21	non-phage	0
DLF006_scaffold13243_2	non-phage	0
DLF006_scaffold40972_2	non-phage	0
DLF006_scaffold39718_2	non-phage	0
DLF006_scaffold38464_1	non-phage	0
DLF006_scaffold28718_4	non-phage	0
DLF006_scaffold20006_4	non-phage	0
DLF006_scaffold11076_5	non-phage	0
DLF006_scaffold31224_1	non-phage	0
DLF006_scaffold42912_1	non-phage	0
DLF006_scaffold10324_2	non-phage	0
DLF006_scaffold36051_2	phage	0.998
DLF006_scaffold16046_4	non-phage	0
DLF006_scaffold34515_1	phage	0.995
DLF006_scaffold41_5	non-phage	0
DLF006_scaffold32854_1	non-phage	0
DLF006_scaffold11076_13	non-phage	0
DLF006_scaffold3576_2	non-phage	0
DLF006_scaffold44730_1	non-phage	0
DLF006_scaffold31260_6	non-phage	0
DLF006_scaffold46852_1	non-phage	0
DLF006_scaffold46809_1	non-phage	0
DLF006_scaffold5280_1	phage	0.999
DLF006_scaffold51_7	non-phage	0
DLF006_scaffold9095_6	non-phage	0
DLF006_scaffold19713_18	non-phage	0

DLF006_scaffold36282_4	non-phage	0
DLF006_scaffold10010_1	non-phage	0
DLF006_scaffold7_4	non-phage	0
DLF006_scaffold12115_5	non-phage	0
DLF006_scaffold46760_1	phage	0.861
DLF006_scaffold19875_2	non-phage	0
DLF006_scaffold14420_1	non-phage	0
DLF006_scaffold42495_2	non-phage	0
DLF006_scaffold41113_2	non-phage	0
DLF006_scaffold756_3	non-phage	0
DLF006_scaffold17977_21	non-phage	0
DLF006_scaffold20006_10	non-phage	0
DLF006_scaffold46678_1	non-phage	0
DLF006_scaffold5514_8	non-phage	0
DLF006_scaffold8762_2	non-phage	0
DLF006_scaffold7233_55	non-phage	0
DLF006_scaffold11084_29	non-phage	0
DLF006_scaffold28982_10	non-phage	0
DLF006_scaffold43316_4	non-phage	0
DLF006_scaffold3774_16	non-phage	0
DLF006_scaffold46848_1	phage	0.999
DLF006_scaffold36098_2	non-phage	0
DLF006_scaffold9366_6	non-phage	0
DLF006_scaffold46709_1	non-phage	0
DLF006_scaffold45997_7	non-phage	0
DLF006_scaffold22006_12	non-phage	0
DLF006_scaffold24687_3	non-phage	0
DLF006_scaffold2090_11	non-phage	0
DLF006_scaffold23225_3	non-phage	0
DLF006_C667838_1	non-phage	0
DLF006_scaffold858_55	non-phage	0
DLF006_scaffold11076_10	non-phage	0
DLF006_scaffold14168_2	phage	0.997
DLF006_scaffold27826_3	non-phage	0
DLF006_scaffold8740_4	phage	1
DLF006_scaffold24857_1	non-phage	0
DLF006_scaffold23730_13	non-phage	0
DLF006_scaffold39429_2	non-phage	0
DLF006_scaffold27575_2	non-phage	0
DLF006_scaffold38598_4	non-phage	0
DLF006_scaffold20006_2	non-phage	0
DLF006_scaffold3736_3	non-phage	0
DLF006_scaffold41_17	non-phage	0
DLF006_scaffold2911_3	non-phage	0
DLF006_scaffold28778_2	non-phage	0
DLF006_scaffold9056_12	non-phage	0

DLF006_scaffold34472_17	non-phage	0.001
DLF006_scaffold17300_4	non-phage	0
DLF006_scaffold33406_1	non-phage	0
DLF006_scaffold25840_8	non-phage	0
DLF006_scaffold46799_1	non-phage	0
DLF006_scaffold13243_5	non-phage	0
DLF006_scaffold9770_6	non-phage	0
DLF006_scaffold25693_5	non-phage	0
DLF006_scaffold234_5	non-phage	0
DLF006_scaffold46677_6	phage	1
DLF006_scaffold42362_1	non-phage	0
DLF006_scaffold46739_1	non-phage	0
DLF006_scaffold12609_2	non-phage	0
DLF006_scaffold41_13	non-phage	0
DLF006_scaffold5702_2	non-phage	0
DLF006_scaffold39508_1	non-phage	0
DLF006_scaffold7466_9	non-phage	0
DLF006_scaffold20006_5	non-phage	0
DLF006_scaffold12807_1	non-phage	0
DLF006_scaffold27662_1	phage	1
DLF006_scaffold52_6	phage	0.999
DLF006_scaffold39217_2	non-phage	0
DLF006_scaffold4305_15	non-phage	0
DLF006_scaffold10687_4	non-phage	0
DLF006_scaffold7447_1	non-phage	0
DLF006_scaffold46851_2	phage	1
DLF006_scaffold34156_2	non-phage	0
DLF006_scaffold38500_2	non-phage	0
DLF006_scaffold46701_1	non-phage	0
DLF006_scaffold92_1	phage	0.998
DLF006_scaffold46785_2	non-phage	0
DLF006_scaffold856_10	non-phage	0
DLF006_scaffold23712_2	non-phage	0
DLF006_scaffold18518_2	non-phage	0
DLF006_scaffold558_10	non-phage	0
DLF007_scaffold594_1	non-phage	0
DLF007_scaffold272_21	non-phage	0
DLF007_scaffold13660_6	non-phage	0
DLF007_scaffold9646_2	non-phage	0
DLF007_scaffold19398_2	non-phage	0
DLF007_scaffold6236_16	non-phage	0
DLF007_scaffold52_4	non-phage	0
DLF007_scaffold910_9	non-phage	0
DLF007_scaffold1310_2	non-phage	0
DLF007_scaffold15529_4	phage	0.989
DLF007_scaffold18947_1	non-phage	0.03

DLF007_scaffold15181_4	non-phage	0
DLF007_C306841_1	non-phage	0
DLF007_scaffold20276_2	non-phage	0
DLF007_scaffold15022_6	non-phage	0
DLF007_scaffold15438_5	non-phage	0
DLF007_scaffold18472_1	non-phage	0
DLF007_scaffold20109_1	non-phage	0
DLF007_scaffold19917_2	non-phage	0
DLF007_scaffold14289_4	non-phage	0
DLF007_scaffold6639_1	non-phage	0
DLF007_scaffold19979_2	non-phage	0
DLF007_scaffold17919_1	non-phage	0
DLF007_scaffold20199_1	non-phage	0
DLF007_scaffold19827_4	non-phage	0
DLF007_scaffold19117_1	non-phage	0
DLF007_scaffold11794_2	non-phage	0
DLF007_scaffold10740_1	non-phage	0
DLF007_C306093_1	non-phage	0
DLF007_scaffold10068_1	non-phage	0
DLF007_scaffold13625_1	non-phage	0
DLF007_scaffold19298_3	non-phage	0
DLF007_scaffold7453_1	non-phage	0
DLF007_scaffold11889_1	non-phage	0
DLF007_scaffold977_1	non-phage	0
DLF007_scaffold4220_1	non-phage	0
DLF007_scaffold5418_24	non-phage	0
DLF007_scaffold18446_2	non-phage	0
DLF007_scaffold3987_3	non-phage	0
DLF007_scaffold13779_1	non-phage	0
DLF007_scaffold7402_16	non-phage	0
DLF007_scaffold16615_3	non-phage	0
DLF007_scaffold1662_7	non-phage	0
DLF007_scaffold19937_2	non-phage	0
DLF007_scaffold9239_4	non-phage	0
DLF007_scaffold7850_3	non-phage	0
DLF007_scaffold3028_1	non-phage	0
DLF007_scaffold6719_15	non-phage	0
DLF007_scaffold3237_10	non-phage	0
DLF007_scaffold6542_4	non-phage	0
DLF007_scaffold77_3	non-phage	0
DLF007_scaffold6571_3	non-phage	0
DLF007_scaffold14424_2	phage	0.999
DLF007_scaffold12_2	non-phage	0
DLF007_scaffold11092_1	non-phage	0
DLF007_scaffold20191_1	non-phage	0
DLF007_scaffold11884_1	non-phage	0

DLF007_scaffold16578_1	non-phage	0
DLF007_scaffold285_6	non-phage	0
DLF007_scaffold20321_2	non-phage	0
DLF007_scaffold20303_2	non-phage	0
DLF007_scaffold16729_1	non-phage	0
DLF007_scaffold910_23	non-phage	0
DLF007_scaffold18927_1	phage	0.979
DLF007_scaffold758_7	non-phage	0
DLF007_scaffold14358_2	non-phage	0
DLF007_scaffold5278_2	non-phage	0
DLF007_scaffold20306_1	non-phage	0
DLF007_scaffold15315_2	non-phage	0
DLF007_scaffold15612_3	non-phage	0
DLF007_scaffold20320_2	non-phage	0
DLF007_scaffold2141_12	non-phage	0
DLF007_scaffold2558_23	non-phage	0
DLF007_scaffold7339_2	non-phage	0
DLF007_scaffold993_3	non-phage	0
DLF007_scaffold14898_1	non-phage	0
DLF007_scaffold3028_2	non-phage	0
DLF007_scaffold19258_1	non-phage	0
DLF007_scaffold11709_2	non-phage	0
DLF007_scaffold7453_3	non-phage	0
DLF007_scaffold14558_2	non-phage	0
DLF007_scaffold3632_1	non-phage	0
DLF007_scaffold171_2	non-phage	0.099
DLF007_scaffold549_30	non-phage	0
DLF007_scaffold14289_1	non-phage	0
DLF007_scaffold19877_1	non-phage	0
DLF007_scaffold2141_17	non-phage	0
DLF007_scaffold18503_1	non-phage	0
DLF007_scaffold14058_1	non-phage	0
DLF007_scaffold20022_1	non-phage	0
DLF007_scaffold20132_1	non-phage	0
DLF007_scaffold18768_1	non-phage	0
DLF007_scaffold13268_1	non-phage	0
DLF007_scaffold18179_1	non-phage	0
DLF007_scaffold79_3	non-phage	0
DLF007_scaffold10297_3	non-phage	0
DLF007_scaffold12662_10	non-phage	0
DLF007_scaffold7_3	non-phage	0
DLF007_scaffold6506_1	non-phage	0
DLF007_scaffold4925_13	non-phage	0
DLF007_C306295_1	non-phage	0
DLF007_scaffold4809_1	non-phage	0
DLF007_scaffold2112_1	non-phage	0

DLF007_scaffold18834_1	non-phage	0
DLF007_scaffold15612_6	non-phage	0
DLF007_scaffold18763_1	non-phage	0
DLF007_scaffold9150_8	non-phage	0
DLF007_scaffold20109_3	non-phage	0
DLF007_scaffold10015_5	non-phage	0
DLF007_scaffold136_5	non-phage	0
DLF007_scaffold17705_3	non-phage	0.038
DLF007_scaffold20304_1	non-phage	0
DLF007_scaffold690_19	non-phage	0
DLF007_scaffold2677_26	non-phage	0
DLF007_scaffold3091_12	non-phage	0
DLF007_scaffold13257_1	non-phage	0
DLF007_scaffold2150_2	non-phage	0
DLF007_scaffold4925_12	non-phage	0
DLF007_scaffold19382_1	non-phage	0
DLF007_scaffold10982_1	non-phage	0
DLF007_scaffold11403_6	non-phage	0
DLF007_scaffold10325_1	non-phage	0
DLF007_C306149_1	phage	0.997
DLF007_scaffold20333_1	non-phage	0
DLF007_scaffold14803_2	non-phage	0
DLF007_scaffold19421_2	non-phage	0
DLF007_scaffold141_1	non-phage	0
DLF007_scaffold102_10	non-phage	0
DLF007_scaffold13847_4	non-phage	0
DLF007_scaffold13274_3	non-phage	0
DLF007_scaffold4495_9	non-phage	0
DLF007_scaffold8813_3	non-phage	0
DLF007_scaffold6312_3	non-phage	0
DLF007_scaffold15463_2	non-phage	0
DLF007_scaffold16959_5	non-phage	0
DLF007_scaffold17682_1	non-phage	0
DLF007_scaffold4809_5	non-phage	0
DLF007_scaffold18162_2	non-phage	0
DLF007_scaffold61_1	non-phage	0
DLF007_C306659_1	non-phage	0
DLF007_scaffold11857_2	non-phage	0
DLF007_scaffold77_1	non-phage	0
DLF007_scaffold18719_3	non-phage	0
DLF007_scaffold4084_1	non-phage	0
DLF007_scaffold2300_3	non-phage	0
DLF007_scaffold20079_1	non-phage	0
DLF007_scaffold5129_1	non-phage	0
DLF007_scaffold15329_1	non-phage	0
DLF007_scaffold7823_2	non-phage	0

DLF007_scaffold15193_1	non-phage	0
DLF007_scaffold11742_1	non-phage	0
DLF007_scaffold4994_1	non-phage	0
DLF007_scaffold14818_2	non-phage	0
DLF007_scaffold1841_2	non-phage	0
DLF007_scaffold14780_3	non-phage	0
DLF007_scaffold285_4	non-phage	0
DLF007_scaffold4068_1	non-phage	0
DLF007_scaffold14116_1	non-phage	0
DLF007_scaffold1764_1	non-phage	0
DLF007_scaffold20067_1	non-phage	0
DLF007_scaffold15660_1	non-phage	0
DLF007_scaffold20275_1	non-phage	0
DLF007_scaffold19556_2	non-phage	0
DLF007_scaffold2138_25	non-phage	0
DLF007_scaffold171_3	non-phage	0
DLF007_scaffold15847_3	non-phage	0
DLF007_scaffold20082_1	non-phage	0
DLF007_scaffold20135_2	non-phage	0
DLF007_scaffold20123_4	non-phage	0
DLF007_scaffold1662_6	non-phage	0
DLF007_scaffold10173_1	non-phage	0
DLF007_scaffold4822_3	non-phage	0
DLF007_scaffold4495_6	non-phage	0
DLF007_scaffold12662_8	non-phage	0
DLF007_scaffold18913_3	non-phage	0
DLF007_scaffold19353_1	non-phage	0
DLF007_scaffold78_2	non-phage	0
DLF007_scaffold70_1	non-phage	0
DLF007_scaffold20223_1	non-phage	0
DLF007_scaffold597_9	non-phage	0
DLF007_scaffold18350_3	non-phage	0
DLF007_scaffold6404_1	non-phage	0
DLF007_scaffold4450_1	non-phage	0
DLF007_scaffold19977_2	non-phage	0
DLF007_scaffold3237_7	non-phage	0
DLF007_scaffold15193_2	non-phage	0
DLF007_scaffold5577_9	non-phage	0
DLF007_scaffold1849_2	non-phage	0
DLF007_scaffold13462_1	non-phage	0
DLF007_scaffold52_1	non-phage	0
DLF007_scaffold7707_3	non-phage	0
DLF007_scaffold14289_3	non-phage	0
DLF007_scaffold79_1	non-phage	0
DLF007_scaffold12417_4	non-phage	0
DLF007_scaffold75_3	non-phage	0

DLF007_scaffold20318_1	non-phage	0
DLF007_scaffold19877_2	non-phage	0
DLF007_scaffold4892_1	non-phage	0
DLF007_scaffold16495_1	non-phage	0
DLF007_scaffold7707_1	non-phage	0
DLF007_scaffold65_2	non-phage	0
DLF007_scaffold7402_1	non-phage	0
DLF007_scaffold4747_10	non-phage	0
DLF007_scaffold16188_2	non-phage	0
DLF007_scaffold19954_1	non-phage	0
DLF007_scaffold13935_1	non-phage	0
DLF007_scaffold11870_1	non-phage	0
DLF007_scaffold9389_3	non-phage	0
DLF007_scaffold12_5	non-phage	0
DLF007_scaffold57_1	non-phage	0
DLF007_scaffold962_2	non-phage	0
DLF007_scaffold1513_2	non-phage	0
DLF007_scaffold15900_1	non-phage	0
DLF007_scaffold2097_38	non-phage	0
DLF007_scaffold19977_4	non-phage	0
DLF007_C306343_1	phage	0.996
DLF007_scaffold1080_1	non-phage	0
DLF007_scaffold3939_1	non-phage	0
DLF007_scaffold14104_2	non-phage	0
DLF007_scaffold1532_23	phage	0.948
DLF007_scaffold20103_2	non-phage	0
DLF007_scaffold15141_1	non-phage	0
DLF007_scaffold16767_2	non-phage	0
DLF007_scaffold14223_1	non-phage	0
DLF007_scaffold11697_1	non-phage	0
DLF007_scaffold9735_7	non-phage	0
DLF007_scaffold1531_1	non-phage	0
DLF007_scaffold18777_2	non-phage	0
DLF007_scaffold87_2	non-phage	0
DLF007_scaffold16467_1	non-phage	0
DLF007_scaffold758_2	non-phage	0
DLF007_scaffold20269_1	non-phage	0
DLF007_scaffold14658_6	non-phage	0
DLF007_scaffold15391_7	non-phage	0
DLF007_scaffold11776_1	non-phage	0
DLF007_scaffold6679_3	non-phage	0
DLF007_scaffold20333_2	phage	0.994
DLF007_scaffold1643_17	non-phage	0
DLF007_scaffold18_1	non-phage	0
DLF007_scaffold20236_2	non-phage	0
DLF007_scaffold20259_4	non-phage	0

DLF007_scaffold5046_1	non-phage	0
DLF007_scaffold6738_2	non-phage	0
DLF007_scaffold20123_1	non-phage	0
DLF007_scaffold20128_1	non-phage	0
DLF007_scaffold13920_2	non-phage	0
DLF007_scaffold1662_4	non-phage	0
DLF007_scaffold1052_1	non-phage	0
DLF007_scaffold19020_1	non-phage	0
DLF007_scaffold4822_1	non-phage	0
DLF007_scaffold4929_2	non-phage	0
DLF007_scaffold19200_1	non-phage	0
DLF007_scaffold12277_2	non-phage	0
DLF007_scaffold910_21	non-phage	0
DLF007_scaffold15660_5	non-phage	0
DLF007_scaffold3460_24	non-phage	0
DLF007_scaffold14573_1	non-phage	0
DLF007_scaffold1310_3	non-phage	0
DLF007_scaffold5942_1	non-phage	0
DLF007_C305789_1	non-phage	0
DLF007_scaffold20022_2	non-phage	0
DLF007_scaffold15181_1	non-phage	0
DLF007_scaffold20266_1	non-phage	0
DLF007_scaffold4179_16	phage	0.998
DLF007_C306415_1	non-phage	0.005
DLF007_scaffold16578_3	non-phage	0
DLF007_scaffold13921_3	non-phage	0
DLF007_scaffold7296_1	non-phage	0
DLF007_C306069_1	non-phage	0
DLF007_scaffold12264_2	non-phage	0
DLF007_scaffold2097_1	non-phage	0
DLF007_scaffold18926_1	non-phage	0
DLF007_scaffold10189_1	non-phage	0
DLF007_scaffold3237_8	non-phage	0
DLF007_scaffold15385_1	non-phage	0
DLF007_scaffold15612_1	non-phage	0
DLF007_scaffold20289_1	non-phage	0
DLF007_scaffold690_37	non-phage	0
DLF007_scaffold18326_2	non-phage	0
DLF007_scaffold5224_2	non-phage	0
DLF007_scaffold11563_3	non-phage	0
DLF007_scaffold3028_4	non-phage	0
DLF007_scaffold6113_1	non-phage	0
DLF007_scaffold18968_1	non-phage	0
DLF007_scaffold19522_1	non-phage	0
DLF007_scaffold10173_2	non-phage	0
DLF007_scaffold20331_2	non-phage	0

DLF007_scaffold14898_4	non-phage	0
DLF007_scaffold20327_1	non-phage	0
DLF007_scaffold13156_1	non-phage	0
DLF007_scaffold19556_1	non-phage	0
DLF007_scaffold20185_1	non-phage	0
DLF007_scaffold17475_2	non-phage	0
DLF007_scaffold3663_33	non-phage	0
DLF007_scaffold2664_3	non-phage	0
DLF007_scaffold4898_2	non-phage	0
DLF007_scaffold14695_2	non-phage	0
DLF007_scaffold15513_1	non-phage	0
DLF007_scaffold20232_1	non-phage	0
DLF007_scaffold17057_1	non-phage	0
DLF007_scaffold743_6	non-phage	0
DLF007_scaffold12318_1	non-phage	0
DLF007_scaffold20277_1	non-phage	0.2
DLF007_scaffold40_2	non-phage	0
DLF007_C306905_1	non-phage	0
DLF007_scaffold19838_3	non-phage	0
DLF007_scaffold52_5	non-phage	0
DLF007_scaffold1052_2	non-phage	0
DLF007_scaffold7850_1	non-phage	0
DLF007_scaffold15022_3	non-phage	0
DLF007_scaffold3025_1	non-phage	0
DLF007_scaffold17449_2	non-phage	0
DLF007_scaffold12617_1	non-phage	0
DLF007_scaffold3708_6	non-phage	0
DLF007_scaffold20081_1	non-phage	0
DLF007_scaffold6457_5	non-phage	0
DLF007_scaffold1266_2	non-phage	0
DLF007_scaffold7850_2	non-phage	0
DLF007_scaffold14424_1	non-phage	0
DLF007_scaffold19821_1	non-phage	0
DLF007_scaffold19110_2	non-phage	0
DLF007_scaffold5426_1	non-phage	0
DLF007_scaffold9150_4	non-phage	0
DLF007_scaffold16767_4	non-phage	0
DLF007_scaffold12617_2	non-phage	0
DLF007_scaffold84_2	non-phage	0
DLF007_scaffold5942_4	non-phage	0
DLF007_scaffold16578_4	non-phage	0
DLF007_scaffold20257_1	non-phage	0
DLF007_scaffold20302_2	non-phage	0
DLF007_scaffold20310_1	non-phage	0
DLF007_scaffold18162_3	non-phage	0
DLF007_scaffold2938_4	non-phage	0

DLF007_scaffold6841_7	non-phage	0
DLF007_scaffold15438_4	non-phage	0
DLF007_scaffold4809_6	non-phage	0
DLF007_scaffold11092_2	non-phage	0
DLF007_scaffold18630_1	non-phage	0
DLF007_scaffold15660_3	non-phage	0
DLF007_scaffold8297_1	non-phage	0
DLF007_scaffold16615_2	non-phage	0
DLF007_C306153_1	non-phage	0
DLF007_scaffold19772_1	non-phage	0
DLF007_scaffold13813_1	non-phage	0
DLF007_scaffold35_4	non-phage	0
DLF007_scaffold2097_35	non-phage	0
DLF007_scaffold4925_1	non-phage	0
DLF007_scaffold15417_1	non-phage	0
DLF007_scaffold285_5	non-phage	0
DLF007_scaffold17540_1	non-phage	0
DLF007_scaffold20281_2	non-phage	0
DLF007_scaffold17400_1	non-phage	0
DLF007_scaffold910_22	non-phage	0
DLF007_scaffold19592_3	non-phage	0
DLF007_scaffold5942_6	non-phage	0
DLF007_scaffold11015_4	non-phage	0
DLF007_scaffold285_3	non-phage	0
DLF007_scaffold15686_1	non-phage	0
DLF007_scaffold977_2	non-phage	0
DLF007_scaffold20303_1	non-phage	0
DLF007_scaffold15612_2	non-phage	0
DLF007_scaffold15181_3	non-phage	0
DLF007_scaffold15391_3	non-phage	0
DLF007_scaffold18336_1	non-phage	0
DLF007_scaffold52_3	non-phage	0
DLF007_scaffold3237_1	non-phage	0
DLF007_scaffold20160_3	non-phage	0
DLF007_scaffold17126_1	non-phage	0
DLF007_C306743_1	non-phage	0
DLF007_scaffold5949_2	non-phage	0
DLF007_scaffold8544_3	non-phage	0
DLF007_scaffold20257_3	non-phage	0
DLF007_scaffold6639_2	non-phage	0
DLF007_scaffold7067_2	non-phage	0
DLF007_scaffold8981_1	non-phage	0
DLF007_scaffold13910_2	non-phage	0
DLF007_scaffold1662_1	non-phage	0
DLF007_scaffold16295_1	non-phage	0
DLF007_scaffold13943_2	non-phage	0

DLF007_scaffold4200_1	non-phage	0
DLF007_scaffold6517_5	non-phage	0
DLF007_scaffold2141_32	non-phage	0
DLF007_scaffold2838_29	non-phage	0
DLF007_scaffold1662_8	non-phage	0
DLF007_scaffold19979_1	non-phage	0
DLF007_scaffold20314_1	non-phage	0
DLF007_scaffold4967_8	non-phage	0
DLF007_scaffold11658_1	non-phage	0
DLF007_scaffold9239_3	non-phage	0
DLF007_scaffold15763_5	phage	0.999
DLF007_scaffold16578_2	non-phage	0
DLF007_scaffold20234_3	non-phage	0
DLF007_scaffold19748_1	non-phage	0
DLF007_scaffold14930_6	non-phage	0
DLF007_scaffold14892_3	non-phage	0
DLF007_scaffold20070_1	non-phage	0
DLF007_scaffold6270_2	non-phage	0
DLF007_scaffold20211_1	non-phage	0.007
DLF007_scaffold13787_11	non-phage	0
DLF007_scaffold7402_7	non-phage	0
DLF007_scaffold18162_1	non-phage	0
DLF007_scaffold18446_1	non-phage	0
DLF007_scaffold52_2	non-phage	0
DLF007_scaffold9677_2	non-phage	0
DLF007_scaffold19977_3	non-phage	0
DLF007_scaffold3237_11	non-phage	0
DLF007_scaffold6312_4	non-phage	0
DLF007_scaffold9400_31	non-phage	0
DLF007_scaffold13274_2	non-phage	0
DLF007_scaffold35_2	non-phage	0
DLF007_scaffold2664_1	phage	0.996
DLF007_scaffold20221_2	non-phage	0
DLF007_scaffold20299_1	non-phage	0
DLF007_scaffold2677_47	non-phage	0
DLF007_scaffold11857_1	non-phage	0
DLF007_scaffold5046_2	non-phage	0
DLF007_scaffold15278_1	non-phage	0
DLF007_scaffold14780_4	non-phage	0
DLF007_scaffold20330_1	non-phage	0
DLF007_scaffold15660_2	non-phage	0
DLF007_scaffold16146_1	non-phage	0
DLF007_scaffold4809_8	non-phage	0
DLF007_scaffold2138_13	non-phage	0
DLF007_scaffold13660_5	non-phage	0
DLF007_scaffold19760_1	non-phage	0

DLF007_scaffold758_9	non-phage	0
DLF007_scaffold16437_1	non-phage	0
DLF007_scaffold182_2	non-phage	0
DLF007_scaffold13268_2	non-phage	0
DLF007_scaffold15612_4	non-phage	0
DLF007_scaffold20160_1	non-phage	0
DLF007_scaffold15196_2	non-phage	0
DLF007_scaffold665_4	non-phage	0
DLF007_scaffold3237_3	non-phage	0
DLF007_scaffold19064_3	non-phage	0
DLF007_scaffold20325_1	non-phage	0
DLF007_scaffold1310_1	non-phage	0
DLF007_scaffold5102_1	non-phage	0
DLF007_scaffold20219_2	non-phage	0
DLF007_scaffold10483_1	non-phage	0
DLF007_scaffold2097_4	non-phage	0
DLF007_scaffold13299_1	non-phage	0
DLF007_scaffold16753_4	non-phage	0
DLF007_scaffold6453_4	non-phage	0
DLF007_scaffold12394_9	non-phage	0
DLF007_scaffold11015_1	non-phage	0
DLF007_scaffold15047_1	non-phage	0
DLF007_scaffold11709_1	non-phage	0
DLF007_scaffold3078_1	non-phage	0
DLF007_scaffold12491_3	non-phage	0
DLF007_scaffold16145_1	non-phage	0
DLF007_scaffold15120_2	non-phage	0
DLF007_scaffold20290_1	non-phage	0
DLF007_scaffold20179_1	non-phage	0
DLF007_scaffold6087_20	non-phage	0
DLF007_scaffold11817_1	non-phage	0
DLF007_scaffold19925_1	non-phage	0
DLF007_scaffold20328_1	non-phage	0
DLF007_scaffold155_6	non-phage	0
DLF007_scaffold20120_1	non-phage	0
DLF007_scaffold7596_26	non-phage	0
DLF007_scaffold5429_1	non-phage	0
DLF007_scaffold5308_1	non-phage	0
DLF007_scaffold15993_1	non-phage	0
DLF007_scaffold20109_2	phage	0.856
DLF007_scaffold11207_12	non-phage	0
DLF007_scaffold6679_1	non-phage	0
DLF007_scaffold57_2	non-phage	0
DLF007_scaffold285_1	non-phage	0
DLF007_scaffold1952_6	non-phage	0
DLF007_scaffold5278_1	non-phage	0

DLF007_scaffold16320_4	non-phage	0
DLF007_scaffold20322_1	non-phage	0
DLF007_scaffold5587_2	non-phage	0
DLF007_scaffold12318_2	non-phage	0
DLF007_scaffold20310_3	non-phage	0
DLF007_scaffold20144_1	non-phage	0
DLF007_scaffold20221_1	non-phage	0
DLF007_scaffold11143_2	non-phage	0
DLF007_scaffold4925_5	non-phage	0
DLF007_scaffold12246_2	non-phage	0.06
DLF007_scaffold18326_7	non-phage	0
DLF007_scaffold12630_5	non-phage	0
DLF007_scaffold13366_1	non-phage	0
DLF007_scaffold20262_2	non-phage	0
DLF007_scaffold13156_2	non-phage	0
DLF007_scaffold16188_3	non-phage	0
DLF007_scaffold7928_1	non-phage	0
DLF007_scaffold7402_11	non-phage	0
DLF007_scaffold977_11	non-phage	0
DLF007_scaffold16767_1	non-phage	0
DLF007_scaffold17626_1	non-phage	0
DLF007_scaffold19739_2	non-phage	0
DLF007_scaffold16272_4	non-phage	0
DLF007_scaffold6679_2	non-phage	0
DLF007_scaffold6457_4	non-phage	0
DLF007_scaffold19911_1	non-phage	0
DLF007_scaffold17404_1	non-phage	0
DLF007_scaffold20259_3	non-phage	0
DLF007_scaffold594_2	non-phage	0
DLF007_scaffold6542_5	non-phage	0
DLF007_scaffold4929_3	non-phage	0
DLF007_scaffold4929_1	non-phage	0
DLF007_scaffold4336_5	non-phage	0
DLF007_scaffold79_2	non-phage	0
DLF007_scaffold20242_2	non-phage	0
DLF007_scaffold13119_1	non-phage	0
DLF007_scaffold10_3	non-phage	0
DLF007_scaffold1531_7	non-phage	0
DLF007_scaffold20252_1	non-phage	0
DLF007_scaffold20301_1	non-phage	0
DLF007_scaffold403_46	non-phage	0
DLF007_scaffold16104_2	non-phage	0
DLF007_scaffold11873_1	non-phage	0
DLF007_scaffold14930_5	non-phage	0
DLF007_C306403_1	non-phage	0
DLF007_scaffold6841_4	non-phage	0

DLF007_scaffold20154_1	non-phage	0
DLF007_scaffold18665_1	non-phage	0
DLF007_scaffold16959_3	non-phage	0
DLF007_scaffold3426_3	non-phage	0
DLF007_scaffold15850_2	non-phage	0
DLF007_scaffold10189_2	non-phage	0
DLF007_scaffold3237_4	non-phage	0
DLF007_scaffold15992_1	non-phage	0
DLF007_scaffold76_9	non-phage	0
DLF007_scaffold15660_6	non-phage	0
DLF007_scaffold20326_1	non-phage	0
DLF007_scaffold594_4	non-phage	0
DLF007_scaffold20308_2	non-phage	0
DLF007_scaffold5942_2	non-phage	0
DLF007_scaffold19977_1	non-phage	0
DLF007_scaffold3122_1	non-phage	0
DLF007_scaffold162_1	non-phage	0
DLF007_scaffold4828_13	non-phage	0
DLF007_scaffold18465_2	non-phage	0
DLF007_scaffold20234_4	non-phage	0
DLF007_scaffold9578_5	non-phage	0
DLF007_scaffold977_7	non-phage	0
DLF007_scaffold18009_3	non-phage	0
DLF007_scaffold6125_2	non-phage	0
DLF007_scaffold2097_85	non-phage	0
DLF007_scaffold13907_1	non-phage	0
DLF007_scaffold16057_1	non-phage	0
DLF007_scaffold14289_2	non-phage	0
DLF007_scaffold12662_14	non-phage	0
DLF007_scaffold1849_1	non-phage	0
DLF007_scaffold19944_1	non-phage	0
DLF007_scaffold15721_1	non-phage	0
DLF007_scaffold2228_46	non-phage	0.002
DLF007_scaffold15904_1	non-phage	0
DLF007_scaffold1826_2	non-phage	0
DLF007_scaffold6113_2	non-phage	0
DLF007_scaffold4224_10	non-phage	0
DLF007_scaffold12746_2	non-phage	0
DLF007_scaffold20289_2	non-phage	0
DLF007_scaffold6407_4	non-phage	0
DLF007_scaffold977_3	non-phage	0
DLF007_scaffold6542_3	non-phage	0
DLF007_scaffold6571_2	non-phage	0
DLF007_scaffold758_3	non-phage	0
DLF007_scaffold11143_7	non-phage	0
DLF007_scaffold20321_1	non-phage	0

DLF007_scaffold9150_2	non-phage	0
DLF007_scaffold14048_1	non-phage	0
DLF007_scaffold12630_11	non-phage	0
DLF007_scaffold20327_2	non-phage	0
DLF007_scaffold4484_1	non-phage	0
DLF007_scaffold18705_4	non-phage	0
DLF007_scaffold5224_1	non-phage	0
DLF007_scaffold20072_1	non-phage	0
DLF007_scaffold7977_7	non-phage	0
DLF007_scaffold17475_1	non-phage	0
DLF007_scaffold11776_2	non-phage	0
DLF007_scaffold12244_2	non-phage	0
DLF007_scaffold2632_2	non-phage	0
DLF007_C306545_1	non-phage	0
DLF007_scaffold17178_1	non-phage	0
DLF007_scaffold20294_1	non-phage	0
DLF007_scaffold674_45	non-phage	0
DLF007_scaffold19592_4	non-phage	0
DLF007_scaffold16767_3	non-phage	0
DLF007_scaffold18401_2	phage	1
DLF007_scaffold1674_11	non-phage	0
DLF007_scaffold20332_2	non-phage	0
DLF007_scaffold20259_2	non-phage	0
DLF007_scaffold20103_1	non-phage	0
DLF007_scaffold8107_17	non-phage	0
DLF007_scaffold6356_2	non-phage	0
DLF007_scaffold9239_6	non-phage	0
DLF007_scaffold17705_1	non-phage	0
DLF007_scaffold2838_9	non-phage	0
DLF007_scaffold20223_2	non-phage	0
DLF007_scaffold18350_2	non-phage	0
DLF007_scaffold7838_1	non-phage	0
DLF007_scaffold19956_2	non-phage	0
DLF007_C306727_1	non-phage	0
DLF007_scaffold18910_1	non-phage	0
DLF007_scaffold7951_1	non-phage	0
DLF007_scaffold5464_2	non-phage	0
DLF007_scaffold1849_3	non-phage	0
DLF007_scaffold17214_3	non-phage	0
DLF007_scaffold4_1	non-phage	0
DLF007_scaffold9908_1	non-phage	0
DLF007_scaffold4336_7	non-phage	0
DLF007_scaffold19615_3	non-phage	0
DLF007_scaffold10712_9	non-phage	0
DLF007_scaffold6266_1	non-phage	0
DLF007_scaffold19556_3	non-phage	0

DLF007_scaffold2097_12	non-phage	0
DLF007_scaffold14294_4	non-phage	0
DLF007_scaffold6595_3	non-phage	0
DLF007_scaffold6553_7	non-phage	0
DLF007_scaffold7453_2	non-phage	0
DLF007_scaffold19909_1	non-phage	0
DLF007_scaffold1906_5	non-phage	0
DLF007_scaffold2068_8	non-phage	0
DLF007_scaffold3237_9	non-phage	0
DLF007_scaffold12277_1	phage	1
DLF007_scaffold20248_1	non-phage	0
DLF007_scaffold20281_3	non-phage	0
DLF007_scaffold20334_1	non-phage	0
DLF007_scaffold1724_4	non-phage	0
DLF007_scaffold15181_7	phage	0.985
DLF007_scaffold2698_2	non-phage	0
DLF007_scaffold8544_1	non-phage	0
DLF007_scaffold1724_2	non-phage	0
DLF007_scaffold13366_2	non-phage	0
DLF008_scaffold659_6	non-phage	0
DLF008_scaffold601_2	non-phage	0
DLF008_scaffold15863_1	non-phage	0
DLF008_scaffold7316_8	non-phage	0
DLF008_scaffold112_2	non-phage	0
DLF008_scaffold7912_8	non-phage	0
DLF008_scaffold17518_2	non-phage	0
DLF008_scaffold4108_18	non-phage	0
DLF008_scaffold8602_7	non-phage	0
DLF008_scaffold17536_2	non-phage	0
DLF008_scaffold60_22	non-phage	0
DLF008_scaffold15861_1	non-phage	0
DLF008_scaffold11485_7	non-phage	0
DLF008_scaffold5686_1	non-phage	0
DLF008_scaffold3748_1	non-phage	0
DLF008_scaffold2567_5	non-phage	0
DLF008_scaffold8686_15	non-phage	0
DLF008_scaffold1760_2	non-phage	0
DLF008_scaffold2372_9	non-phage	0
DLF008_scaffold1838_8	non-phage	0
DLF008_scaffold2857_6	non-phage	0
DLF008_scaffold11270_3	non-phage	0
DLF008_scaffold8858_9	non-phage	0
DLF008_scaffold14455_1	non-phage	0
DLF008_scaffold5673_1	non-phage	0
DLF008_scaffold113_6	non-phage	0
DLF008_scaffold12603_1	non-phage	0

DLF008_scaffold16846_3	non-phage	0
DLF008_scaffold929_6	non-phage	0
DLF008_scaffold15923_2	non-phage	0
DLF008_scaffold9884_3	non-phage	0
DLF008_scaffold2284_5	non-phage	0
DLF008_scaffold4224_3	non-phage	0
DLF008_scaffold1702_9	non-phage	0
DLF008_scaffold944_1	non-phage	0
DLF008_scaffold12115_4	non-phage	0
DLF008_scaffold12621_2	non-phage	0
DLF008_scaffold60_30	non-phage	0
DLF008_scaffold4219_1	non-phage	0
DLF008_scaffold145_9	non-phage	0
DLF008_scaffold7461_4	phage	0.949
DLF008_scaffold3473_25	non-phage	0
DLF008_scaffold7912_9	non-phage	0
DLF008_scaffold17584_1	non-phage	0
DLF008_scaffold15150_2	non-phage	0
DLF008_scaffold3781_17	non-phage	0
DLF008_scaffold2122_3	non-phage	0
DLF008_scaffold8420_10	non-phage	0
DLF008_scaffold17633_2	non-phage	0
DLF008_scaffold17587_3	non-phage	0
DLF008_scaffold1838_16	non-phage	0
DLF008_scaffold7229_1	non-phage	0
DLF008_scaffold6699_2	non-phage	0
DLF008_scaffold9343_2	non-phage	0
DLF008_scaffold3962_23	non-phage	0
DLF008_scaffold5667_2	non-phage	0
DLF008_scaffold3962_31	non-phage	0
DLF008_scaffold9631_1	non-phage	0
DLF008_scaffold11816_1	non-phage	0
DLF008_scaffold16606_1	non-phage	0
DLF008_scaffold13862_9	non-phage	0
DLF008_scaffold16426_2	non-phage	0
DLF008_scaffold4872_5	non-phage	0
DLF008_scaffold1838_4	non-phage	0
DLF008_scaffold10909_1	non-phage	0
DLF008_scaffold15230_1	non-phage	0
DLF008_scaffold4193_4	non-phage	0
DLF008_scaffold17612_2	non-phage	0
DLF008_scaffold15262_3	non-phage	0
DLF008_scaffold17237_3	non-phage	0
DLF008_scaffold4389_3	non-phage	0
DLF008_scaffold138_1	non-phage	0
DLF008_scaffold17544_2	non-phage	0

DLF008_scaffold5600_5	non-phage	0
DLF008_scaffold7912_10	non-phage	0.008
DLF008_scaffold1702_20	non-phage	0
DLF008_scaffold8351_1	non-phage	0
DLF008_scaffold60_1	non-phage	0
DLF008_scaffold13122_4	non-phage	0
DLF008_scaffold924_1	non-phage	0
DLF008_scaffold17558_1	non-phage	0
DLF008_scaffold17620_1	non-phage	0
DLF008_scaffold3839_1	non-phage	0
DLF008_scaffold2103_4	non-phage	0
DLF008_scaffold3132_9	non-phage	0
DLF008_scaffold11457_1	non-phage	0
DLF008_C336313_1	non-phage	0
DLF008_scaffold2609_9	non-phage	0
DLF008_scaffold1838_5	non-phage	0
DLF008_scaffold80_7	non-phage	0
DLF008_scaffold13076_3	non-phage	0
DLF008_scaffold846_5	non-phage	0
DLF008_scaffold16450_1	non-phage	0
DLF008_scaffold3434_23	non-phage	0
DLF008_scaffold8633_3	non-phage	0
DLF008_scaffold3794_2	non-phage	0
DLF008_scaffold802_1	non-phage	0
DLF008_scaffold17152_1	phage	0.998
DLF008_scaffold2840_13	non-phage	0
DLF008_scaffold2857_27	non-phage	0
DLF008_scaffold17576_1	non-phage	0
DLF008_scaffold17578_3	non-phage	0
DLF008_scaffold51_10	non-phage	0
DLF008_scaffold8485_5	non-phage	0
DLF008_scaffold15586_16	non-phage	0
DLF008_scaffold2275_14	non-phage	0
DLF008_scaffold14160_10	non-phage	0
DLF008_scaffold16981_3	non-phage	0
DLF008_scaffold17588_2	non-phage	0
DLF008_scaffold1091_2	phage	0.998
DLF008_scaffold16605_3	non-phage	0
DLF008_scaffold4551_2	non-phage	0
DLF008_scaffold9492_3	non-phage	0
DLF008_scaffold11207_1	non-phage	0
DLF008_scaffold60_18	non-phage	0
DLF008_scaffold7094_9	non-phage	0
DLF008_scaffold17446_1	non-phage	0
DLF008_scaffold5947_1	non-phage	0
DLF008_scaffold6389_2	non-phage	0

DLF008_scaffold1596_4	non-phage	0
DLF008_scaffold10663_10	non-phage	0
DLF008_scaffold2379_1	non-phage	0
DLF008_scaffold7085_5	non-phage	0
DLF008_scaffold534_16	non-phage	0
DLF008_scaffold154_3	non-phage	0
DLF008_scaffold151_8	non-phage	0
DLF008_scaffold84_7	non-phage	0
DLF008_scaffold13939_2	non-phage	0
DLF008_scaffold470_18	non-phage	0
DLF008_scaffold8967_1	non-phage	0
DLF008_scaffold1865_11	non-phage	0
DLF008_scaffold11168_2	phage	0.692
DLF008_scaffold1929_1	non-phage	0
DLF008_scaffold3697_1	non-phage	0
DLF008_scaffold2664_5	non-phage	0
DLF008_scaffold1421_4	non-phage	0
DLF008_scaffold15216_1	non-phage	0
DLF008_scaffold15640_2	non-phage	0
DLF008_scaffold3463_7	non-phage	0
DLF008_scaffold1488_5	non-phage	0
DLF008_scaffold17572_2	non-phage	0
DLF008_scaffold17596_1	non-phage	0
DLF008_scaffold5937_6	non-phage	0
DLF008_scaffold45_6	non-phage	0
DLF008_scaffold134_7	non-phage	0
DLF008_scaffold17625_2	phage	0.856
DLF008_scaffold2154_1	non-phage	0
DLF008_scaffold3627_20	non-phage	0
DLF008_scaffold15308_1	non-phage	0
DLF008_scaffold13439_1	non-phage	0
DLF008_scaffold17343_1	non-phage	0
DLF008_scaffold14113_4	non-phage	0
DLF008_scaffold11136_2	non-phage	0
DLF008_scaffold4219_4	non-phage	0
DLF008_scaffold8845_4	non-phage	0
DLF008_scaffold17603_3	non-phage	0
DLF008_scaffold686_6	non-phage	0
DLF008_scaffold17607_1	phage	0.992
DLF008_scaffold7094_10	non-phage	0
DLF008_scaffold10663_4	non-phage	0
DLF008_scaffold4708_17	non-phage	0
DLF008_scaffold16020_2	non-phage	0
DLF008_scaffold7109_10	non-phage	0
DLF008_scaffold10559_6	non-phage	0
DLF008_scaffold4317_1	non-phage	0

DLF008_scaffold8602_10	non-phage	0
DLF008_scaffold1721_1	non-phage	0
DLF008_scaffold9517_2	non-phage	0
DLF008_scaffold450_21	non-phage	0
DLF008_scaffold14698_2	non-phage	0
DLF008_scaffold17497_1	phage	0.998
DLF008_scaffold12318_1	non-phage	0
DLF008_scaffold17503_2	non-phage	0
DLF008_scaffold1806_45	non-phage	0
DLF008_scaffold2681_18	non-phage	0
DLF008_scaffold1653_16	non-phage	0
DLF008_scaffold3794_6	non-phage	0
DLF008_scaffold8947_1	non-phage	0
DLF008_scaffold16075_1	non-phage	0
DLF008_scaffold17060_1	non-phage	0
DLF008_scaffold12448_4	non-phage	0
DLF008_scaffold3382_1	non-phage	0
DLF008_scaffold17630_1	non-phage	0
DLF008_scaffold12483_1	non-phage	0
DLF008_scaffold4651_1	non-phage	0
DLF008_scaffold2372_8	non-phage	0
DLF008_scaffold17612_1	non-phage	0
DLF008_scaffold17274_1	non-phage	0
DLF008_scaffold8879_2	non-phage	0
DLF008_scaffold13199_2	non-phage	0
DLF008_scaffold13134_3	non-phage	0.012
DLF008_scaffold48_1	non-phage	0
DLF008_scaffold5765_1	non-phage	0
DLF008_scaffold14160_4	phage	0.997
DLF008_scaffold12115_3	non-phage	0
DLF008_scaffold25_1	non-phage	0
DLF008_scaffold8142_2	non-phage	0
DLF008_scaffold51_5	non-phage	0
DLF008_scaffold11958_1	non-phage	0
DLF008_scaffold5434_3	non-phage	0
DLF008_scaffold7109_9	non-phage	0
DLF008_scaffold4108_19	non-phage	0
DLF008_scaffold17620_2	non-phage	0
DLF008_scaffold11857_3	non-phage	0
DLF008_scaffold4384_1	non-phage	0
DLF008_scaffold50_2	non-phage	0
DLF008_scaffold81_1	non-phage	0
DLF008_scaffold17215_1	non-phage	0
DLF008_scaffold682_9	non-phage	0
DLF008_scaffold1196_4	non-phage	0
DLF008_scaffold17587_2	non-phage	0

DLF008_scaffold2857_36	non-phage	0
DLF008_scaffold116_1	non-phage	0
DLF008_scaffold470_4	non-phage	0
DLF008_scaffold3962_6	non-phage	0
DLF008_scaffold17392_2	non-phage	0
DLF008_scaffold8395_3	non-phage	0
DLF008_scaffold1313_13	non-phage	0
DLF008_scaffold15586_9	non-phage	0
DLF008_scaffold10488_1	non-phage	0
DLF008_scaffold15869_2	non-phage	0
DLF008_scaffold17557_1	non-phage	0
DLF008_scaffold142_5	non-phage	0
DLF008_scaffold1106_1	non-phage	0
DLF008_scaffold6032_18	non-phage	0
DLF008_scaffold4389_4	non-phage	0
DLF008_scaffold16295_1	non-phage	0
DLF008_scaffold17397_2	non-phage	0
DLF008_scaffold1154_4	non-phage	0
DLF008_scaffold15216_2	non-phage	0
DLF008_scaffold9604_4	non-phage	0
DLF008_scaffold9492_1	non-phage	0
DLF008_scaffold2681_20	non-phage	0
DLF008_scaffold8096_5	non-phage	0
DLF008_scaffold463_13	non-phage	0
DLF008_scaffold4708_1	non-phage	0.002
DLF008_scaffold17494_3	non-phage	0
DLF008_scaffold5947_2	non-phage	0
DLF008_scaffold17538_2	non-phage	0
DLF008_scaffold12630_1	non-phage	0
DLF008_scaffold12390_1	non-phage	0
DLF008_scaffold4509_2	non-phage	0
DLF008_scaffold12953_1	non-phage	0
DLF008_scaffold22_1	phage	1
DLF008_scaffold17365_4	non-phage	0
DLF008_scaffold16124_1	non-phage	0
DLF008_scaffold3530_3	non-phage	0
DLF008_scaffold2326_6	non-phage	0
DLF008_scaffold7884_1	non-phage	0
DLF008_C335551_1	non-phage	0.479
DLF008_scaffold15262_4	non-phage	0
DLF008_scaffold10341_2	phage	0.999
DLF008_scaffold7912_3	non-phage	0
DLF008_scaffold17462_1	non-phage	0
DLF008_scaffold17377_5	non-phage	0
DLF008_scaffold2326_3	non-phage	0
DLF008_scaffold17628_1	non-phage	0

DLF008_scaffold93_1	non-phage	0
DLF008_scaffold3962_2	non-phage	0
DLF008_scaffold2103_1	non-phage	0
DLF008_scaffold5252_9	non-phage	0
DLF008_scaffold17536_1	non-phage	0
DLF008_scaffold17588_6	non-phage	0
DLF008_scaffold1538_14	non-phage	0
DLF008_scaffold11715_1	non-phage	0
DLF008_scaffold14160_6	non-phage	0
DLF008_scaffold3962_22	non-phage	0
DLF008_scaffold13348_9	non-phage	0
DLF008_scaffold2899_1	non-phage	0
DLF008_scaffold17553_3	non-phage	0
DLF008_scaffold8409_1	non-phage	0
DLF008_scaffold16743_1	non-phage	0
DLF008_scaffold11374_3	non-phage	0
DLF008_scaffold15905_1	non-phage	0
DLF008_scaffold17619_1	non-phage	0
DLF008_scaffold364_7	non-phage	0
DLF008_scaffold11170_5	non-phage	0
DLF008_scaffold12413_2	non-phage	0
DLF008_scaffold1838_14	non-phage	0
DLF008_scaffold670_18	non-phage	0
DLF008_scaffold7863_3	non-phage	0
DLF008_scaffold16449_2	phage	0.843
DLF008_scaffold11123_1	non-phage	0
DLF008_scaffold6603_1	non-phage	0
DLF008_scaffold9517_3	non-phage	0
DLF008_scaffold2095_26	non-phage	0
DLF008_scaffold17060_5	non-phage	0
DLF008_scaffold5600_4	non-phage	0
DLF008_scaffold1410_11	phage	0.999
DLF008_scaffold17598_2	non-phage	0
DLF008_scaffold15534_2	non-phage	0
DLF008_scaffold71_6	non-phage	0
DLF008_scaffold117_1	non-phage	0
DLF008_scaffold17626_1	non-phage	0
DLF008_scaffold4756_3	non-phage	0
DLF008_scaffold5667_7	non-phage	0
DLF008_scaffold124_1	non-phage	0
DLF008_scaffold6992_1	non-phage	0
DLF008_scaffold17633_1	non-phage	0
DLF008_scaffold1313_8	non-phage	0
DLF008_scaffold17404_1	non-phage	0
DLF008_scaffold14591_4	non-phage	0
DLF008_scaffold5415_1	phage	0.998

DLF008_scaffold8485_2	non-phage	0
DLF008_scaffold13022_1	non-phage	0
DLF008_scaffold12663_1	non-phage	0
DLF008_scaffold17598_3	non-phage	0
DLF008_scaffold11149_3	non-phage	0
DLF008_scaffold5686_8	non-phage	0
DLF008_scaffold1029_2	non-phage	0
DLF008_scaffold8303_28	non-phage	0
DLF008_scaffold2395_13	non-phage	0
DLF008_scaffold17595_1	non-phage	0
DLF008_scaffold15744_2	non-phage	0
DLF008_scaffold7166_3	non-phage	0
DLF008_scaffold17185_1	non-phage	0
DLF008_scaffold80_10	non-phage	0
DLF008_scaffold81_6	non-phage	0
DLF008_scaffold12826_2	non-phage	0
DLF008_scaffold922_1	non-phage	0
DLF008_C336049_1	non-phage	0
DLF008_scaffold3684_2	non-phage	0
DLF008_scaffold213_2	non-phage	0
DLF008_scaffold3962_5	phage	0.982
DLF008_scaffold5667_3	non-phage	0
DLF008_scaffold17159_1	non-phage	0
DLF008_scaffold2496_4	non-phage	0
DLF008_scaffold7854_16	non-phage	0
DLF008_C336303_1	non-phage	0
DLF008_scaffold5982_9	non-phage	0
DLF008_scaffold7801_2	non-phage	0
DLF008_scaffold2068_3	non-phage	0
DLF008_scaffold2487_36	non-phage	0
DLF008_scaffold13862_5	non-phage	0
DLF008_scaffold11453_1	non-phage	0
DLF008_scaffold14144_1	non-phage	0
DLF008_scaffold1642_2	non-phage	0
DLF008_scaffold4384_2	non-phage	0
DLF008_scaffold4618_2	non-phage	0
DLF008_scaffold5900_3	non-phage	0
DLF008_C336195_1	non-phage	0
DLF008_scaffold11564_2	non-phage	0
DLF008_scaffold4224_2	non-phage	0
DLF008_scaffold3276_1	non-phage	0
DLF008_scaffold8551_3	non-phage	0
DLF008_scaffold5618_3	non-phage	0
DLF008_scaffold1838_22	non-phage	0
DLF008_scaffold4708_5	non-phage	0
DLF008_scaffold4651_2	non-phage	0

DLF008_scaffold17383_1	non-phage	0
DLF008_scaffold11460_1	non-phage	0
DLF008_scaffold165_2	non-phage	0
DLF008_scaffold14695_1	non-phage	0
DLF008_scaffold9298_4	non-phage	0
DLF008_scaffold151_9	non-phage	0
DLF008_scaffold187_5	non-phage	0
DLF008_scaffold16489_2	non-phage	0
DLF008_scaffold10487_1	phage	1
DLF008_scaffold7371_5	non-phage	0
DLF008_scaffold11540_5	non-phage	0
DLF008_scaffold17516_1	non-phage	0
DLF008_scaffold6032_3	non-phage	0
DLF008_scaffold1538_15	non-phage	0
DLF008_scaffold3463_6	non-phage	0
DLF008_scaffold2326_8	phage	0.944
DLF008_scaffold16981_5	non-phage	0
DLF008_scaffold710_9	non-phage	0
DLF008_scaffold17540_3	non-phage	0
DLF008_scaffold463_21	non-phage	0
DLF008_scaffold7109_6	non-phage	0
DLF008_scaffold8602_8	non-phage	0
DLF008_scaffold2487_33	non-phage	0
DLF008_scaffold1085_6	non-phage	0
DLF008_scaffold4914_1	non-phage	0
DLF008_scaffold8303_9	non-phage	0
DLF008_scaffold17631_1	non-phage	0
DLF008_scaffold10059_4	non-phage	0
DLF008_scaffold2704_1	non-phage	0
DLF008_scaffold16981_4	non-phage	0
DLF008_C335601_1	non-phage	0
DLF008_scaffold601_4	non-phage	0
DLF008_scaffold3781_12	non-phage	0
DLF008_scaffold1838_18	non-phage	0
DLF008_scaffold887_5	non-phage	0
DLF008_scaffold6335_1	non-phage	0
DLF008_scaffold17588_1	non-phage	0
DLF008_scaffold3434_12	non-phage	0
DLF008_scaffold6845_2	non-phage	0
DLF008_scaffold17603_2	non-phage	0
DLF008_scaffold17459_1	non-phage	0
DLF008_scaffold450_11	non-phage	0
DLF008_scaffold2090_34	non-phage	0
DLF008_scaffold15134_1	non-phage	0
DLF008_scaffold16613_1	non-phage	0
DLF008_C336279_1	non-phage	0

DLF008_scaffold14160_1	non-phage	0
DLF008_scaffold5252_6	non-phage	0
DLF008_scaffold9193_5	non-phage	0
DLF008_scaffold11170_1	non-phage	0
DLF008_scaffold171_13	non-phage	0
DLF008_scaffold4193_15	non-phage	0
DLF008_scaffold17455_1	non-phage	0
DLF008_scaffold3620_3	non-phage	0.004
DLF008_scaffold6810_1	non-phage	0
DLF008_scaffold67_5	non-phage	0
DLF008_scaffold7109_3	non-phage	0
DLF008_scaffold6232_4	non-phage	0
DLF008_scaffold5667_4	non-phage	0
DLF008_scaffold1555_10	non-phage	0
DLF008_scaffold2716_5	non-phage	0
DLF008_scaffold7534_1	non-phage	0
DLF008_scaffold15869_4	non-phage	0
DLF008_scaffold4001_4	non-phage	0
DLF008_scaffold8372_30	non-phage	0
DLF008_scaffold71_20	non-phage	0
DLF008_scaffold4389_1	non-phage	0
DLF008_scaffold1850_4	non-phage	0
DLF008_scaffold14312_2	non-phage	0
DLF008_scaffold19_1	non-phage	0
DLF008_scaffold12463_2	non-phage	0.003
DLF008_scaffold15262_1	non-phage	0
DLF008_scaffold10900_5	non-phage	0
DLF008_scaffold14113_2	non-phage	0
DLF008_scaffold14259_1	non-phage	0
DLF008_scaffold12448_5	non-phage	0
DLF008_scaffold10663_5	non-phage	0
DLF008_scaffold4193_6	non-phage	0
DLF008_scaffold12002_1	non-phage	0
DLF008_scaffold13122_6	non-phage	0
DLF008_scaffold17374_1	phage	0.999
DLF008_C335467_1	phage	1
DLF008_scaffold45_8	non-phage	0
DLF008_scaffold16443_1	non-phage	0
DLF008_scaffold14357_4	non-phage	0
DLF008_scaffold16020_1	non-phage	0
DLF008_scaffold1705_1	non-phage	0
DLF008_scaffold17577_2	non-phage	0
DLF008_scaffold7109_8	non-phage	0
DLF008_scaffold13303_1	non-phage	0
DLF008_scaffold1132_2	non-phage	0
DLF008_scaffold8542_1	non-phage	0

DLF008_scaffold17629_1	non-phage	0
DLF008_scaffold710_21	phage	0.999
DLF008_scaffold17571_1	non-phage	0
DLF008_scaffold17588_5	non-phage	0
DLF008_scaffold1538_5	non-phage	0
DLF008_scaffold12122_3	non-phage	0
DLF008_scaffold1952_22	non-phage	0
DLF008_scaffold15869_1	non-phage	0
DLF008_scaffold6935_3	non-phage	0
DLF008_scaffold13338_2	non-phage	0
DLF008_scaffold15586_8	non-phage	0
DLF008_scaffold11374_4	non-phage	0
DLF008_scaffold6980_1	non-phage	0
DLF008_scaffold4941_1	non-phage	0
DLF008_scaffold10869_9	non-phage	0
DLF008_scaffold8642_1	non-phage	0
DLF008_scaffold17349_1	phage	0.999
DLF008_scaffold8473_2	non-phage	0
DLF008_scaffold17119_1	non-phage	0
DLF008_scaffold8155_4	non-phage	0
DLF008_scaffold17060_7	non-phage	0
DLF008_scaffold17376_1	non-phage	0
DLF008_scaffold2725_2	non-phage	0
DLF008_scaffold11270_1	non-phage	0
DLF008_scaffold13348_12	non-phage	0
DLF008_scaffold9649_1	non-phage	0
DLF008_scaffold17605_1	non-phage	0
DLF008_scaffold15216_3	non-phage	0
DLF008_scaffold15326_1	non-phage	0
DLF008_scaffold12390_2	non-phage	0
DLF008_scaffold4528_2	non-phage	0
DLF008_scaffold8096_6	phage	0.987
DLF008_scaffold11857_2	non-phage	0
DLF008_scaffold4753_1	non-phage	0
DLF008_scaffold7094_4	non-phage	0
DLF008_scaffold17365_3	non-phage	0
DLF008_scaffold14062_1	non-phage	0
DLF008_scaffold14658_1	non-phage	0
DLF008_scaffold6777_1	non-phage	0
DLF008_scaffold4108_8	non-phage	0
DLF008_scaffold15531_1	non-phage	0
DLF008_scaffold11169_1	non-phage	0
DLF008_scaffold7166_1	phage	0.996
DLF008_scaffold4001_6	non-phage	0
DLF008_scaffold2617_1	non-phage	0
DLF008_scaffold17578_2	non-phage	0

DLF008_scaffold4201_2	non-phage	0
DLF008_scaffold16661_2	non-phage	0
DLF008_scaffold281_4	non-phage	0
DLF008_scaffold11149_11	non-phage	0
DLF008_scaffold16426_4	non-phage	0
DLF008_scaffold9028_1	phage	0.994
DLF008_scaffold364_5	non-phage	0
DLF008_scaffold8047_2	phage	0.997
DLF008_scaffold7641_2	non-phage	0
DLF008_scaffold6032_2	non-phage	0
DLF008_scaffold6389_1	non-phage	0
DLF008_scaffold14357_5	non-phage	0
DLF008_scaffold17516_7	non-phage	0
DLF008_scaffold12092_3	non-phage	0
DLF008_scaffold57_4	non-phage	0
DLF008_scaffold17577_3	non-phage	0
DLF008_scaffold12826_1	non-phage	0
DLF008_scaffold5365_1	non-phage	0
DLF008_scaffold93_6	non-phage	0
DLF008_scaffold11136_4	non-phage	0
DLF008_scaffold4219_2	non-phage	0
DLF008_scaffold3962_4	non-phage	0
DLF008_scaffold15_1	non-phage	0
DLF008_scaffold5578_3	non-phage	0
DLF008_scaffold6782_5	non-phage	0
DLF008_scaffold17565_1	non-phage	0
DLF008_scaffold15079_2	non-phage	0
DLF008_scaffold601_6	non-phage	0
DLF008_scaffold25_2	non-phage	0
DLF008_scaffold12115_1	non-phage	0
DLF008_scaffold5618_2	non-phage	0
DLF008_scaffold7870_1	non-phage	0
DLF008_scaffold2127_5	non-phage	0
DLF008_scaffold1804_6	non-phage	0
DLF008_scaffold2084_1	non-phage	0
DLF008_scaffold534_67	non-phage	0
DLF008_scaffold16075_4	non-phage	0
DLF008_C336159_1	non-phage	0
DLF008_scaffold14815_1	non-phage	0
DLF008_scaffold13149_10	non-phage	0
DLF008_scaffold11509_1	non-phage	0
DLF008_scaffold2726_2	non-phage	0
DLF008_scaffold1029_1	phage	0.99
DLF008_scaffold8485_1	non-phage	0
DLF008_scaffold12115_2	non-phage	0
DLF008_scaffold17540_2	non-phage	0

DLF008_C336275_1	non-phage	0
DLF008_scaffold135_7	non-phage	0
DLF008_scaffold17573_1	non-phage	0
DLF008_scaffold11476_1	non-phage	0
DLF008_scaffold13288_1	non-phage	0
DLF008_scaffold17615_1	non-phage	0
DLF008_scaffold17048_2	non-phage	0
DLF008_scaffold5667_1	non-phage	0
DLF008_scaffold2567_24	non-phage	0
DLF008_scaffold557_2	non-phage	0
DLF008_scaffold5686_6	non-phage	0
DLF008_scaffold9518_4	non-phage	0
DLF008_scaffold16981_6	non-phage	0
DLF008_C336143_1	non-phage	0
DLF008_scaffold12621_3	non-phage	0
DLF008_scaffold4708_10	non-phage	0
DLF008_scaffold6850_1	non-phage	0
DLF008_scaffold14357_2	non-phage	0
DLF008_scaffold17355_1	non-phage	0
DLF008_scaffold1488_3	non-phage	0
DLF008_scaffold6637_3	non-phage	0
DLF008_scaffold1702_5	non-phage	0
DLF008_scaffold6287_3	non-phage	0
DLF008_scaffold17539_1	non-phage	0
DLF008_scaffold9884_1	non-phage	0.173
DLF008_scaffold13149_9	non-phage	0
DLF008_scaffold17060_6	non-phage	0
DLF008_scaffold17566_1	non-phage	0
DLF008_scaffold7684_2	non-phage	0
DLF008_scaffold12859_2	non-phage	0.001
DLF008_scaffold6335_5	non-phage	0
DLF008_scaffold11149_8	non-phage	0
DLF008_scaffold929_4	non-phage	0
DLF008_C336263_1	non-phage	0
DLF008_scaffold17627_1	non-phage	0
DLF008_scaffold17494_2	non-phage	0
DLF008_scaffold1305_1	non-phage	0
DLF008_scaffold14554_1	non-phage	0
DLF008_scaffold2011_1	non-phage	0
DLF008_scaffold8845_2	non-phage	0
DLF008_scaffold14357_1	non-phage	0
DLF008_scaffold9884_2	non-phage	0
DLF008_scaffold8373_10	non-phage	0
DLF008_scaffold1838_11	non-phage	0
DLF008_scaffold3962_1	phage	0.923
DLF008_scaffold17623_1	non-phage	0

DLF008_scaffold5827_2	non-phage	0.168
DLF008_scaffold1333_6	non-phage	0
DLF008_scaffold1952_24	non-phage	0
DLF008_scaffold17365_2	non-phage	0
DLF008_scaffold60_38	non-phage	0
DLF008_scaffold9604_2	non-phage	0
DLF008_scaffold534_57	non-phage	0
DLF008_scaffold222_2	phage	0.999
DLF008_scaffold601_3	non-phage	0
DLF008_scaffold111_1	non-phage	0
DLF008_scaffold12889_2	phage	0.986
DLF008_scaffold11407_2	non-phage	0
DLF008_scaffold11149_7	non-phage	0
DLF008_scaffold186_2	non-phage	0
DLF008_scaffold16138_1	non-phage	0
DLF008_scaffold7422_6	non-phage	0
DLF008_scaffold974_4	non-phage	0
DLF008_scaffold14160_9	non-phage	0
DLF008_scaffold2716_6	non-phage	0
DLF008_scaffold2122_2	non-phage	0
DLF008_scaffold3830_6	non-phage	0
DLF008_scaffold2953_7	phage	0.716
DLF008_scaffold3537_12	non-phage	0
DLF008_scaffold19_9	non-phage	0
DLF008_scaffold7371_15	non-phage	0
DLF008_scaffold8401_4	non-phage	0
DLF008_scaffold17084_1	phage	0.997
DLF008_scaffold17625_3	non-phage	0
DLF008_scaffold1952_3	non-phage	0
DLF008_scaffold17518_1	non-phage	0
DLF008_scaffold5252_7	non-phage	0
DLF008_scaffold3569_6	non-phage	0
DLF008_scaffold17450_3	non-phage	0
DLF008_scaffold4013_13	non-phage	0
DLF008_scaffold17275_1	non-phage	0
DLF008_scaffold5667_5	non-phage	0
DLF008_scaffold994_20	non-phage	0
DLF008_scaffold84_8	non-phage	0
DLF008_scaffold14031_1	non-phage	0
DLF008_scaffold8573_3	non-phage	0
DLF008_scaffold852_4	non-phage	0
DLF008_scaffold2857_3	non-phage	0
DLF008_scaffold1410_2	non-phage	0
DLF008_scaffold145_30	non-phage	0
DLF008_scaffold8373_7	non-phage	0.477
DLF008_scaffold1049_1	non-phage	0

DLF008_scaffold470_2	non-phage	0
DLF008_scaffold84_10	non-phage	0
DLF008_scaffold5434_1	non-phage	0
DLF008_scaffold13415_4	non-phage	0
DLF008_scaffold11149_4	non-phage	0
DLF008_scaffold2840_26	non-phage	0
DLF008_scaffold15632_1	non-phage	0
DLF008_scaffold14357_3	phage	0.998
DLF008_scaffold14591_5	non-phage	0
DLF008_scaffold16641_3	non-phage	0
DLF008_scaffold71_8	non-phage	0
DLF008_scaffold134_2	non-phage	0
DLF008_scaffold4193_5	non-phage	0
DLF008_scaffold7912_1	non-phage	0
DLF008_scaffold17603_1	non-phage	0
DLF008_scaffold3027_3	non-phage	0
DLF008_scaffold17629_2	non-phage	0
DLF008_scaffold16702_1	non-phage	0
DLF008_scaffold4701_1	non-phage	0
DLF008_scaffold12112_2	non-phage	0
DLF008_scaffold17529_1	non-phage	0
DLF008_scaffold81_3	non-phage	0
DLF008_scaffold15034_2	non-phage	0
DLF008_scaffold3110_2	non-phage	0
DLF008_scaffold12952_1	non-phage	0
DLF008_scaffold13149_7	non-phage	0.001
DLF008_scaffold12834_1	non-phage	0
DLF008_scaffold17_3	non-phage	0
DLF008_scaffold192_4	phage	0.998
DLF008_scaffold13122_2	non-phage	0
DLF008_scaffold1760_1	non-phage	0
DLF008_scaffold1313_11	non-phage	0
DLF008_scaffold17617_1	non-phage	0
DLF008_scaffold17622_2	non-phage	0
DLF008_scaffold4798_1	non-phage	0
DLF008_scaffold11407_3	non-phage	0
DLF008_scaffold1838_24	non-phage	0
DLF008_scaffold978_1	non-phage	0
DLF008_scaffold12418_3	non-phage	0
DLF008_scaffold2372_3	non-phage	0
DLF008_scaffold13758_1	non-phage	0
DLF008_scaffold4553_3	non-phage	0
DLF008_scaffold17067_2	non-phage	0
DLF008_scaffold15126_1	non-phage	0
DLF008_scaffold17632_1	non-phage	0
DLF008_scaffold14072_1	non-phage	0

DLF008_scaffold17570_1	non-phage	0
DLF008_scaffold17377_3	non-phage	0
DLF008_scaffold7085_6	non-phage	0
DLF008_scaffold1975_3	non-phage	0
DLF008_scaffold145_2	non-phage	0
DLF008_scaffold13155_1	non-phage	0
DLF008_scaffold15640_1	non-phage	0
DLF008_scaffold3891_1	non-phage	0
DLF008_scaffold13149_13	non-phage	0
DLF008_scaffold223_16	non-phage	0
DLF008_scaffold463_15	non-phage	0
DLF008_scaffold1333_2	non-phage	0
DLF008_scaffold3781_5	non-phage	0
DLF008_scaffold3812_7	non-phage	0
DLF008_scaffold5178_2	non-phage	0
DLF008_scaffold17624_1	non-phage	0
DLF008_scaffold4201_1	non-phage	0
DLF008_scaffold10663_9	non-phage	0
DLF008_scaffold13119_1	non-phage	0
DLF008_scaffold14160_8	non-phage	0
DLF008_scaffold5686_7	non-phage	0
DLF008_scaffold3328_5	phage	0.997
DLF008_scaffold4219_5	non-phage	0
DLF008_scaffold17621_1	non-phage	0
DLF008_scaffold9522_2	non-phage	0
DLF008_scaffold13076_2	non-phage	0
DLF008_scaffold10793_1	non-phage	0
DLF008_scaffold16828_1	non-phage	0
DLF008_scaffold1488_4	non-phage	0
DLF008_scaffold10053_2	non-phage	0
DLF008_scaffold4453_1	non-phage	0.004
DLF008_scaffold12953_3	non-phage	0
DLF008_scaffold4449_1	non-phage	0
DLF008_scaffold17572_1	non-phage	0
DLF008_scaffold4523_8	non-phage	0
DLF008_scaffold1538_12	non-phage	0
DLF008_scaffold5578_2	non-phage	0
DLF008_scaffold1952_27	non-phage	0
DLF008_scaffold17397_1	non-phage	0
DLF008_scaffold4872_9	non-phage	0
DLF008_scaffold17588_4	non-phage	0
DLF008_scaffold17588_3	non-phage	0
DLF008_scaffold3422_1	non-phage	0
DLF008_scaffold16761_1	non-phage	0
DLF008_scaffold11968_1	non-phage	0
DLF008_scaffold8685_2	non-phage	0

DLF008_scaffold17249_3	non-phage	0
DLF008_scaffold8027_4	non-phage	0
DLF008_scaffold17350_1	phage	0.999
DLF008_scaffold4675_3	non-phage	0
DLF008_scaffold9504_2	non-phage	0
DLF008_scaffold17576_2	non-phage	0
DLF008_scaffold17599_1	non-phage	0
DLF008_scaffold13199_1	non-phage	0
DLF008_scaffold14668_2	non-phage	0
DLF008_scaffold17377_1	non-phage	0
DLF008_scaffold17060_3	non-phage	0
DLF008_scaffold17154_1	non-phage	0
DLF008_scaffold12720_2	non-phage	0
DLF008_scaffold17140_1	non-phage	0
DLF008_scaffold7912_6	non-phage	0
DLF008_scaffold601_5	non-phage	0
DLF008_scaffold113_1	non-phage	0
DLF008_scaffold3879_4	non-phage	0
DLF008_scaffold55_10	non-phage	0
DLF008_scaffold6232_1	non-phage	0
DLF008_scaffold17601_1	non-phage	0
DLF008_scaffold17614_2	non-phage	0
DLF008_scaffold10059_13	non-phage	0
DLF008_scaffold3488_3	non-phage	0
DLF008_scaffold7854_13	non-phage	0
DLF008_scaffold14213_1	non-phage	0
DLF008_scaffold17547_2	non-phage	0
DLF008_scaffold106_3	non-phage	0.003
DLF008_scaffold11149_1	non-phage	0
DLF008_scaffold4333_2	non-phage	0
DLF008_scaffold3962_8	non-phage	0
DLF008_scaffold14113_1	non-phage	0
DLF008_scaffold2605_1	non-phage	0
DLF008_scaffold16652_2	non-phage	0
DLF008_scaffold11480_1	non-phage	0
DLF008_scaffold10663_7	non-phage	0
DLF008_scaffold12052_1	non-phage	0
DLF008_scaffold12389_3	non-phage	0
DLF008_scaffold3571_5	non-phage	0
DLF009_scaffold34424_1	phage	0.996
DLF009_scaffold37433_1	non-phage	0
DLF009_scaffold37412_1	non-phage	0
DLF009_scaffold15167_2	non-phage	0
DLF009_scaffold1880_30	phage	0.981
DLF009_scaffold32625_1	non-phage	0
DLF009_scaffold37450_1	non-phage	0

DLF009_scaffold4543_22	non-phage	0
DLF009_scaffold37429_1	non-phage	0
DLF009_scaffold27654_1	non-phage	0
DLF009_scaffold36221_2	phage	0.999
DLF009_scaffold37483_1	non-phage	0
DLF009_scaffold15785_6	non-phage	0
DLF009_C428906_1	phage	0.999
DLF009_scaffold23041_2	non-phage	0
DLF009_scaffold23276_3	non-phage	0
DLF009_scaffold29258_1	non-phage	0
DLF009_scaffold37383_3	non-phage	0
DLF009_scaffold37482_1	non-phage	0
DLF009_scaffold26879_6	non-phage	0
DLF009_scaffold5907_1	non-phage	0
DLF009_scaffold13685_24	non-phage	0
DLF009_scaffold37264_3	non-phage	0
DLF009_scaffold12854_6	non-phage	0
DLF009_scaffold35832_1	non-phage	0
DLF009_scaffold37380_2	non-phage	0
DLF009_scaffold37496_2	non-phage	0
DLF009_scaffold30115_1	phage	0.999
DLF009_C428242_1	non-phage	0
DLF009_scaffold20630_8	non-phage	0
DLF009_scaffold18235_3	non-phage	0
DLF009_scaffold5_5	non-phage	0
DLF009_scaffold4572_1	non-phage	0
DLF009_scaffold30315_1	non-phage	0
DLF009_scaffold37500_1	non-phage	0
DLF009_scaffold1880_23	non-phage	0
DLF009_scaffold4234_1	non-phage	0
DLF009_scaffold24605_1	non-phage	0
DLF009_scaffold37440_1	non-phage	0
DLF009_scaffold30754_1	non-phage	0
DLF009_scaffold37495_2	non-phage	0
DLF009_scaffold3670_1	non-phage	0
DLF009_scaffold37460_2	non-phage	0
DLF009_scaffold26734_3	non-phage	0
DLF009_scaffold464_46	non-phage	0
DLF009_scaffold11_1	non-phage	0
DLF009_scaffold15935_5	non-phage	0
DLF009_scaffold35735_1	non-phage	0
DLF009_scaffold11451_8	non-phage	0
DLF009_scaffold789_3	phage	0.622
DLF009_scaffold16253_17	non-phage	0
DLF009_scaffold28641_5	non-phage	0
DLF009_scaffold5713_4	non-phage	0

DLF009_scaffold5713_3	non-phage	0
DLF009_scaffold13838_3	non-phage	0
DLF009_scaffold36682_1	non-phage	0
DLF009_scaffold37141_1	non-phage	0
DLF009_scaffold24398_3	non-phage	0
DLF009_scaffold4_2	non-phage	0
DLF009_scaffold21043_10	non-phage	0
DLF009_scaffold37297_1	non-phage	0
DLF009_scaffold23013_8	non-phage	0
DLF009_scaffold35728_1	non-phage	0
DLF009_scaffold772_2	non-phage	0
DLF009_scaffold6185_6	non-phage	0
DLF009_scaffold28641_19	non-phage	0
DLF009_scaffold37414_2	non-phage	0
DLF009_scaffold36875_2	non-phage	0
DLF009_scaffold9093_1	non-phage	0
DLF009_scaffold2982_18	non-phage	0
DLF009_scaffold2665_13	non-phage	0
DLF009_scaffold14937_2	non-phage	0
DLF009_scaffold6675_14	non-phage	0
DLF009_scaffold22875_8	non-phage	0
DLF009_scaffold6859_4	non-phage	0
DLF009_scaffold21043_1	non-phage	0
DLF009_scaffold10619_4	non-phage	0
DLF009_scaffold17_2	non-phage	0
DLF009_scaffold15785_2	non-phage	0
DLF009_scaffold2514_3	non-phage	0
DLF009_scaffold1792_6	non-phage	0
DLF009_scaffold28677_2	non-phage	0
DLF009_scaffold17338_3	non-phage	0
DLF009_scaffold20258_6	non-phage	0.44
DLF009_scaffold13089_2	non-phage	0
DLF009_scaffold2131_3	non-phage	0
DLF009_scaffold8306_4	non-phage	0
DLF009_scaffold37393_1	non-phage	0
DLF009_scaffold23977_5	non-phage	0
DLF009_scaffold23913_4	non-phage	0
DLF009_scaffold12259_7	non-phage	0
DLF009_scaffold8843_9	non-phage	0
DLF009_scaffold21305_2	non-phage	0
DLF009_scaffold26104_3	non-phage	0
DLF009_scaffold304_33	non-phage	0
DLF009_scaffold37400_1	non-phage	0
DLF009_scaffold3930_4	non-phage	0
DLF009_scaffold37424_1	non-phage	0
DLF009_scaffold8641_3	non-phage	0

DLF009_scaffold15935_1	non-phage	0
DLF009_scaffold19370_3	non-phage	0
DLF009_scaffold1792_12	non-phage	0
DLF009_scaffold16253_11	non-phage	0
DLF009_scaffold36895_1	non-phage	0
DLF009_scaffold13313_4	non-phage	0
DLF009_scaffold8765_4	non-phage	0
DLF009_scaffold21043_8	non-phage	0
DLF009_scaffold21242_2	non-phage	0
DLF009_scaffold794_6	non-phage	0
DLF009_scaffold16253_7	non-phage	0
DLF009_scaffold37050_1	non-phage	0
DLF009_scaffold25600_2	non-phage	0
DLF009_scaffold25600_3	non-phage	0
DLF009_scaffold25059_2	non-phage	0
DLF009_scaffold464_1	non-phage	0
DLF009_scaffold37444_1	non-phage	0
DLF009_scaffold21305_1	non-phage	0
DLF009_scaffold30410_4	non-phage	0
DLF009_scaffold1880_3	non-phage	0
DLF009_scaffold2514_4	non-phage	0
DLF009_scaffold37442_2	phage	1
DLF009_scaffold6859_18	non-phage	0
DLF009_scaffold37322_2	non-phage	0
DLF009_scaffold5355_3	non-phage	0
DLF009_scaffold2982_23	non-phage	0
DLF009_scaffold1236_6	non-phage	0
DLF009_scaffold28641_9	non-phage	0
DLF009_scaffold1876_1	non-phage	0
DLF009_scaffold34888_1	non-phage	0
DLF009_scaffold36221_1	phage	0.999
DLF009_scaffold37157_1	non-phage	0
DLF009_scaffold2_2	non-phage	0
DLF009_scaffold18266_4	non-phage	0
DLF009_scaffold7798_1	phage	0.999
DLF009_scaffold37407_2	non-phage	0
DLF009_scaffold27654_4	non-phage	0
DLF009_scaffold32916_1	non-phage	0
DLF009_scaffold37363_2	phage	0.999
DLF009_scaffold5438_4	non-phage	0
DLF009_scaffold13766_3	non-phage	0
DLF009_scaffold19862_3	non-phage	0
DLF009_scaffold11451_17	phage	0.837
DLF009_scaffold5713_9	non-phage	0
DLF009_C428464_1	non-phage	0
DLF009_scaffold37503_1	non-phage	0

DLF009_scaffold5_4	non-phage	0
DLF009_scaffold37475_1	non-phage	0.003
DLF009_scaffold36152_1	non-phage	0
DLF009_scaffold37270_2	non-phage	0
DLF009_scaffold21876_2	phage	0.999
DLF009_scaffold12222_5	non-phage	0
DLF009_scaffold24329_2	non-phage	0
DLF009_scaffold32087_1	non-phage	0
DLF009_scaffold37177_1	phage	0.699
DLF009_scaffold36682_4	non-phage	0
DLF009_scaffold4543_30	non-phage	0
DLF009_scaffold10_1	non-phage	0
DLF009_scaffold37452_1	phage	0.993
DLF009_scaffold794_2	phage	0.998
DLF009_scaffold5685_5	non-phage	0
DLF009_scaffold7058_3	non-phage	0
DLF009_scaffold30226_1	non-phage	0
DLF009_scaffold34888_2	non-phage	0
DLF009_scaffold36152_2	non-phage	0
DLF009_scaffold29321_1	non-phage	0
DLF009_scaffold783_6	non-phage	0
DLF009_scaffold6866_4	non-phage	0
DLF009_scaffold4543_3	non-phage	0
DLF009_scaffold28641_13	non-phage	0
DLF009_scaffold25528_1	non-phage	0
DLF009_scaffold37317_1	non-phage	0
DLF009_scaffold23913_8	non-phage	0
DLF009_scaffold2982_12	non-phage	0
DLF009_scaffold8457_18	non-phage	0
DLF009_scaffold37476_2	non-phage	0
DLF009_scaffold6929_14	non-phage	0
DLF009_scaffold37496_3	non-phage	0
DLF009_scaffold37370_1	non-phage	0
DLF009_scaffold1792_1	non-phage	0
DLF009_scaffold37443_3	non-phage	0
DLF009_scaffold20179_5	non-phage	0
DLF009_scaffold30315_2	non-phage	0
DLF009_scaffold3286_3	non-phage	0
DLF009_scaffold4351_1	non-phage	0
DLF009_scaffold1236_7	non-phage	0
DLF009_scaffold8306_9	non-phage	0
DLF009_scaffold37240_1	non-phage	0
DLF009_scaffold13313_5	non-phage	0
DLF009_scaffold29342_1	non-phage	0
DLF009_scaffold464_43	non-phage	0
DLF009_scaffold37436_2	non-phage	0

DLF009_scaffold17406_15	non-phage	0
DLF009_scaffold1406_1	non-phage	0
DLF009_scaffold29258_4	non-phage	0
DLF009_scaffold35779_1	non-phage	0
DLF009_scaffold37447_1	non-phage	0
DLF009_scaffold34887_2	non-phage	0
DLF009_scaffold18235_6	non-phage	0
DLF009_scaffold30115_3	phage	0.999
DLF009_scaffold2567_2	non-phage	0
DLF009_scaffold37436_3	non-phage	0
DLF009_scaffold15785_5	non-phage	0
DLF009_scaffold37166_1	non-phage	0
DLF009_scaffold26879_5	non-phage	0
DLF009_scaffold37418_2	non-phage	0
DLF009_scaffold304_10	non-phage	0
DLF009_scaffold27793_3	non-phage	0
DLF009_scaffold36515_1	non-phage	0
DLF009_scaffold789_1	non-phage	0.43
DLF009_scaffold30781_2	non-phage	0
DLF009_scaffold2_1	non-phage	0
DLF009_scaffold8843_5	non-phage	0
DLF009_scaffold12603_12	non-phage	0
DLF009_scaffold37420_1	non-phage	0
DLF009_scaffold464_57	non-phage	0
DLF009_scaffold28458_10	non-phage	0
DLF009_scaffold37141_2	non-phage	0
DLF009_scaffold30410_3	non-phage	0
DLF009_scaffold17595_5	non-phage	0
DLF009_scaffold37414_1	non-phage	0
DLF009_scaffold37460_3	non-phage	0
DLF009_scaffold28641_17	non-phage	0
DLF009_scaffold24966_6	non-phage	0
DLF009_scaffold2567_13	non-phage	0
DLF009_scaffold3934_3	non-phage	0
DLF009_scaffold10619_2	non-phage	0
DLF009_scaffold9784_1	non-phage	0
DLF009_scaffold27654_12	non-phage	0
DLF009_scaffold23013_11	non-phage	0
DLF009_scaffold37505_1	non-phage	0
DLF009_scaffold37497_1	non-phage	0
DLF009_scaffold6_5	non-phage	0
DLF009_scaffold18266_8	non-phage	0
DLF009_scaffold10619_1	non-phage	0
DLF009_scaffold8701_1	non-phage	0
DLF009_scaffold2319_1	non-phage	0
DLF009_scaffold26734_2	non-phage	0

DLF009_scaffold25763_1	phage	0.999
DLF009_scaffold8843_8	non-phage	0
DLF009_scaffold26104_1	non-phage	0
DLF009_scaffold37400_2	non-phage	0
DLF009_scaffold26950_1	non-phage	0
DLF009_scaffold8641_2	non-phage	0
DLF009_C428450_1	non-phage	0
DLF009_scaffold23498_1	non-phage	0
DLF009_scaffold28458_13	non-phage	0
DLF009_scaffold8765_2	non-phage	0
DLF009_scaffold7058_13	non-phage	0
DLF009_scaffold7058_9	non-phage	0
DLF009_scaffold14586_10	non-phage	0
DLF009_scaffold16253_8	non-phage	0
DLF009_scaffold56_4	non-phage	0
DLF009_scaffold13354_1	phage	0.999
DLF009_scaffold5_1	non-phage	0
DLF009_scaffold37116_2	non-phage	0
DLF009_scaffold12603_24	non-phage	0
DLF009_scaffold19370_10	non-phage	0
DLF009_scaffold37264_1	non-phage	0
DLF009_C428558_1	non-phage	0
DLF009_scaffold15940_3	non-phage	0
DLF009_scaffold15785_1	non-phage	0
DLF009_scaffold3_1	non-phage	0
DLF009_scaffold18383_1	non-phage	0
DLF009_scaffold28641_8	non-phage	0
DLF009_scaffold17595_1	non-phage	0
DLF009_scaffold19672_1	non-phage	0
DLF009_scaffold13353_2	non-phage	0
DLF009_scaffold5713_10	non-phage	0
DLF009_scaffold2982_30	non-phage	0
DLF009_scaffold35205_1	non-phage	0
DLF009_scaffold19370_2	non-phage	0
DLF009_scaffold16253_3	non-phage	0
DLF009_scaffold26328_1	non-phage	0
DLF009_scaffold9023_5	non-phage	0
DLF009_C429134_1	non-phage	0
DLF009_scaffold7460_3	non-phage	0
DLF009_scaffold1076_1	non-phage	0
DLF009_C429044_1	non-phage	0
DLF009_scaffold35459_1	non-phage	0
DLF009_scaffold15935_2	non-phage	0
DLF009_scaffold6929_13	non-phage	0
DLF009_scaffold13766_4	phage	0.999
DLF009_scaffold23013_4	non-phage	0

DLF009_scaffold37252_1	non-phage	0
DLF009_scaffold24515_3	non-phage	0
DLF009_scaffold4234_3	non-phage	0
DLF009_scaffold8306_6	non-phage	0
DLF009_scaffold28641_16	non-phage	0
DLF009_scaffold16253_9	non-phage	0
DLF009_scaffold37486_1	non-phage	0.001
DLF009_scaffold37491_2	non-phage	0
DLF009_scaffold4543_9	non-phage	0
DLF009_scaffold37120_2	non-phage	0
DLF009_scaffold37369_2	non-phage	0
DLF009_scaffold35492_3	non-phage	0
DLF009_scaffold7_8	non-phage	0
DLF009_scaffold25370_1	non-phage	0
DLF009_scaffold4_1	non-phage	0
DLF009_scaffold37425_1	non-phage	0
DLF009_scaffold10619_5	non-phage	0
DLF009_scaffold7279_1	non-phage	0
DLF009_scaffold16764_1	non-phage	0
DLF009_scaffold37322_1	non-phage	0
DLF009_scaffold42_1	non-phage	0
DLF009_C428536_1	non-phage	0
DLF009_scaffold9960_11	non-phage	0
DLF009_scaffold31590_1	non-phage	0
DLF009_scaffold1236_8	non-phage	0
DLF009_scaffold37019_1	non-phage	0
DLF009_scaffold10136_4	non-phage	0
DLF009_scaffold37476_1	non-phage	0
DLF009_scaffold37446_1	non-phage	0
DLF009_scaffold19408_13	non-phage	0.131
DLF009_scaffold12127_9	non-phage	0
DLF009_scaffold37443_4	phage	0.922
DLF009_scaffold7_5	non-phage	0
DLF009_scaffold8695_1	non-phage	0
DLF009_scaffold12259_5	non-phage	0
DLF009_scaffold21043_6	non-phage	0
DLF009_scaffold37484_1	non-phage	0
DLF009_scaffold20630_6	non-phage	0
DLF009_scaffold8089_1	non-phage	0
DLF009_scaffold37460_4	non-phage	0
DLF009_scaffold6_1	non-phage	0
DLF009_scaffold28641_3	non-phage	0
DLF009_scaffold37498_1	phage	0.998
DLF009_scaffold37469_1	non-phage	0
DLF009_scaffold24515_1	non-phage	0
DLF009_scaffold10619_7	non-phage	0

DLF009_scaffold37488_1	non-phage	0
DLF009_scaffold37261_2	non-phage	0
DLF009_scaffold37453_2	non-phage	0
DLF009_scaffold37448_2	phage	0.999
DLF009_scaffold36152_3	non-phage	0
DLF009_scaffold3930_1	non-phage	0
DLF009_scaffold37345_2	non-phage	0
DLF009_scaffold36682_3	non-phage	0
DLF009_scaffold27654_14	non-phage	0
DLF009_scaffold24398_5	non-phage	0
DLF009_scaffold8843_4	non-phage	0
DLF009_scaffold11273_3	non-phage	0
DLF009_C428180_1	non-phage	0
DLF009_scaffold9023_7	non-phage	0
DLF009_scaffold16253_10	non-phage	0
DLF009_scaffold18266_2	non-phage	0
DLF009_scaffold37472_1	non-phage	0
DLF009_scaffold464_24	non-phage	0
DLF009_scaffold8298_5	non-phage	0
DLF009_scaffold17406_14	non-phage	0
DLF009_scaffold37454_1	non-phage	0
DLF009_scaffold23977_4	non-phage	0
DLF009_scaffold19862_1	non-phage	0
DLF009_scaffold19978_12	non-phage	0
DLF009_scaffold37438_1	phage	0.999
DLF009_scaffold26879_4	non-phage	0
DLF009_scaffold12854_3	non-phage	0
DLF009_scaffold30115_4	phage	0.998
DLF009_scaffold37466_1	phage	1
DLF009_scaffold37470_1	non-phage	0
DLF009_scaffold7058_7	non-phage	0
DLF009_scaffold37398_2	non-phage	0
DLF009_C428632_1	non-phage	0
DLF009_scaffold10619_3	non-phage	0
DLF009_scaffold30226_2	non-phage	0
DLF009_scaffold15785_4	non-phage	0
DLF009_scaffold37441_2	non-phage	0
DLF009_scaffold37436_1	non-phage	0
DLF009_scaffold37505_2	non-phage	0
DLF009_scaffold1406_2	non-phage	0
DLF009_scaffold22875_7	non-phage	0
DLF009_scaffold9324_4	non-phage	0
DLF009_scaffold2514_2	non-phage	0
DLF009_scaffold37480_1	non-phage	0
DLF009_scaffold8765_1	non-phage	0
DLF009_scaffold8695_3	non-phage	0

DLF009_scaffold37417_2	non-phage	0
DLF009_scaffold12603_11	non-phage	0
DLF009_scaffold37383_1	non-phage	0
DLF009_scaffold27654_7	non-phage	0
DLF009_scaffold23913_2	non-phage	0
DLF009_scaffold15449_5	non-phage	0
DLF009_scaffold37317_2	non-phage	0
DLF009_scaffold34242_3	non-phage	0
DLF009_scaffold13685_26	non-phage	0
DLF009_scaffold4038_1	non-phage	0
DLF009_scaffold18071_1	non-phage	0
DLF009_scaffold6929_19	non-phage	0
DLF009_scaffold37504_1	non-phage	0
DLF009_scaffold37020_3	phage	0.999
DLF009_scaffold16303_2	non-phage	0
DLF009_scaffold30781_1	non-phage	0
DLF009_scaffold18235_1	non-phage	0
DLF009_scaffold6361_4	non-phage	0
DLF009_scaffold7279_2	non-phage	0
DLF009_scaffold35026_1	non-phage	0
DLF009_scaffold36056_1	non-phage	0
DLF009_scaffold2987_8	non-phage	0
DLF009_scaffold1236_4	non-phage	0
DLF009_scaffold14586_8	non-phage	0
DLF009_scaffold464_17	non-phage	0
DLF009_scaffold5816_1	non-phage	0
DLF009_scaffold14000_1	non-phage	0
DLF009_scaffold20630_7	non-phage	0
DLF009_scaffold35459_2	non-phage	0
DLF009_scaffold24515_4	non-phage	0
DLF009_scaffold34242_2	non-phage	0
DLF009_scaffold36515_2	non-phage	0
DLF009_scaffold2284_19	non-phage	0
DLF009_scaffold37492_1	non-phage	0
DLF009_scaffold37448_1	non-phage	0
DLF009_scaffold13838_1	non-phage	0
DLF009_scaffold28641_15	non-phage	0
DLF009_scaffold11718_3	non-phage	0
DLF009_scaffold3043_4	phage	0.888
DLF009_scaffold37346_1	non-phage	0
DLF009_scaffold2665_18	non-phage	0
DLF009_C428576_1	phage	0.999
DLF009_scaffold37436_5	non-phage	0
DLF009_scaffold37120_3	non-phage	0
DLF009_scaffold2344_6	non-phage	0
DLF009_scaffold28677_4	non-phage	0

DLF009_scaffold37467_1	non-phage	0
DLF009_scaffold28458_14	non-phage	0
DLF009_scaffold1406_5	non-phage	0
DLF009_scaffold27288_1	non-phage	0
DLF009_scaffold5_6	non-phage	0
DLF009_scaffold2760_3	non-phage	0
DLF009_scaffold1792_4	non-phage	0
DLF009_scaffold25528_2	non-phage	0
DLF009_scaffold1078_19	non-phage	0
DLF009_scaffold8694_1	non-phage	0
DLF009_C428500_1	non-phage	0
DLF009_scaffold2665_4	non-phage	0
DLF009_scaffold37272_1	non-phage	0
DLF009_scaffold37485_1	non-phage	0
DLF009_scaffold34242_6	non-phage	0
DLF009_scaffold12962_6	non-phage	0
DLF009_scaffold19370_1	non-phage	0
DLF009_scaffold464_18	non-phage	0
DLF009_scaffold37424_3	non-phage	0
DLF009_scaffold15935_3	non-phage	0
DLF009_C428738_1	non-phage	0
DLF009_scaffold7460_2	non-phage	0
DLF009_scaffold16253_13	non-phage	0
DLF009_scaffold33944_1	non-phage	0
DLF009_scaffold23913_6	non-phage	0
DLF009_scaffold33259_2	non-phage	0
DLF009_scaffold37426_1	non-phage	0
DLF009_scaffold13685_4	non-phage	0
DLF009_scaffold37481_1	non-phage	0
DLF009_C428922_1	non-phage	0
DLF009_scaffold3286_21	non-phage	0
DLF009_scaffold35584_1	non-phage	0
DLF009_scaffold3284_13	non-phage	0
DLF009_scaffold37464_1	non-phage	0
DLF009_scaffold37270_7	non-phage	0
DLF009_scaffold9023_4	non-phage	0
DLF009_scaffold7058_14	non-phage	0
DLF009_scaffold34888_3	non-phage	0
DLF009_scaffold17926_1	phage	0.999
DLF009_scaffold21278_1	non-phage	0
DLF009_scaffold36682_2	non-phage	0
DLF009_scaffold12537_7	non-phage	0
DLF009_scaffold37261_1	non-phage	0
DLF009_scaffold2665_6	non-phage	0
DLF009_scaffold28458_1	non-phage	0
DLF009_scaffold1880_8	non-phage	0

DLF009_scaffold37345_1	non-phage	0
DLF009_scaffold15175_7	non-phage	0
DLF009_scaffold12461_14	non-phage	0
DLF009_scaffold6_2	non-phage	0
DLF009_scaffold28458_9	non-phage	0
DLF009_scaffold26734_5	non-phage	0
DLF009_C428856_1	non-phage	0
DLF009_scaffold6274_1	non-phage	0
DLF009_scaffold24398_2	non-phage	0
DLF009_scaffold23522_1	non-phage	0
DLF009_scaffold4_3	non-phage	0
DLF009_scaffold30344_2	non-phage	0
DLF009_scaffold5354_23	non-phage	0
DLF009_scaffold1405_1	non-phage	0
DLF009_scaffold9682_11	non-phage	0
DLF009_scaffold36221_3	non-phage	0
DLF009_scaffold900_2	non-phage	0
DLF009_scaffold37386_2	phage	0.999
DLF009_scaffold37453_1	non-phage	0
DLF009_C428544_1	non-phage	0
DLF009_scaffold37257_1	non-phage	0.077
DLF009_scaffold12259_1	non-phage	0
DLF009_scaffold8843_1	non-phage	0
DLF009_scaffold37470_2	non-phage	0
DLF009_scaffold11949_1	phage	0.999
DLF009_scaffold19674_5	phage	0.749
DLF009_scaffold37502_1	non-phage	0
DLF009_scaffold27779_1	non-phage	0
DLF009_scaffold23013_13	non-phage	0
DLF009_scaffold28641_11	non-phage	0
DLF009_scaffold14586_2	non-phage	0
DLF009_scaffold4543_1	non-phage	0
DLF009_scaffold15935_4	non-phage	0
DLF009_scaffold8695_4	non-phage	0
DLF009_scaffold6204_7	phage	0.999
DLF009_scaffold9324_3	non-phage	0
DLF009_scaffold34782_2	non-phage	0
DLF009_scaffold464_33	non-phage	0
DLF009_scaffold13353_1	non-phage	0
DLF009_scaffold30600_2	non-phage	0
DLF009_scaffold36943_1	non-phage	0
DLF009_scaffold5130_11	non-phage	0
DLF009_scaffold9346_4	non-phage	0
DLF009_scaffold37506_1	non-phage	0
DLF009_scaffold15175_14	non-phage	0
DLF009_scaffold37297_2	non-phage	0

DLF009_scaffold3557_3	non-phage	0
DLF009_scaffold37471_1	non-phage	0
DLF009_scaffold37417_1	non-phage	0
DLF009_scaffold2665_19	non-phage	0
DLF009_scaffold23913_3	non-phage	0
DLF009_scaffold36875_1	non-phage	0
DLF009_scaffold5523_4	non-phage	0
DLF009_scaffold36992_1	non-phage	0
DLF009_C428696_1	non-phage	0
DLF009_scaffold37458_2	non-phage	0
DLF009_scaffold22900_1	non-phage	0
DLF009_scaffold3440_1	non-phage	0
DLF009_scaffold37284_1	phage	1
DLF009_scaffold37069_2	non-phage	0
DLF009_scaffold22858_1	non-phage	0
DLF009_scaffold2982_31	non-phage	0
DLF009_scaffold28677_1	non-phage	0
DLF009_scaffold37495_1	non-phage	0
DLF009_scaffold25528_5	non-phage	0
DLF009_scaffold28641_6	non-phage	0
DLF009_scaffold4543_25	non-phage	0
DLF009_scaffold3934_8	non-phage	0
DLF009_scaffold37020_2	non-phage	0
DLF009_scaffold14937_1	non-phage	0
DLF009_scaffold9117_17	non-phage	0
DLF009_scaffold5494_2	non-phage	0
DLF009_scaffold37363_3	phage	0.992
DLF009_scaffold37307_2	phage	0.613
DLF009_scaffold18511_9	non-phage	0
DLF009_scaffold12603_26	non-phage	0
DLF009_scaffold28641_18	non-phage	0
DLF009_scaffold37487_1	non-phage	0
DLF009_scaffold37407_1	non-phage	0
DLF009_scaffold22290_1	non-phage	0
DLF009_scaffold2837_2	phage	0.999
DLF009_scaffold37390_1	non-phage	0
DLF009_scaffold11288_9	non-phage	0
DLF009_scaffold37499_1	non-phage	0
DLF009_scaffold37424_2	non-phage	0
DLF009_scaffold25503_6	non-phage	0
DLF009_scaffold8695_2	non-phage	0
DLF009_scaffold603_11	non-phage	0
DLF009_scaffold1107_11	non-phage	0.019
DLF009_scaffold61_24	phage	0.909
DLF009_scaffold25600_1	non-phage	0
DLF009_scaffold8294_12	non-phage	0

DLF009_scaffold2982_1	non-phage	0
DLF009_scaffold37241_1	non-phage	0
DLF009_scaffold37383_2	non-phage	0
DLF009_scaffold9960_10	non-phage	0
DLF009_scaffold7143_1	non-phage	0
DLF009_scaffold28237_4	non-phage	0
DLF009_scaffold8641_4	non-phage	0
DLF009_scaffold16303_3	non-phage	0
DLF009_scaffold10_3	non-phage	0
DLF009_scaffold10782_1	non-phage	0
DLF009_scaffold5945_13	non-phage	0
DLF009_scaffold10782_2	non-phage	0
DLF009_scaffold20738_5	non-phage	0
DLF009_scaffold37501_1	non-phage	0
DLF009_scaffold35492_1	non-phage	0
DLF009_scaffold37316_1	non-phage	0
DLF009_C429118_1	non-phage	0
DLF009_scaffold34593_1	non-phage	0
DLF009_scaffold3934_1	non-phage	0
DLF009_scaffold17338_4	non-phage	0
DLF009_scaffold1491_4	non-phage	0
DLF009_scaffold7570_14	phage	0.988
DLF009_scaffold20630_1	non-phage	0
DLF009_scaffold32862_1	non-phage	0
DLF009_scaffold304_6	non-phage	0
DLF009_scaffold37496_1	non-phage	0
DLF009_scaffold8843_6	non-phage	0
DLF009_scaffold3557_6	phage	0.988
DLF009_scaffold37322_3	non-phage	0
DLF009_scaffold12962_9	non-phage	0
DLF009_scaffold37386_1	phage	0.998
DLF009_scaffold19370_8	non-phage	0.204
DLF009_scaffold14716_1	non-phage	0
DLF009_scaffold10300_1	non-phage	0
DLF009_scaffold9117_13_1	non-phage	0
DLF009_scaffold37307_1	non-phage	0
DLF009_scaffold9579_1	non-phage	0
DLF009_scaffold12537_1	non-phage	0
DLF009_scaffold32172_1	non-phage	0
DLF009_scaffold37373_3	non-phage	0
DLF009_scaffold10_2	non-phage	0
DLF009_scaffold29258_3	non-phage	0
DLF009_scaffold37455_2	non-phage	0
DLF009_scaffold37507_1	non-phage	0
DLF009_scaffold30410_5	non-phage	0
DLF009_scaffold37222_1	non-phage	0

DLF009_scaffold23041_6	non-phage	0
DLF009_scaffold37289_1	non-phage	0
DLF009_scaffold23450_1	non-phage	0
DLF009_C428590_1	non-phage	0
DLF009_scaffold603_5	non-phage	0
DLF009_scaffold5355_4	non-phage	0
DLF009_scaffold30115_5	phage	0.999
DLF009_scaffold2982_37	non-phage	0
DLF009_scaffold37490_1	non-phage	0
DLF009_scaffold5713_13	non-phage	0
DLF010_scaffold779_33	non-phage	0
DLF010_scaffold28674_11	non-phage	0
DLF010_scaffold23688_1	non-phage	0
DLF010_scaffold43071_4	non-phage	0
DLF010_scaffold6984_4	non-phage	0
DLF010_scaffold1405_19	non-phage	0
DLF010_scaffold44010_3	non-phage	0
DLF010_scaffold33416_3	non-phage	0
DLF010_scaffold17302_2	non-phage	0
DLF010_scaffold14216_2	non-phage	0
DLF010_scaffold16949_1	non-phage	0
DLF010_scaffold45640_3	non-phage	0
DLF010_scaffold20862_1	non-phage	0
DLF010_scaffold14049_2	non-phage	0
DLF010_scaffold28674_8	non-phage	0
DLF010_scaffold7357_3	non-phage	0
DLF010_scaffold44456_5	non-phage	0
DLF010_scaffold4567_24	non-phage	0
DLF010_scaffold36092_5	non-phage	0
DLF010_scaffold11475_3	non-phage	0
DLF010_scaffold44929_4	non-phage	0
DLF010_scaffold22334_6	non-phage	0
DLF010_scaffold4389_21	non-phage	0
DLF010_scaffold54953_2	non-phage	0
DLF010_scaffold38291_1	non-phage	0
DLF010_scaffold2375_6	phage	0.965
DLF010_scaffold4247_61	non-phage	0
DLF010_scaffold9015_5	non-phage	0
DLF010_scaffold9_7	non-phage	0
DLF010_scaffold24711_6	non-phage	0
DLF010_scaffold41685_27	non-phage	0
DLF010_scaffold779_4	non-phage	0
DLF010_scaffold49956_11	non-phage	0
DLF010_scaffold2402_3	non-phage	0
DLF010_scaffold5474_6	non-phage	0
DLF010_scaffold27888_12	non-phage	0

DLF010_scaffold52853_1	non-phage	0
DLF010_scaffold7584_1	non-phage	0
DLF010_scaffold14683_7	non-phage	0
DLF010_scaffold26021_3	non-phage	0
DLF010_scaffold51819_3	non-phage	0
DLF010_scaffold39555_1	non-phage	0
DLF010_scaffold19024_3	non-phage	0
DLF010_scaffold46141_1	non-phage	0
DLF010_scaffold2940_18	non-phage	0
DLF010_scaffold15168_14	non-phage	0
DLF010_scaffold15303_1	non-phage	0
DLF010_scaffold46304_2	non-phage	0
DLF010_scaffold51019_1	non-phage	0
DLF010_scaffold55549_1	non-phage	0
DLF010_scaffold36638_7	non-phage	0
DLF010_scaffold55444_2	non-phage	0
DLF010_scaffold11109_1	non-phage	0
DLF010_scaffold53131_1	non-phage	0
DLF010_scaffold2083_2	non-phage	0
DLF010_scaffold35048_10	non-phage	0
DLF010_scaffold49096_1	non-phage	0
DLF010_scaffold19584_7	non-phage	0
DLF010_scaffold18643_2	non-phage	0
DLF010_scaffold55891_1	non-phage	0
DLF010_scaffold29752_3	non-phage	0
DLF010_scaffold924_1	non-phage	0
DLF010_scaffold353_18	non-phage	0
DLF010_scaffold363_15	non-phage	0
DLF010_scaffold54062_2	non-phage	0
DLF010_scaffold47338_1	non-phage	0
DLF010_C744688_1	non-phage	0
DLF010_scaffold1333_7	non-phage	0
DLF010_scaffold49956_2	non-phage	0
DLF010_scaffold114_2	non-phage	0
DLF010_scaffold26002_3	non-phage	0.114
DLF010_scaffold9004_8	non-phage	0
DLF010_scaffold645_7	non-phage	0
DLF010_scaffold2189_7	non-phage	0
DLF010_scaffold55822_1	non-phage	0
DLF010_scaffold24792_3	non-phage	0
DLF010_scaffold177_4	non-phage	0
DLF010_scaffold2596_4	non-phage	0
DLF010_scaffold9963_9	non-phage	0
DLF010_scaffold24026_11	phage	0.999
DLF010_scaffold38018_9	non-phage	0
DLF010_scaffold45075_10	non-phage	0

DLF010_scaffold3040_9	non-phage	0
DLF010_scaffold106_10	non-phage	0
DLF010_scaffold20354_2	non-phage	0
DLF010_scaffold44057_2	non-phage	0
DLF010_scaffold41359_2	non-phage	0
DLF010_scaffold55916_1	non-phage	0
DLF010_scaffold4389_24	non-phage	0
DLF010_scaffold101_34	non-phage	0
DLF010_scaffold35043_4	phage	0.997
DLF010_scaffold44456_10	non-phage	0
DLF010_scaffold285_2	non-phage	0
DLF010_scaffold19199_1	non-phage	0
DLF010_scaffold21604_8	non-phage	0
DLF010_scaffold41685_20	non-phage	0
DLF010_scaffold5195_9	non-phage	0
DLF010_scaffold8211_3	non-phage	0
DLF010_scaffold52737_5	non-phage	0
DLF010_scaffold49629_3	non-phage	0
DLF010_scaffold55380_1	non-phage	0
DLF010_scaffold44456_4	non-phage	0
DLF010_scaffold46224_20	non-phage	0
DLF010_scaffold28105_1	non-phage	0
DLF010_scaffold44456_22	non-phage	0
DLF010_scaffold55870_1	phage	0.992
DLF010_scaffold15773_2	non-phage	0
DLF010_scaffold55922_1	non-phage	0
DLF010_scaffold5622_2	non-phage	0
DLF010_scaffold51052_2	non-phage	0
DLF010_scaffold42442_3	non-phage	0
DLF010_scaffold28_21	non-phage	0
DLF010_scaffold1532_1	non-phage	0
DLF010_scaffold354_8	non-phage	0
DLF010_scaffold43436_5	phage	1
DLF010_scaffold13138_6	non-phage	0
DLF010_scaffold49956_22	non-phage	0
DLF010_scaffold363_30	phage	0.689
DLF010_scaffold28523_1	non-phage	0
DLF010_scaffold55310_10	non-phage	0
DLF010_scaffold13193_14	non-phage	0
DLF010_scaffold21604_9	non-phage	0
DLF010_scaffold49739_2	non-phage	0
DLF010_scaffold363_22	non-phage	0
DLF010_scaffold14988_8	non-phage	0
DLF010_scaffold19147_3	non-phage	0
DLF010_scaffold9836_14	non-phage	0
DLF010_scaffold2887_3	non-phage	0

DLF010_scaffold9004_11	non-phage	0
DLF010_scaffold31375_12	non-phage	0
DLF010_scaffold53793_1	non-phage	0
DLF010_scaffold25202_7	non-phage	0
DLF010_scaffold7380_1	non-phage	0
DLF010_scaffold48528_1	non-phage	0
DLF010_scaffold41818_1	non-phage	0
DLF010_scaffold9004_2	non-phage	0
DLF010_scaffold55893_3	non-phage	0
DLF010_scaffold1590_1	non-phage	0
DLF010_scaffold11121_9	non-phage	0
DLF010_scaffold36342_7	non-phage	0
DLF010_scaffold38269_4	non-phage	0
DLF010_scaffold23679_22	non-phage	0
DLF010_scaffold49447_9	non-phage	0.002
DLF010_scaffold106_22	non-phage	0
DLF010_scaffold5195_14	non-phage	0
DLF010_scaffold36638_6	phage	0.937
DLF010_scaffold46224_11	non-phage	0
DLF010_scaffold52783_2	non-phage	0
DLF010_scaffold28427_1	non-phage	0
DLF010_scaffold44456_14	non-phage	0
DLF010_scaffold2356_8	non-phage	0
DLF010_scaffold26724_9	non-phage	0
DLF010_scaffold31938_9	non-phage	0
DLF010_scaffold363_2	non-phage	0
DLF010_scaffold55714_3	non-phage	0
DLF010_scaffold27498_2	non-phage	0
DLF010_scaffold2983_27	non-phage	0
DLF010_scaffold29752_4	non-phage	0
DLF010_scaffold9265_25	non-phage	0
DLF010_scaffold433_2	non-phage	0
DLF010_scaffold23679_21	non-phage	0
DLF010_scaffold27950_2	non-phage	0
DLF010_scaffold6880_11	non-phage	0
DLF010_scaffold112_3	non-phage	0
DLF010_scaffold130_15	non-phage	0
DLF010_scaffold31235_5	non-phage	0
DLF010_scaffold36607_1	non-phage	0
DLF010_scaffold24792_9	non-phage	0
DLF010_scaffold15168_7	non-phage	0
DLF010_scaffold2887_9	non-phage	0
DLF010_scaffold3040_5	non-phage	0
DLF010_scaffold6136_1	non-phage	0
DLF010_scaffold9328_7	non-phage	0
DLF010_scaffold16949_2	non-phage	0

DLF010_scaffold6815_13	phage	0.997
DLF010_scaffold20307_1	non-phage	0
DLF010_scaffold433_19	non-phage	0
DLF010_scaffold22641_3	non-phage	0
DLF010_scaffold19024_4	non-phage	0
DLF010_scaffold106_46	non-phage	0
DLF010_scaffold21_1	non-phage	0
DLF010_scaffold177_3	non-phage	0
DLF010_scaffold13546_16	phage	0.995
DLF010_scaffold37959_5	non-phage	0
DLF010_scaffold2887_6	non-phage	0
DLF010_scaffold55459_3	non-phage	0
DLF010_scaffold1854_9	non-phage	0
DLF010_scaffold161_1	non-phage	0
DLF010_scaffold55785_1	non-phage	0
DLF010_scaffold20307_5	non-phage	0
DLF010_scaffold24063_13	non-phage	0
DLF010_scaffold55847_3	non-phage	0
DLF010_scaffold38970_1	non-phage	0
DLF010_scaffold20014_1	non-phage	0
DLF010_scaffold24792_10	non-phage	0
DLF010_scaffold446_2	non-phage	0
DLF010_scaffold55464_1	non-phage	0
DLF010_scaffold2402_6	non-phage	0
DLF010_scaffold24210_10	non-phage	0
DLF010_scaffold3799_7	non-phage	0
DLF010_scaffold363_8	non-phage	0
DLF010_scaffold54834_1	non-phage	0
DLF010_scaffold9328_27	non-phage	0
DLF010_scaffold49956_3	non-phage	0
DLF010_scaffold158_2	non-phage	0
DLF010_scaffold93_1	non-phage	0
DLF010_scaffold363_32	non-phage	0
DLF010_scaffold14964_3	non-phage	0
DLF010_scaffold27888_11	non-phage	0
DLF010_scaffold55735_2	non-phage	0
DLF010_scaffold11496_16	non-phage	0
DLF010_scaffold8871_4	non-phage	0.142
DLF010_scaffold6058_7	non-phage	0
DLF010_scaffold55574_1	non-phage	0
DLF010_scaffold18759_22	non-phage	0
DLF010_scaffold55917_1	non-phage	0
DLF010_scaffold2392_27	non-phage	0
DLF010_scaffold28007_18	non-phage	0
DLF010_scaffold26057_14	non-phage	0
DLF010_scaffold45075_8	non-phage	0

DLF010_scaffold114_1	non-phage	0
DLF010_scaffold19877_7	non-phage	0
DLF010_scaffold130_1	non-phage	0
DLF010_scaffold32926_26	non-phage	0
DLF010_scaffold42442_1	non-phage	0
DLF010_scaffold2402_11	non-phage	0
DLF010_scaffold1059_1	non-phage	0
DLF010_scaffold12509_1	non-phage	0
DLF010_scaffold55821_1	non-phage	0
DLF010_scaffold6880_7	non-phage	0
DLF010_scaffold33582_1	non-phage	0
DLF010_scaffold35043_3	phage	1
DLF010_scaffold3594_3	non-phage	0
DLF010_scaffold55923_1	non-phage	0
DLF010_scaffold1042_21	non-phage	0
DLF010_scaffold42442_4	non-phage	0
DLF010_scaffold6153_20	non-phage	0
DLF010_scaffold3040_12	non-phage	0
DLF010_scaffold28064_2	non-phage	0
DLF010_scaffold1_4	non-phage	0
DLF010_scaffold45761_2	non-phage	0
DLF010_scaffold46634_1	phage	0.992
DLF010_scaffold55858_1	non-phage	0
DLF010_scaffold55900_1	non-phage	0
DLF010_scaffold21921_2	non-phage	0
DLF010_scaffold26952_6	non-phage	0
DLF010_scaffold23900_1	non-phage	0
DLF010_scaffold9727_6	non-phage	0
DLF010_scaffold9836_55	non-phage	0
DLF010_scaffold48066_1	non-phage	0
DLF010_scaffold55919_1	non-phage	0
DLF010_scaffold3519_6	non-phage	0
DLF010_scaffold55797_1	non-phage	0
DLF010_scaffold13248_1	phage	0.985
DLF010_scaffold8644_4	non-phage	0
DLF010_scaffold44719_2	non-phage	0
DLF010_scaffold49739_1	non-phage	0
DLF010_scaffold55043_1	non-phage	0
DLF010_scaffold55572_1	non-phage	0
DLF010_scaffold36092_7	non-phage	0
DLF010_scaffold22973_5	non-phage	0
DLF010_scaffold423_1	non-phage	0
DLF010_scaffold45822_3	non-phage	0
DLF010_scaffold30180_1	non-phage	0
DLF010_scaffold32774_3	non-phage	0
DLF010_scaffold15168_6	non-phage	0

DLF010_scaffold2887_2	non-phage	0
DLF010_scaffold9004_12	non-phage	0
DLF010_scaffold35048_6	non-phage	0
DLF010_scaffold49177_4	phage	0.999
DLF010_scaffold86_9	non-phage	0
DLF010_scaffold12814_3	non-phage	0
DLF010_scaffold35048_1	non-phage	0
DLF010_scaffold10656_1	non-phage	0
DLF010_scaffold44456_3	non-phage	0
DLF010_scaffold26420_1	non-phage	0
DLF010_scaffold4302_1	phage	0.999
DLF010_scaffold16949_3	non-phage	0
DLF010_scaffold50807_2	non-phage	0
DLF010_scaffold10857_7	non-phage	0
DLF010_scaffold42267_1	non-phage	0
DLF010_scaffold37693_1	non-phage	0
DLF010_scaffold5195_15	non-phage	0
DLF010_scaffold40667_1	non-phage	0
DLF010_scaffold2481_1	non-phage	0
DLF010_scaffold2699_1	non-phage	0
DLF010_scaffold24211_1	non-phage	0
DLF010_scaffold38269_1	non-phage	0
DLF010_scaffold2355_16	non-phage	0
DLF010_scaffold363_31	non-phage	0
DLF010_scaffold55906_1	non-phage	0
DLF010_scaffold46487_3	non-phage	0
DLF010_scaffold2824_5	non-phage	0
DLF010_scaffold1059_6	non-phage	0
DLF010_scaffold18237_8	non-phage	0
DLF010_scaffold363_33	phage	0.533
DLF010_scaffold28105_3	non-phage	0
DLF010_scaffold2402_9	non-phage	0
DLF010_scaffold53785_1	non-phage	0
DLF010_scaffold33244_2	phage	0.999
DLF010_scaffold27882_1	non-phage	0
DLF010_scaffold363_18	non-phage	0
DLF010_scaffold55310_3	non-phage	0
DLF010_scaffold4247_70	non-phage	0
DLF010_scaffold140_7	non-phage	0
DLF010_scaffold2887_8	non-phage	0
DLF010_scaffold55816_2	non-phage	0
DLF010_scaffold7975_1	non-phage	0
DLF010_scaffold11846_13	non-phage	0
DLF010_scaffold42442_10	non-phage	0
DLF010_scaffold161_3	non-phage	0
DLF010_scaffold42705_2	non-phage	0

DLF010_scaffold11685_2	non-phage	0
DLF010_scaffold31137_1	non-phage	0
DLF010_scaffold55472_1	non-phage	0
DLF010_scaffold27888_9	non-phage	0
DLF010_scaffold4389_33	non-phage	0
DLF010_scaffold4389_1	non-phage	0
DLF010_scaffold14391_5	non-phage	0
DLF010_scaffold18237_29	non-phage	0
DLF010_scaffold35048_5	non-phage	0
DLF010_scaffold1523_6	non-phage	0
DLF010_scaffold114_4	non-phage	0
DLF010_scaffold55875_1	non-phage	0
DLF010_scaffold363_24	non-phage	0
DLF010_scaffold35048_8	non-phage	0.124
DLF010_scaffold5474_10	non-phage	0
DLF010_scaffold27498_1	non-phage	0
DLF010_scaffold22641_8	non-phage	0
DLF010_scaffold24606_1	non-phage	0
DLF010_scaffold114_7	non-phage	0
DLF010_scaffold55695_1	non-phage	0
DLF010_scaffold19270_12	non-phage	0
DLF010_scaffold38638_5	non-phage	0
DLF010_scaffold3644_2	non-phage	0
DLF010_scaffold26724_1	phage	0.964
DLF010_scaffold11388_21	non-phage	0
DLF010_scaffold130_28	non-phage	0
DLF010_scaffold55460_3	non-phage	0
DLF010_scaffold54721_6	non-phage	0
DLF010_scaffold38970_2	non-phage	0
DLF010_scaffold19584_9	non-phage	0
DLF010_scaffold21921_1	non-phage	0
DLF010_scaffold55266_2	non-phage	0
DLF010_scaffold433_11	non-phage	0
DLF010_scaffold37767_1	non-phage	0
DLF010_scaffold26057_21	non-phage	0
DLF010_scaffold55847_2	non-phage	0
DLF010_scaffold11163_1	non-phage	0
DLF010_scaffold86_4	non-phage	0
DLF010_scaffold54027_1	non-phage	0
DLF010_scaffold19114_5	non-phage	0
DLF010_scaffold18759_1	non-phage	0
DLF010_scaffold33416_10	non-phage	0
DLF010_scaffold50677_1	non-phage	0
DLF010_scaffold40000_4	non-phage	0
DLF010_scaffold5195_7	non-phage	0
DLF010_scaffold55915_1	non-phage	0

DLF010_scaffold38660_3	non-phage	0
DLF010_scaffold9836_7	non-phage	0
DLF010_scaffold6815_34	non-phage	0
DLF010_scaffold43669_1	non-phage	0
DLF010_scaffold44456_11	non-phage	0
DLF010_scaffold6858_12	non-phage	0
DLF010_scaffold42442_5	non-phage	0
DLF010_scaffold25454_7	phage	0.997
DLF010_scaffold45075_3	non-phage	0
DLF010_scaffold55460_2	non-phage	0
DLF010_scaffold10337_2	non-phage	0
DLF010_scaffold7686_8	non-phage	0
DLF010_scaffold9836_56	non-phage	0
DLF010_scaffold2402_16	non-phage	0
DLF010_scaffold51522_2	phage	0.585
DLF010_scaffold7987_4	non-phage	0
DLF010_scaffold55908_1	non-phage	0
DLF010_scaffold2264_11	non-phage	0
DLF010_scaffold49956_17	non-phage	0
DLF010_scaffold54643_1	non-phage	0
DLF010_scaffold28_26	non-phage	0
DLF010_scaffold49956_8	non-phage	0
DLF010_scaffold114_3	non-phage	0
DLF010_scaffold112_5	non-phage	0
DLF010_scaffold55847_1	non-phage	0
DLF010_scaffold38660_2	non-phage	0
DLF010_scaffold24711_2	non-phage	0
DLF010_scaffold15318_1	non-phage	0
DLF010_scaffold363_20	non-phage	0
DLF010_scaffold28626_24	non-phage	0
DLF010_scaffold19147_1	non-phage	0
DLF010_scaffold20862_7	non-phage	0
DLF010_C744928_1	non-phage	0
DLF010_scaffold22656_1	non-phage	0
DLF010_scaffold161_4	non-phage	0
DLF010_scaffold49629_12	non-phage	0
DLF010_scaffold53634_1	non-phage	0
DLF010_scaffold6880_6	non-phage	0
DLF010_scaffold31235_12	non-phage	0
DLF010_scaffold55444_4	non-phage	0
DLF010_scaffold55858_3	non-phage	0
DLF010_scaffold19877_15	non-phage	0
DLF010_scaffold5195_4	non-phage	0
DLF010_scaffold13193_1	non-phage	0
DLF010_scaffold5333_18	non-phage	0
DLF010_scaffold3040_7	non-phage	0

DLF010_scaffold23989_1	non-phage	0
DLF010_scaffold353_13	non-phage	0
DLF010_scaffold2855_1	non-phage	0
DLF010_scaffold45075_9	non-phage	0
DLF010_scaffold50901_1	non-phage	0
DLF010_scaffold7088_4	non-phage	0
DLF010_scaffold5195_16	non-phage	0
DLF010_scaffold38027_2	non-phage	0
DLF010_scaffold10472_10	non-phage	0
DLF010_C744572_1	non-phage	0
DLF010_scaffold433_53	non-phage	0
DLF010_scaffold55734_1	non-phage	0
DLF010_scaffold38291_2	non-phage	0
DLF010_scaffold23_3	non-phage	0
DLF010_scaffold55444_1	non-phage	0
DLF010_scaffold2402_12	non-phage	0
DLF010_scaffold29805_1	non-phage	0
DLF010_scaffold54883_2	non-phage	0
DLF010_scaffold2356_7	non-phage	0
DLF010_scaffold5077_5	non-phage	0
DLF010_scaffold363_10	non-phage	0
DLF010_scaffold17329_5	non-phage	0
DLF010_scaffold3276_2	phage	0.914
DLF010_scaffold54288_1	non-phage	0
DLF010_scaffold98_5	non-phage	0
DLF010_scaffold1405_20	non-phage	0
DLF010_scaffold6173_6	non-phage	0
DLF010_scaffold55427_1	non-phage	0
DLF010_scaffold35048_11	non-phage	0
DLF010_scaffold150_6	non-phage	0
DLF010_scaffold2699_18	non-phage	0
DLF010_scaffold37479_9	non-phage	0
DLF010_scaffold15168_5	non-phage	0
DLF010_scaffold101_38	non-phage	0.028
DLF010_scaffold9004_6	non-phage	0
DLF010_scaffold24063_1	non-phage	0
DLF010_scaffold29955_6	non-phage	0
DLF010_scaffold35043_2	non-phage	0
DLF010_scaffold6447_2	non-phage	0
DLF010_scaffold28674_9	non-phage	0
DLF010_scaffold41685_8	non-phage	0
DLF010_scaffold11496_18	non-phage	0
DLF010_scaffold55460_6	non-phage	0
DLF010_scaffold55310_9	non-phage	0
DLF010_scaffold55419_1	non-phage	0
DLF010_scaffold51230_2	non-phage	0

DLF010_scaffold5426_23	non-phage	0
DLF010_scaffold4389_17	non-phage	0
DLF010_scaffold130_23	non-phage	0
DLF010_scaffold5195_6	non-phage	0
DLF010_scaffold44929_5	non-phage	0
DLF010_scaffold4389_38	non-phage	0
DLF010_scaffold55459_1	non-phage	0
DLF010_scaffold20307_3	non-phage	0
DLF010_scaffold7619_1	non-phage	0
DLF010_scaffold38291_9	non-phage	0
DLF010_scaffold8211_1	phage	0.805
DLF010_scaffold285_5	non-phage	0
DLF010_scaffold1169_2	non-phage	0
DLF010_scaffold363_4	non-phage	0
DLF010_scaffold55713_1	non-phage	0
DLF010_scaffold2867_50	non-phage	0
DLF010_scaffold114_6	non-phage	0
DLF010_scaffold49230_2	non-phage	0
DLF010_scaffold31655_1	non-phage	0
DLF010_scaffold86_6	non-phage	0
DLF010_scaffold18009_46	non-phage	0
DLF010_scaffold55768_1	non-phage	0
DLF010_scaffold15773_10	non-phage	0
DLF010_scaffold33733_1	non-phage	0
DLF010_scaffold26021_10	non-phage	0.018
DLF010_scaffold55310_6	non-phage	0
DLF010_scaffold52897_3	non-phage	0
DLF010_scaffold44923_1	non-phage	0
DLF010_scaffold6447_1	non-phage	0
DLF010_scaffold22334_5	non-phage	0
DLF010_scaffold54953_1	non-phage	0
DLF010_scaffold101_41	non-phage	0
DLF010_scaffold45015_1	phage	0.999
DLF010_scaffold22049_7	non-phage	0
DLF010_scaffold37185_2	non-phage	0
DLF010_scaffold55844_2	non-phage	0
DLF010_scaffold8_2	non-phage	0
DLF010_scaffold6880_9	non-phage	0
DLF010_scaffold55795_1	non-phage	0
DLF010_scaffold11685_1	non-phage	0
DLF010_scaffold29955_1	non-phage	0
DLF010_scaffold177_11	non-phage	0
DLF010_scaffold9670_2	non-phage	0
DLF010_scaffold2699_37	non-phage	0
DLF010_scaffold106_12	non-phage	0
DLF010_scaffold54354_1	non-phage	0

DLF010_scaffold24063_6	non-phage	0
DLF010_scaffold15550_3	non-phage	0
DLF010_scaffold13877_19	non-phage	0
DLF010_scaffold20645_10	non-phage	0
DLF010_scaffold23103_1	non-phage	0
DLF010_scaffold41818_12	non-phage	0
DLF010_scaffold51387_5	phage	0.999
DLF010_scaffold26057_20	non-phage	0
DLF010_scaffold8359_6	non-phage	0
DLF010_scaffold2402_4	non-phage	0
DLF010_scaffold779_45	non-phage	0
DLF010_scaffold46750_2	non-phage	0
DLF010_scaffold3377_1	non-phage	0
DLF010_scaffold10207_2	non-phage	0
DLF010_scaffold363_12	non-phage	0
DLF010_scaffold26562_15	non-phage	0
DLF010_scaffold29955_3	non-phage	0
DLF010_scaffold81_3	non-phage	0
DLF010_scaffold55556_1	non-phage	0
DLF010_scaffold52724_4	non-phage	0
DLF010_scaffold7088_8	non-phage	0
DLF010_scaffold112_1	non-phage	0
DLF010_scaffold363_29	non-phage	0
DLF010_scaffold10208_1	non-phage	0
DLF010_scaffold15168_2	non-phage	0
DLF010_scaffold42442_6	non-phage	0
DLF010_scaffold41606_1	non-phage	0
DLF010_scaffold3594_1	non-phage	0
DLF010_scaffold35579_3	non-phage	0
DLF010_scaffold22426_1	non-phage	0
DLF010_scaffold4389_44	non-phage	0
DLF010_scaffold285_1	non-phage	0
DLF010_scaffold52197_1	non-phage	0
DLF010_scaffold38660_1	non-phage	0
DLF010_scaffold53971_1	non-phage	0
DLF010_scaffold55918_1	phage	0.999
DLF010_scaffold19147_2	non-phage	0
DLF010_scaffold18759_8	non-phage	0
DLF010_scaffold55916_2	non-phage	0
DLF010_scaffold5445_2	phage	0.988
DLF010_scaffold15318_2	non-phage	0
DLF010_scaffold12814_1	non-phage	0
DLF010_scaffold263_2	non-phage	0
DLF010_scaffold43130_4	non-phage	0
DLF010_scaffold26531_6	non-phage	0
DLF010_scaffold9004_13	non-phage	0

DLF010_scaffold2699_33	non-phage	0
DLF010_scaffold35048_3	non-phage	0
DLF010_scaffold2481_18	non-phage	0
DLF010_scaffold24711_8	non-phage	0
DLF010_scaffold45116_1	non-phage	0
DLF010_scaffold38568_1	non-phage	0
DLF010_scaffold41685_28	non-phage	0
DLF010_scaffold10337_1	non-phage	0
DLF010_scaffold32926_24	non-phage	0
DLF010_scaffold28_8	non-phage	0
DLF010_scaffold19024_5	non-phage	0
DLF010_scaffold52677_1	non-phage	0
DLF010_scaffold18414_39	phage	0.987
DLF010_scaffold55735_5	non-phage	0
DLF010_scaffold55381_1	non-phage	0
DLF010_scaffold28497_1	non-phage	0
DLF010_scaffold6621_3	non-phage	0
DLF010_scaffold55310_11	non-phage	0
DLF010_scaffold11496_4	non-phage	0
DLF010_scaffold49956_16	non-phage	0
DLF010_scaffold55185_1	non-phage	0
DLF010_scaffold51522_1	non-phage	0
DLF010_scaffold8871_7	non-phage	0
DLF010_scaffold20354_1	non-phage	0
DLF010_scaffold363_7	non-phage	0
DLF010_scaffold9004_7	non-phage	0
DLF010_scaffold27012_2	phage	0.812
DLF010_scaffold54891_1	non-phage	0
DLF010_scaffold23670_1	non-phage	0
DLF010_scaffold41615_13	non-phage	0
DLF010_scaffold363_23	non-phage	0
DLF010_scaffold2402_13	non-phage	0
DLF010_scaffold363_16	non-phage	0
DLF010_scaffold9702_1	non-phage	0
DLF010_scaffold17611_22	non-phage	0
DLF010_scaffold4241_51	non-phage	0
DLF010_scaffold44216_5	phage	0.539
DLF010_scaffold1405_28	phage	0.999
DLF010_scaffold22334_8	non-phage	0
DLF010_scaffold74_4	non-phage	0
DLF010_scaffold55924_1	non-phage	0
DLF010_scaffold55901_1	non-phage	0
DLF010_scaffold5195_3	non-phage	0
DLF010_scaffold8849_9	non-phage	0
DLF010_scaffold9328_36	non-phage	0
DLF010_scaffold36638_8	non-phage	0

DLF010_scaffold16598_3	non-phage	0
DLF010_scaffold17611_37	non-phage	0
DLF010_scaffold54751_1	non-phage	0
DLF010_scaffold14049_4	phage	0.69
DLF010_scaffold779_25	non-phage	0
DLF010_scaffold36452_2	non-phage	0
DLF010_scaffold17758_1	non-phage	0
DLF010_scaffold17549_1	non-phage	0
DLF010_scaffold50901_2	non-phage	0
DLF010_scaffold3040_8	non-phage	0
DLF010_scaffold16627_2	non-phage	0
DLF010_scaffold49956_5	non-phage	0
DLF010_scaffold86_10	non-phage	0
DLF010_scaffold11059_20	non-phage	0
DLF010_scaffold3793_5	non-phage	0
DLF010_scaffold42442_2	non-phage	0
DLF010_scaffold30646_3	non-phage	0
DLF010_scaffold51370_1	non-phage	0
DLF010_scaffold86_3	phage	0.996
DLF010_scaffold14216_4	non-phage	0
DLF010_scaffold24711_11	non-phage	0
DLF010_scaffold29955_7	non-phage	0
DLF010_scaffold10337_15	non-phage	0
DLF010_scaffold22973_1	non-phage	0
DLF010_scaffold24026_10	phage	1
DLF010_scaffold55874_1	phage	0.629
DLF010_scaffold31584_1	non-phage	0
DLF012_scaffold3746_25	non-phage	0
DLF012_scaffold18020_1	non-phage	0
DLF012_scaffold19209_2	non-phage	0
DLF012_scaffold14817_1	non-phage	0
DLF012_scaffold7457_4	non-phage	0
DLF012_scaffold19174_1	non-phage	0
DLF012_scaffold571_21	non-phage	0
DLF012_scaffold19155_2	non-phage	0.012
DLF012_scaffold19148_1	non-phage	0
DLF012_scaffold3027_4	non-phage	0
DLF012_scaffold18376_1	non-phage	0
DLF012_scaffold18738_2	non-phage	0
DLF012_scaffold3488_8	non-phage	0
DLF012_scaffold2865_1	non-phage	0
DLF012_scaffold9600_2	non-phage	0
DLF012_scaffold7725_1	non-phage	0
DLF012_scaffold19117_1	non-phage	0
DLF012_scaffold3488_1	non-phage	0
DLF012_scaffold3698_13	non-phage	0

DLF012_scaffold19146_3	non-phage	0
DLF012_scaffold18368_4	non-phage	0
DLF012_scaffold1762_2	non-phage	0
DLF012_scaffold18733_1	non-phage	0
DLF012_C256365_1	non-phage	0
DLF012_scaffold3746_31	non-phage	0
DLF012_scaffold6599_1	non-phage	0
DLF012_scaffold1440_4	non-phage	0
DLF012_scaffold1665_1	non-phage	0
DLF012_scaffold18113_1	non-phage	0
DLF012_scaffold6481_17	non-phage	0
DLF012_scaffold16914_3	non-phage	0
DLF012_scaffold3698_2	non-phage	0
DLF012_scaffold19146_1	non-phage	0
DLF012_scaffold2102_1	non-phage	0
DLF012_scaffold1605_3	non-phage	0
DLF012_scaffold1663_5	non-phage	0
DLF012_scaffold5603_8	non-phage	0
DLF012_scaffold19178_1	non-phage	0
DLF012_scaffold3_5	non-phage	0
DLF012_scaffold19121_3	non-phage	0
DLF012_scaffold4165_33	phage	1
DLF012_scaffold11276_1	non-phage	0
DLF012_scaffold1329_11	non-phage	0
DLF012_scaffold2461_1	non-phage	0
DLF012_scaffold19194_1	non-phage	0
DLF012_scaffold17432_5	non-phage	0
DLF012_scaffold11616_3	non-phage	0
DLF012_scaffold3746_15	non-phage	0
DLF012_scaffold1890_1	non-phage	0
DLF012_scaffold4_5	non-phage	0
DLF012_scaffold12333_1	non-phage	0
DLF012_scaffold1762_4	non-phage	0
DLF012_scaffold18542_1	non-phage	0
DLF012_scaffold3440_2	non-phage	0
DLF012_scaffold5065_5	non-phage	0
DLF012_scaffold16018_1	non-phage	0
DLF012_scaffold19143_4	non-phage	0
DLF012_scaffold18762_1	non-phage	0
DLF012_scaffold19092_1	non-phage	0
DLF012_scaffold1705_4	non-phage	0
DLF012_scaffold8336_1	non-phage	0
DLF012_C256065_1	non-phage	0
DLF012_scaffold18836_1	non-phage	0
DLF012_scaffold19171_1	non-phage	0
DLF012_scaffold3191_12	non-phage	0

DLF012_scaffold115_3	non-phage	0
DLF012_scaffold17400_4	non-phage	0
DLF012_scaffold18000_1	non-phage	0
DLF012_scaffold4_2	non-phage	0
DLF012_scaffold1147_2	non-phage	0
DLF012_scaffold19211_2	non-phage	0
DLF012_scaffold19202_1	non-phage	0
DLF012_scaffold7654_5	non-phage	0.013
DLF012_scaffold18545_2	non-phage	0
DLF012_scaffold18938_2	non-phage	0
DLF012_scaffold18385_8	non-phage	0
DLF012_scaffold19210_1	non-phage	0
DLF012_C256459_1	non-phage	0
DLF012_C257035_1	non-phage	0
DLF012_scaffold18081_1	non-phage	0
DLF012_scaffold19213_1	non-phage	0
DLF012_scaffold16450_1	non-phage	0
DLF012_scaffold36_1	phage	0.998
DLF012_scaffold3_2	non-phage	0
DLF012_scaffold3698_6	non-phage	0
DLF012_scaffold17306_7	non-phage	0
DLF012_scaffold3081_1	non-phage	0
DLF012_scaffold17185_5	phage	0.995
DLF012_scaffold16860_1	non-phage	0
DLF012_scaffold19161_1	non-phage	0
DLF012_scaffold16110_1	non-phage	0
DLF012_scaffold14258_3	non-phage	0
DLF012_scaffold19080_3	non-phage	0
DLF012_scaffold15031_1	non-phage	0
DLF012_scaffold3698_10	non-phage	0
DLF012_scaffold14338_1	non-phage	0
DLF012_scaffold13715_4	non-phage	0
DLF012_scaffold19199_1	non-phage	0
DLF012_scaffold19208_1	non-phage	0
DLF012_scaffold5222_6	non-phage	0
DLF012_scaffold4135_1	non-phage	0
DLF012_scaffold2983_1	non-phage	0
DLF012_scaffold15936_2	phage	0.999
DLF012_scaffold1267_22	non-phage	0
DLF012_scaffold12743_1	phage	0.998
DLF012_scaffold5162_4	non-phage	0
DLF012_scaffold19164_1	non-phage	0
DLF012_scaffold15082_4	non-phage	0
DLF012_scaffold1329_7	non-phage	0
DLF012_scaffold16080_1	non-phage	0
DLF012_scaffold9107_4	non-phage	0

DLF012_scaffold5603_4	non-phage	0
DLF012_C256601_1	non-phage	0
DLF012_scaffold19207_1	non-phage	0
DLF012_scaffold123_4	non-phage	0
DLF012_scaffold3440_3	non-phage	0
DLF012_scaffold15016_1	non-phage	0
DLF012_scaffold7124_2	non-phage	0
DLF012_scaffold4228_23	non-phage	0
DLF012_scaffold18_1	non-phage	0
DLF012_scaffold3787_3	non-phage	0
DLF012_scaffold3488_17	non-phage	0
DLF012_scaffold19093_4	non-phage	0
DLF012_scaffold7551_1	non-phage	0
DLF012_scaffold17721_5	non-phage	0
DLF012_scaffold17773_9	non-phage	0
DLF012_scaffold7512_2	non-phage	0
DLF012_scaffold75_1	non-phage	0
DLF012_scaffold12567_1	non-phage	0
DLF012_scaffold3314_7	non-phage	0
DLF012_scaffold17432_2	non-phage	0
DLF012_scaffold183_8	non-phage	0
DLF012_scaffold18545_1	non-phage	0
DLF012_scaffold19151_1	non-phage	0
DLF012_scaffold15462_1	non-phage	0
DLF012_scaffold12838_3	phage	0.999
DLF012_scaffold18743_1	non-phage	0
DLF012_scaffold4_6	non-phage	0
DLF012_scaffold372_1	non-phage	0
DLF012_scaffold18610_2	non-phage	0
DLF012_scaffold18822_3	non-phage	0
DLF012_scaffold7654_1	non-phage	0
DLF012_scaffold14115_1	non-phage	0
DLF012_scaffold19116_2	non-phage	0
DLF012_scaffold11534_6	non-phage	0
DLF012_scaffold19177_1	non-phage	0
DLF012_scaffold10615_3	non-phage	0
DLF012_scaffold19098_3	non-phage	0
DLF012_scaffold8233_2	non-phage	0
DLF012_scaffold5222_4	non-phage	0
DLF012_scaffold571_3	non-phage	0
DLF012_scaffold18729_2	non-phage	0
DLF012_scaffold18301_3	non-phage	0
DLF012_scaffold11103_4	non-phage	0
DLF012_scaffold6031_24	non-phage	0
DLF012_scaffold1705_3	non-phage	0
DLF012_scaffold9930_2	non-phage	0

DLF012_scaffold10_1	non-phage	0
DLF012_scaffold19143_3	non-phage	0
DLF012_scaffold19098_2	non-phage	0
DLF012_scaffold18385_4	non-phage	0
DLF012_scaffold5397_1	non-phage	0
DLF012_scaffold19195_1	non-phage	0
DLF012_C257141_1	non-phage	0
DLF012_scaffold13715_3	non-phage	0
DLF012_scaffold19111_1	phage	0.993
DLF012_scaffold1329_12	non-phage	0
DLF012_scaffold19109_1	non-phage	0
DLF012_scaffold19209_3	non-phage	0
DLF012_scaffold3787_2	non-phage	0
DLF012_scaffold3314_6	non-phage	0
DLF012_C257127_1	non-phage	0
DLF012_scaffold10311_3	non-phage	0
DLF012_scaffold8697_1	non-phage	0
DLF012_scaffold9934_2	non-phage	0
DLF012_scaffold18724_1	non-phage	0
DLF012_scaffold3191_7	non-phage	0
DLF012_scaffold571_13	non-phage	0
DLF012_scaffold5065_7	non-phage	0
DLF012_scaffold3027_1	non-phage	0
DLF012_scaffold18742_1	non-phage	0
DLF012_scaffold19120_2	non-phage	0
DLF012_scaffold7563_11	non-phage	0
DLF012_scaffold8753_1	non-phage	0
DLF012_scaffold8811_3	non-phage	0
DLF012_scaffold13_2_1	non-phage	0
DLF012_scaffold19211_1	non-phage	0
DLF012_scaffold3314_10	non-phage	0
DLF012_scaffold19152_1	non-phage	0
DLF012_scaffold19118_3	non-phage	0
DLF012_C256493_1	non-phage	0
DLF012_scaffold15591_1	non-phage	0
DLF012_scaffold1267_6	non-phage	0
DLF012_scaffold1411_8	non-phage	0
DLF012_scaffold6451_2	non-phage	0
DLF012_scaffold5448_10	non-phage	0
DLF012_scaffold18938_1	non-phage	0
DLF012_scaffold1_1	non-phage	0
DLF012_scaffold571_7	non-phage	0
DLF012_scaffold18743_3	non-phage	0
DLF012_scaffold19156_1	non-phage	0
DLF012_scaffold131_2	non-phage	0.025
DLF012_scaffold19015_1	non-phage	0

DLF012_scaffold3314_2	non-phage	0
DLF012_scaffold13255_2	non-phage	0
DLF012_scaffold17432_8	non-phage	0
DLF012_scaffold19124_2	non-phage	0
DLF012_scaffold2461_2	non-phage	0
DLF012_scaffold18081_2	non-phage	0
DLF012_scaffold15597_2	non-phage	0
DLF012_scaffold19154_1	non-phage	0
DLF012_scaffold10338_1	non-phage	0
DLF012_scaffold18733_2	non-phage	0
DLF012_scaffold1762_3	non-phage	0
DLF012_scaffold17015_1	non-phage	0
DLF012_scaffold19146_2	phage	0.856
DLF012_scaffold3698_11	non-phage	0
DLF012_scaffold5603_18	non-phage	0
DLF012_scaffold17572_7	non-phage	0
DLF012_scaffold19032_1	phage	0.999
DLF012_scaffold12333_3	non-phage	0
DLF012_scaffold18861_1	non-phage	0
DLF012_scaffold18721_1	non-phage	0
DLF012_scaffold18021_1	non-phage	0
DLF012_scaffold8603_1	non-phage	0
DLF012_scaffold17306_1	non-phage	0
DLF012_scaffold19116_3	non-phage	0
DLF012_C256377_1	non-phage	0
DLF012_scaffold136_3	non-phage	0
DLF012_scaffold8907_3	non-phage	0
DLF012_scaffold10849_2	non-phage	0
DLF012_scaffold2461_6	non-phage	0
DLF012_scaffold19040_2	non-phage	0
DLF012_scaffold18183_1	non-phage	0
DLF012_scaffold18573_1	non-phage	0
DLF012_scaffold18155_1	non-phage	0
DLF012_scaffold19048_2	non-phage	0
DLF012_scaffold16860_4	non-phage	0
DLF012_scaffold3285_2	non-phage	0
DLF012_scaffold18944_2	non-phage	0
DLF012_scaffold1440_7	non-phage	0
DLF012_scaffold1329_8	non-phage	0
DLF012_scaffold19192_1	non-phage	0
DLF012_scaffold15936_5	non-phage	0
DLF012_scaffold29_1	non-phage	0
DLF012_C257063_1	non-phage	0
DLF012_scaffold9107_5	non-phage	0.043
DLF012_scaffold15408_4	non-phage	0
DLF012_scaffold19120_1	non-phage	0

DLF012_scaffold1_4	non-phage	0
DLF012_scaffold1762_5	non-phage	0
DLF012_scaffold19093_3	non-phage	0
DLF012_scaffold183_3	non-phage	0
DLF012_scaffold19193_2	non-phage	0
DLF012_scaffold19172_1	non-phage	0
DLF012_scaffold10736_4	non-phage	0
DLF012_scaffold3118_11	non-phage	0
DLF012_scaffold3_1	non-phage	0
DLF012_scaffold3746_11	non-phage	0
DLF012_scaffold6749_1	non-phage	0
DLF012_scaffold18931_1	non-phage	0
DLF012_scaffold14494_4	non-phage	0
DLF012_scaffold15031_3	non-phage	0
DLF012_scaffold17721_1	non-phage	0
DLF012_scaffold6703_1	non-phage	0
DLF012_scaffold19086_2	non-phage	0
DLF012_scaffold2983_17	non-phage	0
DLF012_scaffold18888_1	non-phage	0
DLF012_scaffold5006_1	non-phage	0
DLF012_scaffold19121_1	non-phage	0
DLF012_scaffold16450_7	non-phage	0
DLF012_scaffold7563_18	non-phage	0
DLF012_scaffold18738_1	non-phage	0
DLF012_scaffold3884_2	non-phage	0
DLF012_scaffold11693_1	non-phage	0
DLF012_scaffold19188_2	non-phage	0
DLF012_scaffold19091_2	non-phage	0
DLF012_scaffold131_1_2	non-phage	0
DLF012_scaffold5224_1	non-phage	0
DLF012_scaffold19110_1	non-phage	0
DLF012_scaffold10615_2	non-phage	0
DLF012_scaffold17528_5	non-phage	0
DLF012_scaffold1147_1	non-phage	0
DLF012_scaffold3130_1	non-phage	0
DLF012_C256881_1	non-phage	0
DLF012_scaffold5222_7	non-phage	0
DLF012_scaffold5603_3	non-phage	0
DLF012_scaffold19113_1	non-phage	0
DLF012_scaffold19160_1	non-phage	0
DLF012_scaffold19121_2	non-phage	0
DLF012_C257085_1	non-phage	0
DLF012_scaffold4_1	non-phage	0
DLF012_scaffold18453_1	non-phage	0
DLF012_scaffold19122_2	non-phage	0
DLF012_scaffold9129_2	non-phage	0

DLF012_scaffold17432_1	non-phage	0
DLF012_scaffold19203_1	non-phage	0
DLF012_scaffold6481_11	non-phage	0
DLF012_scaffold16450_2	non-phage	0
DLF012_scaffold5395_1	non-phage	0
DLF012_scaffold571_11	non-phage	0
DLF012_scaffold19150_1	non-phage	0
DLF012_scaffold4733_11	non-phage	0
DLF012_scaffold14258_5	non-phage	0
DLF012_scaffold19144_4	non-phage	0
DLF012_scaffold183_7	non-phage	0
DLF012_scaffold12889_1	non-phage	0
DLF012_scaffold10521_1	non-phage	0
DLF012_scaffold15776_1	non-phage	0
DLF012_scaffold19134_1	non-phage	0
DLF012_scaffold17432_3	non-phage	0
DLF012_scaffold19169_2	non-phage	0
DLF012_scaffold17432_7	non-phage	0
DLF012_scaffold15469_2	non-phage	0
DLF012_scaffold19213_2	non-phage	0
DLF012_scaffold136_8	non-phage	0
DLF012_scaffold14285_2	non-phage	0
DLF012_scaffold14995_1	non-phage	0
DLF012_scaffold18559_1	non-phage	0.412
DLF012_scaffold571_16	phage	0.999
DLF012_scaffold19212_1	non-phage	0
DLF012_scaffold19204_1	non-phage	0
DLF012_scaffold19054_1	non-phage	0
DLF012_scaffold17572_4	non-phage	0
DLF012_scaffold18743_2	non-phage	0
DLF012_scaffold17721_3	non-phage	0
DLF012_scaffold18133_10	non-phage	0
DLF012_scaffold6451_1	non-phage	0
DLF012_scaffold1267_5	non-phage	0
DLF012_scaffold5222_3	non-phage	0
DLF012_scaffold19098_1	non-phage	0
DLF012_scaffold123_2	non-phage	0
DLF012_scaffold12447_1	non-phage	0
DLF012_scaffold6481_28	non-phage	0
DLF012_scaffold19179_1	non-phage	0
DLF012_scaffold19166_1	non-phage	0
DLF012_scaffold17036_1	phage	0.968
DLF012_scaffold115_1	non-phage	0
DLF012_scaffold13715_2	non-phage	0
DLF012_scaffold1705_1	non-phage	0
DLF012_scaffold6481_16	non-phage	0

DLF012_scaffold19125_1	non-phage	0
DLF012_scaffold19007_1	non-phage	0
DLF012_scaffold3698_7	non-phage	0
DLF012_scaffold14059_1	non-phage	0
DLF012_scaffold10849_4	non-phage	0
DLF012_scaffold3118_1	non-phage	0
DLF012_scaffold18964_2	non-phage	0
DLF012_scaffold13255_1	non-phage	0
DLF012_scaffold136_5	non-phage	0
DLF012_scaffold7563_10	non-phage	0
DLF012_C257135_1	non-phage	0
DLF012_scaffold490_2	non-phage	0
DLF012_scaffold18992_1	non-phage	0
DLF012_scaffold19136_1	non-phage	0
DLF012_scaffold8615_11	non-phage	0
DLF012_scaffold629_1	non-phage	0
DLF012_scaffold16204_1	non-phage	0
DLF012_scaffold12743_3	non-phage	0
DLF012_scaffold18088_1	non-phage	0
DLF012_scaffold14753_1	non-phage	0
DLF012_scaffold18535_1	non-phage	0
DLF012_scaffold1329_3	non-phage	0
DLF012_scaffold18629_1	non-phage	0
DLF012_scaffold6323_8	non-phage	0
DLF012_scaffold19210_2	non-phage	0
DLF012_scaffold1440_2	non-phage	0.001
DLF012_scaffold16860_2	non-phage	0
DLF012_scaffold16914_1	non-phage	0
DLF012_scaffold19104_1	non-phage	0
DLF012_scaffold19154_2	non-phage	0
DLF012_scaffold19113_2	non-phage	0
DLF012_C255997_1	non-phage	0
DLF012_scaffold17587_5	non-phage	0
DLF012_scaffold1329_2	non-phage	0
DLF012_scaffold183_2	non-phage	0
DLF012_scaffold4552_1	non-phage	0
DLF012_scaffold15261_2	non-phage	0
DLF012_scaffold18949_3	non-phage	0
DLF012_scaffold19122_1	non-phage	0
DLF012_scaffold136_1	non-phage	0
DLF012_scaffold18507_2	non-phage	0
DLF012_scaffold19076_1	non-phage	0
DLF012_scaffold18936_1	non-phage	0
DLF012_scaffold19072_1	non-phage	0
DLF012_scaffold10736_3	non-phage	0
DLF012_scaffold383_2	non-phage	0

DLF012_scaffold19124_3	non-phage	0
DLF012_scaffold15082_1	non-phage	0
DLF012_scaffold1267_8	non-phage	0
DLF012_scaffold17400_5	non-phage	0
DLF012_scaffold3314_9	non-phage	0
DLF012_scaffold3698_12	non-phage	0
DLF012_scaffold18503_3	non-phage	0
DLF012_scaffold18822_1	non-phage	0
DLF012_scaffold9129_1	non-phage	0
DLF012_scaffold15_1	non-phage	0
DLF012_scaffold3191_11	non-phage	0
DLF012_scaffold17430_1	non-phage	0
DLF012_scaffold3433_1	non-phage	0
DLF012_scaffold19183_1	non-phage	0
DLF012_scaffold18618_1	non-phage	0
DLF012_scaffold19116_4	non-phage	0
DLF012_scaffold19091_1	non-phage	0
DLF012_scaffold10736_2	non-phage	0
DLF012_scaffold2693_3	non-phage	0
DLF012_scaffold2693_2	non-phage	0
DLF012_scaffold18457_1	non-phage	0
DLF012_scaffold19171_4	non-phage	0
DLF012_scaffold10766_3	non-phage	0
DLF012_scaffold19144_1	non-phage	0
DLF012_scaffold1440_6	non-phage	0
DLF012_scaffold15408_5	non-phage	0
DLF012_scaffold10615_1	non-phage	0
DLF012_scaffold19193_1	non-phage	0
DLF012_scaffold18793_1	non-phage	0
DLF012_scaffold29_2	non-phage	0
DLF012_scaffold3027_2	non-phage	0
DLF012_scaffold4952_1	non-phage	0
DLF012_scaffold16450_3	non-phage	0
DLF012_scaffold19200_1	non-phage	0
DLF012_scaffold3698_1	non-phage	0
DLF012_scaffold17587_6	non-phage	0
DLF012_scaffold17741_1	non-phage	0
DLF012_scaffold19205_1	non-phage	0
DLF012_scaffold18424_1	non-phage	0
DLF012_scaffold17741_3	non-phage	0
DLF012_scaffold19094_1	non-phage	0
DLF012_scaffold90_2	non-phage	0
DLF012_scaffold9657_2	non-phage	0
DLF012_scaffold5224_2	non-phage	0
DLF012_scaffold3_3	non-phage	0
DLF012_scaffold19143_2	non-phage	0

DLF012_scaffold16144_1	non-phage	0
DLF012_scaffold16914_5	non-phage	0
DLF012_scaffold18195_1	non-phage	0
DLF012_scaffold19201_1	non-phage	0
DLF012_scaffold7654_11	non-phage	0
DLF012_scaffold5395_2	non-phage	0
DLF012_scaffold383_1	non-phage	0
DLF012_scaffold13715_6	non-phage	0
DLF012_scaffold19188_1	non-phage	0
DLF012_scaffold19191_1	non-phage	0
DLF012_scaffold19155_3	non-phage	0
DLF012_scaffold571_20	non-phage	0
DLF012_scaffold9027_1	non-phage	0
DLF012_scaffold3787_1	non-phage	0
DLF012_scaffold1329_5	non-phage	0
DLF012_scaffold19187_2	non-phage	0
DLF012_scaffold3285_1	non-phage	0
DLF012_scaffold15082_2	non-phage	0
DLF012_scaffold19102_2	non-phage	0
DLF012_scaffold18348_3	non-phage	0
DLF012_scaffold17773_5	non-phage	0
DLF012_scaffold17528_4	non-phage	0
DLF012_scaffold1_3	non-phage	0
DLF012_scaffold18610_3	non-phage	0
DLF012_scaffold1267_44	non-phage	0
DLF012_scaffold18933_1	non-phage	0
DLF012_scaffold19198_1	non-phage	0
DLF012_scaffold18376_2	non-phage	0
DLF012_scaffold19117_2	non-phage	0
DLF012_scaffold10311_1	non-phage	0
DLF012_scaffold6750_1	non-phage	0
DLF012_scaffold3118_7	non-phage	0
DLF012_scaffold17587_1	non-phage	0
DLF012_scaffold19168_1	non-phage	0
DLF012_scaffold1605_2	non-phage	0
DLF012_C257041_1	non-phage	0
DLF012_scaffold19182_1	non-phage	0
DLF012_scaffold115_2	non-phage	0
DLF012_scaffold3_6	non-phage	0
DLF012_scaffold10688_2	non-phage	0
DLF012_scaffold4_3	non-phage	0
DLF012_C256793_1	non-phage	0
DLF012_scaffold19189_1	non-phage	0
DLF012_scaffold19034_1	non-phage	0
DLF012_scaffold10695_1	non-phage	0
DLF012_scaffold1329_10	non-phage	0

DLF012_scaffold11737_1	non-phage	0
DLF012_scaffold16018_2	non-phage	0
DLF012_scaffold19112_1	non-phage	0
DLF012_C257037_1	non-phage	0
DLF012_scaffold19047_1	non-phage	0
DLF012_scaffold17432_6	non-phage	0
DLF012_C256237_1	non-phage	0
DLF012_scaffold15597_7	non-phage	0
DLF012_scaffold3191_9	non-phage	0
DLF012_scaffold5614_1	non-phage	0
DLF012_scaffold19067_1	non-phage	0
DLF012_scaffold19181_1	non-phage	0
DLF012_scaffold4105_1	non-phage	0
DLF012_scaffold19206_1	non-phage	0
DLF012_scaffold14995_2	non-phage	0
DLF012_scaffold1762_1	non-phage	0
DLF012_scaffold17015_3	non-phage	0
DLF012_scaffold14441_1	non-phage	0
DLF012_scaffold4552_3	non-phage	0
DLF012_scaffold2865_2	non-phage	0
DLF012_scaffold3415_37	non-phage	0
DLF012_scaffold123_1	non-phage	0
DLF012_scaffold16914_6	non-phage	0
DLF012_scaffold16596_3	non-phage	0
DLF012_scaffold12743_4	non-phage	0
DLF012_scaffold10024_3	non-phage	0
DLF012_C256323_1	non-phage	0
DLF012_scaffold9579_19	non-phage	0
DLF012_scaffold19125_2	non-phage	0
DLF012_scaffold19184_1	non-phage	0
DLF012_scaffold18618_3	non-phage	0
DLF012_scaffold19209_1	non-phage	0
DLF012_scaffold3027_3	non-phage	0
DLF012_scaffold18998_1	non-phage	0
DLF012_scaffold183_5	non-phage	0
DLF012_scaffold17306_9	non-phage	0
DLF012_scaffold3118_9	phage	0.858
DLF012_scaffold19169_1	non-phage	0
DLF012_scaffold3440_1	non-phage	0
DLF012_scaffold3787_4	non-phage	0
DLF012_scaffold1267_13	non-phage	0
DLF012_scaffold2461_9	non-phage	0
DLF012_scaffold19062_1	non-phage	0
DLF012_scaffold10849_3	non-phage	0
DLF012_scaffold17185_2	non-phage	0
DLF012_scaffold383_3	non-phage	0

DLF012_scaffold17932_1	non-phage	0
DLF012_scaffold3698_8	non-phage	0
DLF012_scaffold18886_1	non-phage	0
DLF012_scaffold7563_3	non-phage	0
DLF012_scaffold15408_3	non-phage	0
DLF012_scaffold7916_1	non-phage	0
DLF012_scaffold19080_1	non-phage	0
DLF012_scaffold16204_2	non-phage	0
DLF012_scaffold6352_1	non-phage	0
DLF012_scaffold18859_1	non-phage	0
DLF012_scaffold18876_2	non-phage	0
DLF012_scaffold13715_5	non-phage	0
DLF012_scaffold16450_5	non-phage	0
DLF012_scaffold17181_1	non-phage	0
DLF012_scaffold15_2	non-phage	0
DLF012_scaffold571_8	non-phage	0
DLF012_scaffold19118_1	non-phage	0
DLF012_scaffold16840_1	non-phage	0
DLF012_scaffold19143_1	non-phage	0
DLF012_scaffold5082_7	non-phage	0
DLF012_scaffold19135_2	non-phage	0
DLF012_scaffold2149_1	non-phage	0
DLF012_scaffold10736_1	non-phage	0
DLF012_scaffold10299_2	non-phage	0
DLF012_scaffold4_7	non-phage	0
DLF012_scaffold5222_5	non-phage	0
DLF012_scaffold3746_3	non-phage	0
DLF012_scaffold4958_5	non-phage	0
DLF012_scaffold1705_2	non-phage	0
DLF012_scaffold11038_1	non-phage	0
DLF012_scaffold19167_2	non-phage	0
DLF012_scaffold6803_1	non-phage	0
DLF012_scaffold1267_52	non-phage	0
DLF012_scaffold13363_2	non-phage	0
DLF012_scaffold18985_1	non-phage	0
DLF012_scaffold17773_2	non-phage	0
DLF012_scaffold15082_3	non-phage	0
DLF012_scaffold4552_2	non-phage	0
DLF012_scaffold18507_1	non-phage	0
DLF012_scaffold19076_2	non-phage	0
DLF012_scaffold14258_2	non-phage	0
DLF012_scaffold19156_2	non-phage	0
DLF012_scaffold19176_2	non-phage	0
DLF012_scaffold1267_38	non-phage	0
DLF012_C256641_1	non-phage	0
DLF012_scaffold19116_1	non-phage	0

DLF012_scaffold17036_2	non-phage	0
DLF012_scaffold7654_10	non-phage	0
DLF012_scaffold1657_1	non-phage	0
DLF012_scaffold136_2	non-phage	0
DLF012_scaffold8615_15	non-phage	0
DLF012_scaffold2692_1	non-phage	0
DLF012_scaffold84_4	non-phage	0
DLF012_scaffold571_14	non-phage	0
DLF012_scaffold8610_1	non-phage	0
DLF012_scaffold18301_2	non-phage	0
DLF012_scaffold19098_5	non-phage	0
DLF012_scaffold19183_2	non-phage	0
DLF012_scaffold6323_10	non-phage	0
DLF012_scaffold18620_2	non-phage	0
DLF012_scaffold10766_4	non-phage	0
DLF012_scaffold17959_1	non-phage	0
DLF012_scaffold19089_1	non-phage	0
DLF012_scaffold15031_2	non-phage	0
DLF012_scaffold13537_1	non-phage	0
DLF012_scaffold11275_1	non-phage	0
DLF012_scaffold19095_1	non-phage	0
DLF012_C257149_1	non-phage	0
DLF012_scaffold6749_3	non-phage	0
DLF013_scaffold22413_5	non-phage	0
DLF013_scaffold21690_1	non-phage	0
DLF013_scaffold42127_3	non-phage	0
DLF013_scaffold4753_3	non-phage	0
DLF013_scaffold26542_1	non-phage	0
DLF013_scaffold44460_1	non-phage	0
DLF013_scaffold24725_8	non-phage	0
DLF013_scaffold37695_1	non-phage	0
DLF013_scaffold47724_1	non-phage	0
DLF013_scaffold47429_1	phage	0.901
DLF013_scaffold43190_1	non-phage	0
DLF013_scaffold3400_23	non-phage	0
DLF013_scaffold54297_1	non-phage	0
DLF013_scaffold17713_4	non-phage	0
DLF013_scaffold3258_4	non-phage	0
DLF013_scaffold13599_4	non-phage	0
DLF013_scaffold54725_1	non-phage	0
DLF013_scaffold1932_12	non-phage	0
DLF013_scaffold26406_1	non-phage	0
DLF013_scaffold51036_1	non-phage	0
DLF013_scaffold43486_2	non-phage	0
DLF013_scaffold18599_4	phage	0.849
DLF013_scaffold113_21	non-phage	0

DLF013_scaffold5156_2	non-phage	0
DLF013_scaffold33635_1	non-phage	0
DLF013_scaffold47104_5	non-phage	0
DLF013_scaffold6386_2	non-phage	0
DLF013_scaffold12815_7	non-phage	0
DLF013_scaffold48840_2	non-phage	0
DLF013_scaffold19423_1	non-phage	0
DLF013_scaffold2829_1	non-phage	0
DLF013_scaffold54265_1	non-phage	0
DLF013_scaffold44968_1	non-phage	0
DLF013_scaffold25083_8	non-phage	0
DLF013_scaffold4224_3	non-phage	0
DLF013_scaffold14499_1	non-phage	0
DLF013_scaffold12787_4	non-phage	0.4
DLF013_scaffold19146_1	non-phage	0
DLF013_scaffold24088_2	non-phage	0
DLF013_scaffold48931_6	non-phage	0
DLF013_scaffold134_1	non-phage	0
DLF013_scaffold15946_5	non-phage	0
DLF013_scaffold54153_1	non-phage	0
DLF013_scaffold18496_6	non-phage	0
DLF013_scaffold11990_2	non-phage	0
DLF013_scaffold5684_2	non-phage	0
DLF013_scaffold35295_1	phage	0.979
DLF013_scaffold36189_4	non-phage	0
DLF013_scaffold25725_9	non-phage	0
DLF013_scaffold51141_3	non-phage	0
DLF013_scaffold54785_3	non-phage	0
DLF013_scaffold9550_2	non-phage	0
DLF013_scaffold20747_5	non-phage	0
DLF013_scaffold6714_7	non-phage	0
DLF013_scaffold50109_5	non-phage	0
DLF013_scaffold12451_2	non-phage	0
DLF013_scaffold54690_1	non-phage	0
DLF013_scaffold857_2	non-phage	0
DLF013_scaffold30936_3	non-phage	0
DLF013_scaffold27683_1	non-phage	0
DLF013_scaffold6385_3	non-phage	0
DLF013_scaffold1813_1	non-phage	0
DLF013_scaffold18517_1	non-phage	0
DLF013_scaffold52665_1	phage	0.995
DLF013_scaffold5745_2	non-phage	0
DLF013_scaffold53396_1	non-phage	0
DLF013_scaffold2829_25	non-phage	0.007
DLF013_scaffold25714_3	non-phage	0
DLF013_scaffold20513_2	non-phage	0

DLF013_scaffold12074_4	non-phage	0
DLF013_scaffold138_1	non-phage	0.102
DLF013_scaffold28417_1	non-phage	0
DLF013_scaffold265_23	non-phage	0
DLF013_scaffold52685_1	non-phage	0
DLF013_scaffold49186_4	non-phage	0
DLF013_scaffold22616_1	non-phage	0
DLF013_scaffold15096_2	non-phage	0
DLF013_scaffold8750_1	non-phage	0
DLF013_scaffold22029_1	non-phage	0
DLF013_scaffold26172_5	non-phage	0
DLF013_scaffold23198_5	non-phage	0
DLF013_scaffold21632_2	non-phage	0
DLF013_scaffold266_1	non-phage	0
DLF013_scaffold21643_5	non-phage	0
DLF013_scaffold31574_1	non-phage	0
DLF013_scaffold54090_1	non-phage	0
DLF013_scaffold25655_4	non-phage	0
DLF013_scaffold25322_1	non-phage	0
DLF013_scaffold13756_4	non-phage	0
DLF013_scaffold46137_1	non-phage	0
DLF013_scaffold12558_6	non-phage	0
DLF013_scaffold54026_7	non-phage	0
DLF013_scaffold22497_1	non-phage	0
DLF013_scaffold19049_1	non-phage	0
DLF013_scaffold16968_5	non-phage	0
DLF013_scaffold28751_1	non-phage	0
DLF013_scaffold3815_11	non-phage	0
DLF013_scaffold23543_1	non-phage	0
DLF013_scaffold70_1	non-phage	0
DLF013_scaffold1520_3	phage	0.998
DLF013_scaffold43548_1	non-phage	0
DLF013_scaffold5156_6	non-phage	0
DLF013_scaffold11388_7	non-phage	0
DLF013_scaffold6963_1	non-phage	0
DLF013_scaffold5284_1	non-phage	0
DLF013_scaffold21690_5	non-phage	0
DLF013_scaffold113_25	non-phage	0
DLF013_scaffold32797_1	non-phage	0
DLF013_scaffold50109_1	non-phage	0
DLF013_scaffold14114_1	non-phage	0
DLF013_scaffold1308_3	non-phage	0
DLF013_scaffold777_1	non-phage	0
DLF013_scaffold27195_4	non-phage	0
DLF013_scaffold43379_2	non-phage	0
DLF013_scaffold34743_5	phage	0.997

DLF013_scaffold22656_5	non-phage	0
DLF013_scaffold48992_2	non-phage	0
DLF013_scaffold35543_1	non-phage	0
DLF013_scaffold156_1	non-phage	0
DLF013_scaffold22539_1	non-phage	0
DLF013_scaffold32542_1	non-phage	0
DLF013_scaffold217_1	non-phage	0
DLF013_scaffold54742_1	non-phage	0
DLF013_scaffold53959_3	non-phage	0
DLF013_C686334_1	non-phage	0
DLF013_scaffold21004_4	non-phage	0
DLF013_scaffold5684_1	non-phage	0
DLF013_scaffold19199_1	non-phage	0
DLF013_scaffold43538_1	non-phage	0
DLF013_scaffold40509_4	non-phage	0
DLF013_scaffold15141_1	non-phage	0
DLF013_scaffold49993_6	non-phage	0
DLF013_scaffold54722_1	non-phage	0
DLF013_scaffold36295_6	non-phage	0
DLF013_scaffold53343_2	non-phage	0
DLF013_C686976_1	non-phage	0
DLF013_scaffold12074_2	non-phage	0
DLF013_scaffold50566_2	non-phage	0
DLF013_scaffold37892_1	non-phage	0
DLF013_scaffold4073_21	non-phage	0
DLF013_scaffold42896_1	non-phage	0
DLF013_scaffold34809_3	phage	0.998
DLF013_scaffold42888_2	non-phage	0
DLF013_scaffold5693_7	non-phage	0
DLF013_scaffold45541_4	non-phage	0
DLF013_scaffold28972_1	non-phage	0
DLF013_scaffold52572_2	non-phage	0
DLF013_scaffold54655_3	non-phage	0
DLF013_scaffold17919_3	non-phage	0
DLF013_scaffold3400_16	non-phage	0
DLF013_scaffold40562_3	non-phage	0
DLF013_scaffold14099_2	phage	0.961
DLF013_scaffold51185_1	non-phage	0
DLF013_scaffold43361_2	non-phage	0
DLF013_scaffold13599_6	phage	0.999
DLF013_scaffold30039_1	non-phage	0
DLF013_scaffold48533_1	non-phage	0
DLF013_scaffold10926_7	non-phage	0
DLF013_scaffold32540_3	non-phage	0
DLF013_scaffold54720_1	non-phage	0
DLF013_scaffold36295_1	non-phage	0

DLF013_scaffold53908_2	phage	0.988
DLF013_scaffold52685_2	non-phage	0
DLF013_scaffold51249_1	non-phage	0
DLF013_scaffold45127_1	phage	0.999
DLF013_scaffold45647_1	non-phage	0
DLF013_scaffold26172_1	non-phage	0
DLF013_scaffold48394_1	non-phage	0
DLF013_scaffold51495_1	non-phage	0
DLF013_scaffold34541_1	non-phage	0
DLF013_scaffold47859_1	non-phage	0
DLF013_scaffold36189_3	non-phage	0
DLF013_scaffold42590_1	non-phage	0
DLF013_scaffold20948_2	non-phage	0
DLF013_scaffold24088_3	non-phage	0
DLF013_scaffold19302_1	non-phage	0
DLF013_scaffold27850_5	non-phage	0
DLF013_scaffold18927_3	non-phage	0
DLF013_scaffold14487_2	non-phage	0
DLF013_scaffold46980_1	non-phage	0
DLF013_scaffold12074_8	non-phage	0
DLF013_scaffold17930_3	non-phage	0
DLF013_scaffold26172_4	non-phage	0
DLF013_scaffold29144_3	non-phage	0
DLF013_scaffold28830_1	non-phage	0
DLF013_scaffold45185_1	non-phage	0
DLF013_scaffold40_2	non-phage	0
DLF013_scaffold48783_1	non-phage	0
DLF013_scaffold15284_26	non-phage	0
DLF013_scaffold53396_2	non-phage	0
DLF013_scaffold27092_1	non-phage	0
DLF013_C686316_1	non-phage	0
DLF013_scaffold39401_1	non-phage	0
DLF013_scaffold42590_3	non-phage	0
DLF013_scaffold26172_6	non-phage	0
DLF013_scaffold10253_2	non-phage	0
DLF013_scaffold113_8	non-phage	0
DLF013_scaffold43191_4	non-phage	0
DLF013_scaffold47061_1	non-phage	0
DLF013_scaffold3815_1	phage	0.999
DLF013_scaffold1100_2	non-phage	0
DLF013_scaffold8750_2	non-phage	0
DLF013_scaffold7820_5	non-phage	0
DLF013_scaffold10030_3	non-phage	0
DLF013_scaffold43506_1	non-phage	0
DLF013_scaffold13599_1	non-phage	0
DLF013_scaffold53748_1	non-phage	0

DLF013_scaffold39498_2	non-phage	0
DLF013_scaffold25725_11	non-phage	0
DLF013_scaffold88_22	phage	0.998
DLF013_scaffold45541_2	non-phage	0
DLF013_scaffold27850_7	non-phage	0
DLF013_scaffold28982_3	non-phage	0
DLF013_scaffold54278_1	non-phage	0
DLF013_scaffold47243_3	non-phage	0
DLF013_scaffold31902_2	non-phage	0
DLF013_scaffold32_6	non-phage	0
DLF013_scaffold6671_1	non-phage	0
DLF013_scaffold22757_2	non-phage	0
DLF013_scaffold53550_1	phage	0.996
DLF013_scaffold113_24	non-phage	0
DLF013_scaffold22628_1	non-phage	0
DLF013_scaffold47104_4	non-phage	0
DLF013_scaffold21495_2	non-phage	0
DLF013_scaffold19641_1	non-phage	0
DLF013_scaffold3883_19	non-phage	0
DLF013_scaffold42596_2	phage	0.999
DLF013_scaffold54725_2	non-phage	0
DLF013_scaffold52424_3	non-phage	0
DLF013_scaffold54789_1	non-phage	0
DLF013_scaffold53189_1	non-phage	0
DLF013_scaffold27850_4	non-phage	0
DLF013_scaffold2098_16	non-phage	0
DLF013_scaffold26229_12	non-phage	0.052
DLF013_scaffold14579_3	non-phage	0
DLF013_scaffold43602_7	non-phage	0
DLF013_scaffold46170_2	non-phage	0
DLF013_scaffold36167_1	non-phage	0
DLF013_scaffold54143_1	non-phage	0
DLF013_scaffold53647_2	non-phage	0
DLF013_scaffold52424_1	non-phage	0
DLF013_scaffold33578_3	non-phage	0
DLF013_scaffold11344_1	non-phage	0
DLF013_scaffold28070_1	non-phage	0
DLF013_scaffold33577_6	non-phage	0
DLF013_scaffold11561_1	non-phage	0
DLF013_scaffold53796_1	non-phage	0
DLF013_scaffold21004_8	non-phage	0
DLF013_scaffold6385_2	phage	0.999
DLF013_scaffold54784_1	non-phage	0
DLF013_scaffold777_3	non-phage	0
DLF013_scaffold36060_4	non-phage	0
DLF013_scaffold48931_1	non-phage	0

DLF013_scaffold40244_3	non-phage	0
DLF013_scaffold3705_1	non-phage	0
DLF013_scaffold35295_2	non-phage	0
DLF013_scaffold42479_2	phage	0.999
DLF013_scaffold42258_2	non-phage	0.148
DLF013_scaffold25554_6	non-phage	0
DLF013_scaffold19199_2	non-phage	0
DLF013_scaffold30143_10	non-phage	0
DLF013_scaffold21160_6	non-phage	0
DLF013_scaffold36470_1	non-phage	0
DLF013_scaffold25554_10	non-phage	0
DLF013_scaffold266_2	non-phage	0
DLF013_scaffold1308_10	non-phage	0
DLF013_scaffold2854_1	non-phage	0
DLF013_scaffold51356_1	non-phage	0
DLF013_scaffold46999_1	non-phage	0
DLF013_scaffold47328_1	non-phage	0
DLF013_scaffold24218_1	non-phage	0
DLF013_scaffold46613_1	phage	0.999
DLF013_scaffold44577_1	non-phage	0
DLF013_scaffold3400_14	non-phage	0
DLF013_scaffold48553_2	non-phage	0
DLF013_scaffold48822_2	non-phage	0
DLF013_scaffold15981_1	non-phage	0
DLF013_scaffold46980_2	non-phage	0
DLF013_scaffold53513_1	non-phage	0
DLF013_scaffold49864_2	non-phage	0
DLF013_scaffold36505_2	non-phage	0
DLF013_scaffold22757_8	non-phage	0
DLF013_scaffold32041_1	non-phage	0
DLF013_scaffold26172_2	non-phage	0
DLF013_scaffold20513_8	non-phage	0
DLF013_scaffold54736_2	non-phage	0
DLF013_scaffold53959_2	non-phage	0
DLF013_scaffold32548_2	non-phage	0
DLF013_scaffold4073_4	non-phage	0
DLF013_scaffold19049_10	non-phage	0
DLF013_scaffold53290_1	non-phage	0
DLF013_scaffold24503_2	non-phage	0
DLF013_scaffold40386_1	non-phage	0
DLF013_scaffold36470_3	non-phage	0
DLF013_scaffold50109_7	non-phage	0
DLF013_scaffold53938_2	non-phage	0
DLF013_scaffold25322_2	non-phage	0
DLF013_scaffold5693_6	non-phage	0
DLF013_scaffold28710_3	non-phage	0

DLF013_scaffold42888_7	non-phage	0.002
DLF013_scaffold43916_2	non-phage	0
DLF013_scaffold36189_2	non-phage	0
DLF013_scaffold26375_1	non-phage	0
DLF013_scaffold3815_4	non-phage	0
DLF013_scaffold23060_2	non-phage	0
DLF013_scaffold7940_5	non-phage	0
DLF013_scaffold34719_1	non-phage	0
DLF013_scaffold45541_5	non-phage	0
DLF013_scaffold14958_2	non-phage	0
DLF013_scaffold11035_4	non-phage	0
DLF013_scaffold33156_3	non-phage	0
DLF013_scaffold7927_2	non-phage	0
DLF013_scaffold22757_11	non-phage	0
DLF013_scaffold26542_4	non-phage	0
DLF013_scaffold36037_1	non-phage	0
DLF013_scaffold26229_4	non-phage	0
DLF013_scaffold43415_3	non-phage	0
DLF013_scaffold3400_25	non-phage	0
DLF013_scaffold28394_1	non-phage	0
DLF013_scaffold4985_11	non-phage	0
DLF013_scaffold19681_2	non-phage	0
DLF013_scaffold54734_1	non-phage	0
DLF013_scaffold33373_1	non-phage	0
DLF013_scaffold88_6	non-phage	0
DLF013_scaffold49788_1	non-phage	0
DLF013_scaffold265_26	non-phage	0
DLF013_scaffold48931_5	non-phage	0
DLF013_scaffold44455_1	non-phage	0
DLF013_scaffold48931_2	non-phage	0
DLF013_scaffold36273_1	phage	0.994
DLF013_scaffold14862_1	non-phage	0.026
DLF013_scaffold2101_9	non-phage	0
DLF013_scaffold46137_6	non-phage	0
DLF013_scaffold266_3	non-phage	0
DLF013_scaffold19681_6	non-phage	0
DLF013_scaffold54043_1	non-phage	0
DLF013_scaffold45213_1	phage	0.998
DLF013_scaffold53796_2	non-phage	0
DLF013_scaffold42888_3	non-phage	0
DLF013_scaffold36189_1	non-phage	0
DLF013_scaffold15507_1	non-phage	0
DLF013_scaffold28910_7	non-phage	0
DLF013_scaffold51161_2	non-phage	0
DLF013_scaffold53817_3	non-phage	0
DLF013_scaffold28276_7	non-phage	0

DLF013_scaffold118_2	non-phage	0
DLF013_scaffold31785_1	phage	0.999
DLF013_scaffold37995_3	non-phage	0
DLF013_scaffold19049_18	non-phage	0
DLF013_scaffold200_1	non-phage	0
DLF013_scaffold39570_1	non-phage	0
DLF013_scaffold17492_5	non-phage	0
DLF013_scaffold43336_4	non-phage	0
DLF013_scaffold9721_5	non-phage	0
DLF013_scaffold54786_1	non-phage	0
DLF013_scaffold27850_1	non-phage	0
DLF013_scaffold15096_1	non-phage	0
DLF013_scaffold28038_3	non-phage	0
DLF013_scaffold51896_5	non-phage	0
DLF013_scaffold9373_4	non-phage	0
DLF013_scaffold29621_1	non-phage	0
DLF013_scaffold6091_2	non-phage	0
DLF013_scaffold15798_1	non-phage	0
DLF013_scaffold37927_2	non-phage	0
DLF013_scaffold51185_2	non-phage	0
DLF013_scaffold53312_2	non-phage	0
DLF013_scaffold16307_4	non-phage	0
DLF013_scaffold31672_1	phage	1
DLF013_scaffold45912_1	non-phage	0
DLF013_scaffold14812_2	non-phage	0
DLF013_scaffold45432_2	non-phage	0
DLF013_scaffold1308_2	non-phage	0
DLF013_scaffold32343_3	non-phage	0
DLF013_scaffold176_2	non-phage	0
DLF013_scaffold51185_4	non-phage	0
DLF013_scaffold53489_2	non-phage	0
DLF013_scaffold139_7	phage	0.974
DLF013_scaffold20037_1	non-phage	0
DLF013_scaffold38734_1	phage	0.99
DLF013_scaffold50572_1	non-phage	0
DLF013_scaffold3400_3	non-phage	0
DLF013_scaffold11775_2	non-phage	0
DLF013_scaffold29509_6	non-phage	0
DLF013_scaffold5055_2	non-phage	0
DLF013_scaffold17919_4	non-phage	0
DLF013_scaffold13718_3	non-phage	0
DLF013_scaffold29837_2	non-phage	0
DLF013_scaffold51185_5	non-phage	0
DLF013_scaffold51141_5	non-phage	0
DLF013_scaffold54022_3	non-phage	0
DLF013_scaffold68_1	non-phage	0

DLF013_scaffold53396_3	non-phage	0
DLF013_scaffold6424_2	non-phage	0
DLF013_scaffold37927_1	non-phage	0
DLF013_scaffold4152_1	non-phage	0
DLF013_scaffold1387_1	non-phage	0
DLF013_scaffold30936_1	non-phage	0
DLF013_scaffold29177_1	non-phage	0
DLF013_scaffold337_2	non-phage	0
DLF013_scaffold14892_2	non-phage	0
DLF013_scaffold186_1	non-phage	0
DLF013_scaffold38790_2	non-phage	0
DLF013_scaffold49609_1	non-phage	0
DLF013_scaffold36714_1	non-phage	0
DLF013_scaffold49692_5	non-phage	0
DLF013_scaffold20747_4	non-phage	0
DLF013_scaffold15507_3	non-phage	0
DLF013_scaffold951_1	phage	0.662
DLF013_scaffold25725_10	non-phage	0
DLF013_scaffold139_4	non-phage	0
DLF013_scaffold777_4	non-phage	0
DLF013_scaffold43602_6	non-phage	0
DLF013_scaffold573_8	non-phage	0
DLF013_scaffold13912_2	non-phage	0
DLF013_scaffold54783_2	non-phage	0
DLF013_scaffold11990_4	non-phage	0
DLF013_scaffold12558_14	non-phage	0
DLF013_scaffold44455_3	non-phage	0
DLF013_scaffold23340_1	non-phage	0
DLF013_scaffold51197_1	non-phage	0
DLF013_scaffold54787_1	non-phage	0
DLF013_scaffold38248_1	non-phage	0
DLF013_scaffold54790_1	non-phage	0.361
DLF013_scaffold35456_1	non-phage	0
DLF013_scaffold33578_4	non-phage	0
DLF013_scaffold30767_1	non-phage	0
DLF013_scaffold10030_1	non-phage	0
DLF013_scaffold115_1	non-phage	0
DLF013_scaffold17241_1	non-phage	0
DLF013_scaffold8750_3	non-phage	0
DLF013_scaffold26304_3	non-phage	0
DLF013_scaffold38735_1	non-phage	0
DLF013_scaffold9035_1	non-phage	0
DLF013_scaffold18364_2	non-phage	0
DLF013_scaffold43379_4	non-phage	0
DLF013_scaffold21495_1	non-phage	0
DLF013_scaffold26664_3	non-phage	0

DLF013_scaffold13065_1	non-phage	0
DLF013_scaffold4073_16	non-phage	0
DLF013_scaffold16307_9	phage	0.999
DLF013_scaffold54775_1	non-phage	0
DLF013_scaffold27683_6	non-phage	0
DLF013_scaffold10557_3	non-phage	0
DLF013_scaffold22413_3	non-phage	0
DLF013_scaffold19614_3	non-phage	0
DLF013_scaffold54466_1	non-phage	0
DLF013_scaffold18744_4	non-phage	0
DLF013_scaffold38372_1	non-phage	0
DLF013_scaffold22_8	non-phage	0
DLF013_scaffold6091_6	non-phage	0
DLF013_scaffold9_1	non-phage	0
DLF013_scaffold42814_1	non-phage	0
DLF013_scaffold3128_4	non-phage	0
DLF013_scaffold23397_1	non-phage	0
DLF013_scaffold17824_1	non-phage	0
DLF013_scaffold45912_2	non-phage	0
DLF013_scaffold53129_1	non-phage	0
DLF013_scaffold7914_2	non-phage	0
DLF013_scaffold176_3	non-phage	0
DLF013_scaffold19715_2	non-phage	0
DLF013_scaffold38907_1	non-phage	0
DLF013_scaffold46613_2	non-phage	0
DLF013_scaffold14183_3	non-phage	0
DLF013_scaffold54759_1	non-phage	0
DLF013_scaffold15616_1	non-phage	0
DLF013_scaffold18445_3	non-phage	0
DLF013_scaffold4753_1	non-phage	0
DLF013_scaffold12098_7	non-phage	0
DLF013_scaffold39102_3	non-phage	0
DLF013_scaffold4985_3	non-phage	0
DLF013_scaffold25725_2	non-phage	0
DLF013_scaffold29443_3	non-phage	0
DLF013_scaffold53889_1	phage	0.996
DLF013_scaffold44575_6	non-phage	0
DLF013_scaffold34916_1	non-phage	0
DLF013_scaffold33643_1	non-phage	0
DLF013_scaffold14997_1	non-phage	0
DLF013_scaffold19199_3	non-phage	0
DLF013_scaffold10926_2	non-phage	0
DLF013_scaffold21004_6	non-phage	0
DLF013_scaffold6091_7	non-phage	0
DLF013_scaffold42888_4	phage	0.943
DLF013_scaffold4073_12	non-phage	0

DLF013_scaffold22885_4	non-phage	0
DLF013_scaffold50685_1	non-phage	0
DLF013_scaffold32343_4	non-phage	0.238
DLF013_scaffold30433_1	non-phage	0.014
DLF013_scaffold136_1	non-phage	0
DLF013_scaffold16776_2	non-phage	0
DLF013_scaffold45326_1	non-phage	0
DLF013_scaffold18744_3	non-phage	0
DLF013_scaffold32276_1	non-phage	0
DLF013_scaffold6714_5	non-phage	0
DLF013_scaffold12295_1	non-phage	0
DLF013_scaffold43415_4	non-phage	0
DLF013_scaffold52386_3	non-phage	0
DLF013_scaffold15444_6	non-phage	0
DLF013_scaffold51124_1	non-phage	0
DLF013_scaffold5055_1	non-phage	0
DLF013_scaffold17713_2	non-phage	0
DLF013_scaffold12098_5	non-phage	0
DLF013_scaffold21493_3	phage	0.989
DLF013_scaffold24088_5	non-phage	0
DLF013_scaffold38035_1	non-phage	0
DLF013_scaffold5808_3	non-phage	0
DLF013_scaffold18927_5	non-phage	0
DLF013_scaffold46137_4	non-phage	0
DLF013_scaffold44575_1	non-phage	0
DLF013_scaffold34613_2	non-phage	0
DLF013_scaffold46806_1	non-phage	0
DLF013_scaffold45360_2	non-phage	0
DLF013_scaffold4985_6	non-phage	0
DLF013_scaffold22885_5	non-phage	0
DLF013_scaffold113_27	non-phage	0.435
DLF013_scaffold22102_5	non-phage	0
DLF013_scaffold21883_3	non-phage	0
DLF013_scaffold32726_3	non-phage	0
DLF013_scaffold35350_4	non-phage	0
DLF013_scaffold573_10	non-phage	0
DLF013_scaffold1520_1	non-phage	0
DLF013_scaffold44732_5	non-phage	0
DLF013_scaffold573_4	non-phage	0
DLF013_scaffold53343_4	non-phage	0
DLF013_scaffold8880_2	non-phage	0
DLF013_scaffold19423_11	non-phage	0
DLF013_scaffold45541_6	non-phage	0
DLF013_scaffold12082_1	non-phage	0
DLF013_scaffold25714_2	non-phage	0
DLF013_scaffold22029_4	non-phage	0

DLF013_scaffold14997_8	non-phage	0
DLF013_scaffold12815_8	non-phage	0
DLF013_scaffold29177_4	non-phage	0
DLF013_scaffold5780_1	non-phage	0
DLF013_scaffold3883_9	non-phage	0
DLF013_scaffold5677_2	non-phage	0
DLF013_scaffold7940_1	non-phage	0
DLF013_scaffold39583_1	non-phage	0
DLF013_scaffold33635_3	non-phage	0
DLF013_scaffold34485_2	non-phage	0
DLF013_scaffold34347_1	non-phage	0
DLF013_scaffold88_1	non-phage	0
DLF013_scaffold43319_1	non-phage	0
DLF013_scaffold19715_3	non-phage	0
DLF013_scaffold31981_1	non-phage	0
DLF013_scaffold28806_1	non-phage	0
DLF013_scaffold28276_3	non-phage	0
DLF013_scaffold4873_2	non-phage	0
DLF013_scaffold36485_1	non-phage	0
DLF013_scaffold51161_1	non-phage	0
DLF013_scaffold32653_2	non-phage	0
DLF013_scaffold90_2	non-phage	0
DLF013_scaffold36505_3	non-phage	0
DLF013_scaffold26542_3	non-phage	0
DLF013_scaffold48931_4	non-phage	0
DLF013_scaffold6714_10	non-phage	0
DLF013_scaffold35531_1	non-phage	0
DLF013_scaffold11119_1	non-phage	0
DLF013_scaffold32343_2	non-phage	0
DLF013_scaffold52129_1	non-phage	0
DLF013_scaffold5693_2	non-phage	0
DLF013_scaffold3400_11	non-phage	0
DLF013_scaffold7820_4	non-phage	0
DLF013_scaffold3400_18	non-phage	0
DLF013_scaffold6386_9	non-phage	0
DLF013_scaffold10557_2	non-phage	0
DLF013_scaffold54765_1	non-phage	0
DLF013_scaffold40873_1	non-phage	0
DLF013_scaffold9715_1	non-phage	0
DLF013_scaffold28276_6	non-phage	0
DLF013_scaffold4873_1	non-phage	0
DLF013_scaffold8750_5	non-phage	0
DLF013_scaffold24725_7	non-phage	0
DLF013_scaffold34050_2	non-phage	0
DLF013_scaffold39401_3	non-phage	0
DLF013_scaffold19199_4	phage	0.999

DLF013_scaffold43486_1	non-phage	0
DLF013_scaffold7914_3	non-phage	0
DLF013_scaffold54779_1	non-phage	0
DLF013_scaffold43231_1	non-phage	0
DLF013_scaffold8478_2	non-phage	0
DLF013_scaffold49186_5	non-phage	0
DLF013_scaffold32808_2	non-phage	0
DLF013_scaffold49433_5	phage	0.999
DLF013_scaffold29445_2	non-phage	0
DLF013_scaffold44851_1	non-phage	0
DLF013_scaffold3400_2	non-phage	0
DLF013_scaffold25714_1	non-phage	0
DLF013_scaffold46980_3	non-phage	0
DLF013_scaffold48931_7	non-phage	0
DLF013_scaffold28910_5	non-phage	0
DLF013_scaffold4865_1	non-phage	0
DLF013_scaffold13718_2	non-phage	0
DLF013_scaffold113_18	non-phage	0
DLF013_scaffold17845_2	non-phage	0
DLF013_scaffold54026_2	non-phage	0
DLF013_scaffold44286_1	non-phage	0
DLF013_scaffold33578_7	non-phage	0
DLF013_scaffold51952_1	non-phage	0
DLF013_scaffold10941_7	non-phage	0
DLF013_scaffold54773_2	non-phage	0
DLF013_scaffold36295_3	non-phage	0
DLF013_scaffold14862_2	non-phage	0
DLF013_scaffold32343_1	non-phage	0
DLF013_scaffold42888_1	non-phage	0
DLF013_scaffold37927_4	non-phage	0
DLF013_scaffold54022_2	non-phage	0
DLF013_scaffold17919_2	non-phage	0
DLF013_scaffold54202_1	non-phage	0
DLF013_scaffold36635_1	non-phage	0
DLF013_scaffold20747_1	non-phage	0
DLF013_scaffold27195_3	non-phage	0
DLF013_scaffold2098_10	non-phage	0
DLF013_scaffold265_24	non-phage	0
DLF013_scaffold49788_2	non-phage	0
DLF013_scaffold36295_5	non-phage	0
DLF013_scaffold2857_3	non-phage	0
DLF013_scaffold53938_1	non-phage	0
DLF013_scaffold28910_15	non-phage	0
DLF013_scaffold25083_24	non-phage	0.024
DLF013_scaffold13304_3	non-phage	0
DLF013_scaffold20948_3	non-phage	0

DLF013_scaffold11990_5	non-phage	0
DLF013_scaffold26229_18	non-phage	0
DLF013_scaffold25725_1	non-phage	0
DLF013_scaffold188_1	non-phage	0
DLF013_scaffold4073_5	non-phage	0
DLF013_scaffold28005_1	non-phage	0
DLF013_scaffold6386_5	non-phage	0
DLF013_scaffold54785_4	non-phage	0
DLF013_scaffold5994_1	non-phage	0
DLF013_scaffold15444_1	non-phage	0
DLF013_scaffold25655_3	non-phage	0
DLF013_scaffold47104_6	non-phage	0
DLF013_scaffold42896_2	non-phage	0
DLF013_C686784_1	non-phage	0
DLF013_scaffold50109_6	non-phage	0
DLF013_scaffold27688_1	non-phage	0
DLF013_scaffold81_3	non-phage	0
DLF013_scaffold36320_1	non-phage	0
DLF013_scaffold11483_1	non-phage	0
DLF013_scaffold11990_1	non-phage	0
DLF013_scaffold10926_6	non-phage	0
DLF013_scaffold54706_2	non-phage	0
DLF013_scaffold14997_4	non-phage	0
DLF013_scaffold6385_4	non-phage	0
DLF013_scaffold30936_2	non-phage	0
DLF013_scaffold50729_3	non-phage	0
DLF013_scaffold3400_27	non-phage	0
DLF013_C686658_1	non-phage	0
DLF013_scaffold51656_1	non-phage	0
DLF013_scaffold38018_1	non-phage	0
DLF013_scaffold33567_1	non-phage	0
DLF013_scaffold54765_2	non-phage	0
DLF013_scaffold12074_13	non-phage	0
DLF013_scaffold777_6	non-phage	0
DLF013_scaffold24088_6	non-phage	0
DLF013_scaffold10885_2	non-phage	0
DLF013_scaffold41029_2	non-phage	0
DLF013_scaffold14065_1	non-phage	0
DLF013_scaffold4562_4	non-phage	0
DLF013_scaffold4985_5	non-phage	0
DLF013_scaffold50663_1	non-phage	0
DLF013_scaffold54446_2	non-phage	0
DLF013_scaffold52685_3	non-phage	0
DLF013_scaffold53817_1	non-phage	0
DLF013_scaffold32726_2	non-phage	0
DLF013_scaffold23543_2	non-phage	0

DLF013_scaffold50519_3	non-phage	0
DLF013_scaffold19049_9	non-phage	0
DLF013_scaffold30930_3	non-phage	0
DLF013_scaffold53343_1	non-phage	0
DLF013_scaffold5619_1	non-phage	0
DLF013_scaffold28910_12	non-phage	0
DLF013_scaffold13718_7	non-phage	0
DLF013_scaffold6975_3	non-phage	0
DLF013_scaffold16776_1	non-phage	0
DLF013_scaffold22029_3	non-phage	0
DLF013_scaffold82_7	non-phage	0
DLF013_scaffold54026_6	non-phage	0
DLF013_scaffold54798_1	phage	0.998
DLF013_scaffold30152_2	non-phage	0
DLF013_scaffold25735_1	non-phage	0
DLF013_scaffold31981_2	non-phage	0
DLF013_scaffold20747_8	non-phage	0
DLF013_scaffold7802_6	non-phage	0
DLF013_scaffold15284_5	non-phage	0
DLF013_scaffold10557_5	non-phage	0
DLF013_scaffold2098_8	non-phage	0
DLF013_scaffold54026_1	non-phage	0
DLF013_scaffold52386_4	non-phage	0
DLF013_scaffold33578_1	non-phage	0
DLF013_scaffold54726_1	non-phage	0
DLF013_scaffold50109_9	non-phage	0
DLF013_scaffold10941_3	non-phage	0
DLF013_scaffold35350_5	non-phage	0
DLF013_scaffold29443_6	non-phage	0
DLF013_scaffold18894_2	non-phage	0
DLF013_scaffold47104_3	non-phage	0
DLF013_scaffold39787_1	non-phage	0
DLF013_scaffold47389_1	non-phage	0
DLF013_scaffold1100_4	phage	0.998
DLF013_scaffold3883_7	non-phage	0
DLF013_scaffold54229_1	non-phage	0
DLF013_scaffold22885_6	non-phage	0
DLF013_scaffold10082_8	non-phage	0
DLF013_scaffold23104_1	non-phage	0
DLF013_scaffold54744_1	non-phage	0
DLF013_scaffold53343_5	non-phage	0
DLF013_scaffold27850_12	non-phage	0
DLF013_scaffold26172_3	non-phage	0
DLF013_scaffold28108_3	non-phage	0
DLF013_scaffold5693_8	non-phage	0
DLF013_scaffold15284_16	non-phage	0

DLF013_scaffold54386_1	non-phage	0
DLF013_scaffold573_3	non-phage	0
DLF013_scaffold34809_2	non-phage	0
DLF013_scaffold39988_3	non-phage	0
DLF013_scaffold22757_13	non-phage	0
DLF013_scaffold22_3	non-phage	0
DLF013_scaffold17975_4	non-phage	0
DLF013_scaffold932_1	non-phage	0
DLF013_scaffold13599_2	non-phage	0
DLF013_scaffold1520_4	non-phage	0
DLF013_scaffold34532_1	non-phage	0
DLF013_scaffold45541_3	non-phage	0
DLF013_scaffold42008_2	phage	0.998
DLF013_scaffold54776_1	phage	0.999
DLF013_scaffold40813_1	non-phage	0
DLF013_scaffold5823_2	non-phage	0
DLF013_scaffold43319_2	non-phage	0
DLF013_scaffold33577_4	non-phage	0
DLF013_scaffold13599_5	non-phage	0
DLF013_scaffold48958_2	non-phage	0
DLF013_scaffold113_10	non-phage	0
DLF013_scaffold30131_1	non-phage	0
DLF013_scaffold15284_15	non-phage	0
DLF013_scaffold265_16	non-phage	0
DLF013_scaffold43415_1	non-phage	0
DLF013_scaffold19681_7	non-phage	0
DLF013_scaffold26229_6	non-phage	0
DLF013_scaffold21690_4	non-phage	0
DLF014_scaffold129_6	non-phage	0
DLF014_scaffold9072_1	non-phage	0
DLF014_scaffold10381_1	non-phage	0
DLF014_scaffold10626_2	non-phage	0
DLF014_scaffold89_3	non-phage	0
DLF014_scaffold12062_3	phage	0.999
DLF014_scaffold4655_3	non-phage	0
DLF014_scaffold2234_1	non-phage	0
DLF014_scaffold134_3	non-phage	0
DLF014_scaffold12731_2	non-phage	0
DLF014_scaffold5731_5	non-phage	0
DLF014_scaffold3197_2	non-phage	0
DLF014_scaffold12200_1	non-phage	0
DLF014_scaffold2504_3	non-phage	0
DLF014_scaffold129_1	non-phage	0
DLF014_scaffold12685_2	non-phage	0
DLF014_scaffold12356_5	non-phage	0
DLF014_scaffold222_7	non-phage	0

DLF014_scaffold4764_5	non-phage	0
DLF014_scaffold2950_11	non-phage	0
DLF014_C195831_1	phage	0.984
DLF014_scaffold5451_6	non-phage	0
DLF014_scaffold9120_3	non-phage	0
DLF014_C195957_1	non-phage	0
DLF014_scaffold12009_1	non-phage	0
DLF014_scaffold12779_1	phage	0.998
DLF014_scaffold7481_3	non-phage	0
DLF014_scaffold12449_1	non-phage	0
DLF014_scaffold11720_1	non-phage	0
DLF014_scaffold12754_1	non-phage	0
DLF014_scaffold33_1	non-phage	0
DLF014_scaffold12420_1	non-phage	0
DLF014_scaffold1686_6	non-phage	0
DLF014_scaffold12724_1	non-phage	0
DLF014_scaffold11683_2	non-phage	0
DLF014_scaffold6560_3	non-phage	0
DLF014_scaffold9942_1	phage	0.999
DLF014_scaffold5796_1	non-phage	0
DLF014_scaffold26_2	non-phage	0
DLF014_scaffold7121_1	non-phage	0
DLF014_scaffold6182_7	non-phage	0
DLF014_scaffold4430_1	non-phage	0
DLF014_scaffold12751_6	non-phage	0
DLF014_scaffold2812_4	non-phage	0
DLF014_scaffold4827_1	non-phage	0.002
DLF014_scaffold3282_1	non-phage	0
DLF014_scaffold6258_3	non-phage	0
DLF014_C196781_1	phage	0.999
DLF014_scaffold10265_1	non-phage	0
DLF014_scaffold12684_1	non-phage	0
DLF014_scaffold1392_2	non-phage	0
DLF014_scaffold8147_2	non-phage	0
DLF014_scaffold12721_1	non-phage	0
DLF014_scaffold12299_5	non-phage	0
DLF014_scaffold7468_1	non-phage	0
DLF014_scaffold12571_1	non-phage	0
DLF014_scaffold12792_2	phage	0.988
DLF014_scaffold60_4	non-phage	0
DLF014_scaffold108_1	non-phage	0
DLF014_scaffold12505_2	non-phage	0
DLF014_scaffold6782_10	non-phage	0
DLF014_scaffold94_2	non-phage	0
DLF014_scaffold12285_2	non-phage	0
DLF014_scaffold12417_1	non-phage	0

DLF014_scaffold9087_2	non-phage	0.01
DLF014_scaffold2627_2	non-phage	0
DLF014_scaffold12678_2	non-phage	0
DLF014_scaffold8775_3	non-phage	0
DLF014_scaffold9269_4	non-phage	0
DLF014_scaffold10460_6	non-phage	0
DLF014_scaffold1585_9	non-phage	0
DLF014_scaffold12747_5	non-phage	0
DLF014_scaffold12739_1	non-phage	0
DLF014_scaffold10265_3	non-phage	0
DLF014_scaffold12773_3	non-phage	0
DLF014_scaffold11064_2	non-phage	0
DLF014_scaffold9194_2	non-phage	0
DLF014_scaffold12764_1	non-phage	0
DLF014_scaffold12134_4	non-phage	0
DLF014_scaffold58_1	non-phage	0
DLF014_scaffold12595_4	non-phage	0
DLF014_scaffold12576_1	non-phage	0
DLF014_scaffold4203_1	non-phage	0
DLF014_scaffold134_6	non-phage	0
DLF014_scaffold12750_1	non-phage	0
DLF014_scaffold12319_1	non-phage	0
DLF014_scaffold11490_1	non-phage	0
DLF014_scaffold339_3	non-phage	0
DLF014_scaffold12785_1	non-phage	0
DLF014_scaffold2320_1	phage	0.999
DLF014_scaffold8630_3	non-phage	0
DLF014_scaffold12633_1	non-phage	0
DLF014_scaffold8109_2	non-phage	0
DLF014_scaffold11004_1	non-phage	0
DLF014_scaffold1854_1	non-phage	0
DLF014_scaffold12772_4	non-phage	0
DLF014_scaffold9953_3	non-phage	0
DLF014_scaffold2249_1	non-phage	0
DLF014_scaffold6782_9	non-phage	0
DLF014_scaffold12798_1	non-phage	0
DLF014_scaffold12453_1	non-phage	0
DLF014_scaffold12618_2	non-phage	0
DLF014_scaffold222_1	non-phage	0
DLF014_scaffold1533_7	non-phage	0
DLF014_scaffold5363_16	non-phage	0
DLF014_scaffold10944_1	non-phage	0
DLF014_scaffold4596_3	non-phage	0
DLF014_scaffold10666_2	non-phage	0
DLF014_scaffold3784_2	non-phage	0
DLF014_scaffold12718_1	phage	0.994

DLF014_scaffold7151_1	non-phage	0
DLF014_scaffold12556_1	non-phage	0
DLF014_scaffold10824_4	non-phage	0
DLF014_scaffold12690_2	non-phage	0
DLF014_scaffold3421_2	non-phage	0
DLF014_scaffold9953_2	non-phage	0
DLF014_scaffold5538_1	non-phage	0
DLF014_scaffold7985_2	phage	0.999
DLF014_scaffold8896_1	non-phage	0
DLF014_scaffold10956_3	non-phage	0
DLF014_scaffold5719_6	non-phage	0
DLF014_scaffold12732_1	non-phage	0
DLF014_scaffold11624_1	non-phage	0
DLF014_scaffold12793_1	non-phage	0
DLF014_scaffold222_5	non-phage	0
DLF014_scaffold5486_3	non-phage	0
DLF014_scaffold5471_1	phage	0.976
DLF014_scaffold11526_3	non-phage	0
DLF014_scaffold12438_1	non-phage	0
DLF014_scaffold6855_3	non-phage	0
DLF014_scaffold7481_2	non-phage	0
DLF014_scaffold12493_10	non-phage	0
DLF014_scaffold12773_1	non-phage	0
DLF014_scaffold8483_2	non-phage	0
DLF014_scaffold12062_1	non-phage	0
DLF014_scaffold1992_1	non-phage	0
DLF014_scaffold12697_1	non-phage	0
DLF014_scaffold583_1	phage	0.999
DLF014_scaffold6560_5	non-phage	0
DLF014_scaffold11456_1	non-phage	0
DLF014_scaffold8789_2	non-phage	0
DLF014_scaffold994_23	non-phage	0
DLF014_scaffold12744_1	non-phage	0
DLF014_scaffold1853_4	non-phage	0
DLF014_scaffold807_1	non-phage	0
DLF014_scaffold6782_12	non-phage	0
DLF014_scaffold4827_3	non-phage	0.04
DLF014_scaffold1009_1	non-phage	0
DLF014_scaffold12795_1	non-phage	0
DLF014_scaffold6504_1	non-phage	0
DLF014_scaffold925_5	non-phage	0
DLF014_scaffold9120_5	non-phage	0
DLF014_scaffold9631_1	non-phage	0
DLF014_scaffold12770_1	non-phage	0
DLF014_scaffold10436_15	non-phage	0
DLF014_scaffold5546_1	non-phage	0

DLF014_scaffold8419_3	non-phage	0
DLF014_scaffold68_3	non-phage	0
DLF014_scaffold12796_1	phage	0.998
DLF014_scaffold28_4	non-phage	0
DLF014_scaffold4892_6	non-phage	0
DLF014_scaffold5363_7	non-phage	0.084
DLF014_scaffold8169_2	non-phage	0
DLF014_scaffold9925_2	non-phage	0
DLF014_scaffold9535_1	non-phage	0
DLF014_scaffold1928_1	non-phage	0
DLF014_scaffold8199_1	non-phage	0
DLF014_scaffold5451_8	non-phage	0
DLF014_scaffold9269_1	non-phage	0
DLF014_scaffold9072_2	non-phage	0
DLF014_scaffold1312_1	non-phage	0
DLF014_scaffold723_1	non-phage	0
DLF014_scaffold12371_1	non-phage	0
DLF014_scaffold3055_4	non-phage	0
DLF014_scaffold11503_2	non-phage	0
DLF014_scaffold4597_5	non-phage	0
DLF014_scaffold8896_2	non-phage	0
DLF014_scaffold9953_5	non-phage	0
DLF014_scaffold14_2	non-phage	0
DLF014_scaffold12766_1	non-phage	0
DLF014_scaffold11998_9	non-phage	0
DLF014_scaffold3505_2	non-phage	0
DLF014_scaffold12591_2	non-phage	0
DLF014_scaffold12748_1	non-phage	0
DLF014_scaffold3686_1	non-phage	0
DLF014_scaffold10220_2	non-phage	0
DLF014_scaffold9245_1	non-phage	0
DLF014_scaffold2444_1	non-phage	0
DLF014_scaffold8630_1	non-phage	0
DLF014_scaffold12682_1	phage	0.999
DLF014_scaffold4991_1	non-phage	0
DLF014_scaffold8_3	non-phage	0
DLF014_scaffold9120_2	non-phage	0
DLF014_scaffold3230_3	non-phage	0
DLF014_scaffold2249_3	non-phage	0
DLF014_scaffold10759_2	non-phage	0
DLF014_scaffold2414_1	non-phage	0
DLF014_scaffold4662_3	non-phage	0
DLF014_scaffold12638_2	non-phage	0
DLF014_scaffold6396_1	non-phage	0
DLF014_scaffold4518_5	non-phage	0
DLF014_scaffold12673_2	non-phage	0

DLF014_scaffold11998_4	non-phage	0
DLF014_scaffold12496_1	non-phage	0
DLF014_scaffold11340_1	non-phage	0
DLF014_scaffold12454_2	non-phage	0
DLF014_scaffold12789_1	non-phage	0
DLF014_C196115_1	non-phage	0
DLF014_scaffold9789_1	non-phage	0
DLF014_scaffold1211_3	non-phage	0
DLF014_scaffold9327_4	non-phage	0
DLF014_scaffold5538_4	non-phage	0
DLF014_scaffold796_3	non-phage	0
DLF014_scaffold2092_2	non-phage	0
DLF014_scaffold12595_2	non-phage	0
DLF014_scaffold7863_2	non-phage	0
DLF014_scaffold5363_15	non-phage	0
DLF014_scaffold12167_1	non-phage	0
DLF014_scaffold12742_1	non-phage	0
DLF014_scaffold12667_1	non-phage	0
DLF014_scaffold1802_13	non-phage	0
DLF014_scaffold12585_1	non-phage	0
DLF014_scaffold4982_5	non-phage	0
DLF014_scaffold2504_1	non-phage	0
DLF014_scaffold8638_2	non-phage	0
DLF014_scaffold2657_2	non-phage	0
DLF014_scaffold3092_1	non-phage	0
DLF014_scaffold2735_4	non-phage	0
DLF014_scaffold8745_7	non-phage	0
DLF014_scaffold68_8	non-phage	0
DLF014_scaffold5330_1	phage	0.988
DLF014_scaffold12493_8	non-phage	0
DLF014_scaffold4324_3	non-phage	0
DLF014_scaffold3405_4	non-phage	0
DLF014_scaffold6331_1	non-phage	0
DLF014_scaffold12650_1	non-phage	0
DLF014_scaffold11433_1	non-phage	0
DLF014_scaffold12554_1	non-phage	0
DLF014_scaffold6155_4	non-phage	0
DLF014_scaffold10436_10	non-phage	0
DLF014_scaffold5731_2	non-phage	0
DLF014_scaffold12693_1	non-phage	0
DLF014_scaffold31_4	non-phage	0
DLF014_scaffold3411_1	non-phage	0
DLF014_scaffold12511_1	non-phage	0
DLF014_scaffold5386_3	non-phage	0
DLF014_scaffold5425_8	non-phage	0
DLF014_scaffold28_7	non-phage	0

DLF014_scaffold5457_2	non-phage	0
DLF014_scaffold1389_1	non-phage	0
DLF014_scaffold6248_1	non-phage	0
DLF014_scaffold6344_3	non-phage	0
DLF014_scaffold129_2	non-phage	0
DLF014_scaffold221_4	non-phage	0
DLF014_scaffold8506_2	non-phage	0
DLF014_scaffold8436_1	non-phage	0
DLF014_scaffold2386_4	phage	0.989
DLF014_scaffold1006_4	non-phage	0
DLF014_scaffold10461_1	non-phage	0
DLF014_scaffold8460_1	non-phage	0
DLF014_scaffold11758_1	non-phage	0
DLF014_scaffold1957_2	non-phage	0
DLF014_scaffold10121_3	non-phage	0
DLF014_scaffold4056_3	non-phage	0
DLF014_scaffold3110_1	non-phage	0
DLF014_scaffold9120_7	non-phage	0
DLF014_scaffold8386_13	non-phage	0
DLF014_scaffold2322_5	non-phage	0
DLF014_scaffold2950_7	non-phage	0
DLF014_scaffold2234_9	non-phage	0
DLF014_scaffold12715_1	non-phage	0
DLF014_scaffold3686_4	non-phage	0
DLF014_C196379_1	non-phage	0
DLF014_scaffold1713_1	non-phage	0.002
DLF014_scaffold4892_1	non-phage	0
DLF014_scaffold11854_1	non-phage	0
DLF014_scaffold12402_1	non-phage	0
DLF014_scaffold4203_5	non-phage	0
DLF014_scaffold12675_1	non-phage	0
DLF014_scaffold7677_9	non-phage	0
DLF014_scaffold9698_1	non-phage	0
DLF014_scaffold10439_1	non-phage	0
DLF014_scaffold10266_1	non-phage	0
DLF014_scaffold6637_1	non-phage	0
DLF014_scaffold58_4	non-phage	0
DLF014_scaffold9120_9	non-phage	0
DLF014_scaffold10436_8	non-phage	0
DLF014_scaffold2735_3	non-phage	0
DLF014_scaffold12741_1	phage	0.999
DLF014_scaffold11967_1	phage	0.993
DLF014_scaffold7259_1	non-phage	0
DLF014_scaffold4892_3	non-phage	0
DLF014_scaffold1494_8	non-phage	0
DLF014_scaffold134_4	non-phage	0

DLF014_scaffold6258_1	non-phage	0
DLF014_scaffold8898_1	phage	0.999
DLF014_scaffold12505_1	non-phage	0
DLF014_scaffold3999_1	phage	0.998
DLF014_scaffold12700_4	non-phage	0
DLF014_scaffold6344_1	phage	0.987
DLF014_scaffold5363_8	non-phage	0
DLF014_scaffold11623_1	phage	0.999
DLF014_scaffold12728_3	non-phage	0
DLF014_scaffold11854_3	non-phage	0
DLF014_scaffold4705_1	non-phage	0
DLF014_scaffold4688_4	non-phage	0
DLF014_scaffold4714_2	non-phage	0
DLF014_scaffold12700_5	non-phage	0
DLF014_scaffold12613_4	non-phage	0
DLF014_scaffold12758_1	non-phage	0
DLF014_scaffold564_1	non-phage	0
DLF014_scaffold9106_2	non-phage	0
DLF014_scaffold2693_2	phage	0.988
DLF014_scaffold5451_19	non-phage	0
DLF014_scaffold9453_3	non-phage	0
DLF014_scaffold1072_2	non-phage	0
DLF014_scaffold12686_1	non-phage	0
DLF014_scaffold8419_1	non-phage	0
DLF014_scaffold2693_7	non-phage	0
DLF014_scaffold12291_2	non-phage	0
DLF014_scaffold8386_6	non-phage	0
DLF014_scaffold10265_7	non-phage	0
DLF014_scaffold9747_1	non-phage	0
DLF014_scaffold2386_2	non-phage	0
DLF014_scaffold786_1	non-phage	0
DLF014_scaffold8_1	non-phage	0
DLF014_scaffold4991_3	non-phage	0
DLF014_scaffold11651_1	non-phage	0
DLF014_scaffold12678_1	non-phage	0
DLF014_scaffold12486_1	non-phage	0
DLF014_scaffold12788_1	non-phage	0
DLF014_scaffold753_7	non-phage	0
DLF014_scaffold5516_6	non-phage	0
DLF014_scaffold12681_2	non-phage	0
DLF014_scaffold10999_3	non-phage	0
DLF014_scaffold12299_2	non-phage	0
DLF014_scaffold5_2	non-phage	0
DLF014_scaffold1312_3	non-phage	0
DLF014_scaffold3055_2	non-phage	0
DLF014_scaffold87_2	non-phage	0

DLF014_scaffold646_1	non-phage	0
DLF014_scaffold12438_3	non-phage	0
DLF014_scaffold12555_1	non-phage	0
DLF014_scaffold12796_2	non-phage	0
DLF014_C196067_1	non-phage	0
DLF014_scaffold12514_2	non-phage	0
DLF014_scaffold6766_2	non-phage	0
DLF014_scaffold4772_3	non-phage	0
DLF014_scaffold2950_5	non-phage	0
DLF014_scaffold222_4	non-phage	0
DLF014_scaffold5425_6	non-phage	0
DLF014_C195523_1	non-phage	0
DLF014_scaffold5710_1	non-phage	0
DLF014_scaffold7677_3	non-phage	0
DLF014_scaffold8629_2	non-phage	0.293
DLF014_scaffold2001_7	non-phage	0
DLF014_scaffold5451_24	non-phage	0
DLF014_scaffold9830_1	non-phage	0
DLF014_scaffold1802_1	non-phage	0
DLF014_scaffold10436_1	non-phage	0
DLF014_scaffold8745_1	non-phage	0
DLF014_scaffold12766_2	non-phage	0
DLF014_scaffold12773_4	non-phage	0
DLF014_scaffold4203_8	non-phage	0
DLF014_scaffold12277_2	non-phage	0
DLF014_scaffold12751_5	non-phage	0
DLF014_C196591_1	non-phage	0
DLF014_scaffold12747_4	non-phage	0
DLF014_scaffold6782_7	non-phage	0
DLF014_scaffold12749_2	non-phage	0
DLF014_scaffold2812_2	non-phage	0
DLF014_scaffold60_8	non-phage	0
DLF014_scaffold8472_2	non-phage	0
DLF014_scaffold12790_3	non-phage	0
DLF014_scaffold11998_3	non-phage	0
DLF014_scaffold8300_4	non-phage	0
DLF014_scaffold7527_1	non-phage	0
DLF014_scaffold12631_2	non-phage	0
DLF014_scaffold2513_10	non-phage	0
DLF014_scaffold12787_1	non-phage	0
DLF014_scaffold1571_1	non-phage	0
DLF014_scaffold2001_4	non-phage	0
DLF014_scaffold1750_1	non-phage	0.01
DLF014_scaffold5363_3	non-phage	0
DLF014_C195605_1	non-phage	0
DLF014_scaffold513_4	non-phage	0

DLF014_scaffold2001_1	non-phage	0
DLF014_scaffold12516_1	non-phage	0
DLF014_scaffold6986_2	non-phage	0
DLF014_scaffold9197_2	non-phage	0
DLF014_scaffold8209_2	non-phage	0
DLF014_scaffold4945_1	non-phage	0
DLF014_scaffold506_3	non-phage	0
DLF014_scaffold5363_19	non-phage	0
DLF014_scaffold3717_1	non-phage	0
DLF014_scaffold6782_4	non-phage	0
DLF014_scaffold8109_3	non-phage	0
DLF014_scaffold5485_1	non-phage	0
DLF014_scaffold3154_3	non-phage	0
DLF014_scaffold10824_3	non-phage	0
DLF014_scaffold12633_3	non-phage	0
DLF014_scaffold58_2	non-phage	0
DLF014_scaffold6455_7	non-phage	0
DLF014_scaffold38_1	non-phage	0
DLF014_scaffold3407_8	non-phage	0
DLF014_scaffold6855_4	non-phage	0
DLF014_scaffold5812_5	non-phage	0
DLF014_scaffold8215_5	non-phage	0
DLF014_scaffold7731_3	non-phage	0
DLF014_scaffold5599_3	non-phage	0
DLF014_scaffold11613_1	non-phage	0
DLF014_scaffold4344_3	non-phage	0
DLF014_C195077_1	non-phage	0
DLF014_scaffold12417_2	non-phage	0
DLF014_scaffold12438_4	non-phage	0
DLF014_scaffold1322_4	non-phage	0
DLF014_scaffold1928_3	phage	0.997
DLF014_scaffold8435_6	non-phage	0
DLF014_scaffold12747_1	non-phage	0
DLF014_scaffold10615_3	non-phage	0
DLF014_scaffold7647_2	non-phage	0
DLF014_scaffold1399_2	non-phage	0
DLF014_scaffold5421_1	non-phage	0
DLF014_scaffold1006_2	non-phage	0
DLF014_scaffold2092_1	non-phage	0
DLF014_scaffold12699_1	non-phage	0
DLF014_scaffold120_1	non-phage	0
DLF014_scaffold3717_2	non-phage	0
DLF014_scaffold12716_2	non-phage	0
DLF014_scaffold1853_9	non-phage	0
DLF014_scaffold12587_1	non-phage	0
DLF014_scaffold12520_1	non-phage	0

DLF014_scaffold12516_3	non-phage	0
DLF014_scaffold564_3	non-phage	0
DLF014_scaffold12720_1	non-phage	0
DLF014_scaffold2735_1	non-phage	0
DLF014_scaffold9473_1	non-phage	0
DLF014_scaffold12523_3	non-phage	0
DLF014_scaffold12767_1	phage	0.831
DLF014_scaffold3019_1	non-phage	0
DLF014_scaffold12319_3	non-phage	0
DLF014_scaffold12276_2	non-phage	0
DLF014_scaffold2264_6	non-phage	0
DLF014_scaffold2494_1	non-phage	0
DLF014_scaffold12617_1	non-phage	0
DLF014_scaffold3775_1	non-phage	0
DLF014_scaffold5421_3	non-phage	0
DLF014_scaffold3230_4	non-phage	0
DLF014_scaffold8745_8	non-phage	0
DLF014_scaffold8109_4	non-phage	0
DLF014_scaffold872_5	non-phage	0
DLF014_scaffold3282_2	non-phage	0
DLF014_C195817_1	non-phage	0
DLF014_scaffold3784_6	non-phage	0
DLF014_scaffold12762_1	non-phage	0.263
DLF014_scaffold12285_1	non-phage	0
DLF014_scaffold12617_2	non-phage	0
DLF014_scaffold4556_3	phage	0.998
DLF014_scaffold94_1	non-phage	0
DLF014_scaffold5731_7	non-phage	0
DLF014_scaffold3019_2	non-phage	0
DLF014_scaffold2414_2	non-phage	0
DLF014_scaffold12784_4	non-phage	0
DLF014_scaffold2428_2	non-phage	0
DLF014_scaffold10217_3	non-phage	0
DLF014_scaffold3410_4	non-phage	0
DLF014_scaffold12792_1	phage	0.999
DLF014_scaffold3154_2	non-phage	0
DLF014_scaffold5363_14	non-phage	0
DLF014_scaffold10220_1	non-phage	0
DLF014_scaffold8386_3	non-phage	0
DLF014_scaffold12134_2	non-phage	0
DLF014_scaffold3628_16	non-phage	0
DLF014_scaffold1009_6	non-phage	0
DLF014_scaffold14_1	non-phage	0
DLF014_scaffold11395_2	non-phage	0
DLF014_scaffold7052_1	non-phage	0
DLF014_scaffold796_2	non-phage	0

DLF014_scaffold12595_3	non-phage	0
DLF014_scaffold12409_1	non-phage	0
DLF014_scaffold12718_2	non-phage	0
DLF014_scaffold8215_4	non-phage	0
DLF014_scaffold12703_1	non-phage	0
DLF014_scaffold4597_4	non-phage	0
DLF014_scaffold6266_3	non-phage	0
DLF014_scaffold3505_5	non-phage	0
DLF014_scaffold10908_1	non-phage	0
DLF014_scaffold12556_2	non-phage	0
DLF014_scaffold7841_1	non-phage	0
DLF014_scaffold222_2	non-phage	0
DLF014_scaffold4925_1	non-phage	0
DLF014_scaffold12618_3	non-phage	0
DLF014_scaffold872_2	non-phage	0
DLF014_C195411_1	non-phage	0
DLF014_scaffold12685_1	non-phage	0
DLF014_scaffold5363_17	non-phage	0
DLF014_scaffold12276_4	non-phage	0
DLF014_scaffold5624_1	non-phage	0
DLF014_scaffold12132_1	non-phage	0
DLF014_scaffold9087_3	non-phage	0
DLF014_scaffold7105_2	non-phage	0
DLF014_scaffold5731_6	non-phage	0
DLF014_scaffold10166_1	phage	0.982
DLF014_scaffold2234_7	non-phage	0
DLF014_scaffold3328_13	non-phage	0
DLF014_scaffold10626_1	non-phage	0
DLF014_scaffold11387_1	non-phage	0
DLF014_scaffold5047_1	non-phage	0
DLF014_scaffold2408_4	non-phage	0
DLF014_scaffold2322_2	non-phage	0
DLF014_scaffold6182_1	non-phage	0
DLF014_scaffold3010_3	non-phage	0
DLF014_scaffold11369_1	non-phage	0
DLF014_scaffold614_1	non-phage	0
DLF014_scaffold12650_5	non-phage	0
DLF014_scaffold6855_2	non-phage	0
DLF014_scaffold1006_3	non-phage	0
DLF014_scaffold1211_2	non-phage	0
DLF014_scaffold2812_5	non-phage	0
DLF014_scaffold10381_2	non-phage	0
DLF014_scaffold6182_8	non-phage	0
DLF014_scaffold5477_1	non-phage	0
DLF014_scaffold12552_1	non-phage	0
DLF014_scaffold12757_2	non-phage	0

DLF014_scaffold10780_1	non-phage	0
DLF014_scaffold26_1	non-phage	0.026
DLF014_scaffold5538_2	non-phage	0
DLF014_scaffold7097_1	non-phage	0
DLF014_scaffold12493_1	non-phage	0
DLF014_scaffold4113_6	non-phage	0
DLF014_C195935_1	non-phage	0
DLF014_scaffold12062_4	non-phage	0
DLF014_scaffold12665_1	phage	0.997
DLF014_scaffold9269_2	non-phage	0
DLF014_scaffold6343_1	non-phage	0
DLF014_scaffold12775_2	non-phage	0
DLF014_scaffold291_3	non-phage	0
DLF014_scaffold10436_14	non-phage	0
DLF014_scaffold10828_1	non-phage	0
DLF014_scaffold28_3	non-phage	0
DLF014_scaffold1686_7	non-phage	0
DLF014_scaffold12371_2	non-phage	0
DLF014_scaffold4597_1	non-phage	0
DLF014_scaffold10460_2	non-phage	0
DLF014_C196047_1	non-phage	0
DLF014_scaffold12564_1	non-phage	0
DLF014_scaffold5681_2	non-phage	0
DLF014_scaffold12577_1	non-phage	0
DLF014_scaffold3055_3	non-phage	0
DLF014_scaffold9366_1	non-phage	0
DLF014_scaffold12774_2	non-phage	0
DLF014_scaffold3598_1	non-phage	0
DLF014_scaffold12056_3	non-phage	0
DLF014_scaffold12402_7	non-phage	0
DLF014_scaffold12688_1	non-phage	0
DLF014_scaffold12613_3	non-phage	0
DLF014_scaffold9631_4	non-phage	0.005
DLF014_scaffold134_5	non-phage	0
DLF014_scaffold11807_3	non-phage	0
DLF014_scaffold10589_1	non-phage	0
DLF014_scaffold4991_2	non-phage	0
DLF014_scaffold9_4	non-phage	0
DLF014_scaffold2093_2	non-phage	0
DLF014_scaffold9120_1	non-phage	0
DLF014_scaffold8_2	phage	0.998
DLF014_scaffold12402_5	non-phage	0
DLF014_scaffold11960_4	non-phage	0
DLF014_scaffold672_1	non-phage	0
DLF014_scaffold11998_8	non-phage	0
DLF014_scaffold4556_1	non-phage	0

DLF014_scaffold8436_2	phage	0.99
DLF014_scaffold7169_7	non-phage	0
DLF014_scaffold12761_2	non-phage	0
DLF014_scaffold3477_8	non-phage	0
DLF014_scaffold12283_1	non-phage	0
DLF014_scaffold12771_1	non-phage	0
DLF014_scaffold4883_1	non-phage	0
DLF014_scaffold911_2	non-phage	0
DLF014_scaffold9453_4	non-phage	0
DLF014_scaffold222_8	non-phage	0
DLF014_scaffold12438_2	non-phage	0
DLF014_scaffold9953_4	non-phage	0
DLF014_scaffold4596_4	non-phage	0
DLF014_scaffold895_3	non-phage	0
DLF014_scaffold5223_1	non-phage	0
DLF014_scaffold10828_2	non-phage	0
DLF014_scaffold5719_3	non-phage	0
DLF014_scaffold11624_2	non-phage	0
DLF014_scaffold9269_5	non-phage	0
DLF014_scaffold738_1	non-phage	0
DLF014_scaffold12725_1	non-phage	0
DLF014_scaffold5486_2	non-phage	0
DLF014_scaffold8584_1	non-phage	0
DLF014_scaffold12595_1	non-phage	0
DLF014_scaffold3642_1	non-phage	0
DLF014_scaffold129_4	non-phage	0
DLF014_scaffold12673_1	non-phage	0
DLF014_scaffold614_2	non-phage	0
DLF014_scaffold134_2	non-phage	0
DLF014_scaffold6182_5	non-phage	0
DLF014_scaffold11526_2	non-phage	0
DLF014_scaffold9372_1	non-phage	0
DLF014_scaffold6330_1	non-phage	0
DLF014_scaffold2408_2	non-phage	0
DLF014_scaffold8459_1	non-phage	0
DLF014_scaffold8300_1	phage	0.999
DLF014_scaffold8506_1	non-phage	0
DLF014_scaffold2812_7	non-phage	0
DLF014_scaffold12638_1	non-phage	0
DLF014_scaffold11525_2	non-phage	0
DLF014_scaffold5468_18	non-phage	0
DLF014_scaffold9120_6	non-phage	0
DLF014_scaffold10999_1	non-phage	0
DLF014_scaffold12088_1	non-phage	0
DLF014_scaffold1313_4	non-phage	0
DLF014_scaffold7985_1	non-phage	0

DLF014_scaffold9459_2	non-phage	0
DLF014_scaffold3999_2	non-phage	0
DLF014_scaffold5731_4	non-phage	0
DLF014_scaffold12791_1	non-phage	0
DLF014_scaffold12652_1	non-phage	0
DLF014_scaffold7586_2	non-phage	0
DLF014_scaffold11540_1	non-phage	0
DLF014_scaffold9453_1	non-phage	0
DLF014_scaffold9096_5	non-phage	0
DLF014_scaffold925_3	non-phage	0
DLF014_scaffold12618_4	non-phage	0
DLF014_C195667_1	non-phage	0
DLF014_scaffold12672_1	non-phage	0
DLF014_scaffold6560_2	non-phage	0
DLF014_scaffold9_2	non-phage	0
DLF014_scaffold3927_3	non-phage	0
DLF014_scaffold12631_6	non-phage	0
DLF014_scaffold5363_6	phage	0.99
DLF014_scaffold12724_2	non-phage	0
DLF014_scaffold11321_2	non-phage	0
DLF014_scaffold10265_8	non-phage	0
DLF014_scaffold3230_2	non-phage	0
DLF014_scaffold12751_3	non-phage	0
DLF014_scaffold872_3	non-phage	0
DLF014_scaffold10436_13	non-phage	0
DLF014_scaffold12554_2	non-phage	0
DLF014_scaffold12493_11	non-phage	0
DLF014_scaffold10626_3	non-phage	0
DLF014_scaffold12545_1	non-phage	0
DLF014_scaffold1802_5	non-phage	0
DLF014_scaffold5194_1	non-phage	0
DLF014_scaffold12588_1	non-phage	0
DLF014_scaffold8775_4	non-phage	0
DLF014_scaffold3418_3	non-phage	0
DLF014_scaffold5927_4	non-phage	0
DLF014_scaffold2627_3	non-phage	0
DLF014_scaffold9925_1	non-phage	0
DLF014_scaffold895_1	non-phage	0
DLF014_scaffold5363_11	non-phage	0
DLF014_scaffold9106_1	non-phage	0
DLF014_scaffold3927_6	non-phage	0
DLF014_scaffold8300_3	non-phage	0
DLF014_scaffold6782_6	non-phage	0
DLF014_scaffold8435_3	non-phage	0
DLF014_scaffold811_1	non-phage	0
DLF014_scaffold12658_1	non-phage	0

DLF014_scaffold7522_1	non-phage	0
DLF014_scaffold12666_1	non-phage	0
DLF014_scaffold5363_21	phage	0.998
DLF014_C196521_1	non-phage	0
DLF014_scaffold7374_2	non-phage	0
DLF014_scaffold5547_1	non-phage	0
DLF014_scaffold4203_6	non-phage	0
DLF014_scaffold12783_1	non-phage	0
DLF014_scaffold12421_1	non-phage	0
DLF014_scaffold6805_1	non-phage	0
DLF014_scaffold58_5	non-phage	0
DLF014_scaffold7677_8	non-phage	0
DLF014_scaffold12637_1	non-phage	0
DLF014_scaffold10759_1	non-phage	0
DLF014_scaffold11998_2	non-phage	0
DLF014_scaffold12649_2	non-phage	0
DLF014_scaffold12720_4	non-phage	0
DLF014_scaffold6782_3	non-phage	0
DLF014_scaffold5193_5	non-phage	0
DLF014_scaffold12633_4	non-phage	0
DLF014_scaffold11787_1	non-phage	0
DLF014_scaffold12413_1	non-phage	0
DLF014_scaffold5546_2	non-phage	0
DLF014_scaffold2513_9	non-phage	0
DLF014_scaffold5363_18	non-phage	0
DLF014_scaffold11944_1	non-phage	0
DLF014_scaffold12276_1	non-phage	0
DLF014_scaffold12631_7	non-phage	0
DLF014_scaffold2386_1	non-phage	0
DLF014_scaffold4991_5	non-phage	0
DLF014_scaffold12634_1	non-phage	0
DLF014_scaffold11613_2	non-phage	0
DLF014_scaffold62_1	non-phage	0
DLF014_scaffold513_18	non-phage	0
DLF014_scaffold8386_5	non-phage	0
DLF014_scaffold1993_1	non-phage	0
DLF014_scaffold12484_1	non-phage	0
DLF014_scaffold12488_1	non-phage	0
DLF014_scaffold1924_3	non-phage	0
DLF014_scaffold3055_1	non-phage	0
DLF014_scaffold12651_1	non-phage	0
DLF014_scaffold12681_3	non-phage	0
DLF014_scaffold12782_1	non-phage	0
DLF014_scaffold2344_5	non-phage	0
DLF014_scaffold10999_2	non-phage	0
DLF014_scaffold4764_1	non-phage	0

DLF014_scaffold4636_1	non-phage	0
DLF014_scaffold12757_1	non-phage	0
DLF014_scaffold1853_3	non-phage	0
DLF014_scaffold2264_3	non-phage	0
DLF014_scaffold10956_1	non-phage	0
DLF014_scaffold12492_3	non-phage	0
DLF014_scaffold12676_3	non-phage	0
DLF014_scaffold454_1	non-phage	0
DLF014_scaffold12516_2	non-phage	0
DLF014_scaffold11967_2	phage	0.999
DLF014_scaffold3628_20	non-phage	0
DLF014_scaffold6855_1	phage	0.998
DLF014_C195115_1	non-phage	0
DLF014_scaffold221_3	non-phage	0
DLF014_scaffold68_5	non-phage	0
DLF014_scaffold12650_2	non-phage	0
DLF014_scaffold1924_1	non-phage	0
DLF014_scaffold1392_4	non-phage	0
DLF014_scaffold9435_4	non-phage	0
DLF014_scaffold12514_1	non-phage	0
DLF014_scaffold12706_2	non-phage	0
DLF014_scaffold12405_1	non-phage	0
DLF014_scaffold12663_1	non-phage	0
DLF014_scaffold2657_1	non-phage	0
DLF014_scaffold3505_1	non-phage	0
DLF014_scaffold6560_4	non-phage	0
DLF014_scaffold5425_5	non-phage	0
DLF014_scaffold753_4	non-phage	0
DLF014_scaffold8629_3	non-phage	0
DLF014_scaffold3620_2	non-phage	0
DLF014_scaffold4982_7	non-phage	0
DLF014_scaffold2932_3	phage	0.997
DLF014_scaffold6699_1	non-phage	0
DLF014_scaffold3065_6	non-phage	0
DLF014_scaffold12689_1	non-phage	0
DLF014_scaffold2408_1	non-phage	0
DLF014_scaffold9246_1	non-phage	0
DLF014_C195621_1	non-phage	0
DLF014_scaffold2513_11	non-phage	0
DLF014_scaffold8712_1	non-phage	0
DLF014_scaffold12545_2	non-phage	0
DLF014_scaffold846_4	non-phage	0
DLF014_scaffold8789_3	non-phage	0
DLF014_scaffold8062_1	non-phage	0
DLF014_scaffold5560_1	non-phage	0
DLF014_scaffold12777_2	non-phage	0

DLF014_scaffold6327_2	non-phage	0
DLF014_scaffold12683_2	non-phage	0
DLF014_scaffold6855_6	non-phage	0
DLF014_scaffold5097_1	non-phage	0
DLF014_scaffold11117_3	non-phage	0
DLF014_scaffold12698_1	non-phage	0
DLF014_scaffold12724_3	non-phage	0
DLF014_scaffold9830_2	non-phage	0
DLF014_scaffold12631_3	non-phage	0
DLF014_scaffold12402_2	non-phage	0
DLF014_scaffold6255_3	non-phage	0
DLF014_scaffold2697_1	non-phage	0
DLF014_scaffold2400_5	non-phage	0
DLF014_scaffold2950_2	non-phage	0
DLF014_scaffold12790_2	non-phage	0
DLF014_scaffold10436_7	non-phage	0
DLF014_scaffold4596_5	non-phage	0
DLF014_scaffold12453_3	non-phage	0
DLF014_scaffold10847_1	non-phage	0
DLF014_scaffold10824_2	non-phage	0
DLF014_scaffold2882_1	non-phage	0
DLF014_scaffold12794_2	non-phage	0
DLF014_scaffold10824_5	non-phage	0
DLF014_scaffold3474_1	non-phage	0
DLF014_scaffold7020_2	non-phage	0
DLF014_scaffold454_5	non-phage	0
DLF014_scaffold12618_1	non-phage	0
DLF014_scaffold1750_2	non-phage	0
DLF014_scaffold12566_1	non-phage	0
DLF014_scaffold137_2	non-phage	0
DLF014_scaffold2735_2	non-phage	0
DLF014_scaffold12679_1	non-phage	0
DLF014_scaffold12595_5	non-phage	0
DLF014_scaffold1767_9	non-phage	0
DLF014_scaffold11502_1	non-phage	0
DLF014_scaffold6185_2	non-phage	0
DLF014_scaffold2779_6	non-phage	0
DLF014_scaffold7731_2	non-phage	0
DLF014_C196185_1	non-phage	0
DLF014_scaffold6155_2	non-phage	0
DLF014_scaffold6258_2	non-phage	0
DLF014_scaffold6782_8	non-phage	0
DLF014_scaffold4518_1	non-phage	0
DLF014_scaffold5957_1	non-phage	0
DLF014_scaffold7522_2	non-phage	0
DLF014_scaffold10901_1	non-phage	0

DLF014_scaffold12772_1	non-phage	0
DLF014_scaffold2029_18	non-phage	0
DLF014_scaffold12747_2	non-phage	0
DLF014_scaffold8233_3	non-phage	0
DLF014_scaffold11490_2	non-phage	0
DLF014_scaffold2093_4	phage	0.997
DLF014_scaffold9327_2	non-phage	0
DLF014_scaffold4982_1	non-phage	0
DLF014_scaffold4335_2	non-phage	0
DLF014_scaffold6152_1	non-phage	0
DLF014_scaffold6182_2	non-phage	0
DLF014_scaffold12606_1	non-phage	0
DLF014_scaffold12720_2	non-phage	0
DLF014_scaffold12781_1	non-phage	0
DLF014_scaffold12759_1	non-phage	0
DLF014_scaffold12700_6	non-phage	0
DLF014_scaffold2603_1	non-phage	0
DLF014_scaffold12753_3	non-phage	0
DLF014_scaffold8435_5	non-phage	0
DLF014_scaffold5719_2	non-phage	0
DLF014_scaffold542_3	non-phage	0
DLF014_scaffold2264_5	non-phage	0
DLF014_scaffold12276_3	non-phage	0
DLF014_scaffold5011_1	non-phage	0
DLF014_scaffold14_3	non-phage	0
DLF014_scaffold5386_2	non-phage	0
DLF014_scaffold781_2	non-phage	0
DLF014_scaffold443_2	non-phage	0
DLF014_scaffold12786_1	non-phage	0
DLF014_scaffold2738_1	non-phage	0
DLF014_scaffold7751_2	non-phage	0
DLF014_scaffold94_3	non-phage	0
DLF014_scaffold8386_7	non-phage	0
DLF014_scaffold5159_1	non-phage	0
DLF014_scaffold8630_2	non-phage	0
DLF014_scaffold7863_1	non-phage	0
DLF014_scaffold1228_2	non-phage	0
DLF014_scaffold222_3	non-phage	0
DLF014_scaffold12797_1	non-phage	0
DLF014_scaffold7677_4	non-phage	0
DLF014_scaffold12493_5	non-phage	0
DLF014_scaffold11489_1	non-phage	0
DLF014_scaffold7373_2	non-phage	0
DLF014_scaffold3183_1	non-phage	0
DLF014_scaffold12650_3	non-phage	0
DLF014_scaffold55_1	non-phage	0

DLF014_scaffold8745_9	non-phage	0
DLF014_scaffold12543_3	non-phage	0
DLF014_scaffold6889_2	non-phage	0
DLF014_scaffold6766_1	non-phage	0
DLF014_scaffold1494_13	non-phage	0
DLF014_scaffold12751_4	non-phage	0
DLF014_scaffold5451_25	non-phage	0
DLF014_scaffold9911_5	non-phage	0
DLF014_scaffold68_9	non-phage	0
DLF014_scaffold6266_1	non-phage	0
DLF014_scaffold129_3	non-phage	0
DLF014_scaffold6886_2	non-phage	0
DLF014_scaffold8215_2	non-phage	0
DLF014_scaffold12631_1	non-phage	0
DLF014_scaffold12383_2	phage	0.998
DLF014_scaffold1006_5	non-phage	0
DLF014_scaffold2234_4	non-phage	0
DLF014_scaffold12737_1	non-phage	0
DLF014_scaffold10205_1	non-phage	0
DLF014_scaffold12633_2	non-phage	0
DLF014_scaffold2513_4	non-phage	0
DLF014_scaffold6855_5	non-phage	0
DLF014_scaffold2203_11	non-phage	0
DLF014_scaffold89_2	non-phage	0
DLF014_scaffold12428_1	non-phage	0
DLF014_scaffold12749_1	non-phage	0
DLF014_scaffold8789_5	non-phage	0
DLF014_scaffold12277_1	non-phage	0
DLF014_scaffold7097_3	non-phage	0
DLF014_scaffold12751_7	non-phage	0
DLF014_scaffold12473_2	non-phage	0
DLF014_scaffold10166_3	non-phage	0
DLF014_scaffold12528_1	non-phage	0
DLF014_scaffold12768_2	non-phage	0
DLF014_scaffold12780_1	non-phage	0
DLF014_scaffold7731_1	non-phage	0
DLF001_scaffold11546_2	non-phage	0
DLF001_scaffold7_5	non-phage	0
DLF001_scaffold939_6	non-phage	0
DLF001_scaffold14791_4	non-phage	0
DLF001_scaffold16868_2	non-phage	0
DLF001_scaffold2555_18	non-phage	0
DLF001_scaffold18728_2	non-phage	0
DLF001_scaffold1831_2	non-phage	0
DLF001_scaffold18262_3	non-phage	0
DLF001_scaffold25877_1	non-phage	0

DLF001_scaffold17779_3	non-phage	0
DLF001_scaffold22842_2	non-phage	0
DLF001_scaffold24118_2	non-phage	0
DLF001_scaffold6692_1	non-phage	0
DLF001_scaffold42_6	non-phage	0
DLF001_scaffold2038_6	non-phage	0
DLF001_scaffold14031_2	non-phage	0
DLF001_scaffold14031_3	non-phage	0
DLF001_scaffold3102_16	non-phage	0
DLF001_scaffold1872_26	non-phage	0
DLF001_scaffold9005_10	non-phage	0
DLF001_scaffold7411_3	non-phage	0
DLF001_scaffold47_1	non-phage	0
DLF001_scaffold9005_8	non-phage	0
DLF001_scaffold18558_1	non-phage	0
DLF001_scaffold39_9	non-phage	0
DLF001_scaffold3102_19	non-phage	0
DLF001_scaffold18396_6	non-phage	0
DLF001_scaffold11546_4	non-phage	0
DLF001_scaffold45_3	non-phage	0
DLF001_scaffold2009_12	non-phage	0
DLF001_scaffold10872_3	non-phage	0
DLF001_scaffold2225_24	non-phage	0
DLF001_scaffold4002_15	non-phage	0
DLF001_scaffold5605_2	non-phage	0
DLF001_scaffold19350_2	non-phage	0
DLF001_scaffold18728_1	non-phage	0
DLF001_scaffold16_20	non-phage	0
DLF001_scaffold15922_7	non-phage	0
DLF001_scaffold1123_7	non-phage	0
DLF001_scaffold2038_9	non-phage	0
DLF001_scaffold4223_4	non-phage	0
DLF001_scaffold60_1	non-phage	0
DLF001_scaffold25_11	non-phage	0
DLF001_scaffold3362_8	non-phage	0
DLF001_scaffold18018_1	non-phage	0
DLF001_scaffold20624_5	non-phage	0
DLF001_scaffold9018_1	non-phage	0
DLF001_scaffold13003_4	non-phage	0
DLF001_scaffold16237_1	non-phage	0
DLF001_scaffold4153_14	non-phage	0
DLF001_scaffold24601_1	non-phage	0
DLF001_scaffold5236_1	non-phage	0
DLF001_scaffold12963_3	non-phage	0
DLF001_scaffold578_22	non-phage	0
DLF001_scaffold17628_4	non-phage	0

DLF001_scaffold11526_1	non-phage	0
DLF001_scaffold11273_4	non-phage	0
DLF001_scaffold717_32	non-phage	0
DLF001_scaffold26036_1	non-phage	0
DLF001_scaffold18648_2	non-phage	0
DLF001_scaffold26036_2	non-phage	0
DLF001_C323082_1	non-phage	0
DLF001_scaffold9721_1	non-phage	0
DLF001_scaffold15_1	non-phage	0
DLF001_scaffold45_4	non-phage	0
DLF001_C322960_1	non-phage	0
DLF001_scaffold11231_4	non-phage	0
DLF001_scaffold55_15	non-phage	0
DLF001_scaffold13_37	non-phage	0
DLF001_scaffold1119_3	non-phage	0
DLF001_scaffold54_2	non-phage	0
DLF001_scaffold25599_1	non-phage	0
DLF001_scaffold1440_6	non-phage	0
DLF001_scaffold8927_8	non-phage	0
DLF001_scaffold23326_3	non-phage	0
DLF001_scaffold4900_3	non-phage	0
DLF001_scaffold2699_3	non-phage	0
DLF001_scaffold5135_10	non-phage	0
DLF001_scaffold26031_3	non-phage	0
DLF001_scaffold5723_9	non-phage	0
DLF001_scaffold17884_13	non-phage	0
DLF001_scaffold46_9	non-phage	0
DLF001_scaffold15255_13	non-phage	0
DLF001_scaffold25653_1	non-phage	0
DLF001_scaffold24927_1	non-phage	0
DLF001_scaffold18424_1	non-phage	0
DLF001_scaffold11035_1	non-phage	0
DLF001_scaffold25933_1	non-phage	0
DLF001_scaffold3038_1	non-phage	0
DLF001_scaffold2414_19	non-phage	0
DLF001_scaffold2555_12	non-phage	0
DLF001_scaffold4776_2	non-phage	0
DLF001_scaffold22_57	non-phage	0
DLF001_scaffold29_4	non-phage	0
DLF001_scaffold6484_5	non-phage	0
DLF001_scaffold20212_1	non-phage	0
DLF001_scaffold26038_1	non-phage	0
DLF001_scaffold717_33	non-phage	0
DLF001_scaffold5528_8	non-phage	0
DLF001_scaffold84_15	non-phage	0
DLF001_scaffold7839_23	non-phage	0

DLF001_scaffold15493_5	non-phage	0
DLF001_scaffold25899_1	non-phage	0
DLF001_scaffold17662_1	non-phage	0
DLF001_scaffold1430_18	non-phage	0
DLF001_C322996_1	non-phage	0
DLF001_scaffold39_3	non-phage	0
DLF001_scaffold9963_4	non-phage	0
DLF001_scaffold20546_4	non-phage	0
DLF001_scaffold25977_1	non-phage	0
DLF001_scaffold7411_2	non-phage	0
DLF001_scaffold13_50	non-phage	0
DLF001_scaffold19350_1	non-phage	0
DLF001_scaffold25972_1	non-phage	0
DLF001_scaffold25_1	non-phage	0
DLF001_scaffold8593_1	non-phage	0
DLF001_scaffold9159_1	non-phage	0
DLF001_scaffold19421_8	non-phage	0
DLF001_scaffold4753_6	non-phage	0
DLF001_scaffold22895_1	non-phage	0
DLF001_scaffold12148_6	non-phage	0
DLF001_scaffold14163_1	non-phage	0
DLF001_scaffold22535_1	non-phage	0
DLF001_scaffold4223_3	non-phage	0
DLF001_scaffold18714_2	non-phage	0
DLF001_scaffold5723_13	non-phage	0
DLF001_scaffold25923_1	non-phage	0
DLF001_scaffold18599_3	non-phage	0
DLF001_scaffold2555_21	non-phage	0
DLF001_scaffold26027_1	non-phage	0
DLF001_scaffold25780_3	non-phage	0
DLF001_scaffold20994_2	non-phage	0
DLF001_scaffold6562_10	non-phage	0
DLF001_scaffold16754_1	non-phage	0
DLF001_scaffold25743_1	non-phage	0
DLF001_scaffold4115_3	non-phage	0
DLF001_scaffold11231_3	non-phage	0
DLF001_scaffold22960_2	non-phage	0
DLF001_scaffold39_18	non-phage	0
DLF001_scaffold22215_2	non-phage	0
DLF001_scaffold4981_1	non-phage	0
DLF001_scaffold8851_2	non-phage	0
DLF001_scaffold38_6	non-phage	0
DLF001_scaffold2225_40	non-phage	0
DLF001_scaffold18728_5	non-phage	0
DLF001_scaffold25625_1	non-phage	0
DLF001_scaffold42_5	non-phage	0

DLF001_scaffold3038_6	non-phage	0
DLF001_scaffold26003_1	non-phage	0
DLF001_scaffold3102_34	non-phage	0
DLF001_scaffold14262_4	non-phage	0
DLF001_scaffold16741_5	non-phage	0
DLF001_scaffold25554_1	non-phage	0
DLF001_scaffold3426_12	non-phage	0
DLF001_scaffold6229_15	non-phage	0
DLF001_scaffold7839_19	non-phage	0
DLF001_scaffold11181_2	non-phage	0
DLF001_scaffold16_15	non-phage	0
DLF001_scaffold8101_2	non-phage	0
DLF001_scaffold23103_4	non-phage	0
DLF001_scaffold9_6	non-phage	0
DLF001_scaffold8487_1	non-phage	0
DLF001_scaffold55_16	non-phage	0
DLF001_scaffold717_20	non-phage	0
DLF001_scaffold24198_1	non-phage	0
DLF001_scaffold12642_1	non-phage	0
DLF001_scaffold5135_11	non-phage	0
DLF001_scaffold14766_2	non-phage	0
DLF001_scaffold10374_2	non-phage	0
DLF001_scaffold24967_1	non-phage	0
DLF001_scaffold3362_5	non-phage	0
DLF001_scaffold22723_2	non-phage	0
DLF001_scaffold25993_1	non-phage	0
DLF001_scaffold23184_1	non-phage	0
DLF001_scaffold9018_6	non-phage	0
DLF001_scaffold4753_15	non-phage	0
DLF001_scaffold6749_1	non-phage	0
DLF001_scaffold25884_1	non-phage	0
DLF001_scaffold18617_15	non-phage	0
DLF001_scaffold9018_45	non-phage	0
DLF001_scaffold22226_4	non-phage	0
DLF001_scaffold19487_1	non-phage	0
DLF001_scaffold25175_1	non-phage	0
DLF001_C323810_1	non-phage	0
DLF001_scaffold23_64	non-phage	0
DLF001_scaffold12803_3	non-phage	0
DLF001_scaffold4799_5	non-phage	0
DLF001_scaffold9493_2	non-phage	0
DLF001_scaffold18617_14	non-phage	0
DLF001_scaffold20885_2	non-phage	0
DLF001_scaffold24609_2	non-phage	0
DLF001_scaffold20_1	non-phage	0
DLF001_scaffold2038_2	non-phage	0

DLF001_scaffold39_16	non-phage	0
DLF001_scaffold26043_3	non-phage	0
DLF001_scaffold39_1	non-phage	0
DLF001_scaffold5683_2	non-phage	0
DLF001_scaffold15747_2	non-phage	0
DLF001_scaffold7396_2	non-phage	0
DLF001_scaffold26034_1	non-phage	0
DLF001_scaffold25840_1	non-phage	0
DLF001_scaffold19182_2	non-phage	0
DLF001_scaffold370_1	non-phage	0
DLF001_scaffold26012_1	non-phage	0
DLF001_scaffold46_8	non-phage	0
DLF001_scaffold9403_1	non-phage	0
DLF001_scaffold12803_5	non-phage	0
DLF001_C323262_1	non-phage	0
DLF001_scaffold28_11	non-phage	0
DLF001_scaffold2816_15	non-phage	0
DLF001_scaffold6708_4	non-phage	0
DLF001_scaffold13_10	non-phage	0
DLF001_scaffold22370_2	non-phage	0
DLF001_scaffold7839_20	non-phage	0
DLF001_scaffold23_24	non-phage	0
DLF002_scaffold36513_3	non-phage	0
DLF002_scaffold2788_1	non-phage	0
DLF002_scaffold8081_1	non-phage	0
DLF002_scaffold7340_6	non-phage	0
DLF002_scaffold27783_1	non-phage	0
DLF002_scaffold72_13	non-phage	0
DLF002_scaffold36472_1	non-phage	0
DLF002_scaffold36266_3	non-phage	0
DLF002_scaffold36496_1	non-phage	0
DLF002_scaffold138_24	non-phage	0
DLF002_scaffold35878_2	non-phage	0
DLF002_scaffold35056_2	non-phage	0
DLF002_scaffold17567_2	non-phage	0
DLF002_scaffold33418_2	non-phage	0
DLF002_scaffold17676_3	non-phage	0
DLF002_scaffold545_3	non-phage	0
DLF002_scaffold21633_1	non-phage	0
DLF002_scaffold138_10	non-phage	0
DLF002_scaffold846_5	non-phage	0
DLF002_scaffold986_30	non-phage	0
DLF002_scaffold7277_4	non-phage	0
DLF002_scaffold36523_1	non-phage	0
DLF002_scaffold17786_7	non-phage	0
DLF002_scaffold17883_1	non-phage	0

DLF002_scaffold30665_2	non-phage	0
DLF002_scaffold36502_1	non-phage	0
DLF002_scaffold27044_1	non-phage	0
DLF002_scaffold35956_1	non-phage	0
DLF002_scaffold28_9	non-phage	0
DLF002_scaffold32436_5	non-phage	0
DLF002_scaffold36086_1	non-phage	0
DLF002_scaffold8249_5	non-phage	0
DLF002_scaffold3272_2	non-phage	0
DLF002_scaffold16149_21	non-phage	0
DLF002_scaffold31953_2	non-phage	0
DLF002_scaffold5170_1	non-phage	0
DLF002_scaffold9607_1	non-phage	0
DLF002_scaffold26856_1	non-phage	0
DLF002_scaffold7277_3	non-phage	0
DLF002_scaffold33848_4	non-phage	0
DLF002_scaffold10147_1	non-phage	0
DLF002_scaffold15360_12	non-phage	0
DLF002_scaffold127_2	non-phage	0
DLF002_scaffold18354_2	non-phage	0
DLF002_scaffold12006_3	non-phage	0
DLF002_scaffold31940_1	non-phage	0
DLF002_scaffold152_4	non-phage	0
DLF002_scaffold20138_12	non-phage	0
DLF002_scaffold35056_3	non-phage	0
DLF002_scaffold124_9	non-phage	0
DLF002_scaffold294_21	non-phage	0
DLF002_scaffold17786_8	non-phage	0
DLF002_scaffold7788_10	non-phage	0
DLF002_scaffold34879_2	non-phage	0
DLF002_scaffold7287_1	non-phage	0
DLF002_scaffold8361_2	non-phage	0
DLF002_scaffold36138_2	non-phage	0
DLF002_scaffold26328_1	non-phage	0
DLF002_scaffold8249_4	non-phage	0
DLF002_scaffold26985_2	non-phage	0
DLF002_scaffold2214_6	non-phage	0
DLF002_scaffold32019_3	non-phage	0
DLF002_scaffold35429_1	non-phage	0
DLF002_scaffold36086_2	non-phage	0
DLF002_scaffold27754_1	non-phage	0
DLF002_scaffold35294_1	non-phage	0
DLF002_scaffold34715_1	non-phage	0
DLF002_scaffold19705_13	non-phage	0
DLF002_scaffold25611_7	non-phage	0
DLF002_scaffold33453_1	non-phage	0

DLF002_scaffold10479_9	non-phage	0
DLF002_scaffold2892_3	non-phage	0
DLF002_scaffold5548_3	non-phage	0
DLF002_scaffold26934_4	non-phage	0
DLF002_scaffold5697_1	non-phage	0
DLF002_scaffold35012_3	non-phage	0
DLF002_scaffold36389_1	non-phage	0
DLF002_scaffold8669_8	non-phage	0
DLF002_scaffold36293_2	non-phage	0
DLF002_scaffold36335_1	non-phage	0
DLF002_C488323_1	non-phage	0
DLF002_scaffold10095_2	non-phage	0
DLF002_scaffold32224_3	non-phage	0
DLF002_scaffold25530_3	non-phage	0
DLF002_scaffold9606_4	non-phage	0
DLF002_scaffold19324_3	non-phage	0
DLF002_scaffold3272_4	non-phage	0
DLF002_scaffold36419_1	non-phage	0
DLF002_scaffold8257_1	non-phage	0
DLF002_scaffold875_19	non-phage	0
DLF002_scaffold11918_5	non-phage	0
DLF002_scaffold26193_1	non-phage	0
DLF002_scaffold5530_1	non-phage	0
DLF002_scaffold13173_2	non-phage	0
DLF002_scaffold29665_1	non-phage	0
DLF002_scaffold255_2	non-phage	0
DLF002_scaffold19705_9	non-phage	0
DLF002_scaffold172_5	non-phage	0
DLF002_scaffold6624_52	non-phage	0
DLF002_scaffold35617_1	non-phage	0
DLF002_scaffold35901_2	non-phage	0
DLF002_scaffold34431_1	non-phage	0
DLF002_C488611_1	non-phage	0
DLF002_scaffold13816_24	non-phage	0
DLF002_scaffold12838_4	non-phage	0
DLF002_scaffold19307_3	non-phage	0
DLF002_scaffold28634_1	non-phage	0
DLF002_scaffold36494_1	non-phage	0
DLF002_scaffold8669_11	non-phage	0
DLF002_scaffold138_48	non-phage	0
DLF002_scaffold21633_2	non-phage	0
DLF002_scaffold13204_2	non-phage	0
DLF002_scaffold36513_1	non-phage	0
DLF002_scaffold34338_1	non-phage	0
DLF002_scaffold25530_4	non-phage	0
DLF002_scaffold5329_2	non-phage	0

DLF002_scaffold31708_2	non-phage	0
DLF002_scaffold128_27	non-phage	0
DLF002_C488603_1	non-phage	0
DLF002_scaffold806_2	non-phage	0
DLF002_scaffold32019_1	non-phage	0
DLF002_scaffold16149_5	non-phage	0
DLF002_scaffold14980_3	non-phage	0
DLF002_scaffold289_4	non-phage	0
DLF002_scaffold128_17	non-phage	0
DLF002_scaffold28935_2	non-phage	0
DLF002_scaffold6624_51	non-phage	0
DLF002_scaffold26181_6	non-phage	0
DLF002_scaffold14419_2	non-phage	0
DLF002_scaffold31141_6	non-phage	0
DLF003_scaffold57115_1	non-phage	0
DLF003_scaffold16416_28	non-phage	0
DLF003_scaffold10073_1	non-phage	0
DLF003_C717722_1	non-phage	0
DLF003_scaffold56951_1	non-phage	0
DLF003_scaffold151_1	non-phage	0
DLF003_scaffold42085_1	non-phage	0
DLF003_scaffold18315_1	non-phage	0
DLF003_scaffold42084_1	non-phage	0
DLF003_scaffold136_6	non-phage	0
DLF003_scaffold56061_1	non-phage	0
DLF003_scaffold39106_2	non-phage	0
DLF003_scaffold11995_1	non-phage	0
DLF003_scaffold46061_1	non-phage	0
DLF003_scaffold10112_1	non-phage	0
DLF003_scaffold36943_4	non-phage	0
DLF003_scaffold20948_1	non-phage	0
DLF003_scaffold50902_4	non-phage	0
DLF003_scaffold50567_1	non-phage	0
DLF003_scaffold36236_1	non-phage	0
DLF003_scaffold108_2	non-phage	0
DLF003_scaffold9508_2	non-phage	0
DLF003_scaffold16606_2	non-phage	0
DLF003_C717664_1	non-phage	0
DLF003_scaffold2315_1	non-phage	0
DLF003_scaffold80_3	non-phage	0
DLF003_scaffold10123_9	non-phage	0
DLF003_scaffold53034_1	non-phage	0
DLF003_scaffold27569_3	non-phage	0
DLF003_scaffold13298_2	non-phage	0
DLF003_C717520_1	non-phage	0
DLF003_scaffold57140_2	non-phage	0

DLF003_scaffold56769_1	non-phage	0
DLF003_scaffold28889_1	non-phage	0
DLF003_scaffold34709_3	non-phage	0
DLF003_scaffold7994_2	non-phage	0
DLF003_scaffold54872_2	non-phage	0
DLF003_scaffold10171_1	non-phage	0
DLF003_scaffold1152_1	non-phage	0
DLF003_scaffold47828_1	non-phage	0
DLF003_scaffold51058_1	non-phage	0
DLF003_scaffold10170_11	non-phage	0
DLF003_scaffold29689_12	non-phage	0
DLF003_scaffold29943_8	non-phage	0
DLF003_scaffold44913_5	non-phage	0
DLF003_scaffold38169_1	non-phage	0
DLF003_scaffold54745_1	non-phage	0
DLF003_scaffold276_6	non-phage	0
DLF003_scaffold158_1	non-phage	0
DLF003_scaffold53366_2	non-phage	0
DLF003_scaffold168_3	non-phage	0
DLF003_scaffold107_4	non-phage	0
DLF003_scaffold57122_1	non-phage	0
DLF003_scaffold36407_1	non-phage	0
DLF003_scaffold1924_2	non-phage	0
DLF003_scaffold133_4	non-phage	0
DLF003_scaffold57030_1	non-phage	0
DLF003_scaffold48433_3	non-phage	0
DLF003_scaffold44053_1	non-phage	0
DLF003_scaffold327_3	non-phage	0
DLF003_scaffold35886_8	non-phage	0
DLF003_scaffold23409_3	non-phage	0
DLF003_scaffold49404_1	non-phage	0
DLF003_scaffold39680_11	non-phage	0
DLF003_scaffold4936_5	non-phage	0
DLF003_scaffold11995_3	non-phage	0
DLF003_scaffold7634_7	non-phage	0
DLF003_scaffold52415_4	non-phage	0
DLF003_scaffold7527_42	non-phage	0
DLF003_scaffold10586_1	non-phage	0
DLF003_scaffold55397_3	non-phage	0
DLF003_scaffold56819_1	non-phage	0
DLF003_scaffold57077_5	non-phage	0
DLF003_scaffold47403_3	non-phage	0
DLF003_scaffold3102_54	non-phage	0
DLF003_scaffold66_6	non-phage	0
DLF003_scaffold37127_1	non-phage	0
DLF003_scaffold3102_34	non-phage	0

DLF003_scaffold154_1	non-phage	0
DLF003_scaffold55257_1	non-phage	0
DLF003_scaffold29221_2	non-phage	0
DLF003_scaffold7634_2	non-phage	0
DLF003_scaffold34709_5	non-phage	0
DLF003_scaffold48930_1	non-phage	0
DLF003_scaffold36783_2	non-phage	0
DLF003_scaffold36782_2	non-phage	0
DLF003_scaffold33974_2	non-phage	0
DLF003_scaffold31129_11	non-phage	0
DLF003_scaffold37834_2	non-phage	0
DLF003_scaffold16938_2	non-phage	0
DLF003_scaffold11984_8	non-phage	0
DLF003_scaffold52984_5	non-phage	0
DLF004_scaffold32013_1	non-phage	0
DLF004_scaffold36596_1	non-phage	0
DLF004_scaffold20406_2	non-phage	0
DLF004_scaffold23723_6	non-phage	0
DLF004_scaffold29477_2	non-phage	0
DLF004_scaffold36554_1	non-phage	0
DLF004_scaffold20398_2	non-phage	0
DLF004_scaffold21102_2	non-phage	0
DLF004_scaffold16505_10	non-phage	0
DLF004_scaffold30280_3	non-phage	0
DLF004_scaffold35759_3	non-phage	0
DLF004_scaffold34696_1	non-phage	0
DLF004_scaffold21400_2	non-phage	0
DLF004_scaffold33778_1	non-phage	0
DLF004_scaffold36424_6	non-phage	0
DLF004_scaffold8390_20	non-phage	0
DLF004_scaffold36576_1	non-phage	0
DLF004_scaffold6537_3	non-phage	0
DLF004_C547862_1	non-phage	0
DLF004_scaffold651_12	non-phage	0
DLF004_scaffold16828_2	non-phage	0
DLF004_scaffold2105_3	non-phage	0
DLF004_scaffold23337_2	non-phage	0
DLF004_scaffold15124_6	non-phage	0
DLF004_scaffold2869_6	non-phage	0
DLF004_scaffold22277_1	non-phage	0
DLF004_scaffold9511_1	non-phage	0
DLF004_scaffold5637_1	non-phage	0
DLF004_scaffold20496_10	non-phage	0
DLF004_scaffold33220_1	non-phage	0
DLF004_scaffold36591_2	non-phage	0
DLF004_scaffold33116_6	non-phage	0

DLF004_scaffold162_4	non-phage	0
DLF004_scaffold29929_1	non-phage	0
DLF004_scaffold8385_1	non-phage	0
DLF004_scaffold7798_1	non-phage	0
DLF004_scaffold8396_1	non-phage	0
DLF004_scaffold5653_6	non-phage	0
DLF004_scaffold5994_6	non-phage	0
DLF004_scaffold18054_2	non-phage	0
DLF004_scaffold3603_3	non-phage	0
DLF004_scaffold36468_1	non-phage	0
DLF004_scaffold28_2	non-phage	0
DLF004_scaffold36594_1	non-phage	0
DLF004_scaffold4632_22	non-phage	0
DLF004_scaffold4725_11	non-phage	0
DLF004_scaffold22822_2	non-phage	0
DLF004_scaffold20406_1	non-phage	0
DLF004_scaffold21375_1	non-phage	0
DLF004_scaffold26019_1	non-phage	0
DLF004_scaffold27726_20	non-phage	0
DLF004_scaffold32113_1	non-phage	0
DLF004_scaffold30930_1	non-phage	0
DLF004_scaffold2870_4	non-phage	0
DLF004_scaffold36578_1	non-phage	0
DLF004_scaffold50_12	non-phage	0
DLF004_scaffold4408_3	non-phage	0
DLF004_scaffold28284_3	non-phage	0
DLF004_scaffold5225_14	non-phage	0
DLF004_scaffold36527_2	non-phage	0
DLF004_scaffold16828_3	non-phage	0
DLF004_scaffold20709_2	non-phage	0
DLF004_scaffold16420_22	non-phage	0
DLF004_scaffold18861_1	non-phage	0
DLF004_scaffold32365_3	non-phage	0
DLF004_scaffold34116_1	non-phage	0
DLF004_scaffold2204_1	non-phage	0
DLF004_scaffold17043_6	non-phage	0
DLF004_scaffold36588_2	non-phage	0
DLF004_scaffold6537_6	non-phage	0
DLF004_scaffold36442_1	non-phage	0
DLF004_scaffold31616_3	non-phage	0
DLF004_scaffold17901_9	non-phage	0
DLF004_scaffold9651_1	non-phage	0
DLF004_scaffold29715_3	non-phage	0
DLF004_scaffold18471_2	non-phage	0
DLF004_scaffold33116_5	non-phage	0
DLF004_scaffold10622_2	non-phage	0

DLF004_scaffold9639_1	non-phage	0
DLF004_scaffold31581_3	non-phage	0
DLF004_scaffold17208_2	non-phage	0
DLF004_scaffold35418_2	non-phage	0
DLF004_scaffold53_5	non-phage	0
DLF004_scaffold26937_1	non-phage	0
DLF004_scaffold17874_2	non-phage	0
DLF004_scaffold9363_1	non-phage	0
DLF004_scaffold17271_1	non-phage	0
DLF004_scaffold36521_1	non-phage	0
DLF004_scaffold29715_11	non-phage	0
DLF004_scaffold3423_22	non-phage	0
DLF004_scaffold2418_10	non-phage	0
DLF004_scaffold5567_4	non-phage	0
DLF004_scaffold26827_3	non-phage	0
DLF004_scaffold53_2	non-phage	0
DLF004_scaffold30047_1	non-phage	0
DLF004_scaffold27435_3	non-phage	0
DLF004_scaffold13080_11	non-phage	0
DLF004_scaffold7789_3	non-phage	0
DLF004_scaffold55_6	non-phage	0
DLF004_scaffold1299_38	non-phage	0
DLF004_scaffold5516_3	non-phage	0
DLF004_scaffold5653_4	non-phage	0
DLF004_scaffold33287_1	non-phage	0
DLF004_scaffold3644_2	non-phage	0
DLF004_scaffold36355_1	non-phage	0
DLF004_scaffold36546_1	non-phage	0
DLF004_scaffold32532_1	non-phage	0
DLF004_scaffold36183_1	non-phage	0
DLF004_scaffold30271_1	non-phage	0
DLF004_scaffold3589_4	non-phage	0
DLF004_scaffold9673_3	non-phage	0
DLF004_scaffold27201_1	non-phage	0
DLF004_scaffold4681_18	non-phage	0
DLF004_scaffold25229_2	non-phage	0
DLF004_scaffold11849_4	non-phage	0
DLF004_scaffold36409_1	non-phage	0
DLF004_scaffold876_11	non-phage	0
DLF004_scaffold3569_1	non-phage	0
DLF004_scaffold10898_13	non-phage	0
DLF004_scaffold6537_5	non-phage	0
DLF004_scaffold80_5	non-phage	0
DLF004_scaffold36508_1	non-phage	0
DLF004_scaffold8390_11	non-phage	0
DLF004_scaffold3669_1	non-phage	0

DLF004_C548008_1	non-phage	0
DLF004_scaffold36598_1	non-phage	0
DLF004_scaffold3669_4	non-phage	0
DLF004_scaffold12768_7	non-phage	0
DLF004_scaffold973_1	non-phage	0
DLF004_scaffold30471_2	non-phage	0
DLF004_C547930_1	non-phage	0
DLF004_scaffold20398_3	non-phage	0
DLF004_scaffold27726_1	non-phage	0
DLF004_scaffold36308_3	non-phage	0
DLF004_scaffold18755_2	non-phage	0
DLF004_scaffold30116_1	non-phage	0
DLF004_scaffold33287_2	non-phage	0
DLF004_scaffold14410_1	non-phage	0
DLF004_scaffold17301_21	non-phage	0
DLF004_scaffold35009_1	non-phage	0
DLF004_scaffold21342_1	non-phage	0
DLF004_scaffold32638_2	non-phage	0
DLF004_scaffold35542_1	non-phage	0
DLF004_scaffold3644_3	non-phage	0
DLF004_scaffold35272_1	non-phage	0
DLF004_scaffold8390_15	non-phage	0
DLF004_scaffold36529_3	non-phage	0
DLF004_scaffold15328_2	non-phage	0
DLF004_scaffold30280_4	non-phage	0
DLF004_scaffold10640_1	non-phage	0
DLF004_scaffold181_4	non-phage	0
DLF004_scaffold14591_2	non-phage	0
DLF004_scaffold7502_5	non-phage	0
DLF004_scaffold7502_1	non-phage	0
DLF004_scaffold118_4	non-phage	0
DLF004_scaffold26804_1	non-phage	0
DLF004_scaffold16828_1	non-phage	0
DLF004_scaffold35320_3	non-phage	0
DLF004_scaffold32483_1	non-phage	0
DLF004_scaffold162_1	non-phage	0
DLF004_scaffold34925_3	non-phage	0
DLF004_scaffold17382_2	non-phage	0
DLF004_scaffold20398_4	non-phage	0
DLF004_scaffold7020_2	non-phage	0
DLF004_scaffold25229_1	non-phage	0
DLF004_scaffold29929_5	non-phage	0
DLF004_scaffold34692_1	non-phage	0
DLF004_scaffold26834_1	non-phage	0
DLF004_scaffold104_7	non-phage	0
DLF004_scaffold19326_2	non-phage	0

DLF004_scaffold2195_2	non-phage	0
DLF004_scaffold3644_7	non-phage	0
DLF004_scaffold33441_3	non-phage	0
DLF005_scaffold21559_3	non-phage	0
DLF005_scaffold7661_11	non-phage	0
DLF005_scaffold41287_2	non-phage	0
DLF005_scaffold36201_3	non-phage	0
DLF005_scaffold41201_6	non-phage	0
DLF005_scaffold41863_3	non-phage	0
DLF005_scaffold56462_3	non-phage	0
DLF005_scaffold56485_3	non-phage	0
DLF005_scaffold56992_1	non-phage	0
DLF005_scaffold56189_1	non-phage	0
DLF005_scaffold8490_5	non-phage	0
DLF005_scaffold50427_1	non-phage	0
DLF005_scaffold36061_6	non-phage	0
DLF005_scaffold14721_3	non-phage	0
DLF005_scaffold55741_2	non-phage	0
DLF005_scaffold51024_2	non-phage	0
DLF005_scaffold35642_4	non-phage	0
DLF005_scaffold27950_2	non-phage	0
DLF005_scaffold52167_1	non-phage	0
DLF005_scaffold119_13	non-phage	0
DLF005_scaffold5492_11	non-phage	0
DLF005_scaffold56978_2	non-phage	0
DLF005_scaffold43084_3	non-phage	0
DLF005_scaffold42045_1	non-phage	0
DLF005_scaffold56732_2	non-phage	0
DLF005_scaffold5492_10	non-phage	0
DLF005_scaffold41874_1	non-phage	0
DLF005_scaffold42922_2	non-phage	0
DLF005_C847520_1	non-phage	0
DLF005_scaffold110_6	non-phage	0
DLF005_scaffold99_4	non-phage	0
DLF005_scaffold33412_2	non-phage	0
DLF005_scaffold3726_1	non-phage	0
DLF005_scaffold2599_7	non-phage	0
DLF005_scaffold49505_2	non-phage	0
DLF005_scaffold51799_1	non-phage	0
DLF005_scaffold13131_3	non-phage	0
DLF005_scaffold56569_1	non-phage	0
DLF005_scaffold1242_44	non-phage	0
DLF005_scaffold26271_4	non-phage	0
DLF005_scaffold13577_2	non-phage	0
DLF005_scaffold28014_2	non-phage	0
DLF005_scaffold24081_13	non-phage	0

DLF005_scaffold56922_1	non-phage	0
DLF005_scaffold55741_3	non-phage	0
DLF005_scaffold41894_1	non-phage	0
DLF005_scaffold39841_1	non-phage	0
DLF005_scaffold3540_25	non-phage	0
DLF005_scaffold56972_1	non-phage	0
DLF005_scaffold35811_2	non-phage	0
DLF005_scaffold56987_3	non-phage	0
DLF005_scaffold55644_2	non-phage	0
DLF005_scaffold51530_2	non-phage	0
DLF005_scaffold267_12	non-phage	0
DLF005_scaffold52894_1	non-phage	0
DLF005_scaffold38532_1	non-phage	0
DLF005_scaffold22020_10	non-phage	0
DLF005_scaffold53686_1	non-phage	0
DLF005_scaffold17120_1	non-phage	0
DLF005_scaffold1767_5	non-phage	0
DLF005_scaffold25448_1	non-phage	0
DLF005_scaffold20201_30	non-phage	0
DLF005_scaffold45447_3	non-phage	0
DLF005_scaffold27905_7	non-phage	0
DLF005_scaffold49998_1	non-phage	0
DLF005_scaffold225_8	non-phage	0
DLF005_scaffold56774_1	non-phage	0
DLF005_scaffold47678_1	non-phage	0
DLF005_scaffold4824_41	non-phage	0
DLF005_scaffold2379_7	non-phage	0
DLF005_scaffold33322_1	non-phage	0
DLF005_scaffold25882_1	non-phage	0
DLF005_scaffold38601_2	non-phage	0
DLF005_scaffold52169_2	non-phage	0
DLF005_scaffold6811_7	non-phage	0
DLF005_scaffold51641_1	non-phage	0
DLF005_C847640_1	non-phage	0
DLF005_scaffold41863_2	non-phage	0
DLF005_C847638_1	non-phage	0
DLF005_scaffold52997_3	non-phage	0
DLF005_scaffold46702_6	non-phage	0
DLF005_scaffold45156_1	non-phage	0
DLF005_C847938_1	non-phage	0
DLF005_scaffold41947_1	non-phage	0
DLF005_scaffold24559_1	non-phage	0
DLF005_scaffold55743_1	non-phage	0
DLF005_scaffold1566_21	non-phage	0
DLF005_scaffold46767_3	non-phage	0
DLF005_scaffold294_1	non-phage	0

DLF005_scaffold1566_10	non-phage	0
DLF005_scaffold30718_1	non-phage	0
DLF005_scaffold1412_33	non-phage	0
DLF005_scaffold906_2	non-phage	0
DLF005_C848044_1	non-phage	0
DLF005_scaffold51642_1	non-phage	0
DLF005_scaffold56380_4	non-phage	0
DLF005_scaffold36201_1	non-phage	0
DLF005_scaffold22507_3	non-phage	0
DLF005_scaffold26496_2	non-phage	0
DLF005_scaffold56904_1	non-phage	0
DLF005_scaffold8242_35	non-phage	0
DLF005_scaffold28276_3	non-phage	0
DLF005_scaffold56981_2	non-phage	0
DLF005_scaffold48200_1	non-phage	0
DLF005_scaffold53277_3	non-phage	0
DLF005_scaffold43945_2	non-phage	0
DLF005_scaffold9574_2	non-phage	0
DLF005_scaffold56986_2	non-phage	0
DLF005_scaffold56786_1	non-phage	0
DLF005_scaffold23988_8	non-phage	0
DLF005_scaffold2503_1	non-phage	0
DLF005_scaffold11809_1	non-phage	0
DLF005_scaffold742_18	non-phage	0
DLF005_scaffold46493_1	non-phage	0
DLF006_scaffold46571_2	non-phage	0
DLF006_scaffold22659_1	non-phage	0
DLF006_scaffold43446_1	non-phage	0
DLF006_scaffold14721_9	non-phage	0
DLF006_scaffold4059_2	non-phage	0
DLF006_scaffold28546_1	non-phage	0
DLF006_scaffold37381_3	non-phage	0
DLF006_scaffold558_6	non-phage	0
DLF006_scaffold2090_24	non-phage	0
DLF006_scaffold9354_1	non-phage	0
DLF006_scaffold3598_4	non-phage	0
DLF006_C667766_1	non-phage	0
DLF006_scaffold474_5	non-phage	0
DLF006_scaffold9366_9	non-phage	0
DLF006_scaffold22006_17	non-phage	0
DLF006_scaffold2040_1	non-phage	0
DLF006_scaffold43843_1	non-phage	0
DLF006_scaffold34635_1	non-phage	0
DLF006_scaffold6687_2	non-phage	0
DLF006_scaffold12579_2	non-phage	0
DLF006_scaffold7466_6	non-phage	0

DLF006_scaffold1108_2	non-phage	0
DLF006_scaffold37381_4	non-phage	0
DLF006_scaffold9584_7	non-phage	0
DLF006_scaffold29588_1	non-phage	0
DLF006_scaffold15815_9	non-phage	0
DLF006_scaffold1668_4	non-phage	0
DLF006_scaffold39940_1	non-phage	0
DLF006_scaffold46245_1	non-phage	0
DLF006_scaffold46657_1	non-phage	0
DLF006_scaffold20006_6	non-phage	0
DLF006_scaffold12199_2	non-phage	0
DLF006_scaffold15815_24	non-phage	0
DLF006_scaffold19474_9	non-phage	0
DLF006_scaffold234_7	non-phage	0
DLF006_scaffold2636_9	non-phage	0
DLF006_scaffold43200_1	non-phage	0
DLF006_scaffold2685_1	non-phage	0
DLF006_scaffold42698_3	non-phage	0
DLF006_scaffold46517_2	non-phage	0
DLF006_scaffold34472_4	non-phage	0
DLF006_scaffold22006_23	non-phage	0
DLF006_scaffold46849_1	non-phage	0
DLF006_scaffold30721_1	non-phage	0
DLF006_scaffold46701_3	non-phage	0
DLF006_scaffold41814_2	non-phage	0
DLF006_scaffold46057_3	non-phage	0
DLF006_scaffold5230_6	non-phage	0
DLF006_scaffold24857_2	non-phage	0
DLF006_scaffold45209_1	non-phage	0
DLF006_scaffold7466_12	non-phage	0
DLF006_scaffold36792_1	non-phage	0
DLF006_scaffold38364_5	non-phage	0
DLF006_scaffold11481_10	non-phage	0
DLF006_scaffold5222_1	non-phage	0
DLF006_scaffold9056_25	non-phage	0
DLF006_scaffold3325_35	non-phage	0
DLF006_scaffold25840_3	non-phage	0
DLF006_scaffold16003_1	non-phage	0
DLF006_scaffold46810_1	non-phage	0
DLF006_scaffold16999_1	non-phage	0
DLF006_scaffold20006_7	non-phage	0
DLF006_scaffold520_12	non-phage	0
DLF006_scaffold43519_1	non-phage	0
DLF006_scaffold5383_3	non-phage	0
DLF006_C667854_1	non-phage	0
DLF006_scaffold40303_3	non-phage	0

DLF006_scaffold20275_4	non-phage	0
DLF006_scaffold11428_1	non-phage	0
DLF006_scaffold38598_2	non-phage	0
DLF006_scaffold3136_10	non-phage	0
DLF006_scaffold46833_2	non-phage	0
DLF006_scaffold566_1	non-phage	0
DLF006_scaffold46844_2	non-phage	0
DLF006_scaffold8915_27	non-phage	0
DLF006_scaffold2524_8	non-phage	0
DLF006_scaffold46775_2	non-phage	0
DLF006_scaffold9165_3	non-phage	0
DLF006_scaffold4305_1	non-phage	0
DLF006_scaffold22006_31	non-phage	0
DLF006_scaffold9366_7	non-phage	0
DLF006_scaffold22006_1	non-phage	0
DLF006_C667706_1	non-phage	0
DLF006_scaffold9895_3	non-phage	0
DLF006_scaffold7233_8	non-phage	0
DLF006_scaffold7086_4	non-phage	0
DLF006_scaffold16305_6	non-phage	0
DLF006_scaffold31124_1	non-phage	0
DLF006_scaffold423_21	non-phage	0
DLF006_scaffold9601_2	non-phage	0
DLF006_C667616_1	non-phage	0
DLF006_C667724_1	non-phage	0
DLF006_scaffold46623_1	non-phage	0
DLF006_scaffold161_4	non-phage	0
DLF006_C667472_1	non-phage	0
DLF006_scaffold43812_2	non-phage	0
DLF006_scaffold46245_3	non-phage	0
DLF006_scaffold91_3	non-phage	0
DLF006_scaffold29532_1	non-phage	0
DLF006_C667648_1	non-phage	0
DLF006_scaffold44721_1	non-phage	0
DLF006_scaffold43541_2	non-phage	0
DLF006_scaffold5925_1	non-phage	0
DLF006_scaffold40836_3	non-phage	0
DLF006_scaffold34641_1	non-phage	0
DLF006_scaffold34211_1	non-phage	0
DLF006_scaffold25_28	non-phage	0
DLF006_scaffold35279_2	non-phage	0
DLF006_scaffold8996_14	non-phage	0
DLF006_scaffold14168_7	non-phage	0
DLF006_scaffold41800_1	non-phage	0
DLF006_scaffold17124_1	non-phage	0
DLF006_C667564_1	non-phage	0

DLF006_scaffold3598_1	non-phage	0
DLF006_scaffold46832_1	non-phage	0
DLF006_scaffold43687_4	non-phage	0
DLF006_scaffold8915_16	non-phage	0
DLF006_scaffold36366_1	non-phage	0
DLF006_scaffold34234_1	non-phage	0
DLF006_scaffold14721_7	non-phage	0
DLF006_scaffold29453_2	non-phage	0
DLF006_scaffold2636_16	non-phage	0
DLF006_scaffold2090_18	non-phage	0
DLF006_scaffold43176_1	non-phage	0
DLF006_scaffold16159_5	non-phage	0
DLF006_scaffold46078_14	non-phage	0
DLF006_scaffold3774_6	non-phage	0
DLF006_scaffold21079_4	non-phage	0
DLF006_scaffold39432_1	non-phage	0
DLF006_scaffold2040_4	non-phage	0
DLF006_scaffold3681_1	non-phage	0
DLF006_scaffold34863_1	non-phage	0
DLF006_scaffold12468_1	non-phage	0
DLF006_scaffold19713_1	non-phage	0
DLF006_scaffold423_27	non-phage	0
DLF006_scaffold5514_5	non-phage	0
DLF007_scaffold9325_4	non-phage	0
DLF007_scaffold10536_1	non-phage	0
DLF007_scaffold5277_1	non-phage	0
DLF007_scaffold11761_1	non-phage	0
DLF007_scaffold9169_1	non-phage	0
DLF007_scaffold3063_1	non-phage	0
DLF007_scaffold13534_2	non-phage	0
DLF007_scaffold17226_3	non-phage	0
DLF007_scaffold3316_2	non-phage	0
DLF007_scaffold13378_9	non-phage	0
DLF007_scaffold5942_3	non-phage	0
DLF007_scaffold8544_2	non-phage	0
DLF007_C306049_1	non-phage	0
DLF007_scaffold19811_2	non-phage	0
DLF007_scaffold272_11	non-phage	0
DLF007_scaffold1662_2	non-phage	0
DLF007_scaffold1662_9	non-phage	0
DLF007_scaffold8981_2	non-phage	0
DLF007_scaffold18839_2	non-phage	0
DLF007_C306337_1	non-phage	0
DLF007_scaffold4747_20	non-phage	0
DLF007_scaffold8813_5	non-phage	0
DLF007_scaffold3240_4	non-phage	0

DLF007_scaffold6312_5	non-phage	0
DLF007_scaffold6457_2	non-phage	0
DLF007_scaffold2632_29	non-phage	0
DLF007_scaffold4645_14	non-phage	0
DLF007_scaffold910_12	non-phage	0
DLF007_scaffold14335_1	non-phage	0
DLF007_scaffold12433_2	non-phage	0
DLF007_scaffold13925_2	non-phage	0
DLF007_scaffold10483_2	non-phage	0
DLF007_scaffold6329_3	non-phage	0
DLF007_scaffold14956_2	non-phage	0
DLF007_scaffold13660_4	non-phage	0
DLF007_scaffold7107_1	non-phage	0
DLF007_scaffold18953_2	non-phage	0
DLF007_scaffold4417_1	non-phage	0
DLF007_scaffold11503_2	non-phage	0
DLF007_scaffold9239_2	non-phage	0
DLF007_scaffold7402_13	non-phage	0
DLF007_scaffold20101_1	non-phage	0
DLF007_scaffold16272_1	non-phage	0
DLF007_scaffold7_15	non-phage	0
DLF007_scaffold11670_1	non-phage	0
DLF007_scaffold8622_31	non-phage	0
DLF007_C306503_1	non-phage	0
DLF007_scaffold13589_2	non-phage	0
DLF007_scaffold11143_3	non-phage	0
DLF007_scaffold102_7	non-phage	0
DLF007_C305925_1	non-phage	0
DLF007_scaffold7_28	non-phage	0
DLF007_scaffold20182_1	non-phage	0
DLF007_scaffold231_45	non-phage	0
DLF007_scaffold84_1	non-phage	0
DLF007_scaffold19992_2	non-phage	0
DLF007_scaffold4747_2	non-phage	0
DLF007_scaffold20155_1	non-phage	0
DLF007_C306329_1	non-phage	0
DLF007_scaffold15438_1	non-phage	0
DLF007_scaffold6236_5	non-phage	0
DLF007_scaffold285_2	non-phage	0
DLF007_scaffold20215_1	non-phage	0
DLF007_scaffold14424_5	non-phage	0
DLF007_scaffold13678_1	non-phage	0
DLF007_scaffold20001_3	non-phage	0
DLF007_scaffold17522_1	non-phage	0
DLF007_C306299_1	non-phage	0
DLF007_scaffold19739_1	non-phage	0

DLF007_scaffold19838_1	non-phage	0
DLF007_scaffold7977_4	non-phage	0
DLF007_scaffold20008_1	non-phage	0
DLF007_scaffold13438_1	non-phage	0
DLF007_C306533_1	non-phage	0
DLF007_C306041_1	non-phage	0
DLF007_scaffold7864_9	non-phage	0
DLF007_scaffold17682_2	non-phage	0
DLF007_scaffold7398_1	non-phage	0
DLF007_scaffold16688_2	non-phage	0
DLF007_scaffold10015_1	non-phage	0
DLF007_scaffold7593_1	non-phage	0
DLF007_scaffold18684_1	non-phage	0
DLF007_scaffold15634_1	non-phage	0
DLF007_scaffold6718_1	non-phage	0
DLF007_scaffold1033_1	non-phage	0
DLF007_scaffold13439_1	non-phage	0
DLF007_scaffold66_1	non-phage	0
DLF007_scaffold6595_2	non-phage	0
DLF007_scaffold35_5	non-phage	0
DLF007_scaffold7816_13	non-phage	0
DLF007_scaffold17059_3	non-phage	0
DLF007_scaffold9400_4	non-phage	0
DLF007_scaffold758_4	non-phage	0
DLF007_scaffold12746_1	non-phage	0
DLF007_scaffold20324_1	non-phage	0
DLF007_scaffold16615_4	non-phage	0
DLF007_scaffold20300_2	non-phage	0
DLF007_scaffold4406_1	non-phage	0
DLF007_scaffold9008_3	non-phage	0
DLF007_scaffold12_1	non-phage	0
DLF007_scaffold6841_2	non-phage	0
DLF007_scaffold2632_16	non-phage	0
DLF007_scaffold16486_2	non-phage	0
DLF007_scaffold11223_1	non-phage	0
DLF007_scaffold19924_1	non-phage	0
DLF007_scaffold6844_3	non-phage	0
DLF007_scaffold16290_1	non-phage	0
DLF007_scaffold18768_2	non-phage	0
DLF007_scaffold96_1	non-phage	0
DLF007_scaffold16091_1	non-phage	0
DLF007_scaffold12612_2	non-phage	0
DLF007_scaffold17704_1	non-phage	0
DLF007_scaffold6529_19	non-phage	0
DLF007_scaffold13131_6	non-phage	0
DLF007_scaffold3475_3	non-phage	0

DLF007_scaffold20316_1	non-phage	0
DLF007_scaffold12491_1	non-phage	0
DLF007_scaffold18263_2	non-phage	0
DLF007_scaffold78_3	non-phage	0
DLF007_scaffold1906_9	non-phage	0
DLF007_scaffold11503_1	non-phage	0
DLF007_scaffold12630_1	non-phage	0
DLF007_scaffold11973_1	non-phage	0
DLF007_scaffold20262_3	non-phage	0
DLF007_scaffold10114_2	non-phage	0
DLF007_scaffold20001_2	non-phage	0
DLF007_scaffold8107_8	non-phage	0
DLF007_scaffold49_4	non-phage	0
DLF007_scaffold7823_3	non-phage	0
DLF007_scaffold15181_5	non-phage	0
DLF007_scaffold18400_1	non-phage	0
DLF007_scaffold20271_1	non-phage	0
DLF007_scaffold14930_1	non-phage	0
DLF007_scaffold20329_1	non-phage	0
DLF007_scaffold11979_8	non-phage	0
DLF007_scaffold11548_1	non-phage	0
DLF007_scaffold14294_2	non-phage	0
DLF007_scaffold3010_25	non-phage	0
DLF007_scaffold7416_3	non-phage	0
DLF007_scaffold5659_10	non-phage	0
DLF007_scaffold4336_3	non-phage	0
DLF007_C305931_1	non-phage	0
DLF007_scaffold20185_2	non-phage	0
DLF007_scaffold10610_1	non-phage	0
DLF007_scaffold19781_1	non-phage	0
DLF007_scaffold1643_5	non-phage	0
DLF007_scaffold2838_26	non-phage	0
DLF007_C306679_1	non-phage	0
DLF007_scaffold18777_3	non-phage	0
DLF007_scaffold7138_21	non-phage	0
DLF007_scaffold20236_1	non-phage	0
DLF007_scaffold75_2	non-phage	0
DLF007_scaffold351_1	non-phage	0
DLF007_scaffold20263_1	non-phage	0
DLF007_scaffold17400_2	non-phage	0
DLF007_scaffold15220_2	non-phage	0
DLF007_scaffold20175_1	non-phage	0
DLF007_scaffold19741_2	non-phage	0
DLF007_scaffold13967_1	non-phage	0
DLF007_scaffold78_4	non-phage	0
DLF007_scaffold16448_1	non-phage	0

DLF007_scaffold1977_2	non-phage	0
DLF007_scaffold20309_1	non-phage	0
DLF007_scaffold18326_3	non-phage	0
DLF007_scaffold6890_37	non-phage	0
DLF007_scaffold15583_1	non-phage	0
DLF007_scaffold16188_1	non-phage	0
DLF007_scaffold2558_1	non-phage	0
DLF007_scaffold13787_24	non-phage	0
DLF007_C306723_1	non-phage	0
DLF007_scaffold2632_37	non-phage	0
DLF007_scaffold20305_2	non-phage	0
DLF007_scaffold18152_2	non-phage	0
DLF007_scaffold13787_13	non-phage	0
DLF007_scaffold949_16	non-phage	0
DLF007_scaffold17704_2	non-phage	0
DLF007_scaffold6278_1	non-phage	0
DLF007_scaffold20110_1	non-phage	0
DLF007_scaffold14413_3	non-phage	0
DLF007_scaffold19891_1	non-phage	0
DLF007_scaffold13534_1	non-phage	0
DLF007_scaffold5426_3	non-phage	0
DLF007_scaffold18610_1	non-phage	0
DLF007_scaffold20312_1	non-phage	0
DLF007_scaffold231_44	non-phage	0
DLF007_scaffold7861_3	non-phage	0
DLF008_scaffold14542_1	non-phage	0
DLF008_scaffold1819_3	non-phage	0
DLF008_scaffold3185_1	non-phage	0
DLF008_scaffold11374_2	non-phage	0
DLF008_scaffold142_6	non-phage	0
DLF008_scaffold17604_2	non-phage	0
DLF008_scaffold2119_8	non-phage	0
DLF008_scaffold936_5	non-phage	0
DLF008_scaffold6002_1	non-phage	0
DLF008_scaffold1806_36	non-phage	0
DLF008_scaffold15934_2	non-phage	0
DLF008_scaffold17586_2	non-phage	0
DLF008_scaffold213_5	non-phage	0
DLF008_scaffold12728_2	non-phage	0
DLF008_scaffold7350_3	non-phage	0
DLF008_scaffold17556_1	non-phage	0
DLF008_C335705_1	non-phage	0
DLF008_scaffold17506_1	non-phage	0
DLF008_scaffold13523_1	non-phage	0
DLF008_scaffold918_1	non-phage	0
DLF008_scaffold16648_1	non-phage	0

DLF008_C335651_1	non-phage	0
DLF008_scaffold17108_2	non-phage	0
DLF008_scaffold3124_1	non-phage	0
DLF008_scaffold7644_1	non-phage	0
DLF008_scaffold501_3	non-phage	0
DLF008_scaffold4001_2	non-phage	0
DLF008_scaffold12441_3	non-phage	0
DLF008_scaffold12356_1	non-phage	0
DLF008_scaffold1984_3	non-phage	0
DLF008_scaffold17_2	non-phage	0
DLF008_scaffold12394_1	non-phage	0
DLF008_scaffold91_1	non-phage	0
DLF008_scaffold12448_3	non-phage	0
DLF008_scaffold45_4	non-phage	0
DLF008_scaffold2857_2	non-phage	0
DLF008_scaffold11492_1	non-phage	0
DLF008_scaffold628_1	non-phage	0
DLF008_scaffold17606_2	non-phage	0
DLF008_scaffold7094_2	non-phage	0
DLF008_scaffold9604_3	non-phage	0
DLF008_scaffold1154_2	non-phage	0
DLF008_scaffold1952_13	non-phage	0
DLF008_scaffold12070_1	non-phage	0
DLF008_scaffold16601_1	non-phage	0
DLF008_scaffold8849_2	non-phage	0
DLF008_scaffold135_10	non-phage	0
DLF008_scaffold17593_1	non-phage	0
DLF008_scaffold14331_1	non-phage	0
DLF008_scaffold15744_1	non-phage	0
DLF008_scaffold1043_75	non-phage	0
DLF008_scaffold7669_4	non-phage	0
DLF008_scaffold1764_2	non-phage	0
DLF008_scaffold8373_3	non-phage	0
DLF008_scaffold10118_1	non-phage	0
DLF008_scaffold7822_1	non-phage	0
DLF008_scaffold16655_1	non-phage	0
DLF008_scaffold7850_2	non-phage	0
DLF008_scaffold16946_1	non-phage	0
DLF008_scaffold15531_2	non-phage	0
DLF008_scaffold259_2	non-phage	0
DLF008_scaffold12780_2	non-phage	0
DLF008_scaffold13813_1	non-phage	0
DLF008_scaffold6667_14	non-phage	0
DLF008_scaffold5892_5	non-phage	0
DLF008_scaffold10318_13	non-phage	0
DLF008_scaffold601_1	non-phage	0

DLF008_scaffold17060_4	non-phage	0
DLF008_scaffold15586_11	non-phage	0
DLF008_scaffold12448_6	non-phage	0
DLF008_scaffold17414_1	non-phage	0
DLF008_scaffold1702_3	non-phage	0
DLF008_scaffold4013_15	non-phage	0
DLF008_scaffold177_3	non-phage	0
DLF008_scaffold6782_1	non-phage	0
DLF008_scaffold7125_2	non-phage	0
DLF008_scaffold16850_2	non-phage	0
DLF008_scaffold145_8	non-phage	0
DLF008_scaffold207_2	non-phage	0
DLF008_scaffold1629_2	non-phage	0
DLF008_scaffold4179_2	non-phage	0
DLF008_scaffold15308_5	non-phage	0
DLF008_scaffold11149_5	non-phage	0
DLF008_scaffold1085_9	non-phage	0
DLF008_scaffold5982_2	non-phage	0
DLF008_scaffold9049_2	non-phage	0
DLF008_scaffold1629_1	non-phage	0
DLF008_scaffold16846_1	non-phage	0
DLF008_scaffold17545_1	non-phage	0
DLF008_scaffold621_5	non-phage	0
DLF008_scaffold5081_4	non-phage	0
DLF008_scaffold13586_7	non-phage	0
DLF008_scaffold16725_1	non-phage	0
DLF008_scaffold7869_2	non-phage	0
DLF008_scaffold13588_1	non-phage	0
DLF008_scaffold112_11	non-phage	0
DLF008_scaffold4559_1	non-phage	0
DLF008_scaffold9641_1	non-phage	0
DLF008_scaffold2485_11	non-phage	0
DLF008_scaffold1952_1	non-phage	0
DLF008_scaffold534_17	non-phage	0
DLF008_scaffold515_4	non-phage	0
DLF008_scaffold79_2	non-phage	0
DLF008_scaffold17610_1	non-phage	0
DLF008_scaffold12446_1	non-phage	0
DLF008_scaffold17618_1	non-phage	0
DLF008_scaffold75_2	non-phage	0
DLF008_scaffold16731_1	non-phage	0
DLF008_scaffold132_8	non-phage	0
DLF008_scaffold1306_6	non-phage	0
DLF008_scaffold17589_1	non-phage	0
DLF008_scaffold1962_1	non-phage	0
DLF008_scaffold1533_5	non-phage	0

DLF008_scaffold7109_12	non-phage	0
DLF008_scaffold14434_2	non-phage	0
DLF008_scaffold684_20	non-phage	0
DLF008_scaffold12630_11	non-phage	0
DLF008_scaffold10845_1	non-phage	0
DLF008_scaffold2084_2	non-phage	0
DLF008_scaffold7859_3	non-phage	0
DLF008_scaffold5491_2	non-phage	0
DLF008_scaffold14718_1	non-phage	0
DLF008_scaffold1804_5	non-phage	0
DLF008_scaffold16075_3	non-phage	0
DLF008_scaffold1975_4	non-phage	0
DLF008_scaffold12023_7	non-phage	0
DLF008_scaffold6335_4	non-phage	0
DLF008_scaffold17516_6	non-phage	0
DLF008_scaffold57_3	non-phage	0
DLF008_scaffold5578_4	non-phage	0
DLF008_scaffold2095_18	non-phage	0
DLF008_C335543_1	non-phage	0
DLF008_scaffold470_3	non-phage	0
DLF008_scaffold16605_2	non-phage	0
DLF008_scaffold15770_1	non-phage	0
DLF008_scaffold8373_2	non-phage	0
DLF008_scaffold3216_13	non-phage	0
DLF008_scaffold16325_1	non-phage	0
DLF008_scaffold10196_20	non-phage	0
DLF008_scaffold11199_1	non-phage	0
DLF008_scaffold8551_7	non-phage	0
DLF008_scaffold57_5	non-phage	0
DLF008_scaffold1653_11	non-phage	0
DLF008_scaffold1410_3	non-phage	0
DLF008_scaffold17504_1	non-phage	0
DLF008_scaffold123_2	non-phage	0
DLF008_scaffold60_24	non-phage	0
DLF008_scaffold10118_3	non-phage	0
DLF008_scaffold2326_5	non-phage	0
DLF008_scaffold5409_12	non-phage	0
DLF008_scaffold8120_13	non-phage	0
DLF008_scaffold45_1	non-phage	0
DLF008_scaffold37_1	non-phage	0
DLF008_scaffold7422_3	non-phage	0
DLF008_scaffold9915_2	non-phage	0
DLF008_scaffold132_7	non-phage	0
DLF008_scaffold12031_1	non-phage	0
DLF008_scaffold10884_7	non-phage	0
DLF008_scaffold16408_1	non-phage	0

DLF008_scaffold2711_64	non-phage	0
DLF008_scaffold509_13	non-phage	0
DLF008_scaffold5434_2	non-phage	0
DLF008_scaffold15364_1	non-phage	0
DLF008_scaffold17192_1	non-phage	0
DLF008_scaffold1091_1	non-phage	0
DLF008_scaffold2001_15	non-phage	0
DLF008_scaffold15876_1	non-phage	0
DLF008_scaffold16848_2	non-phage	0
DLF008_scaffold13029_1	non-phage	0
DLF008_scaffold9911_1	non-phage	0
DLF008_scaffold12942_3	non-phage	0
DLF008_scaffold4914_4	non-phage	0
DLF008_scaffold534_27	non-phage	0
DLF008_scaffold4708_15	non-phage	0
DLF008_scaffold12484_2	non-phage	0
DLF008_scaffold9280_26	non-phage	0
DLF008_scaffold19_12	non-phage	0
DLF008_scaffold534_62	non-phage	0
DLF008_scaffold17547_3	non-phage	0
DLF008_scaffold13149_14	non-phage	0
DLF008_scaffold4914_2	non-phage	0
DLF008_scaffold1508_9	non-phage	0
DLF008_scaffold2160_1	non-phage	0
DLF008_scaffold80_9	non-phage	0
DLF008_scaffold1838_7	non-phage	0
DLF008_scaffold893_2	non-phage	0
DLF008_scaffold13458_1	non-phage	0
DLF008_scaffold17301_1	non-phage	0
DLF008_scaffold7166_5	non-phage	0
DLF008_scaffold9504_1	non-phage	0
DLF008_scaffold17117_1	non-phage	0
DLF008_scaffold15840_1	non-phage	0
DLF008_scaffold16608_2	non-phage	0
DLF008_scaffold5856_4	non-phage	0
DLF008_scaffold670_20	non-phage	0
DLF008_scaffold84_5	non-phage	0
DLF008_scaffold2119_42	non-phage	0
DLF008_scaffold13883_2	non-phage	0
DLF008_scaffold72_6	non-phage	0
DLF008_scaffold12630_4	non-phage	0
DLF008_scaffold16586_1	non-phage	0
DLF008_scaffold15350_1	non-phage	0
DLF008_scaffold2312_1	non-phage	0
DLF008_scaffold470_16	non-phage	0
DLF008_scaffold15420_1	non-phage	0

DLF008_scaffold1653_19	non-phage	0
DLF008_scaffold2665_6	non-phage	0
DLF008_scaffold13998_4	non-phage	0
DLF008_scaffold8685_6	non-phage	0
DLF008_scaffold887_3	non-phage	0
DLF008_C335965_1	non-phage	0
DLF008_scaffold4245_1	non-phage	0
DLF008_scaffold4523_2	non-phage	0
DLF008_scaffold191_17	non-phage	0
DLF008_scaffold3132_67	non-phage	0
DLF008_scaffold470_8	non-phage	0
DLF008_scaffold1391_6	non-phage	0
DLF008_scaffold96_1	non-phage	0
DLF008_scaffold974_2	non-phage	0
DLF008_scaffold14761_1	non-phage	0
DLF008_scaffold17604_1	non-phage	0
DLF008_scaffold8303_23	non-phage	0
DLF008_scaffold2326_7	non-phage	0
DLF008_scaffold7077_3	non-phage	0
DLF008_scaffold4378_1	non-phage	0
DLF008_scaffold10318_11	non-phage	0
DLF008_scaffold3470_5	non-phage	0
DLF008_scaffold17524_1	non-phage	0
DLF008_scaffold1200_16	non-phage	0
DLF008_scaffold34_1	non-phage	0
DLF008_scaffold134_8	non-phage	0
DLF008_scaffold16866_1	non-phage	0
DLF008_scaffold132_6	non-phage	0
DLF008_scaffold1248_1	non-phage	0
DLF008_scaffold3867_7	non-phage	0
DLF008_scaffold1145_4	non-phage	0
DLF008_scaffold99_3	non-phage	0
DLF008_scaffold19_4	non-phage	0
DLF008_scaffold2326_4	non-phage	0
DLF008_scaffold16034_1	non-phage	0
DLF008_scaffold4571_1	non-phage	0
DLF008_scaffold8551_1	non-phage	0
DLF008_scaffold10516_1	non-phage	0
DLF008_scaffold3149_1	non-phage	0
DLF008_scaffold4550_1	non-phage	0
DLF008_scaffold11741_2	non-phage	0
DLF008_scaffold10107_1	non-phage	0
DLF008_scaffold4413_2	non-phage	0
DLF008_scaffold1173_1	non-phage	0
DLF008_scaffold14964_1	non-phage	0
DLF008_scaffold3664_5	non-phage	0

DLF008_scaffold5864_1	non-phage	0
DLF008_scaffold17195_2	non-phage	0
DLF008_scaffold14536_2	non-phage	0
DLF008_scaffold17048_1	non-phage	0
DLF008_scaffold17549_1	non-phage	0
DLF008_scaffold17583_1	non-phage	0
DLF008_scaffold17340_1	non-phage	0
DLF008_scaffold11446_1	non-phage	0
DLF008_scaffold17402_2	non-phage	0
DLF008_scaffold14671_1	non-phage	0
DLF008_scaffold13537_1	non-phage	0
DLF008_scaffold11421_1	non-phage	0
DLF008_scaffold7094_8	non-phage	0
DLF009_C428520_1	non-phage	0
DLF009_scaffold24305_10	non-phage	0
DLF009_scaffold22875_5	non-phage	0
DLF009_scaffold5523_6	non-phage	0
DLF009_scaffold28641_14	non-phage	0
DLF009_scaffold25032_1	non-phage	0
DLF009_scaffold2982_11	non-phage	0
DLF009_scaffold19370_7	non-phage	0
DLF009_scaffold12259_3	non-phage	0
DLF009_scaffold37431_2	non-phage	0
DLF009_scaffold3043_9	non-phage	0
DLF009_scaffold20504_10	non-phage	0
DLF009_scaffold6551_3	non-phage	0
DLF009_scaffold2317_1	non-phage	0
DLF009_scaffold7460_5	non-phage	0
DLF009_scaffold29258_5	non-phage	0
DLF009_scaffold20630_4	non-phage	0
DLF009_scaffold3_2	non-phage	0
DLF009_scaffold603_1	non-phage	0
DLF009_scaffold19862_10	non-phage	0
DLF009_scaffold26950_2	non-phage	0
DLF009_scaffold37152_1	non-phage	0
DLF009_scaffold14287_1	non-phage	0
DLF009_scaffold37237_2	non-phage	0
DLF009_scaffold37461_1	non-phage	0
DLF009_scaffold7058_12	non-phage	0
DLF009_scaffold37069_3	non-phage	0
DLF009_scaffold26879_2	non-phage	0
DLF009_scaffold16253_16	non-phage	0
DLF009_scaffold23251_12	non-phage	0
DLF009_scaffold22875_3	non-phage	0
DLF009_scaffold21290_2	non-phage	0
DLF009_scaffold2179_2	non-phage	0

DLF009_scaffold37418_5	non-phage	0
DLF009_scaffold8343_11	non-phage	0
DLF009_scaffold7143_2	non-phage	0
DLF009_scaffold13838_7	non-phage	0
DLF009_scaffold37237_4	non-phage	0
DLF009_scaffold7460_4	non-phage	0
DLF009_scaffold6580_4	non-phage	0
DLF009_scaffold36875_3	non-phage	0
DLF009_scaffold37363_1	non-phage	0
DLF009_scaffold37457_1	non-phage	0
DLF009_scaffold24462_1	non-phage	0
DLF009_scaffold3930_2	non-phage	0
DLF009_scaffold11718_1	non-phage	0
DLF009_scaffold19408_4	non-phage	0
DLF009_scaffold37489_2	non-phage	0
DLF009_scaffold16253_5	non-phage	0
DLF009_scaffold37373_1	non-phage	0
DLF009_scaffold37270_3	non-phage	0
DLF009_scaffold1406_4	non-phage	0
DLF009_scaffold37424_4	non-phage	0
DLF009_scaffold18235_2	non-phage	0
DLF009_scaffold28677_3	non-phage	0
DLF009_scaffold783_11	non-phage	0
DLF009_scaffold603_3	non-phage	0
DLF009_scaffold22858_3	non-phage	0
DLF009_scaffold28237_2	non-phage	0
DLF009_scaffold28458_3	non-phage	0
DLF009_scaffold13838_6	non-phage	0
DLF009_scaffold14937_3	non-phage	0
DLF009_scaffold37468_2	non-phage	0
DLF009_scaffold27261_2	non-phage	0
DLF009_scaffold1880_21	non-phage	0
DLF009_scaffold304_18	non-phage	0
DLF009_scaffold6274_2	non-phage	0
DLF009_scaffold17406_12	non-phage	0
DLF009_scaffold2982_32	non-phage	0
DLF009_scaffold304_22	non-phage	0
DLF009_scaffold783_16	non-phage	0
DLF009_scaffold8641_1	non-phage	0
DLF009_scaffold36853_1	non-phage	0
DLF009_scaffold2665_7	non-phage	0
DLF009_scaffold9117_6	non-phage	0
DLF009_scaffold34413_1	non-phage	0
DLF009_scaffold28641_7	non-phage	0
DLF009_scaffold30410_2	non-phage	0
DLF009_scaffold16253_4	non-phage	0

DLF009_scaffold2567_11	non-phage	0
DLF009_scaffold15175_4	non-phage	0
DLF009_scaffold4243_2	non-phage	0
DLF009_scaffold6675_4	non-phage	0
DLF009_scaffold6572_14	non-phage	0
DLF009_scaffold17595_4	non-phage	0
DLF009_scaffold5354_3	non-phage	0
DLF009_scaffold6675_11	non-phage	0
DLF009_scaffold28458_2	non-phage	0
DLF009_scaffold34424_4	non-phage	0
DLF009_scaffold35832_2	non-phage	0
DLF009_C428480_1	non-phage	0
DLF009_scaffold27288_5	non-phage	0
DLF009_scaffold1107_3	non-phage	0
DLF009_scaffold13685_25	non-phage	0
DLF009_scaffold13089_7	non-phage	0
DLF009_scaffold26879_3	non-phage	0
DLF009_scaffold36874_1	non-phage	0
DLF009_scaffold30410_1	non-phage	0
DLF009_scaffold22761_1	non-phage	0
DLF009_scaffold6572_3	non-phage	0
DLF009_scaffold6859_3	non-phage	0
DLF009_scaffold37211_1	non-phage	0
DLF009_scaffold21043_13	non-phage	0
DLF009_scaffold35459_4	non-phage	0
DLF009_scaffold28237_6	non-phage	0
DLF009_scaffold30344_1	non-phage	0
DLF009_scaffold2982_6	non-phage	0
DLF009_scaffold37441_1	non-phage	0
DLF010_scaffold55529_1	non-phage	0
DLF010_scaffold11496_20	non-phage	0
DLF010_scaffold3594_7	non-phage	0
DLF010_scaffold49956_21	non-phage	0
DLF010_scaffold46917_1	non-phage	0
DLF010_scaffold45075_7	non-phage	0
DLF010_scaffold40000_6	non-phage	0
DLF010_scaffold26569_11	non-phage	0
DLF010_scaffold22271_21	non-phage	0
DLF010_scaffold7987_6	non-phage	0
DLF010_scaffold2699_27	non-phage	0
DLF010_scaffold44981_1	non-phage	0
DLF010_scaffold66_5	non-phage	0
DLF010_scaffold49642_1	non-phage	0
DLF010_scaffold10065_4	non-phage	0
DLF010_scaffold3644_4	non-phage	0
DLF010_scaffold23679_18	non-phage	0

DLF010_scaffold22334_9	non-phage	0
DLF010_scaffold2867_81	non-phage	0
DLF010_scaffold2071_10	non-phage	0
DLF010_scaffold45363_2	non-phage	0
DLF010_scaffold38247_1	non-phage	0
DLF010_scaffold6621_4	non-phage	0
DLF010_scaffold11388_10	non-phage	0
DLF010_scaffold5474_14	non-phage	0
DLF010_scaffold10332_9	non-phage	0
DLF010_scaffold353_15	non-phage	0
DLF010_scaffold28674_1	non-phage	0
DLF010_scaffold47377_1	non-phage	0
DLF010_scaffold41433_3	non-phage	0
DLF010_scaffold2392_10	non-phage	0
DLF010_scaffold8154_4	non-phage	0
DLF010_scaffold285_3	non-phage	0
DLF010_scaffold1059_3	non-phage	0
DLF010_scaffold135_1	non-phage	0
DLF010_scaffold28782_5	non-phage	0
DLF010_scaffold46012_2	non-phage	0
DLF010_scaffold54036_1	non-phage	0
DLF010_scaffold1708_17	non-phage	0
DLF010_scaffold17548_1	non-phage	0
DLF010_scaffold35835_3	non-phage	0
DLF010_scaffold13269_8	non-phage	0
DLF010_scaffold55619_1	non-phage	0
DLF010_scaffold3594_8	non-phage	0
DLF010_scaffold433_82	non-phage	0
DLF010_scaffold11420_7	non-phage	0
DLF010_scaffold8408_1	non-phage	0
DLF010_scaffold19787_1	non-phage	0
DLF010_scaffold49532_4	non-phage	0
DLF010_scaffold13367_15	non-phage	0
DLF010_scaffold25202_8	non-phage	0
DLF010_scaffold1532_2	non-phage	0
DLF010_scaffold13917_38	non-phage	0
DLF010_scaffold55615_1	non-phage	0
DLF010_scaffold55741_1	non-phage	0
DLF010_scaffold3242_32	non-phage	0
DLF010_scaffold185_2	non-phage	0
DLF010_scaffold4567_33	non-phage	0
DLF010_scaffold8706_6	non-phage	0
DLF010_scaffold46224_8	non-phage	0
DLF010_scaffold32926_7	non-phage	0
DLF010_scaffold29897_3	non-phage	0
DLF010_scaffold2025_69	non-phage	0

DLF010_scaffold17315_1	non-phage	0
DLF010_scaffold22575_13	non-phage	0
DLF010_scaffold36092_6	non-phage	0
DLF010_scaffold24041_1	non-phage	0
DLF010_scaffold14683_21	non-phage	0
DLF010_scaffold433_47	non-phage	0
DLF010_scaffold9328_33	non-phage	0
DLF010_scaffold2392_16	non-phage	0
DLF010_scaffold13193_11	non-phage	0
DLF010_scaffold15974_11	non-phage	0
DLF010_scaffold106_52	non-phage	0
DLF010_scaffold9004_10	non-phage	0
DLF010_scaffold35048_9	non-phage	0
DLF010_scaffold17611_18	non-phage	0
DLF010_scaffold30683_2	non-phage	0
DLF010_scaffold2514_1	non-phage	0
DLF010_scaffold49642_2	non-phage	0
DLF010_scaffold49831_1	non-phage	0
DLF010_scaffold10337_4	non-phage	0
DLF010_scaffold27882_4	non-phage	0
DLF010_scaffold29752_2	non-phage	0
DLF010_scaffold1708_16	non-phage	0
DLF010_scaffold1_6	non-phage	0
DLF010_scaffold8706_2	non-phage	0
DLF010_scaffold28_1	non-phage	0
DLF010_scaffold31655_2	non-phage	0
DLF010_scaffold44719_1	non-phage	0
DLF010_scaffold54839_8	non-phage	0
DLF010_scaffold41023_1	non-phage	0
DLF010_scaffold264_19	non-phage	0
DLF010_C744566_1	non-phage	0
DLF010_scaffold55444_5	non-phage	0
DLF010_scaffold2025_7	non-phage	0
DLF010_scaffold175_1	non-phage	0
DLF010_scaffold27101_1	non-phage	0
DLF010_scaffold9727_8	non-phage	0
DLF010_scaffold33627_1	non-phage	0
DLF010_scaffold54380_1	non-phage	0
DLF010_scaffold44734_2	non-phage	0
DLF010_scaffold3040_1	non-phage	0
DLF012_scaffold19157_2	non-phage	0
DLF012_C256009_1	non-phage	0
DLF012_scaffold7563_1	non-phage	0
DLF012_scaffold8339_1	non-phage	0
DLF012_scaffold17306_3	non-phage	0
DLF012_scaffold12322_1	non-phage	0

DLF012_scaffold14995_3	non-phage	0
DLF012_scaffold17572_6	non-phage	0
DLF012_scaffold19195_3	non-phage	0
DLF012_scaffold13255_3	non-phage	0
DLF012_C255969_1	non-phage	0
DLF012_scaffold17400_3	non-phage	0
DLF012_scaffold4228_9	non-phage	0
DLF012_scaffold1267_24	non-phage	0
DLF012_scaffold1129_1	non-phage	0
DLF012_scaffold14285_3	non-phage	0
DLF012_scaffold16578_2	non-phage	0
DLF012_scaffold11276_2	non-phage	0
DLF012_scaffold6703_7	non-phage	0
DLF012_scaffold9930_1	non-phage	0
DLF012_scaffold16506_3	non-phage	0
DLF012_C256755_1	non-phage	0
DLF012_scaffold3_4	non-phage	0
DLF012_scaffold13715_8	non-phage	0
DLF012_C256127_1	non-phage	0
DLF012_C256861_1	non-phage	0
DLF012_scaffold18610_1	non-phage	0
DLF012_scaffold4852_1	non-phage	0
DLF012_scaffold18364_1	non-phage	0
DLF012_scaffold3698_4	non-phage	0
DLF012_scaffold3027_5	non-phage	0
DLF012_scaffold10024_2	non-phage	0
DLF012_scaffold7457_2	non-phage	0
DLF012_scaffold18933_2	non-phage	0
DLF012_scaffold84_18	non-phage	0
DLF012_scaffold7563_7	non-phage	0
DLF012_scaffold19197_2	non-phage	0
DLF012_scaffold8615_28	non-phage	0
DLF012_scaffold2143_2	non-phage	0
DLF012_scaffold16506_2	non-phage	0
DLF012_scaffold1267_10	non-phage	0
DLF012_scaffold5426_4	non-phage	0
DLF012_scaffold19086_3	non-phage	0
DLF012_C256773_1	non-phage	0
DLF012_scaffold18741_1	non-phage	0
DLF012_scaffold19212_2	non-phage	0
DLF012_scaffold8907_1	non-phage	0
DLF012_scaffold84_10	non-phage	0
DLF012_scaffold19175_1	non-phage	0
DLF012_scaffold19107_2	non-phage	0
DLF012_scaffold183_6	non-phage	0
DLF012_scaffold19186_2	non-phage	0

DLF012_scaffold18729_3	non-phage	0
DLF012_C255999_1	non-phage	0
DLF012_scaffold6481_30	non-phage	0
DLF012_scaffold18822_2	non-phage	0
DLF012_scaffold783_9	non-phage	0
DLF012_scaffold8546_2	non-phage	0
DLF012_scaffold19180_1	non-phage	0
DLF012_scaffold7932_1	non-phage	0
DLF013_scaffold34289_5	non-phage	0
DLF013_scaffold4985_8	non-phage	0
DLF013_scaffold38372_3	non-phage	0
DLF013_scaffold15284_17	non-phage	0
DLF013_scaffold10557_1	non-phage	0
DLF013_scaffold15996_1	non-phage	0
DLF013_scaffold13586_1	non-phage	0
DLF013_scaffold53955_1	non-phage	0
DLF013_scaffold41726_2	non-phage	0
DLF013_scaffold39401_2	non-phage	0
DLF013_scaffold51740_2	non-phage	0
DLF013_scaffold50964_1	non-phage	0
DLF013_scaffold25554_11	non-phage	0
DLF013_scaffold37995_1	non-phage	0
DLF013_scaffold14997_7	non-phage	0
DLF013_scaffold13799_1	non-phage	0
DLF013_scaffold33577_9	non-phage	0
DLF013_scaffold15284_21	non-phage	0
DLF013_scaffold22651_6	non-phage	0
DLF013_scaffold54797_1	non-phage	0
DLF013_scaffold19556_2	non-phage	0
DLF013_scaffold5263_1	non-phage	0
DLF013_scaffold3_2	non-phage	0
DLF013_scaffold51867_2	non-phage	0
DLF013_scaffold5745_3	non-phage	0
DLF013_scaffold5607_2	non-phage	0
DLF013_scaffold42268_2	non-phage	0
DLF013_scaffold15346_2	non-phage	0
DLF013_scaffold48931_3	non-phage	0
DLF013_scaffold2857_2	non-phage	0
DLF013_scaffold4405_18	non-phage	0
DLF013_scaffold2872_1	non-phage	0
DLF013_scaffold16362_2	non-phage	0
DLF013_scaffold38578_4	non-phage	0
DLF013_scaffold33768_2	non-phage	0
DLF013_scaffold22636_4	non-phage	0
DLF013_scaffold27850_15	non-phage	0
DLF013_scaffold54589_3	non-phage	0

DLF013_scaffold43415_2	non-phage	0
DLF013_scaffold48983_1	non-phage	0
DLF013_scaffold14390_2	non-phage	0
DLF013_scaffold13954_1	non-phage	0
DLF013_scaffold24038_1	non-phage	0
DLF013_C686502_1	non-phage	0
DLF013_scaffold573_6	non-phage	0
DLF013_scaffold22757_14	non-phage	0
DLF013_scaffold11990_7	non-phage	0
DLF013_scaffold50109_4	non-phage	0
DLF013_scaffold30697_1	non-phage	0
DLF013_scaffold40284_3	non-phage	0
DLF013_scaffold10082_9	non-phage	0
DLF013_scaffold19715_1	non-phage	0
DLF013_scaffold11388_3	non-phage	0
DLF013_scaffold44460_2	non-phage	0
DLF013_scaffold53409_1	non-phage	0
DLF013_scaffold24663_2	non-phage	0
DLF013_scaffold27850_13	non-phage	0
DLF013_scaffold3128_1	non-phage	0
DLF013_scaffold26664_2	non-phage	0
DLF013_scaffold1095_2	non-phage	0
DLF013_scaffold19093_2	non-phage	0
DLF013_scaffold50979_1	non-phage	0
DLF013_scaffold15284_18	non-phage	0
DLF013_scaffold49186_3_2	non-phage	0
DLF013_scaffold15284_20	non-phage	0
DLF013_scaffold52386_2	non-phage	0
DLF013_scaffold30994_2	non-phage	0
DLF013_scaffold43036_1	non-phage	0
DLF013_scaffold50519_1	non-phage	0
DLF013_scaffold46137_9	non-phage	0
DLF013_scaffold22628_4	non-phage	0
DLF013_scaffold54600_1	non-phage	0
DLF013_scaffold54446_1	non-phage	0
DLF013_scaffold48798_1	non-phage	0
DLF013_scaffold54273_1	non-phage	0
DLF013_scaffold3883_17	non-phage	0
DLF013_scaffold35653_4	non-phage	0
DLF013_scaffold17018_4	non-phage	0
DLF013_scaffold43548_2	non-phage	0
DLF013_scaffold8289_2	non-phage	0
DLF013_scaffold20747_6	non-phage	0
DLF013_scaffold25554_1	non-phage	0
DLF013_scaffold40195_1	non-phage	0
DLF013_scaffold22616_2	non-phage	0

DLF013_scaffold18744_10	non-phage	0
DLF013_scaffold20083_1	non-phage	0
DLF013_scaffold49500_1	non-phage	0
DLF013_scaffold18007_4	non-phage	0
DLF013_scaffold29823_1	non-phage	0
DLF013_scaffold25138_2	non-phage	0
DLF013_scaffold54747_1	non-phage	0
DLF013_scaffold21833_8	non-phage	0
DLF013_scaffold90_3	non-phage	0
DLF013_scaffold30994_5	non-phage	0
DLF013_scaffold3821_4	non-phage	0
DLF013_scaffold5364_7	non-phage	0
DLF013_scaffold13692_1	non-phage	0
DLF013_scaffold54655_2	non-phage	0
DLF013_scaffold21264_2	non-phage	0
DLF013_scaffold29870_2	non-phage	0
DLF013_scaffold52908_1	non-phage	0
DLF013_scaffold24001_1	non-phage	0
DLF013_scaffold17018_3	non-phage	0
DLF013_scaffold46137_5	non-phage	0
DLF013_scaffold6975_5	non-phage	0
DLF013_scaffold44915_1	non-phage	0
DLF013_scaffold45613_1	non-phage	0
DLF013_scaffold24088_4	non-phage	0
DLF013_scaffold34301_1	non-phage	0
DLF013_scaffold5532_3	non-phage	0
DLF013_scaffold10941_1	non-phage	0
DLF013_scaffold45866_1	non-phage	0
DLF013_scaffold4985_7	non-phage	0
DLF013_scaffold5745_7	non-phage	0
DLF013_scaffold3883_12	non-phage	0
DLF013_scaffold15141_3	non-phage	0
DLF013_scaffold35981_1	non-phage	0
DLF013_scaffold13799_3	non-phage	0
DLF013_scaffold53683_1	non-phage	0
DLF013_scaffold5677_1	non-phage	0
DLF013_scaffold6036_8	non-phage	0
DLF013_scaffold9158_1	non-phage	0
DLF013_scaffold54697_1	non-phage	0
DLF013_scaffold36411_2	non-phage	0
DLF013_scaffold40284_2	non-phage	0
DLF013_scaffold10253_1	non-phage	0
DLF013_scaffold2238_1	non-phage	0
DLF013_scaffold49186_2	non-phage	0
DLF013_scaffold54715_1	non-phage	0
DLF013_scaffold34485_3	non-phage	0

DLF013_scaffold2715_7	non-phage	0
DLF013_scaffold22885_1	non-phage	0
DLF013_scaffold18364_1	non-phage	0
DLF013_scaffold34697_1	non-phage	0
DLF013_scaffold42211_2	non-phage	0
DLF013_scaffold46893_2	non-phage	0
DLF013_scaffold38037_1	non-phage	0
DLF013_scaffold13921_7	non-phage	0
DLF013_scaffold139_10	non-phage	0
DLF013_scaffold14028_4	non-phage	0
DLF013_scaffold31876_1	non-phage	0
DLF013_scaffold11388_1	non-phage	0
DLF013_scaffold52865_2	non-phage	0
DLF013_scaffold29509_2	non-phage	0
DLF013_scaffold18131_11	non-phage	0
DLF013_scaffold72_2	non-phage	0
DLF013_scaffold26229_2	non-phage	0
DLF013_scaffold48237_1	non-phage	0
DLF013_scaffold29706_2	non-phage	0
DLF013_C686636_1	non-phage	0
DLF013_scaffold9550_1	non-phage	0
DLF013_scaffold573_15	non-phage	0
DLF013_C686320_1	non-phage	0
DLF013_scaffold13718_6	non-phage	0
DLF013_scaffold22757_9	non-phage	0
DLF013_scaffold28910_11	non-phage	0
DLF013_scaffold54748_1	non-phage	0
DLF013_scaffold16968_3	non-phage	0
DLF013_scaffold13586_2	non-phage	0
DLF013_scaffold32304_1	non-phage	0
DLF013_scaffold21690_7	non-phage	0
DLF013_scaffold34289_3	non-phage	0
DLF013_scaffold35350_1	non-phage	0
DLF013_scaffold49692_1	non-phage	0
DLF013_scaffold47780_2	non-phage	0
DLF013_scaffold47243_2	non-phage	0
DLF013_scaffold18256_1	non-phage	0
DLF013_scaffold25725_6	non-phage	0
DLF013_scaffold19556_1	non-phage	0
DLF013_scaffold23947_1	non-phage	0
DLF013_scaffold49933_1	non-phage	0
DLF013_scaffold11990_9	non-phage	0
DLF013_scaffold35567_2	non-phage	0
DLF013_scaffold53489_5	non-phage	0
DLF013_scaffold777_5	non-phage	0
DLF013_scaffold53586_1	non-phage	0

DLF013_scaffold45866_2	non-phage	0
DLF013_scaffold26229_14	non-phage	0
DLF013_scaffold15284_22	non-phage	0
DLF013_scaffold40244_1	non-phage	0
DLF013_scaffold26726_2	non-phage	0
DLF013_scaffold12451_1	non-phage	0
DLF013_scaffold52016_1	non-phage	0
DLF013_scaffold25083_2	non-phage	0
DLF013_scaffold6385_5	non-phage	0
DLF013_scaffold5156_3	non-phage	0
DLF013_scaffold5745_11	non-phage	0
DLF013_scaffold14958_1	non-phage	0
DLF013_scaffold54489_1	non-phage	0
DLF013_scaffold54215_2	non-phage	0
DLF013_scaffold15141_2	non-phage	0
DLF013_scaffold113_19	non-phage	0
DLF013_scaffold31602_2	non-phage	0
DLF013_scaffold15996_2	non-phage	0
DLF013_scaffold9511_3	non-phage	0
DLF013_scaffold49692_4	non-phage	0
DLF013_scaffold19614_9	non-phage	0
DLF013_scaffold29800_1	non-phage	0
DLF013_scaffold19049_11	non-phage	0
DLF013_scaffold13756_6	non-phage	0
DLF013_scaffold265_9	non-phage	0
DLF013_scaffold46205_1	non-phage	0
DLF013_scaffold19423_10	non-phage	0
DLF013_scaffold16968_4	non-phage	0
DLF013_scaffold32932_2	non-phage	0
DLF013_scaffold20037_2	non-phage	0
DLF013_scaffold113_32	non-phage	0
DLF013_scaffold7820_2	non-phage	0
DLF013_scaffold40_1	non-phage	0
DLF013_scaffold48129_1	non-phage	0
DLF013_scaffold113_1	non-phage	0
DLF013_scaffold19614_8	non-phage	0
DLF013_scaffold5220_1	non-phage	0
DLF013_scaffold37221_1	non-phage	0
DLF013_scaffold34485_1	non-phage	0
DLF013_scaffold2964_3	non-phage	0
DLF013_scaffold17713_1	non-phage	0
DLF013_scaffold54621_1	non-phage	0
DLF013_scaffold26542_2	non-phage	0
DLF013_scaffold6358_1	non-phage	0
DLF014_scaffold2622_1	non-phage	0
DLF014_scaffold12493_4	non-phage	0

DLF014_scaffold12784_5	non-phage	0
DLF014_scaffold63_2	non-phage	0
DLF014_scaffold5654_2	non-phage	0
DLF014_scaffold2472_13	non-phage	0
DLF014_scaffold6783_2	non-phage	0
DLF014_scaffold12543_1	non-phage	0
DLF014_scaffold4597_2	non-phage	0
DLF014_scaffold5516_3	non-phage	0
DLF014_scaffold11946_1	non-phage	0
DLF014_scaffold134_1	non-phage	0
DLF014_C195403_1	non-phage	0
DLF014_scaffold8386_10	non-phage	0
DLF014_scaffold1571_4	non-phage	0
DLF014_scaffold18_4	non-phage	0
DLF014_C195173_1	non-phage	0
DLF014_scaffold222_10	non-phage	0
DLF014_scaffold12493_7	non-phage	0
DLF014_scaffold11836_1	non-phage	0
DLF014_scaffold2950_3	non-phage	0
DLF014_scaffold3265_8	non-phage	0
DLF014_scaffold4_2	non-phage	0
DLF014_scaffold3410_1	non-phage	0
DLF014_scaffold2779_11	non-phage	0
DLF014_scaffold513_17	non-phage	0
DLF014_scaffold6155_3	non-phage	0
DLF014_scaffold4687_3	non-phage	0
DLF014_scaffold1356_2	non-phage	0
DLF014_scaffold5474_1	non-phage	0
DLF014_scaffold291_7	non-phage	0
DLF014_scaffold12403_2	non-phage	0
DLF014_scaffold3477_6	non-phage	0
DLF014_scaffold10626_5	non-phage	0
DLF014_scaffold7878_3	non-phage	0
DLF014_scaffold911_3	non-phage	0
DLF014_scaffold11378_3	non-phage	0
DLF014_scaffold2824_1	non-phage	0
DLF014_scaffold4901_2	non-phage	0
DLF014_scaffold2264_4	non-phage	0
DLF014_scaffold5468_12	non-phage	0
DLF014_scaffold12567_1	non-phage	0
DLF014_scaffold3474_2	non-phage	0
DLF014_scaffold7631_1	non-phage	0
DLF014_scaffold6560_7	non-phage	0
DLF014_scaffold11946_3	non-phage	0
DLF014_scaffold12471_2	non-phage	0
DLF014_scaffold811_2	non-phage	0

DLF014_scaffold1092_3	non-phage	0
DLF014_scaffold7775_2	non-phage	0
DLF014_scaffold3628_25	non-phage	0
DLF014_scaffold6335_5	non-phage	0
DLF014_scaffold9725_1	non-phage	0
DLF014_scaffold9077_6	non-phage	0
DLF014_scaffold5854_1	non-phage	0
DLF014_scaffold11490_3	non-phage	0
DLF014_scaffold11946_2	non-phage	0
DLF014_scaffold781_6	non-phage	0
DLF014_scaffold339_2	non-phage	0
DLF014_scaffold12731_1	non-phage	0
DLF014_scaffold2350_3	non-phage	0
DLF014_scaffold2001_3	non-phage	0
DLF014_scaffold10956_2	non-phage	0
DLF014_scaffold222_12	non-phage	0
DLF014_scaffold4844_3	non-phage	0
DLF014_scaffold1211_1	non-phage	0
DLF014_scaffold222_11	non-phage	0
DLF014_scaffold1585_4	non-phage	0
DLF014_scaffold12119_1	non-phage	0
DLF014_scaffold10460_5	non-phage	0
DLF014_scaffold3265_9	non-phage	0
DLF014_scaffold9774_2	non-phage	0
DLF014_scaffold11440_1	non-phage	0
DLF014_scaffold4755_1	non-phage	0
DLF014_scaffold80_1	non-phage	0
DLF014_scaffold12591_1	non-phage	0
DLF014_scaffold7755_1	non-phage	0
DLF014_scaffold4751_1	non-phage	0
DLF014_scaffold2504_2	non-phage	0
DLF014_scaffold5061_1	non-phage	0
DLF014_scaffold513_1	non-phage	0
DLF014_scaffold8386_11	non-phage	0
DLF014_scaffold12728_1	non-phage	0
DLF014_scaffold8483_1	non-phage	0
DLF014_scaffold135_3	non-phage	0
DLF014_scaffold2234_10	non-phage	0
DLF014_scaffold12499_1	non-phage	0
DLF014_scaffold6619_1	non-phage	0
DLF014_scaffold5193_1	non-phage	0
DLF014_scaffold4763_2	non-phage	0
DLF014_scaffold2693_4	non-phage	0
DLF014_scaffold12613_1	non-phage	0
DLF014_scaffold4769_1	non-phage	0
DLF014_scaffold2738_4	non-phage	0

DLF014_scaffold5927_3	non-phage	0
DLF014_scaffold12765_2	non-phage	0
DLF014_scaffold3169_1	non-phage	0
DLF014_scaffold6452_2	non-phage	0
DLF014_scaffold4044_1	non-phage	0
DLF014_scaffold6805_4	non-phage	0
DLF014_scaffold10265_4	non-phage	0
DLF014_scaffold6151_1	non-phage	0
DLF014_scaffold1312_2	non-phage	0
DLF014_scaffold5425_7	non-phage	0
DLF014_scaffold11998_1	non-phage	0
DLF014_scaffold2950_8	non-phage	0
DLF014_scaffold8278_4	non-phage	0
DLF014_scaffold12747_3	non-phage	0
DLF014_scaffold12734_1	non-phage	0
DLF014_scaffold12052_1	non-phage	0
DLF014_scaffold3708_3	non-phage	0
NLF001_C572570_1	non-phage	0
NLF001_scaffold10423_4	non-phage	0
NLF001_scaffold21827_8	non-phage	0
NLF001_scaffold41265_3	non-phage	0
NLF001_scaffold16053_2	non-phage	0
NLF001_scaffold39270_2	non-phage	0
NLF001_scaffold69_7	non-phage	0
NLF001_scaffold8522_28	non-phage	0
NLF001_scaffold7121_1	non-phage	0
NLF001_scaffold28306_7	non-phage	0
NLF001_scaffold25289_3	non-phage	0
NLF001_scaffold35916_2	non-phage	0
NLF001_scaffold29251_2	phage	1
NLF001_scaffold39084_3	non-phage	0
NLF001_scaffold39512_2	non-phage	0
NLF001_scaffold42208_5	non-phage	0
NLF001_scaffold207_3	non-phage	0
NLF001_scaffold42465_3	non-phage	0
NLF001_scaffold3044_3	non-phage	0
NLF001_scaffold39690_1	non-phage	0
NLF001_scaffold18110_3	phage	0.999
NLF001_scaffold7964_1	non-phage	0
NLF001_scaffold7562_3	non-phage	0
NLF001_scaffold34904_3	non-phage	0
NLF001_scaffold3576_3	non-phage	0
NLF001_scaffold9889_4	non-phage	0
NLF001_scaffold40086_3	non-phage	0
NLF001_C572628_1	non-phage	0
NLF001_C572177_1	non-phage	0

NLF001_scaffold16427_3	non-phage	0
NLF001_scaffold42460_2	non-phage	0
NLF001_C572505_1	non-phage	0
NLF001_C572523_1	non-phage	0
NLF001_scaffold42370_1	non-phage	0
NLF001_scaffold4068_1	non-phage	0
NLF001_scaffold114_2	non-phage	0
NLF001_scaffold15503_1	non-phage	0
NLF001_scaffold7562_12	non-phage	0
NLF001_scaffold39778_1	non-phage	0
NLF001_scaffold42202_1	non-phage	0
NLF001_scaffold36_1	non-phage	0
NLF001_scaffold15397_1	non-phage	0
NLF001_scaffold18524_1	phage	0.986
NLF001_C572395_1	non-phage	0
NLF001_scaffold27204_1	non-phage	0
NLF001_scaffold37207_3	non-phage	0
NLF001_scaffold42452_1	non-phage	0
NLF001_scaffold21827_12	non-phage	0
NLF001_scaffold159_2	phage	0.999
NLF001_scaffold42461_2	non-phage	0
NLF001_scaffold41982_1	non-phage	0
NLF001_scaffold2179_11	non-phage	0
NLF001_C572129_1	non-phage	0
NLF001_scaffold64_28	non-phage	0
NLF001_scaffold15105_8	non-phage	0
NLF001_scaffold33383_1	non-phage	0
NLF001_scaffold63_8	non-phage	0
NLF001_scaffold25594_18	non-phage	0
NLF001_C572425_1	non-phage	0
NLF001_scaffold13029_7	non-phage	0
NLF001_scaffold41911_1	non-phage	0
NLF001_scaffold42477_2	non-phage	0
NLF001_C572207_1	non-phage	0
NLF001_scaffold41852_4	non-phage	0
NLF001_scaffold2_2	non-phage	0
NLF001_scaffold7498_6	non-phage	0
NLF001_scaffold38654_1	non-phage	0
NLF001_scaffold15523_8	non-phage	0
NLF001_scaffold5338_7	non-phage	0
NLF001_scaffold4415_17	non-phage	0
NLF001_scaffold42389_3	non-phage	0
NLF001_scaffold25289_6	non-phage	0
NLF001_scaffold14415_1	non-phage	0
NLF001_scaffold29097_1	non-phage	0
NLF001_scaffold42461_3	non-phage	0

NLF001_scaffold41012_1	non-phage	0
NLF001_scaffold28_2	non-phage	0
NLF001_scaffold23147_1	non-phage	0
NLF001_scaffold19409_2	non-phage	0
NLF001_scaffold27602_5	non-phage	0
NLF001_scaffold32838_2	non-phage	0
NLF001_scaffold31866_2	non-phage	0
NLF001_scaffold27486_10	non-phage	0
NLF001_scaffold22726_5	non-phage	0
NLF001_scaffold42323_1	non-phage	0
NLF001_scaffold16002_2	non-phage	0
NLF001_scaffold2157_1	non-phage	0
NLF001_scaffold48_1	non-phage	0
NLF001_scaffold3192_4	non-phage	0
NLF001_scaffold25017_2	non-phage	0
NLF001_scaffold23727_3	non-phage	0
NLF001_scaffold25289_8	non-phage	0
NLF001_scaffold34904_4	non-phage	0
NLF001_scaffold7965_11	non-phage	0
NLF001_scaffold25733_1	non-phage	0
NLF001_scaffold42247_1	non-phage	0
NLF001_scaffold42395_1	non-phage	0
NLF001_scaffold42439_2	non-phage	0
NLF001_scaffold20518_5	non-phage	0
NLF001_scaffold42254_1	non-phage	0
NLF001_scaffold3979_3	non-phage	0
NLF001_scaffold30562_1	non-phage	0
NLF001_scaffold41982_2	non-phage	0
NLF001_scaffold42359_1	non-phage	0
NLF001_scaffold21827_11	non-phage	0
NLF001_scaffold25962_5	non-phage	0
NLF001_scaffold9912_11	non-phage	0
NLF001_scaffold38450_3	non-phage	0
NLF001_scaffold1094_2	phage	0.961
NLF001_scaffold42474_1	non-phage	0
NLF001_scaffold34524_4	non-phage	0
NLF001_scaffold42258_2	non-phage	0
NLF001_scaffold17369_1	non-phage	0
NLF001_scaffold5734_4	non-phage	0
NLF001_scaffold11201_1	non-phage	0
NLF001_scaffold30907_8	phage	0.976
NLF001_scaffold3044_2	non-phage	0
NLF001_scaffold42470_1	phage	0.992
NLF001_scaffold41586_2	non-phage	0
NLF001_scaffold10463_6	non-phage	0
NLF001_scaffold35870_2	non-phage	0

NLF001_scaffold42185_1	non-phage	0
NLF001_scaffold12897_12	non-phage	0
NLF001_scaffold15921_9	phage	0.996
NLF001_scaffold2452_4	non-phage	0
NLF001_scaffold15397_4	non-phage	0
NLF001_scaffold20431_1	non-phage	0
NLF001_scaffold41911_2	non-phage	0
NLF001_scaffold3482_1	non-phage	0
NLF001_scaffold114_1	non-phage	0
NLF001_scaffold42398_2	non-phage	0
NLF001_scaffold3532_5	non-phage	0
NLF001_scaffold37207_4	non-phage	0
NLF001_scaffold35629_3	non-phage	0
NLF001_scaffold22726_1	non-phage	0
NLF001_scaffold2410_41	non-phage	0
NLF001_scaffold38127_2	non-phage	0
NLF001_scaffold41912_3	non-phage	0
NLF001_C572618_1	non-phage	0
NLF001_scaffold39084_2	non-phage	0
NLF001_scaffold34092_2	non-phage	0
NLF001_scaffold37226_2	non-phage	0
NLF001_scaffold34892_1	non-phage	0
NLF001_scaffold84_6	non-phage	0
NLF001_scaffold19409_7	non-phage	0
NLF001_scaffold14576_3	non-phage	0
NLF001_scaffold7121_2	non-phage	0
NLF001_scaffold18150_6	non-phage	0
NLF001_scaffold22631_6	non-phage	0
NLF001_scaffold38412_3	non-phage	0
NLF001_C572381_1	non-phage	0
NLF001_scaffold10983_1	non-phage	0
NLF001_scaffold36675_1	phage	0.998
NLF001_scaffold10203_1	non-phage	0
NLF001_scaffold10423_7	non-phage	0
NLF001_scaffold20431_2	non-phage	0
NLF001_scaffold13339_2	non-phage	0
NLF001_scaffold38608_2	non-phage	0
NLF001_scaffold38542_1	non-phage	0
NLF001_scaffold15523_5	non-phage	0
NLF001_scaffold42282_4	non-phage	0
NLF001_scaffold42282_1	non-phage	0
NLF001_scaffold33233_3	non-phage	0
NLF001_scaffold41852_1	non-phage	0
NLF001_scaffold25182_2	non-phage	0
NLF001_scaffold38183_2	non-phage	0
NLF001_scaffold31057_5	non-phage	0

NLF001_scaffold42260_2	non-phage	0
NLF001_scaffold2439_4	non-phage	0
NLF001_scaffold15039_4	non-phage	0
NLF001_scaffold200_1	non-phage	0
NLF001_scaffold30495_2	non-phage	0
NLF001_scaffold36072_1	non-phage	0
NLF001_scaffold33646_2	non-phage	0
NLF001_scaffold29958_1	non-phage	0
NLF001_scaffold42418_1	non-phage	0
NLF001_scaffold42461_4	non-phage	0
NLF001_scaffold3597_3	non-phage	0
NLF001_C572658_1	non-phage	0
NLF001_scaffold40967_2	non-phage	0
NLF001_scaffold16705_11	non-phage	0
NLF001_scaffold39688_1	non-phage	0
NLF001_scaffold57_3	non-phage	0
NLF001_scaffold13029_8	non-phage	0
NLF001_scaffold42186_2	non-phage	0
NLF001_scaffold25017_3	non-phage	0
NLF001_scaffold14565_1	non-phage	0
NLF001_scaffold19489_3	non-phage	0
NLF001_scaffold15028_2	phage	0.519
NLF001_scaffold2541_2	non-phage	0
NLF001_scaffold38616_3	non-phage	0
NLF001_scaffold30425_1	non-phage	0
NLF001_scaffold40631_2	non-phage	0
NLF001_scaffold21827_2	non-phage	0
NLF001_scaffold21827_1	phage	0.999
NLF001_scaffold3192_14	non-phage	0
NLF001_scaffold37591_2	non-phage	0
NLF001_scaffold63_2	non-phage	0
NLF001_scaffold32094_1	non-phage	0
NLF001_scaffold37498_1	non-phage	0
NLF001_scaffold37836_2	non-phage	0
NLF001_scaffold40376_1	non-phage	0
NLF001_scaffold37207_1	non-phage	0
NLF001_scaffold25289_9	non-phage	0
NLF001_scaffold42393_1	non-phage	0
NLF001_scaffold25962_4	non-phage	0
NLF001_scaffold57_5	non-phage	0
NLF001_scaffold42439_1	non-phage	0
NLF001_scaffold35629_4	non-phage	0.049
NLF001_scaffold42230_1	non-phage	0
NLF001_scaffold42384_1	non-phage	0
NLF001_scaffold31057_2	non-phage	0
NLF001_scaffold114_4	non-phage	0

NLF001_scaffold19489_1	non-phage	0
NLF001_scaffold17928_1	non-phage	0
NLF001_scaffold32838_1	non-phage	0
NLF001_scaffold42441_1	non-phage	0
NLF001_scaffold12411_7	non-phage	0
NLF001_scaffold42475_2	non-phage	0
NLF001_scaffold25182_1	non-phage	0
NLF001_scaffold31366_1	non-phage	0
NLF001_scaffold41197_2	non-phage	0
NLF001_scaffold7562_7	non-phage	0
NLF001_scaffold22032_1	non-phage	0
NLF001_scaffold16200_1	non-phage	0
NLF001_scaffold38025_1	non-phage	0
NLF001_C572297_1	non-phage	0
NLF001_scaffold41076_1	non-phage	0
NLF001_scaffold28949_3	non-phage	0
NLF001_scaffold42332_1	non-phage	0
NLF001_scaffold17952_7	phage	1
NLF001_scaffold7965_9	non-phage	0
NLF001_scaffold41613_1	non-phage	0
NLF001_scaffold15397_3	non-phage	0
NLF001_scaffold39084_1	non-phage	0
NLF001_scaffold19409_1	non-phage	0
NLF001_scaffold41912_2	non-phage	0
NLF001_scaffold42467_1	non-phage	0
NLF001_scaffold42383_2	non-phage	0
NLF001_scaffold1580_6	non-phage	0
NLF001_scaffold7064_2	non-phage	0
NLF001_scaffold35341_2	non-phage	0
NLF001_scaffold42428_1	non-phage	0
NLF001_scaffold21827_10	non-phage	0
NLF001_scaffold3044_1	non-phage	0
NLF001_scaffold5052_3	non-phage	0
NLF001_scaffold18110_5	non-phage	0
NLF001_scaffold16705_15	non-phage	0
NLF001_scaffold806_1	non-phage	0
NLF001_scaffold37836_1	non-phage	0
NLF001_scaffold20223_1	non-phage	0
NLF001_scaffold57_4	non-phage	0
NLF001_scaffold14576_4	non-phage	0
NLF001_scaffold1094_4	non-phage	0
NLF001_scaffold84_1	non-phage	0
NLF001_scaffold33713_1	non-phage	0
NLF001_scaffold40966_1	non-phage	0
NLF001_scaffold42258_4	non-phage	0
NLF001_scaffold207_5	non-phage	0

NLF001_scaffold34766_6	phage	0.999
NLF001_scaffold26_6	non-phage	0
NLF001_scaffold2_4	non-phage	0
NLF001_scaffold27547_1	non-phage	0
NLF001_scaffold20134_3	non-phage	0
NLF001_scaffold3597_1	non-phage	0
NLF001_scaffold2439_1	non-phage	0
NLF001_scaffold22726_2	non-phage	0
NLF001_scaffold17952_15	non-phage	0
NLF001_scaffold42479_1	non-phage	0
NLF001_scaffold2151_12	non-phage	0
NLF001_scaffold32919_1	non-phage	0
NLF001_scaffold4068_8	non-phage	0
NLF001_scaffold13029_10	non-phage	0
NLF001_scaffold39778_3	non-phage	0
NLF001_scaffold42478_2	phage	0.513
NLF001_scaffold6683_26	non-phage	0
NLF001_scaffold22238_2	non-phage	0
NLF001_scaffold42385_2	non-phage	0
NLF001_scaffold42444_1	non-phage	0
NLF001_scaffold28306_9	non-phage	0
NLF001_scaffold5627_23	non-phage	0
NLF001_scaffold42232_1	non-phage	0
NLF001_scaffold38154_2	non-phage	0
NLF001_scaffold34524_1	non-phage	0
NLF001_scaffold2452_1	non-phage	0
NLF001_scaffold42260_3	non-phage	0
NLF001_scaffold32_1	non-phage	0
NLF001_scaffold33437_4	non-phage	0
NLF001_scaffold25289_13	non-phage	0
NLF001_C572261_1	non-phage	0
NLF001_scaffold17928_2	non-phage	0
NLF001_scaffold39296_1	non-phage	0
NLF001_scaffold42456_1	phage	0.998
NLF001_scaffold42428_2	non-phage	0
NLF001_C572145_1	non-phage	0
NLF001_scaffold42195_2	non-phage	0
NLF001_scaffold41731_1	non-phage	0
NLF001_scaffold15039_1	non-phage	0
NLF001_scaffold42321_1	non-phage	0
NLF001_scaffold13326_20	non-phage	0
NLF001_scaffold27204_3	non-phage	0
NLF001_scaffold26_3	non-phage	0
NLF001_C572375_1	non-phage	0
NLF001_scaffold33233_2	non-phage	0
NLF001_scaffold42465_1	non-phage	0

NLF001_scaffold5627_18	non-phage	0
NLF001_scaffold15119_11	non-phage	0
NLF001_scaffold3979_5	non-phage	0
NLF001_scaffold19409_6	non-phage	0
NLF001_scaffold4106_3	non-phage	0
NLF001_scaffold40459_1	non-phage	0
NLF001_scaffold38127_1	non-phage	0
NLF001_scaffold22726_9	non-phage	0
NLF001_scaffold21408_7	phage	0.999
NLF001_scaffold40893_1	non-phage	0
NLF001_scaffold3597_4	non-phage	0
NLF001_scaffold1_3	non-phage	0
NLF001_scaffold42265_1	non-phage	0
NLF001_scaffold41222_1	non-phage	0
NLF001_scaffold20518_3	non-phage	0
NLF001_scaffold3979_1	non-phage	0
NLF001_scaffold30495_3	non-phage	0
NLF001_scaffold42397_2	non-phage	0
NLF001_scaffold15523_3	non-phage	0
NLF001_scaffold24222_1	non-phage	0
NLF001_scaffold36054_10	non-phage	0
NLF001_scaffold25978_11	non-phage	0
NLF001_scaffold3654_6	non-phage	0
NLF001_scaffold29262_7	non-phage	0
NLF001_scaffold31057_3	non-phage	0
NLF001_scaffold3044_4	non-phage	0
NLF001_scaffold44_5	non-phage	0
NLF001_C572149_1	non-phage	0
NLF001_scaffold41852_2	non-phage	0
NLF001_scaffold36702_1	non-phage	0
NLF001_scaffold3748_18	non-phage	0
NLF001_scaffold42472_1	phage	0.996
NLF001_scaffold4747_7	non-phage	0
NLF001_scaffold7562_8	non-phage	0
NLF001_scaffold1631_5	non-phage	0
NLF001_scaffold39036_6	non-phage	0
NLF001_scaffold21827_9	non-phage	0
NLF001_scaffold17946_1	non-phage	0
NLF001_scaffold28070_3	non-phage	0.002
NLF001_scaffold7881_2	non-phage	0
NLF001_scaffold28306_8	non-phage	0
NLF001_scaffold42429_1	non-phage	0
NLF001_scaffold34766_1	non-phage	0
NLF001_scaffold12485_4	non-phage	0
NLF001_scaffold12796_3	non-phage	0
NLF001_scaffold2452_2	non-phage	0

NLF001_scaffold9889_6	non-phage	0
NLF001_scaffold42257_1	non-phage	0
NLF001_scaffold31046_1	non-phage	0
NLF001_C572369_1	non-phage	0
NLF001_scaffold41025_2	non-phage	0
NLF001_scaffold27602_3	non-phage	0
NLF001_scaffold5052_4	non-phage	0
NLF001_scaffold11064_1	non-phage	0
NLF001_scaffold25962_1	non-phage	0
NLF001_scaffold17780_3	non-phage	0
NLF001_scaffold13029_11	non-phage	0
NLF001_scaffold42420_1	non-phage	0
NLF001_scaffold37207_7	non-phage	0
NLF001_scaffold42383_1	non-phage	0
NLF001_scaffold48_3	non-phage	0
NLF001_scaffold25289_2	non-phage	0
NLF001_scaffold7374_1	non-phage	0
NLF001_scaffold39244_3	non-phage	0
NLF001_scaffold24484_5	non-phage	0
NLF001_scaffold25017_4	non-phage	0
NLF001_scaffold36397_2	non-phage	0
NLF001_scaffold11298_3	non-phage	0
NLF001_C572546_1	phage	0.999
NLF001_scaffold41025_3	non-phage	0
NLF001_scaffold38144_4	phage	0.999
NLF001_scaffold16103_1	non-phage	0
NLF001_scaffold2541_3	non-phage	0
NLF001_scaffold12289_1	phage	0.999
NLF001_scaffold42471_1	non-phage	0
NLF001_scaffold57_2	non-phage	0
NLF001_scaffold42282_2	non-phage	0
NLF001_scaffold34766_5	phage	0.999
NLF001_scaffold15039_2	non-phage	0
NLF001_scaffold2523_51	non-phage	0
NLF001_scaffold16245_2	non-phage	0
NLF001_scaffold29098_1	non-phage	0
NLF001_scaffold41586_1	non-phage	0
NLF001_scaffold8668_2	non-phage	0
NLF001_scaffold9268_2	non-phage	0
NLF001_scaffold13541_1	non-phage	0
NLF001_scaffold42480_1	non-phage	0
NLF001_scaffold806_2	non-phage	0
NLF001_scaffold15397_2	non-phage	0
NLF001_scaffold5321_8	non-phage	0
NLF001_scaffold13029_2	non-phage	0
NLF001_scaffold28949_4	non-phage	0

NLF001_scaffold38616_5	non-phage	0
NLF001_scaffold23483_1	non-phage	0
NLF001_scaffold33713_2	phage	0.999
NLF001_scaffold30376_1	non-phage	0
NLF001_scaffold41569_1	non-phage	0
NLF001_scaffold3654_5	non-phage	0
NLF001_scaffold207_4	non-phage	0
NLF001_scaffold42404_1	non-phage	0
NLF001_scaffold19409_5	non-phage	0
NLF001_scaffold3979_4	non-phage	0
NLF001_scaffold19409_9	non-phage	0
NLF001_scaffold42430_2	non-phage	0
NLF001_scaffold31866_1	non-phage	0
NLF001_scaffold19409_4	non-phage	0
NLF001_C572389_1	non-phage	0
NLF001_scaffold38412_1	non-phage	0
NLF001_scaffold41076_2	non-phage	0
NLF001_scaffold36_3	non-phage	0
NLF001_scaffold7562_10	non-phage	0
NLF001_scaffold41852_5	non-phage	0
NLF001_scaffold28615_1	non-phage	0
NLF001_scaffold23357_1	non-phage	0
NLF001_scaffold42451_1	non-phage	0
NLF001_scaffold42464_1	non-phage	0
NLF001_scaffold27486_12	non-phage	0
NLF001_scaffold7121_4	non-phage	0
NLF001_scaffold42444_2	non-phage	0
NLF001_scaffold8432_1	non-phage	0
NLF001_scaffold22726_7	non-phage	0
NLF002_scaffold24169_4	non-phage	0
NLF002_scaffold12226_1	non-phage	0
NLF002_C494639_1	non-phage	0
NLF002_scaffold8636_6	non-phage	0
NLF002_scaffold1090_1	non-phage	0
NLF002_scaffold24121_1	non-phage	0
NLF002_scaffold38960_2	non-phage	0
NLF002_scaffold112_2	non-phage	0
NLF002_scaffold38967_1	non-phage	0
NLF002_scaffold7918_3	non-phage	0
NLF002_scaffold3316_2	non-phage	0
NLF002_scaffold280_14	non-phage	0
NLF002_scaffold22632_7	non-phage	0
NLF002_scaffold30029_1	non-phage	0
NLF002_scaffold2389_2	non-phage	0
NLF002_scaffold9950_11	non-phage	0
NLF002_scaffold35154_2	non-phage	0

NLF002_scaffold38862_1	non-phage	0
NLF002_scaffold805_4	non-phage	0
NLF002_scaffold103_11	non-phage	0
NLF002_scaffold13115_2	non-phage	0
NLF002_scaffold14983_2	non-phage	0
NLF002_scaffold5482_11	non-phage	0
NLF002_scaffold6226_1	non-phage	0
NLF002_scaffold22063_2	non-phage	0
NLF002_scaffold10644_1	non-phage	0
NLF002_scaffold104_12	non-phage	0
NLF002_scaffold33284_8	non-phage	0
NLF002_scaffold25135_10	non-phage	0
NLF002_scaffold38673_1	non-phage	0
NLF002_scaffold27272_2	non-phage	0
NLF002_scaffold21559_3	non-phage	0
NLF002_scaffold168_1	non-phage	0
NLF002_scaffold25269_2	non-phage	0
NLF002_scaffold14983_7	non-phage	0
NLF002_scaffold10018_3	non-phage	0
NLF002_scaffold24121_2	non-phage	0
NLF002_scaffold15782_3	non-phage	0
NLF002_scaffold2254_2	non-phage	0
NLF002_scaffold38266_2	non-phage	0
NLF002_scaffold25423_1	non-phage	0
NLF002_scaffold95_2	non-phage	0
NLF002_scaffold6031_1	non-phage	0
NLF002_scaffold33406_4	non-phage	0
NLF002_scaffold20226_22	non-phage	0
NLF002_scaffold30595_1	non-phage	0
NLF002_scaffold336_14	non-phage	0
NLF002_scaffold9125_1	non-phage	0
NLF002_scaffold26980_4	non-phage	0
NLF002_scaffold28111_3	non-phage	0
NLF002_scaffold11785_3	non-phage	0
NLF002_scaffold27015_7	non-phage	0
NLF002_scaffold5880_1	non-phage	0
NLF002_scaffold19968_1	non-phage	0
NLF002_scaffold28243_7	non-phage	0
NLF002_scaffold36414_1	non-phage	0
NLF002_scaffold34280_6	non-phage	0
NLF002_scaffold25503_2	non-phage	0
NLF002_scaffold299_3	non-phage	0
NLF002_scaffold2797_4	non-phage	0.001
NLF002_scaffold22175_4	non-phage	0
NLF002_scaffold18165_12	non-phage	0
NLF002_scaffold7830_8	non-phage	0

NLF002_scaffold31123_5	non-phage	0
NLF002_scaffold280_5	non-phage	0
NLF002_scaffold23718_3	non-phage	0
NLF002_scaffold10424_8	phage	0.974
NLF002_scaffold23394_3	non-phage	0
NLF002_scaffold7918_12	non-phage	0
NLF002_scaffold23394_2	non-phage	0
NLF002_scaffold38882_1	non-phage	0
NLF002_scaffold30350_2	non-phage	0
NLF002_scaffold19968_3	non-phage	0
NLF002_scaffold38961_1	non-phage	0
NLF002_scaffold41_3	non-phage	0
NLF002_scaffold336_5	non-phage	0
NLF002_scaffold104_11	non-phage	0
NLF002_scaffold19016_7	non-phage	0
NLF002_scaffold26669_4	non-phage	0
NLF002_scaffold16106_5	non-phage	0
NLF002_scaffold5978_1	non-phage	0
NLF002_scaffold14983_6	non-phage	0
NLF002_scaffold36662_1	phage	0.999
NLF002_scaffold336_22	phage	0.999
NLF002_scaffold8905_3	non-phage	0
NLF002_scaffold2166_25	non-phage	0
NLF002_scaffold8960_1	non-phage	0
NLF002_scaffold5694_1	non-phage	0
NLF002_scaffold38963_1	non-phage	0
NLF002_C494885_1	non-phage	0.105
NLF002_scaffold25510_1	non-phage	0
NLF002_scaffold38018_2	non-phage	0
NLF002_scaffold984_5	non-phage	0
NLF002_scaffold3093_21	non-phage	0
NLF002_scaffold25262_5	non-phage	0
NLF002_scaffold12352_1	non-phage	0
NLF002_scaffold159_2	non-phage	0
NLF002_scaffold23148_1	non-phage	0
NLF002_scaffold38725_2	non-phage	0
NLF002_scaffold8_1	non-phage	0
NLF002_scaffold38286_1	non-phage	0
NLF002_scaffold35171_2	phage	0.95
NLF002_scaffold36001_1	non-phage	0
NLF002_scaffold10337_9	non-phage	0
NLF002_scaffold38930_1	non-phage	0
NLF002_scaffold280_10	non-phage	0
NLF002_scaffold141_11	non-phage	0
NLF002_scaffold27015_10	non-phage	0
NLF002_scaffold38936_1	non-phage	0

NLF002_scaffold16871_1	phage	0.991
NLF002_scaffold2155_1	non-phage	0
NLF002_scaffold32332_2	non-phage	0
NLF002_scaffold17400_3	non-phage	0
NLF002_scaffold2254_17	non-phage	0
NLF002_scaffold24766_1	non-phage	0
NLF002_scaffold38813_2	non-phage	0
NLF002_scaffold510_2	non-phage	0
NLF002_scaffold6180_2	non-phage	0
NLF002_scaffold239_16	non-phage	0
NLF002_scaffold31160_2	non-phage	0
NLF002_scaffold103_16	non-phage	0
NLF002_scaffold35981_2	non-phage	0
NLF002_scaffold2243_3	non-phage	0
NLF002_scaffold103_4	non-phage	0
NLF002_C495327_1	non-phage	0
NLF002_scaffold38830_1	non-phage	0
NLF002_scaffold22680_1	non-phage	0
NLF002_scaffold37_6	non-phage	0
NLF002_scaffold31123_2	non-phage	0
NLF002_scaffold25135_4	non-phage	0
NLF002_scaffold187_7	non-phage	0
NLF002_scaffold33404_1	non-phage	0
NLF002_scaffold26540_1	non-phage	0
NLF002_scaffold8960_5	non-phage	0
NLF002_scaffold38764_1	non-phage	0
NLF002_scaffold38343_1	non-phage	0
NLF002_scaffold36137_2	non-phage	0
NLF002_scaffold717_4	non-phage	0
NLF002_scaffold20207_4	non-phage	0
NLF002_scaffold38703_1	non-phage	0
NLF002_scaffold38856_2	non-phage	0.095
NLF002_scaffold27015_1	non-phage	0
NLF002_scaffold11045_1	non-phage	0
NLF002_scaffold5813_2	non-phage	0
NLF002_scaffold6393_3	non-phage	0
NLF002_scaffold31980_1	non-phage	0
NLF002_scaffold21559_4	non-phage	0
NLF002_scaffold16149_3	non-phage	0
NLF002_scaffold943_1	non-phage	0
NLF002_scaffold12247_8	non-phage	0
NLF002_scaffold31975_2	non-phage	0
NLF002_scaffold5670_9	non-phage	0
NLF002_scaffold32625_2	non-phage	0
NLF002_scaffold7918_2	non-phage	0
NLF002_scaffold9840_1	non-phage	0

NLF002_scaffold28626_2	phage	0.971
NLF002_scaffold1090_3	non-phage	0
NLF002_scaffold95_4	non-phage	0
NLF002_scaffold6690_1	non-phage	0
NLF002_scaffold34193_1	non-phage	0
NLF002_scaffold24169_3	non-phage	0
NLF002_scaffold3501_1	non-phage	0
NLF002_scaffold34060_1	non-phage	0
NLF002_scaffold5482_2	non-phage	0
NLF002_scaffold28243_10	non-phage	0
NLF002_scaffold38874_3	non-phage	0
NLF002_scaffold30583_2	non-phage	0
NLF002_scaffold35902_1	non-phage	0
NLF002_scaffold2166_22	non-phage	0.003
NLF002_scaffold336_9	non-phage	0
NLF002_scaffold563_15	non-phage	0
NLF002_scaffold36194_1	non-phage	0
NLF002_scaffold37572_1	non-phage	0
NLF002_scaffold38877_1	non-phage	0
NLF002_scaffold31316_3	non-phage	0
NLF002_scaffold3075_5	non-phage	0
NLF002_scaffold5450_1	non-phage	0
NLF002_scaffold161_1	non-phage	0
NLF002_scaffold27163_1	non-phage	0
NLF002_scaffold38743_3	non-phage	0
NLF002_scaffold6031_13	non-phage	0
NLF002_scaffold23799_2	non-phage	0
NLF002_scaffold6457_1	non-phage	0
NLF002_scaffold38970_1	non-phage	0
NLF002_scaffold34776_1	phage	0.999
NLF002_scaffold38571_1	non-phage	0
NLF002_scaffold5880_2	non-phage	0
NLF002_scaffold22063_1	non-phage	0
NLF002_scaffold23017_1	non-phage	0
NLF002_scaffold2155_4	non-phage	0
NLF002_scaffold159_1	non-phage	0
NLF002_scaffold197_2	non-phage	0
NLF002_scaffold10077_5	non-phage	0
NLF002_scaffold18165_11	non-phage	0
NLF002_scaffold2717_3	non-phage	0
NLF002_scaffold26709_4	non-phage	0
NLF002_scaffold2254_3	non-phage	0
NLF002_scaffold28890_4	non-phage	0
NLF002_scaffold28836_2	non-phage	0
NLF002_scaffold266_2	non-phage	0
NLF002_C494733_1	non-phage	0

NLF002_scaffold27908_2	non-phage	0
NLF002_C494755_1	non-phage	0
NLF002_scaffold28727_1	non-phage	0
NLF002_scaffold336_1	non-phage	0
NLF002_scaffold17395_2	non-phage	0
NLF002_scaffold32233_1	non-phage	0
NLF002_scaffold239_19	non-phage	0
NLF002_scaffold2199_5	non-phage	0
NLF002_scaffold38971_1	non-phage	0
NLF002_scaffold32309_1	non-phage	0
NLF002_scaffold19661_6	non-phage	0
NLF002_scaffold25410_1	non-phage	0
NLF002_scaffold23617_16	non-phage	0
NLF002_scaffold22680_4	phage	0.945
NLF002_scaffold2797_2	non-phage	0
NLF002_scaffold22204_4	non-phage	0
NLF002_scaffold2899_1	non-phage	0
NLF002_scaffold14032_2	non-phage	0
NLF002_scaffold28111_2	non-phage	0
NLF002_scaffold103_10	non-phage	0
NLF002_scaffold25757_1	non-phage	0
NLF002_scaffold8960_4	non-phage	0
NLF002_scaffold38607_1	non-phage	0
NLF002_scaffold99_8	non-phage	0
NLF002_scaffold38958_1	non-phage	0
NLF002_scaffold35163_1	non-phage	0
NLF002_scaffold238_9	non-phage	0
NLF002_scaffold36854_1	non-phage	0
NLF002_scaffold95_9	non-phage	0
NLF002_scaffold38872_2	non-phage	0
NLF002_scaffold2797_8	non-phage	0
NLF002_scaffold31899_1	non-phage	0
NLF002_scaffold38469_1	non-phage	0
NLF002_scaffold13115_3	non-phage	0
NLF002_scaffold29_1	non-phage	0
NLF002_scaffold280_3	non-phage	0
NLF002_scaffold6663_3	phage	0.999
NLF002_scaffold26758_3	non-phage	0
NLF002_scaffold18606_1	non-phage	0
NLF002_scaffold6533_1	non-phage	0
NLF002_scaffold20226_23	non-phage	0
NLF002_scaffold25503_4	non-phage	0
NLF002_scaffold37757_3	phage	0.999
NLF002_scaffold75_2	non-phage	0
NLF002_scaffold10337_3	non-phage	0
NLF002_scaffold16787_3	non-phage	0

NLF002_scaffold37614_1	non-phage	0
NLF002_scaffold38253_1	non-phage	0
NLF002_scaffold3933_4	phage	0.999
NLF002_scaffold29053_1	non-phage	0
NLF002_scaffold38787_1	non-phage	0
NLF002_scaffold6666_2	non-phage	0
NLF002_scaffold23200_3	non-phage	0
NLF002_scaffold25935_2	non-phage	0
NLF002_scaffold38948_1	non-phage	0
NLF002_scaffold37_7	non-phage	0
NLF002_scaffold38327_3	non-phage	0
NLF002_scaffold27163_3	non-phage	0
NLF002_scaffold159_4	non-phage	0
NLF002_scaffold385_3	non-phage	0
NLF002_scaffold984_1	non-phage	0
NLF002_scaffold14_6	non-phage	0
NLF002_scaffold266_3	non-phage	0
NLF002_scaffold510_16	non-phage	0
NLF002_scaffold14709_2	phage	0.999
NLF002_scaffold20480_5	non-phage	0
NLF002_scaffold2243_61	non-phage	0
NLF002_scaffold6371_2	non-phage	0
NLF002_scaffold25508_2	non-phage	0
NLF002_scaffold12312_1	non-phage	0
NLF002_scaffold27060_12	non-phage	0
NLF002_scaffold22063_5	non-phage	0
NLF002_scaffold238_11	non-phage	0
NLF002_scaffold10424_7	non-phage	0
NLF002_scaffold27060_6	phage	0.937
NLF002_scaffold30771_2	non-phage	0
NLF002_scaffold6170_25	non-phage	0
NLF002_scaffold19661_3	non-phage	0
NLF002_scaffold16156_28	non-phage	0
NLF002_scaffold19680_5	non-phage	0
NLF002_scaffold33404_3	non-phage	0
NLF002_scaffold19490_3	non-phage	0
NLF002_scaffold24722_8	non-phage	0
NLF002_scaffold280_11	non-phage	0
NLF002_scaffold2155_3	non-phage	0
NLF002_scaffold33406_9	non-phage	0
NLF002_scaffold4_1	non-phage	0
NLF002_scaffold33109_4	non-phage	0
NLF002_scaffold197_10	non-phage	0
NLF002_scaffold38537_2	non-phage	0
NLF002_scaffold30049_1	non-phage	0
NLF002_scaffold11755_1	non-phage	0

NLF002_scaffold17400_8	non-phage	0
NLF002_scaffold32332_1	non-phage	0
NLF002_scaffold95_13	non-phage	0
NLF002_scaffold4057_5	non-phage	0
NLF002_scaffold42_1	non-phage	0
NLF002_scaffold25423_5	non-phage	0
NLF002_scaffold26540_3	non-phage	0
NLF002_scaffold6806_3	non-phage	0
NLF002_scaffold336_19	non-phage	0
NLF002_scaffold38537_1	non-phage	0
NLF002_scaffold189_5	non-phage	0
NLF002_scaffold9125_3	non-phage	0
NLF002_scaffold2807_10	non-phage	0
NLF002_scaffold2155_6	non-phage	0
NLF002_scaffold2722_3	non-phage	0
NLF002_scaffold14254_2	non-phage	0
NLF002_scaffold22632_1	non-phage	0
NLF002_scaffold25423_6	non-phage	0
NLF002_scaffold31730_4	non-phage	0
NLF002_scaffold63_2	non-phage	0
NLF002_scaffold33404_2	non-phage	0
NLF002_scaffold141_5	non-phage	0
NLF002_scaffold38951_2	non-phage	0
NLF002_scaffold19661_5	non-phage	0
NLF002_scaffold2797_3	non-phage	0
NLF002_scaffold120_10	non-phage	0
NLF002_scaffold5482_17	non-phage	0
NLF002_scaffold15090_4	non-phage	0
NLF002_scaffold27060_14	non-phage	0
NLF002_scaffold2700_1	non-phage	0
NLF002_scaffold38945_1	non-phage	0
NLF002_scaffold33406_2	non-phage	0
NLF002_scaffold27776_1	non-phage	0
NLF002_scaffold5394_1	non-phage	0.259
NLF002_scaffold1827_2	non-phage	0
NLF002_scaffold3427_3	non-phage	0
NLF002_scaffold30771_1	non-phage	0
NLF002_scaffold37588_2	non-phage	0
NLF002_scaffold15559_1	phage	0.986
NLF002_scaffold35132_2	non-phage	0
NLF002_scaffold38367_1	non-phage	0
NLF002_scaffold280_7	non-phage	0
NLF002_scaffold105_2	non-phage	0
NLF002_scaffold28890_3	non-phage	0
NLF002_scaffold27015_4	non-phage	0
NLF002_scaffold4563_2	non-phage	0

NLF002_scaffold6031_5	non-phage	0
NLF002_scaffold22175_2	non-phage	0
NLF002_scaffold38687_1	non-phage	0
NLF002_scaffold27163_4	non-phage	0
NLF002_scaffold336_12	non-phage	0
NLF002_scaffold103_2	non-phage	0
NLF002_scaffold9125_4	non-phage	0
NLF002_scaffold31123_4	non-phage	0
NLF002_scaffold10424_1	non-phage	0
NLF002_scaffold9044_1	non-phage	0
NLF002_scaffold34056_1	non-phage	0
NLF002_C495013_1	non-phage	0.001
NLF002_scaffold25935_7	non-phage	0
NLF002_scaffold16106_3	non-phage	0
NLF002_scaffold2254_10	non-phage	0
NLF002_scaffold6393_4	non-phage	0
NLF002_scaffold38804_1	non-phage	0
NLF002_scaffold29763_6	non-phage	0
NLF002_scaffold192_3	non-phage	0
NLF002_scaffold17091_1	non-phage	0
NLF002_scaffold18611_2	non-phage	0
NLF002_scaffold32285_2	non-phage	0
NLF002_scaffold24722_7	non-phage	0
NLF002_scaffold37359_4	non-phage	0
NLF002_scaffold20226_1	non-phage	0
NLF002_scaffold32846_1	non-phage	0
NLF002_scaffold21559_1	non-phage	0
NLF002_scaffold3280_1	non-phage	0
NLF002_scaffold168_3	non-phage	0
NLF002_scaffold38916_1	non-phage	0
NLF002_scaffold38907_1	non-phage	0
NLF002_scaffold26980_1	non-phage	0
NLF002_scaffold19765_4	phage	0.974
NLF002_scaffold37749_2	non-phage	0
NLF002_scaffold11941_2	non-phage	0
NLF002_scaffold4313_3	non-phage	0
NLF002_scaffold10337_5	non-phage	0
NLF002_C495391_1	non-phage	0
NLF002_scaffold285_4	non-phage	0
NLF002_scaffold37757_1	non-phage	0
NLF002_scaffold315_1	non-phage	0
NLF002_scaffold27449_2	non-phage	0
NLF002_scaffold13096_1	non-phage	0
NLF002_scaffold5685_1	non-phage	0
NLF002_scaffold30049_2	non-phage	0
NLF002_scaffold510_10	non-phage	0

NLF002_scaffold29551_1	non-phage	0
NLF002_scaffold6737_1	non-phage	0
NLF002_scaffold20480_2	non-phage	0
NLF002_scaffold19743_1	non-phage	0
NLF002_scaffold141_14	phage	0.999
NLF002_scaffold2199_6	phage	0.999
NLF002_scaffold19122_7	non-phage	0
NLF002_scaffold239_14	non-phage	0
NLF002_scaffold2155_2	non-phage	0
NLF002_scaffold30881_3	non-phage	0
NLF002_scaffold84_1	non-phage	0
NLF002_scaffold11013_1	non-phage	0
NLF002_scaffold38925_2	non-phage	0
NLF002_scaffold38607_2	non-phage	0
NLF002_scaffold542_21	non-phage	0
NLF002_scaffold2233_6	non-phage	0
NLF002_scaffold9840_3	non-phage	0
NLF002_scaffold38347_2	non-phage	0
NLF002_scaffold38969_1	non-phage	0
NLF002_scaffold36924_1	phage	0.996
NLF002_scaffold37002_1	non-phage	0
NLF002_scaffold27060_11	non-phage	0
NLF002_scaffold54_2	non-phage	0
NLF002_scaffold34280_8	non-phage	0
NLF002_scaffold336_3	non-phage	0
NLF002_scaffold31503_1	non-phage	0
NLF002_scaffold38429_1	non-phage	0
NLF002_scaffold25135_2	non-phage	0
NLF002_scaffold4057_6	non-phage	0
NLF002_scaffold29_2	non-phage	0
NLF002_scaffold4248_2	non-phage	0
NLF002_scaffold11467_17	non-phage	0
NLF002_scaffold38671_1	non-phage	0
NLF002_scaffold34060_2	non-phage	0
NLF002_scaffold19490_4	non-phage	0
NLF002_scaffold38168_2	non-phage	0
NLF002_scaffold38539_1	non-phage	0
NLF002_scaffold19490_5	non-phage	0
NLF002_scaffold41_1	non-phage	0
NLF002_scaffold11467_16	non-phage	0
NLF002_scaffold2166_23	non-phage	0
NLF002_scaffold35525_6	non-phage	0
NLF002_scaffold31946_1	non-phage	0
NLF002_scaffold11467_21	non-phage	0
NLF002_scaffold95_5	non-phage	0
NLF002_scaffold25262_3	non-phage	0

NLF002_scaffold21559_12	non-phage	0
NLF002_scaffold20226_19	non-phage	0
NLF002_scaffold2717_5	non-phage	0
NLF002_scaffold38774_1	non-phage	0
NLF002_scaffold28111_1	non-phage	0
NLF002_scaffold38848_3	non-phage	0.019
NLF002_scaffold10337_7	non-phage	0
NLF002_scaffold3117_10	non-phage	0
NLF002_scaffold2807_9	non-phage	0
NLF002_scaffold21559_5	non-phage	0
NLF002_scaffold17057_1	non-phage	0
NLF002_scaffold33406_11	non-phage	0
NLF002_scaffold120_1	non-phage	0
NLF002_scaffold29065_10	non-phage	0
NLF002_scaffold19661_2	non-phage	0
NLF002_scaffold563_24	non-phage	0.336
NLF002_scaffold33335_2	non-phage	0
NLF002_scaffold103_9	non-phage	0
NLF002_scaffold38743_1	non-phage	0
NLF002_scaffold20092_2	non-phage	0
NLF002_scaffold38929_1	non-phage	0
NLF002_scaffold36625_1	non-phage	0
NLF002_scaffold15559_4	non-phage	0
NLF002_C494841_1	non-phage	0
NLF002_scaffold25135_3	non-phage	0
NLF002_scaffold34192_1	non-phage	0
NLF002_scaffold23394_4	non-phage	0
NLF002_scaffold28890_2	non-phage	0
NLF002_scaffold563_20	non-phage	0
NLF002_scaffold103_14	non-phage	0
NLF002_scaffold15782_2	non-phage	0
NLF002_scaffold38143_1	non-phage	0
NLF002_scaffold115_2	phage	0.999
NLF002_scaffold4_3	non-phage	0
NLF002_scaffold285_5	non-phage	0
NLF002_scaffold239_12	non-phage	0
NLF002_scaffold30595_2	non-phage	0
NLF002_scaffold32295_1	non-phage	0
NLF002_scaffold7918_18	non-phage	0
NLF002_scaffold11737_1	non-phage	0
NLF002_scaffold33406_5	non-phage	0
NLF002_scaffold38800_1	non-phage	0
NLF002_scaffold24014_2	non-phage	0
NLF002_scaffold239_17	non-phage	0
NLF002_scaffold36509_4	non-phage	0
NLF002_scaffold36422_1	non-phage	0

NLF002_scaffold38736_1	non-phage	0
NLF002_scaffold37911_11	non-phage	0
NLF002_scaffold29375_2	non-phage	0
NLF002_scaffold19204_2	non-phage	0
NLF002_scaffold141_16	non-phage	0
NLF002_C495329_1	non-phage	0.001
NLF002_scaffold22974_3	non-phage	0
NLF002_scaffold38730_1	non-phage	0
NLF002_scaffold34766_1	non-phage	0
NLF002_scaffold15474_3	non-phage	0.252
NLF002_scaffold563_4	non-phage	0
NLF002_scaffold297_2	non-phage	0
NLF002_scaffold17275_1	non-phage	0
NLF002_scaffold2243_17	non-phage	0
NLF002_scaffold104_15	non-phage	0
NLF002_scaffold6534_2	non-phage	0
NLF002_scaffold36001_2	non-phage	0
NLF002_scaffold38855_1	non-phage	0
NLF002_scaffold18043_6	non-phage	0
NLF002_scaffold15090_2	non-phage	0
NLF002_scaffold38496_1	non-phage	0
NLF002_scaffold3093_18	non-phage	0
NLF002_scaffold31123_3	non-phage	0
NLF002_scaffold38347_1	non-phage	0
NLF002_scaffold33277_1	non-phage	0
NLF002_scaffold37757_4	phage	0.999
NLF002_C495141_1	non-phage	0
NLF002_scaffold23200_4	non-phage	0
NLF002_scaffold16542_4	non-phage	0
NLF002_scaffold280_6	non-phage	0
NLF002_scaffold38899_1	non-phage	0
NLF002_scaffold2166_27	non-phage	0
NLF002_scaffold189_6	non-phage	0
NLF002_scaffold95_1	non-phage	0
NLF002_scaffold103_1	non-phage	0
NLF002_C495023_1	non-phage	0
NLF002_scaffold16913_1	non-phage	0
NLF002_scaffold38493_1	non-phage	0
NLF002_scaffold25562_1	non-phage	0
NLF002_scaffold141_15	non-phage	0
NLF002_scaffold38900_1	non-phage	0
NLF002_scaffold26980_3	non-phage	0
NLF002_scaffold16952_1	non-phage	0
NLF002_scaffold25135_7	non-phage	0
NLF002_scaffold7917_3	non-phage	0
NLF002_scaffold38544_2	non-phage	0

NLF002_scaffold20362_1	non-phage	0
NLF002_scaffold25227_2	non-phage	0
NLF002_scaffold2807_4	non-phage	0
NLF002_scaffold14983_11	non-phage	0
NLF002_scaffold37757_5	phage	0.999
NLF002_scaffold38430_4	non-phage	0
NLF002_scaffold10424_2	non-phage	0
NLF002_scaffold17055_1	phage	0.991
NLF002_scaffold16156_11	non-phage	0
NLF002_scaffold8668_2	non-phage	0
NLF002_scaffold22063_7	non-phage	0
NLF002_scaffold336_23	non-phage	0
NLF002_scaffold23617_15	non-phage	0
NLF002_scaffold9840_5	non-phage	0
NLF002_scaffold34280_1	non-phage	0
NLF002_scaffold1328_3	non-phage	0
NLF002_scaffold37503_2	non-phage	0
NLF002_scaffold19968_4	non-phage	0
NLF002_scaffold24116_1	non-phage	0
NLF002_scaffold25489_2	phage	0.999
NLF002_scaffold31305_6	non-phage	0
NLF002_scaffold2254_11	non-phage	0
NLF002_scaffold22786_2	non-phage	0
NLF002_scaffold34060_5	non-phage	0
NLF002_scaffold6031_9	non-phage	0
NLF002_scaffold38187_1	non-phage	0
NLF002_scaffold27449_1	non-phage	0
NLF002_scaffold28111_4	non-phage	0
NLF002_scaffold18611_12	non-phage	0
NLF002_scaffold33406_1	non-phage	0
NLF002_scaffold19016_6	non-phage	0
NLF002_scaffold8905_4	non-phage	0
NLF002_scaffold2243_27	non-phage	0
NLF002_scaffold16106_4	non-phage	0
NLF002_scaffold38860_2	non-phage	0
NLF002_scaffold38931_1	non-phage	0
NLF002_scaffold11467_13	non-phage	0
NLF002_scaffold4057_3	non-phage	0
NLF002_scaffold29492_1	non-phage	0
NLF002_scaffold150_4	non-phage	0
NLF002_scaffold2155_5	non-phage	0
NLF002_scaffold19490_6	non-phage	0
NLF002_scaffold26709_5	non-phage	0
NLF002_scaffold26540_5	non-phage	0
NLF002_scaffold23148_2	non-phage	0
NLF002_scaffold112_4	non-phage	0

NLF002_scaffold38848_2	non-phage	0
NLF002_scaffold38957_1	non-phage	0
NLF002_scaffold95_6	non-phage	0
NLF002_scaffold36590_1	non-phage	0
NLF002_scaffold7918_1	non-phage	0
NLF002_scaffold3093_15	non-phage	0
NLF002_scaffold28965_2	non-phage	0
NLF002_scaffold32296_2	non-phage	0
NLF002_C495405_1	non-phage	0
NLF002_scaffold10697_1	non-phage	0
NLF002_scaffold8029_2	non-phage	0
NLF002_scaffold14739_1	non-phage	0
NLF002_scaffold103_8	non-phage	0
NLF002_scaffold38918_2	non-phage	0
NLF002_scaffold10337_6	non-phage	0
NLF002_scaffold18433_1	non-phage	0
NLF002_scaffold222_3	non-phage	0
NLF002_scaffold2883_1	non-phage	0
NLF002_scaffold336_8	non-phage	0
NLF002_scaffold2406_1	non-phage	0
NLF002_scaffold15090_1	non-phage	0
NLF002_scaffold38327_1	non-phage	0
NLF002_scaffold36509_6	non-phage	0
NLF002_scaffold221_1	non-phage	0
NLF002_scaffold21559_6	non-phage	0
NLF002_scaffold4313_1	non-phage	0
NLF002_scaffold38935_2	phage	0.998
NLF002_scaffold10644_4	non-phage	0
NLF002_scaffold21186_2	non-phage	0
NLF002_C494713_1	non-phage	0
NLF002_scaffold31160_3	non-phage	0
NLF002_scaffold35163_3	non-phage	0
NLF002_scaffold103_5	non-phage	0
NLF002_scaffold22063_3	non-phage	0
NLF002_scaffold16156_32	non-phage	0
NLF005_scaffold11063_2	non-phage	0
NLF005_scaffold48181_1	non-phage	0
NLF005_scaffold3555_7	non-phage	0
NLF005_scaffold37558_2	non-phage	0
NLF005_scaffold10734_7	non-phage	0
NLF005_scaffold48433_1	non-phage	0
NLF005_scaffold19706_6	non-phage	0
NLF005_scaffold7337_21	non-phage	0
NLF005_scaffold25190_1	non-phage	0
NLF005_scaffold48419_2	non-phage	0
NLF005_scaffold26630_6	non-phage	0

NLF005_scaffold27915_1	non-phage	0
NLF005_scaffold13871_1	non-phage	0
NLF005_scaffold16081_6	non-phage	0
NLF005_scaffold7337_11	non-phage	0
NLF005_scaffold174_6	non-phage	0
NLF005_scaffold48623_1	non-phage	0
NLF005_scaffold18384_6	non-phage	0
NLF005_scaffold13278_4	non-phage	0
NLF005_scaffold135_9	non-phage	0
NLF005_scaffold248_3	non-phage	0
NLF005_scaffold21158_2	non-phage	0
NLF005_scaffold13387_6	non-phage	0
NLF005_scaffold32729_3	non-phage	0
NLF005_scaffold28589_13	non-phage	0
NLF005_scaffold135_4	non-phage	0
NLF005_scaffold48622_1	non-phage	0
NLF005_scaffold4391_2	phage	1
NLF005_scaffold48094_1	non-phage	0
NLF005_scaffold48177_1	non-phage	0
NLF005_scaffold48311_1	non-phage	0
NLF005_scaffold83_2	non-phage	0
NLF005_scaffold37521_2	non-phage	0
NLF005_scaffold8024_3	non-phage	0
NLF005_C729885_1	non-phage	0
NLF005_scaffold48409_1	non-phage	0
NLF005_scaffold47996_1	non-phage	0
NLF005_scaffold235_1	non-phage	0
NLF005_scaffold47881_1	non-phage	0
NLF005_scaffold58_1	non-phage	0
NLF005_scaffold48344_2	non-phage	0
NLF005_scaffold32394_6	non-phage	0
NLF005_scaffold25566_4	non-phage	0
NLF005_scaffold39995_12	non-phage	0
NLF005_scaffold48425_1	non-phage	0
NLF005_scaffold134_1	non-phage	0
NLF005_scaffold1317_4	non-phage	0
NLF005_scaffold45763_1	non-phage	0
NLF005_scaffold549_13	non-phage	0
NLF005_scaffold41583_1	non-phage	0
NLF005_scaffold38234_1	non-phage	0
NLF005_scaffold48461_1	non-phage	0
NLF005_scaffold48449_4	non-phage	0
NLF005_scaffold40666_4	non-phage	0
NLF005_scaffold48093_1	non-phage	0
NLF005_scaffold13578_1	non-phage	0
NLF005_scaffold34861_1	non-phage	0

NLF005_scaffold46429_3	non-phage	0
NLF005_scaffold16718_21	non-phage	0
NLF005_scaffold6409_2	non-phage	0
NLF005_scaffold12384_3	non-phage	0.002
NLF005_scaffold3103_2	non-phage	0
NLF005_scaffold48491_1	non-phage	0
NLF005_scaffold21492_3	phage	0.999
NLF005_scaffold235_6	non-phage	0
NLF005_scaffold48484_1	non-phage	0
NLF005_scaffold30572_4	non-phage	0
NLF005_scaffold28003_1	non-phage	0
NLF005_scaffold35783_2	non-phage	0
NLF005_scaffold236_2	non-phage	0
NLF005_scaffold45718_3	non-phage	0
NLF005_scaffold2467_2	non-phage	0
NLF005_scaffold48495_3	non-phage	0
NLF005_scaffold3524_1	non-phage	0
NLF005_C729703_1	non-phage	0
NLF005_scaffold48256_1	non-phage	0
NLF005_scaffold16738_1	non-phage	0
NLF005_scaffold48093_2	non-phage	0
NLF005_scaffold47894_4	non-phage	0
NLF005_scaffold2467_16	non-phage	0.186
NLF005_scaffold48413_3	non-phage	0
NLF005_scaffold32729_6	non-phage	0
NLF005_scaffold361_3	non-phage	0
NLF005_scaffold15318_7	non-phage	0
NLF005_scaffold48485_1	non-phage	0
NLF005_scaffold46950_1	phage	0.974
NLF005_scaffold48565_4	non-phage	0
NLF005_scaffold17372_2	non-phage	0
NLF005_scaffold10674_1	non-phage	0
NLF005_scaffold48499_3	non-phage	0
NLF005_scaffold4043_1	non-phage	0
NLF005_scaffold39584_1	non-phage	0
NLF005_scaffold12496_1	non-phage	0
NLF005_scaffold24685_2	non-phage	0
NLF005_scaffold19849_17	non-phage	0.199
NLF005_scaffold47156_1	non-phage	0
NLF005_scaffold39995_7	non-phage	0
NLF005_scaffold9605_1	non-phage	0
NLF005_scaffold23423_1	non-phage	0
NLF005_scaffold28363_1	non-phage	0
NLF005_scaffold1798_4	non-phage	0
NLF005_scaffold48624_1	non-phage	0
NLF005_scaffold48202_1	non-phage	0

NLF005_scaffold7301_1	non-phage	0
NLF005_scaffold43152_1	non-phage	0
NLF005_scaffold405_9	non-phage	0
NLF005_scaffold48383_6	non-phage	0
NLF005_scaffold18384_9	non-phage	0
NLF005_scaffold9805_1	non-phage	0
NLF005_scaffold47934_9	non-phage	0
NLF005_scaffold15835_5	phage	0.997
NLF005_scaffold20_4	non-phage	0
NLF005_scaffold4074_1	non-phage	0
NLF005_scaffold48610_1	non-phage	0
NLF005_scaffold4105_4	non-phage	0
NLF005_scaffold19706_2	non-phage	0
NLF005_scaffold48560_1	non-phage	0
NLF005_scaffold38915_1	non-phage	0
NLF005_scaffold5148_1	non-phage	0
NLF005_scaffold36523_1	phage	0.996
NLF005_scaffold7337_15	non-phage	0
NLF005_scaffold34886_1	non-phage	0
NLF005_scaffold31720_1	non-phage	0
NLF005_scaffold8024_2	non-phage	0
NLF005_scaffold5099_5	non-phage	0
NLF005_scaffold43586_1	non-phage	0
NLF005_scaffold19104_2	non-phage	0
NLF005_scaffold4621_2	non-phage	0
NLF005_scaffold6498_6	non-phage	0
NLF005_scaffold11063_3	non-phage	0
NLF005_scaffold39080_2	non-phage	0
NLF005_scaffold6489_2	non-phage	0
NLF005_scaffold235_7	non-phage	0
NLF005_scaffold48383_5	non-phage	0
NLF005_scaffold5281_11	non-phage	0
NLF005_scaffold12922_8	non-phage	0
NLF005_scaffold163_15	non-phage	0
NLF005_scaffold48595_1	non-phage	0
NLF005_scaffold14050_6	non-phage	0
NLF005_scaffold47480_1	non-phage	0
NLF005_scaffold28589_2	non-phage	0
NLF005_scaffold28589_10	non-phage	0
NLF005_scaffold48291_1	non-phage	0
NLF005_scaffold48442_1	non-phage	0
NLF005_scaffold6777_15	non-phage	0
NLF005_scaffold505_23	non-phage	0
NLF005_scaffold19655_7	non-phage	0
NLF005_scaffold20259_1	non-phage	0
NLF005_scaffold40686_5	non-phage	0

NLF005_scaffold13387_5	non-phage	0
NLF005_scaffold48437_1	non-phage	0
NLF005_scaffold32720_4	phage	0.999
NLF005_scaffold10674_5	non-phage	0
NLF005_scaffold8350_1	non-phage	0
NLF005_scaffold47934_4	non-phage	0
NLF005_scaffold19918_1	non-phage	0
NLF005_scaffold2467_7	non-phage	0
NLF005_scaffold163_17	non-phage	0
NLF005_scaffold16738_5	non-phage	0
NLF005_scaffold19849_13	non-phage	0
NLF005_scaffold45099_1	non-phage	0
NLF005_scaffold33031_2	non-phage	0
NLF005_scaffold39995_3	non-phage	0
NLF005_scaffold35615_2	non-phage	0
NLF005_scaffold3432_1	non-phage	0
NLF005_scaffold48182_1	non-phage	0
NLF005_scaffold41101_3	non-phage	0
NLF005_scaffold36889_1	non-phage	0
NLF005_scaffold5511_2	non-phage	0
NLF005_scaffold48493_1	non-phage	0
NLF005_scaffold24889_10	non-phage	0
NLF005_scaffold30654_1	non-phage	0
NLF005_scaffold48491_4	non-phage	0
NLF005_scaffold375_16	non-phage	0
NLF005_scaffold28312_1	non-phage	0
NLF005_scaffold21492_6	phage	0.824
NLF005_scaffold45363_5	non-phage	0
NLF005_scaffold48533_1	non-phage	0
NLF005_scaffold9606_7	non-phage	0
NLF005_scaffold24190_15	non-phage	0
NLF005_scaffold1754_5	non-phage	0
NLF005_scaffold35204_2	non-phage	0
NLF005_C729175_1	non-phage	0
NLF005_scaffold24_3	non-phage	0
NLF005_scaffold47999_1	non-phage	0
NLF005_scaffold32012_2	non-phage	0
NLF005_scaffold44182_2	non-phage	0
NLF005_scaffold30199_1	non-phage	0
NLF005_scaffold5281_3	non-phage	0
NLF005_scaffold45690_2	non-phage	0
NLF005_scaffold505_17	non-phage	0
NLF005_scaffold505_18	non-phage	0
NLF005_scaffold231_1	non-phage	0
NLF005_scaffold25196_6	non-phage	0
NLF005_scaffold289_3	non-phage	0

NLF005_scaffold47491_1	non-phage	0
NLF005_scaffold48473_2	non-phage	0
NLF005_scaffold48218_3	non-phage	0
NLF005_scaffold28_2	non-phage	0
NLF005_scaffold48421_1	non-phage	0
NLF005_scaffold47556_2	non-phage	0
NLF005_scaffold41864_1	non-phage	0
NLF005_scaffold3933_8	non-phage	0
NLF005_scaffold2609_4	non-phage	0.225
NLF005_scaffold48494_1	non-phage	0
NLF005_scaffold28043_3	non-phage	0
NLF005_scaffold48591_1	non-phage	0
NLF005_scaffold47894_1	non-phage	0
NLF005_scaffold258_3	non-phage	0
NLF005_scaffold235_2	non-phage	0
NLF005_scaffold48567_3	non-phage	0
NLF005_scaffold137_7	non-phage	0
NLF005_scaffold225_1	non-phage	0
NLF005_scaffold25566_3	non-phage	0
NLF005_scaffold38774_3	non-phage	0
NLF005_scaffold549_10	non-phage	0
NLF005_scaffold1754_3	non-phage	0
NLF005_scaffold43034_1	non-phage	0
NLF005_scaffold47931_1	non-phage	0
NLF005_scaffold37681_1	non-phage	0
NLF005_scaffold26449_1	non-phage	0
NLF005_scaffold516_1	non-phage	0
NLF005_scaffold163_19	non-phage	0
NLF005_scaffold10734_6	non-phage	0
NLF005_scaffold3555_9	non-phage	0
NLF005_scaffold48620_2	non-phage	0
NLF005_scaffold40666_3	non-phage	0
NLF005_scaffold4043_2	non-phage	0
NLF005_scaffold135_1	non-phage	0
NLF005_scaffold13578_4	non-phage	0
NLF005_scaffold28003_5	non-phage	0
NLF005_scaffold219_3	non-phage	0
NLF005_scaffold1669_12	non-phage	0
NLF005_scaffold39634_1	non-phage	0
NLF005_scaffold21492_8	non-phage	0
NLF005_scaffold28589_14	non-phage	0
NLF005_scaffold48443_2	non-phage	0
NLF005_C729183_1	non-phage	0
NLF005_scaffold7046_3	non-phage	0
NLF005_scaffold45170_1	non-phage	0
NLF005_scaffold48556_2	non-phage	0

NLF005_scaffold55_3	non-phage	0
NLF005_scaffold29022_2	non-phage	0
NLF005_scaffold142_5	non-phage	0
NLF005_scaffold39_2	non-phage	0
NLF005_scaffold48474_2	non-phage	0
NLF005_scaffold108_2	non-phage	0
NLF005_scaffold48186_3	non-phage	0
NLF005_scaffold46429_2	non-phage	0
NLF005_scaffold18384_5	non-phage	0
NLF005_scaffold10674_3	non-phage	0
NLF005_scaffold47663_1	non-phage	0
NLF005_scaffold13_2	non-phage	0
NLF005_scaffold48289_1	non-phage	0
NLF005_scaffold25196_3	non-phage	0
NLF005_scaffold48463_1	non-phage	0
NLF005_scaffold24685_4	non-phage	0
NLF005_C729497_1	phage	0.999
NLF005_scaffold19336_2	non-phage	0
NLF005_scaffold142_1	non-phage	0
NLF005_scaffold10734_1	non-phage	0
NLF005_scaffold1317_3	phage	0.998
NLF005_scaffold244_1	non-phage	0
NLF005_scaffold45586_1	non-phage	0
NLF005_scaffold235_10	non-phage	0
NLF005_scaffold289_2	non-phage	0
NLF005_scaffold38902_3	non-phage	0
NLF005_scaffold34258_1	non-phage	0
NLF005_C729937_1	non-phage	0
NLF005_scaffold37681_6	non-phage	0
NLF005_scaffold17945_1	non-phage	0
NLF005_scaffold19849_16	non-phage	0
NLF005_scaffold19003_5	non-phage	0
NLF005_scaffold6409_3	non-phage	0
NLF005_scaffold45578_2	non-phage	0
NLF005_scaffold34460_2	non-phage	0
NLF005_scaffold34673_1	non-phage	0
NLF005_scaffold21214_1	phage	0.999
NLF005_scaffold41101_4	non-phage	0
NLF005_scaffold44912_1	non-phage	0
NLF005_scaffold21492_11	non-phage	0
NLF005_scaffold505_12	non-phage	0
NLF005_scaffold8414_6	non-phage	0
NLF005_scaffold3038_6	non-phage	0
NLF005_scaffold46895_1	non-phage	0
NLF005_scaffold5169_1	non-phage	0
NLF005_scaffold48505_2	non-phage	0

NLF005_scaffold48237_4	non-phage	0
NLF005_scaffold375_17	non-phage	0
NLF005_scaffold33867_2	non-phage	0
NLF005_scaffold36327_2	non-phage	0
NLF005_scaffold48311_2	non-phage	0
NLF005_scaffold25549_1	non-phage	0
NLF005_scaffold12792_3	non-phage	0
NLF005_scaffold3126_5	non-phage	0
NLF005_scaffold1259_1	non-phage	0
NLF005_scaffold11666_1	non-phage	0
NLF005_scaffold19663_1	non-phage	0
NLF005_scaffold13578_6	non-phage	0
NLF005_scaffold4392_1	non-phage	0
NLF005_scaffold66_4	non-phage	0
NLF005_scaffold48437_3	non-phage	0
NLF005_scaffold1317_2	non-phage	0
NLF005_scaffold2418_5	non-phage	0
NLF005_scaffold1745_7	non-phage	0
NLF005_scaffold41594_1	non-phage	0
NLF005_scaffold47798_1	phage	0.999
NLF005_scaffold21567_2	non-phage	0
NLF005_scaffold45363_6	non-phage	0
NLF005_scaffold48290_3	non-phage	0
NLF005_scaffold3555_3	non-phage	0
NLF005_scaffold19104_4	non-phage	0
NLF005_scaffold432_1	non-phage	0
NLF005_C729695_1	phage	0.999
NLF005_scaffold48563_1	non-phage	0
NLF005_scaffold40182_2	non-phage	0
NLF005_scaffold30066_6	non-phage	0
NLF005_scaffold39909_6	non-phage	0
NLF005_scaffold27522_3	phage	0.99
NLF005_scaffold7834_3	non-phage	0
NLF005_scaffold47577_1	non-phage	0
NLF005_scaffold46430_1	non-phage	0
NLF005_scaffold39548_5	non-phage	0
NLF005_scaffold17644_1	non-phage	0
NLF005_scaffold3933_2	non-phage	0
NLF005_scaffold35204_1	non-phage	0
NLF005_scaffold26221_2	non-phage	0
NLF005_scaffold135_6	non-phage	0
NLF005_scaffold47800_1	phage	0.75
NLF005_scaffold48419_3	non-phage	0
NLF005_scaffold19667_2	non-phage	0
NLF005_scaffold3126_3	non-phage	0
NLF005_scaffold549_5	non-phage	0

NLF005_scaffold43586_4	non-phage	0
NLF005_scaffold48626_1	non-phage	0
NLF005_scaffold29618_1	phage	0.782
NLF005_scaffold19706_1	non-phage	0
NLF005_scaffold36349_1	non-phage	0
NLF005_scaffold4420_5	non-phage	0
NLF005_scaffold21492_4	phage	0.996
NLF005_scaffold4183_1	non-phage	0
NLF005_scaffold25196_2	phage	0.995
NLF005_scaffold30066_3	non-phage	0
NLF005_scaffold2055_3	non-phage	0
NLF005_scaffold47605_1	non-phage	0
NLF005_scaffold10734_2	non-phage	0
NLF005_scaffold19470_6	non-phage	0
NLF005_scaffold28363_2	non-phage	0
NLF005_scaffold31720_2	non-phage	0
NLF005_scaffold32729_4	non-phage	0
NLF005_scaffold505_16	non-phage	0
NLF005_scaffold48133_3	non-phage	0
NLF005_scaffold43102_1	non-phage	0
NLF005_scaffold46665_1	non-phage	0
NLF005_scaffold32486_1	non-phage	0
NLF005_scaffold25196_5	non-phage	0
NLF005_scaffold5511_1	non-phage	0
NLF005_scaffold48051_2	non-phage	0
NLF005_scaffold47690_3	non-phage	0
NLF005_scaffold12858_3	non-phage	0
NLF005_scaffold39631_2	non-phage	0
NLF005_scaffold35092_1	non-phage	0
NLF005_scaffold24819_2	non-phage	0
NLF005_scaffold33591_2	non-phage	0
NLF005_scaffold48223_1	non-phage	0
NLF005_scaffold19918_2	non-phage	0
NLF005_scaffold78_1	non-phage	0
NLF005_scaffold10674_4	non-phage	0
NLF005_scaffold9445_1	non-phage	0
NLF005_scaffold3324_6	non-phage	0.001
NLF005_scaffold48594_2	non-phage	0
NLF005_scaffold46924_2	non-phage	0
NLF005_scaffold8678_3	non-phage	0
NLF005_scaffold40967_2	phage	0.999
NLF005_scaffold2418_2	non-phage	0
NLF005_scaffold10309_1	non-phage	0
NLF005_scaffold291_32	non-phage	0
NLF005_scaffold15525_4	non-phage	0
NLF005_scaffold47104_1	non-phage	0

NLF005_scaffold7337_12	non-phage	0
NLF005_scaffold1669_8	non-phage	0
NLF005_scaffold37681_5	non-phage	0
NLF005_scaffold505_2	non-phage	0
NLF005_scaffold40348_3	non-phage	0
NLF005_scaffold35502_2	non-phage	0
NLF005_scaffold1669_7	non-phage	0
NLF005_scaffold48449_2	non-phage	0
NLF005_scaffold37884_1	non-phage	0
NLF005_scaffold33031_1	non-phage	0
NLF005_scaffold3202_9	non-phage	0
NLF005_scaffold13387_1	non-phage	0
NLF005_scaffold19524_2	non-phage	0
NLF005_scaffold46931_1	non-phage	0
NLF005_scaffold18839_2	non-phage	0
NLF005_scaffold11979_2	non-phage	0
NLF005_scaffold42017_2	non-phage	0
NLF005_scaffold33209_4	non-phage	0
NLF005_scaffold30066_7	non-phage	0
NLF005_scaffold2467_18	non-phage	0
NLF005_scaffold15548_1	non-phage	0
NLF005_scaffold10734_9	non-phage	0
NLF005_scaffold48471_1	non-phage	0
NLF005_scaffold25549_4	non-phage	0
NLF005_scaffold46363_3	non-phage	0
NLF005_scaffold235_11	non-phage	0
NLF005_scaffold3933_9	non-phage	0
NLF005_scaffold48537_1	non-phage	0
NLF005_scaffold48587_2	non-phage	0
NLF005_scaffold28043_2	non-phage	0
NLF005_scaffold8350_5	non-phage	0
NLF005_scaffold48481_2	non-phage	0
NLF005_scaffold39909_11	non-phage	0
NLF005_scaffold22405_4	non-phage	0
NLF005_scaffold32729_1	non-phage	0
NLF005_scaffold2609_5	non-phage	0
NLF005_scaffold222_1	non-phage	0
NLF005_scaffold48218_2	non-phage	0
NLF005_scaffold39909_9	non-phage	0
NLF005_scaffold13578_3	non-phage	0
NLF005_scaffold2552_2	non-phage	0
NLF005_scaffold32729_8	non-phage	0
NLF005_scaffold27433_2	non-phage	0
NLF005_scaffold8449_6	non-phage	0
NLF005_scaffold26221_12	non-phage	0
NLF005_scaffold505_21	non-phage	0

NLF005_scaffold29964_1	non-phage	0
NLF005_scaffold46895_2	non-phage	0
NLF005_scaffold34017_1	non-phage	0
NLF005_scaffold505_13	non-phage	0
NLF005_scaffold31392_2	non-phage	0
NLF005_scaffold549_6	non-phage	0
NLF005_scaffold48237_3	non-phage	0
NLF005_scaffold19849_2	non-phage	0
NLF005_scaffold43034_2	phage	0.991
NLF005_scaffold2443_2	non-phage	0
NLF005_scaffold28589_1	non-phage	0
NLF005_scaffold25196_10	non-phage	0
NLF005_scaffold35740_4	non-phage	0
NLF005_scaffold47588_1	non-phage	0
NLF005_scaffold291_42	phage	1
NLF005_scaffold48617_1	non-phage	0
NLF005_scaffold3199_3	non-phage	0
NLF005_scaffold47934_7	phage	0.99
NLF005_scaffold7471_2	non-phage	0
NLF005_scaffold29022_3	non-phage	0
NLF005_scaffold48360_1	non-phage	0
NLF005_scaffold16125_2	non-phage	0
NLF005_scaffold10734_13	non-phage	0
NLF005_scaffold48290_1	non-phage	0
NLF005_scaffold2418_6	non-phage	0
NLF005_scaffold48562_1	non-phage	0
NLF005_scaffold14386_1	non-phage	0
NLF005_scaffold43004_3	phage	1
NLF005_scaffold17_1	non-phage	0
NLF005_scaffold36413_2	non-phage	0
NLF005_scaffold289_1	non-phage	0
NLF005_scaffold48430_1	non-phage	0
NLF005_scaffold25190_3	non-phage	0
NLF005_scaffold39995_2	non-phage	0
NLF005_scaffold48133_2	non-phage	0
NLF005_scaffold14516_2	phage	0.999
NLF005_scaffold21151_1	non-phage	0
NLF005_scaffold11063_5	non-phage	0
NLF005_scaffold6498_9	non-phage	0
NLF005_scaffold33853_1	non-phage	0.419
NLF005_scaffold19706_4	non-phage	0
NLF005_scaffold19104_1	non-phage	0
NLF005_scaffold1745_3	non-phage	0
NLF005_scaffold4335_20	non-phage	0
NLF005_scaffold6409_4	non-phage	0
NLF005_scaffold31897_2	non-phage	0

NLF005_scaffold48625_1	non-phage	0
NLF005_scaffold33476_4	non-phage	0
NLF005_scaffold41101_5	non-phage	0
NLF005_scaffold4420_1	non-phage	0
NLF005_scaffold47106_1	non-phage	0
NLF005_scaffold48609_1	non-phage	0
NLF005_scaffold41314_4	non-phage	0
NLF005_scaffold16034_10	non-phage	0
NLF005_scaffold40686_3	non-phage	0
NLF005_scaffold13387_3	non-phage	0
NLF005_scaffold11797_1	non-phage	0
NLF005_scaffold48616_1	non-phage	0
NLF005_scaffold8350_3	non-phage	0
NLF005_scaffold35740_3	non-phage	0
NLF005_scaffold48237_1	phage	0.999
NLF005_scaffold47583_3	non-phage	0
NLF005_scaffold19618_5	non-phage	0
NLF005_scaffold33768_1	non-phage	0
NLF005_scaffold208_5	non-phage	0
NLF005_scaffold37637_1	non-phage	0
NLF005_scaffold163_8	non-phage	0
NLF005_scaffold40666_2	non-phage	0
NLF005_scaffold505_5	non-phage	0
NLF005_scaffold4183_2	non-phage	0
NLF005_scaffold7337_17	non-phage	0
NLF005_scaffold64_1	non-phage	0
NLF005_scaffold32729_5	non-phage	0
NLF005_scaffold46924_5	non-phage	0
NLF005_scaffold19470_3	non-phage	0
NLF005_scaffold5281_5	non-phage	0
NLF005_scaffold6409_1	non-phage	0
NLF005_scaffold48579_1	non-phage	0
NLF005_scaffold505_15	non-phage	0
NLF005_scaffold48621_1	non-phage	0
NLF005_scaffold34258_2	non-phage	0
NLF005_scaffold26008_4	non-phage	0
NLF005_scaffold10898_9	non-phage	0
NLF005_scaffold19918_3	non-phage	0
NLF005_scaffold508_3	non-phage	0
NLF005_scaffold48413_1	non-phage	0
NLF005_scaffold8350_9	non-phage	0
NLF005_scaffold48486_1	non-phage	0
NLF005_scaffold19849_4	non-phage	0
NLF005_scaffold10734_5	non-phage	0
NLF005_scaffold30170_1	non-phage	0
NLF005_scaffold48497_1	non-phage	0

NLF005_scaffold5_2	non-phage	0
NLF005_scaffold37681_2	non-phage	0
NLF005_scaffold18384_2	non-phage	0
NLF005_scaffold18384_8	non-phage	0
NLF005_scaffold5269_7	non-phage	0
NLF005_scaffold3555_5	non-phage	0
NLF005_scaffold44953_1	non-phage	0
NLF005_scaffold2418_3	non-phage	0
NLF005_C729433_1	phage	0.995
NLF005_scaffold27643_1	non-phage	0
NLF005_scaffold48218_5	non-phage	0
NLF005_scaffold135_7	non-phage	0
NLF005_scaffold48186_2	non-phage	0
NLF005_scaffold48604_1	non-phage	0
NLF005_scaffold16081_4	non-phage	0
NLF005_scaffold37296_1	non-phage	0
NLF005_scaffold3933_5	non-phage	0
NLF005_scaffold7337_13	non-phage	0
NLF005_scaffold8449_1	non-phage	0
NLF005_scaffold291_45	non-phage	0
NLF005_scaffold508_2	non-phage	0
NLF005_scaffold488_3	non-phage	0
NLF005_scaffold4074_8	non-phage	0
NLF005_scaffold9851_4	non-phage	0
NLF005_scaffold26630_4	non-phage	0
NLF005_scaffold43454_1	non-phage	0
NLF005_scaffold14050_4	non-phage	0
NLF005_scaffold48586_1	non-phage	0
NLF005_scaffold4359_1	non-phage	0
NLF005_scaffold11360_1	non-phage	0
NLF005_scaffold31720_3	non-phage	0
NLF005_scaffold505_1	non-phage	0
NLF005_scaffold25566_2	non-phage	0
NLF005_scaffold48563_2	non-phage	0
NLF005_scaffold36413_1	non-phage	0
NLF005_scaffold42612_1	non-phage	0
NLF005_scaffold48442_3	phage	0.999
NLF005_scaffold48419_1	non-phage	0
NLF005_scaffold45136_2	non-phage	0
NLF005_scaffold850_2	non-phage	0
NLF005_scaffold14666_1	non-phage	0
NLF005_scaffold36558_2	non-phage	0
NLF005_scaffold48603_1	non-phage	0
NLF005_scaffold28622_3	non-phage	0
NLF005_scaffold48389_1	phage	0.999
NLF005_scaffold16738_7	non-phage	0

NLF005_scaffold39631_1	non-phage	0
NLF005_scaffold30908_1	non-phage	0
NLF005_scaffold47191_1	non-phage	0
NLF005_scaffold37951_3	non-phage	0
NLF005_scaffold32528_2	non-phage	0
NLF005_scaffold2467_17	non-phage	0
NLF005_scaffold39631_4	non-phage	0
NLF005_scaffold48471_2	non-phage	0
NLF005_scaffold46412_1	non-phage	0
NLF005_scaffold20400_1	non-phage	0
NLF005_scaffold46303_1	non-phage	0
NLF005_scaffold48441_1	non-phage	0
NLF005_scaffold7009_1	non-phage	0
NLF005_scaffold30031_1	non-phage	0
NLF005_scaffold33625_1	non-phage	0
NLF005_scaffold1754_1	non-phage	0
NLF005_scaffold32681_1	non-phage	0
NLF005_scaffold29356_3	non-phage	0
NLF005_scaffold35446_6	non-phage	0
NLF005_scaffold33476_1	non-phage	0
NLF005_scaffold29356_1	non-phage	0
NLF005_scaffold46924_1	non-phage	0
NLF005_scaffold42023_1	non-phage	0
NLF005_scaffold222_2	non-phage	0
NLF005_scaffold29986_2	non-phage	0
NLF005_C729401_1	non-phage	0
NLF005_scaffold46422_1	non-phage	0
NLF005_scaffold12496_2	non-phage	0
NLF005_scaffold26630_5	non-phage	0
NLF005_scaffold1669_4	non-phage	0
NLF005_scaffold2443_1	non-phage	0
NLF005_scaffold13542_2	non-phage	0
NLF005_scaffold38447_2	non-phage	0
NLF005_scaffold39282_7	phage	0.94
NLF005_scaffold25190_2	non-phage	0
NLF005_scaffold48569_1	non-phage	0
NLF005_scaffold38050_2	non-phage	0
NLF005_scaffold41101_1	non-phage	0
NLF005_scaffold4392_3	non-phage	0
NLF005_scaffold48284_3	non-phage	0
NLF005_scaffold28_3	non-phage	0
NLF005_scaffold48213_1	non-phage	0
NLF005_scaffold25549_3	non-phage	0
NLF005_scaffold48587_1	non-phage	0
NLF005_scaffold7722_4	non-phage	0
NLF005_scaffold163_20	non-phage	0

NLF005_scaffold14218_1	non-phage	0
NLF005_scaffold33263_2	non-phage	0
NLF005_scaffold10734_8	non-phage	0
NLF005_scaffold28531_4	non-phage	0
NLF005_scaffold16289_16	non-phage	0
NLF005_scaffold48567_1	non-phage	0
NLF005_scaffold39995_14	non-phage	0
NLF005_scaffold6498_1	non-phage	0
NLF005_scaffold5802_1	non-phage	0
NLF005_scaffold46363_2	non-phage	0
NLF005_scaffold26162_1	non-phage	0
NLF005_scaffold39909_8	non-phage	0
NLF005_scaffold549_12	non-phage	0
NLF005_scaffold134_2	non-phage	0
NLF005_scaffold48202_2	non-phage	0
NLF005_scaffold24236_3	non-phage	0
NLF005_scaffold48290_2	non-phage	0
NLF005_scaffold13578_2	non-phage	0
NLF005_scaffold42653_2	non-phage	0
NLF005_scaffold19706_3	non-phage	0
NLF005_scaffold47583_1	non-phage	0
NLF005_scaffold21592_1	non-phage	0
NLF005_scaffold40666_1	non-phage	0
NLF005_scaffold14583_3	non-phage	0
NLF005_scaffold3126_4	non-phage	0
NLF005_scaffold33622_3	non-phage	0
NLF005_scaffold12858_2	non-phage	0
NLF005_scaffold13387_9	phage	0.999
NLF005_scaffold3933_3	non-phage	0
NLF005_scaffold2418_7	non-phage	0
NLF005_scaffold505_14	non-phage	0
NLF005_scaffold12496_3	non-phage	0
NLF005_scaffold39995_8	non-phage	0
NLF005_scaffold26790_3	non-phage	0.008
NLF005_scaffold26809_2	non-phage	0
NLF005_scaffold28363_4	non-phage	0
NLF005_scaffold29356_7	non-phage	0
NLF005_scaffold5448_1	non-phage	0
NLF005_scaffold43004_2	non-phage	0
NLF005_scaffold40798_1	phage	0.996
NLF005_scaffold11192_16	non-phage	0
NLF005_scaffold16034_1	non-phage	0
NLF005_scaffold46359_2	non-phage	0
NLF005_scaffold6408_61	non-phage	0
NLF005_scaffold263_2	non-phage	0
NLF005_scaffold43505_1	non-phage	0

NLF005_scaffold28_1	non-phage	0
NLF005_scaffold11063_6	non-phage	0
NLF005_scaffold13387_4	phage	0.998
NLF005_scaffold2418_4	non-phage	0
NLF005_scaffold1430_6	non-phage	0
NLF005_scaffold8270_5	non-phage	0
NLF005_scaffold48536_2	non-phage	0
NLF005_C730111_1	non-phage	0
NLF005_scaffold48437_2	phage	0.999
NLF005_scaffold36327_1	non-phage	0
NLF005_scaffold39909_4	non-phage	0
NLF005_scaffold35492_1	non-phage	0
NLF005_scaffold405_1	non-phage	0
NLF005_scaffold4074_7	non-phage	0
NLF005_scaffold42989_1	non-phage	0
NLF005_scaffold35740_2	non-phage	0
NLF005_scaffold163_6	non-phage	0
NLF005_scaffold30003_1	non-phage	0
NLF005_scaffold29852_1	phage	0.999
NLF005_scaffold9227_3	non-phage	0
NLF005_scaffold1669_1	non-phage	0
NLF005_scaffold505_4	non-phage	0
NLF005_scaffold7337_14	non-phage	0
NLF005_scaffold37681_7	non-phage	0
NLF005_scaffold4391_3	phage	0.999
NLF005_scaffold291_44	phage	0.993
NLF005_scaffold1299_9	non-phage	0
NLF005_scaffold6498_5	non-phage	0
NLF005_scaffold9851_3	non-phage	0
NLF005_scaffold48512_1	non-phage	0
NLF005_scaffold7337_7	non-phage	0
NLF005_scaffold41900_3	non-phage	0
NLF005_scaffold48627_1	non-phage	0
NLF005_scaffold48580_1	non-phage	0
NLF005_scaffold25566_1	non-phage	0
NLF005_scaffold48611_1	non-phage	0
NLF005_scaffold38902_2	non-phage	0
NLF005_scaffold28589_16	non-phage	0
NLF005_scaffold22054_3	non-phage	0
NLF005_scaffold46363_1	non-phage	0
NLF005_scaffold36460_3	non-phage	0
NLF005_scaffold38375_1	non-phage	0
NLF005_scaffold39_1	non-phage	0
NLF005_scaffold47075_2	non-phage	0
NLF005_scaffold10734_4	phage	0.988
NLF005_scaffold13_4	non-phage	0

NLF005_scaffold28204_1	non-phage	0
NLF005_scaffold11224_2	non-phage	0
NLF005_scaffold46891_1	non-phage	0
NLF005_scaffold6498_4	non-phage	0
NLF005_scaffold7337_1	non-phage	0
NLF005_scaffold2609_3	non-phage	0
NLF005_scaffold39995_4	non-phage	0
NLF005_scaffold16738_6	non-phage	0
NLF005_scaffold2233_3	non-phage	0
NLF005_scaffold47663_3	non-phage	0
NLF005_scaffold16081_2	non-phage	0
NLF005_scaffold235_12	non-phage	0
NLF005_scaffold40121_2	non-phage	0
NLF005_scaffold48600_2	non-phage	0
NLF005_scaffold48557_1	non-phage	0
NLF005_scaffold375_15	non-phage	0
NLF005_scaffold2416_1	non-phage	0
NLF005_scaffold36214_1	non-phage	0
NLF005_scaffold6784_1	non-phage	0
NLF005_scaffold8449_2	non-phage	0
NLF005_scaffold19524_3	non-phage	0
NLF005_scaffold28589_5	non-phage	0
NLF005_scaffold29964_5	non-phage	0
NLF005_scaffold508_1	non-phage	0
NLF006_scaffold3307_7	non-phage	0
NLF006_scaffold17097_3	non-phage	0
NLF006_scaffold306_3	phage	0.999
NLF006_scaffold11296_1	non-phage	0
NLF006_scaffold194_2	non-phage	0
NLF006_scaffold400_1	non-phage	0
NLF006_scaffold29760_1	non-phage	0
NLF006_scaffold212_3	non-phage	0
NLF006_scaffold8039_4	non-phage	0
NLF006_scaffold281_2	non-phage	0
NLF006_scaffold11957_4	non-phage	0
NLF006_scaffold3721_2	non-phage	0
NLF006_scaffold3878_1	non-phage	0
NLF006_scaffold1220_2	non-phage	0
NLF006_scaffold177_1	non-phage	0
NLF006_scaffold5774_1	non-phage	0
NLF006_scaffold168_1	non-phage	0
NLF006_scaffold8341_3	non-phage	0
NLF006_scaffold2572_3	non-phage	0
NLF006_scaffold149_2	non-phage	0
NLF006_scaffold11004_1	non-phage	0
NLF006_scaffold244_2	non-phage	0

NLF006_scaffold12968_1	non-phage	0
NLF006_scaffold20268_1	non-phage	0
NLF006_scaffold5007_5	non-phage	0
NLF006_scaffold414_6	non-phage	0
NLF006_scaffold197_3	non-phage	0
NLF006_scaffold12572_1	non-phage	0
NLF006_scaffold352_3	non-phage	0
NLF006_scaffold5462_3	non-phage	0
NLF006_scaffold203_2	non-phage	0
NLF006_scaffold105_1	non-phage	0
NLF006_scaffold15424_12	non-phage	0
NLF006_scaffold139_2	non-phage	0
NLF006_scaffold342_1	non-phage	0
NLF006_scaffold24148_3	non-phage	0
NLF006_scaffold14464_2	non-phage	0
NLF006_scaffold31519_1	non-phage	0
NLF006_scaffold19030_1	non-phage	0
NLF006_scaffold13985_5	non-phage	0
NLF006_scaffold266_1	non-phage	0
NLF006_scaffold8289_1	non-phage	0
NLF006_scaffold4_2	non-phage	0
NLF006_scaffold22261_2	non-phage	0
NLF006_scaffold1471_1	non-phage	0
NLF006_scaffold65_1	non-phage	0
NLF006_scaffold31331_1	non-phage	0
NLF006_scaffold9046_1	non-phage	0.135
NLF006_scaffold21535_8	non-phage	0.031
NLF006_scaffold36_1	non-phage	0
NLF006_scaffold91_1	non-phage	0
NLF006_scaffold3_2	non-phage	0
NLF006_scaffold22186_2	phage	0.971
NLF006_scaffold51_4	non-phage	0
NLF006_scaffold137_1	non-phage	0
NLF006_scaffold174_2	non-phage	0
NLF006_scaffold275_2	non-phage	0
NLF006_scaffold585_2	non-phage	0
NLF006_scaffold65_2	non-phage	0
NLF006_scaffold31140_1	non-phage	0
NLF006_scaffold9686_1	non-phage	0
NLF006_scaffold31179_2	non-phage	0
NLF006_scaffold31099_1	phage	1
NLF006_scaffold3721_6	non-phage	0
NLF006_scaffold10442_1	non-phage	0
NLF006_scaffold118_3	non-phage	0
NLF006_scaffold31446_1	non-phage	0
NLF006_scaffold14417_1	non-phage	0

NLF006_scaffold14043_1	non-phage	0
NLF006_scaffold32_4	non-phage	0
NLF006_scaffold3002_2	non-phage	0
NLF006_scaffold89_1	non-phage	0
NLF006_scaffold31382_1	non-phage	0
NLF006_scaffold2323_4	non-phage	0
NLF006_scaffold29560_1	non-phage	0
NLF006_scaffold184_2	non-phage	0
NLF006_scaffold4766_5	phage	0.999
NLF006_scaffold17663_4	non-phage	0
NLF006_scaffold14433_1	non-phage	0
NLF006_scaffold8594_1	non-phage	0
NLF006_scaffold2410_1	non-phage	0
NLF006_scaffold10188_1	non-phage	0
NLF006_scaffold25_1	non-phage	0
NLF006_scaffold306_1	phage	0.999
NLF006_scaffold17012_5	non-phage	0
NLF006_scaffold95_4	non-phage	0
NLF006_scaffold490_1	non-phage	0
NLF006_scaffold398_1	non-phage	0
NLF006_scaffold6048_7	non-phage	0
NLF006_scaffold116_1	non-phage	0
NLF006_scaffold24366_1	non-phage	0
NLF006_scaffold16784_1	non-phage	0
NLF006_scaffold6234_1	non-phage	0
NLF006_scaffold29625_2	non-phage	0
NLF006_scaffold3524_2	non-phage	0
NLF006_scaffold7609_8	non-phage	0
NLF006_scaffold7609_1	non-phage	0
NLF006_scaffold95_3	non-phage	0
NLF006_scaffold15421_1	non-phage	0
NLF006_scaffold18272_1	non-phage	0
NLF006_scaffold1220_1	non-phage	0
NLF006_scaffold3721_1	non-phage	0.004
NLF006_scaffold15157_4	non-phage	0
NLF006_scaffold117_4	non-phage	0
NLF006_scaffold967_1	non-phage	0
NLF006_scaffold6303_2	non-phage	0
NLF006_scaffold237_6	non-phage	0
NLF006_scaffold30362_2	non-phage	0
NLF006_scaffold16981_1	non-phage	0
NLF006_scaffold29664_1	non-phage	0
NLF006_scaffold77_2	non-phage	0
NLF006_scaffold6636_3	non-phage	0
NLF006_scaffold121_1	non-phage	0
NLF006_scaffold31580_1	non-phage	0

NLF006_scaffold513_1	non-phage	0
NLF006_scaffold1877_1	non-phage	0
NLF006_scaffold16788_3	non-phage	0
NLF006_scaffold17574_10	non-phage	0
NLF006_scaffold137_4	non-phage	0
NLF006_scaffold8341_6	non-phage	0
NLF006_scaffold130_1	non-phage	0
NLF006_scaffold24743_2	non-phage	0
NLF006_scaffold9313_2	non-phage	0
NLF006_scaffold16222_2	non-phage	0
NLF006_scaffold3496_2	non-phage	0
NLF006_scaffold20465_2	non-phage	0
NLF006_scaffold17_4	non-phage	0
NLF006_scaffold124_1	non-phage	0
NLF006_scaffold1_4	non-phage	0
NLF006_scaffold16014_1	non-phage	0
NLF006_scaffold4727_5	non-phage	0
NLF006_scaffold29822_1	non-phage	0
NLF006_scaffold4740_2	non-phage	0
NLF006_scaffold6290_1	non-phage	0
NLF006_scaffold13414_1	non-phage	0
NLF006_scaffold147_3	non-phage	0
NLF006_scaffold13018_1	non-phage	0
NLF006_scaffold17033_2	non-phage	0
NLF006_scaffold9628_2	phage	1
NLF006_scaffold26881_2	non-phage	0
NLF006_scaffold2323_3	non-phage	0
NLF006_scaffold17663_3	non-phage	0
NLF006_scaffold571_2	non-phage	0
NLF006_scaffold158_6	non-phage	0
NLF006_scaffold27_1	non-phage	0
NLF006_scaffold10442_2	non-phage	0
NLF006_scaffold19663_2	non-phage	0
NLF006_scaffold16741_1	non-phage	0
NLF006_scaffold28751_2	non-phage	0
NLF006_scaffold8542_4	non-phage	0
NLF006_scaffold4766_2	phage	1
NLF006_scaffold78_1	non-phage	0
NLF006_scaffold5249_2	phage	0.921
NLF006_scaffold203_3	non-phage	0
NLF006_scaffold57_3	non-phage	0
NLF006_scaffold31525_1	non-phage	0
NLF006_scaffold17585_1	non-phage	0
NLF006_scaffold692_2	non-phage	0
NLF006_scaffold3814_1	phage	0.942
NLF006_scaffold11_7	non-phage	0

NLF006_scaffold121_2	non-phage	0
NLF006_scaffold1802_7	non-phage	0
NLF006_scaffold5420_1	non-phage	0
NLF006_scaffold25865_2	non-phage	0
NLF006_scaffold134_3	non-phage	0
NLF006_scaffold7576_2	non-phage	0
NLF006_scaffold31480_2	non-phage	0
NLF006_scaffold118_6	non-phage	0
NLF006_scaffold68_1	non-phage	0
NLF006_scaffold2962_1	non-phage	0
NLF006_scaffold63_2	non-phage	0
NLF006_scaffold197_5	non-phage	0
NLF006_scaffold3380_1	non-phage	0
NLF006_scaffold316_1	non-phage	0
NLF006_scaffold31228_1	non-phage	0
NLF006_scaffold14479_2	non-phage	0
NLF006_scaffold2066_4	non-phage	0
NLF006_scaffold26997_2	non-phage	0
NLF006_scaffold23138_2	non-phage	0
NLF006_scaffold10442_4	non-phage	0
NLF006_scaffold196_1	non-phage	0
NLF006_scaffold19_1	non-phage	0
NLF006_scaffold123_2	non-phage	0
NLF006_scaffold12_2	non-phage	0
NLF006_scaffold13018_2	non-phage	0
NLF006_scaffold130_5	non-phage	0
NLF006_scaffold115_1	non-phage	0
NLF006_scaffold27406_2	non-phage	0
NLF006_scaffold2809_1	non-phage	0
NLF006_scaffold8542_1	non-phage	0
NLF006_scaffold3731_2	non-phage	0
NLF006_scaffold3307_5	non-phage	0
NLF006_scaffold38_5	non-phage	0
NLF006_scaffold27913_6	non-phage	0
NLF006_scaffold251_1	non-phage	0
NLF006_scaffold165_1	non-phage	0
NLF006_scaffold15157_3	phage	0.995
NLF006_scaffold20434_3	non-phage	0
NLF006_scaffold22418_1	non-phage	0
NLF006_scaffold17000_1	non-phage	0
NLF006_scaffold31525_2	non-phage	0
NLF006_scaffold3265_1	non-phage	0
NLF006_scaffold2613_1	non-phage	0
NLF006_scaffold14549_2	non-phage	0
NLF006_scaffold8_3_2	non-phage	0
NLF006_scaffold187_2	non-phage	0

NLF006_scaffold98_2	non-phage	0
NLF006_scaffold8341_1	non-phage	0
NLF006_scaffold6048_1	non-phage	0
NLF006_scaffold28327_1	phage	0.993
NLF006_scaffold10442_3	non-phage	0
NLF006_scaffold183_2	non-phage	0
NLF006_scaffold14841_1	non-phage	0
NLF006_scaffold8319_1	non-phage	0
NLF006_scaffold21648_2	non-phage	0
NLF006_scaffold67_1	non-phage	0
NLF006_scaffold78_2	non-phage	0
NLF006_scaffold136_1	non-phage	0
NLF006_scaffold3307_1	non-phage	0
NLF006_scaffold1946_2	non-phage	0
NLF006_scaffold4466_3	phage	0.999
NLF006_scaffold31289_1	non-phage	0
NLF006_scaffold14746_1	phage	0.98
NLF006_scaffold79_1	non-phage	0
NLF006_scaffold3731_5	non-phage	0
NLF006_scaffold158_4	non-phage	0
NLF006_scaffold28063_2	non-phage	0
NLF006_scaffold57_1	non-phage	0
NLF006_scaffold4315_1	non-phage	0
NLF006_scaffold424_4	non-phage	0
NLF006_scaffold22779_2	non-phage	0
NLF006_scaffold130_4	non-phage	0
NLF006_scaffold109_2	non-phage	0
NLF006_scaffold24743_1	non-phage	0
NLF006_scaffold133_4	non-phage	0
NLF006_scaffold3307_14	non-phage	0
NLF006_scaffold11296_4	non-phage	0
NLF006_scaffold8629_2	non-phage	0
NLF006_scaffold13243_1	non-phage	0.041
NLF006_scaffold7609_4	non-phage	0
NLF006_scaffold7907_1	non-phage	0
NLF006_scaffold135_2	non-phage	0
NLF006_scaffold10484_1	non-phage	0
NLF006_scaffold24366_3	non-phage	0
NLF006_scaffold8956_1	non-phage	0
NLF006_scaffold698_1	non-phage	0
NLF006_scaffold352_4	non-phage	0
NLF006_scaffold923_1	non-phage	0
NLF006_scaffold132_1	phage	0.999
NLF006_scaffold117_3	non-phage	0
NLF006_scaffold14487_4	non-phage	0
NLF006_scaffold147_2	non-phage	0

NLF006_scaffold97_1	non-phage	0
NLF006_scaffold16002_4	non-phage	0
NLF006_scaffold8341_5	non-phage	0
NLF006_scaffold3403_4	non-phage	0
NLF006_scaffold12922_1	non-phage	0
NLF006_scaffold9628_3	non-phage	0
NLF006_scaffold1514_1	non-phage	0
NLF006_scaffold3721_4	non-phage	0
NLF006_scaffold677_1	non-phage	0
NLF006_scaffold74_5	non-phage	0
NLF006_scaffold3677_1	non-phage	0
NLF006_scaffold29417_2	non-phage	0
NLF006_scaffold5891_10	phage	0.845
NLF006_scaffold5844_1	non-phage	0
NLF006_scaffold277_1	non-phage	0
NLF006_scaffold3814_3	non-phage	0
NLF006_scaffold3721_3	non-phage	0
NLF006_scaffold31318_1	non-phage	0
NLF006_scaffold352_6	non-phage	0
NLF006_scaffold109_1	non-phage	0
NLF006_scaffold158_9	non-phage	0
NLF006_scaffold8904_1	non-phage	0
NLF006_scaffold24366_5	non-phage	0
NLF006_scaffold21648_1	non-phage	0
NLF006_scaffold23564_2	non-phage	0
NLF006_scaffold3677_9	non-phage	0
NLF006_scaffold63_3	non-phage	0
NLF006_scaffold400_2	non-phage	0
NLF006_scaffold5420_4	non-phage	0
NLF006_scaffold6343_1	non-phage	0
NLF006_scaffold143_2	non-phage	0
NLF006_scaffold27057_1	non-phage	0
NLF006_scaffold5774_2	non-phage	0
NLF006_scaffold3721_8	non-phage	0
NLF006_scaffold5000_1	non-phage	0
NLF006_scaffold137_5	non-phage	0
NLF006_scaffold31078_1	non-phage	0
NLF006_scaffold457_1	non-phage	0
NLF006_scaffold19948_2	non-phage	0
NLF006_scaffold2572_2	non-phage	0
NLF006_scaffold17012_3	non-phage	0
NLF006_scaffold7104_1	non-phage	0
NLF006_scaffold21648_4	non-phage	0
NLF006_scaffold134_2	non-phage	0
NLF006_scaffold24_1	non-phage	0
NLF006_scaffold208_1	non-phage	0

NLF006_scaffold5144_2	non-phage	0
NLF006_scaffold27913_5	non-phage	0.003
NLF006_scaffold297_1	non-phage	0
NLF006_scaffold158_10	non-phage	0
NLF006_scaffold107_2	non-phage	0
NLF006_scaffold102_3	non-phage	0
NLF006_scaffold6391_3	non-phage	0
NLF006_scaffold17_3	non-phage	0
NLF006_scaffold20434_2	non-phage	0
NLF006_scaffold20465_1	non-phage	0
NLF006_scaffold31572_2	non-phage	0
NLF006_scaffold57_2	non-phage	0
NLF006_scaffold4040_3	non-phage	0.135
NLF006_scaffold30421_2	phage	0.998
NLF006_scaffold22470_1	non-phage	0
NLF006_scaffold18096_2	non-phage	0
NLF006_scaffold4358_3	non-phage	0
NLF006_scaffold585_1	non-phage	0
NLF006_scaffold1190_1	non-phage	0
NLF006_scaffold6289_2	non-phage	0
NLF006_scaffold2006_2	non-phage	0
NLF006_scaffold2891_1	non-phage	0
NLF006_scaffold15735_1	non-phage	0
NLF006_scaffold13985_4	non-phage	0
NLF006_scaffold18985_1	non-phage	0
NLF006_scaffold4929_1	non-phage	0
NLF006_scaffold198_2	non-phage	0
NLF006_scaffold143_6	non-phage	0
NLF006_scaffold3130_3	non-phage	0
NLF006_scaffold31180_1	non-phage	0
NLF006_scaffold67_2	non-phage	0
NLF006_scaffold118_4	non-phage	0
NLF006_scaffold7441_2	non-phage	0
NLF006_scaffold13707_1	non-phage	0
NLF006_scaffold13029_2	non-phage	0
NLF006_scaffold9154_1	non-phage	0
NLF006_scaffold98_4	non-phage	0
NLF006_scaffold4358_2	phage	0.51
NLF006_scaffold12922_2	non-phage	0
NLF006_scaffold130_3	non-phage	0
NLF006_scaffold5820_1	non-phage	0
NLF006_scaffold8268_3	non-phage	0
NLF006_scaffold39_1	non-phage	0
NLF006_scaffold24106_2	non-phage	0
NLF006_scaffold29417_3	non-phage	0
NLF006_scaffold26257_1	non-phage	0

NLF006_scaffold65_5	non-phage	0
NLF006_scaffold5977_1	non-phage	0
NLF006_scaffold2978_3	non-phage	0
NLF006_scaffold8629_1	non-phage	0
NLF006_scaffold70_3	non-phage	0
NLF006_scaffold13810_2	non-phage	0
NLF006_scaffold5462_1	non-phage	0
NLF006_scaffold6450_2	non-phage	0
NLF006_scaffold184_1	non-phage	0
NLF006_scaffold92_1	non-phage	0
NLF006_scaffold2572_6	non-phage	0
NLF006_scaffold28827_2	non-phage	0
NLF006_scaffold8897_4	non-phage	0
NLF006_scaffold571_1	non-phage	0
NLF006_scaffold7709_1	non-phage	0
NLF006_scaffold32_3	non-phage	0
NLF007_scaffold557_7	non-phage	0
NLF007_scaffold34128_5	non-phage	0
NLF007_scaffold62447_1	non-phage	0
NLF007_scaffold882_1	phage	0.999
NLF007_scaffold38312_4	phage	0.617
NLF007_scaffold42371_6	non-phage	0
NLF007_scaffold21756_1	non-phage	0
NLF007_scaffold67330_1	non-phage	0
NLF007_scaffold67075_1	non-phage	0
NLF007_scaffold1651_3	non-phage	0
NLF007_scaffold6460_2	non-phage	0
NLF007_scaffold21776_6	non-phage	0
NLF007_scaffold126_2	non-phage	0
NLF007_scaffold41044_8	non-phage	0
NLF007_scaffold60337_8	non-phage	0
NLF007_scaffold54837_5	non-phage	0
NLF007_scaffold65902_4	non-phage	0
NLF007_scaffold5043_8	phage	0.958
NLF007_scaffold526_5	non-phage	0
NLF007_scaffold67436_2	non-phage	0
NLF007_scaffold404_17	non-phage	0
NLF007_scaffold67178_1	non-phage	0
NLF007_scaffold36067_3	non-phage	0
NLF007_scaffold63405_2	non-phage	0
NLF007_scaffold13004_1	non-phage	0
NLF007_scaffold50009_2	non-phage	0
NLF007_scaffold177_1	non-phage	0
NLF007_scaffold36778_2	non-phage	0
NLF007_scaffold29510_4	non-phage	0
NLF007_scaffold6478_1	non-phage	0

NLF007_scaffold63658_3	non-phage	0
NLF007_scaffold5950_13	non-phage	0
NLF007_scaffold14729_3	non-phage	0
NLF007_scaffold66705_1	non-phage	0
NLF007_scaffold8826_28	non-phage	0
NLF007_scaffold36536_1	non-phage	0
NLF007_scaffold66948_2	non-phage	0
NLF007_scaffold67_3	non-phage	0
NLF007_scaffold66997_1	non-phage	0
NLF007_scaffold54934_1	non-phage	0
NLF007_scaffold203_2	non-phage	0.002
NLF007_scaffold38563_3	non-phage	0
NLF007_scaffold23298_3	non-phage	0
NLF007_scaffold29677_4	non-phage	0
NLF007_scaffold13886_1	non-phage	0
NLF007_C1010371_1	non-phage	0.02
NLF007_scaffold61925_5	non-phage	0
NLF007_scaffold60272_1	non-phage	0
NLF007_scaffold21323_2	non-phage	0
NLF007_scaffold53183_2	non-phage	0
NLF007_scaffold67038_4	non-phage	0
NLF007_scaffold46468_5	non-phage	0
NLF007_scaffold404_30	non-phage	0
NLF007_scaffold13995_11	non-phage	0
NLF007_scaffold9122_18	non-phage	0
NLF007_scaffold3629_38	non-phage	0
NLF007_scaffold67397_3	non-phage	0
NLF007_scaffold66770_1	non-phage	0
NLF007_scaffold34800_2	non-phage	0
NLF007_scaffold11052_4	non-phage	0
NLF007_scaffold262_2	non-phage	0
NLF007_scaffold4208_35	non-phage	0
NLF007_scaffold28253_2	non-phage	0
NLF007_scaffold2328_1	non-phage	0
NLF007_scaffold67260_2	phage	0.989
NLF007_scaffold32534_8	non-phage	0
NLF007_scaffold49129_1	non-phage	0
NLF007_scaffold31610_4	non-phage	0
NLF007_scaffold52152_6	non-phage	0
NLF007_scaffold14743_4	phage	0.999
NLF007_scaffold25109_28	non-phage	0
NLF007_scaffold42263_4	non-phage	0
NLF007_scaffold32500_1	non-phage	0
NLF007_scaffold64602_1	phage	0.998
NLF007_scaffold66908_2	phage	0.999
NLF007_scaffold62482_8	non-phage	0

NLF007_scaffold62416_2	non-phage	0
NLF007_scaffold61894_1	non-phage	0
NLF007_scaffold23842_16	non-phage	0
NLF007_C1010319_1	non-phage	0
NLF007_scaffold62698_2	non-phage	0
NLF007_scaffold26244_7	non-phage	0
NLF007_scaffold34270_3	non-phage	0
NLF007_scaffold33600_7	non-phage	0
NLF007_scaffold47226_1	non-phage	0
NLF007_scaffold53555_2	non-phage	0
NLF007_scaffold29054_5	non-phage	0
NLF007_scaffold35031_5	non-phage	0
NLF007_scaffold44940_5	non-phage	0
NLF007_scaffold66844_1	non-phage	0
NLF007_scaffold110_7	non-phage	0
NLF007_scaffold4801_99	non-phage	0
NLF007_scaffold62332_1	non-phage	0
NLF007_scaffold48956_4	non-phage	0
NLF007_scaffold34161_7	non-phage	0
NLF007_scaffold54406_2	non-phage	0
NLF007_scaffold53288_4	non-phage	0
NLF007_scaffold16814_1	non-phage	0
NLF007_scaffold301_3	non-phage	0
NLF007_scaffold16369_8	non-phage	0
NLF007_scaffold66956_1	non-phage	0
NLF007_scaffold25670_4	non-phage	0
NLF007_scaffold75_1	non-phage	0
NLF007_scaffold35849_1	non-phage	0
NLF007_scaffold34161_4	non-phage	0
NLF007_scaffold26786_1	non-phage	0
NLF007_scaffold51079_1	non-phage	0
NLF007_scaffold52619_10	non-phage	0
NLF007_scaffold11272_3	non-phage	0
NLF007_scaffold67417_3	non-phage	0
NLF007_scaffold5175_3	non-phage	0.003
NLF007_scaffold5059_2	non-phage	0
NLF007_scaffold2750_18	non-phage	0
NLF007_scaffold326_2	non-phage	0
NLF007_scaffold23667_17	non-phage	0
NLF007_scaffold67340_1	non-phage	0
NLF007_scaffold24542_9	non-phage	0
NLF007_scaffold41413_1	non-phage	0
NLF007_scaffold46_4	non-phage	0
NLF007_scaffold526_8	non-phage	0
NLF007_scaffold3629_30	non-phage	0
NLF007_scaffold59474_3	non-phage	0

NLF007_scaffold42568_3	non-phage	0
NLF007_scaffold64146_3	non-phage	0
NLF007_scaffold66974_1	non-phage	0
NLF007_scaffold8274_2	phage	0.998
NLF007_scaffold32119_2	non-phage	0
NLF007_scaffold22938_1	phage	0.944
NLF007_scaffold52647_4	non-phage	0
NLF007_scaffold54983_1	non-phage	0
NLF007_scaffold48996_1	non-phage	0
NLF007_scaffold53748_1	non-phage	0
NLF007_scaffold44768_1	non-phage	0
NLF007_scaffold7804_1	non-phage	0
NLF007_scaffold28538_1	non-phage	0
NLF007_scaffold66217_2	non-phage	0
NLF007_scaffold326_4	non-phage	0
NLF007_scaffold25_1	non-phage	0
NLF007_scaffold13496_5	non-phage	0
NLF007_scaffold1369_3	non-phage	0
NLF007_scaffold44718_5	non-phage	0
NLF007_scaffold62482_3	non-phage	0
NLF007_scaffold19476_4	non-phage	0
NLF007_scaffold7589_1	non-phage	0
NLF007_scaffold11745_6	non-phage	0
NLF007_scaffold12525_11	non-phage	0
NLF007_scaffold67411_4	non-phage	0
NLF007_scaffold66668_1	non-phage	0
NLF007_scaffold31610_3	non-phage	0
NLF007_scaffold41299_2	non-phage	0
NLF007_scaffold28817_3	non-phage	0
NLF007_scaffold4754_2	non-phage	0
NLF007_scaffold23003_3	non-phage	0
NLF007_scaffold67435_2	non-phage	0
NLF007_scaffold67116_1	non-phage	0
NLF007_scaffold3969_1	non-phage	0
NLF007_scaffold27701_4	non-phage	0
NLF007_scaffold58945_3	non-phage	0
NLF007_scaffold465_2	non-phage	0
NLF007_scaffold1651_1	non-phage	0
NLF007_scaffold52871_3	non-phage	0
NLF007_scaffold67442_2	non-phage	0
NLF007_scaffold5223_7	non-phage	0
NLF007_scaffold66524_2	phage	1
NLF007_scaffold47013_11	non-phage	0
NLF007_scaffold404_6	non-phage	0
NLF007_scaffold47325_12	non-phage	0
NLF007_scaffold44425_4	non-phage	0

NLF007_scaffold404_35	non-phage	0
NLF007_scaffold66763_2	non-phage	0
NLF007_scaffold66514_2	non-phage	0
NLF007_scaffold6460_3	non-phage	0
NLF007_scaffold27701_1	non-phage	0
NLF007_scaffold66542_1	non-phage	0
NLF007_scaffold43025_1	non-phage	0
NLF007_scaffold1880_40	non-phage	0
NLF007_scaffold675_6	non-phage	0
NLF007_scaffold388_4	non-phage	0
NLF007_scaffold66078_1	non-phage	0
NLF007_scaffold51_1	non-phage	0
NLF007_scaffold326_1	non-phage	0
NLF007_scaffold27952_2	non-phage	0
NLF007_scaffold38312_8	phage	0.933
NLF007_scaffold36640_2	non-phage	0
NLF007_scaffold4500_11	non-phage	0
NLF007_scaffold11241_3	non-phage	0
NLF007_scaffold61029_7	non-phage	0
NLF007_scaffold5542_23	phage	0.638
NLF007_scaffold19360_12	non-phage	0
NLF007_scaffold2785_11	non-phage	0
NLF007_scaffold11055_7	non-phage	0
NLF007_scaffold11619_1	non-phage	0
NLF007_scaffold25374_3	non-phage	0
NLF007_scaffold66874_2	non-phage	0.012
NLF007_scaffold27701_8	non-phage	0
NLF007_scaffold65783_2	non-phage	0
NLF007_scaffold2231_2	non-phage	0
NLF007_scaffold3903_6	non-phage	0
NLF007_scaffold16474_2	non-phage	0
NLF007_scaffold17554_17	non-phage	0
NLF007_scaffold67331_1	non-phage	0
NLF007_scaffold49977_7	non-phage	0
NLF007_scaffold53114_1	non-phage	0
NLF007_scaffold44643_1	non-phage	0
NLF007_scaffold11102_4	phage	0.998
NLF007_scaffold43895_1	non-phage	0
NLF007_scaffold404_33	non-phage	0
NLF007_scaffold41422_1	non-phage	0
NLF007_scaffold16747_3	non-phage	0
NLF007_scaffold26102_3	non-phage	0
NLF007_scaffold2240_2	non-phage	0
NLF007_scaffold32735_1	non-phage	0
NLF007_scaffold49453_1	phage	0.998
NLF007_scaffold52399_1	non-phage	0

NLF007_scaffold66265_2	non-phage	0
NLF007_scaffold11694_4	non-phage	0
NLF007_scaffold65212_1	non-phage	0
NLF007_scaffold22798_5	non-phage	0
NLF007_scaffold65103_3	non-phage	0
NLF007_scaffold206_7	non-phage	0
NLF007_scaffold13769_2	non-phage	0
NLF007_scaffold11052_3	non-phage	0
NLF007_scaffold23667_31	non-phage	0
NLF007_scaffold460_2	non-phage	0
NLF007_scaffold53298_4	non-phage	0
NLF007_scaffold66684_1	non-phage	0
NLF007_scaffold43402_4	non-phage	0
NLF007_scaffold67434_1	non-phage	0
NLF007_scaffold24900_3	non-phage	0
NLF007_scaffold11694_7	non-phage	0
NLF007_scaffold36741_1	non-phage	0
NLF007_scaffold65761_1	non-phage	0
NLF007_scaffold49682_1	non-phage	0
NLF007_scaffold9391_30	non-phage	0.279
NLF007_scaffold53211_1	non-phage	0
NLF007_scaffold25225_11	non-phage	0
NLF007_scaffold22169_2	non-phage	0
NLF007_scaffold67347_1	non-phage	0
NLF007_scaffold39446_2	non-phage	0
NLF007_scaffold55114_3	non-phage	0
NLF007_scaffold25109_33	non-phage	0
NLF007_scaffold66823_1	non-phage	0
NLF007_scaffold67315_4	non-phage	0
NLF007_scaffold195_2	non-phage	0
NLF007_scaffold23933_2	non-phage	0
NLF007_scaffold1905_24	non-phage	0
NLF007_scaffold47382_1	non-phage	0
NLF007_scaffold40019_1	non-phage	0
NLF007_scaffold64629_1	non-phage	0
NLF007_scaffold123_2	non-phage	0
NLF007_scaffold67223_1	non-phage	0
NLF007_scaffold65730_1	non-phage	0
NLF007_scaffold23509_1	non-phage	0
NLF007_scaffold206_6	non-phage	0
NLF007_scaffold45_1	non-phage	0.114
NLF007_scaffold13185_1	non-phage	0
NLF007_scaffold67414_1	non-phage	0
NLF007_scaffold22798_10	non-phage	0
NLF007_scaffold61029_6	non-phage	0
NLF007_scaffold47325_10	non-phage	0

NLF007_scaffold62482_1	non-phage	0
NLF007_scaffold40797_2	non-phage	0
NLF007_scaffold8776_3	non-phage	0
NLF007_scaffold11032_1	non-phage	0
NLF007_scaffold23805_3	phage	0.919
NLF007_scaffold67066_1	non-phage	0
NLF007_scaffold31937_4	phage	0.998
NLF007_scaffold58275_4	non-phage	0
NLF007_scaffold51844_3	non-phage	0
NLF007_scaffold46564_13	non-phage	0
NLF007_scaffold25181_1	non-phage	0
NLF007_scaffold31892_13	non-phage	0
NLF007_scaffold66760_10	non-phage	0
NLF007_scaffold67436_3	non-phage	0
NLF007_scaffold46564_10	non-phage	0
NLF007_scaffold32119_3	non-phage	0
NLF007_scaffold66822_5	non-phage	0
NLF007_scaffold53298_2	non-phage	0
NLF007_scaffold21756_8	non-phage	0
NLF007_scaffold52434_1	non-phage	0
NLF007_scaffold32686_11	non-phage	0
NLF007_scaffold110_13	phage	0.987
NLF007_scaffold67448_1	non-phage	0
NLF007_scaffold33152_3	non-phage	0
NLF007_scaffold18980_16	non-phage	0
NLF007_scaffold1877_5	non-phage	0
NLF007_scaffold326_11	non-phage	0
NLF007_scaffold507_1	non-phage	0
NLF007_scaffold439_2	non-phage	0
NLF007_scaffold57215_3	non-phage	0
NLF007_scaffold23509_2	non-phage	0
NLF007_scaffold39528_1	non-phage	0
NLF007_scaffold64018_5	non-phage	0
NLF007_scaffold557_15	non-phage	0
NLF007_C1010437_1	non-phage	0
NLF007_scaffold31639_1	non-phage	0
NLF007_scaffold512_3	non-phage	0
NLF007_scaffold13771_4	non-phage	0
NLF007_scaffold27425_1	non-phage	0
NLF007_scaffold40161_9	non-phage	0
NLF007_scaffold23404_3	phage	1
NLF007_scaffold28407_1	non-phage	0
NLF007_scaffold25969_1	phage	0.99
NLF007_scaffold29510_1	phage	0.994
NLF007_scaffold4484_2	non-phage	0
NLF007_scaffold28179_2	non-phage	0

NLF007_scaffold49977_1	non-phage	0
NLF007_scaffold66669_1	non-phage	0
NLF007_scaffold9108_11	non-phage	0
NLF007_scaffold61307_5	phage	0.999
NLF007_scaffold64156_2	non-phage	0
NLF007_scaffold58972_1	non-phage	0
NLF007_scaffold32686_9	non-phage	0
NLF007_scaffold557_18	non-phage	0
NLF007_scaffold404_32	non-phage	0
NLF007_scaffold47013_7	non-phage	0
NLF007_scaffold27711_3	non-phage	0
NLF007_scaffold41299_28	non-phage	0
NLF007_scaffold23509_5	non-phage	0
NLF007_scaffold11052_2	non-phage	0
NLF007_scaffold28817_2	non-phage	0
NLF007_scaffold67351_2	non-phage	0
NLF007_scaffold65617_6	non-phage	0
NLF007_scaffold20950_10	non-phage	0
NLF007_scaffold61925_2	non-phage	0
NLF007_scaffold39880_1	non-phage	0.061
NLF007_scaffold25021_2	non-phage	0
NLF007_scaffold4118_1	phage	0.992
NLF007_scaffold3779_5	non-phage	0
NLF007_scaffold284_9	non-phage	0
NLF007_scaffold67086_1	non-phage	0
NLF007_scaffold15473_6	non-phage	0
NLF007_scaffold25181_4	non-phage	0
NLF007_scaffold2513_3	non-phage	0
NLF007_scaffold60838_1	non-phage	0
NLF007_scaffold27952_3	non-phage	0
NLF007_scaffold24542_11	non-phage	0
NLF007_scaffold55484_2	non-phage	0
NLF007_scaffold526_2	non-phage	0
NLF007_scaffold23139_1	non-phage	0
NLF007_scaffold181_2	non-phage	0
NLF007_scaffold63412_1	non-phage	0
NLF007_scaffold12765_3	non-phage	0
NLF007_scaffold36793_5	non-phage	0
NLF007_scaffold6625_1	non-phage	0
NLF007_scaffold13004_2	non-phage	0
NLF007_scaffold526_4	non-phage	0
NLF007_scaffold404_23	non-phage	0
NLF007_scaffold16925_2	non-phage	0
NLF007_scaffold402_1	non-phage	0
NLF007_scaffold8150_11	non-phage	0
NLF007_scaffold29164_1	non-phage	0

NLF007_scaffold67442_1	non-phage	0
NLF007_scaffold67411_2	non-phage	0
NLF007_scaffold11052_5	non-phage	0
NLF007_scaffold16474_1	non-phage	0
NLF007_scaffold34979_10	non-phage	0
NLF007_scaffold59904_1	non-phage	0
NLF007_scaffold67271_1	non-phage	0
NLF007_scaffold67236_3	phage	0.998
NLF007_scaffold47325_11	non-phage	0
NLF007_scaffold65761_3	non-phage	0
NLF007_scaffold36819_2	non-phage	0
NLF007_scaffold3779_4	phage	0.999
NLF007_scaffold58393_1	non-phage	0
NLF007_scaffold65323_1	non-phage	0
NLF007_scaffold44940_9	non-phage	0
NLF007_scaffold27103_9	phage	0.999
NLF007_scaffold67_6	non-phage	0
NLF007_scaffold46468_4	non-phage	0
NLF007_scaffold51844_2	non-phage	0
NLF007_scaffold67445_1	non-phage	0
NLF007_scaffold33716_4	non-phage	0
NLF007_scaffold22798_6	non-phage	0
NLF007_scaffold262_1	non-phage	0
NLF007_scaffold17151_6	non-phage	0
NLF007_scaffold6478_4	non-phage	0
NLF007_scaffold10248_5	non-phage	0
NLF007_scaffold47382_2	non-phage	0
NLF007_scaffold31892_21	non-phage	0
NLF007_scaffold42116_4	non-phage	0
NLF007_scaffold29054_2	non-phage	0
NLF007_scaffold67235_1	non-phage	0
NLF007_scaffold67413_1	phage	0.999
NLF007_scaffold67035_1	non-phage	0
NLF007_scaffold5542_21	non-phage	0
NLF007_scaffold63984_1	non-phage	0
NLF007_scaffold62482_9	non-phage	0
NLF007_scaffold12791_1	non-phage	0
NLF007_scaffold219_1	non-phage	0
NLF007_scaffold38563_4	non-phage	0
NLF007_scaffold4208_25	non-phage	0.008
NLF007_scaffold35195_1	non-phage	0
NLF007_scaffold2647_15	non-phage	0
NLF007_scaffold66745_3	non-phage	0
NLF007_scaffold25789_2	non-phage	0
NLF007_scaffold47325_8	non-phage	0.006
NLF007_scaffold52871_5	non-phage	0

NLF007_scaffold29925_3	non-phage	0
NLF007_scaffold34270_1	non-phage	0
NLF007_scaffold8069_2	non-phage	0
NLF007_scaffold36465_3	phage	0.989
NLF007_scaffold404_15	non-phage	0
NLF007_scaffold30358_3	non-phage	0
NLF007_scaffold24692_3	non-phage	0
NLF007_C1010321_1	non-phage	0
NLF007_scaffold439_13	non-phage	0
NLF007_scaffold32686_12	non-phage	0
NLF007_scaffold66696_3	non-phage	0
NLF007_scaffold3779_23	non-phage	0
NLF007_scaffold3512_2	non-phage	0
NLF007_scaffold17502_6	non-phage	0
NLF007_scaffold6999_3	non-phage	0
NLF007_scaffold47382_6	non-phage	0
NLF007_scaffold301_2	non-phage	0
NLF007_scaffold8169_12	non-phage	0
NLF007_scaffold36741_3	non-phage	0
NLF007_C1010391_1	phage	0.998
NLF007_scaffold52014_3	non-phage	0
NLF007_scaffold36067_10	non-phage	0
NLF007_C1010357_1	non-phage	0.007
NLF007_scaffold58357_1	non-phage	0
NLF007_scaffold47325_14	non-phage	0
NLF007_scaffold40161_7	non-phage	0
NLF007_scaffold11037_4	non-phage	0
NLF007_scaffold67424_2	non-phage	0
NLF007_scaffold404_8	non-phage	0
NLF007_scaffold44940_2	non-phage	0
NLF007_scaffold28504_1	non-phage	0
NLF007_scaffold401_18	non-phage	0
NLF007_scaffold30847_9	non-phage	0
NLF007_scaffold66683_1	non-phage	0
NLF007_scaffold12475_7	non-phage	0
NLF007_scaffold9108_10	non-phage	0
NLF007_scaffold17554_50	phage	0.992
NLF007_scaffold219_2	non-phage	0
NLF007_scaffold1651_2	non-phage	0
NLF007_scaffold55284_1	non-phage	0
NLF007_C1011091_1	non-phage	0
NLF007_scaffold23925_3	non-phage	0
NLF007_scaffold27903_12	non-phage	0
NLF007_scaffold64205_2	phage	0.995
NLF007_scaffold35174_1	non-phage	0
NLF007_scaffold63378_2	non-phage	0

NLF007_scaffold67075_2	non-phage	0
NLF007_scaffold60368_2	non-phage	0
NLF007_scaffold6220_2	non-phage	0
NLF007_scaffold12989_3	non-phage	0
NLF007_scaffold7051_14	non-phage	0
NLF007_scaffold64847_1	non-phage	0
NLF007_scaffold63231_2	non-phage	0
NLF007_scaffold62482_4	non-phage	0
NLF007_scaffold2750_12	non-phage	0
NLF007_scaffold20753_18	non-phage	0
NLF007_scaffold56850_1	non-phage	0
NLF007_scaffold5950_22	non-phage	0
NLF007_scaffold63286_3	non-phage	0
NLF007_scaffold163_1	non-phage	0
NLF007_scaffold28141_1	non-phage	0
NLF007_scaffold67411_3	non-phage	0
NLF007_scaffold404_3	non-phage	0
NLF007_scaffold61221_1	phage	0.999
NLF007_scaffold11745_3	non-phage	0
NLF007_scaffold58880_3	non-phage	0
NLF007_scaffold28817_4	non-phage	0
NLF007_scaffold56111_1	non-phage	0
NLF007_scaffold61993_3	non-phage	0
NLF007_scaffold57911_1	non-phage	0
NLF007_scaffold47636_1	non-phage	0
NLF007_scaffold44643_11	non-phage	0
NLF007_C1011055_1	non-phage	0
NLF007_scaffold67209_1	non-phage	0
NLF007_scaffold65575_2	non-phage	0
NLF007_scaffold66696_1	non-phage	0
NLF007_scaffold10248_2	non-phage	0
NLF007_scaffold32119_1	non-phage	0
NLF007_scaffold50011_1	non-phage	0
NLF007_scaffold6865_4	non-phage	0
NLF007_scaffold42859_2	non-phage	0
NLF007_scaffold404_25	non-phage	0
NLF007_scaffold26941_4	non-phage	0
NLF007_scaffold8274_5	non-phage	0
NLF007_scaffold41188_2	non-phage	0
NLF007_scaffold1460_5	non-phage	0
NLF007_scaffold52619_5	non-phage	0
NLF007_scaffold66987_2	non-phage	0
NLF007_scaffold54210_2	non-phage	0
NLF007_scaffold17365_1	non-phage	0
NLF007_scaffold29054_1	non-phage	0
NLF007_scaffold326_5	non-phage	0

NLF007_scaffold53366_1	non-phage	0
NLF007_scaffold20753_28	non-phage	0
NLF007_scaffold66524_5	non-phage	0
NLF007_scaffold954_5	non-phage	0
NLF007_scaffold47454_2	non-phage	0
NLF007_scaffold2938_6	non-phage	0
NLF007_scaffold4208_26	non-phage	0
NLF007_scaffold60505_1	non-phage	0
NLF007_scaffold23509_3	non-phage	0
NLF007_scaffold67393_1	non-phage	0
NLF007_scaffold56144_4	phage	0.991
NLF007_scaffold67422_2	non-phage	0
NLF007_scaffold67435_1	non-phage	0
NLF007_scaffold56745_2	non-phage	0
NLF007_scaffold21401_14	non-phage	0
NLF007_scaffold57251_3	non-phage	0
NLF007_scaffold67153_2	phage	0.999
NLF007_scaffold23957_1	phage	0.999
NLF007_scaffold32534_1	non-phage	0
NLF007_scaffold67042_1	non-phage	0
NLF007_scaffold43402_3	non-phage	0
NLF007_scaffold42409_1	non-phage	0
NLF007_scaffold23805_2	non-phage	0
NLF007_scaffold1877_2	non-phage	0
NLF007_scaffold31_4	non-phage	0
NLF007_scaffold67317_3	non-phage	0
NLF007_scaffold206_4	non-phage	0
NLF007_scaffold5952_3	non-phage	0
NLF007_scaffold34128_1	non-phage	0
NLF007_scaffold23632_3	non-phage	0
NLF007_scaffold67315_3	non-phage	0
NLF007_scaffold63324_1	non-phage	0
NLF007_scaffold110_11	non-phage	0
NLF007_scaffold13995_16	non-phage	0
NLF007_scaffold34128_8	non-phage	0
NLF007_scaffold54945_9	phage	0.998
NLF007_scaffold25181_2	non-phage	0
NLF007_scaffold36741_2	non-phage	0
NLF007_scaffold41171_2	non-phage	0
NLF007_scaffold18257_1	non-phage	0
NLF007_scaffold36591_9	non-phage	0
NLF007_scaffold48195_9	non-phage	0
NLF007_scaffold45232_1	non-phage	0
NLF007_scaffold24316_2	non-phage	0
NLF007_scaffold56975_1	non-phage	0
NLF007_scaffold5575_1	non-phage	0

NLF007_scaffold31892_15	non-phage	0
NLF007_scaffold45526_1	non-phage	0
NLF007_scaffold30119_11	non-phage	0
NLF007_scaffold3295_5	non-phage	0
NLF007_scaffold67146_2	non-phage	0
NLF007_scaffold63831_1	non-phage	0
NLF007_scaffold23667_30	non-phage	0
NLF007_scaffold67403_3	non-phage	0
NLF007_scaffold65103_2	non-phage	0
NLF007_scaffold67403_1	non-phage	0
NLF007_scaffold62463_3	non-phage	0
NLF007_scaffold36819_1	non-phage	0
NLF007_scaffold12118_10	non-phage	0
NLF007_scaffold2231_8	non-phage	0
NLF007_scaffold20693_10	phage	0.999
NLF007_scaffold439_5	non-phage	0
NLF007_scaffold30615_1	non-phage	0
NLF007_scaffold64830_7	non-phage	0.055
NLF007_scaffold67315_5	non-phage	0
NLF007_scaffold53298_5	non-phage	0
NLF007_scaffold64018_8	non-phage	0
NLF007_scaffold53748_2	non-phage	0
NLF007_scaffold12118_5	non-phage	0
NLF007_scaffold508_5	non-phage	0
NLF007_scaffold64796_1	non-phage	0
NLF007_scaffold13507_1	non-phage	0
NLF007_scaffold47454_3	non-phage	0
NLF007_scaffold31933_4	non-phage	0
NLF007_scaffold24542_16	non-phage	0
NLF007_scaffold67329_1	non-phage	0
NLF007_scaffold67238_1	non-phage	0
NLF007_scaffold39267_1	non-phage	0.257
NLF007_scaffold46468_6	non-phage	0
NLF007_scaffold219_7	non-phage	0
NLF007_scaffold19380_1	non-phage	0
NLF007_scaffold26941_1	non-phage	0
NLF007_scaffold50009_1	non-phage	0
NLF007_C1010401_1	non-phage	0
NLF007_scaffold20101_2	non-phage	0
NLF007_scaffold54814_1	non-phage	0
NLF007_scaffold3629_43	non-phage	0
NLF007_scaffold27261_1	non-phage	0
NLF007_scaffold66417_2	non-phage	0
NLF007_scaffold5256_2	non-phage	0
NLF007_scaffold136_7	non-phage	0
NLF007_scaffold3779_29	non-phage	0

NLF007_scaffold439_6	non-phage	0
NLF007_scaffold21401_17	non-phage	0
NLF007_scaffold18101_2	non-phage	0
NLF007_scaffold23086_2	non-phage	0
NLF007_scaffold28753_3	non-phage	0
NLF007_scaffold34800_1	non-phage	0
NLF007_scaffold26288_2	non-phage	0
NLF007_scaffold9122_10	non-phage	0
NLF007_scaffold44425_3	non-phage	0
NLF007_scaffold404_26	non-phage	0
NLF007_scaffold67038_3	non-phage	0
NLF007_scaffold45840_4	non-phage	0
NLF007_scaffold27872_2	non-phage	0
NLF007_scaffold67339_2	non-phage	0
NLF007_scaffold64704_1	non-phage	0
NLF007_scaffold40076_1	phage	0.999
NLF007_scaffold8934_5	non-phage	0
NLF007_scaffold15473_3	non-phage	0
NLF007_scaffold67437_2	non-phage	0
NLF007_scaffold17193_1	non-phage	0
NLF007_scaffold57215_11	non-phage	0
NLF007_scaffold41792_2	non-phage	0
NLF007_scaffold66874_3	non-phage	0
NLF007_scaffold41171_1	non-phage	0
NLF007_scaffold6999_9	non-phage	0
NLF007_scaffold40573_4	non-phage	0
NLF007_C1010981_1	non-phage	0
NLF007_scaffold45346_1	non-phage	0
NLF007_scaffold64677_1	non-phage	0
NLF007_scaffold36064_7	non-phage	0
NLF007_scaffold67002_1	non-phage	0
NLF007_scaffold1460_6	non-phage	0
NLF007_scaffold34037_3	non-phage	0
NLF007_scaffold32975_1	non-phage	0
NLF007_scaffold25653_2	non-phage	0
NLF007_scaffold17910_4	non-phage	0
NLF007_scaffold67249_2	non-phage	0
NLF007_scaffold44929_12	non-phage	0
NLF007_scaffold66908_3	phage	0.999
NLF007_scaffold2513_9	non-phage	0
NLF007_scaffold63326_3	non-phage	0
NLF007_scaffold47382_8	phage	0.997
NLF007_scaffold3969_4	non-phage	0
NLF007_scaffold47325_6	phage	0.922
NLF007_scaffold2053_3	non-phage	0
NLF007_scaffold16007_20	non-phage	0

NLF007_scaffold64156_3	non-phage	0
NLF007_scaffold18235_2	non-phage	0
NLF007_scaffold58275_2	non-phage	0
NLF007_scaffold8029_13	non-phage	0
NLF007_scaffold12765_2	non-phage	0
NLF007_scaffold67344_1	non-phage	0
NLF007_scaffold41727_1	non-phage	0
NLF007_scaffold51844_5	non-phage	0
NLF007_scaffold28407_2	non-phage	0
NLF007_scaffold61307_4	non-phage	0
NLF007_scaffold65491_1	non-phage	0
NLF007_scaffold28141_2	non-phage	0
NLF007_scaffold33600_6	non-phage	0
NLF007_scaffold29915_3	non-phage	0
NLF007_scaffold42371_3	non-phage	0
NLF007_scaffold51975_1	non-phage	0
NLF007_scaffold65521_2	non-phage	0
NLF007_scaffold67360_2	non-phage	0
NLF007_scaffold14743_2	phage	0.999
NLF007_scaffold34772_1	non-phage	0
NLF007_scaffold110_15	non-phage	0
NLF007_scaffold66696_5	non-phage	0
NLF007_scaffold30358_8	phage	0.627
NLF007_scaffold11832_2	non-phage	0
NLF007_scaffold60189_2	non-phage	0
NLF007_scaffold2240_1	non-phage	0
NLF007_scaffold51369_2	non-phage	0
NLF007_scaffold4239_1	non-phage	0.282
NLF007_scaffold41167_1	non-phage	0
NLF007_scaffold66850_2	non-phage	0
NLF007_scaffold181_1	non-phage	0
NLF007_scaffold42512_1	non-phage	0
NLF007_scaffold2647_4	non-phage	0
NLF007_scaffold66437_1	non-phage	0
NLF007_scaffold51201_8	non-phage	0
NLF007_scaffold32686_10	non-phage	0
NLF007_scaffold58439_3	non-phage	0
NLF007_scaffold155_2	non-phage	0
NLF007_scaffold34161_5	non-phage	0
NLF007_scaffold3034_1	non-phage	0
NLF007_scaffold44940_6	non-phage	0
NLF007_scaffold19390_4	non-phage	0
NLF007_scaffold5950_7	non-phage	0
NLF007_scaffold19390_10	non-phage	0
NLF007_scaffold8600_2	non-phage	0
NLF007_scaffold578_1	non-phage	0

NLF007_scaffold12749_1	non-phage	0
NLF007_scaffold3779_12	non-phage	0
NLF007_scaffold11620_1	non-phage	0
NLF007_scaffold3779_6	phage	0.999
NLF007_scaffold51975_3	non-phage	0
NLF007_scaffold40139_2	non-phage	0
NLF007_scaffold40161_5	non-phage	0
NLF007_scaffold38944_1	non-phage	0
NLF007_scaffold67332_1	non-phage	0
NLF007_scaffold67364_2	non-phage	0
NLF007_scaffold67441_1	non-phage	0
NLF007_scaffold5654_2	non-phage	0
NLF007_scaffold38563_1	non-phage	0
NLF007_scaffold14743_1	phage	0.998
NLF007_scaffold36536_3	non-phage	0.376
NLF007_scaffold186_1	non-phage	0
NLF007_C1010999_1	non-phage	0
NLF007_scaffold13_1_1	non-phage	0
NLF007_scaffold13_1_2	non-phage	0
NLF007_scaffold66683_5	non-phage	0
NLF007_scaffold67315_1	non-phage	0
NLF007_scaffold4500_2	non-phage	0
NLF007_scaffold25724_1	non-phage	0
NLF007_scaffold66332_1	non-phage	0
NLF007_scaffold54105_1	non-phage	0
NLF007_scaffold62698_5	non-phage	0
NLF007_scaffold62447_3	non-phage	0
NLF007_scaffold1460_7	non-phage	0
NLF007_scaffold11055_2	non-phage	0
NLF007_scaffold57652_6	phage	0.999
NLF007_scaffold54814_8	non-phage	0
NLF007_scaffold4906_5	non-phage	0
NLF007_scaffold50111_1	phage	0.999
NLF007_scaffold11832_3	non-phage	0
NLF007_scaffold5542_8	non-phage	0
NLF007_scaffold22032_1	non-phage	0
NLF007_scaffold40013_5	non-phage	0
NLF007_scaffold58779_1	non-phage	0
NLF007_scaffold60542_1	non-phage	0
NLF007_scaffold58037_1	non-phage	0
NLF007_scaffold44643_16	non-phage	0
NLF007_scaffold58357_2	non-phage	0
NLF007_C1010479_1	non-phage	0
NLF007_scaffold65276_1	non-phage	0
NLF007_scaffold38538_3	non-phage	0
NLF007_scaffold8776_19	non-phage	0.019

NLF007_scaffold67310_1	non-phage	0
NLF007_scaffold21776_4	non-phage	0
NLF007_scaffold8069_5	non-phage	0
NLF007_scaffold404_5	non-phage	0
NLF007_scaffold58945_6	non-phage	0
NLF007_scaffold13886_4	non-phage	0.001
NLF007_scaffold31411_1	non-phage	0
NLF007_scaffold37589_6	non-phage	0
NLF007_scaffold57227_1	phage	0.999
NLF007_scaffold59216_1	non-phage	0
NLF007_scaffold36640_5	non-phage	0
NLF007_scaffold31_3	non-phage	0
NLF007_scaffold48133_2	phage	0.676
NLF007_scaffold41044_15	non-phage	0
NLF007_scaffold6999_8	non-phage	0
NLF007_scaffold6478_3	non-phage	0
NLF007_scaffold29054_3	non-phage	0
NLF007_scaffold177_10	non-phage	0
NLF007_scaffold42859_4	non-phage	0
NLF007_scaffold66524_3	phage	0.997
NLF007_scaffold48440_2	non-phage	0
NLF007_scaffold9122_8	non-phage	0
NLF007_scaffold57215_12	non-phage	0
NLF007_scaffold17151_3	non-phage	0
NLF007_scaffold67213_3	non-phage	0
NLF007_scaffold219_4	non-phage	0
NLF007_scaffold388_5	non-phage	0
NLF007_scaffold66966_2	non-phage	0
NLF007_scaffold65899_4	non-phage	0.097
NLF007_scaffold67_8	non-phage	0
NLF007_scaffold22798_1	phage	0.992
NLF007_scaffold37349_5	non-phage	0
NLF007_scaffold67288_1	non-phage	0
NLF007_scaffold42946_3	non-phage	0
NLF007_scaffold67427_2	non-phage	0
NLF007_scaffold54706_3	non-phage	0
NLF007_scaffold47382_5	non-phage	0
NLF007_scaffold51551_1	non-phage	0
NLF007_scaffold301_1	non-phage	0
NLF007_scaffold63528_2	non-phage	0
NLF007_scaffold38312_5	non-phage	0
NLF007_scaffold48195_2	non-phage	0
NLF007_scaffold29915_2	non-phage	0
NLF007_scaffold56745_3	non-phage	0
NLF007_scaffold55_2	non-phage	0
NLF007_scaffold17910_3	non-phage	0

NLF007_scaffold28518_1	non-phage	0
NLF007_scaffold44643_7	non-phage	0
NLF007_scaffold61140_2	non-phage	0
NLF007_scaffold49948_6	non-phage	0
NLF007_scaffold36793_3	non-phage	0
NLF007_scaffold177_2	non-phage	0
NLF007_scaffold36640_3	non-phage	0
NLF007_scaffold2231_6	non-phage	0
NLF007_scaffold67441_2	non-phage	0
NLF007_scaffold37589_2	non-phage	0
NLF007_scaffold12077_1	non-phage	0
NLF007_scaffold46055_1	non-phage	0
NLF007_scaffold43991_1	phage	0.999
NLF007_scaffold67285_1	non-phage	0
NLF007_scaffold63286_1	non-phage	0
NLF007_scaffold64205_3	non-phage	0
NLF007_scaffold62735_2	non-phage	0
NLF007_scaffold13374_1	non-phage	0
NLF007_scaffold67419_1	non-phage	0
NLF007_scaffold40013_10	non-phage	0
NLF007_scaffold32409_4	non-phage	0
NLF007_scaffold7301_3	non-phage	0.291
NLF007_scaffold125_1	non-phage	0
NLF007_scaffold13150_2	non-phage	0
NLF007_scaffold40797_6	non-phage	0
NLF007_scaffold16814_2_1	non-phage	0
NLF007_scaffold6968_1	non-phage	0
NLF007_scaffold9893_2	non-phage	0
NLF007_scaffold388_8	non-phage	0
NLF007_scaffold62482_5	non-phage	0
NLF007_scaffold48816_1	non-phage	0
NLF007_scaffold578_2	non-phage	0
NLF007_scaffold19390_3	non-phage	0
NLF007_scaffold19360_9	non-phage	0
NLF007_scaffold36860_5	non-phage	0
NLF007_scaffold67392_2	non-phage	0
NLF007_scaffold67427_1	non-phage	0
NLF007_scaffold67404_2	non-phage	0
NLF007_scaffold52810_3	non-phage	0
NLF007_scaffold65521_3	non-phage	0
NLF007_scaffold45232_3	non-phage	0
NLF007_scaffold23214_2	non-phage	0
NLF007_scaffold47304_1	non-phage	0
NLF007_scaffold11776_3	non-phage	0
NLF007_scaffold2750_16	non-phage	0
NLF007_scaffold52815_3	non-phage	0

NLF007_scaffold3629_45	non-phage	0
NLF007_scaffold58945_2	non-phage	0
NLF007_scaffold61726_1	non-phage	0
NLF007_scaffold34128_3	non-phage	0
NLF007_scaffold3517_2	non-phage	0
NLF007_scaffold17742_4	non-phage	0
NLF007_scaffold41167_3	non-phage	0
NLF007_scaffold28817_5	non-phage	0
NLF007_scaffold62416_1	non-phage	0
NLF007_scaffold65880_2	non-phage	0
NLF007_scaffold11694_1	non-phage	0
NLF007_scaffold65103_1	non-phage	0
NLF007_scaffold23219_5	non-phage	0
NLF007_scaffold25181_7	non-phage	0
NLF007_scaffold3672_2	non-phage	0.002
NLF007_scaffold48034_3	non-phage	0
NLF007_scaffold285_5	non-phage	0
NLF007_scaffold3629_10	non-phage	0.007
NLF007_scaffold578_7	non-phage	0
NLF007_scaffold44425_6	non-phage	0
NLF007_scaffold26327_1	non-phage	0
NLF007_scaffold163_2	non-phage	0
NLF007_scaffold404_4	non-phage	0
NLF007_scaffold17594_1	non-phage	0
NLF007_scaffold67371_1	non-phage	0
NLF007_scaffold1762_1	non-phage	0
NLF007_scaffold35174_2	non-phage	0
NLF007_scaffold13601_2	non-phage	0
NLF007_scaffold23298_1	non-phage	0
NLF007_scaffold27903_5	non-phage	0
NLF007_scaffold394_3	non-phage	0
NLF007_scaffold61039_1	non-phage	0
NLF007_scaffold67446_1	non-phage	0
NLF007_scaffold62735_1	non-phage	0
NLF007_scaffold61955_12	non-phage	0
NLF007_scaffold59581_1	non-phage	0
NLF007_scaffold22798_13	phage	0.986
NLF007_scaffold67029_2	non-phage	0
NLF007_scaffold4670_9	non-phage	0
NLF007_scaffold26941_3	non-phage	0
NLF007_scaffold18400_3	non-phage	0
NLF007_scaffold28538_3	non-phage	0
NLF007_scaffold36067_6	non-phage	0
NLF007_scaffold1877_6	non-phage	0
NLF007_scaffold11745_8	non-phage	0
NLF007_scaffold19390_5	non-phage	0

NLF007_scaffold4118_5	non-phage	0
NLF007_scaffold33367_6	non-phage	0
NLF007_scaffold15694_1	non-phage	0
NLF007_scaffold8263_1	non-phage	0
NLF007_scaffold61993_2	non-phage	0
NLF007_scaffold38312_6	non-phage	0
NLF007_scaffold31937_5	phage	1
NLF007_scaffold3779_9	non-phage	0
NLF007_scaffold66078_2	non-phage	0
NLF007_scaffold8851_10	non-phage	0
NLF007_scaffold67062_4	phage	0.999
NLF007_scaffold27261_3	non-phage	0
NLF007_scaffold67315_2	non-phage	0
NLF007_scaffold13995_15	non-phage	0
NLF007_scaffold34037_4	non-phage	0
NLF007_scaffold66966_1	non-phage	0
NLF007_scaffold11102_2	non-phage	0
NLF007_scaffold25969_2	non-phage	0
NLF007_scaffold110_10	non-phage	0.002
NLF007_scaffold4906_3	non-phage	0
NLF007_scaffold27701_6	non-phage	0
NLF007_scaffold67440_2	non-phage	0
NLF007_scaffold29510_2	non-phage	0
NLF007_scaffold21013_3	non-phage	0
NLF007_scaffold66874_4	non-phage	0
NLF007_scaffold42859_3	non-phage	0
NLF007_scaffold20918_1	non-phage	0
NLF007_scaffold6625_5	non-phage	0
NLF007_scaffold404_14	non-phage	0
NLF007_scaffold9122_20	non-phage	0
NLF007_scaffold48820_2	non-phage	0
NLF007_scaffold66441_3	non-phage	0
NLF007_scaffold9589_4	non-phage	0
NLF007_scaffold34404_4	phage	0.999
NLF007_scaffold58145_4	non-phage	0
NLF007_scaffold32046_2	phage	0.999
NLF007_scaffold326_10	non-phage	0
NLF007_scaffold21464_1	phage	0.952
NLF007_scaffold59138_2	non-phage	0
NLF007_scaffold16369_7	non-phage	0
NLF007_scaffold30995_7	non-phage	0
NLF007_scaffold67333_2	non-phage	0
NLF007_scaffold11694_2	non-phage	0.001
NLF007_scaffold23340_4	non-phage	0
NLF007_scaffold67062_3	phage	1
NLF007_scaffold49552_4	non-phage	0

NLF007_scaffold405_1	non-phage	0
NLF007_scaffold27103_3	phage	0.998
NLF007_scaffold25807_1	non-phage	0
NLF007_scaffold52871_4	non-phage	0
NLF007_scaffold65472_1	non-phage	0
NLF007_scaffold46_1	non-phage	0
NLF007_scaffold40161_3	non-phage	0
NLF007_scaffold66822_4	non-phage	0
NLF007_scaffold67333_1	non-phage	0
NLF007_scaffold2513_2	non-phage	0
NLF007_scaffold13995_10	non-phage	0
NLF007_scaffold8029_4	non-phage	0
NLF007_scaffold66217_1	non-phage	0
NLF007_scaffold11055_5	non-phage	0
NLF007_scaffold20393_21	non-phage	0
NLF007_scaffold61433_1	non-phage	0
NLF007_scaffold67131_1	non-phage	0
NLF007_scaffold61667_1	non-phage	0
NLF007_scaffold67426_2	non-phage	0
NLF007_scaffold66952_1	non-phage	0
NLF007_scaffold9391_7	non-phage	0
NLF007_scaffold44643_17	non-phage	0
NLF007_scaffold55_1	non-phage	0
NLF007_scaffold110_1	non-phage	0
NLF007_scaffold29915_1	non-phage	0
NLF007_scaffold29510_3	non-phage	0
NLF007_scaffold13710_3	non-phage	0
NLF007_scaffold28817_1	non-phage	0
NLF007_scaffold27903_19	non-phage	0
NLF007_scaffold32534_4	non-phage	0
NLF007_scaffold44643_4	non-phage	0
NLF007_scaffold2513_4	non-phage	0
NLF007_scaffold29054_6	non-phage	0
NLF007_scaffold394_2	non-phage	0
NLF007_scaffold41299_8	non-phage	0
NLF007_C1010385_1	non-phage	0
NLF008_scaffold15727_3	non-phage	0
NLF008_scaffold3592_4	non-phage	0
NLF008_scaffold11789_2	non-phage	0
NLF008_scaffold3455_3	non-phage	0
NLF008_scaffold1813_24	non-phage	0
NLF008_scaffold19924_2	non-phage	0
NLF008_scaffold11316_1	non-phage	0
NLF008_scaffold20658_1	non-phage	0
NLF008_scaffold2979_3	non-phage	0
NLF008_C316552_1	non-phage	0

NLF008_scaffold37_3	non-phage	0
NLF008_scaffold13558_2	non-phage	0
NLF008_scaffold3034_2	non-phage	0
NLF008_scaffold8081_1	non-phage	0
NLF008_scaffold17551_1	non-phage	0
NLF008_scaffold33_1	non-phage	0
NLF008_scaffold20315_2	non-phage	0
NLF008_scaffold6399_12	non-phage	0
NLF008_C315574_1	phage	0.741
NLF008_scaffold9879_1	non-phage	0
NLF008_scaffold17515_2	non-phage	0
NLF008_scaffold20431_6	non-phage	0
NLF008_scaffold9442_4	non-phage	0
NLF008_C315474_1	non-phage	0
NLF008_scaffold13153_1	non-phage	0
NLF008_scaffold3669_2	non-phage	0
NLF008_scaffold19572_2	non-phage	0
NLF008_scaffold9612_1	non-phage	0
NLF008_scaffold4766_9	non-phage	0
NLF008_scaffold13575_7	non-phage	0
NLF008_scaffold6465_2	non-phage	0
NLF008_scaffold83_2	non-phage	0
NLF008_scaffold10133_4	non-phage	0
NLF008_scaffold9868_1	non-phage	0
NLF008_scaffold16234_1	non-phage	0
NLF008_scaffold8834_7	non-phage	0
NLF008_scaffold7795_1	non-phage	0
NLF008_scaffold19241_2	non-phage	0
NLF008_scaffold8040_1	non-phage	0
NLF008_scaffold2279_3	non-phage	0
NLF008_scaffold3150_19	non-phage	0.004
NLF008_scaffold20667_1	non-phage	0
NLF008_scaffold20351_1	non-phage	0
NLF008_scaffold20439_5	non-phage	0
NLF008_scaffold9194_2	non-phage	0
NLF008_scaffold20191_1	non-phage	0
NLF008_scaffold18979_4	non-phage	0
NLF008_scaffold6889_12	non-phage	0
NLF008_scaffold16729_1	non-phage	0
NLF008_C315244_1	non-phage	0
NLF008_scaffold15612_3	non-phage	0
NLF008_scaffold2458_4	non-phage	0
NLF008_scaffold4012_1	non-phage	0
NLF008_scaffold20710_2	non-phage	0
NLF008_scaffold3567_3	non-phage	0
NLF008_scaffold10026_1	non-phage	0

NLF008_scaffold20633_1	non-phage	0
NLF008_scaffold10577_2	non-phage	0
NLF008_scaffold6328_3	non-phage	0
NLF008_scaffold13441_1	non-phage	0
NLF008_scaffold6557_7	non-phage	0
NLF008_scaffold2996_2	non-phage	0
NLF008_scaffold3812_4	non-phage	0
NLF008_scaffold13731_1	non-phage	0
NLF008_scaffold20081_4	non-phage	0
NLF008_scaffold13026_1	non-phage	0
NLF008_scaffold15265_2	non-phage	0
NLF008_scaffold18579_1	non-phage	0
NLF008_scaffold3277_1	non-phage	0
NLF008_C315436_1	non-phage	0
NLF008_scaffold19700_1	phage	0.999
NLF008_scaffold2617_3	non-phage	0
NLF008_scaffold5503_5	non-phage	0
NLF008_scaffold20546_1	non-phage	0
NLF008_scaffold139_1	non-phage	0
NLF008_scaffold9739_1	non-phage	0
NLF008_scaffold2293_1	non-phage	0
NLF008_scaffold15132_2	non-phage	0
NLF008_scaffold20768_1	non-phage	0
NLF008_scaffold2990_1	non-phage	0
NLF008_scaffold9785_1	non-phage	0
NLF008_scaffold141_3	phage	0.997
NLF008_scaffold105_1	non-phage	0
NLF008_scaffold3150_15	non-phage	0
NLF008_scaffold17697_1	non-phage	0
NLF008_scaffold3520_7	non-phage	0
NLF008_scaffold2773_1	non-phage	0
NLF008_scaffold9515_2	non-phage	0
NLF008_scaffold9584_6	non-phage	0
NLF008_scaffold12997_5	phage	0.653
NLF008_scaffold18404_4	phage	0.996
NLF008_scaffold18699_1	non-phage	0
NLF008_scaffold18693_1	non-phage	0
NLF008_scaffold17016_1	non-phage	0
NLF008_scaffold2260_8	non-phage	0
NLF008_scaffold150_9	non-phage	0
NLF008_scaffold2636_17	non-phage	0
NLF008_scaffold3574_7	non-phage	0
NLF008_scaffold5771_2	non-phage	0
NLF008_scaffold13775_2	non-phage	0
NLF008_scaffold5025_9	non-phage	0
NLF008_scaffold14749_1	non-phage	0

NLF008_scaffold5713_4	non-phage	0
NLF008_scaffold1786_2	non-phage	0
NLF008_scaffold15890_1	non-phage	0
NLF008_scaffold13565_9	non-phage	0
NLF008_scaffold20758_1	non-phage	0
NLF008_scaffold20404_2	non-phage	0
NLF008_scaffold19995_1	non-phage	0
NLF008_scaffold20134_1	non-phage	0
NLF008_scaffold13388_5	non-phage	0
NLF008_scaffold20265_3	non-phage	0
NLF008_scaffold18296_3	non-phage	0
NLF008_scaffold3106_5	non-phage	0
NLF008_scaffold10158_1	phage	0.985
NLF008_scaffold9131_1	non-phage	0
NLF008_scaffold13575_11	non-phage	0
NLF008_scaffold11316_2	non-phage	0
NLF008_scaffold10473_9	non-phage	0
NLF008_scaffold167_3	non-phage	0
NLF008_scaffold4128_5	non-phage	0.015
NLF008_scaffold10801_12	non-phage	0
NLF008_scaffold19936_4	non-phage	0
NLF008_scaffold17964_2	non-phage	0
NLF008_scaffold13141_2	non-phage	0
NLF008_scaffold1586_6	non-phage	0
NLF008_scaffold3229_1	non-phage	0
NLF008_scaffold5918_4	non-phage	0
NLF008_scaffold15728_2	non-phage	0
NLF008_scaffold17_2	non-phage	0
NLF008_scaffold20464_2	non-phage	0
NLF008_scaffold5912_3	non-phage	0
NLF008_scaffold20315_1	non-phage	0
NLF008_scaffold13575_3	non-phage	0
NLF008_scaffold2789_1	non-phage	0
NLF008_scaffold20136_3	non-phage	0
NLF008_scaffold5517_1	non-phage	0
NLF008_scaffold7849_1	non-phage	0
NLF008_scaffold121_5	non-phage	0
NLF008_scaffold790_3	non-phage	0
NLF008_scaffold20763_1	non-phage	0
NLF008_scaffold5248_2	non-phage	0
NLF008_scaffold9347_2	non-phage	0
NLF008_scaffold20496_1	phage	0.712
NLF008_scaffold17551_3	non-phage	0
NLF008_scaffold6876_3	non-phage	0
NLF008_scaffold11801_3	non-phage	0
NLF008_scaffold2979_7	non-phage	0

NLF008_scaffold174_2	non-phage	0
NLF008_scaffold11629_14	non-phage	0
NLF008_scaffold5311_2	non-phage	0
NLF008_scaffold14373_1	non-phage	0
NLF008_scaffold16433_1	non-phage	0
NLF008_scaffold1783_1	non-phage	0
NLF008_scaffold9492_3	non-phage	0
NLF008_scaffold8874_2	phage	0.995
NLF008_scaffold877_4	non-phage	0
NLF008_scaffold11791_1	non-phage	0
NLF008_scaffold4915_3	non-phage	0
NLF008_scaffold13567_1	non-phage	0
NLF008_scaffold18404_14	phage	0.998
NLF008_scaffold10905_2	phage	0.999
NLF008_scaffold8532_1	non-phage	0
NLF008_scaffold5517_7	non-phage	0
NLF008_scaffold10033_7	non-phage	0
NLF008_scaffold10905_1	phage	1
NLF008_scaffold19632_1	non-phage	0
NLF008_scaffold4675_2	non-phage	0
NLF008_scaffold20209_1	non-phage	0
NLF008_scaffold15448_1	non-phage	0
NLF008_scaffold12908_6	non-phage	0
NLF008_scaffold15291_1	non-phage	0
NLF008_scaffold17061_1	non-phage	0
NLF008_scaffold3403_1	non-phage	0
NLF008_scaffold19652_3	non-phage	0
NLF008_scaffold12122_2	non-phage	0
NLF008_scaffold3455_4	non-phage	0
NLF008_scaffold8017_1	non-phage	0
NLF008_scaffold1152_14	non-phage	0
NLF008_C316544_1	non-phage	0
NLF008_scaffold7778_1	non-phage	0
NLF008_scaffold815_2	non-phage	0
NLF008_scaffold4928_1	non-phage	0
NLF008_scaffold8132_1	non-phage	0
NLF008_scaffold7371_7	non-phage	0
NLF008_scaffold20269_1	non-phage	0
NLF008_scaffold12977_3	non-phage	0
NLF008_scaffold19382_4	non-phage	0
NLF008_scaffold1586_11	phage	0.94
NLF008_scaffold7552_2	phage	0.827
NLF008_scaffold20716_1	non-phage	0
NLF008_scaffold82_6	non-phage	0
NLF008_scaffold15649_2	non-phage	0
NLF008_scaffold19077_1	non-phage	0

NLF008_scaffold11252_2	non-phage	0
NLF008_scaffold2458_3	non-phage	0
NLF008_scaffold17778_6	non-phage	0
NLF008_scaffold735_8	non-phage	0
NLF008_scaffold20413_2	non-phage	0
NLF008_scaffold15135_1	non-phage	0
NLF008_scaffold1157_31	non-phage	0
NLF008_scaffold10308_1	non-phage	0
NLF008_scaffold7580_1	non-phage	0
NLF008_C316554_1	non-phage	0
NLF008_scaffold14760_1	non-phage	0
NLF008_scaffold11629_10	non-phage	0
NLF008_scaffold20089_2	non-phage	0
NLF008_scaffold10897_1	non-phage	0
NLF008_scaffold1225_1	non-phage	0
NLF008_scaffold1634_15	non-phage	0
NLF008_scaffold6889_3	non-phage	0
NLF008_scaffold9879_3	non-phage	0
NLF008_scaffold14162_1	non-phage	0
NLF008_scaffold20431_5	phage	0.999
NLF008_scaffold6328_6	non-phage	0
NLF008_scaffold20598_4	non-phage	0.001
NLF008_scaffold20559_4	non-phage	0
NLF008_scaffold1786_7	non-phage	0
NLF008_scaffold1868_4	non-phage	0
NLF008_scaffold18648_1	non-phage	0
NLF008_scaffold9446_1	non-phage	0
NLF008_scaffold1868_3	non-phage	0
NLF008_scaffold11789_1	non-phage	0
NLF008_scaffold7908_1	non-phage	0
NLF008_C316226_1	non-phage	0
NLF008_scaffold15826_4	non-phage	0
NLF008_scaffold12679_2	non-phage	0
NLF008_scaffold7710_2	non-phage	0
NLF008_scaffold7660_2	non-phage	0
NLF008_scaffold17602_2	non-phage	0
NLF008_scaffold1474_3	non-phage	0
NLF008_scaffold758_4	non-phage	0
NLF008_scaffold6839_2	non-phage	0
NLF008_scaffold20568_1	non-phage	0
NLF008_scaffold2201_3	non-phage	0
NLF008_scaffold15651_3	non-phage	0
NLF008_C316094_1	non-phage	0
NLF008_scaffold3150_45	non-phage	0
NLF008_scaffold7698_1	non-phage	0
NLF008_scaffold10874_2	non-phage	0

NLF008_scaffold20553_2	non-phage	0
NLF008_scaffold1474_12	non-phage	0
NLF008_scaffold4766_5	non-phage	0
NLF008_scaffold20190_1	non-phage	0
NLF008_scaffold20446_1	non-phage	0
NLF008_scaffold877_6	non-phage	0
NLF008_scaffold1764_2	non-phage	0
NLF008_scaffold1157_48	non-phage	0
NLF008_scaffold7607_1	non-phage	0
NLF008_scaffold1515_2	non-phage	0
NLF008_C315844_1	non-phage	0
NLF008_scaffold11779_2	non-phage	0
NLF008_scaffold12767_1	non-phage	0
NLF008_scaffold2636_13	non-phage	0
NLF008_scaffold5181_6	non-phage	0
NLF008_scaffold5771_4	non-phage	0
NLF008_scaffold13444_3	non-phage	0
NLF008_scaffold8779_1	non-phage	0
NLF008_scaffold19450_7	non-phage	0
NLF008_scaffold17060_1	non-phage	0
NLF008_scaffold12145_1	non-phage	0
NLF008_scaffold3574_6	non-phage	0
NLF008_C316618_1	non-phage	0
NLF008_scaffold20666_1	non-phage	0
NLF008_scaffold9194_3	non-phage	0
NLF008_C316384_1	non-phage	0
NLF008_scaffold82_4	non-phage	0
NLF008_scaffold9128_1	non-phage	0
NLF008_scaffold1157_47	non-phage	0
NLF008_scaffold12809_1	non-phage	0
NLF008_scaffold2933_9	non-phage	0
NLF008_scaffold6563_1	non-phage	0
NLF008_scaffold735_2	non-phage	0
NLF008_scaffold13388_1	non-phage	0
NLF008_scaffold20270_1	non-phage	0
NLF008_scaffold2933_2_1	non-phage	0
NLF008_scaffold9772_2	non-phage	0
NLF008_scaffold20758_2	non-phage	0
NLF008_scaffold20439_2	non-phage	0
NLF008_scaffold11499_18	non-phage	0
NLF008_scaffold8157_1	non-phage	0
NLF008_scaffold303_7	non-phage	0
NLF008_scaffold11943_2	non-phage	0
NLF008_scaffold20681_1	non-phage	0
NLF008_scaffold9657_5	non-phage	0
NLF008_scaffold18567_2	non-phage	0

NLF008_scaffold5462_26	non-phage	0.435
NLF008_scaffold20761_3	non-phage	0
NLF008_scaffold395_2	non-phage	0
NLF008_scaffold20391_1	non-phage	0
NLF008_scaffold15265_3	non-phage	0
NLF008_scaffold11112_2	non-phage	0
NLF008_scaffold10052_7	non-phage	0
NLF008_scaffold1829_9	non-phage	0
NLF008_scaffold3866_4	non-phage	0
NLF008_scaffold8668_1	non-phage	0
NLF008_scaffold20411_2	non-phage	0
NLF008_scaffold12977_7	non-phage	0
NLF008_scaffold20533_2	non-phage	0
NLF008_scaffold20590_1	non-phage	0
NLF008_scaffold17551_2	non-phage	0.001
NLF008_scaffold12790_5	non-phage	0
NLF008_scaffold20764_1	non-phage	0
NLF008_scaffold3972_1	non-phage	0
NLF008_scaffold19381_1	non-phage	0
NLF008_scaffold20757_1	non-phage	0
NLF008_scaffold3229_4	non-phage	0
NLF008_scaffold5503_11	non-phage	0
NLF008_scaffold12702_1	non-phage	0
NLF008_scaffold11957_1	non-phage	0
NLF008_scaffold444_13	non-phage	0
NLF008_scaffold3739_3	non-phage	0
NLF008_scaffold20378_2	non-phage	0
NLF008_scaffold19473_1	phage	0.998
NLF008_scaffold20769_2	non-phage	0
NLF008_scaffold9226_1	non-phage	0
NLF008_scaffold16727_1	non-phage	0
NLF008_C316004_1	non-phage	0
NLF008_scaffold6482_1	non-phage	0
NLF008_scaffold4395_8	non-phage	0
NLF008_scaffold9492_1	non-phage	0
NLF008_scaffold1923_13	non-phage	0
NLF008_scaffold215_5	non-phage	0
NLF008_scaffold9366_1	phage	0.985
NLF008_scaffold429_1	non-phage	0
NLF008_scaffold8008_7	non-phage	0
NLF008_scaffold14458_1	non-phage	0
NLF008_scaffold9243_6	non-phage	0
NLF008_C316376_1	non-phage	0
NLF008_scaffold11973_1	non-phage	0
NLF008_scaffold2636_18	non-phage	0
NLF008_scaffold769_1_1	non-phage	0

NLF008_scaffold20258_3	non-phage	0
NLF008_scaffold4989_4	non-phage	0
NLF008_scaffold20265_4	non-phage	0
NLF008_scaffold790_1	non-phage	0
NLF008_scaffold2260_2	non-phage	0
NLF008_scaffold11806_1	non-phage	0
NLF008_scaffold7651_2	non-phage	0
NLF008_scaffold20602_2	non-phage	0
NLF008_scaffold1157_19	non-phage	0
NLF008_scaffold20559_1	non-phage	0
NLF008_scaffold20367_4	non-phage	0
NLF008_scaffold11629_4	non-phage	0
NLF008_scaffold3106_2	non-phage	0
NLF008_scaffold7645_1	non-phage	0
NLF008_scaffold15901_1	non-phage	0
NLF008_scaffold1474_13	non-phage	0
NLF008_scaffold5109_2	non-phage	0
NLF008_scaffold20390_1	non-phage	0
NLF008_scaffold20672_2	non-phage	0
NLF008_scaffold20464_4	non-phage	0
NLF008_scaffold19595_3	non-phage	0
NLF008_scaffold1410_9	non-phage	0
NLF008_scaffold13893_1	non-phage	0
NLF008_C316584_1	non-phage	0
NLF008_scaffold15799_2	non-phage	0
NLF008_scaffold19450_2	non-phage	0
NLF008_scaffold98_11	non-phage	0
NLF008_scaffold6876_7	non-phage	0
NLF008_scaffold82_10	non-phage	0
NLF008_scaffold16669_1	non-phage	0
NLF008_scaffold8575_4	non-phage	0
NLF008_scaffold14354_2	non-phage	0
NLF008_scaffold877_1	non-phage	0
NLF008_scaffold19572_1	non-phage	0
NLF008_scaffold13565_5	non-phage	0
NLF008_scaffold20352_3	non-phage	0
NLF008_scaffold15389_1	phage	0.935
NLF008_scaffold16625_3	non-phage	0
NLF008_scaffold5771_3	non-phage	0
NLF008_scaffold20315_3	non-phage	0
NLF008_scaffold75_5	non-phage	0
NLF008_scaffold20125_1	phage	0.711
NLF008_scaffold16820_2	non-phage	0
NLF008_scaffold7580_2	non-phage	0
NLF008_scaffold17138_3	non-phage	0
NLF008_scaffold10810_3	non-phage	0

NLF008_scaffold18955_1	non-phage	0
NLF008_scaffold421_7	non-phage	0
NLF008_scaffold17605_2	phage	0.997
NLF008_C316442_1	non-phage	0
NLF008_scaffold20431_1	non-phage	0
NLF008_scaffold12725_3	non-phage	0
NLF008_scaffold1157_27	non-phage	0
NLF008_scaffold1800_10	non-phage	0
NLF008_scaffold421_1	non-phage	0
NLF008_scaffold10117_1	non-phage	0
NLF008_scaffold4745_2	non-phage	0
NLF008_scaffold8571_1	non-phage	0
NLF008_scaffold13280_5	non-phage	0
NLF008_scaffold261_5	non-phage	0
NLF008_C315706_1	non-phage	0
NLF008_scaffold13280_2	non-phage	0
NLF008_scaffold20567_1	non-phage	0
NLF008_scaffold7370_3	non-phage	0
NLF008_scaffold9880_4	non-phage	0
NLF008_scaffold10033_8	non-phage	0
NLF008_scaffold4675_1	non-phage	0
NLF008_C315766_1	non-phage	0
NLF008_scaffold8834_4	non-phage	0
NLF008_scaffold4787_3	non-phage	0
NLF008_scaffold19764_1	non-phage	0
NLF008_scaffold20446_4	non-phage	0
NLF008_scaffold6835_1	phage	0.998
NLF008_scaffold13615_3	non-phage	0
NLF008_scaffold20730_2	non-phage	0
NLF008_scaffold10766_1	non-phage	0
NLF008_scaffold11112_3	non-phage	0
NLF008_scaffold20526_3	non-phage	0
NLF008_scaffold6328_4	non-phage	0
NLF008_scaffold1800_11	non-phage	0
NLF008_scaffold17_4	non-phage	0
NLF008_scaffold5458_4	non-phage	0
NLF008_scaffold429_5	non-phage	0
NLF008_scaffold12977_4	non-phage	0
NLF008_scaffold9492_2	phage	0.998
NLF008_scaffold8571_2	non-phage	0
NLF008_scaffold12810_1	non-phage	0
NLF008_scaffold18893_1	non-phage	0
NLF008_scaffold2979_6	non-phage	0
NLF008_scaffold2293_5	non-phage	0
NLF008_scaffold12460_1	non-phage	0
NLF008_scaffold18868_3	non-phage	0

NLF008_scaffold9301_4	non-phage	0
NLF008_scaffold18404_10	non-phage	0
NLF008_scaffold15286_1	non-phage	0
NLF008_scaffold75_2	non-phage	0
NLF008_scaffold12194_1	phage	0.998
NLF008_scaffold218_2	non-phage	0
NLF008_scaffold7552_3	non-phage	0
NLF008_scaffold20405_3	non-phage	0
NLF008_scaffold7927_2	non-phage	0
NLF008_scaffold8403_2	non-phage	0
NLF008_scaffold1157_52	non-phage	0
NLF008_scaffold20751_1	non-phage	0
NLF008_scaffold9_3	non-phage	0
NLF008_scaffold986_9	non-phage	0
NLF008_scaffold7710_1	non-phage	0
NLF008_scaffold20731_1	phage	0.999
NLF008_scaffold17589_1	non-phage	0
NLF008_scaffold14781_2	non-phage	0
NLF008_scaffold19169_4	non-phage	0
NLF008_scaffold1829_5	non-phage	0
NLF008_scaffold5986_2	non-phage	0
NLF008_scaffold9768_1	non-phage	0
NLF008_scaffold20431_2	non-phage	0
NLF008_scaffold11984_1	non-phage	0
NLF008_scaffold6103_28	phage	0.994
NLF008_scaffold17715_1	non-phage	0
NLF008_scaffold20157_1	non-phage	0
NLF008_scaffold15135_2	non-phage	0
NLF008_scaffold19681_2	non-phage	0
NLF008_scaffold395_6	phage	0.999
NLF008_scaffold6876_2	non-phage	0
NLF008_scaffold20190_4	non-phage	0
NLF008_scaffold8021_2	non-phage	0
NLF008_scaffold8154_1	non-phage	0
NLF008_scaffold14290_1	non-phage	0
NLF008_scaffold19847_4	non-phage	0
NLF008_scaffold15651_2	non-phage	0
NLF008_scaffold11609_1	non-phage	0
NLF008_scaffold8834_6	non-phage	0
NLF008_scaffold6694_4	non-phage	0
NLF008_scaffold815_1	non-phage	0
NLF008_scaffold18693_2	non-phage	0
NLF008_scaffold18699_4	non-phage	0
NLF008_scaffold8189_2	non-phage	0
NLF008_scaffold20553_1	non-phage	0
NLF008_scaffold9688_6	non-phage	0

NLF008_scaffold6185_2	non-phage	0
NLF008_scaffold15986_2	non-phage	0
NLF008_scaffold3106_6	non-phage	0
NLF008_scaffold303_2	non-phage	0
NLF008_scaffold9879_6	non-phage	0
NLF008_scaffold7537_2	non-phage	0
NLF008_scaffold1533_3	non-phage	0
NLF008_scaffold12947_1	phage	0.978
NLF008_scaffold11660_1	non-phage	0
NLF008_scaffold11827_2	non-phage	0
NLF008_scaffold2383_5	non-phage	0
NLF008_scaffold14699_1	non-phage	0
NLF008_scaffold3781_7	non-phage	0
NLF008_scaffold11629_11	non-phage	0
NLF008_scaffold15826_7	non-phage	0
NLF008_scaffold10027_2	non-phage	0
NLF008_scaffold161_5	non-phage	0
NLF008_scaffold3699_2	non-phage	0
NLF008_scaffold12114_1	non-phage	0
NLF008_scaffold7674_1	non-phage	0
NLF008_scaffold150_5	non-phage	0
NLF008_scaffold20305_2	non-phage	0
NLF008_scaffold10874_5	non-phage	0
NLF008_scaffold3468_2	non-phage	0
NLF008_scaffold6889_13	non-phage	0
NLF008_scaffold4446_4	non-phage	0
NLF008_scaffold2726_1	non-phage	0
NLF008_scaffold8582_2	non-phage	0
NLF008_scaffold1446_5	non-phage	0
NLF008_scaffold7660_6	non-phage	0
NLF008_scaffold11801_2	non-phage	0
NLF008_scaffold6224_3	non-phage	0
NLF008_scaffold16625_4	non-phage	0
NLF008_scaffold9445_1	non-phage	0
NLF008_scaffold5863_18	non-phage	0
NLF008_scaffold15129_1	non-phage	0
NLF008_scaffold1693_6	non-phage	0
NLF008_scaffold8786_1	non-phage	0
NLF008_scaffold19936_1	non-phage	0
NLF008_scaffold10761_2	non-phage	0
NLF008_scaffold18404_15	non-phage	0
NLF008_scaffold17931_1	phage	0.998
NLF008_scaffold141_6	non-phage	0
NLF008_scaffold16176_1	non-phage	0
NLF008_scaffold11499_7	non-phage	0
NLF008_scaffold20464_3	non-phage	0

NLF008_scaffold3034_1	non-phage	0
NLF008_scaffold19461_1	non-phage	0
NLF008_scaffold13026_3	non-phage	0
NLF008_scaffold9935_1	non-phage	0
NLF008_scaffold5835_3	non-phage	0
NLF008_scaffold82_9	non-phage	0
NLF008_scaffold10577_1	non-phage	0
NLF008_scaffold14149_1	non-phage	0
NLF008_scaffold20749_1	non-phage	0
NLF008_scaffold121_2	non-phage	0
NLF008_scaffold18830_1	non-phage	0
NLF008_scaffold5517_4	non-phage	0
NLF008_scaffold10392_2	non-phage	0
NLF008_scaffold790_4	non-phage	0
NLF008_scaffold20604_1	non-phage	0
NLF008_scaffold5181_9	non-phage	0
NLF008_scaffold20081_3	non-phage	0
NLF008_scaffold3257_1	non-phage	0
NLF008_scaffold8287_1	non-phage	0
NLF008_scaffold160_1	non-phage	0
NLF008_scaffold17603_2	non-phage	0
NLF008_scaffold11779_1	non-phage	0
NLF008_scaffold6328_9	non-phage	0
NLF008_scaffold100_1	non-phage	0
NLF008_scaffold9584_3	non-phage	0
NLF008_scaffold3730_4	non-phage	0
NLF008_scaffold4395_3	non-phage	0
NLF008_scaffold16613_1	non-phage	0
NLF008_scaffold12977_8	non-phage	0
NLF008_scaffold17589_3	non-phage	0
NLF008_scaffold10907_1	non-phage	0
NLF008_scaffold20350_3	non-phage	0
NLF008_scaffold10577_4	non-phage	0
NLF008_scaffold19450_1	non-phage	0
NLF008_scaffold11659_2	non-phage	0
NLF008_scaffold17621_2	non-phage	0
NLF008_scaffold7625_5	non-phage	0
NLF008_scaffold14354_1	non-phage	0
NLF008_scaffold68_1	non-phage	0
NLF008_scaffold3718_3	non-phage	0
NLF008_scaffold8102_2	non-phage	0
NLF008_scaffold3150_13	non-phage	0
NLF008_scaffold141_5	non-phage	0
NLF008_scaffold12834_4	phage	0.656
NLF008_C316406_1	non-phage	0
NLF008_scaffold2618_1	non-phage	0

NLF008_scaffold12992_1	non-phage	0
NLF008_scaffold7523_1	non-phage	0
NLF008_scaffold11181_5	non-phage	0
NLF008_scaffold3140_2	non-phage	0
NLF008_scaffold8008_5	non-phage	0
NLF008_scaffold2895_5	non-phage	0
NLF008_scaffold4395_10	non-phage	0
NLF008_scaffold3106_3	non-phage	0
NLF008_scaffold20759_1	non-phage	0
NLF008_scaffold19050_1	non-phage	0
NLF008_scaffold3950_5	non-phage	0
NLF008_scaffold57_5	non-phage	0
NLF008_scaffold1474_4	non-phage	0
NLF008_scaffold20726_1	non-phage	0
NLF008_scaffold12813_5	non-phage	0
NLF008_scaffold20190_3	non-phage	0
NLF008_scaffold15799_1	non-phage	0
NLF008_scaffold9128_2	non-phage	0
NLF008_scaffold3781_1	non-phage	0
NLF008_scaffold10122_1	non-phage	0
NLF008_scaffold6443_1	non-phage	0
NLF008_scaffold11176_1	non-phage	0
NLF008_scaffold19572_4	non-phage	0
NLF008_scaffold12732_5	non-phage	0
NLF008_scaffold401_3	non-phage	0
NLF008_scaffold2990_4	non-phage	0
NLF008_scaffold8318_7	non-phage	0
NLF008_scaffold10523_2	non-phage	0
NLF008_scaffold9479_4	non-phage	0
NLF008_scaffold5509_1	non-phage	0
NLF008_scaffold16324_2	phage	0.872
NLF008_scaffold5912_1	non-phage	0
NLF008_scaffold19610_1	non-phage	0
NLF008_scaffold11789_4	non-phage	0
NLF008_scaffold22_2	non-phage	0
NLF008_scaffold3958_1	non-phage	0
NLF008_scaffold18174_1	non-phage	0
NLF008_scaffold7649_1	non-phage	0
NLF008_scaffold20559_2	non-phage	0
NLF008_scaffold20654_1	non-phage	0
NLF008_scaffold10119_4	non-phage	0
NLF008_scaffold174_4	non-phage	0
NLF008_scaffold18830_3	non-phage	0
NLF008_scaffold37_1	non-phage	0
NLF008_scaffold9935_2	non-phage	0
NLF008_scaffold1243_6	non-phage	0

NLF008_scaffold157_3	non-phage	0
NLF008_scaffold1693_1	non-phage	0.012
NLF008_scaffold3455_5	non-phage	0
NLF008_scaffold2665_2	non-phage	0
NLF008_scaffold17778_2	non-phage	0
NLF008_scaffold20709_1	non-phage	0
NLF008_scaffold10473_6	non-phage	0
NLF008_scaffold20683_1	non-phage	0
NLF008_scaffold107_1	phage	0.935
NLF008_scaffold3669_7	non-phage	0
NLF008_scaffold18173_1	non-phage	0
NLF008_scaffold5863_13	non-phage	0
NLF008_scaffold6876_1	non-phage	0
NLF008_scaffold5912_5	non-phage	0
NLF008_scaffold10052_1	non-phage	0
NLF008_scaffold13929_4	non-phage	0
NLF008_scaffold10810_4	non-phage	0
NLF008_scaffold15545_3	non-phage	0
NLF008_C316622_1	non-phage	0
NLF008_scaffold2201_11	non-phage	0
NLF008_C316338_1	non-phage	0
NLF008_scaffold19864_4	non-phage	0
NLF008_scaffold18473_4	non-phage	0
NLF008_scaffold1161_16	non-phage	0
NLF008_scaffold17_1	non-phage	0
NLF008_scaffold10056_2	non-phage	0
NLF008_scaffold11284_1	non-phage	0
NLF008_scaffold19752_2	non-phage	0
NLF008_scaffold14753_1	non-phage	0
NLF008_scaffold7552_5	non-phage	0
NLF008_scaffold6399_7	non-phage	0
NLF008_scaffold4787_1	non-phage	0
NLF008_scaffold20237_1	non-phage	0
NLF008_scaffold7847_3	non-phage	0
NLF008_scaffold1829_6	non-phage	0
NLF008_scaffold8866_1	non-phage	0
NLF008_scaffold19652_1	non-phage	0
NLF008_scaffold5503_16	non-phage	0
NLF008_scaffold3150_42	non-phage	0
NLF008_scaffold1829_2	non-phage	0
NLF008_scaffold382_2	non-phage	0
NLF008_scaffold13693_3	non-phage	0
NLF008_scaffold98_9	phage	0.999
NLF008_scaffold1813_6	phage	0.996
NLF008_scaffold20270_2	non-phage	0
NLF008_scaffold13558_4	non-phage	0

NLF008_scaffold7238_1	non-phage	0
NLF008_scaffold2260_3	non-phage	0
NLF008_C316056_1	non-phage	0
NLF008_scaffold18382_1	non-phage	0
NLF008_scaffold4874_1	non-phage	0
NLF008_scaffold11396_6	non-phage	0
NLF008_scaffold17151_3	non-phage	0
NLF008_scaffold8433_4	phage	0.999
NLF008_scaffold9619_2	non-phage	0
NLF008_scaffold10572_3	non-phage	0
NLF008_scaffold6999_1	non-phage	0
NLF008_scaffold15393_3	non-phage	0
NLF008_scaffold20586_1	non-phage	0
NLF008_scaffold19654_1	non-phage	0
NLF008_scaffold9301_3	non-phage	0
NLF008_scaffold17338_1	non-phage	0
NLF008_scaffold4982_6	non-phage	0
NLF008_scaffold7580_3	non-phage	0
NLF008_scaffold8909_2	non-phage	0
NLF008_scaffold12963_3	non-phage	0
NLF008_scaffold9584_5	non-phage	0
NLF008_scaffold15135_3	non-phage	0
NLF008_scaffold11181_1	non-phage	0
NLF008_scaffold10337_2	non-phage	0
NLF008_scaffold9175_6	non-phage	0
NLF008_scaffold18296_2	non-phage	0
NLF008_scaffold11629_12	non-phage	0
NLF008_scaffold11828_2	non-phage	0
NLF008_scaffold18830_4	non-phage	0
NLF008_scaffold12853_1	non-phage	0
NLF008_scaffold7370_2	non-phage	0
NLF008_scaffold6328_5	non-phage	0
NLF008_scaffold10392_1	non-phage	0
NLF008_scaffold19681_1	non-phage	0
NLF008_scaffold1669_2	non-phage	0
NLF008_scaffold10921_1	non-phage	0
NLF008_scaffold4976_6	non-phage	0
NLF008_scaffold14149_4	non-phage	0
NLF008_scaffold7229_2	non-phage	0
NLF008_scaffold20741_1	non-phage	0
NLF008_scaffold3520_5	non-phage	0
NLF008_scaffold57_4	non-phage	0
NLF008_scaffold9241_4	non-phage	0
NLF008_scaffold20752_1	non-phage	0
NLF008_scaffold17589_2	non-phage	0
NLF008_scaffold20258_1	phage	0.998

NLF008_scaffold20628_1	non-phage	0
NLF008_scaffold877_2	non-phage	0
NLF008_scaffold3106_4	non-phage	0
NLF008_scaffold12715_1	non-phage	0
NLF008_scaffold20431_3	non-phage	0
NLF008_scaffold13627_1	non-phage	0
NLF008_scaffold18534_2	non-phage	0
NLF008_scaffold13929_3	non-phage	0
NLF008_scaffold15849_1	non-phage	0
NLF008_scaffold12120_1	non-phage	0
NLF008_scaffold19607_1	non-phage	0
NLF008_scaffold15736_1	non-phage	0
NLF008_scaffold57_1	non-phage	0
NLF008_scaffold3106_7	non-phage	0
NLF008_scaffold20721_1	non-phage	0
NLF008_scaffold12714_1	non-phage	0
NLF008_scaffold17033_1_2	non-phage	0
NLF008_scaffold20519_2	non-phage	0
NLF008_scaffold1410_10	non-phage	0
NLF008_scaffold4766_1	non-phage	0
NLF008_scaffold161_4	non-phage	0
NLF008_scaffold5503_10	non-phage	0
NLF008_scaffold7990_2	non-phage	0
NLF008_scaffold8732_9	non-phage	0
NLF008_scaffold18404_6	phage	0.995
NLF008_scaffold2636_11	non-phage	0
NLF008_scaffold20765_1	non-phage	0
NLF008_scaffold14648_16	non-phage	0
NLF008_scaffold5713_2	non-phage	0
NLF008_scaffold19077_4	non-phage	0
NLF008_scaffold9241_1	non-phage	0
NLF008_scaffold8834_3	non-phage	0
NLF008_scaffold10093_9	non-phage	0
NLF008_scaffold16907_1	non-phage	0
NLF008_scaffold877_9	non-phage	0
NLF008_scaffold5222_2	non-phage	0
NLF008_scaffold3699_3	non-phage	0
NLF008_scaffold6876_9	phage	0.999
NLF008_C315970_1	non-phage	0
NLF008_scaffold7847_2	non-phage	0
NLF008_scaffold20368_2	non-phage	0
NLF008_scaffold877_8	non-phage	0
NLF008_scaffold6482_2	non-phage	0
NLF008_scaffold10976_1	non-phage	0
NLF008_scaffold7660_5	non-phage	0
NLF008_scaffold9442_2	phage	0.998

NLF008_scaffold6224_4	non-phage	0
NLF008_scaffold11933_1	non-phage	0.024
NLF008_scaffold10473_2	non-phage	0
NLF008_scaffold7376_1	non-phage	0
NLF008_scaffold20754_2	non-phage	0
NLF008_scaffold20767_1	non-phage	0
NLF008_scaffold4766_3	non-phage	0
NLF008_scaffold20136_1	non-phage	0
NLF008_scaffold15301_1	non-phage	0
NLF008_scaffold7539_3	non-phage	0
NLF008_scaffold3229_3	non-phage	0
NLF008_scaffold1787_2	non-phage	0
NLF008_scaffold3669_4	non-phage	0
NLF008_scaffold41_1	non-phage	0
NLF008_scaffold18993_1	non-phage	0
NLF008_scaffold7607_3	non-phage	0
NLF008_scaffold19936_2	non-phage	0
NLF008_scaffold2260_7	non-phage	0
NLF008_scaffold16779_1	non-phage	0
NLF008_scaffold20296_1	non-phage	0
NLF008_scaffold15727_5	non-phage	0
NLF008_scaffold5454_4	non-phage	0
NLF008_scaffold5863_9	non-phage	0
NLF008_scaffold16669_3	non-phage	0
NLF008_scaffold10119_8	non-phage	0
NLF008_scaffold9128_3	non-phage	0
NLF008_scaffold20755_2	non-phage	0
NLF008_scaffold8938_1	non-phage	0
NLF008_C315408_1	non-phage	0
NLF008_scaffold2895_1	non-phage	0
NLF008_scaffold5517_3	non-phage	0
NLF008_scaffold9175_1	non-phage	0
NLF008_scaffold20692_1	non-phage	0
NLF008_scaffold5528_12	non-phage	0
NLF008_scaffold13615_6	non-phage	0
NLF008_scaffold17964_4	non-phage	0
NLF008_scaffold20727_1	non-phage	0
NLF008_scaffold121_3	non-phage	0
NLF008_C315880_1	phage	0.987
NLF008_scaffold16608_2	non-phage	0
NLF008_scaffold12963_2	non-phage	0
NLF008_scaffold12121_1	non-phage	0
NLF008_scaffold7528_1	non-phage	0
NLF008_scaffold11862_2	non-phage	0
NLF008_scaffold98_5	non-phage	0
NLF008_scaffold20756_1	non-phage	0

NLF008_scaffold1586_8	non-phage	0
NLF008_scaffold19450_5	non-phage	0
NLF008_scaffold20528_1	phage	0.818
NLF008_scaffold150_6	non-phage	0
NLF008_scaffold10542_8	non-phage	0
NLF008_scaffold14699_2	non-phage	0
NLF008_scaffold761_11	non-phage	0
NLF008_scaffold4904_4	non-phage	0
NLF008_scaffold2979_5	non-phage	0
NLF008_scaffold9027_1	non-phage	0
NLF008_scaffold20626_1	non-phage	0
NLF008_scaffold2134_4	non-phage	0
NLF008_scaffold20725_1	non-phage	0
NLF008_scaffold20712_1	non-phage	0
NLF008_scaffold12385_1	non-phage	0
NLF008_scaffold9291_5	non-phage	0
NLF008_scaffold8834_5_2	non-phage	0
NLF008_scaffold3580_3	non-phage	0
NLF008_scaffold20665_1	non-phage	0
NLF008_scaffold18838_1	non-phage	0
NLF008_scaffold3563_2	non-phage	0
NLF008_scaffold16625_1	non-phage	0
NLF008_scaffold1474_11	phage	0.953
NLF008_scaffold20190_2	non-phage	0
NLF008_scaffold17138_1	non-phage	0
NLF008_C315460_1	non-phage	0
NLF008_scaffold6029_12	non-phage	0
NLF008_scaffold15132_1	non-phage	0
NLF008_scaffold6889_11	non-phage	0
NLF008_scaffold19864_3	non-phage	0
NLF008_scaffold18473_3	non-phage	0
NLF008_scaffold17641_2	non-phage	0
NLF008_scaffold12732_6	non-phage	0
NLF008_scaffold14694_1	non-phage	0
NLF008_scaffold2260_9	non-phage	0
NLF008_scaffold8318_6	non-phage	0
NLF008_scaffold10027_1	non-phage	0
NLF008_scaffold17305_5	non-phage	0
NLF008_scaffold11553_3	non-phage	0
NLF008_scaffold11306_2	non-phage	0
NLF008_scaffold19572_3	non-phage	0
NLF008_scaffold20492_1	non-phage	0
NLF008_scaffold12679_3	non-phage	0
NLF008_scaffold20754_3	non-phage	0
NLF008_scaffold215_3	non-phage	0
NLF008_scaffold3150_24	non-phage	0

NLF008_scaffold20766_1	non-phage	0
NLF008_scaffold20499_1	non-phage	0
NLF008_scaffold16607_1	non-phage	0
NLF008_scaffold17139_1	non-phage	0
NLF008_scaffold19451_1	non-phage	0
NLF008_scaffold11586_2	non-phage	0
NLF008_scaffold386_3	phage	0.902
NLF008_scaffold19034_1	non-phage	0
NLF008_scaffold3718_6	non-phage	0
NLF008_scaffold5986_1	phage	1
NLF008_scaffold13447_6	non-phage	0
NLF008_scaffold9820_2	non-phage	0
NLF008_scaffold15393_1	non-phage	0
NLF008_scaffold8287_2	non-phage	0
NLF008_C316060_1	non-phage	0
NLF008_scaffold1446_7	non-phage	0
NLF008_scaffold18741_1	non-phage	0
NLF008_scaffold37_2	non-phage	0
NLF008_scaffold8240_1	non-phage	0
NLF008_scaffold16911_3	non-phage	0
NLF008_scaffold13615_1	non-phage	0
NLF008_scaffold20762_1	non-phage	0
NLF008_scaffold7580_4	non-phage	0
NLF008_scaffold8575_2	non-phage	0
NLF008_scaffold17305_2	non-phage	0
NLF008_scaffold8102_1	non-phage	0
NLF008_scaffold15537_1	phage	0.997
NLF008_scaffold421_5	non-phage	0
NLF008_scaffold141_4	non-phage	0
NLF008_scaffold4766_8	non-phage	0
NLF008_scaffold13565_3	non-phage	0
NLF008_scaffold4200_1	non-phage	0
NLF008_scaffold33_2	non-phage	0
NLF008_scaffold20464_1	non-phage	0
NLF008_scaffold16339_1	non-phage	0
NLF008_scaffold13466_1	non-phage	0
NLF008_scaffold761_7	non-phage	0
NLF008_scaffold2979_9	non-phage	0
NLF008_scaffold20682_1	non-phage	0
NLF008_scaffold20717_1	non-phage	0
NLF008_scaffold19939_1	non-phage	0
NLF008_scaffold20555_1	non-phage	0
NLF008_scaffold2895_4	non-phage	0
NLF008_scaffold9_4	non-phage	0
NLF008_scaffold4787_2	non-phage	0
NLF008_scaffold8834_1	non-phage	0

NLF008_scaffold8874_1	non-phage	0
NLF008_scaffold19077_5	non-phage	0
NLF008_scaffold20760_1	non-phage	0
NLF008_scaffold3718_2	non-phage	0
NLF008_scaffold11943_4	non-phage	0
NLF008_scaffold20404_1_2	non-phage	0
NLF008_scaffold20628_2	non-phage	0
NLF008_scaffold209_3	non-phage	0
NLF008_scaffold2201_9	non-phage	0
NLF008_scaffold8571_4	non-phage	0
NLF008_scaffold2933_8	non-phage	0
NLF008_scaffold20292_2	non-phage	0
NLF008_scaffold13558_3	non-phage	0
NLF008_scaffold5515_3	non-phage	0
NLF008_scaffold19924_3	non-phage	0
NLF008_scaffold14875_1	non-phage	0
NLF008_scaffold6328_2	non-phage	0
NLF008_scaffold15702_2	non-phage	0
NLF008_scaffold1787_9	non-phage	0
NLF008_scaffold7607_5	non-phage	0
NLF008_scaffold20701_1	non-phage	0
NLF008_scaffold3669_8	non-phage	0
NLF008_scaffold1586_3	non-phage	0
NLF008_scaffold8154_2	non-phage	0
NLF008_scaffold261_4	non-phage	0
NLF008_scaffold20610_1	non-phage	0
NLF008_scaffold9175_5	non-phage	0
NLF008_scaffold10916_1	non-phage	0
NLF008_scaffold1829_7	non-phage	0
NLF008_scaffold3277_2	non-phage	0
NLF008_scaffold3257_2	non-phage	0
NLF008_scaffold7154_2	non-phage	0
NLF008_scaffold11984_2	non-phage	0
NLF008_scaffold20770_1	non-phage	0
NLF008_scaffold10473_5	non-phage	0
NLF008_scaffold17515_1	non-phage	0
NLF008_scaffold1786_3	non-phage	0
NLF008_scaffold286_14	non-phage	0
NLF008_scaffold12853_2	non-phage	0
NLF008_scaffold13141_5	non-phage	0
NLF008_scaffold13667_1	non-phage	0
NLF008_scaffold17_3	non-phage	0
NLF008_scaffold4395_6	non-phage	0
NLF008_scaffold11181_2	non-phage	0
NLF008_scaffold2636_16	non-phage	0
NLF008_scaffold220_1	non-phage	0

NLF008_scaffold11770_3	non-phage	0
NLF008_scaffold57_2	non-phage	0
NLF008_scaffold210_1	non-phage	0
NLF008_scaffold1474_2	non-phage	0
NLF008_scaffold5173_3	non-phage	0.491
NLF008_scaffold3750_1	non-phage	0
NLF008_scaffold14317_1	non-phage	0
NLF008_scaffold20493_1	non-phage	0
NLF008_scaffold17292_1	non-phage	0
NLF008_scaffold2618_5	non-phage	0
NLF008_scaffold18404_13	phage	0.999
NLF008_scaffold13661_2	non-phage	0
NLF008_scaffold16324_3	non-phage	0
NLF008_scaffold13758_1	non-phage	0
NLF008_scaffold9128_8	non-phage	0
NLF008_scaffold10473_8	non-phage	0
NLF008_scaffold1829_10	non-phage	0
NLF008_scaffold20672_1	non-phage	0
NLF008_scaffold9793_1	non-phage	0
NLF008_scaffold2636_12	non-phage	0
NLF008_scaffold8318_2	non-phage	0
NLF008_scaffold4769_1	non-phage	0
NLF008_scaffold14359_1	non-phage	0
NLF008_C315950_1	non-phage	0
NLF008_scaffold15183_1	non-phage	0
NLF008_scaffold598_1	non-phage	0
NLF008_scaffold5713_1	non-phage	0
NLF008_scaffold20473_2	non-phage	0
NLF008_scaffold1157_15	non-phage	0
NLF008_scaffold11629_2	non-phage	0
NLF008_scaffold10874_7	non-phage	0
NLF008_scaffold14014_6	phage	0.999
NLF008_scaffold19382_3	non-phage	0
NLF008_scaffold16233_1	non-phage	0
NLF008_scaffold8780_3	non-phage	0
NLF008_scaffold7896_4	phage	0.999
NLF008_scaffold11499_11	non-phage	0
NLF008_scaffold20291_1	non-phage	0
NLF008_scaffold7371_6	non-phage	0
NLF008_scaffold11072_2	non-phage	0
NLF008_scaffold444_5	non-phage	0
NLF008_C316264_1	non-phage	0
NLF008_scaffold7469_1	non-phage	0
NLF008_scaffold8583_1	non-phage	0
NLF008_scaffold12732_2	non-phage	0
NLF008_scaffold20292_1	non-phage	0

NLF008_scaffold19936_3	non-phage	0
NLF008_scaffold6889_7	non-phage	0
NLF008_scaffold9181_10	non-phage	0
NLF008_scaffold15690_2	non-phage	0
NLF008_scaffold20714_1	non-phage	0
NLF008_scaffold3574_2	non-phage	0
NLF008_scaffold18542_2	non-phage	0
NLF008_scaffold14661_1	non-phage	0
NLF008_scaffold3574_1	non-phage	0
NLF008_scaffold6224_1	non-phage	0
NLF008_scaffold17254_2	non-phage	0
NLF008_scaffold13615_5	non-phage	0
NLF008_scaffold11194_1	non-phage	0
NLF008_scaffold972_2	non-phage	0
NLF008_scaffold1152_3	non-phage	0
NLF008_scaffold11862_1	non-phage	0
NLF008_scaffold20476_2	non-phage	0
NLF008_scaffold3812_2	non-phage	0
NLF008_scaffold2933_10	non-phage	0
NLF008_scaffold5517_2	non-phage	0
NLF008_scaffold18443_1	non-phage	0
NLF008_scaffold2996_3	non-phage	0
NLF008_scaffold11629_13	non-phage	0
NLF008_scaffold20598_3	non-phage	0
NLF008_scaffold20633_2	non-phage	0.002
NLF008_scaffold14519_1	non-phage	0
NLF008_scaffold20742_1	non-phage	0
NLF008_scaffold14149_3	non-phage	0
NLF008_scaffold7370_1	non-phage	0
NLF008_scaffold8914_1	non-phage	0
NLF008_scaffold8732_8	non-phage	0
NLF008_scaffold19369_1	non-phage	0
NLF008_scaffold7569_2	non-phage	0
NLF008_scaffold4675_3	non-phage	0
NLF008_scaffold15817_1	non-phage	0
NLF008_scaffold11828_1	non-phage	0
NLF008_scaffold7383_3	non-phage	0
NLF008_scaffold758_3	non-phage	0
NLF008_scaffold2933_3	non-phage	0
NLF008_scaffold261_7	non-phage	0
NLF008_scaffold1225_3	non-phage	0
NLF008_scaffold332_1	non-phage	0
NLF008_scaffold877_7	non-phage	0
NLF008_scaffold12806_1	non-phage	0
NLF008_scaffold7224_1	non-phage	0
NLF008_scaffold13342_4	non-phage	0

NLF008_scaffold9737_2	phage	0.582
NLF008_scaffold12807_1	non-phage	0
NLF008_scaffold20411_1	non-phage	0
NLF008_scaffold10523_1	non-phage	0
NLF008_scaffold15826_1	non-phage	0
NLF008_scaffold14519_5	non-phage	0
NLF008_C316444_1	non-phage	0
NLF008_scaffold12833_2	phage	0.999
NLF008_scaffold4207_14	non-phage	0
NLF008_scaffold7470_2	non-phage	0
NLF008_scaffold19652_2	non-phage	0
NLF008_scaffold18724_4	non-phage	0
NLF008_scaffold10473_1	non-phage	0
NLF008_scaffold8575_1	non-phage	0
NLF008_scaffold18993_2	non-phage	0
NLF008_scaffold5977_1	non-phage	0
NLF008_scaffold104_1	non-phage	0
NLF008_scaffold13141_1	non-phage	0
NLF008_scaffold17408_1	non-phage	0
NLF008_scaffold7552_1	non-phage	0
NLF008_scaffold10052_4	non-phage	0
NLF008_scaffold4766_10	non-phage	0
NLF008_scaffold20640_1	non-phage	0
NLF008_scaffold83_1	phage	0.998
NLF008_scaffold20512_1	non-phage	0
NLF008_scaffold395_4	non-phage	0
NLF008_scaffold8008_1	non-phage	0
NLF008_scaffold8157_2	non-phage	0
NLF008_scaffold19572_6	non-phage	0
NLF008_scaffold3730_3	non-phage	0
NLF008_scaffold19985_1	non-phage	0
NLF008_scaffold9772_1	non-phage	0
NLF008_scaffold13575_2	non-phage	0
NLF008_scaffold20431_4	phage	0.999
NLF008_scaffold6225_6	non-phage	0
NLF008_scaffold174_1	non-phage	0
NLF008_scaffold13153_3	non-phage	0
NLF008_scaffold286_2	non-phage	0
NLF008_scaffold12972_1	non-phage	0
NLF008_scaffold7932_1	non-phage	0
NLF008_scaffold5863_2	non-phage	0
NLF008_scaffold13388_2	phage	0.974
NLF008_scaffold17964_5	non-phage	0
NLF008_scaffold9016_2	non-phage	0
NLF008_scaffold9128_4	non-phage	0
NLF008_scaffold20717_2	non-phage	0

NLF008_scaffold15265_1	non-phage	0
NLF008_scaffold12732_3	non-phage	0
NLF008_scaffold1868_2	phage	0.998
NLF009_scaffold9999_1	non-phage	0
NLF009_scaffold411_17	non-phage	0
NLF009_scaffold18408_2	non-phage	0
NLF009_scaffold3817_1	non-phage	0
NLF009_scaffold29449_3	non-phage	0
NLF009_scaffold18570_1	non-phage	0
NLF009_scaffold28039_1	non-phage	0
NLF009_scaffold40763_6	non-phage	0
NLF009_scaffold7539_7	non-phage	0
NLF009_scaffold52991_5	non-phage	0
NLF009_scaffold127_4	non-phage	0
NLF009_scaffold127_25	non-phage	0
NLF009_scaffold53174_1	non-phage	0
NLF009_scaffold37541_2	non-phage	0
NLF009_scaffold691_15	non-phage	0
NLF009_scaffold54623_1	non-phage	0
NLF009_scaffold127_3	non-phage	0
NLF009_scaffold47311_1	non-phage	0
NLF009_C805905_1	phage	0.998
NLF009_scaffold2332_25	non-phage	0
NLF009_scaffold19140_8	non-phage	0
NLF009_scaffold16884_2	non-phage	0
NLF009_scaffold10839_2	non-phage	0
NLF009_scaffold53941_1	non-phage	0
NLF009_scaffold4105_4	non-phage	0
NLF009_scaffold39848_1	non-phage	0
NLF009_scaffold375_9	non-phage	0
NLF009_scaffold54499_2	non-phage	0
NLF009_scaffold9694_2	non-phage	0
NLF009_scaffold51895_2	non-phage	0
NLF009_scaffold35732_5	non-phage	0
NLF009_scaffold54101_4	non-phage	0
NLF009_scaffold3308_11	non-phage	0
NLF009_scaffold43595_7	non-phage	0
NLF009_scaffold3162_3	phage	0.988
NLF009_scaffold52225_5	non-phage	0
NLF009_scaffold30204_7	non-phage	0
NLF009_scaffold50869_1	non-phage	0
NLF009_scaffold14046_12	non-phage	0
NLF009_scaffold17912_16	phage	0.997
NLF009_scaffold14456_11	non-phage	0
NLF009_scaffold24188_11	non-phage	0
NLF009_scaffold622_30	non-phage	0

NLF009_scaffold1079_5	non-phage	0
NLF009_scaffold18012_8	non-phage	0
NLF009_scaffold5509_3	non-phage	0
NLF009_scaffold38976_1	non-phage	0
NLF009_scaffold54633_2	non-phage	0
NLF009_scaffold19043_7	non-phage	0
NLF009_scaffold411_22	non-phage	0
NLF009_scaffold48654_1	non-phage	0
NLF009_scaffold52866_1	non-phage	0
NLF009_scaffold54624_1	non-phage	0
NLF009_scaffold36768_2	non-phage	0
NLF009_scaffold30773_1	phage	0.991
NLF009_scaffold32398_1	non-phage	0
NLF009_scaffold13083_6	non-phage	0
NLF009_scaffold21679_4	non-phage	0
NLF009_scaffold9313_6	non-phage	0
NLF009_scaffold21762_18	non-phage	0
NLF009_scaffold2174_4	non-phage	0
NLF009_scaffold17744_3	non-phage	0
NLF009_scaffold17251_7	non-phage	0
NLF009_scaffold10259_6	non-phage	0
NLF009_scaffold43907_9	non-phage	0
NLF009_scaffold30204_1	non-phage	0
NLF009_scaffold54553_5	phage	0.999
NLF009_scaffold49152_13	non-phage	0
NLF009_scaffold37415_2	non-phage	0
NLF009_scaffold54270_1	non-phage	0
NLF009_scaffold17903_1	non-phage	0
NLF009_scaffold3758_2	non-phage	0
NLF009_scaffold1100_16	non-phage	0
NLF009_scaffold14474_7	non-phage	0
NLF009_scaffold17903_5	non-phage	0
NLF009_scaffold50103_2	non-phage	0
NLF009_scaffold1738_2	non-phage	0
NLF009_scaffold1838_14	non-phage	0
NLF009_scaffold41546_1	non-phage	0
NLF009_scaffold127_24	non-phage	0
NLF009_scaffold8485_14	non-phage	0
NLF009_C805805_1	non-phage	0
NLF009_scaffold26981_2	non-phage	0
NLF009_scaffold106_5	non-phage	0
NLF009_scaffold9034_1	non-phage	0
NLF009_scaffold9999_5	non-phage	0
NLF009_scaffold10771_4	non-phage	0
NLF009_scaffold18183_2	non-phage	0
NLF009_scaffold11068_3	non-phage	0

NLF009_scaffold17895_9	non-phage	0
NLF009_scaffold24219_3	non-phage	0
NLF009_scaffold7338_1	non-phage	0
NLF009_C805613_1	non-phage	0
NLF009_scaffold2413_2	non-phage	0
NLF009_scaffold3308_10	non-phage	0
NLF009_scaffold54116_1	non-phage	0
NLF009_scaffold53261_1	non-phage	0
NLF009_scaffold47004_5	non-phage	0
NLF009_scaffold10593_3	non-phage	0
NLF009_scaffold47828_1	non-phage	0
NLF009_scaffold43907_11	non-phage	0
NLF009_scaffold54372_2	non-phage	0
NLF009_scaffold53224_1	non-phage	0
NLF009_scaffold52403_3	non-phage	0
NLF009_scaffold16672_1	non-phage	0
NLF009_scaffold16211_27	non-phage	0
NLF009_scaffold52403_1	non-phage	0
NLF009_scaffold21790_2	phage	0.998
NLF009_scaffold19140_5	non-phage	0
NLF009_scaffold54593_1	non-phage	0
NLF009_scaffold48582_1	non-phage	0
NLF009_scaffold9165_3	non-phage	0
NLF009_scaffold54601_2	non-phage	0
NLF009_scaffold15595_1	non-phage	0
NLF009_scaffold6837_16	non-phage	0
NLF009_scaffold12773_18	non-phage	0
NLF009_scaffold10839_14	non-phage	0
NLF009_C805687_1	non-phage	0.105
NLF009_scaffold54628_1	non-phage	0
NLF009_scaffold375_18	non-phage	0
NLF009_scaffold21519_1	non-phage	0
NLF009_scaffold21679_5	non-phage	0
NLF009_scaffold37007_2	non-phage	0
NLF009_scaffold18984_1	non-phage	0
NLF009_scaffold2561_2	non-phage	0
NLF009_scaffold4105_2	non-phage	0
NLF009_scaffold26804_4	non-phage	0
NLF009_scaffold7091_22	phage	0.982
NLF009_scaffold54614_1	non-phage	0
NLF009_scaffold33174_1	non-phage	0
NLF009_scaffold54503_2	non-phage	0
NLF009_C805809_1	non-phage	0
NLF009_scaffold18715_1	non-phage	0
NLF009_scaffold51472_3	non-phage	0
NLF009_scaffold356_3	non-phage	0

NLF009_scaffold41020_10	non-phage	0
NLF009_scaffold18984_6	non-phage	0
NLF009_scaffold54553_3	phage	0.999
NLF009_scaffold30987_3	non-phage	0
NLF009_scaffold106_4	non-phage	0
NLF009_scaffold54372_1	non-phage	0
NLF009_scaffold52832_1	non-phage	0
NLF009_scaffold4933_28	phage	0.663
NLF009_scaffold26530_3	non-phage	0
NLF009_scaffold51895_1	non-phage	0
NLF009_scaffold44813_1	non-phage	0
NLF009_scaffold49562_2	non-phage	0
NLF009_scaffold129_8	non-phage	0
NLF009_scaffold9216_1	non-phage	0
NLF009_scaffold54307_1	non-phage	0
NLF009_scaffold34894_6	non-phage	0
NLF009_scaffold36133_2	non-phage	0
NLF009_scaffold24700_8	non-phage	0
NLF009_scaffold9626_7	non-phage	0
NLF009_scaffold3162_2	non-phage	0
NLF009_scaffold19140_10	non-phage	0
NLF009_scaffold51472_1	non-phage	0
NLF009_scaffold43907_12	non-phage	0
NLF009_scaffold44337_2	non-phage	0
NLF009_scaffold4997_23	non-phage	0
NLF009_scaffold54587_1	non-phage	0
NLF009_scaffold18408_1	non-phage	0
NLF009_scaffold3203_1	non-phage	0
NLF009_scaffold25739_16	non-phage	0
NLF009_scaffold31384_20	non-phage	0
NLF009_scaffold7662_1	non-phage	0
NLF009_scaffold52375_4	non-phage	0
NLF009_scaffold30874_4	non-phage	0
NLF009_scaffold40284_1	non-phage	0
NLF009_scaffold41271_2	non-phage	0
NLF009_scaffold894_1	non-phage	0
NLF009_scaffold24288_5	non-phage	0
NLF009_scaffold9571_1	non-phage	0
NLF009_scaffold5629_5	non-phage	0
NLF009_scaffold16449_22	non-phage	0
NLF009_scaffold16211_15	non-phage	0
NLF009_scaffold127_5	non-phage	0.002
NLF009_scaffold28804_2	non-phage	0
NLF009_scaffold918_16	non-phage	0
NLF009_scaffold6837_13	non-phage	0
NLF009_scaffold9694_1	non-phage	0

NLF009_scaffold21177_1	non-phage	0
NLF009_scaffold33470_1	non-phage	0
NLF009_scaffold28110_3	non-phage	0
NLF009_scaffold8027_4	non-phage	0
NLF009_scaffold46179_1	non-phage	0
NLF009_scaffold52838_1	non-phage	0
NLF009_scaffold16795_7	non-phage	0
NLF009_scaffold15143_3	non-phage	0
NLF009_scaffold17109_2	non-phage	0
NLF009_scaffold1353_3	non-phage	0
NLF009_scaffold11385_5	non-phage	0
NLF009_scaffold9999_7	non-phage	0
NLF009_scaffold27398_2	non-phage	0
NLF009_scaffold37662_1	non-phage	0
NLF009_scaffold16211_22	non-phage	0
NLF009_scaffold31384_19	non-phage	0
NLF009_scaffold13518_8	non-phage	0
NLF009_scaffold2890_10	non-phage	0
NLF009_scaffold30987_5	non-phage	0
NLF009_scaffold2279_3	non-phage	0
NLF009_scaffold45676_2	non-phage	0
NLF009_scaffold47167_1	non-phage	0
NLF009_scaffold54553_6	non-phage	0
NLF009_scaffold13923_1	non-phage	0
NLF009_scaffold9844_25	phage	0.953
NLF009_scaffold15442_20	non-phage	0
NLF009_scaffold52926_4	phage	0.999
NLF009_scaffold2174_48	non-phage	0
NLF009_scaffold41841_1	non-phage	0
NLF009_scaffold9689_1	non-phage	0
NLF009_scaffold52375_7	phage	0.979
NLF009_scaffold17903_2	non-phage	0
NLF009_scaffold49189_5	non-phage	0
NLF009_scaffold51297_10	non-phage	0
NLF009_scaffold9626_1	non-phage	0
NLF009_scaffold54499_1	phage	0.816
NLF009_scaffold15144_2	non-phage	0
NLF009_scaffold19140_3	non-phage	0
NLF009_scaffold19140_7	phage	0.932
NLF009_scaffold49112_1	non-phage	0
NLF009_scaffold11677_2	non-phage	0
NLF009_scaffold54170_2	phage	0.997
NLF009_scaffold9313_13	non-phage	0
NLF009_scaffold29449_1	non-phage	0
NLF009_scaffold54415_1	non-phage	0
NLF009_scaffold691_12	non-phage	0

NLF009_scaffold12342_2	non-phage	0
NLF009_scaffold13518_10	non-phage	0
NLF009_scaffold29942_1	non-phage	0
NLF009_scaffold8496_4	non-phage	0
NLF009_scaffold19140_12	non-phage	0
NLF009_scaffold159_2	non-phage	0
NLF009_scaffold20657_2	non-phage	0
NLF009_scaffold37645_4	non-phage	0
NLF009_scaffold16558_2	non-phage	0
NLF009_scaffold2174_13	non-phage	0
NLF009_scaffold21474_21	non-phage	0
NLF009_scaffold66_2	non-phage	0
NLF009_scaffold15308_1	non-phage	0
NLF009_scaffold127_8	non-phage	0
NLF009_scaffold4704_11	non-phage	0
NLF009_scaffold4933_32	non-phage	0
NLF009_scaffold28886_3	non-phage	0
NLF009_scaffold14489_5	non-phage	0
NLF009_C805739_1	non-phage	0
NLF009_scaffold5_4	non-phage	0
NLF009_scaffold411_18	non-phage	0
NLF009_scaffold1838_9	non-phage	0
NLF009_scaffold46501_2	non-phage	0
NLF009_scaffold19140_4	non-phage	0
NLF009_scaffold25752_1	non-phage	0
NLF009_scaffold21679_1	non-phage	0
NLF009_scaffold47933_2	non-phage	0
NLF009_scaffold42875_3	non-phage	0
NLF009_scaffold35663_1	phage	0.999
NLF009_scaffold13047_17	non-phage	0
NLF009_scaffold54186_3	non-phage	0
NLF009_scaffold9549_5	non-phage	0
NLF009_scaffold42119_1	non-phage	0
NLF009_scaffold27081_4	non-phage	0
NLF009_scaffold3644_1	non-phage	0
NLF009_scaffold29449_2	non-phage	0
NLF009_scaffold2655_4	non-phage	0
NLF009_scaffold4106_1	phage	0.999
NLF009_scaffold8809_4	non-phage	0
NLF009_scaffold37343_5	non-phage	0
NLF009_scaffold13707_6	non-phage	0
NLF009_scaffold52277_1	non-phage	0
NLF009_scaffold1353_6	phage	0.99
NLF009_scaffold37342_2	non-phage	0
NLF009_scaffold27397_15	non-phage	0
NLF009_scaffold9165_20	non-phage	0

NLF009_scaffold46105_7	non-phage	0
NLF009_scaffold4370_18	non-phage	0
NLF009_scaffold54573_1	non-phage	0
NLF009_scaffold15341_1	non-phage	0
NLF009_scaffold3203_3	non-phage	0
NLF009_scaffold13707_3	non-phage	0
NLF009_scaffold14029_2	non-phage	0
NLF009_scaffold30204_4	non-phage	0
NLF009_C805829_1	phage	0.998
NLF009_scaffold27217_3	phage	0.566
NLF009_scaffold37541_3	non-phage	0
NLF009_scaffold8496_68	non-phage	0
NLF009_scaffold14456_3	non-phage	0
NLF009_scaffold16884_1	non-phage	0
NLF009_scaffold2890_3	non-phage	0
NLF009_C805873_1	phage	0.998
NLF009_scaffold7929_1	non-phage	0
NLF009_scaffold29449_9	non-phage	0
NLF009_scaffold17109_10	non-phage	0
NLF009_scaffold41978_3	non-phage	0
NLF009_scaffold52926_2	non-phage	0
NLF009_scaffold37415_1	non-phage	0
NLF009_scaffold12978_1	non-phage	0
NLF009_scaffold17912_4	phage	0.997
NLF009_scaffold27_20	non-phage	0
NLF009_scaffold43595_2	non-phage	0
NLF009_scaffold49562_1	non-phage	0
NLF009_scaffold52649_1	non-phage	0
NLF009_scaffold18051_5	non-phage	0
NLF009_scaffold39406_1	non-phage	0
NLF009_scaffold5429_52	phage	0.999
NLF009_scaffold40330_1	phage	0.778
NLF009_scaffold16783_1	non-phage	0
NLF009_scaffold39552_2	non-phage	0
NLF009_scaffold42957_8	non-phage	0
NLF009_scaffold375_68	non-phage	0
NLF009_scaffold50487_1	non-phage	0
NLF009_scaffold351_3	non-phage	0
NLF009_scaffold18984_7	phage	0.998
NLF009_scaffold3689_1	non-phage	0
NLF009_scaffold38501_1	phage	0.999
NLF009_scaffold30204_8	non-phage	0
NLF009_scaffold8414_14	phage	0.999
NLF009_scaffold17912_17	phage	0.999
NLF009_scaffold30204_2	non-phage	0
NLF009_scaffold41024_2	non-phage	0

NLF009_scaffold3162_1	non-phage	0
NLF009_scaffold30204_9	non-phage	0
NLF009_scaffold47154_1	non-phage	0
NLF009_scaffold47251_1	non-phage	0
NLF009_scaffold31015_1	non-phage	0
NLF009_scaffold52991_2	phage	0.961
NLF009_scaffold18859_7	non-phage	0
NLF009_scaffold5668_4	non-phage	0
NLF009_scaffold31299_10	non-phage	0
NLF009_scaffold31384_17	non-phage	0
NLF009_scaffold5413_1	non-phage	0
NLF009_scaffold52375_10	non-phage	0
NLF009_scaffold52375_9	non-phage	0
NLF009_scaffold54616_1	non-phage	0
NLF009_scaffold27060_3	non-phage	0
NLF009_scaffold37882_3	non-phage	0
NLF009_scaffold52581_1	non-phage	0
NLF009_scaffold53528_2	non-phage	0
NLF009_scaffold25289_5	phage	0.997
NLF009_scaffold129_40	non-phage	0
NLF009_scaffold127_23	non-phage	0
NLF009_scaffold16075_15	non-phage	0
NLF009_scaffold1738_3	non-phage	0
NLF009_scaffold8390_14	non-phage	0
NLF009_scaffold28886_1	non-phage	0
NLF009_scaffold10839_8	non-phage	0
NLF009_C805659_1	non-phage	0
NLF009_scaffold22050_13	non-phage	0
NLF009_scaffold36767_1	non-phage	0
NLF009_scaffold19424_1	non-phage	0
NLF009_scaffold9999_8	non-phage	0
NLF009_scaffold39552_1	non-phage	0
NLF009_scaffold36158_1	non-phage	0
NLF009_scaffold7539_2	non-phage	0
NLF009_scaffold53945_7	non-phage	0
NLF009_scaffold54375_1	non-phage	0
NLF009_scaffold13379_2	non-phage	0
NLF009_scaffold54594_1	non-phage	0
NLF009_scaffold36413_1	phage	0.999
NLF009_scaffold53468_9	non-phage	0
NLF009_scaffold2988_19	non-phage	0
NLF009_scaffold33283_3	non-phage	0
NLF009_scaffold16071_1	non-phage	0
NLF009_scaffold17903_3	non-phage	0
NLF009_scaffold54422_1	non-phage	0
NLF009_scaffold47167_2	non-phage	0

NLF009_scaffold54553_7	non-phage	0
NLF009_scaffold375_24	phage	0.997
NLF009_scaffold7507_6_1	non-phage	0
NLF009_scaffold11825_2	non-phage	0
NLF009_scaffold19474_2	non-phage	0
NLF009_scaffold22486_8	non-phage	0
NLF009_scaffold37415_4	non-phage	0
NLF009_scaffold4105_1	non-phage	0
NLF009_scaffold9752_10	non-phage	0
NLF009_scaffold9313_29	non-phage	0
NLF009_scaffold410_2	non-phage	0
NLF009_scaffold7539_6	non-phage	0
NLF009_scaffold54073_1	non-phage	0
NLF009_scaffold17903_7	non-phage	0
NLF009_scaffold18570_2	non-phage	0
NLF009_scaffold52926_1	phage	0.999
NLF009_scaffold52991_3	non-phage	0
NLF009_scaffold49160_1	non-phage	0
NLF009_scaffold46505_1	non-phage	0
NLF009_scaffold51820_1	non-phage	0
NLF009_scaffold24853_1	non-phage	0
NLF009_scaffold41024_3	non-phage	0
NLF009_C805903_1	non-phage	0
NLF009_scaffold37415_3	phage	0.998
NLF009_scaffold43595_4	non-phage	0
NLF009_scaffold13518_6	non-phage	0
NLF009_scaffold2224_3	non-phage	0
NLF009_scaffold159_3	non-phage	0
NLF009_scaffold2820_6	non-phage	0
NLF009_scaffold16075_5	non-phage	0
NLF009_scaffold53782_1	non-phage	0
NLF009_scaffold52489_1	non-phage	0
NLF009_scaffold9750_4	non-phage	0
NLF009_scaffold35732_6	non-phage	0
NLF009_scaffold21471_1	non-phage	0
NLF009_scaffold622_2	non-phage	0
NLF009_scaffold127_6	non-phage	0
NLF009_scaffold51472_2	non-phage	0
NLF009_scaffold49152_4	non-phage	0
NLF009_scaffold43333_2	non-phage	0
NLF009_scaffold622_22	non-phage	0
NLF009_scaffold6837_4	non-phage	0
NLF009_scaffold54503_1	phage	1
NLF009_scaffold2572_1	non-phage	0
NLF009_scaffold40763_17	non-phage	0
NLF009_scaffold54101_3	non-phage	0

NLF009_scaffold2279_5	non-phage	0
NLF009_scaffold54405_1	non-phage	0
NLF009_scaffold14874_11	non-phage	0
NLF009_scaffold52926_3	non-phage	0
NLF009_scaffold50562_1	non-phage	0
NLF009_scaffold18984_5	non-phage	0
NLF009_scaffold127_14	non-phage	0
NLF009_scaffold18132_1	non-phage	0
NLF009_scaffold17912_6	phage	0.999
NLF009_scaffold2436_28	non-phage	0
NLF009_scaffold16211_7	non-phage	0
NLF009_scaffold46458_1	non-phage	0
NLF010_scaffold1235_8	phage	0.999
NLF010_scaffold8776_1	non-phage	0
NLF010_scaffold8269_3	non-phage	0
NLF010_scaffold12377_1	non-phage	0
NLF010_scaffold9_11	non-phage	0
NLF010_scaffold5743_4	non-phage	0
NLF010_scaffold29472_1	non-phage	0
NLF010_scaffold16009_3	non-phage	0
NLF010_scaffold5851_10	non-phage	0
NLF010_scaffold27106_1	non-phage	0
NLF010_scaffold20315_2	phage	1
NLF010_scaffold27873_3	non-phage	0
NLF010_scaffold29464_1	non-phage	0
NLF010_scaffold12405_4	non-phage	0
NLF010_scaffold12409_6	non-phage	0
NLF010_scaffold11485_7	non-phage	0
NLF010_scaffold16274_9	non-phage	0
NLF010_scaffold83_2	non-phage	0
NLF010_scaffold10760_3	non-phage	0
NLF010_scaffold11410_1	non-phage	0
NLF010_scaffold26024_6	non-phage	0
NLF010_scaffold26791_3	non-phage	0
NLF010_scaffold22985_2	non-phage	0
NLF010_scaffold619_1	non-phage	0
NLF010_scaffold6428_2	non-phage	0
NLF010_scaffold2595_7	non-phage	0
NLF010_scaffold29445_3	non-phage	0
NLF010_scaffold29371_2	non-phage	0
NLF010_scaffold7866_7	non-phage	0
NLF010_C401943_1	non-phage	0
NLF010_scaffold2872_6	non-phage	0
NLF010_scaffold17009_2	non-phage	0
NLF010_scaffold26845_1	non-phage	0
NLF010_scaffold5851_21	non-phage	0

NLF010_scaffold1045_1	non-phage	0
NLF010_scaffold63_4	non-phage	0
NLF010_scaffold27434_5	non-phage	0
NLF010_scaffold280_65	non-phage	0
NLF010_scaffold19640_5	non-phage	0
NLF010_scaffold26024_3	non-phage	0
NLF010_scaffold1672_21	non-phage	0
NLF010_scaffold576_11	non-phage	0
NLF010_scaffold4291_9	non-phage	0
NLF010_scaffold109_13	non-phage	0
NLF010_scaffold20205_1	non-phage	0
NLF010_scaffold23055_2	non-phage	0
NLF010_scaffold12409_9	non-phage	0
NLF010_scaffold1355_1	non-phage	0
NLF010_scaffold1372_1	non-phage	0
NLF010_scaffold1469_7	non-phage	0
NLF010_scaffold576_1	non-phage	0
NLF010_scaffold26791_7	non-phage	0
NLF010_scaffold17124_4	non-phage	0
NLF010_C402191_1	non-phage	0
NLF010_scaffold36_1	non-phage	0
NLF010_scaffold3545_2	non-phage	0
NLF010_scaffold1044_27	phage	0.99
NLF010_scaffold29246_3	non-phage	0.069
NLF010_scaffold2789_1	non-phage	0
NLF010_scaffold28662_1	non-phage	0
NLF010_scaffold23407_1	non-phage	0
NLF010_scaffold28855_1	non-phage	0
NLF010_scaffold25958_2	non-phage	0
NLF010_C401859_1	non-phage	0
NLF010_scaffold134_4	non-phage	0
NLF010_scaffold16344_6	non-phage	0
NLF010_scaffold2495_12	non-phage	0
NLF010_scaffold25630_1	non-phage	0
NLF010_scaffold1417_7	non-phage	0
NLF010_scaffold14021_2	non-phage	0
NLF010_scaffold2891_2	non-phage	0
NLF010_scaffold22231_1	non-phage	0
NLF010_scaffold1668_17	non-phage	0
NLF010_scaffold28589_2	non-phage	0
NLF010_scaffold25721_1	non-phage	0
NLF010_scaffold21096_4	non-phage	0
NLF010_scaffold83_9	non-phage	0
NLF010_C402215_1	non-phage	0
NLF010_scaffold21955_2	non-phage	0
NLF010_scaffold15070_3	non-phage	0

NLF010_C401933_1	non-phage	0
NLF010_scaffold29466_2	non-phage	0
NLF010_scaffold36_4	non-phage	0
NLF010_scaffold802_11	non-phage	0
NLF010_scaffold19251_7	non-phage	0
NLF010_scaffold1584_3	non-phage	0
NLF010_scaffold1868_3	non-phage	0
NLF010_scaffold23000_1	non-phage	0
NLF010_scaffold3566_4	non-phage	0
NLF010_scaffold29376_1	non-phage	0
NLF010_scaffold24082_4	non-phage	0
NLF010_scaffold1372_6	non-phage	0
NLF010_scaffold29250_1	non-phage	0
NLF010_scaffold14576_1	non-phage	0
NLF010_scaffold19_3	non-phage	0
NLF010_scaffold20446_1	non-phage	0
NLF010_scaffold11410_48	non-phage	0
NLF010_scaffold2489_1	non-phage	0
NLF010_C401947_1	phage	0.829
NLF010_scaffold28287_1	non-phage	0
NLF010_scaffold1584_7	non-phage	0
NLF010_scaffold2149_11	non-phage	0
NLF010_scaffold304_94	non-phage	0
NLF010_scaffold19382_2	non-phage	0
NLF010_scaffold6369_16	non-phage	0
NLF010_scaffold137_7	non-phage	0
NLF010_scaffold27873_16	non-phage	0
NLF010_scaffold977_25	non-phage	0
NLF010_scaffold70_14	non-phage	0
NLF010_scaffold5523_2	non-phage	0
NLF010_scaffold17686_1	non-phage	0
NLF010_scaffold13824_4	non-phage	0
NLF010_scaffold29475_2	non-phage	0
NLF010_scaffold7866_1	non-phage	0
NLF010_scaffold792_8	non-phage	0
NLF010_scaffold11485_1	non-phage	0
NLF010_scaffold2057_2	non-phage	0
NLF010_scaffold12405_5	non-phage	0
NLF010_scaffold11_2	non-phage	0
NLF010_scaffold5627_3	non-phage	0
NLF010_scaffold16373_7	non-phage	0
NLF010_scaffold3721_1	non-phage	0
NLF010_scaffold29471_2	non-phage	0
NLF010_C402177_1	non-phage	0
NLF010_scaffold13747_2	phage	0.992
NLF010_scaffold86_12	non-phage	0

NLF010_scaffold8198_7	non-phage	0
NLF010_scaffold3414_11	non-phage	0
NLF010_scaffold12409_8	non-phage	0
NLF010_scaffold7220_1	non-phage	0
NLF010_scaffold1515_5	non-phage	0
NLF010_scaffold17009_5	non-phage	0
NLF010_scaffold2495_3	non-phage	0
NLF010_scaffold22461_1	non-phage	0
NLF010_scaffold155_8	non-phage	0
NLF010_scaffold18583_7	non-phage	0
NLF010_scaffold29397_1	non-phage	0
NLF010_scaffold666_65	non-phage	0
NLF010_scaffold18279_3	non-phage	0
NLF010_scaffold155_6	non-phage	0
NLF010_scaffold24082_3	phage	0.999
NLF010_scaffold29416_2	non-phage	0
NLF010_scaffold29430_1	non-phage	0
NLF010_scaffold29458_2	non-phage	0
NLF010_scaffold1469_5	non-phage	0
NLF010_scaffold8767_1	non-phage	0
NLF010_scaffold29306_2	non-phage	0
NLF010_C402145_1	non-phage	0
NLF010_scaffold20567_1	non-phage	0
NLF010_C402013_1	non-phage	0
NLF010_scaffold11313_1	non-phage	0
NLF010_scaffold16597_2	non-phage	0
NLF010_scaffold109_12	non-phage	0
NLF010_scaffold94_5	non-phage	0
NLF010_scaffold5768_1	non-phage	0
NLF010_scaffold666_13	non-phage	0
NLF010_scaffold1584_8	non-phage	0
NLF010_scaffold17340_5	non-phage	0
NLF010_C402235_1	phage	0.999
NLF010_scaffold29467_1	non-phage	0
NLF010_scaffold3721_7	non-phage	0
NLF010_scaffold26791_8	non-phage	0
NLF010_scaffold5_1	non-phage	0
NLF010_scaffold29458_3	non-phage	0
NLF010_scaffold24405_3	non-phage	0
NLF010_scaffold2978_1	non-phage	0
NLF010_scaffold5357_1	non-phage	0
NLF010_scaffold15728_1	non-phage	0
NLF010_scaffold23055_3	non-phage	0
NLF010_scaffold4700_2	non-phage	0
NLF010_scaffold5851_2	non-phage	0
NLF010_scaffold12883_3	non-phage	0

NLF010_scaffold3803_2	non-phage	0
NLF010_scaffold6978_11	non-phage	0
NLF010_scaffold2346_2	non-phage	0
NLF010_scaffold687_15	non-phage	0
NLF010_scaffold14048_8	non-phage	0
NLF010_scaffold1584_4	non-phage	0
NLF010_scaffold18175_1	non-phage	0
NLF010_scaffold24527_3	non-phage	0
NLF010_scaffold22417_1	phage	0.998
NLF010_scaffold21356_1	non-phage	0
NLF010_scaffold15038_2	non-phage	0
NLF010_scaffold8207_1	non-phage	0
NLF010_scaffold27490_1	non-phage	0
NLF010_scaffold11490_2	non-phage	0
NLF010_scaffold20341_2	non-phage	0
NLF010_scaffold17009_9	non-phage	0
NLF010_C401877_1	non-phage	0
NLF010_scaffold2495_6	non-phage	0
NLF010_scaffold576_12	non-phage	0
NLF010_scaffold3898_4	phage	0.986
NLF010_scaffold8584_6	non-phage	0
NLF010_scaffold5570_18	non-phage	0
NLF010_scaffold11028_2	non-phage	0
NLF010_scaffold18350_2	non-phage	0
NLF010_scaffold21634_1	non-phage	0
NLF010_scaffold26569_1	non-phage	0
NLF010_scaffold23407_2	non-phage	0
NLF010_scaffold17124_3	non-phage	0
NLF010_scaffold19406_1	non-phage	0
NLF010_scaffold29465_1	non-phage	0
NLF010_scaffold12295_8	phage	0.999
NLF010_scaffold28510_1	non-phage	0
NLF010_scaffold16183_1	non-phage	0
NLF010_scaffold1372_10	non-phage	0
NLF010_scaffold134_3	non-phage	0
NLF010_C402185_1	non-phage	0
NLF010_scaffold2057_1	non-phage	0
NLF010_scaffold29250_3	non-phage	0
NLF010_scaffold27728_2	phage	0.993
NLF010_scaffold6_1	non-phage	0
NLF010_scaffold11635_2	non-phage	0
NLF010_scaffold5801_22	non-phage	0
NLF010_scaffold5523_11	non-phage	0
NLF010_scaffold14635_5	non-phage	0
NLF010_scaffold29468_1	non-phage	0
NLF010_scaffold20046_1	non-phage	0

NLF010_scaffold28220_2	non-phage	0
NLF010_scaffold26024_1	non-phage	0
NLF010_scaffold2495_8	non-phage	0
NLF010_scaffold2489_2	non-phage	0
NLF010_scaffold666_2	non-phage	0
NLF010_scaffold14009_1	non-phage	0
NLF010_scaffold19_1	non-phage	0
NLF010_scaffold15070_2	non-phage	0
NLF010_scaffold3581_2	non-phage	0
NLF010_scaffold6369_9	non-phage	0
NLF010_scaffold29316_1	non-phage	0
NLF010_scaffold3178_2	non-phage	0
NLF010_scaffold14083_1	non-phage	0
NLF010_scaffold23533_4	non-phage	0
NLF010_C402245_1	non-phage	0
NLF010_scaffold3566_2	non-phage	0
NLF010_scaffold29158_3	non-phage	0
NLF010_scaffold21032_3	non-phage	0
NLF010_scaffold27728_3	non-phage	0
NLF010_scaffold7525_3	non-phage	0
NLF010_scaffold14058_1	non-phage	0
NLF010_scaffold10619_11	non-phage	0
NLF010_scaffold17009_4	non-phage	0
NLF010_scaffold5793_1	non-phage	0
NLF010_scaffold11485_9	non-phage	0
NLF010_scaffold4204_14	non-phage	0
NLF010_scaffold29475_1	non-phage	0
NLF010_scaffold12795_1	non-phage	0
NLF010_scaffold28874_2	non-phage	0
NLF010_scaffold576_3	non-phage	0
NLF010_scaffold26570_1	non-phage	0
NLF010_scaffold7861_24	non-phage	0
NLF010_scaffold29246_1	phage	1
NLF010_scaffold29453_1	non-phage	0
NLF010_scaffold12405_2	non-phage	0
NLF010_scaffold26024_8	phage	0.996
NLF010_scaffold5523_8	non-phage	0
NLF010_scaffold86_4	non-phage	0
NLF010_scaffold2149_14	non-phage	0
NLF010_scaffold15038_1	non-phage	0
NLF010_scaffold871_1	non-phage	0
NLF010_scaffold71_2	non-phage	0
NLF010_scaffold25231_2	non-phage	0
NLF010_scaffold29407_1	non-phage	0
NLF010_scaffold27201_1	non-phage	0
NLF010_scaffold19640_1	non-phage	0

NLF010_scaffold11307_7	non-phage	0
NLF010_scaffold24331_1	non-phage	0
NLF010_scaffold20232_3	non-phage	0
NLF010_scaffold7623_2	non-phage	0
NLF010_scaffold9747_19	non-phage	0
NLF010_scaffold28365_2	non-phage	0
NLF010_scaffold1534_12	non-phage	0
NLF010_scaffold4834_2	non-phage	0
NLF010_scaffold27434_3	non-phage	0
NLF010_scaffold11028_1	non-phage	0
NLF010_scaffold1584_5	non-phage	0
NLF010_scaffold29173_1	non-phage	0
NLF010_scaffold29422_1	non-phage	0
NLF010_scaffold12883_4	non-phage	0
NLF010_scaffold7527_3	non-phage	0
NLF010_scaffold20517_1	non-phage	0
NLF010_scaffold11485_6	non-phage	0
NLF010_scaffold155_10	non-phage	0
NLF010_scaffold4291_7	non-phage	0
NLF010_scaffold1882_6	non-phage	0
NLF010_scaffold18671_1	non-phage	0
NLF010_scaffold1207_10	non-phage	0
NLF010_scaffold20446_3	non-phage	0
NLF010_scaffold4779_4	non-phage	0
NLF010_scaffold29426_1	non-phage	0
NLF010_scaffold13747_5	non-phage	0
NLF010_scaffold13991_4	non-phage	0
NLF010_scaffold11410_45	non-phage	0
NLF010_scaffold1534_2	non-phage	0
NLF010_scaffold29109_1	non-phage	0
NLF010_scaffold22985_1	non-phage	0
NLF010_scaffold11410_20	non-phage	0
NLF010_scaffold2046_76	phage	0.998
NLF010_scaffold2677_29	non-phage	0
NLF010_scaffold88_1	non-phage	0
NLF010_scaffold29409_1	non-phage	0
NLF010_scaffold9518_4	non-phage	0
NLF010_scaffold3721_18	non-phage	0
NLF010_scaffold28720_1	non-phage	0
NLF010_scaffold20045_1	non-phage	0
NLF010_scaffold10122_3	non-phage	0
NLF010_scaffold29458_1	non-phage	0
NLF010_scaffold28036_3	non-phage	0
NLF010_scaffold26812_1	non-phage	0
NLF010_C402025_1	non-phage	0
NLF010_scaffold3721_4	non-phage	0

NLF010_scaffold86_8	non-phage	0
NLF010_scaffold17667_3	non-phage	0
NLF010_scaffold11485_8	non-phage	0
NLF010_scaffold10620_7	non-phage	0
NLF010_scaffold15038_5	non-phage	0
NLF010_scaffold22461_2	non-phage	0
NLF010_scaffold1051_2	non-phage	0
NLF010_scaffold10014_1	non-phage	0
NLF010_scaffold27289_1	non-phage	0
NLF010_scaffold4291_8	non-phage	0
NLF010_scaffold576_10	non-phage	0
NLF010_scaffold29474_1	non-phage	0
NLF010_scaffold1668_1	non-phage	0
NLF010_scaffold29445_2	non-phage	0
NLF010_scaffold29477_1	non-phage	0
NLF010_scaffold5691_2	non-phage	0
NLF010_scaffold20499_1	non-phage	0
NLF010_scaffold290_4	non-phage	0
NLF010_scaffold12295_6	non-phage	0
NLF010_scaffold29371_3	non-phage	0
NLF010_scaffold557_1	non-phage	0
NLF010_scaffold13991_3	non-phage	0
NLF010_scaffold2642_1	non-phage	0
NLF010_scaffold17052_1	non-phage	0
NLF010_scaffold24245_2	non-phage	0
NLF010_scaffold12409_11	non-phage	0
NLF010_scaffold27873_8	non-phage	0
NLF010_scaffold26377_1	non-phage	0
NLF010_scaffold2495_9	non-phage	0
NLF010_scaffold21301_6	non-phage	0
NLF010_scaffold17124_1	non-phage	0
NLF010_scaffold9138_1	non-phage	0
NLF010_scaffold14635_3	non-phage	0
NLF010_scaffold4779_2	non-phage	0
NLF010_scaffold29433_2	non-phage	0
NLF010_scaffold29217_1	non-phage	0
NLF010_scaffold29158_2	non-phage	0
NLF010_scaffold304_13	non-phage	0
NLF010_scaffold3721_8	non-phage	0
NLF010_scaffold83_7	non-phage	0
NLF010_scaffold11485_3	non-phage	0
NLF010_scaffold2411_1	non-phage	0
NLF010_scaffold21719_3	non-phage	0
NLF010_scaffold22232_1	non-phage	0
NLF010_scaffold5712_18	non-phage	0
NLF010_scaffold12409_3	non-phage	0

NLF010_scaffold1355_2	non-phage	0
NLF010_scaffold29447_2	non-phage	0
NLF010_scaffold7866_6	non-phage	0
NLF010_scaffold5523_4	non-phage	0
NLF010_scaffold22632_2	non-phage	0
NLF010_scaffold9988_1	non-phage	0
NLF010_scaffold9_14	non-phage	0
NLF010_C402193_1	non-phage	0
NLF010_scaffold69_6	non-phage	0
NLF010_scaffold9747_18	phage	0.997
NLF010_scaffold17009_3	non-phage	0
NLF010_scaffold549_7	non-phage	0
NLF010_scaffold21245_4	non-phage	0
NLF010_scaffold29337_1	non-phage	0
NLF010_scaffold23254_1	non-phage	0
NLF010_scaffold83_5	non-phage	0
NLF010_scaffold19640_6	non-phage	0
NLF010_scaffold29413_2	non-phage	0
NLF010_scaffold29158_1	non-phage	0
NLF010_scaffold10484_2	non-phage	0
NLF010_scaffold26024_7	non-phage	0
NLF010_scaffold29316_2	non-phage	0
NLF010_scaffold11102_2	non-phage	0
NLF010_scaffold22414_2	non-phage	0
NLF010_scaffold20591_1	phage	0.998
NLF010_scaffold1668_18	non-phage	0
NLF010_scaffold2891_1	non-phage	0
NLF010_scaffold18583_6	non-phage	0
NLF010_scaffold16373_9	non-phage	0
NLF010_scaffold6368_2	non-phage	0
NLF010_scaffold3721_17	non-phage	0
NLF010_scaffold9518_3	non-phage	0
NLF010_scaffold2146_8	non-phage	0
NLF010_scaffold27873_6	non-phage	0
NLF010_scaffold28_8	non-phage	0
NLF010_scaffold28036_2	non-phage	0
NLF010_scaffold11410_33	non-phage	0
NLF010_scaffold23893_4	non-phage	0
NLF010_scaffold29309_1	non-phage	0
NLF010_scaffold1515_1	non-phage	0
NLF010_scaffold22207_1	non-phage	0
NLF010_scaffold29445_4	phage	0.998
NLF010_scaffold25265_3	non-phage	0
NLF010_scaffold12295_9	non-phage	0
NLF010_scaffold1584_6	non-phage	0
NLF010_scaffold22415_1	non-phage	0

NLF010_scaffold5437_1	non-phage	0
NLF010_C401975_1	non-phage	0
NLF010_scaffold7007_1	non-phage	0
NLF010_scaffold29476_1	non-phage	0
NLF010_C402217_1	non-phage	0
NLF010_scaffold29183_1	non-phage	0
NLF010_scaffold5177_3	non-phage	0
NLF010_scaffold5_3	non-phage	0
NLF010_scaffold19893_5	non-phage	0
NLF010_scaffold28485_1	non-phage	0
NLF010_scaffold20446_2	non-phage	0
NLF010_scaffold7827_3	non-phage	0
NLF010_scaffold4779_3	non-phage	0
NLF010_scaffold26743_10	non-phage	0
NLF010_scaffold12883_5	non-phage	0
NLF010_scaffold29445_1	non-phage	0
NLF010_scaffold7541_5	non-phage	0
NLF010_scaffold6667_20	non-phage	0
NLF010_scaffold6369_15	non-phage	0
NLF010_scaffold29475_3	non-phage	0
NLF010_scaffold36_15	non-phage	0
NLF011_scaffold488_1	non-phage	0
NLF011_scaffold124_10	non-phage	0
NLF011_scaffold13061_8	non-phage	0
NLF011_scaffold3727_6	non-phage	0
NLF011_scaffold10694_1	non-phage	0
NLF011_scaffold10146_1	non-phage	0
NLF011_scaffold4965_2	phage	0.999
NLF011_scaffold803_2	non-phage	0
NLF011_scaffold611_1	non-phage	0
NLF011_scaffold8230_2_2	non-phage	0
NLF011_scaffold9992_1	non-phage	0
NLF011_scaffold5613_1	phage	0.651
NLF011_scaffold2385_3	non-phage	0
NLF011_scaffold42_2	phage	0.999
NLF011_scaffold20601_2	non-phage	0
NLF011_scaffold29592_4	non-phage	0
NLF011_scaffold5411_2	non-phage	0
NLF011_scaffold7993_2	non-phage	0
NLF011_scaffold7616_2	non-phage	0
NLF011_scaffold14427_1	non-phage	0
NLF011_scaffold14419_1	non-phage	0
NLF011_scaffold22134_1	non-phage	0
NLF011_scaffold4378_2	non-phage	0
NLF011_scaffold403_3_2	non-phage	0
NLF011_scaffold108_1	non-phage	0

NLF011_scaffold21929_2	non-phage	0
NLF011_scaffold16170_9	non-phage	0
NLF011_scaffold3575_8	non-phage	0
NLF011_scaffold11451_1_1	non-phage	0
NLF011_scaffold3618_3	non-phage	0
NLF011_scaffold19118_2	non-phage	0
NLF011_scaffold504_3	non-phage	0
NLF011_scaffold25032_1	non-phage	0
NLF011_scaffold555_3	non-phage	0
NLF011_scaffold168_1	non-phage	0
NLF011_scaffold12182_3	non-phage	0
NLF011_scaffold619_5	non-phage	0
NLF011_scaffold514_15	non-phage	0
NLF011_scaffold514_1	non-phage	0
NLF011_scaffold18150_2	non-phage	0
NLF011_scaffold276_21	non-phage	0
NLF011_scaffold12014_1	non-phage	0
NLF011_scaffold12201_2	non-phage	0
NLF011_scaffold41_4	non-phage	0
NLF011_scaffold16119_1	non-phage	0
NLF011_scaffold18970_1	non-phage	0
NLF011_scaffold3171_1	non-phage	0
NLF011_scaffold4255_6	non-phage	0
NLF011_scaffold11816_1	non-phage	0
NLF011_scaffold10609_1	non-phage	0
NLF011_scaffold4287_4	non-phage	0
NLF011_scaffold124_4	non-phage	0
NLF011_scaffold7399_6	non-phage	0
NLF011_scaffold8548_5	non-phage	0
NLF011_scaffold353_2	non-phage	0
NLF011_scaffold11804_2	non-phage	0
NLF011_scaffold12454_4	non-phage	0
NLF011_scaffold13916_1	non-phage	0
NLF011_scaffold5361_1	non-phage	0
NLF011_scaffold22590_5	non-phage	0
NLF011_scaffold7561_1	non-phage	0
NLF011_scaffold83_11	non-phage	0
NLF011_scaffold21824_1	non-phage	0
NLF011_scaffold7252_3	non-phage	0
NLF011_scaffold16470_2	non-phage	0
NLF011_scaffold514_8	non-phage	0
NLF011_scaffold5695_7	non-phage	0
NLF011_scaffold2275_5	non-phage	0
NLF011_scaffold4289_1	non-phage	0
NLF011_scaffold514_18	non-phage	0
NLF011_scaffold43_1	non-phage	0

NLF011_scaffold3531_1	non-phage	0
NLF011_scaffold16988_1	non-phage	0
NLF011_scaffold22392_2	non-phage	0
NLF011_scaffold5166_3	non-phage	0
NLF011_scaffold871_2_1	non-phage	0
NLF011_scaffold3229_1	non-phage	0
NLF011_scaffold21043_1	non-phage	0
NLF011_scaffold6105_1	non-phage	0
NLF011_scaffold2473_1	non-phage	0
NLF011_scaffold2317_5	non-phage	0
NLF011_scaffold4381_2	non-phage	0
NLF011_scaffold12741_2	non-phage	0
NLF011_scaffold30378_1	non-phage	0
NLF011_scaffold38_3	non-phage	0
NLF011_scaffold1756_4	non-phage	0
NLF011_scaffold16366_2	non-phage	0
NLF011_scaffold584_3	non-phage	0
NLF011_scaffold1327_1	non-phage	0
NLF011_scaffold603_1	non-phage	0
NLF011_scaffold36364_1	non-phage	0
NLF011_scaffold2637_9	non-phage	0
NLF011_scaffold174_2	non-phage	0
NLF011_scaffold5525_3	non-phage	0
NLF011_scaffold5761_2	non-phage	0
NLF011_scaffold1105_1	phage	0.942
NLF011_scaffold1469_2	non-phage	0
NLF011_scaffold86_2	non-phage	0
NLF011_scaffold12675_1	non-phage	0
NLF011_scaffold8591_3	non-phage	0
NLF011_scaffold888_1	non-phage	0
NLF011_scaffold3526_11	non-phage	0
NLF011_scaffold7209_5	non-phage	0
NLF011_scaffold543_1	non-phage	0
NLF011_scaffold22074_1	non-phage	0
NLF011_scaffold348_3	non-phage	0
NLF011_scaffold24283_1	non-phage	0
NLF011_scaffold3661_4	non-phage	0
NLF011_scaffold5033_3	non-phage	0
NLF011_scaffold24060_2	non-phage	0
NLF011_scaffold628_1	non-phage	0
NLF011_scaffold32311_2	non-phage	0
NLF011_scaffold4378_3	non-phage	0
NLF011_scaffold7297_6	non-phage	0
NLF011_scaffold743_4	non-phage	0
NLF011_scaffold889_11	non-phage	0
NLF011_scaffold347_8	non-phage	0

NLF011_scaffold16348_3	non-phage	0
NLF011_scaffold12675_7	non-phage	0
NLF011_scaffold6836_5	non-phage	0
NLF011_scaffold34768_1	non-phage	0
NLF011_scaffold22_4	non-phage	0
NLF011_scaffold410_1	non-phage	0
NLF011_scaffold10242_7	non-phage	0
NLF011_scaffold29860_4	non-phage	0
NLF011_scaffold7297_2	non-phage	0
NLF011_scaffold66_1	non-phage	0
NLF011_scaffold31276_1	non-phage	0
NLF011_scaffold2872_2	non-phage	0
NLF011_scaffold5345_2	non-phage	0
NLF011_scaffold18508_1	non-phage	0
NLF011_scaffold27904_1	non-phage	0
NLF011_scaffold1097_4	non-phage	0
NLF011_scaffold9785_2	non-phage	0
NLF011_scaffold5695_1	non-phage	0
NLF011_scaffold23581_7	non-phage	0
NLF011_scaffold514_9	non-phage	0
NLF011_scaffold276_19	non-phage	0
NLF011_scaffold14644_4	non-phage	0
NLF011_scaffold9750_5	non-phage	0
NLF011_scaffold1444_1	non-phage	0
NLF011_scaffold4064_4	non-phage	0.002
NLF011_scaffold18831_2	non-phage	0
NLF011_scaffold7643_6	non-phage	0
NLF011_scaffold5531_3	non-phage	0
NLF011_scaffold2270_9	non-phage	0
NLF011_scaffold1559_5	non-phage	0
NLF011_scaffold555_2	non-phage	0
NLF011_scaffold472_1	non-phage	0
NLF011_scaffold24377_1	non-phage	0
NLF011_scaffold11462_1	non-phage	0
NLF011_scaffold7117_8	non-phage	0
NLF011_scaffold6901_5	non-phage	0
NLF011_scaffold153_2	non-phage	0
NLF011_scaffold7214_2	phage	0.883
NLF011_scaffold16170_7	non-phage	0
NLF011_scaffold8741_12	non-phage	0
NLF011_scaffold26784_3	non-phage	0
NLF011_scaffold1243_9	non-phage	0.003
NLF011_scaffold38_16	non-phage	0
NLF011_scaffold26316_6	non-phage	0.108
NLF011_scaffold4406_2	non-phage	0
NLF011_scaffold17308_2	non-phage	0

NLF011_scaffold8572_1	non-phage	0
NLF011_scaffold100_2	non-phage	0
NLF011_scaffold3653_3	non-phage	0
NLF011_scaffold740_1	non-phage	0
NLF011_scaffold6836_6	non-phage	0
NLF011_scaffold10609_2	non-phage	0
NLF011_scaffold20432_1	non-phage	0
NLF011_scaffold16748_2	non-phage	0
NLF011_scaffold13984_4	non-phage	0
NLF011_scaffold633_4	non-phage	0
NLF011_scaffold68_4	non-phage	0
NLF011_scaffold889_2	non-phage	0
NLF011_scaffold34609_1	non-phage	0
NLF011_scaffold504_2	non-phage	0
NLF011_scaffold9120_1	non-phage	0
NLF011_scaffold11065_2	non-phage	0
NLF011_scaffold8944_1	non-phage	0
NLF011_scaffold726_1	non-phage	0
NLF011_scaffold14725_2	non-phage	0
NLF011_scaffold14897_1	non-phage	0
NLF011_scaffold6024_4	non-phage	0
NLF011_scaffold16042_1	non-phage	0
NLF011_scaffold7690_1	non-phage	0
NLF011_scaffold276_5	non-phage	0
NLF011_scaffold1756_9	non-phage	0
NLF011_scaffold18200_1	non-phage	0
NLF011_scaffold5469_1	non-phage	0
NLF011_scaffold11673_2	non-phage	0
NLF011_scaffold157_2	non-phage	0
NLF011_scaffold9662_1	non-phage	0
NLF011_scaffold16669_1	non-phage	0
NLF011_scaffold10809_1	phage	0.993
NLF011_scaffold4466_11	non-phage	0
NLF011_scaffold4729_11	non-phage	0
NLF011_scaffold5393_1	non-phage	0
NLF011_scaffold4255_7	phage	0.986
NLF011_scaffold1984_6	non-phage	0
NLF011_scaffold853_11	non-phage	0
NLF011_scaffold639_7	non-phage	0
NLF011_scaffold14295_6	non-phage	0
NLF011_scaffold16484_1_1	non-phage	0
NLF011_scaffold4287_5_2	non-phage	0
NLF011_scaffold633_10	non-phage	0
NLF011_scaffold24_2	non-phage	0
NLF011_scaffold2140_1	non-phage	0
NLF011_scaffold356_4	non-phage	0

NLF011_scaffold7209_6	non-phage	0
NLF011_scaffold9508_1	non-phage	0
NLF011_scaffold62_1	non-phage	0
NLF011_scaffold3085_4	non-phage	0
NLF011_scaffold10054_4	phage	0.961
NLF011_scaffold889_5_1	non-phage	0
NLF011_scaffold1016_1	non-phage	0
NLF011_scaffold2270_1	non-phage	0
NLF011_scaffold17089_1	non-phage	0
NLF011_scaffold68_5	non-phage	0
NLF011_scaffold2143_1_1	non-phage	0
NLF011_scaffold2143_1_2	non-phage	0
NLF011_scaffold5703_5	non-phage	0
NLF011_scaffold356_7	non-phage	0
NLF011_scaffold20285_2	non-phage	0
NLF011_scaffold4661_1	non-phage	0
NLF011_scaffold5613_6	non-phage	0
NLF011_scaffold6338_7	non-phage	0
NLF011_scaffold124_14	non-phage	0
NLF011_scaffold29618_1	non-phage	0
NLF011_scaffold294_2	non-phage	0
NLF011_scaffold1786_1	phage	0.999
NLF011_scaffold1243_2_2	non-phage	0
NLF011_scaffold13248_1	non-phage	0
NLF011_scaffold734_1	non-phage	0
NLF011_scaffold6238_3	non-phage	0
NLF011_scaffold4096_9	phage	0.992
NLF011_scaffold5468_1	non-phage	0
NLF011_scaffold32541_1	non-phage	0
NLF011_scaffold2421_1	non-phage	0
NLF011_scaffold649_1	non-phage	0
NLF011_scaffold276_11	non-phage	0
NLF011_scaffold3943_1	non-phage	0
NLF011_scaffold4951_4	non-phage	0
NLF011_scaffold5440_1_2	non-phage	0
NLF011_scaffold12015_4	non-phage	0
NLF011_scaffold19170_1	non-phage	0
NLF011_scaffold181_1	non-phage	0
NLF011_scaffold871_5	non-phage	0
NLF011_scaffold25621_1	non-phage	0
NLF011_scaffold671_1	non-phage	0
NLF011_scaffold403_12	non-phage	0
NLF011_scaffold548_6	non-phage	0
NLF011_scaffold146_1	non-phage	0
NLF011_scaffold2885_7	non-phage	0
NLF011_scaffold3221_1	non-phage	0

NLF011_scaffold7575_2	non-phage	0
NLF011_scaffold2534_5	non-phage	0
NLF011_scaffold20609_2	non-phage	0
NLF011_scaffold242_6	non-phage	0
NLF011_scaffold1491_2	non-phage	0
NLF011_scaffold4287_2	non-phage	0
NLF011_scaffold23581_2	non-phage	0
NLF011_scaffold33453_1	non-phage	0
NLF011_scaffold1756_5	non-phage	0
NLF011_scaffold5497_1	non-phage	0
NLF011_scaffold6890_1	non-phage	0
NLF011_scaffold96_2	non-phage	0
NLF011_scaffold4505_2	non-phage	0
NLF011_scaffold639_1	non-phage	0
NLF011_scaffold2637_6_1	phage	0.999
NLF011_scaffold4101_4	non-phage	0
NLF011_scaffold4255_8	non-phage	0
NLF011_scaffold1756_1	non-phage	0
NLF011_scaffold25518_1	non-phage	0
NLF011_scaffold4287_6	non-phage	0
NLF011_scaffold7399_4	non-phage	0
NLF011_scaffold13048_1	phage	0.995
NLF011_scaffold10242_9	non-phage	0
NLF011_scaffold8548_3	non-phage	0
NLF011_scaffold853_12	non-phage	0
NLF011_scaffold7617_4	non-phage	0
NLF011_scaffold5538_3	non-phage	0
NLF011_scaffold8548_1	non-phage	0
NLF011_scaffold10520_2	non-phage	0
NLF011_scaffold13545_4	non-phage	0
NLF011_scaffold2495_8	non-phage	0
NLF011_scaffold14121_2	non-phage	0
NLF011_scaffold6961_7	non-phage	0
NLF011_scaffold123_2	non-phage	0
NLF011_scaffold72_1	non-phage	0
NLF011_scaffold566_6	non-phage	0
NLF011_scaffold19809_2	non-phage	0
NLF011_scaffold4096_5	non-phage	0
NLF011_scaffold1513_3	non-phage	0
NLF011_scaffold3722_1	non-phage	0
NLF011_scaffold276_6	non-phage	0
NLF011_scaffold5525_4	non-phage	0
NLF011_scaffold4683_2	non-phage	0
NLF011_scaffold16989_5	non-phage	0
NLF011_scaffold38_5	non-phage	0
NLF011_scaffold3934_2	non-phage	0

NLF011_scaffold3151_2	phage	0.998
NLF011_scaffold807_1	non-phage	0
NLF011_scaffold29471_3	non-phage	0
NLF011_scaffold2473_3	non-phage	0
NLF011_scaffold679_1	non-phage	0
NLF011_scaffold36301_1	non-phage	0
NLF011_scaffold25550_1	non-phage	0
NLF011_scaffold26329_2	non-phage	0
NLF011_scaffold9567_2	non-phage	0
NLF011_scaffold160_11	non-phage	0
NLF011_scaffold490_2	non-phage	0
NLF011_scaffold13765_3	non-phage	0
NLF011_scaffold91_4	non-phage	0
NLF011_scaffold8692_2	non-phage	0
NLF011_scaffold193_1	non-phage	0
NLF011_scaffold4413_1	non-phage	0
NLF011_scaffold29407_1	non-phage	0
NLF011_scaffold2147_2	non-phage	0
NLF011_scaffold2270_8	non-phage	0
NLF011_scaffold30369_1	non-phage	0
NLF011_scaffold2762_1	non-phage	0
NLF011_scaffold15782_1	non-phage	0
NLF011_scaffold5538_4_1	non-phage	0
NLF011_scaffold743_3	non-phage	0
NLF011_scaffold8495_1	non-phage	0
NLF011_scaffold25327_2	non-phage	0
NLF011_scaffold888_4	non-phage	0
NLF011_scaffold734_3	non-phage	0
NLF011_scaffold880_1	non-phage	0
NLF011_scaffold68_6_1	non-phage	0
NLF011_scaffold124_2	non-phage	0
NLF011_scaffold663_4	non-phage	0
NLF011_scaffold2872_4	non-phage	0
NLF011_scaffold3588_2	non-phage	0
NLF011_scaffold1978_4	non-phage	0
NLF011_scaffold10212_2	non-phage	0
NLF011_scaffold15295_2	non-phage	0
NLF011_scaffold2637_2	non-phage	0
NLF011_scaffold6961_3	non-phage	0
NLF011_scaffold25432_1	non-phage	0
NLF011_scaffold15_1	non-phage	0
NLF011_scaffold13285_1	non-phage	0
NLF011_scaffold356_3	non-phage	0
NLF011_scaffold7558_1	non-phage	0
NLF011_scaffold22_5	non-phage	0
NLF011_scaffold1509_1	non-phage	0

NLF011_scaffold18440_1	non-phage	0
NLF011_scaffold12015_3	non-phage	0
NLF011_scaffold4064_2	non-phage	0
NLF011_scaffold2670_3	non-phage	0
NLF011_scaffold32594_1	non-phage	0
NLF011_scaffold30362_5	phage	0.974
NLF011_scaffold16989_2	non-phage	0
NLF011_scaffold9530_5	non-phage	0
NLF011_scaffold5703_3_1	non-phage	0
NLF011_scaffold29392_1	non-phage	0
NLF011_scaffold34068_5_1	non-phage	0
NLF011_scaffold14740_1	non-phage	0
NLF011_scaffold1379_3	non-phage	0
NLF011_scaffold13254_1	non-phage	0
NLF011_scaffold276_14	non-phage	0
NLF011_scaffold2106_1	non-phage	0
NLF011_scaffold25120_2	phage	0.998
NLF011_scaffold514_16	non-phage	0
NLF011_scaffold91_3	non-phage	0
NLF011_scaffold3669_1	non-phage	0
NLF011_scaffold486_7	phage	0.938
NLF011_scaffold198_1	non-phage	0
NLF011_scaffold18200_3	non-phage	0
NLF011_scaffold41_1	non-phage	0
NLF011_scaffold18742_6_2	non-phage	0
NLF011_scaffold6294_2	non-phage	0
NLF011_scaffold24096_1	non-phage	0
NLF011_scaffold8522_2	non-phage	0
NLF011_scaffold132_1	non-phage	0
NLF011_scaffold5122_1	non-phage	0
NLF011_scaffold5309_1	non-phage	0
NLF011_scaffold1491_1_1	non-phage	0
NLF011_scaffold19200_2	non-phage	0
NLF011_scaffold794_4	phage	0.998
NLF011_scaffold8271_1	non-phage	0
NLF011_scaffold3727_1	non-phage	0
NLF011_scaffold4453_3	non-phage	0
NLF011_scaffold11670_15	non-phage	0
NLF011_scaffold5989_1	non-phage	0
NLF011_scaffold578_2	non-phage	0
NLF011_scaffold27012_1	non-phage	0
NLF011_scaffold514_4	non-phage	0
NLF011_scaffold8476_3	non-phage	0
NLF011_scaffold12644_2	non-phage	0
NLF011_scaffold9280_4	non-phage	0
NLF011_scaffold8673_6	non-phage	0

NLF011_scaffold2492_1	non-phage	0
NLF011_scaffold368_2	non-phage	0
NLF011_scaffold4378_5	non-phage	0
NLF011_scaffold19318_2	non-phage	0
NLF011_scaffold12182_1	non-phage	0
NLF011_scaffold1507_2	non-phage	0
NLF011_scaffold3888_1	non-phage	0
NLF011_scaffold1423_12	non-phage	0
NLF011_scaffold889_13	non-phage	0
NLF011_scaffold28029_1	non-phage	0
NLF011_scaffold7685_4	non-phage	0
NLF011_scaffold1138_2	non-phage	0
NLF011_scaffold5522_2	non-phage	0
NLF011_scaffold26824_4	non-phage	0
NLF011_scaffold83_12	non-phage	0
NLF011_scaffold276_20	non-phage	0
NLF011_scaffold397_2	non-phage	0
NLF011_scaffold803_3	non-phage	0
NLF011_scaffold242_4	non-phage	0
NLF011_scaffold22115_1	phage	0.998
NLF011_scaffold456_2	non-phage	0
NLF011_scaffold4181_1	non-phage	0
NLF011_scaffold11775_1	non-phage	0
NLF011_scaffold5538_2	non-phage	0
NLF011_scaffold347_5	non-phage	0
NLF011_scaffold11155_1	non-phage	0
NLF011_scaffold6961_6	non-phage	0
NLF011_scaffold1848_1	non-phage	0
NLF011_scaffold2314_3	non-phage	0
NLF011_scaffold19259_1	non-phage	0
NLF011_scaffold4287_7	non-phage	0
NLF011_scaffold4255_9	non-phage	0
NLF011_scaffold230_1	non-phage	0
NLF011_scaffold4378_1	non-phage	0
NLF011_scaffold3035_5	non-phage	0
NLF011_scaffold4500_14	non-phage	0
NLF011_scaffold1379_1	non-phage	0
NLF011_scaffold2885_9	non-phage	0
NLF011_scaffold3280_9_1	non-phage	0
NLF011_scaffold52_2	non-phage	0
NLF011_scaffold803_4	phage	0.955
NLF011_scaffold31097_1	non-phage	0
NLF011_scaffold3583_2	non-phage	0
NLF011_scaffold3093_18	non-phage	0
NLF011_scaffold11962_1	non-phage	0
NLF011_scaffold10565_1	non-phage	0

NLF011_scaffold16_4	non-phage	0
NLF011_scaffold9382_24	non-phage	0
NLF011_scaffold8572_3	non-phage	0
NLF011_scaffold17541_3	non-phage	0
NLF011_scaffold5166_1	non-phage	0
NLF011_scaffold16119_2	non-phage	0
NLF011_scaffold24060_1	non-phage	0
NLF011_scaffold30423_1	non-phage	0
NLF011_scaffold14579_1	non-phage	0
NLF011_scaffold6501_1	non-phage	0
NLF011_scaffold2637_7_1	non-phage	0
NLF011_scaffold35879_1	non-phage	0
NLF011_scaffold524_2	non-phage	0
NLF011_scaffold3618_4	non-phage	0
NLF011_scaffold5361_4	non-phage	0
NLF011_scaffold23252_2	non-phage	0
NLF011_scaffold34218_1	non-phage	0
NLF011_scaffold22134_4	non-phage	0
NLF011_scaffold4255_3	non-phage	0
NLF011_scaffold83_5	non-phage	0
NLF011_scaffold24791_1	non-phage	0
NLF011_scaffold164_1	non-phage	0
NLF011_scaffold24965_1	non-phage	0
NLF011_scaffold33456_2	non-phage	0
NLF011_scaffold514_3	non-phage	0
NLF011_scaffold4497_1	non-phage	0
NLF011_scaffold26894_1	non-phage	0
NLF011_scaffold4500_66	non-phage	0
NLF011_scaffold10943_3	phage	0.999
NLF011_scaffold15540_14	non-phage	0
NLF011_scaffold871_3_1	non-phage	0
NLF011_scaffold1646_1_3	non-phage	0
NLF011_scaffold4378_6	non-phage	0
NLF011_scaffold181_4	non-phage	0
NLF011_scaffold181_3	non-phage	0
NLF011_scaffold27581_3	non-phage	0
NLF011_scaffold152_1	non-phage	0
NLF011_scaffold128_4	non-phage	0
NLF011_scaffold68_7	non-phage	0
NLF011_scaffold10242_2	non-phage	0
NLF011_scaffold22029_3	non-phage	0
NLF011_scaffold13003_3	non-phage	0
NLF011_scaffold20518_1	non-phage	0
NLF011_scaffold24096_2	non-phage	0
NLF011_scaffold6937_1	phage	0.867
NLF011_scaffold6961_2	non-phage	0

NLF011_scaffold5370_1	non-phage	0
NLF011_scaffold1099_10	non-phage	0
NLF011_scaffold500_5	non-phage	0
NLF011_scaffold4466_10	non-phage	0
NLF011_scaffold22017_1	non-phage	0
NLF011_scaffold18508_2	non-phage	0
NLF011_scaffold1978_5	non-phage	0
NLF011_scaffold26331_6	non-phage	0
NLF011_scaffold32109_1	non-phage	0
NLF011_scaffold101_1	non-phage	0
NLF011_scaffold46_1	non-phage	0
NLF011_scaffold142_2	non-phage	0.042
NLF011_scaffold14109_3	non-phage	0
NLF011_scaffold96_4	non-phage	0
NLF011_scaffold93_4	non-phage	0
NLF011_scaffold38_2	non-phage	0
NLF011_scaffold2637_8	non-phage	0
NLF011_scaffold4466_4	non-phage	0
NLF011_scaffold630_2	non-phage	0
NLF011_scaffold26824_1	non-phage	0
NLF011_scaffold21554_2	phage	0.542
NLF011_scaffold9671_3	non-phage	0
NLF011_scaffold139_5	non-phage	0
NLF011_scaffold106_2	non-phage	0
NLF011_scaffold9582_1	phage	0.999
NLF011_scaffold13500_1	non-phage	0
NLF011_scaffold13254_4	non-phage	0
NLF011_scaffold14135_1	non-phage	0
NLF011_scaffold69_2	non-phage	0
NLF011_scaffold20_1	non-phage	0
NLF011_scaffold7399_2	non-phage	0
NLF011_scaffold13119_5	non-phage	0.026
NLF011_scaffold2210_4_2	non-phage	0
NLF011_scaffold11601_5	non-phage	0
NLF011_scaffold7713_1	non-phage	0
NLF011_scaffold76_2	non-phage	0
NLF011_scaffold4494_1	non-phage	0
NLF011_scaffold10052_4	non-phage	0
NLF011_scaffold3375_1	non-phage	0
NLF011_scaffold6498_4	non-phage	0
NLF011_scaffold83_1	non-phage	0
NLF011_scaffold9556_2	non-phage	0
NLF011_scaffold9341_2	non-phage	0
NLF011_scaffold6961_9	non-phage	0
NLF011_scaffold129_3	non-phage	0
NLF011_scaffold681_1	non-phage	0

NLF011_scaffold184_1	non-phage	0
NLF011_scaffold4816_5_1	non-phage	0
NLF011_scaffold38_9	non-phage	0
NLF011_scaffold23953_3	non-phage	0
NLF011_scaffold294_4	non-phage	0
NLF011_scaffold5989_2	non-phage	0
NLF011_scaffold5079_10	non-phage	0
NLF011_scaffold14295_8	non-phage	0
NLF011_scaffold2253_4	non-phage	0
NLF012_scaffold39114_1	non-phage	0
NLF012_scaffold238_5	non-phage	0
NLF012_scaffold5983_18	non-phage	0
NLF012_scaffold13372_9	non-phage	0
NLF012_scaffold24036_3	non-phage	0
NLF012_scaffold31969_2	non-phage	0
NLF012_scaffold7670_32	non-phage	0
NLF012_scaffold23280_1	non-phage	0
NLF012_scaffold8654_10	non-phage	0.435
NLF012_scaffold24777_5	non-phage	0
NLF012_scaffold39142_2	non-phage	0
NLF012_scaffold3418_2	non-phage	0
NLF012_scaffold9865_1	non-phage	0
NLF012_scaffold18682_1	non-phage	0
NLF012_scaffold38288_4	non-phage	0
NLF012_scaffold21589_10	non-phage	0
NLF012_scaffold39179_1	non-phage	0
NLF012_scaffold27493_5	non-phage	0
NLF012_scaffold49_2	non-phage	0
NLF012_scaffold30111_2	non-phage	0
NLF012_scaffold7_11	non-phage	0
NLF012_scaffold15260_3	non-phage	0
NLF012_scaffold96_11	non-phage	0
NLF012_scaffold3662_3	non-phage	0
NLF012_scaffold298_19	non-phage	0
NLF012_scaffold39014_1	non-phage	0
NLF012_scaffold238_2	non-phage	0
NLF012_scaffold34065_1	non-phage	0
NLF012_scaffold17901_1	non-phage	0
NLF012_scaffold12611_4	non-phage	0
NLF012_scaffold164_5	non-phage	0
NLF012_scaffold37436_4	non-phage	0
NLF012_scaffold83_2	non-phage	0
NLF012_scaffold32548_3	non-phage	0
NLF012_scaffold10655_1	non-phage	0
NLF012_scaffold29347_2	non-phage	0
NLF012_scaffold21224_2	non-phage	0

NLF012_scaffold15079_19	non-phage	0
NLF012_scaffold3283_4	non-phage	0
NLF012_scaffold813_4	non-phage	0
NLF012_scaffold9707_1	non-phage	0
NLF012_scaffold38797_1	non-phage	0
NLF012_scaffold26058_1	non-phage	0
NLF012_scaffold128_15	non-phage	0
NLF012_scaffold15501_17	non-phage	0
NLF012_scaffold15197_18	non-phage	0
NLF012_scaffold18954_2	non-phage	0
NLF012_scaffold29849_3	non-phage	0
NLF012_scaffold10340_2	phage	0.998
NLF012_scaffold2172_1	non-phage	0
NLF012_scaffold25219_3	non-phage	0
NLF012_scaffold13215_2	non-phage	0
NLF012_scaffold168_1	non-phage	0
NLF012_scaffold15106_23	non-phage	0
NLF012_scaffold38996_1	non-phage	0
NLF012_scaffold22193_3	non-phage	0
NLF012_scaffold26022_1	non-phage	0
NLF012_scaffold6929_3	non-phage	0
NLF012_scaffold37359_1	non-phage	0
NLF012_scaffold7330_1	non-phage	0
NLF012_scaffold19888_16	non-phage	0
NLF012_scaffold2172_21	non-phage	0
NLF012_scaffold30623_1	non-phage	0
NLF012_scaffold17109_1	non-phage	0
NLF012_scaffold39111_2	non-phage	0
NLF012_scaffold7940_2	non-phage	0
NLF012_scaffold16808_1	non-phage	0
NLF012_scaffold17529_2	non-phage	0
NLF012_scaffold33334_1	non-phage	0
NLF012_scaffold17845_5	non-phage	0
NLF012_scaffold350_7	non-phage	0
NLF012_scaffold29420_9	non-phage	0
NLF012_scaffold35219_5	phage	0.983
NLF012_C512197_1	non-phage	0
NLF012_scaffold30180_11	non-phage	0
NLF012_scaffold21670_9	non-phage	0
NLF012_scaffold13124_2	non-phage	0
NLF012_scaffold284_23	non-phage	0
NLF012_scaffold5906_4	non-phage	0
NLF012_scaffold35219_1	phage	0.999
NLF012_scaffold36627_2	non-phage	0
NLF012_scaffold8855_8	non-phage	0
NLF012_scaffold38237_1	non-phage	0

NLF012_scaffold26330_3	non-phage	0
NLF012_scaffold38072_3	non-phage	0
NLF012_scaffold15511_5	non-phage	0
NLF012_scaffold7046_2	non-phage	0
NLF012_scaffold36403_1	non-phage	0
NLF012_scaffold28161_3	non-phage	0
NLF012_scaffold38995_1	non-phage	0.002
NLF012_scaffold14942_1	non-phage	0
NLF012_scaffold15106_14	non-phage	0
NLF012_scaffold203_2	non-phage	0
NLF012_scaffold68_3	non-phage	0
NLF012_scaffold4480_10	non-phage	0
NLF012_scaffold37862_1	non-phage	0
NLF012_scaffold509_1	non-phage	0
NLF012_scaffold39158_2	non-phage	0
NLF012_scaffold20272_3	non-phage	0
NLF012_scaffold19133_7	non-phage	0
NLF012_scaffold7868_1	non-phage	0
NLF012_scaffold6180_18	non-phage	0
NLF012_scaffold12677_5	non-phage	0
NLF012_scaffold12454_4	non-phage	0
NLF012_scaffold37578_3	non-phage	0
NLF012_scaffold25991_1	non-phage	0
NLF012_scaffold138_1	non-phage	0
NLF012_scaffold26948_1	non-phage	0
NLF012_scaffold38885_2	non-phage	0
NLF012_scaffold730_4	non-phage	0
NLF012_scaffold39113_1	non-phage	0
NLF012_scaffold26933_1	non-phage	0
NLF012_scaffold119_1	non-phage	0
NLF012_scaffold17832_1	non-phage	0
NLF012_scaffold284_34	non-phage	0
NLF012_scaffold3101_8	non-phage	0
NLF012_scaffold37313_2	non-phage	0
NLF012_scaffold3017_2	non-phage	0
NLF012_scaffold6180_22	non-phage	0
NLF012_scaffold15106_7	non-phage	0
NLF012_scaffold83_11	non-phage	0
NLF012_scaffold8622_4	non-phage	0
NLF012_scaffold8036_1	non-phage	0
NLF012_scaffold18241_3	non-phage	0
NLF012_scaffold32493_6	non-phage	0
NLF012_scaffold907_13	phage	0.849
NLF012_scaffold16168_6	non-phage	0
NLF012_scaffold38879_2	non-phage	0
NLF012_scaffold908_3	non-phage	0

NLF012_scaffold1552_27	non-phage	0
NLF012_scaffold29166_1	non-phage	0
NLF012_scaffold8732_3	non-phage	0
NLF012_scaffold39031_1	non-phage	0
NLF012_scaffold14429_1	non-phage	0
NLF012_scaffold2778_4	non-phage	0
NLF012_scaffold19458_2	non-phage	0
NLF012_scaffold35130_3	non-phage	0
NLF012_scaffold15079_10	non-phage	0
NLF012_scaffold1129_6	non-phage	0
NLF012_scaffold20272_1	non-phage	0
NLF012_scaffold35683_3	non-phage	0
NLF012_scaffold32835_3	non-phage	0
NLF012_scaffold7839_5	non-phage	0
NLF012_scaffold28810_1	non-phage	0
NLF012_scaffold39053_1	phage	0.945
NLF012_scaffold2371_23	phage	0.999
NLF012_scaffold38961_1	non-phage	0
NLF012_scaffold14447_12	non-phage	0
NLF012_scaffold16450_1	non-phage	0
NLF012_scaffold28161_6	non-phage	0
NLF012_scaffold21034_1	non-phage	0
NLF012_scaffold37256_1	non-phage	0
NLF012_scaffold1959_8	non-phage	0
NLF012_scaffold22746_1	phage	0.974
NLF012_scaffold3809_3	non-phage	0
NLF012_scaffold238_10	non-phage	0
NLF012_scaffold14430_3	non-phage	0
NLF012_scaffold2787_2	non-phage	0
NLF012_scaffold38962_1	non-phage	0
NLF012_scaffold18050_1	non-phage	0
NLF012_scaffold29704_1	non-phage	0
NLF012_scaffold10004_20	non-phage	0
NLF012_scaffold26030_7	non-phage	0
NLF012_scaffold39117_2	non-phage	0
NLF012_scaffold15319_1	non-phage	0
NLF012_scaffold31170_2	non-phage	0
NLF012_scaffold9029_1	non-phage	0
NLF012_scaffold26150_2	non-phage	0
NLF012_scaffold174_2	non-phage	0
NLF012_scaffold12460_2	non-phage	0
NLF012_scaffold39173_2	non-phage	0
NLF012_scaffold24971_3	non-phage	0
NLF012_scaffold2215_6	non-phage	0
NLF012_scaffold39054_1	phage	0.995
NLF012_scaffold8622_2	phage	0.949

NLF012_scaffold907_18	non-phage	0
NLF012_scaffold19472_2	non-phage	0
NLF012_scaffold32449_4	non-phage	0
NLF012_scaffold28854_1	non-phage	0
NLF012_scaffold37397_1	non-phage	0
NLF012_scaffold17199_3	non-phage	0
NLF012_scaffold38666_1	non-phage	0
NLF012_scaffold23584_2	non-phage	0
NLF012_scaffold3102_2	non-phage	0
NLF012_scaffold30756_3	non-phage	0
NLF012_scaffold3087_2	non-phage	0
NLF012_scaffold23075_5	non-phage	0
NLF012_scaffold16383_2	non-phage	0
NLF012_scaffold11786_16	non-phage	0
NLF012_scaffold39166_1	non-phage	0.183
NLF012_scaffold8509_3	non-phage	0
NLF012_scaffold13845_3	non-phage	0
NLF012_scaffold7601_3	non-phage	0
NLF012_scaffold15106_20	non-phage	0
NLF012_scaffold10275_22	non-phage	0
NLF012_scaffold10275_29	non-phage	0
NLF012_scaffold2172_5	phage	0.983
NLF012_scaffold6723_7	non-phage	0
NLF012_scaffold37063_1	non-phage	0
NLF012_scaffold96_7	non-phage	0
NLF012_scaffold3418_6	non-phage	0
NLF012_scaffold128_1	non-phage	0
NLF012_scaffold26128_1	non-phage	0
NLF012_scaffold17110_2	non-phage	0
NLF012_scaffold32329_4	non-phage	0
NLF012_scaffold27405_2	phage	0.999
NLF012_scaffold38405_1	non-phage	0
NLF012_scaffold3662_6	non-phage	0
NLF012_scaffold11833_29	phage	0.994
NLF012_C511863_1	non-phage	0
NLF012_scaffold3101_3	non-phage	0
NLF012_scaffold8319_5	non-phage	0
NLF012_scaffold49_1	non-phage	0
NLF012_scaffold2371_16	phage	0.998
NLF012_scaffold15106_18	non-phage	0
NLF012_scaffold19888_21	non-phage	0
NLF012_scaffold31308_5	non-phage	0
NLF012_scaffold12333_5	non-phage	0
NLF012_scaffold2255_2	non-phage	0
NLF012_scaffold10275_8	non-phage	0
NLF012_scaffold38863_1	non-phage	0

NLF012_scaffold36240_1	non-phage	0
NLF012_scaffold9016_1	non-phage	0
NLF012_scaffold350_10	non-phage	0
NLF012_scaffold38823_1	non-phage	0
NLF012_scaffold29701_7	non-phage	0
NLF012_scaffold39140_2	phage	0.856
NLF012_scaffold18119_7	non-phage	0
NLF012_scaffold2371_28	non-phage	0
NLF012_scaffold2854_6	non-phage	0
NLF012_scaffold2862_1	non-phage	0
NLF012_scaffold38839_1	non-phage	0
NLF012_scaffold5604_3	non-phage	0
NLF012_scaffold2172_18	non-phage	0
NLF012_scaffold19920_2	non-phage	0
NLF012_scaffold25701_2	non-phage	0
NLF012_scaffold38988_3	non-phage	0
NLF012_scaffold25728_1	phage	0.998
NLF012_scaffold32449_1	non-phage	0
NLF012_scaffold38830_1	non-phage	0
NLF012_scaffold27019_3	non-phage	0
NLF012_scaffold11786_13	non-phage	0
NLF012_scaffold10004_19	non-phage	0
NLF012_scaffold6627_1	non-phage	0
NLF012_scaffold265_2	non-phage	0
NLF012_scaffold23075_4	non-phage	0
NLF012_scaffold37598_3	non-phage	0
NLF012_scaffold16161_1	non-phage	0
NLF012_scaffold20046_3	non-phage	0
NLF012_scaffold33280_2	non-phage	0
NLF012_scaffold21659_1	non-phage	0
NLF012_scaffold7839_10	non-phage	0
NLF012_scaffold12958_5	non-phage	0
NLF012_scaffold14429_2	non-phage	0
NLF012_scaffold6326_1	non-phage	0
NLF012_scaffold3530_6	non-phage	0
NLF012_scaffold38637_2	non-phage	0
NLF012_scaffold6145_1	non-phage	0
NLF012_scaffold34102_1	non-phage	0
NLF012_scaffold37648_3	non-phage	0
NLF012_scaffold7839_9	non-phage	0
NLF012_scaffold35219_4	non-phage	0
NLF012_scaffold907_1	non-phage	0
NLF012_scaffold21248_2	non-phage	0
NLF012_scaffold4829_1	non-phage	0
NLF012_scaffold2047_3	non-phage	0
NLF012_scaffold730_9	non-phage	0

NLF012_scaffold36675_4	phage	0.993
NLF012_scaffold21083_1	non-phage	0
NLF012_scaffold1215_1	non-phage	0
NLF012_scaffold34868_1	non-phage	0.042
NLF012_scaffold10615_3	non-phage	0
NLF012_scaffold16168_10	non-phage	0
NLF012_scaffold10935_2	non-phage	0
NLF012_scaffold40_2	non-phage	0
NLF012_scaffold14025_6	non-phage	0
NLF012_scaffold15106_3	non-phage	0
NLF012_scaffold21675_1	phage	1
NLF012_scaffold6595_10	non-phage	0
NLF012_scaffold29798_8	non-phage	0
NLF012_scaffold290_3	non-phage	0
NLF012_scaffold750_50	non-phage	0
NLF012_scaffold39092_1	non-phage	0
NLF012_scaffold37049_4	non-phage	0
NLF012_scaffold37049_1	non-phage	0
NLF012_scaffold10340_16	non-phage	0
NLF012_scaffold10340_8	non-phage	0
NLF012_scaffold750_44	non-phage	0
NLF012_scaffold34_13	non-phage	0
NLF012_scaffold2172_17	non-phage	0
NLF012_scaffold1375_8	non-phage	0
NLF012_scaffold17066_2	non-phage	0
NLF012_scaffold14506_3	non-phage	0
NLF012_scaffold3602_6	non-phage	0
NLF012_scaffold16161_12	non-phage	0
NLF012_scaffold31995_1	non-phage	0
NLF012_scaffold15106_8	non-phage	0
NLF012_scaffold17179_2	non-phage	0
NLF012_scaffold38647_1	non-phage	0
NLF012_scaffold28532_1	non-phage	0
NLF012_scaffold38672_1	non-phage	0
NLF012_scaffold18277_2	non-phage	0
NLF012_scaffold20522_1	non-phage	0
NLF012_scaffold683_8	non-phage	0
NLF012_scaffold11833_30	phage	0.999
NLF012_scaffold17845_4	non-phage	0
NLF012_scaffold16882_1	non-phage	0
NLF012_scaffold29420_1	phage	0.932
NLF012_scaffold19508_1	non-phage	0
NLF012_scaffold31969_1	non-phage	0
NLF012_scaffold1150_9	non-phage	0
NLF012_scaffold8622_7	non-phage	0
NLF012_scaffold38871_2	non-phage	0

NLF012_scaffold35536_1	non-phage	0
NLF012_scaffold4099_1	non-phage	0
NLF012_scaffold16932_1	non-phage	0
NLF012_scaffold3425_2	non-phage	0
NLF012_scaffold12930_1	non-phage	0
NLF012_scaffold34227_1	non-phage	0
NLF012_scaffold38885_1	non-phage	0
NLF012_scaffold1569_2	non-phage	0
NLF012_scaffold9707_4	non-phage	0
NLF012_scaffold6162_1	non-phage	0
NLF012_scaffold3659_1	non-phage	0
NLF012_scaffold29581_6	non-phage	0
NLF012_scaffold3659_8	non-phage	0
NLF012_scaffold14952_1	non-phage	0
NLF012_scaffold39159_1	phage	1
NLF012_scaffold23249_1	non-phage	0
NLF012_scaffold17264_3	phage	0.995
NLF012_scaffold25918_5	non-phage	0
NLF012_scaffold7531_11	non-phage	0
NLF012_scaffold27546_1	non-phage	0
NLF012_scaffold7034_1	non-phage	0
NLF012_scaffold38889_2	non-phage	0
NLF012_scaffold7315_8	non-phage	0
NLF012_scaffold7670_34	non-phage	0
NLF012_scaffold1802_2	non-phage	0
NLF012_scaffold28390_3	non-phage	0
NLF012_scaffold7_10	non-phage	0
NLF012_scaffold11786_12	non-phage	0
NLF012_scaffold96_10	non-phage	0
NLF012_scaffold37592_1	non-phage	0
NLF012_scaffold39143_3	non-phage	0
NLF012_scaffold14430_4	non-phage	0
NLF012_scaffold49_3	non-phage	0
NLF012_scaffold28659_2	non-phage	0
NLF012_scaffold2255_7	non-phage	0
NLF012_scaffold290_11	non-phage	0
NLF012_scaffold13372_13	non-phage	0
NLF012_scaffold37436_3	non-phage	0
NLF012_scaffold2562_9	non-phage	0
NLF012_scaffold34471_3	phage	0.965
NLF012_scaffold8220_6	non-phage	0
NLF012_scaffold1008_3	non-phage	0
NLF012_scaffold26966_9	non-phage	0
NLF012_scaffold3825_1	non-phage	0
NLF012_scaffold8654_3	non-phage	0
NLF012_scaffold17982_3	non-phage	0

NLF012_scaffold293_2	phage	0.999
NLF012_scaffold6856_9	non-phage	0
NLF012_scaffold14712_2	non-phage	0
NLF012_scaffold18697_1	non-phage	0
NLF012_scaffold13215_3	non-phage	0
NLF012_scaffold34407_1	non-phage	0
NLF012_scaffold38720_1	non-phage	0
NLF012_scaffold6773_9	phage	0.964
NLF012_scaffold479_20	non-phage	0
NLF012_scaffold238_3	non-phage	0
NLF012_scaffold2491_2	non-phage	0
NLF012_scaffold26689_1	non-phage	0
NLF012_scaffold11833_13	non-phage	0
NLF012_scaffold32790_4	non-phage	0
NLF012_scaffold38472_1	non-phage	0
NLF012_scaffold23126_1	non-phage	0
NLF012_scaffold34846_1	non-phage	0
NLF012_scaffold3158_2	non-phage	0
NLF012_scaffold156_2	non-phage	0
NLF012_scaffold39169_1	non-phage	0
NLF012_scaffold38245_1	non-phage	0
NLF012_scaffold3017_1	non-phage	0.001
NLF012_scaffold4830_1	non-phage	0
NLF012_scaffold2680_2	non-phage	0
NLF012_C512573_1	non-phage	0
NLF012_scaffold3101_17	non-phage	0
NLF012_scaffold26294_1	non-phage	0
NLF012_scaffold21589_9	non-phage	0
NLF012_scaffold21659_3	non-phage	0
NLF012_scaffold10048_4	non-phage	0
NLF012_scaffold4760_4	non-phage	0
NLF012_scaffold17240_2	non-phage	0
NLF012_scaffold83_10	non-phage	0
NLF012_scaffold1990_3	non-phage	0
NLF012_scaffold35683_2	non-phage	0
NLF012_scaffold36307_3	non-phage	0
NLF012_scaffold16971_4	non-phage	0
NLF012_scaffold16168_7	non-phage	0
NLF012_scaffold28836_2	non-phage	0
NLF012_scaffold30873_2	non-phage	0
NLF012_scaffold3106_2	non-phage	0
NLF012_scaffold39111_1	non-phage	0
NLF012_scaffold10245_3	phage	1
NLF012_scaffold14025_1	non-phage	0
NLF012_scaffold17023_1	non-phage	0
NLF012_scaffold19133_9	non-phage	0

NLF012_scaffold5014_4	non-phage	0
NLF012_scaffold2486_10	non-phage	0
NLF012_scaffold3326_8	non-phage	0
NLF012_scaffold36967_4	non-phage	0
NLF012_scaffold2854_1	phage	0.983
NLF012_scaffold18119_4	phage	0.999
NLF012_scaffold38597_1	non-phage	0
NLF012_scaffold3602_5	non-phage	0
NLF012_scaffold31367_1	non-phage	0
NLF012_scaffold20199_5	non-phage	0
NLF012_scaffold28960_2	non-phage	0
NLF012_scaffold39074_1	non-phage	0
NLF012_scaffold7733_1	non-phage	0
NLF012_scaffold38971_1	non-phage	0
NLF012_scaffold16451_1	non-phage	0
NLF012_scaffold27237_5	non-phage	0
NLF012_scaffold38072_2	non-phage	0
NLF012_scaffold24777_4	non-phage	0
NLF012_scaffold3437_6	non-phage	0
NLF012_scaffold20199_4	non-phage	0
NLF012_scaffold36373_1	non-phage	0
NLF012_scaffold38476_1	non-phage	0
NLF012_scaffold35316_1	non-phage	0
NLF012_scaffold7839_12	non-phage	0
NLF012_scaffold6845_3	non-phage	0
NLF012_scaffold36403_3	non-phage	0
NLF012_scaffold38705_1	non-phage	0
NLF012_scaffold29151_4	non-phage	0
NLF012_scaffold7449_9	non-phage	0
NLF012_scaffold6975_1	non-phage	0
NLF012_scaffold6555_5	non-phage	0
NLF012_scaffold9845_8	non-phage	0.003
NLF012_scaffold19843_1	non-phage	0
NLF012_scaffold36776_1	non-phage	0
NLF012_scaffold974_6	non-phage	0
NLF012_scaffold6595_6	non-phage	0
NLF012_scaffold3418_3	non-phage	0
NLF012_scaffold350_14	non-phage	0
NLF012_scaffold9181_1	non-phage	0
NLF012_scaffold21248_1	non-phage	0
NLF012_scaffold16383_1	non-phage	0
NLF012_scaffold18940_2	non-phage	0
NLF012_scaffold14639_1	non-phage	0
NLF012_scaffold2215_5	non-phage	0
NLF012_scaffold35718_1	non-phage	0
NLF012_scaffold39184_1	non-phage	0

NLF012_scaffold1008_12	non-phage	0
NLF012_scaffold39031_4	non-phage	0
NLF012_scaffold38962_2	non-phage	0
NLF012_scaffold907_10	non-phage	0
NLF012_scaffold25866_3	non-phage	0
NLF012_scaffold32493_10	non-phage	0
NLF012_scaffold23075_3	non-phage	0
NLF012_scaffold17199_4	non-phage	0
NLF012_scaffold1802_14	non-phage	0
NLF012_scaffold173_1	non-phage	0
NLF012_scaffold39118_1	non-phage	0
NLF012_scaffold9449_2	non-phage	0
NLF012_scaffold32329_3	non-phage	0
NLF012_scaffold34347_2	non-phage	0
NLF012_scaffold39175_1	non-phage	0
NLF012_scaffold128_12	non-phage	0
NLF012_scaffold39160_2	non-phage	0
NLF012_scaffold38032_1	phage	0.998
NLF012_scaffold39037_2	non-phage	0
NLF012_scaffold18265_1	non-phage	0
NLF012_scaffold16746_1	non-phage	0
NLF012_scaffold13124_4	non-phage	0
NLF012_scaffold7839_3	non-phage	0
NLF012_scaffold30623_7	non-phage	0
NLF012_scaffold27405_1	non-phage	0
NLF012_scaffold16168_11	non-phage	0
NLF012_scaffold14370_1	non-phage	0
NLF012_scaffold30452_1	non-phage	0
NLF012_scaffold5980_4	non-phage	0
NLF012_scaffold36643_7	non-phage	0
NLF012_scaffold31452_2	non-phage	0
NLF012_scaffold38498_1	non-phage	0
NLF012_scaffold27645_2	non-phage	0
NLF012_scaffold19472_5	non-phage	0
NLF012_scaffold7670_13	non-phage	0
NLF012_scaffold6478_6	non-phage	0
NLF012_scaffold13768_2	non-phage	0
NLF012_scaffold2486_1	non-phage	0
NLF012_scaffold28520_2	non-phage	0
NLF012_scaffold23794_2	non-phage	0
NLF012_scaffold14917_5	non-phage	0
NLF012_scaffold237_2	non-phage	0
NLF012_scaffold37146_1	non-phage	0
NLF012_scaffold2821_4	non-phage	0
NLF012_scaffold28671_5	non-phage	0
NLF012_scaffold15048_1	non-phage	0

NLF012_scaffold17901_9	non-phage	0
NLF012_scaffold38962_5	non-phage	0
NLF012_scaffold15106_15	non-phage	0
NLF012_scaffold28161_5	non-phage	0
NLF012_scaffold4710_25	non-phage	0
NLF012_scaffold11730_2	non-phage	0
NLF012_scaffold6283_9	non-phage	0
NLF012_scaffold36675_1	non-phage	0
NLF012_scaffold1008_7	non-phage	0
NLF012_scaffold9517_1	non-phage	0
NLF012_scaffold37973_2	non-phage	0
NLF012_scaffold38785_1	non-phage	0
NLF012_scaffold39141_1	non-phage	0
NLF012_scaffold17715_1	non-phage	0
NLF012_scaffold18050_2	non-phage	0
NLF012_scaffold16653_1	phage	0.913
NLF012_scaffold34868_3	non-phage	0
NLF012_scaffold39151_1	non-phage	0
NLF012_scaffold9696_1	non-phage	0
NLF012_scaffold21670_2	non-phage	0
NLF012_scaffold32018_2	non-phage	0
NLF012_scaffold6180_1	non-phage	0
NLF012_scaffold30111_5	non-phage	0
NLF012_scaffold908_4	non-phage	0
NLF012_scaffold174_3	non-phage	0
NLF012_scaffold28854_2	non-phage	0
NLF012_scaffold31736_3	non-phage	0
NLF012_scaffold22895_4	non-phage	0
NLF012_scaffold25988_1	non-phage	0
NLF012_scaffold6581_5	non-phage	0
NLF012_scaffold24922_1	non-phage	0
NLF012_scaffold18971_4	non-phage	0
NLF012_scaffold26294_4	non-phage	0
NLF012_scaffold23584_1	non-phage	0.012
NLF012_scaffold9016_11	non-phage	0
NLF012_scaffold3437_3	non-phage	0
NLF012_scaffold6247_25	non-phage	0
NLF012_scaffold1552_3	non-phage	0
NLF012_scaffold2047_2	non-phage	0
NLF012_scaffold26646_3	non-phage	0
NLF012_scaffold14917_7	non-phage	0
NLF012_scaffold2172_20	non-phage	0
NLF012_scaffold8622_1	non-phage	0
NLF012_scaffold37648_2	non-phage	0
NLF012_scaffold290_8	non-phage	0
NLF012_scaffold7670_22	non-phage	0

NLF012_scaffold17213_1	non-phage	0
NLF012_scaffold96_6	non-phage	0
NLF012_scaffold33556_6	non-phage	0
NLF012_scaffold10615_2	non-phage	0
NLF012_scaffold65_8	non-phage	0
NLF012_scaffold35690_3	non-phage	0
NLF012_scaffold296_29	non-phage	0
NLF012_scaffold1215_2	non-phage	0
NLF012_scaffold3101_4	non-phage	0
NLF012_scaffold7670_5	non-phage	0
NLF012_scaffold28667_1	non-phage	0
NLF012_scaffold21253_4	non-phage	0
NLF012_scaffold26030_9	non-phage	0
NLF012_scaffold32346_1	non-phage	0
NLF012_scaffold16161_7	non-phage	0
NLF012_scaffold38335_1	non-phage	0
NLF012_scaffold38891_2	non-phage	0
NLF012_scaffold38527_1	non-phage	0
NLF012_scaffold9016_9	non-phage	0
NLF012_scaffold38920_1	non-phage	0
NLF012_scaffold12333_7	non-phage	0
NLF012_scaffold36325_2	non-phage	0
NLF012_scaffold37741_3	non-phage	0
NLF012_scaffold6555_1	non-phage	0
NLF012_scaffold16168_3	non-phage	0
NLF012_scaffold10655_3	non-phage	0
NLF012_scaffold22111_1	non-phage	0
NLF012_scaffold5752_1	non-phage	0
NLF012_scaffold284_27	non-phage	0
NLF012_scaffold3418_7	non-phage	0
NLF012_scaffold5906_5	non-phage	0
NLF012_scaffold39140_3	non-phage	0
NLF012_C512229_1	non-phage	0
NLF012_scaffold15197_16	non-phage	0
NLF012_scaffold21224_4	non-phage	0
NLF012_scaffold2327_6	non-phage	0
NLF012_scaffold7034_2	non-phage	0
NLF012_scaffold38599_1	non-phage	0
NLF012_scaffold32790_3	non-phage	0
NLF012_scaffold42_1	non-phage	0
NLF012_scaffold34875_2	non-phage	0
NLF012_C511949_1	non-phage	0
NLF012_scaffold35595_1	non-phage	0
NLF012_scaffold29420_11	non-phage	0
NLF012_scaffold22955_2	phage	0.998
NLF012_scaffold39186_1	non-phage	0

NLF012_scaffold39172_1	non-phage	0
NLF012_scaffold750_17	non-phage	0
NLF012_scaffold173_5	non-phage	0
NLF012_scaffold20761_6	non-phage	0
NLF012_scaffold21413_1	non-phage	0
NLF012_scaffold21799_1	non-phage	0
NLF012_scaffold10004_12	non-phage	0
NLF012_scaffold2778_1	non-phage	0
NLF012_scaffold9009_2	non-phage	0
NLF012_scaffold10272_2	non-phage	0
NLF012_scaffold28161_1	non-phage	0
NLF012_scaffold6694_2	non-phage	0
NLF012_scaffold32352_1	non-phage	0
NLF012_scaffold20761_4	non-phage	0
NLF012_scaffold4829_2	non-phage	0
NLF012_scaffold100_1	non-phage	0
NLF012_scaffold36967_3	non-phage	0
NLF012_scaffold35008_2	non-phage	0
NLF012_scaffold31024_2	non-phage	0
NLF012_scaffold1919_1	non-phage	0
NLF012_scaffold2172_26	non-phage	0
NLF012_scaffold31416_2	non-phage	0
NLF012_scaffold15731_1	phage	0.999
NLF012_scaffold26987_2	non-phage	0
NLF012_scaffold27882_1	non-phage	0
NLF012_scaffold15501_12	non-phage	0
NLF012_scaffold12333_4	non-phage	0
NLF012_scaffold19021_3	non-phage	0
NLF012_scaffold38889_1	non-phage	0
NLF012_scaffold296_9	non-phage	0
NLF012_scaffold22193_2	non-phage	0
NLF012_scaffold11833_1	non-phage	0
NLF012_scaffold38290_4	non-phage	0
NLF012_scaffold2374_2	non-phage	0.001
NLF012_scaffold35778_1	non-phage	0
NLF012_scaffold9557_9	non-phage	0
NLF012_scaffold5604_1	non-phage	0
NLF012_scaffold2313_1	non-phage	0
NLF012_scaffold13034_3	phage	0.999
NLF012_scaffold16161_6	non-phage	0
NLF012_scaffold38871_1	non-phage	0
NLF012_scaffold25640_3	non-phage	0
NLF012_scaffold10340_15	non-phage	0
NLF012_scaffold170_1	non-phage	0
NLF012_scaffold31400_2	non-phage	0
NLF012_scaffold38907_2	non-phage	0

NLF012_scaffold14447_9	non-phage	0
NLF012_scaffold1646_6	non-phage	0
NLF012_scaffold31024_5	non-phage	0
NLF012_scaffold18049_1	non-phage	0
NLF012_scaffold21589_12	non-phage	0
NLF012_scaffold16348_2	phage	0.99
NLF012_scaffold8654_4	non-phage	0
NLF012_scaffold9891_7	non-phage	0
NLF012_scaffold14370_2	non-phage	0
NLF012_scaffold38597_2	non-phage	0
NLF012_scaffold10275_14	non-phage	0
NLF012_C511983_1	non-phage	0
NLF012_scaffold5980_1	non-phage	0
NLF012_scaffold20199_3	non-phage	0
NLF012_scaffold730_6	non-phage	0
NLF012_scaffold14434_3	non-phage	0
NLF012_scaffold26623_2	non-phage	0
NLF012_scaffold128_9	non-phage	0
NLF012_scaffold30873_1	non-phage	0
NLF012_scaffold37974_1	non-phage	0
NLF012_scaffold3102_9	non-phage	0
NLF012_scaffold23075_1	non-phage	0
NLF012_scaffold1833_2	non-phage	0
NLF012_scaffold32504_1	non-phage	0
NLF012_scaffold6145_3	non-phage	0
NLF012_scaffold7601_1	non-phage	0
NLF012_scaffold8622_6	phage	0.916
NLF012_scaffold6720_1	non-phage	0
NLF012_scaffold265_4	non-phage	0
NLF012_scaffold38440_7	non-phage	0
NLF012_scaffold3530_4	non-phage	0
NLF012_scaffold17845_1	phage	0.999
NLF012_scaffold14429_3	non-phage	0
NLF012_scaffold283_3	non-phage	0
NLF012_scaffold3659_7	non-phage	0
NLF012_C512109_1	non-phage	0
NLF012_scaffold36182_2	non-phage	0
NLF012_scaffold7839_7	non-phage	0
NLF012_scaffold23664_1	non-phage	0
NLF012_scaffold39189_1	non-phage	0.141
NLF012_scaffold28810_3	non-phage	0
NLF012_scaffold2976_1	non-phage	0
NLF012_scaffold245_3	non-phage	0
NLF012_scaffold6283_8	non-phage	0
NLF012_scaffold14905_8	non-phage	0
NLF012_C512465_1	phage	0.998

NLF012_scaffold22698_2	non-phage	0
NLF012_scaffold24036_1	non-phage	0
NLF012_scaffold23091_5	non-phage	0
NLF012_scaffold3418_4	non-phage	0
NLF012_scaffold23344_2	non-phage	0
NLF012_scaffold17901_3	non-phage	0
NLF012_scaffold8220_8	non-phage	0
NLF012_scaffold6773_1	non-phage	0
NLF012_scaffold15019_8	non-phage	0
NLF012_scaffold12754_3	phage	0.998
NLF012_scaffold27499_3	non-phage	0.061
NLF012_scaffold18976_2	non-phage	0
NLF012_scaffold9422_7	non-phage	0
NLF012_scaffold7855_18	non-phage	0
NLF012_scaffold25219_5	non-phage	0
NLF012_scaffold16653_2	non-phage	0
NLF012_scaffold39013_1	non-phage	0
NLF012_scaffold20422_2	non-phage	0.029
NLF012_scaffold26128_3	non-phage	0
NLF012_scaffold29151_1	non-phage	0
NLF012_scaffold29849_5	non-phage	0
NLF012_C512703_1	non-phage	0
NLF012_scaffold35503_1	non-phage	0
NLF012_scaffold15106_19	non-phage	0
NLF012_scaffold16691_1	non-phage	0
NLF012_scaffold6617_2	non-phage	0
NLF012_scaffold18940_1	non-phage	0
NLF012_scaffold10394_1	phage	0.833
NLF012_scaffold33945_1	non-phage	0
NLF012_scaffold38907_1	non-phage	0
NLF012_scaffold4829_3	non-phage	0
NLF012_scaffold750_2	non-phage	0
NLF012_scaffold28740_2	non-phage	0
NLF012_C512543_1	non-phage	0
NLF012_scaffold11833_26	non-phage	0
NLF012_scaffold24971_2	non-phage	0
NLF012_scaffold30756_1	non-phage	0
NLF012_scaffold26865_1	non-phage	0
NLF012_scaffold6723_1	non-phage	0
NLF012_scaffold27605_2	non-phage	0
NLF012_scaffold17066_1	non-phage	0
NLF012_scaffold37566_2	non-phage	0
NLF012_scaffold14818_2	non-phage	0
NLF012_scaffold156_3	non-phage	0
NLF012_scaffold38426_1	non-phage	0
NLF012_scaffold1959_4	non-phage	0

NLF012_scaffold1802_13	non-phage	0
NLF012_scaffold3036_10	non-phage	0
NLF012_scaffold39109_1	non-phage	0
NLF012_scaffold23422_2	non-phage	0
NLF012_scaffold6929_1	non-phage	0
NLF012_scaffold96_9	non-phage	0
NLF012_scaffold17110_4	non-phage	0
NLF012_scaffold6723_12	non-phage	0
NLF012_scaffold25843_1	non-phage	0
NLF012_scaffold29151_2	non-phage	0
NLF012_scaffold17390_1	non-phage	0
NLF012_C511751_1	non-phage	0
NLF012_scaffold6326_10	non-phage	0
NLF012_scaffold3210_6	non-phage	0
NLF012_scaffold5271_11	non-phage	0
NLF012_scaffold102_1	non-phage	0
NLF012_scaffold212_2	non-phage	0
NLF012_scaffold15965_2	non-phage	0.002
NLF012_scaffold37883_1	non-phage	0
NLF012_scaffold29798_10	non-phage	0
NLF012_scaffold22846_8	non-phage	0
NLF012_scaffold1734_6	non-phage	0
NLF012_scaffold17431_20	non-phage	0
NLF012_scaffold16807_1	non-phage	0
NLF012_scaffold3101_1	non-phage	0
NLF012_scaffold32276_1	phage	0.996
NLF012_scaffold27019_5	non-phage	0
NLF012_scaffold24690_1	non-phage	0
NLF012_scaffold296_24	non-phage	0
NLF012_scaffold32160_2	non-phage	0
NLF012_scaffold13132_1	phage	0.999
NLF012_scaffold30180_18	phage	1
NLF012_scaffold14395_1	non-phage	0
NLF012_scaffold3101_12	non-phage	0
NLF012_scaffold2282_21	non-phage	0
NLF012_scaffold28041_1	non-phage	0
NLF012_scaffold4660_9	non-phage	0
NLF012_scaffold37993_2	non-phage	0
NLF012_scaffold13731_3	non-phage	0
NLF012_scaffold15501_8	non-phage	0
NLF012_scaffold4660_2	non-phage	0
NLF012_scaffold17431_22	phage	0.636
NLF012_scaffold19133_12	non-phage	0
NLF012_scaffold6145_4	non-phage	0
NLF012_scaffold20211_2	non-phage	0
NLF012_scaffold30623_6	non-phage	0

NLF012_scaffold9517_10	non-phage	0
NLF012_scaffold39031_3	non-phage	0
NLF012_scaffold30312_1	non-phage	0
NLF012_scaffold14206_3	non-phage	0
NLF012_scaffold21589_1	non-phage	0
NLF012_scaffold2880_2	non-phage	0
NLF012_scaffold38385_2	non-phage	0
NLF012_scaffold37648_1	non-phage	0
NLF012_scaffold11833_14	non-phage	0
NLF012_scaffold15704_1	non-phage	0
NLF012_scaffold12958_3	non-phage	0
NLF012_scaffold35951_2	non-phage	0
NLF012_scaffold28616_1	non-phage	0
NLF012_scaffold36015_1	non-phage	0
NLF012_scaffold20383_1	non-phage	0
NLF012_scaffold29304_4	non-phage	0
NLF012_scaffold2215_20	non-phage	0
NLF012_scaffold15106_5	non-phage	0
NLF012_scaffold36127_1	non-phage	0
NLF012_scaffold4830_5	non-phage	0
NLF012_scaffold14555_1	non-phage	0
NLF012_scaffold15079_7	non-phage	0
NLF012_scaffold28161_4	non-phage	0
NLF012_scaffold39153_1	non-phage	0
NLF012_scaffold34267_2	non-phage	0
NLF012_scaffold39160_3	non-phage	0
NLF012_scaffold237_1	non-phage	0
NLF012_scaffold907_8	non-phage	0
NLF012_scaffold28122_3	non-phage	0
NLF012_scaffold119_5	non-phage	0
NLF012_scaffold30180_2	non-phage	0
NLF012_scaffold35713_1	phage	0.997
NLF012_scaffold283_6	non-phage	0
NLF012_scaffold7364_1	non-phage	0
NLF012_scaffold8220_10	non-phage	0
NLF012_scaffold15048_2	non-phage	0
NLF012_scaffold14414_2	non-phage	0
NLF012_scaffold9422_4	non-phage	0
NLF012_scaffold19509_1	non-phage	0
NLF012_scaffold7299_1	non-phage	0
NLF012_scaffold11591_2	non-phage	0
NLF012_scaffold34475_1	non-phage	0
NLF012_scaffold15501_4	non-phage	0
NLF012_scaffold19920_7	non-phage	0
NLF012_scaffold34327_4	non-phage	0
NLF012_scaffold26966_7	non-phage	0

NLF012_scaffold30623_3	non-phage	0
NLF012_scaffold20242_1	non-phage	0
NLF012_scaffold19753_4	non-phage	0
NLF012_scaffold32506_1	non-phage	0
NLF012_scaffold34965_1	non-phage	0
NLF012_scaffold36257_2	non-phage	0
NLF012_scaffold35993_2	non-phage	0
NLF012_scaffold9422_10	non-phage	0
NLF012_scaffold30563_1	non-phage	0
NLF012_C512381_1	non-phage	0
NLF012_scaffold25219_2	non-phage	0
NLF012_scaffold28810_4	non-phage	0
NLF012_scaffold28883_1	non-phage	0
NLF012_scaffold5271_6	non-phage	0
NLF012_scaffold26461_1	non-phage	0
NLF012_scaffold298_13	non-phage	0
NLF012_scaffold34290_1	non-phage	0
NLF012_scaffold19699_1	non-phage	0
NLF012_scaffold21540_2	non-phage	0
NLF012_scaffold38494_1	non-phage	0
NLF012_scaffold27788_3	non-phage	0
NLF012_scaffold9817_3	non-phage	0
NLF012_scaffold1959_15	non-phage	0
NLF012_scaffold30617_1	non-phage	0
NLF012_scaffold750_20	non-phage	0
NLF012_scaffold31308_3	non-phage	0
NLF012_scaffold39171_1	non-phage	0
NLF012_scaffold17565_4	non-phage	0
NLF012_scaffold972_1	non-phage	0
NLF012_scaffold26627_1	non-phage	0
NLF012_scaffold16168_18_1	non-phage	0
NLF012_scaffold1008_5	non-phage	0
NLF012_scaffold19888_13	non-phage	0
NLF012_scaffold39025_1	non-phage	0
NLF012_scaffold11786_15	non-phage	0
NLF012_scaffold29215_2	non-phage	0
NLF012_scaffold10663_6	phage	0.521
NLF012_scaffold24671_1	non-phage	0
NLF012_scaffold23537_7	non-phage	0
NLF012_scaffold34227_3	non-phage	0
NLF012_scaffold189_1	non-phage	0
NLF012_scaffold35670_2	phage	0.971
NLF012_scaffold1552_21	non-phage	0
NLF012_scaffold7_16	non-phage	0
NLF012_scaffold15197_22	phage	0.606
NLF012_scaffold26058_3	non-phage	0

NLF012_scaffold2787_21	non-phage	0
NLF012_scaffold10340_5	non-phage	0
NLF012_scaffold3809_1	non-phage	0
NLF012_scaffold10275_20	non-phage	0
NLF012_scaffold37298_1	non-phage	0
NLF012_scaffold24256_2	non-phage	0
NLF012_scaffold16888_10	non-phage	0
NLF012_scaffold27749_1	non-phage	0
NLF012_scaffold52_11	non-phage	0
NLF012_scaffold21689_1	non-phage	0
NLF012_scaffold17199_1	non-phage	0
NLF012_scaffold15197_10	non-phage	0
NLF012_scaffold39162_1	non-phage	0
NLF012_scaffold14206_5	non-phage	0
NLF012_scaffold38929_1	non-phage	0
NLF012_scaffold15576_1	non-phage	0
NLF012_scaffold6180_12	non-phage	0
NLF012_scaffold39183_1	non-phage	0
NLF012_scaffold37458_1	phage	0.997
NLF012_scaffold750_61	non-phage	0
NLF012_scaffold2787_20	non-phage	0
NLF012_scaffold34524_2	non-phage	0
NLF012_scaffold37847_1	non-phage	0
NLF012_scaffold9105_2	non-phage	0
NLF012_scaffold15197_19	non-phage	0
NLF012_scaffold39143_1	phage	0.999
NLF012_scaffold5980_2	non-phage	0
NLF012_scaffold6929_2	non-phage	0
NLF012_scaffold10655_2	non-phage	0
NLF012_scaffold37359_2	non-phage	0
NLF012_scaffold2477_1	non-phage	0
NLF012_scaffold3101_15	non-phage	0
NLF012_scaffold10340_22	non-phage	0
NLF012_scaffold8747_17	non-phage	0
NLF012_scaffold32493_7	non-phage	0
NLF012_scaffold39112_1	non-phage	0
NLF012_scaffold250_1	non-phage	0
NLF012_scaffold24036_4	non-phage	0
NLF012_scaffold38561_1	non-phage	0
NLF012_scaffold6145_2	non-phage	0
NLF012_scaffold32790_2	non-phage	0
NLF012_scaffold21224_3	non-phage	0
NLF012_scaffold6720_3	non-phage	0
NLF012_scaffold30623_2	non-phage	0
NLF012_scaffold238_4	non-phage	0
NLF012_scaffold16168_16	non-phage	0

NLF012_scaffold4760_2	non-phage	0
NLF012_scaffold16351_1	non-phage	0
NLF012_scaffold25640_2	non-phage	0
NLF012_scaffold12594_6	non-phage	0
NLF012_scaffold38430_1	non-phage	0
NLF012_scaffold33261_3	non-phage	0
NLF012_scaffold974_2	non-phage	0
NLF012_scaffold907_4	non-phage	0
NLF012_scaffold5014_2	non-phage	0
NLF012_scaffold36967_2	non-phage	0
NLF012_scaffold6247_3	non-phage	0
NLF012_scaffold479_10	non-phage	0
NLF012_scaffold33556_4	non-phage	0
NLF012_scaffold7034_4	non-phage	0
NLF012_scaffold34868_2	non-phage	0
NLF012_scaffold29701_2	non-phage	0
NLF012_scaffold24891_3	non-phage	0
NLF012_scaffold25866_2	non-phage	0
NLF012_scaffold38293_1	non-phage	0
NLF012_scaffold34831_2	non-phage	0
NLF012_scaffold52_3	non-phage	0
NLF012_scaffold1375_2	non-phage	0
NLF012_scaffold6326_8	non-phage	0
NLF012_scaffold15887_2	non-phage	0
NLF012_scaffold23537_14	non-phage	0
NLF012_scaffold21989_2	phage	0.984
NLF012_scaffold34843_2	non-phage	0
NLF012_scaffold296_8	non-phage	0
NLF012_scaffold7670_4	non-phage	0
NLF012_scaffold350_12	non-phage	0
NLF012_C511839_1	non-phage	0
NLF012_scaffold38808_1	non-phage	0
NLF012_scaffold39064_1	non-phage	0
NLF012_scaffold16908_2	non-phage	0
NLF012_scaffold22623_2	non-phage	0
NLF012_scaffold974_15	non-phage	0
NLF012_scaffold297_2	non-phage	0
NLF012_scaffold36323_1	non-phage	0
NLF012_scaffold21879_1	non-phage	0
NLF012_scaffold14481_3	phage	0.999
NLF012_scaffold24256_4	non-phage	0
NLF012_scaffold12051_13	non-phage	0
NLF012_scaffold8865_1	non-phage	0
NLF012_scaffold15197_13	non-phage	0
NLF012_scaffold283_5	non-phage	0
NLF012_scaffold1476_2	non-phage	0

NLF012_scaffold541_1	non-phage	0
NLF012_scaffold4660_10	non-phage	0
NLF012_scaffold38894_1	non-phage	0
NLF012_scaffold1959_5	non-phage	0
NLF012_scaffold28281_3	non-phage	0
NLF012_scaffold4362_3	phage	0.994
NLF012_scaffold39181_1	non-phage	0
NLF012_scaffold36643_2	non-phage	0
NLF012_scaffold10340_14	phage	0.998
NLF012_scaffold23584_5	non-phage	0
NLF012_scaffold38908_2	non-phage	0
NLF012_scaffold1150_3	non-phage	0
NLF012_scaffold21499_1	non-phage	0
NLF012_scaffold6683_1	non-phage	0
NLF012_scaffold22412_3	non-phage	0
NLF012_scaffold35219_8	non-phage	0
NLF012_scaffold3102_5	non-phage	0
NLF012_scaffold28740_1	non-phage	0
NLF012_scaffold27605_1	non-phage	0
NLF012_scaffold8732_4	non-phage	0
NLF012_scaffold20272_2	non-phage	0
NLF012_scaffold21813_1	non-phage	0
NLF012_scaffold33018_2	non-phage	0
NLF012_scaffold16808_2	non-phage	0
NLF012_scaffold38234_3	phage	0.997
NLF012_scaffold36627_4	non-phage	0
NLF012_scaffold27373_3	non-phage	0
NLF012_scaffold479_15	non-phage	0
NLF012_scaffold15033_5	non-phage	0
NLF012_scaffold21670_3	non-phage	0.282
NLF012_scaffold39125_1	non-phage	0
NLF012_scaffold38288_1	non-phage	0
NLF012_scaffold19472_1	non-phage	0
NLF012_scaffold750_55	non-phage	0
NLF012_scaffold15197_26	non-phage	0
NLF012_scaffold34267_3	non-phage	0
NLF012_scaffold16451_3	phage	0.996
NLF012_scaffold21306_3	non-phage	0
NLF012_scaffold38924_1	non-phage	0
NLF012_scaffold4660_4	non-phage	0
NLF012_scaffold13372_3	phage	0.999
NLF012_scaffold12181_17	non-phage	0
NLF012_scaffold3180_2	non-phage	0
NLF012_scaffold22698_3	non-phage	0
NLF012_scaffold37376_1	phage	0.999
NLF012_scaffold4070_5	non-phage	0

NLF012_scaffold39148_1	non-phage	0
NLF012_scaffold19920_5	non-phage	0
NLF012_scaffold38863_4	non-phage	0
NLF012_scaffold38428_1	non-phage	0
NLF012_scaffold17264_1	non-phage	0
NLF012_scaffold15106_13	non-phage	0
NLF012_scaffold24630_2	non-phage	0
NLF012_scaffold69_1	phage	0.897
NLF012_scaffold7247_9	non-phage	0
NLF012_scaffold750_6	non-phage	0
NLF012_scaffold37436_7	non-phage	0
NLF012_scaffold32505_1	non-phage	0
NLF012_scaffold2172_7	non-phage	0
NLF012_scaffold16168_9	non-phage	0
NLF012_scaffold18976_1	non-phage	0
NLF012_scaffold16230_1	non-phage	0
NLF012_scaffold189_2	non-phage	0
NLF012_scaffold16511_1	non-phage	0
NLF012_scaffold21659_2	non-phage	0
NLF012_scaffold6326_2	non-phage	0
NLF012_scaffold281_1	non-phage	0
NLF012_C512291_1	non-phage	0
NLF012_scaffold24415_1	non-phage	0
NLF012_scaffold26689_3	non-phage	0
NLF012_scaffold14206_2	phage	0.863
NLF012_scaffold30133_1	non-phage	0
NLF012_scaffold9557_5	non-phage	0
NLF012_scaffold39145_2	non-phage	0
NLF012_scaffold34262_1	non-phage	0
NLF012_scaffold3659_2	non-phage	0
NLF012_scaffold34846_3	non-phage	0
NLF012_scaffold2371_13	non-phage	0
NLF012_scaffold12958_2	non-phage	0
NLF012_scaffold23584_3	non-phage	0
NLF012_scaffold34_12	non-phage	0
NLF012_scaffold7601_4	non-phage	0
NLF012_scaffold11865_1	non-phage	0
NLF012_scaffold32390_1	non-phage	0
NLF012_scaffold36127_2	non-phage	0
NLF012_scaffold3176_1	non-phage	0
NLF012_scaffold4830_4	non-phage	0
NLF012_scaffold354_3	non-phage	0
NLF012_scaffold28532_4	non-phage	0
NLF012_scaffold12958_10	non-phage	0
NLF012_scaffold4646_6	non-phage	0
NLF012_scaffold9589_4	non-phage	0

NLF012_scaffold25176_1	non-phage	0
NLF012_scaffold8654_9	non-phage	0
NLF012_scaffold22355_2	non-phage	0
NLF012_scaffold36766_3	non-phage	0
NLF012_scaffold200_3	non-phage	0
NLF012_scaffold6555_3	non-phage	0
NLF012_scaffold3809_4	non-phage	0
NLF012_scaffold128_4	non-phage	0
NLF012_scaffold20242_2	non-phage	0
NLF012_scaffold15079_1	non-phage	0
NLF012_scaffold22936_1	non-phage	0
NLF012_scaffold3418_5	non-phage	0
NLF012_scaffold24395_1	non-phage	0
NLF012_scaffold2680_1	non-phage	0
NLF012_scaffold17431_21	phage	0.996
NLF012_scaffold5271_17	non-phage	0
NLF012_scaffold28397_4	non-phage	0
NLF012_scaffold29151_3	non-phage	0
NLF012_scaffold17901_5	non-phage	0
NLF012_scaffold14371_1	non-phage	0
NLF012_scaffold7449_12	non-phage	0
NLF012_scaffold2778_6	non-phage	0
NLF012_scaffold37316_1	non-phage	0
NLF012_scaffold31900_1	non-phage	0
NLF012_scaffold16161_2	non-phage	0
NLF012_scaffold13132_2	non-phage	0
NLF012_scaffold25918_2	non-phage	0
NLF012_scaffold972_2	non-phage	0
NLF012_scaffold37049_6	non-phage	0
NLF012_scaffold9107_2	non-phage	0
NLF012_scaffold23155_1	non-phage	0
NLF012_scaffold37854_1	non-phage	0
NLF012_scaffold18443_1	non-phage	0
NLF012_scaffold28836_1	non-phage	0
NLF012_scaffold8375_1	non-phage	0
NLF012_scaffold34589_10	non-phage	0
NLF012_scaffold5014_6	non-phage	0
NLF012_scaffold8622_3	phage	1
NLF012_scaffold10004_30	non-phage	0
NLF012_scaffold19133_11	non-phage	0
NLF012_scaffold3158_1	non-phage	0
NLF012_scaffold26987_1	non-phage	0
NLF012_scaffold38999_2	non-phage	0
NLF012_scaffold20218_1	non-phage	0
NLF012_scaffold38750_1	non-phage	0
NLF012_scaffold24277_2	non-phage	0

NLF012_scaffold33261_5	phage	0.82
NLF012_scaffold26987_4	non-phage	0
NLF012_scaffold36989_1	non-phage	0
NLF012_scaffold8509_2	non-phage	0
NLF012_scaffold15373_2	non-phage	0
NLF012_scaffold6723_6	non-phage	0
NLF012_scaffold15164_1	non-phage	0
NLF012_scaffold39120_1	non-phage	0
NLF012_scaffold7315_7	non-phage	0
NLF012_scaffold2778_7	non-phage	0
NLF012_scaffold14430_5	non-phage	0
NLF012_C512643_1	non-phage	0
NLF012_scaffold7670_29	non-phage	0
NLF012_scaffold16161_10	non-phage	0
NLF012_scaffold2808_10_2	non-phage	0
NLF012_scaffold2854_8	non-phage	0
NLF012_scaffold40_1	non-phage	0
NLF012_scaffold17199_2	non-phage	0
NLF012_scaffold27405_3	non-phage	0
NLF012_scaffold26298_2	non-phage	0
NLF012_scaffold29414_2	phage	0.999
NLF012_scaffold128_17	non-phage	0
NLF012_scaffold25494_3	non-phage	0
NLF012_scaffold2787_12	non-phage	0
NLF012_scaffold33819_3	non-phage	0.059
NLF012_scaffold7839_11	non-phage	0
NLF012_scaffold9707_3	non-phage	0
NLF012_scaffold10275_21	non-phage	0
NLF012_scaffold2172_4	non-phage	0
NLF012_C511719_1	non-phage	0
NLF012_scaffold36427_1	non-phage	0
NLF012_scaffold52_6	non-phage	0
NLF012_scaffold15106_2	non-phage	0
NLF012_scaffold21767_2	non-phage	0
NLF012_scaffold2371_15	phage	0.999
NLF012_scaffold16470_1	non-phage	0
NLF012_scaffold33334_4	non-phage	0
NLF012_scaffold14495_7	non-phage	0
NLF012_scaffold36216_1	non-phage	0
NLF012_scaffold11591_1	non-phage	0
NLF012_scaffold350_9	non-phage	0
NLF012_scaffold31308_4	non-phage	0
NLF012_scaffold7299_2	non-phage	0.188
NLF012_scaffold38462_1	non-phage	0
NLF012_scaffold37493_1	non-phage	0
NLF012_scaffold29236_1	non-phage	0

NLF012_scaffold30180_3	non-phage	0
NLF012_scaffold38696_1	non-phage	0
NLF012_scaffold83_1	non-phage	0
NLF012_scaffold6283_4	non-phage	0
NLF012_scaffold34854_6	non-phage	0
NLF012_scaffold38459_1	non-phage	0
NLF012_scaffold2486_3	non-phage	0
NLF012_scaffold21502_1	non-phage	0
NLF012_scaffold2255_5	non-phage	0
NLF012_scaffold39036_2	non-phage	0
NLF012_scaffold25918_3	non-phage	0
NLF012_scaffold22498_2	non-phage	0
NLF012_scaffold27762_4	non-phage	0
NLF012_scaffold17240_1	phage	0.999
NLF012_scaffold26646_1	non-phage	0
NLF012_scaffold11986_1	non-phage	0
NLF012_scaffold39187_1	non-phage	0
NLF012_scaffold17431_12	non-phage	0
NLF012_scaffold38981_1	non-phage	0
NLF012_scaffold9016_2	non-phage	0
NLF012_C511689_1	non-phage	0
NLF012_scaffold750_25	non-phage	0
NLF012_scaffold10663_7	non-phage	0
NLF012_scaffold11786_14	non-phage	0
NLF012_scaffold284_1	non-phage	0
NLF012_scaffold27467_3	non-phage	0
NLF012_scaffold2662_5	non-phage	0
NLF012_scaffold23629_2	non-phage	0
NLF013_scaffold30305_2	non-phage	0
NLF013_scaffold20688_5	non-phage	0
NLF013_scaffold48874_2	non-phage	0
NLF013_scaffold42205_7	non-phage	0
NLF013_scaffold37695_1	non-phage	0
NLF013_scaffold40472_5	non-phage	0
NLF013_scaffold16278_1	non-phage	0
NLF013_scaffold256_1	non-phage	0
NLF013_scaffold327_2	non-phage	0
NLF013_scaffold23543_12	non-phage	0
NLF013_scaffold20688_3	non-phage	0
NLF013_scaffold6073_3	non-phage	0
NLF013_scaffold11783_1	phage	0.92
NLF013_scaffold48332_1	non-phage	0
NLF013_scaffold48765_1	non-phage	0
NLF013_scaffold30013_1	non-phage	0
NLF013_scaffold48545_2	non-phage	0
NLF013_scaffold30386_2	non-phage	0

NLF013_scaffold33634_2	non-phage	0
NLF013_scaffold21577_5	non-phage	0
NLF013_scaffold48622_1	phage	0.999
NLF013_scaffold48869_1	non-phage	0
NLF013_scaffold43485_1	non-phage	0
NLF013_scaffold20090_10	non-phage	0
NLF013_scaffold43192_24	non-phage	0
NLF013_scaffold48798_2	non-phage	0
NLF013_scaffold26790_2	non-phage	0
NLF013_scaffold15667_17	non-phage	0.459
NLF013_scaffold26037_6	non-phage	0
NLF013_scaffold48806_2	non-phage	0
NLF013_scaffold43192_11	non-phage	0
NLF013_scaffold44450_4	non-phage	0
NLF013_scaffold40943_15	non-phage	0
NLF013_scaffold43358_1	non-phage	0
NLF013_scaffold8291_2	non-phage	0
NLF013_scaffold19278_2	non-phage	0
NLF013_scaffold20110_3	non-phage	0
NLF013_scaffold19443_1	non-phage	0
NLF013_scaffold26525_2	non-phage	0
NLF013_scaffold27842_15	non-phage	0
NLF013_scaffold47762_2	non-phage	0
NLF013_scaffold42628_2	non-phage	0
NLF013_scaffold180_1	non-phage	0
NLF013_scaffold19270_3	non-phage	0
NLF013_scaffold11031_1	phage	0.98
NLF013_scaffold67_3	non-phage	0
NLF013_scaffold15667_9	non-phage	0
NLF013_scaffold203_2	non-phage	0
NLF013_scaffold32505_2	non-phage	0
NLF013_scaffold11235_1	non-phage	0
NLF013_scaffold18949_1	non-phage	0
NLF013_scaffold9406_9	non-phage	0
NLF013_scaffold31504_10	non-phage	0
NLF013_scaffold8272_4	non-phage	0
NLF013_scaffold9702_4	non-phage	0
NLF013_scaffold109_13	non-phage	0
NLF013_scaffold48808_2	non-phage	0
NLF013_scaffold367_9	non-phage	0
NLF013_scaffold1857_10	non-phage	0
NLF013_scaffold43192_17	non-phage	0
NLF013_scaffold19933_23	non-phage	0
NLF013_scaffold43868_1	phage	0.995
NLF013_scaffold44804_6	non-phage	0
NLF013_scaffold35551_2	non-phage	0

NLF013_scaffold29146_2	non-phage	0
NLF013_scaffold69_3	non-phage	0
NLF013_scaffold22615_4	non-phage	0
NLF013_scaffold48522_1	non-phage	0
NLF013_scaffold42889_4	non-phage	0
NLF013_scaffold48788_1	non-phage	0
NLF013_scaffold28200_2	non-phage	0
NLF013_scaffold17809_6	non-phage	0
NLF013_scaffold43186_2	non-phage	0
NLF013_scaffold48240_1	non-phage	0
NLF013_scaffold36748_1	non-phage	0
NLF013_scaffold8291_1	non-phage	0
NLF013_scaffold38007_1	non-phage	0
NLF013_scaffold2043_5	non-phage	0
NLF013_scaffold39173_2	non-phage	0
NLF013_scaffold47580_4	non-phage	0
NLF013_scaffold14354_6	phage	0.999
NLF013_scaffold27101_4	non-phage	0
NLF013_scaffold30746_1	phage	0.998
NLF013_scaffold45578_1	non-phage	0
NLF013_scaffold179_1	non-phage	0
NLF013_scaffold35032_3	non-phage	0
NLF013_scaffold44302_3	non-phage	0
NLF013_scaffold8272_1	non-phage	0
NLF013_scaffold45308_1	non-phage	0
NLF013_scaffold48811_1	non-phage	0
NLF013_scaffold43054_2	non-phage	0
NLF013_scaffold28691_2	non-phage	0
NLF013_scaffold8211_3	non-phage	0
NLF013_scaffold4874_6	non-phage	0
NLF013_scaffold17930_1	non-phage	0
NLF013_scaffold1472_8	non-phage	0
NLF013_scaffold15667_6	non-phage	0
NLF013_scaffold12177_2	phage	0.517
NLF013_scaffold1671_2	non-phage	0
NLF013_scaffold35551_3	non-phage	0
NLF013_scaffold63_1	non-phage	0
NLF013_scaffold37142_1	non-phage	0
NLF013_scaffold14570_1	non-phage	0
NLF013_scaffold31650_1	non-phage	0
NLF013_scaffold4173_13	non-phage	0
NLF013_scaffold3690_3	non-phage	0
NLF013_scaffold30900_4	non-phage	0
NLF013_scaffold30660_5	non-phage	0
NLF013_scaffold43361_2	non-phage	0
NLF013_scaffold258_1	non-phage	0

NLF013_scaffold37369_4	non-phage	0
NLF013_scaffold47914_1	non-phage	0
NLF013_scaffold10053_26	non-phage	0
NLF013_scaffold48785_2	non-phage	0
NLF013_scaffold27842_9	non-phage	0
NLF013_scaffold109_4	non-phage	0
NLF013_scaffold30806_1	non-phage	0
NLF013_scaffold48267_1	non-phage	0
NLF013_scaffold25874_2	non-phage	0
NLF013_scaffold31083_1	non-phage	0
NLF013_scaffold19219_3	non-phage	0
NLF013_scaffold35209_3	non-phage	0
NLF013_scaffold4561_4	non-phage	0
NLF013_scaffold8211_2	non-phage	0
NLF013_scaffold14143_7	non-phage	0
NLF013_scaffold35856_6	non-phage	0
NLF013_scaffold17653_3	non-phage	0
NLF013_scaffold25268_5	non-phage	0
NLF013_scaffold48451_4	non-phage	0
NLF013_scaffold20486_1	non-phage	0
NLF013_scaffold18588_5	non-phage	0
NLF013_scaffold19933_20	non-phage	0
NLF013_scaffold36804_2	non-phage	0
NLF013_scaffold13902_16	non-phage	0
NLF013_scaffold67_4	non-phage	0
NLF013_scaffold42464_3	non-phage	0
NLF013_scaffold5635_12	non-phage	0
NLF013_scaffold30706_3	non-phage	0
NLF013_scaffold25948_1	non-phage	0
NLF013_scaffold48807_1	non-phage	0
NLF013_scaffold20090_5	non-phage	0
NLF013_scaffold48818_2	non-phage	0
NLF013_scaffold26855_1	non-phage	0
NLF013_scaffold47373_2	non-phage	0
NLF013_scaffold47787_2	non-phage	0
NLF013_C659127_1	phage	0.999
NLF013_scaffold26229_1	non-phage	0
NLF013_scaffold2043_12	non-phage	0
NLF013_scaffold48672_1	non-phage	0
NLF013_scaffold31083_3	non-phage	0
NLF013_C659355_1	non-phage	0
NLF013_scaffold48068_2	non-phage	0
NLF013_scaffold12494_5	non-phage	0
NLF013_scaffold15694_7	non-phage	0
NLF013_scaffold29064_2	non-phage	0
NLF013_scaffold48768_1	non-phage	0

NLF013_scaffold35135_4	non-phage	0
NLF013_scaffold1472_9	non-phage	0
NLF013_scaffold7506_3	non-phage	0
NLF013_scaffold39192_1	non-phage	0
NLF013_scaffold179_2	non-phage	0
NLF013_scaffold36281_1	non-phage	0
NLF013_scaffold16278_2	non-phage	0
NLF013_scaffold12375_2	non-phage	0
NLF013_scaffold33257_2	non-phage	0
NLF013_scaffold46702_2	non-phage	0
NLF013_scaffold26790_1	non-phage	0
NLF013_scaffold18704_4	non-phage	0
NLF013_scaffold31504_3	non-phage	0
NLF013_scaffold18588_10	non-phage	0
NLF013_scaffold5635_5	non-phage	0
NLF013_scaffold1857_4	non-phage	0
NLF013_scaffold28098_6	non-phage	0
NLF013_scaffold48836_1	non-phage	0
NLF013_scaffold31650_2	non-phage	0
NLF013_scaffold34744_1	phage	0.958
NLF013_scaffold111_8	non-phage	0
NLF013_scaffold328_1	phage	0.996
NLF013_scaffold26185_3	non-phage	0
NLF013_scaffold48672_2	non-phage	0
NLF013_scaffold23493_1	non-phage	0
NLF013_scaffold36281_3	non-phage	0
NLF013_scaffold33867_2	non-phage	0
NLF013_scaffold24518_2	non-phage	0
NLF013_scaffold109_3	non-phage	0
NLF013_scaffold15667_1	non-phage	0
NLF013_scaffold1671_7	non-phage	0
NLF013_scaffold41429_4	non-phage	0
NLF013_scaffold53_1	non-phage	0
NLF013_scaffold30491_5	non-phage	0
NLF013_scaffold24841_6	non-phage	0
NLF013_scaffold111_11	non-phage	0
NLF013_scaffold17012_1	non-phage	0
NLF013_scaffold22591_1	non-phage	0
NLF013_scaffold273_11	non-phage	0
NLF013_scaffold29131_2	non-phage	0
NLF013_scaffold48405_1	non-phage	0
NLF013_scaffold31409_3	non-phage	0
NLF013_scaffold109_12	non-phage	0
NLF013_scaffold40991_1	non-phage	0
NLF013_scaffold33849_1	phage	0.999
NLF013_scaffold48692_3	non-phage	0

NLF013_scaffold40943_18	non-phage	0
NLF013_scaffold48313_1	non-phage	0
NLF013_scaffold48688_2	non-phage	0
NLF013_scaffold19933_13	non-phage	0
NLF013_scaffold43192_10	non-phage	0
NLF013_scaffold43678_1	non-phage	0
NLF013_scaffold9310_1	non-phage	0
NLF013_scaffold43192_6	non-phage	0
NLF013_scaffold39898_1	non-phage	0
NLF013_scaffold267_4	non-phage	0
NLF013_scaffold48873_1	non-phage	0
NLF013_scaffold46506_1	non-phage	0
NLF013_scaffold48858_1	non-phage	0
NLF013_scaffold17717_1	non-phage	0
NLF013_scaffold327_4	non-phage	0
NLF013_scaffold8039_2	non-phage	0
NLF013_scaffold15696_1	non-phage	0
NLF013_scaffold3922_6	non-phage	0
NLF013_scaffold34354_2	non-phage	0
NLF013_scaffold48750_1	non-phage	0
NLF013_scaffold32342_4	non-phage	0
NLF013_scaffold25322_16	non-phage	0
NLF013_scaffold4636_6	non-phage	0
NLF013_scaffold1030_1	non-phage	0
NLF013_scaffold48845_1	non-phage	0
NLF013_scaffold32585_1	non-phage	0
NLF013_scaffold40943_14	non-phage	0
NLF013_scaffold48798_4	non-phage	0
NLF013_scaffold6494_6	non-phage	0
NLF013_scaffold34385_6	non-phage	0
NLF013_scaffold9406_3	non-phage	0
NLF013_scaffold5034_1	non-phage	0
NLF013_scaffold5635_8	non-phage	0
NLF013_scaffold12239_1	non-phage	0
NLF013_scaffold45794_7	non-phage	0
NLF013_scaffold14068_1	non-phage	0
NLF013_scaffold41354_4	non-phage	0
NLF013_C659033_1	non-phage	0
NLF013_scaffold15128_1	non-phage	0
NLF013_scaffold39011_1	non-phage	0
NLF013_scaffold7410_2	non-phage	0
NLF013_scaffold12168_6	non-phage	0
NLF013_scaffold7701_6	non-phage	0
NLF013_scaffold7750_6	non-phage	0
NLF013_scaffold2043_28	non-phage	0
NLF013_scaffold15458_2	non-phage	0

NLF013_scaffold43248_2	non-phage	0
NLF013_scaffold68_1	non-phage	0
NLF013_scaffold25753_3	non-phage	0
NLF013_scaffold48674_1	non-phage	0
NLF013_scaffold273_2	non-phage	0
NLF013_scaffold1671_3	non-phage	0
NLF013_scaffold21790_2	non-phage	0
NLF013_scaffold36275_1	non-phage	0
NLF013_scaffold166_1	non-phage	0
NLF013_scaffold39130_1	non-phage	0
NLF013_scaffold32342_7	non-phage	0
NLF013_scaffold4636_5	non-phage	0
NLF013_scaffold28200_4	non-phage	0
NLF013_scaffold13950_2	non-phage	0
NLF013_scaffold48711_1	non-phage	0
NLF013_scaffold3064_5	non-phage	0
NLF013_scaffold45836_1	non-phage	0
NLF013_scaffold130_5	non-phage	0
NLF013_scaffold48267_3	non-phage	0
NLF013_scaffold45794_10	non-phage	0
NLF013_scaffold27155_1	non-phage	0
NLF013_scaffold47787_3	non-phage	0
NLF013_scaffold3923_2	non-phage	0
NLF013_scaffold31776_1	non-phage	0
NLF013_scaffold395_3	non-phage	0
NLF013_scaffold47404_1	non-phage	0
NLF013_scaffold48830_2	non-phage	0
NLF013_scaffold3690_2	non-phage	0
NLF013_scaffold36765_1	non-phage	0
NLF013_scaffold23543_4	phage	0.998
NLF013_scaffold43578_2	non-phage	0
NLF013_scaffold39574_5	non-phage	0
NLF013_scaffold35391_3	non-phage	0
NLF013_scaffold12656_3	non-phage	0
NLF013_scaffold45154_4	non-phage	0.491
NLF013_scaffold42889_7	non-phage	0
NLF013_scaffold21577_3	non-phage	0
NLF013_scaffold27299_6	non-phage	0
NLF013_scaffold19270_4	phage	0.998
NLF013_scaffold26037_3	non-phage	0
NLF013_scaffold1727_14	non-phage	0
NLF013_scaffold47100_1	non-phage	0
NLF013_scaffold43192_26	non-phage	0
NLF013_scaffold8530_2	non-phage	0
NLF013_scaffold44302_1	non-phage	0
NLF013_scaffold34358_2	non-phage	0

NLF013_scaffold35887_1	non-phage	0
NLF013_scaffold33066_2	non-phage	0
NLF013_scaffold4799_2	phage	0.999
NLF013_scaffold15667_19	non-phage	0
NLF013_scaffold43192_7	non-phage	0
NLF013_C658587_1	non-phage	0.458
NLF013_scaffold43361_4	non-phage	0
NLF013_C659465_1	non-phage	0
NLF013_scaffold109_11	non-phage	0
NLF013_scaffold67_1	non-phage	0
NLF013_scaffold27842_4	non-phage	0
NLF013_scaffold10491_3	non-phage	0
NLF013_scaffold1671_4	non-phage	0
NLF013_scaffold47514_1	non-phage	0
NLF013_scaffold345_1	non-phage	0
NLF013_scaffold33849_2	non-phage	0
NLF013_scaffold47834_1	non-phage	0
NLF013_scaffold38476_3	non-phage	0.004
NLF013_scaffold40233_1	non-phage	0
NLF013_scaffold11031_3	non-phage	0
NLF013_scaffold11783_7	non-phage	0
NLF013_scaffold5447_3	non-phage	0
NLF013_scaffold30900_2	non-phage	0
NLF013_scaffold48345_4	non-phage	0
NLF013_scaffold1896_2	non-phage	0
NLF013_scaffold1472_1	non-phage	0
NLF013_scaffold32744_2	non-phage	0
NLF013_scaffold45698_2	non-phage	0
NLF013_scaffold38582_1	non-phage	0
NLF013_scaffold28328_4	non-phage	0
NLF013_scaffold46_2	non-phage	0
NLF013_scaffold48790_1	non-phage	0
NLF013_scaffold10845_4	non-phage	0
NLF013_scaffold109_2	non-phage	0
NLF013_scaffold48512_2	non-phage	0
NLF013_scaffold48872_1	non-phage	0
NLF013_C659343_1	non-phage	0
NLF013_scaffold48799_1	non-phage	0
NLF013_scaffold48633_1	non-phage	0
NLF013_scaffold48871_1	non-phage	0
NLF013_scaffold2043_2	non-phage	0
NLF013_scaffold35856_1	non-phage	0
NLF013_scaffold9575_15	non-phage	0
NLF013_scaffold29029_1	non-phage	0
NLF013_scaffold14960_4	non-phage	0
NLF013_scaffold25755_2	non-phage	0

NLF013_scaffold6154_5	non-phage	0
NLF013_scaffold20110_2	non-phage	0
NLF013_scaffold8427_5	non-phage	0
NLF013_scaffold47787_8	non-phage	0
NLF013_scaffold2881_2	non-phage	0
NLF013_scaffold4874_8	non-phage	0
NLF013_scaffold31255_3	non-phage	0
NLF013_scaffold41441_1	non-phage	0
NLF013_scaffold48860_2	non-phage	0
NLF013_scaffold21144_3	non-phage	0
NLF013_scaffold36953_1	non-phage	0
NLF013_scaffold558_16	non-phage	0
NLF013_scaffold8039_1	non-phage	0
NLF013_scaffold32505_3	non-phage	0
NLF013_scaffold9814_6	non-phage	0
NLF013_scaffold20327_1	non-phage	0
NLF013_scaffold44405_3	phage	0.989
NLF013_C659441_1	non-phage	0
NLF013_C659221_1	non-phage	0
NLF013_scaffold3884_1	non-phage	0
NLF013_scaffold47100_2	non-phage	0
NLF013_scaffold12239_2	non-phage	0
NLF013_scaffold26037_4	non-phage	0
NLF013_scaffold21626_1	non-phage	0
NLF013_scaffold41335_2	non-phage	0
NLF013_scaffold45474_1	non-phage	0
NLF013_scaffold19771_6	non-phage	0
NLF013_scaffold48785_1	non-phage	0
NLF013_scaffold48460_1	non-phage	0
NLF013_scaffold17653_2	non-phage	0
NLF013_scaffold9406_14	non-phage	0
NLF013_scaffold48452_1	non-phage	0
NLF013_scaffold45794_8	non-phage	0
NLF013_scaffold39690_2	non-phage	0
NLF013_scaffold17809_1	non-phage	0
NLF013_scaffold25855_2	non-phage	0
NLF013_scaffold48843_2	non-phage	0
NLF013_scaffold6081_4	non-phage	0
NLF013_scaffold215_3	non-phage	0
NLF013_scaffold20110_4	non-phage	0
NLF013_scaffold109_1	non-phage	0
NLF013_scaffold8211_1	non-phage	0
NLF013_scaffold111_1	non-phage	0
NLF013_scaffold48449_1	non-phage	0
NLF013_scaffold386_3	non-phage	0.003
NLF013_scaffold5102_5	non-phage	0

NLF013_scaffold988_18	non-phage	0
NLF013_scaffold15667_14	non-phage	0
NLF013_scaffold9575_16	non-phage	0
NLF013_scaffold40523_1	non-phage	0
NLF013_scaffold4117_2	non-phage	0
NLF013_scaffold53_3	non-phage	0
NLF013_scaffold9406_1	non-phage	0
NLF013_scaffold2881_1	non-phage	0
NLF013_scaffold180_2	non-phage	0
NLF013_scaffold2043_20	non-phage	0
NLF013_scaffold22764_1	non-phage	0
NLF013_scaffold27057_1	non-phage	0
NLF013_scaffold30660_2	non-phage	0
NLF013_scaffold18303_1	non-phage	0
NLF013_scaffold4597_1	non-phage	0
NLF013_scaffold40472_1	non-phage	0
NLF013_scaffold4770_11	non-phage	0
NLF013_scaffold273_13	non-phage	0
NLF013_scaffold166_2	non-phage	0
NLF013_scaffold44450_5	non-phage	0
NLF013_scaffold48866_1	non-phage	0
NLF013_scaffold32342_8	non-phage	0
NLF013_scaffold39651_4	non-phage	0
NLF013_scaffold15694_14	non-phage	0
NLF013_scaffold20110_5	non-phage	0
NLF013_scaffold48345_6	non-phage	0
NLF013_scaffold9268_17	non-phage	0
NLF013_scaffold41335_4	non-phage	0
NLF013_scaffold24608_2	non-phage	0
NLF013_scaffold42205_1	non-phage	0
NLF013_scaffold32342_11	phage	0.991
NLF013_scaffold2043_14	non-phage	0
NLF013_scaffold48859_1	non-phage	0
NLF013_scaffold352_2	non-phage	0
NLF013_scaffold30835_1	non-phage	0
NLF013_scaffold32431_2	non-phage	0
NLF013_scaffold29083_4	non-phage	0
NLF013_scaffold47264_1	phage	0.999
NLF013_scaffold42889_5	non-phage	0
NLF013_scaffold48758_3	non-phage	0
NLF013_scaffold48336_1	non-phage	0
NLF013_scaffold111_12	non-phage	0
NLF013_scaffold7750_7	non-phage	0
NLF013_scaffold32505_1	non-phage	0
NLF013_scaffold42006_1	non-phage	0
NLF013_scaffold11235_2	non-phage	0

NLF013_scaffold38018_1	non-phage	0
NLF013_scaffold10496_1	non-phage	0
NLF013_scaffold558_4	non-phage	0
NLF013_scaffold19219_2	non-phage	0
NLF013_scaffold34522_1	non-phage	0
NLF013_scaffold22430_1	non-phage	0
NLF013_scaffold19933_21	non-phage	0
NLF013_scaffold30176_2	non-phage	0
NLF013_scaffold36747_1	non-phage	0
NLF013_scaffold28_1	non-phage	0
NLF013_scaffold48837_1	non-phage	0
NLF013_scaffold27766_1	non-phage	0
NLF013_scaffold17717_7	non-phage	0
NLF013_scaffold18704_5	non-phage	0
NLF013_scaffold27702_4	non-phage	0
NLF013_scaffold27965_1	non-phage	0
NLF013_scaffold21580_6	non-phage	0
NLF013_scaffold35551_1	non-phage	0
NLF013_scaffold558_17	non-phage	0
NLF013_scaffold7270_17	non-phage	0
NLF013_scaffold47787_7	non-phage	0
NLF013_scaffold12864_5	non-phage	0
NLF013_scaffold7718_13	non-phage	0
NLF013_scaffold20416_2	non-phage	0
NLF013_scaffold35209_2	non-phage	0
NLF013_scaffold44302_2	non-phage	0
NLF013_scaffold146_6	non-phage	0
NLF013_scaffold48680_1	non-phage	0
NLF013_C659763_1	non-phage	0
NLF013_scaffold10031_4	non-phage	0
NLF013_scaffold13819_1	non-phage	0
NLF013_scaffold28200_13	non-phage	0
NLF013_scaffold332_1	non-phage	0
NLF013_scaffold130_3	non-phage	0
NLF013_scaffold8272_2	non-phage	0
NLF013_C659831_1	non-phage	0
NLF013_scaffold48806_1	non-phage	0
NLF013_scaffold48186_1	non-phage	0
NLF013_scaffold10681_2	non-phage	0
NLF013_C659281_1	non-phage	0
NLF013_scaffold1420_99	non-phage	0
NLF013_scaffold27702_15	non-phage	0
NLF013_scaffold395_4	non-phage	0
NLF013_scaffold48711_2	non-phage	0
NLF013_scaffold36696_1	non-phage	0
NLF013_scaffold15895_11	non-phage	0

NLF013_scaffold48831_1	non-phage	0
NLF013_scaffold2043_29	non-phage	0
NLF013_scaffold3923_27	non-phage	0.003
NLF013_scaffold20286_2	non-phage	0
NLF013_scaffold20327_11	non-phage	0
NLF013_scaffold11031_2	non-phage	0
NLF013_scaffold22949_4	non-phage	0
NLF013_scaffold48312_1	non-phage	0
NLF013_scaffold17717_3	non-phage	0
NLF013_scaffold25679_2	non-phage	0
NLF013_scaffold48458_2	non-phage	0
NLF013_scaffold31895_1	non-phage	0
NLF013_scaffold19615_1	non-phage	0
NLF013_scaffold1161_1	non-phage	0
NLF013_scaffold4561_27	non-phage	0
NLF013_scaffold42151_4	non-phage	0
NLF013_scaffold45596_1	non-phage	0
NLF013_scaffold48428_6	non-phage	0
NLF013_scaffold256_7	non-phage	0
NLF013_scaffold47533_3	non-phage	0
NLF013_scaffold11243_2	non-phage	0
NLF013_scaffold48102_1	non-phage	0
NLF013_scaffold1447_6	non-phage	0
NLF013_scaffold27842_6	non-phage	0
NLF013_scaffold5876_1	non-phage	0
NLF013_scaffold19575_2	non-phage	0
NLF013_scaffold48838_1	non-phage	0
NLF013_scaffold22133_5	non-phage	0
NLF013_scaffold23252_1	non-phage	0
NLF013_scaffold26366_1	non-phage	0
NLF013_scaffold48828_1	non-phage	0
NLF013_scaffold17658_16	non-phage	0
NLF013_scaffold48849_1	non-phage	0
NLF013_scaffold37196_2	non-phage	0
NLF013_scaffold40523_2	non-phage	0
NLF013_scaffold7750_4	non-phage	0
NLF013_scaffold48093_1	non-phage	0
NLF013_scaffold33180_2	non-phage	0
NLF013_scaffold20188_4	non-phage	0.353
NLF013_scaffold43420_2	non-phage	0
NLF013_scaffold268_2	non-phage	0
NLF013_scaffold27842_11	non-phage	0
NLF013_scaffold27766_3	non-phage	0
NLF013_scaffold22426_2	non-phage	0
NLF013_scaffold30660_3	non-phage	0
NLF013_scaffold48180_1	non-phage	0

NLF013_scaffold34979_1	non-phage	0
NLF013_scaffold24111_11	non-phage	0
NLF013_scaffold1671_6	non-phage	0
NLF013_scaffold39574_3	non-phage	0
NLF013_scaffold36275_3	non-phage	0
NLF013_scaffold46469_1	non-phage	0
NLF013_scaffold32342_5	non-phage	0
NLF013_scaffold37779_1	non-phage	0
NLF013_scaffold48104_1	non-phage	0
NLF013_scaffold3706_4	non-phage	0
NLF013_scaffold1472_3	non-phage	0
NLF013_scaffold7506_2	non-phage	0
NLF013_scaffold33867_1	non-phage	0
NLF013_scaffold48662_1	non-phage	0
NLF013_scaffold28488_2	non-phage	0
NLF013_scaffold25855_1	non-phage	0
NLF013_scaffold12866_3	non-phage	0
NLF013_scaffold43678_2	non-phage	0
NLF013_scaffold47221_1	non-phage	0
NLF013_scaffold43054_3	non-phage	0
NLF013_scaffold27299_7	non-phage	0
NLF013_scaffold3064_1	non-phage	0
NLF013_scaffold48826_1	non-phage	0
NLF013_scaffold1420_60	non-phage	0
NLF013_scaffold48847_1	non-phage	0
NLF013_scaffold36890_2	non-phage	0
NLF013_scaffold375_4	non-phage	0
NLF013_scaffold33946_4	phage	1
NLF013_scaffold4874_2	non-phage	0
NLF013_scaffold42627_5	non-phage	0
NLF013_scaffold46830_1	non-phage	0
NLF013_scaffold34358_1	non-phage	0
NLF013_scaffold40943_5	non-phage	0
NLF013_scaffold48514_1	non-phage	0
NLF013_scaffold332_3	non-phage	0
NLF013_scaffold111_10	non-phage	0
NLF013_scaffold7048_1	non-phage	0
NLF013_scaffold48552_3	non-phage	0
NLF013_scaffold48741_1	non-phage	0
NLF013_scaffold48143_1	non-phage	0
NLF013_scaffold41994_4	non-phage	0
NLF013_scaffold30761_1	non-phage	0
NLF013_scaffold19726_2	non-phage	0
NLF013_scaffold47107_1	non-phage	0
NLF013_scaffold10053_29	non-phage	0
NLF013_scaffold15694_3	non-phage	0

NLF013_C659075_1	non-phage	0
NLF013_scaffold48739_1	non-phage	0
NLF013_scaffold4834_4	non-phage	0
NLF013_scaffold36927_1	non-phage	0
NLF013_scaffold396_1	non-phage	0
NLF013_scaffold42627_3	non-phage	0
NLF013_scaffold26037_2	non-phage	0
NLF013_scaffold27842_2	non-phage	0
NLF013_scaffold15694_15	non-phage	0
NLF013_scaffold2103_5	non-phage	0
NLF013_scaffold1000_2	non-phage	0
NLF013_scaffold24705_1	non-phage	0
NLF013_scaffold1668_4	non-phage	0
NLF013_scaffold150_1	non-phage	0
NLF013_scaffold28098_2	non-phage	0
NLF013_scaffold111_4	non-phage	0
NLF013_scaffold9350_1	non-phage	0
NLF013_scaffold28200_9	non-phage	0
NLF013_C659719_1	non-phage	0
NLF013_scaffold48852_1	non-phage	0
NLF013_scaffold28488_1	non-phage	0
NLF013_scaffold29776_1	non-phage	0
NLF013_scaffold47616_1	non-phage	0
NLF013_scaffold27299_3	non-phage	0
NLF013_scaffold25322_17	non-phage	0
NLF013_scaffold15366_3	non-phage	0
NLF013_scaffold5635_29	non-phage	0
NLF013_scaffold4561_2	non-phage	0
NLF013_scaffold48428_5	non-phage	0
NLF013_scaffold48572_3	non-phage	0
NLF013_scaffold21580_7	non-phage	0
NLF013_scaffold4240_3	non-phage	0
NLF013_scaffold40943_9	non-phage	0
NLF013_scaffold48783_1	non-phage	0
NLF013_scaffold48528_1	non-phage	0
NLF013_scaffold22552_3	non-phage	0
NLF013_scaffold34385_5	non-phage	0
NLF013_scaffold48796_1	non-phage	0
NLF013_scaffold1931_1	non-phage	0
NLF013_scaffold13962_5	non-phage	0
NLF013_scaffold23156_8	non-phage	0
NLF013_scaffold44450_3	non-phage	0
NLF013_scaffold5635_19	non-phage	0
NLF013_scaffold273_23	non-phage	0
NLF013_scaffold386_1	non-phage	0
NLF013_scaffold327_1	non-phage	0

NLF013_scaffold24111_9	non-phage	0
NLF013_C658603_1	non-phage	0
NLF013_scaffold48767_1	non-phage	0
NLF013_scaffold27842_21	non-phage	0
NLF013_scaffold43732_1	non-phage	0
NLF013_scaffold38007_3	non-phage	0
NLF013_scaffold30305_1	non-phage	0
NLF013_scaffold86_1	non-phage	0
NLF013_scaffold42205_4	non-phage	0
NLF013_scaffold28200_14	non-phage	0
NLF013_scaffold43192_16	non-phage	0
NLF013_scaffold27712_1	non-phage	0
NLF013_scaffold32199_1	non-phage	0
NLF013_scaffold34385_3	non-phage	0
NLF013_scaffold23543_5	non-phage	0
NLF013_scaffold25755_1	phage	0.87
NLF013_scaffold47820_2	non-phage	0
NLF013_scaffold29146_3	non-phage	0
NLF013_scaffold48518_1	non-phage	0
NLF013_scaffold345_2	non-phage	0
NLF013_scaffold26651_1	non-phage	0
NLF013_scaffold26525_1	non-phage	0
NLF013_scaffold21818_1	non-phage	0
NLF013_scaffold27299_5	non-phage	0
NLF013_scaffold7214_8	non-phage	0
NLF013_scaffold14143_3	non-phage	0
NLF013_scaffold121_1	non-phage	0
NLF013_scaffold48867_1	non-phage	0
NLF013_scaffold47787_4	non-phage	0
NLF013_scaffold42628_1	non-phage	0
NLF013_scaffold11031_7	phage	0.999
NLF013_scaffold47184_2	non-phage	0
NLF013_scaffold43192_3	non-phage	0
NLF013_scaffold4117_6	non-phage	0
NLF013_scaffold27842_3	non-phage	0
NLF013_scaffold36890_1	non-phage	0
NLF013_scaffold21878_2	phage	0.998
NLF013_scaffold27842_14	non-phage	0
NLF013_scaffold4173_33	non-phage	0
NLF013_scaffold17658_7	non-phage	0
NLF013_scaffold42565_4	non-phage	0
NLF013_scaffold45055_2	non-phage	0
NLF013_scaffold1198_1	non-phage	0
NLF013_scaffold5221_17	non-phage	0
NLF013_scaffold4561_28	phage	0.997
NLF013_scaffold4117_4	non-phage	0

NLF013_scaffold39584_2	non-phage	0
NLF013_scaffold35334_1	non-phage	0
NLF013_scaffold48822_2	non-phage	0
NLF013_scaffold3328_3	non-phage	0
NLF013_scaffold17658_18	non-phage	0
NLF013_scaffold395_1	non-phage	0
NLF013_scaffold12583_1	non-phage	0
NLF013_scaffold16097_10	non-phage	0
NLF013_scaffold9583_1	non-phage	0
NLF013_scaffold48512_3	non-phage	0
NLF013_scaffold48731_1	non-phage	0
NLF013_scaffold4173_27	non-phage	0
NLF013_scaffold25322_2	non-phage	0
NLF013_scaffold48851_1	non-phage	0
NLF013_scaffold41006_1	non-phage	0
NLF013_scaffold32342_6	non-phage	0
NLF013_scaffold44450_2	non-phage	0
NLF013_scaffold18893_1	non-phage	0
NLF013_scaffold28656_2	non-phage	0
NLF013_scaffold2103_6	non-phage	0
NLF013_scaffold48860_1	non-phage	0
NLF013_scaffold8291_6	non-phage	0
NLF013_scaffold15694_16	non-phage	0
NLF013_scaffold48827_1	non-phage	0
NLF013_scaffold19219_4	non-phage	0
NLF013_scaffold47446_2	non-phage	0
NLF013_scaffold48861_1	non-phage	0
NLF013_scaffold268_1	non-phage	0
NLF013_scaffold47373_6	non-phage	0
NLF013_scaffold7347_12	non-phage	0
NLF013_scaffold28200_1	non-phage	0
NLF013_scaffold3922_3	non-phage	0
NLF013_scaffold48730_1	non-phage	0
NLF013_scaffold43115_2	non-phage	0
NLF013_scaffold45698_1	non-phage	0
NLF013_scaffold5447_4	non-phage	0
NLF013_scaffold32505_4	non-phage	0
NLF013_scaffold39651_7	non-phage	0
NLF013_scaffold5102_4	non-phage	0
NLF013_scaffold39613_2	non-phage	0
NLF013_scaffold45167_3	non-phage	0
NLF013_scaffold48854_1	non-phage	0
NLF013_scaffold233_39	non-phage	0
NLF013_scaffold41335_3	non-phage	0
NLF013_scaffold3923_3	non-phage	0
NLF013_scaffold8984_5	non-phage	0.034

NLF013_scaffold38036_1	non-phage	0
NLF013_scaffold48800_1	non-phage	0
NLF013_scaffold23156_2	phage	0.992
NLF013_scaffold14570_2	non-phage	0
NLF013_scaffold33180_1	non-phage	0
NLF013_scaffold48809_1	non-phage	0
NLF013_scaffold30660_6	non-phage	0
NLF013_scaffold48863_2	non-phage	0
NLF013_scaffold32949_10	non-phage	0
NLF013_scaffold48243_1	non-phage	0
NLF013_scaffold28328_3	non-phage	0
NLF013_scaffold48370_3	non-phage	0
NLF013_scaffold20327_20	non-phage	0
NLF013_scaffold26465_7	non-phage	0
NLF013_scaffold48864_1	non-phage	0
NLF013_scaffold31385_1	non-phage	0
NLF013_scaffold15334_9	non-phage	0
NLF013_scaffold5216_1	non-phage	0
NLF013_scaffold47787_1	non-phage	0
NLF013_scaffold111_2	non-phage	0
NLF013_scaffold24673_2	non-phage	0
NLF013_scaffold15667_15	non-phage	0
NLF013_scaffold30660_7	phage	0.998
NLF013_scaffold43115_1	non-phage	0
NLF013_scaffold20188_2	non-phage	0
NLF013_scaffold38953_3	non-phage	0
NLF013_scaffold27842_18	non-phage	0
NLF013_scaffold1671_8	non-phage	0
NLF013_scaffold42565_3	non-phage	0
NLF013_scaffold67_5	non-phage	0
NLF013_scaffold30660_1	non-phage	0
NLF013_scaffold31776_3	non-phage	0
NLF013_scaffold45698_6	non-phage	0
NLF013_scaffold19270_1	non-phage	0
NLF013_scaffold12866_4	non-phage	0
NLF013_scaffold19771_7	non-phage	0
NLF013_scaffold20562_1	non-phage	0
NLF013_scaffold48526_2	non-phage	0
NLF013_scaffold3706_2	non-phage	0
NLF013_scaffold39574_2	non-phage	0
NLF013_scaffold2529_1	non-phage	0
NLF013_scaffold28943_5	non-phage	0
NLF013_scaffold674_3	non-phage	0
NLF013_scaffold15819_6	non-phage	0
NLF013_scaffold18704_2	non-phage	0
NLF013_scaffold22343_3	non-phage	0

NLF013_scaffold25855_3	non-phage	0
NLF013_scaffold9575_10	non-phage	0
NLF013_scaffold190_1	non-phage	0
NLF013_C659335_1	non-phage	0
NLF013_scaffold48868_1	non-phage	0
NLF013_scaffold1668_2	non-phage	0
NLF013_scaffold109_9	non-phage	0
NLF013_scaffold43587_1	non-phage	0
NLF013_scaffold30135_1	non-phage	0
NLF013_scaffold43192_4	non-phage	0
NLF013_scaffold2043_13	non-phage	0
NLF013_scaffold11031_6	non-phage	0
NLF013_scaffold8291_7	non-phage	0
NLF013_scaffold30013_3	non-phage	0
NLF013_scaffold24953_1	non-phage	0
NLF013_scaffold48692_1	non-phage	0
NLF013_scaffold14082_2	non-phage	0
NLF013_scaffold42889_6	non-phage	0
NLF013_scaffold48407_3	non-phage	0
NLF013_scaffold10053_21	non-phage	0
NLF013_scaffold37657_1	non-phage	0
NLF013_scaffold44034_1	non-phage	0
NLF013_scaffold5221_18	non-phage	0
NLF013_scaffold40472_2	non-phage	0
NLF013_scaffold16826_1	non-phage	0
NLF013_scaffold48743_1	non-phage	0
NLF013_scaffold1857_2	non-phage	0
NLF013_scaffold30884_4	non-phage	0
NLF013_scaffold39856_1	non-phage	0
NLF013_scaffold8765_1	non-phage	0
NLF013_scaffold42889_1	non-phage	0
NLF013_scaffold1727_6	non-phage	0
NLF013_C659857_1	phage	1
NLF013_scaffold4092_9	non-phage	0
NLF013_scaffold42516_1	non-phage	0
NLF013_scaffold28656_4	non-phage	0
NLF013_scaffold17012_2	non-phage	0
NLF013_scaffold7976_18	non-phage	0
NLF013_scaffold17717_8	non-phage	0
NLF013_scaffold20413_4	phage	0.999
NLF013_scaffold27702_5	non-phage	0
NLF013_scaffold48123_1	non-phage	0
NLF013_scaffold42565_2	non-phage	0
NLF013_scaffold150_13	non-phage	0
NLF013_scaffold3922_4	non-phage	0
NLF013_scaffold22026_1	non-phage	0

NLF013_scaffold111_5	non-phage	0
NLF013_scaffold37369_6	non-phage	0
NLF013_scaffold17919_5	non-phage	0
NLF013_scaffold48856_1	non-phage	0
NLF013_scaffold48690_1	non-phage	0
NLF013_scaffold32585_2	non-phage	0
NLF013_scaffold47624_1	non-phage	0
NLF013_scaffold1214_3	non-phage	0
NLF013_scaffold13339_1	non-phage	0
NLF013_scaffold41155_1	non-phage	0
NLF013_scaffold33867_3	non-phage	0
NLF013_scaffold41982_6	non-phage	0
NLF013_scaffold48853_1	non-phage	0
NLF013_scaffold29169_2	non-phage	0
NLF013_scaffold48862_2	non-phage	0
NLF013_scaffold28200_8	non-phage	0
NLF013_scaffold40182_1	non-phage	0
NLF013_scaffold48801_1	non-phage	0
NLF013_scaffold367_2	non-phage	0
NLF013_scaffold22507_3	non-phage	0.002
NLF013_scaffold48824_1	non-phage	0
NLF013_scaffold40561_1	non-phage	0
NLF013_scaffold47688_5	non-phage	0
NLF013_scaffold5635_27	non-phage	0
NLF013_scaffold19359_1	phage	0.993
NLF013_scaffold28495_2	non-phage	0
NLF013_scaffold25126_1	non-phage	0
NLF013_scaffold20327_16	non-phage	0
NLF013_scaffold17717_5	non-phage	0
NLF013_scaffold21113_3	non-phage	0
NLF013_scaffold27702_16	non-phage	0
NLF013_scaffold20411_3	non-phage	0.008
NLF013_scaffold30305_4	non-phage	0
NLF013_scaffold28495_1	non-phage	0
NLF013_scaffold48857_1	non-phage	0
NLF013_scaffold11031_5	non-phage	0
NLF013_scaffold2849_15	non-phage	0
NLF013_scaffold26525_4	non-phage	0
NLF013_scaffold2043_37	non-phage	0
NLF013_C659717_1	non-phage	0
NLF013_scaffold30491_6	non-phage	0
NLF013_scaffold3923_28	non-phage	0
NLF013_scaffold14354_8	non-phage	0
NLF013_scaffold42627_1	non-phage	0
NLF013_scaffold27093_2	non-phage	0
NLF013_scaffold1472_6	non-phage	0

NLF013_scaffold19933_14	non-phage	0
NLF013_C658539_1	non-phage	0
NLF013_scaffold8427_10	non-phage	0
NLF013_C658725_1	non-phage	0
NLF013_scaffold41982_10	non-phage	0
NLF013_scaffold26465_8	non-phage	0
NLF013_scaffold48784_1	non-phage	0
NLF013_scaffold48798_3	non-phage	0
NLF013_scaffold28977_6	non-phage	0
NLF013_scaffold1727_23	non-phage	0
NLF013_scaffold13950_1	non-phage	0
NLF013_scaffold17286_2	non-phage	0
NLF013_scaffold48606_2	non-phage	0
NLF013_scaffold48794_2	non-phage	0
NLF013_scaffold30196_2	non-phage	0
NLF013_scaffold31083_5	non-phage	0
NLF013_scaffold26525_3	non-phage	0
NLF013_scaffold35601_4	non-phage	0
NLF013_scaffold31506_1	non-phage	0
NLF013_scaffold40472_4	non-phage	0
NLF013_scaffold48874_1	non-phage	0
NLF013_scaffold18755_2	non-phage	0
NLF013_scaffold22364_5	non-phage	0
NLF013_scaffold27842_16	non-phage	0
NLF013_scaffold48407_2	non-phage	0
NLF013_scaffold48828_2	non-phage	0
NLF013_scaffold48267_2	non-phage	0
NLF013_scaffold12688_14	non-phage	0
NLF013_scaffold25753_2	non-phage	0
NLF013_scaffold39192_10	non-phage	0
NLF013_scaffold2121_2	non-phage	0
NLF013_scaffold1000_1	non-phage	0
NLF013_scaffold48840_1	non-phage	0
NLF013_scaffold12583_3	non-phage	0
NLF013_scaffold5708_33	non-phage	0
NLF013_scaffold18098_1	non-phage	0
NLF013_scaffold5723_1	non-phage	0
NLF013_scaffold24608_1	non-phage	0
NLF013_scaffold25874_1	non-phage	0
NLF013_scaffold43192_25	non-phage	0
NLF013_scaffold17717_2	non-phage	0
NLF013_scaffold33066_3	non-phage	0
NLF013_scaffold28691_1	non-phage	0
NLF013_scaffold17765_2	non-phage	0
NLF013_scaffold42354_1	non-phage	0
NLF013_scaffold26044_5	phage	1

NLF013_scaffold39129_2	non-phage	0
NLF013_scaffold4874_4	non-phage	0
NLF013_scaffold36804_1	non-phage	0
NLF013_scaffold48865_2	non-phage	0
NLF013_C659805_1	non-phage	0
NLF013_scaffold15694_1	non-phage	0
NLF013_scaffold48855_2	non-phage	0
NLF013_scaffold48674_2	non-phage	0
NLF013_scaffold680_8	non-phage	0
NLF013_scaffold28328_5	non-phage	0
NLF013_scaffold48345_1	non-phage	0
NLF013_scaffold7048_2	non-phage	0
NLF013_scaffold13849_5	non-phage	0
NLF013_scaffold42628_3	non-phage	0
NLF013_scaffold11731_1	non-phage	0.001
NLF013_scaffold159_3	non-phage	0
NLF013_scaffold48803_1	non-phage	0
NLF013_scaffold47831_1	non-phage	0
NLF013_scaffold145_2	non-phage	0
NLF013_scaffold26185_2	non-phage	0
NLF013_scaffold14143_1	non-phage	0
NLF013_scaffold4636_1	non-phage	0
NLF013_scaffold4173_9	non-phage	0
NLF013_scaffold23087_3	non-phage	0
NLF013_scaffold1420_22	non-phage	0
NLF013_scaffold48723_1	non-phage	0
NLF013_scaffold233_37	non-phage	0
NLF013_scaffold4799_13	non-phage	0
NLF013_scaffold29146_1	non-phage	0
NLF013_scaffold17809_5	non-phage	0
NLF013_scaffold41335_1	non-phage	0.003
NLF013_scaffold111_3	non-phage	0
NLF013_scaffold9848_7	non-phage	0
NLF013_scaffold46874_1	non-phage	0
NLF013_scaffold47834_2	non-phage	0
NLF013_scaffold30765_3	non-phage	0
NLF013_scaffold48870_2	non-phage	0
NLF013_scaffold32744_1	non-phage	0
NLF013_scaffold9350_4	non-phage	0
NLF013_scaffold46_1	non-phage	0
NLF013_scaffold43192_5	non-phage	0
NLF013_scaffold34385_4	non-phage	0
NLF013_scaffold17576_2	non-phage	0
NLF013_scaffold31255_4	non-phage	0
NLF013_scaffold32199_3	non-phage	0
NLF013_scaffold35209_1	non-phage	0

NLF013_scaffold18588_18	non-phage	0
NLF013_scaffold43192_14	phage	1
NLF013_scaffold270_8	non-phage	0
NLF013_scaffold27299_2	non-phage	0
NLF013_scaffold20241_1	non-phage	0
NLF013_scaffold16431_8	non-phage	0
NLF013_scaffold48428_4	non-phage	0
NLF013_scaffold36313_1	non-phage	0
NLF013_scaffold20411_1	non-phage	0
NLF013_scaffold41335_8	non-phage	0
NLF013_scaffold28200_7	non-phage	0
NLF013_scaffold15667_13	non-phage	0
NLF013_scaffold20110_1	non-phage	0
NLF013_scaffold17399_1	non-phage	0
NLF013_scaffold48338_2	phage	0.991
NLF013_scaffold109_7	non-phage	0
NLF013_scaffold13902_10	non-phage	0
NLF013_C659749_1	non-phage	0
NLF013_scaffold6154_6	non-phage	0
NLF013_scaffold48782_1	non-phage	0
NLF013_scaffold15667_7	non-phage	0
NLF013_scaffold31957_2	non-phage	0
NLF013_scaffold35032_1	non-phage	0
NLF013_scaffold20688_4	non-phage	0
NLF013_scaffold20688_2	non-phage	0
NLF013_scaffold39173_3	non-phage	0
NLF013_scaffold2043_10	non-phage	0
NLF013_scaffold24111_1	non-phage	0
NLF013_scaffold89_2	non-phage	0
NLF013_scaffold846_9	non-phage	0
NLF013_scaffold48818_1	non-phage	0
NLF014_scaffold5915_17_1	non-phage	0
NLF014_scaffold16966_1	non-phage	0
NLF014_scaffold16106_1	non-phage	0
NLF014_scaffold294_7	non-phage	0
NLF014_scaffold611_1_1	non-phage	0
NLF014_scaffold19355_1	non-phage	0
NLF014_scaffold142_6	phage	0.998
NLF014_scaffold1898_1	non-phage	0
NLF014_scaffold7338_3	non-phage	0
NLF014_scaffold135_4	non-phage	0
NLF014_scaffold8474_1	non-phage	0
NLF014_scaffold1518_7	non-phage	0
NLF014_scaffold4080_2	non-phage	0
NLF014_scaffold6779_1	non-phage	0.001
NLF014_scaffold421_2	non-phage	0

NLF014_scaffold3518_1	non-phage	0
NLF014_scaffold10472_12	non-phage	0
NLF014_scaffold448_6	non-phage	0
NLF014_scaffold5996_9	non-phage	0
NLF014_scaffold562_2	non-phage	0
NLF014_scaffold944_1	non-phage	0
NLF014_scaffold452_3	non-phage	0
NLF014_scaffold6163_2	non-phage	0
NLF014_scaffold7950_4	non-phage	0
NLF014_scaffold7312_4	non-phage	0
NLF014_scaffold382_4	non-phage	0
NLF014_scaffold813_12	non-phage	0
NLF014_scaffold204_1	non-phage	0
NLF014_scaffold8530_4	non-phage	0
NLF014_scaffold849_1	non-phage	0
NLF014_scaffold79_3	non-phage	0
NLF014_scaffold5840_1	phage	0.949
NLF014_scaffold7292_1	phage	0.986
NLF014_scaffold333_6	non-phage	0
NLF014_scaffold105_1	non-phage	0
NLF014_scaffold388_2	non-phage	0
NLF014_scaffold127_3	non-phage	0
NLF014_scaffold1534_1	non-phage	0
NLF014_scaffold342_1	phage	0.62
NLF014_scaffold283_4	non-phage	0
NLF014_scaffold140_2	non-phage	0
NLF014_scaffold274_4	non-phage	0
NLF014_scaffold12181_1	non-phage	0
NLF014_scaffold545_3	non-phage	0
NLF014_scaffold60_1	non-phage	0
NLF014_scaffold9741_6	phage	0.682
NLF014_scaffold6817_2	non-phage	0
NLF014_scaffold5465_3	non-phage	0
NLF014_scaffold4634_1	non-phage	0
NLF014_scaffold2130_6	non-phage	0
NLF014_scaffold22641_1	non-phage	0.122
NLF014_scaffold11664_4	non-phage	0
NLF014_scaffold346_5	non-phage	0
NLF014_scaffold2202_2_2	non-phage	0
NLF014_scaffold7720_20	non-phage	0
NLF014_scaffold283_1	non-phage	0
NLF014_scaffold1307_2	phage	0.999
NLF014_scaffold1484_2	non-phage	0
NLF014_scaffold1263_2	non-phage	0
NLF014_scaffold818_4	non-phage	0
NLF014_scaffold7249_4	non-phage	0

NLF014_scaffold9717_2	non-phage	0
NLF014_scaffold3870_2	non-phage	0
NLF014_scaffold269_7	non-phage	0
NLF014_scaffold7968_2	non-phage	0
NLF014_scaffold3378_9	non-phage	0
NLF014_scaffold677_3	non-phage	0
NLF014_scaffold23190_1_2	non-phage	0
NLF014_scaffold3942_21	non-phage	0
NLF014_scaffold18_1	non-phage	0
NLF014_scaffold104_6	non-phage	0
NLF014_scaffold510_8	non-phage	0
NLF014_scaffold457_4	non-phage	0
NLF014_scaffold301_3	non-phage	0
NLF014_scaffold63_1	non-phage	0
NLF014_scaffold143_5	non-phage	0
NLF014_scaffold1802_1	non-phage	0
NLF014_scaffold232_1	non-phage	0
NLF014_scaffold3732_2	non-phage	0
NLF014_scaffold10919_3	non-phage	0
NLF014_scaffold66_1	non-phage	0
NLF014_scaffold3399_4	non-phage	0
NLF014_scaffold250_2	non-phage	0
NLF014_scaffold1571_1	non-phage	0
NLF014_scaffold9462_3	non-phage	0
NLF014_scaffold12637_2	non-phage	0
NLF014_scaffold813_13	non-phage	0
NLF014_scaffold2_2	non-phage	0
NLF014_scaffold775_4	non-phage	0
NLF014_scaffold843_18	non-phage	0
NLF014_scaffold2994_2	non-phage	0
NLF014_scaffold27_6	non-phage	0
NLF014_scaffold6371_3	non-phage	0
NLF014_scaffold2494_1	non-phage	0
NLF014_scaffold6676_2	non-phage	0
NLF014_scaffold14578_3	non-phage	0
NLF014_scaffold6161_2	non-phage	0
NLF014_scaffold3095_2	non-phage	0
NLF014_scaffold7720_15	non-phage	0
NLF014_scaffold472_1	non-phage	0
NLF014_scaffold14396_5	non-phage	0
NLF014_scaffold1518_14	non-phage	0
NLF014_scaffold5242_5	non-phage	0
NLF014_scaffold668_4	non-phage	0
NLF014_scaffold290_2	non-phage	0
NLF014_scaffold50_2	non-phage	0
NLF014_scaffold601_1	non-phage	0

NLF014_scaffold18778_1	non-phage	0
NLF014_scaffold6160_2	non-phage	0
NLF014_scaffold4655_2	non-phage	0
NLF014_scaffold346_1	non-phage	0
NLF014_scaffold124_5	non-phage	0
NLF014_scaffold2685_1	non-phage	0
NLF014_scaffold2547_7	non-phage	0
NLF014_scaffold142_5	non-phage	0
NLF014_scaffold27_5	non-phage	0
NLF014_scaffold1566_3	non-phage	0
NLF014_scaffold12057_1	non-phage	0
NLF014_scaffold3311_1	non-phage	0
NLF014_scaffold631_2	non-phage	0
NLF014_scaffold101_2	non-phage	0
NLF014_scaffold17676_2_1	non-phage	0
NLF014_scaffold1275_1	non-phage	0
NLF014_scaffold5947_2	non-phage	0
NLF014_scaffold405_7	non-phage	0
NLF014_scaffold6532_4	non-phage	0
NLF014_scaffold35_2	non-phage	0
NLF014_scaffold43_2	non-phage	0.037
NLF014_scaffold5242_2	non-phage	0
NLF014_scaffold9569_1	non-phage	0
NLF014_scaffold883_1	non-phage	0
NLF014_scaffold21515_1	non-phage	0
NLF014_scaffold6197_2	phage	0.996
NLF014_scaffold7611_1	non-phage	0
NLF014_scaffold172_1	non-phage	0
NLF014_scaffold7645_1	non-phage	0
NLF014_scaffold4189_19	non-phage	0
NLF014_scaffold182_2	non-phage	0
NLF014_scaffold283_2	non-phage	0
NLF014_scaffold13513_2	non-phage	0.011
NLF014_scaffold527_1	non-phage	0
NLF014_scaffold7201_1	non-phage	0
NLF014_scaffold1233_3	non-phage	0
NLF014_scaffold1307_1	non-phage	0
NLF014_scaffold636_2	non-phage	0
NLF014_scaffold204_3	non-phage	0
NLF014_scaffold16145_1	non-phage	0
NLF014_scaffold8286_1	non-phage	0
NLF014_scaffold212_6	non-phage	0
NLF014_scaffold6120_6	non-phage	0
NLF014_scaffold202_5	non-phage	0
NLF014_scaffold348_1	non-phage	0
NLF014_scaffold695_2	non-phage	0

NLF014_scaffold395_1	non-phage	0
NLF014_scaffold7249_3	non-phage	0
NLF014_scaffold464_16	non-phage	0
NLF014_scaffold326_1	non-phage	0
NLF014_scaffold1856_1_1	non-phage	0
NLF014_scaffold510_11	non-phage	0
NLF014_scaffold173_1	non-phage	0
NLF014_scaffold19358_3	non-phage	0
NLF014_scaffold3279_1	non-phage	0
NLF014_scaffold3075_2	non-phage	0
NLF014_scaffold12896_2	non-phage	0
NLF014_scaffold4677_1	non-phage	0
NLF014_scaffold1619_3	non-phage	0
NLF014_scaffold1870_17	non-phage	0
NLF014_scaffold2931_1	non-phage	0
NLF014_scaffold10754_2	non-phage	0
NLF014_scaffold10772_2	phage	0.992
NLF014_scaffold5352_4	non-phage	0
NLF014_scaffold325_2	non-phage	0
NLF014_scaffold3922_3	non-phage	0
NLF014_scaffold247_6	non-phage	0
NLF014_scaffold647_3	non-phage	0
NLF014_scaffold497_2	non-phage	0
NLF014_scaffold963_1	non-phage	0
NLF014_scaffold131_1	non-phage	0
NLF014_scaffold5548_1	non-phage	0
NLF014_scaffold6532_8_1	non-phage	0
NLF014_scaffold2139_1	non-phage	0
NLF014_scaffold435_3	non-phage	0
NLF014_scaffold181_1	non-phage	0
NLF014_scaffold600_6	non-phage	0
NLF014_scaffold5034_1	non-phage	0
NLF014_scaffold1691_1	non-phage	0
NLF014_scaffold2258_3	non-phage	0
NLF014_scaffold17661_1	non-phage	0
NLF014_scaffold3214_1	non-phage	0
NLF014_scaffold10232_1	non-phage	0
NLF014_scaffold290_5	non-phage	0
NLF014_scaffold7836_1	non-phage	0
NLF014_scaffold5343_1	non-phage	0
NLF014_scaffold462_5	non-phage	0
NLF014_scaffold19617_3_2	non-phage	0
NLF014_scaffold410_3	non-phage	0
NLF014_scaffold705_3	non-phage	0
NLF014_scaffold3059_3	non-phage	0
NLF014_scaffold249_3	phage	0.525

NLF014_scaffold819_1	phage	0.92
NLF014_scaffold27_4	non-phage	0
NLF014_scaffold10241_2	non-phage	0
NLF014_scaffold1417_3	non-phage	0
NLF014_scaffold8403_7	non-phage	0
NLF014_scaffold15559_1	non-phage	0
NLF014_scaffold123_2	non-phage	0
NLF014_scaffold72_1	non-phage	0
NLF014_scaffold14535_1	non-phage	0
NLF014_scaffold30_1	non-phage	0
NLF014_scaffold105_2	non-phage	0
NLF014_scaffold306_5	non-phage	0
NLF014_scaffold5265_1	non-phage	0
NLF014_scaffold847_1	non-phage	0
NLF014_scaffold782_1	non-phage	0
NLF014_scaffold1513_3	non-phage	0
NLF014_scaffold11097_1	non-phage	0
NLF014_scaffold346_2	non-phage	0
NLF014_scaffold1518_32	non-phage	0
NLF014_scaffold174_4	non-phage	0
NLF014_scaffold245_3	non-phage	0
NLF014_scaffold9408_9	phage	0.997
NLF014_scaffold272_3	non-phage	0
NLF014_scaffold346_3	non-phage	0
NLF014_scaffold12741_4	non-phage	0
NLF014_scaffold425_2	non-phage	0
NLF014_scaffold3828_1	non-phage	0
NLF014_scaffold396_2	non-phage	0
NLF014_scaffold293_6	non-phage	0
NLF014_scaffold6614_3	non-phage	0
NLF014_scaffold464_14	non-phage	0
NLF014_scaffold472_2	non-phage	0
NLF014_scaffold5814_1	non-phage	0
NLF014_scaffold14011_1	non-phage	0
NLF014_scaffold15108_9	non-phage	0
NLF014_scaffold12890_3	non-phage	0
NLF014_scaffold822_1	non-phage	0
NLF014_scaffold2244_4	non-phage	0
NLF014_scaffold1039_4	non-phage	0
NLF014_scaffold747_1	non-phage	0
NLF014_scaffold4634_3	non-phage	0
NLF014_scaffold12234_1_1	non-phage	0
NLF014_scaffold2497_1	non-phage	0
NLF014_scaffold1536_5	non-phage	0
NLF014_scaffold586_1	non-phage	0
NLF014_scaffold3310_1	non-phage	0

NLF014_scaffold544_3	non-phage	0
NLF014_scaffold9439_2_1	non-phage	0
NLF014_scaffold124_2	non-phage	0
NLF014_scaffold268_4	non-phage	0
NLF014_scaffold6884_2	phage	0.993
NLF014_scaffold510_10	non-phage	0
NLF014_scaffold57_4	non-phage	0
NLF014_scaffold3755_1	non-phage	0
NLF014_scaffold64_1	non-phage	0
NLF014_scaffold18249_1	non-phage	0
NLF014_scaffold8648_2	non-phage	0
NLF014_scaffold2824_1	non-phage	0
NLF014_scaffold1518_48	non-phage	0
NLF014_scaffold532_1_1	non-phage	0
NLF014_scaffold418_1	non-phage	0
NLF014_scaffold6482_2	non-phage	0
NLF014_scaffold3297_2	non-phage	0
NLF014_scaffold20839_1	non-phage	0
NLF014_scaffold556_1	non-phage	0
NLF014_scaffold21331_2	non-phage	0
NLF014_scaffold135_2	non-phage	0
NLF014_scaffold484_2	non-phage	0
NLF014_scaffold641_5	non-phage	0
NLF014_scaffold452_2	non-phage	0
NLF014_scaffold11131_2	non-phage	0
NLF014_scaffold665_1	non-phage	0
NLF014_scaffold3797_1	non-phage	0
NLF014_scaffold294_5	non-phage	0
NLF014_scaffold8634_1	non-phage	0
NLF014_scaffold20289_1	non-phage	0
NLF014_scaffold73_1	phage	0.998
NLF014_scaffold299_2	non-phage	0
NLF014_scaffold1598_1	non-phage	0
NLF014_scaffold11444_2	non-phage	0
NLF014_scaffold610_3	non-phage	0
NLF014_scaffold1282_3	non-phage	0
NLF014_scaffold1370_5_1	non-phage	0
NLF014_scaffold2312_1	non-phage	0
NLF014_scaffold27_7	non-phage	0
NLF014_scaffold4326_1	non-phage	0
NLF014_scaffold9455_2_1	non-phage	0
NLF014_scaffold631_1	non-phage	0
NLF014_scaffold12184_1	non-phage	0
NLF014_scaffold1019_1	non-phage	0
NLF014_scaffold424_1	non-phage	0
NLF014_scaffold4277_3_2	non-phage	0

NLF014_scaffold1329_6	non-phage	0
NLF014_scaffold293_5	non-phage	0
NLF014_scaffold11677_7	non-phage	0
NLF014_scaffold52_3	non-phage	0
NLF014_scaffold26_1	non-phage	0
NLF014_scaffold864_2	non-phage	0
NLF014_scaffold7505_1	non-phage	0
NLF014_scaffold510_4	non-phage	0
NLF014_scaffold12696_1	non-phage	0
NLF014_scaffold273_1	non-phage	0
NLF014_scaffold10951_1	non-phage	0
NLF014_scaffold337_1	non-phage	0
NLF014_scaffold515_14	non-phage	0
NLF014_scaffold211_1	non-phage	0
NLF014_scaffold4680_2	non-phage	0
NLF014_scaffold8_2	non-phage	0
NLF014_scaffold6072_1	non-phage	0
NLF014_scaffold337_6	non-phage	0
NLF014_scaffold234_2	non-phage	0
NLF014_scaffold3240_1	non-phage	0
NLF014_scaffold699_1	non-phage	0
NLF014_scaffold209_3	non-phage	0
NLF014_scaffold3557_3	non-phage	0
NLF014_scaffold343_2	non-phage	0
NLF014_scaffold7_4	non-phage	0
NLF014_scaffold12195_2	non-phage	0
NLF014_scaffold3003_1	non-phage	0
NLF014_scaffold2758_1	non-phage	0
NLF014_scaffold6771_1	non-phage	0
NLF014_scaffold17826_2	non-phage	0
NLF014_scaffold673_31	non-phage	0
NLF014_scaffold968_4	non-phage	0
NLF014_scaffold208_1	non-phage	0
NLF014_scaffold11174_9	non-phage	0
NLF014_scaffold204_2	non-phage	0
NLF014_scaffold5003_2	non-phage	0
NLF014_scaffold10232_2	non-phage	0
NLF014_scaffold792_11	non-phage	0
NLF014_scaffold5010_14	non-phage	0
NLF014_scaffold3870_5	non-phage	0
NLF014_scaffold930_2	non-phage	0
NLF014_scaffold643_3	non-phage	0
NLF014_scaffold7338_6	non-phage	0
NLF014_scaffold247_8	non-phage	0
NLF014_scaffold124_3	non-phage	0
NLF014_scaffold83_5	non-phage	0

NLF014_scaffold210_1	non-phage	0
NLF014_scaffold148_5	non-phage	0
NLF014_scaffold7170_1	non-phage	0
NLF014_scaffold8172_1	non-phage	0
NLF014_scaffold269_6	non-phage	0
NLF014_scaffold8642_3	non-phage	0
NLF014_scaffold17938_1	non-phage	0
NLF014_scaffold636_4	non-phage	0
NLF014_scaffold27_10	non-phage	0
NLF014_scaffold445_1	non-phage	0
NLF014_scaffold10025_7	non-phage	0
NLF014_scaffold4634_4	non-phage	0
NLF014_scaffold198_2	non-phage	0
NLF014_scaffold10396_1	non-phage	0
NLF014_scaffold969_3	non-phage	0
NLF014_scaffold544_2	non-phage	0
NLF014_scaffold736_2	phage	0.999
NLF014_scaffold792_12	non-phage	0
NLF014_scaffold417_1	non-phage	0
NLF014_scaffold4298_1	non-phage	0
NLF014_scaffold567_7	non-phage	0
NLF014_scaffold13739_12	non-phage	0
NLF014_scaffold9880_2	non-phage	0
NLF014_scaffold653_5	non-phage	0
NLF014_scaffold1856_3	non-phage	0
NLF014_scaffold13894_2	non-phage	0
NLF014_scaffold15794_2	non-phage	0
NLF014_scaffold8397_1	non-phage	0
NLF014_scaffold467_1	non-phage	0
NLF014_scaffold775_3	non-phage	0
NLF014_scaffold332_1	non-phage	0
NLF014_scaffold979_2	non-phage	0
NLF014_scaffold813_9	non-phage	0
NLF014_scaffold23190_2	non-phage	0
NLF014_scaffold12930_2_1	non-phage	0
NLF014_scaffold52_6_1	non-phage	0
NLF014_scaffold2547_5	non-phage	0
NLF014_scaffold13810_2	non-phage	0
NLF014_scaffold268_3	non-phage	0
NLF014_scaffold153_5	non-phage	0
NLF014_scaffold59_4	non-phage	0
NLF014_scaffold284_1	non-phage	0
NLF014_scaffold6581_3	non-phage	0
NLF015_scaffold32023_1	non-phage	0
NLF015_scaffold9823_2	non-phage	0
NLF015_scaffold33838_2	non-phage	0

NLF015_scaffold4960_7	non-phage	0
NLF015_scaffold34520_4	non-phage	0
NLF015_scaffold25640_1	non-phage	0
NLF015_scaffold3382_6	non-phage	0
NLF015_scaffold4223_16	non-phage	0
NLF015_scaffold18664_1	non-phage	0
NLF015_scaffold9748_4	non-phage	0
NLF015_scaffold27137_4	non-phage	0
NLF015_scaffold32930_3	non-phage	0
NLF015_scaffold26401_1	non-phage	0
NLF015_scaffold13933_2	non-phage	0
NLF015_scaffold3835_2	non-phage	0
NLF015_scaffold25297_4	non-phage	0
NLF015_scaffold34154_4	non-phage	0
NLF015_scaffold17181_6	non-phage	0
NLF015_scaffold13530_3	non-phage	0
NLF015_scaffold5098_7	non-phage	0
NLF015_scaffold26409_5	non-phage	0
NLF015_scaffold32845_3	non-phage	0
NLF015_scaffold20634_1	non-phage	0
NLF015_scaffold15972_1	non-phage	0
NLF015_scaffold23966_2	non-phage	0
NLF015_scaffold26705_7	non-phage	0
NLF015_scaffold6165_1	non-phage	0
NLF015_scaffold34422_2	non-phage	0
NLF015_scaffold34468_1	non-phage	0
NLF015_scaffold15960_1	non-phage	0
NLF015_scaffold10681_4	non-phage	0
NLF015_scaffold169_1	non-phage	0
NLF015_scaffold16356_1	non-phage	0
NLF015_scaffold5739_8	non-phage	0
NLF015_scaffold20251_3	non-phage	0
NLF015_scaffold12313_5	non-phage	0
NLF015_scaffold15868_6	non-phage	0
NLF015_scaffold122_2	non-phage	0
NLF015_scaffold18_28	non-phage	0
NLF015_scaffold34502_2	non-phage	0
NLF015_scaffold16588_6	non-phage	0
NLF015_scaffold11376_6	non-phage	0
NLF015_scaffold19443_1	non-phage	0
NLF015_scaffold19123_1	non-phage	0
NLF015_scaffold19_7	non-phage	0
NLF015_scaffold20500_1	non-phage	0
NLF015_scaffold1205_20	non-phage	0
NLF015_scaffold22635_1	non-phage	0
NLF015_scaffold14044_19	non-phage	0

NLF015_scaffold32239_6	non-phage	0
NLF015_scaffold21261_2	non-phage	0
NLF015_scaffold45_3	non-phage	0
NLF015_scaffold34478_2	non-phage	0
NLF015_scaffold23893_2	non-phage	0
NLF015_scaffold33952_1	non-phage	0
NLF015_scaffold5194_2	non-phage	0
NLF015_scaffold3077_11	non-phage	0
NLF015_scaffold34510_2	non-phage	0
NLF015_scaffold31615_2	phage	0.993
NLF015_scaffold11_1	non-phage	0
NLF015_scaffold34540_1	non-phage	0
NLF015_scaffold27043_2	non-phage	0
NLF015_C420009_1	non-phage	0
NLF015_scaffold72_3	non-phage	0
NLF015_C419735_1	non-phage	0
NLF015_scaffold33276_3	non-phage	0.008
NLF015_scaffold19528_1	non-phage	0
NLF015_scaffold27226_1	non-phage	0
NLF015_scaffold33765_1	non-phage	0
NLF015_scaffold12027_7	non-phage	0
NLF015_scaffold18293_2	non-phage	0
NLF015_scaffold15918_2	non-phage	0
NLF015_scaffold33777_1	non-phage	0
NLF015_scaffold3740_2	non-phage	0
NLF015_scaffold21791_3	non-phage	0
NLF015_scaffold32932_4	phage	0.984
NLF015_scaffold33730_3	non-phage	0
NLF015_scaffold17280_7	non-phage	0
NLF015_scaffold4136_4	non-phage	0
NLF015_scaffold34353_3	non-phage	0
NLF015_scaffold18853_1	non-phage	0
NLF015_scaffold34003_2	non-phage	0
NLF015_scaffold12423_2	non-phage	0
NLF015_scaffold2332_5	non-phage	0
NLF015_scaffold31821_3	phage	0.961
NLF015_scaffold3535_1	non-phage	0
NLF015_scaffold14253_4	non-phage	0
NLF015_scaffold22606_6	non-phage	0
NLF015_scaffold2634_2	non-phage	0
NLF015_scaffold16283_1	non-phage	0
NLF015_scaffold20251_1	non-phage	0
NLF015_scaffold2386_16	non-phage	0
NLF015_scaffold840_1	non-phage	0
NLF015_scaffold23914_1	non-phage	0
NLF015_scaffold12027_2	non-phage	0

NLF015_scaffold3816_28	non-phage	0
NLF015_scaffold3209_1	non-phage	0
NLF015_scaffold24231_1	phage	0.998
NLF015_scaffold21144_2	non-phage	0
NLF015_scaffold12334_1	non-phage	0
NLF015_scaffold13567_5	non-phage	0
NLF015_scaffold2330_1	non-phage	0
NLF015_scaffold31615_3	non-phage	0
NLF015_scaffold6814_3	non-phage	0
NLF015_scaffold19041_2	non-phage	0
NLF015_scaffold680_4	non-phage	0
NLF015_scaffold34513_1	non-phage	0
NLF015_scaffold15653_1	non-phage	0
NLF015_scaffold1205_14	non-phage	0
NLF015_scaffold20763_1	non-phage	0
NLF015_scaffold17082_1	non-phage	0
NLF015_scaffold29081_1	non-phage	0
NLF015_scaffold8485_5	non-phage	0
NLF015_scaffold21346_4	non-phage	0
NLF015_scaffold20500_8	non-phage	0
NLF015_scaffold14080_4	non-phage	0
NLF015_scaffold14142_2	non-phage	0
NLF015_C419393_1	non-phage	0
NLF015_scaffold12313_1	non-phage	0
NLF015_scaffold34530_1	non-phage	0
NLF015_scaffold27664_3	non-phage	0
NLF015_scaffold122_1	non-phage	0
NLF015_scaffold18041_1	non-phage	0
NLF015_scaffold65_2	non-phage	0
NLF015_scaffold12387_2	non-phage	0
NLF015_scaffold6828_5	non-phage	0
NLF015_scaffold6637_1	non-phage	0
NLF015_scaffold27121_1	non-phage	0
NLF015_scaffold17883_1	non-phage	0
NLF015_scaffold22001_4	non-phage	0
NLF015_scaffold34535_1	non-phage	0
NLF015_scaffold34313_2	non-phage	0
NLF015_scaffold9140_14	non-phage	0
NLF015_scaffold31821_4	non-phage	0
NLF015_scaffold18_23	non-phage	0
NLF015_scaffold14044_5	non-phage	0
NLF015_scaffold31378_1	non-phage	0
NLF015_scaffold13663_6	non-phage	0
NLF015_scaffold29126_2	non-phage	0
NLF015_scaffold19427_1	non-phage	0
NLF015_scaffold10113_11	non-phage	0

NLF015_scaffold23148_1	non-phage	0
NLF015_scaffold1725_2	non-phage	0
NLF015_scaffold27044_1	non-phage	0
NLF015_scaffold7994_1	non-phage	0
NLF015_scaffold11305_31	non-phage	0
NLF015_scaffold34034_1	non-phage	0
NLF015_scaffold7335_1	non-phage	0
NLF015_scaffold7465_4	non-phage	0
NLF015_scaffold21631_10	non-phage	0
NLF015_scaffold28079_2	non-phage	0
NLF015_scaffold6508_2	non-phage	0
NLF015_scaffold33239_2	non-phage	0
NLF015_scaffold8643_6	non-phage	0
NLF015_scaffold5153_2	non-phage	0
NLF015_scaffold20358_4	non-phage	0
NLF015_scaffold19055_1	non-phage	0
NLF015_scaffold25292_2	non-phage	0
NLF015_scaffold11305_8	non-phage	0
NLF015_scaffold1773_1	non-phage	0
NLF015_scaffold9033_1	non-phage	0
NLF015_scaffold31211_1	non-phage	0
NLF015_C419895_1	phage	0.999
NLF015_scaffold29248_3	non-phage	0
NLF015_scaffold34544_1	non-phage	0
NLF015_scaffold5144_10	non-phage	0
NLF015_scaffold32044_2	non-phage	0
NLF015_scaffold14491_1	phage	1
NLF015_scaffold12619_2	non-phage	0
NLF015_scaffold34511_1	non-phage	0
NLF015_scaffold24904_2	non-phage	0
NLF015_scaffold33835_3	non-phage	0
NLF015_scaffold10104_1	non-phage	0
NLF015_scaffold16588_2	non-phage	0
NLF015_scaffold31615_6	non-phage	0
NLF015_scaffold350_3	non-phage	0
NLF015_scaffold20500_4	non-phage	0
NLF015_scaffold2100_2	non-phage	0
NLF015_scaffold161_2	non-phage	0
NLF015_scaffold3864_1	non-phage	0
NLF015_scaffold33538_3	non-phage	0
NLF015_scaffold34541_1	non-phage	0
NLF015_scaffold13173_4	non-phage	0
NLF015_scaffold12725_2	non-phage	0
NLF015_scaffold18837_1	non-phage	0
NLF015_C419801_1	non-phage	0
NLF015_scaffold18736_4	non-phage	0

NLF015_scaffold34402_1	non-phage	0
NLF015_scaffold34459_1	non-phage	0
NLF015_scaffold40_2	non-phage	0
NLF015_scaffold34335_1	non-phage	0
NLF015_scaffold22035_2	non-phage	0
NLF015_scaffold32743_1	non-phage	0
NLF015_scaffold34523_1	non-phage	0
NLF015_scaffold12027_6	non-phage	0
NLF015_scaffold6263_2	non-phage	0
NLF015_scaffold21169_7	non-phage	0
NLF015_scaffold3382_1	non-phage	0
NLF015_scaffold21392_8	non-phage	0
NLF015_scaffold16847_1	non-phage	0
NLF015_scaffold34058_2	non-phage	0
NLF015_scaffold17754_2	non-phage	0
NLF015_scaffold6393_3	non-phage	0
NLF015_scaffold26705_1	non-phage	0
NLF015_scaffold5593_10	non-phage	0
NLF015_scaffold15881_2	non-phage	0
NLF015_scaffold34546_1	non-phage	0
NLF015_scaffold34467_1	non-phage	0
NLF015_scaffold8819_1	non-phage	0
NLF015_scaffold26409_3	non-phage	0
NLF015_scaffold24133_2	non-phage	0
NLF015_C419779_1	non-phage	0
NLF015_scaffold18_9	non-phage	0
NLF015_scaffold14946_3	non-phage	0
NLF015_scaffold30109_1	non-phage	0
NLF015_scaffold14142_4	non-phage	0
NLF015_scaffold18301_1	non-phage	0
NLF015_scaffold22095_1	phage	0.935
NLF015_scaffold12997_2	non-phage	0
NLF015_scaffold624_10	non-phage	0
NLF015_scaffold591_2	phage	0.977
NLF015_scaffold2056_10	non-phage	0
NLF015_scaffold8222_13	non-phage	0
NLF015_scaffold21847_3	phage	0.939
NLF015_scaffold19670_2	non-phage	0
NLF015_scaffold34510_3	non-phage	0
NLF015_scaffold25347_1	non-phage	0
NLF015_scaffold11_2	non-phage	0
NLF015_scaffold31818_1	non-phage	0
NLF015_scaffold26705_6	non-phage	0
NLF015_scaffold3816_13	non-phage	0
NLF015_scaffold11431_19	non-phage	0
NLF015_scaffold33913_2	non-phage	0

NLF015_scaffold32392_2	non-phage	0
NLF015_scaffold11481_6	phage	0.932
NLF015_scaffold22611_4	non-phage	0
NLF015_scaffold12313_4	non-phage	0
NLF015_scaffold1768_4	non-phage	0
NLF015_scaffold24591_1	non-phage	0
NLF015_scaffold28110_4	non-phage	0
NLF015_scaffold31748_1	non-phage	0
NLF015_scaffold14590_1	non-phage	0
NLF015_scaffold40_5	non-phage	0
NLF015_scaffold13747_2	non-phage	0
NLF015_scaffold3209_4	non-phage	0
NLF015_scaffold22_1	phage	0.999
NLF015_scaffold169_2	non-phage	0
NLF015_scaffold31821_2	non-phage	0
NLF015_scaffold18_11	non-phage	0
NLF015_scaffold19_6	non-phage	0
NLF015_scaffold21784_2	non-phage	0
NLF015_scaffold5707_3	non-phage	0
NLF015_scaffold1429_1	non-phage	0
NLF015_scaffold23457_3	non-phage	0
NLF015_scaffold34549_1	non-phage	0
NLF015_scaffold12974_3	non-phage	0
NLF015_scaffold20500_2	non-phage	0
NLF015_scaffold15238_1	non-phage	0
NLF015_scaffold34520_3	non-phage	0
NLF015_scaffold15041_3	non-phage	0
NLF015_scaffold31052_1	non-phage	0
NLF015_scaffold33907_1	non-phage	0
NLF015_scaffold1961_4	non-phage	0
NLF015_scaffold3859_2	phage	0.544
NLF015_scaffold4136_1	non-phage	0
NLF015_C419705_1	non-phage	0
NLF015_scaffold15918_5	non-phage	0
NLF015_scaffold14067_1	non-phage	0
NLF015_scaffold23965_1	non-phage	0
NLF015_scaffold21784_4	non-phage	0
NLF015_scaffold34057_2	phage	0.997
NLF015_scaffold20358_1	non-phage	0
NLF015_scaffold24362_4	phage	0.686
NLF015_scaffold27137_5	non-phage	0
NLF015_scaffold30318_1	non-phage	0
NLF015_scaffold16383_1	non-phage	0
NLF015_scaffold21784_1	non-phage	0
NLF015_scaffold13173_5	non-phage	0
NLF015_scaffold27391_1	non-phage	0

NLF015_scaffold2431_1	non-phage	0
NLF015_scaffold22035_3	non-phage	0
NLF015_scaffold26705_5	non-phage	0
NLF015_scaffold20905_4	non-phage	0
NLF015_scaffold12387_1	non-phage	0
NLF015_scaffold21392_5	non-phage	0
NLF015_scaffold2898_24	non-phage	0
NLF015_scaffold14159_1	non-phage	0
NLF015_scaffold28079_3	non-phage	0
NLF015_scaffold22016_2	non-phage	0
NLF015_scaffold19128_1	non-phage	0
NLF015_scaffold12681_3	non-phage	0
NLF015_scaffold33191_1	non-phage	0
NLF015_scaffold3209_7	non-phage	0
NLF015_scaffold34559_1	non-phage	0
NLF015_scaffold7530_4	non-phage	0
NLF015_scaffold1779_16	non-phage	0
NLF015_scaffold6215_10	non-phage	0
NLF015_scaffold15241_12	non-phage	0
NLF015_scaffold34329_2	non-phage	0
NLF015_scaffold1779_29	non-phage	0
NLF015_scaffold28900_1	non-phage	0
NLF015_scaffold19212_5	non-phage	0
NLF015_scaffold18_17	non-phage	0
NLF015_scaffold18852_1	non-phage	0.475
NLF015_scaffold11305_25	non-phage	0
NLF015_scaffold3382_4	non-phage	0
NLF015_scaffold27217_2	non-phage	0
NLF015_scaffold5593_30	non-phage	0
NLF015_scaffold3407_2	non-phage	0.345
NLF015_scaffold25303_3	non-phage	0
NLF015_scaffold14066_1	non-phage	0
NLF015_scaffold34372_1	non-phage	0
NLF015_scaffold14660_1	non-phage	0
NLF015_scaffold28424_4	non-phage	0
NLF015_scaffold14142_1	non-phage	0
NLF015_C419629_1	non-phage	0
NLF015_scaffold15238_6	non-phage	0
NLF015_scaffold10852_3	non-phage	0
NLF015_scaffold26349_1	non-phage	0
NLF015_scaffold2043_4	non-phage	0
NLF015_scaffold33835_2	non-phage	0
NLF015_scaffold1567_2	non-phage	0
NLF015_scaffold12974_10	non-phage	0
NLF015_scaffold8377_4	non-phage	0
NLF015_scaffold33705_2	non-phage	0

NLF015_scaffold34478_3	non-phage	0
NLF015_scaffold9823_6	non-phage	0
NLF015_scaffold3550_12	non-phage	0
NLF015_scaffold13875_2	non-phage	0
NLF015_scaffold34265_4	non-phage	0
NLF015_scaffold24904_1	non-phage	0
NLF015_scaffold34520_2	non-phage	0
NLF015_scaffold154_6	non-phage	0
NLF015_scaffold22035_5	non-phage	0
NLF015_scaffold17616_1	non-phage	0
NLF015_scaffold13263_1	non-phage	0
NLF015_scaffold624_6	non-phage	0
NLF015_scaffold4691_1	non-phage	0
NLF015_scaffold33396_1	non-phage	0
NLF015_scaffold26_4	non-phage	0
NLF015_scaffold20126_1	non-phage	0
NLF015_scaffold27217_6	non-phage	0
NLF015_scaffold3550_6	non-phage	0
NLF015_scaffold11501_2	non-phage	0
NLF015_scaffold12014_5	non-phage	0
NLF015_scaffold34451_1	non-phage	0
NLF015_scaffold26_10	non-phage	0
NLF015_scaffold23043_2	non-phage	0
NLF015_scaffold14_3	non-phage	0
NLF015_scaffold3531_18	non-phage	0
NLF015_scaffold31821_6	non-phage	0
NLF015_scaffold4443_4	non-phage	0
NLF015_scaffold624_14	non-phage	0
NLF015_scaffold24485_1	non-phage	0
NLF015_scaffold12027_3	non-phage	0
NLF015_scaffold25786_1	non-phage	0
NLF015_scaffold22001_5	non-phage	0
NLF015_scaffold34427_2	non-phage	0
NLF015_scaffold27137_1	non-phage	0
NLF015_scaffold34555_1	non-phage	0
NLF015_scaffold1205_24	non-phage	0
NLF015_scaffold34353_4	non-phage	0
NLF015_scaffold4531_1	non-phage	0
NLF015_C419605_1	non-phage	0
NLF015_scaffold1605_1	non-phage	0
NLF015_scaffold17588_1	non-phage	0
NLF015_scaffold19559_1	non-phage	0
NLF015_scaffold12279_1	non-phage	0
NLF015_scaffold18075_4	non-phage	0
NLF015_scaffold33431_1	non-phage	0
NLF015_scaffold16197_1	non-phage	0

NLF015_scaffold6087_2	non-phage	0
NLF015_scaffold13128_5	non-phage	0
NLF015_scaffold5239_1	non-phage	0
NLF015_scaffold29017_1	phage	0.983
NLF015_scaffold33835_5	non-phage	0
NLF015_scaffold27178_5	non-phage	0
NLF015_scaffold122_4	non-phage	0
NLF015_scaffold34527_1	non-phage	0
NLF015_scaffold20358_2	non-phage	0
NLF015_scaffold21979_3	non-phage	0
NLF015_scaffold24118_2	non-phage	0
NLF015_scaffold33613_1	non-phage	0
NLF015_scaffold27217_4	non-phage	0
NLF015_scaffold34152_2	non-phage	0
NLF015_scaffold18075_5	non-phage	0
NLF015_scaffold33913_1	non-phage	0
NLF015_scaffold15709_1	non-phage	0
NLF015_scaffold21767_1	non-phage	0
NLF015_scaffold14815_6	non-phage	0
NLF015_scaffold14555_5	non-phage	0
NLF015_scaffold32983_2	non-phage	0
NLF015_scaffold17280_9	non-phage	0
NLF015_scaffold40_3	non-phage	0
NLF015_scaffold30303_2	non-phage	0
NLF015_scaffold19_1	non-phage	0
NLF015_scaffold8129_1	non-phage	0
NLF015_C419607_1	non-phage	0
NLF015_scaffold2721_7	non-phage	0
NLF015_scaffold25380_1	non-phage	0
NLF015_scaffold30081_2	non-phage	0
NLF015_scaffold19343_2	non-phage	0
NLF015_scaffold31615_8	non-phage	0
NLF015_scaffold23914_18	non-phage	0
NLF015_scaffold115_1	non-phage	0
NLF015_scaffold14514_3	non-phage	0
NLF015_scaffold10104_2	non-phage	0
NLF015_scaffold14946_4	non-phage	0
NLF015_scaffold34186_2	non-phage	0
NLF015_scaffold32403_2	non-phage	0
NLF015_scaffold1779_11	non-phage	0
NLF015_scaffold23965_4	non-phage	0
NLF015_scaffold25848_1	non-phage	0
NLF015_scaffold18664_3	non-phage	0
NLF015_scaffold26705_3	non-phage	0
NLF015_scaffold33907_2	non-phage	0
NLF015_scaffold18582_1	non-phage	0

NLF015_scaffold33773_1	non-phage	0
NLF015_scaffold18_4	non-phage	0
NLF015_scaffold81_2	non-phage	0
NLF015_scaffold15999_2	non-phage	0
NLF015_scaffold31821_5	non-phage	0
NLF015_scaffold30237_1	non-phage	0
NLF015_scaffold28170_1	non-phage	0
NLF015_scaffold11863_2	non-phage	0
NLF015_scaffold28078_1	non-phage	0
NLF015_scaffold12313_3	non-phage	0
NLF015_scaffold34303_1	non-phage	0
NLF015_scaffold2056_4	non-phage	0
NLF015_scaffold12378_2	non-phage	0
NLF015_scaffold14450_1	non-phage	0
NLF015_scaffold7790_3	non-phage	0
NLF015_scaffold23115_1	non-phage	0
NLF015_scaffold14640_1	non-phage	0
NLF015_scaffold14527_2	non-phage	0
NLF015_scaffold31378_6	non-phage	0
NLF015_scaffold40_6	non-phage	0
NLF015_scaffold32555_2	non-phage	0
NLF015_scaffold14590_2	non-phage	0
NLF015_scaffold14_2	non-phage	0
NLF015_scaffold22001_6	non-phage	0
NLF015_scaffold23056_5	non-phage	0
NLF015_scaffold18_8	non-phage	0
NLF015_scaffold18663_2	non-phage	0
NLF015_scaffold27370_2	non-phage	0
NLF015_scaffold4753_1	non-phage	0
NLF015_C419809_1	non-phage	0
NLF015_scaffold8487_2	non-phage	0
NLF015_scaffold169_3	non-phage	0
NLF015_scaffold14686_1	non-phage	0
NLF015_scaffold24943_2	non-phage	0
NLF015_scaffold7530_2	non-phage	0
NLF015_scaffold7225_5	non-phage	0
NLF015_scaffold27137_2	non-phage	0
NLF015_C420005_1	non-phage	0
NLF015_scaffold32016_1	non-phage	0
NLF015_scaffold15206_2	non-phage	0
NLF015_scaffold31211_3	phage	0.999
NLF015_scaffold22135_1	non-phage	0
NLF015_scaffold12014_3	phage	0.999
NLF015_scaffold34427_1	non-phage	0
NLF015_scaffold15880_2	non-phage	0
NLF015_scaffold18075_1	non-phage	0

NLF015_scaffold11812_3	non-phage	0
NLF015_scaffold21064_1	non-phage	0
NLF015_scaffold11111_9	non-phage	0
NLF015_scaffold34154_1	non-phage	0
NLF015_scaffold27043_5	non-phage	0
NLF015_scaffold19192_6	non-phage	0
NLF015_scaffold8589_4	phage	0.779
NLF015_scaffold16338_1	non-phage	0
NLF015_scaffold16588_4	non-phage	0
NLF015_scaffold25034_3	non-phage	0
NLF015_scaffold6297_14	non-phage	0
NLF015_scaffold33851_2	non-phage	0
NLF015_scaffold32527_2	non-phage	0
NLF015_scaffold33835_1	non-phage	0
NLF015_scaffold4117_9	non-phage	0
NLF015_scaffold15238_5	non-phage	0
NLF015_scaffold14298_2	non-phage	0
NLF015_scaffold10852_1	non-phage	0
NLF015_scaffold32540_1	non-phage	0
NLF015_scaffold32845_1	non-phage	0
NLF015_scaffold28196_1	non-phage	0
NLF015_scaffold17391_1	non-phage	0
NLF015_scaffold14946_1	phage	0.675
NLF015_scaffold29979_4	non-phage	0
NLF015_scaffold20215_1	phage	0.995
NLF015_scaffold14044_11	non-phage	0
NLF015_scaffold17280_18	non-phage	0
NLF015_scaffold20500_6	phage	0.919
NLF015_scaffold17906_1	non-phage	0
NLF015_scaffold4950_2	non-phage	0
NLF015_scaffold26689_2	non-phage	0
NLF015_scaffold3816_3	non-phage	0
NLF015_scaffold32866_2	non-phage	0
NLF015_scaffold13173_2	phage	0.988
NLF015_scaffold3998_17	non-phage	0
NLF015_scaffold21261_1	non-phage	0
NLF015_scaffold10328_4	non-phage	0
NLF015_scaffold28903_1	non-phage	0
NLF015_scaffold14159_2	non-phage	0
NLF015_scaffold5553_5	non-phage	0
NLF015_scaffold19446_6	non-phage	0
NLF015_scaffold22035_4	non-phage	0
NLF015_scaffold12678_1	non-phage	0
NLF015_scaffold22901_2	non-phage	0
NLF015_scaffold14478_4	non-phage	0
NLF015_scaffold7592_1	non-phage	0

NLF015_scaffold27796_7	non-phage	0
NLF015_scaffold34451_2	non-phage	0
NLF015_scaffold8914_3	non-phage	0
NLF015_scaffold6393_1	non-phage	0
NLF015_scaffold24362_1	non-phage	0
NLF015_scaffold4833_1	non-phage	0
NLF015_scaffold12027_4	non-phage	0
NLF015_scaffold511_1	non-phage	0
NLF015_scaffold7628_6	phage	0.672
NLF015_scaffold23133_3	non-phage	0
NLF015_scaffold34490_1	non-phage	0
NLF015_scaffold81_7	non-phage	0
NLF015_scaffold6481_4	non-phage	0
NLF015_scaffold34508_1	non-phage	0
NLF015_scaffold4338_1	non-phage	0
NLF015_scaffold34498_2	non-phage	0
NLF015_scaffold31821_9	non-phage	0
NLF015_scaffold34477_1	non-phage	0
NLF015_scaffold2898_33	non-phage	0
NLF015_scaffold972_1	non-phage	0
NLF015_scaffold5731_10	non-phage	0
NLF015_scaffold26409_7	non-phage	0
NLF015_scaffold12348_1	non-phage	0
NLF015_scaffold25566_2	non-phage	0
NLF015_scaffold6637_3	non-phage	0
NLF015_scaffold34516_4	non-phage	0
NLF015_scaffold12974_7	non-phage	0
NLF015_scaffold26705_4	non-phage	0
NLF015_scaffold34557_1	non-phage	0
NLF015_scaffold2979_5	non-phage	0
NLF015_scaffold20576_5	non-phage	0
NLF015_scaffold15960_3	non-phage	0.007
NLF015_scaffold34292_1	non-phage	0
NLF015_scaffold22001_2	non-phage	0
NLF015_scaffold26287_3	non-phage	0
NLF015_scaffold14190_1	non-phage	0
NLF015_scaffold33889_2	non-phage	0
NLF015_scaffold22001_3	non-phage	0
NLF015_scaffold34472_2	non-phage	0
NLF015_scaffold34551_1	non-phage	0
NLF015_scaffold19528_9	non-phage	0
NLF015_scaffold10113_20	non-phage	0
NLF015_scaffold32983_1	non-phage	0
NLF015_scaffold3209_2	non-phage	0
NLF015_scaffold1544_2	non-phage	0
NLF015_scaffold16113_4	non-phage	0

NLF015_scaffold34422_1	non-phage	0
NLF015_scaffold2553_3	non-phage	0
NLF015_scaffold13953_4	non-phage	0
NLF015_scaffold4960_2	non-phage	0
NLF015_scaffold12562_1	non-phage	0
NLF015_scaffold21791_2	non-phage	0
NLF015_scaffold5225_1	non-phage	0
NLF015_scaffold22888_5	non-phage	0
NLF015_scaffold6637_4	non-phage	0
NLF015_scaffold14_1	phage	0.976
NLF015_scaffold20835_2	non-phage	0
NLF015_scaffold18_25	non-phage	0
NLF015_scaffold16995_1	non-phage	0
NLF015_scaffold3531_13	non-phage	0
NLF015_scaffold17748_1	non-phage	0
NLF015_scaffold33305_1	non-phage	0
NLF015_scaffold12496_2	non-phage	0
NLF015_scaffold29319_1	non-phage	0
NLF015_scaffold11305_44	non-phage	0
NLF015_scaffold12423_1	non-phage	0
NLF015_scaffold11572_2	non-phage	0
NLF015_scaffold3732_1	non-phage	0
NLF015_scaffold27217_5	non-phage	0
NLF015_scaffold21784_6	non-phage	0
NLF015_scaffold16588_1	non-phage	0
NLF015_scaffold8759_1	non-phage	0
NLF015_scaffold23965_3	non-phage	0
NLF015_scaffold22863_1	non-phage	0
NLF015_scaffold34553_1	non-phage	0
NLF015_scaffold34152_1	non-phage	0
NLF015_scaffold31821_8	non-phage	0
NLF015_scaffold20649_2	non-phage	0
NLF015_scaffold12619_1	non-phage	0
NLF015_scaffold17181_7	non-phage	0
NLF015_scaffold6124_3	non-phage	0
NLF015_scaffold5779_8	non-phage	0
NLF015_scaffold25324_5	non-phage	0
NLF015_scaffold5098_6	non-phage	0
NLF015_scaffold31398_1	non-phage	0
NLF015_C419419_1	phage	0.998
NLF015_scaffold34022_1	non-phage	0
NLF015_scaffold34189_4	non-phage	0
NLF015_scaffold6553_2	non-phage	0
NLF015_scaffold2384_2	non-phage	0
NLF015_scaffold32555_1	non-phage	0
NLF015_scaffold14736_1	non-phage	0

NLF015_scaffold34081_1	non-phage	0
NLF015_scaffold24720_4	non-phage	0
NLF015_scaffold121_21	non-phage	0
NLF015_scaffold34329_1	non-phage	0
NLF015_scaffold14159_3	non-phage	0
NLF015_scaffold4960_9	non-phage	0
NLF015_scaffold40_4	non-phage	0
NLF015_scaffold11305_26	non-phage	0
NLF015_scaffold3382_3	phage	0.995
NLF015_scaffold361_2	non-phage	0
NLF015_scaffold16383_3	non-phage	0
NLF015_scaffold30081_3	non-phage	0
NLF015_scaffold6498_1	non-phage	0
NLF015_scaffold26409_1	non-phage	0
NLF015_scaffold24144_1	non-phage	0
NLF015_scaffold8487_3	non-phage	0
NLF015_scaffold21963_1	non-phage	0
NLF015_scaffold3003_1	non-phage	0
NLF015_scaffold12014_2_1	non-phage	0
NLF015_scaffold20251_2	non-phage	0
NLF015_scaffold23893_1	non-phage	0
NLF015_scaffold13663_4	non-phage	0
NLF015_scaffold34552_1	non-phage	0
NLF015_scaffold11767_2	non-phage	0
NLF015_scaffold34478_1	non-phage	0
NLF015_scaffold9853_2	non-phage	0
NLF015_scaffold81_3	non-phage	0
NLF015_scaffold10512_1	non-phage	0
NLF015_scaffold12976_1_1	non-phage	0
NLF015_scaffold18919_4	non-phage	0
NLF015_scaffold5483_1	non-phage	0
NLF015_scaffold33090_2	non-phage	0
NLF015_scaffold12141_2	non-phage	0
NLF015_scaffold3077_19	non-phage	0
NLF015_scaffold9724_2	non-phage	0
NLF015_scaffold26409_6	non-phage	0
NLF015_scaffold14478_2	non-phage	0
NLF015_scaffold34269_1	phage	0.916
NLF015_scaffold34006_1	non-phage	0
NLF015_scaffold32403_3	non-phage	0
NLF015_scaffold16772_2	non-phage	0
NLF015_scaffold5225_5	non-phage	0
NLF015_scaffold18273_3	non-phage	0
NLF015_scaffold17280_16	non-phage	0
NLF015_scaffold21631_7	non-phage	0
NLF015_scaffold3961_1	non-phage	0

NLF015_scaffold5867_1_1	non-phage	0
NLF015_scaffold3740_3	non-phage	0
NLF015_scaffold25324_4	non-phage	0
NLF015_scaffold11812_7	non-phage	0
NLF015_scaffold21326_2	non-phage	0
NLF015_scaffold34554_1	non-phage	0
NLF015_scaffold19_4	non-phage	0
NLF015_scaffold20920_2	non-phage	0
NLF015_scaffold8643_7	non-phage	0
NLF015_scaffold20500_5	non-phage	0
NLF015_C420043_1	non-phage	0
NLF015_scaffold4136_5	non-phage	0
NLF015_scaffold32505_6	non-phage	0
NLF015_scaffold27043_4	non-phage	0
NLF015_scaffold28310_1	non-phage	0
NLF015_scaffold31748_2	non-phage	0
NLF015_scaffold40_7	non-phage	0
NLF015_scaffold21593_1	non-phage	0
NLF015_scaffold21011_1	non-phage	0
NLF015_scaffold1826_5	non-phage	0
NLF015_scaffold18405_1	non-phage	0
NLF015_scaffold19225_14	non-phage	0
NLF015_scaffold16133_1	non-phage	0
NLF015_scaffold4223_6	non-phage	0
NLF015_scaffold34521_1	non-phage	0
NLF015_scaffold34484_1	non-phage	0
NLF015_scaffold12027_1	non-phage	0
NLF015_scaffold12871_1	non-phage	0
NLF015_scaffold28110_3	non-phage	0
NLF015_scaffold67_2	non-phage	0
NLF015_scaffold34265_3	non-phage	0
NLF015_scaffold18075_2	non-phage	0
NLF015_scaffold15880_3	non-phage	0
NLF015_scaffold4273_5	non-phage	0
NLF015_scaffold33613_4	non-phage	0
NLF015_scaffold33730_1	non-phage	0
NLF015_scaffold2056_11	non-phage	0
NLF015_scaffold33332_2	phage	0.995
NLF015_scaffold972_2	non-phage	0
NLF015_scaffold4657_3	phage	0.965
NLF015_scaffold10287_3	non-phage	0
NLF015_scaffold11376_1	non-phage	0
NLF015_scaffold6814_2	non-phage	0
NLF015_scaffold2559_2	non-phage	0
NLF015_scaffold34463_2	non-phage	0
NLF015_scaffold32540_2	non-phage	0

NLF015_scaffold8589_1	non-phage	0
NLF015_scaffold32259_1	phage	0.999
NLF015_scaffold11572_1	non-phage	0
NLF015_scaffold19_5	non-phage	0
NLF015_C419583_1	non-phage	0
NLF015_scaffold13163_7	phage	0.999
NLF015_scaffold34334_1	non-phage	0
NLF015_scaffold21169_11	non-phage	0
NLF015_C419645_1	non-phage	0
NLF015_scaffold8589_5	non-phage	0
NLF015_scaffold2043_24	non-phage	0
NLF015_scaffold8416_1	non-phage	0
NLF015_scaffold25229_1	non-phage	0
NLF015_scaffold6258_2	phage	0.996
NLF015_scaffold24063_2	non-phage	0
NLF015_scaffold34543_1	non-phage	0
NLF015_scaffold106_2	non-phage	0
NLF015_scaffold6814_6	non-phage	0
NLF015_scaffold25303_5	non-phage	0
NLF015_scaffold20_1	non-phage	0
NLF015_scaffold22035_1	non-phage	0
NLF015_scaffold34558_2	phage	0.996
NLF015_scaffold34154_2	non-phage	0
NLF015_scaffold20557_1	non-phage	0
NLF015_scaffold33239_3	non-phage	0
NLF015_scaffold33960_1	non-phage	0
NLF015_scaffold9033_2	non-phage	0
NLF015_scaffold14044_23	non-phage	0
NLF015_scaffold33420_1	non-phage	0.23
NLF015_scaffold27909_1	non-phage	0
NLF015_scaffold16738_6	non-phage	0
NLF015_scaffold15241_16	non-phage	0
NLF015_scaffold23742_4	non-phage	0
NLF015_scaffold8485_4	non-phage	0
NLF015_scaffold21847_1	phage	0.997
NLF015_scaffold1205_17	non-phage	0
NLF015_scaffold33835_4	non-phage	0
NLF015_scaffold14142_3	non-phage	0
NLF015_scaffold32845_5	non-phage	0
NLF015_scaffold25297_1	non-phage	0
NLF015_scaffold17701_1	non-phage	0
NLF001_scaffold7067_1	non-phage	0
NLF001_scaffold60_22	non-phage	0
NLF001_scaffold15397_5	non-phage	0
NLF001_scaffold27602_4	non-phage	0
NLF001_scaffold36647_1	non-phage	0

NLF001_scaffold26628_4	non-phage	0
NLF001_scaffold37207_8	non-phage	0
NLF001_scaffold25017_5	non-phage	0
NLF001_scaffold11298_2	non-phage	0
NLF001_scaffold7965_2	non-phage	0
NLF001_scaffold3979_2	non-phage	0
NLF001_scaffold32433_3	non-phage	0
NLF001_scaffold20134_1	non-phage	0
NLF001_scaffold37207_5	non-phage	0
NLF001_scaffold33437_2	non-phage	0
NLF001_scaffold2439_5	non-phage	0
NLF001_scaffold44_2	non-phage	0
NLF001_scaffold33646_3	non-phage	0
NLF001_scaffold15347_7	non-phage	0
NLF001_scaffold2866_9	non-phage	0
NLF001_scaffold1973_3	non-phage	0
NLF001_scaffold7498_1	non-phage	0
NLF001_scaffold42398_1	non-phage	0
NLF001_scaffold35341_4	non-phage	0
NLF001_scaffold7353_3	non-phage	0
NLF001_scaffold29897_4	non-phage	0
NLF001_scaffold38608_1	non-phage	0
NLF001_scaffold23356_16	non-phage	0
NLF001_scaffold3532_1	non-phage	0
NLF001_scaffold1_8	non-phage	0
NLF001_scaffold26628_3	non-phage	0
NLF001_scaffold15105_4	non-phage	0
NLF001_scaffold42226_1	non-phage	0
NLF001_scaffold8668_1	non-phage	0
NLF001_scaffold34618_1	non-phage	0
NLF001_scaffold248_7	non-phage	0
NLF001_scaffold16406_5	non-phage	0
NLF001_scaffold31251_6	non-phage	0
NLF001_scaffold17767_2	non-phage	0
NLF001_scaffold28949_2	non-phage	0
NLF001_scaffold29897_6	non-phage	0
NLF001_scaffold31057_1	non-phage	0
NLF001_scaffold10423_3	non-phage	0
NLF001_scaffold13921_1	non-phage	0
NLF001_scaffold1973_1	non-phage	0
NLF001_scaffold20083_1	non-phage	0
NLF001_scaffold15105_5	non-phage	0
NLF001_scaffold16289_3	non-phage	0
NLF001_scaffold22712_1	non-phage	0
NLF001_C572153_1	non-phage	0
NLF001_C572317_1	non-phage	0

NLF001_scaffold88_6	non-phage	0
NLF001_scaffold5338_6	non-phage	0
NLF001_scaffold18425_9	non-phage	0
NLF001_scaffold39198_2	non-phage	0
NLF001_scaffold42041_2	non-phage	0
NLF001_scaffold42213_1	non-phage	0
NLF001_scaffold33646_1	non-phage	0
NLF001_scaffold42453_1	non-phage	0
NLF001_scaffold39569_1	non-phage	0
NLF001_C572037_1	non-phage	0
NLF001_scaffold31210_1	non-phage	0
NLF001_scaffold8824_2	non-phage	0
NLF001_scaffold59_1	non-phage	0
NLF001_scaffold40718_1	non-phage	0
NLF001_scaffold1094_1	non-phage	0
NLF001_scaffold42381_1	non-phage	0
NLF001_scaffold28306_5	non-phage	0
NLF001_scaffold42126_1	non-phage	0
NLF001_scaffold34033_1	non-phage	0
NLF001_scaffold42473_1	non-phage	0
NLF001_scaffold13339_4	non-phage	0
NLF001_scaffold23313_1	non-phage	0
NLF001_scaffold3192_8	non-phage	0
NLF001_scaffold36484_1	non-phage	0
NLF001_scaffold6875_1	non-phage	0
NLF001_scaffold33233_1	non-phage	0
NLF001_scaffold24127_2	non-phage	0
NLF001_scaffold15039_5	non-phage	0
NLF001_C572041_1	non-phage	0
NLF001_scaffold7562_1	non-phage	0
NLF001_scaffold2897_26	non-phage	0
NLF001_scaffold3192_10	non-phage	0
NLF001_scaffold63_6	non-phage	0
NLF001_scaffold27486_7	non-phage	0
NLF001_C572215_1	non-phage	0
NLF001_scaffold16245_1	non-phage	0
NLF001_scaffold41155_1	non-phage	0
NLF001_scaffold41954_1	non-phage	0
NLF001_scaffold42282_3	non-phage	0
NLF001_scaffold3532_2	non-phage	0
NLF001_C572079_1	non-phage	0
NLF001_scaffold42313_1	non-phage	0
NLF001_scaffold38412_2	non-phage	0
NLF001_scaffold42426_1	non-phage	0
NLF001_scaffold4397_4	non-phage	0
NLF001_scaffold2575_8	non-phage	0

NLF001_scaffold30952_4	non-phage	0
NLF001_scaffold84_5	non-phage	0
NLF001_scaffold3192_9	non-phage	0
NLF001_scaffold7965_1	non-phage	0
NLF001_scaffold41265_2	non-phage	0
NLF001_scaffold42454_1	non-phage	0
NLF001_scaffold25977_3	non-phage	0
NLF001_scaffold7121_3	non-phage	0
NLF001_scaffold7498_3	non-phage	0
NLF001_scaffold42383_3	non-phage	0
NLF001_scaffold42011_1	non-phage	0
NLF001_scaffold7562_2	non-phage	0
NLF001_scaffold42365_1	non-phage	0
NLF001_scaffold44_8	non-phage	0
NLF001_scaffold23006_2	non-phage	0
NLF001_scaffold88_23	non-phage	0
NLF001_scaffold8852_3	non-phage	0
NLF001_scaffold18911_3	non-phage	0
NLF001_scaffold28_1	non-phage	0
NLF001_scaffold42379_1	non-phage	0
NLF001_scaffold33646_4	non-phage	0
NLF001_scaffold34411_1	non-phage	0
NLF001_scaffold38616_1	non-phage	0
NLF001_scaffold42468_1	non-phage	0
NLF001_scaffold40513_1	non-phage	0
NLF001_scaffold41265_1	non-phage	0
NLF002_scaffold2254_12	non-phage	0
NLF002_scaffold38631_1	non-phage	0
NLF002_scaffold38874_2	non-phage	0
NLF002_scaffold33406_10	non-phage	0
NLF002_scaffold34632_3	non-phage	0
NLF002_scaffold1815_1	non-phage	0
NLF002_scaffold4248_4	non-phage	0
NLF002_scaffold18611_8	non-phage	0
NLF002_scaffold4811_3	non-phage	0
NLF002_scaffold25234_1	non-phage	0
NLF002_C495033_1	non-phage	0
NLF002_scaffold9672_1	non-phage	0
NLF002_scaffold141_12	non-phage	0
NLF002_scaffold336_28	non-phage	0
NLF002_scaffold19171_1	non-phage	0
NLF002_scaffold36002_1	non-phage	0
NLF002_scaffold22241_1	non-phage	0
NLF002_scaffold5320_3	non-phage	0
NLF002_scaffold1864_19	non-phage	0
NLF002_scaffold104_5	non-phage	0

NLF002_scaffold22161_1	non-phage	0
NLF002_scaffold38965_1	non-phage	0
NLF002_scaffold37400_1	non-phage	0
NLF002_scaffold33270_1	non-phage	0
NLF002_C494683_1	non-phage	0
NLF002_scaffold22063_6	non-phage	0
NLF002_scaffold38816_1	non-phage	0
NLF002_scaffold104_6	non-phage	0
NLF002_scaffold38018_3	non-phage	0
NLF002_scaffold14270_1	non-phage	0
NLF002_scaffold20479_11	non-phage	0
NLF002_C494587_1	non-phage	0
NLF002_scaffold24722_5	non-phage	0
NLF002_scaffold20287_1	non-phage	0
NLF002_scaffold35486_1	non-phage	0
NLF002_scaffold25423_4	non-phage	0
NLF002_scaffold30029_2	non-phage	0
NLF002_scaffold16671_1	non-phage	0
NLF002_scaffold14254_3	non-phage	0
NLF002_scaffold33406_3	non-phage	0
NLF002_scaffold336_27	non-phage	0
NLF002_scaffold8855_1	non-phage	0
NLF002_scaffold38456_1	non-phage	0
NLF002_scaffold2807_11	non-phage	0
NLF002_scaffold36458_1	non-phage	0
NLF002_scaffold26669_1	non-phage	0
NLF002_scaffold2722_2	non-phage	0
NLF002_scaffold25503_1	non-phage	0
NLF002_scaffold54_6	non-phage	0
NLF002_scaffold37599_1	non-phage	0
NLF002_scaffold38738_1	non-phage	0
NLF002_scaffold10424_9	non-phage	0
NLF002_scaffold120_4	non-phage	0
NLF002_scaffold2254_13	non-phage	0
NLF002_scaffold2807_6	non-phage	0
NLF002_scaffold33284_4	non-phage	0
NLF002_scaffold33299_1	non-phage	0
NLF002_scaffold4313_2	non-phage	0
NLF002_scaffold22161_2	non-phage	0
NLF002_scaffold33766_1	non-phage	0
NLF002_scaffold37837_1	non-phage	0
NLF002_scaffold24722_4	non-phage	0
NLF002_scaffold12172_1	non-phage	0
NLF002_scaffold1072_1	non-phage	0
NLF002_scaffold26607_2	non-phage	0
NLF002_scaffold38537_3	non-phage	0

NLF002_scaffold16149_2	non-phage	0
NLF002_scaffold336_25	non-phage	0
NLF002_scaffold2016_8	non-phage	0
NLF002_scaffold2354_6	non-phage	0
NLF002_scaffold2254_14	non-phage	0
NLF002_scaffold10337_8	non-phage	0
NLF002_scaffold11737_3	non-phage	0
NLF002_scaffold5312_5	non-phage	0
NLF002_scaffold9383_3	non-phage	0
NLF002_scaffold8905_2	non-phage	0
NLF002_scaffold33270_2	non-phage	0
NLF002_C494881_1	non-phage	0
NLF002_scaffold18611_9	non-phage	0
NLF002_scaffold19266_3	non-phage	0
NLF002_scaffold38889_1	non-phage	0
NLF002_scaffold336_2	non-phage	0
NLF002_scaffold37_4	non-phage	0
NLF002_scaffold14860_2	non-phage	0
NLF002_scaffold99_5	non-phage	0
NLF002_scaffold563_1	non-phage	0
NLF002_scaffold104_14	non-phage	0
NLF002_scaffold7918_15	non-phage	0
NLF002_scaffold38865_1	non-phage	0
NLF002_scaffold38899_2	non-phage	0
NLF002_scaffold28812_2	non-phage	0
NLF002_scaffold19490_1	non-phage	0
NLF002_scaffold26540_6	non-phage	0
NLF002_scaffold12743_3	non-phage	0
NLF002_scaffold2717_9	non-phage	0
NLF002_scaffold24121_4	non-phage	0
NLF002_scaffold2233_23	non-phage	0
NLF002_scaffold197_1	non-phage	0
NLF002_scaffold31305_5	non-phage	0
NLF002_scaffold103_6	non-phage	0
NLF002_scaffold1328_4	non-phage	0
NLF002_scaffold17225_26	non-phage	0
NLF002_scaffold6393_1	non-phage	0
NLF002_scaffold11755_2	non-phage	0
NLF002_scaffold30350_4	non-phage	0
NLF002_scaffold10644_3	non-phage	0
NLF002_scaffold25935_3	non-phage	0
NLF002_scaffold19316_3	non-phage	0
NLF002_scaffold29813_1	non-phage	0
NLF002_scaffold38938_1	non-phage	0
NLF002_scaffold31980_4	non-phage	0
NLF002_scaffold26695_2	non-phage	0

NLF002_scaffold24767_2	non-phage	0
NLF002_scaffold103_12	non-phage	0
NLF002_scaffold25941_6	non-phage	0
NLF002_scaffold12312_3	non-phage	0
NLF002_scaffold16149_1	non-phage	0
NLF002_scaffold35116_1	non-phage	0
NLF002_C494809_1	non-phage	0
NLF002_scaffold29829_10	non-phage	0
NLF002_scaffold4313_4	non-phage	0
NLF002_scaffold19344_2	non-phage	0
NLF002_scaffold336_29	non-phage	0
NLF002_scaffold33284_5	non-phage	0
NLF002_scaffold14983_8	non-phage	0
NLF002_scaffold239_6	non-phage	0
NLF002_scaffold171_1	non-phage	0
NLF002_scaffold238_8	non-phage	0
NLF002_scaffold38942_1	non-phage	0
NLF002_scaffold12172_3	non-phage	0
NLF002_scaffold2233_7	non-phage	0
NLF002_scaffold38966_1	non-phage	0
NLF002_scaffold21559_13	non-phage	0
NLF002_scaffold11467_7	non-phage	0
NLF002_scaffold38771_1	non-phage	0
NLF002_scaffold290_1	non-phage	0
NLF002_scaffold20480_3	non-phage	0
NLF002_scaffold16661_3	non-phage	0
NLF002_scaffold36590_2	non-phage	0
NLF002_scaffold36659_2	non-phage	0
NLF002_scaffold12482_1	non-phage	0
NLF002_scaffold1072_3	non-phage	0
NLF002_scaffold141_8	non-phage	0
NLF002_C494687_1	non-phage	0
NLF002_scaffold14314_1	non-phage	0
NLF002_scaffold51_3	non-phage	0
NLF002_scaffold15846_23	non-phage	0
NLF002_scaffold31282_1	non-phage	0
NLF002_scaffold29_3	non-phage	0
NLF002_scaffold18531_10	non-phage	0
NLF002_scaffold239_9	non-phage	0
NLF002_scaffold32673_1	non-phage	0
NLF002_scaffold141_10	non-phage	0
NLF002_scaffold38253_3	non-phage	0
NLF002_scaffold38507_1	non-phage	0
NLF002_scaffold5789_8	non-phage	0
NLF002_scaffold37_5	non-phage	0
NLF002_scaffold33109_2	non-phage	0

NLF005_scaffold48333_1	non-phage	0
NLF005_scaffold12858_1	non-phage	0
NLF005_scaffold48284_2	non-phage	0
NLF005_scaffold48615_1	non-phage	0
NLF005_scaffold5281_7	non-phage	0
NLF005_C729157_1	non-phage	0
NLF005_scaffold48284_6	non-phage	0
NLF005_scaffold48597_1	non-phage	0
NLF005_scaffold48379_1	non-phage	0
NLF005_scaffold13997_1	non-phage	0
NLF005_scaffold37862_1	non-phage	0
NLF005_scaffold22517_2	non-phage	0
NLF005_scaffold38062_2	non-phage	0
NLF005_scaffold36148_1	non-phage	0
NLF005_scaffold29939_2	non-phage	0
NLF005_scaffold163_5	non-phage	0
NLF005_scaffold25190_5	non-phage	0
NLF005_scaffold47934_2	non-phage	0
NLF005_scaffold34974_1	non-phage	0
NLF005_scaffold66_2	non-phage	0
NLF005_C729493_1	non-phage	0
NLF005_scaffold48383_1	non-phage	0
NLF005_scaffold44982_1	non-phage	0
NLF005_scaffold48605_1	non-phage	0
NLF005_scaffold2055_2	non-phage	0
NLF005_scaffold19470_5	non-phage	0
NLF005_C729217_1	non-phage	0
NLF005_scaffold43774_1	non-phage	0
NLF005_scaffold28035_1	non-phage	0
NLF005_scaffold48445_1	non-phage	0
NLF005_scaffold2628_1	non-phage	0
NLF005_scaffold48472_1	non-phage	0
NLF005_scaffold163_10	non-phage	0
NLF005_scaffold3103_1	non-phage	0
NLF005_scaffold19849_1	non-phage	0
NLF005_scaffold12384_1	non-phage	0
NLF005_scaffold48391_1	non-phage	0
NLF005_scaffold367_1	non-phage	0
NLF005_scaffold20550_39	non-phage	0
NLF005_scaffold21567_11	non-phage	0
NLF005_scaffold41540_2	non-phage	0
NLF005_scaffold2861_1	non-phage	0
NLF005_C729811_1	non-phage	0
NLF005_scaffold10734_12	non-phage	0
NLF005_scaffold48546_1	non-phage	0
NLF005_scaffold7337_9	non-phage	0

NLF005_scaffold8350_8	non-phage	0
NLF005_scaffold48237_2	non-phage	0
NLF005_scaffold24236_5	non-phage	0
NLF005_scaffold29964_3	non-phage	0
NLF005_scaffold21943_2	non-phage	0
NLF005_C729723_1	non-phage	0
NLF005_scaffold468_2	non-phage	0
NLF005_scaffold14666_2	non-phage	0
NLF005_scaffold19655_6	non-phage	0
NLF005_scaffold48218_4	non-phage	0
NLF005_scaffold46991_1	non-phage	0
NLF005_scaffold25786_1	non-phage	0
NLF005_scaffold4335_7	non-phage	0
NLF005_scaffold32394_8	non-phage	0
NLF005_scaffold48093_5	non-phage	0
NLF005_scaffold16601_2	non-phage	0
NLF005_scaffold8414_2	non-phage	0
NLF005_scaffold40348_4	non-phage	0
NLF005_scaffold48582_2	non-phage	0
NLF005_scaffold48618_1	non-phage	0
NLF005_scaffold38294_1	non-phage	0
NLF005_scaffold115_1	non-phage	0
NLF005_scaffold222_5	non-phage	0
NLF005_scaffold30199_2	non-phage	0
NLF005_scaffold11920_1	non-phage	0
NLF005_scaffold47894_5	non-phage	0
NLF005_scaffold5169_6	non-phage	0
NLF005_scaffold48577_1	non-phage	0
NLF005_scaffold40572_1	non-phage	0
NLF005_scaffold48495_2	non-phage	0
NLF005_scaffold36394_1	non-phage	0
NLF005_scaffold47156_3	non-phage	0
NLF005_scaffold48419_4	non-phage	0
NLF005_scaffold42875_1	non-phage	0
NLF005_scaffold19407_2	non-phage	0
NLF005_scaffold43692_1	non-phage	0
NLF005_scaffold46473_1	non-phage	0
NLF005_scaffold39548_4	non-phage	0
NLF005_scaffold1317_5	non-phage	0
NLF005_scaffold34975_1	non-phage	0
NLF005_scaffold35305_2	non-phage	0
NLF005_scaffold47997_1	non-phage	0
NLF005_scaffold37558_1	non-phage	0
NLF005_scaffold48449_1	non-phage	0
NLF005_scaffold37681_3	non-phage	0
NLF005_scaffold30199_3	non-phage	0

NLF005_scaffold163_2	non-phage	0
NLF005_scaffold48359_2	non-phage	0
NLF005_scaffold46924_4	non-phage	0
NLF005_scaffold48116_1	non-phage	0
NLF005_scaffold145_11	non-phage	0
NLF005_scaffold42017_1	non-phage	0
NLF005_scaffold2552_1	non-phage	0
NLF005_scaffold30066_4	non-phage	0
NLF005_scaffold19849_10	non-phage	0
NLF005_scaffold46246_1	non-phage	0
NLF005_scaffold163_7	non-phage	0
NLF005_scaffold549_7	non-phage	0
NLF005_scaffold28589_12	non-phage	0
NLF005_scaffold48133_1	non-phage	0
NLF005_scaffold38660_1	non-phage	0
NLF005_scaffold34258_3	non-phage	0
NLF005_scaffold48473_1	non-phage	0
NLF005_scaffold29939_1	non-phage	0
NLF005_scaffold48096_1	non-phage	0
NLF005_scaffold47554_1	non-phage	0
NLF005_scaffold5_3	non-phage	0
NLF005_scaffold42584_2	non-phage	0
NLF005_scaffold28569_2	non-phage	0
NLF005_scaffold222_3	non-phage	0
NLF005_scaffold3555_8	non-phage	0
NLF006_scaffold9313_7	non-phage	0
NLF006_scaffold31160_1	non-phage	0
NLF006_scaffold3878_2	non-phage	0
NLF006_scaffold8628_1	non-phage	0
NLF006_scaffold5360_5	non-phage	0
NLF006_scaffold32_5	non-phage	0
NLF006_scaffold5638_1	non-phage	0
NLF006_scaffold7_3	non-phage	0
NLF006_scaffold14479_4	non-phage	0
NLF006_scaffold6812_5	non-phage	0
NLF006_scaffold130_2	non-phage	0
NLF006_scaffold8268_2	non-phage	0
NLF006_scaffold29537_2	non-phage	0
NLF006_scaffold31555_1	non-phage	0
NLF006_scaffold48_5	non-phage	0
NLF006_scaffold13297_2	non-phage	0
NLF006_scaffold31538_1	non-phage	0
NLF006_scaffold21564_1	non-phage	0
NLF006_scaffold13439_1	non-phage	0
NLF006_scaffold6319_1	non-phage	0
NLF006_scaffold697_1	non-phage	0

NLF006_scaffold4727_1	non-phage	0
NLF006_scaffold24562_1	non-phage	0
NLF006_scaffold28_2	non-phage	0
NLF006_scaffold2006_1	non-phage	0
NLF006_scaffold386_1	non-phage	0
NLF006_scaffold1802_10	non-phage	0
NLF006_scaffold130_6	non-phage	0
NLF006_scaffold5621_1	non-phage	0
NLF006_scaffold135_1	non-phage	0
NLF006_scaffold12888_2	non-phage	0
NLF006_scaffold126_1	non-phage	0
NLF006_scaffold16002_5	non-phage	0
NLF006_scaffold82_1	non-phage	0
NLF006_scaffold9001_1	non-phage	0
NLF006_scaffold4778_1	non-phage	0
NLF006_scaffold14549_3	non-phage	0
NLF006_scaffold20876_2	non-phage	0
NLF006_scaffold18598_1	non-phage	0
NLF006_scaffold45_2	non-phage	0
NLF006_scaffold1006_1	non-phage	0
NLF006_scaffold26936_1	non-phage	0
NLF006_scaffold21535_9	non-phage	0
NLF006_scaffold10643_1	non-phage	0
NLF006_scaffold4559_1	non-phage	0
NLF006_scaffold144_1	non-phage	0
NLF006_scaffold6376_1	non-phage	0
NLF006_scaffold145_4	non-phage	0
NLF006_scaffold2326_2	non-phage	0
NLF006_scaffold131_1	non-phage	0
NLF006_scaffold23958_1	non-phage	0
NLF006_scaffold181_1	non-phage	0
NLF006_scaffold4198_1	non-phage	0
NLF006_scaffold1702_2	non-phage	0
NLF006_scaffold3814_2	non-phage	0
NLF006_scaffold30362_1	non-phage	0
NLF006_scaffold20847_1	non-phage	0
NLF006_scaffold22216_2	non-phage	0
NLF006_scaffold143_3	non-phage	0
NLF006_scaffold8382_1	non-phage	0
NLF006_scaffold140_3	non-phage	0
NLF006_scaffold102_1	non-phage	0
NLF006_scaffold212_2	non-phage	0
NLF006_scaffold55_2	non-phage	0
NLF006_scaffold5336_2	non-phage	0
NLF006_scaffold3575_3	non-phage	0
NLF006_scaffold1647_2	non-phage	0

NLF006_scaffold80_5	non-phage	0
NLF006_scaffold198_1	non-phage	0
NLF006_scaffold5845_1	non-phage	0
NLF006_scaffold6048_4	non-phage	0
NLF006_scaffold1802_8	non-phage	0
NLF006_scaffold1883_3	non-phage	0
NLF006_scaffold3786_1	non-phage	0
NLF006_scaffold13426_2	non-phage	0
NLF006_scaffold5007_6	non-phage	0
NLF006_scaffold19030_2	non-phage	0
NLF006_scaffold11296_3	non-phage	0
NLF006_scaffold1702_1	non-phage	0
NLF006_scaffold9328_1	non-phage	0
NLF006_scaffold4727_3	non-phage	0
NLF006_scaffold87_1	non-phage	0
NLF006_scaffold5393_3	non-phage	0
NLF006_scaffold4298_1	non-phage	0
NLF006_scaffold15032_2	non-phage	0
NLF006_scaffold38_2	non-phage	0
NLF006_scaffold31282_1	non-phage	0
NLF006_scaffold21070_1	non-phage	0
NLF006_scaffold14928_2	non-phage	0
NLF006_scaffold10805_1	non-phage	0
NLF006_scaffold178_3	non-phage	0
NLF007_scaffold56850_4	non-phage	0
NLF007_scaffold66760_2	non-phage	0
NLF007_scaffold17151_1	non-phage	0
NLF007_scaffold8169_16	non-phage	0
NLF007_scaffold54861_2	non-phage	0
NLF007_scaffold57860_2	non-phage	0
NLF007_scaffold10894_6	non-phage	0
NLF007_scaffold6768_2	non-phage	0
NLF007_scaffold39661_3	non-phage	0
NLF007_scaffold13496_8	non-phage	0
NLF007_scaffold5950_26	non-phage	0
NLF007_scaffold67349_1	non-phage	0
NLF007_scaffold66503_2	non-phage	0
NLF007_scaffold9122_13	non-phage	0
NLF007_scaffold3517_4	non-phage	0
NLF007_scaffold64018_3	non-phage	0
NLF007_scaffold33152_15	non-phage	0
NLF007_scaffold41299_17	non-phage	0
NLF007_scaffold65521_1	non-phage	0
NLF007_scaffold42593_1	non-phage	0
NLF007_scaffold1460_2	non-phage	0
NLF007_scaffold49136_1	non-phage	0

NLF007_scaffold51446_1	non-phage	0
NLF007_scaffold14653_1	non-phage	0
NLF007_scaffold29_5	non-phage	0
NLF007_scaffold8274_4	non-phage	0
NLF007_scaffold67213_5	non-phage	0
NLF007_scaffold470_4	non-phage	0
NLF007_scaffold51201_7	non-phage	0
NLF007_scaffold61925_6	non-phage	0
NLF007_scaffold11694_8	non-phage	0
NLF007_scaffold19231_1	non-phage	0
NLF007_scaffold9391_4	non-phage	0
NLF007_scaffold46729_1	non-phage	0
NLF007_scaffold512_4	non-phage	0
NLF007_scaffold29915_6	non-phage	0
NLF007_C1010493_1	non-phage	0
NLF007_scaffold64677_2	non-phage	0
NLF007_scaffold2029_1	non-phage	0
NLF007_scaffold4670_7	non-phage	0
NLF007_scaffold63658_2	non-phage	0
NLF007_scaffold16292_30	non-phage	0
NLF007_scaffold62828_5	non-phage	0
NLF007_scaffold66760_3	non-phage	0
NLF007_scaffold67397_1	non-phage	0
NLF007_scaffold53421_6	non-phage	0
NLF007_scaffold60542_3	non-phage	0
NLF007_scaffold66966_3	non-phage	0
NLF007_C1010875_1	non-phage	0
NLF007_scaffold8826_46	non-phage	0
NLF007_scaffold15727_4	non-phage	0
NLF007_scaffold96_2	non-phage	0
NLF007_scaffold64693_1	non-phage	0
NLF007_scaffold67378_1	non-phage	0
NLF007_scaffold45928_7	non-phage	0
NLF007_scaffold62373_8	non-phage	0
NLF007_C1010885_1	non-phage	0
NLF007_scaffold52662_1	non-phage	0
NLF007_scaffold66368_3	non-phage	0
NLF007_scaffold29925_1	non-phage	0
NLF007_scaffold61029_9	non-phage	0
NLF007_scaffold66380_1	non-phage	0
NLF007_scaffold66556_2	non-phage	0
NLF007_scaffold63326_2	non-phage	0
NLF007_scaffold33367_15	non-phage	0
NLF007_scaffold38563_5	non-phage	0
NLF007_scaffold14577_1	non-phage	0
NLF007_scaffold52070_11	non-phage	0

NLF007_scaffold64433_3	non-phage	0
NLF007_scaffold3903_14	non-phage	0
NLF007_scaffold1905_8	non-phage	0
NLF007_scaffold21756_10	non-phage	0
NLF007_scaffold60542_5	non-phage	0
NLF007_scaffold20085_2	non-phage	0
NLF007_scaffold66760_9	non-phage	0
NLF007_scaffold65907_1	non-phage	0
NLF007_scaffold64274_2	non-phage	0
NLF007_scaffold67411_1	non-phage	0
NLF007_scaffold22798_4	non-phage	0
NLF007_scaffold51793_2	non-phage	0
NLF007_scaffold45526_3	non-phage	0
NLF007_scaffold23509_9	non-phage	0
NLF007_scaffold67443_1	non-phage	0
NLF007_scaffold57860_3	non-phage	0
NLF007_scaffold64529_3	non-phage	0
NLF007_scaffold2513_7	non-phage	0
NLF007_scaffold28214_3	non-phage	0
NLF007_scaffold57911_2	non-phage	0
NLF007_scaffold57221_1	non-phage	0
NLF007_scaffold3912_19	non-phage	0
NLF007_scaffold219_5	non-phage	0
NLF007_scaffold1905_5_1	non-phage	0
NLF007_scaffold195_1	non-phage	0
NLF007_scaffold16474_4	non-phage	0
NLF007_scaffold5575_2	non-phage	0
NLF007_scaffold47537_3	non-phage	0
NLF007_scaffold19360_14	non-phage	0
NLF007_scaffold5059_1	non-phage	0
NLF007_scaffold48195_4	non-phage	0
NLF007_scaffold20917_3	non-phage	0
NLF007_scaffold39661_2	non-phage	0
NLF007_scaffold15286_3	non-phage	0
NLF007_scaffold59738_2	non-phage	0
NLF007_scaffold67269_1	non-phage	0
NLF007_scaffold47283_1	non-phage	0
NLF007_scaffold4754_3	non-phage	0
NLF007_scaffold8851_8	non-phage	0
NLF007_scaffold33152_25	non-phage	0
NLF007_scaffold470_1	non-phage	0
NLF007_scaffold3969_6	non-phage	0
NLF007_scaffold67243_3	non-phage	0
NLF007_scaffold3629_42	non-phage	0
NLF007_scaffold3727_3	non-phage	0
NLF007_scaffold59763_7	non-phage	0

NLF007_scaffold8274_6	non-phage	0
NLF007_scaffold27952_1	non-phage	0
NLF007_scaffold58261_1	non-phage	0
NLF007_scaffold20779_1	non-phage	0
NLF007_scaffold32735_2	non-phage	0
NLF007_scaffold25181_6	non-phage	0
NLF007_scaffold61029_4	non-phage	0
NLF007_scaffold2053_2	non-phage	0
NLF007_scaffold66205_1	non-phage	0
NLF007_scaffold39528_2	non-phage	0
NLF007_scaffold404_19	non-phage	0
NLF007_scaffold47325_3	non-phage	0
NLF007_scaffold40161_10	non-phage	0
NLF007_scaffold64529_1	non-phage	0
NLF007_scaffold61492_3	non-phage	0
NLF007_scaffold13_2	non-phage	0
NLF007_scaffold2619_5	non-phage	0
NLF007_scaffold42859_1	non-phage	0
NLF007_scaffold8826_2	non-phage	0
NLF007_scaffold57860_1	non-phage	0
NLF007_scaffold6048_2	non-phage	0
NLF007_C1010287_1	non-phage	0
NLF007_scaffold59763_4	non-phage	0
NLF007_scaffold10248_3	non-phage	0
NLF007_scaffold31964_17	non-phage	0
NLF007_scaffold52822_1	non-phage	0
NLF007_scaffold10109_1	non-phage	0
NLF007_scaffold27903_3	non-phage	0
NLF007_scaffold52152_5	non-phage	0
NLF007_scaffold65105_10	non-phage	0
NLF007_scaffold48195_20	non-phage	0
NLF007_scaffold65880_1	non-phage	0
NLF007_scaffold42735_1	non-phage	0
NLF007_scaffold66712_1	non-phage	0
NLF007_scaffold67447_1	non-phage	0
NLF007_scaffold27261_2	non-phage	0
NLF007_scaffold13769_3	non-phage	0
NLF007_scaffold56837_2	non-phage	0
NLF007_scaffold20387_2	non-phage	0
NLF007_scaffold12791_2	non-phage	0
NLF007_scaffold41280_1	non-phage	0
NLF007_scaffold320_3	non-phage	0
NLF007_scaffold60392_12	non-phage	0
NLF007_scaffold17579_3	non-phage	0
NLF007_scaffold59738_1	non-phage	0
NLF007_scaffold66763_1	non-phage	0

NLF007_scaffold25517_4	non-phage	0
NLF007_scaffold62463_5	non-phage	0
NLF007_scaffold67432_1	non-phage	0
NLF007_scaffold16920_1	non-phage	0
NLF007_scaffold66229_1	non-phage	0
NLF007_scaffold47368_2	non-phage	0
NLF007_scaffold2029_5	non-phage	0
NLF007_scaffold43402_6	non-phage	0
NLF007_scaffold24900_5	non-phage	0
NLF007_scaffold26244_3	non-phage	0
NLF007_scaffold67348_1	non-phage	0
NLF007_scaffold48793_6	non-phage	0
NLF007_scaffold110_5	non-phage	0
NLF007_scaffold67412_1	non-phage	0
NLF007_scaffold47454_1	non-phage	0
NLF007_scaffold19360_10	non-phage	0
NLF007_scaffold65963_2	non-phage	0
NLF007_scaffold53836_2	non-phage	0
NLF007_scaffold26102_5	non-phage	0
NLF007_scaffold14506_36	non-phage	0
NLF007_scaffold67304_1	non-phage	0
NLF007_scaffold404_31	non-phage	0
NLF007_scaffold29677_7	non-phage	0
NLF007_scaffold4674_1	non-phage	0
NLF007_scaffold1460_8	non-phage	0
NLF007_scaffold44718_2	non-phage	0
NLF007_scaffold64572_1	non-phage	0
NLF007_scaffold48793_2	non-phage	0
NLF007_C1010819_1	non-phage	0
NLF007_scaffold14653_2	non-phage	0
NLF007_scaffold62828_3	non-phage	0
NLF007_scaffold12212_9	non-phage	0
NLF007_scaffold23340_3	non-phage	0
NLF007_scaffold66229_3	non-phage	0
NLF007_scaffold20753_12	non-phage	0
NLF007_scaffold67330_2	non-phage	0
NLF008_scaffold20499_2	non-phage	0
NLF008_scaffold4797_2	non-phage	0
NLF008_scaffold10119_6	non-phage	0
NLF008_scaffold20753_2	non-phage	0
NLF008_scaffold8544_34	non-phage	0
NLF008_scaffold5149_19	non-phage	0
NLF008_scaffold4207_13	non-phage	0
NLF008_scaffold6779_1	non-phage	0
NLF008_scaffold5248_5	non-phage	0
NLF008_scaffold3522_1	non-phage	0

NLF008_scaffold2260_10	non-phage	0
NLF008_scaffold3673_3	non-phage	0
NLF008_scaffold11830_2	non-phage	0
NLF008_scaffold7973_2	non-phage	0
NLF008_scaffold11943_5	non-phage	0
NLF008_scaffold8411_2	non-phage	0
NLF008_scaffold7927_3	non-phage	0
NLF008_scaffold20702_2	non-phage	0
NLF008_scaffold14087_2	non-phage	0
NLF008_C315998_1	non-phage	0
NLF008_scaffold3730_6	non-phage	0
NLF008_scaffold12853_3	non-phage	0
NLF008_scaffold11181_3	non-phage	0
NLF008_scaffold11816_1	non-phage	0
NLF008_scaffold10392_3	non-phage	0
NLF008_scaffold20459_1	non-phage	0
NLF008_scaffold386_2	non-phage	0
NLF008_scaffold20190_5	non-phage	0
NLF008_scaffold19939_2	non-phage	0
NLF008_scaffold429_4	non-phage	0
NLF008_scaffold19077_6	non-phage	0
NLF008_scaffold4186_2	non-phage	0
NLF008_scaffold16676_1	non-phage	0
NLF008_scaffold15177_1	non-phage	0
NLF008_scaffold8732_3	non-phage	0
NLF008_scaffold10473_4	non-phage	0
NLF008_scaffold18081_1	non-phage	0
NLF008_scaffold6029_15	non-phage	0
NLF008_scaffold1243_5	non-phage	0
NLF008_scaffold104_5	non-phage	0
NLF008_scaffold16583_1	non-phage	0
NLF008_scaffold3669_9	non-phage	0
NLF008_scaffold5025_21	non-phage	0
NLF008_scaffold972_3	non-phage	0
NLF008_scaffold9128_5	non-phage	0
NLF008_scaffold12402_1	non-phage	0
NLF008_scaffold7469_2	non-phage	0
NLF008_scaffold13343_11	non-phage	0
NLF008_scaffold4769_2	non-phage	0
NLF008_scaffold3150_3	non-phage	0
NLF008_scaffold10473_7	non-phage	0
NLF008_scaffold7537_3	non-phage	0
NLF008_C316390_1	non-phage	0
NLF008_scaffold15826_8	non-phage	0
NLF008_scaffold13775_1	non-phage	0
NLF008_scaffold5025_4	non-phage	0

NLF008_scaffold3730_2	non-phage	0
NLF008_scaffold8985_2	non-phage	0
NLF008_scaffold9241_3	non-phage	0
NLF008_scaffold14564_1	non-phage	0
NLF008_scaffold9900_3	non-phage	0
NLF008_scaffold8021_3	non-phage	0
NLF008_scaffold11629_1	non-phage	0
NLF008_scaffold5421_3	non-phage	0
NLF008_scaffold19382_2	non-phage	0
NLF008_scaffold20436_2	non-phage	0
NLF008_scaffold9882_1	non-phage	0
NLF008_scaffold18250_5	non-phage	0
NLF008_scaffold6465_1	non-phage	0
NLF008_scaffold8010_2	non-phage	0
NLF008_scaffold7635_1	non-phage	0
NLF008_scaffold5515_1	non-phage	0
NLF008_scaffold8594_1	non-phage	0
NLF008_scaffold10052_2	non-phage	0
NLF008_scaffold10908_1	non-phage	0
NLF008_scaffold19610_2	non-phage	0
NLF008_scaffold2618_2	non-phage	0
NLF008_scaffold6225_3	non-phage	0
NLF008_scaffold3455_9	non-phage	0
NLF008_scaffold18547_1	non-phage	0
NLF008_scaffold11770_1	non-phage	0
NLF008_scaffold2979_2	non-phage	0
NLF008_scaffold10147_1	non-phage	0
NLF008_scaffold108_2	non-phage	0
NLF008_scaffold7644_3	non-phage	0
NLF008_scaffold7479_1	non-phage	0
NLF008_scaffold18692_3	non-phage	0
NLF008_scaffold9061_1	non-phage	0
NLF008_scaffold2131_12	non-phage	0
NLF008_scaffold8410_2	non-phage	0
NLF008_scaffold11959_2	non-phage	0
NLF008_scaffold15341_1	non-phage	0
NLF008_scaffold17305_3	non-phage	0
NLF008_scaffold19847_1	non-phage	0
NLF008_scaffold12243_3	non-phage	0
NLF008_scaffold9821_1	non-phage	0
NLF008_scaffold13614_1	non-phage	0
NLF008_scaffold20643_1	non-phage	0
NLF008_scaffold3507_1	non-phage	0
NLF008_C316168_1	non-phage	0
NLF008_scaffold9784_1	non-phage	0
NLF008_scaffold20706_1	non-phage	0

NLF008_scaffold5480_13	non-phage	0
NLF008_scaffold20673_1	non-phage	0
NLF008_scaffold20239_1	non-phage	0
NLF008_scaffold10123_2	non-phage	0
NLF008_scaffold4665_1	non-phage	0
NLF008_scaffold16041_1	non-phage	0
NLF008_scaffold9624_3	non-phage	0
NLF008_scaffold20735_1	non-phage	0
NLF008_scaffold41_2	non-phage	0
NLF008_scaffold13929_1	non-phage	0
NLF008_scaffold4395_4	non-phage	0
NLF008_scaffold8011_1	non-phage	0
NLF008_scaffold8771_1	non-phage	0
NLF008_scaffold3592_5	non-phage	0
NLF008_scaffold17103_1	non-phage	0
NLF008_scaffold13519_1	non-phage	0
NLF008_scaffold10148_1	non-phage	0
NLF008_C316068_1	non-phage	0
NLF008_scaffold1446_8	non-phage	0
NLF008_scaffold7660_1	non-phage	0
NLF008_scaffold10518_2	non-phage	0
NLF008_scaffold2618_3	non-phage	0
NLF008_scaffold10542_9	non-phage	0
NLF008_scaffold10182_1	non-phage	0
NLF008_scaffold19453_2	non-phage	0
NLF008_scaffold15649_1	non-phage	0
NLF008_scaffold3150_9	non-phage	0
NLF008_scaffold20740_2	non-phage	0
NLF008_scaffold18168_2	non-phage	0
NLF008_scaffold9243_11	non-phage	0
NLF008_scaffold10093_8	non-phage	0
NLF008_scaffold5635_1	non-phage	0
NLF008_scaffold7978_1	non-phage	0
NLF008_scaffold150_2	non-phage	0
NLF008_scaffold13676_1	non-phage	0
NLF008_scaffold14564_2	non-phage	0
NLF008_scaffold19572_9	non-phage	0
NLF008_scaffold12825_7	non-phage	0
NLF008_scaffold2910_2	non-phage	0
NLF008_scaffold18305_2	non-phage	0
NLF008_scaffold14217_1	non-phage	0
NLF008_scaffold13565_4	non-phage	0
NLF008_scaffold16313_1	non-phage	0
NLF008_scaffold9993_1	non-phage	0
NLF008_scaffold20562_1	non-phage	0
NLF008_scaffold9879_5	non-phage	0

NLF008_scaffold13669_1	non-phage	0
NLF008_scaffold429_2	non-phage	0
NLF008_scaffold17036_1	non-phage	0
NLF008_scaffold17845_1	non-phage	0
NLF008_scaffold3036_2	non-phage	0
NLF008_scaffold5509_5	non-phage	0
NLF008_scaffold10424_1	non-phage	0
NLF008_scaffold19008_1	non-phage	0
NLF008_scaffold2201_8	non-phage	0
NLF008_scaffold19864_6	non-phage	0
NLF008_scaffold20187_1	non-phage	0
NLF008_scaffold8017_2	non-phage	0
NLF008_C315798_1	non-phage	0
NLF008_scaffold215_2	non-phage	0
NLF008_scaffold5025_7	non-phage	0
NLF008_scaffold13343_9	non-phage	0
NLF008_scaffold8_3	non-phage	0
NLF008_scaffold2260_4	non-phage	0
NLF008_scaffold13148_1	non-phage	0
NLF008_scaffold9468_1	non-phage	0
NLF008_scaffold5173_9	non-phage	0
NLF008_scaffold3739_1	non-phage	0
NLF008_scaffold7248_1	non-phage	0
NLF008_scaffold17305_4	non-phage	0
NLF008_scaffold6889_1	non-phage	0
NLF008_scaffold4510_3	non-phage	0
NLF008_C316016_1	non-phage	0
NLF008_scaffold20415_1	non-phage	0
NLF008_scaffold261_6	non-phage	0
NLF008_scaffold11679_2	non-phage	0
NLF008_scaffold18987_1	non-phage	0
NLF008_scaffold11531_1	non-phage	0
NLF008_scaffold8919_1	non-phage	0
NLF008_scaffold10874_4	non-phage	0
NLF008_scaffold3066_2	non-phage	0
NLF008_scaffold20706_3	non-phage	0
NLF008_scaffold8964_2	non-phage	0
NLF008_scaffold15305_2	non-phage	0
NLF008_scaffold9688_9	non-phage	0
NLF008_scaffold4665_3	non-phage	0
NLF008_scaffold12130_2	non-phage	0
NLF008_scaffold20566_2	non-phage	0
NLF008_scaffold2910_6	non-phage	0
NLF008_scaffold3066_1	non-phage	0
NLF008_scaffold17765_1	non-phage	0
NLF008_scaffold8081_3	non-phage	0

NLF008_scaffold7552_4	non-phage	0
NLF008_scaffold9099_1	non-phage	0
NLF008_scaffold7940_1	non-phage	0
NLF008_scaffold1474_10	non-phage	0
NLF008_scaffold790_5	non-phage	0
NLF008_scaffold1243_3	non-phage	0
NLF008_scaffold9780_1	non-phage	0
NLF008_scaffold9657_2	non-phage	0
NLF008_C315352_1	non-phage	0
NLF008_scaffold3344_3	non-phage	0
NLF008_scaffold8720_4	non-phage	0
NLF008_scaffold15826_11	non-phage	0
NLF008_scaffold5437_2	non-phage	0
NLF008_scaffold8874_4	non-phage	0
NLF008_scaffold11943_3	non-phage	0
NLF008_scaffold20305_1	non-phage	0
NLF008_scaffold11171_1	non-phage	0
NLF008_scaffold15687_1	non-phage	0
NLF008_scaffold18979_3	non-phage	0
NLF008_scaffold15302_1	non-phage	0
NLF008_scaffold111_1	non-phage	0
NLF008_scaffold1152_7	non-phage	0
NLF008_scaffold10976_2	non-phage	0
NLF008_scaffold15176_1	non-phage	0
NLF008_scaffold20643_3	non-phage	0
NLF008_scaffold11659_1	non-phage	0
NLF008_scaffold11252_1	non-phage	0
NLF008_scaffold20723_1	non-phage	0
NLF008_scaffold6495_4	non-phage	0
NLF008_scaffold12129_3	non-phage	0
NLF008_scaffold6889_8	non-phage	0
NLF008_scaffold20125_3	non-phage	0
NLF008_scaffold20769_1	non-phage	0
NLF008_scaffold4665_6	non-phage	0
NLF008_scaffold99_6	non-phage	0
NLF008_scaffold3574_8	non-phage	0
NLF008_scaffold5528_9	non-phage	0
NLF008_scaffold7552_6	non-phage	0
NLF008_scaffold10033_15	non-phage	0
NLF008_scaffold7248_2	non-phage	0
NLF008_scaffold14544_1	non-phage	0
NLF008_scaffold4982_4	non-phage	0
NLF008_scaffold98_8	non-phage	0
NLF008_scaffold9853_2	non-phage	0
NLF008_scaffold85_13	non-phage	0
NLF008_scaffold4989_2	non-phage	0

NLF008_scaffold4231_2	non-phage	0
NLF008_scaffold17964_1	non-phage	0
NLF008_scaffold12825_17	non-phage	0
NLF008_scaffold9347_1	non-phage	0
NLF008_scaffold1813_9	non-phage	0
NLF008_scaffold15811_1	non-phage	0
NLF008_scaffold8021_4	non-phage	0
NLF008_scaffold20669_1	non-phage	0
NLF008_scaffold20265_1	non-phage	0
NLF008_scaffold7660_3	non-phage	0
NLF008_scaffold20746_1	non-phage	0
NLF008_scaffold10939_2	non-phage	0
NLF008_scaffold16397_1	non-phage	0
NLF008_scaffold12340_2	non-phage	0
NLF008_scaffold8655_2	non-phage	0
NLF008_scaffold150_7	non-phage	0
NLF008_scaffold6368_2	non-phage	0
NLF008_scaffold735_10	non-phage	0
NLF008_scaffold150_4	non-phage	0
NLF008_scaffold877_3	non-phage	0
NLF008_scaffold10142_7	non-phage	0
NLF008_scaffold8919_2	non-phage	0
NLF008_scaffold11943_8	non-phage	0
NLF008_scaffold6876_4	non-phage	0
NLF008_scaffold9492_4	non-phage	0
NLF008_scaffold13669_2	non-phage	0
NLF008_scaffold7390_4	non-phage	0
NLF008_scaffold20047_3	non-phage	0
NLF008_scaffold9241_2	non-phage	0
NLF008_scaffold20525_2	non-phage	0
NLF008_scaffold4326_8	non-phage	0
NLF008_scaffold13758_4	non-phage	0
NLF008_scaffold8786_3	non-phage	0
NLF008_scaffold1800_13	non-phage	0
NLF008_C316312_1	non-phage	0
NLF008_scaffold20598_5	non-phage	0
NLF008_scaffold1787_3	non-phage	0
NLF009_scaffold43907_13	non-phage	0
NLF009_scaffold54360_1	non-phage	0
NLF009_scaffold28954_1	non-phage	0
NLF009_scaffold622_18	non-phage	0
NLF009_scaffold10856_2	non-phage	0
NLF009_scaffold30204_3	non-phage	0
NLF009_C805763_1	non-phage	0
NLF009_scaffold5668_33	non-phage	0
NLF009_scaffold18836_1	non-phage	0

NLF009_scaffold2332_5	non-phage	0
NLF009_scaffold1596_1	non-phage	0
NLF009_scaffold26509_5	non-phage	0
NLF009_scaffold2413_3	non-phage	0
NLF009_scaffold29449_6	non-phage	0
NLF009_scaffold30987_1	non-phage	0
NLF009_scaffold54233_1	non-phage	0
NLF009_scaffold45822_4	non-phage	0
NLF009_scaffold7929_5	non-phage	0
NLF009_scaffold9173_1	non-phage	0
NLF009_scaffold48591_1	non-phage	0
NLF009_scaffold6837_1	non-phage	0
NLF009_scaffold54627_1	non-phage	0
NLF009_scaffold17443_12	non-phage	0
NLF009_scaffold43595_6	non-phage	0
NLF009_scaffold42573_1	non-phage	0
NLF009_scaffold53070_1	non-phage	0
NLF009_C805679_1	non-phage	0
NLF009_scaffold6837_15	non-phage	0
NLF009_scaffold9575_6	non-phage	0
NLF009_scaffold15013_25	non-phage	0
NLF009_scaffold375_72	non-phage	0
NLF009_scaffold7854_1	non-phage	0
NLF009_scaffold1838_3	non-phage	0
NLF009_scaffold8027_2	non-phage	0
NLF009_scaffold11746_34	non-phage	0
NLF009_scaffold410_7	non-phage	0
NLF009_C805545_1	non-phage	0
NLF009_scaffold2279_7	non-phage	0
NLF009_scaffold9165_9	non-phage	0
NLF009_scaffold16211_13	non-phage	0
NLF009_scaffold16558_3	non-phage	0
NLF009_scaffold52230_2	non-phage	0
NLF009_scaffold8485_21	non-phage	0
NLF009_scaffold29612_1	non-phage	0
NLF009_scaffold7539_4	non-phage	0
NLF009_scaffold9165_17	non-phage	0
NLF009_scaffold36161_3	non-phage	0
NLF009_scaffold35939_4	non-phage	0
NLF009_scaffold2642_2	non-phage	0
NLF009_scaffold12510_15	non-phage	0
NLF009_scaffold1838_10	non-phage	0
NLF009_scaffold16804_1	non-phage	0
NLF009_scaffold129_37	non-phage	0
NLF009_scaffold8496_14	non-phage	0
NLF009_scaffold14474_9	non-phage	0

NLF009_scaffold49152_7	non-phage	0
NLF009_scaffold9165_14	non-phage	0
NLF009_scaffold48695_15	non-phage	0
NLF009_scaffold8889_10	non-phage	0
NLF009_scaffold3100_2	non-phage	0
NLF009_scaffold12978_17	non-phage	0
NLF009_scaffold54603_2	non-phage	0
NLF009_scaffold54615_1	non-phage	0
NLF009_scaffold11426_3	non-phage	0
NLF009_scaffold11045_28	non-phage	0
NLF009_scaffold2279_8	non-phage	0
NLF009_scaffold43083_1	non-phage	0
NLF009_scaffold13587_11	non-phage	0
NLF009_scaffold7539_3	non-phage	0
NLF009_scaffold12978_13	non-phage	0
NLF009_scaffold116_3	non-phage	0
NLF009_C805641_1	non-phage	0
NLF009_scaffold12644_42	non-phage	0
NLF009_scaffold11426_7	non-phage	0
NLF009_scaffold2279_2	non-phage	0
NLF009_scaffold17504_9	non-phage	0
NLF009_C805639_1	non-phage	0
NLF009_scaffold46673_4	non-phage	0
NLF009_scaffold220_12	non-phage	0
NLF009_scaffold14474_3	non-phage	0
NLF009_scaffold31384_18	non-phage	0
NLF009_scaffold54173_1	non-phage	0
NLF009_scaffold51579_2	non-phage	0
NLF009_scaffold9313_18	non-phage	0
NLF009_scaffold46673_3	non-phage	0
NLF009_scaffold15144_20	non-phage	0
NLF009_scaffold16783_3	non-phage	0
NLF009_scaffold17149_4	non-phage	0
NLF009_scaffold39552_4	non-phage	0
NLF009_scaffold52060_1	non-phage	0
NLF009_scaffold39552_5	non-phage	0
NLF009_scaffold106_2	non-phage	0
NLF009_scaffold9165_6	non-phage	0
NLF009_scaffold54630_1	non-phage	0
NLF009_scaffold16_10	non-phage	0
NLF009_scaffold54626_1	non-phage	0
NLF009_scaffold1100_3	non-phage	0
NLF009_scaffold22486_11	non-phage	0
NLF009_scaffold375_43	non-phage	0
NLF009_scaffold11746_30	non-phage	0
NLF009_scaffold46104_2	non-phage	0

NLF009_scaffold622_15	non-phage	0
NLF009_scaffold21790_7	non-phage	0
NLF009_scaffold24862_1	non-phage	0
NLF009_scaffold1838_4	non-phage	0
NLF009_scaffold54597_1	non-phage	0
NLF009_scaffold16075_14	non-phage	0
NLF009_scaffold1836_1	non-phage	0
NLF009_scaffold410_10	non-phage	0
NLF009_scaffold21411_2	non-phage	0
NLF009_scaffold50505_1	non-phage	0
NLF009_C805765_1	non-phage	0
NLF009_scaffold5668_8	non-phage	0
NLF009_scaffold5629_3	non-phage	0
NLF009_scaffold48442_1	non-phage	0
NLF009_scaffold129_34	non-phage	0
NLF009_scaffold14874_9	non-phage	0
NLF009_scaffold46673_2	non-phage	0
NLF009_scaffold35671_2	non-phage	0
NLF009_scaffold6815_2	non-phage	0
NLF009_scaffold24156_9	non-phage	0
NLF009_scaffold9165_4_1	non-phage	0
NLF009_scaffold35482_1	non-phage	0
NLF009_scaffold46104_1	non-phage	0
NLF009_scaffold14474_1	non-phage	0
NLF009_scaffold4370_2	non-phage	0
NLF009_scaffold43714_2	non-phage	0
NLF009_scaffold9750_6	non-phage	0
NLF009_scaffold86_12	non-phage	0
NLF009_scaffold54358_1	non-phage	0
NLF009_scaffold6815_33	non-phage	0
NLF009_scaffold53998_3	non-phage	0
NLF009_scaffold29936_9	non-phage	0
NLF009_scaffold8485_9	non-phage	0
NLF009_scaffold50173_1	non-phage	0
NLF009_scaffold38675_8	non-phage	0
NLF009_scaffold54592_1	non-phage	0
NLF009_scaffold21429_15	non-phage	0
NLF009_scaffold21602_1	non-phage	0
NLF009_scaffold8926_5	non-phage	0
NLF009_scaffold9694_3	non-phage	0
NLF009_scaffold24288_2	non-phage	0
NLF009_scaffold26804_8	non-phage	0
NLF009_scaffold8510_1	non-phage	0
NLF009_scaffold25945_5	non-phage	0
NLF009_scaffold8496_21	non-phage	0
NLF009_scaffold30161_1	non-phage	0

NLF009_C805595_1	non-phage	0
NLF009_scaffold9626_8	non-phage	0
NLF009_scaffold11426_6	non-phage	0
NLF009_scaffold16795_6	non-phage	0
NLF009_scaffold15442_19	non-phage	0
NLF009_scaffold15013_16	non-phage	0
NLF009_scaffold107_1	non-phage	0
NLF009_C805813_1	non-phage	0
NLF009_scaffold21771_3	non-phage	0
NLF009_scaffold6815_17	non-phage	0
NLF009_scaffold54415_2	non-phage	0
NLF009_scaffold38926_2	non-phage	0
NLF009_scaffold8027_1	non-phage	0
NLF009_scaffold29936_1	non-phage	0
NLF009_scaffold9165_11	non-phage	0
NLF009_scaffold8414_19	non-phage	0
NLF009_scaffold30204_6	non-phage	0
NLF009_scaffold918_17	non-phage	0
NLF009_scaffold21947_2	non-phage	0
NLF009_scaffold3239_22	non-phage	0
NLF009_scaffold53087_2	non-phage	0
NLF009_scaffold54629_1	non-phage	0
NLF009_scaffold13707_2	non-phage	0
NLF009_scaffold15013_3	non-phage	0
NLF009_scaffold15144_1	non-phage	0
NLF009_scaffold4997_16	non-phage	0
NLF009_scaffold53958_1	non-phage	0
NLF009_scaffold14474_10	non-phage	0
NLF009_scaffold7539_8	non-phage	0
NLF009_scaffold29936_11	non-phage	0
NLF009_scaffold15442_15	non-phage	0
NLF009_scaffold37541_1	non-phage	0
NLF009_scaffold15595_2	non-phage	0
NLF009_scaffold16795_3	non-phage	0
NLF009_scaffold9689_2	non-phage	0
NLF009_scaffold42561_5	non-phage	0
NLF009_scaffold5_25	non-phage	0
NLF009_scaffold51462_1	non-phage	0
NLF009_scaffold54296_1	non-phage	0
NLF009_scaffold43084_1	non-phage	0
NLF009_scaffold9173_6	non-phage	0
NLF009_scaffold14874_6	non-phage	0
NLF009_scaffold51986_1	non-phage	0
NLF009_scaffold16211_44	non-phage	0
NLF009_scaffold54621_1	non-phage	0
NLF009_C805793_1	non-phage	0

NLF010_scaffold14000_15	non-phage	0
NLF010_scaffold28481_1	non-phage	0
NLF010_scaffold7527_5	non-phage	0
NLF010_scaffold2050_3	non-phage	0
NLF010_scaffold8584_3	non-phage	0
NLF010_scaffold26275_2	non-phage	0
NLF010_scaffold16654_1	non-phage	0
NLF010_scaffold29116_2	non-phage	0
NLF010_scaffold22308_6	non-phage	0
NLF010_scaffold1207_12	non-phage	0
NLF010_scaffold5627_8	non-phage	0
NLF010_scaffold2792_2	non-phage	0
NLF010_scaffold813_1	non-phage	0
NLF010_scaffold27133_2	non-phage	0
NLF010_scaffold2595_3	non-phage	0
NLF010_scaffold4291_10	non-phage	0
NLF010_scaffold12295_4	non-phage	0
NLF010_scaffold666_52	non-phage	0
NLF010_scaffold5627_1	non-phage	0
NLF010_scaffold28853_2	non-phage	0
NLF010_scaffold7516_1	non-phage	0
NLF010_scaffold2677_37	non-phage	0
NLF010_scaffold19382_1	non-phage	0
NLF010_scaffold1656_3	non-phage	0
NLF010_scaffold11313_34	non-phage	0
NLF010_scaffold23824_1	non-phage	0
NLF010_scaffold70_1	non-phage	0
NLF010_scaffold16373_10	non-phage	0
NLF010_scaffold5177_2	non-phage	0
NLF010_scaffold5851_17	non-phage	0
NLF010_scaffold20128_1	non-phage	0
NLF010_C401749_1	non-phage	0
NLF010_scaffold6978_3	non-phage	0
NLF010_scaffold29473_2	non-phage	0
NLF010_scaffold9547_4	non-phage	0
NLF010_scaffold29247_2	non-phage	0
NLF010_scaffold5627_5	non-phage	0
NLF010_scaffold28405_6	non-phage	0
NLF010_scaffold21203_1	non-phage	0
NLF010_scaffold3319_5	non-phage	0
NLF010_scaffold5712_24	non-phage	0
NLF010_scaffold29461_1	non-phage	0
NLF010_scaffold4291_5	non-phage	0
NLF010_scaffold6368_12	non-phage	0
NLF010_scaffold26791_4	non-phage	0
NLF010_scaffold29329_1	non-phage	0

NLF010_scaffold666_61	non-phage	0
NLF010_scaffold17756_2	non-phage	0
NLF010_scaffold29210_1	non-phage	0
NLF010_scaffold28255_1	non-phage	0
NLF010_scaffold26308_1	non-phage	0
NLF010_C401883_1	non-phage	0
NLF010_scaffold2677_25	non-phage	0
NLF010_scaffold64_3	non-phage	0
NLF010_scaffold2871_1	non-phage	0
NLF010_scaffold36_11	non-phage	0
NLF010_scaffold18_11	non-phage	0
NLF010_scaffold6368_8	non-phage	0
NLF010_scaffold60_2	non-phage	0
NLF010_scaffold5523_9	non-phage	0
NLF010_scaffold23480_1	non-phage	0
NLF010_scaffold29460_1	non-phage	0
NLF010_scaffold8595_2	non-phage	0
NLF010_scaffold24646_1	non-phage	0
NLF010_scaffold18534_1	non-phage	0
NLF010_C402041_1	non-phage	0
NLF010_scaffold19985_3	non-phage	0
NLF010_C401767_1	non-phage	0
NLF010_scaffold12405_1	non-phage	0
NLF010_scaffold666_32	non-phage	0
NLF010_scaffold9988_2	non-phage	0
NLF010_scaffold14948_2	non-phage	0
NLF010_scaffold28405_2	non-phage	0
NLF010_scaffold1372_9	non-phage	0
NLF010_scaffold1045_5	non-phage	0
NLF010_scaffold1628_3	non-phage	0
NLF010_scaffold29256_1	non-phage	0
NLF010_scaffold9618_4	non-phage	0
NLF010_scaffold1534_3	non-phage	0
NLF010_scaffold2346_5	non-phage	0
NLF010_scaffold3721_15	non-phage	0
NLF010_scaffold11410_30	non-phage	0
NLF010_scaffold18686_7	non-phage	0
NLF010_scaffold25929_2	non-phage	0
NLF010_scaffold13824_3	non-phage	0
NLF010_scaffold12795_3	non-phage	0
NLF010_scaffold36_12	non-phage	0
NLF010_scaffold21634_3	non-phage	0
NLF010_scaffold6368_7	non-phage	0
NLF010_scaffold11490_1	non-phage	0
NLF010_scaffold7861_15	non-phage	0
NLF010_scaffold13546_3	non-phage	0

NLF010_scaffold14948_4	non-phage	0
NLF010_scaffold18_4	non-phage	0
NLF010_scaffold22338_2	non-phage	0
NLF010_scaffold16274_2	non-phage	0
NLF010_scaffold21245_5	non-phage	0
NLF010_scaffold21202_1	non-phage	0
NLF010_scaffold5743_2	non-phage	0
NLF010_scaffold11762_6	non-phage	0
NLF010_scaffold29031_2	non-phage	0
NLF010_scaffold29184_1	non-phage	0
NLF010_scaffold2309_1	non-phage	0
NLF010_scaffold24779_2	non-phage	0
NLF010_scaffold549_46	non-phage	0
NLF010_scaffold5712_22	non-phage	0
NLF010_scaffold18175_2	non-phage	0
NLF010_scaffold10014_5	non-phage	0
NLF010_scaffold13786_2	non-phage	0
NLF010_scaffold4994_4	non-phage	0
NLF010_scaffold22632_10	non-phage	0
NLF010_scaffold16597_1	non-phage	0
NLF010_scaffold5357_4	non-phage	0
NLF010_scaffold2978_4	non-phage	0
NLF010_scaffold28036_1	non-phage	0
NLF010_scaffold10484_1	non-phage	0
NLF010_scaffold27658_1	non-phage	0
NLF010_scaffold1882_3	non-phage	0
NLF010_scaffold1207_21	non-phage	0
NLF010_scaffold21301_2	non-phage	0
NLF010_scaffold144_7	non-phage	0
NLF010_scaffold26791_5	non-phage	0
NLF010_C402065_1	non-phage	0
NLF010_scaffold28844_4	non-phage	0
NLF010_scaffold8584_4	non-phage	0
NLF010_scaffold26226_1	non-phage	0
NLF010_scaffold24847_1	non-phage	0
NLF010_scaffold109_8	non-phage	0
NLF010_scaffold21301_3	non-phage	0
NLF010_scaffold1631_6	non-phage	0
NLF010_scaffold25324_1	non-phage	0
NLF010_scaffold3566_1	non-phage	0
NLF010_scaffold29388_1	non-phage	0
NLF010_scaffold12295_3	non-phage	0
NLF010_scaffold1584_2	non-phage	0
NLF010_scaffold18279_1	non-phage	0
NLF010_scaffold4291_3	non-phage	0
NLF010_scaffold7525_4	non-phage	0

NLF010_scaffold27434_4	non-phage	0
NLF010_scaffold16896_1	non-phage	0
NLF010_scaffold16274_4	non-phage	0
NLF010_scaffold5124_15	non-phage	0
NLF010_scaffold18534_14	non-phage	0
NLF010_scaffold1207_7	non-phage	0
NLF010_scaffold4103_1	non-phage	0
NLF010_scaffold2677_33	non-phage	0
NLF010_scaffold4994_5	non-phage	0
NLF010_scaffold28233_2	non-phage	0
NLF010_scaffold12295_2	non-phage	0
NLF010_scaffold17035_1	non-phage	0
NLF010_scaffold27739_2	non-phage	0
NLF010_scaffold2871_2	non-phage	0
NLF010_scaffold2_3	non-phage	0
NLF010_scaffold2053_1	non-phage	0
NLF010_scaffold26791_6	non-phage	0
NLF010_scaffold20163_1	non-phage	0
NLF010_scaffold14000_6	non-phage	0
NLF010_scaffold2978_3	non-phage	0
NLF010_scaffold86_10	non-phage	0
NLF010_scaffold17340_1	non-phage	0
NLF010_C401733_1	non-phage	0
NLF010_scaffold19985_1	non-phage	0
NLF010_scaffold549_3	non-phage	0
NLF010_scaffold11563_4	non-phage	0
NLF011_scaffold11962_2	non-phage	0
NLF011_scaffold1984_2	non-phage	0
NLF011_scaffold9178_1	non-phage	0
NLF011_scaffold106_1	non-phage	0
NLF011_scaffold35391_1	non-phage	0
NLF011_scaffold343_4	non-phage	0
NLF011_scaffold3588_4	non-phage	0
NLF011_scaffold4378_7	non-phage	0
NLF011_scaffold177_1	non-phage	0
NLF011_scaffold169_1	non-phage	0
NLF011_scaffold619_1	non-phage	0
NLF011_scaffold33097_2	non-phage	0
NLF011_scaffold13221_1	non-phage	0
NLF011_scaffold25086_2	non-phage	0
NLF011_scaffold7575_1_1	non-phage	0
NLF011_scaffold15567_4	non-phage	0
NLF011_scaffold2143_3	non-phage	0
NLF011_scaffold2638_2	non-phage	0
NLF011_scaffold24439_2	non-phage	0
NLF011_scaffold356_1	non-phage	0

NLF011_scaffold288_5	non-phage	0
NLF011_scaffold182_3	non-phage	0
NLF011_scaffold7117_4	non-phage	0
NLF011_scaffold242_1	non-phage	0
NLF011_scaffold7117_12	non-phage	0
NLF011_scaffold5166_2	non-phage	0
NLF011_scaffold20438_2	non-phage	0
NLF011_scaffold17878_1	non-phage	0
NLF011_scaffold6661_1	non-phage	0
NLF011_scaffold22590_1	non-phage	0
NLF011_scaffold16783_4	non-phage	0
NLF011_scaffold32719_1	non-phage	0
NLF011_scaffold83_9	non-phage	0
NLF011_scaffold547_4	non-phage	0
NLF011_scaffold2050_2	non-phage	0
NLF011_scaffold16484_6	non-phage	0
NLF011_scaffold30773_2	non-phage	0
NLF011_scaffold5175_3	non-phage	0
NLF011_scaffold16243_11	non-phage	0
NLF011_scaffold7117_7	non-phage	0
NLF011_scaffold24896_1	non-phage	0
NLF011_scaffold4500_16	non-phage	0
NLF011_scaffold514_14	non-phage	0
NLF011_scaffold10835_1	non-phage	0
NLF011_scaffold110_3	non-phage	0
NLF011_scaffold490_1	non-phage	0
NLF011_scaffold456_1	non-phage	0
NLF011_scaffold36458_1	non-phage	0
NLF011_scaffold529_1	non-phage	0
NLF011_scaffold7827_1	non-phage	0
NLF011_scaffold15295_8	non-phage	0
NLF011_scaffold8_8	non-phage	0
NLF011_scaffold438_1	non-phage	0
NLF011_scaffold9043_9	non-phage	0
NLF011_scaffold2169_2	non-phage	0
NLF011_scaffold22_1_1	non-phage	0
NLF011_scaffold200_2	non-phage	0
NLF011_scaffold649_3	non-phage	0
NLF011_scaffold121_1	non-phage	0
NLF011_scaffold11601_3	non-phage	0
NLF011_scaffold7547_2	non-phage	0
NLF011_scaffold10791_1	non-phage	0
NLF011_scaffold10520_1	non-phage	0
NLF011_scaffold7355_3	non-phage	0
NLF011_scaffold7617_1	non-phage	0
NLF011_scaffold6961_4	non-phage	0

NLF011_scaffold7385_4	non-phage	0
NLF011_scaffold14109_9	non-phage	0
NLF011_scaffold6323_1	non-phage	0
NLF011_scaffold209_2_1	non-phage	0
NLF011_scaffold74_2	non-phage	0
NLF011_scaffold3094_1	non-phage	0
NLF011_scaffold30928_1	non-phage	0
NLF011_scaffold489_1	non-phage	0
NLF011_scaffold1281_1	non-phage	0
NLF011_scaffold1598_2	non-phage	0
NLF011_scaffold12114_1	non-phage	0
NLF011_scaffold5703_2	non-phage	0
NLF011_scaffold15540_12	non-phage	0
NLF011_scaffold8792_5	non-phage	0
NLF011_scaffold342_3	non-phage	0
NLF011_scaffold100_1	non-phage	0
NLF011_scaffold9681_1	non-phage	0
NLF011_scaffold4505_1	non-phage	0
NLF011_scaffold411_6	non-phage	0
NLF011_scaffold11463_2	non-phage	0
NLF011_scaffold11957_2	non-phage	0
NLF011_scaffold580_2	non-phage	0
NLF011_scaffold26118_1	non-phage	0
NLF011_scaffold7399_1	non-phage	0
NLF011_scaffold7575_4	non-phage	0
NLF011_scaffold10111_2	non-phage	0
NLF011_scaffold127_1	non-phage	0
NLF011_scaffold139_4	non-phage	0
NLF011_scaffold17880_3	non-phage	0
NLF011_scaffold36307_2	non-phage	0
NLF011_scaffold1186_1	non-phage	0
NLF011_scaffold3726_1	non-phage	0
NLF011_scaffold13186_1	non-phage	0
NLF011_scaffold105_6	non-phage	0
NLF011_scaffold33873_1	non-phage	0
NLF011_scaffold5873_1	non-phage	0
NLF011_scaffold16267_3	non-phage	0
NLF011_scaffold11349_2	non-phage	0
NLF011_scaffold35_1	non-phage	0
NLF011_scaffold4490_7	non-phage	0
NLF011_scaffold12454_2	non-phage	0
NLF011_scaffold209_4	non-phage	0
NLF011_scaffold12595_2	non-phage	0
NLF011_scaffold1088_1	non-phage	0
NLF011_scaffold627_5	non-phage	0
NLF011_scaffold12915_1	non-phage	0

NLF011_scaffold5196_2	non-phage	0
NLF011_scaffold489_2	non-phage	0
NLF011_scaffold52_1	non-phage	0
NLF011_scaffold6230_1	non-phage	0
NLF011_scaffold5407_1_1	non-phage	0
NLF011_scaffold6675_2	non-phage	0
NLF011_scaffold2729_4	non-phage	0
NLF011_scaffold29664_6	non-phage	0
NLF011_scaffold474_1	non-phage	0
NLF011_scaffold7350_1	non-phage	0
NLF011_scaffold5308_3	non-phage	0
NLF011_scaffold22392_4	non-phage	0
NLF011_scaffold16854_2	non-phage	0
NLF011_scaffold26189_2	non-phage	0
NLF011_scaffold1756_2	non-phage	0
NLF011_scaffold22198_1	non-phage	0
NLF011_scaffold853_4	non-phage	0
NLF011_scaffold13688_3	non-phage	0
NLF011_scaffold469_2	non-phage	0
NLF011_scaffold4208_2	non-phage	0
NLF011_scaffold4333_1	non-phage	0
NLF011_scaffold9341_3	non-phage	0
NLF011_scaffold5531_6	non-phage	0
NLF011_scaffold5033_1	non-phage	0
NLF011_scaffold4287_3	non-phage	0
NLF011_scaffold11725_1	non-phage	0
NLF011_scaffold13688_2	non-phage	0
NLF011_scaffold614_1	non-phage	0
NLF011_scaffold889_7	non-phage	0
NLF011_scaffold2046_1_1	non-phage	0
NLF011_scaffold13545_2	non-phage	0
NLF011_scaffold5175_4	non-phage	0
NLF011_scaffold7117_10	non-phage	0
NLF011_scaffold13381_2	non-phage	0
NLF011_scaffold2270_2	non-phage	0
NLF011_scaffold39_5	non-phage	0
NLF011_scaffold614_2	non-phage	0
NLF011_scaffold134_2	non-phage	0
NLF011_scaffold13258_7	non-phage	0
NLF011_scaffold416_1	non-phage	0
NLF011_scaffold15136_1	non-phage	0
NLF011_scaffold8_6	non-phage	0
NLF011_scaffold6297_1	non-phage	0
NLF011_scaffold11185_6	non-phage	0
NLF011_scaffold2336_3	non-phage	0
NLF011_scaffold4286_3	non-phage	0

NLF011_scaffold16989_1	non-phage	0
NLF011_scaffold443_8	non-phage	0
NLF011_scaffold17688_1	non-phage	0
NLF011_scaffold35824_1	non-phage	0
NLF011_scaffold20345_3	non-phage	0
NLF011_scaffold13048_4	non-phage	0
NLF011_scaffold5385_2_1	non-phage	0
NLF011_scaffold3934_1	non-phage	0
NLF011_scaffold627_3	non-phage	0
NLF011_scaffold2105_4	non-phage	0
NLF011_scaffold9785_5_1	non-phage	0
NLF011_scaffold9382_2	non-phage	0
NLF011_scaffold7209_4	non-phage	0
NLF011_scaffold1097_3	non-phage	0
NLF011_scaffold16132_4	non-phage	0
NLF011_scaffold4453_4	non-phage	0
NLF011_scaffold490_8	non-phage	0
NLF011_scaffold1356_1	non-phage	0
NLF012_scaffold18530_1	non-phage	0
NLF012_scaffold25640_1	non-phage	0
NLF012_scaffold12819_1	non-phage	0
NLF012_scaffold8654_2	non-phage	0
NLF012_scaffold36182_4	non-phage	0
NLF012_scaffold5839_1	non-phage	0
NLF012_scaffold22600_4	non-phage	0
NLF012_scaffold3180_1	non-phage	0
NLF012_scaffold77_11	non-phage	0
NLF012_C512275_1	non-phage	0
NLF012_scaffold28397_1	non-phage	0
NLF012_scaffold21129_8	non-phage	0
NLF012_scaffold7247_3	non-phage	0
NLF012_scaffold9845_5	non-phage	0
NLF012_scaffold25640_5	non-phage	0
NLF012_scaffold3176_2	non-phage	0
NLF012_scaffold20153_4	non-phage	0
NLF012_scaffold10550_3	non-phage	0
NLF012_scaffold3106_5	non-phage	0
NLF012_scaffold11833_4	non-phage	0
NLF012_scaffold3602_15	non-phage	0
NLF012_scaffold20242_3	non-phage	0
NLF012_scaffold8654_14	non-phage	0
NLF012_scaffold907_20	non-phage	0
NLF012_scaffold20136_3	non-phage	0
NLF012_scaffold39182_1	non-phage	0
NLF012_scaffold15501_1	non-phage	0
NLF012_scaffold38689_1	non-phage	0

NLF012_scaffold22895_5	non-phage	0
NLF012_scaffold35_10	non-phage	0
NLF012_scaffold10004_8	non-phage	0
NLF012_scaffold11986_2	non-phage	0
NLF012_scaffold7531_17	non-phage	0
NLF012_scaffold38963_1	non-phage	0
NLF012_scaffold39139_1	non-phage	0
NLF012_scaffold23091_6	non-phage	0
NLF012_scaffold19427_1	non-phage	0
NLF012_scaffold39133_1	non-phage	0
NLF012_scaffold38103_1	non-phage	0
NLF012_scaffold17596_1	non-phage	0
NLF012_scaffold27546_2	non-phage	0
NLF012_scaffold14270_1	non-phage	0
NLF012_scaffold186_4	non-phage	0
NLF012_scaffold9845_2	non-phage	0
NLF012_scaffold39180_1	non-phage	0
NLF012_scaffold36967_1	non-phage	0
NLF012_scaffold10663_4	non-phage	0
NLF012_scaffold1959_17	non-phage	0
NLF012_scaffold2047_4	non-phage	0
NLF012_scaffold8747_8	non-phage	0
NLF012_scaffold39130_2	non-phage	0
NLF012_scaffold4830_3	non-phage	0
NLF012_scaffold6247_5	non-phage	0
NLF012_scaffold38408_2	non-phage	0
NLF012_scaffold1150_2	non-phage	0
NLF012_scaffold13599_1	non-phage	0
NLF012_scaffold21200_1	non-phage	0
NLF012_scaffold10204_1	non-phage	0
NLF012_scaffold19699_3	non-phage	0
NLF012_scaffold14917_2	non-phage	0
NLF012_scaffold15501_16	non-phage	0
NLF012_scaffold3306_11	non-phage	0
NLF012_scaffold8747_2	non-phage	0
NLF012_scaffold16348_5	non-phage	0
NLF012_scaffold17180_3	non-phage	0
NLF012_scaffold39165_1	non-phage	0
NLF012_scaffold25682_5	non-phage	0
NLF012_scaffold21129_10	non-phage	0
NLF012_scaffold39142_1	non-phage	0
NLF012_scaffold9422_8	non-phage	0
NLF012_scaffold479_9	non-phage	0
NLF012_scaffold17135_1	non-phage	0
NLF012_scaffold39091_1	non-phage	0
NLF012_scaffold38358_2	non-phage	0

NLF012_scaffold10550_4	non-phage	0
NLF012_scaffold2808_1	non-phage	0
NLF012_scaffold23633_1	non-phage	0
NLF012_scaffold19920_3	non-phage	0
NLF012_scaffold38414_1	non-phage	0
NLF012_scaffold3176_5	non-phage	0
NLF012_scaffold5296_2	non-phage	0
NLF012_scaffold241_5	non-phage	0
NLF012_scaffold3210_1	non-phage	0
NLF012_scaffold33761_2	non-phage	0
NLF012_scaffold6247_20	non-phage	0
NLF012_scaffold38988_1	non-phage	0
NLF012_scaffold15106_22	non-phage	0
NLF012_scaffold37578_4	non-phage	0
NLF012_scaffold15414_1	non-phage	0
NLF012_scaffold750_16	non-phage	0
NLF012_scaffold2808_8	non-phage	0
NLF012_scaffold36257_1	non-phage	0
NLF012_scaffold12677_9	non-phage	0
NLF012_scaffold2787_1	non-phage	0
NLF012_scaffold9422_5	non-phage	0
NLF012_scaffold38656_1	non-phage	0
NLF012_scaffold34520_2	non-phage	0
NLF012_scaffold52_10	non-phage	0
NLF012_scaffold14203_1	non-phage	0
NLF012_scaffold14622_1	non-phage	0
NLF012_scaffold750_39	non-phage	0
NLF012_scaffold13768_1	non-phage	0
NLF012_scaffold31276_2	non-phage	0
NLF012_scaffold21129_4	non-phage	0
NLF012_scaffold17602_1	non-phage	0
NLF012_scaffold2787_22	non-phage	0
NLF012_scaffold39093_2	non-phage	0
NLF012_scaffold4830_2	non-phage	0
NLF012_scaffold2313_10	non-phage	0
NLF012_scaffold31459_5	non-phage	0
NLF012_scaffold2808_13	non-phage	0
NLF012_scaffold15887_4	non-phage	0
NLF012_scaffold38754_1	non-phage	0
NLF012_scaffold37883_2	non-phage	0
NLF012_scaffold29395_1	non-phage	0
NLF012_scaffold6595_12	non-phage	0
NLF012_scaffold4710_13	non-phage	0
NLF012_scaffold13372_4	non-phage	0
NLF012_scaffold2787_14	non-phage	0
NLF012_scaffold3530_5	non-phage	0

NLF012_scaffold39185_1	non-phage	0
NLF012_scaffold14917_1	non-phage	0
NLF012_scaffold15887_1	non-phage	0
NLF012_scaffold3360_3	non-phage	0
NLF012_scaffold161_3	non-phage	0
NLF012_scaffold37768_1	non-phage	0
NLF012_scaffold24518_3	non-phage	0
NLF012_scaffold36307_2	non-phage	0
NLF012_scaffold38865_1	non-phage	0
NLF012_scaffold36675_7	non-phage	0
NLF012_scaffold350_11	non-phage	0
NLF012_scaffold29304_1	non-phage	0
NLF012_scaffold22623_3	non-phage	0
NLF012_scaffold2215_19	non-phage	0
NLF012_scaffold38520_2	non-phage	0
NLF012_scaffold2215_7	non-phage	0
NLF012_scaffold2787_11	non-phage	0
NLF012_scaffold18182_3	non-phage	0
NLF012_scaffold16882_2	non-phage	0
NLF012_scaffold3602_2	non-phage	0
NLF012_scaffold23865_18	non-phage	0
NLF012_scaffold38830_3	non-phage	0
NLF012_scaffold155_1	non-phage	0
NLF012_scaffold10921_1	non-phage	0
NLF012_scaffold908_5	non-phage	0
NLF012_scaffold6247_10	non-phage	0
NLF012_scaffold38336_1	non-phage	0
NLF012_scaffold15887_6	non-phage	0
NLF012_scaffold750_53	non-phage	0
NLF012_scaffold479_14	non-phage	0
NLF012_scaffold38650_1	non-phage	0
NLF012_scaffold8747_6	non-phage	0
NLF012_scaffold6478_5	non-phage	0
NLF012_scaffold34222_1	non-phage	0
NLF012_scaffold34055_3	non-phage	0
NLF012_scaffold15033_2	non-phage	0
NLF012_scaffold38453_1	non-phage	0
NLF012_scaffold16907_1	non-phage	0
NLF012_scaffold19579_1	non-phage	0
NLF012_scaffold26298_1	non-phage	0
NLF012_scaffold31459_6	non-phage	0
NLF012_scaffold350_1	non-phage	0
NLF012_scaffold148_1	non-phage	0
NLF012_scaffold23264_1	non-phage	0
NLF012_scaffold2808_9	non-phage	0
NLF012_scaffold15019_4	non-phage	0

NLF012_scaffold4070_2	non-phage	0
NLF012_scaffold33635_3	non-phage	0
NLF012_scaffold37173_3	non-phage	0
NLF012_scaffold39152_1	non-phage	0
NLF012_scaffold20522_4	non-phage	0
NLF012_scaffold35_5	non-phage	0
NLF012_scaffold39115_1	non-phage	0
NLF012_scaffold38291_2	non-phage	0
NLF012_scaffold30111_6	non-phage	0
NLF012_scaffold9422_14	non-phage	0
NLF012_scaffold32548_1	non-phage	0
NLF012_scaffold18388_1	non-phage	0
NLF012_scaffold39163_1	non-phage	0
NLF012_scaffold36608_2	non-phage	0
NLF012_scaffold32787_5	non-phage	0
NLF012_scaffold33756_1	non-phage	0
NLF012_scaffold3298_1	non-phage	0
NLF012_scaffold2371_9	non-phage	0
NLF012_scaffold7531_6	non-phage	0
NLF012_scaffold730_7	non-phage	0
NLF012_scaffold245_15	non-phage	0
NLF012_scaffold13215_1	non-phage	0
NLF012_scaffold128_7	non-phage	0
NLF012_scaffold8747_10	non-phage	0
NLF012_scaffold11624_4	non-phage	0
NLF012_scaffold36187_1	non-phage	0
NLF012_scaffold14297_2	non-phage	0
NLF012_scaffold35700_1	non-phage	0
NLF012_scaffold31857_1	non-phage	0
NLF012_scaffold6247_11	non-phage	0
NLF012_scaffold15700_4	non-phage	0
NLF012_C512633_1	non-phage	0
NLF012_scaffold38736_1	non-phage	0
NLF012_scaffold6478_2	non-phage	0
NLF012_scaffold1734_5	non-phage	0
NLF012_scaffold245_8	non-phage	0
NLF012_scaffold39007_1	non-phage	0
NLF012_scaffold2313_2	non-phage	0
NLF012_scaffold10370_6	non-phage	0
NLF012_scaffold6845_1	non-phage	0
NLF012_scaffold3602_8	non-phage	0
NLF012_scaffold14434_1	non-phage	0
NLF012_scaffold353_1	non-phage	0
NLF012_scaffold20211_1	non-phage	0
NLF012_scaffold52_2	non-phage	0
NLF012_scaffold750_46	non-phage	0

NLF012_scaffold12454_3	non-phage	0
NLF012_scaffold8747_4	non-phage	0
NLF012_scaffold39188_1	non-phage	0
NLF012_scaffold750_65	non-phage	0
NLF012_scaffold907_15	non-phage	0
NLF012_scaffold16519_1	non-phage	0
NLF012_scaffold15581_1	non-phage	0
NLF012_scaffold5906_1	non-phage	0
NLF012_scaffold24971_1	non-phage	0
NLF012_C512383_1	non-phage	0
NLF012_scaffold17529_1	non-phage	0
NLF012_C512147_1	non-phage	0
NLF012_scaffold29849_4	non-phage	0
NLF012_C512377_1	non-phage	0
NLF012_scaffold9641_2	non-phage	0
NLF012_scaffold10275_30	non-phage	0
NLF012_scaffold285_1	non-phage	0
NLF012_scaffold15876_2	non-phage	0
NLF012_scaffold4497_1	non-phage	0
NLF012_scaffold750_33	non-phage	0
NLF012_scaffold19458_1	non-phage	0
NLF012_scaffold9422_13	non-phage	0
NLF012_scaffold11161_3	non-phage	0
NLF012_scaffold24405_1	non-phage	0
NLF012_scaffold7247_11	non-phage	0
NLF012_scaffold38072_1	non-phage	0
NLF012_scaffold3102_1	non-phage	0
NLF012_scaffold46_5	non-phage	0
NLF012_scaffold2215_3	non-phage	0
NLF012_scaffold37701_2	non-phage	0
NLF012_scaffold2880_5	non-phage	0
NLF012_scaffold7531_13	non-phage	0
NLF012_scaffold17264_4	non-phage	0
NLF012_scaffold39156_1	non-phage	0
NLF012_scaffold37298_2	non-phage	0
NLF012_scaffold5019_1	non-phage	0
NLF012_scaffold479_3	non-phage	0
NLF012_scaffold18622_4	non-phage	0
NLF012_scaffold174_1	non-phage	0
NLF012_scaffold14430_2	non-phage	0
NLF013_scaffold38007_2	non-phage	0
NLF013_scaffold367_32	non-phage	0
NLF013_C659601_1	non-phage	0
NLF013_scaffold48839_1	non-phage	0
NLF013_scaffold15667_2	non-phage	0
NLF013_scaffold5783_1	non-phage	0

NLF013_scaffold5242_1	non-phage	0
NLF013_scaffold43248_1	non-phage	0
NLF013_scaffold48850_2	non-phage	0
NLF013_scaffold48794_1	non-phage	0
NLF013_scaffold15334_7	non-phage	0
NLF013_scaffold37864_3	non-phage	0
NLF013_scaffold48143_2	non-phage	0
NLF013_scaffold4561_22	non-phage	0
NLF013_scaffold41335_7	non-phage	0
NLF013_scaffold35601_1	non-phage	0
NLF013_scaffold8291_5	non-phage	0
NLF013_scaffold13609_6	non-phage	0
NLF013_scaffold13448_1	non-phage	0
NLF013_scaffold18303_6	non-phage	0
NLF013_scaffold42173_2	non-phage	0
NLF013_scaffold48820_1	non-phage	0
NLF013_scaffold48497_3	non-phage	0
NLF013_scaffold3923_17	non-phage	0
NLF013_scaffold48870_1	non-phage	0
NLF013_scaffold46971_1	non-phage	0
NLF013_scaffold7184_1	non-phage	0
NLF013_scaffold47086_1	non-phage	0
NLF013_scaffold48056_1	non-phage	0
NLF013_scaffold4092_10	non-phage	0
NLF013_scaffold256_6	non-phage	0
NLF013_scaffold47688_4	non-phage	0
NLF013_scaffold14051_2	non-phage	0
NLF013_scaffold367_27	non-phage	0
NLF013_scaffold27702_6	non-phage	0
NLF013_scaffold48115_1	non-phage	0
NLF013_scaffold29083_6	non-phage	0
NLF013_C659045_1	non-phage	0
NLF013_scaffold20240_2	non-phage	0
NLF013_scaffold26651_5	non-phage	0
NLF013_scaffold44405_4	non-phage	0
NLF013_scaffold39161_11	non-phage	0
NLF013_scaffold5242_6	non-phage	0
NLF013_scaffold18303_2	non-phage	0
NLF013_scaffold20530_7	non-phage	0
NLF013_scaffold48855_1	non-phage	0
NLF013_scaffold10995_4	non-phage	0
NLF013_scaffold1671_14	non-phage	0
NLF013_scaffold47688_2	non-phage	0
NLF013_C659699_1	non-phage	0
NLF013_scaffold22_5	non-phage	0
NLF013_scaffold29542_3	non-phage	0

NLF013_scaffold25322_15	non-phage	0
NLF013_scaffold7347_11	non-phage	0
NLF013_scaffold3966_9	non-phage	0
NLF013_scaffold46667_4	non-phage	0
NLF013_scaffold48422_6	non-phage	0
NLF013_scaffold23240_2	non-phage	0
NLF013_scaffold8984_4	non-phage	0
NLF013_scaffold41067_1	non-phage	0
NLF013_scaffold38582_4	non-phage	0
NLF013_scaffold37196_3	non-phage	0
NLF013_scaffold970_12	non-phage	0
NLF013_scaffold4561_26	non-phage	0
NLF013_scaffold9814_5	non-phage	0
NLF013_scaffold24917_1	non-phage	0
NLF013_scaffold36275_2	non-phage	0
NLF013_scaffold8291_4	non-phage	0
NLF013_scaffold43192_18	non-phage	0
NLF013_scaffold48479_1	non-phage	0
NLF013_scaffold39192_3	non-phage	0
NLF013_scaffold10031_5	non-phage	0
NLF013_scaffold45904_2	non-phage	0
NLF013_scaffold20090_3	non-phage	0
NLF013_scaffold19933_19	non-phage	0
NLF013_scaffold28691_5	non-phage	0
NLF013_scaffold39192_11	non-phage	0
NLF013_scaffold36561_3	non-phage	0
NLF013_scaffold1857_7	non-phage	0
NLF013_scaffold41858_2	non-phage	0
NLF013_scaffold165_4	non-phage	0
NLF013_scaffold39192_2	non-phage	0
NLF013_scaffold35053_2	non-phage	0
NLF013_scaffold2625_2	non-phage	0
NLF013_scaffold37690_3	non-phage	0
NLF013_scaffold48734_3	non-phage	0
NLF013_scaffold48821_1	non-phage	0
NLF013_scaffold5397_2	non-phage	0
NLF013_scaffold20530_4	non-phage	0
NLF013_scaffold4092_2	non-phage	0
NLF013_scaffold12549_5	non-phage	0
NLF013_scaffold47294_2	non-phage	0
NLF013_scaffold146_5	non-phage	0
NLF013_scaffold40458_1	non-phage	0
NLF013_scaffold19726_1	non-phage	0
NLF013_scaffold17930_3	non-phage	0
NLF013_scaffold20383_5	non-phage	0
NLF013_scaffold3135_1	non-phage	0

NLF013_scaffold38716_1	non-phage	0
NLF013_scaffold25322_21	non-phage	0
NLF013_scaffold33497_2	non-phage	0
NLF013_scaffold43671_2	non-phage	0
NLF013_scaffold4636_2	non-phage	0
NLF013_scaffold35856_2	non-phage	0
NLF013_scaffold21980_1	non-phage	0
NLF013_scaffold20327_7	non-phage	0
NLF013_scaffold30013_5	non-phage	0
NLF013_scaffold34407_2	non-phage	0
NLF013_scaffold1319_1	non-phage	0
NLF013_scaffold42889_3	non-phage	0
NLF013_scaffold2043_4	non-phage	0
NLF013_scaffold41354_1	non-phage	0
NLF013_scaffold48428_1	non-phage	0
NLF013_scaffold21664_1	non-phage	0
NLF013_scaffold10845_1	non-phage	0
NLF013_scaffold47898_2	non-phage	0
NLF013_scaffold28456_2	non-phage	0
NLF013_scaffold37293_2	non-phage	0
NLF013_scaffold3923_15	non-phage	0
NLF013_scaffold8119_2	non-phage	0
NLF013_scaffold31186_1	non-phage	0
NLF013_C658909_1	non-phage	0
NLF013_scaffold273_10	non-phage	0
NLF013_scaffold16515_2	non-phage	0
NLF013_scaffold39710_2	non-phage	0
NLF013_scaffold1082_1	non-phage	0
NLF013_scaffold17324_2	non-phage	0
NLF013_scaffold18588_8	non-phage	0
NLF013_scaffold27842_20	non-phage	0
NLF013_scaffold48451_1	non-phage	0
NLF013_scaffold2043_32	non-phage	0
NLF013_scaffold29368_1	non-phage	0
NLF013_scaffold39192_8	non-phage	0
NLF013_scaffold19771_5	non-phage	0
NLF013_scaffold48841_1	non-phage	0
NLF013_scaffold2121_1	non-phage	0
NLF013_scaffold48497_1	non-phage	0
NLF013_scaffold20090_4	non-phage	0
NLF013_scaffold17286_3	non-phage	0
NLF013_scaffold18893_4	non-phage	0
NLF013_scaffold17765_1	non-phage	0
NLF013_scaffold39613_1	non-phage	0
NLF013_scaffold30884_1	non-phage	0
NLF013_scaffold36561_2	non-phage	0

NLF013_scaffold273_17	non-phage	0
NLF013_scaffold5645_5	non-phage	0
NLF013_scaffold3923_10	non-phage	0
NLF013_scaffold32611_2	non-phage	0
NLF013_scaffold16431_17	non-phage	0
NLF013_scaffold48567_1	non-phage	0
NLF013_scaffold24_1	non-phage	0
NLF013_scaffold12662_7	non-phage	0
NLF013_scaffold5397_4	non-phage	0
NLF013_scaffold1383_2	non-phage	0
NLF013_scaffold109_5	non-phage	0
NLF013_scaffold48829_2	non-phage	0
NLF013_scaffold42205_2	non-phage	0
NLF013_scaffold24917_3	non-phage	0
NLF013_scaffold1472_7	non-phage	0
NLF013_scaffold4561_15	non-phage	0
NLF013_scaffold15694_10	non-phage	0
NLF013_scaffold48417_1	non-phage	0
NLF013_scaffold20327_4	non-phage	0
NLF014_scaffold4495_13	non-phage	0
NLF014_scaffold17826_3	non-phage	0
NLF014_scaffold204_4	non-phage	0
NLF014_scaffold1789_5	non-phage	0
NLF014_scaffold4138_3	non-phage	0
NLF014_scaffold7131_2	non-phage	0
NLF014_scaffold5065_1	non-phage	0
NLF014_scaffold813_4	non-phage	0
NLF014_scaffold9880_5	non-phage	0
NLF014_scaffold515_15	non-phage	0
NLF014_scaffold16928_1	non-phage	0
NLF014_scaffold3737_3	non-phage	0
NLF014_scaffold397_3	non-phage	0
NLF014_scaffold895_9	non-phage	0
NLF014_scaffold6697_1	non-phage	0
NLF014_scaffold1091_3_1	non-phage	0
NLF014_scaffold210_3	non-phage	0
NLF014_scaffold236_2	non-phage	0
NLF014_scaffold4548_2	non-phage	0
NLF014_scaffold1656_3	non-phage	0
NLF014_scaffold2369_1	non-phage	0
NLF014_scaffold904_6	non-phage	0
NLF014_scaffold61_1	non-phage	0
NLF014_scaffold1024_1	non-phage	0
NLF014_scaffold4458_4	non-phage	0
NLF014_scaffold17988_1	non-phage	0
NLF014_scaffold313_2	non-phage	0

NLF014_scaffold2931_2	non-phage	0
NLF014_scaffold747_2	non-phage	0
NLF014_scaffold10980_1	non-phage	0
NLF014_scaffold463_1	non-phage	0
NLF014_scaffold272_4	non-phage	0
NLF014_scaffold294_3	non-phage	0
NLF014_scaffold159_2	non-phage	0
NLF014_scaffold813_8	non-phage	0
NLF014_scaffold6653_2	non-phage	0
NLF014_scaffold2298_1	non-phage	0
NLF014_scaffold354_2	non-phage	0
NLF014_scaffold743_4	non-phage	0
NLF014_scaffold12999_1	non-phage	0
NLF014_scaffold1192_2	non-phage	0
NLF014_scaffold3803_9	non-phage	0
NLF014_scaffold202_1	non-phage	0
NLF014_scaffold216_3	non-phage	0
NLF014_scaffold323_2	non-phage	0
NLF014_scaffold13714_1	non-phage	0
NLF014_scaffold4189_20	non-phage	0
NLF014_scaffold5011_2	non-phage	0
NLF014_scaffold544_1	non-phage	0
NLF014_scaffold424_2	non-phage	0
NLF014_scaffold17251_1	non-phage	0
NLF014_scaffold6470_3	non-phage	0
NLF014_scaffold1430_3	non-phage	0
NLF014_scaffold15_5	non-phage	0
NLF014_scaffold9006_1	non-phage	0
NLF014_scaffold2159_24	non-phage	0
NLF014_scaffold259_2	non-phage	0
NLF014_scaffold21233_1	non-phage	0
NLF014_scaffold775_1	non-phage	0
NLF014_scaffold17826_4	non-phage	0
NLF014_scaffold382_3	non-phage	0
NLF014_scaffold9240_1	non-phage	0
NLF014_scaffold13739_9	non-phage	0
NLF014_scaffold664_1	non-phage	0
NLF014_scaffold428_1	non-phage	0
NLF014_scaffold438_1	non-phage	0
NLF014_scaffold34_3	non-phage	0
NLF014_scaffold142_1	non-phage	0
NLF014_scaffold10696_2	non-phage	0
NLF014_scaffold4634_6	non-phage	0
NLF014_scaffold4591_2	non-phage	0
NLF014_scaffold452_6	non-phage	0
NLF014_scaffold2879_7	non-phage	0

NLF014_scaffold60_2	non-phage	0
NLF014_scaffold11356_1	non-phage	0
NLF014_scaffold5227_3	non-phage	0
NLF014_scaffold14639_1	non-phage	0
NLF014_scaffold74_1	non-phage	0
NLF014_scaffold13584_2	non-phage	0
NLF014_scaffold1883_2	non-phage	0
NLF014_scaffold10710_3	non-phage	0
NLF014_scaffold1465_7	non-phage	0
NLF014_scaffold1176_1	non-phage	0
NLF014_scaffold79_2	non-phage	0
NLF014_scaffold75_2	non-phage	0
NLF014_scaffold233_2_3	non-phage	0
NLF014_scaffold246_3	non-phage	0
NLF014_scaffold1465_10	non-phage	0
NLF014_scaffold23921_1	non-phage	0
NLF014_scaffold355_10	non-phage	0
NLF014_scaffold7029_3	non-phage	0
NLF014_scaffold709_2	non-phage	0
NLF014_scaffold270_1	non-phage	0
NLF014_scaffold391_3	non-phage	0
NLF014_scaffold105_3	non-phage	0
NLF014_scaffold802_2	non-phage	0
NLF014_scaffold257_3	non-phage	0
NLF014_scaffold4170_2	non-phage	0
NLF014_scaffold10715_1	non-phage	0
NLF014_scaffold1491_2	non-phage	0
NLF014_scaffold4277_1	non-phage	0
NLF014_scaffold5914_3	non-phage	0
NLF014_scaffold19217_3	non-phage	0
NLF014_scaffold651_3	non-phage	0
NLF014_scaffold8373_2	non-phage	0
NLF014_scaffold11852_1	non-phage	0
NLF014_scaffold12624_3	non-phage	0
NLF014_scaffold2688_2	non-phage	0
NLF014_scaffold3095_1	non-phage	0
NLF014_scaffold1518_33	non-phage	0
NLF014_scaffold2675_1	non-phage	0
NLF014_scaffold883_2	non-phage	0
NLF014_scaffold13739_4	non-phage	0
NLF014_scaffold1870_11	non-phage	0
NLF014_scaffold7131_7	non-phage	0
NLF014_scaffold11356_2	non-phage	0
NLF014_scaffold583_1	non-phage	0
NLF014_scaffold523_2_2	non-phage	0
NLF014_scaffold270_2	non-phage	0

NLF014_scaffold11595_1	non-phage	0
NLF014_scaffold6995_2_1	non-phage	0
NLF014_scaffold12655_1	non-phage	0
NLF014_scaffold1829_2	non-phage	0
NLF014_scaffold7700_3	non-phage	0
NLF014_scaffold2758_2	non-phage	0
NLF014_scaffold142_4	non-phage	0
NLF014_scaffold1944_1	non-phage	0
NLF014_scaffold2298_3	non-phage	0
NLF014_scaffold79_1	non-phage	0
NLF014_scaffold241_2	non-phage	0
NLF014_scaffold17796_1	non-phage	0
NLF014_scaffold2796_2	non-phage	0
NLF014_scaffold5_2	non-phage	0
NLF014_scaffold268_5	non-phage	0
NLF014_scaffold1007_2	non-phage	0
NLF014_scaffold547_1	non-phage	0
NLF014_scaffold13458_1	non-phage	0
NLF014_scaffold619_6	non-phage	0
NLF014_scaffold695_3	non-phage	0
NLF014_scaffold464_10	non-phage	0
NLF014_scaffold355_11	non-phage	0
NLF014_scaffold894_3	non-phage	0
NLF014_scaffold14_1	non-phage	0
NLF014_scaffold148_4	non-phage	0
NLF014_scaffold6798_1	non-phage	0
NLF014_scaffold379_1	non-phage	0
NLF014_scaffold11567_6	non-phage	0
NLF014_scaffold6414_1	non-phage	0
NLF014_scaffold10998_1	non-phage	0
NLF014_scaffold3808_1	non-phage	0
NLF014_scaffold13466_1	non-phage	0
NLF014_scaffold305_3	non-phage	0
NLF014_scaffold18131_1	non-phage	0
NLF014_scaffold235_4	non-phage	0
NLF014_scaffold2529_2	non-phage	0
NLF014_scaffold3803_12	non-phage	0
NLF014_scaffold448_5	non-phage	0
NLF014_scaffold4410_1_1	non-phage	0
NLF014_scaffold15_8	non-phage	0
NLF014_scaffold4692_9	non-phage	0
NLF014_scaffold2136_2	non-phage	0
NLF014_scaffold1566_5	non-phage	0
NLF014_scaffold19401_4	non-phage	0
NLF014_scaffold1488_9	non-phage	0
NLF014_scaffold189_2	non-phage	0

NLF014_scaffold2879_11	non-phage	0
NLF014_scaffold6653_1	non-phage	0
NLF014_scaffold6532_6	non-phage	0
NLF014_scaffold313_1	non-phage	0
NLF014_scaffold12027_1	non-phage	0
NLF014_scaffold231_3	non-phage	0
NLF014_scaffold16282_1	non-phage	0
NLF014_scaffold85_2	non-phage	0
NLF014_scaffold3797_17	non-phage	0
NLF014_scaffold456_5	non-phage	0
NLF014_scaffold343_1	non-phage	0
NLF014_scaffold813_14	non-phage	0
NLF014_scaffold11004_6	non-phage	0
NLF014_scaffold25004_1	non-phage	0
NLF014_scaffold17_6	non-phage	0
NLF014_scaffold284_8	non-phage	0
NLF014_scaffold47_3	non-phage	0
NLF014_scaffold224_2	non-phage	0
NLF014_scaffold1900_6_1	non-phage	0
NLF014_scaffold6936_3	non-phage	0
NLF014_scaffold863_1	non-phage	0
NLF014_scaffold9874_1	non-phage	0
NLF014_scaffold12344_1	non-phage	0
NLF014_scaffold1007_1	non-phage	0
NLF014_scaffold1398_1	non-phage	0
NLF014_scaffold390_4	non-phage	0
NLF014_scaffold1774_3	non-phage	0
NLF014_scaffold6066_1	non-phage	0
NLF014_scaffold22060_1	non-phage	0
NLF014_scaffold612_1	non-phage	0
NLF014_scaffold12105_2	non-phage	0
NLF014_scaffold7829_5	non-phage	0
NLF015_scaffold624_13	non-phage	0
NLF015_scaffold34516_2	non-phage	0
NLF015_scaffold12200_1	non-phage	0
NLF015_scaffold33290_3	non-phage	0
NLF015_scaffold16063_1	non-phage	0
NLF015_scaffold34556_1	non-phage	0
NLF015_scaffold15708_1	non-phage	0
NLF015_scaffold34550_1	non-phage	0
NLF015_scaffold7711_2	non-phage	0
NLF015_scaffold27631_2	non-phage	0
NLF015_scaffold19848_2	non-phage	0
NLF015_scaffold14539_1	non-phage	0
NLF015_scaffold4443_1	non-phage	0
NLF015_scaffold30103_4	non-phage	0

NLF015_scaffold34142_2	non-phage	0
NLF015_scaffold11305_35	non-phage	0
NLF015_scaffold34545_1	non-phage	0
NLF015_scaffold5435_1	non-phage	0
NLF015_scaffold12014_1	non-phage	0
NLF015_scaffold3816_12	non-phage	0
NLF015_scaffold18919_3	non-phage	0
NLF015_scaffold27387_1	non-phage	0
NLF015_scaffold1368_1	non-phage	0
NLF015_scaffold14785_1	non-phage	0
NLF015_scaffold19791_5	non-phage	0
NLF015_scaffold8636_4	non-phage	0
NLF015_scaffold11305_4	non-phage	0
NLF015_scaffold33913_3	non-phage	0
NLF015_scaffold27274_1	non-phage	0
NLF015_scaffold20539_1	non-phage	0
NLF015_scaffold22436_1	non-phage	0
NLF015_scaffold17733_1	non-phage	0
NLF015_scaffold13003_2	non-phage	0
NLF015_scaffold11305_42	non-phage	0
NLF015_scaffold34516_1	non-phage	0
NLF015_scaffold17767_1	non-phage	0
NLF015_scaffold18838_2	non-phage	0
NLF015_scaffold23291_2	non-phage	0
NLF015_scaffold17650_1	non-phage	0
NLF015_scaffold28792_1	non-phage	0
NLF015_scaffold30081_5	non-phage	0
NLF015_scaffold24720_2	non-phage	0
NLF015_scaffold31821_7	non-phage	0
NLF015_scaffold7022_12	non-phage	0
NLF015_scaffold17_2	non-phage	0
NLF015_scaffold29559_1	non-phage	0
NLF015_scaffold91_1	non-phage	0
NLF015_scaffold178_1	non-phage	0
NLF015_scaffold21815_2	non-phage	0
NLF015_scaffold30297_2	non-phage	0
NLF015_scaffold2386_25	non-phage	0
NLF015_scaffold14421_1	non-phage	0
NLF015_scaffold34517_1	non-phage	0
NLF015_scaffold17481_6	non-phage	0
NLF015_scaffold29678_1	non-phage	0
NLF015_scaffold12478_1	non-phage	0
NLF015_scaffold19508_4	non-phage	0
NLF015_scaffold11362_1	non-phage	0
NLF015_scaffold18943_2	non-phage	0
NLF015_scaffold33293_1	non-phage	0

NLF015_scaffold34154_3	non-phage	0
NLF015_scaffold34509_1	non-phage	0
NLF015_scaffold34537_1	non-phage	0
NLF015_scaffold624_3	non-phage	0
NLF015_scaffold16495_4	non-phage	0
NLF015_scaffold18663_4	non-phage	0
NLF015_scaffold12404_1	non-phage	0
NLF015_scaffold3531_7	non-phage	0
NLF015_scaffold32403_4	non-phage	0
NLF015_scaffold27386_1	non-phage	0
NLF015_scaffold14372_1	non-phage	0
NLF015_scaffold1817_6	non-phage	0
NLF015_scaffold26280_3	non-phage	0
NLF015_scaffold2367_5	non-phage	0
NLF015_scaffold6277_2	non-phage	0
NLF015_scaffold34170_1	non-phage	0
NLF015_scaffold31748_3	non-phage	0
NLF015_scaffold6556_1	non-phage	0
NLF015_scaffold10051_1	non-phage	0
NLF015_scaffold10932_2	non-phage	0
NLF015_scaffold4960_12	non-phage	0
NLF015_scaffold18532_1	non-phage	0
NLF015_scaffold12185_2	non-phage	0
NLF015_C419335_1	non-phage	0
NLF015_scaffold16407_1	non-phage	0
NLF015_scaffold22359_5	non-phage	0
NLF015_scaffold6355_2	non-phage	0
NLF015_scaffold5282_4	non-phage	0
NLF015_scaffold34187_1	non-phage	0
NLF015_scaffold34516_3	non-phage	0
NLF015_scaffold190_4	non-phage	0
NLF015_scaffold28895_1	non-phage	0
NLF015_scaffold19843_4	non-phage	0
NLF015_scaffold15044_2	non-phage	0
NLF015_scaffold1178_2	non-phage	0
NLF015_scaffold108_2	non-phage	0
NLF015_scaffold13289_2	non-phage	0
NLF015_scaffold30103_3	non-phage	0
NLF015_scaffold680_2	non-phage	0
NLF015_scaffold4960_14	non-phage	0
NLF015_scaffold5225_3	non-phage	0
NLF015_scaffold3041_1	non-phage	0
NLF015_scaffold14121_1	non-phage	0
NLF015_C419535_1	non-phage	0
NLF015_scaffold16018_4	non-phage	0
NLF015_scaffold11305_11	non-phage	0

NLF015_scaffold29479_1	non-phage	0
NLF015_scaffold21655_1	non-phage	0
NLF015_C419497_1	non-phage	0
NLF015_scaffold13128_4	non-phage	0
NLF015_scaffold33838_3	non-phage	0
NLF015_scaffold34486_2	non-phage	0
NLF015_scaffold16772_1	non-phage	0
NLF015_scaffold30189_1	non-phage	0
NLF015_scaffold2354_2	non-phage	0
NLF015_scaffold11305_5	non-phage	0
NLF015_scaffold6555_5	non-phage	0
NLF015_scaffold26_7	non-phage	0
NLF015_scaffold31378_5	non-phage	0
NLF015_scaffold18_2	non-phage	0
NLF015_scaffold2367_4	non-phage	0
NLF015_scaffold114_1	non-phage	0
NLF015_scaffold8636_2	non-phage	0
NLF015_scaffold23111_1	non-phage	0
NLF015_scaffold22718_5	non-phage	0
NLF015_scaffold17619_2	non-phage	0
NLF015_scaffold24133_3	non-phage	0
NLF015_scaffold27274_4	non-phage	0
NLF015_scaffold15990_1	non-phage	0
NLF015_scaffold17766_1	non-phage	0
NLF015_scaffold32760_1	non-phage	0
NLF015_scaffold22329_2	non-phage	0
NLF015_scaffold31128_1	non-phage	0
NLF015_scaffold1779_40	non-phage	0
NLF015_scaffold17711_3	non-phage	0
NLF015_scaffold5593_25	non-phage	0
NLF015_scaffold20763_2	non-phage	0
NLF015_scaffold20500_3	non-phage	0
NLF015_scaffold14044_12	non-phage	0
NLF015_scaffold27189_1	non-phage	0
NLF015_scaffold14122_1	non-phage	0
NLF015_scaffold5707_6	non-phage	0
NLF015_scaffold13768_1	non-phage	0
NLF015_scaffold5167_6	non-phage	0
NLF015_scaffold24885_1	non-phage	0
NLF015_scaffold33979_1	non-phage	0
NLF015_scaffold9140_12	non-phage	0
NLF015_scaffold8589_3	non-phage	0
NLF015_scaffold25272_2	non-phage	0
NLF015_scaffold34252_1	non-phage	0
NLF015_scaffold42_12	non-phage	0
NLF015_scaffold13358_1	non-phage	0

NLF015_scaffold32343_3	non-phage	0
NLF015_scaffold7_1	non-phage	0
NLF015_scaffold8948_10	non-phage	0
NLF015_scaffold21815_1	non-phage	0
NLF015_scaffold28291_1	non-phage	0
NLF015_scaffold25980_4	non-phage	0
NLF015_scaffold19446_2	non-phage	0
NLF015_scaffold21243_1	non-phage	0
NLF015_scaffold12032_4	non-phage	0
NLF015_scaffold18369_4	non-phage	0
NLF015_scaffold3816_14	non-phage	0
NLF015_scaffold6556_4	non-phage	0
NLF015_scaffold21990_2	non-phage	0
NLF015_scaffold12032_7	non-phage	0
NLF015_scaffold30407_2	non-phage	0
NLF015_scaffold19528_3	non-phage	0
NLF015_scaffold34452_1	non-phage	0
NLF015_scaffold33379_1	non-phage	0
NLF015_scaffold20964_3	non-phage	0
NLF015_scaffold23170_1	non-phage	0
NLF015_scaffold12271_3	non-phage	0
NLF015_scaffold28692_1	non-phage	0
NLF015_scaffold8185_2	non-phage	0
NLF015_scaffold33838_4	non-phage	0
NLF015_scaffold34276_1	non-phage	0
NLF015_scaffold11340_7	non-phage	0
NLF015_scaffold5212_2	non-phage	0
NLF015_scaffold14538_1	non-phage	0
NLF015_C419357_1	non-phage	0
NLF015_scaffold228_5	non-phage	0
NLF015_scaffold16355_2	non-phage	0
NLF015_scaffold11305_33	non-phage	0
NLF015_scaffold2354_3	non-phage	0
NLF015_scaffold34539_1	non-phage	0
NLF015_scaffold1567_4	non-phage	0
NLF015_scaffold31128_3	non-phage	0
NLF015_scaffold4273_6	non-phage	0
NLF015_scaffold11305_10	non-phage	0
NLF015_scaffold29723_1	non-phage	0
NLF015_scaffold11431_21	non-phage	0
NLF015_scaffold26_9	non-phage	0
NLF015_scaffold24518_1	non-phage	0
NLF015_scaffold389_20	non-phage	0
NLF015_scaffold2299_1	non-phage	0
NLF015_scaffold33838_1	non-phage	0
NLF015_scaffold84_1	non-phage	0

NLF015_scaffold21631_1	non-phage	0
NLF015_scaffold14940_5	non-phage	0
NLF015_scaffold11673_3	non-phage	0
NLF015_scaffold8612_1	non-phage	0
NLF015_scaffold12168_2	non-phage	0
NLF015_scaffold9268_1	non-phage	0
NLF015_scaffold19528_6	non-phage	0
NLF015_scaffold12403_2	non-phage	0
NLF015_scaffold2043_18	non-phage	0
NLF015_scaffold33760_1	non-phage	0
NLF015_scaffold19599_1	non-phage	0
NLF015_scaffold17955_2	non-phage	0
NLF015_scaffold14044_22	non-phage	0
NLF015_scaffold21794_1_2	non-phage	0
NLF015_scaffold34524_1	non-phage	0
NLF015_scaffold18405_2	non-phage	0
NLF015_scaffold5553_1	non-phage	0
NLF015_scaffold32786_2	non-phage	0
NLF015_scaffold7711_4	non-phage	0
NLF015_scaffold24532_1	non-phage	0
NLF015_scaffold3531_3	non-phage	0
NLF015_scaffold19444_1	non-phage	0
NLF015_scaffold1779_41	non-phage	0
NLF015_scaffold5657_2	non-phage	0
NLF015_scaffold32360_3	non-phage	0
NLF015_scaffold34520_1	non-phage	0
NLF015_scaffold34344_1	non-phage	0
NLF015_scaffold18_15	non-phage	0
NLF015_scaffold25485_4	non-phage	0
NLF015_scaffold34543_2	non-phage	0
NLF015_scaffold18_3	non-phage	0
NLF015_scaffold17427_3	non-phage	0
NLF015_scaffold121_32	non-phage	0
NLF015_scaffold6910_1	non-phage	0
NLF015_scaffold12681_4	non-phage	0
NLF015_scaffold10994_3	non-phage	0
NLF015_scaffold24903_1	non-phage	0
NLF015_scaffold10562_3	non-phage	0
NLF015_scaffold30176_3	non-phage	0
NLF015_scaffold24885_2	non-phage	0
NLF015_scaffold18838_1	non-phage	0
NLF015_scaffold28247_3	non-phage	0
NLF015_scaffold16281_8	non-phage	0
NLF015_scaffold11606_2	non-phage	0
NLF015_scaffold16324_1	non-phage	0
NLF015_scaffold8799_1	non-phage	0

NLF015_scaffold8643_3	non-phage	0
NLF015_scaffold2332_4	non-phage	0
NLF015_scaffold33619_1	non-phage	0
NLF015_scaffold19480_3	non-phage	0
NLF015_scaffold22432_4	non-phage	0
NLF015_scaffold17680_1	non-phage	0
NLF015_scaffold26_1	non-phage	0
NLF015_scaffold25179_1	non-phage	0
NLF015_scaffold12335_1	non-phage	0
NLF015_scaffold3077_22	non-phage	0
NLF015_scaffold228_3	non-phage	0
NLF015_scaffold26_5	non-phage	0
NLF015_scaffold34368_2	non-phage	0
NLF015_scaffold8907_1	non-phage	0
NLF015_scaffold22329_4	non-phage	0
NLF015_scaffold25258_1	non-phage	0
NLF015_scaffold34112_1	non-phage	0
NLF015_scaffold15884_1	non-phage	0
NLF015_scaffold25180_1	non-phage	0
NLF015_scaffold14785_2	non-phage	0
NLF015_scaffold22035_7	non-phage	0
NLF015_scaffold13003_3	non-phage	0
NLF015_scaffold18_12	non-phage	0
NLF015_scaffold34504_1	non-phage	0
NLF015_scaffold25292_1	non-phage	0
NLF015_scaffold22017_1	non-phage	0
NLF015_scaffold34520_5	non-phage	0
NLF015_scaffold2299_2	non-phage	0
NLF015_scaffold27252_1	non-phage	0
NLF015_scaffold4960_16	non-phage	0
NLF015_scaffold12168_1	non-phage	0
NLF015_scaffold34538_1	non-phage	0
NLF015_scaffold20802_1	non-phage	0
NLF015_scaffold11891_23	non-phage	0
NLF015_scaffold25566_1	non-phage	0
NLF015_scaffold6555_11	non-phage	0
NLF015_scaffold32436_2	non-phage	0
NLF015_scaffold10113_17	non-phage	0
NLF015_scaffold31090_10	non-phage	0
NLF015_scaffold16839_1	non-phage	0
NLF015_scaffold5153_1	non-phage	0
NLF015_scaffold28424_6	non-phage	0
NLF015_scaffold19843_3	non-phage	0
NLF015_scaffold129_3	non-phage	0
NLF015_scaffold15241_14	non-phage	0
NLF015_scaffold11305_45	non-phage	0

NLF015_scaffold31453_2	non-phage	0
DLM001_scaffold29465_7	non-phage	0
DLM001_scaffold5235_4_1	non-phage	0
DLM001_scaffold5235_4_2	non-phage	0
DLM001_scaffold1529_2	non-phage	0
DLM001_scaffold58232_1	non-phage	0
DLM001_scaffold37_3	phage	0.988
DLM001_scaffold53074_1	non-phage	0
DLM001_scaffold57794_1	non-phage	0
DLM001_scaffold49546_7	non-phage	0
DLM001_scaffold7_11	non-phage	0
DLM001_scaffold38448_1	non-phage	0
DLM001_scaffold3496_7	non-phage	0
DLM001_scaffold45243_2	non-phage	0
DLM001_scaffold57075_1	non-phage	0
DLM001_scaffold54957_3	non-phage	0
DLM001_scaffold7996_2	non-phage	0
DLM001_scaffold108_1	non-phage	0
DLM001_scaffold31841_10	non-phage	0
DLM001_scaffold17301_3	non-phage	0
DLM001_scaffold54506_2	non-phage	0
DLM001_scaffold53255_3	non-phage	0
DLM001_scaffold14455_1	non-phage	0
DLM001_scaffold14249_1	non-phage	0
DLM001_scaffold55604_4	non-phage	0
DLM001_scaffold58265_3	non-phage	0
DLM001_scaffold58168_3	non-phage	0
DLM001_scaffold33229_6	non-phage	0
DLM001_scaffold57558_3	non-phage	0
DLM001_scaffold43220_1	non-phage	0
DLM001_scaffold22028_1	non-phage	0
DLM001_scaffold38308_3	non-phage	0
DLM001_scaffold48897_2	non-phage	0
DLM001_scaffold23603_4	non-phage	0
DLM001_scaffold334_5	non-phage	0
DLM001_scaffold34460_8	non-phage	0
DLM001_scaffold43051_2	non-phage	0
DLM001_scaffold17760_6	phage	0.981
DLM001_scaffold10290_3	non-phage	0
DLM001_scaffold44441_6	non-phage	0
DLM001_scaffold58066_1	non-phage	0
DLM001_scaffold15122_3	non-phage	0
DLM001_scaffold34133_4	non-phage	0
DLM001_scaffold13671_3	non-phage	0
DLM001_scaffold40530_1	non-phage	0
DLM001_scaffold19755_10	non-phage	0

DLM001_scaffold2098_2	non-phage	0
DLM001_scaffold57834_1	phage	0.999
DLM001_scaffold1255_2	non-phage	0
DLM001_scaffold56746_1	non-phage	0
DLM001_scaffold4912_14	non-phage	0
DLM001_scaffold15619_8	non-phage	0
DLM001_scaffold13982_1	non-phage	0
DLM001_scaffold48857_2	non-phage	0
DLM001_scaffold22190_4	non-phage	0
DLM001_scaffold4466_1	non-phage	0
DLM001_scaffold91_6	phage	1
DLM001_scaffold4307_3	phage	0.997
DLM001_scaffold23682_1	non-phage	0
DLM001_scaffold13799_1	non-phage	0
DLM001_scaffold53855_3	non-phage	0
DLM001_scaffold41942_5	non-phage	0
DLM001_scaffold57337_1	non-phage	0
DLM001_scaffold12192_3	non-phage	0
DLM001_scaffold24253_4	non-phage	0
DLM001_scaffold36534_1	phage	1
DLM001_scaffold58233_1	non-phage	0
DLM001_scaffold32367_2	non-phage	0
DLM001_scaffold9645_1	non-phage	0
DLM001_scaffold15680_2	non-phage	0
DLM001_scaffold30022_3	non-phage	0
DLM001_scaffold35296_7	non-phage	0
DLM001_scaffold4306_2	non-phage	0
DLM001_scaffold23034_2	non-phage	0
DLM001_scaffold32104_2	non-phage	0
DLM001_scaffold55576_2	non-phage	0
DLM001_scaffold21149_3	non-phage	0
DLM001_scaffold22300_3	non-phage	0
DLM001_scaffold19551_4	phage	0.999
DLM001_scaffold2810_1	non-phage	0
DLM001_scaffold26715_7	non-phage	0
DLM001_scaffold16944_1	non-phage	0
DLM001_scaffold56905_1	non-phage	0
DLM001_scaffold27611_4	non-phage	0
DLM001_scaffold57439_2	non-phage	0
DLM001_scaffold8907_13	non-phage	0
DLM001_scaffold57055_1	non-phage	0
DLM001_scaffold43024_5	non-phage	0
DLM001_scaffold17213_5_1	non-phage	0
DLM001_scaffold17213_5_2	non-phage	0
DLM001_scaffold53864_4	non-phage	0.04
DLM001_scaffold65_1	non-phage	0

DLM001_scaffold58270_2	non-phage	0
DLM001_scaffold20121_2_1	non-phage	0
DLM001_scaffold16422_3	non-phage	0
DLM001_scaffold37749_1	non-phage	0
DLM001_scaffold20770_3	non-phage	0
DLM001_scaffold36192_2	non-phage	0
DLM001_scaffold45937_1	non-phage	0
DLM001_scaffold2780_1	non-phage	0
DLM001_scaffold34635_1	non-phage	0
DLM001_scaffold57493_1	non-phage	0
DLM001_scaffold26195_1	non-phage	0
DLM001_scaffold5806_1_2	non-phage	0
DLM001_scaffold35151_1	non-phage	0
DLM001_scaffold20464_2	non-phage	0
DLM001_scaffold55_18	non-phage	0
DLM001_scaffold32326_2	non-phage	0
DLM001_scaffold57559_2	non-phage	0
DLM001_scaffold32500_1	non-phage	0
DLM001_scaffold57468_1	non-phage	0
DLM001_scaffold32176_5	non-phage	0
DLM001_scaffold54640_1	non-phage	0
DLM001_scaffold42239_2	non-phage	0
DLM001_scaffold26899_5	non-phage	0
DLM001_scaffold7_9	non-phage	0
DLM001_scaffold32003_2	non-phage	0
DLM001_scaffold44816_5	non-phage	0
DLM001_scaffold8778_2	non-phage	0
DLM001_scaffold46084_5	non-phage	0
DLM001_scaffold52139_1	non-phage	0
DLM001_scaffold55343_1_1	non-phage	0
DLM001_scaffold787_9	non-phage	0
DLM001_scaffold3954_1	non-phage	0
DLM001_scaffold22697_1	non-phage	0
DLM001_scaffold38563_11	non-phage	0
DLM001_scaffold55479_1	non-phage	0
DLM001_scaffold50716_1	non-phage	0
DLM001_scaffold14338_1	non-phage	0
DLM001_scaffold24698_4	non-phage	0
DLM001_scaffold34916_2	non-phage	0
DLM001_scaffold38880_1	non-phage	0
DLM001_scaffold36801_6	non-phage	0
DLM001_scaffold57815_2	non-phage	0
DLM001_scaffold57433_1	non-phage	0
DLM001_scaffold24171_5	non-phage	0
DLM001_scaffold21297_4	non-phage	0
DLM001_scaffold43209_1	non-phage	0

DLM001_scaffold51591_5	non-phage	0
DLM001_scaffold47826_3	non-phage	0
DLM001_scaffold40181_1	non-phage	0
DLM001_scaffold34460_1	non-phage	0
DLM001_scaffold32661_2	non-phage	0
DLM001_scaffold26672_2	phage	0.824
DLM001_scaffold14143_2	non-phage	0
DLM001_scaffold8593_2	non-phage	0
DLM001_scaffold15625_1	non-phage	0
DLM001_scaffold53713_4	non-phage	0
DLM001_scaffold23997_2	non-phage	0
DLM001_scaffold25207_1	non-phage	0
DLM001_scaffold21921_3	phage	0.995
DLM001_scaffold40252_2	non-phage	0
DLM001_scaffold2435_6	phage	0.982
DLM001_scaffold41297_3	non-phage	0
DLM001_scaffold20575_6	non-phage	0
DLM001_scaffold53955_2	non-phage	0
DLM001_scaffold63_1	non-phage	0
DLM001_scaffold38513_4	non-phage	0
DLM001_scaffold101_14	non-phage	0
DLM001_scaffold58240_1	non-phage	0
DLM001_scaffold23814_4	phage	0.941
DLM001_scaffold186_4	non-phage	0
DLM001_scaffold30759_1	non-phage	0
DLM001_scaffold4912_17	non-phage	0
DLM001_scaffold25895_3	non-phage	0
DLM001_scaffold19369_2	non-phage	0
DLM001_scaffold58190_1	non-phage	0
DLM001_scaffold801_4	non-phage	0
DLM001_scaffold1255_3	non-phage	0
DLM001_scaffold56997_3	non-phage	0
DLM001_scaffold44566_4	non-phage	0
DLM001_scaffold9483_3	non-phage	0
DLM001_scaffold2399_2	non-phage	0
DLM001_scaffold56935_2	non-phage	0
DLM001_scaffold1529_6	non-phage	0
DLM001_scaffold51088_1	non-phage	0
DLM001_scaffold16965_10	non-phage	0
DLM001_scaffold45623_2	non-phage	0
DLM001_scaffold45559_1	non-phage	0
DLM001_scaffold49546_1	non-phage	0
DLM001_scaffold21347_2	non-phage	0
DLM001_scaffold45932_1	non-phage	0
DLM001_scaffold14847_5	phage	0.856
DLM001_scaffold56433_1	non-phage	0

DLM001_scaffold22445_3	non-phage	0
DLM001_scaffold19868_1	non-phage	0
DLM001_scaffold46_4	non-phage	0
DLM001_scaffold42296_1	phage	0.678
DLM001_scaffold4146_2	non-phage	0
DLM001_scaffold27728_1	non-phage	0
DLM001_scaffold3954_19	non-phage	0
DLM001_scaffold44622_6	non-phage	0
DLM001_scaffold20379_1	non-phage	0
DLM001_scaffold50748_1	non-phage	0
DLM001_scaffold9602_1	non-phage	0
DLM001_scaffold46861_1	non-phage	0
DLM001_scaffold1780_1	non-phage	0
DLM001_scaffold25152_5	non-phage	0
DLM001_scaffold27010_3	non-phage	0
DLM001_scaffold28826_21	non-phage	0
DLM001_scaffold34822_1	non-phage	0
DLM001_scaffold11597_5	non-phage	0
DLM001_scaffold9674_13	non-phage	0
DLM001_scaffold56261_1	non-phage	0
DLM001_scaffold56117_3	non-phage	0
DLM001_scaffold41942_9	non-phage	0
DLM001_scaffold8150_2	non-phage	0
DLM001_scaffold45465_1	non-phage	0
DLM001_scaffold15625_4	non-phage	0
DLM001_scaffold17984_3	non-phage	0
DLM001_scaffold58029_1	non-phage	0
DLM001_scaffold30952_1_1	non-phage	0
DLM001_scaffold228_1	non-phage	0
DLM001_scaffold23337_1	non-phage	0
DLM001_scaffold14266_16	non-phage	0
DLM001_scaffold226_1	non-phage	0
DLM001_scaffold43326_1	non-phage	0
DLM001_scaffold1_8	non-phage	0
DLM001_scaffold6827_1	non-phage	0
DLM001_scaffold28890_7	non-phage	0
DLM001_scaffold35595_5	non-phage	0
DLM001_scaffold41842_3	non-phage	0
DLM001_scaffold34678_2	non-phage	0
DLM001_scaffold53856_2	non-phage	0
DLM001_scaffold52764_1	non-phage	0
DLM001_scaffold46950_2	non-phage	0
DLM001_scaffold11369_2	non-phage	0
DLM001_scaffold22953_5	non-phage	0
DLM001_scaffold57167_1	non-phage	0
DLM001_scaffold2582_26	non-phage	0

DLM001_scaffold56746_2	non-phage	0
DLM001_scaffold50409_1	non-phage	0
DLM001_scaffold50948_1	non-phage	0
DLM001_scaffold1291_4	non-phage	0
DLM001_scaffold16500_1	non-phage	0
DLM001_scaffold32104_1	non-phage	0
DLM001_scaffold14163_1	non-phage	0
DLM001_scaffold4307_2	phage	0.955
DLM001_scaffold22953_1_1	non-phage	0
DLM001_scaffold36534_2	non-phage	0
DLM001_scaffold24338_7	non-phage	0
DLM001_scaffold31256_1	phage	0.746
DLM001_scaffold14114_14	non-phage	0
DLM001_scaffold45939_3	non-phage	0
DLM001_scaffold165_7	non-phage	0
DLM001_scaffold51128_4	non-phage	0
DLM001_scaffold52744_1	phage	0.998
DLM001_scaffold55645_3	non-phage	0
DLM001_scaffold51404_4	non-phage	0
DLM001_scaffold58230_2	non-phage	0
DLM001_scaffold24236_1	non-phage	0
DLM001_scaffold6645_2	non-phage	0
DLM001_scaffold21082_6	non-phage	0
DLM001_scaffold58168_6	non-phage	0
DLM001_scaffold57918_1	non-phage	0
DLM001_scaffold27703_2	non-phage	0
DLM001_scaffold54429_2	non-phage	0
DLM001_scaffold24171_4	non-phage	0
DLM001_scaffold32737_1	non-phage	0
DLM001_scaffold35963_1	non-phage	0
DLM001_scaffold8604_2	non-phage	0
DLM001_scaffold33465_2	non-phage	0
DLM001_scaffold32340_2	non-phage	0
DLM001_scaffold41681_1	non-phage	0
DLM001_scaffold31841_6	non-phage	0
DLM001_scaffold38738_1	non-phage	0
DLM001_scaffold58080_1	non-phage	0
DLM001_scaffold20221_2	non-phage	0
DLM001_scaffold16190_3	non-phage	0
DLM001_scaffold26134_5	non-phage	0
DLM001_C705453_1	non-phage	0
DLM001_scaffold14652_4	non-phage	0
DLM001_scaffold9098_7	non-phage	0
DLM001_scaffold80_1	non-phage	0
DLM001_scaffold3881_2	non-phage	0
DLM001_scaffold54273_1	non-phage	0

DLM001_scaffold1293_4	non-phage	0
DLM001_scaffold45970_1	non-phage	0
DLM001_scaffold53029_2	non-phage	0
DLM001_scaffold43284_1	non-phage	0
DLM001_scaffold27011_1	non-phage	0
DLM001_scaffold20331_4	non-phage	0
DLM001_scaffold9646_1	non-phage	0
DLM001_scaffold38308_2	non-phage	0
DLM001_scaffold36_2	non-phage	0
DLM001_scaffold1293_15	non-phage	0
DLM001_scaffold8529_15	non-phage	0
DLM001_scaffold2757_1	non-phage	0
DLM001_scaffold4912_6	non-phage	0
DLM001_scaffold22680_4	non-phage	0
DLM001_scaffold15292_11	non-phage	0
DLM001_scaffold23603_12	non-phage	0
DLM001_scaffold31231_3	non-phage	0
DLM001_scaffold46552_1	non-phage	0.001
DLM001_scaffold57963_1	non-phage	0
DLM001_scaffold204_3	non-phage	0
DLM001_scaffold9176_1	non-phage	0
DLM001_scaffold58212_1	non-phage	0
DLM001_C706771_1	non-phage	0
DLM001_scaffold53015_3	non-phage	0
DLM001_scaffold18985_9	non-phage	0
DLM001_scaffold40426_1	non-phage	0
DLM001_scaffold53_1	non-phage	0
DLM001_scaffold58096_1	non-phage	0
DLM001_scaffold15021_10	non-phage	0
DLM001_scaffold36255_3	non-phage	0
DLM001_scaffold47290_3	non-phage	0
DLM001_scaffold40118_3	non-phage	0
DLM001_scaffold26950_1	non-phage	0
DLM001_scaffold41528_1	non-phage	0
DLM001_scaffold173_1	non-phage	0
DLM001_scaffold36801_9	non-phage	0
DLM001_scaffold57803_1	non-phage	0
DLM001_scaffold3591_3	non-phage	0
DLM001_scaffold26837_2	non-phage	0
DLM001_scaffold209_2	non-phage	0
DLM001_scaffold305_6	non-phage	0
DLM001_scaffold23603_14	non-phage	0
DLM001_scaffold26803_1	non-phage	0
DLM001_scaffold55645_2	non-phage	0
DLM001_scaffold20129_3	non-phage	0
DLM001_scaffold43024_6	non-phage	0

DLM001_scaffold14266_2	non-phage	0
DLM001_scaffold24104_3	non-phage	0
DLM001_scaffold57999_2	non-phage	0
DLM001_scaffold2777_6	non-phage	0
DLM001_scaffold20858_1	phage	0.997
DLM001_scaffold49315_1	non-phage	0
DLM001_scaffold26854_4	non-phage	0
DLM001_scaffold6056_5	non-phage	0
DLM001_scaffold27372_1	phage	0.998
DLM001_scaffold18360_7	non-phage	0
DLM001_scaffold51708_1	non-phage	0
DLM001_scaffold23779_1	non-phage	0
DLM001_scaffold80_6	non-phage	0
DLM001_scaffold45345_2	non-phage	0
DLM001_scaffold8656_4	non-phage	0
DLM001_scaffold47187_1	non-phage	0
DLM001_scaffold6338_7	non-phage	0
DLM001_scaffold57606_2	non-phage	0
DLM001_scaffold33851_1	non-phage	0
DLM001_scaffold8135_1	non-phage	0
DLM001_scaffold36138_2	non-phage	0
DLM001_scaffold4306_3	non-phage	0
DLM001_scaffold17316_8	non-phage	0
DLM001_scaffold33599_3	non-phage	0
DLM001_scaffold29648_2	non-phage	0
DLM001_scaffold39494_4	non-phage	0
DLM001_scaffold57602_1	non-phage	0
DLM001_scaffold787_10	non-phage	0.048
DLM001_scaffold45278_1	non-phage	0
DLM001_scaffold18193_4	non-phage	0
DLM001_scaffold24698_5	non-phage	0
DLM001_scaffold2698_4	non-phage	0
DLM001_scaffold2047_2	non-phage	0
DLM001_scaffold43274_6	non-phage	0
DLM001_scaffold39435_2	non-phage	0
DLM001_scaffold32773_1	non-phage	0
DLM001_scaffold34133_1	non-phage	0
DLM001_scaffold3006_1	non-phage	0
DLM001_scaffold20566_1	non-phage	0
DLM001_scaffold57595_1	non-phage	0
DLM001_scaffold30101_3	non-phage	0
DLM001_scaffold58255_1	non-phage	0
DLM001_scaffold1739_1	non-phage	0
DLM001_scaffold11491_3	non-phage	0
DLM001_scaffold6447_3	non-phage	0
DLM001_scaffold58022_1	non-phage	0

DLM001_scaffold27449_3	non-phage	0
DLM001_scaffold27285_1	non-phage	0
DLM001_scaffold41274_1	non-phage	0
DLM001_scaffold25370_1	non-phage	0
DLM001_scaffold6224_3	non-phage	0
DLM001_scaffold4637_2	non-phage	0
DLM001_scaffold14201_2	non-phage	0
DLM001_scaffold49578_1	non-phage	0
DLM001_scaffold21149_1	non-phage	0
DLM001_scaffold58225_3	non-phage	0
DLM001_scaffold30077_2	non-phage	0
DLM001_scaffold58251_1	non-phage	0
DLM001_scaffold176_2	non-phage	0
DLM001_scaffold58188_1	non-phage	0
DLM001_scaffold1293_18	non-phage	0
DLM001_scaffold51530_4	non-phage	0.229
DLM001_scaffold2234_1	non-phage	0
DLM001_scaffold25152_4	non-phage	0
DLM001_scaffold53544_7	non-phage	0
DLM001_scaffold55116_2	phage	1
DLM001_scaffold38308_1	non-phage	0
DLM001_scaffold15122_1	non-phage	0
DLM001_scaffold58125_1	non-phage	0
DLM001_scaffold17663_5	non-phage	0
DLM001_scaffold13800_1	non-phage	0
DLM001_scaffold27326_2	non-phage	0
DLM001_scaffold47290_7	non-phage	0
DLM001_scaffold63_2	non-phage	0
DLM001_scaffold8974_5	non-phage	0
DLM001_scaffold80_2	non-phage	0
DLM001_scaffold10496_11	non-phage	0
DLM001_scaffold55409_1	non-phage	0
DLM001_scaffold9798_1	non-phage	0
DLM001_scaffold4637_4	non-phage	0
DLM001_scaffold2768_2	non-phage	0
DLM001_scaffold44777_2	non-phage	0
DLM001_scaffold10443_4	non-phage	0
DLM001_scaffold20637_2	non-phage	0
DLM001_scaffold53947_1	non-phage	0
DLM001_scaffold28826_15	non-phage	0
DLM001_scaffold249_3	non-phage	0
DLM001_scaffold27996_4	non-phage	0
DLM001_scaffold23934_2	non-phage	0
DLM001_scaffold37_4	non-phage	0
DLM001_scaffold9674_12	non-phage	0
DLM001_scaffold37205_1	non-phage	0

DLM001_scaffold24253_2	non-phage	0
DLM001_scaffold32682_3	non-phage	0
DLM001_scaffold38907_2	non-phage	0
DLM001_scaffold19551_6	non-phage	0
DLM001_scaffold58241_2	non-phage	0
DLM001_scaffold17622_5	non-phage	0
DLM001_scaffold38515_1	non-phage	0
DLM001_scaffold9128_2	non-phage	0
DLM001_scaffold54783_2	non-phage	0
DLM001_scaffold20332_1	non-phage	0
DLM001_scaffold53458_3	non-phage	0
DLM001_scaffold7234_3	non-phage	0
DLM001_scaffold52168_1	non-phage	0
DLM001_scaffold54593_1	non-phage	0
DLM001_scaffold4146_3	non-phage	0
DLM001_scaffold50352_4	non-phage	0
DLM001_scaffold21002_2	non-phage	0
DLM001_scaffold14266_3	non-phage	0
DLM001_scaffold21149_5	non-phage	0
DLM001_scaffold51924_2	non-phage	0
DLM001_scaffold6827_2	non-phage	0.002
DLM001_scaffold24276_1	non-phage	0
DLM001_scaffold26733_1	non-phage	0
DLM001_scaffold57732_2	phage	0.999
DLM001_scaffold90_1	non-phage	0
DLM001_scaffold35116_2	non-phage	0
DLM001_scaffold36602_3	non-phage	0
DLM001_scaffold4588_1	non-phage	0
DLM001_scaffold1529_4	non-phage	0
DLM001_scaffold58171_1	non-phage	0
DLM001_scaffold52444_1	non-phage	0
DLM001_scaffold1529_12	non-phage	0
DLM001_scaffold12535_1	non-phage	0
DLM001_scaffold17614_8	phage	0.998
DLM001_scaffold4440_2	non-phage	0
DLM001_scaffold27705_2	non-phage	0
DLM001_scaffold30298_1	non-phage	0
DLM001_scaffold39435_3	non-phage	0
DLM001_scaffold8905_5	non-phage	0
DLM001_scaffold6056_1	non-phage	0
DLM001_scaffold3496_13	non-phage	0
DLM001_scaffold5235_6	non-phage	0
DLM001_scaffold2780_5	non-phage	0
DLM001_scaffold50948_2	non-phage	0
DLM001_scaffold54957_1	non-phage	0
DLM001_scaffold4544_1	non-phage	0

DLM001_scaffold57236_2	non-phage	0
DLM001_scaffold6960_3	non-phage	0
DLM001_scaffold15157_3	non-phage	0
DLM001_scaffold24698_2	non-phage	0
DLM001_scaffold20121_4	non-phage	0
DLM001_scaffold787_2	non-phage	0
DLM001_scaffold57716_1	phage	0.994
DLM001_C705975_1	non-phage	0
DLM001_scaffold53015_4	non-phage	0
DLM001_scaffold91_4	non-phage	0
DLM001_scaffold41398_1	non-phage	0
DLM001_scaffold6383_2	non-phage	0
DLM001_scaffold25895_8	phage	1
DLM001_scaffold41770_3	non-phage	0
DLM001_scaffold20183_2	non-phage	0
DLM001_scaffold16604_11	non-phage	0
DLM001_scaffold58089_1	non-phage	0
DLM001_scaffold26876_7	non-phage	0
DLM001_scaffold25742_1	non-phage	0
DLM001_scaffold53167_1	non-phage	0
DLM001_scaffold32340_1	non-phage	0
DLM001_scaffold21223_1	phage	0.992
DLM001_scaffold2405_5	non-phage	0
DLM001_scaffold43274_2	non-phage	0
DLM001_scaffold16773_1	non-phage	0
DLM001_scaffold58254_3	non-phage	0
DLM001_scaffold3496_3	non-phage	0
DLM001_scaffold58246_1	non-phage	0
DLM001_scaffold167_1	non-phage	0
DLM001_scaffold24936_3	non-phage	0
DLM001_scaffold27328_2	non-phage	0
DLM001_scaffold48552_5	non-phage	0
DLM001_scaffold58216_1	non-phage	0
DLM001_scaffold34336_3	non-phage	0
DLM001_scaffold39109_1	non-phage	0
DLM001_scaffold34635_3	non-phage	0
DLM001_scaffold32104_3	non-phage	0
DLM001_scaffold500_6	non-phage	0
DLM001_scaffold58111_2	non-phage	0
DLM001_scaffold58265_1	non-phage	0
DLM001_scaffold43001_3	non-phage	0
DLM001_scaffold51449_5	non-phage	0
DLM001_scaffold26195_8	non-phage	0
DLM001_scaffold39252_3	non-phage	0
DLM001_scaffold688_6	non-phage	0
DLM001_scaffold5571_1	non-phage	0

DLM001_scaffold32359_2	non-phage	0
DLM001_scaffold14454_1_1	non-phage	0
DLM001_scaffold49825_5	non-phage	0
DLM001_scaffold42483_5	non-phage	0
DLM001_scaffold93_6	non-phage	0
DLM001_scaffold45795_1	non-phage	0
DLM001_scaffold8814_2	non-phage	0
DLM001_scaffold3294_5	non-phage	0
DLM001_scaffold5235_1	non-phage	0
DLM001_scaffold46184_3	non-phage	0
DLM001_scaffold43274_3	non-phage	0
DLM001_scaffold3486_2	non-phage	0
DLM001_scaffold40369_8	non-phage	0
DLM001_scaffold54893_2	phage	0.999
DLM001_scaffold6942_1	non-phage	0
DLM001_scaffold48657_1	non-phage	0
DLM001_scaffold53544_4	non-phage	0
DLM001_scaffold1980_1	non-phage	0
DLM001_scaffold32568_1	non-phage	0
DLM001_scaffold54889_1	non-phage	0
DLM001_scaffold53309_4	non-phage	0
DLM001_scaffold6345_1	non-phage	0
DLM001_scaffold57380_1	non-phage	0
DLM001_scaffold26192_1	non-phage	0
DLM001_scaffold26330_1	non-phage	0
DLM001_scaffold58065_3	non-phage	0
DLM001_scaffold48857_3	non-phage	0
DLM001_scaffold32121_1	non-phage	0
DLM001_scaffold15191_6	non-phage	0
DLM001_scaffold34791_4	non-phage	0
DLM001_scaffold2582_22	non-phage	0
DLM001_scaffold26411_14	non-phage	0
DLM001_scaffold37730_3	non-phage	0
DLM001_C706467_1	non-phage	0
DLM001_scaffold54769_10	non-phage	0
DLM001_scaffold30022_1	phage	0.998
DLM001_scaffold45673_2	non-phage	0
DLM001_scaffold20355_4	non-phage	0
DLM001_scaffold27178_2	non-phage	0
DLM001_scaffold57853_1	non-phage	0
DLM001_scaffold17663_2	non-phage	0
DLM001_scaffold51615_2	non-phage	0
DLM001_scaffold48202_4	non-phage	0
DLM001_scaffold26745_1	non-phage	0
DLM001_scaffold40390_1	non-phage	0
DLM001_scaffold57522_1	non-phage	0

DLM001_scaffold33440_1	non-phage	0
DLM001_scaffold58236_1	phage	0.996
DLM001_scaffold19379_1	non-phage	0
DLM001_scaffold42062_1	non-phage	0
DLM001_scaffold32900_1	non-phage	0
DLM001_scaffold3576_11	non-phage	0
DLM001_scaffold16854_2	non-phage	0
DLM001_scaffold32682_2	non-phage	0
DLM001_scaffold57600_1	non-phage	0
DLM001_scaffold14179_5	non-phage	0
DLM001_scaffold58164_1	non-phage	0
DLM001_scaffold58208_1	non-phage	0
DLM001_scaffold35617_1	non-phage	0
DLM001_scaffold17316_7	non-phage	0
DLM001_scaffold2385_1	non-phage	0
DLM001_scaffold17663_1	non-phage	0
DLM001_scaffold143_16	non-phage	0
DLM001_scaffold31471_2	non-phage	0
DLM001_scaffold18164_1	non-phage	0
DLM001_scaffold57142_1	non-phage	0
DLM001_scaffold57596_1	non-phage	0
DLM001_scaffold39237_1	non-phage	0
DLM001_scaffold58085_1	non-phage	0
DLM001_scaffold54889_10	non-phage	0
DLM001_scaffold20129_2	non-phage	0
DLM001_scaffold49546_3	non-phage	0
DLM001_scaffold57265_1	non-phage	0
DLM001_scaffold49077_1	non-phage	0
DLM001_scaffold10804_1	non-phage	0
DLM001_scaffold19576_13	non-phage	0
DLM001_scaffold1529_13	non-phage	0
DLM001_scaffold48318_2	non-phage	0
DLM001_scaffold39171_1	non-phage	0
DLM001_scaffold52078_2	non-phage	0
DLM001_scaffold10958_1	non-phage	0
DLM001_scaffold334_3	non-phage	0
DLM001_scaffold26852_4	non-phage	0
DLM001_scaffold32362_4	non-phage	0
DLM001_scaffold57558_1	non-phage	0
DLM001_scaffold55975_1	non-phage	0
DLM001_scaffold305_2	non-phage	0
DLM001_scaffold49825_1	non-phage	0
DLM001_scaffold31292_3	non-phage	0.245
DLM001_scaffold36801_4	non-phage	0
DLM001_scaffold56543_1	non-phage	0
DLM001_scaffold44566_2	non-phage	0

DLM001_scaffold56831_1	non-phage	0
DLM001_scaffold143_7	non-phage	0
DLM001_scaffold24171_3	non-phage	0
DLM001_scaffold20473_1	non-phage	0
DLM001_scaffold47697_3	non-phage	0
DLM001_scaffold12890_4	non-phage	0
DLM001_scaffold5421_1	non-phage	0
DLM001_C705855_1	non-phage	0
DLM001_scaffold47925_1	non-phage	0
DLM001_scaffold12187_2	non-phage	0
DLM001_scaffold23815_2	non-phage	0
DLM001_scaffold21020_1	non-phage	0
DLM001_scaffold21806_1	non-phage	0
DLM001_scaffold44766_2	non-phage	0
DLM001_scaffold20213_1	non-phage	0
DLM001_scaffold57479_1	non-phage	0
DLM001_scaffold52100_2	non-phage	0
DLM001_scaffold20473_5	non-phage	0
DLM001_scaffold48283_1	non-phage	0
DLM001_scaffold26745_2	non-phage	0
DLM001_scaffold24815_8	non-phage	0
DLM001_scaffold31471_16	non-phage	0
DLM001_scaffold26925_1	non-phage	0
DLM001_scaffold9602_2	non-phage	0
DLM001_scaffold963_8	non-phage	0
DLM001_scaffold55419_1	non-phage	0
DLM001_scaffold35578_1	non-phage	0
DLM001_scaffold3006_3	non-phage	0
DLM001_scaffold54083_5	non-phage	0
DLM001_scaffold34133_3	phage	0.999
DLM001_scaffold3162_2	non-phage	0
DLM001_scaffold10206_1	non-phage	0
DLM001_scaffold10330_2	non-phage	0
DLM001_scaffold44_5	non-phage	0
DLM001_scaffold16944_2	non-phage	0
DLM001_scaffold19765_27	non-phage	0
DLM001_scaffold36901_1	non-phage	0
DLM001_scaffold30101_1	non-phage	0
DLM001_scaffold23322_1	non-phage	0
DLM001_scaffold32694_1	non-phage	0
DLM001_scaffold29631_4	non-phage	0
DLM001_scaffold51940_3	phage	0.997
DLM001_scaffold1980_2	non-phage	0
DLM001_scaffold20559_3	non-phage	0
DLM001_scaffold54893_1	non-phage	0
DLM001_scaffold8529_12	non-phage	0

DLM001_scaffold11541_2	non-phage	0
DLM001_scaffold11491_4	non-phage	0
DLM001_scaffold688_1	non-phage	0
DLM001_scaffold19016_2	non-phage	0
DLM001_scaffold21149_10	non-phage	0
DLM001_scaffold21666_3	non-phage	0
DLM001_scaffold182_4	non-phage	0
DLM001_scaffold23395_4	non-phage	0
DLM001_scaffold15680_1	non-phage	0
DLM001_scaffold49843_4	phage	0.585
DLM001_scaffold23819_4_2	non-phage	0
DLM001_scaffold1255_1	non-phage	0
DLM001_scaffold38118_2	non-phage	0
DLM001_scaffold43009_4	non-phage	0
DLM001_scaffold15021_6	non-phage	0
DLM001_scaffold4545_6	non-phage	0
DLM001_scaffold51202_3	non-phage	0
DLM001_scaffold6387_6	non-phage	0
DLM001_scaffold24171_9	non-phage	0
DLM001_scaffold58085_2	non-phage	0
DLM001_scaffold55299_3	non-phage	0
DLM001_scaffold30244_1	non-phage	0
DLM001_scaffold56709_1	non-phage	0
DLM001_scaffold51928_3	phage	0.994
DLM001_scaffold38452_9	non-phage	0
DLM001_scaffold51153_1	non-phage	0
DLM001_scaffold27263_2	non-phage	0
DLM001_scaffold24123_2	non-phage	0
DLM001_scaffold21013_18	non-phage	0
DLM001_scaffold58270_1	non-phage	0
DLM001_scaffold36133_1	non-phage	0
DLM001_scaffold20211_1	non-phage	0
DLM001_scaffold49825_4	non-phage	0
DLM001_scaffold38452_3	non-phage	0
DLM001_scaffold45559_2	non-phage	0
DLM001_scaffold29714_4	non-phage	0
DLM001_scaffold10884_11	non-phage	0
DLM001_scaffold44518_1	non-phage	0
DLM001_scaffold25465_1	non-phage	0
DLM001_scaffold19551_5	phage	0.999
DLM001_scaffold53856_4	non-phage	0
DLM001_scaffold26967_6	non-phage	0
DLM001_scaffold27326_1	non-phage	0
DLM001_scaffold14517_1	non-phage	0
DLM001_scaffold25674_1	non-phage	0
DLM001_scaffold801_1	non-phage	0

DLM001_scaffold15292_8	non-phage	0
DLM001_C705415_1	non-phage	0
DLM001_scaffold58259_1	non-phage	0
DLM001_scaffold21193_1	non-phage	0
DLM001_scaffold27222_3	non-phage	0
DLM001_scaffold53864_5	non-phage	0
DLM001_scaffold33180_3	non-phage	0
DLM001_scaffold6827_3	non-phage	0
DLM001_scaffold26297_3	non-phage	0
DLM001_scaffold28347_2	non-phage	0
DLM001_scaffold50512_1	non-phage	0
DLM001_scaffold58124_2	non-phage	0
DLM001_scaffold30245_1	non-phage	0
DLM001_scaffold58269_1	non-phage	0
DLM001_scaffold27178_4	non-phage	0
DLM001_scaffold58194_1	non-phage	0
DLM001_scaffold52220_1	non-phage	0
DLM001_scaffold19465_5	phage	0.911
DLM001_scaffold55968_1	non-phage	0
DLM001_scaffold50341_2	non-phage	0
DLM001_scaffold17622_6	non-phage	0
DLM001_scaffold3627_4	non-phage	0
DLM001_scaffold55_14	non-phage	0
DLM001_scaffold32015_1	non-phage	0
DLM001_scaffold8086_1	non-phage	0
DLM001_scaffold40636_1	non-phage	0.003
DLM001_scaffold27200_6	non-phage	0
DLM001_scaffold58127_1	non-phage	0
DLM001_scaffold6224_6	non-phage	0
DLM001_scaffold22825_3	non-phage	0
DLM001_scaffold42099_3	non-phage	0
DLM001_scaffold18746_8	non-phage	0
DLM001_scaffold12778_1	non-phage	0
DLM001_scaffold30286_3	non-phage	0.438
DLM001_scaffold42124_2	non-phage	0
DLM001_scaffold56543_4	phage	0.999
DLM001_scaffold20493_1	non-phage	0
DLM001_scaffold54769_4	non-phage	0
DLM001_scaffold8741_6	non-phage	0
DLM001_scaffold50217_3	non-phage	0
DLM001_scaffold36801_3	non-phage	0
DLM001_scaffold53302_2	non-phage	0
DLM001_scaffold55930_6	non-phage	0
DLM001_scaffold7233_3	non-phage	0
DLM001_scaffold31060_4	non-phage	0
DLM001_scaffold48382_2	non-phage	0

DLM001_scaffold20331_6	non-phage	0
DLM001_scaffold58166_1	non-phage	0
DLM001_scaffold14859_7	phage	0.98
DLM001_scaffold58195_1	non-phage	0
DLM001_scaffold20473_2	non-phage	0
DLM001_scaffold30863_15	non-phage	0
DLM001_scaffold277_6	non-phage	0
DLM001_scaffold50895_1	non-phage	0
DLM001_scaffold20858_3	phage	0.996
DLM001_scaffold19868_4	non-phage	0
DLM001_scaffold25263_1	non-phage	0
DLM001_scaffold594_2	non-phage	0
DLM001_scaffold39863_3	non-phage	0
DLM001_scaffold56720_2	non-phage	0
DLM001_C705523_1	phage	0.891
DLM001_scaffold2435_9	non-phage	0
DLM001_scaffold27200_3	non-phage	0
DLM001_scaffold54957_2	non-phage	0
DLM001_scaffold14201_9	non-phage	0
DLM001_scaffold12187_1	non-phage	0
DLM001_scaffold31841_8	non-phage	0
DLM001_scaffold10383_1	non-phage	0
DLM001_scaffold6056_3	non-phage	0
DLM001_scaffold1293_16	non-phage	0
DLM001_C706477_1	non-phage	0
DLM001_scaffold28190_6	non-phage	0
DLM001_scaffold2757_2	non-phage	0
DLM001_scaffold14114_9	non-phage	0
DLM001_scaffold29714_1	non-phage	0
DLM001_scaffold17051_8	phage	0.977
DLM001_scaffold49221_11	non-phage	0
DLM001_scaffold23887_4	non-phage	0
DLM001_scaffold45453_2	non-phage	0
DLM001_scaffold47503_1	non-phage	0
DLM001_scaffold55454_2	non-phage	0
DLM001_scaffold20826_2	non-phage	0
DLM001_scaffold19551_1	phage	0.855
DLM001_scaffold36255_1	non-phage	0
DLM001_scaffold24359_3	non-phage	0
DLM001_scaffold29214_1	non-phage	0
DLM001_scaffold58265_2	non-phage	0
DLM001_scaffold55343_2	non-phage	0
DLM001_C706737_1	non-phage	0
DLM001_scaffold7351_1	non-phage	0
DLM001_scaffold43024_4	non-phage	0
DLM001_scaffold57163_1	non-phage	0

DLM001_scaffold27378_7	non-phage	0
DLM001_scaffold19427_2	non-phage	0
DLM001_scaffold20560_8	non-phage	0
DLM001_scaffold51128_5	non-phage	0
DLM001_scaffold32693_2	non-phage	0
DLM001_scaffold58011_2	non-phage	0
DLM001_scaffold30584_2	non-phage	0
DLM001_scaffold1114_6	non-phage	0
DLM001_scaffold26838_10	non-phage	0
DLM001_scaffold39_1	non-phage	0
DLM001_scaffold52265_1	non-phage	0
DLM001_scaffold26639_4	non-phage	0
DLM001_scaffold10897_2	non-phage	0
DLM001_scaffold38412_1	non-phage	0
DLM001_scaffold44934_1	non-phage	0
DLM001_scaffold57969_1	non-phage	0
DLM001_scaffold36801_7	non-phage	0
DLM001_scaffold10651_10	non-phage	0
DLM001_scaffold5865_2	non-phage	0
DLM001_scaffold37623_2	non-phage	0
DLM001_scaffold57727_2	non-phage	0.374
DLM001_scaffold37033_1	non-phage	0
DLM001_scaffold10590_2	non-phage	0
DLM001_scaffold58129_1	non-phage	0
DLM001_scaffold56915_2	non-phage	0
DLM001_scaffold26854_6	non-phage	0
DLM001_scaffold26899_4	non-phage	0
DLM001_scaffold33599_5	non-phage	0
DLM001_scaffold36324_1	non-phage	0
DLM001_scaffold9742_3	non-phage	0
DLM001_scaffold7383_2	non-phage	0
DLM001_scaffold2542_3	non-phage	0
DLM001_scaffold57214_2	non-phage	0
DLM001_scaffold33108_8	non-phage	0
DLM001_scaffold43274_7	non-phage	0
DLM001_scaffold688_7	non-phage	0
DLM001_scaffold787_3	non-phage	0
DLM001_scaffold57943_1	non-phage	0
DLM001_scaffold688_3	non-phage	0
DLM001_scaffold2803_4	non-phage	0
DLM001_scaffold4248_7	non-phage	0
DLM001_scaffold58204_2	non-phage	0
DLM001_scaffold24290_2	non-phage	0
DLM001_scaffold20817_1	phage	0.995
DLM001_scaffold14249_13	non-phage	0
DLM001_scaffold25009_5	phage	0.996

DLM001_scaffold22953_2	phage	0.999
DLM001_scaffold14435_2	non-phage	0
DLM001_scaffold13189_2	non-phage	0
DLM001_scaffold106_1	non-phage	0
DLM001_scaffold2136_1_1	non-phage	0
DLM001_scaffold51153_2	non-phage	0
DLM001_scaffold57323_1	non-phage	0
DLM001_C706083_1	non-phage	0
DLM001_scaffold787_5	non-phage	0
DLM001_scaffold9742_1	non-phage	0
DLM001_scaffold26925_2	non-phage	0
DLM001_scaffold53015_2	phage	0.999
DLM001_scaffold43024_9	non-phage	0
DLM001_scaffold36956_2	non-phage	0
DLM001_scaffold58256_1	non-phage	0
DLM001_scaffold26717_1	non-phage	0
DLM001_scaffold45939_2	non-phage	0.002
DLM001_scaffold13932_3	non-phage	0
DLM001_scaffold631_3	non-phage	0
DLM001_scaffold5026_1	non-phage	0
DLM001_scaffold20473_3	non-phage	0
DLM001_scaffold21193_2	non-phage	0
DLM001_scaffold35003_2	non-phage	0
DLM001_scaffold49221_7	non-phage	0
DLM001_scaffold18193_2	non-phage	0
DLM001_scaffold6387_5	non-phage	0
DLM001_scaffold120_16	non-phage	0
DLM001_scaffold58254_1	non-phage	0
DLM001_scaffold31_5	non-phage	0
DLM001_scaffold20908_2	non-phage	0
DLM001_scaffold23771_1	non-phage	0
DLM001_scaffold53302_1	non-phage	0
DLM001_scaffold41027_1	non-phage	0
DLM001_scaffold2435_5	non-phage	0
DLM001_scaffold134_1	non-phage	0
DLM001_scaffold58068_1	non-phage	0
DLM001_scaffold14177_2	non-phage	0
DLM001_scaffold19576_11	non-phage	0
DLM001_scaffold58007_3	non-phage	0
DLM001_scaffold58174_1	non-phage	0
DLM001_scaffold28597_2	non-phage	0
DLM001_scaffold45921_2	non-phage	0
DLM001_scaffold41842_2	phage	0.997
DLM001_scaffold7083_3	non-phage	0
DLM001_scaffold3278_1	non-phage	0
DLM001_scaffold32362_3	non-phage	0

DLM001_scaffold40635_3	non-phage	0
DLM001_scaffold51449_3	non-phage	0
DLM001_scaffold51545_2	non-phage	0
DLM001_scaffold56530_2	non-phage	0
DLM001_scaffold56199_2	non-phage	0
DLM001_scaffold27178_3	non-phage	0
DLM001_scaffold1521_11	non-phage	0
DLM001_scaffold1291_1	non-phage	0
DLM001_scaffold4912_5	non-phage	0
DLM001_scaffold22680_5	non-phage	0
DLM001_scaffold49013_1	non-phage	0
DLM001_scaffold7_3	non-phage	0
DLM001_scaffold58075_1	non-phage	0
DLM001_scaffold8581_2	non-phage	0
DLM001_scaffold38449_1	non-phage	0
DLM001_scaffold15739_3	non-phage	0
DLM001_scaffold15292_12	non-phage	0
DLM001_scaffold51362_1	non-phage	0
DLM001_scaffold32176_2	non-phage	0
DLM001_scaffold155_7	non-phage	0
DLM001_scaffold3255_2	non-phage	0
DLM001_scaffold13349_4	non-phage	0
DLM001_scaffold10496_14	non-phage	0
DLM001_scaffold1521_18	non-phage	0
DLM001_scaffold49221_5	non-phage	0
DLM001_scaffold39319_10	non-phage	0
DLM001_scaffold32056_3	non-phage	0
DLM001_scaffold10804_2	non-phage	0
DLM001_scaffold2047_10	non-phage	0
DLM001_scaffold36734_5	non-phage	0
DLM001_scaffold21195_3	non-phage	0
DLM001_scaffold41942_6	non-phage	0
DLM001_scaffold40952_2	non-phage	0
DLM001_scaffold8604_1	non-phage	0
DLM001_scaffold4559_3	non-phage	0
DLM001_scaffold18188_2	non-phage	0
DLM001_scaffold20858_8	non-phage	0
DLM001_scaffold49096_1	non-phage	0
DLM001_scaffold19016_4	non-phage	0
DLM001_scaffold32236_2	non-phage	0
DLM001_scaffold19551_3	phage	0.999
DLM001_scaffold18811_1	non-phage	0
DLM001_scaffold4248_13	non-phage	0
DLM001_scaffold53000_1	non-phage	0
DLM001_scaffold56726_3	non-phage	0
DLM001_scaffold57887_1	non-phage	0

DLM001_scaffold10496_8	non-phage	0
DLM001_scaffold26297_4	non-phage	0
DLM001_scaffold22662_2	non-phage	0
DLM001_scaffold1382_3	non-phage	0
DLM001_scaffold43_1	non-phage	0
DLM001_scaffold43122_1	non-phage	0
DLM001_scaffold12192_2	non-phage	0
DLM001_scaffold24698_1	non-phage	0
DLM001_scaffold13258_2	non-phage	0
DLM001_scaffold26854_5	non-phage	0
DLM001_scaffold19765_15	non-phage	0
DLM001_scaffold14179_3	non-phage	0
DLM001_scaffold80_7	non-phage	0
DLM001_scaffold58253_1	phage	0.998
DLM001_scaffold16687_2	non-phage	0
DLM001_scaffold31_4	non-phage	0
DLM001_scaffold12260_3	non-phage	0
DLM001_scaffold16201_10	non-phage	0
DLM001_scaffold20770_7	non-phage	0
DLM001_scaffold55604_8	non-phage	0
DLM001_scaffold2698_1	non-phage	0
DLM001_scaffold7383_1	non-phage	0
DLM001_scaffold36324_2	non-phage	0
DLM001_scaffold18050_1	non-phage	0
DLM001_scaffold24489_1	non-phage	0
DLM001_scaffold31471_21	non-phage	0
DLM001_scaffold46490_1	non-phage	0
DLM001_scaffold12907_7	non-phage	0
DLM001_scaffold58242_1	non-phage	0
DLM001_scaffold48826_3	non-phage	0
DLM001_scaffold787_4	non-phage	0
DLM001_scaffold31168_5	non-phage	0
DLM001_scaffold54294_1	non-phage	0
DLM001_scaffold53994_4	phage	0.987
DLM001_scaffold56486_2	phage	0.999
DLM001_scaffold65_2	non-phage	0
DLM001_C705945_1	non-phage	0
DLM001_scaffold39435_4	non-phage	0
DLM001_scaffold34467_8	non-phage	0
DLM001_scaffold49968_1	non-phage	0.202
DLM001_scaffold54221_2	non-phage	0
DLM001_scaffold48552_3	phage	0.845
DLM001_scaffold56543_3	non-phage	0
DLM001_scaffold25916_1	non-phage	0
DLM001_scaffold29518_1_1	non-phage	0
DLM001_scaffold5235_12	non-phage	0

DLM001_scaffold4962_1	non-phage	0
DLM001_scaffold38737_1	non-phage	0
DLM001_scaffold47697_1	non-phage	0
DLM001_scaffold2455_2	non-phage	0
DLM001_scaffold57560_1	non-phage	0
DLM001_scaffold27279_1	non-phage	0
DLM001_scaffold56496_3	non-phage	0
DLM001_scaffold50978_3	non-phage	0
DLM001_scaffold53856_6	non-phage	0
DLM001_scaffold58196_2	non-phage	0
DLM001_scaffold53667_4	non-phage	0
DLM001_scaffold27055_4	non-phage	0
DLM001_scaffold44_2	non-phage	0
DLM001_scaffold2571_1	non-phage	0
DLM001_scaffold15292_3	non-phage	0
DLM001_scaffold45345_1	non-phage	0
DLM001_scaffold49825_3	non-phage	0
DLM001_scaffold56726_2	non-phage	0
DLM001_scaffold4106_5	phage	0.989
DLM001_scaffold24488_1	non-phage	0
DLM001_scaffold40413_2	non-phage	0
DLM001_scaffold58055_2	non-phage	0
DLM001_scaffold56496_2	non-phage	0
DLM001_scaffold23819_5	non-phage	0
DLM001_scaffold23040_1	non-phage	0
DLM001_scaffold19112_2	non-phage	0
DLM001_scaffold10897_1	non-phage	0
DLM001_scaffold7605_1	non-phage	0
DLM001_scaffold18985_5	non-phage	0
DLM001_scaffold40390_2	non-phage	0
DLM001_scaffold2768_4	non-phage	0
DLM001_scaffold51591_2	phage	0.996
DLM001_scaffold8529_11	non-phage	0
DLM001_scaffold34796_4	non-phage	0
DLM001_scaffold9098_3	non-phage	0
DLM001_scaffold50104_1	non-phage	0
DLM001_scaffold54221_3	non-phage	0
DLM001_scaffold44617_4	non-phage	0
DLM001_scaffold57810_1	non-phage	0
DLM001_scaffold2582_15	non-phage	0
DLM001_scaffold5529_1	non-phage	0
DLM001_scaffold40458_1	non-phage	0
DLM001_scaffold58260_1	non-phage	0
DLM001_scaffold36532_1	non-phage	0
DLM001_scaffold36005_1	non-phage	0
DLM001_scaffold57236_4	non-phage	0

DLM001_scaffold57282_1	non-phage	0
DLM001_scaffold48072_2	non-phage	0
DLM001_scaffold24556_1	non-phage	0
DLM001_scaffold24276_3	non-phage	0
DLM001_scaffold32056_2	non-phage	0
DLM001_scaffold27378_6	non-phage	0
DLM001_scaffold50119_2	non-phage	0
DLM001_scaffold48528_1	non-phage	0
DLM001_scaffold54691_2	non-phage	0
DLM001_scaffold6387_4	non-phage	0
DLM001_scaffold58225_2	non-phage	0
DLM001_scaffold38452_5	non-phage	0
DLM001_scaffold16902_4	non-phage	0
DLM001_scaffold7_14	non-phage	0
DLM001_scaffold43274_1	non-phage	0
DLM001_scaffold10420_4	phage	0.995
DLM001_scaffold17630_1	non-phage	0
DLM001_scaffold9128_1	non-phage	0
DLM001_scaffold3496_14	non-phage	0
DLM001_scaffold119_7	non-phage	0
DLM001_scaffold23657_3	non-phage	0
DLM001_scaffold23976_1	non-phage	0
DLM001_scaffold20183_3	non-phage	0
DLM001_scaffold21364_1	non-phage	0
DLM001_scaffold56199_7	non-phage	0
DLM001_scaffold32315_1	non-phage	0
DLM001_scaffold38003_1	non-phage	0
DLM001_scaffold55531_2	non-phage	0
DLM001_scaffold39687_3	non-phage	0.197
DLM001_scaffold10290_2	non-phage	0
DLM001_scaffold48318_1	non-phage	0
DLM001_scaffold20404_3	non-phage	0
DLM001_scaffold16178_2	non-phage	0
DLM001_scaffold57577_1	non-phage	0
DLM001_scaffold25099_3	non-phage	0
DLM001_scaffold44040_1	non-phage	0
DLM001_scaffold26346_3	non-phage	0
DLM001_scaffold48072_3	non-phage	0
DLM001_scaffold58136_1	non-phage	0
DLM001_scaffold40621_2	non-phage	0
DLM001_scaffold56530_3	non-phage	0
DLM001_scaffold27377_2	non-phage	0
DLM001_scaffold58124_4	phage	0.999
DLM001_scaffold58250_2	phage	0.98
DLM001_scaffold11369_1_2	non-phage	0
DLM001_scaffold30370_4	non-phage	0

DLM001_scaffold19191_11	non-phage	0
DLM001_scaffold32222_1	non-phage	0
DLM001_scaffold30_2	non-phage	0
DLM001_scaffold16902_5	non-phage	0
DLM001_scaffold57742_1	non-phage	0
DLM001_scaffold4106_1	non-phage	0
DLM001_scaffold39435_7	non-phage	0
DLM001_scaffold108_2	non-phage	0
DLM001_scaffold26854_8	non-phage	0
DLM001_scaffold19465_3	non-phage	0
DLM001_scaffold2768_5	non-phage	0
DLM001_scaffold247_1	non-phage	0
DLM001_scaffold23040_12	phage	0.992
DLM001_scaffold9492_1	non-phage	0
DLM001_scaffold28179_1	non-phage	0
DLM001_scaffold40906_3	non-phage	0
DLM001_scaffold40118_6	non-phage	0
DLM001_scaffold26871_1	non-phage	0
DLM001_scaffold11723_3	non-phage	0
DLM001_scaffold64_2	non-phage	0
DLM001_scaffold17676_2	non-phage	0
DLM001_scaffold49913_2	non-phage	0
DLM001_scaffold56891_1	non-phage	0
DLM001_scaffold3626_3	non-phage	0
DLM001_scaffold27010_7	non-phage	0
DLM001_scaffold26854_10	non-phage	0
DLM001_scaffold15122_5	non-phage	0
DLM001_scaffold14177_3	non-phage	0
DLM001_scaffold19765_29	non-phage	0
DLM001_scaffold27378_8	non-phage	0
DLM001_scaffold2047_7	non-phage	0
DLM001_scaffold20981_3	non-phage	0
DLM001_scaffold32326_3	non-phage	0
DLM001_scaffold36248_2	non-phage	0
DLM001_scaffold41388_1	non-phage	0
DLM001_scaffold12445_2	non-phage	0
DLM001_scaffold17162_1	non-phage	0
DLM001_scaffold5529_4	non-phage	0
DLM001_scaffold56543_6	non-phage	0
DLM001_scaffold55978_2	non-phage	0
DLM001_scaffold44006_2	non-phage	0
DLM001_scaffold48552_4	non-phage	0
DLM001_scaffold138_2	non-phage	0
DLM001_scaffold55143_2	non-phage	0
DLM001_scaffold12673_1	non-phage	0
DLM001_scaffold21655_1	non-phage	0

DLM001_scaffold44_3	non-phage	0
DLM001_scaffold3752_2	non-phage	0
DLM001_scaffold48989_2	non-phage	0
DLM001_scaffold41942_4	non-phage	0
DLM001_scaffold38074_1	non-phage	0
DLM001_scaffold29648_7	non-phage	0
DLM001_scaffold20784_6	non-phage	0
DLM001_scaffold36956_3	non-phage	0
DLM001_scaffold58114_1	non-phage	0
DLM001_scaffold13671_4	non-phage	0
DLM001_scaffold35116_3	non-phage	0
DLM001_scaffold30298_2	non-phage	0
DLM001_scaffold13893_1	non-phage	0
DLM001_scaffold58157_1	non-phage	0
DLM001_scaffold51940_5	non-phage	0
DLM001_scaffold6960_2	non-phage	0
DLM001_scaffold688_4	non-phage	0
DLM001_scaffold58258_2	non-phage	0
DLM001_scaffold58196_1	non-phage	0
DLM001_scaffold9742_2	non-phage	0
DLM001_scaffold248_2	non-phage	0
DLM001_scaffold27359_6	non-phage	0.396
DLM001_scaffold80_3	non-phage	0
DLM001_scaffold48086_4	non-phage	0
DLM001_scaffold33562_1	non-phage	0
DLM001_scaffold14355_1	non-phage	0
DLM001_scaffold41837_2	non-phage	0
DLM001_scaffold51202_1	non-phage	0
DLM001_scaffold29538_4	non-phage	0
DLM001_scaffold38991_4	non-phage	0
DLM001_scaffold15560_4	non-phage	0
DLM001_scaffold19112_3	non-phage	0
DLM001_scaffold58056_1	non-phage	0
DLM001_scaffold24359_1	non-phage	0
DLM001_scaffold15619_1	non-phage	0
DLM001_scaffold55351_1	non-phage	0
DLM001_scaffold26872_2	non-phage	0
DLM001_scaffold12480_2	non-phage	0
DLM001_scaffold44617_5	non-phage	0
DLM001_scaffold39289_3	non-phage	0
DLM001_scaffold209_9	non-phage	0
DLM001_scaffold17860_3_2	non-phage	0
DLM001_scaffold7533_10	non-phage	0
DLM001_scaffold18340_1	non-phage	0
DLM001_scaffold41423_1	non-phage	0
DLM001_scaffold8593_3	non-phage	0

DLM001_scaffold52194_1	non-phage	0
DLM001_scaffold8599_1	non-phage	0
DLM001_scaffold12890_5	non-phage	0
DLM001_scaffold53105_1	non-phage	0
DLM001_scaffold41027_2	non-phage	0
DLM001_scaffold56684_1	non-phage	0
DLM001_scaffold58091_1	non-phage	0
DLM001_scaffold27010_6	non-phage	0
DLM001_scaffold133_1	non-phage	0
DLM001_scaffold30497_1	non-phage	0
DLM001_C705425_1	phage	0.686
DLM001_scaffold43574_1	non-phage	0
DLM001_scaffold19427_4	non-phage	0
DLM001_scaffold58168_7	non-phage	0
DLM001_scaffold58055_1	non-phage	0
DLM001_scaffold30364_4	non-phage	0
DLM001_scaffold16329_2	non-phage	0
DLM001_scaffold28992_4	non-phage	0
DLM001_scaffold16422_2	non-phage	0
DLM001_scaffold22294_2	non-phage	0
DLM001_scaffold40392_1	non-phage	0
DLM001_scaffold26411_8	non-phage	0
DLM001_scaffold52289_2	phage	0.994
DLM001_scaffold38878_1	non-phage	0
DLM001_scaffold58147_2	non-phage	0
DLM001_scaffold57281_3	non-phage	0
DLM001_scaffold49612_1	non-phage	0
DLM001_scaffold19576_3	non-phage	0
DLM001_scaffold14114_10	non-phage	0
DLM001_scaffold32900_2	non-phage	0
DLM001_scaffold18361_2	phage	0.84
DLM001_scaffold13613_1	non-phage	0
DLM001_scaffold55454_4	phage	0.999
DLM001_scaffold26921_1	non-phage	0
DLM001_scaffold2780_2	non-phage	0
DLM001_scaffold58193_1	non-phage	0
DLM001_scaffold36089_1	non-phage	0
DLM001_scaffold46867_2	non-phage	0
DLM001_scaffold39252_2	phage	0.996
DLM001_scaffold40466_1	non-phage	0
DLM001_scaffold58216_2	non-phage	0
DLM001_scaffold21379_5	non-phage	0
DLM001_scaffold51852_2	non-phage	0
DLM001_scaffold57236_3	non-phage	0
DLM001_scaffold48826_2	non-phage	0
DLM001_scaffold18715_4	non-phage	0

DLM001_scaffold31060_6	non-phage	0
DLM001_scaffold29538_3	non-phage	0
DLM001_scaffold41616_1	non-phage	0
DLM001_scaffold39687_2	non-phage	0
DLM001_scaffold11564_12	non-phage	0
DLM001_scaffold24553_1	non-phage	0
DLM001_scaffold45932_2	non-phage	0
DLM001_scaffold51671_1	non-phage	0
DLM001_scaffold23819_3	non-phage	0
DLM001_scaffold7_20	non-phage	0
DLM001_scaffold15213_5	non-phage	0
DLM001_scaffold38919_2	non-phage	0
DLM001_scaffold143_13	non-phage	0
DLM001_scaffold57389_1	non-phage	0
DLM001_scaffold32831_2	non-phage	0
DLM001_scaffold22300_2	non-phage	0
DLM001_scaffold38465_4	non-phage	0
DLM001_scaffold48673_2	non-phage	0
DLM001_scaffold19755_6	non-phage	0
DLM001_scaffold16604_1	non-phage	0
DLM001_scaffold14177_4	non-phage	0
DLM001_scaffold55964_1	phage	0.995
DLM001_scaffold3943_1	non-phage	0
DLM001_scaffold48321_1	non-phage	0
DLM001_scaffold3496_15	non-phage	0
DLM001_scaffold17213_1	non-phage	0
DLM001_scaffold54379_1	non-phage	0
DLM001_scaffold16944_4	non-phage	0
DLM001_scaffold4248_1	non-phage	0
DLM001_scaffold21872_2	non-phage	0
DLM001_scaffold52929_1	non-phage	0
DLM001_scaffold49276_3	non-phage	0
DLM001_scaffold3006_5	non-phage	0
DLM001_scaffold58229_1	non-phage	0
DLM001_scaffold20332_2	non-phage	0
DLM001_scaffold33465_6	non-phage	0
DLM001_scaffold58156_2	non-phage	0
DLM001_scaffold40252_1	non-phage	0
DLM001_scaffold27359_2	non-phage	0
DLM001_scaffold20404_7	non-phage	0
DLM001_scaffold11681_2	non-phage	0
DLM001_scaffold17192_2	non-phage	0
DLM001_scaffold2435_7	non-phage	0
DLM001_scaffold39468_2	non-phage	0
DLM001_scaffold30659_1	non-phage	0
DLM001_scaffold11475_1	non-phage	0

DLM001_scaffold40943_4	non-phage	0
DLM001_scaffold2385_2	non-phage	0
DLM001_scaffold56809_1	non-phage	0
DLM001_scaffold46454_3	non-phage	0
DLM001_scaffold9859_12	non-phage	0
DLM001_scaffold57789_1	non-phage	0
DLM001_scaffold29631_2	non-phage	0
DLM001_scaffold14249_10	non-phage	0
DLM001_scaffold8905_2	non-phage	0
DLM001_scaffold19112_4	non-phage	0
DLM001_scaffold27611_6	non-phage	0
DLM001_scaffold19192_2	non-phage	0
DLM001_scaffold58137_1	non-phage	0
DLM001_scaffold39008_1	non-phage	0
DLM001_scaffold57167_2	non-phage	0
DLM001_scaffold32597_6	non-phage	0
DLM001_scaffold48226_2	non-phage	0
DLM001_scaffold10695_2	non-phage	0
DLM001_scaffold57960_1	non-phage	0
DLM001_scaffold12282_12	phage	0.984
DLM001_scaffold36901_2	non-phage	0
DLM001_scaffold4135_21	non-phage	0
DLM001_scaffold40647_2	non-phage	0
DLM001_scaffold28054_5	non-phage	0
DLM001_scaffold13671_1	phage	0.902
DLM001_scaffold48528_3	non-phage	0
DLM001_scaffold48189_1	non-phage	0
DLM001_scaffold6_1	non-phage	0
DLM001_scaffold23040_13	non-phage	0
DLM001_scaffold6752_1	non-phage	0
DLM001_scaffold3380_1	non-phage	0
DLM001_scaffold58263_1	non-phage	0
DLM001_scaffold39863_2	non-phage	0
DLM001_scaffold30759_3	non-phage	0
DLM001_scaffold27548_2	non-phage	0
DLM001_scaffold53856_1	non-phage	0
DLM001_scaffold55299_2	non-phage	0
DLM001_scaffold47290_17	non-phage	0
DLM001_scaffold41297_4	non-phage	0
DLM001_scaffold8987_5	non-phage	0
DLM001_scaffold29144_6	non-phage	0
DLM001_scaffold52168_2	non-phage	0
DLM001_scaffold51591_3	non-phage	0
DLM001_scaffold52139_4	non-phage	0
DLM001_scaffold21183_1	non-phage	0
DLM001_scaffold20364_2	non-phage	0

DLM001_scaffold41942_8	non-phage	0
DLM001_scaffold13669_1	non-phage	0
DLM001_scaffold46454_2	non-phage	0
DLM001_scaffold39272_1	non-phage	0
DLM001_scaffold10330_1_1	non-phage	0
DLM001_scaffold17213_3	non-phage	0
DLM001_scaffold10496_6	non-phage	0
DLM001_scaffold10295_4	non-phage	0
DLM001_scaffold21364_2	non-phage	0
DLM001_scaffold47542_1	non-phage	0
DLM001_scaffold4912_1	non-phage	0
DLM001_scaffold32236_4_2	non-phage	0.453
DLM001_scaffold39777_3	non-phage	0
DLM001_scaffold45546_1	non-phage	0
DLM001_scaffold26852_2	non-phage	0
DLM001_scaffold16944_5	non-phage	0
DLM001_scaffold22711_3	non-phage	0
DLM001_scaffold12700_9	non-phage	0
DLM001_scaffold20559_2	non-phage	0
DLM001_scaffold58262_1	non-phage	0
DLM001_scaffold9570_5	non-phage	0
DLM001_scaffold37_1	non-phage	0
DLM001_scaffold56100_1	non-phage	0
DLM001_scaffold47290_13	non-phage	0
DLM001_scaffold57806_1	non-phage	0
DLM001_C706827_1	non-phage	0
DLM001_scaffold12155_1	non-phage	0
DLM001_scaffold20397_1	non-phage	0
DLM001_scaffold20232_8	non-phage	0.277
DLM001_scaffold47891_1	non-phage	0
DLM001_scaffold31471_22	non-phage	0
DLM001_scaffold251_1	non-phage	0
DLM001_scaffold51449_2	non-phage	0
DLM001_scaffold38452_8	non-phage	0
DLM001_scaffold57606_1	non-phage	0
DLM001_scaffold58130_1	non-phage	0
DLM001_C706761_1	non-phage	0
DLM001_scaffold29648_10	non-phage	0
DLM001_scaffold22757_3	non-phage	0
DLM001_scaffold25900_1	non-phage	0
DLM001_scaffold3954_20	non-phage	0
DLM001_scaffold24171_7	non-phage	0
DLM001_scaffold35296_4	non-phage	0
DLM001_scaffold1521_6	non-phage	0
DLM001_scaffold56884_1	non-phage	0
DLM001_C705841_1	non-phage	0

DLM001_scaffold12480_1	non-phage	0
DLM001_scaffold31471_17	non-phage	0
DLM001_scaffold39256_4	non-phage	0
DLM001_scaffold19765_4	non-phage	0
DLM001_scaffold43312_1	non-phage	0
DLM001_scaffold50508_2	non-phage	0
DLM001_scaffold33469_1	non-phage	0
DLM001_scaffold27703_3	non-phage	0
DLM001_scaffold58044_1	non-phage	0
DLM001_scaffold19755_7	non-phage	0
DLM001_scaffold56199_4	non-phage	0
DLM001_scaffold53856_5	non-phage	0
DLM001_scaffold55604_9	non-phage	0
DLM001_scaffold23603_6	non-phage	0
DLM001_scaffold25099_2	non-phage	0
DLM001_scaffold143_3	non-phage	0
DLM001_scaffold14338_2	non-phage	0
DLM001_scaffold9674_1	non-phage	0
DLM001_scaffold50893_2	phage	0.999
DLM001_scaffold38782_1	non-phage	0
DLM001_scaffold54404_1	non-phage	0
DLM001_scaffold58225_4	non-phage	0
DLM001_scaffold38513_2	non-phage	0
DLM001_scaffold55604_10	non-phage	0
DLM001_scaffold22168_9	non-phage	0
DLM001_scaffold47917_4_2	non-phage	0
DLM001_scaffold23040_10	non-phage	0
DLM001_scaffold28826_23	non-phage	0
DLM001_scaffold8907_11	non-phage	0
DLM001_scaffold43205_1	non-phage	0
DLM001_scaffold2970_5	non-phage	0
DLM001_scaffold6056_4	non-phage	0
DLM001_scaffold2582_13	non-phage	0
DLM001_scaffold56574_1	non-phage	0
DLM001_scaffold93_11	non-phage	0
DLM001_scaffold22680_3	non-phage	0
DLM001_scaffold9483_2	non-phage	0
DLM001_scaffold51940_1	non-phage	0
DLM001_scaffold57866_1	non-phage	0
DLM001_scaffold23257_1	non-phage	0
DLM001_scaffold64_1	non-phage	0
DLM001_scaffold6387_2	non-phage	0
DLM001_scaffold25450_1	non-phage	0
DLM001_scaffold29538_2	non-phage	0
DLM001_scaffold31471_12	non-phage	0
DLM001_scaffold9098_5	non-phage	0.003

DLM001_scaffold12700_5	non-phage	0
DLM001_scaffold49320_2	non-phage	0
DLM001_scaffold36839_1	non-phage	0
DLM001_scaffold30107_1	non-phage	0
DLM001_scaffold3997_5	non-phage	0
DLM001_scaffold26876_15	non-phage	0
DLM001_scaffold6752_6	non-phage	0
DLM001_scaffold30663_5	non-phage	0
DLM001_scaffold30497_2	non-phage	0
DLM001_scaffold33123_1	non-phage	0
DLM001_scaffold36005_3	non-phage	0
DLM001_scaffold33465_9	non-phage	0
DLM001_scaffold29714_2	non-phage	0
DLM001_scaffold16141_3	non-phage	0
DLM001_scaffold26838_9	non-phage	0
DLM001_scaffold54280_2	non-phage	0
DLM001_scaffold277_3	phage	0.994
DLM001_scaffold54769_2	non-phage	0
DLM001_scaffold10383_2	non-phage	0
DLM001_scaffold58087_2	non-phage	0
DLM001_scaffold39468_1	phage	0.992
DLM001_scaffold6645_7	non-phage	0
DLM001_scaffold21297_2	non-phage	0
DLM001_scaffold36801_8	non-phage	0
DLM001_scaffold43487_5	non-phage	0
DLM001_scaffold17421_4	non-phage	0
DLM001_scaffold33667_2	non-phage	0
DLM001_scaffold10897_8	non-phage	0
DLM001_scaffold5589_3	non-phage	0
DLM001_scaffold56649_1	non-phage	0
DLM001_scaffold36801_1	non-phage	0
DLM001_scaffold7681_1	non-phage	0
DLM001_scaffold25009_1	non-phage	0
DLM001_scaffold27996_3	non-phage	0
DLM001_scaffold9128_3	non-phage	0
DLM001_scaffold26899_3	non-phage	0
DLM001_scaffold41682_2	non-phage	0
DLM001_scaffold40118_2	non-phage	0
DLM001_scaffold24290_1	non-phage	0.001
DLM001_scaffold45972_3	non-phage	0
DLM001_scaffold52611_1	non-phage	0
DLM001_scaffold58214_2	non-phage	0
DLM001_scaffold26690_2	non-phage	0
DLM001_scaffold57289_2	non-phage	0
DLM001_scaffold29101_2	non-phage	0
DLM001_scaffold31471_23	non-phage	0

DLM001_scaffold51404_5	non-phage	0
DLM001_C706031_1	non-phage	0
DLM001_scaffold54429_1	non-phage	0
DLM001_scaffold15619_3	non-phage	0
DLM001_scaffold55770_2	non-phage	0
DLM001_scaffold8256_2	non-phage	0
DLM001_scaffold24171_11	non-phage	0
DLM001_scaffold48552_1	non-phage	0
DLM001_scaffold38_1	non-phage	0
DLM001_scaffold35702_2	non-phage	0
DLM001_scaffold44441_2	non-phage	0
DLM001_scaffold4135_18	non-phage	0
DLM001_scaffold1291_7	non-phage	0
DLM001_scaffold44039_1	non-phage	0
DLM001_scaffold37336_8	non-phage	0
DLM001_scaffold940_21	non-phage	0
DLM001_scaffold58017_1	non-phage	0
DLM001_scaffold56700_1	non-phage	0
DLM001_scaffold11242_1	non-phage	0
DLM001_scaffold14249_2	non-phage	0
DLM001_scaffold20770_9	non-phage	0
DLM001_scaffold32599_1	non-phage	0
DLM001_scaffold54547_2	non-phage	0
DLM001_scaffold58227_1	non-phage	0
DLM001_scaffold35702_1	non-phage	0
DLM001_scaffold51914_1	non-phage	0
DLM001_scaffold58011_1	non-phage	0
DLM001_scaffold10367_4	non-phage	0
DLM001_scaffold11963_1	non-phage	0
DLM001_scaffold55604_5	non-phage	0
DLM001_scaffold29144_8	non-phage	0
DLM001_C706385_1	non-phage	0
DLM001_scaffold19576_10	non-phage	0
DLM001_scaffold24698_7	non-phage	0
DLM001_scaffold47007_2	phage	0.963
DLM001_scaffold57057_1	non-phage	0
DLM001_scaffold25539_1	non-phage	0
DLM001_scaffold49276_1	phage	0.998
DLM001_scaffold33678_1	non-phage	0
DLM001_scaffold54083_1	non-phage	0
DLM001_scaffold31_7	non-phage	0
DLM001_scaffold12700_7	non-phage	0
DLM001_scaffold35700_1	non-phage	0
DLM001_scaffold30024_2	non-phage	0
DLM001_scaffold19755_8	non-phage	0
DLM001_scaffold57105_2	non-phage	0

DLM001_scaffold57439_1	non-phage	0
DLM001_scaffold21002_1	phage	0.999
DLM001_scaffold18811_2	non-phage	0
DLM001_scaffold57142_2	non-phage	0
DLM001_scaffold5235_5	non-phage	0
DLM001_scaffold1114_1	non-phage	0
DLM001_scaffold42810_2	phage	0.996
DLM001_scaffold55603_1	non-phage	0
DLM001_scaffold20637_1	non-phage	0
DLM001_scaffold37_2	non-phage	0
DLM001_scaffold18552_1	non-phage	0
DLM001_scaffold15122_2	non-phage	0
DLM001_scaffold8529_6	non-phage	0
DLM001_scaffold7532_2	non-phage	0
DLM001_scaffold49834_2	phage	0.902
DLM001_scaffold40426_3	non-phage	0
DLM001_scaffold11970_2	non-phage	0.065
DLM001_scaffold651_4	non-phage	0
DLM001_scaffold26717_2	non-phage	0
DLM001_scaffold43124_1	non-phage	0
DLM001_scaffold33_2	non-phage	0
DLM001_scaffold57275_1	non-phage	0
DLM001_scaffold8256_3	non-phage	0
DLM001_scaffold49947_2	non-phage	0
DLM001_scaffold4440_1	non-phage	0
DLM001_scaffold41942_7	non-phage	0
DLM001_scaffold28068_1	non-phage	0
DLM001_scaffold18193_1	non-phage	0
DLM001_scaffold1380_6	non-phage	0
DLM001_scaffold16773_2	non-phage	0
DLM001_scaffold51708_2	non-phage	0
DLM001_scaffold15680_3	non-phage	0
DLM001_scaffold30022_2	non-phage	0
DLM001_scaffold16868_1	phage	0.993
DLM001_scaffold58206_1	non-phage	0
DLM001_scaffold58228_1	non-phage	0
DLM001_scaffold209_3	non-phage	0
DLM001_scaffold57732_1	non-phage	0
DLM001_scaffold24837_1	non-phage	0
DLM001_scaffold57559_1	non-phage	0
DLM001_scaffold17788_3	non-phage	0
DLM001_scaffold31873_1	non-phage	0
DLM001_scaffold34085_1	non-phage	0
DLM001_scaffold28890_5	non-phage	0
DLM001_C705375_1	non-phage	0
DLM001_scaffold25742_2	non-phage	0

DLM001_scaffold27010_11	non-phage	0
DLM001_scaffold4545_1	non-phage	0
DLM001_scaffold7234_1	non-phage	0
DLM001_scaffold58061_1	non-phage	0
DLM001_scaffold3496_4	non-phage	0
DLM001_scaffold58204_1	non-phage	0
DLM001_scaffold42483_2	non-phage	0
DLM001_scaffold57747_1	non-phage	0
DLM001_scaffold54429_4	non-phage	0
DLM001_scaffold23611_1	non-phage	0
DLM001_scaffold53554_1	non-phage	0
DLM001_scaffold6938_1	non-phage	0
DLM001_scaffold37730_1	non-phage	0
DLM001_scaffold36945_1	non-phage	0
DLM001_scaffold16141_1	non-phage	0
DLM001_scaffold23697_6	non-phage	0
DLM001_scaffold35272_1	non-phage	0
DLM001_scaffold21346_3	non-phage	0
DLM001_scaffold18361_4	non-phage	0
DLM001_scaffold41842_1	non-phage	0
DLM001_scaffold58249_1	non-phage	0
DLM001_scaffold20232_5	non-phage	0
DLM001_scaffold34017_20	non-phage	0
DLM001_scaffold14179_1	non-phage	0
DLM001_scaffold1702_1	non-phage	0
DLM001_scaffold16329_1	non-phage	0
DLM001_scaffold6623_2	non-phage	0
DLM001_scaffold21347_1	non-phage	0
DLM001_scaffold43274_4	non-phage	0
DLM001_scaffold48552_2	non-phage	0
DLM001_scaffold52139_2	phage	0.999
DLM001_scaffold39272_2	non-phage	0
DLM001_scaffold36678_1	non-phage	0
DLM001_scaffold57202_2	non-phage	0
DLM001_scaffold23910_1	non-phage	0
DLM001_scaffold14847_6	non-phage	0
DLM001_scaffold7_22	non-phage	0
DLM001_scaffold29510_2	non-phage	0
DLM001_scaffold21013_3	non-phage	0
DLM001_scaffold8256_1_1	non-phage	0
DLM001_scaffold15191_7	non-phage	0
DLM001_scaffold2582_1	non-phage	0
DLM001_scaffold41942_3	non-phage	0
DLM001_scaffold56836_1	non-phage	0
DLM001_scaffold23997_7	non-phage	0
DLM001_scaffold22668_2	non-phage	0

DLM001_scaffold58016_2	non-phage	0
DLM001_scaffold31841_13	non-phage	0
DLM001_scaffold8987_7	non-phage	0
DLM001_scaffold14143_1	non-phage	0
DLM001_scaffold30863_7	non-phage	0
DLM001_scaffold91_7	non-phage	0
DLM001_scaffold46861_2	non-phage	0
DLM001_scaffold53800_1	non-phage	0
DLM001_scaffold54769_11	non-phage	0
DLM001_scaffold50978_5	non-phage	0
DLM001_scaffold58111_1	non-phage	0
DLM001_scaffold57530_1	non-phage	0
DLM001_scaffold5806_2	non-phage	0
DLM001_scaffold53544_3	non-phage	0
DLM001_scaffold49834_1	non-phage	0
DLM001_scaffold27079_2	non-phage	0
DLM001_scaffold56997_1	non-phage	0
DLM001_scaffold28837_1	non-phage	0
DLM001_scaffold4453_1	non-phage	0
DLM001_scaffold6752_4	non-phage	0
DLM001_scaffold39237_12	non-phage	0
DLM001_scaffold6224_1	non-phage	0
DLM001_scaffold58096_3	non-phage	0
DLM001_scaffold47891_3	non-phage	0
DLM001_scaffold57756_4	non-phage	0
DLM001_scaffold58266_1	non-phage	0
DLM001_C706107_1	non-phage	0
DLM001_scaffold13671_6	non-phage	0
DLM001_scaffold9859_14	non-phage	0
DLM001_scaffold11723_1	phage	0.998
DLM001_scaffold593_1	non-phage	0
DLM001_scaffold58124_6	non-phage	0
DLM001_scaffold17887_2	non-phage	0
DLM001_scaffold38659_4	non-phage	0
DLM001_scaffold3496_9	non-phage	0
DLM001_scaffold43312_2	non-phage	0
DLM001_scaffold31_2	non-phage	0
DLM001_scaffold19300_4	non-phage	0
DLM001_scaffold55377_1	non-phage	0
DLM001_scaffold52289_1	non-phage	0
DLM001_scaffold57558_2	non-phage	0
DLM001_scaffold49995_2	non-phage	0
DLM001_scaffold52368_2	non-phage	0
DLM001_scaffold10130_2	non-phage	0
DLM001_scaffold19755_9	non-phage	0
DLM001_scaffold26718_4	non-phage	0

DLM001_scaffold19049_4	non-phage	0
DLM001_scaffold16178_4	non-phage	0
DLM001_scaffold19427_9	phage	0.998
DLM001_scaffold56199_9	non-phage	0
DLM001_scaffold10496_5	non-phage	0
DLM001_scaffold4912_11	non-phage	0
DLM001_scaffold12260_10	non-phage	0
DLM001_scaffold48072_1	non-phage	0
DLM001_scaffold34027_1	non-phage	0
DLM001_scaffold7455_2	non-phage	0
DLM001_scaffold27611_5	phage	0.993
DLM001_scaffold55811_1	non-phage	0
DLM001_scaffold20858_7	non-phage	0
DLM001_scaffold19379_2	non-phage	0
DLM001_scaffold14114_7	phage	0.996
DLM001_scaffold1702_4	non-phage	0
DLM001_scaffold38513_3	non-phage	0
DLM001_scaffold3576_10	phage	0.997
DLM001_scaffold19576_1	non-phage	0
DLM001_scaffold58076_2	non-phage	0
DLM001_scaffold19049_5	non-phage	0
DLM001_scaffold27549_1	non-phage	0
DLM001_scaffold17367_1	non-phage	0
DLM001_scaffold53228_1	non-phage	0
DLM001_scaffold57030_2	non-phage	0
DLM001_scaffold21346_5	non-phage	0
DLM001_scaffold15560_9	non-phage	0
DLM001_scaffold35296_1	non-phage	0
DLM001_scaffold9483_1	non-phage	0
DLM001_scaffold4545_4	non-phage	0
DLM001_scaffold143_15	non-phage	0
DLM001_scaffold19191_12	non-phage	0
DLM001_scaffold13189_1	non-phage	0
DLM001_scaffold21348_1	non-phage	0
DLM001_scaffold1529_16	non-phage	0
DLM001_scaffold29648_4	non-phage	0
DLM001_scaffold19465_1	non-phage	0
DLM001_scaffold32597_2	non-phage	0
DLM001_scaffold11722_1	non-phage	0
DLM001_scaffold26729_4	non-phage	0
DLM002_scaffold21972_1	non-phage	0
DLM002_scaffold22078_4	non-phage	0
DLM002_scaffold26701_6	non-phage	0
DLM002_scaffold50443_2	non-phage	0
DLM002_scaffold9161_2	non-phage	0
DLM002_C781545_1	non-phage	0

DLM002_scaffold22467_1	non-phage	0
DLM002_scaffold50706_2	non-phage	0
DLM002_scaffold154_7	non-phage	0
DLM002_scaffold2284_5	non-phage	0
DLM002_scaffold52699_1	non-phage	0
DLM002_C781379_1	non-phage	0
DLM002_scaffold58_1	non-phage	0
DLM002_scaffold27657_4	non-phage	0
DLM002_scaffold10382_2	non-phage	0
DLM002_scaffold46747_1	non-phage	0
DLM002_scaffold12593_5	non-phage	0
DLM002_scaffold9840_9	non-phage	0
DLM002_scaffold41770_1	non-phage	0
DLM002_scaffold5402_6	non-phage	0
DLM002_scaffold25143_3	non-phage	0
DLM002_scaffold832_12	non-phage	0
DLM002_scaffold288_3	non-phage	0
DLM002_scaffold29142_3	non-phage	0
DLM002_scaffold27285_6	non-phage	0
DLM002_scaffold51878_1	non-phage	0
DLM002_scaffold82_2	non-phage	0
DLM002_scaffold38087_1	non-phage	0
DLM002_scaffold52676_1	non-phage	0
DLM002_scaffold52363_1	non-phage	0
DLM002_scaffold2899_4	non-phage	0
DLM002_scaffold32671_8	non-phage	0
DLM002_scaffold12502_6	non-phage	0
DLM002_scaffold278_8	non-phage	0
DLM002_scaffold49071_1	non-phage	0
DLM002_scaffold6768_2	non-phage	0
DLM002_scaffold864_22	non-phage	0
DLM002_scaffold1607_20	non-phage	0
DLM002_scaffold11243_13	non-phage	0
DLM002_scaffold3907_2	non-phage	0
DLM002_scaffold14127_8	non-phage	0
DLM002_scaffold48180_16	non-phage	0
DLM002_scaffold52283_2	phage	0.96
DLM002_scaffold14139_8	non-phage	0
DLM002_scaffold22219_1	non-phage	0
DLM002_scaffold6404_1	non-phage	0
DLM002_scaffold52455_2	non-phage	0
DLM002_scaffold52043_1	non-phage	0
DLM002_scaffold17097_9	non-phage	0
DLM002_scaffold52716_1	non-phage	0
DLM002_scaffold4422_7	non-phage	0
DLM002_scaffold13154_5	non-phage	0

DLM002_scaffold93_3	non-phage	0
DLM002_scaffold14044_5	non-phage	0
DLM002_scaffold52498_2	non-phage	0
DLM002_scaffold31967_7	non-phage	0
DLM002_scaffold49919_3	non-phage	0
DLM002_scaffold46552_3	non-phage	0
DLM002_scaffold19426_2	non-phage	0
DLM002_scaffold22629_1	non-phage	0
DLM002_scaffold43881_1	non-phage	0
DLM002_scaffold28661_15	non-phage	0
DLM002_scaffold30489_1	non-phage	0
DLM002_scaffold1276_3	non-phage	0
DLM002_scaffold52344_1	non-phage	0
DLM002_scaffold9323_44	non-phage	0
DLM002_scaffold15072_4	non-phage	0
DLM002_scaffold35924_1	phage	0.998
DLM002_scaffold31454_1	non-phage	0
DLM002_scaffold2354_1	non-phage	0
DLM002_scaffold832_17	non-phage	0
DLM002_scaffold34417_3	non-phage	0
DLM002_scaffold48645_2	non-phage	0
DLM002_scaffold52050_2	non-phage	0
DLM002_scaffold17699_1	non-phage	0
DLM002_scaffold28835_3	non-phage	0
DLM002_scaffold36129_1	non-phage	0
DLM002_scaffold26552_1	non-phage	0
DLM002_scaffold6037_1	non-phage	0
DLM002_scaffold42095_1	non-phage	0
DLM002_scaffold51827_1	non-phage	0
DLM002_scaffold52529_1	non-phage	0
DLM002_scaffold51657_1	non-phage	0
DLM002_scaffold18105_4	non-phage	0
DLM002_scaffold52498_4	non-phage	0
DLM002_scaffold31980_1	non-phage	0
DLM002_scaffold22078_5	non-phage	0
DLM002_scaffold25891_1	non-phage	0
DLM002_scaffold45817_1	non-phage	0
DLM002_scaffold13841_1	non-phage	0
DLM002_scaffold48371_2	non-phage	0
DLM002_scaffold30489_2	non-phage	0
DLM002_scaffold97_2	non-phage	0
DLM002_scaffold36794_5	non-phage	0
DLM002_scaffold52687_4	non-phage	0
DLM002_scaffold6775_3	non-phage	0
DLM002_scaffold34643_3	non-phage	0
DLM002_scaffold44168_1	non-phage	0

DLM002_scaffold982_2	non-phage	0
DLM002_scaffold20586_4	non-phage	0
DLM002_scaffold44407_2	non-phage	0
DLM002_scaffold2606_12	non-phage	0
DLM002_scaffold44485_5	non-phage	0
DLM002_scaffold82_1	non-phage	0
DLM002_scaffold21880_10	non-phage	0
DLM002_scaffold1400_2	non-phage	0
DLM002_scaffold9001_1	non-phage	0
DLM002_scaffold22438_6	non-phage	0
DLM002_scaffold42905_4	non-phage	0
DLM002_scaffold13686_9	non-phage	0
DLM002_scaffold45143_1	non-phage	0
DLM002_scaffold52464_2	non-phage	0
DLM002_scaffold13154_3	non-phage	0
DLM002_scaffold49919_1	non-phage	0
DLM002_scaffold21495_7	non-phage	0
DLM002_scaffold5181_3	non-phage	0
DLM002_scaffold10596_1	non-phage	0
DLM002_scaffold3680_7	non-phage	0
DLM002_scaffold15734_3	non-phage	0
DLM002_scaffold22629_3	non-phage	0
DLM002_scaffold51215_2	non-phage	0
DLM002_scaffold50415_1	non-phage	0
DLM002_scaffold18188_12	non-phage	0
DLM002_scaffold41642_3	non-phage	0
DLM002_scaffold109_3	non-phage	0
DLM002_scaffold29967_1	non-phage	0
DLM002_scaffold25681_6	non-phage	0
DLM002_scaffold2920_21	non-phage	0
DLM002_scaffold1034_2	non-phage	0
DLM002_scaffold5328_2	non-phage	0
DLM002_scaffold2284_2	non-phage	0
DLM002_C781461_1	non-phage	0.001
DLM002_scaffold39104_8	non-phage	0
DLM002_scaffold97_6	non-phage	0
DLM002_scaffold6895_4	non-phage	0
DLM002_scaffold10705_21	non-phage	0
DLM002_scaffold17586_4	non-phage	0
DLM002_scaffold42163_1	non-phage	0
DLM002_scaffold52710_1	non-phage	0
DLM002_scaffold52658_1	non-phage	0
DLM002_scaffold864_8	non-phage	0
DLM002_scaffold32671_10	non-phage	0
DLM002_scaffold5402_9	non-phage	0
DLM002_scaffold52291_1	non-phage	0

DLM002_scaffold1276_2	non-phage	0
DLM002_scaffold52263_1	non-phage	0
DLM002_scaffold41325_1	non-phage	0
DLM002_scaffold52498_10	non-phage	0
DLM002_scaffold11310_15	non-phage	0
DLM002_scaffold39638_1	phage	0.998
DLM002_scaffold28835_2	non-phage	0
DLM002_scaffold50400_2	non-phage	0
DLM002_scaffold52670_4	non-phage	0
DLM002_scaffold48645_1	non-phage	0
DLM002_scaffold41192_1	non-phage	0
DLM002_scaffold20393_2	non-phage	0
DLM002_scaffold13154_4	non-phage	0
DLM002_scaffold21810_25	non-phage	0
DLM002_scaffold23337_3	non-phage	0
DLM002_scaffold18693_2	non-phage	0
DLM002_scaffold52707_1	non-phage	0
DLM002_scaffold21972_8	non-phage	0
DLM002_scaffold52687_1	non-phage	0
DLM002_scaffold52650_1	non-phage	0
DLM002_scaffold58_3	phage	0.998
DLM002_scaffold49919_2	non-phage	0
DLM002_scaffold52693_1	non-phage	0
DLM002_scaffold6275_8	non-phage	0
DLM002_scaffold52394_3	non-phage	0
DLM002_scaffold4794_16	non-phage	0
DLM002_scaffold278_3	non-phage	0
DLM002_scaffold3108_2	non-phage	0
DLM002_scaffold271_6	non-phage	0
DLM002_scaffold49071_4	non-phage	0
DLM002_scaffold52695_2	non-phage	0
DLM002_scaffold3108_11	non-phage	0
DLM002_scaffold2920_70	non-phage	0
DLM002_scaffold7_8	non-phage	0
DLM002_scaffold22629_5	non-phage	0
DLM002_scaffold1782_9	non-phage	0
DLM002_scaffold4822_9	non-phage	0
DLM002_scaffold51622_2	non-phage	0
DLM002_scaffold26446_1	non-phage	0
DLM002_scaffold8357_7	phage	0.999
DLM002_scaffold52541_2	non-phage	0
DLM002_scaffold38216_3	non-phage	0
DLM002_scaffold121_2	non-phage	0
DLM002_scaffold50198_4	non-phage	0
DLM002_scaffold37422_1	non-phage	0
DLM002_scaffold34433_1	non-phage	0

DLM002_scaffold2922_3	non-phage	0
DLM002_scaffold37176_14	non-phage	0
DLM002_scaffold6623_5	non-phage	0
DLM002_scaffold14428_1	non-phage	0
DLM002_scaffold42095_3	non-phage	0
DLM002_scaffold7576_2	non-phage	0
DLM002_scaffold52372_1	non-phage	0
DLM002_scaffold31287_3	non-phage	0
DLM002_scaffold14988_9	non-phage	0
DLM002_scaffold30887_6	non-phage	0
DLM002_scaffold1034_3	non-phage	0
DLM002_scaffold11243_16	non-phage	0
DLM002_scaffold52678_4	non-phage	0
DLM002_scaffold42095_2	non-phage	0
DLM002_scaffold52328_1	non-phage	0
DLM002_scaffold51827_2	non-phage	0
DLM002_scaffold166_1	non-phage	0
DLM002_scaffold15734_4	non-phage	0
DLM002_scaffold52662_1	non-phage	0
DLM002_scaffold10271_1	non-phage	0
DLM002_scaffold14044_2	non-phage	0
DLM002_scaffold832_6	non-phage	0
DLM002_scaffold42905_1	non-phage	0
DLM002_scaffold34299_2	non-phage	0
DLM002_scaffold37454_1	phage	0.989
DLM002_scaffold17097_1	non-phage	0
DLM002_scaffold118_5	non-phage	0
DLM002_scaffold20816_10	non-phage	0
DLM002_scaffold34643_2	non-phage	0
DLM002_scaffold15734_2	non-phage	0
DLM002_scaffold52703_3	non-phage	0
DLM002_scaffold44373_2	non-phage	0
DLM002_scaffold17004_2	non-phage	0
DLM002_scaffold6132_2	non-phage	0
DLM002_scaffold15954_3	non-phage	0
DLM002_scaffold43803_1	non-phage	0
DLM002_scaffold6117_5	non-phage	0
DLM002_scaffold154_2	non-phage	0
DLM002_scaffold11013_6	non-phage	0
DLM002_scaffold17136_2	non-phage	0
DLM002_scaffold8038_1	non-phage	0
DLM002_scaffold52498_5	non-phage	0
DLM002_scaffold34041_1	non-phage	0
DLM002_scaffold52515_3	non-phage	0
DLM002_scaffold52678_1	non-phage	0
DLM002_scaffold51949_1	non-phage	0

DLM002_scaffold9186_2	non-phage	0
DLM002_scaffold9739_19	non-phage	0
DLM002_scaffold48827_2	non-phage	0
DLM002_scaffold98_2	non-phage	0
DLM002_scaffold660_94	non-phage	0
DLM002_C781311_1	non-phage	0
DLM002_scaffold52642_1	non-phage	0
DLM002_scaffold24418_3	non-phage	0
DLM002_scaffold166_4	non-phage	0
DLM002_scaffold49997_2	non-phage	0
DLM002_scaffold45622_4	non-phage	0
DLM002_scaffold40965_3	non-phage	0
DLM002_scaffold3766_5	non-phage	0
DLM002_scaffold52679_1	non-phage	0
DLM002_scaffold15193_2	non-phage	0
DLM002_scaffold30153_1	non-phage	0
DLM002_scaffold1652_4	non-phage	0
DLM002_scaffold37352_8	phage	1
DLM002_scaffold4794_6	phage	0.998
DLM002_scaffold52448_2	non-phage	0
DLM002_scaffold4542_6	non-phage	0
DLM002_scaffold26156_2	non-phage	0
DLM002_scaffold14013_5	non-phage	0
DLM002_scaffold52567_4	non-phage	0
DLM002_scaffold52713_2	phage	0.998
DLM002_scaffold40497_1	non-phage	0
DLM002_scaffold13569_1	non-phage	0
DLM002_scaffold14082_3	non-phage	0
DLM002_scaffold24790_1	non-phage	0
DLM002_C780713_1	non-phage	0
DLM002_scaffold26611_3	non-phage	0
DLM002_scaffold2140_27	non-phage	0
DLM002_scaffold42313_1	non-phage	0
DLM002_scaffold13658_1	non-phage	0
DLM002_scaffold42401_2	non-phage	0
DLM002_C780885_1	non-phage	0
DLM002_scaffold52667_1	non-phage	0
DLM002_scaffold43304_6	non-phage	0
DLM002_scaffold43957_2	non-phage	0
DLM002_scaffold248_1	non-phage	0
DLM002_scaffold52724_1	non-phage	0
DLM002_scaffold2547_3	non-phage	0
DLM002_scaffold52498_6	non-phage	0
DLM002_scaffold47629_2	non-phage	0
DLM002_scaffold14988_2	non-phage	0
DLM002_scaffold21495_5	non-phage	0

DLM002_scaffold5402_10	non-phage	0
DLM002_scaffold38216_1	non-phage	0
DLM002_scaffold22117_1	non-phage	0
DLM002_scaffold34001_1	non-phage	0
DLM002_scaffold7520_15	non-phage	0
DLM002_scaffold39638_2	non-phage	0
DLM002_scaffold9161_4	non-phage	0
DLM002_scaffold37624_3	non-phage	0
DLM002_scaffold97_1	non-phage	0
DLM002_scaffold17174_7	non-phage	0
DLM002_scaffold34643_5	non-phage	0
DLM002_scaffold98_5	non-phage	0
DLM002_scaffold50288_1	non-phage	0
DLM002_scaffold13686_4	non-phage	0
DLM002_scaffold32671_6	non-phage	0
DLM002_scaffold9490_16	non-phage	0
DLM002_scaffold45452_2	phage	0.983
DLM002_scaffold166_9	non-phage	0
DLM002_scaffold52715_1	non-phage	0
DLM002_scaffold1028_12	non-phage	0
DLM002_scaffold43449_1	non-phage	0
DLM002_scaffold7963_50	non-phage	0.162
DLM002_C780893_1	non-phage	0
DLM002_scaffold17174_8	non-phage	0
DLM002_scaffold52516_1	non-phage	0
DLM002_scaffold24305_3	non-phage	0
DLM002_scaffold3907_1	non-phage	0
DLM002_scaffold15954_6	non-phage	0
DLM002_scaffold27414_1	non-phage	0
DLM002_scaffold147_1	non-phage	0
DLM002_scaffold7228_5	non-phage	0
DLM002_scaffold52201_3	non-phage	0
DLM002_scaffold162_9	non-phage	0
DLM002_scaffold18094_2	non-phage	0
DLM002_scaffold43659_4	non-phage	0
DLM002_scaffold47759_2	non-phage	0
DLM002_scaffold52234_1	non-phage	0
DLM002_scaffold11013_2	non-phage	0
DLM002_scaffold8357_5	non-phage	0
DLM002_scaffold3366_1	non-phage	0
DLM002_scaffold44287_3	non-phage	0
DLM002_scaffold7197_1	non-phage	0
DLM002_scaffold16253_2	non-phage	0
DLM002_scaffold25419_1	non-phage	0
DLM002_scaffold26156_1	non-phage	0
DLM002_scaffold166_2	non-phage	0

DLM002_scaffold11243_14	non-phage	0
DLM002_scaffold14013_6	non-phage	0
DLM002_scaffold39181_1	non-phage	0
DLM002_scaffold48724_2	phage	0.989
DLM002_scaffold106_12	non-phage	0
DLM002_scaffold9305_1	non-phage	0
DLM002_scaffold49997_1	non-phage	0
DLM002_scaffold51434_4	non-phage	0
DLM002_scaffold41777_1	non-phage	0
DLM002_scaffold39196_1	non-phage	0
DLM002_scaffold41703_2	non-phage	0
DLM002_scaffold37737_1	non-phage	0
DLM002_scaffold44485_3	non-phage	0
DLM002_scaffold18094_1	non-phage	0
DLM002_scaffold4794_4	non-phage	0
DLM002_scaffold20816_11	non-phage	0
DLM002_scaffold52706_1	non-phage	0
DLM002_scaffold52680_1	non-phage	0
DLM002_scaffold6018_10	non-phage	0
DLM002_scaffold22629_2	non-phage	0
DLM002_scaffold52725_1	non-phage	0
DLM002_scaffold52360_2	non-phage	0
DLM002_scaffold2706_4	non-phage	0
DLM002_scaffold1028_5	non-phage	0
DLM002_scaffold265_1	non-phage	0
DLM002_scaffold7520_16	non-phage	0
DLM002_scaffold154_4	non-phage	0
DLM002_scaffold42905_2	non-phage	0
DLM002_scaffold36659_2	non-phage	0
DLM002_scaffold45574_1	non-phage	0
DLM002_scaffold32671_9	non-phage	0
DLM002_scaffold278_1	non-phage	0.001
DLM002_scaffold11013_7	phage	0.949
DLM002_scaffold52718_1	non-phage	0
DLM002_scaffold36556_1	non-phage	0
DLM002_scaffold13686_2	non-phage	0
DLM002_scaffold44407_5	non-phage	0
DLM002_scaffold41190_4	non-phage	0
DLM002_scaffold52721_1	non-phage	0
DLM002_scaffold30887_1	non-phage	0
DLM002_scaffold52541_4	non-phage	0
DLM002_scaffold98_4	non-phage	0
DLM002_scaffold46457_1	non-phage	0
DLM002_scaffold2145_20	non-phage	0
DLM002_scaffold4877_5	non-phage	0
DLM002_scaffold5537_11	phage	0.999

DLM002_scaffold10705_18	non-phage	0
DLM002_scaffold11242_12	non-phage	0
DLM002_scaffold41642_1	non-phage	0
DLM002_scaffold51215_1	non-phage	0
DLM002_scaffold26552_2	non-phage	0
DLM002_scaffold49009_2	non-phage	0
DLM002_scaffold14014_3	non-phage	0
DLM002_scaffold48827_3	non-phage	0
DLM002_scaffold4794_14	non-phage	0
DLM002_scaffold22_3	non-phage	0
DLM002_scaffold49612_3	non-phage	0
DLM002_C781587_1	non-phage	0
DLM002_scaffold82_3	non-phage	0
DLM002_scaffold27864_3	non-phage	0
DLM002_C781159_1	non-phage	0
DLM002_scaffold52066_1	non-phage	0
DLM002_scaffold16755_1	non-phage	0
DLM002_scaffold93_5	non-phage	0
DLM002_scaffold20286_2	non-phage	0
DLM002_scaffold2507_3	non-phage	0
DLM002_scaffold52717_2	non-phage	0
DLM002_scaffold21810_11	non-phage	0
DLM002_scaffold49612_4	non-phage	0
DLM002_scaffold23629_1	non-phage	0
DLM002_scaffold10690_1	non-phage	0
DLM002_scaffold51888_2	non-phage	0
DLM002_scaffold2881_3	phage	0.996
DLM002_scaffold40070_2	non-phage	0
DLM002_scaffold126_7	non-phage	0
DLM002_scaffold135_4	non-phage	0
DLM002_scaffold4428_13	non-phage	0
DLM002_scaffold4134_8	non-phage	0
DLM002_scaffold41776_4	non-phage	0
DLM002_scaffold19724_6	non-phage	0
DLM002_scaffold52526_1	phage	0.999
DLM002_scaffold24004_2	non-phage	0
DLM002_scaffold52671_2	non-phage	0
DLM002_scaffold25517_1	non-phage	0
DLM002_scaffold37004_3	non-phage	0
DLM002_scaffold9745_1	non-phage	0
DLM002_scaffold33158_9	non-phage	0
DLM002_C781513_1	non-phage	0
DLM002_scaffold14177_2	non-phage	0
DLM002_scaffold4898_44	non-phage	0
DLM002_scaffold13117_3	non-phage	0
DLM002_scaffold5268_2	non-phage	0

DLM002_scaffold15749_1	non-phage	0
DLM002_scaffold97_3	non-phage	0
DLM002_scaffold49334_3	non-phage	0
DLM002_scaffold4822_10	non-phage	0
DLM002_scaffold52662_4	non-phage	0
DLM002_scaffold31454_5	non-phage	0
DLM002_scaffold34417_1	non-phage	0
DLM002_scaffold5402_3	non-phage	0
DLM002_scaffold30910_16	non-phage	0
DLM002_scaffold28661_26	non-phage	0
DLM002_scaffold1400_8	non-phage	0
DLM002_scaffold1034_1	non-phage	0
DLM002_scaffold10708_1	non-phage	0
DLM002_scaffold22438_7	non-phage	0
DLM002_scaffold30910_11	non-phage	0
DLM002_scaffold9238_5	non-phage	0
DLM002_scaffold52705_2	non-phage	0
DLM002_scaffold33309_1	non-phage	0
DLM002_scaffold33902_1	non-phage	0
DLM002_scaffold4422_4	non-phage	0
DLM002_scaffold29503_1	non-phage	0
DLM002_scaffold93_2	non-phage	0
DLM002_scaffold11050_1	non-phage	0
DLM002_scaffold44502_1	phage	0.996
DLM002_scaffold3134_1	non-phage	0
DLM002_scaffold41_3	non-phage	0
DLM002_scaffold3680_4	non-phage	0
DLM002_scaffold20342_2	non-phage	0
DLM002_scaffold1782_7	non-phage	0
DLM002_scaffold51958_1	non-phage	0
DLM002_scaffold126_6	non-phage	0
DLM002_scaffold14863_1	non-phage	0
DLM002_scaffold52526_4	phage	0.999
DLM002_scaffold2284_1	non-phage	0
DLM002_scaffold121_5	non-phage	0
DLM002_scaffold11013_8	non-phage	0
DLM002_C781203_1	non-phage	0
DLM002_scaffold5271_1	non-phage	0
DLM002_scaffold106_10	non-phage	0
DLM002_scaffold52652_1	non-phage	0
DLM002_scaffold13141_20	non-phage	0
DLM002_scaffold97_7	non-phage	0
DLM002_scaffold294_3	non-phage	0
DLM002_scaffold154_3	non-phage	0
DLM002_scaffold28957_6	non-phage	0
DLM002_scaffold32671_11	non-phage	0

DLM002_scaffold51888_1	non-phage	0
DLM002_scaffold52405_1	non-phage	0
DLM002_scaffold2257_3	non-phage	0
DLM002_scaffold19405_1	non-phage	0
DLM002_scaffold19337_18	non-phage	0
DLM002_scaffold32671_4	non-phage	0
DLM002_scaffold4794_13	non-phage	0
DLM002_scaffold50599_2	non-phage	0
DLM002_scaffold4822_8	non-phage	0
DLM002_scaffold660_77	non-phage	0
DLM002_scaffold42057_1	non-phage	0
DLM002_scaffold2207_27	non-phage	0
DLM002_scaffold37691_2	non-phage	0
DLM002_scaffold2871_11	non-phage	0
DLM002_scaffold15440_16	non-phage	0
DLM002_scaffold14372_1	non-phage	0
DLM002_scaffold52448_4	non-phage	0
DLM002_scaffold271_1	non-phage	0
DLM002_scaffold3108_3	non-phage	0
DLM002_scaffold41776_1	non-phage	0
DLM002_scaffold166_6	non-phage	0
DLM002_scaffold50443_9	non-phage	0
DLM002_scaffold5854_3	non-phage	0
DLM002_scaffold41864_1	non-phage	0
DLM002_scaffold52712_1	non-phage	0
DLM002_scaffold5854_1	non-phage	0
DLM002_scaffold35710_2	non-phage	0
DLM002_scaffold6404_7	non-phage	0
DLM002_scaffold5336_42	non-phage	0
DLM002_scaffold82_4	non-phage	0
DLM002_scaffold17136_1	non-phage	0
DLM002_scaffold5402_17	non-phage	0
DLM002_scaffold50443_8	non-phage	0
DLM002_scaffold51657_2	non-phage	0
DLM002_scaffold29503_2	non-phage	0
DLM002_scaffold51776_2	non-phage	0
DLM002_scaffold8544_21	non-phage	0.004
DLM002_scaffold162_19	non-phage	0
DLM002_scaffold4794_2	non-phage	0
DLM002_scaffold52527_1	non-phage	0
DLM002_C780975_1	non-phage	0
DLM002_scaffold27657_3	non-phage	0
DLM002_scaffold7329_4	non-phage	0
DLM002_scaffold32199_1	non-phage	0
DLM002_scaffold2920_32	non-phage	0
DLM002_scaffold9237_9	non-phage	0

DLM002_scaffold32671_7	non-phage	0
DLM002_scaffold30537_1	non-phage	0
DLM002_scaffold51995_2	non-phage	0
DLM002_scaffold20958_1	non-phage	0
DLM002_scaffold2920_74	non-phage	0
DLM002_scaffold3837_1	non-phage	0
DLM002_scaffold19083_1	non-phage	0
DLM002_scaffold6286_2	non-phage	0
DLM002_scaffold12502_5	non-phage	0
DLM002_scaffold43659_1	non-phage	0
DLM002_scaffold4794_9	non-phage	0
DLM002_scaffold121_1	non-phage	0
DLM002_scaffold20393_5	non-phage	0
DLM002_scaffold33851_8	non-phage	0
DLM002_scaffold52726_1	non-phage	0
DLM002_scaffold48180_13	phage	0.999
DLM002_scaffold25681_12	non-phage	0
DLM002_scaffold14013_4	non-phage	0
DLM002_scaffold37006_1	non-phage	0
DLM002_scaffold14044_6	non-phage	0
DLM002_scaffold6275_46	non-phage	0
DLM002_scaffold16005_9	non-phage	0
DLM002_scaffold14127_9	non-phage	0
DLM002_scaffold25891_2	non-phage	0
DLM002_scaffold22919_1	non-phage	0
DLM002_scaffold52530_4	non-phage	0
DLM002_scaffold5402_2	non-phage	0
DLM002_scaffold37616_9	non-phage	0
DLM002_scaffold19724_1	non-phage	0
DLM002_scaffold20423_14	non-phage	0
DLM002_scaffold29693_2	non-phage	0
DLM002_scaffold14127_1	non-phage	0
DLM002_scaffold48293_1	non-phage	0
DLM002_scaffold16329_2	non-phage	0
DLM002_C781459_1	non-phage	0
DLM002_scaffold52601_2	non-phage	0
DLM002_C780883_1	non-phage	0
DLM002_scaffold59_2	non-phage	0
DLM002_scaffold51776_1	non-phage	0
DLM002_scaffold6132_4	non-phage	0
DLM002_scaffold9032_11	non-phage	0
DLM002_scaffold12826_2	non-phage	0
DLM002_scaffold26082_1	non-phage	0
DLM002_scaffold43957_1	non-phage	0
DLM002_scaffold21442_1	non-phage	0
DLM002_scaffold25459_1	non-phage	0

DLM002_scaffold2284_9	non-phage	0
DLM002_scaffold52649_1	non-phage	0
DLM002_scaffold52722_1	non-phage	0
DLM002_scaffold147_3	non-phage	0
DLM002_scaffold41616_1	non-phage	0
DLM002_scaffold40070_5	non-phage	0
DLM002_scaffold7228_3	non-phage	0
DLM002_scaffold51949_2	non-phage	0
DLM002_scaffold1028_11	non-phage	0
DLM002_scaffold22438_2	non-phage	0
DLM002_scaffold36659_4	non-phage	0
DLM002_scaffold37486_1	non-phage	0
DLM002_scaffold52561_1	non-phage	0
DLM002_scaffold40965_2	non-phage	0
DLM002_scaffold29693_5	non-phage	0
DLM002_scaffold155_2	non-phage	0
DLM002_scaffold832_4	non-phage	0
DLM002_scaffold10143_3	non-phage	0
DLM002_scaffold11013_9	non-phage	0
DLM002_scaffold52708_2	non-phage	0
DLM002_scaffold47625_3	non-phage	0
DLM002_scaffold9867_6	non-phage	0
DLM002_scaffold1626_2	non-phage	0
DLM002_scaffold37649_1	non-phage	0
DLM002_scaffold4822_7	non-phage	0
DLM002_scaffold10705_6	non-phage	0
DLM002_scaffold50188_1	non-phage	0
DLM002_scaffold20393_4	non-phage	0
DLM002_scaffold52225_1	non-phage	0
DLM002_scaffold920_27	non-phage	0
DLM002_scaffold45452_1	non-phage	0
DLM002_scaffold35588_3	non-phage	0
DLM002_scaffold42939_1	phage	0.991
DLM002_scaffold52637_2	non-phage	0
DLM002_scaffold52559_4	non-phage	0
DLM002_scaffold2922_6	non-phage	0
DLM002_scaffold48366_4	non-phage	0
DLM002_scaffold41764_1	non-phage	0
DLM002_scaffold11243_10	non-phage	0
DLM002_scaffold30_1	non-phage	0
DLM002_scaffold49971_1	non-phage	0
DLM002_scaffold9323_36	non-phage	0
DLM002_scaffold7875_4	non-phage	0
DLM002_scaffold32192_4	non-phage	0
DLM002_C780607_1	non-phage	0
DLM002_scaffold48502_2	non-phage	0

DLM002_scaffold18105_6	non-phage	0
DLM002_scaffold5725_1	non-phage	0
DLM002_scaffold349_4	non-phage	0
DLM002_scaffold59_1	non-phage	0
DLM002_scaffold44761_2	non-phage	0
DLM002_scaffold20423_21	non-phage	0
DLM002_scaffold18188_11	non-phage	0
DLM002_scaffold6275_10	non-phage	0
DLM002_scaffold8392_14	non-phage	0
DLM002_scaffold52523_1	non-phage	0
DLM002_scaffold3680_6	non-phage	0
DLM002_scaffold46747_3	non-phage	0
DLM002_scaffold7849_4	non-phage	0
DLM002_scaffold3108_5	non-phage	0
DLM002_scaffold37004_5	non-phage	0
DLM002_scaffold50790_2	non-phage	0
DLM002_scaffold49178_1	non-phage	0
DLM002_scaffold15072_10	non-phage	0
DLM002_scaffold37593_1	non-phage	0
DLM002_scaffold21810_21	non-phage	0
DLM002_scaffold34636_5	non-phage	0
DLM002_scaffold52383_1	non-phage	0
DLM002_scaffold27657_2	non-phage	0
DLM002_scaffold50589_1	non-phage	0
DLM002_scaffold52720_1	non-phage	0
DLM002_scaffold51776_3	non-phage	0
DLM002_scaffold37004_1	non-phage	0
DLM002_scaffold15012_5	non-phage	0
DLM002_scaffold29142_1	non-phage	0
DLM002_scaffold52628_1	non-phage	0
DLM002_scaffold41325_2	non-phage	0
DLM002_scaffold18949_3	non-phage	0
DLM002_scaffold155_1	non-phage	0
DLM002_scaffold6404_8	non-phage	0
DLM002_scaffold8782_2	non-phage	0
DLM002_scaffold50206_1	non-phage	0
DLM002_scaffold52584_1	phage	0.999
DLM002_scaffold32192_3	phage	0.999
DLM002_scaffold13740_4	non-phage	0
DLM002_scaffold38105_1	non-phage	0
DLM002_C781113_1	non-phage	0
DLM002_scaffold20393_1	non-phage	0
DLM002_scaffold28835_5	non-phage	0
DLM002_scaffold48841_1	non-phage	0
DLM002_scaffold37616_1	non-phage	0
DLM002_scaffold52663_1	non-phage	0

DLM002_scaffold52601_3	non-phage	0
DLM002_scaffold17174_12	non-phage	0
DLM002_scaffold41350_5	non-phage	0
DLM002_scaffold367_2	non-phage	0
DLM002_scaffold49460_9	non-phage	0
DLM002_scaffold39814_1	non-phage	0
DLM002_scaffold52706_3	non-phage	0
DLM002_scaffold19724_5	non-phage	0
DLM002_scaffold17136_4	non-phage	0
DLM002_scaffold52662_2	non-phage	0
DLM002_scaffold38216_4	non-phage	0
DLM002_scaffold18105_2	non-phage	0
DLM002_scaffold5537_7	non-phage	0
DLM002_scaffold52719_1	non-phage	0
DLM002_scaffold31454_8	non-phage	0
DLM002_scaffold103_25	non-phage	0
DLM002_scaffold9398_2	non-phage	0
DLM002_scaffold36050_2	non-phage	0
DLM002_C781603_1	non-phage	0
DLM002_scaffold18388_1	non-phage	0
DLM002_scaffold5336_45	phage	0.999
DLM002_scaffold52498_9	non-phage	0
DLM002_scaffold35485_3	non-phage	0
DLM002_scaffold8763_5	non-phage	0
DLM002_C781391_1	non-phage	0
DLM002_scaffold22617_5	non-phage	0
DLM002_scaffold40347_1	non-phage	0
DLM002_scaffold37616_8	non-phage	0
DLM002_scaffold52678_3	non-phage	0
DLM002_scaffold28939_1	non-phage	0
DLM002_scaffold27008_1	non-phage	0
DLM002_scaffold52428_3	non-phage	0
DLM002_scaffold43881_2	non-phage	0
DLM002_scaffold52723_1	non-phage	0
DLM002_scaffold12502_7	non-phage	0
DLM002_scaffold5271_4	non-phage	0
DLM002_scaffold13390_10	non-phage	0
DLM002_scaffold19486_16	non-phage	0
DLM002_scaffold864_20	non-phage	0
DLM002_scaffold8357_9	non-phage	0
DLM002_scaffold43568_2	non-phage	0
DLM002_scaffold29142_2	non-phage	0
DLM002_scaffold30887_5	non-phage	0
DLM002_scaffold50001_2	non-phage	0
DLM002_scaffold10143_1	non-phage	0
DLM002_scaffold15012_2	non-phage	0

DLM002_scaffold38216_6	non-phage	0
DLM002_scaffold19292_4	non-phage	0
DLM002_C781437_1	non-phage	0
DLM002_scaffold49997_3	non-phage	0
DLM002_scaffold40829_3	non-phage	0
DLM002_scaffold52666_1	non-phage	0
DLM002_scaffold11890_3	non-phage	0
DLM002_scaffold29967_3	non-phage	0
DLM002_scaffold49912_1	non-phage	0
DLM002_scaffold32533_1	non-phage	0
DLM002_scaffold24095_16	non-phage	0
DLM002_scaffold36184_4	non-phage	0
DLM002_scaffold18643_3	non-phage	0
DLM002_scaffold52536_1	non-phage	0
DLM002_scaffold19724_8	non-phage	0
DLM002_scaffold40070_7	non-phage	0
DLM002_scaffold49971_2	non-phage	0
DLM002_scaffold40337_1	non-phage	0
DLM002_scaffold1816_3	non-phage	0
DLM002_scaffold34643_1	non-phage	0
DLM002_scaffold9165_2	non-phage	0
DLM002_scaffold20957_4	non-phage	0
DLM002_scaffold12386_12	non-phage	0
DLM002_scaffold38240_2	non-phage	0
DLM002_scaffold52711_1	non-phage	0
DLM002_scaffold50188_2	non-phage	0
DLM002_scaffold14059_9	non-phage	0
DLM002_scaffold34226_1	non-phage	0
DLM002_scaffold31610_1	non-phage	0
DLM002_scaffold8357_11	non-phage	0
DLM002_scaffold34006_1	non-phage	0
DLM002_scaffold52696_1	phage	0.999
DLM002_scaffold4422_8	non-phage	0
DLM002_scaffold34242_1	non-phage	0
DLM002_scaffold2324_2	non-phage	0
DLM002_scaffold40070_3	non-phage	0
DLM002_scaffold25488_1	non-phage	0
DLM002_scaffold4794_5	non-phage	0
DLM002_scaffold11281_2	non-phage	0
DLM002_scaffold49258_1	non-phage	0
DLM002_scaffold20342_1	non-phage	0
DLM002_scaffold3837_3	non-phage	0
DLM002_scaffold5854_2	non-phage	0
DLM002_scaffold52559_1	non-phage	0
DLM002_scaffold22438_4	non-phage	0
DLM002_scaffold42480_1	non-phage	0

DLM002_scaffold3680_5	non-phage	0
DLM002_scaffold2920_18	non-phage	0
DLM002_scaffold13889_1	non-phage	0
DLM002_scaffold925_8	phage	0.932
DLM002_scaffold103_21	non-phage	0
DLM002_scaffold36191_2	non-phage	0
DLM002_scaffold121_4	non-phage	0
DLM002_scaffold42401_5	non-phage	0
DLM002_scaffold29693_7	non-phage	0
DLM002_scaffold97_8	non-phage	0
DLM002_scaffold18411_2	non-phage	0
DLM002_scaffold93_4	non-phage	0
DLM002_scaffold18949_27	non-phage	0
DLM002_scaffold51304_1	non-phage	0
DLM002_scaffold9745_7	non-phage	0
DLM002_scaffold52583_1	non-phage	0
DLM002_scaffold50001_6	non-phage	0
DLM002_scaffold22117_2	non-phage	0
DLM002_scaffold9_18	non-phage	0
DLM002_scaffold49368_1	non-phage	0
DLM002_scaffold1400_3	non-phage	0
DLM002_scaffold36028_1	non-phage	0
DLM002_scaffold48796_2	non-phage	0
DLM002_scaffold52498_7	non-phage	0
DLM002_scaffold2706_2	non-phage	0
DLM002_scaffold48511_15	non-phage	0
DLM002_scaffold19724_3	non-phage	0
DLM002_scaffold52429_1	non-phage	0
DLM002_scaffold49071_3	non-phage	0
DLM002_scaffold21495_4	non-phage	0
DLM002_scaffold507_3	non-phage	0
DLM002_scaffold39809_1	non-phage	0
DLM002_scaffold52720_2	non-phage	0
DLM002_scaffold52687_3	non-phage	0
DLM002_scaffold12593_9	non-phage	0
DLM002_scaffold14013_2	non-phage	0
DLM002_scaffold5537_6	non-phage	0
DLM003_scaffold19233_1	non-phage	0
DLM003_scaffold16535_3	non-phage	0
DLM003_scaffold47010_2	non-phage	0
DLM003_scaffold54156_9	non-phage	0
DLM003_scaffold67460_1	non-phage	0
DLM003_scaffold67439_1	non-phage	0
DLM003_scaffold30478_2	phage	0.999
DLM003_scaffold29333_5	non-phage	0
DLM003_scaffold106_1	non-phage	0

DLM003_scaffold62995_1	non-phage	0
DLM003_scaffold482_1	non-phage	0
DLM003_scaffold44375_1	non-phage	0
DLM003_scaffold49606_21	non-phage	0
DLM003_scaffold15125_1	non-phage	0
DLM003_scaffold28718_31	non-phage	0
DLM003_scaffold24118_1	non-phage	0
DLM003_scaffold53021_1	non-phage	0
DLM003_scaffold28376_4	non-phage	0
DLM003_scaffold34695_4	non-phage	0
DLM003_scaffold51098_1	non-phage	0
DLM003_scaffold235_1	non-phage	0
DLM003_scaffold42859_2	non-phage	0
DLM003_scaffold3123_6	non-phage	0
DLM003_scaffold25980_2	non-phage	0
DLM003_scaffold49279_1	non-phage	0
DLM003_scaffold18804_10	non-phage	0
DLM003_scaffold24702_27	non-phage	0
DLM003_scaffold2040_2	non-phage	0
DLM003_scaffold47864_4	non-phage	0.211
DLM003_scaffold16005_6	non-phage	0
DLM003_scaffold33104_4	non-phage	0
DLM003_scaffold26385_5	non-phage	0
DLM003_scaffold21534_9	non-phage	0
DLM003_scaffold50718_1	non-phage	0
DLM003_scaffold36857_6	non-phage	0
DLM003_scaffold35342_9	non-phage	0
DLM003_scaffold11766_1	non-phage	0
DLM003_scaffold35975_4	phage	0.997
DLM003_scaffold287_38	non-phage	0
DLM003_scaffold29970_11	non-phage	0
DLM003_scaffold5569_12	non-phage	0
DLM003_scaffold32272_4	non-phage	0
DLM003_scaffold29970_7	non-phage	0
DLM003_scaffold53890_18	non-phage	0
DLM003_scaffold139_2	non-phage	0
DLM003_scaffold51623_3	non-phage	0
DLM003_scaffold45047_1	non-phage	0
DLM003_scaffold20093_1	phage	0.961
DLM003_scaffold5883_10	non-phage	0
DLM003_scaffold8802_8	phage	0.936
DLM003_scaffold66267_2	non-phage	0
DLM003_scaffold4491_4	non-phage	0
DLM003_scaffold33914_2	non-phage	0
DLM003_scaffold14015_7	non-phage	0
DLM003_scaffold67435_1	non-phage	0

DLM003_scaffold28158_14	non-phage	0
DLM003_scaffold16633_5	non-phage	0
DLM003_scaffold12712_13	non-phage	0
DLM003_scaffold25702_10	non-phage	0
DLM003_scaffold21360_5	non-phage	0
DLM003_scaffold67352_2	non-phage	0
DLM003_scaffold4001_2	non-phage	0
DLM003_scaffold67260_2	non-phage	0
DLM003_scaffold41811_3	non-phage	0
DLM003_scaffold31526_6	non-phage	0
DLM003_scaffold18640_1	non-phage	0
DLM003_scaffold30479_1	non-phage	0
DLM003_C810585_1	non-phage	0
DLM003_scaffold1898_14	phage	0.999
DLM003_scaffold1319_8	non-phage	0
DLM003_scaffold4491_14	non-phage	0
DLM003_scaffold14916_8	non-phage	0
DLM003_scaffold66132_1	non-phage	0
DLM003_scaffold18674_1	non-phage	0
DLM003_scaffold67406_2	non-phage	0
DLM003_scaffold56164_1	non-phage	0
DLM003_scaffold276_4	non-phage	0
DLM003_scaffold29333_1	non-phage	0
DLM003_scaffold67325_1	non-phage	0
DLM003_scaffold53476_2	non-phage	0
DLM003_scaffold4631_32	non-phage	0
DLM003_scaffold65_2	non-phage	0
DLM003_scaffold14016_1	non-phage	0
DLM003_scaffold44857_3	non-phage	0
DLM003_scaffold30928_7	phage	0.932
DLM003_scaffold19233_6	non-phage	0
DLM003_scaffold5007_1	non-phage	0
DLM003_scaffold59810_1	non-phage	0
DLM003_scaffold64540_1	non-phage	0
DLM003_scaffold64192_2	non-phage	0.084
DLM003_scaffold67349_1	non-phage	0.019
DLM003_scaffold23240_7	non-phage	0
DLM003_scaffold23156_1	non-phage	0
DLM003_scaffold67459_1	non-phage	0
DLM003_scaffold29812_2	non-phage	0
DLM003_scaffold11185_1	non-phage	0
DLM003_scaffold46494_4	non-phage	0
DLM003_scaffold1638_4	non-phage	0
DLM003_scaffold58033_2	non-phage	0
DLM003_scaffold46848_3	non-phage	0
DLM003_scaffold14809_5	non-phage	0

DLM003_scaffold21957_12	non-phage	0
DLM003_scaffold62996_1	non-phage	0
DLM003_scaffold36857_2	non-phage	0
DLM003_scaffold6264_1	non-phage	0
DLM003_scaffold59785_6	phage	0.998
DLM003_scaffold53890_9	non-phage	0
DLM003_scaffold24118_9	non-phage	0
DLM003_scaffold13220_6	non-phage	0
DLM003_scaffold27999_3	non-phage	0
DLM003_scaffold67334_2	phage	0.999
DLM003_scaffold39949_3	non-phage	0
DLM003_scaffold35709_10	non-phage	0
DLM003_scaffold66050_3	non-phage	0
DLM003_scaffold55221_1	non-phage	0
DLM003_scaffold31633_1	non-phage	0
DLM003_scaffold51368_3	non-phage	0
DLM003_scaffold12725_2	non-phage	0
DLM003_scaffold32923_3	non-phage	0
DLM003_scaffold16005_8	non-phage	0
DLM003_scaffold6710_1	non-phage	0
DLM003_scaffold3026_1	non-phage	0
DLM003_scaffold62038_7	non-phage	0
DLM003_scaffold32186_2	non-phage	0
DLM003_scaffold4214_3	non-phage	0
DLM003_scaffold8440_7	non-phage	0
DLM003_scaffold67369_1	non-phage	0
DLM003_C810975_1	phage	0.998
DLM003_scaffold17060_1	non-phage	0
DLM003_C810431_1	non-phage	0
DLM003_scaffold39345_4	non-phage	0
DLM003_scaffold61571_3	non-phage	0
DLM003_scaffold38753_1	non-phage	0
DLM003_scaffold27419_1	non-phage	0
DLM003_scaffold3168_1	non-phage	0
DLM003_scaffold28547_16	non-phage	0
DLM003_scaffold229_6	non-phage	0
DLM003_scaffold52764_1	non-phage	0
DLM003_C810657_1	non-phage	0
DLM003_scaffold28042_1	non-phage	0
DLM003_scaffold32272_1	non-phage	0
DLM003_scaffold37839_1	non-phage	0
DLM003_scaffold28158_3	non-phage	0
DLM003_scaffold31125_1	non-phage	0
DLM003_scaffold10552_3	non-phage	0
DLM003_scaffold31156_2	phage	0.999
DLM003_scaffold63034_1	non-phage	0

DLM003_scaffold11294_3	non-phage	0
DLM003_scaffold67455_1	non-phage	0
DLM003_scaffold36510_1	non-phage	0
DLM003_scaffold30928_3	non-phage	0
DLM003_scaffold46494_1	non-phage	0
DLM003_scaffold66421_2	non-phage	0
DLM003_scaffold25694_5	non-phage	0
DLM003_scaffold9719_10	non-phage	0
DLM003_scaffold24702_43	phage	0.998
DLM003_scaffold6359_7	non-phage	0
DLM003_scaffold57972_3	non-phage	0
DLM003_scaffold51167_2	non-phage	0
DLM003_scaffold10664_1	phage	0.996
DLM003_scaffold67418_1	non-phage	0
DLM003_scaffold42835_3	non-phage	0
DLM003_scaffold26499_2	non-phage	0
DLM003_scaffold67056_3	non-phage	0
DLM003_scaffold54156_4	non-phage	0
DLM003_scaffold33382_2	non-phage	0
DLM003_scaffold11408_8	non-phage	0
DLM003_scaffold5186_13	non-phage	0
DLM003_scaffold43292_1	non-phage	0
DLM003_scaffold29479_1	non-phage	0
DLM003_scaffold31321_2	non-phage	0
DLM003_scaffold12946_1	non-phage	0
DLM003_scaffold16743_8	non-phage	0
DLM003_scaffold48836_1	non-phage	0
DLM003_scaffold59785_2	non-phage	0
DLM003_scaffold55894_2	non-phage	0
DLM003_scaffold18033_3	non-phage	0
DLM003_scaffold5766_9	non-phage	0
DLM003_scaffold64681_3	non-phage	0
DLM003_scaffold11253_5	non-phage	0
DLM003_scaffold87_15	non-phage	0
DLM003_scaffold14808_1	non-phage	0
DLM003_scaffold46929_2	phage	0.999
DLM003_scaffold67453_1	non-phage	0
DLM003_scaffold66988_2	non-phage	0
DLM003_scaffold27795_3	non-phage	0
DLM003_scaffold14809_3	non-phage	0
DLM003_scaffold50794_3	non-phage	0
DLM003_C810937_1	non-phage	0
DLM003_scaffold21404_8	non-phage	0
DLM003_scaffold1825_2	non-phage	0
DLM003_scaffold70_10	non-phage	0
DLM003_scaffold49606_1	non-phage	0

DLM003_scaffold52264_4	non-phage	0
DLM003_scaffold6127_3	non-phage	0
DLM003_scaffold65226_1	non-phage	0
DLM003_scaffold36857_5	non-phage	0
DLM003_scaffold12872_2	non-phage	0
DLM003_scaffold38761_2	non-phage	0
DLM003_scaffold1907_21	non-phage	0
DLM003_scaffold24702_39	non-phage	0
DLM003_scaffold30054_16	non-phage	0
DLM003_scaffold28629_2	non-phage	0
DLM003_scaffold61896_1	non-phage	0
DLM003_scaffold12578_2	non-phage	0
DLM003_scaffold28674_3	non-phage	0
DLM003_scaffold29333_2	non-phage	0
DLM003_scaffold44496_1	non-phage	0
DLM003_scaffold64654_1	non-phage	0
DLM003_scaffold21534_8	non-phage	0
DLM003_scaffold25233_9	non-phage	0
DLM003_scaffold41905_2	non-phage	0
DLM003_scaffold1825_9	non-phage	0
DLM003_scaffold30928_1	non-phage	0
DLM003_scaffold63034_2	non-phage	0
DLM003_scaffold33104_1	non-phage	0
DLM003_scaffold17989_5	non-phage	0
DLM003_scaffold59785_5	phage	0.997
DLM003_scaffold30928_8	non-phage	0
DLM003_scaffold64319_3	non-phage	0
DLM003_scaffold40695_7	phage	0.999
DLM003_scaffold8440_6	non-phage	0
DLM003_scaffold4744_1	phage	0.994
DLM003_scaffold30037_8	non-phage	0
DLM003_scaffold16005_10	non-phage	0
DLM003_scaffold61651_10	non-phage	0
DLM003_scaffold67404_1	non-phage	0
DLM003_scaffold56406_8	non-phage	0
DLM003_scaffold815_1	non-phage	0
DLM003_scaffold44857_4	non-phage	0
DLM003_scaffold28718_6	non-phage	0
DLM003_scaffold303_2	phage	0.856
DLM003_scaffold67428_1	non-phage	0
DLM003_scaffold49503_1	non-phage	0
DLM003_scaffold25875_2	non-phage	0
DLM003_scaffold58022_1	non-phage	0
DLM003_scaffold37654_3	non-phage	0
DLM003_scaffold33316_1	non-phage	0
DLM003_scaffold39345_3	non-phage	0

DLM003_scaffold67150_1	non-phage	0
DLM003_scaffold28917_3	non-phage	0
DLM003_scaffold27039_10	non-phage	0
DLM003_scaffold56406_13	non-phage	0
DLM003_scaffold36199_4	phage	0.563
DLM003_scaffold276_1	non-phage	0
DLM003_scaffold28718_10	non-phage	0
DLM003_scaffold27926_16	non-phage	0
DLM003_scaffold179_3	non-phage	0
DLM003_scaffold67452_1	non-phage	0
DLM003_scaffold32038_1	non-phage	0
DLM003_scaffold5569_10	non-phage	0
DLM003_scaffold10552_4	non-phage	0
DLM003_scaffold52764_2	non-phage	0
DLM003_scaffold8440_4	non-phage	0
DLM003_scaffold33382_1	non-phage	0
DLM003_scaffold46920_2	non-phage	0
DLM003_scaffold14184_9	non-phage	0
DLM003_scaffold54492_1	non-phage	0
DLM003_scaffold67453_2	non-phage	0
DLM003_scaffold67461_1	non-phage	0
DLM003_scaffold67441_1	non-phage	0
DLM003_scaffold27039_6	non-phage	0
DLM003_scaffold5883_16	non-phage	0
DLM003_scaffold102_4	non-phage	0
DLM003_scaffold4491_2	non-phage	0
DLM003_scaffold28547_2	non-phage	0
DLM003_scaffold35850_3	non-phage	0
DLM003_scaffold65401_2	non-phage	0
DLM003_scaffold56743_1	non-phage	0
DLM003_scaffold1319_15	non-phage	0
DLM003_scaffold3026_2	non-phage	0
DLM003_scaffold28158_19	non-phage	0
DLM003_scaffold28663_36	non-phage	0
DLM003_scaffold66829_1	non-phage	0
DLM003_scaffold42910_2	non-phage	0
DLM003_scaffold5790_5	non-phage	0
DLM003_scaffold25694_4	non-phage	0
DLM003_scaffold67418_2	non-phage	0
DLM003_scaffold17963_2	non-phage	0
DLM003_scaffold39345_5	non-phage	0
DLM003_scaffold4001_4	non-phage	0
DLM003_scaffold12201_4	phage	0.999
DLM003_scaffold31932_1	non-phage	0
DLM003_scaffold28718_7	non-phage	0
DLM003_scaffold280_2	non-phage	0

DLM003_scaffold33854_5	non-phage	0
DLM003_scaffold30037_22	non-phage	0
DLM003_scaffold61377_2	non-phage	0
DLM003_scaffold225_2	non-phage	0
DLM003_scaffold27886_5	non-phage	0
DLM003_scaffold38239_10	non-phage	0
DLM003_scaffold51623_1	non-phage	0
DLM003_scaffold41913_4	non-phage	0
DLM003_scaffold64319_1	non-phage	0
DLM003_scaffold67059_1	non-phage	0
DLM003_scaffold62419_1	non-phage	0.036
DLM003_scaffold59256_6	non-phage	0
DLM003_scaffold9756_9	non-phage	0
DLM003_scaffold29333_3	non-phage	0
DLM003_scaffold67325_3	non-phage	0
DLM003_scaffold14221_2	non-phage	0
DLM003_scaffold16470_4	non-phage	0
DLM003_scaffold11967_6	non-phage	0
DLM003_scaffold57690_9	non-phage	0
DLM003_scaffold17334_1	non-phage	0
DLM003_scaffold32013_4	non-phage	0
DLM003_scaffold67438_2	non-phage	0
DLM003_scaffold11349_2	non-phage	0
DLM003_scaffold44857_1	non-phage	0
DLM003_scaffold6129_18	non-phage	0
DLM003_scaffold21_9	non-phage	0
DLM003_scaffold6750_20	non-phage	0
DLM003_scaffold482_3	non-phage	0
DLM003_scaffold37326_6	non-phage	0
DLM003_scaffold15117_3	non-phage	0
DLM003_scaffold4215_19	non-phage	0
DLM003_scaffold66411_1	non-phage	0
DLM003_scaffold30680_18	non-phage	0
DLM003_scaffold41913_3	non-phage	0
DLM003_scaffold3026_3	non-phage	0
DLM003_scaffold50466_4	non-phage	0
DLM003_scaffold3141_4	non-phage	0
DLM003_scaffold31156_3	non-phage	0
DLM003_scaffold43383_1	non-phage	0
DLM003_scaffold49641_1	non-phage	0
DLM003_scaffold9756_11	non-phage	0
DLM003_scaffold59982_1	non-phage	0
DLM003_scaffold10071_6	non-phage	0
DLM003_scaffold65702_1	non-phage	0
DLM003_scaffold59785_8	non-phage	0
DLM003_scaffold72_2	non-phage	0

DLM003_scaffold25233_8	non-phage	0
DLM003_scaffold16412_17	phage	0.999
DLM003_C810793_1	non-phage	0
DLM003_scaffold64681_4	non-phage	0
DLM003_scaffold8338_23	non-phage	0
DLM003_scaffold49406_1	phage	0.998
DLM003_scaffold66994_2	non-phage	0
DLM003_scaffold36857_8	non-phage	0
DLM003_scaffold16750_9	non-phage	0
DLM003_scaffold46929_3	non-phage	0
DLM003_scaffold41969_4	non-phage	0
DLM003_scaffold52924_1	non-phage	0
DLM003_C810579_1	non-phage	0
DLM003_scaffold31683_3	non-phage	0
DLM003_scaffold10644_11	non-phage	0
DLM003_C810753_1	phage	0.999
DLM003_scaffold67189_1	non-phage	0
DLM003_scaffold31001_5	non-phage	0
DLM003_scaffold51623_5	non-phage	0
DLM003_scaffold54156_5	non-phage	0
DLM003_scaffold7695_1	non-phage	0
DLM003_scaffold24702_42	non-phage	0
DLM003_scaffold45047_5	non-phage	0
DLM003_scaffold17170_5	non-phage	0
DLM003_scaffold50446_4	non-phage	0
DLM003_scaffold26412_7	non-phage	0
DLM003_scaffold67292_4	non-phage	0
DLM003_scaffold57868_2	non-phage	0
DLM003_scaffold18033_1	non-phage	0
DLM003_scaffold39949_2	non-phage	0
DLM003_scaffold7665_2	non-phage	0
DLM003_scaffold1898_11	phage	0.999
DLM003_scaffold53858_1	non-phage	0
DLM003_scaffold30479_3	non-phage	0
DLM003_scaffold58267_2	non-phage	0
DLM003_scaffold23742_5	non-phage	0
DLM003_scaffold22796_4	non-phage	0
DLM003_scaffold3595_3	non-phage	0
DLM003_scaffold29333_6	non-phage	0
DLM003_scaffold1319_6	non-phage	0
DLM003_scaffold51368_5	non-phage	0
DLM003_scaffold11409_4	non-phage	0
DLM003_scaffold12709_4	non-phage	0
DLM003_scaffold14916_15	non-phage	0
DLM003_scaffold55616_2	non-phage	0
DLM003_scaffold59500_1	non-phage	0

DLM003_scaffold19061_2	non-phage	0
DLM003_scaffold33382_5	non-phage	0
DLM003_scaffold8885_3	non-phage	0
DLM003_scaffold66942_1	non-phage	0
DLM003_scaffold67404_2	non-phage	0
DLM003_scaffold66050_1	phage	0.997
DLM003_scaffold64319_4	non-phage	0
DLM003_scaffold56_5	non-phage	0
DLM003_scaffold5660_9	non-phage	0
DLM003_scaffold20452_3	non-phage	0
DLM003_scaffold28674_2	non-phage	0
DLM003_scaffold50921_5	non-phage	0
DLM003_scaffold13266_3	non-phage	0
DLM003_scaffold28718_32	non-phage	0
DLM003_scaffold17993_2	non-phage	0
DLM003_scaffold16174_12	non-phage	0
DLM003_scaffold66050_2	non-phage	0
DLM003_scaffold56_9	non-phage	0.001
DLM003_scaffold27039_2	non-phage	0
DLM003_scaffold51914_1	non-phage	0
DLM003_scaffold35942_4	non-phage	0
DLM003_scaffold17135_3	phage	0.827
DLM003_scaffold5660_2	non-phage	0
DLM003_scaffold57972_1	non-phage	0
DLM003_scaffold29479_3	non-phage	0
DLM003_scaffold8802_3	non-phage	0
DLM003_scaffold67442_1	non-phage	0
DLM003_scaffold40695_8	non-phage	0
DLM003_scaffold6589_2	non-phage	0
DLM003_scaffold51368_1	non-phage	0
DLM003_scaffold36199_2	non-phage	0
DLM003_scaffold67077_1	non-phage	0
DLM003_scaffold3748_3	non-phage	0
DLM003_scaffold65553_1	non-phage	0
DLM003_scaffold42594_2	non-phage	0
DLM003_scaffold43835_2	non-phage	0
DLM003_scaffold29743_2	phage	0.999
DLM003_scaffold14015_13	phage	0.749
DLM003_scaffold1811_13	non-phage	0
DLM003_scaffold3089_1	phage	0.999
DLM003_scaffold64319_2	non-phage	0
DLM003_scaffold1825_3	non-phage	0
DLM003_scaffold12578_5	non-phage	0
DLM003_scaffold14809_1	non-phage	0
DLM003_scaffold11409_9	non-phage	0
DLM003_scaffold39345_2	non-phage	0

DLM003_scaffold49606_27	non-phage	0
DLM003_scaffold22731_5	non-phage	0
DLM003_scaffold67428_2	non-phage	0
DLM003_scaffold60511_1	non-phage	0
DLM003_scaffold25694_3	non-phage	0
DLM003_scaffold15117_2	phage	0.999
DLM003_scaffold38849_1	non-phage	0
DLM003_scaffold55891_2	non-phage	0
DLM003_scaffold5883_7	non-phage	0
DLM003_scaffold31683_13	non-phage	0
DLM003_scaffold482_4	non-phage	0
DLM003_scaffold49646_2	non-phage	0
DLM003_scaffold42857_3	non-phage	0
DLM003_scaffold40984_4	non-phage	0
DLM003_scaffold52_2	non-phage	0
DLM003_scaffold43107_1	non-phage	0
DLM003_scaffold41913_2	non-phage	0
DLM003_C810575_1	non-phage	0
DLM003_scaffold58082_1	non-phage	0
DLM003_scaffold280_6	non-phage	0
DLM003_scaffold59785_12	phage	0.998
DLM003_scaffold11890_2	non-phage	0
DLM003_scaffold54156_1	non-phage	0
DLM003_scaffold52841_1	non-phage	0
DLM003_scaffold29333_4	non-phage	0
DLM003_scaffold56369_1	non-phage	0
DLM003_scaffold66829_3	non-phage	0
DLM003_scaffold30478_1	non-phage	0
DLM003_scaffold70_4	non-phage	0
DLM003_scaffold35342_20	non-phage	0
DLM003_scaffold102_3	phage	0.996
DLM003_scaffold6141_1	non-phage	0
DLM003_scaffold3123_3	non-phage	0
DLM003_scaffold46929_4	phage	0.999
DLM003_scaffold14015_16	non-phage	0
DLM003_scaffold12455_1	non-phage	0
DLM003_scaffold30928_6	non-phage	0
DLM003_scaffold28718_3	non-phage	0
DLM003_scaffold28718_15	non-phage	0
DLM003_scaffold39255_5	non-phage	0
DLM003_scaffold66942_2	non-phage	0
DLM003_scaffold30596_8	non-phage	0
DLM003_scaffold5007_2	non-phage	0
DLM003_scaffold18387_1	non-phage	0
DLM003_scaffold40695_5	phage	0.993
DLM003_scaffold64192_1	non-phage	0

DLM003_scaffold55894_1	non-phage	0
DLM003_scaffold10358_1	non-phage	0
DLM003_scaffold20663_2	non-phage	0
DLM003_scaffold7927_4	non-phage	0
DLM003_scaffold66009_1	non-phage	0
DLM003_scaffold16800_1	non-phage	0
DLM003_scaffold12712_2	non-phage	0
DLM003_scaffold24688_13	non-phage	0
DLM003_scaffold5660_6	non-phage	0
DLM003_scaffold10125_4	non-phage	0
DLM003_scaffold11409_5	non-phage	0
DLM003_scaffold67292_3	non-phage	0
DLM003_scaffold63374_1	non-phage	0
DLM003_scaffold3168_3	non-phage	0
DLM003_scaffold56010_3	non-phage	0
DLM003_scaffold14808_2	non-phage	0
DLM003_scaffold67420_1	non-phage	0
DLM003_scaffold66829_4	non-phage	0
DLM003_scaffold28718_21	non-phage	0
DLM003_scaffold3164_3	non-phage	0
DLM003_scaffold46_1	non-phage	0
DLM003_scaffold10557_5	non-phage	0
DLM003_scaffold53890_12	non-phage	0
DLM003_scaffold46_6	non-phage	0
DLM003_scaffold53306_2	non-phage	0
DLM003_scaffold28015_3	non-phage	0
DLM003_scaffold31321_5	non-phage	0
DLM003_scaffold16743_12	non-phage	0
DLM003_scaffold31001_2	non-phage	0
DLM003_scaffold73_2	non-phage	0
DLM003_scaffold56_6	non-phage	0
DLM003_scaffold14015_4	non-phage	0
DLM003_scaffold4912_11	non-phage	0
DLM003_scaffold2432_8	non-phage	0
DLM003_scaffold17937_1	non-phage	0
DLM003_scaffold5766_13	non-phage	0
DLM003_scaffold24702_15	non-phage	0
DLM003_scaffold67456_2	non-phage	0
DLM003_scaffold4214_2	non-phage	0
DLM003_scaffold29334_1	non-phage	0
DLM003_scaffold31683_23	non-phage	0
DLM003_scaffold28547_9	non-phage	0
DLM003_scaffold63682_1	non-phage	0
DLM003_scaffold16470_1	non-phage	0
DLM003_scaffold6129_12	non-phage	0
DLM003_scaffold66132_3	non-phage	0

DLM003_scaffold30478_4	non-phage	0
DLM003_scaffold30928_5	non-phage	0
DLM003_scaffold62851_3	non-phage	0
DLM003_scaffold53228_1	non-phage	0
DLM003_scaffold44857_6	non-phage	0
DLM003_scaffold16600_2	non-phage	0
DLM003_scaffold46494_3	non-phage	0
DLM003_scaffold9777_20	non-phage	0
DLM003_scaffold47070_1	non-phage	0
DLM003_scaffold67257_1	non-phage	0
DLM003_scaffold8862_5	non-phage	0
DLM003_scaffold3893_4	non-phage	0
DLM003_scaffold19233_3	non-phage	0
DLM004_scaffold7821_1	non-phage	0
DLM004_scaffold13351_1	non-phage	0
DLM004_scaffold47_9	non-phage	0
DLM004_scaffold10109_2	non-phage	0
DLM004_scaffold18133_3	non-phage	0
DLM004_scaffold14817_1	non-phage	0
DLM004_scaffold1128_1	non-phage	0
DLM004_scaffold18072_1	non-phage	0
DLM004_scaffold941_3	non-phage	0
DLM004_scaffold18375_2	non-phage	0
DLM004_scaffold9772_3	non-phage	0
DLM004_scaffold15819_4	non-phage	0
DLM004_scaffold770_2	non-phage	0
DLM004_scaffold15987_2	non-phage	0
DLM004_scaffold14498_1	non-phage	0
DLM004_scaffold2416_6	phage	0.989
DLM004_scaffold17676_1	non-phage	0
DLM004_scaffold11368_1	non-phage	0
DLM004_scaffold73_6	non-phage	0
[truncated: 3,559,080 more chars]
